# Anoikis-Related Long Non-Coding RNA Signatures to Predict Prognosis and Immune Infiltration of Gastric Cancer

**DOI:** 10.3390/bioengineering11090893

**Published:** 2024-09-05

**Authors:** Wen-Jun Meng, Jia-Min Guo, Li Huang, Yao-Yu Zhang, Yue-Ting Zhu, Lian-Sha Tang, Jia-Ling Wang, Hong-Shuai Li, Ji-Yan Liu

**Affiliations:** 1 Department of Biotherapy, Cancer Center, West China Hospital, Sichuan University, Chengdu 610041, China; mwj1995@foxmail.com (W.-J.M.);; 2 Division of Abdominal Tumor Multimodality Treatment, Cancer Center, West China Hospital, Sichuan University, Chengdu 610041, China; 3 West China School of Nursing, Sichuan University, Chengdu 610041, China; 4 Department of Urology, The General Hospital of Western Theater Command, Chengdu 610083, China

**Keywords:** gastric cancer, anoikis, long non-coding RNA, prognostic model, immune infiltration, bioinformatics

## Abstract

Anoikis is a distinct type of programmed cell death and a unique mechanism for tumor progress. However, its exact function in gastric cancer (GC) remains unknown. This study aims to investigate the function of anoikis-related lncRNA (ar-lncRNA) in the prognosis of GC and its immunological infiltration. The ar-lncRNAs were derived from RNA sequencing data and associated clinical information obtained from The Cancer Genome Atlas. Pearson correlation analysis, differential screening, LASSO and Cox regression were utilized to identify the typical ar-lncRNAs with prognostic significance, and the corresponding risk model was constructed, respectively. Comprehensive methods were employed to assess the clinical characteristics of the prediction model, ensuring the accuracy of the prediction results. Further analysis was conducted on the relationship between immune microenvironment and risk features, and sensitivity predictions were made about anticancer medicines. A risk model was built according to seven selected ar-lncRNAs. The model was validated and the calibration plots were highly consistent in validating nomogram predictions. Further analyses revealed the great accuracy of the model and its ability to serve as a stand-alone GC prognostic factor. We subsequently disclosed that high-risk groups display significant enrichment in pathways related to tumors and the immune system. Additionally, in tumor immunoassays, notable variations in immune infiltrates and checkpoints were noted between different risk groups. This study proposes, for the first time, that prognostic signatures of ar-lncRNA can be established in GC. These signatures accurately predict the prognosis of GC and offer potential biomarkers, suggesting new avenues for basic research, prognosis prediction and personalized diagnosis and treatment of GC.

## 1. Introduction

Globally, gastric cancer (GC) is a malignant tumor of the digestive system with a high incidence and mortality rate. According to recent published data by the United States, there will be 26,890 and 10,880 of new and death cases throughout the whole year of 2024 in this country, respectively [1]. Globally, 62% of GC cases occur in East Asia, and nearly 50% occur in China [2]. Most GC patients have a past history of Helicobacter pylori infection, which lacks specific symptoms during the progress of disease [3]. Therefore, the late stage frequently accounts for its poor prognosis. For GC with advanced stage, systemic chemotherapy is still the standard approach with only a longest median overall survival (OS) of 12 months [4]. For one single GC patient, heterogeneity of pre- and post-treatment shows a big difference in gene expression profile; moreover, heterogeneity of both intratumor and intertumor is also the prominent feature in over 30% GC patients [5,6]. These factors together result in its poor prognosis.

Immune checkpoint inhibitors (ICIs), as the representative and personalized approach of cancer immunotherapy, have been applied in GC for years. Serving as the core drug in both perioperative and posterior-line therapy, ICI-oriented combinational chemo-immune therapy shows better efficacy than traditional chemotherapy against GC [7,8,9]. However, not all GC patients can benefit from those personalized therapies. Studies proved that over half of GC patients have a low expression of programmed death-ligand 1 (PD-L1), which is a sensitive and a positively correlated biomarker of the efficacy of immunotherapy [10,11]. Indeed, another study based on the KEYNOTE-061 trial demonstrated that ICIs had a limited efficacy in individuals with elevated PD-L1 expression [12]. Currently, the connection between the clinical biomarkers and the effectiveness of immunotherapy in GC is still unclear. Therefore, it is necessary to explore more reliable biomarkers for the early assessment of the prognosis of GC patients so as to implement prompt therapeutic intervention strategies to halt the disease’s development.

Anoikis is a unique type of programmed cell death that happens when cells attach to the wrong places or fail to connect with the surrounding extracellular matrix [13]. This procedure can effectively remove dislocated or detached cells so as to maintain tissue homeostasis [14]. The generation of anoikis resistance intensifies the complexity and heterogeneity of tumor microenvironment (TME), aids isolated cells in avoiding the death signaling pathway, then enables them to endure harsh circumstances, and is a unique mechanism for tumor progress [15,16,17]. Studies have confirmed that inhibiting the anoikis of GC cells can activate signaling pathways like JAK-STAT3, PI3K/AKT and PDGFB/PDGFRβ, thereby leading to epithelial–mesenchymal transition in tumor cells, and then induce tumor proliferation and metastasis [18,19,20,21]. Therefore, the anoikis-related gene (ARG) family is overexpressed in tumors during their cell cycles and fails to induce tumor apoptosis. Several studies have proven that both overexpression and silencing of ARG family represent poor prognosis in many solid tumors [14]; in contrast, inducing ARG family can inhibit tumor metastasis, as well as increase the sensitivity to therapeutic drugs [22]. Comprehensive analysis of anoikis in GC is currently limited, and it is unclear how GC prognosis is predicted by ARGs.

Long non-coding RNA (lncRNA) is a group of non-protein coding transcripts with more than 200 nucleotides, which participates in various gene regulations and pathological processes by regulating the transcription and translation of metabolism-related genes and acting as a competitive endogenous RNA [23,24]. Certain lncRNAs have the ability to control immune cell activity inside TME, trigger tumor immune escape, or elicit anti-tumor immunity [25,26]. These functions may offer direction for addressing immunotherapy resistance in malignancies with unfavorable prognoses. However, the function of anoikis-related lncRNA (ar-lncRNA) in the TME of GC and its prognosis has not been studied.

Although anoikis and lncRNA is strongly correlated with the course and prognosis of tumors, its specific value in GC has not been explicitly analyzed. In this study, hub ar-lncRNAs related to GC will be identified and created, which serves as a potential clinical value for prognosis and immune infiltration prediction, as well as pharmacy selection.

## 2. Materials and Methods

### 2.1. The Capture and Pre-Processing of Patients’ Data

The RNA sequencing (RNA-seq) transcription data and clinical information of GC patients were acquired from an online database, The Cancer Genome Atlas (TCGA) bioinformatic database (https://tcga-data.nci.nih.gov/tcga/ (accessed on 24 July 2024)). To minimize statistical biases, those with missing data of OS or OS < 30 days were excluded. Finally, 375 GC samples and 32 normal tissue samples were gathered along with corresponding RNA-seq data and clinical information.

### 2.2. Screening of Ar-lncRNAs

A total of 413 ARGs were obtained according to the previously published literature (Supplementary Table S1) [27]. Via Pearson correlation analysis in the condition of |Pearson R| > 0.5 and *p* < 0.001, ARG and lncRNA filtering were performed and 636 ar-lncRNAs were acquired. Under the criteria of Log2 fold change (FC) > 2 and fdrFilter (FDR) < 0.05, a total of 106 differentially expressed lncRNAs were finally obtained. The above synthetic data matrix was filtered through Strawberry Perl 5.30.0 (https://www.perl.org/ (accessed on 24 July 2024)) and R4.1.3 via the “limma” R package [28].

### 2.3. Creation and Validation of Risk Signature

To construct a predictive model with clinical value, univariate Cox (uni-Cox) regression was used to analyze the ar-lncRNA in GC samples, and ar-lncRNAs with *p* < 0.05 were considered to have prognostic significance. By means of last absolute shrinkage and selection operator (LASSO) Cox analysis, seven hub lncRNAs related to GC prognosis were selected to build the model [29]. Multivariate Cox (multi-Cox) regression was conducted to create a novel anoikis-related signature, and a new risk score formula was generated as follows: Risk Score = Σ[Exp(lncRNA) × coef(lncRNA)].

Then, all tumor samples were randomly divided into two groups of Train and Test by a 1:1 ratio. According to the median risk score acquired from the risk model, the GC patients were classified into low-risk and high-risk groups. The obtained receiver operating characteristic (ROC) curve and area under the ROC curve (AUC) were performed to validate the accuracy of the model. All the analyses above were based on the R packages of “caret” “glmnet” “rms” “survival” “survminer” and “timeROC”.

### 2.4. Establishment of an Anoikis-Related Nomogram

According to the GC patients’ clinical data, involving age, gender, tumor stage, etc., a nomogram was drawn using “rms” R package, which assessed the patients’ one-, three- and five-year OS; and calibration curves were made to demonstrate the predictive ability of the alignment chart.

### 2.5. Analysis of Gene Set Enrichment

According to the median risk score, differential analysis was conducted to obtain differentially expressed genes (DEGs) between high- and low-risk groups. The gene differential expressions were analyzed by Kyoto Encyclopedia of Genes and Genomes (KEGG) in the two groups, using Gene Set Enrichment Analysis (GSEA) tests. A *p* < 0.05 was considered statistically significant.

### 2.6. Analysis of the Immunity Signature

To further analyze the immune infiltrating factors in the high- and low-risk groups, we assessed the immune cell subpopulations and risk score values by Spearman correlation analysis on TIMER 2.0 (http://timer.cistrome.org/ (accessed on 24 July 2024)). Next, Wilcoxon signed-rank test was used with R packages including “ggplot2” “ggtext” “limma” and “scales” to examine the differences among immune infiltrating cell contents. Then, the TME scores and activation of immune checkpoints were compared between the high- and low-risk groups via the “ggpubr” R package.

### 2.7. Investigation of the Model in Clinical Therapy

After analyzing the expression matrix of GC patients, the half maximal inhibitory concentration (IC50) was predicted. Eventually, several drug candidates associated with this model were attained, which could be the therapeutic approaches for GC.

### 2.8. Consensus Clustering

To explore the response of GC to immunotherapy, the patients were divided into groups as per the expression of ar-lncRNAs. Potential molecular subgroups were searched using ”ConsensusClusterPlus” R package [30]. Next, principal component analysis (PCA), T-distributed stochastic neighbor embedding (t-SNE), and Kaplan–Meier survival were generated by “Rtsne” R package. Then, immunoassays, prognosis and drug susceptibility of different subgroups were conducted via R packages of “GSVA” and “pRRophetic”.

### 2.9. Statistical Analysis

The result display and statistical analyses were performed by R software (Version 4.1.3). Kaplan–Meier survival curves and log-rank test were applied to assess the differences between subgroups in their survival time. Time-dependent ROC curve analysis was applied by “survivalROC” R package for evaluating the risk model’s predictive performance. The independent prognostic value of the risk signature was confirmed by uni-Cox and multi-Cox regression. Student’s *t*-test and Wilcoxon signed-rank test were used to explore the differences between subgroups. *p* < 0.05 indicated the statistical significance.

## 3. Results

### 3.1. Identification of Ar-lncRNA

**Figure 1:**
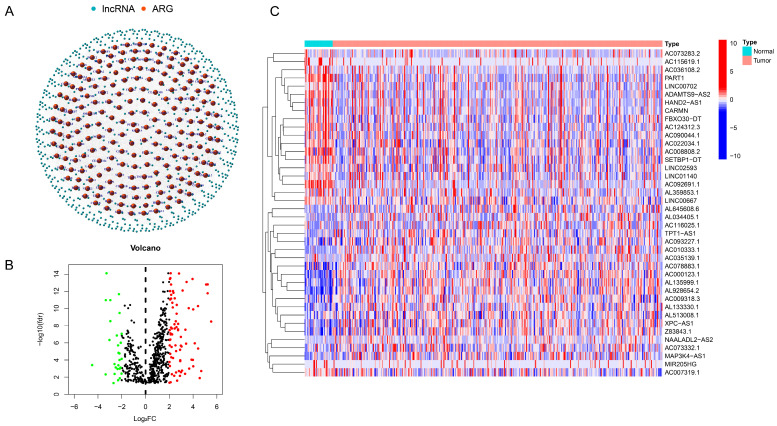
Ar-lncRNAs’ identification and expression in GC. (**A**) The network that connects ar-lncRNAs to ARGs. (**B**) A volcano plot of ARGs with differential expression. (**C**) Heatmap of top 20 ar-lncRNAs with upregulated and downregulated expressions. Red dot: significant upregulated gene. Green dot: significant downregulated gene. Black dot: insignificant gene.

A total of 14,057 ar-lncRNAs were found in GC, which had a co-expression relationship with ARGs. A network graph was generated to express the co-expression relationship between ARGs and ar-lncRNAs (Figure 1A and Supplementary Table S2). According to the regulation of ARGs and lncRNAs (|Log2FC| > 2 and *p* < 0.05), 106 ar-lncRNAs were selected and displayed in the volcano plot, of which 78 genes were in upregulation and 28 genes in downregulation (Figure 1B). Specifically, a heatmap was drawn to show the top 20 upregulated and downregulated expressed lncRNAs (Figure 1C). Also, the heatmap of all selected ar-lncRNAs with differential expression is displayed in Supplementary Table S1.

### 3.2. Construction and Evaluation of Prognostic Model

**Figure 2:**
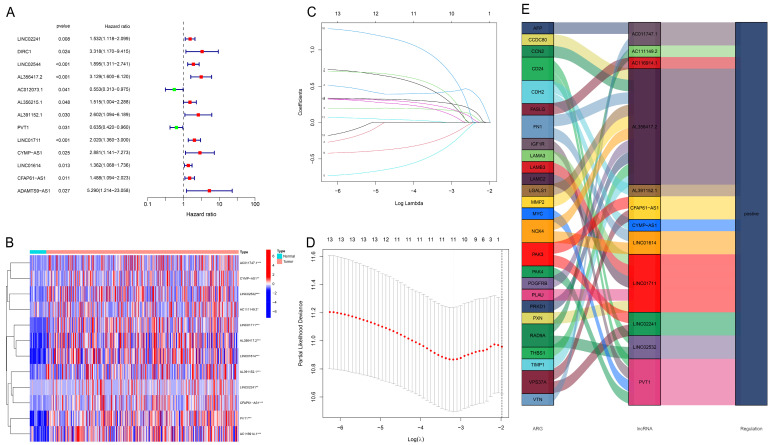
Extraction of the predictive signature of ar-lncRNAs in GC. (**A**) Uni-Cox regression was used to identify the lncRNAs associated with prognosis. (**B**) Expressions of the retrieved lncRNAs in the heatmap. (**C**) LncRNAs were selected for the LASSO model by 10-fold cross-validation. (**D**) The diagram of the LASSO regression analysis. (**E**) The Sankey diagram illustrating the relationship between ARGs and lncRNAs. *** *p* < 0.001, ** *p* < 0.01, * *p* < 0.05.

First, the top 12 lncRNAs were identified using uni-Cox regression and were shown in the forest plot and heatmap (Figure 2A,B). Next, LASSO regression was conducted to filtrate seven core lncRNAs to build this model (Figure 2C,D). The Sankey chart shows the upregulated expressing lncRNAs extracted (Figure 2E). Then, the risk model was built by core lncRNAs using the following formula: Risk Score = LINC02241 × 0.536140892484566 + AL356417.2 × 0.750219726404683 + AC012073.1 × (−0.653621768170201) + AL391152.1 × 1.08857235774243 + PVT1 × (−0.412173266030869) + LINC01711 × 0.743996692181376 + (CYMP-AS1) × 1.27107435020237.

**Figure 3:**
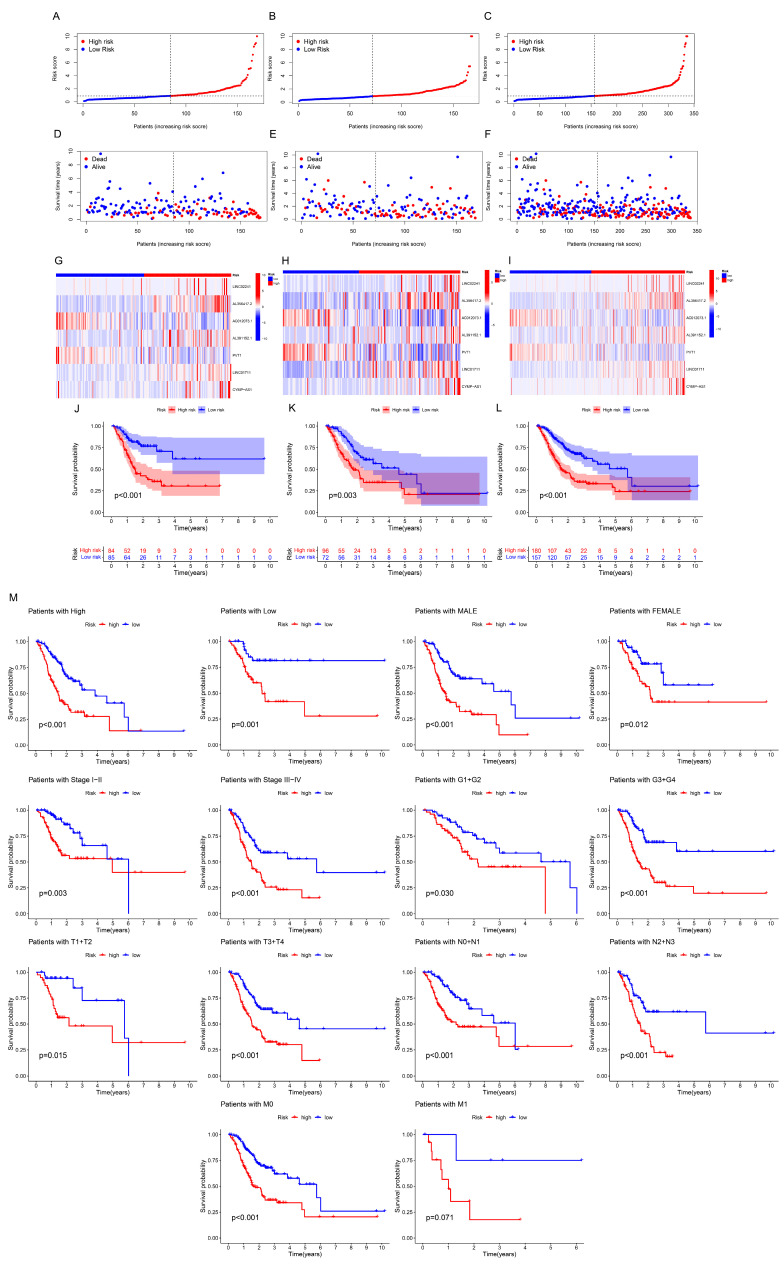
Prognostic values of the seven ar-lncRNAs in the train, test and whole sets, corresponding to the high- and low-risk groups. (**A**–**C**) Display of the ar-lncRNA model according to risk ratings. (**D**–**F**) Time and status of survival between high- and low-risk groups. (**G**–**I**) The seven ar-lncRNAs’ heatmap expression. (**J**–**L**) Kaplan–Meier curves of survival probability. (**M**) Probability of survival classified by age, sex, pathological grade and clinical stage.

**Figure 4:**
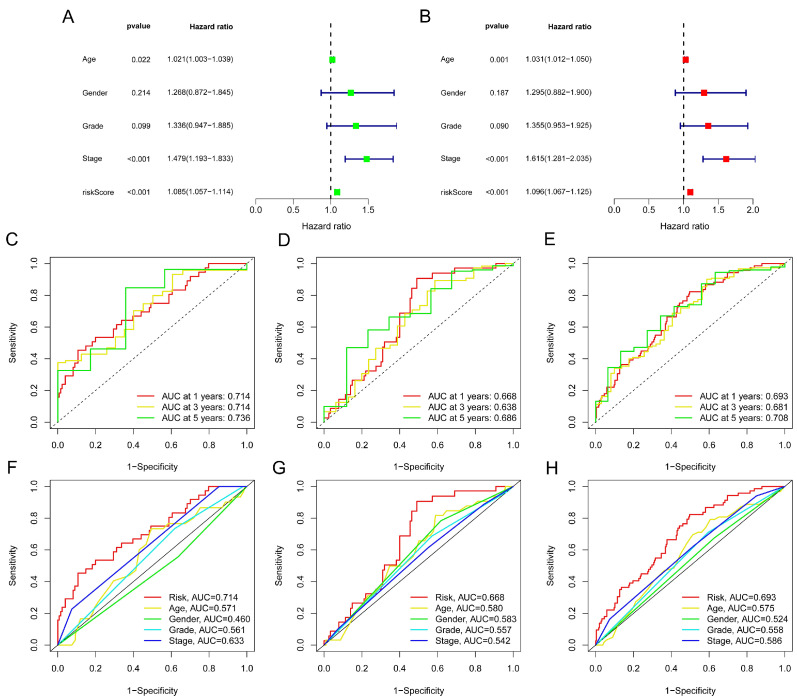
Evaluation of the risk model. (**A**) Uni-Cox analysis of OS-related risk score and clinical variables. (**B**) Multi-Cox analysis of OS-related risk score and clinical variables. (**C**–**E**) ROC curves for the train, test and complete sets for the 1-, 3- and 5-year OS, respectively. (**F**–**H**) ROC curves for the clinical features and risk score in these sets.

The risk score distribution, survival status, and time were compared in the train, test, and complete sets between low- and high-risk groups using the aforementioned algorithm. Results were consistent, showing that the high-risk group’s OS was significantly shorter than that of the low-risk group (Figure 3A–L). The same outcomes were also suggested by subgroups’ clinicopathological features (Figure 3M). Uni- and multi-Cox regressions were employed to confirm if the model was an independent prognostic predictor. The risk scores’ hazard ratio (HR) and 95% confidence interval (CI) were determined by uni-Cox regression to be 1.085 and 1.057–1.114, respectively (*p* < 0.001) (Figure 4A), and 1.096 and 1.067–1.125 via multi-Cox regression, respectively (*p* < 0.001) (Figure 4B). We also found that age (HR: 1.615, CI: 1.281–2.035) was also an independent prognostic parameter (Figure 4B). Also, the model’s specificity and susceptibility to prognosis were assessed using time- and factor-dependent ROC curves (Figure 4C–H). The AUC results in the entire sets were 0.693, 0.681 and 0.708 in, respectively, 1-, 3- and 5-year OS (Figure 4E), and the model’s risk score AUC was 0.693, indicating a better capacity for prediction than other clinicopathological features (Figure 4H).

### 3.3. Construction of Nomogram

**Figure 5:**
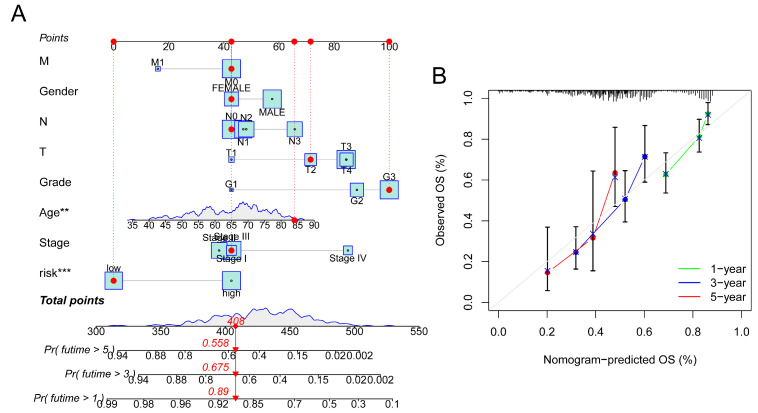
Construction of nomogram. (**A**) Prediction of OS in GC patients using nomogram. (**B**) Calibration diagram of OS. *** *p* < 0.001, ** *p* < 0.01.

To predict the 1-, 3- and 5-year OS in GC patients, an alignment diagram was created using the risk score and additional clinical parameters listed above (Figure 5A). Additionally, the calibration diagram demonstrated strong agreement between the nomogram and the model (Figure 5B).

### 3.4. Analyses of Functional Enrichment

**Figure 6:**
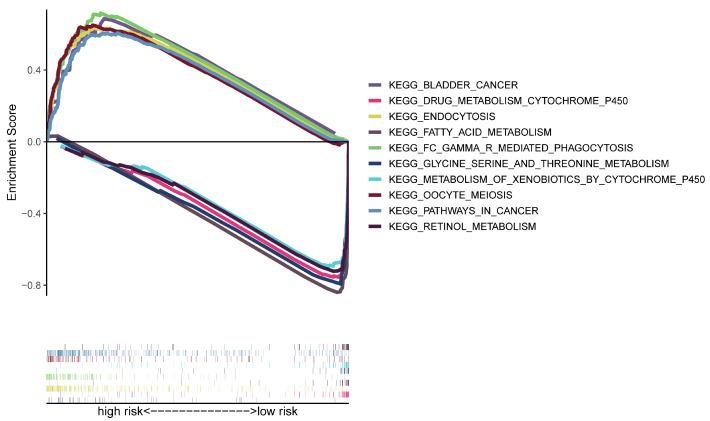
KEGG analysis by GSEA.

Using GSEA software(Version 4.2.3), a functional enrichment analysis was conducted to further investigate the gene expression variations between high- and low-risk groups. The KEGG data suggested that the high-risk group was strongly correlated with the pathways in various malignancies, endocytosis, phagocytosis and other immune-related pathways (Figure 6). As a result, we kept analyzing immunological correlations based on the risk model’s strength.

### 3.5. Analyses of Immune Characteristics and Clinical Treatment in Groups

**Figure 7:**
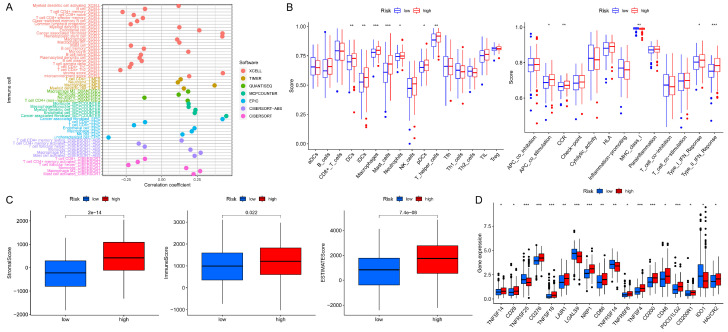
The investigation of tumor immune traits and interventions. (**A**) Immune cell bubble chart in groups. (**B**) Single-sample GSEA analysis of immune cells and immunological-related pathways in the high- and low-risk groups. (**C**) Scores relating to immunity among risk groups. (**D**) Checkpoint expressions in risk groups. *** *p* < 0.001, ** *p* < 0.01, * *p* < 0.05.

In the high- and low-risk groups, a bubble chart was plotted according to the correlation between immune cells that infiltrate tumors and the risk scores (Figure 7A and Supplementary Table S3). It is clear that immune cells applied more to the group with high risk on the majority of these platforms under corresponding software, such as CD4+ T cell, macrophage, myeloid dendritic cell, neutrophil, etc. Subsequent single-sample GSEA analysis revealed that the fraction of immune cell subpopulations and the component levels and functioning of the relevant pathways were dramatically upregulated in almost all high-risk groups (Figure 7B). Also, immune-related scores reached higher in those groups (Figure 7C). Furthermore, the high-risk groups showed greater activity in the majority of immune checkpoints (Figure 7D). Additionally, the sensitivity to anticancer drugs in each group was investigated (Supplementary Figure S2). In high-risk groups, drugs that were more sensitive included mitomycin, methotrexate, gemcitabine, and gefitinib, and in low-risk groups, the sensitive drugs were pazopanib, imatinib, bexarotene, etc. The above findings could provide guidance for GC systemic therapy in different risk classes.

### 3.6. Prognosis and Immunotherapy Prospects of Each GC Subgroup

**Figure 8:**
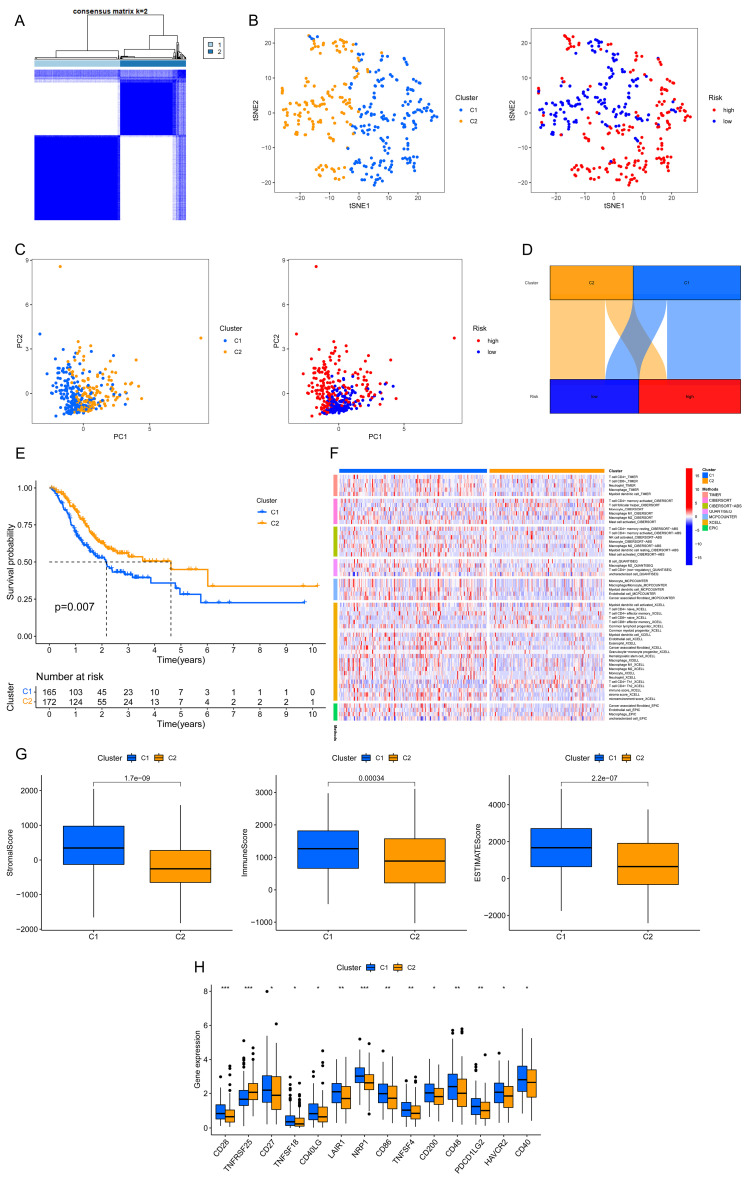
Consensus clustering in GC based on predictive ar-lncRNAs. (**A**) Two clusters of patients were separated. (**B**) The t-SNE of two clusters and two risk groups (C1, cluster 1; C2, cluster 2). (**C**) The PCA of clusters and risk groups (C1, cluster 1; C2, cluster 2). (**D**) Sankey diagram showing risk groups and clusters. (**E**) The comparison of OS between clusters by Kaplan–Meier curves. (**F**) Immune cell heatmap between clusters. (**G**) Immunological scores that differ between clusters (the numbers on the top of columns are *p*-values). (**H**) The expressions of immune checkpoints between clusters. *** *p* < 0.001, ** *p* < 0.01, * *p* < 0.05.

Once again, the GC patients were split based on the ar-lncRNAs that had previously been examined for prognostic assessment using “ConsensusClusterPlus” R package (Figure 8A and Supplementary Figure S3). The t-SNE displayed the distribution of clusters and groups (Figure 8B). Subsequently, the distribution in both risk groups and clusters was shown by the PCA (Figure 8C). Based on the Sankey diagram, the majority of GC patients in cluster 1 were classified into the high-risk group; in contrast, the majority of individuals in cluster 2 belonged to the low-risk group (Figure 8D). Also, the expected survival time of cluster 2 was much longer than cluster 1 (*p* = 0.007) (Figure 8E). Based on the immune cell heatmap generated by several platforms, cluster 1 had a greater degree of immune cell infiltration (Figure 8F). As for the immune-related scores, cluster 1 reached better scores (Figure 8G). A better activity was also seen for cluster 1 in most of the immune checkpoints (Figure 8H). Therefore, compared with cluster 2, cluster 1 could be classified as hot tumors because of its higher degree of immune infiltration and related genes. The above findings revealed that cluster 1, as hot tumors, might receive better immune responses and better efficacy in immunotherapy. Then, the drug sensitivity was performed between clusters (Supplementary Figure S4). Based on the IC50 results, a personalized selection of drugs with different conditions of tumors could be expected to improve the efficacy of systemic therapy.

## 4. Discussion

In the present study, we methodically examined the connection between ARGs and lncRNAs in GC patients. After Cox and LASSO regressions, the prognostic model was structured using chosen hub lncRNAs. Drawing conclusions from the Sankey diagram, all seven lncRNAs have a strong relation with ARGs, which are the key genes in anoikis and anoikis resistance of GC cells. Thus, an original ar-lncRNA-based model was ultimately built, aiming to forecast the prognosis and immune reaction of GC, as well as augment the possibility of the effective therapy.

In this model, the chosen ar-lncRNAs of GC were LINC02241, AL356417.2, AC012073.1, AL391152.1, PVT1, LINC01711, and CYMP-AS1. Previous studies confirmed that most of the above lncRNAs have a strong correlation with tumor prognosis, including breast cancer, gastric cancer, hepatocellular carcinoma, glioblastoma, melanoma, etc. [31,32,33,34,35,36,37,38]. In our study, LINC02241 is first found to be associated with GC, which expresses more in the high-risk group with poorer prognosis. The Sankey diagram indicated that LINC02241 is highly and positively correlated with ARGs of PAK3 and VPS37A. VPS37A is considered to lead gluconeogenesis in liver cells and diabetes through the cAMP/PKA/p-Creb pathway, but there are no reports showing its role in cancers [39]. It is also unclear how PAK3 functions in GC. According to Magne et al., PAK3 may function as a tumor suppressor and be a viable target for glioma treatment [40]. Also, Tan et al. discovered that blocking the production of PAK3 can regulate lung cancer metastasis, as it is a downstream effector of SMAD4, which can mediate the transduction of metastatic signals through the PAK3-JNK-Jun pathway [41]. Many identified ar-lncRNAs have been implicated in various cancers, but their association with GC-specific outcomes and immune modulation is novel. From our study, we firstly reported these hub genes in the anoikis-related hub lncRNAs. More research is needed to explore their specific mechanisms, and the findings may support the application of anoikis as a possible direction for GC treatment.

In this study, GC patients were identified into risk groups of high and low via the above ar-lncRNAs. A prognostic model of GC was established using the selected ar-lncRNAs. The accuracy of the model was repeatedly verified by multiple methods. Clinicopathological and survival analyses illustrated that the model had high sensitivity for survival prediction. Additionally, uni- and multi-Cox analyses revealed that this model is suitable as an independent predictor for prognosis.

GC is a highly immune-sensitive tumor that can respond quickly to immunotherapy, especially ICIs [7]. However, due to the absence of highly specific and sensitive prognostic markers, some patients still do not respond adequately to immunotherapy, leading to poor prognosis [42]. In our further research, the correlation analysis with immune-related signatures was conducted. The GSEA analysis showed that some immune-related signaling pathways were enriched in our risk model. The immunological ratings indicated that immune checkpoint expression was higher in high-risk groups, which further supported the notion that these groups were more effective. The above also indicated that ar-lncRNAs could affect the TME of GC by regulating the expression of related immune molecules, and this model could be a guidance for GC immunology.

This study has some limitations. Firstly, only one public database was verified to support our results rather than using our own datasets, leading to its low volume. External validation is urgently needed, with the aim of further inquiry and validation by the broader scientific community. Secondly, the biological functions of ar-lncRNAs were not verified by experimental research, so there is a dearth of further analysis to support our findings. Moreover, the potential value of this risk model needs verification in external validation. Deeper studies are urgent to explore the detailed mechanisms behind ar-lncRNAs in patients with GC.

## 5. Conclusions

This study first proposes that prognostic signatures of ar-lncRNA can be established in GC. These signatures accurately predict the prognosis of GC and offer potential biomarkers, suggesting new avenues for basic research, prognosis prediction, and personalized diagnosis and treatment of GC.

## Data Availability

The data that support this study are available from the first author upon reasonable request.

## Supplementary Materials

The following supporting information can be downloaded at: https://www.mdpi.com/article/10.3390/bioengineering11090893/s1, Figure S1: Heatmap of all selected ar-lncRNAs with upregulated and downregulated expressions; Figure S2: Sensitivity to different types of drugs. (A) Drugs were more sensitive in high-risk groups. (B) Drugs were more sensitive in low-risk groups; Figure S3: The heatmap, cumulative distribution function (CDF) plot, and consensus CDF plots of consensus clustering matrix; Figure S4: The drug sensitivity between different clusters. (A) Drugs were more sensitive in cluster 1. (B) Drugs were more sensitive in cluster 2; Table S1: The retrieval of all the ARGs; Table S2: The network data of ARGs and ar-lncRNAs; Table S3: Data of immune cell infiltration at different platforms.
