# Supplementary material for: Anoikis-Related Long Non-Coding RNA Signatures to Predict Prognosis and Immune Infiltration of Gastric Cancer
Source: Bioengineering (Basel). 2024 Sep 5;11(9):893. doi: 10.3390/bioengineering11090893 (PMC11428253; doi:10.3390/bioengineering11090893)
Supplement: Supplementary file 1 [file bioengineering-11-00893-s001.zip › Supplementary Table S1.pdf]

ID	TCGA-HU-8238-11A-01R-2343-13	TCGA-FP-7735-11A-01R-2055-13
	TCGA-BR-7851-11A-01R-2203-13	TCGA-BR-7703-11A-01R-2055-13
	TCGA-BR-6453-11A-01R-1802-13	TCGA-HU-A4GH-11A-11R-A36D-31
	TCGA-CG-5721-11A-01R-1602-13	TCGA-BR-8060-11A-01R-2343-13
	TCGA-BR-6454-11A-01R-1802-13	TCGA-BR-7717-11A-01R-2055-13
	TCGA-IN-AB1V-11A-11R-A414-31	TCGA-IN-8663-11A-01R-2402-13
	TCGA-BR-7715-11A-01R-2055-13	TCGA-IN-7806-11A-01R-2055-13
	TCGA-CG-5728-11A-01R-1602-13	TCGA-HU-A4HB-11A-11R-A251-31
	TCGA-CG-5720-11A-01R-1602-13	TCGA-BR-7704-11A-01R-2055-13
	TCGA-CG-5734-11A-01R-1602-13	TCGA-CG-5730-11A-01R-1602-13
	TCGA-BR-7716-11A-01R-2055-13	TCGA-HU-A4GC-11A-11R-A251-31
	TCGA-IP-7968-11A-01R-2203-13	TCGA-HU-A4GY-11A-11R-A36D-31
	TCGA-FP-7829-11A-01R-2055-13	TCGA-IN-AB1X-11A-21R-A39E-31
	TCGA-CG-5733-11A-01R-1602-13	TCGA-HU-A4GP-11A-21R-A251-31
	TCGA-BR-6457-11A-01R-1802-13	TCGA-BR-6802-11A-01R-1884-13
	TCGA-CG-5722-11A-02R-1602-13	TCGA-HU-A4GN-11A-12R-A251-31
	TCGA-KB-A93J-01A-11R-A39E-31	TCGA-IN-A7NR-01A-11R-A354-31
	TCGA-B7-A5TN-01A-21R-A31P-31	TCGA-BR-8676-01A-11R-2402-13
	TCGA-BR-4294-01A-01R-1131-13	TCGA-FP-7735-01A-11R-2055-13
	TCGA-EQ-8122-01A-11R-2343-13	TCGA-BR-8368-01A-11R-2343-13
	TCGA-VQ-A91Q-01A-12R-A414-31	TCGA-IN-A6RO-01A-12R-A33Y-31
	TCGA-BR-4257-01A-01R-1131-13	TCGA-HF-A5NB-01A-11R-A31P-31
	TCGA-VQ-A927-01A-12R-A414-31	TCGA-VQ-A8PJ-01A-11R-A414-31
	TCGA-FP-A9TM-01A-11R-A39E-31	TCGA-BR-6565-01A-11R-1802-13
	TCGA-BR-4368-01A-01R-1157-13	TCGA-VQ-AA68-01A-11R-A414-31
	TCGA-CD-5799-01A-11R-1602-13	TCGA-HU-A4GY-01A-21R-A24K-31
	TCGA-BR-8295-01A-11R-2343-13	TCGA-F1-A72C-01A-21R-A33Y-31
	TCGA-RD-A7BW-01A-11R-A32D-31	TCGA-VQ-A8P2-01A-11R-A36D-31
	TCGA-HU-8604-01A-11R-2402-13	TCGA-FP-8209-01A-11R-2343-13
	TCGA-D7-6528-01A-11R-1802-13	TCGA-CG-5732-01A-11R-1602-13
	TCGA-CG-4462-01A-01R-1157-13	TCGA-BR-6852-01A-11R-1884-13
	TCGA-BR-4187-01A-01R-1131-13	TCGA-D7-6818-01A-11R-1884-13
	TCGA-R5-A7ZR-01A-11R-A354-31	TCGA-BR-A4IV-01A-31R-A251-31
	TCGA-CG-5719-01A-11R-1602-13	TCGA-BR-6563-01A-13R-2055-13
	TCGA-HF-7131-01A-11R-2055-13	TCGA-HU-A4H2-01A-11R-A251-31
	TCGA-HU-8602-01A-11R-2402-13	TCGA-HU-A4G2-01A-11R-A251-31
	TCGA-BR-8369-01A-11R-2343-13	TCGA-CG-4475-01A-01R-1157-13
	TCGA-VQ-A91A-01A-11R-A414-31	TCGA-CG-5722-01A-21R-1602-13
	TCGA-D7-A4Z0-01A-22R-A251-31	TCGA-VQ-A928-01A-11R-A414-31
	TCGA-HU-A4H5-01A-21R-A251-31	TCGA-CG-4436-01A-01R-1157-13
	TCGA-B7-5818-01A-11R-1602-13	TCGA-BR-8291-01A-11R-2343-13
	TCGA-BR-4253-01A-01R-1131-13	TCGA-ZA-A8F6-01A-23R-A36D-31
	TCGA-BR-8059-01A-11R-2343-13	TCGA-B7-A5TI-01A-11R-A31P-31
	TCGA-BR-8367-01A-11R-2343-13	TCGA-D7-A6EY-01A-21R-A31P-31

TCGA-RD-A7C1-01A-11R-A32D-31
TCGA-CG-4465-01A-01R-1157-13
TCGA-B7-A5TK-01A-12R-A36D-31
TCGA-CG-5721-01A-11R-1602-13
TCGA-BR-8366-01A-11R-2343-13
TCGA-VQ-AA69-01A-11R-A414-31
TCGA-D7-8574-01A-13R-2343-13
TCGA-R5-A7ZI-01A-11R-A354-31
TCGA-CD-8530-01A-11R-2343-13
TCGA-BR-6802-01A-11R-1884-13
TCGA-BR-6452-01A-12R-1802-13
TCGA-HU-A4HD-01A-11R-A251-31
TCGA-CG-5723-01A-11R-1602-13
TCGA-RD-A7BS-01A-11R-A32D-31
TCGA-CG-5718-01A-11R-1602-13
TCGA-BR-8289-01A-11R-2343-13
TCGA-IN-A6RS-01A-12R-A354-31
TCGA-VQ-A8E2-01A-11R-A36D-31
TCGA-BR-8678-01A-11R-2402-13
TCGA-BR-8372-01A-11R-2343-13
TCGA-BR-8365-01A-21R-2343-13
TCGA-VQ-A91K-01A-11R-A414-31
TCGA-IN-A6RL-01A-11R-A32D-31
TCGA-CG-5734-01A-11R-1602-13
TCGA-BR-7851-01A-11R-2203-13
TCGA-BR-8286-01A-12R-2343-13
TCGA-VQ-AA6J-01A-11R-A414-31
TCGA-VQ-AA6F-01A-31R-A414-31
TCGA-CD-8526-01A-11R-2343-13
TCGA-KB-A93H-01A-11R-A39E-31
TCGA-VQ-A91Z-01A-11R-A414-31
TCGA-BR-8361-01A-11R-2343-13
TCGA-CD-8528-01A-11R-2343-13
TCGA-BR-8592-01A-11R-2402-13
TCGA-MX-A5UJ-01A-11R-A31P-31
TCGA-IN-A7NT-01A-21R-A354-31
TCGA-CD-5803-01A-11R-1602-13
TCGA-CG-4476-01A-01R-1157-13
TCGA-BR-7717-01A-11R-2055-13
TCGA-BR-6454-01A-11R-1802-13
TCGA-RD-A8N1-01A-12R-A36D-31
TCGA-RD-A8N4-01A-21R-A36D-31
TCGA-CD-8534-01A-11R-2343-13
TCGA-IN-A6RI-01A-11R-A32D-31
TCGA-CD-A489-01A-11R-A24K-31
TCGA-HU-8608-01A-11R-2402-13
TCGA-HU-A4HB-01A-12R-A251-31
TCGA-D7-8570-01A-11R-2343-13
TCGA-FP-A8CX-01A-11R-A36D-31
TCGA-RD-A8NB-01A-12R-A39E-31
TCGA-VQ-A91V-01A-11R-A414-31
TCGA-BR-8077-01A-11R-2343-13
TCGA-MX-A5UG-01A-21R-A31P-31
TCGA-BR-6803-01A-11R-1884-13
TCGA-BR-8487-01A-11R-2402-13
TCGA-CD-A4MH-01A-11R-A251-31
TCGA-BR-A44T-01A-32R-A24K-31
TCGA-VQ-A8E0-01A-11R-A414-31
TCGA-VQ-A92D-01A-11R-A414-31
TCGA-RD-A7BT-01A-11R-A33Y-31
TCGA-BR-4369-01A-01R-1157-13
TCGA-D7-A748-01A-12R-A32D-31
TCGA-HU-A4GJ-01A-11R-A251-31
TCGA-BR-6566-01A-11R-1802-13
TCGA-BR-6456-01A-11R-1802-13
TCGA-BR-8680-01A-11R-2402-13
TCGA-CG-4306-01A-01R-1157-13
TCGA-BR-8682-01A-11R-2402-13
TCGA-CG-4440-01A-01R-1157-13
TCGA-CG-4466-01A-01R-1157-13
TCGA-VQ-A8E3-01A-11R-A39E-31
TCGA-VQ-A8PH-01A-12R-A414-31
TCGA-BR-A4J6-01A-11R-A251-31
TCGA-BR-A4J5-01A-21R-A251-31
TCGA-3M-AB47-01A-22R-A414-31
TCGA-D7-6524-01A-11R-1802-13
TCGA-FP-7998-01A-11R-2203-13
TCGA-IN-A6RN-01A-12R-A33Y-31
TCGA-BR-A4J7-01A-31R-A251-31
TCGA-BR-8690-01A-11R-2402-13
TCGA-RD-A8N0-01A-12R-A36D-31
TCGA-HU-A4GF-01A-11R-A24K-31
TCGA-HF-7132-01A-11R-2055-13
TCGA-CG-4301-01A-01R-1157-13
TCGA-D7-A6EX-01A-11R-A31P-31
TCGA-HF-7133-01A-11R-2055-13
TCGA-R5-A7ZE-01B-11R-A354-31
TCGA-CD-A48C-01A-11R-A24K-31

TCGA-D7-8578-01A-21R-2343-13
TCGA-VQ-A8PX-01A-12R-A414-31
TCGA-3M-AB46-01A-11R-A414-31
TCGA-CG-5717-01A-11R-1602-13
TCGA-RD-A8MW-01A-11R-A36D-31
TCGA-BR-8364-01A-11R-2343-13
TCGA-BR-8373-01A-11R-2343-13
TCGA-BR-7901-01A-11R-2203-13
TCGA-D7-6525-01A-11R-1802-13
TCGA-CD-5801-01A-11R-1602-13
TCGA-BR-4256-01A-01R-1131-13
TCGA-D7-8573-01A-11R-2343-13
TCGA-VQ-A8DV-01A-12R-A36D-31
TCGA-HF-7134-01A-11R-2055-13
TCGA-R5-A7ZF-01A-11R-A354-31
TCGA-CD-5804-01A-12R-2055-13
TCGA-VQ-A8E7-01B-11R-A414-31
TCGA-CG-4304-01A-01R-1157-13
TCGA-VQ-A8PC-01A-11R-A39E-31
TCGA-HJ-7597-01A-21R-2203-13
TCGA-D7-6520-01A-11R-1802-13
TCGA-BR-7196-01A-11R-2055-13
TCGA-D7-8576-01A-11R-2343-13
TCGA-D7-6527-01A-11R-1802-13
TCGA-VQ-A8PQ-01A-11R-A414-31
TCGA-VQ-A940-01A-11R-A414-31
TCGA-CG-5726-01A-11R-1602-13
TCGA-BR-6801-01A-11R-1884-13
TCGA-HU-8244-01A-11R-2343-13
TCGA-BR-4371-01A-01R-1157-13
TCGA-BR-6453-01A-11R-1802-13
TCGA-BR-4361-01A-01R-1157-13
TCGA-RD-A8N6-01A-11R-A36D-31
TCGA-BR-8588-01A-11R-2402-13
TCGA-BR-4201-01A-01R-1131-13
TCGA-BR-8381-01A-11R-2402-13
TCGA-BR-8590-01A-11R-2402-13
TCGA-D7-A4YU-01A-21R-A251-31
TCGA-FP-8631-01A-11R-2402-13
TCGA-HU-A4H0-01A-11R-A251-31
TCGA-BR-8679-01A-11R-2402-13
TCGA-VQ-A8PO-01A-11R-A414-31
TCGA-HU-A4H6-01A-11R-A251-31
TCGA-CG-4477-01A-01R-1157-13
TCGA-CD-A487-01A-21R-A24K-31
TCGA-VQ-A922-01A-11R-A414-31
TCGA-B7-A5TJ-01A-11R-A31P-31
TCGA-VQ-A8PF-01A-11R-A414-31
TCGA-HU-8610-01A-22R-2402-13
TCGA-SW-A7EA-01A-12R-A354-31
TCGA-D7-A4YX-01A-11R-A251-31
TCGA-VQ-A8PK-01A-12R-A414-31
TCGA-CG-5716-01A-21R-1802-13
TCGA-BR-8683-01A-11R-2402-13
TCGA-MX-A666-01A-11R-A31P-31
TCGA-BR-A44U-01A-11R-A36D-31
TCGA-BR-A4J8-01A-11R-A251-31
TCGA-HU-A4GU-01A-11R-A251-31
TCGA-VQ-A91E-01A-31R-A414-31
TCGA-BR-8060-01A-11R-2343-13
TCGA-FP-8099-01A-11R-2343-13
TCGA-BR-8080-01A-11R-2343-13
TCGA-BR-7197-01A-11R-2203-13
TCGA-BR-8591-01A-11R-2402-13
TCGA-D7-A6F0-01A-11R-A31P-31
TCGA-D7-6815-01A-11R-1884-13
TCGA-BR-4363-01A-01R-1157-13
TCGA-VQ-A91D-01A-11R-A414-31
TCGA-BR-A4QL-01A-31R-A251-31
TCGA-IN-A6RJ-01A-21R-A33Y-31
TCGA-D7-6519-01A-11R-1802-13
TCGA-RD-A8N5-01A-12R-A36D-31
TCGA-VQ-AA6G-01A-11R-A414-31
TCGA-BR-7722-01A-31R-2203-13
TCGA-HU-A4GX-01A-12R-A251-31
TCGA-CD-8533-01A-11R-2343-13
TCGA-FP-7916-01A-11R-2203-13
TCGA-BR-8382-01A-11R-2402-13
TCGA-CD-8529-01A-11R-2343-13
TCGA-BR-8371-01A-11R-2343-13
TCGA-HU-A4H4-01A-21R-A251-31
TCGA-CG-4469-01A-01R-1157-13
TCGA-BR-8677-01A-11R-2402-13
TCGA-BR-7723-01A-11R-2055-13
TCGA-D7-6521-01A-11R-1802-13
TCGA-MX-A663-01A-11R-A31P-31
TCGA-BR-7707-01A-11R-2055-13
TCGA-HU-A4GQ-01A-11R-A36D-31

TCGA-F1-6874-01A-11R-1884-13
TCGA-D7-A6EV-01A-11R-A31P-31
TCGA-CD-8524-01A-11R-2343-13
TCGA-BR-8284-01A-11R-2343-13
TCGA-IN-A6RR-01A-12R-A32D-31
TCGA-HU-A4GH-01A-11R-A24K-31
TCGA-VQ-AA6K-01A-11R-A414-31
TCGA-CD-8531-01A-11R-2343-13
TCGA-FP-8210-01A-11R-2343-13
TCGA-CD-5798-01A-11R-1602-13
TCGA-VQ-A8PB-01A-11R-A39E-31
TCGA-VQ-A91N-01A-11R-A414-31
TCGA-F1-A448-01A-11R-A24K-31
TCGA-VQ-A8PE-01A-11R-A414-31
TCGA-VQ-A94P-01A-13R-A414-31
TCGA-BR-6709-01A-11R-1884-13
TCGA-FP-A4BF-01A-12R-A36D-31
TCGA-HU-A4H3-01A-21R-A251-31
TCGA-VQ-A8P8-01A-11R-A39E-31
TCGA-BR-A4J4-01A-12R-A251-31
TCGA-VQ-A8DT-01A-11R-A36D-31
TCGA-CG-4460-01A-01R-1157-13
TCGA-BR-8484-01A-11R-2402-13
TCGA-BR-7716-01A-21R-2055-13
TCGA-D7-A6EZ-01A-11R-A31P-31
TCGA-HU-A4G8-01A-11R-A251-31
TCGA-VQ-A8PM-01A-21R-A414-31
TCGA-R5-A805-01A-11R-A36D-31
TCGA-CG-4438-01A-01R-1157-13
TCGA-D7-6526-01A-11R-1802-13
TCGA-BR-4279-01A-01R-1131-13
TCGA-CG-4441-01A-01R-1802-13
TCGA-HU-8238-01A-11R-2343-13
TCGA-D7-A74A-01A-11R-A32D-31
TCGA-BR-8058-01A-31R-2343-13
TCGA-BR-6564-01A-12R-1884-13
TCGA-VQ-A91X-01A-12R-A414-31
TCGA-VQ-A923-01A-11R-A414-31
TCGA-CG-4443-01A-01R-1157-13
TCGA-VQ-A8P3-01A-11R-A36D-31
TCGA-BR-8384-01A-21R-2402-13
TCGA-VQ-AA6A-01A-11R-A414-31
TCGA-CD-8527-01A-11R-2343-13
TCGA-VQ-A91U-01A-11R-A414-31
TCGA-BR-8485-01A-11R-2402-13
TCGA-CG-5720-01A-11R-1602-13
TCGA-D7-8575-01A-11R-2343-13
TCGA-CG-4437-01A-01R-1802-13
TCGA-BR-4280-01A-01R-1131-13
TCGA-D7-A6F2-01A-12R-A31P-31
TCGA-BR-A4J9-01A-12R-A251-31
TCGA-BR-4367-01A-01R-1157-13
TCGA-BR-A4PF-01A-11R-A251-31
TCGA-BR-8081-01A-11R-2343-13
TCGA-BR-8686-01A-11R-2402-13
TCGA-BR-8483-01A-31R-2402-13
TCGA-BR-7957-01A-11R-2203-13
TCGA-RD-A8N2-01A-12R-A36D-31
TCGA-BR-8486-01A-31R-2402-13
TCGA-BR-6705-01A-12R-1884-13
TCGA-BR-7958-01A-21R-2343-13
TCGA-HU-A4GP-01A-11R-A251-31
TCGA-RD-A8N9-01A-12R-A39E-31
TCGA-KB-A93G-01A-11R-A39E-31
TCGA-VQ-A8DZ-01A-11R-A36D-31
TCGA-BR-4357-01A-01R-1157-13
TCGA-VQ-A94T-01A-11R-A414-31
TCGA-CG-5724-01A-11R-1602-13
TCGA-D7-6522-01A-11R-1802-13
TCGA-BR-8380-01A-11R-2343-13
TCGA-VQ-AA64-01A-11R-A414-31
TCGA-BR-4370-01A-01R-1157-13
TCGA-CG-4444-01A-01R-1157-13
TCGA-BR-6707-01A-11R-1884-13
TCGA-SW-A7EB-01A-11R-A354-31
TCGA-CG-5725-01A-11R-1602-13
TCGA-VQ-A8PP-01A-21R-A414-31
TCGA-BR-7959-01A-11R-2343-13
TCGA-CD-8532-01A-11R-2343-13
TCGA-IN-7806-01A-11R-2055-13
TCGA-HU-A4GC-01A-12R-A251-31
TCGA-BR-4267-01A-01R-1131-13
TCGA-BR-7704-01A-11R-2055-13
TCGA-VQ-AA6D-01A-11R-A414-31
TCGA-IN-AB1V-01A-21R-A414-31
TCGA-BR-A4CR-01A-11R-A24K-31
TCGA-CD-8525-01A-11R-2343-13
TCGA-D7-8572-01A-11R-2343-13

TCGA-BR-8687-01A-11R-2402-13	TCGA-ZQ-A9CR-01A-11R-A39E-31
TCGA-BR-8589-01A-11R-2402-13	TCGA-VQ-A8PU-01A-12R-A414-31
TCGA-VQ-A8PD-01A-11R-A414-31	TCGA-VQ-A8P5-01A-11R-A39E-31
TCGA-D7-A747-01A-22R-A33Y-31	TCGA-CD-A486-01A-11R-A24K-31
TCGA-BR-8296-01A-11R-2343-13	TCGA-CD-5813-01A-11R-1602-13
TCGA-IN-8663-01A-11R-2402-13	TCGA-KB-A6F7-01A-12R-A32D-31
TCGA-HU-8249-01A-11R-A36D-31	TCGA-CD-A4MG-01A-11R-A251-31
TCGA-HU-A4G9-01A-11R-A24K-31	TCGA-RD-A8MV-01A-11R-A36D-31
TCGA-VQ-A925-01A-11R-A414-31	TCGA-BR-A4CS-01A-11R-A24K-31
TCGA-VQ-A8DU-01A-11R-A36D-31	TCGA-IN-8462-01A-11R-2343-13
TCGA-CD-8535-01A-11R-2343-13	TCGA-BR-4366-01A-01R-1157-13
TCGA-BR-6455-01A-11R-1802-13	TCGA-BR-7715-01A-11R-2055-13
TCGA-FP-8211-01A-11R-2343-13	TCGA-R5-A707-01A-11R-A33Y-31
TCGA-HU-A4G3-01A-11R-A24K-31	TCGA-VQ-A94U-01A-12R-A414-31
TCGA-BR-6458-01A-11R-1802-13	TCGA-IP-7968-01A-11R-2203-13
TCGA-CD-A48A-01A-12R-A36D-31	TCGA-F1-6875-01A-11R-2055-13
TCGA-VQ-A91S-01A-11R-A414-31	TCGA-D7-5577-01A-01R-1602-13
TCGA-BR-6457-01A-21R-1802-13	TCGA-IN-A7NU-01A-22R-A354-31
TCGA-F1-6177-01A-11R-1802-13	TCGA-D7-6822-01A-11R-1884-13
TCGA-VQ-A91Y-01A-11R-A414-31	TCGA-CG-4442-01A-01R-1157-13
TCGA-HU-A4GD-01A-11R-A36D-31	TCGA-CG-4305-01A-01R-1157-13
TCGA-IN-AB1X-01A-11R-A39E-31	TCGA-BR-4191-01A-02R-1131-13
TCGA-VQ-A94R-01A-11R-A414-31	TCGA-D7-8579-01A-11R-2343-13
TCGA-BR-6710-01A-11R-1884-13	TCGA-FP-7829-01A-11R-2055-13
TCGA-CD-5800-01A-11R-1602-13	TCGA-IN-7808-01A-11R-2203-13
TCGA-VQ-A924-01A-11R-A414-31	TCGA-HU-A4H8-01A-11R-A251-31
TCGA-HU-A4GT-01A-21R-A251-31	TCGA-D7-5578-01A-01R-1602-13
TCGA-BR-8297-01A-12R-2343-13
BRMS1	10.24632	4.907193	8.393686	13.65227	10.41525
3.763267	13.69712	5.333133	11.81261	12.49721
5.278127	6.664696	15.86757	8.08429	13.1337	2.483228
16.32346	6.718329	13.2437	12.83663	12.59446	4.0979
6.425747	3.650644	4.28268	6.082385	14.59982	3.632872
11.26829	9.153073	11.82391	4.838371	18.89409
9.901557	20.35781	16.08907	3.674209	19.50046
27.28692	15.04656	9.907826	27.93312	11.4767	10.71459
10.10472	21.75109	12.57474	21.623	8.979616	13.91653
18.69297	10.05432	17.2233	22.69702	9.859333	12.90137
21.34884	5.14817	29.34463	21.94494	7.427222	26.4574
6.152868	14.92499	14.33102	6.508437	17.5135	14.52621
21.45442	20.34265	16.91978	19.10832	11.26226
9.823568	13.05941	24.24784	9.956489	14.07497
24.45919	10.73923	25.67371	8.67368	9.786802	8.622447
15.92439	20.30615	7.890761	15.91825	23.46028

9.777735 9.868378 20.33304 11.39905 14.89883
19.04434 13.58497 11.45568 15.07353 9.248505
12.12554 7.105332 13.1879 25.41957 12.81749 14.34745
7.986358 19.90009 11.935 15.03495 13.50596 19.29792
18.96938 13.6713 7.855313 14.75684 33.57258 17.15478
9.431597 16.71412 19.60701 14.71204 10.37039
14.36301 8.626253 17.00422 12.81969 12.71777
23.6514 8.720077 18.10051 15.10818 16.28411 17.74657
8.251558 17.28619 6.975526 18.88192 14.03154
13.37529 6.885292 54.70148 13.65509 21.6918 17.50555
25.83846 6.800101 25.73933 10.09905 9.040245
11.4371 12.89296 13.68025 15.27564 9.643277 6.828984
10.28888 14.14888 10.5312 26.93373 16.1642 17.36906
8.236378 7.168736 14.07382 49.39479 13.38662
23.6925 9.711467 23.12337 12.67138 4.513127 23.63161
11.95558 15.7898 22.86478 29.06452 12.91401 19.39016
21.94921 11.78307 21.59294 18.6248 10.87933 13.70255
20.92163 29.276 6.861184 22.69909 9.041233 17.0178
11.27503 10.1093 18.35716 23.15654 20.16299 9.16422
6.907763 12.52066 15.26354 13.38996 12.51573
11.27513 10.90629 13.67192 21.90159 18.6041 21.77518
9.877985 12.72592 23.85666 7.96858 8.427414 11.62422
11.28814 11.68543 17.53005 20.93488 18.18926
23.66648 12.27651 21.51221 8.304054 12.20474
10.44079 8.667669 19.99345 12.0772 10.99527 19.17839
27.73914 14.69609 19.56945 12.18846 10.81069
6.523724 13.43655 15.95818 10.87523 9.899238
13.07596 11.61285 10.86383 10.11624 12.78988
7.186098 14.91861 12.58576 7.981815 16.04813
30.50143 11.84461 7.636666 29.9845 17.66399 13.82966
9.574768 17.2192 17.34229 21.6542 11.42175 20.7822
22.11366 9.602242 11.25326 11.67097 14.06601
26.34396 19.26349 20.67256 13.64153 15.24206
26.04939 21.302 12.38059 13.20648 14.29964 11.29956
4.454416 16.15911 12.32074 7.831313 10.11545
13.22678 12.19538 11.1908 15.21102 17.61397 18.26319
11.70054 10.19391 15.26257 2.886812 6.058463
13.05248 16.91427 12.21525 11.67924 13.81488 12.269
14.85481 12.07602 14.81472 14.49357 6.916787
16.43076 21.52121 7.880771 15.72364 14.276 9.291012
18.58243 59.22013 27.62728 18.33615 14.95269
6.013413 16.05239 17.63761 9.565302 5.573355
13.74955 14.34395 20.68966 19.13374 5.027702
13.31829 13.01884 16.44824 14.27655 8.838523

12.31879	7.097883	17.95329	21.26691	15.22232
17.38552	13.74433	17.44682	12.73563	11.83159
10.25305	31.80312	10.54561	15.17541	6.313094
11.20312	6.794162	8.952489	19.70668	30.74503
15.35674	9.590827	9.53561	22.24139	21.37216	8.958169
9.928563	21.21209	5.664201	16.30216	17.42729
13.15692	14.0956	15.14803	17.90883	13.05656	8.055878
26.9291	10.7005	11.99367	14.93561	11.84991	14.47575
7.551927	21.88127	18.24026	19.01853	16.80505
12.65023	17.6888	17.67683	9.173444	11.65676	17.4688
15.10527	21.89599	13.43806	21.12911	16.91978
8.390401	12.60764	4.938171	15.8382	11.19562	10.84174
13.13262	8.241001	10.4604	9.596931	23.90176	24.35337
9.259163	18.05105	5.711936	15.68271	19.61399
13.12431
PTK2	4.753359	4.16517	6.429143	7.169574	4.992302
5.984164	3.881196	5.233076	4.855792	4.747397
5.544913	3.277665	4.783673	3.459504	4.205614
5.725604	4.622797	5.797485	3.789604	4.595128
6.991263	5.553116	4.414343	5.609763	5.735664
4.322938	4.998794	5.434737	5.479498	3.449735
6.07998	4.295892	9.106194	7.275643	7.4383	7.140593
4.297048	9.290421	10.40588	11.55424	9.168695
8.00589	12.98152	11.26818	9.58695	8.472634	9.787407
12.2196	12.7868	10.14994	4.90383	7.939994	6.514994
5.582974	5.532703	8.53498	6.044702	6.752062	9.671369
7.238155	10.04904	9.00417	9.477767	7.504631	6.427814
6.379622	6.014115	5.497653	9.468699	5.887701
19.58794	8.623616	12.74383	7.735735	10.8465	6.401561
8.049263	9.664533	9.725444	15.9317	4.762998	7.418866
7.23984	7.894683	6.052573	7.061209	11.29173	10.71439
6.997409	8.588463	8.183161	7.175133	9.61494	8.722206
5.159717	5.242726	8.324519	7.338738	12.81337
11.23286	6.631717	13.1106	4.358008	10.89241	10.69978
10.21457	8.060516	6.152803	12.54476	7.654887
8.040436	6.643508	6.740633	5.437101	6.402793
9.973706	10.99426	6.058314	7.473666	7.387392
9.233029	7.106492	18.19744	6.597053	17.03673
6.700479	14.72755	4.197642	6.747359	10.63079
5.99309	6.20126	8.825435	9.257659	4.901931	11.91362
8.168667	7.91378	6.215601	11.89004	8.610766	7.430846
5.310611	10.25205	9.323198	6.963145	8.01365	7.752657
12.11176	8.959032	7.6867	9.959961	7.790683	6.774028
6.528571	6.290855	10.63998	6.336899	10.61797

9.805199 7.024383 7.001299 7.589868 9.413008
5.222837 13.21848 5.570595 9.00179 10.25599 10.23443
5.934862 6.820777 10.57724 7.801991 6.840931
6.787149 6.064837 5.634559 14.7996 13.77301 6.247068
12.61577 4.789259 8.646183 6.700717 14.67107
7.061759 10.7342 9.665212 10.17621 11.58731 12.01346
9.96456 5.37258 4.866724 9.92871 8.368552 12.84544
6.392684 18.01269 9.66213 5.399764 15.1969 7.462976
11.47011 10.44572 5.638461 6.829011 15.34151
12.07231 8.084132 11.14948 7.836015 15.71726
9.34042 10.83182 14.14172 15.36573 8.007957 14.74139
9.046036 18.29242 4.262459 6.947284 7.21027 7.338448
12.98262 3.269619 11.27121 7.832306 10.36294
6.920442 10.36846 8.958629 14.93989 7.325524
6.825719 6.988064 9.490709 13.12553 9.283444
8.522982 8.92679 8.651821 10.18188 5.773262 8.69941
5.436658 7.285813 6.541932 8.612424 12.65121
10.88623 9.186585 10.97809 10.2915 13.09302 4.802258
10.61391 10.72324 5.413788 8.754467 7.027995
9.333517 8.680241 9.028978 14.27612 6.337429
7.227564 10.11475 8.572505 7.044161 8.024287
9.15542 6.985367 19.28191 8.491465 6.016577 1.14031
10.56527 8.503297 9.468699 5.023851 6.146609
13.13141 9.820616 9.230289 10.90307 9.181402
10.51932 14.74232 5.400645 6.611804 12.81334
12.7824 6.992598 9.159746 10.18304 8.994388 10.19544
8.82508 11.90922 9.748593 9.077158 11.08137 10.03558
12.20207 10.37427 7.420212 11.43192 8.288239
4.403515 7.820822 3.927282 11.96265 6.686981
6.255324 11.04702 8.34926 11.34746 15.55424 6.101496
12.19168 8.986636 8.060595 8.523901 9.168431
8.005726 5.029212 11.61111 8.452737 5.862902
6.783982 6.157172 5.179684 9.757078 9.280671
13.61529 8.438585 12.86587 7.483653 6.914946
5.380249 9.833126 8.499057 7.911948 6.159825
9.517943 10.32199 8.38398 18.96299 7.707975 9.376871
5.018732 9.210968 13.39405 12.28673 16.2202 7.219914
9.814151 13.34927 8.305698 19.13904 9.363866
8.271057 6.867489 13.46848 7.37079 9.562566 9.7334
5.357463 5.201822 12.79679 9.420658 8.45934 13.82328
3.798379 7.130632 6.245518 12.44082 8.648888
15.04592 5.108718 15.72641 28.75416 5.9643 6.476481
5.163273 12.27622 13.94607 7.553627 12.887 10.50014
12.80643 9.007755 12.11618 4.742463 8.44948 6.470154

9.416371	8.708458	7.189328	7.173551	13.61665
10.5462	13.32347	8.700992
NTRK2	0.1034548	0.7320546	0.3799904	0.05384327	0.3032156
2.65254	0.05511849	0.4360993	0.2202984	0.3244908	2.495683
1.146619	0.2468028	0.9224883	0.07909639	1.870651
0.04838953	1.159282	0.4299073	0.09905005	0.3234863
2.059222	1.587543	1.226636	0.6670287	2.230347
0.03275188	2.276972	0.1985421	0.07123406	0.4980155
0.5020483	0.03856757	1.058398	0.2387313	0.007820252
0.03415105	3.736545	0.4509055	0.1919515	0.3998631
0.05031699	0.2948171	0.02981377	0.02424509	0.03149997
0.07554791	0.1817539	0.2121337	0.07750076	0.09224689
1.230528	0.8802491	0.1705754	0.7317264	0.009647979
0.0932346	1.130017	0.3033362	0.02224706	0.2308598
0.199468	1.05879	0.07681091	0.03338295	1.151141	0.7700598
0.5783284	0.02867384	0.02492049	0.01828094	0.02482172
1.475126	0.5162592	0.04219529	0.2645224	0.04043852
1.204531	5.535059	0.005591382	0.01736798	1.700572
0.02827705	0.6947629	0.2540185	0.4310708	0.2481351
0.3570785	0.4301808	0.297764	0.07906848	0.05098676
0.1255554	0.0487856	0.4393615	0.06924512	1.010418
0.03906751	0.02035745	0.4020954	0.4752346	0.08477444
0.02301515	0.1865718	0.4792738	0.8347542	0.05043385
0.6865609	0.04542712	0.0466083	0.08598455	0.02406108
0.2203719	0.7676685	0.7403706	0.02322518	0.07823436
0.03165407	0.1707734	0.3426104	0.02067376	0.1780781
0.1295252	1.196127	1.574667	0.1100353	0.07635277
0.03163829	1.297308	2.595235	0.6710769	0.2274418
0.2976477	0.05679586	0.08134611	0.6092917	0.2266676
0.09064928	0.3439233	2.71399	0.09331797	0.1044263	1.19666
0.02854585	0.3575866	1.855632	0.755771	0.2354565
15.63234	1.133236	0.1279207	0.07132629	0.01551664
0.3552509	1.413511	0.1881863	0.2658465	1.097418
0.04810442	0.4630982	0.8968934	0.4808392	0.5143923
0.1304617	0.2004034	0.09911681	0.5064787	0.5961509
0.2468064	5.862323	0.8722982	0.3359443	0.4597836
0.3826189	0.1963412	0.3352003	0.2974746	0.1253455
0.01235486	0.9800013	0.1485412	0.3718246	0.7558734
0.08176023	0.2988127	0.08022187	1.017714	0.03282191
0.2377197	0.04688089	0.3885381	0.2751806	0.03254187
0.2257878	1.974025	2.026793	0.2707532	0.1367894
0.08069876	0.3566194	0.01482057	0.5220803	0.0293778
0.5131338	0.5089318	0.01548934	0.2037538	0.3081692
0.1629906	0.1834551	0.3422291	0.4072283	0.2039761

3.943228 2.078765 0.1348362 0.1305664 3.315035
1.148856 0.02145134 0.07190652 0.4460387 0.06066094
0.1272657 1.844685 0.02717583 0.07145801 1.525281
0.01740992 0.3451034 0.2857589 2.263368 0.02698734
0.1491734 0.1333806 0.2167212 0.6526171 0.1464266
0.2964746 0.04880924 0.93194 0.3038524 0.4456455 0.1432266
1.747586 0.4063285 0.2375884 0.7005112 0.9168402
0.1119225 0.3918779 0.02415414 0.08371047 2.832689
0.6686888 2.154095 0.3731045 0.2085597 0.03416285
1.81311 0.05621779 0.01998635 16.02 0.1547782 0.03873809
0.1476091 0.01482859 0.06985737 1.840109 0.1217208
0.2790462 0.04750854 0.1244759 0.02086686 0.09535444
0.3396961 0.81061 0.7018429 0.02294851 0.09443127 1.121116
0.03166086 0.364246 0.5095478 0.02134359 0.4220869
0.3729344 1.350457 0.07279173 0.7999342 0.07772109
0.8725233 1.7439 0.1690502 0.09402468 2.776715 0.3652655
0.1476133 0.0786568 0.02922651 0.2124793 0.8971925
0.1794841 0.08410025 0.07827506 0.176576 0.03938146
0.2067874 0.1703629 0.05370854 0.1751913 0.09154796
0.03185169 0.476485 1.629297 0.8095789 0.1195182
0.8408316 2.179104 0.335858 0.2528205 0.2333933
3.048759 0.03092704 1.357358 0.1126911 0.1047806
0.08523639 0.1272861 0.06514609 0.18016 1.482309 0.341813
0.4489703 0.4948684 1.139061 0.1567526 0.4592221
0.239097 2.698145 26.90295 0.3951446 0.1585049
0.05897161 2.468486 0.7342876 0.06152888 0.01814618
0.1135576 0.2008593 0.02620498 0.2151535 0.1003864
0.06801001 0.07644624 0.5393241 0.163923 0.9737064
0.8193628 0.0469214 0.424119 1.909108 0.0714381
0.2216722 0.08264628 0.1088787 0.02964216 0.07583124
0.2366031 0.0302458 0.9341607 0.8454322 4.497088
0.1548035 0.2253387 0.09841105 1.208233 0.1009907
0.02275941 0.3152609 0.696776 0.3733544 1.11318 17.43811
0.1697322 0.0329666 1.990667 0.1899454 0.01054291
0.5826371 0.5992993 0.04950253 0.3283926 0.1944937
0.2575271 0.2151035 0.2906023 0.5054532 0.07155765
0.3863894 1.145774 0.8286043 0.0814427 0.0402035
0.02907983 0.04324156 0.8699077
BCL2L11 3.729259 1.873778 2.681628 4.717228 3.589236
2.351349 1.827362 3.080764 4.285153 5.39959 3.422144
2.803764 5.580336 3.361492 2.746395 1.779907
3.22979 3.920179 2.726676 2.530691 2.774285 1.488653
5.43281 2.391402 2.921841 2.742605 2.687298 2.218939
3.917454 1.22301 2.614627 1.850397 2.3191 4.302297

4.418159 2.023434 3.430904 2.296956 8.679476
3.387236 8.928957 3.862659 4.790013 3.4341 2.384094
2.251152 5.029948 4.08115 4.690433 5.202467 1.622249
5.210812 5.688687 3.130153 4.207821 3.497995
5.699819 5.901051 6.008374 5.54386 6.68163 6.657074
3.911956 7.458726 2.135076 2.324447 3.960022
4.719673 2.959768 2.326158 8.608417 2.731373
3.101969 6.165194 4.790176 8.911083 2.478647
4.44421 13.54451 2.443281 5.528136 5.701634 11.0161
3.486869 3.231707 3.264303 5.759129 3.92616 4.388079
4.89557 3.431814 15.73037 5.074046 5.470929 5.33232
4.902175 5.258356 2.541356 14.10595 2.82451 6.893053
2.354458 2.12176 5.093665 3.134295 8.258266 5.653669
2.9772 4.090878 2.042219 2.915216 5.766226 4.327329
4.253086 5.680242 2.033955 8.90197 8.286038 3.051574
2.449546 4.642827 7.979425 7.770038 2.39023 15.45064
12.39887 4.650427 2.685774 5.639296 4.427133
3.595697 2.87686 3.254997 6.631609 5.570968 4.494587
4.450058 4.144565 5.182471 8.675623 6.503394
13.21637 4.350793 4.457169 3.917376 3.855896
3.161275 2.110007 10.92325 3.421155 6.661093
3.745529 9.204581 15.58847 3.820801 3.083324 3.6403
3.090239 4.009123 3.497074 4.775726 4.799979
5.293196 4.88536 2.276941 5.39072 6.11953 4.225775
4.689513 5.980075 4.607957 4.80319 2.719301 2.398157
3.292526 2.817963 5.638252 2.884627 3.467954
6.979301 1.334376 3.581292 4.401185 11.2221 8.568278
6.926494 4.479362 3.170934 15.38679 8.253882
8.241479 6.008611 6.230385 2.49534 8.573945 10.3466
4.935014 5.215414 5.503222 1.490785 2.301636
4.345003 6.563315 5.923036 9.128616 3.693402
1.527677 9.482935 3.765581 8.420353 5.562768
8.110681 8.332797 8.348909 5.576642 5.275111
4.619122 10.16299 9.423182 4.058606 4.691643
5.350884 4.619851 5.163194 6.886269 2.090261
5.583139 2.769503 2.502606 4.411616 2.584988
2.984118 1.946777 4.002823 4.121187 2.342799
4.491743 2.461242 5.324667 10.52754 9.446421
8.591198 3.498589 4.883423 4.939672 5.865749
5.976668 3.95733 5.109035 5.830103 12.10299 6.005929
2.702457 5.035948 8.60765 9.086354 7.713343 3.313729
3.581156 4.005145 4.740289 3.074676 13.77829
5.044855 8.350094 10.11451 2.445236 4.85231 3.310385
5.491606 5.072908 2.434539 3.112607 3.322616

	7.002266	5.634645	5.556949	3.575229	2.606252	
	4.834333	5.8837	5.885258	2.295631	5.543933	2.813313
	10.36547	3.604134	3.492025	5.634794	5.686695	
	5.845762	1.436041	2.258868	3.959538	7.587101	
	5.241159	7.232105	11.60067	3.007075	1.689044	
	2.702302	4.060634	6.356525	5.801174	1.440074	
	3.351561	4.921725	8.370046	4.534217	4.715245	
	6.358274	8.43272	3.737045	4.391728	6.640369	3.89585
	4.503519	3.06494	4.60935	6.091365	9.717733	5.968441
	6.3164	5.463586	4.045912	6.462324	5.47207	5.440096
	3.49474	6.945663	3.104664	9.695991	7.72741	4.799268
	2.585445	6.589663	3.166727	4.839022	5.985422	
	3.999545	3.334094	3.737571	2.137522	6.553903	
	5.064688	5.951169	2.70559	18.78717	1.678653	3.201091
	4.812767	9.149765	2.644132	6.032104	8.384107	
	1.903249	6.044574	8.377419	2.178449	6.427364	
	7.343744	4.989708	6.53935	4.347907	2.688033	2.865035
	2.426303	4.59679	4.583519	4.854477	10.78972	2.85437
	6.55951	7.557983	8.310248	10.80285	3.311128	4.009924
	1.896347	2.714701	7.071081	5.629557	3.576371	
	12.61005	6.909349	7.259466	3.566222	4.382823	
	4.909796	6.533156	4.926177	4.061234	3.414075	
	4.774619	8.552484	9.583093	8.333098	3.692748	
	2.571532	4.218697	7.628706	5.708913	6.075122	
	3.491051	2.02697	3.356239	3.145544		
SRC	37.51878	8.602368	31.00493	29.23135	17.94302	
	5.652957	17.31113	9.906831	30.53737	12.15488	
	11.72103	7.021141	12.47018	11.97288	21.74501	
	6.961001	28.97658	17.06646	23.69671	23.63718	
	31.04874	6.733534	11.37626	5.562899	8.412249	
	8.580743	21.79213	7.168519	29.08718	7.584199	
	32.61458	10.28889	43.88287	13.63976	26.49584	
	13.45251	9.106193	51.38197	69.53815	27.09875	
	20.85672	20.27666	15.87931	32.44585	24.63379	
	54.58667	30.68463	41.59991	7.771805	30.35742	
	32.40494	9.638457	21.36039	23.05121	10.38842	
	17.98994	14.22125	13.71229	44.02161	49.19528	
	18.18297	40.62257	10.21259	26.59509	19.51741	
	11.88145	26.01814	10.56343	62.44405	39.83545	
	36.33725	22.59869	36.76609	32.94001	34.33095	
	26.46233	14.91461	62.38642	25.78006	18.36231	
	23.18392	24.30582	13.95748	21.98387	25.2976	20.26556
	30.49669	19.38307	27.82521	11.67494	14.78204	
	35.76837	19.92913	24.95935	13.55046	43.29983	

20.73246	43.44798	26.33751	23.15512	9.958504	
50.30143	13.86016	15.80263	20.51941	16.97968	
22.92255	20.0433	31.75767	18.50064	43.56269	27.26846
4.375606	8.136979	12.10788	31.49293	27.6965	21.90286
30.82639	26.54275	21.33909	35.25492	39.5319	8.974364
20.74107	10.72597	8.302321	16.57344	15.25933	
28.77553	42.73506	18.75534	19.8477	12.02092	23.66157
29.38589	20.25884	25.25203	20.84936	24.48364	
33.66497	23.39178	50.69921	25.56329	38.4376	15.23178
24.32731	9.974636	41.87049	11.09376	10.90481	
22.70753	35.83187	35.07301	13.05979	20.41874	
19.06534	16.38915	16.00275	20.4565	19.94507	10.76047
25.11403	43.23888	31.55726	24.32178	73.45951	
36.56275	36.78269	24.18604	6.985175	30.68974	
18.51342	15.21848	33.74317	30.58414	54.90325	
19.01946	45.33377	35.19122	16.04912	31.71746	
16.85909	28.17832	18.85799	43.41256	18.57327	
18.94322	35.95681	35.46908	41.94207	38.14273	
41.49247	34.29205	26.45038	43.04249	8.270039	
23.50277	42.31838	19.84458	14.01502	31.01214	
37.98883	26.91677	6.086015	46.20445	65.44635	
15.10665	21.41637	55.14489	14.76509	23.38752	
34.79819	35.62774	21.37097	34.66951	56.51927	
51.38767	30.59898	42.18117	65.29835	31.71994	
23.39349	15.48278	19.63602	9.686124	47.18782	
12.70593	31.56571	32.00172	20.25596	17.17166	
35.61432	36.80366	25.72267	45.4246	15.04883	22.51423
15.75335	45.2439	33.2901	42.33388	12.33816	15.52598
16.48452	31.15457	24.6239	14.45985	32.23867	26.62415
28.82623	33.13732	26.63458	22.80826	49.17082	
50.77606	38.89485	24.99279	16.37349	25.05343	
22.61339	26.61932	11.21389	12.39556	46.46861	
29.31794	44.29181	21.80726	56.03097	29.96236	
20.74641	24.62404	23.86728	24.52663	31.17716	
55.45021	47.02151	12.06735	96.54511	27.69664	
12.02652	25.8084	14.47732	14.39427	11.14787	46.11303
29.39317	46.11817	22.13446	13.41079	30.48798	
8.862365	9.445178	23.01736	12.68045	12.3589	13.62932
39.64316	20.46794	38.63008	19.69476	26.60578	
68.97996	31.21233	19.10029	39.39064	13.39264	
25.43347	36.48153	24.69604	81.73084	23.8938	18.02438
13.46298	10.0999	16.5876	20.75171	49.28704	22.38743
14.71359	40.03073	18.61634	39.33352	34.81088	
9.385152	40.74346	47.15381	9.507152	32.9731	14.90648

26.15916 28.11304 23.14433 42.29813 29.04072
45.3641 37.88658 39.17306 47.0677 29.10976 20.10506
38.0185 25.55891 40.09219 16.03704 22.37155 45.9546
21.3074 39.624 33.51503 23.83434 26.70277 20.58879
21.28023 40.25637 62.07505 27.94737 24.98357
8.540689 33.77601 50.87207 38.0832 30.87964 23.97705
22.35699 9.311095 22.35049 48.9411 36.69489 42.43792
19.76891 20.86326 17.96029 22.59227 41.09931
37.5055 32.79925 10.93287 41.57795 21.7587 32.51073
35.22151 37.9021 24.15003 16.87595 23.42683 26.02639
20.32532 35.94907 13.03654 20.59491 11.8608 17.35562
28.66194 38.20283 27.22359 29.86978 23.31059
23.21711 28.38074 40.5521 15.86143 23.46258 19.16189
14.40546 38.2028 19.65233
CEACAM6 95.65467 1.069717 227.6693 1.928091 86.12366
5.327176 13.94514 0.77591 64.00395 4.908545 135.016
231.556 4.256355 153.6394 19.58819 0.1481217 58.19001
9.756248 46.95747 118.4675 63.88698 0.5546869
394.6093 0.5923108 15.44771 122.8302 21.73155
0.4742794 49.65698 28.88376 42.49646 32.10568
47.60025 279.1226 117.268 0.3965442 0.024767 121.7527
441.0932 9.120803 1054.21 806.3182 18.84777 91.9032
208.1507 963.1106 480.4051 357.8539 8.22072 1092.851
55.16176 9.764097 15.10648 145.7175 231.6302
36.60982 322.8787 1.600618 198.7111 66.29782
209.8376 80.09795 227.9387 18.79479 384.5724
3.919248 406.3776 139.2663 1041.811 28.24262
46.95469 111.4838 251.2882 1821.168 1375.308
10.01257 105.79 0.5928679 59.75457 165.787 1.837517
171.3949 50.01153 204.1663 119.8055 73.7096 48.45702
46.28981 1.467703 117.6602 310.2523 16.80193
312.4123 176.8782 17.08995 1.692396 670.609 145.5982
2.480808 36.02463 17.17105 116.5087 0.1316207
198.9013 1.306597 213.2531 228.2813 29.86078
1.666998 110.3885 499.3558 13.98956 744.302 102.6846
664.1709 31.93663 7.989507 657.8282 0.3798012
154.1266 278.4823 459.2367 176.6686 1132.492
43.27404 43.89246 3.370056 98.34794 124.5436
1094.702 419.3432 508.5887 211.5915 59.73869
365.6383 118.5098 236.6525 295.3439 804.4416
590.2366 485.7338 32.06983 279.6558 115.4579
489.6107 25.36989 297.1872 415.1756 156.6524
248.0054 2.815634 343.4132 446.2371 23.45166
100.3906 30.97103 23.40306 0.63694 29.18003 238.918

6.88816 9.093316 6.698553 193.6194 238.6129 107.2417
146.7651 825.5243 3.643479 1.998458 14.47727
7.486035 26.40545 817.7567 23.98028 262.5298
108.5031 102.6533 74.95803 685.5711 202.3936
383.455 250.5554 9.796929 26.3619 2.28213 81.75694
627.0797 169.9294 1142.161 337.1217 137.3897
34.17469 57.95869 315.3608 467.4864 10.51341
238.4033 551.8066 284.6518 29.76382 239.6866
156.2147 825.4233 116.198 40.00565 49.78337 0.1644889
466.1029 639.3115 17.11606 659.0707 968.0735
37.90197 14.72384 50.13353 0.4737786 1.817709
0.7580405 159.4103 558.7979 9.55023 6.011004 77.14704
270.4264 7.183266 484.9774 123.3288 0.7840752
1555.634 56.12351 1319.874 1335.046 2050.63 646.8506
77.36261 73.33008 22.02603 30.30554 76.79652
239.308 711.8429 70.82393 637.4039 68.65848 130.3809
14.13993 8.78069 30.1628 84.35206 119.4636 725.5068
12.18987 248.1564 446.9223 0.1977553 417.1569
345.7222 148.2115 85.15311 155.4121 164.2463
157.1277 8.463085 317.6433 349.2383 47.94716
240.6731 8.743481 85.67163 127.8133 17.77663
41.26617 36.80302 254.0486 228.7888 1392.901
46.99855 0.4338199 229.5131 59.29562 657.8775
491.9111 20.15262 65.95426 1.117235 425.2351
290.3373 38.89529 186.1117 335.6712 37.24274
25.96388 111.6302 0.05835669 1.161928 122.9847
49.37238 7.837264 214.5404 211.7225 113.6096
373.2085 530.9734 198.8658 410.5942 38.07355
116.4616 7.409212 2066.994 416.11 25.01325 3.165118
20.88692 41.68124 47.81262 60.5159 11.53947 160.2062
15.15423 1251.115 83.61165 393.9576 1.945546
389.3091 54.88701 189.8513 27.7725 439.6029 14.07851
602.0483 1166.268 31.89054 74.92762 13.70429
51.51405 729.2713 214.6731 134.3542 65.55142
0.9924318 0.495131 366.0296 1104.388 2.208319
478.8033 8.231382 19.12198 45.11224 22.39482
33.89276 337.7643 5.618034 96.81623 2.103959
554.9877 212.0223 195.1034 86.06156 9.715924
119.9192 785.789 1.105796 149.5388 135.4471 303.209
2.144276 123.6103 360.7597 159.1504 151.4311
350.1379 86.31092 30.95725 2.255196 1.508406
27.71368 6.324373 111.6435 10.24402 1.349263
555.2947 295.8883 7.917271 486.6379 123.016 2.179906
137.3774 65.44199 254.5896 15.17715 7.735547

208.0323 86.17076 118.6204 15.93642 2.007085
172.9616 4.893698 849.6025 182.0942 29.48331 775.23
10.93253 51.39304 134.2847 15.39431
CAV1 6.260617 127.287 11.45204 15.2454 22.07723 120.5925
10.21743 43.68439 36.76005 34.60399 25.3729 9.52383
18.8853 19.16146 9.434894 170.1489 7.397908 79.74084
14.50233 9.464103 38.69153 182.7339 27.5437 149.5188
92.58393 16.03305 14.34384 177.6265 17.28429
15.92615 36.33981 107.577 4.775488 27.01556 11.41939
13.35744 4.581257 16.07598 20.59092 6.227208
20.95884 3.620756 13.47701 4.898792 27.53178
13.7914 5.706884 20.04635 73.1553 15.63218 6.783433
41.19626 10.11101 11.30804 61.42202 6.777411
19.40353 111.3514 5.074892 6.354761 48.03757
8.343803 60.93518 11.77832 9.629374 124.9954
38.3172 29.51172 24.03056 4.748854 8.751607 32.25326
51.33798 20.67915 32.42941 15.46789 41.45905
14.48888 7.960212 8.305001 3.781213 27.36265
8.078527 45.37766 35.39447 7.691783 25.26541
13.00995 13.65605 61.03354 7.826903 13.02335
24.42693 8.854326 20.71715 7.599423 10.41556
4.666062 2.356623 28.98016 15.24913 5.009453
7.331488 24.04052 33.51598 21.23695 9.868433
74.76737 13.89576 4.877856 11.52083 6.144309
35.97156 57.74075 34.29879 7.121057 6.483278
6.15269 12.85214 4.989551 14.91344 9.273963 17.14389
16.50634 4.44079 14.74806 7.298662 8.64585 75.51631
19.64344 9.545694 3.804929 13.47195 14.26669
13.42303 39.37213 12.77316 20.48708 18.26117
10.72261 6.769369 6.936979 7.448056 7.086303
13.66248 11.02411 3.331226 96.14445 2.553281
20.98543 13.10799 41.40154 5.069997 13.056 77.98543
8.907088 20.6622 75.30218 159.8288 11.81277 29.59156
21.96543 14.42805 16.04351 15.96905 14.35862
8.675476 10.93753 7.876711 16.53252 147.5496
12.0137 24.83114 21.60042 5.255742 16.91317 24.52474
29.24999 5.485252 19.26659 10.8035 7.674587 18.00831
14.39447 22.27147 53.93711 48.70841 15.52561
10.51718 3.526433 23.04799 8.292666 182.2982
9.157627 4.504463 35.15359 35.21691 10.22497
4.446345 9.975651 12.81218 28.07084 21.96516
4.985868 42.21006 5.585398 16.72595 17.26283
13.08479 5.146958 13.75324 34.10722 19.47186
17.10738 22.62521 12.04815 10.04417 8.334764

27.82748 8.874428 35.76087 13.02015 10.47541
4.568973 16.25667 4.891923 11.00271 14.42199
7.256244 6.334853 17.45138 28.62841 3.514203
6.523241 8.61139 13.96476 21.45088 8.445418 10.12364
3.666799 36.96251 12.64325 18.16709 13.63694
23.01605 13.78541 11.38391 107.6631 18.94277
12.6638 33.20412 15.53551 14.0787 32.72675 4.960395
9.641099 16.22389 8.747454 5.884533 34.79037
24.15385 11.25133 16.87767 28.12315 24.13875
17.54778 6.195173 11.2947 19.21577 7.716992 17.44727
7.061504 10.87402 10.00929 4.461924 6.540256
12.71558 128.2486 7.154896 40.0239 42.8746 3.168823
74.5434 14.67398 11.44706 24.70771 7.985877 5.659815
27.93524 30.30491 10.7123 204.0212 153.8138 22.14433
36.32885 57.25853 12.37951 10.82939 14.15447
19.50933 22.7006 25.98222 6.106617 34.08544 7.980688
10.82906 8.609303 11.23359 8.601635 7.348781
21.62653 563.1024 5.071012 17.9149 9.167746 71.23248
21.15199 12.32647 11.6622 34.70824 42.88525 9.649921
4.996237 10.78484 49.74425 10.4638 11.78533 5.079044
8.448424 33.56397 3.457747 17.39625 9.524114
7.696163 74.0477 13.42754 1.92509 13.11288 11.70658
7.966554 8.764731 9.87029 11.99657 3.029249 96.06206
12.20532 7.886375 4.726344 6.018855 17.26913
8.046013 21.78706 8.021525 36.44576 5.005232
5.648348 21.44301 11.04822 72.62586 6.727354
10.5926 19.68636 59.14995 10.44548 8.196832 18.72637
2.844542 11.50151 11.74053 19.73914 14.52749
17.63336 2.758514 6.163774 10.74429 13.46719
5.354244 11.87885 8.496715 18.58908 10.49871
11.75792 7.098439 3.882587 8.050477 5.981994
40.97088 164.6614 6.19197 7.762621 49.78353 50.29832
7.954214 14.43701 10.02356 15.07175 8.560992
43.16401 16.85284 8.057149 8.651845 5.837671
7.872253 6.989599 7.236347 14.16016 92.82227
AKT1 7.181006 16.62091 7.765524 16.10427 9.277391
10.82891 9.544535 9.068078 15.25789 11.603 8.88013
5.896914 11.84911 7.720146 13.47476 12.20417
13.56102 14.14764 8.045362 7.639375 16.05548
10.38982 9.119069 10.71078 14.15314 6.00353 11.39572
12.09534 12.86073 3.981298 18.12292 10.5112 17.20412
8.608889 13.92184 8.197589 5.857766 10.59495
11.90596 15.34802 9.505263 17.50053 12.53694
14.55218 10.62207 11.50562 8.834313 14.98109

9.957261 14.30699 13.92277 8.678609 8.79448 5.431027
8.258103 18.81861 14.10403 12.25829 21.8729 23.21674
10.02215 17.23963 8.57278 14.50774 8.126701 11.69092
12.72873 9.621767 9.881379 15.98109 16.27934
7.044279 10.03688 12.47182 10.64822 9.241844
11.01233 15.71692 8.036776 6.737391 18.94063
13.00951 8.220338 10.17319 10.56793 10.0746 16.44234
13.70724 9.007193 9.136294 8.223264 14.2416 7.614332
9.367005 18.06672 16.1031 11.66679 14.88339 16.76689
12.08376 9.720903 14.83781 8.922212 7.770642
10.2786 11.51812 16.52374 15.96172 22.32977 12.42279
10.8062 11.14723 12.20245 8.424337 8.901507 26.31799
17.11378 7.60404 11.67042 11.51878 8.99648 6.326572
14.22566 6.940229 12.89957 10.46368 10.9445 13.4047
12.32178 19.05857 10.98268 15.11662 11.96196
9.426207 11.66547 11.32609 15.17494 17.91492
9.990084 8.401109 14.62391 11.64282 8.902445
6.883707 10.1145 6.442094 9.79328 11.53322 11.14991
8.884898 7.550763 14.65218 10.32624 12.74964
11.7463 13.0826 14.34261 7.915669 10.59673 4.764183
12.54824 8.250014 9.329559 6.397669 10.45324
14.62887 12.70935 8.980334 22.48427 8.742943
11.93778 12.57054 5.879861 9.625222 20.61137
5.492254 14.79484 9.637866 10.0725 11.35958 8.430671
14.65235 10.56078 14.20017 11.02159 12.97207
11.28479 8.032389 9.27894 15.42497 12.24856 15.4363
12.94984 18.35677 14.02895 12.70207 9.880444
10.24953 12.84792 7.357095 8.391603 10.86185
13.51981 13.43398 14.06223 15.58498 14.89981
11.28293 13.55854 11.87869 8.762314 10.51975
9.223175 19.06737 12.4637 17.68463 13.04357 11.3591
15.49201 11.0142 9.814217 10.68121 16.33601 14.38724
8.907506 7.969283 8.886111 6.950421 22.15818
11.87611 10.20748 8.131047 8.693941 5.783728
6.689967 10.81886 11.66205 12.83319 9.300492
16.40259 7.256841 13.15635 11.99128 13.52903
6.672296 14.66667 7.843024 10.58838 14.07491
10.12927 8.810575 7.806806 15.33202 11.95654
12.03255 18.29058 11.0198 12.13754 14.07051 10.67331
9.554676 18.94351 5.149915 7.844347 15.30016
11.88618 22.73609 13.31909 10.13242 7.091021
12.50717 15.71213 9.573832 9.930918 12.67164
20.09324 14.37544 11.87349 10.02535 6.569629
10.28903 9.303842 14.04408 11.94295 12.72589

12.84544 9.757837 9.012264 14.16213 12.76227
13.62317 9.71916 10.42323 13.69721 13.40731 18.26794
9.114395 11.96598 12.08416 10.83807 11.01836
6.748052 14.05974 13.10842 10.87574 14.325 9.958167
12.04297 12.18844 15.29033 14.84586 21.84586
8.158541 13.89122 11.89443 12.93016 15.52066
8.938816 10.22333 9.678753 6.861142 7.623689
19.48065 11.84296 5.550812 8.302789 15.92219
19.99943 8.205338 14.14754 6.477012 10.1347 9.695619
10.81378 16.79837 10.11736 9.522798 10.37351
14.15724 14.81652 5.753161 12.14223 10.84159
14.95691 12.09969 9.512606 13.4858 10.14197 10.50326
9.595625 10.13012 12.93006 13.26352 7.455156
8.518077 12.16857 12.04058 8.846094 10.65583 12.138
13.42552 14.18668 12.0486 10.6473 10.46331 11.34559
11.7574 8.615942 14.52331 6.932018 6.948867 6.464476
9.991479 8.23374 22.73535 10.10882 13.1779 11.99815
13.08115 9.313519 13.17846 13.88524 11.87578
15.08365 5.813015 12.7763 16.37344 8.605249 22.15582
13.27503 14.51268 6.845994 7.150372 9.39715 13.05042
7.150156 16.51854 12.23865 12.82341 16.48926
15.84794 8.469051 12.7101 15.68369 11.52435 11.27547
12.79714
ITGB1 23.64556 88.39903 31.59022 36.98116 21.27594
92.44535 13.48123 46.13645 33.88146 35.04283
39.20159 16.73863 22.29408 19.01539 13.18465
103.9124 16.07916 63.07009 14.37861 17.21084
50.54464 76.87175 28.19682 78.59367 152.3346
22.66102 18.53264 91.85513 27.07625 17.31429
34.68436 80.55489 55.0933 81.94777 63.82487 46.53066
49.40754 48.69131 92.27245 37.99953 53.795 47.09671
82.36119 39.55541 44.21257 56.18638 36.20113
62.0208 86.13456 88.98867 31.75199 45.40725 39.61847
61.37514 89.586 45.32637 56.34438 101.9927 107.1843
17.45183 101.825 47.88729 106.886 70.46785 29.58603
180.2855 55.13829 43.00133 93.43769 39.06339
71.50935 53.65603 81.16454 90.30005 105.222 20.34807
59.64527 47.03589 44.3303 38.32574 34.91085 75.49096
99.63662 73.31683 110.3883 51.30259 105.5261
50.63195 24.84845 139.1614 69.2601 77.41809 140.9075
35.54469 36.38073 48.06099 168.7912 37.71606
98.41678 68.73455 51.53545 53.43731 12.48023
28.08496 99.56575 82.01625 50.66251 67.65607
59.62808 37.40091 73.62878 49.27465 40.81774

55.03033 50.20472 35.99621 19.31192 98.87752
69.07017 36.10611 54.44463 49.03318 51.54693
84.58104 93.67686 23.10162 64.21039 25.17651
110.2056 101.885 66.58877 44.84785 48.90096 84.0192
24.85533 91.41895 103.5812 106.9334 65.23929
50.6509 72.45873 48.88209 47.44825 17.08073 73.01518
32.62521 29.145 95.79092 50.07231 42.12721 56.73941
82.16899 70.96647 109.4761 124.9993 34.31138
39.13777 82.40334 132.4997 29.71829 60.93067
35.67562 71.53423 56.69558 36.13011 54.15496
39.51643 71.55567 33.5554 74.23595 95.46042 45.44975
48.27961 49.90372 37.46816 79.46236 117.8976
57.83579 36.86837 159.3008 40.16374 57.69511
28.43743 59.26318 74.20475 73.60554 106.9086
48.14451 93.26585 74.17903 93.90766 93.49842
91.51601 27.26977 42.61304 147.5853 100.5024
35.97642 82.77164 27.55184 49.83646 50.21033
68.41824 56.18608 29.55155 52.77937 40.24658
81.2313 52.59185 113.4286 57.01495 92.80398 84.3955
76.83279 66.52623 68.76989 51.14237 56.37638
72.41143 55.90757 63.81664 88.30877 31.33947
64.65189 44.8881 16.25766 62.73428 11.35857 23.52052
56.15932 73.87124 80.82849 25.0625 91.47282 51.59992
31.79597 17.97902 30.69287 80.36937 35.21403
72.17433 138.9072 53.56956 54.64164 114.2757
68.06534 46.61346 64.27361 90.39532 49.4999 180.6924
45.32021 45.17016 75.69075 64.29245 110.209 56.83118
61.02142 35.22756 62.70481 41.06518 39.16804
82.95261 73.33882 61.94343 80.99244 214.6568
41.10092 74.9115 45.01798 74.36165 39.28603 46.452
50.4985 76.88665 44.90385 61.83524 155.9433 35.42653
116.1948 76.98802 67.21146 82.63159 53.46183
96.62639 59.4592 53.60043 67.31432 57.73841 88.40526
59.62049 255.6349 127.0687 79.72793 80.80412
65.59124 70.50989 75.26416 57.86091 53.11181
68.72888 79.27904 164.6839 119.1254 32.49512
66.68416 50.63285 89.75674 61.40017 63.67238
165.6135 69.14986 65.11512 33.4481 41.42695 123.1932
53.27514 103.9162 79.69731 134.3955 79.42865
88.4199 57.90089 24.47606 121.2811 25.18714 51.19197
46.27912 51.96419 78.34509 63.5756 113.5406 64.6294
45.00945 68.21679 41.37072 30.8729 42.94001 51.46335
72.10356 50.08157 27.79392 65.32619 43.30497
125.5973 27.05174 49.03578 37.55778 31.69528

24.53667	47.17376	88.05309	56.94031	54.1393	44.96789
47.78034	77.34987	85.85634	135.7506	33.78759
61.8059	39.32254	171.1365	34.10968	33.85583	32.51823
47.63175	51.09755	59.81082	30.32633	47.78109
39.72187	71.63725	66.90251	71.99129	55.76448
42.81705	51.64186	34.73681	46.5656	57.24059	109.1962
24.99897	78.40574	64.62285	49.2083	57.44179	82.37044
30.49144	56.36661	127.1747	80.88238	19.30115
62.1301	57.43615	63.91519	46.39253	88.82263	30.87935
62.07268	14.47413	18.55846	60.10975	43.94107
40.52716	31.22107	117.919
CEACAM5	66.51529	0.06488109	126.1777	37.277	158.9451
0.5325417	131.0424	11.78102	303.1673	12.709	227.4786
241.0369	12.29195	120.816	155.1957	0.1256685	114.7315
21.69608	35.98784	27.6608	324.5835	0.2829659	219.7161
0.3500762	28.72807	260.1189	73.48986	1.187037
49.12034	55.63749	305.3992	28.00785	22.73907
180.215	69.93886	0.1580353	0.07060258	9.864641	472.2531
763.7808	361.1588	468.1376	77.07006	112.7639
484.1395	535.9512	756.2234	327.864	4.240262	234.6649
61.48132	168.4345	0.5959824	87.93018	3.877843
98.28303	52.49532	3.76034	218.4053	85.56371	175.4575
108.5636	79.34787	123.7815	1414.27	23.049	116.5712
116.7727	541.1762	28.24039	30.21356	281.5065
113.518	1048.682	1843.516	46.9369	454.8007	0.1449816
13.49643	1049.832	5.544897	529.8306	103.9259
302.2601	33.74272	16.86743	15.56428	63.90648
1.641614	17.08106	933.718	51.49926	124.1327	164.8864
17.06452	0.4808869	7.612833	654.4542	3.6762	91.00612
14.85058	466.4993	0	618.9761	5.933579	266.1424
1732.973	9.845387	5.24632	228.9802	617.1001	353.479
132.7752	50.88699	652.1774	53.53363	21.51332
404.0366	0.1002489	642.2659	309.411	158.1906	115.3258
941.0915	31.71806	33.75777	12.68744	268.229	155.0166
1420.422	290.5978	896.5632	144.2997	217.1338
916.2308	59.47247	102.6279	86.72678	127.0229
810.5033	556.6037	105.6934	146.5099	316.7282
282.6749	3.055681	710.5587	311.3179	239.5694
282.0036	2.284872	291.7585	39.2382	5.04061	112.4231
25.85903	21.42044	3.128278	13.23556	255.486	5.01124
55.5988	36.31122	503.1286	118.8885	481.982	38.89766
232.5236	7.819803	0.8196672	0.5076125	42.84449
429.0315	926.4588	4.007389	262.0252	66.55566
412.7122	214.69	1046.722	536.5389	931.0614	204.9859

48.11355 2.094024 6.743484 132.925 663.246 396.7981
55.05426 321.3984 48.02174 16.21703 174.1497
143.683 708.3878 3.023471 247.5783 96.60771 325.5955
17.77757 239.8434 108.9411 1072.002 172.0674
74.99226 41.96755 3.355592 334.6226 623.8338
85.25844 586.7806 1398.641 42.30488 30.62825
71.62332 0.2949871 1.726782 1.710436 77.92426
370.1104 49.87475 2.203351 175.3994 228.1624
113.6545 595.7894 149.7629 1.960922 999.2421
259.2512 1900.884 238.5868 3113.771 299.4587
142.0967 73.8953 24.08819 163.1805 366.9795 619.2403
285.2668 264.9893 313.3697 17.25722 380.2959
13.14708 2.546547 22.81701 363.3727 46.25598
529.1536 79.55225 204.7679 188.3508 0.08290228
458.7678 203.4366 167.4308 76.42648 242.6515
56.1887 278.5089 8.248886 793.4837 727.8886 75.87424
182.9041 4.767768 71.07523 232.689 54.07191 35.64862
67.032 1256.325 402.9657 719.2417 57.66473 1.027534
48.55726 35.31947 952.841 503.4522 87.77808 69.51572
3.150324 795.4142 72.08316 680.2186 402.3661
641.8616 28.21529 33.60294 197.7352 0.1494602
1.150095 549.2752 22.84961 13.10059 379.6272
354.8082 411.3737 0.3507761 750.2995 138.1658
695.3458 7.646296 73.85035 19.74903 1491.861
618.5526 68.73648 0.3383517 46.51423 96.57251
165.4371 33.07196 3.819346 232.0215 16.33777
1714.784 265.3929 585.914 3.868973 443.0118 170.2048
361.683 28.29259 848.394 45.54324 1237.836 1387.44
33.93019 93.6558 13.1218 13.60936 1253.735 178.2102
218.0284 12.817 0.1364707 0.1374795 1.827237 1304.362
1.954556 427.3398 6.437192 897.7215 8.054385
48.75329 99.74465 816.6707 4.831165 291.2216
0.8519455 540.874 187.6738 102.8468 73.22387 23.30824
47.42714 776.7439 3.541199 206.4093 166.3658
453.7337 28.66684 318.3115 377.1919 142.0659
607.1524 421.5699 25.6726 66.28922 3.578057 1.939202
11.71608 4.707262 91.94268 108.3905 0.6549197
47.23162 471.8921 5.951348 1119.497 390.0602
6.849105 102.4479 240.0892 301.9838 9.538415
10.77798 426.8998 37.94267 158.2623 63.49368
13.81035 302.4336 48.8703 1424.762 321.1892 447.6479
569.8237 17.58266 92.06786 79.43998 30.8577
BCL2 1.031124 3.965707 1.963189 1.730979 2.864103
4.514862 2.102719 2.437499 3.748521 3.699924

3.34026 0.8415963 5.435551 1.796707 3.961552 4.172001
2.340414 4.307114 1.291885 2.481598 2.088649
4.589014 0.9573171 4.72592 3.794637 1.723772 2.808301
4.583251 3.893616 0.4817067 3.192433 3.681835
0.6458602 1.04471 0.5339164 0.7645364 0.3333209 1.103143
0.7125729 1.917316 0.8051322 0.4227414 2.996743
0.6263201 1.03056 0.2677871 3.557501 2.450928 2.719364
1.297411 0.2844505 6.731231 1.726414 0.8379077
4.102188 3.429897 2.657787 8.108389 0.9578965
2.132811 1.990558 1.68796 3.011021 1.097918 0.2921416
2.189894 1.275042 4.113029 0.6665359 0.6090796
3.980756 1.211683 2.266933 1.346761 1.038371
3.177737 1.919021 0.7313365 0.7847211 0.4957584
1.14032 2.675052 1.638459 3.500439 1.12006 0.9212921
3.502736 2.321197 1.038934 2.931147 1.063061
2.829687 1.70015 3.892946 1.889364 1.523947 2.726556
0.8642311 2.510042 2.144115 11.44572 0.4057156
0.5939561 1.05951 1.865867 6.442696 0.9843695 3.272696
1.436459 0.3537731 0.5073635 1.44888 0.9413682 6.170452
1.463415 0.3265612 0.515356 0.4925439 0.9269641
0.8645776 0.9454489 1.013464 1.191248 1.527246
5.409493 9.317536 2.522502 1.260764 5.70254 2.049069
0.8859381 0.8545491 0.8794539 1.560419 1.258498
3.428171 1.871414 0.5959294 1.535306 1.071931
1.351887 1.387106 1.443558 1.57658 1.144976 0.8722033
0.8980955 1.413089 0.2656025 3.912271 1.790274
1.444391 1.234937 4.27615 6.868997 0.4285197 1.454848
2.459818 0.3833856 1.040028 4.696845 5.471912
2.401631 0.886298 0.7588978 5.576276 2.001653
1.268415 3.314495 1.290441 3.780388 1.256886
1.389322 0.2025232 0.1791169 0.5361021 1.76208 0.8816195
0.898963 1.142729 0.3583967 0.7714771 2.031419
2.345118 1.345008 1.971937 4.147811 0.4821391
1.281821 1.273306 1.724665 0.2336965 0.9528868
2.266034 0.9354229 2.201209 2.460597 2.16344 0.8942408
0.5733114 0.1385334 1.865342 1.887962 1.794909
1.746533 1.330004 1.036914 2.781359 0.3187169
1.125933 1.246173 4.031256 2.880095 1.33167 1.1296
1.903813 0.6937307 0.8724204 2.887258 0.7329848
0.7570384 1.234912 0.4277521 1.372754 11.11856
1.662042 1.205562 0.4193344 0.7099613 0.6705586
0.6265685 0.7956967 0.8356193 0.191419 1.251553
1.180677 4.423126 1.398471 1.990455 2.140484
1.580033 3.937337 2.036026 1.465625 1.314897

2.770556 2.591003 5.048014 3.137133 1.121766
4.211992 0.722964 0.5506725 2.316031 1.089973
2.57066 1.157442 2.365579 3.213892 1.758698 1.212161
1.022101 1.46697 1.889503 2.86365 2.929528 0.4349748
1.919769 1.059221 2.183211 2.960096 1.022576
0.7142805 1.94197 0.3984522 1.373531 0.9714963 3.804917
3.883503 1.507469 5.968932 2.246963 1.811379
3.189434 1.362227 3.606622 1.120753 0.5958179
1.787098 2.769753 0.9788467 4.229301 3.555829
2.113042 3.194962 2.981516 1.98799 2.131263 1.395124
0.5758579 2.608354 1.988341 5.39212 1.220231 0.3455122
0.5736912 0.6872205 0.7859203 1.970096 0.5962121
1.719826 0.3314308 1.017215 4.309766 1.561552
4.735743 2.64388 0.7491979 1.135374 2.011338 0.8559727
1.805803 0.3656533 0.8881258 2.9764 1.755441 1.036418
2.722266 0.8002042 1.372433 0.4440179 3.135033
3.731503 2.169157 3.650164 2.839305 0.6853391
0.7518078 2.455437 0.626227 2.004973 0.9114897
0.8460922 0.2688714 6.294045 1.505951 0.4336665
6.551841 0.3142495 0.5593231 2.109386 1.97526 0.8550201
0.7769103 1.218532 0.4489197 4.446494 0.5954758
3.793125 0.4296841 3.629445 3.555602 0.6338784
0.8073169 1.048221 1.68274 0.4952833 1.908162 0.5661069
0.4192259 0.4250892 0.7579071 3.978147 0.7192252
1.179877 1.587488 0.7892126 0.64014 0.9379956 1.58539
1.670268 1.380767 1.017419 1.933728 4.11153 0.9086401
3.089908 0.7288852 2.599191 1.181389 2.927733
1.105141 0.7098735 1.832549 1.636959 1.335761
1.723896 3.229428 0.928573 0.5656733 0.634044
7.155821 1.205448 0.6162964 1.607963 1.794625
3.218269
CASP8 4.599492 0.920679 6.727195 5.081608 5.456806
1.769371 2.913254 1.747724 3.524088 4.852899
3.190512 3.798972 7.784269 2.7872 3.91767 1.61683
4.653454 2.528228 2.288497 3.114577 4.987915
1.618129 2.118054 1.583895 1.643109 4.011042
4.334052 1.773257 3.821997 2.949398 3.820578
1.636215 10.5273 4.681333 8.24184 10.31138 1.690653
3.145536 7.297292 7.279993 4.827157 5.987353
5.747668 6.225785 5.517052 9.337086 10.62212
5.198234 5.04075 6.086388 5.707868 8.607143 4.488956
9.398099 3.774102 7.863601 7.162654 5.459393
5.249552 5.150483 4.991917 8.854935 4.201322
2.243966 9.752426 2.452048 5.290578 4.786515

5.296324 15.16833 10.05974 6.13226 6.845814 5.78695
4.234708 5.361747 6.041092 3.696656 7.384401
10.2551 5.287418 6.461572 11.47353 5.826776 1.905805
8.361043 6.633468 8.794589 8.376553 10.70402
10.44006 17.25956 6.771868 10.4137 6.079143 5.233711
6.814942 4.6215 3.976011 8.317773 9.277193 6.368457
4.968412 6.068172 5.08133 6.208284 6.42974 3.940305
6.27395 7.225631 4.913706 13.01047 8.701923 7.281803
4.965717 7.862517 6.082247 7.155941 6.063652
6.622899 11.43971 10.52874 7.935463 4.509798
4.209144 8.006229 7.461676 7.820918 4.028147
6.432956 8.686475 7.225568 5.922967 7.743281
4.703979 5.504499 6.616758 9.911142 3.198741
6.719456 11.15963 12.90927 8.129856 9.862786
11.28572 8.600801 17.39401 4.928711 4.990448
6.281836 12.38707 5.325317 7.884955 7.982057
4.478206 4.408897 3.946713 5.196769 9.20202 7.22787
5.115622 8.405745 4.631829 6.662269 6.909248
7.136814 8.629893 6.48099 6.012652 0.9606306 2.483845
3.770973 4.641938 5.49687 7.221755 8.092103 5.737824
16.55688 7.711059 5.315384 5.439008 6.440242
3.836799 9.353289 3.910606 9.026401 5.900555
8.714621 6.48475 10.19378 4.245495 5.552902 7.552872
4.636659 8.778242 10.00421 4.386492 8.10188 5.810908
4.291508 3.050922 5.576901 4.96737 8.085914 8.18258
6.595783 4.771202 4.907223 5.338664 7.023082
5.780563 3.622266 7.669086 7.690074 6.109896
6.948028 8.676704 5.790576 6.453491 5.793765
8.275387 6.946667 2.587613 6.196035 9.091948
8.129525 8.182942 3.433349 4.383142 7.282702
2.769976 3.601483 5.023272 7.794261 8.987666
6.646044 4.787739 11.06391 10.44832 6.676796
6.796532 8.990749 6.501194 9.380669 5.794108
5.594258 8.22589 2.872824 10.39811 9.13084 14.34409
10.12494 9.72755 8.374754 11.97468 1.178855 11.2961
4.95275 10.22226 3.056106 6.158472 6.237057 11.17449
8.491528 6.59843 11.62579 15.68555 4.047966 10.52813
8.310429 8.739349 4.298635 8.174883 7.457812
9.874275 7.395922 7.897321 2.469265 6.367882
6.139627 6.529261 14.98077 2.821295 10.35684
6.255202 7.540135 10.78042 7.883338 4.768627
3.082906 7.917988 2.725342 2.099551 8.076143
6.07063 4.753242 8.58065 7.903053 6.560911 5.974264
5.966185 4.018092 7.507456 3.58661 6.087276 7.040537

5.231931 8.041722 5.622484 4.381593 7.414584
2.939664 7.764 4.245516 8.425333 3.556125 7.034332
4.800976 5.289165 6.157517 7.783848 5.843203
6.300268 5.999439 4.432844 3.002591 4.025936
4.921712 7.801732 4.276599 9.03161 4.487093 6.823909
11.64063 2.838201 8.657311 6.894998 8.366568
9.344561 5.883762 4.386715 7.155893 4.783695
9.051402 2.992687 2.779197 5.628994 3.639159
18.06991 5.242154 7.584615 9.778948 2.565698
7.672045 9.883021 3.57871 9.317831 6.509831 4.556943
5.874099 6.486808 2.42845 7.567702 6.6505 6.03366
5.267265 6.033093 11.29679 6.225755 13.52397
9.72332 6.057059 3.944779 9.944366 6.497244 5.476136
10.30634 6.485459 4.778191 3.934963 6.339608
8.084539 5.706496 4.516039 7.885 3.718883 2.673152
7.582987 8.494936 3.988885 6.103939 6.814526
6.357397 8.053762 13.07108 6.092785 6.562939
6.579384 2.623045 7.452229 10.46838 10.29939
8.352884 8.80246 9.920892 3.560635 4.155715
EGFR 3.353879 10.75098 6.297909 5.114132 5.012296
12.41706 2.888861 11.42999 6.897978 2.664197
28.81306 13.31488 2.651942 15.32663 3.687841
9.673954 4.143345 14.00022 3.143225 3.634263
4.961778 8.242158 19.18198 13.53174 28.80106
23.11451 3.475969 10.19934 4.34318 1.546471 6.924674
13.83708 10.63956 21.81118 4.783743 0.6929519
7.203718 6.406079 3.873314 5.798367 7.950565
8.15332 10.28461 4.157262 9.196192 3.24239 9.171981
6.111078 9.701724 34.97951 4.142119 7.020302
2.514578 8.009381 17.73159 10.36939 3.214346
15.5428 12.94834 3.565135 8.800329 5.368799 8.608696
2.232533 3.859547 7.212194 4.365211 5.196754
7.643759 7.215029 8.321476 2.204163 8.318845
11.05115 11.60301 4.755978 8.391562 13.47973
0.419959 5.932905 4.591977 11.29806 3.761803
15.6043 9.202638 9.433808 13.97673 6.676655 246.2036
12.87507 9.737027 2.306779 7.081247 5.437202
7.474377 3.378243 19.28863 5.900635 4.292379
10.20996 9.391946 17.90804 4.062728 4.34171 6.480769
13.38539 7.964079 7.782219 10.64014 4.466798
9.816777 5.796355 5.606497 7.193243 9.992358
15.18478 4.369713 8.101817 2.918196 4.794044
8.188566 205.9553 5.217575 4.59923 0.395445 3.105248
3.883758 5.827826 16.20694 13.1202 14.8292 6.740838

7.783249 9.789525 5.12965 13.76307 13.21898 17.76857
8.145767 13.60373 9.627499 21.56556 12.47159
4.238167 8.477838 4.94146 306.6101 5.033283 0.4781388
5.024827 15.25034 7.337854 240.4966 3.351032
13.94551 6.194762 4.78769 5.80557 36.12883 5.520551
7.27025 8.202157 12.54655 9.756891 167.6443 8.891293
6.946971 5.601858 8.313878 6.904262 7.961842
6.007128 15.79396 9.457275 8.348514 6.350673
9.754957 2.566548 6.282594 6.250807 3.708033
8.501072 15.21597 5.031611 3.400026 4.847554
15.48804 10.29812 9.055065 2.222233 9.185309
8.171852 18.68048 5.857407 2.908633 33.28691
6.515329 8.14652 0.370077 5.571423 5.195046 8.965683
11.36961 4.075756 15.91952 11.49668 2.92968 4.281301
9.542335 197.7998 8.113881 8.997647 9.200923
5.67347 16.59542 8.004839 8.448822 7.519764 7.407754
9.027531 4.014697 7.008886 4.138088 5.252418
11.49791 4.551201 4.285842 17.75869 5.266759
9.079462 6.44219 7.950101 6.474005 8.774132 8.795896
4.878268 3.318179 11.97118 10.70939 1.55085 13.59562
5.252324 7.353834 5.920049 5.988229 3.549317
2.519604 19.04068 9.955734 8.772075 13.7394 9.880531
6.026834 10.28273 2.009866 3.46784 7.150143 7.071581
7.206939 8.741612 3.799971 3.151041 6.343426
17.11234 13.53239 6.670778 8.566562 6.868084
7.875297 43.80215 9.354767 9.532666 29.44097
8.66724 5.235588 9.587504 7.24656 10.9737 0.4723121
10.13932 9.300051 12.88405 5.892412 6.821715
17.06741 4.989856 23.27759 8.452884 5.901522
9.15494 11.08916 9.950058 6.68066 7.169138 14.34277
6.950346 201.2137 8.038875 9.507292 8.620093
8.643306 7.61419 46.56037 10.00222 11.02921 10.94677
7.956029 4.145758 7.291679 3.438541 7.392232
19.26346 1.435251 3.834778 6.386478 16.91515
5.728972 6.12529 7.018753 15.54043 9.480673 9.286199
8.537421 1.105987 6.154402 7.947072 8.225854
1.620221 6.788595 13.17461 6.18388 8.729829 7.660033
8.104913 7.698033 12.92055 2.746559 12.53097
3.154295 5.742212 0.4485661 1.402124 10.81355 3.9489
14.7497 18.85985 21.91994 6.232333 4.382942 1.739435
9.01636 11.02957 6.435805 119.8127 0.8782644 10.67351
9.016997 5.046524 9.179336 19.36011 7.574372
7.158284 39.72153 10.34178 4.589267 4.741242
9.350912 11.6876 9.858221 1.021661 3.731395 19.24743

8.9479 9.586304 3.349898 5.208104 11.06792 6.964614
6.597698 7.724061 356.3285 12.59891 4.362929
0.3626005 10.89837 1.735091 7.971749 10.72332
22.37127 2.258006 7.76369 7.756865 3.099545 6.589418
24.93621 4.504575 20.92371 13.99134 5.307714
12.17864 3.163255 3.846106 10.51221 9.640991
4.497538 7.682888 13.85663
PTRH2 1.284281 2.107347 1.683185 2.608828 3.059529
1.701067 2.140569 1.502457 2.233504 4.101384
1.531257 2.101622 3.805586 1.569107 3.248063
1.675837 3.038731 1.871638 2.456197 2.094859
2.546579 1.917026 1.930209 1.866553 1.286873
2.146634 2.590854 1.781383 2.197493 4.576404
2.026867 1.99744 4.198363 4.604791 5.078032 6.840092
6.362862 4.154127 5.244595 5.075223 2.933146
5.060878 3.120071 3.387216 2.357729 4.727305
4.233457 3.502128 8.847962 6.526654 4.84396 2.755952
5.856459 6.046674 2.396463 4.992556 6.800095
1.856981 7.022764 3.178847 3.483381 3.594854
3.415993 2.650592 5.791349 2.211985 5.085633
3.011132 1.872375 5.556322 4.798475 5.22284 5.330464
12.06954 3.329655 6.635386 2.615372 3.882239
7.121971 9.914474 5.995662 3.405058 6.760086
2.90275 5.646794 4.772144 3.172728 4.261128 4.987643
2.637901 6.96782 3.860557 5.084883 3.570665 4.230102
4.859826 4.605174 5.883862 3.081517 4.585684
3.05473 5.551224 6.193681 5.544397 3.94396 2.277437
4.828499 2.289361 4.065719 4.229324 6.381703
3.285568 7.224086 3.128948 4.450705 6.139823
4.063996 6.974119 5.524953 4.498927 6.077435
4.593429 3.505285 2.890792 5.132493 3.044376
6.363971 4.747179 2.526831 3.100756 4.472311
6.337114 5.127243 6.921415 2.048808 2.133077
3.373181 6.102148 4.15924 7.440381 4.165531 3.937481
6.392667 4.75686 7.411896 5.62189 6.907378 2.79609
6.366905 3.661758 4.58616 2.760645 6.118772 3.049264
2.222003 4.042233 4.488873 3.146628 12.12383
3.866127 4.55528 2.949522 4.077989 3.942756 4.497234
3.175288 4.185414 6.113311 3.268496 3.003724
1.958458 3.091089 4.382124 4.276187 6.018948
4.159781 2.214913 9.132148 3.674064 18.69884
6.791324 2.631302 2.763231 3.920766 3.450285
3.287264 2.886871 5.424455 1.903453 6.097647
3.858774 4.357246 4.560574 3.430083 3.995113

2.046674	4.374554	6.034791	8.161063	2.559356
7.27242	2.873262	2.602263	6.73336	9.134025	3.533859
4.066381	4.529477	2.912754	4.228243	4.779174
3.218726	7.132574	2.444158	5.000529	4.896852
2.98169	7.024767	4.983641	3.946767	5.277209	6.000579
3.651602	5.008418	2.929351	8.077909	6.158649
1.513236	5.482316	6.723599	3.382	3.73895	2.878446
2.188555	5.303942	4.638	3.823544	5.65363	7.119302
2.074933	1.845699	4.223182	2.991644	4.709705
5.346137	3.60597	4.169469	2.371314	4.894395	6.244077
2.653379	3.375854	7.603383	6.273533	5.852651
4.089575	4.387559	3.174024	5.686358	3.098477
2.608882	6.340848	10.62018	4.597488	2.593958
3.310519	4.488442	3.661301	4.217236	2.94415	3.691348
7.676519	7.832983	6.311489	3.923601	2.967613
3.732569	2.318373	4.071627	4.25046	3.129941	2.495763
4.311106	3.753804	5.096726	4.988011	4.04518	4.436178
5.008794	3.995131	4.733806	2.106066	1.946146
3.68605	4.189592	3.267936	4.137456	4.714732	6.191684
3.651611	3.046501	3.972666	5.413117	2.670796
4.009361	5.827656	4.355618	4.505541	3.070118
4.929825	4.243345	6.678206	11.05158	3.289497
6.53387	2.293094	3.624347	5.541248	3.487463	4.032013
4.095387	6.272087	3.493226	7.642745	4.321391
3.19079	7.357894	5.476527	7.375806	3.887354	3.285469
2.593373	3.999568	5.080362	2.656187	4.567952
4.955436	3.956681	4.55016	6.821621	6.883283	5.875137
4.389661	8.031611	3.114412	3.085853	2.559662
3.184378	7.447729	11.4151	5.279833	5.206248	3.003132
4.685683	4.633694	4.742538	3.084847	6.908411
2.000461	5.551584	4.087433	3.715912	1.85673	7.037197
4.493454	4.159668	2.959036	4.319202	3.754184
4.423463	3.813471	10.10001	3.252943	5.438705
4.733329	5.03138	7.685911	4.034092	3.644072	3.15297
2.834627	3.044537	5.294072	4.026977	3.82755	4.354946
3.366124	5.884293	4.870429	4.461386	2.556881
7.081286	8.073048	5.633418	4.463115	7.762297
4.540975	10.97111	2.140054	4.466628	11.5289	3.118686
4.775596	3.202209	5.101657	3.480005	3.544908
STAT3	25.39211	31.20173	26.32607	24.69234	21.23445
26.6488	12.11536	16.07459	23.53945	18.67994	31.42674
18.63815	20.37106	19.4992	19.40135	28.92174	26.09676
28.30222	18.1873	18.22648	29.28816	21.5824	33.12589
39.27332	26.33006	24.67077	16.45733	27.51174

23.90839 5.548089 23.57099 25.17296 37.85656
25.5534 25.07331 19.57838 25.76473 47.1677 48.22623
40.29598 35.30293 22.92338 29.76829 28.52654
22.51404 22.0229 30.86771 96.08468 28.61314 24.62893
18.6102 27.06493 25.48288 23.34776 39.78098 22.30291
26.59705 43.30995 22.18265 18.94089 44.33287
39.7664 41.32588 30.5603 27.84833 43.30769 29.73139
26.55669 55.30578 27.50582 48.72574 28.99482
35.80501 48.14065 34.67108 27.17298 33.37444
55.21294 29.95736 36.86602 79.40013 42.44536
44.11206 28.83415 26.63349 23.62935 35.41151
43.81221 20.25989 28.98123 45.18585 110.0969
31.1728 36.87549 33.86887 36.05355 31.35095 16.52786
37.24245 41.61055 38.09998 36.31046 25.73727
16.92502 32.36641 36.15299 36.33552 32.58426
31.05778 30.46475 37.55841 19.18631 32.55092
25.49324 33.80278 47.17274 16.58619 17.35544
29.68234 14.56476 28.41156 40.06167 27.85712
36.73146 25.58558 29.12139 23.36069 21.45528
41.02614 34.87037 30.96022 22.84952 42.74165
44.31774 24.63969 40.89199 31.65632 31.39103
29.01915 24.45021 30.2516 24.42797 90.92308 14.36464
34.80661 25.07359 10.23474 28.62863 27.09889
29.60142 32.84234 35.73231 26.32745 60.98426
38.25433 22.15227 29.14729 29.42786 69.07726
25.04899 39.55269 22.93494 36.53857 29.91945
15.02833 37.29453 52.16495 28.19151 32.34247
13.04545 30.79534 39.17417 29.34485 27.68261
24.57741 19.61292 35.35627 34.4092 23.82492 46.49229
39.65814 15.04637 36.9021 53.04736 35.38457 32.41888
40.85591 22.14918 49.86384 27.4868 38.76848 77.1274
27.00967 19.08317 24.19732 29.9324 39.40733 31.88644
27.26988 19.44688 20.24677 22.23599 30.95808
38.04402 20.03345 28.72146 30.19731 37.4673 30.34286
28.0693 30.88252 46.5768 37.74805 19.70988 46.07625
59.44167 43.99763 25.94633 50.93358 20.83834
37.20677 46.22413 22.13064 33.40802 31.74703
9.88878 34.96097 26.93025 19.61275 22.45449 24.25025
33.78189 22.96036 47.71228 25.6652 29.72982 22.56665
25.72564 31.73434 20.58832 31.80316 47.09031
35.41582 26.83731 28.31156 51.55066 27.65167
36.72819 41.85191 27.89713 40.95184 22.83434
25.49728 36.29511 69.33367 19.15956 19.69371
45.45325 28.05467 26.53793 28.86711 30.08027

39.42198	26.10448	30.34821	34.47107	18.03406
27.18989	28.45915	33.97468	34.80263	31.81943
23.34965	32.97775	40.37869	40.49798	36.66391
38.82247	12.37544	35.52764	41.49008	25.10411
23.47518	29.58428	18.96224	42.17434	23.89996
15.60059	34.63946	48.31879	33.2486	34.65386	49.34528
42.44125	45.70095	35.30755	24.27714	48.78965
25.43334	24.45838	31.66354	37.86966	46.61582
37.16848	23.76764	24.10586	22.40833	39.72097
34.65967	22.25135	38.14975	20.58271	26.69466
29.96249	31.05613	40.99956	30.04468	39.24868
58.39904	40.1538	50.1686	24.26598	45.54877	28.12397
34.45364	17.15151	37.41743	25.44331	37.31485
38.36696	20.20732	40.74519	47.78146	40.1435	33.68778
29.9238	14.82373	18.96455	44.17125	30.81011	37.98965
20.96591	30.37717	33.62263	44.22762	36.33937
30.22395	24.31841	28.57884	40.64956	32.73612
39.49217	25.59042	20.20676	33.86788	33.39714
32.98606	57.35174	26.6239	34.2107	34.75762	35.78921
22.92645	38.28377	28.28781	11.86	26.64567	26.66492
19.24717	23.60097	23.79657	41.37229	25.85454
34.00664	25.6908	31.63264	28.13518	18.73117	27.06552
34.55376	25.23183	34.29656	20.02827	23.71084
30.02688	30.69581	37.88345	24.75569	39.51653
28.01731	41.25381	20.18111	24.01512	24.83911
44.6543	37.01906	31.61806	50.82761	21.0449	27.74165
23.62597	25.40995	33.39693	28.73851	23.8867	24.4147
38.57474
SIK1	0.1278614	0.582889	0.4130088	0.3891466	1.107677
0.7046634	0.4961801	5.669877	2.451381	0.3092161
0.06381019	0.0292095	0.06428629	0.02787949	0.261776
0.2004665	0.3262297	3.667243	1.171208	0.4064523
0.5976417	0.1599141	1.187828	1.444353	0.8085198
0.4884852	0.6165919	0.1457762	1.580197	0.2968766
2.285615	2.023047	0.05166483	0.2153993	0.1258807
0.006518371	0.02475281	0.04807153	0.1426079	0.09736614
0.5690999	0.4692086	1.197737	0.08793256	0.09492048
0.04375999	0.2525761	0.1246488	0.05752659	0.3111191
0.03089331	2.196673	1.346197	0.2056674	1.74509	0.2522209
0.04365916	1.030295	0.04770531	0.2459024	0.1721718
0.721818	0.1273445	0.1670185	0.1063916	0.4533567
1.395708	0.02404243	0.2599162	1.994097	0.2539602
0.0452583	0.703022	0.05268027	0.05553285	1.156206
0.1315938	2.00898	0.1462851	0.06116976	0.08272361	1.559085

0.1082928	0.05197851	0.08456696	1.767749	0.5813175	
0.2755867	0.08149231	0.5970062	0.09456007	0.6009582	
0.4318228	0.1104454	0.2430048	0.1913791	0.4179335	
0.9236824	0.6887185	0.08740587	0.2866655	2.215967	
0.130175	0.7204921	0.1012523	0.3319685	2.337819	
0.1369587	0.3050205	0.07928398	0.04501898	0.08833969	
0.2243883	0.05900444	0.5370813	0.5996491	0.08983044	
0.06831685	0.0856699	0.2333122	0.8646812	0.2111323	
0.3205991	0.2870657	0.0314411	0.1586302	0.5765846	
0.2026087	1.523127	0.3660362	0.1670367	1.303351	
0.2420454	0.3475649	2.406376	0.05305995	0.3478855	
0.1280651	0.500688	0.05462311	0.5651406	0.1753362	
0.4186863	0.197237	0.3199484	2.207531	0.05404013	
0.08643518	5.849084	0.3543626	0.04671713	1.648957	
0.2775313	0.4432789	0.1108225	1.045719	2.428451	
0.05281319	0.3591951	0.07958838	0.6633226	0.8475749	
0.3496966	0.1352525	0.0439582	2.119697	0.5881813	
1.520178	1.538844	0.05125071	0.6014012	0.1506208	
1.097693	0.526494	1.494512	0.1649058	1.213873	
0.06851053	0.1744952	4.166855	0.07775676	0.1441511	
0.2664012	1.133049	1.087632	0.1028721	0.7899149	
0.1287429	0.2133494	0.9049263	0.4105575	0.2914123	
0.2169956	0.236386	0.8896804	0.07026077	1.861665	
0.05021488	0.07271825	0.03819814	1.118661	1.379084	
4.110336	0.09952379	0.6976349	0.5118186	0.01074897	
2.637163	0.3181773	0.150682	1.099188	0.2212331	
0.08280148	0.2492467	0.4052101	1.420475	0.6841668	
0.4169478	8.336159	0.06473872	0.6659538	0.1222562	
0.5181083	4.130266	0.4407929	0.02650733	0.1473699	
0.1592661	0.3228829	0.1218361	0.07380441	0.1037338	
0.05817571	0.1079174	0	0.187302	0.6724696	0.05295565
0.4297719	0.09715508	2.532155	1.701367	1.068627	
0.03684656	0.08995528	0.1941869	0.3267589	1.050532	
0.1083062	0.1462958	1.467473	0.1040208	0.5426922	
0.5498488	0.06372353	0.6103651	0.0221434	0.188018	
2.749846	0.1406768	0.1416664	1.861655	0.5620421	
0.06450572	0.08774223	0.6423193	0.09784993	0.5111108	
0.2123385	2.11523	0.08674752	0.2505276	2.851153	0.4864616
0.184212	0.1958865	0.1044258	0.3665195	2.610992	
0.01063659	1.040784	0.1151949	0.2217361	0.09864477	
0.8938591	0.4267977	0.909576	0.1499519	2.889846	
0.9556315	0.2303375	0.1109394	0.1371305	0.4460379	
0.8702922	2.993879	0.4589671	0.4948317	0.120519	
0.192858	0.08117389	0.1213587	0.2104604	0.1968181	

1.425334 0.05469543 0.0997635 0.2515335 0.1226852
0.2578121 0.2874167 0.3478722 0.1157995 0.131357
8.191224 2.202278 0.1692912 3.424034 0.2336417
0.2728286 0.003961128 0.2662487 0.05012259 0.2025449
1.519733 0.7791983 0.08239358 0.9368783 0.1579292
0.7330616 0.1748941 0.6378972 1.075019 4.304842
0.1892578 0.01137729 0.2460806 0.02680482 1.284995
0.7333164 0.7031244 0.05623265 0.3932807 0.2557351
2.344995 0.0463233 0.7248047 0.1329192 0.4259386
0.2269262 1.021136 0.2378943 1.934657 0.375558
0.0497811 0.08855849 0.2378646 0.04285153 0.3110506
0.1265328 1.059758 1.2758 0.1964998 0.5508376 0.04444367
0.1912092 0.2633295 0.3977081 0.2772676 0.3946009
1.136848 0.1631223 0.07917631 0.07364866 0.147421
0.04064451 0.6702866 0.1772173 4.042773 0.1167235
1.788261 1.624577 0.02128126 0.8079654 0.3954998
0.03864159 0.9335937 0.3342397 1.042816 0.2573302
1.746885 0.003619435 0.114485 0.007205122 1.188456
0.01120588 0.3939469 0.8847459 0.2408742 2.233676
0.254276 0.1437453 0.2909335 0.3735979 0.3861288
0.06407625 0.2029872

DAPK2 2.989616 0.3531341 2.812798 0.7606117 1.504994
0.2669896 0.7322006 0.6664579 1.425336 0.4880995
0.3014237 0.1887244 0.705449 0.2091523 1.18325 0.1407511
1.705394 0.8405992 1.858206 1.789496 2.057917
0.2072809 0.3313182 0.2400694 0.1725217 0.3221573
1.480002 0.1392548 1.959447 0.9203191 2.235033
0.5501277 2.720962 0.7261087 0.4483706 0.1263468
0.1743675 0.9097313 0.8206721 0.5998069 0.3852824
0.2371073 0.1999652 0.1612469 2.538878 0.9236045
1.70891 1.584342 0.2684377 0.4032255 0.6308462 0.5991728
0.2381721 3.055667 0.4266156 0.4552951 1.690941
0.3588808 2.124412 1.137332 0.4289697 0.7001391
0.8854994 1.170293 0.5170211 0.2278241 0.3684583
1.020496 1.288992 0.4160453 0.2370121 0.290096
0.5057525 0.2149709 1.028231 1.59058 1.000229 0.297136
0.3158603 0.722482 0.35261 0.7345148 0.7671403 2.201595
0.1326632 0.3348335 0.8829729 0.3943662 0.994551
0.8078168 0.4299339 0.5684806 0.5890072 2.741753
0.2923917 0.7883584 0.4355648 1.086823 0.3735083
0.5268696 1.419191 0.3123854 0.4721792 1.962689
0.472476 1.81235 0.5142714 0.4676942 0.3161894 0.315893
0.3477518 1.837428 0.1648337 0.8448277 0.8542988
1.943965 0.9147872 1.831554 0.4131678 1.458313

0.9024087	0.428303	0.6275215	0.3646089	0.6073769	
0.7837013	0.3602343	0.4513628	0.5427689	1.803868	
0.5403862	1.588209	0.6494409	0.417293	1.398247	
2.452822	1.373601	0.2987965	0.355918	0.7772717	
0.7430786	0.5057403	0.3437495	1.744098	0.4765053	
0.1303385	1.107049	0.3850841	0.480669	3.560495	
0.2821141	0.4378777	0.5348492	0.9854464	0.9742084	
0.7741774	0.4739921	0.6217958	0.6746461	0.8998948	
0.4021931	1.097841	1.540111	2.578902	0.2787571	
1.622549	0.6878637	0.5007745	0.5756225	0.7371675	
0.3357026	0.6745841	0.68554	0.4822349	0.8124365	0.497218
1.349485	0.525648	0.7721523	1.081932	0.8362455	
1.176069	0.2557947	0.9237885	0.9086635	0.6369691	
0.4381956	0.1169741	1.008761	0.2694542	0.9281488	
0.5072628	0.1503712	0.5792663	0.4368107	1.269405	
0.2986628	0.5258921	0.07395586	2.309705	0.5419571	
3.172146	1.043242	2.287199	0.747205	0.6454896	
0.7472283	0.2434126	1.773867	0.4667507	1.216419	
0.4140725	1.372139	1.690448	0.5131699	0.3464547	
0.463747	0.2570476	0.355581	2.831696	0.949199	
0.5929912	0.5870502	0.2561395	1.655744	0.5734801	
0.8551862	0.2485577	0.7201312	0.2197308	1.791819	
1.006587	2.515592	2.191994	1.623116	1.114552	
1.025488	0.5114144	2.403171	1.946384	1.484476	
0.4021438	0.6553442	0.2698104	0.2638813	0.6263018	
0.76949	0.3001734	0.2970149	0.468047	0.3214209	1.183978
0.4830202	0.5843615	0.3957104	0.1499718	0.4088973	
0.4370169	1.569262	0.3802633	0.794056	0.09119124	
0.5343487	0.3517233	1.572225	1.138183	0.595243	
1.085678	0.4750361	2.11334	0.7356317	0.4626857	1.66994
1.065245	1.669408	1.239001	0.6179701	0.3596582	
0.06179408	0.6325124	0.5906561	1.343465	0.1126529	
0.3068723	0.1767784	0.3995574	0.5915536	0.9329654	
0.4487162	0.3326011	0.7355002	0.1736659	0.3146737	
2.500722	0.6493125	0.6241636	0.8298661	0.7368782	
0.2898424	0.9081356	0.6767778	0.4473879	0.9147234	
1.098423	1.291662	0.8379642	0.3176685	0.3478287	
1.395149	0.09182378	1.969337	0.145008	0.2179681	
0.5436157	0.2775351	0.9665659	0.9617502	0.1766906	
0.6153747	0.393854	1.181548	0.8728674	1.461302	
0.4582497	0.554286	0.9770051	0.6644519	0.1541332	
0.6947959	0.1890645	0.2814181	0.3577322	0.6462895	
0.9558818	0.3710225	4.153282	1.197715	3.680237	
1.46702	0.09298619	0.6287226	1.200983	0.2525571	0.5054718

0.3961087	0.8147551	0.4893398	0.5001435	0.9626395
0.4742475	1.095685	0.3207661	0.4514322	1.106425
0.7058186	0.4071878	0.8835941	0.6137207	0.7111682
0.5359405	0.8758983	0.319497	1.068685	0.4592757
1.149943	0.2110245	0.7550058	0.6258344	0.5357869
0.6108276	0.3533561	0.5035508	0.3136369	1.605196
0.4586094	0.3660579	0.2137545	0.5751565	0.9211136
1.271971	0.3616707	0.9184224	0.5538178	0.9571642
1.944499	0.2157606	0.3336482	0.2304777	0.8905794
0.1405175	0.7793976	0.1156278	3.026024	0.4551824
0.7808363	1.114185	0.8016217	1.031183	2.272697
0.7411745	0.6415874	1.969289	0.5537954	0.7729164
0.5250619	1.684753	0.4973245
CTNNB1	47.89348	27.85884	50.64129	34.95464	34.92676
34.9109	15.34742	23.2805	27.33681	26.36567	33.94188
30.73586	26.55742	28.35806	20.03308	29.22454
32.63107	33.70859	29.12976	37.45756	39.1343	29.06761
42.27329	32.81143	31.98095	28.4649	20.31315	30.67257
40.48364	23.10268	29.79646	28.37182	24.92072
40.41729	26.68888	22.65143	31.29384	56.49986
76.55514	70.33963	57.44425	46.26329	49.56773
50.14125	45.0653	49.27617	40.51495	48.72114	78.15863
51.52161	38.68837	48.95226	41.37242	82.19293
44.19123	69.41538	86.05316	41.68574	99.39902
35.3308	51.0778	45.00726	43.81003	32.26455	52.84326
36.99649	39.84868	40.35968	63.96344	32.31484
50.42945	45.15651	39.8746	45.8522	66.03344	49.78989
47.25922	49.24014	194.8847	76.00888	24.75756
56.84856	45.97636	49.80272	44.2399	40.24895	59.36897
58.78744	18.12812	48.23827	76.19691	43.45554
42.06712	46.09649	26.92746	52.5143	64.59746	51.65562
218.5528	65.53842	56.75165	33.47916	10.42125
39.93745	42.72594	59.31091	61.58371	27.04689
50.64283	41.15123	55.65117	47.14926	28.59232
23.75584	40.34518	46.2062	46.76729	62.01313	32.14353
57.75079	40.69268	52.95453	84.80625	42.79242
215.9743	30.49389	77.03355	28.67807	40.01034
50.43952	38.57234	72.07736	33.18671	59.90446
37.74862	57.914	78.0765	44.10544	67.13858	39.65454
46.88754	88.93195	28.73402	33.85931	35.22554
31.59775	41.74067	39.76338	203.3005	51.2446	39.06919
47.02078	97.18457	89.40479	44.03889	31.04526
48.75555	36.56486	517.5197	23.73105	44.43292
29.2924	40.53814	93.57724	19.59505	57.51794	34.76436

39.31561 38.05287 27.11911 30.64965 38.64069
52.07606 40.26424 40.53504 35.57199 56.61311
30.7811 28.15642 48.30572 28.54191 58.83106 35.24445
50.01883 29.6622 76.03877 53.40832 27.35515 152.7502
329.2902 56.98906 44.35628 29.44444 28.0847 42.46368
62.86555 42.02881 57.29078 90.68091 46.88349
44.18762 43.76788 63.94211 77.18714 51.15443
46.6356 24.66102 42.92041 37.82984 83.07956 65.07315
49.57852 95.1475 77.31901 33.61709 64.64688 89.91045
95.55579 48.38668 86.67908 55.4341 31.95762 47.44888
54.7764 36.9264 23.6336 114.4106 25.19365 39.77475
32.98124 87.51273 42.31869 44.01366 82.62023
127.9197 30.6713 31.42355 34.9884 55.55543 50.37718
55.03198 64.23861 61.9312 70.85879 64.78271 62.11718
52.8897 25.47166 63.15005 36.83444 71.27844 26.39218
26.94254 52.54228 72.73537 255.8152 48.89988
35.1019 67.48407 48.2818 32.19723 21.75822 49.27984
49.0142 66.64882 66.1232 42.49042 63.97762 33.54478
49.46935 31.41895 19.09842 39.15109 147.7198
59.52837 38.15478 36.87649 33.61658 29.45769
71.35777 51.43161 79.10766 32.58197 54.76161
61.45519 40.30184 66.88712 40.13004 62.89933
67.33244 38.33354 31.60273 37.40621 67.68692
79.35134 71.71225 40.74773 88.53226 28.67179
43.87173 59.96063 63.15137 39.51491 44.4854 44.06614
64.2888 47.51677 41.79356 45.0171 58.21873 54.59151
24.95801 96.0298 35.19631 42.91228 43.78361 50.64749
40.73243 48.61479 72.83781 56.66933 42.44516
65.87579 22.74333 41.64314 30.6972 50.66375 35.71266
40.43593 101.2778 49.48605 80.25429 38.25182
36.36835 33.46873 39.18535 48.91196 33.09852
40.67367 37.18339 112.9946 44.69524 50.52055
72.15544 41.5924 46.85668 34.17727 39.46309 15.42107
29.97005 57.02404 71.45677 30.48899 33.5517 95.83221
47.13764 51.23234 44.98313 40.72676 34.15552
32.86659 36.39138 129.2905 33.86437 79.1364 41.68022
48.97858 36.08247 29.2349 17.40735 44.4993 27.98646
88.42493 72.23497 49.37439 62.62241 31.72534
20.92486 34.43519 42.81988 37.28683 100.0286
34.1308 105.3798 62.46426 56.38774 34.43578 39.55187
66.94825 344.3745 50.46673 52.60716 27.12525
50.85025 44.13951 39.90637 33.11367 70.67759
26.78458 52.95021 41.58509 21.57676 72.55606
45.59024 53.89299 50.39153 32.62894

ZNF304 0.6682723 2.896691 0.6147026 1.800697 0.9385716
3.208327 0.9351118 1.439379 1.52808 1.789077 3.801659
2.33476 1.820576 2.447158 1.387543 2.737 1.163267
1.939075 0.5137644 0.67325 2.147473 2.850531 2.077306
3.609159 3.303226 3.347397 1.247422 3.516969
1.443689 1.237417 2.431888 2.430017 2.126897
2.680569 1.396052 1.195451 4.054149 2.191307
2.260363 3.520565 2.853596 1.24213 0.5183873 2.98498
1.996274 4.165818 3.172723 1.551801 2.680083
1.983939 0.4512222 2.325718 2.559992 0.6209057
2.937825 0.1278771 0.6248215 4.029564 0.1580387
1.491438 3.364081 1.526099 1.918226 1.634082
3.218731 2.836628 1.070983 2.102709 1.833747
1.092203 0.3042222 3.239679 2.450983 6.087252
2.291038 2.816246 2.232042 3.248464 2.621991
0.898581 1.238696 2.534271 2.832888 2.174925
1.799629 2.524089 1.55361 2.064522 2.202944 2.172435
1.65553 4.544004 1.963048 1.979769 2.619633 1.948301
1.472763 2.074567 2.931023 3.086502 2.336432
3.274485 1.597878 0.9490702 3.363243 3.414572
1.132297 2.601646 2.918532 2.004567 1.347743
1.495851 0.8076652 3.005904 2.467226 2.28247 1.312047
3.089202 3.616445 2.776739 1.131947 8.322084
3.625248 1.269944 4.24209 2.950511 1.94904 1.056881
2.546093 2.018948 1.684488 0.1650841 2.148961
3.986285 0.9161045 2.000852 2.113816 3.380467
0.3538528 2.808438 2.269729 3.146579 0.3825441
5.320201 6.557426 0.4767081 2.041906 2.048198
2.733604 2.524926 1.570228 1.780761 1.716706
4.016248 2.927859 1.979583 1.794194 2.015541
3.418189 0.9597272 2.104813 2.382107 3.178464
2.013071 1.114518 1.206122 2.338241 3.968454
2.738915 2.487577 4.119323 4.528909 0.8374346
1.812339 1.38138 2.370014 2.384594 3.354506 2.535167
2.969172 3.522294 1.808305 2.799408 3.553509
0.8762786 3.489751 2.873151 1.774244 3.337982
2.583602 2.392424 1.773834 2.143817 1.508375
2.412079 3.445548 1.11566 4.496604 0.5781645 0.2760944
4.60291 1.454217 2.320838 0.1929972 1.425447 2.160539
1.378793 1.8081 2.112139 2.245205 1.483777 2.382146
2.434372 1.359375 2.105464 2.66143 2.438982 3.228893
4.141181 0.3856325 2.391499 0.9005797 1.660974
3.768801 4.502424 2.214824 3.912179 1.805833
0.8961025 2.170492 0.4658839 1.546886 3.054823

1.295741 2.640465 1.681132 1.621019 0.9997959
1.936305 5.535932 3.031388 5.199225 1.379487
1.906178 3.018687 1.619614 2.907636 3.236894
1.942676 1.607073 3.984097 0.5051191 1.43827 1.520098
3.337133 7.132933 3.651544 1.133825 0.5504677
2.918716 2.241156 0.2560746 1.678765 1.21786 1.804501
3.34707 1.591447 0.4012274 2.078249 2.421613 2.299026
0.514034 2.37137 0.460959 2.196387 1.554298 3.94936
3.057917 1.544488 4.341201 3.417975 1.627501
1.439305 2.271564 2.920938 4.359399 0.8424686
1.605536 1.549783 2.636533 3.599122 3.881026
2.89446 1.550178 1.642319 1.832689 1.423388 2.885141
1.231628 0.9549906 1.329901 3.796982 2.413878
2.426592 1.332763 1.257168 5.605241 2.325543
1.11795 2.959762 4.241093 1.368052 4.679815 1.511289
3.091981 2.35827 2.640214 2.652825 1.659801 3.231781
5.383363 1.542967 1.062699 2.270803 1.614311
3.869884 1.123844 3.716166 0.5881189 2.158662
2.263222 3.317508 1.896266 0.8718737 2.757405
2.752938 0.03416155 1.426472 4.304246 2.989144
5.34117 1.903333 0.9543486 1.80267 4.052016 3.149007
1.1789 2.074742 1.082854 0.8874155 2.76126 3.071565
2.790124 1.134292 2.94473 1.493152 2.061263 0.9585075
3.340457 2.319357 2.304937 1.661762 1.776942
3.402502 2.854367 0.4023764 0.1206228 2.153224
1.195616 0.334072 1.848156 1.852229 2.176309
3.794441 2.732023 0.5914703 1.121645 0.8140946
1.40231 1.998854 4.475883 2.729973 2.451549 2.795468
0.4839565 3.81875 1.996692 2.247182 1.459494 5.63294
2.64516 1.600013 3.138954 3.322594 4.429014 3.654879
3.129351 3.098482 1.544264 2.652437 2.219889
3.649968 2.738383 1.276965 0.1583559 2.067535
3.102692
MAPK1 8.571577 10.52367 11.50486 15.71995 9.328314
15.97393 9.354468 12.77969 11.26628 9.172038
18.80671 14.50089 10.28577 12.38903 9.565451
14.34976 9.469463 11.49487 6.272932 7.193639
13.21513 13.73406 13.34228 15.48376 14.5486 15.7113
9.433505 14.32567 11.58845 7.601907 13.39352
11.0989 17.30501 13.17529 10.1846 8.507237 15.92461
10.80528 13.01995 16.3737 22.76552 23.62778 17.31885
16.84754 17.35076 20.15326 13.01004 13.80612
19.18603 15.86239 13.23563 13.30117 13.86646
13.64377 13.86127 17.81605 12.91843 23.00776

19.74058 6.623378 19.97527 13.52478 16.97302
15.86129 8.866181 16.78043 11.01368 10.40931
12.25742 9.374686 26.53334 11.11067 12.20677
17.82903 13.37 9.31555 18.46753 14.62124 18.1219
24.2911 13.06764 15.67559 16.42994 12.34042 14.51374
14.41212 19.39181 15.21047 10.44515 12.76044
20.36982 14.26199 12.57751 12.50248 13.22072
12.4268 13.95372 13.91268 26.93796 18.8055 14.98415
8.929737 8.377744 24.01949 12.18256 17.43785
12.13048 12.84492 22.98323 10.80882 12.45653
9.045584 13.50636 13.42944 12.00575 13.77258
11.4463 10.88474 14.52672 10.73459 15.88383 19.76386
18.98664 9.586693 19.76566 14.91384 17.50523
8.908804 16.29241 12.1398 16.87003 18.03777 18.29087
19.66463 8.392047 17.61294 19.52689 20.78899
17.7256 14.58516 17.68478 15.03897 12.54251 9.07203
10.71651 9.37904 9.102249 15.0532 19.20042 13.57849
16.56706 15.10175 23.48045 18.14055 15.63363
6.318155 12.51233 12.07519 18.8303 6.093227 12.80188
14.14972 22.43311 15.46244 8.385492 17.40973
12.15444 18.62769 13.33515 10.57001 16.6193 18.2499
8.261885 12.6036 10.06964 9.909163 12.24435 9.842435
13.47866 21.37975 6.071267 12.11693 14.79354
15.23595 13.13072 14.28367 18.94529 12.03619
18.97803 12.83358 16.41523 12.02417 40.14975
14.40514 8.819734 17.33452 21.68221 16.6056 11.43357
6.066242 12.75705 10.97028 14.89042 12.52888
18.24521 16.86315 8.039767 17.33038 19.26376
12.21593 17.04416 15.49111 17.11277 15.02597
14.39404 17.33245 18.38586 45.62229 15.69597
23.37644 20.38679 22.10057 9.702011 19.4997 16.80955
7.760752 20.21354 10.60734 13.02079 8.263806
10.52507 10.29097 11.17662 20.10726 14.18055
7.916943 10.0892 12.98511 18.74094 16.61931 19.8151
14.11924 13.56123 13.15303 18.88206 16.15488
11.96273 10.73242 16.35168 10.34391 17.94977
16.88821 8.76682 10.46723 13.43081 12.12348 10.27164
15.9952 12.93884 14.03986 10.19711 12.13658 16.79868
12.19625 21.29928 19.27032 18.06529 11.72036
8.173069 11.25842 19.4753 9.298597 10.92946 14.34209
14.18287 9.767044 17.91367 16.45821 14.80307
18.25106 17.22356 15.70321 12.66842 17.29399
24.23727 15.25847 13.78108 11.76081 13.29322
17.95748 8.783106 18.25003 18.03033 14.16967

8.358473 10.98804 8.476868 16.56383 12.99265
14.09624 16.63485 12.22159 15.61992 12.77125
13.14346 10.16602 13.04 14.13128 14.50581 10.25847
11.49398 10.1265 14.27103 9.921298 14.98999 14.7764
14.65659 19.40897 15.7882 20.23632 14.62824 17.05908
12.73415 9.235295 22.47793 11.67184 16.76212
12.19337 10.85979 21.10856 11.4178 16.42528 15.65898
14.01212 9.40183 13.56513 10.64183 12.79298 15.75278
12.52003 8.623705 14.8245 12.48152 20.001 19.29978
14.74621 16.02727 25.05858 7.535544 7.092231
10.86194 17.75981 12.41404 8.379146 12.77518
10.39319 19.21123 15.59028 20.45409 8.345257
15.21678 10.05575 14.80872 28.45862 12.611 9.391539
16.25002 11.45597 19.15973 11.13784 11.90508
11.07904 20.03822 18.18029 18.54687 10.71332
13.61074 7.399653 11.38426 9.87232 13.40464 17.72156
6.718907 23.98434 15.72971 11.07013 10.9402 8.056141
13.4071 19.20219 16.75705 17.61592 5.86307 19.63778
16.68968 13.03594 15.25654 15.8029 16.73244 15.52156
7.773334 10.91468 18.19587 19.14657 12.92306
7.301484 16.33534
BMF 6.126718 0.6248765 6.857413 4.583739 3.026265
1.747697 5.406865 3.666595 4.863126 6.118831
0.7944665 0.3563355 8.74041 0.570185 7.557565 1.304189
4.793819 3.410041 1.554467 5.104271 3.119914
1.012078 0.8483208 1.13604 2.077097 0.5304223 5.720868
1.51853 8.618087 1.761215 5.035085 1.652107 6.261555
2.839417 7.198392 1.309734 2.125981 4.192273
6.783903 2.351049 4.355293 1.067493 2.75674 0.9773787
2.559825 3.393425 5.580269 5.493072 2.270479
1.949272 1.425605 6.28374 7.230984 2.438413 5.023709
1.206635 3.105594 6.838402 4.632237 4.689959
8.485081 3.150081 4.051144 5.680556 3.967855
3.663594 4.316354 4.763444 1.197892 1.171984
1.599186 1.859731 2.050162 5.787445 5.830242
6.584543 3.924335 7.106237 4.707728 1.262004
13.07376 4.361605 2.954183 6.258736 4.27048 3.718869
4.34447 3.671793 1.836618 3.847915 2.36985 4.556729
2.340039 5.394831 1.998635 7.280923 3.361078
2.780558 7.744705 2.143936 11.49788 1.204761
1.339759 5.832579 4.293502 11.10029 2.643426
5.070175 3.03089 1.749511 7.834178 16.81202 1.235753
4.469427 2.796132 2.274384 9.553039 2.632958
6.464962 3.418286 1.18138 15.45228 32.48177 3.798126

12.6067 9.541358 1.546723 2.088585 4.963489 8.75895
3.734305 1.985978 2.095175 3.104245 2.426864
5.490443 2.435558 1.289476 2.941302 1.137438
7.351472 1.499757 3.079216 25.15958 16.19514
2.096653 4.49427 4.423942 5.679194 4.231265 1.661435
6.002036 1.974662 8.824613 3.982194 1.943037
2.905247 4.405766 1.865767 3.266155 5.471637
5.504671 6.205419 6.995456 3.680211 5.115529
6.433353 5.586566 5.372486 3.0079 2.228306 2.628464
2.902693 3.053464 1.359977 1.758156 14.15252
2.511951 3.859202 17.04033 2.427781 3.106004
1.613006 7.633832 5.902464 13.10005 5.138615
0.9779994 14.15523 3.21923 6.898832 3.566832 7.20463
5.317311 2.939943 8.034448 4.094731 9.23649 1.750307
3.603952 6.630137 4.430206 8.24738 4.221988 0.7368099
2.254165 8.089561 3.929279 5.665111 8.610375
8.167595 4.014314 7.969552 12.63794 1.825062
2.025615 17.18939 7.49701 7.737469 6.35464 4.378192
10.53343 1.996172 1.887788 7.491564 0.6765716
17.11609 0.6459889 1.399328 3.79309 2.717337 2.171167
5.740246 1.647751 5.747014 4.036005 7.01184 1.766608
2.189568 21.04644 19.1997 9.712768 2.546745 1.976488
3.316661 6.590931 6.452756 3.798703 6.458631
2.27843 4.138421 5.580389 3.094651 2.175964 1.400432
16.28167 4.093942 8.167257 2.248768 11.90032
2.773675 1.176788 3.668757 1.05805 6.475884 13.02864
1.600686 2.678279 6.360787 3.709262 4.897367
2.961297 3.000508 3.529882 3.759665 3.567478
4.610119 2.457241 2.613749 3.199449 7.405915
2.639629 4.531837 2.939672 1.017817 9.017452
2.533905 4.550853 3.882455 3.370241 4.896332
0.9536708 4.265709 6.928994 6.30249 4.643167 5.543844
4.570639 1.234576 0.8148977 4.193401 6.681628
1.946983 3.40035 1.755735 5.220665 2.964671 5.074768
4.462861 1.403691 12.35433 2.637275 3.569686
7.866768 2.547622 3.755374 4.543982 5.450297
4.561732 5.282961 5.685059 3.601512 5.050545
5.378956 3.288715 10.32143 3.483515 12.14359
2.78242 1.660952 0.6437545 7.549236 5.303652 5.268391
3.504137 3.605099 5.050654 4.498655 7.332136
1.204757 7.060943 5.782713 1.656392 8.547245
4.414509 2.36329 9.792231 7.057152 4.616681 3.722786
1.900509 7.301869 3.236535 2.564725 5.586218
1.426095 8.293995 3.7285 4.712922 11.99807 3.904171

5.02561 4.398533 6.322216 3.551424 1.881558 1.253947
3.376357 2.993884 5.380683 8.579668 1.573774
4.887818 10.4618 6.183339 20.10848 1.207473 1.768488
2.430629 4.381372 7.664226 4.706923 3.21021 6.416569
4.616016 5.626766 6.209494 3.242742 1.755215
3.226668 6.012582 0.8668584 6.072944 5.060567
5.316183 10.80899 7.946125 4.204316 3.796588
2.865649 9.546131 6.665001 1.688394 3.322767
1.264089 5.007673 1.642964

ITGA5 2.068943 28.20704 3.90672 9.445957 6.793218
14.43981 3.697314 4.947704 12.24944 6.303823
28.50882 2.316783 8.889088 15.73973 4.176367
78.33624 6.314864 15.38424 8.049676 5.553228
17.44548 21.0384 16.02431 20.91718 20.03404 14.72502
7.184242 46.20561 11.44967 1.962258 13.34069
18.24657 9.045259 8.418512 17.15731 6.847252
2.621718 18.3857 26.58538 6.602732 26.58219 7.667295
19.82181 7.345502 15.96176 21.3515 5.716164 31.10887
27.61685 6.859502 20.2601 16.85528 19.63693 19.61968
18.53591 5.619457 29.32182 143.192 15.8735 6.65471
48.56552 18.55571 40.90221 44.15976 4.625643
103.6709 31.90614 16.58118 23.25485 5.277489
30.75512 15.48732 44.85611 36.31465 57.05751
24.81899 27.95692 21.61584 9.491189 6.382591
14.29619 23.1633 9.782672 36.75955 34.85447 20.56695
11.69959 17.65263 13.28858 59.15456 14.73419
21.60081 12.35376 8.675927 20.76616 14.00215
12.63875 11.66431 5.717209 30.99984 12.84623
3.868071 9.11443 9.883241 32.4601 25.64629 17.46526
69.72053 14.8875 5.25575 16.73656 5.145372 19.63154
37.18635 19.16386 9.69444 13.71793 22.11048 22.51685
5.181515 18.3944 6.722817 27.81409 16.54984 18.71906
10.0395 6.197977 23.14812 23.65764 27.70152 18.18693
11.23161 10.2585 18.43822 21.28716 41.93844 27.76071
26.78969 10.3043 8.305199 17.20522 9.020961 9.761345
8.004738 25.27027 3.126158 4.331056 57.61116
11.18516 15.83458 6.859724 52.50614 11.07672
16.01694 77.12593 7.724668 27.97857 14.23557
38.33135 5.048828 31.83492 11.22769 11.20248
14.41698 17.51564 13.25629 7.304324 19.04827
10.83977 31.32084 76.31018 14.68608 20.05283
16.61051 5.135249 7.53791 30.02874 23.50862 5.301473
32.33474 4.807765 6.50687 10.8527 20.78311 49.02473
45.36957 25.99449 11.32695 17.25729 7.115189

24.90261	26.84776	12.83458	9.644294	4.958939
26.25983	59.65199	16.56567	4.849424	8.668199
9.306924	10.75798	22.71001	11.38604	8.050447
5.151368	24.7637	22.49604	13.97636	10.15032	11.52976
35.07634	12.94632	21.10372	22.81023	19.81286
10.56991	21.39241	25.02359	10.7388	11.68429	20.43076
11.26079	19.13682	11.5336	3.042581	21.32652	4.585875
5.853269	13.78538	36.79765	53.95537	2.415927
10.08149	3.354791	8.583504	13.31301	8.387461
11.85842	4.17602	17.62243	10.90011	33.16581	15.90519
29.67575	29.96089	10.72063	15.02055	18.04967
11.38349	9.559471	11.07427	17.83332	21.10722
5.16114	14.97054	16.09094	23.52506	14.54947	32.53889
11.91279	19.94625	12.952	15.52135	37.06233	18.14301
10.02098	20.60759	11.0066	7.33149	27.7439	18.97191
8.767258	8.350541	6.402564	7.83546	10.51028	205.5236
12.40461	17.27545	49.4513	4.011281	75.59984	16.73138
16.22459	14.6833	5.334762	3.751801	40.40857	49.77622
13.32149	322.9204	47.84432	23.05093	37.53313
61.85693	15.05736	14.2642	9.913484	16.64674	15.40599
23.85802	7.143819	120.1995	6.090797	17.57896
8.22621	18.69559	8.007255	14.19433	20.1482	18.78051
13.92376	25.33429	9.766912	54.1343	20.05939	35.41431
28.7238	38.29974	6.161017	8.792789	6.719252	8.461964
19.61961	9.705109	22.70827	12.19864	11.16324
19.91168	5.370653	50.45526	13.90043	7.346045
91.71624	26.66306	1.885319	5.603793	14.14991
11.14986	6.935251	11.44047	9.369537	15.6086	97.4526
10.60248	8.544588	5.315648	8.992733	15.36166
8.89823	47.31371	6.672708	61.52442	5.036671	6.05602
27.11366	16.20102	51.07808	18.26968	14.91334
26.13809	32.95092	17.18971	16.36448	18.92756
1.983178	16.59208	9.848512	12.60268	8.545234
11.35805	10.08367	11.13974	9.939457	15.67326
6.020882	16.1608	7.678732	12.44443	15.65467	18.55797
4.492042	15.95484	9.042868	6.979777	45.40947
9.239569	12.36299	13.13561	67.24184	16.87467
4.711391	10.51666	10.91658	16.12617	7.112985
24.66672	15.17765	23.69087	17.05036	5.091527
21.26217	4.994934	5.078018	18.14177	63.77962
MCL1	52.09495	67.37512	67.82274	91.39374	94.3419
75.92776	29.04676	87.21702	119.1118	103.9236
99.12941	86.96925	90.72405	82.7132	51.20832	58.91242
64.39351	180.9935	158.4052	85.65044	71.75787

51.04464 101.3046 52.06262 74.22844 100.4739
55.28126 60.85198 87.3105 32.04666 79.76583 105.2254
59.11568 76.15914 136.3583 48.10467 64.72871
70.88895 99.57995 75.62477 110.5724 81.00906
109.506 120.9153 68.55358 76.7857 63.70717 101.2467
104.8366 142.6518 48.76475 86.50347 64.84939
87.27186 85.72062 58.43473 66.4504 107.0932 52.29952
81.04762 122.0633 102.9069 90.21252 52.12346
63.33772 71.1508 64.98675 70.10386 269.7515 72.70402
133.9981 54.87167 79.89729 173.8008 112.8254
67.22454 115.7717 70.76335 80.87985 58.99624
59.37747 135.1925 139.5019 64.42909 82.11927
98.26079 92.05406 105.4705 50.35215 81.92617
105.9896 169.2772 113.4319 81.07281 115.0475
98.51542 141.0869 73.24539 121.9761 99.8099 70.66765
106.0721 65.90186 66.78291 73.16362 166.4226
105.2211 46.99616 93.75479 73.89584 73.42357
48.3812 119.2533 56.86546 112.8091 100.1994 68.69464
225.9277 57.71604 69.91915 69.35152 76.27003
78.84505 56.91449 78.98804 94.60523 77.11427
51.37151 85.4433 149.2615 74.78987 97.337 110.3968
109.3236 119.9829 61.18837 105.505 85.38059 56.6484
130.4611 63.34022 70.96406 66.13516 64.83039
80.02785 45.53014 41.02947 77.12482 50.25243
85.27823 81.94158 96.06169 130.8932 143.7763
79.81021 72.04561 62.72037 60.27156 149.1399
65.74561 86.41709 98.82364 90.27038 120.6875
40.03473 97.24749 111.3 126.0952 106.2965 52.52189
73.90464 82.46933 45.89642 60.54044 55.95813
82.72972 105.0596 57.92515 103.9321 80.94806
59.74625 53.76882 172.7847 120.517 96.79118 82.72084
90.22234 98.89427 133.9911 99.16604 162.8091
142.4776 79.69523 54.62093 67.76919 105.1492
141.9525 86.35965 61.53355 43.19946 41.80486
54.35804 117.655 62.05301 49.65767 89.27425 56.95317
124.6953 91.63752 49.84135 103.1084 102.8093
97.01241 41.5244 123.3722 87.84877 66.77034 51.9448
112.2972 50.43247 53.26619 108.9956 50.13161
94.10003 111.0696 40.54257 79.22985 70.12881
54.05773 78.65533 43.24135 104.4183 70.1588 117.4121
78.95047 75.11277 77.90403 59.89969 121.5471
57.62698 62.53236 119.3854 95.58925 84.67407
108.2897 135.3313 95.50952 43.34292 86.3305 82.65749
87.22964 79.88966 147.8328 102.939 112.4081 76.28535

77.54919 84.97097 182.8357 95.98127 67.26859
75.33497 172.2927 89.49373 104.4005 109.7982
94.09456 72.88809 108.541 128.0171 79.08976 92.67691
97.44489 62.76529 108.2217 83.26023 108.3123
46.04642 110.8899 87.98066 66.94059 90.61476
48.53891 103.1397 78.33583 140.3529 82.79913
55.58263 147.3324 108.0991 96.88351 59.67316
125.515 203.9492 166.8992 77.83436 94.53703 139.4794
103.6284 146.1809 72.32228 84.12225 105.2053
239.9831 71.21467 68.64883 60.38151 127.5081
107.5354 79.80129 70.24647 53.29044 74.00998
70.00084 102.9574 82.18218 107.1666 105.3024
75.44756 103.8005 99.07727 189.0077 90.1586 51.08613
180.8034 80.56199 63.84466 62.37325 64.21863
137.1426 52.98738 97.93355 86.3962 93.11379 50.93087
47.98739 65.11076 53.64878 149.455 85.2996 31.96425
45.98518 83.56517 60.30293 101.4791 130.8738
151.4465 64.09688 45.65055 48.18472 90.89473
117.8995 62.2988 150.4533 76.05194 91.45033 95.68648
73.2301 52.15488 109.0383 90.56451 73.89828 144.5974
133.9934 41.34214 43.71419 61.11692 127.7217
68.54243 62.93547 61.34598 120.775 66.6664 206.1873
77.17991 47.56043 82.82742 53.49454 55.1634 52.20384
133.287 102.6364 41.35263 43.01762 166.757 71.89952
59.58954 85.50011 53.55264 128.3253 174.2105
67.59864 118.5154 109.4222 104.2625 126.7649
49.43962 82.92491 51.23529 87.86405 90.63762
86.39316 106.9947 71.25034 72.08739 66.1778 57.57547
TP53 10.71559 6.17334 14.81039 18.03233 15.30929
7.551193 8.052832 9.157401 11.34021 16.00142
14.47353 13.36527 19.04431 14.85398 12.95021
7.12098 15.41091 13.27359 7.884748 11.77819 18.42647
6.900106 16.61423 11.01393 5.829856 12.61807
17.29484 7.845533 11.46285 6.616812 14.33241
7.37187 4.346911 18.83345 30.835 30.26279 9.490861
27.86452 9.742247 31.53312 14.27822 0.8427739
22.87482 43.28752 12.26959 11.29625 19.95048
33.87491 31.28191 2.181652 25.07223 21.73134
27.22891 14.12517 14.87614 49.80063 24.67039
16.96336 35.82783 4.443696 18.16683 26.00522
14.7015 9.571754 22.81772 13.14153 15.96592 18.0751
4.209286 4.001889 46.84081 30.63253 9.975854
16.36603 28.76944 82.21763 14.66475 26.23072
2.751828 3.066279 4.114015 10.68496 29.45611

17.26211 30.10541 14.31374 23.17937 21.19213
4.678716 18.87725 25.24452 29.09014 19.68874
18.43408 23.69069 28.39158 30.34125 12.98989
43.70771 19.13756 40.35495 11.77079 14.41591
51.96768 19.29543 24.67546 20.63937 15.44382
31.16605 23.90624 4.041592 8.22165 26.34537 21.80692
10.96807 15.54116 36.32403 16.36754 15.404 4.587658
18.87396 2.815499 29.33142 16.70059 6.539263
26.96695 41.59116 31.75821 17.78264 25.80077
27.78265 26.29031 19.46268 22.48395 12.50161
6.125593 33.42814 2.336934 28.35906 3.278955
30.28457 7.069921 18.74227 26.13596 52.73325
29.07213 2.678542 18.78966 26.51569 31.5172 47.36311
27.9228 22.07917 52.6241 19.14663 11.24261 23.74399
18.43992 37.1334 7.113454 29.66549 15.13787 8.09691
39.40885 52.44442 20.84831 12.41248 13.54082
29.24712 35.01863 8.138989 16.63227 75.08665
28.50915 17.13788 38.20433 26.63779 20.54933
29.27808 38.84343 9.448241 38.78603 14.95592
35.01865 22.57779 20.63292 30.23377 26.81421
18.77956 51.27158 23.94091 19.60442 3.71132 10.86294
40.27308 8.137212 18.87238 27.83067 55.6362 16.71259
1.962302 7.162328 38.44904 11.88443 2.47234 21.59625
19.87272 35.37268 24.57611 30.48582 11.70439
18.49831 15.58245 3.704721 3.432528 24.95781
11.49911 41.18148 22.18906 43.6875 22.68918 9.004671
15.50558 40.34031 22.82399 53.60255 52.65985
15.67042 30.44388 41.09005 4.705832 14.50393
31.87182 13.98474 31.64048 25.76723 28.1166 35.69525
32.72287 39.7166 21.52773 25.72883 34.05962 37.31462
33.3846 17.93387 40.94365 14.97302 19.17217 31.87055
9.499867 3.390638 16.12014 41.78346 20.03967
23.91665 5.179954 12.03341 46.40354 11.55058
6.117874 45.12889 6.439561 25.39519 30.89403
37.27895 24.70578 43.95937 7.680858 34.38684
26.86742 28.02971 3.626893 53.7145 8.928555 15.91632
25.16578 14.79582 43.72236 29.14091 31.34357
3.663362 17.88732 29.65084 39.45822 34.10833
5.956943 11.57819 18.62822 21.22398 5.787148
10.54675 12.30509 8.107219 15.89977 10.3486 22.50313
45.03228 4.766502 10.44568 20.84028 11.34354
27.19281 16.25247 14.56292 16.75817 10.91013
46.06368 20.26519 11.35763 37.71595 2.442423
53.64296 26.08026 42.79227 31.09609 12.77479

4.379675 10.63709 20.82876 28.36237 34.10432
7.943454 46.77387 12.22644 9.473916 33.38768
21.4526 6.288594 32.94855 40.64016 9.470096 23.72544
35.69536 17.39741 17.67536 20.29228 10.27849
34.50975 10.96084 16.54765 10.54056 21.93484
8.328164 14.98578 11.9788 18.21577 27.99323 2.085601
37.96657 15.89584 43.56243 13.91863 18.82406
43.67766 20.48944 17.01957 16.56469 17.92969
18.76308 19.71374 20.7358 12.71009 13.06198 36.99194
24.74565 32.62011 22.81598 7.013184 12.7189 24.84649
9.760201 5.223222 5.745124 53.03014 25.05756
30.62492 19.74417 18.19341 7.92842 29.16358 7.620557
5.807501 23.02439 74.30584 47.74968 9.215752
14.18345 61.7984 9.654559 14.74713 33.86845 28.83043
24.45781 25.43438 19.15946 9.919392 20.63216
6.806084 24.59517 23.77112 14.98895 56.84887
32.42737 44.32187 32.75631 25.62291
BCL2L1 43.84889 10.46351 28.15953 25.93969 22.00091
9.030624 17.81444 12.73086 20.47368 20.67448
17.57389 18.34571 19.20661 25.41882 19.79779
6.453558 42.08987 16.34915 56.25812 34.15745
22.53039 8.435313 23.06776 8.187499 15.37344
18.33923 23.5665 8.361286 31.11719 13.99647 20.34269
9.419614 41.08639 29.57582 35.38034 15.01139
16.81933 73.78804 58.16916 18.7471 38.73806 25.38324
21.16324 18.77012 20.39151 64.68534 32.80056
39.51659 50.24584 72.34835 49.80377 15.60599
9.011405 42.00998 42.33705 15.62899 37.26318
14.28685 61.9558 52.48605 30.2868 47.63849 24.72631
32.84279 29.2481 9.518115 58.10792 29.74446 85.94427
32.51761 40.66647 41.63424 27.48246 52.01385
42.21676 48.47561 23.16614 37.53816 46.04508
34.15891 30.23936 23.61372 34.35074 29.01746
67.6743 58.61591 18.19236 35.75 46.67761 47.61469
37.63776 27.74575 49.95521 29.92469 32.17405
65.62234 64.22729 37.95127 21.86449 28.25741
13.40939 21.41051 58.28421 110.9219 16.04097
18.75739 78.91513 24.64033 30.00633 27.38205
41.57654 44.19486 54.64803 10.64218 32.65202
53.31362 62.43396 102.0116 19.36715 34.96255
28.71348 44.73988 68.29961 24.83254 49.62538
15.3338 14.31116 35.95027 18.07058 35.44347 80.06327
18.76676 42.30575 28.25006 31.01913 34.45429
36.22447 33.88719 43.54356 59.95155 41.61599

43.8308 95.24352 43.2733 30.2726 27.68957 59.98324
18.09214 67.1768 17.62643 29.49981 32.37802 11.61988
52.57146 10.46025 16.57389 20.26015 24.28356
26.95931 38.92795 52.24873 14.18177 19.90122
73.29902 33.19343 20.94705 46.15666 82.08723
37.53665 30.51067 10.96222 29.55018 21.02497
40.24387 40.94565 50.01514 44.12272 58.85644
43.89569 28.2617 23.39113 60.86751 23.17337 56.8505
28.53118 40.36579 16.91932 81.4413 25.50402 93.28641
60.00049 61.76712 65.67404 24.24197 68.51755
77.78003 44.60496 17.58098 118.2194 21.66687
40.71306 42.71628 26.94801 39.63581 37.55899
35.23508 59.04347 11.90669 28.91698 48.83776
57.28136 14.70118 64.00981 56.06444 57.84475
39.05486 78.30813 30.10428 64.32725 33.47939
62.81793 21.11218 28.30934 36.55876 31.73726
15.18124 82.25667 16.29407 25.38003 45.35418
61.96441 25.86726 32.21003 46.76397 35.27796
103.5068 21.9636 39.37966 20.25651 43.68394 36.50871
69.91049 13.11587 32.64919 53.00911 27.83422
31.81789 15.29985 24.85281 91.89393 67.79583
43.54527 36.8218 79.85898 53.62089 44.28221 66.57115
52.03267 20.26708 26.49731 61.11742 57.60871
47.17692 32.48031 34.03796 35.92614 118.8555
36.8909 41.26952 38.95973 28.76468 39.72468 51.24476
56.25131 32.17007 20.01509 74.35867 11.48831
28.59643 66.18879 17.16553 21.32544 45.59737
16.55154 24.79308 53.9284 34.83925 56.54534 18.50779
13.96809 69.86175 10.7634 10.50606 21.12593 56.03389
15.93955 57.3958 57.53632 46.0388 23.71249 27.25413
18.93223 146.2188 18.11035 24.67716 70.51992
25.72115 26.63423 45.88992 20.81447 56.30791
97.81229 54.42097 22.43682 22.88373 11.48661
30.35935 47.46742 16.61508 65.33424 80.34218 22.867
23.96358 32.22406 13.5925 29.55437 49.95947 42.78539
43.876 50.30078 34.74379 24.01227 25.12545 64.23931
50.95561 67.66682 34.24661 57.94776 62.47138
81.21672 26.6892 109.3243 17.76194 41.17607 16.07305
24.26671 47.42807 41.13333 60.35616 44.98094
33.15351 19.84377 12.52348 34.60653 64.6902 31.53487
25.77615 44.69226 12.9504 25.93437 57.38999 24.84639
50.72554 40.10762 33.29792 20.11236 18.0987 60.91353
27.5426 29.69099 34.43055 53.31608 53.21388 32.08874
22.04496 50.96833 66.21194 12.12777 23.91746

21.6394 39.23723 37.33649 30.16285 66.49478 59.8333
54.60381 33.19096 69.79417 38.46284 80.5488 24.80748
66.82139 31.77109 60.20517 53.91688 84.25363
30.01407 29.65628 22.33111 38.85493 34.55072
19.25545 54.87158 21.60862 15.30849 28.48888
14.21206
CASP3 37.62205 3.627715 34.02998 24.35819 20.15451
4.040026 9.028578 4.829084 17.93446 16.35047
9.554409 13.14439 15.09916 11.92564 11.7163 3.486352
15.04536 8.369366 10.92796 19.61993 26.33544
3.803237 10.09914 3.393666 4.925579 11.30163
14.45374 3.154374 17.80038 30.44226 12.7604 10.63778
45.49256 10.29128 16.87492 11.51345 4.938439
13.2468 18.60406 29.60928 21.8706 13.29353 26.67052
19.31463 24.18276 16.62403 24.46865 19.47858
16.91114 9.866827 12.49629 11.43786 15.00049
7.904753 5.481752 37.48232 12.72259 6.164976
10.74384 14.83853 19.98258 27.91933 19.08688
11.7348 22.59348 9.885644 6.44158 8.311938 14.70288
23.31252 21.46126 25.68414 17.65421 13.91165
10.26041 22.9763 12.10503 20.69919 25.47812 8.272231
8.252105 10.65311 34.18449 15.23812 4.73516 8.952699
13.87646 11.90917 5.20205 12.00656 38.80476 28.86372
13.80247 24.36392 17.57657 14.223 16.00663 20.66035
18.91722 22.82869 8.684125 27.75932 15.95833
14.55986 10.81827 13.02734 11.31446 9.164629
32.99445 21.86788 15.03547 15.29125 19.32267
7.618715 9.70873 19.42127 13.64852 31.56474 15.1275
18.62845 16.30044 25.58304 13.2037 12.24444 33.12426
16.94556 33.56311 12.04389 8.49972 7.198953 29.08372
18.47492 12.21797 36.8946 14.93449 13.2864 22.55223
10.25789 15.82383 25.88265 15.74174 9.931351
15.11901 12.305 12.11879 8.750282 17.21407 13.10679
19.00952 13.62406 10.83335 18.63176 19.35955
12.9598 8.738434 10.60626 20.23629 7.880513 6.546666
14.99003 5.854233 13.77017 22.54001 22.37158
8.865808 22.74422 19.50674 20.5438 17.69096 9.069731
5.44038 14.61989 10.20207 11.71924 23.40357 12.40379
18.61647 9.663249 18.39786 41.08746 15.54982
14.57721 14.59234 19.81087 12.96005 10.74347
8.300312 13.58865 24.20765 9.25068 17.55251 47.21895
5.943051 15.36932 8.824498 11.21025 23.51649
19.0971 12.66964 13.11586 17.76286 6.430891 25.86794
18.83169 9.842584 26.95505 16.03906 14.63864

18.63287	8.183178	21.25019	9.508138	13.14137
13.98985	20.13244	42.14197	15.98251	31.76566
18.5126 17.66562	19.88526	29.08497	14.37138	30.12445
11.15685	12.02588	21.46921	5.800391	15.01107
22.97845	9.58373 12.73373	16.41355	16.58316	30.70701
9.51952 14.24313	18.6239 33.66489	10.45416	11.0315
11.59052	9.743078	15.76136	28.10039	17.62041
16.02936	4.388819	17.2245 11.06341	13.50625	20.78362
15.65186	10.08489	7.804414	13.21408	22.28044
8.758555	20.56169	12.67748	10.31285	24.05532
34.01854	14.83749	21.88055	22.36042	13.24545
25.31952	16.3167 9.591373	21.54697	4.651466	9.96045
33.70696	12.26541	11.30509	10.70747	6.105149
8.194014	27.27892	9.409087	19.51035	11.60819
18.15431	33.80524	13.9783 20.53756	13.71483	21.65531
11.73925	15.19287	5.469113	7.206018	21.86073
10.82052	6.965734	7.091649	13.64819	15.5146 20.52057
23.33253	13.22317	9.577217	10.97399	25.51989
10.50899	31.97958	28.01458	17.06991	12.16316
34.73331	9.103518	23.12997	8.901192	27.72724
6.204324	24.27456	13.22665	24.56278	22.1417 21.71742
25.03957	22.02974	16.98579	29.02029	15.2375 23.62588
7.506938	7.527983	22.79284	8.40089 12.26947	10.56416
15.28379	6.40407 14.60685	16.92067	27.06107	16.14781
16.30901	13.11972	16.22683	16.43115	15.61741
6.269642	16.09294	13.32726	12.19381	6.799925
13.5647 18.84853	26.4318 20.23506	10.93961	20.37967
16.30458	18.82361	10.7178 7.054546	23.91608	8.449496
7.817553	12.45949	28.85309	18.46427	15.77178
27.08385	11.41905	12.70519	13.25971	25.6794 19.18385
15.16501	17.27863	21.37256	14.06795	11.43712
13.05496	17.88765	6.739157	15.22	20.89627	7.974569
10.43511	15.58652	16.39026	8.856877	7.189877
24.40852	10.37204	14.03878	25.88937	12.79636
25.31935	13.80947	19.76785	14.43668	14.48554
10.74858	20.0417 34.31623	9.679245	21.77563	44.34369
24.44006	14.54553	10.67722
CDH1	122.3843	0.4093354	134.8362	79.16899	65.98433
2.77503 41.02848	29.2658 51.54432	21.99243	81.82205
65.83909	24.42998	56.99066	37.91581	0.7196583
69.98712	31.39861	62.16916	89.80995	72.32004
0.5809258	65.25229	0.4249132	3.619754	58.13248
38.75726	0.4091602	93.20416	18.25262	76.52416
21.58726	153.7115	111.0881	47.21604	27.83222

67.41447 100.296 113.2466 107.8418 93.07431 163.3757
114.656 100.9358 89.00607 101.7776 93.2186 52.44607
43.61627 157.3861 71.75665 11.49057 31.18064
105.5139 3.801734 97.70033 51.39057 7.392526
57.71781 96.26579 90.42862 57.2529 42.31101 25.70944
80.93831 4.905711 95.92317 35.7601 149.7831 57.67763
46.40179 57.58738 66.7261 70.8259 1.821116 69.3958
90.12236 71.3583 83.7127 78.29879 74.13704 45.82493
83.89088 68.10328 62.2 3.354524 118.8856 63.96953
31.81923 31.5997 161.176 92.70255 52.76473 90.91028
53.79897 70.98309 62.60595 117.5698 88.87539
69.84602 3.978468 114.053 30.4732 61.12691 16.80686
72.72764 64.97351 32.14011 117.4802 72.85745
92.76822 55.86728 41.66908 7.299052 48.23078
187.5569 32.05932 247.9195 73.47267 84.41056
29.82171 119.5379 78.25852 38.951 79.3123 23.70133
60.33135 39.20598 16.87362 29.95046 180.8973
114.8022 74.67328 75.13231 52.59175 96.78065
142.4791 29.52622 52.28553 100.7984 85.97472
76.60928 97.19321 25.31145 73.85017 45.90599
36.77045 53.22414 81.05496 17.89328 23.8196 41.58307
113.2801 78.98595 30.24115 30.09542 50.7589 12.94473
218.0412 27.45595 0.9784711 43.12845 65.58776
67.61212 56.89096 95.38582 160.5678 111.2417
66.08566 3.075192 1.009665 50.09061 26.39954
82.28359 59.23975 46.43676 127.0158 0.6164312
56.97285 40.175 31.81727 110.2701 78.79199 91.27408
82.02032 39.17759 58.32464 56.45186 90.8797 77.42184
58.99164 100.6794 97.13816 56.79909 60.66287
103.6331 42.19378 92.37449 70.8719 50.41732 94.91337
50.91164 123.1217 114.788 56.68702 101.9834 46.12849
6.732069 107.4425 99.40826 122.4429 22.21188
68.82088 127.1461 79.59297 100.3065 175.7762
163.5517 45.24061 165.6538 148.4522 115.914 85.67277
55.99116 63.09842 63.32878 121.6839 73.3857 99.39938
48.60451 65.3051 49.50824 74.80204 190.9783 110.0322
57.16834 38.72872 135.0943 113.0835 66.14105
154.4724 121.1202 53.60529 62.52894 70.58038
92.87926 37.85743 7.466953 65.43481 41.53168 41.109
119.7949 57.23732 16.46764 118.9748 91.13101
44.21666 110.4078 31.84957 78.40133 42.22215
44.56575 61.74782 65.27664 128.1725 46.03793
75.02832 85.35596 30.80495 97.52876 85.37051
50.77231 68.00573 124.6163 44.93317 105.1308

128.9352 6.788564 0.2244334 63.84187 38.68812
68.77332 41.95401 66.21534 118.4638 70.82683
85.07033 64.85296 60.96328 69.77467 40.43248
4.531464 5.167822 125.1372 0.1874178 21.75593
38.52083 62.90615 91.8928 70.79545 94.30763 6.068414
85.44323 37.34684 93.20019 140.6946 110.7681
69.91093 48.72015 49.63698 89.76878 43.93788
107.3074 26.39448 75.06543 27.23711 12.21663
117.4118 83.75268 89.67489 109.7326 65.12107
64.43724 73.3823 13.61985 78.30114 162.0446 121.8504
86.98082 82.72539 87.17633 28.5721 73.86565 144.8895
6.513985 94.84495 70.6879 124.3065 77.45334 109.343
120.7548 54.88509 29.21522 61.95998 28.13657
86.30718 122.3551 94.2534 48.83424 43.36681 68.65601
95.47664 82.57262 26.67428 59.65584 91.43149
71.17781 45.7725 1.166019 35.76392 67.13565 1.071739
91.55835 111.0414 128.8456 34.9537 104.4372 83.343
47.46512 85.48429 28.73818 69.32328 57.50403
77.71118 100.0966 40.09075 74.37861 49.00412
73.42028 32.28409 46.34346 92.34697 46.20783
54.64654 36.1196 81.09695 32.95389 94.2248 100.5039
76.43006 26.93314 65.77958 46.29958 83.64779
67.95199 81.2016 58.75352 49.33822 53.72527 125.8768
40.58134 24.23755 68.47704 129.323 100.256 84.09466
33.49225
BAD 20.31284 26.35376 13.02753 28.58605 19.39144
20.10631 35.30101 20.70518 28.93008 33.2403 7.681241
16.42756 27.08598 15.55993 33.08812 17.96019
25.46872 22.99248 25.19644 20.1841 32.00618 20.49248
18.85115 21.46296 19.9014 9.647996 45.72564 17.48774
20.87603 43.37026 32.90813 22.68385 26.3322 20.62874
44.06395 26.68359 7.098628 27.16707 14.94947
23.13313 10.65412 11.69777 12.43034 24.16471
14.16361 14.15811 8.779273 11.56591 16.30048
12.58915 20.37538 10.84238 8.359054 27.10217
18.1938 23.22449 12.85158 14.06517 15.03712 28.71428
9.889113 13.46807 13.35161 27.0843 19.79444 11.85652
22.42379 17.12551 19.96423 13.85105 22.25974
19.40177 12.1087 7.669843 12.50363 68.65772 12.60099
8.045801 28.06977 6.16616 33.15971 20.04215 8.582963
14.43193 17.58826 21.26543 10.29278 15.07342
14.10385 13.80655 9.664364 8.215754 14.12598
14.73458 26.6508 16.51099 15.72373 13.93721 9.836656
12.33809 13.88762 16.40145 20.32443 18.95785

20.63522	10.81187	16.99533	25.0429	24.61969	28.49794
12.85777	20.63338	18.13734	14.74112	19.52138	
23.61025	21.89038	8.902218	25.02877	19.39985	
11.68117	6.707803	13.57656	18.82434	17.64502	
14.22414	12.54952	17.24981	20.13898	23.0599	17.20817
13.61606	39.24226	10.55976	27.74443	9.703478	
16.0928	9.882293	25.25709	5.9378	8.993105	8.11479
6.954701	13.62205	32.76478	15.10644	22.47079	
14.45024	9.985355	11.11757	10.36902	18.81224	
8.648347	10.30517	12.68722	22.19543	20.02762	
20.36563	19.42755	14.25814	18.96255	16.75619	
10.45595	10.09837	21.96421	16.69212	16.9344	10.08868
15.24518	14.75531	15.95409	19.76146	17.17084	
17.58003	52.91988	21.26194	14.73744	16.13202	
19.34491	8.258321	15.51059	15.88471	13.78416	
10.93629	20.59707	13.42135	21.14552	16.49244	12.031
10.03114	25.19626	9.116151	21.98488	24.55195	27.908
9.081855	12.11298	8.280854	9.09229	14.3146	13.07527
17.89228	9.598406	13.99213	29.43648	19.23164	
29.97013	21.35838	8.502384	23.62788	11.77245	
15.27029	9.490452	13.74419	19.28135	26.05546	
34.13661	20.53272	17.15443	10.93534	25.48818	
7.963146	26.49976	12.46727	13.12879	15.1312	7.465381
14.36148	26.79642	34.7001	15.34524	11.2467	12.68126
8.207782	6.044728	25.63537	22.10422	19.02255	
8.625215	23.61513	14.55095	11.94169	13.50258	
13.38648	11.79286	13.87121	23.59523	22.95197	
12.85161	12.84619	12.83085	7.928743	27.59569	
22.19063	11.11976	9.894251	12.90823	27.01618	
13.25702	13.584	16.49092	24.28072	9.125403	9.569128
14.8291	8.66968	15.48372	21.09883	27.94872	21.20469
21.1552	28.06585	28.06576	9.476868	24.08655	11.9799
9.179777	15.91112	15.85028	22.11074	17.30854	
7.123901	28.65133	20.62329	11.16525	16.48244	
11.48762	21.00478	17.07984	10.6927	11.42121	10.00564
12.27285	10.09169	22.00238	15.71592	15.11928	
18.8229	14.85931	18.22063	12.1805	16.18541	14.79023
10.04197	13.96219	19.50831	11.04732	16.10142	
19.86285	8.364184	21.17165	24.79296	20.22318	
26.16003	9.361959	16.21407	11.79045	17.41882	
14.17462	9.02629	9.813014	10.77475	15.50931	27.19721
14.91424	20.61078	14.59767	29.24644	14.45699	
11.76671	22.39327	11.01383	21.12351	27.53308	27.195
18.22605	9.729622	32.80343	11.26604	19.36443	

13.60371 41.18073 16.2252 19.49586 12.48722 14.3696
8.944885 7.939064 30.02159 27.20693 11.93442
10.87981 22.44219 9.973139 18.42741 8.299749
8.988252 14.0971 15.86181 16.09107 22.66431 20.59632
15.7819 16.3035 10.75376 22.05896 19.41827 13.3782
14.56327 13.31848 23.91861 18.69658 14.71416
10.93026 21.2188 15.55903 20.17294 22.34838 8.895575
19.23812 18.46871 7.894418 13.68749 24.80638
10.82901 30.42315 21.47048 26.8727 23.87348 10.33719
14.28283 5.41512 10.86712 15.60281 13.18719 13.30331
10.89635 13.9805 23.85454 20.44482 33.22488 16.94755
16.36779 14.35991 12.31165 18.51331 18.87874
PIK3CA 1.242752 2.416565 1.709726 2.471635 1.636153
3.847646 1.35482 2.925183 2.061595 2.456436 5.438449
2.932907 2.236201 2.362233 1.419492 4.611462
1.13941 3.033779 0.8120273 1.157323 2.54721 3.297905
2.211465 3.884225 4.164893 3.446206 1.441771
4.405542 1.81086 1.005363 2.207509 2.586373 3.101075
2.022681 4.015545 2.544923 2.605572 2.093679
2.328649 2.246083 7.614772 3.162764 3.388791
2.194312 2.861465 3.504451 3.510036 3.272263
7.268597 6.008547 1.595007 4.001105 2.769173
2.750702 4.397635 2.628448 2.425364 5.983692
1.965931 2.019659 5.338747 2.210623 5.336838
1.992584 2.205206 4.336104 2.304471 2.691505
2.052068 2.055491 3.839501 1.945791 4.068393
4.351613 3.572707 2.0577 2.755608 3.200694 3.164121
3.139515 2.104472 4.003381 4.643394 2.843074
3.148183 3.877395 6.263315 3.046629 1.734675
4.137357 4.104528 3.918166 3.241576 3.091201
2.121673 2.741078 3.703668 1.67276 5.121264 4.253141
3.879268 3.243748 1.124033 2.016617 4.019073
5.213626 3.31832 2.486794 2.666189 1.56418 3.40684
2.222553 2.148437 3.034459 2.952441 3.429485
1.034676 2.811813 5.392111 3.587091 2.544973
3.065915 3.801286 2.685562 3.815266 4.155314
3.105061 1.117021 4.944857 4.925285 2.750646
2.857846 3.889334 4.018093 1.228634 4.706712 2.5756
3.030226 3.044608 2.791356 4.149799 4.707602
4.403913 1.601038 28.35247 2.006309 1.79161 3.091123
7.714263 3.563441 4.945082 3.015296 2.550228
4.828366 5.515637 2.244376 2.19192 3.69067 5.57292
1.430728 3.560182 3.680675 5.476658 3.411305
1.179147 3.435092 2.77051 7.057036 4.293233 1.607903

3.432039 3.136856 3.206922 2.788433 2.768624
2.705458 3.508017 3.979313 3.579219 6.566855
2.738931 2.497795 3.217439 3.963896 2.675154
2.844036 5.636877 2.324803 6.327705 2.776205
3.869855 4.530727 2.972964 1.43496 1.738777 6.594405
5.345551 3.050254 2.26995 1.485574 1.545199 2.198577
2.881661 2.02272 2.015456 3.461618 1.529098 6.061652
3.130766 3.479831 3.473422 3.862568 3.91915 1.82868
5.484613 2.875639 2.380703 3.408498 4.496166
2.695284 2.753032 3.590288 1.743582 2.778034
5.313376 1.317405 2.250712 2.210998 1.094331
3.097944 1.978589 2.651534 1.667241 3.361712
3.49837 2.294944 1.610013 2.391934 4.094762 3.485154
4.14974 4.475362 3.23673 2.563418 4.082482 3.402603
3.094765 2.67145 4.869551 4.097103 4.98901 4.238561
2.903799 3.635549 3.149593 7.384649 4.079945
2.781231 2.519066 2.775389 2.434233 2.125509
6.338782 2.629767 4.478868 2.94495 4.021355 1.467868
3.620034 6.522066 4.291234 2.588789 3.096265
2.844486 2.341547 1.809032 3.036082 4.497751
4.550028 8.044152 4.668193 3.079508 2.447647
3.20801 3.854513 5.371932 3.329975 2.151309 3.026496
5.059417 2.675749 4.374209 5.431188 2.581522
2.315966 2.857512 2.455386 4.974961 4.266093
1.95513 3.670345 4.584182 5.614731 4.085959 1.976385
1.630232 3.567184 4.369449 8.146269 2.700881
1.872482 1.844922 4.042755 1.662825 3.527371
3.689934 3.890935 4.016969 3.242072 5.485744
3.13005 4.688792 1.973383 1.564611 6.462935 2.38293
2.107484 2.417854 1.534753 4.901162 2.198022
5.003985 4.134113 2.993739 2.108785 2.749643
1.717156 2.562031 5.05917 6.023286 4.371935 1.359646
1.65899 2.338174 6.034233 3.759334 4.178739 2.022675
2.913707 1.657271 3.447414 4.175584 2.818472
2.376574 3.809602 3.23377 4.042289 3.802122 4.261962
2.247904 4.099129 2.38761 4.267782 3.253909 1.761335
1.738 3.056685 3.349801 3.281666 1.700126 2.756598
2.111599 3.64969 5.65753 2.247949 1.697736 3.549017
1.556462 1.542584 2.7985 3.364503 4.575734 2.480455
2.641336 5.613285 1.564831 2.334617 2.739955
1.522332 2.754446 5.456968 4.015716 2.536934
5.592425 5.538252 4.287766 3.753388 3.434129
1.628719 3.555451 2.737368 2.100291 3.519847
2.190035 1.919832 1.561474 4.007151

PAK1 15.38663 7.820826 16.65247 18.28522 13.35393
5.283726 12.76506 7.914194 17.72938 9.825276
5.969242 5.016301 10.82794 5.910257 13.70239
8.302888 13.91312 10.36943 12.45103 15.09878 21.71
7.80316 6.217801 7.064301 11.27135 4.505193 15.18804
6.064127 16.50494 9.357777 17.98323 8.780682
24.68194 11.71017 15.959 9.819065 5.575265 10.04706
23.99694 20.79178 11.47182 24.29553 11.67445
10.09173 18.75371 12.66778 15.03468 20.37626
10.42984 24.31338 9.497449 11.41599 9.979439
12.66544 8.607461 15.18638 16.58054 9.892781
27.00829 12.11676 13.36024 14.81216 12.59769
10.10511 14.31186 6.433017 12.89075 12.71409
15.33174 8.774744 10.6336 17.90568 11.44258 11.5126
15.66159 22.94671 11.76006 7.080044 31.20606
17.05871 13.12615 9.206167 21.83517 15.00327
15.47598 15.7376 12.36458 12.67779 15.14714 9.324138
16.77007 17.71629 17.64428 15.51798 9.801669
11.04175 12.45083 13.38483 26.12279 12.94491
10.98807 14.03113 5.718317 18.57276 7.201811
13.22998 14.74008 12.9292 16.86168 11.47877 23.37004
16.2826 8.459796 6.555065 11.40347 12.47624 10.58355
13.01774 6.88889 17.49669 9.695528 24.56562 19.73089
10.82094 16.96531 7.827967 18.61549 11.84432
9.370473 15.38967 15.27892 8.804728 16.90684
14.93716 14.63869 18.39445 10.66007 23.00404
11.09782 15.117 15.77809 11.56038 6.730372 9.286402
14.2876 10.01287 25.18061 7.724086 8.531286 9.751181
16.78009 12.31205 19.43119 12.59608 10.41665
8.430082 9.865713 8.114012 20.98721 10.64157
10.07844 10.14709 13.07436 12.89165 14.68463
17.17443 13.74238 10.0542 14.51854 11.77005 22.77253
12.09225 14.89312 8.221785 14.31352 44.56522
13.52344 8.090894 20.17413 12.0558 10.94704 19.43017
8.046114 16.39362 11.86176 7.475149 11.56801
30.8577 20.26594 19.89738 14.63535 11.33128 16.89266
15.58909 15.37271 13.50383 10.8304 13.49524 16.29663
10.69355 8.673663 8.351798 13.99686 15.38509
9.491779 17.41644 8.587881 14.71497 17.1783 14.60144
17.45061 22.20334 13.81744 7.298409 14.91879
14.14825 19.95408 25.36726 10.46526 17.55663
19.42504 14.69888 11.7951 7.44845 15.67574 9.964691
15.32729 5.591498 14.4278 21.41821 14.14038 9.250689
11.25133 19.36413 24.59962 14.99031 10.80784

13.33228 16.43385 13.3295 26.9296 12.59391 10.65656
16.51415 13.61362 13.89087 17.31592 7.636278
11.0155 7.843178 12.31797 20.87184 11.13812 8.931538
19.5541 45.20575 16.93569 11.93749 21.89083 13.18571
16.39227 11.65898 13.43241 17.086 14.03665 16.29083
39.45807 16.5634 11.25558 20.35899 16.54028 13.53093
22.43126 21.03395 12.78298 23.2706 10.82128 8.477349
7.465632 22.34256 9.658137 73.06899 10.51557
13.3224 12.96997 14.62918 20.8443 15.59389 6.075626
9.473153 23.17843 10.65627 7.111539 15.77561
4.470994 5.207555 10.29557 16.60845 11.02177
12.13891 13.15966 9.843503 14.04733 7.420065
14.9879 18.66555 9.20625 22.36079 14.99546 8.335575
17.40933 36.64271 8.844302 11.76839 11.95041
8.731534 16.93718 10.28066 15.7765 14.09392 24.35313
17.44159 22.80255 15.1968 10.38409 22.23888 15.79879
22.0286 9.237931 8.362929 14.78478 11.0569 21.913
37.12222 8.996402 18.33199 16.68605 27.41207
19.82411 16.21131 26.31468 17.66859 8.205623
17.78101 9.291549 10.50786 20.75529 6.440354
11.7136 12.09466 23.25393 14.81677 6.877915 10.77031
15.16377 22.71119 14.09544 11.7234 9.923758 14.53137
16.22527 5.492722 11.71099 10.67173 14.20904
8.283612 9.837218 19.44337 14.08308 9.02096 18.45436
10.65668 9.699242 35.11982 39.70109 8.53524 19.54883
7.142382 8.218544 9.706349 10.21898 13.64704
14.7824 13.87511 21.91116 18.21387 15.79257 8.453326
20.67441 10.38798 15.56371 9.051441 8.533216
13.30428 12.1446 15.08959 12.52243 11.33585 16.17954
14.42839 15.53237 8.062523 12.08365 19.16139
13.84313 12.73629 12.02867

ITGAV 6.819302 14.30381 10.18208 19.16571 7.589269
19.52497 6.938484 12.21472 14.87936 10.70751
18.44308 8.311723 10.33948 5.982298 9.277041
27.28433 7.91323 15.40605 5.636642 6.129681 21.45358
16.5275 8.396344 21.04644 16.77961 9.140293 9.707614
21.46237 9.58085 8.861007 14.40572 17.29765 7.576549
19.01441 28.29863 5.267399 10.43202 8.955024
20.17331 11.31675 40.04882 15.75403 32.72618
18.17716 30.47711 9.115168 23.13952 16.81634
36.33224 3.445454 8.123347 24.50349 8.318133
24.74009 28.72401 12.41359 14.64212 39.01962
6.954889 7.961551 35.33581 17.11572 24.98527
14.27628 11.36151 37.62425 5.987498 16.09848

12.65761 35.67581 23.21814 27.05559 22.88076
40.76354 42.72184 11.35691 36.52752 21.51242
19.65002 12.93392 11.15872 31.15859 24.26364
14.27515 24.48446 26.44677 17.77645 24.83892
14.14161 43.17706 23.8118 25.49456 35.11413 19.85376
18.89593 23.79393 27.44891 19.22257 13.13818
30.56832 20.84865 32.52347 4.615066 10.02216
25.26696 40.64511 12.27259 18.93837 32.81782
17.56922 12.69253 9.369714 12.67231 14.50666
16.65644 27.15799 8.216092 26.43241 19.68287
9.418477 16.5676 22.17465 18.77903 28.04076 15.32453
15.98687 18.27492 9.677604 28.25896 27.31164
24.97391 9.30679 25.09731 27.4554 11.11919 16.2356
23.68174 41.00254 7.317958 14.91459 20.99703
12.03576 3.807323 6.824048 22.88485 8.575627
12.59401 31.90706 16.14735 17.11985 15.17187
39.47412 31.00745 36.28496 29.89014 17.20662
18.31783 18.75131 35.78422 9.235102 23.10409
20.52799 24.35907 9.121914 9.357868 26.45719
22.02745 36.37377 21.3213 13.91317 20.69348 18.887
4.245723 9.442005 6.897721 24.87688 36.42591
10.34222 17.58885 23.14891 13.56233 12.25909
11.31858 32.86732 28.79721 39.38286 33.9713 15.65939
28.3824 15.19986 26.55635 7.012708 26.1172 13.23853
22.21084 32.55222 32.95009 10.70254 9.901936
13.81158 5.849413 20.8594 21.69199 12.58254 9.317022
20.83633 10.81603 27.45183 16.47198 16.20596
17.92489 24.69302 26.54747 11.63302 34.65711
34.74861 17.74652 2.489481 30.20589 19.74035
24.69811 33.49447 10.11086 28.00096 35.35445
6.984718 10.69675 7.730113 8.989243 26.04491
9.340499 33.11467 7.920546 37.54312 16.49444
6.469348 5.273403 10.98703 29.52176 12.19124
25.93318 47.03176 20.43772 17.97472 36.27747
24.46133 30.79512 14.2415 39.92437 14.64907 29.07101
15.65624 16.64418 19.22405 21.75035 14.52588
27.00021 22.84943 15.90295 19.12016 14.02612
12.95146 22.31751 22.98316 38.8913 28.63656 24.07804
5.630426 34.09773 5.678691 27.90845 18.87036
28.08888 9.649592 15.4362 17.05533 18.92725 35.42416
7.178441 43.62617 24.88849 22.67421 20.76819
17.2771 33.29254 22.40755 57.59689 13.43799 26.5397
29.28207 15.01703 38.41161 30.14837 17.01385
24.64095 20.9464 29.29919 21.87303 18.10816 22.70006

28.86541 33.25995 32.90527 50.15686 19.46421
12.24204 19.84508 16.00556 12.50862 18.90542
25.37572 10.42139 23.54271 13.68572 18.80451
24.69417 21.88719 48.69072 41.1835 50.88026 12.03766
15.89186 11.64031 12.23881 39.8904 11.1661 26.24509
15.90021 14.13987 52.23722 17.22745 36.94136
34.40551 9.375018 15.90236 13.05279 5.979291
35.09556 31.00168 35.81504 15.11184 8.520742
6.463079 17.08725 35.86474 10.93294 29.09633
15.08109 9.375565 5.545383 16.92599 25.21043
17.82668 27.96846 15.57552 24.32102 38.6724 32.65916
28.76576 11.0381 24.82203 12.82352 25.43544 13.77704
10.9312 15.61335 19.03368 20.99075 12.36058 14.58916
23.4711 12.20453 10.28774 9.115607 22.19409 12.15223
15.52084 14.00342 6.788363 15.87359 23.59859
25.02057 10.49385 16.26917 5.964297 19.70065
17.1247 16.95809 9.576433 17.94503 48.36126 26.30824
6.688628 23.21867 16.56151 28.98409 20.6369 16.42877
14.20026 25.68681 4.094225 7.533314 13.49823
15.2829 10.67667 10.20664 36.06946
MAPK3 134.4362 27.1757 96.19954 78.26343 73.78053
25.18037 65.14624 29.04428 85.38438 37.43251
38.15418 60.47119 39.49675 70.90527 68.61093
19.99273 104.2576 50.11864 104.744 117.5321 93.4833
21.22081 62.89531 25.45008 29.46156 57.03365
76.81492 21.64548 102.0416 44.25225 91.23415
41.41582 32.96378 52.00657 55.13261 8.940916
9.254436 61.64103 41.93655 60.29998 27.75336
44.72697 28.66097 62.88759 62.64 36.03149 26.01453
48.97267 16.27316 33.92336 66.92199 25.2966 25.00897
61.13035 35.27591 45.37815 15.90733 21.93237
43.1005 63.94748 29.5997 86.57673 32.00413 84.35212
39.83975 15.47974 73.18483 52.6684 102.1362 55.33193
46.61387 28.70019 29.47308 24.78659 24.4014 36.5307
46.85451 15.82327 21.92634 26.3231 42.70188 37.61869
11.93577 31.44202 45.29315 36.04777 20.6247 21.6529
30.29152 33.27075 36.03758 11.62417 21.20609
50.72585 21.68636 19.77788 41.47725 102.5333
19.3668 31.79277 32.00271 69.40903 11.67857 37.23914
24.29118 26.73101 44.32266 40.633 37.73763 45.07309
56.65194 30.00819 27.87342 26.84247 44.93328
88.82157 39.05601 29.22571 18.54051 46.44779
48.60453 19.27255 24.81367 34.5012 25.55596 34.93266
40.26434 89.49673 32.74329 62.13334 61.39127

25.05676 85.87715 15.85266 77.07778 34.95138
57.37496 21.76568 22.54799 20.57025 19.63225
22.09028 34.28555 16.3016 29.40288 24.89834 35.18067
38.46896 19.96509 37.20692 9.423457 47.64161
45.77148 27.08359 26.14519 26.60826 29.54088
23.97826 40.90359 26.93412 32.97027 29.21531
25.89643 19.37683 47.83615 65.11922 53.91997
21.33965 25.89098 16.19486 21.04856 26.51777
36.67586 57.64183 111.7988 24.89691 52.35663
13.40019 15.40531 30.8425 17.10542 66.81206 51.05526
19.99541 27.35298 21.84085 31.20309 47.68895
24.24791 24.66323 52.51408 42.43351 73.67195
79.10429 47.04416 29.77135 17.09872 35.12697
19.41617 32.61126 19.45164 33.50194 34.77563
27.19567 36.03023 27.03939 72.27333 24.89348
27.04721 59.77524 24.23098 34.81936 22.95015
19.74058 35.46275 29.79513 73.2612 40.51432 23.92825
45.64743 47.13604 11.59278 53.16046 25.9894 31.95714
46.57643 33.19115 37.35628 52.77672 62.27192
60.82513 23.6269 48.07426 53.75507 25.1073 50.39589
49.53345 22.25103 37.80484 38.63242 38.77727
34.87444 24.00346 25.26046 25.3053 53.77703 26.29326
24.70626 21.84237 25.44261 26.74342 36.16379
45.44911 26.49677 24.30122 24.4282 21.05138 58.12174
34.34068 35.53707 27.56567 59.572 21.51852 20.24427
58.57264 32.13079 30.55003 79.31241 36.35248
49.36443 27.98869 46.19121 19.40993 32.31861
38.03206 42.56392 51.33327 18.39224 26.53428
25.14239 37.88002 25.67996 40.98564 42.76662
25.68499 22.32755 47.3336 31.94384 27.38808 35.51717
34.95558 11.77388 17.32573 49.68573 19.93908
34.8287 20.16978 32.15578 33.65258 81.4079 37.35807
18.56804 18.42351 24.17086 37.1068 67.80868 19.27681
30.92127 21.74687 48.71797 61.11071 64.07942
20.06235 56.33192 32.62786 31.05305 27.97682
28.85454 30.74227 33.88008 44.28385 27.24933
71.21657 28.10899 35.08101 60.11302 33.12183
40.29874 64.9331 13.86951 37.48183 17.94945 52.98097
56.40325 30.98082 31.2757 22.24493 53.26903 28.87693
20.4891 22.6663 42.11741 14.86087 22.22498 20.46132
39.08361 39.18728 15.60419 58.25051 16.87604
37.45029 39.12521 33.4524 33.78236 22.12873 32.36423
27.04279 44.89898 29.02279 54.28262 28.15265
50.70599 6.026533 32.95196 37.98233 19.69795

38.12837	28.01349	23.98928	67.62754	19.02149
51.78272	21.90117	13.3935	35.20677	17.89609	49.06456
43.23714	27.762	33.60305	29.34332	63.13989	18.64786
22.99729	15.49963	42.1563	36.337	73.17315	55.88022
33.33169	27.59568	13.18269	26.4725	27.91199	36.03875
18.25064	19.09185	26.50982	50.38407	53.57707
67.78352	34.09649	53.78896	32.96896	72.46565
67.40554	33.62626
PTGS2	0.3170983	6.546874	1.397524	0.4161741	2.806608
10.94655	0.1789329	7.233934	11.95341	6.709011
0.8260393	0.8450919	1.848014	0.1938971	0.4854953
41.06231	0.2145115	17.53485	2.049581	1.554744
1.688375	7.411759	1.866212	4.750454	5.357779
0.8040894	0.7419945	9.067948	0.52752	0.2002159	3.919471
15.42318	0.2608398	2.38822	9.396809	1.329802	0.238143
1.604781	13.99203	5.924888	7.800197	5.508053
14.40146	0.4112493	2.606551	3.810378	1.822397
5.148121	5.295196	25.5271	0.6397986	0.6998116	0.9998573
30.17898	5.962222	0.2745631	14.91895	7.318165
1.794389	2.478402	27.38269	2.748846	8.774207
2.003735	1.051088	17.63822	4.033261	0.5295017
6.906559	1.167274	16.40729	1.736697	1.961215
25.46936	18.07517	3.775009	27.56771	7.074717
1.45043	11.47703	0.4794406	1.5572	5.983915	2.897684
3.183265	6.909425	0.9334283	2.811099	4.789166
2.684076	2.997286	5.526567	5.257461	0.5516562
1.425043	0.2338068	1.234828	1.187543	2.184389
6.973301	0.68701	0.6407981	1.391373	2.95362	12.8035
9.154018	4.128925	1.424496	0.8948749	0.2940835
5.988129	0.9564519	13.52505	1.80561	7.343666	1.34199
1.826446	7.431721	0.4277679	1.739477	6.437453
0.9166309	2.081065	2.969144	0.9011752	1.035045
1.102133	3.402737	3.458239	3.48872	0.3554641	1.114434
2.025673	13.7339	8.53132	1.497179	1.056967	2.021823
2.500508	26.91823	0.3024151	0.3396993	5.291814
1.329654	13.37714	3.358261	0.6657736	11.65037
0.7630423	0.4875194	1.707369	42.68726	4.510006
0.602884	2.494617	2.181839	3.013248	2.922129
5.096983	2.490856	2.269243	2.443194	1.700342
2.668961	4.43976	2.278629	2.348854	6.648933	2.230606
1.551362	5.519951	6.47254	0.9061097	2.415864	0.1689395
3.967155	21.51782	28.28698	1.005352	7.274855
3.621873	0.317473	1.723081	1.197117	4.694905
37.22767	5.986709	23.34343	2.428671	0.3671272

3.970307 8.766486 0.2794754 1.635593 0.1564674
83.37484 9.861288 3.615619 1.309313 3.522833
0.7806612 0.9015136 8.26919 1.442867 3.808506 1.019781
1.186149 13.08167 1.952945 8.474305 2.525296
11.2306 3.580633 0.3193231 27.34813 2.619969 7.843389
0.3866401 3.203588 0.4802633 0.4451478 1.511593
2.790068 5.410252 2.56552 2.225655 4.384433 0.6934971
0.6593808 1.788389 0.8119427 15.6635 0.6437671 19.11611
3.747096 1.883541 1.056525 0.6687815 3.206938
0.1998937 1.517897 0.4420865 6.702621 16.70142
13.25172 1.244612 2.433238 1.40192 2.784017 2.752556
28.88671 9.499719 42.02603 4.428964 0.9198578
1.185901 4.857386 1.957877 2.366157 9.66137 3.865274
3.487424 4.567435 23.02663 12.53661 1.392599
2.948962 7.832315 1.046337 6.902226 0.8320709
4.492709 3.729175 1.94049 20.91127 1.043098 1.703775
6.234764 0.1359893 16.90262 3.916597 0.6409286
2.502216 1.804077 9.811827 0.5883128 26.75593
7.323404 21.39705 11.11186 5.506731 3.049109
35.73721 7.687143 0.3380236 1.752687 12.88182
1.823049 37.86157 2.906138 10.33641 3.196665
1.302649 25.13159 3.637078 1.344071 52.48699
7.502228 1.716192 57.38283 1.528217 2.296255
4.847911 2.769358 1.945095 4.812599 2.36082 14.3173
55.18319 28.34665 1.788847 5.81356 8.85429 0.1764389
17.06997 6.463438 4.291383 2.088685 7.536513
3.164734 1.479204 5.552545 1.299208 2.653723
0.9675041 0.6541177 0.3395732 15.85838 2.874158
9.464734 0.308646 0.2532764 18.22897 4.653602
5.124814 0.7436773 1.421884 7.151051 8.52493 5.662935
1.797611 4.083963 0.3432871 15.36939 0.6832056
2.260289 1.918484 17.20598 1.661668 1.004816
2.125007 2.058896 0.2678028 2.043191 9.152741
1.706943 0.5969046 7.596601 1.666403 9.074248
1.148443 12.33987 0.20453 1.619962 0.6506149 1.343138
6.564948 18.98759 1.212392 1.12402 1.192354 5.33959
0.6084756 4.654588 3.665416 0.362309 1.765487
2.675307 3.017846 4.841041 5.605963 29.50224
5.148913 0.3708169 1.444341 15.41381 0.3011349
6.41941 0.6541411 4.062341 0.5756119 1.513868 0.3760649
0.247758 1.019299 9.250732 2.212823
BAX 22.40788 5.370183 20.54964 14.32925 18.87993
5.528269 12.02641 4.879761 13.10927 20.15885
4.290393 6.013227 18.62866 6.250182 17.52407

4.614121 23.62623 9.340681 25.24072 23.23359
15.73688 4.916308 9.223745 5.308007 4.477238
5.429593 19.52162 5.932781 15.57354 12.32273
12.4901 6.931945 26.03707 18.0946 12.78351 36.55159
4.762655 28.4379 19.97458 19.65169 9.977458 17.92167
12.48558 7.946374 13.45711 16.38504 18.97027
37.25085 12.59756 18.29193 25.97756 15.29174
18.03869 21.16146 12.69088 16.8453 23.63244 9.499174
24.10732 12.56244 7.982762 10.55392 11.72357
14.77327 51.37085 9.099009 31.51752 21.69383
23.89597 12.51084 17.9868 52.99648 14.8278 13.45289
22.4101 54.63305 11.9211 13.36237 21.51622 12.46621
22.78145 17.69829 14.22686 17.23758 26.41286
18.2813 13.61998 24.74773 19.83546 9.096416 5.000609
27.26543 27.8494 19.38989 20.68538 68.24571 30.18546
17.9421 11.39583 12.70556 17.98632 19.5671 28.48074
12.77242 23.50422 11.73046 20.43226 11.45141
14.66035 28.05409 25.87857 22.2535 29.13354 16.26407
19.51173 17.16202 26.53615 14.06408 17.8854 20.19355
15.13379 9.280236 8.254563 19.64336 11.15302
22.0724 6.533883 24.49035 17.64509 22.19511 26.94101
30.67096 20.61724 11.2047 22.12167 11.53165 11.75198
22.86911 26.54581 8.231113 14.45727 19.14773
43.4089 25.90467 24.98743 12.28538 11.51867 15.25888
11.06186 16.55634 12.29559 20.4339 14.27971 27.61675
10.87579 14.70686 17.54331 14.5963 23.14457 21.28858
22.78742 11.75776 7.523535 19.74612 17.42785
11.83639 17.58599 8.902047 11.96968 11.17992
6.073325 31.63475 20.91759 14.34987 16.79008
24.60482 25.57185 27.38302 17.94236 14.82985
15.25055 11.75799 8.679253 24.46566 23.36118
16.13317 13.77703 45.25691 6.465686 29.81719
23.5657 10.52237 30.44724 13.64267 106.1883 17.48687
12.97361 16.04462 31.72007 17.47193 20.54457
23.94796 22.89835 10.94341 16.67195 17.1386 20.45695
12.69766 14.21255 23.23618 14.19443 17.42978
17.81225 12.80305 20.00046 27.3883 17.4653 15.70508
53.60471 11.93009 28.9025 5.085765 10.61065 20.27625
16.85596 5.525213 22.2203 7.467733 23.50892 29.98709
13.97093 25.19523 40.99069 6.845722 18.37283
26.02046 26.37228 12.10669 7.996503 19.51347
11.41975 28.45287 19.54984 17.62048 15.25551
20.35494 26.89239 9.948662 12.50241 17.73559
20.59752 10.07657 14.41232 34.38201 52.28936

20.65399	13.80115	21.19568	24.96074	13.71842
33.16736	24.92706	8.076117	9.961075	14.18533
14.75408	16.17058	19.77077	25.86697	17.21674
44.13893	54.65056	25.20791	12.71389	14.33885
16.35136	23.29403	10.43748	19.67106	14.95295
15.83982	15.75175	19.80542	24.24072	13.58352
35.9251	9.873455	23.02499	13.90426	15.98344	12.68391
6.396436	6.511868	17.84878	18.43352	13.89817
25.30524	57.90582	11.51308	15.58589	15.6656	13.80198
20.47797	11.27906	23.51701	14.95998	8.709901
11.54174	16.74738	28.887	43.60203	40.22641	37.86842
21.5547	10.02694	13.99406	12.59882	12.43821	11.49029
11.24104	14.41643	13.42293	20.68435	43.48219
4.897381	21.38935	25.01765	10.88954	17.93485
11.89144	37.59361	13.15755	42.24926	38.77552
15.59932	18.97927	6.943828	22.6195	34.89845	14.29406
12.93398	26.23267	20.14788	9.672549	7.933416
12.12825	15.5622	17.06213	19.76139	21.84855	14.68619
20.6565	12.13988	21.41972	31.38397	24.33208	14.36029
30.80294	15.67021	13.25248	21.87659	27.82212
13.68661	48.77371	26.76974	23.10706	10.30913
28.03366	8.000706	10.72364	9.314251	11.52922
10.50506	5.647392	25.78573	17.27195	30.74383
16.44242	18.81998	19.16808	23.11661	15.37722
18.60283	19.82661	11.06796	50.21119	10.42553
15.68207	31.49604	17.06732	11.08879	6.58984	24.49238
21.99073	14.3378	26.53997	15.91134	15.59597	7.05146
14.14405	18.60947	28.56442	20.2641	15.24534	14.97402
32.00957	11.95155
FN1	2.893137	108.5786	10.75602	4.589601	10.13314
108.3879	4.044838	32.55196	24.35851	7.186493
120.2278	8.578717	6.596665	22.81387	7.884805
298.0121	4.020358	49.52672	5.339723	6.775905
20.0373	85.9457	46.12317	125.9137	241.7511	32.2485
3.377566	148.7303	9.934759	2.745488	33.44555
85.20773	42.62477	26.93701	126.5751	9.301714	57.933
25.11555	107.8278	15.07491	94.47253	10.06926
197.9076	17.18414	38.06488	28.19	8.08701	136.0477
127.9001	11.58258	122.3085	115.757	258.4322	101.1477
160.928	18.65028	105.5678	270.372	56.61794	9.113914
204.227	149.9727	104.0311	277.5955	13.74542	341.597
176.8869	30.6628	29.46657	14.52412	173.8816	80.34742
102.4368	294.371	365.0988	15.23264	16.1772	151.0673
128.2195	27.42373	77.86125	124.9725	57.34644

116.061 294.3935 176.9844 60.97809 66.55413 29.75363
154.5922 203.6231 79.32889 145.9785 33.27589
83.2327 118.4281 135.1765 16.40224 24.85922 148.7267
33.13006 5.04352 6.598442 32.98162 111.8045 116.4379
81.1277 107.9934 94.43837 52.72214 45.62013 20.81822
72.47033 62.21386 41.28543 36.88663 83.19609
68.84456 85.18941 4.670898 66.74442 400.5643
269.5425 287.6895 137.2513 14.53406 24.28717
157.8421 102.9967 172.8588 117.3056 29.18673
14.48838 132.2779 13.99157 127.7025 165.2524
73.02183 20.06811 24.0454 82.9003 25.40904 32.62467
8.291999 59.13049 16.69081 5.840448 172.6011
52.7883 57.8644 44.56789 105.6352 164.5413 142.0691
156.9021 13.10753 100.7981 89.08311 182.3044
47.36496 215.2881 21.68857 66.74097 34.94323
56.70504 48.45423 29.32266 161.5172 73.22587
73.14508 132.0722 27.77934 34.08597 22.06657
9.155983 143.0657 306.6212 86.22421 4.450128
147.0857 29.90393 30.86367 18.30861 43.60876
219.8605 111.5462 131.6519 106.5282 94.41307
27.37375 121.7055 70.45394 122.3331 5.696789
20.37856 158.0693 321.9139 43.64045 22.07082
71.38747 131.9572 57.06057 50.18606 52.49914
25.5284 6.535627 113.3943 114.3585 38.0956 139.2251
57.63513 137.656 15.77383 49.54326 60.25657 113.0683
75.60241 55.61188 71.22722 98.74121 34.96816
226.1606 21.6836 254.6517 20.39244 19.26895 208.0364
5.109734 2.260399 49.28679 125.563 275.118 2.82687
24.82472 10.69161 20.25345 19.39657 14.18891
262.649 29.66273 79.85028 53.1285 179.4726 126.4063
322.7426 178.8251 127.571 105.0195 144.4589 22.83804
49.25405 77.8449 82.25728 97.73588 9.582448 153.7338
99.75578 115.2887 93.03643 740.5591 9.043771
96.84009 62.87584 106.2371 200.5806 227.1977
38.02262 18.36975 69.04752 14.64183 103.7223
100.8881 7.625045 27.57234 4.944085 33.54406
45.4408 215.637 95.00294 58.00832 152.2166 42.32228
301.1166 184.4506 183.8758 46.13966 4.496302
32.51117 61.61525 134.9148 92.49663 608.1204
216.6597 42.40056 449.8209 244.4551 307.7665
121.7242 57.19878 7.766486 24.64154 120.4271
80.69303 681.446 2.493247 36.16496 38.84554 168.1841
32.71855 42.34232 79.3146 132.0692 213.8808 119.1499
33.4757 218.7174 46.85151 496.7454 99.31672 453.3457

61.8682 69.68037 62.80845 71.43649 112.5985 273.2975
127.4988 36.92605 9.746012 182.2768 5.094334
341.2964 131.3341 25.51586 128.8794 39.38295
1.364823 40.18001 22.86167 53.02006 91.28025
46.94179 56.4761 50.90756 231.5045 26.97989 18.12248
58.53116 37.8095 66.02267 33.50985 212.1799 33.13056
183.1287 3.872864 16.45275 79.48339 151.845 135.518
137.2776 46.49225 117.395 65.24657 103.805 27.61584
133.7481 2.191602 9.378929 18.64765 50.52569
77.72483 44.68115 139.6859 118.5196 54.63275
164.0804 11.57605 24.12872 12.1524 35.54369 247.6824
106.3534 35.22958 49.83385 22.11341 26.80083
157.1014 35.03621 63.40833 46.99453 254.9836
144.3138 8.769537 129.6477 10.44572 43.44843
60.75626 72.48397 10.92547 89.81978 32.07753
4.279342 111.6612 5.230259 14.52575 34.58375
138.9449
BCAR1 16.89201 6.504333 12.82668 28.03849 11.28495
7.023353 11.7056 7.928322 22.43701 8.70869 4.447351
3.284153 8.572975 5.534555 18.34378 6.783192
24.31087 12.98668 22.23506 18.77426 21.68301
5.03697 9.026123 6.032012 6.867262 4.557718 14.34272
6.706608 17.34777 5.639341 22.32236 8.349149
24.11209 14.61294 19.11957 3.619335 3.498601
27.34152 21.41153 16.55881 19.1495 21.55293 13.25342
22.48695 12.42432 13.6876 10.4622 11.04096 9.387324
21.46075 18.25181 3.288716 13.63864 11.25357
7.904352 14.35935 8.425367 7.036222 16.88861
16.39791 11.65486 25.2321 6.238543 9.808323 7.212713
8.733614 24.71403 9.559425 34.17396 23.70966
10.10201 8.351339 12.2269 9.448169 12.44622 26.96828
11.15354 22.52394 6.637459 5.421186 9.531635
7.940279 7.136826 7.314984 15.43688 13.65025
11.06926 14.14493 7.076892 9.956048 8.126096
14.96936 8.951998 11.21013 9.195921 10.09122
15.29204 27.63031 8.89117 13.16197 7.253894 37.32834
6.973767 4.317911 9.558741 10.21075 27.17026
14.05809 20.90651 12.77203 12.78171 7.314841
10.4704 3.767647 13.21962 28.7344 24.36626 19.4437
9.819586 12.6773 11.84878 9.974506 11.03984 7.83802
9.940841 7.690944 8.496161 14.51669 9.435502
14.56198 18.88602 14.14917 21.77655 9.391849
17.56678 11.39363 17.69943 17.13622 12.02246
7.219939 14.87469 11.71942 13.71585 5.495056

9.081156 14.84876 8.028551 10.92712 14.49488
7.805718 17.43118 18.97655 17.2763 10.61298 8.186769
12.01376 15.01584 6.699017 20.03375 7.006884
11.30005 7.700385 7.657027 11.73505 9.618051
15.15983 23.29745 9.936263 11.64585 6.678691
6.529997 16.00956 5.718698 12.28741 18.77922
12.19142 22.72022 8.026178 12.85596 14.26578
4.913219 18.37929 9.541032 14.30548 12.49507
9.732524 13.59473 12.50596 12.61246 15.33925
20.06177 21.91925 24.41157 23.04611 12.75535
20.53065 11.45198 9.381497 12.27018 6.753122
7.62985 8.762033 14.5863 13.50407 11.37541 21.32644
16.39827 6.855506 17.22483 20.63509 8.283404
8.855045 8.204794 25.13932 31.21829 19.21396
36.77297 10.27942 14.5374 16.59702 18.69688 10.06303
12.13442 18.9885 15.79541 5.947332 14.80489 3.848589
18.93879 17.80346 11.72643 11.19753 10.33586
25.12603 5.417764 21.30737 8.001323 14.29979
6.227469 18.18171 12.54119 21.14292 8.239522
13.95848 6.696146 31.34005 11.06449 6.69212 11.76913
9.816524 9.610905 6.859545 21.34084 11.80053
16.35808 10.88916 8.742958 26.66419 15.5739 12.51592
9.46345 24.36216 7.009167 9.387974 21.84928 15.84265
16.07705 16.43247 31.72758 9.548628 12.96385
20.74562 15.18061 6.744864 14.33763 11.47735
18.05993 6.033525 7.133233 8.578679 9.608989
9.794182 17.5576 13.70775 9.525181 9.926622 16.06294
11.85984 15.16162 11.73064 17.82113 5.264516
7.85953 16.1333 13.58635 16.57682 9.137548 14.54553
15.84997 14.3292 10.55594 6.994963 15.31853 15.89469
9.04671 13.72633 9.200039 13.50479 11.01839 16.68372
24.34696 17.4699 11.75654 10.90896 13.40196 9.470234
10.94485 27.11371 12.128 11.2295 11.45682 5.898279
15.82614 9.792633 4.372426 12.32509 25.78124
7.965558 13.90905 21.45233 8.923789 9.635569
14.95322 16.4734 12.70783 20.13134 21.24596 11.49576
13.62142 21.21145 9.105386 10.30868 19.49729
8.881128 8.985029 7.738827 15.71149 5.510805
16.03855 13.31287 10.7298 12.61908 18.71834 7.44464
11.29492 18.50778 11.9503 12.3324 5.529295 9.031272
12.44303 14.68408 24.77006 7.543527 13.43496
7.953462 18.71744 13.13066 18.12338 16.41899
7.245644 13.46789 14.57497 4.36459 11.41899 13.19101
22.61176 10.96349 18.56333 8.540122 16.42414

23.12462	12.44523	19.39675	9.209983	17.68441
12.54674	17.98557	18.34658	10.6169 7.370547	6.688071
3.895082	8.524884	13.1819 7.86062 14.16896	10.15461
20.20261	17.91515	13.93079	7.255226	15.54211
18.35681	11.79192	18.86587	9.646443
PTEN	9.237669	9.630277	10.74779	10.25842	7.150765
10.69267	5.343373	10.02307	6.152679	8.67683 10.69911
9.949358	10.50998	9.518524	6.312443	11.21576
5.748001	9.618327	5.163867	6.029343	8.78703 10.61063
7.688395	11.75397	10.24507	10.91354	7.237241
11.38368	6.885333	6.168201	7.199593	10.47596
1.906041	10.08001	14.24181	11.82517	5.347353
4.104168	6.46821 3.618472	8.28786 7.304329	4.950702
10.43078	11.13008	8.245724	11.44173	7.942039
12.63314	6.85177 6.503288	12.43568	3.265126	12.75286
10.93061	11.9226 7.024042	15.23582	3.876225	6.703391
11.83853	13.21466	11.26249	7.931997	1.616688
10.94909	8.028504	10.34749	8.370009	7.432129
21.19297	12.04523	9.350213	10.92763	8.807326
8.559465	8.942276	7.158486	5.145423	4.960877
1.945209	10.3574 6.945794	9.40908 10.12272	10.47497
14.86187	11.6478 8.305196	11.16153	10.73757	10.72647
9.966549	12.08462	7.750552	7.880708	10.46377
5.629108	16.42335	12.51402	11.57518	8.807459
7.081475	7.267123	10.38939	14.02869	8.640905
8.200866	20.11098	6.601962	5.214071	7.785412	6.9481
10.8937 9.944986	5.509334	4.314206	7.855408	14.21819
8.882239	7.47624 6.516376	9.677972	10.96318	12.4459
15.27876	14.44522	7.169538	12.33585	10.40523
10.12062	12.69416	7.975833	12.09009	6.405649
13.57801	10.40532	6.409103	13.07716	5.536611
10.25858	15.89363	8.748509	10.75578	7.008123
8.135554	5.959451	10.60524	10.4929 12.38721	11.88194
10.23248	8.69478 13.31485	12.34065	7.076459	8.904291
11.91761	8.567495	6.806538	10.41789	11.37442
10.65075	7.492434	7.056803	12.97711	7.225931
11.41772	7.461854	13.44631	9.87096 10.00505	14.5559
8.050045	6.738896	8.561263	8.843396	7.821756
12.04242	8.352628	7.776014	8.660338	6.850278
11.93277	12.82518	13.79049	13.6378 7.844767	13.20968
14.27141	8.744747	2.277744	7.731487	6.469852
9.924932	11.53058	7.128834	8.369573	12.00764
7.886955	7.070493	10.68097	9.491078	9.150002
5.841014	15.1573 5.599315	8.395698	7.533904	10.16991

4.014048 10.24616 11.65391 17.03522 12.55699
7.932928 10.87113 1.762667 12.92881 8.516756
10.15026 16.44731 4.813474 9.947172 14.64605
8.335342 6.2232 7.780989 11.46362 9.864208 5.265115
9.871465 8.98416 4.724853 12.47485 6.821961 7.441963
10.97819 12.42558 13.02809 9.016172 16.54711
10.28435 10.14245 9.583237 8.722056 8.637234
10.95484 12.9215 11.12072 14.73785 11.5111 7.802518
14.25577 14.68751 7.73106 8.978428 6.615679 8.82962
9.128333 8.381419 6.599237 11.29337 12.35851
15.33381 9.496586 4.311048 7.742882 8.040803
13.78396 8.288795 2.964357 4.851191 8.265188
5.455419 7.038131 7.798325 11.07425 23.89663
10.42052 14.33779 11.05923 8.55254 11.00384 6.391917
15.94348 5.052294 6.447937 12.239 12.0813 4.67441
11.35274 11.01665 8.389221 8.344358 11.26582
9.615811 10.33791 12.88798 9.039107 12.31753
10.44254 9.977707 10.16748 9.125748 6.385123
5.073698 6.321335 8.867241 7.13066 5.375122 6.861088
6.530761 8.293998 9.785605 11.85373 6.832229
5.355749 8.400596 12.67931 9.30363 6.245288 7.352242
8.987417 13.61195 6.739362 4.943299 1.686208
11.25012 14.78201 3.970745 9.855913 7.771215
14.78098 8.022811 12.29813 4.876376 8.445223
11.33321 9.470878 1.319538 4.929017 6.811 8.072031
13.53282 9.12902 5.699562 5.466145 10.57012 3.031828
6.99159 5.848584 9.46218 8.780239 12.83936 9.335856
13.57428 8.924636 9.360383 5.506619 11.80626
9.445235 18.3028 6.02035 6.222336 7.710932 26.50833
8.691374 11.33799 7.792087 7.928175 5.321638
10.34265 8.130044 1.94101 12.174 2.789717 5.862521
9.173536 14.95064 10.37822 10.06305 4.878625
8.858333 5.7863 6.842794 7.711886 5.793052 10.35443
8.052713 10.43557 11.98432 4.975331 9.65988 12.32967
5.083011 10.43724 10.01056 6.177653 7.528418
5.915737 8.376957 10.1835 13.95068 2.543535 7.229929
11.24078
TLE1 3.174618 25.14612 4.535473 15.72589 6.106231
13.34902 7.732647 12.12049 11.54859 10.20706
8.070858 3.46176 8.853635 6.172346 7.608835 11.8539
9.469351 20.38193 5.166678 3.409136 16.20896
12.97987 5.926232 16.25891 9.999378 4.27137 8.409591
16.25335 6.604048 5.268237 12.56148 12.62236
6.563271 5.605531 9.585796 15.22327 22.54416

17.88152	12.04934	25.76236	7.657507	22.78524	
13.22003	14.36215	9.450303	8.762079	12.81164	
17.88899	6.486066	9.912859	5.172337	8.56926	16.39032
4.524048	11.58266	17.29589	3.808952	14.17407	
0.9739721	12.45778	9.750249	8.658814	5.09366	5.74558
4.56434	11.4342	8.59468	8.557138	9.780021	20.47408
0.8725909	13.10574	19.63366	20.88404	14.42577	
16.70468	10.02704	19.18107	7.450298	9.355632	
21.97657	9.72073	11.84144	8.547582	9.198938	15.03343
16.78948	22.97074	10.48994	7.495621	5.887338	
7.820289	9.294407	8.192138	8.320515	10.56765	
10.44306	19.19976	9.699674	14.60932	8.290533	
12.38263	22.2033	4.355061	7.93271	12.86718	12.15836
9.883496	25.38633	25.51617	9.973198	4.995812	
8.990913	9.287961	6.083083	12.47546	10.23537	
12.84048	12.60339	9.043493	5.298716	6.174339	
10.52299	7.070414	9.991671	9.128575	6.974778	
3.388668	14.01709	7.436149	18.61479	17.66005	
12.39042	11.74217	8.778582	6.918743	9.047585	
12.22181	11.14622	10.21951	1.993128	13.30241	
17.87943	5.627958	14.99863	11.19383	6.892782	
12.53771	25.67072	14.90066	6.727211	15.18788	
26.00574	18.71252	11.42878	8.991856	10.84114	
10.78257	13.18612	7.857037	7.931885	7.299345	
5.769186	3.787796	7.197996	13.84881	11.34247	
9.13647	14.23274	13.65732	9.089364	11.41756	9.807367
7.358948	6.707112	7.284933	12.93149	10.40526	
11.94265	7.765154	10.03739	13.78508	9.146742	
14.38265	21.274	17.1645	13.38765	8.013627	15.8969
12.48162	12.0608	8.769144	10.23826	11.96126	17.02852
13.08779	7.819281	7.669206	11.79268	13.79211	
10.22512	7.665662	30.02249	4.634374	4.499331	
13.53086	17.52077	5.75082	10.25837	8.581649	4.39019
15.49558	10.73955	16.63798	9.205105	21.7566	7.222603
21.54832	15.68292	7.7861	8.588944	13.4277	10.43183
13.17824	12.16535	3.580701	10.72026	5.041258	
32.44571	6.182391	6.100794	8.323416	18.23519	
10.04214	14.18813	11.30009	12.27266	10.74133	
6.356763	15.54192	13.13704	20.167	16.93015	6.385164
9.175105	11.54683	12.04572	16.43174	14.6998	7.200509
7.017285	6.723766	9.02822	8.326852	13.24277	16.47057
14.85646	13.19356	7.534724	12.43775	9.355279	
12.39362	6.066489	13.03822	10.25195	10.60912	
19.06193	8.431166	11.94576	11.17604	10.69664	

10.14525	11.65571	5.729837	11.92909	16.20707
20.88153	11.37267	1.15571	6.098168	10.7553	8.418748
10.41309	9.038322	9.762117	11.534	24.46777	11.47931
11.06687	16.66622	9.907147	9.136673	10.36723
14.02068	4.896425	13.91448	8.202176	11.87416
12.76135	24.21368	13.14801	17.8356	11.6746	9.948472
19.78836	14.39311	9.006213	9.671255	11.87275
23.44687	11.04582	8.959377	12.89417	5.248252
20.79103	11.60796	9.386537	20.62459	10.67953
7.035091	9.187859	3.411767	19.56241	11.37574
5.231908	9.801261	17.17741	11.94316	10.85269
24.57372	8.405742	15.21156	6.499877	6.698894
10.27975	7.855155	7.063483	19.47974	13.1412	12.82637
5.933547	13.73284	7.123633	25.38656	12.66541
5.657238	15.19533	4.446051	7.144665	5.981497
17.93572	17.51722	9.856071	6.499601	18.83474
15.77895	14.93264	16.98538	8.377597	10.18446
13.14429	11.09065	13.71374	16.30226	6.307405
10.60438	17.25062	6.602626	16.23812	7.292189
7.55547	7.260037	8.076855	12.52791	21.80821	10.98816
10.6919	6.942129	9.400638	12.03909	6.615421	15.09149
4.747569	2.172319	10.08802	14.33745	10.19112
7.989052	17.71978	21.10775	11.85825	11.89184
7.162421	8.955335	5.263682	16.81841	19.06035
14.69614	14.20505	13.30431	10.82598	6.633047
13.17034	22.64921	8.51915	15.90191	14.31103
PDK4	39.65711	449.5363	36.81527	8.854181	51.68383
121.0072	15.4684	82.41478	39.02238	74.05172	65.02394
6.234223	16.9945	21.51655	3.868152	166.545	12.73012
234.4983	47.0525	29.52631	59.95553	337.8746	9.730375
365.9345	155.0759	29.55654	6.631046	349.0792
51.16267	17.20197	37.78629	214.1666	9.868718
3.949045	9.262913	6.490973	63.49199	10.97262
9.559744	9.488842	33.89442	4.988118	5.347347
1.314499	27.04115	1.592111	13.02969	5.999078
10.09907	7.308047	2.18228	27.62119	2.769636	9.871302
25.70705	0.3833671	1.151798	188.4715	0.4835586
23.19196	16.35968	2.078247	50.84153	39.73568
3.278411	36.40366	12.7919	13.06248	10.90828	8.684808
0.5656451	3.224974	46.96501	19.98047	5.061109
11.42538	9.00863	34.53171	3.265505	7.498735	0.2012441
48.47096	8.964086	12.69785	6.549446	44.21857
39.19698	3.996742	3.491379	34.04419	2.729486
4.017047	4.556319	44.20796	1.18418	1.35977	7.541638

16.2703 0.47485 9.531903 6.81077 36.45019 1.059306
4.63161 19.76526 20.57048 3.752096 12.89109 1.862188
1.581586 5.593741 2.596948 4.000268 58.99745
12.06943 24.09354 2.694342 1.46966 26.87144 5.001477
6.640815 3.109436 4.307764 26.45385 0.8375013
12.46432 2.314265 1.380445 189.2955 17.54747
2.507593 0.5380579 26.14409 4.143426 24.37215
17.93194 3.767498 8.065619 5.91947 18.95378 10.6154
1.909298 10.97208 3.564407 3.334908 10.13225
5.909663 10.2278 1.745362 29.08734 2.950446 4.417767
2.852125 10.54733 43.10564 67.49545 28.42276
16.16376 1.011559 3.356027 10.17638 7.587011
23.7313 4.936952 5.434053 7.059287 45.47549 24.93097
4.474794 26.07573 379.8717 28.55481 32.22304
1.184306 4.861009 6.849201 13.52886 7.638928
12.13651 5.271633 2.2764 8.039674 10.12808 12.53522
17.31603 1.616759 16.65303 1.174487 10.47057
0.5781559 22.04887 13.24347 0.7005965 7.746147
0.7131578 14.50966 7.326385 6.815363 0.3832915
6.431585 2.66389 54.69849 10.81094 0.2257109 1.15577
31.05436 3.486577 159.7043 7.625717 3.409236 11.498
23.92342 20.84762 5.517426 13.64337 7.181299
3.151593 3.84334 45.65523 2.186552 19.36301 9.886753
12.999 1.837867 23.32492 1.02134 5.049028 1.594727
4.942386 9.812333 2.807448 19.96792 4.335929
10.68145 1.799221 20.79137 10.12822 5.731085
8.578332 4.904302 36.0272 9.793698 18.50367 5.752746
10.43709 9.611721 4.972907 202.2968 37.79117
2.173213 14.65267 18.13648 9.472075 42.82174
2.63882 6.571847 6.969701 16.48295 0.4497483 30.30546
7.62912 2.706309 46.83297 17.14384 15.91885 8.906021
5.072668 3.815873 10.39798 11.95696 23.9933 5.46553
27.27765 0.8521276 9.634125 2.676318 7.772239
134.8323 1.613164 6.260059 14.49136 11.37935
4.083023 4.529916 3.054175 85.74409 22.58375
4.890918 26.46102 43.15023 1.032494 81.61536
129.7682 7.998423 22.268 31.30754 13.11802 4.258457
6.285995 14.38517 7.715451 21.90219 1.590125
17.41772 7.586066 2.118194 182.9678 2.380775
9.971239 1.841045 4.663884 2.163723 0.5679983
6.232386 28.17512 180.5213 19.42685 17.42969
4.386339 13.17496 3.242096 10.16378 5.413296
1.552867 26.36321 9.059535 2.639885 1.565875
5.840699 1.514738 3.961272 13.7572 16.87276 9.001467

108.3636 22.03466 0.886885 12.97511 1.263925
13.4508 23.97189 1.194275 199.3784 3.088226 132.1507
16.07739 23.42234 41.50357 13.62402 1.450324
2.922527 2.941476 29.36161 15.10459 2.007633
4.678099 18.81617 4.114247 23.27863 1.698917
9.136996 12.7248 1.403511 16.29149 1.093207 1.370024
3.261922 7.832271 63.21412 2.571736 2.348479
6.725572 1.305498 20.77702 5.265887 4.542352
2.368824 3.832191 14.50472 57.24438 27.101 22.18014
3.923132 20.0956 7.223102 0.6081279 28.06963 8.483019
0.08868024 1.47106 36.53359 1.624052 2.526291 6.921654
32.50645 9.070798 20.56026 15.4223 47.19477 4.090657
1.754677 28.99308 9.127033 1.76509 0.5972812 5.659314
107.8018
ERBB2 35.47623 5.705939 28.05776 20.36005 16.41637
5.787934 9.839428 8.364533 23.44674 6.313915
34.50598 25.16122 6.799685 34.27587 11.8831 3.537431
21.67753 16.47692 12.8689 23.25228 29.6628 3.719307
27.72751 4.614553 9.493706 29.76275 18.69923
3.948096 29.18021 6.551197 30.69433 6.585414
34.4577 29.92179 21.39826 5.918605 0.7851108 192.7475
2042.134 26.28949 34.82716 683.9207 10.12712
36.24588 26.28159 1260.676 19.48016 81.49931
16.63812 56.22012 1122.338 7.997901 6.973996
18.08637 9.73244 22.54108 11.11134 7.994408 16.62596
40.45382 20.39267 26.55366 11.05185 6.994152
23.87164 6.724783 36.96459 14.20873 15.85305
14.12774 28.92631 12.29923 22.0172 24.27356 14.08677
42.0163 15.63437 22.29373 26.874 12.98697 29.009
16.44527 9.194748 28.34995 40.23301 34.9709 29.9256
37.6501 9.422863 24.88529 15.08719 13.60865 8.356234
19.38951 16.70663 14.84395 18.44247 796.7975
46.15856 26.02978 24.22133 36.11281 4.008891
25.04432 8.652672 19.89711 20.62989 18.16656
31.97416 20.55513 1012.454 12.10529 7.863214
5.311911 20.5449 22.85985 21.45046 79.14734 60.66621
872.5147 54.23581 77.62242 38.66444 9.748066
16.70834 7.562863 13.63242 19.37649 13.51096
20.76528 46.08599 31.51244 30.25623 21.59142
14.08564 53.64653 29.38985 10.71118 22.7531 789.4557
14.75592 25.38465 19.03232 36.70733 19.27588
26.80027 11.24912 10.55102 48.6308 8.694467 17.84376
24.18337 10.13062 21.05494 12.55942 19.51017
24.03926 13.73774 18.97378 20.69904 21.51033

10.19754 16.64114 160.4017 17.79773 35.95852
41.51109 580.7217 15.06258 1.395081 5.970683
15.02741 27.35232 301.574 21.57167 10.71191 34.22585
17.58282 26.23777 60.66207 19.072 21.19472 32.35016
20.02477 14.8391 22.88033 31.6014 15.73394 53.7206
296.4119 25.49006 41.64927 37.1071 23.53254 19.56057
32.47205 8.914814 30.32317 433.1967 277.1176
11.60307 14.15537 23.87459 32.9161 15.14149 30.26091
442.5864 2.331508 80.01964 25.72758 32.9411 12.96589
39.24295 18.99087 27.58405 27.15697 46.34599
56.54233 18.63465 167.4071 23.9759 9.257275 22.20915
27.27111 15.95367 13.99519 44.30833 40.23259
42.21345 35.01597 21.52736 11.20153 26.68317
1245.362 18.15943 20.42945 15.32945 20.385 13.42012
12.99031 24.68101 27.19855 15.99441 24.0578 13.05162
25.45887 25.75336 7.58668 51.96167 16.438 18.47997
25.55597 189.2423 7.86771 49.5452 19.48686 20.90147
18.98214 24.11545 15.18682 13.17062 25.04944
16.5109 15.41258 26.92295 23.87642 79.37486 31.46575
15.70595 42.40925 14.94696 25.34803 42.41386
25.5432 692.9481 40.71417 29.10686 5.392383 0.9387364
15.58457 15.37197 8.96732 15.71654 18.19697 20.73568
26.8778 18.40135 1127.054 18.9907 18.96298 25.40466
4.766111 6.292416 39.26422 9.905246 11.57473
12.98802 20.6936 42.60006 14.18749 21.52144 14.77128
1125.43 7.813889 27.12101 1167.521 16.77827 220.9911
19.06124 16.92794 32.43813 35.43894 11.64857
11.40727 28.41634 15.57048 50.43157 26.04403
20.59155 22.05936 20.39107 25.24559 75.66798
13.19152 6.340402 19.68178 26.05303 24.92592
24.04534 54.95092 23.6681 37.36981 17.32268 23.24367
14.96931 42.90395 720.698 20.7118 32.92399 23.93953
28.75807 10.97535 19.89077 126.0988 10.48619
52.53652 37.75245 1.29863 15.9091 14.3046 22.49278
17.55429 23.51022 5.717812 25.29634 36.19032
21.67403 1166.309 8.210928 25.2641 35.49806 10.90888
18.29326 22.39325 16.15506 11.88773 37.05976
17.56015 66.02848 14.79655 23.5104 21.99818 11.20187
32.29387 10.00965 81.01796 23.27291 6.285157
27.05623 15.97363 14.10779 127.3036 22.98863
25.16473 156.5905 26.66317 19.51023 16.3352 49.04974
24.24757 16.88692 17.42926 21.51153 23.45868
20.1403 11.84762 37.12655 73.0792 15.68466 21.73471
244.4732 4.254717 18.40978 39.1113 19.33889 22.32578

11.76604
ANGPTL4 12.68474 4.326764 4.119035 0.8778979 4.568393
9.016682 1.700298 4.581085 3.054485 2.537534
5.165292 8.809161 2.494888 37.06238 2.328507
11.91271 4.133479 8.188855 11.57864 11.08932
3.741549 3.066412 26.88332 9.545417 9.43352 19.99156
1.52884 8.382334 6.866238 1.062588 4.466318 14.56058
4.452464 5.198969 1.110359 1.231862 19.58246
5.62011 6.570083 1.248461 4.86888 1.532573 5.982926
8.229859 2.947534 2.610186 1.491644 2.007086
2.849014 2.73036 9.203453 1.203959 1.507159 2.983726
5.523932 1.921299 11.26756 6.054632 4.325795
2.277543 4.279858 6.242609 4.354959 36.19411
0.3383257 11.92731 3.713992 1.242394 13.80317
0.7564816 11.42362 2.647374 4.620889 8.782176
9.251248 3.641724 6.135447 2.453898 2.609478
1.473677 2.748052 10.16024 1.404496 2.618279
6.807031 2.023356 1.541883 1.446701 2.125613
1.769479 1.844562 3.53602 2.225196 3.987679 3.375561
2.00332 3.205821 4.388664 1.056143 1.020523 1.033369
1.140436 0.998062 1.762395 6.571606 4.013753
5.091941 1.537524 3.230035 0.7959894 1.024484
0.3196059 5.701333 1.432573 19.01038 15.58756
3.34682 10.50388 7.2086 1.935151 5.797134 0.4786037
3.522421 28.64673 2.396991 0.698361 1.274254
8.508802 7.845692 3.396969 12.24659 3.48541 5.717812
2.583472 15.74245 1.051985 4.270495 12.64939
0.8181467 2.020021 0.5821795 1.17725 0.6648363 0.7950403
3.729327 0.4781204 1.375367 1.916735 0.4488122
1.069255 0.925071 4.913306 0.7585119 1.134415
2.703502 6.83741 8.316336 1.136702 63.37608 2.68307
1.173455 0.9700661 0.7183342 0.4366574 2.769118
1.68677 2.978735 1.214539 1.347254 1.088179 10.62613
1.522973 1.361485 8.613192 1.227883 4.558723
2.638752 1.719567 1.188195 1.903366 1.342305
0.9636425 23.56659 1.668777 26.40491 2.625415
1.190195 11.99879 1.163432 0.4858402 10.42143
3.213767 3.417374 5.458834 0.7247179 1.808321
4.442665 2.157293 0.6083928 3.411157 4.29625 2.302327
5.784561 2.274582 3.355837 12.73964 3.329196
21.91871 2.336405 2.58726 2.695431 3.011266 2.162477
1.601103 25.77874 6.289138 4.641875 2.346446
7.567401 3.186074 7.423419 5.282403 3.545193
15.01607 1.45473 0.5645104 2.81647 21.44253 3.513757

5.937585 1.690641 6.609521 1.725483 4.595243
4.271943 3.965765 4.418308 4.993932 2.082046
1.536032 1.569511 1.079274 4.572187 2.901407
4.506472 1.75395 1.764062 7.16494 2.839941 0.9891973
0.6103901 0.7992741 5.908517 26.43435 0.3721519
3.096849 1.157581 10.62447 1.428885 11.46727
2.198868 5.640685 3.207127 2.503404 7.130173
1.585019 7.588403 4.120159 2.464279 4.5937 1.323459
25.54206 5.996688 0.8827366 3.426697 0.8705594
3.022433 1.920932 1.022965 1.291233 2.218031
0.2927389 2.484715 2.508281 5.116587 1.737135
1.056804 1.239804 7.851161 13.91158 3.896718
1.735006 5.206206 5.642524 27.932 20.79652 5.667985
1.00044 68.83589 1.738421 1.525333 1.091208 0.963388
57.96904 1.170761 3.841932 1.172016 2.881682
0.4881684 0.5901421 5.096771 25.32332 2.835599
5.924661 0.6630853 3.798915 1.486862 3.158925
7.914568 2.224225 2.175579 2.802349 1.129223
1.172008 1.998485 3.004781 15.99811 1.252597
3.57131 1.758427 7.900907 8.579345 1.711506 4.778887
3.725678 3.729107 1.066895 1.957665 0.4391679
2.200686 1.015591 1.336284 2.11091 3.628643 3.223523
6.788017 1.236244 1.944413 3.29962 1.131693 0.386987
2.269514 2.840163 15.01644 0.4507897 2.125213
4.595422 1.442494 1.530815 2.694691 2.725163
10.76638 5.456568 4.124 6.911761 0.966305 0.4031872
2.111082 4.325535 4.504366 0.5438641 8.613106
0.7459225 1.676001 2.181943 2.720398 3.794951
6.759264 1.498842 1.596669 3.211897 4.091893
1.927112 11.77465 1.69061 1.377691 7.776746 5.177229
2.888997 1.848454 3.240847 2.230164 3.15521 1.255267
18.28364 3.367765 0.6811943 1.65875 7.02236 1.755454
2.480722 2.802782 2.941719 0.5081326 1.486956
3.074634 1.528684
CYCS 41.37902 25.52547 49.02425 38.13894 41.52117
20.96285 34.36888 24.97187 26.3097 41.37179 18.97941
29.81796 42.10824 17.99854 22.9897 28.75743 33.18159
23.42432 52.17467 43.66631 29.19681 26.26349
24.90693 20.77109 20.3055 17.17198 27.02215 25.6053
30.19234 53.91407 17.69589 34.47918 40.9302 26.1388
37.02038 126.8877 54.37583 34.98306 27.69891
66.86699 33.21819 79.21032 52.29359 65.36301
26.55464 56.87803 34.01921 36.75095 36.8458 50.90724
66.05925 29.85379 74.05789 52.93491 17.79591

80.17638	45.37748	14.09157	60.59272	35.20336	
17.33113	54.1726	32.31944	32.66331	78.27362	23.66132
22.52376	18.85155	49.63091	80.82179	40.68606	
63.18532	27.39335	69.85125	29.36352	32.99024	
53.66225	47.78571	28.73749	95.06524	51.30561	
28.66033	69.36875	26.59365	39.02613	47.94569	
37.1462	37.09945	67.728	30.37034	72.46586	52.53676
32.02881	40.38714	25.15742	49.626	28.49129	76.93622
17.75094	34.53778	16.72913	79.05209	101.2753	
68.23137	54.42273	16.48712	30.65064	21.50268	
43.85274	84.70331	40.51618	68.24005	48.03599	
22.22607	33.99899	43.36812	31.16976	31.40581	
57.17069	86.74669	115.2975	42.74543	29.31217	
28.07457	37.39713	25.48409	78.39018	54.55454	
14.32709	30.63819	26.73472	41.61711	66.33577	
61.61013	44.8168	26.21676	50.95793	80.87265	50.7338
118.8205	60.45262	58.38808	36.52617	70.79714	
31.34547	41.27994	79.40921	31.27467	21.95964	
58.36301	41.11565	25.48268	80.02812	16.43145	
23.44748	65.59913	37.58824	19.09431	45.14605	
62.15401	14.29281	29.77623	54.98659	43.71918	
53.93187	43.15711	37.59054	44.25211	31.66607	
25.94886	23.26883	51.60704	48.91302	45.5235	119.7799
61.20387	28.13732	47.49914	35.59265	16.13297	
57.16727	35.05545	28.42675	42.86336	25.92997	
21.00011	21.93562	40.04258	21.74323	68.35131	
35.28196	34.01752	23.56684	50.7962	32.26387	34.86601
40.19237	36.3383	36.64685	85.25715	33.61513	43.43958
46.98351	66.36139	117.9187	42.38197	45.36684	
81.65756	37.30806	39.37535	31.6424	30.87028	44.45679
47.89565	26.53389	56.8806	32.84041	29.85483	32.08393
32.32178	43.59334	37.57474	23.08864	25.6584	19.34588
81.62601	34.916	24.24607	65.09309	35.92524	39.97606
29.32421	36.51349	28.83037	39.69598	19.40601	
31.88729	89.89337	66.59252	55.47327	43.90631	
27.40205	46.91131	54.01372	49.94277	32.8959	46.36112
17.738	46.22436	93.13038	32.10704	57.08648	99.25585
29.4118	60.1493	17.48858	48.89477	55.23215	51.33266
21.64079	52.90431	49.5099	48.4378	68.43489	70.58638
48.55126	55.52545	41.05134	27.58134	35.55423	
80.61274	39.14529	55.22322	61.83127	78.05211	
34.40088	33.36543	21.1805	27.86119	50.47007	18.46475
96.12182	23.77686	52.74811	87.32489	20.71942	
57.09314	47.62788	47.76659	41.02138	38.09717	

19.57706 22.01256 58.55977 18.9913 17.7308 46.26113
33.38333 58.8169 84.06494 46.74251 21.83291 52.94279
17.48858 95.31689 71.97929 51.26798 37.9578 48.72404
88.30681 48.3777 30.14763 40.01573 16.67865 69.59652
20.35414 37.51335 53.82707 41.06403 24.42778
43.37622 43.27936 36.72904 63.30171 34.46333
33.05631 38.78823 36.34494 42.24381 15.67954
48.78161 32.89542 46.72757 58.86929 14.1455 29.56581
78.97098 53.78018 27.84099 39.53748 31.67587
34.24391 112.8025 43.47615 17.61447 19.02106
45.66049 65.09855 39.71987 52.40725 44.39384
25.69473 66.66435 88.06249 35.37947 43.34041
27.26152 44.78703 22.19401 107.5472 39.9115 17.63428
28.04292 73.2617 75.9 98.54035 63.31978 32.21645
51.58331 66.74086 39.51522 31.83055 74.51856
65.49754 44.18856 52.20826 44.89287 244.3736
55.86388 38.15461 31.80151 32.68182 33.43528
44.16344 22.72391 36.01153 17.287 24.26029 49.74805
24.60563 17.96683 41.82079 76.9628 46.53482 24.67378
22.99654 52.0077 37.4626 19.11285 84.00947 26.0632
42.86209 51.64842 63.78926 92.67378 43.37554
40.30814

BRAF 1.507449 2.836084 2.11532 2.460178 1.99196 3.769123
1.80359 2.717532 2.14789 2.155222 4.17264 3.148587
2.319243 2.687639 2.039998 5.310205 1.655662
3.04881 1.468988 1.533568 2.140958 3.178221 2.370054
3.905099 3.964942 4.044239 2.016156 4.021942
2.020483 0.9559737 2.179425 3.1479 3.523597 3.531059
2.065902 1.534141 3.641083 1.386587 2.45881 2.121875
3.144513 3.639914 3.378371 2.641825 3.191038
2.029971 4.12372 3.023959 5.720692 3.129063 1.576986
3.916795 5.773624 3.017955 3.615957 2.353825
2.361302 4.865515 2.064503 2.0759 4.050585 2.565447
3.41534 2.006391 2.35183 3.309136 1.890722 2.458637
3.654909 1.992669 4.8845 2.700493 3.572291 3.001777
3.502764 2.192153 2.852635 2.974819 5.191914
4.404018 3.127294 3.727505 3.470786 3.150481
2.580068 2.837822 4.513515 3.097766 2.076207
3.132251 3.358531 3.172619 2.376999 3.777406
1.942942 3.16154 3.127276 2.735488 7.038183 3.301377
4.633426 4.081848 1.528652 2.232984 2.865423
4.222237 2.465393 2.219798 3.241624 2.552687
2.90656 4.077346 2.321238 3.761855 3.063476 1.878046
2.093858 3.244593 4.084292 1.845027 2.786833

4.561064 4.537622 2.019087 3.92462 5.116882 2.345356
1.802957 4.344518 3.319003 2.652127 2.897014
5.238112 4.283303 1.817972 5.733777 2.167413
4.255901 3.666138 3.101945 3.357179 3.112673
2.693804 3.987784 3.644877 2.734747 1.902353
2.589554 4.995797 2.333876 3.77458 2.601805 1.556236
4.160622 3.968677 1.823901 2.967817 3.109139
3.432672 1.873164 2.898718 4.100983 5.06273 3.632731
1.308749 3.758856 2.078717 2.815175 3.734235
3.201648 3.934336 3.230745 2.075753 2.54949 1.908424
2.13575 3.797956 2.148576 4.005868 4.118762 1.680836
2.685879 3.039346 3.477799 2.150242 2.538761
3.923225 2.446763 6.018653 2.713078 3.135288
2.845203 4.962429 2.015698 2.755792 7.353152
3.485567 3.797181 3.437204 1.132763 3.325239
2.236479 4.569393 2.322932 3.169982 4.272525
1.445382 5.74405 3.688387 4.054714 4.441778 3.391899
3.759858 3.150757 4.444724 3.551364 2.282783
3.479505 3.385789 3.292573 3.162159 4.189167
1.771805 3.637672 5.694906 1.741156 4.24451 1.949184
3.304396 1.419758 2.594498 2.543983 2.285656
2.887763 5.154365 3.027015 1.923435 3.655946
5.000582 4.364562 4.994105 3.960799 2.539213
2.452463 2.666156 2.567441 2.90607 2.852123 3.691034
1.929171 6.114912 6.618031 1.404987 3.204812
4.559314 4.742271 4.305932 2.504186 2.119643
6.578595 1.914606 1.895721 3.739598 3.150593
6.314158 4.134928 10.50878 1.632333 2.984568
3.356031 4.209645 1.422727 2.787686 2.704311
2.227206 3.292882 2.842994 3.539012 3.679579
4.449358 3.749986 2.099936 1.742244 2.996242
5.512655 4.08314 2.694691 3.653913 3.151436 3.928878
3.746235 3.326163 4.184163 2.583888 2.981279
2.787673 2.375117 4.403517 4.964998 3.588228
3.273868 3.72857 4.127369 3.917734 2.862054 1.582199
5.103488 4.840816 5.127419 3.794006 3.03204 2.130484
2.738711 1.689453 4.023276 3.288332 3.104749
2.27011 2.922277 6.077803 3.024307 4.262778 1.504731
2.403736 3.552322 4.667636 2.268848 4.779345
2.016692 5.221931 2.855823 3.916452 3.387329
2.881209 1.92563 5.02474 2.781379 2.165141 4.7437
2.878701 4.095016 2.028693 2.442747 4.156942
4.26447 3.597821 3.421603 4.978372 2.452349 1.578519
3.981527 5.598995 2.655617 2.606671 2.490886

2.66788 4.293763 2.424526 3.33891 2.0099 4.055162
2.728013 2.602473 4.957066 2.386647 2.782084
4.130223 2.650976 3.505219 1.424035 2.671161
1.728488 3.997133 4.391663 2.478871 4.135968
1.758787 2.154977 1.85713 3.967187 3.003371 3.569758
2.955123 3.886784 4.571194 1.748174 1.928611
1.850709 5.08654 4.023017 4.240754 3.860618 2.513213
4.737987 4.34476 2.64265 4.434173 2.962329 2.815668
2.210835 2.992275 3.628013 3.637372 4.066035
2.308432 2.201791 3.936385
ANXA5 37.11645 88.28297 34.41423 52.78814 66.27142
54.92629 70.61287 49.30433 62.21759 100.3432
28.58808 24.50754 100.9764 32.16461 59.92511
75.5929 45.5826 82.73145 58.42276 42.64848 60.71277
77.70675 62.49202 60.00644 106.3813 32.14784
63.39084 56.70594 59.87157 74.05034 46.74062
71.37862 75.22833 144.1522 114.997 39.38581 68.40869
294.5 83.48804 49.12075 98.70606 61.38257 175.9892
52.18561 65.75999 96.14461 45.52283 82.25046
160.3287 64.65211 127.1969 100.6464 63.93869
95.64765 137.799 128.9005 68.98054 62.81408 51.01684
79.14258 196.5776 107.5571 160.4374 96.47588
90.60969 133.7692 161.6101 144.3176 175.6371
86.89975 77.47335 122.0332 118.9428 334.5976
88.83102 55.99774 84.40394 153.6023 92.94889
49.13709 79.22389 93.93433 139.7018 71.24942
78.31815 67.14579 46.63075 139.4507 122.1811
155.9836 107.0905 67.93261 167.652 77.79079 126.5236
97.63573 66.89154 71.96534 12.32493 98.06101
81.92798 117.3003 150.4193 139.2005 157.0844
78.67755 148.819 107.4129 162.9588 125.6564 79.28228
114.7606 229.8407 77.98638 163.6911 143.6968
84.32858 91.55436 90.45139 83.51716 96.00788
127.9934 85.40975 194.4751 67.73345 61.17118
79.83698 109.3 75.61307 86.70975 55.50336 56.50226
104.0749 217.6119 87.12922 49.66392 68.39484
259.5247 169.4369 39.16995 89.40619 44.14964
51.38895 69.56676 86.69755 99.24299 81.84273
147.3805 29.57084 110.4015 28.43402 155.3652
35.08267 53.41124 66.66741 71.52667 97.35961
151.7766 108.6551 105.5963 138.0681 68.07279
124.2961 72.75816 179.0952 82.57246 157.8872
175.0247 88.5427 103.1156 52.28745 82.29467 56.35826
69.33075 110.9707 202.3734 100.7345 114.9988

54.18881	58.50376	74.96866	41.6987	43.53446	52.92462
173.4943	115.6591	68.03262	146.6532	51.60036
57.50567	129.4781	129.4446	109.0682	155.8444
130.4383	58.67405	157.716	57.79598	10.45664	71.23422
256.4736	65.35951	36.73034	36.30807	31.45749
56.69067	225.1956	81.58514	45.53425	43.54858
77.72116	73.17932	70.7394	44.88181	161.2366	94.24644
112.6698	75.35516	89.41257	74.52334	113.5961
132.8002	111.5492	149.9782	57.09777	82.47285
79.71705	45.49814	55.0064	182.4818	54.32788	137.4469
14.99618	117.2302	50.49259	129.6423	84.78782
85.14657	133.1124	51.21621	77.97711	45.97863
85.74187	112.3155	258.1201	124.2424	64.15868
80.57339	79.98111	89.97076	47.13568	79.08412
102.5743	96.25743	26.48657	155.96	75.23877	96.6139
73.27756	93.05579	98.72835	77.674	122.1608	89.81254
60.90657	78.43236	101.6882	86.23803	229.2597
58.03751	75.46831	73.12269	128.4428	63.91974
211.2437	33.36691	98.86059	83.48951	99.16404
224.9537	88.23326	62.15987	240.8856	81.48586
61.07327	56.94552	83.97655	76.59093	87.65666
68.38453	75.53632	69.02174	81.08266	51.66908
175.4923	106.866	132.2166	84.90051	72.81319	50.64929
110.4853	120.0147	32.34114	109.1033	51.5046	82.70933
115.3025	144.1862	67.0491	79.85062	115.6404	139.6872
40.71748	132.3303	51.82814	53.46602	108.994	149.6145
78.35985	185.8619	104.8782	90.60215	62.52071
55.26303	191.0746	54.81729	121.3917	86.33897
54.28349	137.9169	98.51513	57.19829	70.45528
43.76193	128.2357	30.21783	28.02823	106.7344
31.55319	233.1947	147.1952	92.79599	250.7651
121.1985	68.5989	60.57966	68.34444	55.4993	98.15901
103.8352	40.82115	82.23081	79.1926	184.3341	54.08149
128.3611	70.30055	87.51001	85.57031	80.43741
85.74819	94.90826	73.50184	69.21997	73.18316
99.52454	20.92601	69.18848	68.87762	123.6609
175.5367	97.93257	60.7433	66.41401	40.75082	91.63041
154.0432	119.6274	92.43567	68.19578	110.5477
96.70202	51.96728	76.79703	41.70909	38.2501	118.5446
85.15915	45.01741	94.96566	97.47061	386.5248
59.31634	149.3859	61.43478	207.783	46.88693	82.23179
46.29953	65.94072	103.7575	73.48692	82.2969	64.1867
65.93644	63.78393	67.56438
BIRC5	1.858444	0.1128893	4.123042	6.784696	3.446695

0.2159796 4.530699 0.3240565 4.115537 9.69542 2.507289
3.66614 5.098805 4.206192 3.799674 0.05078458 5.91016
0.5734753 2.727283 2.920001 4.276562 0.07206189
2.238334 0.191362 0.2373561 3.04129 2.664451 0.07227115
3.319051 6.057595 2.260021 0.1297199 6.45882 6.105104
5.326451 14.2088 0.06736624 6.998097 6.592813 17.00303
6.006221 30.22114 10.15916 10.54449 3.733311
25.35471 7.422378 10.02879 6.173295 10.77365
18.66202 3.697242 30.72774 7.186381 2.04194 23.64189
14.074 0.8783878 17.24868 11.34284 2.585544 13.05146
3.235338 13.46718 15.58073 1.447702 10.36664
2.52879 7.660148 13.20051 21.03102 9.025799 6.27255
6.81086 8.964437 10.95787 2.495526 7.464711 13.1896
11.10594 22.94413 3.207316 8.235993 2.725481
7.112211 11.90637 4.600307 8.922657 16.28171
1.75258 9.361884 13.93906 4.069798 5.838075 14.35578
6.820162 6.596638 6.921502 24.36545 12.8851 3.247063
8.537484 12.57417 4.970106 4.660522 1.809817
11.8831 4.939858 16.23218 10.89542 11.7621 8.048203
17.63053 2.594535 6.824633 11.76175 14.63678
5.533349 13.02296 11.307 9.478153 5.354825 27.98762
3.188698 18.19367 6.896463 24.09024 14.08236
2.275253 13.42234 8.576383 16.52336 6.246416
9.984909 5.30398 2.208559 12.53332 12.94916 14.00268
10.27378 15.73151 10.99938 8.360382 12.51011
17.03775 2.109666 12.08721 4.346622 12.75034
4.173879 7.288972 8.020099 6.974288 5.298742
1.550144 5.046245 8.44586 1.10929 12.39951 6.013961
5.843456 2.629646 4.720143 11.59551 11.58347
12.80899 14.42212 3.242532 5.974788 5.841828
0.6979272 15.37542 4.591369 20.57774 26.9987 8.559655
3.779786 14.00061 5.726266 22.89332 12.68186
4.673173 3.087761 8.419416 6.522567 5.152438
2.026308 10.99579 2.763845 10.79949 7.603089
7.628972 16.74043 10.91638 13.75191 3.862704
10.04811 17.96572 12.72426 2.841903 10.8288 4.340704
10.97651 16.66663 17.84087 11.24395 6.057292
20.81332 6.697885 5.353505 4.349951 6.654459
2.622888 6.821199 9.366976 12.84471 8.86832 46.53033
10.16971 8.386065 10.03235 9.286256 15.10196
9.51013 1.799881 10.22786 15.01282 4.132763 16.56896
17.00071 6.148344 3.321385 6.884503 5.083853
7.137599 1.919073 7.361808 7.499186 13.02525
10.30434 5.764412 6.479756 6.110845 8.975659

6.765537	7.322071	7.75477	0.7661069	6.280854	17.74275
7.059036	4.237352	11.36894	6.02721	16.33618	28.12779
9.254748	6.276618	15.24216	4.0961	9.602204	29.10215
4.819919	8.052962	10.56769	9.697885	16.65103
9.802383	4.686379	1.610465	10.22472	16.90721
5.246139	10.22253	10.53187	5.219032	9.93303	0.6370618
13.44127	7.301736	2.070786	7.907821	3.949576
8.850658	19.71716	9.972018	11.55617	8.507747
8.065434	4.870851	18.03808	0.3946774	0.7683708
7.582124	12.51167	7.397608	6.148323	10.3455	11.69377
11.21899	6.639402	7.296723	6.173526	3.466849
10.57039	13.57702	6.784201	10.43019	4.28255	14.16484
11.69325	27.35948	30.1681	4.903509	13.20171	3.724752
12.10839	10.10363	4.667255	4.044926	7.411874
8.211128	7.870861	17.5217	4.18255	5.577559	17.56275
25.36271	3.765246	9.451159	6.453311	2.559028
6.515413	4.749788	2.057198	5.085379	10.89191
8.606491	9.070599	6.538553	8.504275	14.39352
10.06195	25.46641	0.4673088	5.450744	5.653472
8.273963	10.21324	12.2453	5.587891	8.890384	6.923496
8.390097	9.926862	12.70302	5.394984	16.38759
1.714736	11.40277	8.399038	6.295179	7.122853
17.48137	16.52592	8.881765	8.348103	7.12192	9.152659
6.162813	11.21283	2.038094	12.73704	9.455071
20.42613	21.59801	8.996081	13.11687	6.349843
4.009447	8.386187	6.223783	8.199404	12.62973
6.897492	12.26242	4.500789	6.849155	16.66086
32.40669	3.820998	9.702172	10.19709	8.36504	6.095091
12.50516	7.428346	1.335468	1.08708	14.7029	49.88822
3.554873	12.75337	10.53815	14.47849	11.75063
5.504769
MTOR	3.156519	5.21591	5.849981	6.892927	4.212638
7.401121	3.263684	4.229879	5.641196	3.441088
10.26581	4.26775	4.013621	5.15023	4.979917	6.701992
5.304067	6.072932	2.540079	3.840675	6.018155
4.583507	4.451893	7.320567	6.857682	6.679063
3.725062	7.266919	6.31989	0.9004052	7.216066	3.769731
11.07211	5.79955	3.831712	2.91971	1.723968	3.843884
7.577285	8.389954	9.472035	9.49444	7.041208	11.86012
9.79068	7.024422	10.46067	5.964822	7.249617	9.268669
10.29354	6.479912	6.006198	7.334693	4.808086
7.386347	4.118869	8.99463	11.46304	9.974148	8.076552
8.378924	4.778323	6.040656	2.636316	5.889551
4.277546	3.18299	8.110992	7.311822	11.21669	4.259307

8.010932 18.99105 7.495684 5.144952 6.865439
11.20084 6.54923 8.524736 5.002734 7.096338 8.409737
6.146302 5.393283 8.189098 10.29867 10.04119
7.610071 3.953874 7.614333 13.04738 4.352434
7.628475 8.326232 7.014032 5.367522 8.500813
20.69444 10.24492 6.215508 8.238629 2.256747
2.746616 5.937708 9.33981 7.972636 6.467902 9.999113
3.822991 5.51622 5.559913 4.954609 4.23043 4.165101
8.64624 6.427814 13.53226 9.137002 6.782652 5.270125
9.941208 15.88737 2.917879 8.879007 7.919919
4.477064 2.728035 8.270607 6.021697 8.817742
7.40435 5.981968 9.60838 3.794939 9.349877 13.79987
10.78718 10.37147 8.801063 11.26332 12.242 6.388331
4.561656 10.9101 4.542139 4.712278 4.708573 10.63499
4.46872 10.8367 6.969175 8.696063 11.08159 7.689369
3.09425 7.439472 3.514702 8.331983 2.073657 5.626889
5.974724 12.3443 7.941685 2.597832 10.2285 6.584355
5.517111 12.21562 4.182716 7.161161 7.730089
4.508692 5.144396 11.74206 3.90931 8.337121 4.970106
10.97014 11.88724 9.657929 3.98587 6.361038 12.34726
6.20476 9.716956 6.206874 3.383563 14.7522 9.228739
9.831708 12.56998 8.415647 7.60961 2.201622 8.999572
7.154874 21.20134 8.894261 2.287643 4.018498
5.046269 8.794692 11.40427 8.306375 10.62791
3.513144 7.087682 10.39671 6.260649 8.217589
7.136812 8.049743 6.593892 9.835442 9.527019
7.571979 9.495289 6.604953 11.4445 8.648787 9.222255
4.424335 6.245978 9.505601 4.158185 9.097907
6.197767 6.653614 3.50912 5.096326 4.961508 8.931519
5.800873 6.025202 3.817667 3.301776 5.37208 6.898332
7.419019 7.228801 9.592319 6.387833 5.205323
5.782734 7.400711 8.505145 4.571367 18.0933 6.662388
13.85772 7.431132 6.581754 5.944114 10.78094
10.25919 8.030801 6.709183 9.483754 7.383559
5.038242 5.15318 9.276727 5.632303 12.54111 8.872218
14.42002 4.349512 3.86531 5.091975 8.252741 4.300852
4.487549 6.957543 9.367846 6.830164 6.789672
6.216859 8.358378 12.43904 7.509373 7.015419
3.566254 6.461597 8.503822 8.533813 14.85563 3.5407
5.652555 12.56307 13.50603 7.335274 7.2159 7.530318
10.19924 6.11444 3.614623 7.017569 3.492612 8.643001
7.888638 7.135554 3.577032 9.124265 5.203212
5.574012 10.91171 15.59227 16.97522 7.802078
5.926567 3.518343 8.230529 4.546688 8.658885

9.017719 17.22598 10.23851 10.55985 5.147092
6.847947 9.1442 6.79882 5.250968 2.339882 5.55569
6.860728 14.71849 6.527206 10.79952 3.199778
6.11997 9.80984 8.642138 5.03794 12.1073 6.380275 4.25358
10.47172 4.343165 13.74615 3.499467 6.305175
10.78311 8.77795 7.445927 7.593461 13.74369 3.871764
3.728706 8.110516 6.914235 6.346258 3.359659
9.260378 4.051554 11.51798 7.259539 5.722322
5.62824 7.572457 5.43669 8.798504 9.147266 4.012204
4.437827 7.204882 4.377948 8.29819 4.255675 5.975082
3.934256 7.035248 6.677322 8.60274 8.836841 3.058609
4.545906 6.119689 8.650458 8.170277 17.96281
5.788597 6.751181 6.886551 7.045205 5.70675 3.872432
9.262465 21.31679 9.716617 7.164881 5.490556
6.69284 10.6563 3.730244 15.14974 9.13942 4.892641
9.707071 4.604178 7.76716 8.450588 10.3373 4.629343
8.224751 6.959091
TIMP1 35.70401 139.3233 64.02743 74.60768 118.1863
146.3532 137.3074 70.64368 170.6024 193.0722
21.21133 15.31829 142.2155 14.13462 165.0173
125.0341 119.1966 114.6505 131.6376 98.10423
139.8828 108.9186 224.5692 140.9062 179.8552
48.99177 198.5304 97.09347 106.1268 117.4482
147.1499 124.1302 365.5375 404.7872 401.5745
238.814 60.34357 284.6084 286.3864 158.6549 178.014
64.56141 435.4416 75.68344 163.7133 260.7776
117.3388 291.105 279.0974 104.1878 1552.187 502.942
86.36821 370.7773 843.3108 163.3608 354.4606
233.5031 583.7225 165.2528 1093.416 416.6621
1430.585 1662.235 160.9725 650.1079 824.359 839.6895
118.9652 253.2472 152.9522 345.5184 196.9864
808.0465 515.4999 220.2126 209.3376 251.6996
237.2197 101.0382 315.9928 962.3963 152.0482
458.4048 710.5546 642.9806 429.1955 403.4132
593.3491 688.3807 260.3062 80.73378 698.1865
153.644 965.7357 474.2187 2579.166 127.5221 168.8014
595.5728 879.6941 212.801 517.3177 273.5675 829.1814
275.3872 386.9978 424.7909 218.8523 439.6818
229.7147 161.2671 1364.334 396.2221 627.9385
181.4757 294.401 240.4446 305.7797 99.93605 524.3612
333.6198 266.1006 1877.582 244.2569 157.8666
226.226 436.5396 409.5937 474.1708 449.4217 142.6086
205.6947 358.1542 382.2498 158.1414 274.1342
339.7639 436.3904 97.60592 198.8594 125.5614

278.1181	225.581	235.9131	505.3332	368.3025	500.1143
114.8708	563.0357	116.4695	530.3254	92.7204	389.9299
375.7784	216.6112	637.0627	406.5873	435.082	570.812
1119.444	223.2087	466.311	145.5657	792.2297	211.1532
479.497	365.3755	474.4603	403.102	91.03039	263.1837
334.6233	361.7282	147.2099	796.9683	377.4915	
518.9757	48.9414	214.3876	162.2618	280.5367	81.24669
207.9183	893.5286	217.7324	533.6031	392.8679	
141.0458	79.65349	702.9729	247.0284	293.6703	
174.9077	1373.445	219.8775	612.7568	150.767	336.0588
366.9523	163.4694	460.5771	94.48109	114.17	220.0909
131.3889	642.2417	309.9102	134.207	228.0166	179.5279
646.2721	136.4137	155.9344	351.6335	201.8322	
403.8871	86.01983	632.1461	129.8252	152.2845	
366.1847	203.414	524.132	385.3288	182.4256	136.4597
134.698	268.4422	929.0372	455.2545	1103.577	95.83099
289.4687	304.8806	453.1512	730.0877	177.7872	
821.8461	380.9669	189.806	598.673	968.7306	352.6109
592.6444	419.5829	501.9163	368.0772	274.5164	
359.9183	222.5072	208.9541	262.0025	716.5668	
215.0243	238.2565	297.7891	498.1506	137.0409	
558.214	194.1569	468.4519	325.2481	348.279	214.2819
179.9363	166.7592	325.805	776.0282	314.152	328.3381
376.6196	392.4728	149.4413	339.7218	162.5188	
300.6384	407.7427	371.6167	562.9169	786.0208	
392.233	782.0531	494.8279	231.6076	272.5304	133.6435
274.4443	454.7125	522.24	272.7303	234.2376	275.1011
154.0877	756.3812	919.5994	976.8677	724.2176	
329.2167	146.2102	257.8669	724.244	284.0286	1623.449
165.9874	240.9134	209.0039	382.2127	300.4773	
186.0651	382.299	684.5757	488.7821	1955.357	104.7604
203.8255	216.5851	303.7338	453.8605	566.6071	
310.4062	215.3913	204.3032	346.2056	1418.056	
185.1242	292.2	180.6578	263.8007	416.5395	300.9097
810.8821	606.2153	327.1646	663.2506	59.68381	
39.93157	394.3328	341.6087	300.2479	493.5756	
614.2775	450.4653	393.8419	194.6696	114.8385	
197.1899	102.9046	796.3258	981.2488	98.25596	
259.5993	134.8388	1000.224	198.4988	280.9634	
130.9659	187.294	478.347	290.7194	263.827	2620.308
212.5118	124.2232	210.6814	494.9353	11.53441	
209.8553	126.9045	547.7945	325.6295	383.0893	
376.6344	248.694	356.2803	404.0083	606.4095	1333.71
190.0147	664.5613	819.7153	187.3853	516.0101	

390.2085 252.2114 638.1369 1114.167 1095.544
251.056 113.9842 496.8266 325.4487 144.0261 792.4149
133.4225 379.1893 203.2701 310.7383 40.35052
430.3975 479.9053 390.1705 159.8221 181.2513
241.5598 493.4486 255.0697
ITGA2 7.293697 0.9853077 7.940672 4.251313 4.522057
1.579249 1.61191 1.582739 3.527499 2.114835 13.81119
10.37093 1.602565 11.11452 1.694843 1.113091
9.918043 2.374505 9.125954 5.311599 4.221762
0.6749887 8.127005 1.467915 4.058275 14.90349
2.171821 1.440733 4.305427 1.129457 2.901503
2.109066 6.923418 47.4764 8.535262 2.295043 1.31301
11.22093 9.41555 5.542907 18.3672 27.60507 23.81447
20.17834 11.56759 19.29573 9.27761 9.910701 24.52416
47.83618 9.999373 3.859231 1.7892 23.86153 9.339763
6.525395 15.80608 5.656161 17.87206 5.562796
11.88413 3.241298 7.180065 4.014923 6.025836
4.746956 8.403241 6.285682 41.65997 7.244239
12.95127 9.804573 26.09715 8.225911 41.64049
2.381733 30.69736 9.812439 20.15727 15.09941
5.068313 16.32161 25.77717 9.79266 29.86018 4.778559
17.88593 13.62972 14.25187 13.12449 45.81088
16.9096 47.42105 11.16825 20.84329 2.799624 55.48242
7.744114 20.01944 11.1928 7.579646 16.44968 1.612412
6.536908 10.93373 16.5514 28.53957 3.297085 13.34794
7.791707 10.1511 5.126381 19.44975 1.557096 15.8917
15.69691 2.95526 33.54259 5.280967 9.421392 20.10051
13.20366 3.852349 12.01345 3.490509 2.741819
6.735041 8.897654 6.545009 12.40321 22.80376
24.97626 6.769122 22.22972 7.896073 17.09455
15.18371 8.737222 13.45243 12.98051 16.66358
7.467243 27.63256 3.682067 10.10349 6.822407
4.065273 19.71853 11.58677 6.834017 9.495694
18.26305 10.94494 15.02503 5.695186 6.643951
5.996926 5.402313 18.83869 11.35635 5.041387
4.793086 8.324734 7.818174 7.632239 17.49668
11.0783 49.87065 5.398082 31.75475 1.933238 15.47608
4.366133 14.96923 5.16511 14.51827 15.39773 4.374716
8.447911 12.59301 4.540709 14.53611 11.28236
11.76796 7.002139 11.89862 15.18592 26.32256
13.48607 53.31514 12.43275 8.402175 10.84963
5.447498 6.266188 23.53727 9.50468 5.751044 22.73599
12.9236 4.684357 7.373243 15.67127 10.77475 15.37094
7.606112 2.565111 3.200827 22.89198 18.51698

6.510097 12.0823 8.940391 7.098341 27.07624 8.814439
4.859023 1.037468 4.989527 13.13164 15.11059
7.72215 2.766568 28.37966 8.655218 4.313687 7.352588
11.8867 4.600668 23.06384 16.39129 18.37067 7.103169
36.32997 15.49332 5.802812 1.952569 4.986039
11.54586 5.954376 5.308619 26.32486 6.85422 20.10171
12.073 11.59625 4.703453 1.807028 16.107 4.562869
15.08285 23.94793 11.13069 25.08683 28.74604
2.181341 16.19622 15.85577 18.91198 3.376528
11.27833 24.27486 16.19046 24.1044 13.98967 15.05732
10.23216 9.811072 33.0984 42.8891 11.86715 6.117643
10.62076 20.26188 11.93747 9.987908 21.69417
2.446283 0.8034498 21.20426 8.900208 12.67737
13.28994 20.1586 18.30492 11.42883 15.58539 5.955797
12.85401 10.81058 19.91169 2.383223 2.810403
17.97437 24.45279 7.783318 11.93708 28.03371
30.73516 16.88956 15.1312 9.388627 26.79121 13.09006
13.22811 6.411532 6.225994 16.47962 9.624383
12.79558 7.342015 17.24819 25.07886 2.858961
8.554223 5.909621 5.184308 8.452055 19.22646
23.84276 28.07686 18.60828 9.15576 1.368647 8.484529
4.93178 6.385556 4.711655 8.424554 54.66273 15.41318
14.12287 8.385753 8.134042 3.797893 5.453529
18.41779 4.218803 9.844584 59.43124 16.68146
7.500248 10.09105 12.96153 5.181071 15.97011
6.995286 1.667561 4.100005 3.865636 5.366478
19.49875 12.1524 8.180643 9.830692 25.6711 15.5864
12.29237 4.469243 2.179931 9.111423 3.787576
46.94744 2.944385 18.9699 4.75436 4.395793 18.33487
17.71014 4.285843 6.527994 14.48936 6.715997
11.76078 3.123083 3.439533 19.25778 11.31158
16.48448 6.581439 10.70902 13.08932 10.51633
5.049296 30.09774 13.02976 3.495336 18.84215
9.71829 6.76225 8.962776 41.1507 7.306381 17.88472
9.963825 15.18425 5.812225 9.560615 2.075944
9.56994 2.295936 5.513519 14.71166 11.67562 16.56523
9.558532 3.675531

BDNF 0.03754165 0.02153901 0.06388467 0.1201379 0.1181812
0.1596826 0.08120562 0.06748966 0.1477924 0.1011265
0.1530686 0.2581455 0.04637916 0.1038363 0.04284267
0.03059395 0.04118755 0.0777322 0.2201808 0.1563532
0.1250258 0.0635601 0.2841734 0.0426691 0.02314116
0.1180341 0.1099362 0.1217086 0.1342124 0.08944481
0.1166996 0.07571626 0.07428803 0.02955962 0.6158479

0.07406688	0.01367241	0.161845	0.1626231	0.06644751	
0.9292084	0.03971336	0.4630323	0.1870407	0.1758945	
0.1325962	0.163811	0.09886265	0.116589	0.1394283	
0.06067259	0.08677851	0.1268671	0.1580549	0.6148555	
0.1176832	0.1322907	0.05195426	0.09599076	0.09924534	
0.6031019	0.05631744	0.3832464	0.1559532	0.06974456	
0.1666254	0.2535619	0.2921606	0.3375823	0.2084897	
0.02565064	0.2707351	0.107699	0.1540244	0.3008974	
0.1357905	0.4765637	0.1390438	0.04688276	0.1893944	
0.04895683	0.3055113	0.06232969	0.05404375	0.2471494	
0.1254047	0.1100898	0.07886574	0.3601033	0.1100968	
0.02713299	0.2389368	0.02565601	0.1077498	1.860254	
0.02492928	0.1965058	0.1304539	0.04095609	0.0913264	
0.1175079	0.05411788	0.6444258	0.1958888	0.5868756	
0.7362935	0.2274606	0.05841715	0.1676505	0.0850838	
0.1477786	0.1823694	2.398146	0.05587126	0.6714346	
0.1639339	0.1134138	0.0817909	0.08320078	0.05105779	
0.03108008	0.2786778	0.3649819	0.1568205	0.9400594	
0.1128827	0.06867516	0.1380508	0.1217087	0.2223795	
0.04866676	0.07265943	0.1145963	0.3574804	0.5329312	
0.2320721	0.04463584	0.2728456	0.444147	0.2758537	
0.09845903	0.08696583	0.3986441	0.1581067	0.2498082	
0.3309277	0.07553749	1.129788	0.4449984	0.1375187	
0.03642999	0.6567779	0.03742052	0.03171372	0.1481474	
0.4774133	0.1308249	0.1822542	0.05800523	0.2323667	
0.1669274	0.1182077	0.2572237	0.1451466	0.1364342	
0.2354329	0.1946325	0.7017452	0.07669107	2.391799	
0.0976228	0.9184038	0.150203	0.1470779	0.1578889	
0.1591235	0.4757456	0.7163013	0.04695042	0.4843749	
0.4210001	0.1175387	0.1177191	0.1066762	0.1441829	
0.2852708	0.05410534	0.1219065	0.3343786	0.04219455	
0.2173174	0.1265953	0.6050036	0.2123551	0.02904958	
0.5190972	0.1175206	0.361741	0.07076955	1.784376	
0.09819987	0.07844347	0.213466	0.08581112	0.1029952	
0.1630023	0.4093895	0.1837713	0.06216927	0.07222106	
0.2905172	0.5845641	0.4077042	0.1138652	0.0828633	
0.05748064	0.2290028	0.1085535	0.2458116	0.1148178	
0.08057553	1.484157	0.09063225	0.02823787	0.07038282	
0.09279309	0.1240395	0.2316776	0.07786574	0.9942155	
0.1185933	0.545697	0.04651752	0.08441529	0.07648	0.1287474
0.1135625	0.03183725	0.08478138	0.05174779	0.03795824	
0.07711847	0.126952	0.1209519	0.09399017	0.1748619	
0.1093866	0.02630116	0.2516018	0.0963669	0.03037266	
0.0614284	0.3845322	0.1054915	0.1300657	0.05733673	

0.2811207 0.2081929 0.1533218 0.1178019 0.2700074
0.1314541 0.2780851 0.08144055 0.07311309 0.2826899
0.08777749 0.3001842 0.3304294 0.1901988 0.1471696
0.1504972 0.2357864 0.07205387 0.2952901 0.1199093
0.2333941 0.04807716 0.01207495 0.1768157 0.1125157
0.08594019 0.1205934 0.1141637 0.08902207 0.05461276
0.2841325 0.3660169 0.8716834 0.1450873 0.1014801
0.0894857 0.03864545 0.4271524 3.135239 0.1718122
0.1565624 0.02954856 0.03803979 0.2643142 0.3447211
0.5445495 0.0416177 0.539528 0.1172229 0.1302624
0.3352019 0.1327543 0.08568606 0.2813397 0.1331509
0.03069677 0.01248054 0.1707202 0.1594746 0.1516892
0.2589488 0.1218312 0.1455746 0.250853 0.6101284
0.2173754 0.6134779 0.08523981 0.3686862 0.2762622
0.1612381 0.6234548 0.2607417 0.2073837 0.2722048
0.325835 0.1705829 0.1486133 0.1225354 0.05386575
0.0388356 0.10491 0.1276848 0.05786473 0.02889021 0.1572044
0.6555742 0.03144874 0.03932527 0.1462117 0.09084822
0.5569713 0.06970979 0.1814511 0.09307187 0.4717023
0.1040412 0.2661525 0.1445556 0.03993141 0.3422157
1.206462 0.1408639 0.08278044 0.2542654 0.01872951
0.06390128 0.3395112 0.1145611 0.1134977 0.06772374
0.1905364 0.07238069 0.1902833 0.2185035 0.3569751
0.04462618 0.1245983 0.06721136 0.38253 0.1556137 0.2563716
0.292913 0.1700391 0.1227773 0.22541 0.1007561 0.0541802
0.09986625 0.02981383 0.2110322 0.2276517 0.04253026
2.196448 0.1099652 0.1645075 0.2430917 0.181934
0.2263917 0.2098588 0.1008184 0.09142279 0.06154637
0.1120384 0.1628763 0.01080256 0.06400149 0.05717326
0.1547902 0.2654477 0.0998292
CSPG4 0.6957613 130.1151 1.80712 2.332076 3.39297 39.14637
1.364725 9.428705 13.24136 4.400284 11.23298
0.9399736 1.615711 3.422734 2.068586 56.71941
2.842171 30.09226 2.702743 2.215901 8.853125
39.48767 4.945207 45.99637 45.21507 3.957671
2.499483 56.93609 5.361155 0.3539258 16.33367
39.05074 2.96892 1.4572 3.698195 1.713456 0.5707918
10.08799 13.15261 3.00269 6.90793 1.503228 9.598431
2.661158 4.975468 6.075181 1.588568 7.568896
10.55347 6.868633 3.578893 6.026984 6.014634
3.657598 10.1862 3.787475 4.299825 78.32321 5.412481
2.288012 4.472878 4.380958 8.533569 11.81463
1.791933 38.66466 13.26936 4.956043 6.960492
1.542568 5.349573 9.059187 23.74454 6.155863

11.60832 5.191702 6.825228 7.897554 17.22109
2.37775 2.328101 3.415248 1.710165 19.84089 9.545759
9.695976 14.73445 4.01302 2.305456 16.75714 1.859431
11.84109 5.367162 1.884791 3.653204 2.642647
3.118967 4.002756 2.035484 7.558596 3.302328
3.751279 1.304294 2.850601 11.08537 8.675016
5.508971 31.52715 3.468407 0.7733612 2.419855
1.324167 2.011446 20.53928 4.257875 4.264743
4.070859 6.810324 7.5933 1.76197 9.405484 2.017407
13.06543 2.596449 7.080491 2.796327 0.6684396
1.531538 39.61928 10.57269 5.760524 0.859957
3.520084 2.099707 4.697567 19.80234 7.916484
2.617255 6.82004 3.265394 5.796162 3.441607 2.833053
3.027996 4.446346 2.028162 1.788174 16.38585
3.382492 6.787819 4.601479 17.72011 0.846449
11.26735 41.25224 4.583961 10.63447 11.2328 6.971467
1.606605 11.00478 2.644892 3.708114 5.663009
6.535367 3.471099 1.632681 3.352924 3.914298
3.941934 63.79932 7.707163 4.940109 2.864103
2.769922 2.245158 4.602881 6.95277 3.205049 17.33312
1.72915 3.69755 1.835088 10.50522 5.613852 27.74963
18.21433 1.659855 8.569442 2.577352 12.61433
3.917099 2.845639 3.024902 1.636378 22.58413
5.757136 3.810905 3.10728 1.496118 1.359092 10.02084
14.37907 1.619528 1.399323 4.592813 7.604449
8.788882 4.679292 2.605083 2.064584 16.79814
2.402682 9.498387 2.790212 6.533585 3.079609
7.137645 14.60191 3.732022 4.091844 2.727039
4.494018 3.1449 1.613796 0.6178193 7.50775 0.9305279
2.05056 1.546507 8.743339 10.80736 0.9509172 4.716441
0.9870821 4.535924 2.037255 1.334653 1.872053
1.937952 9.23275 6.560966 10.72342 3.8265 4.669265
28.32993 3.294401 54.42709 9.674686 3.21655 8.593924
3.453042 6.155568 11.3616 2.169582 3.188409 7.974149
6.100273 2.751106 17.86517 3.238683 2.614209
2.949997 5.098125 17.04491 8.096669 2.375145
3.73882 6.439357 2.410015 10.14245 3.408022 2.546322
1.790755 2.319044 1.682808 3.579705 49.77461
2.532856 1.945493 12.73554 1.022115 23.3107 3.11855
2.388623 10.09252 2.311544 1.735181 10.3951 11.72384
4.108076 60.71816 35.84049 19.18784 8.34664 23.08764
4.020659 4.571753 1.934914 4.587084 5.952062
8.809415 3.715286 23.7994 0.9894489 4.921951 2.351964
2.340406 2.349267 5.351251 2.282788 6.602867

4.368031 4.019 4.354029 38.85499 10.77023 8.146423
8.432188 4.512916 1.41712 2.307813 1.843061 5.042768
2.306853 2.164126 4.239421 11.97314 2.133705
4.743008 0.4798549 13.29373 4.864524 3.753555
35.38571 6.645253 0.7804328 1.897382 4.864964
3.260333 43.67007 3.825611 2.75255 3.402109 53.74208
3.798425 2.933261 3.430775 1.499277 2.618467
6.00555 10.75492 3.682298 15.06003 2.039145 6.323055
11.29538 2.394273 27.09276 3.765435 6.391258
5.528776 4.251777 2.489876 6.160056 10.4723 7.363492
2.337753 9.163658 7.634491 2.955293 6.484231
3.351569 1.642536 3.983962 5.346378 2.115676
6.954573 5.265618 5.729396 6.054779 15.38831
4.076427 13.70173 4.769924 1.91055 26.3253 2.058088
3.319983 7.868909 17.20654 4.757126 1.07041 1.596718
2.338975 4.867571 5.341854 28.28587 7.916384
7.327468 5.005097 0.8431785 3.226787 2.144546
1.255834 7.014954 45.46228
BSG 151.9113 158.2175 141.5786 204.4792 141.0579
84.20016 224.9605 132.6888 170.917 162.2857 48.3533
47.04868 138.1026 78.23274 193.0811 78.76416
180.3223 150.5876 216.6756 195.4504 180.3894
85.82457 104.8372 81.452 117.7727 49.09372 222.0988
85.76431 152.6122 123.2521 165.8073 103.8802
272.1849 151.7522 212.7971 280.4553 81.86539
336.9987 112.1651 249.6968 135.2245 137.8905
409.3726 190.3252 117.5415 158.2435 135.4918
134.2974 94.43859 225.2514 343.7479 97.77942
315.385 96.18099 102.4065 462.5105 199.2023 78.31671
356.4404 94.20641 105.6515 247.7366 112.7058
168.103 166.3469 117.5733 190.3693 153.5661 116.2964
149.2022 181.1653 112.9267 201.8664 92.47106
139.5177 247.9418 150.903 209.8556 89.92732 73.68032
178.6089 99.57745 91.00019 109.7066 245.9524
91.06583 140.3382 144.3379 170.3714 114.2246
130.295 117.2203 169.639 120.5671 164.8759 206.4735
143.6116 210.4577 198.9538 142.8374 90.96073
126.8508 135.7364 312.9715 208.1272 110.1488
226.3845 152.7967 226.3032 208.7329 163.9522
132.6702 364.2828 76.82063 147.2743 157.5546
168.3882 249.4829 244.0689 239.4764 107.8036
80.55147 233.1549 215.5506 160.1736 101.032 259.6467
271.1808 84.17321 206.7177 184.4042 191.0634
229.4703 162.1471 243.6727 99.74603 207.591 163.0909

266.418 103.6789 105.2878 292.731 113.3556 206.0276
324.7885 117.7917 157.9848 113.5255 112.5312
149.2583 189.2769 183.5808 201.1442 132.9794
99.49321 128.8306 244.4152 100.8117 119.4547
174.7656 166.7317 91.05637 68.632 119.3592 484.8434
219.1709 104.8181 72.49761 111.4361 398.2855
85.57014 238.8575 141.5584 212.9414 179.6548
205.714 126.9567 177.4351 164.194 88.3158 133.9781
232.4316 91.39487 123.9994 238.8646 157.2053
118.5644 214.8957 68.53608 168.3921 127.848 98.13638
135.5714 200.8779 333.0404 92.25451 140.5512
123.0057 242.0935 191.4053 195.8793 208.1986
204.9749 133.7067 225.3654 195.1931 173.3124
256.5513 103.8054 157.3756 61.05726 100.3572
201.2467 149.5047 223.5526 195.9958 137.7579
380.5641 122.908 105.9497 166.4784 75.3559 354.4106
410.2476 76.50739 268.7132 154.6694 116.1807
428.1068 451.5402 213.1127 161.9424 162.7554
85.62304 126.6385 132.9877 217.8744 166.5836
144.8204 137.0928 174.1391 106.3773 185.4002
256.9207 81.66866 84.46722 154.081 132.3725 190.0713
134.0642 111.8103 63.39468 163.1408 117.1232
121.378 93.36346 192.3203 198.4849 236.6292 218.4977
148.4745 302.3953 98.64057 186.2006 151.8188
171.2262 167.5161 278.522 274.9597 147.0326 211.7148
235.6856 242.4982 160.7447 413.4835 79.4872 254.2513
111.6364 124.4083 161.2688 112.8656 184.8121
224.7114 177.7016 155.4299 98.37082 155.1161
198.7743 272.5274 188.974 151.954 58.30003 98.85005
109.7147 117.538 245.7971 146.394 178.9228 312.5746
121.6618 161.8292 130.7656 138.8669 111.8068
340.7402 255.3953 143.2559 97.5685 199.0719 178.3944
234.2807 258.8593 169.2889 182.6015 139.2699
119.0776 92.093 102.519 119.7337 123.1614 108.808
133.8816 155.5744 243.7271 96.08585 168.8375
286.3133 220.1416 216.7201 229.3435 370.8442
146.7389 251.804 166.264 154.019 100.3275 135.9989
146.6148 93.53759 195.3609 137.3546 275.4868
368.714 264.6339 91.83334 115.2361 229.1299 225.8042
263.6041 197.561 112.2188 140.1159 222.0897 191.1609
113.2149 208.2716 133.9334 120.0197 108.7345
110.1092 242.1487 159.4402 159.6484 113.6757
133.179 227.0346 207.3746 140.9346 88.4427 128.4937
91.14484 181.2112 140.3319 74.51001 211.2082

286.1935	244.2312	387.7106	173.6766	208.3021
297.0232	133.7825	183.1068	158.3913	139.353 157.6617
259.5292	207.3635	265.0388	275.1601	76.61929
175.3362	231.0451	125.6075	97.48059	106.6672
205.8656	119.5058	181.2036	313.5213	115.1338
104.9994	267.2889	248.1108	253.4791	131.1616
135.5616
AKT2	4.824835	8.378103	5.035381	7.130848	5.144745
7.128297	4.332744	4.34874 7.958918	7.47724 6.989934
5.57771 6.556928	8.413246	7.429504	5.846013	7.62355
9.847683	4.523214	5.262836	6.850724	5.295935
7.673576	6.870734	9.15385 6.295133	6.081671	6.999834
7.491856	2.543498	8.552755	6.49644 8.802509	6.84168
4.721738	3.798126	9.075694	13.80729	9.191808
6.122216	5.730901	13.00434	4.249396	4.865356
3.847888	6.307682	6.355087	14.80922	7.316518
6.696146	6.844747	6.31537 8.864679	4.877631	6.929678
6.076138	9.403899	8.438855	10.46358	5.401276
6.577048	7.190053	4.961242	11.51461	8.80988 6.277551
8.886816	6.021041	7.723326	4.1069 6.303697	5.39847
6.052878	6.329811	7.475883	11.27564	4.728878
8.082984	7.98509 7.384444	11.97475	7.855397	6.170067
5.421249	5.317288	7.064914	7.101166	6.628545
4.144584	4.551655	4.629005	8.406379	4.810073
4.777714	8.781297	12.31134	6.623233	6.33621 11.3749
6.410653	8.056451	6.702397	3.569091	4.9448 5.746317
7.207649	7.963951	9.631805	7.418922	4.948229
5.61267 3.005826	5.791253	6.465161	5.913759	4.493541
13.36591	7.284314	7.102203	6.25672 2.911998	6.148117
9.18713 3.623926	10.66157	8.264071	6.308018	6.873776
7.712891	8.760459	6.38101 6.97713 7.553205	5.648469
5.655546	8.254547	6.744643	9.386929	8.11535 4.378194
5.073925	5.121067	6.95122 6.082102	14.49451	4.676337
2.225318	5.461712	8.67541 3.444387	5.86468 8.01071
6.028769	9.179158	6.935275	3.249649	5.560567
6.751912	5.624731	4.519776	9.570719	4.910958
4.69789 5.590031	5.039146	6.490367	17.99685	3.588191
6.799854	3.450006	7.637332	8.891849	5.345688
6.246039	4.699246	4.989256	11.24451	4.952396
4.903725	7.623796	2.439431	5.430175	8.263465
6.156983	4.03997 6.812735	8.691002	3.962864	4.889985
7.096314	6.199517	5.608787	10.68742	7.566726
9.753779	7.025798	6.077728	11.11871	9.449693
2.647449	8.081817	5.187977	6.358581	5.898213

6.283999 5.112722 5.973283 6.937347 8.333522
5.375585 7.65341 7.29223 5.296473 7.337865 7.488321
6.166757 8.797312 6.035386 9.23869 7.501717 7.441185
5.558153 4.524751 4.392197 7.177503 2.546677
5.66916 6.784736 8.01468 12.56858 11.0787 4.412757
9.965801 4.896905 5.50279 5.326669 7.098936 4.875958
4.70606 6.141931 6.411572 10.77336 5.667923 6.15235
4.759975 8.462685 7.431586 6.745725 6.81692 4.806615
8.735205 5.143688 4.623189 6.877704 15.05374
9.118354 4.875725 10.19788 6.435053 6.762304
5.312969 8.250623 4.672376 3.21329 7.429618 8.236322
6.565613 8.379553 8.278961 6.175582 6.52969 10.02326
4.090592 6.321749 3.240072 3.622731 10.63199 6.0715
11.59212 6.556068 7.186584 4.693726 6.766867
7.731101 5.139133 7.677155 4.454412 6.31224 5.116334
7.226136 8.196506 5.870555 5.015039 5.940529
11.62241 10.8104 8.704597 9.311651 4.547135 4.171946
4.474921 6.576643 7.46405 5.6163 5.000581 6.03611
5.602497 5.309217 6.172159 8.865359 22.56969
9.629897 7.069301 9.346108 5.500521 8.023083
6.543659 6.959645 5.264651 5.05858 7.280787 4.889193
21.88463 9.524248 3.854613 7.055472 8.993119
16.52975 4.586175 8.007065 5.773777 8.042 7.513984
6.604352 9.302882 8.035236 5.032402 3.77269 9.430604
5.292026 6.120769 5.795534 5.277007 3.408487
7.820201 6.156111 5.437908 15.12137 4.321762
3.846231 6.3423 8.951076 4.452094 2.676974 10.04792
5.004857 5.834356 8.927752 5.599157 4.203211
7.419108 12.63414 8.712683 12.07109 7.168584
3.392562 6.001908 5.347521 5.180703 3.289595
4.489094 5.17502 22.91193 2.95441 11.62571 7.300672
3.496024 2.438027 3.175781 6.852119 10.43595
8.233516 6.760272 10.68452 7.295225 14.85698
8.860714 3.178552 9.95725 10.05824 6.069223 4.980142
5.283077 6.748159 7.239474 5.551059 4.473017
6.612968 5.524198 6.082838 9.400759 7.805625
4.999484 6.929936 5.121873 11.86141 6.792942
STK11 6.526862 6.582089 5.983549 9.430196 4.888263
4.276639 4.479637 4.857474 7.023067 5.381395
5.515909 3.683994 8.344322 6.926855 6.969499
4.171342 7.376935 8.461011 5.158042 5.639223
8.116195 4.532219 4.516502 4.863109 6.62305 4.719146
6.860108 5.057212 7.250502 2.12172 8.255132 5.087775
12.28715 4.925942 8.416065 6.377862 1.821693

7.278843 6.33405 10.33936 7.837566 9.802476 4.507313
9.375645 6.016142 10.40718 6.383271 6.865444
4.784444 6.271468 7.210309 7.040168 7.512712
3.054671 4.672574 10.26458 6.66945 5.842604 10.21059
5.704404 3.52463 11.245 3.145555 5.844802 4.098667
5.202215 7.170943 6.647621 5.68408 5.970347 5.844446
4.440462 6.296901 3.092369 6.709529 16.16858 6.2296
1.585244 5.165219 2.35418 9.605829 5.162081 4.838567
5.523315 5.914845 5.578657 6.74342 8.572567 5.870712
3.050246 3.24126 8.057758 5.335664 6.820056 7.353867
7.252024 5.470804 6.091849 8.946299 8.102153
8.066532 2.471328 3.99893 5.174343 8.362533 6.525684
8.423335 8.156367 8.875438 7.284423 6.506965
7.389077 5.764 5.393192 4.693383 7.027273 5.701543
18.51973 10.0968 6.418315 4.197754 2.931 8.342395
4.033949 8.346361 7.601496 4.31008 5.045613 4.729365
10.63433 8.200875 10.19937 7.224622 3.865389
5.443942 8.52909 9.957735 3.739217 5.424773 2.698763
5.171349 9.913029 6.871078 9.411113 13.31128
3.368838 7.339662 5.360157 7.709993 7.114859
5.595253 8.18253 8.704681 6.26434 7.019984 3.627803
7.97184 6.312486 5.938644 7.314641 7.617238 5.072371
3.216068 8.056496 8.291358 10.39313 6.545899
3.259911 9.069769 6.737199 4.457881 7.943716
5.982192 6.759759 7.298209 5.711912 4.088306
6.325582 8.812537 22.3504 7.841484 7.519279 6.900677
8.286838 6.385122 8.716892 5.168481 4.739718
11.08593 6.774125 5.643493 5.958167 5.435792
6.800393 9.586461 7.102748 4.169741 6.951382
8.059297 6.070697 4.53231 7.784589 11.78509 6.426458
6.243031 3.988711 5.157312 5.226106 4.888724
8.032954 4.870128 4.840505 7.275887 3.568706
13.10018 9.887815 8.799005 11.30181 10.50417
4.857535 6.989933 2.990964 7.135078 10.93655
9.215351 5.576978 4.960982 7.307978 9.830399
16.5798 6.650245 3.097735 8.743347 3.549339 3.815022
6.735003 9.367791 7.309318 3.849211 6.264124
6.845723 6.071783 6.353886 7.852317 3.691173
6.342519 9.738747 7.43955 8.205754 6.692446 8.586895
4.10872 7.701513 5.972657 7.908468 8.048667 7.389009
9.476306 14.06666 6.248151 5.98919 11.49716 3.872513
6.092628 7.400049 8.409155 11.17897 7.881769
9.275112 5.894661 6.051516 9.589354 8.917087
4.99231 6.810699 6.585197 16.98391 5.076015 6.352201

2.656972	5.944013	6.617003	5.905769	4.001453
4.754814	8.122312	8.630014	7.177668	5.629517
6.812797	8.3809	2.980365	4.908283	5.773492	3.066681
9.686372	7.938961	6.565466	6.628123	6.003463
6.752929	6.474176	7.047212	5.061037	6.212049
8.201822	2.761122	3.684793	9.875192	2.735388
8.185462	7.584308	7.233015	7.324338	7.293333
7.358429	7.083422	1.975793	4.231971	3.15822	3.913829
4.013552	7.280729	11.89629	2.222307	5.24608	8.085053
6.440185	5.247022	9.251746	5.329509	5.577838
7.279432	5.635454	7.218298	8.471295	4.362536
5.642599	7.313029	5.03104	2.971932	9.775114	0.8393243
5.094238	5.252229	7.406211	6.905195	6.295318
9.557935	5.282058	6.417675	7.855474	5.915525
5.955387	7.131644	5.876809	7.414803	6.453978
4.937299	4.803879	7.314114	6.376577	6.969531
5.372223	11.64854	6.55315	5.4866	6.464834	2.171837
4.443378	5.147373	5.336604	3.820704	3.475595
11.6497	7.176282	6.861346	7.504691	1.522393	10.8283
9.455478	5.038129	7.494506	7.060358	7.85325	8.24651
8.231531	5.457215	7.973767	6.025952	5.474208
3.856663	10.04505	5.128431	7.553668	3.544921
13.72367	5.626408	7.372766	8.497969	5.88572	8.265367
9.915059	6.090491	8.002125	9.843665	4.953945
IGF1	0.1455197	0.9637423	0.4808852	0.2539922	0.329905
0.2656346	0.4108122	0.3681846	0.2662605	0.477184
0.05551605	0.06000245	0.6325447	0.0151598	0.5389145
0.2808335	0.3296967	0.3760422	0.4769225	0.3146368
0.2309251	0.3775214	1.044495	0.5308971	0.7653438
0.1567452	0.3526259	0.3906551	0.6318528	0.3791162
0.3858459	0.2109606	0.06589798	0.3281787	0.4943546
0.1260245	0.03738785	0.2339642	0.4377522	0.07790554
0.07253977	0.02375583	0.09536448	0.01039442	0.2848951
0.05023389	0.2006812	0.1583447	0.5652487	0.1119221
0.3463415	0.9169948	0.06938484	0.07833784	0.5849703
0.01545952	0.1371655	2.408899	0.007205643	0.177796
2.355807	0.2548045	9.815978	0.4456822	0.0395567
4.695406	0.3578946	1.152997	0.138986	0.150599
0.01227501	0.07552191	0.7606429	0.1189375	0.1118394
0.6010833	0.4170594	0.1915733	0.09790057	0.05983598
0.0281137	11.09362	0.07697448	0.7112163	0.3273777
0.2591819	0.47692	0.3352277	0.2338709	1.173285	0.1644688
0.1243722	0.1396574	0.2320344	0.1468185	0.1780294
0.9525604	0.0813458	0.1688561	1.267412	5.553565

0.04080879	0.04967311	0.09912922	3.453582	1.979937	
0.06738352	0.7670929	0.1032643	0.007185264	0.3916735	
0.1875269	0.3468091	0.5604859	0.3308561	0.02995358	
0.07035482	0.03273585	0.4260246	0.03383099	0.0167324	
0.2203341	0.2334326	2.47818	4.156686	0.4652651	0.1396729
0.05829143	1.349578	0.5071521	0.08151245	0.02658948	
0.7165707	0.159304	0.1365526	0.8106027	1.064597	
0.06383379	0.2649394	0.04243135	0.07220552	0.08134258	
0.1201917	0.02648145	0.3136988	0.3733905	0.03498208	
1.489874	0.02533259	1.061719	0.08467661	0.5066048	
0.01302359	0.971294	1.368018	0.1015396	1.452773	
1.276346	0.1059857	0.2404284	0.8394422	1.566035	
2.945053	0.03922406	0.08410237	0.3665192	0.1432932	
0.07723797	0.8000642	0.5020693	0.4968314	0.2047545	
0.89705	0.012797	0.01305924	0.8184118	0.3197752	0.3207931
0.0916	0.214125	0.08853743	0.05443321	0.2836817	0.4418708
0.4205599	0.02885686	6.31622	0.0607686	0.5319032	0.08232134
0.4360142	0.0473294	0.1219998	0.4366983	0.1143017	
0.5109736	2.505505	0.1766789	0.0457655	0.1153927	
0.100897	0.3444737	0.0475541	0.007332956	0.0101975	
0.05711011	0.1428748	2.127186	0.285303	0.1281289	
0.34699	1.508499	0.8037665	0.2258846	0.06896328	0.2420627
0.1826473	0.09677949	2.653781	0.1396922	0.3805614	
1.78601	0.2281728	0.00772178	1.411486	0.0334863	0.04451893
0.1380625	0.01972711	0.09156475	0.1864719	0.3133692	
0.03749181	0.02834848	0.04521076	0.1835772	1.81667	0.1416909
0.2556022	0.1063921	0.1116796	0.3546453	1.360376	
0.09350013	2.709961	0.3519712	0.1944298	0.7318041	
1.217117	0.05123997	0.4925651	0.03040997	0.2342025	
3.185283	0.1116513	0.6694933	0.224463	0.5158405	
0.04891434	1.384189	0.3687038	0.06290683	0.2822138	
0.2987933	0.03392772	0.8608733	0.02644794	0.467113	
0.4117233	0.2804866	0.4373049	0.3539956	0.07271563	
0.06075238	0.03751589	0.1470416	0.400719	0.2688958	
0.19454	0.4584172	5.265167	0.1885483	0.43076	0.9436532
0.02097287	1.177804	0.1133087	0.084558	0.2227909	
4.297409	0.1130717	0.9383824	1.257566	0.1163694	
0.4542361	4.549508	0.4650348	0.290887	0.07281514	
0.05672725	0.349993	1.722086	0.8747709	0.135203	
0.04093531	0.2613627	0.2216567	0.2441508	0.3611568	
0.0700338	0.1400414	0.1016987	0.04095437	1.603604	
0.05318987	1.556574	0.3225825	0.1585285	0.1008297	
0.7596857	0.07658345	0.3474619	0.1211322	0.12979	2.368455
0.1483266	0.1825286	0.4929289	0.2055148	0.09678203	

0.0346152 1.449435 0.7151649 0.421168 0.6772782
0.1031089 0.02477948 0.5522469 0.158149 0.1011321
0.2790194 0.1747266 0.3211477 0.02006622 2.118476
0.1854178 0.0600746 0.0692302 0.1048436 0.3016292
0.1319679 0.5693953 0.09651302 0.1070304 0.3007672
0.02293081 0.4077436 0.05436904 0.4412279 0.1111978
0.2882461 5.040155 0.005396416 0.04716919 0.04699093
0.1258702 0.005892529 0.2746138 0.06423678 0.09933741
0.3702907 0.57381 0.1042157 0.2046863 0.2094765 0.6898167
0.05020332 0.06245811 0.1858638 1.654554 0.5598004
0.215738 0.06643147 0.02145742 0.05871414 0.1297026
3.901608 0.4623382 0.01061879 4.507081 0.3187049
0.0393622 0.1194746 0.3656677 0.2261671 0.5010068
0.4309995 2.3275 0.05544049 0.02353852 0.07681417 0.345324
0.117184 0.03693603 0.02894853 0.4137512 0.8128514
IGF1R 1.204534 4.404639 1.946874 4.565591 2.608327
5.493779 3.429695 4.07306 2.759503 1.978064 11.47497
3.188406 2.607476 4.929867 3.646948 6.262579
2.449949 4.321686 1.524458 2.5221 2.208078 3.433894
4.501426 5.338522 3.582075 7.477947 3.670334
5.823564 4.742686 1.037152 4.710816 3.382811
0.8761838 6.723682 1.946371 1.150507 2.10165 1.094399
3.341826 1.049813 12.54637 5.890397 5.703283
1.163607 4.683453 1.347696 5.104501 3.383582
9.162523 9.577683 2.35888 3.390083 1.750298 4.894972
5.27375 1.163442 3.947714 12.45374 8.086868 0.7829564
10.19916 4.050994 3.874276 2.705778 2.339894 7.19
5.124373 3.894624 1.843416 0.5379207 5.712372
3.395448 7.630299 10.80844 7.386401 4.205163
3.263214 22.0384 9.298163 1.508658 5.258418 8.353903
3.166837 4.238811 7.125542 4.70779 6.501266 2.352342
2.855345 8.282903 3.701993 5.505989 2.42636 2.630109
4.025662 8.461186 6.860588 1.810837 11.30964
3.156977 5.301276 2.337733 0.4683453 2.236955
6.379895 8.463168 2.997448 5.606925 3.710028
2.606631 4.817719 3.498216 1.300413 2.991298
4.52117 0.5763649 2.078176 12.404 3.093783 4.012248
1.997765 8.803768 9.85327 2.567729 18.47499 2.708059
3.639646 3.533747 6.030922 4.86358 2.261677 7.230207
3.43392 8.320631 1.976319 12.83054 5.690763 0.8256395
5.206669 4.438122 3.728336 5.522891 1.56787 13.87573
7.780678 5.35853 0.3978904 6.432676 3.233 2.938399
12.5504 6.224701 710.0809 8.575369 5.98806 6.811349
4.112986 9.547044 19.99489 2.96135 9.384192 5.567893

5.55632 4.579776 6.8326 7.214139 3.081244 3.653774
2.422573 5.188924 6.368385 9.594186 13.80501
2.346385 0.264687 2.187228 5.91663 2.005568 0.602153
27.62663 1.136836 4.17967 5.006757 6.614017 1.927561
6.028909 8.932949 4.427978 10.37207 7.319575
5.944308 2.491053 9.015849 4.48696 2.546531 16.30431
6.123235 8.414328 5.368709 2.343643 1.57704 3.0897
4.586461 7.178177 5.363919 2.872649 1.207791
1.491431 7.450053 7.380629 2.968627 6.099554
9.760065 5.14085 5.941805 4.994617 1.491622 1.771304
6.336588 6.054453 1.604765 2.100931 0.6366809
5.640282 5.35866 1.741402 7.886393 5.236968 1.197998
8.3253 4.353538 3.551793 1.715565 3.082687 3.707888
3.193015 1.993801 1.668157 3.344431 3.239424
107.1301 5.446626 4.271536 4.880289 3.863866
3.59435 4.261777 3.099967 6.065502 0.6403237 9.480254
4.975913 2.079003 3.993037 3.795209 9.455877
6.994465 3.548432 4.19843 5.12441 2.898922 3.945095
7.103885 5.526052 4.511452 8.30452 2.889941 3.793161
4.699065 23.98221 4.382732 3.352645 3.008096
3.235829 1.753255 1.464216 2.36517 5.807876 0.7218155
7.469772 8.18143 4.584581 6.589856 4.85225 2.544773
6.095381 1.238762 2.236303 3.960459 9.301583
7.440586 10.34001 6.135095 2.29705 41.93052 8.9307
2.812744 5.611049 3.086165 2.507465 4.54136 4.90066
8.476868 7.836325 1.672723 2.299267 3.029803
2.162541 2.035511 4.660168 1.550092 6.389767
2.375069 4.646183 2.611001 7.212678 2.302014
2.783506 8.607403 7.926414 5.985737 6.869264
1.012179 4.49756 3.207298 6.397023 2.077095 0.5788767
3.520972 2.63566 2.661137 11.26642 3.82869 1.759361
7.840347 7.230044 2.069334 1.961277 5.613736
3.189239 8.524632 0.5941746 7.966756 1.69495 10.01041
6.182386 6.509291 12.21515 0.8758342 1.273086
5.757196 4.96952 3.65275 3.053694 4.58409 9.816512
6.23732 5.359467 8.371636 4.643066 5.719775 7.030659
25.31204 1.162434 1.900822 5.226035 4.671575
7.147298 2.225841 0.83254 2.645109 3.766749 6.135449
1.643007 2.171306 4.070703 6.160991 1.789369
4.216022 4.751051 10.00468 4.296127 4.200056
18.83431 11.40447 2.559175 7.155879 6.941598
6.955409 4.967492 7.753126 7.05733 2.917345 2.242448
5.268935 2.842501 8.03979 5.186653 5.395251 1.567238
8.32173 5.711526 7.356269 5.305896 1.566808 1.529577

6.237064
ITGA6 37.57248 3.785235 55.81452 47.23148 36.72204
8.164219 19.2761 15.20281 23.288 16.55608 21.09944
11.32915 21.06222 11.13857 20.02767 5.044828
42.02763 16.53498 35.88063 27.95226 34.19087
5.364238 19.63187 7.814337 7.696447 18.42761
28.18624 5.347515 40.70624 13.98806 25.89868
8.916693 22.86193 28.78017 24.39638 3.880547
27.47719 37.09895 43.27815 80.31027 52.20128
97.46332 56.96895 40.41362 65.56553 51.67406
66.07893 24.19529 118.5421 116.6655 31.77189
25.39227 15.7941 79.95843 13.86228 50.9314 52.01214
16.93848 71.23005 21.49025 29.54307 55.15207
34.19793 13.75858 21.19617 15.91789 47.6102 18.87579
73.15549 135.2631 46.22084 38.02795 63.20662
48.55656 42.10383 14.92147 84.68819 67.78841
105.7871 92.00474 25.30426 69.23316 77.75679
49.64958 77.44038 37.46213 84.3123 48.9525 56.28521
35.33355 49.85619 72.14107 70.72785 34.71895
39.16671 20.71317 90.24665 28.50384 112.7728
91.2639 40.99971 79.00694 26.38383 11.05448 32.12164
70.11831 125.9617 13.91674 52.89709 43.87023
17.71553 29.52206 14.3895 13.86636 35.55267 16.05504
30.53627 164.413 26.7805 40.16133 82.10675 31.10008
16.59158 32.39418 141.0879 21.46499 29.84605
44.17943 22.6077 16.62094 104.097 51.44253 39.75301
81.15797 32.8871 36.69003 147.851 77.77276 32.72016
59.84288 59.77316 62.91344 71.17473 16.1538 19.92177
44.39541 22.68513 34.50303 49.48166 32.11378
81.62078 63.74086 38.76616 52.14244 26.53902
23.26014 43.53978 17.29518 95.29236 10.0744 26.99365
38.7783 45.68701 50.40944 33.20763 61.88012 32.45312
54.42732 22.72714 43.20083 9.950348 19.82103
24.5315 44.57661 25.99203 54.54283 22.4338 12.03149
51.06134 20.68488 34.80065 44.66924 25.70697
53.1812 16.82183 112.4223 31.69388 47.61954 70.23707
90.76486 33.89991 101.919 22.56056 41.92349 85.1314
45.10149 43.104 38.63574 68.33329 22.03128 22.88872
33.99462 113.9728 62.426 55.94591 66.56696 13.48694
19.4722 75.7101 37.64826 62.59165 101.469 73.07573
73.07779 65.92934 55.70032 19.24174 20.32202
55.97138 79.89857 39.12083 42.98842 25.9432 64.43262
31.49289 48.96703 32.48653 17.29748 34.30138
61.2953 32.203 46.8854 78.13118 116.1167 103.9483

29.70679 28.45598 43.32063 102.4465 12.88385
25.40206 59.34807 31.09 45.89955 57.72146 32.3802
16.26248 9.90456 34.64605 77.06708 42.92508 118.8608
24.05028 38.67169 185.5696 38.69289 59.92117
21.85569 45.23052 17.76572 56.9592 64.02507 75.26229
55.37667 73.35753 41.83432 73.32787 27.95802
62.13415 48.10754 67.95369 48.15066 55.94739
87.15679 51.05588 25.50476 54.54305 11.78174 2.5679
91.8968 40.34335 39.10595 41.75991 39.05822 104.6791
44.03235 63.76807 20.99416 66.1241 60.80664 38.95238
6.808236 20.81952 39.356 11.63419 34.81172 18.46991
39.96889 70.21745 70.46195 87.67014 42.71285
64.99388 30.28677 79.57845 36.35654 30.87279
126.2105 36.57148 36.96696 33.60793 33.98741
80.76953 18.09374 71.55891 38.07352 31.56049
34.24427 55.76007 89.99813 48.94954 65.47798
40.32084 21.02392 69.77407 20.17303 47.13039
10.50738 62.82178 148.7716 45.42455 36.91121
39.33238 36.56088 13.3633 94.00982 26.83735 23.10239
41.32924 70.05626 68.60495 36.08416 49.17762
25.35602 26.68654 23.45898 62.95542 11.0139 8.429975
9.296724 48.40735 31.21812 33.23366 36.50885
47.74882 39.92872 66.54618 33.63486 16.87603
13.37254 43.90237 15.52921 224.5323 21.80191
21.5861 27.4526 52.14124 49.2801 42.09865 9.072895
22.0802 20.13787 35.9354 112.3662 40.72306 41.79979
50.67316 54.31377 37.02867 37.45936 33.66006
90.74756 39.98669 92.66856 45.836 29.33336 18.4345
57.04723 71.26603 28.64933 17.95502 109.445 22.67836
59.53679 52.29087 54.93909 50.42172 53.20361
25.97144 98.42036 12.55973 11.96137 76.05682
71.24332 36.96223 27.04317 25.17981
ILK 2.264363 17.80047 2.266967 3.692731 3.227172
11.46598 2.525672 4.902841 5.85206 8.187067 3.114186
1.859753 3.767225 2.838552 2.569823 16.43258
3.019553 11.01754 2.317027 3.063821 6.826937
10.91009 2.360801 11.1756 14.40624 1.409448 3.439059
15.09874 3.239514 3.103648 7.506698 8.010537
2.008623 3.913884 2.80761 0.8135877 0.7810562 2.000009
4.577301 2.922851 1.653473 3.415104 4.058237
2.254567 2.03541 1.463662 1.329521 4.701912 4.950954
1.495468 2.08442 1.955399 3.028726 2.087984 7.9518
2.071041 2.13686 13.765 2.989703 3.120667 3.555552
3.179032 5.328607 3.015693 1.417445 10.2405 4.517853

3.52768 2.455201 1.843971 2.877745 1.233146 3.799321
2.6791 3.251485 3.763334 2.204871 2.715582 1.84321
1.862763 2.748046 4.058649 2.568407 6.076033
6.052872 3.16551 4.475272 2.519887 1.971429 7.30005
2.849151 3.305974 3.341448 2.235342 4.310307
3.10568 3.217567 1.63789 1.840745 2.97571 3.712185
1.956428 1.277912 2.165913 5.753044 2.761881
2.592788 13.02977 4.288704 1.845733 1.846071
1.724395 3.117573 6.89111 2.850378 1.251499 2.939515
1.734461 3.872347 4.288496 3.025123 2.611013
3.520037 3.197447 4.072622 2.612949 7.655083
2.67904 9.915446 6.634735 2.348548 1.842076 1.934849
2.260661 3.430663 5.554314 4.152917 2.262958
2.092743 1.444533 1.979978 2.34916 1.871808 0.8912901
2.621347 2.360682 0.6550117 5.426273 0.9886015
1.81269 2.027161 4.302538 2.57334 3.995196 11.16577
1.257024 3.744765 6.236341 3.432456 1.311484
6.021489 2.432583 2.473415 2.15476 2.799822 3.144825
3.765141 2.272222 2.373901 3.270292 12.57888
3.005259 3.430319 1.956232 1.814443 1.919506
4.31235 2.606168 2.154285 2.292098 0.4949053 3.24718
1.989441 2.439834 2.169749 3.127546 9.092936
3.492522 3.029866 2.238914 4.180594 1.300745
4.235078 2.594706 2.552761 4.098304 5.727819
1.367013 2.137349 0.6769236 0.8598043 3.540048
2.808602 2.28764 1.364985 2.04341 3.406832 5.110498
1.225322 5.204685 1.674862 7.49942 3.162381 3.444871
2.345119 2.900571 7.131413 2.573482 4.936665
2.805852 3.34273 2.875141 2.534348 2.601918 2.909049
0.7029298 1.730887 1.281873 2.691448 2.225403
3.683806 3.118066 1.283624 2.773554 2.263758
3.624907 2.781181 2.263332 2.076548 3.142297
6.034096 3.639615 3.804703 3.999569 2.684041
3.580336 2.638411 10.06252 4.447334 2.018961
4.378405 1.717619 2.741432 4.573765 1.47157 3.021415
2.594746 3.267885 1.875353 4.670461 1.704016
2.768506 2.098354 3.411767 2.362573 3.273314
1.841715 3.683743 4.151689 4.514307 3.885378
5.66115 0.9952568 1.970432 1.552949 1.485949 1.986942
13.05388 6.464951 3.594966 8.016676 2.634743
14.14652 3.850477 2.479732 4.901605 1.762168
2.824952 2.200589 4.682176 2.537114 20.69249
7.750911 3.05656 3.591231 5.781978 2.630612 4.876458
2.15666 1.690413 1.831776 2.332128 1.863369 3.096717

1.105028 2.308137 1.584136 2.70637 2.734578 1.160351
2.467217 3.016338 3.822307 4.345343 2.539051
10.39459 1.52494 3.044145 2.859593 4.358516 3.710827
2.095963 2.464142 2.014181 4.228275 2.709437
4.286893 1.489074 2.407903 2.699593 2.330098
5.048879 4.1027 3.867554 18.40906 2.903927 2.506128
2.026175 2.240099 3.940379 1.289475 1.678619
2.10706 1.485818 13.41668 1.587565 2.176062 1.153797
1.681404 1.166894 1.860903 5.2268 2.314918 1.291873
2.645934 1.407548 2.738491 1.298062 15.02117
1.429287 3.559242 3.481303 1.870559 2.627719
2.628176 1.352777 1.518218 2.534776 1.420886
1.091413 1.612142 2.287645 2.823531 1.193983
4.559872 3.357836 1.623666 1.356567 2.160715
1.737679 5.557487 2.755878 1.233597 2.610695
1.610079 3.829525 7.129584 2.25736 1.634994 2.33422
5.825276 2.284171 0.4743671 3.086938 2.679943
1.901545 1.724619 7.802865 3.337832 2.677222
1.945565 2.227089 2.429504 1.956035 1.66406 2.858711
11.66491
CFLAR 6.290855 4.451646 6.230276 5.224579 4.693762
7.287251 3.35981 3.424655 5.31148 5.205755 5.044556
4.559818 6.441276 5.167248 5.151618 5.431681
5.531473 5.004603 4.446316 4.170586 4.664602
4.655676 4.972558 7.12518 5.351886 6.727997 4.350624
6.552537 4.841513 1.804255 5.192042 4.365537
5.471086 4.441981 5.992987 2.820288 1.511845
5.194826 4.457655 3.857449 8.127765 2.162544
3.72189 4.878723 6.816615 3.649569 9.387246 3.800611
4.901153 3.724992 3.474332 10.07753 2.365412
10.61389 7.74288 2.644658 5.586489 12.31731 2.087333
5.130204 7.810635 6.723521 4.223622 2.982304
3.772565 7.310802 6.164785 5.738909 4.937848
7.84756 8.537071 4.868926 7.826297 5.545978 6.881494
12.79309 7.426292 8.771212 7.93973 3.623583 5.101906
8.005887 11.77138 6.970627 4.547097 7.846853
6.514036 5.650082 5.986975 9.924579 5.36994 12.85476
8.759768 7.879434 6.195815 6.104607 9.340896
4.117858 3.268746 7.598183 10.68774 5.389597
8.10254 4.209669 7.082839 9.781241 8.602541 4.346279
5.402763 5.179072 3.556661 7.774314 3.421224
8.935616 5.536228 6.366282 5.272425 12.9198 3.544444
2.685164 5.267486 6.114291 5.035297 4.094639
3.398872 10.09074 3.727238 4.457567 7.289954

5.389788	8.92513	3.041775	5.587971	5.517575	5.279403
8.715235	6.968821	4.629272	4.460593	4.551572	
6.376479	5.037794	13.72079	3.291034	12.24475	
8.693834	3.996523	7.137157	3.182114	5.536549	
9.828468	6.447487	2.432723	12.76373	8.260414	
4.805142	4.166708	11.22636	4.308096	5.135091	
6.703041	8.368024	6.54803	4.026251	2.780598	6.616161
10.38193	5.859683	8.139705	2.067399	8.53914	3.798169
5.908236	3.567263	4.479471	4.616127	5.991973	
4.75001	11.82278	4.320702	4.318373	3.921542	4.266038
10.59408	5.537902	13.8357	8.24365	5.105225	6.515081
7.984641	4.237976	6.924855	4.957106	4.27089	6.704471
10.05361	8.052351	4.843026	3.909198	3.080239	
2.340771	6.519743	5.693161	3.540882	4.645163	
5.800562	5.120857	5.479458	3.933834	9.771112	
7.558284	6.493085	7.212992	6.007287	7.640021	
7.778975	3.952412	3.975678	7.842397	3.64178	5.476884
8.910888	3.964598	4.1882	14.82097	2.954886	3.84797
3.207808	2.327366	4.882516	3.163448	6.42975	3.38554
8.454694	5.387973	9.792446	4.255444	7.104176	
6.644655	5.231437	6.699604	11.02628	6.307967	
6.864385	5.184051	8.322563	9.497142	7.033199	
7.824663	9.215392	11.79532	6.126031	9.158743	
13.17819	6.662212	3.743492	6.999723	4.732759	
4.948405	5.468037	5.640227	3.703285	9.640291	
5.533943	7.821757	6.801354	8.8867	3.719712	8.108212
11.85863	7.67883	4.88738	5.403108	4.046792	6.304206
7.755092	7.867847	6.627548	8.206298	3.441777	
9.092346	10.77721	3.929262	5.012065	3.09653	9.380052
6.923094	5.875455	7.910514	5.987792	5.580354	
11.5288	5.40477	7.048459	8.222782	5.642575	7.330642
8.844963	4.505092	5.173249	5.54067	5.747672	6.451725
10.76835	4.677629	4.162528	4.443121	6.964717	
7.467644	3.00328	4.502108	2.464541	4.791901	4.934322
4.507611	6.605573	5.90691	6.339065	9.626228	10.26213
5.785003	4.664091	2.744169	5.518844	3.213042	
4.479377	4.22666	3.227278	4.802174	5.878267	5.121904
5.670721	7.830135	13.3047	5.1591	7.207457	3.317772
5.194204	8.742439	8.808067	4.657738	5.653491	2.7607
5.05936	9.764217	6.812096	7.89766	2.663513	4.665837
5.131469	5.049762	6.35593	3.720685	5.513979	5.253492
5.106109	10.25864	4.762903	6.058148	4.554861	
8.152824	4.599709	7.554026	5.019347	3.794461	
3.315639	3.651505	8.380438	3.711047	5.293447	

6.393215 3.581757 2.569584 5.690089 4.385504
2.218129 6.574648 4.205339 4.673568 6.57464 5.146084
6.261456 4.095394 2.929458 6.441904 3.949978
3.77539 10.43263 4.796409 3.758184 9.389492 3.26861
3.275178 4.909746 13.1105 7.855495 4.497022 8.320518
4.11451 4.076354 4.147331 11.45381 7.007535 7.034394
3.510444 3.641779 5.336517
RHOA 184.348 202.6489 169.5652 220.6657 162.7202
155.5757 171.5995 116.4441 213.3599 229.4028
155.9173 204.1934 188.5069 181.2211 168.6266
140.8927 175.7271 177.0625 149.2573 157.8665
275.0805 162.2441 183.1284 159.4611 215.9248
174.8309 186.5093 133.7283 180.2092 198.4137
222.4109 174.5118 131.1218 147.7309 163.6718
142.3943 170.1365 194.953 180.102 340.3876 233.5747
263.3139 338.6127 254.1542 202.1142 125.4672
181.5257 162.0958 304.8349 180.4557 193.5689
215.5501 154.9961 272.8059 201.4506 203.1161
250.1661 142.1348 148.3601 169.1204 235.5578
225.1004 234.8303 234.1107 240.9047 164.3014
185.0566 198.085 195.1279 128.3302 328.7338 214.562
118.5793 208.4435 188.2626 183.7043 182.5529
80.26992 471.4285 202.6127 191.0381 149.5866
253.5838 156.9141 215.1675 143.8804 190.111 129.527
209.3407 200.9339 349.9993 204.0385 263.0027
194.4596 249.7286 174.0747 249.14 180.675 206.0935
218.0554 167.5375 126.8573 173.3416 133.5062
164.3953 161.6497 358.7441 168.2862 285.4701
172.1237 168.5586 124.8745 287.7126 151.5606
205.3397 152.2198 213.4999 161.8963 154.6419
196.9049 141.0389 131.4582 138.2867 225.7511
223.1678 214.5584 273.9406 246.9905 159.7388
145.4694 170.6313 229.2188 167.8566 319.9239
201.858 159.0877 229.1791 227.8374 267.9723 139.72
166.6476 175.6198 118.9753 131.1308 165.4839
203.1126 144.3134 208.9386 176.4598 181.8623
306.6173 223.5737 286.3512 182.5072 134.9112
182.2836 188.991 158.5449 180.3777 122.3875 182.9577
173.9602 190.7601 177.8903 186.3934 205.606 163.8673
166.9684 150.6809 127.1105 151.1052 240.6254
181.1486 185.9991 236.9245 185.3438 122.5656
174.6475 127.1172 144.36 184.5099 140.7724 193.0525
162.9309 137.1661 210.0191 146.4929 294.6863
173.0971 232.2032 205.5243 124.5978 148.7555

207.2471	127.8449	148.1334	300.1141	147.7611
230.0216	180.0057	170.1294	143.8645	212.955	158.0932
147.9746	255.5437	161.0163	225.9284	110.7763
145.4849	206.0667	189.575	260.8957	123.7232	209.6636
234.7627	125.2338	186.3162	195.7051	246.5368
211.2472	176.9525	222.4578	203.0867	166.9872
151.2663	135.0673	136.5926	184.9618	122.2725
113.1583	155.1022	178.9806	239.8911	188.3457
246.9484	217.7008	107.3961	297.4284	143.0522
141.3692	206.7392	192.355	130.6803	271.0925	255.8806
207.2363	160.5963	130.5048	205.1827	282.4796
270.791	105.8601	169.9579	187.5153	207.1598	116.8199
175.8373	157.5456	137.8356	192.5741	280.671	206.735
185.2965	175.8336	159.151	250.0939	250.276	163.291
122.6588	249.5978	160.8741	218.4361	215.6702
161.9495	113.3276	134.1279	127.1677	225.3733
323.1409	166.9926	161.0963	281.1631	262.119	230.2339
155.624	158.0904	106.75	249.7863	180.6586	148.9693
121.0737	153.5477	153.157	215.3276	164.0203	171.659
229.6929	197.488	221.583	224.2466	219.4531	101.4557
151.3862	239.2819	166.8262	178.78	244.6718	170.836
214.1997	196.8786	164.5795	258.874	235.2725	215.7034
152.2452	163.6028	125.9853	187.709	294.0956	184.9881
171.3763	156.0628	171.6291	333.2791	130.7055
209.6238	114.1664	190.2477	273.334	294.1358	144.9119
179.0678	152.903	176.9387	128.9731	120.9457	140.3725
150.1817	252.0257	178.1591	177.5236	138.8024
217.9847	158.1672	152.9212	115.7418	156.3024
73.04903	148.2643	164.2686	182.3901	156.2767
149.149	195.4228	230.3375	191.1225	134.5028	156.4041
119.3292	163.3414	189.5549	187.9947	188.0037
116.5516	161.5982	276.3757	175.4825	141.964	110.348
123.6273	173.9111	179.2208	224.6663	199.0963
144.4004	272.9376	128.5523	136.6521	136.5093
157.0894	177.2811	106.2421	144.3419	147.1345
244.2867	157.0098	222.7039	167.9235	165.699	167.1646
202.0369	85.25909	228.5957	136.884	284.7368	123.5177
150.3471	146.8589	199.3421	149.4542	145.89	232.4708
215.8704	218.1064	116.0775	140.9851
HIF1A	11.50217	12.89205	24.70949	29.79416	26.63354
22.2061	15.32238	18.78815	17.89671	30.68073	55.13561
58.82703	34.68834	29.51548	21.56606	26.97668
22.7865	18.52055	13.20955	13.9036	26.53201	18.23336
70.16157	20.92864	25.82237	50.08441	20.94372

22.22906	20.42321	12.59535	19.59197	19.45263	
50.34366	39.96633	52.53803	21.14594	24.04287	
22.40403	44.39077	88.69768	98.46623	42.91813	
84.20189	35.01536	39.96489	29.15363	47.14514	
23.47745	35.70122	85.41836	24.31578	37.01361	
28.44671	33.05869	32.97974	43.31376	44.39066	31.973
34.60291	28.77408	149.1347	35.25733	122.2033	
37.50406	31.24582	46.32126	32.732	32.78866	55.54053
53.27155	38.94789	35.38857	23.27014	162.9945	
109.1093	38.01296	61.73086	42.44724	35.72304	
51.73976	75.72632	87.73737	80.55485	29.28102	
30.59509	22.5604	44.57141	44.00381	51.52034	26.68892
71.84322	63.51077	58.05096	47.23876	27.00782	
30.67913	38.76015	23.49006	107.1058	47.16102	
44.35435	38.50315	39.94839	33.88707	43.02446	
60.50354	63.61307	17.28892	34.18813	33.84606	
25.73449	24.22127	46.37532	25.2202	64.58033	103.1184
29.38448	23.50756	27.02291	46.35689	71.34251	
32.69261	42.94124	55.97279	29.19186	55.9007	42.10779
18.79754	39.06099	34.19577	54.34606	22.44925	
30.59328	131.0778	36.42665	25.97398	37.01769	
59.58427	25.31715	49.96989	80.17553	63.06635	
93.23506	18.44468	33.81497	19.78999	31.73724	
42.10587	27.91982	91.59046	27.02876	69.01567	
44.25467	60.06889	32.04545	33.98214	34.5351	26.43407
151.7612	20.20404	31.57889	44.97559	91.16724	
51.23047	27.15846	32.86221	32.93987	96.03995	
46.32936	15.97319	19.20475	110.3978	21.32095	
42.5616	61.6198	34.4352	39.46492	32.33049	44.38868
41.19593	19.47454	20.68687	70.84329	48.43767	
75.38722	73.01055	35.91474	27.06856	39.43954	
37.76101	48.28521	80.62852	40.42882	27.10964	
21.19959	37.3465	75.67556	29.66882	31.04835	20.8175
22.56974	20.57885	45.56982	67.05883	28.60443	
35.53243	25.44916	52.6775	41.41001	23.15465	74.08505
52.04915	43.80791	24.98627	29.72862	49.20961	
10.96607	33.35808	50.03225	55.20008	32.54534	
101.5569	21.81343	54.78222	39.02496	15.81306	
38.86113	44.38247	21.40117	14.09367	16.04762	
86.48119	18.58556	50.01221	46.44138	22.20036	
25.11272	30.30142	61.00912	23.88589	23.15062	
50.40207	50.12894	29.68733	101.7132	55.26712	
32.66668	25.91709	49.35354	34.1031	27.83447	57.85844
26.13785	35.369	30.87339	38.88019	32.62886	42.71806

50.53101 36.29555 32.09572 40.11747 70.19267
45.55819 75.13844 39.13515 39.28067 42.28639
35.70891 35.63418 53.87847 26.37025 24.83626
48.78837 60.58593 27.05489 74.13483 28.02077
48.33581 65.22445 38.66837 37.94002 20.4763 45.9428
36.67875 29.55336 38.5361 17.37667 95.57393 71.83585
58.75037 24.57167 33.90327 49.78771 134.3219
34.29818 34.67967 43.59474 32.2539 24.28237 68.44054
82.61539 69.06578 183.9408 31.14136 44.38702
84.97867 111.0553 33.0476 66.11724 29.11866 24.37206
54.85138 29.49703 31.80178 25.42727 122.8111
39.75825 65.16541 78.67575 35.16873 52.11645
32.23217 15.5097 75.14906 29.54421 43.12057 28.99687
45.41376 33.86088 28.16235 68.90827 46.37189 28.282
15.10739 17.89309 22.7474 12.44593 39.07773 63.32326
40.13547 25.10013 40.53398 83.63915 31.93445
35.54757 29.14622 46.49996 26.41879 31.3263 58.60306
52.96828 26.05253 96.6948 31.99158 34.55702 54.70675
29.13575 22.56477 119.2899 46.03321 29.73815
81.37362 48.28797 31.98327 30.24856 30.64303
47.41866 36.49417 34.23806 29.28143 41.96285
34.9202 42.56412 38.43956 31.97838 52.41695 92.99437
23.5899 27.01533 29.64615 52.08217 20.83911 32.66037
28.05714 31.33885 26.25368 48.21577 39.83898
31.87731 39.81721 54.63984 19.41385 42.52391
67.79035 99.24648 24.10235 42.61585 17.79985
61.6453 16.09973 24.71833 37.76971 26.3206 42.87961
30.66339 27.70623
DAP3 11.51079 14.23786 13.02901 14.51991 11.48886
11.36553 9.090575 9.366529 10.69174 17.73461
8.719959 10.53273 16.58732 9.717643 11.0892 11.25148
11.81856 11.66863 9.180608 9.509223 14.63117
13.18886 10.5509 10.69519 10.71414 10.15536 10.78194
11.50062 12.00625 12.75416 10.81014 12.79044
17.9795 10.23278 20.44626 20.52676 42.85567 31.29652
25.55099 19.68994 12.0906 23.04133 26.50602 22.16716
12.04225 23.97688 13.28326 20.87841 17.74372
18.26598 18.07667 14.57389 39.31285 16.25553
11.16297 21.04837 17.52858 11.12796 26.15764
19.79083 12.87438 15.71088 16.22974 14.05518
20.87807 11.12164 15.50311 16.22656 17.03423
23.44827 17.8122 17.08731 17.50168 14.37391 14.73074
15.72633 16.49486 13.08942 32.02254 38.39706
14.59101 14.26864 24.96822 13.23627 16.66439

17.07496	17.9206	17.72659	14.44977	16.01602	27.11324
19.89985	14.10179	17.6589	16.6791	23.37663	15.7865
14.53175	11.44823	14.57231	13.40082	27.15682	
11.87842	15.85099	21.49062	11.5829	11.489	12.5356
14.81627	31.54441	25.68597	17.63572	14.76568	
12.37887	11.43932	16.13842	17.56341	29.89444	
28.25223	19.44663	19.13127	24.75007	22.2129	7.860305
19.25592	16.42956	29.71118	17.60397	12.59808	
21.27547	17.2732	36.44868	20.08003	17.61356	12.23996
12.63319	16.45479	46.76578	24.64958	35.28486	
18.23663	27.80863	14.83478	17.26727	50.06036	
13.46526	16.66488	15.1367	15.06736	13.99396	26.64925
12.80311	34.21867	14.86708	12.52408	15.13358	
11.97454	14.93453	15.31882	19.51549	18.79615	
12.65776	24.18399	22.84722	22.15443	17.85288	
14.97262	14.69751	18.82027	26.9584	12.34657	20.92177
17.68014	13.41791	19.44428	20.4142	12.38496	20.36964
14.07731	9.604202	22.64459	23.27976	11.09994	
16.09644	15.92629	14.92672	12.27049	13.36774	
11.33684	20.60737	26.02162	17.34234	24.18741	
13.67625	14.97433	16.03735	19.69434	29.5127	17.89866
13.34277	21.97242	16.80134	28.49909	22.30402	
17.06383	12.39894	15.36588	19.27255	18.31638	15.723
17.16179	16.00415	15.66658	22.41535	21.34247	
17.69571	15.38881	25.7537	16.05335	15.81436	18.64924
21.78629	19.07562	13.38162	16.46848	17.60465	
19.30385	8.473166	17.64477	18.67445	12.8901	9.673623
31.36955	10.92555	25.29979	13.90955	14.62858	
17.65171	21.91889	18.92772	15.12671	21.37441	
26.09086	14.67569	23.28884	18.21054	34.72972	
12.45855	19.7233	20.79565	23.94283	25.53236	21.79664
17.81948	22.28378	18.02229	25.92148	20.03943	
19.7797	13.08886	24.77136	16.55249	20.04327	19.62401
14.3451	18.28119	20.97272	18.30668	34.75045	12.57275
19.78417	17.73789	16.27337	21.85396	21.90129	
20.83252	9.458731	11.2282	16.62237	23.0325	12.5278
16.49225	10.63349	19.39084	17.48108	16.117	26.47324
24.07616	13.19864	16.20078	25.90709	10.19986	
11.60736	19.85592	15.52787	16.12955	18.55231	
16.22219	16.87763	22.87875	14.91985	12.32332	
14.30923	8.421401	21.17862	30.39487	27.70895	
29.40874	22.73655	17.22476	24.69168	14.54781	
16.84384	14.72948	18.78762	14.31991	13.77462	
20.6177	11.63301	18.0867	25.74497	17.07301	23.75328

17.74627 21.3654 14.84562 16.9787 17.76376 15.80096
9.986862 15.39484 13.32904 17.41039 22.82457
14.35142 23.4783 20.3786 27.56387 13.85564 26.37393
16.50005 19.86046 10.11397 12.98416 13.23051
9.758505 23.87819 17.97473 20.24839 23.80137
23.89507 17.35095 12.16478 18.27325 23.31577
14.60915 13.05158 19.69599 9.836802 16.40536
18.37397 14.34498 15.03522 22.87801 13.99796
19.98296 17.80232 28.58517 18.95321 13.24019
24.20701 10.95578 24.86807 35.60512 23.29941
20.73719 18.82782 13.05368 12.76592 23.1271 22.64007
14.84559 20.75601 23.98528 20.57806 17.24703
13.39826 16.38303 19.96805 22.8827 13.63123 27.71404
20.83741 22.35177 15.53626 18.9786 17.2236 13.9481
10.79825 16.20414 30.32071 17.99805 20.1108 16.93935
25.52195 24.715 14.54634
MYBBP1A 2.550647 4.759295 4.752691 7.546762 5.373241
5.073741 3.792526 4.202202 5.763809 4.850746
9.718704 5.268794 6.521354 6.891115 6.117165
3.975367 8.087411 7.276234 3.43567 3.621762 7.879629
4.247709 7.732377 5.621212 4.969735 10.908 5.412913
4.696732 5.444974 0.8078274 7.221221 3.289916
6.031982 6.602237 8.178311 8.189387 5.968634
8.925656 14.621 21.66908 7.47685 5.63673 9.475818
13.3783 8.598238 15.65066 10.87139 13.95973 14.60263
11.34026 12.14815 8.448111 18.24753 7.205513
5.137543 15.87634 17.5215 8.077046 16.62064 9.421114
5.126849 10.88257 3.676374 8.792441 8.91534 7.007125
5.669458 6.197404 10.00126 9.218144 23.68876
6.308213 10.4102 4.169193 14.13788 19.21417 7.108502
14.66903 6.839126 4.776941 6.068026 5.733529
9.744322 7.367262 9.843772 11.76759 14.49596
20.07221 9.764925 4.184713 7.177491 23.84346
6.400627 8.399068 11.56014 21.00635 16.77554
10.89874 22.2009 16.77758 8.794535 7.018756 6.77282
10.20412 10.72097 10.73683 11.61641 7.213387
16.20095 13.59239 23.08775 7.872362 8.266002
5.776036 4.537419 8.963182 20.28395 14.44204
10.59448 14.46686 10.72543 10.00604 11.87973
3.652657 14.01735 12.25422 11.07566 8.975121
7.113841 8.10983 12.96153 23.95642 7.931255 8.680431
5.330333 6.627404 16.30775 15.40861 10.94479
12.43136 10.62621 16.74823 8.275781 15.53849
27.17152 9.285155 13.85236 5.833159 20.83573

7.280144 18.0723 9.931391 24.64101 24.65094 9.078479
8.957688 19.41941 6.672652 18.15371 7.577707
14.56259 5.985297 4.601216 27.8869 10.12497 8.27388
7.491272 2.604987 17.48327 7.921039 5.450608
8.342654 7.986624 9.180833 5.776559 8.179585
9.559213 12.98523 12.2846 65.9197 4.619491 11.37583
7.921453 19.11093 11.27001 7.475079 8.229134
5.17184 8.180451 31.0633 9.645439 11.16851 11.51836
12.08492 7.777119 11.8701 9.317642 29.87214 29.89484
4.866057 5.463493 7.47538 13.3593 4.564016 14.26081
15.79553 10.29279 11.94284 17.97723 8.936893
4.020482 9.706372 7.361316 7.512109 11.53084
18.16651 7.226364 13.3002 10.31805 14.15923 14.94124
8.985451 9.563431 15.56369 11.5313 11.04709 27.90776
10.84881 19.94343 14.72528 11.30188 6.384227
13.40506 5.231489 8.580322 9.914331 8.127585
9.542181 10.8169 11.78523 8.592118 11.4346 10.05429
14.04734 6.970792 9.537366 16.92095 7.306727
13.95006 6.114125 11.79696 3.721457 21.68754
12.87007 13.08891 15.97144 15.44972 5.686018
23.28074 24.81213 7.203882 18.40375 4.95358 10.49591
13.27801 22.10121 28.29049 9.31316 7.398139 9.108024
13.68513 5.56998 12.73604 14.22039 7.507742 8.369416
7.218385 5.547189 13.47987 8.188357 9.940484
9.472951 5.387523 12.93935 13.63211 13.76072
18.11272 11.42562 8.833215 14.74217 29.93078
4.616847 6.232101 15.15139 9.503889 14.49874
7.672772 11.34549 14.72757 8.149227 9.596712
9.378371 13.75742 10.04512 9.388876 11.49133
7.46783 8.243456 11.57383 14.36003 31.05605 15.96937
12.62749 10.74847 18.81357 11.65311 7.93676 15.13015
4.249551 5.722948 7.925092 11.5206 15.28782 17.78643
1.654701 11.32516 10.40132 11.90581 4.63782 20.50484
5.835849 8.514988 8.645894 10.89745 9.03625 13.2454
9.557844 8.386365 17.76685 3.291499 4.742045
9.744574 8.736783 8.541505 7.370928 8.134564
13.1249 21.24471 7.309655 12.24212 19.08461 25.89041
9.213284 8.771366 13.37905 7.374211 10.94978
11.79683 4.86823 15.85624 6.545581 9.375415 9.368858
18.85865 10.98945 10.58108 8.578493 11.1378 16.78717
5.068102 8.784673 4.896848 9.256875 9.790075
18.59227 16.29855 5.748804 9.957498 9.117107
12.21639 12.62832 11.49452 8.915067 16.91994
30.41053 12.44887 7.421199 4.725095 29.25 16.71584

8.292671 12.84362 11.33783 10.42563 13.93057
6.093354 35.17354 12.3596 4.671036 23.90574 13.65205
12.43866 12.37483 9.477498 11.602 15.30242 7.299149
TLE5 48.1353 122.8129 45.7145 82.59583 63.84974 65.06149
71.57118 53.51877 96.78884 97.85449 38.87498
42.40044 86.21185 57.28509 83.05705 79.72066
64.59198 91.51794 71.21062 68.61997 102.8245
78.63876 69.35566 72.92059 84.3011 31.31932 93.22265
80.55549 74.24875 65.30513 94.61409 80.64402
88.19744 62.3066 60.9838 50.83899 63.07064 45.47112
43.6491 83.07928 44.21089 39.84655 38.96336 55.24296
40.13521 29.35769 32.12581 51.41753 34.91448
46.07443 42.72367 48.5091 39.27818 35.69575 61.07099
73.00148 46.90756 70.01769 58.95122 46.74266
37.47897 69.02936 36.78105 47.84566 30.13356
44.63278 86.44999 72.38855 37.42135 35.63686
39.13665 30.32422 47.61991 23.28943 38.39581
77.53195 45.491 34.31162 33.10257 17.37785 111.2412
44.29752 29.95261 50.40653 58.01923 39.59669
47.43456 54.82645 33.64128 56.16533 27.58107
40.77685 51.43178 58.26891 70.16142 54.9156 73.78248
42.60704 60.42997 39.55943 62.14823 32.14963
56.05794 55.89054 42.26929 45.37475 75.46458
73.66491 67.15807 59.82551 42.933 46.55676 93.28123
66.47513 54.65947 39.56033 56.80389 47.80931
43.50897 40.44023 26.72131 25.58418 48.31259
50.31625 64.34493 65.52122 68.74578 73.45093
60.58671 76.99671 58.45495 50.62118 51.33007
36.5301 72.4626 41.55522 47.32926 23.20784 46.28686
19.11024 32.46524 61.14324 35.67089 20.7278 47.50587
88.43138 45.70116 56.01752 32.82698 46.82963
43.89516 61.28422 18.08789 57.96069 50.71142
34.70973 67.7688 62.37311 40.56807 37.80057 76.16566
45.61519 34.56665 34.41177 57.99215 55.49579
63.4875 24.45614 39.16178 190.6036 59.0356 52.21668
62.95175 36.54748 51.60047 46.90206 50.40031
43.34735 41.76413 22.86652 37.58983 80.77836
69.92488 41.32994 54.08455 44.60351 53.01932
55.30646 32.09647 48.20738 50.07799 39.67106
37.23638 63.17486 118.2361 35.62151 32.8417 33.60737
59.01571 47.44845 41.95871 70.88142 50.07481
35.19816 38.02187 45.71123 59.67672 55.06301
22.06811 54.48167 26.55988 44.62402 49.9232 30.63542
71.14489 46.53181 41.45863 37.39222 67.44147

27.17683	53.83419	34.0704	53.87824	47.56334	56.26162
47.60744	23.80967	50.80159	67.12959	126.2231
84.06336	30.7601	51.6594	23.20235	37.76864	112.7931
89.05633	64.38648	32.44501	72.60383	43.04484
51.98783	48.68349	74.52072	26.65587	58.31352
56.26084	91.81992	30.50849	43.32077	43.24326
33.60083	45.38624	50.356	29.58384	45.22968	43.81754
105.5617	50.43172	48.17481	45.23997	55.10158
36.0698	38.50382	51.35763	46.10051	50.09779	61.0357
61.62597	50.1181	71.87642	85.92275	57.59107	38.45953
39.55116	54.90766	31.91083	62.03051	73.10576
26.09963	59.12891	34.89393	99.44012	56.60582
35.59703	55.9774	31.82197	61.13377	62.00194	46.40857
25.35384	42.26061	49.96555	34.97191	49.41747
54.95159	39.26208	60.37401	74.0238	45.24205	46.72031
35.84806	71.13423	33.46544	61.40625	56.65557
26.90415	28.94718	52.58334	60.06012	59.29989
63.97424	51.87862	72.00468	47.71521	66.82482
33.48006	43.18877	30.69784	40.89516	22.19618
27.43215	46.68191	83.83085	31.18945	43.62811
56.67053	68.77614	47.90693	83.40514	81.43929
65.67902	55.19176	62.0014	76.0771	42.67576	27.41701
54.35013	45.50987	49.4684	32.24963	116.8362	69.24115
66.9777	51.89915	49.78594	47.05626	58.28172	65.4365
37.03313	37.00436	37.55582	51.12	36.77546	58.34684
46.7979	35.8767	22.06751	49.47116	33.81549	50.0971
72.75228	27.07027	42.25614	63.1754	59.98186	48.99607
29.29002	28.17956	29.97242	38.81867	50.63377
43.48551	9.970855	70.50114	78.34451	71.0043	34.70289
52.17612	42.24988	48.20289	33.67228	63.60236
55.52475	29.19825	63.60345	67.99586	43.51157
67.65547	47.24412	36.18952	23.17218	32.62005
38.35054	37.47043	37.83098	48.38181	40.11236
73.69845	77.9277	69.81267	81.94528	52.41375	65.43849
48.53117	49.98928	51.45222
ITGA3	29.62492	22.94656	22.19604	20.91987	15.51251
17.30748	10.97416	12.08489	29.65376	9.820062
12.1479	5.119313	7.212362	6.999961	12.55095	13.39674
23.40186	25.56423	18.60145	22.27063	25.14364
11.98785	13.5541	14.63163	17.77766	9.63728	12.79384
12.02489	22.68806	4.853203	25.52557	24.44008
81.49076	36.19303	38.01788	4.256465	4.387888
22.30481	35.48781	13.6971	52.23286	34.7798	18.56827
40.34233	27.21222	48.1239	22.98134	15.42349	17.29118

209.5698 25.24814 7.47184 16.73938 19.90428 30.82708
24.7331 6.328614 12.53994 17.75597 35.46635 21.39671
81.48155 8.920785 14.87813 24.55428 10.22053
53.62897 11.63987 49.90606 23.16012 31.14783
10.14551 36.09335 24.3177 32.89939 37.74316 41.7717
16.84912 7.101158 5.029608 51.5234 11.05505 13.68252
22.56431 74.3067 30.74191 22.48761 47.00307 8.951951
35.2911 10.83398 33.9247 49.95079 21.277 31.83003
7.696845 84.31073 22.01313 14.24841 37.3448 11.49277
51.95082 6.359055 5.122239 13.56758 23.10406
63.25934 20.83888 24.1002 18.05106 41.60397 20.31664
18.56983 8.702662 41.48066 65.57359 8.794683
45.01101 22.48193 6.720535 32.06861 48.29381
15.7323 27.29509 5.98972 7.797971 4.944265 20.67016
22.24703 24.00214 50.12311 17.01489 50.95751
35.58357 19.80509 22.72517 21.14338 56.26713
32.88391 7.431289 24.88499 22.71301 43.49659
8.708046 12.80213 23.12492 18.20389 13.80251
35.03151 8.985425 8.339513 38.90728 13.65009
25.96587 16.41544 26.12346 18.76422 14.77052
29.5429 13.0423 17.29414 8.384306 9.929788 13.75218
17.5532 27.84119 27.45115 24.7388 13.61746 4.769976
9.953929 42.77677 14.93322 24.77054 38.27816
37.31906 52.55872 31.16226 17.39326 60.85365
19.90891 28.47252 13.19962 25.69866 17.90997
24.14491 23.65032 38.35224 28.94749 24.9759 46.69845
65.19372 98.34216 24.36841 24.90108 36.49512
8.691798 20.53834 45.95623 14.4951 3.915873 11.7167
9.092078 12.01036 31.52465 37.87358 26.24009
10.35418 33.39448 27.74773 15.88944 16.59526
17.16174 4.753888 54.94133 31.92123 49.53276
36.27029 24.35429 38.16807 30.36999 6.508185
27.82065 56.63167 16.38867 8.535663 15.01174
21.45238 27.82399 77.22394 29.63041 17.04129
13.85951 72.20266 9.088886 27.37029 9.883232
7.510514 11.89302 7.538255 16.52186 45.32253
11.41819 25.2104 21.52474 33.2424 19.80219 31.19252
31.17014 17.53876 11.34144 19.68674 45.68678
40.25258 71.86237 5.476473 14.66435 20.17773
26.03384 18.64846 17.44226 44.63225 14.00237
9.067469 22.04512 16.74389 37.09356 26.003 37.29658
60.40299 11.43184 52.85261 22.21602 13.70928
38.91898 7.656135 33.13672 12.67147 4.921546
25.97989 19.70326 9.187384 44.70122 16.98326

13.15272	23.38478	19.88048	21.4348	18.03689	15.80287
24.41639	12.01852	10.37781	18.80259	159.3166
15.06427	13.91728	28.4824	41.95644	23.93354	31.31447
15.57466	165.1203	9.691177	31.16506	53.01735
17.08435	19.26975	12.9362	16.41561	42.62238	158.0212
11.83312	14.08151	12.62701	20.50568	27.65119
24.03756	35.23998	37.99928	21.3424	10.57842	39.79074
10.13296	4.574207	13.87623	16.8075	16.27514	38.68122
117.9527	23.47231	8.153201	21.2911	28.01318	20.96509
22.99686	34.33271	12.38467	22.15911	32.74031
8.019705	4.701077	31.33859	28.21859	17.05897
23.44578	36.71495	6.366304	33.959	21.49201	16.91151
22.34379	12.38132	14.58604	9.166428	29.86638
30.47205	30.10505	10.73885	13.36913	27.31539
9.85965	130.9596	15.73514	4.755687	4.886519	19.57259
24.42667	20.15157	14.40939	9.133432	35.98487
23.23619	14.5055	15.44544	3.066636	52.80715	9.249665
19.48367	12.9644	14.03609	45.72732	18.88236	8.044599
8.173055	37.80422	18.94191	74.38187	12.34116
23.58827	19.55928	44.9024	19.38472	31.5642	28.29343
10.45474	25.00408	27.93383	12.92166	12.52813
25.62191	2.5522	28.95377	21.1983	19.00149	18.30765
20.46014
PTK2B	15.91866	2.623372	16.1301	13.56021	11.47295
2.681947	7.379708	4.238398	17.35734	14.32266
5.882607	3.565691	18.8809	6.341328	17.95027	1.774697
16.38473	6.445112	9.723616	12.28218	14.25288
1.741857	4.841563	3.231881	2.856138	5.588261
13.51206	2.325714	15.42346	2.788444	13.54939
5.217158	6.966986	6.552556	3.624573	3.34426	3.88378
6.104111	7.043668	14.93218	5.479544	2.994835
7.830634	13.96816	11.99609	22.65327	17.90261
5.561067	6.709532	6.039712	6.044385	14.54204
1.198511	7.275392	5.327655	4.005197	11.16474
10.68814	5.257558	10.78546	7.375118	11.10435
4.924833	5.972778	7.10469	3.539346	4.268155	9.249405
14.82898	9.01945	15.64944	6.320979	3.652393	1.929696
4.611723	10.84736	10.18809	5.091014	1.332067
2.613924	7.483304	4.539196	10.10285	12.94495
2.916787	4.544823	6.798958	13.40915	7.415836
10.49394	7.881858	18.82372	11.36602	20.32688
8.145348	8.894654	17.49491	12.47538	14.44542
8.320137	18.25214	8.144194	4.86541	4.268415	4.983301
16.70925	10.169	7.366154	6.505622	3.420657	7.640606

7.343763 4.149292 14.97262 6.142368 7.587236
4.842164 7.993162 2.275651 6.882219 9.06686 10.18435
7.000533 6.395621 5.00669 32.81887 3.641688 5.855148
5.621854 14.88783 10.72715 4.470348 4.375433
5.235441 9.813839 7.11725 11.23413 8.63893 3.64813 4.0852
11.52889 8.027973 8.062429 8.484308 3.939448
2.766668 7.681417 5.751069 4.68228 12.79832 4.392185
7.014248 6.25153 14.16423 8.151969 3.634378 7.532518
6.074077 2.803977 6.047736 8.924518 12.29802
7.09821 7.988771 3.458731 13.30981 8.332111 4.310014
15.29678 4.527428 4.559797 4.016458 6.528258
3.764537 7.49318 6.31891 8.01847 4.040286 10.59754
5.120085 1.86775 13.24174 5.277182 13.83082 6.591632
7.51945 5.080587 5.287905 10.10147 16.55112 6.470851
7.287406 4.911614 16.41271 8.9028 5.563137 6.92181
13.04969 8.733615 3.161113 5.156875 4.8052 9.905518
4.876891 3.025079 10.16982 4.23925 4.19352 7.236819
9.053767 8.639347 5.469836 10.24722 3.920643
4.75769 8.332214 9.817266 4.091415 6.98197 9.203094
9.529533 10.78314 4.669345 4.946039 27.72691
3.778746 16.54216 5.450577 9.167956 8.109795
8.923985 5.445601 10.35752 15.95358 6.917652
4.006867 17.85457 5.060111 13.16055 3.502429
9.841645 11.74697 5.374702 5.823306 5.528957
9.684186 9.461965 7.546251 10.3422 7.995822 9.616145
11.86278 5.726565 6.537397 12.1459 5.744104 7.384382
5.926776 13.35321 7.311694 6.411455 4.837817
9.629507 6.837275 13.4476 10.11034 10.56727 9.609289
5.50649 7.559197 8.18458 5.602158 3.48398 7.490392
11.80215 7.880513 4.95934 3.973945 25.67905 5.494242
11.56593 11.49252 3.283962 9.864432 7.195709
12.37612 6.693707 7.567084 8.535369 6.112305
10.81143 2.754018 3.204947 12.81031 10.33312
4.88617 9.435596 6.939717 7.005701 17.1985 10.16276
4.658482 16.20303 6.806693 10.39849 14.36307
4.025649 6.935914 8.692941 1.921465 7.636228
5.195165 3.423451 12.73046 6.907465 5.54614 12.58633
1.896533 7.937028 8.668537 4.945255 6.235793
5.07655 5.719555 3.531249 8.612167 5.120361 2.577405
7.008421 5.267484 5.094353 5.235483 10.29894
7.553065 4.629739 8.330009 12.05829 8.210079
16.15488 2.566337 1.404242 6.531011 3.164062
9.002578 5.505885 7.837082 12.21109 6.492821
10.31381 4.811047 6.978728 11.45118 4.511728

4.17666 8.729069 4.303774 18.9227 5.988483 6.097197
6.59593 9.817202 7.851663 8.904568 6.868122 8.608879
3.831833 10.29185 9.99053 4.262866 6.457383 3.809995
5.543621 7.602168 8.04338 9.543858 1.761433 3.478391
2.396743 6.100996 6.193686 7.608346 3.785349
5.509437 3.363121 6.912433 6.986527 4.567422
3.656741 4.912713 4.891326 6.580948 2.313951
4.39099 13.59709 12.91929 4.490766 8.197133 8.731731
6.404052 11.26987 4.693714 19.15283 8.739503
8.279489 8.460834 7.046985 6.168212
CCND1 14.94673 13.4382 19.4843 55.57193 22.08686 8.666714
32.17948 25.21363 38.37193 14.68658 57.49599
34.87865 14.49741 59.1496 34.61179 5.040799 38.4014
22.48052 24.66511 17.00552 49.13193 4.709253
47.02696 6.456188 25.76675 40.92214 37.66252
6.267319 36.4869 15.39133 53.30615 10.13814 53.99436
73.81012 68.24701 22.90737 3.70902 36.52299 61.72289
69.70519 48.24448 59.93037 55.05531 58.12631
40.39387 70.25007 46.22531 49.34157 60.49068
110.1566 92.88292 33.66417 27.17801 60.38481
37.41781 90.37686 45.24741 19.35854 44.37965
34.66645 23.36241 137.2628 18.36747 9.165807
31.37687 11.86075 42.20896 31.98844 46.07315
30.65539 134.2892 84.07728 67.57928 33.60702
59.54497 114.9266 23.89613 64.45978 331.1909
37.62536 16.70516 40.7959 49.49332 21.35723 184.8744
37.93759 71.69348 108.0484 92.01016 29.26676
37.16107 68.84341 50.71332 18.49572 87.73084
63.34955 64.67063 32.41231 254.0065 43.42911
18.61203 19.00286 46.6509 43.78847 38.10877 37.45498
80.06027 19.80093 78.49582 34.07434 65.15087
22.44483 128.2968 7.660031 48.30117 22.60666
36.45648 70.534 26.04562 37.30332 36.63806 62.14861
100.1777 11.34212 150.6228 19.40804 41.3076 42.59543
133.0454 27.90742 47.05259 68.45386 100.1566
53.3363 35.90221 46.99258 56.47584 20.78304 20.96484
59.28619 132.2025 56.31784 37.62492 87.79046
45.39444 72.06088 75.15107 27.17758 74.35889
24.81678 79.0278 41.48345 67.88129 47.49348 24.38447
30.44133 51.74733 29.30568 117.6102 38.3233 29.62433
25.4429 47.81792 38.71751 109.2723 65.54601 41.98594
43.58982 28.24566 36.23219 26.3637 30.71236 27.51017
32.60481 43.38104 149.9644 92.65474 30.39451
46.5256 30.79403 5.618017 54.45859 57.88981 44.47985

27.28875 128.8694 29.03378 159.038 43.2291 77.27482
22.72639 21.76065 72.19793 55.15549 27.10332
54.69119 55.60583 33.66651 135.2732 20.32166
40.66928 13.35918 106.9931 15.29319 130.684 59.04481
15.15417 8.453615 42.798 258.5506 30.61468 37.45741
56.41895 111.6533 64.97622 97.05429 65.49833
10.60412 45.49495 21.85784 71.47691 25.72411
17.71257 113.1441 29.30111 28.10908 66.1629 43.70357
47.98473 35.39041 39.6038 54.76986 29.23679 38.00255
34.22613 42.8421 15.36015 47.31728 29.55313 518.5609
42.28637 70.35685 17.85473 85.10346 51.65249
23.21438 70.4016 19.58039 46.30526 58.43754 62.55667
29.64343 44.72541 58.2181 54.88754 36.30043 64.82349
24.29233 37.69877 25.93198 95.74415 82.23059
39.46488 39.70527 49.8083 89.46187 89.07295 30.97639
130.4388 93.47741 34.09215 82.33278 177.7714
43.1565 12.44762 12.21672 129.045 12.1463 17.6826 61.20238
33.80713 239.4002 42.14211 38.84543 97.91503
35.78242 43.13189 67.14325 46.69425 65.34479
56.64766 14.64545 12.92081 40.19609 25.79383
27.6703 24.22951 58.07276 69.06805 33.0941 31.08428
24.05213 66.48395 45.35664 38.68906 57.85713
34.89885 16.20136 37.99806 86.62443 109.5397
164.3361 47.84828 37.2064 64.44646 28.70594 32.24514
56.92894 42.32517 67.22501 45.21961 49.14836
35.90387 68.03076 17.3656 33.32172 30.60015 73.88823
40.18995 236.4398 33.1332 54.3015 81.00561 97.59843
22.47353 35.21241 18.98713 265.7952 27.12324
67.38052 232.2129 43.74021 48.28789 11.21482
21.47568 43.381 39.23591 3.934121 17.75772 25.29211
47.89804 55.36858 91.09049 47.87612 53.55602
75.63128 32.50641 42.32967 16.82047 14.073 19.96932
20.05919 136.1871 16.50173 21.82758 21.39524
46.06117 76.00241 62.15531 234.466 42.93977 48.51032
224.5329 58.9792 72.65956 67.26163 68.85091 21.54251
50.86772 26.20095 63.83949 59.37816 25.36716
230.931 45.30795 74.81752 79.0383 71.22659 39.65086
83.26438 37.35562 93.45357 26.04725 48.76477
51.45251 36.96605 28.83575 43.95108 69.24783
63.6797 14.5899 6.010358 53.10896 39.38025 32.78522
38.29597 30.47638
CTTN 18.96519 8.994544 18.58149 29.76642 16.25951
10.79648 14.65923 9.123371 20.51239 11.69336
19.9476 19.24398 12.35283 17.98156 16.97448 9.364826

21.92451 15.29167 13.9505 13.51231 28.41829 8.59744
18.33958 9.96297 15.206 21.37207 16.61351 11.79394
17.55898 7.158857 28.38366 11.84063 43.35796
20.70285 35.99617 10.39931 3.08273 17.66247 37.22474
27.63393 28.07783 37.08829 35.5577 26.94464 23.43031
32.08093 31.64057 25.60537 33.34001 43.43203
76.07213 16.72946 10.60934 22.71311 17.75295
24.13654 17.16925 13.36112 18.33527 32.97564
21.18038 23.32912 12.07733 9.551341 18.9778 14.8403
26.60497 17.16469 47.76786 17.37867 31.75068
26.3063 20.48804 32.57047 32.03758 19.21221 23.22581
33.5985 22.0081 16.01195 33.343 25.36397 15.42794
16.61826 34.49176 28.89138 24.91148 26.52858
37.30887 17.75999 20.89113 26.03919 18.44786
19.97894 24.07473 12.45669 27.35519 24.39776
19.30874 22.90229 12.00728 32.77301 27.42981
18.85183 20.64375 16.84528 27.20219 19.31441
27.53125 21.18362 30.14461 27.18059 15.51308
9.984626 19.45926 30.68913 19.3322 64.37867 13.56247
21.24738 17.91693 21.36808 17.76391 15.32838
28.96011 11.42337 18.13635 18.54677 63.95849
24.47322 35.24173 21.53933 33.327 31.30391 18.81741
21.80366 26.31207 35.32483 22.42274 20.78902
68.74384 20.62786 31.26367 13.98705 17.4662 17.36295
35.90159 20.48996 17.05855 17.81089 42.24044
25.83652 34.05073 22.49495 16.35442 29.58144
25.92447 13.50941 74.72201 21.43283 17.71049
16.87368 33.18734 21.65977 35.94086 24.73612
39.13513 19.15371 38.31121 22.75902 13.17248 54.115
25.48321 21.08168 40.17669 79.03916 22.78567
20.00013 38.08667 19.98126 14.85832 29.93378
22.4588 21.26667 21.91167 16.27722 24.36136 42.1663
21.32912 29.15112 23.10813 21.74569 47.31929
27.35726 17.13444 30.45355 22.48639 19.46882
24.96202 19.06184 10.32334 14.45246 154.4958
21.19804 15.73629 35.88987 24.37406 10.89673
25.72931 76.928 24.77978 32.90634 18.39934 14.85022
25.75849 28.96063 31.49892 10.9995 27.35075 22.13537
47.28712 28.31426 14.65075 23.96955 14.03625
14.7392 11.82985 19.86493 22.21286 40.09156 17.83487
18.08852 17.86443 44.57573 17.49563 28.79759
11.34187 26.98779 14.59683 122.9594 18.17527
23.44564 21.59583 28.1668 19.33911 26.44003 24.07519
13.61171 26.91232 23.05494 20.04425 23.58393

41.81018	24.94401	28.02432	12.35117	31.26188
15.80979	25.88311	20.691	53.47656	27.33321	18.27462
19.90456	20.62786	23.45292	101.7904	23.27968
38.03505	47.42823	22.44776	22.07578	32.63563	25.271
26.49269	24.24087	147.7611	13.43429	2.873696
22.01019	18.0376	21.02218	19.87464	12.97406	21.24976
26.01061	33.52164	32.55117	20.43317	23.91037
32.34724	18.60612	11.66504	16.64856	51.61612
19.57285	15.7206	19.46983	23.41696	28.9405	19.65203
21.08805	32.3558	19.02731	25.83449	44.82933	19.41686
31.65485	24.74089	41.52711	29.9092	42.59405	16.4471
12.80274	41.06962	19.41251	23.68059	27.70397
30.16329	25.26761	38.01743	23.82252	26.0989	18.86337
8.047519	15.27287	22.57255	19.65242	17.47086
45.45922	17.12916	23.60608	68.49508	33.32955
16.4292	33.25248	24.36767	67.49327	25.07238	28.65987
22.34084	29.76681	27.13182	20.9494	19.20389	26.41709
34.35105	13.22276	13.78195	23.7669	29.16859	24.47701
51.55773	23.89275	41.57564	117.9939	21.71696
22.2931	13.18421	20.50957	25.87919	13.27833	35.83458
21.98803	12.18725	15.29446	24.57277	35.63382
34.54483	13.78787	30.88861	21.32053	23.2942	45.24061
35.23696	15.17236	26.48593	13.57161	22.44311
22.8978	27.07708	30.24291	11.62652	48.39453	31.85105
21.41583	32.18805	38.54808	24.63315	10.55196
28.91965	28.79308	10.24141	28.33715	26.08677
20.88226	19.94911	21.90257	23.9368	31.37749	22.25112
9.404376	32.71131	32.06511	20.28293	13.80607
18.62395
CALR	182.0413	155.8446	273.1294	430.3138	333.3171
78.87017	319.8901	102.0553	376.1062	365.4102
97.93967	156.9578	311.3004	178.0838	297.8451
69.74072	314.7125	202.9607	262.2496	311.9245
542.5258	83.76609	171.443	78.40493	175.7211	80.89799
316.6092	96.09116	356.5956	191.12	341.8445	109.0369
466.4703	152.1624	300.8369	297.6462	233.7351
962.5256	298.99	364.319	196.9768	217.771	646.5607
227.3288	170.5541	367.6467	193.9811	337.7233	271.16
261.5909	488.4415	168.9442	297.7235	315.9783
133.4221	335.829	278.0453	129.0866	522.2529	500.9152
270.9597	369.813	278.1322	468.1189	343.896	153.0057
332.9821	249.1992	618.7378	226.2125	263.1342
227.3787	182.6821	224.9705	298.1269	519.5745
202.9759	282.5907	284.9434	229.4923	425.2328

264.4368 429.9018 165.5976 238.1514 193.1303
275.8962 278.2205 169.7987 155.9347 398.6876
615.0941 216.9198 228.9667 492.1098 629.5871
202.2564 405.3024 234.4617 227.7435 152.9682
283.5417 126.9826 195.1169 269.8466 133.706 388.1406
261.0801 392.0576 345.6103 213.0413 325.107 512.5002
87.79528 189.3427 261.1607 406.735 637.1966 422.1067
354.814 234.8594 333.7915 465.1618 205.5385 404.2707
214.5545 363.8219 464.733 137.5204 266.8197 339.9202
279.6927 337.5671 326.5931 348.2862 146.5809
262.9478 272.9308 316.469 189.1525 155.4719 333.948
192.6832 201.8684 429.2221 278.2508 213.3807
182.4193 281.5569 124.6945 240.8558 342.4907
262.1548 325.1451 154.791 313.7559 233.5585 151.5628
186.7486 262.01 348.2799 171.7921 219.9399 377.4309
351.2132 275.5057 446.8471 215.8194 331.7668
148.6609 103.6917 435.492 149.6464 254.0985 232.0998
213.3119 255.114 281.1362 274.4423 250.0419 233.4844
215.3105 227.8343 355.3729 258.1748 337.3752
179.8034 193.9233 271.6103 327.9326 534.0611
355.4722 424.6322 598.3109 359.4407 261.4691
344.9562 272.38 282.6385 135.1414 244.0178 189.0317
352.5619 232.95 161.754 335.3155 510.9199 383.4515
207.0855 433.799 200.0499 226.8345 296.077 280.9975
241.474 440.5303 350.0955 491.9842 397.3466 292.654
423.556 276.5436 417.1405 380.7007 129.3545 345.1661
542.4247 158.198 388.3959 424.9222 251.0633 207.2505
328.6256 258.3206 270.4842 219.5989 262.2933
287.3693 289.8896 348.1813 224.3549 311.5854
297.3227 329.5108 289.2524 513.9848 418.415 119.7177
305.3357 337.4872 323.7295 173.3741 469.4713
282.7786 348.5037 498.1722 418.4281 415.5133
280.2203 185.1587 308.6638 397.5497 278.1949
274.5407 243.8868 366.3557 327.0479 357.0241
411.2668 225.8974 351.956 604.1533 249.5553 427.1675
286.0044 189.2252 245.7632 82.06049 377.1578
335.7968 169.2706 310.323 271.1178 260.2231 267.4726
265.6662 345.9145 314.9595 314.3673 220.6796
415.1764 72.62728 101.5407 326.6612 319.9189
261.1725 200.5644 412.1112 301.4795 257.1573
233.1548 224.2741 193.6497 203.8712 283.2337
386.2314 391.2522 338.8761 476.5895 353.5795
791.2829 381.703 446.0362 365.0438 269.8976 145.2195
284.1783 255.5459 280.1082 248.3034 256.6304

288.8994 238.031 340.9278 227.6358 184.3415 452.578
649.8409 281.0677 237.194 293.673 186.9822 465.3674
377.2118 194.7694 162.9535 159.6129 213.6656
220.3163 301.379 625.4929 398.766 253.6597 315.086 138.21
123.9102 330.799 330.5966 304.4895 486.0103 210.8692
373.0482 280.4027 256.6495 426.0835 244.3248
188.1358 279.6906 87.89294 264.2271 293.0387
294.7758 303.7384 308.1752 240.7086 263.1327
231.0422 339.297 234.4567 303.5816 234.6343 178.5438
182.7423 389.7224 429.7977 454.4386 345.5838
243.4167 228.5589 195.6966 313.9545 305.9289
109.2099 360.2873 236.365 351.0313 338.1892 201.6271
355.2882 378.4276 188.6989 291.691 246.2882 306.221
244.4108 570.1744 221.9333 220.2951 185.0114
501.9971 354.1268 189.5777 264.1288 411.0399
331.965 401.2591 135.8745
CDCP1 19.76892 0.2946346 21.2115 12.15099 12.42333
0.4974635 7.727451 4.554448 13.53295 3.773711
33.53216 19.17377 4.365125 20.56581 9.682563
0.208422 16.34555 4.439835 11.60641 14.52554
15.41175 0.113406 31.45299 0.2457581 3.243497
25.48615 8.383278 0.1412399 13.77254 3.424859
17.77435 3.282318 26.16674 25.50566 10.37377
6.813197 2.097949 10.40972 17.04675 25.24886
25.34757 24.99315 16.84152 27.39695 22.48127
22.44411 18.30303 7.814132 15.34132 36.03544
19.24601 7.77325 3.046883 32.83364 5.324152 14.83269
25.74118 2.687111 29.10244 15.15595 15.69116
30.40384 5.748373 20.23828 17.73508 2.065203
8.442002 6.18769 31.98718 19.71178 69.30794 10.96865
14.67263 11.60679 42.41627 13.46683 21.9882 12.96229
16.12834 16.6534 22.24911 9.785027 35.18083 15.39611
20.92676 7.985864 19.44041 27.76496 15.71085
24.40116 24.67268 32.32294 28.45377 15.11429
44.03398 10.45157 45.03417 19.7705 46.02987 33.42461
6.997102 18.27986 5.093097 9.036403 8.598155
16.07284 67.84978 3.830097 19.76413 13.37112
18.54353 7.278683 18.62005 1.464542 29.09982
17.71108 10.61998 26.40078 9.075214 10.88282
13.19807 6.634483 8.036765 15.16747 41.52969
4.493802 16.05379 15.72309 4.258337 13.98018
51.04321 22.55759 17.92944 30.56625 19.86601
11.82449 25.40645 16.52543 14.71644 17.77147
15.49123 25.94114 21.9913 10.46117 12.56855 9.382228

9.019877	8.284647	28.92962	9.688116	23.62064
18.50103	26.07551	23.55031	4.550709	16.5035	12.91801
4.264402	56.37645	6.969698	13.54234	8.870553
9.448367	16.18443	5.575515	21.68751	14.63519
13.08003	9.018927	11.33609	4.021848	18.88491
23.67664	17.21528	24.23173	22.84573	9.499239
10.2694	19.07174	13.89497	10.32764	10.58053	28.14761
25.17625	7.654379	19.84645	12.28536	34.29401
30.73089	58.66816	24.2259	32.38959	16.7374	17.72936
14.18255	27.33824	15.35829	11.44474	28.77803
6.896804	1.971987	7.854303	15.90473	23.36886
41.44694	43.89688	7.272176	23.07579	16.90223
28.14368	11.26091	15.70311	24.79717	9.764087
52.65295	24.997	19.61492	7.570504	9.309699	27.56721
19.38959	17.31021	10.63764	24.4877	9.429156	7.326453
19.19449	28.66529	18.13725	17.66994	18.50727
17.28164	15.21631	57.16028	16.58367	19.91491
4.718194	12.24338	24.66452	13.09263	8.324484
23.06264	10.79559	30.68323	13.75608	15.24162
10.73255	1.593296	16.76604	22.06575	17.52806
20.03081	11.36007	19.82598	40.74498	1.331291
11.20298	10.07236	28.19468	5.171653	11.97742
32.14201	32.09981	22.18843	39.38822	10.77719
25.75647	17.10514	13.77686	33.74658	21.84339
13.17123	15.0454	21.73699	27.70943	10.81096	14.10773
1.896114	1.151435	22.15017	11.01041	22.47059
14.27785	28.49666	22.55961	11.99184	32.51399
4.890438	33.14801	6.365465	15.53007	0.9950031
1.40663	18.68526	25.17811	6.248381	12.12416	35.26817
23.07461	38.89968	30.04664	11.43511	12.86336
17.16562	19.99389	20.96992	7.51041	20.21244	10.09229
17.66443	15.21506	31.31168	33.88158	7.055221
25.23494	5.470639	15.10748	8.336785	24.42686
30.77392	15.29112	11.12365	19.81129	11.89971
9.43452	8.231296	16.68992	4.848195	23.45579	29.26935
20.50031	12.31593	13.27272	23.20558	8.858225
9.796712	11.62561	8.149544	19.09885	17.6308	13.96451
7.226366	7.470371	29.39264	4.627577	33.5899	27.07074
23.79704	7.569318	11.48842	13.69498	25.24192
9.656756	7.933256	21.01119	30.72484	22.48807
17.73248	3.863941	10.61018	16.52881	5.075793
85.99618	18.77551	9.002104	6.739496	33.6458	25.15446
10.65712	3.376578	8.47918	24.16782	62.31227	16.25866
13.09083	8.958801	26.62121	10.12437	13.85582

12.56481 12.81925 30.41326 10.75171 26.92762
38.25199 23.78613 4.974489 45.97469 26.29101
23.92701 7.179386 14.48846 9.233413 16.76623
62.36723 12.20916 12.82769 13.94919 12.63496
16.37576 14.67085 5.304283 44.9426 27.69412 15.91484
13.4448 3.884932
CHEK2 0.9961679 0.3421177 1.616455 1.330229 1.789731
0.6074427 0.8887974 0.4498116 1.093866 2.069895
1.342852 1.350683 1.874174 1.03595 1.578296 0.3560118
1.434359 0.7764553 0.9917956 1.09377 1.49196 0.4366631
1.240544 0.73859 0.4636613 1.290311 1.116005 0.4299024
1.030304 1.544075 0.9020259 0.3961668 1.547327
2.021673 1.60771 3.121824 0.9781662 2.595888 4.417225
4.401755 1.189687 5.553447 2.168604 2.978778
1.596481 4.400631 2.395801 5.666645 3.95015 1.664122
4.364999 2.134552 7.815845 3.881761 1.012177
4.717291 6.623076 0.9816369 14.35346 1.231618
1.752217 3.544617 2.868126 2.236986 2.06113 0.9293464
1.096012 1.595307 1.920676 2.918301 3.762692
2.882939 1.391422 2.855302 2.325529 2.175877
1.500568 1.236314 4.714906 7.366914 3.153141
2.505818 5.083489 1.889947 0.7721944 2.706787
1.927278 2.687398 2.811809 0.7517195 4.759265
2.921783 2.028564 2.054933 2.39013 3.457997 2.070617
1.617775 3.611774 2.805195 2.454934 1.464825
2.982249 6.00827 1.686632 2.066472 3.187853 1.650528
3.45541 3.750362 3.628164 7.452249 3.342527 1.577668
2.484524 1.133447 2.837536 2.281478 3.954226
6.568596 4.083623 3.45283 3.490949 1.204739 2.919889
2.37137 5.139645 3.543298 0.8902357 1.412229 1.919881
3.869614 2.812396 3.870218 1.521248 0.9433321
3.720056 5.238302 3.485835 4.533441 4.054876
4.051665 1.773646 5.691068 5.694385 1.991337
4.603444 1.673359 3.811537 2.014693 4.496215
1.869063 4.227364 2.848074 1.658462 1.354707
3.30658 1.104781 4.719988 2.347108 2.569465 1.592735
1.965701 3.979103 2.592706 2.405773 2.120665
2.173521 2.359834 1.6496 0.7268006 1.429483 1.74395
2.677003 1.901375 2.306532 1.252076 3.485217
3.988544 3.230879 2.656459 2.68882 1.406283 2.872484
2.399202 2.548358 1.481046 2.56752 2.918816 3.320688
2.156804 2.613291 3.501853 1.630179 2.243733
1.412051 2.426874 4.568036 2.408992 1.618043
5.143465 2.468941 3.345911 6.425535 3.165689

4.016863 2.096383 3.466386 2.520069 3.518751
2.45417 2.19589 3.047446 2.680973 2.410173 3.305218
2.338595 17.07829 2.186453 4.211112 2.965544
6.848421 3.38354 4.590733 1.465091 4.238326 7.32305
1.364129 2.884418 1.710627 1.642295 1.680504
2.127388 1.602344 4.888544 1.315855 2.844623
3.828379 7.620377 5.78046 3.709436 1.947221 2.730476
3.007782 3.337082 2.12561 3.609237 0.8074313 2.42745
3.321424 4.874731 4.567808 3.050618 2.225638
4.106775 4.658577 2.139009 1.967027 5.740014
2.411292 3.184789 3.448833 2.826701 3.242988
2.869097 4.508818 4.881307 3.2321 1.692193 0.9719575
2.839641 2.564422 1.948454 5.632623 4.646331
1.95011 2.756638 0.7748699 1.993154 2.003365 1.809685
5.237809 0.6840934 2.385455 5.387252 2.585338
2.065719 4.227593 2.594488 2.497151 4.720987
0.3744242 0.8117 5.961117 0.8761182 2.063102 2.401028
3.46285 3.019629 2.152268 1.989085 2.491715 2.198482
2.022752 2.264504 1.636462 1.495717 5.637581
2.374062 1.508818 2.857261 2.012079 3.584913
2.511131 4.524245 1.940358 1.482387 3.120751
1.667973 1.976487 3.75213 5.954827 7.637079 4.756813
1.756642 2.883394 1.778639 2.362287 2.75624 2.270734
3.17645 3.335413 2.071952 3.274792 0.6296876 3.492795
5.114501 2.716123 2.898285 0.8015698 1.339836
5.562525 1.609233 8.219557 0.7978444 1.88522 1.305645
4.109098 2.861157 4.312743 2.937314 3.889183
1.558334 2.524361 2.237344 1.313283 1.810788
4.074222 1.172557 2.271481 3.602732 1.866621
1.267853 2.696335 4.849478 2.386842 2.695958
2.580302 3.506796 2.223962 5.802487 0.8509367
4.463101 4.309133 3.429719 2.632628 1.996318
3.553844 0.9949171 2.976218 1.829061 2.371079
2.192405 3.149054 3.894949 2.603269 1.486019
0.788554 7.480988 3.597422 2.454276 2.617247
6.034243 3.1745 3.439981 2.701024 2.320405 1.248673
0.3788545 2.951493 2.712782 2.955281 4.549893
3.023502 5.083496 3.207674 1.172184
SKP2 3.566838 1.07173 5.530884 4.376628 4.484812
1.648906 1.953996 1.778899 2.642224 4.236402
6.783193 7.022435 3.917661 5.687338 2.728475
1.281971 3.074092 2.754004 1.781271 3.663444
5.004224 1.49331 4.133072 1.889057 2.650408 5.753057
2.828982 1.6557 3.321972 3.462502 2.931882 1.449285

10.10966	6.95259	6.550531	15.53011	1.25686	9.562961
10.30115	13.90309	8.65343	7.757302	10.75907	9.37648
6.733676	7.408447	7.974612	9.938732	9.727337	
7.237019	8.095145	5.987414	12.90144	7.589259	
4.023551	29.43645	8.228086	3.40413	8.303424	3.408496
2.656696	10.07457	6.622133	9.583885	8.852972	
2.296372	3.637229	2.816618	5.192882	6.043977	
22.52415	14.55058	8.798643	3.174065	10.9019	18.72221
3.610113	2.453422	13.53525	17.74106	14.56977	
6.009741	7.977867	4.648801	4.215738	16.28885	
13.26985	8.36616	9.196753	3.476521	9.180496	14.01557
3.768289	5.264416	6.145218	21.06267	5.009951	
7.802848	20.40314	12.34299	6.532743	6.351514	
5.714485	15.36532	5.172978	5.80962	5.932064	4.228068
14.19371	9.979844	11.99046	21.6317	9.78805	3.552249
4.371687	4.963319	12.01934	8.081446	10.35807	
33.27889	8.412855	11.45481	11.61587	2.432292	
40.55001	6.247254	18.41085	4.828285	4.489342	
5.807063	5.230313	15.68285	10.49484	9.485073	
3.005003	5.091584	26.81375	8.528919	22.9484	34.54973
22.82744	16.36261	5.696765	23.3738	14.76359	2.615072
13.1091	3.259889	18.10986	5.969711	13.38156	5.317267
31.93347	6.8865	4.726149	4.86275	8.468485	2.304496
7.5098	4.39593	3.80101	4.007611	8.85567	21.33921	8.405723
14.3196	12.76898	3.726465	8.214639	8.087848	2.044267
9.249947	8.831137	16.3023	8.174668	14.97526	3.717823
3.762254	7.554818	18.53826	15.80428	6.933238	
5.215705	10.36074	4.33845	4.252705	4.731425	6.803509
23.3736	8.93379	6.481423	6.559768	5.649211	4.463077
10.22224	5.16603	5.471794	16.70797	15.09359	9.347946
17.70565	3.921777	9.48442	8.423086	7.565404	11.13251
6.620255	26.00595	9.8815	6.889806	5.724705	8.946499
8.484295	34.8292	7.140294	16.36551	6.130898	27.9717
5.807412	6.948319	16.04877	5.396901	11.44236	
11.77475	5.048413	12.44609	17.94884	5.766802	
11.16127	4.457579	4.395787	4.44275	4.360867	5.287723
8.952518	2.395687	5.81968	8.705308	9.235139	38.73096
15.12671	6.275657	10.60002	10.17227	7.015091	
7.763731	16.51758	2.645157	4.733281	6.587708	
7.913752	12.48535	3.448244	6.981131	17.41911	
18.75075	13.94508	4.529363	11.47894	5.955377	
4.423188	10.77118	7.766304	9.188055	6.337334	
17.34919	10.61077	9.058262	41.73122	5.664992	
16.41073	2.721957	5.268907	14.48973	6.243883	

12.33972 6.274975 2.145711 4.169209 10.22161
4.895998 15.79697 1.967809 10.27238 16.65907
8.79116 7.239035 5.817529 5.947788 11.48808 13.82833
1.746244 2.828231 7.52897 5.43667 3.279672 4.530351
9.877464 9.932437 3.454326 6.300632 11.87429
13.78415 4.885498 7.052325 12.90716 13.67911
8.858144 8.16365 10.31713 30.4188 5.944469 13.58772
4.890153 20.52494 7.362629 5.016236 12.47015
5.378492 2.613183 13.15492 14.73688 7.974658
10.5903 3.535563 8.916422 8.201186 36.33716 8.296894
6.088323 9.300002 22.0187 8.942859 7.646412 2.366734
6.10296 9.576034 12.39711 9.242068 1.958946 7.062156
10.4903 5.08625 12.33243 2.742447 7.967021 12.46828
79.80223 8.497385 11.77696 6.571064 17.28268
3.690517 20.64519 17.50853 9.338393 5.74443 9.036252
5.872448 9.020108 9.640611 3.217698 5.554148
6.185853 15.86051 9.245127 6.963092 7.166999
6.965833 4.459519 16.40537 3.521981 12.03747
9.081995 16.33551 15.7057 9.833308 17.42336 3.780227
8.700845 6.563161 10.40684 4.381063 9.892843
10.06676 9.414718 3.057732 3.964965 14.26387
9.464427 4.998802 11.84502 13.24648 8.927211
7.920189 6.178676 6.890885 3.929783 1.117363
17.45743 10.59945 11.00442 8.324393 14.10485
14.28063 11.0101 5.788796
HGF 0.1779948 0.3981824 0.4716523 0.9858699 0.6382942
0.8682305 0.3027657 0.4344765 0.4159713 0.5462672
0.2915568 0.176452 0.8170436 0.05205635 0.4046593
0.7101472 0.6612515 0.6762361 0.7540928 0.5371063
0.5323859 0.5480661 0.6190848 1.246459 0.5894143
0.4684982 0.5712532 0.8070208 0.8772698 0.3075469
0.3645618 0.4362046 0.370425 0.2828713 0.3616052
2.903152 0.4061087 0.5030596 0.611374 0.3396127
1.459814 0.1108443 0.9280107 0.4157162 3.627089
1.18477 0.6194365 0.4124786 1.373425 0.07173447 0.1319562
2.421955 0.1979632 0.2725464 1.445496 0.4456509
0.3980095 2.509322 0.06058406 0.4184125 7.352827
0.2828273 9.176027 0.7471538 0.159552 1.918742
1.25891 2.091101 3.624263 0.4254942 0.171546 0.6107149
0.7850858 0.9410068 2.746018 1.305356 5.652111
0.4240315 0.3052766 1.306921 0.1561644 6.76048 0.6576858
2.30209 0.3405744 0.5660376 1.987716 0.6396849 0.9062573
1.392485 0.9662113 1.941893 0.5733348 0.7057888
0.2462281 0.3369885 1.451505 0.4075293 0.2096689

1.087471	5.362816	0.4337084	0.2821924	1.290842	
3.528397	3.653559	0.1813907	1.441265	0.5978348	
0.1415072	0.7881771	0.2189679	0.2149329	0.8388957	
1.370655	0.547124	0.1863673	0.1552462	0.9325089	
0.2716334	0.2653134	1.159483	1.005224	1.91301	0.230745
0.902834	0.5541291	0.1024366	2.282497	1.086704	
0.3363514	0.09481577	1.070047	1.271942	0.9491571	
2.258283	0.374333	0.9916528	1.157368	0.5785248	
0.4460737	0.3937575	0.67201	0.5226744	0.6504252	0.6457775
0.08903229	2.891356	0.05324824	1.893428	0.3199433	
3.394326	0.1435345	1.493081	1.787638	0.4102011	
0.9455142	1.575864	1.128514	0.453469	2.729634	
2.383258	4.251693	1.53308	0.2715828	1.594456	0.4298167
1.549112	1.12086	0.1909877	0.5553992	1.94009	0.8537107
0.6015373	0.210274	0.4152914	0.7898431	0.3832965	
0.3817418	0.6027883	0.1921589	0.501372	0.5394835	
1.407268	0.6854005	2.11048	3.048054	0.03093222	1.335961
0.3331903	1.041912	0.2804933	0.3376435	0.8712718	
0.5503649	1.516262	3.963279	1.854127	0.3377832	
0.3909428	0.3392057	0.7533424	0.8917093	0.1066456	
0.1822923	0.6659763	0.3325387	1.758603	0.5727414	
0.3585859	1.249065	2.761417	3.330096	0.2305343	
0.1379064	0.3200361	0.3879595	0.2664928	2.55759	0.4164758
0.7664974	0.9554522	0.7216131	0.1181494	3.048683	
0.144248	0.7992004	0.1407042	0.3167831	0.3769763	
0.239504	1.931212	0.1086252	0.3955323	0.7338336	
0.3748726	1.364303	0.8087526	1.342361	0.6219196	
0.5442515	1.770513	1.199711	0.489691	1.180516	
1.218546	1.174565	0.7305531	2.945397	0.5495848	
1.277106	1.031945	0.5017892	1.252257	0.468937	
0.7881109	1.059321	1.228899	0.2652334	1.273936	
1.080216	0.1806878	1.814414	0.3576223	2.068132	
1.74882	0.1965629	2.455138	1.037293	0.5386041	1.633141
0.2796046	0.3441522	0.7686217	0.1023011	0.7839988	
0.5486801	0.9083872	0.2853771	3.343192	3.981771	
0.7430411	0.5058209	0.9450201	0.3419508	1.745227	
1.251363	0.1619543	2.290777	3.269752	1.109469	
0.8292572	1.998364	1.356981	0.5580157	2.568654	
0.8892295	0.5177914	0.2104712	2.228345	2.51869	2.813585
0.4233267	2.545236	0.8158304	0.4819621	0.531969	
0.9148655	1.642816	0.3797516	0.6555524	0.1088736	
0.226457	1.90741	0.4897798	1.38357	1.217553	0.3072455
0.6815423	1.379416	0.6765333	1.842332	0.4098318	
0.4381881	4.888359	0.5099813	0.5987872	0.4930712	

0.9229025 0.3295178 0.07120747 1.994932 2.430851
0.6699422 1.439106 0.2483625 0.06113524 0.5461308
1.553489 0.5110582 0.5232278 0.2911684 0.4707678
0.06018986 2.069782 0.1621771 0.2759004 0.2567436
0.4006866 0.2713989 0.5817733 0.9932394 0.399642
1.756988 0.6014465 0.24317 2.586758 0.3341678 1.2106
0.2830077 0.9363407 2.772671 0.1831243 0.6073932
0.5410286 0.5516792 0.4088461 2.176309 0.190049
0.4680215 0.7202435 0.6469848 0.3711462 0.7199374
0.617693 0.6473062 0.184052 0.5798834 0.7222289
1.656778 1.01767 0.8999674 0.2045245 0.120274 0.5163111
0.3725456 2.610543 0.5071935 0.08525963 1.882249
0.8852369 0.2405116 0.52584 1.444256 1.650362 1.922092
1.580198 2.46844 0.3810977 0.3090465 0.1519631 1.182238
0.1767187 0.4240184 0.3467125 1.403162 1.036979
E2F1 1.932892 1.115631 3.73743 3.304696 3.867685
0.7289205 2.995399 1.154793 4.588092 4.530693
1.997961 1.56231 3.890207 1.893899 5.337129 0.5719323
5.129288 1.818075 2.400937 2.020737 6.138378
0.6821309 2.591933 0.645837 1.152898 1.875165
3.19741 0.5094604 3.028551 2.067834 3.854737 0.7960643
11.55884 4.990817 7.178047 7.48185 0.9724179 17.85536
13.3532 9.69328 7.64985 27.96377 6.693956 8.052597
4.154088 8.405526 8.158849 17.13858 5.780249
5.822877 13.15331 4.753527 15.63635 13.79851
2.410591 12.50854 18.18706 2.397963 32.61069
6.979901 2.293315 9.134949 2.653487 19.94215
10.44004 1.604761 9.448641 2.217547 15.7985 15.96773
10.66843 10.85404 6.597564 9.942513 9.652412
23.44111 2.74004 15.42671 10.27566 15.32388 13.27174
6.169272 6.789472 3.867609 8.461857 14.98884
4.700317 6.885902 25.51208 4.196715 4.810441
14.22633 4.849401 7.64875 12.86783 26.31091 3.837215
10.63512 20.32215 6.071765 2.899681 7.236539
15.48729 7.338564 5.845658 3.283893 11.01115
4.068074 8.874801 7.351427 10.38737 17.92487
7.354643 3.589779 5.94101 10.43817 23.12727 12.65985
12.75684 13.79069 10.12144 8.461409 21.2621 1.163843
29.99556 8.996893 10.09257 6.993511 3.38185 22.2147
5.263029 10.2099 9.603291 5.731873 4.995837 2.766961
9.866165 8.918406 21.13167 11.2492 14.23863 10.84251
6.886089 23.57776 23.02097 3.145079 10.01396
3.548218 30.67753 2.948384 14.54285 5.583736
13.49133 11.38166 1.76459 4.210189 8.754841 2.144363

12.81673	5.72894	7.874051	4.027269	4.867434	17.16134
6.178243	8.381521	19.6299	4.166399	7.383301	3.744516
3.095447	23.30112	7.524431	11.382	25.41851	13.87873
7.091807	13.51574	8.917664	13.56104	22.2366	9.737308
4.290252	13.12465	7.855543	7.06429	2.744778	7.947789
9.052245	14.43285	10.1747	11.62786	21.70888	11.00355
10.89934	8.945427	5.072875	10.48839	10.79413	
5.748281	8.413235	4.960792	13.56047	17.51802	
21.4597	13.25382	17.0099	31.5225	7.152513	18.98836
7.617526	7.051652	6.540759	18.78315	9.441787	
8.48106	13.54789	48.4435	15.48214	9.815025	19.62428
7.99175	9.358285	5.384713	3.634149	6.510315	15.32466
3.000829	20.04168	11.87415	6.542108	5.332351	
10.44774	4.735293	5.432505	5.58999	6.630386	9.421662
5.58419	55.60368	9.359797	9.946052	6.396621	6.372697
4.632151	10.46885	11.37503	1.41167	6.985315	20.05253
19.86952	7.545774	13.82581	9.133884	25.46304	
15.38979	12.42004	5.673799	6.730258	6.198323	
13.0772	19.84505	7.361207	7.770993	10.63978	13.97772
25.23325	11.28431	4.183528	2.448623	11.00156	
19.15949	4.350414	9.225453	12.19382	9.675414	
14.95232	1.333371	16.95824	6.069786	2.336109	
12.17144	3.70912	12.3613	8.504096	11.04465	25.78658
13.56245	7.029734	4.349563	15.05331	1.046707	
1.295412	10.83912	9.526071	4.13228	8.720015	17.53051
6.300265	12.24022	3.759731	5.263002	7.216762	
5.194599	7.156673	10.9632	5.515655	11.38172	13.76295
13.44378	11.82738	31.33199	28.32208	5.120788	
9.108175	4.482732	4.753627	12.25878	7.321005	
3.693306	9.139638	9.133436	12.71405	15.12026	
1.343031	7.688544	13.91776	30.80834	7.888983	
5.130322	13.7562	6.999169	8.617608	13.90603	3.504963
8.383947	9.498618	14.091	14.02256	5.259118	6.0343
36.33366	10.10614	13.06373	2.319183	3.628333	
6.772943	26.15684	10.67036	10.81839	6.544902	
7.50753	5.137707	10.56095	20.94133	13.0065	5.085982
16.14419	1.953563	11.09251	14.19852	11.33244	
8.682832	17.73767	21.70361	13.00809	10.31242	
7.701106	13.6328	11.16376	19.32805	2.292654	27.89748
4.617777	18.14577	21.52289	13.95487	10.07545	
5.469661	6.502519	15.16542	15.31164	7.574537	
34.09745	8.364484	9.604831	5.09325	6.902289	20.05903
19.18982	4.363101	4.444103	10.37156	9.479683	
10.54702	7.925867	13.53037	3.120043	1.757654	

13.46105 29.81088 7.235447 10.27417 8.327104
8.489267 18.84025 6.468893
EGF 0.1920708 0.02947684 0.174493 0.004530313 0.04354401
0.05763452 0.007327421 0.002841902 0 0 0.06716911 0.03261052
0.01098543 0.06002805 0 0.00878367 0.03784045 0.02320998
0.09954692 0.04051587 0.002770882 0.002361559 0.1689342
0.09645658 0.06496295 0.03168543 0 0.01312497 0.05040536 0
0.006701003 0.01487879 0.00686672 0.05227813 0.03474833
0.0051981 0.03701104 0.1512096 0.00733698 0.02308365
0.1474472 0 0.002468062 0 0.2026665 0.2896415 0.05518295
0.005097524 0.02414463 0.5208272 0.008211998 0.002237223
0.09412465 0.02852352 0.1063371 0 0.01740809 0.02187726
0.02139907 0.03214686 0.005384277 0.04527321 0.03070158
0.06659479 0.1011583 0.01928166 0.02264852 0.002396592
0.007147283 0.06625833 0.002025216 0.003437277 0.07037167
0.4886437 0.009226018 0 0.06075475 0.02702285 0 0.00232286
0.004123017 0.02590204 0.00761987 0.7217258 0.004995426
0.006887779 0.09515464 0.01172094 0.6498636 0.04546878
1.327625 0.002647715 0.01215384 0.03202731 0.01364684
0.004844952 0.3332827 0 0.001989932 0.00961409 0.02968864
0.02071667 0.01092722 0.002844353 0.01223395 0.243265
0.006841478 0.01771106 0.006290683 0.0189676 0.5729729
0.01332778 0.1165186 0.01568444 0.4366955 0.06777532
0.002653172 0 8.453889 0.009922972 1.079712 0.9158214
0.4938447 0.2509334 0.02051413 0.01380003 0.09326125
1.715731 0.008785705 0.9876213 0.001707745 0.4589391
0.05308052 0 0.02650516 0.003022347 0.009021875 0.0255315
0.06002182 0.07156188 0.003230635 0.147313 0.07747562
0.02496625 0.4789547 0 0.009234539 0.01824567 0.004179547
0.8153355 0.004382913 0.004472678 0.002148722 0.00235663
0.4800022 0.05956519 0.004237588 0.002807739 0 0.1507367
0.003574116 0.02357797 0 0.004314306 0.172352 0.5308261
0.03404377 0.01551353 0.003229361 0.01751575 0.6616381
0.09281469 0.08601799 0.01447775 0.08618419 0.01690776
0.03440787 0.08195098 0.002281178 0.09054902 0 0 0.01327767
0.008836716 0.02818842 0.04101789 0.04802914 0.009624985
0.02346708 0.006150315 0.1647171 3.453334 0.03845421
0.1558807 0.02038733 0.1162098 0.03484247 0.06359937
0.07248681 0.01107682 0.02189154 0.003886062 0 0.001935747
0.01345964 0.007354086 0.00571454 0.03550547 0.02215118
0.0111261 0.04549575 0.005040662 0.02861316 0.0104612
0.104678 0.002074659 0 0.06011238 0.002484194 0.03195904
0.05089396 0.0199987 0.004977921 0.003397314 0.02067718
0.1249086 0.03182852 0.04969855 0 0.06872199 0.002675255

0.04136146 0.2659834 0.04677129 1.758947 0.01659608
0.1707846 0.002010937 0.0107101 0.00726344 0.1012302
0.007128347 0.009433738 0 0.02968353 0.0227728 0.07106596
0.01898585 0.09327875 0.003381571 0.008526354 0.002675865
0.1678096 0.00859763 0.0118572 0.01448625 0.01373426 0
3.312689 0.04578909 0.008690188 0.003321218 0.03936916
0.009798159 0.1455384 0.003607184 0.004106885 0.9467376
0.6399105 0.03922787 0.05836822 0 0.07942927 3.428527
1.444248 0.002366824 0.09002677 0.01708144 0.007626907 0
0.02791971 0.006680916 0.002720374 1.514295 0.005190374 0
0.01869446 0 0.04588182 0.007717185 0.6161052 0.04914948
0.02538603 0.7457463 0.02906881 0.1286185 0.1432194
0.005332507 0.02114384 0.01821261 0.01471192 0.02877182
0.3882389 0.006450139 0.1760989 0.06146043 0.08468949
0.003288301 0.002081613 0.06215611 0.1959304 0.02982925 0
0.2464733 0.009157183 0.3575646 0.8229923 0.08300472
0.4234511 0.01418923 0.2116408 0.008492841 0.002220581
0.004894551 0.02438465 0.008511982 0.04599356 0.01245195
0.061356 0.04330243 0 0.01941579 0.009369161 0.009752593
0.03482869 0.01587752 0.009344665 0.06840194 0.00711614
0.01589092 0.0530849 0 0.01470109 0.1165354 0.01774217
0.06233742 0.08346547 0.001827533 0.02830553 0.07238528 0
0.06566874 0.004044035 0.002825379 0.07542645 0.04708087
0.004411303 0.160609 0.1024548 0.02018081 0.3461514
0.007886755 0.5033379 0.09684641 0.09746489 0.02958578 0
0.002831731 0.1928462 0.1813757 0.2304445 0.04625156
0.01803984 0.02349256 0 0 0.01104386 0.1001035 0.02862076
0.004773418 0.5579283 0.01320041 0.149909 0.02360129
0.2018514 0.01027163 0.003251083 0.2595265 0.1495007
0.02619689 0.01565236 0.09524898 0.9171149 0.03447451
0.01461272 0.14 0.004245331 0.0062995 0 0.03106838
0.02087373 0.01091716 0.007030486 0.003611238 1.245165
0.02235531 0.01317571
PIK3CG 0.9359076 0.2436487 1.334145 5.656764 2.709076
0.8088765 1.665586 1.21857 3.547898 5.810766 0.3743422
0.05294751 5.39143 0.1263416 4.561429 0.4192868 2.580438
1.999649 1.155594 1.721568 2.221636 0.3952819
0.4114307 0.4543367 0.2869426 0.1463584 3.548513
0.8543428 5.89923 0.7616276 3.759524 0.6873964 1.551405
0.6481782 0.7739027 0.5102246 0.3594615 0.6667405
0.5875059 0.6071039 0.6331242 0.2354172 2.170095
0.4110846 2.076332 1.278181 3.754515 1.814436
2.492179 0.9079712 0.251716 5.409016 3.06211 0.6704675
2.31204 0.1686626 3.086466 5.731381 0.2137296 3.152086

4.358726 3.003766 3.621132 1.677174 0.2899555
0.6922968 1.233563 2.728782 1.841258 0.6366733
1.958575 0.5984221 0.7561786 1.178807 1.09052 3.398529
7.91131 0.7299237 0.8789493 0.8376096 0.647518 3.382784
5.306408 1.544309 0.456782 0.9607406 1.73187 3.327311
1.158256 0.6770546 1.891146 8.765102 1.975729
6.277004 2.498555 3.494122 3.21556 1.207384 0.6928853
1.830244 6.791428 1.335665 0.632253 0.9404302
2.037627 6.834406 1.304186 2.015989 1.750577
0.3756229 0.5489795 0.4170506 1.03621 2.652299 1.362286
0.5932833 0.8470656 0.1766322 1.06062 0.3588418 0.573003
1.966294 0.4876732 4.748727 0.4215576 16.77748
2.068367 0.4054223 2.961559 1.449012 1.018104
0.1310983 0.9572755 2.137935 2.733092 2.385783
1.430864 0.7650106 0.9652894 0.9483511 2.566891
1.405979 1.486632 0.3637187 1.463786 0.4557123
0.1113154 1.082931 0.05829834 4.049368 1.40384 1.846195
0.6726917 12.9416 2.460047 0.400036 0.7246194 1.670984
0.254661 0.7213418 3.460202 7.224465 3.683068
0.8915262 0.2746621 4.337117 2.208191 2.287553
4.551182 0.1508136 1.375327 3.099764 1.301766
0.1371215 0.09922414 0.5163194 1.637713 0.2716607
1.70644 0.7201065 0.3622271 0.613659 1.26487 6.660539
2.504739 2.800143 3.756209 0.3779 1.658877 1.226444
2.096899 0.4853797 0.3054374 3.58825 1.272303 1.856803
5.140169 2.594415 0.5417397 0.1201704 0.1300581
0.363372 3.031212 0.2420066 0.1854237 1.607061
0.4221633 2.92069 0.2684029 0.8207946 3.102472 2.841028
2.838116 0.2621116 0.9334287 1.380466 0.5270094
0.5764526 2.918362 1.209067 1.175154 3.414573
0.9617956 0.3492625 7.958125 0.1231016 0.8575628
0.2567479 0.2152218 0.6243543 0.2598282 0.7037117
0.2141036 0.4039976 0.9395183 0.524711 2.766864
1.612328 1.449633 0.582868 0.4587421 5.148245
1.543296 1.001866 1.413359 5.682335 2.888122
0.8042795 3.769433 1.45696 6.030176 1.210965 0.8897354
1.225889 1.32662 2.200222 0.5169406 3.070398 1.856284
0.7498675 2.119017 0.3706076 4.0458 0.2606843 1.954853
3.08597 0.2206526 1.575231 0.6937188 3.245017 3.238723
1.195245 0.2872379 1.334568 0.4860396 1.435481
1.202252 1.278565 46.74057 3.298176 6.390973
1.50655 0.7139299 2.517359 0.874137 3.735181 1.462184
0.09060499 2.131118 1.654513 1.751563 1.109593
1.39621 1.666329 2.729386 1.26662 1.909611 3.54271

0.3773445	0.5540751	2.559791	2.60252	0.9798595	1.075193
0.3928445	0.5981498	0.9784421	2.554951	3.322933
0.2338303	2.284127	0.4962053	1.061174	5.201459
1.42867	1.420622	4.992017	0.4627586	2.532607	2.72443
1.723263	2.503795	0.5093458	0.9659162	3.595289
1.204882	1.163145	0.2187155	1.107732	1.098391
0.2981448	1.404256	6.527019	2.851673	0.8756528
0.543708	0.09172348	0.5742288	3.58314	1.226487	0.7473099
0.6652047	0.3848	0.1286163	2.471098	0.2934157	0.3751365
0.3814255	0.1429798	1.067835	2.18664	2.404917	0.5828842
0.6918601	2.957321	0.2814457	5.542346	0.4413418
1.593448	0.6046859	3.296188	3.161719	0.223233
1.25437	0.5645458	0.4185553	0.1553607	2.759033	0.7454801
0.3002935	0.4971321	0.5435894	0.6710092	1.189377
1.774767	0.541911	0.5134835	0.5353973	0.7625241
0.7644592	2.479459	1.328822	0.4880467	0.104857
0.9552661	1.496414	1.363789	0.5870272	0.2823855
1.159377	1.898229	0.6024083	0.8575405	2.618054
3.099438	4.068692	1.458466	2.23468	0.5386971	0.6099095
0.2711302	4.898272	0.5629635	0.4011047	0.4690961
1.950129	1.12019

YAP1	6.49588	17.23564	11.34325	12.77423	10.02778
28.24238	6.4034	20.63151	8.856445	12.4667	40.95485
31.54929	10.98277	27.29793	6.54597	29.83557	9.588237
22.14218	6.638776	7.555453	14.10811	22.53124
30.68401	24.40002	33.85862	32.93714	8.766506
25.99968	9.814932	6.48759	13.25746	21.24957	16.68818
27.18898	29.40267	11.53354	0.8404101	10.95133
32.31045	17.49744	23.93104	25.21844	21.62112
13.34832	8.725487	14.62651	11.30689	20.48022
33.1865	17.79309	9.874914	14.58051	14.1779	14.4341
24.5929	15.56523	12.69883	36.40121	44.2501	15.9861
22.69074	20.76389	22.97348	7.876777	12.95953
40.60236	11.32886	20.25102	15.2834	10.59726	19.34787
13.82995	25.35002	30.0615	15.26682	10.39166	16.12471
29.15265	38.13368	24.16936	21.52623	27.21071
16.88224	20.49162	26.41894	15.26472	30.06903
20.35173	13.66026	34.68025	19.0396	18.26583	24.28403
15.15331	12.3562	17.29662	36.33688	7.134203	63.96898
18.87578	20.84538	14.59626	7.980747	37.67252
28.4019	22.95287	20.85567	24.94	21.07133	13.26856
33.85246	21.53581	17.15849	17.04417	23.92111
11.57895	24.66283	13.64552	21.99085	29.62649
20.47491	33.378	28.70348	14.53619	40.39056	7.883177

18.69402 10.13286 37.09791 26.88762 10.80903
22.23642 17.4925 20.74574 12.9536 30.84458 29.37031
12.99703 18.13017 22.3154 19.61647 17.49706 11.15142
18.35084 23.1481 17.29946 26.18413 24.93916 24.05842
11.61998 25.357 19.26252 18.17327 32.40076 30.02033
11.79615 23.82755 25.92475 34.76631 22.25063
26.22894 14.24125 18.53889 24.47515 13.03996
15.52297 15.12198 14.78438 14.91829 6.804028
21.72422 9.361746 29.03227 24.77338 17.009 34.07606
23.2939 22.49649 20.39908 12.81456 12.68596 26.77379
26.4705 15.13085 15.93364 18.15077 41.49247 14.05306
53.84052 41.61926 27.50952 16.58677 27.35315
8.25864 19.34408 33.57574 26.07759 21.5362 54.70932
17.04777 24.14089 17.56434 16.09162 36.87513
51.3106 15.83416 10.30203 10.01754 28.09804 18.80079
24.18865 29.31933 17.10118 24.49911 22.20504
26.96334 20.22042 18.84942 29.32684 19.45977
16.22791 21.91895 11.42044 17.93406 12.45762
5.692548 33.17809 24.36497 15.62279 17.57951
20.35373 13.2346 13.88727 11.31342 20.90858 13.54712
7.771525 18.69874 15.38118 10.30112 24.8149 21.60646
18.82617 27.94275 23.0757 12.92239 18.69197 28.22935
26.41886 9.974418 31.98681 17.84808 13.84226
25.05034 37.20314 61.69024 30.45639 19.43009
10.04178 26.43299 15.14423 14.1403 36.61781 24.63407
17.55417 31.15092 32.08507 12.84583 22.42225
16.72299 17.03413 10.36706 14.89581 17.38901
4.532875 6.944429 12.86337 38.61322 3.142057
32.63832 30.3739 17.16906 20.74731 20.65445 17.03383
37.3468 14.25394 26.55561 14.81779 31.56844 33.65025
40.65805 27.23208 16.04667 11.78217 22.72546
15.7184 27.86172 22.75925 10.74452 17.58988 24.8483
28.85188 18.54854 11.30665 28.07051 14.18058
22.34585 27.30491 12.52405 20.1158 9.811654 31.30187
10.14297 17.56433 29.41568 15.88917 25.11145
10.71656 24.96321 31.65018 13.9759 34.41785 12.56524
16.86499 21.41285 14.64471 20.61223 12.31892
23.45939 12.00242 37.34836 17.04093 9.737571
23.31517 27.89626 13.14286 11.83 12.86458 19.18974
33.26257 21.06841 20.58225 19.99319 39.04863
24.99587 29.27148 1.96166 16.04081 14.86724 62.84943
29.6311 16.84584 20.20636 73.91177 9.228875 17.14152
16.25165 30.71429 16.08265 16.88548 11.74667
42.85756 19.09171 25.66951 10.71934 18.04644

18.71961 19.56619 10.95833 36.02188 20.78902
60.18502 22.57619 14.33251 35.79742 12.18757
10.84761 17.89126 19.37317 19.21715 76.13135
4.960091 67.99609 28.85464 17.79606 21.62784
17.07171 10.62061 27.19328 22.56081 20.9007 16.17803
14.48763 42.18403 12.7082 17.64511 29.6303 9.969601
12.50698 12.28092 5.582976 18.03491 17.17791
13.94378 15.66067 31.9397
ITGB4 84.07731 9.183007 87.4816 95.22688 50.67888
17.00085 37.09939 18.55066 85.33296 18.71162
41.91207 18.69408 16.74149 29.07729 52.21333
7.878806 103.8063 38.18067 61.79794 61.05507
78.84941 11.66787 49.84808 15.74179 15.00626
52.60947 47.87936 9.007311 60.12562 9.254694
94.15611 20.40641 126.435 85.8721 76.68834 3.127877
0.8496902 37.44592 90.8914 180.9952 117.6712 195.796
36.54867 156.2058 130.2118 112.4446 99.03389
56.22178 89.07559 318.0098 131.6056 23.82029
3.517978 105.2724 50.11426 70.3338 67.8922 35.86481
109.1642 51.47978 47.23724 266.6924 23.27895
13.65167 51.94273 10.44191 140.7672 37.30814
130.7114 56.02507 146.5252 42.25122 198.9921
31.46842 74.97356 120.1147 98.81002 3.059355
129.0668 29.22214 42.60977 62.62866 77.62513
73.43449 217.7097 104.0357 127.5278 121.0836
79.05567 52.95816 101.4998 124.8208 155.1506
71.4027 140.7207 22.0124 203.3285 55.7007 104.5607
88.58047 60.74799 148.6281 17.94836 14.9322 38.90334
91.26765 234.7542 64.15817 54.27339 60.26731
54.15488 7.590155 63.85696 15.42893 74.59798
91.34321 62.93934 567.2196 32.56064 30.59011
65.94848 8.405763 21.19852 64.17539 156.8456
35.80873 38.4842 77.03321 39.93262 95.7063 410.5423
113.3213 99.69128 58.86616 83.77108 74.7126 187.2702
166.8294 223.0153 26.15199 70.7798 128.6342 211.6144
11.04104 10.93825 96.7049 38.46203 41.09979 150.3419
37.01951 132.0105 82.89933 73.12042 132.0125
36.26914 51.98908 96.55073 18.6559 60.52464 18.5203
90.22771 45.78472 45.02973 67.43324 54.51295
116.6885 56.66027 42.93149 37.74936 71.31582
13.76051 130.2437 59.31445 72.13663 121.5564
85.28715 58.89947 41.849 69.75778 55.9859 21.70555
95.55384 47.05702 119.2101 19.00753 103.3713
58.35404 169.1917 46.25817 219.1383 66.61729

88.57267 163.2967 134.6108 108.1411 131.2024
32.71326 44.78489 269.9313 17.73483 4.822458
69.18465 67.42674 119.861 75.60408 171.6018 39.9379
4.098728 119.2961 98.6781 51.38017 33.75283 78.55397
40.39521 292.7606 123.6493 67.35512 15.12205
61.00194 80.67962 67.42158 33.24503 133.8541
127.8569 82.34696 95.81216 32.9064 45.18829 98.82304
334.1371 98.06165 64.51302 141.452 276.8241 52.32147
114.1702 38.69239 53.38438 55.6481 8.015077 20.60554
192.8969 41.24944 170.0414 24.14724 40.21183
60.76675 30.30746 58.59926 179.1888 97.69394
57.85425 81.96363 181.38 139.3022 6.91574 87.51745
60.30879 161.8725 24.66689 59.64738 138.8861
109.7135 86.83121 109.8777 40.93677 119.4688
107.9018 206.7385 228.3135 74.67577 175.3351
91.81169 90.52397 72.6161 32.76304 77.75632 14.91379
2.227874 89.65614 75.61726 50.43942 151.2989
109.5954 132.2653 75.59175 126.466 72.39233 51.15959
76.29757 50.14923 10.7031 21.13144 54.81613 39.76467
66.10268 43.76782 78.69662 148.544 94.61258 93.52367
45.08397 449.8307 28.24639 95.42671 44.52534
38.17626 42.30929 53.6213 48.11816 46.18836 222.8983
248.5291 57.07984 155.9313 57.26642 63.17335
74.21032 76.35823 97.73227 52.0164 44.04417 41.54295
37.98626 13.37594 19.88614 115.6319 26.48142
72.75142 222.0852 60.09042 48.28332 107.7031
128.2403 79.48539 120.6733 76.41237 58.69846
140.7803 122.3635 141.0857 61.23838 133.2652
61.09874 32.32408 75.73256 333.5664 0.8777644
46.65105 31.31187 72.46896 36.28394 76.99406
28.38242 54.54247 147.5053 118.9798 37.03736
17.40643 13.52011 126.0419 25.16168 330.4313
12.93904 39.22447 36.33567 80.05346 62.01457
78.64158 30.22581 27.97701 55.49206 65.3192 34.82977
12.46882 56.25146 249.7797 26.57459 67.63789
55.76604 74.72285 102.0629 66.00334 129.3062
77.21482 115.2663 69.45416 133.1429 146.7181
212.0822 60.26678 116.1306 39.20151 54.62877
165.434 30.95401 113.73 76.21788 43.51425 135.7353
59.79183 30.44455 129.5172 83.76712 55.17968
52.68204 48.21444
DAPK1 3.492284 7.69111 4.174087 9.585858 4.497276
9.290373 3.593827 5.323204 10.33473 4.44289 1.586361
0.3525078 4.319928 0.6407977 4.940874 8.93515 5.287327

10.09408 3.466141 3.466368 9.556938 5.55898 3.714489
8.055807 8.393355 0.8731207 5.243949 10.83934
5.40901 2.487222 11.16888 5.349935 23.89786 7.166591
8.680733 6.160931 10.68106 6.585172 4.808534
0.8069378 19.01951 13.06351 10.55658 6.66077 11.01518
7.559131 10.11433 7.297057 7.326122 16.70709
3.033469 6.703683 4.009066 6.153614 18.87531
6.081719 2.732644 11.9824 0.4252478 11.3707 10.3224
3.454627 4.220587 6.155667 3.746385 7.370129
7.424793 3.529613 7.174835 1.711461 4.523452
8.028506 10.78523 12.71823 6.75389 2.510432 5.630568
48.15255 5.236962 1.220767 6.401225 6.882959
5.515912 8.649561 6.874285 6.587408 10.09861
14.60755 4.073579 6.365858 7.015244 6.381927
3.600569 7.217951 13.63248 9.276777 7.103913
8.989932 3.750639 5.943791 6.16348 25.06842 2.194871
6.996766 4.584797 11.54144 5.216774 5.443856
13.02942 1.237342 18.39913 2.09816 2.722289 4.66449
6.966893 6.183849 1.936409 23.11217 8.854311
7.057952 2.415524 6.842046 3.511327 11.75286
2.130819 5.407758 5.238176 2.164632 6.947076
9.369279 16.68592 0.3324112 13.85515 22.71968
6.979412 7.426296 3.900484 6.491195 2.597564
5.577567 7.629185 3.067517 13.04824 3.845244
15.27553 7.714247 4.933627 7.653283 2.192227
4.426315 9.097594 6.53028 6.111843 10.38634 7.689657
7.329234 6.007769 5.906234 3.780402 5.009846
8.312473 7.487751 8.276989 9.455252 5.07993 7.61113
11.71692 16.6415 18.64004 3.853619 10.08695 5.344279
9.46207 4.742162 3.843355 4.578584 6.17595 5.764132
11.02042 21.65714 4.475393 6.378727 4.119218
13.29553 4.498577 6.034114 9.729859 5.859489
9.699055 2.986885 11.62022 6.763815 9.501268
5.493573 1.391197 7.401502 5.578208 8.182704
2.892034 4.196488 3.609112 5.560188 2.742486
3.579269 2.215914 4.365721 1.971372 2.943997
11.95727 3.768579 23.62005 4.152212 13.90651
1.030843 20.60169 8.258792 4.276728 11.40497
5.728038 9.755901 5.205722 11.18258 5.63459 12.21531
12.49823 0.5935286 5.145181 1.724541 12.70685
8.321659 0.9900655 4.097083 5.578342 6.936566
3.365981 6.425043 3.045089 3.253511 5.164497
2.326665 12.80182 5.160106 6.067529 5.158217
7.269122 11.14101 8.878109 7.176523 11.18137

2.251698 4.026432 3.059227 4.498882 4.616024
2.317583 5.972954 4.721104 4.587134 2.098045
10.8226 4.425109 17.02955 7.170345 1.674894 8.165052
4.228188 17.81629 2.663549 4.254223 21.11015
5.738573 4.014249 5.003955 4.077321 7.195049
18.82252 10.68725 5.16873 1.50398 5.175449 8.316015
8.019511 11.05568 4.259841 8.191655 7.877923
23.39943 6.405094 3.47176 6.857311 6.392688 7.255461
9.803288 4.199832 6.766156 5.330609 6.070622
3.730515 7.368799 6.240433 7.83095 10.70913 4.163139
5.441192 10.88296 6.343029 18.18139 11.74192
8.502694 7.737797 6.755416 10.85051 2.229491
6.033702 7.067587 7.631669 9.465179 21.79232
12.77165 9.955785 14.0348 7.738843 11.31424 10.71682
4.232045 8.929291 9.229351 0.4004383 7.741456
9.059756 10.1977 9.783603 7.236027 7.022302 6.242434
13.85836 2.645148 5.355189 16.14728 6.838523
4.664965 2.064534 5.50635 12.28863 9.573281 16.9666
14.46994 14.38538 1.71561 5.798097 5.44875 4.366685
8.313513 3.2185 3.063933 13.28994 9.467556 17.1104
4.306529 8.423909 9.775491 7.863809 1.660967
54.7896 1.245407 1.697014 6.787429 7.772153 10.88467
2.305913 6.960924 13.31491 2.429131 9.602393
15.36508 4.499707 2.537369 2.158957 12.81396
4.301473 6.33922 19.00782 11.25178 0.6315413 22.12686
5.968169 4.159656 12.23618 1.738629 1.48819 7.605567
6.141052 7.727449 21.73832 3.660322 6.339674
5.084248 9.24012 10.08932 3.953728 10.26855 30.00151
1.690242 21.8143 1.389931 4.841843 5.160949
PIK3R1 3.099492 12.22818 4.514238 5.050284 4.598251
12.5449 3.490035 8.380302 3.893477 6.15043 10.22287
3.433069 4.569701 5.495832 3.493376 12.20204
3.480311 10.02161 2.165935 1.962617 5.775746
13.55142 4.612649 16.51512 27.39469 8.487161
3.496503 10.89486 4.803158 2.369862 4.667393
5.867514 4.518409 5.393079 4.644614 3.440151
3.260284 4.965239 5.182386 7.824181 6.815948
3.917089 6.001255 2.890385 5.869227 4.299261
5.319508 11.2071 7.438615 4.086791 2.993878 11.72143
3.506138 4.843048 9.718672 7.467861 5.62074 16.96983
3.171488 2.319589 9.213274 6.921015 17.58642
4.332776 5.112096 15.43345 3.809391 13.32967
4.225808 2.201003 5.369293 4.037173 7.071991
7.481317 5.687957 3.323784 4.202156 6.647453

10.09537 7.334845 5.959392 10.56391 14.73123
9.655628 4.740144 9.265734 13.33015 6.448072
5.131075 6.115437 5.523789 9.912526 5.139878
6.394787 3.89221 4.608388 6.344965 3.167156 9.762231
10.2662 18.36762 2.782179 2.984724 5.556616 5.41208
10.50242 4.868406 5.766608 9.67364 6.398793 5.777418
9.246248 2.690103 7.881029 3.493381 2.617253
2.269225 3.93188 4.787789 7.373182 6.671978 7.502258
6.395164 4.852509 2.718661 7.680191 2.643659
1.96582 12.17887 6.665432 8.02511 10.08388 4.547325
6.797039 2.950207 16.03614 16.41702 2.006426
5.034243 6.354488 5.149371 8.345958 6.31295 8.228772
4.597704 3.62403 4.436927 7.357917 4.177636 6.512709
3.673827 6.748581 3.582622 14.07565 13.09725
2.508288 5.89591 7.915273 2.869569 3.178272 6.862256
7.415201 12.7449 4.619216 4.235618 9.203448 4.462185
12.93811 7.709066 1.280811 12.17411 4.127457
10.35844 3.754062 2.354084 2.421391 6.581464
3.39266 2.768874 6.297254 3.0997 4.381435 3.68114
11.98056 4.73078 7.58428 13.32394 2.28579 32.78413
15.67353 5.427249 3.202227 2.274413 2.989135
5.86571 10.70827 9.888616 4.974314 9.22875 8.200599
3.869079 6.093451 5.792845 9.055792 2.43309 4.200562
3.803073 4.754277 6.741496 6.179397 16.38979
10.37503 9.761937 8.917566 2.315358 10.15191
7.370432 3.207991 9.479866 5.226494 6.275808
15.89405 2.683987 4.95783 16.4271 5.505941 10.29466
3.789672 2.136921 4.397458 5.137977 6.17608 3.502372
3.016054 23.9998 3.78329 4.690176 12.13449 5.405929
8.013065 10.12238 11.42286 8.91854 4.467784 11.89867
7.404741 6.227812 8.744458 9.125932 7.754908
11.26623 10.12146 4.204189 9.966047 7.019206
9.118907 8.828201 6.684446 3.343261 5.172205
2.602402 2.385118 7.524015 5.465558 6.643051
9.893921 2.50881 8.665418 4.141151 6.681481 10.71849
3.273112 1.956762 14.79047 1.882281 13.65167
5.890176 10.04642 5.025857 9.35116 12.35205 11.46338
4.145648 4.671663 5.167818 13.49466 4.700997
7.070111 4.597595 18.87573 7.114285 14.03189
12.94363 6.486299 10.74591 9.768685 4.822701
8.781791 7.297942 1.748053 5.337904 8.064303
12.41688 5.380916 2.786513 3.246967 4.332976
4.878269 7.534917 2.781546 6.864298 1.174631
12.27086 5.408643 4.820108 10.6357 5.433772 5.418149

5.505049 10.02037 6.897147 7.091448 3.309445
5.996669 21.63851 3.845957 10.9178 1.390594 3.050455
9.575649 3.597465 8.286467 8.486502 7.855669
6.221087 8.089048 15.86776 2.615231 8.560569
6.85805 33.64622 5.531946 3.177574 2.837975 17.58564
5.769015 3.140694 5.758281 2.927961 2.286106
6.665814 12.77329 3.860385 3.951953 6.689007
2.696377 8.649127 4.855608 10.67239 2.326025
6.058426 6.664659 2.065782 2.783095 3.346252
4.061336 2.717458 5.399824 2.786067 1.670053
4.813097 2.993856 3.790587 10.33655 6.648994
4.237144 3.42005 1.9971 3.707562 7.914029 6.206399
7.242389 3.059758 2.159608 5.645784 5.576233
7.311359 3.384769 8.092656 3.659774 10.15068
4.631788 3.837408 9.55716 5.092601 5.875055 8.408265
8.272416 4.46338 4.142914 3.093441 5.260394 7.793409
5.033692 3.860021 7.465627 8.726232
MAP2K1 7.192806 9.051112 8.813331 12.78944 8.638643
8.63089 6.222493 7.684773 10.09604 12.03833 14.69057
22.52956 10.4689 20.42382 9.224827 9.726959 7.979621
9.96707 6.456873 5.662848 14.67382 9.561496 16.4033
8.429759 10.21542 18.93579 9.57463 9.681912 10.13537
7.1564 10.38714 8.955829 15.06876 9.494938 13.14636
10.11637 5.630457 12.44127 10.54181 19.89011
12.14458 14.11591 13.68164 18.47371 10.51905
13.59144 14.03153 13.92959 13.85678 13.33892
16.97688 12.4968 12.62182 16.73133 10.42962 19.01948
12.86495 12.2746 20.90058 19.51299 12.6464 15.43334
10.06233 11.10792 10.95677 10.0937 15.63132 9.125201
14.77576 9.719828 26.51701 12.78795 9.27562 12.33983
13.38381 22.89928 12.11736 9.038874 11.90883
10.04467 12.74175 12.64464 15.22283 10.19 13.21905
10.9199 12.37601 15.80429 7.176407 16.06052 13.66914
17.47409 13.76809 11.27586 14.3828 13.54739 20.61673
9.279331 19.2826 9.876353 10.81058 16.93427 8.923248
11.76195 11.90066 11.37345 13.47077 10.21936
18.74345 15.07171 7.389437 9.952547 13.58607
11.74282 9.951428 17.11682 12.79202 13.8986 8.791327
11.05088 15.99493 8.149819 10.02263 9.780362
23.63261 17.81854 14.61433 11.51231 10.22828
15.68282 10.03469 17.38996 12.88344 13.28047
9.988353 11.31569 17.82276 13.4417 11.99941 10.77169
13.89642 11.4115 5.893148 12.84298 10.62301 11.70257
15.77437 11.65132 12.1773 10.08046 17.33479 11.59152

17.26293 11.66572 9.936533 17.80362 14.22167
9.807413 12.10648 8.586356 10.3813 9.526625 11.01569
12.83714 11.1799 15.22117 8.533098 14.51075 9.379937
10.04004 8.844485 17.28639 9.674175 16.27874
13.46566 11.81051 12.09903 7.842048 10.7159 10.90383
5.313913 9.048388 14.79014 12.45178 12.67752
16.55196 13.86855 12.9533 7.777556 15.58512 16.28125
9.999024 25.23206 11.96533 8.267731 17.61914
14.10655 11.61422 13.91022 9.799197 12.66421
9.193954 11.28403 13.15079 9.484788 13.63202
11.59182 16.80523 11.58656 18.69361 10.08522
15.65569 15.30449 21.06259 14.53611 19.94129
10.19994 8.729447 12.81548 14.23709 19.11929
12.99397 7.196928 13.72462 15.54376 11.73962
14.45829 13.36605 13.43943 10.3941 12.96826 11.4969
11.56445 13.55765 13.43184 7.996235 10.05476
11.53108 14.68569 19.06092 9.135828 12.91633
13.08033 14.75125 14.39792 14.06054 15.29013
10.19286 22.18097 11.44802 14.98853 11.36181
15.26685 14.74246 14.72418 6.800236 11.32743
11.4502 15.52366 9.247172 9.483208 15.57986 14.19637
12.05378 14.66665 13.63467 18.02035 13.22391
17.0671 13.27947 14.26359 15.41009 15.0828 13.75891
23.02942 10.67458 11.9446 8.139136 36.10526 12.02826
12.87873 12.84155 10.9921 18.32831 18.74189 11.64991
9.488706 7.854854 15.31059 18.86799 16.47301
7.622602 9.513776 15.09369 18.15871 9.682854
10.3307 11.74255 15.12321 12.81104 10.96834 11.14438
28.96762 13.5378 12.46637 14.62272 14.93342 18.36797
12.581 14.85385 17.91114 13.10318 16.50556 11.97186
14.65215 11.04127 11.44667 9.673517 18.8446 11.49837
12.84093 11.00976 12.42918 9.924504 12.62329
7.172933 14.37947 49.8422 11.60613 12.96573 9.018721
15.46054 18.23013 18.9444 8.616302 15.11001 7.03309
14.5532 11.95469 8.161559 11.80539 8.624852 9.856414
15.23464 11.79893 17.78502 13.11133 15.96337
13.16435 10.10736 8.446374 14.17647 14.83109
14.44203 20.66715 9.926828 11.44684 12.58241
8.859615 23.61477 11.92976 8.824994 38.22296
13.61502 7.697652 11.65154 14.67575 10.10353
16.75707 16.23495 8.321298 9.927651 14.80498
11.25014 14.53827 11.23844 14.5592 13.46584 12.8389
12.12584 14.51174 17.32965 7.385668 37.83814
11.53293 13.01992 8.870723 15.16 13.17392 11.78157

10.04241 14.30666 9.449383 11.37886 14.94414
13.75474 8.832994 10.69915 7.775109 15.43396
10.20697 12.47838 16.75933 10.48324 13.62079
12.5139 11.87919
CXCL12 3.54549 35.64496 9.207667 8.781187 11.51494
32.95007 5.673761 24.22598 8.644118 19.95206
8.013741 1.423184 20.37212 2.940918 9.237375
27.4357 6.476267 53.44595 8.714127 9.752753 13.69741
30.62428 11.1319 54.9006 37.75928 5.395138 12.06071
32.36405 6.10765 13.89304 10.91792 19.15012 3.443147
7.394216 1.549292 4.664373 105.608 6.203363 8.665992
2.695503 2.853506 0.771362 3.379204 1.218791
5.238827 3.024038 6.106159 5.771619 13.78317
0.6053114 3.41118 21.41946 0.8720456 4.112423 14.12401
1.14914 8.712473 44.63165 2.085596 3.769441 22.67517
9.810567 40.38092 16.46955 2.506994 33.4843 14.53276
21.73471 3.24274 1.755262 2.505385 7.218758 14.27527
4.527605 8.970779 6.04461 5.306878 5.188263 11.78326
1.726033 2.544047 16.82339 5.695341 14.88761
4.223128 4.768522 7.731583 5.941188 3.046729
8.812621 2.948954 5.12544 3.757843 2.645829 3.904839
3.678265 9.32527 1.976217 0.7824588 12.34428 30.54953
1.058977 3.073778 5.259463 12.939 16.01446 3.624777
16.88359 2.780104 1.912366 5.129689 2.036731
4.803983 19.97244 2.828007 6.924406 16.29343
1.566389 4.201673 1.550715 7.677074 2.175932
5.280908 18.85057 3.945841 6.942873 2.094869
1.665204 16.2832 6.753379 2.60852 0.3647284 4.072177
3.141374 2.645601 14.55654 10.13988 2.007842
3.062619 5.386205 5.882532 5.024678 3.405358
1.960873 6.668018 1.87736 2.156937 19.34215 1.37585
9.02847 1.018574 11.3481 1.217517 12.29352 14.29841
1.486368 11.7877 18.99211 0.9066924 2.791575 10.9403
14.06508 15.45899 6.966209 3.887736 6.822769
2.688703 3.972857 9.598487 2.693129 20.4131 3.638546
13.30475 3.763559 2.539767 4.250354 2.694332
2.699092 1.166073 18.95873 2.642571 3.375577
4.987964 10.67126 5.233717 11.5411 45.37358 1.164501
16.76519 2.577515 6.489124 1.259677 4.337461
5.372791 1.755403 5.789253 35.21162 9.934261
0.7186516 2.135872 2.630206 7.309095 2.657881
0.6791825 4.473702 0.9713118 3.33018 8.260984 2.779498
1.260863 8.800939 11.98113 4.868752 16.90991
1.14946 4.928112 1.929119 0.7765269 12.34784 1.23435

5.936657 13.27245 7.417902 4.065792 19.45471
0.7459303 7.763407 1.508791 2.2285 1.170718 2.206364
6.839091 1.37516 1.056297 0.7649481 3.105576 21.03814
0.650822 4.505558 6.693406 4.970653 7.61103 10.98245
3.809799 22.48572 5.542586 7.161087 14.59103
6.46829 7.352356 9.413175 1.011706 0.5520945 27.31836
1.998152 4.895743 5.800025 5.147192 2.368039 26.947
14.13041 3.492531 4.201359 3.581056 9.867986
7.935634 1.256695 7.188864 5.862164 3.255511
6.595386 4.912244 4.848346 0.7560245 0.7438746
6.080288 1.946963 13.08105 3.46291 4.66653 33.20609
1.98877 6.357711 16.06727 2.091487 12.71053 1.215713
2.266595 7.357335 24.89818 2.432611 23.33739
25.83904 13.74171 30.27515 60.57524 5.677353
4.76885 1.060076 8.424286 8.241195 11.61924 1.77572
15.62721 0.901862 1.860982 2.654737 4.969719
5.414748 2.188543 10.78283 4.439023 7.371158
37.05464 3.182144 10.20572 5.148723 3.605543
2.076125 12.65602 1.633806 5.577184 0.9515117
2.304562 21.27611 3.964892 3.773796 1.550469
1.751638 2.217862 0.8073385 6.05406 6.784768 6.792031
8.404251 4.786438 0.1648771 5.295198 8.131316
1.292949 4.17469 10.16622 4.394736 1.054777 32.80687
3.130877 1.180235 0.9968299 1.474485 4.861336
4.083094 9.293789 1.673735 5.754224 1.004621
3.041358 12.78462 2.139549 9.677371 3.03908 2.930338
29.40518 0.687012 2.151638 3.347027 5.116856
1.547392 6.429527 1.780067 4.441711 3.666385
6.580956 3.282983 2.939415 5.182339 7.90827 0.6769912
11.41664 1.665418 6.439382 17.51193 8.930349
2.518089 2.686509 1.74439 0.9216966 19.68666 5.183734
2.819136 1.818055 7.671609 2.275683 1.343059
8.463612 1.2965 5.126516 14.76048 18.92273 5.399607
3.435199 3.713023 30.51137 2.725463 1.962472
1.144372 5.924602 9.139469
LGALS3 505.3437 27.6868 398.7618 137.2692 309.5108
54.20158 170.1594 61.96379 139.7621 129.4849
79.81585 236.6957 128.2507 156.6038 121.9373
37.45899 252.2331 92.58253 451.9386 511.8089
191.2537 35.96492 151.7829 46.07656 84.94419
118.9384 181.5044 30.89854 271.3407 394.2538
128.755 136.8712 181.2664 161.1563 206.429 47.50076
6.697989 111.9677 144.1725 238.0613 111.6235
313.4994 122.8987 270.6642 248.546 253.6166 205.4448

197.3044	164.6717	224.33	315.9463	96.39257	43.55386
549.399	98.5132	245.7132	156.1756	36.54109	104.1154
138.984	120.4177	200.9371	185.9233	41.86794	243.2243
31.04612	168.4579	202.8794	215.766	143.7559	235.9944
266.7098	95.20935	250.8172	118.8334	113.0711	
238.111	59.42782	230.795	158.9347	239.5701	206.2395
228.6303	229.8411	151.7708	115.0606	231.8379	
172.6372	142.6905	176.844	551.8827	45.09423	112.1348
194.2001	133.6711	82.06415	177.2497	303.8803	
65.9565	90.53316	69.64953	183.0042	35.66814	215.6353
75.32639	118.9216	123.0951	78.71198	183.9302	
246.6668	199.822	162.1068	102.636	76.34698	244.5645
105.5232	66.89571	1482.143	96.38565	192.5589	
231.6154	388.7007	63.28926	206.8841	62.89457	
90.57883	238.68	464.6966	87.57764	377.083	169.7403
580.015	160.8668	183.8907	261.3395	190.4403	497.1819
144.6257	157.8717	151.1398	75.57185	67.88085	
168.6892	88.70497	89.96607	149.1156	123.4752	
233.4696	113.7834	145.8625	165.7068	164.6933	
277.6725	93.35699	97.40216	98.09047	227.1201	
97.2541	51.62697	412.4532	89.90581	140.3439	126.2179
232.467	414.0506	231.6606	109.5578	146.3697	97.73457
41.84709	31.20252	246.3019	73.87686	168.9419	
318.4101	262.8623	130.7353	118.5721	85.31257	
86.74593	84.43063	285.0566	119.1945	93.02152	
108.9881	112.5375	141.3389	258.6383	70.06186	
94.3568	143.1312	159.4245	169.8938	131.905	136.7218
365.7051	93.63821	239.9671	141.1311	285.6063	
55.54687	190.9014	114.7429	372.8951	186.6435	
185.3658	129.8489	38.52939	229.8191	107.1432	
222.9865	111.2254	221.1087	60.10481	144.7488	
144.7523	247.8937	52.59287	150.3651	160.2335	
128.2977	39.60437	109.8219	143.9098	70.44801	
392.4348	146.1364	92.53798	227.9003	72.36025	
84.08314	113.2028	281.1554	288.1984	241.7924	
201.7139	157.61	147.2559	206.2729	14.27119	137.2495
122.8511	129.0484	185.1846	225.5241	182.2259	
95.16575	76.03429	215.1265	190.8912	89.7163	287.5799
71.24919	268.8935	180.6636	13.97455	246.6519	
109.3275	251.0975	137.7936	188.3504	281.0247	
130.7364	254.5969	262.3546	205.5311	300.6056	
194.5403	101.3268	289.0808	123.5948	222.0759	
364.7858	305.0897	141.9917	201.3396	112.5164	
38.44202	171.5775	338.6783	94.43007	178.9132	221.11

270.5696 199.0784 77.43966 200.4538 116.4867
134.9669 257.5524 302.4952 25.15144 37.78068
440.3605 133.7178 56.98905 464.6022 184.6522
275.2515 347.6147 254.7158 132.9274 248.2867
51.29192 417.9698 258.8598 82.99551 94.77777
85.59095 143.6786 275.5738 186.6524 121.4243
182.7511 140.2222 126.4921 94.20682 56.75088
110.7129 112.3864 325.9026 266.5026 377.3837
85.41274 242.7251 243.1409 82.57519 13.88522
353.1874 150.0015 463.5188 94.82102 123.8862
216.0447 63.56459 104.7839 349.4643 212.1829
105.1544 274.1781 255.4043 136.8605 220.2738
102.199 71.3643 138.751 141.5291 6.908968 154.1663
195.7666 381.1419 214.6824 136.7896 138.7271
84.96322 135.3526 125.4149 184.8453 82.20462
67.50993 134.1232 137.3764 72.80752 76.60796
253.2532 310.5319 173.0374 197.1185 176.6285
97.10256 88.88061 806.1927 59.92812 104.1441
100.3782 31.67845 401.3308 89.79733 278.8883
82.32888 54.40207 95.50833 143.5928 72.00091
167.6162 258.0034 103.4861 203.6215 115.7348
61.11972 114.3162 270.669 409.3842 176.4984 116.5268
125.1657 91.65754 101.3689 91.65212 182.1145
142.9853 44.79529 235.9734 284.4125 467.754 168.1464
144.5244
BAK1 44.20325 6.085891 44.07939 23.20213 27.1722
4.363531 25.12665 6.541432 24.14334 26.46479
8.141147 10.28444 20.62035 10.48124 28.26886
3.503493 56.02306 12.39296 63.09446 44.55316
24.32988 3.094016 19.92042 4.353369 6.401377
5.203961 20.46068 3.619895 35.43743 20.10498
22.08765 7.388839 38.6678 10.73624 26.76795 14.25419
3.335206 19.67341 16.61376 29.47598 14.07698
32.84109 31.69292 38.96439 17.71341 18.76165
14.14634 45.59921 15.74977 21.24083 47.61568
14.4325 21.27294 10.81409 6.313447 35.98678 14.8513
9.608093 26.27774 47.4779 9.820778 37.18166 13.97273
23.55712 25.95893 6.618153 21.19511 18.24868
70.7024 20.51389 41.7188 6.217138 10.57776 7.551371
16.00258 21.2033 29.5823 15.25344 15.37073 11.0666
32.81055 19.5364 18.60865 23.44893 12.61223 17.64752
15.956 45.16796 15.90796 9.623572 20.17178 27.45073
21.70258 23.1317 54.38702 17.19772 22.77969 29.75359
18.01839 23.26795 14.62126 20.42832 30.90283

16.82858 8.878316 15.56221 53.70473 17.59537
36.69602 25.61236 17.88341 7.741917 83.00283
7.095886 16.42264 40.06998 31.26999 14.21605
35.18276 34.60431 38.92649 5.165311 21.02687
14.2304 27.81117 11.93881 31.28719 36.05138 7.404061
17.5261 29.45538 13.93846 39.36322 22.15144 46.53133
20.98754 34.42944 26.78854 17.29041 11.99654
12.10286 20.13233 14.49566 15.14468 21.35662
12.79054 15.88435 10.70855 16.87911 24.24562
27.87388 30.58184 23.14031 22.25915 12.46037
17.55072 19.67819 8.181543 26.07189 14.76717
21.64121 12.21268 11.33795 12.09356 22.72891
21.33022 33.01462 13.89184 22.68447 5.27215 5.899231
15.93937 15.85133 17.6782 51.52278 16.82854 14.82327
15.90859 28.46536 16.80433 11.19042 24.90229
17.82247 14.14756 17.89808 23.46963 12.97608
28.43751 19.59788 27.89881 28.36064 20.89568
18.91289 26.70966 33.52101 30.41846 25.23103
23.24704 13.52592 12.79422 7.311835 7.814642
14.43007 19.58133 14.8257 21.99453 26.10937 15.36472
11.04415 8.098997 10.42591 16.39847 16.96674
14.43024 35.94708 30.31321 15.35831 28.7585 15.29635
33.52982 36.86692 12.87916 34.44318 26.1321 12.70327
21.4137 15.52321 17.48277 36.27721 24.22727 26.62473
13.28974 15.96852 15.22488 8.311678 24.67634
29.0361 31.10332 12.86013 4.768189 7.678936 22.00223
21.60116 37.99235 11.77118 31.91126 21.07335
5.955674 20.24871 48.16766 18.3918 14.66806 42.24917
35.24546 35.94918 14.6297 17.74407 24.33963 32.17693
9.918171 19.86449 56.4108 18.88213 22.41072 20.43537
21.90138 24.35521 55.91273 39.7636 18.4581 21.9936
40.05761 19.76427 31.68351 13.70654 12.30532
21.68847 4.925885 15.29501 21.95976 12.05013
31.65351 21.72381 38.30672 10.23147 17.32597
15.14094 14.49652 22.58117 23.55075 24.5859 3.355819
5.582052 24.81003 24.66854 13.47105 12.6252 30.36808
18.19278 30.41951 17.15593 12.14259 27.88146
10.1599 18.52436 23.80597 15.66004 15.07413 35.22929
11.99862 41.36852 17.30142 24.19976 29.5611 41.6568
10.71947 18.45253 13.42602 17.78137 11.20125
17.7179 13.99693 18.78444 23.0611 10.60423 16.55124
34.17277 22.89311 21.8877 20.39631 18.87624 14.35199
17.79628 19.71319 10.83699 23.07013 21.20523
12.13766 16.83971 26.85865 17.60797 22.16263

25.57242 20.39448 8.118571 14.40178 15.90011
8.44859 36.56205 17.37661 22.49517 18.56006 27.20294
14.80678 32.3281 11.90447 17.20411 16.73466 9.648816
17.76053 21.37869 14.77845 30.48149 29.36424
17.63786 15.88558 19.82665 29.7272 8.873554 14.43013
17.77438 13.59587 16.66446 10.80843 14.80422
20.97542 20.85646 19.393 20.74211 11.48318 24.26697
23.11348 12.1279 16.13406 9.10545 30.2627 13.53474
18.20465 33.4904 13.59338 15.45076 13.2954 10.35664
16.63925 25.64421 24.91724 16.32164 22.20667
14.31488 35.1778 22.92895 8.414103 31.84921 25.17689
31.18394 44.84022 10.77132
ABHD4 27.86853 15.8076 8.162864 17.23403 11.85544
10.36822 13.33616 10.78966 16.89666 11.62383
20.00273 13.12862 13.48911 20.99516 19.8682 9.520548
16.10692 18.0212 15.19741 14.49342 17.6738 7.597255
13.16986 11.46702 17.32449 12.95741 18.86131
7.765054 18.81837 9.233944 17.88554 10.98172
26.05663 10.93927 14.26038 6.557558 7.466028
31.96142 8.532362 6.656253 10.36907 4.842957
7.345413 6.418646 8.961712 7.436115 6.687353
10.30597 6.313554 14.10731 9.181132 5.688429
22.51048 5.292289 18.55541 9.820896 6.467697
7.911514 14.3962 35.00342 12.73377 11.36234 16.01642
15.73123 13.39118 12.12349 16.51669 9.102585
12.74649 7.061992 8.353631 7.632381 13.69424
19.16239 12.00834 8.94786 6.299691 17.96037 21.02505
6.339782 12.59983 9.159474 5.349307 14.06564
19.94718 8.028984 8.642528 9.572288 3.803173
12.83217 9.604358 5.919039 12.35316 12.36089
5.731783 12.72736 9.923786 13.73123 15.96384
7.902314 9.582273 30.37301 5.321542 9.677102
12.89193 9.777333 9.464963 13.72221 16.25373
10.7791 10.16909 6.466247 12.62936 7.846869 12.08612
28.81478 29.02512 16.73647 14.42744 4.739111
11.42216 8.875029 16.12205 11.76347 6.917587
8.081335 6.229664 8.555517 13.19298 15.69699
12.77712 3.263436 9.102214 10.74056 12.43316
9.354508 5.921568 5.930559 8.183217 13.52241
13.34764 4.649564 23.40975 5.216705 15.14481
9.229238 5.783117 8.307279 30.76431 9.773953
6.182383 13.00516 5.065537 10.1038 10.32767 13.84277
13.57513 10.19923 10.12844 7.85859 18.61651 9.473063
13.26958 7.845693 29.03748 15.23123 16.34295

17.39217 15.61999 8.30162 9.497681 11.16146 5.396767
15.63419 5.119254 8.532423 19.71424 6.255859
10.30916 17.63807 14.50099 8.34616 22.11503 13.07669
9.081091 15.89143 13.17311 7.273731 9.155701
7.490303 17.01782 18.92135 26.904 20.383 25.06243
8.868978 12.64322 10.37569 7.845875 10.49281
9.603824 10.76418 14.44631 6.612057 7.888754
11.29783 14.87407 8.909724 15.12416 12.87686
12.18657 9.177976 9.304071 8.448985 8.951387
9.413332 38.60663 18.01952 21.2229 13.14919 18.72096
12.94941 10.60746 9.926925 7.977988 4.055836
5.723978 20.3667 11.43691 8.4904 20.08436 6.629229
9.488852 4.706188 9.064555 17.42372 10.8959 5.924379
9.202228 7.632226 17.15084 11.5645 6.5138 6.882807
11.26081 10.55895 11.47096 14.87662 19.23786
3.621986 9.068366 6.739283 7.771432 7.959895
7.224107 11.702 15.10503 13.03221 7.309487 23.52276
5.013774 16.26928 12.79701 8.042547 11.72576
7.510068 7.755979 7.436795 16.42691 13.75216
7.498055 9.25553 13.61142 9.748441 5.692619 8.326802
13.39343 9.914364 9.189097 15.03353 10.03764
8.139022 13.62905 9.477065 5.310656 9.31954 17.70741
9.534845 6.370206 9.26495 21.6001 6.385327 14.37226
8.181049 9.523345 18.76327 6.522853 11.25467
6.458607 7.137652 10.94082 15.52583 24.77549
8.62902 12.12833 15.37073 24.26984 17.96437 8.028465
10.27592 16.72059 12.18725 9.575174 15.19882
9.60374 13.75736 16.79795 23.17669 8.684103 12.70953
9.910046 17.5029 12.89012 9.092463 11.57061 22.64231
16.558 15.59604 10.14564 11.19925 7.468021 12.7885
5.768694 13.26116 16.12554 6.914536 12.97024
12.86836 7.013232 17.72163 17.721 18.89226 20.76266
18.26098 8.402703 22.18868 21.91011 5.345538
19.10407 8.027397 13.84507 10.32518 7.713385
11.16927 12.55595 12.43364 10.41277 5.108279
10.99135 18.06523 7.821168 16.65155 6.133304
14.10548 7.296193 6.375871 5.030677 8.768792
19.62607 6.921502 18.14619 14.63915 10.15003
8.636336 10.989 13.2718 10.27455 6.426756 7.620041
9.334522 18.05641 17.70308 14.63473 16.83388
12.05434 12.72087 21.66172 11.63631 10.03272
17.73019 20.65604 8.016712 6.084766 23.44065
11.70611 25.97689 13.59354 8.01339 16.99203 10.50777
19.00668 4.585298 9.086133 13.97692 7.382874

20.16344 12.46474
CD44 5.119759 22.94631 12.69027 16.33639 18.87292
33.64219 13.22703 19.06579 23.22588 26.19012
64.18686 35.4715 28.12688 41.07571 22.71791 30.56314
17.95863 27.30464 7.580839 11.71535 20.47493
42.19928 48.80766 32.8629 31.43816 51.07969 23.03405
26.99367 20.47408 9.316063 21.77162 46.79131
18.2145 64.19447 56.82401 3.160491 17.03587 13.9998
26.27736 56.50207 31.23011 56.46304 46.89312
9.813606 19.71503 61.30625 26.14043 32.93747
113.6712 49.65606 17.07515 46.51433 3.843552
38.86653 38.32083 14.32258 42.73161 57.47872
42.11063 39.1695 66.56609 39.31572 42.873 23.14258
41.11408 46.32493 38.39617 27.30768 19.21852
42.41902 44.17064 15.19258 41.80911 115.534 58.4265
21.53611 23.19061 93.49491 6.617145 34.35277
1153.567 69.26261 22.30129 31.96405 30.49589
46.15147 67.59546 33.0294 618.1317 43.61414 16.66376
47.81273 43.10501 29.82518 24.49645 24.35872
60.5242 10.35479 18.85038 38.61484 43.1776 17.22031
51.50646 25.43142 214.5628 37.98809 27.69438
41.62808 17.20645 26.11946 12.49219 11.20279
26.4272 134.903 48.63542 57.46846 9.025894 20.36862
15.9953 47.73254 27.44779 12.99122 9.751446 32.00998
6.934875 40.08067 26.98029 15.93061 58.04912
39.54548 71.03261 72.5722 10.52164 56.71436 33.54194
47.10405 44.1716 64.14981 28.43795 60.86964 87.56681
35.25693 144.071 16.63075 70.0097 6.973499 58.93353
37.74295 4.985645 25.39928 4.054685 60.19256
67.47028 55.29976 37.22947 11.36745 30.6365 36.28764
38.18074 12.38294 49.35842 42.5871 27.96002 16.63641
21.10709 43.39211 64.18691 86.47099 38.03998
8.23629 18.82946 39.00126 19.84898 41.65799 17.51592
19.43508 18.84084 31.64318 9.788053 21.25856
6.067508 22.62505 99.68708 59.92989 48.16147
39.33661 48.52432 30.73806 15.50248 27.71112
32.31028 46.31397 18.52518 18.09007 50.16152
18.07377 33.59401 12.48785 38.4745 20.00872 10.45504
38.38302 52.76846 92.0546 26.28914 18.49584 15.09907
19.93038 26.28724 65.83111 56.34918 160.6869
44.95022 7.045625 48.8887 55.27963 12.58596 8.487174
73.62881 28.85053 37.17889 93.70068 11.27098
81.74633 93.95172 13.08296 13.71974 70.31832
18.74818 39.83603 31.96929 32.00046 25.55967

11.91634	41.38379	18.2621 27.79918	46.52264	25.59387
4.544564	29.60267	47.62298	28.88014	43.17409
69.28439	33.57325	21.08483	46.05136	63.73362
73.07542	55.63817	72.3357 6.650602	17.6989 53.64137
9.990376	45.8384 38.6434 20.32287	14.85182	18.15416
71.59516	26.25594	50.94206	54.18685	52.68075
28.43803	25.22267	26.97169	13.04122	73.49362
17.30106	5.964127	50.55257	32.79571	20.20006
38.53809	51.18601	32.16012	67.29054	50.96342
36.05686	31.37319	25.58511	32.79328	534.3811
28.33171	23.11327	43.40636	40.50243	18.74293
34.5365 29.52315	20.02126	50.26182	36.83556	20.53081
48.7014 119.6144	13.89418	42.088	34.90737	48.83641
44.85902	25.76651	212.1264	48.35561	89.90514
28.27778	21.02989	43.08104	42.76968	32.12388
30.57036	46.3822 36.32	51.32362	15.22435	51.57497
109.1307	35.72719	32.0344 32.60476	10.62747	39.6011
11.69383	50.932	3.563557	40.17143	56.55301	35.4716
22.67706	78.8382 86.39158	51.21226	32.92972	35.09893
6.291221	43.65262	10.53894	4.883458	17.95285
75.72557	32.25825	47.28513	41.76277	13.73444
2.839127	14.58885	42.03624	35.54407	28.68368
20.96024	38.99085	6.554186	17.70558	29.49248
52.82301	68.55827	64.70479	39.2379 18.60189	104.0257
29.88847	31.62703	24.45782	34.8927 20.43634	41.23294
20.09219	14.20593	36.87231	3.227047	14.95494
25.92837	7.600551	43.48314	30.85523	50.66938
391.9097	16.50837	58.28418	15.27084	2.823948
31.85341	30.8883 31.13184	70.38489	29.02746	6.967809
32.16322	27.12207	15.95039	43.96415	20.85721
33.5088 19.60599	50.98555	10.2619 24.82895	9.250599
854.2225	63.93265	59.93857	14.89655	23.07527
245.953
ITGA4	0.6288808	0.2830931	1.437715	3.493806	2.561478
0.4934954	2.454713	1.692529	3.664943	6.148993
0.4940442	0.05057705	8.36555 0.091376	5.288177	0.3364874
2.336531	1.997853	0.6584347	1.14679 2.810552	0.2215899
0.4093864	0.3915965	0.2871484	0.167662	4.031324
0.724095	3.43263 0.6425626	3.112665	0.3741635	2.648273
0.9903391	1.604804	0.3869739	0.66395 1.58547 1.385422
1.415784	2.04923 0.6792011	5.998199	1.147843	1.878371
0.8226634	5.013177	2.778948	5.671341	1.815287
0.3629857	11.84765	1.126428	1.401803	2.957853
0.5945099	2.608098	8.38269 0.7393712	2.510348	7.876803

3.327745 5.208501 3.429913 0.6807031 1.010531
1.585714 3.388024 3.258998 0.8208186 4.378545
1.247459 1.098701 3.977899 4.203992 3.39875 3.377901
1.680927 1.597718 2.34891 0.8025177 4.455141 9.771503
2.620653 1.055613 3.108623 2.238288 3.833394
2.192185 1.157349 5.727124 13.0729 2.89189 6.190439
4.726249 4.779059 5.653843 1.063027 1.739114
3.770595 8.709485 0.4915947 1.461721 1.830743
2.430589 7.738609 1.795868 1.497052 3.032643
0.4437163 1.151457 0.7618601 1.18432 4.001573 1.628101
1.195691 1.300314 1.13048 1.170961 1.125391 1.90102
5.22457 2.882386 2.434103 1.085288 13.25384 2.73476
0.7179531 2.871706 4.24123 1.687168 0.3223982 0.9691881
3.535311 3.572285 3.76874 2.697037 2.385773 2.068226
1.691367 3.969593 2.232463 2.486173 0.9506062
2.561727 0.4029998 0.3437338 1.551673 0.3565232
1.821367 2.990965 2.547565 1.169723 10.19379
2.945574 0.6813188 1.117284 1.504529 0.7180393
0.6029165 5.784381 8.410661 6.873096 1.74976 0.5685959
5.580502 2.830663 4.767436 7.340038 0.5795397
1.911152 4.777879 1.259538 0.3311988 0.3150716
0.9454839 2.56506 0.5746038 1.383349 3.468352 0.3606375
0.5812894 2.075765 7.681788 2.843393 4.927999
4.571136 0.6617992 3.213472 2.20505 2.313394 1.366109
0.9132419 3.336879 1.833936 4.974157 6.589194
3.132701 1.399665 0.3822442 0.381966 0.8046125
2.767184 0.616959 0.3061686 2.861422 0.8286835
4.08978 1.321114 1.590999 4.219566 4.131696 4.514544
0.790955 1.099389 2.564489 0.8146954 1.427598
3.093833 2.661348 1.506603 6.242138 1.055774
1.217148 14.6155 0.2116085 2.6201 0.2740608 0.4666483
0.8655346 0.8671761 2.615152 0.2374417 1.009751
2.657512 0.8828674 3.326856 1.847918 6.694135
0.8355009 0.9571632 6.4952 2.960377 1.216773 3.151648
6.777777 3.046337 1.098703 4.171319 2.423016
7.757464 2.581371 1.010232 2.940242 1.958518
2.857846 1.771031 3.714949 2.175275 1.533951
1.175954 0.535706 6.76819 0.869993 4.560014 5.08823
0.8455569 2.688615 1.279516 3.148479 5.634391
1.776662 0.4645299 2.927622 0.7334222 2.094197
1.459096 1.251027 4.692129 9.458727 7.896688
3.020441 1.466153 3.194143 1.764286 3.873393 1.7005
0.6212954 3.407597 2.40276 2.804314 1.659865 2.02313
5.192207 3.107983 1.960514 2.917595 5.384039

0.7751047 1.162823 3.892639 3.54148 1.851883 4.841838
0.409679 1.077749 2.242876 5.372816 6.322944
1.402752 3.283876 0.587546 2.50463 5.461627 2.693697
1.450747 5.860633 2.93678 3.501408 4.518707 4.519461
5.121678 0.7301267 1.343637 5.063992 1.782216
3.410415 1.088729 1.186991 1.981208 0.598582
2.896849 6.206568 4.23331 0.8192626 0.5575831 0.3568912
0.7923411 6.092274 2.60877 2.060864 1.455558 1.052625
0.4395261 3.238762 0.5639771 0.5954708 1.319413
0.3316909 0.8170429 3.255297 4.347421 1.395535
2.182237 4.685441 1.183963 8.683801 1.277276
2.663692 0.6402127 5.04456 3.987601 0.8432475 2.489082
1.534528 1.009489 0.2412499 3.133587 1.321426
1.126908 1.099712 0.733027 0.657501 3.113424
2.628607 1.599479 1.008862 1.452089 1.434721
1.036462 4.777607 6.038095 0.3509438 0.439251
2.551998 3.263141 1.275688 0.7071137 0.5597416
3.040469 4.71408 2.240507 0.4891487 5.340117 3.527411
8.42426 2.953043 3.269749 0.8086252 1.903319 0.4971716
5.945213 1.221235 0.5390793 0.9812766 2.592956
1.338477

FADD 6.408059 2.829847 6.293629 9.581596 6.17972
3.428647 4.755321 3.272497 5.16577 7.296928 3.942509
4.279202 6.934295 5.090647 6.638736 3.016823
11.96393 3.493838 7.678373 7.230804 9.046511
2.507168 4.939566 4.069343 4.221345 3.188364
10.16835 3.020229 8.143656 5.855591 8.875993
1.959492 9.38497 6.089507 9.951922 2.058665 1.47811
8.703891 10.31053 6.78149 5.436118 16.11206 5.3719
5.940849 4.753941 7.235439 7.346137 11.23082
3.206178 9.68158 35.75702 5.427887 8.440942 6.455147
4.569908 4.656642 9.153816 5.42834 9.107973 12.46403
3.825774 11.62335 3.91418 6.519702 5.049946 4.689826
8.905155 6.899287 13.37608 9.652966 8.842991
7.058197 7.978506 3.9442 9.733469 14.08684 6.556079
6.436725 8.977292 4.26733 7.999774 8.499729 4.311125
6.353841 15.41986 6.5178 7.234977 8.435537 11.57098
4.005632 5.285406 12.62733 7.806652 7.048233
10.90027 7.634903 7.733748 6.955459 5.152428
6.583277 7.400588 8.45977 9.01894 6.777828 8.88414
4.637375 7.240543 6.297172 7.690459 7.648733
8.153317 5.094597 11.17648 3.845335 6.70781 7.669103
7.302389 11.81657 8.441738 10.96412 6.811071
4.915741 5.788808 3.867956 11.18156 6.690126

12.01825 10.891 21.63727 9.027979 8.570742 6.271272
7.78894 4.24638 6.386503 6.608514 7.266181 6.57675
9.979194 3.572458 13.28387 5.301562 6.45841 4.69457
7.895517 3.503775 5.870062 5.384114 5.12579 5.311375
13.16449 7.621347 12.62173 8.195996 5.79017 4.509912
5.641218 5.008592 20.17451 3.934102 11.24073
4.786636 6.561209 7.459171 8.302639 7.624086
27.0914 2.094715 7.630783 5.295025 4.286707 14.03655
9.884906 7.452325 9.834106 22.05175 8.695296
5.272966 10.05933 3.96327 2.627196 10.01074 6.946539
7.381739 5.924677 4.953396 7.438938 10.73097
5.388925 6.68646 8.351471 4.149781 10.2623 9.096816
10.58471 8.44647 6.554182 5.628693 5.813935 4.460613
3.369629 4.397266 32.7264 7.9756 7.615113 6.706151
7.626402 11.09843 6.661694 45.50073 3.654115
9.520232 5.926046 6.327758 6.990259 9.424827
9.636267 9.071424 7.879025 7.576271 12.60035
4.609094 7.803025 5.138824 6.737024 3.370417
4.921575 4.41069 7.404249 14.11708 7.817247 12.51737
6.309728 8.654624 4.946042 5.821572 7.27533 8.961346
1.91587 58.66061 6.064246 7.781383 6.342044 11.18723
4.042126 11.3912 8.961229 3.988646 6.312267 8.513761
7.271455 4.43494 10.71408 10.07199 5.479545 4.744005
8.541008 6.765109 7.935263 6.910881 15.29233
12.93235 4.20284 5.60061 7.610101 6.446688 18.90291
14.80972 13.02051 16.15601 9.165923 15.07818
11.00345 6.008142 7.146691 7.364983 28.43361
3.215095 8.909424 7.094996 7.796758 31.6121 10.78287
8.581303 7.68117 7.163675 7.217136 6.367538 4.261676
5.99066 9.182445 3.304185 3.048024 7.358277 38.08062
7.370536 4.81942 7.63122 5.124103 6.539475 4.445793
5.25782 7.079582 3.355945 4.968289 10.8475 2.622093
3.567658 6.671451 9.19204 11.36821 26.11905 12.57809
10.42786 6.978972 5.430629 5.780384 7.188166
7.682591 3.030138 5.96076 6.312489 11.04107 8.372788
3.035165 4.19263 9.534097 8.899483 8.65154 5.851031
5.273905 7.827314 21.78017 17.64873 7.469934
6.567467 5.139245 22.80741 4.705043 6.626655
4.151767 7.440731 7.371583 5.35042 4.558461 4.379798
9.027849 3.187062 5.903398 6.825619 7.492598
9.591961 15.7267 4.033928 14.2556 38.59027 4.771384
6.482475 3.69692 4.793025 6.548873 6.758801 14.33359
6.883681 7.204973 4.764139 5.987888 11.91223
9.903763 4.421797 4.357222 5.474101 9.838742

5.871708 10.88266 7.492077 13.31996 4.585749
6.654051 5.830283 10.72517 10.41817 3.530019
7.359857 6.909719 11.14426 14.03337 8.100347
9.935466 7.221857 7.312463 4.58204 3.397509 5.459427
6.787685 5.204555 2.945857 8.70757 5.266436 6.778323
10.1227 4.414487 8.844458 3.912021 7.405366 13.26452
8.463019

TGFB1 8.130283 23.21164 12.14645 26.64711 26.17798
19.86656 17.94141 15.64851 36.24215 43.24142
23.1666 45.23064 50.24176 89.92655 44.66522 21.20125
24.24635 33.83062 17.56221 20.91992 27.75179
18.98254 54.35937 22.57841 28.35857 46.38105
37.35845 27.24078 28.21452 6.281815 29.59455
24.71109 22.46979 81.31354 36.93768 7.652358
5.042372 27.39915 22.19917 17.97204 21.54114
5.509861 45.41215 8.295783 16.54085 16.5509 16.21614
51.47387 21.02968 18.47399 31.09849 46.81619
76.21901 56.48243 52.20162 30.85539 37.88745
63.3321 44.14494 24.01523 55.15903 57.17645 35.95128
63.82498 11.66387 28.8445 49.39452 60.5088 22.64404
23.77427 18.12606 23.31711 18.8508 19.01538 36.2176
49.45921 28.8692 23.29479 18.20238 5.4898 32.19738
53.70768 25.99647 28.21292 83.77218 31.79226
27.84362 30.05751 22.28801 35.12833 17.87911
36.00725 40.56028 33.8996 45.13307 82.42222 94.53996
14.06553 18.82692 28.17933 90.69273 9.176823
17.66822 24.14963 51.33122 49.41904 32.38333
51.57121 51.60495 15.23565 18.79805 11.46746
89.82928 51.19024 69.02975 11.97316 40.71024
18.62678 25.34728 9.167587 26.25319 24.75826
52.92112 55.32908 20.85778 58.63162 20.34747
45.58738 48.53512 66.91114 22.99159 20.82147
22.67202 16.87315 42.70072 23.45337 29.5505 38.79385
23.1346 5.4753 28.88117 42.03429 16.65314 12.67491
42.25242 18.93774 5.696444 43.59711 11.14323
35.22562 16.30073 88.22123 19.28432 70.47854
35.20607 18.44222 23.85886 39.11322 47.93635
23.23846 90.68513 29.26721 23.22237 19.20896
33.92277 39.13748 34.21323 17.25653 32.63089
75.60608 18.71277 27.55772 14.91212 38.21152
7.031044 48.73625 63.71822 29.74096 9.612812
55.65384 9.291756 30.9872 33.01325 40.71978 36.30309
60.62822 54.58487 42.33207 42.88395 15.48594
52.20073 15.68838 68.407 25.05104 30.94668 38.06526

52.84681 25.04489 8.604555 16.91976 43.20131
27.2402 21.56295 12.85662 28.60968 17.07088 39.35987
34.45509 23.03412 32.35005 18.39984 46.44066
25.7176 15.75671 35.89005 26.03528 32.06613 28.8452
56.16319 23.51952 24.78322 23.28695 24.12285
22.69888 55.43332 12.19588 31.93856 44.37665
9.454182 21.71617 26.2372 33.98261 4.740368 10.93524
11.01192 36.21439 62.18274 18.00588 27.20689
22.7051 24.35704 57.58191 26.99618 30.64663 35.58223
65.57172 45.38438 29.46328 38.1967 20.54612 33.86064
10.16208 21.91808 35.15641 17.31129 23.24464
19.7696 49.3471 26.60669 36.18642 21.95648 30.6242
29.44272 50.22089 26.30507 28.31249 35.65778
27.89657 114.1999 51.77379 60.65905 44.60205
15.29276 13.96744 6.118417 15.17779 14.48403
28.18101 127.8703 43.59755 74.7787 16.06134 62.96471
56.32342 11.12428 33.15828 10.83859 10.6649 35.1575
47.0313 31.98722 22.20411 24.17683 33.21663 101.5865
98.62736 39.82872 61.61114 18.88777 13.00757
23.74594 39.7777 18.15154 53.05135 7.656426 23.85579
34.19658 25.77227 28.2968 9.062223 44.78294 58.61086
41.98788 124.3372 14.42216 30.65588 28.96935
31.92305 29.32617 41.09704 14.52452 14.83289
10.39531 28.76639 17.83352 42.69839 46.00147
17.04697 18.60026 47.08135 15.6661 50.97888 75.98874
41.49849 54.72184 17.28801 14.14129 18.16892
34.31852 13.32662 13.42239 40.2868 15.75407 39.84721
31.98073 31.66025 13.40077 9.316092 16.40133
29.85918 19.35539 82.18158 19.14579 36.00152
23.44417 8.724187 39.32757 16.44978 37.87545
24.1894 41.73667 64.92449 62.45121 25.91742 16.20702
27.96983 3.075804 22.52073 9.465561 21.9939 20.79629
39.12602 28.86994 11.66003 57.54087 34.12779
16.85165 33.99489 13.00922 17.08016 84.80828
53.24604 39.07555 33.89601 18.87567 34.12989
59.7643 51.62983 13.7289 35.59554 40.71689 26.22646
6.60387 30.24106 22.04925 35.92073 24.40854 29.9431
12.80794 31.63455 26.67219 48.22012 22.44251
5.770677 14.15375 52.29022 31.43125
HMCN1 0.1188627 0.708878 0.244564 0.2106555 0.2759168
1.392246 0.1498195 0.4732886 0.306644 0.2547349
1.619715 0.2273772 0.2294431 0.2690804 0.4997505
0.7380758 0.3509736 0.6977105 0.1429013 0.2772483
0.2448941 0.663535 1.137107 0.7493954 7.623568

0.6767428 0.1274955 1.096932 0.3581442 0.1969597
0.4331331 0.5860053 0.288348 0.6280406 3.074922
0.1045683 0.1440253 0.3265875 0.9805413 0.2000024
1.837025 0.1240842 0.5233504 0.07892647 1.259407
0.2583596 0.7015439 0.4925524 1.77005 0.5764852 0.4432356
3.264736 0.3330563 0.512655 2.218428 0.08267259
0.4225269 4.608593 0.1599588 0.2660957 13.74428
0.4276448 5.665991 1.713927 0.05165429 2.452347
0.8866831 3.162191 0.3810544 0.4211729 0.09149921
0.2499474 1.027307 1.797391 1.283962 0.3776063
1.880326 2.544268 0.3521558 0.1348222 0.1957957
6.464586 0.2110826 1.24956 1.089753 1.351518 3.298138
1.745203 0.6991949 3.719577 0.3127325 0.2331353
0.6960741 0.6310793 0.3195332 0.6391091 2.017801
0.1613942 0.1325608 2.267731 4.67704 0.3921546 0.04504486
0.56656 5.981582 12.46778 0.3192507 1.575061 1.082914
0.1084226 1.012972 0.6008931 0.26349 0.8956924 2.412232
0.1142385 0.2047419 0.3934003 1.325221 0.07608346
0.239537 2.760248 2.764306 2.807796 1.051608
1.701311 0.2124295 0.1953712 3.831032 3.81936 1.250263
0.2332561 0.483773 0.8675536 0.2062852 2.678167
0.7542239 0.1709219 1.030151 0.2390757 0.5103275
0.3614757 0.2926081 0.1465544 1.527835 1.646783
0.06598312 2.430229 0.5258348 2.924857 0.3772497
2.755308 0.283443 1.556675 2.35729 0.3732258 0.6113957
2.018547 0.5382192 0.9785036 4.114756 2.825986
3.855333 0.126627 0.4161726 1.992042 0.5763186
1.924372 1.694386 0.5121705 1.238163 0.4573272
1.694517 0.06445555 0.06090407 0.6845914 4.137894
0.7578505 0.05190794 3.700996 0.858066 0.5125692
1.059651 0.5886644 1.033287 0.1256071 5.028913
0.742748 4.85106 0.1257519 1.81538 0.2669938 0.8036879
0.219972 0.4639114 6.434288 2.649282 0.400057
0.223908 0.6502205 0.1173155 1.014346 0.41935 0.2866293
0.171631 0.2103893 0.6633603 4.064833 0.957624
0.8167588 1.068258 2.520068 1.967784 0.6718686
0.2551537 0.4501923 2.541555 0.251516 3.204047
1.345811 1.465304 2.798833 0.5023368 0.7568096
1.20968 0.2382826 0.5143255 0.3460034 0.06583172 1.040163
0.828431 1.235804 0.164213 0.3541414 0.3954577
0.4586434 0.8065238 0.110086 1.974381 1.319835
1.05364 1.816379 1.394795 0.41086 2.522315 2.183919
1.162696 1.694429 4.214333 0.2241507 0.8674493
0.5012313 1.16943 1.487133 0.1128395 0.9306159 1.206734

2.145629 0.4735469 2.65842 0.4399092 0.4742537 1.339267
0.3287793 0.7208599 1.853366 0.2471729 0.5606277
1.221378 0.4325677 1.881385 0.2688534 0.4463938
0.1157822 0.1192074 0.9444456 1.650715 1.187339
2.884106 1.142216 6.40332 0.6438965 1.681217 1.151541
0.2847105 0.7189519 0.4814924 0.7847587 1.18159 7.610128
0.7603465 2.265957 2.514769 0.703808 0.7549243
3.138778 1.66733 0.6664939 0.08937908 0.2355753 1.973676
4.262016 0.7028521 2.597229 0.1940201 0.3432565
0.9318123 0.8046389 1.11621 0.370539 1.259365 0.3511851
0.3089921 2.287078 0.1842117 3.513435 0.9751822
1.137136 0.5529986 2.487068 0.8010809 0.611503
0.5199367 0.3906194 2.406045 0.4659768 0.7764469
0.5050893 0.3801869 1.051745 0.1958733 6.915193
3.583362 0.8201359 1.925792 1.083873 0.03466904
0.7091563 0.7173286 0.2861326 1.490231 0.1806291
1.05447 0.09957161 2.630623 0.9456539 0.3894833 0.3405173
0.6907623 0.7837739 0.206793 2.255464 0.8982104
0.4845132 0.1073518 0.1807714 2.187237 0.573608
2.270885 0.7181617 1.229985 2.243277 0.9301773
0.05018844 0.2279164 0.67172 0.02735871 0.2922953 0.6842161
0.6111144 0.8618471 3.202152 1.273128 1.327904
0.9254429 0.8560837 0.2279926 0.520428 0.3930495
4.427387 1.419383 2.619408 0.6893213 0.2085912
0.3043052 0.2032433 3.320433 1.397148 0.1586924
0.8027288 3.862999 0.3855023 0.5090963 3.130275
0.4949798 0.7504972 6.445735 4.107129 0.1417751
0.7217569 0.3422211 0.3798288 0.2341705 0.1018234
0.09635763 0.7245973 1.548096
MMP2 15.82156 78.09237 42.12377 57.05621 46.66251
94.50953 26.59305 66.51137 66.9071 44.10288 17.48174
1.458132 40.4556 6.119233 45.9313 41.54413 39.33857
119.1741 48.35114 59.41826 56.13521 37.05752
44.67744 51.77615 107.6846 22.82572 60.38641
47.42656 86.27059 21.08458 40.3496 31.37658 14.6979
28.40298 311.2359 2.053092 10.9407 46.6586 70.47876
18.36241 125.3386 7.277436 83.97773 6.975155
54.7841 28.02129 16.91215 124.9744 57.28738 29.61815
55.19715 88.6942 18.34174 116.2995 133.0572 20.34024
144.7263 99.24608 20.2824 26.18864 303.362 53.35145
204.1419 248.4131 15.37708 122.7648 185.7373
134.5217 49.74803 39.03904 28.14739 34.87762
39.71959 159.0588 344.5412 52.77996 46.11294
92.30457 10.11119 13.53397 43.01611 292.963 11.35705

49.54206 239.281 112.0894 42.17732 18.27019 24.19458
30.52597 54.92683 21.30998 82.72889 31.87069
24.41473 31.89048 41.18675 23.85826 14.35495
46.99128 209.8495 17.79813 6.521385 33.64648
172.8897 318.8836 74.15692 92.15658 95.16862
21.53646 88.0386 32.83777 54.24978 25.58473 85.20814
19.10883 44.7575 33.14698 66.93213 9.477742 18.92708
73.09402 165.9238 143.4339 54.81478 33.03209
19.55718 208.7914 123.0572 242.2289 41.50343
41.92346 40.01849 102.7062 51.75771 59.24384
59.22418 26.83087 64.82259 19.44218 42.4873 29.29367
18.55827 17.35273 42.99277 84.07063 6.96593 273.3832
11.17142 184.662 7.074177 229.6489 94.78265 76.66201
80.55797 20.5764 103.2206 91.60528 83.85483 53.3187
248.094 69.63941 118.4839 22.70688 67.46064 68.99599
57.60468 81.14506 49.42764 12.74136 18.06707
64.19394 27.81875 13.57341 12.82087 47.03339
370.2254 133.1879 15.31127 172.6316 60.46935
33.82641 38.24035 58.10749 157.012 35.68767 176.4734
63.17633 70.05269 24.57453 109.509 76.51643 169.9842
51.61299 21.95919 86.80618 179.0454 41.74351
20.02607 51.8019 11.3338 54.04167 55.14564 34.45096
24.75855 14.43644 71.60876 102.0187 48.05724
72.70783 167.8128 247.4393 72.85724 31.03115
15.61306 54.54089 84.62724 22.97496 190.2628
107.2118 60.04639 98.70186 67.1977 73.76936 48.91385
8.365727 40.29568 7.818849 17.6756 90.4476 111.0241
349.4373 5.931166 27.7819 18.92455 36.03574 120.8628
9.888282 42.22185 19.61276 23.99723 41.5682 40.75602
105.18 260.6676 181.0426 108.5586 29.38518 183.2932
25.38515 25.79749 11.53269 124.3595 76.69925
18.37108 37.6836 74.30704 138.3114 74.60303 103.2711
23.84034 58.88163 42.96271 63.36478 91.63743
93.14051 18.04964 70.25858 36.36989 32.27271
50.57257 87.18942 26.4271 14.41443 7.8928 49.66033
102.1813 28.57073 50.36767 60.50393 235.271 23.94592
158.2066 121.3196 81.44293 49.07111 25.68754
13.86712 101.3181 257.5822 32.74326 27.65069
134.405 75.85203 307.1113 293.8396 88.72775 47.76655
28.29087 29.05773 71.6405 256.5542 48.58261 1062.036
20.25171 47.0036 37.33218 85.27019 56.06038 47.1854
61.02217 96.89114 56.11164 288.4898 23.87702
117.8317 65.97815 254.9894 51.16089 168.8227
18.0829 75.74158 40.44967 44.05629 38.99425 40.11112

147.872 19.95097 28.5575 112.2435 9.360274 132.3734
98.89623 47.12921 108.2526 19.91777 4.590363
41.19144 46.01352 36.19214 17.60431 62.47996
100.1284 15.39529 107.6177 9.721237 38.55144
21.50025 34.52388 46.57613 30.50448 226.2841
42.73455 149.8938 9.491559 17.96899 75.93661
146.5735 20.21988 65.30232 42.21498 170.3569
6.921076 18.77773 29.03868 58.71577 6.758795
46.29954 21.08012 76.34975 84.41036 41.64149
14.33441 26.71901 23.06248 162.6393 23.4066 50.19392
27.06998 90.55044 75.83619 87.54014 11.7337 23.27225
19.82162 21.36933 214.5705 84.41036 43.09955
60.1974 47.50629 20.91102 8.602498 125.42 29.18979
88.35263 77.88052 59.04703 31.99234 108.4802
47.01879 9.966301 27.55872 11.43531 21.1157 110.8786
44.89566

CDKN3 2.745877 0.438875 6.187028 5.336766 4.089515
0.488851 3.887975 0.4741921 1.918006 8.853526
1.507939 3.342515 5.877819 2.663546 3.173598
0.5803853 4.97224 0.6970957 3.370879 3.870311 3.689488
0.5910649 2.033556 0.5301713 0.4227422 2.786532
3.206618 0.3441425 2.553735 8.780373 1.632442
0.9646812 5.942687 7.352232 4.284087 2.810185
4.866343 9.914491 11.72811 16.079 3.783055 28.32173
17.82332 9.792722 3.674985 6.655401 7.062289
14.20434 7.51757 6.358199 15.92209 6.76414 7.190519
16.11043 3.733769 21.02168 26.06066 1.038331
31.63543 5.288843 3.440894 7.985833 9.559899
17.00109 17.77195 2.150219 6.780734 3.063148
4.722603 21.27114 11.75969 10.39819 4.986447
17.19219 9.753819 5.896236 4.251553 5.02427 29.46677
21.98389 8.530647 5.739543 15.59622 3.978914
5.817993 5.501202 4.502624 6.845037 20.19592
3.168604 11.69006 6.017188 7.178966 7.468227
22.31533 1.034772 2.83039 7.382699 15.77165 10.16249
3.507266 4.072025 11.7602 11.27652 6.948664 1.850451
9.862996 2.837012 9.229891 10.83445 5.424789
11.30345 10.66532 4.789226 13.92859 4.460696
10.52743 9.322268 6.301779 20.86208 14.60097
11.31401 11.62373 1.748259 18.9098 8.128304 24.56517
11.22021 2.220928 4.855866 4.262847 16.45962
4.460643 16.53741 7.872192 2.168493 10.78594
14.10099 12.19227 29.82041 12.0371 8.215636 4.327592
11.40596 9.463079 1.523077 14.36072 5.500257

6.562421 7.753001 7.934833 4.753925 9.644456
3.546852 1.931458 2.758041 7.205069 1.864597
8.403004 10.32536 4.293845 3.042415 9.759368
10.61813 31.71199 9.617308 4.405505 4.725945
6.045813 6.790244 1.593928 30.14462 4.288933
17.45759 22.06373 20.36267 3.972913 16.62545
4.681764 6.162545 23.33509 6.689334 2.153443
5.271232 8.278062 4.624679 1.781577 16.8844 2.871479
11.88369 3.841009 6.045661 7.787864 6.774655
5.919089 6.288047 13.13395 6.257161 15.3364 14.73054
21.69741 8.027412 8.63444 13.36547 36.95153 7.31955
8.562544 19.14931 8.191872 6.512502 6.730951
7.350963 6.397581 7.802505 5.39936 7.44962 2.47279
13.07686 6.557483 8.245019 5.486589 11.46235
9.560234 5.249186 2.394396 13.42219 11.79002
2.174495 12.53171 4.910939 5.16022 4.104069 7.875095
2.534782 18.47252 2.315032 10.46397 6.106821
25.28688 9.016967 7.272021 3.124201 13.21185
9.197728 21.37182 8.519171 6.429603 1.045416
6.882853 13.15243 8.038167 11.83192 7.515856
5.465739 8.699573 10.36468 12.34224 2.665838
8.826111 6.326123 10.96216 18.56467 12.11734
10.39793 7.051742 9.714422 14.28775 13.86005
4.001551 1.513637 10.04937 13.10836 7.300389
10.76889 6.912777 9.25697 6.361657 1.083062 21.18463
10.06761 2.803635 11.26465 4.148777 8.976569
11.99771 2.68602 11.16088 4.762056 8.880631 5.124921
11.13578 0.5002579 1.505354 12.40285 12.61089
7.65887 14.83902 9.164607 12.7393 8.664259 5.999509
5.19358 2.692439 3.458359 13.7167 7.20505 13.48017
16.99801 3.737295 8.376739 7.852347 27.1458 10.07183
5.490304 9.921442 3.606106 5.908756 9.12365 3.980956
21.30819 11.82749 18.17657 6.469281 11.58069
9.797568 9.856787 9.289631 2.701009 10.7873 6.892961
13.80246 4.021505 4.064614 5.923341 1.988798
3.572743 13.15986 4.066011 6.934299 6.475004
12.81031 37.70047 12.28127 19.06472 1.101273
3.197484 6.970375 7.391677 8.553851 11.90355
13.18045 6.006229 4.743681 15.54793 4.994438
9.853402 4.276497 13.35377 4.354811 9.863174
4.880802 8.988024 10.56159 6.359929 29.66716
12.58454 8.651577 9.279802 11.22339 6.380936
16.4876 10.80437 16.20175 11.34421 15.36586 23.55025
15.93609 28.72916 4.52316 4.388603 6.297351 5.311374

11.59112 9.183526 6.465431 6.832734 3.894912
7.045661 9.182983 12.70545 5.079777 13.28269
8.86312 12.30997 5.148167 8.538834 6.467368 2.50166
0.7020688 9.262056 15.2652 4.590454 11.74261 8.29727
18.66134 11.81924 4.899431
CASP9 4.091947 7.348425 2.508441 3.598062 2.51365
3.270002 1.857494 3.843337 3.733313 4.466916
1.685215 1.982716 2.878474 2.427592 2.625271
2.285892 3.767702 7.071242 3.990214 3.492284
4.446485 2.674714 2.564287 2.249868 2.260578
1.675367 2.87632 3.035004 2.983952 1.670551 3.670121
8.000776 4.088265 1.945314 2.559093 2.17604 4.886235
2.446795 3.058625 2.657415 3.569431 2.481784
2.037671 5.869165 2.512851 1.917363 2.354999
2.089479 1.735117 4.902366 7.368825 2.281542
4.679805 2.699068 2.145109 1.891435 2.08096 2.074752
3.704903 5.31193 2.482506 3.448278 2.131075 2.449009
1.851736 2.333701 2.897025 2.430134 5.493665
2.54643 2.206635 2.104421 2.564036 2.985052 1.821678
2.9472 2.643948 2.233294 5.63059 1.685494 4.041684
3.424634 1.916861 2.381658 2.19866 3.916587 2.37471
2.773618 2.484321 2.780492 2.255936 2.558539
1.500449 3.468219 3.161121 1.935245 2.09012 3.179363
2.459751 1.783153 2.513283 4.210959 1.137241
2.976772 2.737104 2.410704 2.553728 2.783774
3.833088 1.915219 2.008845 2.944632 2.342084
2.176459 1.884744 2.559194 4.387172 2.931619
2.619569 2.555849 2.134404 3.088067 3.194015
2.063043 1.664524 3.150603 2.620403 2.308255
4.525849 3.103353 2.007677 1.902385 3.035704
2.739555 3.495129 2.811603 3.357117 2.591852
3.16205 2.695441 3.014759 2.160139 1.782528 1.132238
4.645565 3.503206 1.49978 2.673314 2.111732 2.488773
2.56615 2.311847 1.401725 2.564997 2.510737 3.471895
1.596553 2.618489 1.564727 1.540641 3.324898
2.013655 2.619598 2.398051 1.929444 3.06006 2.837099
1.60296 2.96663 4.141493 2.260334 5.107561 1.406235
1.759288 3.977237 2.230432 2.734073 2.330777
2.652643 2.316467 2.751071 2.04408 3.90318 2.198995
2.246595 3.481897 2.294153 2.354116 4.830053
1.699751 4.788488 3.908162 3.619207 2.647301
1.839678 2.528923 2.129742 2.588455 1.550945
1.207553 1.198569 2.365626 2.651872 2.847468
1.768608 2.289696 2.95042 3.633269 2.187111 2.406444

3.079027 2.262531 1.883735 1.836868 3.003399
2.13747 4.449899 4.399397 2.807834 4.694002 3.221532
3.063882 2.749548 2.443403 3.094493 1.079309
3.066256 1.675951 2.61061 3.268151 3.017565 1.66094
2.204632 3.158825 2.134589 2.43548 2.561875 2.799061
2.453463 3.488286 3.531435 2.624889 3.016728
1.879251 2.108424 2.748739 2.07973 6.452146 2.695635
3.082863 2.989306 2.850227 3.36352 2.079513 1.985475
4.066426 2.343136 3.998643 2.868383 2.398703
1.995795 2.21295 4.923111 1.865716 2.707804 2.579663
4.126159 2.786756 3.617534 2.106875 2.802898
2.686633 2.582329 2.840172 4.949993 2.898105
1.74565 1.613314 6.010135 2.90971 3.411896 1.975225
2.897636 2.335137 1.412324 4.326773 3.095583
2.210927 2.667659 3.549156 4.634554 1.551702
1.818878 3.42408 2.425319 2.117246 1.411634 2.108288
2.405045 1.69662 2.412171 2.214245 1.123946 2.655168
1.584677 1.872016 2.500802 4.037897 6.494419
7.429763 4.116085 0.9573509 2.257644 3.933707
6.428043 2.722162 6.231117 5.503716 2.273507
2.659217 2.126781 6.313934 2.600254 1.633482
1.953689 3.880042 3.303219 5.585514 2.887683
2.064504 2.046838 1.815226 3.483621 4.000431
2.223009 6.419849 1.654401 2.778945 2.385937
1.951339 3.179054 2.767417 3.014419 2.537769
3.676497 2.163913 8.300793 3.544711 2.987931
1.463665 1.995095 2.050466 2.722784 1.406178
1.864967 1.180982 2.018512 1.271574 1.805888
2.956847 2.592799 2.045139 3.00657 8.402018 1.432155
1.505815 1.908286 1.353361 3.442998 3.003574
3.25981 3.270687 2.286541 1.548935 3.827742 2.196304
1.746543 2.08843 2.179928 2.569982 2.687807 7.854789
2.529285 2.12146 1.854713 3.189116 2.536707 3.246256
2.189364 2.213628 1.815568 1.896333 3.320012
2.328463 3.14842 2.466209 2.722556 2.873962 3.079249
5.073911 3.214411 3.170553 1.798571 2.784128
2.370523 3.219745 2.199691
MTDH 25.71322 15.61213 24.71009 27.75289 22.44406
22.23733 21.65727 16.95189 21.08902 23.15862
27.22911 20.23638 22.72378 14.64927 21.82169
22.55604 20.02962 19.17199 16.39795 19.51337
26.07544 22.60611 18.41959 22.67471 17.91313
22.65377 21.87116 23.71679 23.57734 17.94062
23.09499 22.60873 36.98588 27.6082 30.94217 90.25393

12.8895 30.89354 53.83574 46.46317 36.98765 26.88165
57.67869 34.03805 37.36779 38.73594 45.86729 34.519
44.16961 32.94738 27.98509 50.39868 18.49849
32.61265 22.40563 37.49209 43.27455 29.71953
45.37943 25.28386 38.35522 35.4045 29.97447 26.83039
33.0544 33.6276 23.75708 21.41127 36.66528 34.2777
96.47406 42.22255 53.67495 55.23362 53.48163
32.61512 45.68533 39.85729 31.20763 59.6967 25.06409
32.41828 40.04173 31.18983 37.23266 35.44409
47.1628 50.47277 45.16109 30.08517 43.61639 65.54764
43.2223 42.33551 27.43791 37.82041 29.7546 41.09173
34.01104 66.29522 27.49454 55.21728 34.93168
60.50079 51.68084 33.59685 36.02459 26.01535
62.22005 35.77112 48.00839 19.16037 38.89345
27.58705 27.05165 29.93392 13.24439 52.25971
37.32752 31.73799 55.99952 33.415 22.82591 38.83692
53.93929 38.85865 67.38305 26.539 27.8373 31.83645
34.18312 39.0108 44.55927 44.59476 23.29615 37.89061
40.5842 35.12916 24.50057 48.53413 39.51542 30.47648
25.04491 47.55155 52.58783 26.24369 42.54494
40.87485 53.13347 33.54013 40.57529 41.02254
56.90028 34.8562 28.43279 20.43633 56.24386 35.79767
28.57852 96.89776 27.08972 35.01361 35.06902
36.02948 29.41977 46.40542 24.15536 70.0164 46.94718
33.72434 24.83556 37.28291 38.99855 26.86416
39.06703 37.45797 28.6807 49.77795 64.49962 33.74751
42.7737 41.95838 25.69515 37.59523 38.50922 57.86456
34.1779 41.05576 30.94933 43.91336 28.53741 26.70347
27.43042 33.51662 17.36393 35.11982 59.54918
43.94741 39.90261 70.62337 36.56502 22.65685 46.385
62.40634 69.28397 38.02228 19.09525 22.63319
42.27154 47.68141 33.47705 59.20225 35.46643
39.14594 45.70031 72.31654 27.67808 41.01402
35.47358 45.48223 61.95052 97.06842 26.33351
41.71049 29.798 43.32513 35.41237 18.82001 54.17066
32.86209 27.92495 25.61313 44.70438 29.37509
50.39795 22.48794 31.69154 34.32572 63.10264
30.25134 31.96815 37.38821 46.92354 50.09129
51.44853 54.44708 47.08943 28.48547 40.20508
55.10393 44.82702 52.70472 61.23089 38.09673
54.68532 23.49824 91.98791 22.12686 50.90535
22.3834 55.02558 39.3036 38.74163 39.83278 30.80852
40.41918 75.42565 23.71708 38.25077 38.75447
41.40207 29.45878 34.06045 37.57913 38.4156 48.57821

37.22461 27.61148 38.92587 49.92116 32.05398
55.26182 19.27052 35.89971 64.0142 40.97613 69.94865
47.61003 60.59544 32.92289 50.85687 26.11111
27.75023 36.48911 50.40282 18.71546 29.26957
40.34061 43.69936 57.58788 49.98245 45.52751
31.42195 33.92788 54.65887 91.73526 38.87087
50.83039 44.62218 64.19369 34.1296 18.71678 33.30679
19.11602 54.97358 26.99174 31.9083 53.05892 39.89998
47.44309 55.39729 54.6248 45.18277 31.89871 38.69422
40.20613 24.9948 35.41882 33.1196 73.67227 23.66876
40.7753 40.79912 35.55002 17.81541 24.79389 21.88599
61.19097 24.75771 47.34578 45.40209 26.17818
27.86112 41.99968 31.43995 26.99268 29.09242
38.24179 56.85354 78.96401 55.3442 27.79861 39.32812
53.60725 39.29136 38.14641 53.41963 58.98862
29.01185 80.66192 35.33781 31.5746 45.88739 28.75901
32.20524 41.29311 74.78369 35.12082 42.17657
50.29422 31.29128 32.46038 41.20235 57.28504
42.93025 46.03924 42.55603 51.86811 28.32633
37.95073 26.1906 52.98519 45.67744 58.52535 80.63286
22.85237 25.48596 31.10075 40.14447 56.53119
32.60383 82.06954 53.75242 46.80768 34.36231
37.98623 30.34769 39.68612 15.33279 56.07855
32.87028 27.67524 41.35707 53.7122 90.56617 34.13482
36.75389
TNFRSF10B 5.827784 2.319035 7.891952 5.787689 6.343621
4.549867 2.532857 2.734391 6.235781 5.459419
3.965208 1.986647 7.351047 2.292328 4.050202
3.340903 6.270426 5.533144 7.62005 8.30798 4.979933
2.022556 6.113292 3.545155 2.641045 3.750513
4.137582 2.56578 5.410495 2.16029 6.192784 4.097838
7.267643 6.316384 12.05955 15.03523 4.071073
12.90961 17.90783 14.30588 25.03467 10.2972 21.87357
18.91715 14.44441 39.59115 19.17004 7.493263
23.68151 17.48253 6.633218 12.11827 9.292088
10.11379 6.809969 12.97095 15.02081 8.001171
11.70412 7.304998 16.8433 23.18617 9.310888 3.259806
13.84579 6.277598 13.10578 8.941873 21.56176
12.94121 38.17814 14.57841 7.406245 9.097968
12.30943 20.21855 22.9561 24.54692 15.86531 6.382124
9.300506 12.04263 14.67226 17.84135 20.10994
9.109005 12.76378 31.97737 10.90378 19.45656
15.4148 15.09974 23.11139 9.629907 13.35104 7.447153
21.97077 12.15471 15.68596 23.67413 11.71891

17.4524 16.03036 5.899215 15.88085 17.53341 26.15998
6.132673 24.96553 14.20574 13.37176 7.425012
16.50518 8.03616 11.18272 18.54587 7.021778 41.79648
4.305229 9.680186 31.17764 10.82113 9.289882
9.747561 20.34727 9.229006 20.37352 7.923735
8.562917 8.564063 29.40815 14.23822 9.561039
23.67665 8.463038 7.632629 17.23254 27.85924
9.163308 24.33382 9.580453 10.76619 22.43571
9.193514 6.55602 6.858783 17.70457 13.55548 15.09527
10.74438 6.606798 22.79248 15.28626 14.45027
7.169412 16.08799 17.88214 21.04184 17.25006
10.70558 15.6916 7.611793 9.666911 26.04683 5.430566
11.31187 11.13879 12.89957 19.70958 27.28748
4.204058 10.41549 11.41985 9.83702 11.73803 19.05536
22.49859 11.24353 37.37799 15.45603 5.05396 16.63912
7.072191 15.00519 7.343299 14.70758 7.486986
23.94868 19.21099 33.65752 10.858 16.66956 27.604
7.212824 15.56208 8.824788 21.99867 12.76191 20.777
13.24985 7.359178 6.977252 31.54631 9.073446
6.288697 18.80786 11.24448 3.030158 14.991 13.63657
21.91613 8.119211 9.618753 11.11237 25.20922
28.13618 9.775581 17.88926 18.90336 11.85613
17.41369 14.31579 9.724078 31.12617 18.22721
11.42711 14.50952 4.407954 26.61635 26.37569
14.01744 12.61376 15.62493 26.28111 26.08529
14.04417 10.24487 12.18725 16.45291 2.11673 14.29049
22.05502 11.19105 24.87224 37.15226 8.247844
16.12827 5.657882 23.38752 16.2883 12.33355 18.22588
19.36695 20.71669 28.91194 7.932744 30.78067
6.467188 19.56933 9.145879 11.36019 31.83739
15.74188 19.76281 15.26994 8.145484 25.51413
11.90477 18.37635 10.80185 14.02077 9.35103 10.49656
13.3892 38.34444 6.798121 12.74719 5.415287 13.61979
12.03708 11.10117 12.67977 10.73805 12.83023
24.81391 20.0889 29.0194 19.13811 19.31825 16.82596
20.77587 4.19189 5.64829 15.926 19.87189 6.192413
12.73238 8.494283 23.72549 19.13175 13.74643
9.122151 41.16225 20.31677 18.21542 21.42862
21.15029 13.81571 16.52981 15.45949 6.659396
12.34489 10.41816 7.831776 29.52149 7.373076
8.181639 8.571888 9.740995 39.88705 7.365654
9.11313 6.715703 14.35491 5.923474 6.50142 17.09526
20.04211 9.562958 45.41663 9.069077 13.61657
12.95727 13.5947 5.534618 13.51207 15.97872 7.792591

18.04716 38.99267 12.0243 14.73982 24.94282 15.62665
8.493338 6.741143 13.81842 0.9247861 7.083269
11.96131 9.528048 14.4888 8.934997 22.45946 15.42943
33.58335 28.75726 14.62523 7.257302 10.78306
8.342151 6.393925 7.704971 15.9906 17.12417 15.00103
19.28103 13.71721 13.25673 12.40523 9.355175
12.15903 20.12975 19.19651 13.63885 7.899742
20.90579 9.673621 10.7578 20.73581 16.50459 10.32434
9.471382 15.9688 21.61587 12.08742 6.422244 20.90486
7.145079 17.53777 11.30679 48.90169 11.00745
38.29944 27.05851 12.69548 12.41556 17.41781
4.161424 12.25246 6.776158 8.027255 17.35809
23.17916 8.911896 7.792013 8.064706
CXCL8 2.044484 0.06720285 10.15522 4.467522 5.539356
8.275598 1.125339 9.602052 12.14201 16.32166
0.2193679 0.5146369 11.10943 0.2575825 3.020626
5.187689 1.376409 5.801444 1.632964 0.4366592
8.645378 1.107147 4.641123 0.1452326 3.303772
0.3465112 1.779927 0.7022191 0.6296681 0.9713173
2.563808 12.19278 3.63246 8.833726 31.73364 36.09246
5.172221 1.256674 88.95352 15.26627 45.01102
8.144121 54.76837 10.56394 7.677717 20.06479
5.627665 4.134121 7.227567 26.12182 25.5041 2.73667
2.590901 23.32487 1.781354 9.566262 399.7333
2.927625 48.54722 66.5814 142.8818 8.652069 26.38178
39.41502 5.987481 15.94492 26.21396 0.6263588
74.84081 8.136256 725.7224 20.39338 5.277406
274.3506 250.8204 14.11988 80.89551 119.7562
60.73504 372.3645 26.40594 6.253159 92.04624
5.768096 44.54432 16.93748 4.904126 21.82217
82.88672 15.39299 45.28345 75.56256 31.41524
5.085921 30.29819 15.58007 16.29382 13.41494
24.52939 21.54559 2.343772 7.016372 37.77407 17.458
106.2918 54.14178 39.94535 1.74974 34.90454 16.36235
15.38772 38.97259 409.3611 0.6831986 55.25588
32.03316 140.7923 12.36057 0.28319 12.5259 184.7109
4.660188 17.18006 110.7037 20.57372 4.616815
36.20776 13.57823 1.893756 5.759627 11.93117
6.750121 13.18372 270.2738 43.84866 0.4804562
21.3609 41.32983 35.55754 160.9748 3.934448 29.96379
13.04675 5.388194 36.05509 5.156532 13.05438
25.02821 12.17341 0.8986976 9.872486 310.6401
21.19519 2.349365 1.802222 93.02444 11.89147
5.643558 139.4588 4.317915 4.246828 2.237591

6.925681 49.0954 29.07492 6.963556 11.72061 44.55198
8.196435 0.9438784 1.075948 33.67216 1.561001
38.03971 5.360784 19.10812 8.300418 34.8414 42.79697
32.197 19.46378 4.013563 3.691977 21.23203 134.4492
905.5087 5.544649 36.92899 13.53628 42.38921
53.98581 52.34659 26.86735 4.102331 10.30506
25.73112 372.3345 7.1654 55.02432 9.020671 47.02986
7.957575 109.6186 54.52932 66.67972 10.48194
19.13089 200.2414 16.65712 27.80746 49.24545
47.48062 2.834704 2.057603 40.86533 58.59892
2.782793 1.00722 39.08241 10.52523 7.536035 49.3846
18.05975 187.7134 3.454144 9.475179 55.30721
1.830053 5.448729 1.915475 11.20864 277.0328
4.041617 62.60634 17.42576 16.66118 4.399101
5.011846 25.25708 1.963564 4.048211 14.81455
50.08191 40.07579 297.0866 11.77467 16.53094
0.4220281 12.18473 27.65707 26.58569 245.0242
21.67898 3.845424 8.420501 1.63151 7.601925 30.2457
18.90095 35.63007 38.57554 77.24425 50.14089
25.10628 291.8687 20.80857 47.90796 76.15055
1.729462 23.39811 15.67997 38.2622 48.86606 69.93109
23.97954 9.017224 14.77224 0.5727398 5.329227
356.5098 13.98521 45.08767 33.28416 17.08567
233.6207 22.15751 6.044376 12.39366 311.501 6.788902
49.87265 1.072658 7.965898 58.25386 3.818206
2.035957 6.511063 36.99696 77.59745 8.298539
22.00778 7.731054 16.80233 402.87 38.13599 18.22638
82.85187 91.93036 15.1938 21.01554 6.633726 108.7983
148.5038 9.371979 22.87995 5.194972 14.95552
3.424572 343.3496 215.9163 11.71968 54.44597
40.20622 3.32331 316.7986 12.77448 65.23699 3.581483
40.59809 22.03949 4.775048 23.15039 18.01159
3.99412 0.4066462 0.2138996 1.76246 16.66189 12.60979
270.3106 1.895153 6.697076 90.65452 92.27725
1.55841 3.471177 14.19037 58.84734 8.041785 33.31811
4.552363 57.67457 4.609064 465.6411 26.3874 62.71992
19.53413 42.62951 0.7264508 17.58277 13.72943
13.57865 1.68243 26.10334 125.5578 24.98148 5.176017
50.46307 10.52189 74.32264 12.65687 5.810355
2.964968 16.22953 7.660758 3.223282 42.28363
125.0372 21.60871 5.549676 2.362275 43.88942
7.69716 35.43906 33.84574 38.958 1.550453 26.62861
54.53675 39.3683 11.01249 123.4575 17.1967 40.91678
19.96165 127.1525 1.018026 60.98468 2.908145

29.41003 5.383122 0.4853458 28.47093 2.768189
25.96746 35.56358 0.6947904
AR 0.1034915 3.280952 0.2970936 0.3031024 0.1969071
2.54652 0.1651718 1.238968 0.5214571 0.4702085 1.367668
0.3932415 0.2177733 1.009782 0.1705754 2.495617
0.09074303 2.001994 0.2316967 0.1967466 0.3375505
1.389729 0.4065583 3.390689 2.194996 0.6695446
0.1440154 2.2005 0.2922959 0.04903086 0.613847 1.553609
0.02854226 0.573098 0.3527552 0.05734023 0.02011759
0.1705786 0.3448501 0.05938145 0.3196038 0.02406117
0.1751882 0.01900894 0.09368731 0.1815932 0.1349777
0.2053646 0.4006671 0.08356718 0.08533514 0.6169699
0.06222412 0.1641615 1.507831 0.04333973 0.04508497
1.737096 0.05017553 0.03391941 1.044559 0.08530273
1.712601 0.251523 0.001565044 0.8346003 0.4996716
0.6701153 0.05484638 0.05163936 0.04079505 0.08490021
0.9742747 2.13467 0.1938096 0.1172369 0.3788005 0.4267672
0.09123997 0.0367641 0.03361638 0.4571607 0.03898192
0.6092636 0.4552114 0.241151 1.009865 0.3555577
0.2025921 1.012112 0.08548325 0.01523841 0.2992264
0.08256309 0.09425004 0.1533629 0.245831 0.06347009
0.01622461 0.3850185 0.2705396 0.06756407 0.03930592
0.04228946 0.5703215 0.8141302 0.1542179 1.237348
0.138785 0.01819404 0.08609109 2.087483 0.8721793
1.272538 0.5115241 0.2275394 0.06489669 0.09280248
0.2310409 0.02260593 0.07532207 0.3915194 1.585652
0.7305444 0.08854879 0.1654653 0.07801552 0.08733099
1.19037 0.7157706 0.2031242 0.1152347 0.1689477 0.1512671
0.0127121 0.6305515 0.1882231 0.06734819 0.08135452
0.08207362 0.116208 0.06945508 0.04954396 0.04550131
0.2425918 0.6125351 0.02509746 0.6281115 0.02505684
0.2321811 0.05570519 0.7357813 0.09171856 0.5018354
1.50345 0.2356858 0.4095254 0.9601371 0.2555904 0.3386431
0.6839566 0.5402806 0.5873237 0.08276712 0.2185626
0.2834783 0.1251789 0.52827 0.1641759 0.1722144 4.091457
0.147946 0.3227595 0.03934736 0.04487765 0.1603801
1.154062 0.3521019 0.01094073 0.2474085 0.1212448
0.09418529 0.7909161 0.11549 0.2776679 0.07447459 1.757762
0.1307931 1.722017 0.03736342 0.4574183 0.1522023
0.1533072 0.07476174 0.03520058 0.4657872 0.7817723
0.1344383 0.03824183 0.2045327 0.6807092 0.4240119
0.08362036 0.02785203 0.02582143 0.02468958 0.2590002
0.5986253 0.09271765 0.1248665 0.1309209 1.002072
0.3156317 0.1944196 0.09604331 0.1731342 0.7558014

0.2144262 0.6076348 0.1969037 0.1294748 0.2937411
0.08236716 0.1067245 0.7606303 0.01751134 0.05284118
0.3257566 0.02220074 0.3182312 0.2707289 0.4337759
0.02373357 0.0610148 0.05042556 0.2260533 0.2031362
0.04822315 0.1746463 0.7547818 0.7521129 0.2769242
0.2910763 0.1775661 0.6287777 0.5471938 0.1268676
2.262785 1.062096 0.06487321 0.3915517 0.07956882
0.2350948 0.5855366 0.03249609 1.181114 0.1467976
0.3736986 0.06619976 0.6028252 0.07814792 0.05097231
0.2338437 0.2631598 0.1812154 0.3702278 0.03545455
0.06557321 0.5184361 0.11406 0.224988 0.08809095 0.08730095
0.04261593 0.03878972 0.1112446 0.3465003 1.491018
0.03170206 0.07865054 1.520434 0.04966549 0.8571978
0.2221774 0.1394035 0.4919124 0.08876362 0.7130698
0.1350326 0.5792423 0.2000518 1.70665 1.070495 0.1531471
1.289603 1.5865 0.3914197 0.3503797 0.06084461 0.06308555
0.3104623 1.083865 0.1260748 0.6032408 0.02015488
0.07892217 1.597634 0.1119739 0.1377736 0.1582255
1.23402 0.08011565 0.04536958 0.5349036 0.09076518 0.973046
0.4918363 0.4495187 0.3284268 0.4196588 0.1424096
0.0991155 0.09798088 0.115026 0.8093002 0.1510495
0.1071433 0.0462835 0.04491469 0.2149511 0.06458443
0.8461484 0.2943262 0.2443956 0.8185029 0.189287
0.01792713 0.2921799 0.1160407 0.04801497 0.09547491
0.03840547 0.1727438 0.04064827 3.020801 0.1727415
0.07605844 0.08239104 0.2881039 0.2623032 0.05638953
0.3114535 0.2647491 0.1531236 0.009519782 0.06128964
0.2073385 0.2868117 0.6258656 0.139904 0.2407997
0.8586401 0.03529981 0.01990644 0.02974682 0.1582686
0.0111905 0.02308808 0.6585976 0.09642195 0.1408867
0.4852454 0.1589817 0.1994301 0.105458 0.2176319
0.0762729 0.1948662 0.07059479 0.4510063 0.3068471
0.1220984 0.1763527 0.02603458 0.05445545 0.04926362
1.036899 0.195117 0.05601705 0.08794976 1.006877
0.1273565 0.1632046 0.4602039 0.1101318 0.1600056
0.8012043 1.10378 0.6821721 0.2516557 0.09153116 0.3023771
0.06575169 0.01905179 0.05282023 0.1102814 1.01468
CDKN2A 0.3993776 0.1059039 0.7532482 0.6379867 1.571552
0.1699424 1.099303 0.6958497 1.64246 1.942638 0.9016962
1.991255 1.627368 0.8244267 1.236546 0.08453668
1.051057 1.207868 1.251458 0.9963463 0.6902255
0.09024492 2.237284 0.08833563 0.3723755 1.384938
1.476853 0.07165136 0.8950009 1.081378 0.5026619
0.1323679 0.2786037 10.02053 37.04123 0.3237111

1.428314 7.713502 1.518705 0.7187659 6.764733
10.00581 0.7056149 0.1553425 1.503327 0.07161617
10.34859 1.331116 0.9705083 5.07425 40.23411 1.659208
57.48907 8.689736 0.8899305 0.1196454 1.670479
1.592912 27.51399 1.637953 2.575754 11.90915
0.6584819 44.29553 0.207832 0.3024649 2.271349
1.909689 5.239997 81.03823 0.149051 0.4037876
1.450836 2.897819 0.4700858 1.025769 0.3126856
0.4698854 2.453747 1.624091 0.4376598 3.020797
0.4889062 1.004231 0.2722034 5.303205 0.9565451
2.840883 1.40266 12.97674 2.157176 0.6033335 0.5590583
1.323054 15.96569 0.5108648 1.158124 2.392518
0.04787926 0.6259308 1.138728 27.89902 5.536726
12.08522 1.447548 1.407589 2.435276 3.839452
1.035768 8.559261 40.53737 70.16615 43.13661
1.590913 2.424744 2.350163 2.557248 1.917039
61.11976 1.299104 3.153562 10.90394 12.09877
0.9782356 39.17069 2.063851 0.4758004 0.9292302
1.72843 20.74673 0.1136007 0.164299 1.891832 0.1995243
4.396615 4.470138 0.2043041 0.02890857 35.8494 11.32623
9.368934 0.4488002 10.80616 22.32509 21.28679
6.877596 29.35957 0.9353952 0.04140832 0.5835445
6.901177 1.550937 2.396441 0.9205783 0.6548398
18.07286 0.7257122 1.271648 22.09023 19.97568
1.552985 0.7082028 0.3946911 20.61149 14.95454
3.396828 2.46271 4.959121 0.4616346 36.02503 0.551337
9.601177 0.1969397 24.6813 21.8762 17.38396 0.321412
55.06548 4.93336 8.559368 15.35826 2.413362 3.739687
0.6659947 2.645422 0.8708129 0.6091829 20.65188
15.10677 4.633843 24.53338 27.26396 27.30811
2.216501 1.754382 0.4552466 11.68096 14.93576
0.1402109 7.474224 22.64926 8.376641 0.5889482
6.898659 0.09524971 0.8587029 2.887419 27.34496
0.2194599 5.039105 0.3262522 3.845357 2.890884
2.354175 17.14086 0.5813961 0.2945122 15.14679
0.7946103 0.2226848 0.206701 1.011901 2.624428
1.58916 2.431939 1.623729 17.13215 6.057078 0.1719769
0.5835892 15.90284 2.681153 0.09192542 13.16795
0.7359926 0.2254951 2.668566 3.973235 0.4395941
10.83878 14.99109 1.17369 0.8456223 1.014735 1.144833
3.477749 1.49756 0.6986563 14.27294 1.244377 8.501678
0.9165157 18.06931 0.6936077 3.023149 2.0913 1.013586
3.826733 1.490387 3.804175 1.657365 2.580655
0.9129619 6.455419 3.046217 16.69972 1.017211

0.4527366 0.5629211 1.138175 1.866392 7.689309
22.99443 1.297483 36.51111 5.932598 4.539278
1.176566 21.16472 1.856799 1.401016 25.27288
0.3657738 0.5968906 0.3134351 0.4882222 3.950326
3.384915 1.319428 1.452683 26.50655 0.09043208
0.1879406 3.53258 0.6222759 8.316892 14.18605 5.909537
1.05012 1.38623 2.069741 1.706616 5.380417 1.624985
0.4390014 0.9400242 12.84364 14.82318 12.43291
0.9970153 15.27547 0.7725983 0.8638893 2.511671
10.31225 0.4277871 5.045072 8.412041 0.1917845
10.64376 7.229276 4.912273 12.46778 2.355332
1.639346 6.60194 0.3192826 0.4141576 11.35312 1.477031
0.2382381 8.282444 2.569228 0.4969159 4.633672
6.073876 14.727 19.21716 0.4465141 7.255608 1.873632
20.61348 4.789081 32.32765 0.4620457 2.18284 13.7919
14.99956 26.87709 46.09666 1.362073 1.56627 5.116968
1.487579 0.4326344 0.8329421 1.389176 8.217233
1.572194 10.80448 7.909362 3.270591 28.57639
0.3133039 0.6562876 4.425748 0.3339041 2.272455
14.34596 15.81384 35.62638 19.31527 51.62987
9.572654 1.133912 26.26502 0.1500556 15.26814
3.122015 1.911948 22.40615 0.3763298 0.180146
0.3540804 3.361937 0.431289 1.435631 23.77639
0.4959237 53.74687 0.2104572 1.752524 46.21802
0.6505747 1.781602 1.833917 0.6248923 3.022498
0.4401653 14.90172 56.25062 2.97441 0.1144309 1.170447
3.55329 5.830861 1.041629
MAPK8 3.171581 1.841005 3.250436 2.647732 2.624283
2.562543 1.566596 1.763114 1.770335 2.459425
2.595494 2.875399 2.411587 2.280459 1.707135
2.881745 2.353639 2.453871 2.964052 2.613502
2.191572 2.368218 2.426294 2.624727 2.386143 3.3753
1.65513 2.909906 2.749555 2.057643 1.675711 2.554867
4.386819 2.133708 3.107769 2.801526 4.014556
3.355354 3.347366 2.287367 5.994839 3.963706
2.946155 6.020685 4.193469 2.501386 4.169704
3.063571 4.678155 2.065563 2.217291 3.443592
3.883279 3.570957 3.371384 5.47175 2.2907 2.91251
3.562952 2.397358 4.088994 2.833439 3.266958
2.786138 3.380343 2.883227 2.38406 3.126184 7.456985
3.769161 5.574738 3.030315 3.767321 6.519627
3.692595 3.487386 3.514425 6.605271 4.36351 2.472781
1.621326 3.307868 3.237233 3.856988 2.660509
3.415617 3.769474 3.380392 3.711277 4.120272

4.623738 2.552411 2.581724 3.613487 2.414035
2.242793 3.064048 2.119477 5.161062 3.751565
2.928119 4.07197 2.360566 4.198925 3.601131 4.363746
2.281119 2.054837 4.121556 2.389704 3.87065 2.880967
3.140427 2.548257 3.398594 3.441687 2.241972
3.837135 4.662553 4.364591 3.227472 5.184632
2.850378 2.549128 8.052427 3.008975 6.558068
1.538868 2.872071 4.193725 2.80247 3.428731 3.408256
6.342996 1.720273 5.182864 5.145086 3.487988
4.413702 3.250826 2.588096 3.811413 3.950905
4.716613 3.201427 3.211607 2.464003 3.522943
3.804379 3.883658 3.203027 3.260089 2.980598
3.006578 2.963872 6.073755 3.503981 4.157381
3.933528 2.781529 2.206008 3.722369 4.369776
3.596242 2.053966 4.680846 3.112149 5.082344
3.168602 8.87359 2.819986 4.95224 3.601165 2.504208
2.696541 2.276026 2.457963 4.169506 5.555003
4.783955 5.220301 3.082796 2.779614 2.496552
4.626235 4.189045 2.895093 2.178261 5.013508
1.88891 2.659085 2.232361 3.246688 2.145229 2.153452
6.326268 3.55692 4.575964 2.012538 2.926789 4.679232
2.549383 3.198057 6.783134 2.869095 5.083059
1.99082 6.025213 3.814607 2.578397 5.062261 2.888271
4.617512 2.293175 3.1624 4.310191 1.33214 3.031307
2.816825 4.181345 3.80303 4.426817 1.732615 3.992724
3.699758 3.169426 4.341869 1.792 2.825762 4.549249
1.840169 4.698745 2.288772 5.316487 4.046587
2.312684 2.195162 2.012769 5.763712 9.406858
3.465898 3.501651 2.8333 2.75592 4.777048 2.709722
2.635308 2.19871 4.238309 4.597143 5.917732 2.312228
2.436233 3.613418 2.640687 2.815086 3.030008
1.779853 4.072699 3.416187 3.637112 3.250277 5.3262
3.82495 3.802234 5.637198 2.86019 3.021928 3.906418
2.32137 3.225687 1.209729 3.470509 4.905143 3.338803
4.417177 4.529785 2.746244 2.929627 3.680943
2.876397 6.151361 2.040841 3.072813 4.559888
2.920742 3.678062 2.89647 3.788705 4.256175 3.921538
2.419056 2.79825 3.501701 2.441563 2.040906 2.60665
2.419288 3.08096 5.608983 3.710818 3.941151 4.543204
4.414825 2.304725 2.065139 4.840168 5.346003
4.017344 2.211778 6.274758 2.676259 2.972378
1.829061 4.3662 2.938916 5.537417 4.721573 3.054735
4.50382 5.471159 6.153416 3.608728 1.88112 4.226593
3.016842 2.762155 9.181084 4.109605 3.878175

4.343951 4.785271 2.320577 2.533454 1.787714
4.771491 2.488433 2.873986 3.485089 3.578462
3.073825 4.078306 3.810361 4.676649 3.282977
3.118902 1.927881 5.121287 5.325255 1.995901
4.236644 3.285577 3.560088 2.818964 3.38234 3.644163
4.098137 3.457289 3.139544 3.281508 3.238396
2.451487 6.361737 5.086185 2.65736 2.910733 4.973637
3.643341 6.326939 3.124431 4.651987 2.014129
5.486904 4.514442 3.403635 3.39922 3.118708 2.675255
3.540854 7.18791 2.963473 4.670128 2.64927 5.050441
3.946073 1.686265 1.87423 4.070442 4.539406 3.403308
3.934084 4.885339 3.5014 2.992487 3.898592 4.454149
4.73814 3.074455 2.095298 4.445835 2.168777 3.249304
4.079875 5.516003 5.313036 3.377384 3.63138
PIK3CB 5.963211 2.606865 6.973104 5.892665 5.059136
3.488157 2.442065 3.726461 4.437362 3.197659
7.627207 4.836036 3.595463 4.176811 2.956963
3.863894 3.70293 3.889282 2.751399 4.130758 5.458654
3.056057 4.970454 3.848407 3.771125 4.739582
3.474522 3.335208 4.939193 2.242582 4.377675
4.300143 10.94776 7.169771 10.79131 2.641216
6.309024 4.393356 8.399465 9.711168 7.257761
7.810164 11.29609 6.003755 9.006909 11.79367
7.621382 10.2391 14.02831 22.16 7.800414 6.053984
6.127394 17.31496 5.986617 7.605927 5.937596
5.543817 7.671866 6.403206 6.190252 4.873868
4.503177 4.123577 6.303664 3.749838 5.116342
3.453059 13.37544 7.453462 12.74202 7.177905
7.693268 6.740785 6.767495 5.131246 6.026608
5.681172 4.527327 9.026999 6.477942 7.856246
13.89507 6.738832 6.98112 9.191129 11.1099 7.696014
11.8754 5.955859 11.81938 10.19438 6.615034 8.233592
7.502909 7.668945 7.81288 5.942127 14.54846 7.982262
5.547441 15.35623 3.138598 4.391248 5.503877
7.181578 12.61804 3.792289 9.379619 6.471986
8.336727 6.216212 5.51185 3.62971 5.912351 12.5048
3.63637 13.23454 5.911982 6.068187 5.897164 4.589501
5.346847 4.0202 8.900602 5.617105 6.913481 4.862155
6.423601 9.426613 12.26278 8.990979 6.38773 10.55921
3.798231 7.676091 10.47623 9.567229 10.23111
4.788936 12.77791 12.4275 12.52458 6.525994 5.298236
6.855095 4.687372 5.05869 14.37478 5.172372 13.4339
5.459874 8.237906 12.06628 5.031352 4.875765
6.274286 5.949833 17.72445 3.319008 6.985238

5.954339 9.257103 13.38693 3.99463 6.247045 6.244299
10.13997 11.34246 2.463096 3.954088 8.154071
7.94004 7.311113 13.73604 12.6952 7.832904 7.728926
14.45929 5.270595 6.483389 8.424575 7.380776
10.43193 30.94403 7.839066 9.361708 7.046624
14.59655 16.54051 8.474067 17.66222 6.64995 5.845646
6.508215 13.126 6.38252 6.046877 11.84149 4.476241
2.978756 5.312489 7.747648 5.212499 6.684921
18.44241 5.211805 8.017398 7.633972 9.757376
11.1572 5.281777 12.73831 5.946208 16.58731 8.421932
5.838951 11.25159 10.38882 8.772732 10.32458
6.183184 4.081523 8.428226 7.667513 4.366157
6.550766 4.353122 7.959722 7.120929 7.519112
4.68319 6.404298 9.164325 8.494524 6.867893 3.031219
8.668657 9.341559 6.249331 8.186665 11.84706
6.602266 6.137151 7.759442 6.146384 6.27709 4.17515
10.50818 9.35121 19.06463 9.623986 8.134359 9.488692
12.55064 10.36142 8.277157 4.846038 7.722004
5.673539 6.550465 5.542214 7.152533 5.217545
12.76186 5.568943 10.70304 5.748412 7.202928
16.41301 12.04728 5.007477 5.667404 7.799638
7.992518 6.877606 9.926935 3.764132 3.651096
10.14471 5.72921 7.422868 3.815531 10.50938 16.60696
8.730472 8.899174 5.189321 9.413461 6.54285 4.996928
3.3202 4.787377 6.976858 4.376042 4.344916 3.933735
16.66494 12.7658 9.508331 10.49987 7.159598 10.00295
4.5813 9.866498 5.926673 7.068796 15.18628 14.42425
7.839274 7.86268 3.429441 10.56704 3.154947 11.39751
5.341117 7.542079 6.759337 7.129522 15.77056
7.820525 7.945654 6.237731 5.936659 6.646133
6.796488 6.167689 6.79349 6.883405 14.02446 11.99311
5.11505 8.460817 10.12966 2.987234 4.374571 5.371737
7.446009 13.7004 11.78783 7.27154 3.590035 4.976039
10.05695 6.054317 8.076723 12.59101 4.282073
7.661271 4.921552 5.686197 6.877673 9.971093
6.820793 11.12353 12.52025 8.604217 9.129711
3.442788 5.328241 7.272805 3.114778 5.488922
8.036788 3.932706 5.092671 10.43305 7.934594
14.01896 5.515938 6.311058 5.234469 8.166557
10.47362 5.734865 3.842992 5.649441 9.876771
7.000016 6.493892 7.717374 8.123213 5.655996
5.441573 10.27009 4.478889 2.938426 7.06233 10.21009
7.766422 10.54159 6.886841 6.620497 17.0079 17.75979
8.381344 11.99495 7.663684 3.384022 9.928688

4.331383	3.872772	10.09674	9.332403	7.008293
5.399075	6.242245
CLDN1	1.126434	0.4247825	4.669914	0.5152857	2.575233
1.233164	0.1348201	0.7225429	1.745143	1.058474
39.42872	25.09149	0.6860013	19.49389	0.3621101
0.9520548	0.9335967	0.8451402	0.4162735	0.982662
0.8806079	1.125783	38.18754	1.266996	3.477176
18.81789	0.8915207	0.5362996	0.659164	0.9359943
0.9639383	1.148374	5.172427	26.13332	59.0364	1.347682
38.51463	26.06188	27.36124	19.19337	25.65922
32.74791	23.94391	6.604018	11.15415	80.86655
30.45999	47.91886	138.2456	38.92086	8.41558	11.63432
3.633879	73.93937	17.35364	14.47146	44.95824
4.744957	6.888286	6.554707	25.64046	124.1224
14.09066	3.160728	55.2153	17.07469	30.33391	14.67189
8.416366	19.70808	0.07113798	19.81544	7.466659
10.09656	40.4383	101.1351	12.12391	2.682966	35.17683
83.66787	1.44136	20.675	41.2361	31.69745	17.2253	18.00732
60.27772	28.99134	5.964962	47.50268	29.42309
16.82043	37.85672	56.4741	141.9044	48.1095	11.24942
41.19327	4.460197	30.10003	7.816386	20.11561
0.219332	22.92722	4.297311	20.39899	43.86886
11.61293	5.443513	6.049201	53.60773	41.89175
61.62939	14.93205	75.53121	11.4783	6.670146	25.39285
3.272905	31.44884	7.146033	42.95635	13.80511
11.64197	1.261972	2.06977	25.81545	7.116729	26.16186
54.1368	10.42907	51.60447	19.74285	14.69962	7.031446
6.572017	62.52431	33.19097	30.65596	47.8278	13.15284
44.67056	69.0907	8.519114	5.98979	14.91215	44.86653
29.38304	15.83814	13.99821	5.223465	18.20207
20.20967	59.78635	10.93317	9.191151	20.50941
20.68777	79.62355	34.90053	12.02839	3.212025
8.546232	41.32844	4.578411	12.18499	26.34613
14.41655	18.28463	6.860641	1.44068	3.410903	17.38408
36.64768	1.622806	50.65783	29.09885	29.9228	20.65801
11.30434	10.5234	43.50739	60.56447	14.04928	15.57038
13.62879	43.86402	27.2832	48.64068	28.77747	17.78621
7.899568	21.06925	1.928244	12.11961	28.79615
2.536449	10.30319	28.90522	27.86595	68.95663
42.13696	2.721904	23.90844	20.53237	23.43644
33.54575	0.8131728	26.98036	70.50337	16.07221
30.04521	50.44491	18.56117	246.4077	22.05675
55.88058	15.8018	39.20887	10.23085	34.25885	7.037312
13.78857	4.850095	22.85107	7.730453	108.3448

20.6438 9.658998 11.51872 87.30386 19.14096 16.59592
59.7638 24.78244 48.33538 4.736353 40.62605 23.37394
15.61597 24.46598 78.73811 14.39621 60.54157
27.56862 6.186051 50.2094 8.45533 62.07476 12.34762
194.4632 25.25273 4.85299 90.88086 6.550628 5.039997
79.17454 64.16609 16.76836 13.27308 22.21803
11.96058 26.63448 36.69932 18.5762 57.07821 14.56328
17.06989 46.80935 67.99804 12.92706 20.50856
39.79686 20.35819 4.864637 6.599524 48.31906
7.685775 0.2679037 77.4046 19.26185 38.72412 15.13428
3.22737 70.37465 5.969922 28.10521 26.76237 15.89325
15.83529 48.99876 7.218124 5.396006 15.99295
37.37863 8.228975 28.595 110.2722 80.72616 8.438453
20.82348 12.08396 144.1052 5.171514 26.75771
22.5139 4.129539 0.3355153 27.13411 6.723188 19.53688
21.52452 24.74416 3.122939 4.967804 8.013065
27.96185 18.51486 14.34399 21.44757 36.04518
64.34255 4.546309 22.35865 26.67505 10.45052
39.15115 4.899797 18.7378 7.992738 40.61619 35.16745
15.99281 54.57887 3.864381 22.3238 12.4771 59.62403
32.42767 11.58902 2.758153 31.4871 4.069663 24.13976
12.63047 61.11387 30.77711 5.407592 39.01828
18.86109 10.00796 39.75478 1.829756 13.82336
10.45831 9.765111 18.62375 51.16853 15.75733
2.491799 53.2939 19.55708 15.32135 17.70923 64.5043
16.46025 2.471758 21.74081 20.68332 9.66437 26.93548
84.97921 38.73684 17.6672 7.472389 2.641743 43.06
3.65414 7.669966 13.0225 8.215319 25.66007 1.963409
40.17993 77.24222 19.27144 22.76889 30.16429
24.15479 16.97614 43.07802 7.999814 34.16521
66.18932 231.905 33.38228 8.645528 26.37763 0.221848
17.54183 1.476402 15.23411 20.30312 4.146747
0.676707 13.9049 16.53528
CDKN1B 13.06594 18.03427 19.22112 18.59369 15.7823
17.60818 12.90475 15.48589 13.41312 29.59974
12.79937 16.84586 29.57933 16.60217 18.1162 25.90364
13.71213 15.19636 8.634431 11.23751 19.06084
19.62179 13.5846 22.81741 21.66074 13.29264 19.22591
21.56624 16.48991 15.01156 15.32154 10.33609
8.775684 11.23203 13.90236 18.92118 6.087984
10.51884 14.31289 21.4339 15.83544 18.69421 12.14694
31.32926 9.255299 8.503772 18.08404 19.75505
15.62492 8.780423 19.33124 20.95374 27.55229
18.11517 22.8553 23.05111 21.71889 25.70211 11.95085

13.97876 12.13486 14.06187 16.55981 12.05983
12.3332 18.47099 13.92823 24.18589 9.264066 13.50328
22.30086 19.10851 13.13493 11.60978 10.55347
23.9947 9.779325 10.66518 28.66194 13.49296 16.94899
18.00992 14.8869 17.53806 19.81668 9.308033 21.52747
17.1999 12.65975 15.67625 11.31107 30.99079 14.87918
17.68333 13.30807 21.93903 18.87241 9.039885
26.4362 13.57908 45.7053 4.876752 10.69407 20.95498
21.88724 17.79399 10.28185 17.31387 22.92105
25.1984 19.57833 32.9924 13.5239 21.60594 10.85468
5.91727 11.29418 10.01564 12.84435 28.56059 14.37711
14.20117 17.26065 9.480418 19.89758 26.3394 49.93051
13.86315 27.681 13.39439 10.61597 45.63846 13.55759
14.88445 15.29803 22.97118 31.70907 7.891136
17.95169 14.04498 22.30093 40.2561 8.266581 17.27219
24.14266 18.2616 15.53887 18.36011 13.85624 21.43684
34.74029 11.88876 14.02698 28.6059 37.02246 12.57741
13.44368 28.4575 9.379661 14.05862 24.53953 23.94285
17.24131 24.2709 12.49889 27.50169 23.83583 9.294043
17.78022 23.38416 21.63637 24.36569 19.44925
14.48441 9.483816 13.07054 17.44434 14.39416
12.11718 9.981899 14.53432 19.76808 13.15065
11.51805 18.82456 30.55086 25.09664 15.21685
28.96371 25.41244 22.15323 9.792809 25.97796
14.35707 22.85853 19.50386 16.99174 11.45962
28.00742 9.603119 17.09025 18.80398 19.12285
29.66979 13.09422 12.11418 9.559753 34.4443 10.82391
44.68009 22.4377 23.61082 18.7244 18.97677 24.4728
22.84531 10.17733 32.54719 18.24205 16.85226
22.67335 5.574095 16.34572 10.82976 17.84283
15.15425 17.13031 10.19463 15.72063 8.346917
21.94835 8.510497 20.8643 9.888675 28.15649 12.05307
16.58005 22.76604 18.84468 50.81469 43.60287
26.14412 27.20387 20.54584 15.75439 25.36439
28.20023 28.92121 19.67639 13.38878 28.27629
13.84625 20.2134 19.84549 22.59922 29.86993 22.21244
14.72777 15.35814 17.96477 19.59506 10.89484
14.8057 27.63006 15.1901 27.95084 18.01952 25.47679
23.06755 20.0665 19.79015 14.02602 13.88632 38.41364
14.54176 21.39376 15.17325 19.5714 16.74037 16.67021
25.87454 26.2008 17.13023 16.22693 14.83916 28.94208
10.195 16.33357 14.74185 24.68449 13.4206 14.40881
19.07701 27.06909 12.14134 14.95457 25.85546
20.49136 18.84926 10.2595 12.16616 12.39428 10.57782

6.856132	12.81046	9.373041	20.77517	17.65318
26.99174	13.31859	18.22256	21.47524	12.08168
19.0178	21.97537	29.94316	12.12679	13.84048	12.18855
14.9174	11.567	23.0529	14.25891	22.72373	15.45072
18.38617	19.84471	16.52882	18.28646	11.48184
10.69751	30.50118	23.40815	26.41745	19.26539
21.83236	14.39516	17.63949	11.20907	1.731834
11.07891	17.97056	12.91343	11.94408	26.96195
14.12082	9.214533	47.07962	8.952804	10.67609
9.861668	40.99492	14.00638	12.89557	27.68899
15.86943	13.91305	10.45001	24.36493	16.21926
13.94106	17.96394	11.91804	12.57502	82.23983
17.65943	19.9924	15.56008	10.87442	22.49128	21.47146
9.186519	1.361475	15.43849	28.40026	12.177	17.91902
11.96199	15.81862	25.27746	18.24134	16.30566
10.84438	5.13397	19.86131	18.92581	18.66512	8.301698
22.87635	5.510157	17.06687	24.22741	15.7658	10.36339
15.82066	15.98473	10.40538	16.87962	13.12863
15.02593	14.83217	25.85988	17.89844	18.71781
33.95962	27.71858	20.16035
ATF4	60.99245	164.9491	45.43966	141.9384	80.63102
96.51053	81.86125	92.33939	156.7133	186.7123
54.03632	57.90452	134.3299	66.78209	137.9132
118.6127	157.0787	158.5682	125.4513	74.37842
129.2468	127.5529	63.32375	76.57364	84.66011
64.0899	150.8557	94.81111	117.217	102.5066	132.9373
165.9447	45.51428	61.15667	59.40791	131.8417
95.46329	76.9897	72.60269	61.87909	49.6838	97.41184
69.37749	105.1508	82.89676	157.0756	63.34443
96.80965	82.10523	56.23846	79.87542	38.40161
74.39873	85.45273	72.17032	116.7375	144.8514
79.31973	101.188	108.0324	77.09351	101.2348	49.34382
60.5385	58.11936	73.04174	72.25404	70.96642	80.46424
81.851	99.28771	101.0747	67.97944	164.3279	66.65065
165.0212	65.3117	100.8957	111.6716	102.569	137.8341
61.76367	102.096	65.97865	108.4575	102.4148	67.62151
70.77157	82.31885	64.87201	83.71971	91.35994
86.20132	78.21642	108.878	54.72954	94.09339	113.7178
72.64466	81.51059	49.94716	92.70408	100.0138
86.05533	85.49435	62.24738	89.15439	111.3717
83.2288	94.98089	70.69606	66.48101	121.1147	71.97199
80.96817	60.32236	171.8841	99.8101	67.90673	41.38451
70.7135	86.33286	93.01075	47.65897	104.0196	85.57297
168.3175	94.96811	94.52906	74.47741	67.3417	124.3579

94.42491	121.6071	104.7636	52.72525	105.9037	
87.48091	90.47878	78.37707	106.663	67.10709	83.36397
114.3672	79.90449	83.24632	167.842	69.96214	57.0223
78.99838	99.04188	62.78199	69.6856	70.65036	100.9313
62.98538	76.41942	72.03507	83.43126	109.2243	
99.41481	90.75339	91.75746	55.83262	103.2392	
83.65492	89.94852	132.4281	51.86623	134.0705	
93.83582	102.7163	32.55569	47.22826	68.08425	
91.92318	52.33808	77.34428	92.77267	51.72646	
57.95511	89.32434	82.96379	79.30248	110.77	53.81786
70.60176	104.5268	34.01544	101.2324	84.31741	
58.33556	69.82047	133.4085	84.06051	42.60958	
79.45959	56.86827	53.36351	87.41945	89.71753	
86.05281	79.14852	116.5557	98.08151	91.91375	
98.3539	50.46825	70.12755	66.88976	77.23904	79.62432
74.96471	96.23923	92.26885	128.2722	82.13151	
116.1815	133.1474	80.45339	119.6088	117.2925	
65.41547	157.5227	58.34551	109.3387	82.51757	
52.9038	113.8576	72.39435	90.07385	84.44701	55.85433
74.462	65.07795	102.6954	83.17066	131.282	128.7423
57.83868	114.7044	74.44273	75.36598	104.9544	
97.26552	76.95738	133.0069	107.3785	61.21191	
77.11479	76.80815	91.67259	58.44072	93.63174	
61.99404	114.0923	81.64417	83.69226	104.7876	
71.79463	62.27776	81.91444	89.2765	80.21158	95.62554
85.31033	120.1142	93.49456	123.0324	36.59254	
65.06195	104.1347	109.2282	124.8042	66.03642	
47.46166	99.74329	113.8675	100.0588	61.69329	
48.59002	65.69137	105.8673	105.7909	73.13653	
79.72055	59.20226	107.9374	70.9504	49.36147	116.8591
68.13955	87.90245	86.03804	63.27354	80.19749	
79.53294	92.97431	83.8218	74.66861	72.27687	68.59776
78.64278	56.06265	71.16092	77.1507	76.91883	56.61153
65.85529	84.0386	111.6072	89.40452	78.22148	74.29821
67.25734	103.7109	71.49228	112.1094	75.73219	
69.75627	105.9008	113.2199	68.63362	108.7188	
60.00557	80.63579	115.3974	53.62369	52.14011	
64.90378	103.4263	95.89083	54.52205	65.57149	
124.4288	132.5612	46.53373	64.92675	71.51145	
73.96775	105.4091	168.4301	92.2233	175.4047	82.641
66.92612	37.17973	119.0993	113.7101	102.7905	
85.98499	82.32226	85.71208	125.2472	103.3971	
90.48324	68.39258	80.56763	65.63113	75.20636	
91.46856	76.33707	91.03699	54.3542	90.30715	95.47817

50.73623	103.5894	84.40471	109.397	64.2293	113.4689
197.6759	93.37709	94.77814	74.87844	63.08469
102.4807	75.89143	52.89191	92.83616	59.81582
103.4357	102.1726	61.49282	84.42973	104.0578
72.46463	193.325	87.92097	72.29844	128.4628	73.99251
100.4455	76.1771	149.7438	45.88163	64.55357	197.5006
115.3191	77.71857	101.8109	134.8817	64.85556
87.01358	108.8576	111.3387
KLF12	0.6497921	1.767588	1.202638	1.569322	0.9554138
3.673759	1.113269	1.888822	1.446097	2.746008
1.366307	0.5507947	2.955715	0.3709486	2.302378
3.616306	1.123702	2.134543	0.3623375	0.7051022
1.115994	2.343265	0.7866615	3.614337	2.939449
0.5097207	1.476483	3.61839	1.369612	0.3388832	1.325376
1.689845	0.5020361	0.9613781	0.6569896	0.2268757
0.954233	0.611844	0.6465384	0.32115	1.996338	0.276015
0.8863102	0.2753985	1.087315	0.9447028	2.829236
1.188469	5.375787	0.5315461	0.3417845	5.852555
1.634548	0.6953512	2.056218	1.012989	1.502271
8.663509	0.97339	0.8596077	2.637075	1.513258	3.518962
1.292328	0.2668196	3.38224	1.008178	2.454823	0.5409759
1.63173	4.293183	0.4823526	1.863596	1.789592	1.675955
1.874111	1.892906	2.228773	0.5830234	0.21945	0.2551229
2.530071	1.620782	2.167469	0.7851835	0.8371788
3.446771	1.673938	0.8437356	4.954665	0.9215919
2.217618	0.8292715	3.016902	0.7630077	1.287511
1.701633	0.5297291	7.431924	2.230868	5.685062
0.4051721	0.5312619	0.8705817	1.846561	5.011303
0.7124881	1.97494	0.8804537	0.345814	1.400304	1.581011
0.531087	4.107369	1.763094	0.533584	0.2755748
0.5386654	0.9407014	1.363707	0.704977	4.498788
3.363407	1.51283	2.711259	8.516272	0.4098031	0.4590758
3.298241	1.294987	0.7661174	2.714525	0.6478361
1.263315	0.5564555	2.718411	4.267895	0.8971183
0.7886512	0.7391605	1.187825	2.136765	0.899897
0.7655308	1.831268	0.6441539	0.1513562	2.270321
0.4525866	1.64954	0.6816439	1.593836	0.5045283	2.68861
4.485279	0.880304	1.452703	2.083287	0.5630868
0.8547049	2.636127	4.507118	2.687488	0.4457269
0.4163899	5.161355	1.201654	1.717855	2.624494
3.345049	4.35081	1.304579	2.65431	0.2679552	0.1346825
1.22681	1.73368	1.562031	0.4789157	4.713102	0.246393
0.5451246	0.833329	1.720942	0.9284221	1.120128
3.82412	0.6168426	9.564991	0.5810451	1.316733	0.3050979

0.3664792	1.163634	0.3716984	3.337438	3.648247
1.745156	0.7876007	0.7022377	2.289917	1.184054
1.412774	0.2416982	0.560192	0.6876599	0.5935474
3.085755	0.3660298	0.6048595	1.54667	3.183099	4.570999
0.5158292	1.220768	0.6602151	0.5804246	1.588933
1.65245	0.6736719	0.518347	1.838256	0.4551582	2.157551
7.982897	1.278267	3.011026	0.3050827	0.08791027
2.056791	1.862311	1.255302	0.2887747	0.2553011
1.472588	0.3850979	1.688955	0.6232722	3.128674
35.24667	3.44789	1.931064	2.062194	0.7889602	2.448999
1.677116	2.935963	2.010011	2.424036	0.6688332
2.91353	0.7155269	0.4537978	1.42263	0.408961	6.072949
2.347913	1.190943	0.9175567	1.465993	0.9194207
0.4330377	2.400938	0.6157611	1.29579	2.118448	0.3653042
0.7639144	1.438082	1.39111	1.830928	0.5269598	0.4699144
1.391309	0.2389193	0.7832659	0.6043162	3.876571
7.510238	1.918233	4.574236	1.01689	1.420289	1.202411
1.04571	2.002343	1.643234	1.995174	1.175261	3.490813
0.7674532	7.05599	3.633623	0.9702884	2.20824	1.969669
1.674201	1.184333	0.4131019	0.3711795	1.700385
2.804895	0.6870514	2.091816	0.1967933	1.196115
1.142791	1.286238	2.094239	0.3662932	0.7291392
0.3230091	0.5624492	1.911508	1.19902	2.365109	1.83309
1.043279	1.097551	2.985453	2.230343	1.324743
0.3410576	0.5516446	3.202301	1.509566	0.8216064
1.432646	0.4906281	5.428291	1.554372	2.37921	2.229679
1.014312	1.723241	0.5651678	0.4646464	0.4676597
1.913341	0.792781	1.075983	0.86004	1.118136	0.1638851
5.322589	1.056165	1.385695	6.532595	0.2675875
0.7109582	0.7073127	2.217151	0.5451563	0.7617508
0.5877886	0.2489646	3.483914	0.6721067	4.614732
0.4439524	2.155345	2.633971	5.402962	1.035717
2.576515	1.413261	0.1260431	1.285998	0.47118	0.4515326
2.115403	0.5493906	2.539003	0.900754	0.6314614
0.7358166	0.6906906	0.4652139	0.3783679	1.652216
1.622149	1.102278	0.9688571	1.941542	0.9439271
0.3991323	2.018881	0.9920564	0.2206865	2.339842
3.77144	3.50183	0.9170812	1.72688	0.9134284	1.216161
1.293521	2.534852	1.341706	1.640178	0.247121
4.588473	3.410328	0.7162536	0.9549044	0.7586955
3.249692
MYC	10.25863	38.23376	19.54499	23.62278	46.48291
103.4316	12.62628	77.11673	36.44355	55.78503
42.00517	29.27589	36.08736	44.28373	16.82715

65.94398 27.23312 191.4095 33.49877 21.49669
36.98663 25.54904 90.51909 38.21617 13.61797
40.23759 19.9858 37.40045 18.18705 17.33476 27.37592
163.6123 9.079936 42.15984 21.6064 25.27949 25.72692
32.68927 69.56136 66.15598 38.27066 42.59655
52.27885 32.9591 15.43928 39.38517 45.66852 90.50252
147.7567 57.328 30.39126 21.36281 140.7255 56.72101
16.66033 54.99692 142.5987 41.00814 71.24295
34.43749 31.02175 45.3644 37.67186 11.80049 33.62442
27.51715 39.70731 28.25223 28.65436 30.37634
203.4232 33.34122 48.12617 27.42835 26.25407
95.44445 16.67024 32.17668 34.04735 140.2492
20.52252 44.63853 60.00385 36.23051 56.22158
27.65616 58.62055 75.17041 53.07183 15.51924
63.1171 57.57087 65.32264 18.80741 57.47953 25.47115
43.45479 21.59964 17.99798 65.13762 15.98995
24.10463 45.87967 77.37487 26.10145 35.72684
39.85747 29.51246 41.76243 15.3352 44.25641 1.570446
51.45234 28.04609 43.92749 12.93794 47.32165
27.57311 27.44919 125.5922 129.7087 42.56245
21.31621 12.92481 30.32775 29.73388 153.415 47.53299
28.8657 113.2399 30.18717 98.61589 37.88094 43.87999
31.44043 59.72508 58.71227 86.11289 31.93063
82.37754 25.13013 72.80479 51.65275 151.0669
20.35226 5.492996 18.89143 10.23583 66.10231
27.37185 149.5186 23.55967 348.806 38.6357 39.10793
45.84751 47.17331 25.38892 90.80941 36.99502
40.75433 21.98838 21.96204 97.19975 53.25956
45.24901 13.17516 27.8453 43.0736 53.42108 38.05918
20.25371 21.64761 49.90466 15.97198 41.34702
10.37342 72.32211 108.7543 42.57604 7.108945
51.59397 85.16914 44.69332 11.14718 28.19192
43.07423 53.54209 30.85908 87.6507 21.70557 11.72364
51.65968 19.04345 19.52159 55.31548 56.27494
52.20364 128.0385 11.97465 19.44974 18.65281
26.24646 53.89511 143.436 48.38044 11.66127 57.03835
99.02636 42.62363 28.88152 46.72242 50.50023
24.65796 104.535 41.6669 9.605516 8.978365 33.94597
49.71492 46.73592 48.07779 19.66068 26.52032
39.97397 38.37462 50.14171 38.71537 103.4966
40.93078 25.5892 19.5126 91.71789 12.95236 58.70007
28.98389 46.36086 56.78505 55.35456 14.44386
20.57705 24.12018 40.05525 76.21227 45.83136
30.86747 24.73013 15.11253 48.76747 44.25805

103.4941	58.81122	20.39424	104.8375	54.00869
26.73661	59.49413	17.55905	68.5063	65.69221	28.45872
101.5082	55.67732	81.16301	43.7937	38.87781	87.82693
52.14642	28.08318	21.09362	61.38899	68.34789
31.38607	108.4625	5.931003	34.7341	20.65491	14.53874
23.25343	41.40659	38.02575	45.50682	32.30241
126.2095	44.99109	49.79657	38.65232	35.83979
33.38227	78.61363	202.9863	18.67442	41.50344
51.2511	31.77366	67.4457	49.22602	48.4586	70.70589
44.38508	16.0696	32.09453	25.46798	29.38431	27.71671
32.84765	13.80632	24.52467	20.45492	1.688762
53.93878	94.84109	43.91969	33.29383	36.13896
67.31536	29.87373	10.48929	21.80617	19.64614
67.02662	93.91615	119.3439	49.04552	18.69427
69.42715	24.85743	57.51856	50.32463	22.28337
57.8483	123.0858	29.941	39.03632	29.15432	57.67799
58.28978	39.60472	18.42238	38.76704	37.68947
24.03285	7.893818	10.01447	41.41054	29.09801
13.1725	164.5192	18.98725	26.4372	79.07274	34.91354
19.92297	23.27567	53.52529	30.95154	38.06984
51.69779	21.68837	16.66068	28.95009	41.06014
21.86646	69.33147	60.14306	42.08293	78.347	43.75372
24.44489	7.923663	13.88714	59.77396	53.18871
22.51894	73.45935	75.13189	32.01326	14.79927
17.82644	23.67221	37.57697	61.51292	22.3945	66.61664
323.9005	57.78237	30.92449	43.1194	94.55885	26.4483
14.57225	98.34643	64.64544	64.87736	49.26888
25.88903	42.47938	45.38357	14.62245	21.62287
41.64281	37.45677	55.35547	23.173	54.98759	105.9998
31.9613
PLAU	2.77275	1.200891	4.421439	11.55453	5.090406
8.234635	3.743796	3.20505	7.306753	5.972977	2.477303
5.224916	6.799224	4.950067	6.037892	6.479997
6.248119	5.762464	5.497221	6.942326	8.960505
1.701978	6.747939	2.828265	3.339387	2.547197
7.858117	2.539065	9.241217	2.39149	6.485621	4.043813
15.59769	19.71485	36.66262	2.062072	2.213858
16.81741	42.72123	9.623582	35.02275	2.570195
45.57561	15.28272	12.7242	17.29697	6.446935	26.82292
28.12254	17.43358	12.41101	8.377815	27.97122
26.40708	16.31459	29.77501	77.43107	4.144952
25.41747	11.90463	47.48246	24.75008	16.14741
38.55502	11.2641	22.56983	46.54927	12.45395	35.91885
17.45649	51.24498	17.24437	8.66232	107.8077	148.9613

10.97335 72.44522 29.14095 6.921975 41.60134
19.76409 22.61098 17.58805 13.7998 48.52503 24.89645
13.23623 12.68921 21.39328 46.56268 51.00498
71.79167 18.62085 8.693215 17.11965 29.0561 19.91659
16.57963 15.51524 6.839032 8.071529 2.801088
17.84984 14.31996 29.35789 43.06133 57.45572
5.726195 28.19084 11.81185 15.95662 8.972636
34.43361 2.16629 52.08241 21.85781 26.63777 39.19714
6.727164 23.06204 18.38856 18.08863 41.80369
50.51748 10.46707 5.2117 24.12677 43.8378 6.646638
38.99634 12.54184 12.16723 7.63701 67.63032 38.71107
13.28896 21.53462 14.37048 33.25773 9.518808
22.32372 9.536668 17.22918 19.7666 27.7186 21.00563
4.475256 29.6973 4.336013 9.385919 3.786884 123.8247
27.16944 14.80127 8.321032 9.958967 20.02726
6.758745 193.1568 16.39511 18.35029 9.592728
18.17657 27.1506 21.32023 17.49142 12.23234 76.37713
14.62845 1.794713 1.910854 45.69249 4.261447
24.43652 4.500576 55.37882 48.53318 26.85032
4.434666 30.09051 22.36423 11.48503 10.08768
16.16935 73.40758 61.89204 13.57423 71.87955
15.24747 16.97224 32.74955 60.28033 21.76887
6.896763 10.00152 51.18164 49.2498 13.15545 16.72125
24.17355 16.45928 10.16965 55.1386 23.20603 12.17905
8.79786 21.92762 20.17156 23.22093 34.0386 72.58273
28.70131 16.34729 10.92344 9.47155 13.90502 36.00305
14.47874 17.83139 23.17589 11.98134 19.19495
14.42275 28.04074 4.466327 6.230281 38.56026
7.374593 12.42015 36.81995 22.07545 88.19647
4.714959 17.84297 13.85754 19.91631 9.797114
12.80968 25.23064 4.867683 8.734598 12.98911
14.16602 42.68291 71.92124 35.44509 31.91448
2.257518 17.0774 24.35347 10.20027 25.11496 54.92129
28.91915 11.07151 25.99534 17.73463 54.11877
39.33046 26.88998 16.15392 27.52302 20.18405
23.25019 35.38371 29.95276 28.59612 70.20909
22.36504 18.69326 13.08362 28.58412 14.39368
20.14731 5.3102 10.39686 12.54175 4.221796 17.06043
26.20247 12.34613 13.94183 46.51145 37.00159
31.88048 4.690329 9.780631 7.343206 117.801 15.69562
37.93951 3.184926 6.517682 41.59302 61.7857 19.18356
33.64596 18.5512 36.86438 9.700436 32.01648 62.14248
26.50044 196.0683 23.27074 17.3069 17.52561 49.74706
18.96245 28.91068 19.88619 116.9812 24.32527

37.29923	31.24188	6.659174	29.70538	71.74571
48.50744	50.20268	21.69624	41.03466	16.2113	14.09987
28.97946	19.07559	33.07484	7.034485	45.64795
24.02216	6.937147	50.10859	17.87821	17.35908
9.524427	4.07499	3.823454	5.664936	9.94192	26.16938
4.899441	26.96967	24.52342	12.28893	5.800126
6.018035	7.956927	7.568611	16.55895	23.3529	26.38214
42.26594	17.36126	136.0066	13.90124	17.76187
19.08455	21.12889	4.670256	49.97354	10.68693
22.67046	526.2343	26.60862	65.59947	16.93133
5.858343	24.0136	4.471454	14.76878	9.319972	10.85502
6.748894	13.61321	9.445729	20.85032	17.28113
57.08961	17.72701	5.884046	29.55582	38.47328
9.105615	10.80862	16.63465	25.15238	13.82878
34.74516	22.62323	28.76001	9.017227	19.8127	14.03175
33.68269	24.09347	42.92921	15.84035	11.457	4.663951
62.72068	16.74071	3.015695	13.83536	6.817824
33.90464	56.29409	6.563756
PLK1	3.036969	0.1780362	8.111038	11.3274	4.347816
0.3647492	5.838251	0.6547915	5.682813	11.34231
3.083706	2.63248	4.854831	4.970856	6.926626	0.1628094
10.94705	1.312225	3.199614	3.820091	7.32944	0.3617317
2.838493	0.2811801	0.3825584	5.129315	4.193656
0.2510005	3.792591	1.592934	4.821615	0.4897378
10.92173	10.29562	9.852481	9.78881	0.1492722	15.36518
14.5887	30.69075	11.9766	54.68236	28.74098	14.34548
8.228381	16.43562	16.59276	28.36845	11.7819	12.97165
22.2048	6.698226	32.36498	14.20413	2.005523	16.63697
15.21912	1.386102	27.36093	10.67577	3.586565
19.86345	6.681684	39.36682	21.28293	1.722856
10.49086	3.294627	23.35542	26.74592	29.33723
8.901915	14.04917	13.62684	11.89222	12.33732
5.140043	7.928133	6.96037	17.4643	13.87272	5.204486
16.72295	5.661845	6.069955	13.27808	8.843801
14.98141	28.03302	3.153558	14.22416	16.02488
6.046497	9.146813	18.44431	8.06048	9.192359	19.73723
24.96094	15.88964	4.249482	13.29843	15.79539
5.931156	6.204456	5.145874	20.77834	8.108281
19.41726	9.468874	13.96116	7.320418	16.49256
4.666972	7.91275	11.31446	17.31467	9.391945	14.23011
22.10635	23.92017	12.85879	24.44796	3.020311
21.81386	14.93884	20.75932	16.82261	3.248461
17.93877	7.192456	19.50135	17.1867	12.61903	8.003859
2.322521	20.18018	37.12618	18.86804	18.32656

18.60687 18.83014 15.87297 14.87905 16.87605
3.645786 13.27729 9.370733 8.691148 7.89799 14.80925
10.63348 28.07843 8.23573 2.658769 7.168777 14.87186
1.984129 19.36978 4.830225 5.8749 5.182971 4.547782
11.2871 15.23613 16.43243 11.70497 5.705366 12.26252
10.10061 1.441255 24.87429 9.570858 33.43526
36.88113 14.49202 6.3127 17.04919 11.32236 24.73756
7.892478 17.67896 4.505139 14.83233 16.89705
7.757477 2.655985 17.61322 4.218836 15.95193
6.313526 16.36831 14.85858 22.26719 13.74045
8.275172 15.37725 17.88883 26.84354 6.779639
10.33915 9.563977 19.09983 13.61944 24.81523
19.89531 10.95627 20.39368 11.20548 15.31525
5.856144 14.56615 7.351919 9.994763 12.60697
14.55406 8.094152 29.04976 7.226614 15.23342
17.78737 15.87317 18.03392 12.32522 4.466056
11.09632 34.63472 7.441985 40.41209 27.10472
7.062717 3.946847 17.5564 12.22127 10.78277 3.610216
10.34966 7.351193 17.6735 24.71667 9.592057 11.27669
12.7744 13.54781 11.16316 10.92781 10.75848 1.478517
10.99725 19.40437 16.52011 12.56837 27.56867
5.854305 27.09078 31.33728 14.1201 9.399804 25.54701
14.54321 11.43476 40.2878 14.77772 15.88802 10.89784
18.06171 29.32057 21.35751 8.140524 4.544417
17.27171 15.56643 5.951229 15.1029 13.48263 10.71164
18.10932 1.370521 31.70314 11.33304 4.890812
15.59533 4.142422 16.40628 21.05842 5.813643
25.8078 10.26654 12.86399 9.809276 30.89167 0.5440744
1.379954 18.53231 12.57677 15.76401 12.90449
17.96288 18.77659 32.86186 11.92271 6.376694
4.487206 8.283149 14.57912 18.41243 22.5851 30.28462
10.50601 21.41915 20.66363 51.5156 10.40212 10.23153
19.22065 6.21467 7.281732 16.62735 9.365686 7.51266
29.99068 18.01206 34.48822 12.68859 3.650373
12.79719 18.28503 17.11787 10.87382 11.23683
12.67499 5.428199 13.14523 9.371106 2.112037
7.176411 8.979135 9.814371 13.93509 8.611369
5.111945 11.68441 8.338744 15.5389 1.539245 5.622729
23.47465 10.82327 9.995186 8.804871 11.64194
18.24235 6.564036 7.768174 10.32196 15.11149
12.23644 35.18427 3.768942 9.582943 10.03114
8.283796 10.1034 23.0397 21.06129 11.99009 13.7853
14.29918 23.02535 13.43966 9.775577 4.167402
23.44992 8.318824 23.556 18.83486 17.68406 36.033

6.425986 8.334704 21.76376 8.981149 6.553377
11.8084 12.85646 18.0914 5.754024 12.19765 23.27562
12.51583 8.373797 15.48885 9.176891 8.386889
10.57887 9.727284 15.42636 4.10026 1.512936 26.66939
40.81686 8.863557 27.18933 16.90133 20.48868
26.12397 8.162398
SMAD4 3.002485 4.504555 4.118261 4.304348 3.370468
5.592585 2.302401 3.837268 3.503836 3.272143
5.412079 2.968979 3.475746 2.685355 3.025035
5.898892 2.771563 4.563005 1.952054 2.610472
4.508536 6.289165 2.776073 6.273059 5.037907
3.654894 3.087826 6.900915 3.461604 3.469264
4.167339 4.418141 6.88436 3.664709 4.030677 3.506931
1.984907 2.618102 2.642823 4.945907 6.886847
2.411604 5.351346 5.863191 5.790243 4.101729
4.668505 4.977736 5.825831 5.229462 2.319631
5.257156 8.836684 2.41377 4.969091 4.966222 2.319635
6.940035 4.867844 2.78418 5.285403 4.130895 4.825134
3.056129 0.3001378 5.382695 2.892847 4.163595
4.313624 1.470203 4.751405 2.961716 5.333598
4.669188 4.902839 3.283682 3.7546 6.509319 2.389773
3.866316 2.092471 4.075479 4.424522 4.584341
3.393519 2.732389 6.71072 4.942939 2.844515 5.233555
5.342918 3.570123 3.085986 4.217748 3.317042
2.407963 3.327831 2.470313 9.003712 6.216883
6.157205 4.128184 3.229447 3.806049 4.295356
5.791645 3.499267 3.431686 4.918594 4.429368
4.474682 3.316651 3.660438 4.984462 2.936784
2.204506 0.6119375 2.050665 2.698151 4.215974
3.273258 2.832324 4.212516 2.820021 7.95387 4.882795
4.074793 2.765985 5.648398 3.545429 2.939753
3.244363 2.334087 6.746989 2.328975 6.798701 5.9782
3.070696 4.350968 5.053859 4.709829 2.853745
3.530274 3.705601 1.070028 3.078154 3.739521
4.824437 5.707815 5.130422 1.52043 3.977071 3.703878
4.281218 6.084096 3.54235 4.591083 5.364227 2.708947
2.674344 3.210617 5.458906 5.512565 4.516076
2.216319 6.259215 3.876541 3.93922 3.087887 8.633168
5.209468 3.313797 3.746781 2.170932 2.470647
2.693631 3.629551 3.413299 5.011106 4.991183
0.6046234 3.692105 2.983062 4.632215 3.24568 6.572845
4.904892 3.617518 8.161715 2.286689 3.832898
3.376322 1.549646 2.865469 2.128369 2.087934
5.153295 6.332504 3.209481 0.6141345 2.513501

3.799279 4.529257 4.132088 2.057019 6.362369
1.538138 4.744892 6.395222 3.638346 1.578953
5.503509 5.160763 3.571593 6.116577 6.313581
3.974993 5.250074 3.923758 1.867466 3.095245
6.38314 4.541375 6.222556 5.612819 3.990786 5.210293
2.243031 3.56058 2.460635 2.632998 3.028006 3.3761
0.5328242 2.995717 1.862751 3.313896 5.423011
5.966777 3.388968 6.609414 4.541823 4.435351
5.443634 5.160148 2.994083 4.98806 5.041574 4.937385
2.17167 4.649658 3.745507 3.039102 3.935873 3.461588
4.783139 5.958028 2.337658 5.506865 4.811227
2.680903 3.391258 4.05232 4.746214 6.278883 5.257943
2.173446 3.55786 2.944473 4.310918 5.271661 2.288302
2.518672 6.006338 2.949988 3.086397 3.656837
5.052704 2.885539 3.842764 4.689203 7.116578
3.762084 4.45222 6.408105 4.464421 8.202817 1.820077
3.956212 3.544528 2.253881 5.635883 5.894334
2.930765 4.108906 3.798207 1.578291 3.771888
4.331051 2.962923 4.640595 4.247359 5.554071
4.848526 3.183177 4.47276 8.322716 3.405241 8.355292
3.87232 2.557931 2.378729 2.896058 2.881215 5.8972
5.534958 7.337058 3.728466 1.873115 5.660903
2.738742 4.450303 2.032064 3.537518 5.299697
3.688021 1.999122 5.700328 3.347119 7.045792
0.1964742 5.892276 4.397335 2.752524 3.375097
6.791419 2.860993 3.722563 4.19737 2.035119 7.059027
3.267625 3.961553 3.378318 6.527513 4.81845 3.304589
8.87814 2.431666 1.682289 4.373236 4.879497 6.441322
2.89024 3.230593 3.253277 4.751483 1.557172 5.075561
0.6227233 5.352213 2.791144 5.122412 3.357335
2.624145 4.216247 4.455543 3.458633 2.597907
2.338463 4.566169 2.055517 6.384351 4.877669
4.456116 3.265955 2.307946 1.880491 3.967784
5.206154 3.572451 3.878376 0.627278 5.444592
5.041971 2.10852 3.022136 2.433233 5.404079 2.509408
5.42066 6.871415 2.115772 4.647666 3.703662 5.151289
1.706591 4.542154 3.563836 1.871212 1.887889
3.15639 5.319234 7.287114 4.686522 3.902125 5.397596
CDKN1A 154.7369 71.62237 70.8291 35.05602 84.56187
99.62322 38.41844 45.84296 119.8039 80.59948
30.68699 27.67318 54.72893 45.54634 57.45184
62.89065 111.6483 173.4107 269.4311 187.0657
62.93807 23.90039 84.30769 30.71778 28.77873
28.12641 45.10519 20.47519 132.1311 32.94518

64.93744 207.4488 24.76064 38.66201 48.12229 3.9576
18.751 16.73663 33.2215 15.54715 21.24944 12.98813
58.18676 29.39793 37.62246 70.31237 16.3344 35.20571
58.58678 108.5643 28.98469 13.64005 5.646924
40.47497 37.71707 26.08755 23.31818 24.66958
25.20504 48.49831 38.00278 95.77391 40.10294
85.00547 27.11242 27.54633 54.88304 40.01728
175.4288 13.21049 32.42889 31.26921 17.06291
53.40241 40.97803 51.25007 55.67577 29.08509
21.13061 17.96052 12.03452 23.0716 25.93549 36.45174
125.5589 22.92252 31.26555 84.23454 11.32504
33.10272 95.9341 27.12623 38.14479 40.55716 42.24024
44.98497 76.22322 45.40449 53.26642 37.47744
20.42077 42.38248 31.97188 17.73018 19.3218 46.50757
80.02164 41.19446 69.54171 40.23687 14.81611
24.70433 64.61969 10.53933 64.76518 40.32706
20.96222 89.02488 17.21135 37.14661 47.98873
30.42506 30.60465 48.61355 16.58926 13.61623
29.50515 34.52317 42.90958 59.94723 50.81759
57.07357 35.80861 36.83438 109.6527 24.34651
67.74607 36.16901 45.76083 16.33833 13.46648
8.311121 51.15272 10.67267 9.077473 8.259287
8.230383 33.55772 63.85649 32.93459 6.436181
69.48512 15.53928 29.84864 24.55888 31.87381
42.63581 18.85563 120.6908 26.28052 43.44541
14.44096 20.80544 49.71712 65.77776 26.00838
38.06222 85.38153 16.2572 6.327715 37.25505 60.964
7.664075 39.86885 20.37508 54.48395 60.01949
17.66315 13.70641 34.66566 31.52901 36.71231
126.6695 20.96915 24.60199 47.68541 20.9332 114.9154
33.21146 45.70142 99.72934 43.47069 65.46356
55.56439 58.10715 43.45891 56.67942 10.06006
46.64059 31.63635 4.712855 15.25785 62.0449 13.29523
16.26812 47.40744 37.72793 21.65544 42.66189
28.38092 46.87916 24.37822 42.56586 11.04338
14.52321 59.11442 91.14748 13.19269 66.77626
36.13035 27.92888 13.27263 17.58984 61.83864
27.3773 9.654741 33.92691 32.0366 25.42651 38.94504
59.1215 44.02613 47.45519 109.016 33.58789 30.10519
56.84852 18.47344 24.96817 42.76188 24.12451
38.83052 39.25381 34.86133 79.95366 60.92612
27.36253 18.54546 17.73895 20.28752 10.88496
12.47097 37.38313 70.83852 45.92714 13.08369
24.10981 29.17183 46.99437 29.98663 24.54934

84.72565 46.8194 55.41764 56.13411 24.89104 17.57689
66.73598 105.6907 50.11378 23.50274 94.43071
70.79885 31.8684 37.86917 12.30966 30.50948 18.39616
16.64918 42.2806 21.08346 22.27882 62.11873 34.69542
17.98605 31.34006 38.68855 8.807993 34.78572
76.5949 29.26961 8.313407 51.61268 33.67667 121.4688
37.52097 30.81201 41.64911 19.61566 49.28193
28.78567 23.56071 128.4422 51.61788 33.01997
72.1553 16.49733 39.37004 12.86276 41.72647 50.44702
66.37024 34.6129 54.16088 33.45515 40.17687 39.77236
20.53038 29.47627 78.21518 38.96197 18.68733
53.42277 18.64229 63.37702 28.23097 59.22033
44.37508 35.71804 44.02907 25.40894 27.77046
46.30031 36.70471 39.97374 19.69325 56.88871
32.57891 27.21821 31.99275 96.97551 17.99727
48.58066 43.70266 28.58441 30.76402 34.92143
14.97867 21.79696 13.93525 16.97936 24.74699
24.44066 57.85312 19.33881 39.57436 42.42961
12.21226 26.48535 29.47646 15.6301 54.82583 108.4152
11.40203 38.13209 17.68374 10.32219 43.71635
15.19208 20.17573 27.20598 54.99843 19.31106
38.08011 72.01241 19.99505 36.13127 29.66716
39.95074 10.53362 24.30698 23.32039 12.2674 17.27201
13.42823 71.36678 50.0401 54.96725 34.30742 28.03633
32.87788 50.65902 24.36735 46.59077 20.16675
39.39744 13.42346 26.38941 58.45051 48.28593
33.78541 15.7903 63.11662 23.2251 44.64058 44.22848
18.07768

MUC1 23.60246 3.068932 21.52346 184.3852 76.33194
2.05872 255.3891 101.0225 172.4665 76.10664 20.02456
12.32025 104.2602 50.81724 208.3072 3.249361
160.8366 75.47637 77.90528 2.762909 324.413 1.656435
45.53994 3.135917 6.020627 26.56067 194.2962
2.011696 91.6079 123.0122 323.1904 22.85202 638.9111
163.2922 172.0356 44.17099 1.698627 82.52497
120.0393 110.5857 253.4382 196.6423 171.668 351.3428
258.5723 277.1048 96.83721 31.63642 7.084658
335.3409 243.6421 39.21026 2.018947 216.7224
50.61916 59.99455 8.750724 15.37664 24.72886
185.9151 96.40868 42.29848 15.10182 98.5321 200.6295
10.14126 213.1797 18.39147 413.3555 66.36625
86.99889 187.0281 132.6652 371.9047 88.10046
48.64052 203.2313 719.1464 104.7396 51.99251
231.2543 180.2445 125.6895 45.63799 49.25212

303.7572 107.8822 455.0961 99.32127 15.85942
73.89135 155.0273 81.17861 128.7781 96.8627 231.983
57.17659 351.4092 11.17133 134.5636 8.854095
316.2297 44.31943 10.79927 21.57349 54.95986
272.5586 57.33288 164.7918 244.9077 99.96949
13.2484 319.6637 22.09771 153.5563 396.7672 144.112
84.12434 152.1037 27.83409 26.9527 2.064365 7.073136
124.893 14.26163 63.13226 15.64381 131.5304 42.16418
7.181053 377.2685 84.71281 464.3161 228.0586
304.9155 13.78735 77.10893 81.22365 252.3339
71.08081 67.52739 178.8257 283.2479 13.23433
10.2534 364.7686 38.2278 70.172 96.0724 16.98399 65.82587
133.7497 52.82276 126.9181 11.74491 198.1455
42.82332 70.69029 37.40544 15.80784 79.97612
115.8679 121.9913 48.4488 134.3796 120.731 67.92697
106.9605 251.4584 3.625078 23.99749 169.0092
48.62572 53.09396 375.7492 104.2356 47.07964
120.858 666.6304 49.76575 210.7429 39.77086 57.34154
292.6418 29.97317 65.46456 87.26596 137.502 17.12571
212.3173 206.5318 260.3985 75.39786 162.113 89.62924
13.08927 61.37315 32.64029 90.69274 25.5421 1.184806
192.632 176.3583 90.64837 64.66953 468.209 398.6485
4.736038 87.70146 102.4235 46.83892 53.23372
80.01304 38.59401 111.7452 160.8749 299.8635
7.134661 67.29098 24.09624 243.142 162.7634 198.0445
198.6416 62.11981 45.90067 4.50635 49.05416 276.5234
63.10677 4.420123 60.76318 164.2367 292.8154
32.05634 467.0618 4.809389 98.40878 66.77197
13.57956 16.51928 180.8836 148.7676 56.98287
10.3717 260.6739 86.49345 11.64311 155.8317 377.8045
70.62001 153.3006 664.2953 89.03387 83.29153
0.3575128 13.9379 233.7275 122.0634 16.37086 218.9109
92.36688 105.2328 13.78972 199.9598 30.29605
275.098 17.86299 48.70528 260.5332 159.808 200.5752
333.431 16.45948 404.0433 257.2831 194.8792 8.55201
0.3137132 49.83662 50.34962 20.33899 67.99248
82.72525 138.1141 110.0742 167.8485 0.465973
172.6924 22.94242 14.83483 14.20785 24.53802
52.41285 4.696155 22.15699 26.59643 143.7871
226.3045 242.3261 200.5207 186.9904 17.87868
29.98281 213.5946 94.90751 56.9058 75.04562 279.0775
156.8116 25.74027 38.49006 207.9174 42.40372
237.1156 6.06091 157.2678 323.4941 195.8102 51.93146
20.60961 8.431752 130.6641 199.4387 47.90962

70.06424 182.557 9.833839 186.3974 326.5691 62.98128
17.79322 179.4404 326.8553 33.31146 94.10256
17.81246 50.8075 145.8868 256.9725 52.80505 90.29586
117.3324 146.4401 52.02323 111.4494 298.2141
37.25468 91.96958 95.79336 54.23793 11.06667
111.6997 40.4574 63.22857 446.0109 206.4893 137.5059
2.829711 132.0623 197.676 57.7571 5.467824 93.85252
13.33126 50.42841 272.4778 431.4516 491.3414
8.027171 75.35519 64.12847 17.34868 255.4924
111.4561 31.13922 349.162 60.86762 389.8227 181.411
30.944 37.57479 33.71955 6.164953 16.44063 239.1912
123.3042 202.153 192.3627 38.84254 207.9393 16.21116
25.18351 70.47935 433.8183 139.1568 95.91644
73.84196 124.2472 166.497 107.783 67.07027 457.4188
99.01373 49.15166 31.58061 71.03604
PLAUR 2.865303 1.009922 5.337197 11.77362 10.38136
7.982391 11.66447 5.598356 27.54266 14.34331
0.7163097 0.4418898 7.485351 0.4126002 17.08019
4.525649 9.025288 5.99107 22.30997 15.23308 33.19257
2.210808 6.48592 1.977271 1.355188 0.9121494 9.003944
1.917691 5.732324 5.540079 16.34175 9.54898 9.851819
15.04059 27.64189 3.008375 2.435397 9.040731
23.06687 10.06798 16.47879 9.35064 50.41397 16.01227
11.40933 23.8325 10.09311 19.72267 17.88022 29.21445
20.71525 10.52125 5.221156 26.88407 8.764485
11.0519 24.30885 4.46439 19.935 17.08905 31.13533
36.52405 11.37917 14.07921 13.14803 7.27157 19.12117
10.13411 39.07198 52.58324 37.16145 5.594632 9.5256
29.29491 33.47025 8.206292 23.60046 12.44461
12.1187 18.0768 20.20031 27.39271 47.82173 16.62187
28.22407 11.69368 7.295077 27.66547 38.91708
10.89803 53.33241 24.78698 76.19303 5.319093
21.6305 12.65793 34.89922 11.38906 6.343723 12.02032
4.668975 22.62659 26.0624 6.967811 13.64268 22.38855
48.05445 7.020124 20.7546 24.2146 8.45107 3.447075
40.72505 4.111015 35.3728 12.84972 16.12103 33.84369
7.190586 13.56941 15.18004 8.290277 7.70203 37.30366
6.329469 4.824903 19.81221 42.56058 7.358299
33.20269 16.13051 13.33425 29.84446 33.02056
50.33327 5.118708 28.06949 33.24125 30.60817
7.686122 8.244952 25.14556 23.1395 3.830212 7.611768
37.55238 4.417735 13.5165 4.685527 6.397037 4.362211
52.11513 16.20767 11.24803 7.625256 29.11991
16.8499 5.878437 27.92095 20.99429 25.50006 8.466849

8.247155 13.21391 14.3323 13.34201 7.337149 25.64947
10.65894 2.339652 3.396005 56.05022 6.190641
7.944389 4.745837 22.53275 29.13693 22.75678
14.74508 10.44852 17.10395 11.40751 12.54325
8.372842 29.03298 43.84085 11.48839 81.60236
10.86624 26.15065 17.55013 22.50315 42.51151
14.8955 29.40518 28.59857 34.77161 6.142733 7.025114
15.63454 8.140458 21.82263 21.07259 7.701094
21.42013 26.24958 17.87964 18.29213 11.77902
27.06596 11.37025 22.56545 18.54157 2.926517
22.46403 18.15878 6.708318 3.922627 14.97819
9.442392 10.92343 14.57078 11.33218 30.91245
5.713373 7.81416 18.29943 3.787974 14.69138 38.41082
15.74034 44.90365 6.471068 32.23806 16.70731
23.68144 14.42934 12.48411 27.67204 2.229981
11.16212 38.9064 24.5061 23.58152 29.74735 18.34194
9.829079 2.714367 15.37804 17.48577 23.06421
17.28522 15.12817 36.05413 13.99937 3.946979
21.86347 18.35102 56.0396 15.15737 34.55475 49.56926
15.03716 34.20229 24.04308 9.664632 19.30547
24.77407 20.79562 39.1201 14.27647 49.35133 65.41435
12.489 10.49877 7.148277 12.47491 2.053345 6.007701
35.99175 8.084432 15.14474 31.16272 48.35944
22.8135 4.956981 13.37896 7.89049 40.2752 10.39657
32.68403 1.597921 5.140738 22.63711 18.29698
15.08416 66.18063 41.39562 48.49838 19.5954 10.06768
16.92123 5.1981 41.93181 12.65152 10.8882 6.632521
33.91204 13.07168 26.85617 20.2389 35.56452 15.01364
20.34643 23.85483 4.342399 11.90962 18.68246
45.53134 38.17288 19.24117 22.38494 20.55019
5.476998 23.294 12.9768 25.08906 3.547622 34.31533
25.41228 22.3165 23.9736 14.29969 16.67807 7.676553
3.585813 13.86729 10.02612 35.50068 29.06056
1.970335 11.8667 24.30861 5.814539 6.287746 4.875078
21.50214 2.745937 26.20465 9.475319 10.82908
20.66612 9.6982 24.41886 11.111 23.89155 7.795504
13.5714 2.942893 13.35922 32.57224 13.41679 34.39436
16.68929 11.36429 19.76939 4.768496 20.3669 13.5843
13.11866 7.056413 14.48866 9.436103 12.95031
13.48851 4.47592 11.72042 8.491435 15.08027 19.89263
14.58445 21.58913 11.87479 7.290277 47.11272
62.36005 9.684834 32.93845 21.8587 5.533942 13.01789
20.54933 16.04747 17.34541 31.11341 25.75645
8.027446 16.79726 6.846675 30.36167 10.86308

3.455531	30.31832	7.684795	13.61959	22.24972
13.47314
LGALS1	27.95999	567.1721	51.01174	59.44189	118.9008
418.8761	106.5391	161.3374	145.0071	220.5699
35.90395	26.82935	179.6507	21.60419	129.8736
442.9884	72.38672	170.4261	103.3244	105.4289
117.0021	401.7164	93.57652	438.521	397.5753	25.69716
135.8922	354.3123	83.66534	207.3099	134.6776
374.4613	70.92459	126.158	362.06	105.5733	21.61058
137.0059	139.2684	66.03117	118.2333	125.1272
195.5516	133.0627	79.40466	90.68011	27.82683
132.9301	113.3803	29.14206	233.0327	168.406	73.69211
458.5955	493.5235	59.9835	233.0395	222.1701	104.2826
55.86317	275.9828	164.0428	264.5264	302.4153
72.4603	513.0452	443.7205	261.0637	47.3776	369.3301
106.0424	177.3541	119.8689	238.9919	337.0992
137.3783	124.9744	174.9419	92.06139	59.35033
571.8252	201.1427	77.68413	237.8044	466.5545
199.9863	98.52219	104.3397	234.682	347.5071	146.3759
80.95039	292.4117	81.34271	171.2303	192.8986
254.492	72.15022	98.31509	125.6499	182.4319	21.852
232.288	233.0412	354.3663	136.0407	135.9561	230.0158
100.5953	193.3735	77.44129	97.28421	244.2494
303.7729	232.5987	57.51747	116.9573	95.297	173.1425
67.29829	207.9316	86.21493	219.2463	649.5887
181.6413	85.66835	137.4208	499.9701	306.7674
296.9233	108.061	122.5577	83.98962	135.74	187.7541
113.4665	140.4685	150.0404	186.3865	37.08616
90.90158	66.42624	51.7187	79.65761	101.9918	141.5066
49.66411	524.9925	45.77533	129.8194	42.12601
303.6893	79.0632	120.9256	295.6305	70.60851	198.1179
403.8561	341.929	237.5143	286.0767	88.3482	90.2944
68.80231	231.2123	73.48106	66.72395	132.427	95.56476
180.4437	401.1164	71.42308	80.90395	130.0982
115.6041	325.6645	373.7798	243.9752	28.83108
169.0525	131.8829	98.0406	34.02139	99.30652	379.5955
214.8752	282.6576	336.9845	57.78349	47.74092
229.9174	109.9709	166.845	56.56881	161.6483	82.01435
187.9487	44.53147	43.49667	217.2507	69.51584
219.7162	58.32121	67.80565	153.9788	39.4108	302.1343
130.6744	69.67327	138.5847	85.15604	270.6788
76.00529	111.8468	204.5472	114.5764	201.7007
73.9382	161.5827	86.33208	99.25871	142.0853	91.09676
145.5938	84.41712	111.2805	71.70189	28.72321

43.19379 120.1971 185.9121 416.2818 19.38084
35.81705 41.5221 218.2278 185.2856 79.21773 124.8527
44.58409 211.7856 129.3305 183.2716 244.477 251.9826
201.5362 215.9497 391.7676 142.7593 99.24892
113.7156 85.86877 208.2583 208.4579 54.84802
130.6242 94.53045 187.0638 123.7065 215.4796
132.7365 222.3588 75.89141 168.2053 105.0277
107.6303 88.75697 86.14709 177.8064 70.51645
178.7448 245.5234 89.33966 57.42739 60.00646
85.0959 89.81597 545.1081 963.5435 139.7699 307.1908
54.98524 934.2576 215.0288 230.9244 107.4386
27.00415 104.7109 207.4259 158.4675 71.89672
437.3089 326.5397 132.0625 292.094 408.0053 372.9685
270.2671 225.9728 47.90267 103.5912 179.7701
207.0327 435.4392 39.18128 120.0146 72.35336
186.2216 71.16268 48.26465 71.46486 570.4926
291.8199 343.5264 65.70382 248.2888 109.2782
292.8288 175.7761 198.0022 91.73373 81.40729
49.22244 190.8212 372.438 127.9858 157.1105 109.9274
113.4752 126.344 86.59011 179.0794 159.3128 121.5064
517.1457 36.4948 20.5914 96.49232 57.67127 75.67613
55.97191 254.6097 201.3883 63.04972 176.1457
24.97106 47.25382 27.37682 192.049 399.7416 74.8465
205.1695 67.27658 461.4928 87.35492 46.53379
104.3986 163.4402 490.6845 226.1848 116.6584
254.7355 121.1142 166.4127 140.4538 371.9966
11.14358 108.4573 43.06084 163.1171 198.8671
134.4072 205.9491 61.17732 73.07856 167.6237
142.7322 406.7775 70.74327 82.55083 172.6381
81.26788 163.6341 94.30252 40.84365 190.139 275.2826
328.7151 86.6425 68.85013 219.0192 89.45266 34.24076
113.1084 67.64081 153.6607 73.43 152.3395 53.0347
151.4033 143.2545 62.59856 135.381 27.23981 73.99244
190.7944 338.4408
PYCARD 43.97946 2.952504 34.36337 13.37728 32.12452
4.90749 14.43347 6.82446 13.0581 23.73703 7.192823
14.84744 25.85331 13.97457 26.2099 2.300449 31.80763
10.33973 40.30592 50.55234 12.25506 3.040202
15.57887 3.149907 4.301386 10.26476 26.05733
1.950418 26.18266 16.35439 9.467097 5.457041
38.14631 19.11876 66.92296 26.95927 1.847627
99.9828 15.45367 7.856988 19.7356 61.58578 11.58034
17.21793 17.25749 23.22833 16.8137 30.08552 4.185976
7.468216 23.78927 25.92947 7.094172 69.75814

19.69777 33.34399 13.29228 8.025124 28.46068
38.61013 12.71117 31.94494 39.61143 19.58043
31.95821 6.021921 32.40335 35.02569 17.0754 33.9302
6.193042 18.76526 11.94947 8.286516 13.94648
80.9145 23.59931 12.0542 11.71774 17.62526 41.90009
30.34539 19.3756 26.37976 6.633661 24.98378 8.415022
18.71018 14.98237 19.04145 17.26266 45.7474 41.81366
21.60494 32.32813 30.02458 29.16631 18.91842
3.523366 24.93112 20.73095 22.16899 28.05415
22.54748 9.016547 12.35164 20.2901 21.03676 19.32952
21.78292 13.26398 11.65859 33.75521 25.79407
38.16492 71.16133 24.02675 10.59549 12.23202
25.31117 36.02605 9.240726 8.374825 30.98419
10.16518 17.24822 8.208398 53.01508 13.78969
8.743392 46.94335 15.83367 45.03408 19.98963
19.45637 13.52332 4.459435 14.20221 16.48023
6.645241 23.45261 5.903714 44.9571 9.725132 6.406497
54.36863 18.55343 27.5795 12.18735 21.45451 59.40724
34.16026 14.1005 23.9579 11.27562 29.85454 12.40349
20.68298 74.38036 29.29393 18.774 14.14762 9.231178
8.028794 30.64588 35.21767 43.50603 16.07216
16.37939 7.656411 13.04789 19.12234 46.32553
38.09533 24.62449 20.75481 27.76202 14.66136
8.776508 8.896864 32.65552 35.65428 13.41994
30.08644 46.15104 11.54236 25.88002 60.35694
5.828642 15.3288 16.12012 15.48525 15.85148 10.32332
58.62873 9.943564 24.02297 14.65826 8.654366
25.86408 18.37113 11.58959 17.36469 6.533531
16.40076 14.96072 54.61688 7.848155 17.80535
21.86682 8.301598 24.7854 17.01735 7.457257 27.35647
27.19068 14.68691 16.7898 23.93519 27.80359 16.65125
11.38825 29.21436 15.92167 26.49716 28.67317
13.74567 25.8081 13.57457 62.54399 23.24977 16.83375
17.48946 20.57904 2.630959 30.64606 35.69467
44.41054 12.336 5.921243 21.3107 31.54479 28.32608
25.51451 8.093495 20.78234 63.36602 10.95155
10.11854 28.53368 14.86084 25.98923 28.52315
26.98898 32.50369 4.349693 11.247 24.63446 37.899
10.06669 15.65127 43.4302 14.83505 28.24566 16.12626
13.93135 15.27043 33.38116 20.05306 20.12255
29.08216 65.6363 14.58866 23.00754 9.163225 16.98976
17.16052 9.192315 23.72608 18.30384 20.63517
20.43618 26.19072 27.2602 17.51854 21.71622 7.48895
26.49533 17.92471 13.36512 8.949453 4.260543

5.942698 48.81481 13.53745 14.58904 32.7881 35.83918
32.73628 26.08885 22.69507 13.78749 20.75818
6.804769 31.37192 39.08948 8.605135 11.49895
16.78527 14.06237 60.99623 52.05633 27.34228
44.60647 26.75256 10.5007 11.20471 26.45931 29.77622
9.543402 24.04985 13.93047 24.50792 32.82351
18.29556 16.13883 20.94877 16.67333 20.84586
8.04248 24.86304 12.32816 51.49502 54.90653 15.73093
28.12103 6.006407 40.59151 21.84842 16.87598
8.787265 41.67252 61.95608 6.741126 6.88495 14.29928
16.31008 2.372936 60.55885 34.74105 19.82171
12.02204 17.15501 21.50078 37.39979 15.63023
13.31062 19.75237 18.31963 14.23869 25.77883
27.78305 6.288201 29.72759 36.82385 10.94701
17.10837 27.37772 21.25494 19.52331 10.33138
16.26127 13.47523 10.45095 12.91325 7.086644
39.40988 23.5455 22.78903 22.2828 16.74254 29.16008
14.11652 14.03392 19.63824 68.71872 19.69153
74.15398 22.70503 17.26519 18.27392 3.449607
32.77377 28.76107 30.40874 23.81044 7.375827
20.54171 4.099502 24.6397 31.4041 24.83689 41.429
18.86243 58.3671 49.30108 14.1084
SESN2 22.71648 4.711355 24.98635 5.980706 9.293809
4.329288 3.068418 5.930178 6.677587 4.90257 7.533924
9.588903 4.509275 16.26109 4.129423 3.26677 13.90721
9.104779 8.920116 14.52205 6.086813 3.264281
12.02774 4.485202 7.991107 22.19801 4.895204
3.836021 12.47271 1.26979 6.337505 5.917577 7.712663
5.181435 8.104524 4.408835 1.304691 6.821023
6.584223 16.74503 4.546249 11.57984 7.528063
6.315906 7.508509 13.18596 6.369473 9.248555
4.948276 5.474359 4.580265 3.243266 13.32055
3.485909 3.142006 3.817203 4.3924 3.575661 7.505499
10.47279 6.611268 7.247162 7.446566 11.9889 19.78499
4.413523 7.273941 6.578633 12.32281 11.66576
9.231806 8.084359 6.380725 8.407628 5.199315
7.038749 5.262755 3.873621 2.703655 4.472024
40.89575 5.129199 21.96662 11.36126 4.768767
9.484015 10.13054 6.728033 4.367432 2.059707
7.541603 15.20776 7.14186 15.30482 3.92282 7.539462
9.515044 13.42456 2.517556 5.168948 8.191661
7.274732 7.891425 8.529458 7.276489 7.941075
8.789007 9.674123 20.10681 4.162136 9.16798 4.513542
6.276356 2.096561 4.053178 8.705521 9.001286

4.666752 25.30764 3.90002 4.711225 7.043317 11.21713
4.632224 2.725975 4.803486 7.681475 4.568862
7.541347 10.8573 15.59352 7.573372 4.786363 5.320819
7.656604 8.591286 15.00388 4.552958 4.940798
4.063339 10.92029 3.256325 6.837628 5.307151
7.589141 0.5750529 4.01451 5.068501 2.106197 9.64624
2.410881 10.19817 6.64552 17.21565 8.87998 5.19214
6.060453 3.801304 7.210223 3.392607 7.642 4.109297
5.593632 7.896442 5.784685 5.461 9.524206 6.850338
5.730176 3.372172 3.449088 5.938538 4.982929
5.874287 7.274867 4.450166 9.414418 2.199383
7.318278 10.98399 2.10742 2.613417 10.64436 13.5082
8.660725 6.415223 5.428041 8.607596 23.93951
40.09478 8.284703 18.04581 3.812525 6.612541
15.79412 5.202932 6.951218 10.93398 16.93504
4.309293 5.195429 6.309313 9.870924 6.756228
3.399314 10.17176 5.693539 13.24472 7.132078
10.90951 9.515095 4.369215 9.302838 4.612741
9.048793 22.29747 24.07184 18.24277 14.43604
20.34512 10.70325 12.86799 10.74954 9.86815 8.68809
1.087729 8.984106 7.6721 8.490776 4.10392 3.731119
5.739234 9.417261 7.585371 6.862328 8.263129
9.866981 11.29694 12.78019 12.46293 8.029455
21.80607 5.925682 3.806069 7.123675 8.085649
7.46088 4.240536 7.049658 3.780161 3.502399 7.577893
8.517535 7.002332 13.85595 5.346513 5.438005
13.95214 4.973416 11.48036 3.743171 5.633866
5.71212 7.154275 19.30368 7.424676 6.886453 11.04516
10.95279 6.173173 15.25142 11.06945 8.109877
5.283712 6.006902 10.8982 4.109399 2.593128 3.035943
5.383166 4.85499 12.15394 3.54304 6.948106 5.225024
20.73729 4.995617 3.600023 6.031938 9.058162
17.29794 1.674447 4.27927 10.07321 8.536436 13.15121
3.517459 21.08132 9.85349 9.353812 6.223387 3.508712
23.4761 10.20905 4.999515 6.866968 6.256819 8.081141
5.278171 3.099425 24.45746 5.711039 9.399768
6.352394 2.8814 5.524379 5.864301 4.498283 4.907528
5.744634 4.652152 5.485367 7.316944 13.89582
3.097996 12.97404 38.09287 1.861482 4.463671
5.762684 5.150049 5.101097 10.24077 17.1579 5.3766
4.733072 5.310118 5.662778 9.895259 2.292403
3.87372 3.285074 3.443299 21.70253 4.646698 11.31105
9.904257 6.495273 4.553762 2.768604 3.762946
17.51722 12.15928 7.209198 8.896613 7.936325

8.657117	3.377368	4.77453 9.85719 4.332676	7.123073
9.592639	17.19669	4.328781	3.359807	2.911891
5.813487	6.938119	10.84151	3.617197	5.616579
4.46938 6.394507	12.8869 7.649101	4.624706	14.99352
7.726523	6.322816	15.63721	6.909722	5.194496
1.880327	7.460705	14.29524	7.032067	4.119641
15.8211 2.250587	3.308522	6.550407	7.356111	10.80697
12.13097	8.055017	8.225939	8.036858	15.17538
7.607624	12.89463	2.043683	11.3771 3.085344	7.569457
14.82422	6.863395
NTRK1	0.02334718	0.4241791	0.06442691	0.1115132	0.1924922
0.1418668	0.20613 0.09593572	0.2883521	0.2005815	0.05725925
0.006552691 0.2008725	0.006254325 0.1413758	0.12849 0.09314389
0.4796503	0.08648249	0.1567176	0.2299482	0.2258747
0.1400243	0.2513932	0.1210714	0.03932439	0.1689437
0.1819741	0.1869141	0.03027254	0.235635	0.02391772
0.09658493	0.03151398	0.04616049	0.02193441	0.2776451
0.07488952	0.1315793	0.07674413	0.1077372	0.05586401
0.0381864	0.03430658	0.02060701	0.02699638	0.08149937
0.15057 0.2411232	0.09323715	0.007700476 0.141606	0.143127
0.07522538	0.3186493	0.01640052	0.09794246	0.2837844
0.04013226	0.09947668	0.2423468	0.427564	0.2125971
0.03512623	0.08720738	0.3457917	0.1638341	0.3606931
0.1306906	0.1025164	0.05697197	0.009669512 0.1286771
0.1337299	0.0467172	0.3136648	0.2744936	0.198192
0.0151462	0.01306902	0.02319717	0.4335518	0.06430709
0.2092042	0.003513198 0.08073427	0.2814098	0.1195255
0.6855568	0.1015753	0.0192846	0.1228981	0.01709517
0.2787377	0.1881127	0.2623676	0.1293197	0.073754
0.03078867	0.2062235	0.1461566	0.0407951	0.09990404
0.01600307	0.5713005	0.349716	0.08660689	0.07473533
0.112078	0.0311257	0.07271495	0.0615953	0.07023905
0.165459	0.5669917	0.05560942	0.08956465	0.05813238
0.2537853	0.02442529	0.02761247	0.08970494	0.1690459
0.09907487	0.03847263	0.06470216	0.1433807	0.06853883
0.2286169	0.1203934	0.09848426	0.02658056	0.1085982
0.1142674	0.0447375	0.2083054	0.1142087	0.05027641
0.1468254	0.06126917	0.08179381	0.2353011	0.1634618
0.08779168	0.2581395	0.002697303 0	0.1882007	0.01469702
0.04235425	0.09247282	0.2359168	0.02115623	0.188941
0.1754915	0.05236397	0.09238692	0.1105795	0.08432767
0.323611	0.2262253	0.1584501	0.4399962	0.01820506
0.02465332	0.1694506	0.1330133	0.09975222	0.1726077
0.0164247	0.2202692	0.1881455	0.07828762	0.003393984

0.006927069	0.06341834	0.07420862	0.1440868	0.06417253	
0.08490876	0.02683616	0.02021127	0.1008499	0.279662	
0.070148	0.2019302	0.1507022	0.03158907	0.07701863	
0.0403705	0.06578723	0.07732381	0.04056628	0.1478706	
0.08602836	0.224753	0.1747249	0.1689737	0.03058724	
0.02337041	0.03335788	0.019131	0.07847567	0.04628684	0
0.08275204	0.05626513	0.687646	0.04815187	0.0813779	
0.1535826	0.1311654	0.1609851	0.01839295	0.2112523	
0.1721705	0.0571096	0.06869851	0.2970054	0.03803672	
0.1680708	0.2566815	0.09452393	0.0621211	0.1076101	
0.01351478	0.04132514	0.06213713	0.01220792	0.1199942	
0.03010338	0.02327104	0.03045188	0.07894426	0.03197511	
0.2054226	0.2690461	0.06505586	0.2033702	0.06129903	
0.01249008	0.2740574	0.1769223	0.1074573	0.06958638	
0.2370327	0.1832581	0.1201718	0.3012802	0.09750622	
0.149911	0	0.124229	0.3567473	0.08577222	0.1670821
0.02483761	0.2143517	0.09707322	0.1545729	0.1344565	
0.05138662	0.08093313	0.05168158	0.1181015	0.1319173	
0.06354263	0.2612499	0.1024702	0.1206987	0.1459533	
0.1994262	0.1210327	0.03048324	0.03714613	0.07989826	
0.1076343	0.01868702	0.04291098	0.06760762	0.3498681	
0.04933502	0.05356939	0.09527936	0.08517369	0.2975869	
0.03418347	0.01121311	0.1554726	0.2469451	0.09419482	
0.1901129	0.4889129	0.1421329	0.255545	0.3505138	
0.1630765	0.2550177	0.07570233	0.03984902	0.2483193	
0.264891	0.04550696	0.08467708	0.09288549	0.08087084	
0.08663369	0.09250423	0.2576574	0.0246588	0.1663933	
0.06713088	0.07286457	0.3986831	0.06440089	0.1528265	
0.2556198	0.07540207	0.1463503	0.2029072	0.1155202	
0.08760208	0.04997428	0.08261409	0.1406243	0.05986331	
0.04928987	0.01313587	0.3134098	0.1218154	0.08455763	
0.111059	0.230621	0.2537768	0.2449442	0.03828475	
0.01314389	0.05712577	0.2535694	0.03352744	0.02800028	
0.1621917	0.08615853	0.02607733	0.2395731	0.01298988	
0.09098474	0.02570545	0.115017	0.1244401	0.03062512	
0.1197355	0.03412921	0.07153346	0.1507627	0.0141905	
0.1644269	0.2691658	0.2958035	0.03612717	0.2982717	
0.1996783	0.01669759	0.1508159	0.1370908	0.02560883	
0.01823266	0.09957539	0.08184293	0.04610546	0.08192432	
0.1382437	0.03488956	0.04295698	0.2327259	0.04558978	
0.07456277	0.09110727	0.1236465	0.2484226	0.2060381	
0.1564953	0.0795686	0.01659838	0.06844435	0.1107659	
0.3109546	0.06906183	0.04599931	0.033777	0.1265924	
0.06495707	0.3502214	0.1333493	0.1027687	0.1759703	

0.09554139 0.5434539 0.02573159 0.02427762 0.04194324
0.04990606 0.1137217 0.05841359 0.05374353 0.3413943
0.121785
KRAS 5.829752 4.157144 6.833896 10.62469 5.305714
5.714572 5.392086 4.900348 5.848549 6.207189
10.52577 9.475417 5.982297 7.327705 6.331804
8.453673 5.727152 5.445551 5.729062 4.814722
8.365371 6.798694 6.102852 5.960646 6.094156
11.00536 6.024946 7.279574 5.863541 4.085861
6.651669 6.012215 7.526783 52.41963 10.00565
7.475866 5.182114 7.35176 4.820423 29.23157 20.53443
10.96237 12.57873 19.73391 10.77694 8.08161 17.18543
6.216992 16.53167 10.77449 5.239127 10.31533
13.05068 5.911615 5.714335 10.9333 9.091314 6.681907
5.62211 4.445308 10.04322 12.04765 7.446979 6.16994
10.76886 5.855432 5.128376 5.115843 10.92286
9.002445 21.65964 8.428982 95.31696 13.23917
15.03228 5.152898 10.00567 25.92017 7.686153
7.698856 8.811505 6.737547 10.98867 5.720365
7.24516 10.59563 10.66414 7.838391 7.027437 5.722827
22.19929 9.068811 10.38167 61.88496 9.527116
7.652978 8.397292 7.712197 21.61289 12.25383
6.220351 185.7501 21.18926 4.587149 34.04186
8.744143 12.19013 4.332008 12.72099 8.329074
14.40994 6.698702 8.732872 5.88936 10.83772 23.01468
3.435084 8.647633 10.89899 5.938675 8.217564
10.51003 8.276554 5.865973 41.23495 8.11523 9.250367
9.055342 6.356468 7.115757 9.036141 10.63299
8.312612 27.26644 6.511898 7.699317 9.165524
8.397855 9.301905 10.19747 8.750392 11.72811
25.86261 4.424389 15.24905 6.629382 8.179901
7.700763 7.20337 10.70906 32.42573 9.532514 10.28589
6.357383 7.30062 10.57707 7.065171 5.390373 9.887841
5.385423 5.686081 8.471917 10.80845 7.998544
4.559143 10.76403 11.10885 15.63548 6.468076
222.7557 6.139728 24.67175 7.468137 6.246129
5.86585 170.9981 7.177364 7.478069 14.20647 8.507272
7.058004 8.122404 12.41786 7.040312 7.226745
9.749987 8.601066 9.037248 9.313784 6.166963
14.31941 48.23881 5.666779 7.455441 3.301595
9.716166 10.53379 6.629155 4.012265 5.936795
11.07183 5.916607 13.83956 9.281003 5.37584 31.0263
6.243071 9.06826 8.073399 5.552903 10.41154 8.173595
13.3397 4.274805 8.101707 16.74316 4.980413 13.80306

9.319577 10.20466 9.775587 11.28077 7.372228
15.60153 11.63312 6.54169 8.231869 6.922418 5.624613
37.2487 6.599615 10.59915 5.504308 17.29139 7.665652
8.338605 3.757399 11.62414 19.06301 13.63778
10.85007 9.261032 8.860318 9.176666 12.46803
9.049701 6.980069 5.500089 11.09968 6.378533
9.37576 14.10709 5.991688 8.429727 5.851552 21.4053
259.415 6.911235 10.02385 19.42947 8.061093 12.273
10.54295 6.614189 14.00377 13.76256 11.66307
5.586729 73.96382 175.232 8.628829 4.945261 9.244622
7.750337 6.063389 12.30307 12.51889 6.949806
10.55938 231.7979 6.093154 114.3343 8.619195
7.838253 16.9445 6.228816 129.4131 5.72593 11.08764
9.254931 14.69496 6.474774 7.373438 9.562531
82.14654 33.91444 6.060166 5.749639 10.96424
11.64653 11.84089 10.23621 10.35323 10.0559 14.58981
3.921025 28.95226 7.549075 15.47358 17.48104
7.20268 6.535146 6.332993 3.869972 14.65785 6.421576
9.253948 16.47611 10.76492 18.51577 12.77019
12.19533 7.99337 5.523842 12.66933 12.61326 7.457544
2.915062 8.562421 15.71464 11.46463 11.02174
10.07847 8.839963 4.825247 7.287456 3.771507
9.984946 6.43679 18.5007 4.256617 3.800332 10.51393
5.306956 8.857881 14.37651 10.35287 17.12103
76.40354 4.605921 9.061613 10.56319 19.82699
9.779277 8.364169 14.49829 8.563325 22.66913
5.244067 4.772146 7.088563 5.87979 275.338 19.8019
17.00309 6.603447 10.91583 8.297214 12.13329
5.427488 6.510582 7.521669 8.126658 15.31643
6.629247 3.088043 7.443043 9.007268 5.746508
7.96655 10.7584 54.01262 112.7806 2.639805 10.45107
2.887289 5.21854 47.55514 6.754366 4.376134 8.390769
14.14135 5.442476 9.63994 10.72515 13.49178 6.387015
5.96064 10.18866 18.96666 3.96586 12.21315 17.94824
10.76304 10.91382 5.930211 20.57594
ITGB3 0.1884686 1.672558 0.6386973 0.8970247 0.3122631
1.198788 0.2656515 0.7053699 0.8677845 0.4184069
1.773877 0.08444829 0.4646492 0.2387091 0.3018683
1.38157 0.540839 1.384185 0.3470209 0.5013966 0.8113816
1.389629 0.5552535 0.8861124 3.238393 0.4860044
0.7170352 1.181753 0.8817612 0.120043 0.8876694
2.35034 0.6351393 0.3124144 0.506066 0.06523414 0.1995519
1.00078 1.854314 0.4681873 1.084076 0.2098644 1.211397
0.4506311 2.110976 0.6058149 0.7233036 0.7712174

1.518396 0.4700767 0.3702432 1.163606 0.6172182
0.4524206 1.39251 0.5365365 1.111743 5.284263 0.4177445
0.4437738 2.931813 0.6132535 1.396275 0.9672903
0.4413663 3.257732 0.8346443 0.6483113 1.461707
0.3949703 1.205832 0.685391 2.152238 1.9886 2.814812
9.806954 2.502988 1.29191 0.7307401 0.6218879 0.4139381
1.047418 1.253769 1.873354 1.504576 0.838778
1.554782 1.08277 0.4304311 1.68574 0.8857926 3.669826
1.217383 0.6224338 0.5860985 0.526953 0.5833161
0.7311616 0.3107736 1.561788 0.919033 0.3668694
0.1980801 0.4799065 2.985331 3.314771 0.5771559
1.913966 0.6871195 0.1895472 0.572684 0.1460193
0.2553159 1.450737 0.4566967 0.9686865 0.4624483
0.8775422 1.165133 0.2698138 0.9854523 0.7932481
1.523491 0.3560405 0.5021756 0.5804906 0.2752086
0.2217188 2.894252 0.9272747 0.6715215 0.1693973
0.6997835 1.176105 0.8796204 3.287205 1.295752
0.6871023 0.7496117 0.7332101 1.092415 0.7415774
0.8488238 0.327242 1.148461 0.1417208 0.2060265
2.699656 0.1631831 1.24026 0.412529 3.112118 0.380515
1.616755 2.962387 0.3841423 1.096101 1.413357
0.4923016 0.1769999 0.8970755 1.026498 1.04483 0.9986351
0.415482 0.8792973 0.6223933 1.03525 0.8465689 0.8100621
1.629543 1.035368 0.7443559 0.3162754 0.2231828
0.2279033 0.8444196 0.7142036 0.4485038 1.993404
0.1496479 0.4274448 0.525809 2.381213 1.200344
2.884283 2.256438 0.08947395 1.281632 0.5145605
1.321379 0.5644603 0.3384801 0.6419711 0.2046825
3.168521 2.454568 1.326961 0.5693932 0.3089107
0.2391172 0.8263564 1.474902 0.2321319 0.1689132
0.5024729 0.768947 1.542392 0.946285 0.291668
1.120762 2.312444 1.132496 0.9773362 7.412388
1.058804 0.2618476 0.2697982 1.347482 0.5690446
1.286656 0.655326 0.6559453 0.4974081 0.7871215
0.1179015 1.403145 0.1462994 0.3267634 0.1685217
1.171339 1.603351 0.133692 1.006965 0.4477425
0.4720886 0.3333991 0.3420946 0.4704267 0.3207248
0.9856126 1.18946 1.692535 0.7375014 0.8002212 2.048161
1.679101 2.091437 1.189882 0.7049324 1.108664
0.7798272 1.040657 1.588482 0.5503359 0.3676336
0.7906658 1.089909 0.5618807 2.308119 0.9148796
0.2871289 0.9416845 0.724116 2.115248 1.639735
0.5153981 0.9352436 0.8291453 0.9162149 1.606674
0.5167198 0.5537814 0.2870863 0.2830913 0.32673 0.4299521

5.59732 1.308101 1.344401 2.551769 0.4285304 1.471798
0.6794693 0.7140966 1.00048 0.5604529 0.1889744 2.727775
2.335105 1.339339 8.772365 3.39007 2.79553 0.7600049
3.076901 0.4311099 0.7212585 0.343075 1.162558
1.550883 1.732432 0.7951238 5.051677 0.5385413
0.4865578 0.6906543 0.6786114 0.6588911 0.7555896
0.7912652 0.2121292 0.7474692 0.9580158 0.6852583
4.068593 2.012793 1.094725 2.668883 1.721337
0.4184426 0.7223882 0.390144 0.232049 1.36008 0.4866339
0.8245734 0.7943583 1.310341 0.5343716 0.1575936
2.144213 1.342364 3.613942 2.449843 0.5629792
0.123787 0.4530539 1.531412 0.5473107 0.4395037
0.2009863 0.6013104 0.2400525 6.066177 0.643877
0.6517525 0.6141437 0.4122347 0.4990633 0.7832949
1.729629 0.5695398 2.19048 0.3930214 0.7502816 2.900256
0.552736 4.714484 0.2839597 1.26104 0.9182747 0.7250295
0.5739318 1.000756 0.196752 0.2928147 0.9871425
0.4334935 0.4905856 0.6850378 0.8585663 0.28928 0.625679
0.5099978 0.6204111 0.2063542 1.436899 0.6784502
0.7288386 0.4475615 1.980031 0.4180665 0.4804812
0.8896207 0.6779447 1.894925 0.3423229 0.3843555
1.202412 3.462098 0.905559 0.902701 0.4326418
0.9285633 1.283443 0.7488415 2.283848 1.013985
1.36223 0.4158051 0.2131208 0.5832975 0.2643645 0.2319192
0.9618875 3.173145
BID 1.94107 1.518631 3.787363 5.643845 5.182092 1.568658
2.97887 1.746716 5.271933 10.2191 1.73069 1.842553
8.70994 1.311332 6.094612 1.20805 6.192533 2.544077
3.340855 4.494688 6.407497 1.640241 2.847728
1.470006 1.542068 1.733776 5.089858 1.550351
4.527742 7.718947 4.377202 1.523181 6.273954
5.393016 9.045213 8.173512 3.798146 11.63117
8.139386 12.97038 3.807944 7.037532 9.907748
6.585875 4.660495 19.29159 5.81381 9.553809 4.731498
11.91984 13.24454 4.840467 5.707349 10.73601 4.0331
15.46997 9.010627 3.045038 9.456427 11.66012
7.831844 11.5913 6.83463 7.429853 8.863489 3.228487
7.553247 6.668202 4.970774 8.594566 13.88211
7.529281 4.920052 8.656646 7.058984 4.172397
7.461734 8.259299 17.64675 23.66585 14.57975
7.203335 3.178269 6.582538 8.426212 12.55475
5.69247 9.8369 5.328655 4.983329 7.195551 9.260647
7.682261 5.930856 11.98357 13.09319 13.69014
5.977017 8.997522 7.068399 4.962831 5.438678

6.904424 16.75315 5.536663 6.172391 8.956781
4.861158 13.87063 13.31352 10.70426 8.480266
10.96487 4.139198 6.643023 16.39182 11.99637
4.181059 7.44254 10.00994 11.52818 7.474085 10.65715
10.37428 8.041351 6.33607 16.9082 8.560036 3.89862
6.336368 12.74147 15.19766 8.206087 8.939881
7.371062 5.831104 13.07949 14.30613 9.332481
9.84474 9.106801 7.239578 8.294902 7.662173 9.909911
9.135821 10.5499 5.545697 12.85876 6.435209 4.726347
10.81741 7.814444 9.970298 3.376691 4.237708
7.679323 3.714552 9.354492 5.02923 7.664021 4.187939
12.0705 9.028261 11.29212 10.09352 16.11807 8.82956
8.259867 6.581716 2.408983 11.68846 5.013577
14.42506 9.830745 9.514355 6.287525 22.05547
8.667111 8.939139 5.383475 10.68325 3.564423
9.516961 21.17719 9.771001 5.883149 6.839865
6.847954 12.13438 9.44133 11.27425 30.77264 7.428709
5.113405 8.550752 11.21073 8.676937 11.95726
4.167313 8.474854 7.719742 11.64199 10.10502
8.38398 7.63643 15.70702 5.084938 10.67868 8.705965
5.304196 5.251554 8.981301 8.166946 7.648701
8.394235 11.86565 24.49747 9.109799 16.92249
10.48695 9.918176 6.678246 7.650084 5.71896 9.936196
17.38648 2.290583 11.10361 7.91932 14.38938 6.653792
8.000161 9.552256 8.478097 7.776119 7.724415
11.71688 9.206246 4.043916 7.131869 9.715464
10.33545 10.85215 8.914241 10.0206 7.192817 2.138928
8.707852 9.667414 7.658132 7.124425 7.807971
8.252362 9.024526 5.230409 5.199541 8.945859
9.857502 4.795723 7.110681 12.22044 5.654972
8.813934 6.691942 7.173201 16.47246 9.15062 12.5305
3.578072 9.944374 14.66751 8.319028 15.17201
13.9549 6.18419 7.927768 2.327825 11.05713 10.41901
5.71193 6.226181 6.965508 11.3948 8.399791 6.667916
3.637692 12.87574 10.52195 6.920724 10.13059
2.367252 3.022106 11.50253 4.897276 7.571821
5.695522 10.47923 9.440768 7.284189 6.143139
5.940326 6.019078 5.573334 9.679645 11.3195 4.084849
8.292798 5.82337 8.835837 12.10716 22.42413 11.66754
8.865207 8.328834 3.215173 6.214622 8.17399 7.056012
6.218196 9.698802 7.611451 11.01966 12.37734
8.421241 4.222908 6.329284 8.691513 6.777691
7.372817 4.752043 10.90605 8.350559 8.729265
2.741171 7.820783 6.426029 13.48567 7.704798

5.873357 3.593784 12.47873 6.469522 17.32618
2.943823 3.881712 6.677094 4.734798 13.605 15.52277
6.103053 10.00166 4.274533 8.885274 13.96141
6.358417 7.571624 20.58406 4.607325 8.955151
15.67748 6.559248 9.285698 6.482504 11.58371
11.14642 6.184371 6.911129 8.699056 11.01297
9.984384 6.162759 18.15444 8.521081 8.940583
7.808588 11.15891 3.99249 6.426239 5.404001 5.637441
7.507843 6.404242 21.25819 6.641308 14.51858
6.025994 9.646928 12.14909 8.366577 7.395952
4.761228 12.0954 13.33817 7.491218 16.67023 7.906896
7.292288 2.68494 16.93575 10.84887 7.695448 10.28451
9.266467 10.17802 8.599747 3.571809
THBS1 2.652528 50.86948 5.736385 5.825442 23.11653
141.635 2.740376 36.34573 57.35757 30.43468 24.2449
8.50259 27.41895 3.403249 2.679468 173.563 5.357603
110.18 30.401 8.670672 9.684304 35.70866 92.59165
18.68598 25.58953 70.49777 5.135381 48.61055
9.344289 6.443609 33.53425 197.9091 14.22168
31.91789 132.303 5.939908 31.60404 29.6069 89.79322
12.7186 84.15739 7.819073 85.83504 12.62701 16.19408
21.88893 5.484346 41.26766 95.53046 56.14612
33.97548 34.43181 13.56801 33.42655 101.1189
6.521553 87.82916 76.68928 12.6973 8.72551 145.0485
69.75615 157.4197 73.42883 4.746204 278.3133
69.27768 77.40563 53.70241 16.81287 62.75517
14.42686 48.68326 105.75 252.8975 23.7908 57.67426
57.05915 8.226091 18.60842 19.8745 273.5692 42.04612
27.61263 70.97958 38.94192 65.73137 25.66978
27.78787 80.51483 36.60678 40.81678 110.8135
9.320882 19.98962 40.13539 71.1558 12.93322 10.17535
60.99115 50.02214 5.593947 7.862621 25.37793
172.4102 116.3929 48.44249 46.8797 34.99651 11.87778
35.09028 13.58398 29.62281 28.3829 41.94794 14.90555
14.56258 19.58043 75.42798 17.12642 32.30274
34.49121 63.06366 99.72225 36.73014 22.12917
15.2519 32.8424 95.32668 240.4002 14.0772 24.89414
45.36896 66.53394 21.36095 39.71101 67.05614
23.0411 31.67479 13.14542 21.75125 17.09717 15.49828
8.755835 31.53153 13.90228 3.715162 64.35501
7.439432 98.05265 24.7795 95.64953 43.04357 53.50245
76.14312 13.96281 81.41371 38.05214 42.72207
21.27887 82.34814 62.25116 88.14428 19.26535
85.26617 41.9317 21.10865 76.67898 128.7341 9.550663

127.0846 19.98032 12.16481 20.41826 3.568332
49.58358 160.9972 96.88542 16.95589 89.00522
18.72409 10.49151 21.0887 52.20045 67.1452 132.958
99.27879 17.85224 63.06001 12.02196 90.44382
72.60184 61.68077 9.599464 12.95234 106.9426
172.1053 12.54754 10.76289 12.61681 16.59165
46.23795 34.00639 16.60419 11.69141 11.97927
34.59817 137.2323 36.75056 44.60727 49.23064
133.2034 60.64103 14.61366 25.08703 50.97065
20.7687 11.05092 208.9259 30.36357 16.24764 98.28785
11.55313 43.14786 46.37888 5.213351 27.85788
7.976293 5.284093 24.40916 20.31102 148.6918
2.404415 24.13076 11.8877 25.22674 40.43677 15.73156
82.3753 11.01968 23.87293 35.51069 141.9662 69.30398
102.6938 73.0554 35.55078 19.76772 115.1701 17.35698
19.23241 12.80559 42.77785 90.09505 9.867336
70.91664 39.77512 74.30642 39.65056 214.7425
17.33886 27.75059 55.13817 98.96025 116.9825
100.1046 14.23277 40.67038 29.6207 24.12218 69.32863
31.06782 14.13544 13.85702 6.089023 23.78708
54.84018 66.58991 47.9581 45.95692 80.02677 26.1134
60.38459 69.48518 53.67141 65.94249 12.27832
23.5208 91.00235 259.2999 54.84874 36.3698 226.5807
66.35073 38.12777 130.9357 98.48613 99.09066
42.79783 22.69492 40.5482 105.5963 84.88232 519.5815
5.427563 24.27084 30.02638 94.54856 57.04223
32.60968 35.16137 16.5621 55.02185 44.63601 24.34244
222.9949 68.21468 75.55824 32.08766 121.4589
22.5558 57.74793 21.12284 19.98824 107.9033 16.04793
26.74163 13.27924 24.14987 64.93211 8.312631
363.5523 76.00439 31.17714 62.17107 8.52841 2.755518
15.42789 30.83337 32.8738 5.187487 36.64241 70.08126
7.651296 109.1977 15.2813 10.52745 43.53837 27.89047
14.06568 24.41692 69.57439 32.34264 124.9639
11.24257 11.87274 54.41026 38.2417 39.43897 21.80634
57.27073 100.8206 16.20845 13.49785 17.57828
22.3168 1.994643 25.95303 12.50163 30.54125 23.1957
79.78279 6.381612 58.6192 35.2057 51.6334 9.701363
22.7155 16.43601 43.87329 51.59083 93.75017 18.68685
12.35096 50.35376 26.139 119.9665 23.6843 25.58346
19.31574 65.53993 35.74375 16.42783 41.25867
23.0611 54.5327 25.23149 65.52674 34.48707 15.69325
12.65048 21.68174 17.03888 4.690733 18.93173
21.44539 44.94253

HRAS 6.903836 10.76794 4.115876 8.548747 6.876456
5.634663 6.053817 5.05277 7.065227 9.199566 13.68857
18.41319 9.632681 18.44176 7.512026 6.328061
10.11367 7.988922 9.433909 8.167576 7.078409
7.328287 15.0039 6.422168 10.03657 15.92859 8.906246
6.444243 6.329924 5.263308 6.134197 8.351985
8.199654 10.14383 11.71311 20.37359 2.878412
8.509136 17.28801 15.95063 15.6561 8.427844 9.143234
12.89157 6.552364 11.67659 7.166971 12.11229
10.30911 5.282525 11.70577 7.149469 15.46043
7.541041 11.61204 15.17247 9.987088 5.502742
13.32651 11.93044 5.14237 16.61812 4.550132 14.51604
8.24972 7.588107 12.03978 6.668748 10.26681 13.86609
12.68959 11.18326 6.798519 12.59724 15.59241
14.35655 7.021183 15.28369 7.419724 6.778318
13.03963 5.63246 4.373229 6.212595 11.49 11.21426
6.80528 12.06587 10.5771 11.12829 9.363954 6.851272
7.832244 6.820203 13.20509 10.81566 8.97843 17.23784
9.193557 9.16626 5.415861 15.39056 19.08503 8.083883
10.7544 5.160516 13.37343 9.725831 9.576852 14.29175
14.46012 16.76853 28.316 8.008214 11.6286 40.36
13.49404 8.381221 8.394124 21.94665 11.73443
7.713928 22.61014 6.910593 26.83889 7.25129 11.38924
16.82371 6.830373 18.90125 29.522 9.29634 8.148948
8.085774 10.16077 6.202605 10.36817 18.84096
10.83201 8.081922 9.245789 11.38692 12.20444
17.33475 23.63141 13.87247 15.06562 8.414824
5.808091 9.836372 14.52063 10.11025 6.760135
5.895564 7.221322 8.070762 13.8011 9.612905 21.28787
17.12051 10.37054 5.566896 4.567854 9.388686
13.62164 8.845877 8.700465 6.035009 6.96896 17.35588
7.038349 6.00828 6.98964 18.18918 15.44521 18.52595
6.140302 14.70366 13.1626 7.190865 11.72766 12.14076
19.14894 8.885045 12.44702 9.653362 5.933841
15.33152 6.191294 13.374 8.756616 8.30743 11.91388
12.27965 13.35372 7.886616 6.873687 8.928749
11.76193 10.68898 11.42464 11.75084 7.467379
14.32962 15.84995 9.940086 11.96371 11.98638
6.941074 13.18535 5.773514 6.671573 10.87431
11.29627 13.56664 13.97983 7.094758 30.22678
10.07322 6.410111 10.12075 7.311275 8.529723
19.18178 7.598611 13.78087 9.434301 26.46107
13.57648 18.90485 9.283092 7.545241 6.572115
6.498007 4.310354 9.751591 9.95601 10.43365 7.919446

16.67466 7.870499 8.174411 7.035409 11.53605
8.125663 10.26524 11.41656 8.776234 6.347926
11.57754 7.255691 5.429637 21.81787 11.8198 7.428364
10.33108 9.719571 8.545335 13.59621 8.844733
11.28651 25.6504 7.476038 7.698447 7.482745 9.822762
19.13682 10.94629 10.47611 5.777 12.00218 12.20372
10.22631 8.272204 17.18779 9.13581 14.07993 6.836701
13.50147 2.775117 5.720589 9.896793 17.56675
11.95847 8.296272 9.803786 13.06038 14.45044
9.993649 7.268452 8.150609 4.442366 7.473055
9.717753 18.62461 8.766686 7.482707 8.141633
15.27087 10.19184 8.294428 7.477027 14.15344
7.872737 10.30999 24.34713 5.504253 7.476497
7.869808 19.25589 6.749884 25.64017 11.14265
10.38528 11.36849 6.215142 9.875293 7.657808
9.228034 6.979065 9.656158 5.239115 6.024873
14.96211 2.788244 6.976226 12.6809 13.61055 4.8984
11.41251 6.007762 6.296652 8.568928 6.260648
11.88088 16.9182 33.08979 11.09769 8.7405 9.216466
6.447132 27.05609 19.2876 17.8293 5.558445 13.11593
7.743989 10.30948 13.50132 11.32284 11.22485
5.658558 10.49782 14.41658 6.874474 13.75863
8.178391 8.785969 7.039049 15.26651 19.2203 8.092786
15.33156 13.94516 10.94153 19.34411 9.458609
10.08809 4.241394 8.861023 9.515325 12.70563
14.06964 3.478136 21.4904 15.03489 6.022029 11.57858
4.033348 7.903243 10.19436 7.478399 12.1329 11.69164
7.313922 11.59792 10.43243 25.4751 28.77139 13.15926
6.013273 7.430568 9.819348 7.842149 9.182252
4.31164 10.03127 6.876962 7.088553 11.237 16.11231
7.332948 15.26472 9.404169 13.38475 12.09499
6.512694
CDK11B 15.64943 10.95446 15.22603 8.925868 8.8458
11.05355 6.337856 6.702674 10.38792 6.639163
6.780384 5.055596 7.84168 5.954899 8.31585 8.970061
10.65402 9.615793 9.244838 9.624133 10.03664
10.7371 5.213711 9.660238 9.624319 6.832105 9.28709
10.73449 10.46856 1.80794 8.263121 11.88866 12.61841
19.04294 10.28544 10.63018 2.883921 10.31642
17.53441 12.49997 9.756115 18.0267 5.749544 20.80232
10.81912 10.46615 12.9174 9.359104 6.746513 9.856269
27.66814 10.63578 15.24707 10.16894 7.73035 17.95389
8.273026 9.156546 17.54583 12.81221 6.020512
9.119096 6.743249 6.138416 9.66883 12.69167 9.33717

8.850422 12.95012 11.54725 10.227 6.87178 10.23141
7.113053 7.573629 7.811624 11.47548 8.164563
12.23685 5.732451 8.610595 8.999072 6.059952
13.79067 10.78191 12.68294 9.410324 12.85627
14.96669 9.920212 9.305582 11.14423 11.4569 13.71907
9.868712 12.3032 7.41284 13.26341 9.341789 12.80202
10.46738 11.35751 8.274458 8.350128 12.24573
9.88967 8.755318 8.969416 8.755026 11.26771 11.53895
17.26425 7.272368 11.03204 7.290849 12.14941
9.226369 8.994334 18.46926 5.727237 14.79434
3.674796 12.42936 4.522654 9.077068 12.29872
5.669729 10.84396 9.227095 6.37948 12.75865 15.67699
9.821666 6.457436 7.278255 13.40132 14.32692
10.98838 12.13357 7.528342 10.47098 9.312922
8.134868 12.33459 20.88056 10.99103 14.83166
15.49804 9.931449 11.78254 12.14107 10.34395
9.593235 10.68817 11.65361 17.02202 11.53072
11.19934 12.59458 11.44107 8.343422 10.24322
10.52231 12.31864 9.763997 11.96902 8.957912
5.13435 15.40434 13.01476 9.233638 4.818913 7.028506
6.812134 16.92802 13.65787 13.40172 12.98873
10.40098 16.17669 29.00219 13.26033 6.685787
9.713979 17.09918 11.67699 7.641528 10.36773
9.029654 10.21755 13.07744 17.02552 11.69621
8.147794 6.870061 10.69614 6.79751 18.45038 10.20097
13.19155 9.526207 20.35806 8.56992 15.3957 13.21437
9.801491 12.88526 10.90718 11.77164 9.648571
7.553355 8.780625 11.40844 11.10029 14.39997
11.56404 8.669558 18.55858 10.63569 10.26435
11.62598 7.29051 6.574527 10.26839 13.16423 16.34344
19.18132 6.090238 11.34905 7.421567 9.363378
8.901788 16.62766 14.3923 7.761834 14.58425 8.25915
11.97831 9.332582 17.34893 13.69949 11.92258
10.56112 14.02088 5.690925 10.75665 12.42192
12.06072 11.91393 13.52082 11.76484 6.956379
17.33045 14.15427 9.989008 8.510076 9.510504
8.302525 17.50948 10.45712 19.04294 14.5877 5.521216
10.67242 9.822837 10.84473 12.55833 10.05737
16.40372 6.330371 13.10624 7.537744 15.78686
9.498081 29.27499 17.13472 10.3849 8.775659 12.92812
4.627468 9.385448 9.795797 7.583797 10.14222
8.188874 9.353958 13.45277 14.47643 12.94238
11.57576 13.34127 8.715721 9.792098 20.18065
9.714903 7.812474 10.74893 11.31656 12.77838

12.57117	15.61069	9.85692 6.043467	8.589802	14.8025
12.91234	6.407608	16.54193	19.92924	7.66637 7.928493
6.594077	6.326017	8.975261	14.47186	12.6946 18.52391
16.6365 12.04412	6.45206 7.424732	10.04484	9.792674
12.10259	3.253896	8.361435	6.591625	12.89236
15.69461	8.656991	6.549002	8.336497	12.06918
15.77738	9.693286	17.39085	17.07051	15.82205
13.52293	4.621646	10.81219	12.55477	12.80631
15.86561	9.572771	8.344308	6.578598	9.543114
18.8501 11.92842	18.23746	7.660168	8.094559	12.88226
9.196932	6.844774	12.70198	12.04263	8.010501
12.62995	9.71641 7.848703	10.86726	8.997315	9.748373
13.1171 7.210661	7.967077	7.475746	13.65252	14.72999
9.624883	9.937125	11.98799	8.568238	7.797467
9.012626	10.75444	9.008887	15.65172	8.741318
17.39031	18.97164	8.237672	14.38896	6.853247
7.578879	15.73125	9.948421	5.412049	12.88424
5.429267	18.20056	7.223748	10.70278	3.711426
20.56116	12.204 6.470506	15.07353	11.06009	21.7246
10.73786	11.41718	13.68426	11.80404	10.74269
CDK11A 1.288754	1.323955	2.814367	1.287189	2.334966
1.114241	1.260616	0.9815503	1.507717	0.6543859
0.9019702	0.5828926	1.617906	0.7070301	1.027849
1.021171	2.170034	1.600949	2.32775 1.33335 1.708192
1.2681 0.3341998	1.238669	1.094344	0.9182484	1.428475
1.246316	0.8241776	0.4418014	1.056776	1.235555
2.71175 2.277694	1.919745	1.686948	0.3633926	2.017962
1.843187	1.526173	1.482483	2.021529	0.778447
3.850427	2.418661	1.819284	3.711182	1.338727
1.312243	1.853781	2.979008	2.38809 2.045712	1.329036
1.144901	2.659458	1.520139	1.577747	2.361375
1.512528	0.9719295	1.529951	1.049613	0.5677881
0.8080735	1.576942	0.8771277	1.255676	1.462508
2.03371 1.251144	1.162544	2.198179	1.827035	1.272206
1.190528	1.876423	1.962255	1.829181	1.068699
1.176838	1.718341	1.24472 1.919442	1.015675	2.046782
1.669484	1.477989	2.731552	1.669773	1.715456
1.773749	1.494295	3.240259	1.383787	2.169071
1.168334	3.345469	0.7988157	1.807514	2.696998
1.242039	1.659179	1.386477	1.728437	1.995594
1.391016	1.288831	1.128715	2.459761	1.499172
3.640386	0.7593168	3.430875	1.411961	2.061133
2.766387	2.299104	4.100466	1.189836	1.420025
0.7580691	2.577438	1.514764	1.601242	2.781854

0.8706451 1.146502 0.4379915 0.7351377 1.175207
2.885211 1.968541 1.033902 1.381809 3.072533
1.846095 1.949986 1.826473 0.8637562 1.018969
0.8395883 1.322319 3.81363 2.712821 0.852279 2.475351
2.267055 1.634211 2.155507 1.787989 1.469014
1.159413 1.548031 2.186233 4.72595 1.933056 2.448164
1.788514 1.84053 1.071406 2.349146 1.80465 1.644729
1.047019 3.309579 0.9909432 0.1993055 3.548149
2.575869 0.6250683 0.7791831 1.107917 0.9214568
1.912773 1.816792 0.92381 2.158452 2.099044 2.245883
4.022187 3.096361 0.9310259 1.456931 2.258041
2.852486 1.174922 1.647527 1.725536 1.627238
1.913589 4.041662 1.412727 1.096149 1.073493
1.866214 1.03262 3.524427 1.413975 2.916246 1.612064
3.390923 1.402822 2.109175 1.684078 1.78039 1.556629
1.690842 2.146917 1.638623 1.241272 0.5945574
0.6942173 1.550924 1.874443 1.965797 0.9492238
2.803349 1.110548 1.265625 1.433351 1.257641
0.4768623 2.043455 2.773092 3.626645 0.9668816
0.9284287 1.35744 1.043932 1.227342 1.279417 1.144806
2.145752 1.4555 1.280516 2.127963 1.724575 2.062428
2.019569 2.555419 1.867422 1.959062 2.052697
1.051016 1.590885 0.8799193 0.7052327 2.76468 2.686271
1.98918 1.311323 3.177092 3.036726 0.7152976 1.147935
1.2428 1.220556 2.747801 1.691892 3.729173 1.653554
1.180162 2.356126 2.028181 1.828839 3.144691
1.748335 2.630373 0.1725544 1.651079 0.9155532
2.786503 2.011123 4.377797 2.736197 1.469627
1.437195 1.888686 0.571184 1.465479 1.467214
0.8154816 1.540749 1.283061 2.487721 2.124648
2.368965 1.913587 1.523804 2.376564 1.088994
1.32092 2.902712 1.079766 1.131527 2.785138 1.688866
1.911496 2.938139 2.669108 1.499978 1.01465 2.084867
2.579454 1.81138 1.133895 3.681479 0.8790277 0.9326597
1.055183 0.8280939 1.191824 0.9396226 2.406875
1.495925 3.025044 3.182393 2.068839 1.109603
0.9222107 2.143707 1.655466 1.531023 0.2161141
1.919263 1.007651 0.7992845 1.287901 1.695955
0.8556045 1.17923 1.392077 2.052817 1.180234 3.343526
4.335821 2.198128 4.056157 0.4639325 3.191281
2.542383 2.168331 3.041156 1.8823 1.955942 0.592872
1.957918 2.348773 1.415139 3.643682 0.906606 1.5048
1.557667 1.453898 1.275461 3.80612 1.901767 1.855412
2.135281 2.015962 0.806911 1.386307 1.879833

1.485864	2.49159 1.594849	1.627602	1.867747	2.312676
2.46879 1.8951	1.022774	1.543187	1.24848 1.021537
1.505602	1.450507	2.152892	4.183881	1.221309
1.961326	3.413661	1.396527	3.396563	0.8924949
0.5254209	2.248914	3.065846	0.9730881	2.180287
0.8802781	4.965143	1.471523	1.705918	0.430934
4.487351	1.661235	1.066982	1.961644	1.368051
4.240175	1.475261	1.885021	2.188243	2.031235
1.555331
XIAP	11.03531	4.454888	7.50308 8.113415	6.448669
6.690822	4.840215	4.184748	5.410461	5.38923 7.584894
7.14774 4.984378	5.53933 5.800088	7.260747	6.412761
5.278617	5.839846	6.346987	7.268684	7.006172
4.532227	6.702439	5.00126 7.234018	5.290054	7.969751
6.455098	3.839125	5.577389	6.712899	7.378014
5.577189	8.231727	8.098936	4.547896	7.518393
10.44644	7.156088	5.326138	7.988228	5.143931
11.68608	9.206835	11.64176	10.24995	7.35911 6.732029
9.604664	9.289907	13.34294	5.160395	4.98633 6.964899
8.18698 19.7955 8.325538	20.75518	6.625841	6.581444
8.261296	4.840398	5.903759	5.418721	8.395157
5.519948	9.659658	13.38408	11.28605	13.48642
6.791768	13.70021	9.988113	10.47753	5.048382
11.08265	6.557241	20.00261	11.06718	4.646478
9.274166	11.08934	12.17982	10.24359	5.782725
12.21432	6.777591	6.17662 13.46043	8.983668	14.2381
14.16171	9.992306	12.10715	6.170537	7.809973
6.904327	9.834908	12.04382	15.43902	11.9927 8.798225
13.23899	10.77559	8.128812	11.34217	4.949839
7.981928	10.28224	5.624222	9.588424	5.929942
8.319669	6.128928	8.953192	5.374832	25.02275
9.366462	6.921883	4.889496	6.257271	3.754696
4.892523	12.45373	9.463978	6.823222	5.98242 8.597891
8.562879	9.964821	21.76042	7.390644	8.451268
8.450049	11.1046 13.67685	6.260726	8.635103	6.875521
8.75327 6.326032	7.488893	12.06796	6.386173	12.59047
4.273541	11.64087	12.66217	12.38826	9.03518 7.339137
13.38403	15.5206 8.972592	6.035421	5.15678 14.07121
6.869514	17.29443	6.083082	8.652617	7.268262
14.67988	4.107192	12.2412 11.80768	7.388957	5.250768
5.709806	6.198163	9.725956	10.98034	7.184872
6.817222	10.87146	12.11409	8.994465	10.84691
7.179301	9.939595	5.362083	5.604969	7.593456
7.938867	12.47135	10.60592	5.985442	16.10686

9.223458 11.57183 10.59579 6.128444 6.054241
7.188909 23.48502 5.999957 6.967505 8.521656
19.70795 4.3075 11.35488 8.061491 13.10878 8.275656
5.97493 7.702857 13.79327 8.791914 11.21985 6.906266
10.24908 14.35525 11.96158 9.060792 12.241 4.606713
5.152239 9.142512 11.75462 15.12154 12.54341
5.430809 6.559429 12.7405 6.549125 8.438804 4.539897
4.273858 8.635965 11.09885 10.32968 5.780467
9.387354 13.99974 9.247909 4.366461 11.2384 7.072867
20.71058 10.06029 13.12278 9.14134 12.07663 6.594712
13.54165 10.74816 7.561208 9.674835 13.21876
16.8242 9.96418 16.07835 16.86853 11.97574 5.475904
13.50926 7.956318 7.342364 4.863381 13.46016
5.079947 8.986575 7.649675 12.76483 11.48773
6.782129 6.418569 15.38284 17.90634 11.02916
3.740815 7.237036 6.931303 17.22185 11.77316
9.551286 7.819873 7.538943 6.476382 9.76672 17.9894
4.792551 7.441069 10.01084 10.24166 20.12367
14.17703 9.717009 8.699584 4.746442 5.737487
5.605148 20.08797 6.650554 5.872139 11.31269
8.192503 7.917096 10.10308 8.839205 9.775358
12.13119 7.369769 6.487061 4.976042 5.820934
9.253303 18.4068 8.389686 11.78568 4.906779 13.73569
4.917374 8.790467 7.898019 10.06935 11.50974
9.867872 6.999948 12.04778 12.42897 7.095964
5.981169 6.022544 5.258632 5.262098 5.059439
8.626782 7.997636 15.26225 13.45012 12.97254
11.07521 5.577574 10.3264 7.811072 11.1363 10.14851
8.656469 7.804678 11.39892 8.70628 9.558522 9.997366
9.299511 13.19139 12.26677 8.271593 11.40908
9.143835 9.644569 16.02115 8.888602 9.306091
12.31025 9.321309 7.479415 7.651132 6.687134
7.530567 4.759932 50.2623 6.40341 9.542247 6.58188
8.136631 6.75353 13.41384 15.91699 11.32607 8.701673
17.00664 9.984019 5.542467 7.333443 11.56728
9.922082 8.733968 11.96442 8.540674 9.272747
8.48932 14.92196 10.49177 4.165401 4.412061 6.857143
6.314882 7.235472 9.478625 7.912494 11.11141
6.024701 8.455065 23.54484 6.794601 10.17707
5.107557 9.198488 5.935628 14.2469 8.720162 9.979401
8.60744 5.866933 9.15828
PPARG 12.16583 0.7107714 9.618367 14.11187 9.989944
1.59646 18.35307 5.302025 17.41266 7.265811 1.15587
1.495607 8.267907 1.50831 11.60292 2.146849 10.7515

6.334694 10.59222 8.627992 15.11415 0.8761668
1.868422 1.297424 0.9935944 1.422536 11.90798
1.524743 14.79426 18.3004 14.42984 3.035312 13.36902
5.12321 9.541873 5.691214 2.174628 3.376678 4.024832
23.75445 4.503386 26.63441 25.5232 9.956322 10.59377
8.727369 15.73538 9.645339 36.48263 38.27093
8.530902 3.198182 3.775285 2.780625 1.358253
12.73077 4.998174 1.925192 6.599892 10.86563
6.743027 7.355382 4.670706 2.155601 18.37262
2.992886 14.63031 3.846998 9.124111 17.83245
3.572886 14.41577 14.95125 5.903021 3.487232
2.214516 9.679837 1.917472 7.585324 13.64121
4.276886 3.812609 12.00445 5.959448 10.45827
19.55353 5.224404 8.191364 7.368533 6.176696
11.79291 1.311116 3.766887 10.59121 12.98644
5.124811 6.811733 17.25356 1.365081 9.925567 3.4708
13.23898 0.347497 6.641861 5.231825 4.426034
20.95369 4.058834 24.65381 10.05315 6.771764
11.90204 5.898413 1.409256 6.05347 2.354893 5.954367
9.450549 5.788232 8.437495 4.626437 10.91332
1.46922 9.073266 2.371316 4.604497 7.258503 5.149779
4.28405 23.94938 14.12785 9.652151 26.70134 22.94007
10.62044 5.446448 9.464449 27.07587 6.998773
6.662318 4.694128 4.639334 5.017 5.954645 5.683759
7.67809 11.05683 5.780821 2.332323 5.738508 8.167074
7.345464 11.31051 2.458853 4.733932 20.73511
10.24495 3.452509 0.5245573 6.259289 8.421709
5.074071 10.02866 8.931027 23.29081 7.827547
2.457392 10.01124 3.232512 1.374864 1.325053
9.913089 11.70808 6.193575 11.51486 29.7035 7.138391
6.960012 12.91626 4.458528 6.034883 17.09235
2.549547 10.00286 1.668066 6.380081 2.968034
9.099941 12.62453 7.382611 10.91364 6.663646
12.11965 19.2852 3.124373 8.189382 20.54323 3.691913
15.14451 6.231548 3.837243 5.552309 13.88074
5.651681 3.811509 32.18742 5.356658 1.708142
4.650462 11.63905 11.16112 3.629611 5.806208
9.853573 9.628217 8.4701 23.10309 6.3835 8.272423
16.73037 10.08283 3.001974 7.187713 15.45717
5.723866 5.290476 8.078901 2.023665 22.47939
28.08763 7.235502 3.373945 16.70633 14.70944
7.792961 25.74525 7.360469 14.2791 12.8452 6.687564
4.20893 2.990134 4.680994 7.137216 18.61713 6.648835
6.522827 1.947876 12.17934 6.431764 0.4777351

15.50761 16.99928 5.617997 9.493475 2.680706
10.32189 4.172562 7.309018 3.248581 7.997903
6.980858 3.439506 7.60061 16.35153 6.751631 24.17714
6.910563 19.52986 15.20375 6.364011 19.91886
17.48106 7.338291 6.386259 5.530367 1.230385
1.437057 1.070272 21.51945 4.858333 9.009319
8.358584 6.148155 28.83298 3.956091 33.89234
5.746391 9.013509 3.768866 2.804764 1.276454
1.954677 4.205072 1.276578 12.57493 13.19883
5.38616 20.96944 18.20279 15.07382 6.979151 3.016171
2.467965 22.6096 14.10186 5.914966 4.98457 6.228864
3.354718 12.01092 20.4943 0.4809165 3.181899 13.13209
5.390794 4.525159 1.340768 13.23167 7.527701
19.00009 6.284746 10.75393 16.08402 13.45234
5.349347 2.381355 0.9333934 8.205733 23.78383
34.56104 3.416513 4.54727 20.0965 1.652592 10.30109
11.73114 12.50447 6.846493 14.9198 4.979053 6.325075
5.376364 7.203332 2.579289 5.048639 8.297811
1.079324 2.786164 3.160583 6.258006 7.867164
5.150305 5.994278 5.35636 10.08332 13.23012 4.377884
2.041459 5.264743 3.409591 4.233861 4.012453
6.133444 3.689863 6.706633 19.35874 7.212736
25.08218 4.05708 4.172304 15.60149 6.337862 19.04363
19.17421 6.349532 10.32212 10.52215 12.70833
3.769691 10.42249 8.190122 6.347781 1.751273
6.293542 11.25641 6.621743 5.268461 6.947471
4.572067 3.16434 69.08605 12.63134 15.03532 1.700737
9.58993 11.40479 5.418467 13.60916 12.93296 7.252196
3.046066 6.936202 19.06909 9.400759 10.25759
4.01329
IL6 0.1825733 3.538634 0.1356422 0.2414839 3.757923
85.35175 0.1084947 11.36977 3.609996 10.89175
0.934275 0.634607 2.754337 0.2501851 1.007423
52.01235 0.2241164 16.69142 4.994118 0.1649739
1.173387 7.336038 10.56822 3.049629 1.938198
2.65487 0.4668408 6.107739 0.569929 0.3983422 0.7739124
14.16249 0.3253544 1.349085 0.3430047 1.062138
0.2557381 0.1009086 14.17703 2.094253 7.167707
0.04952174 4.239078 0.7899924 0.6869337 0.8835605
0.5147577 0.9510146 6.406429 2.15928 1.54017 0.6558912
2.162076 1.119197 2.300136 0.448878 28.14693
12.55406 7.618456 1.780195 52.58543 0.5554292
8.770053 1.791319 0.4155234 21.43132 1.63842 0.5606716
1.015944 0.7701363 5.385611 0.3155467 1.347615

33.06578 14.54587 1.219161 1.886383 8.951222
0.6696536 8.804819 2.31983 6.650289 6.573931 0.8881228
10.43655 1.488982 0.5129926 0.6681603 4.426265
0.8712534 1.894719 5.590469 0.7318286 0.6757604
1.44678 0.717376 2.375514 0.2292215 1.750177 1.615703
0.7121356 0.07975367 8.138326 4.017808 4.28949 1.122959
2.664177 1.44233 1.291598 0.7208404 1.369318 0.3608503
22.5392 1.788204 2.064148 0.5686652 8.64262 1.27953
0.2230617 0.4701637 2.434375 0.6421296 1.617742
1.603526 4.198445 0.5925642 1.657066 1.359451
6.36776 5.075939 0.1972309 0.3437653 1.028874 15.52414
4.097211 0.5638613 2.57817 2.132125 0.4482257 1.455789
0.4400815 0.5175764 0.5462647 0.3918471 8.629368
0.1419702 0.5925086 1.374802 1.398605 0.5213083
0.1362823 29.59616 0.1208986 0.1395754 1.231579
2.866377 2.974093 6.842959 21.12732 0.9397507
0.8996529 0.9232053 1.177689 1.226506 9.074591
0.7079364 0.5264781 6.418578 1.817011 0.2334149
6.591005 10.72744 0.179808 3.572787 0.03646002
10.36996 1.541984 0.9505132 0.2094152 14.09797
1.511373 0.1489323 0.8964872 1.635529 16.90055
31.34734 0.8479116 0.4940484 1.389877 0.6981703
10.10492 8.901918 2.939418 0.2254398 0.6761854
2.187722 11.87021 0.5231638 0.6869045 0.60684 0.669891
0.6904756 2.375036 1.180873 4.357861 0.3484465
0.7869037 19.24127 0.8110181 0.1976887 3.682568
9.433917 0.1564304 0.2633223 2.48663 5.504806 0.1953844
0.6007934 6.304544 0.1601624 0.1245029 1.458362
0.9065891 3.46084 1.255257 0.7910084 3.661439 0.3107007
0.2227518 0.7859649 0.6733976 5.568989 0.02747671
1.678685 0.3113516 0.1670982 2.336566 0.3334832
2.140843 0.1649059 0.3891835 0.5769907 5.736284
2.123145 7.244659 1.058775 1.356234 0.6395415
0.5013054 2.964132 0.2588062 4.588072 0.7977197
9.904116 0.5668262 2.977164 0.3369944 4.702135
3.823782 6.406949 2.245339 3.206286 2.780109
2.945085 14.89394 1.249803 2.42629 7.058671 0.5072739
2.693618 1.069096 2.652782 3.655658 1.880425
0.1613002 0.9181735 1.686241 0.4159116 0.8055609
6.380124 0.9620898 0.3363352 0.507524 7.900966
6.64515 3.187126 0.08304079 0.2242731 25.50676 9.47835
5.67746 0.9970039 32.63817 2.741054 1.947616 3.654089
2.026353 2.13183 4.143907 0.509373 0.6796437 2.819444
4.000015 117.1911 0.3437077 0.7221484 4.537089

14.17818 1.910949 1.340371 0.9633762 5.6534 13.43974
4.799026 1.853028 4.457777 0.7303777 0.3789489
17.36942 4.629099 0.4302984 2.992867 5.839382
0.7682033 14.39889 3.428128 1.653885 1.548725
3.318335 2.481308 0.4201382 4.473238 0.4369871
1.27797 0.4240175 0.4164507 0.07609985 0.208891 0.4144409
1.934098 0.09825133 1.280523 2.16223 6.776513 2.206701
0.09571952 0.135943 4.962744 0.3446021 1.309959
0.502921 1.973481 0.2455026 68.87625 1.704612
0.109546 0.4768119 0.6071703 0.4151829 0.885566
1.098949 3.269744 0.2249889 1.454458 21.37582
2.170116 0.07677281 2.171897 0.1280011 4.859913
0.4949443 7.139369 0.0400665 1.822716 5.686266
1.762599 0.2420137 15.48463 0.2966447 1.809368
1.869105 4.272067 0.4020503 22.44909 0.3789305
1.541165 2.734222 0.7477733 8.792173 0.9309327
0.4287548 3.983086 0.380961 0.5955282 0.7541891
5.971814 0.2325791 1.262317 0.9209646 0.6031364
0.5739879 11.04048 0.3122942 0.0802056 1.797084
9.22093 0.234106
CCR7 0.9268656 0.1827982 2.019517 1.604784 5.84315
0.2698669 2.524007 1.464913 8.844877 22.66637
0.2983508 0.1479529 34.30691 0.3150205 15.2806 0.1030001
10.52701 1.980193 1.001376 2.400255 1.631381
0.0750002 1.127564 0.2425724 0.05157859 0.3415013
6.308715 0.1878039 1.959036 0.5520756 0.9167442
0.2388625 1.118352 4.944377 0.3584226 0.2158807
0.3857806 4.053021 3.298037 1.245772 0.2993961
0.6996155 1.055151 0.06330919 0.8709175 0.5924994
8.735752 2.920266 2.925652 4.367705 0.1538069
28.89607 0.5344664 1.803034 3.964463 0.1353043
1.471457 24.98293 0.4414548 6.79321 3.584381 2.133032
3.41554 1.572669 0.7971866 1.311082 1.193546 12.30686
2.566721 2.035489 1.578274 0.7893375 2.56729 1.193748
2.686649 10.35094 6.386678 1.211872 1.004033
0.2837353 0.8964487 3.822006 2.494438 1.155587
0.3600103 1.194697 1.162306 6.958076 2.063887
2.456816 0.5749857 5.808553 1.024361 10.74852
5.840991 4.6516 8.512641 2.555881 13.46599 4.016286
23.35428 0.880622 4.377981 3.126917 1.715532
13.70368 4.144975 6.014227 2.940416 0.2548563
0.6174392 0.9814372 2.409384 33.17597 3.344132
0.4415304 1.425963 0.1652183 0.9158057 0.8181555
1.672553 3.838994 0.9822457 1.505677 0.4677462

43.31026 1.780297 0.3445927 2.945841 2.021341
1.234909 0.07914213 1.072764 1.792017 5.387337
2.185528 1.823833 0.3929499 1.313315 1.824255
7.142608 0.7136664 2.933709 2.092029 2.230803
0.267033 0.1879985 1.4957 0.2246323 4.470346 1.038616
3.370879 0.1364816 8.572479 4.258747 0.6730148
2.117056 1.838281 0.4719391 4.08948 25.03322 11.73772
3.495196 1.976207 2.526317 6.056326 7.706931
2.273957 17.52208 1.355038 5.274723 2.714267
3.096481 0.2593718 0.5714832 0.5277908 1.529852
0.1501536 1.738738 1.072532 0.3087924 0.3042327
8.75802 5.343016 1.334899 11.7241 3.483928 0.141082
1.953986 0.8163644 2.364727 0.2441805 0.5988864
7.740263 4.661832 2.032916 3.264149 5.063981
0.8204893 0.338256 0.5080662 0.906637 1.101757
0.1655149 0.5699487 1.257613 1.298328 2.341959
0.4624369 0.3479152 3.682615 2.690663 3.113303
0.2555652 2.162556 1.018739 1.122091 0.9040669
7.592121 1.123084 0.7459983 1.844332 1.653892
0.3029347 84.7069 0.6009097 1.567093 1.025263 0.3251163
0.4747139 0.2614241 1.14518 0.2493391 0.8958097 0.9232813
0.9811632 56.73408 1.350988 1.053125 0.5855671
0.6898471 8.96843 4.609291 0.6173356 1.927726 7.221236
5.111935 12.20429 4.279049 3.91577 6.332239 1.222436
0.172613 4.515829 0.5958912 3.128445 0.2540408
7.541977 2.431116 1.331157 3.242879 0.9817548
3.625177 0.1136995 3.015319 2.38813 0.4966358 4.342133
1.784556 1.7789 3.881686 1.857812 0.3961738 1.06948
0.7177512 1.856058 1.380085 3.097268 3.968701
1.201867 20.86525 0.4569981 0.5914786 5.562438
0.629773 7.854046 1.360973 0.1363279 4.121799
1.668486 5.78682 2.413433 1.569523 2.794483 8.601643
6.294073 2.054865 6.422419 0.6010651 1.329849
3.939129 3.667811 0.9781002 3.487677 0.3226548
0.5230919 0.6645716 0.9880912 5.242989 0.1051244
8.105516 0.9400532 0.8473632 29.07535 2.028289
1.33683 5.41645 0.6379092 1.54877 2.3456 1.744027 2.386004
0.6835298 2.522991 2.210095 4.429255 0.5564567
0.4791135 3.605288 2.750464 1.092663 1.109919
9.470198 4.872297 3.22381 1.446257 0.188338 0.7363053
9.665841 1.257809 0.1458962 6.502606 0.191544
0.2199907 4.135 1.348042 0.6881783 0.3794936 0.1843998
1.424512 1.71275 2.026141 0.5186741 1.256228 1.784466
0.05750888 16.39141 0.4007131 6.652586 0.1747958

17.88037 8.207828 0.1798067 1.698407 2.453203
1.278757 0.1836701 9.22152 0.653882 1.227861 0.9217892
1.052236 0.07161541 0.2353069 3.483636 0.3599193
3.070318 2.431126 0.4295087 0.2448889 4.546872
0.8660988 0.4145943 0.1383781 1.452941 3.002845
6.457121 4.026876 0.3880543 0.5279879 4.086733
0.3878193 0.7907632 7.56582 4.12064 3.303744 2.784686
2.236617 0.5586464 0.4806969 1.828505 43.79287
0.6870141 0.5469758 0.4447568 3.84636 2.71299
MSLN 5.950024 0.06310066 5.392129 1.313268 8.107403
1.809964 3.313606 0.8897293 7.844444 1.49508 2.42624
0.1080375 3.49022 0.2474838 5.809405 0.18333 4.82652
10.21446 2.23388 7.786886 4.009753 0.3121682 53.50477
0.1184242 0.6628778 0.7304871 3.159758 0.02608956
4.20818 2.64533 1.911437 0.6506669 2.017097 69.6593
204.4104 0.229041 0.08583144 2.306497 17.06606
14.22511 180.8514 47.71568 18.47586 83.51883
26.88621 115.2776 38.20008 8.535157 62.14131
142.8499 54.69509 16.76855 0.2772775 16.09882 3.3774
58.40044 0.07266723 12.92052 4.234758 70.76384
175.7669 122.7282 28.58926 5.064293 0.9459425
3.89026 255.235 23.47092 23.49244 0.3111577 0.2442247
0.1235553 15.03047 61.50569 7.415799 281.0838
90.1514 36.64291 294.0364 1.158181 0.793612 3.628157
0.09424566 16.25674 140.1932 109.0816 27.64044
89.50954 1.16799 90.50953 1.98192 0.1289453 16.65304
15.72283 7.620854 0.117976 92.64236 2.177254
1.836692 114.1713 5.452938 366.1782 0.1520464
0.4419502 0.1588801 14.02909 187.7096 28.25844
66.98588 58.13655 15.08934 0.2781732 6.446573
38.30727 40.50921 17.16713 102.5874 1.964839
0.1990583 7.582497 0.9560479 33.89302 17.26004
53.58894 86.99141 1.445483 39.09135 38.70666
7.895205 0.8194433 310.76 0.9578834 108.5834 51.22777
24.14443 18.07635 38.97846 1.651945 47.53818
5.923961 2.109023 109.1885 6.814698 1.980134
0.9083093 789.686 7.045733 113.6577 149.3886 0.8487227
0.7064196 111.2553 12.77156 7.312444 1.426615
212.2118 66.23103 43.71728 38.51279 31.66367
66.15999 3.572369 4.228946 16.25345 30.53959
8.822355 19.52699 14.29317 12.69409 0.575455
0.2088222 46.75867 11.20621 8.723905 81.23766
19.14338 45.24879 49.27854 61.11798 11.42855
9.916668 207.5711 8.880392 0.8914463 9.237441

78.9594 101.2559 257.0942 71.91407 0.2044371 1.216714
115.8168 27.01544 6.254779 0.2647668 16.29028
2.118427 6.451401 103.7977 5.156865 1.751501
0.2343139 95.23411 16.15195 67.33885 119.9755
5.300982 0.1393444 101.7926 42.21254 44.10943
71.87985 24.16893 103.6643 186.3576 45.63991
121.8836 50.44726 1.417215 18.76832 34.03796
1.484112 5.63356 272.8173 16.00157 6.056384 33.02837
1.792851 118.3181 125.2001 81.14991 1.752146
28.41748 103.711 20.7581 153.2268 9.275783 70.52172
28.88606 91.93152 19.12681 6.832889 21.01704
53.34189 35.56649 105.2435 158.9842 42.7968 82.41012
26.93768 63.74902 51.74797 82.91224 152.52 0.3692556
0.2217252 4.814022 25.91312 23.67185 18.04141
30.57746 30.3542 30.8524 2.789209 33.6606 9.528116
68.24551 12.99985 8.470224 78.73095 0.9876293
112.7574 38.94349 0.5830075 7.674729 10.35745
136.6093 7.605727 19.22618 68.09391 64.33591
0.398959 249.7447 13.43389 99.09558 0.5013129
18.39071 6.433696 162.4152 29.00615 2.733347
9.98477 2.712647 1.602898 79.66507 4.315682 17.41345
204.0966 98.6345 38.43515 52.9717 38.96078 2.499425
22.79655 108.8351 24.44644 64.85412 29.2374 29.22037
184.169 22.616 71.6805 0.6738036 9.566996 61.88938
45.87738 4.039932 444.7821 36.99673 86.05993
7.450073 12.13246 1.447065 0.8286112 4.999009
20.07552 45.93602 0.6497333 59.22228 0.6678039
88.46685 46.01728 12.31966 79.50444 29.78633
12.69804 0.1679502 20.79531 11.40349 247.404 78.82756
1.834755 317.0303 5.276104 11.47958 23.60162
36.57369 0.05933463 48.52454 36.92142 5.655238
1.167563 0.5955301 16.7476 70.13359 5.30323 74.01513
3.280857 0.6377699 13.04893 26.1726 1.170559 30.14003
0.2245942 0.3219194 0.8497294 46.46247 101.5915
175.0694 0.5219795 11.55166 6.630065 3.299797
22.82211 2.221462 0.1093326 108.0756 3.646084
255.7665 173.4324 1.518683 77.30594 0.6165874
0.240826 1.450302 115.0937 18.72814 55.14356
12.69802 29.95102 48.65884 36.33301 5.196851
101.0889 399.8011 3.025655 14.12793 50.93192
3.489455 108.7154 24.89543 0.3987544 229.0011
64.62784 10.3517 73.78096 0.2226184
RAC1 157.636 78.62248 149.965 114.2958 130.6432 86.20268
91.79421 65.41647 121.0151 87.16753 129.2121

176.1375 84.10947 139.7712 103.4451 95.75168
114.9843 83.68682 145.2052 127.8671 151.8025
109.3001 173.5487 95.94174 90.88562 143.0849
126.8332 84.48957 113.4302 108.2907 123.7644
100.8775 145.9473 172.8941 158.2947 219.0139
61.04883 170.0779 126.3871 195.7955 111.3013
200.6271 167.3452 184.5438 176.7898 154.5566
120.8126 142.462 164.3238 291.9378 197.6026 111.4028
119.1614 210.401 144.6747 142.6978 108.8994 77.25134
151.8273 106.4389 114.3455 144.0837 117.5342
129.1297 156.8593 121.0631 146.2349 104.2115
219.1305 212.7437 137.4936 192.1963 124.4625
126.0311 142.0348 121.9269 139.1071 76.79158
141.502 139.7622 194.9874 110.8467 134.8784 156.0848
191.6622 121.6442 104.7873 112.3337 148.5151
252.9061 162.819 103.8577 217.6884 158.1816 121.6138
140.8704 197.6493 159.0886 109.2179 143.6508
92.65685 124.1674 266.4606 229.4545 196.2998
96.47761 190.6692 97.80584 125.2627 220.1444
129.7229 197.7797 207.4704 109.5896 179.7437
126.4848 135.5709 120.0034 111.7269 195.2215
213.662 146.5242 101.8407 172.7961 72.31688 101.8806
135.1951 168.4576 95.62866 212.1856 169.0433
180.094 185.4654 199.2058 185.0201 118.4219 162.4095
197.82 256.4191 98.72107 133.4258 247.26 109.1277
213.9443 127.269 193.4396 194.4628 157.0087 76.28973
148.3955 198.7282 139.4714 188.9044 115.1416
100.1614 100.2779 113.735 140.2648 194.7802 305.3101
90.78007 121.9522 151.462 190.1549 137.594 136.1506
129.5607 112.7029 109.1932 130.5473 85.55784
101.2569 194.1053 261.8995 259.7278 341.5426
147.9601 168.8157 150.3693 96.7577 211.8172 130.2887
151.5807 167.6437 157.0786 78.98753 118.8307
211.4846 101.2228 233.4352 197.584 151.8868 126.4062
113.9233 225.8385 135.4264 145.3959 92.4801 125.7836
331.3126 172.4178 214.6905 167.9241 141.1013
198.3821 200.2346 193.3247 82.14921 162.5719
157.0266 121.2343 101.3503 186.6547 167.356 253.9228
123.7712 148.276 103.5424 123.041 161.1605 184.9883
154.6898 94.12896 196.8534 119.5328 150.3406
141.9365 137.3012 224.8754 131.7134 171.5979
163.2891 125.3034 194.0893 130.7908 242.3702
118.7664 205.0139 151.7929 180.9564 170.1357
189.073 126.4904 137.8166 133.1131 141.7738 181.5109

109.747 102.9887 283.697 148.0245 217.3263 370.7967
178.062 153.0136 96.92611 108.1772 178.2334 161.8007
123.2878 184.929 169.4059 137.8992 243.0106 138.8939
123.3807 122.0569 125.8308 235.0231 226.842 212.8344
209.8079 241.4692 132.1155 234.5321 139.5202
126.3007 88.69376 95.7511 196.6218 117.1989 142.6511
162.6951 133.6722 259.9942 149.1903 132.7189
208.7491 126.9643 105.5635 104.1461 84.59255
91.99396 151.7249 98.70393 98.92481 252.6615
179.3932 254.8268 195.5608 175.9668 113.6841
283.4055 99.17651 245.6902 219.2376 82.78588
161.1794 141.6747 129.7626 158.6429 199.7549
158.668 113.9638 130.3015 78.60123 108.5589 163.9943
114.9296 183.3717 161.5574 79.05607 143.7403
135.7109 137.4821 168.8689 125.5731 110.3849
167.0347 136.8574 228.4895 123.8173 137.5624
149.8986 86.43633 123.9115 151.4383 244.5079
191.7409 118.4873 45.18683 149.1742 72.00004
143.8228 88.82219 172.6136 211.0276 100.0169 191.98
210.9836 168.5145 94.31675 138.5655 235.123 112.5077
251.6555 128.1012 123.7611 121.9068 153.883 215.361
96.64429 151.8786 184.7467 238.7345 162.6487
150.7172 136.9058 140.5162 279.9141 166.9912
348.2798 157.2765 109.4056 103.04 129.0127 220.4199
227.7671 125.6027 202.9379 163.1213 117.5337
175.1926 112.7771 84.12615 169.5089 94.08295
257.6639 162.2389 97.59875 101.3608 119.0272
216.5054 162.2006 152.5513 141.6974 152.0741
128.8882 87.08864 269.4774 147.1671 190.5351
129.9052 170.3365 178.2578 144.8633 110.5921
GRHL2 1.810084 0.08482319 3.429206 6.982821 3.345933
0.2063791 3.163401 3.037102 3.88633 2.375972 13.61116
9.481536 2.932219 12.2912 3.455168 0.09058398 2.754635
2.700463 1.509713 1.81777 7.272738 0.1146081 6.313573
0.1204696 0.4614103 9.816131 4.034044 0.1573453
3.715641 2.322908 5.874003 0.5458567 10.65695
9.33288 5.074697 7.625833 0.07982985 4.517537 7.369881
13.39863 7.908845 11.36829 7.456103 6.092556
6.115121 6.176183 7.632491 10.40311 3.617796
7.303262 5.056399 3.510558 0.8179072 3.446246
3.571293 8.770932 5.941946 1.103499 7.947498
5.687899 5.294101 8.562963 2.320913 1.938574
6.130002 1.008858 4.259201 2.163818 3.278333
5.938743 11.87612 6.352812 7.5727 11.95547 8.523946

5.467551 3.774935 5.017421 7.209184 9.427047
6.394634 4.457269 5.141214 5.64289 4.250127 6.088797
9.1125 9.139722 5.026421 3.915689 4.29491 5.916145
3.084341 3.926246 3.951667 6.554217 5.440282
7.543312 12.33315 8.690024 3.28985 11.75474 4.673318
7.264271 5.212383 4.781646 6.906742 4.345403
16.20588 6.402023 13.10194 4.342842 4.203181
1.69679 4.984123 3.719366 8.444527 7.521524 5.300919
5.164323 3.771153 7.497848 6.864335 2.568234
12.58514 2.081263 12.24046 3.767593 3.160071
10.57344 8.774712 9.209297 5.327708 6.075706
3.928617 8.284173 7.292736 0.3046025 0.06068543
11.10777 5.990492 7.93935 4.127361 6.780076 11.55864
4.978976 7.344821 3.502955 8.200081 4.519873
5.080567 5.711449 10.58432 4.927392 1.660914
3.018715 8.371795 2.830265 6.950604 7.042633
5.246994 3.644665 6.136706 7.624785 4.406647
11.30104 6.260644 13.52275 8.672014 0.593265
1.365615 5.357209 8.28252 7.892222 4.553533 5.694433
5.25767 4.491542 12.6529 7.781762 7.564151 10.51592
11.02149 6.843762 3.042931 9.887964 3.610261
5.430121 7.794165 9.478783 5.138014 7.677795
14.30761 5.955971 4.83987 7.42524 5.35614 7.349541
7.568232 8.47506 2.185691 4.031181 14.75572 8.959828
10.60098 10.13258 2.698195 0.08836435 8.457577
11.4201 6.694352 7.443281 5.803704 14.0064 2.63668
10.9935 7.771478 9.697729 7.778609 8.637349 10.94908
15.02331 3.412345 6.337345 4.074538 6.069671
8.416416 11.21648 10.735 7.278816 6.437503 2.330699
8.661858 6.102095 12.05515 5.936612 3.156458
5.965323 8.308177 10.8614 5.451454 7.437788 5.989888
7.403308 5.912307 4.418417 7.280969 0.9765841
5.239447 7.973009 7.323219 13.82214 10.2424 1.169166
11.05663 3.251503 12.94185 3.444401 7.441421
4.409632 4.127349 5.896538 5.278679 6.981598
9.938115 8.745662 13.47211 4.400013 5.693472
9.791451 8.799575 5.538519 4.781639 7.449983
8.74961 18.55287 10.5836 0.8366497 0.0257041 7.85273
3.569609 8.070275 2.227865 4.447297 7.094971
9.797962 10.94565 10.03485 6.865586 4.277725
5.894496 0.4273587 0.5291289 6.277131 0 1.235385
3.505148 9.968816 7.70177 9.432043 5.354847 4.114902
6.687343 5.634535 9.563894 10.28516 8.474362
7.979177 7.393556 9.695596 6.877315 21.12038

5.558679 1.376821 9.384957 2.990013 3.575106
8.768145 5.881322 6.921998 11.95098 7.911249
9.934707 11.54029 3.025077 5.723051 9.501923
10.60361 4.123652 11.49256 7.556298 5.758265
5.716803 7.481593 1.713736 8.379697 6.443523
10.67414 6.998159 7.744109 8.06419 5.463317 4.51111
12.92848 2.572357 11.65993 10.88187 3.900575
4.553519 6.216709 12.82958 6.066516 5.761044
4.17923 7.561818 7.072209 7.918863 20.96144 2.104463
9.159511 8.226071 5.278756 18.3109 5.808585 9.047991
5.579903 12.38503 4.79559 8.300026 7.584906 6.6262
5.292978 4.881291 6.386993 15.22744 8.53224 6.849199
3.715012 7.378986 6.628776 4.634741 10.47307
6.909139 7.046516 10.88929 5.265293 4.13508 2.616867
12.92363 10.37609 4.20956 11.55917 8.999824 8.530924
7.465232 9.784872 9.958062 5.768325 6.168474
8.310187 9.08097 6.669631 7.502721 17.31564 6.487817
8.576025 4.603488
NOTCH1 2.082239 9.381766 5.984034 4.155156 6.241656
18.62582 2.890009 9.183772 9.591638 5.622961
16.8069 4.928669 5.71798 9.284414 7.398241 21.47364
4.728488 23.12589 3.170181 6.341069 4.80104 12.36771
9.952499 20.33418 11.72329 10.7575 4.685617 21.66344
5.836809 0.6172933 7.708585 14.88572 6.422995
6.340396 7.162612 2.375763 5.228486 8.427537
13.14144 13.05801 29.64733 15.14092 7.796612
19.41919 11.73233 14.42793 10.08329 23.45601
9.212945 12.05269 7.58628 15.49859 2.295385 10.69498
12.58671 24.17268 23.48665 24.96178 28.38698
10.39841 16.5181 26.05836 8.803492 24.11471 3.901554
9.270939 13.66406 9.205686 5.299587 8.238456
18.05135 7.601282 16.40006 18.55466 16.52123
19.96988 14.8674 76.20341 32.66676 12.85216 19.59318
11.1541 5.722459 14.72452 10.96027 16.21565 24.64776
18.88212 6.397744 10.85256 9.587875 17.48522 8.2682
8.211477 15.46185 17.70285 14.71009 6.415228
34.91099 21.29512 32.35578 7.540063 7.010212
9.572694 15.30709 28.43244 15.02123 15.47191
12.69553 8.247541 13.37676 7.850675 5.772903
14.21539 9.327429 6.92099 31.31745 16.58496 9.856235
11.56117 14.2452 5.992627 21.39711 8.621575 62.27116
15.23747 4.803011 11.6642 24.594 28.06043 20.72231
45.44341 5.910178 7.193888 11.0292 23.82777 26.90279
10.81879 11.91591 21.46376 13.0004 17.04002 15.08068

13.71605 22.54991 4.539237 6.297486 10.65109
80.44386 13.09163 21.99428 21.24373 10.57881
27.29101 25.61354 5.019067 15.81999 8.805228
11.40991 5.613605 15.84324 7.939575 14.78987
20.37512 4.675662 30.49419 28.26422 8.758857
22.58201 34.75013 13.81577 25.74378 18.2042 9.555856
3.646572 3.471769 10.62762 6.742282 9.059442
44.59076 8.135851 17.3333 14.44085 14.03432 8.653004
19.005 16.9705 7.650082 33.84037 13.37212 14.82659
16.9372 7.705581 7.960509 5.249388 24.42541 8.672969
15.47387 12.44124 10.58543 5.115835 11.74469
43.93809 35.78864 10.95946 9.832601 8.75679 19.20684
36.5176 13.41589 19.74967 23.80079 16.28236 43.86147
23.53175 28.9095 13.25522 16.81247 15.45328 15.65864
8.549041 9.864261 8.947717 25.61783 26.35723
9.85185 16.85945 18.81741 13.48281 5.841519 14.51658
11.61602 7.648955 8.395157 5.776462 5.497945
15.05626 7.483644 11.14823 13.45453 14.0081 14.88411
25.12099 14.50827 7.025717 24.26222 9.005167
18.08915 13.18461 13.64657 18.76154 14.96627
8.412891 22.94251 13.05393 50.56828 18.06861
10.76907 20.23411 16.33606 5.569684 20.15756
12.1152 8.682957 31.63418 15.08833 9.581251 16.68229
6.564265 6.776544 17.83954 12.23358 2.696843
12.55813 8.051531 21.59525 16.26309 15.97058
6.531803 4.578092 22.48639 16.64289 9.109895
21.89147 15.77089 14.94291 22.56735 7.361255
16.46537 25.71981 12.43871 12.44179 18.04988
20.92763 14.4372 30.73566 8.616592 18.9023 17.55244
5.566883 11.72731 23.75824 24.07631 19.34667
6.439446 10.28714 8.294895 10.36328 9.233771
15.17412 9.824677 5.575407 15.62268 19.72318
20.78612 21.78572 11.42621 15.9593 9.966784 10.33482
9.780859 8.51987 15.01149 16.01736 3.754778 4.550183
23.60171 14.39569 13.11503 10.38565 3.285173
31.96763 17.53375 10.68226 20.09161 32.13414
20.61722 3.478349 18.93478 4.861287 46.66227
10.98256 14.75395 21.25021 23.10559 9.685986
4.989704 3.411321 5.115853 5.586203 20.60456
16.98037 11.53121 8.373455 14.47812 11.05516
18.26445 16.86212 19.67291 15.08281 12.94746
25.04144 10.2123 5.132856 66.9881 10.93362 9.659195
11.5497 7.245624 5.321732 14.65673 4.849139 68.56951
15.43701 13.56163 19.43132 6.241957 15.23111

8.609499 15.13104 18.11863 25.4834 13.81843 46.32632
30.62577 8.941168 17.55849 2.875229 50.17727
77.91341 17.38467 6.286253 5.015731 7.381128
22.20438 5.541692 29.08088 18.70342 3.051756
20.26439 18.10589 67.08073 39.03852 5.02987 11.80151
23.65701 14.06779
RHOG 74.3226 24.66688 51.26609 46.07513 56.79588
26.79331 45.27301 21.3306 53.3932 69.72621 35.91554
40.82909 72.53273 55.79484 61.03566 22.97061
81.05588 33.14247 90.20811 74.61501 49.1405 27.04665
56.7669 28.25801 27.01542 37.17524 61.67502 25.18099
60.23218 25.06735 44.97382 31.19867 71.31688
54.12934 65.89003 39.74839 21.14113 47.03261
42.10938 42.35358 25.98682 51.32367 56.95714
38.09365 35.89895 55.78651 34.06673 54.42031
57.12318 30.83338 39.78323 54.82985 29.5287 47.55247
78.60739 31.93181 46.4824 41.07846 47.25202 43.41604
63.49824 66.31184 52.3823 61.5264 61.74181 30.02637
59.13805 57.97758 56.47175 31.95663 37.65024
41.39948 27.40565 27.16272 49.93607 74.52087
42.96199 32.53908 25.97236 37.72083 63.73553
43.48715 63.51555 42.18532 39.04021 38.34125
28.84946 50.91878 73.8751 59.0115 54.10902 45.44499
69.17339 55.27954 81.84055 58.99523 73.92347
48.54886 31.43181 53.94715 55.73437 39.89566
93.45724 50.54127 40.17076 41.26705 57.46634
49.45726 46.27902 33.59698 38.74405 39.06213
78.67944 60.3627 63.20644 47.00732 60.37155 31.49483
36.11964 51.67152 52.9937 47.01336 38.88056 87.51889
33.54244 67.33697 46.83091 75.34862 45.28881
83.08172 57.77156 31.44776 52.06377 41.71732
72.06715 26.15579 44.10219 45.91114 46.45149
22.24071 44.79776 41.92807 48.05028 49.58162
52.92769 49.88572 35.35987 42.10119 27.08824
39.59725 34.84855 61.13636 35.33628 47.35237
29.88555 53.45314 41.87412 47.42897 91.9359 49.67243
78.91856 39.75009 39.49506 34.52556 53.16261
47.31732 50.64438 35.21184 40.71625 32.96254
27.07659 37.67518 37.40408 67.85629 42.06117
59.79351 33.39316 51.83389 57.99343 33.5767 41.48349
44.85179 48.76571 52.41072 43.83742 43.96127
42.37676 75.11079 26.80126 42.65945 50.42543
26.65953 53.76234 48.59789 67.99427 34.10537
78.37385 37.74925 39.72716 39.54187 30.37734

45.66905 32.34724 31.54766 42.79162 39.22389
62.14706 31.45842 20.63292 62.07951 29.15794
33.69179 66.72421 21.8778 41.68712 36.14413 40.72866
38.37839 54.80973 29.78698 43.60226 41.07607
29.43856 38.82728 65.20719 29.65322 37.37629
52.17448 38.90349 53.55519 47.27903 55.02654
30.32814 39.30898 27.44441 50.36278 83.05409
42.21088 41.54695 16.20792 29.13909 56.33847
40.11221 45.74558 53.93022 45.03763 50.11768
36.40092 37.73778 63.15074 48.67909 44.54811
32.29541 57.74739 36.8254 28.2011 33.16342 52.60836
72.51892 36.60372 40.97936 64.9641 45.52323 37.29343
34.77784 31.89917 30.53576 61.80333 38.05307
38.59141 51.7472 91.95762 36.16291 41.47482 38.99363
35.23661 37.70928 24.95399 207.3395 117.1424
48.90537 62.88258 70.73031 88.79695 27.54815
41.96981 32.93534 32.06951 44.52252 35.58843
43.62984 24.72313 32.45625 43.43646 96.58577
42.26015 53.83088 65.78457 43.2026 36.2917 40.71977
43.82392 38.77382 44.77603 28.89083 54.22002
18.84764 43.50577 39.47168 24.1437 59.76524 68.08004
64.29119 117.2013 32.56586 26.00767 41.69525
26.91016 52.25576 51.9169 52.2951 39.3528 52.42163
49.03911 42.44873 54.7869 47.04239 21.74911 50.0527
46.41506 51.29379 39.09275 57.11493 63.77799
47.51434 32.89511 54.94144 51.17467 48.10256
45.19599 15.3203 86.2209 19.21603 37.70948 26.20664
39.96913 36.72587 14.68466 57.07401 49.66544
43.23995 44.88143 22.81727 44.56005 51.15756
39.33607 40.13256 33.7205 45.09024 37.15364 78.89384
65.70402 20.35918 76.66962 46.82411 40.43918
26.47383 54.47438 25.95071 26.57519 32.94703
57.57714 34.09873 17.34214 62.77204 32.79425
38.37428 42.55722 34.18201 26.67 55.0014 29.59205
32.23855 29.19692 35.05421 78.06157 48.64711
92.77745 37.13021 36.95204 34.25456 26.52684
30.43424 48.45026 50.79517 56.8617 30.44121 32.64071
26.37059 49.82631 48.07535 56.59517 47.00385
28.99854 57.56459 60.68933 29.94439
CCAR2 5.716724 5.927876 8.191421 10.49074 6.737226
6.834376 6.582417 5.151215 8.156246 8.131908
6.857749 4.412145 8.416044 6.888097 8.888548
5.511892 11.90468 8.603571 5.938465 9.54553 10.16189
4.849663 6.837148 6.653274 7.063261 6.162569

7.553193 6.475464 8.632424 2.512621 10.70097
5.148288 8.119573 6.9891 7.590233 12.6196 2.64457
8.092062 14.15896 17.84793 9.243853 9.161698
12.6478 11.59563 9.195308 19.22142 18.32054 9.366412
10.96488 7.224547 11.48654 9.595643 15.3456 9.418218
5.614297 19.19134 13.54871 9.730729 7.226036
9.634166 8.373588 15.6258 6.490855 11.72956 12.61906
7.655632 9.814878 8.283581 14.90543 11.78317
22.07094 10.09175 6.139107 3.980797 7.957497
17.31189 10.65346 17.48433 11.58179 3.670217
15.25214 9.210244 9.012519 10.01665 8.42699 7.261779
10.75763 8.902589 12.18006 8.334192 7.428858
16.4038 8.350041 11.3297 13.35216 7.468958 11.2659
13.05095 19.46964 13.09688 10.04722 11.57609
10.17484 4.787586 13.04355 10.6156 12.80499 11.32655
16.71141 8.842662 11.58996 7.557475 8.264795
7.505709 6.985022 10.28863 7.165172 11.45912
6.215686 9.539822 22.06237 8.621243 9.812859
6.514173 20.45889 11.81677 7.923563 10.50688
8.815139 7.026034 13.35863 9.214595 7.714995
8.563334 7.963276 6.963351 16.45663 10.89801
9.301392 10.74836 7.791618 10.96199 7.972439
8.102586 9.95621 7.115486 12.84178 9.166704 11.98297
9.363548 7.42043 13.15682 11.28937 13.69878 8.914317
5.767019 19.10919 11.90573 16.34083 7.617877
13.17134 8.003571 6.523768 16.16003 8.094508
14.26386 11.78675 4.991767 16.92464 15.11072
6.452052 8.096657 10.72303 7.984912 12.96978 10.309
16.19039 11.38606 18.52661 17.04608 5.280015
14.26017 7.843884 13.69308 5.02219 7.189816 10.02776
10.25893 12.04241 21.62129 9.482895 6.783407
13.10967 11.52711 11.58515 6.398162 7.413736
15.33133 16.39023 9.701318 8.1838 6.806993 20.48869
4.225716 7.391841 12.9369 9.173895 13.12802 6.116232
10.04078 8.78265 8.48203 7.444531 9.699215 14.54013
18.33761 13.85622 32.55076 14.51949 14.09664
21.7662 8.870163 12.01037 8.945438 12.57196 13.8085
21.42938 6.462262 25.06423 15.96145 9.946657
6.376266 15.33615 10.17679 12.03064 12.6117 14.04805
10.02049 10.19111 8.034714 11.28438 13.85661
9.500727 14.87319 11.28923 9.09693 19.20692 8.374193
16.8231 12.63142 8.355038 11.66521 12.25726 12.71794
15.81703 11.08175 19.13784 7.594503 16.53167
11.54574 10.29 18.29986 9.658021 10.272 11.91251

9.538632	17.52621	12.79882	13.27682	7.554592
16.01837	6.91344 7.699541	9.945665	9.938138	7.09097
7.477416	6.39096 12.49425	7.500469	11.39094	8.903797
7.54053 7.827251	14.38381	15.58234	6.542191	13.8781
11.08255	17.79994	12.84817	5.230985	6.335725
12.79575	10.78059	9.128434	9.162129	8.725606
11.59868	14.34565	10.17557	7.932013	10.75671
11.23786	12.50107	13.4543 10.51877	11.52294	14.02671
7.508231	7.262375	12.11153	7.166072	10.82511
16.92742	9.540079	8.622909	8.187205	5.978808
8.743859	4.549718	6.734742	5.049485	26.80827
4.488546	8.237191	16.94956	28.11076	10.14418
15.5071 7.698127	10.8862 12.44035	13.22383	8.553089
12.46594	10.59178	5.584294	15.28312	8.681373
5.572055	16.47747	9.408511	16.2648 9.849116	6.785047
15.05846	10.42956	5.739056	10.13621	8.207532
21.21713	8.864113	8.806564	15.0316 11.92948	15.85737
8.860042	6.982986	10.37882	6.790068	10.986	4.59295
19.96932	18.64158	10.33103	14.50347	7.650046
9.462602	9.831734	7.586905	7.616957	14.77414
11.35386	19.88786	9.449518	10.33937	5.168267
9.16709 15.45144	16.62647	8.418916	11.00169	10.55657
10.23792	12.897	10.15175	6.597049	7.942548	24.51187
9.813664	11.15638	6.638642	14.39634	14.21928
5.002586	11.01314	12.36554	6.973261	21.33517
11.16809	12.05679	11.95561	17.16174	5.709459
9.04695 9.869878
NQO1	203.5346	3.522264	92.82505	233.7399	72.99417
7.677847	166.9977	54.78945	150.9902	74.94238
51.52841	47.34154	73.54072	27.32757	137.6023
4.452269	132.5255	54.6078 124.2495	99.89898	225.6235
4.019736	22.51044	5.539391	7.782585	30.44462
188.3827	3.265671	102.3895	292.1928	165.1114
49.65944	198.5186	88.36667	69.30653	5.304864
5.00364 184.864 136.1527	98.31673	161.8296	169.9737
55.89123	218.6454	140.4668	64.09411	85.48241
91.50919	30.30514	51.23389	130.658 17.77252	57.29272
179.1408	13.42368	192.8118	24.58344	6.535954
75.84408	179.1197	45.37318	46.89722	26.25836
10.32918	153.2687	9.787408	105.7662	45.64469
207.8634	262.6046	32.79061	108.8509	122.1817
216.5545	24.20238	56.65293	63.6596 127.8257	63.45589
85.45422	41.89067	53.01441	48.58082	64.29748
69.09614	66.46227	77.93806	16.94387	73.49469

56.85258	26.22427	27.74884	26.78264	105.16	48.97109
46.5117	50.06058	193.2458	35.2543	14.82142	8.480202
150.1094	29.18565	79.47813	39.59795	20.4976	58.49675
72.24931	161.0263	105.5359	42.1593	4.015208	6.934433
11.94587	65.9231	488.4147	46.31007	172.5718	178.775
60.68127	57.94986	7.815094	27.27061	46.68491
19.91831	65.51931	2.989332	12.72913	19.83886
44.34539	144.4033	52.92111	109.032	27.42127	81.64773
36.78545	83.74367	101.2718	24.19031	105.9036
208.8965	20.24386	83.12721	30.81929	47.2028	80.44439
91.43104	72.2295	42.87426	29.86674	20.85031	60.25281
63.73844	36.43048	7.96782	128.2926	62.46369	15.25221
23.83151	11.44259	31.46159	104.9554	86.56096
21.38096	123.9351	90.70635	106.4583	137.7114
117.5625	18.733	13.52696	58.85882	36.02279	107.7329
108.4986	136.6925	48.69394	40.9234	66.96741	12.41521
51.50675	115.5698	29.09581	54.73739	86.22459
38.80965	56.21561	71.9242	12.42376	33.27081	73.2026
99.20992	67.10221	104.2847	128.3297	44.05565
27.84804	60.33732	51.95582	106.2283	92.13329
144.3777	82.45289	90.88054	104.7355	95.0777	142.2524
2.962134	59.51461	90.99201	44.1972	37.17201	38.32248
183.512	38.22874	126.587	62.77528	11.20192	73.61951
85.92685	212.3027	71.95084	96.88395	26.43818
67.25344	54.93724	7.09483	30.44406	79.24363	30.46643
40.41908	40.03035	97.5849	157.4519	89.74353	90.16581
32.27302	44.14967	15.39185	7.718064	95.12999
122.8879	56.17817	35.24295	53.90736	161.0886
32.7391	13.5711	91.83801	94.3365	49.13044	97.80586
112.1281	20.64045	90.39192	11.70701	63.19919
150.1967	22.03248	72.5041	84.28105	66.58796	69.16779
1.40213	100.7083	20.75639	57.5715	38.91174	283.855
28.3725	164.2388	57.80981	176.4416	10.07109	79.39074
93.72964	177.1915	13.47267	2.43655	79.22794	30.73935
36.18832	55.67351	4.534079	42.89055	63.63491
65.59528	136.7686	45.36338	60.67695	18.8782	7.315619
11.78446	93.1817	19.51953	39.01327	10.88952	33.70645
44.58947	52.48697	97.33117	64.48284	29.41595
19.68308	165.9251	76.46788	176.6656	72.96784
73.13388	75.94043	81.5835	28.52666	84.90722	16.66656
79.15311	6.461095	101.449	104.0375	120.8011	44.19007
80.66378	56.12941	78.8107	61.37867	67.23674	70.66447
103.947	7.36789	99.13521	0.9947236	139.9847	17.40904
131.6131	146.3003	19.15647	99.85687	73.75466

234.4309 67.61174 136.2374 119.2017 114.9226
81.33103 43.34163 33.94817 38.36455 116.9414
1.132117 152.814 81.87556 64.20072 27.0195 137.2945
46.55483 104.9987 103.213 75.81394 77.72651 9.117703
29.36265 92.48132 19.37192 36.269 50.69424 22.52822
7.12627 131.1943 40.03194 87.06879 149.3325 73.44473
38.48731 45.52065 31.97267 24.89048 63.09866
124.6763 108.4905 103.8584 55.17242 66.731 11.71036
62.03452 52.98484 26.57766 47.41668 48.14565
106.7537 23.51379 51.66947 32.05821 15.46176
56.13428 159.9553 34.81368 83.54126 62.70172
26.38374 205.9518 125.695 78.42717 43.30137 96.59619
119.7214 81.27168 98.52796 73.3381
BIRC3 14.29672 6.56566 27.471 21.98832 18.80071 4.798853
20.9188 6.088989 22.70416 30.27984 3.345855 0.9112058
34.3522 1.679638 21.74514 9.616718 14.15515 8.062414
13.7823 10.07921 23.8986 7.034474 3.033823 5.969731
4.520037 1.717983 14.53937 8.109518 11.92418
5.077887 15.04551 6.586086 27.09504 8.752385
13.89367 28.61644 4.878389 9.534022 7.979606
4.116551 19.44818 18.88596 3.572272 28.71934
17.84816 5.941099 30.61302 8.894499 13.21333
10.92026 9.376322 29.23821 1.165154 89.16885
28.02207 1.518043 15.41864 22.01742 6.29769 22.76166
5.671572 39.15261 5.768324 1.820415 12.51784
6.06041 7.892128 11.17993 18.18137 14.36645 12.34248
33.37613 6.161443 16.52939 7.027254 116.5969
10.00178 3.984593 14.69077 13.17015 22.36862
13.18716 54.3232 9.854173 6.619705 34.24913 3.992773
58.08278 23.65434 8.5861 47.13431 64.07671 36.79911
27.79813 11.42438 16.28372 15.32861 18.13946
4.01383 22.65959 23.581 14.47264 58.90056 10.98308
6.873586 16.22196 28.51387 8.944341 21.23848
23.25165 15.76195 5.05541 35.88133 27.95525 24.24206
144.5034 12.73364 8.131249 1.707442 4.015194 14.949
4.956421 4.398701 5.704613 1.937495 39.94082
47.15719 14.71018 10.4395 10.35087 5.213583 1.438896
16.62342 9.769518 29.10101 8.70968 25.28567 10.57442
10.26182 5.281883 9.825963 51.33953 23.84019
3.513915 13.15655 7.632925 6.013226 5.617482
5.676493 5.446093 42.8887 12.1146 7.018649 55.96052
6.743455 29.7091 4.368727 10.66322 13.88174 18.24601
48.38492 19.84322 8.173824 18.3222 2.433104 26.95996
33.71622 6.65031 22.36552 2.148378 10.97265 16.47436

22.55864	3.478749	98.64247	20.14248	11.23722
37.8383	50.46922	2.965233	9.609359	1.377947	5.813041
75.2184	9.816509	13.85618	15.86934	13.64097	17.71785
33.58955	22.76458	23.86121	7.919053	9.341818
7.706136	14.89458	22.63888	10.85977	9.954157
5.371278	2.273255	8.530456	2.98015	2.662972	17.38268
17.44982	10.11083	4.169032	11.67026	24.6147	11.0097
8.199619	19.13599	1.786121	92.25183	18.08141
16.03203	3.530359	25.7504	5.291324	17.93989	40.74214
3.192775	8.308972	46.63634	18.64777	5.439318
9.405742	8.905394	3.617271	4.674504	12.66322
2.180307	17.41972	10.43052	23.91837	14.24425
31.97105	16.57977	1.312165	16.26559	34.57393
29.92536	9.37279	10.36602	9.910677	34.51219	13.272
15.8713	37.94328	31.24174	5.157521	19.01338	55.13359
30.66733	3.470954	5.580566	12.74425	17.27452
6.392415	22.91834	4.352401	37.75908	7.068967
22.28053	7.881531	35.9202	11.20353	15.21533	26.88263
15.62899	20.93282	29.35644	9.613577	6.939764
5.047953	13.40282	10.36495	37.06663	18.60002
17.05718	44.78753	9.934573	38.5443	9.354024	24.42058
7.145477	3.544105	6.902342	4.740584	26.94874
9.11668	9.44216	11.99062	12.91424	3.84235	26.79756
45.24922	4.207681	4.245059	16.37818	11.35328
7.244377	6.714581	4.815961	4.297948	5.071926
13.98099	34.79794	6.47875	10.42733	5.934112	18.11904
12.61967	20.99313	4.02013	39.57883	15.32345	37.03646
12.24232	18.20591	11.27739	5.353161	32.20677
12.41463	11.05769	14.97028	0.9691399	21.67531	20.622
7.807602	6.776646	59.26031	40.42028	5.329996
7.921016	5.12536	5.881207	27.4258	16.40859	4.026163
12.42563	18.64452	8.603754	12.36574	14.90803
35.22884	1.219817	13.45655	43.27746	35.51381
9.867233	9.132654	11.8608	22.21039	16.38941	21.73964
18.04369	15.84711	20.99522	26.78986	3.695828
1.393163	35.13971	10.72328	2.463107	2.9136	107.0564
28.36292	3.218139	9.01474	6.125271	3.116838	39.93262
22.49227	0.9509453	32.62319	15.08789	13.81523
2.954628	4.701482	9.69145	5.564608	10.29239	16.14199
22.42351	3.918698	40.24945	3.388667	2.163453
37.18627	3.963619	12.11442	9.496431	121.5011
34.87479	3.634571	16.94717	21.26787	2.644536
4.214021	24.28502	19.12569	7.264986	16.13759
7.175712	4.884212

MMP13 0.06151849 0 0.07073385 0.053869 0.06724321 0.01557545
0.04752482 0.01228817 0.08705944 0.07065295 0.004400517
0.01007181 0.05541687 0 0.01671551 0.04937389 0.0204524
0.01929965 0.04430931 0.08759377 0.02396216 0 0.1239171
0.06133396 0.007022371 0 0.03245937 0 0 0 0.04346194
0.06893011 0.03958823 0.3552161 0.3756229 0.2472381 0
0.322305 0.5115582 0.1315703 1.771893 0.501637
0.5709366 0.09887051 0.1425331 0.1735236 0.1133383
0.6722598 0.9970158 0.04094576 0.1301962 0.04353111
0.02199934 1.102293 0.1999102 0.06932315 1.467789
0.05255312 0.4420779 0.02780012 0.2968353 0.04893948
0.2859258 3.719364 0.141097 0.03126465 0.8254141
0.08290144 0.07211005 1.009898 0.4203301 0.003715631
0.8114179 1.864286 3.969314 0.09271511 0.2826003
3.517862 0.05432108 0.8085315 0.2495865 0.06159916
0.01647387 0.03162859 4.190369 0.3524231 0.2637445
0.2470673 0.5444299 0.2312983 0.6323499 0.00572426
0.05255227 0.0605866 0.02950395 0.09427141 0.2087094
0.03778786 0.07743883 0.4416876 0.1476271 0.1343659
0.08268482 0.1783321 0.8728277 0.119881 0.09614159
0.1340173 0.5485421 0.2050361 0.2173238 0.0576283
2.995916 0.005651527 3.43151 0.2279319 0.40726 0.2883645
0.07195664 0.0750858 0.1007992 0.2316401 0.2409352
0.3045661 1.685329 0.114368 0.3000964 4.502224
0.08072603 0.1138773 0.420897 0.4260661 0.1669208
9.778723 0.4469646 0.0261368 0.04388614 0.2428717
0.1410488 0.06054029 0.04889157 0.2375143 0.06380927
0.005397608 0.1838706 0.1368144 0.2595414 0.3287203
0.388549 0.0250387 0.04264061 0.3045974 0.5435183
0.005094944 0.2397682 1.49704 0.8291162 0.01821067 4.176511
0.1200636 0.06181682 0.1585879 0.04194085 0.02331841
2.071505 0.08139189 0.08995718 2.079459 0.03490878
0.8772841 0 0.1475455 0.02734818 0.04695051 0.1011489
1.080372 0.7538035 0.3395852 0.355091 0.8305119
0.3351443 0.02588811 0.01722349 0.06209009 9.872652
0.6017511 0.1317933 0.1734071 0.6468703 0.017729
1.477206 0.2917241 2.125178 0.039187 0.008815327
0.1390799 1.631017 0.04583314 0.03134275 0.712443
0.09465735 0.1176212 0.06031052 0.7867822 0.1745953
0.02782369 0.04941847 0.194856 0.6878747 0.5291927
1.285238 0.09807947 0.1475136 0.07915848 0.3886625
0.3812533 0.1042391 0.004061271 0.05907806 1.902749
0.4114195 0.4377688 0.03228623 0.3158292 0.08493622
0.1454102 0.2858346 0.2984621 0.006254731 1.229581

2.990224 4.190907 0.4279411 0.05662594 0.8355049
0.02870408 0.007384594 0.00434757 1.290882 0.1675017
0.1228665 0.3390467 0.26514 2.833253 1.663193 0.6941985
0.3584978 0.06670085 0.09709801 0.04020955 0.8433396
0.3066113 0.2673247 0 0 0.006263744 0.6320357 0.2231889
1.73272 0.1088937 0.1596968 1.084232 3.090315 2.41489
3.557948 0.1325761 0.5682514 0.07250283 0.8343341
0.259728 0.1028214 0.05573237 0.1359474 0.4029467
0.2390873 1.330416 0.09731722 0.06975557 0.1758835
0.531807 0.07682354 0.03249874 0.2587792 2.04084 0.9388564
0.03977443 0.08891674 0.1034107 1.009983 0.3086588
0.09514256 0.02656482 0.02110909 0.1310793 5.451861
0.3910335 0.0638929 0.05764338 0.2077826 0.1181249
0.1717557 0.0395841 4.065639 3.09578 0.1817068 0.09858476
0.05548347 1.421836 0.05400441 0.03790181 0.07992339
1.534855 0.696326 0.1998247 0.07918992 0.01708378
0.1459345 1.390761 3.07603 4.698629 0.1502433 0.0489632
0.0912154 0.1375638 1.386502 0.03680516 0.1941376
0.07403179 0.1047232 2.345136 0.01039755 0.4589397
0.07089519 0.0316271 0.05019887 0.04413418 0.02525351
0.1201544 0.01025655 0.1133734 0.8679295 0.1731227
0.0158916 0.3721915 0.1534316 0.1697118 0.04511229
0.8810855 0.2855789 0.5998945 0.02824341 3.37581 0.08305897
3.87881 0.03433034 0.01454101 0.1621302 1.625334 0.163213
0.5354609 0.1348408 0.07672888 0.9034091 0.08225574
0.3511923 13.51594 0.01120981 0.08570933 0.1437676
0.7039397 0.6186592 0.4624728 4.251159 0.5332953
0.07060094 0.2611841 0.1241573 0.1400364 0.1149144
0.386998 0.1862888 1.749019 0.04280533 59.34218
0.04286023 0.04441374 0.007028717 0.8340475 3.838176
0.1793494 0.07613956 1.054584 0.6459695 0.3912961
0.5596322 4.752636 0.02294561 0.3268627 0.009887686
2.350903 1.044396 0.1770187 0.2431943 0.1522435
0.3498628 0.01380895 0.01627736
FAS 4.723376 1.826991 6.51168 2.216514 5.151723 3.439028
1.777301 2.148213 4.001824 6.94943 5.954865 4.860448
8.005875 3.841323 3.969054 3.175352 2.648215
4.265277 3.555685 4.703314 2.748206 2.942503
3.538272 4.628299 2.663053 6.539757 2.855976
2.93121 4.11088 4.004644 1.569697 2.602521 2.996479
4.429423 4.769023 5.898238 2.672666 3.463863
1.831423 4.291675 4.781473 1.239777 6.207817
4.462316 4.928753 0.9565852 5.234775 2.820074
22.27436 2.721661 2.260169 8.325574 0.5620957

7.617476 7.212394 1.041197 3.505182 6.804674
0.7426209 1.623563 11.32447 9.407114 12.30411
2.659878 5.507954 6.799062 6.423426 6.565558
3.293063 2.542784 15.89418 12.80286 2.618803
4.433679 3.954495 9.974416 4.572613 2.446722
1.285069 1.998986 2.212456 5.753372 24.68956
5.590061 6.282397 3.093398 4.069641 11.6445 10.59397
4.030219 11.09268 12.25489 12.49859 8.386921
8.914614 9.876225 15.03756 1.758851 1.771985
9.85885 8.049472 1.707098 5.418898 3.88902 4.955155
8.867404 9.303055 3.614247 7.549053 8.702429
1.949127 2.10348 11.44866 8.060328 6.878271 1.138485
1.313526 3.526719 2.051707 1.689751 4.431409
4.051643 2.965931 6.158183 2.893084 10.32837
22.34351 4.205302 5.637702 15.43781 10.10304
3.534185 2.068327 12.29764 3.725271 4.719861
6.800963 3.249092 12.6553 2.057283 8.979266 4.961091
10.15606 1.816705 2.647388 3.488517 1.646671
5.206953 0.4721665 4.044393 8.388268 7.679538
1.295818 17.83351 4.891182 2.99347 8.053817 9.371166
3.784867 3.347773 17.21962 7.281433 6.53442 2.057073
1.52388 6.557805 6.40363 12.70248 5.434794 2.555907
3.843954 7.115701 12.63025 0.9340339 0.8959022
3.277101 4.736905 2.479027 2.569484 2.903797
2.682354 1.094138 6.057304 11.45832 4.243554
4.11787 7.835005 17.2269 2.92075 13.90633 3.831102
1.988488 2.503505 3.330474 34.27661 4.306001
10.77919 1.88231 13.57188 3.068583 0.3909685 2.537226
3.302075 2.273657 3.190163 7.08315 1.955714 2.79505
2.475671 2.299431 7.376963 3.165456 3.038648
0.9298055 11.20932 9.550808 5.269824 1.366926
11.84058 4.553887 1.775589 7.953118 1.410033
7.865017 15.91171 4.511054 1.769372 4.935606
10.50673 7.621844 1.368904 8.873424 7.892312
2.33419 15.68734 5.536384 5.578136 18.04721 15.26199
1.693402 1.705376 15.5256 4.576396 4.998727 16.09699
6.620816 11.30138 4.676077 3.633119 7.154419
8.68322 5.459082 2.039552 13.76219 34.79647 2.031213
1.766703 5.112538 6.816478 4.864739 2.584278
8.34269 11.55159 15.52109 5.500675 3.15218 1.391873
5.241396 2.51547 4.439747 6.47179 10.56218 2.413742
5.991167 1.083148 1.478464 2.684683 4.228318
32.5894 10.31986 7.644921 5.532968 4.551087 8.977558
5.495691 25.39884 1.633637 5.688351 5.633722

7.519413 2.349094 3.115089 4.425097 4.378237
16.21438 4.740986 7.315564 13.71385 5.792759
4.612546 7.860357 6.309548 22.09442 5.125742
5.325905 2.882033 2.43023 4.698151 3.72454 1.773989
2.819003 4.430338 2.218913 6.387803 8.666418
3.350878 3.610098 2.551123 3.481289 30.17744
4.360964 3.278095 7.647559 6.602766 8.543782
2.799967 3.289297 0.941252 4.527963 5.956615
0.6032353 3.761062 11.14865 12.72692 9.283651
2.788516 0.3842341 5.014298 13.27249 14.10031
5.456865 2.474435 8.413868 1.306828 7.309968
5.162822 1.539171 0.4888189 2.748347 2.819338
3.609984 3.830542 3.352528 4.097774 7.188752
13.48686 7.270018 3.905866 6.584504 1.74036 6.709187
5.889589 10.19232 1.953716 2.448453 3.622114
3.48438 8.115179 2.220579 1.328737 1.825878 5.186652
1.963049 5.827029 4.600608 2.38397 9.287523 2.475015
3.1484 9.171324 4.885041 4.154222 1.160662 0.8547698
3.11689 6.712079 3.824003 5.558472 6.605106 1.893197
3.955828 6.679662 1.523634 7.054209 8.566175
8.378072 7.264501 5.846768 0.9421613 1.600064
1.327681 9.331516 8.251909 4.595596 4.947549
3.36379 5.472312
MTA1 6.211057 9.387419 7.246017 6.943292 5.810578
9.952705 3.647808 6.146558 6.761746 7.09976 9.361354
4.753156 7.085704 7.436716 6.929632 8.716186
7.721625 10.47265 4.540102 5.131592 7.038492
8.713549 8.684403 11.06402 9.843565 7.30992 5.906182
9.339237 5.404771 1.68478 6.760326 9.013039 8.821956
10.58235 15.74173 11.65868 6.239393 6.689217
11.86439 12.29464 9.729993 13.87026 5.457399
15.12951 8.947207 13.66477 10.52516 10.30099
8.016818 12.11543 8.320812 8.571592 22.39082
6.356694 8.507128 15.67572 12.06846 10.2305 14.51364
10.91416 5.71609 12.80795 5.311442 8.524953 10.80036
8.260608 7.8829 5.619771 4.146961 14.34077 14.08647
5.52354 11.58899 6.736624 9.356384 8.034271 6.167962
20.73772 6.642513 5.681833 5.588331 8.422634
6.304458 9.738598 9.442204 7.526304 9.753572
13.53604 9.124047 6.582283 4.668884 12.91563
6.260779 9.29958 12.95384 10.47882 7.949187 9.63031
15.60882 11.99927 11.02224 11.2864 7.198731 7.963142
9.772652 11.56988 9.889721 9.10269 10.59573 9.840247
11.91449 10.06967 8.506245 8.976189 9.233149

17.52105 13.633 9.532358 11.86536 14.44674 12.06727
6.948994 21.12052 4.758826 9.532912 12.32606
11.97046 7.543167 10.27183 11.75793 9.680779
18.49254 11.85435 7.328242 5.4179 10.44192 11.93066
16.82885 8.804898 10.67291 20.60962 13.24033
9.962046 12.60022 24.35285 6.362179 15.81685
7.571146 15.39206 8.87658 8.375043 8.947836 11.34144
8.919225 10.32002 9.253566 14.92559 9.236188
21.22757 5.86848 8.660733 7.802168 6.277025 6.9917
8.18605 7.683854 7.98825 5.09545 20.18395 18.86735
9.300853 5.712111 5.879056 9.31281 14.43957 6.521915
9.460437 11.34069 8.424991 13.02768 6.418835
10.5195 7.651739 13.17228 9.863939 10.58878 9.872193
7.559898 9.131768 9.720976 7.592693 17.00684
11.82104 7.221985 8.639559 11.51907 5.632195
17.93995 13.02755 6.605553 14.01915 10.00624
9.078439 11.72241 18.08138 11.19024 6.712581 13.945
12.86249 11.17567 8.107271 8.822772 7.910159
10.87815 17.32123 11.76255 6.660278 17.21324
9.620521 9.28273 5.842699 8.685068 7.578958 15.06468
10.79385 13.01639 12.84971 8.956257 12.80204
6.769836 8.510952 5.452119 9.812205 3.111682
7.975415 10.21513 9.045941 9.634304 7.253518
13.49702 10.97196 7.828883 8.646014 9.13859 7.886906
9.950903 8.427976 12.40747 9.875673 8.179289
5.750182 7.980985 10.86789 12.12585 9.767591
22.71554 11.76945 5.94528 14.31787 15.25786 6.949873
11.10641 5.614974 10.09604 7.894736 10.99667
21.05396 7.551119 10.55712 6.208416 8.784541
10.88908 11.02083 9.136355 8.70772 14.8138 10.04644
8.330036 12.33657 4.96377 10.52826 8.072501 7.013082
13.36522 10.10286 11.33518 8.466426 10.01288
8.019781 11.77668 25.97418 7.530632 8.654864
11.78002 12.71354 17.10345 11.50524 8.540942
9.20563 5.768635 7.875274 6.609593 7.676153 11.04775
7.065057 12.12294 15.60212 7.360779 7.04896 14.83236
10.34318 19.16843 6.955427 8.809705 11.79414
11.8328 9.743692 10.18302 5.954939 5.545391 5.27611
6.899739 17.14261 10.03505 1.512064 16.07412
11.62378 21.08301 6.106696 15.61567 6.897124
9.212638 6.106373 8.483612 8.54344 14.01906 13.84314
11.72624 15.2721 13.69465 4.12342 13.92865 5.629059
14.50369 9.506729 11.2786 10.54574 19.38549 15.34999
13.00223 13.48202 15.14795 9.219648 7.142133

5.600655 14.1091 10.10351 8.469881 8.651034 14.46963
7.024528 8.03288 9.251068 18.79785 11.9315 9.223942
8.683232 7.696175 10.32339 5.710422 12.48432
5.713127 10.97804 5.337271 13.56099 8.695043
9.235797 17.8735 6.877792 12.45484 12.17162 16.72081
20.33531 14.46259 6.928816 8.504469 9.602253
8.371905 16.91554 12.94558 10.96554 11.32513
14.5125 8.808636 10.97256 4.045218 27.23963 9.059167
5.398381 8.255621 19.20944 11.68837 12.58387
12.19696 9.777679 10.29488 10.07563

MYO5A 0.434024 1.510351 0.9620667 1.95335 1.247168
2.997553 0.9298417 1.736139 2.120699 2.14851 3.055353
1.184552 3.09729 0.9649035 2.622443 2.856623 1.546341
2.519988 0.4834177 0.7716908 1.528052 2.26152 1.687796
2.841244 2.535942 2.112857 1.699844 3.252368
1.549918 0.2538251 1.978881 1.862032 2.75019 1.951809
1.335061 0.7969546 1.143029 0.8740693 1.501282
0.8840338 2.597103 0.42304 2.336733 0.5184876 2.193191
0.9103607 2.391056 1.977884 2.403304 0.9050571
0.5333143 7.192513 1.483289 1.806438 3.629241
0.7824479 2.227134 6.462894 0.3432254 1.278415
7.014198 1.848934 3.697687 2.494925 0.4695103
3.746032 1.904856 2.080259 1.722929 1.453447
1.951845 1.803121 1.845773 4.034522 3.444419
1.673068 2.963336 2.071525 0.7187959 0.7199362
0.9024822 4.258361 4.958181 3.168894 1.429253
1.537984 2.71288 2.466056 1.778098 3.322162 3.380501
6.453798 1.958942 2.9528 1.691855 3.462643 2.90593
1.011824 13.87265 2.962738 4.544756 0.5726947
1.007771 1.394134 3.400438 6.861045 1.329697
2.152824 1.425431 0.8724137 1.155024 0.7463909
1.56263 3.377656 1.735222 3.679507 0.8460921 1.240706
1.1156 0.4798121 1.156449 2.049447 3.492456 2.674884
1.817201 6.259432 1.47231 0.7150166 4.526935 4.227299
1.57337 0.9895386 1.072183 2.302806 1.517941 3.612905
1.719427 1.3863 1.334493 0.730952 2.550464 1.820976
1.715173 2.145889 2.552951 0.9661546 0.3223684
2.906388 0.3006801 1.775631 1.060683 3.09214 1.553918
6.381295 4.053808 0.6287257 1.375349 2.471576
3.60443 1.302417 3.235558 4.110473 4.12735 1.518292
0.5222868 3.114186 2.210915 3.745976 3.805526
0.637623 3.317925 3.298018 1.754419 2.50539 0.600559
1.025375 2.695541 1.206508 1.331173 3.404326
0.5760311 0.5592333 2.077026 5.467198 2.334257

4.631539 4.31711 0.8793475 2.471474 1.110257 2.424178
1.644411 1.072558 1.479264 0.9136705 3.320597
4.794512 3.158048 0.5471225 0.6401132 1.074027
1.134765 2.150537 1.204478 2.281584 1.287088
1.324983 5.050606 1.218811 1.362553 2.399486
3.539501 3.455504 0.7044709 0.9479027 1.632482
0.7697429 1.77235 3.448906 1.264103 2.774591 3.008346
0.5894233 0.9287414 8.568192 0.4922484 1.061437
1.724506 0.3207259 0.8898491 0.6133482 2.610704
0.2597058 0.7987181 0.9410328 1.020913 2.344484
0.9666702 2.623468 1.05764 1.678954 4.103675 2.090158
1.460995 3.168179 3.813734 2.473391 2.057743
4.780096 1.59603 4.039344 2.509495 1.270775 2.390937
1.646103 2.315288 1.573482 2.105701 1.631003
2.636364 1.272906 0.8460513 3.181549 1.161331
3.185937 2.4538 1.048131 1.532128 1.541876 5.001105
2.937046 1.457786 1.259936 1.173677 1.341604
1.961749 1.424852 2.973421 6.037345 2.966382
4.573906 2.201446 1.493782 2.36367 1.236504 2.551578
1.308737 0.3741924 2.686861 3.379321 1.582291
3.67381 3.950134 2.159427 5.330934 2.662111 2.438694
2.123144 0.5667308 1.075868 2.107059 3.635072
1.145635 5.315787 0.7127323 1.222405 2.780879
3.983568 3.24252 0.9360395 2.020354 1.338223 1.896585
3.287594 1.385228 2.995211 3.033668 2.085921 3.1174
6.703713 2.08323 2.946069 0.5426276 1.085216 2.214384
1.224718 1.410315 11.04629 1.212613 1.497345
0.400436 2.355152 4.50271 2.785735 1.723345 0.9234235
0.1179215 0.8097288 3.157809 1.720432 1.158213
1.027497 0.8900094 1.842904 4.921412 2.026922
1.101495 2.431195 0.7818889 1.845849 1.651617
3.617633 2.215847 1.81268 1.683586 0.544147 5.874668
1.207423 2.612491 1.851238 2.640664 2.537396
13.82004 1.94005 0.8401507 1.566392 0.2996345 2.515405
1.428917 1.237052 1.096663 1.180946 3.832769
2.186569 1.566198 1.815363 0.8679633 1.261136
1.00298 1.764307 2.710237 1.986477 1.216892 3.334557
1.316104 1.422983 1.828142 1.95226 0.5542898 1.203895
4.146056 1.071585 0.965126 2.981571 4.046455
3.372462 2.213991 3.179768 1.083069 1.333723
0.9664165 2.947143 1.716332 0.8722312 0.5449868
1.506566 2.452478
EDA2R 0.08118814 0.7685798 0.2277739 0.2947121 0.2555805
1.319662 0.1379844 0.5108396 0.4574927 0.5187713

0.5784288	0.2950853	0.6331455	0.2892612	0.1323603	
0.5864443	0.1214629	0.9413871	0.2806875	0.3034516	
0.4648687	0.5498241	0.5164351	1.79697	1.29284	0.4786171
0.1863444	0.9308605	0.3686957	0.4789812	0.4473945	
1.077079	0.1684933	0.3324157	0.2445566	0.02669636	
1.600905	0.08021025	0.3328506	0.1580704	0.3573209	
0.03220677	0.3549127	0.03914488	0.4347337	0.2090918	
0.2692378	0.3097946	0.5497414	0.04863382	0.1921311	
1.24474	0.03919497	0.3540196	1.543397	0.0598832	0.1788086
0.6824652	0.08140827	0.1045631	1.636108	0.2712661	
2.280251	0.5985297	3.284756	0.935251	1.334889	
0.3036071	0.1509064	0.05955058	0.1594833	0.1412251	
0.2610214	2.78589	0.7139025	0.1908807	0.2363816	0.2544369
0.227358	0.2266648	0.09881646	1.476605	0.2304562	
1.389988	1.034771	0.3419506	0.3508576	0.09531085	
0.375476	0.4075122	0.2503604	0.1767755	0.2739526	
0.2295945	0.2382969	0.1617373	0.3305295	0.09831511	
0.04087951	0.5431357	0.8182808	0.10285	2.609577	0.2191201
0.376986	0.6736736	0.1932502	0.5760822	0.233333	
0.3409477	0.3195882	0.1662326	0.3775717	0.4698868	
0.4600569	0.1643711	0.1180932	0.1157775	0.2099198	
0.02548116	0.1722366	0.3755787	0.7818338	0.406971	
0.2848523	0.3740577	0.2004992	0.1273231	2.301199	
1.187275	0.1929537	0.06932399	0.1404357	0.5481185	
0.245025	0.4501423	0.2973129	0.2272824	0.2725194	
0.3089357	0.1908065	0.2051712	3.082136	0.06838479	
0.2567093	0.08207218	0.03161778	0.2984708	0.09659379	
0.3529148	0.1012938	0.5627833	0.2243863	0.4316794	
1.029388	0.3951395	0.6311378	0.6729316	0.3079056	
0.09507131	1.566372	0.1390311	1.004618	0.1366372	
0.2350435	0.3834743	1.871454	0.6829222	0.5583727	
0.1099477	1.162839	3.687236	0.2641961	0.09500878	
0.03793921	0.1865339	0.6126005	0.1578312	0.07810437	
0.5542912	0.07349014	0.1516946	0.2591271	0.3403765	
0.382341	0.5717897	1.264963	0.1098487	0.2644789	
0.09125058	0.4107919	0.1326107	0.2016073	0.2420331	
0.1047051	0.5968293	0.7832027	0.3427632	0.3350495	
0.09007306	0.003123067	0.3392861	0.06367515	0.1424962	
0.1843357	0.1699608	0.3228356	0.523668	0.1965012	
0.2133277	2.62734	0.7680045	0.384334	0.1880429	0.1090791
0.3445122	0.09122935	0.1350671	0.5018266	0.3114356	
0.1136438	0.5349435	0.05113114	0.2675345	3.504392	
0.1052725	0.1215506	0.1890677	0.03466928	1.80122	0.1831941
1.996784	0.02117884	0.1120969	0.1410738	0.3920765	

0.3917776 0.03442586 0.6508908 0.302573 0.3374773
0.6955845 0.3229981 0.3923587 0.6944873 2.062331
0.3244267 0.2681452 0.5973467 0.1070968 0.2262462
0.3389863 0.2116788 3.885696 0.1072932 0.6795051
0.3795526 0.2019773 0.2362956 0.525198 0.1301737
0.1535137 0.4977029 0.5325672 0.3689361 0.3334634
0.09139912 0.1059891 0.2595451 0.1804801 0.3811852
0.2758199 0.09631733 0.1855057 0.03673357 0.1377625
0.2311794 0.6888172 0.1044539 0.6243996 0.4127889
0.2459012 0.5215944 0.4900435 0.2754524 0.5511631
0.06080687 0.1160036 0.3356077 0.6440502 0.1474001
0.9592017 0.7822643 0.2318066 7.831577 0.5091799
0.4514456 0.2099643 0.09542791 0.09700046 0.3022294
1.070185 0.1834678 1.527503 0.04796099 0.09163975
1.296056 0.2645792 0.3064676 1.364192 0.4841428
0.1940493 0.1773669 0.4061223 0.4128135 0.7440204
0.1733354 0.6276009 0.2899676 0.9432998 0.2325301
0.2423193 0.1216474 0.1675827 2.007927 0.07285962
0.3112017 0.3250825 0.1050372 0.3298819 0.07409885
0.5118726 1.50369 2.057753 0.7419914 0.1087248 0.04799226
0.164671 0.3411055 0.1360208 9.491037 0.1809532
0.3817032 0.07254563 1.050919 0.8023564 0.07501572
0.128273 0.1238348 0.1583404 0.1118215 2.185951
0.4050018 0.3337429 0.1291248 0.2763401 0.3247293
0.4153521 0.563112 0.3077926 0.2829471 1.235394
0.1097422 0.04736981 0.455054 0.3506456 0.05769652
0.2617773 0.1679157 0.1720858 0.1430623 0.2672307
0.386037 0.3096758 0.1639872 0.6557591 2.136414
0.09072543 0.06999538 0.4208457 0.4757234 0.3712558
0.198528 0.09292867 0.1018155 0.1817046 0.6400473
0.2281497 0.09997455 0.4335234 0.4655763 0.3261193
0.2131272 1.116423 0.7326103 0.4079495 0.7049681
1.002941 0.07829478 0.3013925 0.1276228 0.6634748
0.2286784 0.09582392 0.1000407 0.2788297 0.7411223
CCN6 0.07259771 0.5466819 0.18364 0.0520123 0.4285085
0.4089667 0.07010479 0.2066423 0.2171518 0.1478051
0.1051589 0.1515427 0.3012943 0.04679611 0.05917771
0.4672479 0.09956016 0.2869705 0.1624701 0.2228895
0.1060412 0.6461916 0.2713026 0.5591351 0.1149831
0.08916149 0.1077333 0.5022905 0.05260922 0.2676879
0.06411156 0.9883313 0.1489133 0.1714864 4.236932
0.3381817 0.0519349 0.06520299 2.298927 0.3774559
0.1941995 0.1359953 0.03778093 0.1312613 0.2009087
5.53225 3.294474 0.3755316 0.1570819 4.573481 1.131378

0.3381916 0.2239165 0.2387854 0.5838851 0.1171341
2.251771 0.1441912 0.1546884 0.2398997 4.558975
0.04331496 0.2259508 0.4406904 2.91597 0.3781767 0.1362044
0.06878787 0.186909 0.957225 0.05425326 0.345303
1.373487 0.131156 0.8579799 0.1969429 1.683917
0.1218831 1.16761 0.0666716 0.08678293 0.1090394 0.1409454
0.2612733 0.06213159 0.074685 0.1820776 5.270562
2.642456 1.724729 0.08307677 0.106394 0.348844
1.53976 0.09922983 0.05098926 0.2287045 0.7023444 0.03807718
6.130627 0.2556403 5.962032 0.2822734 0.7129864
0.6554674 1.567591 0.09163777 0.2993614 0.07623541
0.2117169 0.1016692 0.1675885 8.257387 0.660265
2.394353 0.06124132 0.6447564 0.07908327 0.1407813
2.026918 0.0422595 0.2440692 0.2801453 1.937483
0.3096675 0.4489061 1.489889 0.05888855 0.4034729
0.1091887 3.940913 0.2169611 0.6925168 3.438174
0.6694698 0.08674854 0.1035797 1.309295 0.3595343
3.220904 12.14722 3.382586 0.04235684 0.5254998
1.032106 0.5724281 2.043854 1.757284 3.083047
0.1728562 0.07128667 1.664615 3.124787 0.2389977
0.5603724 2.236808 0.5311126 0.9939282 0.1402125
0.6527701 0.5471233 0.6466663 0.2691248 0.0660431
1.967585 0.1404735 1.18704 0.1399436 0.1853807 0.3407484
0.2209949 0.861883 0.232369 0.05078892 2.501961
2.282674 2.238532 4.364615 0.07857027 0.1890161
1.003967 0.1099816 0.07622012 0.07186317 0.1991568
0.04484994 0.1201813 0.1903124 0.7446589 0.1216086
0.6303697 1.056694 0.07358181 0.4370097 0.117033
0.186629 1.988528 0.7617322 0.1895606 0.3603218
0.8133501 0.3532075 0.7828941 0.06296861 0.4550033
0.1547918 0.2296292 1.688033 0.1001853 0.1064485
0.08705573 1.032043 1.48249 1.826589 2.904356 3.787229
0.8643222 0.09705196 0.1045766 2.290899 1.943469
0.1004518 0.03333824 0.2080234 0.1780452 3.662392
0.2108201 0.3275589 2.34168 0.1904467 0.1177388 1.456264
0.112444 3.529741 0.2283694 0.5017521 0.124182
0.3617037 0.4764783 0.1297196 3.048277 0.2273339
0.1805138 0.06746994 0.1514645 0.2396656 0.2039762
1.148913 3.642479 1.119415 0.2202539 6.999376
0.143651 0.6662854 0.2268865 0.03880704 0.1614367
1.50035 1.277984 1.047024 1.272088 0.08261658 0.1506651
0.1968613 0.06426612 1.049152 3.831013 1.510468
3.213745 0.1548158 0.1571892 0.04316125 4.217646
0.07049796 3.227833 0.09510687 0.07321282 1.74321 0.07297011

1.655521	0.2574072	0.9715746	3.440778	0.183974
0.0794539	0.1760161	0.5544611	1.899163	2.645806
0.1181342	0.1977057	0.1786895	0.3624514	0.1121474
0.05562293	0.06152759	1.840044	0.06122224	0.1839026
3.341696	3.47385	0.6256209	0.1044692	0.6664835	0.8577258
3.759535	0.4714258	0.8808991	0.1314439	0.09148861
0.3006363	2.477186	0.2499672	0.5718449	0.4438955
0.3855695	1.520143	0.09265015	3.671681	0.07692776
2.375437	0.6825412	0.9687862	0.1685823	3.261522
0.2931769	1.559004	0.04765342	0.4325417	11.16937
4.03993	0.4730681	1.949649	1.875481	0.1480987	0.1258662
0.2324522	2.981759	0.1543223	0.03648858	0.06348595
0.2512907	0.2391085	0.1875139	0.3123354	0.3225036
5.070807	0.6784119	0.2226676	1.161934	2.574737
1.777808	0.1160734	1.719216	0.08355824	0.05147935
0.1814813	0.9415881	0.3611381	0.2071212	0.2685234
0.2414598	0.7223496	1.55532	0.2373614	3.348826	0.5258386
0.1137884	5.162953	0.7777511	1.826808	1.913855
0.4358035	0.1213726	0.05832111	0.1578699	0.03381172
1.171818	4.158273	0.9270903	0.7254673	0.220113
4.984687	2.361913	0.2000177	0.02620624	1.237963
0.3086984	3.048416	3.634245	0.5091612	12.97346
3.343029	0.1539223	0.2157016	0.1310945	0.6498724
0.1325944	0.1312697	1.862738	0.4850064	3.18571	0.1278014
0.4698849	0.2924507	0.2077726	0.8283812
ABL1	9.25748	89.54746	13.92614	16.11432	14.50813
47.64575	8.91321	26.55408	22.54404	16.50315	31.89699
7.085852	13.29854	12.30444	13.60164	61.54374
16.0421	55.36451	13.30271	14.32843	18.05923	41.43892
17.58791	50.84719	37.04876	19.38237	12.68232
57.38743	18.41299	3.440608	26.97885	46.27219
20.4999	10.1199	24.14967	10.39297	8.683809	14.15278
21.01742	20.61189	32.94321	19.48072	14.72184
14.17365	17.6806	10.18295	17.90194	28.59249	17.13642
15.97865	10.61188	18.52997	33.35359	7.612493
25.25803	14.51921	23.90148	50.7107	30.04362	17.76629
25.32293	18.40231	27.94837	39.81008	7.314362
47.21658	21.378	19.94823	12.39221	10.17823	17.77048
11.84552	34.34132	19.58681	30.46882	21.45662
22.89996	37.31823	17.82054	15.30933	22.87482
30.86543	12.72311	23.7252	21.15568	23.46316	31.61307
27.45097	9.468943	30.94195	13.2243	20.61164	19.73593
13.5894	15.77692	19.53696	18.96123	14.57227	29.70177
27.95338	26.31149	15.42143	13.00404	11.06009

22.60187	40.31211	21.21267	32.2527	21.68301	10.9252
16.91288	9.455541	12.8337	24.36151	13.42933	12.32714
14.83138	17.63212	23.22212	13.42914	9.687939	
15.06734	21.84687	11.77603	27.33032	14.9117	10.48736
17.84661	51.40483	24.34004	15.64668	34.74708	
17.01591	14.80996	12.17202	38.91537	24.67253	
13.2229	16.40711	23.98121	18.52937	19.15673	17.56382
10.60492	24.3952	11.49297	9.426998	28.58113	35.74564
25.69292	18.88688	27.34952	19.98907	34.1651	41.36424
9.762106	26.63803	22.34528	19.06762	11.20724	
24.22944	16.77004	20.39953	23.8624	10.55761	24.22052
13.64872	15.4611	15.62159	32.16206	57.21067	27.72701
20.93962	8.977463	7.517135	9.104169	25.3276	21.67812
19.23032	37.84828	11.05002	19.77726	16.36638	
26.04252	14.84131	22.4161	34.2956	10.91871	27.59884
23.49482	19.1062	15.24181	12.7828	15.60021	16.92976
25.14759	25.20087	20.43203	20.72969	15.56382	
8.25308	13.06775	25.9176	33.79512	10.38067	19.00052
13.65707	41.0031	36.49365	13.46883	13.02266	30.04757
25.75286	22.69036	28.50735	22.35033	16.653	30.29259
36.18907	18.28337	12.45814	17.80092	12.19552	
17.25491	24.10443	10.2601	17.90514	10.54472	17.17515
14.32959	15.67567	20.65815	15.4382	12.28826	11.22691
14.96747	18.20024	11.64764	12.14706	18.9046	21.1923
25.91573	26.34301	16.21897	14.55943	21.28951	
19.81535	56.29341	26.73577	18.20495	17.73621	
13.46399	15.97299	22.71704	22.09635	26.34433	
19.6296	19.12247	20.32578	50.56467	7.614275	23.18617
14.33606	20.60178	15.63734	20.11506	17.90991	
19.39941	13.63808	13.15486	21.49928	14.97727	
9.482926	15.84068	8.322789	31.70882	22.88421	
55.34177	15.81992	13.45356	33.127	14.18438	24.17034
20.02057	13.25059	25.93511	16.20656	11.16041	
21.69383	39.42526	20.5698	42.88206	49.84691	18.05898
53.7102	36.24902	19.26841	21.92207	11.51253	18.53291
21.09872	29.3987	27.11781	51.14427	13.92732	12.86737
10.633	13.1748	14.12984	20.21593	18.19855	13.7102
32.26577	24.86525	12.8554	51.53783	20.29417	18.66654
15.63589	16.71749	14.06532	18.08866	19.32648	
24.03987	11.56482	14.06866	23.6097	30.83453	18.12112
24.10908	8.397242	37.33849	16.56929	11.699	45.06752
29.47986	17.34931	12.01795	20.56601	10.62065	
14.14164	22.39261	14.75632	24.21204	50.94785	
15.19633	13.97041	25.29025	12.77283	10.39587	

26.21297 27.82598 17.50554 19.62255 15.9557 19.51791
23.70338 16.42354 34.84465 12.75237 23.47571
24.95027 24.16146 22.95807 26.4462 10.53132 13.89496
17.47995 18.62228 7.771865 16.32912 15.17157
34.08937 9.907843 24.12941 28.52151 9.57196 10.31306
13.6094 18.64735 21.79991 27.32967 18.89847 19.60786
41.87509 18.22046 33.84962 10.11406 32.16632
19.67778 27.81479 15.35423 11.75693 16.02028
20.54478 14.43824 23.19078 32.86324 21.68601
17.18744 24.38101 44.43577 19.78082 16.18911
8.660626 23.82308 40.66513
MMP9 1.666811 0.14868 2.860441 5.988717 9.357048
0.9258994 3.555477 1.706945 15.33399 29.26002
0.2615934 0.2688174 31.88666 0.03498781 34.65682
0.1013687 9.78855 4.842024 7.364488 4.078013 7.274902
0.3840303 1.42422 0.4464566 0.5196855 0.1319926 17.58282
1.017995 4.963742 0.5221625 6.22183 1.254375 42.53466
3.408366 3.245597 1.526995 1.093709 17.93086
16.38615 3.027259 4.153616 6.908187 45.63733
8.588292 2.100645 9.633407 10.44278 32.2286 18.89074
13.71024 12.48539 48.6744 4.724001 13.64406 5.917684
5.573655 65.0275 27.3261 35.03556 24.24105 32.86236
115.7936 4.441314 18.99523 10.77557 1.643625
64.53809 28.62609 10.11676 7.642253 143.5742
4.052465 4.583613 29.43141 42.6814 16.28719 6.224354
81.80236 72.14332 17.27234 19.55188 1.929423
14.48308 96.58063 10.85526 8.791963 5.298717
35.20772 24.05477 1.024218 5.26222 18.90316 15.53512
46.64799 47.00434 67.86509 45.75743 12.99995
0.9655728 11.81389 28.28986 2.146308 21.49542
27.45404 2.759561 15.55716 5.096163 4.567941 39.137
6.55033 13.7439 2.531878 19.24264 31.21695 17.22492
43.47345 31.37071 7.602849 30.78358 3.142707
27.43114 10.23724 37.1105 22.95488 104.4608 47.69969
34.49586 27.06799 4.378298 25.24193 6.575429
4.283873 4.391845 38.49808 110.4523 79.12131
12.23974 8.230063 22.63552 5.075974 98.83493
4.335329 7.605773 6.934647 28.69372 1.227254
5.958341 29.22396 8.364285 1.828669 2.672765
23.89647 1.076437 79.43343 3.881472 5.99537 6.507598
11.75343 27.72806 6.97084 28.51694 9.599208 4.414014
24.40256 9.508478 14.07587 8.98873 5.132729 39.77588
2.335352 2.726298 57.2943 12.0703 7.366781 13.25939
14.73295 6.419571 0.7881351 1.848812 15.0415 12.24155

27.72615 12.24463 25.4083 81.92487 151.3018 17.2051
3.357583 16.88545 6.457942 55.01181 4.974476
21.19321 8.681 5.101334 9.378103 38.99042 8.754552
7.211989 2.331499 3.899669 9.530076 20.9138 5.82354
3.000733 7.614228 38.11559 30.44499 5.688464
17.56185 20.74733 21.21962 3.902511 43.53764
8.266512 39.82118 4.426147 30.32614 4.515374
27.19769 3.74346 28.22013 18.38992 30.02453 74.89624
5.130996 22.68134 1.28904 8.946423 2.361872 14.18099
47.16218 4.550865 6.830517 12.49885 8.357605
38.15586 1.635066 6.341564 3.391075 9.963713
24.36174 2.713523 17.77202 35.65792 26.60966 33.572
6.398941 4.041372 27.34431 39.81809 10.72414
2.768255 8.478941 1.550546 52.8364 6.273155 10.05715
27.82375 19.13167 12.55902 21.89967 9.317208
2.826911 103.9912 16.27315 2.639086 41.00259
2.236139 176.4228 6.736032 3.515911 1.93575 10.36814
7.840199 12.91853 1.877214 4.773925 15.54335
17.2493 19.28598 2.299908 3.7745 43.19098 30.92108
17.34722 4.378641 25.53036 73.64904 6.897153
25.34343 3.880256 2.279221 42.95752 83.66761 14.137
37.79697 26.68608 6.947291 5.551832 6.420926
7.29947 8.061511 96.29053 1.716314 5.928077 16.12789
45.14195 37.56327 0.7733321 18.44214 25.56034
82.8054 102.6102 20.34325 2.808344 8.607135 5.928054
46.768 44.87673 15.2695 13.04604 4.449743 25.22612
40.71536 8.416802 32.939 13.74158 19.59089 10.10828
2.850797 7.665892 18.84211 55.24572 1.938666
11.3868 2.591905 2.231396 30.8402 22.47784 10.11189
46.11416 3.624536 15.61624 3.480835 0.8417341
7.623826 1.69685 0.5609353 40.5449 26.60065 107.331
5.086026 22.75787 13.08821 0.7320987 14.46857
3.603073 6.53425 56.96866 14.07664 18.52418 10.96625
31.57038 34.46448 17.8001 4.09347 17.42429 2.459276
11.70967 6.382979 2.994874 81.69103 36.39141
30.02959 34.27547 9.906538 5.297482 4.434389
0.6447802 23.09183 3.976677 3.509037 19.19608
8.24395 8.917485 4.894575 4.562541 29.67241 15.25379
10.76126 10.41693 2.231312 10.29302 14.85449
32.4152 7.638546 10.46438 0.8037046 27.24416 30.08841
43.13741 27.65996 5.938799 11.43063 18.06791
3.357065
MAPK11 0.4161844 2.13455 0.9545701 2.215002 1.162687
2.030785 1.085633 1.06236 1.310067 2.58008 0.8768717

0.8070341 3.88956 0.820964 2.220551 1.305396 1.293797
1.733643 0.8742564 0.9696905 1.181077 0.8235856
1.505887 2.560748 2.854163 0.7519737 1.719677
2.611321 0.7729198 0.7849238 1.81763 1.526156 1.179112
1.276738 1.500495 0.6042733 2.260918 2.543729
2.512757 0.7940329 3.2736 0.438354 0.7707175 1.563611
1.363605 3.285121 1.075449 1.440786 1.783135
0.9626854 1.02327 3.314677 1.432245 1.414103 2.06553
3.793956 1.849079 3.302291 0.7804291 1.011198
2.472982 4.002504 1.437299 3.52292 0.4388441 3.402494
3.834986 2.643979 0.8200028 1.491407 1.001728
1.375024 1.539883 5.216257 2.229147 5.405629
2.089846 2.173445 0.8917999 0.338861 1.503674
2.71588 0.9958029 1.611748 1.878776 2.580022 1.55163
1.756616 1.110969 1.786285 0.6979477 2.450281
1.999257 1.236484 2.301879 4.036412 3.546925
0.8309554 0.4762623 2.010633 3.681356 0.7460976
2.316388 1.309644 2.476271 2.609392 2.300173
2.556793 1.409994 1.333065 1.076901 0.4383285
10.5952 2.973288 1.952514 2.544734 4.197043 0.7921834
1.014195 0.5315306 1.085115 0.8722204 1.967423
1.485128 2.68089 2.67373 1.05795 1.017164 3.364376
2.591352 1.307919 0.5415112 0.6709512 0.7298051
1.341226 2.080511 2.087284 3.40448 2.171165 0.5389967
1.093536 1.496799 1.829042 0.6487491 3.06091 2.718809
0.5121951 2.412161 1.619559 1.620879 6.613623
3.874092 0.6905863 3.588281 2.709607 1.289837
1.936653 2.495973 2.444624 1.37435 2.541803 2.060163
1.470278 1.445596 1.18523 1.604698 2.914274 1.020428
2.149404 4.311923 1.891857 1.630263 1.026476
1.177015 2.379819 1.443086 1.636551 1.05074 0.7799541
2.032693 0.4348896 1.18457 1.821952 2.266011 4.700311
3.285708 2.732718 1.68931 1.676039 3.901946 2.044141
3.406045 1.051826 1.693934 1.198943 2.067068
2.755514 2.840294 0.3745116 0.8331969 1.605096
2.192318 1.144554 0.7412702 0.9817524 1.508655
3.041493 4.11502 3.887821 1.227459 1.375787 2.780491
0.8679349 2.73623 5.197372 2.052367 4.413389 0.8135536
4.801754 0.5155334 2.133523 1.361847 2.394137
2.431554 1.81927 0.9505112 0.6920116 1.409736 0.5209623
1.874329 0.8293212 2.972928 0.4864338 0.8059716
0.4080574 1.097039 2.950777 1.233018 0.6774468
0.7726232 1.74879 1.966042 1.906614 2.384356 1.843744
3.036624 2.690785 2.710169 2.141936 2.616838

1.588802 0.5977354 1.530014 5.163902 0.888036
1.109466 1.198563 4.061924 1.496988 3.11548 2.040258
2.838881 1.028532 0.8710191 1.786286 1.685546
0.9782445 1.652299 1.893943 0.894156 2.05479 1.549782
1.018408 0.5631506 3.461306 0.7552882 1.467224
1.829957 3.546478 0.9280424 3.401842 0.5444157
2.232286 1.638671 0.3904193 2.820115 0.9715526
0.7631926 2.709639 3.051947 0.9158764 1.120308
1.201805 2.496793 3.809901 5.478645 1.466447
2.066326 0.8499856 1.111643 1.401729 2.253643
0.7512838 2.798353 0.5108702 3.814126 1.257687
1.416616 2.154142 0.5267516 1.862245 1.298657
0.8214235 3.988843 1.321943 2.080351 1.420255
2.136385 1.924905 2.161577 0.5400132 1.064717
0.3239404 1.126816 1.267273 1.261138 1.302977
5.633998 2.465851 3.63721 0.3398311 2.537352 2.738903
1.144843 3.096139 2.538544 0.4419778 0.7844458
2.10866 1.735015 3.637598 12.26575 4.959408 0.7968887
2.588245 1.047265 0.4328195 0.783144 1.003634
4.848999 1.042211 1.945901 1.590093 2.060845
1.529239 0.5059167 2.000961 0.8645966 0.9587204
2.253997 3.525684 3.739214 0.7360059 1.340272
0.8549813 1.421376 0.8036697 1.69111 1.106508 1.927661
1.194852 1.192624 2.138232 1.328008 1.637585
2.404454 0.8055442 2.348859 1.23486 2.312102 1.752934
1.448564 1.03155 6.019833 0.5586906 0.7492176 3.423644
4.108959 0.63362 0.6170335 2.336898 0.5131681 0.5202508
1.637101 3.264192 1.507307 1.069283 2.417101
2.449814 1.888452 2.718821 2.140065 1.330093
0.7614062 0.6762539 1.87083 1.17127
PTHLH 0.2108091 0.3867072 0.3047162 0.341109 0.4740198
2.945337 0.2260044 0.6238947 0.2656758 0.910947
1.672903 1.758085 0.9766301 1.173817 0.7574992
1.118742 0.326112 1.515982 0.4674642 0.4226782
1.804801 1.379658 5.024456 1.09635 3.211804 3.040096
0.245161 1.177046 0.3658083 1.26908 0.79026 1.160788
0.09551526 1.788598 2.003431 0.06601756 3.017288
0.2975285 0.5757318 0.2512885 1.22294 0.1061924 0.7970634
0.07744125 0.4474474 0.1392907 0.1051262 0.6890208
0.6001475 0.5429187 0.1936908 2.273076 0.2676988
1.254965 1.224691 0.1004866 1.054907 0.7673907
0.2156945 0.2274678 2.867162 0.6277893 0.6427253
0.3675096 0.02960237 1.57425 0.5987684 2.96983 0.3328337
0.189338 0.1543254 0.07795453 0.6766912 1.08313 1.198517

0.3890356	1.53653	0.6128554	0.087917	0.1980787	0.2393767
2.688109	0.2258079	0.4512295	1.060413	0.4248884	
0.8809176	0.5954392	0.5895342	2.135177	0.3109407	
0.1633304	0.8783688	0.4648655	0.3466386	0.2681057	
1.005029	0.122317	0.07942875	0.597428	0.9857305	
0.2894193	0.1685177	0.5057389	1.527115	1.833606	
0.5027149	1.601332	0.7114346	0.4072266	0.4976355	
0.7323394	5.337939	0.8489608	10.13244	0.2425181	
0.3128928	0.1806459	0.635647	0.4005794	3.494956	
0.8238595	1.094228	0.6869084	0.384601	0.6342919	
0.4171147	1.479677	1.342961	1.449426	0.3408258	
0.5387852	0.4552633	0.7499743	0.3077707	0.7293111	
0.4787838	1.334091	1.117404	0.2766093	0.2754879	
0.2536845	0.1271792	0.1675992	0.9948636	0.5010131	
0.1116968	2.810136	0.06445624	1.517112	0.1232569	
1.436342	0.2923872	0.3848138	3.033944	1.458959	
0.3267567	2.154835	37.24921	0.8924292	2.289076	
2.747316	1.016314	0.07436205	0.7208085	0.7342744	
0.8990487	1.129889	0.8847293	6.615408	0.6179735	
0.2327837	0.4039335	8.068483	0.4422945	0.5760327	
6.038802	1.096562	0.01241644	1.145024	0.9908837	
0.4127838	4.447033	0.2966057	1.353327	0.1063144	
1.779684	0.8382202	0.7273583	0.26037	0.7342156	1.623044
0.7500137	0.3157054	0.303313	1.193523	0.9317461	
0.1752224	0.135899	0.6782748	0.430284	0.5182196	
0.08013721	0.2282858	1.660577	0.06337809	0.5806121	
2.319074	5.19542	1.162739	0.4952664	1.31237	0.4113168
0.3036814	0.2601087	0.5984951	1.648272	0.2590266	
1.198903	0.3835886	0.2248243	0.4626246	0.5599594	
0.1571784	0.4614379	0.1708409	0.3331587	0.6512255	
0.09973119	13.7153	0.5436258	1.33077	0.02244568	0.2206325
0.268091	0.2830416	1.766202	0.1532374	0.8938574	
0.6457364	0.6573283	0.6035489	0.7416905	0.5757621	
1.139947	1.127966	0.9168872	0.7535201	0.9464827	
0.251547	0.170166	0.2039062	0.4647056	0.4991666	
0.1075645	0.1892367	0.7119576	0.6823127	1.013837	
0.8598431	0.5084821	0.3675733	7.368148	1.04885	0.4113165
0.9162481	0.2160863	0.5288821	3.540188	0.08451721	
1.008005	0.4501705	0.4563518	0.5426215	0.1916595	
0.4852453	0.4900154	0.6886979	0.1891158	0.4924643	
2.145957	0.2151549	2.507317	0.6382926	0.734531	
0.4255817	2.554022	0.3398998	1.025425	1.36515	0.3367344
0.383446	1.388847	0.6673489	0.4548867	0.8386254	
0.7011759	0.3676477	0.278996	0.2202915	0.8808461	

1.034542 1.372101 1.326306 0.5518669 0.6690579
0.446996 1.03213 0.6949196 0.1156634 0.72438 1.731846
0.2329272 1.548376 0.2602887 0.8351197 0.348566
1.634617 0.3951159 3.423949 0.4241046 0.6677162
0.4310892 0.5594616 2.09264 0.2934277 0.6596334 1.219967
0.3749732 1.983766 0.5889839 1.542929 0.9901784
0.9156875 1.355092 0.2880709 0.03390865 0.2094252
0.3873313 0.2940805 0.1143725 0.8484685 0.622362
0.1105223 1.062276 0.9550734 0.128719 0.9449892
0.7303914 0.7077279 0.2844246 1.36796 0.2091109 1.071371
0.5473945 0.04271017 0.6402855 1.680189 2.152377
1.141713 0.6846133 0.8585516 0.2326132 0.1694361
0.5733699 0.8795005 0.01881468 0.8785469 0.1296995
0.8127778 0.5835063 2.701037 0.1063734 0.4134462
0.3712922 0.7217144 0.2404467 0.5341794 0.2040012
1.000295 0.5823468 3.962001 3.818049 0.2440773
0.1307948 0.0745446 0.9614627 0.4326889 0.271244
0.3921213 1.171442 0.4660722 0.4592806 0.9173739
1.071856 1.037644 0.9897637 0.7124318 0.2240408
0.5699468 0.5085653 0.1485552 0.2933796 0.3538072
0.1794266 1.002411 0.6966631
GLI2 0.2059823 2.620585 0.5395396 0.6558892 0.5841043
3.576087 0.4425254 1.356591 1.023661 0.4104594 1.0154
0.2273923 0.4725902 0.2078419 0.4861266 2.687244
0.4656692 2.915699 0.2107329 0.5907701 1.043022
2.012334 0.7636866 3.635537 2.339207 0.2451727
0.7328385 3.780246 0.9477379 0.1112841 1.502357
1.754949 0.5908087 1.081245 3.892075 0.07740719
0.04899091 0.5585273 2.575155 1.362842 8.961818
0.1763936 1.278187 2.864038 1.553439 1.776949
0.3766371 1.853456 1.004738 1.402323 1.202503
4.753006 0.3156873 1.16513 2.566514 2.15113 1.543867
7.766528 0.3737405 1.337737 4.496741 1.12899 3.571558
3.043944 0.253765 4.608728 1.788062 3.792916
1.555109 0.7811232 1.595043 0.6028574 1.368138
1.344471 2.419567 0.7344073 1.465229 6.282054
6.013281 0.1479715 0.6207985 7.067224 0.3446151
2.511389 2.649078 1.91462 3.047941 2.392676 0.8131708
2.708382 0.6597646 0.6878047 1.6423 0.5249573 1.790604
1.30869 2.500545 0.3904202 0.1942605 2.664509 5.418262
0.3222122 1.089334 0.52475 2.678062 5.376136 2.747915
4.27606 1.592515 0.5099878 0.8874543 0.7340232 2.162027
2.497836 2.216309 0.1946898 0.4653347 7.225354
1.840505 0.215494 0.2131615 1.289501 1.827668

3.973493	0.8787407	0.9304688	0.2440259	1.183917	
4.468269	4.430288	1.702449	0.7459621	1.209621	
1.531967	0.3727723	3.165499	1.294972	0.7033713	
5.563652	0.2471094	1.189357	2.350691	0.8652926	
0.3428669	2.858855	3.533086	0.1171429	3.902813	
4.313541	3.525946	1.140375	4.266396	0.9972574	
1.850213	4.951651	2.993664	1.772165	3.786243	
1.025049	1.122343	5.017815	2.496782	2.297042	
0.4033235	1.48629	1.741041	1.017051	1.380612	3.54529
0.9370727	3.693969	2.827524	0.9606988	1.022078	
0.611146	2.841083	4.026949	1.231668	0.2906324	
5.604735	1.173955	0.7627938	0.494309	1.345157	
1.512235	3.601872	7.272963	0.8679421	3.017316	
0.4240141	2.441099	2.195428	1.443526	0.6418032	
0.9782561	4.419035	1.793432	1.268628	5.02536	0.8911871
0.1207395	1.613904	0.5000361	0.3939562	0.2375507	
2.059451	1.009354	1.369269	1.079552	1.846453	
0.9635515	4.026281	2.134097	1.194331	2.213956	
3.79146	2.233752	0.335685	4.482345	1.960612	0.9299644
2.583083	1.569051	1.297095	4.593035	0.3386278	
0.5003042	0.2284207	0.1460006	5.103123	1.73961	2.646225
0.08443726	1.34036	0.2397874	0.9034323	1.67853	0.2578662
1.745517	2.730116	1.751361	1.946108	2.437622	
1.711352	2.351356	2.579195	2.19494	2.927398	4.224327
0.4084468	3.297685	0.768171	1.932649	5.437764	
0.2816724	1.557986	1.580995	2.037545	1.221112	
3.924515	0.8070114	3.739552	1.00216	0.751835	1.539785
1.84127	0.3754386	0.5762242	2.552519	0.9289757	2.316043
0.9463722	1.470306	0.1649503	0.8056562	1.327578	
2.982898	1.899903	2.315681	1.389432	6.986107	
0.6646382	2.24382	1.735452	1.133618	2.031896	1.158401
0.8408927	1.934139	8.714767	0.6030983	7.184881	
3.069511	1.014887	2.278614	8.276419	4.147	1.407299
0.8078284	0.396773	1.273108	4.922891	0.7745384	
4.060859	0.8443223	0.672484	1.014864	2.570796	
1.343256	0.9440531	1.269224	3.321633	0.462109	
4.210699	2.146465	3.767134	1.273665	6.402046	
0.9328575	2.47297	0.5122061	0.8407955	3.440872	0.7734112
1.454508	0.6478623	2.24136	2.477518	0.6091194	2.204979
1.4045	4.539325	4.454961	1.113431	4.90796	0.4747546
0.07344319	0.82231	1.051846	1.482936	0.4666209	0.5220312
0.7885187	0.2695097	5.102121	0.8614415	2.594597	
0.4792792	0.6582844	1.437228	0.4503208	3.483022	
1.443643	1.481938	0.2955811	0.9014168	2.463399	

3.019307 3.644727 0.7482678 1.088729 7.699181
0.7738988 0.423498 0.3152014 2.681105 0.1394112
0.5599047 5.442311 0.7647257 2.995886 0.8226764
0.8264388 0.6335956 1.068046 1.623503 2.25126 0.7648287
0.8574826 2.918355 9.62536 1.435295 0.54054 0.2792132
0.5576344 2.268897 5.85532 0.9052506 0.1236823 0.906643
4.465866 0.54921 0.4806442 3.298911 0.3367688 0.542147
5.031186 3.25377 0.495658 1.979126 0.7845006 1.149296
4.347711 0.7110452 0.293791 1.540336 3.351061
CXCR4 4.977881 6.277934 9.788636 24.57463 51.22352
4.201054 30.90576 41.36995 73.96549 153.2094
6.100952 1.78397 199.9662 3.956727 41.81239 5.560824
31.69152 38.3801 9.348575 10.5759 23.00013 5.185255
6.798232 3.610838 2.944047 2.079596 38.7772 4.571479
24.74305 17.96572 21.13773 2.75806 12.62424 18.76419
32.00034 10.71172 6.91312 43.81071 32.3054 7.770057
29.36311 4.117538 50.43778 8.873809 10.0904 60.01425
58.3324 27.31442 52.46209 37.72353 12.89546 121.5983
22.0342 40.49856 106.482 4.45701 36.61139 130.7874
5.03723 61.4095 53.05904 27.35938 51.00896 30.16471
25.65705 11.5226 33.52419 90.98218 20.66177 10.16281
29.29164 19.67765 22.88314 42.09118 23.80573
94.22069 32.263 42.90777 28.54348 20.75951 17.07211
64.53684 35.60999 11.09695 39.05004 21.11969
24.46023 43.82637 36.67957 30.55706 25.12432
34.04985 18.17578 80.18772 33.28777 64.06171
55.95174 18.6212 43.81887 45.45711 133.6704 8.309873
57.4533 34.58827 30.65703 125.7527 30.46763 48.52467
17.35938 22.36501 8.739167 17.0211 25.45092 201.5232
52.12544 12.94055 22.96596 9.139853 12.19034
10.27881 19.74019 19.46043 14.67069 94.27256
16.45852 230.2897 24.33662 16.52595 64.08047
34.93563 17.23818 24.97468 25.76377 34.32161
56.05038 13.28061 22.76698 24.96995 14.27821 49.433
21.55635 14.4864 22.40116 28.37203 16.08057 17.22267
18.57168 16.46485 15.98203 45.68348 29.25093
37.80386 24.45984 55.97088 51.79243 7.835722
24.62699 84.14317 6.680086 36.2816 96.87968 68.62245
50.61186 10.85163 31.41862 43.35333 42.21535
56.79394 120.4704 7.648407 34.06652 35.60239
15.33367 4.726421 5.494873 14.13961 29.51817
23.16029 9.081135 13.95451 8.232554 5.839002
35.78915 46.65048 83.02791 24.82817 53.35457
8.087476 15.17132 17.24877 43.80443 10.03694

18.14872 19.23505 139.9994 24.81747 73.86154
21.0515 32.33099 6.911883 9.770222 16.67442 19.64863
4.959577 4.820475 30.59783 35.17829 28.33644
4.676758 18.10296 48.83181 92.19415 52.61446
6.052206 18.92665 21.35338 9.675609 29.41066
88.99019 8.262726 6.797143 61.4795 27.68152 14.10932
245.9899 2.964695 25.07141 4.929006 4.614793
32.9622 7.348073 28.17569 1.567787 10.66898 16.25967
31.59342 319.838 12.02185 52.72678 10.27867 20.27045
51.96271 76.64285 23.29734 118.6918 114.4903
73.15754 117.6927 52.63288 19.19105 24.97537
20.69282 18.66257 35.05529 20.36022 120.2054
33.96155 42.89102 27.20097 21.46382 35.69004
11.86726 107.976 6.802337 37.31152 35.72566 9.690651
19.40879 20.23289 31.19189 82.59826 38.85609
15.46352 15.63687 13.66897 16.21642 26.45121
28.31349 10.758 28.54788 94.72016 37.84184 19.14091
66.52606 17.49746 123.6684 13.76139 15.40661
47.42945 31.1359 18.10668 14.53564 32.21951 19.04719
69.82237 26.89639 78.83091 60.54065 10.81334
10.21892 22.9646 24.03886 11.24727 20.74919 3.677163
24.09262 46.30949 116.9982 77.09199 9.821964
49.18596 16.05758 15.07814 85.65847 24.87436
21.08923 35.5401 23.69629 27.04477 63.48308 44.12102
25.86184 21.5767 35.36912 62.093 47.20495 30.62756
11.1661 16.44953 22.11149 18.44978 20.06924 106.5823
80.18715 36.78128 14.47225 4.479464 9.710737
30.30215 175.4954 25.80329 37.73261 34.7091 44.16608
40.03168 10.36142 6.697086 16.1817 15.31878 16.90618
11.51681 23.63271 20.95641 43.51126 32.98485
23.30063 87.99308 19.77931 62.92856 102.9708
148.0959 102.8258 3.950698 19.85804 8.128546
15.78105 3.164003 35.56597 11.01034 7.374801
14.1065 7.008131 12.9776 56.79893 39.41084 22.75365
102.2288 13.54565 14.80665 19.09454 52.97227
32.93757 16.13782 17.47095 26.02699 21.27993
49.16254 16.36728 10.14221 23.40746 59.37818
12.35693 6.973879 46.86246 29.152 140.7039 27.28012
23.99588 9.500163 21.95903 45.7912 261.5126 20.65662
2.691141 9.027923 21.82843 25.24292
RIPK1 11.92576 7.69662 12.59329 12.89455 9.692761
9.740683 7.279141 6.391244 13.45463 9.357368
11.12404 7.920135 9.640392 8.847326 10.1215 7.102746
12.80208 10.22287 11.74447 9.893547 13.22945

6.75415 7.53133 8.122028 8.537235 10.49132 8.805459
8.161956 13.10557 6.116506 12.01338 10.18524
9.400245 9.711877 8.724893 3.31209 5.256027 9.902986
10.98874 12.52939 6.757813 7.61007 12.99168 15.59605
15.64314 9.942655 10.45244 16.07394 16.30388
9.248118 9.775197 10.20203 8.417686 6.521107
9.179081 9.772224 6.791308 10.90528 7.902052
6.205455 13.92949 13.72568 10.6532 9.651767 9.266636
9.007227 6.414313 9.32191 21.92503 9.060087 14.57874
7.292174 13.73362 13.26538 13.13326 8.191287
12.7598 10.30858 15.78599 9.317678 6.728897 10.76142
14.48093 14.62499 9.089498 13.82443 12.41572
13.09918 7.940372 8.765038 16.28718 13.68047
12.51376 14.76895 16.41059 9.338398 11.76628
12.08057 13.13462 15.02223 10.94165 16.26013
7.342307 6.804533 9.435666 13.17413 12.47823
9.553793 16.26076 10.33231 4.125796 8.334945
11.26293 9.153326 7.503527 11.70401 8.63323 11.13479
8.193635 11.65739 10.86315 15.19427 10.09433
7.790397 14.19332 11.24106 11.16234 8.631058
11.17059 10.65972 19.49483 6.759366 10.30018
16.30052 8.605398 12.00536 16.17091 11.25079
14.53057 9.078913 7.824542 11.51243 7.607826
8.810767 12.10207 8.028284 9.124659 9.244741
8.231387 11.01544 7.667162 12.55562 7.362068
15.44581 9.676212 7.827878 11.70545 9.591566
11.70128 7.375732 12.39593 11.25985 13.45642
14.52576 7.284541 13.43863 13.34921 11.942 8.899391
7.791258 10.46858 8.455775 11.97284 10.98943
10.91153 8.966762 9.832481 9.330817 18.07302
7.505966 9.594172 10.79262 12.69026 13.6892 7.627106
13.05078 10.75649 10.73702 18.47268 10.00486
7.995183 7.747331 4.635889 9.727567 7.410354
8.771899 14.52258 13.88316 9.685202 6.700951
10.08111 5.02816 9.782825 8.126466 9.622148 12.2151
8.825655 11.79409 9.136282 7.205262 13.12836
9.029467 14.44499 10.83538 11.41841 12.23975
13.75846 11.81704 12.48662 8.586824 13.16357
8.452855 10.23626 13.46528 12.45631 7.007119
16.57877 7.700738 8.919512 9.927754 8.848429
8.669006 8.975499 8.420254 12.38195 11.45419
9.576103 8.85855 12.4115 11.99441 9.06823 18.41571
8.392527 12.02485 16.45622 12.98665 10.0568 7.852917
11.13651 18.12841 16.27669 16.0424 11.59407 9.537012

11.10451	11.03015	14.08985	7.784466	10.91293
9.952853	9.81272 8.416669	14.27037	8.10542 11.42142
15.21123	13.36053	11.05385	10.33591	12.99098
9.676461	6.724164	8.248121	12.10788	8.081344
13.75778	10.59905	8.712565	8.780524	13.66305
12.06685	10.98329	7.662194	12.36304	12.09371
11.64239	11.67338	7.209473	9.434824	10.42108
11.41012	8.743774	9.527025	10.32812	13.0495 10.12762
8.427641	10.47119	9.216271	10.86025	11.93486
13.4957 10.78168	12.19569	11.58858	12.60385	22.71081
9.638001	11.42769	8.710157	10.55961	6.289391
12.54419	8.392157	10.75443	9.964013	13.4368 12.79639
9.758774	17.1642 13.86943	9.64625 14.25828	7.364331
10.85842	12.16614	9.33728 11.90108	8.903796	17.88722
10.69742	8.746077	9.626268	6.429466	9.420354
9.883518	13.44425	6.262088	15.89723	16.88159
7.408671	13.84107	8.238826	13.43538	11.43844
13.76483	11.0685 11.09673	7.291637	6.644591	18.3441
14.31023	7.364237	4.737947	8.151755	8.235025
15.13341	9.783256	9.901543	8.156225	10.96921
9.84488 13.23355	12.006	9.760111	6.047566	10.13652
13.93824	12.47783	5.278397	4.935997	10.18482
8.970286	12.82557	9.016585	12.13526	5.232858
4.400023	11.29412	11.29155	10.63122	11.12266
7.344071	6.069928	9.455554	9.504846	7.29734 6.856282
14.67093	13.34408	16.81572	10.10248	6.964978
15.66759	15.17243	12.5093 7.16828 10.91348	10.66295
13.13731	7.224417	9.734623	12.97059	15.71206
10.20375	7.26547 9.71718
HMGA1	94.89491	5.420944	112.3964	96.61353	164.0664
8.093435	145.3737	29.10037	93.92126	141.7892
107.6788	175.3335	94.64158	300.2033	161.5363
11.78112	205.3832	56.81532	153.5718	171.9523
143.0695	6.796266	186.103 5.602292	30.03598	183.6149
114.4932	5.692214	119.7233	77.2956 150.6072	14.10333
145.5207	286.2499	223.5009	92.67209	20.28914
154.5607	258.6575	232.5252	101.8471	574.1607
284.7346	309.106 105.3613	259.8676	208.5156	538.6839
639.0731	735.4421	523.5028	97.75477	420.5819
227.5513	46.9646 352.2758	367.537 45.76872	243.1487
273.1508	72.16002	249.5292	50.61663	193.1052
338.6856	20.97592	246.5681	84.48774	338.4189
92.93838	311.4069	200.6873	226.7627	98.94017
144.1739	201.9947	149.4581	95.39353	271.2412

141.496 335.4479 127.7458 156.5394 121.151 415.1803
307.0227 167.6772 283.1909 248.7288 151.755 188.494
225.2613 132.9699 151.1738 357.9133 128.5309
961.2273 193.4853 331.6917 211.846 62.00468 222.4048
246.4128 167.7504 82.52077 136.0346 504.7151
250.3509 237.2264 154.7445 380.3172 24.40404
290.5778 61.36763 215.2377 271.9222 232.4179
159.6195 64.81251 578.411 467.5643 128.9239 166.2882
96.28957 326.4233 177.4627 320.5607 522.5069
80.55996 94.16954 218.7816 218.8534 430.5027
197.7142 160.1081 72.38462 209.8484 465.987 333.3084
169.9491 292.4075 284.9111 218.2052 272.5359
477.0631 74.20282 235.5029 104.8466 192.7264
85.41385 370.4932 244.7232 525.5169 144.5769
69.8082 158.8546 225.188 85.00227 640.7558 265.6658
428.5803 95.58388 86.54664 292.2764 488.1309
128.1344 189.5034 74.01509 190.0318 212.1245
26.47161 155.7644 163.2962 492.4972 397.2584
363.6049 106.3356 289.8698 196.4617 156.9644
147.7932 380.1269 183.0514 183.3972 205.5558
66.93702 84.6899 464.9695 123.135 292.9135 287.2866
157.6343 743.0491 242.7386 496.4104 244.5934
248.0659 401.3644 326.7172 77.2662 238.747 54.22457
186.4732 67.72667 467.7394 190.4723 111.6293
106.4806 287.7395 209.7469 88.46128 100.2772
153.3197 291.1027 248.3553 246.4445 219.7646
280.4406 167.957 457.7019 350.4962 80.04468 303.9356
186.542 78.27283 207.1569 251.7684 175.4058 644.4932
177.2078 238.6774 51.70804 329.2064 356.7429
227.3085 178.3559 273.817 222.8213 68.63111 252.86
142.8652 133.1345 154.7557 238.5834 130.9713
184.4816 319.5957 46.87794 332.6742 321.3074
294.7114 211.7262 264.5362 171.7061 316.0897
563.0955 376.5794 173.6649 377.4163 143.3854
351.9601 557.1386 138.0102 347.952 289.2447 175.6415
390.6302 601.6663 115.1167 148.6689 375.6998
662.8095 423.1463 309.8164 50.68686 161.1558
141.2289 26.19073 263.7393 252.318 115.6823 208.8697
254.636 305.1402 305.3638 133.4653 192.603 161.1422
116.8314 227.8564 500.4358 11.29109 33.03514
100.1678 78.96839 222.2198 173.0876 225.3081
179.6175 280.3063 174.0355 104.2407 153.4257
38.36514 260.5767 256.5927 119.5822 155.188 188.4825
275.9807 215.9467 1285.489 211.8794 241.1879

173.4859	110.053 147.5435	90.68987	99.58719	60.81012
234.5527	149.391 464.5631	405.2692	39.11362	247.5639
298.8547	262.6326	180.554 149.7764	285.9733	159.6603
175.9519	135.7885	79.12024	372.6787	119.8426
145.8606	120.7776	531.4418	63.96351	197.323 235.577
177.2897	58.02029	158.6027	109.1188	157.2	370.2083
281.3966	209.3057	249.1512	119.4914	110.9115
236.5095	125.9564	149.3454	532.465 30.25615	153.5152
207.5241	184.7434	950.3021	522.8348	274.2471
161.174 305.1327	259.2139	168.5342	137.7971	254.7664
144.249 209.4171	42.17343	322.6608	243.0829	245.3994
217.6182	159.3242	171.2511	145.3827	428.7639
218.3347	183.2825	283.3524	418.2096	176.0559
673.8046	390.6283	185.2479	118.9039	306.2993
108.5006	170.0453	252.4642	121.8095	315.3809
90.74534	56.09157	253.708 319.4154	69.31123	397.1321
264.0531	276.1595	458.8768	121.9115
TNFSF10 66.14223	3.080147	51.65893	18.08256	31.3259
8.829015	19.53254	13.95293	18.81303	30.28379
18.21644	28.89692	22.9398 22.94507	18.83685	4.137427
12.9259 16.87315	13.85322	25.31191	32.02349	6.961747
15.18359	8.823652	9.351882	18.87408	20.26267
5.577005	23.35472	51.04016	21.14917	10.17707
38.43412	44.17724	48.82391	12.82937	12.73236
10.25715	19.60009	22.85712	55.49752	18.63421
15.35258	25.67752	21.90904	5.746987	23.22349
16.43462	17.52805	19.10505	34.40286	20.26321	1.4855
24.63196	17.04693	9.282348	17.1306 10.7038 17.13513
19.23701	68.96631	63.81624	47.9302 12.15972	23.94262
8.634478	34.95587	22.1506 13.63884	16.47296	25.60302
24.03458	11.18163	35.12975	14.95798	13.64085
37.19228	5.039738	4.983565	25.51381	50.93805
26.92402	91.17823	27.24163	13.17519	66.91309
22.26214	44.66418	29.8084 16.38277	27.98915	53.85719
146.1886	37.61265	28.98689	15.24851	33.15345
13.40893	11.50424	34.73612	18.20594	18.6358 39.95281
17.01588	13.1224 15.17219	58.66431	15.13325	26.39451
26.52442	6.776853	18.0241 70.94755	11.9443 48.01728
39.92572	11.47233	34.40138	12.06795	15.68092
10.48077	14.23504	8.134063	20.92301	10.45507
11.94159	45.18828	18.57288	20.55631	17.3034 60.78566
8.025076	18.6436 33.6801 26.86824	27.13971	29.19432
26.88117	17.64209	9.935177	17.80215	108.8573
60.49436	5.263375	17.17564	33.32338	12.87563

8.665901 3.236957 22.60984 47.17446 22.05032
6.838485 75.84981 11.75141 15.04011 13.04166
30.1511 7.87382 15.31178 80.6396 19.587 36.05939 25.43288
8.683748 36.89771 49.07517 34.25385 26.4406 5.701224
16.87331 17.44814 28.2793 19.8201 13.60147 11.70928
20.41601 20.65032 50.71965 6.796417 28.14749
13.11858 32.62055 31.58446 11.6214 17.34644 25.84981
49.5709 20.06528 32.64122 24.45985 30.66872 17.18477
13.06578 34.78722 32.61936 55.91755 6.266622
4.769918 17.42126 5.361832 29.2602 18.98428 19.17527
10.6537 14.90057 22.42626 14.70067 11.51045 18.14847
24.67276 13.1033 32.57046 11.69152 74.36616 17.98194
38.73368 6.220443 21.11959 30.39398 14.02225
62.9551 14.56926 19.89235 23.90422 26.14163 12.32736
24.74446 17.04198 19.66564 19.26083 13.90305
16.0361 19.90214 41.71389 43.08037 16.84238 61.74698
32.39944 8.145446 16.78105 95.07644 19.78831
47.46258 20.01296 16.41565 26.21693 8.528528
22.20958 74.13876 49.64622 37.31722 9.457903
45.94318 34.84681 4.845487 32.04208 40.99095
23.30211 8.037634 18.86487 21.59712 50.76042
24.62784 36.02485 10.51287 8.947929 19.8287 18.35794
23.12746 40.43752 58.89417 25.22297 29.65009
11.03611 13.0234 29.78338 6.422049 3.30102 44.5382
25.26803 17.28762 17.90594 21.97329 4.78288 18.24856
8.449579 13.40301 19.89746 17.83161 8.266675
4.578212 11.61324 24.316 19.22458 15.85507 22.71979
88.40233 23.3813 7.539918 24.61004 35.1527 76.75068
10.07819 23.66743 9.716487 9.104807 16.80015 42.713
3.205289 20.29591 12.8824 70.56513 20.03449 29.61198
6.552046 18.63205 9.774558 46.18868 51.62927
33.3639 21.37561 5.331725 27.80429 47.94891 16.33814
45.79055 8.342158 41.09819 20.87447 15.20845
12.41099 38.26026 69.6171 31.89632 20.17979 3.742249
35.28565 32.76217 37.83251 2.920752 20.47846
7.153842 15.41921 15.80044 43.72912 25.44817
4.755545 28.81867 39.15446 20.11993 8.791845
11.36511 9.754627 32.53811 29.20717 17.94218
8.69924 13.91766 14.4935 53.34317 15.9381 30.17453
10.91028 8.816125 10.20606 30.46967 50.56134
8.76935 15.53636 17.09279 16.42122 10.27567 55.85975
15.69708 17.6065 29.17438 4.903266 9.442042 15.05285
15.28114 15.40661 13.54268 7.140735 26.39291
29.51309 16.38632 32.95295 22.55855 7.702674

20.77923	7.658388	19.66439	15.21496	85.37059
69.49221	16.21484	26.97607	19.20289	10.35914
9.132608	27.32973	47.01375	25.32105	19.62356
17.55591	17.89149
SIK2	2.998863	12.19224	4.976705	16.98416	6.166784
8.562914	11.36585	13.916	6.541608	9.012192	6.987921
3.190604	9.589545	4.287095	12.29267	10.92173
9.011464	10.88321	5.041436	4.792623	7.549271
6.044125	4.556652	7.708003	6.493959	4.743485
11.47291	10.04095	6.775333	3.432639	9.509844
11.67381	5.872498	4.923361	6.803846	2.181555
10.35836	6.218003	8.650233	3.192085	10.36003
3.598041	6.258048	3.752768	6.162352	6.746537
6.751074	6.285721	7.346874	9.308336	3.342	6.208658
6.870422	3.766534	6.497691	6.091344	4.788564
11.63844	16.13248	9.440028	11.04925	4.221189
7.95578	13.3409	5.969867	11.14837	4.927651	7.524833
5.710108	6.134437	4.361214	6.142199	10.08815
7.506525	6.863432	10.55203	4.891589	17.54758
12.81798	5.407525	5.291655	15.53321	4.619764
7.751749	5.948927	10.01472	8.638884	5.608195
4.445692	6.658043	4.67926	6.652306	3.670369	5.116868
3.042068	10.40764	4.900844	5.308313	8.748446
6.764876	9.648472	4.4444	3.882014	4.798036	9.681103
10.0029	5.26967	9.884869	4.869682	3.220929	7.759322
7.346383	2.701679	5.505034	4.900678	7.627972
2.542279	4.833913	5.804707	8.043515	2.121106
11.77849	10.75886	4.011827	7.5787	7.307883	3.410336
2.503947	12.89975	6.513483	4.660229	8.108255
5.04032	5.904588	4.45614	12.23249	7.005556	5.127618
6.839029	12.30485	6.053811	6.740151	4.82271	5.44352
12.26544	5.054328	5.85669	6.772342	6.062184	5.839902
8.77501	6.817455	6.824519	10.19697	8.86034	11.33502
4.895362	6.615329	4.13729	3.63721	7.242905	12.2204
13.59566	7.351853	4.995303	10.43595	8.79164	6.101852
7.657192	4.979224	7.841913	6.138126	8.929343
3.16028	5.028539	5.933816	7.391652	4.860873	8.245386
7.971768	2.019706	8.388212	5.127286	9.827018
5.175293	4.837804	9.016705	2.299654	20.30783
6.569005	11.00702	4.499573	3.514532	7.686396
8.206832	11.84498	6.438277	8.741028	6.939669
4.633168	6.209444	5.068962	6.512443	5.202512
5.954655	6.415406	3.088119	4.725014	9.489186
3.931793	5.814457	9.024688	9.651648	5.612304

4.315447 5.451279 6.718564 16.23287 17.65074
8.272405 6.128372 8.163276 8.571431 4.636493
9.731883 3.074529 6.964543 3.129165 5.511751
4.874844 4.733662 5.844876 5.495743 6.187709
6.368072 3.699494 4.796164 2.578407 3.433101
9.363095 8.582013 5.827249 8.510372 3.649899
5.985936 4.626277 6.785861 12.25487 8.314825
3.361848 8.228647 5.83098 5.076656 8.528664 5.853498
15.46819 9.114227 8.366289 4.781643 10.24282
4.197456 3.442064 6.521521 5.132493 8.299335
12.63442 7.668822 5.601991 5.384125 5.945243
6.007421 3.081598 4.752699 5.377277 4.182105
9.882834 5.927834 8.916179 6.638696 5.406065
10.29185 4.836778 3.991681 5.290533 5.790439
11.92969 9.612505 5.085873 5.208544 11.34898
9.374027 9.500506 8.727977 5.908164 7.422541
13.89698 3.334289 4.395881 2.964718 4.843811
6.553456 11.82148 9.41716 11.39555 3.366862 4.935329
16.16061 4.343748 8.605983 10.59524 7.667248
2.304138 6.344918 6.341718 3.707566 12.29908
6.516356 9.433584 5.495289 7.085039 8.874897
10.87883 11.08884 5.605319 3.805277 10.09411
6.64931 3.278829 4.942384 6.343052 4.372169 9.916615
6.785914 5.007978 7.435582 7.63674 6.136944 3.853111
8.234085 10.96385 5.059223 5.062563 4.21741 7.744365
11.98921 6.170454 15.58558 15.87236 3.273266
4.927326 8.317942 9.509191 8.11425 6.163854 13.82759
4.04478 6.894968 3.471019 7.618932 8.456926 9.76412
7.283345 6.364657 10.49219 3.253065 3.19135 5.318547
5.763266 8.977683 3.127204 10.9209 5.114916 5.686727
7.62894 20.59382 5.175008 3.061397 5.126709 6.324212
7.50276 12.31874 6.141521 3.614258 9.006081 12.42885
5.285211 9.668913 5.049571 9.58589 5.270536 8.325099
5.155891 3.031611 8.130824 6.169272 7.364718
9.24702 7.973412 13.29858 5.257438 5.994417 4.388853
4.177419 6.174108 3.936175 5.570964 7.156606
ETV4 1.188229 0.1540634 3.138146 1.212022 3.624317
0.334493 2.066146 0.4827373 1.417789 1.801605
1.853199 1.883382 1.928232 2.670002 0.8405302
0.1253306 1.864044 1.607345 4.033402 4.937975
1.694425 0.136389 4.447859 0.1397503 0.402773
4.065159 1.600323 0.1688022 1.969275 1.252135
1.343147 0.1696951 0.8903593 10.46593 9.680094
2.11913 5.360587 40.738 55.09536 5.489519 29.5315

16.35476 9.743027 11.59534 4.52927 30.10963 13.2121
45.5846 17.75046 21.2585 13.89344 6.471807 6.957116
8.636433 1.044504 9.175182 37.29381 2.370086
31.45834 7.339046 5.213195 2.856396 2.491212
8.710291 22.33257 1.932405 16.31201 3.179412
11.07634 12.82779 20.77142 3.608318 61.15872
25.3779 6.622859 0.4953511 1.801319 30.58066 10.38643
25.9154 11.80094 5.900171 3.494726 2.277954 13.17789
1.887579 5.773222 12.2992 12.68088 8.682141 5.612669
0.5082197 3.148365 3.264175 11.25674 1.74884 11.01021
6.158495 64.64259 2.568033 5.860007 12.51011
1.020879 18.23421 22.49345 1.63465 9.134278 5.706074
5.606387 0.7893691 36.85763 6.494193 8.298645
2.864041 11.22669 10.81545 8.076164 14.5162 35.74856
25.1148 7.190285 17.01251 49.23693 2.692079 49.10598
1.863584 11.34343 8.076711 4.921111 16.46299
2.219147 24.97844 5.893498 14.06334 10.04008
16.65432 4.310137 8.274778 21.29541 28.58727
38.28762 6.705369 9.227898 14.39776 20.37352
14.93519 33.05076 6.243337 16.28282 1.573821
0.8710648 9.135966 8.215944 3.906996 0.4474132
3.085987 19.08228 28.74959 25.77645 12.169 16.47708
1.588671 10.34715 12.84312 23.87269 3.881714
43.58958 8.952125 5.61718 52.03299 0.2926093 20.30554
9.364171 21.19874 9.494829 14.04738 8.357869
2.204085 5.541112 56.28992 7.71328 37.58858 3.225222
0.8555935 20.8029 8.370916 2.839761 5.122812 13.78898
18.49499 13.06402 12.54691 6.65855 5.529706 32.8923
10.76881 9.490164 33.26202 39.4834 42.64073 14.01598
6.924476 16.51785 19.15671 32.12265 13.81923
13.36162 38.13515 11.02127 12.95355 7.882659
21.0418 20.33869 21.21273 17.91633 1.377924 1.500065
8.382543 8.646278 9.007459 8.795409 0.8025558
19.42698 4.824405 3.410454 1.893253 26.97855
5.665521 29.67259 6.26741 9.102232 3.943832 12.92913
4.430001 6.75759 5.525395 5.128999 0.5636172 1.87384
16.63571 36.34562 5.307562 2.077849 8.734585
12.77769 9.423137 2.481376 1.193255 4.407178
1.705991 18.56387 6.313472 6.871164 2.31478 56.21678
14.35077 38.11055 10.29186 5.20731 9.745741 13.01596
45.49917 6.569093 5.733706 7.563907 16.92364
10.91415 5.512466 11.4417 5.055861 4.685599 2.857207
5.846614 15.64558 19.81672 27.20997 19.04659
4.835873 12.99857 19.3262 3.660393 5.702612 10.57733

2.624208 3.617739 4.867273 22.20322 21.59198
4.711631 3.591792 20.34256 0.4633547 1.356677
8.520562 9.461962 10.73267 15.15663 3.894947
5.681228 5.506731 9.261396 11.56957 10.82644
7.490576 9.745482 35.10172 62.56039 20.7395 5.371053
11.01291 13.24354 23.74866 0.8914278 4.961238
7.103345 0.9530069 8.983163 6.001111 1.861938
11.05899 30.61667 8.843317 55.36876 0.6069284
2.642646 5.075041 4.308122 71.43359 15.67716
3.780743 23.63785 26.89227 4.281094 1.29216 14.20532
30.86933 21.07172 10.43172 5.874657 21.96149
24.2676 18.44997 24.42666 22.84544 3.996245 5.467007
7.234218 5.035212 15.99493 7.329582 23.08571
15.05893 29.11237 9.473831 4.430341 31.89801
7.493222 29.77769 2.207484 6.859109 6.85985 16.52491
6.513254 2.203223 119.1616 20.41152 4.165939
8.451339 40.03212 10.87974 20.0324 6.437405 55.09269
8.695396 5.927976 17.73823 15.00764 18.26429
29.93382 48.3094 29.81842 23.17552 16.67662 37.53238
12.19649 3.291821 20.89685 9.04323 4.067138 15.42636
2.735215 11.57071 21.7253 18.53419 5.833116 6.051501
52.14848 2.728651 0.1398366 22.37619 27.88945
0.9225814 9.02836 1.030545 13.24031 20.84754 17.62009
S100A4 13.84825 67.38958 18.39666 17.20948 38.4667
76.36724 32.93768 23.62981 32.51756 58.10679
14.35693 15.78944 58.09261 13.18336 56.56881
59.82411 26.91704 44.84473 42.63342 40.32399
43.27942 52.56933 39.56309 71.93622 109.0403
18.7619 41.77686 40.75194 20.76239 119.2594 31.40363
28.25716 18.26558 20.75864 87.5268 13.92024 3.495525
53.06067 35.22833 34.90993 56.32784 74.48377
68.29159 71.3209 26.95844 55.72652 12.6831 49.40833
625.8265 64.75614 77.16589 74.44824 6.538741
103.622 40.432 28.19292 34.04774 28.26143 87.50755
27.67102 134.3717 109.6798 87.68047 19.29002
17.57472 65.62941 98.9069 90.75377 23.8681 10.50558
184.59 43.61002 26.16449 53.27664 72.2453 50.6796
73.01822 14.20317 69.49559 30.8109 40.88702 49.484
38.62491 36.56833 32.66735 34.68065 8.881916
51.26834 58.07476 27.67486 45.45763 25.92443
67.52846 49.30911 174.8801 27.29574 38.97877
12.70164 6.684956 58.51762 33.25706 48.33286
37.78654 39.98939 19.05746 30.37638 206.0103
39.87731 32.73963 83.39096 20.24945 19.0747 86.96266

34.82987 92.56789 26.56985 37.28568 19.59543
12.88995 53.30951 79.72212 36.39928 33.05964
66.78811 58.233 34.19232 41.68097 432.6328 82.70129
37.0088 28.01116 580.022 33.28682 53.7883 53.90001
35.31955 37.91195 234.8151 143.0145 18.07774
162.8041 31.22301 23.38732 28.86249 14.9972 61.18886
17.03841 214.4816 32.59097 17.88876 200.5543
66.37139 47.58745 25.13103 21.28626 25.68044
98.78873 81.53391 33.08968 58.2829 110.4544 52.7894
29.78757 52.98355 101.872 27.27469 37.21765 31.09207
19.05276 46.57469 27.75508 22.46962 14.33649
317.5789 21.94629 57.94518 34.25177 35.76029
34.64877 13.9088 53.84451 40.87073 24.52474 31.24422
49.88838 82.45286 34.71667 302.8011 16.48242
30.25668 43.68736 158.2543 39.11971 15.79703
42.26331 28.15818 53.54426 15.92486 21.26031
33.05652 23.48901 282.0951 24.58191 31.85509
27.99862 15.03415 43.9968 16.6687 26.78294 108.3797
26.83276 48.23951 16.78793 21.2923 93.28654 114.9734
38.55926 14.56391 29.14941 27.25953 57.37887
25.05929 21.43907 189.9781 28.35363 33.62216
17.26947 19.70605 15.42586 1198.472 27.68129
59.33776 12.70097 9.941802 23.91601 114.0252
62.84507 22.31005 24.10364 21.92592 22.55892
22.08625 26.57884 148.8735 65.29611 262.2328
56.64015 76.32139 54.49822 53.16162 71.91416
712.9838 57.99455 169.211 17.57753 13.87211 41.6351
75.72576 37.12838 25.5095 48.45275 139.307 32.45765
16.91183 38.34537 32.90386 89.29754 19.25972
25.53993 13.45369 42.50114 54.45268 19.05108
22.20424 17.56215 19.83527 52.76467 30.47277
334.648 345.0833 83.06659 10.05378 426.5964 47.03534
364.0875 37.39029 19.60047 11.53203 55.46313
18.41738 22.16546 20.03675 30.71117 41.95595
49.02221 45.81715 66.97983 50.1081 514.6659 18.88237
79.74525 54.83205 11.85744 60.99575 28.05051
20.13499 21.09319 146.6732 23.74118 10.1269 20.82722
118.6806 50.87793 139.7066 28.48181 15.8472 18.74681
33.29479 42.97263 106.4877 169.7494 33.79355
57.27583 45.90499 105.6474 17.40126 58.3112 64.21648
35.22819 23.28292 145.364 16.68929 29.72451 142.8998
34.30519 48.34927 7.094005 23.89374 29.379 22.03744
51.74271 91.3481 33.36384 17.28828 45.83694 17.3754
10.42743 4.347827 66.60763 36.53805 13.09302

27.05611 20.51119 38.27019 33.62513 9.910314
29.91544 68.77634 22.43427 32.18682 25.81766
32.34048 19.98253 22.82541 37.41013 67.42097
9.96449 36.86667 90.87844 377.7534 95.97062 39.7255
93.68102 22.76342 15.46008 46.07619 29.50885
27.87723 48.74625 25.68006 27.11821 193.557 18.25995
13.04973 9.049344 39.02948 37.95371 121.0461
24.31006 33.41966 47.83405 346.5484 13.59365
340.7425 60.02084 41.80732 16.85015 55.21573
8.474856 23.02843 34.09895 37.37653 167.9115
12.76835 16.82076 58.64459 18.37325
NTF3 0.6719299 0.6377584 1.616131 0.4041392 1.135961
0.8014609 0.3845076 0.5010743 0.6224063 0.8356187
0.5255022 0.557376 1.099122 0.7559956 0.5355491
0.8481011 0.5460631 1.424062 0.2519687 0.2551292
0.5816099 0.6444005 0.4875668 0.404926 0.1465845
0.575098 0.7169474 0.6887351 0.9137375 0.6098689
0.5626179 1.561534 0.08167531 0.5016326 0.2188109
0.01091085 0.005179095 2.472065 0.500512 0.1365486
0.1286203 0.04826419 0.1968582 0.0639943 0.4458994
0.1318466 0.2664076 0.3798406 0.1165634 0.139137
1.752432 0.084527 0.4111555 2.290068 0.4367 0.1284904
0.1096191 0.3316482 0.2046209 0.5330641 0.1638737
0.1357554 1.045956 0.3145115 0.4657602 0.3271513
0.516142 0.457772 0.2300336 0.1043075 0.08076787
0.03607434 0.2166422 0.2181281 0.2711166 0.3330566
0.8772148 0.182318 0.06027345 0.3022933 0.07788815
1.011255 0.05331386 2.170002 0.2254368 0.2746924
1.003772 0.2337222 0.4262712 0.1796504 0.3213572
0.06669086 0.225347 0.2899101 0.08020545 0.08135668
0.04342113 0.1939196 0.0417688 0.1967554 0.3925949
0.08696885 0.08027707 1.044805 0.8011889 0.5706871
0.05744123 0.62579 0.211267 0.03317759 0.1265973 1.566605
0.1659605 1.212618 0.8813719 0.1264541 1.609448
0.07492087 0.07721511 0.4113606 0.02575367 0.8674514
0.3623455 0.06468377 0.3731806 0.2751803 0.1229165
1.232742 1.018879 0.2994367 0.4193944 0.130331
0.2329615 0.1053214 0.4895837 0.260101 1.24984 0.03751354
0.25745 0.1306164 0.1152791 0.1415033 0.1897255 0.2829833
1.099285 0.5474223 0.02584448 0.2978716 0.04825093
0.7292874 0.05519858 0.20654 0.09020363 0.06925214 1.684063
0.09377079 0.4091574 2.062712 0.08389433 0.1665248
0.1200335 0.5168989 1.292846 1.937932 0.3495046
0.525495 0.2699528 0.3442374 0.1762397 0.006127611

0.4108824	0.2521182	0.2283456	0.06077777	0.04651744	
0.0354895	0.2118522	0.2651908	0.2489868	0.4319613	
0.5806871	0.07540277	0.4682145	0.2040316	0.1775031	
0.5534795	0.3644795	0.1279517	2.442353	0.06024457	
0.533553	0.04720488	0.110984	0.213057	0.1968484	
0.2177903	0.1176513	0.3460994	0.09129018	1.214829	
0.4939677	2.960944	0.9173508	2.09252	0.02825187	0.07718137
0.2279023	0.1203885	0.1268058	0.04670753	1.814422	
0.5660505	0.5867322	1.394341	0.1146364	0.06532083	
0.207734	0.3312128	0.2294309	1.011394	0.1254053	
0.03673022	0.1410573	0.02495845	0.3515524	1.750582	
0.8633683	0.7997681	0.1518149	0.06963692	0.03369227	
0.7003317	1.661919	0.05497701	1.228692	1.212268	
1.125621	0.3292357	0.7755798	0.1626241	1.450103	
0.3890236	0.6402478	0.185028	0.1713413	0.2437813	
0.1988905	2.854358	0.2538053	0.1809974	0.3534633	
0.06739991	0.6488509	0.5865106	0.1742184	0.6081346	
0.1194316	0.1908933	0.2757043	0.6391422	0.4149773	
0.1324539	0.1843421	0.12854	0.7284667	0.9994382	0.1206853
2.209213	0.165314	0.5712306	0.09982726	0.1893831	
0.305658	2.229005	0.1074012	1.276771	0.2409326	
0.4860216	0.09605356	0.1305639	0.6659506	0.05258732	
0.07994117	0.1651176	0.07989395	0.6275135	0.180503	
0.1338659	0.6653885	0.2429763	0.7429926	0.1650641	
0.3484048	2.808255	0.1525391	0.5483786	0.07634753	
0.07835076	0.1775242	0.2973318	0.3520367	0.5160792	
0.01697742	0.6047368	0.2268201	0.1622977	0.3609144	
0.3313039	0.4500398	0.1572284	0.7410423	0.1064401	
0.09455235	0.6531533	0.1922099	0.7132113	0.3596617	
0.3520846	0.2641085	0.1985553	0.1524987	2.204547	
0.09788128	0.236295	0.6858595	3.466141	0.3907603	
1.136948	0.1423431	0.09543668	0.1110425	0.10596	0.2064923
0.4145328	1.332147	2.766142	0.5246882	0.1435761	
0.2340103	0.2434926	2.959741	0.1949745	0.2982909	
0.02223714	0.2904793	0.4167708	0.2365127	3.218406	
0.0066015	0.2194648	0.2102274	0.5054101	0.3395382	
0.1067488	0.08332663	0.2188225	0.3379664	0.4160195	
0.8828504	0.1309298	0.2160085	0.4469674	0.009967071	
0.1306808	0.1053894	0.8073097	0.03265014	0.1485956	
0.638584	0.08715023	1.100295	0.3701264	0.01419966	
0.5621459	0.06854507	0.3772753	0.2179026	0.3337167	
0.1930988	0.1202332	0.1130401	0.1385387	0.05343281	
0.04458533	0.196712	0.2012287	0.7711169	0.07361458	
1.186567	0.224532	0.2874761	0.05452588	0.5748959	

0.06633204 0.153361 0.5126935 0.182675 1.018147
0.3359916 0.6666191 0.5382874 0.2520672 0.05902814
0.1212802 0.273627 1.045734 0.699299
LATS1 2.629036 4.148671 4.565988 4.102726 3.717417
7.805382 2.654333 4.321353 3.70584 3.603487 7.771509
5.075945 3.647167 3.777825 3.644824 8.397355
2.94069 6.033369 1.994599 2.588534 4.407824 6.396746
4.195773 8.669905 5.551576 5.22169 2.740644 8.780563
3.251633 2.00631 4.023524 5.020628 3.803374 5.093079
5.851707 4.911998 2.779376 2.299346 5.583451
7.229886 4.975115 9.116292 5.332789 8.640794
7.096939 7.034275 7.806206 4.032801 12.95173
3.944537 3.651998 7.855266 5.251415 4.162844
8.210091 5.389252 5.46933 9.387953 5.127373 6.066386
8.412447 5.469745 6.497471 5.517537 3.541589
6.730703 2.722551 4.388356 4.151308 4.429305
9.629039 4.924524 9.819285 8.891932 5.952981
3.89739 5.863894 7.817192 12.31038 7.118552 3.896087
6.913252 8.601317 5.831885 7.179873 3.963148
7.791903 5.753945 3.973422 8.592859 7.205226
8.037136 5.828605 6.960219 3.937403 3.436291
6.556712 3.97734 12.89623 8.63682 7.793407 5.516059
3.311534 3.837122 5.31978 10.66019 5.424619 4.647564
6.527147 5.810947 6.036695 6.102076 3.947088
6.676755 4.869811 6.155194 2.691463 5.452135
4.758588 6.737167 5.952764 12.61193 6.962978
4.259901 14.33397 8.26647 7.842806 2.936155 7.914439
7.219038 8.565107 3.439043 6.345558 8.025311
3.028858 8.313032 6.857664 5.920252 5.435212
8.988038 5.223921 6.789707 4.43955 6.511282 8.143953
3.731603 4.778828 6.086309 11.08706 5.699433
6.468848 5.109535 7.393727 8.586928 8.048608
4.365072 5.920914 8.83538 6.455345 4.819805 4.808928
8.440753 8.003787 5.632043 4.85199 8.023756 5.306697
14.98645 6.513018 6.462979 7.344953 7.500754
8.922506 3.4245 5.606844 4.741077 5.345023 4.608857
6.686796 10.44351 6.60537 7.19493 4.798495 7.858595
5.230856 6.596433 9.148364 6.39327 12.79844 7.244771
5.542854 8.771918 2.640821 4.008962 2.610126
9.74006 8.138025 9.195757 4.881643 3.30583 5.482036
3.724515 5.53056 5.854002 8.088013 6.826806 1.743965
12.08728 7.179087 6.011674 7.933947 5.815565
5.997761 4.610845 5.792903 8.8655 2.511296 7.277589
6.109043 9.65132 4.341981 11.02795 3.40709 6.899915

10.59187	5.187513	6.793244	4.18468 3.728532	6.113123
2.725244	4.845789	4.064893	7.464088	8.565223
2.729461	2.99524 8.65878 10.19465	8.791117	7.706215
7.679281	5.541751	6.494743	8.483665	6.620482
6.593982	5.136297	6.137385	4.72845 9.944402	6.094785
4.617517	7.575882	7.63463 5.204463	7.752783	3.380339
5.264864	5.552244	4.261492	3.371504	11.56493
5.763351	9.904492	9.502269	5.524305	3.487367
4.38696 8.992584	7.394237	3.791935	5.445375	7.163906
6.878923	4.919414	7.33927 6.748742	4.316344	7.23765
8.922688	7.177535	3.459669	5.245024	10.15158
10.22807	11.68204	5.289632	5.013362	7.44698 7.917208
8.877725	7.03924 6.263661	3.665435	4.17706 7.928841
6.188187	7.441265	5.597579	6.322205	5.850021
7.256101	7.037057	4.106822	7.231072	8.73617 8.091137
9.603529	6.181151	3.34133 3.330476	7.515469	3.306657
10.89491	7.221983	7.623071	9.221005	6.492538
10.71085	10.60746	8.822117	4.363582	4.243485
7.915359	7.337492	6.160738	3.603687	3.999261
7.947448	3.083719	6.175358	6.535505	4.412597
3.730929	9.026935	6.539947	4.767169	8.696913
4.150039	6.049304	5.152096	3.404377	10.69821
8.846929	6.820119	6.84579 10.10904	4.483573	4.068039
6.655488	7.541489	5.822483	3.723398	5.8901 6.174469
9.034368	2.967504	7.339447	4.633884	5.524497
4.313379	9.369757	8.174749	5.548346	4.785135
5.895733	4.402243	5.158377	4.363865	9.352896
4.971421	8.676928	18.81548	5.967999	7.465393
2.215596	4.14508 4.581008	8.716832	5.531086	7.528965
5.941098	5.173192	8.772047	4.66018 3.341792	4.559663
6.127007	6.354344	7.67144 8.556848	10.60523	7.182998
9.104009	6.812051	14.14617	5.841135	3.225916
5.851121	3.786003	7.406366	7.500759	7.167991
7.44873 3.445726	6.70628
HTRA1	8.813114	84.41759	17.28492	20.06507	20.12642
73.63747	11.11789	47.02151	34.16294	36.67171
12.6946 5.358642	22.39358	7.183636	12.62502	67.8558
13.94372	97.15623	20.37593	18.38387	29.87025	80.673
19.44225	69.02803	59.48829	14.59796	20.79535
74.9111 30.6185 20.12219	30.30862	80.19709	8.381403
11.78492	59.56375	13.83618	106.7871	36.14257
42.66888	13.09412	55.27596	5.823815	154.6848
18.22175	26.97865	13.21697	11.14793	32.91938
36.04151	13.73326	38.19503	28.69515	16.36586

40.85718 91.2352 16.14154 26.66483 57.24183 14.10073
26.48792 71.47976 30.03801 115.0015 64.26734
12.42988 78.09043 85.72914 82.5624 32.62322 50.77911
9.119074 60.89079 31.06302 45.85582 58.66094
36.93438 38.32585 88.29604 22.87404 16.88391
41.25287 66.6922 12.73614 32.59149 79.31827 49.49377
23.83384 25.06723 36.38949 55.88751 68.8474 53.58799
33.34603 18.9752 37.94191 43.12029 24.42589 17.2904
4.790659 40.53228 65.52773 10.4299 12.50596 21.37316
135.3405 73.38693 21.32466 64.2819 33.59182 17.34994
22.12493 51.59833 45.64163 31.70044 28.73685
41.83064 35.54079 16.33147 53.57844 9.42083 36.03433
30.6853 45.09756 104.3046 30.35534 14.90974 8.969835
65.00319 69.8965 157.1271 26.32125 21.53413 13.83459
91.98824 19.24253 28.34465 36.54933 71.08777
66.28014 15.47558 38.85059 11.77664 20.06677
49.20237 47.50347 31.21364 23.53942 97.69426
11.04047 41.68546 14.89643 134.0345 39.12306
35.6754 51.64884 19.4098 41.02755 70.11041 17.76975
21.69044 82.46066 29.39973 79.80324 24.5572 49.49052
44.33766 22.36726 50.88196 23.64089 100.4259
45.9606 43.51852 21.77444 18.01998 140.9297 48.83788
109.2006 52.75746 20.69486 52.95759 21.18093
18.66011 15.90207 40.83677 41.06574 69.27295
114.2449 47.67853 39.43288 9.716995 38.0956 15.39678
56.64478 20.95395 31.78216 45.02886 88.15017
22.99645 10.34899 40.32044 8.393636 43.29583
26.43079 18.71917 13.70563 9.968127 73.16392
28.15574 20.56174 30.26475 42.45277 84.90952
23.91786 30.74026 8.28667 93.79479 36.58201 13.24695
76.92893 26.98469 34.23961 72.94845 45.53378
23.28962 28.72017 9.024939 13.21788 7.692372
19.60719 43.37983 40.59937 34.98494 7.231303
13.59845 13.953 23.08999 46.84107 9.043159 46.25956
18.06014 35.69805 28.31346 26.30105 78.84055
101.659 65.56545 37.72281 62.22359 50.34239 27.51116
17.44035 16.78944 40.5601 72.15848 14.3291 13.11263
37.19263 46.77104 42.343 65.48588 34.14447 51.54465
24.07772 35.99167 27.75281 33.1831 14.61702 22.67957
82.02239 13.85267 30.27667 30.7882 16.82469 34.66189
9.56922 11.91588 17.97234 52.27962 14.01167 38.62641
101.8315 70.97796 118.907 43.19617 19.12086 32.36733
48.41516 16.49648 39.40111 129.789 15.27336 47.36545
76.55107 63.70041 407.1833 120.5184 58.047 19.27972

69.87943 25.6689 34.3127 75.47294 16.62617 61.41135
34.5676 20.71927 26.73824 27.4271 28.64846 17.44322
53.01984 40.79427 25.93993 113.7415 18.91374
63.16273 31.28099 84.46679 31.63034 86.53418
9.12015 22.5815 40.92559 55.11171 77.43678 12.71842
40.65628 42.99585 14.20464 42.65047 13.10015
50.06442 45.49741 19.1171 99.83057 13.43849 3.558167
29.75823 16.3845 20.16163 40.33997 21.99503 34.31576
7.876486 61.16267 10.4827 35.04971 9.348683 31.37702
43.75773 12.84948 66.70024 56.61494 104.577 51.68647
18.73853 30.85278 27.00116 38.33768 80.76876
23.26179 61.0409 28.77138 13.56492 12.36843 35.14942
5.604459 11.744 40.31133 35.39882 23.39073 23.11679
44.04912 66.82297 21.62533 116.1559 22.59779
58.01488 16.372 36.9102 130.205 29.97533 21.81787
42.76764 8.03175 15.13985 146.7081 19.05548 17.90395
24.56206 37.98195 28.07841 13.1568 52.96409 8.120637
48.64828 55.62336 39.79864 17.10381 37.2132 72.92678
12.43134 14.67015 22.06343 42.82647 37.907 48.34949
CEACAM3 0.04245375 0.00902121 0.2082698 0.3424593 0.3897966
0.1194287 0.02915267 0.1017604 0.2621651 0.5378091
0.1579128 0.2502192 1.121796 0.09729943 0.1845653
0.01397858 0.2728736 0.1314106 0.1456083 0.2921665
0.1157536 0.009395629 0.2220386 0.09029633 0.06461493
0.1260628 0.08213381 0.04848874 0.1002707 0.02140701
0.1999531 0.1268493 0.07740592 0.1039961 0.1228878
0.1034051 0.009816738 0.004236607 0.270014 0.1502836
2.037965 1.031258 0.4075035 0.3487326 0.1942947
0.1804901 0.8452649 0.07605323 0.2401529 2.000565
0.09801606 0.0534057 0.3036338 0.1560389 0.3402952
0.1159751 0.4848152 0.09187582 0.1324364 0.2302175
1.41919 0.3731113 0.3382571 0.3919083 0.2077241 0.08949902
0.1544722 0.06197757 0.3127958 0.1120357 0.8017177
0.1025659 0.1259904 1.504261 0.7671632 0.1109029
0.3332766 0.1036726 0.3748683 1.27535 0.1804409 0.2009536
0.7427469 0.1115592 0.014906 0.03653803 0.07765734
0.4896424 1.518998 0.01064122 0.5454788 0.3265579
0.3626618 0.1114951 0.1465963 0.2023979 0.6264155
0.2185527 0.01979272 0.5020358 0.0649651 0.119513
0.1956363 0.1414557 0.08761253 0.4376662 1.442623
0.01761618 0.1126257 0.08175259 0.1514029 0.1742267
0.5049686 0.03640097 0.9188302 0.4344344 1.145308
0.05605622 0.02090825 0.3503784 0.1659707 0.1979147
0.1391006 1.287354 0.0362742 0.1235349 0.4400785

0.2448846 0.06553979 0.1746365 0.1562708 0.08592574
0.1535888 1.554277 0.5061729 0.06012312 0.1346031
0.4672628 0.332245 0.4518289 0.1574531 0.3675513
0.08439967 0.08443051 0.2804906 0.04196233 0.07348054
0.1330848 0.08314314 0.06450879 0.5492886 1.570397
0.3077584 0.03281607 0.08273169 0.01481152 0.08851263
0.04468316 0.09276044 0.2998583 0.05687951 0.1198641
0.2170745 0.7209205 0.03486684 0.2246733 0.3988119
0.700982 0.1156342 0 0.08956303 0.6407918 0.1258194
0.2832037 0.07347642 0.07100589 0.1414573 0.645303
0.372109 0.31113 0.08065225 0.6622077 0.5441094 0.1538141
0.1768514 0.4138098 0.1175922 1.17434 0.1322677 0.3874332
0.2993523 0.03479572 0.09562052 0.2163428 0.2027811
0.1816371 1.102959 0.1593181 0.2835874 0.0550874
0.05443566 0.05024817 0.2774676 0.7817031 0.07140017
0.1426364 0.2785124 0.2064584 0.136201 0.1505044
0.8386708 0.6968985 0.3458969 0.02081031 0.3168784
0.231117 0.04038464 0.02989518 0.1877873 0.1075896
0.1716723 0.1641054 0.1287326 0.9495312 0.1686445 0
0.1315025 0.05492479 0.09208263 0.3535543 0.0798277 1.3823
0.2153383 0.882033 0.2713317 0.05282296 0.1087166
0.05600461 0.1012009 0.1685723 0.1660468 0.2694261
0.4503936 0.5962108 0.8266862 0.1223144 0.1570783
0.02360517 0.0962153 0.2690764 0.1950556 0.7399055
0.149341 0.1111704 0.151633 0.01729037 0.1209946
0.1955836 0.1904335 0.09564197 0.5488707 1.136378
0.5843616 0.09745669 1.135799 0.1506901 0.1225465
0.4054038 0.06658585 0.1804569 0.1591152 0.6474228
0.1053383 0.5000993 0.137905 0.1318321 0.1522256
0.04153094 0.111262 0.22498 0.4291749 1.023349 0.3138729
0.1651802 0.2615702 0.1006435 0.2826335 0.03171709
0.8463408 0.2686545 0.1941182 0.01955447 0.05826931
0.1286506 0.07228272 0.06396472 0.06783435 0.265197
0.1185359 0.08051128 0.2224232 0.07284502 0.1246972
0.5517403 0.2428292 0.3036839 0.153156 0.3139858
0.2194689 0.4216655 0.05883192 0.2254878 0.1702587
0.2512818 0.3278925 0.04322807 0.05681021 0.02408001
1.227446 0.9644032 0.5466897 0.3998409 0.4461543
0.07789329 0.4414232 0.06773106 0.8408765 0 0.8736541
0.2024311 0.105238 0.3424621 0.3727588 0.1503554
0.07698247 0.04512131 0.2091286 0.03401775 0.1793098
0.597459 0.0198002 0.2293854 0.1462233 0.3371959
0.0282354 0.1607497 0.04566008 0.08725163 0.0875899
0.2799903 0.08662534 0.5370505 0.04424608 0.2248195

0.1816331	0.2876617	0.4475424	0.3670818	0.1930849
0.01824791	0.4776734	0.3843807	0	0.25458	0.146883
0.1584546	0.03610067	0.8506015	0.1025208	0.704228
0.1863906	0.2127675	0.0314006	0.08412016	0.05630043
0.02930771	0.07908979	0.1991345	0.1586043	0.1092005
0.03856722	0.3526259	0.1800524	0.03755977	0.2903996
0.6674847	0.05820599	0.2930195	0.1254655	0.842495
0.03113702	0.2756036	1.205262	0.2285989	0.3031468
0.3436809	0.02533553	0.4887287	0.1000775	0.1579433
0.2728708	0.03800538	0.237756	0.05028648	0.0715376
0.1461193	0.01123296
EIF2AK3 3.940961	2.024842	5.194098	14.8798 5.842566
2.409848	6.832583	5.476201	8.919862	6.873773
5.097209	4.165442	8.41863 3.321503	9.362765	1.982752
6.138608	4.891768	3.915271	3.471899	13.87793
1.898526	5.969459	2.091987	1.928246	3.942501
9.801644	2.002067	9.589375	8.123398	10.86493
3.197082	12.27692	4.505096	4.986718	4.366457
12.39413	3.097697	5.627482	6.62282 7.356075	5.383229
6.247419	6.930208	12.88481	5.907944	9.018855
5.316542	4.404812	6.918854	3.876551	5.463814
5.578912	5.001057	2.72117 2.458484	5.931625	3.839873
3.683556	7.082579	7.312442	4.557761	4.478902
3.250669	5.713988	3.480316	2.922151	3.564241
6.14079 10.64033	5.393608	6.421926	5.871618	7.843086
7.831089	4.898241	8.07503 4.770589	7.22823 5.15438
3.32856 7.619674	4.716808	5.426951	3.64704 5.723674
6.773009	6.300594	5.495557	2.834787	6.86891 8.120232
3.199842	7.429996	2.434259	7.918372	4.136242
9.977649	5.398363	8.238536	6.216278	15.24854
4.065855	4.358441	6.403242	6.980243	4.084549
4.027236	6.492401	4.309388	4.498023	5.373396
5.846334	3.669572	4.507239	10.07241	3.868051
6.966707	5.627168	4.232032	4.911135	5.068162
4.878377	3.25127 7.077874	9.836483	5.629455	3.614609
5.064519	4.43697 5.262485	3.600967	13.05816	9.087158
5.636382	4.164317	4.862425	3.042843	4.946264
5.03775 6.048602	4.949213	6.786738	3.924139	4.507029
10.00887	4.629968	4.041362	5.638586	3.50489 6.073555
4.521122	4.571728	7.315193	4.298475	12.19822
4.981075	4.280176	11.82977	3.363422	3.626932
11.26608	10.27232	4.217531	2.852399	9.319547
5.830313	7.95635 11.62636	3.74912 2.953168	11.01302
6.506747	4.543541	6.191454	4.530881	5.814215

3.873193 11.04973 4.588167 5.133394 4.728209
6.374641 7.133984 9.270945 7.439469 6.342304
3.487258 8.830972 3.592158 7.658231 9.007441
2.875197 12.35635 5.356049 6.120976 4.634033
7.109936 4.042964 1.864051 3.963739 4.291829
6.605301 5.5111 3.652393 9.444537 2.628873 9.121477
2.929019 4.982882 6.059985 6.857142 9.058234
2.689332 5.539599 9.172162 3.748251 8.421483
7.010913 6.009494 8.300791 11.72837 6.148775
4.838016 8.320819 3.380117 5.65092 3.231445 3.461021
4.552978 2.497605 5.882393 4.505539 9.123336
6.129221 3.963326 3.785824 7.026651 5.793444
7.283417 6.642156 7.493562 5.428327 4.253983
4.802813 9.598073 4.554 2.124656 6.937629 5.890812
6.587804 4.863597 6.687093 3.863094 6.073506
13.7791 9.864453 5.889283 4.16942 4.656899 7.228807
3.609962 9.052129 3.145839 8.896058 9.19596 9.404824
3.915046 2.948236 12.18553 4.667773 3.578771
11.23591 5.36858 5.903302 7.992656 7.582641 2.734897
3.987333 4.903106 5.208562 7.474555 2.177786
4.383099 7.091043 9.547243 8.5853 5.748242 8.373715
7.296091 4.873528 2.261035 3.016479 7.027605
5.834122 3.784148 3.685941 4.449297 4.477646
7.264921 7.809694 5.839399 5.40805 6.603637 8.012219
3.965663 8.131116 6.874 9.536192 8.212279 6.556663
2.14581 6.60537 3.143814 3.978567 3.37233 8.74965 9.019769
8.946123 6.359519 4.687148 8.548119 4.606941
3.581672 8.160349 6.555105 7.087415 4.201457
5.017425 7.79488 2.928227 4.197405 8.786618 8.016335
2.626754 3.094555 2.257422 7.218443 6.646269
8.543064 5.588946 3.340689 3.588687 6.955092
4.641123 7.385377 11.54728 11.53523 4.758933
3.690884 7.188149 5.860406 13.93922 6.36503 5.79653
7.886931 7.067881 6.257288 2.760342 3.955142
6.43813 3.561468 4.235049 5.350666 4.239268 3.949176
9.146478 7.365935 11.56556 7.596314 4.248701
3.784137 4.233361 10.12129 3.2083 4.548273 6.746648
4.959629 8.459555 5.729324 6.400111 3.825152
3.925889 3.305107 5.574011 3.625033 4.417162
3.092324 5.354623 8.285348 5.164396 6.489329
3.799406 4.894514 9.353822 7.032911 5.179252
4.763663 10.34441 7.204615 3.843054 7.638004
4.320262 7.380101 5.600641 3.237208 4.236085
LAMB3 58.44917 0.5808366 41.90498 66.39413 21.28841

1.393525 19.39262 10.90871 65.74843 14.26945
36.2826 13.83947 10.7298 22.17921 29.33452 0.390315
53.14018 15.18585 42.15345 34.42567 59.40931
0.2839532 32.41265 0.2966219 1.178037 30.06468
32.99999 0.2499544 38.90964 9.120721 77.21842
8.89511 52.11784 49.91637 34.47968 2.187564 42.08018
120.9403 59.0904 33.89929 68.03797 64.25909 67.54819
77.79388 49.77749 104.7073 41.01915 22.19521
106.3182 171.5678 34.11926 7.611034 23.35653
76.12357 15.37818 31.90294 7.881142 10.75398
41.94518 55.65236 24.42895 54.03067 6.287364
36.81274 43.81242 3.15003 72.49604 33.19229 100.2121
62.52923 170.1033 26.62848 58.52212 16.8039 78.79167
57.40471 89.77907 30.98365 41.89463 12.68387
38.4045 37.77293 35.16791 29.77012 57.04532 71.06486
29.8056 88.3661 20.34174 27.73777 26.80366 35.9886
103.8026 33.64878 55.40867 37.98644 199.486 47.33847
23.00103 35.27358 7.678853 98.29032 23.24294
22.33808 23.19394 48.19771 106.6184 19.04271
113.0146 25.10378 62.20516 41.31986 23.18531
6.590377 57.42532 40.4561 22.75768 174.0797 37.67504
23.15267 31.21172 35.05123 89.21245 35.23538
10.34608 23.17927 21.82082 35.18427 13.49482
20.60078 50.41515 46.12203 61.30752 47.92244
56.52723 42.66469 66.12196 16.94118 51.06819
12.80521 39.45309 35.01786 83.51297 25.33699
27.48133 27.27838 14.33409 45.7583 51.40463 10.39874
14.66436 108.8623 76.97778 45.8767 11.38273 35.21692
38.11758 10.86564 41.61556 23.85845 58.5302 24.00171
36.43078 47.70254 29.78343 38.50889 133.4364
40.27866 38.01518 11.89254 0.3223551 34.47903
22.28261 40.57138 58.03471 66.4133 147.2901 20.87678
50.88048 27.44491 12.69536 56.46496 30.4724 43.06401
32.148 43.38459 45.86451 78.70519 63.16822 62.76746
70.32405 122.3046 246.6751 59.95316 19.18462
64.43545 35.88157 34.37534 53.68742 7.911622
19.17906 39.29802 65.90277 29.71153 55.08368
63.85608 23.65692 7.877973 64.27291 84.35993
21.10464 26.24954 45.72102 51.97184 98.74359
85.13244 159.8621 85.78956 21.82774 35.77032
72.58059 27.23841 43.48553 105.2001 26.60525
27.40335 78.66258 47.72655 65.0202 125.7988 49.12841
21.34191 56.65932 161.967 33.23137 52.3474 13.86565
63.65507 20.18805 66.48568 62.31251 117.654 26.89466

43.23386 35.75929 29.44752 52.18304 3.557654
91.38812 69.15502 74.64509 31.28625 60.29551
47.22787 44.90755 29.99722 121.4628 33.4319 34.07179
28.27042 40.38221 85.96714 36.45295 47.80704
52.70335 61.47136 87.35084 40.00628 86.77025
144.8798 26.65439 128.7056 82.49033 25.86588
119.3826 15.64297 61.59839 4.345866 1.066578
49.84559 22.75635 32.76333 79.21796 17.05236
53.44783 29.07584 108.7724 28.4439 37.6174 26.54385
60.64665 2.457031 1.850332 39.90015 16.87302
27.26872 42.42178 54.22147 95.03593 91.19208
53.90321 24.56531 92.75567 26.13311 62.64872
84.5625 39.79638 24.10556 40.25086 93.98493 52.06957
314.8349 45.24418 22.29135 61.88262 14.82516
59.45392 3.794657 71.87647 81.5162 31.02927 27.77158
73.09182 19.63865 16.05591 33.00719 63.21436
2.310927 73.62659 233.8023 46.35277 38.23417
45.80265 94.82528 18.24826 50.73961 22.0873 45.31929
60.67005 80.93565 12.66176 10.99731 10.43621
66.96634 15.4016 42.1392 195.7621 46.07657 51.28195
28.57048 26.36189 40.90405 46.54823 19.7187 38.58066
94.79966 53.53434 61.86048 6.300522 32.61437
52.04276 22.32116 464.7077 57.2656 64.05777 34.4317
35.62785 54.24841 47.35548 42.92314 39.85179
61.97121 20.29706 17.6577 32.39399 5.275889 41.11616
30.16801 37.67053 37.38503 26.83069 81.87706
27.15582 52.9499 33.13649 82.14928 19.37126 144.8155
30.22142 60.61004 50.76639 58.78759 23.17526
63.46973 90.25241 21.20936 25.84746 44.97773
46.55141 68.16217 38.07649 10.21605 45.27644
70.83122 26.14667 50.67126 17.49802
LAMC2 5.586734 2.059588 6.405234 11.04053 6.762909
1.187343 4.370806 1.695194 16.49231 5.84781 4.791725
2.477872 3.490717 3.226957 4.097878 0.315848
7.746885 5.915074 5.036502 3.670685 15.19463
2.894738 6.861642 1.902116 3.803561 4.419218
3.824124 1.106145 4.514096 2.786282 8.966914
6.024945 32.97589 48.85641 42.19592 3.660441
1.531879 28.18354 34.11718 21.15421 24.80465
30.7495 116.3846 24.52544 24.54578 78.4759 34.60121
18.66171 60.71338 90.34401 12.24742 7.701151
8.509108 37.6678 13.82811 14.52179 3.644968 8.119411
21.8086 8.064589 41.49775 18.71729 2.485734 10.33629
22.84441 4.146802 66.55241 8.557202 35.56811

24.36689 269.1731 12.29719 24.82358 10.16398
72.58852 22.52871 77.51621 10.59004 7.36269 12.80742
14.97248 17.61413 124.1322 26.95364 42.89327
35.44842 38.77428 88.1269 8.976048 79.9588 42.74651
9.610058 164.5347 11.24476 32.56136 4.797785
311.4815 22.29451 17.78069 57.89496 5.446226
55.72705 1.217664 8.744304 14.93191 66.70639
122.1399 7.507479 53.75287 21.11268 33.064 13.23636
22.33257 3.654673 52.22517 8.589093 8.303148
123.2523 13.44278 12.33523 31.37517 31.02866
19.5271 63.73294 11.80766 5.89272 17.82583 31.43886
9.872538 36.77737 81.68227 11.73498 17.35633
19.08997 18.83837 15.41887 38.63394 71.18672
43.58872 20.68341 15.08986 12.84543 65.99092
11.55853 9.02036 20.99207 14.54783 57.07546 11.29722
3.040974 24.16377 168.0245 43.81968 34.93138
7.042457 16.87287 15.60679 9.52831 140.9641 26.92306
32.15009 6.336644 5.701864 10.65955 19.84325
18.83779 54.8182 25.46616 5.028979 31.20501 4.613791
39.7553 12.67699 30.23269 16.00719 60.69759 40.06694
9.020217 15.49557 23.24071 14.45149 38.5549 10.66047
50.13545 5.488383 39.45255 34.56711 105.7049
23.09578 30.50884 60.15536 58.70866 193.6739
19.29858 24.49772 47.64039 36.10697 13.5721 29.68764
29.30508 11.10229 6.126237 39.9806 12.31893 26.73283
22.44833 9.174654 0.8005107 32.16458 49.27009
6.217221 13.47847 40.54955 4.400889 135.5525
72.22961 45.32304 12.30475 21.30613 19.4273 27.29362
11.40252 3.16511 84.80591 33.7489 9.024451 20.68459
8.19599 11.87416 93.89501 60.91808 21.93765 20.09376
112.2813 27.78274 37.92604 6.966385 8.389136
18.86986 3.085675 26.58558 94.01477 20.92467
26.57423 63.36391 9.365906 40.54969 4.320233
61.17928 27.48268 36.2545 17.257 25.78755 32.29658
13.79985 0.1573566 52.37569 47.70706 23.57407
20.71858 18.94653 40.49294 17.67304 50.13328
19.71679 19.76037 56.00597 38.17456 60.13273
49.67096 15.79998 82.90148 51.11401 11.92488
94.95093 28.19189 29.1372 8.104341 0.1142719 65.51566
19.43494 34.19454 51.25144 19.98728 70.45111
19.14426 90.17101 14.46742 29.07342 11.61484
25.86347 3.778618 3.042816 15.7948 0.2449857 24.67158
24.47466 73.56878 102.612 40.8478 61.57013 25.10799
95.09233 8.613102 58.75218 44.94504 13.44099

14.31227	17.36015	42.47568	18.69004	218.3948
4.361642	13.72122	26.36805	10.60544	10.42953
18.03922	66.29954	90.84826	35.84778	10.82121
68.91955	5.981283	11.24817	20.89145	29.3179 33.85558
25.60075	133.6018	28.52991	13.05542	12.46711
27.43183	12.96237	13.74443	6.761977	45.70781
33.48732	103.74 10.61615	1.808649	17.66459	14.76179
11.86914	15.30697	44.62085	8.59948 24.49733	18.67424
12.24135	52.49273	9.336646	16.29778	4.01109 40.78724
42.30181	69.15087	6.125062	5.646985	21.56867
5.856558	457.396 12.64139	22.57365	7.505852	3.129277
74.1519 22.2758 8.1034	11.78799	24.90378	22.26271
12.08621	27.21798	3.87118 41.01103	10.13484	10.3433
12.2029 26.42939	16.13658	12.78233	26.56066	41.70206
101.2995	8.110231	185.3829	11.16683	35.15949
49.54314	76.93852	13.35228	75.59519	121.0964
15.46967	8.930771	51.68098	12.60827	36.85123
17.41322	6.5172	80.38114	25.84754	16.90066	16.72026
15.24272
LAMA3	10.44788	0.5383638	8.723847	8.590741	4.825486
1.11968 4.581811	2.293401	9.491393	2.724627	7.084951
2.277656	2.064065	4.537282	5.198912	0.4799879
9.539616	2.969769	7.652108	4.952148	10.13333
0.4290916	3.694729	0.9718108	0.860255	6.82131 3.918515
0.5369384	5.48352 2.628177	10.3508 2.693823	12.23093
14.46317	11.19115	0.3815633	0.2258387	1.999483
5.433038	5.588232	6.852828	9.341885	20.51655
4.308045	15.01159	22.21759	11.44689	7.190272
19.49445	38.39319	10.94336	2.929638	1.824692
15.93739	6.76863 3.027325	1.363018	3.043255	10.05604
5.778402	11.20671	19.04569	2.316766	1.02595 13.29397
1.788855	14.6605 3.897395	53.44115	5.132354	121.3825
5.123319	7.579385	2.47259 9.251298	11.94657	28.31211
1.493819	0.7221247	1.79665 5.301935	7.571258	41.24324
8.276129	9.918634	21.09863	8.110202	29.27377
12.61769	14.70293	4.085672	40.02011	49.83345
6.623824	15.0273 2.493875	67.93586	4.957761	9.463876
12.1747 2.57881 11.62205	3.569866	0.4485074	4.326042
14.11306	30.89271	3.845371	27.22506	4.913173
7.199786	3.946915	1.672507	1.749465	11.54891
6.444815	4.678915	32.42216	2.22643 1.4017	14.33784
4.266457	1.768359	12.3336 2.612992	5.322327	1.67361
2.908646	4.664644	6.613122	8.250278	8.041606
12.43025	9.741737	7.220319	5.616181	18.71251

9.529108 33.49569 2.843589 8.728171 18.1717 16.20191
0.6787526 6.244925 4.466217 5.986329 12.2559 1.254649
2.125327 8.007378 36.1784 8.759613 18.63027 3.055929
11.12991 10.3273 1.094113 10.07537 3.601969 7.627751
7.492692 5.240026 4.269436 4.503036 5.870672
20.25736 19.1832 1.218901 2.000028 0.5632283 6.564382
4.944209 8.888884 7.832558 13.48615 7.87017 1.898429
7.065911 3.826885 5.058433 3.913046 3.077934 17.86
1.2635 11.85108 11.32327 27.38549 9.919934 14.51918
9.874006 5.296607 23.65107 20.63594 7.58606 10.36857
9.298204 7.030459 19.06638 2.12557 2.997297 3.532219
5.482662 2.441883 8.178414 10.71397 2.740811
0.3539361 15.09656 9.11382 4.019873 5.878995 13.57935
11.13096 18.45788 21.52352 13.55657 0.9277852
6.096367 5.958404 15.97909 5.574065 2.227368
27.99111 16.80256 1.387951 2.71576 4.731509 9.715034
21.1551 8.358078 3.187293 4.75496 28.34651 6.248551
11.65736 2.270469 7.112693 6.331809 4.271929
6.965617 24.76187 3.78013 10.26778 18.90286 13.44625
7.213011 2.376508 8.756859 10.82294 17.71661
4.344282 9.144422 10.41711 30.8874 0.9648954 16.37318
3.579547 14.12758 5.164091 5.254782 12.775 14.75809
9.861634 7.215949 4.800419 75.43096 5.300878
10.2188 15.65282 6.569736 10.18405 78.78763 2.207519
19.14012 0.8836906 5.724185 1.565165 0.1082834
17.25684 8.333425 33.1336 14.8989 3.672323 28.39754
15.4843 58.79943 1.320263 5.66802 4.405044 8.314278
1.556693 1.844864 7.705027 5.262002 4.831673
7.492754 17.20356 25.68461 18.49633 19.13723
4.290325 23.48207 0.9781118 12.40687 10.13418
2.297785 4.5176 6.572264 4.748123 7.405482 72.08913
2.976953 1.75757 17.92473 3.012985 9.267433 2.366331
28.11519 40.6741 7.902221 1.986965 13.32276 2.752274
3.08157 6.051466 5.51871 2.7844 9.436322 18.26413
12.5607 3.953621 9.110399 27.91833 1.902529 5.514957
1.043729 10.09527 17.4828 15.1201 2.424892 1.687923
3.058629 0.9948706 12.84988 11.902 15.70542 0.9183403
5.175849 2.015301 1.550877 15.42321 3.841641
43.82312 4.061635 13.11673 24.94928 11.36669
2.756099 0.3932005 10.79834 1.393702 184.4854
1.850897 2.54939 3.758756 4.567234 29.10702 12.79155
1.446633 1.387261 23.45242 7.047092 4.799739
2.233338 0.7690317 17.68856 16.45971 4.853907
2.022311 5.536328 7.81668 3.082292 4.923546 5.913972

28.5024 3.233475 75.04466 0.2021639 5.469177 12.21559
23.00745 5.340191 27.76869 4.139208 6.757946
3.862622 16.67058 8.324447 18.71056 3.522492
1.331903 12.70102 6.731739 3.374004 5.341137
34.6214
CDH2 0.09392238 1.347167 0.1619877 2.613105 0.2810386
2.425517 1.188133 4.390018 0.5074998 0.9561051
0.1562032 0.08649552 0.7614614 0.09081281 1.502498
2.265773 0.3805593 1.19556 0.1159689 0.2382108 0.3704115
1.522927 0.6011712 1.524001 18.65907 0.1222726
0.4738872 2.316187 0.3062813 0.6193762 0.7464917
1.297058 0.1898216 0.3605199 2.82437 0.07720911 9.003476
0.1766194 0.4427256 0.1220822 1.813217 0.0724468
0.6843003 0.150949 0.2629455 0.06047188 0.3654276
0.4795297 0.3855254 0.2250478 0.9283694 0.6369124
87.35386 0.8032065 1.133091 0.04329737 0.3749237
1.245643 0.8358211 0.1551836 0.6297988 0.952649
0.5320242 0.4705353 0.08885973 1.752192 1.389677
0.8424712 0.4600309 0.09431506 0.7495233 0.1276376
0.4471364 1.409451 1.655865 0.4034212 0.3121976
4.558524 9.281185 0.09775628 0.6124054 1.510072
0.2609439 0.440632 0.8996596 0.3665982 0.2506606
0.1994839 0.723948 1.3739 0.6561908 0.1376459 0.6569104
1.766578 0.2195928 0.4767594 0.6515128 0.1668948
0.03448331 0.3004777 1.028942 0.03333542 0.04733915
0.1936372 2.534922 2.154251 0.4107091 0.9317004
0.8954438 0.07825889 0.3003935 4.329239 0.262694
1.824902 0.6860599 0.03961509 0.3251197 0.4394808
0.346922 0.2088014 0.03644846 1.04517 7.659808 1.373178
0.8746112 1.340888 0.1395979 0.8475728 1.476247
1.086627 0.1331699 0.4009868 0.07764769 0.6062895
0.08529951 0.5274802 0.6225645 2.531346 0.4070001
0.1078337 0.3418981 0.1730692 0.1187304 1.637842
0.4072308 0.5672968 0.007620204 2.486758 0.09829367
0.4902668 0.1573271 0.5370096 0.4707564 0.2508606
1.625831 0.2764818 0.3383149 0.8584132 0.7503289
1.561367 0.4247003 1.151158 1.392707 0.02670076
0.4049719 0.342502 0.1919648 2.010755 0.3557536
17.59019 1.524043 0.2669363 0.2192053 0.07168093
0.02133537 1.16732 2.504251 1.796282 0.06211901 1.243064
1.366766 0.1422873 0.0920349 0.300792 1.086989
0.3227587 2.903875 0.7545275 0.4332848 0.1700175
1.434734 0.3711543 0.4343296 0.3724301 0.2826318
0.4186827 0.7687898 2.061347 0.2123434 0.5861299

1.506134 0.3270855 0.1870337 0.3090872 0.2998807
0.07282179 0.8311163 12.37978 0.2517493 0.4792532
0.5931702 1.03571 0.2342953 0.4532023 0.1999855 0.5007534
0.1633334 2.429814 1.359097 0.8246362 0.3670168
1.980315 0.1509582 1.095434 2.029585 0.1474733
7.170187 0.04101051 0.007161982 2.572409 0.1655686
1.699717 0.06508001 0.1088378 0.1020006 0.2259649
0.3509129 0.1518347 1.511267 0.1618294 1.22223 0.455871
0.8874422 0.7055811 1.460479 1.575691 0.8503174
2.276588 0.9493243 0.09208392 0.3693806 0.5763101 1.2408
0.7555788 0.05451301 0.566612 0.330285 1.521206
0.4849903 13.29919 0.2635348 0.4275367 0.6259965
0.7883163 0.2771449 1.312678 0.2999212 0.1820041
0.7945042 0.06979836 0.5110798 0.4467146 0.1024123
0.2575224 0.055162 0.257805 0.7159579 0.6977198
0.2485976 0.325471 0.9644935 0.1178402 0.7138504
0.6978137 1.128576 0.4554373 0.168919 0.3848341
0.6247064 0.6782034 0.369459 0.7665352 1.829743
0.5853603 7.960736 0.8457558 2.0288 0.8844614 0.4020875
0.1828501 0.6227849 1.544314 1.098765 4.212806
0.1461398 0.8638121 5.263574 0.9605639 0.4457473
0.2189701 3.111555 0.3839887 0.4376943 0.4309244
0.3362585 2.296065 0.3342047 1.962789 1.56343 1.208928
0.1016578 0.5851819 0.1154406 0.2847432 1.798894
0.2633986 0.8024392 25.66994 0.107922 0.5333079
0.1428686 3.300454 0.7190102 0.3621463 0.7759833
0.09826418 0.02313324 0.1613953 0.1908443 0.3068432
0.8952622 0.416292 0.800654 0.04261781 1.045337
0.2314796 0.1678815 3.963163 0.3425738 0.1144849
0.09163036 2.664286 0.3153539 0.5700422 0.1375849
0.1332015 0.4941492 1.370439 0.727525 0.9355906
0.8260372 0.6247712 0.3644082 1.588335 0.1316071
0.7126936 0.009626846 0.1402022 0.1090616 0.1947495
0.9372142 1.560039 1.141586 0.9828558 0.2856405
0.7622611 0.1640381 0.2696775 0.2302699 0.4815371
1.978451 0.8963201 0.5461589 0.2161773 0.2810781
0.2881839 1.344055 0.5643338 0.2284194 0.2296836
0.2421768 0.3894176 1.237916 0.2560307 0.1007721
0.5124927 0.1681528 0.3378865 6.345925 0.6366564
0.3469545 1.668858 0.3002252 0.05587387 0.09458861
0.1818374 0.7362166
CSNK2A1 17.19072 9.922674 17.99397 19.54298 14.6195
10.88374 14.29932 12.74754 13.08985 13.66337
13.84047 15.02464 14.70374 14.86089 13.85038

12.56306 13.03596 12.7191 11.19293 13.26489 17.84753
11.38016 15.22489 12.38043 12.41081 13.62558
14.59637 12.99275 14.56487 8.057703 15.24278
11.10213 23.52816 14.8164 20.63974 21.40982 39.14322
12.92791 27.80891 21.1522 17.81715 10.91618 25.60231
25.84053 14.17641 30.43638 23.37723 31.80584
20.22199 16.74496 19.40373 15.85641 34.94404
22.84253 13.33682 22.47362 17.21154 14.71622
33.1159 19.65404 18.11229 18.86984 19.43756 17.54371
22.03015 11.20758 16.2473 17.12678 18.69119 20.63727
24.6713 23.71405 29.45751 19.37195 16.0843 19.397
13.13808 26.21865 20.71936 32.80435 14.22042
17.80179 24.94294 17.42211 21.2204 18.03581 21.62382
18.37218 25.22729 19.85631 21.35402 34.39546
19.1944 15.26821 16.9662 21.09418 17.99782 20.36114
28.61877 19.52883 14.94445 16.94373 14.37118
47.12906 25.15107 15.98336 17.32997 13.797 19.90146
15.64068 43.90088 33.99561 21.28034 13.73329
14.3123 13.92706 17.1458 16.56389 22.5343 31.23101
30.13711 45.43533 51.95962 11.9722 55.71907 17.23588
21.89209 14.74417 15.00644 23.08697 17.05594
23.78667 22.11938 23.33826 14.70088 19.44887
13.41019 27.04891 21.10311 47.03288 17.37508
49.47147 18.3654 28.68375 48.06423 15.36736 27.7364
13.94745 32.57864 14.94061 25.52628 16.77238
27.17082 25.81936 15.22617 15.88044 19.80446
12.93853 32.10091 19.99166 18.08165 13.74997
36.14755 25.52013 27.77835 24.783 14.5527 16.37447
12.88874 31.87151 12.78702 12.73447 22.37345
21.4905 25.96528 15.44495 17.2363 26.54012 27.48435
23.40937 14.77178 35.07343 14.61301 21.68567
16.28876 27.41808 17.21415 18.17182 31.58252
33.55138 23.88265 25.13117 25.09701 17.91367
20.25217 22.65162 20.59571 33.12256 42.82135
17.04548 16.28467 25.73336 28.01558 34.9906 23.97054
20.94396 12.24036 28.37336 20.79356 31.39614
22.33892 19.1449 27.07156 49.622 26.05457 20.43667
17.17566 25.50508 24.75276 21.8428 15.13387 24.18675
19.76656 17.25261 18.25228 15.62737 80.05343
9.506102 23.18116 13.58048 29.6057 15.64311 27.21033
21.26297 40.95357 24.89724 15.87506 26.02348
23.54839 46.13201 32.70887 27.66758 16.83897
20.20339 32.77557 18.93921 27.86238 12.97778
21.31089 36.35998 32.42707 34.08875 20.95839

21.44305 35.00041 21.00701 47.36596 18.55657
19.41951 24.0975 24.9274 25.59538 29.8592 24.55638
27.37439 38.78951 22.99073 16.25258 29.5919 14.40823
20.09022 13.52018 19.60696 40.55135 26.51933
21.92856 13.42498 13.06133 20.88827 34.13698
15.8136 40.05227 12.67376 21.07321 22.542 25.41203
37.03118 37.5884 14.82993 23.49303 50.75861 10.49031
11.72126 19.0357 20.07248 15.20672 12.99578 25.34573
18.56644 26.63463 17.74774 15.74233 34.88336
16.26897 17.05615 30.84147 20.61258 26.99761
36.54316 27.66529 21.22692 18.75997 19.16287
10.31402 22.89582 16.28727 17.06429 19.14917
16.68649 20.42246 61.1508 19.75754 19.54764 24.43449
19.40014 23.03629 27.22669 31.03075 25.35789
21.90913 24.43046 32.28282 24.98825 26.44568
9.622261 43.56514 18.49157 33.54453 24.14484
12.74407 29.76785 32.72705 15.51871 23.59127
16.34819 13.7904 10.24139 46.10389 25.63037 13.48695
24.94661 29.30855 29.3609 22.51273 18.98943 23.43276
22.24328 22.43995 15.08527 25.64154 31.847 13.42504
23.46226 15.2203 14.068 24.3876 23.40221 18.51778
17.24215 23.75572 13.19365 15.51766 60.41503
35.7955 31.97623 51.40202 16.86104 14.40872 15.34897
19.33902 60.89628 24.97885 15.46435 63.80368
32.44958 22.47677 14.90577 27.02802 20.83067
37.2073 13.02258 29.07556 26.04417 31.11734 25.06663
25.46397 32.34725 19.72511 13.96683 28.50285
25.82703 23.93729 21.25295 25.23987 23.03287
36.46822 18.25901
EDIL3 3.230832 5.900387 5.703798 11.01001 6.974402
13.32379 6.838328 10.64222 8.439469 8.660432
8.267676 3.178688 9.548363 2.260033 6.69545 18.15428
6.005311 14.88342 4.708268 9.907134 11.43549
8.808149 5.199007 3.883545 3.539453 3.359468
11.47068 9.483991 12.11007 9.53153 9.244633 16.5661
4.225814 7.480182 14.45821 0.4495904 1.014351
5.564707 3.090663 3.455282 19.32257 1.72315 8.238201
3.08131 16.48857 4.575815 5.002648 4.310938 2.302287
4.724122 2.361708 4.166228 3.104811 6.803385
12.67766 4.174012 1.918307 3.060213 1.510625
3.779316 4.254479 3.922645 4.002041 4.213237
4.432146 3.510396 9.777226 17.01267 10.68964
3.843506 1.427258 12.74329 5.986346 8.268057
5.810405 7.111501 23.86953 1.756023 5.848765

3.224436 1.422018 4.50826 3.083714 17.49333 8.033131
5.376305 8.073731 1.464319 3.617059 6.077478
4.479809 6.748559 16.83219 9.661093 1.506746
3.815389 5.139357 4.636531 1.236655 1.965913
7.938016 5.315707 1.516842 10.94904 17.15738
24.34456 2.399787 8.132058 6.72163 1.927456 5.047872
17.52892 2.750523 4.672621 10.76975 4.408543
2.374087 4.699969 3.914946 3.647565 3.010667
18.11718 19.553 8.231084 3.400121 9.389023 2.853191
4.061173 14.57388 12.23777 0.8989949 1.75554 6.688127
6.184483 5.971732 8.074563 3.477694 2.104655
9.178934 3.402054 4.299991 2.794073 8.132948
5.814285 8.479135 10.73791 1.383768 14.80029
0.9349763 15.06996 2.04284 20.12563 1.918112 9.053987
15.46697 7.370036 3.341617 5.039782 3.648987
8.41205 2.381445 14.04284 16.02274 6.645292 6.366409
11.09506 5.214452 15.46484 4.386227 1.072317
2.995279 11.4177 1.955853 2.208096 2.537314 3.86155
9.290449 10.10703 3.717073 8.092465 15.09105
3.644051 5.574715 12.97421 3.241476 7.849342
8.154349 6.33779 12.90487 2.696958 16.27903 2.84163
5.799889 5.775374 3.378621 17.44989 3.545757
8.229808 2.982517 4.624653 3.572578 14.46312
7.134079 2.133122 3.339121 4.214908 4.680195
14.29885 4.152158 5.688327 10.90137 8.4342 16.86053
3.84541 1.467647 3.061555 1.276255 3.167258 10.90221
7.956529 8.520233 5.816427 6.133963 3.487967
3.161707 1.725688 12.86512 2.529565 2.208788
6.928169 3.467937 6.848598 1.393657 4.464822
4.317718 9.90969 1.998695 5.550683 11.37328 5.005112
5.556359 10.04732 10.92951 5.409126 7.303259
10.89629 6.833756 34.35254 14.22102 4.026064
3.054522 3.631593 12.40156 8.152279 4.597767
2.10988 7.406006 10.39078 3.000022 11.97886 8.764325
2.542751 10.60331 3.282582 4.602378 16.28125
1.773856 7.759572 8.014494 10.12363 12.5366 4.142698
6.113681 2.571003 2.374043 3.224809 7.483721
15.1205 1.050571 16.33924 8.011429 3.959281 2.6027
3.509234 2.959561 5.71658 6.994622 2.26005 9.958801
5.086363 6.40507 1.094239 3.202801 15.99512 18.99617
7.523547 9.658785 6.929618 2.668222 9.239631
26.07743 20.49741 2.565084 14.47965 4.647512
7.384103 5.31076 6.292119 5.594619 3.618011 6.049419
2.127679 2.287159 4.428048 3.715633 10.33195

18.42678 9.038609 7.601429 5.838252 1.221013
8.06501 2.005969 3.266482 35.13278 7.243965 5.465423
3.35727 5.310207 2.154694 2.857823 13.38047 15.33914
7.633284 8.404908 4.65506 1.980668 7.125883 13.6597
5.141373 5.161762 3.922987 3.770071 1.303747
5.183346 4.548506 7.428477 2.086076 2.663112
2.031076 6.402787 7.308132 11.88799 5.98859 3.916782
6.086583 16.91491 7.691833 3.143828 3.968924
7.362397 7.562441 2.613784 4.132786 2.273824
2.899128 1.948857 6.038338 7.487676 5.369144
20.12884 7.096312 4.755511 7.447799 2.233993
9.180946 3.955225 7.205636 6.84568 5.290712 6.357758
10.80909 1.739649 2.101789 4.308789 5.604638
13.03196 4.104384 1.441712 8.279969 3.403285
1.778319 2.853676 2.334473 6.123208 14.0299 8.279776
3.089736 10.22611 6.185224 3.419744 0.1981738
2.792644 2.467876 2.191166 7.140797 21.80886
EZH2 1.429729 0.1259618 3.106047 2.771426 2.107112
0.3156171 1.727499 0.5082768 2.088212 3.731528
3.185334 3.553738 3.13567 2.732216 2.440871 0.2332649
2.364885 0.6628256 1.741529 1.701783 2.920888
0.294377 2.534187 0.3805445 0.3212743 3.752726
1.688447 0.1879964 1.750856 1.538256 1.489022
0.3599955 6.869405 5.37321 1.836183 6.366935 0.8792515
2.071871 5.499916 8.649447 3.629784 15.83527
9.212871 8.193643 5.03873 2.870045 8.112449 10.89892
10.07928 3.074221 4.843821 6.256569 10.61966
7.560401 1.89809 10.29665 9.698877 1.26144 14.08155
2.474026 2.487706 7.474973 2.655906 2.594152
4.509799 1.378111 2.858293 1.639845 5.497329
4.704236 10.29011 8.296935 3.953796 4.976075
7.915394 4.584551 3.395015 6.988065 6.944929
7.890322 6.027731 3.379696 13.07758 3.353234
3.039039 5.419053 4.224872 4.986657 7.143223
1.413339 7.408998 11.36153 4.273347 5.644038
6.597422 6.259325 5.605761 6.587028 17.85977
6.698843 3.84159 6.413919 9.046243 7.272314 3.262188
2.988279 6.08558 1.983775 6.520435 6.836144 4.183281
12.34151 4.448685 3.375421 4.426315 4.451248
4.525936 7.05834 4.568959 9.199705 5.196747 10.00304
11.07314 1.354025 13.06376 8.844198 9.800449
4.20461 1.464195 3.55653 5.238618 10.57113 2.602213
12.67455 2.722181 2.940263 9.513345 18.83958
9.614083 6.762007 7.246602 10.17617 3.646602

8.697065	8.712315	2.076031	7.895177	4.430665	
7.916874	4.403203	6.805574	3.320975	4.690219	
5.511242	2.350255	2.219436	5.678484	1.963016	
4.215041	10.67411	3.293024	3.947172	7.507628	
7.184228	1.856326	5.646744	3.98742	3.167979	6.143291
3.033822	1.04235	9.635247	3.900946	4.580419	4.193843
4.625044	3.213693	6.504974	5.844778	9.666305	
12.26954	5.17884	3.054758	9.362999	3.901601	3.318707
2.017362	8.859318	4.385237	9.422134	1.677986	
4.140706	5.171205	4.253625	4.519194	9.374273	
6.167979	8.757708	10.71195	4.787658	11.77563	
7.597896	9.934427	7.797598	11.52571	11.15501	
3.863662	5.158601	7.124074	7.082286	9.636953	
3.677821	4.580691	4.712923	7.513985	6.636819	
3.139187	8.406977	4.826718	5.033061	5.588582	
11.2452	3.675884	7.944999	3.908254	7.238374	17.19047
4.212374	10.24277	4.023149	2.769338	4.023812	
5.958994	4.040592	11.19628	2.639921	4.243901	
7.343019	14.68922	10.47132	4.783757	6.587044	
5.364208	5.613567	6.864067	3.36943	11.16423	0.9968252
3.730737	9.566791	11.85299	14.41907	1.735196	
4.222834	13.34371	8.49887	8.491013	1.991561	9.797406
5.794637	5.589995	6.840034	7.513087	8.612697	
9.131625	8.342939	5.289102	5.515538	2.262294	
1.528093	7.893848	4.448985	3.107562	8.530305	
2.860365	7.34056	5.229897	1.041488	9.934357	4.351935
2.503612	6.852503	1.430137	7.363262	12.33987	
7.116712	6.048734	9.381092	5.123103	4.383786	
7.240811	0.7558438	0.9128257	7.645351	3.441373	
2.951685	6.141914	11.04008	8.907066	7.222086	
4.899689	4.422895	5.805586	4.270701	5.449884	
3.438438	10.29259	12.06528	4.377327	7.117439	
8.294754	4.393284	8.401408	2.212448	12.40735	
2.438438	3.700578	3.020173	3.999838	7.670949	
8.016404	8.565206	4.606373	8.349413	2.226865	
5.870374	7.549395	6.942641	4.712338	7.677208	
5.962401	2.767503	4.439926	4.311099	0.5977462	
5.243012	9.632145	4.622412	15.18918	3.401916	
4.063842	5.641918	7.356739	9.082692	1.09139	4.830029
5.414183	16.28832	4.448839	6.914435	7.375222	
9.998339	2.997226	4.057887	8.251117	7.313223	
5.489886	4.921783	1.848488	2.940682	6.109825	
3.061542	4.693495	10.13208	10.52864	7.086096	
8.216136	7.374333	6.406193	2.901647	5.18793	2.150285

8.39914 6.130615 9.164943 4.950554 4.839251 4.9945
3.833598 5.170889 5.27417 6.735223 7.028593 5.592975
11.6919 7.005068 1.515318 3.259804 17.11026 3.265539
6.049546 10.89153 9.581167 11.10155 6.556375
8.067896 5.073381 1.658711 1.115415 5.364841
8.81201 4.947927 13.48381 9.40186 8.006176 8.79273
3.185339
ZEB2 0.627106 1.738876 2.059695 1.508998 2.064196
5.232802 1.453373 2.181794 1.875012 2.324763
2.635827 0.6667043 3.405789 0.7839597 2.527874
3.81033 1.140083 4.375517 1.012807 1.291331 2.201477
2.726123 1.359963 3.986402 3.504923 1.083589
1.997868 3.7598 1.512255 1.708155 2.38719 2.227753
1.872821 1.56257 2.121846 1.036828 0.4467257 0.8905921
1.337578 0.5988853 1.76766 0.3504687 2.4036 0.4193056
2.125273 0.568528 1.564065 1.838713 2.762273
0.7386255 0.7811949 8.992265 0.4364485 1.458154
3.641028 0.3895196 2.199913 6.599466 0.5929882
1.011589 10.17036 2.394255 8.147754 2.648124
0.3495643 3.714639 2.252904 5.347264 1.41432 0.9648665
2.551417 1.304096 2.307359 3.449857 4.056269
2.062509 4.723552 1.915328 1.118489 0.9078569
1.113388 7.156504 3.85887 4.309748 2.148188 1.50772
3.826594 2.065685 1.998743 2.77415 2.428738 3.50702
2.132088 2.396715 1.748196 3.643892 3.345198
0.7174633 0.7688546 3.152061 7.8797 0.4854622 0.9023139
1.389395 5.560969 9.186513 1.233885 3.276601
1.543148 0.8924785 1.31559 1.174369 1.469695 3.405578
2.017134 0.7074453 0.8881838 0.7468037 1.357556
0.3861644 1.007672 2.221091 1.226496 3.885762
1.214508 3.941536 1.341994 1.006638 5.048326
2.823732 1.257718 0.5128726 0.8709696 1.877701
1.16975 4.558867 1.847349 1.126067 1.27693 0.8002784
2.316206 0.8748527 1.21086 0.4736074 1.620845 1.390376
0.3589438 4.170955 0.3130616 3.498389 1.175624
5.089529 1.258198 5.004335 5.201057 0.604438
1.524982 3.983725 1.1596 1.744005 3.947393 6.359738
5.519406 1.021219 0.9828058 3.243147 1.467839
3.098084 3.575641 0.58962 2.250415 2.132318 2.618427
0.2907968 0.4059787 1.242076 2.372167 1.365992
0.423828 1.993274 1.063172 0.742254 1.612748
3.107348 2.223613 3.249734 6.898759 0.9514113
3.222014 1.275945 2.271454 0.8455661 0.7515983
1.378402 1.068101 2.422159 4.866326 1.229473

0.6464167 0.9556108 0.4727886 2.089586 1.114149
0.6436964 0.5181657 0.6462424 1.343097 2.809191
0.8583883 1.307337 2.821835 4.531445 2.849641
0.9995353 0.6335549 1.323059 0.978348 0.9542895
4.471323 1.379173 1.819794 3.046842 0.9040349
1.304572 8.813379 0.5210764 0.6795984 0.5176029
0.2865135 0.6848788 0.8276619 3.410802 0.2062102
0.5636627 0.748325 1.375049 3.72859 1.139931 2.841387
1.138767 1.081547 3.892995 2.955424 1.795159
3.908532 3.724076 2.927523 2.842878 4.75526 1.730111
4.059064 2.16555 1.222084 4.468356 1.090154 1.868659
1.801322 2.86278 1.306711 3.154477 1.975194 0.7588177
2.434798 0.9703514 3.095025 2.556991 0.5510789
1.570271 2.423414 2.96702 3.151049 1.47784 0.6038141
0.8917628 0.4567741 1.720666 1.673266 2.412247
2.509234 4.413881 8.247566 1.711406 1.730293
2.981474 1.497185 3.575931 0.8297347 0.669405
3.109724 6.153403 1.254362 2.58593 3.682598 2.265891
2.986251 5.088316 2.937555 2.917193 1.068161
0.9766342 3.261996 4.797629 1.44691 5.182243 0.4862373
0.815244 1.293689 3.24553 3.355899 0.7318217 1.819201
1.159177 2.082899 4.48563 1.206609 3.299449 2.938311
1.865486 3.160479 3.913497 2.225943 2.520872
0.6008893 1.269849 4.048888 1.249029 1.914231
0.4611352 1.453095 1.387393 0.374991 2.40994 5.004596
2.332977 2.254143 1.657838 0.129692 1.278496
3.296729 1.079455 1.616038 1.407352 1.161309
0.4632643 6.211647 0.8212048 0.5535841 0.8921942
0.6738237 1.521226 1.065378 4.007912 1.048062
2.390591 1.443014 0.4596939 4.019197 1.395626
3.058246 1.23242 2.333233 5.404697 0.5802085 0.9755125
0.7959167 2.098299 0.1837004 2.423615 1.115848
1.891643 1.1501 1.723325 1.439941 1.534221 1.448491
1.570017 0.6765647 1.023398 1.059062 3.187272
2.978284 1.79075 0.743291 0.6077062 0.8612463 1.446106
3.076381 1.522355 0.4991698 1.05237 3.053839 1.219875
0.4595981 3.459032 1.226444 2.74387 3.426228 5.871947
1.109998 1.123837 0.6363846 4.657411 1.097341
0.5032083 0.5003528 1.904788 2.491542
TLN1 20.84127 275.1288 33.05715 40.04633 32.72892
209.5551 23.52582 80.46378 104.0856 44.73192
132.9748 15.23209 34.78502 38.95145 39.3934 208.5745
40.92035 173.6328 37.19891 34.49773 65.3039 148.2023
31.22665 210.8668 277.3545 39.23262 34.31817

180.729 52.65186 4.387503 90.03945 175.0467 58.46125
26.40775 35.41928 21.18404 21.46886 41.52134
58.52209 43.92841 86.18372 16.82931 35.19919
39.12108 52.23622 53.92603 43.99807 73.81614
53.81186 34.93992 31.48926 71.30166 38.61055
24.82221 115.5666 31.88986 50.35091 235.3983
56.29561 39.32239 64.60382 64.39584 70.17236
107.6995 35.51435 198.3371 64.53991 56.83241
102.8623 63.76339 53.69421 19.97751 117.8321
48.40322 82.23462 40.24766 66.70682 96.8644 33.55044
33.01817 43.01964 64.68249 37.76286 109.1427
62.48368 51.58603 117.4321 90.53751 26.42193
111.5507 35.23082 81.35587 56.03345 47.29899
60.31408 54.04936 54.41128 49.35633 68.4408 97.11871
82.41207 32.12957 25.62826 19.47345 80.41181
133.9987 51.71282 176.426 48.6327 21.05685 31.57369
27.13911 31.10677 109.0901 30.85942 34.19969
29.61925 62.44826 73.14904 12.13578 25.04588
25.33158 44.63122 38.03972 44.29729 71.89 25.85456
42.28559 169.5918 76.04315 78.30276 34.39961
23.79705 30.28332 36.41525 104.5869 66.22298
29.83469 38.1515 37.79962 46.40691 49.58515 126.9301
23.3521 81.75601 30.67265 19.00413 84.65209 96.74576
67.08914 34.72031 72.42396 39.89025 118.0031
214.2435 22.19853 68.79892 109.7128 18.27664
25.92041 95.49671 45.01741 47.62938 44.27312
60.43611 68.88691 42.45604 26.08617 75.05675
49.07248 204.0695 74.34295 37.62011 19.93577
26.44146 23.87648 73.64508 50.18354 43.56299
72.22157 66.58192 34.91117 35.14231 86.07141
38.77639 81.72279 131.7696 25.12584 73.06984
37.42368 59.77878 69.14623 32.06857 52.39337
33.71267 56.93681 76.65257 55.56797 39.39663
24.18914 14.98351 56.78836 66.4868 27.432 132.6574
49.29334 56.33143 62.95843 35.27949 36.04683
25.69893 112.7443 68.01823 38.66673 48.27207
52.03412 51.51268 50.45235 95.43273 55.80735
36.5127 45.72301 56.20776 48.32193 98.00926 19.55179
39.90398 23.45792 37.35881 33.07766 62.30837
55.20146 28.36447 50.69939 22.10346 39.38395
51.81721 25.83427 32.68503 27.62231 82.999 68.4057
63.24441 35.38467 33.31864 73.18762 50.68726
255.1709 86.85847 31.5708 83.37506 25.45655 24.69402
63.60688 40.43018 53.63489 50.74259 48.70266

51.3598 113.8682 48.38717 53.23362 38.5224 53.3752
69.00353 59.27582 39.68848 45.36821 49.55062
73.81505 66.6325 42.75275 17.53061 29.2322 45.28082
47.79202 43.15418 241.5152 80.60116 35.34323
124.6495 45.46572 124.8095 54.91244 37.11113
68.57103 73.36485 21.89657 42.71579 100.3689
77.62851 247.4697 263.5093 59.76372 194.3799
108.8465 36.89379 56.09345 29.4989 50.10931 66.68529
91.99308 56.02345 103.5093 33.28545 28.22006
41.31689 35.97182 47.82006 58.09409 53.15827
31.92744 47.74776 82.25524 29.35786 213.2578
62.85387 67.84991 46.88701 46.24842 35.49271
83.45361 86.87345 40.53385 34.92389 23.03817
50.5949 24.0025 31.75804 74.90328 14.9585 94.16252
67.14277 41.26036 211.2187 41.89208 51.00282
20.27176 64.80951 38.48649 30.14328 43.45745
37.51278 42.13913 234.8729 54.87954 56.88206
41.53089 24.51561 29.28038 55.41071 75.894 42.54597
38.66306 40.21172 28.79656 90.18426 30.18822
162.0733 26.74634 48.84822 72.30577 81.65978
45.41278 37.95738 29.01311 23.508 43.06854 49.99965
21.37248 38.41366 38.6133 54.9154 29.11635 63.76075
46.11934 42.35755 27.99699 34.39712 54.9276 61.09769
57.67202 43.06023 24.47925 56.44576 35.03774
136.6047 26.77695 42.21975 93.36568 178.5623
27.04109 25.58758 33.65993 51.4189 28.25625 63.36796
164.907 37.38544 55.40399 30.81466 40.14464 49.00533
40.74962 21.0419 47.47533 190.7408
EPHA2 13.57495 1.423004 11.60311 31.32449 20.63295
5.119163 6.681912 5.195649 74.30538 14.99026
49.07764 70.13374 7.935377 106.3453 13.81629
3.033141 23.07196 13.76652 74.35667 44.81793
33.32984 1.788219 111.037 3.966232 12.73804 67.38755
14.55382 2.187684 30.75578 3.804776 35.31163
6.416093 53.00441 43.16177 52.41381 10.62136
1.802938 26.00965 34.69345 27.06309 85.78693
46.77731 39.52979 69.07624 24.46805 49.08303
29.55887 24.54145 38.54683 126.4278 49.8068 8.295668
6.457224 39.01086 9.658108 28.41995 16.18739
9.296385 49.08078 57.71575 16.96272 73.43238
6.940954 4.606933 14.018 5.780531 38.99008 13.25401
111.4633 38.72967 142.0279 20.56901 62.78309
16.28329 9.913774 11.12553 49.71503 17.54191
21.66707 16.83974 24.3567 33.97646 43.50264 19.10599

58.1562 69.55776 21.25359 58.58324 27.14152 25.93864
31.2363 33.7167 55.00988 10.32998 32.33591 12.20876
99.28136 42.16611 31.57865 45.26265 4.548651
64.2224 3.783002 10.82767 18.84118 52.48623 157.8441
15.32892 16.81411 27.53306 38.75871 11.09061
88.02793 5.114166 65.97904 44.61728 27.9975 128.5966
18.73548 49.58206 20.21174 11.24817 24.03094
20.69518 42.12504 12.05235 14.51239 31.43441
16.60913 25.4617 70.1817 23.89434 79.00104 38.01598
67.25483 17.11539 62.03215 55.37351 45.75728
41.18423 27.06638 28.41493 44.72243 16.59148
17.49142 30.4966 26.26524 16.85335 45.99173 8.933513
34.39896 41.11603 22.37676 26.29515 9.144249
16.71654 34.01689 10.48561 93.5821 15.86003 22.02498
14.93213 20.60194 42.78403 18.63852 13.93472
26.44733 19.76251 23.8016 29.97569 2.425262 38.70369
9.992316 55.99793 29.74989 35.61065 19.93443
26.9629 44.79693 44.57042 20.64834 34.87529 134.3887
45.12422 12.64335 25.16792 17.70807 88.95707
32.33298 81.96207 39.57451 160.92 41.28751 27.77066
22.36363 45.50535 54.34465 17.16335 90.32748
16.46105 6.584459 20.92168 82.1312 27.97257 29.6636
67.51731 16.58045 2.237101 70.03617 40.90506
22.3279 11.35413 27.37003 15.04146 106.9819 55.49447
34.50188 7.436021 40.69211 13.09959 23.21664
15.06608 16.59555 120.8606 41.75348 6.083791
27.9317 73.37 45.99189 63.28235 29.09365 23.64213
18.54881 91.74576 13.70147 47.51456 16.21299
23.99324 24.65316 2.874849 9.010678 55.89903
13.14259 44.3869 46.56098 19.91785 16.60559 5.003093
31.33625 33.88053 26.51302 22.96644 97.46795
39.68241 48.74797 14.79359 50.4818 25.51752 79.53648
15.67004 49.62813 113.1946 46.3897 38.70265 31.44756
20.81521 102.4515 39.51594 49.95494 58.66782
20.86312 55.69298 86.77109 14.58113 43.11717
17.41648 22.97093 3.405557 1.112353 37.17109
19.7803 18.0138 49.64652 47.44971 49.30406 43.65591
74.04814 20.97523 46.58978 31.21328 74.26302
3.960638 7.151737 35.55501 24.96634 15.99326
23.94563 52.13894 65.25131 2.065959 29.41522
10.39622 45.36873 13.62628 32.18666 45.27439
19.28167 39.94605 57.43458 20.29049 31.37493
74.0767 16.29511 13.31677 42.24705 6.067936 57.49264
23.81947 23.36311 25.37678 22.77442 18.82158

27.03434	10.03717	8.179723	13.35486	45.10699
15.89058	34.14804	161.4562	22.07696	18.40708
25.16552	34.3166	11.5959	44.72953	22.39117	17.39527
64.13763	54.41973	39.07277	14.37325	21.57449
35.47509	12.55128	115.9195	251.2791	1.593844
29.83364	13.73844	48.91551	13.85569	24.33098
25.36935	30.22397	51.61308	25.51711	27.86432
3.781331	10.66503	23.86185	7.656719	131.7864
29.70151	19.96693	16.25404	14.06633	26.3817	56.88353
8.717837	8.628983	103.3729	28.2006	8.333853	10.41388
13.63458	33.83774	27.98489	17.30525	14.78862
47.18667	91.4329	20.78022	30.3848	59.15452	41.53249
17.12234	75.68109	16.21884	18.91057	27.25275
63.18223	41.87141	41.81084	58.07456	17.7811	16.77684
36.04803	9.250732	84.63317	17.55003	7.993284
83.1635	43.13756	10.81084	33.93576	11.61847
SOD2	7.480497	10.80752	8.200001	10.28048	7.94691
17.58503	4.82649	7.320792	7.573044	11.97059	11.7695
6.84383	13.11647	5.585813	7.320976	21.59052	7.113602
8.902772	4.987186	5.401868	9.457508	13.32834
17.04038	12.80512	9.895142	16.99647	6.530384
13.44625	5.80297	3.742083	9.346776	14.03226	9.536557
9.884371	12.99049	8.985091	8.905036	7.220972
26.78574	18.89934	15.19705	7.769538	21.06779
12.68435	9.277442	9.245635	10.95527	17.77877
25.18894	10.33086	17.00751	13.76234	7.838011
4.774126	25.53856	16.60802	37.09621	19.54245
12.9681	16.78524	33.9139	37.20054	21.75552	14.44533
12.16983	33.68991	9.129345	10.72618	10.54438
15.84621	49.4	9.352014	17.01504	72.85276	49.7945
6.306941	25.8733	22.65665	14.75805	36.50061	26.48323
16.7128	40.28086	12.06706	27.66397	9.530978	12.32695
28.02778	23.6234	19.32144	27.25862	70.71614	82.43359
21.51012	42.15371	20.95855	27.00864	9.15826	7.962734
28.1023	9.521965	8.792573	41.67471	16.51215	29.8424
16.64323	22.09485	8.972955	17.31407	19.53537
13.88204	18.46774	56.33464	11.94167	29.27364
36.96906	29.88412	9.622257	11.33724	10.45752
69.80243	12.45558	12.8694	20.09381	11.44095	8.424102
21.88619	19.67965	15.908	15.13347	11.32037	6.702536
13.75743	22.26757	16.65971	14.22422	22.42745
9.462518	11.19292	20.37386	26.94855	33.3665	13.51384
17.80668	21.95071	10.61876	14.91714	18.79118
10.87518	14.0656	22.65873	30.0081	17.89319	43.3233

12.75802 12.55047 29.59466 18.76701 21.55423
22.28033 15.90172 9.500613 12.03395 21.20409
12.98653 22.00031 20.46736 11.61187 15.37047
6.747595 15.7658 12.23707 8.173821 8.794979 12.57038
10.45851 11.50046 14.15404 18.91227 13.62223
13.54167 8.73524 9.356905 24.9167 60.04087 62.63484
13.21466 20.54168 21.73971 21.2475 32.92964 37.78135
7.11186 5.698747 10.67865 18.75344 38.30174 11.07227
8.636902 12.45194 10.36674 8.327508 14.19307
13.29968 25.45472 10.2061 10.50297 21.36505 16.5989
14.11796 20.13204 27.00105 6.468969 11.33028
55.70377 27.4275 7.939885 14.75308 21.65526 13.33215
7.788988 26.12962 13.30839 27.98532 9.22825 15.21792
36.32405 9.324085 7.707525 4.657494 9.754479
38.08689 6.702539 13.13819 6.994442 4.894338
6.951361 9.800998 16.7422 6.430699 19.21791 15.03467
19.24045 25.27185 38.94156 17.7261 18.63852 11.7613
16.22367 21.47463 25.40573 14.29222 7.457978
21.20148 16.84221 6.651768 10.35861 20.66031
22.79164 13.96986 14.61883 21.2516 28.31578 35.53144
33.68699 20.83579 12.90683 15.31692 10.82913
17.43671 17.605 9.928272 10.57726 15.39844 79.23377
6.782914 15.35442 21.37751 6.713933 19.20773
15.40711 22.62365 10.41942 27.64891 18.58726
15.47869 14.59439 7.854713 47.8803 48.78002 31.35166
13.0282 50.4777 18.37663 34.66754 12.21268 17.59845
27.15662 19.35951 7.210011 21.17398 28.15528
23.89429 125.6383 14.06272 10.31358 8.051259
27.09847 18.38503 9.473764 11.44743 11.36053
28.33471 15.86703 21.1224 21.72638 16.29311 12.98771
55.31127 39.49849 19.82302 16.79612 7.845825
13.44723 33.20351 10.38938 15.10849 3.767438
16.72587 14.41234 4.626814 33.34069 17.34694
11.31583 11.39133 8.653186 12.65953 8.862524
10.03682 38.88855 4.395431 17.85546 17.52287
52.18288 17.17657 10.72069 8.413673 15.9251 15.75382
33.32547 13.00231 46.78589 7.837277 63.70685
15.61876 29.05049 19.77739 13.55821 10.98109
38.5121 12.34679 10.99179 4.733023 22.33176 27.86695
15.76697 14.03691 21.80975 5.775698 15.15707
22.80084 15.70195 5.990081 14.98451 9.649515
10.20071 7.238823 23.40493 9.419491 9.959448
8.068605 18.74221 12.96946 10.57594 31.64913
15.57473 12.64831 20.42077 11.5411 12.96565 34.17504

11.56925	21.57701	14.28566	81.23208	39.00263
10.43939	20.06599	5.66338 9.670148	10.61776	8.044529
19.1816 14.05182	17.65893	10.05007	11.92674
SIRT3	2.130976	2.88696 2.096311	3.705766	2.351868
2.276905	2.571846	2.16901 2.630162	2.942816	1.850761
1.503446	3.480543	1.743983	2.923171	1.839439
3.242402	3.116428	1.927695	2.224957	2.643508
2.080549	1.826292	2.131823	3.636348	1.839638
3.470398	2.332071	2.714748	1.368864	3.291193
2.067889	3.758101	2.207869	2.344216	1.695907
1.473417	2.912264	3.1045 2.916563	3.798091	1.972098
1.793572	2.303571	2.550968	2.334579	2.323585
3.326186	2.75848 1.708706	1.837723	2.50592 4.365548
1.528594	3.528657	3.172393	1.951936	2.193092
2.769658	3.013902	2.333741	3.481264	2.39774 3.287793
2.659643	1.864133	3.151555	2.523534	1.484407
3.572287	2.595241	1.935174	2.387085	4.309983
3.081755	2.166892	1.368274	4.59914 2.693018	2.678448
2.53977 2.504694	1.720171	2.653567	2.074002	3.580333
2.089187	2.388285	2.564157	3.244734	2.414179
2.179847	1.200073	2.334067	3.819056	3.098609
1.608084	2.276275	2.88753 2.740319	3.381383	2.086237
4.916438	2.452291	2.740656	2.793584	2.578561
2.419804	2.704383	3.429242	4.52949 4.354983	2.617316
2.687878	2.403584	6.477063	3.996809	1.56877 2.713142
4.394476	2.630112	4.693263	5.343021	1.654062
2.416188	2.641259	3.36942 2.561512	2.064415	3.027
2.492136	2.810839	1.949459	2.400546	2.179031
2.566594	2.289927	3.253727	2.790903	2.75581 3.057393
3.229007	4.973236	3.043485	3.152749	3.451204
2.769905	2.244516	2.581976	3.066462	3.980107
2.180949	2.642155	2.348223	2.500214	1.262823
2.260969	2.853941	3.201144	2.747199	3.477173
2.894305	3.398785	2.871024	2.054074	3.724688
4.170119	1.858532	2.66632 3.892256	2.681396	1.443649
3.081119	3.524106	1.595143	1.672796	2.04522 2.127532
4.48697 2.75028 2.171338	2.885595	1.876638	3.283733
1.445511	2.664036	2.617427	2.074573	2.337875
1.926888	3.03225 1.618716	3.402561	2.250153	3.116826
3.118767	2.483693	4.441789	1.559538	2.142881
1.809192	3.203947	2.655908	3.221576	2.282829
2.45994 2.712463	2.615291	1.838161	4.591819	4.690529
1.926548	2.382404	2.769227	2.881267	2.734351
3.79885 8.343496	2.478257	2.023975	2.363868	2.697023

2.723399 3.23114 3.57759 2.141152 3.988162 2.276391
2.317112 2.151937 2.438842 2.542986 2.185671
0.9805661 2.309344 3.2761 2.964993 2.650368 2.705817
4.51989 3.242236 2.26409 2.133352 3.806911 2.447322
2.647349 2.53433 2.960148 2.258341 3.795674 2.954999
2.436247 4.259852 3.129066 1.386585 2.067931
2.560499 2.07863 4.119376 3.955849 1.934499 3.411076
3.195938 2.077277 2.624006 3.09298 1.874398 2.55831
2.300515 3.225562 2.477439 2.330319 1.980207
3.098827 3.557642 2.835075 3.438184 1.795044
2.787754 1.2921 2.520132 3.415099 1.896828 3.031796
2.602231 3.347819 2.571776 4.060971 2.2525 1.931503
1.30258 1.950861 2.256306 3.334881 3.582121 3.152863
2.570455 1.884214 1.933422 1.267999 2.477895
2.208876 1.609986 1.625775 2.008126 2.199694
1.774317 2.025587 3.057341 1.407855 1.955013
2.220429 2.122248 3.225962 1.898244 2.510585
4.332952 2.586681 0.8692458 2.300728 2.567149
2.250676 2.165581 2.340542 1.286685 3.037742
3.884903 3.703194 1.711565 2.034992 1.158387
1.954471 1.914599 3.862424 2.370148 4.58971 5.659989
2.870125 2.900001 2.811232 2.227427 4.093298
3.004184 4.701061 2.791842 2.65679 3.074551 1.508901
1.866968 1.946691 3.190184 3.045129 2.412525
2.362722 1.571039 2.336932 2.52771 2.78117 2.070494
3.48112 4.187218 2.169016 1.398206 3.76422 2.814235
2.468748 2.222946 3.118467 1.202262 1.685854
1.91188 1.705775 2.557682 1.732137 5.079643 2.880517
2.172275 1.692262 1.991298 2.870138 3.912375
3.347187 2.285353 3.193988 2.498434 1.935695
2.818275 2.974131 4.872584 2.91838 2.198446 2.179124
3.225915 3.346499 4.526519 1.592809 2.085092
2.564727 2.930374 1.646678 4.095228 3.028157
3.295687 2.145827 2.692252 3.02424 1.789068
OLFM3 0.00648165 0.2200266 0.01490518 0.04643748 0.05101064
0.2592852 0.06008714 0.1281747 0.1400917 0.2882883
0.002781857 0.006367058 0.03503266 0 0 0.3049221 0.01939397
0.273293 0.004001548 0.006921725 0.0227221 0.4389517 0
0.2054982 0.1043236 0 0 0.5432696 0.03131362 0.03676868
0.1923261 0.2265914 0 0 0 0 0.01686123 0.005821436
0.03509656 0.01434039 0 0 0 0 0.01668603 0.02623159 0
0.00348344 0 0 0 0.006115308 0.03476809 0 0.1011011 0
0 0.1461781 0 0 0 0.008839393 0.07746539 0.09101634
0.03121884 0.1537235 0.01326607 0.0687848 0.003256109 0 0

0 0.02885348 0.003021893 0 0.005861134 0 0.01055218 0 0
0 0.003540081 0.09025657 0.003332419 0.003413671 0.006275771
0.003334606 0 0.3885791 0.1206306 0 0.1049418 0.05813807
0.01094308 0 0 0.01570702 0 0 0.006569879 0 0
0.01493444 0.00777486 0 0.01222334 0 0.09278984 0 0
0.01570111 0 0 0.2107895 0.2869423 0 0.003626139
0.002567524 0.01915307 0.003390478 0 0.003486547 0.09556769
0.00601676 0.006230455 0.00314346 0.00296423 0 0.3572257
0.002999557 0.00700202 0.003689651 0.006595104 0 0 0.03717629
0.01233036 0 0.04636645 0 0 0.02729972 0 0 0 0
0.008414011 0 0.008568398 0.009497167 0 0.02750802
0.005873397 0 0.1681516 0.01017611 0 0.03069912 0 0 0
0.04654644 0.03314197 0 0.007984971 0.02572659 0.005169806
0.006057894 0 0.0079797 0.2407828 0 0.01383087 0 0
0.002567573 0 0.004666831 0 0 0.003259489 0 0
0.003019326 0.01481756 0 0.08583997 0 0.01603645
0.002801917 0.005558567 0 0 0.004954537 0 0.008508541
0.03105643 0.00321935 0.01981382 0 0.01994639 0 0 0
0.006131846 0 0 0.05972429 0.002752221 0.006082502
0.008290652 0.02411209 0.009024493 0.01429751 0.02799105
0.002835475 0 0.002567398 0.003395194 0 0 0.01024972 0 0
0 0 0.01003861 0 0 0.006477492 0.003656321 0
0.004227029 0.05369548 0.0194183 0 0 0.008245158 0
0.003309026 0.067963 0 0.0386798 0.04015888 0 0.06536043
0.005395954 0.0583837 0.04721697 0.004621656 0.005826564 0 0
0 0 0 0 0.003359343 0.009128053 0.07509698 0.002969261 0
0.01655587 0.03013049 0.01147558 0.004930005 0 0 0.02152807
0.01340337 0.02068187 0 0.06784842 0 0 0.01293913
0.01845619 0.07263051 0 0.003864256 0.1838541 0 0 0 0
0 0 0.005447728 0 0.03427847 0.002615039 0.04702146
0.09074224 0.002762128 0.01489833 0.01098658 0.003106994 0 0
0 0.01608564 0 0 0 0 0 0 0 0.002844979 0
0.02273618 0.02853774 0 0.04631821 0 0.1754965 0.03903088
0 0 0.003232115 0.004317215 0.003869108 0 0.02006842
0.02332887 0 0 0.01701832 0 0.002959112 0 0.001769061
0.003201252 0 0.1163581 0.003100014 0.003192882 0
0.003241921 0.004343685 0.009069016 0 0 0.003619797 0.07274568
0 0.08555536 0 0.0386857 0 0 0.0132964 0.002763529 0
0.02170247 0.004596165 0.00602901 0.2008258 0.07369837 0
0.02983461 0.01077898 0 0 0.004036579 0 0 0 0 0
0.00346102 0.01975402 0.003081924 0.009632318 0.005950868 0 0
0.01609569 0.002794038 0.01304784 0.008832406 0.00257732
0.0154629 0 0.002463162 0 0.07997968 0.01917296
0.009577754 0 0 0.00788961 0.1304483 0.003926414 0

0.04071077 0.002901087 0.002869882 0.1312638 0 0
0.007460347 0.009608696 0 0 0 0.02057998
CEMIP 0.0514726 0.8640759 0.5102898 0.1201927 0.3825848
0.7162774 0.1178195 0.1005305 0.4767888 0.3582752
3.677008 0.9737981 0.3768268 1.27443 0.3543092 0.3530872
0.8175997 0.2827701 0.1694796 0.3460908 0.113612
0.2373256 2.321012 0.3489637 0.4230451 3.597815
0.1478646 0.3120374 0.1694641 0.02162892 0.242431
0.1862654 0.8188871 6.508594 3.299595 0.06686526
0.3927726 5.061292 10.81372 4.95003 15.53363 26.64699
12.76455 0.4154637 4.151931 11.70197 2.781688
6.958776 7.762613 55.4545 2.458197 1.071992 1.153498
1.745687 1.6615 2.388073 22.79579 1.79989 41.75232
1.535186 29.71043 10.70361 18.40966 23.94787
1.616056 5.169828 49.05389 0.9383372 6.855156
1.226524 3.012166 1.01142 3.700083 48.8611 80.72477
18.66278 3.740309 3.274323 7.720815 4.584695
2.138015 9.472964 1.75002 1.29574 51.88848 3.370502
9.166363 1.755071 3.200105 2.010536 14.04104
1.809365 3.393875 0.5664728 1.509685 0.9815805
2.797735 1.70833 41.97797 4.467576 1.508493 4.453662
0.5227121 1.182252 15.79997 4.987188 41.78298
1.72055 4.964755 1.723164 7.108309 0.4071714 3.168278
0.9625409 7.420088 2.226474 3.664582 11.53438
5.654454 0.4926229 2.485775 1.909422 5.755218
10.61795 37.19447 0.98558 1.18222 4.156708 3.559943
23.58741 42.52342 23.68787 1.198773 15.9344 6.997929
0.9039059 28.9042 2.424164 4.068132 10.54607 4.511528
5.674803 10.61705 1.240446 4.779083 1.56793 0.7547962
11.03911 18.15516 4.804512 0.5585062 29.80974
15.43342 2.931012 1.43077 0.7721971 12.13525 1.162519
9.784577 0.5230161 7.266971 0.6150108 3.454609
12.20233 5.068174 2.587827 6.340698 10.9489 1.557772
1.997298 0.5366536 3.364464 1.07394 23.34316 0.3880177
15.02956 8.149415 15.27995 0.5556966 5.314257
0.6039721 5.8397 2.480812 1.941275 7.523931 34.03211
7.176197 14.6328 1.410282 13.19555 5.385341 8.556066
2.950522 6.449726 27.33633 6.182954 8.561942
1.539627 20.35994 2.37105 1.845065 4.673876 6.989906
17.14074 18.47874 6.111955 2.754269 6.758595
8.405718 13.32264 11.37297 22.72644 2.109282
18.69845 2.389332 7.060425 0.7038565 0.315643
9.944055 7.593529 3.207525 3.655782 4.38426 9.005143
23.59732 0.3955076 2.759669 5.331877 0.1279261

22.26191 50.63641 28.40921 6.288409 3.821905
11.00461 2.663189 0.5080264 0.4720819 2.992157
2.071088 1.252192 2.752755 2.002275 21.05298
27.69271 16.61861 3.12652 0.9864304 9.065776 6.229802
10.54965 12.22366 2.176354 1.251103 5.207604
9.673504 23.30143 9.754079 6.214317 2.107529
3.175404 8.899561 55.94739 6.672119 29.71989
6.264089 0.8303982 13.2111 1.651034 2.262833 5.561585
4.303036 2.77783 5.422213 2.591623 1.992272 16.8225
1.388543 6.963623 12.47489 9.784593 6.159432
17.79408 7.998539 5.056125 0.7950058 17.88828
0.6857818 32.31493 25.95736 4.716361 0.9305629
1.686132 1.862024 11.28198 4.598267 2.67936 3.026718
5.597261 0.7500089 10.4014 10.62155 11.24154 284.4842
3.822577 4.228702 5.391268 15.11387 5.768689
6.166958 2.089378 5.273556 13.88321 5.018285
3.354633 13.05044 2.940921 25.68097 18.53738
134.5015 5.820697 14.04732 10.11888 2.065894
6.830157 3.469561 7.958882 9.60046 15.23974 15.65217
9.554137 18.55561 2.280451 7.805438 6.325108
7.164774 4.727406 0.5963261 2.065321 27.8255 97.35984
5.685924 40.75038 3.381361 0.8929293 1.405131
0.4428806 4.556201 0.8015378 1.167134 1.529907
9.937234 1.960506 21.85864 5.518245 2.508998
12.48553 6.588052 0.4898838 4.011906 2.304064
1.461521 4.668917 1.309732 9.153656 3.262323
0.5731769 9.085936 6.271148 5.741417 0.9467033
2.307765 59.77198 4.718009 0.9906575 74.15909
100.3132 8.710721 5.29395 8.226003 5.768849 15.10481
2.862947 321.6074 2.66025 1.318529 5.870481 0.8007636
8.246367 36.08506 1.116822 3.968062 2.885915
4.981991 4.410566 7.019108 0.6689652 3.048867
0.1323686 5.103802 1.021286 1.292412 37.01657
0.294685 2.137656 8.593584 1.010854
CLU 31.66606 197.3993 42.89159 37.02922 64.96742
135.585 55.86103 95.931 131.8795 163.8387 39.58092
8.930636 125.6866 19.89344 130.616 154.8881 86.09336
195.833 60.07213 33.15943 162.084 161.2987 190.481
130.0921 98.41808 26.97359 77.28275 159.2255
60.80683 128.0653 133.7147 163.9986 65.72858 107.68
54.11071 4.005164 131.7801 239.0213 19.24936
35.05715 22.77533 26.04875 15.53552 15.5332 17.01394
223.2318 104.6002 22.89095 180.4547 28.35059
55.67646 85.9327 96.63368 71.40055 133.3557 5.104832

3.546713	162.0507	6.27772	216.2494	46.67939	4.890484
100.34	41.84489	75.64326	168.6369	50.16808	63.82044
48.82406	68.6569	0.6358041	12.04531	37.21011	36.60761
2.115521	146.2546	25.15865	74.82281	7.349595	
2.308209	458.5751	61.52335	2.999086	55.85278	
40.55332	52.13304	19.7302	60.88903	79.92316	54.79008
2.082317	3.969817	29.00924	213.8773	15.65618	
43.98911	26.33948	12.73195	26.71584	48.9581	61.36728
36.47732	66.41474	26.52297	60.47121	36.29074	
13.20383	328.0211	49.75949	14.65269	22.15852	
21.70728	23.23844	214.8976	107.0232	97.33077	
160.6359	8.322982	88.51236	5.69752	10.40132	52.9338
12.30174	22.92472	3.767916	251.7882	3.508957	
3.106585	135.6409	26.54444	50.27818	2.3426	126.8972
12.31848	39.17561	32.19644	40.00817	41.78915	
8.743842	3.665164	39.2217	1.385059	130.8894	58.97461
19.3257	40.27242	4.854684	67.27673	13.75428	34.68829
1.473404	11.66207	12.0648	19.2099	77.75487	91.18382
43.40365	75.2268	18.10006	10.87375	115.5767	88.55178
32.96911	13.71231	94.83184	79.32932	287.8954	
13.8579	108.8007	57.77695	105.8183	32.96106	22.25098
8.176445	218.9508	27.09022	20.04929	26.86958	
56.6106	12.02196	7.363569	3.231426	51.15996	5.886039
62.19609	12.21527	76.78825	3.265344	19.05948	
0.4195858	58.12815	11.8945	4.803168	56.24977	6.3023
13.90419	54.31131	54.99605	4.065791	6.390805	
124.7834	81.15016	3.479025	0.6113014	24.60554	
48.91665	59.37283	14.56384	11.19502	4.366731	
17.63129	33.41658	35.05522	32.66791	13.67922	
28.47968	627.3663	160.1382	29.93748	39.0383	54.7288
44.07893	63.14309	0.6260964	100.1411	2.685471	
25.93436	31.2	35.14638	78.67765	56.1357	6.795194
50.34463	19.58675	2.984197	42.25673	152.9897	
17.87627	9.521759	16.51601	125.2508	33.43209	
39.92569	5.785197	21.02265	35.32723	23.82443	
161.3552	73.92901	19.00431	60.47864	11.88491	
13.95795	19.42768	1.830659	81.48871	7.849526	
275.7338	22.64596	117.018	43.33381	2.109958	28.81127
47.09839	5.754129	22.71254	42.29311	19.88364	
129.7423	7.447336	196.0143	29.88933	58.67517	
1.892628	14.00478	4.221577	10.57792	144.5392	
0.8075579	35.91423	94.85669	5.565313	77.60473	
21.65074	9.209723	48.01195	9.412924	50.47592	
17.56407	35.78758	60.2073	208.271	173.8868	9.397392

46.84803 89.89611 5.515881 47.40837 32.54729
31.4335 72.32993 39.16125 42.0385 13.04192 117.859
14.29738 125.747 16.45352 96.64425 0.5579091 13.15846
1.198904 1.188929 152.9858 26.69052 76.2254 91.8948
14.24331 11.45311 57.16328 4.355837 7.581976
10.30078 4.365074 36.89729 104.0851 61.69451
6.77482 8.424471 8.076517 6.863376 27.28276 72.22677
334.3175 164.4511 26.63731 1.068767 44.25526
118.7286 86.67076 30.4247 34.11568 86.5701 17.93959
88.5161 194.0411 56.50559 1.932785 88.4703 50.41417
7.932398 32.26576 28.58473 9.45098 5.282983 0.803505
26.29883 54.55523 133.9886 23.5637 71.16293 49.55649
1.893125 116.8351 1.791546 17.26772 21.5456 33.50945
4.243114 35.2656 27.92648 35.50323 52.75282 42.75526
16.6998 46.84482 24.51757 17.93563 29.20701 29.47881
21.92445 19.6958 22.11833 77.65748 27.95972 16.67213
116.4122 40.08556 0.7571531 17.30794 74.86184
8.07513 6.921458 53.16551 38.45598 10.32253 69.67888
48.08295 50.8804 9.216651 33.8512 59.7036 5.732804
71.23412 3.278311 22.82653 188.3498

SPINK1 120.8526 0.9762412 76.61715 155.7406 332.4111
4.473734 660.1098 239.3019 283.599 355.2369 0.02527918
0.07714465 443.0184 0 631.1326 2.596333 418.6588
108.5035 219.8126 169.4914 436.4286 0.4986032
3.834018 0.6342097 0.04034068 0.3858042 851.0772
4.152794 233.8448 1337.717 460.9208 30.92129
318.1298 792.6697 307.9102 303.9405 2574.139
209.1679 76.29975 367.1799 272.8935 219.1304
1.920879 31.63599 172.9274 275.9899 88.41013
50.42576 5.6175 18.29203 307.0909 13.83713 67.16953
338.885 452.7196 6.045907 60.59404 4.689457 44.63914
428.4762 416.6369 39.58683 47.81907 190.8373
1052.736 25.46368 434.8004 193.5499 247.7666
64.26176 0.2599077 1117.194 15.33363 23.42375
323.5759 71.54737 80.67581 348.0146 2.325535
167.3914 9.643833 184.4158 260.7003 180.5528
83.92102 292.606 99.18889 29.39288 622.2437 115.9415
100.5018 250.5836 555.5558 345.3357 17.80756
1038.895 16.64258 419.5975 0.5437113 102.9951
41.25986 31.32109 499.7581 526.8122 78.39124
126.5446 298.5647 207.0108 27.09296 467.4229
398.6022 314.2385 82.96788 0.486986 467.0886
90.27683 248.2113 99.15113 2.023297 97.20499
109.217 385.8228 38.148 1376.851 0.04718097 173.6284

22.83312	78.08937	222.8126	126.1475	103.9972	
60.38475	1170.828	94.56437	206.2444	4.754602	
11.01806	8.043537	17.27492	110.6711	68.02216	
1.488462	383.9012	332.0134	101.2992	32.17604	
184.6497	202.0634	0.3027983	325.0432	41.98676	
198.9852	27.47795	133.8053	52.48357	124.4672	
111.4334	47.12232	4.577853	126.5466	224.446	323.5325
280.5065	150.4045	34.9138	81.27032	46.05495	331.4688
8.50276	0.8338975	0.8671091	273.9181	100.3896	109.3331
193.4214	1001.244	126.8321	1060.404	584.201	191.9514
81.27593	9.815301	6.925937	407.0478	3.061033	
128.4238	140.9734	1310.261	54.65527	226.7429	
247.4142	32.9064	622.5529	244.6232	16.01927	3.195831
3.913378	162.3054	37.23062	25.23256	1302.498	
76.19168	12.40517	65.60842	28.95639	60.03739	
591.5538	72.09188	117.2046	171.7598	50.86604	
81.79079	128.6053	84.418	275.9985	330.8923	47.8629
21.78278	92.2598	32.23181	497.3023	142.4228	104.173
16.93356	117.1617	32.148	201.5205	141.2098	13.01896
238.7149	9.701871	164.9182	657.3009	46.64857	
170.2224	1159.749	137.4739	410.2648	11.88268	
46.1469	177.4103	847.4303	128.1896	70.69916	17.19377
28.09435	254.4697	19.92481	284.601	632.7379	251.798
150.9893	1086.141	97.82743	104.4155	1.115463	
68.00216	208.9769	8.156563	277.8201	395.2076	
6.297222	61.97115	29.32709	158.7728	162.3991	
60.05938	112.7859	564.887	511.9193	556.0236	177.6754
1633.706	130.542	25.43975	59.42689	373.5931	20.16945
0.8840822	173.3868	124.8831	376.1016	66.01774	
8.476233	19.28857	214.5883	77.16808	128.7279	
87.56443	7.683519	743.9095	72.69044	15.64295	
175.0551	0.3610229	113.1151	156.1797	605.6511	
37.97325	38.61201	136.16	266.7014	224.9406	31.33117
148.6467	128.1957	85.68526	2.028355	629.7653	
43.18322	215.9123	3.148971	120.7306	20.68793	
38.85724	26.49764	429.8183	15.71384	130.2151	
3.788827	119.6291	149.3212	17.03445	416.4002	
315.5974	296.5316	203.3367	4.072403	72.47179	
0.6453583	193.5142	28.80238	1084.767	463.1854	
34.60466	71.99398	143.7269	512.0243	342.3923	
819.9734	0.06867635	22.39659	154.3423	61.4454	4.274802
200.5861	193.9233	20.20052	557.6778	677.8762	
39.89471	267.2784	103.757	117.5985	456.3858	130.0179
286.5795	118.4936	82.19563	210.9153	410.4376	

105.0517 0.06880309 671.5165 161.3355 80.63969
50.46484 224.4951 231.7507 184.4338 258.9768
372.2278 16.54218 194.6871 439.5338 37.13177
58.17423 43.63546 598.3717 206.7724 96.69728
48.80057 61.56849 0.2051829 143.8192 900.6263
233.5413 322.8441 35.43275 4.401326 22.47703
2.724378 590.5206 35.21612 650.1623 776.3304
8.471197 164.5503 211.3556 9.074778 234.2942
51.92973 5.821052 12.84956 64.95626 259.0021
17.45461
CPEB2 5.22682 3.497846 6.688508 6.052677 4.099705
6.828904 2.915023 7.026072 4.200291 3.918528
10.3077 8.741627 3.135464 6.30894 3.828287 10.85649
3.573671 5.491621 3.649276 6.723115 4.470795
6.017942 6.487281 6.352668 5.898046 8.137227
2.839035 6.710063 3.900176 2.025687 3.403782
5.108025 3.104967 4.762637 1.957865 1.273595
1.592454 3.423547 2.784159 6.522851 6.540625
4.316774 3.691434 4.71358 4.690199 2.433939 4.527237
2.365177 5.299412 4.88446 1.633464 3.854209 2.738556
2.526365 4.562676 4.588845 1.548544 7.69061 1.207742
2.909038 4.072546 3.475713 3.304465 2.049038
1.785676 5.475938 2.369922 2.974838 9.837863
2.437938 5.428825 2.605748 4.784072 7.051992
2.438486 3.470689 6.819631 7.034517 3.493269
2.550169 1.552239 4.311949 3.18532 5.310017 4.20702
2.477479 6.47993 2.771707 2.538156 3.858834 4.459443
3.725061 2.770318 4.509671 2.250838 2.835318
3.818498 2.03144 8.310547 5.827355 4.192091 2.40527
1.571862 3.921578 4.346839 4.926177 4.722097
3.850541 4.011653 3.570473 2.706856 2.057777
2.234565 4.467729 5.097415 2.513684 1.445077
7.301935 1.950077 3.223305 2.218483 2.295419
2.447489 2.341405 2.500453 3.709075 2.788449
2.215687 6.587183 3.608604 3.707441 2.562807
4.861043 4.446772 2.96872 6.440119 4.053161 2.112054
3.711601 2.305571 3.295799 1.242689 2.932246
1.485563 1.326005 2.408171 2.646235 4.709239
0.9259159 3.539579 3.107473 4.076059 5.415038
3.736011 7.1896 5.066007 4.299549 6.083497 3.616843
2.174079 2.47454 6.239835 3.552396 2.581867 1.513763
5.690285 2.086088 4.74888 3.65014 3.04048 6.460566
2.871134 2.9395 7.805114 3.666156 1.780211 4.772292
2.737244 3.719611 1.674869 1.08111 2.711007 9.267608

2.842164 2.641535 4.159513 4.876137 3.223741
4.54267 1.351134 3.86268 2.378539 2.636526 3.237133
2.011978 6.189884 4.393206 1.431772 1.629558
2.361704 4.183175 2.861368 5.592548 1.535215
4.144234 4.760429 1.502578 3.889133 1.887607
3.400408 3.814058 4.107414 4.607314 2.159001
6.032037 3.678647 3.015597 1.65821 2.981781 2.988583
6.014734 4.863635 1.640364 7.816268 4.506579
2.833193 1.841424 5.906867 3.645456 1.582011
2.032639 2.805988 3.860187 5.863118 4.441763
2.252722 1.982862 3.445489 5.131625 4.625792
3.890424 4.626982 4.330139 3.71754 4.597245 3.59272
4.179417 5.085324 5.17608 2.202662 4.19813 2.215853
5.409909 2.946926 2.513268 3.113986 5.338193
3.149639 2.970583 3.246434 3.127027 2.174691
3.322162 5.746536 6.334158 3.704579 4.573635
3.305592 4.356882 7.354979 4.305587 1.983397
3.941381 2.299732 4.001814 2.9783 3.679471 6.400059
2.773722 3.070286 4.545992 4.743881 4.410203
4.646819 6.63619 3.989281 6.208224 2.49428 4.759067
4.183479 2.561238 8.339326 7.005069 3.554159
1.405207 2.642099 2.066882 3.26404 4.785019 2.605169
3.898486 2.735368 2.91563 5.128368 3.433799 3.004258
3.255662 1.945404 12.292 2.741569 1.607959 2.62801
1.665142 2.519709 4.388212 5.966762 4.075897
7.140729 3.933119 8.996172 3.147026 3.533329
1.874855 2.089238 2.845706 3.139203 3.61048 1.339408
1.79469 7.495111 1.41031 5.499666 4.94504 4.137914
3.77612 2.306223 1.533681 3.695289 3.298749 5.610927
5.310767 2.975089 3.970081 2.360536 7.233959
6.222496 3.883365 1.302736 1.508933 0.9921573
4.390385 3.133207 4.260249 3.724165 1.714847
8.28261 3.942925 0.9335269 5.455091 1.943671 2.933794
1.582235 5.896203 1.228355 1.522917 3.534088
5.920369 4.170096 3.518314 2.423345 1.850562
3.77081 2.338966 4.831391 2.049989 2.422401 4.072954
2.282239 3.640596 2.966646 3.02149 3.619016 0.6640325
2.985993 2.672505 1.905771 2.997483 3.107795
2.937453 3.513053 5.745836 3.654202 1.200981
3.575851 4.596098 2.593793 3.86706 4.25928 3.705252
5.227989 1.155747 2.437174 5.179658 5.512245
4.672197 2.865434 6.085217
TSG101 18.24498 10.08204 16.35774 17.27325 16.50027
10.21023 14.33743 11.1849 14.60646 19.18427 15.34275

25.92635 16.23521 19.32759 13.06765 10.68781
15.73236 11.89931 15.77075 16.47287 19.09105
11.87995 18.95986 10.50891 11.65624 15.97999
16.06049 9.794363 15.9942 22.76504 14.88706 10.68248
19.69004 17.99818 17.9314 11.9381 9.970881 12.22279
19.11029 14.45181 13.41041 19.65338 21.39664
16.65818 14.97628 19.63511 11.67386 17.95931
27.94141 12.57733 11.14032 11.84005 21.59947
12.70098 20.6902 13.39861 14.38609 9.632898 13.77111
18.96271 16.32287 15.51946 14.39074 16.00468
16.34785 9.147364 15.75483 9.925701 19.00403
10.70165 18.44952 23.67969 15.21084 18.0464 11.8651
16.91923 15.96378 14.42637 13.79745 23.68938
15.25311 14.90284 21.15243 12.50319 20.09505
15.91393 13.59003 14.42738 14.13151 30.8071 24.92322
12.95607 18.44368 17.35362 14.24861 11.2701 23.2502
18.58238 15.2793 12.90429 12.6275 19.82822 13.10595
24.79504 14.73765 12.0985 15.85973 13.24293 16.57581
15.81715 19.31592 21.19501 23.15232 11.33541
18.79114 20.47993 12.81339 12.18481 15.03557
23.20113 21.23462 31.58609 22.07575 13.9257 21.57951
12.55774 25.73615 13.17161 13.08943 24.96051
15.72637 13.46437 15.27197 24.35242 17.74989
14.19112 16.16986 21.44566 23.46956 16.66005
14.93452 15.78026 16.80759 13.11725 21.21048
19.81706 19.00248 14.56317 11.89685 14.46919
16.2041 13.21664 18.64187 13.37843 11.12611 14.01026
12.90069 10.96082 25.36692 12.61114 17.67176
13.76428 17.83757 12.99998 16.64883 15.90438
14.06633 24.18641 11.92126 30.28917 11.02829
12.49094 15.64227 15.358 12.56019 22.75701 12.18005
16.13657 19.3323 8.705946 15.07285 23.40326 15.32534
14.23023 10.03933 14.69201 15.00649 22.04356
21.45131 16.68037 21.35548 10.15357 26.04111
16.20566 14.63557 16.9539 17.61812 9.770687 12.64086
14.40354 16.06304 15.80918 15.01876 15.33593
16.18268 11.71356 13.48442 21.555 9.01181 31.92378
16.49231 10.41807 19.17983 13.07293 12.15942
15.40669 31.90213 17.87457 14.73299 13.03406
20.10171 16.49347 12.0402 14.60978 13.39162 12.4028
16.12249 13.12277 14.93704 13.91039 13.07375
11.09889 14.13705 17.43442 16.01362 16.62864
13.3941 14.7987 17.28122 24.15463 21.2752 17.73637
13.03861 16.85429 19.44331 14.62892 15.05342

9.874401 12.97942 16.06402 18.57827 23.84731
15.40682 15.36832 12.66283 12.20268 18.88854
11.63134 16.66404 14.02749 15.92212 16.42664
16.94067 16.88486 14.93724 15.49215 16.37324
19.88156 25.43797 20.01083 18.61375 17.57807
12.30708 20.34492 18.79565 11.99332 18.34815
9.626437 20.70535 34.6785 13.84181 24.36153 18.34797
18.16957 14.12108 15.01132 17.37358 15.10879
14.11281 12.67988 11.83315 8.636464 10.69929
16.88862 14.91551 11.71984 16.14911 17.41273
18.83603 15.84158 14.18665 11.19601 19.1051 9.439917
16.13173 13.68516 15.11373 17.3292 17.56131 13.0792
11.9063 14.11076 14.41101 14.15635 21.36191 11.42599
14.05886 17.08646 14.37379 21.60037 32.96595
15.62831 19.02431 11.17066 18.46641 16.0575 15.47451
11.67549 16.7243 11.31679 19.83376 14.55167 18.33247
19.68976 10.19698 18.73375 26.78923 18.7429 13.84074
20.06367 10.00789 12.49387 15.23516 15.08203
10.05229 15.87784 15.39574 14.56319 14.24026
14.2418 17.75 19.39698 14.37035 14.02384 17.91757
19.05349 12.09891 15.18347 12.00557 13.70026
19.98787 10.15785 13.91035 18.45957 16.26706
14.02028 15.27193 11.92672 8.875218 23.41597
20.36165 18.06209 25.7251 12.28674 24.24554 11.36635
11.62955 10.56322 14.52573 11.41986 18.74752
16.14688 9.818401 14.83718 21.636 16.12018 10.03493
25.21031 16.676 18.18732 9.136796 18.92833 13.30581
19.34903 13.41999 19.37734 10.87806 11.63575
15.53274 13.90578 14.20138 14.24821 17.50971
14.48078 21.46897 15.70888 13.58126
SERPINA1 637.7417 1.575749 268.1875 31.82852 184.5649
5.120649 22.31934 27.86613 17.52004 83.17871
0.6894448 0.1339251 59.35312 0.2278657 32.24797
1.313051 202.0811 30.11918 313.5838 224.2624
14.41087 1.012436 61.74645 0.3652286 0.4892123
0.5218352 51.16491 0.9632007 363.2899 12.99303
9.941802 31.32528 48.01734 586.8488 42.3702 2.598838
1451.473 340.1193 37.81904 101.6831 154.0793
11.67364 48.45953 22.10192 52.12742 37.51293
43.83928 20.53497 13.31166 50.60843 327.7449
14.42052 227.1144 151.5768 14.29077 9.757143
24.71746 3.673257 442.0924 186.4527 184.3493
21.90721 85.18992 54.58246 351.1129 13.02611
112.7643 127.4583 231.6573 4.995885 26.63954

3.894547 40.51318 153.3298 363.1595 9.096177
18.97741 48.3163 4.201503 129.5557 211.4574 187.4799
34.5025 71.80403 16.50541 130.2315 36.71999 80.32585
33.57693 122.3579 304.1351 8.984951 42.87929
297.2918 58.05574 689.9193 369.0489 84.65148
106.3134 111.7847 36.96676 89.41362 242.5508
129.891 36.2187 56.25715 213.6633 21.98207 83.44173
46.62092 16.67265 20.22801 26.57864 4.234453
126.6104 846.1505 763.2287 76.35435 2.325243
156.5438 619.9158 500.0034 91.10254 1047.688
4.099643 12.86173 19.33658 96.63982 12.71078
13.35923 8.345922 1.393576 39.55383 167.974 92.7605
17.83039 12.27721 107.9384 9.850604 112.7319
32.44303 3.535848 612.8813 94.13069 5.631174
26.05876 97.982 35.77379 93.91721 19.90692 16.17711
108.2635 10.32377 83.53276 25.26304 65.59081
45.82048 16.37129 9.266961 24.90918 40.96496 8.6511
43.73004 180.8544 9.723216 45.57062 487.3913
63.37868 12.65806 22.98024 7.291126 259.372 10.17586
146.6506 238.8184 47.69713 239.2901 145.4981
32.84628 6.98611 1.225143 12.42837 21.78356 21.6399
592.5292 412.2218 84.20807 14.60873 137.3934
13.50396 104.7571 967.4106 136.3391 60.29464
26.12933 46.71097 170.9773 63.56768 22.43586
20.87464 2666.468 2.654449 54.48032 283.2953
198.3396 66.2618 408.7027 20.38667 28.73126 45.41305
85.16109 181.2443 94.53676 30.63246 7.318327
39.07041 247.8876 525.1891 9.712405 66.68447
25.68552 393.3923 37.74823 81.42061 28.59937
1.621277 306.8294 19.33254 8.465187 30.96976
157.1317 447.3946 40.61714 300.011 119.0424 42.58622
15.33093 14.66608 18.86163 135.5459 216.7637
119.889 11.61827 147.2577 108.1536 228.8195 51.94794
1.014473 91.88502 45.06209 19.23602 25.08085
62.7689 34.47368 207.2808 890.8679 52.64698 227.0265
16.99618 451.6955 43.46912 40.27893 154.7006
4.993592 19.11435 129.573 133.3833 50.2607 22.2843
162.1725 14.01804 85.1994 4.794323 13.88256 218.9466
71.53735 514.987 14.69121 3.905293 35.25829 38.90675
95.29967 112.2369 54.56096 4.632031 11.65264
10.93069 33.24543 462.6152 7.911881 296.4597
7.221312 5.457551 195.8182 56.88393 6.329924
23.67189 26.28711 80.60913 4.929649 113.0114
13.81916 34.97403 249.4925 36.08732 113.0705

59.47026 65.27567 64.90459 24.85643 180.4029
32.88407 145.9745 89.05433 9.904211 13.6298 328.1057
65.07774 407.853 125.9487 119.8871 185.6277 70.84184
6.313456 199.3588 446.3957 95.80662 2.548551
67.80495 53.39566 10.74192 63.92773 86.48201
105.7674 115.3606 9.435011 142.877 162.9997 8.153246
1174.055 33.23341 234.3013 50.28893 321.3433
7.038633 16.16312 62.32536 160.4625 156.7095
226.8541 11.83265 124.8972 86.36849 7.938668
607.3517 66.79474 69.01747 115.8639 9.24714 224.8991
109.3367 76.34741 71.77321 495.5451 69.60419
7.98182 5.032295 97.78232 27.48873 145.717 68.84918
8.895473 228.098 110.5294 84.05502 610.4067 469.9385
46.10273 126.5752 11.97732 20.37403 30.51638
154.4113 447.2433 11.92763 43.01322 125.3312
323.4173 4.078817 14.55446 115.6982 10.33584
74.2346 20.20903 225.8055 654.4243 17.07545 102.6971
8.694616 32.2567 99.90246 18.94656 7.864714 7.993725
38.01489 62.1906 9.960962
AKT3 0.4002078 13.15954 1.265171 1.463297 2.043444
13.50236 1.312501 4.967339 3.799433 3.323566 6.3507
1.189941 2.573915 2.18382 1.189919 17.05576 1.101127
7.642897 0.6834784 1.018508 3.142797 12.48557
1.808331 15.99515 13.07821 2.735932 1.432032
14.71205 2.011975 0.6784724 3.271564 8.384327
1.23886 1.849649 3.46537 0.8907568 7.205476 1.98038
3.271299 0.9268323 8.043785 0.5582609 2.786564
0.4889313 3.031613 1.22871 1.896605 3.33092 4.412264
1.462291 1.212514 5.720244 5.512451 1.911333
13.39379 0.4637045 2.902035 15.2991 0.7995108 1.089278
10.38602 2.542819 8.561287 8.84954 0.5676238 15.35907
4.226315 4.856928 2.030504 0.8697716 1.743083
2.364966 6.284347 7.310456 5.463659 5.027695
4.715631 3.146623 1.504063 0.8697016 0.9224861
4.988011 2.751122 6.286304 5.730531 2.679809
7.880786 3.389858 1.691441 9.640511 2.701123
2.775383 5.196286 2.258731 1.784358 2.667735
3.720269 0.9510212 0.5546055 5.234666 5.863109
0.8600498 0.8055328 1.489831 8.006002 8.675023
1.835975 7.291644 1.976982 1.112061 1.291686
0.9430987 1.485406 8.053009 3.687346 1.178455
0.8492572 1.621755 2.97576 0.5983555 1.626477 2.495881
4.650274 3.877299 3.152754 3.762859 1.127606
1.479301 12.52009 8.528032 2.274882 0.881589

1.653815	3.278669	1.220335	7.567429	2.628211	
1.423155	1.876527	1.368143	2.175784	1.713062	
2.513327	0.674836	5.312925	2.029958	0.4458724	
7.851275	0.505405	5.729197	1.602744	5.644232	
1.614563	5.493704	11.99887	1.13146	4.011701	7.531199
5.810964	1.83148	5.727151	4.861995	5.988493	1.304251
2.068459	2.993163	7.215821	4.441225	3.09063	2.695717
11.46201	3.113903	2.596307	0.875349	0.8885102	
1.697105	5.688537	3.263518	0.8009911	4.843966	
1.136597	1.308602	1.792755	3.222839	3.477345	
3.908527	10.43788	1.365128	3.279057	1.132147	
4.010719	1.365388	1.75308	1.446365	0.9412983	9.573185
7.093299	2.094404	0.734352	1.394958	0.8304938	
4.014387	2.256385	0.9100216	2.457334	0.7882263	
3.163178	11.12833	1.846499	1.94976	3.163607	7.690883
2.70993	2.031108	1.602485	2.396055	1.928127	0.9511399
6.409543	2.209703	2.126307	3.658179	1.034898	
1.532317	7.170802	0.5504923	1.390069	0.5391962	
0.4022237	2.203562	2.408092	4.439894	0.4399946	
1.181959	0.9426448	2.210759	2.387663	1.011689	
4.851437	1.852143	5.944267	4.36677	4.225487	2.409297
4.782711	4.118866	3.027907	8.747491	7.760995	
1.536149	4.692039	2.007201	2.470119	4.945308	
0.8007006	2.218229	3.057687	2.573302	1.779364	6.4978
2.053928	1.067388	4.214283	3.806334	3.78078	3.048685
0.8551396	1.558042	3.636326	2.894971	4.601942	
1.416926	1.662873	0.8969118	0.5176593	1.403473	
2.410131	14.50545	3.205756	5.686117	8.702151	
1.006609	7.411205	2.867001	1.924308	3.652271	
1.321301	1.270726	3.625065	9.66748	1.580005	18.67855
16.82372	3.026718	4.69933	7.264469	4.13376	3.634467
1.277771	1.4499	4.501764	7.596025	2.213724	9.491752
0.8294698	1.171787	4.615144	3.038593	3.012511	
1.477873	1.969798	1.037751	1.517627	3.69168	1.320491
10.51215	4.175235	3.670023	3.093432	12.29052	
2.910771	2.99674	1.200443	1.503402	9.448374	2.708099
2.35101	0.8052045	1.399094	1.835497	0.4944798	7.530609
4.686296	2.48634	7.634938	2.998017	0.2235806	1.62691
3.202745	3.414666	2.012438	1.16612	2.96918	0.7861157
16.24571	1.785603	0.9188881	9.035831	1.557852	
1.718889	1.334734	4.834677	1.822705	4.607767	
0.9626535	0.8546291	6.405435	1.924681	13.52942	
1.339395	3.335395	3.830072	1.52455	1.263696	1.243323
1.646791	0.3436708	2.052704	1.503971	1.685816	

1.172483 2.291741 1.280283 2.102496 1.965284
2.372208 1.051229 2.433156 1.286278 3.603753
3.731814 3.213206 1.946086 7.591195 5.957627
1.224368 5.993685 2.485589 0.5785364 1.082761
12.40653 1.806718 1.025094 3.897544 1.704924
2.731336 3.592146 8.03443 1.416612 2.009034 0.7963807
3.081504 1.704846 0.6585274 0.7034468 2.14982 11.22579
RELA 11.30329 19.38718 11.99432 22.02189 16.77926
18.33008 12.57129 13.84 23.04953 19.58197 16.04988
14.00534 19.59447 18.84981 20.31333 17.06717
23.45959 24.83106 18.50692 19.79134 24.4218 17.46693
27.03136 21.44761 16.74719 17.29064 20.33744
18.98354 16.57109 6.238593 22.93303 19.96143
23.8133 22.77028 32.89702 8.265289 7.616991 18.19278
21.03296 25.07984 17.72986 27.76925 19.56371
23.75675 13.16453 22.17157 16.71347 22.38122
26.60324 36.6215 14.90539 14.19917 12.71968 23.04887
23.29846 16.53634 22.97548 22.25583 27.43233
26.95275 18.60186 28.40757 13.32321 20.59485
17.82724 17.02676 23.22709 15.98999 25.39004
18.23461 22.16452 15.17267 25.20403 18.0546 19.86336
26.88618 17.71295 22.2913 22.20962 15.33396 24.10127
18.94024 15.9639 16.64533 19.20498 21.31954 20.52483
23.7004 17.052 23.51052 17.30091 20.26335 17.64674
18.40342 33.81906 17.89305 25.40029 13.56274
21.89217 21.03037 17.96718 22.15021 25.21081
16.48645 18.0013 20.82776 25.13606 24.61331 27.89854
19.85118 24.67415 16.97962 27.97459 17.0541 22.77031
21.85187 26.74621 21.36046 21.7133 23.26382 18.24891
16.21089 20.99662 16.77922 23.68574 20.59401
21.14169 18.87394 21.68865 31.47645 26.43222
20.67891 28.24015 20.26619 23.93158 18.37558
23.44519 17.31225 22.17841 13.76728 17.1061 20.67089
24.34196 16.57342 21.82666 11.0807 18.17657 16.07214
17.47902 15.84385 20.52639 25.10705 23.0801 21.67975
17.80291 18.90237 21.72987 17.30377 32.80704
12.41014 29.28498 15.03199 15.70593 17.94583
13.10927 21.74301 33.12709 15.45505 27.68416
24.74451 17.6766 31.36385 16.67827 17.66581 23.58335
32.93449 22.40124 15.69488 31.29683 18.93496
14.86367 22.85062 25.12332 23.60562 13.54315
21.22669 22.61786 32.31747 22.33887 21.10357
32.42807 21.00097 26.47313 19.39821 17.63964
20.24934 21.86065 15.65529 19.17992 12.76556

9.727087 14.73739 22.77265 22.31625 29.2348 26.94727
19.56344 21.65252 19.53249 35.95374 18.34542
16.96254 19.20908 15.10309 28.79882 19.2742 34.60474
20.63057 27.60276 18.6738 26.22216 23.29431 17.97385
24.06108 23.49452 12.56275 19.55569 19.19553
27.75675 38.81999 22.35203 11.15909 18.3914 25.41977
15.71529 27.98548 20.42236 17.40817 13.86215
16.32215 20.92033 21.79773 16.38951 20.51393
14.85912 18.99371 23.12311 20.97556 22.75569
19.62871 21.03168 17.62826 26.73521 22.0824 17.43859
11.75741 20.58423 22.40799 22.20052 17.80649
12.37084 24.13662 16.11336 17.33209 22.91362
19.42755 30.69835 24.68037 23.14729 42.4097 24.33065
34.88301 23.22427 20.14862 19.44871 19.97942
14.08513 18.05167 22.06222 28.03014 22.21408
17.85243 23.85463 25.36522 15.79595 21.10935
22.68179 18.06185 16.4146 20.45059 26.82351 14.59399
20.94686 18.51048 27.75958 22.90924 15.63405
22.73425 26.18383 24.70898 17.19323 22.38171
16.70193 16.63784 16.15861 17.17615 13.99737
14.42841 22.10716 18.53076 18.26034 60.26457
26.24105 27.61385 23.02498 21.18618 22.66378
23.67051 16.46719 19.18595 21.82646 21.6339 25.95353
15.29083 11.00849 16.27392 27.7544 31.50722 17.78685
20.47152 16.59656 21.24627 23.36537 25.8745 26.75253
22.10759 17.21887 24.08916 21.71518 23.15595
16.25438 22.5309 15.69364 31.29387 21.53037 17.80674
14.77602 15.64219 15.22468 17.54141 27.33004
23.49019 18.01198 10.8733 27.08298 14.77845 19.00966
17.58822 20.41354 20.74512 23.92913 21.39741
18.64958 18.57797 20.52688 12.46641 16.42211
35.46211 41.60501 10.15204 20.74068 22.6223 24.57798
17.4655 31.13786 20.96446 25.31272 12.87574 17.18154
17.42279 26.04203 18.91502 11.29751 29.90287
30.54836 29.07524 23.80191 22.52632 24.68515
19.07946 23.96234 13.83629 9.744181 16.85151
30.78662 20.54533 18.37061 16.87561 15.62118
21.97904 24.99819 19.14625 21.32109 18.80957
16.27114 29.68755 24.13511
PRKCA 7.660354 10.0221 7.460917 2.639575 6.705397
16.26387 2.395318 7.547634 6.914744 4.465816
2.225378 0.66291 2.581177 1.129867 3.534191 18.55914
6.200574 12.61639 3.764089 6.647319 5.016127
12.6152 2.935238 12.45048 8.805013 0.9451649 3.058218

17.32487 7.411686 1.117418 8.184165 13.58724
8.083493 4.222138 10.41733 1.259442 2.837294
2.514505 4.483476 6.01835 25.76602 7.529265 8.07889
2.458107 6.635185 9.46161 8.783114 5.353387 4.96784
16.15948 3.501109 6.149429 13.68853 4.240979
11.28061 6.431709 4.556358 14.33531 9.47208 17.66175
10.40831 2.27237 6.421402 6.800742 6.861092 11.0755
6.235863 4.179228 6.685991 6.285866 6.889653
2.604198 10.97636 25.99128 12.05068 3.176051
6.284385 15.90917 9.248903 6.439697 18.93497
11.04025 4.399926 11.16424 9.339629 16.11818
11.36057 3.475764 5.859925 5.428207 3.697259
5.892894 3.455564 5.05777 2.696168 2.532756 3.628069
6.742561 12.87343 3.43598 8.343169 8.546674 4.115402
2.706615 6.289914 11.31323 5.47276 9.894402 6.693416
3.20618 7.58114 4.513731 0.6836025 7.769806 5.495185
10.30618 3.569844 6.319675 7.401005 4.283722
5.853832 5.548773 2.39628 4.225436 4.723647 3.936243
2.467789 1.393524 21.3773 4.700185 9.663964 7.208377
6.781694 8.129796 3.599764 11.74639 6.324739
9.499228 7.478492 21.27375 17.07401 5.314272
16.25808 2.082189 2.75192 7.86598 4.168751 9.585476
33.45906 8.112767 2.137107 6.020449 3.994898
7.734846 14.77959 4.000705 7.466428 7.716059 1.4127
1.730417 6.859025 6.333033 11.19245 4.152791
4.895073 7.070723 22.51952 8.562223 3.637187
4.105939 9.474904 13.09474 17.24119 8.544128
9.091257 5.19373 7.278027 2.969744 6.072228 10.20143
5.349891 5.898561 1.403531 6.163377 8.97171 8.553856
14.18841 1.757394 5.563331 5.991026 6.662139
8.577618 2.055927 3.636804 3.427496 6.502093 5.866
9.298458 17.96897 1.910908 4.517288 4.583918
4.578762 10.6543 3.293166 10.40604 3.42061 3.670316
11.55379 12.30501 11.60802 9.414511 5.483967
2.232891 10.7633 5.814295 5.030264 4.114863 4.341545
7.48805 5.986205 5.926354 9.730812 4.354573 8.875733
1.473871 2.488513 1.228716 6.825237 8.587633
4.134567 6.458253 3.532691 13.65 6.073049 7.392893
3.621855 9.045206 3.579075 6.438157 10.59645
6.381076 5.844666 5.130403 5.532615 7.832239
3.605083 5.760508 7.449956 11.55312 6.661286
2.78886 7.766295 4.000896 6.505137 11.86985 9.003117
8.137658 2.341072 5.997406 4.287465 2.640347
6.85829 5.373768 15.17395 4.532414 9.017527 7.8447

4.443871	20.8611 7.214107	6.212236	3.472944	8.334782
7.98469 12.78117	21.45637	17.56708	1.187247	8.108721
9.048803	8.023822	3.510806	5.066228	5.402896
16.36937	16.35639	4.912026	7.884327	7.36786 3.114768
15.5565 7.370608	6.57105 8.963523	4.864778	2.351834
8.44932 5.625602	4.580595	9.937053	14.1017 9.225295
9.448843	5.010119	7.048561	14.89354	10.01229
5.096127	10.07135	5.342682	9.562652	26.9477 3.23302
6.007051	15.7691 12.94144	6.584001	10.59942	9.036493
6.154012	7.857605	12.82347	3.030623	4.675598
4.23228 11.32062	0.8209447	7.169476	3.770827	6.721946
10.89689	7.656897	9.390166	13.57109	6.328086
2.866075	6.066916	11.59867	10.98397	20.2644 3.340084
12.26746	5.794307	17.50881	3.096335	6.542592	11.372
10.00466	4.239824	3.708298	6.317824	4.406992
3.453837	9.232375	9.9568 10.57177	8.011054	11.92449
12.54198	6.46264 5.817116	5.8673 7.711389	3.159333
8.479936	5.854058	7.318371	9.082583	3.476921
1.787818	6.368628	7.175736	6.430271	6.084544
3.487029	10.0103 3.578085	14.07117	4.95794 2.538639
19.31939	7.09705 10.39803	7.544091	2.560185	9.409551
7.923157	5.138691	4.095946	10.86121	5.688174
4.247559	5.448573	23.81093	13.17574	21.42211
16.02326	4.470105	9.224967	6.88911 6.299378	7.481868
9.113704	4.73316 5.690164	16.0423
TNFRSF1A	68.86311	38.24038	50.71973	32.58789	48.00582
30.38609	18.98934	16.12108	33.74812	29.41986
26.05689	24.11054	24.88398	27.85266	24.12943
33.70685	73.91798	38.00122	104.5744	120.0605
47.26908	26.90453	42.27833	34.98977	31.71304
23.9602 26.67247	30.00388	63.88381	20.09287	35.21372
44.84142	23.77974	30.76831	40.53988	14.01982
22.0897 56.56087	24.89008	34.33186	27.23248	17.0032
28.94855	21.08664	27.58296	17.22235	26.16628
26.02321	45.26675	43.61145	38.87837	21.52123
13.01797	52.71272	44.8356 23.28733	30.42388	38.97611
21.0936 26.02086	49.91664	41.50198	39.44264	27.87245
26.38835	38.83632	44.41451	41.9619 82.63327	18.21267
24.65278	24.93569	25.20256	36.39972	31.88987
27.59416	40.09869	21.50596	33.22453	17.48371
45.85586	34.17736	33.73195	30.52006	39.40314
21.16218	28.52448	27.96361	23.94745	28.0449 40.58258
35.94075	51.49132	37.96011	37.89675	27.52813
61.43036	25.9944 33.02123	32.48629	24.53524	17.05408

21.56477 28.20576 33.97319 30.49216 40.58851
38.14532 37.74746 25.9415 36.40232 27.44987 52.71243
23.65472 30.11469 72.60271 48.7436 27.96076 26.61652
25.72721 29.78467 33.4927 24.87474 32.73144 13.75983
19.261 30.03466 30.46953 33.81345 46.5223 35.36639
20.94743 31.61811 36.12581 51.6508 34.77948 29.46038
66.32616 32.99663 19.23482 23.62689 34.85136
29.97839 20.09403 33.02731 20.35105 24.21898
28.08039 16.45503 23.23863 26.62278 43.01178
25.7749 38.59071 35.15484 15.87854 30.73506 28.83062
26.21102 25.24492 40.56027 21.1413 30.47559 34.08007
28.95907 30.84455 51.53169 20.458 23.55088 37.38523
29.75505 38.37527 20.93112 28.1398 26.12847 27.52731
41.69317 22.27437 26.15978 26.8865 10.20489 21.70781
41.48661 31.86367 42.60955 27.68203 38.57532
18.26349 35.79312 33.48971 52.45757 54.99791
60.0972 27.37439 45.38668 28.3245 45.16413 15.00171
21.8755 14.08614 18.86074 27.37693 24.14788 30.1113
19.08791 54.37113 37.39089 17.14765 31.25371
22.93087 35.53369 27.15953 29.9478 21.37725 38.97328
31.52308 61.45474 30.15785 45.9356 29.37666 42.4402
41.96836 33.09277 37.66512 32.10089 12.31207
27.60488 24.49034 38.85979 38.90664 45.14255
29.09445 19.51755 44.27381 22.01411 38.79487
27.9614 37.48603 36.93934 15.43441 37.42319 43.27916
22.24466 27.70834 32.6959 39.10295 24.0149 33.63442
27.07626 17.41576 26.92595 28.43852 22.82794
44.00986 43.37954 17.80907 20.73204 45.70248
33.68708 37.18598 25.80477 51.87115 38.95852
32.07398 31.43649 27.95072 26.14301 32.28213
34.99252 39.1055 32.94614 55.9415 30.71264 25.40984
26.43277 39.70632 39.60665 27.68522 8.031425
38.92047 35.4476 18.98498 40.80994 35.02349 25.06794
31.17763 15.42084 20.2146 25.01059 31.24174 32.23665
26.52729 36.94248 70.77418 56.69441 46.81787
27.33216 42.46987 30.12355 60.78632 25.23918
28.10636 32.25541 39.89169 31.87218 31.29499
17.29445 38.2474 25.71508 42.77775 43.23632 55.60036
35.41117 47.66563 28.39158 30.8656 20.12883 35.01048
30.73218 52.71237 32.1988 35.63456 35.4864 27.32323
37.20384 43.70167 39.87219 8.024589 38.81553
67.44142 29.87498 29.1228 26.4951 35.10649 42.85077
28.17296 13.29154 45.99098 23.03955 32.07289
27.60676 36.62389 34.0053 24.27447 34.57973 33.22274

39.80693 5.024104 32.15479 27.85054 18.37997
30.36573 56.30841 23.3581 32.38214 71.54249 33.20975
33.70349 27.01689 27.29486 37.97533 38.54913
34.44773 37.81766 68.89576 22.50538 24.78029
25.1781 14.22725 21.17171 16.78258 28.08184 23.14095
18.57369 31.46708 24.57176 40.79203 21.15163
22.37733 28.23898 30.55606 20.81185 18.54596
11.45648 22.93391 44.39184 47.17487 25.16334
38.47719 13.73639 34.0057 23.85318 24.46744 35.34926
35.10068 37.89115 21.08494 29.3421 32.14275 45.34322
30.57397 35.8247 21.58629 35.13374 33.84465 40.22897
28.65994

FASLG 0.3770669 0.04415041 0.8352488 0.4263557 0.4037442
0.02337968 0.6658173 0.3320148 0.6771672 0.9641297
0.2708229 0.01511839 1.734981 0.151515 1.104003
0.03420607 0.675406 0.3939909 0.494081 0.5588047
0.9621609 0.06131046 0.05873902 0.202545 0.01581149
0.03629175 1.218086 0.0669328 0.6989206 0.838139
0.82636 0.2207321 1.842153 0.7028552 0.2255329 1.248309
0.04003649 0.2280767 0.04762042 1.573148 0.1429287
0.1560243 0.7608964 0.5194368 0.412053 0.1189099
0.3402549 1.240699 1.935368 0.4363803 0.06218298
1.41576 0.1155781 0.6016756 1.590408 0.1608171 3.886739
0.3313185 0.1851866 0.8658897 0.4193576 4.984858
0.5901385 0.09004817 0.1376667 0.3337252 0.1994993
0.6221999 0.2551406 0.5644373 0.7426695 0.8812265
0.09134898 0.02870159 0.08383362 3.827199 0.2509351
0.1127513 0.2562659 0.33922 1.297875 0.4959432 8.226266
0.5459784 0.3161208 0.1341147 0.1979481 1.721183
1.924425 0.6509859 0.6006623 4.184525 5.857142
1.481089 4.446432 2.688633 3.33425 0.2268873 0.7103573
1.669198 1.792046 0.1075687 1.737607 0.3692235
0.2699736 0.9403761 1.742872 0.7088772 1.401812
0.3590666 0.1118455 0.2162587 1.526025 0.8992274
0.3270413 0.1832889 0.3788471 0.1036407 0.1250656
0.2012647 0.5335511 0.4801647 1.092065 0.2142993
0.7249075 1.052431 1.520311 0.4494307 0.4633145
0.4914428 0.554203 0.2803509 0.01565988 0.7754064
1.014982 0.5100275 0.8051474 0.2899948 0.2964116
0.2019435 1.504478 2.05811 0.6644842 0.623864 0.6028435
0.2240362 0.2996823 0.3947429 0.1559815 0.3382611
1.102327 0.3556147 0.1603814 6.171785 0.5342358
0.1208143 0.4263108 3.070666 0.0432358 1.570511
1.960204 1.810879 1.109594 0.2520167 0.07584031

1.624915	2.504212	0.4171444	1.446244	0.7579024	
0.1842246	0.841603	0.4269329	0.01566122	0.007991079	
0.2865414	0.3945375	0.2881125	0.6588629	0.1068555	
0.0773956	0.03108767	0.6549548	2.846212	0.2885076	
0.5134344	0.7433598	0.8017065	0.203083	2.854166	
0.283771	0.5919695	0.2651849	0.4470473	2.67293	0.774459
0.9635695	0.1146638	0.4469496	0.1168273	0.07104315	
0.07566706	0.2565007	0.5402478	0.2911977	0.6682402	
0.2689027	0.3368072	0.07188575	0.2094194	0.5807343	
0.2944462	0.3714254	0.1188216	1.484361	0.4443615	
0.2552917	0.816892	0.6368812	0.2872095	0.2082487	
1.298009	0.1857769	0.3087022	0.7247045	0.4989015	
0.6197457	0.1075222	0.06572105	0.6306051	0.3472731	
0.1610731	0.1806652	0.1092843	0.6455145	0.1723461	
0.3768803	3.060676	0.5908652	0.09428622	0.04610746	
2.506091	0.5408593	1.048917	0.6341723	0.6355663	
3.587509	0.0616135	0.9193453	2.331972	3.147463	
0.4689256	0.08598649	2.218154	2.187828	0.4983193	
0.1973853	0.7099228	1.640021	0.2228944	0.1974118	
0.5389063	0.9434739	0.3020744	0.5631341	1.21744	0.1466058
0.3934661	0.6964794	0.2784766	1.999417	1.139835	
0.3007276	1.076065	0.2517546	0.9447503	0.3798052	
0.1662997	0.1320058	0.3578469	1.466169	1.15994	0.35313
1.482072	0.6119977	1.403039	0.5884783	0.1034837	
0.2774902	0.2379186	0.7389105	0.3588784	0.5703479	
0.787031	2.110747	0.2217421	1.615665	3.287993	
1.322431	0.08537266	0.6397643	1.281733	0.9777548	
0.06977402	0.2922351	0.2058874	0.2914939	0.9604173	
0.8646808	0.2275715	1.337663	0.2226466	2.075835	
1.919672	0.7033069	0.09616413	0.9520524	0.1346848	
0.6046469	1.189557	1.845198	0.4134187	0.06485672	
2.890881	0.6568112	0.607714	0.4477795	0.1818427	
0.3772696	1.180422	0.327754	0.1470204	0.957761	
0.9969573	0.4772261	0.05152624	0.07581404	0.589635	
5.527052	0.1186104	0.05383528	1.995782	0.970072	
0.07735591	0.4375889	0.1723292	0.05417298	0.04744616	
0.7144515	0.7047213	1.116402	0.7577258	0.1115526	
0.4401127	3.64588	0.2291826	0.7945217	0.3770481	0.6999784
0.1071678	0.5566109	0.7806279	0.06163957	0.6285783	
0.8913805	0.05169917	0.1009595	1.810357	0.3291007	
0.120558	0.180798	0.1876214	0.5415273	0.2363404	
0.9820461	0.1529962	0.3799028	0.3439686	0.2322026	
0.1549085	0.6361593	0.7282527	0.2294762	3.025371	
0.672601	3.066713	0.4431219	0.1593397	0.3790349	

0.4109058 0.2793756 0.3653061 0.4578849 1.137425
1.964612 1.217998 0.3788699 0.4020525 0.5046281
0.01120268 0.1645117 1.169149 0.6844669 0.4101758
0.2334066 1.181499 0.3664992
AFP 1.274988 0 0.008201277 0.04684405 0.005847415 0.01354429 0
0.03739992 0.01376476 0.05026846 0.003826654 0.1051004
0.01376859 0 0.07267836 0 0.05335574 0.003356564 0.04953988
0.1142563 0.2865129 0 0 0.005333551 0.01526649
0.007008164 0 0.007050078 0.008614848 0.07080933 0
0.003996071 0.09897362 0.1825277 0.01088795 0 0 0.04404303
0 0.0552336 0 0.1178971 0 0 0.01377173 0.51173 0
0.0143752 0.004539251 0.007121221 0.07719379 0.05467843
6.093048 0.01340623 0.1651483 0.03836181 0.006545526
0.009139953 0.008940171 0.2417476 0.005061286 0.006079625
0.02219989 0.0260833 3.41097 0.03927082 0.01824848
0.004505651 2.803868 0.003114182 0 0.2843353 0.02205008
0.06235263 0.01040708 0 0.003461225 0.01451532 0.05735942
0.03056924 0 0.004869648 0.6112232 0.009167988 0.01878305
0.5524989 0.004587002 0.02203566 0.4887038 0 0.4124148 0
19.5668 0.05644887 0 0.05009745 0.03024867 0.6759775
0.003741121 0.01807473 0 0.2103184 0.02054344 0.2566777
0.01380007 0 0.01929322 0.01664862 0.003942213 0.01188651
0.3293704 0.2863606 0.08605846 0.02948713 0.2841917
0.03893366 0 0.003531823 0.01646655 0.009327715 0.009226751
229.9877 0.780547 0.008276506 0 0.0129722 0.05300779
0.02410774 0 0.07427009 0 0.04060314 0.2358736 0.2560483
0 0.03409252 0.004240336 1.055996 0.004906196 0.02924741 0
0.04224692 0.1179121 2.055848 0.008415388 0 0
0.003811367 0 0.00435469 0 0 0.1373481 0.004430522
0.003257819 0.013998 0.003983385 0 0 0.01491519 0.07391369
0 0.02735357 0.040555 0.005491964 0.02831109 0.01422291
0.06249818 0.006071279 0.005488339 0.04048182 0.005132173
0.01426905 0.00907284 0.04166444 0.02825512 0.05175011
0.006419578 0.004288671 0.05158617 0.04035306 0.004502418
0.004992469 0.01245993 2.03419 0.01101638 0.006945844 0
0.03676557 0.007708496 0.03440803 0.02348898 0.004518429
0.0340767 0 0.02730966 0 9.286477 0.0408831 0.0989173
3112.391 0.003652946 0.008740923 0.02911402 0 0.01382586
0.225613 0.01026941 0.02650122 0 0.09693754 0.004738284
0.004137957 0.004916826 0.4919929 0.04680491 0.362582
1.656343 0.004670345 0.009243659 0.0707214 0.07049633 0 0
0.0194368 0.003612787 7.429167 0 0.02719532 0.008910278
0.165975 0.01036808 0.005814596 0.007034494 0.04273813
0.05616187 0.006421583 0.02646428 0.03523669 3.381999

0.2036708 0.02680294 0 0 0.00930096 0.01712537
0.007422542 0.004461732 0.09093069 0.003178718 0.004007439 0
1.635546 0 0 0.02723451 0 0.03696824 0.06696706
4.782001 0.01633777 0.03121985 0.01708038 0.01381558
0.02630921 1.837812 0 0 0.07033201 0.009218669 0.02438526
0.01817416 0.3919886 0.02852086 0.009309329 0.1512894
0.02961919 0.007136338 0.004779591 0 0.004771795 0.01256029
0.01022874 0.1232429 0.1496231 0.05188128 0.007029207 0
0.003920861 0.003627125 11.93545 0.01386033 0.007342517
0.04179467 0 0 0.05128685 0.01002524 0.1228665
0.003804461 0.01659526 0 0.2432995 0.02425284 0.007524323
8.613852 0 0 0 0.008988848 3.499362 0.01682392 0 0
0.0229543 0.007427959 7.74599 0.01950636 0 0.1467186
0.01781596 0.01596675 0 0.1104225 0.08251875 2.080356
1.57703 0.005852495 0.01821328 0.008140963 0.01356243
0.007300431 0.02201782 0.009167557 0.007275422 0.01279291
0.0087841 0.614856 0.09364963 0.0179252 0.01247512 0
0.009212802 0.1493791 0 0.008681177 0.1255337 0.0549729 0
0.0378017 0.008186746 1.11113 0.01140433 0.005311782
0.007463346 0.01896711 0.004146679 0.3533443 0.1013777
0.02069478 0.0820795 0.2149957 0.2722809 0.7868181
0.4442094 0.00855723 0 0.01064745 0.0156274 0 0.1190224
0 0.008478837 0.1501664 1.649452 0 0.07059326 0
0.03074728 0.008974143 0.0202494 0.02481709 0.01595278
0.0044371 0.006776529 0.3604704 0.006112115 0.01318693
0.008783281 0 0.01103506 0.02713188 0.358883 0.2700539
0.00392461 0.09520125 0 0.01579095 0.04299126 0 0.1121232
0 0 0.003394608 0.0169021 0.07204892 0.03538664
CEBPB 23.44188 103.4279 27.47952 35.78213 59.63508
80.28547 17.48981 85.11212 84.32917 94.92242
20.82974 19.24791 72.99702 25.25905 27.68196
67.48654 35.15953 228.3329 59.99823 38.75589
60.59192 67.9671 88.82525 56.55385 54.33713 18.95127
34.93122 65.56911 39.67356 22.21383 52.43379
157.4302 24.13355 32.72794 147.3898 28.86904
9.096945 78.5927 105.702 30.69126 52.79063 43.52344
34.29467 26.31522 18.93797 43.46731 18.8845 81.75385
16.68419 64.96714 66.48634 37.0578 30.23277 101.8338
86.53142 38.97095 105.9832 65.78748 80.8316 154.8724
58.52425 85.90914 47.92226 68.84748 31.20653
55.43037 67.78243 46.17626 75.35883 47.53898
52.0256 41.16213 51.24336 68.20671 88.23656 116.4933
65.62776 46.21712 199.8756 35.41306 74.3516 116.6329
18.91187 31.61986 120.292 49.78876 31.16676 49.37227

36.38709 39.03338 17.35762 87.97168 78.87009
51.89822 140.1606 93.61556 89.9406 42.21217 40.46028
35.89744 27.89912 41.27854 83.94556 122.3389
82.24618 79.56244 70.70939 42.10808 51.24619
42.78823 81.06234 99.62291 169.5614 38.38702
69.94759 58.22336 230.933 23.34996 66.68555 61.93257
60.60116 24.82771 104.5886 73.04534 75.98953
27.3053 43.45305 82.32679 82.70586 88.15493 32.63646
35.19551 60.64325 30.08767 122.5949 26.53667
46.22668 21.76125 36.85058 27.42566 57.89471
56.06168 31.99406 111.7622 97.13987 36.51581
98.2533 47.61477 185.927 34.77604 25.71459 96.2506
60.55598 75.53199 47.78841 26.44811 72.30281
34.67868 137.092 106.7351 53.71653 21.29513 27.26162
94.80202 79.76462 30.46047 158.5013 25.64416
48.73741 47.47786 47.75389 57.28314 22.71253
50.27079 49.25695 110.6967 131.6636 70.3156 37.71443
75.38726 41.61813 54.22999 58.65183 44.26071
214.4414 93.51871 56.73592 20.22022 109.5339
74.68525 165.2225 208.1346 118.4068 25.14089
96.50756 53.3155 70.4956 36.56785 63.29638 37.19537
33.44094 67.29705 75.15616 82.74334 68.8632 32.64206
152.1883 81.91618 49.05006 154.246 25.91809 103.7524
50.86747 101.9847 96.54359 55.28434 134.0903
101.9157 133.1263 42.13045 88.32612 26.24791
47.2621 49.86744 33.3498 22.47066 128.0225 20.91253
47.6497 80.70235 51.91413 81.84812 15.63647 39.14667
14.82914 84.14419 56.4712 60.30116 20.8923 62.47725
86.60028 55.61392 73.37355 87.31013 38.13774
134.8209 55.89933 84.6577 52.30157 59.9746 32.93104
21.23977 201.78 109.0988 78.2999 69.15442 46.96706
54.35743 41.10935 65.66959 54.5514 64.79253 35.62064
32.80195 77.10617 102.043 102.98 66.53551 137.278
50.0497 46.39228 132.6104 54.14625 23.23058 131.1282
46.77412 69.3934 45.8488 27.31495 26.95055 41.49393
75.18164 56.98218 75.51103 25.54194 123.1514
33.26723 78.81017 126.1869 79.56679 80.42631
20.69945 54.74614 77.61929 319.7301 73.02312 97.173
129.2693 61.39637 37.16452 24.98664 49.88022
37.4655 190.6901 21.98936 84.57194 10.85246 45.93942
74.17688 76.92093 168.7018 164.6369 109.5739
77.02227 35.9794 85.08592 63.92019 121.0107 59.25703
25.26786 33.95868 20.75928 79.42801 70.86338
24.97718 52.36622 80.38466 20.36044 56.27189

89.19758 35.5302 113.1666 59.98748 78.44258 83.45986
48.47736 48.69289 50.05722 33.5995 22.48922 72.97782
182.4862 42.17402 191.1134 64.45683 30.65685
72.89179 28.16497 145.5676 190.5881 50.48309 86.768
57.2385 175.6068 135.1943 54.69281 37.30175 71.87816
25.89405 244.3993 66.74728 80.10458 154.974 65.94894
85.93741 60.31709 15.08846 40.99784 22.44447
102.4096 101.0666 70.99976 127.0941 63.28604
56.96298 127.0302 66.98767 90.65733 21.72141
23.4228 66.11425 91.51743 119.5737 85.24751 46.87355
83.50482 103.3038 76.21902 42.78341 56.96295
95.59558 15.78591 36.29325 21.98464 58.32143
71.53765 31.52987 56.90893 41.9466 118.5021 190.3175
51.74872 48.47127 17.43162 41.71427 91.5841 56.66022
SATB1 1.36629 7.459704 2.20557 1.86511 2.869381 10.55706
1.352646 5.333168 3.300302 3.639468 3.909007
1.962646 3.100437 1.609152 2.080839 11.46267
1.376757 7.533082 0.7665756 1.585175 2.628433
10.62885 2.547317 11.10537 5.544163 2.219405
1.255436 10.2878 2.085427 0.6859376 2.501574 8.475907
0.575231 2.87333 3.827389 0.5629171 2.814078 1.436345
2.671105 1.047095 4.465014 2.364943 6.970538
1.037155 1.216135 1.685424 3.441827 2.486444
3.852533 5.906294 0.2951828 3.913205 1.791248
0.9742397 5.239141 1.341277 0.8749741 6.916411
0.1977312 0.9356847 4.006514 1.322476 4.791282
3.288898 3.71867 5.829551 2.318139 3.303854 0.801215
1.896006 0.4756482 0.5685578 4.302347 2.799549
2.292477 1.205117 2.550627 1.241816 1.206303
2.343547 0.4822881 2.97307 2.109948 4.597974 2.055454
1.955489 3.157258 2.680514 0.9353717 5.561894
1.456549 1.972016 1.521653 3.353999 1.355636
1.178855 1.718223 1.153477 0.5028227 4.276571
4.269122 2.194244 0.6615719 3.501326 2.340008
3.379619 3.548112 3.7955 3.394683 0.4535677 2.963895
3.380757 3.183056 5.807433 2.359946 0.658085
2.200359 0.4017286 1.09258 3.000456 0.7658214 1.614424
3.131191 2.019616 1.091499 5.048743 1.869072
0.6398338 5.723629 1.814128 1.490417 2.567023
2.293123 6.458968 1.310416 5.147013 0.7750079
1.032455 2.96915 2.631551 2.985575 0.6959377 0.7257222
2.350023 3.862594 0.6317664 4.600722 4.476057
3.078547 3.814433 0.4546113 3.517224 4.032333
3.346755 6.028758 0.8607451 3.323641 5.245964

0.678772 1.421029 2.759986 2.959129 5.01715 3.325657
1.478957 1.905146 1.491617 2.9114 2.679686 3.453512
7.324638 0.7421749 5.460995 1.165403 0.2351567
1.399209 3.158037 5.682433 0.5034517 2.10635 3.199469
3.477664 2.244787 1.41625 1.321706 1.905029 4.111508
1.183052 6.031607 0.562055 1.817499 1.353008
0.7720239 1.202575 0.7471129 2.659739 2.951547
2.230131 0.2682845 2.478797 0.7518879 1.868 0.6776976
0.2379318 0.8446189 0.753551 1.329078 5.607119
2.131973 0.5378736 5.741772 3.825681 3.150898
0.8747499 0.5240582 3.699957 1.797266 1.589668
2.912686 0.9065159 0.5571107 1.521484 0.3229891
4.740074 7.777645 0.4899645 1.737385 2.409662
1.225439 3.717261 0.8459074 2.149489 3.766212
1.628493 3.396654 1.096542 2.860833 1.121011
1.556224 2.63963 5.050757 1.973845 3.240116 1.337252
2.566864 2.748124 2.900859 5.488173 3.351355
1.26084 1.821363 1.128541 1.92501 1.758018 0.6609879
5.239782 2.332739 1.358977 1.004029 2.506515
1.526782 0.4795522 2.425751 1.758378 1.515459
1.669664 1.759281 2.181833 4.354444 1.950406
1.491561 1.076312 1.8903 4.603754 2.355359 0.7613603
1.924141 6.64557 0.8096763 1.653667 4.993945 2.376545
3.341254 1.335933 4.233391 2.460959 2.478061
2.77388 3.795594 4.082491 1.31142 6.634083 7.151654
1.395342 1.852904 2.679539 1.840672 1.040425
2.78421 0.9468497 3.455285 3.167175 0.6560057 2.733106
2.908634 1.853493 1.885615 2.958481 1.056734
2.106052 2.279556 1.613785 0.6051421 2.127139
1.860578 5.434054 2.666762 2.511117 1.791149
6.364648 1.092627 1.813557 2.086129 0.6083217
2.987204 1.54409 1.183897 1.952988 1.077154 5.175097
1.775602 3.449905 1.696373 0.9736952 4.466625
0.5855776 1.351416 0.9913705 1.618209 0.732642
0.4699636 0.8283884 0.8206419 4.57213 7.327075 4.91823
3.342799 4.521839 1.718745 1.521457 7.791874
2.99728 0.6864758 1.291006 0.8670606 0.3150082 2.219247
1.285059 7.211954 1.640545 2.46941 1.549595 4.763637
1.289636 0.7975781 2.749493 2.216386 3.070444
1.961376 0.7428918 2.687978 2.327361 2.060745
3.233232 0.6655011 1.866077 1.601693 0.9309534
0.8948911 1.273778 2.453875 1.869823 3.035891
1.030968 0.8218562 0.5381806 2.757132 2.50633 2.513655
2.675338 3.994427 9.91649 3.949774 3.055986 2.351264

3.242078 0.8894108 4.263933 1.916317 2.471705
2.973091 5.160501 0.6248221 3.958565 1.321744 2.8981
5.543929
EEF1A1 555.043 1017.371 481.829 687.7661 678.4905 678.2708
461.2165 527.2412 631.0063 1198.677 723.2336
655.6276 915.531 665.0557 444.6 628.9197 504.8395
847.8621 403.4904 449.5921 842.6916 694.5638
544.4686 690.2641 847.1311 629.7442 579.8857
672.9038 674.442 1149.077 641.2161 660.8046 337.4728
501.9796 487.4295 585.8073 722.0264 345.8226
511.8355 711.9838 383.0763 543.7055 1382.886
882.4746 482.1403 476.4922 461.1213 461.5619
1374.218 394.8881 495.4259 603.1652 421.3919
388.3518 613.7417 704.7468 942.9848 637.3304
487.1025 683.4294 655.8098 462.9423 892.725 415.4835
329.4178 590.1159 497.3947 832.2631 334.4383
361.507 811.1668 813.7033 508.395 453.9046 469.3101
600.4394 481.359 1083.366 607.5569 542.4876 600.5817
658.5165 853.4889 447.3256 960.2013 486.137 782.9898
820.6356 411.5013 558.0644 829.3603 686.0792
537.8732 692.1563 751.1416 481.9698 834.0412
436.5604 654.642 506.3098 790.1924 438.9193 422.17
550.1569 505.6801 762.0776 365.9662 822.3421
642.5737 746.1523 260.0309 483.5993 626.1799
645.2832 432.3285 303.8502 222.4102 683.6229
945.4841 840.8437 394.3804 721.9896 570.1205
539.9252 510.0813 943.1018 1850.515 660.4765 711.63
336.1934 430.1954 910.7621 415.8754 741.4374
320.8299 687.6911 844.8612 605.1647 614.3888
553.2411 552.2831 796.7888 1451.459 522.8694
466.0533 723.0931 451.5621 476.6663 693.7346
654.5731 1596.998 618.1426 890.3124 627.0824
722.6859 174.1256 731.6348 513.9647 210.7633
342.9489 680.8487 683.0491 572.2004 448.656 621.6775
824.0075 443.1384 471.9947 654.7608 652.9774
487.7697 884.1113 794.7572 358.2706 438.5802
410.3309 367.108 471.5546 553.744 378.5201 372.6183
776.6036 557.7008 629.5847 444.3446 621.9507
609.3703 578.5127 480.1663 890.5867 437.3822
417.0153 614.8887 585.6502 569.069 531.3346 1106.668
683.3599 704.4028 403.0299 387.3454 403.5714
695.2055 607.3809 676.7727 479.4168 681.6171
463.7439 551.7615 444.494 451.7152 473.047 452.0057
551.7536 667.7517 813.6108 237.8873 325.577 739.0463

611.6891	577.0711	852.198	564.4628	1541.084	857.9095
648.9954	410.793	530.5936	937.4674	528.5923	461.4969
421.7417	704.4197	523.3589	1031.686	339.6026
963.0758	530.2945	609.3229	564.7783	610.11	506.3716
613.6494	559.4561	1019.592	537.3742	884.2672
694.3005	687.0639	552.8629	585.344	931.0793	204.311
717.4898	501.8711	667.1358	731.8565	574.2777
620.1407	710.6098	474.114	796.3826	749.4212	928.4575
585.2895	792.7566	406.9127	962.4393	438.3164
1022.946	694.9821	415.3472	437.6838	1452.417
196.1279	460.8284	366.7201	661.2561	606.3097
678.3818	695.0047	497.9967	582.5412	829.1081
885.5246	779.1053	445.0555	437.743	444.4832	805.1737
601.3609	470.8636	635.1212	365.6038	787.2176
627.7294	633.6788	508.4031	506.4399	394.131	597.8544
497.4712	425.8717	358.7895	625.9317	359.5785
585.4948	444.1815	714.891	1028.503	429.0379	447.1814
393.9681	726.9178	703.5927	745.6205	517.6468
436.2845	370.698	666.893	756.4618	849.5421	380.6428
612.5544	1413.005	402.9225	600.7943	316.941	421.6302
788.3367	774.349	616.4787	714.979	438.8282	543.4383
996.7036	829.1761	337.6905	426.6989	655.7661
310.6715	363.3333	700.6572	362.0397	684.2173
538.6316	309.3312	806.3538	322.7202	332.8729
873.1752	620.2144	758.0933	315.3513	962.6168
333.706	538.2436	328.3221	425.213	225.8707	553.1567
710.4119	474.8935	435.2355	863.3195	437.7686
941.3314	445.7332	630.8928	307.4029	602.0572
413.5363	365.3668	558.5837	1058.283	817.2905
329.6804	300.2314	964.5758	652.682	525.4548	609.1305
262.264	438.5731	849.5688	568.9514	599.4134	325.9702
766.7097	373.4332	409.8005	1219.12	211.6417	787.0257
668.8869	516.366	926.706	595.9094	435.6037	499.0604
325.2931	646.9262	755.224	668.3738	974.7234	555.8912
567.4932
ITGA8	0.893564	5.370304	2.32911	3.014285	4.728741
6.948312	2.743325	31.10997	6.408905	5.370397
4.805866	1.160244	4.834094	1.773311	2.447876
7.602228	2.760284	29.11168	2.151815	4.051884
3.242987	5.889192	4.900703	1.560874	113.0068
2.52425	2.337017	2.197512	4.673082	1.265596	3.114183
20.55029	0.6333959	3.549794	1.062127	0.215766
0.2088535	3.102718	0.6897117	0.6012052	0.5454841
0.2654064	0.9159867	0.4739463	4.433755	1.157387

2.373658 1.516404 0.4834193 1.81891 0.358692 1.802655
0.9110088 1.279735 17.44114 0.6951418 0.7849266
1.881478 0.3638132 2.252735 5.532571 1.005414
2.166255 1.987375 1.022513 1.936807 7.900078
5.929743 2.270622 1.151348 0.1285684 1.216948
7.027659 2.710149 0.6412693 2.663148 10.934 0.4241654
1.019567 0.63712 0.2449662 1.231162 0.5891684 2.79813
4.667483 0.6932663 3.346059 0.3601204 0.9189995
0.9229933 0.4444937 3.40053 3.406126 2.730504 0.2310275
1.98347 0.88673 1.812756 0.1538616 0.401024 1.029362
0.9256793 0.4224464 0.7662662 32.85459 31.27839
1.15541 6.421857 1.216843 0.3627842 0.8520342 1.067695
1.334449 5.12747 5.454395 1.222757 1.170396 0.7430875
0.5018572 0.569376 0.6730595 3.648013 1.380104
0.4980427 0.3765954 2.645082 0.2488973 0.2943137
50.6301 5.44454 0.1959794 0.1823696 2.63532 1.258218
2.58869 2.368857 1.226257 0.2119555 1.582362 0.8559313
1.185858 0.7010943 2.342117 0.9184595 0.9654388
5.314411 0.2480261 1.321655 0.08164108 2.775043
0.3264015 7.304241 0.1311625 3.845688 11.66796
3.102707 1.408897 0.7244097 0.1192775 3.841939
0.8581416 4.833745 4.689364 1.165782 3.366633
3.820919 1.490077 3.243176 5.905926 0.1378078
1.87839 2.257355 2.409154 0.3554648 0.3346912 0.2889839
2.52973 0.7059028 1.063854 2.753602 0.4367382 0.3294103
1.947354 2.565802 1.005936 0.789299 6.461988
0.1540633 1.588461 0.3854396 3.859673 0.8684138
1.547279 2.026978 0.4413776 4.261281 0.5129118
3.552489 0.7630232 0.2479226 0.3949526 8.53652 1.335786
0.1827574 0.1095473 1.578663 1.251128 13.57526
0.9047146 0.758849 1.340441 6.145648 2.389717
0.721588 0.5000685 0.658536 0.6258064 0.6039199
17.28499 0.6442798 1.320112 0.9237104 2.475469
0.1534606 1.699734 0.1173028 1.76553 0.9977516 0.6334045
0.3375235 0.2003186 1.144572 0.5084821 1.008012
0.5203707 5.724463 1.859829 1.328994 1.464438
0.9399565 0.7227259 3.053641 3.954206 0.3061123
4.928491 2.211186 1.523126 76.59795 8.902216
0.6220068 2.225862 0.2831235 1.4233 1.595445 0.9609154
0.6767623 0.5445402 4.857269 0.4312439 4.949125
4.673418 0.1919216 4.670789 3.080216 0.5102589
4.471327 0.6818847 1.333232 1.751557 2.524257
1.55009 0.9625068 2.152209 0.4004011 0.7294603 0.6433996
5.260952 18.24471 0.4386633 1.192023 4.970295

0.149533 0.515885 0.6740383 0.2436081 11.26009
2.407064 0.6196378 2.849948 0.9892539 1.499662
0.7720483 1.350948 4.717502 0.384458 4.615818
0.3311941 3.602282 0.6914834 2.869102 17.46281
12.40262 1.64905 1.78666 1.034227 1.33608 2.817707
1.244494 1.48752 1.079222 2.372443 0.08254477 0.5004486
1.038122 0.8148595 18.45646 6.218635 3.45245 2.788668
0.902711 0.3033715 3.575952 0.509664 0.5935638
8.597522 1.143097 0.6167684 1.135073 1.658445
0.6026659 0.7769831 6.091301 4.866983 2.881343
10.25528 9.708586 0.5209818 1.282195 2.150685
0.646677 0.8101036 1.817051 0.482594 0.5539958
5.152286 5.892905 1.122578 0.4492006 0.3046099
0.500769 1.548808 1.209496 4.734706 0.450714
0.7722114 1.47254 11.70629 0.5812814 0.8821513 0.7958687
3.492447 2.269112 0.8405843 0.7516316 0.721147
0.2611723 0.3502647 3.339556 3.796724 0.4126268
1.741606 13.68356 0.3909225 0.7456985 1.2864 2.28787
1.12177 1.869103 2.767029 7.412694 0.7100712 1.643792
7.79944 0.2247449 1.491774 0.6270019 9.991444 2.803043
0.2091338 0.829762 1.356743 0.4416442 1.611084
0.5120699 1.885924 3.21956 1.414926 2.435387 6.208847
1.031123 1.412516 1.694895 0.1745233 0.6715998
0.2725661 2.799411 10.42031
PBK 2.013203 0.08983708 5.413493 5.416826 2.763885
0.1495148 3.545988 0.4503883 2.962997 6.558042
2.81103 4.429849 3.036362 2.573379 3.216466 0.04971598
4.216643 0.6012683 3.049206 4.314115 4.265863
0.06683265 2.313603 0.1204299 0.2344045 3.660667
1.91203 0.09551309 2.37315 4.903145 1.915363 0.1503833
5.525443 4.269365 3.22332 49.45752 0.2374154 6.412873
12.75418 39.93871 6.194154 5.980805 30.6208 22.3167
5.120496 18.0036 11.95474 8.583535 12.85285 6.485393
8.815761 7.002519 26.75268 7.416354 1.151409
59.90785 15.36092 0.9699728 8.787908 3.575371
3.756075 17.974 6.696919 19.58463 20.43671 2.173607
4.926224 1.85838 10.41015 14.45722 66.26079 23.72555
2.768233 4.480258 6.407356 10.4495 4.876768 12.08856
14.04865 10.68891 7.677697 3.789785 35.42314
4.988946 6.086052 4.294864 6.193666 13.13553
37.67915 4.178241 19.75607 15.12107 11.27604
7.692903 10.67481 3.277007 6.608889 7.15823 31.66059
19.481 2.730635 11.43845 16.85374 6.294774 8.648663
3.538408 17.42539 4.602937 22.37216 15.6563 8.197041

11.04057 14.47369 3.513997 5.917861 8.183711
3.23618 15.86974 11.1295 13.13547 18.78506 7.493827
7.488866 2.491744 16.99735 7.355246 36.72944
9.682084 2.822035 5.136573 7.578077 10.0161 4.752386
26.1756 4.560622 1.308657 22.74273 22.66631 10.74569
27.85115 12.63531 13.47503 4.374715 15.39574
4.009353 2.925152 23.72963 8.359239 7.65877 10.93402
4.849864 7.70859 8.057236 6.595954 2.221527 4.909627
18.93012 2.34406 6.409682 6.264111 5.431664 4.114783
7.075349 21.9162 6.737702 13.36044 6.144647 3.060711
8.819296 10.45926 0.6758551 9.231998 5.663395
11.3152 33.43565 15.43939 3.817103 15.00736 21.91094
8.580687 10.39396 9.156459 3.825248 12.47905
4.669841 2.926914 1.06125 24.33344 5.877495 20.84603
6.468596 9.327502 8.821733 9.930916 7.085145
2.20817 17.55169 16.52554 15.54273 5.305591 13.4336
3.403771 22.49328 8.798004 10.2719 24.82899 2.288378
22.37641 6.006685 5.327622 7.79391 4.557728 3.232807
7.660404 12.51297 27.29582 5.709344 13.34374
8.816029 8.327881 11.24695 18.08974 10.79113
13.49873 1.673583 31.97219 17.16284 3.313043
29.90889 9.06031 3.88394 2.871722 13.11166 5.463979
20.33765 2.338971 10.36247 8.707229 43.25213
4.789479 7.081681 6.966543 10.65238 18.27764
24.28446 4.930238 14.33527 0.8193883 9.806406
19.75232 7.908535 21.77169 10.11048 8.941823
21.59578 10.70827 17.81278 2.65027 26.99089 5.921998
9.394691 34.74856 17.34655 19.00824 11.10484
9.105904 24.75609 10.87047 5.104126 0.9366956
17.33202 8.59034 3.643417 14.90995 16.76008 4.032297
5.681546 0.7627114 6.367375 9.201837 3.692082
14.36943 2.378905 15.39808 37.86304 9.228522
12.52279 12.23928 16.23083 7.0215 10.7702 0.3964183
1.912131 11.77064 7.558134 2.832322 7.514396
6.142085 29.80999 17.72475 10.86675 6.225305
6.031542 4.48441 21.48628 18.27434 27.80209 25.8799
7.882174 11.33899 3.143839 11.23597 17.21006
3.348121 36.45382 3.673088 6.28927 7.702935 6.389058
18.51854 4.156879 12.16168 5.712424 36.18062
6.065903 5.516405 14.31171 33.58307 7.859445
13.94583 7.397285 3.62651 6.529305 4.616111 0.591396
4.223771 19.04085 4.313184 9.706922 5.428082
4.544503 12.61814 15.57392 30.52129 1.114679
2.528634 10.96011 8.869911 7.912625 9.946704

8.700465	10.12506	6.906884	14.69644	13.32431
6.62393 6.466758	5.64774 2.105949	12.02471	2.921199
3.448393	3.164715	16.93408	9.68741 15.04536	28.2469
4.567903	7.927623	9.659889	11.86077	3.345952
27.14753	22.63797	30.26352	10.8905 10.00765	10.71523
7.185629	10.7193 9.181076	5.53424 8.276796	12.65712
10.58332	11.77292	2.438169	3.712031	7.04049 26.60929
5.841606	28.06658	8.232647	25.44786	8.211789
4.223358	10.19421	2.876207	0.6730386	18.50317
15.44341	2.301739	27.18848	26.03005	25.37933
6.661011	8.560157
LTB4R2	0.1438032	0.1375086	0.2066808	0.2575679	0.2063054
0.2230025	0.2221843	0.1077163	0.2220073	0.2589939
4.578767	1.854049	0.6662072	2.384784	0.2930514
0.06991453	0.3944221	0.3011366	0.1442661	0.2303502
0.168039	0.1342648	1.789436	0.2957044	0.3293303
3.574649	0.1849471	0.2096499	0.1563143	0.07137878
0.2031898	0.1490437	0.8545481	0.9058246	0.340241
0.08866032	0.02338042	0.3188528	0.3163304	0.2266882
1.320959	1.184491	0.1309653	0.3264511	0.6062019
0.6481134	0.9935065	0.4202335	0.3523332	1.062417
0.1348786	1.233799	0.4242545	0.2094677	0.3767624
0.6629198	0.3497028	0.5159536	0.4100491	0.3228915
0.4234651	0.3248115	0.2506383	0.3312926	0.352498
0.2831967	0.3556412	0.1816754	0.5372902	0.4991371
0.3876456	0.2312518	0.6134764	0.8841473	0.8252746
0.4307453	0.4151982	1.166906	0.2754987	0.6251059
0.2109702	0.6774158	1.352617	0.5452613	0.1372722
0.2871733	0.4577652	0.4275978	0.2771066	0.192615
0.2338489	1.660892	0.08061645	0.7055952	0.4138036
0.5050034	0.3876832	0.6956099	0.6297916	0.4236169
1.097153	0.5025417	0.2795668	0.3827225	0.5518051
0.8474672	0.3176568	0.2013901	0.2662524	0.6230696
0.5252435	0.3969123	0.1735011	0.2774265	1.20957 1.619821
0.2564352	0.5589555	1.576903	0.3761092	0.4138924
0.6381642	1.246495	0.1918905	0.3671739	1.242269
0.275391	0.1312288	0.2872155	0.3868148	0.4207349
0.8492908	0.429816	0.5116867	0.1004622	0.8591674
0.5428536	0.5709531	0.2324456	0.8903752	0.4469438
0.8233424	0.8285263	0.6624049	1.064625	0.112661
0.1458398	0.3573077	2.510907	0.3204489	0.6603469
0.2966729	0.207679	0.879832	0.4728987	0.5785336
0.6344363	0.3299063	2.196641	0.01503514	0.2167509
0.6702547	0.6525739	1.054734	0.4373544	0.5850448

0.4587935	0.6510082	0.9547363	0.232364	0.3746859	
0.4242226	1.011661	2.890074	0.5226606	0.07476616	
0.5390517	0.381801	0.2810054	1.778435	0.8361502	
0.3358583	2.969245	1.080172	0.5218832	0.5774585	
0.4586115	0.1641666	1.015478	0.6566062	0.1734237	
1.160216	0.3917099	0.2335851	0.3554597	0.8573405	
0.3158028	1.156195	0.654811	0.2099217	0.252383	
0.3314091	0.3876936	1.280313	0.3741166	0.5644506	
0.1678631	0.4089038	0.9159202	0.7970344	1.138117	
0.281807	0.7883633	0.3717276	0.3536336	0.6448113	
0.1250388	0.5838483	0.4048798	0.6429418	0.3187089	
0.559031	0.1886775	0.4571264	1.214773	0.3532587	
0.6588758	4.824411	0.1809325	0.4535877	0.2180097	
0.3710267	0.2110089	0.219823	0.7862435	0.3711331	
0.2459826	0.4344558	1.126494	0.8075621	1.006349	
0.6574499	0.5065515	0.2895663	0.7594365	0.6085241	
0.3815939	0.2653592	0.8315049	0.2819768	0.3554907	
0.6136091	0.5774633	0.765806	1.42852	0.2690446	0.4052994
0.2003023	0.5948932	0.7766534	0.1935126	0.1384721	
0.6083602	0.2692493	0.3872035	0.676777	0.3385665	
0.2493308	0.2350816	1.2917	0.3400423	0.2809115	0.3449643
0.4394678	0.8226811	0.3364105	0.524637	0.2050213	
0.4914389	0.06429988	0.5194984	0.3830042	0.1288875	
0.215339	0.1442688	0.8890748	0.6766878	0.2794989	
0.2292388	0.6617892	1.261887	0.3260086	0.3108658	
0.4710988	0.3511961	0.9826192	0.4782185	0.3334936	
0.3606356	0.4640418	0.3122694	1.219416	1.839421	
0.5908238	0.2351299	0.758975	0.9484018	0.6107168	
0.3747708	0.4711791	0.217185	0.2204698	0.4951408	
0.2861002	0.7115213	0.3668971	0.7331029	1.212566	
0.3439289	0.7036383	0.3112935	0.7832976	0.4797485	
0.3895869	0.1432585	0.2097086	0.3943152	0.2890785	
0.3304756	0.4267345	0.1321579	0.3017248	0.2974115	
0.3557894	0.2566876	0.3481863	0.4427371	0.1539374	
1.002467	0.3312715	0.4087018	0.267092	0.9147589	
0.577225	0.262267	1.973349	1.237751	0.609565	
0.4589462	0.3505724	0.297093	0.5300721	0.1877687	
0.1981165	0.4438786	1.210911	0.8903457	0.9455118	
0.4292104	0.5006689	0.2659487	0.6501742	0.09898938	
0.5243939	1.164233	0.724588	0.4176199	0.6761838	
1.23189	0.3354504	0.4559223	1.188799	0.2564107	0.9305053
0.4084591	0.3797221	0.347433	0.5244938	0.6431369	
0.7553672	0.5225535	0.5110548	0.7558107	0.2594215	
0.6182079	0.2855045	0.2094832	0.3057385	0.6684707	

0.2399599	0.4560759	0.3829011	0.7471319	0.8112325
1.558732	0.259675	2.196425	0.6247794	0.1040088
0.2289741	0.4068897	0.9362043	0.3464183	0.6604281
0.302425	0.3631417	0.3531451
CD63	124.6768	181.8076	109.3329	164.8356	136.4224
125.0573	181.456	105.2345	166.4124	199.387	38.27049
61.85694	147.8657	50.27642	164.8206	138.5889
145.5657	139.7231	133.7551	112.6417	250.3185
142.4506	104.062	122.7438	121.1393	52.68376	215.1962
137.3947	129.9398	346.7837	189.6764	175.7315
291.7158	110.9634	229.057	182.1294	162.0869	182.732
126.4183	162.0604	109.4031	80.17251	186.3607
198.5621	155.1866	201.4601	103.9358	103.6676
112.2569	88.13862	229.2166	140.0473	159.9085
203.1801	139.0651	111.6515	162.8125	100.7874
108.5712	207.3544	171.6129	122.9521	207.8652
177.6947	283.2341	176.1013	212.4993	254.0825
202.7244	121.7393	82.15621	312.7155	98.39499
141.7794	202.0875	228.1265	212.5617	158.0995
135.1026	88.26861	296.3	248.5284	164.1934	173.7275
242.4031	244.1581	125.5651	138.419	135.5151	153.6677
150.5399	133.1999	320.0111	157.8589	152.5252
256.6294	322.4416	212.3571	101.1643	115.6857
130.5648	164.2535	175.54	275.2634	210.1881	109.4529
122.2269	129.0676	146.4137	255.4246	250.1176
106.652	246.9982	95.68493	134.919	326.2258	162.1215
92.21283	212.3304	117.8727	97.41052	110.2661
142.7727	281.9732	112.9232	115.8466	175.831	213.9485
170.368	131.5591	194.2456	133.4941	189.6347	152.0902
184.4654	109.7572	119.3505	114.5223	179.5379
79.15718	123.9744	80.66208	124.509	169.9963	128.6396
146.1609	182.0961	210.529	77.26679	119.7556	89.4457
183.57	82.80639	164.0395	109.5676	183.9507	176.1203
202.3135	59.72603	183.8007	152.5002	140.0303
260.2299	103.8325	206.303	120.247	248.601	235.2381
118.312	156.3047	120.2099	163.0777	173.3775	94.42119
301.1873	212.8926	207.4062	185.2812	113.566	105.1368
126.6546	156.4696	73.0533	132.1794	355.911	164.6628
156.7103	173.4751	108.2193	143.4329	222.1657
110.2526	188.0207	150.7712	210.6872	83.81589
172.7607	118.035	136.2573	158.315	197.5137	164.187
117.5116	119.9861	82.40456	144.0656	222.5911
113.5471	92.81538	145.9188	104.5842	157.3648
140.6641	130.2302	148.6192	176.2945	219.2971

157.6292 179.2141 97.3316 177.6662 161.8035 224.7921
151.8935 151.4062 95.32394 106.8873 55.20298
88.40064 371.6345 144.2595 352.0376 102.6656
183.7037 117.4627 266.0936 135.1374 193.0417
127.4347 119.182 128.4935 226.3114 153.059 176.4357
159.6005 205.0011 252.4568 132.0655 150.6316
201.9639 85.21152 104.3229 232.4155 174.4785
153.3273 100.9264 114.096 203.9583 88.26599 153.4287
208.7527 169.1379 88.94836 150.9782 98.18126
103.8581 116.4584 160.4573 315.6745 196.4304
154.6974 221.7716 273.6594 79.83742 172.3402
141.0058 110.9263 130.2428 81.49216 156.9441
144.7515 180.3357 290.0933 157.9492 107.3575
164.6609 87.94377 141.3016 226.4919 115.9947
101.3638 117.9165 125.6354 132.0562 148.3247
136.8342 195.472 221.3599 189.6371 165.8313 159.6774
174.8729 260.8428 183.0908 222.2838 292.8976
153.1016 86.61937 186.0598 266.5224 226.2921
148.3698 199.3781 219.4811 116.0548 96.83087
159.721 125.5449 244.423 168.0471 133.6188 208.8804
102.0697 169.4323 317.654 217.9608 153.7624 106.3047
180.4158 147.3989 324.6031 138.334 227.3792 200.6234
168.7028 47.56667 138.4943 178.2274 140.0087
338.6393 96.54544 157.7991 184.5088 209.2633
116.5589 144.3234 239.9546 58.83576 246.0428
237.9226 98.60576 123.4999 154.5719 187.4898
235.2213 285.6317 150.0349 163.546 111.3764 167.8422
145.1488 233.1594 141.8893 136.8269 171.9221
172.8411 132.16 149.3367 157.7718 208.8241 99.2525
143.7164 201.2358 183.3868 111.083 89.26669 143.4369
144.5332 234.2819 113.8879 124.1156 107.6425
326.0983 98.49952 86.39709 229.9224 179.3879
185.2073 100.1919 119.1671 145.6195 130.3607
119.154 144.7545 112.6305 221.3505 95.35608 149.536
147.5583 138.2947 146.8924 202.3959 102.4297
132.853 147.4432 146.3645 133.7342
NOX4 0.02144973 0.08432226 0.05480635 0.1047271 0.06252206
1.103038 0.05155278 0.137105 0.09566463 0.158259
0.02250356 0 0.06624779 0.02904937 0.007770957 0.1447847
0.01426233 0.2907029 0.06474027 0.09671453 0.07240929
0.2302361 0.2152736 0.4419647 0.745976 0.1442462
0.03395295 0.24122 0.06447852 0.01622382 0.02020524 0.3439525
0.2530601 0.240201 1.699685 0.07314347 0.08183836
0.3767355 0.4350831 0.3353605 1.085495 0.1218567

0.8384471 0.1118467 0.3607647 0.2420108 0.08042218
1.229627 0.6746342 0.11802 0.8363808 0.4069965 0.3732995
0.8242214 1.547405 0.1933679 1.245759 0.6083489
0.2222482 0.09693102 0.9443342 1.508114 0.5198336
0.6107676 0.1311906 0.5135623 1.2455 0.5636543 0.2370598
0.1947912 0.3887832 0.8032309 0.5375444 1.28449 1.07937
0.185342 0.2257507 0.6149862 0.2453207 0.1237376
0.1781913 1.075194 0.301239 0.09557582 1.556457
1.158416 1.152567 0.6921071 0.9307649 1.274224
1.024324 0.3592593 1.461805 0.1931411 0.4087439
0.842439 1.804258 0.1003851 0.0700018 0.6377567
0.4028348 0.1270143 0.08237091 0.2915993 0.8189242
0.706539 0.5157202 0.5666693 0.7060319 0.8165769
0.2742317 0.2009333 0.5228177 0.2890103 1.063528
0.2346355 0.3039997 0.4550461 1.135617 0.07480075
0.4390138 0.5307495 1.014675 3.668098 0.3711322
0.1063383 0.6605084 1.19088 0.6887699 1.557348 0.3587337
0.5996548 0.1988517 1.025449 0.1012318 0.1336595
0.7526235 0.2206874 0.7055642 0.2376679 0.5292714
0.4868484 0.2299007 0.08782624 0.6433534 0.5608745
0.1206595 1.124758 0.126024 2.155796 0.3502135
0.8451396 0.8638596 0.9545514 1.03107 0.1272197 0.5579471
0.6293098 0.8235228 1.618692 1.577016 0.758332
0.5849428 0.2319891 0.5226213 0.3670353 0.4068008
1.180569 0.5290627 0.3315574 0.1455923 0.2030357
0.1398544 0.2497985 0.1336461 0.8005941 2.206385
0.906045 0.09400392 0.6122456 0.4027009 0.3995704
0.1574732 0.53734 0.9109754 0.4976625 0.6145573 0.7577913
0.2967957 0.3193822 1.077128 0.3340297 1.103934
0.08380206 0.6065338 0.9761206 1.431238 0.1633582
0.1797101 1.327628 0.3227087 0.6835191 0.7944117
0.3930094 0.3562395 0.06097974 0.6777448 0.4501929
0.3278855 1.388889 0.6676158 1.666811 0.2455547
0.2707457 0.3110565 0.6151366 0.6427359 0.2133513
1.173509 0.4645272 0.2490918 1.241199 0.09005824
1.072182 0.1309287 0.4287803 0.3125213 0.09435242
0.03489352 0.6073525 0.497439 1.851331 0.1087995
0.2237635 0.2199154 0.3536261 0.2300151 0.1576508
1.902637 0.5110264 0.3391501 1.196488 1.235783
1.074996 1.899462 1.112386 1.849175 0.4198127
1.489633 0.3517724 0.3856371 0.2581894 0.998662
1.637536 0.08342248 0.5358049 0.6685131 0.7139634
0.7943447 1.136742 0.2380119 0.5674782 0.7579054
0.699207 0.6779446 0.529327 0.268305 0.2437218

1.731597 0.105961 1.39927 0.5602972 0.2461523 0.2657442
0.1211042 0.409164 0.572315 0.3605343 0.2682992
1.349843 0.9183826 0.3004914 1.219457 1.27382 0.6399237
0.4299142 0.08831076 0.3805947 0.821688 1.054874
0.3000036 0.2420571 0.3375848 0.5423491 0.3104268
1.39372 3.237681 0.7771455 0.4385509 0.1525435 0.384455
1.125178 1.014148 1.912467 0.160904 0.2371285
0.2888927 0.994812 0.454007 0.1810092 0.3827067
0.9713826 0.4436981 1.312952 0.2822483 0.6055901
0.4331592 0.8099354 0.5763901 1.568753 0.7206979
0.5178525 0.1919411 0.5952524 3.70816 0.1368843 1.320784
0.1220239 0.2531634 0.6811292 0.06767274 2.262384
1.570252 0.4729556 1.124075 0.2462131 0.06809335
0.5693342 0.1788082 0.3529753 0.4201695 0.4120776
0.9259531 0.181016 0.6740646 0.07193657 0.1006679
0.6373787 0.6922706 0.5779852 0.1881998 1.31272 0.243876
1.593098 0.3471301 0.1183007 0.4788435 1.020073
0.8996835 1.067646 0.8149283 0.8917719 0.4605576
0.1181972 0.3651256 0.8760752 0.05732521 0.2362289
0.1420283 0.5293234 0.3792401 0.3515562 0.1835823
1.012956 0.698015 0.3761157 0.1709403 0.4557157
0.1335579 0.3814165 1.210301 0.8604943 0.5827852
0.1968869 0.3151853 0.2408902 1.228623 0.7825389
0.2512176 0.1711457 1.244791 0.5076769 0.6131288
1.07992 0.2307962 1.008935 0.3050848 0.2047196 0.04596736
0.4163517 0.2008074 0.07955195 0.7242894 0.07985124
0.2778595 0.4204912 0.4975475

MAVS 19.87302 12.14236 22.5454 8.173185 13.34019
16.3611 10.27885 9.87256 12.95742 6.852134 24.15388
11.57891 7.83189 14.70394 15.66717 13.41255 22.08233
10.51248 13.2999 17.86781 6.856646 13.38755 12.0327
15.81528 13.52233 20.09792 10.36068 14.83762
10.8215 2.220939 13.5543 16.29808 12.65639 12.33464
13.23874 19.67312 13.03075 9.785176 20.56255
8.746135 21.68799 9.932904 9.913775 9.389956
14.00492 20.70814 26.27782 23.74081 9.503681
9.54959 15.63035 20.5315 13.8188 11.55836 11.70296
13.1808 13.22572 18.0909 31.04195 13.23746 12.63134
10.59436 8.65661 16.14028 14.64815 17.89661 11.6542
11.69249 7.982479 18.10391 8.148643 10.10159 23.399
8.876829 19.06223 11.41334 20.55359 27.72478
15.44934 30.40491 13.04184 16.04545 7.209354
26.52155 21.76759 16.74409 27.27174 12.58167
15.58978 21.21421 11.34109 30.55286 10.51005

11.9175 10.60823 26.29057 8.300679 29.01175 15.25101
14.40861 28.26432 11.93503 21.47428 45.47456
30.27079 22.5967 12.74162 15.03757 10.63417 11.1301
19.1854 52.37485 6.643327 17.91864 8.537136 11.17338
17.50995 16.19311 18.49695 20.40461 13.94802
23.32378 39.14278 5.737892 31.99943 12.62198
7.795267 10.48719 17.38123 10.79554 13.93721
24.31572 14.69627 11.01178 14.17716 24.44567
20.48645 12.24501 11.61129 17.49252 15.0377 29.95024
13.91197 26.8159 19.17309 16.76426 22.25997 18.61657
54.54581 16.83476 17.73658 15.74496 16.32147
23.42807 15.36437 7.328728 13.99428 22.11211
12.73224 14.35422 12.05716 11.88545 18.23397
23.68169 13.18463 16.85747 9.460694 5.419511
20.57466 18.65994 15.22158 7.26663 33.50133 17.03868
19.59882 9.206582 21.54915 20.8823 30.97903 13.78404
12.0836 29.24703 14.72648 21.09977 11.34075 27.17002
16.29324 8.485209 44.94001 15.74953 12.87658
18.46553 12.20229 14.17659 15.69931 27.4437 6.538451
41.34632 16.37256 14.61309 19.47675 28.55125
19.12099 22.8652 14.60977 11.77086 10.77517 28.67142
18.1546 27.24121 16.99369 21.2786 27.89919 57.18167
12.53903 11.30757 8.909341 11.28084 18.66796
17.25455 12.25619 11.83577 12.16646 6.013302
22.48029 8.811301 35.58267 14.1273 18.44723 9.897446
31.922 15.31883 21.41931 15.41929 18.97681 16.38557
16.37468 18.02881 6.122507 58.48932 21.97904
16.6499 18.90019 10.04547 8.760585 21.71939 21.70345
24.08599 17.75966 19.89777 21.50153 20.33953
20.51061 28.33031 14.42983 9.340964 45.32511
11.21787 14.58247 21.93799 22.90643 6.662597
8.321449 11.21385 24.25476 29.23797 19.06616
17.86004 19.39061 10.54629 16.00999 8.69331 12.65478
17.23915 16.74738 25.90466 12.79055 17.075 17.40418
6.085507 23.05014 25.50245 10.09325 15.0159 13.80196
20.2679 25.96754 21.09423 18.19827 18.68873 23.52512
11.01993 10.21227 20.48692 14.97003 20.21831
11.13724 16.31254 7.550924 19.2675 15.49172 17.58479
14.027 19.34536 13.68582 22.81796 9.185144 7.871718
16.97866 18.0971 14.24134 11.3158 14.85504 12.20391
11.90504 21.95682 10.55277 15.48371 21.57531
7.42512 25.34067 9.021102 13.59163 15.80335 2.153937
18.57762 15.08246 18.63902 16.9366 13.89681 17.31926
26.31193 16.23975 18.6278 14.34198 32.99428 15.88478

27.43871 18.29316 4.749489 16.35271 19.25173
8.731114 12.53703 20.70139 18.60332 22.42639
47.91692 19.792 11.08981 16.05687 12.84598 20.79727
15.38013 11.82161 18.67211 22.2819 9.838074 17.62678
14.38345 23.07246 13.93505 21.32422 14.30748
13.59466 15.0682 17.41321 18.43338 9.665867 10.32699
15.19808 12.59257 39.10833 19.59335 14.98767
37.96905 15.57731 14.375 18.70072 17.56394 31.18049
13.30526 15.63694 46.77829 24.28455 11.504 14.73097
20.21206 16.17755 29.96037 14.46148 9.492062
25.96609 19.76144 21.96551 10.99752 20.28675
36.20774 9.21281 8.349939 18.6787 29.74823 8.458897
17.75949 9.533581 18.66963 14.37179
RHOB 26.72972 824.2589 54.38554 112.872 181.7057
923.5645 67.51301 587.5781 671.7934 475.3363
83.74856 44.83691 141.2176 72.82444 75.93061
671.0176 42.75072 1291.798 81.26219 88.57705
404.8474 941.0149 165.0164 1006.532 1071.861
87.69465 96.84726 1002.879 89.12761 146.2936
327.2274 965.3911 26.4401 106.2935 233.8513 39.07247
87.34786 111.7065 181.7194 254.9782 215.1683
111.5856 80.57531 274.2651 107.4306 42.3341 62.88172
113.7643 105.2129 276.8236 92.97935 116.4903
89.99711 139.3246 385.356 17.87522 149.7278 694.0132
114.3024 65.62416 87.23015 189.8487 221.2514
75.70217 54.4678 483.9478 142.1606 131.3072 109.8108
52.8764 102.459 74.23009 177.1445 110.9073 128.8415
81.86158 170.6857 93.43422 60.40922 40.29646
37.51984 160.6622 30.06177 230.5945 257.1464
183.9818 211.1394 75.03102 35.02045 368.6009
106.3209 40.93937 167.5916 37.73958 83.83274
59.26366 191.2712 120.0676 102.1235 213.0545
51.26547 38.34152 79.93485 104.1012 149.0363
172.6482 199.1799 364.7155 107.6615 84.13953
48.63291 41.72965 101.6539 301.753 206.664 54.70363
153.8288 175.5404 74.37918 87.44846 107.1338
41.37519 113.5421 96.13443 54.83192 78.91474
76.58338 242.4717 477.4407 202.8617 102.053 84.29344
201.489 73.34649 238.5704 176.3106 94.86465 19.60669
57.43877 44.04207 24.01245 65.73918 70.89139
50.48209 95.44091 281.0408 69.18251 238.8628
21.98603 105.6318 149.4071 165.9554 146.316 111.1358
458.5732 391.6699 205.6573 213.7177 133.2669
83.73303 280.2136 72.27172 59.24854 228.5483

209.3997 85.81366 93.90676 85.43405 140.0056
71.86269 698.4384 361.8531 88.34625 75.74094
36.86558 64.5011 171.5332 193.4701 79.5651 88.73753
52.04858 107.78 90.82509 89.34549 107.3419 106.9655
253.8113 80.03855 95.21871 63.85878 227.2078
94.90077 78.76188 58.6087 50.77626 127.5265 265.6563
43.29704 163.2459 73.43986 59.36492 204.0906
128.6644 39.34607 84.34461 102.4596 136.6938
295.3283 90.58596 42.97101 43.48162 296.3419
156.2556 89.86781 57.69859 147.7356 101.5083
57.13092 262.7231 64.70797 51.22436 44.49018
73.84482 86.95584 97.50038 46.73891 74.22092
36.1444 61.2037 85.96501 112.8663 116.1338 40.20834
186.2593 28.06811 291.1203 124.8618 37.25393 149.86
77.06115 136.9437 175.5792 233.4033 134.5385
88.88445 127.7525 76.78203 730.043 194.0491 50.63548
139.1588 73.32144 270.3427 271.5166 77.78578
78.88619 51.56736 112.5432 123.3785 256.7586
109.2898 86.2692 163.3496 145.5023 120.0095 103.3129
50.42124 126.9247 78.02302 104.3105 153.214 111.7172
143.6788 42.69608 45.68772 93.34292 162.1412
367.8815 21.87434 34.88062 235.636 46.57706 399.6251
104.9502 53.47609 224.9425 23.75807 111.8663
200.9801 506.2461 89.34753 384.0028 819.7135
193.9196 61.03574 285.3633 105.7685 139.8351
207.0628 171.8574 69.49742 103.0063 43.60271
301.6927 49.67932 63.50733 23.38052 48.93309
136.9769 144.6537 139.4062 86.68183 87.79701
126.7179 42.15839 576.3927 90.4451 91.84597 69.17793
82.07499 40.15236 51.0777 104.3929 43.30187 192.9539
96.21465 165.7935 75.23975 64.8874 51.96105 36.10037
155.2204 122.2031 188.5644 458.8181 130.7184
89.47264 85.01899 72.17748 133.7601 24.3647 94.27043
109.111 62.09843 548.2977 37.51785 132.9147 27.03495
47.63827 58.87634 93.57333 216.4739 103.0702
256.2583 66.05778 66.9476 97.23604 56.65064 361.423
48.22796 105.4828 123.2092 63.51101 176.6304
76.4807 39.26308 26.54526 59.05735 103.7403 78.27388
61.29115 305.0224 48.78796 94.32491 152.2554
134.2676 248.7885 45.11331 56.02757 114.5148
79.03555 142.5902 62.35016 96.15142 110.5818
112.9209 384.4475 46.50923 97.85139 28.46564
214.5514 20.63696 92.70013 27.76117 86.57181
62.75388 41.45115 240.9061 147.7991 106.6183

171.7671 103.0484 200.5603 43.11409 62.90996
105.4175 438.4297
CCN2 9.795481 69.32076 15.66654 69.3634 68.438 143.7141
21.09437 58.8178 74.11671 83.37942 48.74858 9.787047
59.81632 19.13708 24.20233 76.72509 25.5861 148.7878
68.78767 40.07777 60.70853 117.3625 100.1523
45.70311 196.3679 65.46736 71.13581 101.7672
44.25628 32.36074 90.83805 111.357 19.27644 63.05216
269.2261 11.13821 7.911141 77.72346 130.6664
86.50154 166.1372 23.09632 111.2058 32.37642
22.85359 41.43679 25.39047 98.72496 184.9588
74.96729 93.1654 67.59379 52.62371 123.6197 191.969
24.6484 256.6482 269.574 49.69566 93.53887 42.7777
94.01035 161.8042 37.47655 32.63572 128.817 170.1264
209.7057 159.8882 71.24778 65.34222 25.03543
62.14043 64.50968 148.1855 81.23038 179.5513
185.1414 18.2266 32.68712 159.9837 242.3002 79.3219
57.76419 138.9025 109.8849 103.0902 93.82056
38.13185 128.6127 58.28904 16.66644 85.49275
54.9777 31.45095 57.91688 235.1649 63.58864 16.99835
103.2799 129.5091 29.78185 22.39813 63.51035
243.972 212.0669 70.94115 103.5438 56.52097 83.87746
48.3034 37.91948 50.45941 103.9869 79.08461 25.29904
31.35132 73.65255 108.623 68.27759 53.14635 128.2491
158.5834 150.0301 61.8748 72.12351 37.58772 140.1243
138.9477 366.3118 126.182 22.26878 97.12869 164.7104
117.9341 57.4301 74.75633 20.45848 64.70912 69.15498
40.74758 19.60823 46.83728 45.14492 45.59428
43.31575 17.44087 69.7374 17.68151 198.9601 37.12388
113.4827 71.37024 32.97713 174.6056 30.77707
59.48674 109.0946 43.60653 79.82285 267.3615
203.3424 93.9284 22.23532 225.7529 113.8974 148.8471
151.0384 176.6809 29.62475 163.9629 46.95224
26.77376 16.37702 15.19681 132.3379 280.5486
59.10587 61.82289 156.6191 67.13907 60.5567 29.25065
135.0555 143.862 54.75918 134.048 56.29975 164.007
43.35395 156.7855 127.3639 71.82094 35.37267
48.33109 89.32289 306.0312 16.03663 18.14609
58.55364 26.45984 128.4859 81.9281 35.68238 20.37881
35.24348 65.61754 250.4663 99.71847 78.38197
141.7955 114.3862 115.011 35.32939 62.67934 81.74663
84.12852 16.56818 438.3666 31.00733 43.15397
197.6346 35.14835 132.3585 122.3668 20.38188
42.2004 18.18252 24.16756 83.33543 40.03567 363.1041

5.573938	68.75664	36.45103	53.30703	101.0429
37.97443	242.017	25.99661	80.53789	135.7628	201.5069
121.6122	236.2158	126.0294	151.2525	81.05913
106.9137	35.88822	53.16228	24.57502	92.70435
188.5383	20.06025	74.87636	47.5768	166.6637	136.8176
428.1893	53.50087	130.5248	141.7921	160.5359
118.2642	134.2136	32.54823	82.04866	102.7356
133.5394	172.313	126.6552	57.92865	25.13775	27.05745
78.74475	202.3966	137.431	68.01398	73.61914	92.84227
39.7308	72.50714	189.8172	106.2223	316.259	43.16709
115.491	277.0807	228.992	72.94558	276.3358	238.9857
119.4852	117.7878	305.5508	158.4496	177.7349
74.88718	129.336	38.4299	93.25035	63.20699	493.6987
33.2129	44.56455	39.66297	221.0445	190.0238	50.80328
98.50794	73.80877	45.45889	149.8682	58.2729	276.0342
197.9822	46.2221	44.59731	206.447	72.33574	92.20623
48.01188	58.22132	293.4696	45.08473	92.03519
50.58211	40.65415	131.3128	45.18891	334.5271
180.1663	158.1096	118.6848	53.97334	12.32483
82.66292	176.894	61.48191	24.11747	100.8529	35.30032
24.78188	227.4234	26.80418	228.3176	28.86211
92.6663	35.35228	90.39922	128.7658	103.9471	274.7647
32.43419	15.26626	59.80491	48.72231	43.81655
127.0984	104.3244	273.8806	16.46248	36.70332
29.32183	109.6265	5.154416	98.95221	50.60743
49.53768	33.02819	135.0129	37.06587	117.0527
110.663	82.87345	26.49348	50.60859	25.23878	35.66126
221.0349	161.7528	67.06778	93.16797	50.65603
64.00157	188.4634	44.18886	67.81695	41.7054	192.4857
74.35984	22.03448	106.1319	134.2517	129.6505
153.4924	73.44273	66.96935	30.13902	37.31427
27.96832	77.3854	12.4867	32.93498	77.42421	79.50971
PPP1R13B	3.407332	2.982823	4.569591	7.024598	4.05992
3.919937	3.967035	3.867693	5.513662	3.46681	8.117032
6.299107	3.694188	7.194836	5.114324	4.244143
5.266074	4.836628	3.269571	3.772677	5.729873
2.835855	5.77562	3.718537	3.697769	10.11989	4.530228
4.148234	4.109865	1.71569	5.878281	3.047062	14.2229
6.870585	7.723331	1.953738	2.795558	3.290001
4.636734	6.066461	5.143317	4.142496	2.280579
4.566046	8.216702	7.558328	5.582061	4.879758
2.969822	5.83735	4.687367	3.826433	4.104172	1.785178
3.070801	4.793825	4.655643	5.084357	6.066912
6.451323	4.184413	3.468624	2.922498	3.827176

5.725062 4.351767 4.657998 3.012878 2.977292
3.986151 3.099719 4.760757 5.78168 6.241799 4.511709
3.663028 4.860037 5.844429 4.307866 3.08881 7.776281
4.649818 3.75143 4.850165 3.040024 4.296 7.32637
4.117131 4.331784 2.660176 3.306757 5.965574
2.414604 5.345245 1.909118 3.727473 3.51956 5.409003
7.09047 5.824675 4.552089 8.619954 2.246282 3.604727
5.722026 4.583526 4.177385 4.517983 4.004673
5.346817 6.447629 3.532882 1.71089 3.12135 5.161972
15.34251 6.16687 2.617869 4.78708 4.786559 2.855167
2.601282 4.936717 2.29978 2.83301 4.350444 3.054725
2.909185 5.32876 4.737947 6.491658 6.025791 4.976204
3.721923 4.578265 6.69278 4.931842 8.298784 4.305831
3.875597 4.267425 5.205515 7.453882 5.14919 5.930959
2.081622 4.185079 4.207351 9.533206 5.905579
1.867388 5.251112 3.099242 4.702171 4.245248
7.668075 5.482536 3.084009 5.811933 1.813874
4.037336 5.085034 6.004877 5.586182 2.849936
5.104454 3.839182 4.698667 12.39848 4.287927
4.629597 3.804086 4.548501 4.611568 9.10308 1.730834
5.71454 5.295795 6.51094 12.00161 6.84582 7.09216 7.901918
7.111794 3.920457 6.23352 4.14706 2.886253 7.640765
4.374302 4.298958 7.425346 2.297455 6.114165
3.278089 5.628718 2.526242 6.286884 4.378483
3.424947 3.368955 4.687632 4.275682 6.821229
2.772953 8.404498 4.123166 3.188928 9.234188
3.420937 4.646389 4.539773 4.812766 4.446461
5.540934 4.622339 3.360632 4.223559 4.752768 4.2931
3.491887 4.580874 4.820989 5.199803 5.117504
2.812167 4.175903 7.96483 5.586085 3.045017 3.49025
3.453412 4.406435 4.206335 4.013792 9.804738
3.786541 2.996008 2.991846 6.910439 4.919566
4.429925 4.527099 3.855414 2.686814 5.722681
2.60088 3.798219 6.42451 4.988729 3.102007 5.340956
6.609227 3.339383 4.620939 6.229027 6.385414
3.118379 4.528156 5.321468 3.761507 3.32129 3.41678
2.1355 5.818018 3.643972 8.83111 4.718408 2.352379
3.712624 6.093906 3.515342 2.487832 4.297758
4.781603 7.516519 7.091138 2.968007 3.882529
2.282684 5.257231 4.296911 3.117241 2.751485
4.159903 10.05076 4.522788 3.383028 5.41912 5.693774
5.568886 3.709633 5.161577 6.277006 2.884265
5.20288 2.345862 4.909342 4.87464 5.092118 5.645348
4.345858 6.402471 6.812033 5.252725 6.382373

6.759011 4.243206 5.713969 7.56019 4.750265 3.99486
2.982774 3.237751 3.220202 3.859939 8.87374 3.845763
4.864172 3.0452 3.570356 4.294529 11.30201 5.170274
1.434326 6.427438 5.367073 5.824838 4.440882
3.864694 3.762046 2.97083 3.472196 7.263327 4.274262
3.277753 6.579131 2.637929 8.246021 4.236954
2.822673 5.135016 5.169763 8.731001 4.34777 9.949646
7.23119 4.241605 4.058368 4.900001 6.402045 4.53279
5.721859 2.371147 2.906053 6.255989 6.27143 4.571838
3.844975 7.979649 5.363313 6.498422 3.948018
3.660683 6.82682 2.428737 7.870914 4.399462 10.6416
3.252743 4.542537 3.022185 3.217319 6.636715
4.224246 3.292459 3.627919 5.013674 4.894596
7.297919 3.586454 4.655682 8.13635 4.496192 2.891725
3.907114 4.457654 1.760734 7.182328 4.887586
7.931216 1.713431 3.919068 4.652446 6.296722
2.621092 13.65218 3.966122 6.379428 4.388921
8.031646 4.797659 4.249864 4.895123 3.923458
4.879363 4.608661
PLG 0 0 0.008378672 0 0.005973895 0.006918627 0 0 0
0.005706197 0 0.004473906 0 0 0 0.00168707 0.004542485 0
0 0.01459095 0.002661002 0 0.002897055 0.03541796
0.003119342 0.001789938 0 0 0.004400594 0 0.006435273
0.002041253 0.004396279 0.007172147 0.02224692 0.01996787
0.002369558 0.006135776 0.008807538 0.05038242 0.007355851
0.002007455 0 0.001463945 0 0.03351274 0 0 0.006956152
0.001818813 0.005257566 0.00859402 0.1539109 0.003424052
0.08880023 0.002799399 0.006687106 0.004668826 0.006850161
0.003087207 0.005170762 0.009316692 0.009072031 0.005329496 0
0.01543087 0 0.01150777 0 0.003181542 0.001944905
0.001650486 0.07208647 0.01061689 0.005316095 0 0.003536092
0.005560984 0.001723533 0 0.003959518 0.009949958 0.007317703
0.004683146 0.004797332 0.004409762 0.01405866 0.01125615
0.4524675 0.002568571 0.008777819 0.00508544 0.01167188 0
0.005242269 0.004652824 0.03752502 0 0 0.00230821 0.01140453
0.005968544 0.00524695 0.008194678 0.03289666 0.0188956
0.003285089 0.002834788 0 0.04250267 0.002758153 0.003656932 0
0.01255206 0.3064688 0.005423973 0 0.001804108 0.01009364
0.009529475 0.007069745 0.1886402 0.04616702 0 0.02845648
0.02429678 0.05415435 0 0.01265596 0.008430729 0 0.02851846
0.009268292 0 0 0.005804991 0 0.02451904 0.05764165
0.04482006 0.006205048 0.1534604 0.005314522 0 0.01074677
0.007366428 0.005912228 0.02141594 0.00602071 0.004448882 0
0.008590627 0.002063514 0 0.01497729 0.01430078 0.004069546

0.09167752 0.006397272 0.09904574 0 0.01509532 0.004657538
0.004143221 0 0.007230866 0.007265279 0.008513336 0.003101301
0.06167757 0 0.002621591 0.02186654 0 0 0.007216569
0.02202895 0 0.002190717 0.0131755 0.002290328 0.02989873
0.005100456 0.01697259 0.002082354 0 0.005322062 0.003081102
0.005634121 0.001968808 0 0.01199852 0 0 0.003915774
0.01992883 0.00436445 0.01131062 0.05801028 0.002659392
0.5045623 0.009329899 0.002232498 0 0.004308631 0.001765615
0.002743964 0.005245768 0.007735554 0.01282188 0.02621496
0.02904464 0.01690984 0.01004636 0.08960011 0.005977163 0
0.01082412 0.002385683 0.0118045 0.01062516 0.01680494
0.007170781 0.001631297 0.01390006 0.001845466 0.01175629
0.005303083 0 0.03413628 0 0.002648084 0.002970183
0.001796663 0.008186731 0.006375184 0.009840724 0.009655967
0.005142695 0.009300543 0.01534988 0.006845672 0.006039761 0
0.04988624 0.004373949 0.03791546 0.00911648 0.04644877 0
0.00204706 0 0.0763366 0 0.001626714 0.01112944
0.003768465 0.01180246 0.004275973 0.6486065 0.002086394 0
0.05234949 0 0.03225394 0 0.001972013 0.004954739
0.005672629 0.02119066 0.04567332 0 0.06992286 0.002081269
0.01426604 0.002272968 0.01080709 0.007290698 0 0.005430551
0.01950004 0.001603996 0.005224995 0.002098478 0 0.02208478
0.001795312 0.0153117 0.002002835 0.0185279 0.02388744
0.002360022 0 0.009704249 0 0.0192997 0.008732698 0 0
0.01749038 0 0.02763087 0.001941892 0.002064786 0.009608843
0.1047186 0.002464579 0.003157903 0 0.004591639 0.01242571
0.002864637 0 0 0 0.003794313 0.03739857 0.003321381
0.002323766 0 0.01213421 0 0.004265046 0 0 0.1226166 0
0.002989543 0.02791085 0.00623779 0 0.009944453 0.004498813
0.004682926 0.01486558 0.00871308 0.008974101 0.01231677
0.002277983 0.019839 0.01593119 0 0.004706038 0.002543503
0.01022316 0.002217238 0.002003891 0.003510123 0.02114239
0.01544774 0 0.06540037 0.00970917 0 0.0343115
0.003229562 0.01059093 0.1476766 0.09839195 0.01233307
0.3324247 0 0.009120338 0.0166082 0.06239994 0.006556743
0.002489701 0 0.003193084 0.008394374 0.0267513 0.04441745
0.01299335 0.02481706 0.002090732 0 0.002121182 0.08482401
0.003926543 0.02750476 0 0.005432975 0.1466806 0.01133269
0.001730777 0 0 0 0.002243316 0.02725455 0.007515832
0.03603437 0.7651735 0.03310742 0.00200475 0.06007264
0.006115473 0.006049693 0 0 0 0.007863182 0 0 0 0
0.01988363
RAF1 13.45476 11.16264 13.49512 16.47853 9.782144
11.46788 9.188422 11.35896 11.85083 11.7099 15.58923

14.84103 13.42667 13.97084 9.918669 11.70722
10.62183 12.90603 7.665256 8.917148 13.0439 10.74086
9.956485 13.23312 12.78615 17.94104 9.990871
11.33584 13.36346 8.129318 10.80972 10.30968
10.47118 8.367931 8.768085 6.778059 8.029934
8.171474 9.058621 12.34145 12.79864 10.49316
14.2951 11.66097 12.82345 11.74777 13.02313 7.948433
17.75512 15.67513 8.072699 11.85756 18.53421
16.01035 10.00678 12.22694 11.69759 12.62735
6.791102 10.81833 12.57652 11.13263 10.04797
8.538462 10.84645 11.44828 9.170494 8.652789
9.598728 11.51105 14.17639 8.765465 17.94744
12.30068 11.17301 7.262836 10.44925 16.40197
15.90635 8.330359 8.807961 8.008911 12.73821
11.61898 11.51613 15.54072 12.09716 13.09852
8.886169 10.48365 13.21914 11.80874 8.691087
14.87146 12.22832 10.68123 10.70532 11.50862
16.57079 12.17784 12.30222 8.595788 8.092659
5.005893 10.18855 13.83932 14.789 10.11587 15.76693
10.13007 7.753497 12.30041 12.55831 10.41568
9.330922 11.01608 9.798751 13.95688 11.0639 8.437501
6.219394 9.999178 17.48499 7.465993 19.02891
14.15499 12.68408 6.690272 12.50054 12.90721
12.55549 14.16721 13.18727 13.94752 8.166422
13.4442 14.52193 12.01694 12.454 9.613117 9.194533
14.58065 7.882889 8.257594 9.312592 8.555674
7.551693 10.42834 20.07169 9.9273 11.46784 11.44253
12.63338 13.42519 11.4967 12.76671 11.95614 10.67214
9.872065 5.729955 10.76093 12.40416 14.08125
11.25127 5.189074 12.61204 9.779091 10.09366
13.40435 7.811523 11.01087 13.53918 12.64255
10.0213 15.01025 8.925826 12.39953 8.057153 18.21416
17.2897 14.85437 8.169745 13.05037 13.06645 7.046505
10.82973 9.343652 8.076405 16.69098 9.580419
12.57385 10.55338 6.986525 11.78917 4.896558
10.39269 14.0391 12.34483 10.39333 6.395839 7.387063
8.339686 11.73301 8.849521 7.864528 17.48622
9.510663 16.23712 7.110154 9.582147 16.34078
8.745323 10.17908 9.55065 13.87513 12.46197 13.77275
20.25298 10.82534 12.12182 12.60917 22.37064
15.00499 11.12124 14.77259 6.000322 10.7934 11.75361
11.29696 15.19281 7.887537 8.724053 10.9516 13.68747
11.34441 13.89135 9.603863 10.58128 14.75778
10.60354 10.20743 10.79408 9.781965 8.465899

13.0468 13.4159 11.43687 12.61919 12.92917 10.17858
15.41864 11.91758 8.792604 11.9191 12.14014 19.88244
8.626548 9.880023 13.03891 12.89056 8.176368
9.875032 15.23273 8.199402 12.59847 8.798801
16.75655 10.77409 10.24046 12.95816 12.2038 8.778386
12.15572 12.07705 13.90977 7.397686 10.21015
10.30827 12.71424 11.38929 11.84529 8.063424
7.772045 10.87169 12.63627 11.00345 30.87803
6.031624 11.95737 10.73016 10.69536 10.39422
11.95243 8.983594 11.57555 10.84604 12.61336
11.89142 9.835028 12.06534 14.00977 14.01424
6.013856 11.91593 12.71713 12.53901 14.26122
12.52699 11.48953 14.52102 10.62661 10.49489
10.9615 8.583196 10.83702 11.65654 14.06217 10.92061
9.990814 14.50434 10.54645 10.28732 8.851289
8.74406 11.24106 10.295 14.21095 9.36285 9.185585
16.54028 12.21174 7.946872 8.30311 14.12945 10.18586
9.937314 7.062904 7.473891 12.93516 11.79482
14.48647 7.813063 8.180506 21.94349 11.83897
16.95642 11.2859 9.350817 7.725592 9.039901 10.61172
11.70772 7.86404 6.624854 11.76632 11.70317 14.12885
7.980545 10.11758 13.88078 11.47585 9.474146
13.31971 9.668358 6.376372 8.385595 16.19198
13.42985 26.93101 6.700485 11.35159 9.780011
14.96249 20.35543 11.15624 9.488065 10.77029
10.1748 9.286908 11.35841 9.641154 12.39635 9.662155
10.95365 11.6953 8.383751 8.986188 9.091434 14.91972
18.74165 11.26668 13.98854 6.827162 14.59403
15.73075 13.29899 11.62496 11.69503 15.40176
12.11305 12.85942 11.62063 11.82978 14.15199
9.16077 10.99927 10.6316
PRKCQ 0.4585664 0.9781784 0.8071115 0.9898253 1.041035
1.929068 0.6948864 1.167868 1.477159 2.016392
0.6169297 0.6150501 2.281042 0.2728518 1.337048
1.685575 0.8443646 2.038413 0.299436 0.8663941
1.380846 2.586464 0.1795031 1.41905 1.642847 0.2772638
1.137659 2.165132 1.218464 0.5002549 1.582472
1.221295 0.8554933 0.7776827 0.728702 1.991152
5.019377 0.736591 0.218288 0.6321488 1.096703
0.240992 0.5553079 0.5754214 0.5130678 0.1719575
1.349345 1.331766 1.43669 0.4965609 0.1170706 3.885514
3.5525 0.9745922 3.043355 0.1788727 1.281854 3.082672
0.1149524 0.7890508 0.5756887 2.970522 0.8914681
0.2218656 0.1516953 0.7738474 0.770095 2.228212

0.8417164	0.3295769	6.526229	0.6807014	0.4536306	
0.472814	0.3499749	3.851608	0.7736904	0.3230265	
1.358248	0.2073274	3.12034	1.199295	5.001667	0.7889005
0.1068225	0.2348081	0.6442378	1.765387	1.102675	
0.9648473	0.3866636	8.266363	3.005782	2.635277	
5.669016	1.450467	4.256931	0.571089	12.58454	
1.488281	3.433785	0.1348281	1.056588	0.6135273	
0.6324174	1.659716	1.367574	1.443583	0.9046009	
0.3409428	0.7850567	0.3080147	1.671412	2.556795	
0.9525718	0.1820392	0.3749483	0.7824847	0.2703583	
1.333129	0.6844457	1.152697	2.511094	0.4502344	
1.597881	5.230554	0.7743307	0.3719719	1.756214	
0.6611278	0.5398391	2.133475	0.3858366	1.661681	
0.7294318	0.9104411	0.5913551	1.058702	2.693189	
0.2487796	1.910326	2.01034	0.7066029	0.8681353	9.338905
0.3244917	0.1946101	0.6408533	0.6295159	1.003557	
0.6845964	0.3077243	0.2197533	5.118311	1.324335	
0.1661408	2.450595	2.949834	0.6069545	1.25394	2.897661
2.6598	1.190407	0.3810632	0.2715981	1.634604	1.434887
0.9972875	2.510067	14.60233	1.223026	1.807092	
3.415383	0.01794743	0.04578803	0.8104459	0.4265392	
0.8190777	0.6023376	1.954164	1.91135	0.1068774	1.02215
5.188319	0.8668788	1.056914	1.710619	1.539186	
1.843649	3.20982	0.7487061	0.297374	0.2323912	0.9504637
2.342833	1.130615	2.828181	0.8015537	2.808085	
0.03089576	0.1289054	0.415499	0.2939444	5.035688	
1.259742	0.8170696	0.4356699	0.8379696	0.1610146	
0.5627357	1.060303	0.8435728	0.7080452	0.1556194	
2.945094	0.6095315	0.2359346	0.5344387	2.106409	
1.174833	0.2427632	1.07843	0.2221528	2.82697	6.121062
0.2894386	1.912118	0.3696546	0.1022131	2.115103	
0.4128913	0.1179302	1.127209	0.1495892	1.225865	
1.024557	1.835561	1.678948	1.095339	0.1530708	
1.202068	2.75705	0.8361632	0.5026698	0.6853509	1.36353
3.355041	1.13855	1.349058	0.9652044	2.889505	0.2885923
0.2135005	1.494799	3.597019	2.596722	0.07661592	
1.042083	1.817334	1.770997	1.632086	0.9325384	
1.852683	2.45508	0.9628053	1.972005	0.5918432	0.6091998
0.4613164	0.8114975	1.804893	0.5212921	0.4184761	
2.25271	0.1626679	0.6821662	0.6486879	0.7552455	0.2080043
0.6519299	1.935061	3.959849	0.4147964	2.311968	
0.5018738	2.06113	0.4171445	0.9857808	0.6243617	0.6636867
0.9321648	1.814144	1.757476	0.8493088	2.574274	
1.434984	1.327344	3.242437	0.4396315	0.2069592	

0.9574802 1.337519 2.485374 0.347824 0.256753
0.269122 0.2815529 1.082274 1.412815 0.162995
1.306086 0.5269367 3.781889 1.753048 1.464377
1.289364 1.235859 1.00968 1.115859 1.011412 1.879606
1.647664 0.152778 3.312888 0.630266 3.181407
0.3298794 0.324159 0.4803811 1.711054 0.3800705
0.5343295 1.881555 1.623064 1.158545 0.3753764
0.1259779 0.381577 4.432837 0.576204 0.1480658
1.375848 0.6970792 0.07879843 1.576984 0.4464891
0.6091706 0.951515 0.467855 0.6879519 2.34012 2.09396
0.1767157 0.583166 4.945809 0.08129284 2.309073
1.143765 1.303511 1.037079 2.000574 1.319876
0.09712676 1.164117 3.020574 0.1523473 0.5977701
1.326921 0.670819 0.08939563 0.310786 0.2795131
0.0293517 0.2184202 1.024199 0.1369769 0.6201835
0.6898162 0.3535319 0.2485306 1.10956 3.566175 0.9151524
0.2194311 1.095857 5.793646 1.323929 0.5086712
0.2693069 0.2679198 1.440713 0.536709 1.350399
1.404964 2.088371 1.163167 0.6196889 0.7106384
0.4592327 0.1027043 1.752191 3.648978 0.5621423
1.618324 0.1713537 0.5107091 1.008001
BRCA2 0.2758224 0.02150742 0.7665805 0.7666993 0.4611625
0.1282781 0.380436 2.130964 0.3764779 0.5991949
0.7021515 0.4953776 0.7294004 0.3207639 0.7603875
0.07542261 0.5124166 0.1648922 0.2426354 0.2553605
0.5588516 0.03654749 0.487946 0.09205841 0.08837499
0.6997223 0.5040592 0.09828491 0.2516367 0.2014593
0.4951065 0.09019569 1.98141 0.9171793 0.692927 1.038011
0.1244109 1.034501 1.614384 1.167063 1.978852
36.87717 1.376279 1.327215 1.236058 1.396315
1.983511 1.954419 1.053487 1.895712 1.007145
1.408385 2.979386 1.909897 0.7316667 1.366471
3.811683 0.9283442 1.816105 0.9580983 0.7996713
1.372238 1.008047 1.234252 1.620914 0.3569606
0.5976415 0.4510582 0.8325588 1.77132 2.295061 1.739144
2.073921 1.731889 2.145413 1.460105 1.409902
2.539107 1.647674 4.200187 1.22264 0.8935302 4.235161
0.558715 1.4589 0.7874297 1.18881 1.850505 3.098295
0.5794971 2.522234 5.507975 1.47036 1.339067 1.481115
1.626593 1.194518 1.034621 5.871146 2.391402
1.876382 1.236939 1.385609 2.652513 0.7976399
0.9019017 1.519872 0.5305094 1.492771 1.163123
2.703428 2.582537 1.144685 0.8482586 1.211631
1.295136 0.7099492 0.969735 1.784007 2.917791

2.021326 2.767408 1.965775 0.3933994 2.028893
2.267739 2.156847 0.9026291 0.925457 1.195363
0.716995 1.913778 0.457713 1.881102 0.640432
0.7498906 2.187796 1.639134 2.907854 2.806786
4.150584 7.639692 1.549876 1.237655 2.122909
0.3810219 2.604718 0.9128952 4.543438 0.6729981
1.354412 0.8027193 1.834313 2.443571 0.6669834
0.8103193 1.194208 0.4639614 2.956422 1.394136
0.8957143 1.34448 2.102791 2.040738 0.8545923 1.879448
1.452174 3.489566 2.318316 0.5319458 0.3127166
5.086003 2.999654 1.565994 2.413109 1.162016
1.255088 0.5676548 1.675209 3.217733 2.753868
2.630281 0.4878618 2.912705 1.345537 1.094067
0.8502753 1.034686 2.233731 2.755169 1.869995
2.55231 0.7199003 1.103954 0.8814048 1.389251 1.94362
4.033484 2.307543 1.249743 1.509999 0.766309
2.16093 2.325099 2.741515 1.889828 2.145341 4.719007
2.284072 3.548176 1.07658 1.842026 2.745907 1.665978
1.413237 2.195734 0.5650165 2.664295 1.013222
1.719435 3.062166 2.43232 0.8238131 1.657867 1.50813
0.9555103 4.724107 0.554108 1.444301 1.704743
0.882803 1.229287 1.246025 1.698905 1.563294
0.6147614 0.7093639 1.638385 2.717491 6.297323
1.741302 1.459049 2.205647 1.965642 1.544859
0.6934952 1.791638 0.5070851 1.129685 2.989745
1.869702 3.175343 2.982827 1.982978 3.583787
2.976655 3.068773 0.4748794 1.746027 1.18753 1.127975
1.195445 3.232344 1.041888 3.14519 3.639416 2.238888
0.629752 0.890556 1.062409 5.428475 0.9668099
0.5766229 2.12166 1.899335 2.599476 2.432578 0.4064774
2.351797 1.026168 1.239237 5.102989 0.2729744
2.579915 2.548847 2.009098 2.733565 2.551064
1.225439 1.138449 2.747167 0.2465938 0.3129346
1.804979 0.7745729 1.223997 2.519483 2.031202
1.261869 1.209263 1.445412 1.058992 1.156857
1.41576 1.716304 2.407103 1.167162 1.892771 2.265445
2.474439 0.775126 2.135446 2.983066 0.4721886
4.662933 0.8432168 1.252019 2.787576 1.71655 0.9987922
3.420434 4.477278 4.39992 1.935242 0.5989338 1.644519
1.056214 1.766994 1.573441 1.731577 0.9915851
1.160559 2.574869 1.346209 0.2318318 0.8673821
1.275905 1.1621 2.445347 0.7718967 0.6658467 1.074564
1.614616 3.265872 0.4251529 0.6362405 2.114657
4.27528 1.295329 2.316758 2.19784 2.846097 1.194172

1.650288 2.505038 1.655353 1.838868 4.612918
0.9152818 2.114789 1.454076 0.6909913 2.983349
2.241286 4.867175 1.199778 1.924546 2.960233
2.478221 0.7691081 1.778755 0.8485567 3.68006 3.212326
2.379108 0.9767423 2.153526 3.648121 1.478832
1.612109 1.429225 4.137569 1.080215 1.720227
2.332993 1.401616 0.3051287 0.836903 2.424459
3.145299 1.068573 1.825661 3.447323 1.577636
2.371933 0.8119326 2.085469 0.806136 0.1643903
4.079167 1.237087 2.329931 2.484939 2.347276
2.232891 1.189375 0.9048973
PARP1 7.555124 10.37153 11.54272 18.6797 12.88967
9.974635 11.79209 11.84301 17.07959 20.95261
13.11819 7.556926 19.33764 9.45877 19.22888 9.673495
15.66489 15.85085 7.344231 9.812418 16.95825
9.869539 8.765926 9.974854 15.60965 8.855166
14.08912 11.50252 17.45876 5.177935 16.88327
11.94777 31.99004 12.63156 10.18467 16.04211
29.73367 33.68212 38.66116 32.75467 26.12787
41.69916 27.88329 34.72946 14.33672 27.47873
29.27123 34.79841 26.66488 36.24458 23.28705
21.75336 56.81073 19.70326 11.54586 34.90567
20.26066 19.20435 29.53634 25.61162 15.09582
28.94225 13.07719 37.35545 23.09188 15.89959
18.28646 15.36803 19.93795 23.79076 49.0853 15.51827
18.22943 16.51584 24.43257 23.19674 15.35225
15.02339 22.10425 25.52221 21.31865 18.5023 24.23458
14.88089 13.98853 22.78724 19.78052 29.80155
20.66809 12.06945 27.22202 33.71458 17.49741
29.51921 31.53756 31.45312 16.07091 15.76603
32.44764 29.13231 21.78057 15.9403 11.33284 17.25961
21.4385 20.78305 24.57529 17.3392 37.12187 28.12555
22.35649 15.9794 23.73629 19.46145 13.42884 23.08606
23.97695 25.40788 41.29075 14.05886 15.81726
24.28795 29.21962 8.58219 54.29019 52.35062 22.0841
18.53873 17.25504 14.82942 24.51276 41.99895
26.94291 27.48436 11.70238 15.42859 38.1191 27.60299
36.59984 29.23206 24.05443 28.47291 21.3637 20.11482
32.26131 14.58971 13.73526 17.20504 32.42179
10.67018 28.0172 18.27195 39.32671 25.69184 15.76961
14.83954 23.31808 16.69669 17.13086 10.92321
29.74594 17.43954 24.75567 36.68978 18.07308
32.4536 37.08069 14.78531 30.17486 31.52654 15.73446
41.26261 18.31399 21.83499 26.49775 19.92047

18.25963 20.83385 28.16996 47.74364 28.96164
16.61577 15.16745 28.10972 15.15157 19.84922
17.70598 13.7676 24.1326 18.84061 17.58481 30.27853
27.63944 24.24363 14.25289 17.26908 20.74763
40.52492 27.38028 11.51876 21.72814 19.7115 49.04205
33.40048 26.54196 12.19169 14.07064 55.91849
22.99323 16.21085 18.32687 24.76461 20.17855
21.2453 31.50078 33.18909 32.63385 31.53793 21.13779
34.16964 19.01559 28.27645 34.72433 25.11982
25.26536 17.96741 34.29374 11.00102 31.23112
18.61443 17.21897 8.476847 33.50638 14.57262
27.68574 11.15136 21.06222 18.86736 24.64784
62.65859 17.93626 21.22136 31.02474 20.68777
19.23188 20.1265 34.70425 18.53448 33.9227 27.96861
45.67533 26.57417 18.77276 18.13114 23.43798
54.62613 34.45443 30.2144 27.25407 19.84276 27.02531
39.79272 20.38745 24.48353 14.91899 28.56294
24.4995 21.87741 16.22865 17.8003 30.812 24.97573
17.17192 26.77 23.97138 19.82355 17.81966 13.90281
183.5177 23.06734 20.44508 23.80154 11.34355
29.98498 29.14576 19.24146 26.44395 21.80927
16.40172 31.07473 26.75785 12.63951 14.86358
25.02433 35.88054 20.61419 11.74095 27.13498
18.15605 11.97328 19.53933 20.82764 16.52997
10.37476 23.67348 19.0979 56.22174 46.29776 33.89099
31.10352 22.73086 15.54092 25.30145 22.25243
22.13772 13.29415 24.56255 50.02315 15.6032 14.67349
17.05934 21.34944 40.6343 18.34724 10.49314 18.20151
20.88217 46.36225 16.19507 19.15005 20.97274
18.9755 34.61787 24.79684 13.41142 25.71468 15.20618
17.0173 21.46884 31.81398 22.7356 18.14968 14.80495
27.70839 16.48563 11.3894 34.85554 53.31561 20.53579
22.01335 16.90752 27.73353 17.21727 15.36001
26.3248 17.3281 22.14197 41.10057 13.60917 12.22853
26.09513 19.70902 10.26544 33.7938 30.13389 25.4995
16.32522 20.60511 21.86943 13.75508 27.62709
8.590143 26.13508 18.75282 25.8572 21.98962 16.5705
16.54708 13.29269 25.06105 55.15079 31.07639
10.91437 31.84782 30.40229 19.39793 16.34895
11.76765 27.6285 40.37336 17.50088 16.69537 18.54199
21.4103 24.65465 17.99352 31.0599 17.47608 9.263695
25.14536 26.11743 26.6009 28.62205 23.47016 34.38041
32.62488 19.95869
DOCK1 5.942065 10.65411 9.841615 6.419413 5.266228

13.3673 3.126446 7.166387 5.179046 4.140448 13.66732
4.711679 2.876824 5.788731 2.827643 11.5514 3.31385
10.81275 3.754354 4.767704 6.084433 9.057954
5.675268 12.46546 12.17745 10.91207 3.584351
11.60098 5.486192 1.927315 6.197385 8.640544
13.47467 7.676963 10.35781 4.572734 6.151461
9.417152 8.240403 6.221926 19.27187 5.104462
5.263476 9.163309 12.11996 8.275931 8.884521
7.411108 9.251791 10.97836 5.009656 8.885776
6.696351 9.677787 12.66412 9.278861 4.90722 12.9875
7.901293 9.475649 16.81685 6.244343 10.42856
7.145387 2.426126 11.31401 7.710929 6.568266
8.424458 5.637985 18.94955 3.907078 8.947787
10.3126 10.31275 6.318761 7.132169 14.36535 13.11919
7.69292 6.658273 11.38123 8.276201 11.84203 9.153524
10.27651 13.57017 8.778322 3.557722 11.71196
10.27606 7.378053 6.55157 6.981794 5.257979 6.097788
9.39726 2.90303 21.66973 7.676095 7.189408 9.927497
2.207856 5.470929 11.07882 16.28437 6.185723
9.418377 6.942982 6.404023 8.136081 4.61352 5.852797
6.041077 6.45235 12.12717 54.73766 10.16156 10.05666
5.856162 6.304281 8.404442 12.70273 4.869873
10.24176 3.882172 9.613131 3.414033 13.43271
8.972933 14.93167 8.757919 7.945233 12.21052
3.877426 24.66554 12.86878 10.32502 9.320213
4.781085 6.448521 17.28785 10.14729 7.667198
10.25855 7.11865 4.718476 8.831563 21.29918 11.81441
10.39764 8.870487 5.379598 12.80044 14.58403
4.594446 9.405545 8.810683 6.396392 5.247723
11.24516 7.296252 15.89221 11.10821 3.668912
11.19469 5.806869 11.01712 8.836033 19.72523
9.93432 9.950817 10.3511 4.459589 4.662158 5.68877
9.894401 6.267171 7.632124 14.48279 10.08969
9.798472 6.528565 11.36116 10.11337 13.69379
14.05398 4.949365 16.89121 5.953436 9.359184
10.14657 6.697218 3.519091 6.195031 15.47864
10.43311 7.731994 8.471245 5.180516 6.740519
10.75861 6.831273 6.521575 5.834083 14.71789
5.852699 5.986998 15.57657 11.08531 13.86573
10.37247 12.72205 30.3396 11.54182 9.404774 10.42459
7.159954 8.503901 8.223164 3.936431 13.46355
5.111382 8.921045 12.2768 3.230568 9.969362 5.17769
3.646634 10.93316 7.331991 8.391364 9.540052
8.511856 11.17074 7.605708 4.441597 7.048794

11.53925 19.26172 9.499474 11.07709 8.066894
8.022072 8.9182 9.195682 8.556544 9.188447 10.07933
6.662004 20.61536 6.546332 5.556306 12.07278
10.98391 7.203487 7.434017 7.385694 9.713433
9.305142 6.82072 4.121305 7.607043 7.498635 13.3383
10.08426 9.614557 4.678401 8.100312 9.285781
8.960317 7.420064 4.649759 7.343783 5.899891
9.204609 10.84795 11.22733 2.037481 8.823432
12.86581 8.776773 7.388093 10.03217 5.814507
11.06856 7.811911 6.056049 7.377767 11.54931 8.7341
12.94505 15.36651 8.063456 9.586631 12.22852
5.162942 9.903606 6.818637 5.684658 9.76239 13.51435
11.83235 14.05188 4.885078 7.382108 4.111573
7.960483 8.671871 6.326427 14.17382 5.593803
13.97449 7.446844 6.075515 13.69006 10.20602
18.83506 8.29048 11.14391 12.06361 6.927859 15.33167
3.020254 6.121522 4.406928 6.444233 2.818123
7.511371 14.28564 8.443341 13.77304 10.10331
10.29295 9.668287 9.080566 6.872048 7.343042
10.95102 11.9594 13.93663 9.63664 9.316747 9.977293
16.56095 8.886843 6.293487 10.61144 18.12902
7.647153 8.228322 13.02776 4.861525 6.318912
7.450463 4.788003 13.26174 13.11986 9.251888
5.711475 7.338171 9.715469 13.41241 6.168657
10.89271 5.855386 9.571156 7.121562 17.42568
5.906827 15.24515 5.488333 14.50351 16.96082
4.108805 10.13544 4.356469 3.974748 5.570555
13.81515 7.419529 11.57257 6.566345 20.99456
14.3217 4.715046 9.40189 7.289379 10.0074 8.430859
17.86826 6.453062 7.744617 10.61272 16.45994
9.080488 25.1981 13.40768 4.794182 6.764472 4.649525
3.968019 9.237328 13.29955 4.045714 5.315996
9.886625
SP1 14.86474 11.05501 19.98563 15.8154 13.57288 16.64861
10.98633 12.3866 15.50348 11.66977 30.24808 17.26454
12.92085 17.58564 14.19453 16.38429 13.77882
16.97493 7.95856 11.40547 18.50835 13.90181 15.43507
18.22717 15.63822 24.7342 14.5018 17.81558 16.21748
5.065541 18.46373 14.71816 30.82776 24.94826
22.85107 10.90258 8.014311 10.08158 18.22645
28.38597 24.11254 22.62357 17.7129 27.98524 24.43352
22.85318 29.43734 21.67691 36.08767 19.24865
17.78005 19.84131 17.36058 25.07781 18.78088
22.8428 21.05476 23.66238 19.30806 15.90721 26.35321

22.90943 16.31018 14.12586 18.49202 14.79718
15.55405 16.10635 22.40587 14.0817 35.29582 17.19495
27.82877 25.57081 24.23919 17.83013 17.08138
25.70693 22.57725 14.74806 20.07687 22.68515
37.57828 20.6669 15.9226 23.16388 25.82561 26.3806
21.54525 15.9962 27.01914 39.80677 25.03715 23.67922
18.30761 14.70295 27.57869 16.31885 35.15151
24.58648 23.69928 17.83598 18.63249 23.40607
17.91633 27.03592 24.26489 17.03655 26.01801
15.65246 23.3074 14.4597 13.2452 16.02435 20.88608
21.14771 11.91194 43.56878 13.15089 15.88775
9.525447 23.05951 22.02806 16.19456 29.29261
23.3044 17.45789 14.36429 22.27822 21.00316 40.72203
19.85376 17.06355 28.43172 13.64996 25.05406
23.35921 25.60421 24.1234 21.20023 26.93619 30.57156
28.21989 24.39013 22.34471 14.68136 18.22535
17.53235 37.18448 18.03884 29.82258 18.38241
20.97673 37.30257 20.9849 14.70795 21.59038 21.61497
19.48072 16.48952 24.16622 21.01789 24.01507
13.39347 11.86652 26.5494 24.12244 23.80275 21.63201
18.56363 19.43795 29.94826 25.24873 17.87711
17.94481 21.11408 20.73308 21.00941 34.82696
23.28878 21.42762 17.71952 18.90336 30.93956
14.72688 22.57501 23.95974 24.06168 39.90359
39.38772 17.78215 18.1134 17.55845 14.74517 20.66484
25.28762 23.80202 30.19133 34.12682 15.07774
16.21651 22.59039 22.06897 24.5218 15.75273 50.80577
12.85643 21.92624 21.6388 27.54617 26.15438 21.79618
26.27715 13.82146 34.88854 28.22841 19.43174
19.16328 20.22391 21.02569 25.78866 31.11431
15.65927 22.20209 30.83295 15.82938 19.26582
18.87369 18.55047 16.09822 13.83009 18.28265
19.21937 24.75054 28.20393 31.07765 13.84408
24.59325 27.73641 19.35093 24.92226 47.3929 18.92836
23.39106 19.12537 19.31448 29.08314 16.85781
24.90171 36.13309 38.27752 40.86688 21.88869
30.88125 32.65298 28.03161 26.16064 15.8201 20.69594
20.34966 21.69842 17.79885 23.07264 18.863 31.88026
24.24489 22.7129 14.96674 21.51274 30.43206 25.1583
13.947 16.24368 19.30253 17.57989 17.80623 23.49034
14.79371 16.91216 27.11358 26.19264 25.43244
11.19293 25.16826 27.17462 31.72929 28.91369
12.62048 20.06427 21.91301 18.33103 13.19415
18.11791 16.61229 18.7596 13.83199 19.80822 36.72245

35.02393	22.78956	24.47149	28.25302	35.86956
20.64252	16.96016	18.29392	31.44414	20.91513
33.43695	24.73175	20.91011	13.85595	27.81153
15.79643	45.442	19.17031	20.06118	16.25787	20.97745
30.74127	30.55516	23.4567	23.52642	21.83407	19.74258
23.06007	21.74327	8.615186	25.37895	37.31891
34.53937	42.1221	25.57225	32.31847	13.73707	17.11939
21.51746	16.67606	46.24011	28.11176	25.59698
18.81668	18.11203	27.33368	23.65098	40.44633
37.51933	14.33359	31.64745	22.15786	20.13506
27.00343	11.02939	13.39827	36.1671	22.71019	26.91436
21.85422	18.76555	12.48118	21.41404	19.26788
37.03054	26.39373	12.7498	17.83756	26.36247	26.3631
33.44738	14.04829	18.63119	16.40112	30.40501
23.50605	20.15105	9.943637	18.20436	10.35979
19.43703	34.41005	26.73512	24.71637	26.53857
17.15833	31.00001	18.4684	15.91974	20.88995	19.32159
23.19193	27.68321	20.11966	16.52082	21.19745
46.1451	22.50001	33.48174	18.47322	11.38479	22.87244
19.15428	23.4709	32.96633	31.12648	20.97252	18.7079
19.16425
HAVCR2	0.3972072	0.6792942	0.7869432	1.62355	1.036998
0.8045298	1.227416	0.4668922	2.116786	4.450204
0.4917612	0.1500711	3.692136	0.3688375	1.419658
0.7950962	1.889406	1.176148	0.966741	0.6117921
1.651304	0.6466287	0.9912133	0.7265365	0.8030665
0.3602462	2.285237	0.9301618	1.520403	0.7366398
1.597382	0.6504751	5.673801	1.299131	2.568319
2.134972	1.534033	2.53154	1.77853	1.70691	1.252216
1.01006	11.90585	2.420927	0.920295	0.4046902	1.275452
5.624154	9.994501	1.574048	1.221279	10.88597
0.6310005	3.905076	4.981847	0.7183504	7.974216
3.147849	0.6969973	1.719032	9.938466	13.56839
8.273399	8.746357	1.014389	1.534706	3.19455	6.183921
1.945517	2.459908	7.67213	2.328027	1.239247	3.856877
4.095443	3.840472	2.75183	1.523374	1.367291	1.848236
6.840056	6.149487	19.7229	5.67094	1.412073	2.666248
2.519015	4.60166	5.233589	1.645641	11.58989	11.62104
9.040784	4.40089	13.79063	13.19204	9.840273	2.175759
3.958335	3.910002	4.270209	0.690713	4.426445
3.555106	3.672991	3.938371	4.721806	2.786111	5.3228
3.569328	1.466418	0.9506678	6.890295	2.854663
2.204806	2.237859	3.987075	0.6596283	1.946819
1.282609	1.948541	3.508986	2.938839	10.863	2.044905

3.937943 11.23456 5.072579 2.904472 5.348405
4.04616 0.8218174 1.601095 4.65283 4.123965 4.210831
4.057863 3.803867 3.333204 1.167662 6.676086
5.09936 1.30433 1.274735 2.50898 0.948234 1.289065
2.631841 0.289471 2.104167 3.854454 3.724479
3.336297 14.38973 2.696174 0.7195503 1.812136
4.065592 2.682347 6.453035 7.339834 3.025453
7.260825 1.685117 1.279795 4.650888 3.990651
8.010189 6.111698 0.7570257 1.888591 5.126769
1.898907 0.2254162 0.7377001 2.251251 2.165068
0.9679724 0.9883683 1.34796 1.014102 1.00677 2.155707
9.639339 8.249255 5.964853 4.079212 2.449427
1.952886 4.084634 3.370361 2.595958 2.918789
1.033486 8.623081 2.473397 11.94132 0.8840002
0.8017009 1.271181 0.617052 1.796383 2.381378
1.440449 0.3974497 2.055112 5.168189 4.544222
0.8757404 3.713131 4.230628 3.84022 4.530651 0.9183013
2.895556 3.822786 2.983729 3.319158 5.697743
2.059023 4.701004 8.519905 1.05835 3.348841 4.116396
1.501164 1.372333 0.4891829 0.8341055 2.736681
1.206509 3.446464 0.4682645 0.7503192 2.457784
2.224008 3.59252 4.945896 12.37721 0.9320224 1.825002
8.549843 5.302939 3.928149 7.809041 10.11988
7.382929 1.410502 6.326839 4.97275 7.323212 3.516914
1.671507 5.345316 5.601192 3.54189 2.787297 7.807096
5.554392 1.666778 1.697142 3.033107 10.56034
2.379081 4.557564 3.474376 1.448652 3.564989
3.266484 5.105998 6.824583 6.051672 1.49257 3.588406
0.7390703 3.587262 1.827046 1.011853 6.367441
12.50987 4.296634 5.678997 3.737533 9.277477
2.402105 5.170812 0.7979324 0.7383146 4.047738
2.311954 3.405404 1.436822 1.701595 2.649699
13.33795 2.246416 10.64421 10.0061 2.414867 1.039747
1.042628 4.781651 3.794295 3.705433 0.6478543
1.203878 2.686806 12.4306 5.401357 1.067874 7.234517
3.046064 5.916841 10.57477 3.539814 1.705486
2.688777 1.389855 4.828852 6.265863 7.850509
4.956426 0.9943029 4.328053 10.46697 1.041961
3.13255 0.772157 2.392592 3.431506 1.196792 4.296839
8.518675 3.286949 2.929543 1.285981 0.2332936
1.198133 3.843515 3.496288 1.784863 7.186618
2.62833 0.9385019 3.074868 0.4871512 0.6889823 1.021414
2.821574 3.575397 3.570039 5.073084 1.674 3.945511
5.070498 0.7420692 3.31456 2.961158 2.240814 3.903518

3.671706 6.110135 1.667315 6.20609 1.741096 4.765302
0.2797707 6.490293 0.6613897 1.01368 1.166537 1.676163
1.40923 4.726071 5.179157 1.509209 1.540444 1.261419
1.323691 0.8880119 7.626296 3.489929 3.512477
0.5245926 2.975398 7.626893 3.335607 2.270824
2.140834 1.694817 4.758545 3.403023 1.122918
10.11983 5.840368 14.40284 2.677014 2.340444
0.7587369 3.180384 1.844338 3.516797 3.974658
1.395965 1.770245 3.549271 1.149006
VTN 6.022163 0.1717264 1.172859 0.2630475 1.790249
1.442868 0.9249095 0.6026518 1.857588 0.7125106
0.09763091 0.02352165 0.4991922 0.05051392 0.9173764
0.1951357 2.997217 1.081735 5.233115 3.426479
2.04258 0.3219368 0.4417084 0.2936398 0.3362 0.1693914
0.8433352 0.2461398 1.746784 1.602836 1.607095
0.6707456 0.8378653 0.3393693 0.311903 0.06561343
1060.68 4.182912 2.060608 0.1112524 23.52801 0.1477592
0.09345992 0.2501433 1.929418 23.40295 1.211999
2.194127 0.8533492 0.1434367 1.057297 0.7907065
70.49583 0.3690414 0.5368993 0.1177431 0.1669986
0.4479726 0.1500619 36.3251 0.6184675 0.3510426 4.149585
106.1185 2.199526 3.013908 1.119027 1.064841
7.596284 0.8614416 0.1380426 1.414426 2.700347
0.591676 0.2888112 6.918014 0.3671736 90.51769
0.1223303 0.04691278 0.2602155 1.582441 0.2757229
2.48064 0.3341924 0.115922 8.75261 0.1627434 0.7177585
0.34436 0.6576312 0.8154723 10.24286 3.537339 0.2756133
0.5626332 0.6034707 0.5988361 0.130614 0.2609124
4.06226 0.470696 0.7310274 5.801935 0.3829714 1.142456
0.06044997 1.24448 0.1588093 0.215478 0.8664374 1.110324
0.3781969 1.102078 0.6784276 27.00526 0.6363075
0.6734446 1.039238 0.2066683 0.4460319 201.6855 12.572
1.211402 0.1438563 1.492245 0.0876054 0.09064194
0.5933057 0.2825701 0.1164032 0.1363058 4.982461
0.2050881 0.6557445 0.3509783 0.2049828 1.276202
1.179268 0.1413854 0.46488 0.2521315 0.8894617 0.1575692
0.3446582 0.4308617 1.662975 0.6295068 0.458983
0.2105108 0.0497913 0.7395833 0.1084897 0.5592388
1.631738 4.248045 0.7274544 1.538135 0.1849858
0.7811024 0.6857414 0.9060718 0.7774653 0.2232764
0.5604745 0.546486 7.62992 1.644894 0.4076287 4.480836
0.4842927 4.203836 1.30931 0.1949296 9.815666 0.3082727
2.374261 0.4827353 4.094557 0.6095798 0.367264
1.420785 0.9184394 0.8254119 13.77808 3.106509

1.413033 0.1619895 4.606144 0.1552657 1.252627
0.1387814 0.2608978 1.162267 0.720554 0.7177172
0.294476 21.88936 0.5428825 0.02097271 1883.298 1.035
0.0762931 0.08796284 0.1359163 0.1531654 4.594823
0.4619604 0.7015545 1.145991 0.9800938 1.374325
0.4778581 0.4027442 0.3734126 0.2199754 1.787357
64.75656 0.9030797 4.83466 1.033447 0.2271916 31.58675
0.07290088 1.388516 0.0776206 29.08728 0.3066917
0.2483234 0.1794722 0.1553357 0.2157965 1.007219
0.1653048 0.1147784 0.3267971 0.8967885 0.1624526
0.2027837 16.0996 6.962816 2.063498 0.3731114 0.04450736
0.7431206 0.4771695 0.7275956 0.7908462 1.857705
0.1365893 0.5596478 0.4863791 0.3678961 0.6945553
0.6029501 209.9967 3.046215 2.519297 0.1629873
232.9245 0.4278011 0.04192233 0.3822618 0.1051263
0.1907727 5.65507 0.1140468 0.3646949 8.161823 2.277722
0.3765649 0.520628 7.151896 0.2188463 0.1541748
1.457921 1.465917 1.7776 0.1026893 0.7209186 1.941507
0.2782903 0.480734 0.4578606 0.048045 0.8127783
2.732559 1.328276 0.3264282 0.4919245 97.82873
0.4901106 0.4141034 0.8826508 1.779578 1.349532
0.3443425 0.228854 0.06308305 0.1277167 0.4011171
0.9310476 0.1786668 0.7544679 0.9548031 28.49899
45.12475 0.2241369 7.872086 0.12875 7.652755 0.5421918
0.3184158 3.07224 0.1232931 0.6283833 54.94322 1.279108
0.8063382 0.3283586 0.9330146 0.3716321 0.4765007
0.2100583 0.5478791 69.9455 0.1824609 0.7230086 0.9538221
0.08198825 0.2246118 0.3038959 1.158977 4.456324
0.9281034 1.173861 0.04718151 1.780781 0.7605103
1.123273 0.1340137 0.6307231 191.3367 0.8558412
0.5464428 1.474632 0.2528519 6.412953 5.938935
1.776617 0.2638379 13.99935 0.3726369 0.7275369
2.395224 0.2122436 0.6793206 0.6901444 0.5172976
0.1898925 0.8659921 1.015422 0.3236649 6.260698
0.13421 0.1206527 0.2094345 0.543305 3.806611 5.411881
0.1917893 1.03627 0.1309329 3.273766 0.7034921 0.2231589
0.1226737 1.962241 0.5728676 2.464341 3.812192
11.41131 0.2142156 0.1370382 0.4731788 1.910242
4.949068 5.117478 0.08845709 0.3031162 1.131106
0.1165855 3.802911 0.478673 0.1633702 0.5639876
1.596893 1.600921 0.7851157 0.1917242 0.9259451
0.9232786 0.1508629 0.1413077 0.2723558 1.805963
0.3943956
INHBB 0.8116799 1.734415 1.021902 1.481425 1.21335

2.810465 0.6165371 1.29886 1.729129 1.352141 1.035363
0.5523403 1.071378 0.6972519 0.4731262 1.978685
0.3708562 2.789387 1.478115 0.7360447 1.997629 1.2419
4.436202 5.83199 2.81688 0.901751 0.5311525 1.631425
2.497378 0.390992 0.666344 1.146236 1.146792
1.785202 3.089885 2.614302 6.643518 3.192952
4.549385 0.8347002 6.206844 0.8314521 0.8967369
0.7695853 4.384548 0.8441676 0.817828 2.505231
0.8587939 1.637023 3.234979 2.695288 2.247496 1.65
6.498298 0.7971296 3.109233 1.036599 3.000901
2.280698 3.299976 2.003612 2.081966 2.541674
0.1497636 4.446179 5.661328 3.281425 0.9430733
8.768029 1.727275 2.03766 4.216575 13.55138 2.501762
0.7626773 2.834116 5.499774 191.3616 3.176029
1.356122 7.528926 0.5342867 0.76468 4.943543 2.66942
11.15579 1.109489 1.351471 0.9973656 1.083694
1.356943 1.963928 3.341721 0.835098 2.0244 1.542792
0.7544296 0.7154042 0.7859586 1.362564 0.4793588
0.3343375 1.387008 9.638715 2.592778 1.269045
3.894914 0.4450973 1.07606 1.730044 6.080392 3.366147
1.849588 5.106907 3.301367 99.67698 3.358941
2.241046 0.5929897 2.243048 1.214706 8.31136 2.635016
160.325 3.06561 0.6470126 1.407539 5.619894 3.638752
0.7184574 0.6659647 3.84799 1.475847 2.980322 1.572058
1.798574 1.142477 2.64492 0.226096 0.4757009 1.036106
3.231221 1.718744 3.072985 15.50092 1.018346
1.628253 101.8809 1.076354 4.136237 1.197433
1.355968 4.326313 4.304553 0.5125786 3.727623
3.597385 1.745193 1.72952 6.855276 1.598122 1.242766
12.66823 1.776424 0.8711117 1.08503 3.276027 0.7472353
9.819709 0.7486678 8.529925 1.519132 3.377662
0.7534161 0.9376472 3.373248 3.277948 1.422107
1.542668 1.204009 5.944459 3.250029 0.8237983
16.39116 3.143138 5.729777 0.2024641 13.93027
1.254531 6.218098 1.997387 2.702444 0.8041505
0.9200846 4.297724 2.303051 0.810806 0.3326792
1.281521 8.773814 2.303705 4.307675 14.72546
8.279322 0.7383179 10.16294 1.937676 2.695614
4.548898 1.560225 7.880082 1.931926 81.29497
2.114896 0.7815706 1.242294 1.6273 5.914393 1.104774
1.227133 0.9799884 8.162769 1.28959 2.131255 0.4079033
6.175515 0.2006366 0.09958374 0.956171 27.57461
3.517109 0.2661484 1.169883 0.4347178 3.58624 4.069322
1.349774 0.9984437 13.67225 3.644101 2.494568

3.752343 1.685696 2.455031 6.127255 1.245743
3.585275 3.243813 0.5561248 0.9579142 2.179863
2.423653 7.80136 0.4955996 3.385186 1.024296 2.359578
1.149468 2.09274 3.950982 0.9209175 20.19969 1.152325
3.687643 3.097205 0.8285531 1.553923 2.277963
4.665016 2.219946 1.676076 2.664445 0.8951797
1.625535 0.7105932 2.840606 2.046993 1.074422
0.6812596 2.747199 0.6771225 3.313779 1.713233
1.217533 3.280652 1.008132 0.4725887 3.517563
8.640546 1.566023 0.5921269 4.862032 0.7922714
3.946763 11.95196 3.573456 1.825323 1.396787
0.6385118 1.429185 4.271574 1.465522 5.941157
0.952859 3.120248 1.761836 4.395458 1.815182
1.816439 3.810395 1.192186 1.115536 1.832296
0.7413098 2.806856 2.978812 10.84984 3.613863
1.171286 2.585376 0.6712879 0.8110643 4.01537 1.925848
0.927814 3.032296 16.12061 2.605981 2.231652
0.4138574 17.64606 1.245205 2.867415 7.429913
4.684504 0.321656 1.737252 0.3628847 0.9572273
77.08093 34.36246 2.37085 2.020847 4.81782 2.083927
0.8180052 20.69961 6.272902 4.175735 0.8826916
1.525542 2.44762 4.408819 0.9868901 0.3794232 2.804877
1.54873 1.196168 11.42665 2.647777 9.222532 2.760468
0.7407964 2.332597 5.78389 0.3420775 1.337533 0.7057672
3.673207 9.138024 2.377006 30.4527 3.225598 1.919232
40.0852 1.96408 1.75663 1.532479 6.111325 4.218266
3.216692 2.379906 98.79994 2.974267 0.7660539
4.731165 0.8047976 0.6298509 41.08308 1.968027
0.5795502 1.015773 0.4010469 2.443092 1.839874
1.331407 2.019799 2.667484 6.29767 2.132696 2.254701
1.216716 0.4247042 0.7392665 0.9771428 2.058862
RANBP9 10.7912 14.57754 11.98246 15.0438 11.74035 17.68974
9.711401 12.9539 13.75133 13.80843 69.38264 99.46312
13.89592 93.46095 12.42677 15.83815 9.974956
16.02127 7.653662 9.512064 15.12071 20.01143
48.82285 17.58978 25.10121 106.5587 10.67077
18.39057 11.24877 10.60808 11.57988 20.38887
11.48076 22.18874 22.60279 19.47846 8.021862
14.99417 22.87641 18.41628 14.57546 10.96315
24.71809 19.67616 15.22889 12.84976 16.0416 20.49664
17.70171 17.28643 14.27619 16.03777 17.3608 13.06028
27.28626 15.048 19.03743 18.27987 10.14146 13.2702
22.80285 16.98976 22.40736 21.47614 10.28644
28.86992 12.44993 14.34172 18.86681 10.71616

20.44232 14.97873 25.57546 26.77049 17.32146
14.04446 15.55357 20.69171 27.6464 24.99027 11.36664
17.62458 20.93729 17.11295 35.4399 15.55077 16.44082
17.04166 12.89269 27.63889 21.10342 16.4649 23.75425
16.50041 13.94486 13.41901 24.22329 12.8807 49.21367
21.77058 17.63588 18.03657 11.21186 16.43284
20.65303 19.77415 17.76525 14.09267 20.17549
13.31588 12.13442 17.2039 15.93644 18.03583 21.83011
18.35534 11.1174 16.5139 20.94952 27.17929 20.04366
25.46969 15.91652 14.57049 38.08298 17.35517
14.60282 10.28157 21.01252 18.06327 14.36484
16.85699 21.757 31.17327 12.13005 16.38784 23.24129
16.23672 29.83362 25.44833 12.02313 13.8446 9.475357
12.78315 19.58397 19.0582 16.03692 21.62492 28.47723
17.06255 15.20021 16.44901 13.77097 21.08268
18.21139 15.36097 19.5872 20.63642 28.05927 18.0957
17.82247 17.12081 26.47458 19.2547 15.21383 15.33055
18.0175 29.02376 11.1612 17.60861 21.55418 14.26711
16.44573 45.6733 25.54945 17.01631 20.70588 18.55235
14.39437 23.60803 22.69336 20.12274 39.46813
15.09667 18.75916 18.06382 18.82205 20.22125
33.76196 18.77206 16.04811 13.20137 9.085235
12.64186 10.1289 13.30341 25.07204 14.97135 20.85065
16.55631 26.63621 12.15393 15.90726 15.02473
26.11749 16.626 13.7353 29.00827 17.34796 15.69511
24.69101 17.0015 14.94819 21.41951 17.05444 18.65508
15.52951 27.53098 21.15521 11.76381 22.7042 15.33023
15.14507 15.28034 15.51156 9.142761 20.55414
40.88951 17.83183 10.64112 13.13458 14.85044
13.01063 9.911286 17.96704 12.86926 10.53588
13.49164 24.83571 26.05654 19.79907 20.33052
16.54404 17.4492 29.17525 21.63595 18.03956 15.97879
18.99181 21.32244 28.71537 23.10587 26.93582
19.40591 20.43129 35.22181 23.01917 12.24773
16.68875 21.02657 18.52511 16.70122 28.22886
22.36676 10.3802 19.48631 23.43373 12.18359 21.42792
20.43004 17.23823 11.47311 12.74698 17.94006
13.08319 10.80141 16.85518 25.68937 32.75898
27.4163 19.95136 14.00646 18.5103 19.63642 19.08846
21.55401 18.70915 15.60041 17.48922 24.51747
24.52874 21.63395 37.29162 12.05336 13.80883
12.42725 19.76728 17.9224 15.76734 12.94699 17.12847
21.6807 14.02168 21.96605 16.40035 18.78903 71.04229
24.00595 19.87984 19.99949 18.20753 14.32842

18.27345	12.12017	18.28294	25.62532	17.83165
27.66086	12.26536	38.09627	18.87412	19.26785
21.76798	11.7196	20.20781	16.59482	16.12545	14.52578
12.76287	23.05544	17.53296	20.68096	16.01487
11.9303	15.60635	24.44807	15.96445	12.6664	17.42547
29.89854	25.266	23.88211	18.43454	23.03954	19.44826
43.92142	19.94692	30.2163	20.5076	12.98579	26.02694
19.40324	12.54066	19.47305	18.01519	10.07761
17.66877	14.81309	21.0948	26.67948	16.15555	12.01196
42.91934	20.57157	14.84119	14.84805	13.21714
15.63938	18.93239	13.69305	9.572761	28.50132
25.12273	17.33546	18.81869	21.43017	16.68697
14.04549	14.87962	18.63856	18.76664	26.31179
13.66417	18.64269	15.97463	13.84542	14.9321	16.84636
15.44127	25.1089	29.54854	26.04338	9.495846	23.57116
20.71976	22.53111	11.79322	14.68771	12.1899	18.59767
13.82159	13.45484	18.50701	16.9069	16.13678	11.34751
21.05277
PDCD4	28.73771	6.514	24.38552	32.5859	21.8312	7.500804
17.35773	17.52339	16.54972	18.59909	48.16732
59.60429	30.38002	56.73154	13.86668	6.104924
14.32163	16.93182	9.227882	13.88745	31.37066
9.361907	35.55183	6.605712	13.07137	47.65577
23.34645	8.610392	27.35954	23.40375	22.09514
10.67175	31.13252	25.94346	14.03902	13.67248
55.53713	5.934641	8.274956	58.20274	14.25353
5.393991	14.01104	20.65187	20.33693	9.884129
21.60101	5.989611	18.6943	8.258332	4.263913	20.40594
53.87586	10.15643	11.51282	9.277701	9.758145
15.20472	11.46025	19.69638	12.74379	10.39261
14.00209	5.107432	24.3993	8.853258	6.574551	19.43431
12.21511	6.63811	9.761423	14.87229	5.891765	5.53565
6.235837	22.44423	8.184112	9.084995	14.60428
7.377438	5.683471	15.51069	10.97268	13.43118
5.782141	13.60927	19.13424	10.10011	7.377599
13.94396	10.12405	10.18592	10.14833	24.5505	5.663772
15.69121	10.0225	8.411417	16.79359	7.91539	30.43973
12.63169	14.70214	18.08274	14.4178	19.64361	6.490012
9.89616	30.04383	18.81105	14.13915	21.12228	8.870881
15.62192	17.23448	16.26618	12.42705	8.176376
7.869692	10.54305	11.16961	13.10365	10.72076
10.10825	21.07888	30.60165	11.98813	5.653655
12.75903	14.33421	2.057233	15.08926	29.00015
18.51933	13.6575	25.84853	15.61846	6.546934	7.701022

7.886161 9.688096 23.97496 9.694006 23.38426
13.2605 9.679668 4.923428 8.900025 12.35913 17.96854
9.667951 13.81957 1.386879 17.19224 12.55995
12.90228 10.08684 14.56979 7.288641 11.89826
11.83787 26.62754 29.62687 16.641 4.314174 22.29031
10.81082 19.46778 20.99166 53.34086 10.81988
11.03892 21.78383 13.56491 4.661855 9.409338
9.720981 6.437001 50.3373 11.46554 24.98583 13.45033
43.35806 12.69915 8.777085 15.2745 20.29669 1.20184
34.96543 14.34003 9.006921 6.192061 10.59321
16.81349 7.86236 15.33749 9.334804 23.05938 7.48188
5.283738 13.19072 12.46263 15.49006 12.35592
4.890841 21.00813 8.882225 8.762624 7.803978
16.65336 24.74258 14.29079 17.94917 23.56544
7.438942 24.66247 27.11597 12.07665 10.23637
13.49235 7.978982 24.09477 14.29402 5.179803
27.82869 20.3554 12.60588 36.92907 13.09074 15.79656
9.491591 9.110622 15.43654 13.73659 24.55918
10.80576 14.66383 19.8865 16.03719 30.97371 21.32
14.72816 13.81348 5.448085 12.34939 14.36716
7.852327 9.277066 22.16824 7.304238 21.35538
14.04773 5.44474 9.048002 8.803527 11.72369 7.973731
11.05234 7.076665 15.59467 10.0822 6.86508 17.23284
6.611323 24.46477 23.53996 8.873693 20.95365
6.157919 35.95723 11.68682 3.221783 14.34641
10.61521 4.599462 10.61064 13.54117 8.22983 49.53914
5.726228 17.96588 20.15415 5.007285 5.582624
52.60838 14.94225 15.93043 9.254319 28.81884
17.51672 13.69814 5.881049 7.438754 14.19903
17.34728 9.802907 8.212606 10.84285 17.66957
9.429258 14.39225 13.63629 10.18498 8.540794
17.32217 4.843448 17.8715 13.48774 13.9826 12.03699
17.44227 4.292759 11.38106 14.59245 17.15963
11.11095 21.0284 14.21859 14.12135 16.44616 14.94865
18.68797 12.5313 26.55276 19.68187 21.72369 18.44509
4.379262 27.67038 52.38595 21.25914 13.94631
26.55306 39.47689 10.46041 37.52624 10.84466
21.4613 14.3002 3.162661 20.31032 13.10792 3.945382
8.782935 9.39077 40.35664 5.955191 7.609332 13.76473
11.78582 19.75081 16.98631 15.96089 3.769076
14.13807 11.33321 19.03911 7.100341 9.867371
17.29492 13.74086 11.31803 21.04459 7.429109
10.18023 13.33988 63.70137 10.28714 19.87008
11.84606 14.27026 21.55602 13.29667 102.3432

37.2092 12.95155 6.595435 4.86389 18.40147 18.09991
7.271253 10.35475 3.895162 11.55696 13.31542
7.12803 6.632493 6.83347 18.60575 10.42522 9.406707
20.26315 6.973019 10.21983 20.84648 12.06541
8.334778 12.61668 33.35858 14.59661 7.634464
17.46893 2.107843 24.28172 10.54554 10.37966
9.039335

PRPF4B 4.752904 6.091629 8.951266 7.563571 7.048975
8.842396 4.901387 6.123895 6.150395 6.978616
10.88897 7.968505 9.509357 5.78572 5.967431 10.3225
6.069833 6.479353 4.87563 5.306584 7.344759 10.57717
6.190077 9.189769 6.3333 8.945198 5.533555 10.86539
4.665604 5.272842 5.354812 8.353472 9.389869
7.580774 10.03998 9.535581 5.536196 6.691241
11.80327 9.161792 12.62384 6.911422 8.681719
12.54401 11.9282 10.59943 11.25012 11.50703 12.43671
10.23529 7.599415 14.6092 8.629216 5.601042 7.709891
13.12866 8.94641 8.798927 5.898385 4.127744 11.58556
7.197804 9.673597 5.771589 6.031867 9.943855
4.780042 7.60634 9.273889 10.00448 11.74063 11.41844
11.74614 16.07248 11.91119 7.569366 10.67587
7.954464 28.68174 16.1083 6.21016 10.49262 19.72269
12.46045 6.546676 13.13458 11.48902 10.83061
7.219675 8.957963 15.88542 16.75305 9.301609
12.60934 8.36461 10.06813 8.984827 8.401721 20.77921
11.96062 12.1029 11.23951 5.337174 11.64189 8.568738
12.98985 8.240603 5.819998 9.090967 10.55084
5.220441 22.43912 7.49428 12.21357 7.411437 10.23897
5.938533 8.805114 9.699078 15.75651 10.03226
20.73352 11.63204 6.740709 15.39429 15.32759
12.12532 5.010542 9.501933 5.362379 10.95064
14.61071 10.96634 16.24912 5.570972 12.49765
11.12011 16.23432 14.30674 18.82332 10.01039
13.35735 8.998342 13.10127 10.55467 12.13218
12.47655 11.65312 11.96825 10.7356 12.61381 8.647687
10.96069 11.79525 11.39269 10.60527 9.838391
14.02465 14.25057 12.03618 8.325874 15.0853 16.06937
15.08025 6.026076 9.345161 9.015112 17.60281
8.678377 8.072707 9.411489 6.833744 13.07295
8.102242 8.60039 11.28048 8.04211 13.48883 12.35306
8.901191 14.67551 11.67298 9.206606 13.79399
12.22549 11.75427 9.620298 9.547322 17.45982
14.54607 7.636792 8.544365 4.2065 7.170097 5.683796
7.904761 9.872246 17.96927 13.77759 8.991884

17.5534 8.117907 8.806256 10.899 7.740591 12.86136
4.591027 10.3994 11.23768 10.33777 16.94955 8.991335
14.30239 10.55757 9.652877 12.98316 5.16555 18.54226
8.726845 6.257692 13.23511 11.70322 6.871073
13.8098 14.26804 9.589751 22.91097 5.51912 6.60859
5.904527 6.252842 7.50473 10.44299 6.850787 17.70509
10.84769 6.894317 13.81352 21.22301 11.61229
15.34246 11.27035 11.22081 9.786206 11.84086
9.416741 11.2809 8.701528 10.26721 15.99986 18.86664
18.80661 14.31867 9.162792 15.02424 19.79561
17.72541 6.057317 13.09016 9.670561 15.31829
7.182645 24.40787 10.57783 10.19649 16.09432
15.59753 6.644835 9.90861 11.8871 9.960491 5.067059
6.305958 17.48389 8.81593 8.976964 12.89322 9.620762
10.78416 11.64963 9.201355 15.01494 5.128207
10.65577 13.15786 15.17477 10.91192 14.68002
10.17499 11.24472 21.76381 11.46657 10.58837
11.04967 6.266182 5.737495 12.22368 10.29349
11.39049 9.929718 11.92613 14.14782 12.37823
9.351576 8.513506 11.51827 30.66373 14.46902
17.62099 10.16125 8.404154 5.51617 10.62694 4.73901
16.46987 8.829549 11.29207 13.02055 7.94951 13.1234
23.5209 15.12933 10.86674 10.14258 7.573847 11.42069
8.96774 8.426318 8.773781 12.71205 8.175597 9.701713
8.650171 8.21914 4.612992 14.60116 13.6653 10.33382
18.18032 18.03316 23.10622 13.09012 13.47837
13.28516 10.90798 13.19919 9.913565 21.86172
7.252832 7.032142 17.85044 13.69219 8.48259 7.859269
13.0575 7.097172 14.09008 11.88993 8.265 15.83054
12.45756 5.140261 14.16193 7.364237 12.66189
11.18856 11.1184 11.03831 15.10977 7.376827 7.012592
7.234225 8.429697 15.54409 9.698219 8.775235
6.819092 5.412461 10.59558 17.2079 7.910066 13.40214
9.490838 10.13652 14.42433 9.676242 4.014799
5.441063 8.677743 14.27603 18.24323 16.1139 10.22996
14.14171 14.41127 12.80726 8.280937 10.7244 4.891942
8.26488 6.967378 11.46245 13.2505 14.45415 12.94217
6.095014 7.755351
SESN1 2.595916 9.047458 4.783475 5.612695 4.480433
5.232081 4.436033 5.521896 4.905534 9.010744
8.852239 6.053166 8.933184 5.022102 4.15347 6.557732
3.757747 7.672922 2.154354 2.684306 6.756399
6.201923 2.472369 10.23711 9.828284 5.753387
5.348561 6.198326 4.660109 11.02756 4.305337

4.737788	2.702952	3.895844	19.4638	7.730523	2.17866
4.054005	4.665667	7.105446	3.878786	1.723181	
5.149863	1.716249	5.089148	5.0311	5.615292	13.29258
5.482895	1.860361	1.581859	13.47191	4.967237	
15.48506	5.996244	3.794544	2.774268	12.24407	
11.39895	2.288488	5.053746	3.455078	12.5174	7.303731
8.776656	5.179094	2.473636	10.96778	1.864192	
2.061138	1.926334	6.098862	5.032108	2.804135	
8.009487	3.617301	3.41642	5.528667	16.15816	28.44209
1.642678	8.509963	9.192736	4.326281	1.822911	
3.65894	7.546039	5.441108	4.004262	4.966486	3.997053
2.540614	3.327559	5.699944	1.655629	8.982022	
2.893035	3.838862	4.704552	4.940427	16.5243	3.232143
2.333679	8.933175	2.874757	7.120108	2.807612	
4.351082	5.564565	6.550916	6.487633	38.70542	
2.778207	10.15218	4.129622	2.243476	2.314121	
6.709232	3.973751	17.39589	2.190217	17.38214	
12.85248	3.518464	3.929776	12.31445	3.863853	
1.030992	7.234624	4.11997	2.705229	36.37598	2.113084
3.867892	2.492563	5.800185	5.343122	2.303248	
9.107745	7.329811	30.79276	6.315201	2.599885	
55.32365	12.27491	3.033153	4.872444	3.866328	
30.04612	9.231269	2.330922	3.700437	2.23148	15.68814
7.009738	3.349527	5.239892	6.392763	1.89687	6.900694
4.246303	6.579146	13.57517	8.733823	6.074452	
5.274522	4.122936	9.147971	10.46692	1.446009	
10.11533	3.076676	10.20078	3.929171	3.555985	
2.211728	4.415864	1.895632	5.737002	4.967088	
8.82273	6.060183	4.425844	14.48189	3.416696	13.8113
6.433789	1.685142	39.67761	6.264553	7.651868	
5.088423	1.081197	4.970518	9.625218	5.181676	
3.992902	9.376375	6.689509	11.12905	13.46053	
6.311646	8.3552	18.00008	13.14601	4.720538	1.661425
5.509378	17.40504	4.065418	29.23063	2.822452	
10.6152	17.90138	1.527402	8.454431	5.962985	12.90087
11.68373	6.738583	2.033259	6.200177	9.228534	
5.109332	11.04355	1.630115	16.23427	4.796572	
1.73763	3.013867	15.14618	3.906155	5.58391	2.502501
17.60084	3.002275	6.236289	4.13347	5.756716	7.844457
21.96458	15.0645	10.77528	4.851485	8.846129	3.8145
10.5244	7.246257	5.315609	4.181403	7.584683	18.015
4.279679	9.525391	14.4601	18.23675	5.183218	4.755305
2.327996	9.411922	3.267364	1.768278	5.322647	
4.144069	4.256111	21.05247	2.370844	2.820999	

4.836352 6.251026 5.410874 4.984953 7.143301
4.317262 2.342132 5.173658 3.301378 9.069495
2.497258 6.474732 6.158562 4.500259 2.828405
2.345107 4.941092 30.11719 1.863163 11.29304
2.659918 11.83003 6.057339 6.221537 6.932057
5.104416 6.968895 3.683624 6.310188 6.877701
3.714128 2.370311 4.791269 8.887633 14.56423
4.100429 4.929733 2.789551 1.971259 2.616293
16.65671 1.569322 10.04026 1.181309 2.409124
2.757241 5.112835 5.422037 4.075336 6.618642
2.863219 5.146754 3.873838 10.83864 2.343302
12.83064 11.13023 12.79126 7.117059 0.9683759
4.107048 2.030914 3.987578 7.803711 5.209706
5.907153 3.250311 4.47234 7.130292 3.575351 10.31339
3.194122 22.06291 9.849003 4.75784 6.882687 7.306523
7.16829 13.13933 4.87162 1.595467 4.012702 4.209711
19.72667 4.032864 5.824453 15.47916 2.196859
9.861729 9.657059 24.34859 3.696419 6.574188
2.67041 2.539536 3.219572 11.43499 5.146294 1.972742
4.746607 5.479839 6.102321 46.15641 2.618155
2.40928 13.18527 29.96492 8.725355 4.727747 3.501465
4.094508 9.760332 6.368607 2.596915 8.240315
3.094848 15.33337 13.85431 4.325396 6.378033
3.510315 3.732448 13.23545 5.048785 19.30416
5.750504 4.071118 10.98032 7.756105 6.165276
6.248932 1.883363 26.56551 5.652493 4.075418
3.432757 4.372511 3.308977 5.940784
SESN3 3.300619 1.625038 4.608269 2.226638 3.609605
6.349891 1.79962 4.152521 2.54346 5.984954 11.88925
8.279358 6.845848 5.881386 4.282813 6.539406
1.794345 3.831253 1.640894 2.229923 2.210495
3.846173 5.141664 6.40179 4.612519 13.23872 3.021014
6.088594 3.313881 1.614427 2.089932 3.83161 2.29604
7.00098 5.279334 0.7106214 2.33345 2.741334 2.753972
1.879024 4.618012 2.137686 1.326244 1.682511
3.275948 1.651136 7.916245 2.448102 6.601525
3.215682 0.5021933 17.58681 2.386231 2.017217
8.022367 9.698622 1.390806 13.0119 0.1871482 2.49225
12.55148 1.198034 16.42101 5.933058 2.247886
7.198477 2.144577 9.827573 1.935851 1.219502
0.8713394 0.9367546 5.054508 3.241214 1.645657
2.649626 3.733895 4.449306 15.72895 0.5072606
0.3635658 13.68579 2.181099 7.637726 2.724266
2.625105 11.40274 4.685647 1.173015 7.178329

1.421294 3.494281 2.172211 9.474659 1.27929 2.124659
3.989684 0.758063 0.2826384 6.267212 20.25998
0.7662696 2.50913 5.911798 5.176715 17.12883 1.269314
3.907879 3.14956 0.3610771 2.489214 2.691891 2.374084
9.14915 6.590231 2.067896 0.4789022 0.9716917 2.516058
1.024752 0.5576583 8.495226 7.803132 5.452987
6.403414 21.40703 1.5454 0.7118321 10.5874 3.805636
1.972806 10.67405 3.132787 1.842509 1.348996
15.31444 4.223329 0.8068618 1.904259 3.439636
3.365934 0.7166589 3.170254 5.158042 3.40537 1.718214
0.7195694 5.208212 0.5664302 7.196721 4.829745
2.996372 0.9333789 6.310905 9.617349 5.538849
3.191231 7.362752 0.9027858 1.525915 7.705654
13.02807 10.09877 1.969829 0.9508424 5.305977
3.165382 4.28755 7.801378 23.47203 5.663768 1.959658
5.512846 6.184713 1.078383 1.45423 5.85501 1.636853
3.257603 2.823911 0.8186157 0.64204 6.540871 3.112949
1.826056 1.340314 11.57735 0.6455641 9.563066
0.8444365 2.8089 1.432966 0.8363401 2.789003 0.8662964
10.02136 6.480088 7.941763 0.3474767 0.8554749
3.784119 2.86211 0.5791987 0.5682141 0.3239318 2.281649
1.702795 8.590863 3.539504 0.8085803 2.365792
8.184162 5.374441 1.255232 0.8484372 1.86012 1.758602
4.551384 4.288482 4.268666 1.856933 6.170688
1.007525 0.3930648 22.95406 0.5834155 1.786561
7.973935 1.080368 4.965984 1.473997 1.764054
1.35264 2.253126 1.833033 1.133426 6.452428 1.284108
3.525699 9.666051 6.255758 4.257995 4.179785
2.879682 6.271413 4.792476 2.922995 6.043562
8.859094 1.370852 4.301693 0.9438266 1.675224
3.620223 0.8500836 5.332465 3.229163 3.227856
1.339925 5.35871 1.191875 1.13804 6.256685 1.268452
1.454288 7.104495 0.8664703 0.9205822 3.533025
5.760324 3.50418 0.978292 1.000857 0.7426109 0.4444538
1.662641 1.981966 6.573065 2.09284 3.937226 16.51563
1.866206 2.150883 3.532966 0.627653 4.888594
4.856898 3.380369 2.628931 10.9051 3.665417 6.598411
14.17889 3.756768 2.53747 6.929079 4.142405 2.111248
0.5801537 1.189215 7.120296 8.286165 1.60954 1.730487
1.064671 1.078279 9.399781 2.115353 4.569182
2.516441 5.064756 0.4378 0.7144552 5.900246 6.653099
5.757897 5.946058 3.146939 2.076418 18.59166
3.94447 3.67746 9.285854 1.054377 10.97194 5.722565
1.187285 2.870296 1.500219 6.008197 0.7955112

6.233947	6.185174	4.598581	3.40555	3.342455	0.2405662
1.523094	4.671843	1.030722	1.751449	1.325028	1.8111
0.8605164	9.720882	9.229796	1.311454	2.980753
1.498326	2.23711	1.25762	7.215631	2.8564	0.9597769
3.305566	1.227957	7.576104	1.318564	10.2088	1.005399
10.38673	6.695462	1.663631	1.310048	5.208734
2.378549	3.587123	2.590855	7.47441	1.685106	3.316562
2.302678	9.177481	7.844137	1.575721	2.2216	0.840511
0.7074212	2.200734	7.948003	3.756993	5.832306
1.484763	17.86857	14.06612	0.7618818	5.78553	2.21668
0.10783	4.072267	6.910355	2.247936	2.27172	4.360998
4.404273	2.452105	6.071277	6.263211	1.223395
1.397378	0.7976679	9.093686	0.9694444	1.887115
0.891197	2.364404	4.748679
PHLDA2	24.59976	0.7710064	22.29196	37.50859	68.24502
5.685155	10.24831	9.499294	89.41338	35.85278
11.8573	20.62307	15.92833	17.50167	22.47058	2.659883
48.70638	11.50031	233.4504	128.4094	20.63929
2.151452	152.7236	2.857572	1.635874	13.76355
29.06591	2.213414	29.39873	18.91707	27.81561
8.741224	73.14576	114.7551	237.8594	15.94105
1.123939	60.17455	121.3985	83.64841	47.2129	219.1769
90.43004	130.5542	34.96572	141.6416	32.34134
45.63873	69.81564	235.179	111.7114	16.29107	7.360761
85.41457	19.93291	105.0851	25.55357	12.28913
77.95029	44.32202	14.14574	135.375	7.439493	8.990107
59.62442	10.57682	73.19285	32.98115	164.0076
59.92763	50.98527	49.59621	85.33047	41.93232
40.0726	64.32673	158.3353	33.33671	22.0959	38.00211
57.5597	64.87664	19.31031	32.30316	422.9532	237.8157
114.5519	70.76144	85.0023	136.4365	62.36507	27.07722
61.54863	21.74212	112.8923	19.52062	376.1393
179.5633	99.92591	76.02206	8.495123	191.2339
97.69249	36.78904	66.35495	54.93494	122.0223
16.7224	48.98259	115.9305	131.8118	48.47032	281.8795
10.01236	204.471	174.293	20.54552	134.8683	18.51004
187.9806	196.5727	18.69076	86.31686	118.7948
61.8872	27.22511	71.60562	176.0068	43.66995	197.7345
207.3168	86.77375	178.8502	54.83362	201.2704
25.80875	124.0839	112.1884	125.4197	23.55486
50.69777	43.53946	104.6313	28.51135	25.17138
66.52282	97.57831	72.49845	34.13518	38.5337	59.9648
86.61704	131.9555	10.46266	10.83958	138.2915
105.0918	6.502921	322.0293	110.96	36.89514	33.61541

9.179007 125.6364 63.66551 23.91183 58.46237
47.53281 36.40264 245.0731 3.139701 28.42502
23.19461 141.727 78.91136 195.3277 96.61527 71.74617
72.29872 45.98196 41.80186 158.0884 74.81002
37.74388 66.44102 31.86101 65.75253 363.4257
51.00148 49.16544 74.62671 164.3553 91.7148 55.51635
56.60987 68.23278 67.52823 21.29338 128.572 97.76854
44.76834 78.88432 45.2953 58.10937 76.36491 110.6174
68.83443 15.75274 57.28546 172.1587 8.678785
32.64663 145.8987 25.85643 96.83938 52.36312
69.32331 2.723748 22.59988 26.48168 147.6584
18.12319 16.33572 218.5611 46.27136 64.39371
39.53673 39.85642 92.60162 89.21386 78.3864 94.68152
35.2803 266.1269 14.22005 175.7912 12.42528 86.94378
51.87822 14.4305 14.52747 104.2721 48.92364 153.7155
61.75043 58.76288 35.9938 12.60709 67.7799 110.8623
31.30356 29.01319 233.8525 31.05501 47.32785
2.472192 54.76977 59.6878 151.7436 27.42337 82.90575
165.0941 48.61212 161.8857 46.32746 24.16091
184.9011 97.87897 150.1468 130.6191 128.805 171.4246
182.5199 50.11069 193.9902 30.73414 72.06821
5.71083 5.219413 25.63146 39.00033 110.7253 127.4951
258.5692 79.40921 47.41943 102.2952 111.0889
89.60717 34.12559 91.9444 7.473288 9.29608 82.67183
12.58855 24.62642 73.87291 111.7699 264.8804
241.2366 67.82675 13.18991 67.27827 27.28467
79.02172 270.5617 8.248933 32.15149 38.26212
90.34819 32.05431 160.6217 69.98219 40.26387
93.00093 16.70459 76.55279 26.26058 77.15536
30.52672 63.12873 10.55243 90.43738 16.76867
7.05566 60.67213 47.07248 47.09403 133.1546 226.267
174.8406 33.46673 125.1336 92.46249 12.19048
27.05256 167.9123 116.3767 59.48856 68.22595
7.088263 38.67499 127.7536 107.2548 15.41239
39.4458 93.02799 2.825704 344.7575 87.41092 69.95626
40.94523 51.52745 119.4013 19.43297 171.4174
42.31093 101.5731 15.32594 26.95417 133.8486
9.715028 95.64422 21.1255 64.21705 69.88064 50.39729
138.455 29.94986 49.82681 47.55457 193.1754 181.0871
19.29234 31.27302 41.74057 120.9622 107.0265
44.48909 13.45962 34.44064 96.64331 52.42564
66.33664 32.01705 107.7681 14.64231 366.3552
84.78011 51.72402 19.06743 110.4405 68.6371 46.76079
89.27767 12.44101 17.25451 28.4931 6.513945 113.3941

64.26234	13.86818	187.4812	30.11923	139.7287
48.00644	23.02879
ZBTB7A	37.22371	23.81287	36.8309	28.84812	31.55927
26.16324	26.56849	20.00365	34.34777	17.13763
41.32333	33.52929	19.28117	46.09801	34.54621
32.33941	42.86665	31.73373	39.94508	44.04918
29.24525	31.12472	39.24252	26.86399	26.687	38.27878
31.39706	31.58275	35.73104	7.12889	35.37002	40.52773
41.20767	25.53673	44.82224	25.46788	7.937823
25.63937	27.93606	28.2912	29.77924	39.3578	14.0547
50.71317	32.32555	20.41758	30.17559	25.84526
16.3309	33.06692	27.09242	34.03282	7.930755	31.2285
21.58316	39.9007	28.49341	28.91821	45.48781	25.76872
16.23831	43.81465	11.7958	23.69462	23.45781	31.54665
35.3091	25.33072	30.82099	31.00933	28.24802	28.63451
50.65054	17.32852	24.66291	47.00669	46.49976
35.43363	31.16181	12.69584	30.87887	27.66263
11.30001	30.2986	44.95884	21.56142	33.4754	26.66848
20.76055	24.76299	18.67157	26.0751	29.07339	33.24793
41.70924	22.79654	29.65408	34.50689	21.68635
46.21641	34.41051	31.94893	22.69905	26.58089
31.48603	29.4498	43.38577	40.91373	36.02959	23.91616
25.57023	30.70756	25.63484	38.50377	28.71565
31.68544	22.26147	38.22998	24.13918	21.73597
16.28769	13.92657	18.1007	19.82113	24.8087	33.05066
18.41841	40.85217	29.78986	35.19007	58.15248
43.92749	29.07524	21.03766	34.40266	47.04684
34.52038	15.28793	22.76484	12.32049	19.14064
28.70695	27.05622	19.85247	21.51424	48.45495
27.84363	54.0156	23.50576	27.76165	49.92988	35.65884
31.04692	34.3032	35.44765	24.52695	33.99771	51.99368
18.38617	60.8775	32.47879	30.59992	14.47086	31.8798
34.09277	40.77828	40.26391	14.48718	28.45425
34.62326	25.27991	30.16771	46.81963	28.56383
32.08492	36.86554	27.74315	48.03335	45.27744
18.94977	44.24525	35.99271	45.18213	21.13451
30.39751	27.64326	32.26246	32.74979	29.50163
27.42794	23.39127	25.59798	24.36083	33.70669
41.34681	44.3112	20.39062	18.54399	26.39677	41.0555
12.28837	59.81796	27.85971	25.72395	24.35843
26.34642	23.61924	14.36055	20.03639	29.65885
19.8884	30.18014	41.03627	16.53614	47.01048	40.63662
30.21625	12.85461	30.34826	22.29519	51.91241
13.3073	26.81045	26.42731	33.37953	35.28932	17.61343

36.20941 36.83273 48.75187 38.19958 33.27312
34.66235 23.91251 17.61724 52.37491 32.19796
49.74246 12.90638 23.50696 24.46548 31.32676
27.40434 34.66054 13.60194 35.94364 28.78723
42.04001 28.93551 37.56722 33.37491 22.8299 35.20965
39.18391 30.14007 9.748569 42.94528 35.99653
45.28359 15.2869 55.37457 39.82737 17.63129 31.06003
35.42593 34.91027 36.44992 47.7095 46.94658 36.87628
32.33001 57.10524 47.54706 20.23203 45.88403
36.21204 24.53028 36.10675 29.59978 17.14493
32.49878 32.8866 38.78851 31.99859 27.5168 37.04033
38.65781 34.56216 37.94813 27.61008 27.14667
18.16301 22.92403 30.31525 21.33755 26.53631
31.57486 33.03751 32.91422 43.02633 33.42341
23.41602 35.34134 22.62362 33.9921 25.4214 9.629414
9.463129 47.71237 34.37751 29.53637 36.33249
24.48551 33.77377 40.67077 32.85696 24.9953 19.2768
23.03216 11.88524 20.13162 15.35771 32.81004
28.87317 8.959067 20.94315 38.9402 14.539 46.90851
45.57376 27.17108 38.91793 33.78113 37.46767
45.23444 36.98519 16.90891 40.09576 29.10768
23.35198 8.77042 31.20491 49.2848 19.11591 33.10747
49.78396 25.68577 25.93273 27.71819 34.77885
28.66592 28.34102 43.22591 24.11761 30.2328 46.07874
32.58946 16.72786 23.06092 19.15732 34.58605
29.52504 40.55865 13.59994 45.38061 32.35009
45.62746 31.40883 19.30613 21.4926 25.45939 37.52663
24.94168 5.020892 20.0351 17.71613 48.0267 20.50644
32.45954 32.81509 31.97394 4.220031 43.26608
22.66195 33.50896 34.62759 38.71145 34.64369
39.45711 25.81684 22.21032 21.17823 42.02298
18.12479 38.38655 14.22753 41.29979 32.15633
23.33265 38.28547 25.02337 64.53812 34.05809
37.00558 41.9071 30.67535 26.07253
CD24 102.54 3.072804 185.046 364.6128 210.2947 8.53266
177.5799 143.8752 264.9338 218.7036 657.8375
991.4288 207.3276 698.1927 182.0226 3.350346
241.515 62.28578 139.37 62.13032 404.0539 4.611291
835.5283 1.925102 64.46588 659.5351 263.836 6.339414
135.4655 284.8749 230.4206 67.20171 308.1641
500.8443 651.0832 100.0094 137.4366 221.8372
813.978 790.5614 261.6324 618.6874 596.6062 161.37
551.1931 502.2322 388.8218 108.8852 132.3574
302.263 283.3145 273.5871 54.29368 109.8691 174.1528

168.8267 101.8722 25.23296 259.5994 114.0635
228.2739 133.0774 166.3313 365.5208 330.0435
53.99591 131.9824 161.9261 395.0135 1536.328
0.5048339 650.1052 835.1817 283.554 219.5185 595.6139
477.7101 307.5732 240.9741 217.4116 875.6364
362.6877 402.8514 91.74341 101.6345 279.4987
596.1514 224.9557 3339.33 336.163 29.87281 580.1566
363.7649 302.0217 28.15398 86.9703 243.3657 232.1527
110.0418 405.9812 206.6826 539.3032 341.5651
117.9455 272.8865 240.2054 439.2497 294.0704
301.0263 203.1908 113.5028 190.974 71.8306 101.3529
409.1438 294.9857 88.5602 297.2808 1100.29 405.1355
55.2173 60.1033 102.9703 469.4736 588.1862 203.3462
31.43878 21.10683 122.5341 439.0665 858.8632
541.5225 240.3073 477.1815 336.2884 148.9566
591.6846 160.7855 317.0134 462.7245 2054.362
220.4909 534.7259 151.2 126.4976 429.5436 460.3254
244.2417 83.86917 635.2093 7.374691 466.1175
15892.75 267.9783 160.7635 767.798 350.7741 565.311
98.9856 62.27928 101.8706 449.0701 106.0657 96.47849
892.182 327.578 303.379 525.4287 265.8532 325.1334
7.923482 805.153 285.2134 591.3152 502.8013 227.6664
229.2326 88.20496 358.5267 186.967 294.5058 291.0133
871.4177 346.0657 7221.478 275.1916 256.6944
78.60432 208.6676 401.3759 224.2313 592.3339
69.63378 405.668 488.327 120.4335 97.99014 111.1761
307.6095 120.0942 199.7614 129.3592 510.0282
399.6473 98.57719 752.7619 71.19608 6681.729
239.9915 324.8502 130.6065 494.9589 286.9772
364.0854 413.0842 715.2387 190.6285 250.0384
420.6494 240.8596 401.0928 599.6334 112.2689
487.0065 126.0509 119.3489 97.02471 604.3412
223.9634 787.4316 51.84665 257.8677 267.628 373.9981
84.02213 269.1266 171.8902 135.3084 121.5108
281.1776 254.3751 565.0958 379.3396 224.1306
157.6029 82.88841 293.2254 47.05449 215.5285
465.5493 27.43621 397.1599 72.48712 14.82357
322.2477 237.0051 338.5634 592.7546 254.3659
143.6221 347.3805 405.4905 280.4517 248.8853
88.31092 483.4578 299.3302 293.2026 1056.737
215.3306 742.3035 87.50243 484.4697 196.605 404.8022
49.53091 394.0905 78.15648 1.700006 101.6082
218.1648 87.75295 141.1994 7.911064 396.1743
499.782 487.8993 280.0781 434.7644 450.7782 77.05073

	27.48366	29.1988	148.3955	115.4632	73.15565	162.435
	398.0805	443.1612	722.0533	747.3202	257.9617	
	331.0219	61.6769	843.8275	388.4644	143.5521	164.7183
	421.5752	60.56625	177.9075	146.6183	689.7061	
	35.34596	86.62592	69.66851	294.8701	110.4098	
	563.2063	39.46852	243.6038	125.6562	400.9295	
	511.9322	95.55731	208.4264	418.3903	90.63042	
	582.1799	45.97805	355.8166	247.4983	644.6646	
	355.2882	65.87979	134.1946	296.5296	312.6663	
	295.7951	315.5879	52.14311	280.2721	574.9934	
	780.8455	169.8163	564.8708	390.191	1371.949	151.1779
	352.6652	150.6847	110.3523	429.5793	90.36203	
	387.7315	192.8663	296.8068	235.9481	177.4845	
	88.44121	325.1572	25.29228	287.1435	298.7418	
	28.43821	365.3447	420.1568	246.0319	267.7944	
	4900.747	3407.259	1373.659	76.2772	357.3283	446.6648
	208.4757	160.2085	553.4558	655.064	465.9409	582.2154
	197.7185	258.3335	93.99565	209.2164	684.0809	
	116.7506	2172.234	291.8575	254.2461	266.5446	
	72.09157	112.6317	72.14763	161.3718	104.2414	
	499.0201	310.1763	168.9898	275.0406	168.3161	
	54.9079	1049.898	932.4306	299.1466	105.3677	188.2886
MET	6.421158	0.6118377	9.754814	10.58198	7.732065	
	2.132105	3.801319	5.311021	8.039505	3.542804	
	4.579869	1.386828	3.553921	2.258386	4.832067	
	1.636163	7.102191	3.948871	6.615924	5.735211	
	9.866984	1.05246	5.451222	2.506432	1.651146	2.829399
	5.573757	1.401943	5.927067	4.383709	8.504215	
	2.810376	9.762501	32.77644	16.31783	3.434182	
	27.54232	11.2098	17.9489	7.739398	9.859103	27.93318
	27.1458	6.955549	21.52393	11.74914	20.36849	18.94406
	76.77487	81.04357	15.37539	5.865446	6.882078	
	14.89054	8.740849	11.47628	18.4559	8.52904	37.43517
	9.611745	18.17959	9.305786	13.54278	5.279783	
	14.75862	5.377697	14.26737	6.383405	37.82937	
	4.967143	0.3525642	11.23769	9.355534	16.51293	
	36.78685	4.580195	14.80888	1.589393	5.751922	
	34.20248	21.80569	13.51637	28.60645	16.41427	
	21.95406	25.07065	19.45138	26.89335	15.41436	
	13.89409	26.04605	23.3094	36.57385	10.22242	13.52747
	6.543018	62.26174	19.24779	1.120674	25.48403	
	10.04481	41.97434	5.15996	9.613637	11.05991	16.07406
	60.59581	6.539102	10.59353	11.45524	14.16586	
	11.04248	18.49503	3.078595	28.74259	15.39582	

8.955918 23.93941 6.143324 16.69809 13.69147
21.00827 16.75884 13.24707 5.144582 5.179193 17.727
6.951501 69.06363 50.50988 17.62077 52.92133
22.91679 31.15532 10.72804 25.7683 13.62882 1108.639
33.6527 38.2813 22.97876 46.93309 27.29937 10.05735
10.06013 7.756742 15.97434 8.826689 6.392017
8.276444 14.15 18.76513 61.84418 27.7889 5.276187
5.599551 7.295952 5.887494 29.31646 13.24496
12.4006 11.00495 16.17676 20.96787 4.681175 10.98434
14.8428 12.23035 8.311988 19.27391 1.383556 13.14489
7.81764 14.27801 5.186947 25.49333 34.86562 8.830443
14.70153 18.90895 6.572313 16.68083 6.622369
15.95279 8.406487 7.824157 14.85892 52.98764
21.51431 42.72237 14.15349 23.89283 142.9252
14.06698 21.07145 125.8815 36.38454 13.99562
31.71583 20.4789 15.03454 13.7975 14.48413 12.58316
49.71385 16.19679 4.849788 8.720033 46.671 23.39597
27.71552 12.03292 20.72173 11.12046 47.62369
16.69877 18.55654 8.162543 10.78787 19.27595
442.2887 8.914861 7.630347 6.938941 19.80518
5.561699 29.8891 3.023352 13.61362 18.55805 21.22089
25.97221 9.037416 33.99566 34.56366 11.25619
5.26246 7.983541 13.31713 29.38001 8.595024 25.07969
6.634865 12.95189 9.923797 8.087487 8.405866
16.76766 19.53749 15.41627 63.16935 49.02437
13.36518 31.88799 34.35987 6.773746 33.91064
67.08614 16.40563 9.802702 9.686027 13.58438
22.56438 32.24051 21.47745 19.81228 31.48131
12.61261 9.4416 19.34761 33.54124 14.50377 16.12934
19.56216 19.34393 19.28113 27.7854 4.26555 9.927122
50.34853 13.81035 15.44401 23.96564 12.39163
35.37507 19.20048 23.03837 20.0089 9.161955 11.43119
22.53876 3.818247 4.795031 15.48399 27.26349
9.684036 21.18881 43.57076 15.56675 25.61126
19.22917 6.588858 37.40718 7.963139 15.92922
13.43302 9.688173 13.85618 16.89969 25.02052
23.44027 161.0524 17.11511 5.191851 7.811328
5.865359 18.55084 9.180305 20.47028 32.7482 706.7742
20.34656 430.9574 7.9925 9.763631 9.039147 19.08309
11.56482 16.90737 37.5205 33.15678 15.39946 16.34517
10.12739 4.16005 20.1876 12.2314 19.07125 28.9948 28.20075
20.10583 9.12255 23.96971 16.36818 9.818932 10.03623
64.9594 34.7054 10.72034 4.172448 24.78162 14.67753
6.791983 11.81454 9.253745 23.67232 20.86908

31.97847 3.999036 2.743507 22.26654 8.474739
29.98459 10.16099 12.7821 10.96067 9.107688 20.0219
18.36189 7.035944 9.511362 7.138429 11.59632
19.72898 12.37432 12.71623 13.32935 8.267451
12.93495 11.00024 8.045889 43.75434 20.54296
16.02797 38.94395 14.14136 4.677758 84.24286
31.77835 15.9657 12.73198 105.8461 7.874534 26.84203
29.90522 22.36799 21.29226 14.63862 6.994478
12.57201 5.811564 5.445044 21.77044 11.24063
8.56505 18.44145 11.02953
ELANE 0.2261263 0.9595127 0.4159994 0.2565114 0.7229691
0.9446483 0.2620337 0.7113988 0.3272813 0.2124837
0.1455766 0 0.4146716 0.02650179 0.2764888 0.6072797
0.3101086 0.9044927 0.9597682 0.9055475 0.9578572
0.8726602 0.3775754 2.401022 0.7937329 0.4110241
0.581648 0.4917086 0.4642883 0.8337907 0.4792651
0.2280326 0 0.04451196 0.1840926 0 0.1911781 0.08885335
0.05466157 0.06253693 0.1597823 0 0.02941987 0 0.3783833
0.02079876 0.03288968 0 0.1151239 0.04515186 0.1142038
1.466752 0.3942119 0.06375132 0.4133357 0 0.08300338
0.405661 0.04251365 0.01915991 0.8504093 0.09636916
3.603396 0.09922817 0 0.8427534 0.03856796 1.256989
0.1135964 0.0691088 0.01207052 0.04097315 0.3075771
0.09224712 0.0769834 0.1533587 0.2414036 0.1035382
0.04278653 0.01384452 0 1.435727 0 0.4214376 0.01488666
0.06841994 0.1454187 0.08732273 0.8230683 0.2869404
0.02723856 0.01578069 0.04829219 0.2982602 0.01626735
0.07219119 0.1780914 0.05952796 0 0.3581319 0.8139597
0.02469475 0 0 0.3062461 0.5650298 0.020388 0.4398334
0.03749321 0 0.1968538 0.2042617 0.1240115 0.1402216
0.150159 0.0785456 0.1265057 0.02239341 0.08352452
0.04435655 0.01462548 0.1368402 0.1823328 0.02623846
0.01358518 0.2878743 0.03878009 0.06114174 0.2356372
0.1438883 0.02035672 0.06436072 0.08628167 0.05696371
0.4739216 0.630474 0.05377143 0.01521707 0.217753
0.1112654 0.02888244 0.5208491 0.01099437 0.02976035
0.01333937 0.1828709 0 0.4108194 0 0.5660207 0.02612267
0.5731402 0.0384199 0.09832052 0.5680427 0.2218847
0.5430152 0.6024403 0.03970291 0.3309924 0.3408344
0.3278974 0.433586 0.1028551 0.03482162 0.2243819
0.06763494 0.05283568 0.01924737 0.05219795 0.2800076
0.1138913 0.4975985 0.02876302 0.08805734 0.03359074
0.08202997 0.2849218 0 0.1144782 0.02842858 0.1141897
0.2215821 0.06583482 0 0 0.6165585 0 0.4195995

0.01221887 0.08484115 0.05957243 0.2005426 0.1944559
0.1944174 0.09894627 1274.459 0.08423548 0.05760398 0
0.2392062 0.1042262 0.04156615 0 0.05348067 0.07670462
0.102178 0.309286 0.07201289 0.07957545 0.07230933
0.1952791 0.2886024 0.1091124 0.4204496 0.06182604
0.1058722 0.0223923 0.6514678 0.01465228 0.1318843
0.1042952 0.05933798 0.04049678 0.2218291 0.08017366
0.02918487 0.09873638 0 0.08474298 0.1594483 0.295823
0.03686725 0.03345148 0.08468117 0.09891458 1.46577 0.03595627
0.07979182 0.0865819 0 0.05664771 0.0937103 0.07005147
0.4275501 0.19002 0.07059357 0.4384867 0.1647266 0.05038639
0.1143407 0.01594847 0.1842408 0.2818358 0.02019151
0.1208756 0.1987975 0.1611472 0.03980648 0.491235
0.05179453 0.01979484 0.2165952 0.2335927 0.1167689
0.1074962 0.08567129 0.1998765 0.09388163 0.043838
0.2061519 0.1152326 0.2169785 0 0.08853824 0 0.06707125
0.158367 0 0.01685161 1.11945 0.01990952 0.3404887
0.01302363 0.02062349 0.1096505 0.0779948 0.04751394
0.1615906 0.8509135 0.02280782 0.3222303 0.9776547
0.1324989 0.2598803 0.4791132 0.1625913 0 0.04582537
0.04824411 0.1929064 0.4832714 0.02410364 0.1537743
0.09541544 0.03544949 0.01529573 0.09799329 0.06203327
0.009498915 0.04406671 0.1244501 0.06712021 1.909713 0
0.4238705 0.1237888 0.01030662 0.8797308 0.1127593
0.0941346 0 0.05293968 0.0583442 0.3924055 0.1014648
0.02610733 0.05566133 0.1924678 0.06452188 0.04299608
0.1157204 0.1814842 0.1307848 0.3459718 0.05407534 0
0.3185028 0.1131014 0.05682706 0.01977451 0.1336346
0.02920673 0.03157111 0.6767707 0.1100854 0.03730979
0.03267692 0 0.2636485 0.02595389 0.1594566 0.1205146
0.1515563 0.09464219 0 0.1051675 0.3055047 0.2249736
0.1968218 0.1115191 0.4700598 0.02830144 0.02061485
0.1232217 0.04069262 0 0.06750979 0.059451 0.1953652
0.2414903 0.2928937 0.05375979 0.01400184 0.05190222
0.1580564 0.02632907 0.0350958 0.1705831 0.5405524
0.06419532 0.03371827 0.2697283 0.02813329 0.01074159
0.04081341 0.930088 0.2090282 0.01392253 0.104091
0.1049511 0.05160864 2.399153 0.1541041 0.1119774
0.3195637 0.1771187 0.8009762 0.4361354 0.04114915
0.08886419 0.2765372 0.04190251 0.05380849 0.02679181
0.3426178 0.3589885
KDR 1.155976 1.393927 1.851186 7.559066 3.050781
2.952596 3.507543 3.772348 3.841606 2.361503
2.008441 0.5472476 2.968036 1.094716 3.345313

2.051584 2.787441 3.757619 2.361669 2.711849
4.348049 2.5175 1.606466 3.824105 4.999992 1.465107
5.272626 1.831782 5.584655 1.011284 6.723673
1.481469 4.810642 1.871565 3.756823 2.015036
2.461258 7.090397 5.600356 4.874752 8.290975
2.095373 4.790951 1.581781 9.195364 5.622837
4.896837 4.481037 4.230743 3.9879 3.033311 5.146595
3.792665 4.638537 5.37218 3.304373 5.037306 8.966115
3.195699 3.294794 14.61835 4.004486 8.491437
8.901196 2.689643 6.297963 9.470131 4.07274 5.783815
2.046364 4.910664 11.02809 3.490303 11.47579
11.65598 8.228136 15.22701 6.993027 6.048842
4.058861 2.950363 7.279645 8.334379 3.864295
4.645571 8.079784 5.533939 3.473678 3.475014
2.665355 4.795867 15.57458 4.150251 4.038512
3.556175 5.385419 4.17155 3.666405 2.012248 4.352741
4.13269 3.59763 2.775812 5.499123 14.62262 8.712925
5.029592 7.83946 3.768705 2.085758 3.643671 2.896367
1.865717 1.545602 3.26369 8.516241 6.838473 3.580505
2.771108 1.690184 7.626793 2.864328 5.189383
4.460328 3.286672 5.833633 1.609329 1.531425
5.009743 8.13395 2.577806 0.7743132 2.546093 3.449995
4.966099 5.281114 4.106697 3.016661 8.389333
2.716825 9.297757 5.325854 9.3827 2.571059 11.21305
1.819016 1.886302 6.358471 1.830903 8.945067
3.054821 14.36975 2.669223 11.0848 4.853156 3.513116
4.9509 3.226768 1.056393 1.009605 6.990481 5.182904
7.805019 10.26055 2.865331 4.59376 4.350868 8.298258
7.470046 1.237395 2.424026 7.025404 6.159268
4.22338 2.728657 2.839447 2.977508 2.694145 4.660415
21.97451 2.918256 2.78745 3.605548 12.49111 3.538392
13.5774 5.947553 1.052123 6.885906 4.702094 10.22004
4.256207 2.023323 5.990173 1.329161 24.49271
6.628748 10.5147 2.835959 2.249967 3.73951 5.287708
9.776221 1.845655 2.547317 2.492651 5.082169
7.855007 2.929314 3.276151 6.727944 8.834965
5.552388 4.198635 1.18732 5.528123 3.428081 4.019824
10.2233 2.928761 8.928723 4.686228 7.365447 2.208242
9.938383 0.6452321 6.480711 1.027065 1.917309
1.28282 2.435517 6.588937 1.789314 4.895321 2.025053
3.349941 5.884834 1.994118 2.60271 3.621491 2.458259
7.094313 5.18687 3.463155 4.394949 12.35452 4.72284
1.647135 4.927441 8.360048 9.707022 5.917298
6.817226 12.5908 6.075507 5.292252 6.756878 7.781407

4.09276 10.90778 9.085385 2.652958 5.582208 1.668924
11.66876 6.285375 2.134764 4.972242 4.116992
2.402435 9.245654 2.77269 4.674967 2.380328 2.905134
2.784925 2.705534 3.164022 4.855426 5.859364
7.948321 2.398552 3.078431 4.825687 4.752346
11.73762 4.073624 1.732456 7.425132 9.437375
2.891939 3.120124 9.527357 23.05989 7.978284
17.79602 1.864868 5.804691 1.75556 8.282333 12.81171
18.57868 2.460435 9.006023 2.425144 5.715604
5.597237 4.889588 7.761646 6.47768 3.302187 1.603089
5.615655 6.345541 6.014885 3.173419 14.57769
8.816079 9.853749 6.586169 1.465702 6.312893
2.102016 3.440173 8.125481 4.032914 6.326884
6.768135 5.415066 2.464932 0.8803743 5.210264
8.898715 5.52289 4.254195 2.27589 0.6494789 2.720224
12.92901 2.722263 9.535644 3.220175 4.672294
2.053398 5.634178 1.10293 2.061014 1.996514 1.871869
4.046819 6.863041 6.490284 2.953226 8.333334
7.507928 4.02282 11.97456 3.686349 1.889752 2.970465
6.32217 14.5337 2.893584 3.016797 5.799735 4.079162
2.258671 7.875283 3.899266 5.596053 3.321794
2.637335 3.004304 4.229169 4.592629 8.370572
3.37734 8.346643 6.694101 10.51836 5.796906 14.25483
1.306883 3.065841 4.117727 2.000946 8.338199
1.421325 2.652554 3.887264 4.554541 3.345346
1.409626 2.933217 2.554383 11.4041 8.09134 6.154313
7.669144 6.785514 3.491211 4.915986 2.202309
3.231061 1.797555 7.327962 2.78409
MDM2 2.30851 2.230488 3.359874 4.485742 2.999284
3.638219 2.50977 2.973989 3.060359 4.233553 5.016425
4.64722 4.39151 3.241018 3.226405 2.94774 2.783911
2.994988 1.981352 2.141458 3.196414 2.880118
4.389293 3.895998 2.683158 4.701108 3.087583
3.825617 3.217304 1.693917 3.068529 2.920972
4.740923 4.602375 3.246381 7.27053 3.652732 2.208053
3.49523 8.203945 79.15273 4.232198 7.305137 7.309846
6.356012 5.487442 6.328819 2.939006 15.16456
3.449823 4.22671 6.893492 4.961433 8.22599 7.777035
6.36873 7.21616 5.479185 2.949163 2.910013 5.504043
7.360835 9.456175 2.837145 7.144495 4.002338
5.563485 7.532795 5.307449 2.915588 15.32528
12.97599 4.387578 69.39635 7.994727 9.246793
5.496016 7.51655 8.899308 2.267839 3.10609 5.880325
11.32263 6.74421 9.48671 6.307056 8.768045 14.96828

4.466042 2.958217 13.75274 17.99477 9.378684
6.846031 5.106431 13.4078 15.02617 120.1938 9.765425
11.25032 8.084394 4.798764 8.602808 7.065212
6.008789 7.20336 7.547908 2.94733 12.50502 5.863403
3.663781 2.792259 6.398686 24.64307 5.652125
20.21598 2.378446 4.790357 4.172513 3.379323
2.477324 8.3456 8.997578 4.680112 7.617773 7.938436
11.87698 3.381116 8.194345 3.897435 8.695942
9.442396 4.085269 9.130355 2.620769 4.61518 13.83071
3.464993 3.14895 4.91775 5.462653 4.586082 14.25954
3.585355 5.62855 34.96243 2.242228 5.756278 3.077292
5.556805 6.262856 5.199821 5.326394 30.63611
4.633891 39.0273 6.81222 6.702087 7.810522 3.076262
5.736568 5.733406 4.063354 4.595486 1.545353
6.546475 8.887747 45.23154 5.077385 5.260972
4.21897 10.33688 8.476296 3.124362 6.075556 4.973428
5.067783 3.358963 8.572662 21.18686 3.994077
2.552613 4.75745 12.80955 6.413997 5.760122 7.793929
12.02579 5.473235 25.03776 3.906921 5.281776
2.87788 4.268873 8.486906 4.514721 7.806919 6.069566
34.0409 2.289441 2.53478 3.019066 5.606978 5.793892
4.026124 14.59402 3.158898 7.53175 4.564198 3.836564
9.75028 4.182493 5.461094 3.178711 6.864705 13.24008
4.002698 4.208265 10.78163 4.119418 5.650663
4.169255 3.935115 8.858244 14.20277 5.473219
4.096942 3.747118 4.288246 40.81549 2.389599
8.399657 5.682218 4.091788 7.542385 3.856537
3.457769 11.30548 9.400884 10.67118 4.186779
21.27885 5.597297 8.195246 13.51963 8.585347
13.20402 4.295519 6.261062 7.820508 6.173432
3.915636 7.674973 16.32163 36.3867 5.990262 6.073648
3.206462 6.52472 4.005438 4.356501 4.921315 6.767474
8.462468 6.249268 4.9704 4.625739 3.815354 3.424724
12.53082 7.214891 4.617107 4.361684 5.942461
3.989441 2.132675 41.6563 4.320995 7.29486 4.361862
6.99005 19.17402 4.298475 5.659736 9.188462 22.6537
6.24957 2.099427 8.231194 9.580965 7.65679 3.985161
2.886367 4.666456 9.693236 2.673598 5.188221
12.87887 6.151644 5.222733 6.819747 4.549507
17.55201 8.933412 4.568849 2.829777 9.679871
3.866155 5.667395 5.143246 5.049514 2.14668 11.25785
2.911231 16.34894 4.586025 5.347427 8.146966
5.918858 16.63795 3.563727 6.7797 2.780963 6.864034
4.352808 3.288781 4.616283 7.800028 4.43475 11.12488

8.617101 4.545231 15.01185 12.38481 3.420915
6.235124 2.471708 4.342588 9.071823 15.72065
9.131073 3.062139 16.67262 8.158401 4.604436
21.46463 6.350511 7.622899 3.549658 3.282002
4.231347 8.362062 56.15561 5.635595 13.8337 8.539588
10.42595 4.790661 8.590185 3.403633 4.004784
2.828773 13.96497 7.117776 8.248376 6.398926
6.123705 11.92266 8.641893 3.453931 3.084915
3.259933 7.345844 8.606189 6.057915 2.5408 7.611309
2.973695 7.931762 4.665859 4.009386 5.429299
7.288201 2.658302 5.020765 6.061251 2.557269
5.025168 5.688065 3.950329 5.518358 11.06667
4.001738 9.899779 18.25218 11.25591 5.443076
6.80651 2.269519 3.829206 2.31805 4.943763 11.35555
9.811112 9.587712 2.458994 3.683139
PRKCI 6.608892 3.4583 8.250388 41.48615 6.391392
5.274834 7.109737 8.844785 9.246818 12.8613 12.38139
14.99744 9.314689 10.36628 6.991004 5.659088
7.978349 9.2479 5.945863 4.303095 27.67041 5.400275
9.524819 6.418947 6.65254 13.12857 9.898242 6.042022
7.243659 11.76217 8.448993 6.069019 32.43966
12.33069 34.59285 15.34368 9.050996 18.5466 13.54152
23.4721 40.97666 11.07971 20.95722 16.28618 17.04817
41.11136 28.92511 14.03619 30.06298 17.97522
11.70368 7.833506 23.93351 19.13768 9.743107
22.33055 9.327442 11.10961 8.29536 15.8522 28.94273
11.54007 7.758893 4.36015 16.92306 7.439711 10.8979
6.269764 48.76458 47.97071 56.96096 22.69353
37.22363 19.87425 9.418399 4.894316 29.18041
57.89469 18.31372 13.08209 11.54315 15.56044
30.45056 9.754879 48.74532 33.89745 18.85969
17.61852 34.42502 9.923392 11.64202 21.02183
11.82464 11.54426 4.993088 27.83002 12.71827
19.30867 14.1273 21.36725 9.840973 22.98275 7.284268
10.77983 32.75231 10.78683 11.59358 7.530393
69.30397 13.41013 25.63031 12.30072 8.874327
5.294936 11.12601 28.84239 4.83978 18.93736 98.3105
8.956809 10.18549 12.15949 12.6985 6.3219 16.03374
8.494845 12.94966 4.826209 12.26174 15.66171
14.15714 12.65261 37.06235 26.01606 6.767242
12.55135 16.13075 12.68646 27.53301 12.14109
14.38554 19.32623 21.8556 10.5619 48.81558 24.37393
7.636349 10.97306 34.21865 7.500513 21.11181
13.89206 16.12238 17.4673 9.606588 50.75862 19.81394

15.73924 28.6352 8.72142 9.301567 13.89689 35.67744
16.63819 13.0905 18.10644 12.83998 40.6503 56.32709
8.607934 5.059472 20.81484 13.36462 13.36171
24.44011 27.37397 14.11222 17.03155 38.47502
24.18317 23.40818 12.25419 14.65762 28.4501 15.76236
28.20374 24.45021 12.47562 43.32643 17.50926
17.40409 22.07267 10.83555 14.1382 19.85437 16.56315
11.16159 13.84675 15.70905 6.233694 9.120314
10.64129 15.85275 8.713002 7.012277 40.04042
12.20749 25.96191 14.81388 18.79246 15.4592 8.526052
17.21429 7.544227 23.44304 15.41433 12.9767 51.53033
35.309 15.12185 34.64748 49.36891 54.84833 14.89254
8.855872 9.381363 17.00424 7.176839 14.26327
10.70366 8.758297 5.813817 11.74749 16.72994
14.31347 17.74991 4.080966 12.35898 12.54614
18.32391 12.69772 17.59677 16.24866 13.29668
18.49276 16.03114 14.11398 8.043362 82.12547
28.20691 20.44786 12.65064 33.19947 9.789697
18.23378 41.49907 22.91702 10.75931 11.47322
12.25034 22.49754 15.91069 15.26712 16.84551
20.1576 16.7547 49.5611 6.794711 27.62011 35.8816 31.00312
13.23311 19.13894 12.84202 11.57086 13.65245
15.00334 7.378159 2.920129 49.68075 10.89202
14.56972 4.602817 10.12201 20.81257 24.38809
17.73316 11.8076 17.44435 24.07064 17.38513 5.537119
7.155018 10.38351 8.667392 7.125261 8.296145
27.07866 19.28778 25.02673 21.78111 19.38967
21.07088 11.17963 23.9718 8.116955 48.30538 16.11863
71.32743 10.65595 13.96206 8.463896 16.61849
3.729464 21.59649 9.061834 16.70751 24.39927
16.37393 31.08275 8.767484 13.80846 29.68239
21.25049 13.5004 12.77706 12.8632 13.52208 17.12999
23.74472 23.34731 12.95986 18.20867 28.85917
4.814376 11.67988 5.249088 22.80118 21.34869
32.32455 15.03124 6.807163 12.50379 16.06919
19.83178 21.57619 48.71409 13.38844 18.4879 13.51968
11.70274 17.34605 52.61624 19.85694 22.86974
33.71663 36.3081 11.9114 5.32832 10.29196 34.45856
5.046483 17.9614 36.80766 7.983795 14.2257 22.43273
21.0422 42.39345 7.688203 15.61404 13.10545 20.79891
31.90511 22.84576 5.665496 23.37311 27.68426
29.22443 14.33545 24.56681 30.03163 9.624834
10.01912 33.2594 13.558 8.286955 11.4559 6.331644
8.440816 18.55526 21.78704 14.5609 42.74411 14.82896

42.73168 44.86599 13.68507 13.63425 24.30824
7.378883 4.323023 18.72812 20.83432 13.01183
10.58597 20.93858
NFE2L2 27.35871 16.35565 29.25816 67.85874 25.81375
23.52357 41.07313 37.69323 34.26162 33.15407
77.09366 84.22702 43.53971 74.87842 34.687 20.06524
26.72229 30.6198 29.89523 22.55507 63.98179 18.40421
41.32524 22.03063 18.65985 68.30299 37.59061
20.3823 36.24167 49.66248 34.14897 28.20712 35.7769
44.76339 23.35322 28.47691 22.43014 12.84142
27.3752 14.28765 27.05813 27.18099 20.24841 24.98625
27.14143 30.146 24.79953 13.85588 24.79673 29.05762
16.22464 19.37477 17.71635 29.0067 28.80105 29.83863
26.5801 19.32002 20.6374 24.74978 28.47059 22.04372
31.49784 15.54239 22.59503 22.6421 17.45328 20.18762
24.31391 38.79717 30.00951 31.34901 28.85986
36.9686 20.62003 20.32953 21.38867 53.99695 18.05838
26.85759 15.67503 29.37271 32.37399 28.62167
19.82108 26.41912 38.81649 23.31049 19.39579
27.95383 38.04451 30.98809 29.28512 24.98635
16.36203 21.13301 20.67567 23.05422 42.06848
27.77791 22.53775 25.92331 18.91292 13.9034 23.13286
26.36572 20.4611 22.4751 25.59435 22.01252 11.94914
18.45543 33.71425 17.83532 34.4579 20.08927 21.71543
19.22219 20.81777 25.40116 32.55444 21.55392
20.1711 18.94751 54.55657 26.33642 28.77317 21.9578
25.57128 16.41575 20.0692 24.80414 46.18583 33.36102
22.2505 22.65573 29.56382 22.46658 18.79725 31.71824
30.50391 23.59318 22.36814 20.02223 22.27453
42.27956 17.04624 20.67091 39.44648 24.05674
40.70111 22.48612 19.37901 24.00474 24.39886
24.24799 23.33433 19.08473 38.12502 16.49554
22.5945 44.78756 39.74437 18.51556 17.52412 29.22176
37.39441 42.12848 22.2782 29.83476 20.40667 23.00316
13.09505 28.7252 15.18052 23.66963 23.03626 16.52647
33.97767 12.76852 22.97299 22.16345 52.09594
23.74566 17.12728 27.99713 30.11007 23.16874
33.83148 28.46024 27.60518 39.2701 17.11455 34.31051
20.02706 28.39572 27.53161 16.91619 22.17539
14.6882 18.39354 19.88937 26.44272 14.37164 25.19946
29.86189 11.14688 22.3064 17.25368 26.03193 32.09453
24.88965 28.41021 28.05998 20.32749 25.96075
22.15583 17.97768 32.35312 30.33166 46.0563 37.45998
16.08135 19.17038 25.36748 19.88537 21.02251

75.43114 21.57704 20.07301 18.34322 20.55687
22.69599 19.31157 20.33694 24.48312 16.94817
28.29774 29.51509 28.66638 22.51617 28.0139 22.7116
29.5748 25.41195 25.21276 22.71333 20.15379 20.22919
23.23987 27.42018 28.70432 16.35915 24.99524
31.22037 18.34227 28.48423 27.48268 25.88679
17.37541 21.05435 21.1421 55.16221 23.18648 21.43614
20.50803 32.91291 12.90832 59.69347 5.848938
27.04435 18.35631 45.47629 25.18673 20.75379
14.24929 16.07547 18.51756 10.96542 26.72257 25.924
23.28766 16.46144 33.97342 21.39243 28.35929
20.75109 25.68432 19.44175 28.80566 18.64068
16.15492 28.75394 23.35902 20.05129 20.01746
23.74557 24.86896 23.49318 20.22701 23.62121
31.36564 29.0119 32.93003 26.8021 16.40371 31.0404
23.82713 26.35494 18.85036 24.36639 8.18601 26.47119
17.20979 35.81191 22.22179 31.62216 22.97489
46.87219 26.66721 24.98647 26.30647 13.92863
22.44028 33.15442 31.34932 24.3538 9.870001 19.61031
25.44867 32.62818 24.8612 24.76838 38.93913 16.13225
36.59709 13.59549 28.969 23.50305 41.29204 53.57275
17.82646 31.94782 30.3544 22.85396 42.70765 29.348
17.37182 17.57131 13.64034 23.97194 18.64799
23.09321 23.68948 26.16995 18.95045 22.55502 17.509
22.88249 11.33471 24.14012 15.7639 24.49063 37.02143
25.18728 15.74677 32.69843 24.90347 28.06883
13.21884 21.19111 36.22273 20.2865 25.79538 30.8987
23.73926 32.50761 22.96616 32.01494 19.06002
21.71062 28.21186 28.86891 38.56378 20.42623
28.5041 18.97988 20.7154 19.79409 24.61494 22.73643
33.01473 11.72006 26.36637 37.35907 28.80909
14.35855 18.98543 34.13314 26.13145 12.94426
17.41039 22.3556 29.10914 24.74586 18.70003 27.72894
RB1 6.636674 5.076035 13.13219 10.32403 8.402381
8.812903 5.487261 7.663233 7.286282 12.7204 17.36124
15.97678 11.8235 10.61225 6.880385 8.231842 6.370289
8.380273 5.362199 6.523915 12.16942 8.044998
17.46752 10.69014 6.708536 15.34056 7.064386
9.970384 7.518431 7.037976 6.648726 6.320546
12.82609 13.44043 22.94457 10.87267 8.917749
22.33453 15.53777 19.17072 20.05323 28.63795
20.32584 17.64283 13.66324 15.67973 20.16907
17.62367 13.91814 28.67995 7.794502 23.31757
3.751986 18.13431 14.78584 17.85727 16.02051

14.97322	10.29136	14.16007	17.98672	12.75078	
22.74744	5.087375	13.55367	8.59533	10.13851	11.76332
7.453554	9.695051	29.49758	15.3283	17.52439	14.93502
17.36244	19.24766	24.45278	15.65857	27.14187	
52.48759	17.52227	10.7482	28.49349	10.24363	18.19599
16.76929	17.74789	16.02985	11.96483	15.35945	
25.74535	25.15298	16.83113	15.77938	11.0573	21.32904
18.31761	9.207979	47.68422	17.91324	20.57274	16.514
9.029551	10.89315	11.35357	13.85875	12.95108	
7.434911	16.51516	10.43651	13.10577	17.74141	
12.82898	12.15057	12.37777	16.54613	5.649672	
29.31381	11.67414	16.58173	20.73557	17.91302	
11.7629	10.791	4.869498	22.4003	16.57796	6.278749
13.02775	5.370958	16.44386	10.94223	15.76302	
29.83702	8.281754	20.11787	16.38643	19.37833	
17.21266	17.83665	29.3735	20.88018	8.887511	10.55825
5.957287	7.603147	17.34974	10.24264	48.05097	
14.42889	22.82316	13.97969	12.59969	24.72594	
12.04744	7.520671	12.59044	11.43336	14.50906	
8.178916	9.829348	14.43816	31.23481	7.063712	
10.70011	20.56501	11.35331	13.56556	14.3081	15.15235
9.886165	9.898804	23.59189	11.61119	21.40914	
11.33504	14.57619	4.968367	9.15548	23.55687	9.666519
18.78592	15.93814	25.96763	17.61411	15.41375	
14.05195	10.22862	28.06977	14.7132	17.23612	17.12678
6.351875	8.529472	10.347	22.42213	19.87846	13.6182
23.49382	10.37433	11.55251	10.93233	22.84613	
12.34289	12.16488	16.83941	7.114407	23.33103	
26.80645	26.38162	20.78857	17.69993	28.91021	
12.09653	19.29234	23.24282	11.23828	15.70938	
17.16734	22.94663	11.62152	19.15742	15.47976	
18.94605	17.89132	10.43997	25.68582	12.0055	11.29527
9.581465	10.18444	13.69055	15.24521	21.79382	
15.55341	8.531648	9.257959	14.47604	24.68359	
2.130531	22.27954	21.6312	20.1788	15.95747	18.4259
13.63625	29.42883	8.237971	13.87253	17.17561	
26.87417	34.73014	13.32898	15.77171	28.3813	20.3441
23.7544	11.29556	16.68727	11.13513	13.40398	13.11526
21.6921	16.49238	18.7745	17.35065	20.44357	12.86176
10.7477	14.01128	19.10032	14.94261	7.228158	23.78464
7.742561	8.89979	16.13473	11.77192	16.83161	34.75082
15.27146	15.32785	8.618913	17.33598	29.31154	
22.37878	18.97937	18.30178	14.54287	16.38893	
15.21985	11.01019	12.43011	20.42962	6.763009	

8.642599 15.47302 20.46452 17.722 18.2293 18.85793
13.91129 26.06586 15.36853 18.74808 18.90727
7.537026 18.95536 28.56996 12.75814 7.686559
21.09976 23.79026 8.026226 27.45429 11.1248 12.82038
26.33602 18.34398 29.79182 28.01933 19.43429
7.375066 10.9086 26.15613 10.68589 15.20506 23.63986
11.29924 23.43055 16.58663 14.46784 20.87661
19.6993 5.119552 20.31552 9.526992 11.60861 23.3705
18.92034 19.39236 14.76741 14.50123 12.84736
12.1658 15.49201 23.65472 8.523665 9.55836 4.800819
30.9969 17.66058 18.14857 18.24554 23.70008 22.49506
17.90187 7.493834 12.93924 6.381095 20.78255
8.549785 13.17218 12.94746 20.87975 13.86715
14.67926 32.81569 7.763702 7.099288 10.23904
14.67102 14.17013 71.25549 16.6817 10.02032 8.064165
10.33404 13.05764 12.60136 20.61406 15.33183
2.733365 16.40608 10.37827 22.8314 7.410437 12.26796
11.81367 4.583598 21.04123 25.2096 5.555901 22.10465
18.35302 24.53437 26.81226 12.26713 6.26843 26.93461
7.119829 12.98972 20.30197 25.51185 19.89639
11.76131 11.1418
FGF2 0.255189 5.403272 0.6785549 0.6432303 1.229194
10.03349 0.351209 4.700687 2.367117 2.162838
1.951192 0.4128804 1.274956 0.5026539 0.5383949
12.65884 0.3849882 7.11078 0.8367092 0.4900694 1.957076
13.30318 1.544133 9.744207 6.491822 1.161365
0.5193524 10.96923 0.6796206 0.6666349 1.569561
9.394002 0.2587478 0.7496931 0.9986104 0.244448
0.2052894 0.3447566 3.380649 0.2220426 2.375956
0.07183576 0.2678403 0.06479387 1.157107 0.3392578
0.616326 0.6361755 1.576509 2.543466 0.2921112
3.30193 0.2208571 0.4385212 5.75744 0.08699415 1.180731
6.885109 0.5827204 0.2325772 4.143769 0.2310356
3.562476 0.4491802 0.1416509 6.87762 0.9099988 1.723057
1.437091 0.3535339 0.2307705 0.2207046 2.409869
2.775416 2.274462 0.9773308 1.781512 0.8762852
0.1119902 0.3130026 0.11186 3.886145 0.1998408 2.67914
1.606019 0.4464111 2.442596 0.8744904 0.9329847
5.860793 0.1715205 0.4621332 2.084697 0.6661728
0.2764513 0.2278407 1.047644 0.2867798 0.2645417
1.699783 1.511601 0.2547988 0.5188091 0.5684788
2.996083 3.270849 0.464034 2.46396 0.3261697 0.08290822
0.5662214 0.6319233 3.206384 3.633542 1.40503 0.2979516
0.129568 0.1732903 0.750907 0.186208 0.2685216

0.6598131 1.156049 0.5573784 0.3236301 1.08369 0.3412877
0.1113276 7.383253 1.363557 0.4015456 0.3295934
0.6153188 0.5877477 0.4650191 2.68134 0.6241612 0.1062118
2.841492 0.38549 0.4806096 0.3409609 0.8257708 0.3228702
1.299434 0.7856092 0.09743176 2.946264 0.04913729
2.090561 0.1565664 1.264027 0.1127059 1.434317
5.444173 0.4040103 1.205252 5.253586 0.5241136
0.7605184 1.664618 1.87641 1.684221 0.1853289 0.7555597
1.132044 0.3865557 0.7796519 1.612109 1.998535
10.03396 1.041811 1.075343 0.2967749 0.08462169
0.3261996 1.255048 1.26918 0.1114016 0.7518843 0.3127354
0.3508616 0.7108568 0.8191309 1.062834 0.7498429
4.313465 0.09574836 0.926718 0.1390516 1.620198
0.519753 0.1890957 0.4852039 0.2433733 2.539166
2.274195 0.7711453 0.2338084 0.3055298 0.181479
2.49345 0.180801 0.1137891 0.3124219 0.3275469 0.8242909
2.368695 0.4352519 0.2475237 0.7762553 2.621163
1.052972 0.6078453 7.530061 0.630412 0.406201
0.2174516 2.347688 0.2667904 0.3882207 0.5584024
0.4636858 0.2488624 2.335567 0.1946415 0.2391307
0.3645547 0.1360512 15.52011 0.5758121 2.595936
0.08391042 0.4889627 0.1696073 0.5643285 1.019368
0.5237568 0.7119008 0.324057 2.076694 1.151784
1.413375 0.3401348 0.9373178 1.040031 0.6248356
5.728318 2.865015 0.599395 1.085301 0.2274734
0.5151828 3.086795 1.159628 0.4637648 0.7576589
1.333721 0.221467 2.223693 0.7171345 0.1261494
1.040722 2.007018 1.214938 1.588678 0.2061314
0.6088954 0.5822671 0.6962143 1.147597 0.1923322
0.380112 0.1234753 0.1328502 0.5222693 0.9505328
8.813754 0.1954271 0.9051667 3.348977 0.4440807
2.317492 0.6027199 0.5022513 2.391676 0.5410051
0.4902456 1.980368 3.348209 1.366988 7.200663
10.28164 0.7036619 7.669671 1.835622 0.5201401
0.6028094 0.3841688 0.5380412 2.498639 2.270829
0.4681406 4.174635 0.4488117 0.2169491 0.6730575
0.550151 0.8772521 0.1772813 0.5950925 0.1348811
0.2770167 0.9501079 0.3450557 5.74911 1.450987 0.4097334
0.9278327 1.484245 0.5027774 0.5939615 0.8434404
0.5046105 5.515297 0.5080579 0.2158951 0.2871549
0.6220447 0.3289502 0.1674488 2.963917 1.620469
1.029712 3.020258 0.9353797 0.06126893 0.4697462
0.8623586 0.2256251 0.1260198 0.4142292 0.6115696
0.1077845 7.143327 1.236077 0.28872 0.6776221 0.4209487

0.5673368 0.3229239 0.9238103 0.5229854 2.4836 0.2082268
0.07907304 1.563837 0.3368503 4.76444 0.170892 1.097062
1.066297 0.1674777 0.2001908 1.185921 0.2675603
0.1055045 0.9705766 0.3713545 0.5592759 0.5061228
1.322804 0.1325546 0.2400753 0.6536539 0.5169578
0.2357064 1.720052 0.2680771 1.532486 0.3292334
0.8373544 0.6343691 0.4247441 0.2803373 0.2012658
1.731737 0.4884379 0.06548828 0.377086 4.113878
0.3341142 0.562939 0.7066826 0.5550331 0.6896182
1.218972 2.709863 1.232541 0.2341402 0.09977973
0.5430141 0.3147234 0.1730894 0.3394472 1.097501
5.057281
HK2 2.065249 0.2291006 8.791049 13.17599 6.910577
0.8637939 5.441607 4.996283 5.851542 4.298543
12.7631 13.04793 4.791311 8.90906 4.976222 0.4579461
7.895235 1.802128 2.606235 2.016137 8.275822
0.1574822 22.45479 0.2895096 0.9550309 30.54383
5.628008 0.3296388 5.046604 1.391738 8.009644
0.9170358 15.68001 7.976009 6.91654 6.418071 1.229067
1.824759 12.84152 16.51302 40.97311 7.592872 40.393
38.30249 11.23532 17.19235 33.79108 11.59889
12.86374 80.20252 8.781324 4.242894 10.60265
1.862483 2.223579 12.58223 17.20902 2.097476
9.753647 13.83684 5.549019 32.61843 2.009177
16.22449 7.124971 2.62682 7.48301 2.416647 22.88505
47.62756 22.48221 7.855898 12.08747 43.17483
23.32332 14.19066 7.796015 53.38829 19.85434
9.176742 13.02662 5.440981 16.99177 9.652363
22.37479 2.090121 3.473482 27.63793 9.775358
2.213283 36.39146 28.39482 12.78768 10.23184
13.16813 6.978231 16.5191 20.16026 27.16932 15.07366
2.87672 50.1005 11.45477 4.417189 9.315284 9.267816
57.35001 1.873017 14.00235 7.448662 13.74332
3.312696 19.75748 1.384005 24.41203 9.935227
5.685841 9.911992 11.48335 7.574691 11.27872
12.06803 8.118285 19.63826 10.88642 3.30691 14.98917
15.69529 2.188176 19.97538 22.64529 19.79211
18.35189 27.32626 19.48016 5.753259 19.82188
60.47531 2.979535 23.42379 19.47584 37.23105
23.30523 5.103145 10.98115 9.637496 11.47027
6.208574 9.938759 10.43332 17.92857 12.11357
30.42286 10.1483 2.468717 23.32131 6.717852 1.912077
24.97067 2.3647 6.976932 6.535423 4.282815 12.39736
7.986945 6.616773 12.21492 11.60152 11.98794

0.4247453	2.355615	99.43052	2.954996	30.05111
6.618047	12.27539	7.038822	7.931779	15.25368
6.578945	2.667514	13.7079	25.02817	8.303525	14.84751
13.54332	3.236453	23.2816	5.228249	31.52677	14.46078
18.41051	14.65271	15.3581	16.31864	19.04464	10.19705
9.108276	26.36636	2.641288	3.15564	1.13474	14.27857
32.67652	7.076259	52.4964	9.417246	52.74356	7.859911
21.08043	3.147304	16.77644	19.31883	1.136262
18.97341	20.83845	1.128159	3.228546	7.665548
16.09337	10.40751	25.20504	10.21016	38.6632	4.732038
1.73388	12.18833	12.152	11.11962	18.12039	6.376492
15.43766	4.90619	82.3504	11.42704	3.393945	3.009427
9.722304	10.25595	6.83741	7.007209	7.144786	11.35866
11.17483	14.34113	12.68968	3.933279	0.9279822
15.76731	11.56383	14.46232	5.342443	10.82462
6.59774	23.06225	14.54893	11.59717	5.178094	19.61926
7.763234	9.452178	18.28869	13.08702	26.67048
22.08584	5.259243	31.79387	5.424058	5.74343	57.09977
3.97751	16.63715	9.924803	19.49075	20.23107	20.07114
32.64856	0.8782863	3.023377	7.736136	4.177611
25.08927	7.308626	17.7046	31.30496	3.884999	51.70208
6.729788	23.30989	6.738869	22.56101	0.7995276
1.248947	8.259846	6.336753	10.80249	9.320988
9.207933	13.99893	5.882449	15.4207	5.811505	3.538624
17.32476	18.81391	13.44543	11.61669	18.75199
8.225768	23.67791	6.878382	13.33658	12.89191
3.302813	13.87539	2.359294	6.749397	6.532895
25.26762	11.08462	25.15966	7.365423	21.8554	7.373594
5.780073	8.51441	18.47127	13.38967	27.29016	11.55061
8.91434	16.1609	8.006292	4.23472	2.783962	2.814308
11.56142	2.89193	4.824538	14.70571	17.13013	8.433914
9.726777	50.31504	1.566776	18.4103	9.451577	17.87246
3.123712	2.523237	15.54462	15.15506	22.2894	12.85772
13.89831	33.73393	7.759868	12.62657	1.847001
21.73439	11.6488	11.36335	5.625424	7.954015	9.913399
5.355473	17.20449	24.86912	29.90103	6.067455
1.555669	10.14962	3.925697	11.39833	8.653548
15.3741	27.42782	8.216422	11.9721	6.605112	9.679088
16.58048	4.821204	8.928125	5.77065	16.45659	4.066655
6.300435	32.74329	6.482711	6.687758	27.15385
4.817705	9.152285	50.64024	8.099022	6.58214	10.56157
9.26047	12.80062	4.850836	3.014107	22.9728	6.250318
18.57403	4.623997	2.13572
KL	0.5344784	1.934421	0.5339249	0.7383994	0.4786796

2.03127 0.5327742 1.570416 0.5367862 0.8964576 0.5352494
0.1016185 0.5990603 0.2290078 0.3372989 1.973452
0.4127049 1.343581 0.4612465 0.9144549 1.148378
1.35077 0.2814888 2.29652 1.039155 0.2778156 0.4866948
5.723601 1.088381 0.7693992 1.047539 0.798495
0.2635072 0.5068166 0.2807266 0.125984 0.179404
1.811753 0.6468315 0.2721049 5.986959 0.06586166
0.2392692 0.0498772 0.9853463 0.2917915 0.3142994
0.3181319 0.371591 0.1560668 0.07297785 0.9407614
0.6165559 0.18579 1.84329 0.1518962 0.1645457 2.515644
0.06627061 0.1986779 1.167942 0.16459 1.010259 3.096916
0.2016743 1.179985 0.6939944 0.7202554 0.4648217
0.1324846 0.1251648 0.5789888 0.8271997 0.55464 0.5053515
0.3793728 1.222609 0.3017406 2.968696 0.1632645
0.06495301 1.735801 0.1631334 0.6559554 0.2663582
0.2448395 1.572961 0.237913 0.3740732 0.8491932
0.1606084 0.5839605 0.1841044 0.5894508 0.07607303
0.1115537 0.359315 0.2693004 0.06510927 0.649522
0.9462085 0.2560713 0.5098134 0.3171116 1.337254
0.9927662 0.2943195 1.062406 0.1321359 0.08428 0.3915476
0.2261136 0.1563291 0.7666091 0.4274305 0.2828991
3.552781 0.1229334 0.3247891 0.1352805 0.1605794
0.4791692 0.2833391 0.2507392 0.671209 1.1985 0.1682107
0.04661816 2.257113 0.5053268 0.2421306 0.06543 0.3917943
0.2895505 0.4689474 1.036506 0.3115892 0.136135
0.7241985 0.3393417 0.2681756 0.444782 0.3867254
0.1996816 0.6183812 0.4322386 0.1119067 0.6756017
0.02025956 0.6905094 0.1619957 0.6910646 0.07551254
0.5083375 1.400652 0.3248223 0.6419033 0.6362666
0.1291601 0.1922809 0.8402552 1.092102 0.8404387
0.3189199 0.1380606 0.3512891 1.457683 1.297181
0.4109101 0.1839592 3.942536 0.4995231 1.014797
0.2924089 0.1462165 0.1365953 0.4975771 0.579311
0.2349735 0.6317776 0.4595242 0.1596194 0.4859254
0.5461373 0.2653931 0.383451 1.755045 0.06609499
0.4881865 0.09689057 0.4731467 0.2785859 0.09028725
0.3821941 0.02964712 1.096433 0.8573117 0.7193337
0.1346903 0.08389497 0.4487784 0.3673205 0.2732602
0.06568217 0.09242764 0.2629006 0.3324018 5.835056
0.2049861 0.1267394 0.4300374 1.240008 0.648141
0.1806496 0.1847622 0.253927 0.1260822 0.2253668
0.915166 0.6613695 0.5175312 0.4574351 0.2322467
0.05557895 0.7416924 0.0279448 0.2699947 0.03345893
0.08063605 0.3072715 0.3047431 0.4611312 0.1011954

0.4738342 0.1205233 0.3016738 0.7119473 0.1852051
0.1070751 7.998117 2.242559 0.4578314 0.8840796
0.2777397 0.314749 0.9962412 0.2392213 0.8541561
0.7954509 0.3319283 0.4417131 0.2269881 0.3692521
0.666806 0.223744 0.3651406 0.3328711 0.4884947
0.1214035 0.9072367 0.5028557 0.06439589 0.7339789
0.2612209 0.4816188 0.5770095 0.1592587 0.3782594
0.1646364 0.2317449 0.5318015 0.1132422 0.2125622
0.1365668 0.1580162 0.2810819 0.2672874 1.382284
0.8872796 0.2261369 1.282611 0.1457301 0.5043836
0.325704 0.1509561 0.9753822 0.2220137 0.14491 0.5383173
2.403438 0.2017247 1.754062 4.510401 0.6955428
0.2245682 1.061819 0.2176352 0.245559 0.03494003
0.4193402 0.706001 0.9635829 0.1004668 0.445538
0.2134015 0.6823278 0.9050016 0.1713487 0.4061315
0.20086 0.3897498 0.1084437 0.07642358 0.6347018 0.2293365
1.003069 1.286769 1.217527 0.7623938 0.4184093
0.1760853 0.3499232 0.2233497 0.1304897 1.043115
0.6601635 0.1592465 0.1999375 0.5282968 0.08396012
0.08742082 0.4235145 0.5563366 0.5288761 0.6659227
0.228141 0.03397237 0.2434934 0.4110552 0.1540565
4.004527 0.1048023 0.2880121 0.06740083 2.481152
0.2014459 0.1871196 2.905627 0.07622539 0.2923949
0.2374658 0.4037907 0.1764242 0.215704 0.46183 0.1222584
0.898084 0.2650286 1.039003 0.1489572 0.7709285
3.851627 0.08631491 0.3353175 1.513964 0.2426963
0.07225846 0.4255147 0.3465153 0.3071849 0.1994707
0.3432996 0.1502956 0.2362922 0.2664608 0.1856774
0.1846889 0.1498513 0.4360203 1.07882 0.2479951 0.8455354
0.2502162 0.1201229 0.1703528 0.141071 0.7682516
0.3060014 0.0594461 0.1957677 0.6472817 0.1399095
0.8197094 0.2471816 0.3238052 0.5450646 0.1903505
1.213791 4.073566 0.1589646 0.1879084 0.4630402
0.07667762 0.1137811 0.09805292 0.4218422 0.9967772
CRYAB 1.776571 83.45213 3.603077 2.493027 16.30562
75.23632 5.356635 31.6854 37.28176 39.51933 27.69201
65.48828 13.19238 48.61508 5.839498 81.68063
4.088297 75.30055 29.08426 12.07862 12.37757
123.4943 42.11419 63.14597 107.1094 37.42865
6.048773 50.44693 7.782379 9.083798 15.75162
62.62209 0.841263 11.44712 7.851511 2.172938
0.8470739 4.576094 7.052719 0.5466808 3.488065
0.4094704 3.154947 0.674177 1.696268 0.8808969
0.9973791 5.780179 15.36877 2.122688 3.543381

4.662525 1.438441 6.055381 59.36216 0.8594541
2.060065 41.17081 2.919364 1.778777 4.909276
2.625256 27.09408 5.794044 0.935751 52.08318
13.54483 18.33312 1.568446 1.692635 0.5684841
2.609965 16.83551 10.18045 4.747293 6.746402
4.320209 2.471296 1.906384 3.114752 6.386203
5.089548 0.8104304 19.13936 12.29727 3.7092 8.65704
5.047588 1.410792 32.65074 2.667225 0.5667734
14.02282 1.855434 7.72756 2.064449 6.359129 1.669039
0.2330765 8.062136 7.824077 0.5480542 0.5516734
1.803621 31.17377 8.279186 3.875387 46.97936
1.638777 1.353409 1.89948 2.649179 6.017037 40.42644
26.89744 0.6767393 2.255692 1.411272 5.592853
1.958803 1.744336 7.490539 5.516 17.97616 2.669752
3.771526 1.664365 5.681487 46.26294 14.02762
2.872766 1.711859 7.610722 2.904773 3.736098
9.771652 4.96473 0.9847861 2.8142 1.225239 1.063427
0.5748125 1.68751 1.366328 2.458736 5.01501 0.5532434
15.36484 0.3966975 7.970684 2.000339 7.695548
0.9112381 5.244514 35.4771 19.20108 9.785595 32.62563
7.183583 12.88112 13.37446 5.057713 6.880307
0.9148134 15.20826 3.166507 2.818732 6.735679
4.558544 20.11494 52.36256 1.47191 6.457877 17.14265
0.8006039 4.950926 8.134359 9.950424 0.3040433
2.931283 2.961951 2.06027 29.80057 0.9368768 10.16768
3.402087 28.10165 3.485727 2.203658 0.6417119
8.422707 1.155576 5.503884 2.737965 3.227816
3.855448 13.31687 1.460359 0.4440291 3.690892
1.55836 16.63319 1.319175 1.196196 2.545955 0.5346425
16.50249 6.833693 2.736854 1.81546 2.364281 17.81895
1.817934 5.994452 2.141488 3.058453 6.21616 1.229112
16.27416 1.767391 1.443355 3.281386 2.995688
1.941576 4.313442 1.323322 1.67612 16.21429 0.7945693
2.62247 9.816405 4.861286 0.4746811 7.427713 0.6312292
7.514039 9.808679 2.014246 8.521619 1.579268
16.9174 6.089218 9.347655 3.94006 10.07564 6.406192
3.292846 55.96333 7.479043 0.788738 6.809923
1.015908 2.907254 29.22969 0.7183494 2.305231
2.686391 7.778154 1.137445 20.57889 3.694142
1.898087 11.30178 22.43335 1.75779 4.297863 1.277219
3.21947 7.12535 1.361569 6.565878 6.852202 1.951572
0.9234553 0.5066517 1.443462 3.290656 92.55404
2.110092 4.733404 24.19828 0.431736 92.3977 5.27765
1.442978 11.80521 1.23073 3.131253 3.984202 15.24548

2.97524 67.11605 63.33683 2.563048 4.564151 30.92507
7.561137 5.222825 2.503705 1.283192 5.496337
6.713555 7.456429 7.858845 0.9415906 2.113977
3.788482 3.891361 1.925297 1.737982 3.643158
4.554036 1.44185 15.57303 1.091054 27.491 3.528449
4.571234 4.006927 10.46363 4.873603 4.493519
2.852042 2.569914 27.91614 3.609793 2.366267
2.439167 2.778084 1.630885 1.466534 10.63613
4.39902 3.554918 74.48442 8.45568 0.297219 3.228883
1.480178 1.209826 2.056941 5.556375 4.087056
0.8771649 32.09595 13.16686 1.158809 0.7344302
8.618685 3.167194 0.6243648 4.813415 2.278513
9.59699 1.138389 0.8896518 5.803816 1.870137 63.38823
2.189605 3.186588 17.16917 1.380856 0.9150168
0.870802 2.431239 0.1518275 2.584775 2.064717
2.824317 3.257592 22.81357 3.101146 2.376843
3.618289 9.22314 1.449661 3.222567 1.593576 9.991508
4.837441 2.052628 7.019981 2.082809 1.193768
3.38111 36.87778 4.787723 0.7547716 1.750214 12.86886
2.331518 0.7877558 3.40555 7.381616 3.488916 2.216177
17.19099 7.388715 2.230789 3.269898 2.799922
3.187381 0.4594401 0.5219722 4.972438 48.806
EPHB6 0.540219 2.658521 0.6249073 1.746229 0.9054181
1.59432 2.166769 1.425686 2.127134 2.174077 9.702839
5.692623 3.459134 9.669645 5.257598 1.394708
1.551099 3.011574 1.138659 0.6235155 2.387966
1.274383 3.974325 1.410149 1.147367 7.982067
1.744784 1.47134 1.318112 1.132944 3.893673 1.597023
0.6610436 14.02395 0.3176331 0.2093346 1.286076
0.8454119 0.8019904 0.25353 0.817425 0.1058277 0.2129833
0.5261954 0.6237134 0.08833518 1.561954 0.8240695
0.4472752 0.3116199 0.6015742 2.303883 0.8517711
1.456363 1.702296 2.029183 0.789179 2.388562
8.415248 3.451028 0.6535941 0.5134766 1.059767
0.6576949 0.1276576 1.68796 1.16896 1.657283 7.176582
0.2401482 0.2027304 0.1344689 1.014829 0.5164453
0.4161296 1.04115 1.220161 1.394733 0.3820254 0.08552662
0.5408143 1.442473 0.263025 1.405551 3.218762
0.6974131 0.6849888 0.9372935 0.5608048 2.818955
0.186675 0.6945994 1.713081 2.084382 22.70223
0.7497906 1.256223 0.7182482 2.724667 0.5752277
4.307583 0.5768509 0.5846432 1.613462 0.5630602
3.327967 0.8265469 1.735572 0.9457817 0.09457201
1.348279 0.3855677 2.719656 3.082974 6.493412

0.2989348	0.5708674	0.4085314	2.829858	0.2968543	
0.920452	1.494041	2.908999	1.002945	0.4274906	
3.974907	0.306949	0.4042703	1.657863	0.7676793	
0.3281465	0.1397806	0.4663909	0.4756175	0.8600186	
1.064128	0.4204168	7.870045	1.47732	0.08949983	2.957034
0.3447455	0.6261322	1.341524	3.172631	2.354303	
3.463866	0.8234177	0.1731251	0.7515728	0.413528	
1.294297	0.1483405	1.868267	1.997834	0.428352	
0.755751	0.8367289	0.2759296	0.5294458	1.616179	
1.80559	1.450878	1.010191	0.4302317	1.704536	2.084773
3.445055	1.475147	33.82815	1.515818	1.749529	
0.366783	1.815749	0.6431521	0.8322094	5.088641	
0.5304018	0.3464666	2.292106	0.2058069	0.1377782	
6.04986	0.8134093	2.49741	0.4247605	2.038348	4.747315
4.014217	0.2052219	2.503605	0.9487958	4.430112	
2.135611	0.4503912	15.22886	1.023169	3.656194	
4.245268	0.1147059	1.410563	0.755657	0.1979355	
0.1870924	1.140861	0.4886624	1.364356	0.5687988	
0.4217003	25.84173	0.5164991	1.377461	0.8255973	
0.189579	5.317845	0.2339381	0.4554933	0.2550494	
1.077594	0.6675606	1.084617	0.8715305	0.4467565	
0.1211785	5.762271	0.2852311	0.3859419	6.391863	
0.3062504	1.592351	2.170114	0.479082	0.08896605	
0.2561619	0.3171481	0.9776959	3.163754	1.256396	
0.1879281	0.178291	1.869755	0.7709833	0.644037	
0.3178035	1.528399	1.00356	0.7404658	3.24949	0.950047
0.4532864	1.05463	0.1631806	1.1585	1.741084	0.2358293
2.640216	0.1602854	2.692405	0.4610824	3.532556	
0.6024409	0.13375	7.7356	1.420507	0.4830519	0.9214027
0.1417627	0.4066422	4.338433	2.564288	0.6367675	
0.5561462	3.648069	0.2319062	0.7368746	1.53049	0.7717137
1.751397	1.629336	0.1301293	3.07229	0.2363793	6.566931
2.21001	0.05972098	1.476483	4.198759	0.09172643	0.6527022
0.7258969	5.012914	1.221521	1.379584	1.20687	9.202072
4.564559	2.299209	0.6319706	0.3450199	0.342275	
1.364371	0.7704473	0.2698883	0.7965847	0.2141338	
0.2440877	1.048268	1.036694	0.9532598	0.0971904	
3.041306	35.57803	0.2928433	2.935206	0.5092569	
1.761594	1.881938	0.6685426	1.372589	0.3863886	
2.053531	0.657978	2.133444	0.484328	0.44611	3.045926
0.6249678	0.1898374	0.2155062	2.070196	2.503973	
0.6314775	1.350227	0.9959023	1.340261	0.5193569	
0.1290248	0.6837408	1.13266	0.2029553	0.2405027	0.6154755
0.2424515	1.215924	1.816875	3.777574	1.685436	

0.1619139 2.142293 1.082734 0.4359085 1.651107
1.51924 0.1657964 0.4384999 0.08125761 1.857703 0.3342097
1.371195 0.8823695 1.435263 1.379336 0.6337822
3.40267 0.1937035 0.340417 0.05369339 0.7298411 1.645053
0.1986353 0.6060614 2.271723 45.71432 2.216524
0.6988817 0.2576657 2.574474 0.9078904 0.1740657
0.3954488 1.462375 7.709261 12.33769 165.1404
2.87827 0.5633553 1.649662 1.50114 0.1343883 0.2511867
1.19765 0.1328418 0.2817162 0.5454175 1.239399 0.5175298
0.5324347 0.8746246 7.651372 1.354437 2.569876
3.388438 0.2022337 0.7396127 0.1396495 1.554359
1.056876
IQGAP1 12.96534 24.49206 25.07593 29.77493 20.21967
28.29363 13.33135 19.24952 24.27294 18.62428
58.23195 39.95149 20.2269 35.23414 19.31656 29.89926
19.50236 23.42937 11.24717 12.96905 33.11639
23.06618 39.58597 28.92468 40.18828 53.06873
16.29262 26.84558 20.96335 13.08111 23.66132
21.2439 37.61012 37.0982 27.42761 12.88978 9.282291
25.52043 28.44288 51.99563 50.25696 45.94755
51.8041 23.1129 47.67443 29.16057 50.72619 30.18437
69.87691 65.56928 25.08518 329.1474 17.94887
53.56101 34.04846 34.19505 26.29575 47.62255
44.98366 28.22685 51.93392 38.09014 36.81779
30.5996 25.23939 32.25237 33.06404 23.17316 38.29032
35.06558 83.38116 27.1214 36.08137 41.2606 51.33515
19.98738 33.4751 26.1926 51.94136 25.38298 17.42173
35.72069 60.32006 31.58998 26.7901 29.86711 51.48679
41.4243 29.66268 45.94544 48.35437 56.20626 41.28496
34.91853 24.98599 36.58749 45.53563 25.12889
62.95913 43.1638 32.32182 45.2629 24.38903 43.51603
30.65507 42.29409 32.11978 25.67333 49.28352
26.89615 42.3626 21.48496 19.74352 27.38981 28.86504
37.4297 14.76016 41.07683 17.10051 27.81615 24.80832
40.79878 21.03431 26.71484 31.86172 35.81244
41.48667 16.30027 34.88384 55.496 42.46553 52.71657
25.53777 51.48546 69.78822 47.20482 53.9188 32.99809
26.38574 29.04245 32.80407 54.05535 53.29565
40.30547 23.62 27.31106 29.52386 30.8309 35.2532
29.39572 46.35004 40.59443 57.957 50.63794 29.90609
106.2747 28.04767 29.11798 33.72613 14.6414 36.27464
32.72314 43.794 42.91486 28.78189 51.45325 59.96325
35.84597 22.04966 16.87282 28.80169 54.64299
46.18573 33.0125 53.26146 24.55598 34.08823 27.25957

	35.96705	93.05765	13.99152	25.93485	46.06917	
	46.85921	27.27287	47.41976	46.65047	22.07375	
	33.74166	40.16631	32.67982	61.34975	30.32027	
	25.40355	26.20491	62.7705	44.47829	31.95168	58.30057
	24.93858	10.65395	27.4673	43.40578	45.03866	26.94584
	41.35657	17.42419	31.74317	43.67728	53.07582	
	32.30879	41.47594	57.55946	25.63105	46.05432	
	36.89713	35.97839	22.51229	31.07335	52.16095	
	31.61813	39.37996	18.1307	45.14069	56.35662	30.06299
	36.99652	30.85588	20.98808	28.60252	21.62531	
	29.26252	27.61879	67.17464	40.13936	20.46608	
	17.89451	25.66637	48.29015	27.89822	96.43082	
	53.5141	30.48444	30.80939	54.07285	52.21374	32.77988
	24.40806	49.46054	28.33493	59.05249	61.20839	
	27.78256	32.9952	60.47539	27.76687	39.27808	27.42043
	38.12934	26.11944	21.53639	31.428	39.35363	42.66928
	40.04713	39.21344	31.68744	25.37127	33.09324	
	88.27265	39.77268	28.48815	22.85492	43.27563	
	43.33981	52.84027	45.88213	28.92639	55.87917	
	78.55503	40.33423	61.26198	29.9938	32.71604	53.99072
	36.69532	56.91648	19.44291	31.79671	45.41237	
	40.45311	40.56474	38.92802	33.18202	40.28169	
	22.16528	27.92278	37.59572	34.24459	45.99525	
	47.73092	34.51841	55.71774	43.62212	38.53634	
	27.35508	77.49898	65.24628	35.31094	48.98084	
	33.12854	22.76473	25.66566	21.45185	47.30128	
	27.68598	27.2412	54.81062	62.12257	83.30967	51.9512
	144.856	33.8651	17.00338	45.40672	20.15381	36.66528
	21.04254	29.24363	69.27797	25.34434	43.68963	
	47.66996	33.12955	21.74555	26.96478	29.11086	
	23.64203	42.73265	67.94438	21.1157	11.47579	49.83046
	33.9356	41.84345	50.6174	49.45758	20.1292	23.09622
	26.21998	29.24716	44.31015	39.44237	29.67165	
	31.86537	106.4993	49.84084	52.94737	33.47904	
	25.63728	31.02652	19.53372	67.85786	35.83299	
	26.82024	24.83628	36.02902	52.56963	53.31412	
	23.85478	18.5819	40.86417	26.40811	40.93745	28.7087
	23.86709	65.9751	44.5995	27.97695	35.25801	59.71696
	36.28581	17.52013	43.88255	36.27914	29.10489	
	19.82228	24.73366	34.70002	39.39349	43.00438	
	47.49599	13.59953	39.37069	37.94001	36.39642	
	35.54992	38.00119	17.87612	55.08342	25.22748	
	31.34843	46.147	47.81939	31.76426	20.38323	33.13977
LTF	2.428848	1.377163	10.95362	418.3126	110.4859	

0.7898996	142.9202	27.12093	108.3617	603.5459	
1.474093	0.08737127	957.9666	0.08660039	488.9876	
0.1241846	687.152	89.3076	32.27066	2.403791	105.8286
0.7052363	11.65918	0.3397009	1.190241	0.1129342	
355.1205	0.1352496	53.52711	25.41401	289.8935	
0.7267477	0.4325786	27.18347	0.7213181	0.08249031	
1.954241	597.8613	0.931463	0.227059	0.7155025	
0.2261754	0.3168923	0.3540699	39.68265	2.018798	
52.30412	3.357111	1.936694	45.06637	0.8253525	
21.72473	1.904735	27.18979	1.594118	0.09251792	
0.4771668	27.76717	0.09261508	6.58092	1.530239	1.017034
3.672837	0.676521	53.77091	124.4138	0.7515076	
199.0437	59.41965	9.56842	0.06427759	0.5231577	5.847712
0.2328566	1.248483	6.421928	6.347896	0.7964065	
0.3132877	18.89021	0.1487033	20.33192	3.12199	0.830153
0.5260912	3.15989	1.657875	41.14902	4.711117	13.14626
5.389926	0.08403488	0.1870285	161.1706	4.949519	
1.342012	5.133119	89.5119	0.01435403	9.667331	29.92586
0.4453196	147.3649	0.9396898	7.765776	13.21223	
93.28596	99.07881	8.945271	0.8300381	0.3915513	
3.787822	0.2521461	69.63205	58.13013	0.6083909	
160.7685	0.4417623	0.6772821	20.70383	0.08142331	
0.7066155	0.8480078	2.121268	0.5064038	403.8107	
0.5225344	0.1738948	4.673845	20.03279	0.3153542	
0.5530445	18.20454	20.90988	176.8129	7.0287	4.112913
0.1141836	0.8207359	0.2962535	18.99013	1.426428	
6.762169	4.31495	2.153635	1.878474	0.2930917	3.091419
1.196893	4.233854	0.1011692	1.593787	0.1177957	
4.596568	2.827429	18.6259	4.09905	2.83544	12.48367
0.1831264	54.73879	371.7475	27.24875	0.7033232	
6.776663	250.8417	33.69204	45.52261	55.07271	
0.9349664	256.1396	181.4392	101.6814	0.1218382	
145.1202	2.843051	8.008432	0.2660127	0.9445103	
1.919894	23.59918	0.3351349	2.800495	1.351333	
0.7413809	74.64938	15.17451	0.09719943	2.412179	
0.038449	2.168023	30.51208	0.05200928	127.7078	
62.83012	3.652415	21.763	82.56036	5.939816	0.1917617
0.5184739	3.39741	0.1710408	0.1061201	0.5178068	55.72102
0.8573935	1.792788	1.077814	0.1573022	1.378338	
7.188361	4.55659	0.9243854	31.27986	2.259744	0.1830479
0.3197876	2.558876	0.3298367	0.8523448	3.63528	5.508191
0.06616604	10.98946	0.2910944	16.68586	13.0969	9.624662
0.6153693	0.9648745	21.25472	0.1026241	9.778523	
0.1721777	2.116525	48.8335	9.266145	0.2665314	1.487977

1.75762 5.652673 46.30029 0.4493389 1.755759 400.2448
3.873144 16.61902 17.15519 2.114819 11.55341
0.1505548 3.88623 1.55664 8.604304 1.070019 0.2066311
204.9101 9.982157 33.88463 142.217 0.5270546 15.83312
0.2049642 0.3916622 14.4566 0.8650296 2.61256 1.633313
0.495186 2.332813 81.0275 0.2578272 0.3470481 11.84656
13.86643 0.1850769 19.65674 0.5574891 0.334477
26.78907 0.1277078 5.804469 8.401174 0.07987172
20.12491 5.771547 0.2587703 7.251045 0.8377821
18.79248 1.925105 12.69429 15.42652 1.436328
4.395297 1.334816 2.461767 0.5573819 19.90454
89.58384 58.96039 0.1633622 1.147665 0.6091468
263.6208 20.11874 48.79594 1.747787 0.1977349
12.62912 0.2280782 0.1104773 47.31417 31.48562
3.568173 16.18402 10.58273 13.76441 12.28219
0.1367135 1.388597 0.2274524 0.388366 3.180172
46.90938 0.7235671 0.07185602 1.74703 13.84043 0.06938226
0.3379932 247.5964 217.9155 6.040704 2.176065
1.533489 4.267958 4.787476 4.433751 0.1196621
5.498936 0.3358052 127.333 3.102238 19.04228 5.00616
1.766467 0.0998199 168.3839 16.02595 1.824584
0.309212 0.2282603 1.92431 61.88175 23.41965 0.3303029
8.199455 108.4767 305.0451 11.98667 0.04795318
0.7235343 2.667314 7.206756 0.5909392 82.68935
1.170411 0.539092 31.30551 3.444705 0.06831683
0.6337787 0.9296693 0.3698279 95.53181 0.5606733
9.608816 16.79604 0.6837021 0.671973 0.2978792
0.0578829 0.09880138 0.04939508 14.202 15.00676 0.03369992
0.198415 16.6197 0.09160828 1.915786 3.050425 30.43833
121.5449 1.626079 3.526172 9.943184 1.06077 13.12102
6.252677 0.5629164 1.101874 0.6874145 6.841869
7.079173

SDCBP 19.29344 35.50218 28.58236 57.60584 37.28784
36.06652 39.57685 25.7978 45.18796 44.23448 17.69127
15.65772 44.46331 11.91704 33.46163 34.28869
30.89776 34.13127 35.03688 28.73758 52.02952
33.55066 20.50639 33.85097 32.05966 17.07712
37.67772 35.4477 30.87279 37.631 43.94427 35.30818
56.2198 30.14799 32.01071 57.64505 24.95794 50.93054
34.24213 54.48466 47.43863 72.50827 110.6918
57.33778 43.12237 58.48263 47.25912 36.44231
68.23921 56.84226 22.83372 42.69453 16.81636
24.84932 34.32118 60.71633 28.1002 32.30885 20.86415
38.69197 84.93164 31.62364 79.53593 44.81201

25.83497 36.13846 40.27851 40.24094 92.20514
33.68879 46.98763 49.93973 38.55659 66.92484
39.64045 33.85344 75.76906 27.31597 27.31314
69.57914 57.86722 41.81783 67.28145 39.1428 29.06318
31.12848 38.92444 45.63915 33.90665 42.34748
63.62694 43.23864 49.84439 48.41099 32.89968
41.85911 65.73993 73.9223 28.19441 68.3245 36.65838
62.11863 41.63074 56.46563 42.5271 45.17044 40.77724
39.6127 71.1704 37.01934 57.56228 21.84434 70.03342
41.4255 37.09936 22.48573 30.73599 15.24416 32.41884
25.28692 46.69721 45.34652 20.6823 76.5556 15.00319
39.26802 65.38914 26.36225 33.76487 46.77748
36.01075 46.21566 58.71711 79.12715 35.45682
40.68672 27.82073 40.99696 50.61825 73.85197
44.82672 31.61753 26.64702 24.29307 25.41101
40.37553 25.55851 56.40514 28.52906 49.27078
25.65135 54.61606 34.00418 38.44879 30.08068
58.53769 41.5403 35.42497 29.52181 41.90432 34.96994
41.38147 53.94489 34.59126 21.23598 44.58808
25.06798 94.88656 35.6758 26.77359 30.93299 30.02094
33.80167 23.6677 24.93265 57.98336 34.46845 39.14553
60.1721 28.62392 26.35289 33.12412 29.248 39.05448
49.17385 44.08523 42.43515 38.18346 19.43987
30.15637 41.02935 28.76174 39.39041 72.41183
36.19293 36.81858 91.45748 19.84523 27.11732
51.55415 29.15075 20.32662 56.5232 13.68231 32.29304
47.71786 42.48017 33.20182 28.53178 19.3095 36.17131
78.78751 46.4065 20.94045 27.1908 45.1897 19.70032
36.89686 36.65317 38.63362 47.45198 92.93763
29.25816 34.78881 32.53666 22.76699 32.65187
13.00805 61.48048 43.1902 26.63156 36.9507 43.37665
54.75374 82.62128 26.78095 46.94989 35.05223
75.60537 18.64386 27.97544 47.10933 62.69182
40.29437 93.61902 63.58234 42.15794 30.02803
61.98255 31.81983 31.05156 43.97285 71.06307
37.12133 30.48802 35.15738 46.07785 31.34912
42.18004 27.35918 32.09118 44.00738 62.15027
53.20136 30.86558 42.04689 46.56555 35.64699
38.98639 28.38678 46.14496 32.63122 61.92359
37.22593 55.38675 46.8371 43.96128 28.84532 19.37468
63.87708 40.74969 59.11995 36.94949 32.0949 67.45878
38.01199 44.95576 39.42406 78.62615 49.2624 43.51674
27.17687 50.22992 33.09721 73.72722 26.91485
50.90751 25.41708 32.84431 93.0606 51.9295 62.17166

37.49971 44.55443 85.023 69.58417 43.57507 72.11411
43.64882 39.73192 48.71614 16.03487 35.11209
36.46955 43.12268 22.10259 31.65391 26.28622
71.54343 90.21865 61.01515 37.78338 17.31583
40.06891 97.554 28.83801 31.24492 47.79897 26.84196
62.76081 25.05405 26.67026 39.98095 22.21396
26.05784 29.85112 10.8566 37.21825 33.12173 99.10393
24.7274 32.09939 33.07536 56.49416 35.2647 31.15194
37.97183 14.78755 28.82401 31.23468 48.89955
33.25958 39.66886 49.59641 42.74095 60.6581 58.81911
27.98353 30.37678 35.38778 34.66281 54.78465
30.39391 51.18386 24.92837 39.63448 82.97865
38.53461 56.05463 39.02377 27.91082 22.08557
42.96402 66.41435 38.57329 29.6308 22.97349 36.00297
33.8492 30.35771 29.09318 40.80217 29.94492 37.5828
52.88451 41.56103 31.03388 42.59842 22.30427
48.86691 33.94606 103.0094 62.37318 75.20869
30.48472 54.53037 24.35074 44.59366 34.71923
59.09172 35.16436 37.56408 38.05869 63.40517
32.29755 58.16537 37.02809
SPIB 6.342907 0.0356971 2.653442 0.8623479 3.498852
0.2887044 0.7970167 0.6019681 3.368905 11.79502
0.05243615 0.05333991 12.72469 0.04136533 11.14304
0.04777077 4.488307 1.456495 3.130201 6.298798
0.717793 0.6996353 0.2417799 0.117748 0.00697316
0.03734577 4.496351 0.008050527 1.796955 0.4081384
0.5850221 0.2433674 12.53032 1.015426 1.541695
0.4686923 0.05650185 24.55213 0.5066608 0.7868931
0.2137682 0.09872681 0.1165662 0.07854212 0.7164079
0.1023857 16.79868 0.4815106 0.6876641 0.4255624
0.2899092 9.023039 1.427218 0.6072433 0.6219957
0.7092333 0.2092825 6.495273 0.0374324 3.234421
0.1310023 0.3795157 0.9227469 0.4686123 3.946381
0.685302 4.538064 3.045858 0.2250434 1.168773
0.327531 0.3419023 0.8997338 0.07594757 0.07394435
310.2972 2.61385 0.127076 1.464097 0.03656945 0.112117
0.4670972 0.1635843 0.2617245 0.02859797 1.951853
1.026633 13.15587 0.3952881 1.412516 0.09484187
0.492626 0.1942404 8.276576 69.41872 0.7211497
1.756664 0.2823163 0.01993604 5.070471 15.92973
0.4921876 0.907069 0.4559362 0.486759 5.012519
0.5434324 2.995314 6.353311 2.827767 0.07193362
0.1634984 0.1965414 18.46323 1.706736 1.190951
0.2923875 0.247358 0.2105965 0.1420183 0.1650653

1.044203 0.2720845 0.2961311 0.5089064 18.59858
0.3911164 0.1761842 0.7450195 0.204171 5.709505
0.2550074 2.064982 1.145595 1.009055 1.6048 0.6746612
0.3909873 4.567835 0.4542101 2.152337 0.2751582
1.731898 0.8182603 1.0026 0.1729072 0.1277599 0.963292
0.089727 0.3480853 0.1725036 4.484133 0.01845161
1.153507 2.018787 0.2344379 1.467698 0.8157469
0.3622879 0.5109524 6.660124 6.31406 0.7878213 0.1914147
0.7023865 4.45596 11.59083 0.241062 4.011798 0.4428798
0.6415669 0.5157205 0.8183168 0.01036033 0.2079286
0.1667009 14.92446 0.0537574 0.9468034 0.5105236
1.140039 2.810598 1.558254 0.4078714 1.72546 0.2599798
11.92369 0 0.1847244 0.06455077 1.169991 0.2145776
0.2132644 2.272483 0.4435133 0.5524211 1.649941
3.887058 0.3354379 0.05944961 2.950368 1.209683
0.5123741 0.01108182 0.1220024 0.6525639 0.507079
0.2697141 0.04899546 0.5605165 0.5643202 1.392347
1.127738 0.7111768 9.187624 1.368832 2.022958
1.408795 0.9101817 0.2181447 1.45363 1.169936 0.616264
0.1555924 81.96762 1.570427 0.9250807 0.3003222
0.4844501 0.3696808 0.07657691 1.061597 0.5931486
0.04819639 0.9963933 2.071213 13.78575 0.5209305
0.2299257 0.1836537 2.015632 3.244282 0.981119
0.4794132 0.2478192 3.976299 100.6139 4.429148
1.522903 1.493057 2.788382 0.1953157 0.1074446
0.3568448 0.1066693 2.156214 0.08985866 2.564516
1.481608 1.464678 2.2574 0.03327346 0.4897719 0.0280464
0.9413362 0.6504912 0.1880897 0.6091864 0.3015243
0.3087877 0.8044308 0.7286672 0.7199719 0.05272938
0.2693029 0.9112153 0.2866846 1.605359 70.35888
0.5462899 8.627491 0.1219127 0.2452855 1.422955
0.3739014 1.125628 0.2809343 0.02281912 2.09834 0.7262018
0.8653438 0.4677817 1.034084 0.2689988 2.116578
0.736319 0.4132069 2.953555 0.1017878 0.5068393
3.099415 0.5727548 0.225733 0.07385191 0.360867
0.1305252 0.09182443 0.2635495 5.323866 0.03649571
3.391459 0.1579599 0.05157642 26.80494 1.205736
0.7633828 1.857844 5.71958 27.49719 1.634773 2.224294
0.5550792 0.06674035 0.938686 0.3001357 2.716678
1.006203 0.03118733 0.7995621 0.734401 1.882534
0.1834009 17.42863 96.37292 0.6037037 7.19053 0.07355786
4.735786 1.863795 0.3707144 0.3798776 0.9443564
0.03506718 0.03032479 0.7567035 6.347681 0.2896818
0.2327865 0.1035284 2.877733 0.8413645 0.5082185

1.522206	0.08087409	0.1647672	0.04813036	2.427537
0.9194591	2.670284	0.05776587	4.030264	2.184147
0.02378617	2.930555	0.2282605	0.9445834	1.465615
6.411522	0.1308635	0.02814792	0.3044437	0.2565079
0.02259142	0.5312392	2.399189	0.02951994	0.3319271
0.2275447	0.2545512	0.9393659	0.4007971	0.6072596
0.6477007	1.236287	0.1083342	0.1666095	11.70688
0.4216305	0.01671614	2.183978	7.860224	0.4544036
0.6355034	0.7976664	0.3435844	0.6437389	0.7017723
1.145018	0.373099	0.4397125	0.4907977	13.3318 0.09558981
4.519793	0.1093701	2.600744	0.8916748
MGAT5	9.276433	5.760807	15.4376 8.421332	10.73912
4.919403	8.316429	5.023959	15.24162	6.815959
6.351839	3.224373	9.457715	4.408306	11.58062
5.154639	11.65932	6.85571 6.934661	11.0367 6.915168
3.228513	4.749009	4.656556	5.811728	5.32649 10.94913
5.564591	12.53559	3.109133	15.32579	5.175574
17.55224	8.428318	7.098423	6.504899	8.48943 9.148424
20.7802 7.401585	14.67462	14.90383	15.80581	5.758812
15.37578	10.78653	19.51657	8.523589	6.768963
16.16691	11.86411	12.93564	6.317833	14.38367
5.151614	14.95981	31.65214	13.39517	31.76838
17.13388	26.32081	7.123436	10.83808	8.359829
8.791706	8.132537	13.85688	10.15104	7.819286
10.70875	8.107906	12.44634	7.928356	10.11061
15.99754	10.01641	16.31398	10.5621 27.10577	27.53355
17.5018 20.90986	11.18281	20.66728	12.27954	12.75521
27.87652	15.19501	5.262612	6.380764	19.12559
22.81521	4.194423	11.86699	6.776473	7.616779
6.389685	15.55551	68.99929	13.7753 21.11661	14.42882
3.867365	6.771433	7.3 25.00946	10.64558	11.44656
12.47174	5.466604	8.706196	6.146954	4.193979
11.61182	8.745704	10.11765	28.65169	27.37466
9.537992	11.87008	20.78047	7.705767	12.30681
3.858246	40.1856 17.95112	9.83558 9.992626	11.01011
10.9451 25.97053	41.24956	9.099306	15.03562	8.87145
19.66639	20.26169	11.22813	13.58102	19.27237
14.20754	11.66884	17.89671	4.361859	8.442258
6.804766	9.816965	10.39689	30.71746	10.13851
9.571746	15.03264	9.888496	12.24746	8.825003
8.179386	10.30769	9.678452	6.313938	1.915331
7.855213	12.71684	20.90761	13.13059	6.614321
9.484194	17.25384	13.24001	15.43829	23.67907
5.627707	10.42971	13.30176	8.5951 14.10046	5.783393

14.16424	4.3633	18.95778	8.986095	5.830948	21.12228
6.181559	14.96842	8.337074	29.29362	11.52145
5.474655	20.34744	11.45396	9.7971	22.27267	8.214246
20.96832	12.73596	25.4366	11.44797	14.66116	18.07814
11.81236	2.606075	8.109189	19.10347	40.99955
33.0138	9.578474	5.065413	8.961995	23.04676	10.6824
20.96005	17.32867	27.68651	16.07064	6.634868
9.000845	6.003179	6.267375	13.12688	30.45696
20.31286	13.46131	7.614264	3.086631	22.11373
4.97152	6.566664	3.952126	17.70731	7.117469	27.98825
11.31505	11.31066	19.98424	34.93253	5.704177
12.96469	4.225743	6.183027	8.185014	8.449609
13.2058	11.52461	10.36791	9.472005	17.70707	6.125066
7.141563	19.37586	13.57186	30.98496	14.21234
5.936282	7.888141	15.66566	20.65691	29.59805
12.24563	9.814439	7.017015	12.14425	5.738456
11.61579	9.330779	27.15387	13.4519	33.05818	19.30651
4.25847	15.27575	16.74882	6.739107	7.557424	23.21493
7.682039	14.93939	13.55032	7.700024	18.30778
11.27903	15.50596	14.80981	8.477655	9.633074
7.719884	10.0132	11.0949	9.896414	14.95593	11.96274
5.325878	6.352422	5.608508	11.61755	7.111311
7.82028	5.506186	10.14676	3.572402	14.15636	17.97124
7.495445	11.28149	17.40576	10.29411	5.250921
11.9534	12.38486	10.50298	10.70062	8.225187	6.108462
7.681813	11.23188	7.340837	9.894178	13.44795
10.94599	15.78485	10.62478	21.28091	20.85551
21.95469	7.341656	7.856901	6.888465	11.20558
28.69664	14.45753	19.01396	10.50466	28.81051
14.96354	15.22936	10.92932	22.86957	24.73776
9.805067	12.41568	14.95982	43.48595	9.107938
5.33077	14.18066	11.09874	9.990246	11.98745	31.92234
5.717135	3.82599	10.27038	9.13087	17.71824	7.396267
8.78726	9.939598	27.06271	9.877953	10.53026	3.401829
20.63273	8.190767	12.62894	7.374484	16.57075
9.569639	21.91914	11.91524	17.1147	4.35635	10.44754
5.227956	26.50346	21.52036	14.48491	17.73154
9.314488	13.78704	13.21927	7.814585	7.619406
18.73471	1.945539	28.88056	18.15719	8.693998
11.38549	8.552152	21.2266	25.7394	8.33329	13.55308
3.666596	16.25394	7.06762	18.05703	14.19879	17.5842
6.552988	17.89349	4.572944	18.23319	16.71459
19.30935	8.258098	9.083345	6.390068
ABHD2	35.80964	6.638128	44.91435	38.36105	23.46492

7.424365	25.8391	20.78034	44.89188	10.48353	13.20161
8.495457	12.2427	7.355442	30.17231	6.953362	34.19176
16.06543	22.80994	32.67983	35.29353	4.079928	
13.94326	6.248799	9.106165	9.555323	28.21941	
6.106629	45.42877	12.28487	61.09542	13.78482	
45.80584	32.36476	25.36291	3.235674	30.95893	
11.19708	18.06403	52.34016	79.6965	101.3586	33.79422
30.35286	84.13853	36.4957	57.23616	19.61958	35.92891
181.5927	31.39676	32.73891	4.976541	107.3457	
19.35674	15.42068	26.76742	26.78968	117.658	51.58758
82.27913	16.37941	43.77334	39.75248	18.15934	
13.68768	74.13326	15.4504	49.95339	24.52761	33.55751
20.11254	47.94313	60.32508	19.02329	14.9271	57.89886
23.03882	86.14297	28.90457	20.27043	63.65408	
24.88015	24.69992	67.86494	25.48403	122.3697	
42.99481	18.62399	35.32061	32.54451	26.38105	
20.44921	32.21006	13.22308	17.76797	29.35564	
53.3953	98.23033	38.56618	32.67735	83.02234	3.190612
13.43649	11.80044	49.65386	37.84549	26.59482	
47.7767	12.81785	18.04609	6.066796	8.897376	10.68588
20.72106	32.67483	16.61269	110.5425	10.99746	
19.58526	35.59565	17.40096	17.55833	22.25381	
117.1258	19.05741	27.56767	23.51166	39.63882	
19.62783	81.27835	14.13115	33.00972	54.83969	
29.39004	43.49429	51.11321	24.01439	30.47316	
18.34847	41.70346	33.0687	84.55085	5.556835	7.898987
15.40093	19.50903	25.00098	89.93897	22.04174	
95.60789	52.73534	58.49201	44.41846	24.82965	
38.20219	31.31833	9.763065	6.651851	4.299359	
39.67998	28.51541	63.07671	38.28358	24.74621	
54.92026	50.69872	40.75994	11.30181	27.12454	
13.21679	47.28805	66.45704	22.67862	24.33342	
21.98386	36.7128	9.616363	61.88828	52.10156	2.926695
37.41321	14.08114	23.22321	13.56667	37.64457	
84.04828	24.27868	26.23161	45.55591	40.09451	
63.04156	38.94266	56.72495	25.97759	73.11169	
51.76415	14.22448	110.9379	15.91725	8.193199	24.818
45.84715	71.49327	35.63348	50.79043	9.56638	27.37291
33.48908	81.46749	38.16398	34.44872	44.48358	
45.81653	44.22698	25.95614	21.64258	9.119821	
23.43348	112.2243	94.86465	61.65392	15.22185	
22.12523	50.23966	13.41527	13.18843	7.231149	
45.41739	55.98596	62.16014	34.66328	39.48036	
86.13042	37.07777	36.91102	19.18411	15.57843	

53.66855 38.04022 91.21936 67.87241 16.91459
42.57777 34.40078 39.17871 15.04484 15.00603
51.12292 13.78321 49.06965 154.6363 11.02422
26.80476 40.51955 10.68004 39.71072 35.63287
20.59304 10.97385 18.22649 21.99749 73.27829
18.77456 73.37236 22.29563 28.53415 47.06392
19.88047 442.4896 51.75063 32.44983 46.92571
22.91424 30.14065 16.77384 59.19305 10.17522
10.45121 104.8785 66.34812 46.23719 47.45914
23.58124 40.75829 18.83945 92.47976 7.341994
43.9828 45.97142 13.99987 12.72672 8.991111 32.47132
18.29683 18.31568 9.846325 38.09501 29.48891
55.09858 44.21169 17.05918 127.4807 24.53844
32.12719 12.96931 23.91053 26.75157 39.05239
29.10493 20.38745 13.76603 11.53083 18.36768
60.14114 44.4362 25.67393 43.18072 61.88356 105.1107
28.73084 203.8486 14.87464 13.09134 27.8446 15.16442
49.74886 83.75097 46.87747 145.7547 28.12647
41.90201 69.56142 43.74413 20.4491 43.41969 13.80175
28.26992 35.21765 77.07591 67.87005 7.973387
50.18841 22.397 46.02338 27.0982 41.08074 51.4242
11.91019 8.581425 11.6884 18.90652 46.54371 10.53559
27.50655 140.918 67.08026 37.39967 11.36324 10.09219
31.07163 16.76444 64.19637 12.68656 15.03305
15.64505 53.56593 35.64929 97.33306 13.14182
9.403942 20.70075 28.60214 53.03687 79.67606
24.75492 41.80034 19.34881 74.44451 43.6001 32.55049
93.09644 26.11484 141.2363 15.72508 26.9278 20.30063
40.62486 34.48939 49.90446 24.03576 45.42599
7.65363 45.02874 19.43953 29.16574 34.72092 37.98764
51.30314 59.67575 9.233258 10.40359 48.33066
50.6493 14.80664 18.19597 22.57785
TRIM31 13.90391 0.07860064 24.44456 9.002451 23.69287
0.4162267 1.625622 2.122975 12.77254 6.034978
0.2010893 0.1049691 6.448343 0.04624132 8.710507
0.1187488 7.239115 5.932147 10.39291 16.75713
33.72866 0.08595605 1.10847 0.1130939 0.03940871 0.09691488
8.899727 0.08124542 7.640427 7.833695 2.833923
4.439307 22.39894 17.68199 33.45285 1.540633
0.1111916 15.80246 13.55922 4.979362 25.41341
23.30722 12.09317 1.849499 17.58463 10.95367
42.33259 24.2792 4.377345 41.76466 12.10784 38.61355
1.036163 64.10883 2.876801 3.258785 36.32754
0.9563895 7.014074 39.2869 9.63087 43.45518 2.959471

2.957757 24.23533 1.111206 9.000681 6.795725
29.62785 23.85833 8.248929 16.86305 45.95864
3.924266 10.57958 5.87946 37.593 4.392648 34.24823
23.90681 17.02218 21.06896 13.65169 15.61541
1.874515 26.34356 11.34182 38.09607 8.490006
6.879498 19.54146 31.05919 14.67915 29.58736
6.192417 3.862831 6.923935 9.265767 0.1345121
38.22194 4.132038 41.19603 3.712139 12.74883
10.65307 9.713138 33.15473 4.210675 37.76162
10.64614 23.74229 39.35948 1.370407 9.029913
29.3589 29.58633 8.208467 8.322517 2.553428 23.1153
13.32521 31.23385 4.772116 12.2619 0.6518581 11.04249
30.26127 1.751369 9.966547 3.161094 68.82429
5.249978 13.95073 7.475137 42.14516 28.36097
10.46902 5.686406 7.07871 24.99552 42.58672 29.49695
12.51077 16.74212 22.37895 12.0918 20.87138 10.32
22.67783 12.20065 14.88181 39.47818 15.24708
25.01002 1.303502 37.7732 14.5316 6.555152 0.8428473
67.70828 43.76005 15.71535 8.321907 28.4303 4.156864
22.02244 39.30454 17.904 62.71165 0.05059829 0.1255348
13.12983 19.57899 10.08336 8.625501 40.00159
13.65291 35.51608 68.80845 2.829732 12.96298
22.82572 5.739536 16.44954 6.952766 2.620315
7.552972 30.75122 37.3659 40.28757 17.28473 4.110127
0.61235 8.450934 19.94371 19.57724 5.986519 19.42549
9.439525 22.99534 1.561998 93.52206 13.69536
58.7346 15.45142 18.03614 41.99082 0.3171649 9.053837
48.31077 20.26574 4.416388 10.60537 4.406016
16.86923 4.516424 4.341149 18.27868 8.632932
20.10327 21.30497 1.598809 11.21631 3.49474 11.48624
3.457281 26.74723 4.46011 6.929902 24.99291 3.486916
10.15598 18.44587 77.02874 10.53492 21.44141
5.541316 15.58687 22.84662 5.849818 41.26606
32.06156 8.529731 19.83156 5.21775 40.75746 26.07189
1.30394 62.61588 65.89601 26.95546 33.61097 29.75896
18.97737 34.62317 0.0753244 90.30165 4.375263
8.215081 7.249851 11.80874 18.13286 8.807713
3.213948 20.92243 16.20547 40.87634 4.158196
18.82429 48.27805 14.48174 13.00202 54.78689
7.38863 48.15635 44.69016 29.59649 3.046151 0.06168985
37.48434 16.24196 36.11395 16.63944 11.91879
5.586615 31.86314 85.10375 14.80529 5.067858
6.126083 17.87501 1.320411 2.697542 18.15184
0.1070643 2.828381 21.70663 24.04901 5.157279

50.21226	25.12698	12.65752	5.155478	9.234377
21.91698	22.44942	3.54958	21.07643	12.0072	10.98032
43.25661	7.930968	13.97949	7.68421	41.44071	2.674148
14.99789	17.62371	9.453173	24.18344	44.52258
25.02912	21.49553	28.30071	10.3886	10.25354	12.34867
0.09711996	30.26903	0.6266964	42.44114	5.561587
13.73413	9.305418	4.064674	10.57536	39.94876
29.94303	48.12305	8.2105	2.0817	15.57802	6.191762
30.86678	1.992692	11.17272	17.84812	0.1425398
57.80388	29.6785	72.38104	9.379581	6.697367	6.405351
36.4536	35.42127	44.49116	39.12039	4.383389	67.70502
4.983478	6.547753	0.06584188	29.05142	15.34703
21.39527	16.48642	149.5881	49.67049	12.77544
11.66644	21.49925	0.6956988	35.92569	9.022133
0.9243827	8.858753	24.29046	17.35878	26.03662
0.1642819	13.75055	91.62616	0.03681601	28.81945
22.62785	9.083464	26.63673	1.700478	2.2173	26.37984
5.332883	10.71707	13.9821	23.95604	48.80432	31.38261
25.20727	17.87523	94.83994	18.05857	1.078557
14.09254	41.21955	28.51198	20.62453	11.54882
PDGFRB	2.540523	8.681698	6.926351	7.828962	5.353613
26.58635	3.85606	15.93874	9.079352	5.53454	6.83401
0.6918914	7.02596	2.675905	6.371989	12.55969	6.423992
26.07161	5.318593	6.582537	6.905533	9.629961
9.450083	20.73083	21.77108	4.361831	7.037476
15.24664	10.87312	1.639795	7.461256	9.014234
19.08791	8.963164	49.93359	4.074249	4.876156
21.04584	29.90613	12.49909	39.13984	5.433837
35.50883	9.852966	14.70495	13.29992	5.786695
32.58257	19.87172	9.300133	16.43794	38.66784
10.00229	24.71961	37.98403	10.13753	32.79734
52.8526	11.15105	7.401667	93.43317	42.60582	55.48717
60.45072	4.647487	24.94674	55.00814	54.20314
20.4589	10.4122	18.74749	19.89904	21.4768	33.86882
69.31449	23.49587	17.9341	48.35932	17.79689	8.16799
11.45042	76.93826	16.48685	34.78496	67.87398
51.35482	48.98182	22.79852	16.08139	28.67867
30.50044	27.05804	23.1406	8.989125	17.97641	46.66749
43.88851	9.802563	6.624883	23.74544	77.93227
7.590607	7.589102	13.06624	51.1941	90.3444	23.68995
43.40932	31.02739	11.64261	18.18383	16.41555
11.54625	11.08605	22.364	8.625781	14.90303	37.63034
30.22416	5.08993	10.91654	19.0291	46.45339	39.51298
23.81797	13.8236	6.023366	36.02838	52.40639	76.54642

17.84209 9.828938 7.514706 27.31631 13.55244
38.54073 29.86911 14.9308 21.44003 11.08496 32.58663
17.09647 17.30409 7.008632 26.98383 16.0594 4.814758
42.05158 6.6792 61.61371 24.2447 109.6759 29.64526
47.13486 50.55719 9.057422 23.63363 18.06353
34.54114 12.06872 75.96857 19.46989 41.81638
15.61136 18.86382 25.89604 17.3083 26.38509 28.87962
9.934436 9.160352 23.20561 12.65574 6.367747
7.056316 18.07445 63.53951 19.17722 10.42628
66.47746 11.00477 16.51963 14.78635 44.03966
25.47629 26.86013 71.06219 14.42977 36.80023
16.95391 57.00797 29.08214 29.26955 8.598784
13.27717 60.17922 34.78731 9.961291 14.66317
15.36698 5.483014 31.02927 28.01369 12.9272 8.670014
4.637166 20.55057 38.38011 23.25364 31.34065
29.69402 53.83195 19.92702 19.53317 11.55148
28.52547 40.80148 13.07394 110.8632 32.81173
26.33087 35.72563 17.36309 20.99617 84.37595
6.73417 17.93733 3.071909 4.393601 15.49376 28.95174
58.68551 2.381665 13.5683 6.386514 28.20429 30.73654
7.012464 30.85658 31.49159 15.43546 49.31967
32.78359 38.68537 43.99823 39.01024 45.65167
10.66976 50.9565 17.12204 31.40168 10.60382 36.37182
54.07267 8.322236 34.12248 32.46806 39.83698
25.31215 50.40619 10.52661 20.06968 21.81777
16.34156 65.50825 36.43588 10.50833 26.7524 55.85482
11.25216 44.39028 27.92595 8.355963 11.62636
4.286385 17.43963 25.15097 14.54846 27.48455
50.88538 64.6521 16.76331 37.81243 41.68809 15.91185
43.91086 7.73717 16.8837 34.81791 100.6681 16.92861
15.48521 26.13041 46.09868 40.14653 125.1783
43.31602 41.25578 15.10547 15.82633 23.79734
71.02191 17.5953 114.2556 5.09367 17.1377 9.894431
21.46009 34.39856 18.17388 20.88257 23.28017
21.09142 78.3742 15.24567 40.06637 30.99575 67.46075
21.9175 37.61563 14.87148 18.82771 10.42192 16.75715
30.02959 10.2742 53.85718 19.18552 12.83007 42.48667
3.261685 58.38405 61.14095 26.36274 41.26847
18.46751 2.749625 14.07309 30.89164 12.76628
22.34194 20.28514 19.6188 9.877047 58.27522 3.51816
12.24025 16.03737 8.243048 13.65734 21.54411
68.71555 28.26169 45.54927 6.625192 12.79524
37.90441 33.36701 20.83627 21.81678 33.10584
80.99393 19.70166 6.333569 15.30184 19.75645

2.301298	13.86397	11.36052	26.61484	14.6179 16.51779
17.4886 13.56499	26.00865	40.32137	10.43286	17.79912
13.51025	48.23641	51.90471	50.74782	7.226314
11.63203	15.39462	19.42612	90.05727	18.23944
10.18129	23.00312	47.30434	14.61696	3.313192
47.64433	13.34228	29.04452	50.25468	45.7212 4.475934
33.64784	13.29134	22.69481	26.70433	4.702988
10.46765	35.10432	25.36764
TLR3	10.09916	1.675632	10.2277 4.968148	3.477484
3.498969	3.905955	2.732006	4.062961	2.56907 3.444195
3.013965	2.589273	1.705779	3.135103	2.982215
3.154987	3.317178	2.505155	3.45189 5.728919	2.414414
2.740595	3.890538	2.05999 2.74116 2.746426	4.20809
4.171284	4.059564	4.591746	3.772931	10.35124
4.144379	1.838222	1.09415 0.3979546	0.59189 0.5599153
1.776428	4.465152	2.148562	0.52625 5.699269	5.204203
0.7679272	3.105003	0.9150517	1.757873	4.790718
0.5487448	2.354569	0.1390827	1.959073	4.752106
0.6427959	0.5551869	2.815245	0.125661	2.03584 1.758882
1.85641 5.001193	1.046766	1.495643	1.856532	1.244155
2.441496	1.586928	1.258829	8.693698	2.55892 1.938671
0.862311	0.610339	1.672501	3.294792	0.93042 0.1897014
0.3513592	3.46015 2.095884	5.286188	3.388212	0.6714053
0.4790861	2.574507	2.945088	0.8142262	1.803501
3.489744	0.7430913	4.557256	4.871857	5.277493
2.068327	5.028716	1.647271	1.220319	8.269902
2.342763	2.518233	0.174248	1.853146	1.571831
2.425291	1.982427	2.219052	2.732328	2.39953 1.3203
1.821668	6.192182	2.23906 4.116972	0.2641861	0.7011058
5.113177	1.810099	1.019479	3.125954	1.043717
0.5515462	2.635537	1.15272 1.402087	8.610741	1.521333
4.188958	0.9999266	7.880193	0.6518868	2.194134
7.300241	4.328014	3.822557	0.8426376	0.7049187
2.396967	1.505458	0.9964758	3.59473 2.494421	0.8280849
1.078839	1.411891	3.795001	1.431657	0.5769817
1.196732	3.594393	2.082614	0.9652667	4.573947
2.791682	0.9634098	3.131553	4.193299	2.956084
1.200034	4.211021	3.15107 2.538864	0.5936193	0.5030906
2.658612	3.284794	2.508155	1.368327	0.3564531
3.622713	2.146687	4.135738	0.7321737	0.7359589
1.020258	2.842665	2.165571	2.725097	0.7450857
1.158291	0.938363	3.249736	5.437204	0.5512541
0.8169338	3.637529	6.010659	1.496846	6.110501
2.436687	2.802908	0.90666 1.94893 2.953777	2.274786

4.950072	0.742652	0.9467306	1.069895	0.1047263
2.514133	2.527112	2.762235	0.670465	6.94741	0.9685329
1.301589	0.5706697	2.003319	1.039172	1.50195	4.438375
0.5481121	2.359717	6.406759	1.618629	0.7942407
2.571485	1.590483	1.358997	3.73801	0.2177256	4.968565
3.020265	1.094408	0.7339883	3.190147	2.172241
3.338316	1.547958	2.143255	3.348056	5.41768	6.310122
2.495225	1.095573	7.994733	5.848194	1.352389
2.770507	3.531358	1.899749	2.275839	1.848282
1.772126	3.737876	2.294434	3.979112	1.337298
3.711797	2.396834	1.094516	2.358117	1.625297
0.5068826	4.36053	1.40207	1.702001	1.370622	1.039318
1.28922	3.752942	4.861413	2.415152	1.337774	1.040345
1.755919	2.081201	4.919401	4.465647	1.086404
0.943917	2.154498	2.474114	3.245807	1.693994
2.827612	0.1366782	3.058142	3.760616	5.777267
1.373279	3.795074	1.168196	3.449181	3.381376
0.9116542	1.212436	1.944349	0.4149503	4.059821
3.446682	1.123349	1.228376	1.519923	1.682044
3.347887	1.390695	2.292033	2.447881	2.075931
1.398491	1.046187	4.28511	0.6431379	1.650975	0.8060167
3.279556	1.161795	1.507437	0.7148556	3.981534
1.459834	10.48025	3.234776	2.729999	1.103011
5.404166	2.46521	1.171491	0.9647694	0.1821081	2.149711
5.072741	1.814959	1.217384	0.3971236	2.151003
3.10138	1.301546	2.743428	3.282594	3.656875	3.004377
2.188773	0.4512942	3.007363	3.611768	1.177226
0.3476715	0.6858894	1.623128	0.6298916	3.53732	5.922208
2.002144	0.238134	0.7881007	0.9344114	1.932399
2.253907	0.4735199	0.4711379	4.644674	1.676193
3.34032	0.9590534	2.388005	1.436016	1.699278	1.322319
0.9410944	1.068428	3.296784	1.70671	4.183683	3.264094
1.061919	0.2986844	2.000618	2.789475	0.470542
0.9825695	1.414428	0.6685506	5.309404	0.4507127
2.61914	1.26356	2.695066	1.360926	0.9381522	0.09032442
0.3235193	4.136965	1.647111	1.303858	3.431219
0.5052686	3.373115	1.157229	1.236515	1.225132
6.406565	1.669266	1.462256	4.261134	3.175571
0.9027723	0.6928869	0.6267157	4.740643	11.1239	2.67883
1.265879	2.478357
ROCK1	4.0563	7.177373	6.069596	5.761431	4.342191
13.43827	4.073996	7.85475	5.424965	5.217009	14.34443
5.028138	6.115937	4.434758	4.016152	13.36391
4.149132	7.667413	3.375712	3.107497	6.371051

13.88992 4.836307 11.82369 16.3003 7.704032 4.807767
12.06438 3.182126 2.476086 4.931744 16.36492
14.37144 8.300515 12.22884 8.103241 1.997667
3.954488 8.238866 9.569982 11.46323 10.37431
8.039273 6.739904 11.80737 9.435409 19.01846
8.765094 7.335203 14.92036 3.768464 13.1165 12.70421
7.356847 9.780385 5.851689 5.552764 13.3132 4.778318
4.840425 10.34648 6.54003 8.018338 4.752746 6.44221
16.55497 5.065279 6.840285 7.608612 6.595461
3.989246 5.897007 13.79678 6.736841 10.10491
3.391301 10.51344 11.05995 7.444059 4.461058
4.287917 8.102916 7.80085 9.704292 7.884118 4.194459
13.09122 10.38204 15.76275 12.38985 8.291041
7.949989 10.16646 10.71145 4.399872 7.10734 6.286653
7.37556 17.81792 11.1353 11.14201 11.24872 5.095156
13.03428 12.37095 13.54775 5.79665 6.881946 7.457317
8.059944 26.09731 10.74831 5.41553 11.206 5.794676
13.9037 3.41311 5.850816 7.111614 4.831587 6.291758
7.882904 7.278691 6.016784 8.024142 14.20804
6.972214 2.140287 12.5489 6.383199 8.186079 5.352108
9.422378 6.031058 3.684172 12.17958 7.00955 8.358847
11.43847 6.540574 11.22006 8.531197 13.85809
9.473411 23.12374 12.46415 9.545918 12.65588
7.430835 10.52081 7.980505 5.746341 4.77037 9.997986
13.98646 9.751455 7.352159 14.83962 6.226955
6.919609 6.104059 12.67541 11.82937 9.014329
4.799737 7.903558 8.546553 10.88241 11.76734
10.79592 10.73836 14.80284 6.411093 3.573114
13.97444 6.378384 7.67127 8.168878 9.493446 10.72779
15.88545 7.027678 5.270545 10.79279 9.857196
14.13672 10.74334 6.968224 11.58878 4.460606
8.550723 7.10293 9.057071 5.253677 1.876407 10.96553
9.387433 13.19443 5.721517 9.629003 9.80553 11.62196
5.880654 8.391414 5.207618 12.85087 6.033284
13.18271 7.878656 9.354225 7.125017 11.57597
12.57267 7.825139 7.932844 4.913847 4.93115 7.022921
7.99277 5.784593 8.83996 8.924936 3.150866 6.295965
17.14837 8.29421 9.99526 4.583936 4.228007 4.018265
5.050425 9.261283 5.068001 11.42334 8.258318
12.34842 5.421139 10.11717 9.018114 9.748795
11.41835 9.183252 8.583355 8.965816 9.09437 10.51259
7.905935 18.40072 11.36617 9.222749 12.23985
13.72772 10.64284 9.731848 4.479125 11.57278
8.590132 7.919261 7.801533 12.0078 8.880146 3.840286

7.040296 7.280342 4.457577 10.87682 12.71676
4.662829 8.954071 8.139535 12.65267 3.963271
17.59631 6.756865 11.94479 7.845822 6.582086
16.05878 13.25088 5.668015 9.628187 14.20517
6.697826 7.233837 13.50641 11.00311 18.71436
9.577708 10.12407 7.707451 10.48139 19.68262
14.07337 10.04241 4.471128 5.595699 7.156941
9.148471 6.104644 10.09246 12.52052 10.58254
9.165416 12.43632 6.700046 11.24377 6.314335
7.484264 12.95462 6.903555 5.944063 5.243062
7.600929 4.838624 7.305688 11.29103 15.22538
12.75945 9.568855 8.693219 8.644959 6.869733
6.194043 5.030679 4.864027 7.598563 5.472459
8.781571 6.389578 8.070447 9.770281 11.02227
7.952575 9.329833 6.461267 9.251092 6.564323
10.76014 11.32575 7.106916 7.512835 4.775038
8.286323 12.06066 14.57474 8.442326 17.13772
16.00958 6.289151 5.714393 13.38612 9.012011
11.26243 15.29429 7.329449 10.0211 13.8316 6.806879
15.83864 30.78903 10.92121 4.911266 9.1418 6.968253
4.149449 7.624841 7.633478 7.727845 6.971991
9.253967 7.950001 7.559066 10.35471 9.673987
5.114198 6.277297 8.46066 8.925054 6.11453 7.319613
7.258968 10.76881 12.36316 9.176359 7.886541
2.851686 5.736005 5.356487 4.874273 8.134946
16.56442 5.923625 16.23142 5.991464 8.368494
6.995748 9.469711 11.29352 4.391687 8.197221
5.51384 8.740551 4.562981 8.711233 6.990046 4.202085
12.20169
CPT1A 39.20182 12.22512 57.18726 20.48919 29.03357
16.67916 12.51455 12.67533 20.03126 11.4152 13.40568
10.86958 13.45813 8.749983 14.83569 13.75237
27.81563 22.58958 22.64979 40.50692 27.15834
14.0439 9.665327 16.36269 25.60202 15.4537 13.04533
27.89168 41.94476 9.490823 31.5886 14.42569 29.1484
10.75477 20.71557 9.363001 9.148961 15.06022
32.00168 41.93554 24.94849 35.85532 19.39001
20.42842 27.5377 24.83826 31.8896 31.7939 23.01148
36.51778 64.05922 69.12821 14.00058 23.85197
18.30378 19.82981 21.42285 29.97087 20.30867
42.53999 20.92683 22.60821 23.25541 18.60409
19.31126 25.82016 16.81033 22.28421 40.21161
27.76979 33.691 22.80532 24.93086 28.47481 15.93111
19.67158 33.41428 22.26922 15.52859 32.37736

17.10794 34.02238 7.320096 22.59659 20.9983 27.48931
44.83118 13.13495 68.80815 17.02152 27.81571
50.42953 7.724559 30.66769 19.97825 15.17712
12.93097 19.71934 20.39543 17.78157 23.84818
18.15897 10.26447 19.98902 32.36698 28.96365
17.78551 24.24592 25.81668 18.14468 26.35532
8.029994 8.291792 13.26692 12.37573 36.89789
15.3299 13.7281 25.99438 31.07739 38.14743 12.15417
10.23558 15.74837 25.37415 13.4546 4.321593 15.07675
23.12067 41.61712 19.86363 25.16253 23.83282
17.0458 20.67997 43.70399 43.2407 39.2973 27.07828
16.63298 90.34583 18.41828 12.48323 6.752504
21.54373 7.488151 52.04098 19.96859 11.46025
27.73966 44.22849 21.0446 19.07704 19.51969 35.80426
17.49037 20.19553 18.24594 10.40725 16.25963
17.33209 17.82398 34.15991 13.2813 42.06172 33.97253
44.08604 15.89947 21.97741 7.952884 19.47668
30.98022 37.44547 12.51195 34.16845 54.37165
18.58444 11.51394 48.49612 16.24343 6.505975
28.90644 11.14024 22.7106 18.92742 66.0188 21.19004
23.56496 29.8498 11.05903 22.62833 10.26116 9.353478
38.40354 19.31255 40.63248 14.12831 19.92609
26.9385 18.62097 4.877899 21.23488 25.76601 27.65723
23.69964 26.73482 16.49317 49.37103 30.19243
28.79635 30.37333 37.48671 22.7047 18.99441 23.385
27.62825 13.30291 11.10816 36.63854 22.0489 50.64367
17.8292 13.53324 26.2546 22.35633 19.65275 7.06065
8.946725 23.92255 17.37892 8.988368 45.58373
20.43054 19.58002 12.64213 23.90546 31.1321 20.04018
12.31952 20.29133 26.17659 20.88302 23.74825
25.43762 9.744572 33.57622 20.71171 19.99164
28.27044 25.50332 42.56068 18.13318 26.38941
43.68275 22.93058 8.111859 22.46803 19.32214
24.65237 16.4612 85.11663 20.98143 15.21141 12.70164
54.03099 17.18609 24.15292 40.4879 20.66372 26.44664
38.77745 24.44066 24.1571 39.49772 26.50955 22.34319
18.17992 16.5476 4.553185 12.11455 29.84472 34.60682
10.14282 19.4227 24.28502 31.92323 30.67917 20.20734
20.47413 49.09818 12.05179 14.18082 15.0902 38.83598
12.16754 20.47763 8.83032 26.23735 29.04006 30.91847
24.11329 16.99747 27.16392 13.57666 22.95484
34.0015 22.59564 18.80631 34.73412 7.686965 30.5477
36.33284 15.00649 15.87777 28.07439 39.48089
22.05638 13.90207 16.45385 22.94495 31.93348

24.93325 23.98008 21.71627 3.315807 12.92503
19.97238 9.732748 21.21726 12.67364 20.71642
30.2925 77.0659 42.14646 16.60645 26.0991 37.85224
53.89751 16.76724 18.98926 17.33084 19.11102
26.96262 15.09545 30.29867 11.08622 20.5083 35.11328
11.52077 19.94401 21.9988 23.60476 24.74761 26.51116
35.81953 30.94614 21.40218 16.86143 18.35387
9.146669 34.01413 11.99037 11.5179 13.01222 14.58704
12.27242 36.71897 31.72688 37.07898 32.27235
11.36325 13.57024 20.8412 31.64026 86.16931 13.77735
14.57415 18.19037 27.52023 22.54649 33.68899
42.33404 7.686989 51.04241 22.35551 13.26109
52.57018 9.007653 11.40846 6.562545 17.54612
4.385448 10.4183 23.62678 27.02816 12.98812 25.74766
26.7759 22.1698 33.04207 21.22604 18.0138 33.0696 15.51014
28.72295 29.6922 25.80717
NRAS 12.40598 3.794018 11.78097 18.24902 10.90547
4.355547 7.679294 6.3129 11.26273 15.16693 13.32321
13.36394 11.95675 9.20069 9.210287 2.953977 10.57232
6.697673 10.04403 10.01556 14.75249 3.340063
12.06872 4.489092 4.526909 13.84289 9.842026
4.288995 12.82311 12.16711 10.0497 5.550007 23.13399
10.99474 12.85149 16.80847 7.920777 11.07193
11.91288 21.08541 21.40293 19.43156 25.82503
21.12357 18.23261 17.43314 24.2921 18.02768 29.62009
26.47878 16.31927 18.16262 13.40393 22.44941
11.13608 17.54302 12.49993 10.55169 9.011275
14.37495 21.12912 17.85397 18.17761 12.5225 15.06361
7.373158 13.25606 10.25795 32.54416 22.44258
27.90447 18.71939 9.503963 11.72016 16.87322
11.67011 17.07106 18.3177 18.9794 28.46113 14.13839
18.21736 31.65282 10.54712 15.9708 18.01947 17.20702
22.10196 21.65823 8.751779 33.53667 26.3302 16.4198
19.73506 12.37768 10.71236 18.0935 16.83578 26.01246
20.83341 13.04267 19.90269 14.37138 15.83307
14.34451 17.04638 16.14431 9.069398 24.59799
14.9589 11.41097 8.343444 19.90915 9.399616 12.60629
23.09071 8.718089 26.99945 15.29481 17.42016
16.72807 21.23022 25.95533 11.24638 26.70063
18.54878 43.41435 11.80361 9.195507 21.58655
17.64588 14.20218 17.88305 36.69954 9.928528
18.71355 20.92612 27.69635 28.33795 16.43254
22.14991 22.14422 22.20051 15.77474 18.78316
40.30572 12.80225 12.02758 15.41723 13.10417

18.39165 18.95004 25.93392 18.99895 9.999148
21.28705 13.42538 7.408627 32.75993 14.35642
14.08717 15.19269 20.80858 28.03499 8.778179
18.57656 14.38292 16.48436 13.01742 11.11222
6.626301 22.54161 8.780038 14.70444 13.86635
17.52307 16.54693 13.68896 26.80375 31.72313
16.98363 14.23803 18.30746 23.1405 17.16444 21.1912
14.80862 17.52334 24.02843 18.84008 8.998082 5.0724
16.36725 15.95072 11.82029 24.97479 32.89183
22.0751 17.53613 11.97867 14.18223 12.60068 21.65473
19.30601 24.33175 23.61798 8.046927 26.42686
20.99354 15.94455 30.43366 13.35952 28.8687 12.9697
31.95622 24.05715 23.21205 31.09854 16.47621
22.25695 27.4811 30.6571 9.882075 20.05038 14.00443
13.36724 25.99975 8.874779 15.28755 12.47308
10.22752 10.54547 8.433478 25.48385 27.85906
13.14192 10.21701 21.44615 29.62449 20.8428 18.61253
23.76017 12.89219 16.17822 30.36974 19.51336
19.76419 5.167224 13.00777 19.83639 34.69522
32.33679 15.30133 19.58984 26.45814 43.89783
18.70627 12.09466 10.49777 17.21513 13.69416
10.99098 32.8226 18.06127 22.79508 23.50707 15.90708
15.31891 15.3231 23.50684 21.78665 13.03343 22.5383
21.35185 3.034592 9.413448 14.67322 5.122989
11.97399 37.80478 13.01707 19.66192 10.15649
15.44062 27.45544 16.40519 16.88636 6.8617 17.77846
9.591612 19.03804 5.039287 7.239557 14.54798
22.07793 8.186665 9.855563 21.18667 12.14772
26.64795 19.92844 12.85963 14.88996 15.87373
15.22069 13.29957 19.21942 37.03883 30.82585
16.96838 17.185 13.57686 11.33708 9.436267 21.77068
8.874531 31.96031 18.054 13.98254 31.79629 24.2089
39.06084 11.55351 12.07915 27.37576 20.59624
21.69266 16.81398 9.517057 20.88997 14.09418
11.41208 14.27702 18.98796 4.70588 15.11873 17.63071
17.73916 21.31519 25.64852 13.13634 11.85275
13.08554 15.22085 9.480342 13.0204 21.43448 23.08167
9.022016 12.35557 35.59213 25.62705 12.67015
15.98037 17.89376 19.62741 22.01003 13.78875
7.188522 9.115866 15.26322 8.553313 26.14787
36.93012 12.41379 12.05409 15.25592 16.42577
13.43243 14.4965 13.54185 13.49558 15.58328 24.78571
16.80176 21.94507 21.13166 15.72402 13.53358
15.38592 63.59872 14.66795 12.44538 20.27202

22.51657	15.97522	8.368022	15.31069	14.29949
19.86662	15.11335	29.73264	10.01841	26.44471
28.10762	28.8548 23.0645 13.27053	11.14192	16.84352
17.88445	9.783844	19.55306	21.5591 13.09002	13.02287
10.79374
PLAT	5.940532	11.21405	7.655312	10.45615	12.97804
13.98216	9.314141	15.75214	19.93523	10.26759
4.499985	1.328408	6.881975	0.8747834	11.06629
10.42684	10.7201 28.64515	12.05145	16.24649	15.84589
10.42671	26.67749	18.1017 11.36557	1.870515	16.04402
9.79214 19.67172	5.912903	16.9489 15.04614	3.827075
12.38337	23.47359	1.823917	1.726963	13.30901
12.62751	4.524832	15.35979	1.48241 19.02451	3.381469
9.166759	11.81487	3.747366	10.68456	7.316237
15.89636	6.525682	5.128389	5.951521	10.80389
17.1984 9.658737	10.3436 7.230733	1.917307	9.020812
18.95723	6.88293 12.02982	13.26332	5.247598	12.99561
20.43201	13.7365 16.15655	5.533096	3.581177	6.22291
6.0677	16.06702	27.73946	12.33176	12.26361	10.27882
1.990537	6.980599	2.878111	13.91099	2.25275 8.239379
13.64549	11.9458 7.722992	7.129335	4.444683	25.95799
5.618865	11.4156 14.10641	8.647018	4.166812	5.696581
11.35486	7.362695	9.747587	4.601716	14.716	4.50914
4.06178 8.360808	21.60603	14.00484	7.955375	13.05479
11.08883	6.468066	10.02429	9.046227	10.00531
3.930293	38.83578	5.755291	5.963966	9.377917
5.857848	7.537745	4.839004	22.89027	35.69643
23.45171	4.965394	7.414445	3.933583	9.096123
16.83306	24.08596	4.792146	3.31164 13.96984	15.67715
15.87163	14.0913 5.447936	4.914189	18.42223	7.008372
5.391186	5.05544 6.776548	11.29579	15.25241	14.25806
4.679379	11.95953	2.095313	5.498356	3.702686
22.80776	5.500428	6.975175	14.61033	9.657435
6.141762	7.525345	15.35018	10.3196 15.37668	8.685391
17.76485	6.430695	12.44336	9.00805 7.238635	14.88166
8.218877	3.500002	16.87775	24.57811	9.055102
6.40693 3.303308	13.00445	21.89719	8.826316	8.323131
12.43748	12.36017	6.478636	5.455446	15.2189 14.30321
7.125683	16.39932	3.721783	8.469526	5.27835 15.12351
10.25764	6.960041	12.7904 5.94555 13.77895	9.27612
11.07178	5.70202 10.81135	3.273181	17.38432	15.1322
3.234796	3.173413	7.525067	10.44358	7.934616
8.342926	7.198087	21.23708	13.47506	36.74612
6.564052	3.575201	6.797486	22.33775	8.193099

11.39364 13.16664 6.723542 9.329226 8.106529
5.46332 7.393068 2.86257 17.36293 7.029509 7.143396
11.23063 8.051991 17.79849 1.322876 4.78219 7.408253
13.54253 9.410932 4.991332 11.76722 5.971404
8.204593 10.12141 10.21859 10.46244 7.20467 13.36678
10.96923 6.250912 13.67979 4.094938 3.453525
2.33613 16.69297 8.930848 7.982208 7.244888 7.814457
14.81872 5.189847 13.48911 7.126256 5.500837
12.73728 5.624155 9.486262 17.83325 2.680543
29.35192 19.38628 18.75798 8.916235 9.227212
8.410712 4.319826 5.388117 3.703172 12.28543
6.04803 3.088034 10.0229 12.22625 2.917891 36.79847
7.21608 5.567732 7.494277 6.723958 7.893473 20.56117
13.91596 10.981 3.781299 11.98633 13.51274 32.68481
21.03553 17.72757 7.346077 3.338452 6.716451
15.79968 34.21911 43.25573 22.27802 5.126302
7.296124 10.00749 6.569679 7.756878 14.20763
9.326388 8.779181 3.211222 15.29709 5.242104
11.47836 14.53922 21.53142 8.586678 10.66904
3.269546 9.810083 9.905 7.893728 23.08572 27.17972
13.17179 10.35802 9.247077 7.789439 3.949367
12.96139 15.19693 10.89127 20.87307 3.246498
1.994141 6.33073 9.192711 9.61426 11.07638 10.02018
17.20353 2.339239 10.92319 27.44337 16.25052
3.898242 8.414878 8.402924 10.95364 13.02175
14.48367 31.04384 6.259012 11.38577 11.7579 7.741533
6.000733 6.99105 8.889431 21.24772 9.988107 13.7662
7.218705 5.568753 1.732919 8.045815 9.236297
6.366767 10.73302 12.35895 5.880994 5.691874
8.513658 16.72777 32.91879 14.0705 23.1795 5.696439
15.70944 15.49629 4.362625 6.043785 8.701402
7.937314 17.99306 31.50159 1.99828 14.24679 15.88223
3.602114 5.766779 14.01147 18.78926 17.82595
8.212494 7.933641 12.79988 10.63605 8.102727
1.31899 5.07042 3.084207 6.479731 17.79919 10.98533
CASP10 9.864851 0.4790573 11.96391 12.03008 9.282566
1.388479 3.657551 1.757734 6.190449 6.042035
3.894951 3.3848 8.516081 3.510361 5.46789 0.640314
7.918488 3.057979 4.008992 6.852922 8.117681
0.702706 2.889978 1.42067 1.305718 2.520533 6.522709
0.8285707 9.352437 2.156423 6.765388 1.87172 10.47288
3.396641 5.724054 2.833609 0.2785587 2.868046
6.889781 9.490048 5.3212 3.040911 7.544986 4.172131
9.19931 7.295417 14.99405 4.930694 2.655728 5.163625

3.423639 4.42182 2.677076 8.688728 3.489646 5.347706
5.157494 3.101027 4.726263 9.407498 6.692979
11.56787 5.500005 2.038431 4.215516 1.867135
2.963936 4.195681 8.837434 12.03655 14.24086
4.466769 6.507042 1.758027 6.237552 2.545241
6.425429 3.390078 2.328611 7.245329 3.304837
4.758131 12.76446 8.938374 0.7855173 4.587835
5.874945 12.20927 5.902087 4.410688 15.71431
24.24629 5.883754 9.630134 7.919027 4.963782
6.081344 3.51761 1.718606 8.309029 5.542298 8.830711
8.59138 3.961178 4.149391 7.542372 10.6808 3.344401
4.597634 4.899627 3.493696 14.09761 4.889287
2.415744 5.336423 10.08058 5.971367 12.84787
4.69123 4.683082 16.99005 4.669719 1.185893 4.306187
0.8638873 4.147144 11.60089 6.427656 2.995083
5.567821 6.617058 4.314777 4.276503 6.832361
8.562292 5.788374 5.361828 8.035694 3.10821 5.647783
9.224916 15.15714 9.600488 3.013864 6.022619
5.715448 6.304872 3.621092 2.339038 7.088495
7.694313 4.742879 9.488385 10.32475 4.766436
10.35115 3.719596 3.192452 0.5801495 5.597858
3.27701 6.25534 6.591838 7.608662 3.401574 8.099674
8.922151 5.683139 7.817645 0.444926 1.314914
3.224958 4.349568 3.418772 3.669527 3.950934
5.123832 9.081178 16.52269 5.098012 9.727423
5.059786 4.567634 8.821859 3.462113 17.36526
4.388467 8.056492 12.09859 5.419181 3.151779
5.446596 3.24629 5.241467 7.223855 12.43681 5.212575
5.46674 2.727812 3.528262 1.749144 3.570858 12.75301
6.969774 2.130963 8.643425 6.027505 2.38302 9.524032
8.661617 8.381896 4.631808 8.121889 3.287881
6.08867 15.63249 6.486896 4.293015 4.202808 6.111848
5.809937 10.56915 3.546526 6.747591 7.303213
3.00163 6.219167 2.912243 5.575798 5.476745 3.270491
2.694982 5.673957 20.16761 10.37798 5.093578
3.91125 5.242674 5.851288 1.476017 3.79659 8.840845
7.447578 11.27867 3.82987 6.001325 5.568363 1.95204
10.50568 10.02403 7.248624 10.9854 5.298886 5.913932
19.43874 1.541979 9.054816 2.628254 15.08912
2.420379 4.220409 9.014882 9.592522 11.3244 6.366869
7.545479 11.30309 4.214554 4.764864 12.25589
5.829449 5.355451 6.89354 10.29879 7.172827 4.937545
7.245207 1.700374 7.419324 6.810367 4.869241
20.12758 1.461004 9.700252 1.702103 5.199871

13.71386 6.881791 6.777532 5.365877 7.651413
1.089934 2.492763 12.27343 2.393911 4.424199
6.097223 8.028313 8.03225 7.886694 7.251055 5.914272
3.670472 3.086146 6.348291 4.519687 4.49908 7.445901
4.65645 1.751716 5.367445 1.157007 3.372788 2.772635
2.712507 2.121919 5.3951 3.647942 7.529889 5.593302
9.019844 6.665967 5.286053 4.223235 3.376257
3.706577 3.940269 0.6200448 9.267872 3.559984
5.150264 2.705499 6.693959 6.641533 2.081465
5.69759 5.542364 6.345018 9.701773 8.492093 1.027379
3.755598 2.633765 9.239325 3.923041 5.298691
4.462405 0.4583256 5.295512 4.922641 10.06404
10.44783 1.96481 6.035417 7.308438 4.705053 10.42633
4.075573 2.161144 7.309866 7.428519 2.947791
2.467887 5.636901 2.422165 1.622335 16.13098
9.184228 5.901355 5.72884 4.905086 4.205247 1.478366
6.408863 2.958146 1.58723 4.189254 4.9526 4.919269
4.759619 5.491003 6.300177 1.475722 1.246596
10.56566 5.607595 3.222022 6.62347 9.290411 1.049212
3.684048 2.478818 5.635366 4.028701 12.73475
12.54821 5.147112 7.513164 3.304481 7.641297
6.846018 3.367363 5.666524 6.702173 9.725602
6.093208 3.506198
PAK4 13.18909 5.572686 11.20396 15.72975 8.592266
3.778183 8.266 5.298342 12.54044 6.810369 8.483478
7.020543 6.650365 13.26007 9.900464 3.541444
16.35403 9.956603 13.31743 14.16525 14.11386
3.060611 9.65855 3.307841 6.450123 8.51951 10.03291
4.036578 12.18525 3.836474 13.85495 4.716905
30.49238 10.32215 8.468245 8.276649 6.841777
24.01227 14.92348 16.42122 12.60655 22.32458
5.678916 17.40529 9.676614 18.75619 13.47672
19.86986 5.696973 15.70091 18.04454 4.918503
20.84192 13.27307 10.09271 13.13092 15.14368
4.253507 10.01026 12.41301 4.956534 20.71576
4.134544 25.81586 21.1568 3.320835 14.50226 7.107717
19.22555 11.60266 16.1075 13.12155 9.239441 10.09705
12.13501 33.26625 6.695151 16.35079 16.29561
8.171242 23.6711 8.761501 7.92661 6.893255 13.28382
18.77662 7.990329 10.54756 9.800389 4.512028
6.529032 9.416604 8.440939 8.070583 12.66846
17.63578 11.11195 13.18504 19.42374 11.94048
7.700488 16.33245 6.662173 15.64665 10.98602
7.826415 13.56509 9.984766 16.68301 17.55875

14.75652	35.24442	9.811689	4.844322	9.683425	
14.56991	20.37538	12.53586	12.78746	12.03143	
3.67475	20.34671	25.84354	5.641043	10.7053	6.250845
11.08477	16.56149	7.675411	26.34783	18.85021	
12.58628	14.0578	6.845564	12.74983	9.814629	14.77874
19.2049	18.58713	6.066013	8.883003	14.02306	19.79437
19.11511	30.38226	7.948013	7.395561	6.457651	
14.92935	9.303047	7.490088	10.75173	231.7948	
9.408226	5.854028	7.572258	11.11531	5.519956	
9.447875	11.24522	12.21275	5.324133	4.556453	
11.08434	11.44838	17.45627	54.2451	8.120544	11.66043
10.91541	5.466681	18.56641	11.27223	8.966098	
8.151094	15.60338	20.20169	6.192618	10.44673	
13.16836	8.130718	11.70983	13.79158	12.06317	
7.381204	7.962606	8.011064	10.55395	9.484477	
10.81499	9.123562	15.3467	28.39146	14.36938	15.03874
10.47011	6.080414	17.5176	13.76387	8.10549	20.32679
9.574157	14.90796	9.975007	11.61766	14.154	16.45832
9.022013	14.54747	13.51129	7.334147	9.271752	
10.24072	12.10297	15.50054	8.000253	43.47127	
28.8558	13.86866	13.95534	11.6522	6.410758	22.78156
9.446627	7.220298	9.288528	18.10032	11.20339	
22.78832	26.88978	12.13393	6.066923	14.33151	
14.90555	6.729266	13.08532	9.344774	8.741599	
6.213915	16.34595	13.06528	15.81481	8.245611	
9.16482	4.87162	14.75497	8.744461	5.910323	9.032203
11.25343	10.71986	6.163977	8.951844	12.78998	
20.17341	20.03431	10.78125	20.63095	13.81865	
14.0472	7.756601	19.39099	9.09103	8.329992	15.98664
17.46353	16.96328	16.75418	14.76818	12.83875	
12.24931	20.70607	10.99523	14.70993	7.911335	
7.00313	22.9398	3.654489	3.399943	10.21634	6.192098
8.428429	9.637535	7.733782	8.980978	10.55915	
11.2432	13.28373	8.245143	6.992796	19.3948	2.361707
4.681593	11.91099	16.83733	9.878257	21.01337	
19.44723	12.59813	13.16375	9.106476	9.453369	
14.23678	7.82809	11.6319	15.13682	8.212289	9.17182
9.118736	18.40849	31.21697	23.47546	12.97948	
10.4585	10.18031	6.571638	12.28506	17.02381	8.603657
5.313182	13.56009	7.740133	79.11458	12.19788	
3.181282	19.05799	21.82524	35.54091	12.06608	
11.42547	14.19079	10.13324	11.27927	15.79307	
9.314283	17.1378	11.24776	10.22345	21.83809	10.13048
6.079889	21.97249	10.58771	19.19025	4.681322	

13.10814 17.37766 19.67828 11.53208 9.966474
15.69712 12.12052 9.251192 6.372139 10.38558
14.58395 7.908982 23.6769 3.659131 9.635573 15.6078
21.59725 16.34409 16.57241 17.85691 6.949465
10.57093 7.422832 10.58106 7.865936 10.46959 11.645
43.14015 9.13198 18.40258 11.03766 7.21831 6.51086
10.79338 11.39451 21.59073 13.34368 14.98933
22.84212 14.82298 23.06263 9.630735 7.719406
14.45719 27.3586 9.142029 4.923839 7.944086 8.559846
15.68273 8.372464 7.580338 7.480719 9.830241
17.68154 22.31489 16.60015 17.46037 12.61325
11.2026 20.95061 6.950301
VEGFA 6.378859 2.442359 8.957785 6.790721 5.386486
3.72352 4.439424 4.632108 9.880497 4.1562 2.277994
3.704665 5.425913 5.55128 6.552146 3.515239 6.255699
5.663195 6.696492 5.033709 11.66322 1.742609
16.76822 2.03198 2.81055 6.583144 5.925063 2.769531
6.332665 3.728211 9.357399 2.958218 7.844417
5.304804 17.85052 5.153271 2.775067 17.14847
17.45611 8.645086 14.65048 12.28038 9.147907
13.44655 8.031355 23.30945 6.80968 15.15636 8.178358
22.48115 12.18329 6.931145 6.478278 8.976753
4.883809 8.97773 11.58316 4.498718 47.88535 11.78698
11.01744 15.74947 2.504732 7.306547 8.75882 4.646494
6.356131 3.659023 9.670058 6.239553 11.04563
12.25143 6.286299 19.34207 8.201645 19.84816
7.321025 12.91877 41.02273 9.617633 26.54835
13.92146 16.58895 6.259965 8.830445 14.2504 5.60835
5.65815 5.352731 4.493471 6.725266 23.38898 6.052892
6.541441 6.737342 12.42241 5.244377 8.887041
9.754865 3.91962 3.331493 12.34831 7.042598 10.15136
6.040845 6.026898 9.738009 8.26532 10.39494 26.98464
8.891183 17.32826 5.311018 3.549341 15.51015
13.92495 76.98471 27.00365 5.072301 11.28878
50.83725 9.078528 15.65773 5.633499 13.05201
3.322114 8.588676 5.010962 5.076755 17.48177
5.741031 26.55728 19.56169 10.7827 12.16037 7.653031
16.05527 6.850855 10.45603 17.78341 32.24034
9.730516 12.99939 18.63581 9.234406 6.780503
19.32657 4.50594 23.80695 4.554282 14.40308 7.108811
5.293092 16.02896 3.476296 44.76912 6.254786
3.790839 7.970031 11.52311 7.46747 6.653679 8.410456
15.60729 6.138088 3.568421 15.05628 12.07514
16.06377 5.436226 3.49396 5.622384 4.335238 5.421519

9.66013 7.970599 11.79792 6.004882 11.11091 109.3993
15.01529 8.463807 14.33121 16.70405 28.78953
16.79433 5.624225 12.40798 19.24277 22.87979
26.81505 43.52736 15.94699 5.259209 9.235837
113.1424 8.58195 7.015654 14.32617 8.534604 7.210237
15.03071 84.67337 12.54 5.247895 12.03974 9.097165
5.731838 10.24949 54.70104 17.43225 5.748374
5.22379 15.08899 25.51435 19.78724 11.53474 21.62195
18.2034 9.154844 10.15771 14.92592 9.673427 19.84677
4.952208 7.046006 26.05195 8.126082 8.458221
3.462054 8.223472 4.863205 3.395752 21.0278 7.673096
20.83007 3.273118 9.051006 8.869334 3.485783
18.55434 13.67573 8.102726 7.295605 8.435472
14.03701 11.42872 2.590559 3.558453 12.22516
15.35626 14.30688 41.04779 12.82538 19.11284
14.07225 9.814717 10.72698 9.663847 10.76144
8.727775 11.58253 8.277805 3.783543 16.55657
11.83244 18.15421 4.857454 7.632347 8.866737
12.36231 13.30213 21.56334 11.8348 9.415753 6.668434
9.334021 2.217593 0.9746897 4.742914 4.466777
5.686535 3.823034 7.102815 11.63327 52.63842
10.33654 11.95977 11.61813 4.451127 26.29215
2.030407 3.1605 68.3185 6.260381 6.86352 9.400948
16.00219 39.14744 6.118177 4.244965 7.386064
8.766324 19.5432 4.294835 9.553912 12.3419 10.24613
16.14516 6.796376 8.02201 8.071193 57.70413 2.61091
32.75802 2.954043 9.924839 14.17295 12.14836
9.503415 14.12897 8.930423 10.6404 28.9665 4.466303
11.95133 32.06708 16.39025 5.739236 11.90337
9.582632 7.565794 7.146269 11.26472 3.943278
15.12714 14.35436 7.073434 21.93374 12.03569
15.82616 19.49192 7.738633 90.00326 4.95811 5.317852
6.807469 10.94822 7.292098 7.398061 28.25027
10.96864 7.370156 13.23973 12.10843 22.07382
5.330319 20.69496 2.630572 71.79926 14.24513
5.113153 19.01455 11.98011 16.83951 7.442085
6.198078 10.67259 17.29795 18.74519 9.426001
7.366376 16.10446 18.94616 12.76579 16.62238
11.91641 21.54809 5.163553 13.40636 10.48312
23.92812 9.452204 11.13372 17.89227 10.12344
8.275013 3.862527 36.33381 7.880268 8.461454
14.77655 9.042106 5.383946 18.47508 24.73214
14.2763 3.661517 11.64286 21.65778 16.0726 5.749871
8.972754 6.981566 6.11588 9.617543 4.802081

ZEB1 0.9338626 29.53917 2.309729 2.835088 3.780502
34.77516 1.717952 15.53623 10.16466 10.82276
6.146144 1.35953 3.478216 1.521246 2.617769 53.18293
1.54454 22.174 1.366678 1.653743 6.616199 43.75574
2.504315 32.28237 22.24585 2.738112 2.589541
38.86564 2.788104 2.115211 6.607099 34.89163
2.25521 3.840523 7.156933 2.234936 1.677811 2.869216
7.503106 1.73912 6.663836 1.412533 3.517352 1.116477
5.911636 2.139349 4.797977 5.05317 11.38837 2.597156
1.634508 10.0009 1.859572 3.805907 40.73233 1.482497
4.510519 26.68959 0.7915566 1.655407 11.02573
2.966298 17.53617 3.588901 1.273571 27.73756
7.471215 6.411694 2.484044 2.70565 2.375546 5.868856
15.07839 13.5033 7.198964 3.554022 8.540299 7.62766
1.947524 1.648628 1.133127 9.129483 3.715165
16.17237 12.94339 5.446341 14.30269 7.714839
2.66982 22.72272 2.573525 5.005548 11.67647 5.016376
4.162343 4.047801 6.491167 1.945498 1.656181
14.63275 10.49986 2.388945 2.418753 4.547228
16.89617 13.70501 2.694365 14.28476 2.749253
2.35116 2.909102 4.849659 2.284393 19.32526 6.061518
2.384341 1.247858 3.36657 5.546366 1.556852 2.62982
6.832109 7.646112 7.985919 1.593528 7.51526 4.32457
2.020038 31.01916 8.49416 4.292999 1.160718 2.621619
5.309208 2.136508 13.47634 3.850221 7.52137 5.334693
1.877696 4.347837 2.530815 3.817928 2.367913
5.914767 7.290094 1.482636 19.95441 0.8481754
11.12877 2.374959 9.114635 2.299728 9.810222
23.37142 2.942853 9.555544 22.19686 3.755886
5.108118 10.27842 9.458241 12.57137 2.568505
4.219036 4.039964 2.304227 7.396898 6.125746
10.07847 31.86569 3.859492 6.012217 1.223755
1.133717 3.897577 10.2622 10.73885 2.578837 6.227218
4.701272 3.399474 2.759394 7.947689 8.393621
4.152639 19.42552 2.954295 5.838578 2.612396
9.800536 3.18946 1.789198 2.516412 1.891749 11.16533
10.51679 4.291329 1.891175 4.403553 2.249705
16.32974 3.620936 1.124331 2.173702 2.035445
5.77613 6.82515 3.069575 2.879675 6.032654 12.76961
7.628329 5.407384 5.037085 4.90823 5.372628 1.881461
11.19114 2.549314 3.347892 5.722882 1.997467
2.15493 10.58916 2.094832 3.095587 1.392461 0.836089
4.171151 4.642619 9.631 0.5805421 2.825259 2.344875
4.836171 4.180749 2.676438 6.527941 3.023467

11.54808 8.746858 8.103208 3.725524 7.437787
7.087749 5.47003 20.67649 11.89533 3.917426 8.017329
2.289618 5.977582 10.24322 2.618006 3.045451
4.866931 4.127989 3.324146 15.8989 5.561463 1.825538
6.29368 12.87073 3.401914 6.597148 1.355596 2.338866
6.796168 3.247492 7.288722 1.837372 2.780262
1.223464 1.170306 2.850012 4.973911 42.01644
6.086613 5.144967 15.69511 2.373224 12.27632
4.552017 3.700975 8.512696 2.770169 4.895726
6.750966 18.07011 2.793243 42.9706 51.8117 6.124029
7.409872 11.15449 6.167253 5.038706 1.550768
2.87106 10.85014 15.4071 3.23983 12.80935 1.967411
2.443515 2.892128 4.486004 5.100142 2.541128
3.006073 1.396611 2.035687 5.100003 2.743684
25.30896 8.898646 6.630272 5.805133 13.59507
4.369769 4.279154 1.914207 3.720012 10.77787
2.29714 3.076325 1.563348 3.003308 4.137671 1.021965
14.09189 7.641813 5.139547 15.88747 4.092091
0.7347143 3.520247 7.179396 2.381752 2.8906 2.847355
4.986153 1.566475 29.02772 2.336948 2.794925
2.669227 3.48861 5.269644 3.664342 7.194278 3.815495
9.089828 1.824931 2.451115 9.989443 3.865526
23.15784 3.443686 6.116428 6.605889 4.690337
1.768864 1.916291 4.45241 0.8024783 4.304409 3.546826
3.926932 3.802705 4.621359 2.242548 3.101759
2.638475 5.224051 2.210674 5.722506 3.779423
8.547484 4.376123 5.797099 4.900495 1.621583
3.462065 1.94017 11.2769 5.514864 0.5129284 2.622564
26.62112 2.982864 2.210452 8.153949 3.837091
5.865523 8.331084 15.84784 2.905834 3.112693
1.343355 4.881385 3.91417 1.544987 1.703463 2.581072
28.22598
PIN1 7.782507 18.47998 6.664445 11.21222 9.528362
9.665266 11.90654 8.26038 11.78908 18.66879 5.00207
6.951628 15.05219 7.189513 12.9158 8.573123 11.47827
12.0497 10.50429 9.580627 12.43756 10.2298 6.725948
9.991823 13.563 4.607903 11.93151 8.783245 9.331974
15.74038 11.38277 12.44755 11.93432 7.619708
7.736046 11.61051 4.793361 9.84224 8.94647 10.04412
7.768664 15.54003 5.383631 9.386974 5.967909
4.278565 5.263947 7.521093 3.994546 4.281049
14.10359 7.969525 6.060672 18.66185 9.427961
7.792815 9.821061 7.69446 12.95182 6.964848 4.970076
9.906749 6.025426 16.52491 5.280543 8.295991

12.30211	9.764644	9.358776	6.771262	9.272432	
7.670612	6.912513	4.852666	7.419121	22.06679	
6.444732	6.930194	12.61789	3.139173	17.65557	
6.75785	6.408813	8.077648	9.218929	7.916999	6.911886
8.026064	9.823595	6.651813	4.047007	7.060537	
9.410162	6.607242	12.53298	9.470625	7.710167	
7.987454	7.747588	7.289076	7.26017	4.871707	10.31369
5.048973	7.948435	5.609222	10.10795	11.33835	
9.676518	7.624719	5.290657	5.005748	8.59749	9.252379
8.73713	15.43466	7.786509	8.165449	9.647867	6.754969
6.596314	4.288896	8.249855	7.962091	9.39085	9.645554
11.82311	11.79367	9.3378	11.84338	7.244212	8.501995
7.970488	4.952502	11.5206	6.098353	9.005196	4.326682
6.241771	4.071322	5.643701	9.540379	7.214128	
4.63256	6.409344	11.15555	4.774842	8.2763	5.766022
6.735734	8.250146	8.611004	6.202346	7.958866	
7.234588	7.736347	10.27754	9.794803	8.493365	
4.307765	11.52432	6.726117	4.349977	7.337246	
10.68416	8.161591	8.241004	4.605491	8.191614	
14.40313	8.652755	9.819518	8.17446	7.489148	7.378511
7.797874	6.572078	7.448386	6.208503	5.617467	
7.254986	6.158324	6.942536	7.475701	9.189325	
7.314028	7.454691	10.3879	3.922302	6.302485	6.161605
7.108985	8.358447	10.7022	13.36598	6.293308	6.747026
5.172907	7.408591	6.508317	6.716257	12.93393	
7.102967	6.298688	9.930951	6.108038	9.174628	
15.47071	6.503883	11.30691	4.517782	13.13758	
6.70739	6.746183	10.59886	7.368181	6.685338	5.497932
10.16154	8.210239	10.89363	5.965727	8.245603	
7.280816	6.21525	7.355576	6.610955	7.706969	9.502948
19.55498	8.518449	5.674723	6.58888	6.636832	5.162179
10.14617	11.15546	7.02864	5.564126	42.99694	7.076457
6.807753	7.816349	8.012815	4.410802	9.037325	
8.606793	11.99535	7.359944	8.965337	9.879488	
4.301453	6.181093	8.87567	6.601777	6.9219	6.257969
14.49972	7.214165	6.630061	7.273097	14.33343	
6.544997	6.016468	7.061965	5.155338	9.767823	
10.42064	8.824781	6.153359	10.97879	13.44006	
9.047284	6.732228	6.857832	5.080297	5.757338	
9.147712	16.75942	4.980968	8.017246	4.906255	
16.10242	7.895281	4.690188	8.898032	6.619313	
9.229119	8.091856	8.421996	6.577315	5.673535	
7.063583	5.83907	9.017277	12.06533	10.28343	9.672993
8.875361	5.849383	6.422223	7.32212	7.197704	4.413211

7.324336 7.256518 3.970572 5.832612 7.479394
7.397381 8.492619 17.83647 10.56584 13.44093
6.551845 8.186658 6.226995 6.275979 7.626926
4.058348 4.188442 5.742518 6.692595 12.48773
6.185423 6.516261 9.322945 12.87968 5.247322
6.063905 12.4798 7.53677 12.0211 10.26738 13.98251
10.90198 4.973773 8.468522 6.614824 6.981637
3.536306 16.90687 6.198717 5.187845 7.469098
5.581006 6.551872 12.83156 9.563207 7.143735
3.882407 6.433595 6.36795 6.575945 8.47847 6.911787
6.354845 7.760988 7.94526 5.354711 7.876907 11.4091
3.985288 6.260239 8.723293 9.287867 6.058277
7.038716 7.502378 6.422045 7.805409 8.037979
8.870224 4.985894 11.61387 9.469226 10.21649
11.84195 6.394397 7.996453 7.641879 7.718763
7.315022 10.01794 5.042756 11.62746 11.75549
10.1563 7.403591 6.737603 6.450302 4.005147 6.325919
6.38221 5.852194 7.551851 6.596699 8.288088 8.860253
10.41335 16.22754 9.151563 7.941214 5.911578
7.141629 14.75468 8.9787
UBE2C 7.433772 0.2160601 18.97561 21.22802 16.43383
0.4690965 17.87426 1.827889 13.70896 38.71273
4.916664 11.40853 19.57473 11.52461 21.03365
0.3264206 30.04024 1.939426 15.55359 20.34088
16.66036 1.980242 9.773394 0.1351636 0.6577057
8.986657 13.03667 0.2143969 10.04266 17.20118
9.833258 0.7392639 24.18781 25.22755 28.69611
127.7913 2.4922 50.51063 55.70271 51.69033 20.70992
97.38128 38.94514 39.00126 12.31989 49.53738
33.45566 85.90159 19.25675 31.86145 82.60618
18.35478 114.0629 93.8539 11.0798 84.07887 105.0337
3.891317 145.1477 70.59147 7.298213 42.6776 16.34895
86.60511 76.87991 5.044933 26.15957 12.18331
52.02074 91.38944 40.10086 47.8358 29.77267 47.55196
56.46627 74.37222 9.043403 31.14765 135.81 93.39443
57.26121 20.95457 61.48688 15.24127 37.28292
33.9099 12.70552 31.43966 171.4521 20.65639 43.8091
38.66417 47.97477 32.24427 44.71994 53.34532
26.5451 28.09914 61.00897 28.19312 8.967811 20.93474
75.30695 138.1585 35.68345 7.669907 31.84574
20.20957 34.45694 49.73304 97.90565 108.873 76.58945
16.42742 33.48943 35.88761 81.50745 44.67143
83.45957 122.9908 62.61852 112.0731 163.4409
6.816694 80.92644 30.06906 50.88125 68.93869

4.939302 83.60989 23.19675 52.07878 43.63607
45.72905 47.55736 12.02369 38.16952 120.0605
67.82394 103.0109 112.4924 53.11503 19.95026
220.0197 108.2208 12.12405 148.2549 16.06262
46.79549 20.8244 31.93885 18.42212 39.89492 29.29357
5.308617 17.59508 40.33863 5.016432 67.37899
102.6602 23.38009 13.4427 28.67529 94.83057 136.9931
23.09337 135.8856 28.01281 31.40267 54.86954
4.112911 44.89681 24.92687 84.69296 86.20562
208.584 44.29453 138.9012 33.43123 53.74328 109.8762
61.56886 9.096779 34.17591 183.5888 18.95623
6.785671 64.87257 34.45495 72.3185 66.86097 65.87159
83.55553 39.15456 80.5622 38.98418 29.41861 71.39859
76.56524 59.43699 169.2437 64.71813 49.17605
43.38333 123.8932 43.31536 65.92842 140.8597
24.16792 45.47116 19.99966 30.16367 32.29833
51.52331 49.61242 38.40118 28.00107 204.7392
33.02148 36.61392 111.4558 40.2207 43.20003 23.21089
7.408257 63.28336 169.9222 13.65449 87.87143
74.65132 35.43373 13.95199 50.64569 15.97292
55.72426 14.34332 41.12357 53.0206 65.22533 305.8463
64.25588 32.19616 36.25641 45.27406 52.97499
32.40848 70.27533 3.674771 16.11419 103.1272
83.11433 68.04064 78.12049 54.66435 80.59027
154.1726 77.02672 18.66682 52.47187 34.83966
79.07015 82.66229 53.97683 63.82625 29.41621
113.4105 93.05962 39.72298 65.42214 6.506341
40.87904 79.11093 33.53851 60.31082 89.85344
47.9137 67.08344 3.291472 61.77379 48.76432 9.456532
64.80682 16.9788 44.47493 56.71996 28.92523 66.88977
90.54811 29.19286 15.58948 117.7883 1.27621 4.689094
33.13266 33.72354 29.33723 130.9683 89.2771 56.82506
69.33073 21.92517 28.18864 20.36619 21.09168
52.76185 71.67508 87.32575 190.7582 43.67714
59.70551 199.4758 215.3089 121.3019 23.5446 48.96555
11.8215 41.57305 48.40345 18.82601 15.30081 136.9835
77.02839 50.44402 113.7993 11.42029 114.485 71.77064
91.09494 47.32566 24.67461 92.5358 27.72662 32.08378
34.72106 7.135305 33.12241 136.4698 87.06441
50.16667 17.52698 61.11106 171.6365 39.6319 122.2114
4.86896 13.18898 54.23111 43.33509 127.7859 204.2597
46.14126 45.74873 18.30398 113.7067 101.1317
74.29516 17.04491 36.6809 13.82191 100.0738 42.40373
63.03297 67.94982 92.03554 174.4025 78.9582 26.14856

155.0978	36.69631	83.12441	139.7383	21.48513
115.1821	89.0832 86.1218 133.0712	40.86251	160.5577
19.39239	19.69492	56.22216	45.39891	122.5636
61.66519	56.31944	85.8862 23.17216	50.96321	38.49646
89.01181	33.38113	41.03478	104.0769	38.68085
28.13668	65.28222	47.49157	7.443286	3.943956
71.0011 203.0353	31.24716	45.95644	30.61688	62.84754
65.48802	27.76411
YWHAZ	71.83472	55.73142	73.9885 107.9823	68.85147
45.30176	52.16334	43.59494	82.33042	77.74529
133.5417	182.8053	74.48829	124.7373	51.31433
52.96703	59.59156	65.09592	73.39973	60.27927
101.4901	56.31599	133.9477	40.33122	65.71882
153.3938	64.4171 51.51719	68.91449	59.14371	67.59655
72.47473	102.2655	121.2234	116.2291	164.56 28.64691
117.2222	135.8174	185.9564	87.97037	147.3521
178.7521	101.9514	112.6135	154.4477	137.5761
175.9225	100.4483	110.5129	103.1647	101.674 67.17058
102.9022	61.21406	170.4784	77.06652	56.11849
147.8172	66.26779	98.37454	152.8917	81.32906
62.18962	113.1739	53.92999	62.14356	66.17804
212.5909	78.11679	182.4404	164.629 119.991 146.0289
153.4464	89.50857	113.3802	95.3781 122.0899	160.064
62.95499	69.70266	125.4572	90.95733	130.6172
77.28952	116.592 112.5838	131.6979	120.3939	153.2098
83.55515	204.1977	115.1311	72.7204 78.60544	150.7753
143.1925	108.6391	128.0412	73.30737	184.5864
93.05065	181.4705	94.07743	104.7597	122.7349
65.12422	162.3958	104.8715	251.5551	74.59175
93.82694	75.92779	107.8169	79.86577	86.05505
138.3835	112.612 111.2523	134.2323	119.2334	73.07848
116.0207	158.5088	92.36234	197.256 112.514 54.6952
124.871 131.201 136.5599	111.3722	108.1145	78.78966
82.41395	134.487 107.8051	108.3795	114.7083	111.4046
120.0779	70.71317	190.9537	124.6229	113.5034
134.9301	116.1978	112.7354	149.8506	85.99122
142.9578	125.8152	73.64854	66.5628 78.52631	100.1112
63.47119	158.1345	201.2754	64.75896	84.10875
71.30059	123.6052	100.6218	152.7442	75.41827
349.4466	103.6011	124.7204	54.61566	82.52023
70.78615	267.358 124.3566	165.5995	86.72864	93.94456
139.7808	281.9119	188.8495	132.9129	139.4105
82.12834	107.5645	150.6494	65.52018	216.5304
78.55811	154.7652	89.53384	142.2472	132.95 98.51591

73.98131	107.6349	126.199 114.649 129.2683	274.1045
105.4979	72.17002	194.3071	167.6	237.4021	125.8043
116.39	51.3815 166.5853	135.0898	90.07837	109.867
124.3733	160.2	109.2009	143.5538	89.48829	117.1145
95.1758 142.301 157.7908	164.5839	62.30428	99.2847
74.83319	192.8278	138.4057	99.40098	138.6143
138.1955	166.6585	61.09768	101.8304	148.6574
180.6352	84.20473	93.93722	92.60275	166.4778
118.4131	87.10431	129.3227	121.7673	134.4676
163.8681	96.23014	120.1756	67.4995 80.94322	134.2719
135.1029	169.0269	177.8778	116.1988	137.0488
84.98079	173.3654	56.29652	147.6124	79.62137
106.3044	156.8153	107.3413	148.1062	94.47123
148.1802	193.7645	112.0324	122.5853	99.41092
121.217 72.44862	162.9274	130.7998	113.117 147.5393
113.541 50.549	151.9778	185.7488	76.55665	175.9479
111.0845	100.776 204.5204	74.15786	182.2975	155.6167
124.0244	86.77475	169.6365	49.21006	57.67778
133.5673	121.1965	38.94745	92.14565	120.557 155.067
208.1176	135.6935	103.3938	115.1688	75.98034
175.1278	261.2896	107.7872	168.8662	100.5639
163.4965	123.5586	398.4358	83.92381	47.38943
151.9465	58.1919 69.71313	132.224 100.764 156.9142
181.2858	125.3944	160.4288	132.4911	123.6645
96.78922	97.86032	96.69072	92.05494	160.8228
145.4009	94.51672	94.37243	83.11448	38.09771
93.94812	132.1344	200.3915	88.31902	107.6623
90.7391 115.0259	99.66352	129.4391	65.51603	104.4636
96.08119	74.52908	176.854 124.126 257.3532	77.10339
114.7136	280.2172	98.70806	133.8758	142.1303
394.2739	81.89264	260.8804	129.715 79.71601	329.3883
105.5566	115.9497	114.023 161.0617	88.05007	103.6965
138.3444	85.3702 126.2865	134.2689	127.664 147.9235
109.8808	100.7622	120.3516	72.3019 106.9412	65.11032
129.3387	112.4154	143.1058	222.8477	89.30708
60.21219	118.6555	127.6267	151.6386	77.02148
149.7637	178.6245	154.8183	123.0336	123.3875
79.22225	85.39322	61.0123 193.1571	129.3306	94.48186
174.1661	120.3368	205.6264	153.0342	106.9133
TWIST1	0.1572207	0.5603533	0.6113381	0.36864 1.331065
1.997519	0.4637468	4.402331	3.320872	4.727527
2.846322	1.47421 1.997492	2.211133	0.5359323	5.066738
0.07127658	9.739136	1.235343	0.6868442	0.4425928
5.131502	2.454731	0.6326961	2.452189	1.482945

0.6447888	1.32229	0.3866808	0.1783742	0.4644919	5.099097
0.6967227	0.6752331	6.463772	0.5013085	0.1338514	
1.906286	1.592063	0.499631	7.796714	0.491387	
2.268644	0.3859111	0.2134088	0.8203286	0.1829398	
4.946814	0.3201723	0.5821992	2.326415	0.3034116	
0.5903409	5.276004	9.656075	0.6500994	3.955787	
0.6739828	0.996042	0.4553517	5.679445	4.375928	
1.572969	3.796604	0.2065235	1.225164	7.82036	3.546386
2.412512	1.48268	0.8972203	0.8028358	0.6221137	6.350434
2.808317	1.73188	1.603521	1.733524	1.179123	0.4620378
2.012985	3.801662	0.7731401	1.477022	11.07301	
3.771073	0.6838468	0.5651881	0.6365259	0.2257007	
0.7644215	0.5106952	2.33204	0.3317979	0.9870834	4.460784
1.129127	0.2633812	0.3958154	0.4273766	2.057921	
0.2497413	1.119693	1.757308	2.01312	6.070013	2.91754
3.425032	1.801076	1.781612	0.8049792	1.239436	
2.295086	2.717987	3.46742	0.01134774	2.566734	1.624904
0.7391077	0.08224035	1.183275	1.107106	11.37326	
4.444663	4.870431	0.6099889	1.621045	7.242244	
8.287651	5.503155	0.9006821	1.887577	1.003465	
3.60051	1.278089	0.4827594	0.6253672	0.5617067	2.005543
1.200257	1.966753	0.5493162	0.6782442	0.1655337	
2.144955	2.040116	0.3618004	4.52126	0.2330298	1.458984
0.4821323	4.252825	1.502376	2.187523	4.757649	
0.7405033	1.02169	2.352405	7.729276	5.594891	1.131015
0.1026403	0.9866054	0.9686741	4.217064	0.2779775	
1.493403	1.669795	0.5450234	14.08573	0.2064821	
0.765123	0.274488	0.6981222	0.6233749	2.117511	
2.454174	4.003161	0.1306241	4.010717	3.205647	
0.7073247	2.913156	0.8788512	9.763118	1.200866	
5.366845	2.233579	1.219997	0.976211	4.437137	
6.243033	4.418392	0.4697891	1.843284	1.125739	
1.986007	0.1987731	0.3058423	2.59553	0.09346618	3.970269
0.8407281	0.7817429	1.446793	0.07203152	2.454181	
1.983716	3.271173	3.212322	1.800762	3.137026	
0.00663335	0.8748928	1.83113	1.994563	2.217878	0.8888411
2.530537	2.385705	0.9669787	3.186857	1.327706	
3.982867	1.277485	1.181461	0.1992266	0.632405	
0.06103362	9.705718	2.023715	6.407218	0.102532	
0.8626632	0.428196	1.990669	3.602941	0.7757451	
2.412766	0.2480907	0.7279219	1.754463	2.539849	
2.249297	6.239794	1.79816	4.49771	3.440294	2.384322
0.3974601	0.5781711	0.5483826	4.059228	2.837288	
0.3420341	1.231163	1.336369	1.681557	1.100353	

3.794943 0.2029744 2.182048 2.053552 2.16303 7.186615
1.793753 0.5507861 1.788142 1.530968 0.3546716
0.6580283 4.137038 1.915054 0.7968406 0.328313
1.597808 2.089166 7.842055 1.563032 1.005493
0.956178 0.7953231 1.97586 1.98223 1.048055 1.677227
0.4225563 1.825956 7.875525 1.72108 1.49928 0.111094
0.2942605 1.632338 1.993055 4.263895 5.686563
3.455257 1.575689 0.07318487 0.2216919 4.871623
1.255381 16.85383 0.04221652 1.111367 1.624222
3.062249 1.624839 0.9990635 1.593208 5.196138
1.106437 1.894177 0.7083401 6.94796 0.8763247 3.710667
1.597055 2.665568 0.4569579 0.7508011 1.15108 2.020896
3.814121 0.718289 4.191427 1.623058 0.3114325
1.944505 0.3333659 4.205261 1.962438 1.67535 13.77384
3.041971 0.1267321 0.5153698 0.7434762 0.6417479
0.3999646 3.002182 1.809151 0.8700458 1.978422
0.8001906 0.3144324 1.029953 0.1990484 3.308651
0.5511979 5.534169 1.096902 14.62886 0.3828517
0.2432418 0.4187819 5.798168 0.7475618 4.915393
1.729304 2.578924 0.722696 0.2293288 0.801101
4.074146 0.06250583 0.7254065 0.1903914 3.009732
1.160056 3.615472 0.9854198 1.15406 1.371286 4.076036
0.3661204 2.839428 0.172513 0.122281 1.460736
2.284682 1.611106 1.436804 0.5214294 0.9183719
4.771638 3.424566 1.147523 0.638191 0.9670395
0.3566488 1.063068 2.441611 0.6794653 2.711113
0.2558893 0.2721221 0.3170188 3.724514 1.779417
0.5922334 1.271297 0.01088345 1.408935 0.981733
1.820922
ELK1 5.344547 8.197407 6.253305 9.933134 7.823464
7.642591 4.135424 4.950063 8.224655 10.86916
6.338295 4.116046 11.30379 7.396246 9.047754
6.515756 9.944647 12.17202 6.409027 7.542925
9.797609 6.17435 9.69308 9.601559 9.818922 5.393992
7.187554 7.302619 9.658798 4.744192 10.58445
7.266622 11.82435 5.911681 14.17404 8.238687
7.443214 16.78135 11.39386 18.45394 13.28979
11.8992 12.87446 12.32502 5.851313 11.3544 9.875519
21.09896 9.347106 8.523736 17.53668 9.060318
7.967571 7.354678 8.686586 15.44237 17.45848
11.60628 28.21855 13.1116 15.47049 14.58315 12.62176
23.52035 8.078574 7.440166 13.07983 16.0365 14.09023
16.56774 21.8445 6.526306 8.497301 12.30725 13.6046
17.1355 7.53226 11.52008 14.18042 13.89709 15.06187

16.24849 10.97331 12.51678 12.45717 15.11452
14.73347 14.23392 10.1945 13.81699 8.29407 19.06317
8.437206 7.756422 23.8982 14.1742 12.53289 8.69837
26.49083 12.25176 14.05219 12.1552 12.71834 6.795537
10.86639 12.99459 23.7283 13.66825 19.97477 10.93287
11.87721 7.480686 12.92847 7.928085 8.116424 11.776
14.22757 8.481921 16.09935 12.5425 8.62366 5.763575
15.01569 8.684111 42.76389 9.89518 14.33672 11.72264
12.9981 21.36168 11.6735 23.40427 5.681725 10.81819
9.032684 10.76307 12.97741 8.79294 17.18919 8.336759
13.07762 11.95753 10.59375 13.73473 12.30042
11.60423 11.67233 9.928222 13.18085 12.26237
16.95175 18.22639 17.41544 23.63027 10.45163
6.283085 14.79274 9.122965 13.23652 7.309887
21.19615 6.622721 9.824869 18.06332 8.933354
14.60831 25.14056 6.237922 8.152094 11.08036
7.507508 16.647 11.07145 11.82561 10.34967 12.00499
13.02044 13.78547 14.00557 17.32154 8.888641
8.252156 9.935608 10.86277 13.97745 13.49245
15.68501 8.139494 19.80998 13.29292 19.03397
18.1036 14.07119 9.360337 12.756 11.98121 14.90868
12.55504 18.6338 6.196571 6.08543 8.390923 12.74271
23.14473 8.114048 9.567303 17.13555 18.30004
9.07029 12.85152 8.783136 19.62297 6.429274 22.36183
19.10037 5.787106 19.99105 8.403598 19.89256
12.59792 16.15115 20.4235 15.45182 10.68639 13.07903
7.623003 9.782893 4.928683 17.56941 22.44124
23.44852 12.10179 8.827982 9.684942 11.69748
10.49759 11.26463 8.089518 8.810914 23.11157
6.206027 16.54643 11.82773 12.28119 8.822366
20.26003 18.55188 8.712489 14.73244 9.27106 14.65914
7.174634 10.93272 14.98322 15.80895 21.13144
15.52735 15.60064 13.1747 11.45565 7.133516 20.09159
11.42895 9.610985 15.1297 11.14817 14.31056 17.0336
13.12887 11.52056 13.95469 11.62629 8.808058
10.93776 12.54159 12.35916 9.613936 7.504903
28.99886 14.44301 15.0982 27.50214 10.87965 16.97373
9.89856 15.18479 12.23613 12.592 12.61639 18.12778
8.335284 6.092788 6.457662 12.79076 27.51763
19.10395 10.14521 16.5669 10.92104 12.13602 7.889233
20.00644 7.3796 18.43414 9.071178 8.844191 6.626483
17.62751 21.03403 14.38582 22.54697 18.95946
12.70339 23.72096 13.12113 14.57949 10.43917
12.00457 10.62838 9.240406 8.32715 8.810429 10.32224

12.79692 13.67858 5.341146 21.45566 28.4303 8.361465
16.06875 6.54718 22.42909 17.50611 14.09005 11.86926
13.85237 14.26735 7.970645 10.83385 10.98565
14.2441 20.09879 10.13482 20.21042 11.60003 5.54932
10.45586 13.98179 37.27494 12.91416 10.85912
16.91489 19.62609 12.46623 17.91659 12.78844
7.616057 14.8742 9.460544 12.37868 9.9527 22.27682
14.40138 9.610647 11.15156 10.7432 11.85946 9.446007
14.55348 15.75319 13.945 7.385855 28.93772 6.722516
30.29706 29.21615 12.20293 8.929371 10.57199
11.22358 18.64034 18.12345 8.461018 31.54672
19.18926 14.41549 19.21593 8.878542 20.47451
16.93021 11.87133 11.09579 4.025671 13.31296
11.58204 11.47781 12.9252 10.51063 6.943576 17.73344
21.65788 9.488147 12.01761 12.2644 12.05265 19.04326
13.09031

BMP6 1.473766 0.5247229 1.802695 7.064115 3.715376
0.8627046 4.021181 1.164998 8.027888 3.778057
1.211218 0.09240708 5.205456 0.2890983 8.281537
0.4762272 4.409708 2.573429 1.684195 2.902095
7.884013 0.4632248 1.668808 0.6502635 1.632201
0.5052647 8.446192 0.6611857 10.1699 1.23329 10.89932
0.4309835 0.3581703 0.9546695 0.5318321 0.1603894
0.3099688 1.14998 1.317887 0.8695063 1.325191 0.1520322
0.3481281 0.03023731 2.701535 0.2614958 0.3101331
1.247054 0.9152762 0.5175898 0.3016481 0.8530184
0.6167333 0.8093818 1.406174 0.1798865 2.041107
1.248273 0.1676893 3.641705 3.476947 2.480251
1.410554 1.021289 0.5753508 2.121256 1.083898
2.107512 1.995292 0.4417423 0.366006 0.4583244
0.9926151 3.820491 1.264759 2.145514 1.594637
1.008218 0.2452376 0.3634833 0.09086951 5.44039 0.5150119
0.6609806 0.968854 0.7792588 3.26943 0.68456 0.8414594
0.8901116 0.4532575 1.120406 0.6741278 2.18378 0.5774793
0.9396697 2.021253 1.128141 0.1008717 0.651563
2.82014 0.4657182 0.5900365 0.6770337 4.556762 4.115705
0.9273168 2.127376 0.8133778 0.2159861 0.6234915
0.7385425 0.7795776 0.2016457 0.9069317 1.037319
1.005766 0.1904567 1.640821 0.2460353 0.481336
1.894739 1.531483 0.9508515 2.652453 4.724412
0.2294442 0.2487017 3.514442 0.6965359 0.2107726
0.1606471 1.020979 0.990021 1.962786 0.8992516
1.590706 0.2138272 0.3623472 0.6514493 0.3132879
0.8144302 1.260319 0.1155515 1.465009 1.107323

0.09497845 1.478932 0.1335674 2.996642 0.3960498
1.96166 0.06156398 3.833106 2.409883 0.6563956 1.125405
0.866339 5.534929 0.4895831 1.362755 3.429679
2.191221 0.3565704 0.5021829 1.738282 1.634008
1.558139 1.430592 1.679272 1.169166 1.05288 1.254573
0.3563097 0.303914 0.6169258 0.8645015 0.6472089
0.6435351 1.064353 0.1892239 0.5806013 0.7784067
2.556191 1.505362 1.052534 2.100798 0.06363915
0.7671863 0.1988071 1.228025 0.490144 0.2012846
5.337077 0.2066909 1.669362 1.829636 2.26349 0.266263
0.1708898 0.5910381 0.8307771 1.296246 0.08959237
1.680986 1.235867 0.7871624 2.92544 0.4660115 0.5149505
1.062869 3.699431 1.552299 0.2651443 0.2758438
1.947867 0.3355702 0.6044633 4.790674 0.3575999
1.355771 1.272702 1.546928 0.1647258 0.9478895
0.02964695 1.003666 0.2920892 0.255049 0.7102966
0.3183919 1.251915 0.2726585 0.9153673 0.1628315
0.5706019 1.769088 0.8996999 0.590115 0.3521828
0.4031584 3.29922 1.926684 0.5180785 2.611397 3.899784
1.90562 0.4602838 2.824853 0.428538 2.057693 0.2476947
0.9246126 2.30228 0.3173263 3.071384 0.4713428 2.860304
0.5250064 2.780269 1.508283 0.3367117 1.915569
0.1403657 0.882091 2.591725 0.2217592 1.222377
1.310527 2.772012 1.453167 1.136299 0.9336463
0.468091 0.3528649 0.8033224 0.9871152 1.539334
0.4706625 1.508013 2.39982 0.2908079 0.7194705 1.892661
0.3279284 1.571197 2.237256 0.3338281 1.562788
2.478966 0.6705006 0.3141376 2.022797 1.639138
4.973155 3.71168 1.743587 0.9402091 0.2541824 0.6912965
2.133528 1.729414 2.094589 3.51131 0.5027845 0.6685416
6.255669 1.123327 1.637839 1.496346 1.034743
0.2498211 0.1737399 3.27288 0.3430946 2.338048 4.480241
1.383475 1.24258 1.297807 0.1044285 3.119643 0.3425845
0.2481097 1.784258 1.219403 0.738537 0.6449239
0.362975 0.6680568 0.1006954 4.076609 2.606965
1.278911 2.191951 0.9348226 0.1493155 1.022307
1.589278 0.360736 0.8116656 0.8753683 1.582228
0.1984665 1.415528 0.3307513 0.5334674 2.485155
0.2911262 0.7888072 0.8637606 2.315697 0.8066155
1.519391 0.5074592 0.2371753 2.566693 0.6100424
0.3684203 0.4002987 1.931312 2.77244 0.07325797 1.07485
0.3905608 0.2859023 0.06285153 1.747484 1.527891
0.9536069 0.6418387 1.312432 1.923343 0.4245681
0.5949712 0.6688906 0.7496777 1.180986 0.4325412

2.135643	1.362585	1.712338	0.3366267	0.7698401
0.2184638	0.196198	2.493506	0.7265759	0.2213782
9.00213 0.7028789	0.2035633	0.3201125	0.5571892	0.7683384
1.844759	1.160209	1.180125	2.731612	0.2662853
0.2563124	0.8601793	0.139454	0.2706064	0.6241527
2.716896	1.783805

PRDX4	17.50454	13.71755	20.46711	49.13642	35.70133
12.94396	41.14599	22.87198	38.99277	62.09401
12.05187	20.70305	58.1637 16.34058	53.1937 10.48651
42.69531	17.70125	29.75377	32.85539	41.80099
13.75492	31.14188	12.55268	13.13869	13.603 63.46045
10.62529	41.5386 105.7396	31.6122 13.44727	34.47844
29.39202	37.54006	44.9295 85.54566	82.7266 65.43546
79.88725	28.88779	68.46394	68.65971	48.34807
31.24145	41.92566	27.19586	41.73086	43.0361 20.38019
58.21625	32.30325	43.20679	59.0768 27.31558	71.79711
72.76811	10.96679	61.00354	46.68581	36.70598
29.75147	61.35263	122.3739	95.49388	26.72702
49.39324	43.42707	32.78222	72.99478	22.04072
55.02861	18.87111	81.11213	58.32697	47.77608
19.13001	31.47898	40.62911	137.9638	47.80356
46.14356	65.74751	31.18879	31.65186	43.33182
19.70698	39.24511	102.183 19.32243	50.95814	33.8339
49.33149	23.2664 80.69823	52.62699	36.53866	60.1984
23.85313	34.27906	28.37446	97.33823	95.22244
26.57121	41.28168	22.84561	52.80446	25.02016
65.99952	52.91908	33.95097	27.74259	71.70968
17.59788	41.96892	45.78855	38.17253	20.97107
53.15717	91.16514	51.90388	62.16776	46.79624
48.22298	59.13361	28.64314	93.23687	48.13063
76.82227	49.38811	26.47712	37.39033	43.13757
81.31784	57.67487	12.54628	34.15621	55.90946
73.76572	58.20164	30.01343	26.3217 41.6888 68.11263
33.72042	61.75868	63.76148	26.81267	25.72241
24.51537	24.29422	34.34148	60.7232 39.90462	16.09872
49.5489 56.91833	31.12626	41.8221 22.97676	35.2862
35.55073	36.54301	52.74949	51.65506	33.88492
58.41532	61.65361	29.92885	31.62077	14.85349
62.47561	26.63818	59.64148	46.51345	34.59778
21.84938	60.63251	41.81288	33.85344	27.37502
23.39726	21.55152	35.49551	57.14685	22.25042
23.62328	46.63477	18.1994 33.28927	67.84685	39.35278
52.74597	52.26163	52.05153	12.78137	46.34528
44.02472	50.97505	33.6665 43.56507	25.39806	31.59866

60.79038	77.28412	47.00495	48.35433	56.16085
21.73443	24.56828	31.76958	48.54843	26.04336
46.78417	40.08512	52.64162	33.83179	94.24729
42.62423	24.08899	45.26594	91.81052	199.4336
36.56898	27.82635	38.61204	54.76477	23.52719
54.87211	63.11777	50.18971	67.66785	24.49337
31.20939	77.21314	24.63064	30.19383	33.4104	51.30932
32.67612	17.54505	41.97792	34.16221	36.30903
64.63552	39.71885	48.5834	12.99812	21.21184	67.12417
41.75985	76.33504	33.59398	32.25954	47.85855
37.86168	42.03072	33.78203	34.58088	32.12914
38.1019	48.62836	50.95799	49.0372	26.6772	44.9105	42.0791
35.17248	21.52347	17.35715	34.23316	40.80328
52.33418	49.30619	28.27294	50.50403	53.71813
16.85668	34.84302	60.04071	22.03642	53.75809
47.18439	46.34569	45.21347	42.55149	30.66256
36.73586	51.94453	31.67527	40.66183	9.861348
16.67131	34.9692	39.34625	35.43928	45.86799	32.29475
79.60071	36.12174	38.51777	69.31372	24.49223
32.1446	67.34471	44.22557	46.86648	49.41621	54.70707
123.4971	38.81717	53.00535	60.59931	50.7016	34.86001
15.97531	38.62259	61.75158	47.52922	76.13874
12.52322	67.97793	52.16394	47.07819	83.02794
41.41555	37.98104	175.8268	35.99666	39.94341
40.52437	23.95872	44.335	27.96268	20.54753	23.86055
36.39316	29.8462	32.21192	51.93924	229.7065	42.66212
64.38557	72.40181	14.76442	23.22496	51.9652	72.71771
39.69332	40.43978	37.47055	25.61662	74.69128
37.06793	39.70379	77.10496	22.64167	70.4983	27.72914
36.62677	29.9449	48.76628	11.12413	55.55127	38.26388
43.29672	36.2569	34.03483	55.76587	64.10418	37.23292
25.24627	61.00491	29.41525	59.84977	58.30425
62.73294	54.66723	62.97703	37.23428	27.95682
35.11751	22.81849	62.80904	45.94988	46.2091	43.99213
39.40989	37.75786	64.68665	21.43188	68.88062
23.20931	62.54112	20.9589	59.53504	39.80224	16.9335
49.5678	37.8468	51.28976	26.95428	32.09191	47.91848
50.12958	55.17687	31.17532
BNIP3	3.587942	4.783754	1.262782	5.672279	2.351129
6.092474	5.640834	6.563303	2.887584	5.903601
6.015545	16.97771	7.392234	12.02281	4.955123
5.509442	2.810212	4.730396	2.590728	1.86936	3.467593
7.251053	10.06719	7.222496	7.027284	15.05991
4.137361	5.501111	3.666593	5.087511	3.168008

5.943328 0.9374497 2.869924 3.306554 0.4796698
35.67197 2.770201 3.440853 0.5093167 5.272038
0.20082 1.943654 1.011653 1.966157 1.954166 1.248627
3.054305 1.159789 0.4381137 11.63323 2.808234
9.409159 1.73069 4.991023 0.501131 3.234769 3.914666
0.4869027 13.72286 3.19209 1.226336 3.45104 8.263787
0.4867394 5.863875 1.926361 2.878011 1.029961
0.9045637 2.721319 1.101457 2.03708 11.57391 5.035839
1.073353 1.740776 1.359043 4.591744 3.29451 1.214356
2.848578 1.281074 3.109901 4.679136 3.093784
1.693217 1.322348 7.619228 3.039478 1.296942
1.178118 2.806387 5.136549 7.769719 0.9523459
1.62417 1.511878 0.7093527 1.348982 2.908485 0.3221068
8.536366 5.716832 4.317743 2.828609 1.206421
3.805997 0.9674851 8.823379 0.7896331 6.948345
13.25075 5.244083 5.424643 8.181818 1.29793 1.021125
8.195268 0.9689806 1.402066 1.705874 6.958009
4.863734 16.44347 2.974262 0.973276 4.217695
6.483035 5.875959 0.5224121 12.53435 1.646953
1.576627 2.352028 2.070706 1.670742 12.67718
1.052318 6.528853 0.8718879 4.576484 5.689583
9.467808 5.545128 3.849418 0.4786013 2.957317
0.5388952 1.373224 1.019426 1.894032 0.7822537
1.483527 3.690951 4.84713 2.188193 7.325568 6.172291
4.723469 5.385422 5.036955 3.025459 1.428851
0.9674763 1.917843 3.351876 3.885652 2.004347
17.22964 6.455839 5.610909 1.720551 20.31721
1.058311 1.327487 3.882594 2.020057 4.215814
3.49974 3.898018 0.650872 7.146647 1.091899 9.634466
7.210844 4.179837 2.112961 2.222349 0.5908617
1.732561 2.634338 1.482582 2.442452 0.7525201
2.313653 2.393679 7.51542 0.6414044 2.271824 19.27171
1.552286 0.8903921 0.9445192 1.179642 1.301462
5.284093 13.70966 0.6745668 1.358611 1.303567
3.447146 2.985362 1.325686 12.86394 0.9205102
0.8083594 5.718015 2.069406 1.780655 0.9761998
12.27659 7.673697 1.507363 3.954623 0.5781369
8.935003 8.725415 0.3913084 1.950085 0.9299782
2.732723 0.3401341 0.588862 0.6034568 1.153833
1.968876 10.01543 1.445228 0.8416444 4.200336
1.252502 2.746727 1.500579 3.173768 1.643719
1.152858 5.042925 2.375706 0.857053 1.387668
1.051961 4.404382 7.906542 0.7215873 1.252527
1.686518 3.492339 0.91461 6.213538 1.790571 0.6213466

4.249233	2.127238	4.284432	3.073286	0.7475665
1.027186	1.040368	6.099225	1.251564	3.869616
0.8994616	0.9396586	0.5988118	4.23647 0.8961838	4.167318
0.6491625	1.526123	3.089722	0.7642923	4.614834
1.789893	1.76711 1.534881	1.306811	1.143765	3.363903
2.302215	7.355128	3.457474	4.818436	3.231693
10.46323	3.053534	3.856458	1.058293	2.026432
1.122972	1.77044 2.770726	1.658889	5.272595	0.7462267
6.388202	4.781761	23.30234	2.757631	3.181092
9.311059	1.723191	28.65041	2.200408	0.7794116
3.43363 3.721555	5.519475	3.554236	2.511087	5.063125
1.671181	0.7326253	1.373538	3.920835	6.251463
1.749724	4.80472 1.236861	0.9524372	11.01276	1.716382
3.985297	2.434793	5.248911	1.792006	5.356929
0.9484194	1.430265	7.2616 4.806296	2.749228	0.9353627
26.16094	3.61532 3.791134	1.569418	17.21523	1.117146
8.072409	3.192645	8.676145	7.537625	4.328667
0.7677792	1.126516	1.829008	1.464355	3.898968
19.61501	1.710833	3.569079	0.972397	4.136104
3.770774	1.403894	0.2851111	1.349486	2.202365
11.24269	2.509666	3.434861	0.7097689	14.0999 0.7045081
11.81843	4.350038	1.801319	0.7907687	5.985714
0.9939083	0.7580384	1.122681	8.932281	7.470163
1.809128	2.663041	1.108319	0.7972636	3.212153
3.096485	1.204024	9.038401	1.005939	4.364589
6.438744	1.400641	2.083672	6.26109 0.9207324	13.96955
2.201129	0.999799	0.5980014	0.7386955	1.243393
4.418375
BNIP3L	9.094301	15.88608	9.631251	13.95917	9.698709
18.76182	9.110361	13.07845	12.42564	16.26289
16.70723	27.01926	14.92429	24.5144 11.32875	20.50989
7.926558	16.39967	7.434643	7.896212	14.75009
21.85676	21.51726	16.33274	23.51942	26.62323
11.82178	17.92965	11.6097 14.62725	12.79279	22.00064
9.618594	18.09296	17.36706	23.2192 8.947113	7.513559
15.86478	32.01412	13.85464	10.2859 34.55512	48.1159
11.01818	25.36992	17.43126	13.50968	19.05593
10.90622	15.43598	18.3827 10.02431	12.42153	21.7897
24.6277 12.92195	15.51599	8.716452	11.04406	25.33379
12.75204	29.49076	24.59968	15.20281	23.51162
12.82779	13.95432	14.35989	15.46757	21.9331 18.64955
11.17544	20.4544 19.80508	11.457	9.933954	15.53874
33.47605	8.0628	19.24982	17.88205	16.64268	17.08283
28.83395	9.11245 14.97681	18.77891	15.78541	26.95355

22.54012 8.904136 26.45894 15.45943 22.24794
11.36536 18.91891 9.064836 25.14612 16.30946
16.4713 12.41902 8.448552 4.272877 20.5119 17.40738
12.3213 13.59701 34.33702 28.75006 13.16814 14.75604
19.13886 15.55531 19.53288 10.86811 7.091942
15.97854 6.391894 6.412239 15.68778 22.04992
13.0465 33.14808 17.86633 14.59928 31.94273 16.14028
20.00972 12.39173 16.18212 19.4287 12.92605 25.26168
11.60572 10.71864 28.55616 19.42046 10.91445
25.62303 8.01737 9.128411 11.54889 6.854624 10.0196
27.44085 9.191352 17.55471 20.47101 18.44259
8.940978 14.67872 7.415562 17.92228 16.53597
12.32902 21.48772 24.50441 62.86603 20.10028
17.04299 15.40662 18.49 20.63085 9.961053 18.36159
7.920925 15.38843 21.11855 18.21042 16.17348
8.760924 10.69627 25.97446 9.097132 32.70597
19.70301 32.49205 17.21217 13.38865 15.9524 12.03306
20.57323 12.55197 21.57069 15.01281 18.35833
36.61749 24.91801 23.17382 19.80738 10.35486
21.78576 8.09133 16.07891 10.56844 28.11457 8.869844
13.74555 11.2036 15.47248 14.60823 32.94453 14.0578
13.09672 27.18469 12.36063 31.38159 6.903897
16.41959 18.02258 12.77099 11.98581 9.479682
26.1275 27.4711 8.613359 11.62188 19.43311 22.44518
13.82213 29.72973 15.2534 30.1042 18.46654 29.47756
23.20683 17.89484 11.31791 17.49934 8.342165
16.05141 8.245151 10.88518 38.79858 13.5516 15.88424
15.39626 30.42979 8.12842 24.47106 20.58871 13.84063
20.41508 48.45529 13.39893 15.3273 19.15589 19.41775
12.09123 18.33043 18.77415 15.30952 20.63321
14.48451 20.29856 18.01396 10.57727 12.71558
15.43122 12.48601 18.40727 17.18932 15.49966
14.99833 15.25584 18.8561 10.58723 23.10803 14.84046
16.47124 14.52204 12.02439 30.4015 25.98817 10.3568
11.98728 15.74878 13.21143 28.65792 17.69603
6.417614 19.69788 18.5546 47.07822 18.59486 7.124568
14.98104 22.61606 20.07368 12.32476 12.98736
22.06804 10.08262 17.67813 16.3814 21.69338 12.34977
52.69489 8.273502 17.27037 19.82494 22.42265
25.21805 13.05228 16.5556 15.68021 35.77606 20.07125
8.091682 9.513437 26.85232 25.51719 12.28941
13.14395 18.63494 16.158 14.80918 15.93746 45.36275
16.89156 17.01095 5.363858 14.55928 47.3225 12.95649
25.21196 22.7356 9.082034 34.367 22.11196 13.23379

11.74887 11.04349 12.69483 14.6879 18.428 13.91178
16.80787 17.01322 10.92635 18.23088 9.857134
36.57698 19.64134 22.0023 12.66609 16.95063 18.24522
14.99401 14.72482 20.1991 13.22307 19.1037 13.67783
7.309924 17.01569 14.3892 15.7102 22.97208 9.706518
20.34646 10.51081 15.66648 8.600528 22.39394
14.20993 7.943866 10.21195 21.1265 21.14999 20.51601
19.20547 31.04696 13.22738 6.245599 18.5574 7.363126
8.387286 22.22534 15.54899 11.14637 24.6773 7.846346
14.29584 15.35731 17.1167 22.80023 10.82316 18.11479
25.56478 33.45983 6.889304 31.25632 25.19752
16.96246 15.28842 14.91487 11.78902 28.03556
11.58926 15.80607 18.11243 19.12591 16.75412
9.408834 17.35365
KDM3A 2.953781 2.879341 3.637934 2.382204 2.003206
3.514255 1.157246 2.316801 1.881533 2.278228
6.828751 5.12903 2.813056 4.80517 1.619879 4.031805
1.325663 3.252456 0.6441607 1.400897 2.595336
3.144368 4.667965 4.036343 3.72586 7.88573 1.732134
3.503858 2.001678 1.149405 2.104391 2.585623
4.423614 3.927107 3.041799 1.42362 3.422702 1.074191
2.906098 6.139039 6.523152 4.072314 3.187038
9.889664 4.044726 3.366536 4.397921 5.538928
3.784593 3.832277 2.442714 3.768199 5.29963 3.271203
3.678346 0.4736809 3.311898 3.961111 3.50444 2.093924
3.733219 3.13373 2.525736 1.926444 2.244663 2.906601
1.826879 2.385877 1.833478 4.259107 4.860603 2.8916
5.105181 6.432953 3.498719 2.19631 2.398383 4.794684
4.670266 3.3102 2.154859 3.510286 4.554032 4.619889
3.634621 3.309545 4.033779 4.276057 2.810604
4.291292 6.538769 2.915056 2.738707 4.019322
2.931449 2.856157 2.43196 1.842954 12.12782 3.918844
4.039713 2.887934 1.316776 2.87448 3.114894 4.304418
2.796493 2.409124 4.493766 3.914796 3.962377
5.058894 2.936602 3.035521 4.159139 3.217386
2.001873 6.842843 2.602666 2.478711 2.998486
6.348491 5.222517 2.658531 8.960102 4.423016
5.199431 1.823849 4.400282 3.256816 4.803336
5.914876 3.762086 5.121649 1.61393 4.45738 3.535374
4.308479 3.309115 3.586027 4.839033 3.367789
3.586094 7.465505 3.254803 3.266703 2.565624
2.393007 5.724284 2.948767 6.961102 2.373334
2.976715 2.953862 4.009443 3.653502 4.191563
3.971702 8.352655 2.415402 2.962667 4.059776

5.362796	4.601241	1.366741	4.084704	2.520846
4.763647	5.86086	6.126955	4.472938	2.738822	4.586886
4.545509	2.185013	2.399328	3.455244	3.742255
4.271232	2.805538	2.80859	4.19661	4.918834	4.251379
5.468372	5.51655	3.533844	4.321617	7.392839	3.316409
2.52198	4.134632	2.878631	1.932703	3.294291	6.115282
3.289368	3.566423	5.745005	2.234785	3.578151
2.996858	3.839111	5.5056	2.328471	7.609053	1.715044
3.356025	3.749352	5.255304	4.882962	3.884963
5.169999	2.403053	6.138797	4.930025	2.960658
3.729937	3.969866	6.278397	2.811939	8.602481
2.891373	3.67056	6.823825	2.952509	5.57216	5.038628
2.431155	3.830204	2.699928	2.278146	2.889829
4.581496	8.832984	2.86955	1.753999	3.373294	5.422772
6.103718	6.282541	4.406692	2.880767	2.663668
5.706473	3.000969	2.706807	3.933367	3.9881	2.958187
6.190067	5.443838	4.237055	2.94725	4.094005	5.52526
5.823973	3.157167	3.661342	4.543807	2.571402
2.475608	6.411236	3.702047	4.624674	4.937908
4.713705	1.819241	3.363985	6.10251	3.684557	2.371007
3.128583	4.743381	1.955855	5.621059	4.017887
3.72086	2.460149	2.759261	3.721278	3.532892	1.959449
4.386626	6.328854	5.51931	4.638639	4.826939	2.496025
4.070307	5.840212	3.665804	4.589577	3.225958
3.297251	2.043793	4.184926	2.903823	6.383843
2.221483	3.800879	3.795452	4.36758	3.11202	2.627006
2.625306	5.030092	4.816589	5.676275	1.985277
2.714221	3.318151	4.468035	1.613027	4.752484
3.886436	3.783848	4.476303	3.263781	5.875492
7.065109	5.042168	2.963754	2.189461	2.57226	3.395787
4.239789	5.921058	2.138795	6.760832	3.954716
3.79726	3.919507	3.955267	1.709891	5.676767	4.613054
2.806116	6.005922	7.838129	3.636148	2.23841	3.416707
9.702923	4.773262	6.000685	5.995713	5.262886
2.747698	1.477651	4.448424	3.881188	3.436183
2.38716	4.064411	3.969568	4.558706	3.417532	3.66252
2.506403	3.262541	1.816175	4.623202	2.9176	4.424571
3.065541	3.8412	3.347727	3.78131	2.251288	2.681707
2.171874	3.792963	6.189492	2.354152	3.042326
3.137536	2.671684	2.464449	5.834966	3.344133
4.992471	5.610993	2.706161	4.148595	2.566194
2.006333	3.092826	0.21645	5.498553	6.168472	6.973432
2.750403	4.790324	7.601003	3.596285	5.771749
3.425608	2.171092	1.999567	2.089338	3.128203

4.760704 4.685488 3.292193 2.434431 3.601599
LMO3 0.08348154 6.295536 0.2954445 0.07163679 0.2950934
8.150665 0.157404 0.7783659 1.124979 0.8792923
0.270846 0.03335822 0.2337985 0.09817123 0.1430195
10.22575 0.05362681 1.976229 0.2970023 0.1269248
0.7126189 10.00927 0.3924173 8.572523 3.96555 0.2124253
0.1948558 12.08332 0.2324155 0.2568508 1.435477
1.499164 0.1201912 0.481291 0.5897846 0.5955358
0.1869846 0.1029362 0.49034 0.1640383 0.2868016 0.02868854
0.08983548 0.02001162 0.09179233 0.1061977 0.5235571
0.5292618 0.363064 0.05198533 0.04573489 1.888981
0.1533151 0.1893499 2.811209 0.06783661 0.03531766
1.073357 0.01844411 0.03069168 1.800798 0.09069287
3.362403 0.1970325 0.3719076 1.458325 0.1930651
2.292396 0.0199026 0.3508899 0.02537772 0.1292162
2.544676 0.2625519 0.1970876 0.2533376 0.2944172
0.09905209 0.1210132 0.02633532 0.02706262 1.921176
0.04092155 1.444747 0.5603926 0.3479795 1.413665
0.398657 0.5671267 0.9352421 0.06408543 0.01105939
0.114828 0.1875263 0.1188904 0.192253 0.4731795
0.1236649 0.003562226 0.34134 2.983283 0.1100201 0.01304072
0.3615139 1.108155 2.534932 0.05511194 2.837599
0.3290746 0.1452492 0.52013 0.4135453 0.1390553 1.942
0.866929 0.06178653 0.04274557 0.06165384 0.8811682
0.0947379 0.1405687 0.742845 0.4850505 0.5542785
0.0720856 0.1921405 0.2044805 0.01989429 1.895165
0.52908 0.09476936 0.09343211 0.3973595 0.1981802 0.02214219
1.289474 0.3418476 0.05941593 0.6041922 0.2914846
0.2602458 0.4022674 0.2146409 0.01638732 0.8053042
1.169453 0.2479646 0.7971952 0.1172166 0.5390392
0.1111511 0.483068 0.03461832 0.3290556 2.685329
0.1155149 0.456413 2.740956 0.01192479 0.7479703
0.8061623 1.030176 0.8681854 0.1814939 0.1272474
0.4110988 0.2708559 0.3279641 0.289048 0.7420771
0.4894179 0.07655925 0.8559639 0.09790856 0.01763205
0.321727 0.7843017 1.043218 0.04628072 0.3520224
0.3244646 0.2643723 0.1125051 0.3321953 0.2393653
0.1066442 2.355581 0.1148662 0.7946701 0.05260252
0.5132821 0.1505962 0.1936064 0.1384414 0.09975536
0.5745591 0.7484683 0.1433677 0.1167844 0.1305402
0.1132115 0.6875376 0.02358168 0.02772183 0.08031476
0.05375598 0.5183067 0.4139497 0.1045407 0.7502112
0.4995174 1.80769 0.4806905 0.3183564 0.5730229 0.3416786
0.6420325 0.09639943 0.8819916 0.2039047 0.07130077

0.3505432 0.07277398 0.04865293 0.5873004 0.1823216
0.1811577 0.2174739 0.0345265 0.2304875 0.6114008
0.1974458 0.07197516 0.03907234 0.1899075 0.964554
0.5299245 0.1211942 0.230069 0.6443443 1.446539
0.6720599 0.3471333 0.1192267 0.5948416 0.3030284
0.4758855 1.814058 0.8039806 0.08777478 1.032812
0.07664212 0.2503334 1.200485 0.02526889 0.1970847
0.5877235 0.3256044 0.1315149 0.8579337 0.1724181
0.01189079 0.262025 1.10063 0.05010229 0.2690545 0.1139534
0.1262233 0.6344416 0.23115 0.9468273 0.07114317 1.236248
0.02586385 0.1408418 0.1737133 0.5895738 5.930981
0.02730623 1.297404 2.271807 2.625148 0.2824489
0.3350983 0.1620836 0.6723943 0.1639805 0.3258508
0.1792018 1.198427 0.1061803 0.8006516 0.4591025
0.2508361 0.0607096 1.753205 0.3974577 0.1527336
0.05390774 0.0579606 0.618022 0.8927559 0.07963496
0.2668537 0.1946362 0.1597091 0.1561988 0.08829703
0.2235808 0.09414916 0.1919142 0.07119744 0.09811003
1.137975 0.09106944 3.489231 0.1626626 0.4787861
0.06208629 0.4572089 0.524 0.2010207 0.1815303 0.2044433
2.259696 0.299671 0.1594408 0.4606714 0.3102354
0.191208 0.3831123 0.7530614 1.003523 0.5019278
1.637204 0.5400319 0.01812213 1.177287 0.3241311
0.1147352 0.1366036 0.1892184 0.2280788 0.05531401
6.801025 0.4808089 0.3249746 1.606312 0.2083118
0.1919684 0.03118107 0.3860456 0.5176119 0.1466756
0.03790112 0.05016704 0.2448002 0.3425652 2.290977
0.03065251 0.1730566 0.6353223 0.03825159 0.1692393
0.4212057 0.1561707 0.03403336 0.1841792 0.1567373
0.271223 1.372059 0.3415337 0.04574922 0.1233602
0.1234118 0.5274704 0.2583264 0.2635261 0.1634632
1.783059 0.22109 0.1136508 0.3544321 0.03661602 0.1828204
0.05516249 1.87011 0.2301997 0.03205923 0.259229 1.446516
0.2979576 1.116786 0.4628526 0.3537638 0.4088086
1.973381 1.157761 0.1610129 0.2162855 0.1174378
0.135173 0.2349283 0.103433 0.07510473 0.3811314
2.897728
ZNF32 6.414445 29.20749 5.816496 10.94052 10.69618
18.95556 11.34883 14.27181 14.66676 22.77881
9.500936 10.5945 21.82658 7.55087 10.39144 19.93048
9.22308 18.23681 7.432917 5.920784 15.29839 23.70905
7.941603 23.83374 20.39339 6.028002 14.17323
19.83218 10.02986 40.19837 13.75239 21.6751 21.36824
13.98878 20.30354 2.328774 28.30899 28.54187

10.80015 17.97914 14.65756 7.152969 8.498123
17.45065 8.022099 10.6459 8.664553 11.48383 25.03343
5.503025 7.311313 12.55245 6.957498 14.74112
23.45482 17.43729 10.26485 13.38968 17.51002
14.74485 13.25552 11.89362 13.26071 13.68612
12.12694 16.63211 12.5211 17.56753 4.279522 9.187922
24.88612 8.645534 10.35882 12.65092 9.849066
10.52116 7.745272 5.229622 24.36609 5.193352
4.978459 14.61029 17.97903 15.63626 11.57353
13.95938 9.317123 15.54058 15.83951 23.30309
10.9782 3.377816 10.44053 13.90427 12.93807 5.777829
8.286954 7.276464 9.59423 7.475998 12.51528 9.139741
16.10836 20.2065 21.45368 15.04253 3.006634 17.56957
18.1621 12.2473 10.25063 11.1457 18.78523 16.84179
12.39781 14.20184 9.395868 8.231885 23.9248 23.06388
15.07687 15.30365 9.453951 13.99372 11.08687
12.33552 13.1961 10.40632 20.12066 10.82503 12.83145
8.984505 15.38001 7.421784 7.486553 8.743141
15.82093 3.801851 9.26399 8.29712 15.45208 28.31636
10.68044 13.09178 16.92146 19.71061 15.62911
10.31932 16.94128 16.03016 20.03773 10.54371
11.05658 8.536749 13.94436 15.18532 22.08542
18.94009 14.35332 9.681101 18.77242 12.94392
19.47208 12.44374 16.5522 14.96268 13.58941 12.91685
11.72572 34.34058 20.38755 10.34294 20.17498
15.18571 13.6688 13.61052 9.679574 21.83926 15.34915
10.9699 29.98776 23.39975 8.373166 17.36257 10.53767
9.875653 14.65225 14.63356 11.3561 12.94587 12.86733
7.601749 12.47981 7.795571 3.270707 12.48439
14.60127 10.79411 3.041258 16.36433 11.57443
12.53502 7.012561 23.78672 11.50483 13.47675
10.63068 13.23804 4.536612 11.15589 9.175278
13.17307 13.26961 17.80933 14.33103 20.17365
10.81653 17.80949 10.23896 9.723521 9.450743
13.59926 16.11006 15.66224 13.96579 12.61039
9.53756 8.105001 13.04755 15.57083 12.35054 5.259029
13.11704 5.459952 12.58499 19.47295 12.30027
15.47849 17.81038 22.57179 13.61686 11.10133
11.5923 19.33216 17.29843 14.02091 17.50242 24.45404
10.67213 28.79867 20.2452 17.68517 10.60803 18.37849
3.469116 5.908909 10.22154 13.61267 20.47003
12.9725 17.45472 16.60804 15.9578 17.80933 1.564368
6.58727 8.086818 8.585224 11.73615 7.113397 11.33595
13.41877 21.61912 10.99452 8.429876 16.29908

10.22715 17.19196 10.72795 9.759525 15.29137
12.83462 15.97131 5.855045 7.573877 9.498056
5.814787 16.46285 3.505965 13.75526 8.702612
12.94939 19.64666 7.855549 16.2167 11.86733 16.45055
5.005501 13.95868 6.916009 13.69675 15.22238
3.934197 5.192275 7.974698 11.14072 21.15515
11.95376 13.71326 7.344064 13.25736 7.954554
14.34768 15.07776 15.8889 13.67163 18.83124 18.40142
7.340544 16.14437 7.224083 7.162751 10.36519
3.383151 19.83047 10.67755 9.027101 19.29908
17.60872 14.0694 25.60889 12.76313 10.86335 14.46
18.80667 20.28473 16.89534 13.1698 6.882173 18.57097
19.63455 24.44949 9.555158 20.20534 13.77754
10.14649 20.74418 3.298367 33.88314 16.54049
12.16977 12.98212 14.29555 10.45176 3.997381
8.709608 7.011107 14.624 15.82926 16.91586 10.52478
13.37608 11.1432 9.769603 11.48011 26.36349 23.03002
15.98473 9.753248 23.34688 13.90583 10.49614
16.43327 9.742942 6.186412 27.59579 23.97515
10.72807 8.146921 25.20392 10.95591 9.671351
16.56974 14.34974 3.937107 4.294958 17.94964
16.49017 12.34889 9.985197 14.75929 9.285626
14.67842 7.58754 17.84052 22.59724 19.47213 11.49489
12.3717 8.616392 16.62919 15.9305 17.84825 13.63618
11.74754 16.95105 21.93264
TUBB3 0.01639799 0.1473939 0.0543006 0.04386018 0.1118453
0.1760319 0.01013434 0.1375692 0.1468308 0.2465386
0.01126055 0.006443233 0.07596812 0.003074926 0.06416039
0.1895155 0.04579399 0.2000141 0.04049422 0.0770499
0.04982021 0.2874257 0.03337826 0.1608722 0.06289381
0.030934 0.09344343 0.2645116 0.05703885 0 0.2316989
0.155808 0.2659203 0.7023847 0.3310757 0.5607682
0.3139584 1.016212 0.4160498 0.07255978 1.038188
0.1908124 0.2764937 0.2298096 0.1519709 0.2654542
0.1106667 0.3877627 0.05676932 0.2855167 0.8896918
0.210408 0.4749846 0.2958752 0.2685656 0.05241131
0.2696579 0.2185284 0.7366217 0.4623981 0.4617035
0.2594092 0.3625638 0.4720394 0.07221107 0.4600209
0.496717 0.2916895 0.2207693 2.332236 0.05882131
0.08081791 0.2627881 0.4831714 0.9238406 0.01779377
0.2215286 3.219546 0.2358088 0.1638466 0.2052871
0.3403312 0.04918113 0.2765276 0.3039971 0.1651222
0.3138286 0.1702143 0.6179297 1.627651 0.161181
0.4907047 1.081418 0.2519336 0.2642435 0.3082417

1.471872	0.07942884	1.717382	0.1429423	0.1437151	
0.1203409	0.1133483	0.1770272	0.5177643	0.4279874	
0.2933295	0.3511042	0.1305068	0.1923791	0.7428087	
0.1816992	1.37556	0.2277731	0.6736706	0.6145047	0.6568445
0.4131204	0.2253182	0.7102255	0.3326025	0.5786345	
1.178666	0.5236319	2.440033	0.2608476	0.1349862	
0.2482934	0.2794786	1.290063	0.1393539	1.930371	
0.1868722	0.059484	0.08064869	0.1588445	0.3556197	
1.009919	0.1696381	0.357171	0.1027685	0.2141041	
0.07653871	0.2935056	0.6190914	0.3951848	0.03831604	
0.6561109	1.034729	0.1217367	0.04546406	0.5196263	
0.5438454	0.1857849	0.5656134	0.09268068	0.2022004	
0.1669819	5.509509	0.8229447	0.2570491	0.0688433	
0.09390774	0.423656	0.3474616	0.148396	0.1203281	
0.8459911	0.1473923	3.157391	0.1055875	0.2001049	
0.2134442	0.8142986	0.1498499	0.9223932	1.151642	
0.5383837	0.1167361	0.6868984	0.05607425	0.301417	
0.08814694	0.07944168	0.8637023	0.717237	0.3295834	
0.2440538	0.6112661	0.09924036	0.5118813	0.2661126	
1.529065	0.1654558	1.229394	0.8753842	0.2786614	
1.117448	1.092772	0.1263901	2.558451	0.3896654	
0.1864816	0.08031821	1.383761	0.04577047	0.5413972	
1.54119	1.286739	0.2308227	0.1216527	0.5646987	0.9467318
0.7849197	1.252635	0.1779027	0.3123656	0.161083	
0.1546116	0.193807	0.2693179	0.1867023	0.3752223	
0.6554719	0.1515693	0.7787368	2.140101	0.07637397	
0.04001333	3.415149	0.3145055	0.4995973	0.1154952	
0.2691019	0.03144099	0.1147677	0.1936897	0.04450028	
0.1925667	2.052701	0.4544135	1.304675	0.1913636	
0.09753442	0.5371283	0.3338616	0.4641434	0.105035	
0.5208223	0.1239391	0.3832577	0.28497	0.5069386	0.3745704
0.7075143	0.3365964	0.200809	0.4453389	0.1477962	
0.8011185	0.1412249	0.3215433	2.169635	0.1016365	
0.1548383	2.574317	0.2726452	0.1213072	1.661154	
0.08477326	0.2063032	0.2005516	0.1922484	0.3297144	
0.2944879	0.1276659	0.7470799	0.3044977	3.382567	
0.297197	0.5616715	0.103952	0.7299188	0.1662203	
0.09810823	0.1717528	0.5067725	1.736565	0.2336399	
0.477635	0.5583745	0.2005326	0.299791	0.1956622	
0.7186501	1.167392	0.3112724	0.1401294	0.6061362	
0.05597624	0.1709199	0.1989672	0.455858	0.3033138	
0.06088927	0.3482425	0.7027889	0.6367131	0.2389583	
0.2534902	4.703897	0.8911284	0.9968336	0.3195843	
0.08021182	0.3551922	0.07540497	0.7581671	0.1740254	

0.2649335 0.3189272 0.07439256 0.2149853 0.1353901
0.1686284 0.4002707 0.345324 1.084981 0.2099159
0.1706873 0.1529871 0.6981882 0.2753619 0.09441963
0.3800119 0.1097986 0.1938649 0.2660756 0.1279476
0.2791239 0.247793 1.072076 0.6337004 0.1648397
0.3337259 0.08621702 0.2251052 0.5914597 0.6611753
1.629632 0.1144315 0.770326 0.1817785 0.4884621
0.7412214 0.2093019 0.4148776 0.4253619 0.2311983
0.3882232 0.2846631 0.5781205 1.369317 0.2439719
0.8088046 0.1542339 0.06095553 0.05091427 0.2000399
2.321168 0.3572476 0.1319364 2.61667 0.09747558 0.37939
1.650494 0.4185193 0.4397828 0.05654931 0.7427217
0.390296 0.2269095 0.4968206 1.78553 0.2542483 0.5398425
0.4316616 1.639498 0.2035392 0.6702943 0.2191891
0.06786401 0.3558503 0.09138794 0.02887202 0.05355718
0.2407352 0.4414409 0.03795264 0.2243976 0.9444558
0.05662201 0.3889461 0.03745946 0.124343 0.2606027
0.2316915
PTPN11 6.722289 20.18964 10.193 13.93218 9.130666
22.80442 6.532765 12.72325 10.58004 9.593141
22.02401 8.92699 9.492612 9.581686 7.279108 29.10424
7.613948 15.64083 5.100136 6.378113 13.27162
26.48563 9.703951 22.13751 23.0846 13.09458 8.188492
21.96796 11.36516 4.071371 12.38284 17.87246
23.07888 11.75352 24.16648 17.59512 13.89254
10.72474 20.65517 16.62702 18.29652 18.39668
23.25768 20.12178 16.74845 28.4333 21.36843 19.59399
45.28528 18.28845 14.81063 15.48261 22.53765
18.26699 14.27296 23.61442 23.3687 31.46253 14.41808
15.38322 25.05332 20.22416 23.36569 12.80185
12.67166 33.43267 10.94907 11.44741 23.40215
11.87401 34.09853 20.20075 24.02769 23.97473
20.81736 14.29827 16.82507 20.69782 14.59236
22.02771 12.5117 18.36776 29.21827 18.58222 19.40061
24.04846 26.54758 21.98771 18.95953 21.02077
27.69105 30.74308 23.04639 17.6043 15.37229 15.32411
18.46722 13.87947 43.21509 27.14151 13.13045
12.30665 11.09631 11.08515 21.2765 20.35236 17.82223
21.95856 23.07843 12.51404 15.57095 9.944786
13.55017 16.44454 9.804008 15.98014 7.702771
31.81761 26.1112 15.36897 45.80557 29.59146 18.12491
8.663326 47.7057 17.77228 12.43768 10.00619 26.69187
15.02506 16.86759 26.31712 15.47601 26.69292
7.576676 23.6265 24.3753 27.63712 17.56399 30.10207

21.46342 20.404 18.16174 19.11228 25.25396 7.717086
14.13153 20.46061 22.47113 13.58539 22.28246
16.74956 35.99359 26.16824 29.30363 21.45342
17.58006 13.84405 17.4903 12.15215 16.25493 14.79634
25.59295 20.72268 12.3569 19.12149 14.55969 25.46893
19.41863 20.38692 22.43068 27.20684 17.03863
11.47487 16.64849 15.4114 15.3221 21.63314 19.44682
19.42889 18.72069 16.1059 12.4982 23.8673 25.63084
19.79608 26.15202 13.90856 28.98194 24.09319
14.51296 17.64515 20.36025 12.44855 8.099002
29.82914 29.22763 25.18234 28.54405 11.2458 16.09675
12.053 18.07902 24.97347 20.32603 22.50509 12.15645
20.2358 21.95693 17.73034 20.8812 24.26895 21.23462
19.10229 22.84514 26.40584 13.11009 22.39412
25.11756 22.21058 23.09617 28.95598 13.49512
20.4345 14.47798 11.66973 25.34693 7.191347 13.15761
15.32454 12.27238 14.52574 12.93384 21.58515
25.66772 10.10156 8.070838 25.40108 21.20529
31.92272 23.64472 25.11106 17.5456 15.90851 25.09521
17.2357 24.0725 15.8024 25.43654 20.79884 24.6121 26.64075
21.92103 18.46655 24.96403 36.59143 22.02893
18.60965 17.82644 18.577 13.35305 16.17806 29.51664
24.233 22.5409 19.95617 22.1524 10.59582 14.32963
24.4008 19.30749 12.36066 12.33875 22.52941 10.16941
22.79701 14.04215 39.59829 19.71132 24.3088 20.57277
26.62815 16.6177 15.49719 37.30925 25.30416 23.87088
13.391 15.03745 27.30645 33.20908 53.29116 32.62226
14.82516 19.4245 13.88668 14.1596 20.68403 15.52439
18.10896 20.72857 22.7698 25.15043 22.78407 13.78386
21.22489 35.25766 25.4809 26.3321 23.71153 25.62889
13.94763 25.18741 8.313229 20.67755 25.71464
26.43185 16.76666 22.4991 28.90872 35.78124 26.08476
22.89559 13.69134 17.73167 15.25227 16.77204
11.02521 17.59106 23.0519 18.58934 33.27018 21.13091
17.35142 20.33838 21.52939 20.23885 13.77639
17.43359 35.08628 15.04566 11.66964 14.87922
28.3495 36.27272 14.03881 20.46741 54.49797 17.58959
12.23153 21.75331 26.90912 14.30851 12.48844
34.79836 14.67592 21.65605 30.67865 32.78868
16.48831 20.40149 12.27048 34.97836 21.99783
10.38786 12.1741 18.57936 19.88457 11.99862 16.92958
11.08902 10.64814 40.86423 16.88546 19.59838
17.11165 7.940759 10.35529 14.74219 19.6316 17.56978
30.25609 12.35348 22.53822 32.86821 14.57911

13.55862 11.49349 14.76857 28.6002 26.99472 31.49146
14.47208 26.27485 33.42006 15.14626 18.80937
26.85131 11.8199 14.18812 10.74135 10.52199 21.79342
17.57655 22.38868 11.7303 35.308
SLC2A1 4.272776 4.638407 6.13273 14.1265 6.242653 4.115153
9.066498 4.958292 13.27025 9.598294 35.77806
34.97407 7.937041 58.22839 13.9702 3.056251 11.82888
8.54786 15.02254 9.195035 15.45073 3.788382 160.5792
3.845429 10.57476 51.03102 12.62597 2.716387
7.333781 3.225086 11.28298 6.076787 20.09831
51.88788 41.05572 2.902393 5.697932 12.88984
35.22983 12.52356 47.11845 18.36781 44.81913
48.28462 8.933298 25.42345 13.26141 28.19295
27.24787 107.3355 35.33039 5.466849 11.40123
58.77921 10.55014 31.38488 11.75482 6.329836
28.61267 27.05204 7.914257 55.5719 9.700435 14.66504
9.219626 2.647551 13.69207 9.37303 47.69918 10.62702
50.21071 5.477703 10.32923 33.95173 19.39408
44.71148 25.64416 71.69025 98.84026 26.02923
27.05624 9.508581 9.488369 4.872611 49.43893
13.35336 11.26563 21.98372 11.76856 43.50816
21.45701 9.189597 26.43183 9.359522 18.22643
3.669206 47.46232 18.33234 22.98401 19.10813
8.417642 29.52621 5.211232 9.656314 10.50955
16.8985 39.36115 7.829781 19.39015 13.77103 15.2273
10.45809 17.43427 9.303636 89.60413 29.0985 14.14439
74.38769 15.59505 26.28371 39.65185 71.95851
39.28488 35.84739 221.8872 11.25483 16.89468
25.01328 7.279125 31.73026 31.46226 74.08571
28.60573 22.10659 25.25445 14.55651 169.553 140.9979
38.83577 116.9577 10.01557 30.00622 24.41205
31.84483 7.957197 56.31248 11.53726 10.06406
271.8631 4.297618 10.57922 12.71479 27.81076
9.099516 6.266474 32.28641 12.48123 4.745058
69.23653 35.22077 11.20487 7.67872 14.49568 36.72129
34.616 11.58941 11.70807 14.35045 9.03174 7.892276
8.364159 36.40491 6.012241 102.1451 17.8982 34.15048
33.44033 18.37788 10.59534 24.00486 5.2227 43.36196
109.3921 8.425778 22.21068 29.08916 10.34195
57.20084 111.6722 22.67454 40.36007 46.90296
353.0936 17.95559 14.6894 38.17476 18.92139 18.95802
217.7907 11.06448 18.42114 12.55734 33.76251
117.8016 188.8918 24.37886 14.07057 24.50833
68.10932 101.3427 26.7192 10.72514 23.32267 30.13554

56.06491	29.77009	22.11508	42.41305	12.60716
43.48174	17.14595	15.61804	12.42088	121.7827
15.20423	5.713784	177.1412	73.75836	14.14619
61.92287	132.8104	14.40874	10.75372	65.63778
59.13969	18.86327	8.805281	10.22041	15.31307
23.72836	21.15759	14.39004	12.10678	54.07911
90.66479	21.06386	8.753985	12.37162	23.4959 11.52007
15.28952	11.9893 26.24982	23.94391	9.070338	26.61457
13.75861	14.89182	24.64787	18.35788	12.30617
131.4576	25.02494	31.6829 22.41083	39.87369	28.35229
10.48361	38.80393	33.94011	9.008499	52.89829
35.3202 25.39543	28.40352	6.981457	17.37516	2.490002
26.23092	68.26364	8.651524	8.630031	19.76312
15.06216	73.70632	4.752939	30.29162	14.9051 15.90064
27.32195	53.21727	1.638019	3.751848	10.21623
40.80284	8.087182	52.28545	26.81022	184.2317
19.31908	16.08505	7.819419	42.99657	11.52633
11.11866	65.20915	11.56243	33.25326	7.170427
31.49656	34.97532	312.1637	10.21759	9.16062 20.28867
9.259071	12.1521 40.65833	29.92809	36.24304	96.13793
12.16312	29.36766	7.249198	15.89567	9.960603
51.38613	15.49244	27.55621	69.02902	28.34826
41.63015	11.35689	14.29272	7.310006	32.36701
29.17065	13.63113	8.403903	11.76387	86.34637
10.19697	84.91206	55.16445	7.011743	40.44538
18.3871 22.86275	14.48264	11.882	15.29789	43.25706
12.94963	11.62271	10.05603	35.505	20.40955	12.84197
4.539758	20.04712	19.71578	14.58884	203.4534
5.841045	17.74706	10.50824	11.46751	10.68045
15.36884	12.26399	13.56959	33.49612	161.2243
24.09597	11.55476	15.21013	76.13938	12.66811
10.64186	6.185324	8.874939	197.513 18.9023 33.94859
22.87585	34.84143	9.614024	116.2808	23.12577
152.0817	21.55703	82.03382	32.93985	28.70927
59.80058	21.6511 10.22338	10.6905 16.67539	59.5069
33.76719	9.326784	82.44462	4.073134	15.7806 20.65943
6.464945
HMOX1	96.02178	2.021801	66.21978	9.129301	15.36424
4.912695	5.712226	4.257152	11.52727	21.91144
8.068597	33.10099	15.47658	35.16211	19.61841
3.902611	21.82316	9.092367	15.56697	32.20421
9.395694	2.364417	44.69786	3.425817	8.196398
48.15311	11.36964	4.017253	33.46914	11.62405
6.470112	16.40219	23.4999 6.944606	9.68367 12.50113

5.003638	32.27734	12.90199	11.38204	7.268835	
7.040164	15.26643	5.732138	4.093196	8.65469	4.779876
21.70044	7.898024	9.292873	13.68928	17.69146	
17.87429	13.98836	10.98933	5.391467	29.31469	
9.660684	8.365248	17.14937	38.99752	17.39963	
28.20575	36.39153	7.107855	10.2174	20.62348	19.80039
24.34474	6.849074	28.21707	11.83925	7.033732	
19.25329	18.72822	19.80766	7.76711	14.21622	17.90016
10.45073	79.24093	17.59908	20.33131	19.60425	
9.118461	15.13168	10.52959	10.98862	12.27212	
3.918006	22.63967	103.17	10.3314	28.97064	16.61854
45.52731	30.47298	9.796248	15.6599	5.651747	14.25721
1.393339	10.94045	8.920194	11.51944	9.43287	11.88686
10.62597	12.85301	15.55164	16.77021	10.06493	
14.26971	6.499806	19.40508	10.58533	25.45532	
3.359737	8.256077	12.35553	16.58538	18.50926	
8.110998	28.70031	5.609454	11.61356	16.78624	
22.76324	11.77881	25.14396	19.77813	9.553031	
4.819049	9.715142	28.19836	19.88078	22.25446	
10.97947	8.224014	4.319995	16.8682	7.073375	5.46548
5.800707	10.01239	3.854602	4.720883	15.07077	
3.460552	9.21738	9.022087	37.48266	14.45163	27.8515
11.24853	7.492598	9.019823	7.775334	16.68222	
9.539817	16.28055	9.010119	11.15706	16.71207	
26.87352	11.0345	10.14743	10.96465	11.3041	1.740308
4.039444	16.16243	3.789837	22.57894	3.437739	
9.10305	20.00351	5.428256	3.847246	7.500709	6.227691
8.444057	18.76007	23.85762	17.24619	69.17738	
11.31355	6.947532	23.55028	33.55692	12.76001	
38.19571	29.99606	6.640269	22.93624	7.211106	
46.00342	8.635404	5.275351	8.09897	80.28248	10.04372
26.6152	13.69485	2.743192	4.384069	24.58916	16.21215
8.721395	12.73823	13.83138	17.93335	13.18841	
12.85913	6.597396	14.03279	71.08159	17.32907	
17.30914	15.03749	36.12946	12.08762	5.250264	
10.68896	7.517061	3.143017	19.71652	5.191112	
5.37305	6.622732	7.960509	11.64721	12.63055	12.62089
9.476206	12.83631	23.62551	11.54231	12.59892	
3.139581	6.447376	25.94493	16.32674	11.04897	
12.08692	27.55189	34.78139	3.645777	16.70506	
7.882652	10.06263	10.88684	3.622612	16.40565	
14.77147	17.16097	6.558038	32.81876	12.34154	
12.97178	8.152761	8.520392	17.75261	7.332552	
31.35901	10.20056	4.289722	19.99197	17.60661	

11.18048 22.48665 29.10171 5.30338 12.04727 3.898039
10.17218 5.303788 2.436942 16.91091 18.64163
13.83773 20.63293 9.965988 32.23209 6.972953
15.22081 4.98646 14.10494 22.74552 19.77833 18.89851
2.598183 4.392836 29.38363 24.31813 9.686733
9.874575 25.74385 14.91605 5.464724 4.677531
11.15483 24.95884 33.18804 5.81691 11.46044 4.642017
22.46793 13.14647 3.865351 57.45451 16.22456
15.93445 40.55564 8.911648 9.114382 10.64205
4.061863 30.55127 16.20663 12.74713 13.99878
8.050155 37.44562 16.2202 28.99312 15.65663 3.97327
14.22431 7.711205 4.986663 23.47224 26.02313
25.07562 9.198332 8.985613 6.933659 13.2602 10.19462
10.69989 5.181367 28.91146 4.987442 13.22495
7.54842 11.76381 1.96158 3.370827 11.16926 13.33895
5.990655 25.13192 7.191229 19.31933 25.20292
4.780612 13.46907 5.327901 4.739479 18.40579
7.873169 20.20288 4.560662 19.59716 7.37383 7.809803
1.360567 13.6203 4.794379 21.73977 2.774127 11.5327
10.37512 14.03479 31.18689 9.833744 5.492036
12.37619 6.906047 1.854522 22.88482 7.619478
12.42287 7.972822 4.031263 36.05964 10.62643
7.951936 12.35445 4.504911 9.714814 4.823731
10.74133 17.3679 9.306874 29.56528 4.899396 8.232965
6.944255 8.899988 34.37585 11.92468 9.53757 3.900809
10.97671 40.22481 4.473941
PRKACA 11.8045 20.65132 12.82455 11.5881 12.08415 19.03382
9.082783 10.7325 11.41197 12.81364 8.202404 8.706145
10.24296 10.84167 14.27938 20.71765 13.02827
16.5777 11.00454 13.01506 13.21922 19.01211 11.94008
17.2519 19.63579 7.533279 12.73395 20.44448 11.95871
3.992073 13.75251 21.35982 19.06177 8.371147
13.06387 15.89846 17.32491 10.62972 11.52011
14.05044 7.59257 7.704548 7.912864 17.22817 7.468171
16.12459 7.05488 11.28519 9.989891 6.991881 15.14197
12.38446 13.64583 10.88006 13.9746 16.24973 9.402132
16.54193 13.24494 16.00972 11.46631 13.20577
12.20897 19.00448 7.419441 14.92437 15.76815
12.83887 12.20467 12.3775 17.62919 9.762279 9.361606
7.657755 8.987039 14.6431 8.726313 9.237418 21.04014
7.382472 19.99886 13.35689 8.292809 13.23717
15.68707 10.19776 12.30911 9.423862 6.958603
16.70755 7.873071 10.9893 10.93243 10.64157 12.62106
14.48497 12.82453 8.951624 15.68292 12.72979

14.49534 5.166903 9.649281 8.503774 18.05858
10.77215 11.69726 16.29914 17.37698 17.01414
8.948874 11.28398 14.32534 13.04724 8.843741
10.25645 16.97565 16.88127 17.66457 5.974418
7.705922 7.919397 13.25333 9.829565 15.95448
12.59438 6.691905 13.89247 15.59747 17.57479
8.223882 11.61941 11.07276 8.794764 12.34895
12.33021 13.61233 6.21754 11.28931 6.093855 9.257544
11.26441 7.319584 8.44512 10.56141 13.0728 6.900601
14.80684 9.503001 9.703827 10.17519 14.37556
7.591858 16.30506 15.78588 11.79098 15.05041
15.69663 9.771853 10.03977 14.64184 9.02889 9.133618
12.41018 12.308 11.03766 13.07436 7.498823 9.294149
14.23583 17.28773 12.22853 14.52709 9.081306
27.76783 12.11742 11.68689 11.7785 7.215875 11.38308
14.60753 7.89315 9.859197 10.01955 10.68196 18.42451
17.68293 6.613265 9.848403 11.66349 15.05324
8.432524 9.179508 9.786083 17.24469 10.91626
10.16096 22.7643 13.4091 8.350906 9.139899 14.00343
5.83272 12.90273 8.250299 10.39924 18.36415 11.86959
8.916284 14.33959 11.49672 23.31139 9.657657
11.37376 13.49787 10.2577 9.102616 10.30698 13.7194
15.39105 14.42713 9.340972 11.80474 8.451152
10.26462 3.171565 9.82489 8.353788 11.67013 18.06877
11.1783 8.890107 8.312114 8.238821 7.18351 15.94443
12.55881 13.7709 8.394847 23.76782 9.744344 13.54667
12.84769 11.96113 9.442845 15.0952 16.18183 20.79825
13.47077 10.45892 14.79268 11.0501 13.72231 17.04051
9.489919 8.563688 7.501637 16.60662 11.87759
12.62794 10.26288 12.91691 9.108372 8.637606
10.84052 9.521796 11.22173 12.50764 13.96643
14.83111 13.8338 16.95074 10.50516 8.413606 13.55976
7.231885 7.182214 16.7181 21.3647 14.42494 17.54834
8.179799 16.35304 14.04201 9.865481 16.73657
6.114815 13.73349 11.63629 15.4263 10.68332 14.69424
12.506 15.17798 21.58547 17.29541 9.62809 17.4635
11.23082 11.84378 8.682423 11.81597 11.52016
9.982917 9.081915 8.819883 9.658599 9.467058
12.90143 7.163141 13.92602 15.93234 12.68361
16.32669 8.790923 20.37798 9.672529 7.196256
9.881339 9.629056 5.673289 9.931715 10.16648
15.56066 6.845287 6.994466 10.33521 21.88274
13.5769 9.392219 9.762265 13.69414 18.65845 20.89304
17.94117 8.712995 8.967024 9.814099 9.350756

7.848997 8.294726 19.43035 9.130993 7.585535
18.55297 9.799037 8.566913 17.12216 20.86305
15.34733 6.544217 13.16532 10.78786 9.176798
12.07443 7.39472 7.907377 11.09906 11.2012 9.594124
11.267 17.28319 10.57636 8.536844 12.41742 11.22738
9.190864 11.41701 8.914353 11.89409 8.803372
13.71888 15.07331 9.061781 13.13262 11.73011
12.88116 10.99266 9.808108 11.54827 11.90319
16.03173 9.539421 12.20576 9.183989 14.71608
16.64812 9.694825 8.933576 11.25962 9.862969
7.008774 10.55695 10.05589 9.107679 10.56006
10.59455 14.72308 9.331445 11.08138 10.80052
16.55906 7.326529 13.5911 14.39185 22.14059 14.66903
CBL 1.407876 3.453475 2.509608 2.435567 2.441721
4.627762 1.989247 3.464242 3.613399 2.354532
6.007648 2.086304 2.504839 2.700622 4.280605
6.207442 2.641535 4.965265 1.273641 1.948685
2.145657 3.569044 2.703563 4.752962 6.435625
3.485206 3.034903 5.46499 3.323057 0.6095911 3.545266
3.440668 4.406011 3.420398 3.768643 2.39456 0.9322519
2.270585 5.95102 2.961788 6.362099 2.415172 7.24238
3.038646 3.5676 4.646409 6.452471 5.570913 9.103145
5.168929 1.44969 6.470544 5.60653 2.709484 5.116946
3.702888 3.64307 9.149176 7.185529 3.600839 8.571793
4.519171 4.866628 4.48123 2.443158 4.186977 2.251265
2.926537 3.021282 3.471858 5.240465 2.962021
6.000865 5.213259 4.447282 6.380365 5.401476
5.388652 2.790995 6.011894 3.9793 4.412921 6.642998
4.827725 3.410415 2.809747 6.19606 5.507427 3.390448
5.894635 5.806625 9.611844 3.039717 6.209785
4.07095 4.770575 4.409741 2.924965 14.30855 6.227083
6.950841 3.470814 2.102258 3.263543 4.849838
8.345047 4.665245 4.330216 5.121139 2.255361
4.941439 4.356926 2.386222 4.834964 2.881272
1.965927 1.633397 5.967439 3.889682 3.909582
3.674491 8.820525 6.260393 2.176201 9.308753
8.472486 2.454893 1.927002 7.579163 5.968762
4.18169 4.684489 3.824349 4.757734 2.280501 7.458854
5.97609 6.924463 4.945314 5.29088 5.049093 6.463489
3.24033 2.781847 4.498397 2.450322 3.691396 4.25345
8.466415 2.817343 4.569766 5.631405 4.953751
7.221424 7.038027 2.287938 3.605252 4.378404
5.909483 2.75159 4.574546 4.592103 7.354258 4.069391
1.85774 5.516637 2.805811 4.717335 5.192932 4.277782

4.291146 4.205874 4.945701 2.937496 3.500378
2.948637 4.628842 3.029178 4.992142 5.890409
1.75373 6.011414 3.055411 6.512219 2.998925 5.780705
6.309197 2.348947 10.41218 5.427576 3.926409
3.589497 3.306026 3.361222 2.769553 12.60481
7.560583 6.199678 5.301888 1.59236 1.772404 3.343562
5.491107 8.425846 3.502968 5.058026 1.763628
4.419586 6.134129 4.251312 3.843129 5.000994
5.684772 3.450772 4.616337 4.971484 2.879075
4.643009 5.759023 4.303959 3.943738 5.167399
1.340764 3.327006 12.15397 1.629782 4.376662
2.201475 2.651542 3.321772 2.467629 3.302329
2.165216 5.421916 6.119852 2.148095 2.997268
2.112788 4.327023 6.299915 4.107678 4.344047
4.424735 3.206506 4.59899 5.565338 4.612253 5.022373
6.759156 4.23323 8.799859 6.29203 2.506291 5.228713
4.675502 10.57965 5.816199 3.357453 3.623944
5.339378 2.243108 2.896619 5.484766 3.424491
8.405903 8.107955 7.148322 2.864725 3.932175
5.45319 4.896068 2.378137 2.332856 3.374928 1.464251
4.820353 2.217923 5.773254 33.10165 6.471555
7.005875 4.983305 2.366775 5.038854 5.891515
6.390622 5.144102 2.579611 3.867419 5.894418
9.827462 5.007599 5.959879 4.70356 3.273925 5.543898
2.619291 5.357511 4.208536 4.064061 3.888635
4.650518 3.767029 6.342504 1.980563 2.60747 4.124039
5.018843 6.211824 2.562803 4.096413 2.576069
3.902252 3.888962 4.135161 5.196266 4.409245
3.884139 4.825926 6.750004 8.30039 6.15895 7.21394
3.348087 2.962033 4.089443 3.11924 3.11095 2.566186
7.81363 1.746409 5.701251 5.058286 3.245695 3.172443
4.475278 2.866484 1.927114 6.501743 2.139199
2.868821 4.323876 2.279664 4.06078 6.631416 4.07414
8.006544 6.118712 2.342963 2.978618 5.375682
5.728116 2.906772 4.544824 4.448794 3.637482
7.302171 2.81781 4.126631 2.622669 5.699583 3.371803
16.18498 4.450981 3.063611 2.590557 3.081262
3.236955 4.919279 1.392038 4.210162 3.64116 9.168264
5.157446 4.727068 4.728724 1.861471 3.810819
2.413363 4.258824 6.495095 4.294124 2.075373
10.48876 6.853676 3.507632 4.055111 3.01002 4.268116
7.461922 5.711042 6.274819 1.803 4.90679 6.39571
4.191359 6.105189 4.805132 1.830637 4.327907
2.597547 4.213774 4.005128 4.075903 2.18366 3.488489

4.727674
PAK3 0.03804041 0.347586 0.04795067 0.9178835 0.1459931
0.3952357 0.4484287 0.5420247 0.2936391 0.3495101
0.0882843 0.01383995 0.3568164 0.1056782 0.3583201
0.5031043 0.2107815 0.5675317 0.1757014 0.05717337
0.3803076 0.48128 0.2939533 0.4247745 0.7941647 0.06644571
0.4148108 0.6728873 0.1524674 0.3356782 0.3145371
0.348565 0.8119091 0.1242467 0.147964 0.009265543
3.908456 0.7288673 0.223417 0.2119658 0.1399445
0.008694038 0.04839216 0.01630328 0.09430227 0.2031954
0.3065643 0.05148884 0.2194912 0.01350352 0.0422869
1.458212 0.1995173 0.04448744 0.3818358 0.003463957
0.05585342 0.7163695 0.03249265 0.5596431 0.9725365
0.05572066 0.8517477 5.615373 0.7910515 0.6635182
0.1787845 0.5596179 0.1344771 0.7371694 0.01443968
0.02552875 0.2787479 0.1234903 0.05152846 0.4153317
0.1947114 0.4610353 0.1407574 0.0124214 0.0122487
1.480519 0.08300304 0.5215404 0.09497898 0.2646456
1.035067 0.1549521 0.2654605 0.2606233 0.03665795
0.09124399 0.1372057 0.1320166 0.02270362 0.09355732
0.2785992 0.1513252 0.01655279 0.1970753 0.9701926
0.1083197 0.3019031 0.08788034 0.9522321 0.5197022
0.2073123 0.6331485 1.223469 0.02817457 0.1254248
0.06900722 0.3387372 0.5917629 0.2494884 0.04138813
0.02364621 0.02902109 0.9637961 0.03684909 0.02916018
0.1773402 0.3128989 0.2668018 0.1286587 0.4072396
0.1314431 0.0213332 0.7699697 0.2634112 0.05885128
0.08982539 0.3354543 0.0908591 0.1102386 0.8565796
0.08978764 0.04702653 0.5302888 0.1275581 0.1074932
0.09197838 0.1030264 0.0356016 0.4521312 0.1435639
0.04572344 0.3806355 0.03973326 0.2848846 0.09765604
0.1116148 0.03319392 0.2744432 0.7320455 0.1327177
0.2266033 1.047662 0.01583188 0.3535343 0.3397767
0.7829552 0.8443097 0.01666208 0.04512763 0.2538832
0.1370976 0.5109157 0.8672815 0.04683237 0.3384556
0.1184038 0.2690719 0.007168441 0.05705965 0.1038082
0.4620311 0.0649229 0.1314728 0.6765858 0.02834036
0.1252189 0.1009803 0.1404493 0.06828232 0.06615061
1.461977 0.007625069 0.3137231 0.03410665 0.3540188
1.063037 0.04284007 0.6763299 0.02422676 0.3329074
0.2079208 0.08817279 0.09905852 0.07075034 0.06286777
0.1904884 0.08839928 0.01265162 0.007997207 0.05898851
0.3242572 19.31096 0.06341399 0.2710391 0.1369614
0.5855167 0.1987792 0.105666 0.1892912 0.05670344

0.1764087 0.1160786 0.4339481 0.137304 0.1748618
0.3980627 0.7394231 0.01412989 0.8354206 0.01941032
0.04655089 0.026248 0.001718959 0.045056 0.06358141
0.1032167 0.0477787 0.02112019 0.07935328 0.6212281
0.2699201 0.3010954 0.151134 3.25257 0.7207108 0.3487141
0.1961809 0.4155128 0.1410946 0.1610158 0.5606502
0.6711999 0.5562797 0.03114262 0.1557806 0.01589898
0.2650083 0.3448161 0.0211353 0.3821588 0.08393532
0.3490423 0.02513254 0.3536799 0.1484472 0.01776008
0.7809216 0.08004831 0.07815866 0.1993224 0.04880311
0.04904769 0.3650022 0.2287069 0.2170723 0.06318127
1.158216 0.01416442 0.03726681 0.1504717 0.07221217
0.7882502 0.05135835 0.1646333 0.675618 0.04267263
0.06142105 0.1038657 0.02158747 0.2951377 0.03665488
0.2249908 0.1016101 0.844834 0.05002149 0.6117978
0.4815091 0.1681116 2.23883 0.4489688 0.1553331 0.1774289
0.0274099 0.2609121 0.2377624 0.3465649 0.04205047
0.1392449 0.1581359 0.01766974 0.361384 0.09573542
0.2807571 0.006628607 0.1405759 0.03899151 0.02542653
0.3203487 0.1759209 0.5458005 0.2992562 0.06986754
0.4974464 0.1475373 0.1839313 0.1244711 0.03166524
0.08724471 0.8736528 0.1820699 0.2797829 0.0647367
0.06907341 0.1955378 0.0285751 0.3914592 0.5859058
1.041583 0.4621636 0.09568588 0.01388062 0.1612973
0.1310724 0.06703668 0.1655905 0.05899738 0.07424606
0.01888388 1.003572 0.7723221 1.456766 0.5168649
0.0373735 0.2723878 0.06468339 0.1864188 0.2294686
0.1024027 0.08491365 0.2817349 0.1625038 0.2274023
0.6728264 0.4392947 0.635539 0.7099307 0.01410681
0.03288141 0.07545842 0.06761058 0.00924222 0.1682507
0.3546113 0.0545325 0.1098381 0.1614504 0.05091331
0.4927312 0.1539299 0.1015425 0.1850439 0.2903912
0.1481897 0.5034227 0.2291061 0.1826341 0.1512513
0.02944828 0.05032881 0.07933918 0.4848492 0.1125249
0.01387932 0.06226079 0.6916905 0.1149016 0.544915
0.2901822 0.0620166 0.2601672 0.7579857 0.6138396
0.4565211 0.06563433 0.1311109 0.2302731 0.02715211
2.098456 0.01335433 0.148007 0.771667
PIK3R2 0.4110661 0.1105517 0.3367582 0.1717954 0.1895563
0.2666924 0.3076367 0.2270839 0.1338641 0.1891092
0.3776604 0.3407052 0.2578841 0.2198055 0.2617803
0.318813 0.2242124 0.1571701 0.2736045 0.3772447
0.1388497 0.2974452 0.3023337 0.3919028 0.1319721
0.3660193 0.2440049 0.1675983 0.2234148 0.2696224

0.1724323	0.24181	0.5021904	0.3843535	0.4915239	2.604784
0.3141183	0.2999712	0.3477848	0.6650479	0.45644	0.09342385
0.2774338	0.660652	0.4332119	0.3875448	0.3998379	
0.5143287	0.4218282	0.2641943	0.2595081	0.372683	
0.565029	0.3573306	0.2567237	0.6119198	0.3112076	
0.1613139	0.4894645	0.265579	0.1677183	0.3766492	
0.399809	0.3156702	0.159099	0.3481836	0.2935905	
0.3732649	0.1484236	0.3544565	0.3757657	0.6051781	
0.2477972	0.1497253	0.2848882	0.1742405	0.2169256	
0.3450662	0.9479429	0.2327984	0.4969702	0.1718919	
0.2717557	0.3137107	0.3923971	0.1616913	0.3932218	
0.3373236	0.4730706	0.4057025	0.2568637	0.3083863	
0.5349609	0.2954965	0.4029154	0.4166664	0.4233592	
0.2739155	0.5282258	0.4361926	0.4262078	0.2272638	
1.183927	0.8667448	0.2982389	0.2810114	0.2316409	
0.2238756	0.579338	0.9290649	0.2411616	0.9179838	
0.259251	0.4283793	0.3980785	0.4615013	0.4743125	
0.1704651	0.5954814	0.3998103	0.2858111	0.2625761	
0.292983	0.2921493	0.2932645	0.3551067	0.1674299	
0.3820683	0.2825957	0.1872595	0.4024366	1.060302	
0.3855847	0.3203642	0.100511	0.327461	0.48569	0.09681877
0.3781729	0.2106926	0.2690843	0.6391108	0.1623882	
0.9771842	0.2637091	0.5038448	0.5586297	0.3322216	
0.3311391	0.5113362	0.5401688	0.3785933	0.4888941	
0.169158	0.1713214	0.433607	0.6226927	0.3992743	
0.2886974	0.9132916	0.2081433	0.305132	0.2003338	
0.5258704	0.4233244	0.2600331	0.09733611	0.2581287	
0.4023739	0.3400174	0.2757163	0.1146106	0.3734755	
0.3039191	0.3334915	0.5546581	0.2702782	0.4254574	
0.6178947	0.5462764	0.63307	0.3502929	0.2049991	0.3350998
0.6078873	0.3531511	0.2902116	0.4953463	1.681809	
0.4497969	0.2754096	0.2064361	0.1822793	0.2700299	
0.5135688	0.2726162	0.274923	0.3413486	0.6250236	
0.7313327	1.213115	0.5184074	0.1574197	0.2988676	
0.3524239	0.3610456	0.441145	0.2293342	0.4090918	
0.3375331	0.373806	0.3106163	0.4143454	0.5738015	
0.570157	0.4383253	0.2577601	0.7327094	0.1513991	
0.4761148	0.3116715	0.260692	0.1853984	0.4785137	
0.5964804	0.8536464	0.5139608	0.2841908	0.3017009	
0.5070842	0.2065215	0.2912893	0.3015878	0.2331055	
0.2347563	0.3865977	0.2312991	0.6699756	0.3009795	
0.8951772	0.4329454	0.2027372	0.3619985	0.4854982	
0.3350114	0.2744933	0.4651935	0.3921255	0.2807343	
0.2953952	0.3724075	0.2500568	0.6519058	0.4250078	

0.4909341 0.2354304 0.3746724 0.1564582 0.6964902
0.4030888 0.385448 0.4587993 0.2255801 0.5407511
0.2274316 0.4885326 0.444967 0.1746863 0.2079964
0.2025489 0.3718276 0.3316716 0.6185484 0.3698256
0.6683902 0.2051499 0.2529968 0.3007413 0.1859286
0.2259249 0.4056258 0.2556111 0.2284261 0.5090153
0.3959947 0.3363682 0.3949687 0.7827616 0.3954315
0.2743554 0.6193301 0.1364577 0.1877738 0.3257144
0.4281361 0.4245934 0.375618 0.2455475 0.5466833
0.3151757 0.2948874 0.2231724 0.8462166 0.3115709
0.43634 0.5934054 0.2606766 0.2404865 0.2960155 0.3216109
0.180241 0.1656348 0.3355417 0.2795632 0.37619 0.2514949
0.3164494 0.29275 0.1474698 0.2274004 0.1967921 0.1571958
0.2405924 0.7225446 0.1287972 0.4726502 0.2669596
0.3414977 0.2711567 0.4105212 0.4136012 0.243671
0.2537792 0.1948217 0.1781964 0.7188291 0.5188871
0.7440067 0.2987662 0.2883894 0.3010587 0.8459135
0.5541662 0.3873951 0.1441728 0.5002998 0.245862
0.512343 0.6303995 0.4901687 0.3243663 0.2404601
0.2464636 0.639026 0.456998 0.3962425 0.3405391
0.2452862 0.2775157 0.370218 0.3927818 0.149538
0.1639907 0.2342183 0.4759995 0.7058305 0.5301787
0.3298191 0.5426196 0.9618554 0.9843107 0.32103 0.2473734
0.3818009 0.4481672 0.3711274 0.5354625 0.3189963
0.5454371 0.7628458 0.1983868 0.2528423 0.5746035
0.2397299 0.7981543 0.2967723 0.2994072 0.5794756
0.4998569 0.2572249 0.2411329 0.5824491 1.146534
0.4474454 0.2431402 0.2138116 0.6180809 0.2644801
0.1176862 0.2571367 0.3028925 0.4250823 0.3046917
0.4988633 0.5814011 0.7309618 0.3110004
CASP6 35.30088 3.660856 21.17741 15.25719 14.08413
3.17293 6.65455 5.206673 9.211983 10.71743 2.721426
3.408182 10.60019 3.077727 7.874975 3.495509
11.02265 6.812269 10.14663 16.32154 14.819 3.872373
2.577947 3.09531 4.414567 2.627201 11.06672 3.985065
16.45822 21.6159 10.94573 6.15971 15.32333 9.796939
8.218079 33.40595 10.02146 26.74221 12.99677
25.90709 4.546904 9.096466 14.04002 14.22867
12.65076 9.518619 10.19712 17.32819 10.88759
6.653056 15.45097 6.12356 7.067285 8.01637 4.621588
20.30525 12.82327 4.606807 12.53614 9.498461
10.44185 13.75626 13.68049 7.463681 18.91299
4.970581 7.794896 11.22265 13.42516 10.80727
9.732654 19.18505 7.855542 4.4274 8.873383 27.01923

11.43108 6.622274 15.58022 25.40254 13.0274 5.211105
17.1642 13.93201 6.53726 11.96033 12.06387 9.665166
4.252217 10.33179 34.13327 11.3967 7.818414 12.6495
13.12523 12.61193 12.4956 9.023311 12.57509 11.08837
11.13133 9.093566 5.616586 15.43616 6.812905
10.59281 9.729747 7.018428 16.44476 23.55591
13.87927 20.90564 11.63955 5.66304 5.402324 5.111487
11.50385 11.04814 8.545444 21.74711 13.29393
25.94306 13.27819 7.565804 13.99432 8.007057
23.04824 22.4258 5.270798 19.12895 12.34816 26.52642
10.19512 12.5526 9.193868 12.28492 22.88036 13.61429
12.26147 16.95402 13.07552 5.487783 4.857103
18.33572 14.56684 4.734854 21.61669 12.85596
5.008272 19.00536 7.670628 13.96297 10.84792
10.53762 12.5844 8.264571 10.99081 6.631528 1.743495
22.39048 7.44981 7.261526 10.78185 18.95802 12.15943
25.31019 12.54833 12.43964 5.784157 6.600421
5.049312 7.041516 9.174519 6.832838 13.71277
11.18461 9.736137 9.658517 6.45248 7.908445 11.63418
13.27627 4.20369 11.15314 6.208524 15.09637 4.986322
10.66337 11.7044 13.34323 11.27962 7.950543 10.79975
8.230601 17.46084 9.606293 11.2212 13.96439 8.35071
20.94013 21.31943 6.868476 22.25567 11.35368
5.560063 14.95765 12.51493 5.12441 11.2841 7.17132
20.54418 5.825546 11.32707 7.026451 12.48596
14.19651 15.11597 12.03685 11.96972 12.90743
16.25111 16.42011 4.363831 9.914875 8.456806
39.63676 19.60247 2.315943 17.78064 21.08026
17.02399 9.947312 18.27682 13.50393 23.68287
8.718778 16.16718 9.733791 31.73811 13.75023
9.201275 11.14925 14.12042 13.50937 14.98431
9.450341 17.64144 4.834456 8.824275 13.84102
8.328174 24.6727 12.67213 8.485905 13.78628 6.760717
12.53493 8.432943 19.94454 7.428866 9.824114
12.76663 9.100824 18.01309 14.06817 15.62896
11.77203 30.69202 22.72438 5.527845 13.63383
9.002814 6.658326 39.33661 13.38168 12.41922
4.960939 5.494139 4.160731 10.66333 7.915705
14.68398 7.01709 26.30802 16.95094 7.744837 9.255237
14.17818 15.47444 4.56284 13.9134 3.076483 4.131893
13.31307 5.671756 4.930353 7.942845 11.28311
15.14721 7.823393 11.77432 6.713889 15.76236
4.013697 15.56116 10.12801 11.06265 13.29336
9.938043 6.005681 19.67183 9.838959 16.71899

13.6111 14.15886 9.830103 14.22039 11.90938 7.378478
10.12704 18.72653 17.94332 9.131019 19.20031
24.04471 10.62921 15.8157 10.60172 7.484553 10.46645
10.11542 6.595993 8.922421 6.858041 6.003919
10.07076 15.17561 30.89181 10.95064 11.79088
15.03429 6.494596 9.294777 15.26709 4.151316
5.067216 8.060426 8.373477 12.0994 10.08138 15.07736
20.1019 4.81401 6.993933 12.7937 10.60453 9.737332
6.012861 6.138483 7.324108 8.368503 6.752168
7.980977 14.82245 33.38862 11.66904 16.2767 10.05462
7.43369 12.48632 18.07909 3.600631 9.649342 11.20842
23.93857 8.232346 8.525253 3.583709 9.889029
5.49411 8.823946 9.609608 6.680495 9.652744 20.28315
15.39617 6.622708 3.229297 34.34818 13.4583 5.423177
18.38539 17.49597 11.469 7.965141 17.74342 7.898392
11.38245 9.907827 12.71435 28.46358 8.434339
13.20207 16.77389 41.36967 16.22383 6.113475
PPP2CA 16.83071 14.45956 14.84179 21.32908 16.87198
14.67722 14.89366 11.6236 22.00969 21.12885 22.93521
26.3754 18.84522 20.55921 16.29267 16.72612 18.58863
16.44624 20.42016 16.70177 22.33936 17.1428 19.417
15.17436 13.79669 23.93902 16.11163 16.0115 19.79341
22.86304 17.58034 18.81358 38.08638 19.58529
22.78671 29.5421 28.21932 16.13748 15.67344 14.15448
25.89002 29.21689 20.49365 22.74524 21.97459
19.37747 19.33911 23.23819 19.43449 19.56758
27.02838 17.96668 18.11408 38.79391 24.77448 31.051
22.86176 13.87539 25.1035 21.66567 21.36069 25.711
19.30852 21.31368 26.3224 17.61333 18.83291 17.15588
28.98799 15.68712 22.42341 25.34776 18.39373
25.64171 17.59442 20.36685 20.36641 19.02329
21.60962 20.26091 23.41875 17.89706 33.09844
15.69134 17.51751 22.59539 23.65809 21.35651
26.92985 18.35016 34.20643 23.48129 31.70409
26.94888 19.83049 12.70567 24.35218 23.91146
33.96015 24.47113 13.50209 23.01844 27.15416
16.69467 17.04648 17.19903 24.0318 16.5684 24.89648
23.91282 16.33343 21.11804 28.56846 15.6175 24.99556
55.09325 10.19696 15.37541 18.65223 24.41427
29.27587 25.74936 25.72443 18.99698 17.56242
25.65758 27.22282 26.15851 16.50987 21.35227
21.93959 34.256 21.61164 33.37049 17.63677 14.429
22.18502 36.29343 17.41282 16.14047 11.96353
21.70809 29.77788 21.22802 21.30691 20.28602

19.21315 19.24295 9.343491 29.92314 17.91934
21.27557 16.69316 17.0407 15.00216 16.34275 19.02904
14.32521 33.41735 17.22467 20.96634 21.55209
22.50022 12.63553 17.31024 26.19781 17.80489
29.64974 16.8713 13.28296 18.03604 27.63989 17.19589
28.61516 44.96831 24.75932 14.14136 35.50097
30.55584 18.4958 19.68955 13.15446 27.53705 24.29768
23.21252 12.23382 16.14865 28.3142 14.40537 21.01709
14.55923 24.86508 24.29548 25.58544 14.04694
21.99731 29.38149 22.72311 19.92725 22.41276
25.08955 16.05412 17.81864 28.20415 23.68207
23.42667 20.01449 21.02613 16.0194 18.65784 15.99999
13.49715 19.73886 9.44849 19.7798 22.66996 21.36523
15.6593 22.39138 13.8652 23.96369 27.49449 10.61914
21.80997 21.00743 21.6638 15.72444 23.76306 21.04501
28.26403 16.71848 19.5151 13.09512 25.5233 21.28559
20.45539 20.80964 23.7475 32.02506 20.11981 15.23314
23.18141 19.44382 19.48203 28.21019 19.51089
22.98932 15.69665 17.3796 24.22876 23.5735 24.88071
16.65676 23.92875 24.20169 25.97964 18.74456
18.34274 30.72152 16.48063 18.7523 24.36428 33.18658
23.63527 20.535 16.52613 14.0305 22.51763 20.23205
20.25996 28.92151 18.21912 23.04651 25.73838
28.91888 13.68693 26.00852 13.85128 30.16556
23.83238 15.83698 26.06187 18.82297 22.87905
27.13692 19.5285 17.2125 13.66278 22.16132 19.10413
33.46947 13.6712 19.48665 20.18492 29.33016 14.85154
20.07242 18.29555 21.43016 26.81684 25.69967
24.45642 26.61034 19.13054 25.42926 20.81638
28.16212 21.31833 23.66741 34.95591 19.32136
23.1772 26.72469 16.47925 26.85876 13.46798 26.8992
20.99951 17.37906 24.22351 21.88744 22.77766
16.74671 23.06514 36.83787 19.41109 20.81974
16.77546 12.28873 22.10716 26.80679 15.15533
19.31241 22.18218 15.24582 10.97845 35.11545
13.44849 21.64754 27.02139 13.6455 23.73328 34.39216
20.63344 14.38664 21.27519 19.24386 17.74438
13.80643 19.07238 18.99361 17.83442 15.3302 29.02919
25.0148 17.75105 18.79932 13.92858 18.34332 27.08215
16.58567 16.86708 36.86201 25.8228 23.03474 25.43223
19.72279 19.91222 15.08368 17.77162 16.85053
19.12562 19.44674 29.27089 26.76257 11.30858
12.49563 33.44636 17.33813 18.59887 18.118 13.47675
19.03213 9.155002 23.47159 22.69384 15.02487

34.64818 21.02989 12.31481 18.69208 27.83733
20.12168 26.0969 22.63439 27.49041 15.65677 15.16564
19.56223 19.08081 26.36164 13.45824 27.59863
20.13662 29.78727 22.82677 15.93079
CD36 33.41798 3.841599 15.85842 1.793312 6.919955
7.35317 2.427842 3.086536 3.128846 3.019698 0.8568848
0.3442718 3.413631 0.2181662 2.093443 4.226659
6.884971 4.679509 11.94274 14.7926 3.778118 2.679282
0.8880713 5.691481 4.337384 0.5927234 2.139458
6.48172 13.13719 4.43577 5.191494 6.79292 1.231184
0.9499993 0.8466073 2.616542 3.108747 1.652541
0.842187 0.5783693 0.3224575 0.06964071 1.665418
0.2400783 2.607583 0.5474753 0.6267411 0.4353719
2.919195 1.034783 0.2918248 5.178074 0.7057471
1.123048 2.47286 0.506759 0.5398871 5.586371 0.4522348
1.018409 7.755704 1.7316 8.837862 3.738056 0.1423161
9.478933 3.559096 1.701389 1.047703 0.3582043
0.570435 2.539091 10.51454 1.837537 1.855386
3.691293 2.736673 0.9435365 3.454842 1.528041
0.4095821 6.103313 3.375553 3.721886 1.021241
1.126484 2.150356 0.7685505 0.9939524 1.028774
2.304601 1.541263 0.9227032 4.651158 0.9522842
0.8628172 3.127062 1.22813 0.8437587 1.061348 1.170721
0.3388174 0.2018792 0.7546395 3.521478 3.502905
0.8412556 1.84525 0.3861288 0.6931889 0.6941384 1.002216
0.7360413 1.447258 1.215781 0.9214301 0.2121394
0.2822078 1.175685 0.5529827 1.196556 2.320963
0.9569787 15.20789 0.5577942 1.699693 0.4545597
0.3075886 2.714118 1.239013 0.3589504 0.1259154
0.5173659 1.418377 1.997252 3.194639 0.8333906
2.360004 1.609204 0.5691711 0.3962734 0.5293454
0.5519817 0.5051051 0.8825587 0.1986314 0.3859635
1.600087 0.1696235 1.595277 1.88231 1.874804 0.4672579
1.191955 4.725535 0.5321898 2.149738 1.816394
0.3712252 0.4421148 0.9807286 5.060582 4.936847
1.281834 0.5803902 1.017068 1.315191 3.188293
1.220628 1.600324 3.489603 1.433636 1.932446
0.5963372 0.7681548 1.688727 2.328748 0.6122329
0.6079864 2.649355 0.1170139 0.8196203 0.7286719
2.696422 0.8011969 1.167759 2.879077 0.2876223
0.6846799 0.3688201 1.394383 0.417755 0.2926186
1.756693 0.1802998 2.624625 3.702598 1.045864
0.4083419 0.8001207 8.380605 0.9933543 0.3140152
0.158294 0.2092593 0.6058284 0.7546051 2.003448

0.4513235 0.4892848 2.250596 1.780074 2.835776
0.6811956 0.1915995 0.6936936 0.3289462 0.8488575
6.873747 0.2546411 1.868457 2.787709 0.7734235
0.5401906 3.366685 0.1303704 0.376694 0.2642471
0.3171912 0.370339 0.3159962 1.895756 0.1761026
0.2277809 0.3459713 2.21564 4.975946 0.678478 1.417513
0.7978146 1.095129 1.216204 1.230487 0.6941432
5.448038 2.001548 1.042696 0.9185923 1.642747
0.6626355 2.823802 3.496201 1.029854 27.21376
0.7787676 4.834923 1.764882 1.810468 0.2521746
11.14433 0.7237921 0.2917073 1.99583 0.6484073 3.678883
1.378731 0.2077213 2.137626 2.567804 1.64401 1.010909
1.153557 0.5673326 0.5369161 0.3899242 1.324708
0.4253518 1.10366 1.210408 3.329392 1.732703 1.774547
0.9837506 3.256074 0.7367409 7.06803 0.755302 0.09657801
0.8691372 2.767341 0.2433886 2.451684 3.314375
1.481263 0.7945596 26.37693 2.272099 0.3649991
0.2596439 0.8053357 1.245829 3.670986 0.5842493
1.954837 0.6084987 1.01238 1.543643 4.037452 1.775352
0.3784315 0.4635304 0.4535217 1.638527 2.041641
0.515854 1.536466 9.523671 0.745802 2.447731
2.38222 1.479061 1.524458 0.7384497 1.153306 16.53718
2.856423 0.835796 0.4978098 1.613762 0.3042652
0.6044813 1.183137 1.373403 1.611265 0.9728027
0.5358351 0.1825473 2.018583 1.044575 1.039573
1.10333 0.5025136 0.7955089 0.5807586 4.369746 0.6782797
0.4550209 0.5557134 0.2305134 0.74295 0.8994647 0.9266753
0.3245733 1.6173 1.752991 0.1222221 3.145034 0.4119144
1.819349 0.5156392 1.15605 7.899226 0.2444864 0.9763227
0.3291805 1.37579 0.2465481 1.27445 0.9022837 0.4394619
0.592099 0.6671345 0.33465 2.998313 9.257427 1.599207
0.3491992 0.3673834 1.549147 3.923659 2.153003
0.6739431 0.2175893 0.09006546 0.5971496 0.8254355
3.198129 0.6415625 0.3834551 0.6955625 1.102186
0.449326 0.5153085 2.822603 0.5336143 3.395719
1.026045 2.09743 3.662352 0.9451409 0.7532169 3.117982
0.5216791 0.3171802 0.2750113 1.2342 1.14698
CDH3 0.4915873 0.03787288 1.579419 0.4762706 1.182641
0.1840381 0.6119442 0.1088969 1.625756 1.304785
9.247306 7.492903 1.755729 5.491583 1.306597
0.03624658 0.9666549 0.5525654 0.5983482 0.7115625
0.6016613 0.0719287 11.9803 0.195116 0.3350941 9.042814
0.3687855 0.11606 0.4592255 0.1585959 0.3291938 0.1399221
2.473788 27.4359 5.821034 0.05617966 0.4533394 5.314917

22.86431 1.410294 18.75783 6.660506 28.25277
7.560641 4.291963 4.508689 5.503168 16.81328
3.150362 31.80315 14.55819 1.402399 0.7648299
2.932111 1.394559 29.30487 2.004566 1.191771
6.695304 5.514734 19.24033 29.02442 4.680206
0.6352237 1.673601 3.233217 11.75576 2.945731
9.814911 6.021768 0.4795458 0.3883776 2.346206
2.632961 1.774879 0.9227592 11.95484 14.96501
4.13854 2.044904 0.9519732 0.8932704 11.56443 3.375462
4.61117 1.157223 6.304678 11.66002 4.908503 6.561826
10.74069 20.62418 29.00168 3.841002 0.7106401
10.28217 26.55565 5.198511 63.24323 14.88224
2.447338 9.354955 4.428692 2.107155 4.33445 12.27402
18.10878 1.450124 13.08254 1.38528 18.43652 4.581316
23.60373 1.810709 29.65482 4.322844 1.405063
46.49859 9.851687 19.2675 7.736896 28.30528 29.96296
16.05585 5.137429 2.727582 3.49903 6.952108 1.309927
12.74836 27.82789 32.5854 5.694129 12.91183 3.338568
1.591662 10.5794 9.494758 5.943422 14.54216 7.806862
17.56483 1.000453 8.850374 27.40596 7.674073
4.497246 9.232275 7.404006 2.00271 1.539503 10.16008
13.085 2.621076 2.858617 4.747763 1.900646 2.571073
82.4604 9.408368 0.807678 1.487762 1.586774 12.06178
6.782186 7.481065 2.452905 7.904255 7.028006
0.1606228 0.5942977 5.278429 1.203088 26.08097
7.449223 9.542802 4.608946 0.1476173 1.539776
8.211859 2.055 15.12045 17.47065 3.707324 9.412282
10.99352 5.199935 14.5098 21.03365 3.146291 7.66233
3.32914 29.35666 2.012399 6.169541 14.35591 6.714221
15.52704 0.4249448 7.291748 5.319867 1.082442
6.201195 12.20647 14.56004 9.351659 1.159426
0.4186183 24.87229 0.8001952 10.07848 4.056143
10.68944 8.592695 32.62527 8.279962 3.690053
33.95492 2.753191 9.065061 3.36986 2.561756 1.318101
33.32592 3.91891 5.179007 11.35902 15.76662 5.471836
19.58801 9.015734 4.391667 10.85752 21.77841
16.14571 3.098139 4.758791 1.254625 5.545567
6.28724 6.690048 12.01143 5.413005 12.39671 5.551012
6.969736 8.797797 1.940015 19.78246 1.988494
4.666168 4.75603 2.100005 4.008268 18.9062 3.953939
0.7807358 3.844679 12.387 5.013995 7.255412 18.76973
13.03253 5.802611 0.7177225 7.279649 3.930187
10.21181 5.459271 2.416114 0.5437439 5.800116
6.25628 11.67086 7.490716 2.032447 8.00059 1.493675

0.08742527 16.68452 5.675868 5.382594 8.876451
6.872339 4.214012 0.7637029 4.584576 12.23066
9.270055 6.8525 7.948327 0.9030302 1.295081 6.634115
1.349494 3.309323 11.78217 10.38953 24.35499
3.682242 11.86736 1.991674 3.067516 1.21678 7.612916
10.99418 16.39473 14.75518 0.8896741 3.455917
15.61475 25.94918 1.06 4.586003 1.091651 2.389328
1.206534 4.128664 20.40069 13.47651 24.37881
4.95657 9.959765 0.5001359 2.28803 4.005973 20.84748
9.273603 20.98185 44.98127 11.43274 7.692882
2.02979 2.397927 1.25473 2.636398 18.10328 0.6657627
1.484583 10.61851 1.528853 0.5507391 8.92647 9.737572
2.485811 11.23784 7.040263 2.718522 7.793198 26.429
11.01709 19.70516 1.342992 1.868244 1.119422
4.298684 13.11528 8.350057 0.8900816 2.957987
12.44279 2.615254 93.03883 0.9855196 7.028298
4.331228 7.661956 14.69298 4.777721 0.6333808
6.551151 15.90793 15.26968 4.960307 0.7764609
13.41278 24.08014 4.125073 5.302722 5.142566
4.501817 13.16978 4.099072 15.66633 1.701684
18.57281 3.194257 14.35526 2.928571 17.68697
3.858149 13.95538 14.72315 10.18698 15.80329
0.9979903 0.4984496 8.648641 0.1392997 9.313618
12.08559 2.003144 22.01452 3.837288 4.74123 2.438066
2.600177
LRP1 31.38862 24.19599 38.40726 10.87965 17.73074
46.55133 7.01035 21.26846 21.10687 4.909044 29.13725
7.682739 6.094809 12.85451 12.43455 23.70771
24.74356 44.57401 22.95072 34.13772 10.56726
16.82168 19.38702 36.96447 43.57078 22.17324
11.74649 26.83295 28.98789 1.893625 27.77197
20.6875 35.51829 14.52303 27.68746 1.995992 2.041433
16.94227 24.72679 13.24383 29.94542 5.137406
24.62492 11.71944 24.5772 36.01159 12.42323 34.46534
15.53667 27.56479 20.50278 42.9372 7.953702 12.96952
22.7567 13.47719 19.76875 51.33162 12.87555 26.55642
65.04096 21.27867 68.03743 51.06855 12.44565
39.64649 36.2841 27.76267 30.47123 7.649707 10.17438
8.218318 21.61457 22.85703 41.18536 10.8133 27.74808
26.60037 9.122905 8.523317 13.62671 72.0681 10.5663
34.38698 20.38972 22.75084 61.62685 21.61422
8.997023 12.61991 25.64104 19.59333 8.98299 15.27128
11.86704 28.19998 33.67425 21.13141 14.18331
18.79612 56.33568 10.52104 2.274647 26.42494

29.55851 73.79521 12.43512 38.29996 18.01227
7.418972 26.33557 16.65844 7.772974 8.871713
11.38769 13.68323 14.79724 14.21502 30.9487 8.031183
5.196123 37.25099 51.26269 43.84251 14.17466
8.720093 2.519479 20.74212 39.82941 33.85661
27.46009 8.115177 12.75195 11.0688 18.80905 51.57809
20.87975 23.92751 17.60594 7.059492 20.47468
4.635159 14.36492 34.07743 19.67931 9.42764 7.992356
28.04451 21.03982 23.61735 3.602412 34.18247
15.73585 40.75214 46.80876 6.335372 20.82865
22.72245 12.29981 10.82083 38.22352 18.81812
34.99819 17.954 9.589455 21.13882 41.31663 26.9601
28.14176 4.84864 16.86675 19.2845 13.65324 11.42141
9.038249 36.09444 75.28606 12.83054 29.41971
44.82967 7.489509 25.14521 18.41289 28.09884
21.90415 30.98623 67.18552 6.539899 136.0028
17.40601 35.07609 18.49718 19.58155 12.3148 16.73701
36.37989 37.10331 21.91065 29.32215 7.65095 11.58134
15.94277 33.81861 18.05883 3.436298 24.3974 15.92882
25.56875 16.65797 30.95456 24.09282 45.65855
35.50624 12.25075 11.87591 16.42562 55.45353
32.03082 35.89442 48.77419 27.22212 27.96572
19.74454 20.33789 42.22949 2.92897 35.18355 21.28831
5.751839 26.92539 27.13581 31.68247 26.2536 22.73894
17.29265 14.03641 25.61486 4.886663 27.43988
19.5618 33.75212 35.93611 22.29938 16.75832 20.86366
38.80149 20.48593 19.29817 55.91134 7.068023
13.66252 13.97106 24.23958 32.30201 35.19774
43.45651 21.2023 38.5933 11.53922 58.16235 7.425627
7.335402 9.549509 8.89956 38.4375 34.92456 15.44983
20.7197 20.48685 44.65242 26.85154 18.51913 7.258066
8.388667 17.0702 23.78324 27.05627 13.19113 10.40489
27.17436 49.08216 137.9869 24.0238 25.89383 9.160389
33.5244 20.71839 15.64195 16.02182 56.45043 27.04345
22.14503 37.60198 22.86676 42.24525 106.9989
30.60039 21.16274 8.871694 19.24395 15.13247
38.18178 22.64123 81.0711 11.55191 27.0245 8.856878
23.88802 31.86399 7.119711 41.84146 15.74177
16.67195 70.53768 5.649465 44.40889 23.81792
30.74384 15.37557 27.76513 23.39596 23.47792
25.2543 11.42771 22.70717 24.80636 30.15208 9.029124
12.18816 21.54761 13.54202 52.56245 44.96677
17.46688 38.32677 14.04008 25.53303 15.29965
28.59561 23.98981 11.49028 10.58625 9.158351

18.90087 48.3641 27.71903 39.01828 5.701544 11.36731
6.716972 8.904631 55.50453 21.80765 9.282317
27.81177 27.51401 37.9215 18.04669 15.42157 16.54201
18.49079 78.77769 45.05928 13.42161 23.93838
10.19955 17.68242 15.356 22.22391 22.61466 16.49559
13.02586 14.35727 28.76776 18.4564 13.78512 7.763097
5.013834 15.5779 25.36686 42.51419 34.36963 11.25841
11.2914 19.2037 21.22674 72.84975 9.606176 10.79591
19.3469 33.73644 8.192656 15.42597 26.05896 15.21039
13.78585 53.76789 48.08686 7.461607 26.52486
15.44999 5.325094 10.06976 10.02622 2.972403
27.8697 19.28523
PTK6 61.21542 0.193281 42.74025 20.45294 13.67014
1.462874 16.81853 6.511161 14.42288 5.799932
42.58866 70.72439 7.430422 75.77338 14.08132
0.3506267 23.80833 9.20186 26.99059 21.54312 16.55665
0.2356718 32.6451 0.4423662 6.604537 64.0625 16.00536
0.3352483 21.45459 7.103467 20.1036 5.638819 20.18675
22.54532 24.60711 0.4863231 0.1898058 7.708813
19.65865 14.49806 13.63161 21.37346 22.94185
18.61972 21.40795 27.28325 17.92638 8.361881
6.711503 33.31581 15.51391 10.89341 0.5077391
7.813066 3.748774 17.47835 4.220047 2.248942
34.89995 32.32827 6.358336 46.41751 5.774207 2.5268
7.191397 1.239381 14.59047 6.636911 36.64391
17.8152 10.40006 2.922844 28.7835 25.17738 17.65463
6.009386 17.4733 7.985902 18.32814 20.66489 3.685966
22.38627 2.033082 10.25013 36.19973 14.92635
17.02374 8.614294 13.57863 15.1808 8.998024 0.5284578
8.848221 18.30721 9.533209 6.079033 5.538548
55.47396 1.758308 12.20286 5.67866 41.44748 20.43519
1.507965 8.739517 4.347333 16.18676 7.855451
12.09783 13.74961 13.35749 0.9698254 16.42106
7.326144 22.20906 8.380182 9.526326 24.49679
13.38062 19.2173 19.24397 8.3163 5.969598 8.859855
10.146 6.532016 5.528292 13.77791 5.543743 23.37599
16.94303 12.12349 31.17106 10.97354 24.95196
43.67766 11.09948 15.66438 13.71053 8.131243
2.958704 13.00363 8.709649 4.168085 2.545281
29.17229 20.82472 8.813309 9.771392 4.021128
10.31518 16.51034 19.56244 4.032357 3.966601
13.25086 8.228741 8.470165 14.51432 36.94716
2.474461 9.068719 11.12677 7.328257 16.82332
14.85584 15.70511 20.96485 17.67137 2.88903 1.473262

19.15488 14.03869 34.73558 19.64863 19.87668
19.72013 17.16592 22.12476 3.468495 12.40582
59.8885 48.62365 4.873198 9.787122 22.73898 11.56789
24.48674 6.659772 8.980663 14.70453 9.169444
13.49135 22.20365 11.53762 40.27511 3.618835
9.823986 6.384855 28.11315 0.8382143 27.88594
15.91561 28.09532 22.41474 25.36547 24.74789
0.1589929 12.00746 27.59633 15.5945 18.96854 28.96636
50.77419 9.282484 11.93072 3.645649 0.7772036
2.721848 9.435172 72.14063 6.829278 12.10881
27.2888 13.5029 12.5132 1.796865 49.61965 15.75903
14.15964 33.65582 28.2516 35.12813 25.09193 8.170563
26.72704 9.324189 11.96146 7.59304 3.136006 8.514564
8.526605 10.90502 14.25841 12.07536 33.31745
4.941438 3.532814 13.12994 13.15758 10.70701
25.4632 31.66606 11.29701 5.715713 0.2288955 27.76304
11.82514 12.60819 4.531445 19.07923 17.74591
7.454735 5.342293 18.74272 5.557177 39.55448
11.32727 13.72578 20.87355 8.777739 18.72488
13.91292 9.09264 26.68098 14.18168 27.05094 2.955504
3.631213 10.63391 5.519728 12.37951 13.98121
12.17984 9.348705 6.416323 37.98079 37.3998 14.16993
10.02381 9.073858 1.139361 2.013728 25.83669
0.9971924 2.849545 6.985595 12.56114 6.426063
30.78017 13.68463 2.893037 10.69084 8.287534
22.8451 41.29772 5.095499 9.509692 15.2382 33.40671
4.361737 25.83004 3.245111 4.855309 7.628038
7.824178 9.785929 19.80984 11.38459 9.189348
22.3685 10.29412 10.17527 0.732678 5.804834 8.830778
19.8305 1.449751 26.97622 13.9094 17.60799 18.46102
13.35291 16.45419 4.42512 16.97204 44.90817 13.67457
9.744912 16.14248 34.71038 10.74158 25.05779
19.65257 7.015927 47.27646 34.7971 0.3989534 10.23319
3.728897 13.98763 3.362671 47.62601 13.8806 7.320848
63.93232 19.73222 7.019345 6.995699 14.6812 45.46245
8.362502 47.34789 5.565884 6.699259 7.272459
31.52065 9.790661 19.67714 24.53368 2.585082
28.74581 32.3536 16.69438 1.294509 9.176865 32.68711
18.91457 25.91416 14.64818 9.0379 14.39659 21.46408
10.04434 16.80521 4.790721 8.94919 20.09542 17.5517
22.17179 13.623 9.763414 12.63107 15.58327 13.16789
7.58637 4.298414 17.19208 12.789 19.15545 6.894052
27.73632 22.18834 8.266299 17.4392 10.75636 11.92384
EEF2K 5.010743 6.322721 5.275607 6.667619 4.569337

6.584433 3.168408 5.023957 5.808783 3.940151
9.393728 4.299323 3.975242 4.382114 4.660388
5.772301 4.578429 7.167663 1.954942 3.544086
7.288339 4.097221 5.79617 7.165217 6.379863 7.293296
3.094048 5.775564 4.410909 1.660672 7.064679
6.164096 10.36597 7.862211 7.587878 3.975138
2.490098 5.710862 5.769148 9.877261 6.191059
15.04808 7.462861 8.962642 7.786276 5.065355
8.210241 6.449011 6.77509 9.760696 6.190031 8.36292
7.649451 6.698001 5.789077 4.986148 3.473743
8.187741 3.404046 5.717354 7.245753 8.098335
4.517781 5.829552 6.573903 5.701936 7.38083 4.300813
5.482116 7.062624 5.975263 7.322489 10.21468
5.337779 4.331919 11.02128 4.047908 9.096859
3.72525 3.1978 6.246134 9.413274 6.939007 5.971969
5.508874 9.133117 9.970375 6.760565 6.447944
6.162675 2.87519 12.39346 4.444472 5.378317 4.057686
6.27753 5.245097 5.325927 9.460445 9.854925 6.713601
8.646187 4.516276 3.422453 6.054538 6.988355
5.34425 6.345868 9.400267 6.598044 7.173747 3.544776
4.317284 6.452504 5.062044 4.973562 6.581444
7.259183 5.916478 5.778468 4.467379 5.371172
6.620765 2.754488 6.314107 5.953134 2.863189
3.203423 9.363201 5.336888 5.495217 3.93512 10.13054
9.001025 3.798716 6.582867 8.281215 10.07637
6.280083 6.574756 4.549929 8.003834 7.392946
3.860445 4.44557 6.595516 5.759121 5.320072 5.239759
4.091241 5.817832 5.369769 8.51143 13.28863 7.790826
7.735974 6.856749 9.172082 5.587833 3.47897 4.633658
6.90889 5.728121 4.484179 3.483699 5.979448 7.631976
6.010553 14.56341 3.093338 8.202645 8.164754
9.219392 5.179998 6.522092 4.49366 6.627681 3.626029
6.929136 7.596814 3.039065 6.420061 5.481642
9.873253 7.096601 9.154657 9.103996 4.772196
10.04418 8.466924 5.796322 3.592723 3.877853
6.486111 8.008773 5.192058 5.003107 6.098656
11.05148 3.044314 3.298882 7.842126 6.26387 6.810073
6.803715 10.40222 4.105949 5.262238 3.616849
10.26625 5.489315 9.340333 5.108386 9.220757
10.56825 9.898117 5.191743 6.489602 7.025794
6.111411 7.662979 6.371054 4.053953 6.004526
9.997695 2.118453 8.759326 5.096143 8.940311
10.09109 4.150961 3.932986 4.572045 6.371664
4.024809 8.122056 3.209647 6.71357 4.823374 11.63813

7.3361 9.577362 4.038348 5.005885 6.381736 7.115629
7.498316 9.989991 8.129113 5.178429 11.91218
9.009088 9.66228 6.587994 13.3755 5.951827 5.871301
6.450234 2.61922 7.734359 4.297093 5.371452 4.826147
4.521213 9.514978 7.638973 6.221695 2.650693
3.801147 11.36938 6.863754 5.324753 3.104854
2.620931 5.3188 4.489689 10.2289 7.667378 26.89989
4.209903 7.996693 3.507715 3.215085 3.437217
8.276829 9.19194 6.966717 6.209076 6.667867 6.119695
9.697236 6.402016 5.680962 3.827496 11.5285 7.40942
5.053913 8.701741 9.832867 8.532248 7.409007
5.557112 6.579289 7.017018 5.197881 6.066106
4.691717 5.386044 10.45609 9.518936 7.626821
4.598721 6.712403 4.19667 6.01636 7.014805 5.908545
6.913306 6.298119 7.595281 9.751846 6.663416
10.14644 7.411226 2.533547 5.384366 7.114502
7.110862 5.75068 7.770855 5.757385 5.898583 10.26774
10.26885 5.84487 5.014834 1.956085 5.193009 9.497535
6.142897 6.448247 4.891616 6.077907 3.864666
10.95467 7.464561 7.906823 10.34423 5.324418
3.145946 5.861749 9.312784 8.088315 3.639216
9.853562 6.782564 8.094935 13.86767 4.309074
3.959478 5.796405 4.892467 3.304803 8.902254
7.655819 2.800972 9.615742 6.697712 11.64778
9.188679 3.014158 5.518819 10.70158 3.323877
7.041992 3.730017 8.747607 3.778793 11.61202
13.79556 6.666753 7.04917 4.773324 4.938571 3.727139
5.459012 6.030457 3.777832 7.241839 3.148792
7.913112 9.353854 3.477856 7.719031 8.305724
4.558108 8.815248 7.226918 4.930003 4.947462
5.663392 9.568447 4.261925 10.94026 2.687232
3.813425 7.507633
GLO1 90.12438 49.59303 73.10984 36.22484 56.00344
41.80117 28.49847 29.47215 38.94677 46.26537
37.92752 48.04583 39.43456 28.83654 27.5731 45.85017
32.0492 36.32859 50.9414 72.07373 57.23306 57.08963
41.29646 56.48425 40.64472 36.54143 31.21085
46.26012 56.41419 83.22996 46.259 55.73618 57.43216
36.49769 58.28482 55.03119 57.40513 33.83132
77.34536 117.2927 40.53528 98.38733 82.16701
68.51295 34.26584 55.73457 77.13761 82.96804
47.85847 32.77941 71.4292 42.82999 80.18582 85.56135
49.01871 132.0224 65.42861 35.84078 42.8183 49.60105
35.12845 39.77021 42.75754 75.03757 38.36326

36.9016 34.49802 26.80814 52.505 33.71389 104.7072
39.91319 70.44353 57.94401 48.98116 38.40775
24.98223 32.39175 106.4316 63.94187 28.48388
40.12079 65.20028 41.2798 66.50381 45.04834 50.8852
51.93707 54.85014 45.58117 61.74679 48.16415
78.22894 49.81393 62.14184 44.66005 49.72132
57.96849 63.46535 58.17297 31.61635 41.94876
32.30845 78.27487 39.59484 39.74663 54.43781
38.0943 70.50379 47.26977 29.29844 62.29596 64.95607
37.71215 47.25413 73.19221 95.80801 99.00641
104.8466 182.5428 156.8638 53.33004 89.17655
32.2829 84.0309 39.63412 158.0399 47.62799 53.99022
49.72879 48.24175 92.0569 81.06725 62.54227 36.42888
30.80022 51.58131 84.38121 82.44138 80.48478
51.43773 64.37292 39.63465 61.01819 97.80918
51.07915 75.01503 35.32555 68.91972 45.9669 115.5914
37.41266 100.4352 43.47683 38.52359 35.02925
60.34464 45.4578 125.8797 51.0473 45.529 36.71612
51.42025 101.5373 69.11245 35.88934 44.3423 40.45919
51.63618 24.82968 47.4341 68.29683 41.90265 84.82053
177.6975 66.01368 41.08781 85.69592 26.54518
30.55026 94.0604 37.39466 55.76367 43.00673 48.44208
32.30756 44.58005 51.01571 84.01593 83.98562
135.8947 109.5207 87.0511 38.27464 37.4952 51.1449
56.82297 75.42005 70.46483 55.01264 63.2011 32.20714
183.9098 105.7243 132.1158 53.89968 37.5809 76.66192
46.57779 63.49747 44.38074 30.3195 94.2272 59.899
80.95867 58.80119 28.60399 172.4587 48.94942
86.60962 75.81826 36.30506 49.56205 62.29559
47.17444 57.05821 64.30286 33.7042 61.21919 31.02636
39.2917 22.1313 44.00665 97.96911 69.664 34.02443
40.0496 89.78834 48.46927 56.01361 68.10567 51.74691
30.52221 43.72213 108.6883 46.91306 39.93623
46.29929 44.95365 73.90792 76.77891 48.86443
55.24154 53.33839 65.38593 90.31201 50.89674
26.95677 62.01953 38.86783 76.4092 66.38548 69.18675
70.52384 52.40014 52.65952 74.54718 34.2791 58.11341
33.63304 54.99712 40.29223 119.0966 55.98197
45.21627 41.30964 45.68474 39.72632 51.92891
90.14533 41.88002 38.18416 40.415 78.21788 107.3614
35.56071 57.51883 52.53049 40.32269 47.12237
98.87976 34.72315 38.80875 36.15283 55.15601
34.11262 101.8118 65.17784 58.08444 46.2475 44.58707
56.45179 47.14454 23.4398 64.04123 46.01741 87.51163

95.57176 70.91182 84.72034 31.10563 59.16513
85.26376 32.05521 59.79557 41.83345 54.75422
45.33145 47.25039 46.53666 51.5944 63.223 82.11311
42.05078 70.99268 69.62591 84.05238 96.02327
38.02353 45.80953 46.74322 58.77216 31.17972
50.87486 32.47102 72.34074 140.9095 57.7998 45.20101
76.26409 50.93286 74.85443 58.98951 127.5091
36.69762 29.88578 41.45827 52.50635 81.01992
61.98547 58.64742 74.97222 50.48267 113.9058
56.79514 34.0344 43.43794 61.76135 48.36446 64.42381
44.76969 29.37954 24.56652 87.06588 74.39203
69.74374 41.24379 30.53957 69.63422 38.73823
70.45419 40.98787 113.2323 80.06603 64.54493
94.01357 53.32127 152.9654 31.68165 58.63024
41.20621 64.22071 36.31793 47.93118 94.84594
38.9058 43.42121 35.59423 37.18049 82.71661 44.98975
64.89602 49.87922 34.02177 49.06213 44.60233
57.56293 36.44182 28.47443 117.5247 60.20247
31.40421 82.62602 63.33018 44.65032 37.81178
56.07878
PAK2 12.14889 12.4192 16.16466 17.53309 14.59496
14.8638 10.33948 11.87482 14.04448 16.37959 18.50173
14.25355 16.61045 11.79046 13.53155 16.96926
14.70315 14.08641 12.23682 13.86209 16.93658
14.87312 13.65458 17.17412 14.48962 14.53976
12.97688 16.31604 16.09768 7.623901 14.26192
14.02226 25.3508 14.4135 34.40103 20.54677 24.65138
28.75303 16.97593 23.74861 47.00518 29.89656
32.61199 18.47139 18.74262 31.77672 25.61139
28.59495 29.77795 45.53702 24.22391 24.67562
26.23112 21.31238 20.42545 18.91696 23.40722
26.80127 23.68289 17.90266 28.9618 26.20312 21.92177
18.56563 21.60695 16.88234 19.44769 17.08674
21.48363 17.79576 25.94306 9.979242 26.21387
29.39105 25.3122 13.04135 20.40139 29.20375 36.6345
25.68599 17.17555 22.13979 20.39482 17.78889
23.35552 20.48822 32.44204 21.18348 21.40886
27.36213 35.9126 22.73486 16.67283 26.1231 21.07824
20.65878 19.93845 21.994 20.62582 27.28961 22.41721
22.48117 14.17505 15.8486 21.41844 26.6103 35.32589
14.80913 25.50888 17.00277 24.18959 14.49736
18.49024 17.07653 25.23486 33.72741 15.34855
25.56295 18.35548 19.60664 24.60031 25.10303
20.04208 17.13461 21.04858 29.76653 20.48825

15.55053 20.76791 30.64932 25.05531 27.67073
32.03203 27.05654 14.65977 23.88133 21.26667
31.60564 23.91253 21.3377 36.19132 39.4871 40.40137
20.15423 16.79882 18.16949 14.60673 19.14892
56.91571 22.15707 23.93742 25.02646 46.27354
21.26251 18.79606 19.47014 13.75 17.82309 44.67473
15.55165 21.74144 20.4674 33.5918 26.98182 14.97235
26.03163 15.43234 20.80995 31.04587 17.38727 14.884
25.48877 20.51465 24.64268 19.4569 26.26103 21.19602
29.62698 27.12431 39.4504 19.84428 25.59065 18.16997
20.06167 19.61242 27.61677 21.95235 16.20249
28.34952 12.23845 18.89397 17.15856 18.61715
18.30386 12.09287 34.2341 29.40843 20.57357 13.19866
14.10392 15.70192 13.04608 26.54667 18.81573
18.64171 29.05446 15.9848 37.6982 22.32881 18.39819
22.14015 18.198 22.5986 18.31636 32.71742 23.23942
16.76813 20.95967 18.90808 29.9305 22.09885 29.51419
14.77558 19.86255 29.95277 13.64816 22.78625
10.89476 15.795 32.70663 26.20776 14.89812 13.47942
27.36059 23.96644 17.20036 15.87119 19.39725
27.88622 28.66321 18.54864 23.44911 25.47094
20.7597 27.32657 27.40287 15.00015 14.58949 32.47013
36.19361 34.95646 27.44271 19.12552 19.20173
20.11298 66.77037 25.63607 25.7388 24.30898 16.02311
24.2234 21.91342 32.20341 24.47052 29.55696 22.45018
37.92492 17.87018 18.9288 29.86635 33.16174 34.53012
20.85329 25.06396 17.79217 17.49667 23.90021
17.60064 32.46261 47.17672 23.45837 27.85893
17.71246 24.74164 36.90893 15.70809 27.2359 18.33026
25.39517 23.50209 27.16907 18.95205 20.37422
21.29139 23.25728 18.35395 16.54777 24.53807
24.8315 27.34643 27.44739 24.10787 24.60663 18.96582
23.10209 19.46096 18.21679 33.08419 42.00494
26.26552 20.8314 21.62851 27.18385 17.35038 16.2979
17.47048 29.21839 31.93432 21.79627 32.25605
24.80223 27.59742 25.20597 10.96246 27.89604
13.59137 14.27098 42.27759 17.45685 33.83917
22.15638 18.6947 27.2879 22.30297 12.60027 15.68385
19.56661 20.7377 20.24032 39.34146 27.61328 17.34341
15.85984 27.78871 23.66572 16.07046 29.23177
19.21577 32.21772 15.14109 27.91038 28.3945 19.77284
23.84624 20.4205 27.8803 28.8213 39.16302 15.42873
18.70225 35.51696 17.59096 23.69272 32.01794
15.25315 14.76241 30.15229 25.24192 29.27442

15.45824 20.91286 15.37818 31.36314 26.71479
13.2325 21.66138 35.1797 18.99443 18.80649 17.47807
24.68783 25.30667 19.23165 25.07641 38.82083
13.71773 16.08165 19.69914 23.40881 27.15031
24.86837 31.25119 21.5412 35.29687 26.12935 44.30219
20.71972 18.39361 11.38298 20.00374 26.51405
17.31178 24.74625 19.70701 21.06939 14.83931
19.31833
LPAR1 5.487968 11.40074 7.200933 4.234663 5.099816
12.29731 4.6741 4.936076 5.705842 6.945851 5.118564
3.802142 6.991332 3.845188 4.510511 11.74954
6.243575 10.62868 6.167993 6.058292 7.604836
11.60603 6.759549 16.1951 11.55851 4.286202 3.638455
8.75626 7.729449 9.04427 8.371333 5.564371 4.791606
3.190648 1.638109 0.2603649 2.090099 3.062173
2.685979 0.5162823 2.821011 0.1416561 3.210522
3.227106 5.331028 2.300566 3.349354 3.030133
3.048319 1.989342 1.048479 17.95582 1.952533
1.050515 5.407996 2.945918 3.53394 15.01889 0.6164871
2.306357 20.75822 1.848434 21.21601 5.359085
2.499781 6.351009 3.012436 8.568855 1.867198
5.417419 1.193411 0.7190546 6.911392 7.33546 9.07109
2.609225 5.288833 3.853021 9.909479 1.995035
0.7349527 18.19023 1.249775 4.755842 2.697152
4.562773 6.563343 4.800277 2.010483 4.373673
2.70654 1.731862 3.061741 5.378786 2.758323 2.123417
3.599457 3.582813 0.5481997 5.633488 11.92709
7.095808 0.5553768 2.338173 7.616492 7.63393 1.93513
4.961786 3.679148 0.759115 2.739618 1.018182
8.398793 10.21678 3.476488 3.838523 0.7817256
0.357013 6.229676 0.3654607 1.413484 2.841169
3.067848 3.73564 0.8663419 6.688595 7.620442 1.930647
6.833914 2.618918 0.7421709 3.094174 6.717896
7.096316 4.584137 9.28345 2.561829 2.779583 2.82571
1.233006 2.279725 0.8901504 3.807263 0.9195012
3.260888 3.873167 1.87285 5.584931 1.142174 4.139172
0.171107 5.5052 1.221874 3.593277 6.157434 5.232146
4.241966 4.041199 3.091271 1.764823 6.687 7.224849
8.352234 1.026461 2.586412 3.959955 3.611013
8.02189 5.566234 1.229992 5.47919 2.521531 6.455312
1.109081 1.048374 2.598772 3.277912 1.267659
2.725455 2.215182 1.985079 1.044318 5.825128
2.519015 3.954641 1.985464 9.228226 1.819695
2.880957 0.5134333 4.532644 1.700715 3.21845 6.211016

0.4264889 2.396789 9.728075 2.186193 0.5339345
0.762879 3.249229 1.245168 0.2226056 0.8098898
0.2974292 1.530298 2.672908 4.566744 1.254884
0.8129873 4.092927 6.345394 4.581667 1.136586
2.296397 1.283674 1.368262 2.534944 7.041246
1.528346 2.340571 5.630904 4.763061 1.559024
10.24113 1.704254 1.825083 2.54627 2.523139 2.604327
2.108521 8.250372 0.2004784 3.006928 2.227064
1.408273 8.926705 1.12871 3.692057 0.7882652 3.092515
2.9684 5.656362 1.861358 8.421572 5.022238 2.157854
5.52403 5.430531 2.022569 3.730601 0.9539785 1.268606
3.160154 0.5240375 8.715668 2.058004 3.367574
2.09902 5.969862 2.582865 1.154698 3.707428 1.685234
3.005805 3.36912 2.044976 3.728134 6.642481 6.046292
2.200643 1.670979 4.73421 3.815294 1.76048 1.154125
2.970836 4.00111 1.411977 4.102815 12.58984 1.983216
4.416409 6.12597 1.763772 4.32968 3.6381 1.186169
5.44121 10.30589 3.427611 4.293708 9.763876 2.206185
21.97402 9.266734 2.133338 1.57116 0.5889976 6.021988
5.951532 6.40805 1.611588 27.51545 3.098386 0.931716
3.10019 2.984085 2.796751 4.817875 4.542039 1.362269
1.921171 11.57842 2.005554 5.366558 3.514884
5.994355 4.391722 5.389605 1.35418 4.228912 0.6248257
0.6777971 16.55702 4.689875 2.65544 0.7566943 2.221669
4.337906 0.3896767 4.631804 4.223584 1.601968
5.501498 1.249727 0.1789642 1.958699 1.747239
2.013291 1.950217 1.014527 1.516025 4.092979
10.41322 4.833237 4.623317 3.569995 1.626279
1.255951 1.186803 4.643644 4.456311 5.764816
0.9064852 5.548728 5.497892 1.782108 5.465453
1.297317 3.588006 9.486663 0.1853782 5.100568
5.464908 2.863232 3.166165 3.358202 1.812357
1.590329 1.816824 4.75684 0.7408096 3.675478 1.853779
2.556755 1.21372 3.539317 4.12001 4.859219 4.36038
2.836442 1.225062 0.5145841 0.4248081 0.4943143
9.808824 2.149326 0.6882595 1.144926 3.029364
5.791386 2.488197 3.931801 2.149657 6.595506
2.389098 6.091013 5.161006 2.715666 0.5535177
16.42885 1.035725 3.032424 1.251607 3.006371
4.505969
SFN 67.86625 0.4209898 41.3676 99.51597 96.01991 3.901337
96.62652 9.932944 231.8243 87.2216 763.1913 889.455
34.02781 1296.198 113.0103 0.2853961 256.8336
19.11017 618.4178 266.9764 93.72182 0.3069239

1155.271 0.1580186 43.06693 658.568 83.29613 0.4351554
97.90368 60.86371 109.1122 15.80542 24.37224
275.5204 109.4264 38.62868 0.1603401 73.30036
55.80897 141.5309 56.2364 216.8553 92.62112 200.2574
95.58976 106.2172 65.61048 110.0473 144.5945
159.1246 121.6071 21.49579 13.29578 90.16214
14.35678 87.88012 56.33552 8.541227 236.8184
63.08811 52.22082 293.1497 27.02142 47.17804
43.00104 13.11161 138.2569 32.74958 156.3995
178.2093 108.7059 90.6466 66.91802 41.25722 52.76786
35.65102 183.8234 53.12006 52.71482 47.97964
247.4883 82.11617 72.29403 49.9559 46.76839 218.2218
49.11665 192.6199 83.81961 51.03708 105.5774
92.70205 192.5976 48.74193 162.5409 11.63788
168.3122 126.3764 384.0856 128.365 13.06389 140.7973
90.89116 18.93244 43.15728 73.99286 312.7952
62.08139 127.0654 93.92534 47.39197 5.965371
329.0436 6.60071 439.4681 56.39907 184.1483 196.1357
42.63075 70.98863 148.4364 11.43846 19.5958 72.27574
129.4354 37.11995 154.3295 389.7049 14.17116
152.8477 325.7749 225.743 145.0519 82.88051 235.1806
52.94442 190.2032 91.73758 150.154 31.28141 48.26312
111.9425 103.1584 31.05718 26.74011 65.14716
76.85452 104.9407 84.45819 39.19984 77.31745
185.2683 125.5384 41.392 22.51336 96.28477 151.9565
11.06467 1211.389 110.4004 56.40551 38.99751
24.58249 73.69409 175.0645 73.99616 43.5608 29.09149
61.81719 36.0169 1.635499 57.77987 14.08003 955.7644
72.63552 59.49671 34.08209 66.04507 78.17466
44.10586 39.78528 82.48217 2006.369 117.073 33.89795
31.49617 15.53687 342.7838 30.85339 181.7633
32.07385 144.9705 200.1784 203.6531 168.7463
47.0729 96.7972 32.38531 258.5131 54.78258 12.19359
181.0545 66.96525 134.1025 174.5764 134.3135
89.28311 12.95617 241.3709 85.54257 39.18021
22.27401 153.552 45.55897 189.7996 99.62104 157.6246
20.08956 104.9995 91.13979 88.96733 41.26173
43.94733 130.498 14.60763 179.3138 15.52379 1238.671
181.8114 190.5988 218.3146 30.82017 82.31665
280.7495 45.37268 167.5632 116.6257 78.48112
72.0213 31.19712 30.69863 76.7628 72.58635 124.2223
68.29347 46.2454 29.57785 17.09644 66.96585 68.38801
72.7317 46.67615 87.81253 74.16777 149.8977 0.9413648
41.98384 108.1577 292.2136 26.2059 122.8361 365.4359

297.5123	197.6552	60.70773	18.95347	155.0091
250.531	127.3278	24.01215	86.7966	341.7516	237.6938
74.53061	102.8082	27.20136	70.72522	6.157906
0.4838213	89.75389	38.43051	61.40096	151.1587
105.9095	112.5658	39.00394	117.7863	51.24954
110.4336	46.66529	76.76044	1.80227	11.07635	44.85886
19.34526	56.17435	44.88825	104.5513	523.0826
158.7696	94.1689	24.62162	513.0471	6.985859	117.2029
50.53117	15.08076	20.78848	20.3968	47.13036	45.76388
228.6348	106.0816	72.60409	86.4685	21.56688	68.21873
31.11138	57.3033	94.17123	37.72578	28.43564	106.8753
112.413	15.6875	25.12822	86.0555	57.1765	107.3374	147.8321
72.71787	20.28337	31.88796	82.15062	28.65031
105.5041	132.7703	58.48927	90.03082	33.60523
61.1694	119.5947	71.2855	74.04435	4.776489	450.6994
39.98163	25.27874	91.0935	72.66682	71.3504	64.51863
32.60534	84.50963	41.49659	103.3821	90.4416	42.39795
8.309804	24.65293	135.6882	11.47651	393.0759
30.60006	150.4023	80.89636	59.2773	42.21837	66.70246
73.02091	39.3771	841.9114	57.34797	14.60108	45.97868
70.25841	149.7258	102.769	62.60939	71.31106	72.94203
50.47928	64.77759	121.2711	100.4938	219.9203
59.06399	133.0309	198.6165	72.6138	28.30744	98.7254
66.89755	100.6254	247.8509	44.39606	33.24303
48.52895	82.38805	171.08	78.27239	7.347707	299.4909
78.04462	123.8346	74.29639	34.05584
ADCY10	0.0236661	0.02715621	0.01959209	0.03617166	0.01552103
0.1342177	0.007313098	0.01418173	0.01461455	0.01779062
0.01015724	0	0.04385584	0.01553241	0	0.017533	0.03304567
0.01781893	0.04967617	0.0657096	0.02212372	0.04006802
0.01806471	0.1359078	0.02269259	0.02604288	0.01123838
0.04116929	0.009146698	0.02148024	0.006687904	0.006364162
0.06624854	0	0.02119358	0	0.004925159	0.3996033	0.0677344
0.06492676	0.1165805	0.1669009	0.00985295	0.03194972
0.02436992	0.0679153	0.06884386	0.02289402	0.01927795
0.005670645	0.008195946	0.08038258	0.4366971	0.08896177
0.1199722	0.04945802	0.02432368	0.09461616	0.03084934
0.04170923	0.03761626	0.07100456	0.04242678	0.2437036
0.1074741	0.03046961	0.02906261	0.04305433	0.007133313
0.04298373	0.01617005	0.03945142	0.1802675	0.04854818
0.04419832	0.02996062	0.01286218	0.06935151	0.0573182
0.01622824	0.04114957	0.05687311	0.01267496	0.06327091
0.04237812	0.08249178	0.06574752	0.01462254	0.6404859
0.0427105	0.01824482	0.02378286	0.01819512	0.05593824

0.0245163	0.04593707	0.1238765	0.02990458	0.009930212	
0.06716708	0.04740897	0.0599609	0.08179396	0.1021965	
0.08302822	0.01963738	0.01707026	0.03240682	0.03139193	
0.08203225	0.04729611	0.05510715	0.02076623	0.06783306	
0.5615656	0.01503174	0.007943957	0.03749865	0.03321797	
0.04951788	0.004898189	0.7179855	0.07414999	0.05272481	
0.06597189	0.02754611	0.08658507	0.01535764	0.03068986	
0.01095212	0.04090576	0.1320239	0.01926428	0.0381552	
0.01058134	0.09652606	0.02926378	0.03312607	0.1745044	
0.2391084	0.02579456	0.4335993	0.04234419	0.0946863	
0.03797343	0.01148342	0.08602055	0.08295667	0.006257066	
0.03236473	0.02187173	0.01562377	0.01286713	0.02822428	
0.06571997	0.007431095	0.03171979	0.100881	0.01329682	
0.150442	0.01426852	0.08889823	0.03388266	0.0236823	
0.04373264	0.03381628	0.01510099	0.0331783	0.06446098	
0.06118528	0.06251785	0.02179606	0.09594983	0.1083709	
0.07618535	0.03187446	0.07325997	0.0340795	0.07057829	
0.0356011	0.06188623	0.02868228	0.005300686	0.02866319	
0.1320102	0.03216534	0.04240427	0.006404114	0.01951767	
0.006138293	0.03653226	0.08479295	0.05277119	0.01447219	
0.01220849	0.1636184	0.02419086	0.1575122	0.08440264	
0.02487413	0.3004203	0.108597	0.02320139	0.03477534	
0.02686666	0.02935884	0.03422022	0.07632383	0.02009808	
0.09105571	0.08475939	0.01760783	0.05931116	0.1774927	
0.3020643	0.02484724	0.03292477	0.2268556	0.02727272	
0.03435015	0.04196066	0.1372223	0.01242048	0.01186736	
0.1279479	0.03068663	0.2028156	0.01102253	0.005774851	
0.03311128	0.008010078	0.09081735	0.1512524	0.04667986	
0.1162776	0.04637819	0.03409015	0.02207707	0.07749646	
0.06765959	0.09925986	0.03794354	0.03452276	0.02932599	
0.01234396	0.009091316	0.20096	0.0568462	0.05861626	0.06918669
0.05956782	0.02670635	0.4804076	0.02574247	0.006762299	
0.02313269	0.05874607	0.03434421	0.008887671	0.667214	
0.0368611	0.009944177	0.1813486	0.01222376	0.1201138	
0.2736101	0.03074143	0.005149248	0.04126724	0.07096153	
0.1812351	0.01286411	0.1023956	0.01081487	0.3195844	
0.1086611	0.1190523	0.03977878	0.005074666	0.008465609	
0.04813069	0.03834018	0.02443551	0.03925544	0.03798834	
0.05049389	0.1044843	0.06365118	0.01457022	0.007702101	
0.05537927	0.04169539	0.02338745	0.0383238	0	0.03209177
0.2609212	0	0.03261297	0.09088503	0.01761979	0.05743116
0.01816312	0.05793778	0.05592194	0.0929998	0.06403329	
0.01640937	0.03531824	0.06680651	0.02398222	0.0565648	
0.002247909	0.01537447	0.05788213	0.01577307	0.08550681	

0.0793907 0.04829981 0.03304349 0.1387163 0.03390496
0.06205472 0.01465495 0.01460219 0.5861787 0.1136662
0.01242761 0.08701967 0.02809156 0.01679968 0.03875567
0.03039025 0.082735 0.01158687 0.05206701 0.0303108
0.07040162 0.03787856 0.06978339 0.07616039 0.04155848
0.03668088 0.0634405 0.02479049 0.02534708 0.08538487
0.02006356 0.06277823 0.09231151 0.02173042 0.1869115
0.02623485 0.03947799 0.1228236 0.04027615 0.02421474
0.371748 0.1291636 0.05309996 0.5540046 0 0.05450067
0.04487651 0.1650717 0.05678452 0.01552464 0.115874
0.08130167 0.1635734 0.1642815 0.04904622 0.04276088
0.1594372 0.006518425 0.01176319 0.03086235 0.2174465
0.08161377 0.5264316 0.01074976 0.001882086 0.2117207
0.03768825 0.05396166 0.01366874 0.01297891 0.02800208
0.09558668 0.02614568 0.02733809 0.1296311 1.308786
0.04874343 0.03125176 0.3389107 0.05508142 0.02724448
0.02738723 0.01378118 0.09226011 0.6510255 0.03508371
0.04865641 0.03813435 0.01274949 0.02817847
TRAF2 2.689163 3.037953 3.155841 4.046508 3.1319
1.494257 2.733172 1.706532 3.894424 3.935734
3.819966 3.475405 4.336508 4.562953 5.366988
1.63965 5.063575 3.538838 2.7572 2.888687 5.22803
1.36674 2.200022 1.561205 2.539968 3.915717 4.50783
1.655943 3.134861 2.033988 5.929341 1.37236 7.549983
3.747608 8.385798 4.34736 3.22331 8.613671 5.795625
7.258942 7.824925 15.33201 3.736075 7.820279
3.557402 3.960441 7.790374 12.08203 4.543433
6.053863 7.747095 4.774972 11.41651 7.418985
5.018946 7.104083 7.867691 4.854737 15.20036
8.038791 3.251214 12.63023 2.006744 13.84965
3.253096 2.58284 7.268233 4.678168 4.658491 8.342616
9.295362 4.924395 6.497524 5.02979 8.740746 12.05816
3.944813 7.425778 12.7943 4.386599 7.710239 4.406218
4.596895 4.372061 7.908682 10.19224 4.689174
11.35147 7.894403 4.706425 4.754784 10.03557
7.779381 6.254439 10.86551 9.048136 6.539734
7.33819 5.971338 7.594359 5.2192 8.385834 7.420731
4.581637 5.664265 4.837953 7.095003 4.315348
7.656694 7.164919 9.52151 5.426756 6.410737 4.770386
5.295753 9.774662 16.82847 10.33499 5.587672
6.365288 7.006478 5.585563 5.424089 3.556654
10.99936 7.155709 4.734284 4.84744 4.691573 11.43161
7.261218 7.979095 4.721819 4.240429 6.871842
6.890507 6.949241 10.24513 8.018654 7.639958

7.862522 4.606547 10.21303 5.362031 15.5173 3.70799
6.034378 3.316401 11.65402 4.24247 13.71297 6.475088
6.602378 10.42625 3.39995 4.919383 5.868946 5.043964
5.649194 8.638875 9.5713 3.067418 4.482006 10.59731
7.383115 7.304695 13.37553 5.471544 9.658176
9.751388 2.694726 13.95411 6.57616 5.440647 4.814875
4.242173 4.803766 6.408358 8.726424 8.3027 6.622004
6.577583 5.601159 11.47739 6.278205 8.987845
3.142414 5.387949 6.823434 11.4911 6.170239 4.856771
12.16879 5.426977 10.18948 6.157711 5.630359
9.14482 6.308871 10.90367 3.014831 5.619345 8.247369
13.23318 6.979206 6.953128 9.826194 13.30314
8.299576 9.189781 6.610509 10.64883 8.924132
8.040403 11.00034 9.681927 9.406105 18.93137
7.645647 6.743808 5.973831 5.532004 7.264812
8.714259 10.80471 4.336125 6.789981 4.017921
8.666967 4.768418 6.488498 5.894493 4.614577
7.059751 4.311023 7.367454 5.919013 7.874282
4.254692 6.746943 6.33038 7.742256 5.817396 6.713735
3.475558 6.559347 10.13698 3.139984 5.351073
9.805128 7.644325 4.337096 5.488237 12.21668
10.19427 5.795996 8.703457 6.582297 6.903963
5.660013 5.810702 13.58273 4.655209 5.506742
7.935146 5.436307 11.79489 7.456213 7.53534 3.297173
7.45611 12.87107 5.043112 4.658168 5.176615 12.38351
5.631891 3.558758 4.737218 6.370379 4.572407
5.386343 6.220255 7.178432 7.124862 5.062124
6.497845 6.000531 5.030303 4.792216 10.59346
2.178591 3.501525 10.54027 12.56287 6.494436
11.79163 10.43586 5.584728 6.671557 5.167727
8.045765 7.708202 6.160503 4.759754 7.498 6.533429
8.459387 8.673305 13.89423 8.771345 10.29824
12.07445 9.822314 7.740175 3.773575 6.044866
7.011406 5.298047 3.971479 8.744469 8.9875 9.943193
15.82544 2.186212 3.160783 12.92094 6.940946
7.12998 6.442719 4.705975 10.28198 6.127572 5.554541
9.677135 10.3424 9.702674 9.721619 8.292405 5.371882
7.403172 18.28041 9.151619 13.54733 3.685864
5.236676 7.168994 6.425528 5.029186 5.011238
7.771828 6.464424 7.650476 6.956576 8.472892
12.66202 6.094778 10.50578 4.040803 12.28714
10.26548 9.907161 8.570105 10.72621 15.5666 4.191196
3.928794 10.52937 6.198322 3.509 8.382658 4.646659
14.80072 5.544577 7.446899 8.403305 5.201929

9.376662 4.082898 7.720881 7.808156 6.422114
9.391246 9.060024 11.27749 8.521813 4.883322
5.408688 11.26859 6.182104 7.69075 3.828517 6.969091
4.939932 8.813194 6.880735 5.34354 5.86911 2.807525
6.963609 10.12034 15.49889 9.301924 4.071242
4.579051 13.13033 5.034256
PIK3R3 1.111704 4.97961 2.169618 3.230852 2.682343
4.157936 1.048158 2.143056 1.861572 3.00923 2.211468
0.4853554 2.14973 0.9173196 1.790026 5.00554 2.14718
4.25604 1.353288 1.616412 1.963489 3.128174 1.648772
7.61432 7.564412 1.274518 1.809382 4.716168 2.530989
0.8986901 1.81737 3.351584 5.278448 1.247392 4.488554
7.237964 0.691623 3.301809 1.776004 5.713908
2.479518 3.014357 4.224313 2.844846 5.46136 1.950559
4.809226 2.87668 2.07028 4.766891 2.018934 3.930953
4.379374 3.496452 2.649231 2.398227 1.93455 3.782681
1.993517 2.642133 5.237801 4.481434 3.544244
2.833516 2.010137 2.452587 2.437193 4.876804
2.257533 1.991474 6.25281 4.450778 2.940394 6.298236
2.28705 2.666552 4.332423 1.892856 2.755712 3.578208
4.172787 5.690564 7.346421 3.207601 1.354803
4.035997 9.229669 4.378147 1.927719 2.328748
4.313552 5.392192 2.338176 2.949429 2.827762
1.756664 2.19131 2.417647 14.94669 3.670665 4.119191
3.211818 3.736069 2.448295 5.108432 8.332728
3.95984 3.368233 9.941775 1.976116 1.919999 1.709697
2.246882 2.33654 1.735982 2.808247 2.650098 1.905942
1.768539 1.183552 2.246118 2.923541 4.959684
1.612564 4.12936 3.554412 2.465842 0.8842779 6.091291
3.68205 5.04246 11.96913 2.142626 2.406359 2.060099
2.791433 8.06403 1.378539 3.159018 2.742463 2.262565
6.245168 3.404392 3.018077 5.277884 1.796509
1.348961 3.892325 1.496397 8.283416 3.830606
4.317718 2.823149 7.098723 5.645331 2.019324
2.202185 1.634403 1.29439 0.8462226 2.594734 1.873742
5.020688 3.242683 1.033803 5.474005 3.772003
3.774935 4.09344 1.875548 4.252003 4.042621 5.194177
1.54825 3.699509 0.9988131 1.502178 1.665807 5.488856
4.052107 3.494329 1.354466 2.197939 5.551256
1.904064 3.037893 3.922439 0.965628 3.804778
6.872101 2.44987 1.915314 1.74481 2.631771 2.248774
6.353866 3.242969 5.753462 5.641988 1.836366
1.134338 2.633227 2.496908 8.081345 1.713893
3.333781 2.416888 4.008482 2.051373 2.695507

3.114805 5.568314 5.021157 2.032726 1.738598
2.730985 5.426094 1.85925 5.153661 2.260398 3.688575
4.871929 1.41797 1.49584 3.763059 0.6578228 1.904988
0.7168537 2.736631 1.75368 1.232112 3.773395 2.682633
2.031111 2.904091 0.9990026 5.176912 2.291276
4.195391 3.725608 2.099912 5.102338 5.312031
2.308106 3.311393 3.673698 3.30369 3.642064 4.162058
4.115816 6.057767 3.679478 2.654588 5.42772 7.552029
2.215937 2.061971 2.390079 3.068619 3.492674
2.583198 2.644559 4.402114 2.750514 3.052451
3.853545 1.526412 3.60479 1.292665 2.803668 5.412504
1.590646 2.917133 2.720211 6.485444 2.816155
5.350313 4.580846 3.958286 1.991847 4.795502
2.583923 1.754498 2.66501 2.393333 7.12681 3.39913
1.018414 4.28178 4.439579 1.6422 3.6921 3.243852
4.525255 2.403571 5.397207 1.734973 3.915381
2.412641 4.137932 3.96567 4.786144 6.306656 5.422223
2.331388 1.454076 4.923038 3.531916 3.619669
2.968999 3.426582 0.4094304 6.039558 3.09518 3.011
4.31884 3.72448 4.130844 5.011903 4.898296 2.468232
3.153037 2.440176 9.931552 3.917591 1.822889
2.323454 2.133839 2.574009 4.380724 3.016255
1.713502 3.435769 4.255769 4.082959 2.923885
0.9387927 3.154905 4.836665 0.8001956 2.246801
2.682904 1.898312 5.229608 5.492529 1.86704 2.374078
4.938293 1.061008 2.503816 1.937134 3.004521
1.914344 2.934243 3.50567 2.833481 8.817857 3.368045
2.688597 2.028182 3.457814 3.67144 4.181509 1.675918
2.007408 1.597633 3.515553 5.710754 3.418681
4.217272 1.298214 2.253968 1.111268 5.415678
6.527226 2.677578 1.338704 1.431454 3.155929
5.719098 3.186535 3.113845 1.632438 0.8859745
3.251608 6.253793 4.026888 1.19475 7.007454 1.527103
4.109162 3.448609 1.458311 2.715144 6.605735
4.701818 4.702014 4.206956 2.219803 5.988407
2.926728 3.540809 3.357121 3.099439 2.081445
4.040209 3.926655
RBL2 6.732387 9.390833 8.389002 14.47395 7.21396
12.56486 5.697754 12.4384 7.847323 12.45597 14.4678
9.307839 12.99873 8.204788 7.442284 11.40755
6.283188 13.43918 3.313312 3.802458 12.93467
8.634121 7.711439 12.99142 12.17194 10.22946
7.672106 11.78786 9.581055 4.310004 9.76507 8.157289
12.91101 9.507974 6.713814 3.076103 5.095021

5.830937	7.366384	11.56305	17.59727	10.61173	
11.69255	10.59933	10.46022	5.834536	12.71592	
13.03275	19.44971	15.85408	3.331415	15.40404	
3.257841	9.860297	12.00813	9.54006	7.606035	18.45214
7.773585	8.177228	16.62543	9.108255	12.55045	
4.698526	4.747076	9.457552	5.917389	7.261718	
13.83338	4.636429	14.68678	4.173199	13.41998	
11.62057	8.567207	6.938588	8.718987	10.58394	
6.121484	9.021481	3.59945	11.65765	10.03259	10.80033
8.22667	10.57534	14.81017	11.01495	6.126237	11.61452
10.50238	10.50698	6.373402	13.81092	13.71181	
8.605411	8.90379	5.912566	14.16274	8.640685	16.81354
10.38337	4.797246	5.956725	8.054847	17.65639	
8.950467	7.931133	11.21363	5.569932	6.774371	
6.180225	6.116439	11.41061	7.985287	13.0588	3.142379
8.708135	7.447049	8.440713	7.61814	11.15039	9.145106
7.115454	11.29265	22.44537	12.7295	2.773463	15.12682
7.776121	8.053351	8.367518	11.68093	13.2217	5.337808
18.76331	15.18608	9.330325	11.02213	11.28182	
11.21666	11.13082	6.5698	4.09796	8.489993	9.059277
4.25896	8.425065	13.19761	8.590773	17.89173	8.27177
8.069992	18.23031	14.79451	4.07584	8.202332	9.98104
11.16826	3.340181	10.53945	14.51859	15.99622	
9.454038	2.406003	17.53461	10.64777	12.01684	
14.41805	6.741645	11.01876	9.864081	8.404817	
7.733425	5.274322	5.373106	10.07429	7.336782	
8.586553	12.20014	2.75278	10.84266	10.03789	10.87659
5.7915	7.268759	15.68475	5.507839	11.74455	6.532746
10.55996	6.044567	9.55552	6.766524	5.348059	17.39225
9.807949	11.73713	6.06326	3.14537	6.501859	8.216368
13.932	7.688146	9.170533	13.92381	5.256731	12.82026
10.27678	14.27465	14.91249	11.43177	10.42555	
12.43606	7.655125	11.94041	7.903611	6.308291	
8.966047	15.24362	14.62269	14.82688	5.758281	
12.77357	29.68886	5.391625	10.12159	6.956239	
6.458332	3.76272	5.866827	7.007179	8.335827	11.30611
15.31977	7.487401	6.209766	10.20187	11.64052	
11.69524	15.57283	13.37111	9.746598	5.386212	
8.921699	13.74894	9.474441	10.95128	13.0214	7.429927
17.89717	15.66711	7.680959	8.286659	9.534836	
8.305836	9.878243	8.898117	9.259415	9.382321	
6.077324	5.145654	16.15703	7.442655	12.71367	
14.61337	7.744805	7.667452	7.965068	12.89578	
12.93223	5.796191	7.678906	12.66718	4.741521	

9.01195 14.66961 11.20291 10.88878 15.25032 16.24386
8.622976 5.018536 8.941985 14.73061 14.59056
8.21972 9.341811 9.091294 13.56001 6.90741 11.54983
11.74888 7.822028 8.181376 7.125513 8.553182
9.529411 8.578634 4.026202 12.56787 10.29205
7.602814 7.594463 6.897585 5.783271 15.0624 11.26549
17.02804 6.714025 7.715641 5.353083 8.81347 7.506871
15.22988 13.58046 9.963517 11.4738 7.783455 13.98481
15.45682 11.29808 6.906835 3.756808 13.98323
7.081967 9.661068 8.116567 5.427551 14.89854
9.03523 11.50602 17.41947 14.67936 5.636126 14.14987
7.139247 8.652499 14.61493 13.90359 17.63166
6.965335 5.188664 5.065568 18.67016 13.93727
10.78326 5.006658 5.041206 3.281853 13.71573
11.66097 12.94742 4.216624 10.66293 8.579624
13.73003 6.387008 11.57718 3.808329 11.94444
6.624491 18.36253 7.321686 6.659915 6.008114
10.40725 8.618123 12.53761 11.43737 5.850202
6.324829 19.47489 10.65777 6.170139 6.476589
9.739426 4.450665 8.052707 9.285437 12.8163 19.87562
5.989755 10.50758 7.265024 3.986411 7.447999
8.882921 11.22894 14.01707 11.85346 6.921621
3.344988 13.3651 18.89366 12.62087 12.5916 12.14858
8.563463 8.931244 4.330502 14.50643 10.03925
13.45095 7.566518 6.648065 12.12804
SIRPA 26.18477 7.412344 16.35426 6.539489 8.185969
9.871011 4.269819 5.050538 9.404709 8.864332
6.388098 4.162127 11.12056 3.830157 10.21652
7.095675 9.521204 12.46075 12.88432 13.0537 8.378051
6.166855 6.086914 9.30495 8.238583 7.406648 7.042634
11.00528 13.00749 1.951468 9.032874 6.208375
23.54604 8.532279 14.47453 5.096555 2.20741 18.50976
18.48529 2.590293 12.29282 2.572631 19.04549
11.05942 9.242685 5.235592 4.753364 24.0345 11.75767
3.02007 14.32605 22.90077 6.513867 23.66016 11.9055
7.686073 17.39633 12.83937 28.02358 26.46653
34.16766 21.20138 25.83338 41.87207 14.57691
12.07884 17.34398 16.81929 14.1939 6.52844 13.02794
3.210143 10.50977 19.90372 23.2469 8.539007 9.187921
20.24109 2.306412 22.97703 10.48252 22.78129
18.66065 27.51142 7.168603 22.32478 15.2853 12.36698
10.12517 9.687629 17.67482 17.77183 11.22337
12.26688 20.3525 24.03561 15.87524 14.3478 4.917342
13.06138 15.41934 4.172595 11.49979 29.7551 15.87298

21.32637 7.088299 16.02327 14.5487 15.97911 19.36783
31.06303 14.44884 10.764 9.563957 15.86174 33.66349
16.92919 11.27717 18.03667 4.010913 19.04736
14.8414 22.95484 18.31947 10.71647 7.286729 13.757
13.30084 17.21472 44.27311 2.157341 4.334536
10.09294 21.09753 17.61541 15.21536 15.18632
5.46122 15.53514 20.47565 4.319565 31.86387 31.58969
12.05965 7.073319 18.78558 13.78395 15.29512
8.398107 3.211048 15.47293 18.1918 24.47063 9.561376
3.888639 8.109815 9.035997 14.65717 19.65253
20.09245 7.015344 30.95811 17.96848 6.87962 12.3229
18.20533 13.2385 13.30148 6.486855 7.731508 18.05576
6.946064 8.162228 18.53744 8.230819 17.56809
10.42768 8.869242 13.63399 13.15048 17.56289
10.76457 18.01191 23.73707 16.41747 20.24243
2.812083 34.30729 4.932198 17.56085 24.18261
10.2306 6.262784 6.432979 12.22036 26.36356 14.68864
3.210308 10.07369 6.437844 15.29462 5.859778
18.00813 4.659256 3.989739 19.03676 36.28451
14.60963 20.15766 17.0691 21.47508 14.72826 27.0649
4.388028 7.047973 21.76671 40.12932 13.00423
14.54837 12.92102 14.92059 8.762351 6.898061
11.6465 2.241906 45.09779 5.768013 3.934815 6.514776
6.449138 15.08796 5.466567 2.368085 3.631185
7.102645 14.03593 10.76508 11.718 16.60524 14.34674
13.65071 10.54202 8.440345 19.4473 22.23984 9.703709
5.775572 19.46328 7.595833 24.47636 8.928626
7.468994 16.15255 7.925129 17.53779 26.99123
23.53486 7.258886 16.43276 8.924909 11.25063
14.06635 5.061017 38.63114 29.8942 12.9563 19.27674
8.824086 15.5346 11.11525 14.94374 1.888524 6.814794
2.04336 10.55854 9.50433 5.855633 8.980875 12.4491
19.14759 14.04379 14.24387 17.40372 4.812883
8.852181 9.70253 16.91914 19.62853 15.88391 23.86281
7.671078 13.91231 16.072 27.32806 12.56589 14.36014
26.09655 8.377371 3.562068 6.947248 18.71059
5.823054 27.70871 3.807938 20.27995 9.289207
32.86876 29.26031 14.68201 28.9475 7.345894 10.89725
33.50367 4.59432 7.991222 15.1881 13.77711 17.94404
14.19509 19.41517 13.73106 7.538326 6.804904
12.23126 16.32094 21.47914 2.593033 16.2174 9.50691
1.375071 14.54849 19.81079 24.57725 10.20847
30.54787 6.484656 15.36578 9.084891 7.225431
6.826874 21.64657 7.058844 16.81031 12.51795

6.19375 6.220871 3.296441 14.44853 13.58848 12.05049
23.68197 13.44494 11.32326 9.757054 16.55931
14.17799 10.54299 11.42805 19.84868 7.830525
22.79284 2.092486 19.47345 7.298379 8.666854
0.7177395 23.44375 10.50148 4.430426 6.729208
6.500259 8.745576 19.3244 7.972265 37.42514 7.599709
6.107998 4.592088 12.18518 22.98549 10.71059
8.654762 23.78223 11.83763 12.389 21.75489 5.291465
13.39454 14.25456 20.49839 5.202778 11.17282
13.41345 8.280385 20.6202 34.38213 13.89636 3.367249
15.59173 20.81565 6.873065 19.21326 2.680623
9.072875 20.82982 7.106555
TNFRSF12A 15.29907 4.991736 11.18727 6.653924 16.13841
10.67692 3.206794 6.859404 11.67605 12.20413
2.058265 2.33229 6.725919 3.162232 2.563407 21.428
65.20738 29.43715 97.02365 63.47065 6.127591
8.088071 26.25456 6.659354 4.991504 4.072322
6.645372 5.097534 18.27661 6.208034 7.598219
31.43044 12.68091 49.62101 79.77464 12.52925
14.65966 85.54415 33.07063 11.31301 16.87511
42.91364 30.13725 23.73908 8.694688 24.69011
14.78299 88.89778 40.80998 41.4618 124.072 6.223247
30.74292 44.44746 21.9659 15.11542 20.01594 10.59796
55.56024 63.22454 36.41694 46.81046 26.81784
11.44446 20.12327 37.22733 46.07347 35.61327
119.9208 41.83217 37.76265 39.30553 53.60037
77.0604 22.45206 15.39844 42.52634 17.40676 6.586972
42.99685 32.16495 32.79666 22.90557 27.49369
119.9105 52.67565 15.62854 29.40769 30.11064
81.7778 28.78158 27.16926 52.33076 15.37703 59.10003
41.55581 148.6632 35.5562 24.96521 22.88646 9.380567
29.08836 20.63238 17.89887 48.21123 24.32665
49.93609 24.79967 40.28802 37.51186 37.06039
37.16836 80.18791 8.562895 34.38652 125.3243
37.93942 45.13149 57.93908 43.91244 22.3735 12.23857
23.26313 30.63669 27.60618 8.746576 23.96702
72.27497 10.95243 44.80882 34.01722 28.85673
38.4772 25.43722 31.47763 17.81649 47.91872 57.07886
47.53526 22.52955 6.367745 8.965271 30.05362
17.19237 35.37298 21.03203 47.33372 37.64013
4.870802 16.51118 18.32415 37.44337 32.86318
20.51786 12.22591 26.19171 30.74058 10.0444 114.0953
67.28399 29.55364 15.9645 17.52676 25.23222 84.48429
18.68533 51.72014 50.00191 17.31301 18.55434

7.599877 16.75291 7.188481 19.55225 38.14242
95.76452 119.3565 49.48455 9.287599 22.64335
36.73631 49.75292 24.45851 22.55207 72.86852
28.19929 25.55181 89.56469 20.897 25.54341 42.15878
82.8718 189.0258 25.33614 101.9448 46.53074 59.71347
9.395082 40.43924 30.61941 16.87505 35.81148
17.72709 31.28738 67.63679 7.942533 123.4928
12.11585 46.82911 56.59408 25.04951 23.49735
23.8771 18.60788 38.49318 31.89552 130.0386 16.06243
64.14419 32.56215 53.64392 19.51859 28.643 31.69609
10.64654 29.19945 15.79653 6.609283 39.40369
38.87526 32.55196 23.35831 9.577427 36.78859
17.41987 49.25741 19.68223 41.83011 24.15506
7.858682 21.24456 73.82432 25.09485 50.59998
35.07676 46.78902 27.00438 7.286129 41.12059
21.25704 12.41006 6.023931 40.81989 54.27397
33.1964 18.03962 26.60581 43.20767 23.54585 27.48568
33.09791 77.26712 26.43684 58.55523 25.60188
28.46368 74.1134 55.15716 126.4609 51.96672 25.78096
227.7945 56.32309 29.03594 42.49882 11.77857
18.37154 9.828989 9.895579 53.91083 25.99454
24.17164 129.0487 42.21512 17.28313 15.78273
17.49104 66.44731 21.73934 15.48601 28.15983
22.15358 7.78219 51.67466 87.96807 47.76033 28.66378
82.21106 42.22684 71.42693 18.29138 11.95005
122.5049 29.99642 31.7602 60.65134 9.614793 29.25083
29.82844 13.21536 41.37845 175.9201 40.9517 36.96702
11.72924 11.16725 16.46635 33.31642 14.66518
31.94292 20.18144 20.30178 62.33644 43.99348
16.38763 17.94873 49.85715 11.53892 23.52228
49.12903 16.18433 32.99738 24.37687 16.01366
45.32984 19.05623 13.97289 42.25997 31.22192 39.446
6.754067 57.26139 34.30562 16.18365 19.75302
4.355553 31.77527 1.829861 127.0575 85.39704
30.11674 32.55364 13.34086 91.7076 24.21775 50.61955
15.80293 51.96926 27.08553 31.69054 42.10143
19.34978 133.6776 20.15999 22.76686 30.85338
8.036697 30.03393 7.407955 36.87054 13.03055
104.5532 21.45708 16.75393 29.53607 30.76333
20.40224 62.5126 15.66387 7.311683 96.24663 22.63308
32.83143 42.06694 20.77014 71.80002 33.55468
68.69679 59.81295 9.271787 34.28315 61.4749 20.41791
36.33769 15.7664 22.28 9.458598 24.63833 8.627894
37.34557 28.70086 1.72834 32.27187 11.47609 24.08433

47.01231 37.23103
CEACAM1 45.25859 0.2011971 41.39592 5.112777 24.42117
1.632774 6.136098 3.01343 12.06346 5.150422 27.64449
26.30613 4.719503 20.08025 7.623731 0.6780777
27.93178 4.940787 40.83999 35.56889 11.58039
0.204309 69.75037 0.5380756 4.546627 20.73509
5.686919 0.3514644 23.97928 5.466616 7.083158
5.85148 12.28254 31.27038 5.804772 0.5016007 10.23269
47.87455 43.62313 16.76794 11.50957 9.301943
21.28114 9.963872 13.51446 28.38109 31.36845
16.6255 16.43237 51.50181 11.61324 6.082004 2.35886
10.76849 2.487098 7.093627 38.72097 2.165012
22.8858 5.391189 8.522079 11.88666 8.976884 12.12297
83.64743 2.368548 27.78006 6.36374 67.20918 10.79217
7.457677 11.06957 12.57701 10.54784 63.97549 44.498
12.44817 4.380291 100.2731 43.11387 2.359712
11.87391 106.4965 13.80067 7.970336 12.85498
4.118834 12.29513 6.153853 6.529481 107.1311
32.41574 63.05528 16.98685 3.672122 19.57954
34.09575 24.94483 15.48759 8.51219 4.333516 36.36055
4.678334 20.51594 3.522617 10.52297 141.2523
5.317086 4.225963 8.331087 19.04167 11.37207
13.34842 3.13138 43.91889 5.974219 19.75791 24.74154
10.39483 17.23015 5.495229 24.50918 5.324836
37.54949 15.6291 3.142923 12.95907 22.6229 4.380266
56.33919 17.31264 16.88505 12.13356 11.39053
48.42516 8.860041 12.72853 133.9098 14.85754
27.35628 12.74577 5.824016 22.36505 15.07529
36.30536 19.21942 11.03126 7.883578 38.57902
8.583499 2.000446 22.96656 8.436799 19.92822
4.878085 9.563246 8.340441 7.483499 11.26022
11.13141 15.07611 6.892438 3.924178 9.821737
14.27588 10.53942 25.84461 12.09897 5.4158 5.514248
3.727935 9.658789 17.2716 24.10862 2.010164 10.19977
27.49831 7.290677 10.20426 6.424691 13.12831
26.09279 34.72346 14.58683 12.58796 8.874853
9.545933 43.83421 10.66094 36.19585 24.21759
16.57949 7.810625 15.39987 34.86896 43.45611
6.902489 9.622435 24.37444 20.61892 13.93742
7.91149 14.69714 18.6171 13.41112 12.44125 8.065536
1.560103 12.86756 17.1362 9.375852 13.46848 21.19759
14.0224 3.235785 7.046055 18.90877 4.152556 12.65541
8.251132 18.56699 5.401257 4.483305 15.47586
7.898573 6.97194 25.0868 27.63532 2.589574 43.59995

17.9046 82.72753 17.53643 88.68865 55.0648 16.6553
4.580355 20.64729 10.27849 10.11873 21.39042 24.771
6.958244 31.9717 24.03985 17.79474 8.745032 1.40038
5.230531 5.344752 14.69159 12.40609 5.476071
34.53516 42.53206 10.31859 26.15809 9.443811
22.95945 11.85125 17.18359 18.172 27.91981 18.30668
19.25619 23.26149 5.022099 14.24337 7.886015
18.49446 8.00854 8.106002 5.440161 10.88914 11.27739
4.977355 56.71213 3.519755 0.2594234 12.41243
4.946343 33.42744 13.54178 31.63601 4.083601
24.65001 68.43219 17.30419 20.66377 5.407523
31.2987 2.139699 1.494496 20.17892 8.491727 4.618568
24.21377 17.14643 4.890788 13.92501 14.62734
27.7175 5.811114 21.02392 20.73519 56.77636 12.76936
23.14546 9.620943 40.24568 64.22776 3.950399
78.67001 6.349213 19.40961 6.214074 7.904476
6.137701 28.98834 11.4731 161.3669 4.286064 55.2865
9.315891 12.77676 25.35893 8.92263 3.680688 25.63831
71.43882 39.54846 25.9083 11.74285 20.41474 6.726016
12.87343 30.81693 13.06364 30.74036 39.05652
46.96388 21.2097 6.141874 29.52333 1.523148 73.73629
10.20425 14.17094 13.48257 10.40161 18.41657
22.03728 2.922292 2.836087 13.99764 20.22042
13.37703 24.23097 5.131688 8.489546 19.87135
12.15052 2.435901 3.736617 53.02673 7.315302
5.48359 51.15177 7.969519 7.140491 10.26893 21.32813
12.51067 6.178118 4.645906 29.37788 30.92485
2.436146 7.593859 6.938422 4.202799 10.29706
18.23984 41.03863 42.4423 38.73959 8.773327 12.63456
18.932 105.7246 11.86839 3.982548 15.30444 3.5115
52.66855 54.96006 8.423957 11.37509 4.174848
36.14834 7.699796 5.751719 18.80082 7.071605
13.82334 15.12533 4.997976
BAG1 7.468649 9.738542 7.046634 13.0334 11.23837
7.009143 10.47553 7.339629 14.15183 9.795189
7.316007 9.088963 10.45432 7.498762 9.987379
7.532942 11.95112 8.474295 14.5858 10.48302 16.55455
8.4516 11.47467 7.238851 7.105444 7.110474 12.56936
6.867009 10.17204 14.43862 15.36608 9.336038
6.396661 9.473448 16.43103 7.570239 5.96206 5.146089
6.088662 13.88656 8.272214 4.524978 8.530746
6.438141 11.67818 29.28403 10.93495 6.534231
15.8275 13.34219 5.505626 9.464126 9.187505 4.879125
6.03598 10.47244 9.609842 4.755045 22.34991 11.89263

6.465656 14.45466 5.458262 7.558433 6.892253
6.388795 6.253878 8.434475 7.688646 13.1753 6.429381
24.471 10.62454 7.712919 10.97164 9.893025 13.74558
11.10281 18.9811 6.335415 8.02264 6.011027 7.207694
9.289221 19.03788 9.393921 8.700298 11.83773
6.314181 15.40101 8.86706 6.979576 12.00961 7.834764
4.866322 7.128997 8.839953 13.50631 4.731166
9.376418 6.536714 16.3475 16.02212 3.473214 9.056033
6.933275 8.619371 6.522134 11.83215 8.022775
7.134094 10.79767 16.56752 5.334304 11.34749
6.94208 4.652732 7.105248 9.439216 12.10403 11.27519
3.484367 2.415944 7.405282 13.60219 7.991486
10.88697 8.212531 7.427179 5.904582 7.661352
13.05808 15.61044 19.70076 14.00133 7.78482 9.797872
4.974565 11.29145 20.1011 5.602761 8.152522 28.68951
8.100216 9.404216 31.09722 7.92863 11.4831 40.73162
12.80468 9.561346 12.62092 9.389709 5.245586
6.834751 14.41088 16.73217 10.03388 3.622223
5.441976 7.071618 7.813468 9.102598 10.27236
17.86307 13.77824 8.110054 8.227474 8.242899
13.34066 6.742426 28.36321 7.879103 7.053162
9.228417 6.962919 4.870634 9.554402 15.27223
6.826449 4.005535 9.860676 9.691333 6.777338
4.280858 10.6773 5.307321 11.81381 9.823134 7.646129
9.00731 10.06462 3.667928 19.62145 9.511673 5.988915
7.579427 4.285047 9.101162 16.86908 4.101545
7.51636 25.52181 6.69416 17.95102 23.50519 5.466162
5.907502 8.980342 7.683706 5.56198 8.687521 14.53069
4.074723 15.49576 11.51717 5.068983 5.667407
10.0315 7.427465 6.304049 8.448849 10.76713 9.748959
5.128146 11.26072 3.386307 7.704178 15.47464
9.63869 7.48328 11.6315 10.41838 10.84524 8.766442
15.61446 13.32206 8.524201 7.261204 8.890187
6.658904 9.04017 11.6685 10.30058 8.554245 8.777962
7.362489 7.879837 7.433884 9.983529 5.450034
21.38041 11.59915 10.02432 6.543817 3.035972
14.00975 6.955953 8.767428 6.36268 29.66843 19.77383
5.803979 10.61982 7.563601 6.928777 10.7712 16.23466
7.711663 20.41362 7.434858 15.23086 20.0124 9.558278
17.10539 8.358268 9.28439 7.793331 3.255549 9.002838
7.58535 6.84104 11.05087 6.074629 21.35004 6.896416
33.25714 6.244654 23.51995 8.695187 6.474976
4.707677 6.115256 7.41564 4.925432 5.645719 8.126027
7.926816 16.30411 22.42759 11.65353 13.85305

11.28974	6.799738	15.09934	6.560598	16.6794 4.55088
7.706239	16.67309	6.213697	8.73205 4.772713	8.960495
13.8437 10.08314	7.738653	15.2565 6.72262 13.45635
6.878726	43.43866	14.03605	10.9875 8.777268	5.523368
13.85892	5.027191	14.96589	24.51449	11.25825
18.27965	5.310554	5.143394	11.70523	6.307566
11.02416	13.63471	7.025562	12.43681	9.635167
5.432121	21.77517	9.611001	6.623286	24.68881
5.799023	10.8146 9.450593	6.674402	15.62936	8.062977
11.40441	6.926874	9.359754	10.75729	7.2607	5.765844
5.381032	6.679608	7.852386	10.8436 15.78885	6.912012
7.167129	13.37526	13.82224	11.03715	28.76847
5.208727	8.131041	12.32578	13.35184	9.735617
9.879394	8.724614	10.46677	5.465052	19.22013
10.99735	6.827609	14.00633	10.52721	20.31183
9.051314	11.87578	7.631561	7.815502	16.11197
14.0473 5.113762	14.46353	8.790056	10.53899	4.981203
15.90193	11.38856	8.890166	13.57732	19.37175
5.208756	5.082565	14.14352	15.29841	12.15953
7.237969	5.954407
APOBEC3G	0.9856231	1.839109	1.330517	3.600704	2.187295
1.764339	2.518868	1.845311	3.758842	8.363795
0.7820139	0.6960558	10.53509	0.6943333	5.619642
1.428819	2.391176	2.302534	1.042656	1.058232
3.536137	1.016085	0.8811212	1.612636	1.142123
0.615589	5.371979	1.228009	2.625349	2.961814
3.643493	1.057806	4.479288	1.996787	1.099518
3.527072	0.2300652	1.31588 1.611376	1.933118	0.5614602
0.784328	2.243042	1.642286	1.108199	1.495581
2.960132	3.467417	4.757546	1.231891	0.6734486
7.248317	0.7973309	3.713006	8.860713	0.5337746
5.088515	4.877134	1.079112	3.145499	1.853918
7.080401	3.674513	1.664574	2.071194	1.139004
2.148138	4.358867	1.199031	1.775259	4.741347
1.587045	0.9354868	0.5812308	0.8602509	5.80045 1.611152
0.9909509	1.223809	0.4984128	3.446044	3.328839
8.15495 1.917394	1.372095	1.397945	1.343056	5.326155
2.84721 3.043733	1.612099	7.415259	12.99137	5.972571
18.1302 5.919043	8.737916	1.105186	8.416586	3.753149
9.598731	0.3304768	3.185516	4.978329	1.852632
4.372525	5.764265	3.485209	3.698358	1.974552
0.8227429	1.7261	6.105388	8.46633 2.89632 0.7212008
3.683974	0.4051999	2.615333	0.6019609	2.778744
1.848455	2.06939 2.230452	3.285262	9.78491 10.39165

2.264531	3.89858	1.62232	2.653248	0.4003251	1.355237
2.01584	3.358709	1.762156	2.092905	0.7399949	2.764558
0.4788586	5.091882	2.403824	2.368616	2.78226	2.705364
1.953934	0.8714194	1.555502	2.382857	2.2144	2.838539
1.487278	0.6324327	11.96373	2.672802	0.2760255	
1.74948	7.825575	3.756679	5.66833	9.845139	6.392291
2.574327	1.291638	0.8959001	4.163721	8.052617	
1.478876	5.906141	1.442988	2.524518	3.281363	
1.887171	0.4364237	0.260028	1.281047	2.221297	
1.108597	1.301834	1.300813	0.6831929	0.734476	
2.893719	8.740836	2.920649	2.593607	3.650313	
3.308676	3.332699	5.264839	2.7665	3.318034	3.65034
2.471983	8.336706	2.562035	4.446858	2.156649	
0.6487366	0.5070788	0.6107227	2.208986	0.8539733	
1.813625	1.486849	2.813057	2.079979	3.325936	
0.714865	2.997264	2.323789	3.612776	2.611073	
0.5112147	4.460933	3.812964	3.520698	5.953191	
4.629825	1.626666	1.5437	5.70367	1.308569	1.566685
7.203205	1.569445	1.287217	0.9367224	0.263256	
1.450865	0.4027196	0.9478919	0.1563512	0.3257645	
2.451636	2.949475	5.751922	10.72836	3.669663	
1.16412	3.686974	8.050664	2.935596	4.898568	2.0508
5.106341	9.974964	3.353925	3.178598	4.211016	
7.152722	0.7815745	1.513093	8.055222	5.841915	
1.975638	0.9191282	2.816489	3.926541	1.658916	
1.845116	6.503205	3.806936	2.451856	2.266952	
3.699745	0.5259568	1.524341	3.083411	1.950027	
6.355492	2.591882	1.669354	2.011008	0.4060789	
1.930347	1.825499	1.752615	7.668391	2.938064	
6.61799	2.435474	1.549421	5.881616	1.632636	4.7587
0.9009545	1.715014	1.384644	1.684041	2.087137	
1.598454	2.514021	3.017327	6.041319	2.618871	
7.183957	11.95108	1.716687	0.4023793	1.880807	
2.706245	2.744084	0.7221905	0.6901525	0.9488035	
1.473225	4.732095	3.800021	0.4458116	7.271847	
1.363859	7.828555	8.050492	4.701223	1.418109	
3.146956	1.068457	2.59598	3.411186	2.533702	1.202143
0.4565101	6.042871	3.265319	2.381019	1.860083	
0.7868515	1.309614	1.653956	1.531682	1.926692	
5.996775	8.175931	3.225755	0.6268338	0.8345716	
1.416783	6.864389	1.424573	0.4398107	12.17309	
1.544166	3.546616	3.041544	1.579556	0.6891709	
0.2299407	3.098569	8.624153	3.028107	3.005525	
0.4384045	1.942499	10.05656	0.3853447	4.863959	

1.143987	3.755806	1.935282	2.778108	4.788801
1.965731	3.155108	1.383576	1.089136	0.2504674
4.29774 1.075743	0.6063604	4.039672	1.376908	6.971463
1.340685	3.384855	1.271097	3.580551	1.157634
0.567261	0.6729858	3.954244	4.417007	2.06537 0.9226728
1.370677	5.861865	2.852409	1.93054 1.131028	1.155107
3.868435	0.9889606	0.9044112	4.550601	8.318154
3.644104	2.003058	2.087665	1.489969	0.4692392
3.272874	8.026982	3.98455 2.223153	0.8130287	2.902445
2.13142
GDF2	0	0	0	0	0	0	0	0	0	0	0.006379061 0	0	0
0	0	0	0	0	0	0	0	0	0.01778214	0	0	0	0	0
0	0	0	0	0	0	0	0	0	0.005748552 0.01315355
0.006001321 0	0	0	0	0.00546832	0	0	0	0	0
0.00701149	0.03986326	0.01117414	0.1159172	0	0	0	0	0
0	0	0	0	0	0.005035751 0	0	0	0	0.006347057 0
0.02205459	0.006929488 0	0	0	0	0	0	0	0
0.007641553 0	0	0.007646567 0	0.4073365	0	0	0	0	0
0	0	0.01440708	0.007825415 0	0	0.04652233	0	0	0
0.01533655	0	0.01072065	0	0	0	0	0	0	0	0.4211109
0	0	0	0	0	0.007690532 0	0.02739322	0	0.007143512 0
0.01359452	0	0	0.006878268 0	0	0	0	0	0	0	0
0.024536	0	0	0.1017264	0	0	0.007014258 0	0	0	0
0	0	0	0	0	0	0.04666956	0	0	0	0.02486374	0
0.008210414 0	0	0	0	0	0	0	0	0	0.008555372
0.02378662	0	0	0	0	0	0.007149247 0	0.01494863	0	0
0	0	0	0.005789387 0	0.006128848 0.006425061 0	0
0.007532256 0.01136122	0	0	0	0.007382274 0.007572493 0
0.01143474	0	0	0	0	0.005761955 0	0.01711918	0	0	0
0	0	0	0.09271328	0	0	0	0	0.007704625 0.02080467	0
0	0	0.01296052	0	0	0	0	0	0	0	0	0	0.0089056
0	0	0	0.01678281	0	0	0	0	0	0	0.0371203	0
0.0108273	0	0	0	0.02767988	0	0	0	0	0	0	0	0
0	0	0	0.008771531 0	0	0	0	0.03073518	0.00677507	0
0.04148876	0	0	0	0	0	0	0	0	0	0	0	0	0
0	0	0.006046444 0	0	0	0	0.01259663	0	0	0	0
0	0.01639479	0	0.006738278 0	0	0	0	0	0.004994826 0
0.01869707	0	0	0	0	0.016273	0	0	0	0	0	0	0
0	0	0.006864025 0	0	0	0	0	0	0	0	0
0.007321581 0	0	0	0	0	0	0	0	0	0	0	0	0
0.01524495	0	0	0	0	0	0.09638635	0.03379947
0.005749725 0.03909347	0	0.00744089	0	0.02776877	0	0	0
0.005210198 0	0	0	0.007067143 0.007362605 0	0	0	0	0
0.02243995	0	0	0.008864471 0	0	0	0	0	0	0

0.006131842 0 0.2991303 0.009003638 0 0.04667683 0 0 0
0 0 0 0 0.01131767 0 0 0
MNX1 2.136765 0.006612405 3.024547 0.961132 2.095242
0.04201888 0.8226853 0.6547236 0.6672322 0.6757811
0.08013297 0.02717137 0.6994539 0.3339024 0.7102381
0.01793064 1.013854 0.8799133 1.331973 2.052919
0.5979592 0.01721712 0.1803455 0.03722951 0.002368087
0.05978955 1.050812 0.00273396 1.623612 0.3059746
0.6741876 0.0836807 2.196068 1.088965 2.471427
1.788745 0.4425242 0.9626636 2.642446 2.854863
1.616651 6.668953 1.299137 2.413899 1.007586
1.269536 1.488567 4.184653 1.397665 0.9472113
3.76384 0.9753763 0.7418626 0.4367011 0.2089827 2.924272
2.218474 0.1559534 5.051306 2.015575 0.6673261
2.178448 0.7231501 1.043856 3.625655 0.2811488
2.198465 1.684352 1.205427 0.5386135 2.979575
1.904539 0.7251106 0.6512432 1.560501 3.601777
1.269753 1.390343 2.080421 1.930589 9.210128
1.703344 1.203654 2.086942 1.474991 1.466304
2.348016 1.657777 2.214959 0.8540835 1.012896
3.443721 1.246426 1.202512 2.869387 1.720205
2.433174 2.588616 2.225489 1.506983 2.701262
1.40766 2.891866 4.690702 0.5458582 1.369279 2.02506
1.373017 3.470278 2.98234 2.491724 2.987197 1.929551
0.6365406 1.493919 1.383539 1.063873 1.764059
1.951436 4.619176 1.939306 1.060115 5.70968 0.6419118
2.655518 0.3823186 1.938586 4.382708 0.8551056
1.52647 1.850136 4.755157 2.209347 0.7420871 1.654116
6.354794 2.818444 3.034071 3.645345 1.94627 1.73587
2.268135 1.051682 5.540638 4.343607 1.624567
3.025141 1.560785 0.3473729 0.9760754 3.288062
1.966287 2.036505 2.34695 1.068798 0.6079704 3.006031
0.9498096 0.8838953 2.475545 1.06314 0.3476139 0.7389879
2.406214 1.337475 2.805084 2.239304 0.6204483
1.153651 1.804825 0.02568846 3.279872 2.58964 1.808442
1.633664 1.016271 1.582047 0.8663313 1.955818
3.200749 2.46552 5.50339 0.4026957 3.211574 0.8441712
2.063404 1.13398 2.194035 1.243243 3.312133 1.260186
0.7797155 1.152953 0.7823075 6.331867 3.564444
1.002833 2.795795 6.024565 1.917964 2.617472
3.110813 2.223614 2.001183 2.937313 2.468995
3.674614 10.58519 2.431232 4.555458 1.81324 1.157604
2.243321 4.659984 3.540411 2.637879 2.03879 1.572236
3.66933 2.383767 2.093992 1.476245 2.391637 3.148061

1.459246	4.217035	5.722666	0.1288288	4.0708	2.674426
7.493495	1.588157	2.142109	0.8893009	0.9405454
2.41506	2.027052	3.451174	1.471861	13.33751	2.315073
2.52573	2.475986	2.07367	0.7898965	1.211996	3.183509
0.3771884	0.9470406	2.411122	2.949589	2.977012
1.500309	2.156249	4.396387	4.25832	3.172712	2.465787
2.395664	1.725895	1.650436	3.138081	1.015623
1.614413	3.885108	2.903348	0.9760947	5.164472
0.3100641	1.197599	2.354643	2.76743	1.737255	3.671969
1.780972	3.986024	1.51288	0.4538555	0.3410412	1.030667
0.6809699	3.268288	0.8448899	2.338649	2.212427
3.97688	1.049459	4.928604	2.96493	1.187144	2.787124
0.1003318	0.1850788	2.283801	0.1642445	0.4981546
1.276185	4.260924	5.482163	1.138961	1.115487
0.4919899	5.292417	0.3887419	1.34222	4.324714	1.837337
1.313754	2.482819	3.622907	3.021296	2.431342
3.628964	1.666655	1.2017	1.552589	0.855533	1.028759
0.8785296	0.5379279	0.9948786	1.576838	1.214198
4.760259	0.2808895	1.737603	5.566304	6.214022
1.530307	0.4640785	1.598856	2.764988	1.55739	2.723207
0.4401301	5.295021	4.128556	0.5018063	3.524433
1.151589	0.604718	2.970401	2.115007	4.4759	0.388052
0.5925017	2.695705	4.599364	4.223576	1.430736
5.536765	2.886763	1.836812	0.7374314	3.507797
2.446859	1.180306	1.858543	0.7548173	1.995614	3.4558
2.874949	0.1730954	2.647748	10.67585	2.50209	1.648156
1.478178	1.849565	2.424809	1.982856	0.5774559
3.225549	1.308536	2.844271	4.210491	3.349469
0.5752637	2.614235	2.443028	0.4460246	2.614939
3.13442	3.014614	3.382082	4.448234	0.6399612	1.933008
9.77205	4.195422	1.320878	1.325711	3.376428	1.373987
3.180835	1.628746	3.25294	1.105309	0.740221	1.817085
3.44365	2.745938	2.639699	0.7134889	4.80772	4.470394
0.8178694
VPS37A	8.005303	5.305998	7.903345	5.003993	5.46083
6.799407	2.872122	4.437783	3.838569	4.912923
6.185898	5.179404	4.58706	4.463669	3.163238	7.720406
4.259156	5.396959	5.394375	7.473935	5.868443
6.733414	5.44906	8.147833	6.882176	4.452444	3.295038
7.486571	5.71531	5.831391	3.764369	6.530795	3.940152
3.912119	4.524399	6.164124	2.736532	3.581482
5.065903	8.860444	13.21299	3.702185	7.342462
10.09581	5.019242	8.651415	5.878478	3.910972
6.474301	3.716916	5.127414	4.574542	4.831733

6.691458 6.468005 10.51759 5.587666 6.586182
2.673522 3.82953 5.801361 6.860541 5.558451 7.971769
5.186977 6.318494 3.678999 3.571413 9.140423
4.347486 13.55167 5.799005 17.75768 3.778185
4.249585 4.562573 6.010861 8.057404 6.19019 2.542657
2.874713 4.994522 4.854048 6.85082 6.056879 2.981802
5.929815 6.293271 6.41218 8.428056 6.399753 4.708462
8.472255 6.085225 5.255467 3.233672 5.332992
4.834962 6.060213 6.987651 4.845186 6.876054
5.097322 4.74217 7.040514 5.151779 7.49193 4.758759
10.24935 5.159186 3.38893 3.791668 6.656071 4.769043
5.460972 5.155375 2.360281 7.214242 2.55824 4.225572
17.08945 3.442144 3.021476 5.789045 7.174233
4.761079 9.684413 3.346979 7.615471 4.317157
4.775138 3.57683 8.681482 5.714306 4.845103 3.985284
7.001295 4.399635 9.300375 6.665468 2.892962
4.160519 3.315361 3.734012 2.837983 5.001831
5.852051 5.967104 3.799313 5.999774 3.449787
5.610893 6.177019 4.878294 6.477702 5.682263
8.182547 7.703827 10.78121 3.417792 4.867792
5.347299 5.427034 6.154497 2.585546 6.291935
4.366043 5.062015 5.144904 6.5967 6.935601 3.87572
5.447267 4.83145 9.425025 10.28541 6.175762 4.474787
8.298712 4.557363 2.821654 5.273245 5.216427
4.018393 4.136027 2.987454 6.972137 8.177073
7.308534 7.159988 7.294995 4.003559 6.338242
4.086021 2.739841 3.61159 7.265034 3.380454 3.190415
2.498519 4.799518 4.316948 9.859478 2.260524
4.988066 6.509038 3.718581 47.56018 2.989979
4.125951 4.925284 4.600298 4.261959 3.975278
6.609606 8.459923 5.85116 9.287115 4.545555 6.376713
11.21948 5.703049 4.16314 5.001329 5.241985 6.024147
5.065942 3.600812 6.879763 7.088431 3.757142
4.592272 6.217893 6.343013 8.810446 3.489398
5.512711 5.577511 9.825224 2.720217 6.472036
5.354269 5.498319 5.976307 7.539978 4.417869
5.587534 5.555677 8.330906 5.007165 4.683899
7.41208 5.3484 5.365606 4.208591 4.335813 8.509254
3.973656 7.336918 4.261594 4.253931 7.723899
6.430244 8.136886 6.713738 3.231781 6.049626
4.515225 6.977935 4.165101 10.00033 4.676785
4.713712 5.024456 7.01383 3.971676 3.486084 7.221633
4.422273 5.727671 5.804222 6.706679 4.852414
7.544726 9.878567 4.980548 3.457839 6.296339

6.579283 7.848007 5.776934 7.570641 7.636624
6.178534 4.509061 3.140014 5.839193 3.472392
8.193987 8.871384 5.625773 8.126343 5.031314
6.070142 7.670282 4.347551 7.20375 5.087343 8.4582
4.636305 2.871351 4.307774 6.173753 3.085841
8.319973 6.173253 5.716527 5.136739 4.792542
6.674515 2.387877 4.538289 2.366522 6.003088
7.529701 4.804772 6.729365 7.205899 4.333954
7.878464 4.489059 7.097183 6.215192 6.020335
4.372341 7.354294 4.730438 5.150701 5.00447 5.033232
2.837678 6.54366 4.748867 7.534904 7.297295 4.764676
5.133529 6.590623 9.947342 3.984249 3.708698
5.511346 4.087023 7.257188 4.090889 7.16484 7.183311
4.073877 7.173705 5.322106 3.106567 3.44395 2.474524
7.915787 3.08624 5.358494 8.838917 3.337525 4.683069
7.40699 3.533585 4.701995 10.55364 8.119904 3.906889
3.98381 5.238507 3.095176 3.548695 7.313838 21.91848
4.504815 3.808828 5.239377 4.86684 3.599425 3.737821
4.162052 2.2785 12.35832 6.771383 6.722949 2.398686
6.774634 7.701709 3.465039 4.131339 5.734937
4.551874 8.947878 2.620456 2.955501 5.11144 6.844269
6.114124 3.790008 9.817251
COL13A1 0.2521892 0.634225 0.338681 0.4866308 0.6351139
1.593698 0.4675762 1.021588 0.7903531 0.777275
0.2446156 0.07927379 0.5023838 0.06620618 0.4029189
0.8463582 0.5483311 3.328613 0.4110294 0.3124016
0.7337825 2.049457 0.1860834 0.3590459 0.3195417
0.1486695 0.7824168 1.06087 0.6627855 0.1430602 1.004868
1.064733 0.1777055 0.2382831 1.042978 0.005528339
0.06822812 0.9988715 1.244595 0.3325432 0.8329503
0.2512158 0.6142171 0.4523262 0.9400748 0.4360845
0.7453472 0.4526846 0.2028606 0.4612607 0.5473126
0.3878348 0.3381907 0.9631565 1.189932 0.6076362
0.2999273 1.008243 0.6498834 0.3008647 0.8303198
0.5330832 0.1858656 0.5548005 0.3262252 0.6818455
0.7467094 0.4180112 0.2812501 0.2889179 0.4996999
0.7548916 0.4290966 0.5643665 2.557047 0.5792369
1.319703 0.3982492 0.8073887 0.2025755 0.2455575
0.4958571 0.2404176 0.5445651 1.089122 1.164733
0.3113842 0.2648936 1.088561 0.51202 0.5370834 0.8447779
0.4954959 0.5364769 0.6618307 0.5281573 0.3055658
0.852435 0.08042144 0.4396698 0.3852123 0.2313445
0.1975644 0.6655123 0.5959119 1.036781 0.4693098
0.3767252 0.680184 0.3227619 0.3741772 0.4029608

0.3098028	0.4698449	0.7323847	1.693906	1.622494	
0.8391405	0.3223037	0.2110677	1.60241	0.263171	0.9109993
0.2809214	0.4605897	0.5161325	0.2352789	0.6900707	
1.212367	1.202085	0.2996794	1.228853	0.2360752	
0.421834	0.3044418	0.8035897	0.7076342	0.2932581	
0.4274173	0.1720708	1.750581	1.064838	0.4551492	
0.2177295	0.6379188	0.3650672	0.2913627	1.520045	
0.3600512	0.8917688	0.1048806	2.228575	0.3656371	
0.4210665	0.6782055	0.3721778	0.705315	0.4986814	
0.7084637	0.1940632	0.3725158	0.8302908	0.4229539	
0.7112014	0.4287393	0.3363275	0.3982725	0.5916111	
0.6147799	0.1955996	0.3289356	0.447104	0.2098722	
0.6826237	0.720182	0.3296686	1.058781	0.5592599	
0.1213051	2.629327	0.8015073	0.399882	0.2146424	
1.083133	1.323699	1.860243	0.9351654	0.460641	
0.4014071	0.4142664	0.8564439	1.219813	0.365519	
0.3315679	0.1777969	5.118061	0.2867815	1.52816	0.3957387
0.5684109	0.2987898	0.8865169	1.171904	0.5908545	
0.3483253	0.1974877	0.6107976	0.3776115	0.6103783	
0.5845478	0.3838632	0.7773303	0.5079631	0.6897998	
0.5639017	1.15398	0.3427542	0.2057791	1.476887	0.2876028
0.6495275	0.3190384	0.3520624	0.2438875	0.404631	
0.2105068	0.4374539	0.2701525	0.5015315	0.4536493	
0.2702955	1.263956	0.2763028	0.3800342	0.1511061	
0.6107052	0.5812308	0.2245628	0.2107246	0.3398952	
0.4268682	0.3714796	0.7056592	1.275007	0.6077098	
1.232776	0.4534849	0.3255965	0.5658348	1.336064	
0.5372813	0.3272745	1.592146	0.7726547	0.4305584	
0.1756349	0.6364395	0.6927413	0.8097554	1.234497	
1.78145	0.7417655	0.4895605	0.4011947	1.413897	0.4641984
0.6595374	0.3731234	0.4062446	0.2842178	0.7242245	
0.48664	1.038344	0.2212699	0.3756621	0.2391331	0.438039
0.5207768	0.735438	0.2195127	0.8881047	0.1207911	
0.9576497	0.5112694	0.5667325	0.5625275	0.3698076	
0.3094633	1.643562	0.6442859	0.5860592	0.2482919	
0.5212849	2.734021	0.3593935	1.252481	0.6213615	
0.9868034	0.8933521	1.224593	0.9606997	1.084899	
0.3397855	2.684516	0.3256232	0.7105814	0.259292	
0.9652297	0.3542174	0.2017048	0.288977	0.3870368	
1.159375	0.6520532	1.045313	1.00848	0.3396187	1.697514
1.026805	0.6815958	0.09742547	0.1987127	0.3377167	
0.1197266	0.2930522	0.3258991	0.8548576	0.4006018	
0.4018272	0.591787	0.1304286	0.4061026	0.9740191	
0.3474681	0.8231418	0.2388195	0.1217446	0.5183264	

0.8905288	0.2704078	0.08115754	0.9368041	0.7713295
0.9464429	0.7057135	0.1080408	0.2596466	0.08163275
0.3244521	0.4490731	0.5835369	0.8225685	0.2623583
2.869658	0.8824017	0.2217472	1.449691	1.086659
0.3498312	0.8604676	0.9651307	0.6961878	0.6287429
0.2243909	1.002017	0.987529	0.4907836	0.6444894
0.2227787	0.9807619	0.2774037	0.3566272	0.3837185
0.3747759	0.5209592	0.5139355	0.5121029	1.477959
0.6261749	0.5025909	0.4856959	0.7901972	0.2887839
0.3212889	0.1399222	0.2221253	0.8402031	0.5893281
0.6384787	0.4295267	0.445301	0.4727343	0.8959371
0.3208161	0.4373702	1.013748	0.5350322	0.5516096
0.4426271	1.266617	0.5645105	0.1770638	0.6243409
0.3456596	0.3370438	0.4890972	0.284259
RAD9A	3.199154	1.246307	2.936293	3.621956	2.962043
1.665825	2.035302	1.670648	3.265727	3.182974
2.440966	1.732193	4.240706	2.417899	4.988295
1.01981	3.600908	2.621277	2.504309	2.478537	3.774972
1.707575	2.549256	1.688589	1.654074	3.15558	2.813314
1.584471	2.31664	1.795026	3.190809	1.643128	4.72629
3.556174	6.397702	3.009933	0.6636012	2.998489
4.741755	3.651569	4.812357	4.881557	1.537935
3.080464	3.649562	4.592661	5.401261	4.805869
4.336185	3.579463	3.049389	3.251782	4.209747
4.957015	2.976096	5.282221	5.56706	2.279225	7.21237
4.011089	1.606347	6.149836	0.9718323	5.10563	3.179576
1.622317	2.444111	2.444384	3.819975	4.744696
4.056795	4.503518	4.667789	2.300643	4.924659
4.922316	2.013053	4.93876	7.184811	2.673011	4.084049
2.973042	3.288654	3.010064	4.059884	3.887106
2.251858	2.971444	10.72073	2.362408	2.182875
5.253165	1.905277	4.686324	5.375054	3.318114
2.526751	3.449452	2.918669	3.066083	3.176418
3.866692	5.420002	4.88668	4.138928	2.131953	3.845894
3.179899	3.223475	3.595986	6.135108	11.49213
2.642026	3.008123	4.011204	4.526561	6.745707
4.834344	5.09742	7.99533	2.556922	4.022833	7.286726
1.475144	6.069946	4.434846	4.153299	2.653455
2.014268	3.522221	3.975522	5.003077	4.524589
3.009403	3.287675	2.749483	3.676075	3.343258
5.354054	3.35634	10.2484	6.704148	3.039047	8.354729
5.644349	1.502149	10.30085	1.966424	2.675059
2.345465	5.771475	2.356529	4.139987	3.231387
2.421921	3.534444	2.967547	3.519831	6.255823

3.847647 3.650644 2.918429 3.731315 3.328788
6.156436 3.56869 9.761928 2.866231 5.984774 3.260671
1.913116 4.348937 5.818897 2.8618 3.579083 13.03448
3.374556 4.170229 5.836788 4.403606 4.02723 4.360834
2.75142 4.454521 3.626483 6.643963 2.589977 5.780764
4.85139 3.814396 4.993973 3.521804 4.75075 3.937172
4.781996 3.998282 2.059391 4.275353 2.730738
2.559893 2.573844 2.427251 2.15922 5.612957 4.602391
5.701212 3.489541 5.559268 3.965747 3.793561
5.264358 4.481867 3.105343 4.047303 4.949658
3.808247 4.70284 18.57169 3.979495 3.370917 5.079669
3.740033 3.076309 7.262154 3.637487 3.674279
4.807236 3.473449 3.733112 7.20915 4.108634 4.453673
3.445413 2.994216 2.777152 4.756157 3.428588
3.901113 4.533171 6.924364 4.437111 2.971756
2.047304 4.067892 3.275481 4.377642 3.841166
2.451948 2.655441 5.215751 4.874488 3.692715
7.872449 6.109296 3.574823 3.691654 5.605567
2.040984 4.773633 2.735512 11.36672 3.387449
3.565014 2.04839 4.137978 3.938883 4.952301 3.298366
5.156554 7.177613 3.466198 4.560492 4.907683
4.223384 4.215988 4.567166 15.04678 1.327715
2.929269 2.426697 3.260211 7.238803 1.507253
4.823508 2.743563 4.86681 3.250226 5.459692 3.006789
2.067009 6.273567 1.040153 1.449302 3.240451
2.782217 2.782212 5.268766 3.683369 3.562233
3.571812 2.529712 3.263611 2.615149 2.170804
2.990962 7.310559 3.6321 5.272448 4.041278 3.571948
6.530498 8.837896 4.256822 3.192163 4.84485 2.113603
5.162384 4.826169 2.357734 2.630992 4.888424
5.03007 5.521828 4.071414 0.7310764 5.629362 5.063169
5.37108 3.312645 2.733326 2.360724 2.910524 5.810709
4.787211 3.099611 5.576946 3.975834 4.633684
5.103643 2.44285 3.037771 8.096746 3.881799 7.162076
2.221582 6.128001 2.858109 3.126325 3.841014
6.483364 5.401398 3.363571 2.6121 3.994549 5.618396
7.126852 2.544618 3.79673 1.88613 7.457858 5.522633
2.973046 3.768456 3.980185 4.292858 3.394859
2.849953 9.218107 5.53642 66.9644 7.696469 3.772553
5.438352 3.507843 4.764111 4.577262 2.921523
2.760622 2.869277 8.341176 4.878623 3.9299 2.905661
5.768248 5.469528 3.673305 5.002541 4.412044
9.238319 3.371997 4.724364 2.714828 6.043954 4.0997
7.473538 3.064391 2.876262 2.681764 2.352506

3.607129 10.39958 5.459137 4.417102 4.787167
4.462606 4.242942 3.325036
IFI27 87.66919 6.167444 64.52866 65.63142 112.0151
13.26602 257.7977 34.60633 133.9777 117.3501
6.200949 19.78843 182.2627 2.565402 207.3985
9.730503 123.339 36.31223 143.7754 65.96608 125.98
9.437997 8.508428 12.74627 11.08086 9.784076
212.2178 6.42137 62.93526 175.5738 127.161 30.48062
116.6908 148.4965 82.87834 135.4513 1.173842
37.19853 117.6037 57.95994 50.26027 195.9615
14.60767 77.17917 68.80764 53.96325 54.50297
93.99507 16.49143 74.41745 163.7408 57.03455
6.186504 99.18162 23.03341 30.45315 65.29187
10.7383 108.0745 130.1424 27.42891 123.8786 143.352
84.24294 310.3877 10.76362 85.69655 39.00249
72.11625 72.76677 67.01246 65.45826 33.49595
5.314977 20.43289 64.29156 55.50848 8.49262 16.37091
89.41519 36.86006 37.91317 71.13558 34.28063
6.889707 8.945032 8.882187 72.18653 47.62311
69.48973 131.7112 43.22624 135.9205 128.8966
119.0439 37.73798 112.0215 201.3251 82.48554
110.2295 28.30956 175.919 194.1049 156.7621 40.43035
20.94901 174.0592 30.26246 20.36667 49.47124
44.27473 8.02556 311.8575 27.90806 165.4435 23.51779
85.36841 65.86938 88.78259 62.46937 58.39972
5.600786 18.91879 186.9582 59.39154 33.46838
119.8798 147.1879 20.31768 86.92406 126.2591
37.08596 143.9009 117.0468 163.0472 27.02447
7.421144 102.9234 30.49031 27.74247 53.30613
58.12312 59.37548 31.2244 59.72557 35.12086 137.1364
48.48688 40.01457 43.74278 30.27967 44.00384
17.89543 40.9461 15.48165 55.025 99.4284 27.636 236.3251
82.04973 98.46189 66.96517 25.97831 19.98307
80.81647 58.0138 60.38564 30.99509 36.73371 4.472702
53.43497 44.87761 48.03911 32.62005 183.9927
42.03652 51.42234 202.5043 44.29294 29.78068
100.8308 45.27624 80.3675 76.46478 51.00807 54.61974
44.34779 209.2732 15.21213 67.09394 101.1905
136.3992 56.88888 57.85514 113.2743 63.14988
92.41667 42.83417 38.86867 156.4172 4.131912
69.03264 103.5707 79.74759 93.47741 46.32158
140.3897 22.58505 37.83876 82.49726 6.5515 32.79438
119.6664 40.39794 65.84347 67.78409 68.57355
7.83777 56.9958 50.04018 47.0188 59.9844 46.8013 116.0806

32.17175 74.68176 25.77702 204.5685 33.30179
144.5137 91.5928 25.35235 19.34732 54.56326 128.5611
78.64525 69.36954 148.4404 86.15993 4.693197
19.16505 67.04545 87.3303 185.2765 16.1975 74.21245
47.22516 23.69583 73.97604 84.77405 52.00477
61.87176 158.4148 77.58747 52.42525 7.388233
172.4276 20.11502 69.04381 13.81439 139.0822
82.13735 87.45497 94.84164 76.95872 31.47038
44.10634 36.00952 113.0744 38.567 17.6497 128.4188
70.91058 60.10706 54.4865 43.87527 68.75638 9.53399
4.169308 72.79445 47.06634 58.09179 145.1102
66.60245 4.043875 43.87057 33.16909 130.1907
53.46645 42.36048 20.60256 7.149052 10.65478
91.57825 36.14596 56.50952 98.07491 61.23194
115.9565 41.45071 98.37793 24.66297 51.75291
10.15557 89.12868 64.98119 18.24926 59.83497
41.9035 9.665988 84.92356 128.8249 68.52372 52.73389
114.9586 7.880496 95.36443 53.85492 158.2592
12.36173 74.35007 39.26618 101.5868 104.6032
61.48041 58.01336 24.82239 87.20726 139.8071
52.41023 116.1328 38.63803 55.28695 68.88945
63.59038 3.364589 42.51653 151.9984 59.88992
82.85892 3.980347 138.9475 39.06736 146.0558
19.91157 31.50524 10.34008 16.989 162.9962 249.582
66.11283 41.78796 22.14886 80.00591 41.18554
16.00372 54.39176 75.40341 12.49219 132.7689
79.59764 73.09496 44.86856 87.23651 10.31563
42.76341 40.35998 87.45714 65.54419 78.39054
63.0096 68.80433 107.415 130.4371 46.89994 40.34296
116.182 53.75667 34.63815 38.92982 41.61667 36.78542
220.8316 40.88136 44.97356 193.4315 33.48877
78.44943 85.63255 110.4148 57.08274 5.675947
79.45419 10.87027 90.49133 25.6926 61.19863 48.07799
124.7293 5.936671 342.5527 5.750204 96.67901
98.41212 162.8676 52.45467 48.28803

ITPRIP 1.112496 6.695211 1.698553 4.203486 3.235792
5.934867 2.306737 5.340674 5.162082 5.220407
19.62426 17.924 4.732427 24.63423 2.912695 5.721848
2.008792 8.872985 3.112378 2.009492 4.006289
2.938749 26.51996 5.440238 9.371446 17.52629
3.086623 3.404245 3.740242 1.215258 4.59306 6.179809
4.129793 8.473035 9.876824 1.409759 0.8004288
5.298617 7.009216 3.444064 8.108942 3.197333
8.157085 2.537717 3.969077 2.378217 2.851235

6.394533 6.53976 5.200838 3.714299 5.246138 2.205097
5.997705 9.744808 3.274109 7.232068 9.085365
5.075788 3.697627 10.69129 6.187136 5.189883
5.446835 1.431355 5.35542 8.01591 4.758793 7.155214
2.639118 4.441698 4.589121 3.722954 11.10612
8.838807 4.947685 6.81207 5.993974 2.288123 2.844002
3.131648 7.962197 5.470953 3.29148 8.083013 6.739253
6.766004 4.011825 3.662372 3.745844 3.895704
9.03254 4.981396 3.913595 4.681728 5.441831 7.281987
2.588975 13.27341 5.091145 5.44982 1.57354 1.619938
5.293191 7.93315 14.44101 5.676733 6.154783 3.183575
3.776777 3.244654 2.28132 4.543405 4.2674 14.94714
2.902626 5.286781 5.006434 2.780228 3.699283
4.491666 2.157883 8.403502 5.221102 6.034651
6.223723 3.60681 4.484119 6.334182 9.678558 2.740189
2.808577 3.884042 6.871853 6.698362 2.857021
5.328262 4.330476 5.718914 3.103986 3.989247
3.73416 3.39903 3.33543 4.506551 1.555222 2.230667
5.628984 2.983236 5.209365 2.732805 9.672953
5.42461 9.143101 5.795202 6.051433 4.55455 4.164864
10.64804 3.009499 7.879261 3.506809 5.324694
4.159196 4.468209 5.239692 3.12711 8.551958 7.161479
1.07431 3.635994 4.418303 3.501282 7.951817 1.700687
4.7313 11.38 2.763317 2.910337 10.57625 5.032983
4.391989 32.31397 8.21848 7.52954 7.974047 5.96681
2.526424 5.237178 4.984351 11.34409 6.983905
3.883641 2.873608 4.380673 6.661319 15.60826
2.543018 2.745503 3.414635 1.024521 6.020252
7.466297 1.73364 2.934358 3.439028 4.507436 12.56122
6.053203 4.245806 4.551978 10.68153 6.001918
2.091368 4.216928 6.192889 3.392431 2.640698
15.08634 3.239326 2.755538 3.998073 3.559603
7.276187 8.418891 0.9551982 3.123612 26.00367
1.79126 5.470051 2.283956 8.04101 2.222404 4.814468
3.15202 3.363422 5.689207 1.788704 5.213892 3.696979
3.252328 5.812195 7.818069 3.689816 9.426608
9.264199 5.18506 7.065505 7.170272 3.052098 3.450016
3.628561 9.049836 8.2098 4.051816 3.686218 3.232057
5.422948 6.15039 9.467457 3.633771 6.171923 8.501067
5.996069 12.13515 6.542916 3.257279 6.19537 4.402442
5.116314 6.790412 3.931216 4.072265 3.486882
1.578992 3.126932 3.868459 3.147981 21.39189
5.271499 5.672919 3.920946 4.317574 6.688427
4.233551 9.65811 9.56301 1.574614 11.26312 10.79451

3.64244 3.728875 11.02122 7.950186 49.821 13.98161
6.764099 7.41706 2.688657 4.897025 4.115697 7.898905
2.962421 36.05136 1.642114 3.813987 2.640811
7.057046 6.914339 5.323016 4.21993 7.613859 3.767184
6.8699 3.675988 7.628222 7.758725 5.748495 5.344907
7.312841 3.757324 4.431349 4.759794 3.312183
8.480305 2.456962 3.936366 9.474203 2.866757
4.144351 2.480717 10.86436 7.467696 5.328205
5.90234 5.07948 1.053256 1.576989 5.216847 2.928117
1.643384 2.759706 5.098101 2.425125 7.79789 17.78616
2.91689 1.984149 1.404231 3.976771 4.503728 5.601484
3.635912 19.22059 4.060764 2.082162 7.518692
5.718559 4.711793 4.278062 6.578377 12.11492
22.32146 3.770294 3.18856 3.762857 1.216304 3.948573
3.154654 3.92943 2.391626 11.25557 3.838507 6.275049
4.632343 3.862011 2.563708 6.731979 2.287281
8.042227 6.48281 11.04434 2.313632 5.01981 6.020959
3.012652 9.046791 3.052252 4.253373 3.417217
6.765077 4.762196 1.42221 3.387141 8.712553 4.501616
3.931659 4.975242 6.51686 4.31382 3.320723 4.964825
5.431988 1.884777 2.553885 5.149432 3.735836
BCL2L15 33.39087 0.04902139 28.88456 11.80547 13.92831
0.9518333 6.146543 2.736165 6.637623 6.19605 0.4576537
0.6177379 5.473076 0.3364624 5.070371 0.1848356
13.77129 4.405466 13.05226 19.16844 11.08649
0.1361493 0.8435058 0.1349347 0.07022371 0.5963765
7.26008 0.1648493 15.90367 6.653849 6.258519 5.070193
12.50328 5.941797 4.412873 7.581967 0.2809467
4.791601 4.224651 18.55292 6.049829 7.809267
2.426036 3.864738 10.86419 4.12433 7.341138 4.110703
0.1252795 13.85332 14.38471 2.931095 0.355656
10.39085 3.884921 1.079761 4.275406 0.6656728
4.064375 10.69841 7.496547 10.17009 3.376648
0.3839344 11.18429 0.9726781 6.006403 5.968903
17.88673 15.09834 2.087056 10.25266 3.519525
2.501649 5.430713 1.217658 9.40409 8.87742 11.43329
11.65759 8.462172 10.80785 5.721924 11.37575
2.836779 9.345002 15.31476 7.534495 5.900917
4.552721 8.052581 7.933824 9.17621 11.52011 0.5822112
2.730379 6.310976 7.823887 0.3126234 4.555444
4.009467 6.795931 11.22151 2.19328 7.201269 4.336341
7.765283 3.514231 7.97275 7.476983 0.7782262 6.355578
3.406767 1.141608 6.802499 20.3782 3.678724 13.90377
4.855179 3.167906 9.358407 1.169231 0.3558911

8.331151 0.1018425 3.315015 9.662479 4.882954
2.624387 3.903461 19.29973 17.60684 3.533158
3.695217 7.739746 9.230646 16.78076 4.791197
6.033127 4.605546 8.730638 5.106558 7.39124 1.273836
0.0451612 53.57043 5.603432 4.993627 5.608358
13.33728 0.4548332 8.680221 6.952698 6.137709
4.555595 5.065255 3.514964 1.829161 0.220827
2.584226 1.483604 5.803562 8.279823 10.19481
3.90723 9.300381 15.33634 5.98603 7.530988 0.2650786
0.3893495 6.074938 7.992961 6.858252 12.58505
10.76581 12.38486 7.141133 26.0959 0.9926595 8.535007
3.748598 1.155887 7.294789 3.972188 6.452461
6.525767 5.567524 5.997996 17.32123 2.236326
5.877701 3.865849 7.894874 8.403945 3.756653
1.670318 1.867276 7.247139 6.741267 0.2445315
6.886444 14.3204 40.99916 7.766259 6.81018 12.34638
0.06298377 10.25427 15.7507 3.090692 1.075242 10.13879
3.611134 4.198211 7.643007 4.096415 0.6389734
1.82963 15.07317 10.1643 4.676739 7.626724 2.678427
2.884851 6.143236 1.944381 0.4457035 3.24829 3.685326
7.391696 2.893295 16.52923 12.37951 25.68358
9.93161 2.505839 9.066133 3.149057 0.4082853 1.108358
13.59954 10.91833 6.267885 2.288896 8.405874
5.383157 1.04669 6.229211 18.74009 5.649915 8.338285
10.51477 4.944341 14.07473 0.03340663 3.710205
3.046705 2.284623 1.178032 4.565449 2.326432
3.378395 7.216426 8.02757 2.360374 6.96108 10.43297
5.539524 26.44279 11.63128 3.90591 16.94334 16.11787
20.6067 6.877227 9.984747 0.5334249 0.05496361 4.042548
3.504617 7.667296 6.3793 3.184088 1.812878 4.14317
5.709533 1.913098 4.094037 3.400808 1.456095
1.016547 0.4278109 18.52296 0.02618569 1.529375
2.44759 4.642214 3.044708 10.58749 10.92875 4.84057
6.860583 1.620714 15.11339 6.709479 2.060286
8.583152 7.515614 5.343007 3.72443 0.8985566 10.37185
1.496465 2.560474 1.267047 5.163835 3.172829
9.559409 1.452025 4.803234 7.20167 3.082121 8.006921
5.500317 4.158983 7.79707 0.07627518 5.333899 0.6865335
3.996123 2.455141 9.435813 12.52082 2.63823 7.976845
12.26984 7.597458 9.077046 7.594872 2.362299
1.089519 2.210698 15.75038 1.263254 1.690463
47.46414 0.08428919 5.806772 5.396153 10.63207
1.896483 5.11177 4.479466 17.84033 22.98449 12.20109
6.895356 2.984467 7.702704 9.389411 0.32965 0.06159607

2.672065 4.427151 3.991968 14.00852 4.693606
6.225139 4.179543 2.857877 2.928994 1.430053
12.23024 12.86506 0.2675544 17.91048 7.290382
9.275657 5.125574 8.324003 2.723414 6.380032
0.8810332 2.911898 11.94236 2.834916 7.198083
6.605703 3.63104 4.029757 0.8777799 6.549413 1.66042
4.043794 18.48247 1.707153 9.012937 4.864741
9.448391 1.633211 0.3028987 8.339537 6.131379
9.044601 8.248548 1.991263
SNAI2 1.177824 2.191656 3.566461 3.265914 2.91446
4.300605 3.993737 2.884251 2.486726 2.887556
7.323061 8.442978 4.906013 9.294185 4.234811
1.820646 3.301961 5.570186 3.223035 3.399441
4.292656 3.411248 12.15745 4.044639 5.123608
9.047767 7.316459 2.874543 4.847441 4.622864
2.320928 1.906112 2.931424 5.23353 8.977214 1.367738
1.331583 2.435919 3.565563 1.870605 3.876585
1.105648 5.930773 2.308388 3.632 2.806156 0.9926862
4.920286 3.033882 0.4169721 4.358274 7.568538
1.728042 8.290167 8.081093 2.363615 8.843097
12.4461 4.175056 1.648558 26.85273 6.346918 22.28185
9.945863 1.498194 8.373767 7.479581 11.53736
3.102371 1.178579 4.246729 6.455569 2.588515
11.32095 17.80056 4.479034 3.667773 5.55559 5.946202
2.818991 3.099598 7.615184 4.862371 4.883729
10.24703 8.445216 3.052696 2.997504 4.383899
10.36246 10.01271 4.450183 6.760293 2.225016
5.093064 7.661909 12.20067 1.702834 0.6945665
3.639649 7.197974 1.540749 1.540286 4.864689
4.657738 11.19957 3.783985 3.415883 6.750377
2.767011 2.228544 2.837167 7.474733 4.091839
8.523137 1.268747 2.73545 3.671969 2.511555 1.445352
5.672711 4.000013 5.667497 16.14606 6.217798 2.254
2.108015 6.314292 6.575424 9.021638 2.677738
2.689133 3.213116 7.236437 4.241398 4.747145
7.091301 4.284385 3.061928 2.80689 3.352507 2.862524
2.243604 1.468012 2.920446 2.697946 0.9421738
8.420537 1.307337 6.921833 2.547728 15.48536
4.084564 4.739204 5.759919 3.878256 5.734315
2.193724 16.9554 3.748954 7.485064 5.008137 7.66316
2.050295 3.890255 2.855511 1.716382 9.127885
2.263025 3.817145 2.785556 4.192432 2.289141
6.983944 1.401611 7.12214 4.66224 3.419668 0.8145975
6.792552 2.650959 2.957824 7.229729 5.706085

10.85772	7.976557	12.33997	7.683797	4.132232
3.043922	7.114271	3.736956	4.684819	1.927176
1.85016	6.552307	9.184113	2.207226	2.231662	4.468501
2.943756	4.251768	3.601404	3.393861	2.842866
0.8835965	4.357444	3.769183	3.292712	3.638502
10.20382	5.142845	4.810376	3.39157	2.144553	5.146961
2.882051	2.007379	4.28873	4.60061	5.988681	13.18884
2.312205	7.082873	9.81535	2.357911	2.419096	10.74908
0.8855186	9.295659	4.474922	24.37231	0.4899371
1.260797	2.555872	2.530965	4.035186	2.175885
11.88334	2.873265	4.277205	5.741724	5.597683
9.151509	24.40089	5.178836	8.660509	1.847865
4.61007	3.227679	3.525464	5.259054	5.963941	12.45382
1.214305	2.979319	6.52169	4.791197	3.389168	6.958421
2.152424	4.226763	7.651289	4.38017	7.830225	4.890935
2.90553	3.033681	7.623131	6.484003	6.262769	4.533493
2.701314	2.397345	0.7799711	1.950907	2.562181
3.07278	4.225227	12.99639	12.81856	1.614399	9.064267
11.99196	4.691709	6.001564	1.154444	3.135712
6.321849	9.28259	1.859687	4.84303	2.291211	4.60143
103.2472	15.11902	13.98969	6.929613	4.314493
1.689749	2.741328	8.525613	4.506176	41.39614
0.934879	3.188548	2.087807	4.017124	2.134988
1.784381	2.282895	6.719494	8.538773	12.26742
2.688108	5.404834	2.558202	15.26137	6.860589
15.4039	2.586763	4.286897	1.091063	2.61516	13.51321
2.696776	9.173489	1.872224	2.592429	5.243497
0.8328653	6.870726	6.358053	2.867256	2.68069	2.009698
0.6335157	3.368905	2.43287	2.95675	16.24831	4.669798
3.532684	2.261333	7.231207	4.959151	1.848813
2.00197	2.296836	4.113722	2.05775	14.5036	2.383316
14.79257	1.417601	1.742633	6.508289	3.624109
3.605042	5.536617	3.248681	10.30181	6.578633
1.550866	3.861851	8.77458	0.40372	2.501383	1.803295
4.030789	4.909016	3.640085	7.961608	2.888871
2.139363	7.784704	1.933306	4.047367	2.974985
2.467132	7.808087	3.483453	1.199889	9.334063
2.346863	4.927369	6.703807	4.55751	2.220234	3.49925
6.902675	7.416356	0.9470712	10.11234	3.990665
4.758602	4.861417	4.989538	2.873391	5.588892
2.089656	3.95709	3.737513	0.9598945	2.649195	5.479183
3.229304
GLUD1	59.37783	31.66216	36.69303	43.96669	26.60035
32.88925	20.08096	29.12941	26.31137	32.39669

46.14187 38.95838 31.76987 35.46577 22.8176 29.67229
23.84205 33.36734 26.22786 24.8664 39.47837 31.42615
41.11582 31.45957 41.38632 35.85929 21.89955
29.89061 33.42926 28.84478 24.55956 33.61458
29.38731 30.01141 38.75426 23.98386 47.25218
27.7031 25.91319 47.99584 48.28396 42.37159 43.47898
42.41338 37.66744 123.9577 27.69509 47.9804 82.98439
41.3511 26.24649 37.76856 14.86014 34.2115 40.34564
52.84456 30.39443 33.046 24.36695 26.1499 37.87835
40.43778 32.10597 29.72172 27.81696 27.74139
28.73597 33.74088 51.92167 32.31337 102.6213
41.90794 120.7407 43.42479 29.2968 28.01521 36.57789
26.74458 14.03031 28.93752 48.6211 25.0297 44.61285
32.90538 49.21992 44.98367 37.34498 40.42228
38.21608 39.33183 38.25802 47.43679 45.28162
39.83597 37.38046 24.31152 38.85509 29.25401
39.09232 42.05744 23.7574 39.12534 22.64997 43.49656
27.12439 37.05781 32.10834 30.66748 53.01536
55.83952 36.6092 21.23281 49.87775 22.75252 45.26943
35.27364 12.62968 29.21219 34.24524 61.15223
32.07807 28.89742 25.75343 29.18832 32.94746
28.68483 93.40569 24.43864 33.15617 43.21845
37.93448 41.87116 35.9512 52.25602 20.62845 45.45547
53.10144 54.31759 51.07122 24.72454 53.47051
84.6546 32.64443 42.27817 29.27673 48.07626 28.90903
28.89819 34.05679 46.0038 44.87131 37.39542 32.48843
34.37639 33.28483 23.77555 31.3682 35.10022 68.44186
27.61256 34.13918 29.73082 32.10057 32.14642
24.69347 51.10477 27.72719 39.56712 26.85404
55.85527 35.15958 33.5228 30.21782 30.48006 16.94786
35.19856 25.92029 21.62889 44.35313 26.83985
16.3563 45.28774 41.61704 38.00287 35.46932 38.5346
36.20297 37.14187 39.90902 48.73408 25.51389
19.73913 48.00976 27.53834 44.59816 30.04677
40.39755 46.11871 39.97014 34.74867 352.6155
29.12805 31.05282 40.09052 50.43959 50.33708
23.53601 29.9871 35.45789 38.767 17.2263 26.15805
37.06912 43.14032 52.71582 41.13606 35.8282 26.38485
32.49582 37.18766 37.62563 38.83314 19.28876
53.47574 36.76107 38.09594 33.96651 35.19894
30.12246 51.26521 28.3063 25.32536 28.23349 19.70061
30.99643 28.05201 25.32287 31.57107 46.01754
45.77196 27.66878 36.46028 25.71649 48.5816 41.57432
28.04853 41.94594 33.06076 39.04444 37.86686

78.8959 37.18349 22.82052 32.90296 48.43271 44.83011
32.99993 31.30104 49.29366 82.21568 33.04535
41.43838 49.0265 33.17272 40.44163 32.50807 40.26848
27.11523 25.84853 58.61029 41.82853 30.35817
7.784904 37.83184 25.58003 28.82999 47.39257
28.05221 26.44805 57.08028 32.52997 59.31587
40.57757 38.22072 43.48032 33.32133 21.66155
25.87264 29.83732 49.89996 40.75975 27.46961
33.83689 31.60929 30.77859 26.50192 27.76231
30.10045 45.3983 37.22088 39.90337 39.38392 55.55983
24.48616 33.21832 34.83002 16.39426 28.58083
36.50432 24.4802 30.97599 56.02368 31.5802 25.78592
51.43454 27.33773 40.43841 10.45972 25.5899 47.87446
43.22355 27.53842 47.33507 23.50367 46.27769
40.78568 15.96648 24.94865 37.31675 57.11903
71.32201 40.78711 27.29544 41.30065 27.61857
45.50705 31.11672 30.44018 35.03907 53.153 4.009028
29.81423 28.24131 40.63574 35.46315 47.0809 22.07099
24.90833 44.59045 14.89972 40.18392 37.2485 29.69957
30.01279 48.69447 55.49016 43.46071 52.23307
31.50862 20.13362 38.12157 25.82262 83.27182
37.54832 37.82724 28.57352 47.80636 26.34797
24.93659 21.63779 28.52712 32.1665 66.98867 32.06824
51.87718 41.67349 18.16341 20.78178 24.42488
45.89385 39.55442 47.93042 30.77449 46.01557
42.76363 35.03502 21.3798 30.37277 40.18767 27.44317
27.24582 56.05388 30.70782 64.86121 56.71859
27.71566 31.39225 32.05634 33.07819 34.74022
22.24751 20.63803 44.04894 45.44989 45.61762
34.35257 32.79833
MYH9 93.17516 88.34766 135.4194 114.0203 80.44069
116.4788 69.28449 89.23067 121.7472 55.71766
163.9627 44.62016 66.77935 80.62006 98.23334
101.7409 131.678 155.0758 78.59174 101.576 101.7021
81.11286 112.1351 98.19041 101.6529 99.71646
101.2977 87.80146 137.7998 12.37497 157.5098
104.7147 133.3568 112.5238 158.5861 41.42934
67.36599 126.7428 190.6592 176.5992 149.1252
148.2798 130.0503 136.0472 191.9875 220.1727
157.1406 174.1286 99.04275 236.1593 136.7022
151.694 86.50969 117.5933 113.1352 79.97302 193.944
215.0347 156.0788 94.84339 176.5409 204.2854
109.0337 216.976 43.9146 142.2064 235.8005 139.3571
170.289 180.4604 217.7726 82.91498 125.946 156.0636

242.9781 132.0636 230.8203 222.3168 136.7834
96.36695 156.7614 192.6175 91.41796 174.0749
177.2926 200.7555 261.7625 189.365 86.16529 98.33954
124.9945 193.213 149.3889 154.57 124.4329 177.5021
186.4089 170.1025 177.8249 224.2917 181.2729
105.961 57.67702 99.44812 171.7619 274.2296 182.435
155.821 172.4276 80.20903 132.3273 104.5153 77.60024
85.34706 102.9377 153.7981 119.7888 250.4046 107.91
55.08402 103.169 92.62618 170.6275 133.005 102.5929
197.1404 67.53936 172.6635 152.3707 181.8146
213.2485 172.2659 105.8978 114.9326 131.6506
169.4806 224.7438 117.8815 141.6906 72.10666
156.498 159.7974 171.8722 74.91157 168.8081 86.95172
90.27078 171.3145 169.4624 168.3991 156.4516
265.5252 188.0353 278.6842 199.0349 46.15925
144.587 105.2036 170.2696 84.23357 220.7313 113.9308
93.65445 176.1596 97.53272 180.1895 162.0241
123.9974 185.3604 54.34229 104.228 114.7373 89.15597
92.68875 54.47307 116.6216 221.5614 89.37659
222.4209 218.1705 68.46035 132.65 148.5499 253.8397
157.7938 223.946 182.5689 107.8734 304.9305 140.5783
186.2615 147.6959 222.2559 139.3982 101.4224
157.1507 145.9238 142.4951 156.4185 57.95531
70.84004 178.1078 209.739 248.381 58.23055 162.3192
153.0621 96.06313 196.8192 182.1291 100.6214
205.268 176.6194 229.835 169.1264 197.2847 210.8684
130.1416 246.349 281.2386 267.2468 129.75 131.0137
241.5674 280.2446 47.90191 207.6635 128.2537
154.2128 106.4938 133.6588 174.8013 108.4868
265.1597 96.94582 183.3176 124.3459 82.91758
117.0447 110.5217 147.7322 214.8068 154.8358
151.9513 114.1997 210.6842 188.3957 179.9672
230.8398 107.1906 230.8612 96.8659 117.0833 170.7335
129.5457 175.8303 112.1054 171.6402 200.0529
204.0507 86.53837 156.19 96.34136 118.5204 242.0923
200.2924 111.9494 175.2996 193.3196 144.7001
177.9587 122.9195 94.81982 99.08704 179.2137
127.0644 211.5501 129.3379 343.4695 110.5132
216.8135 118.6292 170.6475 145.578 202.3828 176.5925
142.6258 140.6476 143.6126 246.7894 147.9855
173.354 166.7062 228.1407 175.1127 324.2446 151.8237
160.3308 138.0929 180.9947 187.2242 180.8965
172.5758 269.2483 117.6478 106.8409 67.98763
92.89313 162.7276 106.641 212.8063 161.9068 137.8929

203.8688 114.7338 217.7573 182.1112 255.6213
160.2637 136.2867 102.6226 107.6956 121.4806
138.2791 64.98666 69.69827 204.4689 79.77183
128.5065 144.9114 69.53391 229.4459 210.2239
175.1489 164.2777 147.1501 102.5828 110.0988
224.7612 72.8766 92.37203 148.3226 100.3923 167.9214
184.1127 127.3122 131.5671 141.1429 69.9898 123.7705
159.5714 236.939 119.6589 181.1009 101.408 88.27392
278.0776 182.698 113.953 90.05493 204.2303 166.3174
202.5725 70.47859 118.0006 96.56368 80.51896
133.8831 176.5854 97.8614 100.8463 151.3451 129.9568
114.4539 150.6255 113.9979 108.2515 103.9587
133.0344 159.6198 185.3323 227.5527 83.09677
141.3931 182.7171 126.5218 220.216 83.46755 156.7905
124.5303 237.3719 129.6644 60.09568 97.67708
162.732 79.87175 211.0331 217.6912 93.03242 207.4295
125.4726 122.4416 168.7259 155.9443 74.21639
136.4468 137.1144
NOTCH3 2.155939 4.615606 6.893959 8.381241 3.470945
18.60894 3.06342 6.663544 5.415213 2.687592 70.24233
30.41523 4.427953 57.57777 4.447721 7.994145
4.849715 11.01007 3.926172 4.323866 3.762669
5.434062 52.59078 13.49634 10.91675 46.50586
6.955057 9.119308 7.734739 0.3719907 6.027003
2.890501 16.68445 35.6412 43.22476 3.464917 1.718734
21.19693 34.0921 10.15441 34.95545 11.86956 15.4341
10.34913 18.30352 30.36466 5.146812 26.24875
10.02186 11.51262 18.6052 32.64752 27.24242 19.50138
20.76449 15.92281 20.58478 32.84382 17.30823
10.19834 53.83 45.36815 19.44208 46.90955 5.322929
19.7862 55.53401 31.52806 15.77187 12.10894 15.49543
20.07713 29.38246 23.0737 48.80985 15.7278 20.11187
49.54129 35.02283 5.049585 18.40712 37.20556
8.221819 29.53497 44.05658 48.89507 70.27194
23.13474 9.234788 14.87155 14.86057 25.06776 13.834
7.745905 12.80114 30.7215 32.44138 10.82829 13.70824
22.10839 29.66708 10.6474 4.600481 10.71168 40.01291
75.61247 23.42868 36.88232 18.45502 13.29485
23.26913 11.92391 5.808292 9.542681 23.10906
13.33141 19.66673 31.98452 20.69916 4.847534
12.65506 17.6917 68.34459 29.64346 40.70738 12.38649
3.45301 20.02065 27.82755 70.06933 25.19491 10.44139
19.64552 10.97274 9.22106 34.02377 26.41752 9.651659
15.56718 7.362385 25.31997 22.33318 18.45844

7.334137 46.25942 10.84767 5.424711 24.20913
46.03131 41.42612 22.01871 92.05782 16.4362 39.015
40.0175 9.144294 23.8383 13.55145 37.94706 11.90119
35.63128 13.94455 17.12714 29.85123 22.67488
21.04809 14.3291 17.28641 20.87524 7.840813 12.09707
22.56926 15.54385 21.09982 15.00846 18.14663
36.31511 13.73677 7.6356 96.20779 10.45757 18.2316
49.09022 48.2969 18.29468 47.52077 45.88266 15.65019
60.65692 10.74785 46.6536 39.2613 27.5152 7.067197
9.52074 55.17734 18.78083 38.15492 27.28711 15.45698
8.863196 37.28883 35.93281 11.99163 9.267391
12.93861 28.4979 22.88322 34.88821 51.50675 14.6943
41.39474 12.30988 24.28154 12.11856 19.6923 32.10248
46.79453 66.75604 23.60237 22.97335 17.64805
12.55021 35.98258 80.50299 7.453739 43.05808
70.42618 4.517332 13.17618 23.51209 36.9027 2.442854
14.62917 6.668383 32.34324 18.30359 5.718503
17.73068 24.34232 24.59484 48.52304 27.07899
26.25167 21.87072 40.54717 22.25362 10.38473
37.8214 13.11637 35.07803 11.53621 25.99427 52.16458
5.599098 64.63954 29.97417 27.73118 16.04491
75.78936 9.49241 12.57035 15.82329 9.483654 49.76926
39.76525 9.762202 17.97271 49.07063 17.70669
35.63277 22.92724 7.045581 9.084619 11.81289
13.99057 22.16446 16.78197 15.1837 34.45698 46.04629
11.60718 21.65883 26.31338 18.3133 28.79214 6.325722
26.76715 24.66113 62.21586 33.67337 12.5357 30.71652
51.27439 19.70356 120.0964 30.79754 30.18898
36.03844 11.73749 19.71871 64.77577 15.19182
71.88821 2.579041 22.17921 19.19053 16.19257
27.49029 30.02125 19.01911 27.24726 17.54002
40.66726 17.11133 26.62488 33.39864 50.04357
24.74147 33.59254 9.878942 12.45775 14.61787
17.43022 10.5509 18.69821 38.89473 16.95967 9.66959
65.70574 6.627956 42.003 41.21887 21.23899 23.37238
15.95075 9.616106 10.15932 31.94623 14.47604
21.12454 17.17257 13.19046 19.72907 30.88305
65.05584 13.76199 16.23214 11.34156 15.91289
23.07358 60.01069 22.66771 36.97543 5.473591
14.5114 54.48193 23.58952 12.27795 26.66947 37.61589
70.2096 42.63892 25.42642 15.1275 17.94879 14.95092
16.66111 21.45618 24.25817 15.37867 36.96517
21.22151 10.3549 21.47652 33.53956 43.43024 21.84526
13.26569 35.15897 44.90376 79.3599 15.31552 15.19785

19.54217	17.06564	55.02958	12.15082	16.56723
49.8036	38.94935	10.94884	3.13969	23.04539	26.65895
16.41081	48.71067	52.54741	3.305093	30.32781
76.88615	13.58759	34.72796	6.813995	7.24758	27.55334
19.31947
PTPN1	7.338013	12.52437	9.964285	17.29589	10.49747
12.72922	9.598954	9.720916	18.18352	20.6262	16.54476
9.726411	19.02695	14.86916	19.48326	13.12876
16.39463	14.83714	9.37566	8.769208	13.90609	11.02114
17.89775	13.64411	14.63918	13.82472	13.76661
13.12276	12.51981	3.459026	14.73217	13.86112
20.46878	11.5613	14.14612	8.819292	14.95691	16.75574
25.46096	14.09849	12.6878	14.09751	18.7741	13.52342
10.75126	20.56895	19.43628	23.51135	14.25161
28.98279	12.91179	19.73522	15.59039	16.75305
24.28953	9.944412	24.70506	22.78445	25.841	45.23685
18.78056	25.02061	12.51121	13.26514	10.5088	14.94397
16.29183	14.00804	42.12977	16.17932	19.42493
11.00175	18.09245	19.56475	21.44728	22.91381
16.43868	17.43078	31.74213	20.86398	15.30957
19.72678	21.56619	14.21094	25.74061	16.64316
16.14762	19.41081	20.31009	12.64038	15.01923
33.89032	15.35032	20.24652	14.7199	26.94498	22.53406
22.20411	18.07878	16.14764	22.29102	14.85731
14.57979	17.3756	16.40864	22.1901	14.31489	13.32837
18.83435	7.031535	31.26835	12.58651	14.70749
17.64542	13.0387	16.90427	20.94652	14.09652	29.26382
18.75396	14.1346	31.50019	34.30435	9.71193	32.42043
32.43178	15.55439	9.052428	16.26823	27.39317
13.44104	11.09672	20.2154	16.38223	16.00261	20.52964
17.00305	29.37397	14.51737	23.0592	21.78282	21.03344
20.49838	17.62863	25.40665	10.35382	10.18375
11.87411	32.25567	12.20064	11.74909	16.08575
15.33499	40.2193	15.3071	11.43592	12.05343	13.79732
26.44212	10.66959	22.27253	18.49422	20.93145
21.42632	9.323422	17.86638	43.24396	27.1105	29.21184
12.38024	17.34792	14.01884	13.13419	15.58541
14.77354	17.37531	27.73679	16.15313	16.26992
21.86675	6.834256	15.49401	20.19211	24.87396
43.31817	23.67726	16.96477	9.252271	25.75471
18.43274	25.81012	18.31076	25.81073	17.92395
14.9055	29.9663	17.53905	29.18255	23.12038	7.209156
16.70118	13.0981	18.88537	19.59991	24.04698	17.26629
19.77657	24.32455	13.83125	18.86685	19.99531

19.48123 18.51142 19.18828 21.26418 16.03006
20.04976 20.65242 23.10406 16.54979 41.81384
15.93811 11.35154 11.30395 26.41315 3.376925
35.08406 11.05183 18.24352 18.09433 15.92482
18.4835 11.06693 16.10906 15.35963 17.23674 17.04376
12.18381 15.94645 28.08655 21.93506 25.97538
13.39763 12.42985 18.76768 42.83584 19.892 17.46981
22.64976 20.52405 30.71953 16.8048 17.06049 23.35364
22.83719 24.65699 19.86611 18.38795 14.3357 15.36047
15.53137 14.44731 23.69308 11.04548 22.47405
27.66343 22.26738 13.56052 25.03513 27.71658
16.0346 15.34116 12.06321 18.26985 19.37518 21.45983
25.56633 14.25598 90.15667 19.23971 21.00058
19.56038 11.62328 19.4049 12.44723 22.30147 25.06522
16.77702 18.32443 20.44434 38.79019 14.18505
14.57903 22.94814 41.44095 16.06415 19.12303
24.49005 9.667089 16.16653 14.69542 16.76751
21.18225 30.78668 10.86822 20.8438 13.03525 34.9551
25.81423 17.61111 46.02644 20.62368 19.26729
17.9939 14.907 16.65965 28.22375 22.9707 15.69513
17.54454 32.56331 20.55157 24.75992 15.49539
11.12494 15.73234 20.77796 33.9883 16.77451 17.17549
16.05681 23.5252 23.22972 23.02701 15.47849 15.55157
17.0057 17.29067 21.33814 14.01787 26.3346 18.13424
9.209452 18.42014 17.29186 12.68272 27.43944
18.93925 16.20359 16.84071 15.7337 29.96245 18.24458
15.57185 23.047 23.75062 24.98138 14.78198 15.50159
13.66633 26.69581 22.9593 36.82993 30.64223 19.2379
8.850872 11.58795 18.09709 15.7843 12.09021 11.28069
13.92898 43.27797 34.95355 20.5645 20.0595 11.9089
14.96351 10.44271 10.22659 25.93985 24.94673
16.84544 22.32952 18.80835 15.84388 16.0893 30.07589
13.27107 23.26927 24.90996 13.80717 11.09284
22.25419 20.24419 28.48021 18.98859 17.74961
12.0519 22.07709 19.16117 25.26356 12.44518 10.78652
11.39915 15.61122 17.05761
TPM1 18.3957 325.4088 15.3153 14.81185 26.02222 238.2906
7.802376 60.77869 66.41282 68.92193 44.59913
11.36988 15.64156 18.71978 8.665103 240.6478
14.22605 134.1293 10.91673 15.7709 66.26977 310.524
11.41071 227.7824 311.8714 10.32463 9.545458
258.0105 20.2365 16.35145 63.28806 225.8313 11.47235
11.6447 30.23928 11.10576 3.104155 10.93768 23.37199
13.29297 12.39566 14.27266 21.43347 18.096 17.20623

15.62079 19.8081 25.40206 33.93415 9.726688 24.04579
12.11089 5.367696 21.17218 84.03848 14.10427
14.06414 124.2453 8.971477 11.78125 19.72185
14.84902 48.92912 11.34087 10.62572 152.7377
39.02737 23.91602 12.4903 9.131121 15.94271 16.30766
51.03457 28.51603 36.34396 24.7421 22.9928 13.82771
14.58846 7.688012 22.71371 19.60143 12.51389
74.08507 29.75923 13.69553 46.09047 22.44194
9.275367 91.59255 22.00738 5.256876 31.76791
12.14861 12.03878 12.36876 19.58554 16.64828 5.9683
35.18205 14.21203 16.26977 11.06204 9.393078
28.28003 27.35387 16.43053 121.6502 22.42686
11.20094 10.18322 9.747493 15.12417 90.18536
13.37438 14.78993 9.015486 11.55905 22.89634
15.81883 4.525422 14.3064 17.52329 23.95237 19.37314
9.572992 18.58836 13.48824 116.5326 32.85971
36.52206 12.28768 11.41026 19.54769 9.456266
45.25565 15.88573 13.96888 14.87572 27.3774 10.67585
9.581507 34.45312 9.07009 11.17382 16.56135 22.82767
80.55613 6.126302 22.88069 6.94892 33.57617 20.22946
19.09836 139.7341 15.44387 23.08508 124.7384
7.861024 10.49451 36.66071 17.44037 19.05481
7.323664 13.75327 15.5586 19.37476 29.69677 15.75681
33.38612 250.2634 9.799535 26.54841 7.61696 9.663172
13.49702 26.0883 43.97816 17.99348 11.41656 12.49157
15.91495 7.747092 9.640296 27.77899 11.20584
70.49626 14.07744 10.13359 10.16885 23.09581
13.18984 10.86263 11.19272 9.757467 29.64704
40.42962 7.1353 12.57387 12.7021 7.670855 34.27009
14.81115 7.916376 5.514537 10.07792 32.20455
12.45003 7.818545 20.24898 14.70375 44.55479
16.97035 27.6173 19.51882 27.53533 13.04884 5.476571
31.46967 14.43072 12.1544 32.88413 8.597884 15.68899
14.84936 10.82306 10.03671 2.700302 15.34769
17.16931 26.35177 27.57776 9.989131 16.96374
17.47735 28.24398 10.59799 8.673631 25.95126
18.17245 77.29321 23.07887 30.26676 17.49881
30.21356 21.67435 14.74021 142.2103 28.49766
8.21159 27.36671 10.50347 15.64684 20.47311 5.943592
5.135586 24.07649 26.18731 13.9996 34.09178 15.57555
16.2669 13.37112 35.63337 10.64976 13.74514 17.5558
13.13592 17.46603 12.93425 31.8005 12.80178 18.91085
13.78978 14.37618 17.93073 20.39335 221.1503
4.16725 19.00838 36.75938 9.461803 109.4144 9.419601

16.85027	22.15027	8.380597	12.51802	26.71205
25.38179	13.47626	329.7123	148.0685	13.75054
14.91334	26.51697	27.17046	16.30213	17.49692
22.64864	21.31118	20.33386	21.98669	22.81712
11.98116	15.79487	10.601	14.44608	21.21488	9.182525
14.95332	10.69926	8.116232	13.43225	12.69258
132.6531	18.64968	18.42881	17.03903	38.55882
27.77461	37.37302	13.89412	9.998706	52.2534	7.063939
26.10572	12.84225	18.82904	16.28266	16.22765
27.8584	15.31508	17.19032	146.9158	20.93282	5.368207
32.23989	14.30783	13.20162	11.81906	7.770269
13.26852	11.29511	169.8871	12.14276	15.11008
6.423295	13.13806	21.48726	11.77902	17.35816
13.72776	19.16111	8.463999	21.14397	21.52378
27.31632	155.9434	13.25094	11.77588	14.23128
19.68937	6.928307	6.53466	19.11664	20.29024	14.13057
19.03626	6.790976	11.87156	12.7434	7.31625	17.48653
15.61541	14.90011	16.39642	8.941641	11.4578	28.80307
15.07818	12.5068	21.68059	13.85741	12.10752	16.81001
56.50044	16.6132	11.66803	7.490902	94.27639	28.00056
6.177405	22.65786	10.92172	20.37199	10.27578
74.94475	22.27918	15.55315	10.53638	21.1814	15.62583
14.86052	14.6551	11.43305	136.0101
FASN	9.014248	10.40673	18.05678	36.51039	15.92836
13.93524	31.04508	16.52611	22.62952	11.82501
74.7986	23.22201	12.82338	51.92532	25.31875	11.65657
24.7948	21.73676	28.64556	15.80361	20.96293	7.39014
41.53834	10.48072	18.42414	49.27215	15.33933
11.4075	17.51649	1.777028	32.54293	9.672758	31.59529
36.00427	29.07654	19.0298	8.57499	26.24137	48.73361
58.18208	58.73348	70.42803	38.75523	58.20342
32.67059	77.70573	37.10354	56.43351	26.66077
74.69924	53.09804	13.95477	107.4896	37.39686
11.07347	52.14833	57.35776	16.08782	62.54558
48.87791	13.14949	77.68948	9.491886	57.18911
40.89314	28.22663	39.05617	10.37474	34.78342
29.83021	83.83992	15.07684	51.04794	25.1661	38.66857
38.21486	22.93715	44.85297	45.26431	46.48159	49.55
19.97073	15.60911	18.6728	58.83977	27.49038	56.71611
51.56323	29.78304	8.537772	29.38275	67.47563
15.00693	18.06819	46.15496	28.45517	24.38906
64.92613	136.6805	63.57323	15.29932	56.04106
17.65677	23.33351	21.54973	31.67852	44.1469	34.64308
73.35293	34.51229	55.0703	36.08168	27.94735	9.362984

23.39205 160.3682 47.43003 58.37404 34.99937
37.73894 28.6785 16.52319 86.83472 12.3165 163.686
19.10497 34.61648 34.17614 15.62773 26.51813
50.69581 78.2234 29.35841 27.93913 23.92098 31.50666
71.81119 81.98758 69.8354 35.24586 40.37961 49.61526
54.52479 50.92808 84.64749 13.46234 24.97024
13.97338 164.4633 10.67359 62.97362 31.0711 61.9758
33.92273 19.86164 20.91719 75.37108 8.096664
36.99178 16.92287 33.99052 18.33777 12.70644
51.28218 27.54215 61.62103 37.1016 9.964419 51.35094
32.51601 42.04125 59.66059 19.53026 42.28124
58.28358 28.00627 28.36564 50.03522 50.97733
127.6408 28.51992 52.57511 45.11658 38.23113
34.5489 40.91247 23.00962 35.24282 41.06993 50.74339
30.28641 63.61567 55.46316 37.33034 15.09142
24.37991 33.92757 123.4955 106.8001 39.41563
38.08227 14.50232 49.64149 69.35363 69.22322
86.7899 32.40037 46.91446 80.84763 42.79981 17.9626
29.22625 23.39367 69.74401 58.12466 104.3133
33.89791 69.97053 26.85948 97.43262 61.7186 27.75871
51.29653 63.66658 17.04849 40.50182 79.85845
57.79871 74.36826 56.88706 41.99047 15.61154
54.32309 63.09276 13.78836 8.34175 21.95348 25.09897
21.88456 54.1779 26.94225 26.08817 47.73258 55.88038
14.34064 27.54683 21.14576 17.36344 35.96496
20.92079 27.26666 15.168 40.77697 35.46166 75.74561
187.5853 37.19285 21.27411 38.96441 77.70189
22.81538 52.11582 13.21518 66.29974 55.99042
58.54028 55.30565 34.13901 15.27086 35.17137
25.76771 51.32702 26.20577 34.07985 60.12632
33.10529 70.64015 12.13246 23.76143 22.687 25.97595
32.96053 27.08716 72.18104 39.51183 33.13267
66.86848 38.68542 49.90555 50.59418 106.795 8.755128
19.77849 31.51301 39.98626 70.93637 20.1383 22.12719
20.86671 29.44959 25.90456 18.8602 50.94654 26.0151
36.12721 79.58082 44.07943 22.18034 23.12744
162.1445 83.69558 49.46775 149.8441 25.37506
46.30411 29.66719 49.08468 42.07403 44.10373
12.17389 30.31254 20.22932 38.87242 40.83022
5.542569 27.09582 134.7432 78.86767 21.29733
120.9647 12.7528 24.504 33.32633 19.87645 30.43111
28.43086 35.51721 30.06189 30.67111 43.8077 111.4576
100.0384 15.68605 87.46685 16.0632 38.40478 36.13183
67.49677 32.02187 31.18653 57.3162 62.64823 40.26427

17.31688 45.67174 53.74088 41.36535 58.56789 8.831
62.61574 36.61364 34.15217 34.68163 86.86176
28.77743 41.00274 58.71652 40.55873 31.74895
21.25484 48.8265 18.15528 25.50754 22.63043 60.61543
64.38276 63.29031 17.96491 22.93514 26.46668
48.80483 68.94387 17.40145 91.8715 57.17084 37.51945
30.72319 22.14621 130.4881 337.0082 34.21696
51.44653 15.8152 21.70515 33.66447 12.18335 103.8476
33.3327 23.62137 59.36272 54.60388 9.015813 81.08552
42.26473 53.94289 33.99813 20.70174
RPS6KB1 1.699132 3.1718 2.362645 4.354783 2.826157
3.828181 2.388721 2.92433 2.561891 3.554359 5.473835
4.322188 3.72424 2.980626 2.50817 4.897978 2.201761
3.657257 1.507552 1.590459 4.116754 4.237066
4.491563 4.375472 3.312585 5.22931 2.471173 4.420685
2.929203 1.972776 2.9109 4.069431 5.015264 4.452609
4.497605 3.637727 3.321702 1.960623 4.767593
6.68722 5.732741 5.721499 4.548151 5.34858 3.793208
3.47127 4.719496 3.378411 9.045019 6.076001 3.072
5.173065 4.37161 5.09263 4.963814 5.687743 5.747144
4.894122 3.30893 2.91077 4.757229 5.335494 4.335131
2.132048 4.788334 4.013776 3.133352 2.926468
2.907274 4.337175 8.760362 4.547929 5.521169
8.009859 4.553405 3.630095 3.618682 4.938547
8.188096 4.269464 4.387199 4.643169 6.411585
3.702256 5.038784 4.706737 5.32269 5.299416 3.263276
3.835187 6.25083 5.520775 4.599871 5.87775 4.516276
3.367417 4.82377 8.325017 5.700426 4.419639 5.799955
4.567337 3.179022 3.668199 4.035042 5.105473
4.520593 2.588077 4.552759 4.858017 5.183875
3.863751 5.094711 4.290077 5.120683 9.020005
2.453095 9.039566 4.511253 3.104362 4.512258
4.422487 5.131044 2.803634 6.100738 5.936037
4.999448 2.737125 5.138256 3.64558 4.020911 6.061554
4.526995 5.667707 2.099945 4.138648 6.060194
4.226723 4.420092 4.848325 5.595497 5.373334
6.661942 3.755054 3.740848 3.576651 4.139044
4.189968 5.180249 4.189085 5.135363 3.286923
4.502584 4.759803 5.133896 3.815966 4.485469
4.675049 16.4968 3.528503 3.783186 5.594067 4.998259
3.811866 2.30976 6.351004 3.657185 5.924703 3.78892
2.83643 3.945878 5.052322 4.94651 3.583056 5.725151
3.977065 3.848661 5.173876 4.141941 21.17512
4.398704 2.428065 5.219584 4.329925 4.106434

3.456193	5.104772	4.904767	4.01371	4.586797	3.732834
5.154492	4.863822	3.210177	2.08649	4.584996	5.946731
5.350876	4.982246	2.052717	4.578874	2.971979
3.66506	6.319745	5.410558	6.006723	2.15899	6.267358
4.333702	4.765756	4.75285	5.092447	6.491053	2.563997
7.256998	5.04191	2.866091	5.795997	4.930328	3.693625
4.698548	8.970325	2.331025	8.44041	5.270608	4.21367
5.097265	3.463913	2.575756	6.984827	2.185647
3.925338	2.300004	3.841282	4.174249	4.000169
2.254239	5.072982	11.99605	4.048718	3.455367
5.662781	3.691954	4.9698	5.646746	3.617552	4.742347
3.923193	5.526591	5.639946	4.41741	3.401856	7.47616
6.075031	4.917168	6.39902	4.059784	3.322707	4.388265
4.270109	2.824321	4.727916	10.59423	4.537447
4.399184	4.269585	4.196542	3.071887	5.180509
12.78819	5.553464	3.338903	5.386616	5.785052
4.842732	2.676945	3.706235	4.207014	5.386084
4.12635	4.744081	3.742208	2.495715	4.819226	7.926568
6.778348	4.263062	3.675874	5.485584	4.887017
9.926685	4.638329	4.060846	4.004579	4.503588
2.838429	4.510006	3.544773	8.607241	4.630776
4.144068	5.030637	4.435445	5.991151	3.384623
3.683555	4.737455	5.464729	4.184427	4.723018
3.78487	4.524516	7.644079	2.173273	12.29852	4.936334
6.270038	5.070868	4.308632	7.375789	4.098924
5.309391	1.837194	3.503323	5.187477	3.215882
5.148059	4.622548	3.346091	7.199752	2.414984
4.024674	5.365602	4.12962	2.769887	4.163604	3.727853
3.373453	6.190032	10.86745	4.249697	3.464471
4.75346	10.89296	6.058251	5.478415	2.275223	6.961345
4.477146	4.378441	5.900485	5.296228	4.772153
3.423035	4.088119	3.830361	4.498815	4.970794
4.246457	5.281833	4.424398	3.039386	3.970272
8.198935	3.51232	3.368547	4.572908	3.809917	3.862626
3.121261	3.267856	7.087348	3.62167	7.546168	3.421914
3.546359	2.639024	3.313569	3.729838	3.793286
3.088878	6.07843	4.071557	4.010642	8.281407	2.535273
2.661273	5.819984	6.274573	3.029411	4.778258
6.717695	6.486345	5.326202	6.875184	6.467379
5.385291	10.48736	4.184846	4.055689	5.036443
3.880929	4.393315	5.686226	5.3941	2.242589	4.800836
SIRT1	1.835631	6.038165	3.045144	4.293801	3.429457
7.342257	2.515547	5.696982	5.204513	5.075657
7.020625	3.330391	5.117223	3.343372	3.164363

8.705883 2.20335 7.501812 3.113989 2.93187 4.834639
9.038922 2.67237 8.681473 6.323319 4.632159 3.122926
8.161071 3.127758 2.796129 4.03263 8.235719 5.956635
3.569368 5.626786 5.052693 6.401221 3.440672
3.696751 7.38796 10.10069 4.654736 5.117028 5.582226
4.109177 4.489051 6.548899 5.160813 7.521856
3.40395 3.501001 7.418475 6.78807 5.390561 7.593776
7.923461 4.635732 7.029522 2.692089 6.864088
5.762826 3.839927 5.220733 5.144036 4.243828
7.521576 3.066589 5.194706 6.273785 5.894117
8.223253 4.803446 23.42318 4.866971 4.873987
5.629219 3.970726 26.06424 7.659362 3.411842
3.796752 5.398164 7.823902 5.288312 3.049937
3.764838 6.063706 5.519012 4.243312 7.075677
5.46981 15.06405 5.890565 5.947629 4.016274 4.213694
5.322483 2.551462 11.35377 6.785591 6.387165
5.162587 4.619345 7.953549 11.61172 9.533827
3.518474 4.597988 5.52752 5.268933 4.935749 4.69598
4.549194 7.390573 6.037508 5.614148 2.803987
5.511041 7.275543 5.206776 2.956782 6.590712
7.034004 3.064186 7.86196 7.521376 5.615043 2.703497
7.14126 5.398307 4.359155 6.71296 4.391676 7.327878
2.904664 7.531679 5.423246 5.29344 5.920008 4.603548
8.222533 6.891027 5.801358 6.604758 4.980747
6.023176 4.309911 5.524192 4.237066 7.415605
5.865301 4.049496 5.409572 7.398794 7.106151
5.292703 5.562428 9.298928 7.775489 5.030686
7.068569 6.979926 6.764411 10.25462 3.20919 6.568696
4.242102 7.617359 5.012876 11.64921 6.870461
17.05284 5.524618 3.687115 2.53939 4.883902 4.434677
7.856444 7.711643 5.330613 9.26633 5.510174 5.773849
7.066728 4.720397 7.715342 6.791946 5.243708
7.274442 8.572719 5.494677 3.663528 4.507296
2.869739 5.182072 9.409528 5.314917 6.665962
6.125325 3.821378 6.265368 4.758318 5.17029 10.31752
5.774456 6.519669 2.661941 6.596527 5.03451 3.493113
6.247583 6.127966 7.30433 6.0948 12.01868 6.477217
2.565725 5.834486 7.274209 3.888923 5.2074 8.154666
3.159881 5.458825 9.256014 5.205326 4.459764
2.29721 4.362341 5.988293 3.183893 4.276595 4.076428
2.841119 5.673411 4.298844 4.575544 6.113129
6.214582 11.41832 5.652283 6.79139 3.657778 5.759767
7.866023 6.153255 5.623618 7.818348 6.679046
7.784946 14.35163 7.104152 3.109395 5.168223

10.24389 9.369146 6.836166 3.219801 7.684875
6.728633 7.054846 3.590492 6.935186 6.704446
6.266248 6.716321 5.354922 3.452161 8.281099
12.60566 5.247464 2.863218 3.858587 4.919259
4.642085 4.709573 5.766224 6.142673 4.754124
4.465044 7.275033 8.927042 3.933453 4.786364
7.476933 8.873259 6.901148 4.851161 5.733433
6.716894 5.224058 5.783242 9.264637 5.302951
3.859716 4.696931 4.759186 4.745412 5.923495
5.928291 5.304212 8.345725 5.926486 6.035245
3.265259 2.679839 10.13026 4.342918 7.73762 3.345079
3.157901 2.86334 8.629874 3.836743 5.321713 6.792629
6.696888 7.894605 2.619162 10.85046 8.81161 4.718386
8.60396 5.594505 7.872394 2.532585 4.99791 4.270638
4.264945 5.179773 6.536273 5.283649 5.969423
4.982794 4.897377 7.518196 4.126469 3.827464
7.769739 12.27583 5.377973 8.120026 9.163714
8.126256 8.350631 5.532815 2.49179 7.213901 6.321639
8.776105 6.15571 4.833062 5.556086 7.097893 11.06856
5.269999 6.229933 8.534083 5.698879 6.043951
7.960173 4.555087 5.571372 4.684514 5.193002
5.214831 5.322687 6.000643 8.019383 6.61655 5.918556
4.043552 6.15979 6.619736 6.761761 6.561322 4.508074
3.884521 3.388069 15.59697 6.937146 8.497399
5.209294 4.801105 8.722756 4.097896 4.766471
3.937832 4.151244 5.19627 6.711666 7.383139 5.531052
4.923641 5.303297 9.69678 6.99003 5.303453 4.248511
3.783046 4.713149 6.617663 5.399854 5.574054 4.4118
3.280562 7.835314
PPP2R1A 37.4341 45.95395 34.72083 37.49857 35.58178
30.89918 23.79073 22.68731 37.43708 31.56909
34.61613 35.49038 27.70324 44.19569 31.87418
28.72874 42.67078 41.06737 35.61496 37.79355
41.32784 28.65643 37.87108 35.7504 45.71542 34.24559
32.92376 29.6355 39.34083 15.44391 37.971 30.29115
47.83894 35.21857 29.16829 27.69476 25.87524
85.44939 48.05455 49.45428 25.53182 57.566 28.35874
44.66029 24.14592 43.31713 39.29112 31.4803 26.33654
40.82396 48.5852 19.75666 42.98295 32.29085 39.1295
38.47728 33.60551 27.57821 38.81081 29.00562
25.97859 36.1609 20.73881 55.87935 79.37963 26.48603
50.08094 36.31787 46.09963 106.2171 58.20497
37.20437 27.78594 33.42796 34.17968 43.62638
26.64186 43.15818 22.6665 29.73885 46.6885 26.64449

28.94001	34.09087	32.91577	43.78839	35.2811	35.72122
25.79923	31.99272	25.82688	26.49662	36.50067	
33.63862	45.88718	37.52838	42.07075	52.09617	
38.43604	31.06065	25.95355	39.7195	23.28479	18.64354
21.12554	32.14594	39.93655	43.29733	53.3428	32.60214
29.74835	19.35328	38.09603	26.63375	33.8602	31.31057
53.54616	28.85678	38.62228	54.34996	19.44016	
62.69309	27.14052	30.82126	74.24641	34.21562	49.706
45.52394	30.07264	40.93591	50.3161	35.61467	42.8997
21.96837	36.96899	34.42137	46.47299	46.33838	
41.52658	25.58969	24.18037	34.68964	27.31255	
46.76051	53.09613	29.19274	75.85846	29.11819	
27.34525	31.79588	24.44565	51.78111	25.5657	36.93544
27.45605	18.71522	36.87879	30.11048	34.99726	
39.68689	46.42376	21.38671	19.25667	35.97898	
39.46918	40.15463	30.28773	21.61977	24.93441	
44.71263	29.62693	40.70001	33.70732	40.60534	
27.63849	40.70516	50.98501	51.93358	29.2785	31.35877
34.28789	43.93332	44.9412	33.75993	34.23626	28.56935
30.52291	44.03296	23.71568	33.71555	29.80101	
31.39016	56.0886	38.71503	35.80945	30.95817	28.53536
42.86234	39.56552	29.4326	41.0351	34.72077	39.25009
34.27485	44.36618	29.55884	37.22336	17.54854	
38.84785	33.13244	22.78896	32.3729	39.06599	45.20832
39.52386	37.23525	56.86331	38.91024	48.04218	
65.75305	36.58412	27.32545	31.18665	41.69926	
36.68822	26.74616	73.52257	41.08081	75.56094	
55.7559	50.95031	22.39234	76.12522	20.11715	31.73538
31.68795	48.44352	27.28946	22.20889	60.12935	
35.24847	46.54714	28.28238	32.46246	23.69474	
35.03702	32.12331	38.40981	34.13191	27.4622	38.09896
18.15424	22.54575	26.693	55.77172	70.62867	33.80767
52.09721	36.30275	42.46348	40.59225	56.00175	
15.61343	30.35771	29.75423	48.49964	35.38435	
49.95017	41.29135	26.42806	40.12939	51.62909	
37.19338	36.29671	31.06797	37.22977	40.08087	
30.35461	48.18154	48.06171	33.89623	27.12557	
36.30729	31.99543	26.17834	25.85362	26.68463	
36.45099	27.91008	33.81095	40.49137	22.29286	
31.05141	36.25043	28.25917	36.24921	33.72693	
48.83048	27.41502	42.87793	32.16287	32.36642	
35.99502	29.88577	42.72268	39.2237	31.91106	26.29703
32.0969	44.27483	68.61329	55.9596	31.14027	45.43422
34.26315	35.18045	33.89912	40.94904	23.64685	

23.87847 52.85481 32.98786 39.64383 31.83172
16.91526 49.36189 49.05385 32.38637 26.70816
40.41071 44.75863 39.23868 37.08313 36.9215 41.47203
66.78658 22.58203 34.15532 38.8577 35.954 36.02331
44.74713 28.39956 27.68525 27.43018 30.58209
34.71689 51.78729 38.50587 28.48891 48.42115
37.60851 27.41484 23.6983 28.20191 28.16545 33.48615
50.85527 30.23575 37.21209 43.53719 52.84443
32.5756 44.95443 44.48687 25.96619 31.02104 22.47547
23.59872 20.91079 26.29354 35.40624 36.33249
14.57389 42.52583 33.51207 21.95292 16.52324
27.64507 47.99736 55.13331 37.78667 30.98295
55.12571 30.67271 65.16551 43.93942 25.17202
59.17968 80.45919 32.1108 20.88048 35.37497 26.37122
36.71301 29.40912 66.44611 37.52447 23.84313
50.84464 51.89287 37.8766 37.50379 31.10351 38.491
69.10249 31.4248
CD151 61.14711 127.7045 47.0047 72.21602 56.42978
63.98815 57.52685 34.28081 98.42174 77.79396
16.09711 14.14854 48.95109 21.59168 67.2804 65.64975
78.86998 92.8624 82.76147 72.32738 112.572 53.7619
37.15417 68.78222 127.3225 13.33374 88.91809
58.1319 82.30954 73.72743 127.4457 81.45946 79.90692
56.34383 114.8524 59.46773 35.79819 69.23326
60.86668 35.48082 77.84679 46.58596 76.17739
48.32686 62.77564 92.80811 43.80867 81.47978
57.59215 54.92908 92.26104 34.93001 29.6532 45.69904
97.93698 36.51855 37.4364 52.98595 77.65289 91.66971
66.56114 86.02621 56.48327 81.51718 67.98684
94.21147 118.2489 80.46324 107.9662 67.54139
51.27248 62.61638 62.27362 80.77375 91.38041
74.86972 63.40706 84.47828 41.96291 55.82637
95.22861 57.87096 26.65116 69.52549 88.25349
102.8066 57.98541 63.4985 69.86705 143.0638 50.21808
42.23301 64.64926 50.18213 58.50445 82.60245
61.34903 101.314 33.59543 59.76189 24.96653 102.2024
59.86154 61.07538 56.88614 52.89087 80.11524
145.0229 92.2566 67.39829 80.46011 77.99787 42.48599
51.05897 52.35606 76.87547 151.1873 53.24569
45.78789 75.93966 66.79843 53.41571 103.1365
76.34397 58.36954 28.67809 60.26457 79.36119
79.52161 108.9761 94.27114 48.32843 74.12485
46.63282 95.47533 51.74485 55.85955 89.81105
72.54055 29.61663 35.87367 31.64115 70.27006

29.74296 58.86995 92.653 55.53118 71.94418 29.24641
43.18125 57.00872 77.99869 78.82417 59.46492
73.75985 39.23647 62.34454 67.06905 59.39777
70.67874 81.37741 32.4125 44.30099 45.78919 146.7816
65.98367 109.2603 55.3909 54.38468 88.78297 78.29989
39.47838 34.46579 41.81096 40.72153 89.57264
59.76183 64.69272 70.53694 38.43094 38.38231
106.0112 26.64504 43.32573 73.90648 63.52204
52.13351 95.26714 46.84441 35.64796 86.92562
55.68092 183.4114 77.45716 96.08608 71.83198
43.72642 40.78659 28.8453 59.00076 26.96724 109.6326
52.105 65.00288 42.21086 59.89872 169.7125 29.16764
38.95402 93.68911 49.13443 45.35764 76.61246
54.55788 49.4826 49.75471 131.4125 31.6296 100.6735
56.57442 78.53694 61.75607 106.234 78.1289 50.40539
44.57285 49.73042 22.39827 73.88345 122.9495
108.2791 49.62466 60.50164 77.70656 31.02314
102.717 65.36128 52.31894 46.65775 40.46866 103.011
77.70551 40.2216 59.70598 41.19576 71.93997 54.12972
100.4599 27.88094 70.32214 57.08301 54.07695
86.39303 63.59311 33.21469 36.80113 75.83436
89.20851 65.30259 61.59557 58.74435 73.8107 40.0831
61.90079 43.94208 54.36068 102.4439 61.35614 139.38
37.69569 56.82547 105.048 76.19816 45.96222 93.67012
56.38217 97.20479 102.8478 13.84419 47.10503
53.63572 76.40558 164.633 49.20824 44.29965 75.85321
61.95623 80.90074 43.77257 35.09619 70.29466
65.32449 78.32914 44.16301 93.77855 103.6273
48.20205 53.83706 78.9707 46.07179 60.73114 55.66743
87.90781 52.55284 58.26255 98.64098 29.57936
35.25543 61.28612 83.67351 111.1003 94.33825
62.13817 76.99309 43.01274 64.70247 87.53619
55.1096 43.50934 48.10111 70.65367 35.03398 40.54355
59.91691 32.04349 51.02722 112.6181 66.47557
38.70219 111.6805 46.93487 41.16191 62.11692
68.30453 175.4753 73.13232 72.20454 137.3671
69.70411 60.88569 49.27704 96.42658 55.94336
67.30025 72.20218 36.12648 140.4058 22.18417
69.89576 39.76801 55.42763 29.56926 58.91807
76.05806 40.30164 70.78191 54.01492 65.50203
103.9075 63.05988 136.8586 74.37291 56.7471 48.23375
55.78139 55.70875 50.71438 53.04576 37.09668
42.56745 46.7679 52.13141 43.09075 34.99785 126.997
67.08421 48.28356 37.70925 58.70018 56.00177

57.51662 46.22238 47.47939 82.5145 38.67628 128.7931
108.6765 164.7348 94.75304 54.01565 78.74839
46.94178 36.91236 63.84677 50.39146 39.04561
28.33616 75.32859 68.41764 91.1498 51.38282 51.16501
62.43692 52.57606 53.38444 69.89665 86.36202
CTNND1 46.00537 15.36482 57.94791 69.86758 40.07241
17.76194 34.51124 26.21805 48.22393 19.37123
105.6786 76.21408 21.38943 83.77952 34.48973
20.74105 47.97147 32.91744 35.00256 37.51153
69.39257 14.34805 67.31003 18.09047 21.27127
92.05398 40.6003 18.86504 55.82419 15.24243 62.5733
20.70471 90.80677 64.85836 73.88148 23.83339
13.93341 23.07057 49.16124 72.29976 61.19326
77.32675 50.69652 47.44213 71.4194 65.8136 61.53697
40.18717 62.54 104.2234 31.18503 21.3022 26.78809
47.51194 27.9745 40.21317 32.38058 28.67258 40.2554
38.95571 33.39469 43.34161 34.89614 29.94872
43.72164 25.22468 38.29006 18.81995 106.1874
37.15523 50.19168 60.95607 56.88856 48.85133
40.9921 43.03786 29.47035 66.4836 60.58395 41.50094
30.87862 50.04147 60.54909 41.77845 36.66565
64.04506 60.64574 47.89105 29.00187 44.90649
55.24801 43.30116 45.03711 53.7148 16.66604 32.14337
48.60384 67.17358 60.29902 50.53285 27.30079
60.0052 46.28515 29.58289 30.43441 52.08001 47.31346
33.04325 51.23215 32.2891 38.09467 43.35678 19.6169
18.73811 60.65257 55.08401 25.11 62.05063 29.08564
50.84687 39.23259 61.10514 47.82277 26.08146
49.86369 24.52283 21.31027 33.71636 42.96254
43.81811 44.79353 25.25955 43.14546 47.78072
45.45943 52.29589 35.75359 58.40014 40.24747
49.64529 44.29527 51.32555 36.02174 25.99403
45.27748 22.53054 43.40385 32.86504 45.96583
35.48546 43.21952 52.3619 37.8174 52.54494 38.6526
40.83701 39.18365 24.77523 126.2512 27.2622 41.71223
39.12076 50.0864 42.05968 21.62468 53.90052 39.70108
88.50567 48.4678 23.52399 23.4374 36.53421 24.48072
42.12817 35.52391 44.71253 35.19106 38.75185
70.34751 51.70721 32.73166 59.53574 91.592 53.55259
27.06117 49.79712 48.3598 48.85315 72.92911 55.45438
53.09793 44.94237 58.92891 59.20805 34.23976
83.49343 38.10522 72.81856 48.40996 27.08983
24.34415 27.31009 36.63631 58.77661 21.7037 84.69699
27.29927 18.19465 40.95928 45.80092 50.03638

31.0677 57.37907 27.69766 52.45265 46.93286 60.40748
58.98415 42.03817 53.07103 77.69167 68.24886
30.27183 42.8231 43.68787 27.14315 51.93721 83.90045
48.08049 47.73632 29.86729 34.07837 36.11033
92.9825 63.36591 57.4073 20.66969 46.45523 56.23976
45.14632 48.62058 69.72338 25.13436 36.96032
47.36888 44.90896 39.95619 17.88913 44.02179
32.08225 57.03303 78.38356 62.44908 54.49065
47.47903 62.41846 58.34457 34.29532 39.23783
50.90106 31.90588 44.20222 51.84179 41.54689
71.9248 63.25856 50.99807 41.5101 39.57983 93.77882
39.48087 42.58432 66.5514 48.36061 45.37642 52.02197
39.75686 23.36265 4.117691 79.10582 39.53754
59.31672 31.5768 29.75495 70.54182 63.90231 49.29034
44.64724 37.70931 47.16275 81.57677 25.4381 31.73276
46.79686 23.98202 30.34182 37.29025 52.30596
29.91013 75.43396 54.24927 53.05621 77.21377
38.04652 44.92095 40.47286 42.57749 50.00703
61.51915 40.11308 42.49013 34.89128 52.99524
22.72137 68.54513 36.3198 53.09969 54.62055 54.98725
65.64187 82.3923 58.84373 49.26043 42.45912 37.89837
43.32004 63.21426 50.20853 46.67211 51.52642
40.6188 46.62351 61.94596 69.63762 27.53039 55.96234
35.26972 56.87848 86.86717 58.06439 48.24409
36.67754 34.57596 55.30375 37.44724 106.7506
41.02346 30.73963 25.50688 39.18888 73.78925
48.13565 41.88355 24.87993 45.9778 33.14512 46.57466
53.06017 24.91482 30.09401 57.72747 24.53034
108.9942 57.6574 33.17836 22.34388 49.24977 44.45046
53.58198 27.61629 41.26175 55.14954 41.19071
56.07638 76.02235 32.78172 54.83581 23.30622
43.36126 42.39832 44.55205 45.40028 19.53921
56.54382 79.953 50.87894 26.35321 38.826 48.12374
50.18497 62.18349 47.76013 23.13477 29.21795
60.99323 54.51017 38.84886 36.2855 54.38424 69.7232
36.41064 25.87584 31.84721 45.51584 43.48782
37.11426 26.14235
MMP11 0.3957757 0.9743115 0.9553078 1.249504 0.4381279
1.213816 0.6678755 0.8982019 1.269686 1.157015
0.2023898 0.1874961 1.222359 0.1000064 1.079959
1.364154 0.688692 1.069388 0.2807292 0.7134044
1.157834 1.112591 0.3392398 1.13843 0.8016696 0.09045826
2.221548 1.733425 0.7539673 0.7006082 0.8804737
0.7397256 3.57106 25.45173 263.1581 2.381273 0.9463214

4.696642 23.19118 4.570706 21.24831 2.377911
24.09674 5.969207 1.771813 3.744589 0.5062444
16.39466 8.299862 10.15128 5.002977 1.215557
9.910216 48.05071 46.24526 5.538169 5.489577 4.5377
3.360549 1.206289 3.123037 30.71169 1.786372
18.39705 2.14381 0.9529156 61.47103 5.900801 1.934628
8.470672 2.342175 3.377118 5.903281 17.69557
9.193414 2.350375 1.216058 15.97564 4.352992
1.619541 3.870276 4.574635 1.942283 10.49441
204.1135 80.99477 6.80447 10.61049 3.894403 16.52046
22.19921 1.131442 74.45457 7.904605 5.198424
35.96212 21.50257 9.455274 7.575441 6.10849 2.677928
5.294515 2.050183 8.642968 1.926069 5.382324
18.08391 3.567572 73.81454 17.38577 6.559785
28.74258 14.20647 1.859713 20.91581 1.071933
7.889303 24.78164 9.822594 9.411599 2.495181
26.51334 29.55233 44.65992 5.442146 1.295953
12.26427 101.1446 3.106978 198.2629 26.77292
28.87593 2.119191 36.51433 3.777553 11.53792
14.1343 7.824596 18.00958 1.664738 7.608262 3.401872
3.854021 5.943182 6.665686 84.57745 3.753054
8.615199 3.500333 13.32276 11.62675 31.05463
84.20044 10.41086 2.512773 7.024485 21.23345
1.718308 62.452 52.28993 27.46211 1.119502 2.706852
4.340937 50.65195 11.67361 12.32245 55.65827
4.113226 3.213764 1.334688 8.120521 0.6229129
2.427852 1.536115 49.54866 374.2898 42.97457
1.647186 26.64055 43.06899 15.8612 0.67584 6.511542
34.94353 4.498201 6.271396 37.81852 11.75735
9.423172 51.34982 33.56334 148.3025 0.5750192
14.63917 41.60739 18.77327 2.038262 7.762493
51.5498 4.675269 39.6778 4.631285 19.81151 5.448953
1.442899 26.70958 8.305573 15.37747 51.16674
15.73637 6.491874 3.574622 26.28037 7.227228
44.0406 102.7147 15.50555 5.25489 41.66353 8.806224
13.52622 2.625121 7.36942 10.41954 11.51703 10.46246
1.117766 2.243135 55.33659 31.21185 6.877376
0.6370286 1.82482 5.597176 90.56494 1.071465 10.31433
18.23715 29.83209 25.98619 26.53488 13.3632 99.7134
23.78799 36.89323 53.82009 1.011337 39.39437
2.401725 3.371037 1.900506 37.33417 10.97112
3.097892 6.876286 29.84216 26.67494 13.9197 16.15763
1.728192 8.149862 9.299002 28.72054 17.68506
29.06549 4.846869 2.348246 52.77908 8.593434

16.06014	57.89836	3.490604	5.356551	2.962009
1.787477	36.25164	1.116585	17.23788	20.77744
8.127166	2.661183	133.5995	43.40588	13.48407
2.635087	1.83894 42.77181	4.191884	8.632654	8.858096
1.477768	2.052665	21.80834	5.785131	15.539	100.4122
70.5269 17.93417	1.065966	3.413335	28.8875 6.371761
7.586884	0.6869506	5.292174	4.256056	9.48984 11.02673
4.788114	27.00662	48.96418	4.295144	40.94831
7.334834	1.669659	3.257587	131.6254	8.587178
31.99687	7.396128	3.58566 9.999095	20.42908	2.407334
5.531675	45.19895	19.05854	2.366179	76.40079
5.89219 27.03272	17.38449	28.86981	3.792963	7.966282
3.520349	30.38891	5.722437	20.87601	2.513533
19.80719	8.695278	6.55247 2.873072	0.1667126	5.345123
6.20221 54.58157	6.773861	13.25783	43.44192	69.32375
26.6322 2.495274	7.463996	4.300155	116.2471	6.836311
81.70799	20.48754	3.640965	41.90114	3.062532
6.086767	7.726315	0.804035	1.407845	3.744946
15.92404	7.673943	6.706811	4.358957	9.199189
7.823959	25.07477	20.33893	9.716654	5.752227
1.858998	29.09752	24.43198	16.63697	2.874781
8.902578	29.58657	4.525733	66.82233	10.44946
6.638747	62.75736	4.274225	1.252637	42.74701
1.151523	17.91214	26.88053	5.090593	0.4006194
36.85021	19.14235	1.049994	18.81653	1.748369
12.87172	6.320808	2.528863
COL4A2	17.29216	137.5686	15.58057	27.25348	16.54822
120.271 9.859804	41.06478	44.65067	18.03968	38.8256
4.445786	12.39568	12.25128	15.27366	116.1287
21.44119	82.90351	14.7204 19.03293	29.88657	100.4954
24.07106	109.655 118.1797	11.61918	22.93935	134.9225
33.51616	2.94982 44.70719	83.78466	43.25799	17.85972
76.99034	20.9874 9.053996	101.1585	128.7706	43.21752
122.2676	27.78502	44.13048	34.78832	69.00203
68.76418	21.61998	64.19242	36.95806	24.31168
82.70424	52.33114	48.87755	55.55459	125.5921
50.65919	99.67368	168.9082	59.1338 26.51749	119.2278
90.91087	77.34393	141.5664	27.17923	279.9869
218.9991	54.32172	101.6669	20.98021	93.94169
150.5504	84.76089	101.6021	204.8598	55.53279
95.79431	101.1462	83.94754	29.6577 51.82628	118.8368
36.73214	99.17964	118.9633	136.4057	102.9073
47.74622	50.3717 98.16867	36.03399	165.2647	88.6525
26.24053	43.74522	78.24284	90.35164	40.32731

19.84017 77.23824 60.89945 25.5218 30.02263 52.43017
160.6444 123.5373 69.60289 127.254 58.77522 17.59369
46.78086 36.00314 30.84448 59.21626 47.80197
64.83227 73.60566 83.64007 67.84436 20.64487
99.30935 23.95695 120.6728 77.09619 65.87204
25.2221 11.74548 42.15696 130.7152 129.024 47.73769
16.03343 22.47154 30.57751 66.5444 80.3762 83.30602
23.21676 57.84685 28.65213 83.11431 54.01948
55.16614 32.35244 111.2198 17.64172 22.53842 146.21
23.71969 71.76495 65.58374 265.1314 28.55942
144.3393 183.5857 28.77287 87.13766 100.4181
36.03273 32.56207 155.6396 28.05946 40.33689
102.9052 80.06133 46.53167 26.95642 44.72291
53.18391 38.21785 151.2061 81.5904 32.1788 58.26731
38.74034 53.72855 66.91289 55.52607 31.67652
227.442 28.92582 56.96595 24.41129 136.5986 89.68346
302.9495 132.9901 20.08643 79.399 35.41346 134.6333
54.24487 36.0443 28.32654 32.7047 182.4937 71.81773
61.04488 40.63084 34.12483 29.40213 81.74432
185.1339 26.52293 38.28451 23.915 121.9804 94.09559
68.85361 48.90267 35.62157 167.3547 51.91476
81.52404 33.545 75.81305 68.3823 38.26104 165.2209
40.81212 73.72179 51.89068 64.21898 66.96727
63.83639 20.44437 170.8507 7.115719 27.30352
48.82126 78.68986 129.0066 19.4591 61.57025 13.84477
72.73702 39.34626 20.6256 49.92976 50.58951 69.12902
91.20025 98.17323 51.00294 41.52296 145.1047
67.10478 93.25243 86.21824 63.33943 77.57689
45.27747 74.07599 160.5955 39.03776 69.16052
69.87078 69.56354 44.01858 158.3273 59.24904
44.76755 37.48082 47.57809 211.1574 92.50472
37.54634 117.3003 114.9829 21.846 124.8685 51.33547
37.60189 28.59163 24.25572 28.19521 49.77873
148.2875 48.9576 64.86049 92.1466 21.2898 132.0687
56.43964 46.27267 82.09348 33.39001 31.62033
143.1495 156.0302 55.20658 184.7165 186.455 185.9726
73.60164 237.6424 87.40526 115.0585 37.2117 71.71785
73.19494 159.4104 51.88522 299.0677 21.10376
70.88889 28.19161 41.89395 54.70789 86.24751
72.04462 54.974 59.7283 80.2946 51.73237 200.2893
120.2753 106.5508 63.93616 43.17413 26.3612 32.3594
30.56471 55.47226 39.85329 39.11768 87.4296 68.80052
53.03457 34.53257 8.789901 159.6698 104.9867
52.61775 173.2241 39.36309 9.356675 30.05382

71.45235 29.70118 61.40022 67.65575 45.97828
36.00779 178.8447 13.91525 34.17657 48.98333
30.32788 56.24608 73.45287 143.3708 41.78871
249.6509 27.14201 52.69014 146.3057 49.26003
129.0478 73.27638 78.98894 153.1145 44.16047
31.29883 81.60652 49.35819 13.99774 48.77375
50.78812 70.23472 54.05207 34.71901 80.73136
27.52299 61.92478 76.60836 36.43503 112.0273
53.57581 68.54639 121.8555 156.286 30.59531 28.13537
62.44777 41.19716 172.7376 27.17975 50.81988
60.13377 178.0551 26.24652 17.26905 32.24628
30.98703 56.72247 65.89281 112.8739 21.20457
85.27318 59.47034 21.02301 34.83518 24.80858
27.34425 113.5604 136.211
ARHGEF7 3.066714 3.237057 4.592449 4.005079 3.46059
4.717408 2.28138 2.934114 3.760516 3.372029 4.766727
3.073134 3.996064 2.976033 3.647078 3.39881 3.74823
4.037071 2.553342 3.520154 3.654251 3.887817
3.048599 4.829987 3.611441 4.025267 3.202995
3.965387 4.089217 1.353073 3.729604 3.760437
6.555638 4.771026 4.877721 2.427629 2.510209
3.917508 5.341305 5.424707 7.461476 2.90042 4.880409
5.10333 6.68324 6.993199 7.613704 4.96302 5.394167
4.335734 4.118799 7.7716 4.323117 3.991139 4.583202
4.370944 6.614547 6.53175 5.721732 4.729944 8.189898
4.815313 5.137253 5.400245 3.277307 5.295562
5.212036 5.115913 3.593465 5.688613 5.386013
4.888335 6.919877 6.0702 7.774976 4.030184 8.983464
7.654774 7.806996 11.13732 5.02315 7.526469 4.98687
5.468918 4.277122 7.603763 6.819038 5.988078
3.840283 4.88366 4.510227 6.210282 3.764038 6.296895
3.889395 4.126843 5.1698 4.446083 6.718827 6.026705
7.51228 6.975382 3.086417 4.96401 5.345363 8.546468
4.28866 4.79044 5.211998 4.127944 6.026919 6.281866
3.425425 5.4427 4.061967 4.737302 2.047193 5.805966
4.584831 6.651724 5.010812 8.469894 6.496258
3.607725 9.255392 8.493837 3.937585 3.450331
6.132953 5.385433 6.712088 5.851373 5.265978
5.609303 3.030456 11.41162 4.81114 5.739897 5.917179
7.579703 9.045945 7.058853 5.123838 6.29785 4.506092
3.538414 8.010053 5.613203 14.39258 6.27952 5.218843
5.882287 4.067704 6.115256 5.392523 4.275858
5.644811 4.053702 5.653279 4.394931 4.631682
5.476394 8.572632 5.246093 3.210685 6.185097

4.594678 4.708953 9.454313 4.456425 4.557753
6.563522 5.363686 3.82796 4.232045 3.464475 4.396201
3.46853 6.336345 16.23477 5.19526 12.75317 3.416409
7.820259 6.222743 6.37017 6.08231 4.057435 8.973187
4.982556 4.417161 7.037085 2.695348 3.867329
2.793775 7.501482 5.457197 9.737664 6.531571
4.058623 3.307265 5.139119 7.512609 6.432068
4.748918 5.401619 2.301372 4.867478 8.408603
7.561917 6.997236 5.772405 4.372965 5.1062 4.831588
5.150248 3.156389 5.077036 5.947024 6.768416
5.399811 7.069956 4.110517 4.563527 8.616752
3.349728 12.42989 3.358066 4.246204 4.853749
6.394664 8.237784 4.711678 8.072014 7.31156 5.020268
4.520634 4.275919 6.337358 3.029145 10.21468
6.642112 6.550528 4.987298 5.907561 7.441229
8.240819 4.023181 5.30803 5.60843 4.815465 10.82924
4.439771 5.884161 6.885152 6.287223 7.279446
3.919832 6.269602 8.019795 3.868481 2.994956
5.354583 4.346396 6.346111 7.108523 12.84124
4.057509 6.354813 6.453971 4.876931 3.697654
3.665068 8.827133 5.119251 6.953786 8.372576
5.326181 5.1384 5.957515 7.084501 5.795037 3.005518
5.644059 6.11455 9.499557 5.230651 5.474649 5.053468
6.864662 9.076329 4.815969 4.730747 4.336232
2.771534 6.441206 5.294261 4.686505 5.549297
5.502941 5.799962 8.911754 4.868115 10.99942
5.817877 10.47307 7.211294 6.577702 5.398358
5.209377 3.228744 3.916812 7.179052 3.886678
9.459077 5.592172 6.184048 7.044264 5.233316
6.656239 7.502828 4.05773 6.193461 3.809775 4.489253
5.739125 5.322967 5.056818 3.41811 9.001241 3.819797
5.705415 5.587802 4.493747 3.340599 6.490475
6.67883 2.825702 8.675905 4.64397 15.16669 4.762878
3.815574 13.37587 6.601081 6.369777 5.844863
44.70449 1.501562 2.592052 12.73496 4.779055
6.837688 4.925013 4.61741 6.303245 8.061562 4.239759
5.887385 14.88859 5.444048 4.38751 9.229049 4.991878
7.082775 4.80362 4.030464 6.038891 4.88004 4.587403
5.509886 3.816813 5.464046 4.343365 4.001208
6.59059 2.122891 4.025412 4.912133 8.867466 5.997
5.928265 5.822823 6.266876 9.490344 5.693748
4.714096 4.462688 5.894969 5.150006 6.448204
5.956968 5.678087 9.351496 6.407108 7.419362
12.09271 5.601949 3.488426 7.521714 10.80113

6.290782 5.732213 6.512031 5.717628 4.813672
4.936203
PPP2R5A 17.54449 9.457907 17.41685 29.07071 16.81974
15.66375 20.52851 22.42435 18.37181 18.55493
36.54266 34.08059 23.32749 27.53587 20.3358 11.69213
16.41123 14.03629 12.40953 12.52561 27.98463
12.02557 26.57234 18.27728 20.36447 26.76563
21.31399 12.51227 16.14083 27.1836 18.88087 13.99355
29.17019 22.5382 16.90855 62.4652 35.63448 31.6172
21.26044 21.45285 25.32091 26.91764 28.22344
28.41548 23.31521 34.62346 17.88647 22.54458
16.5725 17.53801 20.41097 17.67098 24.0379 19.46387
13.93035 22.68824 10.62411 16.12787 19.95722
17.04445 17.33133 17.73114 14.83684 13.88534
20.3907 13.70821 16.44479 16.56883 21.80984 19.49427
21.11553 17.25835 28.35371 14.64155 17.58348
11.11469 18.03347 13.46195 14.27054 18.48507
13.68426 22.58752 15.57045 24.1254 17.544 20.52515
24.10606 17.53581 20.41604 14.53666 22.9537 17.1641
11.90167 22.58905 14.72047 18.46292 14.63151
22.01411 13.02107 21.6526 17.97623 25.55877 13.95033
17.54581 21.88952 17.78725 15.19997 16.38374
21.29187 33.00085 16.72062 18.96692 12.65173
15.64376 19.85538 27.80157 21.91264 47.31976
24.12588 19.95209 14.24099 13.33776 12.94741
14.32926 20.28932 19.46003 28.84939 12.45572
18.81588 13.47162 22.5434 41.47068 15.21965 20.04008
13.90035 19.62658 18.36426 16.44882 36.83598
13.16251 16.4925 21.09335 22.87037 18.70811 17.26209
19.71143 18.80577 24.40409 20.75427 22.11715
20.07564 19.55184 39.09984 16.95694 14.61709
15.34962 18.57169 24.86679 15.01337 15.14809
26.87947 21.72074 24.92183 22.24927 15.95172
32.84483 20.09819 21.47398 19.98804 25.97634
15.80027 18.80718 25.49045 21.48636 31.3734 24.73313
17.02336 21.14019 24.24468 15.05382 20.36552
23.20504 29.06563 17.269 21.53454 14.38441 21.83757
16.08055 16.29851 16.11748 25.97771 27.08189
15.64182 20.13815 11.48875 22.30456 16.13201
14.99455 14.45202 19.31346 20.89088 25.67423
28.4606 16.71918 16.06224 19.00702 15.51556 14.95779
12.91238 19.7184 10.00351 17.87646 25.31415 31.99341
34.95687 26.3567 20.35682 21.33121 14.37307 25.02942
25.20493 23.19299 21.38967 18.30662 19.32961

16.73612	22.7545	29.15445	22.93941	15.02795	14.05151
12.79586	36.88393	20.21932	32.4813	19.77325	15.87788
21.39186	19.1201	31.40619	21.83384	21.18113	27.62336
17.95145	15.988	14.18271	19.59869	16.02718	26.93374
21.47451	30.31684	25.63196	22.42378	17.19427
16.2175	23.79855	43.28648	21.73355	20.16989	14.60693
23.0101	18.46581	13.77192	29.73415	29.05579	25.01915
29.7547	17.48742	22.44023	28.48059	23.88303	23.90862
28.36748	20.36037	17.71433	17.26156	19.7961	13.6443
16.41966	25.64325	20.17397	25.37371	12.98122
18.7751	19.84852	17.32805	22.15356	18.61192	16.43279
22.22001	16.00181	9.660061	15.64943	24.30555
7.845916	19.10237	12.49755	17.5875	19.30216	17.26099
23.2125	27.36764	11.51797	10.7522	26.34888	18.89271
16.83395	28.37494	40.43948	22.7107	15.35482	8.24229
16.8846	12.37231	20.11506	11.45832	21.72066	21.01867
20.85665	17.08065	20.9783	15.27739	43.80437	17.96983
22.15487	21.1475	10.18804	12.11216	20.28228	17.54194
27.3982	19.57069	25.93349	29.10378	12.08156	27.2352
16.81718	19.08543	22.28855	24.21238	15.20496
12.45751	18.01079	22.38341	16.85303	26.4576	39.77835
29.25933	24.16153	15.55648	25.50875	15.91878
24.89117	17.18438	13.95793	23.33573	20.7851	26.35631
14.80492	13.63579	25.95046	14.65456	17.26997
20.64911	15.98597	21.8365	24.45974	11.99759	22.14543
13.08099	20.55787	15.22491	22.70202	22.34534
20.00656	18.86065	27.84112	11.81865	16.14748
34.86669	23.24326	16.6319	12.7676	42.22469	31.28811
12.27424	12.63904	18.9657	23.23653	27.67457	16.26887
10.39806	12.8626	18.50868	28.50315	22.30634	30.20353
17.30896	27.76529	18.87248	19.18281	22.88669
20.98044	23.52592	29.67693	23.26077	21.90423
BST2	9.244304	152.931	36.16277	29.76647	44.61896
56.38246	51.31443	59.33264	76.18874	142.7592
18.45584	17.65158	162.6253	4.187615	73.70351
36.23426	36.25869	120.173	47.35624	54.35119	56.62099
51.01096	11.64492	26.2051	24.67667	9.082775	108.1025
16.26198	37.45947	30.551	48.21176	87.18209	207.68
58.88467	139.8108	401.653	4.350245	30.40044	21.42229
25.38197	81.44656	123.5317	44.29787	119.7169
28.04963	10.42306	83.98262	232.7647	138.1512
13.87506	200.0606	111.2277	8.009166	67.03957
135.5102	152.6014	190.0233	44.7339	216.3981	112.1937
66.81914	194.6999	146.4446	94.98734	299.7765

29.62266	114.3906	97.07919	20.04986	47.97931	
217.9304	73.5413	23.9152	28.87395	20.3783	253.1743
20.19134	9.437836	23.46629	6.85535	234.2861	94.12905
269.566	131.476	37.1578	32.94904	29.17668	151.3457
164.437	28.38154	165.4926	206.138	429.5969	91.71492
362.4477	177.3275	189.144	71.1341	90.88122	261.0873
75.34559	120.3406	283.0613	92.60323	72.55928	
37.13871	241.2049	54.88819	94.73293	136.9856	
11.43256	32.32803	277.8898	77.37695	126.59	33.40919
66.41855	39.92058	38.98949	13.05338	82.5245	23.91321
51.18474	249.3388	171.4932	69.12574	137.316	229.7755
99.22955	71.59723	144.9254	6.636976	127.6431	
246.9251	79.99401	18.88199	40.01286	61.66392	
45.91027	13.18599	40.8176	207.1325	60.6421	109.6204
100.0464	30.18738	147.9811	59.54205	3.981955	
122.3499	223.2683	96.40415	43.67229	197.7598	
61.39778	76.84785	50.0712	74.17744	87.28268	147.9978
196.0307	55.77095	40.56283	17.55163	176.6319	
75.97091	120.8313	26.18676	103.9002	17.02334	
108.3931	111.3854	115.419	11.33587	95.64637	30.06701
30.01803	364.8438	59.32948	77.31386	98.91698	
67.2956	157.2811	172.5643	94.22082	146.5131	81.21023
356.0823	51.91085	178.9857	120.2528	130.1018	
181.7732	26.77649	447.4995	38.05289	263.3058	
55.20147	83.95675	177.023	4.514933	151.3493	121.1638
68.55457	18.20322	135.8801	179.2577	37.65524	
59.80558	195.4318	24.16353	105.8727	301.8171	
11.64206	172.4328	205.0528	258.4679	96.37655	
225.2086	35.91319	156.574	146.6907	32.98004	177.0048
159.3746	91.69889	20.09521	217.6361	51.37186	
35.53919	18.17836	21.63558	2.692595	87.22516	
264.8131	86.32706	119.7512	222.6041	209.8956	
84.27351	78.22133	142.8785	137.2956	290.1754	
109.9109	138.8307	215.6785	123.0184	122.9183	
155.7619	218.6861	178.426	250.3566	206.3562	148.1733
41.84837	104.2285	46.02768	179.6407	51.48427	
143.7347	127.5865	177.4911	291.918	102.2311	45.13072
125.9381	98.49202	153.2824	104.5889	49.44188	
242.5113	13.5003	94.1836	8.947188	29.28817	73.91557
62.23777	119.7414	237.1255	100.9235	117.6074	
67.69871	190.7502	91.1224	79.42833	12.04778	105.3492
80.39118	23.6416	20.06252	13.94012	38.11788	60.34177
284.5655	88.18415	264.2461	279.7143	308.8363	
61.51724	152.4726	93.40568	92.40468	17.50558	

120.6914 22.62296 91.29484 204.337 36.62085 5.358033
117.5022 141.2786 304.8684 147.9103 208.413 52.37434
62.73187 54.31109 142.9832 42.47166 77.46818
52.82617 37.89842 339.6868 72.60618 36.95912
35.31676 16.312 82.16633 193.4729 201.9874 55.06132
115.964 141.0417 182.0143 12.60574 17.72902 19.44043
98.33187 137.4316 13.42497 105.8954 147.0236
131.9377 75.81268 24.42362 14.80709 11.77767
210.3661 144.8183 60.18038 90.80995 33.00386
113.4426 237.6224 16.01967 95.26241 45.41083
30.08644 78.98635 54.12778 84.63932 86.13764
119.0657 27.65338 89.12201 121.9641 207.8746
77.37307 113.5136 51.84397 71.77968 109.1473
197.617 225.3542 100.7708 174.8424 159.4675 46.35283
159.6155 132.4928 85.85573 46.92585 6.151209
21.49263 345.0231 116.1875 138.9909 204.3124
49.33109 84.07495 188.5721 24.58536 39.54576
205.2392 101.9198 34.08878 128.4728 56.0434 8.637939
219.3437 44.59298 256.607 141.1005 163.1105 84.14457
136.5247

PPP2R2D 3.19757 4.130542 2.655221 3.446391 2.933694
3.619594 2.541123 2.388695 3.551269 3.492228
2.702921 2.439963 3.375801 3.078807 3.127171
3.975948 2.955649 4.303122 2.921207 2.676526
3.714582 4.033337 2.619011 4.226732 4.455748
2.831393 3.065933 3.846648 3.149295 2.452533
3.387894 3.828376 3.732054 1.988209 3.82014 3.096179
6.100908 3.453982 4.031109 4.056541 4.346017
2.52155 3.267685 3.073182 2.868302 2.962097 3.020983
4.753739 4.554755 1.76544 3.526888 3.19181 5.930358
3.495385 3.400295 3.82027 2.186544 3.51216 2.575134
4.85299 3.092231 3.95197 2.986887 3.724195 3.227409
3.174584 2.706748 3.795933 3.758442 4.0094 5.242733
2.350129 2.933375 2.65144 2.512995 3.116739 3.089092
5.222444 4.816127 2.24786 3.449222 2.173045 3.354847
2.881524 3.243803 2.829389 3.477058 3.11803 3.869542
4.172935 4.043501 3.426062 3.001961 3.938985
3.952161 3.436971 4.154425 2.846313 4.373262
3.282867 3.051249 3.811531 2.904724 3.215386
3.299439 3.474636 3.783189 3.479143 4.094823
2.642146 2.943802 3.382667 3.37612 2.872467 2.799613
4.7413 2.102211 3.017052 5.114595 3.172771 3.671515
4.086903 3.992709 2.3405 5.711549 3.444904 4.303903
3.42389 4.112208 3.871478 2.986831 4.892261 4.071213

3.557992 3.023485 3.793834 3.788001 4.406946
3.547907 1.976151 2.925985 4.372853 2.494765 4.763
3.456507 3.173685 2.78669 3.13574 4.662019 3.139938
3.041688 3.515611 1.727978 3.051268 3.247045
3.405581 3.374344 4.243086 4.846601 3.634991
4.116577 2.576579 4.407841 3.007708 3.299972
4.429796 2.533967 3.235375 3.380202 7.218248
3.610778 3.8364 4.704646 3.349127 3.164879 3.297598
2.511539 2.748699 4.961071 4.855432 3.547569
3.666453 3.004035 3.432775 3.336772 4.846543
3.220653 3.104738 4.045976 3.686026 3.029741
2.848439 3.914918 3.299032 3.952256 3.272091
3.705033 4.021184 3.145611 3.106373 3.28435 2.91177
3.353317 4.220538 2.49514 4.100055 3.065216 3.003317
3.327944 3.951659 5.383847 3.113898 3.213176
5.617468 4.193941 2.809102 3.61038 3.346344 3.875812
3.503459 1.828438 4.336647 2.833297 2.443576
3.789565 4.285484 3.113931 1.928118 3.83269 5.249112
2.793874 2.659686 2.536083 1.550333 3.046155
3.125517 3.308104 3.116791 3.784025 7.095861
3.02338 3.421877 3.14738 2.915427 3.982433 3.171604
3.589835 3.226494 3.083951 3.811997 4.411789
3.705581 3.408299 4.30604 4.277961 3.449128 3.123147
2.308698 4.42309 3.476126 3.689926 4.037811 4.50956
2.738111 3.827202 4.129017 5.580216 3.717051
3.407912 2.337718 3.246421 3.960857 4.015786
4.072918 2.494357 3.135517 3.762161 3.219023
2.817701 3.042103 3.859749 5.238134 3.277243
4.517425 3.381327 3.913107 3.058461 2.471815
3.245594 3.534352 4.230697 2.647972 2.792159
3.248921 3.157476 3.991156 2.489055 3.268912
4.707204 3.550489 2.870624 2.95475 3.581323 2.588468
2.118306 2.77372 1.986789 4.228984 3.030647 2.87558
4.568899 3.466292 6.124202 3.370853 4.972094
3.41767 2.684641 4.375509 2.991504 3.125458 3.942681
2.549451 4.594224 3.646151 2.694911 3.516758
4.100494 4.724055 3.482139 4.164773 4.348192
3.384557 3.445327 3.694186 4.289063 5.162236 3.034
3.470087 3.35418 2.729096 2.982658 5.819745 2.909158
5.128124 3.389013 3.122475 1.635191 2.177091
11.42124 6.515586 2.500877 2.659139 3.193065
2.84352 3.339871 2.537055 3.648578 2.980239 3.122431
3.844973 3.081843 3.08711 6.15938 3.75151 3.016775
3.460832 4.276226 2.825306 3.430334 3.963539

3.580866 2.827074 5.246119 6.23191 4.007165 7.050569
1.603527 2.888697 2.642084 4.892681 3.742087
3.971545 2.397516 4.689038 3.824106 3.33226 3.64949
3.169466 5.124473 3.135251 3.684734 3.718865
3.409376 3.622095 3.179436 3.628479 4.148502
3.267843 3.009551 6.329383 5.903277 3.249176
3.626675 4.562999 4.188468 3.688418 3.657809
CCN1 3.988712 32.07479 6.058701 26.05561 52.4972
170.3842 3.072252 111.8716 119.7681 103.4184
16.93118 4.755316 40.08307 5.293406 20.22224
107.1265 8.251633 280.6187 69.34442 17.4173 31.80371
105.8467 32.38138 35.56416 92.06931 39.75766
43.07224 103.0841 13.78058 10.13847 79.12052
117.1133 6.003813 35.65606 262.9851 8.562192
5.431315 23.41334 56.40267 11.72633 96.16417
13.25622 61.5036 23.64096 8.09279 10.55993 6.404043
32.61923 93.18949 55.59063 30.85161 18.36967
44.11846 60.75794 91.13189 14.59368 120.3187
89.79205 20.74532 20.77146 26.70736 23.69549
45.32523 17.46507 14.89532 115.0549 93.20881
40.61853 68.08033 31.26591 26.23727 13.09078
32.1341 53.05185 108.7867 82.59378 117.1755 79.1523
6.194566 18.15688 28.29725 71.14108 26.61124
25.64315 102.3806 27.53836 20.97737 7.971232
13.90762 47.77402 15.7286 7.196073 17.65358 13.76317
11.06909 20.9488 33.19668 16.07141 7.171365 69.6129
80.63718 4.829066 20.27622 53.23327 110.5828
175.6279 28.7192 33.9618 32.88585 51.18012 15.14204
34.91606 31.07387 38.49814 66.79652 11.85918
24.24823 11.49146 21.43083 24.43685 47.65265
36.02265 39.3091 65.7683 34.45029 31.35284 13.82452
91.75874 53.8958 170.8017 5.579034 25.05698 68.03157
72.77497 90.66457 12.99649 25.76405 11.29747
21.1215 21.71138 6.837229 5.994604 18.26309 23.67736
29.09587 20.00996 10.12525 28.4702 6.915748 32.15297
7.596814 59.54113 26.75945 4.611092 91.12657
38.74405 32.42555 43.92901 51.43802 37.54049
46.47322 46.55875 23.56026 18.4121 123.7207 48.70735
61.5138 69.76335 100.2855 6.67374 85.74366 19.92783
15.66511 13.94712 4.732021 91.42703 219.6791
37.97384 28.40332 83.72412 99.32786 7.913464
13.66586 16.69487 59.34876 28.39285 81.92939
48.97142 69.31931 7.070995 62.81023 33.32678
84.92839 16.44804 10.49169 33.66908 149.226 6.202088

9.630283 37.51382 22.66906 30.08 39.93355 8.865048
12.91034 23.69929 28.08883 149.1541 19.66575
21.80318 55.37958 49.40204 32.76644 13.96791
10.54644 38.99899 8.068539 20.36574 263.2088
7.607964 7.593421 79.4524 20.62303 86.12296 155.536
8.057208 22.26018 8.999342 13.38649 24.94599
11.69089 214.0235 0.8104789 21.83751 7.176934
26.57457 85.22914 6.971034 75.53742 4.435203
12.72964 16.70517 71.88767 37.95711 81.65056
40.11387 58.32219 27.36984 37.05798 14.75218
4.473998 4.320808 86.04263 137.6808 10.91533
37.62151 16.45782 31.4633 67.47391 303.1138 40.34024
70.09042 38.23612 71.9116 63.71081 54.10521 8.666076
55.36795 34.28966 95.09945 26.38262 65.10026
32.6507 14.85786 9.767008 36.33358 50.49163 24.44625
16.18579 28.25382 30.93786 8.603637 29.70998
116.9934 36.22728 77.52707 11.43543 10.17673
183.2758 85.77903 22.77313 62.41731 339.4203
73.9124 48.27143 182.3157 62.05201 86.94416 62.44512
63.66032 11.15459 69.61314 38.69186 359.683 16.45605
29.02006 17.60705 103.3304 45.40543 27.40134
38.51472 39.42636 17.05456 111.5075 19.47905
105.1631 88.21017 33.24003 28.89035 70.04571
5.873232 25.80939 18.85373 23.74682 137.9271
43.65549 18.35502 28.15543 14.54712 60.33056
18.1781 55.07557 52.09695 68.2548 22.83171 11.03864
7.258878 12.51001 96.68498 37.85088 4.247807
62.40891 25.91526 10.13974 114.011 8.026618 36.15683
16.66139 11.65606 30.81148 30.69085 53.42239
41.12812 285.1995 12.05749 7.385766 19.06826
29.25246 7.522929 78.40569 46.19131 132.8219
18.62364 23.34149 22.12059 33.70985 2.691595
38.70008 24.88605 25.79092 17.53817 52.03056
12.15606 53.14308 23.56987 82.53893 23.24062
50.90666 6.265388 15.97551 85.99224 59.12607
13.51108 51.45114 23.34412 15.60326 74.27374
18.73097 15.04692 11.75753 21.84163 45.2379 10.71934
24.90824 16.46849 56.84832 10.92362 28.21016
25.47635 9.907643 23.19718 93.60439 17.80934
6.002424 21.17535 50.55613 21.12962
CCDC178 0.07467222 0.1024487 0.08361821 0.151967 0.1277546
0.09863874 0.1103567 0.1984422 0.1754276 0.2033829
0.1086864 0.07016284 0.2055581 0.02739604 0.1376163
0.06253659 0.05828592 0.07577883 0.1523307 0.06240689

0.1024322 0.02586685 0.1280401 0.151486 0.04669602
0.1990491 0.2775113 0.06674651 0.1882176 0.1915389
0.06422331 0.110588 0.01253554 0.1533798 0 0 0.5472804
0.08164581 0.03767068 0.03447846 0.0419489 0.008586074
0.02365424 0.01461001 0.1604722 0.01672267 0.01511088
0.010469 0.09256217 0.01555847 0.003747852 0.0612624
0.2089814 0.06834331 0.3608167 0.003991098 0.01430069
0.1031732 0.01627711 0.03961284 0.2137865 0.05313117
0.2166441 0.1025764 0.004467808 0.1715981 0.1417577
0.1739101 0.03588123 0.009071839 0 0.07765212 0.1766421
0.09990076 0.06821233 0.04697289 0.1688869 0.01321381 0
0.03180372 0 0.2978984 0.003477612 0.1468886 0.03761751
0.03143495 0.08351418 0.04413164 0.5894717 0.08422618
0.01564315 0.01450061 0.03050772 0.1041451 0.02242168
0.003316762 0.08496956 0.04444311 0.005449079 0.1316325
0.05284312 0.1248037 0.0224417 0.01168314 0.2747042
0.1836779 0.01873418 0.07274795 0.02583889 0.01731314
0.04522139 0.02346157 0.05697603 0.0357909 0.3909392
0.0103106 0.02179573 0.01543268 0.01678889 0.003396539
0.003359775 0.03842058 0.02393463 0.2772657 0.01248319
0.157454 0.05642106 0.007022761 0.1293123 0.03605903
0.01636726 0.06653244 0.03964137 0.02617148 0.1306435
0.03310466 0.03396912 0.03146108 0.07503355 0.02555995
0.02432795 0.07862701 0.02525635 0.01709142 0.03677195
0.02625576 0 0.07771952 0.005722477 0.1807688 0.009001377
0.09798114 0.005883897 0.03549269 0.08303982 0.0305829
0.1595535 0.1614585 0 0.08689806 0.09297734 0.2726041
0.2921707 0.02953492 0.01199887 0.03350436 0 0.01820617
0.03095063 0.003996982 0.07236397 0.05232638 0.07966968
0.02973357 0.0101143 0.04887109 0.04710995 0.08415313
0.02810972 0.02254114 0.009795949 0.02623174 0.1236191
0.03932141 0.0326569 0.03209151 0.1138148 0.008785443
0.01874263 0.002806926 0.04454804 0.003421255 0 0.06948752 0
0.03977751 0.1721528 0.09030295 0.06285592 0.06445534
0.03247082 0.05320648 0.01591434 0.002650351 0 0.03524127
0.01564826 0.1196621 0.02481427 0.009140063 0.06229105
0.03795816 0.03917605 0.0107423 0.07477623 0.008521631
0.006948866 0 0.1666619 0 0.03332621 0.06503096 0.00340779
0.01395442 0.1868487 0 0 0.06804537 0.01584439 0.01946721
0.01098857 0.1132611 0.04658044 0.02305349 0.01167181
0.01363363 0.1823888 0.04129949 0.01466386 0.01988965
0.06322132 0.01626644 0.1291632 0.04023068 0.1422455
0.02806169 0.04865041 0.1234748 0.1229836 0.03472438
0.02626641 0.003663693 0.07255531 0.1353728 0.02087284

0.03173444 0.01611808 0.02355744 0.006096247 0.2445015
0.1219573 0 0.04146366 0.07378397 0.04981641 0.02469409
0.002811495 0.06004369 0.08087457 0.03356832 0.03551802
0.02647132 0.1087515 0.008901787 0.0542375 0.05184906
0.01232612 0.07016177 0.02784659 0.03096931 0.427443
0.02058133 0.02607243 0.1106964 0.01421293 0.1700257
0.0486319 0.005457467 0.05139789 0.1637742 0.01047885
0.1312225 0.1951772 0.03873893 0.02984993 0.3301866
0.07158861 0.007301065 0.02105407 0.04433068 0.2376874
0.0931114 0.01937986 0.01766257 0.05753711 0.04614639
0.05621994 0.01350665 0.04560104 0.4997002 0.01012303
0.06534573 0.00616756 0.1434213 0.037613 0.1136005
0.07464671 0.04735288 0.02319093 0.05504418 0.01729973
0.0155041 0 0.01340287 0.444041 0.04661713 0.01199479
0.03835967 0.08842769 0.01185761 0 0.03190003 0.0256558
0.0333822 0.05298458 0.04037223 0 0.02926667 0.07794519
0.01305435 0.009085229 0.01754207 0.07715799 0.003626269
0.1457515 0.1169612 0.005713887 0.332791 0.05167315
0.1321429 0.005962146 0.04329055 0.008305409 0.007736804
0.0326119 0.01381315 0.07549735 0.1918274 0.1255112
0.002511891 0.2647218 0.3887368 0 0.004735658 0.05661313
0.06231958 0.007099126 0.03489389 0.06373324 0.1495981
0.03120487 0.1622726 0.01543717 0.03538164 0.03874979
0.02017157 0.03931416 0.04031115 0.1623439 0.0359457
0.02949398 0.002581928 0.04647162 0.02261979 0.002467565 0
0.1468915 0.1440543 0 0.01494491 0.01339414 0.03161486
0.8806798 0.05113463 0.05716337 0.08972381 0.031969
0.1466256 0.07514199 0.01417922 0.004082788 0.07847368
0.004812937 0.02719403 0 0.04809836 0.05927322
SHC1 24.2824 19.59435 22.51132 28.63623 20.94396
20.32864 15.66469 13.33395 27.85371 20.32824
15.05604 12.8058 22.33257 16.04074 22.21301 17.73178
29.14125 27.76833 24.71459 25.63449 27.20965
14.21934 23.25939 19.89207 19.92203 14.77841
20.55577 18.13173 30.96613 8.602345 28.73615
18.03812 30.09601 20.55013 32.11416 8.912078
20.14891 28.9645 31.38061 23.82889 30.63658 20.20834
28.30726 37.20499 21.91387 31.04499 17.27128
32.07412 26.95659 26.27075 27.18149 19.43732
24.57158 17.46328 23.93009 20.0279 22.791 25.91794
37.12125 27.6575 35.67147 32.47187 37.42776 39.19022
17.01883 27.45324 39.59307 25.66473 43.47539
20.15098 24.21686 17.35938 25.8184 44.02043 41.97014
18.30568 30.07083 22.95 22.97742 20.3813 27.79837

33.58251 16.35441 20.7023 34.00462 31.80819 25.83891
20.34764 17.21711 24.57368 25.83763 28.73133
18.99395 28.69804 29.78683 22.72182 33.59207
25.48137 29.02624 18.34523 24.15589 36.8632 13.78495
21.80225 33.70493 29.27651 28.61933 29.68228
24.28394 16.49182 35.95393 13.36629 21.22774
15.20491 20.85673 24.2495 40.11581 56.93074 28.99515
16.57524 22.24084 21.08384 27.75125 22.28296
27.34897 17.8245 16.13184 27.42431 26.07508 42.99204
24.89083 28.25101 24.69722 25.51996 44.72467
26.54434 26.50229 45.73693 23.74826 19.77033
21.68143 20.76269 25.90243 17.41414 45.75906
21.52353 11.76253 24.54211 42.11887 22.20636
10.7738 37.60648 25.8635 25.9185 23.44252 17.28329
28.15226 16.8187 23.19908 19.14342 41.04854 16.52824
26.61492 27.10342 20.07892 24.75315 35.21273
27.22009 25.53717 20.71241 17.74741 35.58556
22.56955 25.45523 18.69737 32.37341 33.97414
22.73793 20.60786 23.44068 15.32238 33.15197
24.82537 23.08727 24.87268 28.52262 30.72246
12.78502 24.6454 24.30619 39.30405 69.72414 67.83525
30.35495 20.39692 44.85541 34.43347 35.04713
20.61749 15.1108 14.57548 17.81356 41.32273 24.04843
16.01735 21.88325 31.96084 26.73894 26.41547
21.85448 27.9266 32.60901 24.96172 41.22972 21.85162
26.47386 30.7918 18.16042 32.50251 28.49811 22.98441
26.16011 36.27725 21.10538 23.08822 13.336 22.31497
15.89606 22.83303 44.80517 33.31108 28.48222
28.74325 25.61817 20.17719 23.0581 22.639 16.48099
18.79306 22.85627 21.07857 33.40198 33.52838
27.5563 22.28708 37.95432 32.86439 18.86752 28.40191
35.65203 27.88612 19.14389 41.81323 43.69991
19.44702 39.95916 37.51497 43.08834 28.19488
30.60396 20.50635 30.32213 28.58032 21.5101 41.44117
29.50222 35.96229 31.48383 61.96376 27.21365
24.76699 33.22512 17.64369 24.42852 30.16675
26.07738 30.55236 18.84368 22.81615 27.76461
29.92421 24.82718 31.85372 26.60698 19.9119 23.91616
30.22066 22.87539 27.05705 37.05774 27.88787
14.86968 27.22216 33.33783 91.83134 55.14004
21.17527 26.69765 15.45238 32.28755 20.0334 28.11731
59.62086 50.70004 18.64547 32.26799 28.46039
28.3094 31.51206 30.25945 40.56394 21.22704 21.38637
35.15811 23.931 24.44188 21.39476 38.98167 23.52911

26.52973 28.64331 19.36256 30.79568 17.63973
28.7661 24.29879 29.18715 21.77482 20.44643 27.77792
19.06306 26.17387 26.70938 25.55928 35.21095
22.0591 18.36517 19.68145 23.01868 33.89342 28.1561
21.43313 18.50429 29.1326 26.31283 15.57296 36.46413
12.17209 38.63276 24.88883 23.71882 34.60531
21.87888 27.72886 20.35697 21.91434 25.84845
20.05483 15.6977 17.5497 23.75825 39.43563 36.9168
25.52126 24.48917 18.60699 15.76357 30.60428
21.22813 18.35364 22.46961 16.38983 30.27996
37.81132 23.96204 27.3007 35.27905 16.48542 21.9156
26.576 47.92153 24.82195 17.18237 39.79138 20.9937
20.95877 35.0539 21.30994 27.88635 58.97381 19.17433
19.7536 14.92655 23.98298 44.86033 24.76992 21.63801
21.06748 23.50415 34.73151 36.11175 25.85766
23.53692 21.12982 24.03421 46.72805 22.36116
BUB1 1.228381 0.04376004 3.757512 3.768762 1.636503
0.1463556 1.354594 0.2395928 1.279146 4.161409
1.697404 1.921198 2.064313 1.680157 1.633509
0.08565126 1.888189 0.3808353 0.5947924 1.198609
2.803263 0.08155755 1.280833 0.1095023 0.260645
2.411941 1.223837 0.03809052 1.442887 1.442844
1.150305 0.1554493 3.506033 3.664002 2.388335
5.089888 0.3884707 3.27733 10.88442 12.45473 5.80016
13.32564 9.055039 6.935298 3.511993 5.89647 7.726775
6.782926 5.525459 8.883854 6.411703 3.390506
19.95345 5.895954 1.502773 12.98356 9.187698
1.049363 16.3552 2.873478 1.812999 8.770232 2.612352
6.454328 8.382528 0.8176875 2.770486 1.226906
3.937258 12.60227 14.27841 4.922891 4.38887 6.605133
6.638644 3.524013 1.991602 5.677907 7.125978
14.53417 4.703069 4.007011 20.07982 3.494573
2.788218 5.715942 4.929317 6.229574 11.94778
1.912598 10.73025 14.27004 5.351667 6.099698
6.953063 5.078737 5.08031 4.615983 21.63974 7.460846
2.101888 4.282239 3.73492 10.32582 2.701548 2.122121
7.428723 2.21277 6.832781 5.156952 7.021887 9.934724
5.803559 1.765737 4.154234 3.820772 11.2784 9.107798
8.788096 8.229709 10.31412 11.02561 14.70242
1.019542 14.53049 7.595079 14.72061 6.825129
1.421159 9.358515 5.417283 11.81596 3.328119
9.875469 3.031485 1.983186 10.0437 14.45539 10.97143
13.1409 13.52805 11.04495 9.029407 13.33146 11.13489
1.377132 9.17049 2.254851 4.95435 3.326823 7.361284

3.87301 7.119521 6.901167 1.577096 1.709218 5.036063
0.5989114 7.77453 3.610188 3.630406 2.485406 5.933659
8.164137 6.281619 7.21783 4.902666 3.745868 4.654635
4.160271 0.8470322 6.516167 2.52353 7.52935 12.51344
7.676799 4.079803 7.214277 6.49948 12.68142 2.909377
6.672592 2.076965 8.814322 6.006191 5.184179
1.63244 7.397606 5.722787 9.110469 6.349567 16.44213
7.133303 4.543868 4.89547 7.040244 8.612375 16.14547
9.925113 4.036394 6.902524 4.079498 9.560886
9.878359 12.96525 8.205694 2.272477 14.99735
6.48206 9.615174 4.775269 6.986307 7.391147 7.363785
5.801733 9.004634 4.098385 13.75738 6.17456 7.241607
6.27089 12.64526 5.020926 7.11214 3.100018 4.508971
14.34199 2.041687 8.904094 7.938435 2.326085
2.18467 6.741745 5.301882 8.682133 0.9878471 2.237819
5.045243 12.69281 12.82022 5.759978 5.268754
5.493859 5.366347 7.515143 4.762768 7.659652
0.6315792 5.646269 10.95367 12.57958 8.689473
8.271429 4.659075 15.036 16.46835 11.06679 2.576567
9.673978 5.353329 5.907505 9.506592 14.35304
8.738272 8.975019 16.7701 14.70477 4.564553 2.983948
1.930025 8.107014 6.349766 3.50959 7.134552 12.49208
3.579705 5.374658 0.7325749 15.05505 7.570397
2.358989 16.11368 1.226869 8.927004 14.62486
4.625063 10.40021 6.919343 4.291844 5.712472
14.37221 0.3494648 0.6823331 8.186654 5.292648
4.401074 12.41617 10.46466 7.569611 9.666986
5.185436 4.067583 2.635184 3.85023 6.144671 6.226729
11.27687 22.08467 5.41497 7.885403 7.102623 7.732316
11.57619 1.937123 12.07942 2.339705 5.258335
6.76955 5.598936 5.572912 14.56689 11.33822 9.186869
5.463829 2.96729 6.666097 6.722932 8.31294 3.975508
7.483948 6.028156 4.505722 6.074046 5.284952
0.5070735 3.707039 10.00202 4.416273 6.811388
4.64705 3.221777 6.962519 6.667415 15.56575 0.7100511
2.087188 11.92854 10.14291 4.935645 8.520766
6.068599 8.142705 3.277965 5.300663 9.052592
4.491887 4.713778 12.6762 1.424094 6.352717 6.040604
2.563509 8.541986 9.780955 11.39699 3.920596
6.040878 5.102608 7.296657 4.330569 13.01554
1.69128 11.14555 11.12472 8.230649 16.15601 6.533241
15.0965 3.8499 2.574604 12.17453 8.447231 5.208781
10.59846 7.7399 6.819967 1.486028 3.206115 6.660276
12.33127 4.378153 9.824431 13.94809 7.333241

8.763649 8.332603 7.757634 1.699925 0.4738418
10.29482 12.5034 3.670493 12.38286 7.92679 9.369386
4.933992 2.919453
CDC25C 1.487669 0.02279728 2.114027 2.086119 1.119512
0.1496954 1.090331 0.1142916 0.7950132 2.096873
0.4256615 0.3372388 0.9572265 0.7197667 0.7928978
0.02119496 1.654973 0.2836181 0.9655353 1.181323
1.398515 0.01899477 0.4488867 0.2110717 0.0359231
0.7195938 0.7774004 0.03016239 0.8384958 0.908832
0.6602544 0.08975619 2.370341 1.111294 1.12185 3.052129
0.1190767 2.209765 2.954373 5.464583 1.574869
7.620645 2.506241 3.485246 1.306053 1.954265
2.052826 4.156478 1.611885 1.199629 3.671376
1.124669 2.849261 3.742476 0.9575678 7.948271
3.332448 0.249285 10.22199 2.139646 0.5034493
2.464495 1.040008 4.352101 3.510499 0.3327942
1.392294 0.6554029 1.293478 3.464091 4.495891
2.322358 1.669765 2.569823 2.693744 1.431482
0.6219436 1.533118 3.132474 2.582995 2.445753
1.42191 3.677344 1.309083 1.798047 3.818026 1.800557
2.728092 6.337812 0.54858 3.069385 2.497006 0.9531329
2.1655 4.434543 2.25536 1.539091 2.369865 6.290226
2.682357 0.7820545 1.483014 1.845714 4.278194
1.26938 0.6833917 2.772046 1.222745 2.968415 2.612632
2.873159 4.000842 2.083589 0.7043654 1.992658
3.34276 3.531822 2.644289 2.856706 3.312266 3.759981
4.206352 8.445216 0.5488462 2.502527 1.489224
6.999762 2.810574 0.7640788 3.993944 1.43884 4.00082
0.9509201 2.138055 1.609581 0.7414462 2.000096
2.407823 2.639519 2.414997 4.761758 3.685196
2.455552 3.99615 3.429344 1.438315 3.039141 0.9702186
1.86997 2.897073 2.859914 1.389542 0.9678403 1.170479
0.5923629 1.93138 1.823509 0.2088978 1.768141 2.616282
1.818296 0.8217959 1.550607 4.021018 3.201372
3.728334 1.810287 1.056176 1.662387 1.121208
0.2204282 2.684245 2.279102 3.153832 5.466433
4.385824 1.655909 6.248269 3.027461 3.922969
2.834218 1.868484 0.3577685 2.856389 1.774587
0.9308459 0.694622 4.064384 1.309476 3.231972
1.647913 2.974594 4.794144 0.9549281 1.590624
2.474485 2.083592 7.976393 4.55253 3.318767 3.415232
1.93011 4.001409 4.129899 4.71834 2.720959 1.941974
9.660356 1.810036 3.45882 1.122207 1.946096 1.141872
1.125409 1.789162 2.795097 1.00014 3.883135 2.088032

1.473133 2.100177 2.900434 3.228146 2.005023
1.022826 3.864153 4.056726 0.8550024 4.200231
5.103435 1.269558 0.6986354 4.508888 1.290354
2.748417 0.754205 2.376458 2.139093 4.118798
4.595878 2.085565 2.040191 1.61242 1.536212 2.268164
1.556936 2.0165 0.2386082 1.702003 4.756435 4.170534
3.045483 1.90918 1.953586 3.389086 6.390988 3.34958
0.8748305 3.464927 2.803656 1.983355 4.708276
3.769457 1.792922 2.431271 2.248565 4.707201
2.998243 1.441162 0.8381062 4.168758 2.352077
1.098067 2.693178 4.012687 2.679476 4.661485
0.274783 1.881267 2.018322 0.637975 4.550831
0.7165977 1.36651 3.656416 1.387275 4.206475 2.051878
1.622947 1.462567 3.693564 0.1235391 0.3416222
3.880999 2.301565 1.503286 1.705083 3.495622
3.296975 1.859607 1.964323 2.277393 0.7684844
1.686118 3.649695 2.168744 3.612343 10.20926
1.938012 2.93235 3.166729 5.458323 6.209282 1.077965
3.173267 1.302942 3.529057 3.466827 1.391575
1.455139 3.080645 3.529384 3.219417 4.05495 1.534745
2.926862 2.655648 2.416241 0.8896095 1.162436
3.781246 0.8537152 1.82275 1.789486 0.3657362 1.226909
9.334199 0.997199 2.771244 1.930019 0.7338701
5.680808 2.517645 7.152478 0.214059 0.784598
4.283986 1.657361 3.984234 4.107872 1.926397
3.090918 0.9148352 2.744096 2.961552 2.792814
1.490224 1.889644 0.824077 2.287247 2.445587
1.229067 3.27509 4.117396 7.114863 3.528332 2.549203
2.294735 2.229741 1.647813 7.959017 1.150925
3.64111 3.509899 5.003718 3.276539 1.807676 4.345521
1.656661 1.223813 2.122512 1.528163 3.162292
0.9301805 3.979162 1.831366 1.183265 2.665737
3.60275 1.966626 1.624858 2.884551 5.407318 2.351185
3.987788 2.773235 2.79575 0.7135871 0.1655368 3.998288
8.028936 1.234832 5.301418 2.585125 3.882271
3.955841 1.419327
BUB3 5.575138 4.64651 6.66589 8.504414 6.653782 5.350082
6.052726 4.285951 7.023462 12.21559 6.714841
7.249366 10.42054 6.239824 7.556651 5.145735
7.500158 5.565731 5.746945 5.989182 9.12473 6.033511
6.090553 5.403093 4.989737 6.26163 7.156922 5.27899
6.311978 9.30617 7.019271 4.986472 11.16355 7.630058
9.583487 18.93274 7.724638 9.148274 13.80159
21.68286 13.85717 12.4758 13.5732 14.53209 9.331507

10.95074 10.78884 19.48082 22.63215 8.663876
9.660867 11.68107 18.60056 12.77038 8.436749
24.33071 16.89844 7.448571 27.37382 11.68019
9.34039 14.72644 7.942749 9.915939 14.72529 5.979289
8.987644 7.892163 16.80781 16.22147 38.36767
13.68532 7.873563 11.35839 10.73347 18.74738
7.466632 14.97673 28.83108 15.63652 12.33563
5.969483 21.68224 7.311226 10.89867 10.74187
9.60881 12.27331 20.34032 12.01815 19.72401 14.80161
13.11987 11.79605 16.31988 10.74562 13.53827
8.06038 25.58917 14.31475 9.393171 10.39764 13.54751
14.35971 9.74446 9.413239 12.58037 7.872835 14.65254
13.76605 10.4257 11.36484 16.26571 8.434676 12.19409
16.62983 57.14076 25.83418 14.93037 14.22711
14.31222 14.99368 15.83223 6.567796 26.65404
14.48094 20.03383 11.59954 6.665518 26.12447
12.84345 20.65396 10.29105 20.27683 7.517227
9.794419 15.72565 22.44697 21.02688 10.42289
11.76676 23.36183 9.353606 19.59283 11.47255
9.150417 14.88123 8.928331 16.95379 13.31306
16.67867 10.17253 13.72831 11.19985 7.703251
7.062777 13.21646 8.271957 15.04731 11.09213
11.58252 9.249838 12.30849 12.17863 12.49647
14.11084 9.019187 12.79022 10.40807 38.92791
6.346524 16.81039 12.53149 12.40625 9.065018
12.95409 5.098555 14.25428 12.52016 11.02903
19.47308 11.99328 7.727486 14.49781 11.21418
14.28525 7.287318 20.33004 8.82474 15.93127 9.057927
5.983857 11.289 10.74968 14.80898 7.927474 15.33163
15.58519 17.27635 12.95823 10.68053 8.69922 12.05535
14.66107 14.28769 16.95258 7.445532 12.41743
13.18404 12.69105 16.06241 15.17602 14.03152
17.46609 12.12171 15.74074 14.25179 15.25097
11.37515 11.73426 6.439095 16.82047 9.335487
17.40965 10.45872 17.35186 12.7834 7.068383 16.29716
17.05755 7.842145 9.99031 10.38813 11.55524 14.6961
6.267666 12.34748 12.18296 22.064 23.89763 10.08951
11.39243 11.89437 16.32871 21.3534 10.34027 15.51953
7.139 10.07363 19.68169 22.86015 18.35453 9.15051
14.82908 19.84657 11.27324 15.03834 6.065706
15.7505 11.51099 13.11554 21.70979 23.8711 13.9035
11.54724 13.70542 19.84836 12.95108 10.10161
5.697433 12.5447 12.14325 7.554028 20.62647 10.46394
8.482524 12.03372 5.700847 27.00772 13.46012

9.055341	24.19543	7.149052	13.76574	20.10386
12.07674	18.26997	6.8794	12.13302	13.0926	11.64977
4.185104	6.155299	9.477927	14.21886	9.702215
9.578885	13.71448	16.70365	10.41793	13.52316
11.64871	16.1165	6.797758	11.47057	10.01498	16.40413
19.50659	10.37534	9.14252	12.04832	17.7413	34.47817
10.86893	19.61909	8.707487	7.395731	27.67391
23.3977	15.77792	22.80982	14.1165	17.07572	16.30241
8.377468	8.822011	14.71582	12.86872	16.29102
13.38237	29.98142	12.59071	13.56103	12.29819
6.456139	10.50004	17.49719	15.92764	14.40045
12.56696	10.25962	22.57843	14.40273	18.48963
6.540714	8.230232	6.732827	15.72882	28.8652	25.41394
12.66847	16.00792	8.373703	13.55688	19.40276
11.58327	11.01475	13.19299	7.706442	9.623675
10.82419	10.0807	27.87053	11.5101	16.33871	14.19937
18.85829	10.07137	18.62063	11.06611	18.84697
8.130199	30.25596	24.25111	20.15294	18.76482
7.058431	12.09281	8.8555	21.07879	23.23582	10.41681
9.980434	17.47283	17.72897	12.4529	13.19959	12.07476
15.14696	17.09349	9.057252	18.7989	13.52177	13.89294
12.5684	14.99191	13.10115	6.900964	4.686039	16.30895
21.19816	10.84738	21.8389	15.8028	19.86838	12.36275
8.449298
FER	0.4471493	1.963382	0.6194118	1.1591	0.828675	3.444416
0.6034821	1.313535	0.9143875	0.8784501	3.057765
2.079857	0.8668497	1.526888	0.5880584	3.309538
0.5066469	2.110028	0.4427416	0.3965944	1.121088
2.138399	1.376669	2.821677	3.26939	2.430085	0.6773897
3.883373	0.7156088	0.3414367	1.260603	2.041391
1.583303	1.310837	1.075584	1.010626	2.164283
0.6536835	1.182948	0.6053854	2.646596	0.6143188
1.702787	1.750129	1.59673	0.9203278	1.239275	1.061043
5.031109	1.215465	0.5913314	1.74087	0.5369534	0.9820344
2.709131	0.6703793	1.692259	3.896786	0.1690719
0.5888371	2.57344	0.7553405	2.457351	0.7967311	0.6638045
2.624292	0.9727035	1.267343	1.583662	0.5128981
2.459095	0.5615434	1.741898	1.863803	1.871804
1.76791	1.348081	1.540189	3.273641	0.2331256	0.6847051
1.998053	2.746896	1.827077	1.306203	0.7801301
2.628764	1.805378	1.217415	2.148645	1.172375
0.8341256	1.258066	1.132395	1.584579	0.844935
1.630283	0.6056994	1.009309	2.009815	1.858573
0.9528685	0.597514	0.780864	2.062512	2.423894

0.9775878 1.497199 1.550516 1.244891 1.336515
0.8800195 3.161345 1.625878 1.526929 0.6447864
0.2113493 1.469425 1.307121 0.4644481 0.9122156
2.11709 1.268627 1.273962 1.243299 1.275268 0.6998647
0.6532834 2.893353 1.93991 1.071391 0.8346896 1.051114
2.402308 0.5654197 1.560163 3.769995 1.299574
0.8749833 0.922787 1.218412 0.6910768 0.9198514
1.377545 1.4978 1.512912 0.5056488 1.840286 0.6273172
1.487578 2.398604 1.441262 0.8432549 2.153027
2.699931 0.6835887 2.575583 2.018012 2.029637
0.5748483 1.709874 1.749685 1.809447 0.7659846
0.5009003 1.727359 0.8256557 1.780381 0.9548698
0.6004208 3.12043 1.71017 1.892359 1.064236 0.885459
1.324029 1.768422 1.628962 1.043074 1.663411
0.3692249 0.4354374 1.74847 0.9882786 1.374017 0.8478488
2.865728 1.365941 1.784875 1.604493 1.179692
0.8389412 1.361795 0.9707403 0.4514271 2.009829
2.540306 1.270013 0.9830335 0.5944778 0.6781679
0.8893297 1.099585 0.7875519 0.8298815 1.505921
0.6853945 2.666082 0.396048 1.34096 0.865428 2.026956
1.501702 0.43432 1.304755 4.032663 1.059837 0.8648718
1.808948 0.8818015 1.386244 1.782087 0.3102844
2.655814 2.574913 0.5128563 1.06552 1.493612 1.453071
1.865304 0.7103614 2.431091 0.5804358 1.622494
0.2263588 0.894797 0.5380328 0.7466458 3.110167
3.327762 1.600953 2.029672 1.377011 1.377947
2.204254 1.031043 1.778629 2.365788 2.187637
0.5440723 1.258634 0.699685 1.208367 1.468428
1.38059 2.148846 1.059621 0.9978009 0.7033589 1.535255
0.6609643 1.174849 1.579641 1.178486 2.093473
1.134769 0.6690879 0.6605991 1.702258 2.64377 1.051721
0.4495658 2.056957 0.6651929 0.8238104 1.140821 1.1841
2.743592 1.715106 1.299196 1.979607 1.820591
1.170439 1.063078 2.092998 1.831898 1.191347
0.8646601 2.667498 2.359124 1.170255 3.711701
3.829139 0.827691 1.828274 1.382003 1.091661
1.286348 1.468031 0.7112835 1.38249 1.829312 5.679552
1.698193 0.8501744 0.7111388 1.694368 1.365952
1.885844 1.08953 1.039844 0.7732626 0.9122906 0.7903454
1.480886 2.232466 1.543482 1.061544 1.689037
2.73848 1.226867 1.049835 0.3837602 0.8568891 2.586654
1.095617 1.275481 0.846207 0.421467 9.999457
0.9372012 2.451911 1.475811 1.377102 1.210435
0.9095454 0.3827078 0.8052042 1.710204 0.9890847

0.6286303 0.4137234 0.6891923 0.6925376 3.289775
1.884883 1.766435 0.6949987 0.5902378 0.4922762
0.6271333 2.214384 0.9937329 1.052857 1.00408 0.454046
1.572368 0.5967134 2.329765 1.173281 0.8785765
1.027564 5.787147 0.829688 0.7802513 1.131785
0.9345762 1.025912 0.8626698 0.5163243 1.114618
1.137051 1.047333 1.319493 1.342313 0.4270048
0.7137356 0.6201237 1.054387 1.944197 2.132865
1.242489 0.7854147 0.5470224 1.931236 1.009303
1.253923 1.480523 2.805912 0.8763859 2.546539
3.17735 0.8908652 3.318982 1.990793 1.105977 1.466399
2.287925 0.8253784 0.9337116 0.4630024 1.659462
1.904776 2.565445 0.3176209 0.7588899 2.044068
SETD2 5.694568 6.597593 8.898205 8.933345 6.248262
9.748374 4.265909 7.262814 7.275821 6.228076
14.22988 6.774777 6.198485 6.628093 6.997724
11.36727 6.418701 9.019604 3.742128 5.15981 8.413191
7.720602 6.360926 11.16066 8.268248 9.675887
6.01391 10.97326 6.433976 2.07297 7.73687 6.686428
9.199181 7.179563 8.038376 4.374078 5.140859
5.308584 7.438063 9.056397 19.90405 7.937134
8.245382 10.92382 13.02098 9.904164 14.63922
7.89152 14.68563 13.66679 5.189348 11.22838 7.174877
8.185231 8.083409 11.3607 9.613074 14.44462 6.623144
7.564795 11.13259 9.122634 8.298631 6.636518
6.457608 9.546175 4.123472 5.447661 7.127377
7.808107 12.97697 6.994491 11.63665 12.02734
11.22319 6.994376 8.68837 13.93926 15.68149 8.361165
6.911107 8.346142 13.48092 10.33624 6.904059
6.73521 13.76881 11.47744 5.757569 8.778484 12.00652
14.36697 7.587379 12.28282 6.138995 8.173084
7.141961 7.660795 20.51117 13.80479 12.27146
7.643227 2.435739 5.331353 8.784805 15.43773
12.31927 6.540113 10.40355 6.422205 7.465428
8.716317 6.392644 8.032992 5.798196 8.640587
5.368904 14.12956 8.95676 8.3014 3.949705 10.12944
12.69757 4.835188 16.56939 15.19486 8.594711
3.884835 10.94707 5.961836 13.32801 13.51454
8.593249 11.83711 5.313283 13.9194 12.52409 9.31188
10.32545 9.637334 9.885197 13.56657 7.902189
10.50884 7.897324 6.682142 8.047362 8.012626
18.70574 8.957959 13.05985 7.50816 11.50955 13.22705
11.24352 8.168616 9.282562 7.613352 6.99865 3.963792
7.71555 11.94199 11.99844 9.509976 4.221351 12.34018

8.022278 7.813316 10.83418 6.466203 9.375619
14.59392 14.07138 4.356017 9.961228 6.858694
6.19328 6.009761 9.057912 16.48305 8.693868 7.193895
8.514422 14.6313 4.858547 9.904597 11.87744 6.439333
32.3998 10.792 7.835861 7.808632 4.90607 6.743831
3.613119 11.51281 10.60002 13.68473 10.52801
5.349685 5.173157 6.063575 9.853334 4.79077 5.693164
19.07531 4.464426 10.76962 8.045266 8.473746
12.54789 10.3913 14.57048 7.030764 12.85858 12.97787
4.542295 13.30978 8.469424 13.65837 9.744392
8.31179 5.471928 10.43988 16.96058 4.762983 13.44388
6.186573 6.93184 5.161966 3.736671 6.743409 8.10836
12.93287 11.93308 12.0592 6.751083 6.493158 11.41438
13.05762 10.30683 9.908589 9.290158 7.874054
8.42281 8.765299 10.48824 8.526511 12.59183 7.218211
11.53379 12.84242 7.367175 7.991822 10.48928
20.5987 8.34743 5.937393 10.25579 9.78102 6.373271
6.741058 9.133965 9.232093 15.5754 11.72893 12.0917
6.564245 5.744719 15.63955 10.37649 3.773651
6.29236 8.480798 10.09083 8.274805 8.310521 9.512778
10.04657 8.104107 11.55597 11.35091 3.946089
10.31749 17.71366 11.72604 14.45623 5.526005
9.372807 9.087114 7.427786 10.67767 11.32932
7.626825 8.510588 6.056442 6.513199 10.97011
8.223648 11.57207 11.91189 12.25256 5.850248
9.157828 8.63365 8.375481 9.866941 11.82221 17.13948
13.6069 8.41558 5.895725 11.81099 5.9952 11.23353
11.97459 14.7812 8.799552 9.69977 10.29846 10.18605
9.726061 9.847996 6.480288 4.062068 7.323999
7.651832 7.098939 8.011834 17.5755 8.802642 8.632029
8.956795 7.710067 6.35659 8.609735 8.216193 5.003961
12.58828 9.908377 9.830335 6.07619 8.945324 15.94087
13.0938 12.83148 9.53598 16.34051 3.125401 7.451503
12.28442 11.45868 8.157459 4.488894 9.39274 10.86888
15.36329 7.878377 8.052819 7.200608 9.520094
6.156624 13.5393 6.620538 5.808631 5.736819 13.30958
10.68639 7.852698 4.031809 6.28745 5.817787 13.9845
15.27888 13.04198 7.085195 7.899173 4.25999 11.00544
12.66609 9.639071 16.47031 7.332273 8.564594
13.78338 7.910493 5.199041 5.524836 10.48821
13.34098 12.12894 10.81125 5.735396 10.10553
16.82438 9.583452 15.10588 10.70064 8.326241
6.94115 9.646754 9.842479 13.01001 12.81982 7.923919
3.970485 9.010527

CDK1 1.585545 0.2261843 5.589007 6.001945 2.879597
0.2732368 3.426236 0.5448275 2.413572 9.890897
3.803817 5.743558 6.392324 4.341966 2.771688
0.1670247 3.995862 0.7930874 1.857326 2.454938
6.266575 0.1435516 2.727108 0.2122458 0.3138862
4.610335 2.492221 0.2279501 2.156929 6.524592
2.015775 0.2054027 12.7077 5.517536 8.039754 38.82454
0.4884146 71.6536 9.119958 24.92038 11.42218 27.72962
15.39494 14.42456 6.112153 11.6968 10.74697 12.99435
11.23713 8.498601 10.02202 8.351405 20.82037
14.48769 4.251613 33.30777 18.58059 1.579906
11.65144 7.826444 3.478538 11.62802 6.253966
22.30339 24.49972 1.625371 4.937071 3.436582
11.84184 17.50213 33.9174 17.55374 6.869865 10.17328
11.31707 7.633319 4.29856 13.12001 15.50816 15.83242
9.035355 6.265715 32.86257 4.336217 3.994246
13.8238 7.773044 11.25351 25.08196 6.432432 18.53809
13.09442 12.03223 9.84339 11.3329 10.59478 9.049466
7.507292 30.57848 13.0256 2.725527 9.51902 13.37829
18.80611 10.51425 3.10578 10.84999 4.37541 15.13221
19.15228 20.1598 17.34559 15.91035 3.528422 10.22283
9.524944 4.12706 17.51274 18.56074 28.04815 12.86255
11.55067 10.62628 3.266144 29.82471 11.33536
26.89843 9.777422 3.060547 17.19617 9.781944
21.97713 9.604577 19.11611 3.486726 3.424709
18.39286 16.58229 21.59836 14.99957 15.15399
18.86283 8.151052 21.21164 11.55356 3.745119
15.71267 6.344915 14.22418 7.375762 10.78673
6.406606 11.02851 7.368297 2.64418 7.438843 12.86296
2.078716 10.36199 10.58483 5.06381 6.280079 8.802652
13.63043 14.61096 11.11368 8.967454 8.359355
8.994687 23.26018 1.855035 24.41849 8.584473
19.42893 15.27522 20.46754 4.172364 35.14221
13.03456 20.38303 37.30576 11.23926 4.39977 10.13365
13.53214 6.982285 2.375365 20.04248 6.132684
17.11759 6.719461 13.14465 10.66489 11.23838
10.5752 11.175 13.44446 16.66457 21.80149 12.49104
18.60445 7.66508 12.85922 38.95713 21.55909 22.55503
6.849398 19.31796 10.86616 10.72055 6.538025
6.992338 13.36896 15.03747 9.179237 16.99924
2.677704 32.48817 7.530953 7.91636 11.53664 15.33988
4.953974 11.17018 3.43875 15.36231 18.73697 4.596077
13.52327 14.90712 5.366469 4.332215 11.72851
8.520486 18.40255 2.741929 5.185402 6.613416

30.31894 24.18934 8.857888 10.31055 11.50325
13.14405 16.62871 9.019158 14.13497 1.202176
11.29177 36.19348 27.24677 18.10502 13.46315
10.28494 21.10274 15.63807 16.64831 3.321537
15.65639 7.454445 15.01785 17.66246 23.69053
16.23389 9.84143 16.83875 31.72405 10.03582 14.37773
8.380273 14.84575 11.61689 5.096489 13.25823
18.94232 7.012825 13.26365 1.215818 20.67646
12.69182 3.145069 24.85091 3.230937 13.41894
24.99242 7.347924 11.63766 9.679402 12.5603 9.093385
15.64189 0.6128493 1.783585 11.93532 9.644879
4.873913 9.499547 12.5004 19.22208 33.02966 8.576019
9.670172 8.121889 4.715055 12.61021 9.440391
23.20002 21.06484 11.56656 12.07259 6.496617
11.66508 20.53663 3.875238 26.5425 4.258366 9.610477
15.92381 11.27668 10.24701 21.49577 21.97825
14.25618 14.15147 8.559134 10.58713 10.40489
18.8792 13.00088 11.12618 19.12596 7.737024 5.969024
7.459767 1.519988 7.60802 19.08371 10.64472 15.18793
8.089286 5.85386 23.07995 16.00519 28.5912 1.482233
5.682142 7.629915 18.76565 12.05403 34.39828
11.9591 9.06778 7.633152 14.09637 15.47493 20.04376
9.072377 17.61904 3.23582 15.56714 9.672551 4.394599
28.05772 17.42375 23.60626 14.53993 26.48729
12.56563 16.99556 13.04843 15.47628 4.559653
29.03841 16.36019 21.65234 19.90437 13.29904
14.7673 5.929859 13.273 12.59084 12.06555 7.587128
18.34509 12.48901 13.08529 2.979547 8.866368 12.197
19.22479 8.514323 18.38187 18.86096 10.30783
9.175441 17.80029 11.07674 2.687032 0.9623327
11.53575 11.164 7.065849 20.86946 19.30612 24.20345
9.069345 11.19806
ITGB5 10.42491 17.40947 10.56349 10.6237 8.090314
20.23436 6.566789 12.38877 15.70808 7.586605
17.30319 13.06086 6.382422 20.30716 7.112744
12.24773 8.307274 21.61678 9.92805 9.712848 16.81656
13.47503 20.7137 12.86061 26.18946 14.74814 7.969031
9.15912 12.50716 5.277142 13.29721 20.3721 31.70493
20.64695 33.63819 7.16807 10.80076 16.78911 22.25022
15.06933 19.83092 18.50113 29.89414 7.462412
11.60979 29.27593 7.386726 17.01875 37.40461
41.48256 22.55989 11.69823 25.18699 19.7446 28.87211
12.99828 12.83486 14.83811 14.09574 12.32437
26.28872 19.53998 18.42234 27.49394 15.81612

19.74323 37.63994 19.8792 21.30136 18.43592 14.78164
8.029466 21.21171 23.83516 25.01533 6.076735
13.55645 14.1964 14.18735 12.28951 23.49846 23.50372
10.43522 18.86464 34.74678 21.70402 23.02296
13.59535 10.55336 37.5842 18.60017 9.348035 18.29419
11.38367 15.52705 24.91939 31.40095 14.09558
7.014315 9.257954 18.87585 19.17255 4.320094 10.564
19.00037 20.44845 26.57001 20.82675 25.71926
12.13459 25.40199 15.4872 7.565585 8.213171 13.65575
21.09648 16.38962 16.01824 13.98756 7.105837
6.564836 13.11122 21.18849 28.55462 21.65128
4.948749 6.224865 24.5926 29.80815 44.39996 16.66151
14.19571 23.01248 16.8287 13.38574 20.86462 15.1611
15.01228 21.07104 8.347473 24.8309 9.717022 20.30963
12.08181 25.47315 18.70764 12.78689 21.54788
24.58329 18.7883 16.78642 27.0598 28.31125 23.73141
19.93346 9.4961 14.93104 18.86541 20.85362 11.45284
31.1105 8.442712 16.37165 18.51429 14.87223 15.63525
20.83237 21.75931 13.14483 22.88872 11.23092
12.09225 9.958884 25.87176 11.34521 33.73678
58.13191 17.71845 17.39347 20.77305 9.925115
29.45145 20.68445 16.31961 18.13222 17.17712
36.13168 16.54127 37.60907 24.52384 49.2448 48.72544
33.82059 9.646554 29.56685 26.16876 21.15348
14.65845 17.65622 15.66683 9.601481 15.52611
12.61508 14.45793 11.23099 14.07947 19.32584
8.656412 14.76298 29.63428 15.46463 24.73078
16.21326 20.98 26.42118 16.57001 55.92922 38.48509
34.81132 25.52011 15.68169 20.36023 17.36455
21.1479 14.91893 4.65338 18.04684 15.2716 9.939375
17.98528 40.69728 16.51742 8.882612 16.68289
9.608558 22.54812 9.520963 7.052046 20.4766 10.99404
16.99464 26.77294 10.51652 22.42934 24.11735
21.65329 13.88668 24.91804 21.73866 9.070738
14.80242 19.03471 28.73582 23.56074 16.5923 50.15397
19.8231 38.8657 11.79329 27.30432 8.531388 16.39417
9.940891 15.24811 18.41445 17.48694 20.20993
11.27257 36.25312 18.73825 19.3779 22.56671 14.45571
8.681383 21.58765 11.20399 29.84058 16.82882
5.21639 26.67979 16.34971 10.8589 38.56563 15.97374
14.34607 19.44245 11.41145 24.26306 13.14476
24.19899 13.49798 12.35711 12.1762 11.67581 48.00702
33.05677 22.62893 19.37429 43.58283 14.06806
14.68077 22.33236 51.4711 31.69814 15.11168 18.10893

17.60202	18.43487	27.24107	14.77228	25.89307
20.00565	11.76642	27.87157	8.20661	23.81346	15.66753
37.08404	13.7931	40.15591	13.41906	15.59643	20.64144
10.81964	22.08975	13.42046	21.60971	9.609123
7.776788	47.92689	21.02018	24.7553	20.79334	14.63731
35.34339	11.73095	8.843095	13.2045	20.1152	16.851
18.64443	17.66785	23.43934	16.98267	20.89481
15.84648	28.07822	6.021563	21.24548	7.15441	13.33823
25.93451	24.15903	20.058	10.34116	15.20337	15.72549
32.778	16.62455	20.21654	16.00987	19.499	24.4412
14.74931	8.875367	10.03138	5.99942	8.821678	16.34932
16.05562	14.36537	15.53564	16.29149	12.33762
22.30593	26.42646	10.35715	11.45535	8.601079
10.08915	35.016	29.19805	16.59351	28.13149	15.55213
14.56459	27.97736	17.10515	11.36197	10.0686	19.84199
12.93631	8.889464	42.05176	12.35979	17.85715
28.99474	16.809	9.453575	21.66245	12.05562	3.138524
15.01004	8.207609	8.544122	15.48204	21.16887
TP73	0.137488	0.0123253	0.3161669	0.5602339	0.188792
0.02393165	0.2389803	0.03604505	0.5936585	0.767084
2.725452	1.608026	0.7132518	2.116907	1.148746
0.01909834	0.5656513	0.04367205	0.1803708	0.4588214
0.2786439	0.03209212	1.202516	0.03598235	0.05738242
2.617285	0.8399193	0.00169867	0.5479823	0.03899666
0.400675	0.03081046	0.4126574	1.691494	0.0961906
0.08476677	0.01788292	0.1832961	0.2692036	0.4163556
0.9558854	0.1931644	1.008419	2.753792	0.5574589
0.7208188	0.8398906	0.3209612	0.194679	0.8287367
0.6794956	0.5310299	0.1590227	1.763658	0.2387483
3.69985	1.473012	0.1211216	0.5406728	0.7630419	0.1243873
4.321294	0.1818632	4.703393	0.1182519	0.06259506
1.383542	0.3343669	0.209363	0.9829475	0.1816414
0.0685087	0.6056615	0.7952414	0.05850875	0.784806
0.1617881	1.017735	0.1073111	0.06734131	0.3399108
0.572575	0.2070987	0.386569	0.466142	2.088335
0.3359831	0.4937692	0.5593122	0.244734	1.204844
0.9474949	0.2587556	0.9575091	1.023696	0.5157459
0.3539991	0.3958702	0.06309788	2.495407	0.820349
0.3828764	0.8860153	0.2448023	0.7026908	0.3678536
2.823233	0.10697	2.08967	1.134135	0.04163127	0.1776665
0.6220564	0.331554	0.7988647	0.4161668	0.2595958
2.215924	0.568147	0.1708063	0.14228	0.6864063	3.539631
0.1914403	1.664387	1.835748	0.2181043	1.305774
0.3681263	1.769605	0.07581018	0.1443001	0.2929043

0.6688834	0.4898568	0.3340509	6.318075	0.1225916	
2.026146	0.3946303	1.513167	1.447697	0.6935413	
0.3867746	0.9124344	0.3648359	0.04740797	0.3012101	
0.8027376	0.6463279	2.189863	0.131692	0.2842115	
0.8326538	0.251184	0.1011818	0.497161	0.1577092	
2.564868	0.265935	0.9454967	0.3797453	0.5689937	
0.08012596	0.09527417	0.861198	3.296694	0.1606242	
0.3306006	1.520736	0.2092639	1.375058	0.4836167	
0.4809289	0.1026185	0.7624829	0.6400675	0.05568317	
0.2066653	0.437513	0.7216479	0.1583848	1.643164	
0.3862751	0.404672	0.5202473	0.3698556	1.171365	
0.7193037	0.2358785	0.1123808	1.46921	1.876894	0.8571818
0.4617521	1.564179	0.3595761	0.9048225	0.1291511	
0.3537388	0.17519	0.2094764	0.8908666	1.979936	1.46733
1.300853	0.6316108	2.348154	0.9997543	0.8688781	
0.8243456	0.2283316	0.3489546	1.774646	0.7359938	
3.356886	2.154156	0.2501726	0.2408118	1.926527	
0.3227549	0.1789152	0.3968617	2.114467	0.2997228	
1.727027	0.3349334	4.102263	2.225239	1.159311	
0.06059178	0.1573815	1.527077	0.04915252	0.175057	
0.697641	0.8138472	1.717983	0.9266281	8.379015	
0.7208118	0.2604723	1.105358	0.8571698	0.6678194	
0.4518238	0.3612593	0.129003	0.1877927	2.850646	
1.363379	0.7781759	0.03200601	0.3777713	0.1703396	
0.3648457	1.734865	0.7192605	3.586064	0.2115635	
0.4841866	1.287803	1.733957	0.9342757	0.2890597	
1.222217	1.977535	1.869658	0.722436	0.7574214	
1.582459	1.404181	0.215879	2.591693	0.09570223	
0.3516563	0.6565624	0.1031672	6.511207	0.3048191	
0.6898394	1.127303	0.283423	0.5919115	0.1677141	
0.5208521	1.241439	0.1805572	0.8559032	0.7375983	
1.147534	0.06456474	0.05130479	1.265177	2.748731	
0.4842989	0.8444104	0.6425272	1.26426	0.07333277	0.9249905
0.1492876	0.2802836	0.4441106	0.7360519	0.517291	
1.732129	0.6166666	0.3300247	0.07941278	0.2696064	
1.159333	0.2917918	0.4996219	2.480513	0.493962	
0.6915002	2.357798	0.629152	0.7348695	0.4006462	
0.4385674	0.03017632	0.2305816	0.1104575	0.3932868	
2.339376	1.991089	0.6582554	1.469172	0.8278371	
0.2615032	0.5347487	1.92171	0.3330633	2.424795	0.04867882
0.6758532	0.4577321	0.1900341	0.2645101	1.169444	
0.3462831	0.7510311	0.1446631	0.8115687	0.8109846	
7.33296	1.350566	0.2185936	0.4891904	0.9122288	0.4030102
0.1049468	0.4963153	0.8073667	0.2557734	0.232189	

0.3468531 0.4969648 0.680877 0.2679403 3.826557
0.861721 0.9338308 0.8659593 1.193142 0.3360716
0.4864791 0.4395038 0.1445353 0.927082 1.605735
1.206763 0.1617311 0.9289718 0.1941024 0.3147465
0.4556137 1.448714 1.381721 0.8593414 1.204365
2.300681 1.040068 0.2109286 0.1767208 0.4511769
3.51513 0.9038432 0.2534746 0.7661654 0.3270839 0.6246498
2.479386 0.5721034 0.242304 0.4832001 0.1491618
0.05003854 2.671818 0.3263866 5.031771 2.798882
1.826494 0.9692504 0.09719842
MAD2L1 1.059062 0.1740208 2.27383 1.888148 1.634476
0.5861895 1.593355 0.445305 1.045782 3.692489
1.744387 2.075712 1.955531 1.412433 1.326871
0.4119645 1.810056 0.6089932 1.154733 1.63572 1.943457
0.6670766 1.701047 0.3689299 0.3237267 2.177471
1.148273 0.4616826 1.216227 3.859891 0.9499977
0.5120463 2.154368 3.566409 2.12154 26.14723 0.675934
3.112358 5.279007 12.48403 3.853709 13.84589
10.37849 7.55257 3.347156 5.856748 5.517456 4.588704
5.667207 4.114303 5.152872 2.997613 6.608278
4.195786 1.389751 21.51423 9.055281 0.6918196
7.263729 2.326695 1.518043 4.812047 2.809394
4.69985 6.901006 1.120139 4.469696 1.261979 2.481107
7.488243 15.86034 9.954864 6.020938 8.534138
5.694086 9.485314 2.14299 5.211581 5.151809 15.11858
5.216713 1.595853 12.60851 1.873902 1.938229
6.563686 3.414768 7.449413 7.351416 1.208819
10.09613 8.385039 5.909736 3.620874 6.726374
4.221904 4.152844 3.696916 14.73556 8.226586
2.208904 5.75032 7.926272 7.164374 2.545526 2.604489
4.907905 1.230272 7.937036 10.53707 7.148406
7.042394 11.09828 2.599744 3.401654 5.773512
3.014767 4.463511 6.421441 16.59307 7.962163
6.833449 9.88061 1.736266 11.89065 4.56989 18.82096
6.683948 1.334512 3.321983 4.904014 12.96955
4.048073 9.57895 2.118961 1.349862 11.00876 8.597199
9.257544 13.16997 6.814984 4.074698 2.925835
8.172676 6.035764 0.9607242 6.77884 3.222003 5.952054
4.634353 6.323423 2.953521 9.869334 4.240284
1.778775 3.595273 8.674895 1.209119 3.976369
6.807572 1.676303 2.454618 2.840717 7.965076
4.88249 5.148621 4.370745 2.688351 3.535234 3.752804
0.8158525 4.216356 2.516072 7.253205 13.2052 5.621046
2.009877 8.58258 4.52762 5.873063 6.464693 4.178901

1.488911 6.553529 4.971632 3.566502 1.084961
6.43962 2.115801 7.753376 5.14595 11.48259 5.062231
3.659359 4.824413 3.760406 7.387598 9.769704
6.168427 7.783884 11.66943 2.214056 5.501079
6.794719 11.91767 8.511178 2.287296 12.83289
6.439566 3.626701 3.685662 3.156651 2.569021
3.808055 6.005916 8.507461 1.771746 12.5669 4.239435
3.558617 6.090119 12.93163 4.929409 8.609687
2.237116 9.79739 11.30557 2.083702 8.221515 6.408396
2.532266 3.047997 5.123163 3.968474 7.042326
0.934051 3.84844 7.311583 17.16153 6.147435 3.769101
3.097599 7.360817 7.385458 8.682746 4.383948
6.640905 0.7184382 3.40637 8.533743 6.275642 9.483902
7.423281 3.949909 7.11035 9.057934 8.271512 1.621799
10.70013 2.786951 5.400436 12.48276 9.852091
8.240482 5.158226 7.374761 13.43071 5.782744
2.971873 0.9146899 8.449961 4.241969 3.273772
10.32387 8.024626 4.669857 4.894101 1.163271
7.717885 4.270588 2.280746 7.737432 1.522366
8.266359 15.33454 3.848546 12.20335 4.886132
6.858106 2.698136 9.37368 0.4426971 1.040765 4.976315
3.505234 1.739444 4.596843 6.371888 9.465952
7.459087 5.695689 3.995324 4.823797 2.860181
7.457211 4.258338 7.700609 9.838868 3.954019
13.71127 4.947362 7.866349 12.19617 1.983572
12.49079 3.314453 7.712667 7.398949 2.708231
3.89716 7.421082 10.48921 4.61091 6.526815 7.597602
4.010367 5.376539 11.8395 2.846689 6.122808 5.657001
4.027439 3.552849 1.892006 0.8951288 1.17262 6.823158
6.22487 5.701655 3.419925 4.028152 3.04211 7.41472
18.26588 0.8359113 2.507786 4.902183 7.468758
4.964952 2.173286 5.950684 5.929147 5.069363
6.598576 8.810121 7.620981 3.228037 10.0206 1.75886
5.941072 3.849309 1.689762 4.158076 13.06254
15.96149 9.000046 7.524867 3.969254 7.550849
2.480743 10.92689 1.152416 10.01367 5.121001
10.25246 12.55394 2.92653 6.301808 4.003073 1.948764
3.154979 7.539932 3.527514 5.195016 9.849365
5.492698 1.372317 1.315903 6.974411 7.362681
2.075124 10.94978 6.007065 5.653009 6.513087
5.947016 4.977776 1.79063 0.424128 7.031823 8.982667
2.885976 11.5146 7.883465 15.3019 5.858611 3.418823
BCL2L2 5.677572 28.6874 7.165314 8.763174 6.509948
17.75187 7.116341 11.48906 11.35673 8.873253

17.64526 13.49838 7.118008 12.58459 8.300518
21.8739 8.841678 14.84565 6.243728 4.659329 8.750003
23.97498 10.32924 15.94633 23.8755 12.96007 8.328278
18.56909 6.601731 4.462205 12.76111 21.42034
9.250101 8.837064 8.284363 5.930301 7.073488
4.76907 7.36708 5.592393 9.626376 6.05982 4.245227
4.880685 6.678562 6.645555 6.727412 7.560225
7.045325 4.723053 8.868268 6.969896 5.272408
4.464003 15.32337 8.65508 7.439928 12.60388 13.43081
6.629721 7.64294 5.600187 6.987965 5.730615 6.81562
20.10342 7.183749 6.747801 3.592658 6.480997
6.353837 7.996239 9.462554 11.8105 9.346184 8.392592
8.116094 6.764372 10.96464 9.835788 11.2348 8.975045
4.75154 14.61827 11.5041 8.307974 9.92796 9.493225
5.465471 19.95134 5.307469 8.86838 8.073581 5.990232
6.1518 9.290298 5.550465 9.188707 10.26844 10.81364
7.568768 8.169159 5.306221 3.648552 10.60032
8.51849 7.069678 9.887043 6.235216 6.921821 7.307236
5.101791 6.50049 10.1742 7.787574 8.822651 8.327844
5.061305 7.322661 4.143672 6.062091 5.264788
8.917101 3.045665 8.841413 5.379164 3.600528
4.913963 15.9344 7.826168 11.83465 8.580318 7.777887
6.210241 3.931026 12.74019 7.082822 4.227272
5.126192 8.946949 9.750568 4.948412 7.500636
4.276099 6.265563 6.916722 6.505779 12.03873
10.20289 8.483314 8.474846 9.479241 4.201934
7.390157 16.5126 5.793643 11.41374 12.62403 13.10471
4.804001 6.597289 10.1619 8.803158 5.985298 8.447115
8.152279 5.012574 7.027638 6.409646 9.231465
17.31336 8.165234 5.284162 7.467976 10.43387
5.924202 10.19276 7.401652 6.506258 9.030044
8.933415 8.522399 10.38217 8.129785 7.291283
7.640718 11.96941 7.799151 8.73257 9.547435 8.212165
8.859326 7.146589 9.427428 7.073145 9.284782
7.094796 6.432368 6.37193 7.38784 2.985813 9.219303
7.55179 12.14062 11.63479 4.72743 6.527845 15.73132
7.814101 6.968307 5.274714 10.10057 5.862325
9.241784 8.520694 10.73902 7.475256 5.627059
14.43582 5.889817 10.72577 6.669205 3.775151
3.977724 5.954901 5.28695 4.519287 10.07176 5.103233
6.36714 9.461479 7.420958 5.188681 3.625707 7.073904
11.81663 6.564649 6.641406 5.400518 9.147092
10.03454 7.625033 10.33483 10.52946 6.346019
8.640204 10.30777 15.19337 10.1668 3.286631 6.113902

8.665216	6.975806	11.0222	7.36822	5.254667	9.36401
8.01351	7.401414	12.08527	5.54183	6.339359	4.500326
9.408421	7.233497	7.344209	10.6292	5.263508	5.880702
9.047519	9.166392	5.533183	8.059741	6.219244
6.436226	7.595217	12.33482	19.55414	3.837465
5.03286	10.06063	5.342719	13.59998	6.686153	8.677257
10.87786	12.06658	4.844788	9.416556	11.1633	5.432028
17.79587	24.44338	7.035245	2.806136	10.44668
4.542024	9.195656	10.70596	6.600426	9.911303
10.92623	7.973614	9.707826	7.92611	7.951314	5.217565
8.45047	6.743901	6.645903	5.850469	7.076699	10.21899
5.362465	6.777194	20.33298	6.090857	7.916686
8.925814	8.048993	5.969315	8.889991	5.521448
9.865058	5.560326	7.245881	6.486946	5.345436
5.360889	8.781519	4.705941	9.517221	6.16667	12.30673
16.02404	7.414802	6.29406	5.608909	7.091558	7.049513
14.58739	11.57179	5.683802	10.05651	18.98013
11.53898	8.661573	11.61428	7.396945	5.993624
8.82772	6.232561	10.54343	6.599624	6.642531	5.302403
8.213586	5.348022	14.42547	14.25684	4.72542	6.240697
10.37628	4.507551	9.387102	6.204733	5.103906
6.167146	8.617561	4.714446	8.037524	6.566219
10.28716	4.528802	10.41572	6.667187	4.348439
12.86733	6.703881	5.533071	6.017963	7.436125
9.060108	9.078811	7.618144	5.804156	9.743332
5.427168	9.303545	5.811354	12.75469	5.67392	4.248807
6.752589	7.575256	6.670262	8.631055	13.86692
11.7465	8.43704	9.119973	6.015901	9.912881	7.471306
6.624145	6.035076	17.93609
SLCO1B3	0.004101812	0.00784453	0.03018403	0.0117549	0.01614065
0.04984849	0	0.004915961	0	0	0.1056273	0	0.006334251	0
0	0	0.008182117	0.00308838	0	0.01752123	0	0.01634022
0.4487737	0.03925927	0	0	0	0.01297358	0.01585307
0.03722959	0	0.003676792	0.007918764	0.1808628	0.003339341	0
0	0.03315605	0.225276	6.552218	0.6061724	0.1120933
1.238093	0	0.02111899	3.193286	0.3388696	0.03086218
2.050697	1.857562	0	0.03869978	0.1804201	0.3762204
0.03998768	0.166399	0.0120451	0.0294339	0.004112933
0.005560808	0.004656898	2.5564	0	0	0	0.01389736	0.12313
0.01658263	0	0.02005755	6.740244	1.099982	0.01623065
0.1453393	0.1564011	1.772963	0.7420302	0.1769613
0.037254	0.00803623	0	0.004480571	1.911239	0	0.2203495
0.2899213	0.06752812	1.099921	0.1686214	0.097159
0.01976372	0	0.003503979	0.02077548	1.520258	0.0125713

6.270119	0.02591533	0.09982414	2.507061	0.02567791
0.007167195	0	0.024601	0.02539494	0.6435812	1.710083
0.06127368	1.342078	0.06562081	0.01738836	0.04610915
3.203293	0.3481832	0.3341201	0.02279641	0.04130543	0	0
0	0.008489549	0.07501785	0.01511963	0.8605206	0	0.02387148
1.069244	0.01330895	0.0455929	0.1328756	1.081193
8.307711	0.1085139	2.091382	0	0.02091239	0.08583389	0
0.01805679	0.4574801	0.01955939	8.521489	0.003190914	0
0.1122737	4.405215	17.09221	0.00701369	0.03614919
0.09616217	4.548974	1.408116	0	1.830362	0	0.01287959
0.1246141	0.0145706	0.06913821	0.2058524	0.006182555
0.08610272	0.01677871	0.01119442	0	0.003256135	0.5888937
0.03066916	0.01117239	0	0.006772252	0	1.185984	0.2379161
0.02555701	0.3379685	0.674551	0.1771999	0.003946013
0.1186613	0.5280557	0.03728414	0.004593579	0.003821468
0.1725379	0.005068093	0.1054496	5.999345	0.04059367
4.741403	0.1055296	0.2420571	1.184863	0	0.007053257
0.06102432	0.6000893	0.03667169	1.951884	0.01437063
0.1483175	0.003361081	0.7680624	0.006696964	2.576615
0.04452419	0.0444829	0.03779559	0.01393361	1.020043
1.101788	0.3400568	0.01522936	0.00904796	0.05904567
1.60777	0.07023416	0.2372117	0.008594385	0	0.01913849
0.008648505	0	0.1851167	0.6152039	0.07645503	0.1524667
0.009552137	0.005004491	0.06148771	0.1527139	0.7202461
0.02140008	0.1779923	0.2949257	0.05741626	0	0	0.04168458
0.008376265	0.05836981	0.6781892	0	3.090334	1.30079
0.03151417	0.04097696	0.1231574	2.713152	0.01462372
3.628255	0.004628749	0.09930606	0.550275	0.005860214	0
1.208249	0.008503633	0.1386371	0.00792066	0.03758101
3.291935	0.03666993	0.5805029	0.04841429	0.06863731
0.5008422	0	0.003405926	0.1314727	0.02991589	0.005574023
0.06869892	0	3.434777	0.01637666	0.2258078	0.1510216	0
0.01467261	0	0	0	0.0340188	0.7871047	0	1.749482
1.013568	0.1010125	0.3003591	0.02978796	0.01700389
0.006755862	0	0.4588381	0.03476344	3.743669	0.04612121	0
0.01050147	0.2748479	0.5248461	1.822363	0.007438359
0.07269299	0.4629851	0.008878608	0.07963408	0	3.575679
0.2813701	0.7688258	0.00389608	0	0.4382461	0	0.2110735
3.224628	2.360715	5.878462	0.08742664	0	0.007682382
0.01693332	0.03796282	0.02944829	0.003788585	0	1.307129
12.91739	0.02495763	0.3448131	0.01215518	0	0	0.07454825
0.0121234	2.562424	0.06565118	6.399285	0.01721756	0
2.097987	1.365277	0	1.633457	0.05414239	0	0.0326422
0.6608467	0.04519583	0.3029192	0.02098628	0.009774759

0.06180333 0 0.2365527 4.161442 0.0746222 0.0126942
0.1402542 0 4.189124 0.1435939 0.1379423 2.889582
0.008969111 0 0.008627276 5.050181 0.01314152 0.06000494
0.03120557 0.1909975 0.06025464 0 5.681475 0.02037181
0.05304492 0.05779986 0 0 0.1418891 0.004082583 2.596917
0 0 2.353863 0 0.2076971 0.8427301 14.97346
1.20598 0.009939081 1.964406 0.07728955 0.01835908 0.668347
0.04746759 2.979724 0.005158237 0.004721165 5.174688
0.003123384 0.01166374 0 0
DLG1 4.392526 4.752122 6.312085 6.706546 4.361861
5.650851 3.228073 5.441651 4.330573 4.667759
16.78679 18.08197 5.172028 9.565434 3.531028
6.453605 3.646012 5.537635 3.145552 3.429924
6.333461 5.771398 7.135552 5.188858 5.91651 12.89179
4.160073 6.160196 4.810732 3.176512 4.24342 5.707193
11.10993 6.418058 10.75278 5.338525 3.594955
9.815326 4.979189 7.108434 17.20587 7.581386
6.938908 8.17088 9.325104 11.0579 9.562918 7.231092
6.328318 12.87719 4.51116 7.919701 10.26776 5.661429
5.968786 7.326872 7.05245 6.884258 4.179314 6.377467
7.387469 5.394185 5.373102 3.540542 5.194187
5.865233 4.115794 5.196299 5.457505 10.10391
6.043516 7.42113 9.292768 8.441569 7.878233 3.085892
6.374746 9.535479 17.4173 5.460773 5.867076 7.26067
8.801811 6.39477 5.837055 9.034119 11.89971 5.578152
6.85426 7.564371 7.314023 6.793682 4.557842 6.839601
5.662446 12.26689 4.516365 5.547356 8.741094
7.023846 6.944729 8.527836 3.091642 4.771491
8.826452 7.403927 5.81651 4.188983 7.489512 6.720458
8.593762 10.18634 2.948086 4.89039 8.503251 14.49501
3.369116 3.966477 9.153356 5.131441 5.093665
8.222153 9.464764 2.721587 5.545095 7.413068
4.836627 1.948288 7.030295 3.860164 7.161989
10.13552 8.436252 8.987942 3.002598 9.128506
9.597229 5.164613 8.634886 8.782259 7.651524 9.7871
10.75565 7.26672 5.323851 6.838628 6.502682 7.653049
22.7705 7.626826 5.998906 6.169099 9.523573 9.230978
6.262633 11.54483 4.938556 8.510028 7.141904
5.421576 5.02826 8.132923 16.97388 9.524897 2.684503
7.560919 4.179771 7.556516 23.1065 3.817607 5.047781
8.486788 9.298314 8.103689 7.719808 7.379606 5.9445
10.44834 12.38357 12.75299 10.65936 6.949214
12.77706 11.6236 6.745965 8.979673 5.996071 7.063523
13.71912 7.243609 6.106879 5.056054 3.58334 5.528065

3.265615	8.487955	5.738119	6.739293	8.666412
5.347178	6.083031	5.799348	7.62467	7.051938	4.107992
9.516716	5.958032	11.48	5.490004	6.019451	9.91255
5.227367	6.340108	6.376947	6.317982	9.180287
3.896562	8.497529	7.949994	7.436112	6.653765
7.031028	6.829931	5.630985	6.765733	3.697794
7.756188	10.10305	2.766601	7.243151	6.639358	4.704
7.3639	7.485509	9.646094	8.812445	3.028568	5.492433
7.902113	9.761474	8.378876	8.773984	8.220938
4.722963	7.140542	9.483302	6.310834	5.534248
9.872716	13.78245	10.71199	8.651317	10.22173
6.354266	9.423709	26.60539	8.986821	5.74142	6.417823
5.875709	9.520269	3.655106	11.16909	5.877273
7.231266	7.92156	13.9973	4.238394	6.267118	10.58806
9.674182	6.423132	9.725005	9.365319	8.141172
7.01415	8.812167	5.952277	4.198243	9.053046	6.243978
5.648375	3.402939	5.247731	9.694698	9.078318
9.480004	6.970784	8.079662	7.161678	6.683758
5.723855	6.066604	4.726355	5.385151	3.68915	5.220238
11.25606	6.797992	6.925756	10.05378	10.19009
12.04465	5.769396	10.41969	6.217777	9.912376
8.036259	16.39557	8.433431	4.362873	3.168172
9.31535	2.759191	8.054738	5.982064	10.73615	8.353079
8.192436	10.78321	8.193066	7.952845	5.331634
7.365699	7.473634	6.646522	6.096628	7.635554
6.017902	10.42469	5.102623	5.436362	9.220468
8.420494	3.390308	5.115028	7.075525	9.725301
11.96265	14.02352	9.265121	4.247576	4.66566	6.140832
7.768829	15.5103	16.15157	4.769613	11.05985	5.182709
8.369672	7.20782	11.60397	5.260522	11.0107	10.93443
8.430757	11.10145	6.982604	6.656962	12.3628	2.868317
9.543247	6.613251	5.574701	5.229794	11.85149
7.753175	13.34792	5.192036	7.10698	6.99378	7.663562
12.61706	6.192311	3.659615	13.43596	5.929451
7.895011	9.481726	8.316735	7.725857	6.800635
5.157989	11.5362	4.202865	3.612323	7.004483	7.046244
6.701829	9.365942	6.795077	8.484604	13.7812	10.35107
19.67074	10.36923	6.276711	4.546745	4.685408
4.872792	6.458807	6.294366	10.66278	6.123777
4.019822	6.611719
PDCD6IP	27.7423	8.025403	30.3743	26.90959	19.373	12.43771
15.225	14.981	18.77412	14.62384	31.68268	37.45271
14.35323	34.52052	15.6875	12.55703	17.75162	17.13113
16.61307	18.33035	25.69871	12.39766	24.26663

12.55669 14.8688 37.89333 16.46311 11.89993 21.15469
14.20366 19.91071 13.9209 22.01376 21.0717 14.69921
15.79047 14.49888 12.82352 14.9141 23.54997 34.40848
33.48274 27.22529 34.49526 32.53512 18.26315
24.08977 15.94628 36.995 31.15036 14.52441 19.35698
11.92358 23.71384 14.74974 21.57131 17.98977
15.0501 15.07413 20.22159 26.42844 23.23778 18.84864
13.90862 20.88715 14.68129 18.05166 12.85777
27.63894 20.33417 22.85096 18.05426 24.81032
24.30632 31.11344 14.74542 26.6265 22.99453 40.04502
24.91337 14.3079 14.0563 28.36274 20.78732 17.36247
15.19556 22.97104 22.23237 17.2972 21.53146 35.89847
18.27415 20.77258 27.01899 11.67712 13.02456
26.74009 23.62511 25.21561 27.01846 17.34895
15.90781 8.952244 15.341 14.68627 22.29116 30.57101
11.14413 30.62659 17.96965 16.23801 18.22123
19.27069 13.37597 21.55988 26.38318 12.76125
20.83886 13.67204 17.12173 11.87096 18.6791 14.89497
17.75129 18.95194 20.86845 25.11286 15.21619
15.90243 18.64103 29.77737 23.03217 26.28476
29.88677 20.29335 27.65805 27.20733 24.69014
26.44124 17.32755 16.8539 37.18222 19.28055 13.19135
12.56584 17.98688 15.9323 20.97236 22.01111 17.91186
35.22559 23.77468 29.44185 19.53061 17.75726
27.92793 18.61814 11.84302 50.21667 11.8429 17.77475
22.80366 22.8173 21.95891 10.94138 25.57014 11.57273
23.54275 15.69243 12.46593 12.17281 25.31255
22.21379 22.92548 22.93622 16.76641 15.41979
13.31488 15.99644 16.97005 19.11174 19.81507
29.95038 22.80601 8.684473 16.56681 16.63675
26.58255 32.97607 24.75091 14.60045 13.58735
11.70322 23.25564 7.897255 18.65548 24.25286
18.58895 23.83942 16.26294 10.731 13.95585 24.43808
18.62771 19.74845 35.01281 13.57053 14.1715 17.05753
16.38847 25.42226 17.96734 31.47436 12.73688
18.99335 27.1992 22.63745 19.88272 14.78616 32.72661
27.14692 35.68992 38.48742 14.90791 20.87608
14.3157 23.72977 22.83155 11.17332 14.73296 16.38948
16.87982 21.24563 49.20162 26.9363 24.05858 11.91582
23.95768 29.2255 15.13145 14.10797 17.89766 18.8618
20.53699 24.62878 15.59396 18.85851 12.69061
30.54256 16.23606 28.3444 33.34696 15.17807 16.79181
16.4886 19.47199 16.05957 13.78189 22.40894 14.68091
13.96659 21.14163 23.27483 19.62973 30.06937

17.5186 28.32982 21.17267 15.54224 27.56715 22.04417
11.11892 21.64174 30.47825 16.18444 11.676 15.6467
13.62167 35.97563 36.3739 17.93095 20.47831 17.36668
18.82182 40.02096 16.73725 36.26454 13.30276
23.22196 14.50723 18.42146 15.19188 14.87173
16.63392 17.5533 11.72466 16.24342 17.55439 21.40418
30.49749 29.25209 22.92708 16.28233 15.28095
34.15629 22.1577 19.55498 20.81395 20.51964 21.30275
13.01051 15.5687 25.26218 12.56502 28.85797 16.77907
19.82813 16.9678 25.81608 33.80312 21.01153 20.4216
19.39062 11.66973 23.33313 15.75897 24.4954 16.36782
20.72923 29.8236 22.41949 15.77014 16.65839 25.1846
10.55145 15.49345 17.29426 14.43724 17.6242 26.68722
21.38082 14.19233 17.63999 21.86356 18.63571
31.70871 20.21411 23.46501 8.660527 14.37511
30.75443 21.40814 17.37709 12.79083 18.97833
26.65741 31.23567 13.82999 14.97319 13.75053
15.82802 12.62588 26.61681 14.16244 12.1314 14.23406
41.06577 24.47201 18.38833 10.93136 10.57846
23.41408 23.74814 27.78604 15.94377 10.97036
21.47514 16.88559 22.31283 16.83515 16.20248
25.89515 11.38974 15.71488 31.07449 24.34833
10.59002 23.68493 21.231 33.52951 17.19907 23.69747
12.03786 30.72677 33.56358 12.28354 15.67921
19.50778 18.93434 21.05706 13.27126 15.46051
29.89997 27.50089 25.97411 16.78742 13.90077
SCRIB 10.52105 8.680671 12.10787 17.87051 9.71302
8.383337 7.750787 7.809116 17.08307 7.696197
38.07582 24.62985 9.054806 33.89201 11.88884
6.967077 16.17547 14.80975 11.16259 11.12012
17.54474 6.367694 22.82044 8.014492 12.83852
30.70732 12.03727 9.063207 12.65043 1.975239
18.43986 7.117328 23.44376 19.75322 17.45539
24.10774 2.817493 26.10382 33.39945 28.39385
23.85739 25.00908 18.42117 32.58581 24.48026
22.55171 27.73048 55.67329 15.1494 28.12928 22.53824
17.89364 20.49257 14.30609 7.964758 25.79801
20.4396 12.16114 34.94958 19.89996 9.47275 31.02579
8.662771 26.36652 14.43002 11.52705 23.68178
10.06199 21.81084 26.43328 68.08841 21.38671
37.57626 9.637466 40.28337 17.95829 19.96795
29.00819 25.62746 19.02908 18.40777 10.91588
7.348871 25.67078 16.08201 23.0613 27.80009 28.2061
19.61277 10.32422 8.745866 29.14138 13.78409

20.80719 23.48003 27.13456 14.66743 46.50385
31.64646 33.4697 18.95403 30.31216 21.48446 20.35161
28.96125 25.86762 39.40958 19.02939 28.52548
15.74363 57.16615 13.97349 8.474219 10.36722
17.20967 21.66162 81.04101 26.68986 6.250458
25.31043 21.59337 6.202095 34.58247 11.98806
69.0858 16.62289 19.26768 16.80971 11.92093 33.49809
22.76185 38.56559 15.79519 8.007844 16.12956
37.66686 30.02653 15.30209 18.80911 15.22737
34.40312 23.27818 19.64198 51.2241 40.45209 12.49897
35.48435 16.28282 46.32574 22.35825 28.05977
33.48505 36.87622 20.43903 15.51262 10.80074
36.7903 14.53033 16.25465 24.24935 22.41849 9.728804
7.452768 41.21949 26.16819 18.46211 21.46572
6.445091 33.62584 16.79748 11.9278 26.93354 18.90805
34.41766 3.53788 16.57412 19.62904 18.05282 49.54849
21.34345 11.50671 40.67881 33.6419 28.5818 13.11807
39.35229 14.0418 21.20348 26.16783 38.2023 15.2263
40.39427 47.57982 20.50015 27.7141 25.28546 13.42643
54.4849 28.01135 38.37834 11.28757 11.63578 54.89159
33.98733 42.58763 33.78477 29.57331 24.75237
47.33034 36.1942 11.61168 26.38519 25.21363 63.35045
30.14153 40.44584 29.66742 48.04607 28.14789
31.32191 33.37316 13.96839 15.56757 25.49981
21.10257 16.78072 49.71754 44.75639 52.02405
39.44972 35.2418 17.5005 27.17909 24.64889 16.21545
13.93559 25.45016 17.10257 11.28917 73.04104
18.08815 18.77411 22.82647 35.56452 12.20893
17.66195 35.5858 14.44216 28.82139 22.4053 35.44321
10.75527 29.74881 24.46296 58.28988 27.48436
31.51736 12.25565 45.83793 25.84846 11.95471
35.7851 6.433714 25.08417 19.73776 38.75922 71.70934
28.52771 24.11005 22.8748 26.0734 33.14764 14.99863
14.59305 42.12784 41.98466 24.90725 9.745015
15.64964 10.20047 15.54374 31.8491 15.84942 17.38834
19.22534 21.63575 44.11084 37.55223 20.53326
25.76064 55.95035 6.429353 11.9685 25.70914 41.5563
13.65748 31.18201 14.02501 19.40029 37.6027 21.12873
26.47949 4.51392 17.98994 35.52726 43.15922 15.38881
12.82641 21.83965 44.19115 34.29185 24.45116
20.92497 15.67577 37.65171 20.21806 16.24596
32.37595 16.1856 10.73146 18.34136 8.050733 54.55383
29.6838 2.521447 18.66454 31.49777 49.12171 13.81675
32.36085 25.98318 14.57164 19.6124 17.92045 16.34316

25.76129 33.96034 28.84603 23.31421 17.41288
22.9907 33.44714 22.34141 22.91565 15.95483 40.81133
31.18365 47.0478 20.56128 13.48946 65.47348 26.53085
25.59413 16.07539 19.32645 49.69464 30.46549
79.19775 15.19089 35.34144 41.24716 30.40815
53.38342 33.22555 33.21396 19.82607 41.26787
35.52382 27.11973 29.26687 12.94984 17.75722
48.17842 11.04183 42.94849 54.98286 13.98774
24.14555 19.539 38.45718 37.24884 41.01976 21.57019
59.28062 88.3317 19.30784 23.1106 10.78569 54.2908
39.01244 17.40276 17.05909 29.38864 21.89177
22.7693 10.34557 6.542693 25.27009 10.78872 18.64005
64.33911 15.93108 17.92042 24.3844 26.99265 51.45893
22.08721
TDGF1 0.1130293 0.01662796 0.3838846 0.9412984 0.4789841
0.06163692 0.7254147 0.6668996 0.1968693 0.8424182 0
0.0056939 0.8861583 0.01630391 0.3212926 0.02576543
0.5376498 0.3207738 0.2934357 0.2537869 0.6908732
0.02020445 0.03318351 0.1109566 0.1290236 0.01594626
0.9129256 0.006874986 0.6216662 0.8154564 0.3931255
0.02338096 0.01958287 0.1643027 0.0990971 0 0.009047144
1.793455 0.4775151 0.1051589 0.5382992 1.285099
0.2111564 0.1527782 0.2417347 0.3369458 0.1180302
0.5171154 0.07082426 0.1967569 0.9367761 0.9433622
0.1803349 0.0871552 0.2994904 0.007125539 6.999985
0.07130367 1.464647 0.3261121 0.02961352 0.04742908
0.2540096 0.3628799 0.1675094 0.153182 0.03163603
0.3046334 0.1514165 0.008098241 0 0.05251396 0.1261477
2.51864 0.1759094 0.2096586 0.5940465 0.07785155 3.930796
10.05308 0.01007849 0.8072804 0.01862635 0.2592686
0.1617963 0.03086745 0.5278236 0.05013959 0.4070673
0.1209529 0.06702871 0.01618048 0.06437033 0.2275274
0.03669484 0.06809849 0.09832478 0.2655068 5.649859
0.06756569 1.059555 0.06330093 0.01335549 0.6813805
0.1196208 0.3803997 0.04180903 0.2056451 0.03844307
0.1275044 0.04738874 0.8889416 0.02543065 0.1629439
0.4003042 0.05292332 1.715423 0.4087009 0.1691401
9.681237 0.0419888 0.07483035 0.3231599 0.06456765
32.61975 0.1967782 0.1033826 0.1159778 0.01610713
0.06169585 0.01252346 7.034666 0.05308052 0.06716786
0.9524189 0.181005 0.08270052 0.003120516 1.279016
1.703657 0.0256656 0.134274 0.07891035 0.2532687
0.01641278 0.173441 0.6809617 0.3667154 2.411134
0.4756124 0.00803534 0.4482618 0.07878648 0.09505073

0.1101329 0.6825178 0.1553782 0.6829045 0 1.02783
0.03494691 0.5027057 0.5305208 1.413175 0.6569498
0.5176495 0.73047 0.2925408 0.5249506 0.08206452
0.002392511 0.1768332 0.3277679 0.8611578 0.2798934
0.1262864 0.3168073 0.14607 0.02230485 0.06036601 0.9589953
7.841608 0.02271957 0.02430097 0.1828634 1.718845
0.4334938 0 0.3824264 0.1829149 0.4772052 0.03970307
0.01468737 0.4120568 1.086418 0.1394979 0.1814501
0.0719746 0.212628 3.604582 0.7848523 0.5010861
6.415609 2.11276 0.2193422 0.06067121 0.2374709 0.04339557
0.152597 0.005439428 0.2038884 4.811592 0.5568561
8.512217 0.1446275 0.1039634 0.02481243 0.06887877
0.1093045 1.796808 0.04597667 0.03971963 0.824399
0.01038068 0.1465782 0.06811254 0.1346591 0.01349837
0.01060796 0.03475595 6.225616 0.7650339 0.6653021
0.09375045 2.757609 0.1541592 0.893393 0.05407187
0.01636265 0.3906117 0.2322573 0.4007715 0.4688917
0.1005566 0.2237258 0.3228677 0.2219715 0.037708
0.4306945 0.07439455 0.02344747 0.06541004 0.08096075
0.1050821 0.2629287 0.1593487 19.11242 0.2583591
0.005441992 0.06715734 1.054168 0.08118532 0.7476775
0.1287369 0.5849524 0.141081 0.05019526 0.5139269
0.1106991 0.03595888 0.1083295 0.08664476 0.06876515
0.905894 0.875535 0.04917735 0.4786423 0.4105872
0.01553629 0.03801278 0.8841242 0.1653527 0.06317314
0.1762671 0.006343791 0.2585854 0.08911024 2.221525
0.05352879 0.3631364 0.03040132 0.114136 0.09785556
0.1407958 0 0.1965004 0.08057669 0.09776262 0 0.04699302
0.510667 0.585027 0.007414284 0.05255669 0.1907738
6.523177 0.1913355 0.3818083 0.6894764 0.1227185
0.2010648 0.03281218 0.1706752 0.3765565 0.03357634
0.185916 0.4601026 1.426644 0.2040631 0.04335599
0.2355074 0.2941039 0.9879117 0.005982229 1.004377
0.1872636 0.2195037 4.234702 0.3275908 0.0132313
0.09404873 2.865051 0.6384042 0.04171943 0.06621751
0.282771 5.959041 0.04702633 0.2493285 8.149572
0.8312949 6.345988 0.125776 0.1586176 0.355632
0.02257487 0.08416101 0.2390007 0.03843961 0.9535197
2.719687 0.1843049 0.2594923 0.01035972 5.8418 0.1602992
0.08896127 0.1545339 1.080868 0.035877 0.9147545
1.778463 0.01450922 0.1521872 4.209039 0.05284981 0
0.03114904 0.07924428 16.16405 0.6933032 0.07419521
3.265963 0.5369356 0.01596514 4.062319 3.612078
0.1727276 0.07246051 0.1779424 0.002632866 0.09219331

0.1832221 3.193254 0.09692073 0.1464658 0.4887461
0.1114483 0.03997068 8.706341 0.1458713 0.04938836
0.6593637 0.07724845 0.0561314 1.408936 0.1219353
0.3593048 0.5701603 1.818459 0.1421402 0.2268344
0.06874253 0.02868928 0.7691755 0.1053893 0.115026
EDAR 2.071296 0.02824988 2.433658 0.4076412 1.132381
0.08310897 0.6390402 0.4878286 1.140234 0.2755503
3.305142 2.960117 0.385253 1.929721 0.7278083
0.004863764 0.7595577 1.008389 0.6484931 0.89038 1.058677
0 2.38035 0.09425419 0.1393907 1.563579 1.065179
0.01038238 1.42726 0.439458 0.4359876 0.1942004 0.858685
2.822416 2.915565 0.01439167 0 0.6928279 3.892566
2.745239 14.50005 2.911072 0.2220782 0.0126615
0.8687072 0.560373 1.134404 1.524226 7.319834
9.024191 0.2122033 1.046797 0.2641188 5.310824
0.3008093 0.03631755 0.05783606 2.44973 6.12541 0.28926
1.255925 3.930468 0.1667339 0.5224014 0.3478298
0.2357792 5.755479 0.8824948 0.2473532 2.882387
22.15082 0.1379904 3.82199 0.6427702 0.3780441 0.1424788
0.5836309 4.732147 13.21971 0.2057972 0.01141515
3.198419 4.788946 2.11296 0.1279325 0.1207753 1.509765
1.273704 1.248222 1.266272 0.6358168 0.04398342
4.343602 1.025271 1.337525 0.1408462 0.5154615
0.1451751 20.56385 1.78673 0.3493379 0.7571123 0.03025354
1.181248 0.2100026 1.069697 6.757412 0.9929701
2.963731 1.422266 0.3737279 0.05798543 0.04608545
1.389586 6.591662 0.1563712 1.186327 5.960552
0.1673227 1.655256 0.1121 0.8757991 1.361228 0.3412778
37.27096 1.120748 0.6845483 0.6354956 0.6476386
5.128459 0.03782504 13.43138 0.340682 0.3043041
1.210824 1.962247 1.536167 0.1024969 3.901589
1.287839 1.142655 6.463491 0.7047926 0.5356998
1.440688 1.250337 1.606467 1.473374 32.46719
0.1282597 1.167966 2.48903 0.01784711 5.327384 0.1607218
0.4019792 1.088176 0.5247192 0.3411975 4.096477
3.596993 0.5403644 6.522416 1.866369 4.529171
1.292472 1.408588 1.113668 1.935713 0.0363711
0.01625891 0.5781835 0.5603596 2.575376 0.07158389
0.3224792 0.5048939 0.7326745 0.2621041 3.000775
0.24761 1.405674 1.918929 1.186586 0.06003353 6.440688
0.9538428 0.9859803 0.8906562 5.25314 0.1745347 0.5154105
0.4957317 0.5670729 0.4515614 4.217132 0.5095934
0.3195594 14.50988 2.576089 0.2929473 5.320378
0.5213331 0.2277739 53.27016 1.254735 0.003955376

1.001923 8.237552 5.985251 2.599002 3.994841
1.255325 0.6770168 5.76166 3.615837 0.03864188 0.2496443
0.12724 0.8405892 0.3675836 2.214768 0.29976 1.758911
5.372693 0.06118475 0.640577 3.478156 0.2963663
5.360265 32.27891 0.274836 1.507076 0.344451
3.127272 2.784483 0.1087534 0.520567 0.09266379
1.283679 1.84634 9.874492 0.07835587 5.629568 4.34885
0.403518 2.918548 0.2036893 0.7843308 1.687564
5.683242 1.829901 0.7274724 5.373694 5.733231
0.1042787 11.5488 0.466157 0.3944793 0.9444627 0.6105225
1.029869 16.86131 0.04408231 0.2750864 1.712769
1.205271 0.1821253 1.591788 0.651647 5.57522 1.583559
2.04334 0.2850107 0.1782234 0.1179519 3.716966 1.142898
0 6.1411 2.772246 0.6219633 0.3765869 1.146444
3.594964 1.018714 0.4140657 0.3034837 0.4648152
2.796289 0.5800689 0.1871061 0.4703674 0.1314919
0.0301804 0.9347579 10.11448 4.731795 7.187077
1.367055 1.53967 1.658352 1.816682 1.071487 1.55962
1.081351 1.730139 0.9331725 1.351479 0.08163144
1.023511 0.2023368 14.2489 0.07676954 5.032559 0.1859608
0.6612883 2.805599 0.7402765 6.295401 3.988006
6.184082 1.183488 0.7656456 0.08776602 0.6716485
6.260864 0.185303 1.662964 0.09291384 3.218961
2.591006 1.494783 3.476433 5.067833 5.193457
0.3363362 1.65116 1.277348 4.232439 37.3541 3.45414
3.832774 0.05866264 0.6631428 4.292374 1.634932
0.04300813 0.4223155 0.2059757 4.310129 0.3063887
1.953785 0.8878489 7.396931 1.364019 0.8885182
0.7994569 0.06718274 0.5638134 0.6604973 0.02729448
13.25855 0.110126 26.67788 0.6174933 0.2404536
5.201869 2.53843 0.03327594 0.3400425 0.648273 4.307828
0.7772572 0.06630256 2.586071 3.840404 0.1304238
1.267849 2.174011 2.084454 13.78873 6.166926
0.7939231 5.25672 5.550236 0.526563 4.86468 9.25485
1.471749 2.898078 0.315653 4.578532 0.2942965
0.5606234 3.888466 2.009899 0.9360029 0.2595774
0.1194683 0.7554219 0.2720313 7.601037 0.2924478
0.04667077 2.41828 0.7191532
SH3GLB1 12.46155 15.5765 14.86138 11.3776 10.71508 19.77923
6.798533 9.891703 11.01763 12.13959 23.91994
24.38291 11.6565 18.93528 8.261315 21.20009 9.931308
12.54468 10.18043 9.907443 14.02882 18.20382
18.43486 21.60567 19.71051 21.21319 9.084016
21.39682 11.6367 9.304102 10.57304 14.95179 16.40463

14.05134 15.32253 12.54788 8.895783 9.244681
13.0644 18.43713 16.20839 16.0853 18.3655 15.23225
20.39838 11.4332 15.0111 14.54507 16.90461 15.46248
9.266933 17.02227 6.007192 18.18179 19.69859
10.34856 11.71532 24.21799 17.26524 13.16432
22.94065 15.40506 21.37363 10.21894 4.832014
26.83202 10.73736 13.48889 24.50605 13.46185
20.45842 11.99767 13.54303 16.65191 18.12077
10.4552 17.50461 14.11051 15.09352 10.84424 9.08629
17.06027 17.55934 17.10598 15.92177 20.38826
21.04945 18.0814 11.93473 20.52663 27.26062 13.63392
23.47665 16.82197 14.23179 13.38436 17.82213
8.237412 18.52324 19.64873 14.03193 11.72466
7.929826 12.82801 16.12875 20.83107 14.24569
12.13002 15.40937 14.90752 12.46189 8.961551
17.11519 15.01322 15.81547 18.06102 10.21521
13.97083 11.0121 7.691426 18.46981 17.32473 11.53806
18.10622 15.60953 14.37885 22.77088 10.25792
18.70673 21.98697 15.10123 20.01663 13.15231
23.06903 9.652729 21.16334 17.68327 17.89152
17.90169 11.70238 25.0756 13.89967 13.57876 13.18492
13.19075 11.54284 10.66751 18.6093 23.63566 24.3579
14.95689 16.2051 11.69391 17.56778 20.28708 13.51148
14.47606 13.70791 12.28412 11.93846 12.59624
15.55916 19.17597 19.72063 9.998708 19.65225
11.76158 21.30847 16.28969 17.98008 18.35565
13.94745 11.04282 13.37044 11.28961 16.12731
17.19175 12.82466 21.62023 18.3243 13.82869 11.9609
19.1954 14.27134 19.31512 18.74948 21.11501 15.02089
31.31035 16.09691 16.03569 9.992403 13.59562
10.01618 9.203747 20.26833 24.32323 11.37011
12.14932 14.96225 10.89481 19.62301 18.7372 28.42272
12.56403 15.13136 12.2146 16.12845 14.74632 21.38908
10.59483 16.72348 25.3563 21.73396 19.83702 18.22015
16.23274 7.384839 14.62121 16.69335 14.28475
22.52623 8.098095 18.32573 17.6087 11.14439 13.84777
15.15902 8.140367 13.54867 11.95818 18.27219
11.31864 25.18764 20.59093 14.35785 8.972159
15.03733 23.99627 14.27385 16.24535 19.6426 16.15603
15.50983 22.17772 14.34222 16.2978 15.40085 18.52798
16.9009 24.92094 25.77553 11.29312 22.07854 10.14164
14.23263 11.53573 17.51557 15.7317 14.56771 11.54694
12.46201 24.52516 15.66267 15.05994 15.12171
14.65305 11.03935 14.44815 21.06679 14.86026

15.03478 18.4535 16.52624 21.50471 15.39932 13.96621
19.13719 16.07923 28.19185 18.21812 19.65497
15.66249 14.80475 21.53833 13.69079 16.83241
11.02742 16.10213 15.45929 17.91961 23.26703
22.77818 12.0641 14.29742 12.59415 17.87183 12.89132
16.50953 11.79691 19.86982 17.56072 14.6453 22.41247
13.77367 10.91691 10.71957 16.97935 14.11638
10.42619 13.02202 8.879225 21.45094 10.91018
15.26385 16.99923 14.83191 15.80427 18.07042
24.09804 17.37394 18.3283 11.28024 7.622528 28.57602
11.20215 13.45427 11.75553 8.856262 22.39611
10.68653 22.46782 13.3814 11.03511 12.12924 19.14245
15.45155 15.77102 14.79332 15.658 9.695851 10.76377
10.76231 15.73702 21.01455 23.24907 7.823992
27.19993 11.23254 11.77351 16.64969 16.25115
13.5646 16.65699 15.47541 15.74466 20.70213 10.94178
18.8266 10.34714 13.98102 11.6785 21.9314 10.24648
7.710713 13.03008 14.28686 15.16645 12.33829
19.04351 23.98279 16.1053 31.0098 25.18147 11.67175
21.28593 12.98049 8.850527 12.25626 15.82197
13.67617 18.06245 13.8192 24.20522 13.79933 12.77709
11.7241 12.09154 12.38213 14.63531 23.16192 17.53807
8.93848 16.25912 22.59354 20.41228 12.2571 18.64878
9.0365 12.29204 8.998495 14.86325 15.13067 18.10021
14.00514 10.04384 17.96162
DYNLL2 13.08323 19.6988 11.73988 11.65967 11.50124
12.30062 9.388059 10.74422 9.68382 7.901687 8.527239
8.05762 7.866583 7.91893 9.356009 15.28244 9.586533
11.76851 10.30995 8.417569 10.27363 16.05759
8.53134 12.45734 14.82296 7.427487 11.18951 16.69615
12.67021 4.52476 13.5268 16.58663 17.33181 11.32358
11.14834 10.31048 8.448627 13.08518 15.28293
14.27656 21.39796 9.338885 7.475354 12.3046 7.841997
5.631988 11.90699 10.52176 9.236919 11.53411
10.59534 9.271925 10.84759 7.226174 10.50669
15.20673 13.4198 12.89972 14.06921 12.36198 8.258273
11.30869 7.776103 7.400035 11.69638 15.30913
13.16449 10.1784 5.849579 18.92315 13.30927 9.914231
9.231603 21.70729 8.358308 13.14352 6.98357 7.853536
18.68616 11.25815 19.11427 8.859783 8.443213
11.89431 10.37849 8.183954 12.07786 12.04906
7.248124 12.57056 8.597884 12.36299 10.00912
9.914297 14.67088 13.72796 10.3661 7.938231 20.68483
11.20763 16.93923 9.641536 7.226388 8.420444

9.962007 8.078263 9.541598 11.17316 12.38512
11.28843 13.76084 11.85247 12.94135 11.53983
10.28334 19.95442 8.735949 5.402441 24.03665
6.236155 8.07316 9.084114 16.15072 7.134701 29.65297
10.21136 5.897385 12.70467 15.50629 9.647071
9.199447 29.51562 9.879904 7.329517 7.822745
11.3992 8.569767 4.330888 9.786245 10.57805 11.5914
6.505899 10.47344 10.41892 14.74058 13.55201
7.704749 14.26846 16.56717 8.565212 12.36157
9.604523 8.094531 14.38178 14.44911 7.260236
10.71742 16.0275 10.38808 9.11456 11.34995 9.189728
9.255127 14.66578 8.279778 9.762147 14.75378
7.849946 7.376611 9.612185 12.51888 17.85637
13.69004 8.985661 8.930356 8.912336 13.05486
12.53747 8.505193 15.05539 9.611258 7.542613
10.17352 7.200443 12.09256 8.921515 13.71662
9.090658 9.846109 14.48252 9.771713 9.600038
12.57028 8.548289 16.49845 13.33304 7.936868
13.44615 12.26107 6.928886 13.02531 8.427061
5.981991 18.36302 13.91621 7.030249 8.767395
9.209233 11.29748 8.230444 11.3358 11.8635 8.035872
15.09617 9.826444 13.32412 8.974708 17.49872
14.07324 19.45403 16.594 7.425296 11.24523 7.3519
8.104005 12.11493 14.12808 5.732582 10.56782
10.89214 11.53051 6.43516 8.849723 8.29425 8.066883
10.99773 7.036463 11.39219 8.772743 17.73148 10.488
15.40779 8.912859 12.70411 7.293993 12.20597
10.47229 14.62839 9.232144 13.11485 8.419587
7.762642 15.74519 6.71921 18.27398 12.87339 7.113915
11.55443 8.847689 7.043634 8.573544 12.04798
10.3668 11.28768 10.93664 14.59232 8.140497 12.23571
8.686638 14.80328 10.5495 10.6883 13.77866 8.751516
8.151358 10.17132 13.61152 13.23327 13.8668 6.193677
11.84457 10.33425 11.9895 12.60329 11.37935 15.0736
10.57715 13.56946 9.383115 11.94761 7.953872
14.17189 9.26226 13.9748 12.72643 9.868662 6.582593
12.56958 10.24376 8.035038 9.606094 9.931115
19.47145 5.389355 10.17446 12.94707 14.98229
8.073772 8.587659 12.85435 13.02766 14.63454
21.12878 8.541013 14.16187 15.54737 7.844734
12.42154 10.80822 7.265013 5.941316 6.196357
5.230269 14.74988 6.531656 8.437966 6.026301
19.10131 8.542303 8.591027 6.475733 9.444586
16.27877 13.31187 14.85005 9.386081 7.559648

9.447812 10.9199 13.70389 10.80486 15.61075 5.629874
13.42559 15.93613 9.007277 4.708121 15.89835
17.92755 13.99779 7.774818 11.75293 12.77122
6.583559 12.40636 10.10095 7.808545 17.66975
10.36079 9.370839 10.72571 8.826514 11.30194
7.822847 17.8274 6.960733 14.42308 9.866946 10.87298
18.45344 6.178941 6.111169 15.20906 6.354042
12.19806 14.28101 17.59607 6.134137 10.95007
7.224652 7.742637 17.05528 11.66117 14.97476
5.010844 9.602756 9.846996 9.176254 11.33 8.206248
7.305394 6.374758 8.683351 7.527134 10.50478
7.469218 11.40783 12.04893 8.355038 7.978509
15.93465 7.917629 10.3211 10.68098 12.23706 9.891471
13.75258
TSC2 6.036934 12.69715 7.302663 10.99332 5.958118
12.28053 5.211724 8.603612 10.44943 5.457066
12.47667 5.941754 6.209671 9.370353 8.600268
10.00568 8.636204 14.16734 5.142464 5.418617
10.02663 8.506437 9.652954 12.33358 12.86674
10.03764 6.987541 10.7526 7.035988 1.200571 12.33077
9.382416 11.72272 7.603753 10.57719 3.316326
3.813089 9.697681 7.461962 6.766972 13.80037
3.807373 7.939599 7.92902 10.86571 9.222667 9.958331
9.465806 9.755861 7.136045 6.56629 10.5607 3.33847
7.31914 7.715431 9.414286 6.832489 12.20974 9.227323
4.982099 9.465819 10.18201 7.359203 12.14124
2.570868 8.845442 8.698928 6.377423 12.85141
8.967292 8.371634 4.336647 11.54458 10.88303
9.078201 8.723047 8.811304 12.16772 9.328079
3.569239 6.62152 8.980403 4.641687 9.215283 6.416704
7.083534 12.47433 8.88395 5.773324 9.701933 5.082323
9.646591 4.888103 8.438777 8.49149 7.587417 8.075422
12.00811 15.25547 8.824442 11.24029 11.41135
5.008839 4.870443 6.814308 14.35341 9.385411
11.28969 7.684805 5.9147 9.234439 6.164806 4.255194
8.1976 6.574664 12.53176 7.802755 13.60815 11.37855
8.16627 3.279821 4.4483 8.027524 5.197682 6.776207
10.19053 4.865692 4.95098 11.15469 8.684152 10.92626
12.26609 6.292813 6.395066 6.953828 11.2413 9.004076
7.785705 9.200205 5.499109 6.867687 11.26894
10.33877 6.336881 8.140671 7.992502 6.193395
8.550007 8.392112 8.892131 7.034081 9.503094
9.034885 10.93739 11.7982 5.6568 8.919552 9.954268
9.35495 4.139227 7.990698 7.603504 9.019269 5.123456

4.337375 14.21198 9.755236 6.191704 14.09291
7.993516 11.92356 7.763541 10.39669 4.397636
10.23989 4.641018 10.11527 6.099019 10.42257
10.49291 5.213701 15.63656 7.882854 11.62232
7.237036 9.799627 10.94474 5.864881 9.967138
9.238051 9.48049 11.83949 9.430396 9.56673 5.36674
11.45125 5.223842 10.5248 7.669017 4.959937 4.774113
10.20715 9.58521 10.76611 5.222947 10.02887 8.535222
10.48339 9.856366 12.1076 8.00088 10.8057 9.39663 9.998211
11.79879 8.844625 8.972901 6.545003 7.552161
10.2733 11.78878 5.341012 9.160064 15.19833 15.09424
6.910294 6.508402 8.837947 8.664511 6.180591
9.008502 7.451568 10.12324 12.39036 6.093109
9.159898 7.017381 5.19386 5.450697 18.51194 9.94694
10.29735 7.274656 5.51704 4.476862 12.68696 8.600621
11.43415 10.92724 8.12286 12.35444 6.64735 14.64649
8.722771 8.427057 13.49755 7.788347 9.639117
10.09811 10.43516 5.345544 4.934945 6.1583 4.66269
14.39128 10.55874 8.195302 8.41943 6.316649 11.05463
7.509341 6.691133 4.726159 9.616494 8.824466
17.27379 16.73729 9.073097 9.548266 4.846082
10.6458 6.021759 6.726445 8.283859 5.472289 11.14943
8.847285 8.309816 7.445553 12.10247 6.158353
8.608222 11.92052 10.01878 8.686223 10.58486
5.41315 6.451605 5.092723 10.85461 8.427973 9.49363
6.748871 12.77296 5.271045 7.630738 6.64393 4.907775
13.26324 10.12138 7.047028 6.226887 6.895614
9.035256 6.90668 13.43168 10.66036 12.26533 5.033861
6.38031 5.386283 7.736018 12.80661 6.258814 3.033504
6.865558 9.051091 11.56128 6.472472 9.152748
5.447659 8.36571 10.52242 8.758137 10.32768 12.11286
4.691431 5.688131 14.16238 6.498793 4.714183
5.631148 3.59074 4.770063 11.58126 12.78239 7.994394
11.12883 5.832396 6.019653 9.399749 17.46131
11.92993 5.36409 8.739761 6.49765 12.311 5.27338 8.90368
7.349269 14.94571 6.90006 9.253219 6.724828 10.55233
6.2617 9.322614 8.945688 6.045067 6.856443 4.957061
7.784696 3.553144 4.153404 8.201708 6.178565
6.761095 6.436445 9.185043 23.63591 10.87834
6.907957 7.550045 9.079915 9.226289 6.407929
9.674003 5.804356 13.73194 7.465419 11.6191 4.695847
6.246205 6.095291 12.48898 4.130641 16.57594
10.13197 8.299927 8.029683 6.238018 12.69402
8.74414 8.143327 6.000738 10.2156 10.15122

BAG4 2.513581 1.626444 3.445901 2.715695 2.203273
2.579242 1.792444 2.313679 1.856313 4.023611
5.269537 5.140089 3.055838 3.552067 1.861772
3.075288 2.073089 2.238307 1.410048 1.845416
2.940737 2.515348 4.239339 2.826525 2.679592
3.751984 2.214146 2.333931 2.294403 3.489312
2.860077 1.860457 3.514052 4.548236 6.660445
10.6891 1.637838 5.16089 4.440215 12.00202 4.813555
18.61078 9.521492 11.33071 3.680287 9.187795
6.324107 5.848386 9.757688 6.821499 2.995807
5.512629 10.63086 6.382379 5.030462 10.80375
5.482676 3.65448 2.317431 2.314089 4.393793 6.695141
4.928608 3.765058 5.317449 3.451547 3.355516
3.90533 3.724409 5.791056 22.40444 7.488654 2.857114
2.997331 3.48406 4.758978 3.769427 7.126801 9.269774
11.94448 4.680431 4.92705 8.044898 4.380188 5.276187
4.595441 5.666895 9.307176 5.992443 4.474271
6.006089 7.762132 6.924301 5.503577 5.998522
3.626523 7.520261 3.324418 11.32524 8.770297
5.340945 6.207981 4.615155 2.649073 6.992282
4.478126 5.016356 3.561529 9.508186 6.677881
3.668332 4.004976 5.498057 4.406266 5.424709
2.225923 1.696923 3.250336 45.03622 4.295308
7.218922 3.50371 3.251756 6.225864 9.978308 5.166235
15.66507 3.773967 5.174241 2.757071 5.55478 3.391378
4.746275 7.677532 2.093704 3.146006 7.730048
4.534528 4.082938 6.199887 3.897903 6.397264
3.652245 4.402329 3.216495 3.950689 6.286669
4.326385 4.185476 5.843216 12.18458 3.892077
6.66337 6.335296 4.64297 4.894917 9.094585 7.580202
10.73622 5.144891 4.453491 4.562738 4.146148
4.784899 3.066793 6.326609 3.09884 4.173305 5.772546
8.230987 2.43664 4.987467 5.550558 3.870513 6.180432
5.694179 4.573766 6.390838 8.184364 6.348612
3.345421 4.537183 4.327285 5.167693 2.909035
2.982532 5.396705 12.19341 5.312 11.21583 5.275026
3.855386 4.458394 2.738872 3.984546 4.053125
8.955548 4.618711 7.400573 2.222741 3.723494
4.366881 5.739984 4.69579 7.270969 7.795521 1.809259
5.852898 2.583609 4.978411 5.313435 4.880837
4.932662 4.544947 9.527043 10.20298 3.676061
12.72547 4.652602 3.363936 8.728357 5.353928
5.444793 9.340719 4.818347 7.665757 5.74275 3.88359
6.454161 4.190639 2.372289 2.842145 6.07228 3.611146

7.859863 3.915973 3.54674 5.81847 11.52696 2.2796
5.604727 5.764049 3.910258 7.479819 10.33716
6.751589 7.429554 3.468139 9.300612 5.652563
4.733625 6.586649 6.869765 4.945721 7.298789
7.976894 11.24397 2.668136 4.926865 4.477479
4.957444 7.485456 6.543825 8.15799 5.384487 4.820997
7.610608 3.089474 6.324145 5.075995 7.315329
3.136577 4.773191 4.97564 4.529731 2.430492 2.884714
2.867872 5.652177 9.488457 5.332514 4.413447
3.036169 6.194414 12.87531 7.014282 7.890915
4.961625 5.595893 7.991145 5.870141 2.473484
3.582672 4.915455 4.71742 3.410521 5.53207 5.829238
9.631818 4.731063 5.25207 4.945403 7.811242 3.96104
5.507072 4.306767 11.53552 5.689374 5.325455
3.979866 4.622844 3.272191 6.835392 3.466042
8.851278 3.558922 3.612038 3.410559 2.950328
7.463229 2.503272 3.3832 1.78238 9.42883 5.57666 4.51146
5.159501 9.04682 4.941281 11.1113 3.694819 5.156853
5.616341 6.302825 2.598354 5.279784 4.577762
4.079654 3.835111 4.381265 5.710834 5.084839
4.707526 8.324677 4.077899 4.777099 5.843427
4.947858 2.768854 5.215773 3.811515 7.932828
5.768091 5.270004 9.716067 3.444813 6.185147
4.279751 3.634357 4.733301 4.815472 3.069747
2.667998 7.509105 3.243655 6.051391 9.357498
3.135197 5.587545 5.089445 3.949113 4.07706 9.670524
8.997306 6.082791 3.217279 4.859999 5.97932 4.097085
8.036921 3.897266 3.637544 4.54783 3.981226 4.082617
4.466951 3.164568 3.279739 2.267835 8.830906
5.152564 13.59898 4.277333 9.985145 7.146506
4.045577 3.220968 4.633678 2.033598 5.563649
2.047783 2.799232 8.388537 5.37163 6.191074 2.30706
5.791434
MAP3K7 2.816465 3.68318 3.561509 4.284376 3.132612
4.639868 2.503491 3.204428 2.982554 5.00721 5.087131
3.941668 4.901939 2.815989 2.871016 5.398882
3.019612 4.160983 2.04286 2.417316 4.714712 4.582836
3.841398 5.173485 4.195348 3.931434 2.994412
4.824462 2.921405 3.215939 3.317741 3.418431
4.736807 4.349792 7.721634 4.406913 2.97879 2.94125
6.378856 6.177053 7.185335 5.68516 6.892384 5.208256
4.65775 8.527896 7.024575 4.706271 9.976908 5.475265
4.200451 6.974172 7.045967 3.503373 5.801074
5.708728 4.95884 5.34577 5.396231 2.529161 6.614326

4.164481	5.578125	3.258131	3.776262	5.540221	
2.770468	4.394813	3.651809	4.288835	6.890175	
4.812635	5.596165	8.5703	6.301205	3.872058	4.35316
5.47053	5.159527	6.677912	3.638779	6.27385	6.451459
3.303293	3.519358	5.529922	5.11126	5.470282	4.180332
7.01349	6.071392	6.949636	6.012348	6.984989	5.176282
3.458529	4.809277	4.32055	7.699698	6.622491	6.370482
6.43876	3.562016	4.427371	4.6134	5.823201	4.297916
3.736311	5.301198	4.934062	6.945551	6.09511	5.538942
5.748805	4.727519	3.00066	1.73874	3.953002	9.140861
7.798504	2.979121	9.418773	7.180514	4.26972	10.28045
6.961243	5.422473	2.35908	4.606872	3.970655	4.845834
6.781529	5.445157	8.037944	2.701001	5.416755	
6.36944	7.811833	5.502334	7.293549	5.129978	6.045568
3.887825	6.248266	8.847812	5.300016	6.216208	
5.18712	12.2938	5.225506	5.361904	4.976876	5.834814
6.233555	5.465077	2.588071	4.726062	4.913107	
6.596298	4.856152	4.336088	6.465409	6.829496	
5.192017	3.915488	6.258936	4.877689	6.230414	
7.586527	5.256803	4.701541	5.311416	5.763255	
4.150702	8.118715	6.11596	5.565779	6.556936	4.604775
4.615314	9.337146	5.626115	3.703459	5.967144	
8.494801	5.452173	6.239369	3.217758	10.23443	
4.819653	4.687624	8.001426	3.272347	3.989225	
3.266721	8.566123	7.987143	8.367446	4.479983	
4.070934	7.904798	3.452716	4.438355	5.761947	
5.637027	5.946386	2.209204	11.40376	6.927972	
4.835168	7.899336	4.196516	6.491823	5.201627	
6.281621	5.872904	3.179753	7.687429	5.583224	
8.238858	3.479943	8.549435	2.095352	5.933252	
7.178522	4.095755	7.526458	3.113241	3.340623	
6.513292	3.404362	4.752606	3.521453	4.970821	
5.733619	2.740844	3.217233	5.842551	8.097045	
6.999472	11.38877	5.674718	5.131958	4.807836	
10.02845	4.612352	6.855023	4.261434	5.76706	4.604196
6.706318	7.754006	4.619959	6.283127	6.521169	
12.35012	5.9267	4.101003	5.418771	7.447554	6.113167
3.958881	8.878254	5.941446	5.369683	8.174421	
6.077461	3.551726	6.30482	6.838494	5.49091	2.404041
5.858543	6.408663	4.123857	4.753581	5.880769	
5.475625	8.860628	5.879956	6.220942	5.397824	
3.369525	5.989253	7.282601	7.416454	3.388522	
6.204385	4.872894	7.177206	9.521972	6.064862	
5.945066	3.875673	5.772936	4.191296	7.834506	

4.023567 4.540066 4.208399 5.844689 6.348715
3.337748 6.16469 4.711288 7.237653 6.320501 4.315512
8.640347 5.965403 4.35486 4.476159 5.279752 3.468709
5.770791 5.042856 6.99708 4.772021 5.384983 7.919442
9.99706 5.848939 4.439154 3.610123 7.587042 5.340135
3.910443 3.301602 3.285296 9.678986 3.932555
6.846499 6.010948 3.664871 3.047896 3.926721
5.864513 4.166268 6.248191 3.746623 3.861992
7.24234 4.397531 8.725649 5.920409 5.680314 6.635654
8.292225 5.370048 4.125276 11.42241 8.43969 5.888457
4.12477 5.785431 3.278719 6.320383 6.153416 5.67336
4.443713 4.988901 3.620651 7.791781 6.377854
6.334971 3.845358 4.59583 4.215668 4.046468 4.750168
7.810476 3.666299 8.28173 7.879098 4.979697 7.201936
2.932462 3.419643 3.759262 6.918125 4.66981 8.11203
5.687956 5.260368 7.431854 3.581979 3.222051
3.55013 5.158944 8.580284 7.139239 7.678726 4.425904
7.886487 6.820788 4.462277 12.8796 4.68911 3.109645
6.233695 5.253363 6.673213 7.033268 6.404829
4.889492 4.25623 4.975558
F10 10.4463 7.866219 4.736386 1.655302 5.063459 12.11562
1.276728 5.653835 4.103222 4.828833 2.461394
0.7632605 3.323487 1.214178 2.473143 6.560441
4.206928 20.22051 11.4593 11.30043 5.000922 5.944929
1.757317 6.344889 7.699544 2.859798 2.235322
4.905361 4.885854 2.854511 2.892814 7.948793
0.1309554 1.320698 1.727001 0.2162904 26.84111
2.298485 1.187762 0.7749483 0.9162968 0.05979761
0.8472324 0.3131823 0.5778536 3.584697 0.5453322
0.4904938 0.3013939 0.2167136 1.694249 2.49015 3.843708
1.520651 2.416716 1.175011 0.7696132 1.251665
1.298507 3.895804 1.484246 0.3363922 2.622514
66.13541 5.363266 0.9067664 2.633654 5.266515
0.204459 0.3790843 0.06846793 0.3352111 0.9638429
0.3967543 0.858954 1.394072 1.503379 8.663962
0.3407122 1.528327 0.1608344 1.54256 0.1651349 2.130553
0.9093754 1.606138 2.582453 0.2209903 0.6760126
0.8972774 0.3506098 0.2341115 0.8639301 1.82197 0.1632535
0.5417894 0.5319948 0.493506 0.853876 0.3812858
3.875847 10.45726 0.2557554 24.43231 3.35972 3.149212
0.1423353 2.533261 0.539864 0.07399053 2.095061
36.51694 0.5194589 0.9313458 1.223027 1.689121
3.650006 0.2735874 8.149898 2.451534 1.697499
30.16573 30.01033 0.5609862 0.8891489 1.417587

0.2707364	2.927926	2.993095	1.38123	0.2709102	0.2878476
0.6902045	0.2547651	1.723229	2.389409	0.2580846	
0.2655882	0.4343441	0.3560239	3.448845	0.3246622	
0.8107278	11.74531	1.193183	1.635747	5.46748	0.4481349
6.755274	2.391425	0.1481763	1.209689	0.24587	0.4596478
3.104955	4.376063	0.6887655	1.022251	0.5543578	
1.176015	2.072746	1.819063	1.948634	5.536951	
2.560158	0.6951269	2.065794	0.8818132	1.772033	
4.259051	0.9112329	44.14293	0.921109	6.093146	
2.094015	0.6742101	2.368261	0.4528827	4.063707	
5.144909	0.4775664	6.15958	1.174014	0.4998305	8.531767
1.013373	1.695816	0.05840492	18.29833	0.3465469	
2.464406	3.320657	0.237509	1.819508	0.1802652	
3.124682	0.4057802	6.646475	0.2324942	0.9073989	
47.93591	1.677608	1.070064	0.9363413	0.8400733	
0.2916562	0.6018786	20.32087	0.8588566	0.7002144	
4.432905	1.913873	0.94445	6.726517	0.3905836	0.3075347
1.623442	22.42817	2.086703	1.355373	0.8171454	
0.4290701	3.44999	0.2120409	1.82828	0.4747611	19.12694
0.1866883	0.5341848	1.078471	1.008802	1.262089	
0.1849935	0.403822	0.6281358	1.622804	4.49471	0.355615
0.6406104	10.87392	20.51109	0.5808554	1.0304	0.2598089
1.871974	1.676006	0.4620343	10.78215	1.464465	
0.2154553	0.1718453	0.9464023	0.7694502	1.352716	
0.1982299	3.948115	0.9286483	2.480159	0.2315849	
20.7614	0.4915424	0.2245656	0.6694286	0.343994	0.3857649
19.37139	0.5500375	1.663749	1.059934	1.160429	
0.4272665	0.4944178	0.920928	2.485492	0.952926	
0.2338957	0.6379854	1.243813	0.1454529	0.4338218	
2.950519	2.545343	0.6650115	0.8126167	0.1754748	
1.638661	0.5688153	3.037221	0.9816781	1.088758	
19.42591	0.6007439	0.6754228	1.671339	0.2929352	
3.96155	0.6680551	0.3882952	0.2499389	0.3736462	1.086576
2.067859	0.5574119	1.140645	0.1561233	6.336631	
1.655159	3.377857	1.163887	0.3688772	9.426404	
0.07757378	0.2284366	6.289588	0.7858658	2.193696	
2.032243	5.16269	0.6733201	0.8425599	1.511524	0.7951116
0.2483177	0.2673036	1.636096	15.73885	1.395457	
0.704319	0.6550442	0.2364853	0.9943116	1.858127	
0.9197933	1.528093	2.807834	1.221033	2.582054	
1.050637	0.7217416	0.487645	2.648878	31.84512	
3.064897	4.497704	2.657675	0.6964913	0.8465333	
1.891563	0.9160506	3.994972	0.6341739	3.485131	
7.950772	0.2792113	0.09291485	0.4197883	0.8948167	

0.4532259 2.566271 5.529705 0.8758697 2.030515
0.01852327 1.034418 2.941749 0.4498066 0.06067855
0.4050301 2.589718 2.483448 7.487866 1.142675
3.629995 13.53246 0.3849929 11.68577 0.5859001
0.4747168 1.956472 3.463429 1.176442 1.078908
3.140886 24.55709 10.23152 0.3739728 3.779272
1.039747 0.1093474 3.213346 0.595317 0.1276051
4.219693 0.5678101 0.5645975 0.9760528 4.797047
1.643705 1.950577 2.648322 53.47759 1.298894
0.2011178 0.295828 0.6195776 2.126157 1.184578
HSP90B1 87.32356 73.20319 131.8097 188.9126 169.2016
67.06495 133.086 48.0882 180.7244 139.6698 52.87699
41.36619 192.5095 40.99361 132.2024 69.09263
104.2156 80.72676 98.71774 115.6017 203.8712
71.21053 58.57617 64.50785 66.48587 39.48212
162.5079 88.30498 176.0606 72.45945 148.8493 75.789
151.7646 103.4832 162.8058 220.5296 185.7732
184.102 229.853 202.7539 156.2751 122.7428 464.641
209.0356 150.9249 291.8994 197.3066 187.7516
275.8272 197.4244 217.9776 135.829 243.8721 253.8881
76.0812 299.6035 257.6615 65.14659 180.45 213.9083
168.114 182.4042 124.4015 253.2249 387.1426 117.9308
112.9516 107.254 257.1157 139.5547 167.2129 262.7471
120.858 206.516 208.1183 182.1067 141.6826 215.7301
119.1303 121.1404 119.7669 171.6167 213.7329
110.0621 137.01 194.0803 164.5925 235.4834 163.3603
90.59608 270.9216 318.0077 140.8532 175.072 136.9967
287.7446 151.2862 203.1938 197.943 220.3335 99.71645
180.4191 159.9585 141.9169 178.5962 115.1069
186.0099 101.8299 277.8994 204.4413 156.8661
229.3756 271.1697 64.42444 112.7104 166.9792
107.4532 292.0029 160.3502 189.0472 505.7055
311.1368 321.5566 118.7785 263.1204 151.0124
154.6222 232.8591 101.1581 92.5859 174.8884 124.0059
163.1924 265.3097 145.7694 76.59973 187.9955
268.3794 181.6906 182.0626 149.2094 139.4188
207.712 273.4046 328.0823 298.3554 172.6004 131.5385
162.3093 106.936 152.6611 163.5624 344.9692 183.2511
101.1332 226.0928 230.6162 112.1205 164.7994
202.325 164.6693 177.4723 151 191.1231 159.6607
144.7386 162.7033 179.4118 227.7788 133.1758
72.35338 225.1198 115.5958 124.7227 150.8555
182.7943 156.1941 252.8643 202.4754 189.3458
119.1176 126.7238 89.85193 182.2201 207.562 137.9538

101.2704 144.2397 185.1188 155.0618 122.7322
205.0765 225.2747 388.9422 108.1799 155.5314
176.7172 324.3584 217.8728 77.27766 256.3233
124.8623 200.6828 244.1865 128.7319 292.2122
161.2815 254.9436 139.3602 171.9858 143.5385
168.7165 218.7469 105.2528 133.3058 325.1041
130.2584 410.5564 196.8328 187.6777 199.0681
237.8076 185.7536 340.1098 94.98978 229.0846
266.0314 66.25279 197.2423 119.2422 101.4071
119.3518 204.9146 193.1838 188.555 86.82632 105.6218
206.9552 258.3714 200.6152 196.6026 215.6317
153.9744 243.635 240.0796 280.3258 231.5084 64.85351
156.4705 347.7484 217.628 118.9514 165.5399 162.2917
241.0153 413.6149 461.188 137.1392 118.4802 141.1456
216.8663 163.0229 233.754 203.4416 151.9135 253.6595
186.9181 134.2769 188.7309 148.1322 157.3044
137.1242 173.0606 261.31 182.52 185.0399 162.0749
81.54947 140.6853 174.6351 98.49586 268.6183
131.0735 147.5996 322.1251 162.9978 472.7941
156.8738 220.0486 162.3872 276.8135 60.58428
99.07062 181.9728 198.1357 105.6446 171.9957
179.8578 335.1254 180.3054 232.3603 134.5246
176.3991 178.1547 206.2852 298.03 393.5096 171.5797
247.3272 166.4051 429.1983 128.6325 205.989 102.2036
132.8091 79.49354 260.7462 182.8148 214.8862
211.5793 244.3782 220.4078 199.5173 134.6047
156.824 161.654 185.1233 210.0177 154.9429 204.8798
229.435 104.8077 206.0507 158.005 77.36797 64.03518
157.3437 115.5583 169.2716 300.3944 145.762 116.8386
174.3126 239.7752 74.46196 88.71713 409.8997
393.625 191.8346 218.6567 203.8664 239.4133 149.2984
214.1251 200.8778 218.6998 155.2326 280.6789
65.90177 179.4797 149.8435 156.7171 330.7319
243.4376 116.7708 225.5877 163.3223 184.1367
111.3131 229.4413 150.898 128.136 256.8446 173.5189
213.5215 133.0063 196.8483 177.8681 136.4518
162.7473 226.3012 171.1603 83.07552 238.0247
151.0362 138.868 152.0085 184.5098 217.778 391.2191
153.7465 320.3252 204.7645 193.3792 193.1014
215.9828 189.6627 131.7607 84.37592 198.1521
217.8154 100.8336 198.3388 283.0181 336.2877
110.6587 85.60082
F3 11.0367 10.0752 14.21463 16.96811 16.24627 12.02166
5.371797 15.23806 27.21988 28.95348 11.17841

13.51225 9.161658 11.99203 10.72661 5.406089
9.59651 49.02973 14.35115 19.89164 30.13969 15.16883
48.6671 6.257536 6.645337 17.49315 10.54284 6.065718
14.47246 7.097895 10.29639 55.38596 14.33632
22.05948 29.30039 1.01916 31.75522 10.90195 9.995352
18.6491 11.9203 7.043044 34.19371 10.24635 23.43755
15.11925 27.52476 5.969486 35.54022 38.15001
7.700369 36.91718 6.727961 7.773182 8.64105 16.02756
7.983683 9.376463 5.269819 16.39424 23.60324
8.690373 6.069804 2.791136 11.8848 3.701679 18.08632
15.67987 53.36713 19.10859 9.132653 63.00112
9.693139 7.944519 24.94302 13.06005 93.23078
17.93493 4.497898 13.15299 3.242287 44.707 8.271032
13.64537 10.4117 17.80044 27.67782 21.42561 9.52149
31.46397 6.90535 19.73791 42.68121 14.4614 3.629583
4.259155 52.04557 20.05059 9.656312 22.22531
8.592115 56.15429 4.808834 20.22785 19.07959
45.00806 42.69048 5.479256 17.91524 9.236523
10.6745 14.42479 14.28706 11.76024 70.80138 14.19906
5.897388 22.93234 3.833979 13.83023 12.77582
23.07831 26.84672 91.20203 3.871498 9.759106
5.911713 10.28068 31.1746 12.41335 19.50733 29.76176
36.29144 43.26723 28.59319 12.03826 81.98785
15.16977 43.51312 29.63291 4.296181 14.44139
37.95972 15.83953 9.7111 21.09997 7.721122 41.9082
1.273724 8.56946 4.362041 72.23721 11.96547 2.546299
19.7499 37.52699 13.30245 3.578228 34.28426 10.26276
2.985828 17.08981 16.03581 15.7729 9.805162 11.99857
11.35031 25.48086 5.84298 2.855618 2.966084 111.1637
9.397618 19.06775 11.32455 19.32551 15.07617 7.0587
24.51162 13.99922 13.73301 10.39641 19.89806
25.77657 20.34633 28.366 10.26713 319.4417 21.77997
4.801576 15.95253 13.07883 111.3662 10.54549
11.01843 45.16667 6.944348 16.19937 10.87531
3.163224 3.040268 23.313 68.73462 10.29682 7.094569
19.48094 5.163654 12.72366 10.93487 8.014378
27.84616 76.48524 68.32861 5.595838 5.514015
23.3404 2.931153 9.295788 21.44657 8.607915 16.12306
9.621427 10.70938 17.98084 15.25997 2.432626
22.79112 9.226294 9.322285 104.1046 3.352375
55.19157 10.03184 46.17378 32.28957 23.98686
8.480365 17.62226 15.23544 2.951394 13.77297
10.69984 21.62577 45.76264 31.21328 27.65343
5.679406 22.38522 31.71792 13.36098 11.57933

7.065544 31.90383 20.63133 10.42537 5.822476
7.129215 6.469224 33.84231 8.889769 30.50231
139.7824 31.23046 5.731877 21.2878 26.21396 7.378195
33.91473 44.78498 38.29911 30.987 7.86294 67.4769
10.5489 25.60093 5.704777 14.72996 16.49046 1.164865
18.21524 12.71689 8.973439 29.10331 5.462421
28.99921 13.19323 58.20273 10.13414 48.59673
5.636979 18.50026 4.703003 1.431594 27.33221
4.468103 11.90634 38.59327 5.363934 7.197357
29.73109 47.8447 18.85761 44.67032 54.61779 17.93288
9.379873 11.82993 12.62805 10.84204 16.18084
13.59411 60.2326 2.553584 7.944661 96.43737 23.57621
19.75217 6.099154 32.7453 7.791609 13.37619 35.15498
5.186652 18.83825 34.62553 19.11971 20.50916
6.46833 36.23363 16.2003 15.94274 12.4712 18.99352
35.32249 8.387367 10.40248 5.137993 29.51457
26.90852 16.08956 2.510091 9.133394 44.9689 3.561595
12.06923 23.42574 18.16557 1.589042 2.788253
11.92106 14.82978 23.24703 22.48101 36.9464 11.95425
30.22965 38.29523 5.090484 3.41307 4.324253 15.5248
15.58074 93.58109 27.62709 9.49803 50.84833 6.005645
23.27014 29.14772 4.442828 12.80657 60.26824
2.215408 18.04368 5.42834 5.023597 11.58437 17.33022
61.26034 9.837074 8.497576 31.59602 2.296607
3.465493 30.73039 14.61535 9.464306 65.35371
7.26321 17.87923 10.93283 25.56419 12.35486 8.908199
27.47135 31.5371 15.4755 19.57373 13.60636 17.11181
7.638648 56.09409 44.2894 11.13229 12.54477 11.86288
16.18963
ADAMTSL1 0.4268581 0.8638089 1.156615 2.075681 1.487269
2.707698 0.9816119 1.623318 1.71475 1.220243 2.305424
0.1776927 2.009898 0.4622683 1.64715 1.690685 1.562143
4.051896 1.149443 2.096628 1.533773 1.039169
0.6693359 0.9224149 6.920617 0.6866447 2.384948
4.156718 2.751734 0.412961 2.138258 0.5882783
0.2587203 0.3612863 0.3035109 0.04835827 0.04590883
0.3576221 0.5042443 0.2450088 0.8559833 0.03500396
0.2433818 0.04892634 0.5837987 0.4098645 0.2502682
0.3592262 0.6850884 0.1480016 0.1311478 0.9553182
0.4177077 0.1907248 1.470988 0.2806752 0.459934
2.262527 0.1094923 1.290463 1.252257 0.5986769
1.788413 0.8660594 0.08500096 0.8669963 0.842803
1.336624 0.4510351 0.1456259 0.2995674 0.1399003
0.5586537 0.9492877 0.7038082 0.353079 0.5163923

0.9427328 0.1786497 0.08859987 0.1169879 2.207271
0.1039693 1.637736 0.2625712 0.5777652 0.8886348
0.3707384 0.6234653 0.4317079 0.2263994 0.3768677
0.2826699 0.8836146 0.1675839 0.2952276 0.4030717
0.2624888 0.1240336 1.384092 0.9404454 0.07612778
0.1575678 0.48424 0.6897081 2.120834 0.302322 0.9982148
0.338456 0.1382245 0.4335129 0.129303 0.199375
0.397614 0.4062608 0.2504559 0.3455569 0.1179683
0.2289664 0.1200083 0.09930491 0.4378636 0.5783495
0.7310514 1.857552 1.435745 0.2128685 0.09782122
1.16471 0.7431996 0.3503142 0.10925 0.5409482 0.3423185
0.4019243 1.654691 0.4395892 0.1401375 0.4916234
0.07381086 0.1788262 0.3925563 0.3234843 0.2729087
0.6746085 0.2087285 0.03293199 0.7081958 0.0699887
0.6475366 0.08970397 0.9310154 0.1699125 0.869282
1.69995 0.197412 0.4848976 0.5942433 0.1177463 0.1254701
0.8528687 1.713347 1.543055 0.3010221 0.2214869
0.8020377 0.4803457 0.8205816 0.9103019 0.1140651
3.251545 1.024725 0.7590428 0.08642445 0.04467045
0.2403103 0.6764753 0.3859627 0.3087794 0.4045994
0.08874759 0.1425898 0.3816862 1.274233 0.2339977
0.5151494 1.155713 0.04924803 0.7986719 0.1687895
0.757674 0.09066117 0.1967582 0.5311665 0.1137988
0.5473107 1.195801 0.5193541 0.1371178 0.1945039
0.2019613 0.5784367 0.6715086 0.111652 0.1648676
0.1744597 0.3575195 0.8499124 0.3409587 0.1366291
0.3625842 1.343502 0.5702611 0.3029123 0.1788876
0.4719018 0.1451879 0.3582564 0.9440689 0.1909691
0.4395036 0.2337243 0.4723613 0.3081531 1.301323
0.04916293 0.3234354 0.05907792 0.08612648 0.2612415
0.3023902 0.7080108 0.107898 0.1079089 0.2233803
0.2933495 0.9850686 0.1412445 0.2341465 0.2961918
0.3320914 0.6587339 0.8425219 0.3553628 0.8963299
1.003143 0.3801505 0.5078015 0.7183277 0.2839173
0.476919 0.1481178 0.1417354 1.37973 0.4183837 0.8854163
0.4764024 0.586528 0.3230937 1.992517 0.2890223
0.2162826 0.4521827 0.2734588 0.4048842 0.8892836
0.07068225 0.9431533 0.2637695 1.561257 0.6214373
0.4196852 0.1477872 0.105849 0.1243783 0.4172549
0.1748334 0.6338734 0.1395421 0.3287932 3.138119
0.1227522 0.326471 0.5793595 0.1440544 1.255828
0.3000047 0.09826713 0.5151202 2.785588 0.09612113
1.953562 3.174642 0.7210348 0.5273368 3.333503
0.2770503 0.4067911 0.1081864 0.1892003 0.7144403

1.006182 0.1580168 1.322134 0.1033221 0.3485956
0.1838368 0.3242676 0.8172192 0.1408539 0.3284395
0.2428156 0.1236251 1.672426 0.1689599 1.211121
0.8356776 0.6587818 0.2431169 1.116529 0.09550674
0.4648391 0.07333665 0.393872 0.4434958 0.7522816
0.1762463 0.2678832 0.2208091 0.3172408 0.06263787
1.560612 0.812785 0.4706568 0.7632322 0.277483
0.03260027 0.2187446 0.9213102 0.2476225 0.6991098
0.3024533 0.6427965 0.02956736 1.759504 0.2555984
1.099697 1.671263 0.1492194 0.2843266 0.3706765
0.6754068 0.4382955 0.5730063 0.5355058 0.2315125
0.7397216 0.4053295 1.224038 0.1493414 0.7194837
3.059571 0.2319205 0.4247423 0.2582785 0.3228759
0.06270751 0.3543239 0.5420849 0.2215918 0.4228785
0.8726644 0.3346028 0.380282 0.165065 0.4440746
0.1099337 0.1259953 0.7046404 0.9003606 0.7184457
0.5157779 0.221033 0.2129774 0.3931722 0.2548204
1.500154 0.3327947 0.0890991 1.289639 1.871152
0.07249972 0.957435 0.5599214 0.09418915 0.6844682
0.5450255 1.581349 1.421082 0.266551 0.1983509
0.5662144 0.2174719 0.1763768 0.06481941 0.6432307
0.6365117
SERPINB1 127.0385 8.904056 85.6176 101.156 83.44554
17.57644 47.20226 35.66883 63.83106 75.58797
256.1324 492.8877 57.35879 374.211 43.85026 13.42829
86.07205 34.52834 72.32976 69.21095 70.62491
11.48563 414.1339 10.83709 30.69473 377.1414
48.34374 10.50044 56.04269 89.66245 45.30341
46.64611 58.3538 121.1693 68.26109 80.28069 57.21204
57.44571 80.33254 79.60986 29.78271 76.32793
105.3933 102.4468 92.90232 61.92857 63.67625
96.70071 115.6268 107.3616 135.3006 40.00594
63.96322 60.15619 26.72505 37.08841 60.25184
17.34372 46.55265 30.21937 60.73303 97.80594
56.88341 32.068 53.56355 29.59195 59.59993 56.33897
181.648 69.58085 40.55019 92.61654 109.5523 47.09101
65.42903 51.86304 150.1854 43.13907 65.48282
139.0005 49.29057 71.33375 120.4897 110.3447
141.1591 72.45441 135.928 97.65364 71.8581 32.40421
140.7936 118.1614 277.6531 79.86168 112.3414
34.64052 293.8705 67.22719 57.35983 108.5583
43.02983 64.54037 148.5085 55.07019 42.89578
59.47996 167.2103 27.51473 82.98666 103.9319
21.75171 29.62779 77.22375 25.4068 195.5404 82.00873

31.26151	66.53899	58.51408	111.147	128.1765	31.12903
15.24049	67.11899	17.36894	58.6413	121.9944	89.30854
36.01861	23.19979	125.78	45.10469	90.47495	135.6843
78.63717	52.78266	137.3931	77.31072	97.16569	
137.4615	39.98165	97.87508	54.73575	13.64552	
27.74486	95.4007	50.53311	82.51657	20.53447	86.9405
16.48221	127.6762	126.3887	70.02529	33.31011	61.691
59.56799	54.75608	131.7042	78.34877	58.10846	
68.65147	83.17633	134.6635	63.8994	64.34604	54.6372
75.34155	31.60436	29.56417	17.07565	29.54847	
104.0928	246.6772	82.2528	95.18239	37.64363	68.47804
100.9499	6.955164	27.03793	65.81679	322.8744	
66.70262	36.82904	57.03302	62.87227	138.3263	
55.98652	63.2962	34.14491	48.89934	24.86307	99.25401
87.10009	18.83766	172.621	32.32118	77.38963	133.6405
44.5039	37.53099	63.81879	48.40729	179.3897	62.70542
46.79337	30.08226	97.25013	51.37179	117.8016	
46.92013	57.20702	24.17574	48.29006	77.16423	
27.43278	32.50154	38.53374	60.88305	91.83223	
47.60789	69.38342	80.10948	40.74196	79.23336	
49.34726	274.6279	56.36311	78.86597	70.96888	
61.62717	89.38362	68.73549	198.9428	78.44209	
65.96292	77.17956	82.22217	30.91052	47.14436	
78.01999	55.3906	129.7699	224.6348	71.1715	67.01914
25.04455	70.62402	213.1353	44.76531	166.3242	
67.76514	35.84772	119.4661	14.2287	74.71609	43.15007
112.9099	40.22816	86.73788	108.237	150.7368	93.30971
56.28359	43.19523	120.9754	139.7372	91.16467	
49.40597	113.8476	84.62644	69.61506	142.9477	
39.90628	56.00948	73.28142	22.81007	93.68163	
147.9885	65.43198	50.62503	101.1108	146.0509	
144.7784	49.74795	121.6525	37.08317	63.43614	
71.00884	45.1234	12.35117	22.72275	51.57158	50.35456
39.77318	44.46455	63.49656	122.8756	106.9922	
112.2932	39.97153	66.83158	25.85703	156.3877	
48.66403	128.7651	56.51567	86.71429	32.74424	
31.90733	178.4044	92.31314	42.18905	99.83276	
32.89245	43.27858	29.50494	69.88266	118.2815	
96.25743	72.63238	83.40073	43.08362	93.50112	
56.48494	63.78225	21.44049	100.8341	110.3788	
147.738	34.60903	58.75463	44.04792	35.02946	115.1451
48.17339	56.76	60.87266	227.9834	46.4776	86.58808
79.36023	43.87838	34.41078	306.0999	63.22578	
15.87309	41.32975	39.85744	123.6845	46.84912	

43.37317 45.77873 101.5059 57.97793 66.30346
66.44568 23.67402 73.71495 48.55162 27.02698
184.4039 60.84329 73.55367 26.17543 105.7071
89.75017 67.70653 36.8869 21.1273 177.6069 27.31995
53.87243 116.4241 28.57409 76.98232 55.09723
92.15317 58.47928 36.53706 44.90574 63.22048 14.124
48.50927 143.5379 31.07656 97.18953 53.71528
25.5193 46.31983 185.8765 40.33329 98.177 131.6897
63.85954 18.44488 83.32752 59.26762 78.9561 32.99197
29.65599 131.9144 89.7235 143.2646 79.33794 40.87815
MAP3K1 2.257622 0.4285191 3.898694 4.701749 2.729093
1.7696 2.57757 3.107411 3.130736 4.387915 12.90745
10.0287 6.780788 6.216016 5.752635 1.506556 2.554059
2.377498 1.05783 1.705127 4.616142 1.177644 4.009966
1.70982 1.498537 9.517983 3.639179 1.468023 3.260226
2.25162 3.788571 1.068388 4.930983 8.575233 5.077004
2.46832 2.025892 5.338893 3.874312 4.52582 18.52185
6.380542 2.880906 3.901337 5.103233 4.263202
10.07394 5.029344 3.80572 5.326785 2.464883 11.414
9.009323 6.293732 9.174424 4.925664 4.180117
9.87692 7.185827 3.803475 6.934799 6.144855 4.671396
2.897413 5.210836 2.462499 4.06704 4.670079 3.520641
3.029215 12.61595 3.845751 4.000981 4.187396
7.062314 4.229409 4.055351 6.920167 18.02477
3.506395 3.064408 6.478885 11.64148 5.936197
2.302663 4.472096 7.367962 7.939026 4.973272
3.655114 4.663245 11.74222 4.751474 10.2474 5.801845
5.449563 6.437332 3.545108 10.1018 7.359374 15.98835
4.607748 3.735412 4.944073 4.802868 11.51622
3.115581 3.872212 5.642919 5.675671 8.304762
4.564345 3.122661 7.632505 5.348173 6.810962
3.134902 6.304063 5.561189 6.78226 3.801616 7.527447
5.155138 3.352415 3.431663 15.28839 4.470881
1.79006 5.882862 3.60748 5.249305 3.365592 3.2455
5.069622 2.441429 7.35341 3.282879 3.274194 6.738877
5.623178 5.128099 11.26142 8.901448 5.424067
7.119463 4.149463 3.219107 2.37929 10.88244 4.366466
8.932227 3.459253 3.808291 20.47032 4.394951
2.826091 5.089438 4.846988 2.956889 4.20191 9.632437
12.05308 9.181024 4.534094 2.324275 8.69327 8.031787
7.692173 9.323869 1.840575 3.248671 4.669164
4.333274 4.023692 3.275141 2.04968 9.44979 2.887674
5.481302 3.878336 8.137508 4.815059 7.953883
12.1498 6.399936 8.079895 7.456023 2.438071 9.084254

10.1354 3.707501 4.510095 5.610828 6.019129 2.978749
7.039497 5.75116 7.105817 9.742852 3.395143 3.043524
4.869365 5.966104 5.664242 4.007932 5.33887 1.767439
3.446128 4.838701 9.661783 5.192589 5.887839
6.111864 10.74493 14.62208 13.0162 5.408381 12.24801
4.544893 4.085783 9.876605 8.704362 3.257098
6.316632 21.97356 1.544241 5.846045 9.142457
2.726886 2.875257 2.62779 3.019427 5.071445 4.479013
10.42912 3.708389 5.603567 8.450338 4.522273
6.565495 9.994325 15.03959 5.103864 5.954411
5.52142 6.023471 10.47107 2.8324 6.1697 6.374628
16.86525 6.74202 3.380382 9.595448 10.65821 16.26986
8.952175 4.563262 2.939853 7.435515 4.457767
4.29346 5.02591 5.485159 10.12321 5.983597 2.944801
2.814531 3.531895 16.33205 6.188332 2.883693
1.555775 5.38471 3.530912 6.068617 7.375675 2.616299
4.697635 7.141773 10.90766 9.680439 2.110469
5.54375 5.1972 8.213742 4.083749 6.070724 3.752611
4.418246 9.234541 2.238247 3.274923 6.375863
2.792983 3.730985 3.314283 11.68681 4.621711
2.248034 6.911195 5.906904 7.252764 2.958708
4.010688 3.471348 6.072779 9.167651 12.66974
8.695506 4.687988 2.200461 10.36424 5.149769
5.657143 2.407909 6.153703 3.463078 6.684598
6.035443 14.21456 5.705307 4.452292 4.780504
3.009008 7.820233 7.031324 1.871238 3.189918
6.247739 3.836087 6.859707 8.987177 11.63564 2.3938
6.186812 8.53516 3.503352 12.77994 6.387442 10.40911
8.161903 3.797814 4.016929 3.953212 8.880653
6.491827 4.372401 3.296541 10.22161 4.71302 10.98719
3.578909 3.161138 13.69669 4.833378 9.808003
8.490497 5.215329 4.811829 9.719378 3.666747
6.029175 8.346643 3.675013 2.65471 4.728645 7.861024
6.846069 2.210682 5.212135 3.649717 4.114797
6.034765 4.123018 3.474537 3.758622 2.272439
3.331089 7.368539 7.452841 5.928303 4.761494
3.017291 9.606963 4.901957 3.229542 4.795555
5.156604 3.068297 7.865991 5.07205 3.025031 4.074309
13.00248 6.282231 10.45199 6.066011 3.199471
2.82886 3.620472 9.964936 5.685937 7.11297 3.217871
5.457335 3.232868
CTBP1 11.07066 11.55146 11.29409 16.71874 11.69706
8.721524 9.031644 7.657594 14.96117 12.13953
8.023185 6.274727 14.485 9.214877 13.90387 8.980533

15.67424 13.04155 10.6076 11.37881 14.49996 8.753207
8.641416 9.733612 10.30536 6.722673 14.30843
9.862648 14.96637 6.781832 17.13779 8.742878
25.85264 11.8432 14.42243 14.62354 6.624989 17.03626
14.25065 20.34671 22.63304 13.58266 10.24941
16.37762 11.24781 10.82993 31.14368 15.94872
9.552645 14.30345 26.9297 12.55329 10.40221 8.353057
9.225591 19.21233 12.49398 14.62101 13.70379
19.78826 10.65322 22.05291 6.931217 16.37685
11.71007 10.34297 16.63019 15.04746 25.48432
12.00755 17.33717 10.76435 11.54627 14.43992
11.01755 18.27435 10.79984 16.09946 16.40806
10.14516 21.54543 10.89711 10.72108 11.26741
12.3603 18.14545 16.12372 15.70319 9.2209 9.498116
10.24133 17.12089 9.47346 13.02283 19.997 11.08972
14.39452 19.64255 20.09304 14.62951 17.38743
11.70328 10.79417 27.44896 11.63852 11.61615
16.45676 14.3221 20.45855 17.9212 12.39326 12.61159
16.84842 13.05106 8.209579 12.31033 30.62214
12.73268 12.61864 14.06877 12.43835 8.32638 14.10101
7.913539 18.71429 16.23266 14.07379 17.8935 10.90636
10.68878 16.52286 19.56555 12.67714 10.83486
14.55961 13.73174 19.92644 11.05741 15.26833
8.92638 16.66081 9.164943 9.61655 13.23493 16.35508
8.877083 22.47817 12.92933 7.4227 13.42301 18.9771
15.99638 18.15985 14.5116 12.07121 9.597498 14.41496
12.73817 17.52474 11.61583 15.81697 10.56268
8.984542 14.62535 13.67901 18.04956 25.67099
8.485716 14.29602 15.50548 8.635549 11.04848
8.218598 10.18148 20.83439 12.48374 40.65391
11.97832 15.16533 10.84401 9.250431 14.05212
12.27144 16.4011 12.72133 14.53743 11.22891 13.02737
12.06788 13.57206 12.77098 23.78094 21.58199
16.19948 16.9509 21.83881 10.54616 17.38924 12.59802
11.10632 12.00497 10.00266 14.52783 14.62787
16.4553 14.57085 18.30005 14.30826 8.2845 12.81697
13.19582 12.4279 10.53719 13.9771 10.91099 17.73763
18.1571 34.7189 17.14668 19.44385 16.00948 12.65659
11.60383 16.3366 16.1585 15.28334 13.80611 7.49836
19.78873 20.43746 18.66345 9.600179 11.6994 12.58691
9.120651 15.62782 16.34487 15.20049 12.43585
15.18212 17.97943 14.65423 11.56563 17.91064
9.380679 18.32851 17.37368 11.94428 11.21787
15.97096 11.38105 6.184657 15.33914 12.71421

14.17803 17.80028 14.01419 15.04553 19.1611 12.38332
11.0043 18.44099 12.94921 10.61407 17.5811 14.27155
20.41682 23.57704 22.14941 9.482738 16.62589
10.76772 17.97605 15.76077 24.46795 15.88387
16.37101 8.670385 14.40485 9.671798 12.86919
17.73879 13.38442 17.036 10.73487 15.43995 15.71462
12.51048 14.38891 10.11151 15.06309 7.867932
9.287222 14.36123 21.64266 17.39254 10.72178
15.64643 13.18288 11.65283 10.44894 9.217627
13.87995 12.01372 10.51582 24.50102 9.939172
8.626691 18.572 11.35558 10.22121 22.7728 14.5443
17.76598 16.67323 14.0249 10.83478 22.28529 7.0963
10.96466 9.638842 8.978601 15.63631 20.51628
5.401873 10.96577 18.50372 14.92602 8.147608
19.67246 5.180716 11.81685 11.69587 11.56526
16.80245 12.34324 8.417703 13.22685 14.90378
11.65197 29.85651 42.15156 13.70872 29.84658
10.6639 10.72665 12.88727 11.14506 20.38065 11.55485
16.33513 15.54536 13.29425 12.44759 17.29173
7.918025 13.20208 10.32214 9.388903 40.0826 10.21048
14.44289 35.32952 10.23992 9.081538 14.41598
12.21197 13.42183 9.091933 9.850006 9.404837
7.530706 12.78017 10.61853 23.28758 14.41088
19.86674 8.828677 11.04255 11.8512 20.78209 18.02698
26.39199 21.37859 10.48766 17.54321 15.08784
14.18662 24.86389 18.27259 10.61993 12.03506
10.44291 11.02737 13.18651 7.216435 15.49593
13.32577 10.95933 44.40602 28.70919 15.50634
15.90677 15.76028 17.08815 20.61808 10.24146
CEACAM4 0.07456035 0.1222228 0.1665599 0.4680471 0.5309058
0.2373163 0 0.2808441 0.3946571 0.4670799 0.09143009
0.02092633 1.266544 0.1398144 0.2083802 0.09469363
0.5099306 0.4090111 0.06575858 0.2502427 0.5725446
0.1591195 0.1355074 0.06371717 0.1021333 0.1841904
0.3203459 0.1263354 0.08233369 0.120846 0.8428132
0.2291471 0.2673222 0 0.1474158 0.2334953 0.133001
0.1913306 0.2059827 0.1319694 0.1806336 0.2629118
0.7760462 0.04793239 0.1425872 0.1881038 0.136333
0.5037504 0.3036772 0.02552206 0.07377553 0.4622751
0.1828332 0.1441415 0.2284457 0.01309396 0.3284232
0.8407651 0.09612323 0.2310426 2.11626 0.9732437 0.7001559
0.5110299 0.01465795 0.1732239 0.2906735 0.4413787
0.06421027 0.1339327 0.4548569 0.1003602 0.1159052
0.3078897 0.6465058 0.4237978 0.231557 0.06069246

0.03224674 0.1878145 0.2963252 0.6399267 0.4677822
0.1752403 0.04487827 0.134071 0.4164688 0.644959
1.459573 0.1682 0.2258165 0.6065617 1.173776 0.1888223
0.7723891 0.935818 1.09442 0.3140493 0.2860367 0.3778764
0.8401646 0.1395869 0.4172171 0.7154923 0.2637797
0.6267132 0.4302407 0.1193354 0.6028233 0.1846026
0.1354609 0.1881551 0.8972472 0.09393799 0.9430809
0.1353077 0.7150726 0.1265786 0.04721217 0.05571669
0.1543181 0.5844132 0.2061269 0.6328008 0.04095474
0.3306072 0.52609 0.2534424 0.3255848 0.08872663 0.2071191
0 0.4768686 0.8157002 0.1666833 0.1493381 0.314074
0.1949659 0.2227247 0.2515706 0.3047479 0.4486241
0.01657217 0.04485871 0.281496 0.03445587 0.04148095
0.3278334 0.05632277 0.05202322 0.246097 0.8739577
0.1737346 0.8786238 0.2101652 0.1337815 0.1332448
0.2648558 0.1795363 0.8552833 0.3692581 0.2353572
0.3921346 0.3779019 0.03936579 0.1775644 0.3058448
0.06968577 0.5077129 0.01311327 0.2374111 0.3433439
0.1136433 0.03251655 0.04424386 0.1772133 0.07212704
0.2607506 0.1639505 0.2218584 0.02142565 0.09681848
0.2624267 1.141201 0.6915432 0.2237318 0.3319136
0.1152928 0.3074534 0.1105073 0.1644219 0.0448977
0.2159173 0.113987 0.5677872 0.2516819 3.885534
0.1587135 0.09768183 0.01243909 0.09833539 0.02618391
0.1461926 0.0173905 0.02015327 0.2560141 0.5262215
0.3435132 0.1899578 0.04997774 0.9128257 0.622664
0.2669437 0.03524326 0.3782097 0.2982154 0.1595845
0.1434486 0.7476422 0.1546009 0.2186727 0.213353
0.2571458 0.1526052 0.2600656 0.03452808 0.1319738
0.09921897 0.1819375 0.09580182 0.01201707 2.57633 0.05557117
0.1344599 0.06382129 0.04472912 0.1841168 0.027099
0.3247363 0.09788071 0.1755058 0.5977081 0.6073856
0.7655332 0.355564 0.6035344 0.3014891 0.0319812
0.3258905 0.4177194 0.5553476 0.4086737 0.2182023
0.48275 0.1826118 0.1952139 0.06169342 0.5630914 0.1300034
0.1131249 0.1463841 0.2088617 1.305923 0.1540444
1.169205 0.4860971 0.0922393 0.7184371 0.1945773
0.165196 0.4272665 0.6513516 0.40139 0.6230381 0.1037995
0.08505298 0.1112085 0.2131353 0.159878 0.6223237
0.6726726 0.8702971 0.3910319 0.3140947 0.1010309
0.7850784 0.058782 0.03580962 1.274061 0.2859871
0.06875788 0.2318151 0.2280648 0.04539084 0.6528761
0.3249821 0.459523 0.431159 0.103611 0.09998982
0.05286799 0.03524759 0.3451556 1.912258 0.1528116

0.2760763 0.1152787 0.3545001 0.2057103 0.02147702
0.3902359 0.2947804 0.6879723 1.563839 0.1782451
0.4259422 0.2332384 0.09321296 1.065184 0.6267483
0.2412165 0.6866873 0.1296712 0.2198601 0.4162303
0.1529411 0.6198003 0.02796669 0.3046185 0.1361582
0.1836264 0.5116582 0.7259778 0.2628481 0.2607471
0.1630189 0.04197563 0.1536286 0.2876872 0.2641097
0.04471012 1.007158 0.08804846 0.01189703 0.5419375
0.08296761 0.09373037 0.1231374 0.09889183 0.1264472
0.1956056 0.371456 0.05449663 0.3680512 0.5171322
0.04531805 1.258271 0.4911966 0.07266638 0.04944595
0.9385371 1.257651 0.05332456 0.2330506 0.2786038
0.255572 0.02329076 0.9158367 0.02240309 0.176688
0.1706278 0.2726837 0.03038768 0.09497437 0.4205069
0.07941445 0.02976498 0.1322525 0.09183045 0.1715351
0.3386727 0.1694153 0.1270531 0 0.5828809 0.4767748
0.2774694 0.4726121 0.05246465 0.4608927 0.1757735
0.2074435 1.006604 0.4903814 0.5251151 0.7225326
0.1906975 0.4716158 0.06163131 0.263608 0.3482642
0.4168332 0.2052732 0.09732868 0.09086429 0.5020931
0.2113729
PXN 29.66391 12.49888 27.0867 20.67304 16.44211 16.76825
10.0734 9.683485 19.4131 9.977467 8.059865 4.82562
9.359452 6.225528 15.52941 16.37583 33.33057
18.04586 26.22986 28.63637 18.54464 10.61851
14.89733 19.1589 14.1328 6.863339 14.16764 14.71991
22.86867 4.549919 21.19648 11.70781 27.70793
19.38028 26.06446 3.247053 5.781894 13.85014
20.53114 12.59089 17.645 34.35434 9.594644 27.32852
23.29292 20.26801 18.71822 19.17311 29.52867
19.49842 24.88687 15.22576 22.79446 20.31722
15.01238 22.09759 20.14034 21.95063 26.54672
18.17696 19.29007 20.24598 12.76316 20.85492
14.96526 15.24784 23.21571 15.58374 32.75648
11.25677 19.12079 10.9955 18.20302 11.23113 19.29579
25.62356 20.50794 17.16714 13.70492 7.116082
14.76455 17.85611 12.35322 17.56008 17.28074
25.7122 17.07303 19.89349 19.27158 15.85205 21.36917
19.31926 18.09166 18.00262 26.41057 11.13993
51.90594 17.35828 9.575661 24.27377 13.74132
16.4618 6.866661 11.44024 14.16833 23.35123 25.2751
21.41086 20.54652 15.34873 22.51874 16.75825
17.93347 12.01232 16.56087 14.78311 13.40634
41.95391 16.65811 15.86923 17.57887 14.2083 23.72563

12.18838 14.5563 9.911872 8.788233 29.49037 18.04247
28.37436 26.27896 33.64204 17.96959 14.18714
19.79325 49.3828 23.82773 37.54907 18.57108 12.29717
14.31825 16.05303 17.37164 15.54776 21.67549
8.014032 8.363758 19.9555 20.79616 15.23093 12.48592
23.32098 28.70432 14.396 20.42839 26.33511 20.08702
13.36173 30.67181 14.66957 18.45765 10.64578
15.30472 18.73764 13.16167 21.23391 12.59988
17.42633 13.65941 19.45059 16.50754 15.1339 22.24268
12.3529 25.29799 12.05661 19.37634 13.802 16.61258
24.00761 9.348617 24.73149 9.299924 15.61296
12.28397 19.13885 20.63249 25.48057 25.42417
11.90706 15.26022 29.31879 85.36073 19.23002
14.54724 48.32098 18.38729 24.2274 26.06194 9.987848
8.166392 11.00756 15.07947 17.06208 19.28632
26.43582 15.24698 13.80736 26.91121 21.57843
14.34074 22.07655 23.39137 8.524665 22.35436
21.24891 35.04854 26.23219 16.12242 22.312 22.04966
7.703935 20.91687 15.79917 26.65349 10.31828
16.82327 5.885113 15.45249 35.84944 32.98925
21.62468 12.17502 38.0588 10.76115 22.14554 13.33248
11.18179 12.31502 13.07258 19.26219 15.24138
14.3143 21.73275 18.34079 25.62634 14.17755 12.04109
28.68901 21.90567 24.70752 15.22364 21.03013
27.35297 17.54457 21.68632 18.86656 15.34782
28.44391 23.23969 12.29156 26.19505 11.78805
13.69591 25.13432 19.23069 17.84361 27.44555
19.22258 28.34106 14.14133 22.51088 11.84878
17.31462 11.48136 12.31333 18.288 14.35768 18.67893
16.90305 18.55359 21.91682 23.91693 27.19768
19.84715 22.73212 20.94068 12.77065 15.64692 22.906
29.85479 18.62317 13.19581 18.16095 27.20707
20.24601 17.72995 14.85224 13.17102 29.04021
17.99377 15.84774 42.45206 21.77636 12.80777
27.84538 18.95366 19.90432 15.67108 12.78729
27.59429 50.18076 10.09736 22.68745 16.53668
19.52436 17.60839 13.23108 21.23948 22.97194
24.13224 26.11423 31.92017 7.184167 9.781395
35.16221 16.95043 9.77454 32.12761 16.04437 29.05117
14.45826 16.58873 22.88982 16.79287 19.15409
20.57399 13.25486 14.00547 26.27341 5.779316
9.253853 14.33451 22.58569 19.56595 14.41256
21.57786 10.46255 19.47617 12.89413 12.2262 23.17518
12.79537 8.90902 12.85402 21.39795 24.13507 22.29125

16.18803 13.31482 22.00561 23.13907 157.0935
19.9595 16.05724 7.503221 15.93813 24.86773 11.33351
16.75988 11.3297 14.90922 18.29119 5.912893 9.354932
7.46765 11.38281 9.303317 17.69659 20.77327 21.90574
26.07041 10.29089 12.33339 26.64178 16.62093
17.1297 45.10286 21.14449 13.37751 19.89616 23.41238
13.08188 19.7451 36.85709 8.459725 15.75621 16.60887
16.36625 11.20045 16.55143 15.21699 22.01385
18.65386 25.35012 22.06891 15.34905
IKBKG 1.008646 1.055039 1.162579 1.557362 0.8370055
1.067352 1.005019 0.7351764 1.588954 1.862057
1.000088 0.8816219 1.595754 1.459078 1.530286
0.7991044 1.716362 1.329796 1.28099 1.081525 1.645282
0.8159208 1.005604 1.236297 1.20119 0.8769529 1.283792
1.04822 1.392254 0.5137926 1.582249 0.7454479 2.8851
0.8168809 2.202029 0.3158704 0.3041548 1.423567
1.573188 1.067519 1.243408 1.578716 0.7455925
0.9422013 0.7927599 0.969391 1.111371 1.880678
0.7461684 1.282395 2.129128 1.200228 0.8038393
1.15139 1.244185 1.14884 2.309089 1.582625 5.217899
1.936709 0.9254624 1.83032 0.8856605 1.58497 0.9008096
0.8982871 1.477386 1.285727 1.687626 1.665157
1.153297 0.5550009 0.761578 1.0288 1.031565 1.652919
0.9013877 0.9416851 3.162675 0.7985176 3.32862 1.641433
1.001033 1.507047 0.9496923 1.462919 1.025125
0.8852135 1.184698 1.365237 0.6863439 1.549164 1.0621
1.310201 2.734224 1.152408 1.600241 1.508626
0.8084447 0.9430894 1.963864 1.284057 1.23316 0.6765505
1.057772 0.9813502 1.79359 1.470861 1.656472 0.7189994
2.991842 1.275978 1.177663 1.048398 1.093536
1.915415 1.750432 1.898255 1.350356 1.391171
0.7881761 0.4650524 0.6108071 0.8178309 1.381123
1.489477 0.8510155 0.9127948 1.193596 2.274828
0.9428581 2.399785 1.56668 0.6969297 1.13204 1.217387
1.538954 1.347554 1.431742 0.6968509 1.177886
0.8843322 1.111326 1.369735 1.278418 0.8356794
1.052825 0.8905001 1.17192 1.081788 1.247966 1.611321
0.7237327 2.340365 1.269615 0.82733 1.103586 0.8433329
1.676995 0.3994473 1.613386 1.09162 0.8083448 1.471871
0.8926333 1.421637 2.390522 0.6079472 1.23349 0.4206799
1.039971 1.355502 0.9092367 1.336401 1.406539
0.9263139 1.644799 0.5394865 1.08123 1.181456 0.5092977
1.014538 1.461529 1.292579 1.407976 1.424294
1.690201 0.9079515 1.246063 2.121381 1.684102

1.323203	1.827655	1.497949	1.380452	1.441156
1.083614	0.9978726	1.15363 1.52409 0.4560934	0.9074615
0.8435412	1.629996	0.9191583	0.836308	1.785871
2.114866	0.6782693	1.356052	0.8162205	2.0041	1.719615
0.9081291	1.106246	1.0842	1.771134	0.9686495	1.850287
1.84387 2.258978	1.271673	1.616166	1.002723	1.687273
0.353656	0.9819316	0.8532719	1.371259	1.978969
2.136581	1.235152	0.5423425	0.8217809	0.7054956
1.152555	1.28687 0.6563759	0.6694102	2.000895	1.227198
1.761537	0.9499659	1.101926	0.6098441	1.771294
1.542296	0.9559258	1.11565 1.511792	1.373009	1.663196
1.456743	2.142036	1.358696	1.247481	0.8958994
1.779539	0.9199218	0.783059	1.037285	1.15325 1.009509
0.650687	1.472357	0.920623	0.926941	1.540701
2.433953	1.821859	1.351174	2.735738	1.057264
0.8052136	1.398459	1.064295	0.9104662	0.8962869
2.295233	0.9277795	1.136931	1.441215	1.3508	1.206425
0.5436911	1.613034	0.8536204	1.432527	0.9885003
0.9914867	0.6909689	1.258656	0.769615	2.600035
1.596078	1.570117	0.9788342	1.388733	0.6607735
0.9240204	0.6896509	1.17163 0.6424585	1.056405	0.6323267
1.056741	0.6015137	0.9420018	1.817876	0.7471008
2.756673	1.854051	2.103813	2.760107	0.9168176
1.337632	1.036718	1.0328	1.041869	0.7883246	0.8884556
1.155037	1.723337	1.351158	0.3979611	0.886703
1.395533	1.821393	0.9811552	0.9510405	1.335977
1.231517	1.777127	1.981081	1.424383	1.098715
0.9693885	0.7459517	1.34257 0.618007	0.8525287	1.712825
0.6551114	0.7541282	1.336881	0.8858773	1.786035
0.7678506	1.348721	0.5794982	1.035773	1.579293
0.6986091	0.4365791	1.667945	1.442147	1.102873
1.257778	1.338786	0.9269049	0.9853959	1.293976
5.564854	1.122957	0.9332583	0.616397	0.8777097
1.061945	0.7648834	1.813532	0.7474294	0.9887099
1.902722	0.5832606	1.693345	1.130585	1.219478
1.359716	0.9725232	1.102243	2.172956	0.8839938
0.5156289	1.684123	0.7916451	1.545566	1.490144
1.083843	1.42353 2.099783	1.477663	0.3858632	1.102349
0.977618	1.022065	0.9670925	0.7997192	0.9442366
0.9687298	1.762056	1.858629	1.615848	0.7811987
1.332328	0.6516733	1.874112	1.209132
TFDP1	12.58292	12.96797	18.90105	22.35566	19.14842
10.96146	12.81689	9.681276	15.77452	24.50392
21.39849	21.95127	19.24535	21.31224	14.84087

12.67108 18.91787 15.87491 12.0552 15.204 25.29561
13.4095 21.07215 12.61023 10.64759 18.46217 16.00786
13.40125 18.13932 20.03916 18.11954 11.55413
33.95257 26.92168 25.1577 42.38839 9.24605 18.61566
28.01343 46.77332 35.70582 35.74545 47.34207
36.26384 18.96781 37.53271 26.20976 45.83442
33.5966 21.96111 41.15126 29.26164 36.48524 31.65576
17.40829 67.51151 52.11796 14.82986 36.24673
35.67041 18.94623 32.4399 19.14234 48.3311 38.27132
13.6739 27.98454 19.80621 20.88705 27.2705 55.65746
36.76949 31.14946 16.27396 26.41893 29.61169
33.72883 29.64335 39.3433 28.30454 52.38043 33.8233
35.87944 13.70129 24.04542 60.97641 28.51946
31.39592 31.65096 21.33746 35.44878 47.63992
24.89849 25.11247 32.4086 17.94908 30.68051 20.74176
50.11201 35.72752 25.23584 32.01607 22.82708
34.4727 17.87276 20.17581 35.7438 19.80704 42.45467
41.92232 37.90344 28.17709 44.07582 17.43783
18.03177 26.54804 14.26581 18.19912 32.7725 63.50804
30.97023 25.22306 27.11686 14.31213 72.58286
33.64633 59.88593 30.31377 14.55828 34.66533
28.54837 45.89924 35.18502 36.59722 23.25912
28.65551 66.89466 42.35484 51.39568 34.05537
40.17287 42.20569 22.38691 34.29416 25.14577
19.28719 51.91669 26.63213 84.378 20.03411 41.84245
30.63458 43.95797 29.18692 17.87703 23.90115
32.18916 12.6413 31.24646 22.42674 23.61959 18.64292
44.02059 34.53487 41.0068 29.49437 38.8377 13.62959
38.93791 32.72736 11.23384 63.77336 32.31954
45.81875 59.53398 25.18387 21.93263 18.48309
27.93114 45.95532 53.41825 72.24903 21.53302
35.8092 47.0512 18.2439 19.3085 29.79879 34.63252 43.12574
17.60853 38.94851 21.98009 27.53095 26.50523
28.42006 27.95684 43.68283 63.74799 36.01559
18.93086 22.34923 46.17186 55.22453 44.24263
27.83786 12.04537 20.46459 43.34039 42.63655
24.29615 22.78954 19.06945 29.94904 37.34439
42.49716 19.87878 35.68266 33.61482 52.89807
38.69075 34.30143 31.80825 43.567 16.65144 38.46324
46.41055 21.29866 43.34105 32.9857 34.28982 18.58832
23.42254 31.88784 30.4741 18.9941 29.78917 36.86094
34.73552 23.70088 33.47306 31.79045 48.60743
45.84302 26.48335 53.64462 77.43277 13.41359
18.46274 37.95139 25.40132 55.99991 15.73086

26.43741	53.55396	61.50135	44.21987	25.03548
37.45354	21.31308	19.98992	41.03545	23.13793
34.97531	29.30483	29.44465	65.73223	32.8238 31.68317
19.35851	37.33174	35.16409	15.96301	56.3014 24.62974
28.5379 29.08733	13.1518 50.93547	32.03983	18.1203
44.49559	15.26428	68.02223	47.71205	34.37967
29.6992 35.24599	22.91926	31.75911	49.22122	11.05625
14.18421	19.25744	20.45989	22.36192	21.71023
35.53688	43.78406	32.63244	22.67835	33.79106
34.24022	16.89187	34.63593	71.10799	39.28272
37.55897	31.5152 51.61215	25.31432	35.65385	62.18999
18.99283	58.15073	22.01731	18.62391	32.22461
16.09379	22.82853	29.40619	27.89329	35.20118
37.86892	22.19306	30.36328	32.50229	87.11712
18.65387	51.48481	34.19265	29.6601 36.68939	25.93179
12.10565	30.384 22.41369	16.74476	27.78892	17.01848
25.61538	30.55256	16.7338 66.95329	12.99478	19.62891
22.3162 226.2087	14.68542	18.90253	54.63069	31.51675
43.28333	22.63261	51.39633	31.44392	22.16936
26.43322	20.95736	55.39829	23.89261	14.64131
27.58513	43.26218	61.98394	46.74466	31.50109
30.24774	20.3147 18.54636	20.92408	18.3364 39.65759
20.2538 57.28833	36.95646	20.07	31.25644	21.31474
24.71235	34.12715	40.68189	26.82659	39.91039
43.9746 58.50426	18.00785	23.40083	48.13252	40.2987
18.71704	35.06185	29.28655	41.10924	28.37731
28.68585	61.60003	18.85659	15.75987	61.34315
89.23768	17.67895	35.93679	45.54442	65.45361
40.27482	22.98486
CRYBA1	0	0.03499411	0.05049363	0.03495873	0.02400091	0.05559299
0	0	0.1977427	0.0229254	0.125653	0	0.1130273	0	0
0.1220045	0.1095002	0.02755426	0.02259312	0.1172422	0
0.05466978	0.09311442	0.1094587	0.1002589	0.08629574
0.02896406	0.01446864	0.03535993	0	0	0.09841206	0.07065054
0	0.05958663	0.1604471	0	0.03286839	0.2972378	0.145741
0.04432966	0.03226086	0	0	0.07536873	0.05385675	0.08516522
0.01966784	0.03726302	0.01461465	0	0.1899019	0.1374128
0.02751316	0.07135333	0.04498782	0	0.1313034	0.01834762	0
0.08309688	0.02495403	0.01822404	0	0.1259033	0.1859866	0
0.07397439	0	0.1022581	0.03125566	0	0.1448085	0
0.09967121	0.03309252	0.09944701	0.02978932	0.04154712	0
0.03181577	0.01998764	0.05879968	0.1505213	0	0.0354336
0.09413752	0	1.065635	0.3095872	0.01763302	0.06129428	0
0.1235714	0.04212302	0.03738667	0.08868336	0.09633933

0.01535556 0.09273541 0.2978249 0.1278902 0 0.1755904
0.2454524 0.1242255 0 0 0.01618095 0.0243943 0.08864997
0.4260741 0.03211181 0.3025774 0.3888249 0.08716612
0.04094706 0.01449649 0.06758753 0 0.07574303 0.157483
0.03372402 0 0.0703555 0.1597345 0.06694531 0 0.05084699
0 0.03953408 0.1041606 0.03723659 0.01843787 0 0.1865786
0.08702302 0.0197017 0.04027529 0.2641034 0.0124648
0.05780131 0.05693812 0.09632759 0.05181187 0.1183824
0.09501262 0.3910971 0.032252 0.08936986 0 0 0.01658088
0.1091114 0.05348732 0 0.06539976 0.4766579 0.05140377
0.5509791 0 0.04043164 0.0748491 0.08322969 0.04508391
0.0726274 0.08756766 0.01710173 0.04983954 0.06758122
0.09063218 0.04213036 0.07809042 0.01861989 0.1520117 0
0.2124101 0.02634937 0.01760299 0.04234744 0.01840339 0
0.04098352 0.06818962 0 0.1356513 0.02850945 0 0.09054331
0.04745967 0.0470763 0.07712904 0.03709209 0.08392138 0
0.2081735 0.04675935 0.1454141 0.1118707 0.1282135
0.0563096 0.1049555 0 0.1344369 0.138484 0.02837436
0.06614544 0.08430226 0.04661792 0.1545407 0.04680982 0
0.06793754 0.02018128 0.1931602 0 0.03916395 0.07247886
0.05750877 0 0.0512256 0.09645152 0.01920637 0.0524316
0.1914696 0.1186305 0.1133579 0 0 0.07314508 0.02064394
0.1063903 0.09546483 0.04330998 0 0.07683944 0.02635761
0.01551765 0.1239687 0.5604924 0.1781583 0.1283492
0.02426554 0.2267409 0.01908805 0 0.09139822 0.3113266
0.02665915 0.01304716 0.03289734 0.2477838 0.1703847
0.199034 0.07842648 0.02235699 0.2119643 0.01896717
0.05153786 0.01766686 0.06705892 0 0.023369 0.05670654
0.107987 0.05567056 0.01584564 0.03981264 0.06077476
0.2081109 0.1334535 0 0.2043086 0.01672355 0.08915773
0.1461112 0.1042054 0.3368505 0 0.08727183 0.01958599
0.05155412 0.06297633 0.01686182 0.06675362 0 0.02885162
0.09227518 0.04827992 0.01488766 0 0.03792681 0.04520642
0.07797621 0.08411742 0.06203128 0 0.04114895 0.1334938 0
0.1816422 0.1816538 0.2964687 0.2322754 0.01544192
0.07649469 0.1188213 0.02537459 0.03212605 0.07379006
0.01426341 0.09207246 0.01738025 0 0.1413251 0.07622077
0.02003383 0.1200968 0.03734415 0.1642395 0.04875081
0.06553607 0.05140616 0.03776942 0 0.06568377 0 0 0
0.1002446 0.05566746 0.1997658 0 0.05644283 0 0.07001191
0 0.08247377 0.2562587 0.02452486 0 0.04943374 0.151257
0.08175089 0.02738194 0.01781609 0.1288143 0.07051192
0.07280773 0 0 0.07507275 0.07801575 0.06540717 0
0.05190078 0.08510092 0.2636934 0.08322161 0.1274136

0.4090905 0 0.03664216 0.08007079 0.06837273 0.1404938
0.06001623 0.08740563 0.05131455 0.1517648 0.1954125
0.178453 0.08700419 0.10877 0 0 0.1533983 0.04543889 0
0.110504 0.06649148 0 0.34922 0.03642446 3.894029
0.1056831 0.02508739 0 0 0.1010758 0.01509792 0
0.8646147 0.06650672 0.0161087 0.09194276 0.1310385
0.08101813 0.03529182 0.1065525 0.06903203 0.3369747
0.08137743 0.09753298 0 0.02464394 0.05809827
ANKRD13C 3.904862 3.634163 4.380012 5.820259 3.952352
5.497992 2.901817 4.588974 4.036854 4.868597
4.787586 4.398001 4.980334 4.135491 3.329941
5.825647 3.948235 5.792097 3.52142 3.619615 5.065448
5.863282 4.24772 5.390514 4.443708 4.781654 3.563481
6.035316 4.215951 3.034811 4.521189 6.243765
4.208765 4.491184 5.137885 4.304261 2.178013
3.84505 4.351066 6.39382 7.994498 7.832262 4.462499
8.47813 5.95823 4.076141 6.850416 4.139414 5.560007
5.573469 3.569676 6.446535 4.484308 6.394371
5.572982 9.134142 3.115056 6.009282 3.170405 4.6294
5.438415 6.101436 4.931414 3.618514 2.680296
5.696968 3.274508 4.052058 7.941858 6.802892
10.27515 5.541192 6.483929 6.356399 4.470557
4.014227 5.916239 5.567011 8.062239 4.364117
0.5992443 6.051047 4.504435 4.938564 4.799201
5.519707 6.031669 5.770925 8.242885 5.620604
5.533216 7.00094 5.102189 5.503659 3.988733 4.04414
3.44181 4.611247 5.402296 6.864834 5.668859 7.461169
3.125355 3.428579 5.761686 6.084341 5.541899
4.262695 8.603327 5.492471 5.506967 3.658357
5.465576 5.116891 4.837506 8.045619 2.922185
4.764401 7.019013 3.483153 4.351804 4.741781
5.783688 4.291047 6.163693 6.768516 7.400575
1.904762 6.176687 5.495863 9.52518 6.450695 6.530868
4.884139 3.449393 6.141744 4.811928 5.954269
7.21345 5.40166 4.856986 5.829584 6.805927 6.005542
6.058136 6.556169 5.861173 6.668816 4.093393 5.5347
5.817903 4.789008 6.813252 6.187275 5.584734
7.733968 3.946822 5.166521 5.071846 5.553758
4.980206 6.930555 5.273701 6.250331 2.933064
7.650335 4.423409 5.138951 6.480604 5.110984
5.126343 7.491099 3.538737 3.986423 7.000178
5.365688 5.413799 4.753416 7.844638 8.319987
6.379163 4.31739 5.120148 5.281698 6.091922 4.949761
7.714639 7.169934 6.372962 6.424012 5.501716

3.368558 4.303901 4.886945 1.669134 6.021102
5.528639 5.195016 3.798516 4.065414 3.320398
6.655891 5.359462 8.208014 5.342271 6.09932 2.240221
8.71479 4.212981 6.575143 5.423388 5.571915 6.353934
5.771407 7.494744 9.253481 5.312404 4.648944
4.027925 5.979125 7.638935 5.916603 3.345345
5.986394 5.988437 4.115161 5.54336 3.585853 5.913779
5.027247 3.652814 4.551847 5.299337 7.226869
6.782307 4.673711 3.62083 6.789294 6.458543 4.597201
6.524081 7.0622 4.889039 5.438189 6.63298 5.329126
5.290754 5.607458 6.039264 5.239465 9.689308
5.808728 5.929594 5.840974 6.796239 5.605518
6.441065 5.955147 6.005318 4.600528 4.296083
6.819938 5.673505 6.058858 8.262553 5.853575
4.991505 4.306558 6.442773 7.607351 5.113622
5.650119 8.084574 4.872154 6.789708 3.581989
4.315518 5.286676 5.067932 8.467571 6.467572
3.997632 3.819614 5.023157 10.73197 6.034668
7.712506 3.624791 3.551168 4.705167 4.645915
5.33137 5.439416 4.748244 5.634811 3.960593 5.325929
3.327665 3.46009 7.414958 6.944403 6.089495 3.545569
5.148925 5.301932 5.073075 6.265652 4.362717
8.290821 5.208647 5.981747 3.376855 5.50495 3.413842
7.183692 5.924174 5.452296 12.07784 5.656001
5.798982 6.414854 6.36054 3.64195 3.648271 4.73088
6.18571 5.221648 7.338751 5.584776 6.336934 4.104168
4.893044 5.459395 7.656202 4.004228 7.972459
3.903167 6.857668 4.247776 7.496687 4.375319
4.194111 5.547513 6.632283 6.344946 4.704038
6.749941 7.06197 2.423924 3.705256 7.798052 6.46021
6.438454 4.494481 4.425259 8.645416 6.623196
4.843592 5.392652 4.4865 5.734159 3.990915 4.245364
7.803478 2.930013 3.456414 3.218341 6.807852
6.601463 4.819467 4.653038 4.617699 5.24988 6.744299
4.142452 5.017277 5.542845 4.004358 5.65359 6.689513
6.244541 7.573674 4.816205 3.839352 5.421448
4.597375 4.014222 7.033318 6.456913 7.865315
5.901261 5.330385 4.581674 8.599944 9.052028
5.640023 5.343747 5.107073 4.323697 8.474075
3.757476 4.731029 6.53402 7.822366 6.188703 4.756618
5.594333
SERPINE1 1.216032 4.751378 3.887704 6.534696 9.659788
7.504404 1.365149 5.207325 19.8703 6.704782 6.380181
0.9842593 10.49354 1.861805 0.8316045 20.51815

2.462027 11.80319 11.75872 2.033006 3.496557
1.442391 37.54004 2.337585 8.14772 8.903151 3.179274
3.179935 8.783585 1.560497 4.182928 6.356463
6.88897 14.91807 64.92764 12.64965 12.27905 43.64991
86.43718 11.12444 101.6064 4.412385 31.71789
8.567005 21.23574 12.2422 5.675728 16.46806 29.52655
45.64494 62.43886 13.02424 13.54416 20.5717 92.69856
1.914789 93.03771 32.14486 23.14441 5.309997
73.1818 43.28535 27.99175 32.42999 3.046023 65.32194
39.71622 16.30881 141.6654 13.69654 100.0887
17.36641 33.49768 105.672 94.56625 26.64612 68.26425
35.26405 9.193323 19.72426 19.42539 102.9074
19.38703 6.41591 38.2971 50.49177 15.6098 6.883134
22.63898 25.6703 9.870658 19.56877 32.38167 8.273704
16.24579 25.65102 45.82025 27.30264 35.51441
16.95611 9.602616 6.828014 5.782139 29.68659
81.30644 75.00521 32.00991 9.11101 19.46886 12.39864
20.52618 5.613391 24.61757 30.52661 85.82738
12.68125 24.15975 15.40708 12.84583 18.6063 59.43299
10.57855 71.48753 26.39816 18.18588 12.22349
9.35204 12.24071 34.61827 128.3789 5.779444 3.945917
20.41805 32.65702 58.31024 6.182316 25.91435
104.0784 9.743944 19.26665 14.17543 26.21295
3.603246 6.195453 19.32269 6.003639 5.805807
18.77594 6.27358 23.6636 21.96733 60.68419 7.197537
9.197552 22.58242 28.08673 20.51051 2.858181
234.6135 11.03217 85.16431 16.43881 9.790145
18.63207 52.10148 11.78993 14.93741 30.77996
48.4113 14.69048 13.77955 5.578747 2.33729 31.07925
5.018017 53.97272 90.00587 6.440382 6.545864
39.10488 12.74338 15.74053 18.70847 83.87853
125.2286 63.74078 137.3731 8.738004 27.51705
4.099058 77.38188 82.48744 213.3221 3.425676
3.853121 40.26422 45.19533 6.840661 3.633764
12.6002 22.87349 13.08052 52.44583 8.700049 46.68833
12.3699 36.67583 93.93596 60.3876 8.603478 10.20636
49.02252 15.55706 7.283608 42.0596 29.91769 11.33694
4.192541 84.22891 7.087422 10.23416 65.29439
5.822674 116.1385 18.34013 2.306833 20.92149
2.248507 4.295337 18.14685 8.961256 150.7713
1.30094 42.02394 2.832609 13.66202 28.31504 15.47272
52.93891 8.072871 14.91668 6.813725 51.6158 11.71621
75.58003 59.70877 30.42336 4.439726 24.78373
6.160458 2.571107 12.33482 41.69675 81.75773

7.26816 30.40005 9.692492 33.97691 37.96209 111.8857
19.79571 22.37125 51.95978 16.35861 167.9032
46.74511 13.31109 61.28517 44.79486 26.18223
25.14524 30.48745 30.12404 10.01091 6.552866
9.669204 115.7267 14.19863 18.77992 19.76178
13.87428 4.972387 32.41587 39.74516 18.08727
40.0618 5.587012 6.446226 119.2608 46.49391 40.66376
4.932446 16.11287 33.20018 1071.545 79.41441
65.77031 65.82591 25.67338 35.75034 14.19633
15.57879 54.37297 516.3039 6.706973 7.943382
9.202738 34.74956 20.56239 9.143483 20.61602
42.58569 19.31556 22.79415 5.657507 66.88615
62.29105 57.64257 33.26239 38.98103 13.02608
15.3497 24.32356 8.112989 78.66466 14.94287 20.98691
19.19884 27.55111 41.28169 6.332095 132.6516
23.84371 23.19922 14.70206 5.066656 1.305732
16.40594 25.02136 23.73355 4.275932 41.08986
7.18612 8.617389 34.96322 1.361384 14.41599 23.54591
5.738649 8.086892 12.17773 22.6659 19.69408 384.7731
5.299221 4.850803 63.79552 31.5831 2.641047 62.78942
47.85119 39.72565 265.7849 17.13302 19.15111
25.47513 1.005839 24.15405 8.710733 27.40343 8.4145
48.4983 16.80907 43.98936 36.71744 20.57765 7.708376
56.47017 6.659417 19.51463 65.02462 55.44894
4.020135 51.13309 37.66516 42.46255 42.78609
73.97563 32.72101 6.855567 62.2123 23.10635 8.783554
36.55059 31.3583 33.36034 5.340846 10.39337 17.04141
7.783975 15.76759 7.800277 20.30908 3.28076 17.013
11.23125 16.05513
FOXO3 7.244551 9.367336 8.347945 12.27164 6.691203
10.10953 7.576522 9.72213 9.374507 5.295094 20.13525
12.30687 7.145437 15.24858 8.565078 10.45263
9.188658 10.95038 6.500862 5.968099 8.834913
9.222027 14.13112 9.747992 12.27567 20.5234 9.313959
8.683404 11.73794 2.245893 10.52349 7.242793
7.717722 8.344474 21.08227 8.460278 5.793745
7.770774 20.84078 9.125711 10.01091 9.617696
18.25546 7.653669 11.69186 17.37407 11.40303
15.25018 12.69348 12.13907 6.905457 12.33157
8.696643 9.387801 10.6397 10.53659 9.216826 14.01394
15.77556 6.234679 12.61085 8.927751 16.31212
11.28709 5.510431 11.86207 5.294255 12.11266
12.27362 9.265788 15.30129 4.44198 21.17769 11.81011
17.126 5.349699 9.220168 10.79705 13.24635 20.48097

3.520478 18.75399 6.381335 5.841414 6.588369
4.910108 19.55321 10.74516 7.019957 8.878234
6.518875 7.809769 6.542307 10.25665 4.024224
7.80092 7.554908 9.958709 8.067677 10.6638 17.30911
13.24055 4.790891 11.09382 10.71208 17.42071
10.44007 8.650617 13.38514 6.264031 13.97893
13.42581 4.755259 8.293444 7.423948 9.937923
3.735023 24.15639 15.61768 12.0852 4.071774 23.37342
25.07579 9.243739 18.87639 10.81048 3.483515
3.524888 10.89359 12.98374 12.23051 26.43385
7.398164 10.76976 7.184932 13.56526 8.025585
7.360647 12.15402 14.12291 68.39874 14.88001
6.057321 22.32809 22.12069 9.275083 10.54753
10.03706 28.50355 12.17554 6.737004 10.13146
18.57045 12.67182 14.8512 8.667762 6.205934 12.93178
6.026245 19.06948 6.991332 10.642 11.30336 16.07046
8.220103 14.03227 8.789021 17.85412 11.61654
5.842474 10.796 9.535911 14.70191 11.21691 7.35306
10.05094 13.01508 11.8467 9.603264 9.29327 15.79513
13.18533 11.56696 12.11563 11.80047 11.99458
14.91119 7.584442 43.31857 8.262827 15.32065
18.08025 8.849449 9.145108 4.908517 20.44776
13.18268 13.06968 4.948802 14.00908 9.076887
7.845148 17.5602 15.68985 11.06185 10.65478 4.568784
14.62735 20.17643 11.99877 19.15393 13.41406
12.85158 21.10237 7.504942 14.24089 4.728262
18.6918 12.59287 12.55374 7.766578 10.43252 12.5321
12.3474 10.56043 4.150891 17.85318 8.886761 4.272492
8.388886 6.693658 11.5688 9.220863 20.06902 13.73939
6.500624 6.499832 7.838469 9.565626 14.22147
25.97518 13.59871 10.78753 9.690638 10.46447
8.208011 10.09146 8.359868 14.78234 6.353057
17.30258 15.90876 8.375395 15.56929 7.063682
31.40588 16.15361 7.934699 7.517123 12.56497
18.20915 5.318545 10.14624 6.449636 14.9651 24.45131
10.5967 7.224197 15.59184 17.54913 6.990603 6.566573
12.47228 7.529241 11.34687 2.866124 14.80788
9.619515 4.095412 10.04496 12.43941 14.10767
7.268522 6.433175 13.21043 17.34025 10.83102
19.42766 10.60344 19.47576 18.94907 7.695207
10.37937 8.182377 11.92881 9.884376 15.18063
8.306652 8.944571 12.3891 10.76636 12.82944 5.877722
19.99801 9.075373 12.78611 6.712389 9.688819
24.73666 9.879974 9.884749 9.458934 3.487066

6.662248	6.879126	12.5867	13.4923	10.79693	9.710297
7.410868	15.91491	17.1273	15.62491	6.424254	12.72872
10.96961	12.21299	3.16447	10.49377	6.559829	6.331455
20.80512	11.79547	6.013336	6.209407	10.71866
11.17139	7.913059	8.263463	9.068027	7.369335
7.150067	6.241337	12.44862	15.22817	13.53954
14.04824	15.31307	12.89311	11.82015	18.93317
27.51484	12.06317	12.08929	4.62456	5.470722	15.52512
16.56866	11.72475	8.037815	10.00509	9.879097
14.43201	7.007298	8.378854	6.658665	9.379161
9.777221	13.93261	13.32313	31.47562	12.97907
7.118392	16.65523	12.49061	10.80267	5.910343
10.24739	7.851866	16.09119	14.53363	14.65478
12.2574	7.467174	22.99716	6.925677	9.349546	27.21844
8.387957	11.60347	15.91277	12.81278	13.33379
8.05899	9.245265	6.138701	23.24698	12.2858	14.13243
6.651187	18.3533	8.949206	10.58623	8.677333	9.1979
7.496442	10.4272
ACTG1	908.5268	585.6439	908.1984	1970.539	1043.842
664.4561	976.5893	788.0719	1484.557	1295.969
1206.923	1100.316	838.4896	1302.898	1351.878
454.3423	1925.721	1045.454	1512.983	1177.976
1275.594	426.0607	1865.239	502.7709	662.1395
988.9911	1268.044	423.6276	909.5103	532.8525
1448.82	745.0485	749.4428	1629.186	1497.253	1301.454
2263.272	809.5398	1221.313	1811.862	1296.348
1620.595	1592.145	2967.552	1158.73	2069.328	930.7911
906.31	1454.119	1312.168	1427.511	932.8122	1033.666
1304.414	1135.328	1995.105	1880.803	717.2177
725.762	1139.24	1023.305	1311.998	738.8541	989.5653
1525.998	625.9016	1583.903	1044.221	1745.26	748.7364
2618.072	1463.981	1312.322	1298.378	1518.938
1287.356	1522.261	2121.75	1060.976	1025.409	1508.157
939.7857	1303.794	1027.223	2022.364	1734.928
1451.343	2071.964	1218.948	611.9151	1816.919
1037.655	1373.807	1131.833	1470.031	1045.035
1336.079	2016.681	1617.977	1148.074	1253.176
1479.041	1430.337	800.5747	1198.704	1245.039
1864.142	811.8683	1598.745	1479.814	1186.796
340.2235	1811.762	819.2704	1797.778	2085.898
744.973	1195.948	1588.87	954.9951	863.8198	777.8645
1346.492	1354.738	1427.815	1541.885	2484.655
1814.816	1161.56	1208.867	2097.195	2216.554	2245.425
1428.849	1553.359	840.2071	1998.731	1372.234

1759.257	856.0238	819.4732	1182.589	2246.171	
763.0061	1265.773	1092.841	702.3007	1235.012	
1675.398	1437.091	2107.181	1531.361	1525.144	
997.1207	926.2787	1863.179	1557.377	671.1315	
2047.189	912.179	1362.423	1360.439	680.9709	957.0554
854.7767	1449.033	999.6389	1200.69	1041.553	878.1332
493.3802	1639.742	924.3586	1446.044	1299.113	
1155.599	1264.09	1497.582	1136.684	1405.689	1378.254
1305.822	1862.951	956.1183	1595.398	972.0121	
1067.943	1541.067	515.2579	879.3835	1387.881	
1925.103	1616.13	2296.813	674.8502	621.7335	1727.949
1467.221	2242.417	1064.674	1007.943	871.9296	
1171.663	795.5622	1222.441	1432.499	1190.071	
2303.658	1484.01	1178.611	481.4225	1271.957	656.2313
421.4468	2414.131	1689.036	856.0121	1037.194	
1293.29	854.2425	1868.066	1505.896	1660.933	2324.247
1093.238	1517.855	1457.075	1517.501	1653.334	
2738.424	632.6949	1144.74	1351.979	2172.171	1117.469
1198.568	1325.436	1189.773	1491.39	676.6086	646.9182
1348.018	1839.679	1628.82	1636.849	939.8139	1240.854
1078.222	1055.495	1206.589	740.2165	1130.838	
928.7689	1331.687	742.8221	1090.957	946.4957	
972.9447	2272.604	1379.987	1106.784	2523.138	
1468.157	926.6433	1033.816	1166.931	2576.422	
2100.926	807.9415	1026.602	1343.295	1560.78	1582.916
2093.336	953.5578	917.8506	1060.294	528.9596	
3834.759	948.7737	1277.18	934.9341	1538.506	2782.159
1225.552	892.5051	1500.763	1373.607	1602.224	
1650.122	1835.089	403.6687	846.2324	1336.545	
1283.733	1248.574	1225.927	1299.272	2781.656	
2367.684	1186.455	1071.953	1023.21	1043.765	1208.15
1351.487	1048.521	2365.812	1344.527	1645.354	
1180.486	3134.406	1368.621	1755.413	2571.81	1145.6
1460.325	1153.995	1147.691	772.188	875.6482	1268.782
926.6688	1049.179	1422.599	778.1054	2238.174	
1433.669	979.4318	2278.286	1198.781	984.0253	
1461.977	1292.093	660.9887	1154.793	866.642	857.5038
1416.067	1888.725	942.0577	650.0186	1616.789	
1086.63	654.1518	1199.979	653.5682	1121.197	1426.702
1005.867	1478.595	1451.863	1696.472	795.8626	
1171.609	1404.741	1131.014	1621.062	690.4103	
1074.319	1098.077	1238.222	565.8349	1677.015	
665.9516	1328.232	1543.557	839.4936	1926.792	
632.9869	833.4661	931.9643	744.5648	1055.9	1683.06

687.0238 1349.126 1054.411 1602.12 631.3009 963.0956
1443.493 862.6645 981.8278 1862.321 1686.333
980.9426 1602.914 1606.043 917.817 810.4824 2196.273
524.3957 1208.377 1047.133 1032.596 955.0005
951.9349 679.3944 1378.247 1466.058 929.2826
2302.173 1356.187 2386.189 1691.847 883.69
ARHGDIA 116.339 61.52875 94.06346 138.1503 116.5001
45.77287 100.7086 43.27591 141.1841 97.34688
52.83555 53.20323 81.5978 89.22522 130.8557 39.39822
164.003 87.92306 160.4926 159.4368 147.4788 39.56495
80.06421 45.71543 60.04354 61.31998 125.777 41.63893
145.6111 44.23206 131.1478 64.00342 122.9853
90.8861 113.1009 73.6482 49.97772 80.06799 75.88933
115.7361 89.38264 163.0438 53.94472 105.3425
96.80903 100.9325 82.08649 112.1003 61.61971
120.0895 156.9301 68.35788 70.73269 112.1244 109.24
84.95113 158.185 64.70975 143.6477 188.8424 73.55866
129.9856 47.64147 131.9561 115.9316 57.5331 163.8154
83.20718 128.1511 86.81466 84.27348 65.11032
77.01996 43.46941 109.5662 169.0482 102.0535
94.62717 86.99184 59.50741 149.7342 67.05057
54.4547 74.7425 139.8717 111.8345 67.36594 120.7644
112.5558 53.51476 71.39208 72.53487 90.75532
96.38352 160.454 72.7196 100.6907 109.4465 78.13917
104.7181 77.23485 118.3218 122.8436 69.28917
80.46235 73.09191 178.9048 110.8146 119.0899
94.2099 104.0637 63.33106 124.3396 64.18157 96.90035
138.1854 120.5339 88.75907 75.04157 77.84056
93.86123 38.99202 107.993 95.0796 108.3522 103.8342
103.3704 185.6544 60.19023 165.811 144.6127 105.4719
123.7122 63.39487 132.4574 88.90899 97.03946
78.91626 98.50791 53.70998 105.2367 95.83565
109.417 83.59768 66.05137 77.68885 93.69583 90.74397
86.07334 99.04043 72.14902 135.0287 75.41318
75.66212 61.72844 63.21985 112.9193 58.64315
103.112 83.25249 126.4281 75.35237 55.7094 73.01379
89.41758 141.1281 111.5701 52.24763 76.57563
84.54651 43.16973 120.5026 72.54956 144.7201
176.0515 97.38068 88.33061 161.1589 71.80973 118.17
73.78628 76.07614 99.80296 83.97878 80.81087
73.40632 67.3052 147.8607 35.23165 71.20348 131.4023
156.5239 168.337 200.6658 93.90648 61.16408 88.2196
89.73111 109.6471 187.9278 43.64714 66.83668
72.93392 99.2582 128.8852 78.37895 104.9425 73.84749

93.41847 103.7597 77.07382 83.01375 75.31125
62.14174 132.0205 79.25051 119.4091 44.52404
123.5112 94.57803 111.6281 64.83265 128.8332
104.9372 77.40559 92.03895 96.78064 89.57822
144.726 220.4853 106.2752 91.76024 66.1251 116.479
44.37475 127.5203 132.9858 81.54024 69.32227
50.07301 46.64405 94.91836 81.58706 108.5651
59.09919 94.47404 80.85297 63.63284 66.65205
115.317 73.69163 59.93249 75.43921 136.9474 71.30126
86.29296 67.62712 128.0743 134.665 77.545 86.37374
196.6566 77.70979 73.50618 95.14583 63.96638
108.9034 183.2972 94.11252 66.44685 102.6679
189.1347 146.0856 103.1314 105.7671 72.5826 99.16056
52.20681 190.0377 82.51767 86.04363 72.02927
162.2334 107.8165 58.95076 78.192 101.8124 108.7935
88.84797 76.49111 167.4445 36.6491 59.85201 86.64849
138.1492 126.8477 88.12524 99.25482 98.93917
102.0899 93.69211 77.18712 82.49981 81.38153
85.88954 130.6877 49.56612 73.04568 64.0426 90.65231
138.9876 242.3966 163.8354 174.4297 94.2574 59.20592
95.16717 91.27644 81.96228 61.62684 58.4889 67.20598
76.79631 89.17131 48.10494 69.93844 165.1025
137.304 80.39685 88.27653 60.4863 62.52496 114.5971
98.59021 106.7508 90.88579 86.16812 105.1266
96.20767 114.1634 108.4894 107.2389 42.86484
83.94949 46.86765 78.95044 53.23119 77.88348
92.79099 85.67259 100.9598 97.56331 58.48347
81.58817 54.90489 110.795 95.71078 83.00616 60.54681
82.14277 111.9299 123.8432 49.83943 122.6483
112.7711 71.59332 87.83692 86.93536 67.57175
78.15723 79.84494 80.37573 71.72274 42.39331
117.0356 92.00906 112.4747 86.00175 80.44822
62.66437 106.4296 58.74177 87.61133 176.3106
86.13265 128.8097 126.9338 153.6069 135.5557
130.6653 66.25266 66.3765 43.95041 68.16498 83.46522
89.16597 77.85871 74.18928 83.15775 133.2426
202.4983 69.92519 107.5856 82.34581 107.1699
142.3633 70.82382
EZR 176.9909 32.52014 134.9914 238.4821 128.5217
18.83008 158.5971 99.87864 224.7196 121.5098 115.96
110.2453 119.3628 149.4947 162.8386 15.9488 216.1439
57.95531 254.1028 151.1487 146.8309 19.37462
181.0547 14.64627 74.98159 129.2604 149.3046
14.4581 174.1757 97.50952 170.7885 46.72296 86.90597

186.2782 252.1066 76.05773 26.81573 155.3746
284.219 240.4278 97.18876 345.5244 204.4069 139.1102
184.4928 194.4454 213.1221 100.8515 259.5532
320.3469 285.1028 121.624 112.6642 158.9123 111.1222
152.1588 99.03808 49.00345 183.4601 115.1431
104.8467 257.3561 48.23296 79.27739 117.3523
35.2849 109.957 61.49934 335.0789 274.3967 519.4118
144.844 196.541 138.0455 83.42166 183.6452 199.4815
197.9977 239.7369 71.57816 262.7385 124.9528
171.6744 87.3914 290.029 148.5173 176.1634 118.1915
141.2573 134.5127 200.6211 144.6244 174.2975
157.0563 169.9505 56.31449 214.0334 164.7516
162.1519 160.5405 60.51435 232.1334 138.2694
62.17423 62.88615 111.9359 314.1447 97.9845 343.4542
159.6761 107.3645 52.05195 195.5668 79.29618
164.1401 154.2692 78.94026 365.9951 190.9441
78.05327 167.1034 98.65446 41.08671 68.33678
121.7871 162.5908 162.8237 191.5186 40.36446
91.78331 733.8749 48.19175 151.0318 329.7694
186.0521 109.2735 242.4945 171.6112 174.4469
97.83288 96.6991 114.5937 159.9998 58.15722 92.78963
155.8402 113.8993 93.98444 107.0605 64.49643
142.101 199.0405 238.2779 147.0262 56.94611 112.2605
163.2909 62.05852 162.8798 92.98468 111.2557
151.3544 104.7942 122.8117 193.8395 134.4543
153.579 97.98086 143.2109 26.41764 28.95417 79.40479
97.58825 100.1671 145.4182 144.4521 100.5555
121.5344 206.4649 86.05175 127.1383 157.0956
264.4655 164.874 91.16136 89.92245 115.2619 324.6593
133.4788 305.7298 101.6298 368.8629 353.4355
212.4485 120.3831 130.2733 179.3524 139.124 238.511
94.75989 43.41883 88.35026 122.6367 96.62508
276.3794 189.9514 81.09456 60.44168 210.1446
182.6016 112.8689 62.70164 143.5801 71.70017
188.255 258.869 110.9534 149.5496 219.0964 287.0086
187.3455 148.9303 160.954 348.1899 103.6561 103.645
68.12853 130.86 200.2518 134.3578 75.46271 77.25494
89.98022 569.9696 112.6834 165.646 124.6033 131.5901
190.3484 53.46941 85.44571 269.9022 78.88334
206.159 170.6741 103.5696 135.8465 70.76776 167.5591
223.4688 232.5794 152.1716 112.6893 124.2071
150.9532 27.49297 93.80436 163.4828 217.0783
63.35036 199.64 455.2792 257.4157 145.8844 182.305
111.293 256.8165 208.4384 184.2056 225.3678 201.1679

217.2046 231.7244 97.9637 426.0063 79.3896 159.4869
35.62713 238.6421 147.8611 121.4926 70.97917
233.2086 108.1399 292.3631 200.6594 192.8448
91.44792 118.2986 147.4947 252.8455 22.29256
26.55346 198.3961 50.84135 60.26914 130.5634
111.7525 313.7006 307.2231 168.4554 60.22422
115.1031 50.54902 232.8467 89.72034 58.61924
149.1102 214.9487 122.9493 125.1256 367.8792
201.0774 84.34112 229.8524 64.72051 134.4991
150.717 144.1617 268.8591 190.5525 124.2191 165.3259
188.0633 67.25003 99.10926 178.6853 36.53715
224.5517 365.7753 112.9555 89.71754 164.9659
164.0825 76.50961 211.126 110.1195 165.7639 177.0638
123.5021 36.11266 157.1542 110.876 155.2008 93.8283
159.3518 236.1328 52.4115 112.2629 114.7689 122.0187
117.5434 142.9988 106.3477 137.6268 294.1675
158.4158 75.00324 34.06712 83.92863 150.7973
41.39626 314.5308 136.829 51.28819 94.46209 138.9329
79.85057 127.3486 72.37766 90.81962 330.5715
84.38588 308.2972 198.1313 51.68113 159.2473
176.4844 94.44103 126.6833 80.34455 152.4751
83.54408 51.06178 208.45 221.563 63.90057 337.5213
211.0709 80.85851 101.7703 217.6281 91.0074 178.1279
219.3258 159.1879 168.3953 118.355 224.073 336.3095
73.80901 84.46747 303.0192 267.6544 112.2529
87.57268 66.94513
SLC39A6 1.613852 8.610281 3.066965 9.45344 4.388947
10.38984 5.803931 8.569024 5.62874 7.993992 11.62092
6.969208 9.329003 5.678883 5.480817 10.71381
4.895768 9.726893 3.259411 2.966484 8.18428 10.84606
10.76263 7.836554 10.52364 8.557099 7.114099
10.72056 6.412748 5.613847 8.719883 8.708069
14.89255 11.68191 12.45436 30.63778 15.86504
9.951355 9.791648 14.96677 25.23047 8.643886
23.86142 11.99077 7.321882 14.45055 8.700581
14.08093 37.72683 30.30536 5.639133 11.35836
15.13782 16.01327 15.70233 21.91532 10.59062
10.32275 14.76188 20.89999 19.24983 18.37134
15.31482 16.3916 5.201987 14.63365 13.5031 9.651026
17.18593 4.997381 40.50117 15.06302 11.23392
27.92214 24.33981 17.74771 9.702384 17.50828
23.90062 17.35535 7.869418 11.12033 24.95803
8.96273 18.43734 8.771905 10.78345 20.7524 10.63827
15.66202 15.54442 15.04177 19.78966 7.544541

17.25423 24.52924 13.9261 5.792061 29.86157 17.37809
12.34584 8.959635 7.236972 12.71316 14.91005
13.46565 16.98568 8.060576 20.20665 22.88307
20.64854 5.756768 24.2157 10.50942 17.13286 19.41085
19.48775 6.971149 14.61855 4.924774 16.60709
8.791451 14.54838 16.41684 33.16537 10.76238
11.85945 7.26778 13.00429 14.38925 15.08405 21.90004
13.14484 23.84016 6.139512 9.621071 21.52456
9.436021 13.31428 13.94812 19.64626 8.891334
12.94445 9.181417 14.19703 12.8025 4.650974 14.40149
26.02538 8.179056 24.97972 12.53726 8.4276 18.55668
9.602224 20.82737 14.74473 10.39096 23.4113 6.732859
10.05718 12.40689 15.34984 9.813943 6.120037
16.46352 11.97208 14.63719 7.217001 32.51825
44.50227 14.44992 5.557102 17.32184 12.23117
10.1193 14.77208 14.20519 11.79789 25.06266 5.913175
8.691646 10.28282 15.05022 13.42975 14.9577 12.55705
16.75846 11.32781 13.68336 17.56584 13.20196
11.31812 8.692647 9.83157 11.59231 21.1927 16.42747
20.393 5.478997 10.56424 9.929414 13.18727 6.268384
13.61015 15.10108 6.547105 18.43376 13.44402
10.68275 11.44182 20.26929 25.20584 9.713587
26.64705 22.39864 15.27783 14.82312 14.10453
10.08806 8.343772 23.96063 10.20109 30.12628
9.819952 9.522866 19.76778 9.133804 7.307965
11.34493 11.75703 18.05566 6.412659 9.666406
12.19488 5.247147 5.604277 12.47494 17.03788
11.4783 11.37194 18.7971 11.99337 18.51174 28.13023
11.19794 11.93079 8.925088 16.97069 7.602176
16.03977 6.910669 7.759989 10.47974 19.57584
19.11101 12.56011 9.370535 15.61491 9.311022
10.25996 20.42095 13.44506 13.95261 14.31202
12.62326 6.318057 6.288764 12.51266 8.33911 12.96108
17.91472 6.928098 12.84574 13.20298 6.293713
10.64388 8.871514 12.69572 13.39957 12.38781
15.49199 15.14672 15.50089 20.67697 12.02365
39.62892 26.50488 12.49698 14.85786 6.351934
9.777607 13.93057 8.585778 22.03331 10.63444
13.61089 14.56143 17.88318 5.424869 14.11588
13.02703 39.8563 19.14463 7.595621 15.26173 34.4929
17.50451 17.52382 15.94949 11.85726 11.56545
15.41153 8.233597 15.87792 10.97303 16.89618
19.60487 10.52934 35.51558 9.114601 13.27036
12.45261 12.77095 18.95761 7.393759 11.59326

28.13853 5.900243 29.90367 11.42013 21.76312
15.13075 8.552534 6.394521 11.17979 4.377299
11.78431 7.937787 17.98663 30.43157 8.160285
13.39769 15.042 11.15996 11.94455 6.441023 19.25922
5.380047 7.24051 10.15921 17.71073 12.48984 13.48874
16.83852 9.647568 13.27546 17.03071 9.089757
12.16268 18.21957 10.69597 24.58994 18.52148 6.2122
14.28106 14.00337 14.04211 6.985196 68.17058
7.606175 14.03159 31.87272 28.50241 20.67851
18.75961 9.537888 7.407749 6.98063 10.6359 10.4635
9.922365 10.25588 34.29756 14.62317 9.258392
9.537717 53.24753 9.842175 28.71669 12.10496
28.34717 15.56501 14.34175 14.36738 23.9013 8.664494
10.8006 6.651539 8.817564 24.89985 6.326523 19.24488
10.21873 10.68916 8.247807 11.36964
BIN1 4.478731 12.43503 5.145779 7.533189 8.160095
8.81033 6.796792 7.13682 9.452253 11.02832 3.443086
1.00926 10.232 1.275888 9.051841 9.112245 7.336235
12.85878 5.871773 5.893434 7.958136 10.75642
2.512799 5.516397 8.491628 1.430566 7.633738
8.382717 5.591656 4.400965 11.00938 11.29592
7.004982 6.05576 9.718069 3.419754 3.499627 40.54171
19.17943 11.86113 9.690514 12.26043 6.830897
5.668143 5.665588 8.413285 7.309607 13.33772
3.569721 9.463756 14.30273 7.402702 19.95949
4.606178 7.48553 14.51702 9.728489 7.686441 16.3488
14.82701 6.465535 9.956791 9.973761 9.576457
9.282566 4.657462 8.099516 9.754664 8.628931
13.96181 5.743115 4.840289 7.902762 3.186874
3.052779 7.125356 6.152059 6.735 7.525427 5.422559
6.795764 9.894529 2.922005 10.75435 6.159825
8.610007 7.51659 6.7057 5.674618 8.589889 3.540062
7.592688 4.857683 8.221736 9.103556 5.850035
10.0058 10.99766 4.261767 6.956191 14.43352 12.09957
2.362468 28.51405 4.400731 7.56042 6.346039 12.29396
10.18506 9.003867 10.04165 18.24846 6.583018
10.36942 4.307058 9.360832 23.08777 7.561622
9.928978 9.6782 15.27368 14.95346 14.26374 4.88545
1.320168 9.721516 4.501147 8.928239 9.153589
8.108186 5.424212 8.58904 11.72564 3.160766 8.357746
7.92938 2.319497 11.22496 6.345873 3.901283 12.03387
3.249931 8.281333 20.73648 7.474892 4.583457
17.8393 4.296973 5.17225 5.243376 5.778536 8.125519
4.503041 6.22866 9.186075 6.353414 7.636604 5.673098

3.174926 5.022912 10.55899 5.663959 4.582274
10.92791 18.34777 5.204851 13.58999 4.00171 9.694686
10.15263 4.564479 5.356393 4.742677 7.883907
7.860389 13.95903 7.324388 3.80746 9.785156 5.303924
5.841542 16.23834 2.99861 9.960777 20.29147 15.00293
6.914441 3.722116 16.62836 8.627185 7.018262
9.052611 16.16858 9.802543 21.3014 7.316077 3.4989
14.25738 9.330357 7.725592 10.41444 10.22633
5.023915 6.453084 1.273487 9.419428 15.23512
5.605371 8.705658 10.03311 4.771257 6.050688
8.709491 29.78616 8.83162 8.858928 24.16472 12.32523
12.21123 17.6755 10.30574 4.462984 12.66824 4.190707
13.30519 7.318652 13.29936 0.9619328 15.33909
15.74303 9.852752 3.274598 6.897289 7.52876 4.068049
9.685606 16.36421 4.128936 3.05659 26.12124 12.94488
5.977605 6.006115 5.544651 3.622196 10.55781
8.874461 15.16825 6.931556 5.738135 3.057254
2.434765 11.74986 5.789299 11.55723 11.57213
13.37466 11.13248 5.159672 25.68433 7.613258
8.490294 2.70733 7.561564 9.001085 22.34682 14.52068
11.02219 5.360717 7.861037 5.535904 9.425671
7.392307 7.897344 11.62948 3.392551 7.761642 8.7387
2.980904 4.845268 7.289732 14.66865 5.152589
5.997581 3.691578 4.132063 6.336201 20.39704
4.37247 6.64824 23.0996 3.050938 4.978212 7.961406
7.023681 9.834218 4.991318 5.634776 6.21405 6.840868
7.074338 4.042904 1.777303 3.943065 7.484996
14.15994 2.693665 10.03446 8.054796 4.19542 20.82133
8.975975 4.385541 13.10123 4.618149 10.80072
9.765879 14.9073 4.031099 2.904606 10.35159 0.963762
16.69614 12.11415 2.253254 19.59968 12.97373
5.665004 5.605328 3.932421 4.922448 6.620173
7.824922 16.37439 13.95466 17.59784 11.97524
18.44028 8.410948 6.739158 3.772848 21.98169
6.826907 22.98181 7.982359 3.158295 5.297334
2.616311 13.94132 9.764626 10.34263 14.19437
5.215533 12.4568 8.544132 6.417195 9.11207 6.206542
4.014839 10.91139 11.39412 6.276509 8.013518
3.563106 4.912475 5.28057 7.581089 6.40015 8.227838
8.59688 19.683 3.678278 4.84337 5.585969 3.134028
13.41575 7.476529 8.169597 10.88427 8.140038
6.645947 5.172594 2.115921 9.988166 4.870174
10.74185 11.24328 10.55022 13.12052 4.007496
8.489522 3.129062 12.29655 5.45922 13.21856 10.01691

10.0681 8.596925 6.579782 10.66233 22.22306 7.365661
6.931664 3.541318 6.0299 9.403819 7.541318
TIAM1 0.3204232 0.4619775 0.6032194 1.026107 0.7796032
0.6439622 0.5899826 0.6546289 1.038055 1.245967
20.97037 13.0535 1.461373 13.12069 1.077246 0.3948219
0.9107291 0.8712732 0.4284354 0.3616809 1.216398
0.539022 11.63993 0.9633518 1.615809 17.15646
0.9408174 0.6183175 1.331442 0.2373338 1.262067
0.380978 0.7067355 4.941331 0.2757286 0.1674143
0.283318 0.5144348 0.2953761 0.3129548 0.9344772
0.1624545 1.292551 0.5005651 0.8120591 0.08673638
1.529996 0.7370034 1.12079 1.531557 1.192088 3.978626
0.4096563 0.554187 1.437242 1.293949 2.176821
2.404821 0.7557852 0.9003032 1.396709 1.012071
1.479894 0.9014407 0.1256587 0.6446261 0.5119653
1.542055 0.6171798 0.3722852 2.868516 0.6786625
0.6618687 0.8158321 0.4121214 0.810693 0.8056317
0.4162403 0.2626206 0.1821509 0.5513643 2.537126
1.712548 1.027702 0.2483254 0.9050166 1.786847
1.274044 1.012377 0.6778962 1.371104 1.295802
0.9814281 1.742055 2.186142 1.763937 1.56906 0.5961435
1.142463 1.230144 2.681453 0.9312047 0.5546741
0.4361318 0.6252873 2.47843 0.6394718 1.107767 1.3639
0.2545359 0.8304843 0.4759027 0.9002975 1.718595
3.451467 0.5259351 0.341801 0.904928 0.4734201
0.1893209 0.4260859 1.180614 1.216296 1.719919
0.1691635 3.367232 0.5497074 0.2477949 2.042231
0.7560199 0.7329488 0.6332003 0.4966495 1.057282
0.5789956 1.536283 2.052912 0.2520493 1.037815
0.906228 0.8436979 2.281339 0.393918 0.3216336
2.112325 0.4269599 0.08189695 0.9368097 0.04096826
1.404435 0.5063339 0.5323617 0.3129203 2.808285
1.69371 1.230282 0.3619669 0.593682 1.762989 0.8276405
1.844278 2.300126 1.565382 0.2930035 0.1861212
1.789753 1.125574 1.450823 2.306253 0.163515
1.785823 0.6765989 1.347986 4.333385 0.2568834
0.3630303 0.9861094 0.3155782 0.33221 3.058462 1.036937
0.129111 9.378149 1.447761 1.258555 0.7159141
1.756392 0.2246306 0.9105202 0.3861167 1.06499 0.5161106
0.2238029 0.8629648 0.7022887 1.47763 0.6756327 1.217126
0.2892838 0.1279644 0.1315596 0.5169559 0.6705809
0.1558607 0.8858321 0.4672675 0.4301104 3.631341
0.6950905 0.3770324 0.7411313 1.379922 0.9477368
0.2160695 0.8205308 0.5723123 0.2789452 0.5800197

0.970532 0.8485697 0.5685841 0.7806148 0.6953142
1.748289 4.360502 0.1103273 2.672449 12.60788
1.101919 0.4877926 0.1816884 0.3841916 0.2100477
0.5999223 0.2825144 0.3927466 1.614257 0.5969729
0.8754688 0.3932777 0.9785889 1.317598 1.138265
0.4484863 1.991693 2.380491 1.271561 0.7643425
1.828651 0.5847976 1.293936 0.4421468 0.5256204
0.9661487 0.4482973 1.90071 0.2555115 0.8880044 0.649986
2.309863 0.7514262 0.1464968 3.405246 0.3138526
1.620578 1.108723 0.5607087 0.5581003 0.5583173
1.757781 1.404594 0.4331725 0.298916 0.4491413
0.1560118 0.7291366 0.7390515 0.7414917 0.4592372
0.4632258 2.365301 0.7647931 0.5504485 1.493197
0.260439 1.519946 0.2827202 0.1227946 0.5855345
1.726315 0.4635166 0.6744739 0.914677 0.865977 1.992
1.364851 1.30994 1.019258 0.5706204 0.2054413 0.8096215
0.9925769 0.4572888 0.8761148 0.1260975 0.3747899
1.660266 0.6837829 1.192184 0.2209401 1.075442
0.2046721 0.4667781 1.671752 0.4723056 1.337496
1.005199 0.83178 0.7640679 0.708666 0.7165718 0.7472455
0.1570284 0.7573478 0.9646177 2.461336 0.7038508
1.876596 0.3278398 1.120255 0.5993672 0.5537263
2.373009 0.9491287 0.8724504 1.003672 0.05724822
0.2783695 1.149263 1.141528 0.6922413 0.5494426
1.121931 0.09828137 2.506791 12.8059 0.5011078 10.92603
0.2972712 1.061474 0.4207432 2.050273 1.248659
0.1305602 1.045077 0.5533177 1.584435 0.3947717
0.9061085 1.94988 1.844973 2.12317 5.178119 1.445739
0.3536068 0.3059364 0.477382 0.9080457 1.516658
0.18819 1.69324 5.377596 2.191404 0.7681349 0.8352989
0.1258447 0.5273456 0.494734 0.2948564 0.5146836
1.248818 1.353329 0.5822229 0.05122567 0.3091506
0.625675 1.03569 0.8741586 0.4268684 0.2506701 1.001377
0.2445243 2.150074 1.357722 3.879039 1.290955
2.83712 1.007084 1.629876 0.2997009 1.334114 2.763175
0.4282474 0.219971 0.1494965 0.3734129 0.6114825
PDPK1 4.390765 2.132344 4.702957 3.090488 3.123796
2.98016 2.580462 2.605635 3.481671 2.109162 4.376172
3.108804 2.663315 3.443506 3.516245 2.711072
3.350089 3.209827 2.106504 3.586949 2.803117
2.31752 2.50875 2.93678 3.037304 3.793014 2.892048
3.486596 3.993207 1.143549 3.799073 2.754586
4.679845 3.224775 3.647113 3.384259 1.62164 5.473405
3.294587 3.299716 4.295289 2.041989 2.161841

4.391306 3.838076 3.398163 4.771731 5.517003
4.248741 1.571844 3.281874 4.515208 3.372375
4.314707 2.291901 4.800961 2.612305 4.3034 4.41867
5.422544 5.951147 3.360279 5.730479 3.683104
2.312988 3.004107 3.44551 2.781961 4.125568 4.684424
3.292351 3.056099 4.481624 5.9602 3.272881 4.153187
3.543238 4.412966 5.572544 5.235133 2.959455
5.021346 3.536631 4.372659 4.484877 4.059463
5.772469 4.027472 2.42343 3.727155 4.903934 4.899727
1.589156 2.898301 2.822342 3.754777 2.760902
6.09231 4.499305 3.804922 4.580022 3.767503 1.755582
3.057681 2.657742 5.968213 2.994398 3.560578
4.324889 2.84172 2.919147 2.779205 2.632286 3.836261
1.789558 3.800653 2.777771 3.13934 2.050116 3.644767
1.670555 2.971252 3.005842 0.9553911 3.966241
3.581717 2.62708 1.894068 4.149582 4.877404 4.918523
3.772601 1.867146 3.669967 2.131407 9.201336
4.483733 4.385407 2.798053 5.154079 2.776356
3.155947 4.803908 2.144859 3.100018 2.708848
2.998337 3.759569 4.180123 3.682421 4.797659
3.307002 3.746172 5.539867 3.88113 2.12106 3.680924
3.205688 2.820338 3.93727 2.256803 3.925429 6.917475
1.767331 1.862565 5.43622 5.49391 4.275212 4.798983
2.521069 3.483057 2.502893 4.49309 1.978262 3.747624
1.983721 4.347804 2.144607 3.272249 3.195899
2.828573 6.706833 2.686427 4.184824 3.443558
4.030227 4.01308 2.122988 5.752978 6.279347 2.630801
5.700306 3.308736 3.622583 2.908467 7.49307 3.443058
3.500432 4.411729 2.969133 1.862909 3.920375
4.703455 7.295738 2.883257 3.012559 3.185329
4.320576 4.699947 7.409689 6.854429 4.884476
3.575671 3.90259 3.359967 3.943965 2.77492 3.013828
3.920092 5.936235 6.337106 3.908329 3.328841
2.642402 6.121102 2.935436 2.258529 2.594967
3.196049 1.916239 3.451982 2.803426 3.843373
3.790478 5.005145 2.659834 3.074258 2.809047
3.034496 8.70701 6.177658 4.978072 3.662568 3.839239
3.09194 7.327633 3.701793 2.784122 5.056455 4.994832
5.081374 6.111747 2.179227 4.298201 5.145524
3.923646 6.207648 3.451407 3.550537 3.674416
2.653467 2.141815 3.875387 3.107161 8.602422
4.545252 4.004965 4.025905 2.488723 4.801043
5.582503 2.68519 1.447607 6.606436 3.436728 10.28884
8.299657 3.17484 3.793663 2.422684 3.982471 3.948783

2.207415 3.988548 4.197113 5.122263 5.00789 3.017565
3.23487 4.800374 1.9249 2.738252 2.740937 4.703266
2.428847 3.040549 1.938997 3.684078 2.474855
4.829675 3.620117 2.942336 3.176125 4.653951
2.74484 3.513239 4.395126 3.61975 6.022158 3.170951
3.362509 2.119365 4.385256 2.757399 3.108445
4.183065 3.796914 4.198906 2.99742 4.532798 3.376246
5.013379 5.875045 2.829816 2.328724 2.610987 4.4227
4.042991 3.384538 4.367263 2.311268 3.470317
5.13444 4.442184 3.300734 7.518517 2.283431 3.335941
4.754058 4.121427 3.622461 3.534906 2.118699
3.222935 4.306067 3.717106 4.289691 4.027582
2.389763 1.560524 3.079283 4.159886 5.345166
2.180127 3.842355 3.745264 4.926448 2.226715
2.951313 2.64917 5.665977 2.609378 3.686663 2.808541
4.874288 4.140065 4.902237 4.603487 2.890957
2.131019 1.777936 2.428314 1.828512 3.567515
4.282698 2.265369 2.75221 3.097347 3.80608 5.67348
4.132552 2.72092 3.624304 2.780664 3.585784 2.691862
3.259734 2.143696 8.103262 2.41781 4.119898 3.805272
1.982703 3.499157 4.225975 2.151457 5.320786
4.206133 3.218674 3.40509 2.310983 5.003242 3.299875
4.695154 4.473113 4.162033 3.000816
SMAD7 6.949496 3.367804 6.196949 6.976898 4.944838
5.459818 6.432696 5.404803 6.154188 4.614787
3.357594 1.453462 6.860821 2.501534 7.403441
3.052382 7.653448 8.432424 5.927819 6.690129
9.196746 2.915801 2.382993 4.392591 5.393107
2.298189 8.047867 3.217415 8.425778 4.884301
7.973429 4.031829 7.099432 4.089989 7.900248
0.9489067 0.7083899 4.930391 3.430579 5.112391
6.762442 1.651246 6.479608 1.335727 3.75233 8.964786
4.487322 8.912098 4.664014 9.997946 2.584795
5.468877 15.34167 3.550182 7.181562 3.608798
4.772514 5.349093 8.791312 11.42732 3.833283 5.592
3.648826 5.240315 1.413404 5.261098 3.221657
7.620364 6.81226 3.117308 10.22726 2.050688 3.91227
3.900493 4.305423 6.874891 7.598507 4.349999
7.985007 2.320629 4.146426 5.218419 3.452204
3.961406 5.102545 4.473137 5.211108 7.172668
2.979276 22.91673 3.302964 3.985334 4.955025
6.889623 5.725262 4.66341 6.35486 2.710006 4.620002
8.148971 7.533278 1.904653 1.224051 6.45265 5.075663
9.496009 3.882954 7.798708 7.332082 2.895918

5.631332 6.989257 2.182281 5.951934 3.080022
1.580905 4.077205 8.956889 4.032105 3.194697
3.160117 5.588258 9.192764 6.955153 14.55188
7.080417 1.205768 5.077497 5.019226 8.58103 4.562838
6.213965 2.618644 5.154814 39.21812 7.949866
3.473529 6.478432 3.650859 3.748666 4.361458
1.487544 3.939857 7.482681 3.821925 1.896716
4.909119 5.134025 8.312918 7.238194 3.491325
6.320312 5.737497 5.780321 7.413659 3.645111
6.058432 5.726573 4.267181 9.755952 5.575486
6.399675 5.094706 5.45214 5.589587 6.413251 6.038879
5.910369 4.357058 7.05383 2.758022 3.547201 2.456136
2.959241 2.549942 8.096582 11.17658 6.097354
2.04806 9.744729 3.233543 6.001301 3.834046 5.140023
4.5988 11.83456 6.480711 4.541654 14.89948 2.589089
6.958707 3.669949 5.488183 6.06416 3.207413 8.778306
6.868279 6.915173 2.802858 4.080884 2.972985
4.072577 13.32516 6.341354 3.290945 5.336355
4.172728 3.562547 11.39246 5.336139 3.58885 6.169299
6.983876 6.10229 4.425999 6.45212 22.61885 8.638467
7.325868 3.667721 3.396772 4.77085 6.670782 10.61346
5.70993 1.199093 8.134358 1.521232 7.172943 4.467476
10.11358 3.029345 6.497944 3.7319 3.607528 2.963494
6.331666 2.709822 6.318578 6.673857 6.743379
7.306833 5.876072 5.949062 4.76188 6.923539 10.17529
5.97464 5.704639 2.54216 6.604408 1.86953 4.675628
7.64768 2.943181 4.567681 6.479576 6.204269 6.336188
11.02594 6.295056 4.894366 3.930119 2.947194
4.918769 7.806062 2.743245 4.366666 15.50452
4.65058 4.190267 4.999968 3.328343 0.9926505 4.51416
3.786424 3.458668 3.080399 3.586182 4.344941
7.670336 5.773664 8.573138 3.934532 3.726819
4.60256 3.573975 9.876026 5.53071 5.958425 4.515281
2.5326 4.394377 5.691579 7.77278 10.71903 6.722876
7.008785 7.320826 2.988861 4.321443 5.560451
8.16779 7.139719 4.758897 7.162729 2.167802 2.777637
6.418475 1.626599 8.052141 9.781726 2.672519
9.648017 1.985697 6.701053 4.097351 4.70356 4.433232
5.898652 3.774359 4.453754 2.984955 5.190389
3.196906 7.077088 4.917696 3.688603 2.07932 7.836536
1.025586 7.002732 7.074541 3.552555 6.666198
4.494434 3.132574 10.30507 5.302456 1.416151
16.42994 7.362105 2.895106 2.971242 5.712086
3.329592 4.290465 3.736475 4.561697 3.924111

3.410955 8.451967 3.486476 7.03322 4.657744 1.601713
6.398298 2.767438 5.056074 4.003666 5.004444
7.990431 26.09938 1.871239 6.126754 2.817505
4.039911 4.65706 2.014013 3.009992 3.193926 4.116043
10.76636 4.584837 6.940404 4.068406 2.65751 2.814356
2.676803 4.059845 23.28695 4.575334 2.905551
16.07028 4.794392 4.886554 7.386142 21.01084
1.333546 3.28958 7.925813 5.110896 3.264345 4.648445
2.764535 4.602206 4.635789 3.763525 8.166671
3.064899 4.315237 4.280238 5.063622 7.958667
4.00597 10.49383 4.532413
RHOC 99.72013 38.20482 51.26288 29.33528 58.02177
22.41013 44.53845 15.00452 49.38093 33.09307
7.347029 6.873688 24.89228 9.032808 36.23445
21.0968 59.62414 30.32135 96.05884 92.52022 54.7249
24.4591 15.39044 25.61133 28.09813 6.288263 45.19562
18.72663 62.83596 63.90387 58.76213 34.75008
40.6658 40.74851 40.07642 14.42999 11.1244 47.20615
33.66224 51.89641 19.74266 54.45757 39.72292
39.68791 39.29596 38.88202 23.02444 32.08157
20.93408 46.4334 74.24406 17.36914 20.28416 48.37672
38.40497 26.40449 18.08019 19.14569 41.28085
43.73986 38.99276 46.31184 36.7926 31.72299 48.43992
28.53259 74.80236 42.96905 101.2885 24.60179
33.35014 36.30897 18.15084 20.90256 32.14283
23.53572 43.57189 19.3984 18.21133 22.10437 55.00759
27.39852 19.68471 39.58452 53.54321 50.25825
25.37031 31.07997 32.43545 33.86123 63.88566
18.89275 60.71206 40.59938 46.42373 14.95674
60.65305 54.58228 22.28397 31.22958 20.23595
42.63912 45.41803 27.25727 33.77921 24.22614
70.6087 39.39127 43.68464 34.70373 19.25056 19.10577
52.03165 18.7521 40.96687 47.59755 36.4791 78.10255
31.48992 20.62575 51.62586 19.51445 30.19378
53.82573 18.3064 15.90438 39.64489 82.80758 28.20129
57.10975 50.62309 27.89909 33.76438 26.31725
79.78747 26.24138 33.47087 56.11028 36.33316
9.063191 27.70093 23.94978 40.29571 18.83621
32.34849 18.81138 27.81242 42.9162 18.26833 36.03652
14.99043 55.98179 22.45736 22.30743 26.94637
18.12095 31.59089 23.59649 40.25729 35.47897
44.67251 18.76112 21.05803 46.37391 42.17745
31.71066 29.66075 28.34754 16.09724 16.97175
20.04501 22.19858 15.78187 37.25411 61.71202

62.47008 64.38956 29.17466 29.20619 30.79823
26.12291 33.82746 15.84229 26.705 47.11905 25.50512
28.66935 89.36126 28.13426 45.73474 31.44268
34.2121 94.81316 46.29721 73.22989 29.48852 40.21238
19.78894 33.70644 47.2698 21.69448 46.11247 27.88907
32.48339 39.76633 28.59223 70.29978 13.28999
32.08608 34.99533 27.46464 23.08259 37.97596
28.35218 45.92975 27.8465 81.4885 27.88831 38.74692
49.72359 35.25408 25.60229 30.50729 39.85043
16.27017 20.48186 20.28565 13.2294 34.18002 43.22987
56.47833 32.2754 21.17971 65.93049 31.7088 45.08134
42.50014 29.72276 33.98336 10.97478 35.72912
45.96686 24.05963 29.58822 34.32657 43.53179
23.88378 22.73893 24.59892 40.04125 32.14804
16.36654 28.88418 47.9631 27.43278 13.59123 23.82494
35.87016 17.37892 34.74833 33.38704 63.23478
21.65055 37.38669 40.15897 18.3372 24.37888 63.35588
85.10543 30.53965 27.78282 61.13135 71.29389
29.27665 44.18301 15.09556 26.09048 23.89534
8.17892 35.7006 27.25335 20.01476 95.49188 37.98777
22.60628 24.51424 27.31864 17.61937 24.14449
19.56242 28.12641 17.83807 23.19261 42.15256
39.80603 42.22196 31.84779 33.79974 55.6811 47.14475
32.30536 25.62633 51.04268 28.60654 33.51958
80.31189 20.51773 40.94008 21.63881 21.6825 60.16272
67.08903 36.22306 55.10725 20.31778 16.65633
26.92871 18.43377 24.8517 44.01909 25.46589 20.85043
27.04211 25.96856 41.94313 48.69591 49.01928
35.61798 30.52341 31.646 44.64621 23.28019 20.16581
28.97586 50.09365 29.1196 39.28803 43.81977 30.85669
35.91918 8.720737 37.69616 27.00195 34.45333
22.41055 14.35669 22.73845 4.717429 36.90344
30.73776 29.78716 25.04251 18.54952 36.37136
17.62032 63.27681 28.84647 28.02723 31.09503
21.74469 27.28952 50.30758 23.02664 40.09552
17.21095 19.97441 27.93159 35.57012 17.68378
40.18038 23.55097 35.70433 18.57375 35.34866
27.76724 29.41994 52.8357 42.63495 26.80208 21.18718
84.49305 15.56135 40.70925 31.31555 18.8526 75.77737
43.57736 65.66993 35.18751 22.42454 25.90997
38.65315 14.70774 28.95084 40.31105 31.81175
26.68919 27.25173 27.97996 36.31529 55.90004
9.021277 31.69728 23.10382 42.00698 51.46371
29.93356

CASP2 2.528921 0.9284872 4.670728 3.859016 3.331363
1.926346 2.519052 2.249274 2.799515 3.756307
5.330525 3.854443 3.961254 4.310777 3.343194
2.213096 3.226943 2.614731 2.098096 2.344668
3.998743 1.40904 5.060989 2.037297 1.868548 4.026833
3.42668 1.77335 2.835585 1.294831 3.404356 1.424108
7.526167 8.141782 3.639822 5.535807 2.765309
3.929268 6.381516 13.0612 5.687816 8.502176 7.189462
8.28369 5.946969 4.128264 8.939572 9.085472 10.36093
5.168278 5.491561 7.376734 6.91871 5.872668 4.083083
13.93404 13.67421 4.187651 11.73252 3.703509
5.746419 8.140334 4.221888 4.209997 5.491964
3.01239 4.130397 4.258702 5.291006 3.047267 13.02737
6.608228 4.827782 3.885092 7.935039 6.9227 4.934003
6.823013 7.772706 5.581067 10.73781 5.54467 8.157603
6.173146 9.836594 7.715879 8.214515 10.405 5.858382
5.512897 8.180413 8.315575 5.913066 7.432079
9.19339 8.892551 10.48317 6.396072 23.81758 9.91552
10.43103 8.853518 6.048137 6.399444 4.878772
6.75878 6.478433 4.025364 9.884057 11.26643 5.340541
8.221203 6.240669 7.788537 5.69079 4.353665 5.225427
4.855242 5.913801 5.596529 4.538618 4.788451
11.19988 3.830723 9.009705 10.66398 7.520842
6.765395 4.70677 14.97783 10.0276 11.11651 2.580561
7.374598 2.992805 7.446421 11.46033 9.479513
10.39304 4.126456 6.671254 9.93803 5.524276 8.344588
7.919955 6.706857 5.063041 4.781095 10.55775
8.019681 9.38387 5.10837 7.60037 10.23878 4.768488
2.724655 7.117357 4.41873 4.091637 5.51544 5.684901
6.306463 6.269069 8.14766 3.296235 9.57891 5.005143
3.467496 7.440832 5.816457 2.643988 7.138685
5.699787 5.292446 3.564858 5.53132 8.32403 4.416451
6.69038 8.517069 8.601574 7.091523 4.751298 9.866502
3.606279 5.580088 5.66336 5.980564 5.514362 7.824779
4.983811 3.817984 9.029963 4.079437 10.32636
11.37972 5.315584 7.360137 11.28591 5.316442
9.665294 7.346334 8.26362 6.218536 10.76598 12.29838
4.837205 7.150551 10.09364 11.67214 9.581391
5.743602 6.638548 9.090887 11.39577 9.50663 6.888074
6.460899 7.860842 5.318843 7.62917 8.922531 4.287835
11.25768 10.22833 7.970256 9.385928 4.433814
10.94718 4.183803 9.054041 6.079592 5.96138 4.090557
8.633585 6.554953 4.535257 8.477226 13.11802
11.06474 4.473298 9.499305 5.736809 9.625666

8.483155 5.485472 12.76765 3.152921 6.872796
5.879823 12.16622 11.85471 2.591002 8.571617
13.31209 8.061195 7.707488 5.63527 9.856091 5.348636
5.327798 7.109499 7.186008 12.31918 9.209216
6.547691 3.498331 6.473997 5.263242 4.504673
13.31132 4.113575 4.748303 7.289929 5.487771
7.086223 6.444434 2.557726 19.68062 5.054475
7.226885 5.028703 3.655679 13.42167 15.81876
8.960033 5.101499 13.85851 5.617897 7.11164 7.405215
2.333638 3.350694 7.309143 5.399212 6.518938
5.807658 14.42142 13.60536 5.628903 7.062834
6.438337 9.372281 4.957247 5.858318 4.740802
6.732702 5.863352 7.529973 7.99576 8.089994 4.75824
11.13457 4.620338 10.0226 4.501971 5.125102 5.260612
4.961594 7.360413 5.678321 5.480395 4.293705
7.919766 2.345394 4.987691 7.273457 10.93503
4.667648 12.13266 7.770817 6.543323 8.598202
7.560538 2.344452 7.799505 8.029588 6.153226
14.41212 3.900618 6.126342 6.369623 7.620314
8.081402 3.654266 6.990088 8.27347 13.51494 7.921753
6.915551 8.100657 8.0084 4.424166 5.137209 8.325032
6.499958 10.13889 6.926003 3.967149 2.62657 10.34443
3.720564 6.235957 6.981228 12.67396 9.55912 7.250923
6.30182 9.220687 4.008361 6.028315 3.467205 12.94801
6.906197 10.52141 9.377942 7.893771 5.16885 4.090366
6.923306 4.777483 9.037286 6.640301 8.014584
12.28703 6.607314 3.28347 4.361923 18.57573 7.920345
6.824947 7.975693 5.430782 10.55907 8.09521 7.607467
9.697421 4.796556 2.326989 5.021305 5.276113 7.646
13.13221 9.33598 8.520155 6.406944 5.166621
TNC 1.949513 33.66044 5.137006 5.50478 7.159126 48.41322
1.849664 14.86626 17.11089 4.197442 16.22817
4.407717 4.28648 5.479303 3.923066 139.3894 4.114372
28.69512 1.744374 1.796886 12.80337 84.28695
19.52476 42.15633 26.36481 13.45821 2.589482
98.76477 6.252661 1.341115 15.96095 40.85764
3.315377 11.73399 3.029408 8.32576 2.161924 5.890625
46.14446 3.632043 23.70138 1.658762 9.732825
1.93468 27.11742 6.840808 3.547376 25.84884 35.99947
4.313577 19.9533 27.54011 4.605112 6.801199 39.31306
2.960013 42.17228 194.7105 7.900832 3.734464
125.1912 10.60174 49.37572 29.09735 1.927554
281.0463 14.60256 13.54255 11.45533 3.984446
17.01468 3.60292 51.75408 100.8852 120.859 8.316745

49.15918 19.75846 2.823209 4.018858 5.578762
17.07247 8.285043 78.98602 46.06769 5.176637
35.08897 30.97342 13.01619 60.79661 6.122729
11.43963 78.39623 3.333397 33.00487 17.24541
15.0855 4.993189 15.55924 47.42366 2.314365 2.497522
2.549922 6.671802 34.63162 29.99403 19.14052
103.0839 20.71147 2.506563 11.9934 3.52656 24.81411
48.41662 17.42383 5.226415 6.169378 5.706131
23.06527 2.343968 11.50717 4.337394 13.08415
1.763499 15.90909 4.720776 4.128912 9.074847
89.70692 21.28552 32.69418 3.457138 7.299415
32.30877 7.061478 94.70929 44.08509 11.69015
4.818691 4.150596 6.506623 4.829469 5.295715
1.952583 8.225448 1.487581 1.004066 118.6283
9.414347 5.748352 4.0061 62.42566 5.802479 59.62185
110.0827 5.283453 62.63649 54.51193 59.50646
5.876818 39.77929 4.987315 11.65198 6.94997 13.16926
26.28729 3.790551 12.86432 17.14361 28.41495
82.52672 8.165 22.8573 11.68958 2.25775 1.698612
50.17078 31.21927 1.657893 58.43177 1.296545
3.327245 8.891679 12.70406 56.26037 40.33056
68.78223 10.74467 18.9216 2.347691 21.45894 13.2891
11.01972 3.277741 1.936699 38.57262 57.31247
9.208184 2.67785 4.928436 3.043757 16.69115 16.93207
10.88427 7.773734 2.52624 27.28985 24.09724 15.62763
4.493706 6.865634 66.69768 7.571306 27.17052
33.53124 33.20591 9.615095 2.708994 17.46837
4.547212 9.05817 9.53705 11.43423 10.88291 14.80406
4.632162 11.14802 12.05386 4.13163 12.91609 46.68694
24.75131 1.099307 7.138616 3.216127 7.428982
4.187326 4.3947 20.43329 1.771934 48.74141 11.97341
42.5246 11.47902 16.39374 21.27976 5.821581 50.36158
24.68867 11.78284 28.72278 16.10408 7.320238
9.419223 3.948459 5.979785 18.68832 17.39019
17.40292 43.9429 10.83848 14.67264 7.790937 45.74301
47.02625 20.93835 19.08368 13.3403 4.650994 3.583973
25.76888 5.786091 4.002496 5.160645 2.590179
3.973023 7.948197 272.3866 34.89721 22.05523
87.70431 4.131205 96.99494 24.52713 22.17563
21.3457 5.81382 1.530821 48.80762 92.38403 13.1941
399.0183 239.7036 14.82933 42.22268 58.58103
2.37637 24.85005 12.04478 10.83924 25.62571 23.66465
29.04844 111.9952 4.803325 13.9677 8.532677 17.95203
3.822506 6.209964 6.100957 7.581019 8.38077 8.699851

9.411176	112.3287	17.21757	45.00104	39.93196
72.29448	9.492253	8.303597	15.87873	2.77606	21.96883
6.137547	14.0723	3.440158	17.96441	10.49857	0.6375877
35.29393	10.0947	5.173297	139.333	2.656594	0.6313116
1.779136	4.550401	3.394998	13.33906	7.883886
8.42687	7.487307	131.5644	7.859855	3.017091	11.40297
7.02719	11.78772	2.508575	95.73832	2.960942	58.69533
0.9869562	2.161816	48.35514	6.333759	147.8251
3.402777	8.564015	8.740712	62.22757	4.721811
17.33541	34.65308	1.257754	19.51067	5.509455
9.141387	11.01689	7.580121	12.75524	1.758158
13.77907	15.31817	2.383327	13.15314	5.631093
14.18175	5.293942	21.85764	6.041055	4.967236
5.085717	4.495616	73.07908	18.13866	7.745843
6.558754	144.0123	17.25511	3.607041	17.94736
12.95888	5.121391	7.027814	52.24913	7.665546
13.0754	8.560731	19.70032	25.21918	4.099841	5.250582
23.39505	124.2162
IRF6	17.51711	1.037357	18.34794	22.41192	14.39238
0.4789782	11.29914	8.211961	16.83937	7.618128
41.47802	32.7481	6.655456	42.89727	9.638201	0.2627917
15.75532	7.533805	19.61056	18.34027	17.5195	0.1000926
48.34592	0.3182877	3.123892	27.23792	13.42416
0.1519281	16.30852	9.195826	17.63406	2.09325	20.19874
30.38761	10.49956	6.47343	13.53064	42.06131	19.00127
16.42842	9.526905	12.11441	27.23157	37.92616
17.2223	30.12934	13.09774	19.80072	13.34568	21.66164
17.70662	5.948732	10.4999	17.10301	2.449459	16.55563
6.614398	1.763591	23.05391	16.70686	11.78291
13.37159	8.310986	20.3771	13.10252	1.564366	14.86291
10.11592	24.69782	11.49545	17.88692	11.17926
14.78455	5.697244	12.40967	16.50837	14.0709	8.866761
12.80384	15.77265	12.34047	11.34645	10.72739
12.35564	9.944932	10.04019	24.81875	14.68794
7.716343	19.49494	29.40558	7.700349	9.017297
13.87893	9.132064	11.63544	11.98839	14.85046
28.85721	13.23427	7.871947	21.87656	8.418264
12.35693	9.049789	15.36775	16.01232	9.151527
15.57927	6.033378	19.14727	11.53033	14.37468
2.949108	33.60507	13.42658	13.5902	26.16085	14.38624
14.55937	11.44372	12.75856	8.321805	5.378163
13.61891	7.251068	17.89303	23.43427	5.03527	9.37871
18.94686	29.0901	11.54963	18.31523	18.02724	17.68151
11.1032	2.208819	15.88194	24.22204	7.555185	13.7885

9.658049 10.95209 9.248867 9.712722 5.149568
11.44069 18.74887 15.34692 18.62577 14.87293
25.74115 10.03831 7.400741 22.29461 12.65962
6.496195 41.95225 12.34253 12.16342 9.336895
19.14099 18.44984 16.2414 19.28854 16.36247 19.77555
15.45465 7.918823 2.091264 11.62465 10.41702
17.56657 12.61612 14.10759 14.42906 10.77782
22.66143 12.43365 9.017106 30.78872 62.98636
9.782012 6.922475 22.98775 8.446663 16.45785
11.26518 17.47294 25.17509 16.0359 17.70662 11.86706
17.93769 24.47463 9.632982 9.422868 21.54519
16.72182 11.01372 33.5131 58.78744 10.46556 13.57395
20.54447 8.309796 8.855571 24.79691 11.87525
16.92743 9.334361 23.19304 14.75572 13.17956 13.433
21.24536 18.61682 9.6062 30.22622 11.44475 14.61846
22.20616 17.50776 4.827846 19.0613 16.63389 38.20485
26.60509 18.84702 14.95392 6.668539 45.49872
24.65823 36.56725 20.66785 17.30015 10.34063
18.17088 30.49873 20.13771 21.09866 21.58356
14.78353 14.41585 7.630801 23.7225 3.473221 20.59458
12.16997 21.61118 26.45887 15.4085 7.121936 14.7832
10.50279 30.4265 26.5349 23.38703 6.972361 13.29495
15.48436 19.97445 20.32155 28.14212 15.01893
17.20445 26.10258 8.97114 19.58185 13.46894 18.04805
15.81779 20.47023 10.71591 17.80605 22.96157
3.390412 2.795247 17.52789 6.926094 17.50417
5.361483 13.2766 14.18501 11.06567 20.44958 17.67439
13.22525 10.19018 14.31115 0.5973761 1.77948 16.98296
0.1041812 3.772878 9.482253 10.5894 18.48397 15.6832
15.97393 8.74822 51.54591 5.403345 22.30907 10.11085
24.58257 23.00454 42.81362 15.49666 15.33445
10.81709 10.04881 7.681293 22.41198 5.18385 16.05573
7.790656 12.48791 15.98717 23.9455 21.97884 24.13557
14.50329 13.05886 13.35921 18.05786 16.19774
16.32773 24.70146 21.49296 13.483 16.72508 21.8136
5.798604 25.60338 16.75707 18.63667 14.8174 30.58389
11.6427 12.23031 10.38794 11.90571 7.758732 49.55841
22.58609 23.24435 18.14849 8.747774 22.58216
15.14146 15.06581 7.991634 11.20889 23.48045
14.15742 15.73596 3.132485 5.166382 24.59216
6.65597 41.84724 11.3743 21.49059 14.74405 26.44905
8.302592 21.49305 10.12009 12.2011 27.12033 13.04604
16.63717 23.80371 8.400503 22.93613 7.325803
12.94872 28.28275 6.506634 13.4183 6.611155 16.82891

26.88134 12.89843 8.044756 10.61508 35.68917
23.01218 7.299967 12.80197 12.92255 17.50542
17.63839 10.76472 22.60562 7.486438 24.24176
14.99266 13.41198 8.009044 18.2119 30.94046 29.12501
25.10107 8.109124
NTRK3 0.05648509 0.1558056 0.1473785 0.01815893 0.126095
0.8877671 0.03188812 0.1555719 0.07546433 0.1272493
0.1123561 0.0202739 0.1249702 0.04226615 0.01416725
0.1947491 0.03033531 0.3606816 0.1341229 0.04408013
0.05585021 0.215822 0.06633228 0.1864915 0.09336918
0.1511256 0.02665127 0.2559592 0.1647813 0.0320425
0.1473449 0.1226858 0.009436799 0.04276476 0.01989748
0.02262155 0.002825753 0.0126829 0.03823163 0.01297776
0.02193006 0.0009575746 0.01074073 0.002444104 0.03747164
0.003596826 0.05940553 0.01459464 0.04811317 0.0095435
0.02445211 0.3284667 0.03437777 0.03593271 0.116486
0.001335339 0.0526319 0.1798362 0.009802773 0.01693521
0.04254716 0.02962766 0.08817166 0.01715998 0.002989673
0.2164036 0.02075028 0.2228662 0.008730997 0.01062338
0.007885773 0.006298377 0.3449338 0.02228316 0.03592422
0.1178712 0.1332532 0.02387377 0.0008221411 0.0005320435
0.00283309 0.1085698 0.0186166 0.08544685 0.02288372
0.05048394 0.3503943 0.03758501 0.4260806 0.05636069
0.04605812 0.0006064505 0.03201366 0.03392785 0.008752139
0.02385899 0.04053775 0.009150616 0.003646299 0.03413218
0.1373617 0.02135288 0.00625711 0.01433277 0.1905459
0.3654514 0.003134036 0.1622665 0.02209319 0.02751495
0.1450521 0.02354928 0.006672053 0.2359055 0.3058417
0.003880929 0.002430801 0.01118749 0.02808609 0.003409236
0.001124111 0.09232037 0.05205224 0.08369234 0.02558178
0.1480332 0.02483859 0.01351061 0.2359465 0.05579882
0.005084994 0.009893506 0.05305274 0.01806016 0.004856737
0.1716802 0.03874558 0.0245612 0.01135688 0.02209224
0.01664924 0.02687887 0.01774554 0.003431063 0.01179051
0.1475819 0.002820188 0.05572147 0.00574387 0.3082435
0.03915178 0.05839378 0.01131963 0.07826786 0.1377264
0.03069723 0.06939831 0.08360345 0.001525779 0.05633147
0.1080604 0.141612 0.1794014 0.0009881776 0.05085128
0.03406076 0.01646163 0.1025388 0.04733918 0.006017887
0.3891774 0.01500628 0.04461955 0.00110536 0 0.01118771
0.156044 0.02189904 0.0005224964 0.2419662 0.03878402
0.006033913 0.09731861 0.02327624 0.01390624 0.002013217
0.2492131 0.01396229 0.04210461 0.008921827 0.04657768
0.004578726 0.00605537 0.05895307 0.01774468 0.03850027

0.03469806 0.05934797 0.008301434 0.06850195 0.002089244
0.2002702 0.02129844 0.002660259 0.001027629 0.002526644
0.04908362 0.06881271 0.01475973 0.03058077 0.04724029
0.1056411 0.026215 0.02156492 0.01980638 0.01853256
0.06858596 0.006884273 0.1815099 0.02083417 0.0435874
0.04580636 0.01254194 0.004279791 0.1259787 0.005281828
0.01065493 0.03035544 0.01060241 0.04070831 0.01715724
0.03094744 0.005667221 0.004713637 0.0169223 0.1604139
0.1674235 0.01013317 0.04906227 0.03715523 0.5585088
0.09796348 0.05690019 0.02422864 0.1019837 0.1001479
0.05064088 0.1886225 0.2571736 0.007745377 0.04491747
0.004903186 0.03944771 0.2048029 0.00387979 0.05176124
0.05482659 0.08050731 0.02600591 0.1709518 0.002488074
0.000760714 0.07421994 0.02075919 0.01474437 0.05287767
0.001411003 0.02127111 0.3427469 0.06682605 0.0485247
0.01697545 0.05078883 0.007445887 0.002646401 0.01301072
0.03402356 0.1730171 0.00873459 0.021371 0.5034545
0.006886089 0.01931588 0.08408345 0.008321868 0.2475642
0.01027657 0.006390846 0.04346938 0.0910312 0.04426337
0.06135352 0.1373137 0.02036768 0.02413567 0.6195731
0.03592811 0.01587811 0.02421464 0.02132119 0.06200279
0.2749866 0.01482081 0.04973857 0.01191712 0.005903389
0.5296201 0.03464603 0.06865727 0.007300848 0.01354784
0.01434781 0.001031769 0.2208759 0.0118854 0.1221698
0.05054515 0.0293101 0.0343622 0.03899997 0.05571079
0.0609514 0.001017233 0.03363243 0.3250601 0.03314391
0.01203963 0.009269258 0.01775165 0.0282671 0.0005507784
0.06403856 0.05364939 0.04355909 0.133843 0.07117528
0.001605274 0.1248483 0.06900031 0.02911811 0.03039731
0.01100478 0.04994734 0.001213275 0.217819 0.05764155
0.007646997 0.02553401 0.0417812 0.01934288 0.001994812
0.03286783 0.02964074 0.002588575 0.020004 0.0007702657
0.04445736 0.1588883 0.1506825 0.04622349 0.1085702
0.3965126 0.0500306 0.02931239 0.01555916 0.02971246
0.0005938054 0.02140376 0.03541279 0.009509963 0.01102054
0.1277873 0.04958361 0.0306711 0.06033657 0.03374496
0.01062415 0.01618474 0.006555492 0.2378005 0.1504882
0.01036632 0.05701098 0.01135218 0.01609913 0.00705805
0.5666785 0.04016467 0 0.001500078 0.03719564 0.03305526
0.1178629 0.06777636 0.01434427 0.08801243 0.1196026
0.07551072 0.05132947 0.01027883 0.06966687 0.20817 0.01449279
0.004135711 0.01287008 0.03584288 0.2306501
GNE 1.599554 6.417461 2.896302 3.167476 5.546178
7.648285 1.803346 4.689281 4.504774 4.523274

5.845028 6.675963 4.088195 6.058609 3.995938
7.06693 2.354678 6.696842 1.408389 2.113242 3.019132
6.728725 11.75744 6.820159 7.30905 6.534538 3.329741
7.27419 3.936864 1.354305 3.268372 6.163251 9.061786
3.275014 4.299697 0.7094301 8.90174 4.874519 10.56539
0.9531099 35.77399 8.025479 2.647767 3.719275
2.349132 4.871946 9.090031 6.694721 1.836685
25.01106 2.639611 5.273256 3.292609 10.0592 13.75763
0.7761035 3.789649 7.2034 6.436842 4.405344 4.9692
4.83309 4.639241 8.8063 2.818137 6.057566 4.766864
5.327176 21.92102 11.09047 1.04216 4.381613 6.242753
30.05008 34.91369 3.439749 4.48775 4.253775 19.69146
15.01432 4.307915 8.582706 9.402173 4.647884
11.2017 5.349982 3.11154 9.729076 3.135136 17.08164
1.917193 6.664368 11.89444 5.761631 14.38097
5.693648 3.604939 18.31261 4.576848 3.183501
6.921387 8.037188 8.410141 2.415073 3.388616
7.221588 36.3958 3.731445 1.39103 7.263846 5.225771
6.666674 11.87087 6.433475 12.86127 11.0906 4.232432
1.658126 5.36743 2.958313 8.087107 3.027867 2.490929
13.69095 3.174572 5.691399 1.264083 2.848902
8.312349 33.04425 0.9571188 13.53879 6.574988
3.579062 2.520313 6.522014 1.889834 14.88912
12.6262 65.36293 8.986313 5.516471 34.24508 0.6563362
5.603325 28.90275 4.411113 7.987516 33.87917
2.681879 9.965487 3.217242 7.694335 7.982747
7.393487 2.6737 2.254107 6.53633 2.690171 1.437757
4.330143 4.832699 4.462418 10.48035 6.421432
3.007021 12.31383 12.34659 8.25844 9.153889 7.914019
12.1206 3.605661 24.95794 3.782119 8.790966 5.524789
3.495177 4.899655 4.79157 46.52081 1.875363 5.466065
5.12313 5.370444 16.37468 7.751463 7.691413 6.753354
9.416732 37.08513 33.69791 4.783051 2.78603 7.87749
9.255632 2.408132 7.504715 6.492559 2.555725
2.408143 5.566657 6.379204 12.16999 12.49229
4.452785 7.537029 11.55523 5.967485 19.38671
4.410908 24.36559 20.24687 5.816247 19.50749
13.00618 5.887664 10.44841 4.821588 4.009711
3.265039 3.724955 9.491834 5.389111 8.572388
0.3525558 2.440371 3.86088 0.2297863 6.237704 1.221042
51.7393 2.120907 36.97911 8.131445 10.38935 2.613604
5.704965 3.501493 0.9506778 4.158242 6.714383
7.211433 8.382003 2.039421 51.25658 3.215331
6.931895 7.053212 8.814131 12.94817 1.418887

6.965581	20.44302	4.513683	5.205191	4.084687
3.129123	6.908589	7.926799	6.153724	15.8052 9.523109
2.625156	6.584437	10.32627	4.382945	1.223586
6.779687	12.22426	3.804053	4.60326 9.396499	3.260833
27.39216	31.40984	16.92196	7.806937	7.488299
3.770232	7.186469	0.7922222	13.45713	2.190233
11.06692	4.862201	15.62834	7.629418	42.50108
8.095639	3.339094	4.942922	6.522985	9.47292 27.16526
3.956773	10.29454	9.192617	4.679024	1.285829
7.504803	10.15042	1.24413 54.90613	2.079672	6.96416
11.90569	8.721843	4.228837	15.17595	4.238917
6.787322	7.057433	3.043307	1.519518	4.959113
4.678829	13.03073	5.530173	6.738166	12.44216
55.51291	39.90234	0.9855769	37.10159	3.637468
12.33623	3.858535	9.840466	1.196505	5.191768
55.7046 3.298919	4.942236	5.948213	4.361798	0.7892523
10.67929	3.579648	9.304393	20.98886	7.263149
8.658706	31.54847	7.292075	18.84925	4.225037
6.180284	3.574602	10.30193	3.721742	12.08317
8.794604	3.546294	10.81814	5.492556	4.029287
19.13291	4.349298	9.120636	10.0471 14.24431	6.066315
15.08634	4.609073	1.680307	0.9860618	6.563673
15.02505	6.099255	5.796099	11.91651	15.74101
3.245656	1.159266	4.19942 16.85404	4.630851	6.753389
3.93285 3.094203	9.824278	4.679776	3.870569	1.062502
0.7392316	3.90241 2.816953	3.425872	24.09432	6.244487
6.712836	4.832672	2.339664	22.77127	15.29162
2.647656	9.210577	2.338126	11.14107	5.881112
40.73577	0.7668229	1.151347	1.807804	1.482641
5.933651
XAF1	0.9312372	0.382232	2.264705	1.665853	1.163857
1.503613	0.80085 0.9107584	1.661458	3.409408	0.8615686
0.6386154	5.745494	0.4756861	1.317672	1.083673
0.9988936	2.19773 0.6020325	0.4784686	1.896621	0.6802634
0.6510338	1.616018	1.034933	1.146296	1.213321
1.161835	0.6875703	0.4634788	0.9247921	0.7244953
2.726384	3.347999	2.211817	0.7077502	0.07998824
0.5641636	2.993514	2.485996	5.902651	1.107472
1.030981	2.108023	2.840609	0.8646186	5.287537
3.269618	4.213273	1.534924	0.6921628	5.456047
0.3204487	2.797143	2.338124	0.6047886	2.299263
3.845531	2.47535 1.369665	1.954931	6.502661	5.147037
2.032935	4.116563	1.223356	0.9080349	1.664843
1.849191	1.040227	6.963014	1.356258	2.298689

0.8314652 1.596281 1.692116 1.834833 1.791385
2.928979 0.2495738 0.7255824 2.80937 7.436505 2.771042
0.6153784 0.7868242 2.198853 4.05753 1.23394 3.65405
3.786412 6.802927 5.062 6.287042 4.88413 2.710469
4.128009 2.14621 1.782308 5.955113 5.895669 3.388665
2.682092 2.987545 2.198297 2.629273 7.008468
1.211198 1.388675 1.054099 1.221018 2.289035
2.193147 2.578609 4.970434 0.8840871 2.909612
7.563853 1.117909 0.7628478 0.5409415 1.398801
1.896442 2.589258 3.964827 2.662912 5.64692 0.8836576
3.775832 2.120217 6.758867 0.3850746 1.979996
5.692099 1.335511 1.979164 1.077965 2.431014
1.123964 0.8443832 2.907025 5.853279 3.133525
1.36203 4.62071 1.586132 3.452678 1.56979 2.748563
2.171162 7.110351 1.760656 0.6567684 9.001773
2.826447 0.5517096 3.911237 4.899544 3.060323
2.682107 9.434775 3.120491 1.558751 3.5245 1.279804
2.090453 2.851921 2.526903 3.61328 0.2406498 5.5499
2.632125 6.439396 0.268194 1.078794 0.8508843
2.662169 2.558642 2.074859 2.579609 1.071347
1.011498 4.560155 6.380064 2.604866 4.928096
4.607718 5.690699 3.401664 5.476327 2.40338 6.691087
1.54713 0.6346054 2.239562 7.023301 5.679886 3.309731
1.125703 1.574858 0.2196628 2.253212 3.341747
1.53476 1.475226 2.736545 1.93458 2.274205 1.257136
7.584709 1.789522 2.173328 7.644898 0.7315539
2.402815 3.645289 2.578421 1.75559 3.198293 1.858055
1.994203 4.371513 0.4910386 15.37944 9.455238
1.621494 2.299477 4.705514 0.9995107 3.035554
0.5079689 1.775037 0.8565353 2.424237 9.164502
3.212698 1.164245 6.295544 8.193919 0.6211821
3.265231 7.669877 2.743668 10.32566 2.281979
3.528828 5.265864 3.794013 4.931052 3.570601
10.28235 2.748615 6.450343 8.982679 2.729929
0.9392302 11.47076 0.7558425 4.552971 1.314746
3.026461 1.618084 5.511799 3.239893 3.98443 2.906736
0.6694886 1.130167 2.777498 5.282049 1.876162
1.725104 1.888287 2.147845 0.5625996 1.256142
5.215753 1.418644 0.284925 2.380502 3.681527
4.119697 0.8541741 4.748136 0.8108602 6.611587
1.227657 4.947196 1.649658 2.298042 2.575032
1.254176 1.50304 4.093846 2.753015 1.712495 11.01659
4.460024 7.101879 0.4273488 3.594696 2.577712
1.713708 2.387729 1.156582 0.7033164 1.129086

6.255904	3.786687	0.5107559	4.667021	1.226714
3.961608	1.403987	14.82025	1.515041	2.493637
2.918287	5.334088	3.896742	4.546671	3.691921
2.04031 2.121665	1.061531	1.955242	2.446488	0.2162504
2.192428	5.061621	0.7617228	3.382088	3.458176
5.072101	5.602368	0.647073	0.4608944	1.696748
11.85312	0.6726411	0.3441812	2.744264	1.275418
3.534981	4.847824	3.333874	1.403142	0.4671886
1.806087	4.108387	2.339342	4.280225	0.6910816
2.172065	4.040971	0.460995	4.51489 2.383334	1.833002
6.749525	3.772284	1.285669	3.131495	2.107994
0.9711373	2.032052	1.534401	8.185851	5.9545	2.351935
1.953836	1.617191	1.098618	5.883762	1.35506 1.607489
1.117021	1.13444 1.255408	4.160419	2.99287 3.203366
3.196168	0.124603	3.694139	4.411266	0.7738899
1.253878	3.959428	1.581223	11.01641	1.047974
3.889825	2.213901	15.16275	2.43066 6.393669	3.395839
2.562831	0.3325274	2.632164	3.172573	10.45149
5.144349	2.677172	1.41689 2.243739
SFRP1	3.967066	48.71332	15.79201	2.952959	9.578396
66.57438	3.423005	32.32674	13.86956	11.14494
11.08539	4.328591	9.043168	8.523357	5.253578
45.05029	2.319215	53.06159	13.51167	8.055702
22.24758	47.67474	22.9167 73.88738	26.32202	11.14636
0.7921833	66.93309	5.566802	8.925479	16.10892
25.24492	0.3181277	9.832123	0.4720248	0.1939958
0.6287166	0.4905957	5.259231	0.261959	0.4903978
0.01883069	1.391169	0.07847064	0.3770811	0.04266346
0.2982661	1.817141	8.230994	0.1438012	1.000451
9.224682	2.075585	0.5322563	19.96772	0.1425512
0.3808462	35.65882	0.08567617	0.4012916	10.40058
0.944689	64.882	3.388792	0.05879188	16.29442	4.02641
8.8085	0.2238166	0.2963091	0.0755821	0.4003251	8.232115
3.767365	0.4583015	0.7064186	0.3388067	1.269328
0.1847702	0.07772238	0.2016269	35.43708	0.4282007
15.55425	2.070037	2.795109	11.05557	2.420958
0.5122453	13.26212	0.9763088	0.2453316	1.465036
0.1906259	0.7692294	0.1932862	4.167787	0.2088667
0.09987403	8.552499	41.25946	0.2532755	0.06328079
0.2232868	2.849672	4.06289 0.6515254	16.31192	0.5828812
0.05695591	3.542689	0.2229722	2.302796	8.47414 2.539762
0.3198105	0.235594	0.09428654	4.528964	0.1149301
0.3821117	1.375562	3.709168	20.25393	0.4810655
1.826264	0.5889317	0.1122152	27.82235	6.922628

1.063697 0.08338099 11.89217 0.2306187 0.4297845
20.07372 4.191348 0.2332836 0.4500242 0.06406541
0.2847923 0.8161547 0.1020784 0.07389769 2.024816
2.882453 0.05545895 10.37582 0.02151488 9.896496
0.1156289 0.6820806 0.08572187 3.114666 17.44339
2.136767 8.501873 30.16761 0.8744147 0.5513263
4.438597 4.200797 15.34125 0.3414568 0.845856
4.244113 2.09808 1.103756 4.546568 1.104522 26.90953
0.3337422 3.50326 0.5496329 0.1362629 3.171956 2.343887
2.847526 0.1614625 8.42892 0.1442507 0.7181049 5.385893
0.9126108 2.491888 0.04524559 71.71555 0.2848903
2.803578 0.03165972 5.896097 0.1382766 0.2288787
1.628168 0.2046467 2.488974 18.27883 0.9882262
0.06529903 0.3385544 0.08686533 6.201276 0.1136834
0.04982282 0.1385712 0.1041049 2.07754 13.21568 0.8266937
0.06586438 1.37005 13.7198 1.668351 2.312213 1.341259
2.224034 1.178925 0.468992 15.06081 0.1138945
0.3901294 3.921616 1.028189 0.166138 9.672648
0.09150194 0.07877035 4.953175 0.5212407 2.81786 1.421887
0.386795 0.06368332 0.1107508 0.08776533 0.4015255
8.659534 0.2769058 1.464442 0.5608456 9.82088 4.177034
7.62418 1.580624 8.318085 1.85218 0.2743674 10.10006
11.31949 0.2763388 1.388045 0.08953386 0.2671042
10.97037 0.09591499 11.16317 1.44176 0.8129391 0.09168059
27.1976 0.450137 0.2991886 2.794355 3.499107 0.5834975
13.68966 0.1717693 2.562894 1.768646 0.1546039
2.439843 1.065742 2.112519 0.1450288 0.05522766
0.399013 0.4576342 12.82792 0.238836 1.255323
30.34149 0.1160699 7.411383 5.328881 0.2271053
2.929887 0.1323199 0.7130184 0.4401609 35.18432
0.8396592 35.35108 31.42569 0.2522819 0.1636647
26.11611 3.329302 0.8440851 1.125518 0.2890695
1.628236 8.835959 1.571753 1.626952 0.09013462
0.635306 0.8785104 1.366859 1.529628 0.08614265
0.5185636 0.1343569 0.1014487 36.67114 0.1571268
31.75833 0.2606038 2.410126 0.6196807 11.0688 0.8658722
2.695958 0.5172432 0.3716333 28.77007 0.3505342
0.2057549 0.1081661 0.4197343 1.694086 0.08045923
9.186688 1.124344 1.650424 12.94347 0.4203361
0.02104512 5.575989 0.4731563 0.2147277 0.6447297
0.6306773 2.358574 0.09543614 31.47254 13.45368
0.1100982 0.366893 0.9228123 0.584798 0.2521796
3.671493 0.6947777 1.163527 0.1021762 0.07357243
0.9111536 0.2242806 15.58613 0.4887266 1.364494

23.27298 0.293322 1.157307 0.2660615 0.1962294
0.07339939 0.8673186 0.05134688 0.4555812 0.4236606
6.048903 1.349415 0.8465218 1.697827 1.380276
0.1051725 0.8411492 0.1657461 4.026724 4.471597
0.2378291 0.33852 0.05467111 0.1043701 0.1586245 31.98147
2.184465 0.05110482 0.1208055 10.02127 0.1188628
0.3631534 7.579149 2.347979 2.840522 2.138913
11.22538 1.153592 0.3598412 0.1343135 4.162152
1.022383 0.1370969 0.101236 0.7891029 13.6835
MAP2K2 86.6889 19.72992 48.62692 28.4941 34.11721 15.07049
25.05415 14.09681 27.7715 24.71766 18.61132 18.40937
29.47178 27.77457 27.53099 11.25893 48.37556
25.05157 66.55498 58.40366 29.18158 13.44128
21.43078 13.89966 17.58476 19.67059 31.27234
13.81161 43.13784 12.71199 31.79647 18.49513
37.45482 18.03765 20.17257 23.08366 25.8902 26.86756
21.66551 26.02951 17.39945 22.27625 15.95357
32.15296 23.9754 17.34665 16.11427 21.35455 12.07263
14.63579 28.12036 17.13956 22.42425 38.76775
16.27898 35.8056 28.61632 15.50511 39.65303 23.47315
11.56458 29.51212 10.65344 22.07934 15.85423
15.06065 31.38663 23.96934 22.98854 13.98315
22.68176 20.86311 21.69835 9.792963 26.55598
48.28041 19.14348 20.67806 20.72171 7.996758
37.06275 16.28846 16.33508 19.7982 20.76317 17.17287
16.4565 16.68144 18.07969 11.0387 13.24925 21.18598
23.98072 27.76134 39.69278 23.60174 22.39388
28.82674 25.23688 19.94643 21.24046 19.5051 23.84077
22.50644 18.2612 20.92048 28.56827 25.51238 23.2588
20.01644 19.41688 18.74831 26.98685 19.24051
19.82118 23.61191 21.25138 34.22947 28.11954
17.71341 19.70399 11.68383 23.05644 20.70894
16.58744 26.19263 23.81347 31.70895 15.13584
33.8384 20.38116 28.50713 21.41774 11.11955 40.46282
19.16159 27.78103 15.73875 18.92392 8.330253
20.27232 31.73271 24.12406 22.43982 23.07042
26.78633 17.99553 19.94374 18.68031 19.84594
26.62588 26.60333 23.60487 18.98644 16.62136
14.34975 26.82156 18.70122 14.56885 24.77776
30.43472 17.72516 8.785134 22.72313 33.80033
38.17464 26.16401 8.441348 19.07964 36.23758
14.9371 29.11035 21.66758 28.5039 29.75651 28.77167
18.40287 21.24905 20.69289 18.26965 21.57238
21.70461 25.03338 21.04797 29.40249 20.47793

16.01113 23.39294 17.69367 23.45231 15.75764
21.55872 23.80107 35.15782 40.561 18.27541 17.13646
22.88613 26.19296 19.16393 27.10469 28.21855
21.81929 17.36011 30.03007 20.59085 17.79738
16.88101 14.69698 23.01311 11.12312 15.51285
24.22051 12.02101 39.5672 23.76475 21.36823 18.31959
25.0635 12.84746 33.1848 13.1255 33.98599 29.07629
21.44472 26.19259 18.24991 30.97112 32.62163
60.48413 35.04593 21.05717 24.44723 12.77404
14.10526 20.03388 34.28569 20.47227 8.030275
18.16048 19.31255 16.30181 16.45924 21.66851
10.23516 19.91406 25.40049 17.02905 14.84869
24.34746 24.96312 12.10527 20.09128 25.76538
20.39018 22.37917 23.4826 46.1117 33.63717 16.83245
20.68116 34.72483 14.25289 17.42468 21.2815 24.92929
25.41518 37.39496 20.58171 19.37402 22.83771
48.82254 26.78382 16.27605 21.75384 17.68378
16.36294 13.16553 28.41347 12.17208 20.10736
21.93869 27.07711 25.51854 14.45722 18.77782
29.65245 21.82882 20.29217 19.8985 28.60277 8.020208
13.68986 16.91141 23.93408 21.35293 30.9165 23.13142
19.82047 23.23224 19.72542 17.88376 16.549 16.46164
23.84503 33.1454 11.33955 12.6133 20.39039 31.70604
38.09799 40.0014 22.52897 37.77519 17.3733 19.29081
18.12154 25.06952 13.40706 8.55095 11.88376 12.30331
25.44924 28.15041 9.487603 23.84532 24.41899
19.95846 27.64178 20.85623 36.63915 23.76636
22.32715 24.21378 23.6789 27.27981 17.12941 24.08257
16.52925 17.91447 6.037118 41.0345 27.15166 15.2234
13.1004 22.80755 15.61233 24.29787 30.94353 22.25335
12.97851 18.84088 16.88686 20.55114 28.6618 20.26618
20.27104 20.40943 13.83144 16.82457 18.43549
28.2679 14.43859 19.41932 47.03365 19.68566 21.73624
17.97629 19.66182 15.88988 25.33311 19.56514
14.2279 3.502258 30.61622 33.29701 31.71087 24.17175
23.31459 19.63932 22.67147 13.56661 26.93173
22.41158 19.93149 30.31727 25.9302 20.51957 22.8708
20.35476 13.86971 9.926456 23.69248 10.60295
24.18335 13.00829 32.68035 18.99383 17.87481
35.36152 38.36591 32.77405 14.82478 15.02678
28.67816 30.77101 14.33634
CSK 16.89707 4.465716 20.64253 21.09124 19.42785
4.781119 16.36658 7.452631 26.35119 29.10053
29.50212 30.72332 36.04966 31.22907 33.94803

3.681261 27.43371 12.44944 15.30109 21.11676
23.56746 4.590906 25.06356 4.012704 6.819604
26.73533 25.45321 5.153306 19.11242 6.095612
21.34607 5.539845 51.06686 19.96544 22.94415
20.50307 4.055666 20.4497 17.28307 32.01175 13.03677
21.78406 13.77839 28.93959 20.4441 25.49594 29.42705
31.70751 14.70602 17.56601 41.12171 31.82411
17.56128 22.28523 12.645 22.62418 26.94257 19.56368
38.8714 21.12297 13.63613 32.53403 11.48409 20.68032
30.40578 10.00646 27.28935 20.06266 12.08017
13.0273 27.27968 12.89419 13.32498 6.646106 15.88469
26.80442 24.42877 12.1576 23.31807 10.37048 40.99364
18.83586 17.67361 17.31578 13.7497 20.83599 17.15051
33.32604 17.09872 9.653196 16.14207 38.70339
15.90901 41.85481 33.67255 25.10235 26.397 24.13644
39.63169 25.55688 41.17679 20.15898 18.25406
16.89728 12.86577 29.14925 29.59924 18.62417
30.37617 22.07736 18.5404 17.96811 18.73483 34.65787
14.6482 17.19646 37.57729 10.21563 16.14397 28.23717
16.53849 11.51914 22.87167 12.42417 36.91249
71.0342 15.66432 23.82802 11.37262 20.61777 19.99943
37.32336 13.76721 11.90715 25.11648 20.19548
28.32494 13.34973 14.02999 9.41076 31.52113 25.53809
14.56923 42.89805 27.09868 15.38998 36.60162
14.11999 49.22692 24.02381 25.72588 23.85116
23.39491 26.74546 14.89898 13.93541 24.55189
16.97339 14.25385 13.94439 27.88838 22.70021
15.81662 36.73539 31.71957 28.97188 56.50033
11.1542 24.89956 12.41096 5.789499 19.86961 19.65941
20.89844 29.78806 11.93861 19.66703 13.44555
30.30239 25.05236 10.92245 23.60584 36.80612
41.36515 15.02096 22.11885 17.03929 15.48076
19.05043 28.54671 15.58668 31.85308 26.32879
26.63885 20.47811 17.7114 15.09855 34.51447 28.40986
15.63778 14.19317 20.96001 19.17708 26.87129
14.77312 26.38189 23.3139 14.35389 20.74817 25.83584
14.19991 15.39814 25.56953 30.86975 19.08038
23.28946 49.69604 19.12817 21.73523 31.0941 16.99905
16.02032 14.44566 18.12347 46.21518 20.83779
32.60507 35.01822 27.80291 15.45018 25.87456
12.41785 20.52131 13.36145 11.17012 22.40612
45.60642 20.41729 17.22366 35.16751 15.36248
27.9892 23.28463 28.15122 11.48135 24.8099 26.31794
13.60852 18.57818 25.66965 27.28648 13.75819

21.60422	24.03347	27.76743	12.91156	20.43112
24.82088	40.59871	17.43153	19.86193	32.86135
11.51465	16.38765	21.26094	25.55906	24.19931
30.55983	13.85356	15.0514	29.65837	27.42014	21.68863
26.48145	31.53408	14.56182	22.56768	8.458135
149.7986	12.13779	27.71667	21.04024	15.75229
30.12804	14.48577	26.02196	12.52431	12.34468
25.64844	21.13776	32.65314	4.790546	8.762718
28.53964	29.96016	19.00599	16.47665	33.20846
16.09883	20.18525	23.43638	16.2328	27.59798	13.78156
17.66962	33.05304	7.930821	11.70931	25.63925
15.29903	36.33711	14.70509	19.48596	42.73504
28.94925	11.88403	24.6887	19.59302	19.76318	10.01271
13.5902	12.74133	17.95509	46.03988	5.97946	17.74569
30.88464	50.56715	22.27081	20.8884	17.26088	16.03147
29.58171	29.61229	15.79323	27.60923	15.71374
19.45897	34.6833	6.996551	23.72216	26.03237	13.36321
35.6651	10.40441	35.23494	21.88247	11.01598	20.46307
28.13823	16.38785	23.73876	16.73993	13.06855
31.63592	17.98958	30.29567	16.11298	13.04208
27.28025	27.77822	22.85521	26.6174	28.8713	24.10686
19.79011	25.95014	22.798	16.34654	13.02281	12.46693
17.00222	13.1965	10.51983	42.82741	26.52727	32.69572
16.184	14.55116	18.62959	21.56823	15.59681	21.84268
52.39957	21.05055	31.46255	18.38947	14.95233
37.37498	70.60142	15.98542	11.04027	18.61055
14.34745	24.60105	18.27061	24.23248	17.43764
9.228153	28.27802	26.53817	45.79806	21.83009
17.84609	25.97805	30.60974	9.829128
PIK3C2B	6.859964	2.342007	9.234406	10.2608	7.006089
4.818575	6.738559	4.993977	12.86597	5.137535
10.3846	5.891232	6.284631	5.443559	10.78369	5.306293
8.672152	6.684969	3.725369	5.092574	11.09631
2.244012	4.786708	4.431369	2.791566	6.437741
8.877501	4.644542	9.501749	2.328757	15.90121
3.761427	16.33765	8.267991	4.932709	1.897934
2.498753	8.523131	8.801876	12.90266	11.67027
12.33411	6.3883	11.89869	15.00996	15.54867	15.58003
8.897986	3.50114	20.76004	5.813241	8.469971	3.864348
5.740987	3.830531	6.725545	7.416529	10.12996
8.003585	9.971634	6.398849	12.82595	3.842652
4.520261	9.72275	4.842967	8.13257	5.479562	8.038193
7.859262	9.894899	8.114015	10.3311	5.748976	16.41312
6.171133	9.644549	12.26856	12.71436	9.299133

11.09058	11.23253	10.7865	10.52602	6.406527	14.30921
11.25545	12.6556	6.714437	5.183974	15.47891	13.10349
7.823182	12.2384	10.73821	10.1315	7.720623	12.65302
4.89169	10.21649	11.94519	6.972173	6.727823	4.548438
10.37651	13.02002	18.7058	7.997855	10.55081	4.913252
8.399427	5.604798	5.191982	9.166827	6.804848	
12.99922	6.808726	10.636	10.38973	5.54603	5.652096
5.583633	10.1615	3.819332	6.488781	15.20285	4.812087
6.853552	10.80003	8.696135	18.07135	11.21514	
7.138642	6.035327	7.676516	14.09007	17.21315	
5.719536	7.485108	7.962348	8.026767	13.79005	
10.33335	5.199684	7.291975	6.036977	4.086936	
9.283607	18.45121	6.027124	7.512247	7.931004	
11.6311	14.73202	8.127022	4.661775	8.639399	6.187531
6.754741	2.881198	8.062522	8.84581	10.72572	14.33723
3.547722	11.27841	12.50394	6.411476	11.25301	
5.815866	4.601773	7.707278	13.62219	12.03277	
10.27286	7.557463	10.20718	4.524748	12.87366	
6.900501	3.601074	11.82724	5.986919	12.80091	
5.045186	13.32133	10.64618	8.275163	15.02128	
16.5772	6.464026	11.43461	6.449276	10.35059	5.08994
13.92069	3.316735	13.43738	13.37318	5.078901	
3.008676	6.684679	14.03152	10.89779	6.756926	
13.97488	4.63976	3.720444	7.765444	12.52311	4.85216
9.98633	12.81949	12.00859	9.694985	8.938781	13.56287
7.094801	6.856894	8.969685	10.20069	7.186423	
10.91659	7.158453	13.8922	3.963463	9.434998	7.450307
9.437247	5.649478	6.308226	6.871692	20.76745	
13.01883	12.55572	11.32849	7.500624	3.422504	
7.590933	16.47002	8.46097	16.95549	9.954737	6.460049
5.089	10.40465	9.6308	4.439156	9.785466	7.251829
12.07802	12.56882	9.254707	11.02628	20.11084	
8.645677	17.24805	10.51028	8.445891	6.9005	8.967453
5.012701	4.121359	8.322018	14.03526	10.59281	
7.023101	7.412556	6.50604	22.12424	12.08873	7.923282
7.422638	10.34606	5.673139	23.10941	12.5345	5.882271
29.29688	5.686158	15.17464	10.24871	4.396217	
9.568344	8.798822	12.92265	13.05406	6.685353	
8.441355	12.27912	5.600097	5.231703	5.035465	
10.32411	5.150731	9.495847	5.697816	14.25377	
8.169173	5.708108	11.45373	8.305756	7.252736	
8.814408	7.682914	10.56797	10.88855	6.658023	
10.81533	7.798216	9.580779	3.539181	7.235647	
6.208041	13.65601	6.228074	8.870937	5.826576	

13.26653 7.153975 8.599754 7.416027 12.75714
9.483429 3.927946 8.182479 7.309698 3.359199
12.42543 4.526177 12.13222 10.98201 11.89074
15.59866 8.351854 8.475118 6.940939 8.503065
14.45325 9.41472 11.4446 4.826038 4.115854 9.222159
8.960706 10.20903 17.12791 4.163005 5.166446
4.501735 7.759878 9.135235 10.85707 3.258473
8.960306 11.83441 13.87865 10.3641 6.461097 8.919494
10.84634 12.02407 4.972599 4.454972 16.97147
4.341394 10.90168 17.72783 13.25232 6.279217
9.537887 6.257521 6.815672 7.603658 11.14123
6.188529 9.420298 4.537595 6.840289 10.28482
5.404763 10.50476 5.491344 4.641986 7.447197
9.602145 6.045218 4.012755 12.71297 13.26771
7.758803 2.297453 4.910198 6.157187 14.062 7.240932
12.45297 12.16049 7.768923 10.60903 6.586998
15.91414 11.43243 10.48249 10.89947 13.07188
7.144421
FOXC2 0.1758913 0.4148737 0.415265 0.1120146 0.3383761
2.161322 0.2536447 0.4918738 0.2715457 0.470128
0.5988929 0.04607509 0.633783 0.3023429 0.2294032
1.320463 0.1286488 0.7990179 0.513989 0.1252223
0.2192374 0.4671273 1.461949 0.7996582 0.7991079
0.3087679 0.1020872 0.5655966 0.2605904 0.1995569
0.08284311 0.4046756 0.5036916 0.4247138 0.9212242
0.2313469 0.1525199 3.143706 2.961537 0.1867931
1.728165 2.393019 0.3112238 0.290225 0.1328229
0.1941386 0.04093292 0.6932159 0.4835621 0.131119
3.45179 4.010456 0.6038366 1.137233 1.326055 0.4829019
0.6284206 1.430454 0.7877777 0.1828155 1.025096
0.9594915 0.6540063 1.680899 0.09682053 1.942757
2.81599 2.471022 0.9130573 0.1515405 0.1251866 1.27908
0.8873901 1.924373 0.9991599 0.4877608 0.1502197
1.317222 2.445065 0.1091247 0.2446656 0.947856
0.1004831 0.645076 0.8954826 2.815699 4.204794
0.362259 0.562389 0.6679322 0.2203488 0.07855957
0.5058598 0.4751364 1.241728 0.5510536 2.278955
0.09878086 0.2263303 0.7844562 1.358028 0.210015
0.135091 0.4641667 0.9800723 1.220665 0.355235
2.561811 0.5184694 0.2579417 0.4438312 0.2542143
0.3601239 0.7820774 1.142049 0.1489586 0.7675343
0.6781642 1.217088 0.2698864 0.1577521 0.4415308
3.846879 5.0942 4.260681 0.272971 0.3753854 0.1965764
1.721561 2.045813 0.3758024 0.1335004 0.8351935

0.1890515	0.05242845	1.165777	0.5967153	3.112216	
0.3806974	0.2230995	0.491258	0.2839837	1.865459	
0.117288	0.835611	0.9577843	0.2663843	1.438619	
0.5425442	1.145434	0.7965201	1.06719	0.2072008	0.367095
1.44391	0.276147	0.7491545	1.221845	0.7576559	1.255428
2.341861	0.7190077	0.7614642	0.4160275	1.17733	0.6841747
2.562667	3.687858	0.9980981	1.602422	0.8808901	
0.3712334	0.1939182	0.4892267	0.4627215	4.013321	
0.9812038	0.2532858	0.04512277	1.702904	0.6840297	
0.9592766	0.4202216	0.7647254	1.527984	0.6737128	
2.740497	0.3173112	0.5947431	0.1723462	1.478249	
1.105936	0.2852409	0.2151205	0.9980956	1.113425	
0.779096	0.2096709	0.3763786	2.129424	0.320258	
1.825618	0.9599019	0.06700747	0.4548231	0.06364193	
1.278723	0.6010193	0.8564051	0.6547366	0.4274654	1.4956
0.9686993	1.092835	12.67105	0.5437498	0.8094041	
0.1579208	5.884337	0.4437311	0.4103423	1.038407	
0.2953968	1.230609	1.227013	0.6556991	0.2724157	
0.08192171	0.1001464	9.269382	0.8202259	0.8863282	
0.03823601	0.2266637	0.0843121	1.386976	1.165479	
1.914282	1.343929	0.6944255	2.178029	2.144406	
2.138168	0.4940353	0.5749217	0.8671292	0.9078595	
0.6630786	1.101933	0.1505004	0.4901544	0.2977305	
0.6223784	3.454448	0.1005176	0.8811253	0.6694728	
1.02709	0.2532108	2.768136	0.1665242	0.4105952	1.557481
0.3391713	0.5397783	2.265419	0.1116996	0.4209729	
5.325983	0.6122696	1.256108	0.5656846	0.253675	
0.06430266	0.7958199	1.340134	4.112469	1.210733	
0.2514396	0.6361722	2.1212	0.3758062	0.9887639	0.7185804
0.2010582	1.023493	0.04622314	1.369928	0.6909861	
4.126308	0.4494373	0.540786	1.293996	5.741579	
35.30827	3.985147	1.725624	4.324659	0.3802137	
0.05503879	1.134934	1.332258	0.8349507	1.499145	
0.01484369	0.3186353	0.1205635	0.47157	0.5713069	0.855117
0.4615979	0.870304	0.5680352	3.846962	0.1207556	
0.9964436	0.2952847	0.9791373	0.2692305	0.3508371	
1.155933	0.748966	0.4172792	0.8834507	1.760541	
0.4209275	1.635427	1.293104	0.1756599	0.3586771	
0.5172716	2.009879	1.51736	0.9826397	2.372974	0.6673875
0.02310523	0.628944	0.5747724	0.2671804	3.56851	0.380149
2.156725	0.4322108	0.9212398	1.843887	0.6088011	
0.2891956	3.616	0.56676	0.3876117	1.070438	0.4199628
2.046873	0.05889357	0.4157508	0.5998962	0.4478106	
1.119965	1.315499	0.5474545	4.494857	0.5283392	

0.08552086 1.321781 0.5008138 0.02564051 0.2450567
0.5014868 0.6159601 0.3068087 2.66601 5.832047 0.7493189
1.631024 0.75041 0.2512206 0.4076664 0.2476828 0.8556852
2.865547 0.881247 4.168162 4.318312 0.3832294
0.2709035 5.208943 0.5029498 0.5371466 0.3346622
0.6917871 0.8349853 0.06156435 0.5682683 0.3355003
0.7438719 1.453811 0.5607318 0.1017737 0.657224
2.219295 0.6275949 0.5910318 0.107148 0.1444898
0.6554014 0.9307915

ARHGDIB 15.55848 15.20791 22.18217 44.62681 53.76136
13.39065 54.99758 22.0512 73.30727 206.8119 13.87124
10.72758 191.239 14.04963 121.8775 10.27193 60.80231
34.27948 28.61526 33.84967 43.75141 11.53687
16.17941 13.16471 16.00065 11.72947 92.23056
12.8599 56.35193 36.08782 38.73269 11.51146 55.20918
32.6534 33.01922 70.36424 13.22626 88.2746 26.88773
27.32392 26.43459 11.61333 62.93323 17.64907
23.69369 15.92396 72.68795 46.36589 53.64197
31.4722 19.18924 134.2184 13.61412 34.19198 107.4738
18.97324 61.71222 70.47868 11.80272 55.63151
96.90459 68.63087 74.69684 66.97719 30.78914
25.39907 86.14261 116.0323 28.09011 24.76926
32.30223 54.31868 19.65756 43.4063 39.51031 183.4782
42.13145 19.15172 27.08754 30.59712 56.90498
47.9956 125.6905 44.92432 22.51113 39.18321 25.62387
70.09744 48.46913 56.81047 55.18323 78.18004
83.14606 143.1126 97.76734 104.2083 126.4314
26.32509 18.03326 45.40518 103.8972 11.12307
67.95439 57.16535 49.25363 84.20837 45.41753
69.70232 38.61025 32.02993 40.74019 22.08253
88.72926 180.2877 50.79356 28.32162 50.60854
11.00643 26.70971 23.47175 37.30852 55.30053
33.15262 87.93858 16.02519 254.7042 56.35346
49.72679 38.36221 42.50331 26.87519 43.64165
23.53172 46.76059 80.62686 32.4372 27.92778 27.57272
48.02873 22.37372 68.01751 38.27431 38.18128
38.91156 57.33051 21.91703 23.36416 43.55118
4.166492 51.02885 28.994 53.77827 16.72897 88.76388
46.95424 14.15029 28.00794 66.16041 9.533761
66.4148 129.131 88.74065 66.61157 36.40614 44.65212
67.60273 74.87623 71.63415 73.8268 15.67408 35.18786
53.8216 48.03671 20.36871 14.92207 40.18246 34.27365
22.97174 23.69802 27.68131 32.13035 20.32074
39.66214 93.81576 61.2444 57.44892 40.3031 17.71226

18.93619 32.9502 40.31861 22.55001 61.78851 56.47898
86.71423 29.9537 134.6337 37.52273 14.31826 17.05571
13.93194 30.05689 29.67248 11.92803 16.71314 25.541
103.3733 31.00805 18.5484 18.49207 56.80255 56.69928
42.86907 15.19301 18.10157 32.59497 28.15964
40.71085 70.11494 15.19869 26.36078 57.95059
26.36441 23.43954 164.515 16.75369 30.80848 19.89458
16.65493 31.31803 14.94867 41.69844 21.73544
25.13943 36.42776 41.53475 182.328 41.35922 62.85017
12.97831 14.52574 78.26385 68.08471 37.51386
59.95311 80.33868 107.1176 51.70902 51.19643
48.09107 73.91899 38.66325 17.66047 69.21776
42.17043 40.86549 18.98718 73.19329 49.40897
33.00664 53.37777 33.68928 77.56549 17.34312
31.00081 40.40674 16.07695 60.6283 55.52098 51.41151
57.7385 46.69988 26.31654 29.94927 26.09086 37.25418
34.79608 32.53481 866.7588 58.96734 82.82425
33.20287 63.08682 70.9541 23.28373 82.04697 16.37467
13.81444 55.53179 32.90928 36.87627 22.08399
30.30433 78.49592 108.8158 71.75871 104.0209
93.36932 16.9037 22.11954 49.28964 59.64351 31.10284
46.49523 41.85733 33.47747 24.03485 65.28263
58.41861 17.04442 65.58563 99.3724 52.14687 209.8627
36.91469 22.80846 69.55277 20.89302 55.46562
55.74556 67.32492 58.52978 45.16611 75.22909
85.21474 31.93187 28.15982 19.09143 31.82466
38.69267 16.78702 32.90373 70.39058 66.50562
54.99009 23.64271 6.591652 34.50536 70.30993
31.07115 33.8481 141.0362 19.78305 13.17813 36.75948
23.31193 12.75441 9.306218 30.86648 66.23968
43.80341 33.8732 15.90754 60.60532 79.73839 22.52588
76.1723 88.77915 40.06487 36.87724 70.63748 107.403
21.7166 47.13269 29.14985 44.89015 5.56177 82.28464
41.49001 26.60607 21.53161 30.59482 17.1093 29.73277
54.32639 26.96009 45.24782 35.47774 25.38505
20.34192 63.03536 28.85783 21.97997 9.658685
27.59538 66.00813 65.5198 43.11931 19.98419 12.45938
48.52131 20.94047 20.47876 65.2417 38.56563 130.229
26.19676 33.92084 14.99734 35.75086 35.64568
175.028 27.21439 23.91551 20.91199 67.03331 24.13487

ENDOG 19.38802 3.08199 13.75167 14.49439 12.2763 2.309987
7.89112 6.89362 6.411213 10.74771 3.894547 6.60008
8.327324 9.670169 7.881819 2.029638 14.07015
5.021505 14.20421 15.27676 10.27033 2.135822

9.478166 2.580611 4.601774 4.452944 10.7727 2.440732
11.40279 11.78591 7.882112 3.200239 8.017208
5.739309 9.234276 7.880666 3.779444 9.430282
7.182232 18.79271 7.858428 18.82886 5.173932
9.96157 5.833649 3.949671 3.894565 13.93738 2.8779
5.594092 9.416158 2.795293 56.9866 6.188302 2.223643
15.57431 7.062086 2.287406 18.4959 14.55381 1.914064
16.53379 1.938372 11.57583 3.292435 2.469623
8.964746 3.833855 6.127808 13.08474 8.586425
5.130278 6.671074 1.548906 5.160816 9.886927
7.093394 19.86869 6.858513 2.64728 13.44993 3.805052
2.19094 3.900517 8.944445 11.28252 5.625022 12.77582
7.813944 2.922341 3.798803 5.149138 6.819748
5.661324 16.00941 11.00524 5.022747 9.150494
5.326335 8.896142 4.190582 4.841141 14.7955 5.338241
7.13758 2.300361 10.67615 3.348678 10.40311 8.164099
7.965203 3.583294 9.354618 3.334478 7.990373
7.09618 15.72891 12.66909 16.10851 13.42278 8.58226
1.587024 3.746934 12.45091 7.626248 6.204385
15.61302 13.00514 3.972949 8.260964 4.945987
9.08892 8.249175 3.197744 13.607 5.893502 15.77659
4.09765 10.68227 4.7496 8.672586 5.077605 4.137074
3.51096 7.078745 9.193045 5.664439 4.620873 8.739961
9.481471 14.7331 7.868157 24.29725 4.385929 4.928136
4.51524 11.99603 4.638356 6.303277 3.193569 3.60617
3.793709 1.812823 11.51402 10.5374 9.880004 3.658365
2.386712 3.005086 8.485403 2.466273 8.990806
6.758519 13.71196 13.81273 3.682944 3.945324
5.337457 5.795831 6.957157 2.543436 6.685652
5.525291 6.594582 5.554485 4.763497 4.625804
7.430239 6.123854 10.38533 4.805211 2.521205
6.99841 13.0197 3.964821 3.395622 4.347841 4.950344
11.54527 13.49246 5.846229 5.536005 9.965473
12.29547 9.405389 8.333129 12.9805 8.367 6.737588
5.781623 1.363635 9.381188 4.153841 7.109597
7.982525 9.934874 6.553546 5.116488 5.454501
7.454159 8.386808 4.025156 7.000363 5.479125
3.286133 12.11564 4.582224 10.10391 12.67433
6.937548 8.20963 3.156959 9.297028 4.499015 1.604376
8.433194 14.05315 8.78871 1.959665 11.07514 1.81975
5.391103 9.090922 11.55156 2.702719 7.809812
8.276634 4.82625 6.754774 10.33292 4.022822 2.629919
5.102061 7.551066 3.84321 4.195522 5.149099 7.285822
13.41922 5.804891 7.030401 19.30734 3.467186

3.316687 8.866805 5.581876 11.15368 13.08144
4.981975 3.6401 12.748 19.55789 7.88959 7.160419
3.779444 7.176527 4.44758 2.559893 5.236531 2.453664
5.214721 8.687991 9.926688 13.02274 6.846894
4.905083 12.74414 5.563752 7.839116 7.110634
7.422037 1.47743 1.68444 8.240994 4.976899 6.786974
7.891319 5.714859 10.44064 8.050716 6.399011
6.221661 5.401806 4.023937 7.678745 11.81569
2.347869 1.976923 4.592325 6.865414 7.401929
12.00752 23.66709 11.58055 11.90419 5.897017
4.533183 5.089677 4.705745 1.397032 1.882563
3.433523 14.02341 15.42945 2.54957 5.028395 11.59865
20.61932 9.639618 4.606774 8.27178 9.006624 6.99193
5.506354 6.83804 7.233269 6.97365 6.380795 4.650234
3.671907 4.579049 18.03441 5.584766 7.36609 2.059112
4.743489 3.948896 5.741956 10.4241 6.895597 5.85061
7.4764 9.302247 5.214008 10.45974 7.542131 4.277904
6.770666 3.946636 9.188227 7.955788 7.85441 10.00412
14.46558 11.93532 9.910714 6.668151 8.142073
9.534107 5.380744 3.362629 7.556082 21.91505
2.82474 10.56184 14.55683 9.664881 8.126893 5.365092
4.03016 6.182601 9.257103 5.456301 8.895928 5.841519
12.78017 5.234787 3.006867 12.41951 5.592227
2.066148 2.187936 3.536221 3.379245 3.317622
2.756282 4.627409 5.905918 6.479421 9.546674
10.97476 9.60252 10.53004 7.702585 14.06474 12.80555
7.576818
TAGLN 19.13163 2053.929 28.72045 27.29307 106.4832
1054.478 37.77745 237.9715 309.9384 280.5048
147.5451 51.89863 102.9406 103.046 32.88328 1167.894
42.69841 640.6307 50.89035 65.934 288.3219 1070.563
119.3175 893.4424 1553.386 43.73461 37.54628
1048.761 108.1316 98.96773 400.2878 1075.739
10.74813 60.2089 124.82 32.42605 6.534183 76.04386
136.6915 8.416357 23.5101 7.631566 42.54724 10.87795
26.60994 7.783888 13.53891 142.2089 167.3352
13.28375 74.03156 28.17781 22.47851 67.12583
666.519 9.86709 62.59599 758.6017 41.45765 22.27094
49.73686 55.93001 449.4568 93.35627 7.94225 793.812
252.2705 212.6563 18.52126 16.57232 19.10218
23.4743 244.717 106.2882 71.7414 51.7862 97.78898 53.7968
20.48196 7.375873 51.12968 74.51328 14.31081
427.0821 192.3504 37.22746 166.8438 81.14163
12.52863 538.6659 31.96517 14.02752 210.7288

20.11096 83.39218 37.36584 103.6822 25.22701
2.53371 167.4454 76.00237 7.142098 4.050908 18.80214
235.6421 151.0428 50.00271 901.8918 35.80424
20.77342 21.191 25.0973 57.87384 561.2456 66.96834
8.507675 31.81475 23.01454 152.3294 11.49761
18.1249 53.21128 102.4316 100.7516 32.26663 26.2595
54.94924 92.23064 642.0641 189.2469 75.77049
18.96459 34.2985 27.32838 29.60213 218.381 104.3342
13.73019 34.54209 12.69762 14.85189 6.744903
13.09984 12.48114 47.9373 66.25827 7.342739 491.0276
5.220336 80.00671 23.34737 133.3825 21.15107
146.7094 767.4414 16.61961 232.3576 846.1056
20.13117 104.7002 302.5944 59.93956 81.47564
8.921475 138.9735 48.57384 36.70804 49.92287
52.26169 140.4343 1223.191 24.87726 90.77374
7.424178 6.705831 74.04934 175.4925 217.8454
6.194456 43.25092 30.5116 45.20015 30.98739 21.80147
99.18447 42.65756 389.2011 51.19362 56.58136
5.605468 169.2263 13.96555 74.41351 24.16183
28.0951 98.06177 310.0008 16.30409 9.271065 41.17629
17.3212 203.9069 20.03594 9.522776 7.796873 8.834546
274.2177 120.2353 17.05363 42.87882 33.92662
269.158 20.42292 132.8755 26.76049 64.51125 165.714
10.72803 255.5221 32.15163 27.39924 76.51994
49.79856 28.07441 34.16433 15.33004 11.76108
13.86343 13.4811 44.99981 217.0036 76.03297 2.873669
9.101103 7.107303 75.64141 80.08397 22.05641
79.69735 18.40188 370.8608 79.17766 160.0274
71.3051 117.3819 113.8068 50.14019 978.4757 186.4047
10.54722 115.1882 7.370856 45.40368 115.7376
9.742537 25.09766 62.25534 125.8583 26.51807
235.8316 42.62845 56.86527 36.81154 166.2718
22.62513 50.33006 14.03969 45.10472 134.9561
18.35446 120.6276 91.03944 17.22848 11.22418
5.344391 25.97096 37.46882 1307.24 20.05841 51.59003
356.8219 5.410662 983.6899 47.87586 35.53608
126.4619 10.8985 53.17759 45.34149 202.6979 23.82055
1242.461 877.8503 40.54305 45.34461 341.1799
125.1338 65.31117 25.39432 19.97029 73.35001
112.6968 58.41966 99.98551 7.81291 32.27097 41.23489
55.94677 25.79787 13.26 53.32677 55.60836 25.46484
110.1378 14.26644 764.0171 38.17727 98.75154
50.33976 144.8069 65.67637 52.3589 53.09861 41.95539
395.074 24.2324 53.69038 23.43136 22.23049 30.81896

14.53397	232.3025	66.61571	46.1428	1404.391	95.29114
2.70049	27.15328	22.54423	23.73815	13.20788	66.7467
64.36454	10.36542	715.974	48.4618	18.00059	10.61985
63.69801	61.61359	15.79584	83.07347	48.87243
59.58341	10.18997	12.36697	74.23572	38.90257
993.9191	52.11133	36.38464	183.37	22.23078	7.563572
11.48959	44.04626	3.24012	17.33673	40.77496	17.83608
50.82132	122.6418	16.01022	30.46346	59.14122
90.57657	12.31699	17.14119	20.63203	72.93763
79.15428	40.52646	109.3823	14.06549	13.08337
34.36187	716.2384	31.34618	15.39026	16.7051	411.5223
31.81144	7.094895	28.60736	11.06621	51.71869
24.11858	374.1032	115.7067	44.20743	45.18876
18.43431	41.14579	10.12251	11.15531	65.05111
962.1326
FBLIM1	49.38374	28.36566	36.96915	28.80784	26.12824
17.3874	20.03906	9.492601	39.05694	13.17346	9.583362
4.751201	12.38352	6.109068	24.4292	20.77613	55.49786
21.22397	50.30587	41.09711	35.97034	13.71758
17.60138	17.78011	18.21974	7.504235	27.16198
17.69012	40.75633	15.81232	48.8053	23.89243	54.78213
22.22906	40.49492	1.978729	9.947087	27.66357
20.17959	42.55602	23.10692	19.47477	17.05854
58.63622	35.27627	24.51621	21.98477	14.96393
6.957549	12.87122	44.65324	13.24532	21.4728	13.2064
15.78543	16.73917	21.48566	23.66989	43.5156	19.96368
26.24607	21.84274	19.33307	19.58587	19.19218
21.54979	31.8755	29.87847	53.26592	21.65834	9.224806
16.25735	21.63455	13.52129	29.37145	18.50314
25.8802	31.17334	24.64338	9.104843	38.94783	31.03031
14.24566	23.84386	16.82865	32.46538	22.64545
14.03621	45.3072	21.8743	10.67265	27.90788	12.74269
29.82375	11.66273	16.49168	26.85788	31.67113
10.91259	20.76557	20.94036	26.73465	6.033547
21.32596	25.44991	35.26729	12.17712	34.0473	34.19235
25.53383	28.45709	32.13486	9.4018	14.91946	17.36971
27.17423	32.33272	33.28101	19.06478	26.97623
12.23586	7.715666	24.68173	18.23947	25.19784
13.98157	17.63929	19.57848	24.72346	50.31795
32.26866	21.66145	31.63471	15.29157	32.3348	24.6081
31.54459	17.33152	30.4834	9.701508	48.41286	21.81826
39.73511	13.34742	40.48148	17.34265	19.13595
26.2674	17.79154	23.43053	24.02406	50.98174	8.760064
21.9954	25.60428	31.89302	20.38668	17.05346	29.86666

19.75884 31.39915 17.30729 22.00593 10.85712
31.5156 24.36016 22.37028 27.13451 23.06723 27.30763
19.3282 19.65524 16.64807 20.80634 47.88477 36.52291
30.51095 13.08697 57.75349 16.47684 35.27895
33.74317 16.24286 34.6083 21.11131 29.40477 20.84613
19.29062 52.10449 3.798129 35.92286 54.47817
8.496236 40.58393 17.89469 36.66096 15.00901
23.10828 17.45053 17.99947 15.2659 34.36936 23.54258
27.98594 7.058791 22.4337 33.01214 11.44696 21.56309
24.46648 20.95963 21.31307 28.58398 18.93244
77.06301 23.85146 40.04721 36.14702 25.82283
27.98516 33.3423 38.41792 26.91038 10.16933 35.2129
15.56644 30.09429 10.25197 18.11681 20.68292
37.62419 32.57869 22.28085 41.68964 19.51476
83.89161 16.44148 20.60034 16.58957 12.33366
18.71243 54.30096 17.68987 14.90344 15.5737 23.53294
18.74781 26.1987 28.25457 26.57078 12.94116 23.34101
26.31123 35.81099 16.96729 9.346237 23.69104
36.67586 17.08226 31.544 30.24938 13.03965 21.17928
12.48785 45.74709 28.96684 21.98818 41.49142
55.30005 19.15314 42.64547 17.72952 34.25182
17.25416 25.1818 16.44491 18.86016 26.3445 5.925482
22.12818 26.26504 18.2493 26.99141 21.43375 16.4694
29.59636 18.89828 26.71109 31.06191 29.72996
55.62065 23.84016 19.44633 24.6891 23.09393 41.11714
11.60426 37.96523 19.15405 19.95734 24.46163
21.35768 19.42457 40.58147 21.30576 9.584461
18.75479 32.26749 66.57469 10.43783 37.15973
11.72308 28.10606 26.34183 19.6855 22.89108 28.38679
48.49172 27.95995 22.29047 21.07772 16.97742
14.04978 12.21559 17.02188 24.00848 44.09033
6.337539 30.52602 20.70867 28.78871 28.40826
34.83729 54.11576 30.12091 36.87398 13.50027
22.85559 32.4385 11.69975 13.02195 20.4866 21.51248
19.2728 21.77353 12.09127 60.50832 10.36866 21.31689
10.62158 25.71318 14.37108 26.37764 22.42441
21.7649 17.55936 36.04887 10.63289 20.77196 19.88576
27.13703 28.21353 17.40019 37.66131 15.03778
18.2579 21.30031 18.61558 31.42176 13.10228 24.08298
20.89997 20.18241 9.504365 16.17861 33.24365
15.20326 20.63267 34.91306 33.16614 36.12811
54.09811 16.80088 28.48561 4.994347 23.9273 43.01506
24.31308 26.66724 19.14176 30.34304 6.487339
13.42471 17.25873 19.55702 14.12915 47.64021

25.5022 29.01993 56.10577 15.57407 26.49481 22.75728
51.85735 15.07917 29.76999 27.89468
RACK1 118.424 151.5622 139.088 159.6342 226.7814 107.2939
186.4758 117.1568 170.1735 322.8059 129.697 157.7804
261.62 202.4231 201.2469 97.5005 219.2545 173.6148
184.3698 209.8028 173.2694 119.4355 209.9711
129.3294 119.1642 137.4189 221.2364 101.8797
206.4906 259.7938 191.2957 117.9023 67.04424
211.9702 145.2981 310.2728 591.3045 115.4259
149.0545 207.5417 99.15893 176.755 188.9722 277.8976
102.3696 90.89122 83.95639 169.6281 276.2665
71.71251 217.0065 120.406 154.6137 135.8393 172.5836
254.4581 258.4041 119.2877 290.5649 263.0733
140.8303 167.2802 163.7151 142.9795 140.7084
90.42345 205.8644 287.415 93.90174 182.0234 211.0625
198.7974 99.88107 119.6491 121.1469 220.2752
91.72875 186.2326 121.1393 131.3565 283.4838
140.5213 186.0177 118.1073 323.9996 188.6852
142.4326 175.5822 203.0658 110.7318 213.0782
140.4456 125.4059 164.7184 305.6482 144.4754
223.7444 150.5707 137.6144 121.7938 198.7503
86.36396 191.3694 224.9204 199.3741 129.5776
131.6945 197.6622 176.8539 254.747 137.5784 102.4899
217.6888 161.1188 181.3191 134.709 97.81 104.4681
153.6543 182.5337 249.8595 243.797 161.258 146.6366
85.33683 215.332 375.1879 339.7196 171.6989 134.0167
111.184 282.0894 117.4885 161.0462 174.6786 121.1888
191.0057 240.5885 155.1655 102.6563 193.546 155.4862
210.4703 155.9791 136.1237 213.2045 216.9719
100.2098 148.202 229.8596 247.4747 166.5497 156.1936
118.8295 120.1378 78.66506 158.7319 114.1315
136.0168 207.0369 253.8923 133.7917 121.9658
141.0918 402.8764 196.9314 118.1369 187.1989
175.4407 301.4188 74.7291 206.0479 216.9848 123.3999
147.9423 165.485 98.82239 175.9528 157.2609 72.5804
107.1088 119.7142 168.0638 140.0453 124.3551
66.5033 140.9415 229.4176 59.81148 256.888 122.5506
56.33049 350.1537 169.8062 188.4959 101.7062
177.8497 144.5668 170.2066 169.6069 127.1823
118.8527 157.9179 278.4721 247.5026 92.92842
199.8107 122.9907 156.9507 141.9658 112.987 164.1551
76.44467 129.9678 223.371 177.7181 92.72007 79.54335
169.2822 94.29805 135.9189 203.0958 243.8959
349.4441 157.6296 314.9889 73.50753 195.3654

349.5555 271.3485 195.2835 127.1734 204.7935
107.537 224.2838 190.4024 339.6965 176.8528 172.2765
107.491 143.1889 113.1331 157.8819 189.091 228.5659
109.5104 263.7525 155.3291 144.0971 202.3982
119.2494 221.6449 99.53303 148.3505 114.4574
209.4108 125.4561 138.4885 179.8113 140.7948
109.4518 299.9165 153.5015 212.2238 139.165 144.6061
112.0189 272.1548 121.2119 224.7706 174.4129
284.8524 119.2182 335.7376 57.49572 95.43655
108.2676 104.7706 294.7868 133.2778 188.077 127.0371
185.3096 284.8218 185.2039 155.1745 89.667 99.22166
125.2722 213.1375 164.2444 67.94979 81.44938 120.19
206.4371 207.0346 121.0421 148.6779 189.8436
67.9219 113.0135 137.5526 115.7166 83.74854 168.6516
95.64574 155.1217 118.5671 97.92293 316.8273
218.8135 275.6957 153.057 306.8816 182.9707 163.8487
104.9428 113.512 84.64963 141.589 137.9858 173.5042
161.9281 314.6283 317.3567 228.6022 195.9579
121.2634 84.75148 163.3563 345.8936 157.531 162.9593
181.5518 181.0476 199.2427 342.6851 89.11751
77.61973 252.6271 134.3154 199.7871 197.5152
71.59086 87.52036 103.3908 72.46583 149.367 248.8665
173.3132 137.7369 229.6043 219.2587 113.3487
260.5692 176.1711 95.68111 87.65685 110.1042
135.778 111.3593 273.1492 75.9598 149.512 391.1542
248.1637 176.4005 114.2668 97.40919 102.6149
196.1176 140.4232 131.6651 163.009 336.2887 176.6716
139.2332 184.9399 187.9102 148.2233 149.2164
120.4368 172.3601 68.66207 80.19072 275.4084
215.6548 211.1143 241.9386 60.44072 77.92439
313.247 103.555 203.7349 121.0082 146.9192 140.9943
105.0303 104.8732 155.0248 146.6701 200.2832
166.845 153.6874 287.0826 306.9875 131.8999
CCDC80 2.245261 36.21488 14.51727 1.85388 6.874184
42.73488 1.61088 28.81407 7.775141 11.63106 11.01606
1.539529 7.21602 3.102476 4.305535 37.9782 2.849105
51.78281 8.027302 5.65241 8.981508 37.54638 14.11517
29.75998 59.21926 10.97248 1.560611 28.15996
7.163396 4.925726 7.494615 29.81456 2.218798
11.51397 9.915893 15.98895 1.952323 4.060077
11.61587 1.252216 6.927708 0.4134948 5.986948
0.5409192 3.828591 1.183478 1.61668 11.39052 14.68364
1.131018 11.67509 57.79241 4.790077 6.434472
32.31056 0.7255563 9.620638 51.48081 2.192279

1.595782 43.18084 9.889317 104.7442 17.14677
0.5078282 105.4174 23.50025 65.55581 1.612598
3.042174 2.649538 1.102071 17.13102 10.81098
20.5941 6.989502 5.200373 9.279408 2.517056 0.4518666
4.456701 94.62981 2.984831 26.36739 14.12684
8.869046 17.13919 11.02129 3.579309 16.76862
5.935081 1.247316 13.92557 2.664759 4.866779
8.577792 13.75646 0.9328324 0.5596655 23.39367
59.98976 0.8089171 0.5916035 3.165215 33.44208
42.0502 3.426601 35.85919 6.463644 3.203729 8.682057
4.206261 4.291401 18.88985 9.715377 0.5233146
2.617921 1.468827 22.21302 0.3953664 0.6314903
3.71652 10.02263 44.3898 5.144534 6.173325 4.086937
6.738104 34.53794 17.33781 4.6317 4.146882 8.776337
3.86404 1.053575 31.33639 16.1896 2.479734 6.205621
0.4885824 3.124866 1.948688 1.705147 0.5313103
4.762945 9.756603 0.546855 54.03256 1.704463
41.12523 2.570154 14.93168 4.184951 21.00299
44.25513 2.453561 18.52181 47.40668 3.306759
11.29403 30.08441 48.6723 31.30303 1.022283 10.39721
9.136762 6.227141 4.769347 14.07793 4.877016
23.17985 3.277076 8.616777 0.4988011 0.3208806
26.99064 25.8348 13.07208 0.2908487 7.267036 2.28055
4.932662 4.62434 7.204303 11.79537 1.138515 66.05561
12.78462 14.00134 3.422056 14.48893 2.807616
11.95304 2.99072 2.715961 8.720554 40.49848 1.092004
1.279839 7.976568 1.045829 17.79595 0.5459346
1.209533 1.289174 0.5308704 11.1433 21.1457 7.024784
9.281209 11.20469 43.88425 7.112672 9.368444
2.40111 6.886211 6.084345 1.581464 43.96418 5.491012
6.36759 35.24049 3.591085 4.320406 33.62964 3.198664
0.993871 2.578099 0.6861991 2.85143 9.049005 19.06366
0.160923 0.6243935 1.44521 4.085727 41.53246 1.650531
19.09359 3.939261 6.850488 15.78587 12.87404
12.13438 35.45772 10.54874 14.42595 37.40823
34.22956 3.414312 14.47907 1.712127 5.709084
40.28506 0.7558927 12.75372 10.88281 17.88274
3.308081 30.36982 3.066331 4.648643 6.67758 8.462351
2.347057 11.49108 1.76916 6.518865 16.08598 2.044512
14.04782 6.20647 1.261243 1.126605 0.340428 8.414018
6.643309 25.87113 2.683704 15.27714 72.55479
2.82476 17.25941 20.80301 4.779986 21.67143 1.013877
4.454322 5.886073 53.99259 1.894227 23.46952
76.43414 5.134558 13.75368 62.06815 21.32722

9.076047 3.177867 0.8556087 6.674119 26.86065
17.64696 37.51039 0.5093459 3.754519 1.852573
10.34576 13.5161 1.045891 3.224863 4.225479 4.288521
63.39628 1.349247 39.87208 3.234674 13.38525
3.138437 29.78504 3.704681 12.3411 4.344683 4.928176
39.70754 1.627908 7.7778 1.682465 2.736786 8.888455
0.8329833 32.41024 13.74954 6.472108 36.52278
3.370411 0.1850672 8.403576 4.979049 2.844709
1.919134 4.591373 13.79828 1.200523 69.2723 11.2867
1.003537 2.049967 4.275293 9.617391 1.163487
20.91698 3.457632 7.833765 0.710841 0.4256173
7.271375 8.995764 22.97891 8.450099 4.584144
43.81008 3.872952 0.4750422 0.8791178 12.20087
0.1494735 2.814018 1.692591 8.278641 4.76239 12.09761
2.004104 6.130427 10.95194 12.5647 1.184448 1.479463
1.312843 26.74965 23.36784 4.775989 4.237381
1.022226 1.584841 1.786291 54.05863 16.33102
1.379663 2.303472 20.72521 4.458009 0.9823402
16.64599 2.067624 8.750421 15.81743 40.62271
2.872701 2.608436 2.476879 9.117919 5.165733
0.6407093 0.9007876 6.594285 20.449
ANGPTL2 3.306204 61.38278 7.487816 11.8409 10.20213
62.99726 5.437284 33.36953 21.12046 14.17279
8.507517 3.677116 7.580559 4.126157 6.894424
59.76806 7.85447 56.96515 11.74865 12.02426 18.38286
69.81636 12.94633 52.67354 33.42913 7.608308
6.971976 68.19602 16.44552 3.857005 20.99832
83.51723 5.091816 7.742083 42.33608 4.373165
1.788166 7.29106 21.31803 6.559389 29.8217 2.354694
30.01811 4.027546 26.25911 6.399495 4.677321
32.11995 33.80918 4.621918 17.16408 19.42449
3.623097 21.08151 59.32047 3.398437 40.06925
61.56564 8.282301 5.317871 65.22396 23.37473
59.28599 38.98579 3.691255 89.98368 36.1816 25.90169
13.2459 7.800391 16.63932 15.31183 26.47537 44.60561
64.79259 13.87755 34.64384 26.67241 4.294969
3.557126 9.899425 41.7383 10.39222 32.95928 99.87209
20.6142 20.73031 18.67373 15.85672 46.01895 20.04872
9.177067 75.16547 7.909299 15.57473 22.91067
34.03583 6.384615 3.969007 32.46696 17.77394
4.628136 4.243621 9.746588 46.61274 52.69777
19.55766 54.06252 24.50275 12.40637 15.42511
3.692561 13.41383 37.5515 22.42617 6.904973 10.35802
12.96342 16.17525 3.204834 11.22695 14.93489

24.37735 21.81987 19.09717 7.721864 7.534446
44.76044 63.41879 73.04055 20.3913 8.710559 7.366755
41.87496 16.26894 23.89855 28.90708 7.529949
11.94366 5.612668 9.70873 9.071066 9.398655 4.550893
20.8038 9.873287 1.711901 92.89903 4.434828 20.18035
15.81278 55.65731 28.30065 40.8185 52.61933 5.278676
34.47585 39.95753 21.68242 15.16128 61.55711
12.39451 15.90052 6.457203 19.92332 17.40809
7.818778 16.28126 10.52149 24.6198 63.7246 11.11323
11.57888 11.41603 2.672937 20.99935 86.67585
33.18159 4.168954 56.24152 7.841566 11.08144
7.685479 16.57063 31.80439 45.96501 55.17684
19.28339 12.65971 8.313705 24.48655 11.55837
37.38376 9.172325 6.46889 30.44114 76.3732 12.54712
5.618677 11.42617 6.239716 13.77227 23.81702
14.7728 10.18559 4.727144 24.20478 26.72104 15.16499
22.05829 25.01055 57.36476 15.52803 15.15867
19.15905 19.74211 14.38868 6.052255 30.46871
14.96184 19.53649 16.25384 10.92469 20.05531
9.297265 4.326344 12.19291 2.723049 3.299963
9.56504 30.25371 53.36039 2.611745 8.58303 3.885901
9.559203 8.168839 6.092391 23.83795 10.1159 32.85114
18.66882 20.58961 40.8209 57.23316 35.83121 22.54173
39.94323 33.78396 10.63647 19.4107 9.514934 21.91561
29.35267 4.107075 7.79165 29.8634 20.76404 21.47242
32.99652 13.18455 13.73002 17.3956 27.02194 28.63495
19.9985 11.1968 14.5254 13.34803 4.098265 33.05355
24.65741 6.272561 8.232055 2.951477 9.434014
17.26125 67.90613 14.17883 47.92571 53.97562
5.770648 81.08859 41.45239 18.08813 13.32563
13.45098 5.400633 41.33082 54.38358 9.561853
93.30348 94.8064 16.85616 9.731867 69.25032 28.62018
25.06078 11.43492 13.76327 24.05895 49.1821 15.79799
124.2541 8.874875 13.0157 7.657826 18.44818 7.854443
11.27142 79.55848 23.39415 18.34193 45.47609
9.093731 80.51914 21.42456 30.45611 25.62177
63.00299 21.22031 16.81892 8.032456 14.5742 31.47527
7.617088 31.68635 7.567517 13.84846 30.00047
2.504172 22.81734 26.50306 9.749036 89.37354
2.930275 1.497987 11.80702 8.251488 8.714915
8.656081 9.87063 21.82935 5.758298 71.04343 3.157292
5.059358 10.7395 11.84727 10.87822 5.906917 49.99373
8.486509 44.42395 3.089633 3.014662 36.41733
25.91181 86.90891 12.1772 14.82765 23.01173 9.815163

5.062338 9.93441 22.26595 1.915591 16.21238 8.876354
13.91922 13.28953 18.08946 10.88533 5.163148
11.42499 23.63349 7.227652 24.16674 8.731576
18.64676 16.86049 24.60265 11.5593 11.2702 8.396051
9.067671 55.80398 16.66446 7.478197 11.50124
62.31535 10.85902 3.707016 20.01906 9.590262
48.05517 15.03215 37.91885 8.45911 17.26751 20.94833
4.824504 28.52705 3.689572 6.841232 21.19747
72.99671
PRKD1 0.2856987 1.21062 0.3246311 1.006046 0.7935709
2.204063 0.8655313 1.460259 0.9297052 1.228257
1.062697 0.368694 0.9472654 0.459579 0.6118966
1.832307 0.4358054 2.073082 0.3631364 0.4367066
0.8542602 2.513363 0.5701396 1.293516 2.501583
0.8784464 0.6960868 1.971161 0.5683358 0.3050716
0.7282144 1.862967 0.2730745 1.367368 1.817404
0.5096293 0.9326564 0.352193 1.174325 0.2007837
1.296081 0.04691414 0.6588645 0.08823181 0.8277846
0.2967884 0.4367283 0.8761004 1.252036 0.1610739
0.6337461 0.7373013 0.7482257 1.006567 3.030969
0.1446167 0.8965389 1.854875 0.2724305 0.2354301
2.26099 0.8059858 2.496732 0.8882391 0.09636322 7.899449
1.383509 1.370159 0.3123736 0.3502396 0.2774987
0.3958684 1.266264 2.684879 1.569309 0.4913668
0.9677384 1.37028 0.2565129 0.1262155 0.3116927 1.545102
0.3420289 1.091569 1.410269 0.6454558 1.377606
0.9380014 0.4701754 1.62074 0.7638666 0.3283917 1.382998
0.5698351 0.3868788 0.5980498 1.338515 0.2919934
0.06111435 1.283271 2.05153 0.3034403 0.0838986 0.3863785
2.549158 2.55657 0.5414475 1.614381 0.609317 0.3958207
0.6411869 0.4093198 0.4620584 1.324667 1.359026
0.2668591 0.3071306 0.2773812 1.053219 0.08204865
0.2927577 0.7894948 2.036529 2.012455 0.6650269
0.8639478 0.4073442 0.6240536 2.501067 1.599539
0.9037173 0.5038426 0.3305995 0.9116279 0.3130849
1.506567 1.222872 1.26062 0.6874131 0.23154 0.4502996
0.3332722 0.7604538 0.1356572 2.773242 0.8426444
0.1745287 4.324806 0.1258928 1.455583 0.2847457
1.846488 0.6776776 1.205344 1.907704 0.5980577
1.306444 2.998174 0.385563 0.9746086 1.24966 1.182113
1.386361 0.1885577 0.5348445 0.8004556 0.7014979
1.379605 0.7476604 0.5827681 1.269427 1.702564
0.6753835 0.07125599 0.08144214 0.6856993 2.454858
1.254397 0.1185614 2.080829 0.4929922 0.3903849

0.4767899	0.6132399	1.267839	0.4914341	3.447626	
0.6632061	0.9840541	0.2445846	1.573355	0.3512442	
0.8516807	0.4324909	0.2504525	1.644784	2.580346	
0.7206435	0.2083494	0.6214989	0.1810114	1.124624	
0.3789435	0.2995377	0.148389	0.1346456	1.022646	
1.487252	0.3734526	0.8358567	1.071229	2.491809	
0.7903649	0.6889026	0.5349118	0.6371646	0.9562059	
0.3106516	1.927891	0.6185312	0.5410537	2.666436	
0.4145422	0.61198	1.643758	0.1679741	0.205335	0.208729
0.1025211	0.9629147	0.9480213	2.087826	0.06210643	
0.2187793	0.1980367	0.4508842	0.613273	0.2755422	
1.511796	0.4146873	1.917402	0.9402515	0.8728951	
0.8746508	2.384273	1.350367	1.072627	2.363193	
1.742523	0.3195509	0.9089547	0.2623457	1.048482	
1.797561	0.1480633	1.362074	0.7949376	1.358791	
0.5417222	2.623224	0.5235172	0.3334632	0.6975559	
0.9259043	0.6248384	0.6817417	0.2158759	0.3473762	
0.8558849	0.4720565	0.8043681	0.483398	0.6724026	
0.1561574	0.9670455	0.352264	1.132538	2.901001	
0.2912933	0.8950621	3.127045	0.2249119	1.95694	1.004057
0.7868099	0.9561813	0.3246099	0.5414592	0.8252668	
3.559685	0.3367572	3.172785	5.983469	0.7257214	
1.21037	2.459318	1.442006	0.6172898	0.2406737	0.4398241
1.88378	1.779626	0.5684685	3.060318	0.3120179	0.3723597
1.724882	1.009896	0.6294649	0.8772771	0.4519578	
0.3770143	0.4363189	0.9825968	0.2776278	2.597184	
1.288005	1.785273	0.7860127	2.667735	0.6455089	
0.7222992	0.3986959	0.3584563	2.664342	1.186436	
0.9468702	3.081436	0.3585609	0.62147	0.1221386	3.6802
1.167575	0.6998388	2.121018	1.095821	0.04139298	
0.3635905	0.6192231	0.8747153	0.6544842	0.3178169	
0.8422148	0.3222359	1.978383	0.6545276	0.2760556	
0.9433617	0.9324686	0.5795211	0.3497746	1.628957	
1.344699	0.891086	0.1922585	0.123143	1.096859	
1.061596	3.681614	0.6783002	0.6704188	0.885021	
0.4459154	0.7190677	0.247699	0.6048586	0.06737122	
0.3278006	0.6441079	0.4543028	1.172581	0.9526695	
0.3942177	0.9601479	0.3908826	1.030239	0.3470037	
0.4868891	0.2728747	1.350413	1.753192	0.6303871	
0.8051938	0.1449675	0.2533334	0.2709721	2.281109	
0.4722659	0.1434823	0.2991287	2.976716	0.6955177	
0.4558753	0.8551633	0.2860373	0.9605599	0.8048561	
1.334439	0.9562061	0.6238773	0.2923558	0.3965397	
0.5024263	0.1898225	0.2070825	0.5205871	1.816475	

LDHA 206.2853 74.73402 166.6108 58.81091 152.6839
51.83172 60.85782 32.6292 63.5652 97.91976 73.80018
74.77391 68.2471 124.0472 46.50213 66.67656 104.4951
80.45744 195.8898 228.8482 79.72173 54.10015
131.9303 54.90319 95.87764 111.2171 45.66534
52.28474 122.4349 80.6386 37.25547 128.6319 83.17371
159.7561 112.8283 170.5268 57.80298 79.43137
170.538 125.8512 88.82378 145.1688 268.7811 166.3763
81.14429 139.1805 110.0995 185.5544 201.7169
85.95951 226.6062 80.1412 89.43307 113.1672 161.5384
168.2709 151.1677 42.63729 163.7928 128.3119
97.95905 186.3458 125.6737 98.3052 164.0106 102.0635
126.937 58.34315 193.8481 138.3353 200.5621 183.7138
82.24347 212.4372 150.5187 114.0634 76.44696
91.33339 84.52506 196.4634 246.2428 72.91851
359.1111 83.03665 205.7247 130.9988 82.31841
196.3702 271.0339 151.0129 386.0948 153.3175
326.1679 158.6677 226.068 76.18916 133.4476 136.2174
149.8202 144.5343 40.97049 101.8208 234.7924
89.61193 110.669 68.10563 200.2736 94.89735 174.0063
120.9335 90.14434 172.8464 293.6029 69.59538
198.7088 117.5788 64.06585 92.16324 69.20906
197.3954 170.4111 199.6763 59.01045 183.2219
166.3746 81.14208 313.1519 272.6976 69.90101
184.0617 152.0505 222.0743 93.07292 188.1823
131.1363 59.23051 211.0318 384.0053 94.7668 160.7685
110.1569 102.9209 149.4004 164.3263 94.22578
117.3659 191.9864 90.12703 75.45107 132.5289
99.01425 105.6955 235.1402 80.66813 66.39105
106.2177 142.2798 54.42082 266.2676 192.548 96.37151
58.02786 74.95285 92.48833 195.5383 106.7855
90.43958 153.9671 79.07509 71.57767 72.61088
134.9696 59.14573 210.4593 101.2856 165.8465
80.43184 269.2056 115.206 38.23158 71.57669 93.39216
138.4823 131.3328 133.6094 84.6105 55.0694 471.2813
63.45582 228.4584 89.43775 74.18626 186.6447
104.0635 106.5001 77.36453 144.1777 92.93641
200.8297 222.4196 131.8814 95.6211 102.7974 268.2018
181.2729 151.0856 194.8408 128.9111 66.10578
262.5728 94.27309 76.36737 108.2279 51.35659
312.6724 186.3873 223.4393 79.39986 142.3683
115.9088 137.2538 210.6056 57.78003 105.4058
72.81868 255.1031 22.4798 165.3655 123.6018 88.07439
116.8321 88.27454 66.74225 179.9036 209.4637

91.47975 88.71627 75.76531 220.1467 95.96951
73.80752 101.9819 107.5995 161.6406 217.5252
84.32627 90.16934 81.65786 101.8181 143.5158
167.7011 129.3861 110.5724 147.383 179.6088 49.42678
98.71295 82.05118 229.8564 85.2921 108.423 364.8846
115.0409 189.7454 68.9634 108.1536 193.7805 146.3967
140.2275 54.66395 83.94851 192.7775 80.30725
312.5472 153.0504 82.48594 147.1139 82.43056
124.2021 249.1565 69.28709 150.3269 187.4946
230.6731 238.6421 63.1944 92.23404 78.74869 113.404
113.4492 138.5945 59.49795 131.3682 99.42388
283.1677 91.86328 181.9741 123.055 312.0311 111.6405
130.5766 80.85727 309.3946 102.6099 117.6867
124.2884 75.58415 226.9781 53.69431 162.0326
122.8441 248.0202 189.9322 81.33733 144.8488
67.8128 81.80121 102.0979 129.5894 205.8312 343.2964
139.2756 133.3142 171.4004 143.0412 68.59662
202.0503 61.11129 129.4832 108.9283 181.106 89.14951
112.1105 115.0845 72.13597 56.16683 443.8742
87.49149 78.72914 135.2697 40.41744 111.6929
183.7467 194.5895 49.3397 97.1995 75.81544 120.6513
199.7603 283.8184 84.97407 113.8067 56.47823
178.9817 119.649 217.4168 87.00117 182.5048 69.91921
123.6171 129.038 60.16535 78.73385 103.8582 193.8048
126.7984 73.05497 95.97549 134.8825 87.24212
110.2368 120.7545 88.43522 91.30967 244.0627
103.9867 134.3377 138.3473 106.4037 68.13405
84.37726 78.04896 98.02497 64.02443 145.9458
217.054 89.06306 292.3073 192.8296 60.56109 103.0754
268.9762 106.5561 149.4082 261.9005 136.148 48.45151
74.79083 32.20465 148.4565 124.504 44.05711 216.1836
64.44881 217.3599 97.69779 72.74579
ANXA2 76.05955 30.71374 76.43549 70.66077 110.7424
22.48728 51.69247 14.04195 77.2075 62.26981 127.2631
206.1706 47.61784 233.4082 40.76312 17.70661
104.9611 38.22962 125.5868 136.1611 74.09437
15.50232 143.8503 22.49777 55.49793 158.3773
46.59958 14.93037 76.04071 75.68092 39.43319
22.36406 106.8355 138.3276 162.7093 19.84412
7.231657 67.61867 94.44271 109.4921 86.46531
175.1678 138.3105 162.5508 111.7237 194.1251
153.3738 86.59027 144.9902 201.6174 203.1755
58.01438 41.79431 189.7192 75.32859 94.23543
41.46708 24.53115 81.27312 71.64939 121.8674

151.5315 74.77804 36.70148 98.16362 44.44706
176.1441 80.99452 147.6041 93.00638 172.3057
96.93681 97.61526 138.7938 142.898 74.35384 125.3227
82.1814 97.90974 92.12842 116.4404 63.62102 168.4554
91.4842 157.6898 160.9399 81.30318 149.124 113.9768
115.7301 154.816 104.0641 178.5107 88.82559 168.0224
34.84054 196.3804 86.92025 55.90308 133.7274
29.92812 195.3765 86.10164 45.34389 54.16741
68.42574 246.1313 74.99807 164.8591 141.7472
89.21652 38.27019 121.3726 46.57179 123.1491
96.4277 42.34035 249.3701 49.62142 75.31372 139.8949
70.68657 54.79943 111.0349 73.45375 44.60166
220.7596 115.0603 33.24113 87.94048 163.746 99.1505
92.07833 183.5135 101.1412 77.59731 156.9591
241.2385 87.64031 91.40698 121.5922 98.06791
120.7124 39.55955 53.14205 93.08885 145.3165 143.52
73.56811 72.69828 85.36618 187.751 126.6272 46.05481
36.01124 87.1599 93.39166 41.42059 141.3987 78.1792
65.81247 44.43593 86.82841 71.14106 163.9205
108.3253 104.0304 168.7005 53.63871 47.23989
14.24723 79.5004 61.7965 190.7838 117.0041 123.981
93.53612 67.83808 79.26404 32.3302 46.10389 90.46145
189.9627 77.1105 78.14369 80.53215 97.69817 224.8775
23.20882 121.8391 58.14494 121.7505 203.6997
131.2937 103.4497 124.0639 158.4605 77.4054 102.3629
79.80067 62.06376 85.47711 82.4948 101.5668 107.084
93.43355 90.187 14.78069 166.4092 135.1109 112.1138
59.54401 115.9893 33.55874 189.384 122.2467 73.71598
77.30531 65.72791 132.0721 97.04886 92.94568
56.24683 233.9391 93.44899 138.2626 69.86765
169.4329 116.6467 189.0553 155.8398 111.0874
105.5079 295.0322 195.5104 118.9742 75.37521
166.9872 118.7648 11.54774 55.31441 107.6046
73.08254 115.2868 291.5796 114.335 66.87905 27.25244
87.85512 131.5604 95.83087 175.6557 108.0597
121.1843 79.1203 29.22833 113.326 105.7285 178.8519
61.78518 90.16585 365.2922 143.7423 184.7433
78.08676 67.87156 147.5639 166.9065 230.1964
99.6864 101.6121 147.2617 221.2753 128.1065 153.4215
35.27271 119.7079 22.08181 46.52352 261.8171
64.52265 53.97866 218.5052 117.325 174.3264 42.56089
104.4978 81.17103 63.25037 76.99059 116.7672
23.15193 34.4051 63.4904 128.9008 72.922 104.6417
64.95576 127.7278 152.9342 143.6947 78.73834

176.2564 49.62842 138.2226 149.5733 62.27594
140.2397 64.84846 107.5067 161.3281 249.8194
52.48941 75.66113 155.0017 26.47651 74.80405
59.19339 99.26316 181.4218 300.4158 150.1509
98.61365 56.0457 97.6346 51.59734 106.1109 75.92134
114.1175 150.6538 137.5355 62.0696 71.19008 95.98652
45.28499 124.6747 73.87675 136.837 77.63185 101.902
30.23889 41.54785 69.43341 50.05089 26.79467
167.6997 147.6543 6.214091 103.2322 62.10606
59.82575 63.36533 60.42117 122.7471 36.25302
111.6651 101.0137 146.3805 28.26136 46.92717
81.98832 48.14691 319.4293 119.9715 39.52241
72.05878 42.28312 118.2196 129.6687 64.22302
50.26443 157.0025 61.5375 90.93241 44.1132 56.31301
178.6409 104.6742 94.46776 77.90124 96.2714 98.87656
91.71135 174.5356 93.25559 227.8895 62.40609
244.7753 88.59745 69.87072 79.39576 281.8066
52.1517 259.3579 187.1771 98.7094 51.82036 86.26741
35.3828 133.0522 70.82502 51.51262 172.6595 168.2272
127.8514 127.6575 49.76988
SPP1 0.6134444 5.961694 2.625582 2.804425 2.241485
7.518459 0.7543771 2.760766 11.03606 4.564417
0.1665674 0.1844691 4.098591 0.1702006 2.061416
9.209965 2.934316 3.704475 3.841298 0.3743398
0.9508984 21.813 3.153534 8.380227 2.212219 0.1672871
2.140221 14.25495 5.419196 3.721371 3.838595
4.702042 39.09355 31.07069 38.49051 7.025663
5.569022 32.07372 30.47592 5.877599 38.90304
11.37837 1542.81 138.1599 12.07942 16.54942 4.836328
56.42982 163.5307 0.8699272 144.7592 295.5225
11.12748 64.90575 29.25484 42.69972 59.28991
4.677898 20.94504 18.03309 37.08294 154.1202
21.42117 475.7523 8.045615 10.56597 150.7422
83.45982 1.553422 13.84408 144.3577 9.894935
30.37924 484.026 279.1466 0.633962 2.789658 101.9278
26.91945 19.82447 1476.499 5.435931 56.35556
232.6041 67.28656 22.71592 7.104175 35.94578
27.19094 36.85595 659.5976 2.36244 35.23339 71.58407
256.8515 19.08231 36.96994 21.25733 384.0185
6.50981 5.062856 3.292222 14.84116 52.1544 7.873593
9.953688 17.87057 25.58215 38.55962 165.7095
12.73339 2.115995 77.2483 33.92364 32.78811 53.61953
68.23171 10.1514 21.35019 6.293292 74.97414 14.55271
7.441225 321.8414 55.64565 4.972622 251.9747

370.0881	6.905596	79.71454	62.97396	54.46104	
6.776835	629.4807	27.24578	287.6222	67.81617	
379.904	7.391861	52.71405	13.79873	7.955529	51.86554
2.899871	3.338119	15.45501	12.77377	113.9747	
4.507067	1.907748	5.87759	23.05321	35.32658	10.09677
11.21191	7.449284	172.3326	34.25038	90.10511	
67.18501	11.24651	2.136954	21.89264	21.13789	
26.99784	17.07375	8.497622	189.5294	16.77701	
3.028605	11.46658	72.1708	147.1288	9.395383	11.4145
182.6295	92.70741	10.5914	0.4877721	17.00751	21.49348
35.60554	7.97049	2.857589	121.9729	319.4167	29.58058
96.58479	4.801019	24.75398	47.25066	21.4777	138.5636
3.14842	4.283968	7.461129	187.2502	7.194409	2.379136
74.59304	42.98577	17.21884	29.19853	129.6255	
11.50017	49.65946	349.4208	44.44135	13.81603	
105.0946	10.78499	88.56069	7.838033	22.1684	46.4715
40.96576	277.6215	7.542511	21.31941	38.34102	
29.92509	114.4217	5.565115	155.9716	18.88601	
68.53414	11.87285	0.3061211	5.850084	24.57834	
71.73749	158.677	0.7103102	32.12126	69.79182	6.895774
27.91594	38.90595	448.192	9.52953	51.7439	6.115683
57.29414	43.65453	89.77316	301.9861	6.242933	
3.514597	33.02328	6.552202	22.87371	87.79569	
29.88986	4.266869	0.5634137	17.89677	186.0547	
1703.064	147.6226	37.93043	19.39041	12.36201	
21.91263	36.30192	32.3831	14.09298	21.21661	38.91137
35.07907	209.4188	14.45508	100.834	9.872349	55.57404
7.394149	25.56668	10.46864	4.549257	32.32112	
331.4222	17.14594	3.112816	491.2493	73.35004	
37.89407	1.359837	0.7402473	150.4367	143.4768	
11.28604	27.24979	21.81018	15.71743	11.42765	
105.6561	18.59979	849.4618	10.45907	49.08233	
4.465896	14.59482	54.20222	2752.59	189.9094	3.438964
18.71038	77.05512	159.9554	13.74865	3.656047	
13.72094	44.36413	78.74454	148.9891	19.03239	
16.21844	6.579312	37.7885	361.3404	78.71564	479.2655
189.2728	40.55271	60.8306	318.9769	14.13364	81.8101
77.21351	48.8803	110.7266	17.64063	41.02028	11.55022
8.148292	12.13623	9.813778	0.4625277	33.30354	
3.844717	90.74833	59.41685	7.305544	5.50428	147.3973
4.337011	3.333843	2.17028	11.99331	61.06386	58.55645
16.55324	76.77613	29.68319	41.20056	20.85431	
6.489431	11.15009	34.35117	7.900316	220.6347	
7.59832	22.44346	16.09502	206.8715	11.67156	752.766

3.846176 6.697809 6.797939 27.15302 11.33098
6.448161 53.21137 431.8788 15.23539 139.7692
21.01398 5.098553 15.64495 3.98189 108.3123 2.996805
32.60676 12.81571 17.75055 125.9951 18.43466
18.84953 158.0094 247.7529 56.07541 103.8063
4.392872 23.84362 2.099572 60.94817 3.182751
8.564186 0.8451181 288.9558 58.09398 6.505945
23.05046 13.44151 44.71863 9.762566 2.90176
SMARCE1 3.377093 5.53589 3.245815 3.946251 4.049251 6.126
3.625172 3.358612 3.479836 5.818214 3.80174 3.752455
5.842051 3.156449 3.623153 6.785053 3.702171
5.157884 2.594327 3.873835 4.425284 7.703291
3.380707 6.076303 5.554047 3.20774 4.095811 6.953105
4.004277 6.975012 3.091757 6.171308 4.845209
4.516878 5.361316 7.605329 5.291633 6.477019
67.28165 5.828029 6.338916 2.717917 4.206449
4.710659 3.135475 4.878746 4.770048 14.63865
6.904132 3.871168 2.611153 5.475772 4.308852
5.111542 5.620735 6.807344 5.304904 5.157748
3.485378 3.06829 5.78857 3.846932 6.881796 4.291149
2.022563 5.427106 4.419107 6.005725 4.198232
4.476037 4.630389 7.330561 5.230181 7.555282
4.094702 6.002687 3.803028 5.209306 3.47782 3.743335
6.34408 5.135996 6.501947 4.010633 5.982828 5.237846
5.25834 3.878372 4.452425 18.01701 5.106378 4.427276
5.08177 4.818101 5.428626 3.825764 4.900017 2.195838
8.571787 4.922155 5.492386 3.299378 3.820743
10.34225 4.510228 4.175169 4.00276 6.020445 5.767582
4.830846 6.021335 4.12456 5.766962 6.145298 4.439596
4.080466 3.10607 20.83889 5.75888 4.123257 16.61733
25.74826 6.465854 4.884629 9.62841 6.177933 6.311563
3.642516 5.45162 3.246462 3.725113 6.80567 5.582157
5.461652 2.357425 5.470364 5.832624 6.857892
5.653263 149.3149 4.415151 4.208291 3.703829
4.402243 5.272651 5.80437 4.289787 6.552088 6.061649
5.619089 3.937238 4.485302 2.348287 5.225681
5.465631 9.271245 4.912657 6.333682 3.832617
7.007718 5.312727 4.590264 7.106603 24.61586
4.514363 5.710111 6.322895 7.370875 3.193597
7.628781 5.555038 4.540324 5.642334 42.61421
4.209128 4.255579 3.220975 6.732736 2.545037
9.967882 11.09643 3.308233 3.371595 4.766087
4.381465 4.858319 5.439374 4.542503 6.120313
4.27133 3.850223 3.74488 3.469632 3.318983 3.304725

4.167831 6.366347 6.04163 29.81261 66.71623 9.925037
3.781458 6.169269 4.835809 4.155034 3.577044
3.460955 4.852283 9.254216 3.698552 7.175772
5.141752 6.33904 3.631627 4.583036 5.481532 4.914674
8.875019 5.250311 34.33418 4.349488 6.891175
5.025177 5.421017 4.624117 6.234213 5.893162
2.056665 3.774515 5.796127 4.27851 3.156083 3.282082
2.200607 6.168372 4.027882 5.456986 5.846389
4.185394 5.56928 5.65195 5.135483 6.242231 4.511424
8.057853 5.920677 6.465607 5.682014 4.998971
3.558415 6.025344 7.592109 3.014525 5.70412 8.196725
6.986553 3.998409 4.52126 4.92919 4.222755 4.916519
4.264781 7.753971 4.462193 2.546891 4.617955
5.66747 5.063476 4.18763 4.114578 4.464358 4.279303
5.223026 8.006614 4.672989 15.38962 5.871508
5.599103 5.134286 6.010244 5.110391 4.192928
4.609259 4.410531 6.41876 7.509091 3.839854 5.827462
4.654692 5.059928 4.553357 5.211598 6.173368
5.304912 5.782906 4.365816 4.56327 3.577883 5.230358
3.162117 4.543798 6.768335 7.753691 3.119434
4.525247 4.589149 6.090167 5.209594 4.919019
4.21001 4.837487 6.597871 5.378737 4.51282 5.840351
5.048216 5.022307 6.263688 4.016451 5.735619
6.403947 4.672286 8.861719 5.150519 7.948735
4.973028 3.99093 9.848126 4.383657 5.956446 5.003341
8.012728 5.05847 4.446864 4.291657 5.720589 8.272383
5.39582 5.292832 5.708258 8.083696 5.92032 4.52874
5.936263 5.66117 4.615218 1.969573 8.07542 6.493192
4.797279 5.049514 4.598213 5.25927 4.598702 5.479416
3.548135 4.334248 7.850997 4.733129 4.156881
4.989091 4.425803 3.522571 4.320321 5.679006
4.74921 4.441608 3.774754 9.588939 5.570865 5.636462
5.12607 6.980837 5.827033 6.525421 26.29083 3.521417
2.44844 3.828615 5.681115 3.429565 26.15979 5.488947
5.519449 55.88201 5.875464 4.713278 5.735618
5.883623 4.742534 4.264559 4.583995 7.42805 4.853277
3.320002 6.321987 5.418398 3.699087 3.449959
3.974145 14.82922 5.769839 5.594537 5.163147
5.974675 4.832349 5.070453
RBFOX2 4.284303 21.88226 6.980925 5.719697 6.099141
21.58114 3.64487 8.840054 8.439687 6.165747 14.31918
7.351082 4.247903 7.582244 3.983125 24.30872
4.451055 16.51671 3.878351 5.357603 8.152918
17.45589 7.015647 20.00653 26.96348 9.026138

4.772192 24.32876 5.68581 2.5744 9.118321 17.9807
5.281106 6.977523 8.067193 4.538464 3.82247 5.652634
9.547157 4.482311 9.875568 8.934853 8.420136
5.875953 9.174517 9.168222 6.550085 9.687261
11.76205 5.528542 5.45656 8.78515 6.870277 6.256516
14.0816 4.972452 6.720795 22.10534 5.451002 3.670207
17.66422 6.137555 16.42637 7.62961 3.605767 21.66388
9.195342 9.938845 5.278233 6.629259 6.428528
8.728814 9.666171 10.85233 10.26352 9.836899
9.652432 11.78148 7.191303 6.419681 9.485129
13.42801 10.45569 13.01775 10.19163 9.026907
13.34725 8.761635 4.249508 12.66291 7.036662
8.650732 8.963155 4.526607 5.397265 6.720186
11.15917 4.357085 14.11479 12.38522 11.08779
4.129631 2.771242 4.576985 11.19196 17.38693
6.239104 14.75917 6.893404 5.154642 7.667724
5.809508 6.571517 11.12225 7.584065 2.99951 4.706021
11.06654 7.921596 4.715886 5.341971 12.53818
10.36421 6.071245 7.713555 4.245652 6.012667
5.388447 19.19448 9.104329 8.378832 9.704078
6.448155 10.03316 3.772077 13.62678 7.587205
9.416506 7.431013 8.332413 6.695878 7.118939
6.777493 4.89425 7.391861 6.603347 5.260235 15.88689
7.634606 9.075808 5.355268 14.29315 7.510775
13.47733 18.52344 3.42579 8.301955 12.81894 9.728823
4.854065 10.37418 7.204999 11.21363 7.189167
6.059045 9.251692 8.139688 12.0777 6.476881 8.439554
17.63238 6.423731 7.183932 5.108179 3.225644
5.586247 10.72822 7.441785 6.208179 12.49227
4.179109 6.442865 7.751782 9.860444 6.220825
10.55309 18.49416 7.944239 15.11916 9.425135
8.218013 9.480529 15.64903 5.028237 8.417357
10.28696 12.83166 8.213495 9.349102 3.048081
5.266151 9.636662 10.35181 7.751729 4.31524 6.646959
6.158915 9.513347 8.367164 7.373812 9.278758
13.57927 8.743959 6.712644 7.56306 7.843414 9.090678
7.988019 15.83471 9.536301 9.742488 11.02623
4.612725 6.201576 8.939335 2.867682 7.776145
5.82477 4.630614 5.731454 7.004027 8.999076 4.753527
7.222856 10.59683 5.77368 6.50367 5.150366 8.723555
9.828841 12.02448 12.055 9.460259 7.694609 10.86976
9.469132 9.964451 14.88347 11.60464 5.845936
11.77039 8.42836 5.590284 8.35089 8.522259 16.21455
8.438031 8.125507 6.001538 13.09737 5.456606

9.566963 7.602467 8.739122 7.145999 11.22717
6.138698 5.503714 7.496801 6.420351 7.676803
5.31138 5.325561 9.719532 6.846041 5.597007 9.446612
19.33582 5.349333 12.1458 14.8245 7.130948 10.69738
7.175448 9.369363 14.32366 5.775087 7.665155
7.883265 15.85432 8.136246 24.14463 20.08323
7.863101 9.568632 15.5446 6.460814 9.213749 5.038265
5.238572 9.863723 10.972 12.9027 11.36716 4.628135
5.428343 6.17875 7.371695 6.215271 5.167178 6.467795
7.698201 6.417636 7.347881 5.475766 19.64973
6.69219 10.7391 7.597875 14.29568 8.314482 8.300206
10.78902 8.690617 11.89241 5.452776 8.167549
5.411144 6.176361 11.59258 5.533456 13.78092
8.375123 7.002345 13.13862 9.95603 3.674905 5.822559
10.12877 4.983886 6.903955 5.800936 6.629141
7.232231 23.32181 8.900919 5.198702 10.9144 4.383519
6.562073 5.555129 13.53437 5.827015 6.790293
8.42101 4.211867 10.90002 7.708774 16.0147 5.151151
8.818879 8.668937 13.7044 5.017615 4.877277 5.917917
4.936282 6.613243 9.076205 4.606071 6.749214
6.913729 7.803555 5.894683 9.45869 7.16986 5.800304
4.006318 6.05395 11.33917 8.797721 10.35764 4.248577
6.392718 10.04552 9.211171 12.77434 4.720772
9.005608 7.61215 16.07944 15.17686 4.362093 11.88591
10.74927 7.541556 9.875611 14.10548 4.460415
5.603426 5.086688 5.94317 7.265329 8.356374 5.22841
6.065194 15.1095
QSOX1 27.92285 26.24849 27.06956 74.41477 31.20997
15.45158 48.76214 33.38283 74.3641 29.00596 31.49978
43.71503 30.01656 48.85077 55.50702 11.81547
51.64361 40.62401 28.81097 26.99428 101.1521
9.681403 45.47057 17.649 27.78095 71.926 57.11206
13.88788 50.58828 27.71605 98.57042 15.02612
114.7385 34.6615 26.39851 15.95345 43.27993 35.60623
74.87773 4.29461 25.21919 17.99743 112.7372 53.07561
78.54083 105.1972 27.35967 8.139376 33.54933
60.87535 60.34799 25.29793 21.10233 15.73595
14.86794 15.35119 10.4838 22.64825 36.81978 63.08089
56.94057 45.64276 32.34162 26.40638 23.19935
22.88718 28.05285 23.83869 71.18906 21.89838
25.41936 36.27461 19.3441 19.80494 55.14968 24.03199
43.67644 39.09011 1.697073 18.86056 20.8068 52.92163
12.99673 24.06066 48.07672 19.78253 44.18564
50.98763 7.970057 40.62463 22.20311 31.95029

17.40832	40.95705	24.86783	47.37775	47.43059	
56.23594	103.9038	33.09522	24.08237	61.58834	
17.23931	6.360018	32.62027	33.52689	59.01392	
28.85089	75.40263	33.12984	25.2578	12.87465	11.06019
7.377775	31.95252	73.63756	49.03158	39.21581	
72.63353	9.600293	21.79274	10.72844	23.47254	
38.4449	20.21115	20.31107	29.70285	22.84868	20.65382
31.46494	41.71968	58.6075	73.04934	43.59355	68.38435
17.58715	55.13751	39.93866	42.39884	40.39546	
9.49207	17.48164	51.5889	3.877188	15.30346	35.18274
9.250741	39.22744	34.2853	8.361824	15.33202	48.03296
22.83517	35.80381	16.75769	24.821	36.51678	28.01149
12.33073	8.305906	34.11442	32.39234	27.12368	
50.61051	23.8343	54.07402	102.1238	36.0929	46.76587
16.83378	16.34363	52.49454	30.03298	50.19893	
54.99659	55.31582	48.33403	20.19114	64.83417	
15.81705	7.642772	68.96062	29.36755	26.29354	
27.9663	53.48339	33.08319	75.62758	15.23893	24.54594
104.5645	27.18363	45.80407	67.72135	48.24807	
41.72698	28.21303	17.34021	36.27997	10.64009	
7.079095	18.42448	46.01434	23.05955	15.36093	
59.37064	90.74571	13.17908	18.48412	20.21002	
11.17086	23.66977	56.35825	32.16834	32.38511	
39.40121	37.31808	22.17006	22.52121	48.05098	
44.52741	28.43365	87.79275	70.15426	18.17677	
10.24905	14.03758	32.51826	54.51606	68.99278	
40.08657	36.07764	97.20737	55.28716	27.75088	
19.55272	14.09506	11.85125	8.215813	12.62618	
8.25539	37.55555	30.50082	32.89732	28.90383	28.05415
9.524301	13.11185	30.32678	31.3676	20.84084	10.85548
27.44648	9.79609	50.76863	39.14813	27.75858	20.5576
22.43751	14.34688	28.23071	37.61229	18.57691	
29.83613	31.28177	25.09441	28.96258	20.41269	
27.26564	66.2083	29.86052	62.3381	35.42433	30.60715
205.7971	45.54488	63.50055	10.85683	6.464216	
29.48579	29.43068	17.1236	40.78054	16.25977	13.40913
15.85306	56.94816	25.12773	21.58455	35.63125	
11.70181	13.5692	20.56256	20.62795	16.82344	36.49661
10.17984	24.69632	71.18637	64.42523	34.1643	19.85897
10.97217	54.48025	65.73683	52.53331	80.27506	
19.34464	38.87305	43.20629	17.37511	47.36431	
6.743595	47.13214	13.7649	21.9523	35.20829	26.20638
58.17996	82.06814	24.40513	12.67499	81.51024	
30.06299	17.3185	17.25174	46.18091	64.84424	52.75689

86.97004	68.44167	26.44958	45.48738	11.23238
37.62985	13.47496	20.37983	33.29558	22.09296
67.23543	18.23447	22.32732	12.00046	40.8852	23.6583
43.19453	74.67402	20.73141	12.20426	11.89221
20.42237	26.75659	44.65114	20.71717	16.69777
75.8593	47.7933	54.23539	14.26095	15.6013	67.83567
28.78777	28.94233	33.70565	21.91108	6.990723
104.4924	63.37573	25.02343	19.90883	24.90086
41.48715	36.49417	62.72497	72.96624	28.71389
61.7734	18.78127	28.74273	22.69441	30.07275	29.9829
6.363593	41.43866	7.201071	38.29949	23.46641
34.08648	36.29507	46.50391	19.35988	51.75075
3.118524	37.24403	28.91178	40.69593	11.34338
30.26081	61.10543	27.67691	14.08108	57.29355
20.56174	57.91841	14.60515	17.07488	18.02272
RPS6KA3	10.50723	5.626552	13.04489	8.509034	8.602158
9.114215	4.739973	5.369312	6.337773	6.65739	9.075638
5.581442	7.913631	4.111086	5.242682	11.10333
7.467451	7.766385	4.989693	7.668861	9.79786	6.857225
6.77669	10.18886	5.799039	7.323303	5.394789	11.5206
9.667831	3.633548	8.190966	6.360543	8.861689
8.88033	9.310309	5.683546	13.80222	7.145287	21.16948
9.386029	13.39748	14.6076	12.75524	6.980931	18.91714
14.3924	14.48916	13.0539	13.88609	13.3925	9.529296
17.2782	6.051533	23.13833	10.82828	10.71771	14.56563
16.0396	14.7477	6.390124	16.31554	7.611953	15.8355
8.009002	18.11785	12.50971	5.231346	13.22333
12.7537	18.50891	14.7099	9.883064	8.110712	9.779027
11.56015	4.546247	12.39193	5.92054	5.82595	17.6819
8.75027	15.863	17.41845	19.12669	6.137328	5.703583
18.55054	10.45276	8.042729	9.192346	19.46494
11.13568	13.94335	15.57726	16.84206	6.308528
13.10921	12.75554	12.3565	16.01954	16.32736	12.94877
4.716199	9.11105	8.682401	16.44475	12.55824	5.922908
10.87916	6.130509	9.901683	17.18358	9.452012
8.768528	9.642259	10.41242	5.139665	22.5945	8.357258
13.04265	10.41237	20.93458	15.47768	12.03825
12.85616	14.83365	11.70068	6.333737	33.7694	30.0165
9.987369	18.4499	7.634862	16.93577	7.126583	19.70607
10.41018	18.84719	14.43324	10.30941	16.31216
9.607825	10.76761	10.91913	8.880704	8.853639
13.4071	10.55321	6.594003	15.40926	6.566904	11.52665
22.14415	25.06291	12.81109	5.606288	7.54202	8.291009
11.59269	4.835008	9.852764	13.22689	15.21734

29.47885 3.807721 13.12024 9.383794 25.27062
8.855359 7.578029 9.23038 8.055314 9.919414 10.91457
3.983064 8.974409 13.55985 8.37377 6.777242 17.74033
7.23587 8.098005 7.105091 12.43128 9.548004 10.68702
17.12147 6.388608 24.16447 9.978293 13.22935
14.67602 7.936838 6.271011 5.02084 18.23812 13.52085
20.16642 11.64637 6.597073 23.22386 13.35316
16.25645 14.17951 15.48807 13.41251 6.103599
9.697274 12.67469 12.89492 15.00853 10.90224
13.89305 13.50711 9.207798 10.23301 7.081708
5.000059 8.059007 16.78237 21.36678 14.91439
3.535925 9.257022 18.73599 5.505316 24.90948
4.644137 10.35231 21.6434 10.37973 9.798092 11.37801
8.455968 26.0477 6.084712 5.617413 7.524784 14.53207
22.15167 9.142708 14.41184 10.46082 8.650187
11.41882 11.22453 11.28614 6.124341 11.73223
11.21169 14.26326 30.29286 6.836988 11.68363
17.44616 14.07139 14.45235 7.651817 12.87784
10.0702 5.680267 6.044629 10.70633 13.47302 13.93679
27.99104 9.359556 10.03389 6.257827 17.62773
13.00395 4.186799 17.66175 13.06964 15.22734
12.7473 10.12275 10.73516 28.44239 17.60379 15.25036
39.87554 8.840293 12.96256 15.88212 14.22562
14.87205 7.027901 10.42693 20.86464 12.91849
12.8249 12.33155 28.56957 9.701834 6.99193 8.166159
11.99376 10.70429 9.230655 13.6235 15.5417 17.48966
10.52138 12.73678 6.637953 5.854024 11.99908
9.925262 14.8523 15.96055 5.15387 7.642501 7.484919
8.296019 11.45326 10.82898 10.00652 11.82259
20.63333 11.86606 13.07417 9.776498 5.487391
13.94636 9.023629 5.117415 4.797998 7.225126
15.14965 14.6583 11.72599 12.3943 8.203769 4.682018
10.77254 11.80632 6.187539 17.92639 12.49962
7.93391 7.723936 4.737942 14.15802 12.44396 9.852721
13.18268 24.60566 4.51816 5.874695 22.06426 18.3437
12.11412 8.01008 10.29401 12.36696 15.9486 16.16317
13.93791 4.323547 7.922124 7.122281 9.748441
12.02411 8.403973 6.726638 5.112022 6.579145
10.57323 16.09047 14.23755 8.966964 14.23996
17.18244 9.09887 3.930636 8.794935 8.981855 6.192675
10.28616 6.953999 11.76724 8.785627 10.17488
12.6118 8.042749 5.590141 9.152562 10.54032 9.404004
13.71734 12.77963 6.278115 9.780058 28.16704
11.18334 11.44114 13.32428 5.837722 13.53001

8.396108	8.08396 8.11601 12.81819	13.42348	9.346329
9.633696
CDC42	69.65499	58.22717	73.31618	81.92548	75.43243
54.80596	59.91838	41.40631	55.91211	90.72548
41.49974	78.38758	76.48041	60.03034	55.57426
60.8566 65.50138	54.93235	64.51138	71.52963	90.76523
61.3275 74.94485	57.99651	54.39974	47.83431	65.70065
60.50251	62.45653	132.2887	68.7289 64.41017	52.91206
79.05325	59.11015	64.15781	35.79863	71.76087
62.02058	93.0085 43.13795	148.038 78.78642	134.8195
69.23582	53.85989	64.74099	60.91558	64.48581
54.92079	190.3194	62.16361	56.47194	71.2679 74.51065
75.15366	43.35148	61.68117	58.65264	70.27306
68.09234	60.15087	82.15824	55.58668	67.83211
55.7257 67.69778	72.46052	134.1249	67.93818	80.93112
78.59527	63.38885	54.74208	41.60246	59.03581
67.98176	52.67298	51.27965	75.66227	65.4221 77.91739
93.00624	63.91319	89.62621	89.79433	79.41936
65.54694	151.9426	67.27158	100.1212	62.89282
81.08604	65.41272	74.02574	50.77722	85.06114
65.27666	55.98705	71.70414	67.12387	56.20879
66.15094	73.82163	69.44079	51.59895	61.98536
61.0539 93.84014	73.46575	71.49094	65.02007	85.70969
64.09242	67.32845	60.06013	71.89139	46.29997
72.24871	69.69048	71.58962	62.07765	77.51004
90.08037	50.84351	70.01807	161.2845	63.26423
63.10963	68.46466	66.57228	62.63217	86.44601
75.94889	60.33699	60.49977	83.32266	79.7331 108.1344
61.15886	64.16304	51.94885	42.65189	45.5716 70.76346
65.58533	66.60759	75.14542	27.65787	75.92566
54.54041	55.21568	79.17061	70.9834 54.15733	78.42039
58.54462	60.13406	63.86497	54.85017	59.41474
70.87938	63.0434 61.15468	61.93274	70.48478	56.20142
65.16527	45.93523	72.73908	59.15493	46.48625
40.78585	85.31934	115.3425	61.85496	54.81164
64.61329	59.95874	57.15964	109.4216	48.33895
60.81298	60.07522	95.88175	58.54342	56.68249
109.1976	63.30625	64.37946	84.3979 67.92959	72.04392
59.88009	47.19482	53.29317	100.6647	53.26892
67.1539 76.58692	57.85063	82.58687	64.17418	85.79121
58.50786	54.25052	101.0248	45.00692	53.47129
45.59294	70.44994	49.30475	68.68736	51.03789
91.24725	73.34688	77.20027	56.34543	86.94133	85.787
94.27092	83.41065	63.72052	56.42359	60.76005

71.04041 48.47607 43.87657 72.44073 56.46304
55.5108 46.3456 62.6322 66.87429 82.30042 87.66093
64.62767 83.15486 88.35462 55.76287 61.96751
88.80338 71.11273 58.97333 114.3265 59.85242
71.19866 50.82 60.34876 75.20528 77.552 96.35154
51.73784 56.82835 52.58736 66.35826 49.73279
75.87729 65.06653 47.70048 78.30224 69.6213 80.0938
82.5625 61.98282 46.24793 86.57696 75.46106 101.5363
50.14232 93.02606 53.67097 121.4234 86.015 176.43
53.90314 39.16578 49.55918 95.96676 126.7097
72.10296 57.21143 77.18208 83.64888 68.59079
72.0169 70.03296 45.8238 68.78432 75.77401 61.94929
50.88727 52.9696 73.75885 49.402 58.54079 55.823
79.4105 63.42559 60.26379 59.95131 49.81036 43.35555
41.87055 69.6192 64.6835 52.35948 303.992 70.4698 42.51986
74.98895 50.86199 70.97866 55.99551 75.44742
56.58335 66.34636 41.7801 59.90823 93.46941 62.92494
117.4683 45.35796 86.60779 104.4629 87.12928
90.04792 34.3987 46.87967 54.37904 74.37912 46.33002
98.48011 61.40659 44.7984 60.03136 66.35697 88.13973
68.23246 52.45138 32.17596 67.59728 62.26876
128.7786 63.42308 49.69964 38.80574 62.60276
68.9224 55.83869 59.0859 53.24607 67.91859 61.35384
64.97187 37.30935 61.4629 45.16233 71.964 50.8019
61.50011 47.87223 51.88867 94.68107 39.0839 58.79115
62.86614 41.26295 44.92797 74.62205 71.23965
73.6074 53.57931 50.68667 67.20225 56.4738 47.01996
65.43862 60.91557 63.16741 64.43009 67.67738
52.98716 49.14081 57.40613 72.03567 47.33483
126.8144 60.5789 29.79977 47.20135 71.02824 54.49858
86.99788 57.78033 66.16998 51.79095 62.45469
56.23811 98.88141 59.10738 66.79187 56.92756
72.37704 88.49334 73.47249 68.69182
MAOA 125.9243 19.72064 75.48184 26.80069 47.87968
24.21571 11.07759 16.05475 16.93587 9.229805
30.77609 17.46277 11.19361 26.78058 8.920767
18.24033 33.0231 25.5053 50.96437 55.9698 21.86029
22.85558 15.46398 30.13558 24.27553 21.62491
14.5107 20.0169 68.78771 13.20938 14.73538 25.02866
15.59703 13.74901 5.956766 16.17788 92.86439
9.545793 4.403476 19.15381 13.02724 3.600389
4.084094 5.634731 36.63313 7.437755 11.87163
11.48338 2.403128 7.040505 14.48404 2.854068
3.98122 34.19477 11.92943 3.93195 44.15326 13.51412

12.6416 11.0133 14.80886 0.5770574 24.45589 6.178581
19.95004 10.46821 10.7547 22.14541 18.32036 3.686584
0.2783382 19.28486 11.91534 2.377123 9.620363
1.578049 11.17606 0.5369783 8.169526 17.13884
11.96824 6.666166 8.217511 36.03739 5.099317
26.14432 24.28137 8.703966 2.66504 9.776759 55.15404
1.731423 9.887565 23.21772 0.8470323 6.231813
4.611086 9.254487 1.779884 7.618932 13.09315
7.366041 2.815869 14.14869 4.716024 24.42878
1.455898 11.42477 5.147901 4.439331 6.382421
5.056225 4.123393 8.535528 13.84096 11.32006
15.21499 10.14639 6.038227 10.64901 14.56991
2.556022 3.284139 9.978303 0.3082126 5.988136
54.53681 25.19606 23.13678 14.67787 2.118034
15.09848 20.9817 6.165188 16.58049 81.74952 3.232326
9.318539 4.489034 9.048938 4.157191 14.37931
8.699518 4.012372 7.474112 6.112776 6.280982
6.172424 2.172611 15.24195 20.23045 7.996168
2.831666 3.685515 22.73064 12.65924 7.578227
8.218735 1.97871 2.699501 7.201822 10.40957 15.05912
26.54851 6.006036 46.36659 5.739557 8.766757
3.643714 1.669579 36.08946 6.142055 16.03933
5.399654 7.557225 5.063922 9.732363 3.580255
14.13606 16.4926 4.686612 8.424983 17.07397 8.917655
6.275006 5.991306 8.969838 0.2595861 11.81067
8.168736 19.97852 10.84559 6.038865 11.21006
8.582132 15.10203 3.431957 23.51 6.478187 14.32428
6.128594 10.01627 4.35442 19.07778 8.423748 12.37723
12.42999 4.107821 32.98819 4.170581 39.05291
8.450119 7.450258 8.014867 36.96265 0.8737961
14.58336 11.20774 6.288708 22.06521 19.6989 21.17102
8.935293 0.9338288 14.82776 74.13109 4.531786 19.873
12.78138 9.460657 29.81151 14.44675 12.48121
18.51078 28.30458 16.9118 14.0302 10.39084 28.0206
10.24827 12.41475 4.55347 6.893959 1.582773 4.090562
10.67164 5.434887 22.31884 8.634662 3.150185
4.271512 19.80868 7.219464 7.52334 9.835861 2.83548
3.742553 13.36542 23.25374 7.102558 3.595082
1.858954 15.65157 2.519488 32.94728 4.977571
14.82097 77.62994 2.038274 30.14962 7.14257 1.417881
21.9063 40.58253 5.945615 12.04876 3.085753 18.82085
0.5128359 13.67889 6.973882 36.69655 9.356002
36.73787 11.55736 8.982179 8.955244 4.426738
16.13441 8.771512 5.963403 13.32735 15.6722 19.52592

16.81007	18.38072	5.009074	7.789806	2.797279
5.717146	13.2177	6.087988	11.49055	6.846416	14.05078
3.017063	7.460943	5.302912	18.79421	5.70737	19.8989
1.534052	1.700026	32.05091	2.050185	25.34848
9.196512	8.92581	7.206307	3.260678	17.82759	27.38899
22.39689	12.08128	36.28904	15.55719	7.083418
3.533111	9.62083	0.5927329	7.827744	10.99366	8.509999
19.12716	10.75298	64.08927	30.1007	13.48301	12.29619
5.761155	0.2905079	8.736903	0.9423399	7.042311
16.16043	27.65477	7.798557	5.335324	12.60135
2.808007	39.99677	4.094799	16.0733	6.062642	6.538358
10.02276	7.600417	9.745218	17.62517	3.330732
6.691575	3.863674	13.59434	11.5506	17.09101	6.313648
22.92419	4.61056	15.88512	14.43535	8.136789	12.92795
2.166914	53.73755	9.403325	0.7772694	8.250125
2.662811	7.617821	6.986307	0.8523671	10.72853
7.009764	0.5781065	10.0376	9.809763	7.765245	11.11721
47.13654	1.48565	11.15936	0.4542706	3.27025	3.187358
9.180884	7.071792	12.50445	10.56624	30.98709
13.44108	24.47742	6.679267	0.9389711	4.066583
15.83365	19.47525	18.0185
PIP5K1C	5.779759	54.83389	7.41667	12.17852	9.200976
43.11436	7.518288	17.43314	21.85	10.87707	18.61344
7.666754	8.496422	13.70739	14.35165	51.36544
12.14832	31.4526	8.184273	8.33928	15.56454	41.12307
13.90245	48.6744	35.55287	13.22131	11.21015	48.72575
13.03129	1.768013	21.7184	34.36342	12.99757	10.05534
15.39895	7.474514	4.982046	13.09553	12.60671
16.17859	17.86003	6.465005	9.087999	9.065099
14.06672	8.486654	10.76595	14.30985	11.96247
12.30263	9.575918	14.90333	8.161159	12.26726
20.96404	9.621116	13.1452	43.95757	20.19151	7.691417
15.45734	27.18362	15.20484	17.67159	3.688661
34.27476	17.51992	12.78207	8.822828	8.980167
8.801246	9.422661	22.84873	9.222342	22.21085
19.59865	16.40624	22.38422	7.525143	4.22245	15.6197
15.66629	11.79146	21.22827	14.98343	10.93597
20.04073	13.0937	8.461291	25.98888	7.799271	14.48009
14.32078	9.08017	16.02584	17.0731	14.31223	9.559965
21.71266	19.12406	14.41034	11.63692	10.18168
7.003222	17.1168	21.85668	22.67465	36.20972	15.65558
9.811852	9.893394	7.040496	12.67602	23.31881
9.715936	13.79407	10.43987	22.29996	15.49328
6.770981	6.412444	8.398833	16.23653	11.05278

10.86118 11.33854 5.725194 11.48578 33.94331
25.1323 20.28896 14.9148 8.254941 8.024045 13.08105
19.99792 13.45385 5.325774 9.8482 5.809601 12.52138
20.84461 12.56684 10.02726 14.56097 13.445 7.762073
21.64162 10.16634 13.9778 12.20349 20.03521 8.161886
20.69442 32.80811 10.29062 19.9867 20.46167 11.79806
8.779176 25.08493 9.011206 9.060129 10.94627
10.96786 15.0566 10.43173 8.026769 14.67567 19.00607
48.56948 14.95923 14.19261 7.646082 8.334674
9.433255 16.42487 15.68011 11.83871 18.9894 10.18538
11.36562 13.8909 17.82442 13.08894 18.37489 25.16764
9.85808 13.8685 12.02433 14.56838 11.88169 9.729781
12.69827 14.67832 17.37953 13.87822 12.29143
12.94872 7.581126 6.026208 16.48268 11.96449
7.273174 7.2398 15.56248 13.35347 14.75162 9.370698
13.25306 8.009171 18.29103 17.44022 9.883902
18.89152 15.15725 13.69923 8.691552 21.09609
7.265753 14.6352 9.026531 8.973589 10.32216 15.2712
7.350442 10.48772 11.3874 11.14291 33.23128 13.35741
15.20379 7.629165 8.998585 6.653111 13.73815
11.95838 8.453902 9.320133 11.28866 16.20338
16.5093 13.06426 13.52259 8.193794 15.28691 14.94262
38.65885 17.35066 8.390931 18.18562 7.950143
9.915002 16.15585 10.74744 15.16325 13.5353 19.12526
15.4967 22.57935 9.268521 12.36328 9.837413 11.92774
13.836 14.55365 12.34086 12.71013 12.45847 14.25884
15.49502 16.3278 11.04758 7.151029 10.24598 10.53712
11.56914 40.47275 18.0293 7.750786 22.57421 8.06266
27.63846 13.29628 7.870385 18.37564 11.55163
12.35337 14.63543 21.47663 12.06822 42.15674
39.76712 12.39187 15.19237 25.73744 13.12756 16.956
11.34256 9.753772 14.58613 20.51479 12.51512
19.85249 8.571996 9.742928 6.309337 7.948811
12.35965 12.57152 16.58675 12.54556 12.32592
18.91344 10.3987 34.00978 13.70879 17.00868 9.519054
11.13234 6.910104 8.615601 11.93276 16.43253
5.186984 8.54672 13.11847 11.29467 10.75888 20.62584
7.307235 20.00447 15.92054 13.61741 42.50481
10.96177 5.933745 8.184054 15.28257 7.865893
4.716118 12.10816 10.95308 8.426497 38.86079
16.98194 10.77562 13.55418 9.053399 11.18957
10.3749 15.84806 10.50484 13.2846 9.240141 11.47505
15.65398 11.63528 29.0362 10.12403 16.08705 18.75786
14.47957 11.01392 9.829249 9.765387 8.632666

13.75249 10.80981 9.512729 8.277078 12.76103
13.85548 3.003924 16.4813 14.67155 12.99335 7.540154
10.47486 19.83788 15.91753 11.39533 12.76583
11.38004 11.6692 13.69186 32.38165 14.26548 13.78639
13.8013 26.03136 6.570287 9.07423 8.627507 19.31921
10.54851 18.7215 22.63698 13.32433 14.49961 15.42919
12.23576 13.80948 12.17032 7.10973 11.57551 30.80561
JUP 148.6993 6.752363 117.4905 185.446 88.26942 11.15281
76.74502 31.63252 134.9321 47.85686 568.9727
488.7457 46.17023 672.9262 92.01163 6.150576
154.8979 56.16274 140.8472 124.5725 140.6922
5.460378 611.7263 8.8704 54.10265 564.4415 106.048
8.652108 121.6542 22.24692 151.1019 29.45899
251.0978 339.891 273.833 44.73133 27.9841 418.3653
576.2384 144.4957 137.8329 169.6341 84.43677
180.6137 135.0549 495.7355 148.7646 848.5458
70.80006 292.2727 142.189 50.01754 136.7748 235.3845
73.43195 146.3941 102.1832 26.94522 222.3375
92.28046 66.44286 217.5115 45.32454 142.5415
300.5206 18.32521 218.2036 61.70989 354.9981
172.9047 193.0171 114.6826 183.9542 73.591 136.2043
150.188 137.6578 140.3202 117.8469 122.589 306.4019
84.94498 65.76039 93.41103 167.0285 182.9724
107.5593 135.1021 74.50607 100.4405 118.9515
82.94116 167.0952 106.2184 79.48593 63.45158
156.9182 160.9458 92.42425 107.7154 74.60865
335.0676 80.39007 1443.173 78.92518 105.5229
219.4829 128.0033 185.1188 88.48948 257.562 103.3358
72.81181 23.68111 279.1162 278.1093 77.72742
600.1124 311.3974 5684.196 1071.944 377.7591 165.18
95.98877 161.6694 70.00156 102.0257 137.2238
60.04683 294.7949 288.8686 141.1616 243.2692
65.08167 124.6797 134.7477 162.702 86.06263 144.3404
2749.999 158.1546 169.6223 435.0576 133.5733
182.9206 155.6363 84.52656 98.37967 263.3301
120.7808 89.96289 192.971 135.3364 131.9939 41.30022
125.6059 97.8341 41.12433 352.317 133.1081 140.1032
60.94125 83.06036 1822.383 129.1996 150.7255
262.3858 111.658 87.84523 40.47082 18.71127 136.6997
115.9497 2509.493 267.0109 149.8731 222.0731
164.8608 152.3276 455.2081 170.0549 186.2824
684.5904 153.225 116.2004 115.3533 111.7466 216.8085
571.4871 177.0663 178.6412 386.331 427.5388 162.566
146.3875 212.4511 66.00133 366.1879 309.7683

402.9557 128.9207 110.7676 185.5611 214.705 150.2053
163.5353 166.7545 51.06206 3691.923 274.3007
98.9716 98.62988 443.2702 202.2771 165.7263 114.1508
304.8111 312.6794 116.2893 1842.482 266.2244
66.74238 218.8611 126.6653 82.95545 110.5886
434.4257 745.6209 216.868 258.8824 221.8164 102.3943
176.8747 301.8296 84.94903 152.9378 96.31849
79.98687 39.33662 94.28317 213.3139 221.1254
76.17067 110.6714 93.93171 181.7745 76.79929
25.49492 123.6719 131.0757 206.0041 128.8645
118.4938 119.7399 269.2934 1209.38 130.3648 153.4212
186.1072 124.7799 133.0904 197.607 124.3432 91.4591
164.5436 152.5306 246.695 184.4895 140.8451 270.4201
137.8075 204.1914 172.9876 125.7669 154.2719
725.731 210.5533 17.09239 57.29522 88.9764 66.83715
99.27027 121.5476 120.6761 108.7761 132.6784
157.8194 1945.13 94.8195 124.4269 233.2403 11.55016
26.63353 341.4918 20.39355 125.504 108.1542 151.4547
168.2756 227.1212 142.1336 133.0325 332.8005
63.5636 177.8597 197.7104 185.6528 157.816 104.0505
176.8883 295.7164 488.9432 83.22952 90.51075
174.5206 50.67603 162.4611 171.2577 142.2774
116.7698 143.7379 68.3209 437.7518 71.36173 36.39587
171.9611 216.1458 161.378 274.3694 321.3617 199.031
438.1464 152.3081 197.523 53.452 276.201 9285.661
140.938 245.479 108.8259 54.23459 75.08562 89.32934
170.6651 45.37861 420.289 259.4786 107.804 191.2278
112.0029 182.9905 180.524 134.9016 135.4833 83.47696
198.7582 178.3102 367.0417 24.04719 153.0279
293.6212 70.53254 289.1256 288.6229 167.8254
63.45341 155.4488 99.86265 109.0773 1653.241
162.6172 475.1201 112.4312 69.68019 98.99968
210.4908 158.2381 90.99924 117.2958 200.0341
102.819 1140.237 116.7411 142.7646 3004.264 196.3757
82.14025 284.7803 204.9922 155.5905 131.6081
61.62043 182.8908 107.3722 184.9082 75.72427
194.302 82.85633 88.96975 197.3705 572.0855 58.65348
250.3006 113.8135 166.7204 184.7172 76.52246
ATF2 4.873188 6.263897 7.173467 7.792392 5.74916
9.228308 4.449698 6.149269 4.929771 8.048973
7.490011 5.494293 8.231574 4.545378 5.222343
11.14157 4.550495 7.269869 3.596522 4.418996
8.035458 8.56935 5.730407 9.297021 8.7326 5.256168
5.626402 9.82012 5.667882 7.394744 6.503733 7.411741

7.686326	7.009748	9.503902	9.464359	15.50683	
5.763141	8.936409	6.152686	14.30514	9.484855	
11.48269	10.33227	8.569035	7.286909	10.12672	
5.490538	11.48027	9.03948	5.648872	9.543502	7.859667
8.698676	9.693631	8.602443	7.069821	10.08701	
6.130166	7.229884	9.246916	7.000701	9.651625	
8.377865	5.708438	8.961574	7.12848	7.220678	9.815184
12.26683	11.95108	7.620635	9.276605	14.57891	
9.412456	6.186224	8.179126	10.2491	14.81448	9.087203
6.066152	9.026494	9.311411	9.190579	6.758974	
7.692776	10.53269	10.24267	4.751263	11.87957	
9.959406	10.89684	7.842447	11.3965	7.854005	7.360605
7.498156	4.796302	18.34253	11.09536	11.51004	
10.3955	4.994264	5.666691	7.507469	10.55735	6.734826
6.39974	9.577112	9.78726	4.641745	9.529656	8.410256
9.070684	6.73713	11.01149	10.4953	9.989126	8.18358
7.106849	12.7187	11.5453	9.968576	7.748151	11.36721
11.16883	16.4196	5.133982	10.40377	5.789394	8.369226
11.71453	12.73938	11.47805	3.874263	9.347509	
10.64739	12.92579	9.058096	10.10354	12.90695	
6.958751	8.647149	5.553776	9.211065	14.92636	
9.067626	7.650156	14.09536	9.838852	13.4434	7.819532
8.888985	10.04785	9.56308	8.6305	7.796786	8.534204
8.431183	5.7644	7.912591	10.66741	8.931062	8.05055
5.097798	9.331269	7.519886	9.449181	9.62307	17.93626
7.983259	6.177273	5.471557	4.110576	3.612418	
10.7859	8.643828	7.235728	6.948288	7.986473	8.707411
8.503977	6.648508	8.899018	9.432164	14.7736	10.58614
7.034426	11.32353	6.73589	12.53752	14.48484	8.621276
6.615853	7.493488	14.82817	12.88564	8.474706	
8.425356	4.50799	9.671191	8.683928	7.056036	6.529261
7.279751	8.873019	4.888789	15.91636	8.482921	
9.404589	10.61822	13.10065	10.28948	11.0337	8.296253
10.05907	7.834874	10.73913	8.580311	9.606921	
11.17197	13.65994	8.029313	10.24842	11.96838	
4.545026	11.38575	3.800478	5.982609	11.01507	
5.865636	7.170954	5.93766	9.668856	9.911634	6.227295
5.527655	10.07577	10.97103	16.15762	10.40077	
9.171502	7.675243	8.621264	10.7865	9.139378	9.707993
8.361394	10.88366	8.268099	13.52806	9.621495	
8.336837	8.628477	11.27534	12.6677	13.95285	7.442242
8.763886	6.763733	6.422981	5.32523	10.38576	7.818633
9.231658	13.25074	15.02072	3.963085	11.56244	
12.74935	9.776758	5.537431	9.548222	9.979077	

8.29828 10.22748 9.212416 8.251882 7.532388 11.81405
9.769913 12.66597 5.871819 8.771877 12.98931
11.35341 17.12836 12.84595 7.376673 9.24123 8.406634
8.336168 10.57281 6.420619 10.73861 6.367499
7.726031 9.487513 8.881476 5.607567 10.03749
10.50559 8.31337 8.99525 6.274349 6.197243 13.04183
14.24438 11.4966 6.81046 10.6812 4.388747 11.44354
5.255037 10.16237 7.928227 10.46628 16.07428
7.274895 10.32486 11.02486 6.565906 4.570652
5.642151 12.38415 6.665344 9.193449 4.958191
6.23055 13.81699 9.138195 9.322125 8.559322 11.12532
4.67533 12.19614 5.122166 8.259819 9.749672 13.94514
10.96825 11.07351 7.011465 12.29976 9.696806
6.323997 6.724759 20.13799 8.112004 7.968842
10.64719 9.74547 5.863778 8.792509 12.56966 4.578802
10.6566 5.704866 9.283775 4.933326 9.658122 5.52037
8.4737 6.190693 7.611577 7.797587 8.75832 6.778906
10.4339 9.335767 13.00399 5.411064 9.535169 10.77727
8.403187 8.770931 10.23869 5.094023 6.854163
8.337683 6.279126 12.21109 8.027687 12.79847
6.704904 6.026049 5.434448 8.040755 6.784122
6.547135 11.02473 12.13104 4.611267 8.851558
10.90212 12.47175 8.297243 8.08719 5.486426 9.484883
5.289575 7.164652 8.265368 11.81133 7.559557
4.497992 9.134163
NKX2-1 0 0.0395052 0 0 0.006021082 0.01394655 0 0.02750763
0 0.01150254 0.007880609 0.00901849 0 0 0.05986952
0.003400793 0 0.3940128 0.005667912 0 0.01072808 0 0
0.01098391 0.003143981 0 0.02179855 0.003629732 0 0 0
0.008229508 0 0 0.1307988 0 0.009553098 0.008245655
0.007101686 0.008124862 0.007413955 0 0 0 0 0.2735974
0.1335332 0.7253051 0 0.00733272 0 0.008661904 0.01477396
0.01380439 0.1342525 0 0 0.2211681 0 0.03733911 0
0.006260189 0 0.03760116 0.006317043 0.006221101 0 0
0.05995664 0 0.003920536 0 0.02724595 0.1241287 0 0
0.003564023 0.246616 0 0.008993469 0 0.03008565 0
0.004720138 0 0.008889188 0 0 0.1415301 0.00517772
0.004423577 0.005125609 0.007842713 0 0.005283677 0
0.004449579 0 0.003852232 0.004652886 0.01149462 0 0
0.01101254 0.03789315 0 0 0 0 0 11.33103
0.007371636 0.6283565 0.01012097 0.1463161 0.6195725
0.01027234 0 0.003391122 0 0 0.07407672 0.4272455 0
0.02206251 0.06678735 0.02519174 0 0.1658271 0.004248661 0
0.005226131 6.454977 0 0 0 0 0 0.005051909 0.04818569

0.06879467 0.09183682 0.07499101 2.049242 42.94101
0.02969846 0 0 0.0364096 0 0 0 0 0.004562108
0.003354576 4.612397 0 0 0.2321209 0.03071634 0
0.03550064 0 0.01670379 0 0.003643991 0 0.03432233 0
0.005651342 0 0.0105692 0 0.009342302 0.3193806
0.003636786 0 1.156792 0.5431733 2.794021 0.01846735 0
0 0 0.004197605 0.02268712 0.003576067 0 0.1097867 0
0.08660652 1.751109 0 0 0 0.016069 0 6.680375
0.00467748 0 0.2083633 0 0.004500264 0 0 0 1.952541
0.04758483 0 0.004307718 0.510826 0.00487901 0 0
0.1806157 0 0 0.003636538 0 0.01903639 0.004283636
0.2419669 0.154185 0 0.01200844 0 0.02843796 0.05344971
0 0.02293728 0.005178922 0 0 0.01100186 0.06425541 0
0.007785791 0 0.004687003 1.182682 0 0.02434987 0 0
0.004408498 0.01528598 0.004594245 0.1538229 0 0
0.005180103 9.523409 0 0 0 0.003798232 0.06185755 0
0.3013805 0 0.006429416 0.01172511 0 0.02167247 0.07681306
0 0.02496938 0.003811624 0 0.01255475 0 0.1793914 0 0
0.08247318 0.008713965 0.003674143 0 0 0 0.003233331 0 0
0 0 0.007237974 0.547859 0 0 0 0 0 0.07824721
0.02110244 0 0.01320251 0 0 0 0.005696046 0 0.03131569
0.004162191 0 0.007676055 0.158979 0.006365694 0.008059426 0
0.4508588 0.01154906 0 0 0 0 0.05025863 15.02076 0
0 0.01223006 0.01096064 0 0 0.02360265 0.9722028
0.004239864 0 0 0 0 0.002505751 0.009068697 0.009439832
0.014983 0 0 0.00827604 0 0 0.006422812 0.0868097 0
0.01025438 0 0.008939006 0.2908396 2.087331 0.03044199
1.385709 0.008429891 0.04708354 0.4110062 0.131269 0
0.006510145 0 0.1256897 0.07307199 0.003551568 0.03018473 0
0 21.08494 0.01143506 0 0 0 0.003218306 0 0
0.01119207 0.004365329 0.9959756 0 0 0.004275874 0.0113992
0 0.004620337 0 0 0.02737763 0.01827553 0.006977791 0 0
0.0543143 0.009044144 0 0.003787599 0.005587539 4.137261
0.01112298 0.008082341 0.08649592 0.004109185 0 0.008853618
0.01336535 13.08663 0.05811881 0 0.003495427 0 0 0
OCLN 6.900073 0.06575436 7.580368 7.985181 3.340071
0.1501611 3.107663 3.384086 5.80564 2.110778 4.155792
4.242899 1.994377 3.249839 2.669522 0.08915197
3.772124 2.219844 2.629417 4.901663 8.238747
0.1112856 4.762277 0.1542557 0.3017148 4.16018 4.830114
0.1325354 4.073709 3.452225 6.819562 1.234709
6.816047 5.028607 4.174158 2.162192 6.193794
2.480054 6.888331 6.374598 6.554351 5.599637
9.250677 4.65686 7.864266 15.13693 6.431064 3.910418

3.929741	10.03703	4.375857	2.331546	9.670981
8.462245	5.476075	10.90735	4.615963	2.20066	4.524896
6.525647	3.91326	4.967295	1.097922	4.144032	7.041027
0.2227878	3.539045	1.691876	10.21001	3.794849
8.992993	7.728198	10.92426	3.97538	3.688814	2.172457
4.157682	4.264556	3.786275	5.561511	2.701409
6.011468	8.792789	5.278781	10.21968	4.452548
9.237847	5.013505	3.430481	1.015583	17.19585
12.82496	3.812726	4.941338	3.032421	2.904399
4.75124	6.734047	4.558818	3.397893	2.031301	12.00973
0.7080317	5.962055	5.73184	3.353808	4.553849	2.835543
8.530277	4.873066	9.924187	3.745903	1.451894
0.4927403	4.771774	11.31489	3.392574	9.923535
8.986681	8.94975	7.965588	7.901787	4.174356	2.884422
5.215657	1.967604	21.60068	3.0632	2.4622	9.522896
12.27405	7.921728	5.877312	6.052046	5.510117
9.408233	9.181497	2.496518	12.74934	4.074354
2.956965	9.42865	8.762938	2.778361	10.80033	4.261153
2.597052	2.408557	6.84612	2.733012	7.634018	3.524311
4.796027	7.654138	1.50755	4.163175	3.636685	1.473236
9.568278	2.832686	10.03754	4.845559	7.18155	3.76117
4.042408	9.47598	5.196743	11.01806	6.057926	0.4920705
1.075012	6.867429	4.97516	6.75905	10.34522	3.364089
12.25842	3.592627	15.80011	5.823628	4.467328
5.393188	4.435209	7.603662	4.222803	3.92956	5.817324
7.794936	9.041805	15.71192	9.378279	8.94694	10.28677
4.664974	2.072956	11.36434	6.768071	10.12463
9.251271	1.977508	2.417015	5.229656	5.603797
7.906297	7.273767	7.42422	2.809428	1.136061	7.989451
15.95095	3.724396	6.465981	10.16058	7.757188
10.60805	7.704696	5.90556	8.297293	3.194514	5.001539
29.4997	2.555393	5.950175	5.173819	2.988745	2.906512
4.497423	2.151823	14.02394	2.297833	2.594095
4.537926	8.442051	11.99676	9.723687	8.792276
1.680797	3.970935	3.13982	11.17679	9.825575	9.741529
5.388787	7.141644	4.561793	3.037815	4.694182
0.8667263	7.573415	5.784187	8.188489	7.337872
8.720217	6.151313	5.854661	3.877961	14.07505
1.650556	4.563597	9.222332	3.845106	3.918729	3.0216
5.669468	15.96132	3.504289	7.42493	4.24304	4.337683
51.66431	15.12793	5.265045	5.341585	6.966253
3.696096	8.881948	9.072216	0.3250728	0.01382342
5.906016	3.199502	12.5039	2.45295	12.62392	9.283451
6.527166	6.883298	4.533326	4.932821	3.937352

11.90875 0.3563246 0.6317665 8.673879 0.1514719
2.644399 2.365049 7.567634 4.52725 7.652704 5.572916
3.70722 4.500524 2.620638 7.745339 4.272506 7.439894
6.010553 6.172345 6.70731 3.921338 2.700504 6.190673
2.22244 9.153229 2.180521 4.152582 3.232941 8.839241
12.7065 7.872164 7.260309 5.964605 4.785984 3.571042
8.415742 4.529292 1.393611 5.776802 11.93933
7.454917 13.36994 9.447899 11.90929 1.195876
4.721527 11.19732 4.993882 6.700342 17.66256 4.2725
2.371511 3.375039 2.577783 4.694903 5.479709
8.668189 10.85116 3.690928 1.734689 4.553974
12.31432 8.212847 2.908654 6.741811 7.094739
4.957075 6.165668 1.055527 3.612789 11.75072
2.115568 3.593165 12.78545 3.864901 6.197565
11.5473 5.67898 4.06172 2.320949 5.211696 5.637488
3.388163 7.715348 9.239155 1.546074 5.924878
2.476026 6.213742 10.72149 5.452385 6.140213
2.81956 2.190144 10.44949 5.395785 2.689898 7.443443
8.603079 4.269301 1.923785 2.908628 2.606163
7.737535 6.844449 3.833147 7.559813 2.346307
6.851039 5.887524 5.742459 1.434547 5.692681
10.44287 9.357184 6.309227 4.225118
ID2 50.00348 24.93749 39.57678 19.41118 29.78207
65.48725 24.31055 33.61646 39.6105 51.22367 6.996239
3.974087 44.95323 3.230959 27.09106 40.84276
21.05562 35.22322 17.97148 27.7371 50.6236 68.20571
11.27434 38.42181 18.349 4.63007 30.97618 59.47242
25.28731 60.47651 31.5981 49.90158 31.9852 23.71961
37.66705 25.9701 50.50914 34.71382 52.30834 57.57053
41.51366 20.25395 26.05341 52.13015 12.84381
41.58896 16.92063 20.18429 17.23551 12.33027
20.24006 20.73215 123.2715 22.44979 52.95466
50.98044 39.52926 34.06943 10.45197 23.9483 18.94837
19.1845 29.06446 17.59478 29.82241 37.26325 15.20284
23.28117 7.745419 15.47913 71.03512 26.98189
16.82302 28.85143 12.25923 81.43606 11.08574
21.8077 115.5567 9.242838 67.34168 18.05098 90.56491
34.98695 27.15762 15.6174 17.49817 21.04759 17.24507
45.40188 22.3121 21.47557 40.36279 23.77803 43.00158
26.38668 41.91664 11.56595 52.78912 28.73147
22.04276 8.634764 29.42204 88.21162 20.70931
17.49535 23.03099 42.17594 48.87359 25.4565 36.0679
113.667 33.32504 32.31614 25.69224 13.20899 70.6269
19.22022 19.4117 54.7665 10.0506 65.39161 32.63014

39.12022 65.25498 16.0989 20.6893 27.66418 35.46556
24.3022 16.86474 35.42936 16.02322 37.20004 26.24868
21.75961 32.10705 12.60674 69.75102 38.51598
27.5726 56.12071 14.13162 37.0523 44.07399 19.01759
87.10671 36.24412 21.82204 33.46207 54.01877
18.3129 12.44875 37.87555 28.81339 28.71649 39.72859
82.03616 5.664066 36.96899 27.06291 23.44008
29.08845 54.99539 34.70893 25.67648 38.39583
40.89642 13.74721 73.59016 48.39346 23.40088
33.30068 49.01245 28.99745 20.416 36.80295 38.85403
15.83401 23.75782 55.39416 31.02371 11.25281
27.58078 26.52867 52.6731 43.98463 28.04638 31.49528
21.12867 37.84758 11.8176 17.35679 15.11973 66.21248
38.09766 42.34237 62.27836 24.15002 18.59821
116.3165 27.95639 27.17538 16.78733 19.64751
39.20906 29.21718 20.02009 12.45012 61.9679 15.37247
35.08606 31.6193 35.17272 24.54033 32.30606 39.03592
70.14358 28.64659 11.65383 16.45371 35.59263
16.81289 70.23409 28.4404 50.42294 37.13859 3.636544
16.39948 17.54168 17.08262 9.815062 27.87034
12.39844 30.56501 13.38265 16.0495 29.51456 49.83311
44.83275 64.10874 43.54333 38.97056 44.82436
46.34067 22.33096 65.83427 32.22713 16.6651 53.54022
49.392 24.41781 14.23411 40.42205 34.33121 108.473
57.87174 30.76188 18.49026 59.50073 25.7712 25.8309
81.54937 40.65522 21.18462 47.09914 8.712862
10.73062 50.5585 23.09477 49.58113 17.72858 20.28683
18.86691 31.00413 13.65972 15.11568 35.2868 7.481048
30.89154 39.18499 15.72384 42.54997 33.08516
12.85037 39.26056 6.882845 58.94547 21.97483
39.78329 21.38358 26.26941 32.41574 35.29468
13.25688 19.54478 36.41664 61.87727 36.02036
8.243333 17.44749 22.34178 18.50805 15.92749
23.59317 30.07366 20.95822 21.58195 19.90903
21.54917 29.72273 14.85319 46.76951 28.50779
21.08536 26.365 15.71208 10.08587 26.59872 34.55155
39.49811 15.25277 21.13326 41.83488 32.15082
80.96119 18.73206 27.38595 32.46469 68.24766
47.92172 33.2514 30.79165 40.6965 30.64635 27.80733
48.54321 36.61174 34.99252 9.599486 82.04111
89.27905 35.13754 45.59905 37.34961 10.93186
39.44451 30.00671 29.79169 32.56167 27.35821
31.89894 20.72121 17.16978 62.09271 4.251745
11.23411 24.20366 51.0943 23.38884 13.24054 25.79373

3.562516 34.84918 25.23589 34.90112 25.37165
24.36871 8.208803 19.09075 53.67536 18.69141
50.9604 15.90183 20.96108 44.13909 56.24386 14.69319
11.84968 21.78583 47.92184 17.68489 36.56207
79.33278 24.65375 51.88867 28.91698 19.78269
34.9598 23.72442 33.283 49.54885 47.85407 27.93986
16.81592 22.13252 18.80352 18.21617 16.57156
23.3089 41.47108 39.74691 43.21253 56.28346 38.89602
17.55288 39.52956
CRABP2 0.5283134 3.018497 0.8382861 0.5046769 1.957645
3.865667 0.9387159 1.56711 1.264218 2.316716 404.3913
689.6633 0.9382293 563.7122 0.9905003 3.023567
0.632314 5.280573 2.038516 1.495086 1.635984
1.841543 577.7348 2.022638 45.03144 375.2547
0.5226697 2.642263 0.5870392 2.457525 1.306361
2.356015 2.42235 70.01019 9.559131 0.4922081 16.78073
299.967 16.63286 0.9350977 136.1406 0.5705186 43.60582
1.307595 6.065881 30.69164 2.38211 9.838246 2.299704
61.42751 24.6848 22.75439 10.69803 3.395975 3.347769
1.0229 3.975484 4.481606 1.536263 1.647334 13.31575
3.17016 20.50778 11.86985 3.744226 54.8271 17.01254
26.17742 3.728905 14.38407 0.5301812 0.9189904
11.36714 11.25645 30.92585 1.887056 1.804821
3.171614 2.888985 10.72588 3.421815 22.3481 0.7781158
6.491777 10.47593 1.66248 11.40204 5.957316 24.56899
4.096871 1.820081 0.2949555 0.9139078 0.9254092
1.915525 1.011989 2.957413 2.489513 0.842378
3.869022 7.342238 56.83048 1.217292 2.122968
73.32195 8.369031 4.17272 5.310692 2.779769 0.7219383
10.74218 4.061755 10.84772 2.038454 129.0104
14.72283 10.21171 2.019519 48.61028 3.440617
12.15101 13.10094 37.93796 12.40764 6.500336
2.216308 1.473831 25.77002 3.193099 4.15635 1.6456
19.53307 20.96488 3.793006 2.066873 2.020137
1.846755 2.17582 4.317109 1.282451 2.168354 2.475508
0.7911552 4.68639 15.45815 163.7553 2.400364 15.70723
26.13189 5.044587 0.3661924 11.97061 14.63733
0.643195 4.246893 34.50492 5.546791 42.60093
124.5587 15.24543 37.148 1.692689 6.307704 2.006562
16.72679 4.141479 171.4199 1.888684 35.29156
15.20352 32.21933 29.14843 12.09074 239.8937
2.784266 35.06495 124.101 18.90534 4.968109 22.7114
9.803518 4.041849 444.2426 0.5906461 9.179018
31.78992 31.69111 30.48688 9.356773 0.2169626

16.72975	23.95337	325.5554	4.66435 0.6132102	2.161481
7.433822	11.12603	0.524877	5.737904	11.26891
2.121246	0.8287031	1.822177	24.66519	2.068594
36.74772	3.17946 20.88522	3.408482	5.727096	3.20072
2.427408	7.312742	1.939296	0.7857977	16.59405
27.99982	4.676892	24.01791	3.105948	26.0102 5.531541
3.11284 5.228804	1.81963 2.495625	583.5693	7.025905
5.200548	7.927423	6.35858 24.72083	1.896557	35.31155
1.164746	7.515033	0.6384525	23.26555	4.059222
3.294085	2.594053	7.584128	2.160387	7.894854
29.83383	2.133124	3.503007	36.88895	1.101868
0.6648851	5.2166 54.77829	18.14985	61.61018	17.59007
5.048989	0.8899052	2.653669	18.8861 10.60055	2.589885
39.25223	5.621302	1.465404	6.69064 7.42305 0.4023248
7.435682	6.459383	1.228197	7.143445	6.507276
0.8570668	6.003465	8.068126	15.73297	2.082588
2.237379	3.93715 4.212983	1.535024	11.07647	2.239495
2.303194	1.767666	3.3321	35.78957	5.19255 14.20645
1.960975	2.012155	8.353488	3.241995	2.732287
28.67858	9.864019	0.5346371	1.531026	61.43011
2.048635	1.646297	7.230828	10.79016	18.54737
4.892072	123.1047	42.40116	0.8927829	9.720499
20.5191 23.95868	2.483985	12.14978	0.7480808	2.695859
0.723039	94.92342	6.145897	9.91877 3.712463	1.103786
50.5135 0.5725157	32.09486	1.706823	6.61199 49.36512
6.853045	67.52241	7.714903	3.749059	1.226376
0.6382828	18.34338	37.16913	0.4294107	3.262302
0.8720118	31.74942	0.3696036	2.497752	48.69432
2.345619	5.474844	129.3585	1.417953	1.017936
61.12023	66.90648	0.5578672	5.771115	24.31603
13.45544	0.08844743	19.63057	3.145082	38.35325
1.021205	30.26817	0.6079479	68.24405	26.0787 1.965083
30.4835 9.532629	18.45466	1.025228	35.81746	82.67115
9.323558	167.4377	33.62377	9.382202	5.675083
6.532103	53.62815	86.62115	117.7983	26.18912
29.93673	19.83108	0.7089562	6.204873	0.7528878
0.4767748	17.63771	52.15747	2.394067	28.34431
3.302081	11.26989	4.969642	6.448774	78.49759
11.24903	3.925318	3.286596	0.4585369	2.230434
45.8589 1.565822	0.8419358	3.45971 1.339542	10.67306
3.166197
CEACAM8 0.01111175	0	0.1328732	0	0.05101226	0.0562662	0
0.04661045	0.0600412	0.04176544	0.04292144	0.04366122	0
0.02083659	0.01811539	0.008232129 0	0.00418319	0.006860015

0.05933096 0.03246117 0.01106636 0.1342949 0.2060586
0.01141572 0.1004419 0 0.02196578 0.01073644 0 0.01570059
0.01992076 0.02681478 0.1574855 0.01356935 0 0
0.009979923 0.1332279 0.1524227 0.3364986 0.2057046
0.01734816 0.06071872 0.1773543 0.1512623 0.2262659
0.1254079 0.01131428 0.1198122 0.03206814 0.07338603
0.2920612 0.1002468 0.1624891 0 0.03263001 0.03417259
0.1392735 0.02259623 0.1766166 0.03030746 0.06640102
0.05201099 0.259953 0.01505911 0.05306597 0.01123053
0.07256702 1.517517 0.03321586 0.03221445 0.2308352
0.3419165 0.419365 0.06028787 0.08627252 0.004522508
0.04205024 0.1687178 0 0.1699293 0.03570703 0.06284191
0.02340878 0.03765582 0.1028997 0.01373121 0.5138924
0.07520069 0.2891142 0.03101826 0.05220728 0.0562805
0.006394966 0.03405547 0.3177408 0.2164632 0.009324905
0.05631504 0.05564889 0.0825173 0.05120541 0.0599794
0.0515959 0.1047748 0.2484608 0 0.02456534 0.08147604
0.09420966 0.03122726 0.0780016 0.07349795 0.7477135
0.03528867 0.01864931 0.06602415 0.008208719 0.1278735
0.08049326 0.2211537 0.07679791 0.4744796 0.01602168
0.0269448 0.08638889 0.1021523 0.06175528 0.3599583
0.05601795 0.259338 0.1469813 0.0615818 0.2111473
0.1416285 0.09512299 0.07776708 0.6114452 0.2551515
0.09461808 0.3861083 0.09940764 0.02924823 0.1153664
0.02695859 0.1009714 0.09499996 0.2154409 0.1573866
0.03080777 0.09431622 0.04027601 0.01104327 0.02842089 0
0.004964379 0.01973577 0.09364725 0.5018861 0.008374229
0.06752006 0.005681662 0.2729297 0.02053344 0.01764165
0.02658842 0.1402015 0.03026585 0.02051988 0.02751891
0.01279216 0.1778311 0.2204908 0.02884734 0.06162371
0.08061836 0.08000537 0.06413818 0.2507325 0.1508726
0.1234472 0.07466366 0.03623307 0.0609656 0.0205941
0.06492305 0.248067 0.08705777 0.120086 0.1286452
0.07611147 0 0.07644387 0.05732154 0.4278717 12.56849
0.09382405 0.1754994 0.03244153 0.03419489 0.1001564
0.1797438 0.2585233 0.1156328 0.03446156 0 0.1023878
0.2830947 0.2033358 0.085278 0.2421127 0.2681649
0.0183831 0.2985807 0.02430484 0.005945731 0.05721811 0
0.1267212 0.1918299 0.1112862 0.005831687 0.05571985
0.1598753 0.004502514 0.1262036 0.07762983 0 0.4552896
0.1128272 0.4845539 0.6014649 1.411469 0.2729745
0.03888497 0.008003036 0 0.02509399 0.1021104 0.249668
0.1781533 0.03683908 0.0963844 0.07534489 0.1173857
0.1387575 0.03336318 0.04856757 0.06734627 0.06492664

0.2131667 0.2172844 0.02014443 0.08334501 0.1629196
0.08274771 0.08638586 0.06259436 0.08582785 0.05090323
0.007781683 0.09933836 0.03443594 0.1967305 0.08451707
0.01924503 0.01813264 0.03690643 0.1378676 0.2127346
0.03019991 0.1008066 0.0253891 0.1198868 0.1275469
0.1265608 0.02223454 0.01191334 0.0397479 0.08325733
0.5909206 0.07648673 0.07679706 0.02026859 0.04310544
0.3153708 0.1027319 0.1563666 0.1310911 0.1703565
0.0460633 0.01830154 0.03788183 0.01702721 0.06592158
0.1065289 0.08745923 0.1215993 0.09482782 0.09651693
0.1409544 0.06159101 0.1612033 0.1031508 0.1161315
0.02405203 0.06934103 0.01950905 0.2763294 0.06063175
0.04193434 0 0.02165596 0.05006277 0.03240039 0.06691221
0.008103408 1.547762 0.02770472 0.421867 0.03316484
0.1821004 0.01146804 0.09712739 0.09971876 0.04618452
0.007293798 0.393444 0.1166773 0.3155128 0.4458176
0.02744018 0.02856316 0.009067151 0.01594344 0.2846317
0.0300501 0.05557756 0.04467933 0.03886845 0 0.05166744
0.4468007 0.008314057 0.1514674 0.02444517 0.06422921
0.08105834 0.01884447 0.1428407 0.2564387 0 0.03309961
0.1767258 0.1654672 0.0826862 0.2161782 0.1642473
0.008597103 0.67952 0.2771825 0.03894018 0.1296647 0.3667637
0.05865547 0.0060743 0.07961767 0.07011353 0.05120087
0.1186673 0.2031907 0.0634016 0.077061 0.8059432 0
0.005175198 0.3587153 0.03831946 0.1286185 0 0.08394968
0.39763 0.03870882 0.1266809 0.08022215 0.01523471 0.08217245
0.04378541 0.1381047 0.04584226 0.1217293 1.614045
0.04038725 0.0489113 0.3978151 0.0696283 0.09347912
0.01071576 0.1132349 0.02096039 0.08952702 0.2223798
0.05076723 0.02633076 0.2993084 0.008820273
PITPNC1 0.8807475 1.07673 1.461338 3.74167 2.641961 1.819678
3.307275 4.228932 2.022371 4.693283 4.119805
1.102461 4.720179 2.168985 3.289789 2.596237
1.957027 2.379756 1.310031 1.09532 2.870425 2.192874
5.051621 2.220829 2.381092 1.591966 2.852992
1.583856 2.574542 1.796119 2.08462 1.287496 1.065396
4.43011 2.651796 4.557969 4.296163 1.977589 1.966256
3.930125 2.136744 3.089277 3.444424 1.085821
2.62324 0.9570783 3.063666 1.541854 8.368242 3.009716
0.9287785 4.607138 1.879793 3.525893 3.528682
4.403938 2.125275 5.190553 3.892131 2.745755
5.30557 3.575502 2.866589 1.915586 0.4500378 2.830454
3.176798 3.408532 3.653484 1.682218 1.131508
2.627474 3.332507 2.728835 3.834551 2.742138

5.130087 2.559176 0.867709 1.152188 1.970796
4.331369 3.023297 3.23088 5.280575 1.689352 3.034163
3.719754 1.661382 4.040807 2.859572 4.962827
7.147609 3.886856 2.803893 3.208063 7.017144
1.485062 1.032269 3.437223 4.84592 3.181798 4.679469
1.635973 6.194841 4.183904 9.998283 2.949173
2.352969 0.7332455 1.314319 0.7310591 5.086963
4.365137 5.718628 2.733639 1.295239 2.10655 3.250481
0.7114748 3.774612 0.8975514 1.746541 4.205173
1.023439 9.699942 5.292344 1.503216 3.287355
3.236639 3.253154 3.693576 2.869994 3.310262
2.434192 2.024917 4.870939 3.237065 4.482482
2.325943 3.004937 7.58154 2.045356 1.023179 4.465146
3.083499 0.7935723 4.238378 1.057861 6.256503
2.201494 3.851785 2.417861 5.66926 3.093242 1.61614
2.712923 2.68419 4.823211 3.120798 4.556726 4.393133
2.147209 1.514557 1.078156 4.308552 2.217141
2.709155 2.695026 2.461423 2.808593 4.404441
3.962961 5.148658 2.19004 0.8744333 3.007178 1.621482
1.024261 2.270546 0.9474909 2.604764 2.515792
3.444932 3.794135 1.710402 2.81545 7.05445 1.065024
6.048362 2.863509 1.846456 2.237366 2.525355
6.502067 5.449113 2.250692 2.416158 6.713874
1.534162 3.097781 3.578032 4.253509 1.057251
3.480519 3.204056 1.898645 2.857129 2.019812
5.187484 2.086752 3.72079 3.818534 1.026729 15.10528
2.580003 0.7874463 0.8331574 3.904928 2.082441
2.691081 2.301687 1.314542 3.639158 6.472987
2.672846 0.9746157 2.656845 2.117406 6.623652 3.1508
3.100653 2.561697 1.72549 4.517943 1.937709 4.558164
2.210818 1.890406 2.308917 1.113325 4.8522 3.112165
5.802294 2.746634 3.704593 2.156347 3.323236
3.576172 2.946591 3.457565 1.69423 2.609162 6.969568
4.327115 2.787924 2.057122 3.919551 5.409737
2.256885 2.439563 4.895339 2.693427 4.940714
2.633989 2.510024 1.142451 3.567837 1.806714
2.852974 2.529118 6.696373 2.046176 3.033048
2.751045 1.034896 3.499049 2.461368 19.10081
2.255439 4.974261 0.9305555 4.382768 6.451856
5.711702 4.469041 2.458111 5.384807 4.688621
3.693338 1.379379 1.750449 3.615862 2.158526
9.934247 3.914904 6.496713 3.037823 4.160175
1.969463 4.150195 3.166648 4.229052 4.7393 1.663742
1.738444 1.321227 3.347167 2.053631 1.913263

2.475339 8.300222 1.830223 3.985875 2.743473
2.845235 2.258168 3.546189 2.601453 3.255938
2.811898 1.603861 2.47784 3.988806 2.936886 2.541279
3.598925 8.67433 1.872543 4.102374 1.522512 3.563186
2.591625 2.539111 2.216709 4.271053 0.4192141
1.266301 3.468419 6.0169 1.559908 1.551141 5.086006
1.303351 3.9206 4.627882 0.6017157 3.594476 1.58959
2.152262 1.047569 2.056426 2.058215 3.568933
3.036103 3.120074 4.291511 1.558445 2.311334
1.932155 4.242184 3.930539 5.434405 2.268546
1.453418 3.384796 1.375325 3.625774 1.608854
1.568532 1.238244 2.475844 1.272248 1.859296
1.85608 5.398287 4.823746 3.83902 1.731712 4.591426
1.944589 3.206606 2.265162 1.374109 4.952748
3.495901 3.566586 6.837496 2.701757 3.44357 2.207745
2.169011 1.957603 3.498361 2.651629 2.728609
4.595779 3.542628 4.397318 3.835739 2.051602
4.970301 4.925151 2.19591 2.842611 1.656675 2.035409
AFAP1L1 0.5447078 1.374258 0.9115022 1.546315 0.9607076
2.621086 0.5991086 1.519568 1.083238 1.391908
4.023179 0.2962577 1.592042 1.840878 0.7224669
1.582265 0.6629854 2.252224 0.5661729 0.4206579
1.068042 1.357739 1.068824 2.356388 3.140551
1.485228 1.013233 1.557379 1.165612 0.6773522
1.121872 0.9379129 1.256557 1.308488 1.981764
0.856766 0.6052234 2.517986 2.951841 1.236464
5.191562 0.7596096 2.065997 0.5084456 2.192925
2.717365 1.113651 1.729995 1.87072 0.737388 1.971671
2.404105 2.205423 1.587162 2.766124 1.089542
2.937029 3.227305 0.7714472 1.380958 4.479188
1.951552 4.125497 3.18703 0.7665352 3.234363 4.606408
2.233224 2.071612 0.4965996 2.252506 4.766537
2.054917 4.711802 4.99782 2.081555 2.785985 2.988529
2.061339 0.9043951 1.102291 4.269251 1.32845 2.452427
2.269104 4.2997 2.799184 1.665676 1.844948 1.805018
1.120999 3.927638 2.245091 1.18462 0.8926403 2.307647
1.369214 0.7680128 0.5939911 1.671964 1.822491
0.8415465 1.255067 1.901098 7.338552 4.092672
1.429518 2.40201 1.115769 0.9108102 1.369817 0.8772067
0.9019194 1.119555 3.106234 1.871594 1.642471
1.73348 1.204922 0.8596208 3.178335 2.257951 3.136541
2.633894 1.203979 1.543239 0.9767336 0.8620584
3.506191 3.446491 1.021734 0.4519701 1.365252
1.531878 1.382835 2.286793 1.879255 1.643508

3.214115 1.542521 2.285064 1.869729 2.317421
1.820973 3.290974 0.6172134 1.302343 3.225682
1.993428 2.774657 2.53126 4.992062 1.079208 3.648763
2.92137 1.169235 2.785557 1.89849 1.106602 0.4427388
4.582891 1.536796 2.080245 4.087405 1.982804
1.468221 1.227295 2.444811 2.284163 0.3940258
1.821532 2.143419 2.219581 2.533449 0.9587264
0.8314035 1.708804 1.808077 1.33225 10.38416 0.9811684
1.526081 1.768003 4.822484 2.161238 8.274048
2.908079 0.4913316 3.905982 1.058944 2.256492
1.462585 1.185281 0.837448 0.6879353 3.872821
3.996983 3.109023 0.7181036 1.121305 1.536575
2.080403 5.798706 0.7612111 1.589603 0.6704848
2.940616 3.516231 2.846073 1.334225 1.484003
5.16153 1.828169 1.771763 1.066151 2.145946 1.258074
1.852855 4.146105 1.282597 2.736814 1.745452
2.07057 1.005274 2.761344 0.3641209 2.608736 0.6378302
0.6420525 1.623849 1.465182 4.544878 0.4736439 1.5855
0.7154473 2.059099 1.884002 0.6080907 0.9521339
1.391998 2.067482 2.98817 1.742626 1.227926 2.324271
4.767215 2.00858 1.592371 2.174576 1.977093 1.986286
2.14271 1.97727 5.054647 1.116765 1.763489 2.205155
1.744924 1.5111 3.913197 2.684232 1.366377 2.016248
0.941001 5.78634 3.810716 1.327167 2.152724 3.416498
1.692779 2.558712 1.810803 1.340102 0.6244073
0.8739924 1.400699 2.333847 1.807973 2.006063
1.401729 3.478532 0.6307872 2.139514 1.939756
1.861426 3.593406 1.094216 0.4655782 3.773068
4.291644 1.405495 1.652086 4.62771 4.343491 2.452192
8.799993 1.041477 2.269965 0.7883012 1.62305 2.443955
6.683967 0.9683627 7.419592 0.5350015 4.932532
1.42552 1.835075 2.679943 2.707527 0.9931391 1.103319
1.423546 3.174785 2.709634 2.051067 4.363351
2.919797 6.15196 2.909577 1.188875 2.219129 1.596578
2.007307 2.629853 1.668098 2.347857 1.405953
1.747646 1.109924 0.5367785 3.557981 2.228223
2.351055 2.697012 1.430654 0.2152663 1.093021
2.465867 1.453959 3.875319 1.488206 1.949273
1.704907 3.702599 1.276464 0.7068076 0.8206446
0.9510397 2.797412 1.797168 5.129348 1.031315
4.957552 1.380762 1.178404 4.273764 2.430336
1.210706 7.176341 2.153122 6.15158 0.8288767 1.247913
1.767051 2.023225 0.5147844 1.128247 3.767136
3.576268 3.483732 0.9341565 1.284758 1.240905

1.788499 2.764821 1.364494 3.400746 2.547055
3.323633 2.560873 6.179659 1.259099 1.255838
2.586919 0.7287454 3.181363 1.174312 1.106547
1.102128 2.612878 0.9964188 1.098691 1.129727
1.267923 3.602964 1.575765 2.346399 0.8131695
1.689007 2.465218 1.13348 1.035607 0.8904787 1.018021
2.743813 2.288913
HSPB1 82.85046 606.5159 85.18925 97.0144 281.2791
457.4997 202.2203 253.3953 490.3232 369.1464
1479.874 2572.253 201.3328 3507.203 203.1999
926.2546 175.7132 579.9767 258.7094 182.6864
621.4027 1078.456 2191.448 610.0097 1254.519
1133.587 400.443 894.5604 120.1383 328.639 552.4359
685.8937 509.9669 2699.263 555.4864 84.2998 151.8254
1067.482 305.3631 276.9183 476.3974 153.8083
196.0746 243.0564 253.1049 545.2973 97.93485
360.9421 113.2774 90.14072 624.3996 216.7021
536.772 576.0568 924.537 233.6031 177.9005 691.8849
464.9816 393.2526 204.2662 425.3112 343.3455
368.4576 573.6306 970.6437 660.6925 257.2651
250.1166 183.5475 164.143 70.83006 205.1255 171.0141
339.9141 527.5354 291.3988 75.62645 260.2801
283.1626 1550.349 167.0546 141.3275 444.072 405.4956
388.7576 216.8891 122.3786 202.9237 455.5801
187.5775 22.90418 839.587 266.0502 447.5662 122.4098
272.6001 187.9192 183.5563 325.6477 144.8094
183.6927 713.8853 169.0367 430.771 183.3058 262.0756
853.1056 328.8265 562.6041 243.0682 90.61198
474.499 663.3585 1821.596 234.7588 966.6341 325.5755
260.0397 192.6543 349.5957 56.4466 374.0241 552.5035
153.1255 148.7867 108.4968 680.7742 568.3778
850.8816 365.7243 417.2125 272.1542 81.9965 494.9435
256.667 211.0777 261.9988 126.3976 97.41733 100.6725
99.08176 247.0371 173.442 310.6965 164.8734 115.6532
531.1336 205.2408 229.7981 343.8443 448.7296
177.9641 105.3769 479.5364 1709.112 228.0325
631.4686 2834.591 564.6213 348.8901 174.6298
86.70842 95.84257 592.5502 171.5586 266.9239
158.2002 154.6819 430.7646 869.0631 85.65551
299.5079 1734.437 155.8483 944.6441 620.4265
670.522 121.4115 96.62232 227.4831 258.0324 2619.543
59.6761 1880.765 129.9978 459.9893 377.437 165.8474
27.92784 248.4814 716.3897 841.7271 162.003 86.38427
195.7379 319.2938 132.4967 14.72268 901.0742

258.5317 823.5982 230.8658 277.1933 431.7499
196.5311 683.6317 243.5442 796.8942 554.1098
87.41295 298.8617 308.8896 622.1289 170.0594
174.9106 1117.845 215.3726 175.3356 229.1737
381.5408 153.8493 496.9364 108.5608 206.9901
257.6534 121.22 5503.396 287.4114 1110.708 1298.443
295.7207 88.23124 333.2393 86.12524 453.9695
338.382 311.69 338.3949 168.9974 471.7144 115.3183
225.9078 175.6472 179.0791 283.3531 567.5042
591.3134 297.509 136.6635 175.0264 209.3025 298.787
236.8883 83.91037 203.3921 162.1418 830.8218
98.20387 317.5501 154.0237 167.2263 511.25 601.6466
201.8026 95.91169 198.6158 134.7637 313.3263
327.9335 626.6695 1219.136 220.2121 54.80428
63.35191 256.7144 260.2011 1288.647 148.0929
122.229 414.5644 189.3322 1407.25 170.3115 155.3158
119.7572 87.69321 597.3416 236.7309 198.1105
63.35847 1414.698 682.7016 197.546 433.5714 446.0939
444.6002 809.2285 1021.993 301.4927 196.4763
139.6529 229.5503 203.1789 482.7292 574.6726
76.88793 269.4132 140.3569 542.3874 440.1558
520.5637 391.448 420.3491 78.66523 376.1233 159.2146
225.5339 204.2199 176.8987 70.78332 56.24987
262.1098 110.2102 175.1986 320.7691 278.304 1326.293
108.8455 305.3465 541.4721 281.2438 208.6986
510.0495 1139.139 265.4362 460.3945 180.3019
291.7291 276.8847 76.49823 1073.512 247.7115
296.0585 378.1569 1753.833 295.6627 62.39373
1008.939 911.8595 77.23333 182.3253 93.96307
433.2436 266.5264 581.7208 259.4213 642.0716
1185.319 189.104 245.6539 342.1882 170.4324 717.9235
209.5435 227.9853 96.73477 229.5426 60.13693
517.5834 210.849 1386.025 259.9132 198.1407 325.4227
496.7047 146.8189 548.0882 80.88028 214.5446
277.1374 91.13901 184.129 84.68336 74.78931 249.439
505.2502 1004.601 38.37358 242.9727 540.1384
126.0431 65.86817 76.76417 408.507 122.5161 107.873
307.0756 95.52073 288.3299 541.0365 152.6914
257.5979 97.64441 216.7709 482.5599 794.762
PCNA 31.1906 13.20322 54.56429 37.27696 50.66679
13.70336 38.63848 17.32947 35.93933 71.784 28.34179
45.75501 50.13383 28.31804 44.76325 13.24402
46.55489 22.42094 33.51204 43.8134 61.81499 15.52176
33.18434 17.02755 13.9621 32.17885 44.2283 16.13489

38.4205 77.20641 33.80019 16.84782 77.78578 65.26234
75.40617 189.1656 43.55482 46.37584 102.6279
132.6141 44.9204 142.7464 138.4093 102.9238 46.06185
65.26986 79.72267 176.8322 80.26259 55.42482
134.6064 74.585 88.47556 165.1403 47.96957 162.1863
125.6274 20.84693 149.998 57.0539 38.29334 84.03563
81.03531 83.6648 108.7203 21.64404 64.66024 40.52801
51.27355 89.64481 153.5077 234.5041 52.14456
85.64818 58.0342 236.8007 35.65779 94.90834 97.91122
297.8692 95.20802 40.05846 192.9473 50.71825
60.37127 79.53528 44.00363 89.76777 211.74 56.99413
122.7125 161.7403 153.7592 61.78919 111.7395
146.8611 98.36981 96.2538 192.748 82.0713 35.62614
60.19516 146.3275 261.2325 62.208 35.74672 87.01278
41.99973 115.4369 135.2675 97.60304 233.5343
157.9552 54.97413 63.34744 58.65511 118.0134
113.7406 40.47274 356.4911 121.7824 105.9474
148.4833 38.96527 200.094 100.4459 180.0607 78.52441
33.32814 91.18556 67.56011 140.9277 64.83986
145.302 58.9973 25.81935 151.8333 123.7095 148.2732
216.3852 71.4189 270.7375 59.30014 172.1334 136.0567
61.04763 159.1626 31.44576 181.0584 55.18549
103.747 47.44021 108.5169 151.6839 30.20607 36.81192
153.9378 25.54139 107.4054 84.95766 67.42271
47.69578 125.6781 110.0568 111.5268 85.31142
70.15894 37.66239 85.1981 68.62773 25.62615 64.94732
73.03676 113.8212 110.7998 81.12857 46.5393 133.6922
77.21908 84.83684 157.0101 105.9955 83.23262
155.5264 80.08282 56.08678 37.73264 135.21 39.63557
276.6663 71.1235 75.10387 170.9506 68.68709 120.7183
45.25739 86.16676 78.62734 250.3635 113.1051
68.38688 134.5559 76.26139 206.0197 195.3387
137.289 54.81639 153.2907 57.05268 115.272 65.32649
71.31065 73.02943 174.9234 59.96951 118.568 43.03998
123.758 95.57661 77.9443 56.4797 84.62081 54.31273
93.90254 55.76559 140.0723 231.0321 40.06679
195.961 32.85842 72.59636 57.05864 121.3893 49.85615
152.1254 55.99197 83.07155 163.6995 172.6839
297.4235 69.00899 132.6295 85.43683 110.051 143.8627
78.06413 186.844 24.55426 55.84673 245.2251 221.8377
125.9441 74.9174 115.9912 404.3005 41.97114 176.7661
43.76698 138.1283 76.80313 123.0403 173.4169
121.1813 172.1214 72.35878 100.6349 114.858 94.09231
61.50811 17.6463 110.8694 103.6685 71.75055 192.1004

82.32738 82.95745 47.72544 19.72367 134.0696
125.386 42.6048 149.8153 42.27554 137.7783 130.953
132.7783 129.8728 182.3112 69.20667 79.97321
86.41391 14.56562 23.59578 98.57274 106.6085
32.42785 82.57769 213.4248 94.63328 103.4211
58.42243 61.41266 126.5457 40.24539 78.87474
91.91033 109.3092 104.3667 95.44289 104.4663
46.70948 120.4427 187.1385 56.51484 151.1672
45.9634 39.05885 75.55657 60.11888 68.40929 193.6065
79.96957 67.81476 198.302 67.49114 75.09169 115.8281
68.98019 88.84059 64.73228 184.9127 63.47796
88.91703 145.2927 18.10129 67.94686 66.56693
233.2056 155.2549 30.61893 90.91722 214.8365
106.0962 84.60169 25.9831 24.37801 62.02942 313.2273
103.449 53.33178 101.6076 76.0698 50.63363 138.3711
194.0718 87.81121 58.47504 130.8472 58.93561
86.27304 113.9662 71.90835 80.16145 148.9063
153.9819 165.9062 92.87615 84.83934 33.41071
122.1123 73.27528 38.93872 145.3854 106.3739
168.1913 168.5448 133.6294 62.07657 50.34963
88.27701 164.2417 65.05971 63.09973 158.5839
89.88522 177.3512 42.47934 94.83394 111.5118 102.65
31.63593 89.63704 121.8412 103.3511 89.39301
106.9108 133.9282 35.49213 15.83007 106.9036
114.8643 82.42956 118.9343 123.8593 151.2468
123.802 61.44137
GSK3B 7.12639 5.122551 8.474761 8.201315 6.512515
8.152584 5.562821 5.913581 6.401675 5.2703 11.0551
7.173315 5.133974 7.437099 5.591595 8.594943
6.168339 6.286601 6.726542 7.069559 6.898178
6.705095 8.084963 7.933299 6.543896 8.656856
6.580964 9.503585 7.813247 2.343006 9.000139
6.116315 13.42855 10.40793 9.681306 5.744393
7.840246 7.670174 10.107 11.26926 10.09858 11.33899
15.1407 12.24835 9.715294 19.27133 10.55578 9.319823
16.40469 14.74057 10.53307 8.456303 11.92255
9.389233 7.540022 11.21001 11.82765 11.29388
7.591523 8.714038 12.15163 9.552848 9.167034
7.721206 10.54363 7.965927 7.534876 7.732625
12.95436 8.148707 12.2659 6.974908 10.06021 13.95157
10.71247 6.386716 8.58761 13.53842 17.4117 17.62577
13.19882 12.87297 11.00597 9.639782 10.73117
7.109124 13.78202 9.687653 6.975211 10.08098
12.24136 10.9947 7.583696 9.139366 7.527992 7.443502

10.89377 6.867784 23.07646 8.054813 10.62422
8.894368 5.130463 6.782865 7.30474 11.33589 10.76478
7.477453 15.03495 7.957144 14.71767 10.17353
8.066866 5.82361 7.770232 15.97262 5.872134 8.446476
6.350456 10.66396 9.02738 10.09734 13.96036 6.930269
20.96928 9.281514 7.832764 6.518148 10.75625
11.72037 12.09572 12.35904 11.56266 12.59348
6.259775 12.1641 14.39233 7.64572 9.947872 10.81279
15.34129 17.59298 14.16377 10.49913 9.847029
5.973054 7.380514 9.074676 23.046 9.031714 14.67148
10.04634 12.61927 12.39206 9.009928 7.715312
7.955155 7.273571 19.05123 6.866891 9.540638
7.809149 13.92408 19.25081 5.309579 12.23479
10.30859 14.28603 10.36272 7.973633 7.018522
11.13936 11.1194 15.35665 12.50919 12.95966 12.02863
11.07219 12.40906 13.24418 10.76119 13.43074 7.932
10.13567 11.69402 12.32546 11.7353 9.028274 23.17807
9.474874 11.66116 16.21847 9.202095 6.99683 9.230407
14.01051 11.62245 9.047707 13.6781 8.239743 6.2128
7.546543 10.45923 9.340412 7.69117 13.7452 6.391641
12.66023 9.13422 8.908203 16.47585 10.39437 12.46315
8.320198 12.73055 12.3145 9.809317 10.67007 10.30046
13.13598 11.21229 12.83517 7.432545 9.83828 9.60533
7.494465 16.12713 6.199599 7.616428 5.551283
17.44159 6.130319 9.375473 9.040162 17.14514
10.87967 5.967835 8.816773 12.03239 14.43043
10.14606 16.20415 9.221899 9.678478 13.22498
12.25715 9.106776 5.628515 12.46881 8.468966
13.6312 13.8598 10.66264 7.915469 14.04058 21.1217
9.648956 11.36925 9.118558 7.152162 6.562489
6.173865 11.37032 10.24301 13.0958 13.60138 12.01099
9.105109 8.528735 14.56963 11.13753 7.763123
9.001586 13.99832 10.7042 9.424367 12.59448 7.801343
9.891565 14.39799 11.49544 9.255649 6.341484
10.14908 15.30885 11.30938 11.44837 9.712736
11.93536 11.13275 9.790337 9.070651 7.615759
8.048859 14.01801 7.843788 4.782646 12.42248
12.37289 9.017968 10.18718 9.211993 16.70679
10.03714 10.39403 11.35534 10.03619 12.92747
17.67819 10.17714 11.62645 6.115717 9.175237
6.165722 10.28074 10.41815 9.326354 12.70604
9.423885 16.84417 10.72379 11.81407 9.183626
6.783653 10.10899 9.24369 12.41684 14.84933 7.683453
29.22729 10.76015 8.388356 11.9707 11.62093 5.881745

5.749196 6.781393 8.688145 12.84527 11.75758
14.74213 6.086604 7.106729 12.94394 11.67445
9.216265 14.50309 12.75814 13.66648 8.881911
12.07726 14.20952 11.52697 6.975469 9.36343 11.84392
11.19473 11.85468 8.719881 8.56462 9.107177 6.47635
15.66621 10.65162 8.903331 7.759249 15.44185
8.80937 12.15926 7.930398 8.814096 7.099184 12.62896
14.86854 9.071965 7.685049 5.622187 6.737113
8.209119 7.35552 11.44117 10.65647 5.444049 9.009371
9.602407 7.294905 6.167893 9.517461 15.01671
16.06219 11.7901 14.41095 11.95083 21.45628 12.89255
9.939144 14.75351 8.539623 6.310658 10.87386
9.025698 6.844766 11.86656 12.83317 10.28782
12.77275 8.049419
NGF 0.2358992 0.8757552 0.178697 0.198834 0.4004278
1.672321 0 0.6652276 0.5141473 0.9040535 0.09528989 0
0.5571479 0.1170939 0.1357357 1.398125 0.1384005
2.350797 0.8224156 0.2370972 0.7945391 1.506352
0.5472584 1.726583 1.691714 0.1199788 0.197686
0.8448747 0.2681546 0.9446074 0.4313539 0.5846134
0.227708 0 0.3840976 0.09125719 0.08663483 0.2367968
0.429357 0.09824331 0.1008531 0.02446525 0.2888599
0.1873346 0.1286019 0.03063199 0 0.1342372 0.6216909
0.0997481 1.105294 1.139016 6.267364 0.5216201
1.731562 0.1364673 0.04074855 1.081098 0.6261322
0.03762442 0.7719597 0.283861 0.4698913 0.4059478
0.09547966 2.087456 1.420052 1.192104 0.0836512
0.1454029 0.07110886 0.5229848 0.3294496 0.7245848
0.1727689 0.9034543 0.1616065 0.4179324 1.890453
0.1631194 0.07238316 1.894721 0.104046 0.4708642
0.7015926 0.5374266 0.4854511 0.1543285 0.4753718
0.4852073 0.3476754 0.1394489 0.1896633 0.04685562
0.2715265 0.2126434 1.371975 0.0584477 0.05822499
0.4078941 0.7123203 0.03636992 0 0.04993509 0.7302439
0.4815038 0.1401261 1.433748 0.2576897 0.3514923
0.3193346 0.1337033 0.3896355 0.4742209 0.3931575
0.03305153 0.3881562 0.1429157 0.522806 0.07258602
0.1723208 0.3582851 0.6649462 1.391165 1.093769
0.2557311 0.01269211 0.0900483 0.6426697 0.6550121
0.1399111 0.03159638 0.4235795 0.09787747 0.2481714
0.2652997 0.3035749 0.6872825 0.3970598 0.05462311
0.09452771 0.04383404 0.1295383 0.1607116 0.2488487
0.1009982 0.288214 0.3915005 0.09783414 0.7997365
0.3077835 0.3010001 0.1131681 0.1792817 0.9025156

0.08714325	0.781143	0.6736616	0.03898242	0.417839	
2.154304	0.9505103	0.5676235	0.378707	1.042786	
0.2203101	0.1328151	0.3890765	0.207879	0.4270892	
0.9278761	0.07987466	0.3849328	0.3812542	0.1440989	
0.6266422	0.4027068	0.09991119	0.04004807	0.2729731	
0.3489085	0.1541616	0.3885018	0.1551363	0.1015123	
0.01714535	1.156689	0.244075	0.2288806	0.2279454	
1.416126	0.05849136	0.506323	0.1060706	0.2624728	
0.667911	0.7712613	0.0551379	0.0706983	0.3241054	
3.074601	1.046089	0.0544158	0.02265582	0	0.05379475
0.6353837	0.5274326	0.2239025	0.05208759	0.1597435	
0.5604571	0.03864069	0.321397	0.2397017	0.3035204	
0.1336512	0.7475222	2.093385	0.07193191	0.1165419	
2.516178	0.4369588	0.3876784	1.101118	0.08996427	
0.2149144	0.03231487	0.01693019	0.4437608	0.2504877	
0.1775001	0.01809911	0.07663701	0.03325778	0.3884783	
1.099366	0.02353584	1.378851	0.2125267	0.5820022	
0.7508662	1.159323	0.4814617	0.3618891	0.4664292	
0.5776042	0.6249621	0.4447775	0.07915523	0.2744273	
0.4227944	0.2325824	3.874098	0.1189506	3.407872	
0.2066717	0.7335785	0.09119625	2.893923	0.1271365	
0.2137919	0.3012748	0.1576806	0.1801641	0.2955272	
0.09613326	0.3472103	2.235315	0.1147799	0.6072326	
0.4902798	0.2711431	0.2156012	0.05795435	0.1108045	
0.09219575	0.5664392	0.08926468	0.2647329	1.054576	
0.05864466	0.6527002	0.6010024	0.04049847	0.5921338	
0.1531588	0.1166292	0.4027477	1.343529	0.1679544	
0.2444776	1.131326	0.40211	0.1913732	2.187447	1.104182
0.1248224	0.2137204	0.1302637	0.05165611	0.4438885	
0.3313272	0.1761477	0.02342098	0.3132556	0.1501816	
0.423346	0.3532638	0.09326537	0.08653406	0.2618393	
0.1054435	1.586587	0.05358745	0.7283124	0.1671208	
0.1517938	0.1274409	0.4290131	0.2403085	0.3810314	
0.02598949	0.5155688	1.39844	1.344918	0.2050685	1.512018
0.07559005	0.1393722	0.0703597	0.5756765	0.3837956	
0.2568229	1.630528	0.1327351	0	0.300214	0.1110489
0.1115916	1.553253	0.5623265	0.5018426	0.4494737	
0.9344381	0.2161754	0.09768728	0.149725	0.2576666	
0.7059927	0.3822436	0.2704268	0.05916381	0.132272	
0.1858494	0.1574373	0.1677959	0.2799633	0.2840027	
0.5797502	0.8942118	3.299936	0.2223028	0.04048151	
0.1382692	0.2929973	0.03034249	0.08285586	0.0875582	
0.2173958	0.6075892	0.6597391	0.2903131	0.4261802	
0.9045461	0.6379956	0.142182	0.2067535	0.1555241	

0.530743 1.512729 0.1655319 0.3310419 0.662946
0.1371066 0.08014553 1.541041 0.2052349 0.02733975
0.06387628 0.1831939 0.0168907 0.3642704 0.4034868
0.02443229 0.5229409 0.3602307 0.5652543 0.1070552
0.1414085 0.5060591 1.181906 0.06171316 0.02113281
0.05261123 0.3364003 0.4185628
TP63 0.01364372 0.05964116 0.03585721 0.05585705 0.06902758
0.06908726 0 0.02102374 0.03911803 0.1343107 41.05881
29.8608 0.1655454 18.83568 0.1588803 0.05775964 0.02721591
0.1423513 0.009626477 0.02497727 0.0478295 0.09317479
21.23313 0.1189273 0.662134 29.32932 0.09872805
0.1171312 0.04143194 0.0132681 0.09363694 0.05066712
0.06773125 17.53229 0.2221509 0 0 0.09978249 0.04372339
0.06209731 0.1589738 0.1769759 0.0263729 0.03257837
0.1685938 0.1104338 0.179168 0.06285054 0.1032007
0.2117183 0.02250018 0.2390623 0.282289 0.2344561
0.1862136 0.4576457 0.06582143 0.3416686 0.03908776
0.0871989 0.07966331 0.1515119 0.09900246 0.01368481
0.02950467 0.06867915 0.07446606 0.1477453 0.03329102 0.3649
1.093692 0.06074514 0.09062185 0.0218092 0.09555278
1.20908 0.3662184 0.1888029 1.18311 0.003818665 0.1762288
0.08516331 0.06889675 0.1002095 0.004106114 0.1490883
0.2587102 0.5876935 0.5809115 0.3385662 0.007513078
0.2111065 4.470591 0.2204773 0.4554247 0.06770128
0.377862 0.4166398 0.03271348 0.3812975 0.390453
2.275018 0.0179638 0.03039382 0.04424638 0.8130632
0.2755526 0.0703639 0.04653705 0.02078785 0.2030244
0.006260056 2.36702 0.2191676 8.449195 0.04023471 0.04143597
0.07103162 0.07199423 1.180644 1.405875 0.1279101
0.09339934 0.1809306 0.04309203 0.3384077 0.672098
0.008432212 0.1480434 0.02886399 0.0547452 0.02440941
0.3807783 0.0942721 0.07843167 0.04720157 0.06859572
0.02098625 0.1758951 0.0537071 0.07169852 1.093893
0.1243335 0.02873028 0.160051 0.1592024 0.03289242
0.8915167 0.3693144 0.8986574 0.2647943 0.09374893
0.08477738 0.2227654 0.0826133 2.943785 0.09578775
0.1869391 51.08121 0.1565074 0.1498295 0.6287895
0.1215871 0.06383251 0.04081987 0.389908 0.08394935
1.06568 0.4432938 0.02639542 0.141594 0.0605843 0.3535231
7.532917 0.7792535 0.166773 0.1791219 0.01684041
0.01687564 0.1037495 0.03724624 0.01181114 23.39942
0.09261036 0.6897582 0.3516063 0.2550934 0.007911495
0.04340103 0.07920133 0.09861983 0.09858942 0.05333916
0.05959536 0.08714094 0.06652391 0.01992322 0.04453233

0.1588862	0	0.04648526	0.08784173	0.03248413	0.006364541
0.6011159	0.06800482	0.02818324	0.4736885	1.209985
0.02011983	0.07229963	0.5510585	0.1809176	0.04084449
0.3479116	0.04092761	0.05631854	0.2285252	0.05513251
0.02626953	0.04547125	0.2301379	0.03887139	0.01396253
0.5268797	0.1184673	0.03823712	32.02715	0.02140236
1.028466	0.01539295	0.05666346	0.2872718	0.1168722
0.1518217	0.03819637	0.07861314	0.1057882	0.1474575
0.2686664	0.05839207	1.353503	0.2222898	0.1497451
0.1219956	0.1291588	0.3050531	0.09558606	0.2356978
0.07782784	0.3574309	0.02859342	0.08348711	0.06006979
0.05430092	0.1047845	0.06450981	0.258609	0.1555447
0.08092058	0.0839316	0.01364979	3.250981	0.4912842
0.1564378	0.09784546	0.0303818	0.09753936	0.1942118
0.04836648	0.08173894	0.02648671	0.5848793	0.01603253
0.1600936	0.0583641	0.5827479	0.1466453	0.008358833
0.01626834	0.3150313	0.004118658	0.1520534	0.118544
0.1848755	0.1625634	0.2028361	0.1015679	0.2725672
0.3314391	0.05819145	0.1474586	0.09309748	0.02824048
0.2538719	0.1453666	0.1494892	0.1621779	0.06793885
0.05655448	0.1862295	0.2601468	45.16249	0.01944012
0.3026567	0.205335	0.02320421	0.07568122	0.1095061
0.06943103	0.05925418	0.377591	0.04443225	0.5571359
0.1329765	0.05520944	0.1899263	0.0696492	0.06165743
0.05637209	0.1298234	0.05119328	0.02007787	0.02413919
0.084183	0.01399327	0.2358346	0.342879	0.114138
0.1868659	1.065369	0.07341272	0.09819048	0.1282622
0.03180923	0.4213582	0.007681079	0.7889024	0.2105744
0.03918582	0.5127048	0.07635238	0.04027975	0.1132054
0.1020853	16.40432	0.073752	0.006008743	0.1189173
0.09255381	0.0214762	0.1059569	0.01662047	0.01161195
0.06852481	0.05804894	0.2773874	0.1938115	0.1861601
0.05127237	0.4331999	0.1847573	13.75461	0.04548874
0.1966427	0.6827961	0.008523904	0.1862089	0.4331825
0.02694331	0.1040767	9.143237	0.07599502	0.06179294
0.1395803	0.02421995	0.134351	0.4162529	0.02184515
0.0411981	0.08145072	0.1426053	0.1906444	0.06207893
0.0340722	0.3883789	0.1068923	0.6630349	0.01728082
0.3068478	0.6867145	0.06880229	0.6071695	0.103903
6.173801	0.3133998	0.07153588	0.1553409	0.003759284
0.0680998	0.007353291	0.3589454	0.4594211	0.1706799
0.04618657	0.0656268	0.236715
CTNNA1	50.46185	30.40495	53.30306	69.15259	37.35593
31.3285	32.42312	24.14458	56.82608	24.65723	61.64067

51.40743 24.26789 54.76382 31.96923 28.95177
44.87633 38.93704 36.2842 35.11356 76.04168 30.54769
47.63561 31.63808 45.97719 71.67576 33.62631
27.08746 48.17398 25.79101 52.97893 34.65822
88.84756 39.74183 42.1466 26.82798 19.45622 36.88969
36.74059 59.16968 56.49578 76.60023 44.69241
67.46778 85.61481 49.71657 43.8121 37.22259 44.09712
61.6078 47.26069 28.36653 19.61291 49.78685 43.57845
58.623 41.47273 32.87208 43.05234 41.78176 51.97989
43.88813 37.24972 33.34178 38.79814 33.78928
47.7256 34.4066 98.19425 18.85805 29.2492 50.74124
45.10409 80.46668 57.9234 30.64975 65.97408 56.96742
47.99322 30.72729 40.96233 41.96522 45.82688
42.29005 37.22578 56.7688 79.18758 50.01579 35.41494
36.54428 73.34391 45.96329 46.45657 51.63573
40.40184 28.15034 62.99036 67.87557 82.18144
26.86939 28.25975 79.39155 30.57534 23.99369
33.92013 50.18259 52.11471 39.74812 67.08144
37.1566 45.81801 30.69391 7.331405 21.51063 50.37645
83.61099 22.99218 84.21746 25.15486 7.288192
38.74192 60.19127 43.1144 41.34251 34.5628 31.3921
56.37064 36.42383 39.42454 54.28908 66.67395
55.60524 44.78406 66.33943 44.11023 43.6377 52.51943
70.69533 41.16292 21.53977 40.91304 42.35472
65.31606 29.81927 34.89096 40.11447 28.0636 48.06867
46.75433 54.5851 47.55276 60.0516 35.13608 33.60068
36.4497 28.38948 47.02577 25.59022 38.72533 20.20312
43.96586 40.63557 46.30069 47.15266 26.08127
61.03339 32.71971 67.61397 32.80313 18.68055
30.76652 54.04331 38.24503 46.25795 51.8243 48.73226
50.12336 51.28705 54.59807 34.76053 19.4974 42.17724
55.03578 57.86141 31.22601 27.30898 40.46628
55.08977 37.56183 41.16393 31.96582 44.01788
48.42591 64.68663 20.47275 55.24298 50.95386
46.82749 42.09563 6.02585 25.09041 30.30706 62.63519
64.04171 34.25704 61.5778 38.98813 25.69796 56.36451
55.05914 26.23367 37.6066 43.6793 17.1291 46.48919
55.95766 44.17559 23.84178 48.51993 39.66754
81.89286 65.22089 28.73813 66.43466 48.17628
5.383005 48.54692 46.74257 50.15046 60.40061
45.00272 37.75399 46.69627 121.7786 49.46697
45.74752 27.9529 48.25707 50.38978 35.14048 31.06425
61.61873 42.15139 38.20844 46.1114 44.58922 40.91216
35.19924 45.59733 34.54047 40.7663 47.7164 32.254

36.22709 42.9665 38.15456 50.96912 37.96707 51.99554
39.52638 29.61025 52.7763 49.97238 40.96101 47.08123
35.94513 51.66655 51.81844 40.35427 57.07782
64.20127 44.19076 32.74504 46.90333 43.53644
39.24282 68.26366 32.00187 24.17121 11.98517
39.65664 57.50806 41.02377 44.82423 75.22615
38.90653 78.15246 31.37543 40.46442 49.86423
45.75241 34.03244 46.17833 39.20509 39.03476
30.73979 28.18541 31.76573 50.795 55.79432 69.3863
53.00346 56.02812 44.19258 70.14531 40.83053
48.92964 39.8636 48.83512 87.6849 41.80043 40.02797
50.41579 28.31089 60.23194 39.39306 86.75092
42.96998 58.38745 56.55199 46.21305 33.30359
34.8689 31.99271 40.46281 35.29034 63.73117 21.94183
32.79765 88.99136 51.81024 30.81567 57.32521
51.54556 30.91776 34.15792 52.78072 31.24403
53.98474 66.69027 20.0151 34.19851 58.81474 32.80612
37.18912 66.89301 59.59349 20.17668 54.65154
25.38418 37.89371 45.33531 32.78078 38.60591
38.15936 64.15197 67.54871 42.60246 34.74893
42.58231 36.38324 31.68302 3.988464 36.12492
62.17189 43.29906 61.61955 38.26042 44.31748
21.54071 28.46078 34.97561 27.12244 53.05527
43.59379 21.30442 53.41223 27.73099 49.34236
9.565635 35.63461 34.28815 38.93273 18.90839
39.62742 47.45135 31.59354 55.13412 34.16326 27.889
47.99852 59.27432 23.32691 64.77697 66.21699
31.97272 42.41761 49.17371 44.62845 58.19187
31.41363 20.2335 67.22847 76.76462 55.97603 40.83868
39.99808
KRT14 0.5923549 0.006090602 0.2167766 0 0.2005092 0.1419112
0.0196822 0 0.01966653 0.03192068 97.58703 182.4608
0.078688 88.74852 0 0.0141563 0.1397596 0.03836577
0.2280707 0.3264894 0.0818716 0.006343388 472.6306
0.1676479 19.15979 105.8873 0 0 0.2461709 0.01445278
0.6299853 0.03425654 0.04918591 121.1461 0.2540857 0 0
12.97438 0.6306522 0.09018917 0.2777551 0.08422332
0.3049555 0.008189362 0.06558831 0.1452904 0.1037589
0.02053871 0.3242748 0.6257324 0.02941102 0 0.3211605
0.2873141 0.08693157 1.487694 0.2057436 0.02611759
0.2107603 0.05180986 0.02169408 0.1997854 0.1078422
0.1341603 0.008765208 0.06905668 0.07821805 0.1995622
0.2495784 0 0.005439937 0.0230822 0.1575213 0.01187825
0.06938969 0.2303855 0.02967154 0.2229432 1.070207

0.006239441 2.403241 0.02087266 0.1228065 0.03929654
0.06038202 3.60775 0.06553721 3.290042 0.3491201 1.012992
0.04910345 0.02844814 2.88377 0.04839111 1.407622 0.2798018
0.2037424 0.01341402 0.1817355 0.01936832 1.531137
8.591912 0 0.7945832 0.04600629 16.28782 10.27269
0.01585791 0.07322216 0.02547445 0.03085843 0.01534276
0.1229568 0.01404334 526.1622 0.01517095 0.02850677
0.2623985 0.06116953 7.216603 139.5202 0.3083553
0.04695637 0.177377 0.2571476 0 0.2097287 0.08955483 0
0.05895227 0.03211024 0.07251515 16.57813 0.3273228
0.04271735 0.024355 0.07270104 0.6172219 2.649694
0.4345901 0 0.2883899 0.004954939 0.02011857 0.1743417
0.535705 0.01653661 0.1415837 0.09542689 0 0.2119132
0.3724363 0.01731506 0.01899045 0.167567 18.87979
0.1422825 0.6259756 521.1599 0.2344124 0.1824084
0.436293 0.02605444 0.9328877 1.412405 0.07584326 0
0.07738887 23.63773 0 0.2891939 0.09532433 0.06795681
2543.938 8.843283 0.03532354 0.7455461 0.5503219
0.01225496 0.4864479 1.671989 0.01929861 425.186 0.01186816
3.925635 0.6531997 0.1389351 0.07756109 0.01050583
0.05506792 0.01092462 0.04698413 0.2001282 0.05842488
0.02190499 0.1839464 0.109867 0.126544 0.1557656
0.07438369 0.5880292 1.179535 0.02497735 0.005199615
21.28264 0.02469229 0 0.1027076 206.3688 0.1075891
0.4480896 0.1286275 0.4079379 0.007024962 0.2567257
0.04458197 0.04089808 6.640373 0.0200184 2.264994
0.1307626 0.1611557 0.4011362 0.009125535 0.3665708
0.005161804 0.3419786 537.5118 0.1554222 0.2673435
0.00718601 0.03703371 0.631382 0.2261385 0.1450235
0.4190405 0.07339918 0 0.1294579 0.01951034 0.08586783
0.05105993 0.07601996 0.09471229 1.222573 0.5015947
0.2121005 0.03824845 0.05567917 0.02270811 0 0.07187649
0.0593097 0.02309411 0.6005935 10.94193 0 0.2640936
0.1076398 0.07379652 0.04668547 0.5352679 131.6501
18.31793 0.09773277 0.08720339 0 0.0138585 0.1798198
0.1909833 0.04645423 0.02596649 0.1066776 0 0.02660158
0.2225137 0.07254634 0.06117666 0 0.007594677 0.1159017 0
0.2703666 0.05869481 0.1161823 0 0.01506457 0.3533237
0.1232433 0.06218746 0.1901618 0 0.02098138 0.03257156 0
1.964931 0.128234 0.01432366 0.6144135 0.08153488
0.09484253 0.2037489 143.2995 0.02887623 7.331805
0.340829 0.04825432 0.04416357 0.005591425 0.02996677
0.09929982 2.003111 0.04839953 0.9185981 0.04099522
0.02122553 0.02789453 0.1068346 0.2729839 0.616172

0.186668 0.2205222 0.4712119 0.02629453 0.1899491 0
0.01176604 13.20329 0.3903359 11.06735 59.45648
0.03476853 0.006291632 0.0392947 1.839884 1.748594
0.2886584 0 0.01911468 0.05122158 12.2896 0 0.2500947
0.2134268 0.01906292 164.6788 1.227478 0 0.2449906
0.2376419 0.08187822 0.0979961 0.01629404 0.01517852
0.09596992 0 0 0.523202 0.05793771 1.966262 0.3266859
0.1588847 5.471843 0.01858138 13.1059 0.006113111 0
0.02281896 0.03572446 0.07630747 0.06802172 78.75037
0.169599 0.1325172 0.05847817 0.2611869 0.08306181
1.360258 0.02745654 0.7436696 0.3298193 1.823536
0.1975366 0.02535819 0.1016613 0.2023314 0.1746548
7.253763 0.1756887 0.1114152 0.04729933 1.248231
0.8917519 0.1774873 219.5435 0.5280763 0.01140339
0.03948258 0 4.988627 0.1842266 0.007331156 0.05665382
4.345669 0.1690433 0.06004866 0.0050559
SPHK1 1.292968 0.7864958 0.7474872 0.8171286 0.7379302
2.47226 0.3253269 0.6860838 1.178371 1.957952 3.561144
8.570812 1.95603 5.759087 1.201458 1.662299 0.9450345
1.577937 0.8815104 0.6675405 1.05723 1.156624 7.893872
1.916906 1.48945 5.64008 0.8644881 1.436013 0.8582952
0.4628491 0.8553551 2.076162 1.743489 6.693809
6.264848 0.8510311 0.5888265 1.914762 4.041352
1.193753 4.981565 2.987271 5.232367 8.02442 1.934456
1.145072 0.9800172 5.145304 1.869277 2.070646
6.145043 3.504467 4.447244 7.26203 4.265769 4.283042
10.51619 2.731834 3.133969 2.261337 5.838149
10.53041 1.897207 5.794077 0.57952 3.896937 5.180438
3.158904 2.419634 1.852398 5.675988 1.528486
2.219626 4.481971 11.19292 3.117831 2.554378
5.672153 3.486094 0.8024929 4.650766 3.320663
1.836043 2.148192 10.76027 5.1605 2.244387 3.785045
3.877122 1.770115 3.024591 1.943327 6.084696
7.870805 9.084712 6.977593 5.316174 2.726464
2.305385 2.434388 2.356162 5.23418 2.857853 2.529649
5.486011 3.955923 4.983426 2.268935 9.405916
3.096596 2.85314 0.4992756 5.681346 2.027442 4.567518
1.460157 10.74161 7.006215 3.045363 0.9155503
3.442114 0.7645201 5.102522 6.957065 4.421164
1.803001 3.051333 10.7247 2.413574 6.866625 5.914069
2.55452 3.193594 3.11955 2.57423 1.752829 4.831733 10.156
3.026958 1.027424 3.758459 5.088968 0.6398447
0.6997187 3.465482 1.011049 0.8157294 6.489088
4.517369 2.024664 20.53281 9.063335 9.07792 3.776523

1.747901 1.900812 5.541393 2.567207 26.5073 3.852584
6.91274 1.682921 1.413082 2.044186 3.740974 2.737053
2.167524 2.773562 3.136415 2.195308 1.529083
1.443051 0.7301171 5.570855 4.342341 3.82117 8.287553
3.520064 0.4367743 7.397099 1.356666 2.176418 4.9703
2.127204 27.7622 6.72763 1.878686 6.285391 2.669879
1.817589 4.387357 5.983981 9.923747 0.895288
1.63496 2.513551 8.260235 3.183221 0.6470143 4.625944
12.63285 2.509853 1.93201 2.237239 3.236937 1.984573
4.087227 3.452145 3.352774 4.763847 2.297688
4.706754 2.934706 2.92103 3.978202 8.054028 7.059311
7.039752 6.283346 2.182977 2.235057 2.396672
1.65414 8.055576 1.741401 0.9465119 7.789255 8.389462
0.7305514 3.80737 3.422275 8.917964 0.7895737 1.170671
0.4336842 1.174342 2.625471 4.266039 2.35156 1.219401
1.286248 3.276973 4.816709 8.414552 5.477545
8.913297 5.395655 1.254538 4.366728 3.997393
3.513489 0.8687583 3.032893 4.025977 0.9400759
15.35163 3.329297 4.835821 6.035609 6.702446
3.647316 6.921275 3.319393 5.346038 4.741423
2.467368 2.911737 2.018652 5.389913 1.017102
2.684445 13.35884 1.923987 1.136611 1.044281 2.279
2.020388 1.213952 5.798929 4.103337 2.824304
1.098432 9.617162 6.312141 4.253633 1.186937
1.449918 1.957755 8.348002 4.555933 5.38907 0.8660469
2.243368 2.335762 14.1917 8.409565 10.74929 8.382627
15.17211 1.372958 1.926904 5.439953 2.376293
22.51638 1.990728 4.852341 1.762542 5.337972
1.903286 1.835338 10.57629 14.79162 5.465591
8.78437 2.384338 2.49971 1.347184 11.00308 2.971169
5.869982 1.187716 3.134393 1.888625 2.444749
3.112599 1.677019 6.91622 1.572164 1.393422 7.693264
0.7073062 7.165647 3.110079 1.630519 3.280611
1.84417 0.3727524 2.2006 1.869349 9.795899 4.888711
6.163982 9.277279 3.303571 2.190865 2.870198
1.001709 2.200917 2.482904 4.056309 1.848792
6.435236 1.528974 12.95588 1.619333 1.0125 2.163591
5.561459 1.331734 11.29671 3.699373 8.305369
23.08865 2.351475 3.749507 4.569062 0.6186818
2.974176 1.383959 3.974867 4.381373 4.796853
2.077446 0.997403 2.600717 2.927161 2.059391
7.246742 0.9672225 1.27822 5.867022 2.789108 13.44099
3.333514 10.44223 2.517751 4.059657 5.342828
3.876263 11.16458 5.74956 2.434831 2.608246 4.846958

2.751537 3.281478 4.358751 2.677436 0.552058
3.649639 8.332595 1.919891 8.661979 4.376609
1.562334 3.447316 1.410225
EHMT2 4.973097 7.776384 6.380389 8.958551 5.60538
6.386866 5.167649 6.612339 8.877709 6.60534 9.240803
4.197922 7.578163 8.514401 8.136842 6.038138
6.476902 11.77346 4.082961 5.074478 8.510724
6.173608 5.479795 6.014201 9.090645 6.085131
7.313503 7.335111 6.463325 1.424687 8.787075
6.762628 8.111639 8.914362 11.10746 9.219492
1.982795 13.2403 16.69496 16.63249 9.255365 7.632877
13.06588 9.591118 8.302374 11.2347 11.3622 21.42657
12.13267 17.47689 12.35151 10.09301 24.1015 11.41574
8.75973 16.22827 13.32474 8.269202 89.17884 13.103
6.716472 15.95355 6.300923 18.34691 6.829972
7.374552 8.6677 7.324835 8.697401 9.516976 16.80058
7.249284 12.89933 7.746538 11.17838 9.361692
5.410254 15.46724 19.02487 12.42605 13.50249
7.859975 7.314651 8.751617 6.720602 8.26968 8.93989
14.50028 8.589993 8.57482 7.014717 15.11197 6.048046
10.05184 14.58688 10.79437 8.25577 10.56363 25.16243
12.03931 11.98374 9.234672 8.055439 12.21277
9.864059 11.13987 12.08535 12.45596 14.86762
11.12612 7.815934 14.93852 8.479103 7.888459
7.454513 11.84832 18.13605 15.09008 25.39437
22.69605 19.19339 11.4101 24.67557 5.380565 24.9537
10.90212 9.104608 9.586722 8.252853 11.65752
11.10789 23.88179 11.04917 8.013759 6.18486 9.681959
17.8253 12.70509 13.37011 11.20426 15.4111 16.20266
7.636633 24.22014 32.99255 8.03876 19.90017 9.139132
21.36584 8.492964 9.623121 10.7714 10.37829 14.50246
9.140231 14.27604 15.55543 12.52051 12.06127
13.89103 16.60196 6.278286 8.406163 25.98769
13.50784 12.26741 14.43865 5.581286 19.10766
15.18475 6.785821 16.10849 11.78361 13.68689
16.00824 10.66085 10.61653 12.3192 12.21266 25.57656
15.4242 16.3845 8.23456 15.71761 10.40196 11.64894
8.565164 8.718955 22.2312 13.39506 14.50221 10.05481
16.06498 7.508351 10.52225 14.32115 6.110992
22.29499 18.70178 9.050265 10.31565 6.374677
13.88965 9.834517 14.47428 14.90101 10.63503
28.2359 12.53795 13.55724 9.068665 10.55882 9.030487
18.02665 10.91272 14.46407 13.26568 33.37781
10.86891 21.05532 13.70093 5.515958 25.56847

14.92971 10.67706 12.2829 29.28174 8.884297 15.8485
8.477724 11.12017 4.774762 11.80038 4.470912
5.563031 10.66645 12.23404 8.465309 5.383624
18.06418 10.62907 11.28885 8.40033 12.12729 8.084263
12.25688 16.53712 9.403932 10.24537 16.73656
18.96056 9.665171 12.71746 11.2633 18.38047 42.37553
19.62776 7.702488 16.65325 12.42633 12.23008
15.01964 6.659328 9.370683 6.542672 19.00844
13.58602 10.32385 13.3746 10.38276 9.956207 10.3605
9.450282 13.31858 9.040545 15.03273 9.593679
6.801069 15.50403 9.157263 9.517665 10.99023
6.219816 15.48956 9.818055 16.88723 11.45566
17.89194 7.659138 12.25056 22.03753 5.465862
10.05068 9.11751 15.36027 13.06156 10.59793 12.81969
12.45573 6.909545 9.205674 12.3102 9.560933 7.75508
8.773875 14.04554 25.53292 8.19275 14.32008 14.78464
14.76977 11.0576 13.22437 10.50667 17.24978 10.62263
10.80243 16.33107 5.593425 6.959526 11.63262
9.616134 20.50064 19.3165 4.228592 12.39249 11.7015
20.14875 9.468035 16.07754 10.24197 10.11439
7.889328 7.109587 11.22586 19.97377 23.45882
8.852806 14.56145 13.47917 12.34919 24.51736
9.381376 33.93114 8.181681 11.65796 14.72824
19.97969 18.40794 18.96697 21.46454 19.59802
10.58385 9.305101 14.80939 8.387141 9.943164
13.80785 6.853469 18.47338 12.04505 14.492 13.19982
15.44704 19.45399 12.18211 6.178073 11.14695
12.00121 11.98932 15.77545 5.6665 8.652936 10.74062
21.64341 19.31673 14.35378 7.008798 8.243333
20.84667 13.26556 16.7962 13.16218 15.31755 15.40688
11.29346 14.88283 10.68951 21.33403 17.28068
13.99421 9.367553 9.163713 7.326767 15.05392
12.16876 19.0721 8.380607 5.239893 17.10566 17.01719
9.901734 15.06086 12.71284 10.20213 13.0896 9.086457
RAC3 1.541346 1.273722 1.426542 2.926597 1.722219
1.011742 3.175293 3.945427 0.7931909 3.218564
1.306726 2.018795 2.98264 3.140089 3.164322 0.4370252
1.840971 1.977194 1.973636 1.137973 1.745527
0.8528056 3.594967 0.4894747 0.7102988 1.555546
2.786203 0.9027978 1.765079 2.892961 1.74768 1.014905
1.919478 3.326242 2.006181 2.315136 0.7623241
2.563605 2.207941 11.52721 3.180936 2.264598
3.574968 13.42582 1.156098 1.169171 0.9410283
12.52778 5.153961 2.325365 5.985948 0.6104175

76.22805 5.779674 1.335667 6.292579 0.8242176
0.7510174 6.010377 5.75284 0.42128 6.889959 1.032887
5.733833 4.451716 1.244312 4.413972 1.13471 0.9846136
7.822865 3.794871 0.9723274 0.9694438 2.484087
1.747291 0.4129739 0.5835825 5.049631 4.464706
2.43261 2.828916 2.348829 0.9478031 0.8707217 18.62075
4.560075 0.9006624 2.022273 27.18361 1.663432
1.155258 1.232362 0.2925995 0.8754591 8.202623
1.603807 1.13438 1.40263 3.289609 0.9933316 1.500962
1.346616 62.17199 1.027152 3.082727 1.284703
1.866671 1.231787 5.780191 6.874944 8.349076
27.54063 7.613968 0.9125273 2.156573 4.086723
22.47307 1.047753 14.85878 20.30583 5.47443 1.494438
27.45199 2.790933 15.60266 1.116665 4.107557
5.875814 0.9782468 3.592915 2.64496 3.41213 4.53072
1.859919 1.552717 1.467366 13.43916 5.98271 4.837624
5.505558 2.69625 2.494579 0.8733868 10.51844 22.51354
4.99371 1.025135 2.456592 2.817393 1.171044 5.917752
1.669012 1.612244 1.06851 1.077715 4.33191 2.745998
1.396971 28.62634 2.514787 2.466641 2.070804
1.196777 2.882266 36.07793 4.274924 2.914105
0.8892449 2.306458 9.874471 0.9660903 1.632048
1.512535 3.011057 4.870968 6.580618 4.3995 1.507108
3.011369 3.324954 1.674624 5.400418 1.907277
1.311902 2.853712 3.409197 2.357043 7.093152
8.615667 2.311486 6.331758 2.3662 11.63965 1.658203
2.748581 0.6994277 2.534696 6.360818 2.578863
13.13353 7.949389 3.134113 1.837549 10.50885
5.958665 3.858151 3.049592 10.01629 1.680647
2.839285 3.444093 2.123664 1.616153 47.62058
13.53177 3.354753 5.518706 14.70551 0.9369624
5.869164 1.38507 1.243776 9.048051 4.791483 0.5558722
42.38513 8.035912 2.304323 6.268493 1.730525
0.9338848 1.792373 2.395092 1.208576 1.014754
6.579173 2.549179 1.734786 5.210557 16.54415
12.22104 1.706591 1.400536 2.310827 3.721983
1.279244 1.188117 0.5808696 4.519032 2.238779
0.7269946 1.599777 5.173981 3.053028 3.146835
23.58723 0.7636386 1.153905 1.822287 7.422535
9.859176 3.238265 3.268691 2.083671 16.14886
12.69336 6.451387 1.397353 3.160128 0.8755351
3.677794 9.800484 3.293289 13.16545 3.357766
2.089284 2.095118 0.395999 0.5100421 2.756778
1.293394 1.527987 2.696091 1.91136 1.277333 2.971192

1.980275	5.293868	4.627558	1.672097	15.82289
0.2760932	0.2664031	4.022122	3.542847	3.951194
2.088842	1.155404	6.424585	1.388464	0.7910117
0.5667314	3.967487	0.9230824	2.360642	2.100133
12.13025	5.554724	0.9354634	1.106303	4.093962
31.94477	10.26634	1.05077	1.481954	1.735917	0.4062655
3.358271	1.572856	2.88463	1.153386	3.691679	0.4811374
5.145446	1.311083	1.502769	14.17493	54.30951
2.29343	1.607174	1.283256	0.8154028	2.763102	1.985935
2.189387	30.58876	3.102706	7.530465	1.456208
3.022292	8.999256	11.64402	1.317382	6.992619
0.8115598	1.528544	1.490307	3.534443	1.148363
5.776565	0.8561556	7.319224	2.182459	3.446348
0.7964324	2.010535	0.8230546	0.6444342	1.103463
25.69831	2.665211	6.503078	1.143178	14.75257
1.813153	2.940386	2.434136	2.340605	1.174018	4.4104
5.903506	1.507852	7.247432	2.460253	6.691274
18.69199	2.968961	5.835877	1.279637	1.647164
6.733253	4.343202	0.9306244	18.85453	0.5712809
1.593621	5.778839	9.597172	8.285608	10.3274	1.19328
5.466348	2.447697	3.81552	1.758981	1.243004	1.516039
2.83938	3.963777	2.83948	11.51028	2.069763	3.38513
1.26062	3.417947	0.9995077	3.919693
SIRT6	11.45295	3.937999	8.420219	9.222531	8.9369	3.1993
5.734718	2.773978	8.1043	8.75196	2.671412	2.662833
8.734471	4.990305	10.02847	2.40219	12.97752	5.793335
11.87562	11.0782	8.290621	3.057912	4.07201	3.548756
4.177366	2.868268	8.383719	3.14511	8.824683	5.500381
7.557325	3.355097	12.32835	4.974799	7.342069
6.548023	2.847805	9.232915	7.464052	9.106885
10.06131	9.719408	4.785591	10.99549	7.552815
7.357195	6.338728	7.491548	4.267638	5.013007
9.393802	6.76766	6.140658	11.15396	5.163349	10.74686
10.50903	4.189647	18.36813	7.809294	4.367819
9.95296	3.218992	6.88706	6.700349	4.192999	10.49879
8.203394	9.668573	6.074178	8.38809	8.414255	5.87885
2.90678	7.297706	28.14218	9.434513	7.527793	4.864814
3.28356	12.58101	4.680764	6.226043	6.914824	6.012673
5.304831	4.333987	4.792768	5.928997	4.968577
6.826998	7.363304	8.74165	7.916671	13.9147	6.609097
11.62207	11.74162	7.082654	7.017092	4.947325
5.756757	7.182767	8.254072	5.508171	6.362534
9.889096	7.612063	10.99451	7.943615	10.32333
8.342149	12.4968	5.867518	6.941833	8.492926	8.900836

16.97415 9.207881 7.307982 5.605079 3.701444
5.471628 6.184368 5.051055 9.121709 9.216702
13.79647 3.77602 37.19038 10.69237 11.89364 5.484587
4.848374 14.89369 5.501557 10.65575 5.399608
5.513351 3.262746 4.765641 9.743126 7.315823
8.254905 11.8038 7.090987 9.866483 8.704231 5.039841
7.566994 6.812291 9.778751 7.543903 7.149594
4.826708 5.319819 9.297372 5.586197 4.686283
8.860126 10.54465 4.578698 2.558888 7.217827
8.778971 10.20072 7.49257 4.282648 7.560964 10.55092
3.511598 7.483679 5.962331 5.79273 8.222961 8.594618
5.410995 7.53681 8.520468 7.673046 10.08757 9.987585
4.839687 9.159747 7.350599 8.225212 4.491563
7.518772 4.036958 10.74918 5.820706 5.910902
6.240002 10.15323 23.12709 6.482756 4.582584
6.669846 10.49628 6.605349 5.165613 10.42698
7.74772 5.53652 6.697193 8.310033 8.781513 4.75541
5.356739 8.693035 4.754228 5.47426 14.06141 3.030002
8.859862 7.11968 7.899567 5.265445 6.706482 4.837092
10.33246 4.176728 7.786348 9.507691 6.986764
11.10236 7.153341 4.698109 11.9572 24.06859 11.29805
7.007666 9.11112 5.951395 5.840965 8.719957 10.91076
8.594206 4.644313 6.083846 6.461663 7.046776
6.470671 8.345953 4.247136 5.602186 8.462616
4.481713 5.088674 9.404157 6.569954 4.217881
7.077364 8.392138 9.19797 5.547726 8.268759 10.82348
11.42325 5.199281 6.968437 16.74219 4.220976
5.207411 10.2324 7.224329 8.387627 12.74317 10.50117
6.89915 6.344609 13.50924 10.38255 8.414004 7.554839
5.177261 6.580732 3.801117 14.41975 3.2633 5.607835
7.718264 11.32712 7.686135 3.278654 7.603695
18.88466 9.849484 7.02405 6.701422 10.30146 2.431153
4.065485 5.838093 5.352085 7.289844 12.10448
7.139365 8.312018 9.849589 7.225425 6.049247
9.744269 5.180472 6.796938 11.86405 2.866092
4.185632 7.779645 7.75725 11.57709 14.57783 8.536438
12.33391 8.159905 5.324482 5.256626 8.774215
4.484393 3.545708 5.770805 5.38965 8.757898 13.29971
2.884096 7.616159 9.321676 6.699944 11.65702
7.273049 10.52531 6.120897 7.281168 10.23239
8.72026 5.81924 6.394168 9.365623 7.582258 6.850743
3.005102 14.45659 11.58104 6.375733 3.2686 6.507048
7.055277 10.49822 12.73589 7.328975 6.892729
11.6403 4.676399 8.161745 9.390598 8.139818 7.704983

5.664403	4.279538	6.666989	5.799952	11.63407
4.681954	6.417691	12.84964	7.66495	6.252268	8.055587
4.56485	6.413473	6.045967	5.096731	5.165608	1.430609
9.940737	7.617861	9.211472	6.239622	7.289789
9.859097	8.079204	3.590195	9.17802	5.728167	8.974142
16.22095	8.769675	5.92137	11.6779	6.397488	5.12286
3.896061	11.39475	4.673686	6.815071	4.996497
8.278394	5.137962	5.198141	9.911291	12.61954
6.35414	9.018558	7.808028	12.259	11.53536	4.129689
OGT 3.082815	5.737166	8.497542	10.57304	7.426918
9.697608	3.870899	5.788731	6.386926	9.895391
15.78329	7.284241	18.78654	6.100155	9.766924
7.943189	7.082814	7.479619	2.923396	3.241525
6.685678	6.349844	5.502391	10.72294	7.65064	14.60645
6.431415	8.983714	3.885744	3.134735	7.172011
3.327472	23.71039	11.78001	15.12246	9.419598
5.280129	7.668069	7.115069	9.419118	16.46053
12.41075	7.557825	11.96909	25.06968	23.77267
25.75939	13.75574	22.25194	10.40247	4.016852
35.85985	6.28894	11.68193	13.61222	12.4759	19.02084
18.39837	14.91246	6.277068	14.93301	10.43783
6.137758	5.684936	11.07056	12.44759	4.434725
9.028544	7.819256	17.93784	15.69146	9.093103
16.75101	27.24203	22.84618	8.108652	12.21133
24.7182	29.53447	23.47031	5.424628	15.2588	27.83358
21.99168	4.777271	8.739634	14.3838	13.43772	11.35734
16.32736	13.65531	20.71798	5.993046	19.53914
13.16591	12.84576	11.7061	9.893005	20.31265	16.66549
25.83747	23.43541	20.1017	7.154235	10.60037	20.91253
14.869	5.53343	9.393518	11.64079	18.2552	33.67715
6.122136	18.95136	13.52437	16.11629	7.090869
37.91965	13.70381	20.58874	15.80727	20.02837
27.23261	4.901457	16.21725	31.09116	21.48502
2.235624	12.56738	10.56766	12.98961	12.4325	16.50951
19.41114	4.260194	23.93251	11.74327	23.97011
10.14078	19.20724	22.98351	19.22862	23.82082
27.82176	9.07297	8.36903	34.92907	10.7996	56.13318
22.3698	20.64827	8.318703	9.315749	20.68295	15.2593
12.65555	16.55411	16.2808	22.73671	9.229293	7.780122
31.10319	22.89049	19.14373	3.883562	14.65397
9.57567	18.56633	29.86698	17.37243	12.78452	13.03354
14.66682	7.273063	10.51888	8.814651	9.475164
18.11753	21.42527	27.73661	9.45361	10.44274	8.090456
21.04977	16.5147	13.75428	12.90664	12.56937	19.81556

12.78118 9.295123 28.68696 7.335766 11.54701
8.050866 34.55436 10.52945 31.74028 16.79582
7.675923 8.350056 12.18037 9.037811 30.02069
12.59869 20.64874 6.874492 12.41918 18.11121
15.79323 22.05701 11.43047 30.13094 6.259657
14.59914 14.15332 4.642876 13.4416 11.91654 6.690028
12.45922 41.14788 4.216559 22.17305 45.63105
16.31475 17.45176 10.98594 9.135743 12.06439
11.64697 16.77597 9.606212 11.48841 31.48679
10.54771 6.351754 11.54624 36.93677 15.15034
24.31782 16.96064 10.28533 10.82175 17.50066
16.82112 14.08211 9.260637 15.15067 19.43187
20.47418 28.40223 18.57762 16.79598 14.49829
14.65055 19.2095 5.837029 21.50316 12.5321 13.98001
4.301267 15.03007 7.695364 15.83757 20.67765
15.16133 4.954961 9.435133 22.15152 9.168551
5.241644 13.08168 28.86707 11.13547 17.1633 17.39331
10.61892 13.36687 7.489412 16.3897 8.665929 3.288701
12.70059 15.03318 28.38697 8.406128 15.60111
13.21175 10.53897 23.18956 12.35461 10.01239
28.53859 7.725823 8.239761 14.49222 10.51227
23.30458 8.57752 14.56276 48.6118 7.834836 20.99114
8.763308 8.284765 31.14355 23.65248 24.88546
20.64638 7.739003 4.969567 10.26696 5.265567
22.97726 8.167514 16.53425 14.13702 12.35519
24.89601 27.41761 21.69212 11.79873 8.230753
4.469048 12.68742 8.201667 10.33168 6.06557 30.23044
7.603908 12.83849 9.41195 12.46451 5.28728 24.50593
39.6194 7.223814 48.41449 12.1268 30.45708 18.60113
6.992054 15.28982 13.87523 22.79619 11.89098
20.83459 4.654441 10.09221 28.36922 13.17816
9.675515 16.79064 13.39354 15.97214 28.15468
8.031781 19.3109 11.26133 13.52986 5.732468 10.13631
12.45779 14.65642 13.71947 18.81606 21.86788
21.75568 11.8348 29.48974 7.797951 22.9506 14.4159
8.658303 8.064484 6.609337 14.72996 9.940625
30.14541 15.84849 15.42474 11.80887 19.58476
53.5597 6.659881 4.614427 16.21462 14.45093 20.18759
18.05004 26.44033 28.41906 22.53903 22.89141
18.62079 40.88961 11.75639 5.737423 10.42229
10.21151 26.55093 14.07909 15.43772 12.23838
9.307024 11.84162
NDRG1 81.64157 21.15933 55.85989 41.42674 43.73366
24.39886 36.16106 23.47997 35.87881 23.32467

62.8276 52.89109 24.50752 83.7312 38.79247 16.70683
46.74202 45.35754 37.84654 91.43501 47.62532
13.9876 150.1613 26.42255 23.84805 63.58841 48.55853
16.84095 79.42564 25.12028 43.31825 21.73626
63.72749 59.32321 18.96469 6.176513 5.605105
28.98408 32.36866 33.72451 31.93704 7.773458
66.89899 114.218 27.47845 26.16103 27.52726 38.07806
15.41088 30.05612 48.16702 21.00006 15.49768
16.11444 14.95235 41.72947 18.79541 20.52621
57.63729 39.58325 37.26973 22.10487 19.89223 20.402
48.14464 14.15047 23.20629 25.71292 38.05538
21.8904 23.65484 18.10795 21.53422 36.36616 48.14791
21.77897 27.63592 52.41016 16.40857 9.94933 31.79896
32.6128 9.22047 23.2917 26.32892 56.37596 57.92429
22.74763 14.55192 9.841166 22.07346 8.191373
10.25561 36.82084 17.34762 19.73965 16.68216
40.82816 13.98957 21.60909 20.38091 52.13825
5.953555 29.56067 28.78034 28.37317 13.10063
45.17713 130.7708 13.93477 68.2142 21.53798 17.05582
11.60429 70.04998 24.66582 43.96877 23.1294 18.92434
12.8197 21.89969 44.97796 15.27615 21.72569 55.99022
21.76153 21.60977 33.79275 27.46027 28.04119
15.60395 41.56646 46.17426 28.05726 41.7706 55.61221
74.77142 68.86138 15.31052 64.77463 15.66219
5.379168 13.38248 18.74923 34.14747 12.54607
5.725393 21.39671 25.07497 20.42465 17.60293
46.85312 13.17862 17.24222 23.71179 38.30031
32.79626 17.3186 45.33524 17.01771 30.05792 22.64152
30.86612 23.29066 20.23604 43.57573 20.43227
26.81748 36.98626 12.43661 13.79704 18.6379 33.28846
40.79462 25.89683 22.3963 37.94692 16.86406 35.50611
25.78526 27.66562 20.97413 140.3122 15.20848
70.56029 31.69372 27.06333 17.33007 37.13498
6.764497 45.57755 35.13861 21.93318 48.30928
6.190343 22.40166 19.23434 36.6546 6.934158 38.83409
17.57579 16.30795 80.53436 42.67326 17.95478
108.8596 29.0961 30.51233 34.20609 34.35755 46.56185
37.25873 23.54729 15.55413 48.58282 44.01575
46.39101 37.90226 26.34755 77.85815 29.15397
102.9401 36.1243 51.78514 21.35 19.44886 75.18227
127.8811 75.05766 57.53177 56.7446 25.31379 45.76264
23.81228 88.36383 22.68045 70.5069 25.9302 36.89541
22.4743 47.97968 18.66827 31.34906 17.25363 24.22046
21.81072 15.80493 19.05416 21.74703 10.90784

20.96579 28.74596 24.10155 15.28902 6.833731
13.99623 18.48185 36.21866 24.68955 45.39549
16.06883 26.51826 30.15681 9.702015 34.54458
35.53114 74.4038 51.45876 19.04195 29.26831 52.06144
39.30348 20.88866 61.76536 20.98689 86.66523
11.77675 12.9794 10.13285 39.86353 26.75048 9.826154
18.9177 18.52935 80.04367 19.58527 31.23727 49.40621
51.39853 33.56346 31.23009 10.18409 19.93171
24.10218 28.00619 29.27693 21.77708 17.42876
152.8999 53.29955 33.11584 42.82439 30.76065
41.82396 61.12843 57.71572 45.57291 70.21344
33.91773 3.575719 51.0306 27.996 7.305259 37.36345
27.11345 23.89597 25.2343 12.30265 41.02064 22.18941
91.23387 29.10704 37.58375 41.84649 33.79593
52.21132 92.99294 118.4376 24.36805 18.9647 89.83082
19.06662 45.0581 44.48712 31.77022 48.8254 38.92951
38.67853 15.22448 28.14161 13.5073 9.44199 16.84467
94.30416 29.8353 90.20419 32.68765 16.6962 31.48332
14.1845 50.62606 36.01163 38.83025 17.85741 8.869505
32.75384 29.81946 17.29034 12.79785 58.89387
38.78184 71.26569 45.55711 49.7991 13.89944 10.57263
68.28646 17.63792 44.3264 10.83744 11.34421 44.63655
12.75575 63.00461 15.47081 9.424678 27.3184 18.79769
20.33206 22.70842 20.82505 41.02697 14.26734
9.093207 58.13114 11.7608 55.23406 18.92212 88.75905
19.63257 19.36858 29.86554 19.60471 26.2625 52.63387
26.37917 18.16252 29.36068 50.97422 39.20506
49.81183 18.51666 17.58025 56.59228 35.23147
90.76288 28.31293
STK38 17.10444 9.125801 23.54393 20.1271 14.90539
10.55792 10.82203 9.538803 20.72362 14.85338
25.25251 21.23204 15.25843 19.02345 14.50561
10.02006 16.08708 12.87846 12.3556 12.60815 23.77567
9.24965 16.44158 10.1055 12.46898 18.81266 17.20951
13.17148 15.12136 6.97871 16.41823 10.88908 37.8879
23.95401 30.71429 35.06392 3.280591 16.95316
20.60063 23.17675 18.70455 31.50316 17.18542
13.90287 28.5366 26.41251 28.1755 23.67768 23.365
59.12019 16.8842 29.46568 11.86425 26.4027 18.51254
16.94618 29.14043 15.49329 13.45585 15.60949
22.38966 21.5699 19.17584 13.13591 20.99423 20.64509
12.68377 14.81121 25.85113 20.77788 20.084 23.87529
25.10721 20.2639 25.0202 11.20528 22.2259 22.37947
48.65545 25.31224 15.14573 19.30961 31.10722

23.64961 20.42177 23.22101 21.19453 27.60811
10.28554 25.47026 24.96699 21.19003 28.87245
25.22171 14.61378 21.41289 22.4673 28.0647 24.92333
26.13356 24.77757 27.91347 17.87983 20.56583
14.86571 23.8466 16.9734 14.38885 18.54218 13.36228
12.1447 24.97537 12.03539 19.25465 24.18764 20.65717
12.59202 16.65697 23.24861 28.15055 23.19997
31.94223 23.38109 22.08538 27.50545 23.95583
13.61194 10.84033 17.58583 17.30137 26.95133
42.33236 33.27812 18.71307 14.01447 26.20265
23.05915 16.74114 25.83057 31.06875 16.66945
21.74928 17.39897 27.21859 21.36309 22.96006
12.20943 22.98764 40.06634 27.53457 9.852514
19.82123 13.89785 33.54984 20.34673 15.27028
19.27311 26.42029 24.63028 34.29516 17.04858
23.54289 23.62832 41.46536 12.76806 17.59979
26.78619 29.74343 31.82075 8.573114 15.05244
20.88848 23.14305 27.85679 27.27164 22.85992
23.57739 25.22737 22.63019 21.45684 36.51029
19.03518 22.16713 23.57381 22.36528 27.02216
21.81042 21.02069 39.3466 27.44029 32.77246 22.7297
25.18087 16.92547 15.73291 35.23536 17.62646
29.04227 18.27345 21.24186 15.07519 14.33521
15.09969 20.53335 23.6532 31.17931 13.36486 14.77796
27.97834 18.68946 30.71266 18.44325 28.93597
24.69909 23.95516 21.80423 19.72207 30.56364
18.31927 46.39229 20.44651 16.83702 16.46098
12.52355 26.71949 13.86483 30.71938 20.35129
12.53602 8.737564 22.27675 22.94685 20.23746
44.82829 21.3833 23.64101 14.2914 23.69452 16.40061
11.95129 19.93091 36.63228 20.58685 18.61743
20.41132 24.21467 19.86751 10.98691 22.03158
36.46083 38.26768 19.99863 40.88071 25.51391
26.38433 12.70301 25.66986 17.49412 19.39631
14.07456 25.8593 12.57024 25.03574 15.23497 13.46548
26.42133 17.47188 14.3824 26.10785 40.6631 19.73455
16.11036 41.57028 19.74694 18.67914 14.71646
28.00388 13.43345 19.71914 25.55888 21.46129
18.10321 13.28851 21.52759 23.61985 25.42994
37.92407 23.62995 18.38101 21.37066 24.95774
14.37062 15.93946 18.19996 17.81171 15.83225
23.36633 39.55208 16.80611 20.51201 28.14604
26.03761 44.66165 18.98272 24.5972 22.51134 31.9405
19.02061 20.80735 17.66472 20.11037 11.22853

31.62481 12.48422 17.83909 12.2054 28.90902 38.34244
31.58738 20.99286 21.28082 22.83592 21.49638
19.33849 22.97057 19.19274 18.68402 22.46551
22.98372 16.17777 30.33598 20.70277 17.10828
13.71037 10.41649 25.58343 40.60749 30.45673
36.35415 25.44634 28.57658 27.58832 18.13982
22.57608 16.96256 47.81388 32.24092 16.31686
25.03562 23.83989 27.23272 24.25733 22.72684
16.79796 24.44548 25.80186 26.66526 36.76315
21.37001 18.49877 30.04961 14.3507 29.04913 20.44537
34.6536 14.11948 16.86152 28.252 42.20499 13.33286
28.90113 20.49002 10.76401 17.32419 18.13206
18.19491 34.11669 14.57869 19.58012 25.34144
18.84559 24.05494 19.17645 10.57041 19.97258
25.39546 10.47773 15.91177 18.11057 29.63898
32.31933 10.05389 30.51698 24.38995 30.88129
35.26109 20.51942 20.68007 11.02532 16.84482
12.19054 17.52201 22.3811 18.81271 13.60155 13.18712
15.87332
ACP1 15.24806 9.955295 12.4677 16.02713 12.63882
7.636223 11.72021 9.551669 8.880211 17.16574
6.781141 7.959251 19.17839 6.955038 10.48783
7.394737 12.25425 9.087819 9.663742 10.84561
14.14902 9.658933 8.135279 8.569849 8.596831
6.997375 12.70942 7.842839 10.72414 18.39326
9.484572 9.880098 17.07505 14.97044 15.61232
19.4844 28.82553 12.93896 12.85664 20.58142 25.25326
30.44566 22.26106 20.16849 11.79593 10.87559
17.94562 15.68383 18.3033 19.75474 21.29917 11.09118
42.0724 19.97599 10.72487 33.27743 18.46027 7.496899
23.55395 17.39531 13.03718 15.86985 13.19334 10.217
26.02512 7.965843 12.59487 12.16012 10.49871
29.12993 22.13544 22.32077 16.11004 17.80343
9.476562 14.18622 10.07348 15.33731 32.04211
27.79035 16.55372 11.32658 21.63386 13.56023
16.12752 24.77305 11.39145 20.80195 19.765 13.94118
21.14902 13.95295 13.22856 14.89719 14.75308
20.73313 12.13298 12.87885 14.36114 15.08038
10.12269 20.71173 17.48614 14.15134 15.99691
9.795004 13.27059 9.803802 23.36979 20.26769
16.96463 18.09011 24.30774 10.90349 14.21183
14.13035 19.68693 15.01308 24.57473 18.21018
19.71897 19.39872 35.95889 11.01619 29.67011
13.94985 22.54597 22.59183 9.892559 12.15726

13.2125 23.74148 16.55261 23.68037 11.10234 12.28593
19.05463 21.70111 21.29727 23.53605 20.80522
22.47273 17.71405 25.97518 25.28311 17.8925 30.65204
10.77603 15.43257 12.83074 21.71568 11.63686
16.50716 9.166727 9.621868 15.42948 13.32512
12.77407 23.51745 17.93611 10.38605 13.1652 22.68276
19.75859 24.51122 16.49585 24.51333 22.78361
15.2012 20.47488 8.248523 23.5809 14.97556 16.62155
20.549 15.7798 8.969859 12.14045 13.10995 11.21469
25.49072 16.40173 9.019152 13.7146 17.2455 20.62229
7.612613 19.6338 11.3684 16.80939 10.73436 18.37582
18.37863 13.88199 22.79065 16.40978 18.61924
47.70654 18.48749 13.08411 23.54789 11.78762
15.43142 15.74914 25.39435 15.91128 17.19508
13.69694 12.84241 22.53203 19.21784 14.39156
16.04981 14.01709 13.97952 18.98159 19.84997
26.67138 12.69973 19.72614 9.852152 21.45047
15.30851 18.19091 10.43358 23.50003 17.62981
6.970061 21.22422 17.79661 12.25012 9.178413
14.33532 14.43034 24.00633 9.694219 11.53552
20.41556 26.7176 25.37889 17.92024 14.149 16.10356
15.93459 21.41137 12.68842 14.51404 9.028569
12.26791 21.68261 18.07086 21.99929 15.81799
12.66923 26.19558 21.30601 21.10392 16.66005
16.77173 14.1826 19.20524 24.74177 27.11437 18.87225
13.94539 14.94488 11.04808 19.16811 15.13484
18.11285 16.00575 19.68624 15.2131 31.10871 11.20213
18.55917 15.50032 8.075771 13.40119 22.80367
10.1831 20.21361 11.84371 15.75698 20.5639 12.03754
6.41264 19.47315 16.3965 11.94298 20.29864 6.417298
6.802008 12.21593 16.40832 9.903846 16.08157
13.72864 24.49698 24.53519 14.76475 11.7228 15.48659
7.391001 21.32392 11.10782 23.73721 21.19838
15.37843 20.66118 13.80796 18.02157 20.00988
10.2461 26.71332 10.80037 11.41394 12.25325 13.32525
18.58566 22.32697 31.57162 9.393026 20.89853
23.43428 16.00944 18.17394 28.13768 12.13776
17.33151 11.80791 12.5938 10.27009 14.48248 9.763523
27.79139 16.09258 17.23682 13.46448 22.52004
21.87725 21.72452 16.74445 25.37115 7.693061
8.65421 16.51271 17.1647 27.44091 14.17343 24.09592
12.47679 15.61716 20.25315 15.16682 16.43416
11.37972 25.69344 11.30156 27.46409 11.97858
9.29272 11.05358 20.21907 21.72388 18.23447 20.50449

11.98653 16.06979 17.29952 23.16174 8.353336
24.9988 23.09943 16.01306 29.34136 16.15519 12.15914
16.47307 15.60412 12.42124 16.25355 17.3842 8.565071
13.51627 15.32006 9.648452 11.26059 17.53952
16.65209 11.10596 24.66061 14.76668 16.90395
15.2838 22.98879 14.83761 9.958531 14.65751 19.07119
23.72966 12.90758 22.37145 18.99064 23.35109
15.43746 14.38791
FOXA1 10.96396 0.02093319 15.68976 32.85628 14.18008
0.1847515 16.86667 21.92502 7.643665 12.06813
11.03667 11.08194 13.5055 11.59334 18.40384 0.09190339
18.55894 9.963812 7.532373 9.429034 22.6391 0.06177237
25.08834 0.09166824 0.4897883 14.68342 21.0685 0.05770016
12.00025 9.255872 15.60003 1.033483 40.963 21.01756
18.61817 25.36223 10.16331 0.7242023 6.265517
0.1679043 10.95179 42.39167 13.86863 78.53595
15.78349 21.32207 17.73316 4.490362 12.60524
33.02865 18.5701 3.208276 10.34927 10.20954 2.738825
19.60492 1.323197 0.1346479 5.784034 40.46118
10.96887 25.71973 3.60112 58.56027 29.3776 1.485882
13.01848 6.265187 13.00985 10.53908 6.961471
17.04596 17.33187 11.49228 0.5536375 56.02836
15.10722 9.11479 13.23016 7.970298 0.1459115 8.146322
20.5335 10.16333 19.61168 8.77165 15.71864 38.98653
6.249551 7.041433 25.92682 10.22974 3.76821 18.18417
9.138346 15.26371 14.78317 12.81678 7.645456
29.10883 7.053168 24.8985 0.7145708 12.158 8.56118
13.57133 23.24318 7.980164 49.57421 17.37964
36.21265 9.723298 30.06212 2.216236 18.47813
45.57506 9.152666 10.16321 35.70266 24.02073
13.54357 24.07724 6.784998 11.69152 0.5681621
8.185609 54.36264 13.61409 9.608158 10.64415
21.07648 2.762323 25.30397 18.09555 9.339263
9.686798 22.29405 5.975195 0.5782165 5.055504
11.0677 29.96226 2.511919 12.59625 7.011405 8.403426
9.363695 14.71708 5.974363 23.96448 13.22474
19.03904 23.4739 3.836398 7.857606 17.49396 26.00935
6.018032 2.767438 5.871607 7.231711 14.443 7.839202
12.18794 12.47308 18.69009 24.0842 22.94614 15.44345
10.34021 14.17432 17.03658 14.05284 20.40902
26.66206 17.84955 26.51007 10.82323 16.41622
12.40418 11.1849 13.05938 12.77692 12.90679 13.34881
9.908766 13.43012 17.77659 11.16946 0.9431786
14.89386 8.770058 16.37118 18.31782 16.74502

15.24985 12.06718 15.41093 1.126489 6.297616
19.46932 11.25619 25.76105 4.980025 5.605505
6.342361 17.64718 10.60688 9.639419 19.3484 5.581583
1.628747 13.8649 20.09229 105.8549 28.53528 41.75571
35.18505 4.766489 32.89021 36.56019 26.75964
16.18048 10.8337 8.650618 25.00882 9.323841 11.72538
59.63686 29.7095 0.9261778 2.117151 16.56558 6.79069
15.98508 2.288022 6.45392 12.54998 32.06059 65.40234
8.853745 14.35007 17.73788 15.67031 5.412283
26.79503 42.72444 0.6938094 21.61921 9.620596
13.66045 9.700323 20.92134 5.900111 6.984986
5.933496 15.5596 8.566236 13.36289 7.67954 10.55001
18.57584 8.448064 21.87553 11.99781 7.764841
34.79012 23.89498 14.35112 30.66987 21.84038
23.04529 20.85306 17.24671 23.56068 21.72681
5.859474 1.305377 0.03520597 18.26315 7.373384
29.63139 7.312539 20.27071 15.52269 3.064294
22.17756 20.43562 8.997929 16.78423 17.35225
1.504938 1.162811 12.85218 0.09504578 10.32801
7.170703 5.095298 14.85597 9.253905 17.83331
10.46393 22.30815 3.751522 21.71673 11.32981
12.3952 6.815313 0.2594368 32.12701 20.39209 17.93215
3.995807 7.338335 19.82452 7.206994 23.68452
25.97715 24.96007 5.880248 3.591828 0.6882329
3.782342 22.96991 0.5477925 16.52862 47.35463
13.8619 10.92139 43.30508 39.99309 7.167904 12.9024
31.97041 2.292461 1.088894 5.061195 119.9238
7.697443 90.93554 1.613187 5.9191 0.03393027 51.27042
1.332212 23.47479 13.2889 24.50638 13.153 19.26828
14.04383 17.04629 8.073631 1.230297 4.239124
15.99419 17.35578 15.15874 0.7384403 6.227276
3.459593 0.1031487 14.76613 36.75882 12.0246 2.496754
51.81344 38.848 27.82332 10.8706 10.4932 15.13613
0.06245424 5.841419 34.5397 1.804448 25.33301 9.912069
4.671176 4.377461 19.26419 16.63481 26.22572
9.332875 11.56369 43.96865 9.519485 12.4223 35.45381
10.80603 9.675699 42.24399 11.58285 11.02453
42.01014 21.29852 11.47704 18.19999 39.95661
11.50486 11.33302 3.187413 0.1947173 46.11076
34.28914 12.54527 9.201099

RHOQ 1.975313 18.76216 3.101578 5.859125 6.106073
20.42195 4.29609 7.422577 8.036613 9.722068 5.853489
3.6027 10.28778 2.786905 6.676556 21.55964 4.216339
12.26764 3.639053 2.700606 7.939108 24.14886

6.018348 21.54786 22.21942 3.530522 5.611203
21.38941 5.549512 4.710562 6.613215 21.51548
7.053054 4.537194 11.39262 6.019477 7.108857
4.141474 8.501198 5.75655 7.432245 4.125925 9.382793
3.803524 5.50297 3.71242 6.23289 9.574639 7.247782
3.183006 4.967222 11.32149 9.647521 6.985437
17.95676 7.153345 8.580777 15.19665 4.819207
6.937868 20.43004 7.009148 19.04675 6.985944
3.692229 17.70333 7.489231 9.047505 3.890252
9.606398 4.094078 4.848998 10.63838 12.73473
14.58782 5.318804 7.670685 7.455804 8.913085
7.247034 6.590252 12.13885 8.362344 11.30065
11.36414 8.673569 9.586285 7.182976 6.217388
13.9785 6.810867 9.444131 10.43886 8.865778 7.512424
9.019901 9.329667 3.822187 3.457876 8.066194
10.04224 3.421285 5.884853 6.697022 12.35604
9.376863 5.862493 11.77016 6.717283 8.197996
7.813701 7.314054 8.522845 15.33333 5.146563
4.345285 5.447989 6.60859 12.50352 4.419343 10.29241
9.068826 8.423207 11.79016 6.78172 9.869678 5.742397
5.742426 18.43087 9.598355 4.179451 4.601603
9.959832 7.866566 5.055793 10.34342 7.832607
5.730289 5.472845 8.192461 12.76396 5.716494
4.788577 9.004559 7.727779 11.31702 11.37153
10.97716 5.607833 7.09443 6.558007 8.872663 4.623076
10.81914 14.50417 6.636999 8.102767 14.26446
17.17765 8.692511 11.07968 10.00126 11.08124
7.448263 6.614509 7.390856 4.475689 9.454281
8.665876 8.205561 17.61009 5.399025 5.664776
5.005949 7.56481 8.549963 10.34535 7.346724 3.325499
11.58892 4.103083 6.226417 3.518984 8.005925
14.55673 10.68366 14.97175 5.170767 11.13883
5.202247 13.99708 7.905309 5.968012 7.929477
3.391235 7.245035 16.32893 10.81723 2.070476
5.131584 9.781181 6.806879 4.054987 6.260114
6.320283 4.006879 5.976995 11.02437 4.722657
5.991962 7.676404 14.12152 5.868382 6.296481
7.316949 5.514984 9.704511 5.641444 11.78373
4.415389 6.025975 12.09006 7.174269 4.375774
8.104919 4.086131 6.063415 1.875172 2.018581
7.474971 7.228633 10.82305 2.264618 3.144241
4.067087 6.247667 6.741887 7.65562 8.818815 3.68049
11.84252 8.683386 11.54175 7.686158 14.37827
10.20715 9.468637 17.25754 11.39711 4.987962

8.929259 8.263179 7.345655 11.4958 2.927713 8.397255
6.045837 10.01931 6.056801 10.80836 6.945222
3.841614 10.19138 8.876977 9.652671 9.115136
8.133402 4.314978 10.88588 10.68666 7.34669 6.509824
11.71265 4.910084 5.92901 11.21444 5.346963 19.28712
7.419075 9.48871 10.38323 7.882825 12.42773 8.13616
5.785624 9.004969 4.537993 11.90177 11.84027
11.69796 9.654714 16.08703 17.8265 5.466154 8.511927
14.25656 8.925917 7.292727 6.593555 4.769984
6.377512 10.33582 4.707332 34.15827 3.997595
6.085513 5.552256 10.72581 9.078867 3.114024
10.04773 3.442402 6.277034 8.108146 4.700811
15.67291 9.732145 9.207439 10.08489 13.15637
5.564453 6.346267 4.748227 5.518636 18.91253
5.419658 5.781369 9.047318 3.259778 6.9301 2.905836
13.6259 7.720884 4.99676 12.54059 5.337502 3.60089
8.149015 5.723423 6.648303 6.068973 11.88813
4.039888 8.308759 21.72243 3.843516 5.704594
4.579278 7.296567 7.35067 6.099744 9.865801 8.649969
22.09329 3.638715 4.09951 8.513924 4.813892 17.66752
12.82254 4.943232 8.327436 3.804439 5.909371
4.599412 7.036908 3.683066 7.591337 4.000279
13.64701 5.710496 5.510146 7.681353 8.778437
10.9593 11.23706 6.460398 8.240484 3.763939 5.489698
7.220774 7.111579 5.635359 12.6823 5.531935 4.736988
12.1224 6.894427 2.206191 6.156468 12.67032 7.151929
5.532195 9.401258 8.085666 11.94056 7.616383
11.65125 3.991834 5.33918 7.922876 7.486871 5.325021
4.35192 4.132191 5.180946 16.21142
ONECUT1 0.02641567 0.003608484 0.0485963 0.003604835 0.06929718
0.003821718 0.0349832 0.009045368 0.2738168 0 0 0 0 0
0.04921741 0.00838715 0.03763774 0.01420655 0.04193519
0.1611953 0.1543381 0 0.004800834 0.004514817 0.002584597 0
0.06570705 0.002983923 0.01458483 0.04281403 0.09597751
0.003382648 0.5026832 1.093445 0.006144388 0 8.677997
0.01355714 0.008757209 0.02671708 0.9690809 0.04657295 0
0.007277897 0.3108715 0.8413613 0.004390986 0.008112345 0 0
0.01306881 0.0178019 2.420973 0.005674143 0.07357733
0.004639003 0 0.01160537 0.007567797 0.179058 0.4155816
0.07204909 0 0.01766347 0.8153169 0.002557115 0.2729008
0.007628009 0.01895732 0.002636135 0 0.04923163 0.1269237
0.6439294 0.00293651 0 0.07031771 0.1197995 0.01713684 0
0.006561486 0.02473275 0.00404216 0 0.0119248 0.1242293
2.341369 0.009326521 0.2973358 0 0 0.0589911 0.01289464

0．3344852 0．03040516 0．007710394 0．06950012 0．01986842 0 0
0．0141742 0．8538997 0．01738989 0．0271595 0．00389389
0．01423305 0．005443862 1．536131 0．03003352 0．02515464
0．1394049 0．01818018 0．4039749 0．01248033 0．3474849
1．680812 0．012667 0 0．2258091 0．01973963 0 0．1299133
0．4555546 0 0．06892097 0．2891632 0．01380638 0．1755006
0．0943773 0．003492731 0．04348406 0．05155544 0．5529198
0．003802512 0．1349794 0．0096197 0．003589417 0．00812631
0．7558574 0．0198062 0．03084795 0．07947055 0．2671433
0．0039732 3．946458 0 0．004898703 0 0．02993153 0
0．006975093 0．02491282 0．3214365 0．05625612 0．05515446
0．01184922 0．01011573 0 0．03180358 0 0．0227518 0．2960121
0．01543641 0 0．05578695 0．01797387 0．6080001 0．02468867
0．02055719 0．009291687 0．01246094 0．05213222 0．04428842
0．00384005 0．007837484 0 0．2518853 0．01630239 0．003630332
0．3755393 0 0 0 0．04570476 0．314019 0．01398794
0．2998602 0．03574078 0．003112181 0 2．508087 0．08350972
0．149168 0．06346054 0 0．02972243 0．0433951 0．1686898
0．01153576 0 1．277423 0．02473756 0 0 0．02142005
0．02340701 0．5911283 0．04781143 0．01281892 0．00708256
0．01930753 0．01604371 0．07355782 0 0．05794348 0．003301671
1．482116 1．30044 0．7116151 0．03129879 0．04577928 0．3580481
0．5307744 0．1027251 0．026325 0．003058201 0．2610568
0．008787966 0．009208263 0．1093661 0 0．01316476 0．01476605
0．005954653 0 0．2165741 0．005435829 0．1504124 0．00426109
3．282826 1．50078 0．3062949 0．01000875 0．009352326 0．07085882
0．007248258 0．0188494 0．01888414 0．005498017 0．005381528
0．2781662 0．03406759 0．01405563 0．01368251 0 4．518551 0
5．362901 0．003542949 1．730664 0．08297888 0 0 0
0．3607833 0．01148115 0．1307163 0．04105355 0．01253381
0．4877215 4．534352 0．07179342 1．527406 0 0．005253524
0．01506653 0．1038716 0．03020432 0．08091785 0．4229624
0．06462869 0．03455465 0．008658563 0．006954954 0 0．02927811
0．267758 0．1458986 0．01327593 0．08903978 0 0．01173268
0．01243079 0 0．005782617 0．2110836 0．02532482 0 0．4619078
0．2898406 0．009365187 0．008325145 0．003217992 0．02395152
0．006369291 0．01262063 0．126609 0．005233096 0．04969107
0．007609001 0．008824788 0．2468499 0 0 0 0．003143859
0．08676468 0．2531842 0．1309276 0．01129057 0 0
0．003533896 0．09347195 0 0．3928401 1．017766 0 0．06680884
0．01033691 0．02678786 0．00823969 1．79297 2．964426
0．006158597 0．7796962 0．003717842 0．2279189 1．887472
0．01264464 0．0105601 0 0 0．2023175 0．1355299 0
2．327834 1．273874 0．0400411 0．2367918 0 0．2399793

0.2349064	0	0.03790604	0.05351846	0.4598279	0.2936654
0.06865243	2.741566	0.1141453	0.03137802	0.007556852
6.808984	0.01880102	0.01448728	0	0.04957148	0.06878815
0.07650872	0.01612026	0.07360606	0	0.183195	0.003464642	0
0.02460571	0.0562262	0.02928079	0.003798277	0.06856394
0.207074	0.1350393	0.02629184	0.002868144	0	0.02069546
0.06697588	0	0.09032936	0.04670552	0.01837357	0.2509587	0
0.009966471	0.1801362	0	0.02673388	0.01455673	0.01098736
0.1613497	0.0304043	0	0	0.00357688	0.01016482	0.5062321
S100A7	0.02710563	0.02591915	0	0	0.03555359	0.2470569	0	0
0	0	203.2132	13.41971	0.2092903	2.007708	0
0.2208929	0	0	0	0	0	0	407.8721	0	52.94663
4.580705	0	0	0	0	0	0.1046577	37.73352
1.213689	0	0	0	2.054786	0.09595219	0.4158938
0.2867364	24.99614	0.01742531	0.05582348	1.715277
0.1892384	0.320483	0.02759966	0.562882	0	0.02557362
0.0581587	2.608412	0.3699457	1.766024	0	0	5.789153
145.665	0.123095	10.42427	0.1349803	0.1268738	0.2238072
0.09183668	0.03698494	0.2739537	0	0.05680478	0.02315018
0.05893712	0.2949527	0.1769217	11.7485	0.2451069	0
0.0220641	22.85391	0.2389729	1.932333	0.0592171
0.4355126	0.08361513	0.05710257	8.739473	0	5.79471
1.857143	0.183442	0.1044823	0.4539893	3.866889
874.6664	1.934358	0.0553825	0.7094007	0.2283386	0	0
0.033937	1.989218	2.248357	0.06502742	0.1118767
17.82955	36.24789	0	0.2636649	0	0.9849077	34.30039
5.422821	0.3286954	4259.875	44.8487	18.92488	0.02147427
0.02002407	16.70245	832.7309	1.283075	3.472001
0.6038761	0.9119306	0	0.1487535	9.23457	0	0.05017542	0
0	1.213524	0.05462564	0.1817878	0.03454836	0.02578219
0.05836995	0.1789846	0.426794	0.5170101	48.69128
1.834502	4.19521	4.170147	0	0.9500376	0.4634793
192.8251	20.78483	0.05010095	0.2300717	0	0	0
22.89486	0.07265962	0.03209518	1391.656	0.6348138
0.04085561	0	0	36.88889	0.3005314	12.80275	0.08647851
0	5.869443	0	0	4.493486	0	12589.87	1439.444
0.2147468	0.7079676	49.45414	14.26361	5.488971
24.07211	0.1095028	278.9655	0.2020245	16.33413
0.3014192	0.04223228	0.5134403	8.897007	0.09373876
72.6186	9.568822	0	0	0	0.1660488	0.05194998	0.6462245
0.1933387	0.0949641	0.06256039	1.088326	0.1594403
122.2985	278.5832	0.8406434	0.1959682	0.218541
126.6968	14.70226	0.06934141	0.4897671	0.0251597
0.05979081	0.3381613	0.02371536	0	2.297634	0	1.348884

0.02529423 14.28778 0.1707074 0.0388346 0 1.054395
6.604929 2198.115 0.06614144 0.5959413 0.2752269
0.2206408 1.095975 0.5560268 0.1299287 0.1517674 0 0
0.1224268 0.08302817 1.481976 0 0.2516191 5.844332
2.51656 0.02603152 0.315915 0.02712833 7.58234 0.3092371 0
1.559975 32.86224 0.1474189 4.14363 0.09935513 0.02242799
0.1123876 0.6362105 2.852605 0.5215207 0 118.7381
9.688213 0.2559454 0.1237008 105.9329 0.1769286
0.1350424 0.8687994 0.4448036 0.07366862 0.4918082
1.932319 181.695 0.3246612 0.1029093 0 0.05812196
0.1292795 7.137341 0.1527387 0.09328956 1.198951 0 0
0.2136958 0.04556374 15.25742 0.04410746 1.04984 0.02809131
0 0.2079172 0 3.675585 0 0 0.02197224 0 0.2690744
0.02989908 46.80647 0.09830847 3.842964 55.07104 0
569.0895 0 0.09109034 0 0.1704886 0 0.07043574 0
0.06774548 11.30693 0 3.291511 0.3514262 0.108325
0.7119304 0.3553675 0 0 1.946004 11.89199 17.22289
0.1107408 0.5939865 166.1622 0.04438826 0.0535493
0.0278704 0.04423619 0.2592792 5.207406 1.075113 0
2.270609 2.730654 0.3295275 0.1680477 1713.944 0
1166.832 0.9540913 0 22.64918 2607.612 0 1.584721
0 0.4844524 0.09075763 0.4997386 0.05042547 0.5468687
0.2465596 3660.068 0.3564727 4.327356 1.248431
0.9093612 6.549665 0.02601494 0 0.03236943 0.01900361
1.174038 9.1184 227.1759 0.3093194 0.8324827 0.02488595
9.532006 0 1.07697 0 0.1091296 0 1.573603 0.0969965
0 0 0.03913823 0.2973042 576.1696 65.15327 0.2245919
0.4025737 0 2.134652 0 172.5021 0 0 0.1440188
0.8364687 0.3156816 108.4639 0 0.1607305 0.1238398
109.0888 0 0
SRSF3 13.40397 25.45101 18.16097 29.23712 21.91604
20.93765 16.97916 20.05427 22.75424 43.70404
17.49944 19.7134 29.33879 17.74218 17.15712 23.1798
20.80545 39.20719 19.07304 17.54216 29.16748
25.19621 17.0031 20.54686 14.40042 17.0543 18.43545
23.52333 17.15786 35.78273 16.05251 35.92448
29.78995 20.34631 23.94835 55.78231 15.4576 21.30367
32.91319 38.16274 22.56849 35.80107 29.01782
33.22919 20.98659 28.02856 28.75658 33.18291
29.18364 29.80563 33.93676 31.63616 42.35385
25.86897 22.37767 37.39142 47.76744 18.82513
23.80656 21.17403 19.62544 28.1865 17.88306 18.70168
33.21473 18.42148 19.6429 22.92325 25.56037 30.06666
40.07745 42.39851 29.18061 25.89336 26.90273

37.98831 23.45247 24.69446 39.76252 34.28876
26.70616 24.05276 53.23283 19.84412 21.67815
23.50189 26.36348 26.68819 29.73839 27.58568
38.38271 36.60216 33.21249 28.8089 29.50108 22.94563
29.59233 22.11195 52.11794 32.55611 21.7143 22.70243
22.55531 32.57773 25.02932 20.34996 24.83087
23.24138 32.17333 29.21144 28.90356 35.3574 33.67809
24.83919 24.41161 23.36814 20.90577 21.32251
32.86668 57.66547 23.80505 31.58537 36.62352
16.96905 56.44302 35.71339 59.68128 31.37608
22.98817 22.1428 24.83539 55.63142 29.64892 34.68644
17.34396 18.88734 29.78188 36.63327 34.04744
36.02454 27.60134 38.19577 21.52788 43.19988
39.84016 20.50955 38.5202 25.43231 23.48547 29.00992
30.75131 23.66 40.05238 24.67097 24.92619 22.87384
31.94579 24.44868 31.27322 34.7458 22.04936 26.48993
26.45887 40.60503 31.10957 28.66943 28.25561
30.57799 31.5808 28.11633 22.80952 28.64385 28.45833
25.73837 31.27014 33.03871 16.59762 38.77719
26.20597 24.88724 48.05756 26.05257 24.10853
29.97693 29.63929 25.01767 19.91427 35.74404
24.04123 35.8883 33.24758 24.73967 23.81577 26.45497
20.74282 24.76649 31.60457 36.55938 38.04007
27.15708 30.55506 21.99021 31.14404 35.33603
32.6254 26.23084 17.51633 31.26752 24.46668 28.03019
24.52823 24.97675 26.2721 23.70498 27.61891 34.8359
17.69092 37.29558 31.66444 27.79312 26.03164
31.74764 20.28673 30.05914 21.92586 37.71574
33.1443 16.93585 27.7319 24.87128 17.86047 16.45805
26.86192 35.62006 33.91588 23.37965 27.79023
28.89501 40.84014 36.48362 27.51832 30.40955
32.25125 34.94923 39.15881 21.67227 38.69458
26.32376 25.62506 44.99764 36.81014 34.21581
30.8393 37.75801 37.83508 37.75986 36.0963 18.8027
40.42282 23.31149 33.27238 39.44318 43.03777
30.92964 20.51152 35.23215 32.17476 34.16927
30.4459 20.25561 31.93154 27.77028 35.83483 39.76461
24.2298 17.5473 25.29172 16.87937 45.60962 30.82349
20.88652 35.73672 19.55497 32.86318 45.49656
37.93517 27.3672 32.13858 25.30664 29.17502 53.30622
17.31612 22.8787 26.77624 24.27107 21.52303 31.90205
35.44009 32.11491 30.55615 28.51096 25.59912
27.01515 16.88215 28.46289 28.86149 40.35934
29.90978 32.68066 46.75566 25.81649 24.8983 35.96935

21.04609 39.96841 24.26383 25.37544 26.33619
18.72053 24.77558 38.39546 32.59233 24.56941
47.06886 32.40602 29.49812 27.08719 25.1211 24.82246
25.75983 31.52823 19.14822 25.36967 22.55891
16.27231 23.98554 39.91867 26.40526 29.7919 24.0865
20.6652 38.43428 26.21135 33.54484 24.23976 19.7046
24.92843 40.98228 37.20762 36.31488 40.29079
31.43895 25.2634 25.76722 43.24363 22.83354 22.22087
35.09741 19.26268 26.09936 24.48093 18.01388
28.84455 35.20896 50.26351 32.64637 31.01655
25.4839 30.41757 20.42212 29.23536 20.95864 29.10966
32.95146 40.37266 31.20185 26.65561 25.29751
22.50679 34.93337 25.17331 27.00075 27.0298 24.10424
30.47739 38.13073 17.75338 18.92812 36.43639
24.68997 27.66041 39.95548 33.28108 29.41516
23.97059 26.91187 25.5434 22.05132 16.12882 30.17788
34.46698 28.11886 39.07261 33.31653 45.0256 27.11875
23.43604
GKN1 1771.91 5.32008 247.7978 5064.547 2544.943 41.92547
12819.02 5492.447 7232.195 1146.41 0.02170768 0
2977.533 0.1185546 14217.79 30.15469 4570.129
2755.305 9241.318 131.034 13572.96 0.5163099 0.04825904
0.04538393 0.08660313 0.3876174 24338.5 17.5571 6818.849
59325.72 17980.48 2433.275 2.782856 1326.712
0.03088237 0 11.05216 4.247374 0.234745 0.01119025
1.337657 0.111467 20.09662 0.02438634 5475.041
0.04652117 29.02885 0 0.3090009 0.3029773 1.69322
0.04771963 2.699495 815.8107 27.26716 0.1398968
0.5569678 1.464727 0.01267886 60.82051 0.4593833
1.931342 0.2896496 0.9321749 0.03480146 0.1627963
16.33878 15.34846 1262.951 16.97703 0.0863951
188.0938 0.1626102 1.108296 0.1279132 513.6864
71.8139 0.0514637 0.0478509 397.6088 0 0.1104974
3.318335 0.3120467 1.824698 0.5386894 52.17201
0.01562537 0.779709 0 4.069806 2.68258 0 2585.623
0.08732552 0.06458881 0.08579672 5035.505 0.7427867
0.0128167 4.32196 0.4750247 0 0.04550221 4.410025
22.15735 337.0748 75.58636 56.63487 0.01685733 0
0.02030568 0.1331424 0 142.8097 0.07027405 339.211
0.8615127 0.2241859 193.7574 0.8243729 47.0402 1.048703
0.7746853 0 1836.883 0.057827 0.3555693 182.4786
4.447228 0.009106486 0 41.40248 1470.185 2317.573
188.8699 0 0.0544583 0.417475 2.273015 94.49143
0.1597711 14.23351 137.8977 0.03580385 0.02045161 0 0

0.2228728 10.52352 0.01168586 0.09540157 0 0 25.74379
146.4271 12.89147 0.1048052 0.07104368 0.2115257
0.01905884 2353.571 10245.75 2.174055 0.9346377
2095.635 0 3505.757 5.080033 0.06226805 0.06263007
1731.006 0.148399 0 0.1575682 1.322347 992.6075
0.07283343 3.150553 1.46318 0.0254348 5.606278 0.9912375
0.02356073 0.1271885 0.09373988 5.614799 0.3079493 0
0.01093211 0 243.9358 0 4608.851 7.457828 1.914382
0.01615619 6.519049 0.2834577 0 0 0 2.181747
0.1135453 1.10052 1602.949 0 2.009828 2.953009
0.09492726 0 0.2687914 103.8004 0.06972993 32.46004
0.2323238 0.02706369 0.03005129 0.02649374 2.254793
14.59607 0.1199726 0.02654458 1.168485 0.2535987
0.01024724 0.02611144 0.4122466 0.3393994 0.02527293
1.055662 1.823283 0.0329848 63.07981 0.04545809
2.19475 0.05464214 1782.065 10.50846 0.09037473 0.02841087
7.488295 585.182 0 88.09952 0.1092916 0.3579035
0.05062066 93.05167 36.65926 0 0.599296 1.60129
0.02292327 0.6142157 1.48315 0.2092498 18.02212 0
39.84832 2945.945 9.014527 44.73221 0.02612416
1.104419 308.9746 0.3065973 0.1788277 0.3779778
26.50064 0.09222115 19.67446 6.600377 2.692681
50.08108 23.08382 5.388723 0.1518102 1.843716
0.01507699 901.4193 1.033146 5.120723 0.03495636
0.009225827 8.706671 192.6561 0.02125515 1.879457
0.3600766 1.489634 0.02620878 7.747333 44.41147
0.01937605 0.04286583 0.03636724 1.023674 45.85799
22.4019 91.34806 2.036364 1.628181 306.0135 8.173923
0.1585817 1625.855 6.365112 2.530828 0.271955
39.38666 2.704078 1.705473 3.088628 238.0479
0.1580138 93.15687 16.00808 4137.731 289.9932
0.2526641 0 5.435094 3.236402 3389.408 8076.076
0.011679 0 211.8912 0.09236344 2.564547 0.3106019
191.7617 1.300134 6.913012 0.01209518 55.1619 578.0617
0.2909236 7.76198 0 12.29777 0.3005065 0.0141232
4.69263 49.49245 5.741487 0.038981 0.1509382 5.082226
1060.026 4.124297 72.03677 0.723179 0.1058444
1.775332 341.0851 0.9475521 0.7476184 0 17.79323
0.1261675 0 47.17956 0.3622346 0.9587263 6.594264
1.389209 378.1208 0.1165276 122.9377 4.778548
0.0120246 0.3382377 0.208964 0.0157124 132.7873
15.82555 459.4927 0.6618059 0.8959857 0 27.90303
0.0503412 0.5670138 3.615024 0.8148051 2.206784 0
74.58491 13.81356 0.07695632 0.708124 332.8316

490.6178　　564.0861　　0.06791421　38.54105　　22562.76
0.2945262　1.939946　　0　6.541971　　4.544603　　11.63762
93.80036　　18.25729
