# Supplementary material for: Anoikis-Related Long Non-Coding RNA Signatures to Predict Prognosis and Immune Infiltration of Gastric Cancer
Source: Bioengineering (Basel). 2024 Sep 5;11(9):893. doi: 10.3390/bioengineering11090893 (PMC11428253; doi:10.3390/bioengineering11090893)
Supplement: Supplementary file 1 [file bioengineering-11-00893-s001.zip › Supplementary Table S2.pdf]

ID	TCGA-HU-8238-11A-01R-2343-13	TCGA-FP-7735-11A-01R-2055-13
	TCGA-BR-7851-11A-01R-2203-13	TCGA-BR-7703-11A-01R-2055-13
	TCGA-BR-6453-11A-01R-1802-13	TCGA-HU-A4GH-11A-11R-A36D-31
	TCGA-CG-5721-11A-01R-1602-13	TCGA-BR-8060-11A-01R-2343-13
	TCGA-BR-6454-11A-01R-1802-13	TCGA-BR-7717-11A-01R-2055-13
	TCGA-IN-AB1V-11A-11R-A414-31	TCGA-IN-8663-11A-01R-2402-13
	TCGA-BR-7715-11A-01R-2055-13	TCGA-IN-7806-11A-01R-2055-13
	TCGA-CG-5728-11A-01R-1602-13	TCGA-HU-A4HB-11A-11R-A251-31
	TCGA-CG-5720-11A-01R-1602-13	TCGA-BR-7704-11A-01R-2055-13
	TCGA-CG-5734-11A-01R-1602-13	TCGA-CG-5730-11A-01R-1602-13
	TCGA-BR-7716-11A-01R-2055-13	TCGA-HU-A4GC-11A-11R-A251-31
	TCGA-IP-7968-11A-01R-2203-13	TCGA-HU-A4GY-11A-11R-A36D-31
	TCGA-FP-7829-11A-01R-2055-13	TCGA-IN-AB1X-11A-21R-A39E-31
	TCGA-CG-5733-11A-01R-1602-13	TCGA-HU-A4GP-11A-21R-A251-31
	TCGA-BR-6457-11A-01R-1802-13	TCGA-BR-6802-11A-01R-1884-13
	TCGA-CG-5722-11A-02R-1602-13	TCGA-HU-A4GN-11A-12R-A251-31
	TCGA-KB-A93J-01A-11R-A39E-31	TCGA-IN-A7NR-01A-11R-A354-31
	TCGA-B7-A5TN-01A-21R-A31P-31	TCGA-BR-8676-01A-11R-2402-13
	TCGA-BR-4294-01A-01R-1131-13	TCGA-FP-7735-01A-11R-2055-13
	TCGA-EQ-8122-01A-11R-2343-13	TCGA-BR-8368-01A-11R-2343-13
	TCGA-VQ-A91Q-01A-12R-A414-31	TCGA-IN-A6RO-01A-12R-A33Y-31
	TCGA-BR-4257-01A-01R-1131-13	TCGA-HF-A5NB-01A-11R-A31P-31
	TCGA-VQ-A927-01A-12R-A414-31	TCGA-VQ-A8PJ-01A-11R-A414-31
	TCGA-FP-A9TM-01A-11R-A39E-31	TCGA-BR-6565-01A-11R-1802-13
	TCGA-BR-4368-01A-01R-1157-13	TCGA-VQ-AA68-01A-11R-A414-31
	TCGA-CD-5799-01A-11R-1602-13	TCGA-HU-A4GY-01A-21R-A24K-31
	TCGA-BR-8295-01A-11R-2343-13	TCGA-F1-A72C-01A-21R-A33Y-31
	TCGA-RD-A7BW-01A-11R-A32D-31	TCGA-VQ-A8P2-01A-11R-A36D-31
	TCGA-HU-8604-01A-11R-2402-13	TCGA-FP-8209-01A-11R-2343-13
	TCGA-D7-6528-01A-11R-1802-13	TCGA-CG-5732-01A-11R-1602-13
	TCGA-CG-4462-01A-01R-1157-13	TCGA-BR-6852-01A-11R-1884-13
	TCGA-BR-4187-01A-01R-1131-13	TCGA-D7-6818-01A-11R-1884-13
	TCGA-R5-A7ZR-01A-11R-A354-31	TCGA-BR-A4IV-01A-31R-A251-31
	TCGA-CG-5719-01A-11R-1602-13	TCGA-BR-6563-01A-13R-2055-13
	TCGA-HF-7131-01A-11R-2055-13	TCGA-HU-A4H2-01A-11R-A251-31
	TCGA-HU-8602-01A-11R-2402-13	TCGA-HU-A4G2-01A-11R-A251-31
	TCGA-BR-8369-01A-11R-2343-13	TCGA-CG-4475-01A-01R-1157-13
	TCGA-VQ-A91A-01A-11R-A414-31	TCGA-CG-5722-01A-21R-1602-13
	TCGA-D7-A4Z0-01A-22R-A251-31	TCGA-VQ-A928-01A-11R-A414-31
	TCGA-HU-A4H5-01A-21R-A251-31	TCGA-CG-4436-01A-01R-1157-13
	TCGA-B7-5818-01A-11R-1602-13	TCGA-BR-8291-01A-11R-2343-13
	TCGA-BR-4253-01A-01R-1131-13	TCGA-ZA-A8F6-01A-23R-A36D-31
	TCGA-BR-8059-01A-11R-2343-13	TCGA-B7-A5TI-01A-11R-A31P-31
	TCGA-BR-8367-01A-11R-2343-13	TCGA-D7-A6EY-01A-21R-A31P-31

TCGA-RD-A7C1-01A-11R-A32D-31	TCGA-CD-A489-01A-11R-A24K-31
TCGA-CG-4465-01A-01R-1157-13	TCGA-HU-8608-01A-11R-2402-13
TCGA-B7-A5TK-01A-12R-A36D-31	TCGA-HU-A4HB-01A-12R-A251-31
TCGA-CG-5721-01A-11R-1602-13	TCGA-D7-8570-01A-11R-2343-13
TCGA-BR-8366-01A-11R-2343-13	TCGA-FP-A8CX-01A-11R-A36D-31
TCGA-VQ-AA69-01A-11R-A414-31	TCGA-RD-A8NB-01A-12R-A39E-31
TCGA-D7-8574-01A-13R-2343-13	TCGA-VQ-A91V-01A-11R-A414-31
TCGA-R5-A7ZI-01A-11R-A354-31	TCGA-BR-8077-01A-11R-2343-13
TCGA-CD-8530-01A-11R-2343-13	TCGA-MX-A5UG-01A-21R-A31P-31
TCGA-BR-6802-01A-11R-1884-13	TCGA-BR-6803-01A-11R-1884-13
TCGA-BR-6452-01A-12R-1802-13	TCGA-BR-8487-01A-11R-2402-13
TCGA-HU-A4HD-01A-11R-A251-31	TCGA-CD-A4MH-01A-11R-A251-31
TCGA-CG-5723-01A-11R-1602-13	TCGA-BR-A44T-01A-32R-A24K-31
TCGA-RD-A7BS-01A-11R-A32D-31	TCGA-VQ-A8E0-01A-11R-A414-31
TCGA-CG-5718-01A-11R-1602-13	TCGA-VQ-A92D-01A-11R-A414-31
TCGA-BR-8289-01A-11R-2343-13	TCGA-RD-A7BT-01A-11R-A33Y-31
TCGA-IN-A6RS-01A-12R-A354-31	TCGA-BR-4369-01A-01R-1157-13
TCGA-VQ-A8E2-01A-11R-A36D-31	TCGA-D7-A748-01A-12R-A32D-31
TCGA-BR-8678-01A-11R-2402-13	TCGA-HU-A4GJ-01A-11R-A251-31
TCGA-BR-8372-01A-11R-2343-13	TCGA-BR-6566-01A-11R-1802-13
TCGA-BR-8365-01A-21R-2343-13	TCGA-BR-6456-01A-11R-1802-13
TCGA-VQ-A91K-01A-11R-A414-31	TCGA-BR-8680-01A-11R-2402-13
TCGA-IN-A6RL-01A-11R-A32D-31	TCGA-CG-4306-01A-01R-1157-13
TCGA-CG-5734-01A-11R-1602-13	TCGA-BR-8682-01A-11R-2402-13
TCGA-BR-7851-01A-11R-2203-13	TCGA-CG-4440-01A-01R-1157-13
TCGA-BR-8286-01A-12R-2343-13	TCGA-CG-4466-01A-01R-1157-13
TCGA-VQ-AA6J-01A-11R-A414-31	TCGA-VQ-A8E3-01A-11R-A39E-31
TCGA-VQ-AA6F-01A-31R-A414-31	TCGA-VQ-A8PH-01A-12R-A414-31
TCGA-CD-8526-01A-11R-2343-13	TCGA-BR-A4J6-01A-11R-A251-31
TCGA-KB-A93H-01A-11R-A39E-31	TCGA-BR-A4J5-01A-21R-A251-31
TCGA-VQ-A91Z-01A-11R-A414-31	TCGA-3M-AB47-01A-22R-A414-31
TCGA-BR-8361-01A-11R-2343-13	TCGA-D7-6524-01A-11R-1802-13
TCGA-CD-8528-01A-11R-2343-13	TCGA-FP-7998-01A-11R-2203-13
TCGA-BR-8592-01A-11R-2402-13	TCGA-IN-A6RN-01A-12R-A33Y-31
TCGA-MX-A5UJ-01A-11R-A31P-31	TCGA-BR-A4J7-01A-31R-A251-31
TCGA-IN-A7NT-01A-21R-A354-31	TCGA-BR-8690-01A-11R-2402-13
TCGA-CD-5803-01A-11R-1602-13	TCGA-RD-A8N0-01A-12R-A36D-31
TCGA-CG-4476-01A-01R-1157-13	TCGA-HU-A4GF-01A-11R-A24K-31
TCGA-BR-7717-01A-11R-2055-13	TCGA-HF-7132-01A-11R-2055-13
TCGA-BR-6454-01A-11R-1802-13	TCGA-CG-4301-01A-01R-1157-13
TCGA-RD-A8N1-01A-12R-A36D-31	TCGA-D7-A6EX-01A-11R-A31P-31
TCGA-RD-A8N4-01A-21R-A36D-31	TCGA-HF-7133-01A-11R-2055-13
TCGA-CD-8534-01A-11R-2343-13	TCGA-R5-A7ZE-01B-11R-A354-31
TCGA-IN-A6RI-01A-11R-A32D-31	TCGA-CD-A48C-01A-11R-A24K-31

TCGA-D7-8578-01A-21R-2343-13
TCGA-VQ-A8PX-01A-12R-A414-31
TCGA-3M-AB46-01A-11R-A414-31
TCGA-CG-5717-01A-11R-1602-13
TCGA-RD-A8MW-01A-11R-A36D-31
TCGA-BR-8364-01A-11R-2343-13
TCGA-BR-8373-01A-11R-2343-13
TCGA-BR-7901-01A-11R-2203-13
TCGA-D7-6525-01A-11R-1802-13
TCGA-CD-5801-01A-11R-1602-13
TCGA-BR-4256-01A-01R-1131-13
TCGA-D7-8573-01A-11R-2343-13
TCGA-VQ-A8DV-01A-12R-A36D-31
TCGA-HF-7134-01A-11R-2055-13
TCGA-R5-A7ZF-01A-11R-A354-31
TCGA-CD-5804-01A-12R-2055-13
TCGA-VQ-A8E7-01B-11R-A414-31
TCGA-CG-4304-01A-01R-1157-13
TCGA-VQ-A8PC-01A-11R-A39E-31
TCGA-HJ-7597-01A-21R-2203-13
TCGA-D7-6520-01A-11R-1802-13
TCGA-BR-7196-01A-11R-2055-13
TCGA-D7-8576-01A-11R-2343-13
TCGA-D7-6527-01A-11R-1802-13
TCGA-VQ-A8PQ-01A-11R-A414-31
TCGA-VQ-A940-01A-11R-A414-31
TCGA-CG-5726-01A-11R-1602-13
TCGA-BR-6801-01A-11R-1884-13
TCGA-HU-8244-01A-11R-2343-13
TCGA-BR-4371-01A-01R-1157-13
TCGA-BR-6453-01A-11R-1802-13
TCGA-BR-4361-01A-01R-1157-13
TCGA-RD-A8N6-01A-11R-A36D-31
TCGA-BR-8588-01A-11R-2402-13
TCGA-BR-4201-01A-01R-1131-13
TCGA-BR-8381-01A-11R-2402-13
TCGA-BR-8590-01A-11R-2402-13
TCGA-D7-A4YU-01A-21R-A251-31
TCGA-FP-8631-01A-11R-2402-13
TCGA-HU-A4H0-01A-11R-A251-31
TCGA-BR-8679-01A-11R-2402-13
TCGA-VQ-A8P0-01A-11R-A414-31
TCGA-HU-A4H6-01A-11R-A251-31
TCGA-CG-4477-01A-01R-1157-13
TCGA-CD-A487-01A-21R-A24K-31
TCGA-VQ-A922-01A-11R-A414-31
TCGA-B7-A5TJ-01A-11R-A31P-31
TCGA-VQ-A8PF-01A-11R-A414-31
TCGA-HU-8610-01A-22R-2402-13
TCGA-SW-A7EA-01A-12R-A354-31
TCGA-D7-A4YX-01A-11R-A251-31
TCGA-VQ-A8PK-01A-12R-A414-31
TCGA-CG-5716-01A-21R-1802-13
TCGA-BR-8683-01A-11R-2402-13
TCGA-MX-A666-01A-11R-A31P-31
TCGA-BR-A44U-01A-11R-A36D-31
TCGA-BR-A4J8-01A-11R-A251-31
TCGA-HU-A4GU-01A-11R-A251-31
TCGA-VQ-A91E-01A-31R-A414-31
TCGA-BR-8060-01A-11R-2343-13
TCGA-FP-8099-01A-11R-2343-13
TCGA-BR-8080-01A-11R-2343-13
TCGA-BR-7197-01A-11R-2203-13
TCGA-BR-8591-01A-11R-2402-13
TCGA-D7-A6F0-01A-11R-A31P-31
TCGA-D7-6815-01A-11R-1884-13
TCGA-BR-4363-01A-01R-1157-13
TCGA-VQ-A91D-01A-11R-A414-31
TCGA-BR-A4QL-01A-31R-A251-31
TCGA-IN-A6RJ-01A-21R-A33Y-31
TCGA-D7-6519-01A-11R-1802-13
TCGA-RD-A8N5-01A-12R-A36D-31
TCGA-VQ-AA6G-01A-11R-A414-31
TCGA-BR-7722-01A-31R-2203-13
TCGA-HU-A4GX-01A-12R-A251-31
TCGA-CD-8533-01A-11R-2343-13
TCGA-FP-7916-01A-11R-2203-13
TCGA-BR-8382-01A-11R-2402-13
TCGA-CD-8529-01A-11R-2343-13
TCGA-BR-8371-01A-11R-2343-13
TCGA-HU-A4H4-01A-21R-A251-31
TCGA-CG-4469-01A-01R-1157-13
TCGA-BR-8677-01A-11R-2402-13
TCGA-BR-7723-01A-11R-2055-13
TCGA-D7-6521-01A-11R-1802-13
TCGA-MX-A663-01A-11R-A31P-31
TCGA-BR-7707-01A-11R-2055-13
TCGA-HU-A4GQ-01A-11R-A36D-31

TCGA-F1-6874-01A-11R-1884-13	TCGA-BR-8485-01A-11R-2402-13
TCGA-D7-A6EV-01A-11R-A31P-31	TCGA-CG-5720-01A-11R-1602-13
TCGA-CD-8524-01A-11R-2343-13	TCGA-D7-8575-01A-11R-2343-13
TCGA-BR-8284-01A-11R-2343-13	TCGA-CG-4437-01A-01R-1802-13
TCGA-IN-A6RR-01A-12R-A32D-31	TCGA-BR-4280-01A-01R-1131-13
TCGA-HU-A4GH-01A-11R-A24K-31	TCGA-D7-A6F2-01A-12R-A31P-31
TCGA-VQ-AA6K-01A-11R-A414-31	TCGA-BR-A4J9-01A-12R-A251-31
TCGA-CD-8531-01A-11R-2343-13	TCGA-BR-4367-01A-01R-1157-13
TCGA-FP-8210-01A-11R-2343-13	TCGA-BR-A4PF-01A-11R-A251-31
TCGA-CD-5798-01A-11R-1602-13	TCGA-BR-8081-01A-11R-2343-13
TCGA-VQ-A8PB-01A-11R-A39E-31	TCGA-BR-8686-01A-11R-2402-13
TCGA-VQ-A91N-01A-11R-A414-31	TCGA-BR-8483-01A-31R-2402-13
TCGA-F1-A448-01A-11R-A24K-31	TCGA-BR-7957-01A-11R-2203-13
TCGA-VQ-A8PE-01A-11R-A414-31	TCGA-RD-A8N2-01A-12R-A36D-31
TCGA-VQ-A94P-01A-13R-A414-31	TCGA-BR-8486-01A-31R-2402-13
TCGA-BR-6709-01A-11R-1884-13	TCGA-BR-6705-01A-12R-1884-13
TCGA-FP-A4BF-01A-12R-A36D-31	TCGA-BR-7958-01A-21R-2343-13
TCGA-HU-A4H3-01A-21R-A251-31	TCGA-HU-A4GP-01A-11R-A251-31
TCGA-VQ-A8P8-01A-11R-A39E-31	TCGA-RD-A8N9-01A-12R-A39E-31
TCGA-BR-A4J4-01A-12R-A251-31	TCGA-KB-A93G-01A-11R-A39E-31
TCGA-VQ-A8DT-01A-11R-A36D-31	TCGA-VQ-A8DZ-01A-11R-A36D-31
TCGA-CG-4460-01A-01R-1157-13	TCGA-BR-4357-01A-01R-1157-13
TCGA-BR-8484-01A-11R-2402-13	TCGA-VQ-A94T-01A-11R-A414-31
TCGA-BR-7716-01A-21R-2055-13	TCGA-CG-5724-01A-11R-1602-13
TCGA-D7-A6EZ-01A-11R-A31P-31	TCGA-D7-6522-01A-11R-1802-13
TCGA-HU-A4G8-01A-11R-A251-31	TCGA-BR-8380-01A-11R-2343-13
TCGA-VQ-A8PM-01A-21R-A414-31	TCGA-VQ-AA64-01A-11R-A414-31
TCGA-R5-A805-01A-11R-A36D-31	TCGA-BR-4370-01A-01R-1157-13
TCGA-CG-4438-01A-01R-1157-13	TCGA-CG-4444-01A-01R-1157-13
TCGA-D7-6526-01A-11R-1802-13	TCGA-BR-6707-01A-11R-1884-13
TCGA-BR-4279-01A-01R-1131-13	TCGA-SW-A7EB-01A-11R-A354-31
TCGA-CG-4441-01A-01R-1802-13	TCGA-CG-5725-01A-11R-1602-13
TCGA-HU-8238-01A-11R-2343-13	TCGA-VQ-A8PP-01A-21R-A414-31
TCGA-D7-A74A-01A-11R-A32D-31	TCGA-BR-7959-01A-11R-2343-13
TCGA-BR-8058-01A-31R-2343-13	TCGA-CD-8532-01A-11R-2343-13
TCGA-BR-6564-01A-12R-1884-13	TCGA-IN-7806-01A-11R-2055-13
TCGA-VQ-A91X-01A-12R-A414-31	TCGA-HU-A4GC-01A-12R-A251-31
TCGA-VQ-A923-01A-11R-A414-31	TCGA-BR-4267-01A-01R-1131-13
TCGA-CG-4443-01A-01R-1157-13	TCGA-BR-7704-01A-11R-2055-13
TCGA-VQ-A8P3-01A-11R-A36D-31	TCGA-VQ-AA6D-01A-11R-A414-31
TCGA-BR-8384-01A-21R-2402-13	TCGA-IN-AB1V-01A-21R-A414-31
TCGA-VQ-AA6A-01A-11R-A414-31	TCGA-BR-A4CR-01A-11R-A24K-31
TCGA-CD-8527-01A-11R-2343-13	TCGA-CD-8525-01A-11R-2343-13
TCGA-VQ-A91U-01A-11R-A414-31	TCGA-D7-8572-01A-11R-2343-13

TCGA-BR-8687-01A-11R-2402-13 TCGA-ZQ-A9CR-01A-11R-A39E-31
TCGA-BR-8589-01A-11R-2402-13 TCGA-VQ-A8PU-01A-12R-A414-31
TCGA-VQ-A8PD-01A-11R-A414-31 TCGA-VQ-A8P5-01A-11R-A39E-31
TCGA-D7-A747-01A-22R-A33Y-31 TCGA-CD-A486-01A-11R-A24K-31
TCGA-BR-8296-01A-11R-2343-13 TCGA-CD-5813-01A-11R-1602-13
TCGA-IN-8663-01A-11R-2402-13 TCGA-KB-A6F7-01A-12R-A32D-31
TCGA-HU-8249-01A-11R-A36D-31 TCGA-CD-A4MG-01A-11R-A251-31
TCGA-HU-A4G9-01A-11R-A24K-31 TCGA-RD-A8MV-01A-11R-A36D-31
TCGA-VQ-A925-01A-11R-A414-31 TCGA-BR-A4CS-01A-11R-A24K-31
TCGA-VQ-A8DU-01A-11R-A36D-31 TCGA-IN-8462-01A-11R-2343-13
TCGA-CD-8535-01A-11R-2343-13 TCGA-BR-4366-01A-01R-1157-13
TCGA-BR-6455-01A-11R-1802-13 TCGA-BR-7715-01A-11R-2055-13
TCGA-FP-8211-01A-11R-2343-13 TCGA-R5-A707-01A-11R-A33Y-31
TCGA-HU-A4G3-01A-11R-A24K-31 TCGA-VQ-A94U-01A-12R-A414-31
TCGA-BR-6458-01A-11R-1802-13 TCGA-IP-7968-01A-11R-2203-13
TCGA-CD-A48A-01A-12R-A36D-31 TCGA-F1-6875-01A-11R-2055-13
TCGA-VQ-A91S-01A-11R-A414-31 TCGA-D7-5577-01A-01R-1602-13
TCGA-BR-6457-01A-21R-1802-13 TCGA-IN-A7NU-01A-22R-A354-31
TCGA-F1-6177-01A-11R-1802-13 TCGA-D7-6822-01A-11R-1884-13
TCGA-VQ-A91Y-01A-11R-A414-31 TCGA-CG-4442-01A-01R-1157-13
TCGA-HU-A4GD-01A-11R-A36D-31 TCGA-CG-4305-01A-01R-1157-13
TCGA-IN-AB1X-01A-11R-A39E-31 TCGA-BR-4191-01A-02R-1131-13
TCGA-VQ-A94R-01A-11R-A414-31 TCGA-D7-8579-01A-11R-2343-13
TCGA-BR-6710-01A-11R-1884-13 TCGA-FP-7829-01A-11R-2055-13
TCGA-CD-5800-01A-11R-1602-13 TCGA-IN-7808-01A-11R-2203-13
TCGA-VQ-A924-01A-11R-A414-31 TCGA-HU-A4H8-01A-11R-A251-31
TCGA-HU-A4GT-01A-21R-A251-31 TCGA-D7-5578-01A-01R-1602-13
TCGA-BR-8297-01A-12R-2343-13

XPC-AS1 0.06513452 0.3010365 0.2795951 0.1278976 0.1993481
0.5020585 0.08945476 0.1517887 0.1620076 0.158679187
0.5746318 0.2346043 0.2626371 0.210351 0.1769804
0.2091043 0.1443639 0.34329244 0.05808374 0.1081996
0.147995459 0.2162282 0.06444956 0.4242691 0.2354462
0.6570298 0.1374693 0.4206105 0.097898141 0.04105455
0.1636146 0.1556945 0.2689559 0.1253652 0.1502429
0.1507164 0.1129597 0.1625 0.389076 0.11848841 1.025688
0.2870935 0.165718525 0.1721439 0.6744422 0.3860852
0.913686 0.2061425 0.729538403 0.3034678 0.09189938
0.8603439 0.104816 0.266607 0.5150413 0.2624532
0.2815913 0.7122198 0.1669062 0.20603937 0.525859439
0.251678315 0.1261385 0.06351592 0.1095531 0.4242008
0.1530615 0.2852666 0.061806052 0.4777544 0.1267119
0.1626068 0.8054182 0.452134 0.4927705 0.1439751
0.4860431 1.072178 0.6189607 0.173692661 0.1006694

0.3438869	0.4379929	0.4874321	0.2096368	0.2102188	
0.3462666	0.3040702	0.23553104	0.6081532	0.188302259	
0.4888988	0.08037072	0.4734723	0.1124574	0.3696764	
0.2735909	0.2933995	0.3613662	0.3741198	0.9967249	
0.1485868	0.1500771	0.09983288	0.3024432	0.7506469	
0.2401265	0.1171195	0.124796819	0.1736704	0.230098	
0.54333	0.1397084	0.594383	0.3075736	0.1867432	0.2064895
0.3927519	0.20049	0.2460693	0.0711494	0.5255481	0.640244
0.2082608	0.3617482	0.8985269	0.1919655	0.05479153	
0.4055686	0.1373169	0.3413946	0.3007401	0.3092816	
0.3682707	0.06471617	0.6641546	0.4164707	0.1948088	
0.1553132	0.394089	0.387008	0.4229357	0.2308298	
0.2552645	0.1571089	0.2897125	0.3147249	0.3929011	
0.4145767	0.3039866	0.3544876	0.2559536	0.1803455	
0.535846	0.6399429	0.4772151	0.3201005	0.505593	
0.2236416	0.3632025	0.283618354	0.735604	0.384852997	
0.3785655	0.09361508	0.3159789	0.09813103	0.2468867	
0.9412659	0.1960164	0.4182097	0.233285723	0.3783544	
0.08100927	0.2442504	0.2178811	0.3605505	0.1667459	
0.7310394	0.4982865	0.1237404	0.1936959	0.1864112	
0.5866006	0.2150815	0.5454657	0.3777449	0.2545918	
0.6475895	0.3472653	0.1799882	0.2516731	0.1026938	
0.1770257	0.1742251	0.4876831	0.2774116	0.7332998	
0.2396699	0.1605835	0.1809551	0.2550002	0.2483271	
0.3013083	0.1711648	0.6705466	0.1482492	0.3959477	
0.2581344	0.2750556	0.6387354	0.4230704	0.3022924	
0.219506	0.2986471	0.2754408	0.1045575	0.3267993	
0.2956939	0.18757836	0.2532573	0.755334332	0.520356412	
0.4899253	1.110699	0.196479	0.3549433	0.3117922	
0.1192029	0.2929173	0.1428879	0.5133656	0.165191	
0.2540924	0.3729262	0.5723693	0.1407358	0.205606	
0.6537563	0.2142947	0.5122235	0.3408457	0.1727537	
0.2331676	0.3095355	0.3023417	0.2470218	0.6482691	
0.3954053	0.2709194	0.757917	0.4777629	0.256082	
0.478888	0.3256994	0.1901152	0.1736591	0.135033039	
0.3533242	0.2760079	0.1856605	0.05575096	0.388199	
0.2242839	0.3758541	0.4348682	0.3540978	0.1850222	
0.4176505	0.7183536	0.2144314	0.137686	0.3888861	
0.178590024	0.4281982	0.3539602	0.4190191	0.4952681	
0.20562	0.142384592	0.5577545	0.165673	0.1079355	0.2700999
0.2825033	0.4772733	0.9043456	0.249392	0.3659975	
0.4180706	0.3270238	0.4500207	0.2324387	0.3238295	
0.2384339	0.3741498	0.4024218	0.2604016	0.3343939	
0.2810175	0.3906437	0.6067092	0.182059	0.7021404	

0.2290327 0.1603512 0.328970672 0.481731613 0.3938982
0.2042965 0.1410354 0.09559254 0.2027883 0.2115704
0.6381511 0.4069791 0.332796 0.279724 0.1329322
0.6026795 0.1735359 0.410409761 0.2067087 0.2315458
0.09302913 0.3377275 0.2473271 0.156766697 0.1429104
0.6575029 0.1688002 0.191601 0.1751456 0.2381236
0.1948816 0.3288293 0.1925129 0.1696228 0.7999777
0.2206749 0.7037658 0.2443984 0.3066016 0.5577589
0.7147811 0.4298404 0.254741177 0.3653413 0.2399723
0.2638813 0.372133 0.3266179 0.09874093 0.2414499
0.1999154 0.3387059 0.7808852 0.4015364 0.7076843
0.3639583 0.3997466 0.07823012 0.3768074 0.211129
0.1577483 0.2326895 0.3797988 0.5660902 0.5682814
0.2100893 0.4057669 0.3484944 0.4026159 0.329822719
0.1760797 0.179861 0.2494278 0.1797185 0.2807747
0.6264555 0.177514856 0.5841813 0.2330815 0.3781702
0.4482948 0.07837394 0.1885272 0.6315306 0.345778
0.2431944 0.5463907 0.2730866 0.3166386 0.5304746
0.611641 0.290919272 0.7936152 0.4390033 0.06281328
0.121161962 0.1956739 0.7913449 0.3057683 0.5400631
0.178354 0.1851947 0.4681939

NAALADL2-AS2 0.04199022 0 0.06437371 0 0 0.05669986 0
0.05032466 0 0.035072774 0 0 0.02161456 0 0 0.03110841
0 0.02107715 0 0 0 0.05575826 0.03561313 0.1507114 0
0 0 0.02213507 0 0 0 0 0 0 0.1025545 0.3068281
0 0.01257105 0.01082699 0.111482026 0.2260613 0.01233869
0.072841115 0.02699413 0.129717 0.03089759 0.1140049
0.01504457 0 0.1900467 0 0.1980847 0.1501593 0.1262743
0.08187073 0.01720632 0 0.02869664 0.01403469 0.01897532
0.111236148 0.019088149 0 0 0.09630752 0.009484482 0.0190982
0.05658542 0 0.009777573 0 0.07101223 0.1661531 0.03915361
0.06535007 0.7847199 0.1521405 0.03418023 0.127123
0.054844562 0.04867384 0.06115677 0 0.02878466 0.04422974
0.1219694 0.1152142 0.01729629 0.23974676 0.09472534 0
0.01562866 0.01195674 0.04726188 4.704305 0.05719657
0.189943 0.02947724 0.1409516 0 0.08762153 0.02445685 0
0.05036806 0.04332797 0.04223293 0.02019159 0 0 0.1866
0.06781125 0 0.02456337 0.01543008 0.6939912 0.0111127 0
0.02217767 0.06203989 0.01464308 0.02896915 0.06023194
0.0644915 0 0.0269086 0.0407287 0.07681297 0 0.01296484
0 0.0201606 0.191222 0.05696696 0.04231119 0.03129039
0.05352001 0 0.01507047 0.01540394 0.01836557 0.08581258
0.3242368 0.05444228 0.01473682 0.6605431 0.05659659
0.09084781 0 0.0123353 0.01367238 2.354262 0.02640087

0.01268326 0.04173142 0 0.08789883 0 0.1160126 1.061651
0.2341455 0.042193852 0.1701011 0.014313628 0.03819906 0
0.01111101 0 0.117735 0.1334336 0.08615846 0.03466375 0
0.0597339 0 0.0436045 0.1441576 0.0812397 0.06046648
0.05386041 0 0 0 0 0.01304011 0.0895934 0.01729401
0.02180781 0.03787558 0.03462976 0.0726069 0.060017
0.07374814 0.01418647 0 0 0.1347404 0 0 0.05704903
0.2615325 0.08614609 0.08028387 0.04116571 0.03427839 0
0.09767013 0 0.01612139 0.02377304 0.1050784 0 0
0.02598382 0.0771866 0.09402558 0 0 0 0.04399033 0
0.02612275 0.044267346 0 0.02005332 0.01220511 0.04537216
0.05780742 0 0 0.01398776 0.01579122 0.01627628 0.1277923
0.02208614 0 0.2547003 0 0.05934978 0 0.0142913
0.01048301 0.09817845 0.0556845 0 0.04380322 0.02688419
0.02330449 0.02801693 0.06117736 0.07984155 0 0.04738447
0.3649318 0.07612381 0.02999549 0 0.03474394 0 0.05256398
0.02702792 0.1025911 0 0.1966328 0.13013 0.1321645 0
0.1212085 0.03045398 0 0.08683134 0.07656221 0 0.2344235
0.025584776 0.08768527 0.0279413 0.1461352 0.0224059
18.99815 0 0.2546934 0 0 0.03869447 0.04084961
0.05429708 0.03310432 0.09411238 0 0 0.03388213 0
0.01152662 0.02385862 0.02144805 0 0.02683748 0.09442852
0.06807584 0.0358345 0.034736 0.1080745 0.05967856
0.05076429 0.1181203 0.02340531 0.015148377 0.097049235
0.03686139 0.1317036 0 0 0 0 0.01801736 0.1049468
0.09194715 0.01020733 0.05713151 0.02791825 0 0 0
0.1155642 0 0.2009746 0.01292791 0.01837503 0.07624545
2.517671 0.01419395 0.04584221 0.02765166 0.04317496 0
0.02677719 0.05515873 0.06308687 0.1960206 0.1031793
0.03916802 0 0.01446268 0 0.04189067 0.08176866
0.086217495 0.02157475 0 0 0.02570386 0.0143564 0.01193536
0.01667736 0.1405956 0.03970059 0.03905788 0.1613661
0.06365892 0 0.5154086 0 0.01401439 0 0.1743351
0.01343353 0.06121116 0 0.04906521 0.06449435 0.08968644
0.1535675 0.05324187 0.083201697 0 0.0521779 0.3520182
0.1737884 0.03620137 0.04226406 0 0.03339343 0.2504341
0.05572451 0.01063811 0.02021011 0 0 0.04136519 0.03865807
0 0.08518575 0.7103625 0.05087316 0.04928826 0.263737553
0.06264726 0.04957873 0.02699586 0.040752725 0 0 0
0.03197406 0 0.01885095 0.04444126
AL359853.1 0.07252222 0.50855 0.04447248 0.1385552 0.2219585
0.636529 0 0.3186949 0.3732059 0.666324344 0.06225157
0.04749338 0.4479717 0 0 0.5551893 0.3857711
0.782660161 0.5671212 0.7228308 0.169489452 1.08339 0

0.4049062 0.09934143 0.05700401 0.114796 0.7837141
0.140145366 0.1097065 0.1366291 0.7800916 0 0 0.09840222
0.4239435 0 0.02171173 0.3365916 0.085574666 0.3318701
0.1278625 0.050322181 0 0.099572 0.07115185 0.9563729
0.1039352 0.049229343 0.03861574 0.05581244 0.9123103
0.4149493 0.145394 0.4713357 0 0.07098791 0.3717193
0.09695847 0.131090733 0.027445477 0.032967552 0.02407635
0.5374722 0 0.1638085 0.1649245 0.3420546 0.17001655
0.1688706 0.02064642 0.05256289 0.04782775 0.04508202
0.0376225 0.1748783 0.5255303 0.3542004 0.07318561
0.04736161 0.08406563 0.1056252 0.02589404 0.0497146
0.07639013 0 0.1492416 0.3584736 0.41407177 0.4635395 0
0.0539852 0.1239044 0.6326094 0.05565004 0 0.2108923
0.1018215 0 0.1470188 1.815996 0.2745596 0.05569973
0.02899725 0.5238286 0.2370596 0.03487332 0.331024
0.064131477 0 0.1903172 0 0.2121196 1.46573 1.112992
0.01919298 0.1622894 0.07660706 0.03571681 0 0.1751164
0.05201394 0.4455386 0 0.06971164 1.313077 0.02211089 0
0.5374045 0.111872 0.03481981 0 0.04919443 0.02435886
0.3782962 0.3081185 0.04598754 0 0.07981343 0.1268783
0.04940292 0.1018175 0.1316398 0.05090456 0.387885
0.1563987 0.1255242 0.1860087 0.02130456 0.1416832
0.1340471 0.02279875 0 0.1922006 0.317987 0.3795299
0.04320084 0.4293598 0.2037334 0 1.748973799 0.6142783
0.123606872 0.06597442 0.05956177 0.1919007 0.03856279
0.02259363 0.1975338 3.482063 0.818203 1.224514376
0.2321273 0 0.05020685 0.03830425 0.163696 0.1740549
0.9302348 0.2237861 0.09725318 0.02441494 0.1624338
0.09008745 0.04421105 0.1493443 0.2448208 0 0.2392392 0
0.02073133 0.0254744 0.02450176 0.2586994 0.04156845
0.1269344 0.2007695 0.5042916 0 0.05646229 0.07439238
0.03961714 0.02369939 0.03946863 0 0.03748626 0.2621604
4.232226 0.0615884 0.06805621 0.03092096 0.7708187
3.298475 0.1599728 0.06959726 0.04230094 0.07761109
0.03830164 0.1013022 0.325812063 0 0.025485028 0.076122436
0.03463452 0.4637537 0 0.1497606 0 0.05898804 0.09663426
0 0.02811111 0.0630608 0 0.02896913 0.06767666 0.7312591
0.1025043 0.08188951 0.7158013 0.07242171 0.04844746
0.03205797 0 0 0.3250259 0.08049938 0.7500239 0.3169822
0.06894803 0.304232 0 0.2251005 0.04382502 0 0.08860957
0.02000234 0.125290639 0 0.5368257 0.4429682 0 0.1852411
0.2247504 0.05706603 0.1470963 0 0.4996784 0.08029145
0.09997885 0.1101935 0.03285057 0.06747963 0 0.1850971 0
0.06883453 0.270884 0.07775384 0.115297562 0.5175135

0.06810977 0.0832 0.08910673 0.07055224 0 0.09529196 0
0.3614434 0.177017 0.07802475 0.2505315 0.3782496
0.02060336 18.33648 0.04097574 0.04635159 0.2718159
0.01959587 0.1237811 0.5399396 0.1599924 0.06184327
0.1095951 0.1836073 0.02021191 0.340120122 0 0 0.06499091
0.0565315 0.09122987 0.09184636 0.6909968 0.1555908
0.4833488 0.2646732 0.2291808 0 0.0241091 0.03220312
5.281487345 0.0452762 0.3991869 1.814734 0.1735538
0.1339684 0.190415456 0.1975277 0.02207271 0 0.1583501
0.1432733 0.04971226 0.2367115 0.04624746 0 0.04358347
0.04836449 0 0 0 0 0.1620055 0.9043785 0.0470748
0.127635503 1.378702 0.06412572 0.2869784 0.08878737
0.07438573 2.59734 0.2592344 0 0.1028516 0.1798872
0.2090242 0.2748667 0.2805502 0.4450867 0 0.04840912
0.2115681 0.09032936 0.139208 0 0.1732115 0.05084495
0.04455583 0.1548994 0.02946998 0.1839104 0.071849719
0.04438892 0.09011757 0.04503542 0.3001537 0.1042069
2.068196 0.197648959 0.115349 0 0.0481215 0.1102398 0
0.1988624 0 0.02381424 0.2225573 0.05983903 0.02942523
0.296144 0 0.1064085 0.091101358 0.1081994 0 0
0.070384906 0.09120039 1.057321 0 0.05522309 0.04582692
0.260463 0.03837773
ADAMTS9-AS2 0.08236633 1.61556 0.1737022 0.08060036 0.2977613
1.692716 0.2296901 0.6163658 0.3969854 0.548700742
0.5225017 0.05525571 0.2326734 0.1393836 0.07860358
2.827802 0.1081981 1.17830653 0.1265052 0.19308
0.281701218 1.736632 0.1712355 2.124686 0.8338114
0.2684412 0.08903886 2.981633 0.186343727 0.2005723
0.3803681 1.961043 0.01357424 0.1834887 0.1095786
0.01761546 0.10452 0.0505206 0.2315438 0.051558382 0.1411414
0.01416765 0.048092057 0.002582956 0.1985933 0.038434
0.1332414 0.06693918 0.1554618 0.02888171 0.03246722
0.24261 0.19828 0.05739264 1.034071 0.009878409 0.03244615
1.470409 0.07453213 0.087152136 0.282818961 0.049314571
0.3381376 0.05877037 0.01658748 1.253754 0.1891387
0.2964397 0.042386593 0.03227735 0.01544199 0.04950544
0.8207598 0.1910687 0.16258 0.06903122 0.2869945 0.06214071
0.02280725 0.011807658 0 0.3664713 0.01291121 0.6734224
0.1417773 0.09336597 0.7462117 0.08440553 0.51615833
0.8701319 0.032910753 0.02916111 0.1046843 0.09157643
0.06243316 0.03078507 0.2492552 0.03384665 0.006743536
0.2015918 0.2993138 0.03159235 0.004628808 0.05783412
0.4747025 0.4364381 0.02608266 0.4576505 0.01065902
0.05356492 0.0754297 0.08226581 0.0282044 0.8437858

0.5122682	0.05901471	0.04046014	0.04615544	0.1469244	
0.01261021	0.03326325	0.1664166	0.1092253	0.2088628	
0.05020806	0.1364006	0.0514494	0.0239004	1.222564	
0.172922	0.04485121	0.1120706	0.1594397	0.06072873	
0.07185714	0.6683044	0.04968206	0.0757066	0.2144581	
0.205607	0.04242375	0.1734572	0.1031452	0.01692126	
0.08722238	0.2615683	0.01564713	0.5547652	0.01416376	
0.1883885	0.04084557	0.2121999	0.007281648	0.1836829	
0.96894	0.1072361	0.2854141	1.087082	0.01693083	0.3629514
0.218017129	0.3085093	0.279400754	0.03106843	0.1187941	
0.0924955	0.0608889	0.1445749	0.09849374	0.08656352	
1.713143	0.094822439	0.2636361	0.02044274	0.03546483	
0.03819832	0.16713	0.2516817	0.02898944	0.06044114	0.06667666
0.03652113	0.1079897	0.07299363	0.09368867	0.0645369	
0.6729602	0.06251671	0.1110049	0.0138949	0.2015715	
0.04445691	0.07941051	0.05835352	0.01036337	0.1406482	
0.196364	0.09578991	0.1043993	0.0680366	0.1020066	
0.3374606	0.05711514	0.003279957	0.02660723	0.03115216	
0.08956585	0.1827967	0.05118173	0.067868	0.1336203	
0.3715321	0.1435829	0.1484519	0.1658003	0.1001869	
0.09459567	0.01750637	0.2714968	0.054151839	0.08436074	
0.220259427	0.042173289	0.03597785	0.4940033	0.01628052	
0.04978216	0.1497065	0.009804152	0.03412999	0.07932731	
0.1074613	0.06550667	0.03328496	0.07703738	0.312139	
0.1273271	0.0340736	0.09300513	0.05538273	0.3144641	
0.2375413	0.2024723	0.02987261	0.1425058	0.1524169	
0.12376	2.038766	0.4068396	0.03008134	0.1715602	0.02720414
0.261891	0.2330871	0.01435072	0.05154597	0.1296556	
0.054142465	0.016975	0.3646527	0.07730506	0.008441275	
0.1693348	0.2926126	0.1090741	0.0794568	0.01391752	
0.02841165	0.3002603	0.3178013	0.1721591	0.07370937	
0.112155	0.018360756	0.05313818	0.03408823	0.1010595	
1.329771	0.03661555	0.153304945	1.051518	0.005660118	
0.3410985	0.07590151	0.03664434	0.4636949	0.03484376	
0.1013088	0.178455	0.5524654	0.0324204	1.730133	
0.8768328	0.06163916	0.06772507	0.4494866	0.1328923	
0.06324831	0.06188199	0.0942935	0.194439	0.2925082	
0.05653289	0.105649	0.0593378	0.04199169	0.082620653	
0.075218525	0.06701515	0.03915676	0.03601747	0.04296163	
0.007632696	0.1722715	0.0284461	0.9154882	0.1737613	
0.03809117	0.0512501	0.1342369	0.1391609	0.069553535	
0.02633808	0.08293395	0.3284757	0.05769128	0.02968843	
0.005274691	0.07386805	0.02934889	0.03259585	0.3037617	
0.1289863	0.134265	0.5147351	0.2286762	0.0118753	

0.04889581 0.1185672 0.08616256 0.08432605 0.02170928
0.1266244 0.01795083 1.497117 0.303184 0.026517193
0.02477277 0.09059354 0.1464993 0.01106773 0.2122373
0.07708802 0.06941674 0.08744457 0.01994362 0.1438855
0.1737051 0.6487189 0.09636673 0.4280038 0.2873136
0.0382179 0.02930319 0.1326171 0.02892147 0.01098195
0.07437092 0.07042269 0.05924348 0.0772355 0.3232733
0.08023827 0.238836731 0.0553327 0.0349488 0.03742573
0.1596393 0.09699083 0.2608427 0.138701663 0.3307113
0.2995375 0.06998311 0.02748374 0.02610659 0.3580641
0.4278603 0.01385322 0.0240437 0.5453499 0.1760631
0.6187762 0.08762113 0.04951998 0.345732508 0.1690437
0.7507352 0.1278645 0.026321347 0.01263169 0.5110126
0.02978138 0.02600545 0.009520871 0.1055205 0.7749998
MIR205HG 0 0 0 0 0 0 0 0 0 32.2305 28.77881
0 18.5588 0 0 0 0 0 0 0 0 29.60046 0
0.548731 42.0663 0 0 0 0 0 0 34.70243 0
0.02715926 0 0 0.01597274 0.032893218 0.003335015 0.00364058
0 0 0 0.01215528 0.04324834 0 0.004205065 0.1352374 0
0.01558552 0.08417968 0 0.05636467 0 0.08489089 0.01693411
0.01242298 0 0 0.005632035 0 0.004832602 0.3921397 0 0
0 0 0 0 0.02993206 0.02042672 0 0.006427267 0.05228204
0.00641281 0 0 0 0.1866983 0.004511138 0.004423626 0 0
0.2639088 0.0042493 0.05613675 0.35369121 0.05124 0 0
0.1340596 0.006972404 0.03802806 0.03797117 1.485151 0
0.05545111 0 0.01034123 0.2200908 0 0 0.004261364
0.2429894 0.440863 0 0.007303964 0 0.01750698 0
0.1014653 0.004552708 9.755585 0 0.004620803 0
0.003050853 0.1684994 0.4401944 0.02221459 0.03805695
0.02300154 0.1389411 0 0.03399596 0.00893316 0 0 0
0.009403471 0.8908415 0 0 0.005263764 0.003928157 0
0.04090495 0 0 0.08697033 0 0.008696317 0.144223 0 0
0.02824615 0.00363958 0 0 0.007789682 0 0 0.03017974
4.408933 0 0.004889999 7.448257 0 0 0.04562638 0 0
0.005087638 0 0 0.3203633 0.5174359 0 0.02386458 0 0
6.345688 0.1629653 0.006543734 0.03595515 0 0
0.004778833 0 0 32.23567 0 0.06797543 0 0.01286896
0.1117534 0 0.04641641 0.01062495 0.004351937 0.6027517 0
0 0.007228309 0 0.02051215 0.03366509 0 0.03177219
0.006768022 0 0 0.1172075 0 0 0.00475668 0.7996338
0.01550191 0 0 0.1878324 0 0.04359555 0 0.008839163
0.1504956 0 0 0.007707623 0.008707505 0 0 0.003601168 0
0 49.01939 0 0.2600102 0.004659263 0.009604763 0.1777551
0 0.004948962 0.005780795 0 0 0 0.01265011 0.006186107 0

0 0.01023492 0.06462158 0 0.01375217 0.008266507 0 0
0.003712406 0 0.02307312 0.01497376 0.02655087 0.02522946 0
0.004280823 0.003877303 0.01993674 0.01135122 0 3.512684
0.2559692 0.01949783 0.01256465 0 0 0.01371665 0.01280997
0.03011998 0 0.2132667 0.003774445 0.005749309 0.004122096
0.007839596 0 0 0 0 0 0 0 0 0.008010285 0 0
0.01452881 0 0 0.004279972 0 0 0 0.02800048 0.04355178
0.009287171 0 0.003524371 0.010249 0.004555402 14.06559 0
0.09061484 0.124305 0.004469589 0 0 0 0 0.4208036 0
0 0 0.003440551 0.004521563 0 0 0 0.01100288 0 0 0
0 0.4447378 0 0.276502944 0 0 2.198691 0
0.004079367 0 0 0.5846531 0 0.007445613 0.004131191
0.002767587 0 0.005578509 0 0.004612721 0 24.33526
0.029072934 0.006365715 0.02738741 0.01400747 0 0.01270774 0
0 0.006913884 0 0 0.1428352 0.0939141 0.05112319
0.141211 0 23.22631 0.01807169 0.1337395 0
0.004515149 0.009863568 0 0 0.02205195 10.75374
0.003927307 0.012274497 0.0644573 0 0 0.2666403 0
0.01662691 0 0.07882292 0.03940885 0.00822087 0 0.01788922
0 1.197176 0 0 0.006815095 0 0.1590033 0.01000689
6.635107 0.010375576 0.003696865 0 0 0.150303185 0
0.004753373 0 0.08490668 0 0 0.006556286
AL135999.1 0.1791997 0.06854228 0.7953279 0.457913 0.2996895
0.3493519 0.1107496 0.391952 0.3734821 0.336776495
0.642204 0.5280947 0.7264173 0.3486338 0.5550807
0.1958208 0.5451263 0.360924259 0.1216949 0.1817983
0.219871145 0.2810866 0.2849709 0.2036749 0.1933068
0.8697784 0.2694739 0.2904793 0.199119065 0.06099315
0.2532041 0.168663 1.3276 0.5926144 0.554378 0.471397
0.07924829 0.4063903 0.4297144 0.313213042 1.259001
0.9754756 0.167864741 0.4348859 0.8580601 0.7581961
1.35013 0.5874758 0.748109926 0.8802316 0.2068657 1.411746
0.4325587 0.4311159 0.3319261 0.6443537 0.6248928
0.5465103 0.6783131 0.279381314 0.467935649 0.586523551
0.1427803 0.1729991 0.5610247 0.3885742 0.2322878
0.2762007 0.166541475 0.7291844 0.271663 0.3799018
0.9262406 0.7811646 0.9273141 0.2430662 0.5321802
1.378466 0.9324508 0.390583593 0.2882158 0.6802206
0.7150123 0.8291898 0.2312275 0.325327 0.6176908
0.5148568 0.64458833 0.6114356 0.444669759 1.150536
0.1109842 1.22153 0.4022144 0.7139783 0.4603112 0.7878139
0.7293583 0.6856873 1.884649 1.119404 0.4541856
0.3385516 1.030866 0.9698923 0.3683797 0.1505768
0.380319629 0.7405999 0.4802147 0.816557 0.1965524

1.037141	0.7615836	1.056395	0.6065291	0.5998229	
0.728103	0.4874342	0.4172523	0.9205569	1.329348	
0.1247601	0.3660417	1.803341	0.442545	0.1598961	
0.5602106	0.2778137	0.9324418	0.9639819	0.9937335	
0.6635929	0.06009145	0.9307497	0.7755488	0.6174292	
0.4437361	1.016953	0.9826859	1.066101	0.9340099	
0.8254529	1.403847	0.2644804	0.6397165	0.5094127	
1.109447	0.5163886	0.9398524	0.4689878	0.3694218	
0.596619	0.6940652	1.026896	0.7525711	0.9919717	
1.321472	0.5246072	0.202577331	1.133445	0.723864406	
0.912914	0.2207624	0.6188043	0.3144479	0.7913622	
1.537511	0.6453038	0.3402456	0.438386994	0.4875414	
0.3510277	0.6420067	0.4898055	0.3467029	0.9676875	
0.6981924	1.202704	0.8200545	0.5701035	0.4063851	
0.9224102	0.4793075	0.653173	0.4711576	0.7819328	
0.4950892	0.720427	0.3534618	1.647619	0.4449907	
0.4999744	0.4313992	0.7292368	0.3062427	1.263887	
0.4838877	0.5441134	0.2516048	1.06091	0.3294018	1.239793
0.7289731	1.017741	0.291505	0.9649269	0.8674411	
1.177151	1.633147	0.4666428	0.6279142	0.4446976	
0.8125689	0.4821173	0.1725967	0.876621	0.666461	
0.473752487	0.5267565	1.374376195	0.169286157	0.7798534	
1.664186	0.6026816	0.6475928	0.6468355	0.2459654	
0.5551628	0.3639139	0.3490441	0.7537836	0.4312212	
1.143517	1.423516	0.413009	0.5850867	1.79582	0.4940214
0.902586	0.5072791	0.4990485	0.2442621	0.5421172	
0.6066477	0.6041921	0.4797607	0.6788178	0.3162457	
0.8296054	1.359933	1.793794	0.6659836	0.569646	
0.4160069	0.6746517	0.273985862	0.8496304	0.389292	
0.4186685	0.1568694	0.4863317	0.2499074	0.6874143	
0.5043138	0.8690234	0.1998243	0.5393924	1.11633	0.3349095
0.5722664	1.413119	0.843469253	1.297262	0.773594	
0.8929598	0.2976192	0.6244123	0.096152408	0.7336866	
0.3723565	0.1284901	0.7265922	0.4674277	1.13833	0.9854123
0.7756654	0.461004	0.3499219	1.073634	0.2878601	
0.2619454	0.8018349	0.2471387	0.4404355	0.5755287	
0.5843329	0.6318899	0.2638029	0.6782035	1.309548	
0.4889998	0.8977203	0.5217398	0.6367717	0.96487171	
1.025076929	0.8219533	0.782877	0.2095309	0.1352554	
0.7616974	0.1979064	1.384053	0.3433719	0.7946071	
1.303573	0.3657263	0.7729558	0.6027633	1.315733051	
0.7887245	0.4716111	0.1428168	1.33478	0.7075787	
0.305831218	0.3355576	0.6544902	0.2271558	0.3252492	
0.2876438	0.7047782	0.1242924	0.7499348	0.754746	

0.319041 1.299637 0.4353295 0.8963715 1.319239
0.5693827 1.185917 0.4893962 1.400203 0.796341108
0.9460556 0.3684016 0.3494914 1.57961 0.5100572 0.1833695
0.3950112 0.4650104 0.787842 0.9084359 0.8263857
0.5807036 1.192354 0.7541436 0.3129094 0.4216504
0.6273323 1.177383 0.8513454 0.8620524 1.214448
1.63955 0.5614886 0.8611889 0.5734514 0.3024838
0.537052461 0.485349 0.2839134 0.3338426 0.7342521
0.9153822 1.591751 0.492453146 0.6519905 1.410771
0.9497652 0.5243671 0.3493114 0.1474145 0.5168275
0.8870749 0.4083237 0.8612846 0.6162058 1.270128
1.101828 0.8637301 0.821644542 1.303364 0.4919347
0.3197049 0.260877758 0.6647907 2.119301 1.188801
0.8971865 0.4416228 0.2956512 0.3733929
HAND2-AS1 0.06208179 18.02414 0.1400438 0.02965214 1.004312
22.82563 0.04567617 4.637856 2.539855 4.002053621
0.1370307 0.04356016 0.2419582 0.04157672 0.09639181
23.93509 0.1208895 8.034279773 0.1204568 0.2588735
2.311067735 20.95092 0.05077289 26.60448 3.888558
0.06157794 0.04211562 29.04343 0.402755737 0.1442235
3.738534 9.933351 0.02568261 0.04189894 0.1889295
0.3207882 0.01384271 0.009293035 1.102232 0.039243851
0.04655313 0.00651519 0.073847507 0.0123532 0.1126352
0.01631483 0.006879763 0.7514999 1.390692632 0.001180591
0.1262693 0.4657943 0.03964425 0.2000296 9.617266
0.0163538 0.1388991 13.06766 0.05039295 0.028054671
0.431289656 0.104822661 2.833914 0.1141593 0.01423889
9.344084 0.8128033 0.530348 0 0.06195414 0.01641168
0.08034972 3.459632 0.7332468 0.173684 0.1176234
0.2673996 0.3429151 0.01454368 0.014479768 0.05911279
0.634549 0.02216628 6.061418 1.910406 0.114495
2.623572 0.7123701 0.48105459 5.496951 0.037034913
0.037961 1.319523 0.0661325 0.270519 0.01963095
0.7407538 0.01245188 0.01240443 2.199443 0.6088702
0.009039752 0 0.04964551 0.6085664 0.6879619 0.2004409
3.98741 0.036599331 0.06700057 0.06176591 0.0629034 0.09857335
7.015029 0.2565134 0.007041397 0.03473147 0.007026276
0.9074209 0.004639184 0.004588969 0.03339444 0.480834
0.216795 0.0909346 0.2351317 0.1933336 0.06394721
10.56035 1.072587 0.8644055 0.06226535 0.0541444
0.05808803 0.01982669 3.909318 0.607378 0.04137978
0.1431537 0.01745561 0.0342354 0.05291838 0.0448455
0.01867554 0.4673734 0.5199931 0.0153505 5.138344
0.01172412 0.4317212 0.07786571 0.2077124 0.07634729

1.705541 8.680445 0.004641316 1.927004 7.716767
0.02076233 0.4549793 1.216465638 0.4931845 0.636384868
0.009412771 0.2895342 0.09504443 0.007073834 0.04697102
0.1026657 0.7297268 26.95004 0.032331805 1.001435
0.01955382 0 0.1768313 0.7321078 2.139181 0.008531963
0.08894298 0.08027914 0.300066 0.01489817 0.1060377
0.5244423 0.01643714 5.827823 0.09199732 0.4571382
0.01916929 1.2296 0.02024941 0.08839231 0.05423426 0.07879354
1.112479 1.79421 0.0543287 0.0271112 0.07250082 0.02274383
1.952465 0.002898227 0.004826665 0.03356077 0.01948302
0.872742 0.4818111 0.007531718 0.06658139 0.01512544
2.084813 0.2099193 1.177056 0.1461077 0.5289425
0.5425775 0.005854941 0.7928554 0.090415098 0.1269009
0.358408322 0.012412137 0.03811946 0.4730379 0.1042166
0.015262 0.003442229 0.009017146 0.02215785 0.8088081
0.01890758 0.02313534 0 0.0460547 0.05379568 0.2214372
0.1266072 0.4072508 0.06640676 5.285138 0.9716503
1.036947 0.3077162 0.1742414 0.3591497 0.3076364
4.164443 1.761614 0.03372694 1.558619 0.01334421
0.008258343 0.6886846 0.02217389 0.1011376 0.7203791
0.08427067 0.03191862 2.069372 0.06094249 0.04554687
0.03398003 1.655207 0.04885071 0.01124286 0.02560065
0.06432238 0.217244 0.08864235 1.235721 0.07632936
0.08458468 0.020264267 0.007202283 0.1431121 0.08558153
13.21638 0.09984039 0.082836765 5.034507 0.02498767
3.262664 0.3432545 0.0733373 1.21276 0.01748005 0.4348233
0.08190247 3.708958 0.008349021 20.20256 10.84595
0.07180887 0 1.152523 0.2649973 0.2227126 0.03954064
0.01387589 0.2512803 0.6081659 0.1915931 0.02814529
0.009979354 0.07538802 0.012798059 0.2234275 0.1154858
0.04073255 0.1175262 0.03904813 0.060372 0.3937063
0.04947121 10.35767 0.01456524 0.1552253 0.1523439
1.205867 0.5434657 0.024705766 0.2131699 0.1678086
1.617323 0.1061205 0.1105863 0.005821533 0.02214291
0.05938455 0.08843891 0.7261808 0.2117129 0.2978892
5.302258 0.02827829 0.001456271 0.06662343 0.1049833
0.03169843 0.03515916 0.06189653 0.2688125 0.02476481
12.83816 0.03310179 0.002601449 0.04898602 0.3136804
0.5189036 0.02714478 0.8505432 0.01260444 0.1532267
0.01484772 0.01467415 0.2213611 0.1768026 12.86063
0.04574509 0.1419072 0.1868188 0.03404005 0.02371683
0.03498053 0.1035622 0 0.06001634 0.07772361 0.08581836
0.09313557 0.06487053 0.07590582 0.297279521 0.2849896
0.8173599 0.004130576 0.03670616 0.04205384 0.2767266

0.316905052 0.008228598 0.4725253 0.02353934 0.03819718
0.02561167 2.440018 0.05684103 0.007280678 0.02721679
4.948042 0.1169495 0.131929 0.2077368 0.02082052
0.103981663 0.1958312 4.170318 0.3136013 0.002151863
0.02416484 0.1003784 0.2936288 0.01238104 0.01260951
0.07365852 8.490103
AC010333.1 0.02580572 0 0 0 0 0.05226861 0 0.03092777
0.1593583 0 0.1993598 0 0 0.07258572 0 0.01911813 0
0.019429913 0.03186313 0 0.030154873 0.0257003 0 0
0.03534885 0.06085153 0.04084808 0.06121546 0.049868185 0
0.07292548 0 0.02490965 0 0.02100879 0 0.02685218
0.04635435 0.059885 0.137025865 0.1458758 0.04549755
0.026859352 0.04976892 0.05314633 0.01898857 0.2402174
0.02773759 0.210208421 0.1030553 0 0.2921662 0.08305435
0.2328112 0.05031487 0 0.07577928 0.1851774 0.1035027
0.069969388 0.439468871 0.035192715 0.0257014 0.2717759 0 0
0.07042248 0.234734 0.025927416 0.03605371 0 0 0.2042237
0.02406243 0.08032371 0 0.3005364 0.2520716 0
0.050558309 0.04486985 0.1691316 0.1934924 0.07960517
0.08154612 0.02498604 0.1858673 0.03188908 0.53042374
0.407504 0.074603668 0.02881448 0 0.08713647 0.02970309
0.07908975 0.1250703 0.05434702 0.1515918 0.05231397
0.7108083 0.1352729 0.1783777 0.03095444 0.1331392
0.1751954 0.07445424 0.06424849 0.022820022 0.03440333
0.04688371 0.0414409 0 0.5689672 0.4569671 0.04097685
0.02887386 0.1635554 0.1334464 0 0 0.1665739 0.07134158
0.04790965 0.07441687 0.2753347 0 0 0.0239032 0.09553828
0.07433998 0.02937958 0.2100593 0.07800891 0 0.09867454
0.02454575 0.05557068 0.02840016 0.0338605 0.05273739
0.1086897 0.1003749 0.0543404 0.07307038 0.2086937 0
0.3088764 0.1137126 0.0252077 0.04769824 0.1947005
0.0467681 0.1282333 0.0942916 0.08102929 0.1614085
0.4277837 0 0 0.077792573 0.08553123 0.184729702 0.0234758
0 0.1229119 0.08233122 0.04823719 0.0702888 0 0.1065156
0.029708265 0.1651966 0 0 0.04088962 0.2745988 0.03716057
0.1489532 0.02986134 0.1038173 0 0.02889957 0.09616795
0.02359755 0 0.1005173 0 0.1064112 0.1338649 0.1991754
0.02719381 0.05231105 0.07890299 0 0.1129183 0.1813482
0.05126944 0.1314762 0.03013662 0 0.2748923 0.05059799 0
0.04882605 0.06002462 0.03109501 0.1486146 0.06574534
0.04843313 0.2310559 0.2468537 0.09581244 0 0.04952984
0.02257803 0.165699 0.1022171 0.1081396 0.107016286
0.1204059 0.081615462 0.108347149 0.05545829 0.9676075
0.0627393 0.0266448 0.1201906 0 0.07736748 0 0.06001698

0.03365856 0.2443206 0.03092442 0 0.07434437 0.2188457
0.08741669 0.1317439 0.01932746 0.2068698 0.06844347 0
0.02691992 0.2726143 0.1288991 0.4390646 0.1503898
0.09200214 0.09279036 0.05824166 0.6247639 0.09356603
0.03686834 0.09459032 0.1708192 0.05349888 0 0 0.1655033
0.03614405 0.1647867 0.02665778 0.5787185 0 0 0.08422176
0.04285538 0.2401357 0.09410488 0.03506783 0.1440684 0
0.1616648 0.02575761 0.2694287 0.4750608 0.05533459 0
0.331466 0.03635344 0.02960521 0 0.0376571 0.05005361
0.0406895 0.04337862 0.06808927 0.1469726 0.0624683 0
0.02125157 0.131964 0.1581746 0.3061899 0.04948012 0
0.04183702 0.04404525 0.03202129 0 0.06601742 0.4211723 0
0.06472837 0 0.429429935 0.1812297 0.05203313 0 0
0.122557 0.2682313 0.03321851 0.06449658 0 0.1505536
0.1316663 0.1544182 0.06875337 0.15404274 0.2899928 0 0
0 0 0 0.10543 0.02356252 0 0.04225951 0.3313779
0.07960143 0.168459 0.09873793 0 0.09305033 0.1032578
0.06917492 0.03610693 0 0 0.1441168 0.193084 0.1256304
0.204375498 0.0994431 0.03422696 0.1750561 0.04739006
0.1058752 0.08802061 0.4612193 0 0.03659788 0.240036
0.2975099 0.293419 0 0.1696886 0.343319 0.2067061
0.1505653 0.03214206 0.04953466 0.084641 0.06163416
0.3980295 2.663536 0 0.2202135 0.02454044 0.102265674
0.07107746 0.0320667 0.3124882 0.1922477 0.1779847
0.2857141 0.140659591 0.04104486 0.0307816 0 0.05884025 0
0.07076158 0 0.05084319 0.09503157 0.06387789 0.1570565 0
0 0.0908725 0.097250295 0.1848039 0.182816 0.09954423 0
0.129808 0.5049383 0 0.01965014 0 0.1390216 0.1229042
MAP3K4-AS1 0.229588 0.370471 0.3101764 0.3221209 0.3293755
0.8065239 0.155193 0.3353486 0.3544443 0.665187629
0.8190872 2.043857 1.407092 1.39919 0.2807215 0.6006304
0.429349 0.632033471 0.3720669 0.2604996 0.377270964
0.4930269 1.369127 0.7982865 0.5601876 1.71438 0.4883413
0.7261615 0.42980219 0.8953946 0.5677023 0.3472852
0.2146904 0.949061 2.996413 0.3931946 0.1866392
0.4768432 1.975742 0.54605089 0.6720887 0.9993059
0.672080687 0.2813521 0.2364159 0.2481267 0.2754937
1.449806 1.220000366 0.1661812 1.755845 1.218441
0.6927348 1.747636 3.231407 0.264595 3.318291
2.368259 0.3309276 0.340431305 2.71246698 0.675126623
4.851879 5.868526 0.1184796 1.784235 0.9985387
2.102882 0.555051561 1.057513 0.4595739 0.322403
1.142682 1.806286 0.7927851 1.128872 0.3732192
1.530126 0.4724267 0.175705728 1.883714 3.942075

0.2382379	0.258209	3.597264	3.66093	0.4724634	1.507212
0.76193296	1.359554	0.656819286	0.4566348	1.642555	
0.4663523	1.255245	1.077456	3.31731	0.2493119	0.4937134
1.054482	3.224836	0.2883372	0.4132783	0.266789	
2.109912	1.342194	1.179907	0.9288586	1.167393449	
2.257334	0.9254728	0.5184717	1.397601	0.980759	
1.651626	0.5069696	0.5860182	0.3580954	3.418627	
0.330261	0.3192615	0.9262334	1.619835	9.483887	
8.068994	1.190002	1.168088	1.489866	2.252886	
2.835494	1.462286	0.3022252	0.3942114	1.894121	
0.6255297	0.4297987	1.46723	0.3244505	0.8448663	0.2918357
0.5816045	0.4834949	0.1116268	0.5136711	1.821611	
0.6730551	0.2514667	2.471981	0.4868698	1.934308	
0.4972983	2.408854	1.11173	0.9982567	0.9909418	0.2478096
1.69886	1.002447	3.748896	2.520446	3.352379716	1.173136
2.509274435	0.1109565	1.414188	0.7347096	0.5035825	
1.562397	0.6351157	1.757735	0.5389743	0.404721929	
0.6736214	0.8614986	0.4246755	1.847353	4.192049	
1.250118	0.1380422	1.560812	1.226711	0.5869351	
1.044523	0.300792	3.339397	0.327997	2.101562	
1.29108	1.04139	0.3721776	2.0489	1.738927	1.548911
0.4606765	0.592181	1.393896	4.230636	0.3919896	
0.2266326	3.443656	0.7727624	1.087613	0.2883836	
0.814116	0.4343951	0.1613205	0.9682603	5.040859	
0.316833	2.154497	0.6791006	2.470731	0.2930205	
0.8704361	0.5645913	1.606976	2.119147	0.880985	
2.097068	0.795897297	0.3883196	0.915208251	0.602463655	
0.5447967	0.4191688	0.8663393	0.2666849	0.8521072	
0.2188384	2.896689	1.829349	1.460043	0.1778003	
0.3849205	0.103173	0.1004288	1.064483	0.2859692	
1.814698	5.32572	0.5803393	1.445062	6.355678	2.791612
3.510185	1.605444	2.843079	0.4164768	6.355436	
0.4553038	0.6449503	0.1214446	0.9219451	1.261663	
0.430513	0.9204456	1.074506	3.153290047	1.010397	
2.285961	0.3812589	1.668124	0.8521549	2.082637	
0.8214268	1.036847	0.4784054	0.3590412	3.818711	
0.3931637	1.497859	1.277214	0.4205731	0.157374803	
0.7591018	0.3652238	0.4358274	0.5168295	0.9999848	
1.283219167	4.200765	0.5963221	6.272007	2.975175	
0.6962287	0.7236393	0.1640339	1.591963	2.221178	
8.359202	1.047853	0.6097132	0.7031068	0.9661502	
1.945958	10.53159	4.470926	0.677647	0.3722152	
0.1530708	0.4540381	2.081389	0.5383979	5.379925	
0.199807	0.3179308	0.63672554	1.263566657	0.8250762	

0.3134407 0.5145251 1.56139 0.5383671 4.157552 0.572605
1.82305 0.3927619 1.742323 0.7540937 3.184128 0.4205329
0.402580242 0.6382826 0.4738989 3.548825 1.107445
1.053653 0.433269605 0.3517457 1.690148 0.2764772
4.033888 3.600205 0.413115 1.276274 0.5147172
0.1484383 2.276585 0.3373215 0.3846467 0.2710427
0.9013052 0.8303078 0.2884898 1.213216 0.4889968
0.321988131 4.373856 2.673979 4.380293 0.2635121
2.325439 0.4649662 4.086274 0.4203983 0.1424516
0.2736173 1.085636 0.5547286 1.848473 0.7170998
2.076063 3.484082 0.3976783 0.652349 2.52025 0.1411937
0.471235 0.1006019 1.295926 1.233598 1.075807
0.9415529 0.632620511 0.6916451 1.791982 0.3007365
0.801744 0.2041221 0.7221415 1.57079138 0.861567
0.7274338 0.6712533 0.1308723 0.3833034 4.210122
0.7003528 0.3604594 9.135103 1.107017 0.3667909
0.6403605 1.886251 0.6126714 0.612860953 0.687208
0.4129724 0.2629197 1.034032474 0.7759314 0.6771527
0.4998918 0.2403818 0.5372386 1.3528 0.5068608
TPT1-AS1 0.9735096 1.47314 1.639342 0.478987 0.8427315
1.995857 0.561081 0.9708857 0.6317915 0.708721561
0.8308448 0.6805223 0.9015779 0.733347 0.8334004
1.894634 0.8246869 1.426008401 0.9243676 0.868079
0.716495664 1.662255 0.8191464 1.961788 1.571717
1.146152 0.9683609 1.998987 0.631583777 0.7733031
0.7933538 1.534939 0.5055806 1.680414 4.62452 1.653368
0.3139242 2.255494 4.039651 0.618034687 2.095668
6.040559 0.245682254 0.9544689 1.351956 3.450127
1.550438 1.55382 0.913033466 0.6827212 1.154441 2.38912
0.5424269 1.373479 2.132307 0.9512142 1.419117
1.723884 1.019565 1.07930572 0.924478806 1.038103903
0.8082105 0.9561277 1.222441 1.668578 1.35501 1.396116
0.409763841 1.053744 0.6668284 1.216802 3.827255
0.6746225 1.104254 0.8739963 2.087508 1.495065
3.427175 1.332640615 1.187558 0.9688104 0.5026858
1.720554 1.563892 1.632286 1.256053 0.8319182
1.15792015 3.325705 0.479159902 0.845282 1.270699
1.199092 0.5080193 0.9602969 1.88729 0.9706915 1.188522
0.9810982 3.140709 1.161684 0.9654549 2.620301
1.408063 1.487669 0.5681985 0.7789372 0.448346963
0.9906122 1.76613 1.709132 0.9951635 2.84083 1.607644
0.6465002 1.089254 1.105432 2.676518 7.352811
2.575693 3.178028 1.865011 1.796999 7.291577
1.786918 1.028394 0.7417125 1.768545 0.5532899

0.8660783	1.30233	1.901515	0.57703	0.7618708	2.403307
1.01366	0.5504126	1.423388	1.88582	1.791593	1.773648
1.13761	2.857309	1.216784	1.14987	4.14659	2.367942
2.463074	1.18016	0.6738052	0.9075863	0.2784415	1.018869
1.965852	1.46042	1.000908	3.969164	3.213531	5.598261
1.132614101	1.796172	2.750974196	1.257903	1.712057	
0.7639297	0.4634357	0.6709756	1.563578	2.555214	
2.135383	0.40198407	1.897011	1.013375	1.4185	1.016928
1.091714	0.8447404	1.025215	1.461064	3.066586	
2.778942	0.5724848	1.137296	1.610548	1.297759	
1.47653	1.632765	2.051506	0.8018169	1.063648	1.137735
0.7856841	0.7644305	1.407405	1.251658	0.5130739	
2.532112	0.9891305	2.237898	9.257237	2.032608	
0.5874246	1.234833	1.088781	0.9648892	0.6715142	
2.104218	1.722273	3.654239	1.232706	1.249973	
1.085123	2.549473	1.270682	0.8224182	0.5709834	
1.700665	1.008175	1.046935021	0.9592842	1.061641775	
0.932408361	1.2847	1.608885	1.633339	13.19548	0.9107313
0.4106848	2.259829	2.330582	1.328584	0.4681879	
1.237514	0.9908657	2.744945	1.287626	1.095649	
0.8059081	19.24104	5.607008	1.0063	1.129857	1.27383
0.7401658	1.171013	0.8650936	2.99846	0.8587414	1.283726
1.260573	1.954416	2.455478	1.980097	0.9518391	
2.320897	1.111766	0.622550659	0.7618771	1.635776	
1.646944	0.4069038	1.250437	0.8108711	0.6660191	
5.723964	1.285721	0.7673367	1.556394	1.54955	0.8798766
0.6870828	1.185234	1.34674259	0.9265258	2.266823	
2.333218	2.337583	0.6843439	0.348067956	2.052673	
0.8116354	0.8812972	0.8456166	0.4494145	2.262109	
0.4878065	5.355408	1.174375	1.382995	2.350955	
1.498167	1.254895	1.792754	0.6506417	1.960265	
2.304481	0.8135087	1.120876	0.9404541	0.9445541	
1.03224	1.492382	1.775525	0.9099599	1.792559	
0.317443099	1.607610028	0.7307641	0.301339	0.4670724	
1.579549	1.935597	0.8293297	1.193822	1.149646	
0.7279047	1.349611	1.499381	0.8566704	1.659665	
1.734187756	0.9247344	1.052293	0.7684249	2.737502	
0.7237217	0.867300726	0.7817651	1.082732	1.220931	
1.229022	0.8388327	0.5845014	0.8661823	2.615943	
1.093961	1.494515	1.584409	0.3884439	0.7953833	
3.331868	1.350844	0.9344649	1.753597	1.476883	
2.126297204	2.563041	1.796932	3.408549	1.618483	
1.054396	0.6514833	1.571013	1.099003	0.8755275	
1.425196	0.9983541	2.741073	0.8990841	1.263754	

0.7061109 1.536924 1.049213 2.809557 1.172948
0.7283992 4.431743 1.318042 1.007845 2.677445
2.208398 1.492932 1.872230158 0.8719892 1.204496
0.7558889 1.803577 1.070498 3.455512 0.983357303
2.194862 2.347436 1.480527 1.831192 0.9720669
0.7353435 2.978834 1.075972 1.118713 1.758634
0.7667504 6.895497 1.401129 1.068523 0.663212981
1.14402 1.626453 0.5576324 0.919063664 2.583722 5.697373
0.8551384 0.8242497 0.6420813 0.8408553 1.186289
AC093227.1 0.6516512 1.358621 1.002299 0.6939307 0.6866026
2.78044 0.9903333 1.459563 0.7091736 0.923522868 1.494694
1.741994 1.765179 0.7011283 1.323623 2.857078
0.6819072 0.892818775 0.2638079 0.4106924 0.823893209
3.723709 0.4756709 2.914049 1.331638 1.11679 0.9131347
3.057862 0.454167163 0.8484103 0.392457 2.67167 1.835511
1.581353 2.565621 11.17048 0.7447728 1.31447 1.98325
2.609335177 1.207766 0.05650394 0.378045386 2.266316
1.716079 1.831545 2.958427 1.89462 1.598994404 0.8703022
1.011231 3.910658 2.326519 1.301089 4.894786
1.300115 2.274354 2.190229 0.3106414 0.680683811
0.84899354 0.670162456 0.8937284 0.3875249 2.116952
2.93899 1.282725 1.349625 0.418594318 1.029837 2.445204
7.889831 2.430599 1.603743 0.6899718 4.327723
1.998906 1.200028 5.336361 1.192990213 0.5386691
1.540341 1.361703 2.306794 2.295523 2.327284
1.934581 2.059378 2.12260498 3.747432 0.916217151
2.481096 2.50047 2.59718 0.2951089 3.241342 2.267764
1.597363 1.111652 2.912792 3.651421 1.147979
0.9107316 4.933477 2.601462 2.675394 0.9554778
0.7979095 0.935234981 3.645944 1.132161 5.16375 0.6936622
6.100375 1.929543 1.170461 0.9203755 1.819629
1.665178 2.35816 1.017073 3.620228 3.868864 1.269324
1.345213 4.113659 1.426575 0.5314836 2.157159
0.2966253 1.692601 3.636523 2.804408 0.8826861
0.1432916 1.82456 2.042405 0.6556319 2.069202 1.345656
1.586439 1.619797 0.5568004 4.487818 5.666644
5.909284 3.66106 3.050512 1.685238 3.057527 1.954821
1.118329 1.510128 1.688097 3.177363 1.475925
2.625007 5.59098 2.280815 4.146028 0.821197854 4.355112
1.081541951 1.622955 1.25025 2.255764 2.385789 1.79719
1.542167 3.774577 3.077784 0.65181244 1.67547 0.9457535
0.9318538 3.275394 1.167761 3.876613 1.387401
1.817172 3.706794 1.780226 0.8853042 2.169683
1.025713 1.465134 2.271973 2.746258 1.532978

3.675938 1.566602 0.6754462 1.624143 0.8002561
2.277829 2.309195 0.5732833 3.41707 1.719902 3.592995
5.309291 3.300122 1.288184 1.752886 3.436133
1.26727 0.9525591 1.230441 0.8437157 1.954865 1.954
1.203573 1.655952 2.038339 1.404517 1.056171
0.7431071 2.50504 1.779474 0.708825521 2.163252 1.71184431
0.773706197 1.836648 3.036812 6.190046 2.117792
1.467784 0.5995527 1.387874 1.09677 1.155305 2.772797
0.9271307 2.393937 3.304736 1.508043 4.00433 2.074775
2.050633 4.480561 2.290817 1.940851 3.309417
1.53788 0.8515371 3.094905 4.426386 1.618681 2.216616
3.47633 1.82033 3.004132 2.750085 4.380311 1.357464
3.093744 0.708702813 1.193531 2.011294 2.554575
1.152118 1.596275 2.659564 0.6178447 1.966358
1.581931 0.8600115 0.940266 2.054455 1.908876
0.290341 2.624164 1.279031887 1.420286 1.599434
2.565318 2.98412 1.282786 0.891648612 2.652865 1.896206
1.127523 1.407739 2.1123 4.01982 0.7158808 4.579156
1.531491 1.616668 3.111825 1.793547 2.577674
1.875603 0.7857556 0.8872754 5.049137 1.561542
3.368603 1.212524 2.690942 3.676531 2.168122
1.268902 2.181716 1.348714 1.052121582 0.918485374
2.269469 1.507813 0.374729 0.2687703 2.475867
0.8744389 1.993965 1.237086 2.514687 1.70614 1.406254
1.182605 1.480017 0.522907143 3.991613 3.28555 0.9118086
2.799384 0.6314903 1.598789774 1.280251 1.5119 3.325831
1.271245 1.371807 0.560195 0.8194104 3.821775
3.820492 4.670562 3.932602 1.353069 2.959547
5.166044 0.6843825 1.586959 2.190112 1.747444
1.175075629 2.461758 2.04033 0.905851 2.020663 2.344864
1.94943 1.463806 2.217689 1.151434 2.404702 0.5080364
2.963791 1.429885 3.540403 0.6395578 1.422605
1.978965 3.020432 3.506507 1.635148 2.041178
0.6516004 4.213605 3.160194 1.49766 0.3555656
1.037207042 1.275039 0.7832056 1.940414 0.7427922
0.9210054 4.161208 1.475132984 0.9685077 2.472079
2.615651 2.395217 1.604207 0.629804 1.485207
2.13633 1.711302 1.94801 1.573406 5.159998 1.501357
2.652107 1.905581392 1.405749 1.485229 0.3914796
1.539643941 0.846353 3.848604 1.425302 1.033091
1.437853 0.9352011 2.170825
AL645608.6 0 0.1187407 0 0.07908042 0.05429266 0 0.2558126
0.04960779 0 0.103719485 0 0 0 0.03880889 0 0.03066527
0.08256711 0.093496076 0 0.08840483 0 0 0 0.09904298

0.05669915 0.06507011 0 0 0.079988001 0.09392251 0
0.1113094 0 0.1303655 0.06739571 1.270321 0 0
0.06403657 0.073262642 0.3342616 0.03648878 0 0.7982878
0.04262306 0.03045745 0.2408162 0 0.168585956 0 0
0.0781052 0.8881215 0.1244755 0.08070448 1.373859 0 0
0.3320344 0.168345151 0 0.169346143 0.2473485 0 0
0.1963369 0 0.04183452 0 0.1734892 0 0 0.1228397
0.849109 0 0.1497177 0.06427426 0.10108 0.09398408 0 0
0.09042839 0.4433708 0.08512385 0.08719936 0 0.04258985 0
0.14179894 0 0 0.09243619 0 0.3144733 0.09528682 0
0.3611005 0.087172 0 0 0.103648 0.03616269 0.2861157
0 0.1281323 0.09367047 0.05971187 0.05152692 0.073206111 0
0.1504019 0.1661768 0.1452808 0.0912617 0.146594
0.0657264 0.8336402 0.09837787 1.10081 0.04330345 0
0.3562436 0.9154486 0.3073861 0.2387276 0 0.07571877
0.0895352 0.07668093 0 0.08943036 0.09424903 0 0.08341694
0 0.1582728 0.3543399 0.1337021 0.04555354 0.1086237
0.02819673 0.5665956 0.0644001 0.1307421 4.102142 0 0
0.1061645 0 0.2425972 0.3060297 0.03903718 0.4876008
0.08227391 0 1.819581 0.110956 0.1470343 0.2325618 0
0 0.2743822 0 0.03765492 0.05099234 0.06571642 0
0.07737191 0.05637122 0.3567108 0 0 0 0.1263606
0.04298335 0.03279324 0.1201237 0.5364462 0 0.574767
0.1665218 0 0.1854183 0 0 0 0.1612287 0 0
0.3578631 0.1419889 0.08723712 0 0.06327975 0.355878 0
0.1586621 0.6167669 0.3374182 0.1450164 0.1910676
0.3391724 0 0 0 0.5455799 0.04987605 0.8581538 0
0.0388431 0.1588333 0 0.07684103 0.09130449 0.3177812
0.07242982 0.2657793 0.131164 0.04336369 0 0
0.218183785 0.043446898 1.512226 0.1082813 0.8721537
0.04273796 0 0 0 0.1400965 0 0 0.03265729 0.09920483
0 0.05962376 0.4914365 0.7945537 1.648263 0.4340144
0.2073855 0.1646738 0 0.1295377 0.03975184 0.1378351
0.1242802 0 0 0.111626 0 0.1541714 0.1500788 0
0.1517218 0.03424901 0 0.1554456 0.5195366 0.1137709
0.1739239 0.1057264 0 0.146567 0.06296642 0 0
0.03436977 0.1283916 0.07547158 0 0 0.075660963 0.460993
0.04131491 0.07857464 0.2981709 0 0 0.3987508 0 0
0.07628653 0 0 0.130531 0 0.4004528 0.1683874
0.1335979 0.04289727 0 0 0.6342757 0.2104822 0.515876
0.09308338 0.03355305 0.03532404 0 0 0.03529706 0.03753087
0.2794504 0.1038236 1.119944078 0 0 0.02782018 0
0.1041389 0.117948 0.16134 0.1065642 0 0.135956
0.2113004 0.04223826 0.2064041 0.05513981 0.494165599

0.1550484 0.08543851 0 0.5943352 0 0 0.0563695 0 0
0.09037837 0.04088665 0.04255993 0.06755157 0.03959363 0 0
0.2484364 0.08321683 0.2316604 0 0.2993898 0.04623234
0.1238818 0.08060386 0.036423997 0.09570336 0 0 0 0
0.1764801 0.04931937 0.1385928 0 0.1155045 0.1193006
0.2823844 0.4483475 0.1088714 0.4818448 0.04144427
0.1811288 0 0.07945302 0 0 0.05803953 0 0.08840886
0.10092 0 0.082016488 0.3800248 0.2057385 0 0 0.03568568
0.08332402 0.150410921 0.03291774 0.04937334 0.08239607
0.3775164 0.4183665 0.05675038 0.1224393 0.1631038
0.8383625 0 0.05038337 0.6519507 0.1504454 0 0
0.7410582 0 0 0.060258303 0.7807896 0 0.5522537
0.4097417 0.1177008 0 0.03285615
AL133330.1 0.02063724 0.1381373 0.4176244 0.07885576 0.4331074
0.9405008 0 0.09893371 0.06372067 0.051712414 1.204591
0.2027239 0.4461688 0.2515412 0.471026 0.6421411
0.2469976 0.248614565 0.229333 0.2203842 0.096461326
0.2466353 0.2888002 0.07407121 0.05653807 1.395033
0.2940017 0.4569131 0.159521524 0 0.174959 0.9989384
0.3784922 1.234954 0.6384403 0.04523971 0 0.05560547
0.5268009 0.456590683 0.7332864 0.6367395 0.193318663
0.106136 0.7862864 1.397062 1.176647 0.3992795
1.870190579 0.8076668 0.3573502 2.628562 0.04427992
0.3413352 0.9254649 0.1775868 0.3939123 0.9308471
0.5380231 0.279778162 0.890340628 0.478450963 0.5138455
0.1207464 0.5963952 1.62217 0.2815899 0.7091664
0.103672835 0.6487364 0.4406426 0.2243629 1.79653 1.1161
1.3329 0.0746462 1.297856 2.318235 0.07809757
0.181945278 12.73851 1.172229 0.2652667 0.5092921
0.2825928 0.2397808 0.7007362 0.3570309 9.0493504
1.024218 0.159097973 0.8295623 0.3878467 0.662002
0.2850484 0.2951635 0.4400912 0.1303865 0.3290539
0.8367263 2.73887 0.3966595 0.7132572 1.113964 7.900338
0.7939371 0.3274823 0.4367346 0.054748604 0.7978739
0.1499746 0.8285237 0.3983871 9.896514 1.315598
0.8356308 0.1847271 0.3106449 0.1981928 0.9931499
0.1708522 0.1998178 0.05705299 0.4214552 0.1190247
1.160999 0.226511 0.1562415 0.6117047 0.1719078
0.787724 1.221757 0.1679878 1.018954 0.0922711
0.789116 0.6674073 0.7110521 0.4088171 1.137309
1.757289 0.4128743 1.509103 0.4345688 0.9154902
0.1335165 2.545029 2.399559 0.1455005 0.4233389
0.01907252 0.8758413 0.3553113 0.7588716 0.3317886
0.1944012 0.3319224 3.592107 0.1159505 0.1380926

0.186635876	2.120421	0.590925125	0.2816096	0.483051	
0.5570027	0.7900991	1.080129	1.180433	0	0.3918383
0.190065377	1.034864	0.3360044	0.3643206	0.4414511	
0.4990933	1.069844	0.2183871	0.5253729	0.2905853	
0.3960145	0.6471204	1.499687	0.4340407	1.784919	
0.8199302	0.2513023	0.4765528	0.4103734	0.3008693	
1.196103	0.06275097	0.1577499	0.4968138	0.5418152	
0.3427912	0.4510108	0.1892585	0.457914	0.07938534	
1.674134	0.2023201	1.634164	0.07809394	0.5120282	
0.09946871	0.213929	0.8061898	1.54931	2.217349	1.84252
0.6129819	0.1138064	0.812001	0.2888962	0.0441707	
0.09809355	0.8864302	0.299539194	0.3273879	1.348896459	
0.086646939	1.212257	2.231455	0.2843171	0.2770076	
0.6728286	0	0.433104	0.04656616	0.7679445	0.2960901
0.2605161	0.6182687	0.4622009	1.189088	0.4900403	
0.5126624	0.1475008	0.4791509	0.4756316	0.4378827	
0.8183286	0.7534901	0.4162086	0.4466915	0.7848719	
0.6013456	1.706953	0.9090226	1.723341	0.1921668	
0.9353281	1.002463	0.02521513	0.9391721	0.171135622	
0.5618896	0.4981353	1.077753	0.3468597	0.2899217	
0.7887901	2.265335	0.7534505	0.3752987	0.2469628	
0.6683066	0.4480941	0.5456145	0.280443	0.1728207	
0.377230085	0.6464297	0.3501791	0.940217	1.205813	
0.3097636	0.418322157	0.8835954	0.6541295	0.1420546	
0.3803491	0.7378171	11.28808	0.3091304	0.3815963	
2.069179	0.3861909	0.5162213	0.1497139	0.1529569	
0.2990122	0.6008501	1.224325	0.3957004	0.9281894	
3.111569	0.2641776	0.6914142	0.7284502	0.4575582	
3.929546	0.1915763	1.294108	0.781733693	2.346716662	
0.3079811	0.194188	0.06434741	0.3115293	0.1176129	
0.268136	0.6375688	0.3438596	1.107153	0.1505001	
1.242489	0.740944	0.329899	0.443485548	0.1932599	
0.2981852	0.06366681	0.2222425	0.4384103	0.027092729	
0.08431404	0.8102629	0.3976323	0.3830168	0.8561803	
1.167073	0.5725571	0.8488446	1.504564	0.2604484	
1.754759	0.1106406	0	0.2230136	1.620642	0.829818
0.4941195	0.9444048	0.090801299	0.1113367	0.2189748	
1.294955	0.909566	0.4656856	0.1759787	0.860637	0
0.2634112	1.113371	1.576242	0.9855378	2.379071	
0.7599349	0.1715985	1.715051	0.4816379	0.4883859	
0.574398	0.4738213	0.5175431	0.8247137	1.445406	
0.3085519	0.8050662	0.03925075	0.12267523	0.2652616	
0.02564425	0.5766964	1.229948	0.6938938	31.57318	
0.131235665	0.590836	0.4184811	0.123243	0.674462	

0.3575811 0.1980621 1.770326 0.2439606 0.09499782
0.8173467 0.7033632 1.047381 0.675081 0.4542012
0.337014635 0.4433718 1.334083 0.03980355 0.180261343
0.2595238 32.20956 1.10137 0.8642989 0.2738549 0.08338332
2.833983
AC009318.3 0.2599554 0.9114471 0.4516654 0.3862835 0.1894307
1.170069 0.3570188 0.3807866 0.4013262 0.180942043
0.3471071 0.1702397 0.5352505 0.05416271 0.5650706
1.604897 0.3456985 0.630680288 0.2139834 0 0.438774382
1.093106 0.03674595 0.7602484 0.8902217 0.5221779
0.2743237 1.621581 0.279083382 0.06554036 0.08162434
1.346334 0.6691434 0.5458246 0.4702961 0.5698585
0.540994 0.6485462 0.7373118 0.690169042 1.11961 0.8911821
0.180379508 0.5570557 0.7138303 0.4250722 1.344358
0.3415087 0.382334849 0.253766 0.4001186 1.035554
0.65073 1.30291 1.633183 0.5326092 0.3816835 1.273206
0.2606602 0.039157818 0.688646847 0.236343921 0.4315076
0.1351976 0.6359742 2.094241 0.4335253 0.817395
0.377261943 0.4842523 0.4933796 1.297945 6.943249
0.4309232 1.213718 0.2611875 0.9643055 1.199094
0.6339722 0.254651045 0 0.536368 0.433146 1.217711
0.3042443 0.4754302 0.8024335 0.8566308 0.89054244
0.7493283 0.612353232 1.128806 0.8142467 0.7314787
1.263356 0.4131116 0.8119376 0.3649786 0.3151095
0.7026505 1.66352 1.413149 0.4658624 0.4157618 0.8047115
1.132984 0.2916741 0.215737 0.153252554 0.4620853
1.311905 0.7421464 0.6082729 1.846825 0.3068856
0.3439856 0.06463612 0.5720781 0.5974577 0.4834835
0.747266 0.932218 0.479109 0.5362452 1.193875
0.5883394 0.4491192 0.1561971 0.6153531 0.2673362
0.4992454 2.071699 1.293139 0.8731415 0.1937143
0.809929 0.329684 0.7463927 0.5721814 0.7200911
0.6886616 0.7907557 0.9212543 0.5778124 0.9541762
1.448243 0.8248909 2.074323 0.5854732 0.6489353
0.3737152 0.2179253 0.3140808 0.7463536 1.519763
3.809182 0.516177 2.838669 0 1.256286 0.261216017
1.27645 0.561219431 1.129872 0.1067494 0.4815067 0.3686082
0.8908537 0.3540292 0.6045138 1.788319 0.299267949
1.510033 0.3527044 0.4199209 1.281479 0.3073542
0.5407122 0.666884 0.8690065 1.01676 0.4667476 0.3558151
0.3498277 0.6074845 0.7494531 0.2700179 0.7816075
0.9051931 0.4744709 0.6935722 5.387456 0.2634792
0.2207872 0.2980036 1.263876 0.4797713 0.5164657
0.3826141 0.6408975 0.9333064 0.8283763 0.2831677

0.9903252 0.3825516 0.7838206 0.3480416 0.3992207
0.7604065 0.6234201 0.7758518 0.7060985 0.6702585
1.083129 0.6929747 0.4548823 0.216375 0.4576399
0.3933768 0.359344946 0.5121202 0.791707843 0.090953447
0.3517504 0.8563476 0.5383772 0.4473467 0.53811 0.281923
0.577308 0.6843282 1.511461 0.4897555 0.9571247
0.796104 0.8490529 0.3744562 0.6613665 1.271974
0.4718685 0.6273548 0.984071 1.187419 1.431668
0.3917035 0.6934839 0.8175568 1.040692 0.883727
0.8650031 0.6750822 0.7170787 0.2151657 1.413814
0.8459562 0.2117469 0.4301894 0.209581597 0.569478
0.8087419 1.958307 0.2831883 0.5533296 0.8354537
0.7500269 1.054531 0.4752433 0.3456497 5.108523
0.7466114 0.2896578 0.07850175 0.8868944 0.659965158
1.588329 3.834402 1.480421 1.34088 0.8361243
0.068880552 0.8038435 2.339665 0.4970497 0.9315905
0.2318195 1.400606 0.4782027 0.4369771 1.117765
0.6580166 0.7458111 1.347043 1.260687 0.8862314
15.09286 0.3427136 1.024573 0.2598192 0.3746201
0.468342 0.7526594 0.9558194 1.009861 1.361856
0.5118858 0.2173483 0.500167471 0.16021806 0.93817 1.087144
0.2476672 0.4723519 0.2743522 0.2626987 1.15261 1.15504
0.8854712 0.695113 0.9137067 0.8353828 0.6541138
0.862088098 0.730315 0.3577207 0.4455411 0.3110512
0.3468175 0.379190487 0.629366 0.6065827 0.3807817
0.8671741 0.4279686 0.3563865 0.6127988 1.077529
0.398393 1.614321 0.6934488 0.2709924 0.04041392
0.5072118 0.3581454 0.2903538 1.080579 0.5062179
0.381257142 1.3134 0.2681679 1.5675 0.7956443 0.474018
0.7635307 0.688314 0.4352036 0.655415 0.8328724
0.9157448 1.050942 0.2011261 0.7217331 0.1441018
2.140106 0.6319702 1.654901 0.9425372 0.6947405
1.000299 0.7695142 0.7186953 1.203011 0.3169048
0.302145 0.801250541 0.1060745 0 0.6995263 1.004172
0.5478426 1.453615 0.472314213 1.722782 1.894935
0.08624558 0.7903077 0.04170595 0.1980058 1.110717
0.3414479 0.1063673 1.04863 0.3164236 2.123052 0.139977
0.635701 0.544253639 0.4136961 1.099849 0.05570915
0.126147103 0.3269071 3.025319 0.342551 0.6378286
0.848709 0.3890116 0.664896
AC115619.1 19.01846 0 8.051695 0 2.30343 0.3941965 0 0
0 0 0 0 0 0 0.02951511 0.0737686 2.925191
0.361226894 5.543747 5.800018 0.010577691 0.009015121 0 0
0 0 0.01432864 0.01431539 4.040814381 0.1848606 0

2.904862	0	0.02850984	0	0	0.08477258	0.04065031	0	0
0	0	0	0	0.3635309	0	0	0	0	0	0	0	0.8351663
0.01361087	0.07059756	0	0	0	0	0	0	0.012344859	0	0
0	0	0	0	0.136421824	0.00632344	0	0	0	0.008440591	0
0	0.01405624	0	0	0	0	0	0	0	0	0	0.01862809	0
0.21707165	0	0.017446257	0	0.06959492	2.208367	0	0	0
0.08578702	0	0	0	0	0	0.1411561	0.01868097
0.006828315	0	0	0	0	0	0.02907318	0	0	0.256471
0.007186905	0.5064169	0.08605765	0.006687165	0	0	1.207566
0.04170852	0	0	0.01756029	0	0.02937089	0	0
0.006519223	0	0	0	0.0202364	0	0	0	0	0	0	0	0
0.1524917	0	0	0	0	0	0	0	0	0	0.07276609	0
0.5176564	0	0	0	0	0	0.02470444	0	0	0	0	0
0	0	0	0	0	0	0	0.5604274	0	0	0.01047473	0	0
0	0	0.008277521	0	0	0	0.06718821	0	0.007762946	0	0
0	0	0.03960933	0	0.06294481	0.009223815	0	10.25819	0
0	0	0	0	0	0	0	0	0.0115785	0	0	0	0	0	0
0.1290801	0	0.009384762	0	0	0	0	0.0078934	0
0.1028108	0	0	0	0	0.09473708	0	0	0	0	0	0	0
0.009242595	0.1830509	0.01814138	0	0	0	0.1564811	0
0.009059679	0	0	0.008137223	0	0.03371599	0	0	0.2765023
0	0	0	0.9613856	0	0	0	0	0	0.3717961	0
0.01969547	0	0	0.0412625	0	0.02526807	0	0.3150478	0
0	0	0	0	0	0.006376008	0.01038488	0	0	0
0.007136507	0	0	0	0.4090344	0	0.007454599	0.3394613	0
0	0	0	0.1834442	0	0	0	0	0	0	0.1210952
0.039187576	0	0	0	0.4092576	0.01138715	0	0	0
0.007541345	0.3667001	0	0	0	0	0.021613972	0.008476946	0
0	1.202279	0	0	0.1232754	0	0	0	0	0	0.1181837
0	0	0.9547225	0	0	0	0	0	0.09099562	0	0	0
0.02092952	0	0	0	0.1207011	0.007718942	0	0	0	0
0.1826303	0.1029252	0.01400712	0	0	0	0.07922269
0.01127475	0	0	0.01080998	0	0	0.07733709	0	0
0.035872632	0	0	0	0.0224788	0.3043621	0	0	0	0	0
0	0	0	0	0	0	0	0	0.09505075	0	0	0.022742229	0
0.072144	0	0	0.05691737	0	0.01341925	0	0.01716011
0.06095728	0
LINC00667	5.15447	5.185731	5.546896	3.258929	3.830253
6.104866	2.807632	3.970912	3.081889	3.420544492
2.770931	2.651818	4.941163	1.994551	2.435529
4.791153	3.176066	5.084713516	2.659019	3.078753
3.904856569	5.20302	2.108953	6.17465	4.371008	2.465249
3.491798	5.799315	3.663527388	5.188851	3.012002
4.446133	1.964896	2.565822	4.441703	1.737988

2.990829 2.703123 2.495361 3.299445628 6.467736
5.820393 2.889330643 0.2372902 3.319601 5.056001
1.428259 3.002586 3.028958928 2.928918 4.54589 4.290279
5.916415 3.3957 3.811321 1.294137 2.773912 4.522697
6.629189 3.188966339 3.09462193 0.637415623 2.318837
2.985159 3.023662 3.899773 3.376505 2.843188
3.427649606 2.511343 0.4961906 3.055185 4.43785 4.741095
1.811745 2.413456 2.560583 1.278285 3.243299
2.672274919 2.734281 3.514248 3.843789 3.786463
2.710089 3.952418 4.06986 1.819106 2.55142163 3.333173
4.001079632 3.767866 2.113916 4.679374 0.9980406
2.371856 2.000655 3.523389 1.334336 1.516472
4.298713 2.717225 3.688751 4.220604 3.621663
3.300025 3.043636 3.556287 2.902894126 0.4920887
5.855532 1.818587 3.097022 3.245662 2.869756
1.286674 2.781015 4.171846 1.965224 4.988054
3.745186 10.78981 8.002469 2.518087 3.042108
4.118325 0.8450235 1.535437 5.32676 2.286316 2.647297
2.399546 4.733559 1.109199 1.889588 4.509777
2.081614 2.198789 0.7908113 4.96934 2.809025 4.017701
2.968276 6.006509 5.0609 4.149048 3.439144 3.579604
2.232816 3.277031 4.636492 2.352336 1.211232
2.496231 4.352555 3.847344 2.759522 4.145586
5.767546 2.499119 3.153768467 4.968393 5.871991646
4.72088 1.829653 2.279946 2.40403 4.350024 4.164284
6.708781 4.576053 0.814663865 2.707772 3.107081
7.52997 2.358496 2.294521 3.305531 2.89537 7.865114
2.530571 1.226458 1.917624 3.253681 2.04514 4.509386
4.648446 1.781935 7.17617 1.166971 5.946934 2.791817
0.6242646 4.020185 1.438417 5.623323 1.470677
7.157593 0.7277482 5.399725 4.42931 2.997714 3.111179
5.625273 1.458755 1.087175 2.539652 24.59787
2.767379 2.955521 7.509387 3.101414 5.589267
3.500117 3.552735 3.88872 4.340469 7.580239 2.18059
2.280213486 3.795017 2.703182956 3.200361747 1.716359
4.837496 1.019523 4.697404 0.8188846 2.145122
0.4430874 1.650957 3.776689 2.820245 3.020765
2.271838 6.680095 3.098921 4.449049 3.181405
8.03491 6.628274 3.166881 2.256294 1.867442 2.071065
3.475639 2.843751 3.637382 2.911627 2.796987
5.54059 4.75929 5.65384 4.866271 1.664731 5.929604
1.955376 4.906025054 3.428543 4.875459 2.197188
0.3579141 2.714071 1.179999 3.918443 4.355786
1.560386 4.457507 2.602456 3.820944 5.111333

1.092227 5.993058 2.078009235 3.823554 3.655904
1.857442 3.823194 2.610945 4.13033758 4.771522
0.5015212 2.154878 1.720834 0.4430144 3.041698
1.766672 0.9398788 2.456881 2.292368 3.649847
4.067547 5.002051 4.42662 2.433141 1.928798 3.197392
2.382148 1.241095 3.127643 3.38228 4.787052 2.803044
1.810046 0.8994756 3.170146 4.001917792 2.85916499
5.521894 1.917159 2.817666 2.535944 2.340094
3.013701 2.977366 2.884037 6.437351 2.108847
2.391446 4.19091 2.249053 2.62577105 3.268428 2.019699
2.919817 3.912253 2.693113 3.478035249 2.114797
1.615336 2.128491 3.920046 2.614803 4.129881
2.092321 6.411757 3.046931 3.431717 1.822153
3.104456 5.164561 3.770445 3.107619 4.361825
4.735882 2.932547 6.728752971 4.543219 3.064857
2.348349 4.03498 3.552988 4.751065 3.674575 0.8044302
2.90546 2.236762 2.713156 4.460163 2.355615 4.414816
2.19382 1.906935 5.027223 3.218212 0.798657 2.435656
3.375063 2.509734 2.540131 3.601372 2.77973 5.360761
2.639890297 2.925648 3.981443 4.431026 2.147787
3.340431 1.738888 4.031790503 3.864692 2.506239
1.445616 2.390407 0.5298135 3.156198 2.338753
2.947674 4.327194 3.301497 0.842755 6.157759
0.8917486 2.561137 4.601070517 3.290496 5.514139
2.476963 0.499196375 4.336904 4.286175 1.680422
2.118806 1.086856 2.473856 5.03637
AC124312.3 0.1057909 1.663523 0.09731054 0.3593169 0.1387626
1.285655 0.0363229 0.5071553 0.3447928 0.279816037
0.635661 0.09237385 0.3267367 0.1322517 0.2682866
1.332376 0.1406847 1.177097438 0.08708223 0.02510525
0.384596614 0.5502068 0.2093568 1.603198 1.32837 0.2771797
0.1302446 1.552195 0.295295507 0 0.3653944 0.9166813
0.03403915 0 0.08612582 0 1.492208 0.07390068 0.2000366
0.052012919 0.02847714 0.02072421 0.036703436 0.007556608
0.2904989 0.08649324 0.2188388 0.2147868 0.179531767
0.02816512 0 0.310525 0.8953429 0 0.2062665 0.02889994
0.01725879 1.265229 0.03535925 0.063742339 0.293595547
0.064121355 0.1756054 0.08252958 0 1.075292 0 0.2613642
0.01180998 0.0656901 0.03011771 0.008519497 0.7907058
0.5261028 0.2469662 0.08503386 0.2646633 0.1339555 0 0
0 0.3595187 0.07554522 0.5318175 0.1733405 0 1.003859
0.1597808 0.40268145 0.2121357 0.011327364 0.1050003
0.1004134 0.3175264 0 0.1801275 0.07975763 0.06188789
0.03945733 0.2263764 0.6475486 0.020539 0 0 0.3517418

0.4876776 0.06782805 0.4975099 0.051972785 0.01567077
0.412875 0.1132584 0.06188537 0.5701641 0.2081492
0.01866502 0.03945627 0.01862494 0.1128865 0.0614867
0.01216423 0.1643952 0.1408169 0 0.033897 0.4446546
0.1397673 0 1.088795 0.3155044 0.05079297 0.06691224
0.1913647 0.1065996 0 0.6142676 0.2012513 0.05062507
0.07761792 0.3547408 0.04804394 0.3341814 0.07315351 0 0
0.1520965 0 0.5326262 0 0.03444644 0.06517987 0.1773728
0.01065147 0.3971913 0.7902795 0 0.2310686 0.3897124 0
0 0.159455894 0.6363403 0.372640717 0.04277306 0.01448084
0.2332773 0.05625301 0.2087351 0.3201666 0.1012989
1.309987 0.189450279 0.3511542 0.04784528 0 0 0.2615314
0.3046806 0.02261614 0.5848812 0 0.106845 0.1053104
0.2299745 0.04299489 0.05809445 0.5585869 0.0477122
0.21327 0.05081308 0.1209663 0.06193412 0.02382777 0.2336128
0 0.3806149 0.2403037 0.5020962 0 0.01372728
0.009043249 0.2600596 0.1382848 0.01919146 0.1779228
0.01822754 0.0991468 0.2301602 0.06987659 0.04412274
0.315739 0.1998973 0.2291243 0.0518574 0.07896323
0.08227465 0.0628968 0.1955522 0.6403512 0.097492183
0.02193804 0.099135933 0.086366528 0.04210224 0.7994942
0.0190519 0.03641025 0.1642411 0.01434135 0.2114461
0.119354 0.2050337 0 0.04637017 0.01408612 0.04936126
0.1185239 0.1794322 0.06636407 0.7921263 0.1408591
0.2826886 0.2026447 0 0.0367862 0 0.1174275 1.105853
0.4623939 0.0335257 0.3064297 0.07958751 0.02189084
0.08523896 0.02519038 0.9048069 0.06808238 0.158397372
0.03310765 0.1815856 0.1400045 0.03292735 0.1351091
0.07285597 0.09711844 0.2324561 0 0.03836315 0.04880174
0.1093822 0.1071623 0.01597346 0.04921755 0.021486244
0.008182066 0.04693053 0.1227252 1.072547 0.5545099 0
0.5536054 0.008279525 0.1348522 0.07582363 0.01715287
0.09119801 0.0278012 0.03951806 0.1654121 0.4303689
0.01896961 1.132925 0.551767 0.09016472 0.07204879
0.6774258 0 0.05286775 0 0.05015671 0.2042005 0.3241486
0.04009472 0.287767 0 0.1081076 0.534311185 0.13040402
0.1960573 0.4108204 0.02748821 0.02957344 0 0.07636237
0.1664419 1.145754 0.2316533 0.3171704 0 0.1055066
0.01565864 0.098233404 0.09906916 0.04852575 0.1934042
0.1687797 0.06514159 0 0 0.05366383 0 0.1154957
0.2206093 0.1087757 0.6714167 0.1799011 0.08106459
0.04238458 0.1998948 0.04726393 0.4605094 0.03175598
0.02429169 0.01312909 1.706231 0.4463537 0.010343708
0.03623719 0.03118087 0.03986917 0.06475875 0.5425464

0.03007015 0.1400574 0.03935768 0.03334079 0.3280103
0.2032743 0.5880725 0.1182276 0.3246324 0.2345735
0.01176937 0.08572843 0 0 0 0.1965212 0.07416945
0.01083255 0.0376596 0.2149449 0.01117821 0.081518815
0.1187116 0.5404381 0.02189829 0.1167588 0.03040212
0.1419744 0.138819788 0.102828 0.07010535 0.01169944
0.01786789 0.06789026 0.3545521 0.06954076 0 0.09739582
0.3103625 0.0286158 0.3085689 0.1281707 0.09313329
0.221488334 0.06313384 1.113776 0.4307543 0.034224376 0
0.1894119 0.05227644 0.008950669 0.02228315 0.06332455
1.184974
AL513008.1 0.2707196 0.2588695 0.4980366 0.08620257 0.5918237
0.6397223 0 0.4326045 0.6965738 1.017546125 2.013961 0
0.5574141 0.423041 0 1.403936 0.5400197 0.611499178
0.111422 0.1927335 0.210896812 1.617682 1.492433
2.483149 0.4326391 0.8511655 0.8570484 1.141675
0.348767556 0.4095255 0.5100254 0.5662259 1.306596
2.273704 0.2203965 1.582547 0 0.5673369 2.582742
0.55902581 0.5829855 0.7955006 0.375697278 0 1.5797
0.5312082 8.085142 0.678968 0 1.081121 0.1041717
16.26165 0.5808646 1.221174 1.759458 0 0.5299843
14.06099 0.8143608 0.489351121 1.024517493 0.861456466
3.505128 0.1055969 0.2483657 0.4280388 0.24626 2.736134
0.45332718 0.6303802 0.0770714 0.1308087 8.480514
0.2524313 0.5617671 0.6528065 0.6305664 0.5876452
0.8195879 0.088398534 0 4.140048 1.256584 1.948596
0.09505272 0.5242412 2.135577 1.003615 0.3091393
5.292852 0.086960383 2.51903 0.3083502 4.418252 0
1.567221 2.624146 0.6651603 1.060201 1.372023
15.70461 0.551874 0.2079226 0.4329776 1.582951
9.257668 0.5207172 0.4493404 0.319196832 0.2406096
0.4918421 1.666516 0 32.92818 0 0.6448127 0.605813
0.7149199 1.199952 1.510511 1.307391 0.6795734
1.66316 0.502605 0.5204559 13.3919 0.5777671 0.09759893
2.173262 0.1670436 0.06498977 0.5136865 3.12186 0.2727889
0.4034711 2.300363 0.6008372 0.1943248 1.390373
0.9472529 1.844171 0.4750957 1.123202 0.8551018
1.788636 1.167647 0.8200003 1.234409 0.7952822
2.203716 0.6671828 0.3404236 0.08177155 3.318296
3.758896 0.8500526 0.7256936 7.906977 1.014027
2.113418 0.272032415 3.78852 1.015111838 1.149293 0.2223393
0.6447148 1.007663 0.4217009 4.792954 0.6665775
2.905296 0.103886666 2.3107 0.2754818 0.7496724 0.8579201
1.047538 0.7796796 0.8681228 1.984017 0.4537978

0.546834 0.7074111 2.354024 0.3300726 1.003482
3.022887 0 2.530339 0.8582045 3.637254 0.2852816
0.2743893 0 0.3103433 4.501441 0.2882525 3.675318
0.0919517 0 0 7.838042 0.8846792 0.0736666 0.1707395
1.259399 0.4349439 0.4157515 0.919618 4.234139
0.8079781 1.438702 2.345322 0.9952756 3.550613
1.105342 0.2897159 0.6433962 1.228997 0.467780817
0.7578843 0.761068409 0 0.6464393 22.19026 0 0.7453925
0.630441 0 0.3607279 0 0.5246826 1.294705 0.6407725
0.5406971 7.578946 1.819821 0 0.4075826 0.2764168
2.568272 2.622332 1.077028 0.6709278 0.09413611
1.126631 1.802986 8.489651 1.183269 0.6434436
2.839183 1.527487 8.066708 2.617524 1.031398
0.2205149 2.165346 0.093539968 0.5930588 1.045527
1.488208 0 1.152484 0.3728778 1.49116 0.2745492
0.5470192 0.3926861 3.371867 7.557562 0.3290748
0.1226285 1.637324 0.494850893 1.005022 0.9007164
2.912142 3.394703 0.8707464 1.18359035 7.53416 0.6356207
0.1035263 0.3326282 0 4.025741 0.3557172 3.033809
0.7936699 0.5873689 1.01941 0.8416924 0.4458869 2.461142
2.62732 1.988469 0.7786203 0.8117334 0.2925993 0.9241295
0.2239501 0.4976989 0.3078079 3.191056 0.3807728
0.3017977 0.781317509 0.375417784 1.188265 0.06065146
0.4220551 0.1135179 0.08571376 0.3517412 0.6969697
1.503585 1.580806 1.447792 0.8287618 0.8999733
0.4808465 0.754139676 0.3380248 0.1862666 0.371192
0.6478623 0.1666977 0 0.245785 0.4119782 0.09151132
0.6896262 2.852415 0.9278593 1.178166 0.3452761
0.2667147 0.406734 2.798382 1.874705 0.8838348
0.9751649 0.1864877 0.7055457 2.700777 3.778118
0.6352707 0.2781936 0.9575045 1.530382 1.988616
0.5553518 0.3077988 0.9677011 0.1510748 1.279789
10.82801 1.560541 3.078166 4.538176 7.71398 0.3001375
1.174598 0.9213943 2.58514 0.9526961 0.09866022 3.017394
2.151068 0.249485 1.156454 1.540127 0.1716306
0.625821463 0.662801 0.2242677 1.597079 1.792719
0.3111968 3.996448 0.409893114 0.7894124 1.0764 0.2694505
1.028791 0 0.989783 1.601597 0 0.4153942 5.286524
0.109842 2.368889 0.8746391 1.271086 2.153802081
6.462396 2.557154 0.1740478 0 0.1134812 12.77547
0.1337757 0.3435722 0.2566023 0 1.647501
Z83843.1 0.2365582 0.7992522 0.8703814 0.3201309 0.6050575
1.361558 0.2558473 0.4252673 0.24347 0.464331164 1.912117
0.8675371 0.6879942 0.6912617 0.6813305 1.629861

0.4876053	0.676825323	0.2239325	0.1768336	0.128999027	
0.9737799	0.3661532	1.169809	0.9478143	1.645567	
0.4992666	1.30001	0.167616646	0.1163008	0.300825	0.7775043
2.595511	1.080318	1.83277	1.884133	0.1148705	0.4709589
0.8661359	0.373341253	2.798627	0.5352413	0.315978233	
0.3852578	1.973106	1.433145	2.702095	0.8814607	
1.27259522	0.645543	0.236669	3.942988	0.3806752	
0.7113851	2.336904	0.5428326	2.153448	2.117835	
1.205765	0.336735985	2.094853298	0.403259811	1.079842	
0.3137244	0.889803	2.351019	0.4518888	1.530156	
0.22182892	2.134483	0.4411161	0.9944299	3.042157	
1.569778	2.178277	0.4064318	2.231538	1.277311	
1.879937	0.567741682	0.1645268	1.248942	1.672368	
3.42974	0.2782452	0.4924453	1.732222	0.998773	0.985974
4.193595	0.94603929	1.576031	0.5691926	1.787249	
0.5717985	0.6605622	0.9363134	0.4317675	0.9528864	
1.458651	3.346805	1.856603	0.8993429	1.177587	
1.509318	2.468478	0.5289483	0.3631914	0.373053176	
0.8620159	1.253519	2.361614	0.193734	2.520903	
1.424249	0.7637818	0.2999747	1.543024	1.115523	
1.77362	1.007773	1.870533	1.431491	0.7246514	1.466669
2.351875	0.4868296	0.2515854	1.606866	0.9816137	
0.9086222	1.885236	1.339891	0.6118072	0.1762791	
2.733718	0.6112724	0.7344001	0.6725482	1.257102	
1.651747	0.4525077	2.481274	1.905357	0.6772709	
1.476254	3.930661	2.53481	1.375957	2.27225	0.3643705
0.8514978	0.400137	1.383173	2.258863	1.1018	1.278809
4.355608	1.118663	1.54334	0.368443361	2.87487	2.286086453
2.209388	0.2185683	0.7824445	0.3836501	1.473951	
2.276632	1.223174	1.835664	0.354032225	2.0065	0.525571
0.8515953	1.236942	0.9878174	2.168786	1.247859	
1.555731	1.605964	0.856113	0.9581245	1.421518	
0.9842388	1.500398	1.434362	1.184247	1.320122	
0.4465374	0.6525615	1.283806	0.2557492	0.4460321	
0.5762616	3.850608	0.7959367	2.733725	0.3173768	
1.399713	0.5459815	1.479638	0.5643221	2.339879	
0.8727872	1.44285	0.4038133	0.9173047	1.386165	1.232048
1.649065	0.9386766	2.305544	0.6566118	0.8550988	
0.6036637	0.2151838	0.6246753	1.003696	0.396490259	
0.8462071	1.945216501	0.128289199	0.7569217	3.080418	
1.316396	1.306738	1.276222	0.2453246	0.7407403	
1.169853	2.264863	0.606804	0.8709791	2.055236	
1.870879	0.3918665	0.6152148	2.181424	1.034581	
2.465655	1.264235	0.8313235	0.7035183	1.295553	

0.8254359	0.9584123	1.80724	1.50498	1.44498	2.5199	1.904226
2.393652	2.115684	0.982924	0.6647762	2.224857
0.449550292	0.9883241	1.12296	1.524376	0.09939859	1.389736
0.8552915	1.261113	1.379452	1.007198	0.2830857
2.026455	2.547799	0.5175896	0.2303819	1.579289
0.864814045	1.14715	2.247049	1.324724	2.186883	0.5791061
0.789780481	1.768246	1.374648	0.3573273	0.8283662
0.9492962	1.919464	0.9324906	1.033883	1.789279
1.100281	1.460233	2.055262	1.37017	2.523568	0.4591571
0.9489692	1.28136	0.8422973	1.364676	0.7267645	1.286665
3.453071	0.8909515	3.002877	0.6122124	0.843887
2.479145898	1.246570423	1.415577	0.6015275	0.3749437
0.3422172	0.7190186	0.4405435	1.684958	0.9229808
1.523778	0.802188	0.9655769	1.537428	1.307779
1.929860825	0.5242824	0.7405675	0.3527331	1.514345
0.6664065	0.429601727	0.6120948	1.130374	0.6676974
0.7747768	0.5140733	0.7053745	0.5083141	1.591503
2.276207	0.9382806	3.332655	0.665825	1.842502
1.288819	0.6966341	0.8499098	1.77588	3.174688
0.843069729	0.866005	0.6798022	0.6017702	2.172098
0.7562179	0.4135237	0.7986124	0.8316699	1.129479
2.783486	0.6136292	2.447664	1.656365	1.078492
0.2884904	1.8988	0.7361153	2.759821	1.036813	1.012974
1.191195	1.843696	2.020163	3.065256	0.788244
0.6411347	0.921834166	0.4850498	0.5487104	1.068696
1.028016	0.8123842	3.131027	0.974221941	1.019021
1.377938	1.616752	3.338177	0.3358772	0.3081153
1.411159	0.3612074	1.179674	1.981151	1.33894	3.663839
1.347027	1.016977	0.742902525	2.082303	1.518744
0.2205236	0.74041674	1.130439	1.992163	0.7539724
1.660205	0.5493447	0.4885184	1.007725
AC035139.1	0	0	0.1468569	0	0.06980485	0	0	0	0
0.266707246	0	0.3136651	0.5752799	0.04989715	0.3470458	0
0	0.080139493	0	0	0	0	0	0.1910115	0	0	0.0842398
0	0	0	0	0	0.1541113	0	0.08665162	0	0	0	0
0.047097413	0	0	0.166173796	0.06842467	0.1096021	0
0.371545	0.1144049	0.487695086	0.2975392	0	0.7531573
0.2854676	0.1600399	0.1037629	0	0	0.4364411	0
0.072147922	0.120840525	0	0	0	0	0	0	0.1075745	0
0.03717627	0	0	0	0.04962325	0.3312985	0	0.04131917
0.04332	0.08055779	0.104264937	0.09253378	0	0.1140096
0.109445	0.2242269	0	0.05475838	0	0.18231292	0
0.153852985	0.2376931	0.1818475	0.3593981	0	0	0.05158578
0.05603914	0.1339815	0.1618284	1.665772	0.1859795	0	0

0.2745692 0.04014449 0.383862 0 0.094122143 0.070949
0.03222897 0 0 0.99736 0.753912 0 0 0 0.07862931 0
0.05507323 0.05725344 0 0.09880269 0.2046237 2.16802
0.04867635 0.1151167 0.04929489 0 0.0383273 0 0.1082998
0.05362517 0 0.1356624 0 0.1146018 0 0 0.3262764
0.1681108 0.1656002 0.1120646 0.1506909 0 0 0.09099813
0.04690126 0 0.04918335 0 0.04822425 0.1057807 0.03889097
0.1671044 0 0 0 0 0.24064406 0.8231467 0.054423245 0
0 0.08449254 0.1697893 0 0.3623864 0 0.08786563
0.12253299 0 0 0 0.04216274 0.05148157 0 0.153591 0
0.05352487 0 0 0.2974866 0 0 0.0829176 0 0.2194487
0 0 0 0 0.1627193 0 0 0 0.3171944 0.05422793 0
0 0.1308236 0.05217339 0 0 0.123787 0 0 0
0.04994113 0 0 0.1975912 0 0.102144 0 0 0.7167177
0 0.055174148 0.09932387 0.112208804 0 0 1.392188 0
0.6593857 0 0 0 0 0.2475425 0 0 0 0.07449391
0.613273 0.04513192 0 0.32603 0 0.0533277 0.2117235
0.9232425 0.2220647 0.204438 0 0.1065259 0 0.07589334
0.4783972 0.3002752 0.1982204 0 0 0.1950709 0.1761378
0.110329192 0.149894 0 0.2925537 0 0.2718679 0
0.1256289 0 0 0 0.1325691 0.165075 0 0 0.2228311
0 0 0.05311917 0.05051227 0.6389375 0.9129193 0
0.7405371 0.03748532 0 0.3432894 0.03882958 1.032241 0
0 0.2808371 0.08659926 0.214711 0.1103073 0.08765298
0.04535759 0.3261989 0.09020666 0.05102071 0 0 0
0.06603657 0.05870295 0.09076387 0.1447619 0 0.04449582
0.115194248 0.073800077 0.1401543 0 0 0.06694646 0
0.2765828 0.2740223 0.08867296 0.1748006 0.03881028
0.162919 0.3715275 0 0.127071154 0 0.1098495 0 0 0
0 0 0.1457769 0 0.1452509 0.1051371 0.1094398
0.173704 0.05090609 0 0 0.3194183 0 0 0.1437743
0.05498997 0 0.1592766 0 0.140492559 0.04101572 0 0
0.09773115 0.05458586 0 0.06341062 0 0 0.7425289
0.2300797 0.1210219 0.1235243 0.2799551 0.2655063 0 0
0.1325713 0.2043078 0 0.381319 0.07462225 0.1961762
0.4546741 0 0 0 0 0 0.0991439 0.2643112 0
0.05356544 0 0.211614 0 0 0.04044818 0.07684283
0.2188943 0 0.1572786 0.7349282 0.04391118 0 0.4656791
0.1289532 0 0.066852156 0 0.09425406 0 0 0.06692482
0.3062705 0 0 0 0.07167504 0
AC007319.1 0.2270097 0.4058319 0.172497 0.1319977 0.2330306
0.469796 0.2134958 0.1419484 0.2133257 0.160758155
1.499578 0.911376 0.07620896 0.7033057 0.0965457
0.3802326 0.3150115 0.245236649 0.7677671 0.274043

0.161467872 0.176934 0.2511306 0.2361691 0.7233185
1.280073 0.1406095 0.3121767 0.095366129 0.1567715
0.3625962 0.5927678 0.3143997 0.621716 0.3937292 0
2.824313 0.4698259 0.1526959 0.026204335 0.1833216 1.105
0.123275669 0.01903526 0.2337608 1.162018 0.298599
0.06365325 1.28638414 0.1024812 0.102544 0.4376703
0.2753056 0.1780879 0.538834 0 0.07245879 0.2327118
0.1484512 0.575369873 0.280141502 0.040380772 0.2752425
0.1501455 0.5433 0.7758203 0.6329651 0.1795588
0.475993539 0.2757913 0.06743748 0.1359184 0.3807863
0.1196419 0.1843298 9.06789 0.4214723 0.2249579 0.2614571
0.048342948 0 0.345004 0.02114445 0.162383 0.2495134
0.1529037 0.2234231 0.08537695 0.23668478 0.3228513
0.038045167 0.2644981 0.0168629 0.5832283 0.2612943
0.05041613 0.1817768 0.05196565 0 0.05002168 0.5313722
0.3794134 0 0.2012669 0.906414 0.297811 0.3274823
0.1965864 0.087280384 0.05263336 0.1255222 0.7291009
0.1558906 0.3481832 0.3145995 0.117544 0.2871301
0.1329304 0.8312165 0.2994469 0.2349217 0.7539018
0.1819081 0.3115104 0.03794991 0.1723222 0.04513806
0.09607395 0.2285582 0.045676 0.0639743 0.01123689
0.3816244 0.05967258 0.2647779 0.1887016 0.03755233
0.2975599 0.08689832 0.1942608 0.3899653 0.2702107
0.09213764 0.1247024 0.7638965 0.1197295 0.3459376
0.7341361 0.07828559 0.05784755 0.02736492 0.3164876
0.0983814 0.1667556 0.1803199 0.0309915 0.09701112
0.5492811 0.2218184 0.231155 0.104137409 0.381656
0.161495065 0.3771119 0.1215918 0.1096911 0.4723418
0.378213 0.04032533 0 0.2362881 0.045450416 0.294855
0.2812211 0.6457139 0.5786492 0.4392021 0.5258777
0.1424264 0.3654768 0.1786829 0.09968328 0.7074111
0.03678163 0.2075847 0.0365853 0.1999148 0.04006269
1.155862 0.02559985 0.431682 1.955368 0.2300869
0.0603565 0.0509157 0.2332161 0.1071939 0.4412062
0.05028609 0.3112139 0.7061844 0.3477669 0.02902854
0.008057285 0.2987942 0.07652596 0.09514398 0.2842052
0.7292284 0.7872852 0.2651178 0.157358 0.1923897
0.04354331 0.2557416 0.04317742 1.573821 0.07037146
0.2895233 0.26605034 0.5157824 0.072836625 0.02071992
0.02828172 0.4217231 0.01599738 0.4076365 1.080287
0.01204206 0.4241371 0.1336246 0.1492066 0.373331
0.1012331 0.0236555 0.8565788 0.3980858 0.06696203
0.1225932 0.2519424 0.04435338 0.1483539 0.1439777
0.2201482 0.05148069 0.1327042 0.5423044 0.592695

0.3738801 0.2181676 0.07097958 0.4232412 0.9190585
0.4473308 0.02820228 0.07235646 0.3348353 0.143233075
0.07413236 0.1334136 0.4973612 0 0.1512635 0.01019588
0.06989813 0.2402306 0.3333398 0.1181126 1.081807
0.2959462 0.125974 0.2816624 0.757656 0.045103597
0.7969511 0.2462896 0.2060983 0.426595 0.02116398
0.282447697 0.3697655 0.2224672 0.3396957 0.1000483
0.09361832 0.1914415 0.77035 0.2820494 0.1302115 0.1284869
0.3026374 0.3580115 0.05689703 0.1009453 0.3932337
0.3011383 0.3690336 0.0665875 0.07200685 0.04211528
0.1592145 0.2504048 0.2945818 0.1789855 0.1665881
0.7014439 0.053410377 0.068435534 0.07797987 0.2454489
0.3231359 0.1614083 0.03749977 0.3334213 0.03811553
0.2466819 0.2161258 0.08637397 0.3726551 0.344521
0.447037 0.070700595 0.2125859 0.1629832 0.1116476
0.1771498 0.1185117 0.194360885 0.2150619 0.1532044
0.1301177 0.09159098 0.1559914 0.2435631 0.09664647
0.1227349 0.01944795 1.174444 0.1382265 0.08598696
0.01380992 0.01333233 0.04079419 0.3417487 0.1476988
0.2883012 0.104224099 0.07606856 0.09163618 0.2008626
0.05437623 0.3239552 0.09258011 0.2704859 0 0.06998848
0.1285301 0.5120525 0.2020046 0.05345447 0.1817236
0.3282754 0.1680013 0.2447454 0.06146733 0.1420925 0
0.1768004 0.2214334 0.3456406 0.7800039 0.6617732
0.00938605 0.088006143 0.07249386 0.02452928 0.2574239
0.147059 0.263788 0.1788183 0.089664119 0.07849271
0.470925 1.326202 0.3075656 0.1425142 0.1623863
0.1751747 0.009723069 0.109041 0.1221578 0.04805587
0.6736529 0.263075 0.3301845 0.322361825 0.06184715
0.3321303 0.2284378 0.12931792 0.06206004 0.2499267
0.02926344 0.03006257 0.009355291 0 0.1723608
AC116025.1 0.1390298 0.09970811 0.127885 0 0.2735415
0.03520003 0 0.0416563 0 0 0.05967016 0 0 0 0 0
0 0.052339908 0.3862451 0.07423468 0.040615295 0.06923095 0
0.1663353 0 0.0273201 0 0.1374175 0 0.07886794 0
0.06231194 0 0 0.2263722 0 0 0 0.2150894
0.030759793 0.1122735 0 0 0 0.07158226 0.1023021
0.04044329 0.1120784 0.035390951 0 0.2006177 0.1639649
0.3728836 0.05226185 0 0.08545533 0.1530995 0.07126102
0.1394068 0.18848213 0 0 0.1384678 0.3253798 0
0.2119714 0 0.03512899 0 0.2428019 0 0.02519162
0.3438335 0.03240945 0.0811404 0 0.05397192 0.08487817
0.2104524 0.034048239 0 0.07593389 0.07446084 0.4288776 0
0.1346138 0.143053 0.08590217 0.2381408 0.03920451 0

0.1940497 0 0 0 0.07101679 0 0.1097992 0.08750464
0.07046116 1.087932 0.03036628 0 0.04169222 0.1075943
0.05243754 0 0.04326782 0 0.0463375 0.08419626 0.1395408
0 0.1915841 0 0.02759565 0 0 0.05135359 0.03636247 0
0.0373928 0.3202975 0 0.2338731 0 0.2861191 0
0.03219499 0 0 0 0.1414634 0 0 0.04430125 0.09918127
0.0374238 0.03825189 0.04560637 0 0.2195897 0.05407759
0.1829763 0.1312237 0.02810874 0 0.1782954 0.09189505
0.06790399 0.09636641 0.03278002 0 0.1727162 0 0
0.03105706 0.1646222 0 0.232577 0 0.07680077
0.071088737 0.09485796 0 0.02759146 0.1108911 0.0324851
0.1420069 0 0.05738592 0.040013764 0.2595853 0 0
0.1376845 0.1008694 0.0500512 0.06687453 0.1206598 0
0.3510378 0 0.03238191 0.1589165 0.2576725 0 0.04702734
0.2579835 0.1202009 0.2682674 0 0.03522861 0 0 0 0
0.3107443 0.3187512 0.2435443 0.4813258 0.2848074
0.03407496 0.2269918 0 0.02694885 0 0.2802344 0.05903449
0.3914046 0 0 0.06452441 0.345013 0.2334894 0.1216405
0 0.02753503 0.03641305 0.144139161 0.3567818 0
0.145931687 0.1991899 0.2424671 0.08450293 0.07177524
0.1618836 0.04240648 0.2431461 0.03921361 0.1212544
0.09066875 0.02742274 0.1666072 0.04865272 0.05006684
0.02947611 0.03924688 0.3548891 0.3123836 0.06965771
0.09218582 0.08613985 0.03625817 0.1335205 0.05787096
0.03478657 0.2025585 0.1239168 0.0312446 0.1176677
0.2589195 0.1890347 0.0744864 0.08493517 0.172556 0
0.09789727 0.1006758 0.1592248 0 0 0.4308611 0.04102474
0.05287372 0 0 0.1443037 0.3953111 0.06337446 0.04723251
0.2910665 0.222367208 0.02419386 0.7979313 0.1319603
0.1390988 0 0 0.1488161 0 0 0.06405879 0.05071997 0
0.08220641 0.1752787 0.2751263 0.1131177 0.1402299 0
0.05724704 0.05924699 0.05326095 0 0.03332214 0 0.394449
0 0 0.03833949 0 0.2206061 0.02933226 0.05812133 0 0
0.03051206 0.0700829 0.02709365 0 0.03301415 0 0.08948335
0.0289566 0.03805468 0.05069477 0.2482761 0.03466404 0
0.082991448 0 0.1434876 0 0.2495355 0.03210327 0 0 0
0 0 0.03433304 0.1429525 0.05672392 0.09974184 0.06848656
0.06266428 0.1390768 0.02329275 7.732497 0.1408507
0.071829 0.07764379 0 0.1015262 0.122342802 0.1339389
0.2304997 0.2947266 0.1914876 0.1426023 0 0.04141411
0.0581891 0.14788 0.161651 0.2504457 0.07904062 0.2420249
0.4113933 0.1734048 0.1044039 0 0.1731673 0.03335887
0.07600141 0.04150722 0 0.7687479 0.1855951 0.1271157 0
0 0 0 0 0.1726243 0 0.3148571 0.031575498 0.1105658

0.2072971 0.03459452 0.05283423 0 0.04765403 0 0.1712004
0.1599963 0.1433942 0.04230756 1.399043 0.08422063
0.06119763 0.08732363 0.3422519 0.2462331 0.06703756 0
0.04370928 0.360051 0 0.05293314 0 0.04681169 0
AC000123.1 0.1970936 0.457704 0.9323708 0.3227585 0.1846582
0.9980163 0.3480241 0.3037035 0.6085571 0.423320223
1.184268 0.4978521 0.3043632 0.5015824 0.6426401
0.4797688 0.336989 0.27559599 0.2085923 0.4810867
0.361916914 0.5327832 0.2507413 0.4042336 0.443539
2.434454 0.4456873 0.5788579 0.217641769 0 0.3182717
0.5804913 1.929677 1.241504 1.535799 0.4320568 0
0.7586482 1.328573 1.046548318 2.114593 2.060131
0.791257785 1.194647 1.710622 1.056626 2.391641
0.8776582 0.86008242 1.101933 0.4225412 2.258012
1.298878 1.397093 0.7136716 0.865318 1.240225
0.7504504 0.8752099 0.572569262 1.278661367 0.34558428
0.5608484 0.2635828 1.201156 0.8013284 0.2689293
1.024459 0.678936521 0.727747 0.6733292 0.612215
0.6406231 0.9188957 1.84044 0.4582929 1.289783 1.810625
0.5327586 1.379085653 0.3426978 0.6458795 1.176222
1.071224 0.4745268 0.6542856 1.129869 1.600511
2.3149501 0.8574846 1.302383407 1.949221 1.467203
1.735088 0.5833548 0.8054075 0.9006517 0.9487557
2.599137 1.369896 1.762619 3.025682 2.011127
0.6079307 0.929704 1.507986 0.4467976 0.3505029
0.323681671 1.651627 0.818466 2.125131 0 0.9622273
1.794924 0.7377024 0.4725577 0.8922644 0.6656062
0.7953233 1.515154 1.453971 0.9859701 0.3659146
1.461513 1.857102 0.5665701 0.4263333 0.8867348
0.2345409 0.8922238 2.115672 0.5156835 1.475316
0.3147231 2.009697 0.9105712 3.395412 0.836649
1.847235 1.112459 0.6818912 1.051369 2.341955
0.8504108 1.092972 1.608218 1.035104 1.637726
0.8526162 0.5724713 0.5310872 0.4082239 0.8674635
1.358018 0.8840987 0.6038071 0.6334444 0.8700785
0.8478245 0.212195828 1.49315 1.957971178 1.844215 0.10406
0.8716977 0.3593215 1.052618 0.8819474 0.415965
0.6275751 0.680699257 0.5106901 0.7449399 0.2631481
0.8699742 0.9260696 1.054179 2.275289 1.498738
0.3681391 1.222782 0.3468508 1.180434 0.9268898
1.217619 0.5044957 1.066682 1.462906 0.5598892
1.255611 1.008806 1.36982 0.6887191 0.9683192 2.833679
1.043295 1.314574 0.9467818 0.526106 1.429675
1.522726 0.9385143 0.8734346 1.544928 1.615469

0.7124735 0.4864535 1.673788 0.6077138 2.016814
0.7780887 1.515823 0.9005701 0.8106192 1.502708
0.5725071 1.583691 0.8259271 0.554628496 0.7619643
1.246692001 0.384201931 0.7664565 3.31454 1.57444 3.052534
0.4589839 0.3778779 0.2813817 1.20711 1.538865 1.175179
1.643877 1.990729 0.7882495 0.1622321 1.408798
1.557858 1.1787 1.687033 0.8746362 1.381537 1.325819
1.145505 0.5408099 1.359519 0.9017541 1.353727
1.806879 1.82236 2.605411 1.52065 2.39908 1.066002
0.5848346 1.584216 0.466974722 0.6873046 0.5708862
0.3353596 0.4337986 1.402412 1.948756 0.598198
0.685309 1.267897 0.4288346 0.958557 0.4366809
0.7957425 0.153048 2.357864 0.95214043 0.9211485
1.57381 0.9888071 0.8563744 0.4226233 1.376476374 1.537045
0.9122882 0.2584145 1.141637 3.081533 0.9284169
1.664839 1.656542 0.4209835 0.3894449 0.8178989
1.488186 0.8115545 1.583824 0.8197644 0.4772562
1.862552 1.329685 3.811587 1.369628 1.677022
1.149144 0.5762451 2.221085 1.140547 0.8239487
1.554117748 1.288497861 1.408877 2.138434 0.4170111
0.1770967 0.8023206 0.4389948 1.63099 0.8209982 1.633844
1.314134 0.4884412 1.291706 0.6751421 1.042057342
0.9492208 0.4068264 0.5211794 0.9096438 0.4160982
0.628383306 0.728543 1.439688 0.5996119 0.5840439
0.584061 0.6948156 0.3676062 1.319711 3.522943
0.5330107 4.393858 0.8113641 1.930391 0.6085331
1.338302 1.446642 0.7162818 2.275419 1.337946576
1.562413 1.195025 0.7162542 1.758024 0.9241514
1.200475 1.610335 0.5185145 1.317737 1.964248
0.9332498 1.152523 0.8278044 1.518187 0.3277665
1.099478 1.108887 1.017022 1.189024 0.8619385
1.311338 1.974019 0.7524841 0.8118709 0.5491925
1.740421 2.14792599 0.5170105 0.5947876 0.4458593
0.5593563 0.995257 2.097149 0.613886432 0.7837101
0.8732192 1.541333 0.9843959 0.6911387 0.1158104
0.4997232 1.581016 0.1036875 1.579781 1.439441
1.133333 2.967798 2.20608 1.061083645 1.487071 0.5984037
0.1629169 0.65583455 0.4603027 1.587977 0.7930619
1.693758 1.040831 0.5308953 0.5363957
AC073332.1 0.3164291 0.190192 0.2827476 0.3540904 0.6522214
0.3937059 0.642556 0.3033885 0.4187245 0.521050031
0.2095325 0.4795738 1.130871 0.1949622 2.034008
0.3951755 0.6131649 0.32674173 0.379542 0.4054959
0.517661778 0.270117 0.4370641 0.4542911 0.3096049

0.198976	0.3577702	0.335993	0.419302376	1.23087	0.204391
0.2998496	0.1483573	0.7688077	0.8905921	0.6342005	
0.103482	1.242351	0.5664667	0.816101151	0.189824	
0.5339804	0.724567386	0.4126557	0.502724	0.5255474	
0.3261134	0.583059	0.359018847	0.9170577	0.4174645	
0.6738578	0.1454872	0.8564178	0.581705	1.166968	
0.3185842	0.4170566	0.8793385	0.441238192	0.277136126	
0.715110719	0.2251072	0.2856438	0.7216052	0.6493831	
1.184255	0.6487605	0.245253672	1.831515	0.1853167	
0.5831853	0.7959735	0.3287734	0.4221119	0.4251164	
0.4281817	0.264934	2.552308	0.611088557	0.2043574	
0.4641559	0.2324179	1.087673	1.123716	0.4551906	
0.4093078	0.5921209	1.33178057	1.499042	0.357203165	
0.3230378	0.2625869	0.3510673	0.4994998	0.7204205	
0.3680663	0.8377625	0.2958931	0.4581949	1.131937	
1.437551	1.603993	1.496561	0.8582551	0.2045945	
0.4956048	0.4501792	0.375756444	1.964629	0.6022626	
0.7186657	2.348178	0.6378665	1.152683	0.2009829	
0.6474069	0.2148764	0.1736499	0.870165	0.9449497	0.1848
0.1749578	0.8392388	0.2346424	0.648922	0.5623073	
0.6844679	0.360095	0.4853309	0.2734666	0.5969887	
1.435057	0.3826174	0.2223234	0.4263616	0.3611753	
0.2725626	1.363119	0.759217	0.7082519	0.7425329	
0.4149538	1.408793	0.2901293	1.038222	1.384867	
0.9429943	0.3665128	0.3885781	1.203171	0.4263239	
0.5161225	0.2336128	0.396412	0.7096996	0.5412471	
0.9741596	0.279378	1.69389	0.545080509	0.9089445	
1.562492392	0.7484354	1.537005	0.2511905	0.3317072	
0.1182967	0.2955016	0.7346041	0.5149735	0.197753149	
0.7523795	0.9383868	0.46942	1.568627	0.2711192	0.7681162
0.2870153	0.04184676	0.3728089	0.6848185	0.1518711	
0.3116482	0.5952398	0.3351186	1.014206	0.685013	
0.2758744	0.6331304	0.5349767	0.3239228	0.2107579	
0.2349659	0.6373915	0.6487846	0.2137051	0.3951607	
0.3592811	1.24586	0.3547295	0.7334101	0.6204315	0.4059227
0.6158098	0.5677874	0.4684376	0.1770243	0.4146007	
1.128384	0.4047432	0.8167864	0.7804361	0.7877356	
0.8068865	0.6723533	0.3386329	0.2650011	0.3220301	
0.393669432	0.615876	0.362182368	0.313158321	0.252582	
0.5281996	0.7693079	0.326718	0.4000247	0.3640043	
0.3614013	0.5303964	0.6623342	0.4363046	0.1640587	
0.2058485	0.8099301	0.4297586	0.5673647	0.2347975	
0.3415504	1.110481	0.6522771	0.5035514	0.3136841	
0.3489538	0.347303	0.6021173	0.6424367	0.1844077	

0.6059661 0.8289638 0.4488993 2.87913 0.7047729 0.4779119
0.1104633 0.6582971 0.684116514 0.3904519 0.2793277
0.4969955 0.3925464 0.5080394 0.5603608 0.4801963
0.4263458 0.4697489 0.2557225 0.9909274 0.8973832
0.9643408 1.486575 1.526816 0.198309879 1.214571
0.3699831 0.6435835 0.7380981 0.3004837 0.927079933
0.687082 0.4457651 0.6223174 0.2749306 0.9432905
0.5611486 0.4205299 1.109408 0.5327516 0.3898583
0.3939351 0.6371326 0.2159143 0.5239691 0.2770762
0.2911649 1.837507 0.5286117 0.4690328 0.5092201
0.684323 0.4687103 0.3700591 0.3033064 0.9003014
0.6878715 0.273971634 0.150447451 0.5555587 0.3159764
0.5990276 0.4549193 0.3349077 0.3523978 0.6517194
0.5724289 0.2870559 0.3032853 0.3413492 0.387711
0.5419622 0.151109506 0.5587821 0.3732286 0.3718849
0.2920823 0.2505134 0.795214599 0.3570536 0.305433
0.9076533 0.4194833 0.6787132 0.6228263 0.5459192
0.1643123 0.810545 0.8149866 0.5064578 0.1635855
0.2529956 0.8670745 0.4670897 0.3433334 0.4870475
0.2288708 1.161533376 0.1811634 0.4316814 0.5979631
1.012767 0.3523794 0.7015495 1.432717 0.3632564
0.71802 0.2354653 0.7556695 1.089649 0.6715049 1.260321
0.420977 0.5340818 0.4088078 0.6981648 1.431712
0.4843376 0.5722159 0.329601 1.391392 1.544817
0.6392436 1.26384 0.286623893 0.3818221 0.7414651 1.47374
0.6735248 0.2883945 0.4913888 0.895235129 0.2660253
0.560773 0.719876 0.1305568 0.2871923 0.2603031
0.4813761 0.516563 0.3995229 0.3655257 0.2861223
1.281588 0.328602 0.668566 0.340708874 0.3480022
0.3922951 0.2092473 0.881826076 0.3865565 0.8324763
0.3752713 0.2684866 0.385622 0.4992276 0.717642
AL034405.1 0.08286284 0.05282381 0.02540681 0.0263852 0.03622951
0.08391794 0 0.1324132 0 0.138424103 0 0.1627957 0
0.05179438 0 0 0 0.103983278 0 0 0.032276033 0 0
0.1321828 0.01891768 0.04342131 0 0.1965645 0 0
0.07805522 0 0 0 0.1349195 0 0 0.04961502 0.04273163
0.073332283 0.08922116 0.1217449 0 0.1420527 0.05688476
0.1219456 0.09641804 0.02968871 0.028124368 0.02206089 0
0.2345383 0.1185288 0 0 0.1358187 0.04055488 0.1982032
0.1107833 0.074891189 0.062717611 0.113004745 0.02750929
0.03232147 0 0.01871653 0 0.139581 0.11100483 0.01929491
0.1179515 0.0400384 0.1912656 0.05151007 0.2579216 0
0.2787865 0.2023521 0.1881468 0.054114692 0 0.0603429
0.08875845 0.1420079 0.02909408 0.1069744 0.1705214

0.06826446 0.56773491 0.06230983 0 0.03084135 0.1651666 0
0.03179247 0.0564354 0.08032083 1.221568 0.0231793
0.02799693 0.06916437 0.7239412 0.2545668 0 0.1425045
0.1250127 0 0 0 0.03682334 0.03345441 0.1330678 0
0.03044948 0.1956445 0.02192962 0.09271473 0.02188253
0.04080951 0.05779282 0 0 0.0509066 0 0.1858535 0
0.1515815 0.05974687 0.1023384 0.05112933 0.05967691
0.4402469 0.05620884 0.1391604 0 0.1408207 0.07881705
0.02973982 0.3343768 0.07248465 0.01881569 0.08725141
0.04297421 0.02908141 0.1303505 0.02233736 0.03585552
0.3306035 0.07302686 0.02698086 0.02552672 0 2.052373
0.2470563 0 0.5203744 0.07404098 0.1962321 0.1551886
0.09241179 0 0.4272225 0 0.05025428 0.2041633 0.08770518
0.08812258 0 0.07523307 0 0.09120653 0.09539403 0.2062864
0.02810683 0.1720969 0 0.1068781 0.1590981 0.1062873
1.182589 0.1111201 0 0.06186485 0.07719946 0.02525745
0.03412773 0.2151759 0 0.02277927 0.1432813 0.1184366
0.1455334 0 0.0422266 0.04749551 0.1450334 0.01764587
0.1371896 0.1125796 0.064513 0 0 0.02707858 0.04509628
0 0 0 0 0.07037001 0.07776004 0 0.05871509 0.02563802
0 0.3710972 0 0.0295591 0.02188145 0 0 0.1031004
0.058237649 0.057984263 0.0395729 0.1445123 0.1566892
0.05703812 0.06432256 0 0.2208258 0 0.546029 0.1080785
0.2615066 0.1654986 0.3093055 0 0.09369592 0.2495087
0.08460665 0.248244 0.08303305 0 0 0 0.1326321
0.09197743 0.08293223 0.08048428 0.03938951 0.07448808
0.2805233 0.1028786 0.2002953 0.07892348 0.033748
0.1142719 0.028631055 0.05186446 0.05333646 0.07591936 0
0.2469295 0.02853295 0.3260141 0.1260525 0.04783818
0.1502435 0.09173985 0.05711719 0.1510866 0 0.07710125
0.025244295 0.6152416 0.2481251 0.05243289 0.1326464
0.05922694 0.098802997 0.05913034 0.03891062 0.03168771 0
0.02015299 0.3750215 0.04355169 0.1392899 0.3401012
0.134838 0.1337249 0 0.02274645 0.04708221 0.04232524
0.09363657 0.2912836 0.1863437 0.1567297 0.09428698
0.03427373 0.060935 0.02355374 0.05008872 0.1165483 0
0.149467822 0.19151541 0.02424723 0.07425768 0.04306139
0.06949195 0.1311779 0 0.8533241 0.2761337 0.03024117
0.2417157 0.0281856 0.1652803 0.2943585 0.560586717
0.2069277 0.05701315 0.1988276 0.09915003 0.07653518 0 0
0.1008798 0.08403035 0.04523214 0 0.05680051 0 0.1849459
0.05442468 0.02489892 0.02763029 0.01851021 0 0 0.1141617
0.09255258 0.04133319 0.2689349 0.121528734 0.1277258
0.1099037 0 0.05072358 0.1416534 0.09421218 0.1645542

0.04624153	0.03917226	0.1798445	0.4378514	0	0.2137018
0.4358996	0	0.1106231	0.2820237	0.206418	0.05301903
0.1207931	0.03298482	1.510462	0.05090885	0.2359811
0.1010159	0.1576	0.08209445	0	0	0	0.1371805	0.1190653
0.3336129	0	0.2855583	0.1647342	0.1374573	0.06297921	0
0	0	0.0272098	0	0.09116161	0.03362084	1.160125
0.03346409	0.09726467	0.069394063	0.1236271	0.02445947
0.0532732	0	0.1736737	0.222541	0.04094656	0.1261942	0
0	0.02192492
LINC01140	0.03028147	0.2006235	0.0576977	0.05785339	0.1078094
0.4118147	0.1069409	0.1788672	0.1502658	0.140917674
0.1745243	0.1614789	0.19707	0.09734289	0.1198928	0.2852322
0.051775	0.311055055	0.09347371	0.0646749	0.154177281
0.2197209	0.1485922	0.2096093	0.3422081	0.2159171
0.1540697	0.2839102	0.054337539	0.279754	0.1589226
0.1492908	0.08351426	0.1362462	0.08804486	0.009483031
0.1260377	0.07187788	0.05019392	0.021056056	0.0558944
0.03432129	0.042774229	0.001390497	0.04677305	0.0302398
0.08053737	0.05114751	0.18059554	0.02764098	0.07240993
0.6203765	0.3967987	0.04227942	0.2994248	0.01329475
0.06669186	0.4878044	0.04771419	0.067443323	0.594272005
0.050145821	0.954319	0.1290838	0.0208358	0.4704794
0.09739313	0.3257263	0.034770606	0.02115344	0.02955723
0.08935769	0.1027047	0.1714318	0.1178189	0.1369125
0.06381495	0.1197249	0.09986071	0.025425929	0.03760864
0.6969933	0.05560452	0.2869081	0.1572042	0.1549752
0.6031248	0.09889556	0.4742253	0.2439703	0.043771483
0.04347267	0.02956342	0.08581652	0.06224072	0.1922433
0.1132169	0.04099693	0.06716028	0.1205823	0.6093199
0.02456608	0.03986963	0.04929576	0.1874772	0.190897
0.04368381	0.3984994	0.028690649	0.04902103	0.07466354
0.07120592	0.03795857	0.2813665	0.3983376	0.02060738
0.02904151	0.02056313	0.1949403	0.03394264	0.03805193
0.1093671	0.1973284	0.4417217	0.1122734	0.1153889
0.04550228	0.01637546	0.4067103	0.1361317	0.03738581
0.03693771	0.1320494	0.07192336	0.1015435	0.4135311
0.1049251	0.05123556	0.0666518	0.0340571	0.06335763
0.06832551	0.06730515	0.1389171	0.3041861	0.03673344
0.03930923	0.1405411	0.1887152	0.1394473	0.1319317
0.09587559	0.03723972	0.221412	0.4172919	0.1290413
0.1198265	0.2356227	0.03645788	0.02894661	0.456424791
0.2317972	0.387088068	0.09444842	0.04796332	0.1459468
0.08971002	0.08894832	0.1944165	0.04260592	0.2124826
0.191734851	0.1038474	0.08143742	0.04043008	0.1662218

0.106711	0.1463906	0.02913129	0.1551794	0.01305251	
0.008738055	0.1598705	0.05239342	0.06724793	0.0133625	
0.6032357	0.03219171	0.06956886	0.04675076	0.1131504	
0.07293787	0.08549901	0.06282755	0.04835109	0.09085911	
0.3053837	0.1181746	0.02865191	0.0075779	0.01664055	
0.102797	0.006361471	0.03708006	0.08184924	0.04695688	
0.08079519	0.15446	0.03857412	0.03044647	0.1161987	0.245988
0.1385301	0.03816927	0.1577552	0.04731068	0.1296253	
0.09938355	0.235663	0.038121664	0.06862619	0.150497114	
0.124868598	0.04028577	0.5073599	0.02979891	0.03126609	
0.04533319	0.01847273	0.05403937	0.03904431	0.1710354	
0.01692699	0.008532609	0.02073598	0.1029407	0.2772944	
0.01284012	0.1563098	0.08392227	0.2689156	0.2037361	
0.1204715	0.09112844	0.2346606	0.1080171	0.09723563	
0.1472046	0.1953814	0.07402906	0.1730477	0.06346147	
0.0402815	0.3999645	0.015451	0.1585659	0.03579397	
0.15246026	0.04467586	0.4782336	0.04359778	0.009088477	
0.08563415	0.07596892	0.04850655	0.1052909	0.02060382	
0.06823923	0.1760088	0.09616471	0.1104265	0.0676036	
0.1086787	0.033606444	0.07226822	0.05397325	0.03490062	
0.1021425	0.02550894	0.167638056	0.4769337	0.02437634	
0.1960326	0.1514828	0.01893787	0.3398236	0.02728383	
0.08726097	0.04375405	0.4786746	0.0925012	0.2129536	
0.390093	0.0442434	0.04308769	0.6122692	0.2094378	
0.05350528	0.02629996	0.02584224	0.07246622	0.2171144	
0.04980054	0.07060295	0.01460281	0.04159435	0.030432067	
0.173969097	0.1215213	0.02616762	0.2242436	0.1033948	
0.05752533	0.3737697	0.03619566	0.2180388	0.06394003	
0.07098173	0.05076514	0.2286578	0.1469492	0.085215426	
0.1093787	0.07143407	0.8363287	0.147522	0.09389622	
0.03123509	0.08247722	0.03752394	0.02851469	0.2078016	
0.1281931	0.1734711	0.3918232	0.06620748	0.01278579	
0.2534745	0.06707451	0.09856679	0.1240816	0.06427784	
0.03352448	0.3213137	0.5016965	0.09898183	0.068520712	
0.03834118	0.03155688	0.1467271	0.01986051	0.1153641	
0.04795466	0.1030883	0.01086335	0.02454025	0.04023828	
0.1402675	0.1328052	0.1472655	0.06542497	1.154638	
0.01949121	0.9875119	0.005388114	0.04982226	0.00236479	
0.05166002	0.07885496	0.01594644	0.1016366	0.1265669	
0.1336992	0.079287489	0.1409945	0.09138317	0.1813284	
0.0268561	0.05034884	0.1654572	0.184705148	0.1100884	
0.06450068	0.08180726	0.01150759	0.05621641	0.4477932	
0.05118498	0.004261533	0.2449323	0.2569951	0.05792174	
0.02271199	0.3354281	0.06474175	0.081512455	0.2768791	

0.4156398 0.05423286 0.034637086 0.06528085 0.2365081
0.04168417 0.0691748 0.004100339 0.09321917 0.1115994
AC073283.2 0.1073015 0.1231255 0.33558 0.04100034 0 0.02173355
0 0 0.03313099 0.053774799 0 0 0.09942061 0.04024201 0
0.01589883 0.08561612 0.048474332 0.1059908 0.09166941 0
0.08549051 0 0 0.08818936 0 0 0.05090739 0.041470884
0.1947817 0 0.1731296 0 0.1351796 0 0 0 0.09637186
0 0.037984028 0 0.03783622 0 0 0 0.09474651 0.0249709
0.02306686 0 0 0 0 0.09209178 0 0.1673694 0.05276267
0 0 0 0.058187246 0.048728897 0.029266616 0.04274709
0.0251124 0.02953241 0 0 0.06506906 0.064684499 0.01499132
0 0 0.1273758 0.02001056 0.133596 0 0 0 0
0.126134392 0 0 0 0 0.02260486 0 0 0 0.36758809 0
0.041360773 0.04792482 0 0 0 0 0 0 0 0 0
0.01874904 0.04944688 0.4118723 0 0.01618825 0 0.05342969
0.075909439 0 0.01299632 0.2757014 0 0.04731589 0.1520074
0.0851919 0.1920943 0 0.04756086 0.1347076 0.1776661
1.639207 0.4548508 0.03984215 0.02062861 0 0.01962872
0.02321038 0.03975629 0.01986265 0 0 0 0 0.04797552
0.08205874 0 0 0.02361786 0.02815874 0 0.1129843
0.01669456 0 0 0 0.02785821 0 0 0.04192595 0 0 0
0 0.03136554 0 0 0.02541066 0 0 0 0 0 0.07809084
0 0.05110738 0.06846747 0 0 0 0.05314767 0 0
0.04367561 0.04457063 0 0.04151985 0 0.02064517 0
0.04316777 0.02167412 0 0 0.1373677 0.07954736 0 0
0.1415882 0 0.1104242 0 0 0.03280826 0 0.9202588 0
0.234499 0 0 0.08255137 0.05275458 0.0210389 0.03503788
0 0.03327805 0.1292946 0.02471787 0.01822483 0.04027748
0.1372489 0 0.01991965 0.07100711 0.04118952 0 0
0.1190066 0.06744751 0.022248955 0.1001308 0.022624079 0 0
0.01871331 0.1043493 0.2215809 0 0 0.02144652 0.09684661
0.09982144 0.1119632 0.01693162 0.02571706 0 0 0 0
0.5916217 0.3375312 0 0 0 0 0 0 0.3866088 0 0
0 0.02421718 0.3197292 0 0 0 0 0 0.02014823
0.04144015 0.03932406 0 0 0 0.1266495 0.1632291 0
0.02334655 0 0.02218881 0 0.02916276 0 0 0.04481404
0.5355071 0 0 0 0 0 0.06046377 0 0 0 0 0 0
0.07549829 0 0.05194927 0 0 0.6401651 0 0.0363758
0.02057408 0 0 0 0 0 0 0 0.03622122 0.1973721
0.046452038 32.468000617 0 0 0.2007411 0 0 0 0
0.08939334 0 0 0.02189901 0.02140262 0.05717599 0
0.4220317 0 0 0.07703532 0 0 0 0 0 0.05857239 0
0.04413156 0 0.02052786 0 0 0 0.02876328 0 0 0 0
0 0 0.037769048 0 0 0.0363946 0 0.2861525 0.1097982

0	0.03592769	0	0	0.1237061	0.1952082	0.01660371
0.08466884	0	0.02148736	0	0.08018899	0	0	0.1025112	0
1.305282	0	0	0.08163229	0.063783871	0	0.02666699
0.01998987	0	0	0.04320049	0	0.08533328	0.05119658	0	0
0.06197368	0.05884604	0.1269607	0.02114085	0.0197573
0.01770717	0	0.7886966	0	0.01889262	0.026958107	0.03842119
0.01900396	0	0	0.1079496	0	0	0	0.02034119	0.1156117
0.1192431
AL928654.2	0.4897287	0.468292	1.561634	0.7796974	0.856482
1.024993	0.4035515	0.3521595	0.4032302	0.531766302
1.541367	0.5131419	1.613369	0.2754995	0.7451737
0.7498175	0.7163857	0.958702456	0.1612489	0.2091916
0.228905978	0.4877282	1.370665	0.5468508	0.7602782
2.386611	0.5684767	1.032638	0.315458295	0	0.3690521
0.4682498	2.489677	2.262208	1.860576	0.572562
0.1358901	0.909014	2.247684	1.675821192	3.875707
2.906884	0.951485017	3.735987	2.958519	2.066042
3.722964	1.614265	2.759221713	1.981812	0.4899579
3.357558	5.113784	0.6381817	0.9548519	3.812844
2.061282	2.175463	2.357071	1.150799884	1.556806132
1.780988648	0.4552325	0.4966612	1.617447	1.460141
1.425541	1.286905	0.393630612	4.150876	1.589403
0.82821	3.262014	2.800759	3.023285	0.8266437	1.419522
9.354783	1.433204	0.447753472	0.5109112	1.462197
2.937606	2.316421	0.06877972	0.9799581	1.713261
1.896218	1.00661313	1.914942	1.038248345	3.973614
0.3067911	1.791438	1.540756	1.901175	1.075998
2.097123	2.904237	2.349603	3.229277	2.624192
2.181552	0.3916258	1.31386	2.241145	1.177464	0.2844981
1.12597579	4.004393	1.759702	5.059468	0.6302573
1.871583	1.156281	2.436603	2.922424	3.983309
2.894265	4.303682	2.567774	1.826449	6.107534
0.4242965	1.412248	2.565084	1.493106	0.6709104
0.937489	0.5741421	1.787	4.497585	2.724045	3.092425
0.6568873	2.621638	2.515415	5.659656	1.509102
4.54096	3.625212	2.612706	2.006461	2.646866	3.328068
0.6864849	2.585304	1.507297	1.9278	1.275679	1.327619
1.354809	1.065052	2.173972	2.218883	3.485534
4.930187	1.584997	8.621509	0.1092328	0.590524299
1.55103	2.170203348	0.9207261	0.2815465	0.9848602	0.8853844
2.929353	6.847393	1.085248	1.347602	1.165164828
0.8360062	0.9302413	1.864705	0.7501178	1.200157
1.316403	2.135777	4.382437	1.083608	1.714642
1.608764	4.684244	1.642018	3.832276	1.06824	0.9718279

1.642462 2.625107 1.035961 4.403808 1.125099
0.4991279 0.7859706 4.228675 1.647768 5.708074
2.362022 1.83014 0.82889 2.461248 1.632382 3.384856
2.285608 4.151456 0.9048299 2.519501 1.608126
3.400822 3.967263 1.110441 1.909197 1.836451
3.352503 2.656547 0.2795162 2.767487 1.881205
0.981603881 0.639802 3.64842513 0.651117672 5.355859
3.302457 2.592942 1.685508 1.444584 1.832341
0.8809461 0.5525153 1.936256 1.234929 0.8242846
3.325591 4.707178 0.8465234 1.412071 3.575964
1.400097 3.790137 1.79936 2.337998 1.537354 2.588426
1.81858 1.141554 1.60112 1.664849 2.444363 1.614187
3.536905 2.979319 3.610492 2.052365 1.515854
3.403813 0.507638251 2.360241 2.553318 1.525553
1.509034 1.626167 1.38279 1.618495 2.930273 4.721574
0.4972554 1.54525 2.768066 0.7441158 2.44017 2.77963
2.894414383 2.95437 4.985921 3.222796 1.045273 0.7700834
0.50607857 1.397868 2.161682 0.3745558 2.828088
1.333992 4.432838 3.011526 3.89657 1.464455 1.40787
5.848437 1.048911 0.8872648 4.118265 0.9005289
2.04759 5.195857 1.174733 2.990595 1.142356 1.539468
0.9003317 1.447735 4.14442 0.8265765 1.610549
12.013854049 3.214532355 2.178218 3.532913 0.2544976
0.6981989 1.147409 0.3393578 3.067972 1.523182
1.251101 3.404752 1.032797 3.321205 1.478738
2.806416411 3.882917 2.223897 0.1007223 2.226754
2.382279 0.814364829 0.5335468 3.666699 0.8939319
1.247526 0.3224992 0.772104 0.9057982 3.279153
4.438852 2.148469 6.205328 3.413199 1.507485
1.631758 1.147004 5.615827 1.612273 3.306026
2.126015484 3.396931 1.082572 1.716435 2.518169
2.879926 0.6403252 1.594955 0.6012438 4.722854
2.733174 1.882001 1.781878 5.759286 1.588666
0.3800618 2.844008 3.524074 2.765229 2.130769
2.462958 3.820903 3.708139 1.233596 3.242618
0.8756226 0.9003858 2.296553818 1.468774 0.7708258
1.36852 3.324085 1.914035 4.994947 2.432093885 3.349399
5.452148 3.087075 2.034771 0.848552 0.3581013
1.255484 1.672455 1.292483 2.828571 6.040569 2.914
3.995086 4.023916 1.599494177 7.569535 1.619052
0.3778207 0.808002904 2.83295 3.832992 2.371588 5.817412
1.64014 0.6595714 1.39945
LINC00702 0.1498899 0.501651 0.4018058 0.2311398 0.1825626
2.591417 0.1786541 0.2104364 0.3603294 0.217306461

0.8105732	0.2439977	0.6778722	0.554112	0.09076387
3.117214	0.298994	0.443365694	0.1692103	0.2332402
0.760658334	1.531162	0.6292737	1.006801	12.20189
0.3029572	0.155915	4.891544	0.351723213	0.3012442
0.7684959	0.6065335	0.03100398	0.5664992	0.1534059
0.4600115	0.02450929	0.1480846	1.373124	0.047375069
0.1608151	0.03586494	0.095834645	0.02753122	0.3704343
0.04884414	0.1171038	0.2992062	1.670119071	0.03762544
0.2496589	0.4626395	0.1768848	0.1159081	1.010346
0.01579381	0.2137903	7.950546	0.08373653	0.107408424
1.120717949	0.233615313	0.9746115	0.425967	0.002946712
19.80151	0.5113025	0.8007442	0.068844263	0.08376577
0.06949489	0.190892	1.374736	0.1996632	0.2999265
0.2478452	0.5519534	0.2213629	0.07130878	0.023073516
0.02233903	0.3929537	0.1100945	1.252826	0.6563471
0.1285428	1.225006	0.4392469	0.77022842	1.507117
0.206346671	0.06216453	0.5908303	0.1138778	0.2144269
0.1509408	0.6621151	0.1285225	0.01078171	0.8551503
0.2627329	0.05051051	0.05427132	0.05137022	2.859097
0.5524142	0.210052	4.827362	0.102251188	0.07707665
0.1426435	0.1306686	0.1653439	2.171719	0.4322632
0.05780242	0.04312567	0.1204458	1.047189	0.0246418
0.1462505	0.08753827	0.1223409	0.5525815	0.1337895
0.178618	0.08029984	0.1042158	2.120276	0.3071904
1.122671	0.06094585	0.2483793	0.1359321	0.0526564
5.387528	0.6069474	0.08761085	0.1508201	0.09271839
0.04959465	0.241252	0.1049432	0.02029055	0.1172213
0.1575829	0.03335594	3.029795	0.06793597	0.2970161
0.2255983	0.4362038	0.08731539	0.4064646	4.284449
0.255496	0.2315113	6.599772	0.2406166	0.1504467
0.855288357	0.3714169	0.343792885	0.04869888	0.2506027
0.2175761	0.04098966	0.3001939	0.8223634	0.8013994
3.319689	0.054232361	1.050912	0.04793695	0.04447209
0.1866097	0.555136	0.3422661	0.01441967	0.2056582
0.0387651	0.1946358	0.1031142	0.125681	0.4346888
0.4391889	5.618257	0.2723358	0.3266988	0.0333232
0.2993225	0.0383599	0.2691186	0.1473108	0.03682039
1.041904	0.8098429	0.08933803	0.06982095	0.1400365
0.07742674	0.3719749	0.1154581	0.05593667	0.04456591
0.02324314	1.481021	0.5943838	0.1418394	0.204961
0.07395054	2.005079	0.2067063	1.492576	0.1931627
0.2435499	0.1397838	0.0186597	1.101451	0.06881928
0.2477754	0.352155382	0.112379228	0.0582891	0.3977122
0.09717733	0.05085093	0.08477116	0.06270033	0.05135787

0.5773792 0.1518912 0.03072181 0.03040954 0.04875439
0.3566815 0.1850665 0.1234825 0.6987623 0.207703
4.998851 0.4441565 0.812132 0.3396335 0.4132416
0.5387854 0.2281745 2.466683 0.6426643 0.04275083
0.3426269 0.07974 0.1754623 0.9588346 0.03518115 0.07587208
0.3827005 0.368452347 0.05226959 0.576812 0.2570032
0.08697478 0.3144913 0.7653474 0.1592256 0.2117286
0.05562907 0.1048272 0.5831869 0.1948298 2.184443
0.09311453 0.1673614 0.058711123 0.02384798 0.05770691
0.2743743 4.911975 0.1079004 0.324256339 3.497605
0.01055777 3.802749 0.2229737 0.0749923 0.5440831
0.07596672 0.3239491 0.2354106 0.5836356 0.06565693
21.25947 2.183082 0.1533 0.1214051 1.687735 0.3920962
0.1203842 0.1683686 0.1790827 0.1195666 0.3897236
0.5477938 0.4950922 0.03433409 0.1199518 0.208572047
0.100959811 0.1729362 0.0676418 0.2069739 0.04848561
0.04067772 0.2225707 0.03307653 2.679439 0.2930519
0.2076879 0.1201783 0.5146627 0.3365926 0.342558361
0.1002616 0.09281757 0.8543707 0.09992452 0.172066
0.050599715 0.1837139 0.07234058 0.03040037 2.759674
0.6641534 0.1409088 3.27091 0.6923078 0.01687686 0.1138855
0.1306625 0.08609907 0.119842 0.2371799 0.1924933
0.03348353 3.021666 0.3377522 0.04522266 0.07096295
0.4032879 0.1815707 0.04325522 0.5139358 0.2063295
0.2678947 0.04301791 0.03036788 1.724856 0.2437794
23.85526 0.1623566 0.4111441 0.3810221 0.3344617 0
0.04267291 0.1109766 0.00234109 0.1610982 0.2762288
0.1243196 0.08003705 1.198166 0.1160688 0.186685725
0.1730023 0.1676306 0.07379901 0.06912605 0.1126111
1.043141 0.647770067 0.1123909 0.406113 0.01278748
0.1074127 0.1576835 1.644046 0.247026 0.04218823
0.02562771 3.883431 0.1172889 0.3297708 0.1271192
0.1055648 0.156011355 0.2971059 2.377828 0.2890964
0.068579899 0.1023254 0.0591506 0.2475985 0.05380691
0.02232585 0.1067044 4.619802
PART1 0.1802669 2.162537 0.1452607 0.3856752 0.7113051
5.96873 0.5524419 1.385559 1.212104 1.030345447 0.7685715
0.4829451 0.450927 0.3352401 0.4566186 4.462353
0.4011939 1.798121665 0.3587052 0.1368226 1.042795206
8.722809 3.222053 3.983682 4.250884 0.3395963
0.3749587 6.933856 0.451999462 3.154022 1.178832
3.221078 0.05177079 0.5865245 0.01698024 3.563064
0.01550223 0.4736728 1.348334 0.019776856 0.06015475
0.005253306 0.004651912 0.001915498 0.2776749 0.08550698

0.08667605	0.2882411	0.335248697	0.01427895	0.1049087	
0.04779058	0.04475215	0.1097649	4.543053	0.001831436	
0.1334335	2.176304	0.05228469	0.030295913	0.032136987	
0.030476054	1.341343	2.044943	0.3341808	4.528731	
0.2845929	0.1821938	0.046401886	0.3996372	0.00254481	
0.09826076	0.9461626	0.3139515	0.001159307	0.01347183	
0.08212566	0.04487031	0.3315078	0.140103337	0.01295207	
0.08625099	0.004787423	1.835234	0.6371223	0.4226489	
0.846172	0.2835159	0.38277861	0.3680126	0.008614	
0.00166351	0.7699663	0.1597197	0.1131775	0.01369797	
0.109752	0.277673	0.8876685	0.2506747	0.1137822	
0.1171431	0.03089416	0.0500375	0.316678	0.03259064	
0.1504431	2.952505	0.018444157	0.3833297	0.1344317	
0.01794342	0.3058985	2.824883	0.5698406	0.01064549	
0.2800457	0.01062263	0.4897601	0.07481302	0.02929295	
0.0544941	0.2924258	0.2046772	0.557076	0.1387248	
0.06404494	0.009667819	3.17808	0.3267988	0.3283209	0.2204975
0.5032743	0.05254189	0.24646	1.25896	0.2635748	0.006416385
0.05574609	0.04300617	0.05683294	0.0517675	0.01970239	
0.01568583	0.1265544	0.02289167	0.1837265	1.443116	
0.01312966	0.08295121	0.08123423	0.1573656	0.00675001	
0.3820017	2.597695	0.4911861	0.300851	3.067683	
0.02511152	0.199379	0.511985536	0.5267058	0.289472287	
0.04608016	0.2367599	0.228252	0.06892032	0.2575956	
0.1805763	0.524563	4.318061	0.240115407	0.4657278	
0.4654171	0.09437209	0.01534409	0.1470012	0.9375157	
0.4371326	0.01379158	0.2082761	0.2964167	0.375395	
0.0194318	0.3078861	0.1104462	2.197032	0.06450345	
0.1388386	0.01030434	0.3807362	0.04238855	0.06493018	
0.230038	0.6148311	0.3024801	0.1979696	0.07103698	
0.02277106	0.6211229	0.01719256	0.8240199	0.1372922	
0.2104015	0.08738321	0.08085762	0.5475267	0.3637826	
0.01644757	0.005592259	0.04764022	0.8614115	0.2337025	
0.8084267	0.06719694	0.1512024	0.4496063	0.02714538	
0.3964363	0.041703101	0.09175644	0.028270819	0.009382605	
0.004268939	0.02728128	0.03259844	0.01845901	0.2012257	
0.03271804	0.00893312	0.359694	0.01039465	0.1437944	
0.05759564	0.09997796	0.05004964	0.1867033	0.05559112	
0.2456069	0.02890207	2.557432	0.3926246	0.3753793	
0.03322991	0.3776545	0.1745543	0.03720785	2.056167	
0.3212439	0.05948818	0.6870288	0.003362393	0.1415003	
0.2079666	0.001064237	0.005460865	0.5658146	0.086480347	
0.004196171	1.126284	0.1146575	0.008346637	0.09132889	
0.9988111	0.03868576	0.06119081	0.02967321	0.03241513	

0.06803819 0.02156537 0.9765529 0.03644149 0.3118996
0.084420318 0.02592554 0.03717579 0.2771545 5.569882
0.04951572 0.007105602 0.3731542 0.001049374 0.5281306
0.08649119 0.006522031 0.4739079 0.07047227 1.156997
0.03406791 0.1612147 0.006010674 3.852243 3.023057
0.08634314 0.009131697 0.5580843 0.05284661 0.04020377
0.02053025 0.1640112 0.2384752 0.08052393 0.1549929
0.02701668 0.3281471 0.1706509 0.106417656 0.035121632
0.04969786 1.641166 0.06619496 0.0112447 0 0.02129251
0.02684867 2.865854 0.05872097 0.02281576 0.3040531
0.1545237 0.5160028 0.012450419 0.2497318 0.06457827
0.6296694 0.3903981 0.127972 0.121261634 0.09332875
0.01224275 0.0256836 0.6839336 0.1088997 0.08731506
3.47441 0.1724345 0.01320993 0.4472155 0.02831594 0.01996794
0.06253548 0.5252436 0.2801714 0.14477 2.833587 1.156106
0.191405264 0.02181589 0.1402947 0.07579721 0.008207733
0.151281 0.09146851 0.03017726 0.01496496 0.05282149
0.04434463 0.1696107 6.450597 0.05186957 0.144987
0.02725306 0.01939194 0.1238667 0.3804016 1.805915
0.08307004 0.01957036 0.0752038 0.45582 0.09387095 0.3323623
0.3612744 0.059039761 0.1326774 0.6609022 0.3635853
0.02219759 0.1566994 0.3448907 0.058197088 0.01540234
0.6681804 0.06227865 0.04416036 0.01936042 1.546244
0.1057659 0.2509652 0.5527486 1.323914 0.04896259
0.1981524 0.05595421 0.06295474 0.334994291 0.03067352
1.154375 0.6551423 0.04337708 0.08243447 0.005144289
0.1479736 0.4129349 0.01694543 0.08828554 4.085809
LINC02593 0.150465 0.6926255 0.3926796 0.3409486 0.3488225
0.7583606 0.1405675 0.4948592 0.8427335 0.29373448
0.1712068 0.3299829 0.5079422 0.4002588 0.4563753
0.1788733 0.6561222 1.509650869 0.1901044 0.2989412
0.527470604 0.6795538 0.2670984 0.8247261 0.4577514
0.690359 0.3821833 0.2683877 0.595055242 0.1508596
0.5043134 1.110359 0.1047081 0.3747067 0.3361507
0.3451816 0.04005191 0.1225677 0.343757 0.108385303
0.4916834 0.04010074 0.127471951 0.2834383 0.446803
0.1107164 0.2931554 0.2181463 0.256533253 0.0614857
0.1211823 1.327168 0.206469 0.1946729 0.730014
0.5807134 0.09761693 0.6958934 0.1192949 0.621441745
1.239488504 0.057264447 0.6935231 0.08598819 0.03370763
1.019582 0.1002655 0.7320739 0.126564756 0.3079935 0
0.1293437 0.2907679 2.646131 0.1198085 0.2151651
0.6737651 0.6038507 0.04237433 0.037705637 0.4258961
1.532742 0.06371854 0.6512528 0.2395777 0.1998943

1.38617 0.3199814 0.55141755 0.2210258 0.033720187 0.1054935
0.08369717 0.3544641 0.08055317 0.09294442 0.2849145
0.191602 0.01761895 0.4433521 1.686714 0.1589695
0.02418753 0.1091308 0.9098873 0.9687178 0.1514369
0.8232766 0.198037341 0.02798999 0.1229079 0.1770071
0.1412394 0.4667601 0.3469956 0.1944723 0.3602 0.1275216
0.4833941 0.03294692 0.007242289 0.6926673 1.270483
0.3637995 0.3498119 0.580384 0.04160703 0.03784544
0.9820866 0.511712 0.1638049 0.1434165 0.1566591
0.1833485 0.09387118 0.9410601 0.266267 0.05651426
0.1655924 0.1101934 0.1334862 0.2431776 0.7540255
0.1399998 1.261637 0.2914725 0.02725434 0.5414849
0.03700591 0.9023773 0.09378241 1.072535 0.07927035
0.4416575 1.181397 0.9998492 0.509644 1.015111
0.05898062 0.08195092 0.369196209 1.03607 1.044894853 0.06048185
0.1077692 0.3305526 0.307007 0.2616333 0.509907
0.3963289 0.3553035 0.290041963 0.283736 0.7441947
0.03270338 0.09702914 0.6736125 0.5089262 0.04712786
0.6883499 0.1478122 0.1590323 0.4114638 0.2445022
0.1535887 0.02161752 1.335728 0.08995451 0.6781662
0.1300874 0.2310654 0.2728681 0.1312249 0.4065632
0.2166124 0.6124565 0.6549969 0.5874438 0.1034014
0.06538312 0.08883816 0.5017742 0.2401333 0.02570879
0.1390343 0.8383353 0.3204464 0.4594595 0.1099503
0.1576177 0.4207246 0.810783 0.6625873 0.2084038
0.06044502 0.1745067 3.418932 0.2799793 1.217066
0.137855638 0.1273484 0.616053899 0.378308216 3.007998
1.45546 0.2183535 0.1228408 0.3748431 0.02561547 0.3077308
0.4776845 0.3784235 0.04107609 0.09938764 0.04612595
0.5828718 0.776229 0.07418722 0.4464806 1.793558
0.707603 0.4943986 0.4547568 0.0693766 0.3175733
0.1579446 0.2505233 0.7459507 0.8360906 0.05489107
0.3114075 0.01579482 0.7689633 0.9134857 0.07748834
0.6284834 0.2316263 0.522309877 0.1412657 1.388559
0.1570926 0.02940621 0.2189774 0.08313863 0.05782196
0.558918 0.04545318 0.1370429 0.2847426 0.2713479
0.1914055 0.1711837 0.2441911 0.031980971 1.366429
0.3457736 0.6210747 1.008266 0.03001288 0.070929383
1.550633 0.1700653 0.3171366 0.1644515 0.02042481
0.705862 0.314491 0.1764607 0.4801015 0.9565964
0.121411 0.4533039 0.5705677 0.2684094 0.4664951
1.358249 0.3958528 0.09442852 0.03120143 0.1015311
0.334334 0.9456519 0.125325 0.2569942 0.2067105
0.1228779 0.825586522 0.082491849 0.2242401 0.1317036

0.2345767	0.04842009	0.05982622	0.9683896	0.1261215
0.7696095	0.5018782	0.2398722	0.2713747	0.3385088
0.2190851	0.158747128	0.1867807	0.2527967	0.3382478
0.6657284	0.1971506	0.091875152	0.1381949	0.1469707
0.01774244	1.138415	0.4597089	0.3849767	0.5539326
0.2845077	0.04136905	0.2523475	0.6545689	0.07972942
0.4896002	0.1465272	0.2133245	0.01563348	1.717518
0.3304817	0.471117027	0.03505896	0.07889842	0.2136342
0.08353754	0.3158407	0.4028183	0.07087877	0.193319
0.04466317	0.3417564	0.3126469	0.6604613	1.407796
0.2162876	0.9659774	0.1821871	0.244995	0.04358377
0.04030059	0.08416534	0.1211827	0.2011674	0.09996624
0.403589	0.3796531	0.156398	0.104002122	0.3887291
0.2956747	0.1694902	0.1390308	0.2986613	0.4015086
0.562655302	0.233754	0.5509551	0.1149318	0.1329763
0.181891	1.343311	0.3726259	0.06549489	0.5025549
0.3782263	0.1022229	0.2449526	0.4578584	0.06777136
0.127473151	1.068136	1.186791	0.566913	0.147728628
0.272825	0.9021701	0.1400585	0.3224052	0.06633424
0.4147209	0.4110817
AC078883.1	0.3978003	0.1646454	1.567414	2.002105	1.261622
0.2585555	0.623798	0.2775157	1.274101	1.859702653
0.4535859	0.2099646	2.70478	0.205971	2.032707	0.127561
1.409377	0.317397589	0.8137402	1.521705	0.971314702
0.1064352	0.4003311	0.2912398	0.2399229	0.4386847
2.330761	0.07981109	1.514509214	0.5523657	0.8221476
0.2288506	0.6246957	0.9723872	0.7733843	0.1301543
0.2100549	0.8212127	0.6337972	1.124448319	2.042534
1.004925	0.85280365	0.8015501	2.683998	1.065998
2.652895	0.7402887	1.795523103	1.403675	0.6168563	1.6077
0.4840934	1.544448	0.3472898	0.510916	1.987604
0.4138789	0.571528	1.046395481	0.781932517	0.906870394
0.7214248	0.409916	0.7108416	0.740278	0.4212686
1.212158	0.638289116	3.898722	0.7504934	0.8563505
1.615188	0.974376	1.007198	0.7301722	1.839302
3.233279	2.606359	0.796815063	0.9910606	1.420339
1.704414	1.324812	0.631652	0.7818272	1.350117
0.7190226	1.44412396	0.7969394	0.772408631	1.968982
0.6238515	1.593828	0.6150614	1.395087	1.628715
0.6252017	2.087691	1.113355	2.371346	1.177497
1.559547	0.5697539	0.8332002	2.369247	0.9764246
0.3843357	0.787556547	1.836382	0.4134976	3.842454
0.9898625	1.302521	1.093434	0.2734078	0.8901928
3.65956	0.3552779	1.161548	1.15512	1.168911	0.5744936

0.6944452	1.352611	2.078978	0.9286302	0.7448948	
0.8909341	0.1923357	1.137414	1.405994	0.8457743	
1.758914	1.314043	0.9837882	1.99919	1.534269	0.5292736
2.344954	1.472222	1.169081	1.108513	1.312764	
0.6892863	0.8114661	1.672506	1.040603	0.8843005	
1.380335	1.108404	0.7727352	0.3389493	2.112459	
1.106414	1.360941	1.363439	0.9420532	1.084164	
0.5959371	0.223729152	2.374578	1.505791339	0.8858061	
0.431549	1.211296	0.757703	0.9544538	1.714219	
1.915105	0.3774057	0.567322237	0.9628705	0.3987563	
0.2342918	0.9595943	0.6088164	0.282144	1.850624	
0.5221524	0.758382	0.6955913	0.4255452	1.853061	
1.693932	1.833999	0.6105473	1.47009	1.229038	1.550233
0.4633499	3.247221	0.2467299	1.252613	0.9596926	
0.836555	0.7206901	1.285759	0.7985932	0.7349789	
0.557274	1.478997	1.641447	1.216568	0.7863648	
2.789686	0.5294158	0.923208	3.716085	1.521072	
3.842766	1.299986	1.7415	1.532326	1.190852	1.028551
0.1207248	0.4985787	1.492831	0.510907647	0.5429732	
1.464675686	0.529725463	1.535419	1.413409	2.112308	
0.6682107	0.4009711	0.2897574	0.8840929	0.3081321	
1.470612	1.34747	1.883126	2.853122	1.695429	1.017746
1.082557	3.908553	0.387985	1.712025	1.701561	
0.7401237	1.10359	0.9847938	1.454021	1.552039	0.6655365
0.7958307	1.557938	1.409033	4.455535	1.415328	
0.904153	1.87465	0.5803498	1.596631	0.363112445	2.084795
0.2923588	1.664579	0.3908491	1.872939	1.367739	
1.527723	1.264475	2.036065	0.5619488	1.755092	
1.528579	0.83358	0.895584	2.684901	2.740352261	1.438225
0.8474537	1.589186	0.7793628	0.732047	0.79997991	
0.9278655	0.8029549	0.5176738	1.203687	1.711151	
2.072919	1.123409	0.6187879	1.812013	0.8163935	
1.806152	0.2707419	0.2640336	1.108212	1.228245	
0.784986	1.423034	0.6809504	1.48718	0.9728474	1.812376
1.617654	1.134122	1.3997	0.7966819	0.5857737	
0.713267531	3.573347265	1.08412	0.3192443	0.4350485	0.5975115
0.9925562	0.5322835	2.91192	0.5886225	1.625139	0.5455644
0.8724522	1.752723	0.8621144	2.048535522	0.4058853	
0.8946428	0.7632038	0.724642	0.7622658	0.28060406	
1.309883	1.788999	0.6141393	0.4667025	0.7858858	
1.184338	0.3778968	3.504157	1.731448	0.4763458	
2.726151	0.8833159	1.254417	2.406031	0.9141099	
1.23348	0.4975523	1.121504	1.807743646	0.434713	0.2677452
1.157949	1.635509	0.7368757	0.3949057	1.414883	

0.9045349 0.8757169 2.628801 0.4877089 0.9451282
1.061138 1.272755 0.6615414 2.43737 0.5889091 0.8800253
1.874776 0.8243995 1.389703 1.232136 0.4377287
0.7418648 0.7961832 0.2992489 1.935264001 0.4633445
0.5533373 0.76874 1.164773 0.8650751 2.856546
0.701190264 1.001011 1.211049 1.010522 1.146203
0.514379 0.5616827 1.668467 0.88319 0.2787742 0.8426209
2.551144 2.03658 2.014136 1.186514 1.260465034 1.854902
1.319692 0.5038639 1.140943065 0.3061257 1.8588 0.7833561
1.591412 0.6303027 0.6876937 0.4995692
REPIN1-AS1 0.4532324 1.276102 1.042252 1.274812 1.122927
0.9945076 0.3112313 0.4828376 1.010696 0.441661653
0.6051816 0.2473442 0.8554422 0.6610291 0.6568008
0.429048 1.40636 0.910006864 0.1554501 0.2688916 0.82384994
0.3009207 0.7047325 0.9639955 1.138208 0.5541672
0.7971392 0.7964022 1.800353838 0.5713483 0.7115606
0.5642633 1.069431 1.110253 0.1434933 0.4415772
1.441036 1.243815 2.064597 2.963708756 0.610013
1.420596 1.048306097 1.003601 0.9334228 0.815225
2.373187 1.975714 1.461394808 0.7843278 1.395214
0.9502574 1.998966 1.627997 0.3436584 4.395395
3.955823 1.084106 8.155056 1.263026269 0.771850527
2.472394986 0.6520227 1.060727 1.178123 0.9725457
1.065064 1.145193 1.138424755 1.125726 1.354827
0.69349 0.9216168 0.5400076 2.018151 2.322441 1.681268
1.168288 3.868658 3.132556014 1.751246 1.54026 1.078843
1.294561 1.326126 2.242941 2.409464 1.555767
0.86258973 0.7952332 1.819838167 1.827497 0.6668005
1.126545 1.043366 1.594862 1.19595 1.14011 2.049661
1.301639 2.11221 1.143919 1.50843 1.117525 0.987307
1.500513 1.561926 0.78362 4.386467031 2.61835 2.165318
2.46656 0.5744507 1.387905 0.7134072 0.7796609 3.52166
2.533443 1.097472 1.264431 1.276802 1.029371
2.575592 0.0467472 4.743936 1.880579 1.681232
3.349656 0.6297272 2.377115 2.919584 9.230688
0.2562032 1.420835 0.6191919 2.310729 4.598435
1.572448 1.246998 1.189402 1.921091 2.465722
2.056729 1.643679 1.972563 0.4479861 0.4576085
0.9902552 4.571288 2.976125 3.467298 2.184729
0.9354836 2.001951 1.21445 0 1.484926 0.5664772
0.3536793 0.4212184 1.631958762 0.945834 2.780961816
1.947027 1.116706 4.497361 2.450174 0.6589351
1.680292 0.9919721 1.122457 2.116083158 0.9402655
0.7174305 0.6275421 0.558565 2.0217 0.4351072 1.526061

2.301807 0.683764 1.525828 0.8741493 2.017444
1.174275 1.897786 1.098479 0.6472983 2.844922
1.589172 0.95012 1.06136 1.582296 1.385794 2.771038
1.718784 0.6273613 3.876973 2.463094 0.3528649
1.569106 1.671232 5.035772 2.117181 1.095751
2.791752 1.638389 1.363081 2.822606 3.497091
3.188503 0.8296432 1.425687 3.693559 3.552114
2.379263 2.155714 1.496054 3.376506 3.524165012
2.138218 1.539613691 2.325802066 1.893944 1.734564
2.591521 1.611895 1.524567 3.686497 0.8807199
2.102176 0.9662525 1.346517 0.5761153 2.082012
0.7049164 1.994864 3.416572 1.876504 1.696825
1.225803 1.614803 2.838269 1.372863 0.4728015
1.015635 2.18004 0.75602 1.137241 3.106037 2.738803
3.26764 0.2344635 1.688972 5.755806 2.368911 1.250059
1.278919938 1.631169 1.410042 0.5075326 1.410683
0.9968883 3.589514 4.249936 2.413131 0.6541477
3.971949 0.961758 0.5727558 4.407436 1.813499
0.7731505 3.54400146 0.8763461 1.156101 1.147163
0.7859928 1.808731 0.990769738 1.37455 1.02867 0.2888689
1.554619 2.222976 1.074461 0.6352357 1.396761
1.218015 2.130612 0.9549242 0.4958092 0.7672294
2.425019 1.19611 1.536484 1.327688 3.114341 2.857533
1.547156 1.812172 1.222079 3.049006 0.9817216
1.848695 0.8842097 0.92654507 0.907856063 1.282035
1.760049 3.513346 0.9185704 3.08525 1.766631 3.111601
1.573292 1.378411 1.267018 0.8993015 1.230483
0.4360536 0.992013889 0.8960293 2.390797 0.5437609
0.6327043 7.558457 2.346972248 1.508788 1.12655 1.429924
1.621877 1.16899 2.200651 0.780765 1.854587 2.282252
0.5674538 2.64475 1.383678 5.601705 1.224449 1.87328
4.302973 0.9043158 0.8825913 2.681046038 2.85269 0.7013264
0.8540432 1.387206 1.627075 0.8159069 0.3300209
1.391093 2.428273 1.897116 0.9071602 2.633957
1.34421 2.549799 0.5443569 0.302536 2.60769 2.195353
1.909145 2.064683 1.593676 0.6002112 0.4408865
1.075616 0.8287857 0.885965 0.798274864 4.392349
2.628247 1.383805 0.4376939 1.454454 0.6335947
1.555462674 1.281567 2.132466 3.884539 1.186527
0.9816438 1.139237 0.1489643 8.97933 3.593124 1.329664
0.9194746 1.454178 3.325182 3.458037 3.985408101
1.374939 1.939886 1.165547 3.189094955 3.039802
1.622968 4.40463 2.166589 2.124121 2.272111 0.7994806
AL157392.3 0.1701138 0.4700235 0.5723773 0.2009906 0.3065141

0.8523323	0.2130353	0.3744934	0.3013292	0.256882617	
0.8599939	0.3025504	0.3843708	0.337463	0.3561567	
0.4974815	0.2839704	0.471888124	0.1326599	0.1912251	
0.245864328	0.4556478	0.2881773	0.6759148	0.5617083	
0.7776473	0.3245467	0.7461921	0.181669918	0.09548528	
0.3162711	0.4317791	0.5738598	0.8375063	0.3382121	
0.3258085	0.211483	0.4937408	0.777762	0.34071395	
0.991272	0.2225755	0.255338475	0.375855	1.171815	
0.5481327	1.303292	0.4388375	0.970912045	0.363274	
0.2542567	1.220637	0.4869891	0.4307971	0.9706023	
0.2476451	0.4062086	1.224379	0.1984057	0.149297779	
1.083588089	0.22222572	0.4351578	0.2147793	0.3536154	
0.7389584	0.3787156	0.6542474	0.186208602	0.4403143	
0.2775796	0.4256945	1.232008	0.4533248	0.7127374	
0.3643297	0.633973	1.055313	0.7115268	0.234176852	
0.224175	0.71786	0.750927	0.8801325	0.2716093	0.2141236
0.8374115	0.5299656	0.64717885	0.8392186	0.423633772	
0.5768418	0.2447495	0.777724	0.2030196	0.3585545	
0.5415506	0.4016294	0.6657047	0.6153009	1.013371	
0.6062214	0.5611232	0.6024984	1.216724	1.237978	
0.2557377	0.3878665	0.256525906	0.360477	0.8892312	
1.186906	0.1586969	1.159752	0.6912602	0.2942924	
0.3085501	0.4333973	0.5483191	0.3381416	0.2964933	
0.7359009	0.3605563	0.2742692	0.7186324	1.123758	
0.2153764	0.09974013	0.6427291	0.3662772	0.5171386	
0.644218	0.6613918	0.6125801	0.1100862	1.192533	
0.4198492	0.7789279	0.5547526	0.9774328	1.308872	
0.9427547	0.4436749	0.7682819	0.8399949	0.6132857	
0.8310165	0.7738879	1.269594	0.5719803	0.2714041	
0.3918008	0.1452255	0.6362196	0.9631212	0.7056444	
0.7624148	1.103614	0.7168398	0.4193754	0.245612061	
1.257243	1.09598093	0.5009169	0.2371441	0.5408756	
0.3899127	0.7916138	1.229099	1.680958	0.7864209	
0.233977073	1.305836	0.3644344	0.4537211	0.6001	0.6573822
0.6485158	1.340227	0.8682074	0.4835644	0.679097	
0.2346266	0.9542597	0.5100645	1.043198	0.7365548	
0.5487653	0.7812195	0.4079401	0.2457046	0.7151702	
0.1660675	0.2641745	0.4372385	0.7286975	0.4507305	
1.20692	0.4196674	0.4307864	0.5200579	1.485643	0.3475911
0.7981434	0.2270004	0.905918	0.2697124	0.3815603	
0.4227549	1.269542	1.353651	0.427281	0.9108411	
0.9084872	0.6959726	0.2906231	0.160013	0.4376317	
0.4464801	0.236701072	0.3818249	0.923124341	0.165401835	
0.6202144	1.676228	0.4012487	0.7774611	0.4837253	

0.1867955 0.5440151 0.5202141 0.8537449 0.4636136
0.4082976 0.7886041 0.9336873 0.3533774 0.3690154
1.118146 0.4671449 0.8932011 0.7625989 0.4298142
0.2795842 0.6117658 0.2983703 0.5262274 0.814542
0.6496178 1.039966 1.436651 1.271026 0.4818819
1.123219 0.6587657 0.7023129 0.7837952 0.237588174
0.4463565 0.8255532 0.6488664 0.1542454 0.7055171
0.2931929 0.782016 0.540715 0.4551245 0.2551964
0.7635122 0.7757618 0.3232343 0.2311707 0.5273408
0.543348944 0.8413506 1.722096 1.186333 0.8363006
0.2639791 0.369379228 0.949733 0.4799216 0.1838617
0.3019728 0.4141678 1.003768 0.4644601 0.9572007
0.689026 0.6366783 0.6798267 0.6959208 0.7034115
0.2411357 0.3237867 0.6207065 0.4806832 0.4671206
0.5588453 0.3201498 0.50661 0.7732966 0.4420649 1.271303
0.2954339 0.3578257 0.904079982 1.205592277 0.6413569
0.4049897 0.2275219 0.1768283 0.4456249 0.2303323
0.7399209 0.5206442 0.4950369 0.4675028 1.341223
0.7572044 0.7573696 0.857266313 0.2138043 0.4841989
0.3130438 0.8741969 0.4200985 0.309131265 0.4096872
0.6768965 0.7354404 0.4017401 0.3316512 0.3691596
0.3316883 0.9326572 0.8132879 0.6125899 1.488552
0.3966069 1.186344 0.8320782 0.3265747 0.8280281
1.169663 1.32071 0.595632345 0.7204026 0.2553154 0.5724384
0.8845853 0.5776373 0.4214379 0.3189757 0.3297633
0.7517057 1.447426 0.6038477 0.9895202 0.7425922
1.136266 0.2382308 0.8050257 0.5119425 0.5876984
0.4863688 0.762548 0.8938568 0.5084495 0.5338448
0.9217469 0.4191286 0.5440681 0.359248594 0.3008582
0.3782713 0.3711252 0.6358813 0.5789274 1.647348
0.453860573 0.4236584 1.631867 0.5738934 0.3756319
0.1771121 0.3375751 0.5137968 0.5433174 0.4855046
1.266223 0.5198457 1.610773 0.8157261 0.9679235
0.483624492 1.614175 0.7080207 0.276297 0.260684837
0.2938679 1.566402 0.4579142 0.893796 0.2240427
0.3050794 0.5536343
AC036108.2 0.1871368 0.8188103 0.04694607 0.0108342 0.03719111
0.959084 0 0.2514662 0.07003802 0.071049062 0.116825
0.02228222 0.008757188 0.03190145 0 1.155334 0.03393566
0.435512165 0.0490135 0.02422334 0.039759235 0.3727446
0.02885747 0.8548546 0.8738905 0.1604656 0 1.004424
0.021917087 0 0.08012694 0.1677461 0.0328434 0.01786038
0.02308342 0.03729363 0.005900772 0.05093189 0.09650477
0.010037153 0.05953301 0.01499714 0.005902348 0 0.03503675

0.146046 0.06598477 0.06095341 0.265611714 0 0.03927784
0.02675149 0.006083737 0.008526718 0.5860052 0.02091354 0
1.087078 0.03411712 0.038439466 0.038629348 0.015467212
0.3727606 0.006635868 0.0390192 0.5264443 0.007737677
0.08023998 0 0.02772984 0 0.02877076 0.2692691 0.1216176
0.01323836 0.03076752 0.08365438 0.03231246 0.02146007 0
0.02958047 0.01858335 0.006074284 0.5306286 0.05973259 0
0.1867171 0.07708395 0.05828036 0.3070255 0.054647235 0
0.02906579 0.05744477 0.03263629 0.01158666 0.03298107
0.005971384 0.009517809 0.2644081 0.05680009 0.009908746 0
0.04761571 0.0234059 0.06416537 0.008180666 0.1200082 0 0
0.003434235 0.1365997 0.02985571 0.3938466 0.1205023
0.01800934 0.006345036 0.013478 0.02932486 0.01186535
0.005868459 0.1830233 0.05225775 0 0 0.06050485 0.03630777
0 0.4097132 0.0262432 0.03675651 0 0.01154014
0.005714158 0 0.1951537 0.0485454 0.02442334 0.04368659
0.0148817 0.0656713 0.0119423 0.04411482 0.3462981
0.01605723 0.01375814 0.01472288 0.2763509 0.00999535
0.04431517 0.03144509 0.02674093 12.80038 0.06763031
0.4144119 0.03561241 0.08107324 0.2081552 0.01593075 0
0.119664532 0.1190382 0.162377552 0.005158812 0.05588856
0.02250826 0.02713845 0.0636008 0.08495288 0.01396292
1.390366 0.013056794 0.1694093 0.005770567 0.0883323
0.008985502 0.08228611 0.09799252 0.005455417 0.1246787
0.00570347 0.01718194 0.0444548 0.01056646 0.05704123
0.007006708 0.2650645 0.07672688 0.09353551 0.009805616
0.03890563 0.03585507 0.005747681 0.01733895 0 0.06451591
0.2028793 0.09013175 0 0.01986759 0.004362785 0.04646741
0.07783243 0.009258644 0 0.008793615 0.04783195 0.06531616
0.00481585 0 0.07253512 0.1265743 0.0421096 0.06254464
0.03809467 0.1190767 0.06068731 0.004492445 0.1960506
0.029396062 0.01058369 0.017935014 0 0.01624929 0.01977972
0.004595657 0 0 0 0.01700152 0.03838709 0.03956623
0.007396475 0.01342237 0.01359129 0.007937876 0.03267438
0.01923656 0.03201639 0.06948181 0.8197123 0.0568246
0.08272256 0 0.07690355 0.04901486 0.009441867 0.567556
0.06609638 0.008086996 0.1325396 0.07039239 0.03168277
0.04112231 0 0.01385749 0.03753756 0.017634584 0.01597231
0.09855379 0.02597813 0.007942662 0.0434543 0.1288771 0
0.06038583 0 0.0370155 0.04708739 0.03517991 0.03618924
0.007706163 0.0237443 0.082925833 0.02368392 0.1132048
0.02152982 0.7579964 0.01215979 0.013523387 0.1396095
0.01198301 0.07806901 0.01045143 0.01241273 0.05499646
0.02235381 0 0.009975087 0.1245752 0.04118227 2.585892

0.4389834 0.05316504 0.008689725 0.03844874 0 0.01275264
0.04596846 0.03387633 0.02814674 0.0187657 0.00967156
0.02570908 0.004785669 0.07586172 0.07978618 0.047183656
0.02489079 0.03049144 0.02652259 0.007133639 0 0.01473597
0.02189932 0.8456638 0.04346136 0.004135522 0.06944088
0.1357337 0.06043426 0.033850922 0.005310499 0 0.03498941
0.08142531 0.005237771 0.029778738 0 0.01035574 0 0.1145339
0.02240626 0.02332324 0.1943492 0.07594188 0.01676076 0
0.03403637 0.0190015 0.007934504 0.02298032 0.06445546
0.01266788 1.298366 0.03865021 0 0.02185264 0.07521381
0.02885146 0 0.04653221 0 0.02702748 0.01898754 0
0.05274796 0.03268893 0.7737466 0.0350998 0.04474692 0
0.02838981 0.1406187 0 0.01632788 0.02479984 0.2844265
0.03578198 0.005226006 0.06661721 0.09678392 0.01078553
0.18540144 0.03123857 0.02818665 0.02112903 0.01408215
0.01955609 0.03995455 0.005151662 0.01803923 0.03382131
0.03386536 0.008620105 0 0.1010742 0.03354894 0.0055864
0.02088321 1.04343 0.02761056 0.09924308 0.02061137 0.03494619
0.078359495 0.06599236 0.4268473 0.0328123 0 0.042788
0.0522166 0.01681334 0.01295436 0.08600149 0.01527501
0.342104
AC090044.1 0 0.7267791 0.1344465 0.02792478 0.2300611
2.101945 0 0.3853836 0.09026027 0.293002327 0.4265756
0.05743163 0 0.1096332 0.0953154 0.8229645 0.116624
0.880405702 0.1443778 0.1873044 0.034159343 0.9607362
0.3718956 1.433931 2.262433 0.2067972 0.2313628
2.565753 0.05649052 0 0.1652195 1.414995 0.02821757
0.09206901 0.2855842 0.128164 0.486689 0 0.3618002 0
0.118034 0 0 0 0 0.04302038 0.2040881 0
0.238123422 0.1167407 0.03374575 0.882573 0.1881674 0
1.48191 0.1437438 0.3004488 1.078809 0.02931189 0
0.331885948 0.039866195 0.7860905 0.2052446 0.160913
0.2575123 0.1196615 0.295451 0 0.0612623 0 0
0.4337698 0.6541882 0.1819809 0 0.06808933 0.2141594
0.06637508 0 0 0.6386392 0.187875 0.7214118 0.2155421
0.1415205 0.6918066 0.0722477 0.6008623 0.2637824
0.028170265 0 0 0 0 0.2090494 0.2550227 0.03078206 0
0.1777833 0.5490009 0.02553944 0 0 0.5127869 0.6615359
0 0.6186341 0 0 0.08851619 0.1408324 0.1539041
1.579084 0 0 0.06541643 0.06947813 0.06478612 0 0
0.1572454 1.212233 0.3799033 0 0.2835439 0.05347543 0
1.651726 0.29762 0.06315907 0 0.05948863 0.08836824
0.06535096 0.3353346 0.1390267 0.03147515 0.1930297
0.2301424 0.03982717 0.2154659 0.09096352 0.09233494

0.08277389 0.0472815 0.0379477 0.2749169 0.07728799
0.1998864 0.1891134 0.02756952 0.02648938 0.3486295
1.580836 0.1835794 0.1306021 0.7615018 0.08212192
0.1956081 0.220307942 0.0968895 0.657678092 0.05318662
0.2160762 0.0232057 0.04663227 0.1912503 0.3583032 0
2.364947 0.033653427 0.3118903 0.02974687 0 0.06947945
0.08483582 0 0.08436687 0 0 0.0295239 0.03273734
0.02723469 0.2405811 0.03611909 0.8198332 0 0.1446507 0
0.1253474 0.06161012 0.02962889 0 0 0.2814096 0.4482123
0.08711678 0.05957434 0.03413867 0 0.5988406 0 0 0
0.02266523 0.03522433 0.4377102 0.02482537 0.0548649
0.1495654 0.1553528 0.162804 0.1612066 0.1402681
0.07672897 0.1251355 0.02315824 0.6125012 0.060613852
0.02727909 0.092453733 0 0.1465869 0.7902136 0.02369029
0.09054944 0 0 0.02921388 0.03298046 0.1019806 0
0.04612759 0.03503108 0.3682727 0.1684341 0 0.1320338
0.02984782 0.2846231 0.6737316 0.03876627 0 0.4879168
0.05614848 0.1460165 4.183767 0.1703611 0 0.3678942 0
0.4899673 0.4239652 0.02088217 0.07143441 0.02418793
0.121206719 0.1097815 0.1975703 0.05356618 0 0.1866699
0.2113849 0.1725185 0.2668155 0 0 0.2427322 0.03022499
0.2931547 0.1191742 0.2040003 0.026717302 0 0.05835627
0.3884465 0.655136 0 0.139424215 0.6883866 0 0.06707338
0.1077528 0.02132892 0.1701017 0.09218586 0.09827832
0.02571043 0.6897448 0.02358796 1.211826 0.6499901
0.02491473 0 0.5450515 0.2242037 0.3286948 0.09478567 0
0.5803778 0.3546981 0.07478432 0.1325285 0 0.04888273 0
0.040538071 0.07698616 0.05894297 0.02278701 0.183867
0.2498979 0.2278887 0 1.071569 0.3200575 0.1492283 0
0.3498488 0.07788359 0.069799648 0.02737525 0.2413595
0.06012265 0 0.08100102 0.038376824 0 0.1067662 0.02964451
0.6063716 0.1732541 0.06011483 0.3816596 0.055925 0
0.05270356 0.3216677 0.0979514 0.08180362 0 0 0.130604
1.268605 0.3415528 0.051447979 0.2027679 0 0.198303 0
0.3897892 0.04985473 0.03483118 0 0.1243739 0.135956
0.3370182 0.7977219 0.2035541 0.3460008 0.3402968 0 0
0.1092313 0.08416905 0.03196035 0 0.1229691 0 0.09365646
0.3207306 0.1945953 0.08688467 0.1073551 0 0.05445933 0
0 0.2648094 0.265564553 0.09299096 0.4533009 0.08728678
0.1555261 0.08441888 0.2003962 0.1729424 0 0 0.3618034
0.1067479 0.6139093 0.1416669 0.02573502 0.073443214
0.3140178 0.2847535 0.1691451 0.042556668 0.07352305
0.3028195 0 0.04451922 0 0.07874159 0.5569018
AC092171.2 1.121711 2.790498 2.31023 0.9690697 1.318906

2.244833 2.016206 1.333642 2.200882 1.069433709
3.790037 2.396918 1.511371 3.368817 2.914288
1.612374 2.79024 1.086837956 1.186365 1.918502 0.78332175
1.927159 2.933663 2.561058 2.11196 3.825336 2.702255
2.69269 1.563124476 0.3244963 2.172197 1.954882 2.152581
2.364624 2.281182 3.350438 1.646168 8.019502
4.514726 1.269544003 3.836277 2.946803 2.725740957
2.953395 2.130668 6.113122 4.602026 3.934088
1.588096816 1.781117 3.735055 2.622591 11.25721
1.290165 1.246017 3.252297 1.627285 3.536704
4.194296 2.974753574 2.278109659 1.602876087 1.108275
4.941874 2.767471 3.530958 2.54278 1.626026
1.620906123 11.65697 1.339705 2.474622 4.168878
3.358659 5.522373 3.588528 2.449638 2.073523
4.671075 2.644179335 4.071714 4.012701 2.221136
4.760704 7.164537 3.171705 2.621025 1.783758
2.40360322 4.098131 1.490069235 7.070857 1.412521
1.837217 3.636741 6.620006 1.693762 3.877612
6.788092 2.822066 3.787999 1.147884 3.346524
2.133523 6.958517 3.907105 3.113852 2.731529
1.873207244 1.924396 2.644154 6.724503 0.933287
2.630813 1.582728 1.656978 2.780168 9.839088
5.48365 4.156372 3.616537 4.745308 5.098395 0.8130935
4.334437 2.002634 0.7357601 1.512858 2.251886
2.998796 3.447016 7.631823 0.8366836 0.9051289
1.318759 5.029629 4.63334 3.132479 6.157672 2.972986
2.916449 6.700851 3.483489 6.333289 8.128179
3.350275 2.250902 4.241028 3.391043 2.152145
4.964417 4.206289 5.64108 3.473194 2.331807 0.7437198
1.880791 4.132746 3.063291 1.779275 1.603158735
1.866322 4.414761438 4.919231 3.462946 3.508571
4.519759 1.78767 5.29501 5.138723 2.781673 4.141566074
6.770639 9.304355 9.810597 2.843086 5.16613 3.481538
2.906275 4.912746 3.069884 3.777797 1.561483
5.458389 2.4029 6.046309 2.847841 1.330247 10.4708
4.90694 1.881768 1.379841 1.784642 2.111122 4.364853
10.3993 2.058483 6.912077 3.296911 4.096903 3.823233
4.255168 2.422813 4.042214 5.343922 1.729021
4.001022 4.431859 3.878699 2.675622 4.201437
4.441198 11.12946 12.54904 1.54823 2.459394 1.276942
3.735058 3.015104 3.229342353 3.77831 1.856260129
2.120251399 0.8707742 2.692776 1.318291 2.247154
2.112653 3.34235 3.175398 4.033538 2.421706 3.019373
2.143748 1.869039 1.638958 2.079276 1.970756

0.6358213	10.66833	3.922779	2.897362	2.945438
2.126496	1.995303	4.862698	7.812847	2.817808
3.196917	4.623669	4.085594	3.540237	3.562114
4.601803	4.887539	2.440756	7.746798	3.465035747
2.874082	4.608231	3.357484	1.395832	1.141494
4.565745	3.064653	5.200685	2.465204	4.176261
9.785363	2.555234	2.216371	2.635664	1.440828
1.078290904 5.387811	3.229502	5.196114	2.73279 3.526435
1.774442253 3.482415	1.775358	2.671144	2.392678
2.63137 2.314398	4.721158	2.93727 2.833897	4.50143
1.903986	2.116586	1.939514	3.66032 2.294104	6.764501
4.648597	5.668151	1.133878	2.726883	2.561959
3.401376	3.837587	3.954837	2.051652	5.365612
3.351812457 0.923397645 1.785017	5.199328	4.333583
3.507986	3.357654	2.88	1.225325	3.477393	2.774281
4.709343	3.402005	1.698101	3.929157	1.915389511
6.122683	2.545968	0.5330961	4.78055 1.667593
5.761292662 3.803771	3.125662	3.081716	5.149711
2.313145	2.426191	3.362223	1.940758	3.601538
3.553186	3.26345 1.623227	1.61325 4.032491	2.465871
1.906775	3.403968	3.568041	11.019093694	3.988454
1.630018	2.743578	1.526482	3.020726	3.639311
3.935067	2.58119 4.31613 3.383703	3.967195	2.550841
2.97355 2.680025	2.623457	12.90923	3.304946	5.571848
5.318538	2.174258	5.683718	2.472072	2.170414
4.209442	5.268219	11.66584	0.872223023 2.634129
5.258909	2.730773	4.649918	4.161097	3.944845
8.78956588	1.986699	2.249547	3.77192 1.525074	3.67165
2.983925	2.895043	4.006227	9.438277	2.488998
2.551233	3.574198	2.983317	2.899555	1.599948331
4.128499	4.911199	1.198098	1.56141477	2.51774 6.866921
2.530752	2.147268	1.503753	3.105727	2.412213
AC008808.2	0.63834 8.130504	1.127366	0.7561284	1.641089
5.947506	1.420232	0.9180492	1.103745	2.431301384
0.02191751	0	3.469878	0	0.7492896	4.048139	1.578933
3.152904568 0.6305427	2.399515	3.282058478 5.187576	0
7.973134	2.343399	0.02006995	1.495442	6.299276
1.430927695 11.81938	2.958416	2.35745 0.5175866	0.8846068
1.018575	0.05597321	0.1328451	0.3898572	0.6912911
0.225968254 0.06185896	0.02250889	0.23918513	0.04924412
0.3681014	0.3569784	0.1782632	0.6586825	0.779969294
0.1223623	0.08842691	2.264502	0.02739285	0.1919635
1.642882	0.03138869	1.012233	3.79537 0.1024113	0.5192368
2.232151732 0.104464844 3.356811	1.523824	0.1054136

3.252794 0.1393598 6.090342 0.076962048 0.3745721
0.06542265 0.1665569 1.03561 0.3809391 0.3576452 0.877388
1.030873 0.3325514 0 0 0.3995703 1.561916 0.2735029
2.074165 0.9682348 0.173058 1.182261 0.8203733
0.34988701 3.888067 0.147633971 0.02851064 1.003357
0.8190675 0.470238 0.7825576 1.63352 0.2419827 0.02142762
1.164652 3.005064 0 0.3529934 0.7963287 2.344888
1.73348 0.03683456 3.464619 0.338690838 0.306365 0.2319466
0.963592 1.523533 2.758541 1.446875 0.2027238
0.142847 0.08091537 0.8299607 0.1869887 0.1056941
0.6592698 0.8235417 0.3792356 0.2208965 1.857486
0.8874666 0.1656952 1.821138 0.8744104 0.2022792
0.1453489 0.1039221 0.231559 0.5137344 2.538485
0.582886 0.08247705 1.152128 0.0670069 0.3478753
0.2688591 0.1787697 0.2419532 1.470097 1.238958
0.1657294 2.357635 0.02250271 2.294654 0.259574
1.348532 0.06941241 1.522573 2.7616 0.1603497 1.779586
5.321144 0 1.025137 3.540724197 4.005788 2.532832973
0.0464565 0.3145573 1.054004 1.303409 0.7159281
1.077988 0.6915693 3.414715 0.587899936 1.062455 0 0
0.182063 1.012712 0.7353745 0.04912751 0.5318362
0.2568065 0.2063041 0.02859483 0.1665193 0.3035333
0.03154866 3.202528 0.103642 0.5896189 0.19868 0.919684
0.05381411 0.02587972 0.3122839 0.8342181 0.8491295
1.435487 0.2282796 0.2861976 0 0.03928807 0.502142
0.1501933 0.1042208 0 0.0791889 1.138383 0.9999226
0.04336804 0.09584483 0.1632996 1.438365 0.5451122
0.2816158 0.1960302 0.1787196 1.284288 0.08091138
1.497991 0.132359774 0.3335816 0.457610772 0.482420919
0.03658233 0.8906086 0.08277029 0.1845469 0 0.06230547
0.1786205 0.3456862 0.4156888 0.03330364 0.02014536
0.4895732 1.143724 1.103406 0.4763837 0.8361173
1.199263 1.472522 1.637507 0.8465218 1.012484
0.1598163 0 0.6376994 5.647651 1.971653 0.2548896
0.986978 0 0.23776 1.203532 0.1276785 0.3743715
0.6549447 1.93211817 0.2636975 0.5177105 0.7953965
0.3576292 0.9130749 0.5011569 0.09041305 0.6603175
0.06633449 0.5277798 0.6360522 0.8184121 1.280298
0.3469805 0.1425492 0.070009661 0.2488268 0.611664
0.8967038 1.634961 0.1642533 1.88761765 7.62532 0.08992525
2.90001 0.517649 0.2049301 1.634352 0.3422137 0.5150544
0.3593135 3.3655 0.06180959 4.207476 4.226522 0.9357693
0.1956334 2.164009 0.6609378 0.8038871 0.02069793
0.1307424 1.425763 1.295592 0.2395113 0.09260692

0.1292888 0.1707889 0.303979198 0.212450838 0.537956
0.05148445 1.094698 0 0.3880468 4.080569 0.1643411
1.999583 0.5870721 0.1862075 0.2866114 1.145924
0.6122554 0.548706196 0.1434675 0.527046 1.680476
0.9165714 0.212254 0.201124234 0.5215913 0.2098265
0.4142938 0.06968982 2.799621 2.415369 2.416898 0 0
0.5754321 0.8173515 0.1026682 0.1786309 2.000459
0.3166031 0.08555827 4.814409 0.04972224 0.224689329
0.1574312 0.812785 0.6495381 0.1875614 0.7595014
0.5007816 0.1825423 0.128241 0.03621197 0.8075165
0.4047624 3.600029 0 0.436538 1.656031 0 0.03724441
0.06360626 0.1960493 0.05583232 0.7318109 0.4296353
0.3294312 0.4908323 0.5291648 0.1214083 0.177077778
0.3516398 0.7297569 0.880011 0.4438477 0.1761079
1.927509 0.440725145 0.02030602 0.3045702 0 0.1164396
0.4055519 2.660586 0 0.3269959 0.2585811 1.685448
0.09324022 0.04468555 1.082731 0.04495713 0.898098213
1.508555 2.600269 0.3939782 0.371716419 0.2247686
2.116011 0.3028175 0.1361005 0.09680835 1.960167
2.574039
MIAT 0.09218506 0.04347117 0.622607 0.9397167 0.4273477
0.1688133 0.6204539 0.178588 0.6842952 1.098020172
0.1929465 0.01240483 1.714139 0.055648 1.082901
0.04397087 1.246903 0.263373691 0.08107996 0.1726142
0.33349376 0.05156367 0.09478571 0.1223768 0.01470334
0.07047423 0.8795207 0.07688691 0.217187758 0.05157778
0.3925489 0.09961245 0.5424371 0.4374978 0.1089754
0.4761396 0.003942054 0.2279703 0.08009999 0.200044264
0.2467871 0.1313596 0.224757101 0.1794118 0.5747611
0.09292103 1.091755 0.5171487 1.042804145 0.3298146
0.08163509 2.642603 0.1923762 0.3664643 0.9356248
0.07606675 1.84116 0.9721911 0.06964281 0.376636608
0.296776136 1.408730286 0.09684324 0.1019622 0.1876829
0.7333396 0.194707 0.3024849 0.120532624 0.635147
0.8045839 0.3432236 0.2398496 0.1518976 0.2623716
1.214997 0.8275038 0.4851839 1.250148 0.129889371
0.4874491 0.5338341 3.799613 0.2363269 0.2793336
0.3203469 0.2481761 1.040854 0.67486681 0.6167563
0.116823961 3.925562 1.352761 1.129972 1.077064
2.206057 1.888739 0.8895976 0.7746703 1.217279
2.113893 0.08274513 1.108577 0.377176 0.2723354
1.416488 0.9290763 0.210649 0.551651473 0.213809
0.2041897 0.2494342 0.2393439 1.638534 0.6306026
0.1483857 0.2430273 0.0850385 0.1987056 0.2562977

0.6677862	0.9374038	0.4980661	0.3563595	0.9079742	
4.174357	0.3545949	0.1406765	0.4421501	0.1776572	
0.4210806	0.109265	0.2929602	0.7545698	0.2060842	
0.473209	0.8960596	0.09381809	0.1570439	0.1739821	
1.148419	0.8190889	0.5550415	0.4002037	0.7306382	
0.2246745	0.1114716	1.123904	0.09237163	0.3120868	
0.7317482	0.2465297	0.1087089	4.403885	0.7871795	
0.1625727	0.2414704	1.454892	0.4079685	1.161874	
0.517724621	3.186554	0.495897233	0.261925	0.1073432	
0.8831618	0.9568634	0.2726376	3.599541	0.1725685	
0.3805028	0.424504122	0.2802431	0.03084056	0.06163376	
0.2361113	0.2748592	0.2018493	0.6936758	0.4179238	
0.1536799	0.07269731	0.2206163	2.376531	1.458452	
1.43079	0.2980817	0.3758917	0.2645287	1.184587	0.1559472
0.2448554	0.06399635	1.025132	1.708938	0.8178011	
0.4654983	0.9897541	0.2740808	0.08111087	0.0932669	
0.2017788	0.1436095	0.2010224	0.05495424	1.199406	0.2039
0.2821726	0.1790944	0.3270721	1.122601	0.7247907	
0.3821213	0.1002802	1.216725	1.063982	0.05000254	
0.4711901	1.054399	0.13354014	0.1178419	0.973173643	
0.063623932	0.5472961	0.9051579	0.2681276	0.4354926	
0.2705517	0.04005855	0.1438679	0.08975673	0.6931191	
0.0971783	0.1335074	0.205808	0.2227242	0.4620336	
0.9317064	1.273346	1.509869	0.2496897	1.248953	
0.6832568	0.438149	0.5519625	1.124236	1.806107	
0.2780506	1.081826	1.623474	1.199885	0.2807319	
0.3198404	1.407943	1.099636	0.3301878	0.1859896	
0.307612749	1.37767	0.2401926	0.9001399	0.1238103	0.8483201
0.1591496	0.9315701	1.302444	0.1487246	0.1634827	
0.3439307	0.4752667	0.5836895	0.4049885	0.2943385	
0.466276713	0.1564642	0.5533397	0.7575128	0.3062568	
0.06634138	0.081309585	1.497681	0.5585948	0.05215458	
0.8913888	0.3058981	2.006047	0.3594027	0.2695884	
0.7074873	0.3986501	0.4921612	0.1609738	0.2547879	
0.4498858	0.8553046	0.4901738	2.6574	1.337561	0.6633267
0.1541086	0.6581272	0.686725	0.3316727	0.477469	
0.1907604	0.1087508	0.240541209	1.346663783	1.144039	
0.09930391	0.3583097	0.1302619	0.6249244	0.290412	
1.022475	0.2945746	1.164152	0.153794	0.5669949	
0.9974568	0.8966294	0.447764287	0.1229875	1.511827	
0.04545126	0.4805049	0.2204453	2.141910449	0.2080894	
0.7517806	0.2074575	0.1488945	0.6299315	0.8829382	
0.1751761	0.1751515	0.1542719	0.3244327	3.660858	
0.07108692	0.06007465	0.9757158	0.3170778	0.08039726	

0.5631373 0.32829 0.181132214 3.319771 0.1440418 0.4475951
0.5032326 0.9636718 0.05276455 0.6936477 1.397439
0.03940038 2.786203 0.4713385 0.4451145 0.3312126
0.8536258 0.3528084 0.1314978 0.8694182 0.470291
1.170787 0.09664499 1.256199 0.3231523 0.1990025
0.4922425 0.1770376 0.07445532 0.152634074 0.4683967
0.02981465 0.3434748 0.8968662 0.111049 0.2351432
0.40266788 0.7110232 0.3238556 0.2300107 0.582591
0.4813747 0.1973759 0.4445169 0.09330089 0.1871783
0.9346382 0.3489262 0.5834406 2.099096 1.252905
0.671014356 0.359476 0.424942 0.1022956 0.027575833
0.2096222 2.042149 0.5354052 0.2653972 0.1986948
0.1904855 0.1022439
LINC01355 0.06404975 0.2909191 0.6161571 0.1147204 0.3045435
0.648653 0.1896744 0.1663191 0.07416131 0.244085632
0.9873307 0.6396601 0.6758799 0.4103591 0.3654691
0.8501669 0.2555273 0.1647688 0.1021505 0.1424968
0.11538504 0.507647 0.2716122 0.6736979 0.2577239
1.06982 0.2872571 0.8440927 0.06704358 0.1090012 0.2111678
0.2463977 0.904201 0.7312586 0.6083441 1.591268
0.1194093 0.5177314 0.9496092 0.337736203 3.318916
1.964422 0.202772323 0.9007133 1.830239 0.9013536
2.583616 0.5134664 0.49184581 0.1939689 0.2033299
2.573297 0.5840655 0.9871383 1.186374 0.725036
0.5329049 0.8097891 0.433508 0.260495876 1.033193146
0.254765781 0.3349019 0.231095 0.4627431 1.009083
0.1820714 0.6392521 0.168923497 1.784112 0.3852013
1.384928 1.631529 1.020266 1.106882 0.212366
0.7231385 1.911682 1.375527 0.57252827 0.1345683
0.8191626 1.106285 1.13334 0.2108306 0.447027 0.8567414
0.8310609 1.6547811 1.88739 0.68922972 1.001245 0.3670437
1.44407 0.4239073 0.3462525 0.5742851 0.7868538 0.6002094
1.195639 2.098359 1.776661 0.6763978 1.210055
1.068459 1.139428 0.2694932 0.2292304 0.257236646
0.7542697 1.947502 8.614211 0.131137 2.165336
0.9640629 0.4809774 0.8868529 0.454573 0.9305527
0.5751469 1.253837 2.345677 2.107621 0.3616897
1.631539 2.23134 0.3685866 0.04906838 0.6427169 0.202545
0.703451 1.37029 1.477206 0.9896031 0.01789829 1.214346
0.5432257 2.091883 0.8752404 2.311147 2.623337
0.8008731 0.7515434 1.812353 1.43829 1.221563 3.564832
1.569768 1.460564 1.243489 0.6733258 0.3624348
0.2635946 0.6736818 1.349582 2.245779 1.302001
2.060317 2.264145 1.017888 0.14481079 1.943028

1.768495556	0.8740035	0.2465785	0.5253934	0.1830599	
0.5911404	1.900851	3.876941	0.7953179	0.322594431	
0.882677	0.3557548	1.233245	0.6554432	0.2865624	
1.433447	1.414622	1.284674	2.783421	0.962233	
0.4154286	1.158635	0.9444265	1.104638	0.5197581	
0.9604842	1.027837	0.3553257	0.4829097	0.8380629	
0.3976226	0.3427149	0.5047924	1.149078	0.3392844	
2.478737	0.5601892	1.118869	1.396155	2.119011	
0.3558217	1.692776	0.9341435	0.9269931	0.4180463	
0.645507	1.047071	0.4858527	2.102762	0.3460566	
1.273255	0.8889105	0.9732196	0.6444444	0.1970643	
1.334053	0.5368049	0.379053692	0.4457807	1.910342518	
0.089639155	0.7723548	2.645949	1.846998	1.176605	
0.938443	0.2018748	0.3120422	0.358296	1.402726	
0.737941	0.5621864	1.685398	1.367245	0.4420854	
0.9211338	1.793	0.5422555	1.744935	0.6016998	0.5273253
0.4762059	1.425391	0.3383142	0.448787	1.193926	
0.5987829	1.366289	1.988784	2.963392	0.7604213	
1.035362	0.7568405	0.6586688	1.589899	0.210241558	
0.7566838	0.479264	1.3008	0.1682053	1.438318	0.5293166
0.5260421	1.043353	1.758721	0.2322648	1.156741	
0.7257052	0.3746823	0.2466086	2.249759	1.085402653	
2.907219	2.559878	0.9701529	1.153468	0.3319059	
0.222748368	1.194055	0.7650683	0.2051319	0.5336624	
0.8606557	1.263035	1.363382	1.314418	0.4811733	
0.4646677	1.074557	1.26673	0.4725194	1.032643	0.2985313
0.4704533	0.9978278	0.486123	1.261216	0.3598458	
1.205398	1.960553	0.2548858	1.130042	0.5810632	
0.7095628	1.366175428	6.402468497	0.4802679	0.7443841	
0.1997086	0.2215726	0.7528609	0.1421653	1.569953	
0.4424445	1.294404	0.6909071	0.8033726	1.061965	
1.084312	1.325424029	1.487008	0.6224738	0.05488787	
1.609421	0.5398233	0.17517709	0.2871179	1.116034	
0.6468156	0.3700217	0.2715237	0.1372018	0.2351896	
2.379196	2.934254	0.6712007	2.869865	0.2020958	
2.68852	1.773621	0.7197282	1.788488	0.8146982	2.211283
0.126814922	2.141143	0.6619119	0.7105705	1.930962	
0.3613255	0.2821866	0.5660131	0.4959328	0.7607506	
1.829507	0.9307157	1.365498	1.377214	1.14066	0.1420195
1.619296	1.105538	0.7445812	1.175661	0.7381937	
0.6979525	1.902847	1.487943	5.640279	0.4457145	
0.3908348	0.386022864	0.2891232	0.4908019	0.3828259	
0.9012993	0.8421007	3.763287	0.470338043	0.991142	
2.584863	1.019992	1.509095	0.1656976	0.1097688	

1.563058 0.5888993 0.186729 1.973002 1.19543 3.208618
1.626352 0.7565169 0.754297154 3.74591 0.7467955 0.09265074
0.56334429 0.6544348 3.845776 0.7675149 1.296512
0.5387988 0.3091085 0.8219347
LINC02241 0 0 0 0 0.009894457 0.08403413 0 0.009040671 0
0 0 0 0.01164897 0 0 0.01117706 0.01504728 0 0 0
0 0 0 0.09927439 0 0 0 0 0 0.02131726 0 0
0.0237582 0 0.04960866 0 0 0 0.120164426 0.01218337 0
0 0 0 0.4940084 0.008777413 0.05675693 0.007680902 0 0
0.02135119 0.267059 0 0.1176626 0 0.02215147 0.03866447
0.01512774 0 0 0.092586443 0.06010338 0 0 0.005111574 0
0 0 0 0 0.03827139 0.1119333 0 0 0 0 0 0 0
0 0.01647994 0.008080122 0 0 0 0.02328511 0 0.8269396
0.03403424 0 0 0.01933192 0.01273568 0 0 0.2339841 0
0.006330381 0 0 0 0 0.6243442 0.07005363 0.005690262 0
0 0 0.04022653 4.079475 0 0 0.1081067 0.4541674
0.02395635 0 0 0.0891622 0 0.03122532 0.1136151
4.004018 0 0.3335493 0 0.02069882 0 0 0 0
0.1374098 2.225882 0 0 0 0.01435021 3.013296
0.3320725 0.4058162 0 0.1747444 0 0.05559593 1.488053
0 0 0 0.006647998 0 0.01394294 0 0 0 0 0 0 0
0 0.4795241 0 0.08334039 0 0 0 0 0 0.007050244 0
0 0 0 0.09657911 0 0.02350024 0 0.408645 0.02172524
0 1.099846 0.02276058 0 0 0.0140557 0.07587727 0
0.01175312 0 0.006221131 0.006521803 0.09056771 0 0.2828898
0.01153229 0 0.03960933 0.0192767 0.1648555 0.1998493 0
1.021408 0 0 0.006158008 2.654707 0 0 18.19366
0.02562452 0.03539441 0.1447313 0 0.02100561 0.008319801
0.1520227 0 0 0.2270854 0 0.015641269 0.09855032
0.071572438 0 0 0.039467 0 1.690147 0 0 0.01507715
0 0 0.0787114 0.005951561 0.07231754 0.04223642 0 0
0.02555327 0.09242595 0.1073447 0.00755891 0 0
0.007869114 0.02897798 0.07535845 0 0.8352647 0
0.006781019 0.01702495 0.4074016 0.01367541 0.005388597 0
0.01248328 0 0 0.1529477 0.006911316 0 0.1541432 0
0.02671081 0.183603 0.00653242 0 0.00626365 0.07799492
0.06189375 0 0.1052836 0 0.1207683 0 0 0.006037728 0
0 0 0 0 0.01390269 0 0.1024202 0.04162962 0.03804075
0 0 0.006086822 0 0 0.01285838 0 0 0.1880296 0
0.02445923 0.1480638 0 0.07488741 0 0.006839737 0.07002582
2.863396 0.024492235 0.041843034 0 0 4.580627 0.09489296
0 0.009801026 0.00971029 0 1.032377 0 0.007697626
0.01504628 0.5024422 0 0.4944885 0 0 0.02707836
0.03483689 8.051182694 0.184913 0 0.0076497 0 0

0.03102499	0	0	0.02229547	0	0.01509193	0.02022091
0.08443697	0	0.02338359	0.438127	0	0	0	0.01744127
0.06003046	0.03837873	0	0.07737249	0.006432451	0	0.1136591
0	0.01403326	0.1848045	0.1543878	0.1342349	0.3472184	0
0	0.0440126	0.253682	0	0	0.01801663	0	0	0.2175106
0.1379397	3.14919	0.209257021	0.006925686	0	0.02107966
0.5245054	0	0.05314826	0.006852834	0.005999027	0.1889568
0.06006442	0.00573331	0.1198126	0	0	0.00743113	0	0
0.1285482	1.518172	0.03655683	0.01328173	0.473796435	0	0
0	0	0.123321	0.0781419	0.01118271	0	0	0.03047864	0
AC012254.3	0.02745133	0.01968729	0.06312698	0.01311159	0.0450088
0.06255197	0.02120695	0.03290001	0.05297515	0.00859838
0.04712731	0.09438103	0.03179393	0.05147632	0	0.1525296
0.05475873	0.041337896	0.008473755	0.02931516	0.024058375
0.0136696	0.0174617	0.09031766	0.03290265	0.09170367	0
0.05426595	0	0	0.03878795	0.04306206	0.1788623	0.02161469
0.04469701	0	0.01428226	0.03698276	0.06370384	0.042514481
0.4655347	0.06049863	0.085716479	0.09264966	0.1484056
0.1969447	0.2235939	0.08851921	0.230601219	0.2082915
0.00792236	0.2719478	0.0515379	0.02063812	0.06690428
0.02530963	0.03022938	0.2110565	0.07569584	0.065127387
0.140248072	0.084233107	0.02050527	0.04015374	0.009444232
0.04185358	0.01872832	0.09017049	0.08963758	0.05273516
0.05861358	0.01989627	0.1018345	0.05759298	0.1548707	0
0.1172239	0.3966327	0.2077683	0.020168395	0.02386559
0.1499309	0.07351117	0.08468155	0.03614428	0.01993454
0.1129828	0.06784527	0.37616572	0.1238544	0.052907403
0.0842929	0.01758776	0.08110648	0.07109378	0.03505553
0.0332615	0.01445318	0.05759241	0.07651876	0.1976265
0.08394116	0.04743818	0.05762467	0.134548	0.1863675
0.06930188	0.0170864	0.024275243	0.1372396	0.2119622
0.1598029	0.09635059	0.05295937	0.3645807	0.01089748
0.007678783	0.06524446	0.02534932	0.01435948	0.03551013
0.5020561	0.08853949	0	0.1187436	0.4326823	0.03766267
0.007422498	0.06992562	0.0317596	0.1383911	0.03906638
0.02793184	0.1313905	0	0.1924394	0.1305551	0.1773432
0.06042245	0.1260692	0.2103766	0.07226302	0.1388085
0.1661913	0.09716255	0.01665015	0.1870856	0.1290824
0.1935425	0.04022277	0.1712473	0.05177912	0.01243762
0.08866694	0.1053197	0.3878842	0.1287761	0.04875678
0.1156767	0.1377665	0.020688341	0.2047174	0.294764729
0.09364815	0.01690913	0.1688853	0.04379072	0.06414157
0.1121566	0.3802042	0.08498104	0.015801373	0.1317983
0.006983558	0.01425334	0.03262285	0.006638861	0.07906057

0.2310757 0.301773 0.02760943 0.08317456 0.02305686
0.1406632 0.01255118 0.3815793 0.1229664 0.03714205
0.3395909 0.03560036 0.06474011 0.02169596 0 0.02098365
0.02360192 0.1561547 0.03069065 0.2999637 0.0349651 0
0.04751871 0.08435251 0.2018422 0.07843388 0.01298492
0.2873356 0 0.07114123 0.0815942 0.06440211 0.158008
0.160475 0.216585 0.04541503 0.08561855 0.1381025
0.02203319 0.07067802 0.06470756 0.11384066 0.05123367
0.166405073 0.028814098 0.1769845 0.3949683 0.1668504
0.2125795 0.01598189 0.066985 0.04115058 0 0.1436495
0.08951237 0.08663359 0.2220511 0.1825224 0.02965698
0.05238031 0.2169795 0.04204355 0.2929795 0.08252316
0.1183131 0.03401649 0.05727318 0.04613617 0.05713288
0.1098973 0.1499813 0.04404108 0.1295537 0.1626337
0.02556172 0.0870911 0.01960971 0.06708153 0.2612114
0.021341429 0.05798922 0.03313058 0.03772658 0 0.08764755
0.1488781 0.1458055 0.1565981 0.03565831 0.02239813
0.08547796 0.1561077 0.1126191 0.01865205 0.02873542
0.075267826 0.1719741 0.1849511 0.1498189 0.0659159
0.04414743 0.03273209 0.1101886 0.5462375 0.02361984
0.04426925 0.07010234 0.1331138 0.4544846 0.1153621
0.09053911 0.0558375 0.03876359 0.1564727 0.06216861
0.01169827 0.063098 0.05816348 0.1118503 0 0.1223886
0.03514049 0.05961072 0.09841132 0.05267049 0.1617887
0.08108284 0.04590404 0.155977474 0.142754519 0.1807375
0.03228823 0.06954505 0.04316576 0 0.008916758 0.1943526
0.09719679 0.1277357 0.06506268 0.0910407 0.1642653
0.009142217 0.09831958 0.0706946 0 0.02117215 0.1478118
0.03169383 0.018019161 0 0.2569172 0.02087859 0.07117777
0.1084645 0.02822586 0.01120009 0.5186073 0.02028393
0.08042458 0.1853592 0.02759481 0.2784685 0.2317571
0.08509548 0.1763031 0.03080953 0.09354921 0.042273859
0.1322307 0.009102399 0.08147096 0.03780907 0.0985485
0.07022523 0.02453155 0.04595757 0.0681305 0.3319459
0.08901054 0.1248521 0.06371684 0.1714841 0.07989018
0.03435744 0.1101147 0 0.0724535 0.2551091 0.09834681
0.07217244 2.112392 0.007329123 0 0.1696849 0.10198791
0.05670753 0.008527894 0 0.08521134 0.2662519 0.1726897
0.105987478 0.02728892 0.04093066 0.2117505 0.135617 0
0.0564555 0.04060103 0.08788883 0.07581876 0.08493919
0.05012157 0.04804171 0.1496637 0.1389594 0.094830905
0.479185 0.1397783 0.01323651 0.029972599 0.06041254
0.276468 0.03052134 0.4755481 0.04553468 0.03697173
0.04902814

AC007406.3 0.02454158 0.035201 0.04514859 0.3282105 0.2092377
0.0994163 0.1516727 0.07353181 0.03788797 0.061495843
0.02106599 0.0241077 0.227391 0 0.04000993 0.1090896
0.09790898 0.01847811 0.03030226 0.0524157 0.086033075
0.04888267 0.09366493 0.1468078 0.1008517 0.02893531
0.3302001 0.02910837 0.189701272 0.08353074 0.3467656
0.02199864 0.40272 0.07729442 0.4395521 0.1344964 0.1149155
0.683296 0.5884983 0.456097071 0.7134679 0.1514407
0.434241322 0.291874 0.6444217 0.8126274 0.3141187
0.2901671 0.387327657 0.0392028 0.4532875 0.2546995
0.9083368 0.4612638 0.406726 0.6637223 0.324302
0.2767383 0.5782909 0.715324707 1.170234339 0.619171835
0.5010688 1.206159 0.3546134 0.5155265 2.243587
0.2604411 0.628761736 0.1714378 0.9222531 1.378518
0.5462422 1.155627 0.9548617 0.7323397 0.6287912
0.4994239 1.829594 0.721224524 0.6187415 0.3619045
0.4206031 0.1135584 0.7884399 0.5227653 0.1515105
0.1213078 1.30313703 0.1107262 0.413869727 1.507163
0.2096469 0.2382454 0.3531004 0.1880385 0.2854646
0.1938178 0.1853561 0.2736321 0.2458139 0.2680132
0.5937385 0.8389855 0.5191305 0.5831462 0.3717367
0.3055059 0.510000512 0.1472312 0.2675222 0.1477907
0.3230166 0.1217465 0.6518728 0.9547536 0.384432
0.4569087 0.1178443 0.2695859 1.99366 0.7128633 0.5314666
0.1366882 0.5307859 0.1666299 0.2020233 0.252158
0.1591259 0.329361 0.2209323 0.09779133 0.2996539
0.4945835 0.7406626 0.1720415 0.4435234 0.3038787
0.6752234 0.5152288 0.6352837 0.5685098 0.458198
0.4263472 0.961291 0.02977057 0.09557435 0.8497642
0.1189565 0.3236336 0.3515529 2.256672 0.07783492
0.5121968 0.2690177 0.3852998 0.3508609 0.2760619
0.1378871 0.2873819 0.480881626 0.2033534 0.363909488
1.127453 0.4081541 0.3701544 0.2544688 0.5390221
0.3175166 0.06042735 0.1620765 1.299636227 0.1963803
0.6368191 0.407761 0.3208143 0.2017956 0.1236907
0.365946 1.533521 0.1357561 0.3222195 0.1374194
1.337559 0.3029615 1.243241 0.09559335 0.03320511
0.5161122 0.4774026 1.168081 0.2198243 0.1989941
0.3189107 0.2954028 1.417506 0.3449292 0.4875793
0.4126177 0.01433017 0.3964976 0.3117006 1.010507
0.1702918 0.4411252 0.1617386 0.5175061 0.3957365
0.2605196 0.1496968 0.7690805 1.108594 0.2391872
0.3112758 0.1295348 0.4831203 0.1969775 0.2430246
0.8355934 0.432539222 0.6870461 0.297533395 0.669757421

0.1406442 0.1391012 0.05966592 0.7095079 0.1714544
0.583877 0.159418 0.3184119 0.9275005 0.1760536
0.4453417 0.3382097 0 0.3535125 0.07284383 0.2494033
0.488632 0.1929971 0.4057679 0.146454 0.5169846
0.4736223 0.6363652 0.4290467 0.04912427 0.1430227
0.6562146 0.3309183 1.592422 0.9140906 0.9788082
0.2016082 0.5997112 0.5482735 0.839489607 0.1843291
1.457248 0.6857964 0.3609215 0.4701432 0.1014076
2.447694 0.4479976 0.2550292 0.5873709 0.6113407
0.2410596 0.8725764 0.5836247 0.411033 0.560747423
0.3160323 0.2817021 0.1979962 0.08839356 0.4604595
0.907140577 0.4203048 0.1642199 0.2111622 0.5540761
0.2417338 0.3808133 0.1644591 0.2268951 0.8849688
0.2795459 0.2574355 0.1526041 0.2728421 2.300825
0.3384589 1.934339 0.9881814 0.8278446 0.09946892
0.450292 0.4415638 1.109902 0.1988143 1.691171
0.1346212 0.4001241 0.252327941 0.612590506 0.3985638
0.5938106 0.3634759 0.4939563 1.037325 0.2710348
0.50546 0.03066856 1.222571 0.5458679 0.9641667 0.3181831
0.2778879 0.336942473 0.1608758 1.063796 0.1640424
0.616673 0.6006891 1.627028237 0.5681703 0.1008372
0.08710579 0.5157635 0.3636287 0.3911272 0.1602068
0.2464904 0.1329819 0.1769842 0.7978712 0.3618245
1.922938 0.5138353 0.2916233 0.3015255 0.0734502
0.01194762 0.280747799 0.2269721 0.04882545 0.4162018
0.6309602 1.233438 0.1988084 1.213531 0.3081467
0.5220762 1.164215 0.6012388 0.3627591 0.8259645
0.4034403 0.6938146 0.1474352 0.2684806 1.05458 0.3886421
0.1878211 0.6447641 0.4903703 1.741478 0.301404
0.29918 0.4550966 0.340396134 0.3717759 0.5489256 0.2286004
1.340754 0.3914273 0.2964196 0.758025307 0.9660967
0.1610055 0.5740236 0.3917051 0.1948978 0.4374189
0.2177848 0.2659391 0.8246838 0.09112311 0.2389806
0.472444 0.297333 0.2376577 1.233151263 0.2416576
0.1955931 0.02366698 0.696685731 1.033887 0.05649448
0.03638158 0.3550633 0.360558 1.15685 0.3603908
AC093515.1 0 0 0 0 0 0 0 0 0 0 0 0 0 0 0
0 0 0.024582114 0 0 0 0 0.04153531 0 0 0 0 0
0 0 0 0 0 0 0.02657966 0 0 0.05864606 0.02525487
0.086680406 0.02636536 0 0.067963215 0 0 0.5285223 0
0.2807418 0 0 0 0 0.9807257 0 0.1909704 0.0401352
0 0.06693732 0.0327371 0 0.074133625 0 0 1.451779 0
0 0.04454816 0.03299757 0.032802551 0.04561402 0.02788426
0.09465257 0.06459434 0.1826582 0.101623 0 0.07604584 0

0.04942084	0	0.05676792	0	0.06994299	0	0.06877972
0.09483465	0.03359334	0.04034506	2.01322627	0	0.062924142
0.03645517	0	0.1102423	0	0.1000619	0.06329401	0	0
0.06618599	0	0	0	0.3133006	0.1684436	0.2462797	0	0
0.028871174	0	0.07908772	0.02621486	0	0.03599197	0.1156281
0	0.1461212	0	0.1205944	0	0	0	0.3610365	0
2.887263	0	0.08958637	0	0.06048316	0.4532701	0.02351315
0	0.1993203	0.03289814	0	0.04161331	0.09316353	0	0	0
0	0.3093994	0.101593	0.06874976	0.1848927	0	0.08476407
0.1395645	0	0.1913519	0.03017315	0.06158225	0	0.0648947
0	0	0	0	0	0	0.049210358	0	0	0.05940169	0	0	0
0	0	0	0	0.601375395	0	0.0332229	2.64449	0	0.1263323
0	0.09422547	0.1511185	0	0.1318955	0	0.03041717	0
0.161359	0.02543427	0	0.02692561	0	0.2239915	0	0	0
0	0.02857213	0	0.3243224	0	0	0	0	0	2.162061
0.05062751	0	5.565465	0	0.06127608	0	0.2082077	0	0
0.1253272	0.1142601	0	0.3362368	0.1026112	0	0.1523338
0	0	0	0.02846946	0	0.2359712	0	0	0.03262763	0
0.3416922	0	0	0.0782492	0	0	0	0	0	0	0
0.04329626	0.2427402	0	0	0.1087194	0	0.8086409
0.02327964	0.02934886	0.07368552	0.06080243	0	0.02332233	0
0.02701439	0	0	0.0630449	0.08973839	0	0.0416966	0	0
0	0	0.03551824	0.02710964	0.0337569	0.2976463	0	0	0
0.1363555	0	0	0	0.1050114	0	0	0	0	0	0	0
0.02573954	0	0.0861444	0.1593816	0	0	0	0.05565223	0
0	0	0.1468417	0	0	0.04051232	0.07202654	0.02784105
0.177618	0	0.02729743	4.80554162	0.045275104	0.1146431
0.02194356	0.05089953	0.04107052	0	0.1696789	0.04202701	0
0.3574575	0.07142836	0	0	0	0.116934017	0.7643538	0
0.06714821	0.1171976	0.0904663	0	0	0	0	0.0356436
0.06449983	0.03356974	0	0	0	0	0	0	0.0913627
0.04410156	0.06747084	0	0	0.09536614	0.603328718	0.02516246
0.04330287	0	0	2.310638	0	0	0.05465853	0.04630249
0.0911058	0.14115	0.1484898	0.07578008	0.2576216	0	0
1.523924	0	0.03133484	0	0	0.06866924	0	0	0.1592041
0.03104779	0.032345828	0.1199	0.04056978	0	0	0.02814758
0.09858449	0.059319363	0.1038573	0.1557757	0.03249553
0.02481428	0	0.04476266	0	0	0.03005774	0.2155101	0
0.2856863	0	0	0	0.029226	0.08673492	0	0	0.7800878
0	0.04839976	0	0.03094604	0.2198571	0
AC016717.2	0	0.008106981	0	0.005399189	0.003706813	0.005724027	0
0.003386954	0.01745161	0.007081412	0.06064513	0.02220854
0.04800527	0.002649664	0	0.01465562	0	0.008511216	0	0
0.026418508	0.03658831	0.1438103	0.01014319	0.03871115

0.02665583 0 0.03128446 0.005461153 0 0 0.02786523
0.0109116 0.08010595 0 0 0 0.005076347 0.002186038
0.107542604 2.658716 0.02740384 0.029414152 0.1380738
0.002910074 0.3597486 0.03945994 0.01518796 0 0.004514308
0.006524659 0.04799345 0.07579524 0.004249261 0.4793761 0
0.04564298 0.002897015 0.002833692 0.003831236 0.006416935
0.023124099 0.02251685 0.3703798 0 0.02872464 0
0.005712475 0.002839357 0.03948306 0.002413635 0.02867562
0.03634295 0.0131756 0.9566076 0.01022193 0.006582454
0.2898505 0.04277821 0 0.004913776 0.006173967 0.01513549 0
0 0.01094506 0.008723422 0.2374716 0.41629476 0.009562819
0.021786612 0.05048839 0.04345452 0.02385617 0.07481526
0.01443543 0.02191468 0.01190328 0.02371584 0.008593493
0.007076533 0.06172487 0 0.01016963 0.07581762 0.03624014
0.008153615 0 0.007497183 0.0376757 0.4877602 0.1089184
2.07307 0.09346291 0.3603116 0.0381433 0.01264811
0.006716715 0.05636808 0.01182611 0.01169811 0.161136
0.0989614 0 0.01358254 0.08497491 0.06979058 0.01833895
0.01570609 0.01046257 0 0.0321741 0.06901191 0.005695263 0
0.02881606 0 0.3742671 0.05598274 0.01112437 0.02117634
0.06248973 0.01099224 0.01190183 0.6561695 0.02056897
0.2237814 0.04590617 0.007471723 0.002760539 1.209244 0 0
0.03651195 0.004130416 0.2484625 0.002525161 0.0167312
0.2461102 0.0756407 0.008519202 0.0624445 0.005780022 0 0
0.02467721 0.02254059 0.1584762 0.01924363 0 0.0163306
0.003253405 0.003015162 0.0948995 0.008804021 0.09179684
0.005467601 0.04883424 0.03534295 0.5068761 0.2472805
0.005708375 0.1107695 0.04739185 0.005168419 0.03491769
0.008806265 0 0.009322621 0.002443298 0.1914613 0.002978042
0.002864338 0.05184483 0.01457847 2.668557 0.001805433
0.04210955 0.03167603 0 0.006522538 0.02547256 0.005541076
0.006921021 0.01604108 0.01972021 0.06129486 15.43851
0.04079937 0.007955997 0.007229526 0.3484302 0.02098518
0.02805202 0.08678561 0.03461585 0.003024331 0.008955178
0.005921282 0.005859771 0.005274347 0.143005657 0.005932644
0.01012223 0.03942862 0.009160922 0.00291792 0.2303401 0
0.05366011 0.01594173 0.279334 0.02948807 0 0.04402564
0.007911627 0.004070792 0.004793236 0.009573156 0.3491457
0.05926437 0.03115018 0.003747683 0.1120606 0.01474024
0.0189983 0.02823193 0 0.02882154 0.01209038 0.005080818 0
0.042104 0 0.006056276 0.03452918 0 0.005858757
0.007959747 0.01091421 0.02071378 0.0158328 0.05413826 0
0.006671209 0 0.02447275 0 0.03754535 0.1198006 0.01288199
0 0.2090477 0.002582863 1.630754 0.01974533 0.01341164

0.03845308 0.06362778 0.026957337 0.02117467 0.01990565
0.006484238 0.06510546 0.01030974 0 0.4611938 0.0617561
0.01491315 0.02299319 0.004560676 0 0.004654593 0.01926881
0.04330495 0.0383216 0.005418655 0.006355236 0.009163291
0.01205868 0.01052012 0.09975276 0.01927916 0.0358737
0.01907938 0.04725679 0.021409863 0.007837939 0.01240424 0 0
0.00711005 0.002684286 0 0.02910254 0.007063135 0.02165882
1.343722 0.02595422 0.008455303 0.02258791 0.003373899
0.002646469 0 0.008718427 0.04057803 0.005220451 0.278252502
0.01924304 0.05934859 0.008597543 0.1203258 0.002791522
0.01162306 0 0.05136158 0 0.005095053 0.04523176
0.001893867 0.007908267 0.01145216 0.1255645 0.02840847 0
0.5585749 0.019894686 0.3201716 0.01499302 0.02396338
0.01037954 0.02898646 0.1807367 0.03030537 0.0425807 0
0.09988971 0.2280658 0.04498597 0.006559451 0.1375134
0.0281981 0 0.1896198 0.02815947 0.008136942 0.003089729
0.05399733 0.003962629 0.005208725 0.01509021 1.457294
0.01074987 0.142791009 0.01037842 0.007023362 0 0.04912456
0.01461856 0.0768004 0.012836567 0.006742342 0.08427369
0.002812781 0.5004607 0.004080547 0.003874613 0.16719 0.01948775
0.2341592 0.006995381 0.01031972 0.1335351 0.01369548
0.4527982 0.071000345 0 0.01751795 0.01090126 0.057597607
0.007107752 0.0422857 0.004189436 0.002151921 0 0 0.02467566
ARLNC1 0.005724436 0.03831704 0.05265556 0.1203032 0.05255992
0.01159465 0 0.006860651 0.1237256 0.695692927 0.04422363
0.02249292 0.27404 0.01610156 0.07465998 0 0.1484451
0.012930308 0.01413628 0.061131 0.033446007 0.05130947 0
0.1369743 0.03528617 0.01349859 0.1359188 0.02263219
0.055310852 0 0.01617693 0.03591898 0.04973096 0.0721171
0.04660341 0 0 0.02570676 0.1505541 0.12158481 0.1525513
0.03027792 0.017874486 0.07728106 0.04126275 0.05054641
0.1132349 0.05537682 0.058287697 0.05029327 0.01982463
0.1566259 0.2026618 0.06025144 0.1004513 0.1477791
0.008404991 0.1291009 0.08035939 0.03880296 0.019497319
0.062453897 0.1254285 0.04689033 0.02363291 0.01939498
0.03905423 0.1735688 0.057514323 0.1559558 0.06844718
0.0663836 0.1359077 0.04803953 0.0267271 0.06211693
0.07555644 0.1071735 0.1776366 0.072899185 0.02986019
0.01250605 0.03065862 0.05297601 0.05426769 0.03879826
0.06479095 0.05659119 0.43143081 0.0516548 0.005516399
0.03195932 0.04401097 0.6427002 0.02635593 0.05848112
0.127623 0.03013926 0.0816664 0.06382593 0.1935131
0.1200293 0.01318973 0.06866567 0.05316127 0.211589
0.04129012 0.01425213 0.045559122 0.03052652 0.1109347

0.1424878 0 0.1640769 0.3243782 0.05453892 0.03202518
0.0090703 0.09303538 0.2096071 0.118479 0.1539617
0.1160542 0.05313856 0.1980931 1.554687 0.1151892 0
0.08483848 0.02119309 0.04122676 0.03910332 0.05824636
0.121132 0.01279725 0.06566639 0.1197887 0.0677993
0.08189944 0.3605383 0.02729683 0.2832978 0.06679797
0.1747864 0.1404786 0.01388824 0.2526557 0.1174586
0.07567402 0.04473423 0.03703285 0.02159504 0.02593621
0.06826984 0.01254992 0 0.02557497 0.05422543 0 0.09576163
0.46592786 0.1011906 0.064394407 0.05207595 0.1198863
0.1090613 0.09131694 0.04815167 0.1247363 0.02818991
0.09451267 0.065901313 0.04275281 0.01165027 0.1070011
0.09070471 0.071989 0.03297305 0.04405604 0.152354
0.1324203 0.04047031 0 0.01599957 0.1151613 0.03536483
0.01337855 0.007745243 0.339912 0.0494917 0.03436434
0.09048531 0.005802033 0.1050174 0.01968687 0.145281
0.04022815 0.1478491 0.1049945 0.09359215 0 0.06566955
0.02244812 0.04673098 0.08664796 0.05326062 0.02069325
0.05934042 0.1361189 0.1235541 0.1317978 0.1277712
0.06376171 0.1136449 0.1153647 0.1101858 0.03675671
0.0861636 0.05997106 0.100891733 0.03739321 0.084488189
0.018025841 0.004100738 0.09983379 0.02319558 0.2186911
0.03999252 0.02095261 0.02288305 0.03229174 0.1131644
0.05226493 0.0948451 0.1166184 0 0.1484251 0.06796462
0.1034213 0.04091433 0.01714951 0.01147239 0.1138702
0.1134955 0.07763077 0.1044543 0.05718691 0.06302153
0.07506158 0.05714428 0.08233408 0.01937947 0.1918942
0.1037779 0.004089219 0.05595414 0.06157535 0.065271585
0.02687225 0.03316191 0.04720282 0.01603554 0.1023522
0.03548069 0.1621593 0.08708122 0.01982891 0.01245518
0.06654573 0.03551258 0.08350047 0.007779035 0.1038649
0.047086876 0.1235241 0.02285506 0.3423012 0.1374546
6.702026 0.013651268 0.04901901 0.06854586 0.02626908
0.08440212 0.02923693 0.07772312 0.1399042 0.07698081
0.06545119 0.06054783 0.06928616 0.04152821 0.03299938
0.07318334 0.09649088 0.0485154 0.3512347 0.1287323
0.05104347 0.02931144 0.1136516 0.08208698 0.04881509
0.0934279 0.0821257 0.2010201 0.37172612 0.16670443
0.1005047 0.03462725 0.3123562 0.07201097 0.07068514
0.1413155 0.3021209 0.07153581 0.1190821 0.2713508
0.05841461 0.03996336 0.2287715 0.157186717 0.06432859
0.08271175 0.03532028 0 0.0317238 0.052605583 0.1091409
0.06272198 0.02322037 0.04374697 0.03392722 0.08828921
0.02802671 0.03285429 0.05075777 0.02064119 0.0801692

0.02685366 0.02402861 0.1546509 0.1892799 0.07672601
0.02569889 0.005573671 0.050373612 0.02205928 0.2277752
0.1262052 0.0210249 0.1526598 0.02928818 0.04092458
0.2012545 0.04871066 0.1437662 0.1814892 0.07810634
0.02657379 0.3387754 0.04759865 0.05731655 0.06679927
0.2281605 0.1098818 0.006258588 0.08203318 0.03210697
0.0211017 0.1895147 0.1186347 0.1034315 0.107755705
0.02627831 0.0284532 0.01066441 0.355383 0.004935253
0.1498059 0.046803398 0.07283925 0.3550678 0.0512784
0.06091133 0.041328 0.09418148 0.01693309 0.02819613
0.03162102 0.07084959 0.006967912 0.8815976 0.04161256
0.03527665 0.100673342 0.07174074 0.06589986 0.02208172
0.225007066 0.2303606 0.08565436 0.08486167 0.06538432
0.08138882 0.2081626 0.07270296
AP000873.1 0.2870691 0.511786 0.5102123 0.2463384 0.4531237
0.4631881 0.6615488 0.414024 0.1802787 0.841253249
0.3758861 0.2580961 1.059432 0.1414198 0.4124801
0.821862 0.3882258 0.406642175 0.5466991 0.3221477
0.198995904 0.5863301 0.3528276 0.5413697 0.3832319
0.4245138 0.4467023 0.765617 0.084622269 0.4416182
0.2612471 0.61496 1.244606 0.551674 1.11704 0.8212841
0.4050309 0.4151478 1.35117 0.391842271 1.210193 1.424015
0.187376816 0.3409432 0.4910073 0.8198725 1.115319
0.4602248 1.065165702 0.6878477 0.3201552 1.441441
1.753876 0.870599 0.872776 0.5084105 0.8072676
0.7431814 1.853933 0.712394281 0.469541925 0.477753485
0.2519873 0.4839561 0.9909676 1.088016 0.3385869
0.7671437 0.16132132 1.736839 0.1994668 0.6735596
2.276648 1.084316 0.8026722 0.5191736 0.6459821
1.037678 2.515192 0.834102039 0.3891625 0.9247863
0.411729 0.9355784 1.132643 0.8385636 0.8410711
0.5591696 1.7501674 1.668384 0.459497964 0.7334376
0.1828828 1.729182 0.3976294 0.9245502 0.4386173
0.4047543 0.2694885 0.5474308 1.730082 0.9181883
0.9304989 1.406561 1.973091 0.6790011 0.2807618
0.3513011 0.391539918 1.09624 1.567584 3.473118 0.3159346
1.534058 1.154538 1.062331 0.5226304 0.3006686
2.24291 1.318377 1.072489 2.706232 3.053424 0.3071288
1.78662 1.425259 0.9301161 0.1894452 1.063621 0.1621207
0.742877 0.7256639 1.604042 0.4951798 0.4568439
1.09768 0.5322219 0.618182 0.6265061 0.8044194 0.636381
0.8607092 0.4314979 1.705908 2.254863 1.743137
1.623249 0.9858791 0.6689319 0.5228107 1.974038
0.2982697 0.6922102 0.6866537 0.8249157 1.833335

0.5521417 3.001598 13.14924 0.9604513 0.300684189
1.198745 0.80606914 1.265916 1.036975 0.4094181
0.3803201 0.5047701 1.550567 1.162083 0.73906
0.750586378 2.486589 0.5446287 2.339368 0.898168
0.6448299 2.017871 1.465081 1.829832 1.326166
0.8059056 0.3051392 0.7524836 0.431576 0.5470723
0.5571946 0.8228997 1.20782 0.5132087 1.105752 0.6409124
0.3205497 0.6694599 0.7278933 1.362582 0.2579985
1.319501 0.3222621 0.3920688 0.9882278 0.7615022
0.6105649 1.616595 1.611047 0.535692 0.3986743
3.424137 0.661123 1.310428 1.076673 0.9774116
0.8219623 0.7673888 0.5323048 0.5363833 0.4269736
1.102397 0.5301233 0.332929602 1.339425 0.589885364
0.275784497 0.4182588 1.366172 1.936058 0.9595431
0.6005306 0.2908806 1.001666 0.2580009 0.5714577
0.8948149 0.3646876 0.9504029 1.634575 0.4765907
0.9449137 0.6043442 2.056741 3.035557 0.6630779
0.5226436 0.3617527 0.4771118 1.074738 0.4860696
1.12002 0.7372425 0.4336672 1.307774 0.9224262 2.654965
1.455428 0.3510456 0.7847259 1.409073 0.322784938
0.4202654 1.10867 5.224622 0.4225198 1.174445 0.4071248
0.8154938 1.162053 2.688198 0.5663768 1.676649
2.787036 0.7185976 0.2644768 1.684143 0.613674768
1.080394 1.257836 0.4571986 0.96581 0.5946893
0.406109214 1.166606 0.5346366 0.28468 0.8967372 0.4792579
1.207992 0.9129518 1.014182 0.6376205 0.4156677
1.260266 0.4487836 0.3045251 0.655209 0.2162188
0.5525687 0.8489668 0.3720218 0.7257167 0.5979297
0.4226252 1.116339 0.6929028 0.4411699 0.4434613
1.468585 0.695100456 0.775938857 1.029378 0.3891565
0.34514 0.4284484 1.002874 0.2402269 1.434281 0.3972465
1.289169 1.18513 0.5511201 0.4609877 1.354655
1.219857546 1.663097 0.5925472 0.1400982 1.257538
0.5123192 1.277513832 0.7487554 0.3687385 0.4391379
0.7011739 0.8410791 1.355775 0.7702372 1.089077
0.9826881 1.048273 1.10486 0.5640964 1.456877 1.393349
1.493182 0.8966999 0.7426686 0.7153525 2.804440375
0.6374882 3.884932 2.500221 0.9113937 0.7386092
0.3319197 1.298622 1.28702 0.2070121 0.6924468 0.9185468
0.7634594 3.263797 0.7934561 0.412664 0.545632
2.143337 0.6302686 1.181458 0.5638765 2.080143
1.364493 0.9192065 4.598608 0.8897249 1.318699
2.694637157 0.8264203 0.7557582 1.273548 1.123677
0.6753628 1.60142 1.113876988 0.6732813 0.8531534 1.670755

0.9282086 0.3091212 0.893904 1.223368 0.2444508
0.7794275 1.023736 0.6810866 1.072902 0.8665474
0.6082462 0.886250037 0.9887022 1.128871 0.3096839
0.651661457 3.395886 2.363308 0.4616293 1.100375
0.6594948 0.2817794 1.008032
LINC01605 0 0.0365116 0.1317081 0.01823734 0.03756253
0.05800373 0 0.05720214 0.1031588 0.047839079 2.154987
1.687856 0.07370538 2.935604 0.03112468 0.2545902
0.01904143 0.179682011 0.03535928 0 0.022309058 0.09506747
1.299411 0.05710264 0.1307582 1.560656 0.01511003
0.02264409 0.036893283 0 0 0.1026796 0.06449995 0.9019375
0.1165698 0 0 0.8487688 0.7310141 0.185852207 0.2004252
1.396882 0.168903326 0.1104595 0.1671037 0.7234745
0.06664375 2.441965 0.068038057 0.03812099 0.8925773
0.1891306 0.2355389 0.1004719 0.3722375 0.105612
0.7288155 0.06849861 1.71332 0.064705593 0.140887984 0.05207227
0.3422576 0.1675535 0.07881777 0.5174713 0.2865483
0.2894333 0.009590759 0 0.04076378 0.06918593 0.09442996
0.4005399 0.460542 0.03452754 0.1111708 0.0932433
0.8742002 2.823990479 0.116184 0.4170876 0.01022491
0.1766795 0.2614262 0.08318283 0.08839773 0.07077616
0.65402664 0.06460243 0.110385979 0 0.09785357 0.136988 0
0.06826384 0.04626451 0.08041359 1.618165 0.1741622
0.1553699 0.1501155 0 0.9389241 0.07879883 0.1224117
0.06885305 0.08318111 0.067530473 0.0381782 0.2138928
0.1609579 0.05025645 0.1262793 0.8451789 0.03031533
0.1922521 0.2268767 0.02115552 0.8388693 0.8791815
1.550695 0.8356777 0.4784984 1.385546 0.3148045
0.4889383 0.1238904 0.3271535 0.4329196 0.1237453
0.4455775 0.01942566 0.02885612 0.08535986 0.1703357
0.09987636 0.1541703 0.1785926 1.540608 0.6827795
0.2412312 0.03712949 0.2311609 0.198215 0.09263695
1.313509 0.4080567 0.5972977 0.8205586 0.00882198
0.1170346 0.09514942 0.03794762 0.4045989 0.2697605
0.1364715 0.1582406 0.6167777 0.7664957 0.129492434
0.02109246 0.097618568 1.172325 0.176396 0.0833545
0.03045498 0.1605899 0.1820027 0.09401575 0.03152078
0.021978651 0.06110755 1.855309 0.07930175 0.1436913
0.1292789 0.06872997 0.01836633 1.038319 0.06720501
1.600382 2.095275 0.02667997 0.1047471 0.3538342
0.0446186 0.03874657 0.7951194 0.03301181 0.1227943
0.1106513 3.618502 0 0.08207173 0.3258011 0.1402627
1.090486 1.390936 0.3455811 0.2056299 0.6413968
0.7580224 2.470251 1.914482 0.08141316 0.2300459

0.4837687 0.8349773 0.4389372 1.025633 0.3043772
0.2923948 0.3263745 0.1465719 0.01670359 1.052203
1.792237 0.09000388 0.385965276 0.08017042 0.171077877
0.060117719 0.1367631 0.116534 0.09283107 0.4730941
1.600541 0.05823228 0.08585656 1.529279 0.3552122
0.261462 0.03765673 0.6177163 0 0.1787538 0.04857167
0.2910252 0.3118921 1.294038 0.04782675 0.2784957
0.212916 0.846421 0.8984125 0.07946801 0.2197359
0.1529836 0.1225164 0.1458766 0.1723525 0.3022145
0.3461083 0.1091032 0.9097327 0.1579685 0.079158658
0.0358485 0.5806385 0.1136961 0.08021984 1.036251
0.1577748 0.8112225 0.4937196 0.04959832 0.1142325
0.05548392 0.009869791 0.08702537 0.07783124 0.2797833
0.095968127 0.04651198 2.78216 0.3352329 0.7945999 0.09210918
0.068292216 0.1839179 0.1613691 0.06570723 0.1671337
0.09054282 0.314759 0.2332962 1.716933 0.1259338
0.3261981 0.1771577 0.0593572 0.02358337 0.7484896
0.05851006 0.161803 0.2562433 0 0.06964131 0.04887807
0.3079682 0.05264752 0.1628025 0.1125186 0.04027886
0.5985886 0.061986918 0.542736048 0.008379791 0.01924747
0.200906 6.424353 0.226674 0.07441568 0.02457558
0.2624358 0.01045129 0.4246436 0.01948177 0.218962
0.4577836 0.444456826 1.15316 0.0788145 1.884738 0.9594491
0.1146184 2.042670753 0.0909986 0.1133075 0.2323257
0.2032177 0.06600426 0.3631575 0.3271502 2.511022
1.043903 0.04302508 0.009548969 0.02558836 0.9349369
0.3481473 0.2860418 0.6610435 0.1428466 0.7993128
0.13440024 0.08092656 0.1772516 0.3237733 0.4031892
0.5580884 0.01627977 0.3070957 0.3356005 0.2301435
0.09767036 0.7015769 0.499275 0.3766599 0.2761843
0.8413514 4.444367 0.05569532 0.6301492 0.2382025 0 0
0.07361716 8.568273 0.4179671 0.4305676 3.32244
0.633633952 0.07011233 1.696227 0.4801509 0.4977977
0.04937847 0.1825519 0.112734072 0.1746024 0.9792271
0.1045109 0.1958893 0.01378324 0.143964 4.291972
0.08463286 1.450059 0.07876315 0.01161929 1.169399
0.09252096 0.5210242 0.203850761 0.7006944 0.1267971
0.0184111 0.389105518 0.7922809 0.0439484 0.1556615
0.0508812 0.6876446 0.128563 0.1288125
AC114760.2 0.4080677 0.05382145 0.5759779 0.1411385 0.6090769
0.2351328 0.2174097 0.2360994 0.3910259 0.546523832
0.2234525 0.05529011 0.6736207 0.02638629 0.8258514
0.1094594 0.3929635 0.291354599 0.1563685 0.1803202
0.07399261 0.1611589 0.06265496 0.2525236 0.05300615

0.1161336	0.3118301	0.1168282	0.108768187	0	0.1590588
0.119826	0.176575	0.4210207	0.1031007	0.06169251	0
0.1642942	0.3646363	0.217925316	0.3636248	0.1860662	
0.146458261	0.2351953	0.4419381	0.09318652	2.063019	
0.1436847	0.422669075	0.5394609	0.07309672	2.110883	
0.1886989	0.1904204	0.7956328	0.08648989	0.8574068	
1.565085	0.09876617	0.133534797	0.647008844	0.307037268	
0.4414542	0.1152617	0.1645949	0.3909352	0.1439995	
1.137737	0.11310129	0.3981011	0.3425105	0.2447674	
0.8908701	0.4133028	0.9690482	0.39445	1.026956	0.2348091
0.3035254	0.110273425	0.09786624	0.8761263	0.655652	
0.3689593	0.3334901	0.05449739	0.5284648	0.5129587	
0.84358351	0.6507379	0.169499051	1.18625	0.5649602	1.86491
0.07288404	0.8553311	1.227567	0.2815255	0.3129262	
0.5491194	4.615826	0.2335777	0.1783201	0.5823182	
0.5807836	1.443569	0.3044872	0.1926832	0.087102864	
0.590921	0.2684291	0.6496589	0.02469421	4.855343	
0.697688	0.106133	0.1731872	0.3567329	0.1559259	
0.2502583	0.2912347	0.386024	0.3695597	0.1828687	
0.3787272	4.565441	0.7336104	0.1369693	0.5800086	
0.1367493	0.2837519	0.2082607	0.3293049	0.3261138	
0.3145707	0.2421229	0.3613752	0.3333165	0.2864909	
0.6369874	0.4888616	0.3926384	0.3393402	0.1333379	
0.6707023	0.6998463	0.3561938	0.8180577	0.03100253	
0.2955221	0.3901323	0.1526136	0.1593852	0.8600512	
0.7043879	0.3313764	0.2640377	2.132674	0.05929481	
0.6590997	0.296930297	1.484654	0.237432938	0.256017	
0.0953421	0.4244676	0.3928176	0.5129027	1.1019	0.1039409
0.2090904	0.218690235	0.630547	0.05011591	0.07306129	
0.4347765	0.8099185	0.2634172	0.5888521	0.4721996	
0.1485996	0.2131726	0.1733414	0.6292597	0.4117515	
0.8779999	0.5700234	0.1332707	0.4873992	0.437962	
0.2534141	0.222423	0.07844124	0.2151205	0.2661588	
0.8189062	0.3236245	0.5661114	0.2724264	0.11503	0.1786228
0.5419203	0.13795	0.06317848	0.05324759	0.7746132	0.03391088
0.4538034	0.298746	0.8583102	0.3689702	0.9197939	
0.8163197	0.2560726	0.5536549	0.4124291	0.06023472	
0.2229471	0.3169434	0.10941314	0.27575	0.318939261	
0.118158731	0.3729626	3.82828	0.1254381	0.225197	0.09830606
0.1630955	0.08437364	0.06350136	1.186316	0.1651796	
0.5939514	0.2782299	0.6992327	1.084402	0.2565635	
0.7706074	0.1436742	0.3003572	0.7896115	0.7277546	
0.3138577	0.1834856	0.587846	0.445144	1.422392	
0.7380389	0.4565178	1.081502	0.4843083	0.707546	

1.288243 0.6935712 0.1719269 0.6112578 0.175030719
0.4161464 0.1018946 0.5285801 0.02956286 0.4043459
0.4215415 1.253947 0.4388134 0.2680791 0.1913513
0.2336812 2.116877 0.525962 0.1529739 0.2454919
0.102884254 0.4260697 0.3300591 0.3338947 0.6532324
1.810365 0.067112673 1.52877 0.5005244 0.1452878 0.5121911
0.4106721 0.6823289 0.9484988 0.1774007 1.13858 0.2862179
0.4030743 0.1531214 0.08111615 0.2638428 0.4959329
0.5605044 0.7554523 0.7436303 0.6729783 0.234165
0.6722299 0.543251 0.5039703 2.768633 0.1543743
0.1411799 0.159905236 0.604910464 0.8090941 0.2884542
0.115171 0.09735604 0.200483 0.1919672 0.8060434
0.2461802 0.4390755 0.2770659 0.6174354 0.5332723
0.5717184 0.43678017 0.2635447 0.6244665 0.0144702
0.9597146 0.1559613 0.018472912 0.3257693 0.4625329
0.1070217 0.3264453 0.8131196 0.5932009 0.1607498
0.2086287 0.0901104 0.5708063 0.5771184 0.1367335
0.1082858 0.2185857 0.1308575 0.4400692 0.8001625
0.5274771 0.2476479 0.1735174 0.2146271 0.3579537
0.3100893 0.4329862 0.02999734 0.6371155 0.329804
0.2195164 1.806233 0.3345906 0.4959847 0.1796337
1.628484 0.2574061 0.2888254 0.4105001 0.7799237
0.3106171 0.2076881 0.4957148 0.374882 0.4214464
0.3831978 0.4545798 0.1338137 0.188201031 0.1356495
0.1049117 0.8847333 0.4542569 0.2668909 0.2195275
0.134222627 0.4252366 0.5706745 0.1190454 0.2673694
0.2133365 0.2315086 0.1248703 0.09010201 0.07125067
0.8243393 0.3083023 0.7510851 0.2471967 0.4831205
0.433066003 0.4282706 1.040469 0.08141899 0.153636786
0.1681061 6.081597 0.3024692 0.2571551 0.1467127
0.1326596 0.2736527
CARMN 0.1287577 10.02612 0.2847511 0.05931777 0.5282222
12.0918 0.1213381 1.155715 1.072846 0.856937423 2.375067
0.5220719 0.3398527 0.2756919 0.2977483 17.26491
0.3096656 2.965435775 0.1229003 0.3705668 0.644514445
8.571519 0.6621893 11.0028 12.176 0.7873994 0.1618929
18.21128 0.338815295 0.1450456 1.963822 4.838474
0.08373931 0.3192439 0.4133462 0.2222006 0.01140247
0.2599908 1.660687 0.115564795 0.2175432 0.02093002
0.153974488 0.05400861 0.5632593 0.09743129 0.4962146
0.7086702 1.467254117 0.07585286 0.2371853 0.748698
0.08425153 0.236167 5.594236 0.08082538 0.2654752
14.72318 0.1501668 0.167128325 0.879166086 0.315073235
1.896274 0.5278781 0.07539952 14.07244 0.8921391

1.219199	0.045874089	0.1518222	0.08267131	0.154212	
6.145465	0.8046546	0.7191219	0.2444229	3.036631	
0.5515499	0.1064365	0.03041443	0.2064126	0.4997462	
0.2005201	5.56348	0.9897731	0.1732973	2.192087	0.4355796
0.46299002	5.202589	0.140798206	0.218204	1.021126	
0.4864157	0.4530197	0.4328677	0.6975072	0.1807764	
0.03448486	4.045821	2.383823	0.1260534	0.06732981	
0.2223603	1.918457	2.176046	0.2792759	2.054136	
0.135663669	0.1631336	0.1703294	0.445801	0.1201922	
8.075662	0.8570359	0.0594512	0.0950224	0.2322299	
0.6597597	0.03248167	0.09261025	0.5383623	0.2852721	
0.4051904	0.1641459	0.1922057	0.1620363	0.1876525	
5.893896	0.8992849	0.705668	0.2890205	0.3512222	
0.2705257	0.136777	4.294854	0.826897	0.1258531	
0.3386795	0.1198206	0.2046593	0.1403847	0.2621319	
0.04807298	0.4068182	1.588502	0.1351377	8.462998	
0.04667722	0.3585894	0.1797588	1.219491	0.1042626	
1.54283	7.382681	0.1146939	1.395284	21.15506	0.2462728
1.545943	1.157556916	0.8666322	0.409026093	0.08556494	
0.2137448	0.3262071	0.2185063	0.4779454	0.4091572	
0.5362576	12.72639	0.118793847	2.222355	0.1263765	
0.07396595	0.1873791	2.379807	1.989572	0.1976603	
0.3127802	0.266346	0.269304	0.3078194	0.3164838	
0.5402682	0.7886788	2.933782	0.3756039	0.9933453	
0.07500279	1.061135	0.08756885	0.2276862	0.2205762	
0.1994214	1.503215	1.056519	0.4844043	0.07816184	
0.3081986	0.04988054	1.668639	0.07609575	0.06336439	
0.1745063	0.1069112	0.4555429	0.7089073	0.1690591	
0.5013103	0.5933637	2.22556	0.423809	2.150289	0.3067208
0.5337044	0.4749447	0.05136296	1.674176	0.270766003	
0.4218143	0.45728085	0.141859175	0.08634901	0.9873944	
0.2042517	0.1027726	0.2360488	0.04790786	0.1259373	
1.303267	1.268967	0.04645135	0.09149993	0.07113005	1.4981
0.3341104	0.1618537	0.503189	0.3766866	2.391027	
1.102636	0.8610133	0.5114682	0.5210739	0.8384016	
0.3390562	22.39975	1.524689	0.224639	1.358561	
0.06698157	0.8367118	1.574371	0.06588449	0.07587044	
1.137915	0.168489528	0.2109068	2.079877	0.6341852	
0.06267165	0.2169219	1.040489	0.662868	0.3333935	
0.1439235	0.1351077	0.7900986	0.9526724	0.7142951	
0.3288464	0.1096088	0.064264263	0.08835397	0.2533895	
0.6214533	11.71272	0.1272765	0.158970772	5.41509	0.04116553
2.060681	0.2406694	0.1179311	1.944805	0.1029501	
0.4267355	0.9790375	1.948834	0.09578997	11.17772	

3.641279 0.2591711 0.2518764 4.908257 0.3965858
0.5832133 0.2886912 0.08650282 0.8521084 0.9186439
0.4656683 1.290838 0.09710065 0.1504104 0.16900104
0.411559513 0.1739552 0.05646571 0.1025029 0.2067724
0.06071616 0.4105195 0.2456771 5.336044 0.2909425
0.4828111 0.5013317 1.683012 0.6276997 0.355407875
0.2839111 0.7181507 0.4094311 0.1311198 0.3002655
0.085116467 0.2549382 0.1792662 0.0555625 2.101561
0.4365799 0.672281 4.107254 0.9232881 0.05397998
0.3704321 0.4466937 0.1309606 0.08688357 0.1862605
0.3510095 0.1366745 14.89638 1.084732 0.216160738
0.1034571 0.3124829 0.6937987 0.04863215 0.9937749
0.2709829 0.4232569 0.03669095 0.071229 1.432096
0.2895158 4.460574 0.3433681 0.3746929 0.6362973
0.3346436 0.07592382 0.1569607 0.3900115 0.04193214
0.2028349 0.2605705 0.2709784 0.433973 0.7313908
0.1068134 0.200845028 0.176063 0.4141764 0.1471549
0.5533092 0.28106 1.050566 0.265464403 0.2483665 1.486833
0.02817573 0.1117422 0.07383866 2.207283 0.4565039
0.05037662 0.3421689 5.583233 0.2923344 0.3308107
0.6892593 0.1567636 0.286779363 0.4316117 7.945831
0.3910005 0.106351549 0.1768481 0.3132159 0.2896989
0.1342026 0.04760555 0.1894005 4.512247
AC006033.2 0.1107364 0.0352964 0.1833473 0.07757356 0.2227158
0.1794343 0.2509381 0.06193411 0.1595607 0.129491247
0.05069538 0.05801529 0.2052066 0.03460854 0.07221304
0.1367316 0.103083 0.105610659 0.145845 0 0.189785699
0.08087498 0.04695938 0.09715573 0.1365187 0.02321098
0.1635999 0.06421195 0.256790746 0.2847742 0.4381087
0.07940973 0.5487084 0.1860093 0.1141926 0.5016827
0.03840899 0.2254357 0.02855289 0.104533282 0.03577007
0.05857116 0.322721671 0.09491837 0.2432633 0.05432198
0.1546216 0.2142475 0.413433927 0.165098 0.1107883
0.8358209 0.09503983 0.2109063 0.5181818 0.127054
0.8454704 0.3178502 0.04441466 0.120099894 0.796239586
0.503391735 0.5587965 0.2937181 0.1422298 0.185092
0.1007313 0.2462242 0.267020766 0.1701834 0.2206795
0.1605198 0.04381781 0.3992554 0.2929795 0.7476757
0.1375626 0.2043172 0.2346737 0.101245069 0.1540349
0.1612823 1.059629 0.3795538 0.05443314 0.1501067
0.265862 0.3740324 0.55638791 0.3663868 0.42684822
0.4781037 0.504516 0.3988442 0.3144029 0.6410637
0.8229348 0.0699635 0.07434338 0.2619021 0.3789631
0.05804773 0.2211291 0.1948179 0.3275577 0.5624512

0.3194949	0.09190015	0.267659843	0.3641544	0.08941574	
0.07705944	0.3498033	0.4720289	0.4183292	0.1406705	
0.2230242	0.02339474	0.09271306	0.0540633	0.09931651	
0.3971085	0.0816368	0.3289407	0.1277338	0.5943327	
0.2363326	0.2155806	0.211983	0.2391491	0.2605205	
0.05042898	0.1051631	0.2975543	0.2970679	0.38579	0.2949232
0.1669238	0.3087363	0.174361	0.3268843	0.6607403	
0.1033739	0.2487288	0.2160056	0.0597025	0.05750002	
0.2398411	0.01301224	0.2019178	0.197858	0.1253236	
0.1271032	1.474722	0.2265873	0.02318064	0.09894704	
0.9353266	0.06221737	0.6915858	0.45622103	0.4404337	
0.460523529	0.1611814	0.0636627	0.2754379	0.3415198	
0.3656884	0.4926466	0.027266	0.2498679	0.424942916	
0.1811595	0.05258605	0.03833119	0.1754638	0.14283	0.06378477
0.1065303	0.09396915	0.05197461	0.05964788	0.2397577	
0.7084201	0.2632782	0.1824308	0.3795755	0.2696903	
0.1217673	0.2744526	0.1709387	0.09335436	0.1421675	
0.06771704	0.4696939	0.3036514	0.5801081	0.0806686	
0.1128371	0.1120782	0.03975721	0.114936	0.1447493	
0.09642539	0.04190406	0.1202018	0.2579717	0.3231158	
0.01880825	0.1662674	0.4343704	0.3374029	0.342622	
0.03256898	0.1912864	0.2325262	0.08690496	0.1754519	
0.4331081	0.068883578	0.0688908	0.365790437	0.02324674	
0.105769	0.2896854	0.02991381	0.1524494	0.0171919	
0.03602827	0.09590999	0.03331563	0.4979152	0.05777369	
0.0757191	0.2742499	0.1033376	0.3615599	0.4194654	
0.3751189	0.07537787	0.08293721	0.6140002	0.2741217	
0.2744395	0.4004611	0.3544943	0.528543	0.09605193	
0.6561023	0.2526693	0.6039035	0.3998787	0.1512337	
0.3078218	0.3902461	0.1443207	0.2137953	0.298443829	
0.2079322	0.135428	0.2434975	0.07237993	0.4337039	
0.1830284	0.4008251	0.3032179	0.06393009	0.07228179	
0.2635889	0.3511198	0.6461103	0.1705459	0.1545551	
0.215910552	0.1438846	0.1621106	0.0560563	0.1299952	
0.1899596	0.677797929	0.7981094	0.3535963	0.2964282	
0.2653167	0.07540988	0.4009376	0.1338639	0	0.3376324
0.2042211	0.1250953	0.1530177	0.3222182	0.1761755	
0.5203768	0.3003222	0.5662054	0.4482476	0.1077176	
0.09450257	0.08244505	0.4560217	0.3651309	0.2342819	
0.1682134	0.06789682	0.207735969	0.440212961	0.4471691	
0.1240458	0.1611301	0.03714712	0.1262187	0.3453069	
0.2090673	0.1230067	0.3394756	0.08613989	0.3239339	
0.5742818	0.304866	0.361358595	0.06913363	0.2895271	
0.387177	0.1590028	0.1431923	0.029075124	0.1809667	

0.1415547 0.05240511 0.1732826 0.4812907 0.3871267
0.03614421 0.04943168 0.05091252 0.2462311 0.679413
0.173157 0.1859287 0.2792202 0.1296791 0.04947424
0.4750377 0.03593998 0.064963531 0.07965554 0.3622875
0.5007957 0.2711443 0.2120195 0.1259035 0.3430553
0.4449337 0.06281893 0.3914123 0.2340547 0.2518215
0.2113345 0.1262145 0.368309 0.007391738 0.1292199
0.2390729 0.325927 0.04035644 0.7141013 0.1449219
0.1428707 0.09460826 0.0359989 0.04212276 0.365698518
0.2168922 0.05504132 0.08251916 0.293321 0.1209288
0.06687515 0.442634576 0.111549 0.2377597 0 0.09538619
0.106596 0.3340141 0.218375 0.05818031 0.06116933
0.3715716 0.1887072 0.2196358 0.6529239 0.2014733
1.159212315 0.2048642 0.1568985 0.1139094 0.139714754
0.09283779 0.5353197 0.2736013 0.07307893 0.07697199
0.1391982 0.1347803
TMEM75 0.02013203 0 0.05555466 0 0.02640655 0.04077675
0.06221031 0 0.04144051 0.033630983 0.2304123 0.07910444
0.01036301 0.01887564 0.06564216 0.1889207 0.1204755
0.020210699 0 0.05733043 0.01568332 0.006683265 0.06829824
0.01605732 0 0.1107695 0.05311193 0.02122513 0.012968032
0.04568148 0.05689198 0.126322 0.07125425 0.08454189
0.01092651 0 0 0.03013568 0.08305529 0.124715689 0.2763796
0.02957866 0.083816067 0.06039702 0.1451153 0.1283855
0.2108282 0.266883 0.12982676 0.2251128 0.01549343
0.4431974 0.1439864 0.08072222 0.07850514 0.08249498
0.3842692 0.1307056 0.2153239 0.054585818 0.099044578
0.082365584 0.1136204 0.05496889 0.08311361 0.05911483
0.1556613 0.0678241 0.020226977 0.229703 0.1260909
0.06809302 1.181644 0.181463 0.334206 0.03640942
0.1927782 0.125638 0.05079047 0.065737478 0.1166823
0.0952944 0.06469323 0.1449071 0.03534293 0.006497524
0.2416704 0.05804844 0.3908149 0.04541566 0.038800799
0.31471 0.1261174 0.3285629 0.05406922 0.3153605 0.07805775
0.07773004 0.1464204 0.06802024 0.3780879 0.09966871
0.3710914 0.1207438 0.5054863 0.202484 0.04840385
0.03341515 0.047474078 0.1610362 0.3901417 0.06465929
0.0117768 0.2071409 0.2614316 0.01598381 0.195222
0.1860773 0.05948952 0.09828784 0.06944569 0.03609743
0.4390665 0.07475233 0.2064194 0.2994171 0.1227588
0.0217738 0.09323906 0.05589982 0.04832957 0.2444815
0.1502191 0.06761973 0.04500614 0.04276656 0.1085115
0.06502925 0.06646819 0.1320794 0.1554271 0.07772697
0.443736 0.1059826 0.4370385 0.016281 0.1480924

0.2008053	0.2424784	0.03933099	0.1178356	0.03797335	
0.1337805	0.1867409	0.1127928	0.04214272	0.05396613	
0.2542709	0.2073721	0.2918761	0.030344493	0.08155425	
0.020587824	0.1343054	0.05786983	0.02663564	0.03211488	
0.1379829	0.1827833	0.05783163	0.07201735	0.015451042	
0.3293504	0.08194472	0.01393733	0.08506552	0.0454417	
0.06764426	0.4970956	0.2562558	0	0.07455296	0.09018266
0.1875608	0.03068227	0.2653293	0.08887311	0.1997522	
0.2379777	0.3771199	0.06330476	0.0919314	0.07481809	
0.08207368	0.06923594	0.09983748	0.03429739	0.1933201	
0.2598432	0.1567383	0.005162797	0.1814611	0.1513149	
0.1972155	0.0888792	0.2133254	0.05660298	0.05410531	
0.2108608	0.6549323	0.09441961	0.1854475	0.09343372	
0.4292787	0.1932007	0.05871332	0.02872626	0.02126493	
0.06327293	0.236548009	0.2191777	0.205163303	0	0.07691583
0.3920632	0.0380686	0.04157328	0.06251024	0.09824983	
0.04023824	0.1665627	0.1872862	0.3851223	0.04235642	
0.1367102	0.1409019	0.1063313	0.06829199	0.3485631	
0.1233342	0.0753905	0.1344893	0.1868839	0	0.04900296
0.1417846	0.279331	0.2417867	0.4301913	0.0861293	
1.158229	0.2196101	0.09997998	0.2311492	0.5368984	
0.008199284	0.8051286	0	0.1449091	0.03239605	0.1783024
0.0845921	0.03428173	0.1109161	1.093058	0.07145889	
0.3312435	0.08760619	0.1225882	0.3538628	0.1162402	
0.09119257	0.084295	0.061332559	0.1448056	0.3416066	
0.11465	0.1611362	0.08633729	0.056011183	0.2154912	0.06144823
0.02309617	0.03091983	0.1566814	0.3904875	0.04761517	
0.06768267	0.5075822	0.2238581	0.2544994	0.006954708	
0.03315833	0.08007238	0.349628	2.229459	0.006433558	
0.1358201	0.1740729	0.1832608	0.1165782	0.05921812	
0.05722526	0.4867745	0.1076013	0.02244315	0.029051298	
0.2047313	0.1060382	0.09471713	0.02092405	0.1857184	
0.05099276	0.03487626	0.2937038	0.1621302	0.04408367	
0.1810728	0.03423933	0.1137749	0.169851	0.745083033	
0.2827933	0.6094745	0	0.1927128	0.04338761	0.061668809
0.1736389	0.1409289	0.05444184	0.2197885	0.1789759	
0.1863004	0.03285533	0.1155439	0.09255966	0.2359245	
0.1543978	0.1034349	0.3474104	0.01812951	0.09707698	
0.3297978	0.1807587	0.2025521	0.01771569	6.330479	
0.04450295	0.1138067	0.123236	0.06194802	0.07439063	
0.2558684	0.02246932	0.1903428	0.3932488	0.5318934	
0.2289075	0.09864828	0.3441896	0.3124758	0.1746977	
0.2153467	0.3092616	0.128813	0.1320634	0.1282218	
0.1176205	0.04947446	0.5661638	0.07362731	0.2042127	

0.066484497 0.2279622 0.05003294 0.1312684 0 0.06364083
0.4322842 0.115830304 0.09072524 0.2721577 0.2538103
0.3519272 0.0193793 0.1748121 0.05955131 0.1520481
0.3892234 0.09966722 0.08985228 0.6341839 0.1138243
0.1063397 0.042149281 0.2643165 0.4694636 0.0258861
0.05861616 0.1603414 0.3475781 0.03979287 0.2248375
0.06996807 0.06326605 0.1171894
TSC22D1-AS1 0.4076586 0.6510302 0.6494501 0.3813909 0.2535752
0.4937168 0.3246681 0.4432413 0.1557166 0.331725335
0.5122624 0.4045798 0.3309898 0.3506945 0.2740628
0.5075071 0.2934154 0.54742669 0.2075666 0.4398249
0.368322102 0.2469444 0.3903021 0.7793511 1.729931
0.2213268 0.2727498 0.4486242 0.284250098 0.1239712
0.2969124 0.192127 0.3569926 0.4368018 0.5782248
0.2579593 0.04373091 0.4416267 0.5168957 0.453753666
0.6889541 1.43747 0.122479269 0.1404913 0.4197822 0.281412
0.4743459 0.722766 0.115540143 0.2584641 0.475447
0.6185607 0.4147993 0.4928971 0.3441555 0.1859895
0.4689679 1.171839 0.9186653 0.746377242 0.314912435
0.320959192 0.3181115 0.4426087 0.3238741 0.4955196
0.4186135 0.47573 0.152009507 0.8337721 0.3625276 0.2162676
0.8398011 0.1959379 0.4480365 0.2812242 0.5090253
0.5199903 0.7983889 0.292300735 0.8257375 0.5830238
0.2025757 0.8470033 0.3143036 0.2848423 0.6572907
0.3064101 0.47511053 0.5593645 0.121498018 0.3472574
0.2082278 0.5037759 0.1741458 0.5967917 0.3747841
0.2655254 0.6454132 0.3919084 1.052371 0.2203026
0.07746726 0.5797353 0.7719071 0.3709156 0.2000702
0.470852 0.345627289 0.4426257 0.4377621 1.464529
0.1106311 0.7551859 0.3869878 0.3536905 0.4138058
0.1931132 0.6364607 0.6814939 0.3783755 0.5063874
0.4105219 0.1248395 0.9129933 0.4198697 0.2344829
0.195452 0.7084946 0.7468402 0.3874195 0.5502404
0.2907836 0.1566873 0.5543206 1.965889 0.5556484
0.1764775 0.5873995 0.1654336 0.7357657 0.393847
0.3498227 0.3849649 0.460137 0.2956906 0.4364479
0.4922506 1.244477 0.344843 0.5010385 0.503373
0.3998693 0.6056307 0.7309522 0.2243365 0.4168312
0.9753518 0.3896099 0.7592897 0.405412498 0.4596716
0.704841638 0.4702562 0.2329835 0.3703178 0.3888406
0.2160348 0.6467607 0.6984892 0.4128566 0.416087583
0.9281736 0.3207447 0.5106154 0.2863456 0.5610404
0.2481277 0.2708835 0.8024209 0.8749621 1.06538 0.4047611
0.3328115 0.4265785 0.4258019 0.9527369 0.147843

1.168036 0.1199053 0.497372 0.2347226 0.3109534
0.3405245 0.2890672 0.7098401 0.2174776 0.7556421
0.4453686 0.8539901 0.481759 0.8884805 0.5891805
0.5077604 0.1590342 0.2737137 0.2228191 0.8713103
0.9386604 1.656422 0.5590638 1.147993 0.7138747
0.6674709 0.3226532 0.4338875 0.4767421 0.3762192
0.4490908 0.65792418 0.5961156 0.248112264 0.644049681
0.3703048 0.6816363 0.2690633 0.5467542 0.3523321
0.2409944 0.3947972 0.96252 0.5082606 0.5755643 0.2652637
0.4129756 0.5412176 0.3874423 0.5381756 0.1471105
2.840715 1.312562 0.5011446 0.5629015 0.760333
0.214822 0.5246967 0.4828216 0.5383918 0.5755662
0.1738061 0.3929029 0.9247991 0.9313835 0.586662
0.1651183 1.432645 0.4346761 0.614246689 0.2327975
0.4950401 0.2964889 0.135386 0.2254294 0.1259015
0.8829635 0.8630789 0.3894169 0.4526341 0.6490787
0.5692388 0.735635 0.4740193 0.1935672 0.572315981
0.4680606 1.136799 0.5704219 0.6929432 0.2883737
0.055122374 0.6477825 0.6542094 0.4628587 0.2865876
0.09199139 1.336867 0.304824 1.208039 0.5581403
0.6394242 0.6884042 0.7883444 0.3426377 0.5731037
0.23828 1.146906 0.6768914 0.4725523 0.4905714 0.236713
0.3441848 0.5980161 0.1541043 0.5411089 0.4858956
0.8187256 0.109162961 0.244776305 0.6345661 0.290941
0.4422605 0.6661337 0.419147 0.5569659 0.1785267
0.6967509 0.225466 0.594582 0.3731066 0.2430995
0.4254873 0.506759175 0.5391823 0.4077174 0.1685504
0.4073268 0.4308729 0.529660117 0.3033387 0.468156
0.43045 0.5872894 0.4566484 0.6870775 0.5212628 0.7075314
0.393347 0.3523302 0.6348172 0.180251 0.3645787
0.4144169 0.2562116 0.2581763 0.817577 0.5565094
0.832104466 1.35391 0.2898549 0.2209469 0.5788387 0.8319568
0.7812498 0.1151736 0.4291888 0.4052975 0.4378279
0.32705 0.6403274 0.2568748 0.4697974 0.489232 0.8836729
0.2145567 1.376697 0.2783154 0.2067669 0.7126706
0.2563427 0.3330796 0.4174039 0.3330184 0.6554433
0.778610967 0.3897095 0.3133391 0.3327501 0.4800723
0.3043554 0.6387412 0.870486343 0.5614965 0.4411466
0.359735 0.3513622 0.5643513 0.5992525 0.1864747
0.8280218 0.4991219 0.6415198 0.1381207 0.8605296
0.3309625 0.5734733 0.17949717 0.7787542 0.7219965
0.3566546 0.391565667 0.4545158 1.175451 0.2492091
0.3136178 0.2668951 0.9678929 0.5003981
AC092691.1 0.1048998 0.9027723 0.07236813 0.1252583 0.2063905

0.8233249 0.0810381 0.5343135 0.2429207 0.328569848
0.2701313 0.0515226 0.2429883 0.1229415 0.6840687
0.7577199 0.05231238 1.994301671 0.4209499 0 0.09193426
0.4962385 0.2001792 0.4392574 0.1796155 0.1236802
0.08302334 1.078306 0.202713272 0.2380274 0.2964408
0.329106 0.05062863 0.2477886 0 0 0 0.0235537
0.02028595 0.023208653 0 0 0.054591392 0 0.05400972 0
0.06102995 0 0 0 0 0.4948542 0 0 0.4090581 0 0
1.209765 0 0.035553037 0 0.035764438 1.044757 0.03068789
0 0.3731818 0 0.2385474 0 0 0 0.0190074 0.2853694
0.02445334 0 0 0.1425287 0.04269444 0.03969725 0 0
1.919318 0 0.08089839 0.02762363 0 0 0.03240714
0.53904073 0.05916059 0 0 0 0.06641404 0 0.02679153
0.05084086 0 0 0.1063277 0.2298404 0 0 0.03145731
0.05412085 0.1186944 0.03783189 0.2938151 0 0 0.1111725
0 0 0.2312842 0.3715126 0 0 0 0.0581204 0.02743594
0 0 0.1933348 0 0.1008344 0 0 0 0.7044542
0.07281776 0 0 0 0 0 0.3008327 0.09977802 0
0.05772307 0 0 0 0.02040111 0 0 0 0 0.1793682 0
0.128086 0 0.02473294 0 0.05212661 0.1916468 0
0.04686589 0.1552619 0 0 0.118584528 0.02897357 0.05363735
0 0 0.02081811 0.04183437 0 0.1785767 0.4842914
0.5845285 0 0.0559601 0 0 0 0.02536908 0.03776427 0
0.03034645 0 0 0.02936905 0 0.0239809 0 0.8580624 0
0.04325595 0 0.02249012 0 0 0.0400924 0 0.09180214
0.3350805 0.05210233 0 0 0 0 0 0 0.04961925 0
0.06320033 0.09061732 0 0 0.1006327 0.1393688 0.02434224
0 0 0.02294483 0.02806514 0.1038776 0.247267
0.054377409 0 0.05529423 0 0 0.1600764 0 0 0 0
0.1048325 0.02958716 0 0 0 0 0 0.2644325 0
0.05922454 0 0.4910362 0 0.2086661 0 0.08207174
0.02518573 0 0.1312345 0 0 0.04714889 0 0 0.1426291
0 1.890497 0 0.027183998 0.02462158 0.2532038 0 0
0.1004783 0.08127254 0 0 0 0.02852999 0 0 0 0
0.1464089 0 0.1460366 0 0 0.1049518 0 0 0.3368508
0.03694402 0.09025849 0.07249975 0 0.203467 0.04135052
0.04408333 0.02306514 0.5334295 0 0.3533217 0.3455489 0
0 0.1333561 0 0 0 0 0.06508298 0.2024934 0.0223633
0.07133575 0 0.1534865 0.028382698 0 0.02302174 0 0
0.03298986 0 0.2725888 0 0.3277218 0 0.1147495 0
0.02615446 0 0 0 0 0.05393674 0 0 0 0 0.04789061
0 0.02863069 0.05180943 0.02696486 0.513587 0.05017099 0
0.04728099 0.02623381 0 0.1100805 0 0.05419589 0.02929161
0.9811037 0.02553426 0 0 0 0.04447499 0 0.02689881

0.06708791 0 0 0 0.02439355 0.1889644 0 0.04058019
0.1379562 0.3052843 0 0 0.03266423 0.02516969 0 0 0
0 0.08402033 0.223791 0 0.025981757 0 0.03258764 0 0
0 0.02639597 0.071472337 0 0.03128166 0 0 0 0.6112446
0 0.02583458 0.02414385 0.02163854 0.03192159 0.1376863
0.06354553 0 0 0.07042728 0.3251255 0.3034841 0 0
0.09055434 0 0 0 0.03532001 0.0624504

AC026310.1 0 0 0.05507135 0 0.02617682 0.04042201 0 0 0
0.100015217 0 0 0 0 0.06507109 0 0.03980914
0.015026155 0 0 0.023320321 0 0 0.02387643 0 0
0.1263597 0.03156072 0 0 0 0 0.01926392 0.0628548
0.04874154 0 0 0.01792411 0.03087478 0.229599886 0.01611619
0 0.041543449 0.01282963 0.06165121 0.02936968 0.02322156
0.02145091 0.060961886 0 0.3455694 0.07531573 0.3425612
0.1500374 0.1556444 0 0.5567383 0.04091636 0.160088
0.162332824 0.385179173 0.108865378 0.09938111 0.02335315
0.02746352 0.1217088 0.08169202 0.1008511 0 0 2.249892
0 0.7501993 0.3907831 0.9473068 0 0.03098937 0.146205
0.09062751 0.13684773 0.1041005 0.1089985 0.5344202
0.02052093 0.1681702 0.01932299 0.04106879 0 0 0
0.038463246 0.04456745 0.01704821 0.1010807 0.2986221
0.1223284 0.2127913 0 0.05024305 0.0404571 0.02498658
1.970221 0 0.1196933 0.06177806 0.03010837 0.02878965 0
0.035295803 0.1330294 0.01208586 0.01602419 1.996313
0.02200059 0.07067925 0.03168951 0.08931859 0 0.01474299 0
0.08260984 0.1717603 0.1839071 0.03705101 0.1151008
0.3290745 0.2190436 0.1079219 0 0 0.01437274 0.1590452
0.04061243 0.08043776 0.0892292 0.07631012 0.01898249
0.02148784 0.3074864 0.2880468 0.2854919 0.4202769 0 0
0.09418183 0.04841807 0 0 0 0.01949441 0.05533127
0.188215 0.01808409 0.07933555 0 0 0 0.02363052
0.056064 0.2003102 0 0.1102429 0 0.05446515 0.04917119
0.01584235 0.03183549 0.1119129 0.05435796 0.09827745
0.01647481 0.068924807 0 0 0.02072412 0.07905514 0.05791676
0.02873819 0.03839774 0.02309332 0.06021548 0.02015574
0.02234953 0 0.9672078 1.898683 0.01554705 0 0.1152106
0 0.1540326 0.1051519 0.02022742 0.03050988 0.03431681
0.1397208 0.02549926 0.05947395 0.08134189 0.09322483 0 0
0 0.08145826 0.03775971 0.06189352 0 0.2758352 0
0.03745584 0.1786875 0.1696931 0.03704835 0.08804361
0.3447359 0.05238228 0 0.01580995 0.08362998 0.020690305
0.1117394 0.462861316 0 0.9435527 0.2436328 0.04851954
0.06181741 0.09294964 0 0 0.02251551 0.417728 0.0520598
0 0.0956618 0.05587043 0.1437359 0 0.09013845 0

0.01494693 0.01999789 0.02646544 0.04945942 0.7911054
0.01916607 0.3655092 0.01997361 0.1163042 0.05692001
0.01793989 0.8332638 0.111499 0.03617972 0.0142561
0.02438386 0.2146679 0 0.01873675 0.01926853 0.03656922
0.1118082 0.1019505 0 0.2355542 0.09107643 0.0172822
0.04342203 0 2.104706 0.09097021 0 0 0.018239696
0.06945765 0 0.0378842 0 0.6204999 0.594898996 0.02136165
0.02811399 0.02289524 0.1287335 0.3931495 0 0.01573364
0.06709385 0.333494 0.01623736 0.03220664 0 0.0493048
0.05102729 0.2446492 0 0.1913276 0.1346385 0.04853209
0.0510937 0.02476371 0.02201361 0.06807291 0.2895238
0.0673675 0.05005779 0.129593529 0.083025087 0.01751929 0 0
0.2761541 0.3980745 0.1296482 0.05137917 0 0 0.01455386
0.06109462 0.6767107 0.1329263 0.047651683 0.03737774
0.1235807 0.9029959 0.07163862 0.1290305 0.078598688
0.1902471 0.03644423 0.02023808 0.1307259 0 0.04103993 0
0.03817957 0.01966166 0 0.05989092 0.01337413 0.0558467 0
0 0.04458119 0 0.01943129 0 0.07690448 0 0.1015349 0
0.6550304 0.01701772 0.07133694 0.2338754 0.05660607
0.03712645 0.1150399 0.09076643 0.1544054 0.3149495
0.09956486 0 0 0.2982854 0.05746156 0 0.1191622
0.04197502 0.2574813 0 0.1459735 0 0.158174655 0.03664525
0.02479884 0.09294741 0.0991167 0.08602797 0.08034816 0
0.1745816 2.47572 0 0.03033614 0.02881606 0 0.05903323
0.03931966 0.0183732 0.01646669 0.3400877 1.641519
0.1208936 0.0351382 0.250695586 0 0.05301791 0 0
0.02509681 0.06891085 0.08875505 0.01519646 0.01891619
0.02687814 0.03168271
AC016705.2 0.03005506 0.3161342 0.05529161 0.1818326 0.191855
0.3733698 0.1547894 0.3169808 0.1484795 0.150622835
0.1926299 0.09841236 0.1299557 0.02066489 0.1829278
0.2389912 0.1518798 0.437501289 0.04700591 0.06847076
0.194332789 0.2893455 0.06372643 0.397934 0.4254195
0.06772191 0.2505585 0.3247912 0.104543688 0.1136629
0.1868546 0.2532438 0.4313034 0.08834866 0.06687985 0
0.9298757 0.4588927 0.05889669 0.024825033 0.9271507
0.001766316 0.00834192 0 0.103163 0.05749994 0.515249
0.04307341 0.07344684 0.01440301 0.03006913 0.1512339
0.08168366 0.0421785 0.3281604 0 0.2647729 0.429286
0.1446555 0.059760091 0.104641795 0.040987792 0.07782729
1.120745 0.03033069 0.2796922 0.6643518 0.09922934
0.090590413 0.06298585 0.001711281 0.4240503 0.1645147
0.3717946 0.0171509 0.3442517 0.05289263 0.06687087
0.2699369 0.007851145 0.00696779 0.1138118 0.4829018

0.226633	0.08231086	0.05044072	0.3051245	0.0916124	
0.27456311	0.1785424	0.005792562	0.00447457	0.05990736	
0.1031764	0.2029527	0.6550273	0.07574594	0.09072452	
3.235142	0.3493226	0.2634084	0.02100638	0.3185509	
0.04806881	0.04135009	0.1209151	0.02601432	0.5213024	
0.035436967	0.008013685	0.01456104	0.001608828	0.1301035	
0.2562275	0.4683487	0.4899703	0.03138654	0.168264	
0.04736627	0.006288586	0.01244104	0.2026255	0.5169993	
0.05951871	0.7376654	0.2954081	0.0403186	0.01950363	
0.6403032	0.07603469	0.01731626	0.04106091	0.08562721	
0.0605696	0.02687582	0.1327999	0.02096426	0.008629512	
0.5865607	0.09201782	0.1460467	0.02953705	1.006928	
0.1455635	0.04727925	0.2090304	0.01300511	0.0839389	
0.7593075	0.06263162	0.1518437	0.03401419	0.001815642	
0.1413838	0.3938817	0.3586141	0.1002598	0.6263407	
0.08443234	0.02011113	1.120450159	0.2722821	0.084010397	
0.2497189	0.03455749	0.1415608	2.863869	0.1779042	
0.1500822	1.734134	0.5160697	0.223748181	0.9470307	
0.004077835	0.0228877	0.09524558	0.1395562	0.07213282	
0.3431067	0.3408295	0.00201521	0.02023635	0.07853619	
0.007466909	0.1722286	1.26755	1.921496	0.002710995	0.04626854
0.01039387	0.2268181	0.1161297	0	0.1010853	0.03789946
0.3349177	0.1804888	0.07961576	0.02450016	0.002339942	
0.04007914	0.08537546	0.003928654	0.2339024	0.314659	
0.03107053	0.05070147	2.347052	0.01020952	0.1447817	
0.03844331	0.07453763	0.1004306	0.05303744	0.2095916	
0.01928365	0.03216402	0.009523908	0.1007573	0.074782999	
0.2748563	0.547093359	0.296542607	0	2.051206	0.04221844
0.004137643	0.02799642	0.02200154	0.9130878	0.01582389	
0.2679492	0.002613401	0.04110197	0.4922275	0.1514535	
0.04329322	0.07646471	0.02941216	0.1022919	0.541742	
0.08432704	0.1222279	0.08441729	0.3469703	0.1116078	
0.4837345	0.1985295	0.1722348	0.02857383	0.1693095	
0.02261071	0.08209293	0.2869629	0.007156556	0.2007473	
0.04642109	0.08100089	0.02069285	0.07931693	0.1743985	
0.1936406	3.562066	0.04760607	0.02364962	0.06400848	
0.08502146	0.008719138	0.01663742	0.6919443	0.03470693	
0.02178258	0.1370299	0.018312645	0.08647195	0.03399891	
0.05325001	0.4057443	0.004296424	0.019112904	0.3345745	
0.1905284	0.3402048	0.009232028	0.01608126	0.03497747	
0.1042574	0.006736219	0.01762252	0.2217113	0.02101804	
0.3073269	0.372915	0.04610823	0.5925767	0.0475479	
0.0403395	0.1036357	0.01299365	0.0393285	0.1019373	
0.1922843	0.01879492	0.03633522	0.02536385	0.02680426	

0.067224446 0.030564285 2.050919 0.01346695 0.09215053
0.0151232 0.003806348 0.08070336 0.0541639 0.3154916
0.1206561 0.2893188 2.324747 0.03197257 0.06405982
0.014352679 0.009381808 0.01654333 0.1318702 0.04315508
0.1369492 0.397195629 0.09823266 0.6073938 0.4206037
0.5403071 1.516071 1.351493 0.3760473 0.04791533
0.00394806 0.2835751 0.0701522 0.009399336 4.547282
0.2950139 0.006211114 0.2372253 0.2848462 0.09364321
0.015868626 0.09111023 0.4650684 0.2786388 0.007359152
0.1191991 0.2972926 0.01432445 0.1509498 0.06251571
0.07082237 0.3147374 0.2004848 0.02170321 0.2582374
0.02332471 0.004012395 0.09936979 0.04242611 0.01538437
0.0438127 0.138781 0.1966668 0.02031158 0.09843121
0.03663934 1.02131 0.035731635 0.005518771 0.25147 2.993678
0.02985393 0.03627626 0.6473728 0.054607192 0.0541774
0.6381356 3.63158 0.006091493 0.008679393 0.1840572 0.02963467
0.001973846 0.05349539 0.3537966 0.04146153 0.2594855
0.02670297 0.008819683 0.412785098 0.02690437 0.1685615
0.1082068 0.630057023 0.03779577 0.2006407 0.008911002
0.309722 0.05887473 0.01349282 0.2846944
AC007879.3 0.02653629 0.02537473 0.09763639 0.1774435 0.2262442
0.147808 0.3280008 0.07950837 0.1024186 0.049870602
0.0341673 0.1042686 0.143426 0.01244011 0 0.1277859
0.1058669 0.029969975 0.1146782 0 0.077521431 0.05285578
0.01687965 0.1428661 0.03634959 0.1147195 0.1050113
0.1154056 0 0 0.07499004 0.10704 0.4738748 0.6686144
0.3240533 0.1745137 0.01380619 0.2264167 0.2257947
0.105678852 0.4714463 2.479637 0.027619754 0.0170593
0.3142429 0.4100491 0.4477202 0.1568757 0 0.2119457
0.01531656 0.2253282 0.3416226 0.09975092 0.2845662
0.06524261 0.03896232 0.1360142 0.06652059 0.395726409
0.195827864 0.144756155 0.1189306 0.03105223 0.2008473
0.1438523 0.2353526 0.1206897 0.026661434 0.1482976
0.02266392 0.02884959 0.1443787 0.3464111 0.1342213
0.1919668 0.1442209 0.1836056 0.1606742 0.37692489
0.02307007 0.2318931 0.0710608 0.1500745 0.1118063
0.2312407 0.1774772 0.0819797 0.45453358 0.4340064
0.089501691 0.02963023 0.01133433 0.3248122 0 0.06777402
0.4244167 0.02794281 0 0.06724376 0.2491812 0.05795937
0.03057127 0.4615462 0.2190535 0.07006038 0.03828104
0.06606739 0.070398205 0.1238206 0.07231651 0.1171888
0.02328473 0.5558212 0.6578657 0.2317531 0.05938259
0.06306965 0.03920694 0.083285 1.63394 1.141932 0.09781506
0 0.05101577 0.2959991 0.145629 0.08610097 0.1351895

0.02456075	0.1051113	0.045317	0.3780109	0.09358695		
0.1186463	0.2198475	0.08834228	0.3285775	0.2920418		
0.4178293	1.283453	0.4051546	0.2167548	0.2654243		
0.1502781	0.3219028	0.8095162	0.1361232	0.09354552		
0.09072469	0.0490486	0.02502658	0.07213819	0.05274546		
0.05817662	0.6249245	0.03556672	0.1885262	1.602765		
1.065394	0.179988571	0.2931755	0.339213379	0.3862466		
0.1471094	0.2317179	0.7196275	0.1860106	0.2168361		
0.1960164	0.06571867	0.030549321	0.1840295	0.148517		
0.6613548	0.2943306	0.102681	0.09553152	0.3573971		
0.01535336	0.3469585	0.2948076	0	0.03708394	0.2669217	
1.081989	0.1343719	0.2333759	0.09848136	0.05735613		
0.1251642	0.3914915	0.188272	0.08113677	0.2053367		
0.2090071	0.2458175	0.3954067	0.1351984	0.4958369		
0.02041544	0.3587793	0.2081218	0.03249404	0.30125	0.07201128	
0.1119136	0.09169315	0.09014216	0.3112769	0.1697123		
0.1551258	0.2093655	0.05853493	0.3437914	0.1276948		
0.1277925	0.08408872	0.02780028	0.288870674	0.1981035		
0.44760553	0.125341326	0.0380189	0.3702329	0.129031		
0.2739913	0.06179665	0	0.1060771	0.02993843	0.09257413	
0.1038344	0.09421403	0.1748995	0.09286227	0.1720105		
0.05626034	0.1648011	0.4470631	0.8943585	0.09306779		
0.123167	0.1315304	0.1799332	0.1146813	0.287188		
0.1460718	0.1546474	0.06622474	0.2027618	0.3443704		
0.8895479	0.09621493	0.02843408	0.06484548	0.2634828		
0.082520191	0.07474166	0.1665364	0.06078171	0	0.2033423	
0.09594366	0.1409453	0.08073503	0.2872475	0.04330306		
0.1101716	0.1920598	0.1209613	0.03606062	0.6111067		
0.036379449	0.3971328	1.946781	0.1007478	0.0637187		
6.159548	0.063282041	0.2130312	0.3925175	0.1369951		
0.146721	0.1258504	0.1801473	0.08368287	0.3568535		
0.05834743	0.043181	0.06423681	0.1100051	0.09833944		
0.1470083	0.1829842	0.08995952	0.2416844	0.05967537		
0.02151072	0.0226461	0.1646391	0.08781318	1.470872		
0.108274	0.3247175	0.5213934	0.028719662	0.69917991		
0.1863604	0.01783541	0.04137039	0.3004337	0.1890399		
0.1034344	0.06831788	0.1105375	0.08716086	0.2225477		
0.1624726	0.2646497	0.353499	0.427690172	0.1988018		
0.1369357	0	0.1428847	0.1225495	0	0.03613826	0.04845917
0.2556467	0.0724265	0.2096981	0.2046375	0.08661406		
0.444208	0.1437905	0.2033298	0.106181	0.07113329		
0.3712913	0.7706697	0.1233885	0.2667543	0.2581154		
0.1033496	0.210161471	0.01022584	0.5631351	0.2475165		
0.1218292	0.1088726	0.06788439	0.2371383	0.2887667		

0.2258039 0.1974652 0.4206572 0.1810355 0.6672569
0.4187821 0.06619472 0.4649707 0.3677163 0.2313641
0.101874 0.02901241 0.2059819 0.1209289 2.115344
1.105232 0.2426227 0.1514111 0.433788576 0.08527132
0.1318981 0.346053 0.4942258 0.2516574 0.1068374
0.072320867 0.3904134 0.4906222 0.2773248 0.3327833
0.05747422 0.05457365 0.353229 0.1176358 0.1221524
0.08758175 0.1453526 1.811173 0.1285999 0.2452935
0.416681249 0.154404 0.1879917 0.02559059 0.173841076
0.2502805 0.1832588 0.1376852 0.1111354 0.06288123
0.1429573 0.1474476
LINC02605 0.2426289 0.004640168 0.7811278 0.4913604 0.4710079
0.1670885 0.3598806 0.02907871 0.359594 0.303987468
0.03748817 0.05720144 0.5170602 0 0.2848001 0.136611
0.2516725 0.087687702 0.03594978 0.1969178 0.674778228
0.00966551 0.02469367 0.02902813 0.009970641 0.06102767
0.122899 0.02302225 0.178169665 0 0.2194097 0.1652911
0.5433531 0.09169999 0.3515989 0.1489254 0.3585044
0.2484231 0.1388849 0.515334414 0.5094308 0.1454432
0.368701579 0.4273799 0.7945057 0.3427836 0.9316546
0.1616914 0.212463914 0.3255639 0.08402624 0.5860238
0.03123554 0.8390872 0.2838404 0.149133 0.6768629
0.1392851 0.1751664 0.203937161 0.347083531 0.390446367
0.08216036 0.06814066 0.3539253 0.2301745 0.1390456
0.1128023 0.399787938 0.2576263 1.835993 1.041052
0.1920139 0.4615261 0.2492208 0.3949218 0.832635
1.295606 0.1358908 0.351763571 0.4387474 0.09011126
1.496976 0.3492807 0.02555693 0.03288911 0.2646289
1.523114 0.61507776 0.1806236 0.991359982 2.99635 1.276759
0.544814 0.8992574 0.5056566 0.4844825 0.117525
0.06108374 0.5459681 0.249098 0.4748255 0.1229896
0.8207268 0.2103013 0.7247729 0.5250217 0.1087332
0.347582321 1.28739 0.4672555 1.106558 0.9197233 0.2139803
0.7561782 0.897679 2.074077 0.2268211 0.02867842
0.2386029 0.3816497 0.7360911 0.1922855 0.2342356
0.1026194 0.5554013 1.264951 0.1102144 0.08540166
0.1437223 0.4263624 0.07734464 0.1777506 0.2493732
0.2820525 0.2474004 0.09692865 0.08882215 0.3097458
0.5921517 0.7966564 0.4240951 0.6153178 0.9349761
0.2427463 0.1805193 0.447248 0.3816815 0.07270165
0.6730977 1.071832 0.5217214 0.03517761 0.699287
0.09220043 1.66083 0.4205877 0.3447498 0.0954249 1.607298
0.087769978 0.6808693 0.094286364 0.4944188 0.01195612
0.8975427 0.4180083 0.3174729 0.5551266 0.08363759

0.1121651	0.201111261	0.3468816	0.0246897	0.1259783	
0.1230239	0.17838	0.6288999	5.886679	0.5278296	0.1512963
0.2450464	0.2445459	1.790285	0.1730566	0.7194864	
0.2079171	1.286859	0.3881911	1.329938	0.16646	0.2505663
0.07869371	0.1112785	0.2336385	0.331242	0.337912	
0.1783556	0.3461241	0.8103772	0.04106614	0.03578642	
1.098935	0.6575864	0.3856182	1.674267	0.2163461	
0.08942685	0.4862754	0.236795	0.2048281	0.1495725	
0.1531431	0.1819686	0.8335787	0.500985	0.1817579	
0.2075888	0.3050232	0.125772731	0.1630185	2.941545922	
0.020373896	0.9281391	0.3258203	0.4483115	1.172425	
0.1469064	1.101209	0.135785	0.03832299	0.2144294	
0.1898774	2.193765	0.3198312	0.4007593	0.04892972	
0.3909473	1.813668	0.2824178	0.392514	0.80232	0.3668035
0.5652316	0.1468008	0.1770909	0.5574864	0.1019895	
0.2757272	0.9134578	1.025103	0.3395096	0.3343728	
0.9061123	1.109252	0.02371603	0.09636396	0.12072092	
1.161754	0.0843336	0.4045821	0.3398313	0.5825547	
0.7268563	0.5842121	0.1919277	1.827965	0.1847684	
0.225642	0.4264712	0.4468175	0.1648563	0.7653214	
0.412458385	0.4357319	1.772733	0.5112469	0.1281716	
0.005202634	0.219870218	0.2960664	1.14503	0.1002068	0.3890384
0.08851432	0.2353055	0.5317699	0.08972739	0.499344	
0.08685958	0.7948608	0.1005808	0.1039013	0.4259887	
0.2082051	0.0575768	0.4512623	1.495024	0.03146859	
0.3312956	0.5118165	0.2408703	0.4220793	0.9371811	
0.5200843	1.160369	0.068273946	0.282629154	0.3663485	
0.04892227	0.1059132	0.1770258	0.4424822	0.1197925	
3.092017	0.08489681	0.6109848	0.3255708	0.6882974	
0.1790628	0.9696429	0.168006401	0.05453105	0.9014704	
0.2345366	0.3483829	0.07619434	0.095557523	0.1718197	
0.4253531	0.2116008	0.1721762	0.1965263	0.2295166	
0.1029518	0.1903113	0.3920249	0.2930821	2.077606	
0.8064862	0.006789646	0.3408517	0.2105928	0.6070427	
0.1888018	0.1842662	0.473986418	0.04861884	0.1802117	
0.2551147	1.354526	0.4628859	0.07034446	0.6764858	
0.2924615	1.087351	1.773885	0.8461617	0.1103506	
0.4430207	0.3382323	0.03227932	0.2040653	0.3043617	
0.7917748	0.2142366	2.997537	0.6026726	0.1190741	
0.4069477	1.471768	0.2188791	0.05076116	0.072113696	
0.2984984	0.01205982	0.3390066	0.2410052	0.2175468	
0.2002532	0.136658419	0.3396003	0.2720481	0.00482983	
1.349867	0.6025771	0.1064496	0.2153116	0.5210578	
0.116155	0.2882826	0.08860001	1.358775	0.05291216	

1.050907 1.109424262 0.2562888 0.7047333 0.2433414
0.120093952 0.1830709 0.1954853 0.7121748 0.790744
1.550041 0.2940975 0.1925936
ZBTB46-AS1 0 0.05213007 0 0 0.0357537 0.1932369 0
0.03266854 0 0.034151538 0.1169893 0 0 0.02555707
0.08887759 0 0.05437346 0 0 0 0 0.02714685 0
0.4239523 0 0.02142552 0 0.06466099 0 0 0 0.04886753
0.02631169 1.116056 0.08876508 0.05975377 0 0 0.1054261
0.313599845 0.0440247 0 0 0 0.02806884 2.045849
0.1585863 0 0 0 0 0.2057405 4.825099 0.1639434
0.1594406 0.03350875 0 0.05588576 0.1093284 2.956305084
0.061893927 0.223041261 0.2171841 0.09569096 0 0.03694143
0.03719311 0.1652974 0 0.1713735 0 0 0.3235776
0.07625036 0.1060559 0 0.1481443 0.1331297 0.0412613 0
0 0.0595504 0 0.1121143 0 0.02639238 0.2243758
0.03368397 0.46689894 0.122983 0 0.09130892 0.02328535
0.6212765 0.09412479 0.05569422 0.1056879 0.02870298
0.02287488 0.1105169 0.4095361 0.09525781 0.06280589
0.09809014 0.140633 0.1028091 0 0 0.072313353 0
0.08253761 1.553956 0 0.1502479 0.193075 0.04328324 0
0.02159514 1.490121 0.02851691 0.02820824 0.2932493
9.218679 0 0.1048072 0 0.02493179 0.05896221 0.0757458
0.02522892 0.03926211 0.09309965 0.3328238 0 0 0 0 0
0.2999867 0.2503644 0.01856858 0.5166331 0 0 30.79608
0 2.512308 0.0466088 12.53979 0.02662652 0 0 0
0.1625411 0.1195186 0.5135402 0 0.4841374 0 0.729585
0 0.1505756 1.03138687 0 0 0 0.04348263 0.07642835
2.078861 0 0.04500435 0.0627608 0.05816491 0.4715408
0.254755 0 0.4482663 1.570087 0.02622285 0.8200944 0
0 0.03052618 0.1015808 1.670025 0.1010386 0.02123499 0
0.1573608 0 0.9818015 0 0.1933938 0.04167203 0
0.09541911 0.03482826 2.382826 0.05555056 0.06366574
2.159996 0.04467148 0 0 0.05157423 0 1.116737
1.098855 0.06944583 0.6139104 0.2789268 0 0.1012053
0.06012734 0.6016529 0 0.0291709 2.094625 0.1142263
0.056519859 0.3052392 0.201154807 0 0 0.1901525 0
5.375602 0.0634778 0 0.1089628 0.09225866 0.3169752
0.1066591 0.04301203 0.26132 0.2670879 0.07852886 0
0.03077898 1.196769 7.227021 0.08194256 0 0 0
1.806285 0.680771 0.05456205 0.1588545 0.0583083
0.09801308 0.0307599 3.654991 0.1482486 0 0.6994005
0.1353254 0 0 5.289916 0.1997928 0 0.3133112
0.05631643 0.4504255 0.539054 0 0.1186163 0.02263375
0.2536518 0.1988032 0 0.03804433 0 9.866375 0.9522583

0.02587214 0.08726952 0 0 0.262592 0 0.03127155 0 0
0.2643544 0 0 0.1198695 0.02217786 0.04398956 0
0.02244771 0.1161597 0.08353875 0.3234239 0.02613256 0 0
0 0 0 0 0.3954472 0 6.518094 1.445547332
5.367605621 0 0 0.02124793 0.1371586 0 0 0.03508822
0.04541786 0.1790641 0 0.1112617 0 0.3631158
0.065085225 0.969998 0.1125288 0 2.73974 0 0.035784768
0.3340924 0 0 0.0446381 0.0538507 0.05605454 0.08897036
0 0.1342748 0.07371576 0 0.09133555 0.03813921 0 0
0.487131 0.08158071 0.0265403 0.959461376 0.04201611
3.398423 3.883092 0 2.907696 0.06973115 0.2923075
0.09126846 0 0.02535465 0.1571276 0.1859605 4.492067
0.7169581 0.04533034 0 13.63767 0.3055607 0 0
0.03255163 0 0.05024025 14.14757 0 0.0777651 1.59332031
0.05005205 0 0 0.4738262 0 0.05487191 0.024762774
0.02167754 0.8453679 0.1085216 0.06215209 0 0.0373722 0
0 0.2509511 0.1799287 0.06635858 3.100741 0.1320984
0.09598728 1.095723148 0 0.04827647 0 0 11.96322
0.2509924 0 0 0 0.03671161 0.02163697
SETBP1-DT 0.09146345 1.755925 0.2718098 0.3158813 0.2768531
1.325294 0.2391507 0.8010516 0.9123937 0.881490052
0.5797686 0.3870308 0.9343772 0.323232 0.6882095
1.344787 0.308757 0.810495521 0.2084913 0.1051868
0.49328407 1.604582 0.644451 1.969684 1.667284
0.3318103 0.5345658 1.324052 0.217536374 0.2075392
0.2783528 1.242407 0.9847453 0.5096566 0.790439
0.1542313 0.3001593 0.2085272 0.3210977 0.099623001
0.2443104 0.01240442 0.07322913 0.01809195 0.3549995
0.07765543 0.3683963 0.1361224 0.358194131 0.09552954
0.2111683 0.8894915 0.717056 0.4125775 1.138578
0.06919192 0.1652832 1.377564 0.2045871 0.247993195
0.607070026 0.278252524 0.6166345 0.4610466 0.3388719
1.134666 0.3647987 1.685273 0.282753224 0.1376152
0.0420627 0.1886749 0.716872 0.5445101 0.1697203
0.39445 0.4970906 0.1889927 0.1011751 0.041352534 0.03669984
1.429469 0.1356521 0.4702423 0.7114455 0.1703044
0.535704 0.2782149 0.60255965 0.5396363 0.128819279
0.1571191 0.420715 0.3682307 0.1133752 0.1581284
0.6683419 0.1259456 0.03542561 0.3993595 3.224031
0.08605494 0.1134764 0.7764242 1.197866 1.30558 0.1319444
1.278716 0.105767764 0.04689849 0.289733 0.8869256
0.2963305 1.3263 1.196037 0.1228909 0.1338265 0.06131347
0.5041604 0.2797005 0.08008954 0.2497803 0.5121968
0.6008543 0.1690746 0.8189131 0.1287036 0.07609408

1.36856 0.5795565 0.126675 0.06408021 0.3579401 0.1063414
0.1730139 0.7532714 0.2877618 0.1136306 0.2710049
0.2677193 0.1581611 0.2222481 0.2189291 0.5111287
0.398437 0.7282954 0.04566587 1.070693 0.03100253
1.697534 0.07802646 0.5175593 0.04462787 0.440514
1.568163 0.5743858 0.3143306 1.54119 0.01976494 0.682639
0.307534951 1.570157 0.518035502 0.1088072 0.4073708
0.2848401 0.6060615 0.2827541 0.4024332 1.359893
0.958331 0.14579349 0.5329624 0.07159415 0.09497968
0.2508326 0.4968408 0.3343372 0.2368945 0.3093722
0.3608846 0.1350093 0.4491118 0.2884107 0.1994422
0.1043168 1.189376 0.04759668 0.5338182 0.1094905
0.603367 0.07414099 0.1140963 0.430241 0.2903551
0.3140167 0.395541 0.2935392 0.1290441 0.2136271
0.005412811 0.8301757 0.07587248 0.01723049 0.1064952
0.0981904 0.4154083 0.4943215 0.1374231 0.1386501
0.395968 0.6954539 0.3787723 0.5431843 0.303835
0.1538914 0.2334095 0.362289 1.555234 0.204237862
0.2954464 0.267018916 0.199392859 0.07056049 0.7791532
0.1311398 0.1452884 0.4587616 0.05150384 0.2179652
0.1349404 0.3927116 0.02752993 0.04440758 0.05901847
0.3643889 0.9323828 0.190931 0.4687199 0.3520019
0.7061029 0.5640082 0.597132 0.1918019 0.63853 0.5608186
0.2108577 1.866009 0.6355335 0.1505004 0.4869921
0.04763688 0.353773 1.084165 0.1055434 0.3696428
0.2910751 0.590728676 0.09247697 1.093669 0.3029667
0.04927143 0.3594186 0.2907183 0.1162599 0.5779494
0.01827812 0.2755459 0.5725189 0.2546071 0.5067195
0.2103391 0.2160329 0.045011861 0.06366559 0.2738788
0.9616167 1.734445 0.1885797 0.360730618 2.432476
0.05946824 0.7264388 0.3695556 0.05133402 0.7369152
0.1220291 0.2720144 0.2598933 1.305154 0.1362505
1.334344 0.8401315 0.4017606 0.1940607 1.168711
0.5126284 0.3639042 0.1197673 0.07805501 0.541276
0.7062263 0.2519851 0.338105 0.06531222 0.1588274
0.418799427 0.107322824 0.827623 0.1134902 0.2467949
0.08850549 0.04677937 0.9141296 0.1449067 1.207455
0.8473387 0.2821968 0.2010255 0.4420547 0.3280351
0.386382458 0.09224066 0.2614046 0.8899171 0.8081807
0.2599355 0.027709368 0.3066064 0.07066474 0.0356739
0.8026715 0.5629289 0.5497959 1.343409 0.6595359
0.1802208 0.4185913 0.2674451 0.1367335 0.2067275
0.2946155 0.07996846 0.03143351 1.547683 0.2397623
0.092867963 0.363302 0.2332903 0.4176126 0.1033631

0.4546355 0.2579771 0.1592789 0.4240337 0.05986811
0.5300901 0.1419475 1.519953 0.2885025 0.5274069
0.9945236 0.1338459 0.1642 0.1226846 0.148556
0.007692153 0.2352545 0.2170369 0.1620948 0.2554652
0.5746575 0.1137417 0.376402062 0.142109 0.340963
0.5898222 0.3144855 0.1455768 0.7010717 0.409059433
0.2014279 0.8476195 0.07702935 0.1016004 0.1523832
0.9356805 0.2497406 0.03465462 0.058296 0.4876373
0.09420347 0.6156435 0.2131006 0.1176832 0.57447531
0.4723573 0.9781655 0.6649217 0.112666977 0.2388876
0.8259959 0.1147297 0.0107148 0.08669387 0.2747948
1.502298
FBX030-DT 0.1781632 0.7178027 0.255655 0.1679231 0.155794
0.9959836 0.1394714 0.432746 0.2420469 0.428580578
0.5689056 0.2240172 0.2347774 0.171499 0.1161832
0.8869863 0.2748367 0.559829919 0.126124 0.1927965
0.349759053 1.000736 0.2296802 0.9265118 1.35366 0.212861
0.1880107 1.234088 0.206574635 0.3773179 0.3490789
0.7601833 0.1972 0.1645982 0.2649491 0.1353937 0.279318
0.07467401 0.1580288 0.13875101 0.1745692 0.1800932
0.121152544 0.2504504 0.249508 0.1118699 0.2321852
0.1429874 0.372496127 0.2049109 0.1206593 0.5087629
0.2293636 0.08929632 0.569695 0.01752142 0.09068485
0.7135074 0.145299 0.273739785 0.318243678 0.110146984
0.6553563 0.1445482 0.02942135 0.5247604 0.200962
0.3313245 0.085921659 0.09458794 0.1399912 0.07059092
0.5639859 0.2347936 0.1848522 0.1589588 0.2102575
0.1953012 0.2247412 0.053521872 0.06608691 0.5604904
0.101781 0.4738737 0.352811 0.1012024 0.5157369
0.231905 0.33365411 0.5921573 0.135061589 0.1511912
0.2069869 0.3088173 0.1367138 0.1795856 0.2394741
0.09005113 0.2511824 0.3515454 0.8060092 0.1182974
0.1423094 0.225108 0.2353142 0.4139359 0.1165143
0.6003021 0.252079905 0.2501892 0.1452991 0.1430545
0.1459103 0.7908701 0.3196976 0.09995979 0.06910653
0.1449127 0.7072202 0.1317158 0.2433722 0.2887854
0.2210975 0.1675898 0.1758255 0.4216579 0.2542138
0.06936929 0.5720979 0.3034153 0.07014347 0.1568608
0.212704 0.1053214 0.06903737 0.2997505 0.2214335
0.07928999 0.4758095 0.2150718 0.1359307 0.3576889
0.1570779 0.1775792 0.188339 0.3957468 0.1387672
0.4691477 0.2721592 0.232046 0.3117465 0.1411428
0.1894276 0.4155128 0.5537766 0.1118856 0.2271187
0.7482018 0.1067746 0.3655977 0.264960284 0.4120444

0.364393705	0.1577554	0.1258384	0.2300612	0.2008405	
0.3663337	0.3299873	0.5176439	1.308007	0.153146203	
0.572795	0.07251893	0.2886196	0.1486795	0.1723488	
0.2702405	0.1896783	0.2666379	0.2102488	0.1511483	
0.2208057	0.1726259	0.1064398	0.3257982	0.7180317	
0.1285639	0.3565575	0.04518343	0.4991145	0.1952565	
0.1059393	0.06900141	0.1184592	0.4448861	0.2792411	
0.1651838	0.1210286	0.1414835	0.08224093	0.3523211	
0.2817926	0.339365	0.1303438	0.1307698	0.1717445	
0.456929	0.06052097	0.1560456	0.4679298	0.3231826	
0.2888512	0.2200802	0.2735641	0.1267818	0.2516777	
0.240882	0.2886848	0.278297463	0.1396559	0.558466299	
0.299212451	0.06466507	0.4536447	0.152085	0.08584628	
0.08297989	0.1072365	0.1186993	0.2867671	0.1464067	
0.2075923	0.05435227	0.395692	0.06982891	0.1847791	
0.1591498	0.2789635	0.4899513	0.6404987	0.323733	
0.4410329	0.3061373	0.2998472	0.1916356	0.3638793	
0.8297472	0.4533886	0.1422814	0.3245842	0.2037315	
0.1769593	0.5253987	0.07805889	0.150928	0.2319368	
0.238851157	0.1516586	0.2339444	0.1175285	0.04325348	
0.3367566	0.1545986	0.241131	0.2059793	0.03085703	
0.1628113	0.2347271	0.4961426	0.2382241	0.1355811	
0.1956148	0.264875225	0.2033851	0.09958542	0.1330283	
0.7415269	0.2470469	0.17278139	0.6967055	0.1455713	
0.1580655	0.148856	0.1715904	0.3225332	0.2715571	
0.2635485	0.1650538	0.4252086	0.1130919	1.083233	
0.5477595	0.1437485	0.4404565	0.390576	0.3006184	
0.1228682	0.1810087	0.0810905	0.1591745	0.3118186	
0.08913143	0.2067757	0.1623825	0.1092388	0.131119156	
0.227300859	0.5067417	0.08941032	0.1499897	0.08068384	
0.3136344	0.2654354	0.15901	0.821305	0.260086	0.2096174
0.1115073	0.3695836	0.1645539	0.158818166	0.07786011	
0.1078738	0.4910131	0.1193821	0.1075113	0.102913389	
0.2652758	0.2255776	0.06263348	0.3916076	0.2323038	
0.2613511	0.5001091	0.6725997	0.0585092	0.1734538	
0.2328787	0.09392508	0.2027502	0.2470798	0.2282767	
0.05837249	1.084219	0.3700714	0.407625973	0.1775895	
0.1638372	0.132945	0.06107398	0.2217261	0.1438215	
0.08491382	0.09544687	0.104438	0.2739927	0.1985548	
0.6104324	0.1304922	0.3717667	0.2844338	0.1879019	
0.07276544	0.1124342	0.1231158	0.2727029	0.144678	
0.1415639	0.7049161	0.3374093	0.1969227	0.3817771	
0.449514991	0.213736	0.1357854	0.2124234	0.08258645	
0.2560021	0.5953594	0.338811911	0.2833746	0.3343599	

0.2695386 0.2347132 0.06860069 0.2670683 0.1756712
0.1146673 0.279936 0.3802524 0.1619253 1.184832
0.1928289 0.3680663 0.295424008 0.4678263 0.5153845
0.2336669 0.089914531 0.1971634 0.697221 0.06690974
0.3255959 0.1463562 0.1567686 0.7900786
ST7-OT4 0.03272211 0.04693467 0.06521462 0.02083876 0.0286137
0.1049394 0 0.03921697 0 0.006832871 0.06085729 0.05357267
0.008421891 0.02045333 0 0.125251 0.03263633 0.028743726
0.02020151 0.0698876 0.031864102 0.03801985 0.048567
0.1892189 0.05976396 0.05572727 0.01726537 0.1638693
0.031616879 0 0 0.0391087 0.05264314 0.01717654 0.0221996
0.1075972 0.01134968 0.07837087 0.04218626 0.072396361
0.07927421 0.08172993 0.028381786 0.02103596 0.09546989
0.0321038 0.06980415 0.08792941 0.099955524 0.06098213
0.04406962 0.1029089 0.1696732 0.1558046 0.0531668
0.04022559 0.03202983 0.05031606 0.1148379 0.059148296
0.049533729 0.059499996 0.09233788 0.04467254 0.03752523
0.1293436 0.04464849 0.07716773 0.071232266 0.04952649
0.03260491 0.02371644 0.2319844 0.04068213 0.1782409
0.02958948 0.09315425 0.06215053 0.07017055 0.048081635
0.02844789 0.06553003 0.02920855 0.1289799 0.04021187
0.01584137 0.1402875 0.04043593 0.4670742 0.1291806
0.052554886 0.1461491 0.05590583 0.06905664 0.02510937
0.1782884 0.07929571 0.02871375 0.06407372 0.05527921
0.1843604 0.1000582 0.05026357 0.08504337 0.3939202
0.06170859 0.07080698 0.04752314 0.024113499 0.2108496
0.05614664 0.280255 0.009570861 0.08417042 0.2124622
0.02597969 0.07322515 0.0259239 0.0725196 0.02282209
0.03950636 0.07627344 0.1708734 0.0405002 0.1310582
0.0687679 0.1247057 0.08257807 0.3687679 0.1211443
0.03534916 0.06208973 0.07768805 0.08243059 0.08534384
0.159877 0.01556223 1.761616 0.1800596 0.3506418
0.04829642 0.384749 0.03818317 0.04019435 0.09265456
0.1102614 0.0778754 0.1865052 0.05286955 0.03196381
0.04032148 0.03600389 0.09389609 0.06504086 0.03188358
0.03424887 0.05847681 0.3809977 0.04596238 0.1642183
0.024660596 0.09640456 0.050194412 0.1488387 0 0.06493937
0.01739958 0.04587422 0.08170018 0.0134283 0.05402549
0.050227487 0.2618403 0.07214508 0.06229682 0.1382634
0.2268552 0.1256541 0.13641 0.05048629 0.05485094 0.03304815
0.03664517 0.07621419 0.04488318 0.0404306 0.04248593
0.03689457 0.1889038 0.05658105 0.088863 0.04022928
0.02211045 0.008337534 0.0562672 0.1622734 0.02438893
0.1300211 0.1333714 0.0700586 0.08391483 0.1519401

0.09623875 0.03561659 0.07223103 0.04228461 0.04600054
0.1130676 0.06484044 0.2303028 0.2022978 0.1159314
0.08605676 0.09623986 0.2355178 0.01908624 0.07003643
0.0475248 0.04570767 0.084811612 3.928886 0.040246063
0.062968646 0.0976696 0.1331567 0.008839395 0.07883421
0.03810098 0.05988482 0.1144539 0.1538222 0.07610261
0.09958587 0.03442254 0.1633863 0.145045 0.03142333
0.1757502 0.1354784 0.3675181 0.15113 0.09836798 0.01446459
0.09461156 0.0398241 0.06285089 0.2179285 0.1582893
0.1668599 0.03499811 0.1274648 0.2707887 0.3656363
0.2175129 0.07012458 0.03331729 0.2166019 0.231778275
0.06656329 0.1053115 0.06495703 0.0152771 0.1950224
0.04507006 0.3733498 0.08296251 0.06611868 0.05339736
0.04075605 0.2086364 0.1491584 0.02964443 0.03044689
0.06479748 0.04555421 0.03810463 0.04141096 0.1396836
0.04092973 0.045519599 0.134264 0.05377962 0.03753994
0.0603076 0.05172904 0.07404703 0.03869626 0.1466797
0.1151179 0.0488096 0.05280729 0.03391203 0.02694737
0.0743701 0.06685609 0.1386623 0.1202549 0.07358619
0.1503086 0.08842943 0.06767261 0.07820449 0.1395188
0.1977978 0.07363894 0.01823928 0.094438528 0.105879841
0.1340515 0.05131696 0.03826062 0.05488403 0.08288237 0
0.04914194 0.07723934 0.08956564 0.09147513 0.05008658
0.01631708 1.351298 0.130219313 0.7609682 0.2026278
0.02243315 0.03915384 0.07052116 0.057277122 0.1485413
0.01493885 0.04977474 0.09526368 0.1023547 0.06168314
0.06230263 0.0939011 0.06447608 0.2015654 0.1254772
0.105989 0.2518133 0.06630133 0.06198757 0.06091423
0.122417 0.06372062 0.508705413 0.05884463 0.0217002
0.04624464 0.08012193 0.1062826 0.06045635 0.07797792
0.09130272 0.100548 0.1268208 0.1257493 0.1860303
0.05485333 0.31558 0.09069472 0.07098732 0.1511446 0.2377475
0.1360901 0.05366316 0.06512769 0.08794165 0.1558032
0.06989078 0.01994533 0.09853943 0.091852925 0.04005667
0.02710744 0.1625603 0.2573165 0.05172012 0.2360378
0.07927062 0.1040912 0.3447794 0.07599367 0.06632045
0.04724795 0.02990898 0.06452882 0.1450577 0.1305436
0.06299869 0.2389806 0.6012924 0.1255406 0.08642098
0.3904979 0.09275746 0.1448838 0.03155597 0.031757754
0.07544116 0.2636409 0.06467836 0.0664446 0.03101574
0.1028311 0.09523842

AC022034.1 0.1825968 6.204264 0.6158517 0.7849237 1.109713
4.567569 0.8275596 2.093565 1.456473 1.715803583
4.837958 1.303412 0.9305219 1.101395 0.7541377

6.371528 0.6556251 3.936588224 0.1878817 0.4939856
2.325740361 2.339815 1.749982 6.138694 8.933536
1.37784 0.6744125 4.634706 1.129147746 0.580061 1.496424
2.056868 4.142025 0.5942636 1.164461 0.1200833
0.04433366 0.4045276 0.6826857 0.237006334 0.3244032
0.4077816 0.304083428 0.1330368 2.945759 0.3090276
0.7790459 1.341154 2.094752892 2.027181 0.175656
1.590686 0.1371249 1.143981 4.082355 0.01496453
0.0625568 5.447035 0.1220612 0.841655227 1.948686351
3.104419152 5.498194 0.8618066 0.5193101 1.810602
0.755751 1.59327 0.415837953 0.09779211 0.9045154 0.4234979
2.87204 3.172541 0.4262675 0.6714718 2.835391 0.2972692
0.8430219 0.333892409 0.1799117 3.005162 1.030099
3.604943 1.205302 0.4243126 3.582258 6.137453
1.56382783 2.203763 0.099711172 0.6388437 11.418 2.147693
2.452029 0.7399491 1.764052 0.8331919 0.1634495
2.770065 1.98134 0.5583476 10.92478 0.0876113 2.14792
3.778644 8.244816 5.743255 6.523409734 0.1054872
0.2432767 0.1075172 0.5233942 3.006016 0.9915822
0.3382693 0.6878317 0.1012629 1.515286 0.08277908
0.06928567 1.224485 1.368571 0.5876007 0.7196333
0.3837405 0.7014534 0.1184925 5.993015 1.391459
0.7408071 0.02078849 0.3220411 0.2391903 2.109054
2.986762 0.93788 0.137623 0.2076531 0.1677138 0.2570663
1.192058 0.3645876 0.03204194 0.7985294 1.668649
0.06320907 1.217667 0.1072815 0.850208 1.468145
1.957436 0.02757692 7.954494 7.379133 0.2102281
1.89262 2.270193 0.08549349 0.4887333 5.752169299 1.116266
1.811288599 0.1661107 0.6373509 0.9663369 1.320473
1.052397 1.160487 1.146474 2.949425 0.462463221
1.175397 0.06193629 0.8027101 0.4966796 0.6123438
1.11312 0.4274423 0.401458 0.1224323 0.2335938 1.274644
1.888298 0.4229962 0.1128059 5.395966 0.9388131
1.982761 0.4946516 1.43021 0.3335258 1.042572 0.3442874
0.1883902 2.407624 3.554043 0.1753406 0.06822223
0.08529662 0.07960484 2.513655 0.1611102 0.09440559
0.1842585 0.703154 1.107449 1.661482 0.1498986
0.3826867 0.4437618 2.102502 0.6497043 0.3692151
0.3738289 1.027778 0.6969608 0.1060797 4.106453
0.25240959 1.107563 1.42449241 0.306658291 0.2921299
2.409587 0 0.1822496 0.8221 0.2302066 0.1642317
0.8034272 0.06370046 0.09526486 1.70476 0.1021141 0.5282296
0.8767466 0.4439075 0.6803999 0.8886947 1.030242
2.40913 1.590099 0.7542199 1.460352 2.85254 1.185688

2.966637 3.023909 0.8506286 2.500432 0.1167639
0.30605 3.442697 2.191342 0.5354438 0.5640541
0.611987295 0.2514351 2.250745 0.1896023 0.2983739
1.67127 0.9116916 0.2801782 0.7684963 0.5059985 0.2648616
0.8288486 1.390793 1.520403 2.911438 1.265759
0.094568279 0.07202412 0.5832205 2.154845 10.27434
0.06525633 0.776543815 2.423576 0.1114666 1.23594 0.4430979
0.07105471 1.841688 0.9549076 0.2660143 0.2248344
2.164093 0.1129594 8.515656 1.824519 0.840379
0.2704773 3.38394 0.6243678 5.023344 0.0592063 0.1869939
1.737091 1.336052 0.8564011 0.2152325 1.962152
0.4122061 0.395241262 1.190107405 1.260978 0.118635
1.058026 0.2679822 3.358924 1.178316 0.6816404
7.241018 0.6997151 0.941007 0.9875471 1.881762
0.7459467 0.581322582 0.4103879 0.3392127 2.340904
0.1747898 0.3597934 0.671200522 1.218474 0.5279604
0.06789549 2.641352 2.693483 6.126864 4.400417
1.321618 0 0.7461958 2.745967 0.5221011 0.4087782
1.027712 0.251567 0.8905778 9.800417 3.324631
1.108696409 0.07036422 0.1049464 0.3922445 0.1676619
0.3558486 0.5553467 0.3118463 0.4993 0.1553762 2.451434
2.1139 4.706013 0.2495843 0.936536 2.479875 7.97126 0
0.1592022 0.04673316 0.106472 8.271604 0.6315537
0.09535545 0.5655093 1.795632 0.3762284 1.23617436
0.6091064 1.179872 1.128234 0.2720623 0.2256405
0.7106453 0.939989488 0.5711717 0.8204 0.1151025 0.1896676
1.537983 4.856758 1.062252 0.04796768 0.5267345
3.138812 0.2074435 1.821474 0.6194292 1.800395
0.825751562 1.264052 4.182559 0.7043578 0.40759554
0.2372789 0.385308 0.3428739 0.6071456 0.04038413
0.6148079 7.536955
AC124067.2 0.02848749 0.05448103 0.235835 0 0.1120984
0.1442511 0 0.06828366 0.08795952 0.035691705 0.0733592
0.1679031 0.08798399 0.2403868 0.0928858 0.1266295 0
0.278838296 0.1406976 0.06084332 0.066577229 0.1134845
0.2174496 0.2385771 0.1951118 0.1343506 0.04509307
0.1802055 0 0.5817671 0.08050401 0.2042854 0.1374915
1.076666 0.6261854 0.06244855 0 5.270671 2.446009
0.605063229 0.6671469 4.093397 0.326156804 0.2014503
0.6746979 3.207169 0.464067 10.10464 0.087020104
0.09101199 2.663731 0.6988118 0.183371 0.5568448
0.2777184 0.4202391 3.680793 0.05840609 5.884333
0.115861074 0.452796555 0.271949982 0.8511699 0.3666903
0.5880424 0.9458818 0.777409 0.6622159 0.085865501

0.09950118 0 0.8878318 0.1409043 0.5578237 1.39657
0.5667256 0.3096507 0.1855115 5.088409 8.064892899
0.2476639 1.182484 0.03051434 0.6444376 0.06001368
0.08274788 0.2638066 0.07040609 1.36627447 0.8354404
0.137260996 0.03180893 0.2190193 0.3366714 0.09836963
0.1164118 0.3037492 0.209982 5.737559 0.664131
0.3566712 0.09955375 0 4.510608 0.3821357 0.5587168
0.1232875 0.1418506 0.100766059 0.1898929 0.9661108
0.4346013 0 0.2198333 0.9080205 0.09047045 0.7649875
0.8124855 0.06313471 2.563055 4.834781 2.084025
2.231406 1.533766 2.21806 0.1105265 1.172527 0.5237809
0.1847108 1.661102 0.2461965 1.621638 0.5217503
0.2296419 0.1273703 0.1452386 0.2980624 0.5827839
0.2508124 6.541385 2.07644 0.7499059 0.110806 0.9598006
0.6721997 0.5529154 5.805923 1.363901 1.531464
6.12201 0.3159307 0.5373353 0.2065133 0.05662381 0.9159966
0.08944998 0.3054553 0.6408967 0 3.717128 0.472322949
0.03147325 0.145662216 3.731824 3.404189 0.09045672
0.1363308 0.5857509 0.2715766 0.1402862 0.1646188
0.065591189 0.1215761 5.159973 0.9762278 0.9027787
0.4409245 0.574313 0.08221634 2.109733 0.6303341
3.337474 0.2871257 0.1592428 0.1823489 1.161547
0.0443853 0.1156319 1.362647 0.0492588 0.3908872
0.4202777 0.5774729 0 0.1469567 0.4986116 0.3275905
3.565638 6.124885 5.090076 1.249245 4.365149
2.848667 6.97666 12.28923 0.06626248 0.3089381 0.3937412
2.007983 0.3742647 5.866555 0.3936213 1.004809
0.5341312 0.08201559 0.1246219 1.524322 3.70114 0.1790665
0.679291124 0.2126699 0.030032356 0.059803378 0.102036
0.6458659 0.2539506 1.470689 0 0.2085401 0.5978534
2.89258 1.788859 0.4830333 0.06742769 2.526222 0.1595046
1.395196 0.1449531 0.2573364 0.2617829 3.669793
0.05709201 0.5666717 0.2824036 3.0609 3.118883 0.0474315
0.2851134 0.2075232 1.23907 0.3072998 0.7715307 0.1061062
0.7746717 0.6918959 1.844759 0.7778555 0.177175703
0.2407121 2.145381 0.339305 0.3192016 2.473998
0.1765686 1.613962 0.6933714 0.1973559 0.4648711
0.6386712 0 0.1558266 0.2322728 0.4373606 0.234326453
0.3569306 12.96608 1.24379 0.4332247 0.09162756
0.305708096 0.9757666 0.5618388 0.196091 0.1575093
0.2078524 0.4972974 1.055573 6.847783 0.1753855
0.5562729 0.2988271 0.2066639 0.2111406 4.006142
0.1309593 1.110603 0.4096663 0.06406327 0.2078315 0
0.3888389 0.1885401 0.1457561 0.2583007 0.3606142

2.762928 0.030831402 2.330780088 0.05001585 0.1723215
0.4663295 1.54095 1.677637 0.5551993 0.1100119 0.2847967
0.3430891 0.4154983 0.1744192 0.1988765 0.8728307
1.56447015 3.174616 0.1764054 7.177288 2.965558
0.3157451 8.71387175 0.5819322 0.1820782 0.7799994
0.4354111 0.05627927 0.08787373 0.2324569 4.223708
4.153777 0.1284003 0 0.03818184 4.464232 1.924039
0.9419458 2.068217 0.1705197 0.02773721 0.02506828 0
0.2267033 1.401049 0.9939833 1.460975 0.07287589
1.765053 1.192306 0.9292274 0.9539313 2.257939
2.137816 2.115899 0.4121104 4.07422 0.1140936 0.1246592
2.483762 1.531107 0.09343703 0 0.4793382 42.42484
2.220882 0.2430984 11.26974 2.709438805 0.0523093
6.938228 1.645206 1.627069 0.09824057 0.8028514
0.983421989 0.3851376 5.165032 0.1134158 0.4546852
0.04113351 0.4296338 0.8426704 0.2245076 3.750439
0.1410324 0.03467561 5.13507 0.1035418 0.07523709
0.644140189 1.963586 0.2018146 0 1.285628611 1.755399
0.1311558 0.2111558 0.1518455 2.430174 0.5755084
0.1582893
LINC01419 0 0 0 0 0.02505815 0.009673644 0 0 0 0 0
0 0.02950153 0 0 0 0 0 0.01179418 0 0.011161863 0
0 0.01142804 0 0 0 0 0 0 0 0 0.1844067 0
0.06221142 0 0 0.01715812 0.09605486 0.101440581 0.03085492
0.06736389 0.019884044 0.006140676 0 0.9207521 0.04445837
0.1129381 0.019452226 0 0.02205343 0.009012138 14.19287
0.04308768 0.03724822 0.07045429 0 0 0.03831166 0
0.010844662 0.039079879 0 0.02235515 1.03845 0 0 0
0.019194129 0.02669065 0.008158117 0 0.05669523 0 0.00743298
0.01727512 0 0 0.007229545 0.037428439 0.01660863 0
0.02046327 0.01964397 0 0.01849723 0.009828428 0 0.39267398
0 0 0 0 1.653001 0 0.02927517 0.02777696 0.06034985
0 0 0 0 0 0.126036 0.7885063 0.01441084 0.05511865
0 0.067574872 0.05093774 0.05206218 0.4218335 0 0.03159059
0.1014882 0 0 0.007567528 0.007056476 0.009993105 0
0.1644201 0.07922151 0 0.09181832 0 0.008736781 0 0 0
0.01375852 0.05437444 0.9524831 0 0.021354 0 0.1817128
0.02056956 0.03153706 0.02506701 0 0.05028955 0 0.6738245
40.20099 0 0.2355951 0 0 0.00933066 1.633139
0.00900858 0.008655635 0 0 1.319696 0.01707015 0
0.1341703 0.5752497 0 0 0.009768275 0 0.01176746 0 0
0 0 0 0 0 0.01019129 0.0194401 0.07935388 0.01513534
0.4804946 0.06877516 0.009189203 1.613769 0.009607028 0 0
0 0.02620399 0.02360445 0 0 0.03151058 0.2477514

0.04095835 0.02013164 0 0 0 0.008359366 0.0915358
0.7780752 0.04866609 0 0.04409253 0.03130822 0 0.01559543
0.09036511 0.007406063 0.02301971 0.1540276 0.02433572
0.01792758 0.01221795 0 0.008866273 0.02107027 0.009166766
0.04178643 12.51207 2.656071 0.01000701 0 0.05348209 0
0.010026207 0.03421326 0 0.007741009 5.10883 0.04448872
0.104887 0 0.01077665 0.01110768 0.01245876 0 0.01144671
0 0 0 0.1833586 0.1170364 0.06438676 0.03828655 0
0.02367288 0 0 0 0.0573601 1.002005 0.006810941 0
0.07545378 0.8360834 0 0.04776402 0 0.01580724
0.019802676 0.008968017 0.9406995 0 0 0.03659761 0.2072156
0.01127439 0.2179607 0.08271822 0.0103916 0.03965743
0.01975256 0.008708259 0 0.03999533 0 0.04654256 0.02860263
0 0 0 0 0.01022438 0.006728135 0 0 0.006969411 0
0.0225919 0.0321133 0.2940388 0 0.007707573 0 0
0.04070553 0 0 0 0.2792502 0.05420108 0.1059721 0 0
0 0.008660969 0.03224427 3.889391 0 0.119215509 0.0754677
0 0.357401 0.01201603 0 0 0 0.01591566 0.02091632 0
0 0 0.9161691 0 0.6351021 0.01971658 0 0.06857714
0.01764519 0.188099423 0 0.04360848 0 0 0.00943538
0.02946457 0 0.01827398 0 0.02583202 0.009555247 0
0.01336502 0.01290282 0 0.160035 0 0 0.025216613 0
0.7348108 0 0 1.606784 0 0.07966975 0 0 0
0.1238891 0.1520532 2.830502 0.1256209 0 0.009564063
1.114639 0.01189742 0.01833531 0.01044332 0.09125584
0.06696869 0.02640834 0 0 0.1816734 0.037853764 0
0.01186953 0 0.02372024 0.03294063 0 0.104130637 0
0.06836308 0.009507238 0.01451986 0 0 0 0.01881967 0
0.04728896 0 0.3844837 0.03471817 0 0.11999105
0.008550672 0 0 0 0.1321336 0 0 0 0.01810781
0.102918 0

AP001025.1 0.01681553 0 0 0 0 0.1362372 0 0.02015316 0
0.02106802 0 0 0 0.03153222 0 0 0 0.012660927 0 0
0.039299059 0.03349368 0.02139261 0.181063 0.05758507
0.01321737 0 0.01329641 0 0 0 0.06029258 0.01623163 0
0.08213852 0 0 0.01510272 0.1040594 0.059525896 0.01357938
0 0 0 0 0.06186669 0.05869895 0 0 0 0 0
0.2705995 0 0.2295034 0 0.5431726 0.03447582 0.06744449
0 0.019091125 0.04586458 0.01674756 0.01967719 0 0 0 0
0.016894832 0.02349333 0.01436168 0 0.06653815 0.7055806
0.1701068 0.2128799 0 0.06843958 0.3690833 0.131779296 0
0.03673653 0.01801194 0.01729078 0 0.01628141 0 0
0.40324073 0.113802 0 0 0 0.04258493 0 0 0.01629967
0 0 0.05113327 0 0.05876437 0 0 0 0 0 0

0.014869991 0.1569254 0.4277053 0 0 0.01853753 0.2382152
0.01335067 0.0188148 0.013322 0 0.05277608 0.01740161
0.09045248 0.1084711 0 5.075449 0.01631034 0.1384234 0
0 0.03112735 0 0.03828867 0.03421974 0.01694407 0 0 0
0.01810549 0.2035674 0.08825678 0.01145492 0.3010039
0.02616254 0 0.1110997 0.1903847 0.2619445 0.1150115
0.08891697 0.0328517 0.06216229 0.01585885 0.04571257 0 0
0.158401 0.04507588 0.199109 1.60613 0 0.025345613 0 0
0.03059462 0 0.06674324 0.0536487 0 0 0.04140393
0.05552619 0.116151064 0.07176365 0.051334 0.01746199
0.06661127 0 0.04842917 0 0.1945826 0.03382475 0 0 0
0 0.04155367 0 0.02275165 0 0 0.2739943 0.1063202
0.1363478 0 0 0.07357984 0 0.2171534 0 0.1374635
0.01293687 0.02755776 0.03297068 0.2059084 0 0.01303776
0.02026214 0.05810416 0.042841 0.03156002 0.02150868
0.1072366 0.06243334 0 0.2743346 0 0 0 0
0.034866996 0.01569179 0.088637163 0.017650302 0.01204592
0.02932617 0 0.1215361 0 0 0.3697043 0 0.273758
0.1754608 0 0 0 0.04844431 0 0 0.06867762 0.07556501
0 0 0 0.0526247 0.09689512 0.1679865 0.05048884
0.02449927 0.02398019 0.01511602 0.09487863 0.2818446
0.06096953 0 0.1438196 0.04174098 0 0.01578745 0
0.01540648 0.0471044 0 0 0.09923809 0 0.01456185 0
0.3071798 0 0.07665082 0 0.09387792 0.046105899 0.0585245
0.1007051 0.04788142 0.1884274 0 0 0 0.01184432
0.01929136 0 0.01226907 0 0.05302821 0 0.1478945
0.01368148 0.05427416 0.05228105 0.01384795 0 0.02576745 0
0.03224225 0 0 0.04305117 0 0.4266155 0 0.4269136
0.08514503 0.05623777 0.072796365 0.163231421 0 0.0452078
0.05243122 0.1057661 0.01597212 0.04369624 0 0.0140091
0.07364286 0.4659929 0.03431858 0 0.1344033 0.020075477
0.2047123 0 0.2247989 0 0 2.163404616 0.04580022 0
0.2046295 0.1652229 0 0.01728997 0.05488565 0 0
0.03031675 0 0 0.3999762 0 2.345666 0.03756378 0
0.01637266 0.088783492 0.01295983 0 0.05703506 0.03088033
0.1207333 0 0.02003599 0.08445501 0 0 0.4119599
0.2676769 0.09107059 0.2211451 0.05592841 0.2188776 0
0.3141663 0.0161389 0 0.02008104 0.01178928 0.06198623
0.3232449 0 2.702487 0.016659599 0.01543851 0.6059641 0
0.1252725 0.02899461 0.01692519 0.076380546 0.5081676
0.1604633 2.058614 0.0127805 0.0242802 0.02305484
0.1989639 0.03313046 0.5108772 0.02774942 0 1.795128 0
0.01480359 0.211234244 0 0.0446725 0 0.097919742 0
0.05806378 0 0.01280443 0 0.02264732 0.01334781

INE1 0.230613 0.6027504 0.961641 0.3671591 0.3024877
1.167747 0.04750805 0.4974952 0.5696426 0.500816941
0.8050105 0.2718431 0.6410262 0.5765892 0.6015461
0.3075288 0.3986812 0.706117981 0.2088131 0.2626886
0.395236248 0.4134077 0.9779478 0.6621731 0.4843727
1.546809 0.4867189 0.875284 0.207968802 0.1744275
0.3041263 0.6339334 1.513716 1.065273 1.001308
0.4381311 0.1759741 0.8837248 0.868153 0.966020262
1.87473 1.070685 0.43205187 0.6325481 1.836445 1.821355
2.432932 0.8593076 1.408896916 1.240219 0.5146852
2.59644 0.4783169 0.9477921 0.9892063 0.4346918 0.835216
1.229309 0.8632894 0.479382858 0.715644441 1.048333265
0.3674892 0.1978963 1.459838 1.771039 0.1887993
1.010005 0.648761564 1.804288 0.7221876 0.6017199
1.079819 0.9174788 2.11755 0.7229227 1.706941 2.728197
2.373769 0.858447984 0.5079077 0.9908368 1.004552
2.039324 0.2429125 0.5358911 1.04406 0.7789373 1.21136807
1.994244 0.874112885 1.012837 0.2364018 1.103152
0.4954915 0.8010243 1.087887 0.7770761 1.00635 1.994685
2.232875 1.974483 1.239835 0.8114321 0.9994318
1.217716 0.5544674 0.3827714 0.720557292 1.496235
0.7914003 2.789872 0.2158457 2.42365 0.9256364 1.440347
0.877307 1.144931 1.010823 1.302815 0.6204917
1.008933 2.125149 0.428145 1.536951 2.266671
0.3937376 0.3990712 1.1535 0.4268893 1.240101 1.557802
1.126318 0.8985196 0.3093279 1.195337 0.5703185
1.423609 1.082873 1.331417 1.141565 1.327453
0.9089614 1.359717 0.5514172 0.6962639 2.075599
1.616733 1.62591 0.7959497 0.7956773 0.7539752 0.9473386
1.161237 1.359455 0.7241189 1.552327 2.530391
3.109683 1.33738 0.324423844 2.157167 0.943336254 1.664347
0.1136401 0.3783388 0.4414522 1.451276 2.700987
0.3785502 1.586463 0.672569969 1.853552 0.6727199
0.4949226 0.7673619 0.8626023 1.217648 2.011473
1.956945 0.5102704 0.6366228 0.3960005 1.532607
0.913812 1.063774 1.006067 1.185686 1.407398
0.8772757 1.212991 3.305041 0.3739825 0.1880312
0.6344796 1.049459 0.3535897 1.893768 0.7362947
0.7720397 0.8989276 1.259783 0.7385433 1.004048
0.9890246 3.087341 1.20415 0.7791492 1.475363 1.399461
1.651866 1.274583 1.098827 1.746524 1.696725
0.4976963 0.3290601 1.315388 0.7408996 0.589750334
0.8608069 1.588377956 0.516397419 2.290789 2.815313
0.4609955 1.365173 0.5012395 0.2250909 1.198418

0.7805376 1.716295 0.7419486 1.394933 1.510748
1.85076 0.3321895 0.717096 1.51032 1.240122 1.093894
1.078405 1.427173 1.066858 0.6735962 0.9449581
0.7935365 0.8770632 0.6495805 0.942764 1.644623
1.006256 2.720024 1.783794 0.9664577 0.5071843
1.233943 0.462156729 2.020793 1.009385 1.028272
0.538336 1.07992 0.3652821 1.415419 0.9121136 1.105037
0.418138 0.8553163 2.225455 0.8129367 0.4805221
2.017034 0.997643838 1.391211 2.532014 1.897016
0.7137169 1.582393 0.348302345 1.382336 1.440269
0.5996857 0.79338 1.121743 2.057601 0.9939002 2.351764
1.216961 1.12579 1.327391 1.465861 1.000218 2.148942
0.6125291 0.8078508 1.636064 0.6569027 2.180948
1.141471 0.9538616 1.967201 0.8128409 2.690427
0.350311 0.7712608 0.815319289 1.087321139 1.525086
1.167653 0.3715127 0.4641621 0.7447573 0.3795331
5.97673 0.8837738 1.043625 1.704203 0.9413097 1.287962
0.942103 0.513932223 1.180583 0.4125459 0.01581003
0.9933888 0.8946112 1.211002001 0.5862428 1.403778
0.592451 0.6126362 1.442714 0.8220146 0.777808
1.250027 1.332915 0.9839949 3.598779 0.5357579
1.333702 1.869066 1.112019 1.459621 1.587457
2.065762 1.691114132 0.9597679 1.284652 2.972341
1.468139 0.820001 0.5899477 1.337259 0.5405121
1.65709 2.016379 1.262882 1.503361 1.082439 1.98146
0.5369127 0.6619242 1.569783 0.919161 0.8705781
1.37832 1.064869 3.028834 1.98356 1.806067 0.7122067
0.8918436 0.685423507 0.2823041 0.7832758 1.059738
0.9926354 1.537543 3.636498 0.58660259 1.173753
1.448755 1.622025 1.99814 0.5327769 0.3372594 0.8640716
0.6058141 0.5803211 1.737907 0.7672667 1.291483
1.546006 1.475284 0.637323891 1.926751 1.143616
0.2668733 0.447633106 1.276033 2.123475 1.139571
1.158984 1.311523 0.4141224 1.232575
CNIH3-AS2 0.03605116 0.03447311 0.03316124 0.1033147 0.1418615
0 0.2228045 0.1296203 0 0.180672651 0 0 0.1670168 0
0 0 0.07191329 0.054288044 0 0 0 0.1077117 0 0
0.02469156 0 0 0 0.209000905 0 0 0 0.03479933 0
0.6750441 0 0.8252881 0.03237903 0.1673214 0.031904699
0.262018 0 0.225138692 0 0.03712331 0 0.04194863
0.1162501 0.073416465 0 0.1664679 0.2380949 0.07735252
0.3252425 0.210873 0.08863605 0 0 0 0.048874399
0.163719421 0 0 0.1687453 0 0 0.442718 0
0.072442358 0.02518392 0.03079031 0.07838783 0.03566313

0.1680788 0.112214 0.06519966 0.1959328 0.293458 0
0.035315543 0 0 0 0.4448408 0.7215044 0.3839666
0.07418878 0 0.4940092 0.08132747 0 0.04025447 0.03079676
0 0.331967 0.0736601 0.1397808 0.037962 0 0 0 0
0.1661317 0.7351487 0.03719969 0.1087786 0.1560213
0.1346349 0.286920725 0.04806222 0.04366506 0.05789386
0.5061394 0.198715 0.8937505 0 0 0.1142453 0.05326501
0.1131477 0.03730767 0.2327075 0.4983289 0.5354468
0.103962 0 0.1318972 0.272938 0.1669665 0.6673456
0.2077092 0.1641757 0 0.1089802 0 0.2757011 0.03429096
0 0.07935132 0.04730388 0.02455844 0.0379605 0 0.07591475
0.2041619 0.3207047 0.09359804 0.3082195 0.4765773
0.1408629 0 0.2380009 0.1306722 0 0 0.3395992
0.03221303 0.1280622 0.2025538 0.4824675 0.380372869 0
0.03686736 0 0.2664761 0.08585532 0 0.3706364 0.1472925
0.04438337 0 0.04150311 0.1153917 0 0.07487423 0.1142474
0.6626176 0.2076566 0.06936366 0.1251509 0.1812939
0.2184622 0.04037334 0.03358719 0.06593261 0.1336317
0.08425498 0.1463332 0 0 0.1236678 0 0.03653985 0
0.3719499 0.2208491 0.09212635 0.2148736 0 1.010437
0.02773562 0.1181633 0.07068652 0.1765805 0 0.05590383
0.1303213 1.079613 0.09184771 0.1691554 0.09222579
0.1915889 0.03346303 0.1590465 0.1383886 0 0.5787129
0.1142396 0 0.33638432 0.03364196 0.076012415 0 0.05165098
0.2200555 0.467457 0.03722339 0.08395451 0.08796976
0.4323364 0.1626927 0.1257676 0 0.05688688 0.0432021
0.3532453 0 0.1528662 0.08141537 0.1840492 0.3240108
0.07225043 0 0.1786921 0.5265082 0.1384903 0.1800749 0
0 0 0 0.04068245 0.3356958 0.3267846 0 0 0.1491489
0.149478261 0.1015411 0.417692 0 0.05049405 1.197096
0.2979321 0 0 0.03121946 0 0.2394797 0.186375
0.06573331 0.1469716 0.3019002 0 0.02509438 0 0.2737439
0.05771049 0.1159555 0 0.1157663 0 0.5376688 0.06644311
0.07891172 0 0.02842207 0.0606009 0.1902445 0.05866401
0.1163595 0.07472428 0.02968891 0.1843566 0.8286505
0.244431 0.5184362 0 0.02922362 0 0.0894689 0.07953303
0.153713 0.1307527 0 0.2109963 0.351156659 0 0.09494323
0.07269145 0 0.1360525 0 0.4215657 0 0.1501719
0.03947111 0.3680717 0.03678816 0.1797713 0.09604999
0.04304023 0.03376054 0.4464851 0.519024 0.2588234
0.1331927 0 0 0.1975042 0.1096773 0.1771124 0.2136657
0.07413665 0.2941762 0.1034543 0.03551784 0.09749504
0.03606335 0.04831945 0 0 0 0 0.2157941 0
0.031724126 0.2500636 0.286895 0.3668359 0.1324099

0.4807078 0.4611254 0.08591116 0 0 0.1341343 0.4675814
0.1639652 0.1115703 0.04741175 0.1798591 0 0.1051716
0.1347095 0.1730026 0 0 0.176927 0.2325637 0.1540025
0 0.2742683 0.392885435 0 0.3583832 0.705201 0.2685743
0.1243243 0.03628627 0.098252295 0.02867029 0.2580155
2.906455 0 0.1041096 0 0.319922 0.03551453 0.06638061
0.3272091 0.1316469 0.31546 0.1747108 0 0.045286945
0.2581751 0 0.1390655 0.209932151 0.6800426 0 0
0.05490336 0.03417119 0 0.1430832
SUGCT-AS1 0.008203624 0.00784453 0.007546009 0.0156732 0.02152086
0.05815657 0 0 0 0.082225949 0.00704182 0.06446871 0 0
0.02674859 0.006077645 0 0.024707037 0 0.03504246
0.028758656 0.008170112 0 0.02944446 0.02809345 0 0
0.04540752 0 0 0 0.02206075 0.3325881 0.0516751
0.3673275 0 0 0.03684006 0.2347947 0.058080604 0.1126222
0 0.256157235 0.06328606 0.03379038 0.03621874 0.07636498
0.3527106 0.142003444 0 0.1136418 0.2786384 0.2024225
0.3823879 0.5118423 0 0.5058941 0.07568717 0.09048453 0
0.223531089 0.369195628 0.08987509 0.1919943 0.01128935
0.1167379 0.2350664 0.09949577 0.016484625 0.1432682
0.6235777 0.1129711 0.1379605 0.175937 0.1085232
0.01483651 0.07006293 0.08681122 0.12418 0.048217382 0.5277717
0.08961142 1.133562 0.6073546 0.4752636 0.1906332
0.03376406 0.1317878 0.95552147 0.2128245 0.300408595
1.016772 0.6237083 0.1177277 0.1227537 0.8716098
0.373742 0.1641305 0.0894975 0.09146822 0.2259656
0.02866878 0.09451028 0.196808 0.2116245 0.0309414
0.2011862 0.02042456 0.23214315 0.164052 0.07948964
0.08563127 0.3599206 0.2351367 0.5229706 0.03907956
0.03671594 0.07149199 0.08484506 0.02574734 0.07640594
0.1412101 0.1058374 0.3807613 0.06308556 0.0795716
0.1875867 0.3194147 0.02279645 0.3189015 0.2717754
0.2334939 0.03338888 0.09919597 0.2567544 0.1359305
0.3355325 0.3003202 0.1986247 0.182992 0.352069
0.1814002 0.07020011 0.08637392 0.2787484 0.1194185
0.06389612 0.2805476 0.07229838 0.1602703 0.06065298
0.0541583 0.2750498 0.5054898 0.0239802 0.05151834
0.05864191 0.145706 0.5531057 0.6312806 0.222571976
0.081571 0.167787081 0.08209239 0.1313823 0.06512271
0.03925958 0.5673794 0.1117239 0 0.05417801 0.141663635
0.1137845 0.0250438 0.09370905 0.1364873 0.2460127
0.1417599 0.0315681 0.07594324 0.1072613 0.05799754
0.009187157 0.2598598 0.6001319 0.2128599 0.09586323
0.2219924 0.02706245 0.1985928 0.2884476 0.1296735

0.1247224 0 0.1692782 0.2153799 0.5188545 0.122239
0.1003109 0.3065734 0.2524552 0.1478876 0 0.1942119
0.06208711 0.1017696 0.1482763 0.2929159 0.04180082
0.1616671 0.2308509 0.1569493 0.1142202 0.03619184
0.05510929 0.2799243 0.07901343 0.2339623 0.08594385
0.017010212 0.1913849 0.259455139 0.008610876 0.1645482
0.07868887 0.0731309 0.04235185 0.03820855 0 0.204959
0.12032 0.3720476 0.04280016 0.05177959 0.1572937 0.06889951
0.1536212 0.09044222 0.2037913 0.1172677 0.1843257
0.1972914 0.152307 0.426954 0.7102998 0.4727125
0.3687926 0.01642099 0.227092 0.3334208 0.1917371
0.06480248 0.2597236 0.07436149 0.09376342 0.2004681
0.1289704 0.068029067 0.1386371 0.02376198 0.01503241
0.06894105 0.4086078 0.2372858 0.2614372 0.06239755
0.05683316 0.08924685 0.1226133 0.5683015 0.1794954
0.3232934 0.1373978 0.074977408 0.01713106 0.1801433
0.163516 0.0459631 0.1671129 0.332579107 0.1317161
0.2022429 0.4611626 0.5065021 0.2573802 0.2386803
0.006467585 0.1792705 0.2958224 0.03337323 0.1191519
0.04250972 0.02702345 0.03495941 0.07542544 0.09733762
1.03816 0.5534546 0.1595996 0.04900687 0.0610773 0.1085889
0.07695198 0.2157124 0.02076943 0.04801327 0.177572161
0.6484489 0.3168706 0.04962392 0.1598693 0.1960764
0.6311649 0.1385647 0.3168044 0.03417239 0.1257459
0.1615305 0.2427686 0.4581682 0.4371332 0.137116305
0.04609429 0.3894664 0.3796282 0.02944829 0.1212347
0.032309342 0.234613 0.1348292 0 0.1029961 0.2350001
0.06748068 0.02677651 0.1726381 0.01616453 0.1479033
0.09027038 0.07696735 0.0459135 0.1108142 0.2627782
0.08246638 0.01227626 0.01597513 0.01443797 0.0695484
0.2284954 0.222601 0.09039166 0.3449913 0.06995427
0.2052699 0.192277 0.0232689 0.04578439 0.5438249
0.1305889 0.450644 0.2805084 0.1227835 0.07392572
0.1794924 0.2554486 0.2440792 0.008969111 0.2841053
0.01725455 0.3250865 0.1839813 0.1300107 0.1170209
0.211316338 0.08285013 0.1529098 0.0305661 0.2444617
0.01414531 0.04128562 0.238483266 0.05219255 0.5871273
0.0244955 0.06235096 0.4264321 0.02249507 0.194133
0.0727336 0.06042097 0.2030675 0.2096984 0.5024916
0.5466494 0.06499872 0.32976874 0.06609269 0.06538177 0
0.131370585 0.1444306 0.01888466 0.2675515 0.05622091
0.06220661 0.06629233 0.07163048
AC021755.2 0.05044659 0.08039735 0.03093514 0.06425284 0.04411279
0.08514822 0.05196193 0.06045949 0.05192055 0.126408122 0 0

0.07790249 0 0.2193137 0.01245776 0.1341716 0.113948342
0.02076266 0.1795723 0.019649529 0.01674684 0 0.3017716
0.05758507 0 0.1064698 0.01329641 0 0 0.04751973
0.03014629 0.01623163 0.05296099 0.08213852 0 0 0.1812326
0.3381932 0.119051793 0.3259051 0.05929426 0.017502101
0.1081015 0.06926247 0.09898671 0.07826526 0.07229752
0.119854078 0.01343059 0.09705808 0.09519071 0.6313989
0.1264204 0.06557239 0.02067149 0 0.1723791 0.1011667
0.068390217 0.038182249 0.114661451 0.05024267 0.1180632
0.02314056 0.03418365 0.06883309 0.1189669 0.016894832
0.03524 0.01436168 0.01218761 0.2994217 0.03135914 0.03925543
0.06082283 0.01305571 0.164255 0.02545402 0 0.02923811
0.03673653 0.01801194 0.1901986 0.141699 0.03256282
0.2249277 0.1038979 0 0.09483498 0.032408846 0.0187761 0
0.1987297 0.05806541 0.05153649 0.1303974 0.01770681
0.08466885 0.05113327 0.2315886 0.05876437 0.03874484
0.3630698 0 0.07610726 0.0485159 0.08373125 0.074349956
0.08967165 0.2342196 0.1215168 0 0.0556126 0.5955381
0.06675337 0.2069628 0 0.07453403 0.1055522 0.03480322
0.108543 0.3873969 0.06243781 0.1293108 0.1141724
0.1999449 0.01818684 0.07787907 0.1089457 0.02422072
0.03828867 0.1368789 0.1016644 0 0.1285967 0.03198902
0.01810549 0.2035674 0.2206419 0.2520082 0.3541222 0
0.1947512 0.2698135 0.08159342 0.1964584 0.02875288
0.07409747 0.0657034 0.04662171 0.07929427 0 0.06684755
0.04915386 0 0.04507588 0 0 0.4500797 0.126728064
0.1114678 0.309532208 0.09178386 0.3521659 0.0266973
0.2145948 0.03143234 0.06870242 0.1035098 0.06940774 0
0.1614682 0.051334 0.1920819 0.05328902 0.130134 0
0.08088413 0.07783303 0.03382475 0.1868138 0 0.04699875
0.03075329 0.2077684 0.03929949 0.0455033 0.1802832 0
0.2595735 0.07088016 0.05113042 0 0 0.1765916 0.1611412
0.1336328 0.06853807 0.2749269 0.1811162 0 0.03297068
0.09609058 0.09544815 0.06518878 0.04052429 0.1162083
0.2856067 0.1893601 0.1935781 0.05361829 0.06243334
0.6491178 0.2904719 0.05884923 0.2159457 0.1332135
0.070466 0.331236463 0.1412261 0.195001758 0.105901814
0.02409183 0.161294 0.0136274 0.05208689 0.1566374 0
0.01680474 0.1707426 0 0.219326 0.03980107 0.1209059
0.141228 0.04844431 0 0.037975 0.4807433 0.4282017
0.1685007 0 0 0.01754157 0.03229837 0.2799776 0.08414806
0.02449927 0.05995047 0.04534807 0.1328301 0.1252643
0.06096953 0.02402416 0.123274 0.08348197 0.139443841
0.03157489 0.2435328 0.01540648 0 0.2362325 0.05211226

0.1587809 0.1534807 0 0 0.1117018 0.1043182 0.1379715
0.04570183 0.1642864 0.015368633 0.0351147 0.1342735
0.04788142 0 0 0 0.21599 0.02368864 0.01929136 0 0 0
0.0265141 0.4239959 0.07394726 0.01368148 0.01356854
0.08713508 0.05539181 0.1003222 0.02576745 0.1710168
0.0644845 0 0 0.04305117 0.04173144 0.074194 0.01433943
0.09148149 0.1419084 0.1687133 0.018199091 0.489694263
0.02952324 0.0226039 0.05243122 0.04230644 0.07986062
0.04369624 0.06493757 0.04202729 0.03682143 0.04905188
0.01715929 0.03354067 0.1344033 0.040150955 0.09448262 0
0.01729222 0.06036217 0.04659433 0.24283113 0.06870033
0.01535382 0 0.08261147 0.0996612 0.1037398 0 0.2251888
0.1325342 0.07579186 0.08410608 0.02253789 0.2352801
0.2044291 0.05212591 0.07512755 0.02516349 0.01637266
0.473511957 0.1036786 0 0.4562805 0.03088033 0.1034857
0.01433901 0 0.3659717 0.02384793 0.07820617 0.2907952
0.07647912 0.5334134 0.2653741 0 0.06734694 0.09811143
0.2932219 0 0 0.02008104 0.08252496 1.828594 1.095441
0.06149812 0.6236509 0.283213185 0.06175403 0.2716391 0
0.5428475 0.01449731 0.01692519 0.076380546 0.240711
0.2206371 0.2008404 0.07668302 0.0485604 0.1383291
0.09948193 0 0.01548113 0.1109977 0.1023412 1.647986
0.1018641 0.07401797 0.380221639 0.07526373 0.0446725
0.03243252 0 0.4652205 0.03870918 0.02492812 0.07682657 0
0.2264732 0.01334781

GIHCG 0.2657674 4.70148 0.4889261 0.8779916 1.699418
4.508287 1.266097 1.964196 1.572813 4.495196144
0.7794415 0.7179392 3.300405 0.3426245 2.238604
4.585977 0.8835688 2.301200666 0.6289567 1.300804
1.293993399 5.425976 0.6762151 4.915197 3.731512
0.5483595 2.103427 3.931523 1.134159128 1.608137
0.8762174 4.387388 0.6199692 0.9067955 0.6490946
0.3155746 3.606604 0.7658184 0.7966257 0.529199738
2.038894 0.1171423 0.345773219 0.2135663 0.4333128
0.2525972 0.8890743 0.5356188 0.744181069 0.3714704
0.6519463 1.91194 0.8672385 1.282088 4.490909 0.08167758
0.699139 2.066027 0.3553173 1.681398517 1.269792603
0.468154314 1.566101 1.917806 0.2590613 2.77638 1.359873
1.72357 0.189139709 0.3248956 0.2931885 0.6340532 0.7996749
2.2613 0.9823431 0.9012163 0.7823872 0.5408396 0.6872586
0.379669008 1.136007 1.088656 0.4270147 1.912951
1.108103 0.6433143 0.6152805 1.012624 1.63122818
2.98522 0.384163398 0.4327663 1.825717 2.131324 0.586319
0.8371537 1.50275 0.3964598 0.3903027 0.8530527 1.053702

0.1257701	0.7399319	3.719251	1.11979	1.010742	0.4153434
1.847193	0.440659498	0.3838384	0.3621338	0.9158218	
1.807317	3.16178	1.019677	1.046237	0.3345362	0.2631908
1.169821	0.6255897	0.710495	6.564203	2.908311	
2.096997	0.6173802	2.921539	0.4557843	0.6587184	
1.989905	0.5739579	0.3828056	0.6807912	1.014073	
0.5244395	0.3713349	0.9174275	0.4845166	0.9657736	
1.096826	0.4359024	1.848154	0.5247055	0.8614495	
1.480714	2.487549	0.9940467	0.6181252	1.552656	
0.5660324	1.200689	0.2149147	0.7937163	0.1806204	
0.990485	1.602296	0.6954191	1.029049	3.055109	
2.146499	1.000329	1.619028289	1.957483	1.302300145	
0.6547995	0.7639523	0.7296175	2.720382	0.63133	0.6786459
6.625638	3.912565	0.815890399	0.7679585	0.2028319	
1.103938	1.719546	1.681823	2.248413	0.2876319	
0.8713503	1.492414	0.6486707	0.471248	0.8769279	
1.397399	0.3694223	1.630449	0.509415	0.301374	
0.2106264	1.452979	0.5834647	0.853005	0.711029	
0.4760411	0.7365163	3.990012	0.8910214	0.270809	1.0475
2.675092	1.252198	0.2605488	0.2169571	0.7961772	
0.5409079	0.9607221	4.336555	0.2539113	0.2494011	
0.8781835	1.106368	0.7708994	0.4274678	1.774712	
0.523184	1.066561	3.263405	0.8816843	0.470704447	
0.299675	0.980629684	0.848506001	0.222115	1.149085	
0.4666554	0.5373843	0.2836664	0.2567014	2.512104	
0.7246149	0.9915381	0.1299908	0.1223155	0.3450241	
1.023042	2.153408	0.4132047	0.6251981	1.956056	
1.252352	0.6324941	0.7342545	0.6037668	1.224487	
0.6806291	0.97719	3.513284	0.9680198	0.5527141	1.015354
0.3748863	1.959164	1.365123	0.1740287	1.596548	
0.4581328	1.090467838	0.5822102	1.603753	0.9232612	
0.3257097	2.885074	1.029535	0.4836042	0.6232812	
0.728803	0.4336907	1.213735	0.4007346	0.8783062	
0.4965894	1.035519	0.35422825	0.73227	0.7737099	0.4309328
2.791943	2.611944	0.646988932	2.026882	0.382196	
2.058059	0.5714513	0.2908667	1.030982	0.2531774	
1.154096	0.7109758	0.9099853	0.2680614	2.696937	
3.656873	0.5096506	6.074833	1.35145	1.964025	1.170424
0.1974822	0.3213087	1.277898	1.135983	0.3021773	
0.5723171	0.3177363	0.7962445	1.270385347	1.21314502	
0.7485222	0.1488549	0.5351821	1.072623	0.8204218	
1.913576	0.3563647	1.715943	1.976233	1.631274	
0.7119013	0.9939492	0.5015537	0.568478765	0.3940617	
0.6628648	3.017703	1.629779	0.5011732	3.227338792	

0.5579807 0.2932205 0.6513206 0.9127561 0.6672439
0.9222723 2.078292 0.5296251 0.1418277 0.379329
0.7421849 0.4007347 0.4958098 1.002201 0.50346 0.7792193
1.905674 0.3018959 1.22781073 0.964401 1.70373 1.558726
0.4880598 0.6474166 0.7365353 1.055555 0.6674006
0.1884568 0.793125 0.3510821 2.744854 2.081931
1.106804 1.491652 0.5100299 1.049912 0.3310241
0.6589393 0.08474853 0.7141013 0.3571289 2.234906
1.229907 0.5129843 3.001247 0.438838221 0.284671
1.045785 0.6704682 0.8524641 0.4105213 0.7467725
0.834969769 1.153651 1.492603 1.311586 0.176745
0.639576 1.776347 1.343006 0.1527233 0.8563706
1.452784 0.5526425 1.104639 0.8720558 0.5459276
1.015469988 0.3865986 1.127708 1.089258 0.564232659
2.116702 1.567722 0.426818 0.1770759 0.3043892
0.6114777 2.637006
LINC00926 0.5504164 0.1476734 0.7685473 0.2420908 1.412763
0.3408714 0.1590723 0.4555966 1.167639 2.946973324
0.2515287 0.2722876 7.337994 0.1373701 2.788852
0.08214179 1.658769 0.324981045 0.2738023 0.4905275
0.189715162 0.1340843 0.3627123 0.4074287 0.1139083
0.3610513 0.796042 0.3005881 0.329043035 0.2965125
0.1342831 0.3123579 0.5886389 1.0102 0.4223115 0.1128465
0.01236122 2.151674 0.3063074 0.536171116 0.7386827
0.195482 0.168981857 0.5193114 0.693191 0.4166669
12.15486 0.5022401 0.90720059 1.581361 0.3519804
14.53312 0.3780871 3.745105 0.6871429 0.1947142
0.2209351 9.896562 0.3772038 0.778407054 0.229280816
0.334815187 0.9780683 0.4819062 1.182496 0.8801103
0.6375644 2.561381 0.151183194 0.5117436 0.2604127
0.2009013 1.214335 0.2326166 0.4806944 2.22722 1.371202
0.6285473 2.005019 0.760290033 0.09639251 1.427408
0.3520502 1.164519 0.1209597 1.081203 0.3585488
1.844778 0.52905033 2.277901 0.095398076 0.5173177
0.09471535 9.393058 0.3372826 0.3762206 1.093931
0.5337236 0.3123678 1.513177 15.00235 0.4428229
0.2645927 0.3989908 0.6701014 8.375657 0.5998041
0.4042104 0.154074023 0.2481184 0.4963999 0.7153892
0.1250864 26.88176 2.061545 0.1854903 0.3633117
0.454887 0.2135464 0.4888369 0.1147396 0.5325085
0.3247672 0.419043 0.3425732 21.70472 0.3839183
0.5481926 0.660221 0.5167699 0.2481089 0.3200845
0.7010709 0.4628515 0.5577007 1.907815 0.64407 0.2217072
0.5796064 0.5039945 2.368387 0.4461419 1.540231

0.9922713	0.3176872	0.1921415	1.12574	1.235692	0.1535508
1.578171	0.3952363	0.7394402	0.1112352	0.4210904	
2.207945	0.6589896	1.11455	0.9096171	0.1557384	0.5564354
1.128055924	7.616631	1.117656772	0.6952458	1.000042	
1.395681	0.8780314	0.3145804	3.602411	0.5021284	
0.8237672	0.273520085	1.901179	0.1934154	0.6743782	
0.4580328	0.5209615	0.2394928	0.6361739	0.5315302	
0.4460552	0.3599357	1.024387	0.4980407	0.6047057	
0.9002503	0.9131106	0.09108102	0.2090056	0.1027064	
0.4176947	0.2295057	0.08829717	1.162319	0.1702278	
1.354976	0.4022336	3.90999	0.153328	1.183846	1.063214
1.716467	0.7259481	0.1034424	0.2247675	1.240367	
0.3483167	0.2645331	0.1984067	0.7209	0.7952055	1.393111
2.466295	0.7118838	1.200839	0.3880296	0.8348251	
0.4548646	0.759166	1.362977391	0.2438836	0.784819385	
0.083128162	0.2127488	19.0189	0.3850883	0.4538331	0.2489804
0.1062878	0.368028	0.4199458	1.496539	0.9813181	
0.3467867	0.9822738	0.8425182	9.656856	0.2451442	
0.5052568	0.8248053	1.257481	0.6586821	1.517611	
0.3631068	0.5122195	2.186676	0.5735992	5.750526	
0.8480793	0.5279955	0.9183818	1.1216	0.346602	0.9404164
0.2149799	0.3386758	0.3243715	1.17803251	0.4609986	
0.497022	1.338738	0.1386556	0.6574402	0.1881666	
0.4627108	0.4879254	0.4080653	0.254165	0.4833407	
0.4790278	0.4765262	0.6833974	1.387213	0.285909354	
0.4988987	1.241069	0.6915597	1.423077	0.2929385	
0.325788676	7.205553	0.5829382	0.1362856	1.218776	
0.1560168	1.943196	0.5837873	0.2729105	1.863253	
0.4188345	1.051223	0.545809	0.4826289	0.3577427	
0.2791228	0.4429936	1.632412	0.2493386	0.3274097	
0.7333132	3.316677	1.42831	0.4018324	0.843754	0.9056145
0.9468913	0.394278923	0.565599448	1.595559	0.151703	
0.5679549	0.09962596	0.1993446	3.28247	2.125577	0.7620582
1.565106	0.415838	0.2626508	0.9833478	0.1793509	
0.978593348	0.5339852	0.3105966	0.2972622	1.876311	
0.3547722	0.530246583	1.326596	1.381166	0.5621873	
0.3350392	1.228204	1.93806	0.3942073	1.037852	0.2028651
2.141755	1.810254	0.1618748	1.041619	0.7969893	
0.1800326	0.6899695	0.6458973	1.673305	0.756147876	
0.5096615	0.2415944	0.5238085	0.5744802	0.5483134	
0.4794832	0.2406285	0.2187682	0.5952817	5.10137	0.473642
2.863558	0.4871282	4.436933	0.7638824	0.2973618	
1.293819	0.2170137	1.337773	0.2900648	1.546322	
0.7551308	0.1496185	1.370157	0.3089488	0.4443498	

0.286387091 0.2435825 0.2706311 0.4573752 0.570333
0.3687034 1.343165 0.341745172 0.440877 0.7793559
1.872102 1.07745 0.1600945 0.6080597 0.6910862 0.1794408
0.08384872 0.712273 0.3036597 1.469155 0.9211244
1.512944 0.795885034 1.166212 0.564561 0.08401164
0.7378809 0.7569127 19.26559 0.2641606 0.3950004
0.6005337 0.6506429 1.122133
AC007497.1 0.1035909 0.07429231 0.04764343 0.07421719 0.06793841
0.07868242 0.08002693 0.03103803 0.0799632 0.032447005
0.1333804 0 0 0.0242815 0 0 0.05165964 0 0 0
0.060524754 0.02579192 0.1976814 0.09295211 0.01773743
0.08142462 0 0 0 0.05876435 0 0.1160713 0.1999876
0.3262624 0.3795063 0.5109427 0 0.0232598 0.04006566
0.068757185 0.271878 0.06848963 0.080865323 0.03329757
0.1866753 0.1333939 0.3917449 0.05567295 0.474655111
0.04136909 0.05979193 0.2443398 0.3889687 0.584103
0.1009885 0.1273452 0.4562966 0.07964467 0.1817755
0.035109416 0.117609495 0.03531818 0.05158606 0 0.1069168
0.1930371 0 0.1046982 0.078059547 0.434187 0.04423706
0.2252427 0.2561897 0.09659286 0.1410676 0.1405105
0.4021438 0.1686468 0 0.076107829 0.04502981 0.1697346
0.05548062 0.3195559 0 0.3510516 0.2664713 0.2560222
0.53231473 0.1752672 0.299478537 0.08675162 0.3318474
0.06558534 0.05961796 0.1322862 0.100413 0.05454077
0.1086659 0.3150028 0.09727395 0.09050341 0.2386848
0.06212958 0.05344555 0.05860666 0.03735983 0.03223877
0.091605508 0.06905196 0.3136723 0.2911205 0 0.1427489
0.4585962 0.3495449 0.05795359 0.1641385 0.09565865
0.3522169 0.1876024 1.142313 0.8114204 0.1442414
0.1742584 0.07535898 0.07106227 0 0.09595368 0.09587888
0.2051638 0.2653589 0.05270205 0.1826697 0 0.03300877
0.1231663 0.3067284 0 0.5776807 0.9526573 0.6817327
0.2014655 1.172484 0.09777451 0.6073692 0.8404637
0.2878363 0.04564721 0 0.1196707 0.1953947 0.1408045 0
0.09462775 0 0.1851245 0.1226597 0.1455066 0.7798172 0
0.3719566 0.211872314 0.1177975 0.0957129 0 0.1652495
0.3146596 0.6348545 0.1912994 0.06413721 0.08944253
0.1105237 0.2371796 0.2151466 0.5334601 0.3006303
0.2983444 0.2242264 0.449517 0.1562812 0.2354018
0.05800519 0.241277 0.7814954 0.09599565 0.141226
0.1751999 0.02135811 0.08956145 0.044419 0.2183261
0.1574926 0.07918428 0.04453233 0.06799249 0.1158148
0.1800827 0.1847229 0.06048813 2.988624 0.403198
0.05077838 0.1268484 0.1470004 0.3614317 0.09361761

0.1193155 0.1319595 0.1944232 0.2318796 0 0.1682695
0.02856317 0.2733853 0.02265853 0.02771495 0.1025815
0.1085252 0 0.3866726 0.191114995 0.027183353 0.111312
0.2935754 0.2938272 0.3743571 0.2412383 0.03159698
0.0258811 0.08765395 0.4216166 0.2026713 0.1021632
0.1241387 0.1450042 0.03730471 0.3953266 0.263185
0.3173126 0.4073237 0.2335582 0.06868748 0 0.2971748
0.09948589 0 0.1555164 0 0.2031263 0.6285679 0.5552683
0.3858408 0.0938996 0.2034988 0.2214976 0.1499997
0.080534411 0.1702005 0.3000532 0.07118286 0 0.3968981
0.3210338 0.1834047 0.3545649 0.1345609 0.05634801
0.04300816 0.02677687 0.1416606 0.07038571 0.2891641
0.236693386 0.270402 0.1292472 1.056976 0.1243707 0 0
0.3326477 0.2553813 0.02971076 0.3818406 0.3023308 0
0.04083456 0.4353327 0.1594414 0.2107094 0.5642191
0.02683947 0.04265466 0.02207241 0.07936926 0.04389736
0.4717369 0 0.3358893 0.06630341 0.03213544 0.02856668
0.04416852 0.3052644 0.04371082 0.1948774 0.112114188
0.071826813 0.09093791 0.1914683 0 0.2280476 0.1967902
0.06729689 0.06667387 0.2157551 0.1134179 0.925428 0
0.1291406 0.4829893 0.12367353 0.07275667 0.1069124
0.1864231 0.2788926 0.07176026 0.203992316 0.1410745
0.02364652 0.4202017 0.05654689 0.1534889 0.05325681 0
0.2477248 0.1786022 0.3968738 0.2331582 0.08677691
0.03623565 0.6646679 0.02675982 0.02892612 0.1162634
0.3530191 0.159525419 0.5189476 0.1030469 0.08784009
0.3329131 0.5578267 0.04416721 0.09257272 0.1734263
0.03672836 0.2649809 0.2239279 0.117786 0.1402584
0.3746458 0 0.1555822 0.4910818 0.645133 0.2237006
0.1698855 0.3401964 0.2723513 1.933175 0.6914329
0.284141 0.1477675 0.384863467 0 0.06436204 0.07236975
0.128622 0.06698221 0.1824662 0.070580526 0.453103
0.2162394 0.07732893 0.1771501 0.1495764 0.03550692
0.6128512 0.02551222 0.09537036 0.1068427 0.4098027
0.407905 0.2196342 0.09119647 0.195193997 0.139097
0.02293347 0.04994956 0.075403438 0.06513539 0.2980814
0.2303518 0.1183211 0.07364165 0 0.04111411
AC119424.1 1.328331 0.11908 0.381828 0.07930637 0.05444778
1.177089 0.513087 0.1989981 0.4485935 0.104015826
0.1425264 0 0.3846157 0 0 0.2460231 0.165606
0.375052829 0.1537623 0.3546297 0.097012534 0.1653631
0.1584275 0.8442707 0.4264586 0.3915361 0 0.1312926
0.320866151 1.036099 0.1173058 0.03720913 0.1602758 0
0.2027648 0 0.1295809 0.03728214 0.1605488 0 0.100565

0 0 0.1067425 0.256469 0.03054447 0 0 0.33813526
0.03315437 0 0.3524776 1.024258 0.1248312 0.1618701
0.05102904 0 1.063825 0 0.056275379 0.282766828 0
0.1653702 0 0 0 0.1132796 0.1258621 0 0 0.03545284
0.09025799 0.2463813 0.1935307 0.03230161 5.780602
0.09668685 0 0.09425261 0.040663326 0 0.2720603 0.04446376
0.2987847 0 0 0.1281346 0.05129587 0.14220408 0.09364277
0 0 0 0.1051239 0.2388977 0 0.04023691 0.5682369
0.03483518 0.1683015 0.05197208 0 0 0 0.08566558
0.2191889 0.1796474 0.1550224 0.036707636 0 0 0.4332942
0.07284793 0.1830449 0.4410385 0.06591419 0.04644567
0.03288632 0.09199629 0 0 0.3126038 0.3825267 0.0770661
0.3192129 0.1207897 0.07593511 0.1346865 0.3076001 0
0.02989529 0 0.08447386 0.1254829 0 0.5819918 0.1579344
0.08938941 0.09136738 0.05446704 0 0.1311264 0 0.1748208
0.07835928 0.03356986 0.2694287 0 0.1097489 0.04054838 0
0.03914871 0 0.04125449 0.1820097 0.2606828 0.2967281
0.04915148 0 0 0.062567456 0.04586103 0.042450131 0 0 0
0 0.1163895 0.2261291 0.05110428 0.9937602 0.047787866
0.2657305 0.04224055 0.08621233 0.03288694 0.1606225
0.05977544 0 0.0480341 0.0834988 0 0 0.309386 0 0
0.09701359 0 0.5135099 0 0.03559865 0 0 0 0
0.07265484 0.02651923 0 0 0 0.2874203 0.1020424 0
0.1016599 0.1570804 0 0 0.09562285 0 0.1558163
0.1061914 0.1323606 0.07706058 0 0.278853 0 0.04442311
0.3617316 0.2609255 0.129107506 0.1162089 0.043761433
0.174284129 0 0.3981657 0.639164 0.1714403 0 0
0.04148371 0.1404968 0.0482708 0.1082844 0.03275059
0.09948827 0 0 0 0.09374399 0.2543034 0.4663441 0
0.2752405 0 0.08660522 0.1594617 0.4146868 0.3323608 0
0 0.03731498 0.1405288 0 0.1505076 0 0.1521553
0.1717343 0.043028385 0.2338346 0.8015708 0 0 0.1590427
0.04288094 0 0.189439 0 0.2709534 0 0.04291949 0 0
0 0.037938569 0.05778877 0.1242989 0.275797 0 0.04450482
0 0.3554578 0.08771566 0.0952442 0.2677657 0.03028707
0.1610296 0.03272598 0 0.03650882 0.1350948 0.368444
0.6022777 0.1367386 0 0 0.1407224 0.1591846 0
0.03364891 0.1416999 0 0 0 0.2634667 0 0
0.044925757 0 0.07288024 0.3068964 0.06471511 0.1566547 0
0.05393365 0.05343434 0.380407 0.04544816 0.03027202
0.3812304 0 0 0 0.0777457 0 0 0.5960333 0 0
0.05653056 0 0 0.04531829 0 0.08536306 0.06774457
0.1191203 0.04089626 0 0.08304875 0 0.1161611 0.2242879
0 0.3709155 1.056004 0 0.182640326 0.03199226 0.05505651

0.07039756 0 0.2554618 0 0 0 0.05887031 0.1930575
0.2392829 0.09439709 0.03211632 0.3275475 0 0 0.06054877
0.1034056 0 0 0.1487144 0.1746161 0.03825436 0.3103151
0 0.1973752 0.123376231 0.1143332 0.1031632 0 0
0.03578764 0.1253431 0.1131305 0.06602358 0.2970864
0.04131574 0.06309917 0 0.1707376 0.1227891 0 0
0.06850144 0 0.6538134 0.1508752 0 0.208578728 0.1114763
0.07351817 0.40031 0.060430469 0 0.09555639 0.06153684
0.1580432 0.5114939 0.05590653 0

PDCD4-AS1 2.875845 0.7857033 2.353297 2.550954 3.331253
1.096902 1.731174 1.141425 2.623523 4.866550219
1.330461 3.705521 5.075477 2.661352 5.540912
1.258971 3.576069 1.743497513 1.890737 2.393066
1.898477925 1.450647 3.04094 1.050072 1.547602 2.054977
3.901884 1.255127 2.526099198 2.754296 1.372086
1.355885 1.586277 2.411427 2.022001 1.760278
4.372108 0.9056973 1.184512 3.073923412 1.839814
0.6914107 1.535505682 1.104469 1.326848 1.319144
2.129459 0.822965 1.464130021 1.416944 0.6467212
3.400437 15.20588 2.442872 3.313344 1.101912
1.754807 4.479552 1.554171 3.31648602 1.802123636
1.884574427 2.622435 1.398548 1.747499 1.379297
2.242295 4.076769 0.86306969 1.030566 0.9729026
1.015112 1.163819 0.5920348 0.7701733 2.938259
1.754365 0.8816566 2.077676 1.079302921 2.175495
2.81502 1.380205 1.516966 0.6294494 1.771953 1.383459
1.799971 1.85523315 2.190615 0.899782881 1.68898 1.292158
3.609976 1.590604 1.945933 3.02294 1.789435 3.087257
1.249282 4.489105 0.5220814 2.323495 2.128018
1.271771 2.00031 1.670244 2.045711 2.080728446 1.916991
1.232696 1.919278 1.999104 5.31136 1.851833 1.571604
5.516169 1.139185 0.7725481 1.230809 1.352763
2.451006 2.426439 2.669578 1.579653 5.089831
0.7857039 1.37341 2.110303 0.9679084 0.4707164 1.211851
2.546159 1.561816 2.129114 1.808949 1.225615
0.8243818 1.274211 1.127146 1.755518 2.438255
0.9587999 2.988582 1.163303 1.359191 0.7514912
1.596561 1.250781 1.368117 2.105528 2.183874
0.4399688 2.783883 1.214568 2.521386 1.26815 2.365968
3.40996 0.5623103 2.927320542 3.961261 2.940953835 1.953651
0.5981441 1.941971 1.81716 0.9948456 2.950142 12.36881
1.757425 1.07491987 1.514231 2.033302 1.47381 1.36113
1.083892 0.9680855 2.604926 0.9291943 0.8827476
1.47111 2.384103 2.296535 2.100394 1.592072 2.47314

0.3789992	2.710558	1.69525	2.418238	0.5116491	1.305994
1.741494	3.78914	1.274728	2.362188	3.54316	1.446171
0.4143592	3.117619	1.117045	1.226611	1.234815
0.7066598	2.171842	1.350115	2.107882	0.8880973
3.417244	1.52872	2.26272	1.802702	1.833073	1.469534
1.846262	8.713324	3.165892	1.819436	1.277799219
1.097861	1.456839496	1.744521406	0.5083439	5.829652
0.9836952	1.52324	1.73952	1.982203	2.463428	0.9059453
1.889262	0.5845687	0.7808785	0.716111	2.248031
5.433733	1.932087	1.623651	8.828164	5.720407
1.88998	1.683991	1.804951	1.636371	2.044512	1.336983
2.168035	1.632445	1.225024	2.115155	0.9272252
0.8346658	2.369818	0.9871522	1.779708	0.7571321
2.380940845	0.8766294	4.651789	2.036031	0.8631366
2.170313	0.6366001	2.005783	2.102172	1.115835
2.661357	1.131949	3.012088	1.923798	1.421104
2.293614	1.041116244	0.7669267	1.472521	1.205283
1.449846	10.63139	0.489866443	4.95722	1.670506	1.392548
1.462731	1.076398	3.29614	1.604751	0.8161648	2.907097
1.656127	2.230124	1.509565	1.076509	1.527933
3.405278	1.456061	1.718703	2.057765	0.8931235
0.8287074	1.413505	1.25653	1.321734	1.76096	1.087405
1.233472	1.778551809	1.39840712	2.491792	0.5899095
2.518316	1.714879	3.724913	6.599593	1.538469
1.400189	2.862417	1.715933	1.295813	1.48993	0.8955612
2.140283295	1.189169	4.278624	2.611703	4.692422
1.793822	3.775431045	2.822895	2.472399	1.666494
1.906223	2.988299	7.258054	1.828581	1.804156
0.7359224	2.137915	2.802095	0.550643	4.232864
3.027022	0.6174709	1.355772	1.620814	1.309139
0.936677252	0.6332659	1.312723	1.551823	1.371753
2.566658	0.6051134	0.4672661	1.281806	0.6621018
2.43183	1.130289	2.165794	3.409779	2.529581	4.223529
1.776297	2.151894	1.535135	1.523445	1.551679
2.319282	2.003147	2.960042	1.615393	1.661872
1.278626	2.46065277	6.635145	2.018842	1.774268
1.576694	1.191388	1.240543	1.475931324	2.494979
0.6905286	1.617049	0.8515955	2.15713	1.869042	1.933376
1.453311	0.9455824	1.510025	0.681924	0.4248573
1.380114	1.874158	1.900130763	1.019717	1.554465
1.224599	2.256433331	1.808261	6.276261	0.3045203
1.151806	0.849624	1.458744	1.304448
FRGCA	0	0	0	0.2959894	0.1354743	0	0.1063865	0
0.1063018	0	0	0	0.1063314	0	0	0	0	0	0

0.07353103	0	0	0	0.123569	0	0	0.1089927	0	0	0
0	0	0.03323254	0.108432	0	0	0	0.2164484	0	0
0.08340699	0.06069934	0.179168429	0.3319894	0	0.2533309
0.04005995	0	0	0.0549954	0.07948643	0	0.03693491	0	0
0.126968	0.3032967	0	1.001119	1.866955699	0
0.046951419	0.4457549	0.6043043	0	0	0.1879045	0.104388
0.172951839	1.827804	0.9409286	0	0.1362298	0.3210225
0.2411139	0	0.02673017	0	0.130286	0.809412167	0
0.1128212	0	0.1416041	0	0.03333446	0	0.3828957
0.11794177	1.009655	0	0	0	0.0871881	3.130579
0.2110309	0.1001155	0	0	0.06979328	0	0.1203138	0
0.1651881	0.213149	0.0519405	0	0.1714308	0	0.09179657
0.0416991	0.05528727	0.7854456	0.1138609	0.2438602
0.02733408	0.9245109	0	0	0.900445	0.1425118	0.03703836
0.03172615	0	0	0.03339367	0.06297936	0	0	0	0
0.07839197	0.4904288	0.1734558	0	0.3510507	0	0
0.03788932	0.6776113	0.07035818	0.1087541	0	0	0.03249496
0.3897923	2.234598	0.05886846	0.2123893	0.03363017
0.03181766	0.1298773	0	0	0	1.621546	0.3999149
0.1222963	0	0	0	0	0.07041491	0.5011138	0	0.1639796
0.38444	0	0.1875478	0	0	0.039634486	0.4040531	0.2802691
1.215553	0.3818625	0.3330443	0.5949214	0.06624065
0.07967741	0.1038788	0.4172525	0	0.03207497	0.03148204
0.1276151	0.0268205	0.2329079	0	0	0.2361996	0.1813995
0.06978938	0.1578995	0	0.1506469	0	0.1709994	0	0	0
0.3667402	0.1350079	0.7307308	0.3908398	1.17451	1.286022
0.07930805	0.08771238	0	0	0	0	3.113656	0.2643157
0.06024376	0	0	0.0360679	0.285545826	0.5140364	0
0.939564913	0.1973019	0.09006332	0.3627085	0.03554745	0
0.04200452	0.1720296	0.07768383	0	0.04490467	0.3531166
0.08251397	0.04819156	0.04959228	0.08759015	0	0.4921354
0.3094226	0.03449872	0	0.1706467	0.07182898	0.1653187	0
0	0	0.04909688	0.1237938	0.07770155	1.859373	0	0	0
0	0.107061148	0	0	0.2208018	1.639501	0	0.2133886	0
0.1571176	0.2086969	0.03745407	1.457946	0.2135804	0	0
0.2402563	0.031465637	1.078404	0.06872765	0.06535474	0
0.1845579	0	0.03685137	0.02424998	0.355473	0.0317258
0.1004784	0.06677769	0.4342784	0.8680864	0.06055965
0.05602274	0	0	0	0.1173708	0	0.1750693	0	0	0
0.2056663	0	0.493689	0	0.0936493	0.1452711	0.5181337
0	0	0.03022285	0.4627907	0.161021	1.169342	0.06540244
0.0894634	0.04431758	0	0.07538795	0.5272496	0	0
0.2751764	0	1.096178	0	0.1062119	0	0.03179898
1.355920487	0.3281987	0.2829178	0	0.1127588	0.03400761

0.03539937 0 0 0 1.303476 0 0 0.5780531 0.232526
1.422963 0.3076313 0 0 0.636211563 0.1061355 0.2739779
0.5254794 0.06322418 0.6003151 0 0.9024743 0.05763755
0.04882609 0.03202376 0.7442153 0 0.05327352 0.0452771
0.2290149 0.5170713 0 0.08576295 0.03304267 0 0.1233414
0.168961 0.2538204 0.5515078 0 6.940873 0.136435011 0
0 0.2565525 1.966363 0.1484084 0 0 0.1368972
0.04106645 5.174258 0.3140006 0 0.09440466 0 0
1.267838 0.02840699 0.08381309 0 0 0 0 0 0.03048739
0 0 1.039079 0.07925293 0 0.1835099 0.1305307
0.8809915 0.2459539
DIRC1 0 0.01339697 0 0 0.07350713 0 0 0 0.04325878 0
0 0.05505018 0.04327081 0 0 0 0 0.010548738 0 0 0
0 0.03564747 0.01676186 0 0 0 0 0 0.03179055 0 0
0.01352375 0.2647539 0 0 0 0 0.05418717 0.012398837
0.0339419 0 0.131240443 0 0.0144269 0.02061825 0
0.1204726 0.114124726 0 0.1779055 0.1850574 0.01503041 0
0.2731656 0.03444583 0.2674198 0.1148972 0.1404823 0
0.302217841 0.114639337 0.7814019 0.3114954 0 0.332277
0.248516 0.12744 0 0.019574 0.07179458 0 0.04157832
0.4702964 0.6650331 0 0.01087766 0.1254484 0.0106038
0.027448725 0.2679643 0.3519906 0.1350635 0.05762483
1.195354 0.05426091 0.01441566 0.03462595 0.57594708
0.1264223 0.027002163 0.03128747 0.3351115 0 0.03225234
0.200381 0.1086435 0 0 0.05680379 0.2280357 0.01224022
0 0.06722217 0.07228292 0.0739789 0.08084423 0
0.037167808 0.09338995 0.00848458 0 0 0.01544496 0.7938977
0 0.03135194 0.02219906 0.0206999 0 0.01449854 0.09043504
0.01291074 0.7022899 0.02693455 0.04076798 0.05125803
0.1212222 0.01297734 0.01296723 0.03027006 0.1276043
0.02851095 0.4094026 0 0 0.1066095 0.06034001 0.03083759
0 0.07635141 0.118018 0 0 0.0132236 0.0339907
0.05456125 0.203627 0 0 0 0.2378369 0.02539097
0.02784777 0.1945298 0 0.1877799 0 0.118075 0.04687426
0.084469082 0.01547865 0.171929173 0.0127453 0.1035582
0.01112173 0 0.09165997 0.114482 0 0 0.016128981
0.02989575 0.01425669 0.01454885 0.07769817 0.2032951 0 0
0.1296967 0.02818185 0.01414983 0.01568993 0.01305269
0.4740218 0.05193207 0.01091442 0 0.03466316 0 0.2643294
0.01476385 0.04260046 0 0 0.06130471 0.223764 0.0278348
0.02855202 0.1145308 0 0.05740092 0 0.05718574 0
0.0108627 0 0.0484108 0.0713879 0.01314747 0.1254431
0.0297822 0 0.01545221 0.02689036 0.1348368 0.01499333 0
0.1320983 0.043575339 0.03922192 0.177240136 0.044117246

0.2207992 0.01221688 0.04541591 0.02893158 0 0 0
0.0632258 0.5865111 0 0 0 0 0.2219936 0 0.110739
0.07152539 0.03147935 0.01403902 0.1300558 0.2083305
0.6722971 0.215281 0.3965584 0 0.1428848 0.05993891
0.05037702 0.04743017 0 0.4571833 0 0.1027085 0.2086647
0.087135592 0.118383 0.2434858 0.05134501 0.1962305
0.07157179 0.05789134 0.06614597 0 0.01213254 0.03048335
0.01163336 0 0 0 0.09777064 0 0 0.02796824 0
0.06728253 0.1351882 0.033410721 0.2849318 0.05921018
0.4500449 0.2065695 0.1022225 0.02717472 0 0.1884063
0.073933 0.5357546 0.01130494 0.101638 0.04615094 0
0.1073437 0.1187388 0.2686336 0.09451958 0 0.04782541
0.01738475 0.1390869 0.1792083 5.780007 0 0
0.030325969 0.194285642 0.03689694 0.01883295 0 0.1938671
0.3326884 0.2002358 0.0541042 0.105048 0.04601791
0.0715202 0.1715598 0.3912324 0 0 0.01312006 0.05783783
0.4034073 0 0.207046 0 0.05723923 0.07675429 0
0.2523753 0.01383916 0.01440553 0.06859383 0 0 0 0
0.009388977 0.01960289 0.1513998 0 0.09389133 0.02096553 0
0.024657325 0.01079778 0.07432894 0.07128008 0 0.3305156 0
1.101767 0.0703656 0 0.05212739 0.1009511 0.1274406
0.1083965 0.0368504 0.4193822 0.02805581 0 0.01745032
0.3899481 0 0.01673097 0.04911252 0.05164523 0.05984862 0
0.02664661 0.069401609 0.01286294 0.174094 0.02610056
0.03479121 0 0.01410161 0.050910543 0.07799314 0.05013513 0
0 0 0.1920866 0 0.05520678 0 0.3352409 0.03410716
0.02451888 0.2036889 0 0.052798376 0.3386214 0.09925307 0
0.183564106 0.03523718 0 0 0 0 0.01886913 0
AC015911.3 0.1540136 0.1309085 0.2675944 0.2288582 0
0.1906357 0.15864 0.2666199 0.5812166 0.600326912 0.2790933
0.06724033 0.4492469 0.1604467 0.3905798 0.01267787
0.2730842 0.36076931 0.126777 0.182745 0.199966942
0.1022563 0.08708227 0.4913652 0.02344099 0.09415621
0.4063157 0.324752 0.496038661 0.3106412 0.2901558
0.1073762 0.5781444 0.4311736 0.3204273 0.3376193 0
0.2920215 0.2117959 0.287743556 0.1796509 0.06034186
0.267169884 0.154016 0.3348094 0.1385114 0.7964804
0.64378 1.254565451 0.2733576 0.1185275 3.971774 0.1285109
0.5146161 1.234522 0.1262202 1.130664 1.964756
0.01715902 0.463990168 0.407996895 0.910160718 0.8692159
0.1201491 0.0470988 0.6957521 0.4436451 0.8474817
0.154739949 0.3825346 0.6576941 0.4092971 0.4401393
0.1914791 0.1997449 1.021308 0.637746 0.167157
0.1942781 0.150870996 0.7736217 1.476731 1.833017

0.4047143	0.288405	0.2816742	1.144508	1.099629	
1.05522461	0.4246462	0.214379365	9.038006	1.608033	
0.6067223	1.240917	2.080399	2.12322	0.3603931	0.1723295
1.248881	6.29909	0.2093091	1.577175	0.6568612	0.9005481
1.755577	0.6418499	0.2130265	0.590175767	0.1825119	
0.1865408	0.5221358	0.3603784	1.528069	0.666666	
0.08151931	0.268061	0.1355737	0.2781199	0.1969313	
0.2833449	0.8100451	0.3153931	0.603639	0.5099323	
1.759441	0.5165197	0.2776224	1.141272	0.3484503	
0.492973	0.3117212	0.2437703	0.1034606	0.5355854	
0.3926061	0.7161923	0.1842538	0.3766617	0.2469942	
0.8626405	0.8829281	0.7721185	0.2882793	0.9368042	
0.1799092	0.1110719	0.5266959	0.04524397	0.568346	
0.9489084	0.2098076	0.1395606	5.000102	1.062974	
0.1611996	0.1223261	3.160978	0.2403686	1.030571	
1.10911685	2.098588	0.927502871	0.2802164	0.1054082	
0.9509143	1.883581	0.3358706	2.214014	0.1685418	
0.7628475	0.689518682	0.8946366	0	0.05331151	0.2440373
0.6290575	0.123212	0.7408186	0.2376245	0.2237453	
0.05184939	0.4216137	3.427753	0.7198226	1.501218	
0.8931954	0.1620754	0.5362923	0.7101622	0.631048	
0.2885298	0.1214122	0.2877775	1.589002	0.9734379	
0.6450201	0.4589792	0.278996	0	0.03949631	0.2383795
0.1174362	0.1536673	0	0.5837966	0.2474416	0.8081201
0.05813055	0.2729997	0.700438	0.7821071	0.9848142	
0.3397301	1.330218	0.6138619	0.2930146	0.460928	
1.003954	0.088707552	0.3034116	0.883991266	0.107772871	
0.1838811	4.640789	0.2357589	0.1236834	0.2391072	0
0.3591346	0	0.09949811	0.04464021	0.09450995	0.4306471
0.7665239	0.690203	0.8417183	1.198024	0.2271458	
0.2947836	2.760792	0.9758233	0.7209773	0.1785149	
1.002505	2.307886	0.4453008	1.271538	0.9273469	
3.168917	0.4441527	0.4143016	2.481869	1.100188	
0.5854424	0.3115087	0.549891611	0.8997171	0.4130592	
0.4233242	0.07190495	0.9834798	0.4242637	1.030112	
1.457795	0.02963826	0.1675507	0.7815176	0.9908386	
2.137339	0.2558011	0.1910731	0.109481146	0.1548521	
0.6490675	0.5035163	0.4109068	0.183471	0.754968167	
2.894113	0.5785721	0.1177932	0.8357823	0.3371176	
2.423031	0.2563342	0.0862974	0.7976897	0.5569281	
0.5109059	0.4788427	0.4509637	0.9480217	1.520917	
0.5511213	2.428081	2.924726	0.3884091	0.1314354	
0.8281406	1.415716	0.5691184	0.2327444	0.04332468	
0.1430784	0.24076819	0.806846075	1.306951	0.218531	

0.8670605 0.1076348 2.064298 0.6892581 0.528679
0.2993887 0.6557597 0.1871945 0.6635735 0.7509318
0.7522785 0.326882686 0.1121772 1.589519 0.3519547
0.5528577 0.1580585 0.067396754 0.4660941 0.6093784
0.2082448 0.3643078 2.298899 2.111454 0.9216134
0.03273819 0.01685947 0.3856547 3.081314 0.1605526 0
1.132667 0.3182812 0.210251 1.45966 0.2499289
0.210821578 0.1055104 0.1815762 0.6094488 0.5028147
0.7898555 0.1313311 0.4281897 1.375154 0.1698849
2.451307 0.3699162 0.9728793 0.225079 1.710397
0.9675812 0.1884762 0.2745733 0.5328617 0.1149682
0.05612825 1.532687 0.4559077 0.4888808 0.4203326
0.4380926 0.1464622 0.118677568 0.4084931 0.1275869
0.6854242 0.7649148 0.1917948 0.2755876 0.652932212
0.5715822 0.1837107 0.0340648 0.3121514 0.6671478
0.2580839 0.2530989 0.0842895 0.2048104 1.454344
0.333278 0.8984479 1.161034 5.815144 0.558912374
0.8578469 1.348699 0.1980332 0.024912443 0.06455999
5.377157 0.3551596 0.2736438 0.2433037 0.1382847
0.3667582
AL031714.1 0.1844801 0.2568704 0.5745708 0.2195103 0.3056547
0.4916556 0.3000341 0.24437 0.1998635 0.308177876 0.8556667
0.2543419 0.4898017 0.3884174 0.569854 0.2493662
0.2711527 0.246122327 0.2357758 0.2073742 0.109676678
0.3094341 0.3088084 0.4336799 0.3280694 0.8674879
0.2049228 0.3224551 0.206393631 0.07343907 0.3018228
0.2465965 0.6623114 0.6319925 0.561228 0.5463031
0.05388381 0.5871792 0.706 0.272103229 1.513292 0.2796037
0.343601064 0.5763357 1.029818 1.04548 1.852842 1.127126
1.242396623 0.7289677 0.2690038 2.155818 0.6770715
0.4623119 0.4164847 0.4853954 0.6415242 0.5905664
0.4997713 0.22377267 0.624661256 0.304551439 0.6704693
0.2878332 0.3919409 0.6469689 0.2870473 0.4481391
0.19835715 1.051312 0.3510535 0.3166771 1.79613 0.9294991
0.705181 0.5443569 0.871955 1.902123 1.139052
0.526295214 0.2532363 0.6999992 0.7488216 0.762104
0.2147738 0.3603745 0.6293981 0.5919193 0.60980809
0.883442 0.333719428 1.051629 0.3428326 1.16388 0.3986063
0.6116873 0.6431273 1.117837 1.645922 0.6626708
1.843741 0.7408316 0.596579 0.6366864 0.5577137
1.44775 0.2521227 0.2981422 0.260445131 0.4918856 0.7232457
1.629392 0.136316 1.698334 0.6418916 0.4137073
0.4671468 5.848695 0.3825494 0.9886963 0.3784705
0.7903882 1.306334 0.2884185 1.371979 1.475451

0.4055567 0.196024 0.5276277 0.3504791 0.5500894
1.18648 0.6059386 0.6685526 0.1881188 1.018919 0.581831
1.167399 0.5129111 1.210311 0.7165385 0.78041 0.9643014
1.601583 0.9103223 0.3350256 1.327637 1.029339
0.8271714 0.5595839 0.4187542 0.5280585 0.3577984
0.7430219 0.823078 1.249988 0.7490086 1.425601
0.8455685 1.375204 0.234157552 1.03338 1.237853019 0.3385923
0.1634731 0.4393367 0.3614027 0.7985733 2.01874 0.7052595
0.504968 0.23473434 1.253474 0.1514976 0.6688225
0.4512888 0.5385094 0.699089 0.8624565 0.8501468
0.7258946 1.281346 0.3878233 0.9950459 0.8967437
0.6478263 0.4336688 0.5867889 0.7740589 0.7359192
0.5134802 2.056583 0.377242 0.3364582 0.46192 1.767411
0.4714267 2.221597 0.6694715 0.6576618 0.1518879
1.564696 0.4981518 0.8216887 0.4837684 0.9184343
0.4445846 0.7641947 0.992224 1.837497 1.610377
0.6019964 0.7089779 0.9459442 0.835503 0.5323567
0.3047966 2.98702 0.7256004 0.197970594 0.4439704
1.187378614 0.264978654 1.23807 2.466613 0.5088373 0.6583208
0.7989238 0.2843095 0.4948658 0.1497088 0.6209932
0.5445579 0.8018022 0.6205551 1.499553 0.6340387
0.4830694 1.231578 1.701871 1.330779 1.151315
0.5064573 0.2566733 0.4051481 0.7211121 0.5442629
0.874585 0.537554 1.135408 1.056108 1.044548
1.084935 1.719153 0.8831729 0.76716 0.8114246
0.348904704 0.8386584 0.5749739 1.02006 0.2311883 0.7688188
0.3176189 0.6685141 0.6203504 0.7371172 0.4049111
0.7551626 1.047411 0.298011 0.417822 0.5285103
0.709921977 0.7028885 3.763486 1.477594 0.5828579
0.7703329 0.096477271 1.046221 0.5653612 0.1633728
0.3519309 0.533684 1.902115 1.000224 1.09897 0.5209155
0.3949922 0.6581089 0.2683349 0.2532056 1.161302
0.37692 0.729631 0.9215437 0.2620186 0.9497254 0.6573623
0.6907581 0.4676751 0.5713029 1.666842 0.3195641
1.109471 1.082361459 0.875194278 1.108059 0.6308346
0.09334603 0.2809246 0.4334569 0.3322044 1.291518
0.423325 0.5811371 0.8072089 0.3335684 0.8004931
0.698454 0.738011939 1.175971 0.7348587 0.05990835
0.360156 0.6187944 0.497120503 0.4760203 0.8895022
0.173951 0.3003384 0.2493639 0.3793702 0.3538895
1.33432 1.811133 0.5514149 2.40229 0.4489705 0.475487
0.494018 0.5584869 0.9254295 0.7071116 1.301468
1.016747996 0.5138432 0.3906326 0.3897036 0.8023795
1.248191 0.2069877 0.7905492 0.3630305 0.7757137

1.655762 0.6016721 0.6035189 1.066728 1.187533
0.3175548 1.062908 1.454034 1.314166 0.5125331
0.523697 1.062877 0.7987191 0.6144218 0.5357411
0.7417585 0.08925634 0.34950644 0.3981752 0.3056516
0.7386099 1.253785 0.6361892 2.348729 0.740930427
0.5070535 1.266263 0.8504287 0.8830932 0.1962753
0.2085567 0.6510094 0.3857864 0.3635185 1.065519
0.910032 1.246099 1.246931 1.028581 0.333382128
2.126551 0.7337077 0.2746614 0.395779861 0.8343613
1.769464 1.237865 0.8403858 0.3650585 0.4140994
0.3802208
AL050343.2 0.09584575 0.2596759 0.4261193 0.1831153 0.1361943
0.4367977 0.0246812 0.1052972 0.1479693 0.090063181
0.329088 0.1726106 0.1973473 0.1497737 0.1822992
0.2366907 0.1433917 0.174399222 0.0493099 0.1194121
0.046666282 0.3897714 0.1524179 0.1911165 0.2024057
0.4771331 0.0758575 0.6441926 0.092608427 0 0.2708546
0.2362647 0.3623607 0.1257787 0.1430538 0.01750894
0.0664883 0.1578188 0.2224206 0.141369913 0.6127517
0.2041887 0.09975909 0.1283668 0.5346042 0.2644725
0.7342048 0.1717016 0.325308799 0.1594836 0.09220251
0.8741428 0.325612 0.3122494 0.4983357 0.2160108
0.3635448 0.6304583 0.1361496 0.086625102 0.326448784
0.098032704 0.2466006 0.09346396 0.05495723 0.4762785
0.02179651 0.3229009 0.192595517 0.518894 0.2728641
0.2952364 0.5530824 0.4021693 0.3480547 0.08667002
0.3968821 0.7346776 0.3445739 0.101714201 0.08332619
0.322813 0.2395519 0.492773 0.126197 0.2397369
0.3205126 0.2862304 0.84822113 0.3513534 0.277087619
0.5083483 0.08187638 0.4315148 0.05516054 0.2692704
0.3329112 0.2186728 0.2345965 0.307643 0.9200116
0.4047279 0.2024359 0.3065834 0.5439458 0.5121236
0.09217754 0.1193136 0.155386913 0.2875014 0.3192422
0.4040317 0.05606767 0.5635247 0.6506066 0.1839002
0.05362062 0.3480273 0.348126 0.1169836 0.2066381
0.5069715 0.3312147 0.2669139 0.3762027 0.960651
0.1388039 0.05183099 0.3921098 0.1626357 0.1495586
0.3091721 0.6339023 0.4104581 0.0714226 0.4886532
0.2810954 0.3353946 0.2461243 0.3458462 0.2230781
0.1850235 0.3293114 0.2606935 0.1658511 0.3617204
0.2384718 0.2936303 0.1548592 0.2496657 0.3616963
0.1506548 0.1085642 0.4207096 0.3969059 0.4765093
0.2640619 0.7944219 0.4038827 0.1068908 0.120388177
0.7765364 0.359390887 0.3197045 0.04919822 0.3487233

0.1019296	0.2762037	0.6744093	0.2359956	0.3758321	
0.441361271	0.5539096	0.07314884	0.2322377	0.2974107	
0.2240679	0.2530351	0.6147016	0.5360595	0.2329612	
0.1048675	0.1878392	0.3795039	0.4236143	0.6414663	
0.3235564	0.1080672	0.3754636	0.283123	0.2054898	
0.08416776	0.08904956	0.0610533	0.06867135	0.4263828	
0.1734903	0.3411715	0.08952527	0.1772246	0.110607	
0.4188658	0.1487759	0.3716539	0.1813462	0.5883117	
0.1251151	0.4875767	0.3052333	0.5621465	0.4597347	
0.2716582	0.3410322	0.1585656	0.4215755	0.2795257	
0.08547602	0.1898237	0.267763	0.099368064	0.1490677	
0.639941798	0.016767302	0.1830926	0.5223574	0.3042229	
0.2474056	0.1488012	0.02923459	0.0718382	0.08110038	
0.3808059	0.2083537	0.2583678	0.4211433	0.3801278	
0.1725778	0.3793165	0.7756168	0.2528122	0.4546363	
0.4241883	0.2647999	0.1385625	0.5332482	0.2224486	
0.3590609	0.2478089	0.3840149	0.3644883	0.5959319	
0.5137529	0.6247375	0.4923147	0.3252177	0.204937	
0.2445255	0.206980962	0.3074516	0.2313495	0.09513224	
0.0783088	0.4284274	0.3300347	0.3299571	0.4131068	
0.1383336	0.2259187	0.3647655	0.4459474	0.2257301	
0.06512322	0.6577131	0.262796021	0.5448474	0.4305018	
0.3487263	0.2493226	0.2226464	0.190472334	0.5557082	
0.202532	0.03665252	0.2723291	0.3263478	0.6351773	
0.3211428	0.2550968	0.4636359	0.2404451	0.3802473	
0.2566054	0.19075	0.2246435	0.1223918	0.1624613	0.268006
0.05388499	0.4985367	0.2522008	0.2378624	0.5109964	
0.2792524	0.673513	0.1348091	0.1202047	0.233396592	
0.58703316	0.4347172	0.2254674	0.09339042	0.05023745	
0.3338077	0.05188779	0.5654816	0.2861275	0.2973244	
0.3378351	0.2526633	0.4620094	0.1595993	0.219318245	
0.07479658	0.1154054	0.1067764	0.3440544	0.2582028	
0.19922614	0.1957903	0.495914	0.1619936	0.08283855	
0.2603573	0.246375	0.1042797	0.3208847	0.1967247	
0.4896013	0.5353197	0.1605778	0.1452813	0.140257	
0.09903636	0.3479244	0.3227125	0.6221431	0.2741111	
0.5663282	0.1165297	0.311545	0.1906805	0.1966171	
0.2996766	0.161786	0.2139465	0.4530975	0.6166378	
0.1381237	0.2906123	0.3893155	0.5462125	0.03984784	
0.3998609	0.3611622	0.1790696	0.3756219	0.3667622	
0.1907643	0.5431753	0.2281802	0.392372	0.2628968	
0.1974837	0.37982787	0.08066387	0.1091749	0.1562375	
0.2380109	0.323643	0.8039233	0.239446082	0.4065223	
0.2191264	0.294139	0.2974576	0.1153276	0.2190147	

0.2126365	0.2911255	0.2205997	0.6722104	0.2430533	
0.531166	0.3193346	0.6468983	0.341133433	0.6434862	
0.3465741	0.06162001	0.127903962	0.2511061	0.5791694	
0.2841723	0.8514699	0.1438421	0.129086	0.240921	
AL121906.2	0.1808542	0.192153	0.2957451	0.2495463	0.07907355
0.3256124	0.745147	0.2649179	0.1861384	0.201413771	
0.1724905	0	0.1861902	0.1695675	0.2620844	0.2828585
0.2805911	0.226951055	0.2729299	0.3004297	0.093926381	
0.06003846	0.07669383	0.1682909	0.08257842	0.2053353	
0.1272336	0.2701213	0.388323489	0	0.2271482	0.2701908
0.4655313	0.3164474	0.3926289	0.4405084	0.794572	
0.6858264	0.419692	0.302322395	0.1947317	0.1594302	
0.062746122	0.06459175	0.1862327	1.020261	0.2338215	
0.6263785	0.225072744	0.288897	0.3015647	0.2843872	
1.121023	0.8762353	0.3918018	0.4199485	0.1180186	
0.2265963	2.31718	0.21794061	0.13688574	0.493282125	0
0.7289542	0.3871482	0.1906342	0.1370949	0.2234068	
0.524931556	1.066851	0.2402752	0.1747734	0.6957516	
0.318536	0.1876442	0.4724495	0.3744442	0.7687951	
0.1672995	0.275588237	0.2795211	0.3731578	0.1721971	
0.3099427	0.1481666	0.7198951	0.4755572	0.04966405	
0.48188104	0.1359957	0.058093866	0.2916918	0	0.3562291
0.3006873	0.9853948	0.1168707	0.6559603	0.2529526	
0.1425791	0.5283469	0.7198025	0.04630088	0.3856675	
1.078225	0.2425327	0.0579775	0.07504543	0.231009325	
0.830486	0.5232862	1.629636	0.0705305	0.1993746	0
1.116803	0.8094266	0.3184014	0.6828678	0.1051142	
0.291134	0.8647407	2.351772	0.03730724	1.313497	
0.2338943	0.1654188	0.3260047	0.1116805	0.3533793	
0.2460263	0.2287788	0.4498262	0.1619881	0.04492314	
0.5890902	0.4013892	0.2812737	0.08846081	0.4746091	
0.410666	0.3385467	0.3282802	1.56565	1.422497	0.1787607
0.547801	0.5154043	1.576155	0.2355508	2.377127	
0.1516133	0.1638824	0.2396526	0.2496445	0.25239	0.4668439
0.380703	0.2822586	0.4033909	0.363462397	0.3552168	
0.123299137	0.6763823	0.1485337	0.3030863	0.1923339	
0.09390574	0.4105041	0.0989571	0.1658874	0.161936753	
1.007669	0.1840356	1.105974	0.302487	0.5248557	
0.5787387	0.6186126	0.4418074	0.3031595	0.5885588	
0.09001635	0.1872149	0.09187705	0.7200338	0.2504731	
0.1631322	0.8949136	0.1563609	0.2412633	0.1270549	
0.2240403	0.2150461	0.3800954	0.4572331	0	0.79847
0.4095221	0.07040215	0.5874735	0.7080399	0.2167036	
0.8694289	0.228125	0.6076352	0.532701	0.4397593	

0.3925018 0.9240144 0.4369564 0.2562999 0.4290025
1.108156 0.2506977 0.3516302 0.4516042 1.257624
0.2315729 0.541668188 0.5813126 0.720278089 0.16873982
0.3022975 0.2453177 0.9933866 1.016667 0.1871849
0.147103 0.3413943 0.521438 0.1635732 0.3931487
0.1109806 0.7224252 0.2812841 0.2315678 0.1533736
0.3176663 3.590614 0.9632187 0.1006809 0.2398361
0.2988088 0.2096254 0.2894792 0.5687843 0.7843577
0.3513256 0.802391 0.2709594 0.1360583 1.160124
0.2914394 1.033537 0.2209724 1.09739 0.229127638 0.1509306
0.5626517 0.3498101 0.1407268 0.07699164 0.2491009
0.6641128 0.5196689 0.5742566 0.2186116 1.151316
0.3324331 0.2381582 0.2730732 0.3085117 0.257121695
0.5874792 0.3409766 0.4386813 0.1608391 0.2154452
0.023960524 0.172075 0.4812431 0.0922143 0.351836
0.278574 0.3897674 0.4752736 0.5404628 0.4064951
0.4577903 1.686327 0.08330258 0.1489373 0.7535759
0.4310969 0.3065529 0.2119159 0.3615189 0.2443386
0.7717057 0.3241546 0.2438243 1.062427 0.2915261
0.3730836 0.8232649 0.39146925 0.222931354 0.4586516
0.3646637 0.3446102 0.1516712 0.2099572 0.07832688
0.9829554 0.1506704 0.6160332 1.084433 0.1845514
0.0400818 0.1338456 0.047981224 0.3575442 0.2488707 0
0.7934745 1.039382 1.160752955 0.1094644 0.05504439
0.2649145 0.3510131 0.4168402 0.3719137 0.2295632
0.5958759 0.7127149 0.4890934 0.4422374 0.09426637
0.7591444 0.5157381 0.685207 0.5611187 0.2405672
0.3913134 0.8311019 0.8363123 0.8528809 0.6134227
0.1476105 0.1030563 0.1370833 0.4788686 0.3364183
0.3134864 0.3177572 0.2606298 0.3655765 1.725753
0.7399643 0.1336712 0.1810822 1.025895 0.3253773
0.7907389 0.4174296 0.2639698 0.6903333 0.129631
1.394916 0.1714802 0.4968505 0.656982698 0.221392
0.7740804 0.3930788 0.4491097 1.091449 0.5056489
1.113571307 0.2077506 0.3355748 1.040037 0.3512782
0.05803068 0.2204081 0.05944148 0.7126485 1.054515
0.4476754 0.1222999 1.793541 0.4138809 0.4776462
0.201943443 0.7914871 0.3736919 0.503848 0.058508062
0.8339222 0.3700662 0.2383169 0.5814592 0.3618932
0.2977042 0.1595091
LINC01409 0.07636939 0.1007262 0.1647183 0.06792144 0.0587211
0.192021 0.05696316 0.08205921 0.02439334 0.052790341
0.1921407 0.06725861 0.1138673 0.04197438 0.08586506
0.09754857 0.05253046 0.083277092 0.08779253 0.06186896

0.058467768	0.07868009	0.07370503	0.1606823	0.106415	
0.1738741	0.05002168	0.174914	0.035622715	0.04780395	
0.0520934	0.05665338	0.6456642	0.3317622	0.2251105	
0.06927408	0.09590759	0.3311265	0.2688909	0.226062458	
0.5699352	0.782336	0.090451316	0.3673687	0.4447261	
0.1666465	0.5239815	0.1754954	0.187699698	0.09885615	
0.1185597	0.3552958	0.3390219	0.3088536	0.2259199	
0.3010685	0.1198637	0.2375626	0.1452312	0.06426226	
0.137530842	0.107740613	0.09704294	0.04622375	0.2536779	
0.1748775	0.0646784	0.1197709	0.058208623	0.4856567	
0.1192043	0.2347657	0.3933675	0.2062644	0.2090207	
0.1762175	0.1656137	0.2572346	0.5600715	0.036115696	
0.16484	0.2761533	0.1579644	0.2003813	0.04160818	0.1198397
0.1869646	0.276609	0.64052408	0.2376289	0.111659843	
0.2675824	0.02024648	0.3756921	0.08790326	0.3739548	
0.07402658	0.1774725	0.1966859	0.1895184	0.5934822	
0.4118299	0.09708331	0.2400722	0.3369482	0.3039324	
0.07977818	0.08851199	0.100135928	0.2949071	0.2232719	
0.3700349	0.07856537	0.4383688	0.279796	0.03554372	
0.1885777	0.2190633	0.3521216	0.2727482	0.245269	
0.2266477	0.3227591	0.06355823	0.280982	0.477656	
0.1710157	0.0512673	0.1365996	0.06337182	0.331899	
0.2848227	0.4983912	0.2043239	0.01177431	0.2114608	
0.1753395	0.2608609	0.2231603	0.649616	0.3874865	
0.4991217	0.1106253	0.3632198	0.3753207	0.3918614	
0.338434	0.5538565	0.2529709	0.218654	0.4697157	
0.1192131	0.1145424	0.1727349	0.3541002	0.2811424	
0.3835494	0.5581548	0.4290822	0.2819422	0.019846483	
0.5382457	0.554767895	0.1700921	0.06488422	0.1672392	
0.04200877	0.1255242	0.677834	0.4571318	0.1695678	
0.124298691	0.3427797	0.1098698	0.2051	0.1982039	0.1885138
0.2161537	0.5092138	0.359581	0.8899274	0.1383026	
0.05603383	0.210996	0.1155882	0.3091105	0.1661734	
0.110455	0.2779922	0.1525442	0.191963	0.2247815	
0.05605161	0.08454504	0.07245281	0.2512037	0.156462	
1.169342	0.1341691	0.1230155	0.2573023	0.8804096	
0.1833019	0.3891097	0.2092697	0.2613506	0.09836891	
0.1364927	0.275077	0.3805736	0.340209	0.1791358	
0.2175491	0.212026	0.3715008	0.1797157	0.06200071	
0.2566043	0.1600141	0.106478004	0.1523614	0.252637603	
0.022113266	0.3225869	0.4937131	0.4204264	0.4214535	
0.1471829	0.04819442	0.1210599	0.1039868	0.195988	
0.1786094	0.1350509	0.2366836	0.346504	0.0758671	
0.3282924	0.3151987	0.403327	0.5798659	0.2797167	

0.1606443 0.3328492 0.2252646 0.08851756 0.3113095
0.3505391 0.2493889 0.2403496 0.4639854 0.4219855
0.2550242 0.3771557 0.216335 0.1480092 0.4575847
0.051864919 0.1854317 0.1906945 0.2847056 0.07376872
0.2051575 0.157782 0.1460888 0.3445177 0.2782193
0.06016276 0.3236253 0.2722824 0.1872631 0.06441498
0.49619 0.175698826 0.6965657 0.4599906 0.3574313 0.2508267
0.1157593 0.0659403 0.2677853 0.2188784 0.02416927
0.2135525 0.1806135 0.1787759 0.1930815 0.3231499
0.3288904 0.1799795 0.5439811 0.1883141 0.06289185
0.2334226 0.06860111 0.1606944 0.1136105 0.165819
0.3757066 0.164058 0.09149602 0.4531523 0.09880874
0.2722059 0.1689011 0.1299065 0.32206157 0.642730852
0.5270838 0.1380571 0.03489713 0.04306561 0.2701452
0.05132348 0.4203463 0.193065 0.2277763 0.5722986
0.3144097 0.1523283 0.2981867 1.172696081 0.09371197
0.09784312 0.0135404 0.3970314 0.1118872 0.200516644
0.1362799 0.4833071 0.06409276 0.1121252 0.1872915
0.3926205 0.1332298 0.5088395 0.3969539 0.2635035
0.5242292 0.04764944 0.3721494 0.4268666 0.4517012
0.3411992 0.193098 0.4051232 0.183070691 0.7448625
0.2340176 0.2634964 0.7640992 0.1620657 0.130244
0.1851287 0.1410798 0.1232467 0.5217487 0.2998085
0.2994289 0.2995078 0.2978424 0.07444956 0.2610381
0.3687581 0.1344816 0.2375814 0.2015408 0.1635311
0.3489473 0.2281255 0.5793448 0.1187827 0.1853188
0.206111582 0.0507733 0.1341663 0.1692563 0.2877385
0.3042306 0.6361442 0.282296516 0.1989563 0.2293081
0.2149287 0.63448 0.04562935 0.1263691 0.2259035 0.1764075
0.1478915 0.3389685 0.1859171 0.5668674 0.2105745
0.282838 0.125706774 0.9122995 0.2075486 0.05587071
0.40254119 0.1987001 1.030561 0.2225231 0.7680151
0.2071764 0.05674761 0.1651383
BET1-AS1 0.3198123 0.2184381 0.1470878 0.08728688 0.2097438
0.3701538 0.2117694 0.05475576 0.1763339 0.228965655
0.2941289 0.04487975 0.4938724 0.08567246 0.5213872
0.2538563 0.0455677 0.171997498 0.05641176 0.2439473
0.106774801 0.1365025 0 0.2186421 0.2972693 0.3591133
0.03615954 0.4335133 0 0.5183459 0.1936653 0.1842905
0.4851115 0.6475231 0.836883 0.15023 0.1663906 0.2462028
0.2827275 0.242596106 1.346664 0.3423404 0.166435076
0.5580484 1.082063 0.4538447 1.116388 0.3683081
0.697802718 0.2006986 0.7120035 0.840552 0.2695784
0.1030446 0.3563179 0.1965741 0.3018661 0.4449332

0.2290568	0.247753241	0.466832027	0.0623066	0.3185196	
0.05346256	0.1257449	0.4024641	0.1246788	0.4155826	
0.114757352	1.069168	0.273143	0.3808055	0.6327401	
1.980951	0.7110417	0.2065681	0.4434014	0.7066063	
1.314004	0.17902093	0.1191591	0.2994374	0.1957524	
0.4228086	0.2406209	0.3538903	0.5171051	0.2258309	
0.23477088	0.6956951	0.198122004	0.5101431	0.1561144	
0.4242423	0.1051751	0.09334912	0.2657154	0.4089271	
0.5367685	0.370475	1.201242	1.257336	0.4737105	
0.2192119	0.4007156	0.4997231	0.2306794	0	0.101003714
0.2740907	0.1521755	1.045503	0.2405356	0.6547596	
0.566323	0.6529235	0.07667917	0.6515214	0.320637	
0.2150874	0.2600392	0.4915154	0.168408	0.1696424	
0.1097922	1.196502	0.3760937	0.1235332	0.6982667	
0.1691448	0.1645181	0.6241775	0.4648719	0.6905506	
0.2553414	0.8734869	0.2390123	0.3935383	0.3268251	
0.359688	0.4824034	0.3126966	1.315038	1.082322	
0.1078056	1.089965	0.1186164	0.3124841	1.147532	
0.5801734	0.2955641	0.1077205	0.3933006	0.38595	0.3338753
0.7172876	0.5919398	0.5950729	0.3208694	0.7642862	
0.137727103	0.8833296	0.864354091	0.4571875	0	0.4896183
0.2186438	0.8967112	0.9333159	0.2812342	0.6600284	
0.236685186	1.827942	0.09298234	0.1897756	0.3257668	
0.397768	0.1973717	0.5713777	0.7401481	0.2986789	
0.3460703	0.1790773	0.3830841	0.5431147	0.2822514	
0.4626958	0.1854474	0.527505	0.3357498	0.3134473	
0.3851601	0.3241475	0.1047698	0.314247	1.539346	
0.4086296	0.5446168	0.1862167	0.4001632	0.4745146	
0.3182141	0.4479036	0.2237797	0.0864436	0.8147368	
0.3578371	0.578849	0.2715958	0.5573618	0.262974	
0.4370397	0.6361132	0.2267538	0.3288361	0.4197171	
0.04889336	0.3981323	0.2871822	0.331565397	0.1918544	
0.409403348	0.167844385	0.7200277	1.075662	0.7960459	
0.4245573	0.05319759	0.05574184	0.4794108	0.1546348	
0.7969235	0.1489762	0.6488325	0.656998	0.7034755	
0.1974334	0.8523972	0.6190641	0.4198405	0.5474899	
0.274688	0.3029377	0.1132278	0.3812809	0.4607089	
0.7226591	0.2514916	0.5990764	0.6026719	0.6160492	
1.08269	0.6806813	0.4141327	0.1795013	0.2791109	0.4158347
0.213112285	0.2788123	0.1985022	0.3139433	0.09598632	
0.4376176	0.4247641	0.3774792	0.5560054	0.9297606	
0.124258	0.341428	0.330669	0.2499106	0	0.5420121
0.27141586	0.3498219	0.6156311	0.563736	0.4936697	
0.1224582	0.081714428	0.4401305	0.4344407	0.3406927	

0.4210153	0.3333483	0.6203185	0.3782012	0.5375948	
0.5223746	0.2787924	0.2580583	0.757582	0.3762463	
0.1362867	0.07000967	0.3097663	0.6789123	0.3082289	
0.8147693	0.2924231	0.3968424	0.5291572	0.4869996	
0.393543	0.5783436	0.1145977	0.370849407	0.665245036	
0.6617663	0.5373757	0.07122732	0.1436823	0.08679192	
0.1780828	0.9115764	0.2664371	0.3251402	0.5997287	
0.2331074	0.9568584	1.156375	0.327268161	0.2139229	
0.09430477	0.516809	0.1640029	0.1054966	0.029989436	
0.09332875	0.6048825	0.3938152	0.2119842	0.451296	
0.2348826	0.2982465	0.7429408	0.3600929	0.3088876	
1.325385	0.2755576	0.2876632	0.833145	0.3540628	
0.2296352	0.2392906	0.4893263	0.261324995	0.6514148	
0.8786534	0.07748159	0.2097532	0.2811687	0.5259439	
0.2721871	0.5354129	0.3239718	0.7011995	0.2962819	
0.5194808	0.4065037	0.5407623	0.1139673	0.6633038	
0.233246	0.2276224	0.9646797	0.3996049	0.6274377	
0.4484375	0.5262982	0.8050627	0.05569641	0.4779211	
0.38474244	0.0629192	0.2270887	0.1276712	0.340363	
0.7483893	0.9886866	0.788593105	0.1816687	0.2997334	
0.2728398	0.2604329	0.03298442	0.1879187	0.6757263	
0.1575259	0.1682477	0.3581246	0.4727005	0.7196059	
0.6088781	0.9049744	0.487831537	1.431431	0.2427487	
0.08811856	0.133023046	0.0861815	1.078014	0.2370523	
0.9393136	0.389745	0.1230647	0.3807903		
AC245060.2	0.1109055	0.5302543	1.989295	0.7151194	0.9091949
1.403971	0.4283884	0.7975114	0.7704851	0.347381099	
8.663105	5.610654	0.2568998	2.677591	1.356062	
1.499496	1.216762	0.77241319	0.2054077	0.3553064	
0.550786809	0.1656787	1.516752	1.326874	1.481211	
5.230444	0.7899893	1.600442	0.107159497	0	0.9402377
0.4473617	4.014541	1.0479	3.047276	0.3646804	0.08655216
0.3486307	1.265394	0.932420912	6.000634	1.417631	
0.519451676	2.887547	3.311909	4.753629	4.097276	
1.221883	2.06091505	1.306561	0.3840833	4.185485	
0.713887	1.750971	1.892089	1.567877	2.849677	
3.63812	2.585544	0.714181813	1.259139819	0.680616368	1.408329
1.427573	1.068352	0.995762	0.7944705	0.8406823	
0.278571282	0.774742	1.515541	1.105258	3.017073	
1.034133	1.898644	1.103168	3.939435	4.942696	
11.03811	0.488891128	1.639119	2.392642	0.9800695	
3.620769	0.4672821	2.093964	2.481997	4.145763	
0.28495092	1.72006	0.935156025	3.374539	0.1894823	5.570498
1.34038	1.954453	1.773803	2.423265	3.978789	3.203831

5.311269	2.7857	1.661	3.12628	1.230217	3.932015
0.6799633	0.7938489	0.980738357	1.515519	1.343284	
2.782828	0.3406058	8.313871	1.669325	1.078653	
0.6514802	2.086775	2.908534	1.392322	2.811888	
2.893375	11.62545	1.956067	4.290947	9.089962	
1.44552	0.1199498	1.951852	1.437085	2.216474	2.588431
1.579855	0.8381492	0.2479346	4.064048	3.402069	
1.940467	1.006959	1.200562	3.437525	2.423169	
2.458879	1.196889	1.98889	2.668292	1.187748	1.469689
10.92253	2.058372	4.50985	1.229001	1.281348	1.846217
3.282427	0.7835409	4.831033	2.692075	3.115618	
1.205939	0.835824788	3.400193	1.786308011	2.446635	
0.5123563	0.6602995	0.8845893	0.9328926	6.192658	
1.194709	1.762427	1.787485072	2.041155	1.015708	
2.101837	0.8566922	0.9387526	1.716833	2.587304	
8.951393	0.3346326	2.968279	1.987231	2.789788	
1.090216	2.911929	2.375969	0.7502835	4.093053	
1.294454	1.379108	1.869938	2.191973	1.186858	
1.144243	4.367609	0.2834116	7.326344	2.994748	
0.3237962	0.6612617	3.703258	1.114459	2.512397	
0.8393608	4.256479	0.50114	2.267393	4.379593	5.411941
0.8511525	2.269159	2.470644	1.773642	2.474554	
3.711544	1.186877	8.346688	0.9295057	0.948595222	
1.785271	1.636878047	0.465644619	5.779834	12.28207	
1.460525	5.410675	2.00161	0.6089052	0.6372974	2.001989
2.224694	1.012583	2.078163	1.26259	2.095781	1.717369
1.59891	1.784535	1.89676	5.606808	1.444733	1.066294
0.6871461	0.6652405	2.289979	0.7386279	1.637226	
2.504536	0.9094166	3.265061	1.095088	4.543938	
1.809538	2.02023	2.879529	2.890643	1.178353675	1.197436
2.677002	0.7366883	0.2718391	1.451821	0.7446881	
3.436214	2.488495	4.081765	1.447843	0.55254	3.440112
1.440801	0.9042683	1.470515	0.912263288	2.200164	
4.898419	5.052769	2.95155	2.110581	0.198360215	3.353616
2.2459	0.2544692	1.53301	1.355404	3.979644	1.267819
3.262503	2.58488	2.436348	4.675864	2.614851	2.123494
3.379227	0.7222754	2.161861	2.312582	3.304638	
2.921805	0.5205577	2.614746	3.578303	2.577157	
4.600611	0.8423508	0.9040972	0.540137915	1.653318907	
2.920773	2.217598	0.497096	0.8719648	2.844257	
0.6484389	4.425669	2.656381	2.033892	3.255397	
2.546387	0.7742519	1.920633	1.158554273	1.869458	
2.460923	0.2566113	2.687268	3.073092	1.637972649	
0.3775896	3.696168	1.462085	2.694017	0.9311856	

2.53727 1.312228 4.455643 1.93945 0.8747885 6.074128
0.5016832 1.551771 0.8988638 0.6589351 4.521417
1.410692 7.261962 2.513047238 5.1499 0.7722621 0.6582978
1.680267 2.616371 0.9693606 0.7268018 1.578213
1.887445 7.504927 2.277526 2.837317 3.067599
3.281725 0.7838516 2.554043 3.639859 5.387353
1.88936 1.940067 2.483304 6.473124 4.21601 2.931412
1.419621 3.744102 1.400932397 1.858278 0.5512535
1.652903 1.377041 1.649373 3.013976 2.342492214
2.491634 1.455194 5.05014 2.971321 0.4403798 1.216451
0.7381407 1.584192 1.378411 2.951183 0.8437291
0.6793227 1.645999 2.904668 0.870736579 8.165712
2.749916 0.4278122 1.695282248 1.673631 5.010328
2.548377 2.723531 0.6044518 0.4481058 2.99317
LINC01484 0.005161143 0.004935227 0.03323193 0.04437212 0.04061824
0.03136116 0.01594853 0.006185553 0.06374331 0.084062524
0.004430218 0 0.07173117 0 0.08414166 0.01147088 0.06177135
0.03108786 0.04460838 0.06613875 0.036185847 0.00514006
0.02626389 0.01852438 0 0.004056769 0.06535692 0
0.019947274 0 0.04375529 0.004626357 0.1245482 0 0.02521054
0 0.1664835 0.04171891 0.023954 0.095918105 0.104197
0.03639804 0.010743741 0 0.03720243 0.03797714 0.03002717
0.04438014 0.089338579 0.01648885 0.03574772 0.02434718
0.132887 0.03880187 0.05031487 0 0.4016302 0.01058157
0.07245191 0.139938775 0.093753359 0.246349007 0.04626252
0.1872234 0.07812712 0.02448108 0.05633799 0.02608156
0.036298382 0.09734503 0.07493585 0.02992568 0.1072174
0.01443746 0.02008093 0.01866818 0.05610012 0.03781074
0.1523444 0.126395772 0 0.03382632 0.1382088 0.02653506
0.05980049 0.02998325 0.1008994 0.1084229 0.35361582
0.1164297 0.019894311 1.089187 0.06613372 0.2831935
0.09504987 0.3321769 0.4952785 0.05978172 0.1385982
0.02092559 0.1421617 0.01803638 0.05945922 0.05571799
0.05325569 0.08954431 0.02233627 0.04497394 0.077588075
0.04816466 0.096893 0.1450432 0.1449195 0.05689672
0.2193442 0.01229305 0.4215584 0 0.02668927 0.07019313
0.08545643 0.3886725 0.1331709 0.04790965 0.09426137
0.295359 0.02360328 0.05023831 0.07170961 0.009553828
0.003716999 0.07638691 0.0630178 0.1092125 0.2538357
0.06578303 0.2847307 0.03889948 0.05112029 0.06772101
0.05273739 0.1141242 0.2127948 0.0543404 0.2386966
0.05426035 0.06029842 0.0220626 0.1728432 0.03529078
0.07631718 0.009735027 0.01870724 0.6103906 0.06034662
0.01620586 0.05534005 0.03666718 0.01449897 0.05180313

0.038896287 0.142552 0.042223932 0.07512256 0 0.0327765
0.1893618 0.04823719 0.2178953 0.2350978 0.02130312
0.041591571 0.0825983 0.04201547 0.02143823 0.0327117
0.03494894 0.03716057 0.1241277 0.06569495 0.0311452
0.1407393 0.02889957 0.1057847 0.5569022 0.1020316
0.06835179 0 0.02553868 0.09816762 0.0221306 0.03807133
0.02092442 0.03156119 0.1153727 0.0451673 0.02308068
0.1743161 0.105181 0.05424592 0.02382406 0.004229112
0.06071759 0.03791933 0.03906084 0.05602298 0.1243801
0.3685641 0.05697929 0.1501427 0.6205502 0.2468537
0.02874373 0.1707702 0.1040127 0.1174058 0.0110466
0.09403971 0.1243606 0.016052443 0.07224356 0.223082263
0.04333886 0.007394439 0.063007 0.008365239 0.01065792
0.01201906 0 0.01031566 0.1979765 0 0.02019514 0.02443206
0.0371093 0.01444891 0.03717219 0.100669 0.08158891
1.159347 0.09663732 0.6516398 0.06159912 0.02558185
0.05922382 0.3023541 0.3866973 0.0154964 0.1203118
0.0515212 0.1391855 0.0174725 0.06728227 0 0.03686834
0.3089951 0.0213524 0.032099328 0.07752954 0.03986498
0.08511599 0.00722881 0.2900246 0 0.05482596 0.3219004
0.01787771 0.1010661 0.111424 0.04802714 0.08939964
0.03506783 0.07923763 0.250003711 0.03233297 0.06696978
0.3331118 0.1321908 3.641016 0.233851308 0.02209773
0.03998878 0 0.4328007 0.09790847 0.3003217 0.05696529
0.02602717 0.2405821 0.008398436 0.06663286 0.005348825
0.01275094 0.0615832 0.1344484 0.2537002 0.2622446
0.1392779 0.0125511 0.02202262 0.06404257 0.3301963
0.06161626 0.01871877 0.02613332 0.03020657 0.07820114
0.837388372 0.1812297 0.02428213 0.05632399 0.03895499 0
0.06705782 0.03321851 0.03009841 0.06215824 0.1129152
0.0895331 0.2573636 0.1168807 0.141719321 0.08216464
0.06391954 0.005307452 0.09263395 0.1001075 0.020326765
0.03514333 0.03770003 0.05233862 0.01972111 0.06627558
0.1432826 0 0.05924276 0.294918 0.02326258 0.09293199
0.01037624 0.02166416 0.4113273 0.005332953 0.02882336
0.03089344 0 0 0.6563244 0.06160853 0.2450785 0.1895602
0.2488068 0.004401031 0.09224386 0.1814508 0 0.1008151
0.2305701 0.1760514 0.1717054 0.2579266 0.1115787
0.01550296 0.1279805 0.3728479 0.05448812 0.005642734
0.2157196 0.07960591 0.01426894 0.1267717 0.01887544
0.07852939 0.076699256 0.02369249 0.01282668 0.05288262
0.05126605 0.04894579 0.1714284 0.04688653 0.08208971
0.0923448 0.06164338 0 0.02980901 0.07783773 0.03053371
0.01016864 0.02850947 0.1234973 0.03769356 0.3883912

0.04377079 0.09995975 0.162083825 0.02772058 0.1005488
0.03981769 0.015027115 0.0713944 0.2613798 0.02295335
0.007860054 0.01956801 0.1529237 0.004096805
MIR1-1HG-AS1 0.05559966 6.465793 0.06294486 0.1389087
0.258054 4.729839 0.02643221 1.040537 1.082858
0.889508316 0.994895 0.2940898 0.3566498 0.3248088
0.01394517 3.729364 0.1109078 2.920727127 0.1056164
0.1096147 1.004538085 3.492729 0.1142618 4.513089
5.108631 0.1311075 0.1895582 7.139059 0.198357056
0.07763744 1.087764 3.369853 0.008256774 0.2020531
0.1601665 0.02812666 0.004450332 0.06530137 0.3374503
0.01513994 0.03108426 0.003770256 0.004451521 0.01374738
0.1805677 0 0.1244134 0.3355865 0.361452359 0.01707982
0.02468595 0.06859784 0.06423652 0.05144646 1.559376
0.01051526 0.05651658 3.178636 0.03430794 0 0.063123789
0.040828523 0.3620667 0.02001895 0.01177123 2.480785
0.1517286 0.5273588 0.038673599 0.02987672 0.003652781
0.01549911 1.07887 0.08374746 0.06323394 0.06961412 0.5943902
0.02436989 0.003237014 0 0.01487296 0.08409281 0.009162388
2.022976 0.3333701 0.004141048 0.9813469 0.1585536
0.48350224 1.326629 0.012364399 0.004775553 0.1242205
0.05415569 0.08368803 0.03058509 0.0580397 0.06305022 0
0.4985381 0.2998684 0.04483875 0 0.01026043 0.5207519
0.6226591 0.06169817 1.607877 0.011346193 0 0.01813058
0.05494549 0.06004539 2.022681 0.2272056 0.04074766
0.009570788 0.006776692 0.2116881 0 0 0.211654 0.2088864
0.0397014 0.2631134 0.06222607 0.03911876 0.03237975
2.594838 0.3720987 0.2094519 0.03408447 0.05222111
0.01292877 0.009561209 1.090252 0.1830634 0.009209978
0.0329482 0.05611853 0.01165387 0.1396057 0.04657964
0.004503038 0.03229408 0.1902325 0 1.499179 0.09423052
0.07520012 0.02371573 0.2016787 0.007751086 0.412302
3.13485 0.06714673 0.3745133 1.711693 0.08410425 0.04292782
0.367447965 0.2315327 0.109343141 0.007781499 0.1211837
0.1052488 0.02729024 0.1159212 0.1164928 0.2948613
6.655297 0.226489445 0.4563129 0 0.01776527 0.02710728
0.1323942 0.5481321 0.004114447 0.05938864 0.08172903
0.0388756 0.1341103 0.02390752 0.06648577 0.005284423
0.9695647 0.05208032 0.105816 0 0.2457428 0.009013903
0.05201846 0.03923083 0.02206297 0.407975 0.3552031
0.07647402 0 0.01498403 0.01645195 0.4766186 0.01257875 0
0.00809216 0.0331605 0.2370617 0.2561578 0.01089627
0.08428392 0.03829393 0.445488 0.05160813 0.3396301
0.07798362 0.1160007 0.1693492 0.003388179 0.6272864

0.02217036 0.06784841 0.027052991 0.07182743 0.01225513
0.04102384 0.04159226 0.02207981 0.04979933 0 0.05128983
0.342591 0.0248672 0.01673515 0.003374362 0.03075145
0.1496676 0.07392855 0.04352434 0.06278115 1.820998
1.018626 0.2528552 0.3800053 0.09539538 0.1338468
0.1232225 0.007121008 4.490222 0.4984955 0.006099171
0.3037266 0.04343693 0.07964994 0.1783318 0.01527589
0.005225626 0.1627862 0.048766348 0.004015412 0.5409482
0.1097184 0.01797095 0.09831903 0.3225222 0.0656251
0.07807317 0.007407384 0.009305633 0.05326961 0.07517544
0.1949553 0.02905974 0.04178493 0.00781778 0.02679344
0.04695816 0.09742608 4.65215 0.0183417 0 0.6454903 0
0.5495397 0.007882418 0.00624108 0.7797872 0.04720557
0.06470399 0.04890054 0.2296652 0.01725525 4.782585
2.542969 0.0911291 0.006553746 0.471215 0.07380505
0.03847191 0.0381361 0.0437989 0.2706586 0.2783422
0.06564817 0.01938966 0.0397026 0.02503135 0.046287945
0.023723781 0.02628151 0.04599298 0.1066837 0.03228092
0.008124766 0.01667069 0.01651635 2.711521 0.135796
0.08109372 0.05237196 0.37109 0.1082506 0.051060384 0.08010302
0.01765612 0.03518511 0.07676319 0.0197515 0.028073714
0.1223137 0.003905122 0 0.2428004 0.07181943 0.0659635
2.373154 0.1840981 0.02949539 0.01156624 0.06845349
0.01146467 0.01795249 0.03466328 0.0441927 0.004777024
5.267311 0.2831694 0.022581377 0.00659246 0.0453807
0.01450642 0.03141667 0.1886322 0.09117531 0 0 0.01819659
0.1989112 0.2157209 2.888601 0.01323605 0.1237423
0.09957463 0.04710519 0.02495386 0.02663524 0.0082096
0.009351955 0.07661179 0.1019493 0.003941427 0.1918345
0.1668433 0.2765693 0.025423421 0.1335065 0.1116057
0.003983851 0.04248276 0.08112005 0.3185543 0.066051072
0.02040764 0.3877195 1.004616 0.009751858 0.006175476
0.8033428 0.0632561 0.0126397 0.03543751 0.8928175
0.02602968 0.3068793 0.03108998 0.04141694 0.155804687
0.03445692 1.935344 0.2639666 0.006226277 0.03227049
0.03445881 0.1077846 0.07816092 0.01621547 0.08064233
2.912832
NCOA7-AS1 0.02899924 0.05545973 0.05334923 1.96683 0.1521495
0.02936849 0.627277 1.842023 0.492468 0.435994481
0.02489234 0 0.5373872 0 1.323762 0 0.2892321
0.196509716 0.4296752 0.1238726 1.592670838 0 0 0
0.03972335 0.09117608 0.7803531 0.2293034 0.168118492
1.118634 1.475103 0.1039776 0.1399614 0 0.04721735 0
0.09052561 0.02604541 0.06729592 0 0.1639283 0

0.030183264 0.01864265 0.4479249 0.2987383 0.1349724
0.1246807 0 0.02316173 0 0.05472041 0.09333253 0
0.05654146 0 0 0.02972765 0.05815573 0.825596877
0.230465312 0.039547902 0 0.06786864 0.2394424 0 0.03956872
0.08792765 0.058271997 0.1012886 0.04953491 0 0.5737421 0
0.02256599 0 0.1801219 0 0 0.085222539 0.0504226
0.06335402 0.1553125 0 0.03054589 0.02807812 0.2387072
0.1075063 1.29147416 0.1962573 0.083836058 0 0.1486359
0.3671994 0 0.02962577 0.08432885 0.3664356 0.04867191
0.05878796 0.3630784 0.1266777 0 0.06957025 0.1496155
0.02187514 0.08366813 0.0360997 0.025644057 0.1159825
0.07024758 0.1629926 0 0.09590675 0.1027036 0.02302392
0.09734122 0.1148724 0.7069575 0 0 0.2807818 0.02672342
0 0.02787538 0.196896 0.1856697 0.03136413 0 0.02684035
0.02088493 0.1980923 0.2360547 0.1753254 0.2593168
0.1478477 0.02758335 0.03122385 0 0.03805083 0.05926378
0.1221404 0.1127966 0.06106516 0.136855 0.046904
0.07528944 0.1239645 0.02555697 0.08498164 0 0.1093976
0.02627788 0.0864615 0.1907287 0 0.1554713 0 0.1629325
0 0 0.5126183 1.660723704 0.105524 0.07145035 0.4834289
0 0.7588934 0.197468 0 0 0.267077895 0.4022202 0
0.06022817 0.1148745 0.168317 0 0 0.03355676 0.116665
0.1757291 0.03247596 0.1350862 0.05303561 0.03583071
0.1129566 0.2354183 0.4544033 0 0.3233011 0.09167733
0.02939233 0.2216686 0 0.1522706 0 0.2592637 0.02954935
0 0 0.07128713 0.1990087 0 0.0548684 0.2023585
0.1397724 0.1002036 0.1231358 0.1360671 0.185464
0.06164498 0.1345868 0.1599195 0.1948075 0 0 0.02297334
0.1519028 0.030064955 0.4059194 0.061143719 0.06087769
0.103869 0.3287341 0 0.08982649 0.06753227 0.1061432
0.05796127 0.03271714 0.1686106 0.07564778 0.04575931 0
0.3247399 0 0.1229642 0.1309796 0 0.152035 0.1743528
1.19216 0.1437383 0 0.05570019 0.04828356 0 0.1267507
0.02067753 0 0.1308984 0.5940677 0.1051451 0.1450081
0.03543204 0.2879378 0.15029875 0.02722625 0.3639867
0.1859848 0 0 0 0.2738258 0.3087994 0 0 0.1926354
0.2098857 0.07931295 0.03940757 0.647589 0.05300798 0 0
0 0.04642181 0.06218238 0.17288881 0.2483238 0 0 0
0.0634759 0.449983 0.04572492 0.1462405 0.05101032
0.117972 0.04679927 0.03005378 0.0238815 0.1235791
0.08887456 0.2949272 0.1390085 0.1956423 0.1175361 0
0.035984 0.03198783 0 0.1051763 0.09789129 0.04849245
0.37662311 0 0.2036573 0.01949079 0.09042032 0.07295961 0
0 0.1119881 0.0241594 0.09525064 0 0.2367366 0.2313704

0.1545235 0.173105914 0 0.1197162 0.2683918 0.8327811
0.02678477 0 0.1579696 0.05295688 0.2940783 0.09497846
0.2291611 0.05963487 0.09465309 0.02773925 0.08571072
0.2614139 0.05801809 0.03886774 0.04057525 0.07834409 0
0.06478064 0.2169786 0.08470645 0.535890778 0.02234989
0.1153879 0.04917993 0.1597641 0.08923318 0.1483701
0.03455309 0.194196 0.04112696 0.2157928 0.2507456
0.1318921 0.08974621 0.2669632 0.2411285 0 0.3806958
0.1083592 0.05566469 0.06341036 0.06926154 0.04066243 0
0.1238783 0.07070442 0 1.149213066 0.106498 0.07207007
0.108049 0.2160388 0.1500081 0.1751301 0.158066566
0.1614349 0.0691818 0.08658988 0.3085688 0.1256173
0.0795185 0 0 0.08009397 0.1196382 0 0.7105091
0.1054019 0.1787069 0.218570523 0.2595923 0.1284 3.244029
0 0.1458721 0.1668899 0.04298981 0.02208189 0 0.2343387
0.1841518
AC004540.2 0.1317788 0.2047671 0.3030381 0.09441234 0.3457002
3.73679 0.2545074 0.4540631 0.7120532 1.341473948 0.07069764
0.3883472 0.5341885 0.04633306 0.1611284 4.381065
0.2628667 2.12083445 0.3864398 0.2814521 0.40421889
3.264608 0.06286807 0.4532733 0.3158952 0.1424239
0.3911134 3.829367 0.127327838 0.6354145 0.8844488
2.392016 0.1908045 0.3631611 0.1475141 0 1.54263
0.207123 0.2166135 0.204088788 0.1995337 0.05808417
0.222883901 0.02117906 0.1187357 0.02424164 0.2875051
0.2478772 0.2851339 0.131565 0.3232628 0.590571
8.235062 0.1238404 1.220449 0.1012481 0.04837158
0.3883807 0.3138233 0.200983496 0.112209059 0.336964264
0.639825 3.60454 0.0453366 0.5022904 0.1348567 0.4162108
0.04965012 0.1265763 0.2954404 0.07163332 0.06518023
0.1382362 0.1538172 0.2085354 0.2685746 0.02681714
0.1620746 0.01613624 0.1145656 0.3238813 0.7057739
1.117902 0.3470179 0.4944233 0.1186432 0.3053325
0.5643019 0.3530184 0.063494883 0.3862512 0.1547869
0.09733697 0.8721661 0.3365648 0.1916043 0.1907999
0.2073523 0.3506282 0.3506053 0.1439127 0.07590825
0.4346958 0.6798856 0.3106419 0.1901031 0.6561796
0.101965655 0.1756832 0.08978071 0.2248474 0.4336186
0.8171647 0.9334148 0.09154748 0.1105849 0.03915038
0.07301293 0.1378641 0.1363718 0.6911308 0.9411372
0.1834907 1.47256 0.9107159 0.1657314 0.1068941 0.7934129
0.3506591 0.05931606 0.8064062 0.2011282 0.1161879
0.5523712 0.1469676 0.2506895 0.05320798 2.048515
0.2809808 0.5161727 1.300857 0.1537717 0.2774934

0.2332122	0.2264634	0	0.4224913	0.508097	0.3861751
0.04567025	0.155352	0.04477966	0.2455624	0.5778086	
0.1551683	0.4415596	0.7216685	0.2776503	0.05511179	
0.471738752	0.6187599	0.370596385	0.2847173	0.1826358	
0.1699909	0.2364922	0.3540949	0.1346007	3.609746	
0.4895371	0.208597829	0.8084395	0.1676212	0.06842248	
0.104403	0.2390216	0.6167307	0.06338675	0.3049784	
0.06626889	0.1164554	0.3504973	0.1534653	0.5723878	
0.2442338	1.321746	0	0.1222643	0.1281734	0.282529
0.1215088	0.1502608	0.100731	0.1132999	0.1585721	
0.3367521	0.1472688	0.03356967	0.2693162	0.1900928	
0.2564559	0.08074453	0.2554945	0.841502	0.07663008	
0.2381836	2.011116	0.08393339	2.349611	0.4003248	
0.5252404	0.152898	0.03633546	0.1738879	0.1152964	
0.1939104	2.635995	0.2933686	0.085388562	0.06148621	
0.19102213	0.051870276	0.1770012	0.6607367	0.06674645	
0.1870876	0.03836017	0	0.9218602	0.5761111	0.3831016
0.04297	0.0389888	0.3158358	0.1152882	0.7355625	0.3073269
0.186	27.11224	0.6908801	0.3136177	0.4368897	0.4082365
0.1890193	0.2531138	0.5759539	0.3132369	0.4079878	
0.2583988	0.444226	0.09294233	0.2147388	0.5972526	
0.2353387	0.1811372	0.1226674	0.119523292	0.09279152	
0.4453171	0.1660126	0.1615008	1.935441	0.8337961	
0.09721282	0.2255226	0.02852934	0.1612818	0.410333	
0.3065678	0.1351557	0.156692	0.5517722	0.150549875	
0.2063885	0.08220824	0.0938085	1.450287	0.1059639	
0.196411097	1.005015	0.1624364	0.2267719	0.3035893	
0.04807472	0.7029071	0.0649325	0.1107581	0.347703	
0.1742295	0.1329163	0.6828545	0.4476563	4.492561	
0.302899	0.1954478	0.2052976	0.2593037	0.3204659	
0.08434515	0.3883579	0.2907194	0.2107019	0.1045503	
0.02780246	0.04131754	0.196104494	0.137057286	0.2747469	
0.05535649	0.1669239	0.1657722	0.2503386	0.7704807	
0.3604698	2.429006	0.1442799	0.204216	0.2017092	
0.197137	0.1755471	0.078663095	0.01542573	0.03400104	
0.3049077	0.3547817	0.1521448	0.843376393	0.3813569	
0.1353643	0.4176112	0.3057187	0.2928819	0.3726165	
1.209724	0.2205931	0.04868602	0.2375843	0.2636468	
0.04415587	0.09219139	0.4895173	0.1531863	0.2023844	
1.133898	0.1122697	0.159447904	4.913098	0.2621739	
0.05587108	0.1512506	0.3885994	0.449484	0.1570168	
0.1654629	0.09344494	0.1378982	0.1424303	0.3745916	
0.03823372	0.3466111	0.1369675	0.06597251	0.3604093	
0.8411964	0.2529525	0	0.1180273	0.08084077	0.1366227

0.2110987 0.3413773 6.030909 0.065278429 0.1663578
0.163751 0.3835925 0.1636212 0.1420144 0.5139732
0.179572221 0.2488984 0.1571886 0.8853373 0.1377164
0.04756937 0.2935964 0.09745168 0.01622716 0.166817
0.122324 0.1604042 0.2594498 0.2394845 0.2030207
0.248308009 0.3833842 0.481369 0.03177064 0.431646209 0
0.568788 0.1709357 0.0878018 0.09368019 0.2884067
1.216013

AC009226.1 0.1470966 0.03245949 0.2706102 0.2053689 0.1781002
0.2521024 0.1398602 0.06780503 0.06987438 0.15594273
0.058276 0.04446031 0.2096815 0.04243589 0.1475756
0.1173591 0.1805673 0.110753529 0.1397114 0.1208337
0.224775925 0.06761341 0.02879005 0.1759864 0.08524743
0.08893928 0.179108 0.01789424 0.196792908 0 0.09592767
0.1419975 0.4587333 0 0.7737909 0.04960866 0.04709588
0.264227 0.4463858 0.280383662 0.2832633 6.802769
0.082439804 0.09456354 0.314594 0.7576646 0.4871464
0.4500014 0.115213533 0.4518703 0.1436822 0.2135119
0.4370056 0.2041631 0.419173 0.01390978 0 0.2203875
0.1361496 0.582914751 0.102770914 0.077155369 0.07888569
0.05296291 0.1712834 0.1226778 0.2779056 0.09148858
0.090947883 0.3082677 0.09663937 0.03280405 0.5037 0.443131
0.1584892 0.16371 0.1581327 0.3223696 0.2397912
0.399032634 0.1180454 0.3707987 0.06060092 0.2326984
0.07151163 0.3834501 0.337634 0.08389511 0.77525588
0.5998534 0.130846931 0.227419 0.01933192 0.5922091
0.01302402 0.1155959 0.2961361 0.2382973 0.02848671
0.2293829 0.2691701 0.2570255 0.2607129 0.3528902
0.1984853 0.1792433 0.1142617 0.07042815 0.160095607
0.5581432 0.2329823 0.2907318 0.0595719 0.2868986
0.6812511 0.341378 0.1266042 0.1255007 0.2173329
0.1065386 1.405139 1.156439 0.2085426 0.1680569
0.06525965 0.428032 0.2483859 0.1223787 0.2515421
0.125673 0.1711296 0.09017518 0.8059227 0.2508355
0.3035461 0.1586426 0.02152532 0.2436627 0.1867908
0.6384182 1.302649 0.4289182 0.5457457 0.2382683
0.2669952 0.3385746 0.4406543 0.1838035 0.2592719
0.1436878 0.3137162 0.1814134 0.358865 0.1124538
0.1157642 0.4974088 0.07077334 0.2411638 1.366844
0.07571433 0.18760491 0.212518 0.335567628 0.3602731
0.2090924 0.368273 0.8122514 0.1586305 0.4314769
0.1532332 0.1027494 0.039078863 0.434606 0.4375384
0.9752602 0.2330772 0.3064837 0.1792332 0.478955
0.2225879 0.2048452 0.4456867 0.1140452 0.4111292

0.1241628 1.426029 0.2380006 0.07654762 0.4012629
0.1369579 0.2911105 0.4173318 0.4702087 0.2421781
0.2529395 0.207949 0.151804 0.5732474 0.1383572
0.1321411 0.1741037 0.2132507 0.3993458 0.1847402
0.1070447 0.1491422 0.1908808 0.03909813 0.08648277
0.7857559 0.2894626 0.06013266 0.2835757 0.1497564
0.3691979 0.1682978 0.3753822 0.3137364 0.2252278
0.59827855 0.2111793 0.596436983 0.47507356 0.2188529
0.5229378 0.0916984 0.3972234 0.1054008 0.08283134
0.3166202 0.08936061 0.05263171 0.2361342 0.1696195
0.3932266 0.2534185 0.3096814 0.07676644 0.242756
3.084716 0.8305089 0.1927522 0.3151118 0 0.1298404
0.2064681 0.1507169 0.169869 0.2637678 0.1694301
0.1627445 0.3702927 0.8428998 0.2871836 0.1293263
0.1244261 0.4868481 0.117289212 0.2549599 0.2184967
0.1451376 0.07924104 0.375724 0.3272844 0.4140176
0.1204895 0.6663068 0.1354064 0.2254914 0.4211726
0.4332562 0.1845158 0.4895689 0.155122652 0.527705
1.897397 0.1825759 0.09056591 2.935793 0.242852226
0.3027897 0.6854209 0.1038488 0.1355512 0.1155815
0.06584155 0.2408571 0.6276724 0.1592285 0.1749184
0.2282558 0.1055393 0.04659125 0.6268461 0.1733884
0.07671781 0.5640887 0.1526741 0.08254991 0.08690704
0.2527287 0.1497748 0.1736813 0.1025961 0.3533121
0.6906208 0.134707292 0.894394862 0.3973222 0.08365555
0.1234829 0.4412522 0.1934568 0.1323139 0.101958
0.1225469 0.1858278 0.4868514 0.103918 0.5529509
0.6180039 0.43227944 0.4874244 0.163491 0.1629024
0.5280279 0.5434554 0.178254967 0.1232754 0.08265233
0.229491 0.1358843 0.6147326 0.407203 0.0738648
0.6710565 0.1337728 0.3060008 0.2037411 0.144074
0.6332773 0.5808079 0.3273702 0.4297015 0.2201218
0.1432225 0.836390279 0.008720633 0.5252666 0.1727043
0.436365 0.2205116 0.1061354 0.2561611 0.1326023
0.2728024 0.2525986 0.6848636 0.437432 0.507758
0.3869005 0.1317192 1.540797 0.3465993 0.2254951
0.1846169 0.1484514 0.3242994 0.2776541 2.127229
0.5437764 0.2344975 0.5595372 0.582930272 0.2804903
0.1687248 0.8748059 0.3371821 0.4097183 0.1708337
0.154188765 0.6568935 0.3644168 0.3266003 0.3439986
0.08169039 0.04654062 0.4351172 0.2675207 0.2187613
0.1587163 0.09641116 0.574265 0.1782145 0.2191485
0.32691954 0.19245 0.41082 0.1964138 0.263559679 0.2988162
0.520946 0.2516109 0.09477671 0.3539273 0.2285898

0.2784328
AC108449.2 1.052798 2.038597 2.469431 1.684542 1.795199
3.465162 1.87063 1.892645 1.99104 1.516897464 4.179614
1.809825 1.544502 2.048224 1.201458 2.963865
1.575057 2.615857594 1.462414 3.035553 1.445521902
1.310622 1.171943 4.062108 3.3169 2.689446 2.249752
3.246638 2.644820631 0.05972246 3.272663 1.580714
1.702204 1.657909 1.714195 1.096244 0.9585547
1.583816 2.585651 1.933297592 3.740824 0.8352757
4.821448404 4.128536 3.60466 2.866311 7.074499 2.54613
6.137124337 2.522615 0.7899684 5.314125 2.145972
1.068528 2.617194 2.879628 1.468498 6.77225 2.216871
2.06954745 6.723396046 4.881586638 3.460191 3.079908
1.23148 2.764417 1.47243 1.596078 1.375092446 2.096014
2.809895 1.793169 2.994217 2.871406 3.072164
2.713227 2.493073 3.963544 3.745061 1.082882039
2.105144 3.248787 2.255408 4.16783 1.136672 1.248714
5.416318 2.667013 3.78695642 3.235934 2.916796172
3.79114 0.8543869 2.199604 2.24183 1.15621 2.372666
1.690616 6.074069 3.734952 4.349837 3.173276
0.8490173 1.262851 4.345356 7.226881 2.430014
3.473026 4.07308457 1.193023 1.833023 1.415935
1.108555 3.394786 2.143937 0.9194551 0.6773326
9.028843 1.458274 1.239091 1.879374 5.068485
2.352678 1.759117 4.07329 3.957029 2.912909 0.6262598
4.290798 1.729598 2.217776 0.5993009 2.03533 4.296426
1.117951 5.568795 3.855372 3.117294 1.216577
5.836459 1.398496 3.575095 1.556102 1.1916 1.515372
1.809245 1.640001 2.610262 5.219039 2.879523
1.994599 2.804949 1.669503 3.191244 4.289209
1.65288 4.068588 3.552797 2.366063 1.408945
2.618311998 3.663897 7.05195118 2.322531 0.9403099
6.685931 1.259578 3.517689 5.089953 1.036898
3.324329 2.939126917 4.156051 1.365931 2.049885
1.543065 2.036879 1.212835 4.988089 2.862902
0.68826 2.870878 1.237969 4.021458 0.9145761 2.373979
3.916279 1.032727 7.293329 4.369041 1.376868 1.9415
1.280483 1.327843 1.13146 3.570222 5.212566 4.967655
2.681925 0.3688461 1.23519 2.652734 7.663533 1.160249
1.294775 4.489523 0.7294372 3.425619 1.966961
2.000631 4.780537 2.685577 2.882791 2.177165
4.142382 3.523278 1.126673 1.251048 4.025753
5.157283509 1.522786 4.661544003 1.326074894 1.395231
7.413147 1.130482 2.337116 1.654908 3.88557 2.156852

2.256772 1.285472 3.364271 3.613245 8.358277
1.215789 1.061562 1.584768 6.508584 1.719926
5.204129 2.822021 1.919703 3.000538 4.036086
2.224375 3.111403 2.081018 3.796321 1.820409
3.217741 6.771859 3.529185 2.672056 2.914235
5.949309 5.858256 1.636949432 3.854883 2.5158 1.639785
1.36398 5.579941 2.365443 6.710221 3.363228 3.122568
2.577003 2.163615 3.211186 3.455286 1.251833
1.102045 3.391790499 2.271769 2.679631 3.047757
3.813022 2.144616 2.259581577 3.324368 1.946588
1.419173 2.255635 5.012181 4.747748 3.050275
1.548507 2.615804 3.511976 2.484813 2.70043 3.164558
1.772151 2.258573 5.844313 2.775637 1.775667
3.691018 3.63876 2.514773 3.193568 1.885324 4.725218
4.975431 1.848511 4.44374333 3.284905614 3.373351
3.148822 0.8411792 0.7946254 2.149987 2.462188
5.149831 4.078474 2.276525 1.650702 2.148642
5.669832 3.120494 3.173671135 1.306325 3.694287
1.948758 2.834398 2.260838 3.213433298 1.971401
5.911888 1.174395 1.997043 1.793902 1.434316
3.006779 5.513628 1.192808 1.281212 5.581718
2.645753 4.713786 1.173245 0.7070993 2.175433
5.947337 3.408361 3.914193949 4.01642 1.117089 1.249812
2.51339 3.158563 1.975042 1.411231 2.467555 1.866359
6.757013 1.213754 2.992661 1.038544 1.834543
2.319813 1.739308 1.804397 1.27852 1.288305 6.330697
1.697284 2.564937 1.625117 2.080011 2.14976 2.853359
4.954419916 4.252973 1.667991 1.372928 3.00654 2.518749
6.622901 4.973369786 1.611717 2.103465 2.436282
4.320921 1.444144 3.752927 1.479253 2.203896
9.740994 3.561578 6.888009 1.888531 6.47324 4.448802
3.074835866 6.644151 3.402879 2.741253 3.371845024
1.092257 3.029414 2.224022 7.154892 1.147582
1.807847 3.175618
CNN3-DT 4.091557 9.788012 3.314584 2.358816 2.146706
11.22422 1.685785 5.677935 3.280229 4.370823128
2.021002 1.128202 3.458501 1.332579 1.591541
12.02918 2.548729 6.366694724 2.942553 3.495485
3.321624795 11.42386 3.360584 8.519263 10.99297
1.354127 2.113404 9.864783 2.829773122 2.280312
4.178731 11.51755 4.157351 2.306001 2.115473
4.626252 1.180143 1.263614 3.109444 2.109051919
0.8695096 0.2404581 0.597700215 0.1476675 0.7687313
0.4964986 1.987876 3.086218 3.172106346 0.424258

2.469342 2.113008 1.986821 2.115469 9.181171
0.1764838 2.634865 8.329794 3.166959 2.160373984
3.047939541 1.390077478 6.448537 5.191045 2.153444
12.17965 4.289961 3.163136 0.331753073 0.7421294
2.243829 0.926066 3.181207 3.025351 2.837562
4.154223 2.06208 1.203671 2.151418 1.659481566 7.588502
5.64552 0.7996447 4.960063 2.903429 1.292731 2.777094
1.809548 1.57380007 5.019882 0.954587838 1.090053
4.709348 2.108711 1.801175 3.197299 1.669911
0.5593393 7.072024 2.255523 2.33669 0.4640759 0.165393
2.565884 5.925485 2.869815 0.662731 5.39719
0.787110369 1.435455 3.651555 3.446659 0.9825832
5.270228 2.389685 0.7636797 4.080059 0.8075346
1.962042 2.943779 3.47647 1.606264 4.273187 1.812424
2.221795 1.671004 1.011092 0.7763551 9.893659
2.391761 0.6823925 3.43236 1.66527 1.287481 0.5135087
3.897547 1.365539 0.4173567 1.54837 2.392352 3.149062
1.375618 1.507707 1.859199 4.458039 1.683469
4.454092 6.812046 3.150402 2.440115 1.738087
2.274649 0.5203644 3.852321 7.333445 0.8564924
2.065295 8.669505 1.492233 2.641772 3.895009584
2.410876 5.622964543 4.349031 2.900517 1.333387
3.160396 0.8989897 1.290411 3.146044 9.516707
0.628042115 2.757086 0.9641865 1.595184 2.149675
3.944139 3.638505 0.8977179 1.943672 1.140683
1.696428 1.382667 3.303659 2.205485 4.23946 6.55385
1.398552 4.676729 0.5833663 9.221529 1.5885 2.939292
1.40466 0.6665328 2.550458 4.622522 2.567018 0.5558898
0.7041607 2.153759 6.493609 0.8866898 7.676395
2.36319 1.012925 3.684667 11.2938 2.060432 4.47279
0.918157 4.760796 2.385299 2.549263 3.34789 1.695695
3.79484 4.390042 6.166471 3.512608681 0.8574045
2.134018521 2.004181373 0.8021724 2.340994 0.5119163
3.794726 0.5014872 0.2802515 0.3873726 1.230966
1.419028 0.7490027 0.4077643 0.77418 2.672727 2.336815
0.864419 1.248222 8.839054 10.53729 3.193655
2.969987 2.88194 2.590883 1.571769 2.342516 8.592282
3.723114 1.904008 2.503644 1.263648 2.031955
3.825898 0.4307257 4.929009 1.35419 1.815525734 1.36134
4.879127 1.420562 0.6434498 2.310206 5.29444 0.6778001
4.608059 0.4848579 3.092403 2.777533 1.098434
2.002495 1.170545 4.949184 1.627009756 0.8394218
0.9600818 1.212744 12.73178 0.5848953 2.088401154
6.669225 3.468467 5.105729 1.508167 0.7856083

4.037675 1.222374 1.78581 1.25003 3.749483 1.946147
9.060946 7.259174 2.728582 1.473939 4.282855
1.899362 2.259939 1.01246 0.8086133 2.868088 1.884469
3.183014 2.837734 0.4603889 2.31664 1.118704614
1.353779283 6.124964 0.2605256 2.126262 1.264177
5.399967 2.835247 1.034894 12.43875 1.776161
1.748418 1.450333 3.421944 1.33872 1.028372285 0.8335384
1.096433 6.289173 2.473656 1.312745 2.28050105
0.9775541 1.101105 0.9608688 4.349347 1.333018
3.941293 8.083159 1.538048 2.39033 0.9576736 1.622815
0.586876 0.4820917 4.305131 1.705939 8.739146
9.001569 1.020411 1.680218812 2.246097 1.599468
3.798129 2.847337 3.651859 1.114022 1.43689 3.845541
0.3257646 2.123251 1.179272 8.031215 7.908485
1.944678 2.100963 4.527951 3.685577 1.055003
1.460538 0.1726554 0.9772243 0.4126714 1.058421
6.117377 2.502706 4.996792 2.005473325 1.028095
4.870178 2.714652 4.919793 0.4827087 2.803293
2.647536888 2.694441 1.369964 10.78823 1.898587
0.3109401 6.082104 1.783597 1.753688 2.326207
5.366059 1.467888 3.316445 1.165357 2.464535
2.741202648 4.767853 4.983544 2.658185 0.689695573
3.881574 1.041017 0.7874526 0.4700693 0.6803848
1.894856 9.469875
AC116535.1 0.03733264 0.05949748 0.02861666 0.05349359 0.04080669
0.4599973 0.05768108 0.05219971 0.1248761 0.187094848
0.1175004 0.03667262 0.1825621 0.029169 0.1420138
0.2166531 0.03723476 0.10306591 0.02304789 0 0.138144186
0.1859007 0.03957861 0.2679887 0.09375392 0.1222677
0.02954707 0.1918784 0.120238922 0.05647418 0.01758332
0.1338573 0.3123147 0.2939505 0.05572052 0.1363973
0.09064192 0.08382494 0.182895 0.132155138 0.3969482
3.356842 0.045333066 0.2719991 0.05766434 0.1922928
0.325798 0.06019117 0.16472328 0.139149 0.05746171
0.5342095 0.3204084 0.05613392 0.327553 0.03824446
0.1827141 0.2806494 0.1372575 0.160270351 0.134218373
0.110310699 0.08675736 0.07280982 0.017125 0.1728653
0.1528183 0.1320609 0.031257249 0.0956234 0.09034023
0.08117421 0.2769811 0.09282842 0.1210446 0.03375862
0.1545885 0.1924632 0.05180193 0.01219029 0.0432749
0.135933 0.06664803 0.2111327 0.1900647 0.07229365
0.1408475 0.2152889 0.49025389 0.3368733 0.035975901
0.1597939 0.06909817 0.3203991 0.1432363 0.1525568
0.132687 0.1506937 0.09398786 0.1513632 0.7400736

0.07610426	0.04300926	0.1343436	0.2182913	0.2816132	
0.0448798	0.1471662	0.115546519	0.1078364	0.09797055	
0.2148271	0.1419521	0.6927877	0.4627599	0.05434039	
0.09746628	0.1183062	0.08273756	0.04556599	0.07726762	
0.3882445	0.3325607	0.05775832	0.2452198	0.3802159	
0.1991874	0.0336476	0.37462	0.06910671	0.07169749	0.2904364
0.1392824	0.05015734	0.1112786	0.09516705	0.09469298	
0.02679768	0.1985822	0.2612552	0.2839839	0.4389599	
0.1403701	0.1375731	0.187928	0.1408928	0.03230836	
0.3032167	0.1206377	0.1033246	0.1955117	0.06454927	
0.2931869	0.1731452	0.1455038	0.03907451	0.03335808	
0.3757404	0.7166588	0.4787999	0.037513678	0.4880713	
0.292696838	0.05094299	0.06132184	0.1185429	0.1091814	
0.1395677	0.2965826	0.7583568	0.1489576	0.093119825	
0.4182269	0.02532624	0.05169047	0.1183085	0.132419	
0.1343987	0.07781504	0.1295995	0.206512	0.1131138	
0.06968075	0.1333275	0.1422422	0.2536999	0.1502637	
0.07576739	0.1282858	0.04841496	0.0800398	0.07868153	
0.0630645	0.09512287	0.09629281	0.3267134	0.05167559	
0.2781394	0.1521633	0.2034577	0.09573838	0.8055598	
0.1463983	0.1168254	0.03531786	0.06271524	0.03748714	
0.1289987	0.02113612	0.2043629	0.1989668	0.09258628	
0.3003217	0.0617625	0.2866155	0.1306528	0.08656323	
0.1380171	0.1173327	0.064507676	0.1219323	0.150869182	
0.039185939	0.02674348	0.7161866	0.06050916	0.0899419	
0.1014286	0.03795697	0.06839926	0.1474163	0.1230027	
0.1460796	0.02945449	0.2087762	0.2090296	0.1971797	
0.07387332	0.161593	1.461201	0.2190253	0.218221	
0.1072672	0.1079423	0.1038521	0.1374375	0.1657563	
0.2802293	0.1631746	0.1641539	0.3523749	0.09829995	
0.301278	0.2030403	0.02666836	0.1216373	0.2368242	
0.07739581	0.210301	0.1742172	0.2451312	0.03485927	
0.1112505	0.1349786	0.08812853	0.1893038	0.03232918	
0.09476601	0.5631491	0.1929998	0.1928646	0.1606512	
0.09552631	0.113734467	0.1948979	0.02484203	0.1771715	
0.1942261	0.07338053	0	0.3196837	0.2936377	0.07138216
0.06307116	0.08171672	0.1448232	0.1913103	0.1359692	
0.1094483	0.09112393	0.1204956	0.1934511	0.06148847	
0.1537884	0.1048796	0.1160131	0.8052968	0.1399241	
0.07565602	0.111509	0.2161814	0.2127636	0.1061179	
0.1184754	0.04725823	0.1456642	0.094276749	0.051770675	
0.1584014	0.2090982	0.09700338	0.07827145	0.09456045	
0.1455169	0.2082452	0.09330598	0.1771213	0.217803	
0.06349305	0.1799561	0.1243302	0.133710418	0.1514959	

0.07705931 0.1407668 0.08934118 0.0517227 0.081684289
0.2287853 0.04544993 0.05678797 0.159633 0.1475071
0.2111228 0.1523165 0.1071317 0.0613006 0.1065695
0.2053989 0.07922528 0.1479997 0.1092623 0.08358004
0.1876419 0.4283075 0.07269881 0.312092035 0.09590829
0.1237887 0.3587713 0.05713191 0.1148758 0.0265287
0.1186199 0.1562508 0.01764849 0.2488664 0.3586682
0.311288 0.07221014 0.72009 0.1138209 0.1682087 0.05445499
0.3564947 0.1433217 0.03401348 0.215482 0.1788537
0.04587247 0.1727664 0.1441189 0.2070961 0.098630535
0.04570063 0.09278057 0.08693668 0.4326327 0.101922
0.25677 0.203489549 0.4453411 0.31914 0.06812232 0.1986205
0.03593677 0.119431 0.1104313 0.2022731 0.1603938
0.1591522 0.09088426 0.8929072 0.1281524 0.1259859
0.132874029 0.172665 0.1928474 0.03600219 0.036232401
0.10172 1.06708 0.03689575 0.09475826 0.0589764 0.07541994
0.09384053
N4BP2L2-IT2 0.1842074 0.2642163 0.5520499 0.1248791 0.1714715
0.6138194 0.09181004 0.2243307 0.1467791 0.160065254
0.6707341 0.2159735 0.3303448 0.2005682 0.203437
0.5832985 0.1540915 0.152117541 0.09171237 0.2094055
0.114570017 0.4024173 0.1133939 0.5865107 0.2767475
0.6468887 0.2116331 0.5332923 0.08612205 0.04719174
0.1595264 0.2103952 0.5592445 0.3836585 0.4619914
0.3972954 0.0309157 0.4189476 0.4527543 0.231384098
2.233749 3.486066 0.201005727 0.2998722 0.9423095
0.7454975 1.272219 0.3161575 0.614123472 0.2634043
0.1063233 2.164041 0.5163637 0.4958784 0.79942 0.350629
0.6499894 0.8314806 0.1638529 0.346398549 0.671257995
0.275525135 0.3876392 0.1703586 0.4129524 0.4227868
0.2067526 0.6606261 0.122389068 0.4981163 0.2258397
0.5426554 0.9111236 0.748002 0.8901108 0.2364254
1.044969 1.073804 0.8410134 0.462763613 0.09815393
0.4121704 0.5887583 0.626287 0.1345706 0.2589044
0.6572686 0.3524624 0.69211801 1.142767 0.274858829
0.6037841 0.2335012 0.8452193 0.1983486 0.43708 0.4089518
0.3503994 0.4537835 0.5661677 1.420995 0.7138248
0.5408114 1.047779 0.6192798 0.97492 0.1885869 0.1553394
0.186540848 0.2970717 0.9536216 1.22143 0.0729969 1.411673
0.7681345 0.3514749 0.1163516 0.7532244 0.3665428
0.4009687 0.3751061 0.9461219 1.076003 0.1875433
0.6140279 1.219012 0.5543729 0.08354788 0.6577389
0.1264954 0.4600451 0.7204847 0.6046186 0.6945611
0.08634613 1.060331 0.3956433 0.6877866 0.3465981

0.8147779	0.9472024	0.7852389	0.5870681	0.594355	
0.5019641	0.4589255	1.238049	0.6909158	1.547483	
0.6471986	0.3130233	0.2689973	0.166921	0.5226412	
0.6904459	0.7183437	0.520336	1.255926	0.4089806	
0.4970205	0.183607973	1.155435	0.832508712	0.7405775	
0.1720288	0.3018924	0.2511944	3.812608	1.622557	
0.7973939	0.5763823	0.386505135	0.7702925	0.163261	
0.2684218	0.4072196	0.4943494	0.316602	0.5459232	
1.481788	0.9114005	0.9602211	0.2129467	0.8442474	
0.5488047	0.7562259	0.3934771	0.2251157	0.5831681	
0.2722833	0.2955635	0.4320646	0.1716476	0.1771445	
0.2452283	0.5824273	0.3929076	1.437332	0.4026507	
0.4406541	0.5120143	1.088244	0.1864158	0.5311678	
0.4553404	0.6173657	0.7589727	0.2327018	0.60051	1.212834
1.276903	0.4357884	0.6260187	0.4882549	0.6386818	
0.415916	0.1240032	0.7084668	0.4077517	0.271064156	
0.5822329	0.895811757	0.099794593	0.314997	2.075214	
0.5586064	1.368192	0.6019487	0.1341224	0.4394389	
0.5229123	0.9570253	0.4882763	0.5930603	1.039642	
0.8525668	0.4236945	0.3477096	1.41239	0.7614368	2.013831
0.4971908	0.488566	0.5596095	0.6942586	0.4736566	
0.346279	0.8861255	0.5107872	0.8135021	0.5288197	
2.095479	1.067897	0.7594634	0.6027559	0.3484947	
0.9956377	0.123189651	0.5188355	0.3843934	0.4682056	
0.1040343	0.6488553	0.2209815	0.7434455	0.4338889	
0.5042868	0.1325215	0.4415989	1.158124	0.4794297	
0.1816858	0.5266378	0.863509749	0.3991443	1.396773	
1.742766	0.8965248	0.191125	0.230270924	0.8745607	
0.7952408	0.1124816	0.3312848	0.4595707	0.6166216	
0.4872089	1.203628	0.4520673	0.4689642	0.9901205	
0.5942719	0.2789301	0.4406086	0.3551166	0.4230305	
0.843125	0.3407537	0.6839882	0.2256621	0.4424045	
0.8258748	0.3749717	1.263455	0.4688723	1.078109	
0.713850981	0.902307816	0.6050997	0.4133602	0.122747	
0.2541501	0.3612248	0.1582715	1.327117	0.349007	
0.5204698	1.053021	0.5730151	0.6874398	1.001346	
0.986087748	0.4201289	0.4292892	0.06416156	0.7252348	
0.3540026	0.241828927	0.392477	0.792144	0.3374503	
0.4362697	0.2670676	0.3054915	0.2521373	0.9463849	
0.7054396	0.3481776	1.417688	0.5833857	0.7940054	
0.4575198	0.3254192	1.035378	0.751385	1.281527	
0.358184198	2.342501	0.5477504	0.4081331	1.353127	
0.4357827	0.3166897	0.7965229	0.4078711	0.7163156	
1.265731	0.6465288	0.925632	1.395319	0.8166359	

0.1877548	0.7585827	0.3423668	1.69858	0.8725697	0.3540671
1.192148	0.6165721	0.4791574	0.8884276	0.1847207
0.5227012	0.756488889	0.1882172	0.1033741	0.2103311
0.3246325	0.4661907	1.531117	0.296900084	0.4843755
0.8682741	0.4879313	0.8490645	0.2144999	0.09369035
1.151305	0.1551237	0.5798869	1.032074	0.6437325
1.289502	1.087083	0.9416175	0.597157912	1.148958
0.7577333	0.1203386	0.333047269	0.1382429	2.650273
0.3567626	0.6696638	0.6646121	0.1440536	0.3372492
LINC01857	0.206708	0.02823712	0.8963646	0.4513371	1.471861
0.2691514	2.555006	0.9555548	1.86914	34.407674186	0	0
9.941118	0.2491815	10.49496	0.02187705	2.415088
0.289039697	0	0.3153465	0.069012981	0	0.30054	0.2473055	0
0.06963295	3.786168	0.0933992	0.342387661	0	0.4172464
0.05293982	3.791076	2.325117	1.177987	1.229932
0.06145438	1.405658	0.434004	0.418133126	0.166927
0.02603163	0.276618575	0.2657714	0.1824475	0.06518637
7.456198	1.142653	1.052377269	0.778319	0.5113304
12.25871	0.6335989	0.9768291	2.993939	0.1452046
2.774877	7.144062	0.1480489	6.565460875	0.972250442
1.36922552	0.7940793	0.3455507	0.1219112	0.2601293
0.9670209	5.282629	0.178013844	1.547122	0.580072
0.7276897	0.671873	0.3304182	0.1838303	3.204325
2.751252	0.3605597	0.3128982	0.4628346	2.105144
3.870776	2.656981	3.552623	1.119767	1.601138
0.7899898	4.050497	8.29523787	2.498092	0.682957153
2.802677	0.9333546	33.22871	1.563517	3.589957
6.383111	0.4975182	0.04956225	0.8680185	23.88392
0.1289949	3.470025	1.381436	2.407168	6.905342
0.7241885	2.720244	0.496149953	2.086506	0.2682472
0.3319484	1.088277	27.573	13.49112	0.1641156	0.2643249
0.02339474	0.2181484	0.7414396	0.3361482	0.5400675
0.2721227	9.045868	0.2270824	39.84177	1.539538
1.053949	1.094106	0.5466266	0.6167423	0.5379091
1.922982	0.208288	1.914438	2.145369	1.039253
0.1589751	1.267444	0.1937344	5.069221	0.7462478
1.699927	0.6529131	0.8640226	0.549263	0.07666669
1.06035	0.05204896	1.932641	0.682269	0.5848436	0.6422059
3.96194	2.913265	0.1854451	0.9235058	1.853171	2.156869
2.469949	10.370682925	13.34351	1.600508004	0.188045
1.127739	1.007986	2.119778	1.021168	5.228087
0.763448	0.82883	1.325821898	2.362949	0	0.09199486
1.052783	0.3427919	0.08504635	0.681794	0.1025118
0.2375982	0.6561267	2.513322	2.283451	1.458156

0.5108061 2.737545 1.038807 0.1461207 1.480768
1.164915 0.1555906 0.23944 1.309196 2.792774 0.6460669
0.7357469 3.842759 0.6017977 0.5172841 0.2044656
1.524414 0.0868496 0.2892762 0.05587209 0.5265981
1.103051 1.122402 0.1003106 0.9698932 1.246454 2.9817
1.068981 0.1628449 1.133549 1.007614 0.5056289
1.427009 1.948986 0.244919387 1.405372 0.747146426
0.247965224 0.3173071 10.14543 0.3589657 0.1524494
0.5501409 0 0.3246184 0.2332094 0.6867796 0.1155474
0.1630873 0.1769354 4.381514 13.9945 1.001708 2.234041
0.9045345 0.375982 2.39682 2.819537 1.536861 1.601845
4.197213 6.735851 9.664302 2.581386 2.147689
4.937409 0.6664645 0.3299644 0.3747396 0.8859642
1.551448 0.2687712 3.489497082 2.079322 0.4276674
7.277869 0.3722396 1.998809 0 0.6273784 0.6288963
0.1278602 1.702638 1.078876 2.137251 3.419001
2.086679 0.2060735 0.188921733 0.3083242 1.886378
0.3083097 3.899857 1.677976 1.373200999 8.818713
1.809581 0.9485702 5.605665 0.3447309 3.03567 0.3724908
0.1985542 1.142756 0.7928585 0.7386581 0.7650885
0.7052324 0.2265114 2.624509 0.4004296 2.972578
6.109448 0.5266192 0.3024082 3.371086 1.726368
0.7806247 0.2409756 0.2741255 0.2468975 0.351553178
2.579852702 3.758813 0.05954198 2.232803 0.297177
0.8695068 11.70207 1.672538 0.4428241 2.715805
0.4522344 0.4520009 0.765709 2.753628 0.810853432
0.2212276 5.851496 1.062839 2.968052 0.6273188
0.310134659 2.050956 0.6471073 0.4192409 0.3223862
3.383619 2.368304 0.3855382 1.01688 0.0872786 0.5590112
2.89489 0.375998 0.2479049 1.316324 0.2746145 0.06596566
0.883791 0.3162719 0.155912474 0.06827617 0.5874933
1.702705 0.5422887 1.847598 0.4532525 0.5981477
0.6921191 0.08375857 4.01026 0.5532202 5.170735 0.8224911
5.980626 6.531347 0.1182678 0.7322463 1.140194
1.076976 0 4.654883 0.1863281 0.08164041 0.3153609
0.2879912 0.08424552 0.965444086 1.220019 0.2935537
1.815422 1.173284 0.4327976 0.208056 1.555927602
0.281808 3.980274 0.1175651 0.8079772 2.259835
1.902868 2.00905 0.1745409 0.1359318 1.291363 0.3234981
3.204099 1.466843 0.9358759 1.706360528 0.5286818
0.8367922 0 0.42989155 1.299729 29.70599 0.7004193
0.2023724 0.2798981 1.352211 1.359523
LINC01215 0.03923624 0.02143929 0.2577929 0.1392145 0.2205639
0.0113531 0.1558858 0.3090152 0.6576603 2.893341459

0.03849094	0.04404864	2.925671	0.03153222	1.133121		
0.008305176	0.8273913	0.160371741	0	0.04788595	0.144096549	0
0.03565435	0.04694225	0.01535602	0.0396521	0.408134		
0.01329641	0.075821959	0	0.09503946	0	0.06492653	0.5649173
0.05019576	0.01228733	0	0.3322598	0.06503715	0.059525896	
0.0452646	0.02470594	0.029170169	0.05765412	0.1096656		
0.1979734	2.987124	0.1204959	0.365269571	0.2238432		
0.05823485	3.860512	0.08418652	0.1854166	0.3497194		
0.02067149	0.658391	2.763811	0.01686112	0.676303261		
0.076364498	0.152881934	0.3461162	0.01311813	0.1157028		
0.1595237	0.1223699	0.9574004	0.219632821	0.1370445		
0.2154253	0.1096885	0.3715047	0.03135914	0.1003194		
3.639233	0.6832488	0.06387694	0.1315124	0.032944824		
0.1266985	0.1714372	0.3602388	0.1152719	0.005904124		
0.2062312	0.1095801	0.9212279	0.9216931	0.6006215		
0.043211795	0.4568851	0.1388587	3.184406	0.1548411		
0.3092189	0.6248208	0.1770681	0.01411147	0.4317921		
5.459876	0.07835249	0.4261932	0.1411938	0.3701599		
2.143688	0.1132038	0.2930594	0.094176611	0.1345075		
0.04412833	0.09451307	0.1770609	8.56434	0.8337533	0.4049705	
0.07525918	0.01776267	0.08695637	0.08209613	0.09860912		
0.2954781	0.02066117	0.06243781	0.03771565	6.399088		
0.06664829	0.006062279	0.1921017	0.05187891	0.06055181		
0.08295878	0.1596921	0.1468486	0.175429	0.3715015		
0.1706081	0.01810549	0.1665551	0.08825678	0.7292966		
0.2301795	0.4709257	0.2065543	0.1110997	0.1495879		
0.02910495	0.2204388	0.01481949	1.84517	0.1450453	0.2167377	
0.07618762	0.6071986	0.5611732	0.1232008	0.2253794		
0.1061915	0.03149274	0.2062865	0.768816921	2.82385		
2.292831172	0.1274776	0.131199	0.5828909	0.3308337		
0.1204906	1.343514	0.09660916	0.2313591	0.174226596		
0.275094	0.02851889	0.2735711	0.05328902	0.1138672		
0.1049299	2.054457	0.1297217	0.06201203	0.02830513		
0.3640766	0.3812121	0.2203986	0.554049	0.358062	0	
0.08783027	0.09692125	0.230732	0.0590668	0.005681157		
0.4884405	0.08674526	0.1716863	0.2363404	1.263944		
0.08567259	0.01309176	0	0.1745325	0.05495114	0.009151484	
0.0848428	0.265101	0.1756052	0.1484884	0.004760111		
0.02630001	0.3799866	0.4527767	0.4110195	0.03709245		
0.2420599	0.2010682	0.01199698	0.03108314	0.4639012		
0.063922826	0.09415076	0.189092614	0.041184039	0.2047806		
6.344229	0.204411	0.08681149	0.03915934	0.06154829		
0.005601581	0.0063238	0.1108068	0.03655434	0.04422341		
0.08732092	0.09415203	2.874362	0.1853856	0.037975		

0.03433881 0.02518834 0.6234526 0.7433193 0.111131
0.3800673 0.5275401 0.4013012 2.131751 0.4899853
0.3756896 1.168972 0.09487863 0 0.08129271 0.07607651
0.1438196 0.03246521 0.371850241 0.2789115 0.0811776
0.7703238 0.0235522 0.243391 0.04053175 0.1455492
0.1364272 1.761984 0.1829354 0.0465424 0.4172728
0.2197324 0.09140366 0.05476212 0.097334676 0.0936392
0.1734366 0.07448221 0.4755549 1.442286 0.093568064
2.80187 0.04737728 0.01929136 0.4442101 0.05725565 0.6631911
0.08396133 0.009422131 0.6162271 0.1322543 0.2532794
0.3253043 0.1292476 0.2340851 0.3693334 0.142514
0.2740591 0.1764706 0.0545237 0.1530708 1.098928
0.2534962 0.1290549 0.03557613 0.1750203 0.08435665
0.060663638 0.108820947 0.482213 0.003767317 0.2009864
0.05640859 0.1064808 1.172516 0.4401324 0.1774485
0.583006 0.08584079 0.2287906 0.3633573 0.08960219
0.180679297 0.07873552 0.1272678 0.08069702 0.4627766
0.07765722 0 0.2290011 0.1791279 0.06820982 0.03671621
0.6810182 0.4322493 0.02744282 0.06433964 0.01656677
0.06063349 0.7962042 0.09390789 0.01568534 0.2044291
0.005791768 0.006260629 0.3858402 0.08732085 0.172634568
0.04319943 0.2676358 0.8460201 0.08234754 0.2759619
0.09081371 0.04007199 0.2345973 0.07949309 1.892589
0.2665623 1.376624 0.04336695 1.658588 0.7084265
0.03928572 0.2371026 0.1745369 0.4895465 0.0245128
0.5488817 0.08252496 1.213897 0.1496504 0.07516436
0.0213214 0.038872398 0.1543851 0.02786042 0.2088448
0.05567667 0.07248653 0.00564173 0.147669055 0.03566089
0.02005792 0.0111578 0.08946352 0.0647472 0.3842474
0.3481867 0 0.09804713 0.3514927 0.04775923 0.2158078
0.5093204 0.2467266 0.401345064 0.1906681 0.382198 0
0.040799892 0.09868313 7.174102 0.09971247 0.05548586
0.04781593 0.2340223 0.2847533
PANK2-AS1 0.6588942 0.693058 1.90914 0.6608864 0.821038
1.000926 0.7126208 0.789675 0.2543047 0.949350794
1.357395 0.2588981 1.424503 1.173771 0.8593516
1.342388 0.7228835 1.686745528 0.8135574 0.4221782
1.039420004 0.6233925 0.7963289 1.537188 2.301522
0.8804384 1.251563 1.615107 0.190991757 0.5980372
1.768898 0.7087454 2.035248 0.4150412 1.743348
3.61097 0.9255782 0.946848 4.45969 1.195377185 1.330225
1.887736 0.994405097 0.8895207 0.6106405 2.133265
3.143389 1.38103 0.771538787 1.841909 0.9127421 1.771713
4.099859 2.427272 2.31243 0.8909832 0.8706885 2.195195

2.973063	1.741856967	1.458717763	0.943499938	0.7874769	
2.351625	1.541444	1.451061	0.7192355	0.9989059	
0.761301836	3.49811	0.6471551	1.528178	4.432255	0.983013
1.102356	1.370376	1.151034	3.432594	2.194241	
1.64589651	0.7446766	1.69138	0.8469287	1.253405	0.5552286
1.626812	1.762698	0.9770642	0.90288303	4.013261	
0.825433475	1.728934	0.788006	3.142594	1.289289	
2.355954	1.085758	1.630472	0.8017621	1.001795	
2.30987	1.957213	0.7590825	2.726728	4.827188	1.615338
2.186186	2.091572	1.398386119	0.9662578	6.085137	
9.575855	0.8094214	3.304977	0.816738	0.7585363	
3.317548	1.226712	4.794516	3.308737	1.261439	
3.26072	12.50802	0.3669814	4.9402	2.045116	1.084787
0.3919449	2.319207	1.372144	1.423585	1.312754	
2.078325	2.655723	1.104742	1.889584	0.8774131	
1.206048	1.486533	3.631136	1.526076	6.868526	
0.9995158	2.70556	6.685415	1.758421	3.164718	1.971626
5.603584	0.7079876	1.552789	0.7146194	1.552362	1.5716
1.396371	1.655129	0.8536819	3.861902	6.015756	
2.094248	1.440044612	0.7643505	2.661555859	2.217798	
1.298744	0.8891834	1.629169	1.170053	2.422812	
3.731423	3.453956	0.872318193	2.741664	2.011455	
3.797448	1.252836	1.784694	2.372041	2.947484	
2.935417	1.954932	3.227478	2.619506	2.148514	
0.9941471	2.198104	1.308914	1.248091	2.037738	
1.424149	4.746487	4.582619	2.404173	0.8058482	
1.529549	1.989359	0.9892093	4.221707	1.208508	
1.346581	2.331805	6.397899	1.905571	2.474262	
0.9350023	0.6896707	0.8733397	3.908394	1.594734	
4.761054	1.222044	1.085497	3.424915	6.685724	
1.896959	1.297085	1.762822	4.149735	1.725698	
1.912703786	1.260467	0.833551114	1.590688478	0.8732061	
2.585491	0.6140674	8.401934	0.9973644	1.527402	
0.7243187	1.338064	0.6895828	1.76177	0.2599253	3.00044
1.982957	0.5694678	1.061675	1.7484	6.794879	7.47631
1.749656	0.9174684	1.061415	2.508802	1.676879	
1.042202	1.912393	1.775947	1.597374	3.761113	
2.936978	6.932994	1.732032	1.435566	2.173647	
3.870837	0.614691215	0.6804711	2.799136	2.082704	
0.9690048	2.314114	0.7827474	1.788716	3.207432	
1.626173	0.9676908	6.592683	0.9537664	1.50173	0.6715371
2.804842	1.204399	2.499593	3.025263	0.5941205	
1.344811	1.942671	0.785644388	1.939502	0.8817976	
0.3779532	0.7589732	0.673046	2.492125	2.623273	

5.814801 1.15901 0.5896997 4.386238 1.331566 1.275142
2.44283 0.7572475 1.898635 1.989808 2.963471 0.8278701
1.152717 0.7358361 1.598956 0.9270881 1.493575
1.028691 2.313782 1.533180587 1.827430485 1.908768
1.527839 0.6676956 0.9946331 1.032647 0.8988941
1.696328 0.9606237 1.442799 2.402541 1.243874
0.6571234 2.677094 1.101283336 1.573425 1.360042
0.4404222 1.892169 2.160456 4.108756788 0.8524449 0.6317
0.8018134 1.690444 1.236612 3.692291 1.451669
5.892986 1.233379 3.563764 2.372821 0.3974028
5.761962 5.340189 1.906319 1.987047 2.662195
1.443467 3.768768638 4.06251 4.10739 2.402456 1.331005
1.554397 1.236081 1.609422 0.4963887 1.214784
2.206372 0.9970122 2.397386 9.736853 5.155839
1.260101 1.286464 3.027438 1.641359 2.497906
0.6123198 0.983561 0.5774341 5.768515 4.327523
1.365509 2.756987 1.534043087 0.937653 2.865643
2.424304 1.799833 0.9372953 2.321169 3.112585172
1.59819 1.964857 3.67251 1.477322 0.3329856 1.264723
2.631195 0.8762668 7.218628 1.060141 0.882223
7.379904 2.394845 1.943198 2.772772771 4.983995
2.946562 0.6354127 2.493955874 5.468714 6.484183
0.4883876 1.555346 1.092936 0.7542945 1.882858
ANKRD44-IT1 0.09513594 0.1039675 0.3625402 0.09087961 0.1960935
0.3716254 0.08399459 0.3094802 0.1888373 0.289473417
0.1049949 0.08010342 0.5456818 0 0.2658845 0.4530944
0.1355522 0.163727459 0.08390524 0.08708165 0.031762757
0.1894946 0.05187057 0.1300808 0.02792526 0.08546158
0.2366437 0.1504522 0.131317999 0 0 0.1340086 0.3279731
0.1712191 0.1217095 0.5958608 0 0.073239 0.1261562
0.144332202 0.1975552 0 0.127311823 0.008737107 0.4478411
0.1500079 1.439085 0.07304146 0.083031343 0.1736805
0.01568909 3.56471 0.2916096 0.3269665 0.4769793 0.03341471
0.5786943 2.410276 0.0136277 0.202675593 0.216020807
0.203880716 0.5820442 0.1431336 0.1496235 0.5617759
0.1668993 0.8241675 0.054619776 0.3702673 0.02321515
0.187158 1.210011 0.03801819 0.4124574 0.4670101
0.759747 0.1659452 0.164582 0.026627061 0.2126805
0.2969164 0.3785035 0.3493741 0.05726282 0.1184324
0.3076509 0.5710204 0.46558865 0.9351137 0.065484666
0.6221933 0.3831301 1.273484 0.06257376 0.6248016
0.645522 0.2146682 0.03421603 0.6887925 6.891527
0.05936905 1.064704 0.2119322 0.5188818 1.322515
0.1960605 0.1861042 0.048073611 0 0.1728426 0.2946413

0.1431063	7.835914	0.9626659	0.2373897	0.06082688	
0.09690542	0.1405618	0.08531064	0.182839	0.321669	
0.3005823	0.4794102	0.07838484	3.256086	0.09944729	
0.1763902	0.4154328	0	0.1859717	0.232096	0.2489174
0.08216841	0.030383	0.2771625	0.2973273	0.1609678	
0.3440166	0.1426639	0.6480763	0.3434555	0.3806172	
0.4865219	0.4489716	0.2418035	0.5822079	0.8482234	
0.04791036	0.252242	0.2386474	0.05127053	0.1724164	
0.7834108	0.5263923	0.04267492	0.315742	2.719654	
0.03818016	1.727902	0.184366216	1.771806	0.138985463	
0.2349117	0.06697217	0.1402545	0.1951227	0.1397254	
1.129059	0.1505879	0.4151219	0.06258467	0.9135264	
0.08297956	0.1834734	0.1292097	0.2760934	0.3718491	
0.3268657	0.1572679	0.09568384	0.2196203	0.1369823	
0.696408	0.6338227	0.4533976	0.1905786	0.01838861	
0.1569192	0.2232543	0.1398637	0.1861847	0.05510032	
0.1869979	0.4206618	0.4162871	0.303892	0.4860285	
0.1523353	0.1111024	0.07319194	0.5345534	0.03997194	
0.08875826	0.1285737	0.5268766	0.08188256	0.1565388	
0.08079276	0.2550782	0.278144	0.722267	0.6055274	
0.1798758	0.3782384	0.2021463	0.01454452	0.05383369	
0.2705262	0.056361243	0.1395087	0.243573928	0.057062156	
0.08762306	3.22352	0.1321692	0.08419658	0.379798	0.03316353
0.04074639	0.04599987	0.2054557	0.2127196	0.05361416	
0.04886	0.5707251	1.487861	0.265092	0.2301945	0.1526455
0.1119691	0.4766568	0.3965111	0.538918	0.2693755	
0.7570327	0.2489155	2.434799	0.2376131	0.8624792	
0.4886901	0.1840415	0.3037278	1.084106	0.1844624	
0.04981698	0.2474003	0.126790845	0.3445178	0.2230751	
0.7222164	0.03807128	0.3818613	0.1123168	0.5935348	
0.2480959	0.1530016	0.1478542	0.3385535	0.7447676	
0.2973679	0.110813	0.6449387	0.111792788	0.3027288	
0.2170482	0.438592	0.7614653	0.7868485	0.081026468	
2.807668	0.1723133	0.2182865	0.9142609	0.03966501	
0.7644765	0.1714364	0.2969954	0.8486849	0.07740467	
0.2851297	0.2112761	0.4588867	0.1158337	0.3540433	
0.2764425	1.029339	0.2139438	0.3525424	0.104386	
0.3878799	0.3298128	0.3476876	0.5915061	0.1720423	
0.1136329	0.147090878	0.282704693	0.2505471	0.03653839	
0.0211883	0.08548355	0.2194561	0.2472169	0.3848872	
0.1924842	0.4464039	0.1784039	0.2496364	0.1084346	
0.2353629	0.292061577	0.03818194	0.3646922	0.1677135	
0.2927198	0.08787107	0.035684343	0.3886806	0.1861417	
0.2894292	0.09644445	0.389322	0.2515375	0.1552612	

0.1040027	0.06694897	0.306287	1.155613	0.118403
0.1331126	0.1101508	0.2949088	0.04554037	0.4067588
0.4763847	0.143515317	0.2304401	0.07210394	0.5301219
0.1497508	0.2927416	0.06953546	0.4372307	0.02275308
0.07709863	1.833053	0.2937875	1.081725	0.126182
1.376271	0.2712188	0.06803994	0.3171872	0.2200634
0.3782752	0.01485902	0.9088877	0.2286834	0.1878722
0.2757711	0.198819	0.06462238	0.053859284	0.06238948
0.06755305	0.5950025	0.9112432	0.1991922	0.2462307
0.086426481	0.0864668	0.6484581	0.01352714	0.1756034
0.01962403	0.07453464	0.3216179	0.05355419	0.08758639
0.5831273	0.2150601	0.6897626	0.2469894	0.4785895
0.478033567	0.3893155	0.5054804	0.026213	0.079141868
0.08545593	4.458255	0.2619201	0.1655832	0.1159391
0.03660858	0.1294575
AL731577.2	1.335062	3.754774	1.252121	3.175827	1.716822
4.109207	0.9707051	3.702082	1.333656	2.164653679
3.662673	2.674348	2.263804	3.239808	2.475281
5.372058	1.93207	3.961706686	1.195929	1.900943
3.793102669	2.216014	3.363612	3.883556	4.518156
3.827177	2.196154	5.671604	2.17524125	0.2969982
4.438599	2.909687	1.819347	1.5665	1.598371	0.05738515
0.6265025	1.410676	2.531175	1.343676451	1.691183
1.453831	0.790148915	1.716535	1.401723	1.174999	1.2184
3.939238	0.666370163	1.756285	0.8461366	1.531284
2.415211	1.062752	2.347846	1.029775	1.422125
8.318922	2.099893	3.513408793	0.505244032	2.427599842
1.199306	2.603771	0.9006054	3.228415	1.714502
1.904941	2.551211017	1.353216	3.621939	1.138389
2.693177	0.5125948	1.650001	3.456052	1.686939
2.493124	7.509074	0.589801388	2.548931	3.71734	0.2523619
1.938063	2.840107	1.824926	2.558844	1.746832
0.44839124	1.299188	0.327942489	2.922982	0.8721309
1.568967	1.566824	2.085974	1.243357	1.212869
4.437563	1.936984	2.818666	1.623802	0.3618977
1.099022	3.268412	3.001506	1.359494	2.672158
2.060257387	0.3489924	1.553611	0.6515928	0.5512816
2.39525	1.11253	1.621133	18.8628	7.092779	5.24075	1.670577
1.489954	0.506925	2.677687	1.215006	5.5359	2.259819
1.795763	0.8493745	6.037691	5.184975	2.186935
1.907397	2.130872	0.4484224	1.931209	3.837047
3.187144	0.6482746	2.996192	0.2060915	0.8202961
2.61859	1.26259	0.9095408	2.297833	0.9526583	0.135928
2.775197	3.32213	1.022842	2.830568	2.172577	1.636757

3.408141 3.386037 0.6575782 2.339072 1.952775
1.764955 0.5254984 3.038185454 1.706361 0.749569888
2.04802 1.064224 2.285866 2.129709 0.293595 2.067757
3.061653 3.760174 3.40542362 1.787497 3.010115
2.283462 1.431026 2.86154 0.7539244 1.913937 2.877718
0.2632845 1.824258 3.254091 1.755975 1.532013
2.911003 5.730502 0.672957 6.066511 1.290048
2.312309 1.158603 2.440994 1.840928 2.295702
2.840739 1.070326 2.496406 1.920548 0.3974233
0.7048846 1.458625 2.155748 2.521654 1.436366
1.603433 1.135556 3.3468 2.42318 2.186342 0.3683216
15.05254 1.79808 3.464635 2.210727 2.290348 7.031658
2.322667 3.510349 2.415434713 1.856551 0.772724411
1.923405927 1.53771 2.76205 0.3818619 2.108252 2.74327
2.874471 2.511446 1.122286 1.339406 1.19503 1.549014
0.4705526 1.026003 2.111648 2.353203 0.2660302
7.350361 2.607606 3.357627 2.082901 0.908271
0.4096193 2.790579 0.7845426 2.515163 1.754412
1.157271 4.235755 0.4726494 1.316291 3.60676 1.66429
4.797689 1.906096 1.546696004 0.9093569 3.589015
1.031318 1.429937 0.4346213 1.000555 3.275179
2.907006 1.677525 1.62328 1.26072 3.464492 2.81611
2.347839 0.7307254 0.693831478 0.6377589 1.88128 1.465948
3.478123 3.031139 0.655482223 3.334418 2.304841
2.492652 2.074581 1.088697 3.300382 1.898697
0.9240818 2.8319 3.897669 2.217906 2.631574 2.004884
1.628705 2.52716 2.617944 1.12935 2.295889 1.103442
1.965926 0.8445542 1.645904 1.294734 1.803916
0.4639254 1.860406 0.283315583 0.435619916 1.83842 1.337173
2.979227 2.305129 4.575107 2.686965 1.213104
5.321336 1.519033 3.398102 3.445957 0.6526834
0.7671885 0.562547434 3.088816 2.107329 0.242278
2.161292 3.143229 4.880010765 1.782495 2.390216
2.44228 4.47263 3.102965 2.53013 2.905082 3.029876
0.59318 1.156297 2.19967 1.070124 0.7691752 0.4596892
1.352456 1.666613 3.486436 2.141013 1.059642974
2.138582 1.458254 0.7547126 1.153756 3.463694
3.080484 0.4678675 1.183283 0.5940067 2.118415
1.244918 3.393193 1.093691 2.168895 3.221476
4.010237 3.207448 1.695479 0.6532327 2.089286
1.000361 3.945904 0.9408505 1.341903 2.170052
4.406268 0.596503701 2.499536 3.350479 1.072898
1.365132 1.602383 1.844388 3.067773005 4.246922
0.7806325 3.986411 2.62629 1.889918 2.620027 0.4646075

1.392554 2.265433 2.570347 0.4779611 0.183251
0.6660259 1.705374 0.394608404 2.999483 3.013583
3.281821 2.172230381 1.876427 0.4519559 1.125403
1.395337 1.513568 2.855766 4.716899
FENDRR 1.468898 11.70091 2.786867 3.347616 3.34299
15.55161 2.200158 5.906612 7.595392 4.551362104
4.335048 1.176186 4.688595 0.9141421 3.116288
19.51054 2.738166 14.678760039 2.043376 4.580307
5.976371834 14.98245 1.523267 12.87729 16.08221
0.6571219 3.604459 19.933 5.032569546 2.061941 7.220481
14.8382 0.7079142 1.676794 2.623211 0.374967 0.09789289
0.9102439 3.620441 0.671735944 3.641504 0.1734063
2.43907428 0.582803 5.330366 2.166432 2.19433 2.256832
3.328791101 0.8641117 0.3924489 2.662818 0.6675027
1.170235 12.33977 1.067141 0.5431072 15.15877
0.04931041 2.043554047 8.794762723 0.247850977 9.005616
1.899018 1.35055 15.49538 2.532328 4.382485
2.597194475 1.356203 0.1113934 1.974297 4.041994
3.263676 3.339259 1.871571 9.877378 1.308819
0.0258923 1.204340584 0.2602385 3.940103 0.3412487
7.402562 4.031383 0.7970344 4.345011 1.646073
0.90826446 12.19837 0.319366645 2.170169 4.241142
2.933002 0.8687508 0.4608932 1.446634 1.751638
0.4665197 5.522123 2.264744 1.690546 0.1872068
2.436495 4.264684 8.669171 0.8667317 9.204005
2.100627537 0.133973 1.753228 1.485027 0.7542092
8.172032 2.347446 1.049098 0.69378 1.051927 1.467383
0.7515873 0.1460352 5.403284 4.715015 0.04763467
0.1459242 2.455494 0.9426235 0.742312 16.76296
5.10373 1.744662 0.4357301 1.766554 2.880536 2.40429
6.635929 1.18567 0.3706471 2.273091 1.506561 0.9219769
0.5876084 2.107419 1.868486 1.79004 2.930879 0.3247418
10.38303 0.06218299 1.957004 1.126331 8.009499
0.3913723 3.710172 12.45005 1.235318 4.504961
9.428114 0.3423741 1.681092 4.914698954 4.35123
4.757913185 1.062018 1.972897 1.427439 1.599655
2.302096 2.096561 1.39512 22.16223 4.632504412 2.078199
0.6287905 0.8082009 0.2337659 5.106767 4.199675
1.966416 3.295584 1.819278 1.287025 2.183761
2.70912 1.648213 1.595661 7.292319 0.1996118 2.606217
0.432564 4.464894 0.6849558 0.8950193 3.435462
0.8566526 4.513261 4.306887 4.666352 1.05013 0.3046305
0.386564 5.044055 1.903304 0.1937448 0.1982287
1.952866 2.256908 2.945381 1.521622 0.8025859

5.291989 5.488241 5.747506 2.808611 0.5314417
1.679892 1.857991 0.8977352 5.676106 2.449464202
1.430593 0.520693436 2.049024775 0.142445 0.7569649
0.2928357 1.933907 0.3037312 1.275639 0.8205166
2.453266 5.860337 0.5326572 1.169044 1.703892
3.343083 1.318198 0.9682739 1.518597 7.012212
6.584865 2.361063 3.246583 1.128679 1.884731
2.745404 1.028834 18.80351 5.569868 0.7211208
2.569764 0.376398 2.914759 0.3837447 1.339498
0.3605151 2.030989 1.939285199 0.3693635 4.516872
2.384065 0.2605404 3.04198 3.070137 0.429024 3.304611
0.4480808 1.521247 0.2254748 2.761185 2.70948 1.048902
1.133995 0.513943375 0.443515 1.216464 2.408911
15.4467 0.3025935 2.827331237 9.749588 0.8915714 7.18957
0.4295286 0.3634894 3.471201 4.072566 0.812275
4.50759 8.729473 1.385393 23.78975 27.91219 3.870594
2.824249 2.761638 0.4140674 1.692515 0.2426485
2.58193 7.956722 5.266497 0.968168 4.362031 2.165278
0.9725037 2.036369842 0.652307968 1.465917 1.181271
2.230028 0.04841426 0.1116991 2.7197 1.3046 14.62973
4.571905 1.679329 2.098934 3.827635 1.239002
1.799613663 0.9871244 0.9003295 4.599776 2.624915
1.437698 0.853315317 2.274117 0.7008662 0.6114491
3.481572 3.151142 2.556809 11.5081 1.779353 0.636164
1.385817 2.292854 1.291018 0.1854817 0.6498408
1.261516 0.1815002 14.04303 1.111693 3.174098033
0.6294872 0.6380721 0.8339961 1.437101 3.651469
1.943572 1.551502 1.109661 1.585903 5.699956
1.124665 13.81851 0.1191069 1.900852 0.4195742
0.3168428 1.615525 0.8868229 0.6217857 0.7714227
2.310782 2.304016 0.7487609 2.361043 2.749902
0.05896566 0.421543022 0.9913362 2.696741 0.7428789
1.348629 4.070152 2.636295 0.355457861 2.741023
1.780187 0.03830599 1.142425 1.253437 6.569439
1.47365 0.1221658 3.909364 8.473461 0.3279256 1.216111
2.390986 1.456909 3.198897818 2.367607 7.109732
3.876438 2.153974931 1.309451 0.3027007 0.7733989
0.9263962 0.6404168 3.248256 14.52638

CASC8 0.04962807 0 0.05135603 0.2311127 0.2115606
0.006282499 0.1533565 0.07434813 0.1915429 0.435249827
0.287548 0.3290671 0.5173098 0.1803072 0.5663575
0.009191724 0.4331072 0.158807596 0.2144707 0 0.253715529 0
0.5287682 0.04453128 0.02549283 0.2291762 0.4222421 0
0.167831356 0.9712678 0.1928384 0.01668215 0.299405

2.559495 1.100979 0 0 0.3900139 0.8493596
0.581940617 0.3707136 4.626471 0.200160666 0.8055822
0.7218438 0.8901196 0.6785217 0.2000375 0.530592263
1.283281 0.6087059 0.1170578 0.1996567 1.520414
1.124866 0.3126672 0.1184092 0.06995264 2.83647
0.504604166 0.964892499 0.575285189 0.3151001 0.05807376
0.4951407 0.1219052 0.7702722 0.2758721 0.679371365
0.476688 0.3125966 0.6969109 1.804201 0.6189346
0.2992933 0.07853468 0.3949491 0.3282294 0.2864067
1.981078328 0.4530283 0.1151977 0.225926 0.3380784
2.88819 0.4204525 0.2297888 0.5519453 1.76388918 1.329471
0.035868373 0.08312153 1.181757 0.3875192 0.2284931
0.1647759 1.004205 0.4572622 0.03644159 0.911753
0.0854366 0.8996819 12.42111 0.7069163 0.7041257
0.1778219 0.3579653 0.1776161 0.422404261 0.5706517
1.562844 0.03486733 2.166464 0.4581984 2.438706
0.4087974 0.4581105 1.095977 0.5865975 1.323954
1.970855 1.421533 1.4749 1.497228 0.08348331 0.1744969
0.6411686 0.1207693 0.06895391 1.182786 0.8756691
0.5720739 2.638456 0.4438159 0.9846453 0.3320891
0.06490684 2.98569 3.065411 2.865217 0.1901654 0.7773149
0.4102 0.2873869 0.8431486 3.918157 1.159625 0.2704882
0.2952256 0.6847517 0.2579921 0.2574254 3.31098 0.1418013
0.05440086 7.616234 0.532134 0.5876351 1.01078 0.871717
0.093503883 0.2604405 0.716867078 0.8295818 0.4967508
0.1871319 0.2078143 0.8291051 0.76881 1.145591
0.005121118 0.249957883 0.3110779 2.038981 2.763617
0.7028144 0.222039 3.340993 0.8713106 0.2656027
0.3369187 0.469899 0.4446239 0.2369604 0.3630516
0.8278088 0.3237929 1.091147 0.06139316 1.501741
0.7501253 0.9871167 3.621658 0.4078058 1.600083
0.2280158 0.5469166 0.2957953 1.54237 0.4491669 0.5965764
0.5032409 0.1459607 2.45613 8.967398 0.5050321 0.9717523
0.0857421 2.260073 0.2968959 0.1745674 1.305521
0.552783 0.4105193 0.6429574 0.1031244 0.1526926
0.009828901 0.142978 0.282985423 0.5557387 0.268136879
0.123717918 0.177757 0.9033764 0.1457934 0.1601305
0.2744832 1.05205 1.177911 0.8258634 0.4977539 1.367426
0.3132422 1.115101 0.3299743 0.3038215 0.2420011
0.09106216 0.1330152 0.1533241 0.3978401 0.3290664
0.7379632 0.2847396 0.4468263 0.1859185 0.3538961
2.765676 0.6590763 1.700843 1.540097 1.652076
0.4948373 0.5672246 0.09095534 0.3952383 0.225063073
0.2737389 0.05390581 0.471746 0.8080556 0.301063

0.5126671 0.4320035 1.104117 2.492651 0.6816257
0.1751361 0.5451985 0.2771212 1.264508 1.445928
0.04535781 0.7038534 0.439628 1.224718 0.1886803
0.01330204 0.628733559 0.3054478 0.126717 0.832675
0.02858302 0.6246223 0.2406506 0.7091561 2.002159
0.3055392 0.2927439 0.3554007 0.1414401 0.06641341
0.5710176 0.8270229 3.007325 2.069672 0.2511105
0.1659458 0.5399963 0.5388381 0.2121277 0.275082
0.03937376 1.136043 4.839233 0.161134226 1.608696732
0.2940727 0.1917953 0.0870421 26.82149 0.5479898
0.2256831 0.1756808 0.299754 0.6995757 0.5971679
0.348168 0.946586 0.7850713 0.977612321 1.858992
0.7426803 0.0765524 0.623519 0.0572979 0.024432043
0.9208543 0.4814622 0.9121826 0.7348259 0.1593217
0.03827122 0.06074449 0.6883408 0.6233973 0.5368472
0.3164859 1.384377 0.8159056 2.798811 0.6281817
0.2078679 0.1113984 0.6100528 0.704202454 0.3824865
0.9955778 1.725371 1.013909 0.09544368 0.4126107
1.433968 0.9139316 1.328478 1.009801 5.480162
0.1269645 0.2063837 1.4114 0.1341136 1.888245 1.194429
0.6954049 0.4167722 0.596848 0.829719 0.8568018
1.766528 2.073626 3.403141 0.07669148 0.190525492
0.1196056 0.2775098 3.022132 3.034782 1.042915
0.8741551 0.146525412 0.6364143 0.4957783 0.216105
2.663933 0.4568244 0.3742327 6.385873 0.04888935
1.496341 0.1842694 0.6720435 4.179778 0.5787202
0.2348348 0.319503068 2.082446 0.2526991 0.0598243
0.388334143 0.1482233 0.1142433 0.3126765 0.3259389
0.3469207 0.4929419 0.3594676
AC112496.1 0.8531039 0.09888016 2.045022 0.09878017 0.9494454
0.4188929 0 0.2478624 0.07982092 0.194335618 0.2440956
0.1523675 0.2794509 0.09695317 0 0.383043 0.1031354
0.097322427 0.3191982 0.2208548 0.030208529 0.1802222
0.2302179 0.1855734 0.141647 0.08127974 0.08184152
0.1226488 0.099913837 0 0.07305524 0.1853836 0.1746778
0.08142046 0.1894155 0.4533631 0 0.1625289 1.859738
0.366052488 1.064701 0.2051033 0.322885739 0.1661916
0.6655112 0.2853354 0.6316926 0.1389347 0.210582457
0.3097161 0.2387422 0.9512296 0.2773404 0.6219346
0.1512132 0.1271186 1.821939 0.1060039 0.3369824
0.175234721 1.320752533 0.176276679 0.6179312 0.3025106
0.5336327 0.5430457 0.3174651 0.3919195 0.363629702
0.9029467 2.340392 0.1873683 0.537041 2.145367
3.077849 0.187014 1.42507 0.9048637 1.193531

1.595420513	0.4494968	0.8471628	0.9691833	0.691139	
0.4084561	0.150183	0.4787948	0.4472416	1.15129636	
0.08747767	0.473330624	2.251529	0.5079273	0.4582805	
0.2380475	0.8979454	0.7266986	0.7349903	0.5423627	
0.7599023	0.9710087	0.6323966	0.3573901	0.4651427	
0.586855	0.6825277	0.3356402	0	0.182885017	0.1033936
0.4227042	1.017109	0.5444151	0.5699796	0.5493362	
0.7388957	0.8966824	0.9421169	0.1909769	0.9465888	
0.9898482	0.6118579	0.5002796	0.3839592	0.4224459	
0.6017991	0.3073886	0.2236787	0.09578295	0.1674895	
0.1861806	0.4709097	0.3682579	0.9117234	0.3467555	
0.3624504	0.5409673	0.8072087	0.1991549	0.9497811	
1.215118	0.8710651	0.4223248	0.4354967	0.3904021	
0.1045325	1.443025	0.9282781	0.22783	0.227273	0.3344818
0.9752349	0.4685132	0.5395382	0.4156212	0.1623469	
0.2771926	0.3061035	1.452477	1.297383	0.038965497	
0.7425896	0.608049035	1.105326	0.09554259	0.1436523	
0.3711497	0.1932921	1.021001	0.2864386	0.1707282	
0.148805633	0.4137264	0.1052256	0.2147638	0.1638495	
0.5751829	0.7073072	0.3979154	0.628204	0.3120062	
0.6527304	0.2026569	0.3853563	1.867524	2.17203	0.2215316
0.3497763	0.7675237	0.7599178	0.6429294	1.035203	
0.2096165	0.03952169	0.1333593	0.4524767	0.3137952	
0.385205	0.5268407	0.2717122	1.173434	1.143892	
0.3801602	0.9918775	0.1467388	0.320701	0.5918565	
0.6848434	1.383109	0.921867	0.595201	0.9891716	
0.2399573	0.1710741	1.091595	0.3845095	0.02766564	
0.2662373	0.5687434	0.08040503	0.3136125	0.572324797	
0.108539938	0.6666837	0.4283083	0.335205	0.6673054	
0.240809	0.03154076	0.1291752	0.233328	4.990273	
0.2360292	1.672495	0.7744861	0.9046636	0.03723833	
0.2192351	0.2919074	0.633496	0.2517041	0.2590474	
1.028479	1.345437	0.8360024	0.5213716	0.2152141	
0.4139725	0.5649653	1.86175	0.7668826	1.079388	0.6258756
0.4217963	0.2216037	0.03158621	0.3850272	0.294767407	
1.019386	0.6239986	0.8763598	0.3620836	2.013975	
0.2937573	2.349506	0.432586	0.5372857	0.3374865	
0.1502607	0.6415013	0.4242255	0.2107814	0.2525683	
0.567053337	1.061689	1.032138	0.6625017	0.4138314	
0.1940157	0.123298639	0.2490418	1.602398	0.08897367	
0.9052578	0.679034	0.8022828	0.6725713	0.5649255	
2.182734	0.2103345	0.6257946	0.1339586	0.1916044	
1.344021	0.2376841	0.0876385	0.5452497	0.8720356	
0.5029375	0.5736071	0.1924696	0.6843803	0.4849892	

10.66524 0.3272478 0.5835919 0.363722764 1.147184119
0.4538805 0.8340115 0.241818 0.3252025 0.3683251 0
0.3327761 0.1507598 0.4811683 0.05655804 1.371766
0.9539397 0.8265085 0.24690694 1.162035 0.1600832
0.5316896 0.1855976 0.4775505 0.20362934 0.774529
0.3776711 0.05243175 0.7620247 0.4085754 0.1860672 0
0.247284 0.7131376 0.7457272 2.301573 0.7796025
0.1085135 0.5587244 0.6678052 0.4042451 0.2707986
1.107514 0.113743975 0.5977203 0.5828937 0.3945771
0.6646414 0.4242545 0.1763545 0.9856853 1.255104
0.9899011 0.8175745 1.043137 0.1763647 0.6200393
0.9179488 0.1719649 0.1553054 0.3770831 0.4185903
0.5458508 0.1130555 2.006675 0.5437333 3.764175
0.3312972 0.2836354 0.1966728 0.281731014 0.1424079
0.128495 0.0963213 0.5135727 0.3788905 0.7285662
0.070454937 0.5550915 0.8942548 0.5403393 0.6287462
0.03732757 0.07088749 0.2294103 0.1782678 0.3570025
0.2772967 0.4405407 0.9953269 0.3758458 0.8648249
0.941758936 0.4165486 0.618102 0 0.451615607 0.260078
0.3570612 0.3449129 0.196851 0.7106026 0.2785379
0.1436433
AC026369.2 0.02639168 0.1009458 0.1456565 0.2773202 0.05192568
0.02672772 0.08155333 0.0948901 0.1222326 0.033065885
0.01132703 0 0.2445332 0 0.4732873 0.02932836 0.1579349
0.069548852 0.06517326 0.02818356 0.231296913 0.1051355
0.03357532 0.09472503 0.09037875 0.03111663 0.4595316
0.04173697 0.229502356 0.05988519 0.1118721 0.01182852
1.210075 0 0.5478885 0.2892712 0.3295428 1.540724
1.173858 0.805789246 1.278756 0.2210206 0.590588614
0.3138772 1.521887 1.301122 0.6448887 0.3120416
0.389654052 0.1264744 0.4569928 0.4731004 1.840372
0.9127073 1.260705 1.265298 0.6199998 0.1893822
1.084993 0.536686175 1.618011822 0.88179967 0.5782699
2.05372 0.7445332 0.5275646 1.836549 0.1467058
0.768968067 0.3595056 0.9016164 2.22844 0.8615512 1.636486
2.567112 0.6682225 1.178215 0.9345079 2.047419
0.788521344 0.734219 0.3315297 0.6219272 0.2713758
1.292665 1.77596 0.1493546 0.1141461 2.39589977 0.2381469
0.534083659 1.738657 0.2254513 0.3230421 0.4556631
0.5257565 0.2430295 0.1945337 0.2989941 0.5483939
0.3304311 0.507263 1.550638 1.582865 1.307159
0.9356838 0.5710863 0.4435246 0.548447417 0.2111071
0.631319 0.2754823 0.3936832 0.04364149 0.8879522 1.582
0.6644135 0.804983 0.1852181 0.2761038 2.485348

0.4542834	0.8633778	0.5879702	0.6849596	0.3199848	
0.1206961	0.2426233	0.3300205	0.3786171	0.437161	
0.3755837	0.2685363	0.5584616	0.914498	0.3363837	
0.8786085	0.6393657	1.365117	0.6925872	1.591079	
1.069893	0.8622945	1.097592	1.444772	0.04268648	
0.5995461	1.150742	0.348884	0.9280828	0.4146411	
3.497072	0.1315326	0.6032673	0.144649	0.4972152	
0.5541753	0.2343735	0.1112114	0.176598	0.656361591	
0.2332623	0.242902659	1.644607	0.6014873	0.2828313	
0.3578529	0.8509856	0.3594241	0.308668	0.1851882	
1.640673336	0.2675005	1.141377	1.164767	0.5122716	
0.3063644	0.4370502	0.7235934	2.947051	0.7299509	
0.6796933	0.133001	3.061194	0.7360678	2.152186	
0.1130797	0.2142499	0.6420817	0.8898796	1.74275	0.4171693
0.3076184	0.5850359	0.9076344	2.725381	0.463665	
1.219081	0.6319696	0.2773883	0.9035364	0.659584	
1.086685	0.3447142	0.3994778	0.4399443	0.9699328	
0.5927575	0.7620329	0.1857483	0.9958459	1.206192	
0.3919521	0.6985913	0.1772907	0.623449	0.2259486	
0.5122366	1.009181	0.38306193	0.7265257	0.68166084	
1.094222562	0.4726464	0.3106817	0.1176338	1.771238	
0.1843797	0.5312942	0.2505601	0.6699437	1.856738	
0.1721142	0.937006	0.363706	0.09235621	0.2280974	
0.179052	0.327806	0.3637855	0.2273127	0.8859373	
0.349988	0.6540682	0.3992021	1.077199	0.5053328	
0.118862	0.2499326	1.590141	0.3795893	1.757142	
0.8847003	1.698508	0.3959077	0.7739052	1.222886	
0.861740137	0.631842	2.191397	1.487082	0.4805419	
0.4550269	0.2862624	2.928146	0.9233931	0.559937	
0.6747175	1.183369	0.204657	1.275202	0.4841654	
0.4236017	0.518597144	0.6062309	0.3687947	0.5009936	
0.4013526	0.4527284	0.755246158	0.5367381	0.5019152	
0.2422196	0.5593653	0.6547069	0.3839262	0.3016973	
0.332727	1.75249	0.4724025	0.5749807	0.09572988	0.5976882
3.565205	0.4650779	2.191997	1.480157	0.9199276	
0.2032382	0.6644192	0.9497031	1.644802	0.4276042	
3.445886	0.2672672	0.8164436	0.442728847	0.658771262	
0.4633621	1.002209	0.2262972	0.7137915	1.328602	
0.4972085	0.951238	0.05496757	1.704822	1.135544	
1.440819	0.4474527	0.3164154	0.59865402	0.247148	
1.307419	0.325678	0.8526362	0.6094082	1.836301399	
0.5031784	0.3373656	0.09367235	0.3961749	0.5213883	
0.5698624	0.1722841	0.3155623	0.2860138	0.4758159	
1.056024	0.645554	2.0679	1.871612	0.831742	1.208591

0.118481 0.1541796 0.615436522 0.457655 0.1400166
1.141323 1.308585 1.272282 0.2588054 3.349014
0.5743861 1.048009 2.761722 0.817713 0.2700734
1.092422 1.006542 0.5486165 0.3831619 0.8854089
1.413492 1.063848 0.2164073 0.6776119 1.02692 2.419997
1.409242 0.5469478 0.602344 0.313763349 0.3028808
0.8198707 0.3072921 2.195511 0.7281045 0.371893
0.743243064 1.742039 0.5823901 0.7355028 0.6017632
0.3048587 0.43421 0.6635743 0.3639837 1.020489 0.2286492
0.5461172 0.5542469 0.6075207 0.3601267 2.03889969
0.4370628 0.3622489 0.05090227 1.133414342 1.211394
0.1518834 0.1760591 0.5225044 0.4502775 2.114902
0.2409152
TM4SF19-AS1 0.02720715 0.03902433 0.05005236 0.03898487 0.1159819
0.1171027 0.06305493 0.07336657 0 0.008521896 0.4028574
0.2271722 0.1365482 0.1530553 0.1774223 0.09070373
0.04070373 0.061455286 0.02519514 0.04358159 0.047688746
0.02709601 0.2163297 0.1871662 0.1024885 0.33682 0.03229981
0.06991822 0.092008669 0 0.05766439 0.09755203 0.315149
0.2570691 0.1661228 0.08946281 0.08493133 0.2321407
0.2946402 0.060194727 0.2746391 0.5276523 0.4389291
0.02623586 0.1400814 0.1000989 0.2057761 0.1681526
0.235475749 0.2227365 0.08637079 0.8085863 0.2991797
0.2556818 0.05304733 0.150507 0.349539 0.1952339
0.1705057 0.036884612 0.162167305 0.064931878 0.03387146
0.2706167 0.09360225 0.05991742 0.2413025 0.2681052
0.027335459 0.5654238 0.1161844 0.1331052 0.6594005
0.1522151 0.2434719 0.04920498 0.1003383 0.210394
0.1287001 0.159911954 0.1182665 0.1040181 0.2331433
2.552819 0.06448099 0.3556299 0.2099584 0.1260783
0.34951845 0.7748764 0.190083346 0.2278448 0.005810437
0.2239297 0.1252647 0.4377706 0.05933814 0.1504084
0.1769484 0.07583811 0.2639968 0.07725203 0.06268828
0.1631774 0.1544066 0.09235488 0.1766197 0.05927043
0.048118623 0.1813584 0.271864 0.1256129 0.01193669
0.3374247 0.6744971 0.1512076 0.07610479 0.03772073
0.1909411 0.07115876 0.2252433 0.2927002 0.2757917
0.2146738 0.1176874 0.3496643 0.1182042 0.1029906
0.06930363 0.1573855 0.1322615 0.139388 0.08305014
0.05483023 0.04561717 0.9536383 0.2005604 0.2490014
0.1871406 0.08032358 0.472612 0.2076987 0.2433998
0.0859372 0.109138 0.2090258 0.1765918 0.2093468
0.4495802 0.03322082 0.1320076 0.08339263 0.1664143
0.2230755 0.0994123 0.192217 0.04254352 0.3221539

2.71333 0.2048115 0.082017264 0.1953817 0.153027159 0.3588852
0.07541424 0.08099179 0.0868024 0.127142 0.3797932
0.1507289 0.1123002 0.06264327 0.8926108 0.332229
0.127139 0.1185531 0.07237788 0.14692 0.05889092 0.2125104
0.1915469 0.3503475 0.09140705 0.2598133 0.1679337
0.6471167 0.2384463 0.1380437 0.4711982 0.08820922
0.2158252 0.07167657 0.1999257 0.124782 0.09356792
0.3095314 0.0217269 0.1824313 0.0415849 0 0.2145485
0.139337 0.1600375 0.2165508 0.05147765 0.06855809
0.2212899 0.2428623 0.1270789 0.3127633 0.1305021
0.2530301 0.1136427 0.1350332 0.3263729 0.07736359
0.1019069 0.3825771 0.09263508 0.126931536 0.6664604
0.157754231 0.092812826 0.1656652 0.2075898 0.08268311
0.04916066 0.5227112 0.04979187 0.1155562 0.1841718
0.3322007 0.1330742 0.06976367 0.1548682 0.2951507
0.09797725 0.1038288 0.06912302 0.4375304 0.1833937
0.1772097 0.07216044 0.1011417 0.2057685 0.4245966
0.1245743 0.1157272 0.2279258 0.1503477 0.1712017
0.2149161 0.4560183 0.0739855 0.160341 0.08310604
0.2757719 0.05640425 0.1979639 0.2823886 0.1371003
0.0285802 0.3127245 0.0983692 0.1445085 0.1345111
0.1236939 0.0887956 0.1694353 2.43331 0.1922299 0.2495628
0.09493272 0.068381783 0.1467716 0.2715643 0.335708
0.0707737 2.384633 0.194645604 0.232978 0.4311865
0.07022922 0.1190906 0.064516 0.09235079 0.03753682
0.171504 0.3050948 0.04980674 0.08781437 0.32426 0.03920994
0.2434785 0.07295952 0.207526 0.2543153 0.03059201
0.02205453 0.02902326 0.1181609 0.1500553 0.1798068
0.2035206 0.2066441 0.4435833 0.125144313 0.792314313
0.3582596 0.02742945 0.1113427 0.4962688 0.4910082
0.1855863 0.1576013 0.113332 0.07447031 0.1736106
0.1596393 0.1288866 0.3805576 0.203010446 0.2101973
0.1403975 0.2238274 0.1220808 0.08167097 0.294671484
0.08336669 0.1117896 0.09656672 0.1076734 0.04703113
0.3147163 0.02220094 0.1691627 0.07371283 0.2881794
0.2721635 0.1276303 0.1617881 0.2940104 0.09136676
0.2127207 0.05089246 0.1788115 0.215474542 0.1205701
0.3247716 0.2191684 0.1374001 0.2023204 0.1102009
0.1377756 0.1708079 0.1446953 0.1075555 0.4214897
0.3093537 0.1999752 0.4114789 0.1696705 4.814928
0.3968552 0.1270785 0.352517 0.05949174 0.2111893
0.2145914 1.353947 0.3777243 0.1492539 1.306594
0.168467856 0.1436302 0.0676163 0.6462456 0.3378135
0.1290097 0.0616153 0.086507404 0.2109602 0.592272

0.3046456 0.1964464 0.03928482 0.08392999 0.3822789
0.08710704 0.1815988 0.08979589 0.09107218 0.8689624
0.2142579 0.2275429 0.307595034 0.1583075 0.1746745
0.0524751 0.168333938 0.2566078 0.5010447 0.1209994
0.3780903 0.05802383 0.07328571 0.1727716
AL031429.2 0 0.3853127 0 0.8126153 0 0.5894513 0.4842308
0.5097601 0.2073622 0.252426697 0.07686326 0.04398077
0.2419899 0 0.5839354 1.094594 0.04465494 0.421380618
0.248768 0 0.026159004 0.6465467 0.02847953 0.3749593
0.1839883 0.017596 0.2834819 0.9912692 0 0.2031852
0.126524 0.8227289 0 0.1410117 0.3827225 0 0.3959972
0.02010593 0.03463303 0 0.03615588 0 0 0.04317396
0.09220766 0 0 0.02406204 0.205147548 0.03575972 0.1292114
0.1900881 0.6724544 0.3029416 0.2182378 0 0.03286883
0.1606393 0.04489371 0.030348818 0.2287405 0 0.2452524
0.07858749 0 1.077022 0 0.3393817 0.022491734 0.06255236
0.01911941 0 0.1993069 0.2296127 0.01741997 0 0.1216655
0.07288973 0 0 0 0.1222664 0 0.1381131 0.07074032 0
0 0.05532682 0.07668941 0.3282547 0.021572607 0 0.09561706
0 0 0 0.1735953 0.02357271 0 0.06807265 0.05605618
0.01955794 0 0.02685262 0.2309935 0.3377333 0.09688228
0.1114696 0.059388317 0.05968899 0 0.07189901 0 0.4442153
0.1585655 0 0 0.0177353 0.0165376 0.09367958 0
0.4094194 0.1031467 0.5402939 0.02151859 0.2822769 0 0
0.3939793 0.04143918 0.01612227 0.07645934 0.09111202
0.06767183 0 0.171198 0.1064658 0.09641385 0.07391044
0.0293736 0 0.07071532 0 0.04713967 0.04225847 0.03620787
0 0.1913905 0 0.5248173 0 0.02111256 0.04057079
0.04449637 0.3762656 0.2811679 0 0.9807575 0 0.07489769
0.134968317 0.0989298 0.183143864 0 0 0.1066246 0
0.5021425 0.06097472 0.05512017 0.4989657 0 0.04776871
0.1822397 0.02324678 0.07094255 0.0866223 0.06447274 0
0.1036175 0.04503017 0.09043685 0 0.02085615 0.0409412
0.05531953 0.2964719 0 0.09231046 0.05806314 0.2111785
0.1415419 0 0.479132 0.07698809 0.1763195 0.4719524 0
0.04562164 0 0 0.2201224 0 0 0 0 0.1078983
0.1031371 0.05703332 0.04201518 0.2863405 0.02379363
0.04155809 0.1728314 0 0.01958618 0.119785 0.03546885
0.07035742 0.092835392 0 0.330402194 0.023497475 0.01603648
0.3318512 0.05442561 0.02311406 0 0.02731264 0.0223718 0
0 0 0.01766211 0 0.03133565 0.06449288 0.03796923
0.3538872 0.02285727 0.335327 0.02243214 0.2671826
0.1109598 0.4437017 0.06449721 0.07454565 0.246454
0.1630768 0.04788648 0.04024728 0 0.08338084 0.2840861 0

0.109408 0.05556889 0.232048302 0 0.3458241 0.08204126
0.06270909 0.1715407 0.06937595 0.02642272 0.2383798
0.01938589 0 0.1115296 0.09258442 0 0 0 0 0
0.1340666 0.02124784 0.2150138 0 0.133462702 0.1677334
0.04730428 0.4109149 0.1650328 0.04900065 0.08684186 0
0.03763045 0.03937777 0.07285546 0.01806351 0.1392013
0.3318388 0 0 0.227671 0 0 0.05443969 0.03820874
0.0277781 0.222239 0.01908978 0.1217874 0 0
0.096912264 0.062087584 0.01965184 0.03009209 0.06980058
0.05632167 0 0.1454297 0.05763334 0.2424502 0 0.1469289
0.09137521 0.3125639 0.05964275 0.053452119 0.02096379
0.1848315 0.8747891 0.08035888 0.06203005 0 0 0.1022011
0.06810472 0.1344187 0.06633842 0.1381066 0.4018746 0
0.02205499 0.02018002 0 0.06000844 0.09396703 0.06047825
0.06939412 0.02500393 0.03349963 0.04359315 0.03939853 0 0
0 0.08222066 0.1607297 0.05726765 0.0266735 0 0
0.2082284 0.0322608 0 0.08660025 0.08832173 0 0 0 0
0 0 0.02673346 0.01569482 0.04126048 0.07172151 0 0
0.133071436 0.04110592 0.05563498 0.1668184 0 0.05789988
0.2027893 0.081347046 0 0.1068107 0 0.01701441 0
0.1227697 0.06621909 0 0 0.2585956 0 0.07835463
0.08136569 0.01970771 0.028121169 0.06011821 0.0396477
1.468009 0.032589621 0.05630347 0 0 0.01704627 0.02121878
0 0.1066179
AC009318.2 0.2454912 0.9176413 0.8827207 0.511654 0.263457
1.062273 0.0689633 0.481447 0.3100877 0.307573679
0.9003687 0.4823022 0.7237393 0.2720199 0.1455353
0.9093594 0.4006598 0.319265401 0.2204478 0.2859917
0.339022295 1.00018 0.3690964 1.52193 0.8101184 1.017433
0.3885901 0.970577 0.474398879 0.202561 0.2522706
1.06026 0.6031884 0.3514462 0.9447822 0.831688 1.277232
0.6815015 0.656006 0.434511613 2.433024 0.6492312
0.371657523 0.6025786 1.424828 0.4598092 1.376315
0.6716672 0.386310445 0.6060476 0.3091546 1.494977
0.7422159 1.610725 1.653512 0.3292196 1.769464
0.9151185 0.5370695 0.21178906 1.545589293 0.365225783
1.489221 0.3656149 0.1842713 0.8317497 0.5785786
1.150353 0.38118479 0.5144715 0.1906067 1.261671
5.563448 0.7075315 1.736646 0.2018085 1.056971
1.398817 0.6925371 0.765170104 0.3104359 0.6338322
0.8844941 1.055614 0.2585843 0.6266468 0.8726013
0.7446174 1.68198372 1.334158 1.440924192 1.221052
0.5910045 0.7912554 0.7963256 0.8891826 1.276332
0.9165111 0.5805864 1.289407 0.7544335 1.559828

0.7199041	0.5889426	0.782965	1.498296	0.547313	
0.4167269	0.335499896	0.3867865	0.9055301	1.236448	
0.7049799	0.8118927	0.5532741	0.8859434	0.04994158	
1.096211	0.5440641	0.7471342	0.3233331	2.256894	
0.6169786	0.7872343	0.7508369	1.558577	0.6940306	
0.2655111	0.9509143	0.7023018	1.109019	1.321224	
1.044569	1.43923	0.4490243	1.365377	0.5519211	1.033265
0.6631503	1.493451	0.8969677	1.151468	1.250015	
0.5874356	0.6951227	1.570203	1.100891	1.068494	
0.7670625	1.591415	0.7631352	0.5051447	0.1213384	
1.020272	1.043783	4.064409	0.9372459	1.057021	
0.3761714	1.344017	0.370022587	0.739694	1.278068473	
1.015121	0.02749357	0.9212412	0.7120197	1.668666	
0.6990551	1.126492	1.82392	0.822156838	0.8571953	0.7494291
0.2549289	0.6895648	0.474959	0.8355706	1.48141	1.058816
1.279417	0.6987323	0.2499302	1.351485	0.8979394	
0.7996688	0.9040693	0.8454808	1.343592	0.3280137	
0.4976156	5.6678	0.4523982	0.5458972	0.4221336	1.093729
1.083581	1.041967	0.4320741	1.511646	0.6696172	
0.9692202	0.3281874	1.348178	0.6756145	1.107428	
0.5916172	0.8482672	1.137162	0.6073485	1.198935	
0.4744107	1.532925	0.5907442	0.7710222	0.8396185	
0.3582509	0.6541579	0.607891	0.416475824	0.6039532	
1.576352712	0.187402289	0.6554755	1.907147	0.8500484	
0.5530332	0.6236621	0.3267448	0.4906675	0.7050017	
1.323554	0.5821742	1.39102	1.123255	0.7185058	0.6750948
0.5867149	2.016	0.6608243	0.7688827	0.9168923	0.5919151
0.1659284	0.9545199	1.007352	0.9289579	1.295492	
1.333121	0.8115691	1.083338	1.108112	0.4571857	
0.890099	0.7333459	0.4908235	0.6093799	0.370136646	
1.173364	1.07738	0.5316299	0.3125822	0.3420274	0.4841397
0.6321969	1.120338	0.4638319	0.2185108	2.668488	
0.6230249	0.6103807	0.06065496	1.027898	1.22382479	
0.8544038	5.479842	1.56751	1.48261	1.363857	0.718484464
0.7166488	1.949237	0	1.213378	0.6350515	0.3895878
1.249218	0.1875742	0.9029062	0.9442113	1.134505	
0.8557709	2.297356	0.9510462	24.04139	0.3026288	
0.7274565	0.6524416	0.488452	0.419005	1.218481	
1.181634	0.9134945	1.67955	0.734523	0.4105098	
1.304296163	0.897504166	0.8424328	2.279973	0.2957411	
0.4491891	0.4875546	0.4059522	0.9480286	0.9296359	
1.099552	1.220646	0.660435	1.11287	1.486488	
1.412129439	0.3970883	0.2303296	1.377003	1.281792	
0.3504238	0.117193742	1.063747	0.7743419	0.9279051	

1.084228 0.529077 0.4359941 0.4734836 1.088734
0.6596171 1.126609 0.8929973 0.3589445 0.811879
0.5124858 0.4842665 0.3240525 0.9017112 1.368966
0.471329874 2.184418 0.1480014 0.5298741 1.229521
0.4349261 1.027651 0.824338 0.7472517 0.9495211
1.390844 1.575923 1.674787 0.2762694 1.115305
0.8165032 2.212207 0.5208496 2.223777 1.370839
0.9759936 0.8528426 1.063969 0.884375 0.5005082
0.9522289 0.5518017 1.39295745 0.225388 0.4159805
0.5404942 0.2771005 1.115959 2.066589 0.648777058
2.271779 2.236134 0.08885106 1.323047 0.4833662
0.6425608 0.9902348 0.6375704 0.3492885 1.123276
0.9507829 1.40605 0.4866943 0.9037696 1.037286684 0.9389583
1.00791 0.2152204 0.292405496 0.2806525 2.876966 0.3970118
1.189573 1.226908 0.1502864 1.310915
SSBP3-AS1 0.1494942 0.2276618 0.6875524 0.1692514 0.137987
0.7065225 0.2224224 0.3649689 0.1880537 0.076307342
0.5608177 0.1414123 0.1197038 0.3218594 0.415225
0.4307044 0.1877584 0.337676185 0.06152838 0.1419059
0.142339274 0.3584232 0.0986147 0.5630616 0.2654531
0.9400448 0.1752856 0.4640773 0.128395373 0.05025427
0.2503478 0.6154254 0.5451454 0.2964524 0.1893193
0.1577873 0.02880674 0.3978277 0.8865673 0.259699871
1.712492 0.3855935 0.167123723 0.2562796 1.220117
0.4318597 1.043696 0.2440041 0.349539593 0.4245376
0.1086577 1.013433 0.6118189 0.3746362 0.5397727
0.2722586 0.1219428 0.3348779 0.2442806 0.165137354
0.540604771 0.135915769 0.3529241 0.1943723 0.09905287
0.2851417 0.1057679 0.2406282 0.139073316 0.6962049
0.156052 0.1926238 0.8161066 0.4956274 0.6290443
0.1001351 0.8253755 1.167312 0.4651567 0.09220533
0.09627181 0.4959432 0.3854994 0.8198361 0.05832122
0.1018581 0.5640072 0.3626289 0.20864546 0.5808064
0.277451343 0.4142191 0.1087863 0.518809 0.1465797
0.4807978 0.2522474 0.699635 0.5157572 0.2693851
0.9219895 0.3482864 0.08930213 0.5512461 0.5598951
0.885443 0.1996843 0.2205604 0.097924333 0.3321678
0.2883658 0.9780621 0.01943352 0.7141471 0.6863214
0.479159 0.1672681 0.4342648 0.3885768 0.4170598
0.2234621 0.9530603 1.168427 0.1953085 0.3299791
0.6068638 0.1721852 0.08383689 0.8769943 0.1486141
0.378818 0.2899666 0.6873159 0.4407523 0.07426698
0.733942 0.2475248 0.4948099 0.5301328 0.6901788
0.233848 0.4197644 0.3445798 0.9851993 0.5748537

0.1925406	0.4240626	0.837866	0.9076037	0.4489064	
0.2763189	0.2193164	0.1103791	0.5062479	0.8254252	
1.321296	0.4650511	0.8719517	0.1244347	0.5186894	
0.191946653	0.9970929	0.79836661	0.4079904	0.07503103	
0.3648088	0.1236537	0.4812598	2.352639	0.1976785	
0.3793726	0.248591606	0.8979208	0.2084661	0.4542246	
0.1973968	0.3856409	0.1913543	0.5219986	1.851619	
0.3953783	0.1677597	0.1550158	0.4281471	0.4050433	
0.9509203	0.3838889	0.1872849	0.9452183	0.2249873	
0.3276324	0.2217166	0.07856625	0.1438987	0.2284997	
0.6492972	0.2971286	1.435534	0.1805393	0.381497	
0.1490895	1.184143	0.2388364	0.6237517	0.1361882	
0.5280291	0.1734637	0.4655419	0.6206721	1.044367	
0.5382415	0.3413262	0.6886702	0.5129614	0.5579186	
0.2761249	0.1185068	0.6711034	0.2668255	0.355898484	
0.3306758	0.858050973	0.151103808	0.4481963	1.26496	0.2871725
0.4916494	0.3481361	0.1891484	0.09406557	0.1186869	
0.5150847	0.3321987	0.5023671	0.5374414	0.7827822	
0.27117	0.2441664	0.5814342	0.8253877	1.521898	0.5881086
0.3597847	0.1372197	0.1270694	0.2871404	0.285782	
1.462945	0.2581386	0.2960968	0.9008781	0.7685169	
0.5671264	0.5219587	0.4943997	0.2706014	0.5543406	
0.172179132	0.2703119	1.031747	0.4819221	0.1473449	
0.325278	0.2001873	0.5293505	0.4042905	0.3787858	
0.2409393	0.6528423	1.133506	0.1817185	0.1203853	
0.6027649	0.237838968	0.9673672	0.8013424	0.604357	
0.500777	0.1543422	0.283882899	0.8178645	0.3314965	
0.0571683	0.1479651	0.2464287	1.299467	0.5194501	
0.1116867	0.4821	0.4549926	0.5897351	0.2983845	0.4103723
0.8918885	0.1018128	0.3472475	0.3397232	0.2863804	
0.5879584	0.1559292	0.3778731	0.6534954	0.2832912	
1.154675	0.2009215	0.2036904	0.467405929	0.468366115	
0.9429436	0.2418893	0.1424275	0.1253715	0.1104414	
0.2086229	0.7982569	0.3459563	0.3819101	0.5935577	
0.502851	0.276097	0.3466621	0.793226183	0.2022159	
0.4914342	0.233445	1.272022	0.393779	0.094494154	
0.3468529	0.7178846	0.0505335	0.1541409	0.257053	
0.3586615	0.1987931	0.6779202	1.194626	0.8784462	
1.434522	0.1521308	0.6739914	0.2168943	0.4691332	
0.4390839	0.8036974	1.110546	0.838029546	0.4779332	
0.1468733	0.3849867	0.5083957	0.3350665	0.1794124	
0.2638887	0.1205029	0.8480555	1.184537	0.406936	
0.6169627	0.4412322	0.8810743	0.1565313	0.4601357	
0.2907448	0.3999874	0.4357502	0.5750793	0.4760675	

1.051978 0.5255601 0.7805181 0.1484954 0.2053485
0.504663954 0.5388416 0.1582438 0.2939745 0.5087286
0.3532393 1.766618 0.357125624 0.7793752 0.7132792
0.1543042 0.5428596 0.1678887 0.2201456 0.3439406
0.1581777 0.2701645 1.717757 0.2493631 0.8333242
0.3555305 0.2875869 0.521645542 2.493065 0.6668188
0.181543 0.153148865 0.5639896 0.8093524 0.2380331
0.9486208 0.1889312 0.2162543 0.3735759
AC027601.1 0.08705563 0.0647461 0.2342997 0.1663216 0.15648
0.1240828 0.01992681 0.1313847 0.1841762 0.177745642
0.210342 0.2407132 0.4630582 0.1813838 0.1787217
0.06927247 0.1993814 0.237910786 0.03981123 0.08952313
0.146939756 0.1156 0.1107516 0.1234411 0.0993745 0.31426
0.178631 0.1478717 0.074769077 0.0292648 0.1457862
0.04335285 0.6286894 0.2132544 0.2467437 0.1060212
0.006710065 0.1476889 0.2369396 0.239688829 1.033696
0.3410802 0.063762648 0.2093513 0.4880652 0.8730865
0.7916151 0.2425962 0.187133778 0.3090287 0.111662
0.6722923 0.3493653 0.3054291 0.2954687 0.1506183
0.4355375 0.2908639 0.2489429 0.253526452 0.208655061
0.37815377 0.07064748 0.1396006 0.190794 0.1660479
0.07039123 0.1368674 0.142537494 0.2770399 0.1597188
0.08412862 0.3955077 0.3126727 0.4967824 0.1865989
0.3304433 0.8792344 0.5637168 0.154766097 0.1569748
0.1444025 0.2970171 0.2320787 0.07811368 0.2934557
0.272042 0.2231245 0.38659564 0.1818407 0.108748725
0.4284251 0.06335001 0.6450681 0.2263855 0.289867
0.2312774 0.4243976 0.3192838 0.2647219 0.7548986
0.5915561 0.3491679 0.1508359 0.2062742 0.5594036
0.2232636 0.1043575 0.205289041 0.1332537 0.3905242
0.3158466 0.06224246 0.639804 0.4225069 0.5478221
0.5411446 0.6258326 0.4287444 0.2327489 0.2235562
0.3156563 0.9121726 0.08380468 0.4710976 1.019537
0.1533532 0.09415503 0.2150334 0.2506767 0.2786512
0.543281 0.4658623 0.2079314 0.0360402 0.5054826
0.3066857 0.3020315 0.3087147 0.3469161 0.5864431
0.6111084 0.4866023 0.6042686 0.5995198 0.1095155
0.39344 0.1791791 0.8212092 0.274012 0.3456587 0.2402268
0.1577723 0.2595572 0.3557928 0.6074499 0.400461
0.1947079 0.5072386 0.2481132 0.257573524 0.4666507
0.359403576 0.2786514 0.06752581 0.3762506 0.2417406
0.2049171 0.746487 0.1905352 0.2422155 0.233848746
0.2442449 0.1443641 0.3883955 0.191585 0.1465957
0.2042924 0.3318942 1.234965 0.4085993 0.3386665

0.1985963	0.5857634	0.2741998	0.4183033	0.2411345	
0.130875	0.2925008	0.1700445	0.2986305	0.4552941	
0.1895434	0.1725237	0.1718734	0.4119687	0.1647888	
0.6437864	0.2398376	0.5874033	0.2629406	0.3434628	
0.2370728	0.3895534	0.1952175	0.414986	0.2641903	
0.2376778	0.410726	0.4962193	0.7547226	0.2535981	
0.3531516	0.2631539	0.5105498	0.2115751	0.0690106	
0.2171149	0.34792	0.213937574	0.2828284	0.288926464	
0.081224294	0.3603189	0.9756127	0.2508458	0.6791409	
0.3979548	0.177023	0.5993319	0.2218972	0.2212144	
0.2144781	0.3281601	0.1816003	0.3475225	0.2554453	
0.1230461	0.3058226	0.362135	0.3066873	0.3553996	
0.23517	0.1598156	0.1446303	0.2724933	0.2308417	0.2710666
0.3429245	0.5034882	0.4695427	0.3311024	0.7145568	
0.3916339	0.4122815	0.5200159	0.2507794	0.120319053	
0.572132	0.3362115	0.2097414	0.126448	0.2017741	
0.1099145	0.2549798	0.4904841	0.3518113	0.1297843	
0.38285	0.343375	0.2821893	0.3461412	0.6030187	
0.309419084	0.3590958	0.3475734	0.5263769	0.1083897	
0.3767996	0.05382344	0.2553916	0.16806	0.05548513	0.2347258
0.1717343	0.4878061	0.360959	0.6991698	0.2098487	
0.1678942	0.5281432	0.1370028	0.2044555	0.4231966	
0.2025713	0.3661718	0.3369337	0.1522676	0.3110247	
0.1733512	0.3480769	0.3841096	0.2144614	0.4151384	
0.1931919	0.4475058	0.502498478	0.563376622	0.3707899	
0.2990577	0.2211746	0.2433606	0.4440718	0.1717594	
0.485605	0.2068347	0.3918467	0.5478658	0.2204432	
0.218662	0.193283	0.38108656	0.2294758	0.1930046	
0.009947056	0.3009267	0.3811925	0.253971791	0.1053832	
0.3267849	0.1242491	0.1179221	0.1624305	0.2287525	
0.2052182	0.5243124	0.4193092	0.1482331	1.048246	
0.2765769	0.7849771	0.4965096	0.1465911	0.471773	
0.1640486	0.662406	0.229820384	0.4646901	0.1667824	
0.289808	0.5032959	0.1719709	0.1787125	0.1575133	
0.1619377	0.5212884	0.7077919	0.5436438	0.2639599	
0.5587926	0.5597238	0.2734609	0.2647246	0.3527308	
0.4176614	0.2042398	0.2608606	0.5044057	0.7708399	
0.401136	0.4717395	0.1768787	0.1349125	0.188468647	
0.2812234	0.172282	0.183205	0.2642232	0.2668585	
0.6944963	0.225541276	0.3769322	0.6538187	0.5872777	
0.5170743	0.1722569	0.1193572	0.4387272	0.3811547	
0.3324629	0.4230032	0.1923088	0.7561239	0.4882965	
0.539316	0.405029709	0.6984794	0.1970111	0.08084379	
0.15489834	0.446017	0.7051154	0.3441474	0.2798901	

0.1466949 0.2258098 0.2456992
AC137932.3 0.1032088 0.1480366 0.354182 0.09100731 0.1405824
0.1889453 0.07359854 0.142724 0.1225665 0.094495252
0.2862211 0.1481767 0.1287307 0.1451516 0.1164879
0.1382199 0.2375496 0.107597123 0.1323364 0.1610852
0.106687281 0.1225539 0.2020023 0.223212 0.1250634
0.3650593 0.1508031 0.1098589 0.076709804 0.01801464
0.1009599 0.1743543 0.467471 0.187534 0.3037779
0.2349499 0.01239162 0.3743494 0.4360261 0.249423264
0.8142284 0.41992 0.194187248 0.370026 0.5681793 0.4118496
0.6466507 0.2218704 0.468862416 0.4597225 0.1787142
0.6891183 0.442896 0.220842 0.1934923 0.1805536
0.3263892 0.4598283 0.3940527 0.113012031 0.256885165
0.194886873 0.1186056 0.1393532 0.1365672 0.3227834
0.1841567 0.16048 0.247273875 0.5573697 0.2915657 0.1006977
0.7146852 0.218383 0.3953858 0.2010145 0.4006604
0.513768 0.315463 0.12832279 0.1380422 0.2298141
0.3656719 0.355113 0.1254384 0.2229219 0.4574569
0.2207404 0.20398126 0.6089338 0.175964097 0.3767533
0.04408312 0.7070494 0.1827632 0.3568691 0.203933
0.3720172 0.2664987 0.4224782 0.8399314 0.624251
0.1463412 0.166655 0.4259873 0.5839049 0.1546146
0.07906426 0.129880842 0.2963574 0.26684 0.8956387 0.06269699
0.5470092 0.4779926 0.3372249 0.4219449 0.5220478
0.5278476 0.6146251 0.0985901 0.4911125 0.4682295
0.06632738 0.5074919 0.7007562 0.1779081 0.0944523
0.169138 0.0624588 0.4431201 0.7185716 0.3877489
0.2479944 0.06211912 0.5110137 0.3889026 0.4616011
0.2140643 0.6250316 0.697661 0.5224754 0.2563071
0.3176389 0.3859088 0.2150856 0.3607105 0.4717358
0.6996745 0.275308 0.6713527 0.1759553 0.2302115
0.4931377 0.3684122 0.1994295 0.3050381 0.6063358
0.6690917 0.6507709 0.077781946 0.4385617 0.328814543
0.4188955 0.05868299 0.1827671 0.145643 0.400685
0.9244418 0.1612717 0.2293869 0.123386704 0.2625844
0.08886673 0.1772535 0.3113476 0.2572812 0.2572304
0.4353423 0.6109255 0.3433489 0.4289758 0.1155825
0.4918689 0.4428474 0.4070897 0.207192 0.1289012
0.4255865 0.3500605 0.2314885 0.3346473 0.2212857
0.1395785 0.2184274 0.573199 0.2612072 0.9069507
0.2305576 0.3059608 0.4306069 0.6277747 0.2023643
0.3078499 0.2703843 0.7386637 0.1674118 0.4160639
0.3944181 0.8493266 0.4112741 0.1898615 0.5858499
1.173337 0.5676107 0.3160493 0.1062028 0.9497028

0.2994227 0.22634968 0.2778219 0.581692279 0.062499431
0.426544 1.284198 0.3924691 0.2910035 0.3327902
0.1113923 0.2419883 0.06269892 0.4200614 0.3727823
0.3069249 0.3425006 0.6056599 0.1315144 0.3097085
0.5737337 0.7741431 0.7492085 0.3182177 0.2526798
0.1967566 0.2318938 0.2592341 0.3767305 0.5800422
0.2544711 0.4868371 0.4852987 0.6943249 0.8279763
0.4893555 0.4480309 0.1503538 0.3777217 0.201622078
0.5329428 0.39093 0.2909551 0.1111973 0.6996142 0.45107
0.4779046 0.3140063 0.4468818 0.1511466 0.7548109
0.2914069 0.2750382 0.2211669 0.2936378 0.268472384
0.3481536 0.851866 0.8062918 0.2637101 0.2170522
0.165661491 0.3569146 0.4976945 0.07286441 0.2267961
0.2056376 0.5620638 0.2190674 0.5471632 0.3386542
0.2519187 0.7238927 0.1316454 0.1372991 0.8356578
0.158153 0.2489556 0.8791035 0.2856592 0.8269726
0.2201962 0.2462839 0.1882825 0.2606486 0.5506886
0.2009981 0.3551277 0.524133829 0.462399828 0.2752921
0.5122562 0.1670923 0.1697806 0.2639328 0.1083094
0.6744991 0.2976359 0.3433447 0.6368703 0.1660794
0.2573284 0.4864958 0.293826005 0.2750838 0.3113603
0.01224629 0.3277369 0.2786494 0.182394769 0.1027126
0.3117077 0.1288159 0.1798492 0.1764493 0.367341
0.08421798 0.4936222 0.6765761 0.8194467 0.7902111
0.202176 0.1221914 0.3538969 0.06152566 0.5010146
0.4930395 0.6029433 0.489036939 0.3242932 0.7634202
0.3971884 0.1895343 0.4926598 0.1049333 0.3500058
0.2259518 0.5123004 0.5501612 0.2974692 0.3430277
0.4576137 0.5533702 0.2112444 1.033389 0.347411
0.5092585 0.2362099 0.3602176 0.3365714 0.3645786
0.6365275 0.856334 0.270995 0.3133197 0.232032712
0.131202 0.1529126 0.4363144 0.4928754 0.1334703
0.5953228 0.274068423 0.4135493 0.3740637 0.4029978
0.3741126 0.08597579 0.119734 0.3874903 0.2346288
0.449511 0.5830109 0.4252026 0.451556 0.4184109
0.2970426 0.319136851 1.201061 0.4323712 0.06890582
0.19070271 0.6140078 1.119393 0.500197 0.5894235
0.2031785 0.1336563 0.1260383
AL022328.2 0.4086174 1.107072 1.941954 0.9107841 1.071941
1.402391 0.2805944 0.707376 1.121484 0.796371169
1.812201 0.2229963 2.173479 0.6172433 0.6661653
0.8577171 1.72075 1.16227309 0.3363551 0.6787833
0.371376106 0.8817211 0.7797605 1.113537 1.010618
2.462395 0.7546044 1.525764 0.263210515 0.2575531

1.218881	0.9156935	1.994056	1.286952	1.570899	
1.443147	0.2598368	1.100988	2.124764	0.683059661	
1.723223	1.941146	0.425301059	1.240464	3.296028	
3.390914	3.32823	1.244421	1.063276891	1.613685	0.6027307
4.219328	1.850901	1.570142	2.036023	1.19998	0.733283
3.071795	1.047076	0.461633968	0.773190545	0.990674935	
0.6556668	0.3984631	0.9059528	2.215101	0.5265731	
1.422504	0.593008617	2.331126	0.6979779	0.9871967	
2.200749	1.460552	1.554515	0.8211082	1.674395	
4.896852	2.57722	0.644894928	0.986786	2.107759	0.3890579
2.310913	0.4064989	1.011076	1.821914	1.683146	
0.77767855	1.152245	0.765658995	2.255949	0.01939233	
2.414565	0.7316241	1.669782	0.7921642	2.175282	
1.314484	1.541668	3.325401	2.300625	0.9414996	
1.116439	1.967631	3.270709	0.9169504	1.045594	
0.542011182	2.360606	1.553487	1.859207	0.1593548	
2.552618	2.492327	2.631418	1.574798	1.708547	
1.995649	1.899939	0.7517496	1.758396	4.079289	
0.6321828	1.200166	4.954264	0.6851957	0.319179	
1.724243	0.4202192	1.536805	3.46321	1.247309	1.349595
0.1014982	2.546214	1.770592	1.368775	0.9743474	
0.7744532	1.577342	2.079583	2.4547	2.41403	1.949799
0.7159823	2.210157	1.222716	3.561125	1.352669	
1.552503	1.070473	0.658261	1.556712	2.355699	
0.7128045	1.785005	3.467483	1.658093	0.5316566	
0.547465236	2.758827	1.021456287	1.755366	0.6152545	
1.441654	0.8328961	0.8062395	4.297336	0.7825342	
1.780309	0.627215744	2.470464	0.9009117	1.650158	
0.5575364	1.010165	1.863312	2.031	1.36597	1.07309
1.375629	0.7118324	2.580231	1.349301	3.421945	
1.92764	0.8907272	1.666233	1.13834	0.9734007	5.836981
0.368139	0.7288047	1.444309	1.27146	1.160216	5.276827
0.6245532	0.6627703	1.711548	4.985198	0.8902084	
2.131152	0.901985	2.587343	0.9847402	2.405512	
2.197743	2.087695	1.684129	1.399437	1.833199	
2.078104	1.786402	1.866992	0.4615839	3.291039	
1.189114	0.776662338	1.292219	1.603446274	0.524213981	
2.845848	3.365179	1.287789	2.836131	2.061739	
0.4154509	1.179693	0.2048911	2.191042	1.243579	
1.379107	0.5984841	2.319671	1.013697	1.135843	
0.7433605	2.225155	3.247406	2.138274	1.595535	
1.181462	1.136694	1.613304	1.209503	3.158077	
0.9591463	1.731969	1.591717	1.562651	3.086199	
4.280061	1.264872	0.8598359	1.465109	0.423560666	

1.982114 2.498647 1.351918 0.9220686 0.7827885
0.5393619 1.232537 1.381326 2.378679 0.6914958
1.753019 2.300216 0.9933947 0.4010336 1.805976
0.850653841 3.792388 1.17825 2.585597 1.526262 2.652904
0 2.38129 0.7355323 0.1302167 1.108717 0.4637708
2.28964 0.9843361 2.09878 0.9184249 1.05279 2.857534
1.529221 0.4860632 3.579352 0.591363 1.423715
1.893426 0.7147059 2.355424 1.046143 1.267593
2.003238 1.103419 2.140667 1.226088 1.670262
0.835338289 1.448095891 2.072532 1.785143 0.5839528
0.6282507 0.9918689 0.6193942 1.98709 1.210386 2.485446
2.913682 1.297242 1.222558 0.5140926 0.785954943
0.5527233 1.874307 0.04668899 1.54829 1.677396
1.341090106 0.834709 1.388753 0.6445828 1.140038
0.9417983 0.9336584 0.6668606 1.563453 1.856307
2.025917 4.064847 0.5172446 2.25516 1.134581 1.595053
2.281999 2.649716 2.939711 1.717960569 1.679594
0.9333799 1.424451 1.417407 1.490194 0.7743063
0.7844091 0.4940618 1.545346 3.125119 2.12644 1.755196
3.495159 2.418221 0.6040268 1.13648 4.403977 0.8199742
2.135176 1.414695 2.385627 2.960288 2.008354
1.284898 1.632775 0.6044616 1.169503859 1.021257
0.4513388 1.37446 1.578433 1.937565 2.924673 0.84553264
1.949759 2.24749 2.101293 1.138743 0.2950044 0.4668606
0.8729539 2.146854 0.8777799 2.453743 1.022389
2.304238 1.705205 2.138379 0.627365705 3.556211
1.68862 0.6567586 0.760101996 1.398833 4.598651 2.120136
2.558325 1.312547 0.6726254 1.063153
AC011451.1 0.7533357 0.7546632 0.7259447 0.4112182 0.6116973
1.344207 0.7759649 0.9458551 1.052256 1.258463081
0.6774405 0.6343005 1.274138 0.5045156 1.052706
1.116216 0.5724653 0.783289345 0.9301673 1.302498
0.41918996 1.250431 0.5932883 1.373396 0.8107978
0.5639409 1.02211 1.475016 0.34661467 1.058194 0.9123788
1.061149 2.458551 0.7908842 1.547855 4.561057
0.5225898 0.6766018 0.5272345 1.746092962 1.30362 2.2769
0.597405055 2.859644 1.551479 0.7391008 2.087074
0.3855868 1.534132197 0.4870827 0.9938748 2.098426
1.231529 2.049697 1.538765 1.763967 1.422125
1.434194 0.4676151 0.680862609 0.610915986 1.467666571
0.8217468 0.7975822 0.4442987 2.284987 0.6852716
2.066625 0.973142347 0.902144 1.348083 0.5720054
1.774351 0.668995 0.8095341 3.373639 1.949652
0.759223 3.203813 0.351411455 1.808864 1.410683

0.3458292 0.9959491 1.587028 1.31988 0.9966026 0.8865952
1.47470895 2.589627 0.795097031 1.001392 1.869325
1.332435 1.569056 1.759112 2.538403 0.8688143
1.685851 1.781711 2.066051 0.8148659 1.405146
1.979402 1.48064 2.218949 0.7245041 0.7591633
0.888234146 1.626046 1.129685 2.217907 1.38501 2.886912
1.524578 0.2563329 0.1605529 1.705216 0.8745326
0.6380042 0.259864 1.00342 1.884298 1.531808 1.000003
1.217838 0.8202867 1.435548 0.8307101 1.06248 1.627633
0.6534599 0.2190063 1.156715 1.122746 0.7315722
2.081419 0.4635007 0.9869933 0.7060542 1.026361
2.11529 0.865108 0.9064783 1.862242 1.131429 1.257334
1.533487 0.6955283 1.261505 1.094056 0.8796378
9.719508 1.319125 1.179693 0.5632036 1.314212
1.274298 0.8062142 2.160382 0.811059609 2.219447
0.660335378 0.9790278 1.237414 1.28147 0.8583792 1.07289
1.025956 3.223986 1.776838 0.991155744 0.8803007
0.5110585 0.7450448 1.904194 1.249286 0.9814979
0.6902112 0.830219 2.020465 0.3260751 0.9641751
1.637645 0.5576596 1.063774 1.5091 2.863675 1.212982
1.457696 1.138278 0.7938578 0.3635941 0.7677942
1.850566 2.00922 1.237564 0.7127084 0.8041801 1.633851
1.683518 3.115864 0.1758436 0.2928475 0.5429939
1.501949 0.4322591 1.322193 0.5483648 1.413889
1.101244 1.029471 1.931271 1.226524 2.960662
1.443768 0.6142454 1.420944 1.052292 0.483489012
1.740743 0.832007501 0.564809677 1.618971 1.939438
1.918738 1.370464 2.255578 0.6565151 0.5019016
0.8499187 1.293181 0.9357911 0.3113328 1.762539
1.807719 1.395196 1.429846 1.498746 4.468624
0.9672321 1.222192 0.9514487 4.267432 1.384614
0.9646447 1.971042 2.082384 0.9407718 2.071888
2.386316 1.133483 2.471882 1.690888 1.640049
0.8327841 1.127934 0.669330434 1.785034 0.7273513
2.136365 1.205873 1.649332 1.000555 0.4657574
1.20056 1.025154 0.7024719 2.114887 1.446546 0.9484262
0.3412403 1.40191 0.459009841 1.747932 0.6445126 0.9193233
1.349499 0.9230629 1.069349306 1.689522 1.718216
1.358112 2.347082 1.910703 1.669937 1.15955 1.929652
1.104279 0.8464276 1.447311 0.9294409 1.093065
0.3668925 0.9344995 1.094507 0.5846595 1.532773
2.733455 1.591937 1.691515 1.543235 2.814352
0.8456955 1.12013 0.8098238 0.815319289 0.547213906 1.700539
0.4339949 0.615193 0.9927912 1.465176 1.39828 1.431513

0.4781771 1.374667 1.124923 0.3660649 1.323739
1.051332 0.171310741 0.2687506 1.110701 1.032922
1.545272 0.4638725 0.894795923 0.6839499 1.604986
0.4729214 0.8420251 1.027618 0.811476 0.5854469
0.9264909 0 1.487542 2.404312 0.745253 0.7528963
3.052808 1.853366 0 1.556781 1.012922 0.410377029
3.760079 1.237072 1.277585 0.7905364 1.030258
0.7341571 1.453277 1.501422 0.5596313 1.534926
0.9305448 2.039443 1.7208 1.651217 0.3579418 3.879184
1.046522 1.027673 0.9296004 0.6667482 0.8139514
1.835984 1.090958 1.340868 1.53062 0.3411424
1.066214344 0.6257742 0.8023801 1.470267 1.692571
0.7731897 1.660925 0.554013557 0.7132177 2.011141
0.8569191 1.581374 0.5697753 0.7869386 0.6366843
0.459409 0.7596073 0.9767797 1.309968 2.82512 0.6953921
2.779128 1.532152383 1.541401 1.302451 0.4843257
0.156671587 0.6315721 1.651592 1.648579 1.201909
0.9520683 0.5314571 0.6834078
LINC02487 0.1022742 0.02173275 0.02613215 0.02713847 0.01490553
0.005754248 0.05267318 0 0.03508749 0 11.30541 24.60302
0.008774312 16.07781 0.0185263 0.004209429 0.03400202
0.059893067 0.01403124 0 0.053115975 0 7.907952 0.06118044
2.992604 30.61954 0.04496963 0 0.021959944 0.07735659
0.04817017 0.05093155 0.1261459 1.324253 0.3469284 0
0.04729848 0.82671 2.303063 0.01005678 0.1789483 0.4007058
0.088708342 0.03287435 0.3510526 0.97415 0.07933655 0.2992557
0.011570917 0.04991956 0.1770959 0.1447405 0.2072515
0.1366943 0.1883315 0.08381774 0.7591709 0.02329848
2.694825 0.138652674 0.090311397 0.077487285 0.08488398
0.3922818 0.0312764 0.02695121 0.3178651 0.4421829
0.331104357 0.07144473 0.01455827 0.07000848 0.3203824
0.1907303 0.8312263 0.4624152 0.05734913 0.06012619
0.5547524 2.076103695 0.158071 0.05585907 0.03651697
0.3563916 0.1316687 0.04951281 0.09938743 0.02106401
0.33090116 0.2563549 0.197114735 0.05709932 0.01456131
0.3309533 0.7521026 0.06965589 0.01101519 0.1076951
0.009536421 0.01727773 0.3699225 0.09431715 0 0.8655742 0
0.02143028 0.01639333 0.08487735 0.035171571 0.3635963
0.2546303 1.605904 0.02991409 0.05011006 0.9659037
0.5864481 1.131625 1.152372 0.1091339 0.6300932
0.446875 0.9474694 5.05797 0.09493868 4.484055 0.01653359
0.09874237 0.2027936 0.02105202 0.4890779 0.5442416
0.3493148 0.06937626 0.04007732 0.08891513 0.3186502
0.0270224 0.2263576 0.08754403 0.7828172 0.6734794

0.08375953 0.1149228 0.6341271 0.09653177 0 1.158006
0.2866067 2.273381 0.2497603 0.1417796 0.1714766
0.1493122 0.06211567 0.06228334 0.1070461 0.005076986
0.1883788 0.2394286 0.8364406 0.034256722 0.06277419
0.273095292 1.302563 0.2449906 0.4781081 0.453192
0.1699338 0.0773809 1.217149 0 0.058870465 0.0788081
7.036512 0.4897278 0.08552919 0.4891856 0.02454603
0.03279651 0.5325642 1.028632 0.837823 11.62539
0.03705494 0.7897474 0.2176327 0.04426379 0.03843846
1.180852 0.05403634 1.067124 2.47285 0.09214273 0.01737285
0.009770292 0.4823292 0.01451972 0.8522817 0.0752659
1.04177 0.7300098 0.2933171 0.5793144 1.6559 0.8492907
0.01762162 0.2259344 0.07853265 0.9988304 0.2772641
0.3851878 0.09662616 0.5168513 2.205876 0.07088559
0.2038204 0.1945791 0.9902704 0.03571527 0.854152771
0.1855768 0.05990028 0.500973151 0.02849187 0.1833201
0.0736743 1.220263 9.72536 0.03466153 0.02839127 2.698761
0.4162577 0.23715 0.2644895 1.348169 0.02386019 0.07366111
0.05300397 0.0128316 1.264721 0.6893941 0.09679071
0.3164674 0.04224457 0.5038141 0.8403387 0.113524
0.02843329 0.05794742 0.03646264 0.2400592 0.1602955
0.09523416 0.6695442 0.01623534 2.249304 0.5030466
0.076565957 0.1173593 0.7296269 0.2602893 0.04774916
2.902618 0.01760852 1.31446 0.9248455 0.004920391 0.4388732
0.1887179 0.04112348 0.05180001 0.01544246 0.1665351
0.638738903 1.115321 0.5331029 0.264256 0.02273888
0.03045891 0.088073901 0 0.3802041 0.02607389 0.06283122
0.07047637 0.07714559 0.5285815 0.9073545 0.03498107
0.07396667 0.8298406 0.01177704 0.02807501 1.036324
0.008706717 0.1059408 0.3595194 0.07666547 0.004605835
0.02909364 0.126908 0.05640717 0.01938094 0.1133412
0.03836022 0.6555868 0.178332589 1.678295148 0.1047457
0.5155493 0.1062978 0.08577106 0.1403198 0.05905914
0.05851238 0.03786894 0.3794753 0.1616007 0.1275572
0.1246658 0.7190602 0.352737995 1.250407 0.01172816
0.2161899 0.3671304 0.07872019 0.589280109 0.1238057
0.03112796 0.126763 0.8653366 0.1010254 0.06426433
0.1112738 1.358757 4.814147 0.2407318 0.04547057
0.1370783 0.2703007 1.37384 0.06458149 1.218494 0.02550793
6.760416 0.384994665 0.8977102 0.8138976 0.6648811
1.711232 0.3846414 0.1744233 0.1489418 0.04756168
0.2095111 0.02114044 0.4667281 0.7623386 0.3428923
0.2166997 0.09448992 0.02275626 0.07459078 0.8846295
0.3599157 0 0.7938803 0.0318684 1.036773 3.31917

9.399471 0.8753363 0.686763796 0.1564983 2.132254
0.1058517 0.1693163 0.03429007 0.08578431 0.118719922
0.3795646 0.3727619 1.051879 0.2504719 0.08204181
0.1168521 0.1008436 0.4477859 4.289428 0.1969048
0.02766455 0.8949343 0.1720973 1.750726 0.228400641
0.1627604 0.1358519 0 0.115803402 0.7931259 0.3269919
0.2526933 0.01297969 0.3985347 0.2907927 0.06314239
LINC00649 0.3020039 0.2306399 1.251941 0.5653677 0.3296643
0.2186105 0.4384288 0.3145783 0.6242635 0.473607216
1.278771 0.1991038 0.575922 0.3667733 0.472528
0.3205928 0.4609152 0.347949402 0.2402537 0.3116862
0.339875454 0.153413 0.3880753 0.2861441 0.1589502
0.7718882 0.7940671 0.2307878 0.538563181 0.3058443
0.4353146 0.4206013 1.349979 0.5394219 0.8852809
0.6309324 0.02109065 1.143222 1.057521 0.459199772
0.0581064 1.432989 0.483104874 1.243722 1.184458
0.9828519 1.433931 0.7461733 0.427209465 0.3780051
1.316134 1.096712 1.88417 1.158102 1.229043 0.8035578
0.1755832 1.337054 2.083177 1.808066374 0.566085935
1.558986226 0.5753234 0.849106 0.6791856 0.3062794
0.6139666 0.697527 0.739224341 2.419053 0.1402188
0.05949623 1.755426 0.327906 0.469225 0.6579862
0.4839063 0.9206345 1.715078 1.199250421 0.1110134
0.7317376 0.5612247 0.9024399 0.4163198 0.7418218
1.068831 0.3819639 0.82628346 0.3132095 2.135837019
0.8056959 0.8155169 1.286673 2.97339 0.4244858 1.172922
0.7448718 1.054581 1.475104 2.856181 1.38211 0.9643832
0.9992522 0.2342418 0.8890789 1.084785 1.117756
0.528747681 0.6214966 0.6984308 0.8780132 1.39969 1.141796
1.112646 0.8520889 0.9354901 2.832591 1.32065 0.834404
0.1227047 0.941998 0.7275106 0.5512785 0.5854705
1.230702 0.2428589 0.08111005 0.4064662 0.3311102
0.4714931 0.9864887 0.4743405 0.1429655 0.8926405
1.502256 0.3875098 0.6950813 1.293776 0.8590261
0.2457694 2.186507 0.9539399 0.840813 0.2640034
0.09015345 1.078767 0.7104785 1.395977 1.086966
0.1554751 1.103925 0.2856015 1.314382 0.841321
0.6459791 0.4274159 0.5519941 1.469052 0.8069788
0.969978864 1.269688 0.788685303 0.4720313 0.6517103
1.796436 1.516414 0.5995648 1.555466 1.197756
0.4149592 0.439844374 0.2432836 0.7229157 0.6945817
0.1308733 0.983303 0.4465646 0.7214575 1.192644
0.9030754 1.4657 0.4573796 1.322784 0.5532503 0.8890445
1.080824 0.05896131 2.102014 0.7211006 1.263683

0.3598988 0.8474182 0.6166332 1.101355 2.741407
0.547736 1.146655 0.1394092 1.034395 0.4943155
1.051316 0.6974624 1.374163 1.819693 0.6278203
0.5397509 0.8194243 1.463957 0.1017599 0.6766591
0.6322889 1.611386 1.948223 0.5767294 0.1205884
1.39256 1.590444 0.5871249 1.73467197 0.5182495
0.631421074 0.995666166 0.9568438 3.740757 0.6241836
0.5462148 0.9912207 0.09026166 0.28358 0.04802135 0.4171841
0.6754556 1.174576 0.1335902 0.3106702 0.6218822
0.2595526 0.5767452 2.583804 0.5950745 0.4153467
1.927221 0.1280922 0.2949568 1.539724 1.589498
0.5395994 0.8622867 0.502219 0.8035111 0.1052136
1.066354 2.820181 0.8238464 0.2154543 0.1064954
0.742701861 1.200762 0.585131 0.6954579 0.7821118
0.3792279 0.08165804 0.2272729 0.8556204 0.7652774
0.1797095 0.9803512 1.409344 1.265765 0.1267002
0.6350904 0.603904948 2.250319 1.513274 0.529047
0.295259 8.390288 1.039214183 1.635835 1.154266
0.1046384 0.7779327 0.4769326 0.6250906 0.6024265
0.807485 0.6979103 0.2861201 0.765411 0.1753982
0.2436993 2.003884 2.677284 0.6630718 1.513731
0.6745948 0.0985808 1.024003 0.5482841 0.6025365
0.8927256 0.3105878 0.30618 1.776856 0.292851002
1.314023349 1.125409 0.2867618 0.9092663 0.5290666
0.6208807 0.9454189 0.1200185 0.7902631 0.8421669
1.472213 0.6939172 0.4709919 1.887346 1.087705502
0.6928023 1.399455 0.5002392 0.8694578 1.029652
0.027939377 0.1435346 0.4191798 0.7050125 0.5847343
1.117184 0.8805149 0.4283653 0.09015449 1.062344
1.637104 1.514585 0.3538399 0.5317435 0.7857743
0.1361324 1.566611 0.8113542 1.596554 1.415284169
0.8490135 0.9704785 0.5431062 0.8951849 0.9511213
1.00245 0.6013481 0.7414321 0.7674987 1.080293 1.019406
0.6176372 0.7825217 1.210177 0.3960555 1.148656
0.7243383 1.296357 0.07975785 0.01994401 1.592677
1.043035 0.5575648 2.268486 0.6127848 0.831713
0.512059554 0.1386364 0.08185553 0.1935194 1.459648
0.7365473 3.069315 0.379311245 1.222629 1.253575
0.9088253 1.803934 0.9409119 1.06711 0.9952634 0.8655701
1.041244 0.8278372 0.4477885 2.167318 0.6814434
0.5709957 0.423930154 0.1950473 0.9764132 0.3870185
0.86603236 1.389148 3.862148 0.730149 1.044876
0.9413762 1.504126 0.6081596
AC080038.1 0.06294895 0.4093159 0.1505475 0.02405306 0.2146771

0.5865045 0 0.3772169 0.1166188 0.283925349 0.464693
0.8409703 0.3110699 0.4249473 0.2052502 0.6808805
0.05022713 0.862610674 0.09327005 0.08066749 0.088269636
0.5140728 0.2402497 1.204994 0.5949725 0.5343756
0.1195709 0.5773916 0.194632847 0.2285393 0.1423121
0.4175548 0.1458315 0.2775633 0.6764683 0.1103943
0.02620065 0.05653704 0.1266026 0.033425287 0.2236714
0.06659043 0.157245915 0.0404678 0.1426063 0.09263921
0.02929861 0.1759198 0.179469538 0.05027742 0.1744018
0.4870069 0.1080522 0.189302 0.4909406 0.04643023
0.240307 0.516241 0.03787177 0.06827169 0.471686433
0.154524687 0.1630057 0.1767878 0.05197601 0.7166126
0.3779259 0.4835259 0.012649164 0.2550472 0.05376298
0.1551228 0.2366313 0.3521789 0.3428897 0.1593832
0.1954962 0.1742185 0.09528716 0 0.0875623 0.7151209
0.08091325 0.2977491 0.3182701 0.1340889 0.6217971
0.01555767 0.51755384 0.2982125 0.084925781 0.0984037
0.1720776 0.1594167 0.1449119 0.308683 0.3539035
0.05302837 0.05282632 0.2424621 1.087631 0.06599534
0.1450413 0.07550844 0.5845897 0.4463552 0.09080965
0.3604652 0.100198485 0.08392147 0.1143654 0.1010885
0.1104713 0.3608554 0.4458795 0.03998261 0.1267798
0.07979349 0.2046134 0 0 0.2437984 0.2784423 0.350604
0.1331206 0.109904 0.08060703 0.2587133 0.6647121
0.2913131 0.08160327 0.272334 0.1793423 0.2917786
0.1407254 0.4172149 0.2514769 0.02711117 0.1246998
0.03303893 0.1972549 0.2651314 0.04896974 0.03976643
0.130712 0.3767156 0 1.259346 0.1331443 0.1844705
0.02327046 0.5343087 0.1140834 0.3128048 0.6624271
0.1976581 0.2699865 1.505637 1.02567 0.5897058 0.3225965
0.3894611 0.566492222 0 0.1395882 0.1898886 0.06025019
0.2824008 0.2400417 0.1084969 0.4676903 0.072468601
0.2686473 0.05124503 0.02614759 0.1496156 0.5358723
0.2175536 0.08478115 0.218526 0.1392853 0.1144371
0.3383804 0.1407519 0.1726874 0.09333373 0.8728981
0.2725467 0.1349781 0.03265423 0.1619523 0.2653399
0.2424486 0.07698853 0.1515411 0.1762856 0.3941116
0.08754456 0.05131447 0.1764324 0.02905751 0.2372737
0.03702773 0.06166546 0.04764136 0.07809096 0.3034054
0.1885113 0.0962252 0.1772174 0.2898639 0.508491
0.1519175 0.12497 0.3262145 0.1982719 0.1077857 0.07978955
0.4220632 0.13052463 0.105736 0.331813296 0.079288707
0.1262629 0.1427173 0.2244624 0.06499577 0.3518232
0.03072081 0.1132354 0.08522334 0.5709674 0.03284189

0.03973209 0.04526113 0.193852 0.2538918 0.01067679
0.4833421 0.08998315 0.1225803 0.3153913 0.5843495
0.3120144 0.9850031 0.2901816 0.3563527 0.8064214
0.2384539 0.1077238 0.03395211 0.01420713 0.5627123
0.9357819 0.08993447 0.2153556 0.2083432 0.169652645
0.177301 0.2431107 0.08074382 0.1058012 0.3215769
0.0910384 0.2377586 0.114911 0.06541477 0.02739275
0.3554323 0.1041374 0.2065985 0.2737357 0.08785802
0.023012996 0 0.1005306 0.3943371 0.3426123 0.1484779
0.120093302 0.9567945 0.07981061 0.2455385 0.2436343
0.1286019 0.04883914 0.08933002 0.1693044 0.4539875
0.5224097 0.08127014 0.2870474 0.3317749 0.07511122
0.5594707 0.6402015 0.4465859 0.2548097 0.1428767
0.04297656 0.1405995 0.4305052 0.1073593 0.8561526
0.1062468 0.07368414 0.258887593 0.296799098 0.1547284
0.05077063 0.04906908 0.2058865 0.08370832 0.4416577
0.08103128 0.4405217 0.09648874 0.2387135 0.08993015
0.4017905 0.08385647 0.075152567 0.07073916 0.2338824
0.3754539 0.1355795 0.1976827 0.082639868 0.08572658
0.1954219 0.01276718 0.3917244 0.3233375 0.06472502
0.3081967 0.1685989 0.03721063 0.1021417 0.2266929
0.04218531 0.1056925 0.2040748 0.1951334 0.04218601
0.7535965 0.3677464 0.099708324 0.03881211 0.133586
0.2989151 0.02312011 0.5423583 0 0.4050257 0.1053858
0.1071297 0.2927649 0.3265777 0.6584892 0.3311822
0.2980284 0.4815465 0.415987 0.01836401 0.1411298
0.3020798 0.05505822 0.1503468 0.07943972 0.08121593
0.2016779 0.2762619 0.1795875 0.074838269 0.1040293
0.1564433 0.08209012 0.1563192 0.08683309 0.3167973
0.354553992 0.1702081 0.1501737 0.1378385 0.04784382
0.01817859 0.4142679 0.7448214 0.1984382 0.1506794
0.228536 0.03064915 0.6830229 0.5338595 0.08866762
0.158151115 0.3042898 0.2564217 0.02428224 0.183281319
0.1108261 0.1593987 0.1866369 0.06710675 0.08353286
0.2034726 0.2998052
AL731566.2 0.1650127 0.3801296 0.4415564 0.3510852 0.3935307
0.6684553 0.1854212 0.2247333 0.1621143 0.159755851
0.701779 0.3389267 0.6486378 0.3234942 0.2201062
0.3667484 0.3141983 0.361434494 0.08335078 0.144177
0.219117106 0.4108466 0.2385546 0.5115005 0.3082303
0.8489684 0.2018363 0.6405343 0.07247235 0.3063513
0.1483734 0.1277443 1.049819 0.2834794 0.7510777
0.2959616 0.2341421 0.4311409 1.073365 1.141717142
1.265932 0.8066713 0.437182068 0.6364872 0.8804951

0.6843728	1.239314	0.9271401	1.344161848	0.3774155	
0.3723182	26.87002	0.4908511	0.9022386	0.7897123	
0.6823191	0.6057061	0.7073861	0.5490267	0.386402963	
0.323593083	0.521677044	0.1419349	0.2984188	0.6296334	
0.5336674	0.2251556	0.3411338	0.384333451	1.000765	
0.8840333	0.6306099	0.8903814	0.6364432	0.5544804	
0.1085202	1.106466	1.227206	2.213987	0.536369627	
0.3782086	0.7537722	1.269409	0.6170051	0.1185092	
0.806119	1.142208	0.3707497	1.0791946	1.565144	
0.628835354	1.298139	0.2883321	1.241009	0.3108013	
0.4214445	0.6398022	0.3238235	1.22112	0.6158167	1.202039
0.9108628	0.4320539	0.9087053	0.7120371	1.041062	
0.3462487	0.606911	0.570418086	0.9199561	0.6540967	
0.8792874	0.3159125	0.9674352	0.7172283	0.7920251	
3.038031	1.0399	0.7203289	1.216275	0.3027179	1.024798
1.036792	0.3202799	1.766424	1.214964	0.8575532	
0.1865817	1.21583	0.4512421	0.4105394	0.7429227	0.9005603
0.8086964	0.05030371	1.233253	1.041617	0.734913	
1.007069	1.0137	1.083208	1.705929	0.4084436	1.074015
0.6937851	0.7096985	0.9639342	0.7438639	0.6478039	
0.3736647	0.755575	0.4032095	0.3534287	1.006337	
0.750935	0.6358938	0.9248816	1.474291	1.116765	
0.3764218	0.282635641	1.143566	1.012491519	0.7915114	
0.1386033	0.6609131	0.2991254	0.7360717	1.368799	
0.9326464	1.077386	0.509457634	1.352432	0.381626	
0.553014	0.4872768	0.3555338	0.5832503	1.277176	
1.301905	0.6563083	0.7954077	0.4031915	1.362648	
0.6721586	0.9916247	0.5200436	0.497271	1.329947	
1.361801	0.5081584	0.916868	0.2356698	0.401338	
0.3740302	0.8402005	0.2923002	1.11018	0.718429	0.9985693
0.5308859	1.106294	0.6323835	1.071531	0.3405976	
2.244783	0.1807588	0.8379958	1.000051	5.173431	
2.033919	6.608918	0.7240591	1.596603	1.223671	
0.5840566	1.67762	0.6357934	0.6993487	0.435468441	0.41996
1.628910354	0.11022099	0.4567125	1.608958	0.6139295	
0.7124906	0.384275	0.4575608	0.2473603	0.3892614	
0.5494946	0.6358983	0.5385164	1.024673	1.406885	
0.2809119	0.9732138	2.015714	1.064521	1.365086	
1.119882	0.2984022	0.3903643	0.7041984	0.4610147	
0.7243282	0.6155624	0.8851609	1.187298	1.58449	1.472759
1.103516	0.6662897	2.05747	0.430727	1.222621	
0.225471403	0.873208	0.5648653	0.5841258	0.178593	
0.9483447	0.9142916	1.177455	0.9013918	1.058738	
0.4487914	0.5667552	1.488998	1.107761	0.3159726	

0.345463 0.829477737 0.7883677 1.197855 1.025162
0.75043 0.2653746 0.420342055 0.8751107 1.146447 0.1376788
0.8363359 0.8482569 1.105676 1.028917 0.8195357
0.8773802 0.6651869 0.8836293 0.4508532 0.6176888
0.8757972 0.2528589 0.2542738 0.5033586 0.9277102
0.7843307 0.390461 0.4653584 0.8769984 0.7867218
0.6596869 0.5380357 0.4076294 1.006596718 1.133747496
1.336639 0.3680106 0.4268124 0.2453206 1.724099
0.2338888 2.819303 0.7248568 0.6898173 2.565386
0.7271217 0.9948957 1.618668 0.922331901 0.9693127
0.9289287 0.1465509 0.7269637 0.5957917 0.659736635
0.4698719 0.7875867 0.5932881 0.6264313 0.6594001
0.8637666 0.4039493 1.671701 0.8350269 1.487508
2.280946 0.4574133 1.353814 1.084097 0.5347682
1.466093 0.6285547 0.8179399 0.283813827 1.156148
0.8953434 0.7886562 1.088161 0.769331 0.2878165
0.6881541 0.7910954 0.5956934 1.472101 0.8214928
0.767555 0.7137881 1.213295 0.1247345 0.8936943
1.225355 0.6446168 0.7774665 2.000913 0.7434442
1.461895 0.9815394 1.089387 0.6674943 0.4565002
1.151808733 0.6542038 0.5499017 0.3842658 0.4842749
0.5884544 3.140589 0.933506703 0.9484286 0.4741829
1.702115 1.031836 0.3898871 0.3599269 0.3993658
0.6428369 0.4626595 1.373919 0.4930131 1.443918
0.9178179 0.8914255 0.876256788 1.651714 0.5845017
0.2169982 0.8517046 0.764021 1.217271 0.500364
1.593487 0.7180536 0.3838703 0.9287925
LINC02256 0.4315431 1.390795 0.4410555 0.4580401 0.9853312
1.683406 0.3951165 1.072707 0.4194769 0.300375735
0.8231725 0.8478273 0.5430035 0.6443813 0.5732556
1.563018 0.4144706 1.38392706 0.1578785 0.06827304
0.728394436 0.9869028 1.281015 1.931338 3.262166
0.5778998 0.3288967 1.642963 0.308864557 0.5077395
0.4516727 1.490003 0.8948291 0.7047483 0.3903613
0.3153342 0.3159931 0.3158113 0.2349064 0.212171512
0.6969851 0.3804226 0.232899248 0.3287997 0.5595859
0.04704319 0.5393329 0.1374369 0.325487736 0.3191427
0.2214077 0.9801816 0.3086442 0.2162916 0.8414041
0.137537 0.0469348 2.34299 0.2083434 0.28168644
0.580672621 0.283361368 0.5730649 0.3179522 0.06598495
1.494604 0.3707446 0.8238501 0.160584546 0.08932119
0.3412678 0.0695056 0.9486627 0.07451677 0.410433
0.4335885 0.4095098 0.3122471 0.2661331 0.125255568
0.3890694 1.117373 0.1369621 0.9039142 0.5555722

0.2476064 1.036072 0.1975088 0.43803236 0.9735139
0.061608896 0.3569318 0.4369142 0.9309655 0.2023666
0.7021209 0.4183045 0.1683024 0.05365155 0.6480256
2.421362 0.4747695 0.4419213 0.421784 1.517289
1.13332 0.2075135 0.7560683 0.40988174 0.3835461 0.04839664
0.1925019 0.112197 1.145289 0.6226616 0.2157257
0.08941685 0.126625 0.8737455 0.08360568 0.6285255
0.1031696 0.3829476 0.2670607 0.1229093 0.9766823
0.482426 0.103719 1.835787 0.532555 0.3913685
0.4731115 0.6505138 0.2093683 0.2143857 1.527881
0.1824324 0.05162755 0.2990296 0.230691 0.283084
0.2187844 0.1492042 0.1177973 0.2111994 0.2843652
0.04149616 1.27082 0.1408586 0.2810284 0.5169933 0.5275817
0.4200125 0.7624592 1.354895 0.05018646 0.2427849
2.857707 0.3592043 0.6951725 0.674544625 0.9005714
0.490348053 0.4652805 0.1969011 0.4694495 0.4079426
0.2838247 0.6965473 0.2754796 1.21388 0.220802022 2.148646
0.1626424 0.2821581 0.3545566 0.7266887 0.299206
0.1998879 0.3514046 0.3697279 0.2259926 0.6622738
0.4318301 0.2776931 0.6911898 1.69338 0.1513773 0.1977211
0.01381847 0.5482741 1.145311 0.4373929 0.2687823
0.6596075 0.6574104 0.541179 0.1587717 0.1628627
0.3546441 0.2090393 0.9691599 0.1410231 0.3392392
0.151205 0.1239232 0.07703627 0.5522772 0.2036008
0.6899457 0.3884339 0.679519 0.2670416 0.934289
0.3681228 0.2796795 0.4618244 0.1139569 0.7534978
0.248556804 0.522024 0.724542986 0.10065915 0.4236346
1.324033 0.06476389 0.03300555 0.2605453 0.03900089
0.1277826 0.2885155 0.5389973 0.2918556 0.1008819
0.3639147 0.4698279 0.4834838 0.3795252 0.07219009
1.860415 0.3950329 0.6726682 0.3391302 0.4753328
0.3001171 0.660038 0.7983519 2.415466 0.9547437
0.239327 0.488502 0.2344723 0.7143784 0.5795124
0.1027568 0.2343426 0.2512725 0.447324795 0.1950766
0.632703 0.1903691 0.0447725 0.5919632 0.6769434
0.1131904 0.413334 0.09688678 0.2260429 0.4645024
1.090693 0.6119923 0.3257952 0.3792296 0.102254668
0.4561435 0.2712058 0.2275553 1.688651 0.1713607
0.228692525 1.847677 0.2476753 0.4584085 0.2356574
0.06997013 0.310013 0.1890114 0.1343353 0.4498336
1.378443 0.1031746 1.805508 1.224104 0.4086674
0.1714428 0.7585695 0.5209837 0.5032032 0.207298
0.04091993 0.2181604 0.5994288 0.2862207 0.7246059
0.2158131 0.1603612 0.051894438 0.221643796 0.1683702

0.1503943	0.2118014	0.2412724	1.138607	0.1868988
0.1645942	1.797603	0.5249788	0.5245152	0.2283375
0.4144432	0.2767909	0.267142987	0.01496754	0.1319644
0.1807979	0.7458613	0.1476256	0.020982708	0.4788617
0.321062	0.03241655	0.5322033	1.089366	0.6409272
1.382466	0.412793	0.09447981	0.360199	0.2398272
0.2677769	0.06708977	0.3886177	0.3963633	0.1428167
1.482902	0.8092308	0.253164809	0.147819	0.4451764
0.7318558	0.2054612	0.360663	0.3952455	0.07617645
0.5084025	0.1586714	0.4014067	0.161233	0.8723163
0.4575427	0.6936511	0.4518572	0.1760356	0.1398816
0.1592606	0.2761189	0.6640299	0.267217	0.3585809
0.5597173	0.3413807	1.032681	0.77517	0.158348665	0.1907649
0.1390263	0.8635008	0.6350448	0.3307108	0.6595797
0.26135759	0.673673	0.4956888	0.5090608	0.04859122
0.09231284	0.8108	0.04727854	0.2046872	0.4267273	0.5802652
0.2334596	1.006973	0.2323711	0.37991	0.481865048	0.4435344
1.188908	0.5548852	0.395556977	0.4220902	1.39813	0.0947762
0.5111629	0.1363464	0.3659448	1.129145
AL161630.1	0.06710678	0.01069489	0.0514395	0.5876242	0.146703
0.07928813	0.03456129	0.3351103	0.1554019	0.196180734
0.009600511	0.02197346	0.2245319	0.05243234	0.1458715	0
0.1338617	0.033684498	0.08285885	0	0.013069433	0.01113878
0.09960161	0.04014329	0.01532055	0.06153859	0.1770398	0
0.086453543	0.4060576	0.1580333	0	0	0	0	0	2.04829
0.3314925	0	0.37612668	0.4877286	1.656405	0.034923361	0
0.3224784	0.06583874	0.03904226	0	0	0.0178661	0.05164476
0.03165696	1.391869	0.03363426	0.2616838	0	0	0.0229308
0	0.136464545	0	0.015252886	0.2896206	0.2486688	0	0
0.1373482	0.02260803	0.044948838	0.02343908	0	0.01621262	0
0.04171562	0.3220214	0	0.1389393	0	0	0.109562463	0
0.1954757	0	0	0	0	0	0.3455264	0.38315186	0	0	0
0	0.1038561	0	0	0	0.1648819	0	0	0.7701791
0.02931433	0	0.1744076	0.01154078	0.06749466	0.01613462
0.01392298	0	0	0.1083727	0	1.197308	0.06164907
0.3961085	0	0.02502846	0	0	0.1053084	0	0.2887795
0.525643	0.1661163	0.5375506	0.09763601	0.02045981	0
0.196838	0.7349792	0	0.01273341	0.1138024	0.05634978
0.07501023	0.3848991	0	0	0	0.01467549	0.7009459
0.05888407	0.01740141	0.03532753	0	1.13967	0.02903773	0
0.08871162	0.4807121	0	0.01054815	0	0.03334658	0
0.1404757	0	0	0	0	0	0.07414023	0.137252157	0.04069861
0	0.3995346	0	0.06271949	0	0.02753887	0	0.051503474
0.07159791	0.07966847	0	0	0.0216389	0	0.150635

0.2847287 0 0.0112959 0 0 0.02045484 0 0 0
0.00922394 0 0 0.01178608 0 0.1538882 0 0.009787988 0
0.1999863 0 0 0.05162797 0 0 0 0.04232343 0.06070235
0.0134769 0.2189977 0.009498236 0 0.05722401 0.3328544
0.1141968 0.02467119 0.08586698 0 0 0 0.02343442
0.289887265 0 0.330147843 0 0 0.03901126 0.8338836
0.0692888 0.1302297 0 0 0 1.300599 0.02917593
0.01764851 0.02680592 0 0 0.028455 0 0.7537091
1.013583 0 0.05932821 0 0.1050063 0.01074126 0
0.01119383 0.04888537 0 0 0.1766978 0.1458041 0
0.01597912 0.01366547 0.009254352 0.173902249 0.01050066
1.123063 0 0 0.08570432 0 0.01320119 0.3062524 0 0
0 0.01156414 0.03058952 0.06079504 0.2653731 0.010222093
0.03114098 0.4800354 0.1167732 0.03580805 0 0 0.08380212
0.70114 0 0.06183966 0 0 1.07575 0 0 0 0.02707441
0.02318236 0 0.02859728 0 0 0.02144519 0.02515186 0
0.03817933 0.01387836 0.04934844 0.009537543 0.1014114
0.00943871 0.009351314 0.193675318 0 0 0.0225517 0.05231014
0 0 0 0 0.03727131 0.8694279 0.008156433 0.01141311
0.0111544 0.04469763 0 0 0 0.1725228 0.6825247 0
1.159960923 0.04569445 0.06127344 0 0.5922079 0 0 0
0.8130867 0.1432471 0.1109047 0.02237649 0.7570233 0 0
0 0.02498468 0 0 0.00984205 9.076789 0.01483432 0
0.06161802 0.7686143 0.1335216 0 0 0 0.01040341
0.1934158 0.457815 0.8480291 0.07354478 0 0 0.5709949
3.413022 0 0 0.01335644 0.1411446 0 0.05972192
0.1090775 0 0.177291993 0 0 0.01041812 0 0.02892765 0
0 0.008894632 0.01334106 0.4230172 0 0.03229883 0
0.03308406 0 0 0 0 0.137015 0.05420204 0.00984627
0.154547364 0 0 0.4098632 0 0.1547154 4.415529 0 0
0 0.03012669 0
DGCR5 0.00585202 0.02642761 0.03020933 0.015917229 0.0256939015
0.102328665 0.02411123 0.025912905 0.030368175 0.0456245185
0.077452745 0.04455551 0.06138627 0.08086551 0.022261165
0.0137897045 0.039728895 0.035743157 0.0097354615 0.04497115
0.0056985745 0.054077595 0.05366804 0.15375595 0.01887278
0.078525705 0.023287805 0.040939519 0.003769574 0 0.011024985
0.04073107 0.95188035 0.0750155 0.08762464 0.0153259645
4.33736215 0.059206775 0.030495445 0.075610092 1.38578015
0.028019465 0.011166754 0.031719525 0.03892484 0.20689785
0.103580755 0.04752837 0.1502278365 0.029346965 0.0878214
0.11051867 0.228397 0.222309175 0.1722858 0.12272105
0.018857445 0.07308075 0.057051745 0.034601049 0.0417157075
0.0615210385 0.0048569715 0.092360845 0.049560321 0.03662774

0.122335365 0.04362213 0.0150285345 0.03339119 0.026383435
0.10228852 0.06642007 1.4495085 0.05592152 0.019551475
0.018428835 0.3539264 0.008182183 0.005812924 0.17541717
0.04395002 0.097353805 0.0422062095 0.010273555 0.00849921
0.06297997 0.03676567 0.222770115 0.2270342 0.017313071
0.034584577 0.0125677795 0.060318535 0.062783205 0.21219468
0.103455905 0.04083127 0.059558015 0.039386925 0.05750725
0.03202964 0.20450285 0.031883265 0.1400776 0.058376505
0.01266312 0.02833364 0.0317021855 0.07998462 0.02077249
0.032635265 0.1740428 0.1144342 0.13875066 0.1758017
0.26018774 0.16467125 0.110851815 0.008248772 0.142570155
0.37087605 0.2253941 0.05296877 0.057899085 0.0909948
0.10788399 0.023207265 0.232509 1.1606182 0.08612163
0.12419885 0.03390875 0.038254831 0.0174433 0.042476
0.048015345 0.02669531 0.2120188 0.0373285 0.0393118875
0.21955315 0.21367925 0.48938865 0.311927945 0.120953185
0.0311316765 0.04113973 0.46496605 0.01540392 0.037108385
0.051751425 0.03056955 0.03821109 0.08850172 0.033945295
0.039657005 0.03773261 0.55485585 0.181396 0.0876009145
0.084149765 0.0354965345 0.0294293 0.018225275 0.05575275
0.47481545 0.02233747 0.09222223 0.8549123 0.03187499
0.860055965 0.62501815 0.044420585 0.016545871 0.04372696
0.06377008 0.067888165 0.048018725 0.13008585 0.031390525
0.04696 0.06517845 0.077856425 0.464976275 0.133088575 0.17832825
0.03882433 0.38661995 0.03506926 0.042524815 0.0486522
0.47155935 0.0224916 0.110987605 0.939992 0.032197225
0.111193045 0.11083464 0.01708538 0.10831157 0.04243197
0.0353067905 0.01930979 0.44936271 0.08004513 0.056422115
3.637982 0.46309815 0.5198546 0.139118 0.017884615
0.131370675 0.15697419 0.33674825 0.15185345 0.071327715
0.047204335 0.04573198 0.070952619 0.06075896 0.5799659575
0.0862536225 0.257870955 0.1521096 0.06567805 0.04633308
0.059627235 0.032829585 0.5833293 0.104721285 0.011437159
0.068706515 0.02097098 0.081050355 0.012287295 0.01826418
0.218649155 0.19919131 0.83252925 0.19604205 0.125042905
0.040204853 0.021754705 0.02365789 0.04332383 0.24794355
0.060776175 0.07472865 0.04016684 0.3499169 0.04384985
0.10506158 0.223979955 0.09773558 0.033768075 0.01472991
0.115178411 0.0540346 0.042146995 0.020778345 0.020720835
0.33871898 0.080366715 0.047586455 0.0612059 0.01372689
0.031395205 0.24321387 0.27114645 0.0466112 0.079535655
0.05423419 0.009955412 0.166368215 0.03456414 0.101225165
0.033378165 0.006362074 0.0116296045 0.058638955 0.03653226
0.030211385 0.018874465 0.021056065 0.14159952 0.03371067

0.41140805 0.028812805 0.3979139 0.04617549 0.037323583
0.028449965 0.188775765 0.26653553 0.4595003 0.18688735
0.11151162 0.03110013 0.02379566 0.024713795 0.27636947
0.755023625 0.026976635 0.0365623 0.02059259 0.215296788
0.0178103905 0.046098615 0.07910532 0.5318661 0.031292105
0.00571425 0.0305202355 0.066447205 0.04774306 0.0171755405
0.2752174 0.07324077 0.102871 0.062911505 0.139261355
0.0121040835 0.02634108 0.011032845 0.063609035 0.07087265
0.010351068 0.079371935 0.121863965 0.03997886 0.1272586
0.04279572 0.05758425 0.030644505 0.030168395 0.05910004
0.038249815 0.316995535 0.09849435 0.1269148 0.2323196
1.29835965 0.0144367845 0.011676295 0.10598639 0.187212803
0.019045195 0.3966317 0.12020593 0.027318555 0.1650832
0.09606419 0.0094924165 0.070899145 0.045879165 0.053457915
0.18625535 0.055635855 0.062766285 0.5484109 0.0328487
0.0468837 0.027628635 0.08585444 0.12764748 0.065146035
0.13960375 0.73031215 0.04689828 0.18151107 0.087797035
0.63737475 0.1569623195 0.01522293 0.1346308955 0.5389299
0.1871277 0.269491875 0.077810195 0.107450552 0.64376035
0.24424705 0.34197085 0.037993065 0.05281751 0.030868706
0.303661235 0.04788777 0.20723642 0.076121255 0.080839855
1.4849589 0.048845665 0.1142788 0.133454901 0.14088408
0.03340142 0.003762312 0.038456352 0.5817619 0.4863658
0.24935006 0.21597906 0.0179535825 0.120420335 0.044975505
LINC00957 1.381374 3.48473 1.147038 1.406859 0.8037242
2.656398 1.129437 1.661998 1.493652 2.592699328
2.316121 1.309433 1.909087 2.076297 1.752562
2.225972 1.115068 2.950262959 0.7897647 0.9069099
1.476004606 2.360691 1.456502 2.57226 5.684011 1.208311
1.301743 3.055844 1.1425589 0.8049607 1.594891
2.394572 0.6692982 1.472798 0.5032238 0.2827859
1.280788 0.3862005 0.8648136 0.732545105 0.2951593
0.8860576 0.285317273 1.637863 0.5700893 0.5537104
0.9506493 0.710618 0.963241399 1.644226 0.4529521
1.338798 2.035533 0.6061446 1.62439 0.5219951 1.341627
1.989113 0.4527239 1.420937703 0.488190355 1.092198042
0.476441 1.666774 0.2219028 2.086986 1.320128
0.961544 0.415826641 0.7359333 1.36342 0.1246627 1.222943
0.1954638 0.9745454 0.8845975 0.659364 0.1968872
0.2196787 0.21587818 0.3084117 0.9217975 0.2187821
1.68018 1.489019 0.4839971 1.122699 0.8568289 0.6812959
1.521738 0.341857576 0.5581566 0.5142592 1.728731
0.965136 0.5875503 0.6668937 0.8546436 0.1127665
0.5393681 2.012164 0.6996938 0.780229 0.9155322

2.418159 0.8838916 0.4574818 1.699531 0.793770367
0.6592508 1.601504 0.6862126 0.5942644 2.589411
1.237344 0.4224799 1.738061 0.1873656 0.8537083
0.4104937 1.490706 0.248649 1.04512 1.24737 0.6303363
1.892506 0.3146405 1.877705 2.11596 0.9054223 0.6851696
0.6486551 0.8313003 0.1678986 0.5527391 2.295044
0.5828321 0.3414523 0.8518157 0.4866367 0.5272569
0.6904794 0.6606549 0.3791665 0.7914201 0.6129015
0.9489311 1.217769 0.8242336 0.6773065 0.8395013
0.5677505 3.613982 0.9561945 2.180009 0.995764
0.5811336 3.32859 0.7096877 1.492606 1.271950308 1.241115
0.500198552 0.3911765 0.7350028 0.7168268 0.9603177
0.2310853 1.032138 1.713874 2.187523 0.340331641
1.284577 1.438492 0.7981737 0.8303861 1.128309
0.5805051 0.4343505 0.2487893 0.2757036 1.666566
1.378444 0.4657101 0.6242133 0.4715258 2.31976 0.5381612
0.3280283 0.1440587 1.963655 0.7023508 0.5393379
0.9778512 0.8225877 0.8278834 0.4464021 0.9504098
0.1916936 0.4833344 0.5789285 1.973148 0.3899396
0.1842891 1.789891 0.6501224 0.9455967 1.23818 0.3286537
2.365635 0.4881348 1.428234 1.386976 0.551323
1.036801 0.7994612 1.069902 0.600392 1.137467
0.69656596 0.7373227 0.249325222 0.953468944 0.2772299
2.254437 1.450524 0.5771759 1.652255 0.4983977
0.9668789 1.516027 1.287578 0.511777 0.3392588
0.5088514 0.8953325 1.509785 0.5287598 0.8618343
8.989527 1.722983 1.028733 0.7341784 0.4928742
0.3644596 1.352445 0.7517445 3.824824 0.9867141
0.5020669 0.9373612 0.5822873 2.632635 0.9451969
0.1612632 0.4728466 0.6626671 1.119884162 0.5551013
1.03792 0.9799955 0.2936052 0.4805213 1.204886 1.941322
0.6868302 0.7680125 0.4560999 1.660277 2.645352
0.8526362 0.6646801 0.585148 0.658275569 1.309494
0.5096728 0.7040323 2.047815 1.867128 0.230723091
1.835316 0.5376086 1.233277 0.7875463 0.4117831
1.282339 0.6017319 1.210718 0.8272901 1.053945
0.3946773 1.002682 1.761718 0.4764309 0.6918603
1.17529 1.262493 0.7010697 2.121886 0.2935698 0.7269886
0.8715531 0.453769 0.6091997 0.485355 0.6111878
0.610810788 0.320510214 0.9625691 0.9717921 0.4692624
0.8519513 0.2552703 1.536401 0.3390303 1.880733
0.7120717 0.6977235 0.4168508 0.5092524 0.3078894
0.693038459 0.4127454 0.5103552 0.3095326 1.119078
0.372341 1.129014074 0.74663 1.246573 0.3488467 1.261636

1.008779 1.376136 2.096498 0.8791895 0.7519586
1.284003 0.5968265 0.5475132 0.5339631 0.7986575
0.3610052 1.230724 2.469318 1.753201 0.808806772
2.746506 1.518484 0.7748159 0.6909516 0.8214536
1.269599 0.5443742 0.3509429 0.7317952 0.9399323
0.4879938 1.821239 0.7111216 1.434964 1.957556
1.237808 0.6664173 0.5556665 0.9440448 0.5465185
0.6868116 0.3655331 1.971451 0.4419952 1.107376
0.6593778 0.399386616 0.3849175 0.5142891 2.293074
1.601708 0.6487585 1.379563 1.064478618 0.8634605
0.7629578 0.2835394 0.257369 0.3414858 1.333854
1.892034 0.4394841 0.5344339 0.886996 0.3009581
0.9218481 0.703303 0.7239795 0.330847686 0.5100224
1.732559 0.839718 1.901447063 0.3785226 4.206885
0.2470125 0.9290814 0.5094705 0.6225625 1.84742
AP000721.2 0.0164537 1.793617 0.09080848 0 0.3237269
1.516352 0.05084384 0.3352318 0.2286151 0.267990945
0.1271116 0 0.07622622 0.04628059 0 1.730938 0.1312845
1.065410547 0 0.07028334 0.249947355 0.9668028 0.02093229
1.751984 0.3718835 0.01293296 0.0260447 2.328845
0.158979548 0 1.022939 0.5162082 0.03176474 0.259107
0.04018555 0.03606881 0.01712093 0.01477775 0.2036407
0.043683784 0.06643592 0.04351379 0 0.02115508 0 0.06053547
0.07658119 0.05305639 0.033507175 0.0131416 0 0.0776187
0.1412143 0.07422011 0.2887264 0.06068006 0.0241584
1.23129 0.03299662 0.044612418 0 0 0.1638719 0.07701515
0.04528526 0.211838 0.08980262 0.116407 0 0.03448172
0.01405266 0.0357761 0.1953192 0.01534218 0.05121433
0.0892711 0.03832435 0.04018015 0.1245316 0 0 0.05391908
0 0.3214558 0 0 0.1692983 0 0.05636628 0.2041476
0.015855744 0 0.0140556 0.06944767 0 0.03361837 0.03189789
0.06930322 0.01380783 0.08338835 0.02060049 0.01437498
0.07582229 0.03947304 0.1867568 0.1489392 0 0.4506126
0.014550025 0.04387107 0.04982168 0.05284536 0 0.4534663
0.1748171 0.0130634 0.03681989 0.05214139 0.09724032
0.1032809 0.03405434 0.07080493 0.06064977 0 0.1739765
0.03191875 0.03009885 0.0177955 0.7315517 0.1827454
0.02369955 0.0187324 0.1339336 0 0 0.1887444 0.06260139
0.01771591 0.01810792 0.04317885 0.05604219 0.03465024
0.02559959 0.1385896 0.1397688 0 0.2135901 0.3235432
0.02900123 0.0160724 0.01520618 0.06207044 0.01490965
0.01635229 0.4689378 0 0 0.1558597 0 0.1651481
0.148801423 0.05453463 0.033652426 0.07484074 0.02026989
0.05224567 0.02624716 0.015378 0.02240804 0.04051302

0.9915482 0.018941963 0.1228841 0.01674314 0.01708625
0.01303559 0.07958362 0.2843226 0.01582874 0.07615825
0.04964538 0.03323529 0 0.04598745 0.06018311 0.1626382
0.6280797 0 0.02713907 0.02845072 0.155215 0 0.05003021
0.02515424 0 0.01439932 0.02102317 0.1307574 0.0670633
0.1345056 0.0126585 0.1483063 0 0 0 0.02551443
0.0396523 0.1326591 0.04191917 0.3396902 0.06313759
0.279811 0.04581744 0.1270301 0.04737028 0.05758294
0.03521651 0.09124292 0.2068492 0.017058372 0.01535414
0.017345982 0 0.04714688 0.04304272 0 0.05096611 0.1915836
0.02007464 0.08221573 0.07425272 0.05740015 0.04292133 0
0.07886953 0.1612207 0.02370095 0.02790717 0.01857893
2.133595 0.1355549 0.032975 0.174558 0.1223322 0
0.06320678 0.02739531 0.3128821 0.1438326 0.05866048
0.01479076 0.1671067 0.09192667 0.02982881 0.03526083
0.1206214 0.08168564 0.017055419 0 0.1270894 0 0.02304541
0.1050679 0 0.1359438 0.05005937 0.1424852 0 0.1229605
0.136098 0.09000178 0.1117961 0.02296447 0 0 0.08211514
0.04685113 0.6189662 0 0 0.7220866 0.04635784 0.07550502
0.09097364 0.02401014 0.1914848 0.01297179 0.08297451
0.04341366 0.2677418 0.09293604 0.9037575 0.5826491
0.08414012 0.05042599 0.3904528 0.01577424 0 0.01333762
0.01404161 0.08166697 0.05444815 0 0.02983768 0.02777097 0
0.053422474 0.045634023 0.08666393 0.04423504 0.03847727
0.08279222 0 0.06413401 0.06354027 0.3838143 0 0.08399371
0.1007404 0.0820474 0 0.039287005 0.04622479 0.03396253
0.1015208 0 0.07598623 0.021600545 0 0 0.06674212
0.008981542 0.03250558 0 0.4027848 0.06295521 0.2431544
0.07416101 0.06583706 0.0330794 0 0.1555791 0.01700143
0.01837775 1.058748 0.06408144 0.028957696 0.08876676
0.02182308 0 0.1208634 0.01687649 0.0420914 0.01960487 0
0.04666955 0.04591402 0.189692 0.2245004 0.1654918
0.1514706 0.02736248 0.04942335 0.07200023 0.06148125 0 0
0.01964894 0.06921362 0.01516311 0.193288 0.06017483 0 0
0.04531893 0.0613371 0.03065264 0.04085898 0 0.06624401
0.059789618 0.07851049 0.01962632 0.0491297 0.0125055 0
0.3834989 0.09734132 0.04862635 0.04544403 0.1086093
0.02002782 0.1727706 0.01993444 0.01448506 0.1033445
0.01472885 0.34969 0.06346931 0 0.1034568 0.05681439 0 0
0 0 0.3918179

AC012254.1 0.1452862 0 0 0.1387861 0 0.3678403 0 0 0
0.091013848 0 0 0 0 0.05381754 0.1449053
0.109390408 0.0896947 0 0 0.1446927 0 0.1738204
0.0497535 0.2283961 0 0.114881 0 0 0 0 0.07012066

0 0 0 0 0.1957312 0 0.128575936 1.055932 0.0640378
0 0.1867994 0.2992139 0.4810754 0.6762119 0
0.443802528 0.5802014 0 0.8224478 0.6234613 0.1092273 0
0 0 0.2978711 0.07284005 0.295445739 0.494841949 0 0 0
0.1999344 0.1476734 0.09911965 0 0.291942704 0.3552192
0.1861274 0 0.1437224 0.270943 0.1130556 0 0.6204073
0.4730544 0.3298841 0 0 0.3174037 0.1556232 0.2240885
0 0 0.1494904 0.2693033 0.24885714 0 0.140006216
0.08111276 0 0.2452892 0.3344567 0 0.1408292 0
0.06096157 0.07363191 0.5457069 0.1903965 0 0.08713674
0.3747869 0.1643917 0.314383 0.1808595 0.064238362
0.9684538 0.1759702 1.224889 0 0.7207393 1.543635 0
0.08127992 0.2302042 0.107329 0.1519951 0.07517496
1.953774 0.1338844 0.1348657 0.2094835 0.3523033
0.1993297 0 0.2018626 0.06723507 0.209267 0 0.1478293
0.2195951 0.1623971 0 0.6218665 0.2346472 0.4796787
0.3812693 0.2969115 0 0.3390665 0.9178093 0.1371287
0.1174945 0.3772001 0.2484249 0.3841213 0.1419193
1.409841 0.06851025 0.4607827 0.8663442 0.1592585
2.052877 1.038548 0.3440603 0 0 0.218986094 0.401284
0.22286319 0.1982531 0 0.1729985 0 0.06789385 0
1.967515 0 0 0.07750474 0.07392096 0.2263074 0.2877607
0.07027234 0 0.4891872 0.5884177 0 0.07336689 0
0.0676782 0 1.436095 0 0 0.6590044 0 0.1245953
0.1531011 0 0 0 0.127146 0 0.5772938 0.1480422 0
0 0.1190495 0.1424333 0.5337145 0 0.4505848 0.08753246
0.33468 0.06169104 0.06816964 0.3716699 0.231631 0.134856
0.08011969 0.2091398 0.1906715 0 0.4603857 0 0
0.06778854 0.459495052 0.076249306 1.144844 0.6967899
0.1766111 0.3750256 0 0.08862953 0.2177895 0 0.3378956
0.09474884 0.1146271 0.4352612 0.2033684 0 0.3696304
0.9843119 0.07417183 0.3808477 0.1455846 0.09633419
0.1800323 0.3788979 0.139529 0.1209503 0.07270392 0
0.05179721 0.1959037 0.3279006 0.6764271 0.1316942
0.05189219 0.2662718 0.7212842 0 0.2046053 0.3506872 0
0 0.1855499 0.07504165 0 0 0.1258144 0.07902809
0.06031895 0.6008729 0.3973579 0 0.3041644 0 0.3539562
0.2900307 0.4136955 0.05814332 0.2336503 0 0.0777564
0.9210144 0.1666773 0.1338829 0.05300237 0.1409009
1.088139 0.3663324 0 0 0 0.07528471 0.1196463 0
0.1113154 0.2462642 0.2785731 0 0.1766568 0.1859811
0.09013992 0.3205181 0.06194634 0.1976 0.1226088 0.2429472
0.393100371 0.302211316 0.6377021 0 0.05662572 0.09138192 0
0 0.4675505 0.1210386 0.3181372 0.05297603 0.2965126

0.07244785 0.1935407 0.173452125 0.27211 0 0 0.2607646 0
0 0 0.530628 0 0.158614 0.2870243 0 0.118553
1.598197 0 0.1309683 0.5813412 0 0.406564 0.6868818
0.3753065 0.08113776 0.2174126 0.2121897 0.063924114
0.3359188 0.4817445 0 0.133403 0.3725485 0 0 0
0.103023 0.8784117 0 0 0.05620356 0.4776734 0
0.0727347 0.8476827 0 0 0.158843 0.2602503 0.1527891
7.43091 0 0.2656719 0.414488 0.287877873 0 0 0.5413257
0 0.5010269 0.2924673 0.065992791 0.1733119 0.5199012
0.795328 0.2208471 0 0 0.4297619 0.1431236 0.06687847
0.4795101 0.2652684 0.6356519 0.2640317 0 0.547519161
1.235529 0.9005976 0 0.105753321 0.1827048 0.6688947 0
0.7744118 0.2065648 0 0.05766254

EPB41L4A-AS1 1.650979 5.400856 2.106106 2.925964
4.906651 3.920403 4.202204 5.118518 2.964688
7.593559674 3.065555 3.456975 5.649235 3.091818
3.229892 4.525566 3.769964 5.194735906 3.503029
3.526147 2.954762727 5.880304 3.078693 4.173978
2.996518 3.838475 5.075398 4.47981 2.611115473 6.615049
2.9344 6.048153 1.367185 2.599903 3.112618 10.06703
22.70831 2.797833 1.680386 1.472625107 1.652526
2.091187 1.641512861 4.063891 2.232472 2.097192
2.239547 1.900664 4.459259855 1.33252 3.039358 2.93907
2.428404 4.383462 3.710331 5.645392 2.385801
2.850441 4.060223 5.766375177 2.722814552 3.187698058
2.881862 1.911606 1.913248 2.293412 5.009336
7.166314 1.038910938 1.869575 2.133638 8.889494
2.578751 3.309815 2.075842 7.991072 1.942989
6.090009 13.90301 1.557706938 6.594941 2.928201
2.522361 3.301464 4.773187 3.348518 3.057758
3.151536 3.944207 3.200068 2.114328326 2.299489
2.865241 3.311844 3.565605 1.242794 3.647072
1.706462 2.935052 1.809971 4.090626 2.668436
3.794028 4.148376 4.308256 3.293733 2.055774
3.612863 2.808507349 5.965953 2.817944 3.314095
3.392962 4.602807 4.216068 1.883403 1.468094
3.24241 4.095449 2.527192 3.426036 6.394168 4.071782
2.274648 1.708109 4.214154 5.92505 4.722487 5.102905
2.561527 2.20594 6.544065 3.510061 4.841949 2.15623
3.527487 2.96304 2.736615 3.294444 2.337327 3.903771
3.50886 2.517988 2.941345 1.964254 5.46715 4.698082
2.830439 1.236754 5.729399 7.263619 2.388844
1.913366 1.990554 3.730637 3.151346 4.798316
4.275032 3.136767 4.113707 4.34173985 4.492845

3.678842131 1.221296 7.118647 3.252344 2.190099
3.561179 1.92301 22.16021 2.245224 2.130732911 4.088468
0.9195909 2.350601 3.98941 1.471012 3.466046 2.499436
1.628908 3.657442 4.361644 2.238161 3.323194
2.022213 1.170263 3.503107 3.521172 0.6772066
6.881501 2.235561 0.9110983 8.666239 2.510715
4.773895 2.627328 2.670163 2.783751 3.492982
5.916097 1.627817 4.040705 2.444879 3.067936
3.395031 1.542822 1.512971 2.867399 2.021931
4.203522 1.405988 3.588064 1.867175 1.169211
4.323723 2.512637 1.711034 1.882708 3.545721
1.558504677 2.501527 5.024582055 1.135530938 6.940523
5.114541 12.20363 1.875128 3.95603 6.48289 5.301454
2.534179 4.036761 4.567438 1.220312 4.441122
5.218004 7.416151 6.51422 2.874834 2.462043 3.800667
2.007013 3.186173 3.747083 4.45069 1.760802 5.584548
6.83556 2.259794 2.850086 3.354889 7.241464 1.917622
3.717683 2.427015 3.381481 5.568527 1.787916898
4.123923 3.20904 2.786434 3.024373 2.480287 8.603758
2.933269 1.394544 1.316837 4.192459 1.976961
3.333191 3.822272 6.801482 5.79101 6.305698676 1.315541
4.102408 2.416523 3.310552 2.995158 1.67846909
3.301857 2.601217 2.502154 3.867549 4.101978
4.955128 1.66468 3.512877 2.304182 3.312227 3.547816
1.913636 2.250524 1.321949 2.563183 3.387605
4.315217 1.866149 6.011825 1.40524 2.258889 2.496859
2.397086 1.859394 4.088184 1.95433 3.329127548
2.006307185 3.295303 4.464865 1.524018 6.640492 2.6824
4.995798 3.411553 4.386925 2.050196 1.574706
1.303449 1.772199 2.940152 3.584193141 1.346716
8.367123 3.77533 6.441134 4.663001 2.578087094 1.567945
3.11014 9.979358 3.775621 2.15869 2.863517 4.077032
5.111769 5.611617 2.19325 1.99923 2.620434 3.130689
4.002507 3.699194 2.863115 2.737162 2.791969
2.091297277 3.874183 4.765582 2.335709 3.48667 3.190727
4.879241 2.650503 5.862552 3.851041 2.299486
1.020566 2.884983 4.178291 2.976888 6.965094
4.128303 3.415827 5.379009 9.498937 3.909323
2.288085 2.391137 2.99091 4.282608 2.341048 3.235088
1.321448968 7.997738 2.456449 1.570233 1.467307
4.258879 5.409421 2.987831524 1.24041 9.359466 1.682159
3.199773 4.403894 3.967198 6.939935 9.420304
1.271971 2.226194 6.922575 2.912137 3.431991
4.111716 2.183090752 2.197413 2.262267 4.240126

1.429440825 2.338446 4.385621 3.99971 3.986527 5.394735
5.839758 3.176268
SEPTIN4-AS1 0.3320827 0.03528295 0.254552 0.3172255 0.1814926
0.06539383 0.1140193 0.07738815 0.1993749 0.161802396
0.03167254 0 0.3133906 0.008648839 0.09023192 0.0273359
0.165606 0.07639965 0.2277961 0.1182099 0.183245897
0.02756051 0.02347075 0 0.07581486 0.03625334 0.2190235
0.01458807 0.267388459 0.0418626 0.1564078 0.07441826
0.6411031 0.5520049 0.8260788 0.2426572 0.2975562
0.09941904 0.1712521 0.204088788 0.4097092 0.2358217
0.182421901 0.1482535 0.2279725 0.1696915 0.1932034
0.05949053 0.187852922 0.4199553 0.1064866 0.174063
0.326575 0.2774026 0.1618701 0.2721549 0.1354404
0.1985807 0.08324577 0.662798907 0.282766828 0.125799992
0.2480552 0.09714911 0.07616549 0.08751015 0.5538114
0.1025543 0.064876156 0.2770871 0.1102977 0.2473737
0.4745121 0.1720273 0.3301942 0.1334627 0.2076977
0.112632 0.1675602 0.081326651 0.03207838 0.1914498
0.09880835 0.1802194 0.1651805 0.4019183 0.1518632
0.1937844 0.22120634 0.2601188 0.231121373 0.1957006
0.260042 0.2413957 0.1274121 0.1413572 0.2235384
0.1457018 0.06967036 0.112201 0.05774676 0.3223645
0.1275259 0.3983394 0.2474783 0.09045891 0.119765
0.06889885 0.236560319 0.1721696 0.1675906 0.4147774
0.2590148 0.07118412 0.4573733 0.183095 0.2477102
0.102313 0.2112507 0.09650484 0.3150189 0.2480982
0.272019 0.3596418 0.336947 0.1878951 0.1771819
0.1197214 0.1538 0.2646714 0.09965098 0.1365265 0.1689477
0.1022453 0.2062186 0.1646037 0.1491602 0.09932157
0.1827348 0.2299719 0.4398689 0.1651221 0.2081043
0.1165472 0.1567186 0.2685589 0.1436953 0.1735031
0.2194979 0.1351613 0.1875525 0.1043966 0.05851209
0.06417365 0.08763431 0.4634361 0.1895763 0.1638383
0.1813982 0.2777634 0.208558185 0.1732528 0.179233888 0.1007
0.2613722 0.1025176 0.206011 0.3621005 0.2010037
0.1817041 0.06092017 0.159292887 0.1476281 0.09386789
0.8525441 0.2046298 0.2052399 0.2125349 0.1952312
0.1494394 0.1484423 0.1397465 0.08264358 0.2836039
0.312102 0.2849395 0.2443305 0.1747327 0.136936
0.1754551 0.3876298 0.2819005 0.3926816 0.1410234
0.07930995 0.1372369 0.2651923 0.2932286 0.1127941
0.04309059 0.05677438 0.2116436 0.08139048 0.1731984
0.08726688 0.2145642 0.1333828 0.1806209 0.06267027
0.5020748 0.5899523 0.1078494 0.239744 0.142435

0.2390169 0.225981 0.2171796 0.8330789 0.1932782
0.124325746 0.1549452 0.398715283 0.193649032 0.1519851
0.2976188 0.1794145 0.1047691 0.1074085 0.05627272
0.2212464 0.124886 0.8045133 0.060158 0.1164465
0.1658138 0.4261052 0.09301307 0.2659774 0.291648
0.2543034 0.310896 0.3235214 0.1712608 0.1371675
0.2020789 0.1683207 0.2303816 0.06462571 0.4703859
0.342026 0.04146109 0.1873717 0.9448505 0.4013537
0.03953691 0.0901661 0.251877 0.25817031 0.1212476
0.285003 0.2535466 0.1292006 0.5419234 0.06670369
0.1959811 0.1403252 0.3754462 0.1806356 0.2680842
0.4768832 0.1597841 0.1378889 0.1029975 0.092738723
0.2825229 0.598476 0.2188865 0.06644951 0.1285695
0.208981407 0.0691168 0.2469033 0.2222365 0.17001 0.1211483
0.393628 0.1527212 0.2480982 0.3164098 0.2101475
0.09676307 0.04779982 0.06077273 0.2673074 2.007211
0.1094507 0.4068051 0.2696759 0.1719833 0.07084993
0.1716951 0.1221021 0.02359861 0.3429249 0.1089856
0.2930791 0.339439051 0.434928455 0.2915209 0.03719956
0.1510019 0.1044365 0.1664752 0.2277198 0.2849832
0.09221988 0.09089633 0.2085406 0.2635667 0.3219905
0.2089011 0.143166834 0.05183047 0.1142435 0.3794407
0.1655648 0.1874424 0.15743026 0.2512469 0.2611026
0.0935449 0.09063659 0.1913495 0.4932088 0.0602174
0.09706095 0.09088058 0.2494635 0.5075201 0.3276366
0.5937125 0.1370648 0.1143791 0.6800117 0.04141192
0.05388944 0.30034187 0.02843757 0.391513 0.3598098
0.1185805 0.5960776 0.1651854 0.2088323 0.1698752
0.1177406 0.2659904 0.132935 0.08390852 0.3283002
0.3154161 0.1227229 0.1015977 0.2556504 0.1148951
0.08853337 0.1916201 0.2203177 0.2004851 0.272031
0.1674716 0.2361528 0.1403557 0.383837164 0.09316036
0.3324146 0.7131751 0.4581394 0.08748089 0.1671242
0.217880962 0.2640943 0.1870544 0.2203506 0.1051653
0.1465137 0.2023556 0.1364324 0.05452327 0.1358801
0.1750592 0.46036 0.5488804 0.1676392 0.09744994
0.533034527 0.1156051 0.1470363 0.1067493 0.228292884
0.3132082 0.1061738 0.1777731 0.1264346 0.06994788
0.1615078 0.2562779
C5orf66 0.09175969 0.1211691 0.1768467 0.07930637 0.1862687
0.2146196 0.1552763 0.07331509 0.09781363 0.098541309
1.170217 1.128864 0.08097173 1.302788 0.1353479
0.1828987 0.1786802 0.179301572 0.1213913 0.1446516
0.079141804 0.1349015 1.036728 0.1019398 0.2064957

1.518061	0.1625385	0.1589331	0.168876922	0.04461672	
0.1420018	0.2624223	0.4998074	0.5367139	0.147627	
0.05747144	0.0181868	0.2119195	0.2011085	0.098607109	
0.5557539	0.2965961	0.197834277	0.3061824	0.5084386	
0.3617108	0.3635274	0.2888422	0.3003175	0.2809396	
0.09331587	0.3194972	0.08203438	0.1872467	0.3365195	
0.2390308	0.2822864	0.1410968	0.4819492	0.24583455	
0.339816276	0.148973674	0.1936572	0.2812211	0.2405226	
0.1095522	0.1729004	0.4570783	0.151458997	0.1953515	
0.1679345	0.1266779	0.2463813	0.2363112	0.261813	
0.1738527	0.2714017	0.471276	0.2695294	0.169073827	
0.1747427	0.2768333	0.3159267	0.3661798	0.1541855	
0.112114	0.3799079	0.2213822	0.32931471	0.1429284	
0.252642796	0.3537248	0.1381084	0.2471335	0.1735152	
0.09151021	0.300718	0.1886455	0.5976984	0.2812407	
0.3911583	0.334029	0.2617636	0.3275817	0.3516798	
0.3444397	0.2237714	0.1903784	0.162286389	0.1922344	
0.2804938	0.3578624	0.09201843	0.1734109	0.5184137	
0.3868122	0.09044682	0.2302042	0.1404154	0.26742	0.3391351
0.5241402	0.3885666	0.1500761	0.346514	0.2606514	
0.1558668	0.1086944	0.3500975	0.2466971	0.2564701	
0.261169	0.2356376	0.3478298	0.1758285	0.501237	
0.272229	0.2023024	0.2765065	0.2666018	0.3333743	
0.3680741	0.3093238	0.4738564	0.1629048	0.1113106	
0.2836091	0.1643714	0.2002437	0.4503004	0.3715155	
0.3317338	0.2058922	0.1910734	0.3783886	0.2126623	
0.2830629	0.129346	0.196401	0.285073	0.141600031	
0.3210272	0.364177443	0.2245875	0.07266984	0.2480078	
0.139406	0.359378	0.3243168	0.3792475	0.07033875	
0.246484784	0.4009268	0.464646	0.1883059	0.1159697	
0.2219127	0.3020233	0.4623896	0.3286544	0.05273608	
0.2956741	0.8196395	0.3846971	0.2157632	0.2348501	
0.3301866	0.2335243	0.3657632	0.2795529	0.116164	
0.2716682	0.2015077	0.1636614	0.1502715	0.4225452	
0.1521366	0.3971608	0.2248461	0.1760477	0.1596779	
0.2810642	0.2977178	0.5154336	0.2066847	0.3421735	
0.09213943	0.1233032	0.3803508	0.4571979	0.3129852	
0.3204519	0.3873308	0.1614442	0.3522354	0.1835034	
0.04442311	0.1575004	0.1625062	0.167613253	0.3098905	
0.377730268	0.094021701	0.3646598	0.4591288	0.1983002	
0.196254	1.042993	0.2025818	0.36462	0.2538801	0.3937881
0.2621622	0.3378482	0.3534452	0.3119335	0.2234412	
0.1167254	0.2590294	0.3836858	0.2732613	0.3283863	
0.3186996	0.1137046	0.1435823	0.2077198	0.2764579	

0.2689499 0.2196313 0.1573389 0.1924667 0.4215864
0.4841794 0.2099186 0.2996481 0.1548247 0.3290068
0.144937718 0.3117795 0.1814081 0.2041717 0.09486047
0.3208757 0.16701 0.3481242 0.2625558 0.3254147 0.2709534
0.1360578 0.5059982 0.2708805 0.2434499 0.2043009
0.377388918 0.168804 0.3445477 0.7257816 0.2867821
0.1756769 0.062520753 0.308687 0.4201118 0.08521849
0.2033409 0.2327322 0.3262969 0.2635303 0.2350404
0.2747769 0.2417487 0.4530627 0.1200027 0.1151483
0.2178597 0.2711743 0.1814578 0.2953293 0.1768724
0.393161 0.1510222 0.3171841 0.3060586 0.542147 0.2912
0.1622493 0.2831339 0.224628784 0.239345303 0.2685061
0.563867 0.1106969 0.1731447 0.1431871 0.124899
0.5343434 0.3057817 0.2583369 0.22943 0.3121185 0.1830262
0.2648452 0.320821224 0.2127777 0.2164614 0.05392052
0.2431188 0.2865446 0.140539969 0.1368635 0.4029581
0.2348469 0.2039323 0.2460208 0.2695675 0.3636814
0.3030252 0.7899435 0.05120567 0.3759049 0.1830145
0.1100474 0.06787661 0.2054309 0.1415335 0.225586
1.218894 0.201865624 0.2407839 0.2492032 0.1852567
0.2968969 0.2554618 0.09128664 0.1275555 0.3182113
0.2912531 0.5608829 0.2581737 0.2484134 0.2298852
0.244224 0.08356461 0.7459408 0.2262612 0.8462933
0.2788801 0.1839234 0.2791657 0.3339149 0.1812049
0.3406466 0.4980515 0.3261885 0.242423472 0.3269528
0.1275965 0.1282087 0.2712668 0.2486299 0.4991735
0.194505069 0.187646 0.2684202 0.1804846 0.4051631
0.2618318 0.1857146 0.2197279 0.275486 0.1307399
0.2938351 0.3217792 0.4855807 0.3890993 0.4981487
0.230534383 0.3970122 0.2515095 0.08427579 0.108138734
0.2280375 0.3545645 0.3886537 0.3260681 0.2319324
0.4119429 0.2913475
AC090948.3 0.1845741 0.2017084 1.01867 0.4030088 0.830057
0.5874764 0.488876 0.4424186 0.366365 0.59464583
0.2489686 0.2072125 0.447904 0.09888872 0 0.273483
0.3155832 0.436767511 0.1302282 0.6194756 0.18486962
0.2626002 0.0670897 0.2208245 0.1083561 0.2279816
0.2086884 0.229345 0.254771218 0.05983085 0.1490274
0.1181778 0.6872079 1.41178 0.3649276 1.271638 0.05487396
0.2841833 0.1223784 0.653380438 1.533114 1.185455
0.603774845 0.3898716 1.276139 1.358148 1.687462
0.2550751 1.396112007 0.2948392 0.1217542 3.134556
1.131509 0.5154099 1.54232 0.5834536 0.890441 1.270412
0.2115137 0.39321272 0.658591826 0.287673302 0.2888726

0.1542749	0.3265716	0.2322751	0.5396714	0.6395898	
0.264920784	1.178845	0.3828393	0.9746552	0.4955945	
0.4179701	0.08207305	0.8106767	1.06455	2.253662	0.9180079
0.129148493	0.2292352	0.8352728	1.440432	0.5151461	0
0.3318926	0.4069611	0.782006	1.1742805	0.4163789	
0.330323196	1.383775	0.3829194	1.469064	0.6676995	
0.8619969	1.226824	0.805194	0.2434037	0.7750728	
2.343932	0.4146567	1.154329	0.9172288	0.7618173	
1.054175	0.4184166	0.6564773	0.16321907	0.2109155	
0.3832381	1.312646	0.8329255	3.575355	0.6536868	
0.8792546	0.3245296	0.4804625	0.4674947	0.1655119	
0.3820143	2.467925	0.6074608	0.2447653	0.3041503	
3.017915	0.2894079	0.08555405	0.781561	0.2684522	
0.3608053	0.1500971	0.7512195	0.6110934	0.176839	
0.7393726	0.3762048	0.3690761	0.2321494	0.4151753	
0.7184792	0.7773979	0.758951	1.193763	0.6470685	
0.5330968	1.30069	0.969353	0.7668483	1.571158	0.65795
0.2984113	0.1672533	1.624723	0.5780708	1.904262	
1.201587	1.654737	0.3703684	1.146845	0.516664107	
1.689617	1.024658345	0.5756897	0.03248333	0.8163267	
0.5047457	0.6160967	2.549595	0.5843139	0.6530122	
0.394618496	1.603546	0.1609894	0.5476282	0.313351	
0.4591296	0.7593976	1.242944	0.6712569	1.750292	
0.3994568	0.2952896	1.793288	0.7956786	0.5212686	
0.6984039	0.2140555	0.9568122	0.3875443	0.3618012	
0.1667163	0.08017546	0.3627962	0.3627243	0.6691893	
0.2526787	1.30965	0.5642263	0.9237887	0.9940024	1.361183
0.3877496	0.258301	0.2494471	1.451521	0.2224055	
0.8503666	0.06717718	0.3216716	0.8768982	0.9808936	
0.9300414	0.3489787	0.6579109	0.5306043	0.05643589	
0.7102139	0.6077213	0.492061273	0.5167185	1.084108107	
0.083030098	0.2833305	5.288292	2.884755	1.034553	
0.3070206	0.3538747	0.7114719	0.1487413	0.6745647	
0.4814823	0.3952658	0.9479373	0.8119972	1.253398	
0.7826415	2.113918	0.7538335	0.9479226	0.9247662	
0.94411	0.3920848	0.4676053	0.4811344	0.790238	1.081982
1.114072	0.7896489	1.01922	0.7736311	0.7365812	1.195047
0.941782	0.6443358	0.458166	0.27332005	0.9654697	
0.4073318	0.8455387	0.1107936	1.212304	0.4630519	
1.182644	0.4813331	0.7991841	0.344224	0.5911476	
0.6815708	0.8653892	0.07166312	1.21445	0.481978184	0.3303713
0.6842829	1.126213	0.8230814	0.735016	0.125760136	
1.495858	0.668613	0.2117498	1.239206	0.5771583	
1.125162	1.496724	0.13297	1.089964	0.4719738	0.3829726

0.4645517 0.3908591 0.5618249 0.6868826 0.6704107
1.415616 0.5929631 0.5984743 0.7875786 0.7852479
1.017979 0.3372759 1.004138 0.8233262 0.3086443
0.428058481 0.950695006 0.879589 0.1772211 0.1233228
0.1658474 0.5509948 0.274073 0.814607 0.4613086
0.7505958 0.2884357 0.6995759 0.3155623 0.7025072
0.566631263 0.395078 1.687217 0.1084608 1.609074
0.3409589 0.103847268 0.3949957 0.3852109 0.5080456
0.2734725 0.5990524 0.9217971 0.3872875 0.6053298
0.4935755 0.7130763 1.318832 0.1413629 0.2951463
1.353462 0.9808374 0.3534131 0.7102408 0.6931786
0.44085596 0.6502965 0.4546409 1.028494 0.3873772
0.4868148 0.2023596 1.036781 0.7504387 0.3365544
2.305478 0.6079784 1.199237 1.326037 0.6935366
0.526194 0.1584059 0.9999887 0.3284207 1.518404
0.6918749 1.637388 0.7024785 0.2672945 0.957415
0.6750283 0.451348 0.626957255 0.3873356 0.1310603
0.7859538 0.1309564 0.9320374 1.725079 0.38326122
0.3984182 1.006463 0.5773706 0.5811765 0.3045821
0.1807567 1.013957 0.753282 0.6311579 0.979036
0.1925724 1.199779 1.181993 1.253496 1.093050956
2.525583 0.6304416 0.1017122 0.460631526 0.4973814
3.004563 0.6645085 1.124373 0.6748034 0.2485862
0.3558124
ZNF710-AS1 1.276636 11.65701 2.763056 5.222415 2.909073
20.19446 8.694366 9.210176 2.5815 2.659536284 5.89174
2.645833 2.721379 2.04188 8.798545 22.31703 3.09584
2.819573589 1.508305 2.288229 2.462921579 18.62258
3.203672 20.24511 11.59315 3.447191 4.231787
26.60236 1.934926825 0.9769624 3.522813 11.93718
1.377284 2.286338 0.8559162 1.82181 0.8751849 1.497324
2.79605 1.151955321 1.156278 1.734449 0.557558323 1.342097
2.706535 0.5636307 6.402109 3.035001 1.274415715
0.4038618 2.866551 26.645 1.852982 0.7301891 6.091047
5.126642 0.8673882 15.87893 3.278053 3.603990067
1.295933429 1.249438409 1.854858 1.997716 1.708605
13.05754 1.468692 2.18083 0.472822436 2.07389 0.3719983
1.26637 6.151041 1.727238 1.550522 1.557333 2.479919
2.534802 2.910074 1.814576381 1.070707 2.70154 0.9599085
8.900737 3.749407 3.727647 4.110728 1.701319
2.31534896 8.860084 1.013139401 3.326126 1.030694
1.267863 3.019561 1.416738 1.281148 2.161427 1.5587
3.420256 5.610012 0.568609 4.510316 12.11267
1.932051 4.826386 1.726109 5.328562 0.624232191

1.328203 1.200624 5.877025 0.254792 9.44318 0.7446894
1.22425 1.327591 2.689153 1.235282 3.247311 2.875403
2.138244 2.172968 0.7435736 4.215657 2.908748
1.392056 1.196646 7.878807 0.7691951 5.155959
1.43064 0.6826024 1.019026 0.6043725 5.602597 5.214364
0.9648943 1.564769 1.931309 1.596079 2.851916
1.172284 5.202606 0.8127548 2.457587 3.035018
8.076805 1.191279 4.719234 8.374558 2.511885
0.9254669 3.174406 8.509885 2.358003 2.643789
9.253587 0.2953503 0.8877519 1.441293729 5.354141
6.988473565 3.611641 1.289024 7.256954 1.637191
1.773382 1.88863 5.725898 26.51251 3.798163799 12.6486
3.387832 6.74815 0.7060152 2.184206 2.645815 3.515868
1.871211 2.230615 1.587678 0.7400759 2.546678
1.62062 3.092899 6.950078 0.928004 6.469895 0.6752291
1.266561 0.6489125 0.4262395 3.635533 1.024443
4.328746 1.340126 2.466728 0.6937886 4.513598
3.616689 2.453191 1.546055 2.292778 1.449286
0.6560031 1.634824 1.620349 2.7508 2.165831 5.852969
3.565191 1.998211 7.49293 1.974648 1.489279 1.00725
0.5354245 1.830465 6.37900224 0.7381523 2.50700838
0.283767233 0.7603124 3.213065 3.732649 0.6099659
2.04028 0.7757375 0.7454774 2.722468 2.060907 1.070904
1.232379 2.219802 0.9811018 5.588957 1.808667
0.5031229 10.75517 9.302777 2.197314 5.311327
1.737045 1.206415 2.009756 2.43401 8.913872 2.151748
5.101186 2.344723 2.163782 0.5314451 3.104026
0.9155342 0.2813807 2.274208 1.193585458 11.55341
4.050667 1.073336 0.6941974 1.425831 2.384163
1.630924 1.858269 1.933605 5.854941 0.9103963
1.351032 2.998669 0.4626247 0.957283 4.07689269
0.9548488 7.295741 2.623018 17.37087 0.4884488
0.585009979 6.837847 3.917785 3.354224 1.826934
2.903989 1.427459 1.645885 0.8836413 1.87136 3.336056
0.706948 21.21047 14.12915 2.261471 1.089372
2.401543 1.17592 1.159629 0.3855363 0.8758584 2.683697
1.573874 0.98192 2.010954 1.626616 0.8120563
1.322074302 2.763154237 0.8570083 0.8378554 0.9522175
1.045444 0.6419671 1.892882 1.540242 13.71381
1.381298 1.464052 4.265816 1.403022 1.914066
6.580661269 0.5672864 0.8783797 0.4736471 2.677734
1.267006 0.762405464 0.9204234 0.4936925 1.142315
2.798428 2.057238 1.28177 6.495495 1.905979 12.9277
1.927067 2.579176 0.489838 1.106773 1.731237

5.762805 1.347636 12.83349 2.261797 1.079352721
3.403174 0.7038582 2.063168 3.916588 1.304305
1.097156 1.366038 1.768491 3.216364 2.337712
0.7286916 16.75857 1.034208 0.921768 0.9491255
2.526415 1.036965 0.6859263 2.585071 1.160396
2.714303 1.916444 1.093453 1.534466 1.934711
2.704217 0.838238465 2.137345 1.412183 0.7461416
1.243228 1.238755 2.620314 1.450840831 1.059062
1.361561 9.058996 1.487788 0.6867397 2.587735
2.339848 12.54674 2.295346 6.939842 0.8409612
2.716087 1.492116 1.190001 0.981081887 2.366279
8.880068 5.127334 0.962056402 2.940149 8.050016
1.83317 2.981146 3.084474 1.42945 11.88222
AC090152.1 0.8418763 4.136714 1.27276 1.457135 2.536523
5.039602 1.056052 2.117851 1.080948 4.166899058
0.5867052 0.04912826 3.063539 0.2579021 1.956837
4.483251 1.330169 2.529221722 1.101243 2.616996
1.63635599 5.387582 0.3393361 5.724207 3.134214
0.2620724 1.609691 6.129624 1.804066714 2.17509 1.907987
4.438198 0.7482755 0.5775574 0.2035791 0.6029885
1.942847 1.938971 0.3675214 1.482717337 0.599082
0.2057439 0.060730183 0.2143423 0.7381638 0.1778694
2.822399 0.4390114 0.678986465 0.4327377 0.03848913
1.918887 0.2861555 0.350931 2.389047 0.3381442
0.4773051 3.930599 0.05014805 0.666716337 0.359609754
0.647945741 1.975808 4.691639 0.9979514 1.892162
0.4208178 1.322648 0.309864603 0.2794938 0.1210238
0.2235305 0.7668493 0.9637673 1.543731 1.326587
1.177845 2.23907 0.05046994 0.081653264 0.2173986 1.129032
0.5267795 1.791337 0.3511988 1.501141 0.5832098
0.9785356 0.48543561 2.228247 0.289169466 0.6980444
1.160647 2.462743 0.7579484 0.4513213 0.7756513
1.123484 0.06295515 0.7519496 2.055924 1.871559
0.6529939 0.4499315 0.8170914 1.314127 0.4088362
1.950751 1.039312575 0.1333499 0.2574435 0.7295192
0.5119834 2.076714 1.476032 0.3441303 0.1772023
0.03962207 0.4002516 0.3052111 0.120763 0.5380444
0.7450822 0.773756 0.3605568 4.446742 0.7700247
0.667122 2.046035 0.5400407 0.3001535 0.635816
1.865889 1.402654 1.472103 0.9349266 0.3964216
0.09872325 1.55031 1.093716 0.4088283 0.3598516 0.5835912
0.3247174 1.117171 0.7347621 0.4003558 2.54411 0.05142186
1.775009 0.2542127 0.880453 0.02265959 0.8035513
1.656849 1.308649 1.832206 3.07937 0.02341629 1.19918

1.708669469	1.998362	1.176328943	0.1213253	0.2053736	
1.329994	1.223301	1.036131	0.828686	2.391023	
4.452035	1.189898677	1.983196	0.01696408	0.3981694	
0.1320761	1.12081	0.3480899	0.577354	0.9452493	0.4024039
0.1346954	0.6907709	0.5901943	0.4649516	0.1338872	
1.090916	0.4849509	0.302469	0.2234026	1.908602	
0.1668917	0.2703488	1.057676	0.4873262	0.583573	
0.6762936	0.5299312	0.1783641	0.3796385	0.06412769	
0.5054311	0.8171735	0.04082727	0.09462673	0.7884589	
0.8537303	6.710881	0.05662976	0.1095095	0.9488991	
5.368843	0.8123856	0.2206394	0.3279683	0.7074062	
0.2497685	0.5216648	0.4715522	0.993799407	0.1322324	
0.500883877	1.53111057	1.116601	0.8794824	1.020014	
0.1979481	0.116467	3.854342	0.941297	0.3855667	
1.938586	1.108937	0.6247603	2.417285	1.58681	2.737562
0.4665444	0.7435517	1.34471	1.492051	0.5930297	1.558591
0.5990747	1.252124	0.9446023	0.7633124	2.152337	
0.850095	0.5467978	1.071494	0.1034683	0.2638958	
0.8764501	0.3513069	0.2546106	1.013853	0.578895944	
0.4617217	0.6679757	0.5269492	0.1167476	0.5961441	
0.5080272	0.1967681	0.7100792	0.09383748	2.230741	
0.4983321	0.1896042	0.8814976	0.3511411	3.815871	
1.851219306	0.8819169	3.926979	0.5538092	2.581924	
3.31552	0.22859412	1.784427	0.299431	2.228102	0.2995658
1.848849	0.6628729	2.365734	0.2522082	1.708143	
1.539484	0.2421319	3.602263	3.26745	1.534507	0.7408253
0.7064377	1.278591	0.9934769	0.675681	0.9674287	
1.913469	2.44572	0.2203484	1.791211	1.104394	0.1742306
11.465991328	0.254299061	0.4317203	0.4537898	1.643868	
0.1048559	0.6017175	1.147985	0.6974362	2.416605	
0.8213524	0.8996504	0.5698893	0.5569715	0.6884409	
4.090007087	0.1170869	0.6021871	1.628623	1.196854	
1.03935	1.422562591	0.5789274	0.3653205	0.2113213	1.56066
0.7410265	0.7799235	1.727623	1.698301	0.0739089	
0.4733795	0.6753964	0.2401973	0.04665106	1.013349	
3.092026	0.1210317	2.307592	0.7547768	1.261611892	
2.04288	0.3869433	0.6643946	0.5663697	0.4360292	3.26959
0.3774078	0.6419163	0.05910674	0.8761245	0.1681707	
3.753137	0.2708605	0.6577258	4.879345	0.09180513	
0.8510871	1.474257	1.016	0.5376784	0.5076597	2.694043
0.06145279	0.9791927	1.260023	0.01585343	0.834069569	
0.2066262	0.3418056	0.4347998	0.3104861	2.213372	
2.919639	0.318037549	0.629743	0.7854695	0.522669	
0.1330404	0.3971756	0.7771188	0.3205339	0.2299174	

0.7904167 1.485573 1.542199 0.9481899 0.4645423
0.5503573 0.554953443 2.425021 1.409836 1.977435
1.565367577 0.2935016 3.8472 0.2224223 2.329392 0.4661436
2.278921 2.964179
AC073534.2 0 0.2187821 0 0.03642681 0.3501235 0.07723681
0.1178349 0.09140332 0.1177411 0.143328894 0.1636623
0.3745869 0.2355477 0.1430123 0 0.4520109 0.1521315
0.028711393 0.1883353 0.4072191 0.044559563 0.3038167
0.04851237 0.04562216 0.2089386 0.119893 0.1810824
0.4522875 0.073689733 0.08652703 0 0.1709081 0.03680874
0.1201005 0.8692454 4.012442 0 0.1027461 0.2654745
0.033746965 0.9238254 0 0.079379609 0.1716005 0.3534022
0.4770068 0.1331126 0.1229627 0 0 0.1320601 0.5036863
0.1636381 0.1720114 0.5204486 0.04687707 0 0.2345442 0
0.258482712 0.129879777 0.052003934 0.03797871 0.04462229 0
0.1291981 0.05203131 0.3083237 0 0.07991433 0.06513646
0.4422089 0.4149466 0.07111364 0.05934679 0.2068934
0.2368532 0.06208062 1.298756 0 0.06630373 0.2915782 0
0.3921059 0.1204999 0.2584514 0.2354179 0.04712219
0.91443567 1.247341 0.073494077 0 0.09772515 0.1931411
0.04389196 0.2337403 0.3326674 0.04015403 0.03200082
0.1546077 0.2864603 0 0.6150362 1.052045 0.4721725
0.4027093 0.05501015 0.1898787 0.1348837 0.4066998
0.1616524 0.4286577 0.2676827 0.7146438 0.1350512
0.3633065 0.1279999 0.1208421 0.3662144 0.07978746
0.1183858 0.3281929 0.03514025 0 0.2932402 0.6287827 0
0.1237278 0.1766077 0.105882 0.9612004 0.564381
0.232802 0.07684867 0 0.3888278 0.03627101 0.04105813 0
0.2501767 0 0.7628964 0.08899383 0.3211931 0.2159508
1.017669 0.148504 0.7824406 0.06721283 0.3352425
0.3876577 0.1078902 0.03455438 0.3031826 0.3344011
0.8381534 0.1362924 0.5418273 0 0 0.287383326 0.2106478
0.077992367 0.4509695 0.5637264 0.3027095 0.1824901
0.1425593 0.2077302 1.220601 0.2518353 0.043899615
1.098492 0.2328219 0.07919768 0.5135887 0.4426604
0.7138537 0.2567912 0.3088807 1.073869 0.07702561
0.213523 0.1776331 0.3138288 0.1884639 0.0891201
0.2063772 0.2515884 0.3296849 0.06540435 0.1205521
0.03864976 0.1165942 0 0.4338314 0.194892 0.9848845
0.1554249 0.3117282 0.6160802 0.2812193 0.03738408
0.09338837 0.2164493 0.2365275 0.1837952 0.3074488
0.09715125 0.5725534 0.2438779 0.08106073 0.2831622
0.4626334 0.329354 0.06672668 0.2040431 0.1510452
0.2396954 0.276739623 0.2135072 0.040200792 0.240155296

0.1639004 0.1662586 1.69967 0.6693371 0.08880228 0
0.4573007 0.2581305 0.04434325 0.1492108 0.03008585
0.04569671 0.4270202 0.3295739 0.4527407 0.1291748
1.67422 0.9710401 0.03821118 0.6068295 0.2835154 0.1988965
0.1831089 0.3174549 0.2289887 0.1111148 0.2990915
0.1028366 0 1.136255 0.7604389 0.10896 0.1863669
0.1577612 0.118582164 0.1432058 0.2945405 0.3144377 0
0.1461023 0.03939194 0.3150614 0.1740253 0.2311551
0.08296912 0.1583175 0.8279744 0.2433513 0 0.6918876
0.41822044 0.5839547 0.1903088 0.03619383 0.7325142
0.1226511 0 0.7755231 0.05371912 0.08749467 0.2811189
0.2225821 0.3698187 0.03006324 1.089703 0.6372274
0.1861543 0.153848 0.3161563 0.1256129 0.2925029
0.2921664 0.2585451 0.1827907 0.08575398 0 0.423054
0.09463509 0.168251 0 0.2074541 0.0643616 0.2550626
0.165081521 0 0.1004255 0.1537777 0.08917437 0 0.579524
0.2972721 0 0 0.1670011 0.3058984 0.1167372 0.1901518
0.2539905 0 0.2142598 1.416799 0.5489939 0.2737686
0.1761046 0.150183319 0.1557929 0.1740905 0.4640416
0.1665239 0.1506689 0.0784175 0 0.2188561 0.3005499
0.7562476 0.03814575 0.1533287 0.1600646 1.236233
0.1182068 0.085184 0.1141273 0.1113857 0.167779827
0.8816766 0.05057685 0.7760361 0.07002783 0.2737889
0.6178192 0.4997955 0.06384001 0.324482 0.2482892
0.1648603 1.127314 0.2360255 0.6017932 0.06341489 0
0.3893556 0.7599362 0.9881579 0.08338213 0.3187667
0.4010212 0.7731197 0.04072376 0.9762221 0.2901053
0.226675491 0 0.0947693 0.2486404 0.4734709 0.1315031
1.151446 0.06928377 0 0.5003424 0.3036327 0.3477907 0
0.1568455 0 0.4507829 0.1404272 0.1258557 0.6034102
1.001027 0.04619977 0.1342814 0.574823266 0.477895
0.3376819 0.1470955 0.055513554 0.191816 0.4389073
0.1130598 0.2322945 0.03614433 0.0513577 0
LINC02544 0 0.1021519 0.02456614 0 0.07006149 0.05409416 0
0 0.04123102 0 0.02292475 0 0.16497 0 0 0.03957172 0
0.020108531 0 0 0 0 0 0 0 0 0 0.0633535 0 0
0.2264175 0 0.2062372 0.168229 1.174098 0 0
0.07196001 0.3305417 0.118176412 0.1078362 0.02354331
0.138987274 0.01716906 0.05500255 0.03930355 0.03107591
0.1148255 0.271937824 0.08532374 1.880643 0.6299385
0.4297757 0.4015708 1.562166 0.06566236 0.3921299
0.1642671 0.4552503 0 0.242569583 1.420453032 0.1063962
0.6250402 0.03675265 0.3076529 1.639847 1.241655 0
0.07462589 0.02280973 0.1161408 0.1320978 0.5727655

0.8728559 0.2898029 0.06220661 0.58697 0.1010674 0
0.185748 0.05834626 0.08582159 0.3020801 0.815811
0.4137394 0.2473186 0 0.45745797 0.5422329 0.669147357 0
0.04562902 0.1578147 0.1537025 0.1909882 2.692323
0.05624517 0.04482468 0.2707056 1.571582 0.1633304
0.06153592 0.03203557 0.2755786 0.3021906 0.1541093
0.06649246 0.118085225 1.495407 0.08086865 0.06433243
0.1406072 0.3533036 0.4729273 0.02120401 1.045881
0.04231695 0.1578368 0 0 0.5746393 0.246111 9.321598
0.3594079 0.0518093 0.1709936 1.704214 0.4700231
0.5685318 0.5770231 0.3952742 0 0.161467 0.05970483
0.1702015 0.5080609 0.2875579 0.1763525 0 0.05457932
0.08436442 0 0.05623832 0.1764524 0.1511878 0.1386765
0.2968312 0.1176842 0.6782909 0.5183238 0.125938
0.532417 0.1592545 2.556724 0.1677187 0.5011377
0.06324639 0.07502682 2.501913 0.442802764 0.02950618
1.119778283 0.04859145 0.03290131 0.2968111 0.08520676
1.173166 0.2182312 0.6575918 0.1102358 0.122983479
0.2279551 0 0 2.581383 2.196011 0.3461262 0.02569261
0.1545215 0.4297732 0.3506506 0.1495446 0.02488169
0.1709521 0.131994 0.2496673 1.662209 0.1982299
0.02309006 0.9619489 0.6191591 0.2977595 0.04082939
0.4133157 0.3038415 0.6824802 0.02653005 0.02721365
1.996108 0 0.7221753 0 0.06540619 0.05053137 0
0.3539916 0.03076103 0.02268053 1.052619 0.1708042
0.4541785 0.04957941 0.1472788 0.1794091 0.04673321
0.5430398 0.02115743 0.2238332 0.221507975 0.09968904
1.013592026 0.0840985 0.3443715 0.6287877 0.4978009 0 0
0 0.9074561 0.5423588 0.4658487 0 0.08428461 0.2880405
0.07476779 0.03847048 0.1358935 0.8443852 0.08180716 0
1.043713 4.427123 2.250404 1.253706 0.2051897
0.5336043 0 1.011676 0.01904309 0.2640858 0.2411033
0.09947457 6.245791 0 1.827355 0.5966505 1.937859254
0.05014836 4.796164 0.02446911 0.3366579 0.1364337
0.02758884 0.1891361 0.04062723 0.06938295 0.1162178
2.927243 0.05522729 0.535654 0.68956 0 0.024409005 0
0.1866006 0.2788389 0.4489006 0.5154051 0.28660134
0.7146728 0.1504925 2.144745 1.132098 0.07794467
0.5698199 0 0.5836179 0.1409348 0.6301529 0.0646501
0.3321384 0.02199381 0.06828652 0.8184955 2.580342
9.703969 0.3603559 0.368035 0 0 0.6775658 2.23189
0.3148039 0 0 0.086713317 1.851785027 0.07033479 0.08975078
0.3330925 3.225244 0.2029399 0.798099 0.06875742
0.2002477 0.08772164 0.4284826 0.163518 0.3995286

0.213464	0.382614982	0.225091	0.05512668	5.355502
0.09586933	0.1480055	0.070122359	0	0.4877095	0.08124994
2.186774	0.7914272	0.1098422	0.4358566	0.05109325	0
0.1926005	0.1068642	0.3937502	1.793665	0.3607572
0.3311528	0.05966012	1.878381	0.02600364	0.023501513
0.02058326	0.5313358	0.3170478	0	0.1369664	0.1366423
0.5409718	0	0.03787612	0.04968392	0.5003389	0.1822003
2.35559	0.2458613	18.07639	0.4545919	0.03895601	0
1.127824	0	0.03189341	0.01872415	0.4184071	0.4848673
0.03255783	2.158791	0.767321536	0.3187598	0.3318668
0.0497542	0.4642451	0	0.3494554	1.019006338	0.2761096
0.4459937	0.2126546	0.02029845	0.07712534	3.808118
0.3160014	0.1841664	0.07376301	0.2203631	0.1625419
0.3739129	0.3559251	0	0.201293809	0.5976825	0.9933061	0
0.116639692	0.03358544	0.522574	0.1979586	0.04067289	0
0.2158156	0.1695957
FAM222A-AS1	0.08162145	0.117073	0.2127226	0.4028437	0.570988
0.2204287	0.4624028	0.1793405	0.08400628	0.187481709
0.05838513	0	0.1890667	0.02550921	0.310489	0.1713293
0.2713582	0.133153119	0.3359352	0.2033808	0.174858734
0.09483603	0.2769021	0.2115792	0.07453708	0.1176197
0.1937989	0.0645399	0.184017339	0.3086779	0.2691005
0.109746	0.3939363	0.1713794	0.09967371	0.1192837
0.8068477	0.2565765	0.2946402	0.192623125	0.1318268
0.3117945	0.368134084	0.1486699	0.2941709	0.6606529
0.5065258	1.18438	2.105430222	0.2607647	0.2355567	0.3337023
1.576166	0.1022727	0.1060947	0.50169	1.717735	0.1115622
1.268563	1.014326821	0.046333516	0.11131179	0.2709717
0.4616402	0.2808067	0.3134142	0.1484939	0.0962429
0.177680485	0.1425438	0.4182639	0.0690175	0.4844575
0.2283227	0.2117147	0.6150623	0.3591053	0.1882473
0.2471042	0.812885768	0.1655731	0.5052305	0.3497149
0.2377968	0.1003038	0.0658574	0.3779251	0.03362089
0.9320492	0.01534409	0.196637944	0.07594828	0.02324175
0.3674744	0.172239	0.05558992	0.09230377	0.2005446
0.4338089	0.2344087	0.5109615	0.3208931	0.1567207
0.440579	0.5334046	0.1436631	0.1373709	0.06773764
0.108266903	1.124422	0.04942983	0.4915287	0.5729612
0.07498328	0.7226755	0.04320218	1.217677	0.06466411
0.5627738	0.3415621	0.6898076	0.2195251	0.5515835
0.02525574	0.6538186	0.1847283	0.0746553	0.1618424
0.1638086	0.1007267	0.2449287	0.9912033	0.1107335
0.2193209	0.03041145	0.2947609	0.3105451	0.7909456
0.2994249	0.7675365	0.1575373	0.1718885	0.0846608

1.188798 0.6676681 0.07700952 0.3708428 0.2209772
0.4795522 0.09301829 1.05606 0.2437631 5.608778 0.05407892
0.129236 0.8970125 0.9724234 0.2094 0.7643182 0.5916778
0.061512948 0.330646 0.431258358 0.5197648 0.6703488
0.1943803 0.108503 0.03814261 0.3149504 0.3014578
0.07861012 0.266233899 0.6531298 0.09690014 0.7769603
0.03233266 0.171075 0.3721972 0.3140849 0.3463133
0.3830937 0.4121736 0.06093804 0.08871674 0.4478232
0.1008494 0.06358569 0 0.5497313 0.02352246 0.2099921
0.4300594 0.1654557 0.5199249 0 0.2142909 0.1738152
0.9459403 0.1247547 0.2224128 0.2825761 0.3789966
0.1600375 0.7662568 0.3860824 0.7383179 0.04917554
1.190809 0.7393683 0.1404243 0.5394086 0.4626837
0.2146584 0.1500369 0.4830319 0.07141255 0.05823254
0.06466092 0.0570062 0.80389973 0.3427511 0.301167169
0.028557793 0.03898005 0.1186227 0.3968789 0.1264131
0.06335893 1.477159 0.176733 0.1074335 0.4429343
0.2129188 0.1824588 0.2282268 0.01904198 0.3135272
0.2307306 0.1843281 1.95847 0.3158448 0.06815759 0.01804011
0.3034252 0.5534463 0.4311288 0.2491486 0.06807484
0.376573 0.1357979 0.02445738 0.0921069 0.2533435
0.2466183 0.03887055 0.1662121 0.5965678 0.084606375
0.1149468 0.2101497 0.486083 0.1333743 0.3300981
0.3934768 0.1445085 0.2483282 0.3180701 0.3107846
0.0677741 0.4782228 0.02480386 0.01848613 0.1518924
0.820581397 0.1041605 0.8690058 0.1936777 0.25043 0.160434
0 0.1892946 0.2108023 0.1092454 0.3510038 0.1588086
0.07915782 0.4826163 0 0.4187575 0.1106816 0.6915382 0
0.02240568 0.3362322 0.541985 0.4611689 0.2869198
0.1529601 0.3969816 0.3250605 0.3882431 0.07502764
0.01160044 0.29603 0.2410848 0.2274786 0.161951464
0.792314313 0.2149558 0.1462904 0.1908732 0.3422544 0
0.07069954 0.1576013 0.169998 0.2085169 0.1388885
0.3609235 0.1899382 0.4530448 0.535947578 0.4713515 0
0.1538813 0.6348201 0.1759067 0.553625212 0.4816742
0.4595795 0.110362 0.8910901 0.1074997 0.5455083
0.5328225 1.652589 0.4556793 0.1716813 0.04082452
0.06381516 0.6852202 0.05512695 0.2108464 0.2734981
0.1628559 0.1192077 0.083795655 0.8492329 0.270643
0.830533 0.3247639 0.1534844 0.3828031 0.08104447
0.09109754 0.2315125 0.1012287 0.6077294 0.4021599
0.0947251 0.3935885 0.2714727 2.070351 0.3373269
0.4913703 0.3916856 0.4015693 0.24368 0.2575097 0.1504385
0.3922522 0.2155889 1.798184 0.390845427 0.04995832

0.5747386 0.2280867 0.4729389 0.2580195 0.7941528
0.494328025 0.5409235 0.2271728 2.03097 1.106303 0.03928482
0.16786 0 0.04020325 0.4884382 0.1122449 1.142542
1.142745 0.1977765 0.04790376 0.393038099 0.5236326
0.2770699 0.2623755 0.079215971 1.197503 2.286017
0.0604997 0.4764974 0.6576034 0.3297857 0.09718405
AC012615.6 0.3019395 0.3736413 1.056484 0.3958855 0.3261542
0.8873735 0.2012413 0.2980106 0.5027026 0.289286069
0.7673719 0.16284 0.6765517 0.2275871 0.6370285 0.1710582
0.42515 0.485882418 0.1900621 0.1517364 0.276726869 0.389149
0.2259561 0.9703912 0.4460379 1.186654 0.2811426
0.8707363 0.205934877 0.0134339 0.2676906 0.4139402
1.703012 0.6526245 0.5109056 0.4931756 0.1293697
0.5795899 1.149488 0.639212071 2.576961 0.8193916
0.227998032 0.9438924 1.481438 1.455032 2.156218
0.4327245 0.675168204 1.26727 0.2118666 2.055561 1.556113
0.4272949 0.681055 0.6477402 0.5911031 1.832866
0.7064357 0.200655895 0.638549106 0.549029304 0.18279 0.2424769
0.5621627 0.7582252 0.4362234 0.646236 0.368794777
1.153873 0.3134991 0.3475705 1.581298 0.3643487
1.690767 0.4068735 1.098595 2.660208 0.8603335
0.318976779 0.3808817 1.047665 0.6785525 1.114051
0.2993347 0.4815158 0.9563973 0.7535518 0.60845266
0.6944981 0.387955286 1.394849 0.1011499 1.464338
0.3884278 0.6169258 0.550921 1.10345 1.023479 0.6361029
2.045845 0.7965517 0.3546716 0.4047917 0.6719896
1.683664 0.5124417 0.4274593 0.371713425 0.8997859
1.172417 2.08688 0.1454584 1.553346 0.7967688 0.8930916
0.4305782 2.603165 1.180881 0.916678 0.4043641
0.7706817 1.718565 0.2637959 0.5747849 2.664527
0.3628114 0.1664829 0.6416169 0.6575557 1.20239 1.091929
0.5421611 0.8888799 0.2514707 1.494111 0.9235363
0.567335 0.4821547 0.5748559 1.18571 1.976158 0.9119148
1.496022 1.827266 0.3112121 0.9606767 1.093312
1.283535 0.5551857 0.6675226 0.4131833 0.5257504
1.017913 1.094605 0.8365432 1.121497 1.100601
0.731802 0.4753898 0.312327355 1.517488 0.423809381
1.050241 0.2115122 0.6955657 0.4722135 0.5311988
3.071956 0.7871808 1.065451 0.449836883 1.623367
0.421717 0.6393905 0.4268333 0.6357155 0.6308818
1.50931 3.658343 0.7085829 1.357317 0.2519467 1.77607
1.142308 1.653206 0.8532505 0.8651191 1.083937
0.6705339 0.654963 1.565949 0.3120331 0.3620406
0.4173953 1.284931 0.3744463 1.405595 0.3740268

0.6983125 0.1821916 0.878075 0.6964956 1.135768
0.5712887 2.047278 0.2639528 1.02968 0.955281 2.016755
1.120765 0.792869 1.401315 0.8619233 1.119276
0.5646073 0.2154177 1.125637 0.7628935 0.270070033
0.5193254 0.611661438 0.304500083 2.447118 3.655088
0.3982266 1.253142 0.7169318 0.2889308 1.248399
0.4141238 0.9225348 0.5405395 0.7567068 0.6810933
1.566285 0.4519889 0.592453 0.9827075 1.063915
1.631761 0.9551395 0.7929709 0.5282121 0.6176004
0.6083781 1.035027 0.9006517 0.9229455 1.10602 1.309218
0.788351 1.664882 1.502623 1.027694 0.4846566
1.087511 0.38048735 2.012147 1.274707 0.7485513
0.2819354 0.5141561 0.5320802 1.10409 1.35093 1.379139
0.3864454 1.165085 2.173083 0.6638823 0.5792623
1.132044 0.578972857 1.7432 1.305965 1.584653 0.7107985
0.6728316 0.091770564 1.419514 0.554627 0.1630096
0.7583422 0.6047541 1.699538 1.498274 1.174334
1.010167 0.9393055 1.361498 0.6687884 0.3802939
0.943582 0.4808244 0.9633807 1.560872 0.4926137
0.7390695 0.8437609 0.7713685 0.7118271 0.5351518
1.819788 0.6045505 2.08396 0.724047954 0.722482908 1.195367
1.050501 0.2307487 0.3202463 0.7029297 0.2923054
1.585175 0.8927459 0.9009997 1.049159 0.453106
1.033282 0.4574312 0.848176652 1.103298 0.7943275
0.006088214 1.317637 0.7491549 0.582924252 0.370881
1.216293 0.3902469 0.5397014 0.6082013 0.821802
0.3091847 0.5889673 0.7174344 0.6030734 2.274196
0.4602359 0.331348 0.5917947 0.7646833 1.34899 0.9213899
1.763924 1.813006662 1.081401 0.3455054 0.7329493
1.489504 0.8501515 0.2928102 0.7830203 0.3369941
1.217469 1.894387 0.8617209 0.6462392 2.084158
1.346984 0.4036691 1.161858 1.105373 1.172478
0.886416 1.501694 1.739244 1.257677 0.8620482
0.8725241 0.4041734 0.4166274 0.615875645 0.4892006
0.514975 0.6948557 0.9703259 1.051462 3.694575
0.774487527 0.6450348 1.22878 0.760149 1.223928 0.2137132
0.3084501 0.7705593 0.5598966 0.9102448 1.206591
0.6557845 1.83391 1.018541 1.626151 0.54290816 2.69227
0.9489356 0.4681702 0.46541804 0.4318206 2.998305
0.7986749 1.451628 0.5050485 0.3348924 0.6203305
AL592429.2 0.07947821 0.1823982 0.10235 0.1214758 0.2084981
0.2092746 0.09823875 0.09525346 0.1717809 1.334338688
0.05457796 0 0.56458 0.01490363 0.2591453 0.2708542
0.2219556 0.203461372 0.0392537 0.1018493 0.111447659

0.1266457 0.121334 0.1521404 0.0544349 0.02498863
0.4780655 0.1633975 0 0 0 0.1852315 0 0.1501913 0
0 0 0.04282959 0 0.098471724 0 0 0.099267935 0
0.01636837 0 0.6288623 0.05125688 0.097112151 0.0507835 0
0.5099056 0.0682124 0.04780186 0.3099264 0 0.04667805
0.6029119 0.04781622 0.430993055 0.036093505 0.043355577
0.09498833 0.07440304 0 0.09694095 0 0.4819666
0.015970607 0.08883256 0 0 0.3773892 0.07410913 0.0494773
0.08624331 0.2591715 0.01293912 0.1804618 0.031142591
0.05527729 0.2083613 0.06810641 0.06537959 0 0.123126
0.130845 0.2553569 0.27227258 0.1075765 0 0.07099585 0
1.677306 0.05488896 0.06495628 0.2465281 0.01673817 0
0.06444806 2.627035 0 0.03662532 0.01906712 0.04920616
0.3117567 0.04586185 0.1780892 0 0.02119155 0.01925276
0.01276325 0 1.244165 0.5066635 0.02524066 0.01778554 0
0.02348556 0 0 0.1710086 0.04394454 0.05902218 0.06111845
6.506432 0.159929 0 0.1914087 0.01471227 0.01144787
0.05429115 0.06469552 0.04805144 0 0.1215619 0.1209563
0.03423008 0.13995 0.08342873 0.2923636 0.2678001 0.148388
0.01673613 0.1350283 0.2056797 0 0.06794992 0.0280176
0.09316368 0.01469043 0.0149913 0.02880792 0.189572
0.3368707 0 0.04261002 0.09410841 0 0.2659114
0.359386369 1.317125 0.130044166 0 0 0.2523683 0.1774986
0.103995 0.5195527 0 0.1180995 0.018299511 0.1526352 0
0 0 0.09226128 0 0.04587564 0.0551814 0 0 0.1780137
0.05923693 0.02907094 0.01964025 0.1981313 0 0 0.02748576
0.01363187 0 0 0.02430108 0 0.06955468 0.0304652
0.4737094 0.08098591 0 0.03668749 0.1432762 0 0 0
0.1232453 0.01915371 0.1647768 0.0404974 0 0.1626564
0.5744314 0.1918083 0.03506332 0.198309 0 0.03402207
0.02518521 0.1165696 0.049439417 0.01483338 0.100545963 0
0.01138695 0.5960208 0 0.0164125 0.03701714 0 0 0
0.07393776 0.02073279 0 0.01904863 0.1112518 0.503736
0.05392129 0.01794879 0.1136111 0.08333647 0.1752112
0.08431876 0.03939437 0.08290982 0.3816438 0.02646615
0.8431746 0.06947713 0.09067345 0.4858296 0.01793767
0.08880881 0.05763422 0 0.01942172 0.02630504 0.115338669
0.0447714 0.04604209 0.4660375 0 0.1624069 0.04926148
0.1125712 0 0 0.0345856 0.03959669 0.197223 0.07245767
0.1728069 0.08874236 0.014527898 0.03319377 0.01586601
0.01508736 0.4580218 1.670148 0 1.08893 0 0 0.1171841
0.02319579 1.418259 0.01253183 0 0.2376668 0.0646652
0.1154365 0.08236839 0.09163285 0.02709545 0.1461471
0.05388713 0.3657414 0.07149293 0.05154101 0.3255686

0.1380699	0.2279396	0.013555	0	0.0402436	0	0
0.088172521	0.04186228	0	0.06195374	0.01999604	0	0.3510998
0.3478494	0.09269914	0.05221069	0.1159213	0.0162206
0.01585292	0.1270508	0.075909026	0.02977133	0	0.04903877
0.3423605	0.0146818	0	0	0.04354168	0	0.04338458
0.1570155	0.1470972	0.1037663	0.03040999	0	0.0716457
0.1749112	0.03195745	0.02224092	0.04294353	0	0	0.07136081
0.123816	0	0.01225087	0.02108291	0.3234899	0	0.1956491
0	0	0.05322329	0	0.6357802	0.2061656	0.4337722
0.02459674	0.9825229	0.4493854	0	0	0.1385907	0.01525602
0	0.1518601	0.04457741	0	0	0.05813389	0.03023253
0.06299297	0	0	0.04441951	0.236839	0.02740848	0.04799792
0.014440436	0	0.2844099	0.01582113	0	0	0.1089682	0	0
0	0.131157	0.03869707	0.9180093	0.11555	0.02798754
0.09983938	0.05691716	0.2674485	0.09197495	0.023140771
0.03997916	0.8964945	0.02356443	0	0	0.04281682	0.07570574
AL590226.1	0.1043348	0.09976778	0.1439567	0.2491672	0.3763446
0.6339797	0.2418049	0.2188264	0.2416123	1.013080533
0.1567279	0	0.9264382	0.04891174	0.4252402	0.3091852
0.3121837	0.432062655	0.5153017	0.5013839	0.457195513
0.1558629	0.199101	0.9986093	0.2322425	0.1640187
0.5780333	0.4124992	0.10081073	0.5326772	0.2211331
0.07014289	0.1510678	0.3286054	0.2123509	0.1143582
0.05428286	0.3514026	0.262297	0.184669208	0.2317027
0.2069444	0.162892087	0.01676834	0.08057824	0.2495105
0.1214025	0.224291	0.079677294	0.02083309	0.1505533
0.2460945	0.5596601	0.235319	1.220565	0.09619477
0.07659558	0.427822	0.07846325	0.141446195	0.562656914
0.106715433	0.2338043	0.1220904	0.2153692	0.7953683
0.3914959	0.2899876	0.078620118	0.07288417	0.02227737
0.794011	0.3612412	0.1459298	0.5683227	0.4717318
0.2632706	0.1061612	0.1579338	0	0.1814127	0.6553217
0.08381857	0.4559551	0.3846478	0.3030623	0.2683849
0.3223259	0.17871249	0.1765259	0.025135766	0.05824974
1.09182	0.1761502	0.1801383	0.2664723	0.1264176	0.3021284
0.06566776	0.2643875	0.6204926	0.4329784	0.3605983
0.4693182	0.592123	0.4131927	0.1128844	0.5519949
0.092263357	0.3477392	0.09477748	0.0418873	0	0.5463413
0.8314011	0.1863821	0.1751093	0.1033233	0.1348838
0.1091527	0.2969208	0.2525524	0.3365136	0.6779603
0.1253641	0.1771004	0.1908602	0.1692649	0.70066	0.337986
0.03757039	0.02969606	0.2123221	0.1051323	0.5831136
0.1994749	0.2232914	0.05616929	0.2870609	0.136901
0.1421479	0.2471862	0.1217474	0.1373144	0.29543	0.3164126

0.1015799 0.9143106 0.09195004 0.764377 0.1205301
0.4919946 0.02363594 0.1296146 1.315241 0.2457064
0.1864539 0.4015067 0 0.08726859 0.196576386 0.6339855
0.293416528 0.2847442 0.1285337 0.1035299 0.1248271
0.5850817 0.1420919 0.1926732 0.107663 0.060056564
0.1669761 0.1592552 0.08125937 0.1859854 0.02523244
0.2253652 0.1003718 0.211281 0.1049357 0 0.233687
0.2430097 0.1908139 0.2578267 0.3454404 0 0.1075574
0.02255116 0.3131659 0.05497348 0.07931181 0.3190117
0.3139649 0.2054423 0.2999482 0.2331977 0.0797355
0.1523063 0.1404707 0.6839469 0.1278576 0.06387966
0.444168 0.04044747 0.2200098 0.09012926 0.06645355
0.09790971 0.2001813 0.3049601 0.2421113 0.1726097
0.400507 0.2966762 0.1674839 0.2066363 0.3552397
0.027042266 0.2190653 0.137491039 0.164271396 0.09342615
0.3411735 0.06341512 0.1077273 0 0 0.1042678 0.08828342
0.6066348 0.06804226 0.02057937 0 0.292091 1.202323
0.04424062 0.1472639 0.1597957 0.09767829 0.3397846
0.5188556 0 0.3265188 0.4258514 0 0.2610554 0.3040196
0.2417823 0.2579222 0.1766071 0.0971529 1.229461
0.09316372 0.127479 0.2374065 0.540751698 0 1.057728
0.5257564 0.1461336 0.03331236 0.02694494 0.2155088
0.03967902 0.09035146 0.1986343 0.5847798 0.1348458
0.2853557 0 0.3276457 0.095357264 0.1270938 0.1301753
0.1485442 0.2505278 0.1677919 0.093304087 1.312226
0.0918626 0.3291653 0.6009105 0.1332196 0.4553351
0.06169171 0 0.5505818 0.5942233 0.04209414 0.3514187
0.4725707 0.4223875 0.03996962 0.7074016 0.4251115
0.2346303 0.1057192 0.6677956 0.2589298 0.6329801
0.04448571 0.4020587 0.08804944 0.3271281 0.141149146
0.108513938 0.2289774 0.05259363 0.1626592 0 0.09910172
0.2711207 0.2350344 0.2824958 0.4283714 0.1711972
0.4524878 0.2601359 0.06949398 0.186842505 0.07327916
0.2153603 0.7778705 0.2808954 0.1927349 0 0.4973065
0.02381634 0.02645121 0.1993354 0.1030608 0.1072785
0.425684 0.1746527 0.02569783 0.3291843 0.1565551
0.06992007 0.1824794 0.140935 0.242568 0.05826769
0.3903278 0 0.114765017 0.06030858 0 0.3538836 0.2874033
0.5083247 0.1556954 0.3729501 0.04366794 0.1479685
0.2183595 0.2631253 0.05931594 0.06054244 0.6174593
0.867543 0 0.2282808 0.2274181 0.3004095 0.02851759
0.2803414 0.1462971 0.6490192 0.2507034 0.03179795
0.02480478 0.180892739 0.1915816 0.1944727 0.2429649
0.7772743 0.1799019 0.1575228 0.213262163 0.08297398

	0.1866791	0.05192284	0.1189482	0.03766264	0.4291429
	0.07715654	0.1027817	0.2161243	0.2797859	0.1587483
	0.730372	0.1580082	0.2296284	0.294893623	0.09339728
	0.3926689	0.05030826	0.113917402	0.2624126	0.1200888
	0.116003	0.1191708	0.09889399	0.2810382	0.3726843
AC023590.1	0.1617286	0.02577488	0.2231463	0.06008058	0.3653423
	0.04549664	0.6108178	0.1722927	0.1941963	2.048796575
	0.1079746	0.1588693	0.2913755	0.02527258	0.7031058
	0.03993881	0.4839137	0.047355155	0.2662551	0.4029882
	0.073494344	0.02684466	0.1714584	0.1074956	0.01846141
	0.1059351	0.4551137	0.01420916	0.329894852	0.1834887
	0.1269544	0.08053925	0.6157782	0.7640532	0.08046222
	2.166582	0.009349275	0.2340221	0.3892093	0.19878778
	0.4788809	0.1900937	0.196387214	0.1675072	0.7124139
	0.2115634	1.578664	0.1834935	0.155527582	0.3803423
	0.1866973	1.822575	0.1638658	0.3512565	0.297813
	0.132543	0.1846915	0.2855289	0.2342413	0.365424539
	0.265221412	0.208305181	0.3310983	0.1892515	0.234926
	0.280065	0.09807757	0.3904814	0.18054589	0.6213747
	0.2992773	0.325606	0.9954798	0.4105196	0.202759
	0.08124756	0.9975627	0.219413	0.346817	0.132023784
	0.4374324	0.1570333	0.3368467	0.4988984	0.1892825
	0.130493	0.2681027	0.5551501	1.56978527	0.4763214
	0.086583931	0.501625	0.1381569	2.958033	0.3102567
	0.4681311	0.3396622	0.4068296	0.1131012	0.1912517
	0.3824785	0.3139914	0.579663	0.8945392	1.288692
	0.2101069	0.1036926	0.1901429	0.087399132	0.5989201
	0.854277	0.2741455	0.04730385	0.9310725	0.8591659
	0.1712057	0.07037222	0.1921928	0.1261132	0.1503972
	0.1487692	0.3383158	0.1490364	0.3669814	0.2677381
	6.056914	0.2958511	0.1749176	0.09987016	0.04989615
	0.0905918	0.2864191	0.7862286	0.09053601	0.3213796
	0.3893688	0.1281935	0.06771925	0.5141887	0.3301033
	0.7283544	0.2081372	0.2935641	0.5013801	0.7038767
	0.4723033	0.6065061	0.768166	0.2929806	0.2457478
0.15777	0.169475	0.1058428	0.3571817	0.1510182	0.3385491
	0.2007089	0.9362186	0.05048187	1.503049	0.176055611
	0.7842037	0.147013442	0.2779058	0.1771014	0.1854447
	0.2006601	0.4618629	0.9054954	0.1327384	0.1335101
	0.093093246	0.8531752	0.1828595	0.06531237	0.2135515
	0.2694425	0.6210435	0.3889641	0.3327037	0.5783467
	0.2177867	0.1408697	0.351575	0.271131	0.3774523
	0.1749884	0.07294036	0.2148887	0.4272447	0.1464014
	0.340856	0.02732015	0.2060407	0.2780998	0.3145231

0.1492424	0.8389861	0.2929717	0.9968196	0.1313368
0.7288745	0.1585529	0.1466954	0.1530004	0.3343858
0.1299183	0.248371	0.2594305	0.5986449	0.1723887
0.3151442	0.4253344	0.1585547	0.3449017	0.1729447
0.1346155	0.1067685	0.2070838	0.298083707	0.2515345
0.246276466	0.264066862	0.1222918	0.6032813	0.1092215
0.3061434	0.460322	0.1425088	0.1346874	0.1520528
0.1985163	0.6093927	0.1843107	0.3553153	1.370881
0.6471225	0.3124067	0.2232	0.4587002	0.4575959	0.1710642
0.2144731	0.1113372	0.2436944	0.3969284	0.4637551
0.3956676	0.1570862	0.3587685	1.066142	0.2737574
1.690022	0.2931967	0.2246415	0.08782412	0.5129727
0.05588102	0.5904915	0.1474751	1.66287	0.05033791	0.2409738
0.1856318	0.5302518	0.2870287	0.2489834	0.3714369
0.3954118	0.5202362	0.2375464	0.1587269	0.6395542
0.065694491	0.5065898	1.094117	0.4349303	0.726342
9.430783	0.160699988	0.4135467	0.4556657	0.07215469
0.2897898	0.1376685	0.8713724	0.2125064	0.7702723
0.7981365	0.1023446	0.355249	0.1582981	0.1183884
0.375231	0.2202902	0.2132157	0.7063386	0.4849316
0.254916	0.5750806	0.6577929	0.3964355	0.398418
0.2606968	0.5156092	0.2479053	0.165311226	0.510849886
0.4416984	0.1449334	0.0840456	0.09042119	0.1792197
0.1751092	0.5782937	0.1422222	0.3934906	0.2096763
0.275058	0.1702547	0.5027032	0.311076786	0.2860773
0.6676568	0.2587096	0.3870346	0.07468925	0.023590948
0.6362747	0.1886896	0.3371261	0.1324236	0.3550084
0.129338	0.1759599	0.09453989	0.2478561	0.7127529
0.6111813	0.3733178	0.5280052	0.2063255	0.3435088
0.1806406	0.06722715	0.551142	0.237195229	0.3046882
0.3336758	0.7161657	0.3465013	0.1751001	0.1838798
0.05352844	0.2256312	0.3567898	0.6685975	0.3237053
0.4086454	0.4101435	1.323424	0.3137802	0.2518951
0.4587028	0.5483631	0.4225456	0.2554061	0.5472176
0.4094533	0.2318446	0.412602	0.4600379	0.2136095
0.293752932	0.1732321	0.08931875	0.4686803	0.6693596
0.4880132	0.82296	0.187734595	0.1857806	1.286088	0.1430848
0.6077734	0.2205489	0.1970997	0.2657773	0.1150653
0.2398857	0.2298208	0.2515429	2.86181	0.3918838	0.2847563
0.970658582	0.4021513	0.5410428	0.06931775	0.130801882
0.3841659	1.35475	0.09323763	0.3215598	0.1532949	0.1089088
0.1711689
AC243967.2	0	0	0.0707089	0.04895455	0.134439	0.05189987
0.07918009	0	0	0	0.1539638	0.1006826	0	0.1681719	0

0 0.05111297 0.038585682 0 0 0 0 0.4237774 0
0.07019894 0.04028149 0.08111981 0 0 0 0.07241101
0.0459372 0.1236696 0 0.06258173 0 0 0.207123
0.1585668 0.11338266 1.800238 0.5647072 0.080009606
0.2306165 0.0263857 0.5090745 0.5068608 0.302961
0.052181367 1.800978 0 0.04835084 0.05497895 0.03852813 0
0.5039905 0.112867 0 0.2312382 0.069475776 0.11636495
0.104833326 0.05104017 0.05996858 0.0705236 0 0.1748142
0.1035902 0.720846183 0.03579937 0.08753789 0.3157172
0.4309137 0.9557071 1.61508 0 0.1989441 0.02085778
0.05818062 0.100403273 0 0.2239179 0.1372338 0.1317393
0.1349514 0.02480977 0.2636515 0.09499235 0.43890147 0
0.296309452 0.1144448 0.3283358 0.1297826 0 0.0785318
0.5961024 0.134909 0 0.1818072 0.06416307 0.1343186
0.05903975 0.461041 0 0.07731531 0.147858 0.03189762
0.045318069 0.03416063 0.9931298 0.04114855 0 0.1129907
1.088984 0.02034388 0 0.6699064 0 0.4289104 0
0.7167282 0.2833531 0.428145 0.04926126 0.04970769
0.07031029 0 0.2373457 2.774781 0.3321699 0.7584803
0.4171549 0.07745858 0.05728294 0.1959568 0.1706081
0.08276797 0.6485956 1.412108 0.296737 0.3237689
0.2392004 0.620506 0.8464737 0 0.2328396 0.1095348
0.02258209 0.1001195 0 0.2658246 0.3018481 0.05093147
0.03745056 0.1609153 0 0 0.07198341 0.3429178 0
0.02830928 0 0.3030324 0.1262668 0.1423856 0.0408752
0.2873814 0.03489647 0 0.02115284 0.029498683 0.05467706
0.4171906 0.02660874 0.2842081 0.04957484 0.1106953
0.09860161 1.334281 0.2319411 0.2329108 0.4017396
0.04774476 0 0.06331988 0.1596932 0.2080151 0.4015098
1.905195 0.5273875 0.2700197 0 0.3525586 0.2203054
1.031519 5.205626 0.2545387 0.9399507 0 0.2759866
0.3149458 0.2009642 0.6275303 0 0.03973411 0.03087565
0.02951323 0.5875338 0.8175547 0.1966508 0.8170406
0.7848762 0 0.1229511 0 0 0 0.02684419 0.02656533
0.54996 0.135066153 0 0.1835568 0.7820313 0.1245934
0.8466187 0.1193427 0 2.381472 0 1.162075 0.5681589
0.6267089 0.8904815 0.2510721 0.1845497 0 0.05786666
0.2093032 0.7292623 0.770289 0 0.2540138 0.08019002
0.2214745 0.04266325 0.05129025 0.07466443 0.07308248
0.1382036 1.1277 0.04771972 0.09290595 0.5674278 0
0.5300442 0.34528951 0.04811412 0.1731789 0.0704296
0.1435563 0.1963491 0.07940915 1.240004 0.3118337
0.04437898 0.1393793 0.04255305 0.1324676 0.3971243 0
0.2861041 0.023418869 0.1070162 0.3069108 0.6566595

0.08203643 0.1923048 0 0.0548546 0.3248728 0.1763781
0.0708375 0.03739145 0 0.3838232 0.5168712 0.04507261
0.1042398 1.1992 0 0 0.1528719 0 0 0.2456553
0.1728691 0.04154187 0.1749381 0.2861585 0 0.3059079
1.974838 0.1297448 0.1285435 0.027731949 0.142133482
0.0224939 0.9816551 0.0599214 0.03223348 0 0 0.197905
0.08538877 0.05610885 0.01868643 0.2091799 0.2299932
0.5461467 0.122364815 0.7438632 0 0.1581003 0 0.1420018
0.100916833 0.5932219 0.1637741 0.2078776 1.958198
0.02531078 0.2898128 0 0.1715724 5.452835 0.2309847
0.3588526 0.05151518 0.03585221 0 0.2382899 6.496745
0.07668874 0.6985668 0.022548188 0 0 0 0.04705574
0.6833341 0.04369983 0.1221242 0.1286934 0.2543779
0.4290167 0.2215583 0 0.09912445 0.6065694 0.340897 0
0.1495031 0.4787297 0.2705186 0 0.1529984 0.8982308
0.3778209 0.4104697 0.2811342 0.04873462 0.025386056 0 0
0 0.127261 0.1325468 0.0773723 0 0.3260424 0.7335467
0 1.421679 0.184992 0 0 0.07572676 0.1179514
0.02114242 0.1247588 0.9865497 0.09313287 0.2030207
0.418444979 0 0.113454 0.04942099 1.342899317 0.1288923
0.02949271 0.3418713 0.1170691 0.09714983 0.06902041 0
AL080317.2 0.8114477 0.2768874 0.8424109 0.08684185 0.260567
0.6410566 0.1664709 0.1493062 0.1247537 0.156084003
0.4971085 0.4432018 0.1715841 0.2083538 0.1536975
0.4390214 0.2350723 0.139431575 0.1953952 0.4170905
0.047213516 0.3118522 0.1584887 0.4592243 0.2121586
0.4419715 0.341098 0.4392901 0.260262232 0.1528009
0.1807842 0.5221351 0.2730074 0.381761 0.5509658
0.369047 0.03853895 0.3386929 0.8073951 0.289035129
0.9217487 0.4244452 0.133170101 0.3549837 0.5478075
0.5202824 0.8227375 0.1302863 0.380549097 0.1425292
0.3187194 0.9943073 0.4154001 0.2379458 0.6958727
0.5918895 0.4350416 0.6834122 0.4186404 0.04564631
0.31727989 0.202037994 0.3185723 0.1930599 0.4077457
0.5954848 0.2710572 0.4117622 0.104869337 0.5221564
0.1524103 0.4026573 1.475528 0.3327921 0.6366748
0.2861985 0.640471 0.5892624 0.8409561 0.125335395
0.1405055 0.4597388 0.1947544 0.4258461 0.3511113
0.2836248 0.564703 0.4493589 0.69207083 0.6228383
0.243348 0.4097933 0.129432 0.6508832 0.2441573
0.2889388 0.2904704 0.4325469 0.5396829 0.4743841
1.285752 0.4559516 0.2249805 0.5856235 0.5975745
0.8229108 0.1117159 0.2179535 0.25903766 0.5745636
0.5790908 0.8921559 0.1063597 1.239742 0.5485299

0.280689	0.1167869	0.8055814	0.8506725	0.4544002	
0.2090613	1.427183	1.284545	0.1687774	0.9741913	
0.7413473	0.3818753	0.08375639	0.4646971	0.1745151	
0.3225089	1.195991	0.2466676	0.2985611	0.07527098	
0.6608945	0.4163391	0.2682718	0.692931	0.5169004	
3.612481	0.5991601	0.3457457	0.9536141	1.045547	
0.7515293	0.9134974	1.004638	0.9050367	0.6643732	
0.5943377	0.2159303	0.3783285	0.37478	0.4847265	0.7929228
0.5295031	2.61135	0.2648456	0.3379509	0.12687491	0.8927755
0.447620158	0.425758	0.4189408	0.347467	0.1665037	
0.456297	0.6419656	0.4601161	0.4030314	0.484523557	
2.069188	0.4317053	0.409084	0.5281726	0.3810829	
0.3442456	0.822735	0.4013046	0.7009836	0.5508893	
0.1131202	0.3011405	0.5388062	0.6281875	0.3488592	
0.1275569	1.50225	0.2212365	0.181912	0.4683511	0.07849082
0.2110449	0.2315887	0.736651	0.3140516	1.143885	
0.219576	0.4285966	1.675971	0.4800606	0.178248	
0.4700151	0.6179466	0.3837542	0.09331386	0.8027666	
0.5490003	0.417075	0.3574578	0.339976	0.2687744	
0.8244065	0.2875773	0.7423595	0.09008162	0.7601966	
0.2963001	0.397944339	0.1948037	0.472096113	0.240322263	
0.3328526	0.930717	0.6303155	0.2503068	0.2587511	
0.2095067	0.3533085	0.2203232	0.5638118	0.3425451	
0.4595709	0.3389288	0.3016356	0.2473522	0.2998161	
0.4106052	0.8250864	1.119658	0.3475137	0.6161816	
0.2419901	0.3512379	0.2522192	0.3251503	0.778429	
0.2698042	0.605001	0.5327005	0.5091387	0.8088908	
0.7202741	0.312676	0.2920864	0.4791846	0.216388402	
0.4552053	0.6274172	0.620058	0.1744882	0.5977157	
0.2191251	0.5206102	0.8451217	0.3644682	0.3955983	
0.8974453	1.006097	0.3714199	0.2013211	0.7002009	
0.310806117	0.5648299	0.4133693	0.6743125	0.7330235	
0.4295772	0.100367664	1.034349	0.2419041	0.1390587	
0.4188688	0.4987014	0.7379747	0.2946476	0.5376838	
0.5537664	0.4876251	0.5868401	0.5269057	0.332736	
0.390275	0.2270163	0.3196013	0.6520482	0.2952998	
0.6141024	0.3476819	0.5097136	0.5905258	0.4536511	
0.6105833	0.3693893	0.5939959	0.178557759	0.37353272	
0.6532197	0.7218999	0.1128578	0.1016531	0.4093602	
0.1881116	0.5027658	0.5413777	0.5566479	0.2185338	
0.5806561	0.2451306	0.3947065	0.482369758	0.3531439	
0.2223975	0.107336	1.450371	0.195923	0.061883185	
0.2613638	0.3658443	0.3414443	0.7480407	0.3425666	
0.15579	0.291231	0.3832645	1.104626	0.5979319	0.6635239

0.1331276	0.2779522	0.8505008	0.4975095	0.4926557
0.6751631	0.8851481	0.358508358	1.222231	0.647536
0.5310407	0.5441236	0.3626196	0.3129526	0.3490303
0.2423849	0.2674063	0.7829679	1.174233	0.8499019
0.570502	0.5446473	0.139983	0.4483761	0.6630195
0.738098	0.578442	0.4159734	0.4221904	0.710537
0.4437151	1.459883	0.3488931	0.6595946	0.296884481
0.1854768	0.4100227	0.3167665	0.8612013	0.6124953
1.247138	0.201878296	0.4685915	1.128561	0.3552292
0.532285	0.06320167	0.133873	0.2689124	0.431195
0.5827648	0.5361845	0.397544	0.9369053	0.301867
0.397196	0.262233974	1.552229	0.5933419	0.1039043
0.431346107	0.1947722	1.790437	0.3394152	0.6025055
0.6319017	0.2131315	0.4142616
AP000487.1	0.1557649	0.1895685	0.2170887	0.1082153	0.0990603
0.3154966	0.1750297	0.20931	0.1311677	0.189242569	0.239049
0.185468	0.1312042	0.115065	0.4001513	0.2063181
0.2259732	0.181251164	0.250609	0.1512188	0.049640916
0.2726503	0.08406919	0.3501256	0.3200518	0.322782
0.1120734	0.2463336	0.109457264	0.08568369	0.02667774
0.2115529	0.3371624	0.1486624	0.4073302	0.734653
0.02455779	0.1441382	0.6973808	0.246458097	0.6632454
0.6615991	0.19160195	0.2215132	0.3548183	0.461938
0.8567987	0.2333815	0.495036392	0.380769	0.7138026
0.6590983	0.5620874	0.4613237	0.5429854	0.2727185
0.3881042	0.285484	0.2887097	0.345550572	0.235792135
0.225299693	0.2397545	0.287218	0.2143546	0.5341446
0.2962641	0.2433008	0.147014682	0.8737872	0.2378497
0.3934247	0.8638286	0.2244654	0.5656454	0.239023
0.2785218	0.5571222	0.5537366	0.319044611	0.1066934
0.3248283	0.2477432	0.3057738	0.4424985	0.2376515
0.3059745	0.297476	1.00254337	0.2874997	0.282013821
0.3689339	0.1774165	0.5339303	0.271651	0.2603949
0.3340003	0.1739617	0.3406559	0.334908	0.5023293
0.4165053	0.6090417	0.5039097	1.11535	0.3702997	0.1702312
0.2526627	0.288008252	0.2328316	0.4687965	0.6291397
0.2319395	0.572387	0.7689755	0.4721923	0.2693487
0.8526082	0.3033666	0.5876355	0.3663497	0.451945
0.5698132	0.2979488	0.6669715	0.5448224	0.3367493
0.0459457	1.036202	0.1965938	0.1971652	0.671731
0.6435712	0.1521993	0.1371776	0.330892	0.2469328
0.3659216	0.5818066	0.5326374	0.9292553	0.4075512
0.4296164	0.6261857	0.5524355	0.2290342	0.9313583
0.4439041	0.3702274	0.3043105	0.4536756	0.2225804

0.4619376	0.2720813	0.3380406	0.5780247	0.4555036	
1.31901	3.142644	1.04229	0.184978507	0.5058421	0.410296105
0.3692824	0.3023756	0.1798555	0.2710671	0.1235236	
0.6171165	0.6450296	0.3623815	0.271698395	0.992101	
0.2881909	0.3823256	1.417301	0.3333256	0.6525194	
0.499495	0.4260334	0.7358366	0.424279	0.111007	
0.4089716	0.3841464	0.2274517	0.3125573	0.402345	
0.2802783	0.269339	0.3926492	0.2437283	0.2726956	
0.2092672	0.2840781	0.5535269	0.1779149	0.5251536	
0.432872	0.5567443	0.5447103	0.5762955	1.286435	
0.3660593	0.4644027	0.4684443	0.1933791	1.283049	
0.3808089	2.139437	0.3501764	0.3210826	0.3373591	
0.3852409	0.5028052	0.2312671	0.252568	0.3701932	
0.291754	0.176170086	0.3876148	0.298567285	0.198178834	
0.2434543	0.448639	0.390174	0.3070385	0.4067075	
0.1612493	0.3585012	0.2236628	0.8507767	0.4371129	
0.1973761	0.2545387	0.495537	0.4079521	0.3282401	
0.213193	5.238777	0.5408864	0.2223027	0.3129766	
0.1988661	0.2117298	0.2810525	0.4636822	0.5952368	
0.3163414	0.6865907	0.449768	0.2609995	1.213086	
0.6503417	0.2764886	0.1730161	0.8006458	0.141890224	
0.274757	0.4238325	1.548216	0.1586674	0.3255261	
0.297436	0.3231332	0.2943956	1.103635	0.2464814	
0.6702106	0.8345456	0.3356499	0.3463718	1.264881	
0.215700113	0.4566965	0.6266095	1.621808	0.3173514	
0.1619409	0.230754324	0.4092442	3.597349	0.1137175	
0.2392319	0.2961796	0.7049624	0.4614385	0.4998688	
0.2822969	0.2649887	0.4798978	0.2299143	0.268213	
0.2735602	0.694365	0.3600354	0.6516329	0.2653693	
0.3481865	0.5397761	0.3221375	0.4633871	0.2898075	
0.3295474	0.3824057	0.4972602	0.219666224	0.333826665	
0.2486168	0.3933872	0.1508548	0.2850118	0.345222	
0.1533202	0.5589947	0.2752664	0.5012882	0.4440486	
0.1830324	0.2494955	0.4653026	0.417006409	0.3934013	
0.2143457	0.1359108	0.3219316	0.2136255	0.607271471	
0.5463886	0.2068725	0.1579596	0.2653874	0.3683385	
0.388266	0.2388007	0.7675607	0.5998915	1.378614	
0.6327138	0.2562203	0.4028656	0.4973246	0.385305	
0.3690476	0.3672987	0.6618001	0.427822207	0.4256281	
0.1940751	0.8004921	0.4767489	0.5035094	0.4185984	
0.4555552	0.6558846	1.405773	0.403928	0.6053983	
0.483026	0.5331852	0.7697401	0.1255936	0.604941	
0.4612958	0.3939021	0.3533569	0.2631901	0.8342438	
1.006019	0.5306892	1.335827	0.4545826	0.4443828	

0.341375645 0.3250212 0.2639408 0.6155425 0.550906
0.3906643 1.235242 0.265857899 0.5180213 0.7657198
0.5120843 0.746202 0.1294944 0.4206498 0.9634039
0.2185448 0.217279 0.475148 0.3274919 1.404299
0.3431211 0.3282762 0.373551084 0.6126731 0.5266652
0.1001425 0.199275264 0.4214438 0.3857336 0.3428709
0.1617402 0.2058042 0.2797143 0.4421169
AL513217.1 0.02165219 3.457637 0 0.04136696 0.02840048
2.148933 0 0.5449463 0.3008452 0.434045176 0.7620173
0.08507756 0.1671827 0.1218056 0 1.989084 0.2159542
1.613956847 0.05346927 0 0.35421864 2.242629 0.02754577
1.891042 2.713827 0.204229 0.1028203 2.122988
0.209208556 0 0.4283149 3.085973 0.02090034 0 0.03525468
0 0 0 0.1172413 0.057485515 0.0874261 0 0 0
0.1337767 0.03186457 0 0.09309248 0.220468221 0.01729363 0
0.102142 0.04645762 0.03255656 0.8865465 0 0 1.57592 0
0.029353774 0.073746937 0 0.194082 0.0506739 0 1.657933
0.206807 0.1750691 0 0 0 0 0.7282506 0.1615159
0.101093 0 0.1681093 0.2114997 0.01638769 0 0
0.1655608 0 0.8237731 0.1596489 0.02096444 0.5792474
0.05351283 0 0.5372946 0.020865308 0 0.05548924 0.2558903
0.02492226 0.02211998 0.06296387 0.04559966 0 0.4608853
0.02710913 0.0189167 0 0 0.134052 0.4736571 0
0.7547043 0.019147053 0.02886599 0.05245013 0.05215625
0.03799817 1.360561 0.3834165 0 0.04845301 0.01715382
0.2239353 0 0.02240684 0.04658775 0.2992944 0.08039682 0
0.1890152 0.05941272 0 1.343745 0.1202415 0.2962797
0.02465083 0 0 0 0.165585 0.06178505 0 0.0476581 0
0.07374852 0.04559786 0.101063 0.09118821 0.0817459
0.03502072 0.05621462 0.7404617 0.09541016 0.08460169 0
0.1837831 0 0.2367061 1.708884 0 0.2128173 0.9486015
0 0.07244203 0.195814689 0.0956862 0.154996754 0.05909184
0.05334817 0.1203169 0 0.1011831 0.05897555 0.3465342
3.002883 0.024926607 0.2541139 0 0 0.05146242 0.1256733
0.2806149 0.02082977 0.05011009 0.02177689 0.04373585 0
0.1008617 0.2375932 0 0.9277256 0 0.03571356 0
0.185686 0.04563372 0.04389139 0.09930488 0 0.09474364
0.2074902 0.150561 0 0 0.03331581 0.6741999 0 0
0.1229019 0.01678781 0.1565409 0.1496334 0.03677558
0.1015941 0.02769523 0.6213649 0.0602933 0.1910455
0.08311565 0.07577605 0.02317152 0 0.1587848 0 0.02020523
0 0 0.06204276 0.05664191 0 0.08942489 0 0 0.04327659
0.07328445 0 0 0.01708302 0.02594702 0.121233 0.03118918
0 0.1466933 0.04421569 0.5189323 0.02169666 0.05742723 0

0.06776117 0.08317673 0 3.098856 0.283914 0 0.2335662
0.09773489 0.08064704 0.1570124 0 0.07936565 0.08957827 0
0 0.3553909 0 0 0 0.1789369 0.05111279 0 0 0
0.1258518 0.1567105 0.09869793 0 0 0.019789119 0.09042951
0 0.04110238 4.592542 0 0 0.06952882 0.01525111
0.6210035 0 0.03159605 0.1259919 0.03414037 0.10919
0.03808669 0.281867 0 1.279055 2.353698 0.1107239
0.0663579 0.2569075 0 0 0.03510319 0 0.1343367
0.2149525 0.1292472 0.07852956 0 0.07241346 0
0.150129814 0.03801503 0.01455273 0.03375602 0.02723753 0
0.08439692 0 2.002278 0.07111859 0.07895087 0.06628448
0.04318799 0.1442181 0.077549385 0.1419351 0.04469287
0.08906395 0 0.03999752 0 0 0.03954009 0.02195726
0.1418307 0 0.02226309 1.236768 0.248537 0 0
0.1082975 0.02902048 0.03029538 0 0.04474594 0.02418413
2.430096 0.3373109 0.076213549 0.03337494 0.172308 0 0
0.08883422 0.01846334 0.1031958 0 0 0.2618217 0.1248123
1.329437 0.2177783 0.05695062 0.07201512 0.02167949 0 0
0.08312373 0 0 0.1062616 0.05986152 0.04624666 0
0.1029528 0.214514063 0 0.1345272 0 0.05376823 0.03733435
0.1525537 0.019669983 0.03443853 0.07748156 0.04310137 0 0
0.4452917 0 0 0.09966984 1.179123 0 0.1515712 0
0.1334309 0 0.09691186 0.6519122 0.2088055 0 0
0.02492156 0.0641964 0.01648737 0 0 1.546834
LASTR 0.0625335 0 0.05752072 0 0.02734107 0.1055496 0
0.1998546 0 0.339506463 0.01789249 0 0.03218926 0
0.1359304 0.01544262 0 0.455139433 0 0 0.048715046
0.1037968 0.3182189 0.09975348 0.01427647 0.08192112 0 0
0.040280901 0.04729813 0.1178107 0.9529168 0 0.3939021
0.3224261 0 0.06506934 0.4493113 0.2902318 0.01844705
0.2019957 0.3675053 0.086782298 1.179221 0.2146441
0.1993936 0.1212719 0.2912646 0.212244155 0.06659414
0.2165635 0.157331 0.1118116 0.1880527 0.8941176
0.2818677 0 0 0.04180204 0 0.094661301 0.085280482
0.2283627 0.09756725 0 0 0.3981851 0.04213463
0.083771221 0 0 0.1057542 0.2474417 0.03887274 0.3892877
0.603167 0.01618383 0.05090255 0.04732914 0.081676694 0
0.06830782 0 0.3000707 0.197606 0 0 0.2575833
0.71408073 5.478169 0.020086975 0.02327482 0.6232257
0.0527882 0.6478 0.08517939 0.9092271 0.131696 0.05247768
0.08451296 0.1304895 0.2731658 0 0.200027 0.1290515
0.1257899 0.4811218 0.1297414 0.036865631 0.1667352
0.063117 0.03347378 0 0.1148955 0.9596964 0.1985937
0.04664558 0.08256966 0.1385884 0.06542114 0.08628402

0.06727484 0.0960433 0.773978 0.06011004 0.02021826
0.1143929 0.04508875 0.4440783 0.2122197 0.1651318
0.4271631 0.2120936 0 0.09319778 0.07970411 0.07930707
2.042362 0.4817434 0.3555596 0.28399 0.4609178 0.06486208
0.02194666 0 0.2191433 0 0.8197665 0.5511066 0.04072291
0 0.1965861 0.1322189 0 0 0.06545121 0.01862533
0.1974521 1.639611 0.06973975 0.031418378 0.06908763
0.170531374 0.05688755 0.3851861 0.03309392 0.03325142
0.9156416 0.0283878 0.1796348 0 0.047993553 0.1779162
1.61205 0 0.6440555 0.1814781 0.09004908 0.02005277
0.09648169 0.0419291 0.4420961 0.0466871 0.01941986
0.01906086 0.3348141 0.08119263 0.2256233 0.03438134
0.07208605 0.2323872 0.1537601 2.15496 0 0.1075293
0.2189027 0.1731169 0.04141276 0.04247984 0.5111984
0.04810956 0.5124082 0.102176 2.195096 0.3943912
0.1616158 0.1758184 0.02400861 0.3363357 0.2542913
0.2666212 0.5981863 0.2708729 0.0689696 0.2400456
0.1094241 0.04461431 0 0.04367477 0.129663205 0
0.065924685 0.065637853 0.3882346 0.1272344 0.08446251 0
0.1941674 0 1.458179 0.2351691 0.6544606 0.05437523
0.2795782 0.4746033 0 0.09007722 0.08838604 0.188295
0.1489822 0.07805855 0.06266196 0.2211402 0.2066368
0.4566344 0.1000925 0.2429418 0.4172392 0.03036925
0.1932177 0.730774 0.07056683 0 0.07557773 0.01489016 0
0.1379788 0.021606793 0.1174205 0.422636 0.03819568
0.1751716 0.1597273 0.08613102 0.04920615 0.6024721
0.05415254 0.04535328 0.3634714 0.04310422 0.01900325
0.05665191 0.2909273 0.228611173 0.4062625 1.331561
1.622335 0.1501549 0 0.198834534 0 0.073411 0.1434812
0.05762534 0.1672959 0.3638761 0.01643342 0.7007794
0.3116606 0.1695954 0.01681954 0.0648075 0.0171659
0.1243592 0.7665909 0.03533202 0.6994302 0 0.1520719
0.07115488 0.1551907 0.09197076 0.924307 0.0378001
0.05277282 0.06971224 0 2.196849359 0 0.09806914 0.01624841
7.210912 0 0.02708289 0.4293146 0.1736565 0.04564378
0.1520116 0.8082839 0.4573466 0.3609798 0.497710546
1.132165 0 0.5358851 0 0.05775826 0.136824114 0.2554824
0.2474234 0.1691056 0.3982418 0.02058998 0 0.1020542 0
0.02053614 0.1127418 0.0417031 0.0838138 0.05833056
0.08446999 0.04307679 0.04656399 0.03119262 0.4465014
0.201769084 0 0.1935272 0.7070056 0 0.04276023 0.05332382
0.07450975 0 0.1773711 0.1163331 1.081408 0.1422051
0.5160729 0.2741311 0.2079863 39.46666 0 0.1817387
0.2800805 0.02278952 0.04978484 0.1169118 0.7107518

0.04452153 0.5336307 0.4360945 0.041302421 0.01913755
0.05180359 0.5824882 0.7246754 0.07188335 0.3776479
0.056808716 0.0828847 0.4475477 0.06224036 0.03168538
0.2106838 0.1428937 7.152422 0.1642738 0.03838075
0.1031944 0.1776068 0.6201482 0.1010164 0.2202061 0
0.07463743 0.0369173 0.0402033 0.060690572 0.02621302
0.1439515 0 0 0.07903006 0.05614716 0.1820051
RAP2C-AS1 0.2670702 1.976247 0.3892789 0.5769673 0.4095902
3.129139 0.5332564 1.068574 0.6343235 0.59714968
0.7406458 0.2663847 0.6154649 0.3428594 0.4956886
3.433608 0.434388 1.218886339 0.2942473 0.3510192
0.556944127 2.389721 0.8258902 2.487363 2.307572
0.4618303 0.5138009 3.460076 0.468459136 0.3076653
0.673447 3.314728 0.4124745 0.4529243 0.3980559
0.2702101 0.9534118 0.4686627 0.4989909 0.276350994
1.174586 1.405354 0.235209233 0.3882849 0.359632
0.4293157 0.6071755 0.3577255 0.514590036 0.413492
0.5549439 0.732667 0.7405366 0.3459687 1.73841 0.3636685
0.2654418 2.160745 0.6839054 0.345355125 0.69971995
0.487492757 0.8757229 0.5721522 0.1300478 1.353116
0.4933557 0.7350264 0.189894858 0.6142284 0.3052995
0.2561053 1.052718 0.3907831 0.3453056 0.09660096
0.7815702 0.6087096 0.7183563 0.442742656 0.2214687
0.929052 0.409303 1.280144 0.6664981 0.377935
0.9681363 0.3351059 0.39411764 1.728657 0.439499152
0.4266678 0.698475 0.7283757 0.335781 0.3693837
0.4580135 0.3244914 0.2310195 0.7413168 1.867379
0.2584568 0.2366766 0.4337123 1.059918 1.143675
0.4978916 1.028076 0.421473418 0.3177054 0.5076063
0.3925928 0.3749526 1.617043 0.5093063 0.2577103
0.1563075 0.520824 0.7922192 0.3868654 0.4464576
0.5083348 1.378221 0.5721112 0.1974769 0.6655503
0.4509721 0.2621867 2.051356 1.156078 0.3580502
0.2619569 0.3177326 0.4015973 0.3031168 0.9636023
0.3634589 0.2079264 0.4567077 0.2102588 0.512206
0.4456172 0.3515961 0.2206272 0.4731252 0.8838672
0.3680261 1.514015 0.6481685 0.7746163 0.3835216
0.4882518 0.3350876 1.151532 1.591384 0.1806202
0.3561198 2.661213 1.200429 0.494884 0.71220025
1.130314 0.953807393 1.184884 0.1822226 0.6555938
0.46536 0.5875429 0.5539716 0.4147887 2.64566 0.189205353
0.6487953 0.4306481 0.3925392 0.3027347 0.7869866
0.4792516 0.4268925 0.4754506 0.2438137 0.3112283
0.5153538 0.5397413 0.4020192 0.6244332 1.696457

0.2168099 1.379138 0.1420927 1.247362 0.3290636
0.2956772 0.2386949 0.4168483 0.9960247 0.5801081
0.3306052 0.07536087 1.386719 0.1548912 1.010035
0.4833711 0.4393954 0.2332217 0.2229987 0.3168596
1.202046 0.3140381 0.5051031 0.5360624 0.8952557
1.117444 0.3534692 0.6111739 0.3307273 0.7079305
0.5338183 0.7877206 0.566549244 0.4294297 0.58909622
0.263077358 0.3973518 1.046188 0.2364138 0.6660523
0.1435252 0.1904933 0.2299436 0.8112205 0.4682376
0.1446956 0.1620858 0.4234441 0.6211478 0.3551121
0.3763206 0.668085 1.006857 1.40633 0.6381679 0.6157106
0.3869472 0.5957783 0.4814065 0.4925578 2.615367
0.8620194 0.2373062 1.97972 1.251883 0.7039739 0.8491594
0.2436115 0.316273 0.2923758 0.477011509 0.1928783
0.9282897 0.4893822 0.1381161 0.7136533 0.5008436
0.4510169 0.7000384 0.1529983 0.5319198 0.5288143
0.722203 0.6068248 0.1172531 0.4989123 0.244090055
0.4547433 0.4552235 0.3431863 2.463198 0.2863361
0.514412661 1.87794 0.2112684 0.6976314 0.3445515 0.4556765
0.7252253 0.2656209 0.3867763 0.4733963 0.8591474
0.2486542 2.554911 1.444824 0.7844195 0.950714
0.7730578 0.5672302 0.3603559 0.2131605 0.3576559
0.6831872 0.6662353 0.3013227 0.7637256 0.2947328
0.2095557 0.226788676 0.712225011 0.6203889 0.1629322
0.3651206 0.1964091 0.1483023 0.4644456 0.4072555
1.903209 0.8772164 0.5453415 0.343807 0.7744708
0.3229328 0.873147011 0.3462938 0.1781016 0.8239234
0.3244808 0.3036011 0.593343033 0.3972806 0.3263902
0.2416665 0.885924 0.7914272 0.8533891 1.30757 0.4676997
0.2914552 0.437055 0.3658044 0.1844844 0.2701995
0.3774076 0.348135 0.3074791 1.979832 0.5440761
1.258234827 0.5794979 0.3651231 0.4529255 0.324453
0.771226 0.3748904 0.3475928 0.2063607 0.2855277
0.6344254 0.3375067 1.747254 0.7375128 0.3836517
0.4168058 0.6006372 0.2397293 0.2405292 0.3154752
0.3593732 0.4416011 0.509873 0.3900084 0.6186995
0.435774 0.4493412 0.244240277 0.3885474 0.5156699
0.5358145 0.3673148 0.4038254 0.3887433 0.694268054
0.774414 0.2891608 0.2167441 0.3622492 0.09492349
1.092862 0.2916936 0.3764279 0.484188 1.278106
0.4251096 0.352341 0.4878413 0.6872618 0.624526946
0.5112484 1.258915 0.6181258 0.31103918 0.542534
1.130273 0.2436413 0.3910855 0.3037718 0.3209566
2.155822

ANK3-DT 0.06695215 0 0.03079258 0.1918702 0 0 0 0.9227734
0.05168128 0.503302384 0.1436759 0.06576848 0.2584783
0.1883215 0.1091515 0 0.1335533 0 0.04133396 0.07149791
0 0 0.1277641 0 0 0.05262582 0 0 0 0 0 0
0.1292547 0.4217355 0.16352 0.1467685 0.1741679 0.8418548
0.02589497 0.059251584 0.2433024 0.118042 0.034842893
0.02152066 0.03447164 0.04926528 0.07790459 0 0 0.08021218
0.115933 0.06316803 0 0.5536866 0.06527021 0 0
0.1029508 0.1678342 0.13615011 0.038006294 0.273919337 0
0.07834605 0.04606783 0.02268408 0.1370318 0.1691696
0.033633952 0.02338507 0.200137 0.242629 0.09934728 0 0
0.4843403 0.1039644 0 0.1266836 0.032793004 0.3492404
0.2925379 0.1434315 0.0344222 0 0.1296511 0.06888958
0.1241029 1.26148779 0.2643143 0.225816478 0.1495166 0
0.1130365 0.1155956 0.307794 0.03244912 0.03525043
0.2809289 0.2714541 0.1676519 0.05849356 0 0.08031036
0.06908515 0.1515131 0 0.1250181 0.088808796 0.223146
0.04054612 0 0 0.03690421 0.1185587 0.1860481 0
0.05304245 0.1731113 0.1751096 0.1039285 0.03601426
0.03084893 0 0.03217872 0.03247035 0.4899039 0 0.09302422
0.1239356 0.04821821 0 0.1362482 0.06746393 0 0
0.0318416 0 0.1473667 0.08785006 0.09121706 0.07049807 0
0.2819691 0.1579824 0.1082899 0.04345623 0 0.08850721
0.1962018 0.1856275 0.09471463 0.03033461 0.2328882 0
0.6306842 0.0598242 0.277468 0 0.2240028 0.050457625
0.2219082 0.136935908 0.09136089 0.1649614 0.1594456
0.05340147 0.1564374 0.1367716 0.5769838 0.1105406
0.231231611 0.2142988 0.03406496 0.5214455 0.1591303
0.06476713 0.289236 0.03220456 0 0.269351 0.06761926
0.1124686 0.2495049 0.2448926 0.2895353 0.05215784
0.1358808 0 0.08682714 0.02870859 0.1763838 0 0.1023557
0 0.05859261 0.1497053 0.133017 0 0.1172829 0
0.1371538 0.09845623 0.1093117 0.3166943 0.07786605 0
0.0385576 0.1421453 0 0 0.0355808 0 0 0.1606296
0.2636011 0.1074753 0.05303983 0.1753532 0.138825275 0
0.211748872 0.035137929 0.2158273 0.2043372 0 0 0.3897888
0.0408431 0.3680006 0 0.1167842 0.04366306 0.02641177
0.5215111 0.1405772 0.1928842 0 0 0.3076251 0
0.1006345 0 0 0 0.1285981 0.947537 0.06700823 0
0.2148271 0.09027818 0.1133297 0.06234351 0.1213771 0
0.04090196 0.02769909 0.069400621 0.06285877 0.0323214
0.2146967 0.09377466 0.3420274 0.3458141 0.07902462
0.3055467 0.05797899 0.03641847 0.02779675 0.2768999
0.2441523 0.09098244 0.0934453 0.214169338 0.2796231

0.100241 0.0635477 0.05358831 0 0.159663214 0.1433298
0.2357948 0.07680984 0.3084859 0.07327517 0 0.1055677 0
0 0.02723686 0.1080481 0 0.02756828 0.08559416 0 0
0.03209367 0 0.08140866 0.1142741 0.1661565 0.2215563
0.2283736 0.09105992 0.08475266 0.1119572 0.072460898
0.185690517 0.146936 0.02249973 0.1043792 0 0.2861734
0.1739795 0.1723688 0 0.2565622 0 0.5124065 0.03338611
0 0.039965928 0.03134907 0.5527911 0.06885013 0 0.06183949
1.670010824 0.04558916 0 0 0.03654701 0.03306731 0.6195706
0.05463272 0.03202157 0 0.03017704 0.334874 0.04486806
0.4683917 1.175703 0.03459046 0.1869534 0.1001901
0.06518884 0.058916234 0.02580021 0.08880082 0.2270889
0.06147605 0.1030088 0.08563757 0 0.1681316 0.1424282
0.3113831 0.2412126 0.1522534 0 0.1320756 0 0 0
0.2084791 0.06425809 0.03659976 0.039977 0 0.3085029
0.9295152 0.08161962 0.8595372 0.033165654 1.721145
0.04159805 0.436553 0 0.028861 0.3032495 0.152057123
0.1064896 0 2.565574 0.2289889 0.09667323 0.1835888 0
0.3957334 0.03081957 0.1104862 0 0.05858543 0 0.02947075
0 0 0.02964442 0.4519629 0.048734249 0 0.0770616
0.09925296 0.2294176 0.03173039 0.09017182 0.5048793
SACS-AS1 0 0.1243748 0.005982087 0.01863735 0.008530315
0.210759 0 0.03117696 0.01004014 0.057036431 0.01116478
0.0383306 0.04017174 0.02439019 0.06361471 0.2553563
0.05189088 0.132208641 0 0 0.037997299 0.1748748 0.04136798
0.2022977 0.08017574 0.01022364 0 0.2314076 0 0.04427055
0 0.2390112 0 0 0.01058903 0.02851277 0.0203014 0
0.01006125 0.011510827 0.005251828 0.005733017 0.006768942 0 0
0.009570783 0.007567277 0.04893189 0.046353591 0.01558285 0
0.03067919 11.12825 0.009778627 0.3296818 0 0 0.4333397
0.006521043 0 0.081218464 0.044345342 0.07772546 0 0.04474808
0.1322054 0.008873737 0.01971878 0 0 0 0 0.141535
0.03638446 0.02024273 0 0.0100986 0.005293804 0.03445522
0.006370709 0.01130784 0.03551966 0.01393224 0.1471187
0.05480211 0 0.07360762 0.01607301 0.44558126 0.1613808
0.006267064 0 0 0.01646973 0 0.05315133 0.06934293
0.006848114 0.005457616 0.03955161 0 0.01704535 0 0
0.06710598 0.00490575 0.01876353 0.09714923 0 0.01734026
0.02363073 0 0 0.1218797 0.3685192 0 0.01455325
0.09274118 0.03843474 0 0 0.07696154 0.1018816 0 0
0.03154013 0.065432 0.02110129 0.0963832 0.006019254
0.01873474 0.1036571 0 0.01310624 0 0.04144566 0.00618588
0 0.06441523 0.05973332 0 0.2122831 0.01517755
0.006847279 0.116627 0.005259379 0 0.1000816 0.005731443 0

0.09015479 0.01840025 0.005893116 0.03877996 0.1853501 0
0.01743311 0.04620327 0.01826974 0.1087928 0.009802421
0.07185031 0.006650647 0 0 0.01032518 0 0.006078232 0
0.04803893 0.1717981 0.014973817 0.1110184 0 0 0
0.03145584 0.13111 0.006256391 0.04515291 0.006540864
0.006568209 0 0.006058925 0.01189384 0 0.05572998
0.008799207 0.01072686 0 0.0390406 0.02741292 0 0.02982702
0 0.01707421 0.03739283 0.05168253 0.02650712 0.007594861 0
0.01598695 0 0 0 0.005042355 0.01567278 0.0674154
0.01104584 0.006102922 0.07486637 0.06221066 0.01207305
0.007172756 0.1373045 0 0.006959753 0.0154561 0.006813185 0
0.05461924 0.020568266 0 0.004658761 0.0113419 0 0.01342974
0 0 0.01299848 0.05136035 0 0.02544732 0 0.09352076
0.01820666 0.03747169 0 0 0.03320136 0.1022867 0.01303354
0.01724874 0 0.006784205 0.02498281 0.03248442 0.1692303
0.07580077 0 0.0233845 0.0220166 0.06055748 0.04715996 0
0.08740643 0 0.020223726 0 0.05651182 0.01191692 0
0.06644579 0.047027 0.03070428 0.01978623 0.0112636
0.00707503 0.05940094 0.02017254 0.04743156 0.008837596
0.03630731 0 0.009053746 0 0.006172717 0.2082124
0.006972554 0 0.1392236 0 0.09699219 0 0 0.02522845 0
0.0327961 0.01143965 0.04233052 0.02623818 0.3976542
0.08033549 0.02217121 0 0.02204693 0.04987879 0 0
0.01110003 0.008069823 0 0.02218311 0.0117935 0.01646493
0.01087498 0.007038503 0.054111247 0 0 0.005069447 0 0
0.01689953 0.01674308 0.2438109 0.05696278 0.009485413 0
0.03891559 0.06930733 0.007764195 0 0 0.00668777 0.04669016
0.01201358 0.05122636 0.0797096 0.01187619 0 0.04615001
0.02569599 0.0066869 0.03184055 0.01244169 0.01281441
0.01172501 0.03903365 0.03486614 40.21963 0 0.006719902
0.0435834 0.1557118 0.01899639 0 0.08520769 0.01725137
0.02205832 0 0.04002312 0 0.04649355 0.06532599 0 0
0.09372094 0.3401506 0.02012661 0.2736893 0.01081516
0.01953483 0.01897231 0.1701055 0 0 0.01553269 0
0.01797989 0.08334335 0.03171255 0.0742145 0.090203452
0.02985423 0.008081268 0 0.1776469 0.02803418 0.06545822 0
0.01034389 0.2249647 0.01294585 0.004942862 0 0.01783293 0
0 0.005987329 0.112687 0.1108254 0.6373591 0.04727514
0.005725293 0.269593141 0.005821653 0.1209397 0 0.009467621 0
0 0 0 0 0.01751771 0.1290567
AC009061.2 0.4163689 0.4574413 0.4726286 0.4485162 0.5809977
0.5383029 0.3832509 0.3609874 0.5333887 1.176520472
0.4334455 0.2784734 1.080755 0.1578149 1.097638
0.6431853 0.4947985 0.366858077 0.3937816 0.3973366

0.434781782	0.2911499	0.270486	0.5087427	0.41259	0.3412014
0.4206854	0.6304446	0.342387661	0.683458	0.6258696	
0.2302882	0.9491943	0.4464224	0.3245473	0.6020192	
0.07374525	0.4853498	1.014199	0.791839607	0.7297094	
0.2186657	0.396486625	0.5296445	0.5473424	0.6518637	
1.298822	0.3237518	1.064404438	0.5165208	0.4601974	
1.847164	0.7983346	0.6526995	0.6390909	0.6860916	
0.4682605	1.525681	0.9504738	0.372309672	0.412368291	
0.447011861	0.4764476	0.3939278	0.1097201	0.5582774	
0.8582311	0.5998917	0.186914536	0.4827021	0.242117	
0.4430346	1.077918	0.3386787	0.3240009	0.8331244	
0.639666	0.3172926	0.3151332	0.216953719	0.4775082	
0.5322317	0.6452667	0.6831968	0.3265988	0.2744808	
0.4922244	0.6349428	0.88010451	1.548817	0.273182861	
0.8210194	0.2573032	1.046966	0.479252	0.6968739	
0.6354488	0.438438	0.2825048	0.4220366	2.650893	
0.5959567	0.2245311	0.5206952	0.7221504	0.9155146	
0.4217333	0.628597	0.383863385	0.6731935	0.7510922	
0.5334884	0.8239811	3.017729	0.6902432	0.4993803	
0.2973655	0.7228974	0.5693673	0.5375437	0.3758748	
0.7719788	0.5061482	0.4605169	0.8089809	2.156783	
0.8021803	0.2874408	0.4513187	0.2131844	0.3955658	
0.3832602	0.5588667	0.6427173	0.5347223	0.9936446	
0.7499474	0.2480011	0.3899827	0.7555641	1.104366	
0.5130454	0.4203873	0.2052013	0.7023667	0.9098662	
0.6210002	0.5907665	0.5387067	0.3288375	0.4339231	
0.3425513	0.112386	0.9156484	0.8934014	0.1112671	
0.7678291	1.699322	0.945704	1.007739	0.440642751	
1.037466	0.49827136	0.459367	0.1637041	0.4078826	
0.9044386	0.31463	1.025509	0.6434776	0.5558037	0.22436986
0.7183366	0.5318703	0.5151712	0.5895585	0.7284328	
0.548549	0.4431661	0.4920566	0.1960185	0.8499823	
0.2480252	0.676782	0.4536485	0.5254006	0.5866167	
0.1797935	0.7086854	0.428912	0.7141437	0.4481009	
0.493845	0.5281929	1.172965	0.6589882	0.3508947	
1.196829	0.3881595	0.2069136	0.3475916	0.9582033	
0.2866037	0.5206971	0.5866569	0.6799985	0.4910358	
0.6428305	0.4363513	1.072425	0.6912154	0.6685286	
0.567382	1.338585	0.6971325	0.5890664	0.7963655	
0.5123195	0.3990782	0.514330713	1.099497	0.691110444	
0.195272614	0.3871147	3.028178	0.5169106	0.7957861	
0.4538663	0.3458713	0.4426615	0.6496547	0.504783	
0.5892916	0.2376415	0.6263515	0.9052373	0.7018517	
0.4132047	0.5701807	0.9045345	1.207566	0.7279231	

0.6343959 0.5708341 0.2680009 1.003928 0.6490017
1.347683 0.6840673 0.4611215 0.8839555 0.629809
1.418847 0.722712 0.6265033 0.3788419 0.5864099
0.587704772 0.4075471 0.4704341 0.4139457 0.2109358
0.701469 0.4026625 0.5541842 0.2425743 0.7901759
0.269852 0.5149179 0.5770578 0.5168883 0.4333871
0.3462034 0.380542349 0.5549836 1.114142 1.017422
0.8366965 1.348713 0.126757015 2.01977 0.5491143 0.2337548
0.457161 0.4912415 1.460558 0.2793681 1.116867
0.6700704 0.331559 0.3931567 0.4223289 0.4085484
0.2718136 0.5430019 0.6757249 0.9002666 0.4582086
0.4595949 0.1587643 0.3297802 0.4104195 0.3626127
0.4980163 0.3887597 0.5481125 1.045071719 0.810810849
0.6065946 0.3691603 0.1381115 0.6352158 0.8077999
0.6330626 0.8552753 0.4502045 0.5237624 0.9884552
0.6147212 0.2473829 0.4366468 0.349019521 0.4894661
0.8045807 0.1184306 0.3816067 0.2863847 1.290935517
0.591158 0.3235536 0.5300402 0.4400572 0.7788158
1.175043 0.5204766 1.110094 0.3491144 1.485373
1.187496 0.3502718 1.698148 0.8376607 0.3386913
0.3364248 0.6230727 0.6037917 0.818540487 0.4711056
1.151487 0.691098 0.3253732 0.563366 0.09820471
0.3483331 0.593245 0.3015309 1.244279 0.4212985
0.7453916 0.9458648 1.200009 0.4714356 0.6120359
0.5556457 0.6841164 1.0458 0.3293086 0.6982324 0.3478125
3.028859 1.182603 0.2051937 0.6908133 0.149204995
0.479874 0.3412562 1.691643 0.8579639 0.4582563
0.740085 0.490921985 0.8031527 0.750264 0.5554952
0.2154606 0.2558304 0.340087 0.8123549 0.3316278
0.8889942 0.3800994 0.3881977 1.209289 0.8479066
0.3665514 0.890275058 0.8643947 0.8080274 0.3246417
0.786701536 0.62387 2.192262 0.4465173 0.7959982 0.3358778
0.4891821 0.7664898

LNCAROD 0 0 0 0.01179152 0 0 0 0 0 0.030930789
0.02119127 0 0.01906195 0 0 0 0 0 0 0.05272743
0.014424124 0.01229335 0.01570366 0.05907237 0.008454291
0.1164296 0.03907811 0 0.023853686 0.02800918 0 0.01106474
0 0 0 0 0 0 0 0.021848078 0 0.01088153
0.012847762 0 0 0.07266314 0.01436304 0.7297319
0.150824988 0.2168977 0 0 0.01324259 0 0.09626941 0 0
0.01265383 0.02475448 0 0.056056862 0.033667797 0.01229387
0.01444443 0.06794712 0.008364394 0 0 0 0.008622872
0.01054248 0.01789312 0.2564291 2.359529 0.9509352
0.0223241 0.0383352 0.02009577 0 0 0 0.01348359

0.02644404 0.08884847 0 0.01195169 0.01270097 0.1220293
0.54972689 0 0.011895176 0.02756593 2.604537 0.0312603
0.04262405 0.01261046 5.384293 0.01299803 0 0.02502359
0.09272844 0 0 0.1184527 0 0.07449071 0.4273686
0.01536614 0.021831219 0.03291262 2.586478 0.02973393
0.08665004 0 0.5683164 0 0.01381137 0.009779278
0.009118862 0 0.01277399 0.7967811 0.03412516 0.1833348
0.01186539 0 0.0338708 0 0.04573493 0.09139856 0 0 0
0.0497525 0 0.01573315 0 0.172779 0.190187 0.1295732
0.02522613 0.07798512 0.08642307 0.2079432 0.2097126 0
0.01602379 0.05276654 0.01087854 0 0.01140786 0.02328301
0.05592702 0.03680307 0 0 0 0 22.08912 0.04129873
0.037210891 0 0.037869701 0.02245858 0 0 0.01969094
0.4499338 0.01681079 1.914782 0.01019002 0.113683964 0
0.8164592 0.02563663 0.03911785 0.1671729 0.07110078
0.01187492 0.1714046 0 0 0 0.02300024 0.03386259
0.03050329 0 6.780727 0.04072013 0.03201613 0.05292918
0.01300774 9.721121 0 0.1485798 0.1728407 0 0.3555973
0.188669 0.02883079 1.168077 0 0 0.01007674 0.1634864
0.009570621 0.01487383 0 0 0.03475088 0.07894434 0
0.03437281 0.02722844 0.02369185 0 0 0.01955759 0 0
0.06911321 0.078079023 0 0.8223565 0 0 0.02549027 0 0
2.269797 0.01392633 0.05741641 0 0.03895567 0 0.01727854
0 0 0.05575258 0.03781062 0.02773499 0.0494765 0 0
0.09013713 0.01185463 0.0205523 0 0.0539525 0.02640469
0.1775394 0 0 0 0.008817704 0 0.0102136 0 0.01158909
0.09534402 0.02261885 0.05186687 0.04729393 0 0.02913907 0
0.02137883 0.01342873 0.2767394 0.01276281 0.01125341
0.03354833 0.03445647 0.011281647 0.008592212 0 0.0468644 0
0 0.073591583 0 0 0 0.01137492 0.1350953 0.2394238
0.0097316 0 0.02171298 0.01004316 0.09960253 0 0 0
0.0189151 0.04184608 1.266241 0 0.0600363 0 0.03063379
0.01361589 0.3789411 0.0223846 0.02083413 0.08256488 0
0.051352815 0 0.07466777 0.05773226 33.99066 0.04689861 0
0 0 0 0 0 0.08617417 2.532309 0.029473598 0.04623789
0 0.05077477 0 0 0 0 0.02254154 0.1251769 0.05390451
0.02438609 0 0 0.02361489 0 0.0111273 0.07408767
0.02481657 0.0345424 0.03334782 0.01275468 0.8548073 0
0.01201867 0 0.009513418 1.899146 1.276965 0.02266831 0
0 0 0 0.01750604 0.1033358 0.3735619 0 0.04775154
0.1136351 0 9.936907 0 0.03074933 0 0 0.02948176
0.01730831 0.8986687 0 0.03009593 0.05869272 0.024458613
0.1019965 0 0 0 0 0 0.011213728 0.02944977 0.1325152
0 0.1407267 0.03564672 0 0.07302667 0 0.0454569

0.01018501 0.4207032 0.2160244 0.01495507 0.04346748
0.170566717 0.05524882 0 0 0 0 0 0 0.009399343 0
0.01662471 0
AC016924.1 0 0.8213272 0.05043012 0.03491475 0.1438243
0.7032922 0.3953029 0.1314138 0.05642686 0.160275958
0.09412124 0 0.1693277 0.03426898 0.1191742 0.3655531 0
0.288955796 0.3610353 0 0.106774801 0.7826146 0.02324932
0.4372841 0.4881475 0.02872907 0.02892763 0.6936213
0.035315457 0 0.2582204 0.4586786 0 0 0 0 0.2091768
0 0.5795914 0.016173074 0.0442739 0 0 0 0 0.1344725
0.04252905 0 0.093040362 0.05838505 0 0.01724209 0.2548741
0.05495711 0.2494225 0 0 0.1873403 0.03664908
0.049550648 0.02074809 0 0.1274078 0.04277005 0 0.2476702
0.1246788 0.01847034 0 0.06383095 0 0 0.2169395
0.06816175 0 0 0 0.02975185 0 0 0 0.09981247 0
0.2442894 0.09624836 0.01769452 0.1692344 0.09033235
0.37563341 0.3091978 0 0 0 0 0.1472451 0 0.03542873
0 0.03067249 0 0.02288079 0 0 0 0.1508576 0
0.02636336 0 0.048481783 0 0.04426923 0 0.03207141
0.2014645 0.2588905 0 0.4294033 0.01447825 0.1215045
0.01911888 0.01891194 0.03932123 0.2357712 0.03392847
0.1932342 0.05317785 0 0 0.2708428 0.01691448 0
0.04161184 0.1115693 0 0 0 0.01738271 0 0.02011232 0
0 0.1154572 0.08529975 0.05772386 0.6554582 2.039528
0.02372328 0.03124841 0.67644 0.03570298 0.0675575 0.03447057
0 0.05448706 0.3205203 0.114766 0.0653175 0 0.7700866
0.3057145 0.110181683 0 0.112132423 0.083125 0.0225136
0.04352163 0.0291525 0.01708021 0.04977685 0 0.5129363
0.462851031 0.1754824 0.01859647 0.1328429 0.0579141
0.01767858 0.2105298 0 0.02114709 0 0 0 0 0.01671122
0 0.1566047 0 0.07535785 0 0.06268945 0.03851601 0
0.05587721 0 0.04797961 0.1050762 0.1452312 0 0
0.3093132 0.1946721 0 0 0.2074647 0 0.06606223
0.1052453 0 0 0.04675094 0.03884797 0.05088906 0
0.1578413 0 0.01955734 0.1447754 0.03829096 0 0
0.01926604 0.019182215 0 0.0478072 0.08886094 0.03773843 0
0.04459348 0.0547898 0.2267977 0 0.02383619 0 0
0.07674278 0.2369201 0 0.02063547 0 0.1779342 0 0 0
0.1334483 0.01755081 0 0.3292253 0.1597537 0 0.0821399
0 0.1701703 0 0 0.02232887 0 0.018943314 0 0.1411571
0.08371821 0.02559635 0.9335842 0.0188784 0.02157024 0
0.06330285 0.03976256 0.07587289 0.1322676 0.04998212 0 0
0 0 0 0.01734572 0.3217995 0.03918663 0 0.1369295 0
0.1677256 0.05052184 0 0.3544677 0.04322299 0 0.01607306

0.1040825 0.08847712 0.5113678 0.1354487 0.03115125
5.432751 0.2168364 0 0.04109719 0 0 0.2267671
0.02015837 0 0.01657023 0 0.3361533 0.03955727
0.050685336 0.0802141 0.01228287 0.02849093 0.02298916 0
0.04748875 0.02352455 0.5937742 0.2000863 0.0133273
0.03729718 0.01822588 0.02434475 0 0 0 0.03758611 0 0
0.143949294 0 0.01668641 0 0.05985435 0.01805184 0.05637183
0.4771945 0 0.01800464 0 0 0 0.6136815 0 0.07553339
0 0.21878 0.1601431 0 0.01408464 0.02423872 0 0
0.2436795 0 0 0.09178507 0 0 0.1843532 0.1246754
0.1272533 0.07210164 0 0.3293647 0.05331338 0.04552448
0.01753963 0 0.04364784 0.1153125 0.08420772 0.1951667
0.02227856 0.2954421 0 0 0.02270887 0.0851141 0.1815269
0.01575556 0 0.03320392 0.05813397 0.1743904 0.3092184 0
0 0.1252791 0 0 0.1682477 0.03015787 0 0.5756848
0.08856408 0.1608843 0.045913556 0.01635921 0.04854973
0.1057423 0.079813827 0.2298173 0 0.02709169 0 0 0
0.5657456
AL354733.3 0.4842873 1.424888 1.473465 0.6405514 1.514765
1.546816 0.5755783 0.267882 0.345072 1.120170435
2.174442 1.097828 3.279096 1.886112 0.6073302
1.021153 0.8174144 1.121952908 0.4599728 0.7956435
0.740031507 0.6307117 0.7108928 2.495883 1.428818
2.254679 0.8255501 1.23718 0.503924404 1.183424 0.6316468
0.5008922 3.056541 2.463908 2.577886 1.061625
0.5039259 1.907125 0.691595 0.494522832 2.978271
1.149396 0.659156058 0.8621509 2.071481 1.069056
2.81755 1.88196 2.086251202 2.737361 0.430042 4.147386
3.516961 0.6721677 3.050629 0.5953388 1.258033
2.482259 0.5976619 0.505035452 2.072415 1.727330656
1.075966 0.479518 1.07657 1.615572 0.6099671 1.731949
0.336856966 2.966666 0.922683 0.7830073 2.505929
1.979968 2.840885 1.145341 1.966792 4.215036
3.214256 1.459709122 1.489794 1.749789 2.474009
3.102764 0.4316369 1.334575 2.683161 1.93346 2.80761897
2.941343 1.400062163 2.703759 1.050169 2.893154
1.157735 1.446193 1.913832 1.333732 4.189154
2.529917 2.985063 2.245703 1.63086 1.385251 1.652906
1.882777 1.128554 0.5101165 1.054167998 2.0859 1.872503
4.097921 0.3922581 4.106776 3.562234 0.6802682
0.7919582 1.003454 2.586904 1.208166 1.85046 3.286347
2.471711 0.7607807 2.972159 7.154466 1.771819
0.4029084 1.518282 0.5861519 1.019506 3.520201
2.046867 2.890397 0.2498418 4.273367 2.444945

3.088519	1.598929	4.985829	1.903279	1.294453	
1.304102	1.608127	2.531608	1.235199	1.982719	
1.401371	7.583933	1.455583	2.409984	1.475605	
0.6751395	2.443535	2.014552	3.158272	2.196929	
2.117294	6.488474	1.869562	0.393051964	3.539531	
3.581049548	1.389466	0.6883966	3.34168	0.7131151	4.178083
5.631485	2.843494	2.091202	0.771957839	3.49765	0.7581637
1.779511	1.328126	1.11715	1.180182	1.36184	1.163903
2.060708	2.069323	1.543607	2.498887	1.737324	
3.728323	2.147566	2.318568	4.086135	2.641029	
0.6389502	3.062023	0.1132735	0.6834213	0.5124643	
3.260153	2.165737	1.702277	1.176746	1.914217	
0.6591833	1.129444	1.716504	2.067954	1.339211	1.5886
0.9875457	2.660277	3.63819	3.356044	9.339398	1.306637
2.213022	2.383047	2.109273	0.9452091	0.1594676	
0.9148691	1.75623	0.656572357	0.486687	4.477131273	
0.156408834	2.321712	5.002594	0.7245584	0.5384983	
1.821813	0.4545104	0.6328925	0.8826079	2.382597	
1.16614	2.821589	2.812457	2.45085	0.3756297	1.927133
2.229424	0.912884	2.873796	2.650387	3.556955	
1.384864	2.176234	1.967716	2.853187	1.491363	
2.062461	2.603142	2.478097	2.732505	2.081314	
3.174168	1.836185	1.228947	1.664502	0.579228261	
1.503936	0.5035509	1.023938	1.356607	3.901305	
1.269936	2.770117	1.983442	1.290404	0.8915989	
1.515707	5.007274	1.290564	1.214966	2.703684	
2.723794662	2.852394	3.383691	4.702692	1.938111	
1.75737	0.5330295	2.631755	0.7347123	0.08547556	3.844841
1.223132	4.046385	2.320188	0.8140721	1.572688	
1.970133	1.563096	1.968985	0.5215352	2.317773	
1.255866	0.757736	3.142875	1.005301	2.959379	
0.8583741	1.479219	3.040813	2.191948	2.060445	
0.5344488	1.401618	4.072116669	3.047943192	1.635133	
3.455267	0.1451942	0.4217627	2.618445	0.3388139	
6.857407	1.241421	1.549899	2.635218	2.66101	3.343747
1.935407	3.113243276	1.046577	1.691682	0.03830892	
3.878037	1.410733	0.831399451	1.319046	2.65314	1.322221
2.318205	1.287929	0.574559	0.9727426	3.313987	
2.27551	2.585785	2.38499	2.945876	3.231663	4.025681
3.464368	2.371719	3.177569	3.083098	1.344045478	
2.583991	1.185833	1.579432	3.010119	1.681245	
0.8894596	1.198461	1.683903	3.909592	3.707694	
1.556873	4.151051	1.325828	1.665733	0.7434176	
5.259278	2.282223	1.995198	2.574278	2.158635	

2.936157 1.880481 1.510557 2.625743 0.8174519 2.6924
2.768056471 0.8550558 0.7869497 2.186123 1.202616
2.986891 1.312353 1.082958628 2.399703 2.266236
3.040415 2.180157 1.23717 0.1021507 3.08547 1.321141
1.406163 2.059434 5.804164 1.760267 3.565556
1.73817 1.263505755 1.200514 1.84738 1.149608 0.433859779
1.452269 6.774703 1.822437 3.971343 1.412409
0.4515527 0.9166865
AC243829.4 0.03406475 0.008143413 0.1566704 0.07321661 0.03351124
0.01724925 0.05263202 0.0816522 0.01314752 0.085358826
0.02193036 0.08365627 0.1841166 0 0.02776773 0.05047366
0.03397545 0.06412099 0.09463685 0 0.169174846 0.0254442
0.1408451 0.08151017 0.0174983 0.03346953 0.1617641
0.1616146 0.032914171 0 0.02406626 0.03816881 0.34526
0.05364396 0.02773258 0.03733735 0.04430767 0.06118991
0.03952551 0.052756832 0.02750899 0.007507362 0.283644838
0.2573145 0.1315418 0.01879935 0.1288211 0.2013821
0.485598703 0.1768492 0.0688168 0.1767668 0.03654521
0.1664659 0.2490675 0.06281417 0.6126973 0.2793633
0.02561784 0.150089668 1.479304771 0.743296552 0.1611535
0.02989641 0.03515845 0.2539128 0.04648049 0.1721444
0.13690162 0.06543982 0.7418913 0.07406869 0.07582073
0.03970442 0.2717046 0.6314736 0.2049731 0.006932215
0.0386734 0.025027252 0.3405742 0.2046565 0.9760655
0.1751376 0.1166151 0.07421117 0.140202 0.1052377
0.87523026 0.1248753 0.057446865 1.122076 1.01122 0.1869144
0.4901183 0.913519 0.4787862 0.1434812 0.1143476
0.1294817 0.1172875 0.007440271 0.1766002 0.3064598
0.158175 0.08351277 0.2702785 0.07420964 0.21839537
0.2724841 0.09283304 0.01367598 0.3437431 0.04694147
0.2412872 0.04056852 0.05717227 0.0607221 0.04403886 0
0.09694308 0.05497136 0.08632637 0 0.04093073 0.5782234
0.2648382 0.175003 0.1735434 0.03941094 0.1962645
0.02908682 0.06932204 0.2831824 0.1142301 0.3039284
0.2673127 0.05501693 0.1780753 0.07822054 0.2610594
0.3855905 0.09274973 0.0537989 0.1527225 0.02754854
0.03316531 0.02912367 0 0.08318836 0.4486179 0.06425346
0.08488712 1.244164 0.0746816 0.02674062 0.09131432
0.121006 0 0.1994491 0.218215921 0.1599491 0.13063493
0.0697256 0.02098278 0.2298534 0.2581176 0.1751072
0.3827361 0.03145339 0.09139364 0.225493742 0.136292 0
0.008843586 0.06072324 0.07414432 0.03679028 0.04096359
0.02956378 0.008565234 0.008601043 0.05722317 0.4205093
0.3971609 0.273582 0.1326876 0.1267478 0.02809355

0.3681417	0.1095504	0.1346141	0.06905304	0.01301946
0.5857593	0.1117931	0.2502694	0.05075854	0.06074418
0.07956351	0	0.01395657	0.08348965	0.07647336	0.08056584
0.2178971	0.03078516	0.1863693	0.04339347	0.007991751
0.4139349	0.1900841	0.09485768	0.009392695	0.3187359
0.09686321	0.0273413	0.2226367	0.2408896	0.017658315
0.1430473	0.134670297	0.035755829	0.1708176	0.05198262
0.09662195	0.06155168	0	0	0.0340429	0.01921605	0.128741
0.01110772	0.02687622	0.0510271	0.107287	0.1104053
0.4333299	0.2211721	0.06086785	0.02551316	0.4522851
0.2258715	0.08442311	0.5685689	0.2290042	0.5104585
0.06818657	0.0868532	0.2611114	0.3291855	0.624664	0
0.3396568	0.1399203	0.1144584	0.07751198	0.114759175
0.3518028	0.08222444	0.02340773	0.09542368	0.4459287
0.1935424	0.2915017	0.2072798	0.09587264	0.01852945
0.02828556	0.06163702	0.6211143	0.2661743	0.04754427
0.132317985	0.01185584	0.1105041	0.1697461	0.03408166
0.2373938	0.507721534	0.1549659	0.2879295	0.03908027
0.4316271	0.1304865	0.1817011	0.006714005	0	0.3894845
0.006928956	0.1443069	0.05295525	0.05610613	0.09435761
0.3653963	0.01443518	1.061386	0.9575694	0.2692308
0.05814179	0.0211348	0.1690893	0.09440826	0.2470965
0.0359346	0.07832411	0.221205367	0.448770224	0.2317557
0.1259247	0.1062147	0.149982	0.4044524	0.2434285
0.3398374	0.05675901	0.0839166	0.04968444	0.3997532
0.4671315	0.5332013	0.10167182	0.01595017	0.3867269
0.1751521	0.09171087	0.08652453	0	0.09278169	0.0933109
0.02590854	0.0418384	0.2691904	0.06129528	0.02779672
0.008146168	0	0.1074769	0.2640911	0.085607	0.01191571
0.2415762	0.2199921	0	0.140184	0.04145949	0.022482103
0.006563478	0	0.08665585	0.2189499	0.3843408	0.02904783
0.1420606	0.5275221	0.01207773	0.1109006	0.1718181
0.1936634	0.0461225	0.1231983	0.1416241	0.1108501
0.6459482	0.1060726	0.1062556	0.01862168	0.5390099
0.02388262	0.06278559	0.0636637	0.0830549	0.02429589
0.084372178	0.1251008	0	0.03966337	0.04229599	0.05873704
0.008571726	0.185677256	0.5147208	0.2031658	0	0.2071251
0.08607658	0.03502822	0.3274856	0.04194712	0.06272306
0.1756704	0.08292878	0.2086548	0.887328	0.3898569
0.1711666	0.03811715	0.1885357	0.01642538	0.111580292
0.04283816	0.3626774	0.1893719	0.1037564	0.1049372
0.09175749	0.01351994
GAS1RR	0.04305266	0.9151993	0.08224926	0.05694439	0.1650685
1.029651	0.06140202	0.5199488	0.1993976	0.672178863

0.147822 0.09108942 0.0869411 0.1490431 0.09718391
1.001028 0.0594551 0.960002445 0.05315847 0.07073192
0.139315925 1.075218 0.06741085 0.7963964 1.050188
0.1327581 0.02621093 1.545015 0.076797232 0.1127199
0.2058935 1.202278 0.02877073 0.05215215 0.06740339 0
0.4858916 0.1606183 0.09478493 0.011723359 0.07488314
0.05254982 0.031022644 0.00212901 0.0204614 0 0.02312099
0.05339502 0.114651516 0.005290189 0.02676122 0.2374669
0.1421156 0.02987753 0.9879354 0.004071156 0.04376267
0.4447357 0.009962167 0.040407441 0.112797344 0.090328237
0.4452781 0.06975603 0.004557429 0.6126409 0.1129697
0.1305386 0.003327362 0.01156727 0.03111316 0.01200148
0.167081 0.151313 0.09535122 0.05989392 0.06685285
0.04582815 0.01503917 0 0.02303326 0.2279055 0.02837897
0.1498351 0.205814 0.02244586 0.09200457 0.036832
0.39708228 0.1083285 0.035105274 0.01479148 0.138624
0.03913892 0.1029215 0.02706635 0.1412465 0.003487277
0.002779192 0.05706599 0.410493 0.005786689 0.007630622
0.01191749 0.191366 0.1748715 0.01433248 0.4411207
0.014642891 0.02649064 0.02607263 0.02127306 0.0813665
0.6863662 0.2580348 0.007888067 0.02593843 0 0.1370053
0.006929342 0.003427169 0.03562842 0.03967396 0.202898
0.04456757 0.1060042 0.04543644 0.05014543 0.9601547
0.156325 0.01192541 0.03770391 0.08761251 0.02002235
0.007403562 0.1646225 0.1165517 0.02139478 0.04373638
0.04345444 0.01127998 0.04533281 0.007728892 0.006973705
0.03750957 0.03481716 0.004299067 0.22651 0.03210496 0.1326349
0.01530323 0.0312333 0.01500481 0.1810232 0.5542162
0.02079758 0.05918328 0.7646657 0 0.03324044 0.069883869
0.19026 0.179496225 0.006025473 0.0938366 0.04995017
0.005282936 0.07119027 0.09020423 0.2201666 0.6944128
0.022875432 0.1095348 0.006740001 0 0.1652968 0.07368424
0.1335307 0.00955786 0.02682552 0.04663142 0.0167237
0.04450546 0.01851239 0.0393687 0.01636762 0.9468426
0.08961672 0.05462456 0.008589692 0.1902869 0.04187859
0.05370617 0.02531478 0.04555758 0.05216839 0.1629116
0.04934698 0.01687283 0.04641054 0 0.2035266 0.01298686
0.01081406 0.01253206 0.01797409 0.07981077 0.07247458
0.02531203 0.01243121 0.1397892 0.1548783 0.02766592
0.02556817 0.06038531 0.07243807 0.01772064 0.02361222
0.190822 0.041201392 0.03708514 0.164092907 0.020856888
0.0450754 0.3754178 0.005367711 0 0.1002593 0
0.003309619 0.04483598 0.08857531 0.01295858 0.005225761
0.01984323 0.00927141 0.1478838 0.01685117 0.02243702

0.02367006 0.2083507 0.1161491 0.1361459 0.1723582
0.3454733 0.05724918 0.02205613 0.9578953 0.2653761
0.007084186 0.1161043 0.01121155 0.07401071 0.2461574
0.002365726 0.04855651 0.05206443 0.051492825 0.01243707
0.1950483 0.06068473 0.0231925 0.1141975 0.2702663
0.05472459 0.01511366 0.008603675 0.03963114 0.04399832
0.04109001 0.09661463 0.05850501 0.07395534 0.015133917
0.002305228 0.02975013 0.0502935 0.5142377 0.01420259
0.031590533 0.3863892 0.01866149 0.1253784 0.08239885
0.02899606 0.4817674 0.0156655 0.005566939 0.01165086
0.218255 0.01603358 0.4324538 0.3136386 0.02258057
0.8728628 0.2526074 0.08889954 0.06702767 0.04563735
0.02260996 0.05753175 0.2045705 0.04800948 0.1321237
0.005589644 0.03322733 0.017921148 0.036740225 0.03779406
0.004451737 0.01548914 0.03332826 0.05347585 0.2452647
0.008526109 0.7311426 0.02900726 0.07245411 0.04055335
0.1618393 0.04852856 0.039537754 0.009303965 0.02050758
0.2554219 0.05944029 0.03364733 0 0.04961081 0.03628639
0.04701767 0.1482374 0.0719687 0.08172437 0.4053556
0.02217496 0.006525503 0.02686836 0.02319004 0.03107116
0.06950604 0.02236744 0.05475181 0.01479604 0.6591264
0.08706209 0.084513303 0.01531428 0.02635484 0.1291772
0.01824523 0.1053021 0.01976802 0.03156799 0.03326607
0.009393485 0.04004612 0.143177 0.4895222 0.03843417
0.0653303 0.1652227 0.00331592 0 0.01649964 0.01589242 0
0.02768409 0.01625292 0.03051978 0.09195569 0.1130433
0.07873413 0.075463769 0.05472981 0.09053525 0.003084828
0.04111976 0.0142759 0.08666686 0.063179787 0.02633719
0.1461617 0.009888655 0.005034126 0.01912751 0.2134058
0.00979626 0.01304979 0.01829363 0.1557561 0.05240467
0.1390987 0.06820969 0.01749301 0.083203276 0.03261028
0.2140858 0.3257595 0.028927282 0.03331749 0.247767
0.04909481 0.02269597 0 0.02676173 0.3995763
LINC01018 0.02477877 1.409801 0.01139623 0.3077123 0.2870968
2.308674 0.1914232 1.009698 0.6311933 0.491547217
0.304864 0.1135899 0.3698929 0.1510105 0.2289141
1.679695 0.09885522 1.542286601 0.01529756 0.1058446
0.415019509 1.011779 0.1208396 1.521793 1.629222
0.1136137 0.1045934 1.440095 0.111728549 0.04685446
0.6768927 1.428924 0.01594557 0.09104835 0 0 4.353129
0.03338225 0.1373656 0.01827401 1.540777 0 0.004298413 0
0 0.02734937 0.01922149 0.08434025 0.079896229 0.006596947
0.01906951 0.01558552 0.07974917 0.006209622 0.8535221 0 0
1.058382 0.01242298 0.072783677 0.009377335 0.140800872

0.06580961 0.3237843 0.01704955 0.2938356 0.501515
0.03339151 0.004149271 0.05769824 0.00705429 0.005986412
0.2246939 0.2695567 0.01285453 0.02240659 0.05130248
0.02689337 0.087519 0 0.007180706 0.03157796 0 0.2930095
0.1435518 0.03998619 0.2082157 0.02041336 0.42442945
0.2468836 0.003979711 0.0046113 0.1023086 0.01394481
0.03327455 0.01265706 0.1000776 0.0391382 0 0.0837202
0.02585308 0.003608045 0.04757752 0.04458389 0.03835227
0.02803724 0.0655337 0.3804321 0.003651982 0.005505707
0.002500997 0.006631958 0.2971483 0.6646954 0.1170085
0.3869039 0.004620803 0 0.1006781 0.004320498 0 0.1999314
2.085521 0.6133743 0.5795829 0.05608011 0.03021863
0.00446658 0.9754586 0.2599195 0.05056196 0.3996475
0.04202082 0.01664545 0 0.1368579 0.04320973 0.01778641
0.009089989 0.01083767 0.002813261 0.09131884 0.04176487
0.02608895 0.5496064 1.436118 0 0.08473845 0.9681284
0.0484091 0.01145002 0.01557936 0.003742246 0.08208681
0.7333678 0 0.06642221 0.2885099 0.591684 0.02763423
0.205416177 0.1186286 0.11402892 0.0262985 0.0661393
0.03606184 1.982959 0.1428125 0.01687291 0.2694668
1.776207 0.399364203 0.2379338 0.07984641 0.008577122
0.1799527 0.07590531 0.1546209 0.0119188 0.5925753 0
0.008341887 0.09249833 0.007695076 0.003776413 0 0.8815239 0
0.1430471 0 0.2656238 0.01740775 0.03767198 0.05682226 0
0.1517945 0.02110683 0.1681996 0.004208136 0 0.006354437
0.1455125 0 0.003371325 0.03906917 0 0.06469142 0.1522138
0 0.02712834 0.02112962 0.2326406 0.1188328 0.05465811
0.1823087 0.01445302 0.05745456 0.07524781 0.4629364
0.02140782 0.05395336 0.570341593 1.348125883 0.01183362
0.5185681 0 0 0 0.01007726 0.1444501 0.0512519 0
0.01077304 0.009774907 0 0.02890398 0 1.015661 0.1585494
2.403521 1.237221 0.1117335 0.08214968 0.04093969
0.3532646 0.01586458 0.02750434 2.302222 0.2647425 0
0.1299342 0.004660326 0.02307312 0.2171195 0.002950096
0.3178912 0.01366845 0.068493166 0.003877303 0.4027221
0.0189187 0.01156856 0.0210972 0.1365169 0.01462337
0.06910556 0.02145783 0.004492785 0.05143742 0 0.1129499
0.0224482 0.06340362 0 0 0.02061048 0.03527818 0.5619309
0.004427711 0 0.18124 0.03199785 0.2653192 0.0646961
0.006026421 0.1842366 0.03581439 0.05553647 0.007264404
0.184805 0.006664706 0.7917948 0.83664 0.0351979 0
0.1890032 0.1900442 0.04643586 0 0 0.09736546 0.1229959
0.09860703 0.07863559 0.01742593 0.1070404 0.581046598
0.051542578 0.0326283 0.04163538 0.0386304 0.03636574

0.01176798 0.01073154 0.1966955 0.5401665 0.5742385
0.3132182 0.01264266 0.01647478 0.04401154 0.009860837
0.07734791 0.03409769 0.573327 0.1037721 0.01525772
0.113854153 0 0.003770807 0.004187982 0.4305753 0.0734286
0.1358821 0.4380867 0.01185108 0 0.003722807 0.004131191
0.005535173 0 0.02231403 0.004267272 0.1429943 1.038241
0.06433645 0.018170584 10.3411 0.005477481 0.04202242
0.007584026 0.0677746 0.04930205 0.004920722 0.006913884 0
0.02304837 0.166641 0.3756564 0.1853216 0.0706055
0.3227886 0.004135003 0.01807169 0.03600679 0 0.02257575
0.01972714 0.1939903 0.01522345 0.0926182 0.05034525
0.01570923 0.081829981 0.04170767 0.07697649 1.477182
0.0102554 0 0.008313454 0.183834382 0.01313715 0.2019704
0.9659522 0.006277632 0.01192615 0.5379026 0 0 0
0.2010453 0.0251344 0.1951404 0.01000689 0.00727135
0.036314517 0.003696865 0.2413686 0.3823313 0 1.698251
0.1045742 0.006122198 0 0.007828873 0.02781024 0.7474166
AC092145.1 0.05969029 0.022831 0.2745271 0.03991385 0.203564
0.0544053 0.01844499 0.04292276 0.1013667 0.149570826
0.03074215 0.011727 0.1751366 0 0.6617254 0.03095504
0.1666947 0.01797706 0.08107163 0.08924021 0.041850107
0.04161252 0.0379688 0.08569619 0.01226463 0.02815071
0.0755874 0.01415953 0.092278679 0 0.06747255 0.0321032
0.4090852 0.1127978 0.1069083 0.1439344 0 0.07505443
0.1246661 0.084519925 0.02892173 0.09471491 0.018638228
0.01918646 0.1290775 0.1054123 0.6876025 0.1603971
0.170179003 0.2479086 0.07579622 0.6194827 0.2177248
0.215403 0.1396579 0.05136448 0.1928106 0.3059481
0.1556155 0.194212483 0.264295207 0.236068801 0.09511842
0.1047726 0.09857078 0.07685002 0.06515671 0.0904925
0.089957694 0.6588172 0.04078388 0.02595751 0.4723826
0.01669739 0.07431759 0.05397589 0.3290425 0.04372935
0.01355317 0.029236163 0.06227212 0.1825658 0.1918116
0.1411678 0.01886215 0.0520149 0.1842527 0.1180184
0.55210704 0.06732738 0.086281563 0.4665483 0.1070801
0.3124061 0.06870517 0.2378216 0.1909352 0.1194228
0.02504584 0.1028548 0.9715406 0.1303729 0 0.09307951
0.1170247 0.6168628 0.137774 0.08916655 0.036948935
0.2307737 0.04337802 0.04313497 0 0.7896349 0.5496371
0.05686927 0.03339356 0.07093392 0.07055317 0.06244664
0.06177071 0.07063766 0.06050649 0.09973632 0.02295081
0.694764 0.147409 0.09683707 0.1216373 0.02762328
0.0859766 0.04756982 0.2064994 0.03608793 0.05337622
0.1293363 0.1476174 0.07069623 0.08539884 0.02349646

0.2114407	0.2954025	0.2228868	0.03142322	0.1577488	
0.01930888	0.02324569	0.1479935	0.03156297	0.1282755	
0.04413165	0.1688831	0.06490659	0.3618666	0.1003272	
0.09371301	0.06400256	0.2332373	0.01676853	0.1997067	
0.116960527	0.4814089	0.15870838	0.09774189	0.02206037	
0.1279364	0.1333062	0.08368182	0.4227147	0.0146972	
0.08869591	0.034358573	0.2865828	0.03644419	0.06818354	
0.04256116	0.1732268	0.1031458	0.2928577	0.07597834	
0.01200681	0.08439906	0.01336931	0.3002977	0.0491244	
0.06637661	0.06975094	0.1372951	0.1378361	0.1238553	
0.08702218	0.1383823	0.03629965	0.1551316	0.1129043	
0.2507398	0.06101384	0.2549675	0.1033984	0.1324451	
0.01377665	0.2690108	0.0526664	0.07796432	0.04517513	
0.1804931	0.01438496	0.07562613	0.08617484	0.3304855	
0.3283034	0.1839852	0.1551343	0.05925038	0.2978709	
0.08878158	0.01916362	0.04728695	0.1000536	0.167086542	
0.1448235	0.182489133	0.025061399	0.0470355	1.202349	
0.4692215	0.04930493	0.08340263	0.05826099	0.0178956	
0.006734301	0.1665878	0.1323525	0.07064117	0.08583623	
0.5598061	0.1375707	0.111365	0.2359005	0.02437858	0
0.287102	0.1662299	0.04437936	0	0.1203824	0.2087064
0.1851949	0.1478411	0.1915262	0.3058479	0.1481884	
0.733676	0.1190334	0.1364462	0.05834495	0.197558	
0.160870298	0.1737268	0.06339457	0.2679739	0.02508106	
0.1600882	0.05549506	0.760896	0.04540101	0.1447331	
0.1493546	0.09417092	0.2221798	0.2122289	0.05677992	
0.1332958	0.190939796	0.04154903	0.3455583	0.1529688	
0.1003295	0.06399624	0.028469119	0.3130701	0.1009054	
0.01369576	0.08800846	0.07403783	0.3241763	0.02823523	
0.06020254	0.3884874	0.04856532	0.1348604	0.006186089	
0.04915625	0.07631032	0.09146704	0.3844716	0.06867043	
0.3087355	0.3290239	0.1833831	0.09628751	0.06584184	
0.1323422	0.4275651	0.09067211	0.05988837	0.051681232	
0.248324829	0.2043581	0.008023734	0.01861158	0.08259664	
0.02267858	0.2326636	0.1306222	0.09945652	0.1110997	
0.108825	0.1400943	0.09524779	0.1351764	0.163902994	
0.1006158	0.4681918	0.04910592	0.128561	0.1653965	
0.015672376	0.1544487	0.05450164	0.02421253	0.0195498	
0.2771187	0.319147	0.07793131	0.07422585	0.04116509	
0.08609258	0.5672496	0.06800258	0	0.1854476	0.0185032
0.03333515	0.1429171	0.08136552	0.126062345	0.0276022	
0.09500302	0.1417206	0.2082709	0.08571371	0.08652889	
0.09957082	0.0299791	0.1015839	0.7162323	0.1548359	
0.1900352	0.05080026	0.3768007	0.1191179	0.03585934	

0.08706684 0.245344 0.05728844 0.1435721 0.2779994
0.1088062 0.033005 0.1848631 0.1382571 0.03405817
0.112359893 0.08220339 0 0.03336025 0.2668088 0.08233803
0.2102785 0.021690318 0.0332288 0.09255979 0.02376419
0.1270279 0.04309386 0.1309409 0.1059397 0.1705252
0.1263932 0.1132778 0.07265637 1.149083 0.12294 0.105097
0.119971331 0.2457914 0.3065774 0.02302523 0.017379346
0.0900763 1.147344 0.1238827 0.06817806 0.03960432
0.1125481 0.108976
AC011747.1 0 0 0 0 0 0.1041672 0 0 0 0.032217291 0
0 0.03970959 0 0 0 0 0 0 0 0.02560933 0
0.03076468 0 0 0 0.04066585 0 0 0 0.02304991
0.02482147 0 0.06280326 0 0 0.9699955 0.3779291
0.022756803 0.5399065 0.1360095 0 0 0 0 0 0.0276394
0.02618304 0 0 0 3.365588 0 0.651778 0.03161091 0
0 0.02578409 0.209165125 0 0 0.05122085 0 0 0 0
0.1039569 0.025835638 0 0 0 0 0 0 0.7440839 0
0.04186322 0.506017 0.100758683 0 0.1404441 0 0.2908524
0 0 0.02645847 0 0.79281919 0 0 0 0.04393308 0 0
0.02626993 0.04985104 0 0.08631727 0 0 0 0 0.6168973
0.1592015 0 0 0.03201053 0.022739243 0 0.1401532
0.04129421 0 0 0.6374893 0.0612477 0.9782362 0.04074411
0.07598513 0 0 0.9405777 0.09478539 0 0.4449207 0
0.0940789 0.05562275 0 0.02380002 0 0 0 0.02591092 0
0 0 0 0.05659926 0.2024439 0.1926859 0.2436862 0
0.05414804 0.8251995 0 0.03338054 0 0.06798607 0
0.04752939 0 0 0 0.2254988 0 0 0.03044782 0 0 0
0.02840949 0 1.450347 0 0.0204128 0 0.04806644
0.07003999 0.03165752 0 0.088809309 0.02743531 0.1046668
0.05340587 0.06111732 0.1741262 0.1110871 0 0.1190225
0.02586246 0.02597058 0 0 0.1645981 1.27088 0 0
0.9331036 0 0.08820905 0 0.7297623 0.07862368 0
0.5850965 0 0.6641434 0 0 5.183174 0 0.07562833 0
2.821886 0 0.2168946 0.0296177 0.06551261 0.6032711
0.1973469 0 0.1432099 0.2268876 0.07403178 0 0.02751874
0.3463078 0.02693921 0.106637468 0.04799189 0.081326558
0.188936334 0 0.02242285 0 0.02655049 0 0 0.07709361
0.08703339 0.02990226 0.2683153 0.04057595 0.03081495 0 0
0 0 0.1312776 0.2118495 0.05153438 0 0.1274565
0.05364925 0 0.2568856 0 0 0.09167647 0.2080393 0
0.1915546 0 0.01836892 4.367171 0.3829828 0.026654752 0
0.6206854 0.4711927 0 0 0.02656342 0.1821063 0 0 0
0.2348702 1.302777 0.02344294 0 0 0 0.03579835
0.2566643 0.1220341 0 0 0.153304945 0 0.01811238

0.02950041 0.07108825 0 0 0.02027273 0.3890256 0
0.04184354 0.1452434 0 0.02117634 0.3725745 0 0.3486927
0.1972199 0.05782702 0 0.065834 0 0 0 0 0.08680272
0.02149975 0.083490344 0.035659152 0.02257352 0 0.0200445
0.1940855 0 0 0 0 0 0.05625773 0 0 0.102765
0.092098474 0.866899 0 0 0 0.02375074 0.101274061 0
0.02347911 0 0.3649526 0.02540038 0.1321994 0 0.467345
0.3293416 0.1390814 0 0.05169754 0.1079373 0.243144 0
0.08616399 0 0.02503713 0.022628005 0 0.1023174 0.1308273
0.09444462 0.07912535 0.08770904 0.03063911 0.2582978 0 0
0.407628 0.1754282 0.01989507 0.03381758 0 0.05149359 0
0.3523111 0 0 0 0.03605642 0.04739478 0.3020766
0.06269542 0.171175 0.050951836 0 0.09585957 0.07185739
0.06385572 0 0.05176412 0.210242521 0.04089956 0.2760538
0.1023753 0.03908798 0 0.3172999 0.4563843 0 0.2840855
0.1060863 0 0.4050172 0.03115418 0.04527541 0.355322169
0.02301871 0 0 0.037434804 0.2263599 0 0.03812016 0 0
0.1385295 0

DOCK9-DT 0.5400973 1.256709 0.3146414 0.3955491 0.3778285
1.221576 0.500689 0.2157662 0.3335268 0.676682884
2.534392 4.810308 0.6394375 2.29564 0.2935053 0.9069623
0.3591209 0.623538884 0.2445209 0.230707 0.273486387
1.470236 2.611011 1.636971 2.342792 3.650941
0.3704672 1.608619 0.41748384 0.5719147 0.8648944
1.161921 0.9036615 0.5670173 1.348415 0.3551906
0.09366652 1.164201 0.7937917 0.366449385 1.366621
0.2063176 0.093691546 0.1041632 0.6488529 0.2516986
0.4399148 0.9094974 0.29330196 0.2300675 0.415654
0.7303852 0.92708 0.2706995 1.123262 0.4647626 0.3700696
0.9227729 2.54534 0.658986779 0.674505754 0.441936774 0.8248016
0.4213406 0.3220752 0.6831648 0.7123841 0.9279799
0.108528886 0.7294287 0.1230086 0.7176647 0.7836166
0.4196762 0.5743842 0.2604755 0.2655793 0.6154982
1.457976 0.264538363 0.250426 0.3933131 0.2892624
0.8330429 0.2465246 0.5752362 0.4445811 0.1779782
0.55507168 0.9138002 0.242885629 0.3819436 0.2614481
0.4711254 0.2279445 0.2207065 0.3315681 0.1706173
0.7403016 0.2554763 0.40573 0.5662351 0.4977775 0.5614759
0.557304 0.6654434 0.2597133 0.3585814 0.461688354
0.5280293 0.8177053 0.5059436 1.137403 1.170966
2.104087 0.5574527 1.470492 0.1996815 0.5053906
0.320188 0.1676765 0.2324192 0.7797478 0.6016312
0.9344988 0.6810323 0.09880033 0.2141855 0.6837146
1.183071 0.2074518 0.840358 0.8792818 0.761916

0.08049424	0.5966121	0.1198696	0.1744589	0.5349577	
0.2834716	0.4782961	0.5687019	0.1680627	0.2085069	
0.2378937	1.397704	0.4206693	1.046648	1.697686	
1.143093	0.3660411	0.203748	0.2610205	0.7872604	
0.5657262	0.1130595	0.5308564	0.959276	1.871301	
0.4818697	0.56985229	0.5171442	1.436045338	0.3275558	
0.6875436	0.5430766	1.14876	0.3701771	0.29422	1.263358
1.292997	0.310887606	0.7107002	1.227436	1.00955	0.4706867
0.3657297	0.5184982	0.363708	0.4374853	2.227152	
1.363697	1.491962	0.9225033	0.7737482	0.3114208	
0.4908783	0.1217932	0.2524068	0.2334757	0.6638906	
0.3414895	0.2189674	0.5229401	3.776805	0.4569062	
0.2990396	0.6080542	0.1467581	1.408654	0.4847719	
1.40145	0.9001364	1.01408	0.2725062	0.2931314	0.6074124
0.352513	0.5809813	1.081253	0.368446	0.5931897	
0.384348	0.9530966	0.3628199	0.236272	0.5587293	
0.385081	0.7355707	0.52261609	0.2688022	0.436529789	
0.925951924	2.231137	1.051818	0.218849	0.4461271	
1.844711	0.06589556	0.5037674	2.721726	0.2512235	
0.3991897	0.2698779	0.4099114	0.277206	0.5445932	
0.2748182	0.162629	1.985268	1.779851	0.6494481	
0.66849	0.803118	0.5070752	0.6224341	0.7493824	0.8108244
0.3147564	0.4364573	0.6635316	0.4469524	0.3352798	
0.4242935	0.5144207	0.3299526	0.3575139	0.223939551	
0.1690254	0.8343488	0.296904	0.1260787	0.7357615	
0.5765282	0.4037404	0.7942196	0.1870846	0.4700555	
1.091272	0.6142752	0.3774982	0.611623	0.8040696	
0.477170347	1.015063	0.2695452	0.8373019	1.051911	
0.2895295	0.450796324	0.7322783	0.2028945	0.4337329	
0.2488529	0.2889844	0.7333133	0.6954778	1.694723	
0.3008471	0.4833784	0.3922266	0.7463168	0.8302595	
0.6751367	0.4413993	0.6103202	1.380783	0.2834026	
0.08756223	0.2304598	0.6478458	0.5560413	0.5833856	
0.4733912	0.6836924	2.001981	0.389690579	0.399453206	
0.4741279	0.5445103	0.2105045	0.4755936	0.5301083	
0.3742608	0.6257181	0.6749364	0.4730664	0.7352312	
0.09185643	0.5206909	0.5276203	0.171947584	0.9104051	
0.1486439	0.5924353	0.3231283	0.6651385	1.205374668	
0.2206583	0.4273955	0.07302762	0.658435	0.1422673	
0.4072463	0.352574	0.7749459	0.3547383	0.4544131	
0.7203733	0.108584	11.00796	1.094342	0.7627047	
0.7037972	0.511876	2.576777	0.50695704	0.6105091	
0.8834967	0.274786	0.6612293	0.2769877	0.2609806	
0.2788653	0.09042024	1.378747	0.4688881	0.233501	

1.269155 2.521151 1.183825 0.239515 2.072984
0.2100825 1.614511 1.123123 0.07873257 0.9029748
0.5679891 0.08295556 0.4614351 0.9876277 0.65058
0.499416384 0.396695 0.2460831 1.157109 0.536483
0.2638618 0.7610673 0.490652724 0.4581562 0.9663592
0.5196466 0.574695 0.337937 0.6664478 1.171594
0.7094105 0.4640885 0.7575902 0.02191395 0.6301382
0.305365 0.713214 0.407077443 2.095074 0.5898763
0.7986358 0.497971029 0.8376799 1.13969 1.014176 0.1233795
0.1365154 0.460692 0.3001024
CTBP1-DT 1.280079 1.920456 1.546599 2.491761 1.6303
2.141188 1.452489 1.378291 2.053664 2.027444028
1.759026 0.9636176 2.197687 1.128773 2.562436
1.739779 1.860421 1.969586895 1.183695 1.579281
2.394609248 1.866943 1.697052 1.867125 2.221729
1.258807 2.482079 2.259425 1.813078396 0.838925
2.913895 1.48218 4.027903 1.737879 1.97687 2.696148
1.154132 2.58472 1.705883 2.597825822 5.575227 3.763654
1.574061096 1.104792 2.073835 1.844191 6.638896
2.240594 1.420697779 3.76018 1.970989 2.177068 2.031033
0.9777376 1.825696 1.99842 1.55488 2.833965 2.31188
4.264191277 2.223202959 1.890136833 1.508051 3.106722
1.260456 2.115015 2.770787 1.889005 3.852747705
1.774149 2.356345 1.578168 2.029022 2.608952
2.174369 2.808988 1.456897 2.333513 2.015021
2.058385859 1.508596 1.822433 1.536331 2.196946
2.62038 2.012932 2.657182 2.321105 1.89666053 2.150071
1.296221235 4.099202 1.660103 2.565813 2.187655
1.905611 1.727048 3.104733 2.047124 2.150579
2.872754 1.639391 2.14038 4.896149 2.235302 2.344649
2.554194 2.104631 2.114444306 1.882417 1.328777
1.385851 1.959535 2.394272 1.533107 2.399925
2.92483 2.730363 2.282457 2.22356 2.530185 1.846841
3.716917 1.141702 2.337714 3.366253 2.280481
1.507042 2.338715 1.968904 2.253233 2.677817
2.109686 1.930115 1.690425 2.514838 2.140041
1.004202 2.353411 2.086742 2.133814 1.259842
1.466976 1.275387 2.728546 1.17795 2.047589 2.242813
1.072461 1.947307 3.461501 2.35493 2.627993 2.527761
2.452011 1.376752 2.136527 2.939032 5.741183
0.9323901 2.058245261 2.329705 1.875242698 3.025346
1.780676 3.13993 4.264784 0.977602 2.56311 1.854981
2.423271 2.361282802 1.72453 1.98509 4.952523 1.009738
6.949952 1.524962 2.412871 2.485242 0.9585812

2.499353 1.562546 4.024343 1.088997 2.178475
2.492331 1.485619 1.89075 1.458489 2.707 2.905398
1.928257 2.03934 1.868906 4.074524 1.613027 3.054416
0.9503479 2.000448 2.624272 1.481268 3.369587
2.249215 3.43086 2.00372 2.265546 4.361111 1.188065
2.257792 3.025165 2.397272 1.971105 2.124913
1.199924 3.299771 2.934652 8.543923 2.522503
3.212828718 3.701497 2.234799037 1.872105732 2.515409
3.782852 1.574895 3.554591 1.362865 2.78809 1.916102
2.429348 1.205535 2.273003 1.342691 1.999307
2.153312 1.768973 1.958442 1.260809 5.063033
3.691592 2.148397 2.111468 2.389687 1.854758
3.202743 2.632448 2.097443 2.00305 1.714209 2.119367
0.9539306 3.553405 2.185921 1.327166 3.48673 2.330492
1.444367146 2.651322 2.60818 1.407718 1.691421 1.494839
2.00367 3.747636 2.184685 2.257259 3.595677 2.21838
2.791139 3.467142 1.191661 3.19982 2.001962953 3.511111
2.852092 2.458191 1.748761 2.101458 0.937061771
3.412531 2.317585 1.941727 2.186297 2.463051
2.306307 3.617853 1.433475 1.544155 2.019512
2.716429 2.058295 1.949225 2.372016 3.379319
2.220157 1.823905 1.984569 1.338857 1.877251
2.173963 1.828019 1.739567 2.200048 1.600814
4.427106 1.548272563 1.195448004 2.749784 1.227478
1.12817 2.145488 1.884694 2.401841 2.63311 2.93774
2.666738 4.347828 1.148891 1.154348 1.118675
1.508279351 1.66676 1.507116 1.001127 2.160811 2.937803
2.539039137 0.8703682 2.335917 0.4879706 1.794042
1.776467 2.412707 2.507502 1.759385 1.484451
2.170524 1.536986 2.648243 3.31958 3.912491 2.357424
5.71492 2.438246 1.628054 3.595118341 1.327352 2.079756
2.158288 2.03687 4.785009 3.18436 1.600502 2.006185
1.075023 2.566282 1.485956 2.239215 9.298743
1.910693 2.165846 3.84694 3.070411 1.455546 2.747789
1.57218 3.099485 2.133576 1.571755 1.410703 1.184539
2.448765 3.276359424 2.683126 2.52565 4.3801 2.057673
1.834002 2.382374 2.489497885 3.693775 3.75632 2.174624
1.6267 1.966361 2.419874 2.165297 3.534164 3.046306
2.411347 1.017654 1.976861 1.508109 2.983314
1.141248378 2.752458 3.214775 1.537252 3.919093668
4.812939 2.987359 2.795519 2.484229 1.914203
3.828382 1.924542
AC008115.3 0.2117103 0.1518324 0.9250115 0.6825548 0.5900989
0.1340038 0.4088808 0.1268658 0.2859886 0.928374404

0.8632066 0.7278858 2.043344 0.5210571 0.3451494
0.4509302 0.3695217 0.418433257 0.1633783 0 0.402010044
0.1581341 0.2020023 0.2849515 0.2356268 0.6032319
0.5864563 0.209255 0.357979085 0 0.5982812 0.04744334
0.6641665 0.8334845 0.6678804 0.1740369 0.2478324
0.4040596 0.5117677 1.007061067 1.902003 0.8631689
0.66106297 1.718282 1.417042 0.5062927 2.032331
1.052462 1.6976053 1.07797 0.5498897 2.421901 1.618274
1.352906 0.7223712 0.9109009 1.476523 1.193655
0.2918909 0.251137848 1.021531649 0.505262262 0.553493
0.2167717 0.5098509 0.3048509 0.216655 0.9361331
0.558360363 1.12768 0.8588758 0.5562347 0.8900841 0.8389855
1.009058 0.7179088 1.047881 1.701789 2.583792
0.959180448 0.5061547 1.214112 2.324426 0.6530814
0.4460033 0.6918266 1.252561 1.504305 2.1758001
0.5074449 1.045583765 2.275294 0.2260673 1.362718
0.2741449 1.189564 1.000426 0.9474596 1.732241
1.341201 3.710939 0.4855285 1.402436 0.9205703
0.7918995 1.696818 0.5344702 0.09883032 0.608450791
2.15212 1.185956 3.421063 0.2322111 1.400343 1.687033
0.5042615 0.4145424 0.7338021 0.5669474 2.270237
1.424079 1.252693 1.268121 0.3930511 1.424539
2.720885 1.791183 0.1144876 1.274663 0.3918984
0.6670625 3.645583 0.7001021 2.133286 0.828255
1.349211 1.762018 1.082768 1.514468 1.423683
1.928933 3.037324 1.358736 0.9752072 1.973255
0.4494326 0.8244811 0.8597583 1.026189 0.749665
0.9782917 0.4991639 0.4556269 1.762145 1.566477
1.412625 0.8276226 0.6580389 0.4460611 0.354161
0.199440887 3.333069 1.596712594 0.9389037 0.2282116
1.638601 0.1688615 0.9151448 4.000508 0.6841829
0.589852 0.974907289 1.15763 0.6463035 0.3572551 0.733816
0.7424036 0.9527051 0.8401342 0.6737023 0.6121724
0.9889162 0.4741845 1.035513 0.8953745 1.602201
0.659716 0.1432235 2.37892 0.9380706 0.6354587 0.7808437
0.6973853 0.3236603 0.63708 1.459052 0.9636769 1.708748
1.779743 0.2781462 1.119782 0.5421198 0.544827
1.771487 0.951352 2.174954 0.2869917 1.066831
0.8989587 2.756586 3.486521 1.54702 2.014243 0.6129375
1.422201 1.15769 0.1699245 1.844897 0.4713136
0.576162821 1.160678 0.836967307 0.222220201 0.9857905
3.046076 0.7720705 0.8743767 0.3081391 0.258301
0.2380213 0.6867024 0.7385696 1.035506 0.6263774
3.551858 0.3704342 0.343081 0.7181647 4.452413

2.32378 2.814487 1.511534 1.123021 0.6558553 1.463139
1.296171 1.233737 0.4237752 1.42658 0.7359166 2.022078
2.448802 1.182823 1.487257 1.380011 1.002359
1.686062 0.493768353 1.192599 0.6132236 0.7273877
0.3335918 1.318114 1.421554 0.7184187 2.495948
1.512523 0.6045865 0.7251459 2.024799 1.037425
0.2157726 0.9972605 2.805657343 0.6078873 2.060327
1.733151 0.2965415 0.3688468 0.157772848 0.9631029
1.137055 0.03036017 0.8779204 1.48677 2.925811 0.9597237
0.8452115 0.5586049 0.6244138 0.9395658 0.1919829
0.348696 2.368261 0.6488328 0.8074236 2.866918
0.5356109 0.9224338 0.3613475 0.5910814 1.050879
0.1128349 0.503898 0.6923268 0.265516 3.265095982
2.715681532 2.230215 0.8893338 0.4332022 0.4993547
0.9551854 0.1031518 2.725249 0.2866123 0.8112787
1.157946 0.9991771 2.401732 0.9165863 1.390144539
0.5947759 2.021111 0 1.139954 0.5621874 0.208451164
0.5405928 1.014862 0.617243 0.9100805 0.7842193
0.7074716 0.2159435 1.569465 1.981495 0.5248276
1.111855 0.1241431 0.6294707 0.9294263 0.4922053
1.27101 0.4752188 1.906744 0.628761779 2.019184 0.5264967
1.256641 0.5831826 4.750163 0.293362 0.9144296
1.24052 1.125935 1.846178 0.495782 0.5416226 0.9623196
1.32252 0.08801848 0.8744062 0.5018158 4.680564 0.7873665
1.330925 1.200912 1.558504 1.633998 1.07395 0.4516583
0 1.573103131 1.287723 0.460382 0.8627687 1.905785
0.8898019 2.903364 1.105890129 1.262746 0.3472322
0.6584932 2.554424 0.3821146 0.1814152 0.8610895
0.8342358 0.4629111 1.048109 2.834684 1.945164
0.9298019 1.491037 1.362980303 1.397684 0.4218256
0.1531241 0.385258001 0.6988707 3.86838 1.059241 1.531494
1.254188 0.7128337 0.4411342
AC245041.2 0.1648972 0.0112628 0.01625129 0.1968997 0.03862328
0.005964172 0 0.0423486 0.02727565 0.16232709 2.143385
8.075953 0.03637765 4.963967 0.03840434 0.01745198
0.02349498 0.035473177 0.04362936 0.1509368 0.020645148
0.01173025 1.768155 0.007045822 0.1371398 2.059916
0.1025424 0.04191038 0 0.02672623 0.033285 0.01055792
0.2103335 1.020149 2.085593 0.02581983 0 0.4178562
0.3052185 0.005211834 5.673681 0.150555 0.073555649
0.1135789 0.2304444 0.3466742 0.2946606 0.1139411
0.473725169 0.188148 0.08837912 0.01666899 0.568621
5.003113 6.108678 0.4198984 0.008646886 0.02414845
0.1535339 0.455084641 0.447972039 3.73460758 0.05865379

0.05513123 0.1864001 0.2434286 1.341952 0.1964205
1.118305048 1.888304 2.545075 0.1067096 0.9321274
0.07687883 0.3895309 0.4792849 0.1874688 1.270363
0.06685937 0.005769017 0 0.1736907 0.7065178 0.260392
0.7630033 0.1482552 0.1939073 1.237172 0.38332271
4.809298 0.022700643 0.1315164 3.908966 1.426794
1.884454 0.2526897 2.917054 0.05581199 0.01482649
0.4417318 0.01474684 0.07717736 0.05427732 0.416787
0.1154593 1.017314 0.6966466 0.293246 0.213520296
0.7223166 1.736876 0.2364332 2.263394 0.03895361
2.106568 0.4114627 0.7116523 3.401274 0.3872023
0.1786728 0.4509888 0.1647284 0.1790914 0.492011
0.0226438 0.07997155 0.01077312 0.4012752 0.01091002
0.05450755 0 0.05363828 0.2636598 0.5518806 0.06582778
0.2402 0.2408707 1.559876 6.604413 0.0618191 0.05215309
1.556473 0.1786731 0.1798167 0.5058255 1.643114
0.02293475 0.241678 1.204109 0.04026896 6.438672
0.5443052 0.5603357 1.849512 0.02151851 2.256011
0.2157503 0.08367904 4.698559 2.265906 0.390571064
0.136635 0.252945655 1.125068 0.7762949 0.07947504
0.3194131 1.100833 0.3208155 0 0.004861637 1.749186996
0.03141659 0.5153792 0.6421368 2.262893 0.6665475
3.833188 0.07365144 0.4702162 0.7166952 0.1843838
2.499596 0.3072541 0.764706 4.365915 1.770914
5.08368 1.568769 0.6822753 0.2424229 0.4964772 9.144531
0.06302324 0.05063364 3.566478 0.4740568 0.8599736
0.3780568 0.1306735 1.191597 0.2895408 0.09237671
0.1778808 1.225698 0.08219018 0.5606051 0.01356627
1.680437 0.8621373 0.2109193 0.2190808 0.6122364
0.2468219 1.441174 5.703914 0.1827704 6.904853
0.07403643 0.048844888 1.198046 0.15521384 0.259624716
0.4725007 0.102707 0.02863577 1.124925 5.485799 0
1.900986 0.5647586 0.1575111 0.06145041 0.3670668 0
1.739389 0 0.1148696 0.1329971 0.09019679 1.579055
1.168454 0.007809825 3.225548 0.681924 0.2092652
1.147238 0.07662351 1.604498 0.121777 2.731693
0.05981161 0.8445056 3.213616 0.01682763 0.02878225
0.3947036 0.183136621 0.3483349 0.4776283 1.300364
3.167432 0.4738403 0.03041818 0 0.9854615 1.162776
0.1281363 16.42084 3.099354 0.0859036 0.3201166
1.002785 0.016147344 0.1147811 0.09405129 0.4304088
0.0329958 0 1.200774806 0.8825209 0.08296306 0.2905199
0.2062241 0.1675795 0 0.04178632 0.0494977 0.0984125
0.06229039 0.7935863 0.1281702 0.1066972 0.02509652

4.295592 0.479152 1.264701 1.311129 0.696984
0.09549088 0.9573028 0.7470527 15.98 0.02135928 0.08448927
0.4333066 0.280444529 0.236836324 0.2533231 0.04354022
2.465181 7.571327 0.02237522 0.04591028 0.01516175
0.3238162 0.3804234 4.964758 0.5048045 0.2995412
0.7688282 0.12655607 0.09926995 0.07293616 0.03028066
1.099291 0.1305472 0.061850933 2.317822 2.317599
0.555411 0.7072373 0.1687009 2.19809 0.009611107 0.3661648
0.005802064 5.229178 0.03534705 0.165759 0.04120024
0.8591491 0.7058866 0.1578684 0.1498182 1.71449
0.689250684 0.009077659 9.779393 2.726586 0.06489 0.7369423
0.3113546 0.2596314 0.1676091 0 0.9038566 0.6365198
0.01339237 0.4920944 0.9139109 0 25.47336 0.6872175
0.04401131 0.1526097 0.006438709 0.9845976 1.28408 0.07055425
2.000004 2.369186 0.06720518 0.163368067 0.2487183
0.02927208 0.8886782 0.102371 0.2995601 0.130407
0.609905004 0.4823977 0.02809897 0.01172315 0.1074246
4.047658 0.06459468 10.38257 0 0.05421846 5.758202
0.2652326 0.3091943 0.6706925 1.736827 0.503057734
0.04744635 0.3650578 0.02271723 0.282923356 0.2443963 0
2.086565 0.1748918 0.01116416 0.06345289 0.7526282
AC027807.2 0.03560936 0.8512661 0.06550971 0 0.04670766
1.803139 0.2200741 0.1707091 0.2198988 0.803063363
0.183398 0 0.32994 0 0 1.714775 0.071032
0.563038867 0.131904 0 0.083221536 2.695256 0.09060398
2.087549 0.4877794 0.1679383 0 1.126285 0.068813206
0.7272088 0.10063 1.500221 0 0 0.3768708 0.1561214
0.03705338 0.06396446 0.1377257 0.126054839 0.4888576
0.09417324 0 0 0.07333673 0.02620236 0.04143455 0.07655031
0.072516753 0 0.08221391 0.7391279 0.03820229 0 1.388592
0 0 0.2920305 0 0 0.080856528 0.097124994 0.2482579 0
0 0.3136853 0.04858806 0.3239099 0.035777292 0 0
0.1032363 0.2113565 0.4648531 0 0 0.05529477 0.1159447
0 0 0 0.1166925 0 0.5492366 0 0.1034349 0.4396776
0.220019 0.48795517 0.8033081 0 0 0 0.1202398 0
0.0727574 0.2071017 0 0.08964937 0.1443763 0 0 0
0.469855 0.5144134 0.1611683 0 0.4432831 0 0 0
0.1715531 0.06249209 0.5888393 0 0.05654403 0 0.0282113
0.05261226 0.07450741 0 0.07661857 0.09844438 0.1983319
0.102688 0.2417767 0.162851 0 0.5607293 0.06591673
0.02564547 0.1621638 0.1449306 0 0.1592129 0.2723224 0
0 0.2351366 0.04672418 0.02425748 0.1874765 0.05540302
0.07498442 0.2016599 0.7775373 0 0.1522211 0 0.9043879
0.03290945 0.03358346 0 0.1769494 0.780679 1.565375

0.1272731 1.433585 0 0.1191387 0.214692249 0.6294651
0.291324431 0 0.1316052 0.08480317 0.113609 0.1331252
0.1454875 0.6137523 1.264037 0 0 0 0 0.2539065
0.1722361 0.2563897 0.03425681 0.3708515 0.3581444
0.1078925 0 0.09952676 0 0 0.5270755 0.1927199
0.08810219 0 0.1221522 0.03752479 0.07218412 0 0
0.4362852 0.2502427 0.1414936 0.1451394 0.4574414 0
0.2626092 0 0.02906942 0 0.02760937 0.2145403 0.2050735
0.03024071 0 0 0.1513928 0.03305294 0.03927436 0.1708658
0.09346642 0.03810806 0.08462973 0.2238332 0.073835992
0.09968904 0.48802579 0.037377111 0 0.4347174 0.1154321 0
0 0 0 0.04017473 0 0 0.02809487 0.04267267 0
0.3590578 0 0.2412529 0.03635874 0.5067301 0.28546
0.4250038 0 0.0742937 0 0.1185787 1.033536 0.518808
0.1015632 0.1280416 0 0.1326328 0.129112 0 0
0.08839267 0.036911605 0 0.0343811 0.1305019 0.04987525
0.2728674 0.07357024 0.04203025 0.05416964 0 0 0.3252492
0 0.06492776 0 0.09940015 0 0.0495737 0 0.1689933
0.5130293 0 0 0.1143476 0 0.1225569 0 0 0 0
0.1197165 0.1252754 0.08691764 0 0.3690427 0.8211022
0.06069913 0 0.06035887 0.2731108 0.08007909 0 0.06077812
0.1767449 0.5499085 0 0.03228758 0.03005119 0.02977294
0.077078504 0 0.06251981 0 0.08327312 0 0 0.2313331
0.09167657 0.1483316 0.1169622 0 0.109012 0.07102731
0.1897458 0.042512776 0.03334681 0.1470045 0.2929505 0
0.03289012 0.046748239 0 0 0 0.2332559 0 0.1098422
0.4649137 0 0 0.06420017 0.1424856 0 0.1494721
0.04810096 0.07358952 0.03977341 0.266437 0.1386861
0.03133535 0 0.04722985 0 0 0.1095731 0.2732846
0.1272875 0 0.05050149 0.03312261 0.1026336 0.4048895
0.02755077 0.5619687 0.1184366 0.03565426 0.1038827 0
0.03417648 0 0.0850491 0.1497932 0 0.1140864 0.303873
0 0.176395756 0.03269331 0 0 0.1768553 0.06140036
0.2508911 0.032349408 0.08495681 0.4247559 0 0 0
0.5858642 0 0 0 0.3525809 0 0.3739129 0.1725697
0.06269757 0 0.1275056 0.3468688 0.06868064 0 0.08956116
0.08197239 0.05278895 0 0 0 0.2261276
AC099850.3 2.489327 0.2596759 4.121602 6.009743 2.493403
1.054246 2.237762 0.5424403 2.166106 5.613938278
4.234723 3.378984 2.935541 3.649487 2.213633
0.3017806 4.604467 0.92010624 1.22946 0.9666696
3.279084083 1.12689 3.685126 0.9205444 0.5579832 5.122903
1.647794 0.1431539 2.011660839 1.745905 2.430168
0.5274192 6.990221 5.844518 4.384815 8.631907

0.09419176 3.98373 13.19902 16.182142757 9.100976
21.42573 7.443136576 5.819294 3.448882 10.79048
11.48086 8.46489 10.092705476 17.24337 6.583259
6.405356 17.723 9.527611 3.17689 17.74888 20.73378
1.670305 14.65878 3.497488504 4.264992914 6.295878094
2.884962 10.00999 10.33929 1.1041 5.18757 2.378703
8.139835516 20.74088 19.63712 6.429593 12.80517
19.28675 12.50304 7.203242 3.022092 7.626344
19.2518 11.483272462 12.59151 6.179978 14.01093 2.932
8.533721 9.684853 6.892392 5.369287 11.78388931
3.828851 11.55814558 21.02362 3.866384 7.832443
8.647947 10.63482 6.317571 13.10635 24.99234
10.59749 5.780073 7.592136 3.02427 8.197911 7.378982
4.33598 7.704506 4.056661 5.443250642 8.145873 22.03739
7.995125 4.050889 1.896026 5.610303 12.54115
4.760319 11.76121 22.50231 7.244627 7.775104
8.375054 21.56331 2.016683 26.14737 9.702058
18.38055 5.384664 2.305803 7.959291 6.29105 25.24905
4.05264 9.212504 1.821276 3.345917 7.318609 15.05836
17.53343 15.32204 22.63064 9.197911 11.09096
7.529277 18.62559 2.34257 7.402993 5.417365 10.92931
6.410686 11.83756 5.207633 17.30755 12.27996
2.64604 4.121387 3.073585 2.679598 9.027527 4.54286
3.75209819 3.950335 5.322796853 12.51692 10.92856
7.185814 7.942014 3.891735 7.150188 4.346251
1.270356 14.485231724 11.29976 12.25108 15.22816
11.25942 6.655075 18.90096 5.224964 21.00182
7.465469 5.94249 2.534339 9.909269 8.070583 10.62531
3.173342 4.531619 4.479214 11.50446 8.849759
19.36419 13.71633 5.466305 7.471442 7.604992
8.269704 18.254 19.27777 1.057129 7.486458 4.005404
7.543199 18.8435 16.09952 8.843254 4.853825 26.95164
8.648277 12.18984 7.815489 11.49737 13.06549
11.78084 10.07693 9.226679 2.615566 23.55712
11.2851 8.962447294 18.33036 9.065842141 5.22580916 5.706388
2.683756 4.768317 18.97326 1.26481 9.663656 8.910597
3.880804 4.526327 7.379194 7.35613 7.918771 2.787604
3.390196 5.258501 8.125939 20.14886 7.152546
11.29301 7.622705 7.403197 7.601564 7.650186
10.85162 1.13246 7.45144 15.16803 10.78182 6.486506
19.55527 8.615508 15.42217 23.6686 9.025415
3.518676349 11.51569 6.336405 6.966607 5.705355
11.84976 10.33284 8.707725 14.66529 15.48184
6.844177 13.98047 6.645167 13.82294 8.303211

2.716318	10.424242186	12.25536	5.375958	8.677725
1.159244	21.44827	6.422092186 3.827262	12.49698
1.401959	5.38034 11.39303	8.691085	12.95276	6.314765
8.120653	7.364984	14.02404	0.9850336	1.379101
11.03249	8.600067	6.060707	3.427924	16.28524
7.301081	10.19709	7.301053	5.242118	6.831466
6.648225	7.257221	9.422717	12.931900071	15.628373375
7.787514	14.17581	9.031891	17.30848	17.884	3.587176
13.69151	4.675634	7.185341	10.66122	6.373635
5.913189	11.99832	11.239265445	5.510015	7.287026
1.489393	4.874104	3.595167	9.685186562 6.287043
10.33154	5.140598	5.385959	12.69702	7.399461
0.369324	7.273386	12.3071 9.139224	5.342544	10.94962
4.622924	5.196702	7.903652	23.81052	3.860597
4.935668	12.864479057	10.36011	10.08512	11.12983
7.56366 12.34865	7.101426	5.716437	11.06282	8.408734
8.925151	9.97944 1.543878	5.777936	9.22609 3.913943
21.6618 13.79795	12.4586 7.384676	8.75863 7.080536	11.836
3.211698	10.82719	4.358894	16.78612	9.057839618
20.02908	14.56657	8.010271	13.1501 6.009201	3.325563
9.251325897 13.82176	5.776681	9.325002	7.946367
5.293537	2.916545	8.970109	5.439587	12.62565
5.340338	10.02676	7.920896	8.334956	7.889347
11.48482559 12.76248	15.2304 0.6110651	10.278827481
14.57085	4.844798	12.07732	11.20088	7.421748
6.827215	6.933875
AC004147.4	0.05431259	0	0.09991762	0.05188267	0.1424802
0.02750208	0	0.09763925	0.08384926	0.272190947 0.2564144
0.1067047	0.4193629	0.1273077	0.3541814	0.02011871
0.1083404	0	0.2682458	0.05800018	0	0.1081815	0.1036442
0.1299592	0	0.2774906	0.6877747	0.04294618	0.524781088
1.232404	0.2302264	0.04868485	0.02621333	0.08552953	0
0.1190608	0	0.04878037	0.02100639	0.144197312 0.04386012
0.04787873	0	0	0	0	0.03159869	0	0.055302496 0
0.06269771	0.2049714	0.1165348	0	0.1058963	0	0	0
0.05445985	0	0	0.111103731 0	1.461776	0	0.01840167
0.03705407	0.05489315	0.05456873	0.09485159	0.02319345
0.03936485	0.026864	0	0	0	0.08433744	0.06631603	0	0
0	0	0	0.05584761	0	0	0.05588426	0	0.93030705
0.03063081	0	0.1212901	0	0	0	0.138715	0.2632321
0.2287654	0	0.02752595	0.03400043	0.04745085	0.0625711	0
0.02802145	0.02048494	0.1175264	0	0	0	0	0	0
0.0598745	0.5770597	0.04312143	0	0	0	0.0284103
0.393439	0.08764592	0.1001005	0.05041707	0.1305193	0

0.02483859 0 0 0 0.01955767 0.06183441 0.05526327
0.02736387 0 0 0 0 0 0.1068979 0.03699832 0.1143782
0 0.05718438 0 0 0 0.162521 0 0.02652698 0 0 0
0 0.07938119 0.6821606 0 0 0 0.0908572 0 0.3000254
0.249940026 0 0.2341835 0.04311488 0 0.2284279 0.07396747
0 0 0.03126309 0.0869212 0.08290201 0.1128012 0.02151482
0 0 0.02612482 0.2828176 0 0 0.2737086 0.02530026 0
0 0 0 0.04479214 0 0.06986652 0.02861704 0 0.1245487
0.09339303 0.0712968 0 0.02697635 0.02767145 0 0
0.06675673 0.1064922 0 0 0.02105537 0 22.86459 0
0.02548398 0.208413 0 0.07562019 0 0.05212206 0
0.02906185 0.1290801 0 0.028154285 0.1267076 0.085886926 0
0 0.1657614 0 0.0280393 0.6956455 0 0 0.09191377 0
0 0 0 0.03801278 0.07823529 0 0 0.08318336 0.08135598
0 0.03601278 0 0 0.1825613 0.2712904 0.02717904 0
0.03872688 0 0 0.05057399 0.04923147 0.0387979 0
0.02246991 0.056298822 0.101984 0 0.02488074 0 0.1387289
0 0.1282119 0.04131068 0.02351671 0.02954321 0.02254914
0.1403909 0.0742725 0 0.1137063 0 0.01890287 0
0.07732626 0.9781119 0 0 0 0.01912802 0.03115464 0 0
0.1580197 0 0.0456489 0 0.0441899 0.1095628 0 0
0.04629017 0 0 0.02603486 0 0 0 0.03369717 0 0
0.1477383 0 0.02270534 0.029390682 0.037658731 0 0.01825212
0.02116849 0.03416146 0 0 0 0 0.267592 0 0 0
0.1447033 0.129683832 0 0.05605405 0 0.6823746 0.05016512
0.10695298 0.07396521 0 0 0 0.08047409 0.05584499 0
0.5974567 0.02675456 0.04896013 0.02716548 0.01819882
0.1139899 0 0.0561206 0 0.08127573 0.02644108 0.04779373
0 0.07203656 0 0 0.08356228 0.1852546 0 0.04546363
0.1155399 0.05051972 0.3522155 0.0617551 0 0.357139 0
0.02719054 0.03961134 0.03382426 0 0 0 0 0 0.02900141
0.03310553 0 0 0 0.03374496 0.02529559 0.1348728 0
0.08200018 0 0 0.0323926 0 0.02063991 0 0.1116975 0
0 0 0.04481402 0 0.5227792 0 0.1673498 0.170566717
0.3403327 0 0.9951632 0.039533952 0 0 0 0 0 0
0.02155609

AC007014.2 0.06066229 0 0 0.05794829 0.07956879 0.1228694 0
0 0 0.076003213 0.2343206 0.1191796 0.1405172 0.02843825
0 0 0.06050325 0.045674492 0.1498033 0 0.035442992
0.03020724 0.1543483 0.03628819 0.0207739 0.3814548
0.04801141 0 0.058613337 0 0 0.1359415 0.2342235
1.528461 0.222237 0.9308614 0 0.7082828 0.1407735
0.29526833 0.1714574 0.5347624 0.031569552 0.2534856
0.3123318 0.5356442 0.2823432 0.163009 0.216187731

0.04845106	0.1050416	0.343402	0.976192	0.5472765	
0.3548301	0.5965816	0.1336025	0.09327904	0.1216535	
0.082239593	0.068871531	0.330914383	0.5135451	0.2484502	
0.08347992	0.04110602	0.1241582	0.2145875	0.030474186	
0.4873273	0.15543	0.2418185	0.7501169	0.7919001	0.2124218
0.3839838	0.3061414	0.2222072	0.06886934	0.386259147	
0.4219077	0.2981872	0.2274242	0.1559419	0.4472836	
0.2055741	0.1560442	0.2248879	0.83125557	2.0185	
0.087686566	0.2709403	0.4663877	0.3328559	0.03491198	
0.3098645	0.3822086	0.4152044	0.2545368	0.2459521	
0.07595085	0.2914909	0.2096589	0.2182966	0.3129745	
0.2059186	0.04375546	0.03775772	0.134109316	0.6874202	
0.6428973	0.243541	0	0.1003117	1.181627	0.2889766
0.203624	0.1922373	0.06722067	0.09519527	0.3452712	
0.1957852	0.6149177	0.168934	0.262401	0.1765194	
0.05548494	0.1968279	0.05619	0.1122924	0.1747533	0.1726587
0.7406894	0.122252	0.3390337	0.1932977	0.1442511	
0.5225268	0	0.5969769	0.1446333	0.6068132	0.1415727
0.03193491	0.4866782	0.2452913	1.141838	0.337111	
0.3207694	0.2073978	0.1121257	0.02860553	0.137424	
0.150721	0.04433083	0.2857171	0.05420398	0.7901177	
3.749148	0.7103554	0.091434695	0.3015913	0.031017841	
0.1103706	0.18683	0.02407773	0.09676927	0.1417408	0.4130751
0.336072	0.07511686	0	0.550138	0.4629705	0.7244355
0.1682109	0.6161667	0.1310318	0.1750745	0.2456859	
1.159221	0.2757002	0.1358702	0.1412906	0.859808	
0.5246693	0.1417735	0.4514232	0.1500864	0.4457973	
0.1820808	0.3835519	0.1844538	0.04636992	0.1043116	
0.1858083	0.03875461	0.06026032	0.6181304	0.03542154	
1.400099	0.4473677	0.05947113	0.544733	0.6886614	
0.2116526	0.073096	0.4192234	0.489407	0.1992432	
0.4267609	0.03223814	0.1689219	0.2676232	0.523941	
0.2653744	0.06491895	0.4325127	0.2224313	0.157229043	
0.3962587	0.095927986	0.031836871	0.1086396	0.1322433	
0.7374159	0.3444912	0.3531698	0.03700607	0.4546753	
0.1026594	0	0.1978055	0.1675135	0.14539	0.5943961
0.3932181	0.2572237	0.2739908	0.2477556	0.4997703	
0.4559017	0.4022304	0.1503401	0.2847667	0.2330338	
0.2020047	0.3035654	0.176763	0.2162723	0.05453129	
0.1711381	0.8472991	0.6048585	0.3683371	0.4076533	
0.3513563	0.12576146	0.313244	0.08785484	0.2778955	
0.3823422	0.5810538	0.5013221	0.7876065	0.138421	
0.4990551	0.2309798	0.6296341	0.4390512	0.05530381	
0.1648701	0.6349989	0.110885169	0.6122714	0.454119	

0.5469878 0.07283088 0.3251918 0.144663539 0.06493227
0.1281857 0.1043908 0.05590099 0.1991738 0.3529877
0.04782502 0.4078858 0.4001488 0.04935615 0.611859
0.09430235 0.07493506 0.4394667 0.04647824 0.1028243
0.2907861 0.204628 0.02458689 0.5694618 0 0.133828
0.2327838 0.3575226 0.3327589 0.887594 0.39392104
0.504737063 0.2396375 0.3261757 0.1891465 0.5723293
0.1440492 0 0.1952194 0.07580705 0.1992508 0.4423886
0.03095121 0.241997 0.4040516 0.181056498 0.1704238
0.3130367 0.09357293 0.1088787 0.02801497 0.318551675
1.032656 0.387724 0.2768265 0.04967036 0.1198431
0.1559346 0.09900042 0.203093 0.4781191 0.5468407
0.3640968 0.1219587 0.3395107 0.6145676 0.2507267
0.1355119 0.1815554 0.3248554 0.053381306 0.1870112
0.2413751 1.131652 0.2228025 0.2488842 0.1293205
0.5782413 0 0.08603177 0.169278 0.8304966 0.4828243
0.6570771 0.3988922 0.1008813 0.2733245 0.8848463
0.680016 0.2037746 0.0994841 0.1811066 0.12759 0.1677122
0.4858796 0.4806867 0.1442199 0.150249412 0.05569466 0
0.5368052 0.7532042 0.1307482 0.2442316 0.082663204
0.6512765 0.6874129 0.1811337 0.09221173 0.1313868
0.1663414 1.34581 0.08963898 0.139621 0.6757188 0.4061173
1.751692 0.293981 0.1602126 0.457218506 0.4344241
0.3491729 0.1170008 0.264935251 0.3814296 0.9425969
0.2248214 0.1616726 0 0.1225508 0.1685335
AC021078.1 0.7267673 0.8107807 1.962196 0.7585347 0.9444509
1.572564 0.6602222 0.8064999 0.6804742 0.682920026
4.411661 3.077121 1.280791 1.752426 1.140959
2.051498 0.6913036 2.011486437 0.7519565 0.5461527
0.43052401 0.9985739 1.836336 1.094932 0.7466482
5.537996 0.8441636 1.061545 0.377118045 0.4046411
0.684601 1.844299 3.223472 3.491746 1.935258
1.618991 0.3816206 0.8401001 1.929895 1.484370177
4.449306 2.059947 1.241473732 2.172765 4.776127
1.752876 3.762388 0.6437459 3.134779323 2.328599
0.8234338 4.036368 1.198409 1.930069 2.568348
1.108505 1.642623 1.66078 0.8636893 0.82790492
2.079986423 0.90624502 1.755957 0.4094752 2.405417
2.103268 0.8906536 1.695214 1.176424977 3.023817
1.770179 1.89971 2.815867 3.603265 2.903629 0.7758502
2.484346 4.132926 2.896736 0.91464231 0.5791826
2.252993 2.731875 1.909787 1.128801 1.407366
2.343793 1.528006 1.93068876 2.085438 1.666586271
2.765122 1.155456 3.124341 0.8016738 1.531343

1.593216 2.4305 3.809039 2.414608 2.755074 2.846982
1.577645 1.925161 2.190742 2.684024 0.9076675
0.5382224 0.81822833 2.002847 3.172598 3.498603
0.363143 3.596092 3.342516 3.196296 0.6268229
2.519025 1.331049 1.522415 1.880244 3.248748
3.308351 1.055688 1.576705 3.28316 0.8893974 0.4348667
1.958787 1.004322 1.730161 1.899998 2.550551
2.26308 0.6619244 1.951286 2.035444 3.579303 1.153466
1.909437 2.195779 2.003489 2.902769 1.826195
2.41198 1.825818 1.519982 2.563068 2.209127 2.131419
1.209616 1.083661 0.672286 1.800704 2.130577
2.678217 2.271373 3.095587 5.156174 2.566642
0.49953984 3.95708 2.432673696 2.335451 0.4849705 1.51978
0.5957764 4.245331 3.908687 1.060431 2.551222
1.022602788 3.033418 1.67084 1.631705 1.622069 1.985462
2.902251 3.533037 3.313329 1.534663 1.325294
2.541552 2.410585 1.775287 1.743984 1.470482
1.864223 1.571965 2.53518 1.040218 3.171584 1.064005
0.6506984 0.7694976 3.852953 2.038842 3.949008
1.479394 1.149334 3.145115 2.106388 1.1743 2.231707
2.447787 4.244451 1.242645 1.052172 3.048835
2.683844 2.25085 1.294587 1.88636 1.00196 2.436251
1.371822 0.4482948 0.9755742 1.355336 0.420341906
1.186849 3.642901174 1.251986064 1.653465 4.311477
1.754659 1.530327 3.51422 0.4926144 0.8759326 0.7895757
3.220103 1.623764 3.812054 2.84462 3.136715 0.6227977
1.603615 5.516523 1.061561 3.543121 2.189838
1.572849 1.275819 2.59209 1.68514 1.288493 2.471731
2.007419 2.592259 2.48925 3.588073 4.235894 3.516522
2.715988 0.9455381 2.165968 0.530132025 2.114918
1.848458 2.201064 0.5066068 3.549425 1.492839
2.428488 1.732575 1.668104 0.6149476 2.574521
4.699972 1.081277 0.7567156 2.627451 1.516049096
2.395932 2.767314 4.782376 2.26083 1.118289
0.802383453 1.946611 2.697016 0.4651371 1.230929
2.760591 2.34291 3.281314 1.73636 2.658895 1.609687
3.169721 1.862067 1.892507 2.177999 0.8764991
1.066498 2.054783 1.509522 2.811999 0.9016219
2.208616 2.65552 2.113177 6.020745 1.48221 1.492398
2.399751226 3.889040177 2.107459 3.041633 0.5455373
0.5460059 1.728984 0.4502798 5.182252 1.267009
2.884453 1.38145 2.415431 3.560314 1.873553
1.419993662 0.9767726 1.288315 0.9930331 2.427683
1.322338 0.75754233 0.8820626 3.047609 1.868109

1.065263	1.211444	1.330215	0.6314773	1.361414
4.047479	1.592366	5.649498	2.340517	1.626539
1.122608	2.042544	2.113941	1.714428	8.019767
1.71875629	0.9996657	2.465621	1.686167	1.637415
1.282091	1.067316	1.914324	1.337824	2.860691
3.786932	1.78437 2.10415 3.053579	2.409385	0.4448368
3.508716	2.635512	1.716143	2.040901	2.113369
2.005016	2.908583	1.727116	2.928518	1.98936 1.494256
2.461762497 0.9715319	0.6229688	1.585866	1.671074
2.034914	3.606736	0.874198163 1.297766	2.500374
0.9360149	2.256804	0.7870428	0.4105663	3.976144
0.7971565	1.00519 3.199602	3.604079	2.529064	2.20536
4.179509	2.590931337 3.347775	1.768361	0.6813985
1.109454713 0.6558416	3.231778	1.937812	2.970722
1.749279	0.6684054	1.328722
AC012363.2 0	0.05006365	0.02407925	0	0	0.4771982	0
0.03137357	0	0.032797783 0	0	0.04042508	0	0	0.03878742
0.1044362	0.078839934 0	0	0.030589538 0.02607076	0
0.31319 0.1075751	0	0	0.02069928	0	0	0	0	0.05053741
0	0.04262323	0	0	0	0	0.34750253	0.06341937	0	0
0.05048631	0.05391241	0.03852456	0	0	0.053309613 0	0
0.04939626	0.2527546	0.1574447	0.1020803	0.03218047	0
0.05367046	0.2099893	0	0.029720237 0.214199986 0.3128625	0
0	0	0	0.02645751	0	0	0.0447153	0.0758926	0.1035837
0.04881855	0	0	0	0.1278525	5.765542	0	0	0.08578477
0	0.08075263	0	0.0506924	0.2424168	0	0.62774773	0	0
0	0.02236233	0.02209812	0	0.02674326	0.2283716	0.0275652
0.6810121	0	0	0.06861137	0	0.09420188	0	0.01974675	0
0	0	0	0.01585317	0	0	0.2597259	0.2781324	0.06235126
0	0.02073912	0.03867712	0	0.05418015	0.506925	0.09649323
0	0.9310377	0	0.3112655	0.02831248	0	0.04845771
0.01885289	0.1490154	0.0532718	0.05275558	0	0	0
0.08455755	0.4897621	0.824366	0	0.2756411	0	0
0.02470788	0.06351056	0.03398199	0	5.190828	0	0.02419289
0.02468838	0	0	0.1147809	0	0.1403443	0.09298928	0
0.5254984	0	0.05784274	0.026770353 0	0	0	0.08351798
0.02446625	0.03565099	0.09668376	0.08644078	0	0.1396482	0
0	0.02073951	0.02532337	0	0.02518338	0.03029177	0.0526569
0.02643852	0	0.04877708	0.04787539	0	0.02039325	0.03541879
0	0.09052968	0	0.05517158	0	0	0	0.1832735	0.01672384
0.02600423	0.1867199	0.03057103	0.1208374	0.02145036
0.02566367	0.06410985	0	0.04059323	0.03154323	0.2412108
0.06669302	0.07369691	0.7031593	0.02782355	0	0.02887196
0.3768284	0.02290348	0	0	0.0274246	0.108558864 0.07328491

0.303570304 0 0.01875256 0.06848058 0 0.05405775 0.06096156
0 0.0261609 0.1181354 0.1826463 0.2390061 0.04130705
0.1568509 0.0732859 0.075416 0 0.05911783 0.1336429
0.07842423 0.02623146 0 0 0.02730795 0.05028071 0.3050999
0.02619961 0.0381394 0.03733132 0.09412788 0.05908118
0.780024 0 0 0.0319846 0.02166019 0 0.02457721 0 0
0 0.5349185 0.02704204 0.09269374 0 0.06800779 0
0.02173656 0.1623981 0.1193267 0.03557332 0.07307254
0.095700894 0.03644337 0.02612889 0.09938631 0.04190509 0 0
0 0 0 0.04824608 0.07639982 0 0.04127601 0 0.02302358
0.08519495 0.04224583 0 0.04311579 0 0 0.04437193
0.9787702 0.05886895 0.02122004 0.04468014 0 0.0288755 0
0.04747148 0.08836673 0.02188713 0.056663117 0 0 0.01759439
0 0.06586084 0 0 0 0.1090438 0.08598301 0.1527237 0
0.02610733 0.8369329 0.125010541 0 0 0 0.09671473 0
0.1782495 0.02390216 0.02654653 0.1428955 0 0 0 0
0.05158087 0.0235979 0 0.1578871 5.457481 0.03536071 0
0.02923883 0.03917344 0.07646474 0.023035717 0.0403506
0.2777626 0.2219743 0 0.1342517 0 0.03119117 0.1753012
0 0.0243496 0.1131744 0.05952969 0 0.6196844 0 0 0
0.3260537 0.1758703 0.02862035 0 0.03670608 0 0.3913884
0.09573761 0.09957669 0.155609661 0 0 0 0.6500627 0
0.02634841 0 0 0.7181819 0 0.07958454 0.03779836
0.03589078 0.07743458 0 0.3615052 0 1.306427 2.519701
0.2854397 0 1.841505885 0.02343346 0.06954421 0 0
0.03291978 0 0.03880701 0 0 0 0
AL049840.5 1.576471 3.271885 3.575813 1.831087 1.997314
5.497194 3.210748 1.567325 4.065552 1.907811854
11.87135 7.144679 4.011354 6.500226 3.212634
3.676316 2.894532 2.576272259 3.096734 1.951338
2.051498093 4.210539 4.23906 7.201439 3.460046 12.36324
2.94912 4.433756 1.765555117 1.991829 2.632511 3.661269
5.620028 8.011934 5.585824 6.087001 3.336723
4.666014 6.11115 4.058623882 9.982099 7.375007 2.70365381
6.806314 10.88386 7.764171 10.92278 3.504513
7.67944367 5.551619 2.647065 14.19762 9.301951
3.824974 6.252259 4.14021 8.338104 7.657968 6.915763
3.400115373 7.871098818 4.641880092 3.996615 2.410759
4.733342 7.465609 4.204335 3.802247 1.241926423
7.758884 3.901562 5.99865 9.126638 8.769732 9.033298
4.535838 8.025445 13.32835 3.891358 3.562428212
2.959141 5.459656 6.02536 8.307763 6.208197 4.561847
5.732615 8.235169 5.40059876 7.739096 4.83375346
8.841391 8.202647 6.548102 5.505546 5.252528

10.99197	7.564455	10.0425	9.73285	9.779211	7.935146
5.820038	4.104345	5.989197	13.17566	5.840572	
2.676096	3.786182146	5.087069	4.513169	6.084531	
3.175307	7.939093	6.598357	10.8096	4.12914	7.933672
6.008307	4.685423	4.783018	6.745458	7.775528	
3.093097	5.940962	9.018268	2.539753	3.487567	
6.289018	3.962807	7.766878	11.11552	6.088159	
5.126593	1.922334	8.311373	6.066159	14.29294	
4.928881	6.252628	9.262468	7.054849	6.090097	
6.978821	6.831074	4.882325	7.116106	8.086827	
6.773039	7.384705	3.228453	6.622939	2.467464	
6.569688	8.522588	5.456339	7.859543	7.106055	
6.139803	4.79493	2.619192498	10.07416	6.668491668	4.889103
3.862151	5.347096	2.886313	9.091581	14.58965	
6.903173	6.891547	2.206726991	4.262283	8.258001	
8.96672	2.639876	5.493547	7.403752	6.652326	8.72728
4.864757	6.151601	4.89518	11.95008	6.110311	8.920257
4.214688	7.538175	3.619686	7.217538	2.47347	9.608938
1.779414	4.738022	4.897952	9.610416	6.523534	
12.67057	7.904105	7.803528	11.14989	6.532309	
2.651965	5.601114	11.82945	8.181087	5.072781	
4.41563	8.550029	4.623095	9.16572	3.522543	7.10031
4.327056	6.481356	5.689581	1.706264	6.216059	
6.606253	2.135872214	3.510627	7.082229527	2.76415488	
8.534035	12.43459	6.896036	4.957182	7.467604	
2.229399	4.90541	4.163509	8.395309	4.579722	4.409821
6.139813	6.269062	4.670724	4.724828	7.707004	
3.694873	6.131667	5.223813	4.513809	7.4144	7.942372
4.834481	5.816353	5.791213	8.534807	7.523688	
6.602589	9.784455	6.539073	6.36549	8.074962	2.867374
7.796866	2.859716345	8.022748	8.336929	4.989646	
5.796106	5.35372	3.109	4.207793	5.28685	11.13869
2.981824	6.426088	4.019402	5.863192	3.359507	
5.025652	3.945891804	7.46943	9.029932	14.65703	6.352032
4.129454	2.392388878	7.037385	4.16406	5.179123	6.388739
8.286931	8.547873	6.299045	5.601137	5.483075	
4.868245	9.020084	6.757986	4.499646	5.618748	
3.184361	8.016491	8.020568	4.834368	6.113647	
4.020799	7.846952	5.947962	4.216323	15.99967	
4.656415	8.103231	8.259470197	3.974830303	4.340463	
8.645114	2.695133	4.845157	4.88357	4.166391	6.549166
4.149042	8.218216	6.140255	6.178868	10.98778	
5.130858	6.245132011	7.113109	3.586837	1.621162	
7.395296	4.186251	3.927543789	3.098376	9.928828	

5.195721 2.630529 2.583572 3.002443 4.326965
5.072281 9.883683 6.007426 15.69836 6.999141
5.113382 9.389118 4.331528 7.983715 8.042389
13.34362 4.792338031 7.220942 4.300653 8.111778
6.875766 3.711704 3.666259 7.10801 4.168808 8.892248
15.18037 4.85352 5.947831 6.652949 8.080985 3.932505
9.972994 4.99098 8.523631 6.103726 4.328519 8.707098
9.344436 3.615532 9.661474 3.734475 3.305001
4.383836907 3.8487 2.715824 5.006095 8.096527 3.938405
9.574608 4.345271345 3.91786 13.33409 5.884054 3.839635
5.121655 2.898258 4.133345 4.111935 4.106717
8.307172 6.934267 6.897333 9.788597 7.570094
3.555611483 15.44311 6.31386 2.280434 3.181727543 4.077628
9.752996 8.338962 6.575066 3.362091 2.509245
5.602722
LINC02532 0.01318785 0.008407058 0.02628317 0.2981488 0.06919234
0.04897118 0.20376 0.1870311 0.1425184 0.143198792 0.01320688
0.008636466 0.2036543 0.02060805 0.3655007 0.008141837
0.1052262 0.054612458 0.04885037 0 0.066778673 0.01751197
0.01398125 0.1025567 0 0.01382124 0.1043758 0.006951953
0.123176681 0.1047357 0.04969083 0.003940453 0.05728465
0.6091876 3.924158 0.05300096 0.2813142 1.046269
1.848133 0.320953547 6.338435 5.69268 0.009150872 0.04097717
0.3327113 1.923008 0.7340121 0.02835025 0.035808575
0.5916124 0.1446268 0.3193569 0.4833947 0.18177 0.5185477
0.04323186 0.03227211 0.1058997 0.1851305 1.269388653
2.425549039 0.050957561 0.02189091 0.5658458 0.9981596
0.04468181 1.232623 0.1399526 0.245125871 5.573571 0
0.200725 8.679876 7.728638 0.02223484 0.2305562
0.09044584 2.43326 0.02828054 0.038756271 15.97871 1.05161
0.03531539 0.04068173 0.01389122 2.140929 0.4839779
0.05703852 1.4908839 1.579248 0.004236194 0.2086107
0.02065386 0.6382714 0.03541902 0.02245463 0.1470078
0.2245042 0.001844526 0.1448131 0.1926348 1.048477
0.5165666 0.07382235 1.154412 0.162485 0.04756173
2.77172 0.03692977 0.05860537 0.01197982 0.1041258
0.003857303 0.1768834 0.4359231 0.3821726 0.08361625
0.2820959 1.453244 0.1609631 0.004549165 0.09694973
0.06076446 5.394627 0.05704543 0.5244575 0.0402077 0
1.022037 0.05696163 2.364939 0.1025974 0.608314
0.2325515 0.09827361 3.9193 0.03345054 0.3715543 0.5273321
6.889957 2.525919 0.2129234 5.336493 0.2429904
0.01452195 0.6879029 0.1341029 0.3983818 0.009685358
2.104099 0.01015662 0.9680571 26.42604 0.3779061

0.06264329 5.024355 0.08641475 0.9707603 0.01234935
0.2500291 0.341234005 0.9179144 0.182066751 0.08997873
1.153507 0.1308612 1.886357 8.060983 0.3532198
0.08929718 0.0127013 1.647271606 0.1266341 0.0335496
2.154656 0.04701688 1.235347 0.006330228 0.5391949
1.213206 0.08179345 0.08657515 1.309513 0.272351
76.49066 0.4915529 2.157488 0.06542563 0.1722058
0.01140181 1.274227 12.36159 0.02004993 0.2755397
0.04535426 0.04231787 0.008425171 0.1048037 0.1657356
0.005133712 0.1927731 1.689386 0.1659208 0.8020521
0.008317418 0.08691311 0.2913335 53.11083 0.1399948
0.5053422 0.4638846 0.8059778 1.5689 0.2278744 0.8120909
0.1153837 0.4633848 1.166636 0.350006 2.397245766
2.014171 3.5499063 0.175338798 0.1464317 1.592717
0.03740629 0.1066639 0.0614227 0.05095193 0.01098283
0.002479771 0.6977722 0.08887184 0.03641707 0.04214329
4.165821 0.05382375 0.06710386 0.009927503 1.923306
2.06927 6.122923 0.1894621 0.0544727 0.02063589 1.517721
0.2817904 0.08359302 0.227365 0.1034377 0.04346828
1.036781 0.851418 0.01195409 0.05024357 0.5156246
0.1800483 9.498467183 0.05778062 0.4923415 0.1872831
0.1354554 0.5249293 0.08401032 0.085612 0.1237133
0.6814154 0.0956467 4.110084 0.1931702 0.9297733
0.02986867 0.1472506 0.014061951 0.2555033 0.05923471
4.399817 0.2269437 0.0070696 0.049795116 0.439953
0.009289101 0.1008637 0.03443281 0.01443332 1.342929
0.6272892 0.7869774 0.1314538 0.1663138 0.05143318
0.3052391 0.03801169 0.08242592 0.00673618 0.2347147
0.07796674 1.196171 0.1140296 0.2175875 1.453694
1.110418 3.205091 0.0398588 0.3116231 1.547364
1.32976162 0.076200534 0.1929507 0.01034103 0.05140005
0.06359407 0.2630544 0.02855791 0.02263482 0.007324574
4.500125 0.18754 0.6123141 0.01096034 0.1903199
0.068226252 0.2449401 0.08166422 0.002260284 0.1893601
1.613954 0.005771038 0.5687265 0.0140484 0.01560261
0.03959352 1.220179 3.030647 0.03228372 0.4856718
0.1537477 0.6578138 3.783995 0.6466363 0.0738089
0.8728906 1.033371 2.113755 0.5788898 0.03638149
0.806546312 1.844758 0.08745739 2.680113 0.0242184
0.0879237 1.087075 0.04975959 1.552848 0.2711953
0.572455 0.1805482 1.88937 0.8468797 0.7457775 0.02193138
0.008802989 2.645001 0.123195 0.05906689 0.03364299
0.3858476 0.9523312 142.819 36.06885 2.459776 0.03553351
0.635857329 0.2885716 0.03277498 0.1515057 0.6822708

2.315639 0.5398035 0.561088484 0.3216277 0.5925249
0.03937809 0.04176382 1.196481 0.138622 5.402907
0.006495775 0.01416488 1.969546 0.1765781 0.8308673
0.09586627 0.03095989 0.414159728 1.361552 1.452012
0.3984932 0.019198788 0.1741362 0.1087839 0.1042682
0.1221787 0.03750042 0.02664229 0.09595883
AL356489.2 0.04941707 0.6103636 0.2083387 0.01573539 0.2106611
1.19277 0.05090148 0.5132887 0.2479471 0.283773335 0.3181394
0.1132679 0.2289379 0.04633306 0.1208463 1.577306
0.1642917 0.555013694 0.07118627 0.06156765 0.476400835
1.16476 0.4191205 0.75874 0.6148675 0.103581 0.07170412
1.507651 0.135285828 0.1214763 0.4771369 0.5758556
0.003975094 0.1167304 0.003352593 0.009027426 0.1456929
0.03328763 0.1720166 0.021866656 0.01995337 0 0.012858687
0.002647383 0.03392447 0.006060411 0.05270926 0.04869016
0.213850425 0.0131565 0 0.09713338 0.1590463 0.04953617
0.8992785 0.005062405 0 0.797869 0.004129254 0.039080124
0.056104529 0.022464284 0.3404197 0.5927252 0.01700122
1.046438 0.3259036 0.1748085 0.02482506 0.03739755 0
0.002984722 0.2362783 0.1267165 0.009613574 0.02234308
0.2909558 0.03687357 0 0 0 0.05398021 0.02646652
0.372634 0.1735089 0.01993642 0.1355922 0.203555
0.46554906 0.9522206 0.019842151 0.004598229 0.4186282
0.07300273 0.05214037 0.00420706 0.03991757 0.03035454
0.003455871 0.2462745 0.05155961 0.06835855 0.03795413
0.0197589 0.2167135 0.1273632 0.02376289 0.4101122
0.010924892 0 0.01995127 0.04629212 0.06504279 0.3359455
0.4958766 0.05231285 0.004607705 0.003262531 0.08822395
0.03877427 0.004261619 0.04430326 0.2580538 0.137618 0
0.09187046 0.03013298 0.004453919 0.7667115 0.1143454
0.1186321 0.009376817 0.02514103 0.02904697 0.06444331
0.335926 0.1214277 0.05320798 0.02266056 0.113473
0.1009903 0.02601714 0.04485007 0.0390225 0.2098909
0.06993722 0.005345807 0.3732007 0.01451706 0.1367703
0.01141756 0.1398168 0 0.06139061 1.291041 0.02586139
0.1067102 0.6777833 0 0.01377795 0.031035444 0.2411344
0.218988773 0 0.02536609 0.1013408 0 0.1693497 0.08412541
0.03548908 1.968347 0.033186018 0.1274171 0 0.01710562
0.05220149 0.08365755 0.1423225 0.07923343 0.2573255 0
0.004159121 0.02767084 0.01534653 0.02635996 0.005088205
0.4202639 0.02228733 0.2818871 0.01068112 0.1377329
0.008679203 0.01252173 0.03147844 0.007081246 0.07928603
0.2131009 0.06545282 0.008392417 0.07213826 0.0475232
0.2969489 0 0.01008531 0.3116674 0.00957876 0.1190918

2.89335 0.006994449 0.01159348 0.01580229 0.2013422 0.06498164
0.1544257 0.09089597 0.03963315 0.03966349 0.05219793
0.09491339 0.012808284 0.02305733 0.017365648 0.012967569 0
0.1220926 0.006674645 0.1020478 0.5274523 0.00502435
0.04938537 0.1207975 0.004788769 0 0.01299627 0.01973974
0.2709273 0.1067752 0.01746175 0.05114999 0.01681901
0.5027386 0.1155433 0.1419892 0.04082365 0.06873431
0.09491766 0.006856594 0.490463 0.1619951 0.0176181
0.09995084 0 0.1227079 0.03732829 0.02059214 0.01509477
0.05451884 0.034149512 0.01159894 0.7514726 0.1131904
0.01730366 0.03681545 0.3233087 0.02916385 0.04385161
0.003566168 0.1164813 0.01709721 0.08089983 0.09761248 0
0.06897153 0 0.01433253 0.02466247 0.03517819 0.80425
0.008830321 0.009820555 0.5554028 0.0232052 0.4204729
0.01897433 0.006009339 0.2076771 0.0389595 0.006922382
0.08330385 0.3082521 0.01661454 2.116849 1.353143
0.1965495 0.006310396 0.1047042 0.003948031 0.009260847
0.01335274 0.02811505 0.2810485 0.2271245 0.01755849
0.02240363 0.0938333 0.03098816 0.00445692 0 0.08314709
0.005535649 0.0898821 0.03108228 0 0.03210336 0.005301026
1.420351 0.2389636 0.03003176 0.04622503 0.184816
0.03291509 0.117994643 0.01928217 0.0425013 0.3599605
0.2365212 0 0.010812518 0.08412285 0.01880059 0 0.1820824
0.08542388 0.05504562 0.4032415 0.122114 0.004057169
0.03341029 0.1318234 0.01379871 0.02880981 0.01112539
0.008510352 0.01379894 0.9058858 0.08019262 0.166695536
3.951426 0.06008151 0.02095165 0.01512506 0.08870203
0.02458115 0.01472032 0.01378857 0.02920154 0.1800338
0.2077109 0.8428311 0.006372286 0.08665276 0.02739351
0.004123282 0.006006822 0.05642171 0.007904765 0.004502351
0.004917805 0.05196907 0.01138523 0.05717256 0.1305266
0.01174852 0.004079902 0.03780859 0.03582054 0.0153437
0.01022633 0.06035613 0.05802923 0.12719699 0.1015244
0.02947286 0.01639514 0.01251968 0 0.2653659 0.1096331
0.004056791 0.007582592 0.5028875 0.005012631 0.1801734
0.03492483 0.0362537 0.031038501 0.02949109 0.4011408
0.06354127 0.029975431 0 0.0995379 0.02441938 0.003135778
0.003903341 0.07764796 0.5557047
AC093278.2 0.3507916 0.6820543 0.9303697 1.016462 0.6365023
0.7342004 0.8129899 0.6096068 0.8935767 1.135384017
0.7728547 0.2526992 1.616108 0.5262392 1.334416
0.8922667 1.061278 0.625088049 0.2310045 0.3746088
0.7173462 0.8384607 0.4611506 1.105176 1.445554
0.7214926 1.082778 0.6241021 0.847357794 0.2122611

1.024361	0.3668506	1.360087	0.460345	0.3902984	
1.396988	0.565776	2.053143	0.918068	1.081383126	
1.194504	0.5308566	0.535500909	0.1503415	2.058977	
1.200269	1.43542	0.6598421	1.178710941	0.2007941	0.8503929
2.250561	0.6648581	0.7472287	2.143069	0.4887288	
1.158874	2.571161	0.4103665	0.53104935	3.119727914	
0.605966158	3.173477	1.744579	0.5229672	2.254223	
1.531668	2.079975	0.587409867	0.2532175	0.6441432	
3.080636	1.255041	1.515536	1.979042	1.934974	
3.250131	0.9994106	0.9204011	0.618540727	0.2338107	
1.462484	2.072888	1.232412	0.7697932	1.635977	
1.750571	1.307683	1.62232821	1.27275	1.132444647	2.59822
1.658142	1.56452	1.049804	1.517102	1.813495	0.6587362
0.2747564	2.020803	1.581123	1.006254	0.9295019	
2.089879	2.569967	2.527067	0.9108884	1.419219	
0.677281728	0.3975142	0.6762637	0.9294936	0.4411917	
1.830449	1.056008	1.768543	0.9550799	0.6114076	
0.5182889	0.4098058	1.258462	1.251673	0.9105214	
1.13971	0.6856334	1.55382	0.6630953	0.4868949	1.954994
1.152601	0.6947498	0.2928736	0.5948863	0.8012054	
0.5750885	1.684125	0.8285993	0.6924532	1.660055	
0.5216562	1.740447	1.021924	1.628247	0.5170757	
1.633405	0.567378	0.5388573	1.519541	0.319457	
4.431767	0.6754051	2.343409	0.4185322	3.126044	
1.50393	0.6976019	1.211988	1.502235	0.2627901	1.232331
1.295410699	1.666499	2.218168839	0.8616233	0.5762032	
1.01641	1.231092	1.207609	1.544685	0.4894494	1.597546
1.366329133	2.052238	0.892406	1.026048	0.4354045	
0.650408	0.6819451	0.9730312	1.887537	0.4821762	
0.8089549	1.145807	4.030734	0.914208	2.968989	
1.056674	0.3164173	1.133097	0.6015124	1.228404	
0.5544911	0.3792498	0.9474391	0.6032025	2.415006	
0.9748618	2.04434	0.4408501	0.9968492	1.236942	1.154565
1.169272	0.2386383	0.6747819	0.6074283	1.458287	
0.7138044	1.062526	0.3072434	1.420871	1.802092	
3.147545	1.031722	0.6620655	1.391352	0.6882453	
0.8614864	2.229504	0.400051426	1.99683	0.96151882	
0.797779463	0.443949	4.720889	0.2369029	0.8753113	
0.3812244	0.442256	0.3447238	0.5276874	2.318358	
0.5337963	0.6042715	0.7076279	1.08845	1.145352	0.352029
0.6667706	0.4715955	0.8494906	1.939175	0.8993775	
0.8259026	1.469849	1.965197	1.460165	1.749575	
1.056239	1.800917	2.102252	1.72857	0.8275003	2.893562
1.081696	1.057229	1.480302	0.896929717	0.6641782	

1.631366 2.024801 0.46676 1.344023 0.5314821 1.39395
1.191776 0.5923656 1.144874 1.063167 0.6649498
1.84421 0.3336877 1.142402 0.566406797 0.3011527 0.6244121
0.7879914 0.9873835 0.4701213 1.129336144 3.197869
0.6918418 1.294516 1.034427 0.6825255 2.99379 0.6821754
0.3931133 1.295806 2.116803 0.6651806 1.987395
2.397741 2.14765 1.021324 3.72617 0.5717193 1.801248
0.3317499 1.172516 1.81368 3.308366 0.513519 2.714184
0.3552449 1.026537 1.126307704 0.737792885 1.421678
0.9352284 0.9935137 0.34567 0.894079 1.618009 0.609603
0.8864798 2.067571 1.773684 1.575037 1.323595
0.8567195 1.361093137 0.3996786 1.206797 0.5771774
0.8604706 0.6750085 0.322365321 0.9315599 0.4057115
0.3083029 0.9701945 1.357157 2.013847 1.650678
0.950725 0.1670411 0.7325794 3.257616 0.5289376
1.546088 1.682159 0.9786666 0.3134497 2.799679
0.6660279 0.344701461 0.4145477 0.7521805 1.160072
0.6871463 1.217342 0.5334457 1.797289 1.321373
0.6384527 4.97599 1.011055 1.023743 0.637803 1.714626
2.051504 0.4214807 0.6651836 1.106877 1.127865
0.2301145 1.982859 0.9099711 1.217674 0.9240771
0.7056074 0.5003879 0.608192689 0.8158987 1.264112
0.8277818 1.901924 0.8417656 3.136521 0.924164644
1.064746 0.4602747 0.3957001 0.9775927 0.8357469
1.531027 1.037655 0.2591775 0.4521361 1.866905
0.7330019 0.9106321 1.048335 1.044842 2.100475921
1.172333 1.936324 1.105081 0.85113337 0.6249459
2.166842 0.5633654 0.5030672 0.343582 1.070886
0.9699372
AL606489.1 3.109005 0.02858573 5.609572 5.65425 2.784007
0.6357734 3.048433 1.468942 5.584344 1.38580756
2.386438 3.288965 1.200275 5.773901 4.288801
0.2436185 6.798026 2.070764934 3.358937 5.810162
6.392646897 0.1190886 2.091722 0.3218897 0.4299685
3.947586 4.590029 0.1654665 3.870530476 0.8139951
8.025554 0.7235109 6.463797 4.048574 3.601926
0.458727 0.3110654 6.578073 10.0822 5.053087207 3.621168
16.23345 4.947260609 5.015909 4.586714 10.09664
4.313286 6.394314 3.40918266 7.855402 6.453284
2.087145 1.282842 4.270201 1.049158 3.16044 1.624031
1.930646 4.706127 6.241240585 1.59516952 5.952203315
1.04207 3.673076 5.759427 0.3241117 5.629017 2.779672
7.749096463 6.139591 6.204248 2.405023 5.352624
1.449489 4.88512 3.027625 4.224247 5.280494 5.633824

2.782007354 10.65566 6.824832 2.561698 3.442787
1.63741 9.001812 5.875034 3.915796 2.96989998 2.697528
3.658598656 3.571632 1.430085 3.835798 2.821549
5.130744 5.099987 6.106883 0.4264801 2.78771 3.742846
5.641379 0.3443986 3.299004 5.583245 1.804024
5.088778 4.614522 5.049188152 5.539715 5.847569
4.248588 1.783725 1.05458 3.07033 4.272216 7.291779
4.618294 5.786049 5.129053 3.310173 3.183927
7.107441 1.165506 2.068973 2.522665 3.992062
2.651237 5.261164 6.308475 4.822615 3.573609
4.684302 2.16884 5.613728 3.08632 3.58277 4.506256
10.85693 2.078937 4.500511 4.438332 2.627882
1.542272 1.862242 2.49011 2.794075 3.11809 5.163771
0.9636498 2.735141 4.341802 11.21482 4.307953
1.638462 2.909885 3.739628 1.451283 0.4199032
1.500266 2.658473162 2.807337 4.341093687 8.566493
2.983052 4.6038 14.21095 2.346948 5.740469 0.9568908
0.3454963 6.642120089 6.729835 6.327391 7.916101
6.892047 8.386411 1.894119 3.911197 2.871174
1.894186 9.661484 2.544351 4.344773 3.225678
6.205348 7.196173 3.761607 6.040257 6.332188
5.204289 8.600126 9.089852 5.301436 4.009559
7.90084 2.291785 4.602909 8.346414 1.989947 1.264938
4.507224 11.22469 4.856387 4.072454 4.612468
1.512907 2.720136 4.899741 11.95073 1.873645
6.386534 2.885808 7.880084 6.971325 5.884923
10.10946 2.86557 2.81864 8.678007916 7.783129 2.867904644
6.463932969 2.76253 2.893516 1.162872 4.876876 2.993514
6.856935 5.556768 3.069151 1.529568 9.708832
6.580443 3.474925 5.439895 0.6889857 4.690098
1.316466 19.16869 5.440681 8.387591 7.016935
2.370795 1.06029 4.737095 4.628963 1.346369 5.792715
3.943409 9.835492 4.284297 12.30374 4.335611
5.59496 1.132294 14.66809 8.05675523 2.890857 1.212252
4.108394 3.265905 1.14537 6.979182 9.279864 4.365672
5.332874 6.536891 3.524811 16.90728 7.849044
12.2684 3.337881 2.158439134 5.847248 6.266095 4.369623
0.741746 0.6730667 2.49514838 4.767779 6.443311
2.675068 5.454487 2.442908 6.320247 5.703479
5.678404 3.470591 3.721363 3.545912 0.2478509
0.4431345 6.318704 1.878161 2.837612 4.556905
8.739495 7.269827 3.495117 3.746556 1.384955
3.033586 2.060028 4.818578 10.29776 3.558933404
3.60663711 6.377021 3.596532 5.010095 5.151985

4.855526 2.757718 10.81331 2.390886 2.749333
2.550698 7.10776 3.577672 4.300905 1.748797149 14.22138
5.059858 0.2766755 4.292421 0.05522292 0.941890445
6.106696 2.975229 6.335935 4.683357 11.60499
11.58812 2.634511 6.805705 2.73904 6.278935 5.681833
9.335696 1.965896 3.311247 0.1853366 4.440873
2.37096 3.81301 11.916717586 0.7833497 3.885676 4.664201
2.580223 7.665607 3.492345 1.104206 7.006638
4.324424 4.560289 5.729743 1.42761 5.319679 5.150223
2.585135 1.885714 3.183171 7.968189 2.29531 6.406012
9.103404 5.365432 3.361031 3.607572 3.243456
5.003421 2.872855317 6.065618 1.597331 7.824718
4.00872 2.989667 3.971778 5.431505465 9.081639 2.389121
4.046562 2.567461 5.050281 1.967347 2.299138
6.626091 5.201658 3.157268 1.346356 1.883413
3.150995 8.079472 2.215612515 7.519682 3.997361
4.670284 5.048306691 6.842031 3.406408 2.171516
5.303879 1.95514 7.488714 3.654334
AC012065.2 0.0992844 0.7595078 0.06088374 0.06322831 0
0.167581 0.1022668 0.03966363 0.2043706 0.16585667
0.08522367 0 0.3066412 0.0930883 0 0.5148831 0
0.149508531 0.2043159 0.1413672 0.154689689 0.5273538 0
1.10865 0.4080014 0.2081058 0.1571581 0.8635703 0.06395396
0 0.187048 0.4449839 0.4472388 1.563495 0.6466303
0.1450969 0.0688737 0.1783428 0.8448014 0.029288368
0.6414168 0.3792672 0.034446049 0.1489289 0.2044742
0.1948167 0.4621038 0.07114471 0.06739598 0.1585972
0.4584502 0.2810186 0.03550463 0.0995237 1.355063
0.3661537 0.09718391 0.3053348 1.493304 0.134599426
0.413307626 0.045133254 0.3625712 0.1161806 0.3643452
0.5382173 0.135471 0.2675885 0.066501755 0.5779681 0
0.1439193 0.2619087 0.09257732 0.2317769 0 0.2312556
0.4849076 0.0500963 0.226936555 0.05754379 0.1807538
0.1063484 0.4764221 0 0.2243052 0.3064723 0.04089648
0.4534982 1.157203 0.127568306 0.1108603 0.02827128
0.3073099 0.3047442 0.1014294 0.7378294 0.4181873
0.05554585 0.2012717 0.1243068 0.1445684 0.3812704
0.476374 0.5122372 0.4493622 0 0.2471884 0.029265769
0.7059344 0.3006324 0.9034872 0 0.7661617 0.5860421
0.07882676 0.2962366 0.4195065 0.1466911 0.2077382
0.4452275 0.8188937 2.348313 0.55298 0.3181222 0.2568042
0.1513513 0 0.4904785 0.122524 0.2145106 0.1883907
1.48166 0.03334777 0 0.1265462 0.06295789 0.178168
0.6191753 0.1302742 0.2254454 0.4181708 0.2317083

1.149875 0.3123662 0.3479337 1.074032 0.3961217
0.08749916 0.8405245 0.1529277 0.2496957 0.05997822
0.03289082 0.1692953 0.2078337 0.2069999 1.058044
0.09297174 1.217983 0 0.4021981 0.507661025 1.655873
0.2853945 0.1839012 0.05279326 0.09279342 0.5408555
0.6518996 0.8196122 0.419096321 0.3177871 0.5388316
0.1374684 0.9439076 0.5442505 0.6195404 0.2547021
0.1531839 0.3994248 0.7687647 0.03706254 0.09249867
0.7868359 0.6133663 0.1546914 0.04477774 0.354817
0.02861274 0.1135265 0.1394999 0.1006302 0.2023799
0.3983564 0.1737758 0.190286 0.2301285 0.4383939
1.816504 5.779687 0.4338934 0.1946697 0.08105004
1.565437 0.1026389 0.7178061 0.1143553 0.4215789
0.3416246 0.5079771 0.07035111 0.09215673 0.219006
0.1588001 0.2316434 0.2125023 0.0786536 0.1386848
0.137244121 0.06176633 0.453563832 0.06947545 0.1659538
0.3463027 0.08046065 0.8201016 0.2312096 0 0.2976621 0
0.3463622 0.172663 0.1305548 0.118978 0.2779522 0
0.3929253 0.3736947 0.5744515 0.6940276 0.06632556
0.175552 0.4921156 0.3452372 0.09535009 0.05510265
0.6955729 0.09643447 0.3303694 0.3867498 0.5228483
0.3081672 0.4199815 0.4491807 0.768286 0.2464525
0.068610181 0.1864285 0.2556262 0.242573 0 0.04226648
0.1367502 0.1171868 1.208267 0.1146373 0.3600368
0.3572421 0.6159289 0.03017144 0.0449731 0.646667
0.03024715 0.2073287 2.576582 0.5654153 0.5562686
0.0354822 0.276228272 0.1416973 0.1631764 0 0.1829834
0.02414687 0.3209588 0.05218266 1.724572 0.1455363
0.2154132 1.04147 0.7202637 0.3270514 0.592335 0.2535658
0.1121933 0.5393783 0.3721215 0.2146172 0.2259451
0.2463961 0.1460219 2.652828 0.3900987 0.1955038
0.1660231 0 0.229469489 0.1452624 0.06673042 0.1805832
0.6661096 0.2200442 0.08599898 0.2982099 0.3308572
0.1087029 0.2896184 0.1350854 0.231041 0.6613009
0.67168249 0.3719043 0.2732475 0 0.356398 0.03056758 0
0 0.120872 0.2684888 0.1083923 0.1307628 0.03402855 0
0.6014804 0.74992 1.760167 0.132424 0.1330711 0
1.117608 0.5471439 0.2957185 0.3466716 0.1933391
0.17473562 0.3570891 1.272947 0.5051306 0.425431
0.1357808 0.1411037 0.8675266 0.2770278 0.2346766
0.3694039 0.4292376 0.6020771 0.1280263 0.3046664
0.1651098 1.192915 0.09654701 0.1648837 0.3811573
0.1085487 0.07904336 0.5800648 0.6709763 4.559299
0.7262101 0.5979705 0.327879126 0.1519234 0.1644971

0.1233088 0.4109166 0 0.9326976 0.150325265 0.2105536
0.4342379 0.4282155 0.2766877 0.1433583 0 0.9789565
0.1630109 0.03046855 0.4369112 0.161135 1.679627
0.3207674 0.3496211 0 0.266629 0.3809882 0 0
0.5410392 0.5332873 0.2453063 0.2520052 0.06273799 0
0.2889694
LINC01705 0 0.02472004 0 0 0 0.07854241 0 0 0 0
0.8210487 0.7110485 0 0.07271488 0 0 0 0 0 0
0.090625588 0 1.743079 0 0.3541174 0.9956768 0 0 0
0 0 0 0.3992635 0.3256818 1.873109 0.05667039 0
0.06965525 1.079848 0.137269683 0.2922708 0.02278925
4.385864623 0.1661916 0.02662045 0.3804471 0 1.139265
1.65833685 0 2.864906 1.317087 0.1386702 2.526609
0.9072792 0.09533895 1.176669 0.7420276 0.75173 0
1.731653321 0.564085372 5.12368 6.745986 0 0.1051056
1.234586 1.41091 0.103894201 0.2889429 0.1766334 0.01873683
0.1022935 2.892629 3.258899 0 0 1.473034 0
0.075972405 1.753038 0.9318791 0.1107638 0 12.55322
2.402928 0.02659971 0.4791874 1.0627351 2.332738
1.968058912 0.0577315 1.810871 0.06546864 1.844868
1.029996 1.503514 0.1088874 0 2.096282 0.2265687 0
0.05956502 0.2170666 0.1333761 0.2535103 1.230681
0.0321814 0.73154007 5.30754 0.3131142 0.2698452 2.585972
0.05699796 0.8240043 0.02052488 0.08677571 1.351733
0.2291722 0.02704539 0.02675265 0.305929 0.4049883
5.567408 0.3478967 0 0.3546791 5.312369 0.6225891
3.014811 1.899043 1.177274 0 5.287996 0.1733777 0
0.4917885 0.8072087 0.1991549 0.1017623 0.387429
0.4083118 0 0 0.2684015 0.083626 0.03355873 2.14388
0.4556599 0.2777781 0.09556622 2.316183 2.389417
0.1798461 0 0.1623469 1.801752 0 2.033467 2.508273
5.688962594 0.1713668 2.062079338 0 1.210206 2.360003 0
3.044351 0.4928971 2.068723 0 0.059522253 0 1.394239
0 1.065022 2.750875 0.4094937 0.02486971 0.7179474
0.2340046 0.5221843 0.5211178 0.04816954 3.09678 0.06388323
0.3826455 1.399105 0.04264021 0.2682063 0.5985895
1.11693 1.205295 0 1.111327 0.6787151 2.180051 0
0.1317102 2.324649 0.1789984 0.571946 0.02534401
1.076293 0.04891293 0.02004381 0.7476082 0.4764128
0.1317246 0.7520494 0.5621342 0.3297239 0.04799146
0.2566111 0.6698427 2.171348 2.40691 0.06143938 0.2437472
0.938058685 0.4583567 2.861623987 0.054269969 1.796342 0
4.671919 0 0.7826293 0 2.350989 1.662462 1.052166
0 0 0.5266506 0.03618654 0.2606683 0 2.422832

0.1847697 0.07744741 0.1813331 2.74261 2.242395 5.339628
1.886869 2.109098 0.02587328 2.297525 0.3317971
0.02323887 0.2333812 1.251751 0.1405988 0.01846697
0.3158621 0.2994656 0.88430222 0.8980304 0.449279
0.0236854 1.231084 0.5282558 1.602313 1.281549
0.353934 0.2686429 0.02812387 1.030359 0.1069169
0.4242255 4.426409 0.03608119 0.661562227 0 0.645086
0.1226855 0.2482989 11.22519 1.263811048 0.8024682
1.311053 10.76581 1.739048 0.9808268 0 0.0815238
2.650804 0.7275779 6.246935 0.1251589 0.3215005
0.5961027 0.1321988 0.4753682 6.835803 3.469771
0.8138999 0.4610261 0 0.1283131 0.9410229 0.3968093
1.218885 0.02181652 0.2377597 0.027978674 0.86038809
0.09077609 0 0.5440906 4.585356 0.4174352 4.836754
0.2994985 0.1292227 0.08491205 4.14759 0.2374211 3.815759
0.688757 0.12345347 0.2178816 1.334027 4.466193
0.09279878 1.265509 0.033938223 0 4.366823 0.1310794
0.4797933 0.4596474 0.02658102 0.3797071 0.3214693
0.0254692 1.631278 0.1034415 0.242543 0 0.3492027
0.1602733 0.08662394 0.1547421 1.057173 0.068246385
0.1195441 1.234363 3.200458 0 1.405343 0.08817723
3.727123 0 0 0.09618524 1.527451 0.1175764 0.9600608
0.4079773 2.192553 0.6471058 0 0.1931955 1.662364
0.02826387 0.06174383 0.01812444 0.6194212 0.2208648
0.03151505 0.3933456 1.203759789 0.403489 0.5782277 0
0.1925898 0.0891507 0.05204045 1.315158832 0.02055894
1.079273 0.4116871 0.01964832 0.3359481 1.453193
3.517624 0.2546683 0.1904013 1.727772 3.964866
0.04524213 10.64896 0.2958611 0.259795568 0.4628317
0.04578534 0 1.505385355 0.2925877 0 1.226357 0.0393702
0.1715248 0.3829896 0.0820819
AC008268.1 0 0 0.03582837 1.860404 0.4427836 0.01314889
2.006037 2.863154 0.2605771 1.382694739 0 0 4.471143
0 1.651026 0 0.5438804 0.078205833 0.4168118 0
0.257919828 0 0 0 0 0 1.233108 0 0.275990769
7.424159 0.3302175 0 0.01253274 0.2044607 0.03171031 0
0 0.151594 0 0 0.03145465 0 0 0 0.0133697 0
0.06043002 0.01395555 0.013220212 0.02074 0 0 0 0.0195223
0.2278333 0 0 0 0.01301878 2.429044506 0 0 0.142242
0 0.03573448 0 0 0.05248943 0.039134411 0.08162831 0 0
0.01284382 0.09685182 0.01010327 0 0 0.01056869 0
0.02543729 0 0 0 0.0534021 0 0.2262811 0 0
0.40030639 0 0 0 0 0 0 0.03979225 0 0.1230457 0
0 0.04876737 0.01134326 0.2094091 0 0.02679442 0

0.05618999　0.1293008　0　0.01730927　0　0.0312751　0　0
0.1839303　0.4226401　0　0.07200311　0.9495595　0.1765806
0.0134361　0　0　0.02410468　0　0.2518701　0.03562639　0
0.5291601　0　0　0.01478168　1.136132　0　0.1161016　0
0.0246993　0.02795916　0.02857782　0　0.008844549　0.04101362
0.03030085　0　0　0　0.03370868　0　0　0　0　0.01224491　0
0　0　0.4076809　0　0.07686781　0　0　0　1.391405
1.341029621　0.1063019　0.01599492　0　0　0.6795453　0.1060927
0.01598436　2.079329　0.074735269　0　0　0.04044814　0
0.03767954　0.03739304　0.1623754　0.01502407　0.4701005
0.01311294　0　0　0　0.08021084　0.05057307　0　0.01070768　0
0.08907617　0.04104588　0　0.4565303　0　0　0　0.02579508　0
0　0　0.03191676　0.01272863　0　0　0　0.2190267　0
0.03307831　0　0.04982171　0　0.02410295　0.01431987　0.01245992
0　0.02778925　0.05142825　0　0.107685736　0.01211592
0.013687669　0.054512462　0　0.1698245　0　0.04021722　0　0　0
0　0　0.03386911　0.01024371　0　0　0　0.01101074　0
0.01325681　0.02917255　0.01301024　0.3099223　0　0　0.01246908
0　0　0.07566529　0.01851553　0　0　0.5561331　0　0　0　0
0.336459669　0　0.02507147　0.03568685　0　0　0.01341227　0
0.05925258　0　0　0.01078087　0　0　0.0176436　0.09060603
0.035599193　0　0.05183747　0　0　0　0　0.01389748　0　0　0
0　0　0　0.1091249　0.0114192　0　0.01047651　0　0
0.05532896　0　0　0.01244741　0　0　0.04432078　0　0.02864326
0　0.141269　0.021914　0.02171109　0.028103691　0　0.04559085
0　0.01012077　0　0.03699709　0　0　0　0.02843049　0.1704323
0.013249　0　0　0.031001274　0.01215862　0　0　1.025348
0.05996054　0　0　0　0　0　0　0.4138468　0　0.1738723
0.0127915　0　0　0.008700956　0.0181664　0　0.01341578　0　0
0　0.125677436　0　0　0　0.02384326　0.01331719　0.02214281　0
0　0　0.01207688　0.1871069　0.0590509　0　0.08537504　0　0
0　0　0　0　0.01550493　0.02730814　0.1196517　0　0.0949676
0　0.334442569　0.01192035　0　0.06046981　0　0.01119363
0.02613649　0.011794958　0.1548811　0　0　0　0　0　0.03840589
0　0　0　0　0.1136107　0.04719065　0　0.032619551　0
0.01149748　5.133556　0　0.03265501　0　0　0.1186384　0　0
0.02061217
LINC01088　0.1163744　0.8659006　0.1237715　0.01042199　0.1287939
0.8065785　0.0449513　0.1263974　0.09544544　0.205037454
1.420359　0.2857924　0.101088　0.4807737　0.1067199
0.5765684　0.01450866　0.172505525　0.07184557　0.08543956
0.097741038　0.2499073　0.6986145　0.430744　2.139587
0.294428　0.1669403　0.4457211　0.126499171　0.3465846
0.04110843　0.8149685　0.007020842　0.05726946　0.02072481　0

0.01135252 0.04572778 0.08158051 0.099771064 0.03230498
0.6187382 0.030281483 0.04208246 0.1011111 0.08027959
0.02538968 0.02736266 0.029623865 0.1161855 0.0293871
0.1200904 0.1794695 0.05468198 0.1418136 0.4291804
0.02135857 0.4175414 0.1166899 0.285954992 0.123865319
0.05951489 0.467238 0.02553356 0.09008308 0.19468 0.1637521
0.1323207 0.007307702 0.01524273 0.05280211 0.005271639
0.47128 0.2373718 0.04810878 0.3288543 0.1157661 0.04440435
0.01376238 0.284999528 0.06955668 0.06753269 0.007790896
0.115924 0.1379038 0.2218347 0.2507098 0.03145804
0.38621133 0.6111978 0.021027216 0.004060714 0.02795991
0.09516852 0.02092971 0.03715271 0.1022289 0.01148837
0.01831136 0.01474481 0.145704 0.006354495 0 0.05670977
0.00750512 0.04663594 0.01049255 0.185613 0.051455009
0.08726993 0.08809521 0.02336043 0.09573257 0.3167204
0.4507903 0.01443677 0.04882899 0.01728692 0.2095535
0.02663244 0.2370975 0.2464836 0.09048505 0.3578413
0.0629237 0.08818605 0.0698527 0.02359964 0.367176
0.1582002 0.04190579 0.1283509 0.2368228 0.09161247 0
0.2827517 0.1141516 0.01174704 0.1480861 0.08589295
0.06936606 0.1799774 0.02546182 0 0.06178517 0.1382289
0.004720903 0.08083914 0.07692041 0.2024882 0.05377543
0.0411576 0.009886273 0.01445714 0.2896817 0.04567659
0.06824002 0.3229603 0.1123806 0.07300416 0.14251911
0.1084823 0.048347459 0.02977519 0.1120045 0.207858
0.07541705 0.2957079 0.02971659 0.2731105 0.3422471 0
0.135803 0.159129 0.3285564 0.06050538 0.04573419
0.01571069 0.04548138 0.08416488 0.1463058 0.02938349
0.07738139 0.02371702 0.05320819 0.01797365 0.841432 0
0.06598292 0.01572089 0.7235571 0.1188019 0.1806139
0.02223899 0.05002781 0.1304877 0.1835436 0.3684854
0.06670238 0.1274112 0.008393584 0.1400582 0.01426116
0.03859429 0.3302816 0.01127872 0.08325991 0.7874823
0.04632619 0.09214419 0.06047195 0.06957628 0.06076116
0.05214297 0.2024215 0.03181836 0.1751349 0.0489772
0.1943062 0.184747077 0.03393669 0.118851453 0.034355132
0.1667312 0.149046 0.02947203 0.3529653 0.127035
0.02662214 0.09085918 0.2461771 0.0211449 0.04743371
0.01721558 0.2658416 0.06108687 0.09953213 0.006168217
0.1190865 0.1225367 0.2723745 0.1384785 0.04822738
0.2163091 0.1251928 0.04540367 0.1029364 1.193837
0.2384309 0.02333792 0.05884465 0.09028551 0.128682
0.3032757 0.02597857 0.03554733 0.01504556 0.263878707
0.04438663 0.3862388 0.01332785 0.005093642 0.1439811

0.08264913 0.07726412 0.03319331 0.06928453 0.06725795
0.3684061 0.1428859 0.1259875 0.05436173 0.1624241
0.073123148 1.716305 0.09074802 0.03106599 0.2299535
0.3509141 0.030353995 0.02335611 0.002561575 0.225296
0.03351261 0.0451084 0.2892084 0.01720264 0.1895086
11.15964 0.1775339 0.01467236 0.09422367 0.3324341
0.04959248 0 0.2219152 0.1812991 0.07360461 0.008843895
0.0341392 0.05415164 0.04011491 0.06202387 0.4253717
0.02455246 4.074458 0.019679618 0.050431592 0.02234751 0
0.02551347 0.07319703 0.06217734 0.08032671 0.01404407
0.2544992 0.1473226 0.04243387 0.01484418 0.1632117
0.08720233 0.078151145 0.09876331 0.03753306 0.9162496
0.06527273 0.02351294 0.128905782 0.09905229 0.03320576
0.01475176 0.138961 0.1005842 0.07478616 0.3145586
0.3548263 0.03582901 0.02950476 0.01818965 0.03899422
0.1272103 0.04421195 0.05636644 0.3818247 0.2884322
0.3611739 0.115207415 0.1009018 0.04823474 0.01233499 0
0.201428 0.05271873 0.004333191 0.05479534 0.0103152
0.05074102 0.2463207 0.3721537 0.101293 0.2056566
0.211674 0.02184772 0.270537 0.1313601 0.1116917
0.01988022 0.03040053 0.01529803 14.16991 0.3262386
0.09310153 0.3423805 0.079265497 0.03338891 0.02259518
0.0169376 0.08127818 0.009406012 0.09883125 0.09250554
0.1590681 0.3036553 0.2461363 0.01934831 0 0.568413
0.4733298 0.05015594 0.07365838 0.1080248 0.07082678
0.7255501 0.0749026 0.07683787 0.173598065 0.1562622
0.2479743 0.01402839 0.164122801 0.09604006 0.02511494
0.05391212 0.07476889 0.0310235 0.05877533 0.2424858
LINC00589 0.02802048 0 0.02577431 0.06691714 0.1194491
0.0425659 0.411286 0.05876872 0.3352555 0.008776649
0.006013049 0.05505018 0.03245311 0 0.3426115 0 0.06986754
0.089664269 0.08649441 0.1196918 0.171900222 0 0.07129494
0.04190464 0 0.005506173 0.2106807 0.005539104 0.121833218
0.4927535 0.3761253 0.01255853 0.04733313 0.06618847
0.0627326 0 0 0.05033269 0.1083743 0.068193602 0.4638726
0.2593624 0.123949307 1.540149 0.5193683 0.3298921
0.3830998 0.08282493 0.221116657 0.15666 0.03234646 0.08591949
0.2480017 0.04213202 0.1365828 0.08611458 0.07199763
0.02154323 0.02107234 0.028490428 0.159062022 0.315258176
0.1325593 0.1065642 0.05784023 0.0332277 0.2198411
0.04955999 0.598243246 0.5382851 0.2213666 0.1929333
0.1316647 0.05878705 0.08721746 0.1520277 0.3480851
0.03421319 0.03181139 0.020586544 0 0.08417165 0.09004233
0.136859 0.1475746 0.04069568 0.09370178 0.2683511

0.33596913	0.1106195	0.094507571	0.0469312	0.1496033	
0.1064418	0.1128832	0.07872112	0.8012461	0.2139161	
0.01763594	0.3834256	0.1403296	0.8935357	0.225968	
0.008402771	0.1011961	0.08454731	0.1313719	0.01744065	
0.192033677	0.07471196	0.07636122	0.04499755	0.909721	
0.05405738	0.6202326	0.07786391	0.04702792	0.6160238	
0.2639238	0.0146572	0.02899709	0.06782628	0.05809832	
0.1430591	0.01346728	0.1223039	0.4997658	0.1136458	
0.1687054	0.04538529	0.05045011	0.0478516	0.2138321	
0.1764666	0.2035837	0.01785721	0.07995712	0.2941575	
0.115641	0.3033242	0.09543926	0.2581643	0.03269687	
0.03687758	0.0198354	0.09630699	0.04546771	0.4611552	
0.1172984	0.05474227	0.05179191	0.158558	0.1523458	
0.05569555	0.04095365	0.2859467	0.2253359	0.1327137	0
0.1640599	0.168938163	0.1083505	0.200584035	0.0637265	0
0.1390216	0.01117466	0.1964142	0.2575844	0.07761739	
0.1850516	0.104838376	0.1345309	0.02138504	0.007274425	
0.1331969	0.09487105	0	0.3234741	0.03242417	0.01409093
0.2476221	0.03137986	0.02610538	0.0384342	0.06924276	
0.08185814	0.05686816	0.04044035	0.2301436	0.1501872	
0.07381926	0.07810085	0.214187	0.02409126	0.1226094	
0.00895056	0.0139174	0.1213461	0.04908463	0.01616797	
0.05740092	0.2609676	0.005718574	0	0.1575092	0.05064559
1.113448	0.01784698	0.04601615	0.0896022	0.1116832	
0.1235421	0.1931526	0.2218455	0.09806313	0.02998667	
0.01109898	0.007338793	0.087150679	0.07844383	0.258475198	
0.139704611	0.09032696	0.07940974	0.1192168	0.007232895	
0.01631324	0.1025607	0.6580588	0.01580645	0.4317373	
0.1279155	0.4255682	0.9653816	0.2745571	0.1513593	
0.07128841	0.458776	0.1502033	0.04721902	0.1474097	
0.0371588	0.05208263	0.5846061	0.4641996	0.1049713	
0.02804394	0.1837091	0.054944	0.02518851	0.2687709	
0.3000544	0.06349768	0.05004069	0.02567712	0.3187932	
0.043567796	0.09207567	0.06763495	0.1989619	0.04905762	
0.1610365	0.02170925	1.265042	0.1385322	0.09099403	
0.09907089	0.05816678	0.1448585	0.1596584	0.0856744	
0.2835348	0.03201181	0.1706635	0.006992061	0.937497	
0.03924814	0.5482633	0.183758965	0.02249462	0.054276	
0.05625561	0.03227649	0.3373343	0.1222862	0.3810667	
0.1177539	0.1355438	0.7352377	0.0169574	0.08711827	
0.0173066	0.01791122	0.2146873	0.02374775	0.1074534	0
0.07381994	0.2510834	0.1216932	0.04636231	0.1493403	
0.1079782	0.1655278	0.09371153	0.189537305	0.165142796	
0.03689694	1.426596	0.02184213	0.08812142	0.01330754	

0.08191465 0.1442779 0.1458999 0.07669652 0.1890177
0.04288996 0.3004106 0.07465409 0.551969155 0.1443206
0.1012162 0.5042591 0.1257303 0.08411243 0.009196375
0.372055 0.07675429 0.2486337 0.09177282 0.08303498
0.1440553 0 0.1407158 0.02070447 0.08209202 0.1121198
0.05633386 0.01960289 0 0.1809578 0.03912139 0.04193107
0.163695 0.067807643 0.1349722 0.05574671 0.02376003
0.02572864 0.0359256 0.1254421 0.1252008 0.117276
0.317911 0.09773886 0.2523777 0.1433707 0.07587752
0.184252 0.09319604 0.04208372 0.08174376 0.06107612
0.1411881 0.1301991 0.0585584 0.3781664 3.247194
0.07481077 0.06831806 0.04663156 0.104102413 0.07717765
0.04352351 0.1044023 0.06958241 0.2053389 0.1128128
0.108184904 0.1281316 0.2590315 0.00697228 0.04259346
0.4349363 0.07683465 0.207214 0.02070254 0.1741291
0.04046011 0.4945538 0.6252314 0.3988907 0.0246679
0.123196211 0.06897844 0.117863 0.1891532 0.387524224
0.02642788 0.1048172 0.01038471 0.08534639 0.05311857 0
0.05004463
RABGAP1L-IT1 0.6899615 0.131952 0.4351907 0.03766245
0.2327144 0.1796778 0.6700763 0.4252673 0.3043375 0
0.1692139 0.232376 0.27398 0.03696586 0.7713176 0.1460449
0.07864602 0.029685321 0.7302147 0.1263097 0 0.1570613
0.4765007 0.165094 0.05400651 0.1394549 0.5616749
0.2961655 0 0.04473107 0.05570834 0.2650583 1.046577
4.035671 0.09629265 17.54491 0 0.1062313 1.250408
0.348917059 0.5412589 0.3301813 0.164144537 0.1140566
2.679527 0.3046158 1.468031 0.04237792 0.100362399
0.2046844 0.1137832 2.008692 0.2114862 3.141951
0.5765388 0.0484672 0.2604977 0.3031252 0.217433
0.133625391 0.17904729 0.215071899 0.2945025 0.2998836
1.085126 1.349164 0.7531479 0.3187825 0.039612307
0.5783759 0.1178555 1.028721 1.345569 0.07352591
0.3374795 0.819994 0.780579 0.1123263 0.2984027
0.096554708 0.3427642 0.3014689 0.2745049 0.60811 0.04152914
0.1336092 0.3651054 0.3897651 1.89090904 0.511414
0.037993546 1.210638 0.3368004 1.830516 0.6126413
0.9666763 0.6496869 0.5189514 0.3308633 0.4395935
1.160028 1.050582 1.498905 0.8039754 1.261155
0.6096842 0.2275047 0.1963197 0 0.5519004 0.2984569
0.8863967 0.1729768 2.673027 0.9774259 0.2034665
0.2867405 0.3123531 0.3058221 0.1237409 0.1836023
0.3605335 0.1816613 1.976322 0.2652889 1.491433
0.198338 0.1918872 0.2738976 0.03649122 0.383325

1.122165 0.8825627 0.01986387 0.08813956 0.3517651
0.09375344 0.08490175 0.5423776 0.1551978 1.275739
1.681336 1.165493 0.6434213 0.3907332 0.3507299
1.944858 1.769648 0.08686597 1.039843 0.346152
0.2230999 0.08931628 0.6269339 0.3025263 0.2475956
0.4403613 2.380879 0.2768968 4.023236 0.178279046
1.785904 0.24191391 0.466267 0.7042755 0.04694667
0.3773608 0.1842438 1.10073 0.2669626 0.1464626 0.13616624
1.808794 0.2607795 0.1228262 0.4529205 0.2669777
0.9367793 0.8913277 0.1368679 2.954181 0.7366554
0.2428426 0.5693417 0.5047378 0.7307132 0.03071439
1.20025 0.1788343 0.4772155 0.2366803 0.4777919 0.09990203
0.4520595 0.7796481 0.7590804 0.1637212 0.2741558
0.5222657 2.141009 1.076797 3.505257 0.09663049
0.2574833 0.4102846 0.9476344 0.2137835 0.5449335
0.6863861 1.109953 0.2521506 0.3352416 1.354049
0.5000686 0.7378065 0.1552278 0.1054823 0.06246753
0.1239131 0.143063495 0.3863119 0.228604503 0.062075419
0.2541901 0.6360239 0.9106136 0.7531044 1.606755
0.16836 0.07880216 0.200165 0.7335589 1.054193 0.4821492
0.3071042 1.545269 1.618579 2.624694 0.1780754
0.7447375 0.4576964 0.8494082 0.7581252 0 0.2056433
0.1703882 0.1641117 1.164052 0.1723259 4.104419
0.6025078 0.6896145 0.6975367 1.036399 0.6477723
0.02408609 1.321213 0.388247979 0.1665715 0.247432
2.943996 0.3037179 0.1510582 0.1221845 0.5118908
0.1199523 0.2731385 0.2573506 0.7038575 0.7133837
0.395381 0.1875201 1.623311 0.054050878 2.319 2.006997
0.9168286 1.26227 0.4649757 0.070516114 0.4431165 1.957832
0.1809252 0.1998252 0.1294495 1.108854 0.5284114
1.358628 0.7455328 0.1924689 0.2226934 0.1634403
0.3571532 0.2016166 0.0604154 0.2673153 1.322939
0.7536344 0.2237173 0.3196418 1.467678 0.3696613
0.4370705 0.4826052 0.9482638 0.4615007 0.192016464
0.136685353 0.1903588 0.105996 0.03073309 0.07439505
0.4306622 0.179291 0.9896591 0.114962 0.9928296
0.5319167 1.227087 0.2359225 0.2363455 0.117674441
0.09230324 0.6917389 0.1621761 1.132221 0.109247
0.025879622 1.825546 0.2159951 0.999547 0.08608631
0.2920871 0.3648489 0.4504049 0.2451367 0.6020684
2.132456 2.149464 0.1849514 0.4413178 0.1065139
0.2851719 0.286239 0.05899935 1.266804 0.312248048
1.291411 0.3399011 1.136677 0.2172098 0.2426368
0.01680991 0.4932606 0.6270527 0.2516167 1.411913

0.9374891 0.6724351 0.1830238 1.840695 0.196698
0.3158087 1.092671 1.104911 0.8513979 0.1293158
1.035822 0.3455203 0.508674 4.568411 0.7690185
0.2811994 0.117182309 0.2171865 0.073488 1.872972
1.174876 0.4418826 2.182592 0.143267932 0.1724497
2.492516 0.1569662 0.7641249 0 0.1621659 1.107934
0.07767901 0.09074419 0.455437 0.2399533 2.483959
0.2149512 0.4338638 0.222870757 0.6352789 0.9775822
0.1901065 0.114793293 0.371855 1.157179 0.555251
0.4353158 0.5979262 0.07964974 0.1564791
AC116158.1 0.0136483 0.07177984 0.06904828 0.04563189 0.04475511
0.152043 0.08434967 0.02453593 0.06321187 0.068399322
0.181589 0.06703504 0.07376769 0.03199136 0.2225071
0.1162803 0.05445008 0.020552449 0.008425994 0.01457496
0.063794057 0.1155367 0.02604492 0.195946 0.009347769
0.1126425 0.10802 0.1402962 0.039561937 0 0.05785399
0.05505344 0.09222069 0.08597144 0.1111127 0.08975695
0.01420176 0.04290336 0.04222985 0.060392643 0.1598144
0.04211034 0.042616671 0.06141829 0.1756775 0.1405993
0.3811434 0.08068525 0.284889677 0.08720736 0 0.6116529
0.1464212 0.1231308 0.4789957 0.05033395 0.1001967
0.2798225 0.04789857 0.018502943 0.178195786 0.167516664
0.1155416 0.007985483 0.075128 0.07861116 0.01862276
0.1793245 0.027425336 0.09057458 0.05828321 0.009892061
0.1552661 0.08908411 0.1115156 0.04936677 0.3020045
0.1666467 0.03098958 0.013369811 0.2254452 0.2012658
0.4678197 0.06315318 0.05031678 0.1057183 0.1193676
0.2276872 0.25715627 0.1231563 0.059185343 0.5943443
0.09327266 0.2995547 0.1492409 0.1533749 0.1852145
0.05030099 0.06872135 0.1798431 0.5297296 0.02981001
0.1257888 0.1145998 0.05633246 0.2831233 0.05906679
0.1274257 0.08448446 0.09097734 0.095052 0.06027325
0.02395188 0.2708273 0.3866942 0.05418028 0.015271
0.05406393 0.07561931 0.04283564 0.07061997 0.3450535
0.08175184 0.02533878 0.04591778 0.6155792 0.162285
0.01476132 0.2085945 0.08842564 0.137611 0.1165385
0.0416616 0.2681762 0 0.156563 0.1168373 0.0881718
0.03004094 0.05372512 0.3021645 0.201196 0.1698783
0.1580696 0.1288199 0.06070643 0.1860311 0.151692
0.06615523 0.07999211 0.3468708 0.03861545 0.04947006
0.4001433 0.2044653 0 0.06097629 0.2343295 0.05751234
0.1826533 0.072001064 0.3845089 0.118637051 0.1365764 0
0.2275227 0.05442987 0.1084261 0.4275104 0.2184354
0.1521037 0.086417701 0.2257066 0.03472098 0 0.1027234

0.1386303	0.06878808	0.2232083	0.08686298	0.02745381	
0.02067644	0.08406529	0.4831886	0.04368152	0.2445206	
0.1329057	0.04616587	0.3770724	0.1769985	0.05852292	
0.1510157	0.03458328	0.03129806	0.08214112	0.1791629	
0.13079	0.203368	0.04172163	0.007969431	0.04725087	0.02236722
0.02676061	0.1002752	0.03873518	0.2962982	0.01644574	
0.4558816	0.04056715	0.1088665	0.2094899	0.108798	
0.1140164	0.03010604	0.1309784	0.05373551	0.007303	
0.08649802	0.1143873	0.063674439	0.07641734	0.446041854	
0.007162922	0.06843937	1.249634	0.03871232	0.09159855	
0.2065936	0.01665186	0.03409887	0	0.2063242	0.008900784
0.0538408	0.1144886	0.4489579	0.009829939	0.05787231	
0.300518	0.2369039	0.2044408	0.2119832	0.1809942	
0.0845619	0.09254433	0.1572896	0.2726921	0.1707467	
0.06959679	0.1459762	0.3128569	0.1155122	0.3304294	
0.321658	0.1803674	0.07504136	0.1072835	0.141474258	
0.185801	0.05271015	0.1187941	0.02867417	0.2004531	
0.02114842	0.2335847	0.1868585	0.09455287	0.06681567	
0.8046304	0.1834511	0.1679766	0.03709383	0.1333428	
0.068606618	0.08075227	0.14304	0.1165887	0.1583989	0.3219231
0.056958296	0.4017475	0.100941	0.03914451	0.1383477	
0.1394144	0.1985452	0.09684062	0.04588476	0.150048	
0.1832252	0.1762062	0.1697354	0.08991738	0.06397785	
0.17777	0.09253704	0.1570163	0.1381167	0.02765881	0.04076616
0.07621036	0.1204389	0.08147006	0.1299389	0.08638481	
0.06846796	0.07385634	0.340679737	0.1377844	0.1788776	
0.0638336	0.03433797	0.1101919	0.053199	0.1141974	
0.07959325	0.2913891	0.06967256	0.1601641	0.2654266	
0.1272696	0.211825047	0.03834335	0.07042958	0	0.1224822
0.05042431	0.017917597	0.1486947	0.1433119	0.0207609	
0.06332641	0.229188	0.2385675	0.05568483	0.130553	
0.06050879	0.1107295	0.6348607	0.0731714	0.1718683	
0.04609017	0.05641057	0.1295765	0.1123315	0.2059771	
0.108091504	0.1472636	0.07240875	0.1273042	0.08772392	
0.1469896	0.03491471	0.07317984	0.1485202	0.04839034	
0.2983369	0.2753608	0.4034821	0.718054	0.2153905	
0.04539422	0.01366551	0.159264	0.09349732	0.1375407	
0.06714827	0.1629875	0.2966313	0.07546657	0.05101469	
0.04991486	0.06489556	0.060847836	0.0563879	0.06783862	
0.03813943	0.1694621	0.1000171	0.178585	0.111589502	
0.1628106	0.06511993	0.0339608	0.06223967	0.03941398	
0.0561373	0.1211166	0.02016772	0.07539141	0.444825	
0.04153255	0.4299384	0.2728369	0.3123982	0.20573759	
0.3970701	0.1329474	0.01316191	0.049672767	0.01716344	

1.005384 0.06069862 0.239032 0.04527803 0.01838167
0.08125299
KTN1-AS1 0.189943 0.3323419 0.1784342 0.1930266 0.2385404
0.4788575 0.2122995 0.52794 0.237087 0.455702159 1.07886
0.5716644 0.424379 0.5039542 0.3821366 0.4940136
0.3143981 0.413827415 0.05488966 0.1035775 0.14167338
0.5795757 0.5809744 0.6575683 0.4622439 0.8259107
0.2302945 0.5783996 0.210861275 0.4493389 0.2626737
0.4890491 0.370596 0.7764273 0.4441649 0.6644386
1.497059 0.2177816 0.350126 0.708151193 0.6200809
1.813365 0.147222125 0.415687 0.2621763 0.6809845
0.3714933 1.090315 1.148084427 0.6875266 0.2285981
0.5910033 2.870177 0.401057 0.5988519 0.7849529
0.6942499 0.700142 1.406147 0.460221458 0.624000974
0.56767465 0.6963257 0.4161603 0.5338999 0.3861279
0.7830314 0.522821 0.178657566 0.3895907 0.6765133
0.2519022 0.9115079 0.4597363 0.3459282 0.3727534
0.2698446 0.1809318 0.8227986 0.415682118 0.3162107
0.8122708 0.4025856 0.5817783 1.06422 0.4773828 0.8150241
0.6292483 0.76145438 0.7384624 0.521859054 0.4512532
0.3624937 0.6072528 0.7489226 0.4954384 0.5797697
0.1276664 0.2238366 0.5489111 0.9310159 0.1906614
0.2514157 0.2860121 0.6088333 0.5030065 0.4955504
0.3219752 0.382392477 0.9805763 0.5433294 0.2466166
0.3758912 0.6326378 0.901706 0.1860999 0.2848754
0.2721468 0.6836813 0.7060691 1.254658 0.6043383
1.012978 0.6677633 0.4933594 0.313594 1.393552
0.5376221 0.9096449 0.4600786 0.3085161 0.9570144
0.8059676 0.4601622 0.2077961 0.6696328 0.8841249
0.7223261 0.4269741 0.7370853 0.4542457 1.004269
0.6256304 0.3318921 0.8620611 0.3529738 0.5770795
0.3316939 0.4879427 0.5250423 1.919751 0.400199
2.006826 0.5382018 0.4902534 0.2791735 0.5199963
0.7895682 10.49032 0.9194395 0.596957921 0.4286316
0.467010486 0.6397041 0.7617362 0.5004685 0.3932547
0.32861 0.203642 0.7015288 0.4804136 0.390810366 0.3794391
0.7731372 0.6588828 0.528295 0.2853898 0.9136746
0.2293824 0.6500303 0.3007815 0.3755079 0.443534
0.3087406 0.3732483 0.6940793 0.8815328 0.3444834
0.3866204 0.7162736 0.454019 0.2384886 0.9175314
0.1173888 0.6810287 0.5941731 0.4001859 0.9675092
0.6218186 0.6843403 0.5067942 0.5364679 0.1148976
1.055717 3.287983 0.144137 0.1460903 1.349891
0.5182391 0.6712671 0.4497258 0.6614961 0.2550823

0.6552208	0.5235766	0.8203206	0.5535898	0.8420128
0.5969715	0.842161615	0.3922119	0.421789616	0.139984818
0.7498156	1.060735	1.067691	0.4756931	0.4705656
0.4536254	0.7915945	0.7614312	0.2819713	0.5218434
0.05101622	1.234955	0.5034711	0.2328561	0.7642799
0.6114872	0.9119318	0.572066	0.3685174	0.7556673
1.502356	0.6913964	0.5938229	0.4575589	0.7806319
1.11872	0.2823992	0.9845135	1.372713	0.413947	0.366326
0.4070542	0.4888438	0.4012708	0.477561214	0.8005882
0.632115	0.3258373	0.8830208	0.6193598	0.9184513
0.5199358	0.5102645	0.4164649	0.2901727	0.785237
0.4303888	0.9395109	0.3624619	0.8122333	0.783043611
0.1715981	0.6050709	0.5178386	0.4075693	0.4116231
0.284307112	0.8651616	0.236267	0.4033638	0.6815178
0.7017839	0.4468068	0.5065927	0.570643	0.2452539
0.7496918	0.909813	0.2848045	0.4526258	0.6027702
0.8236409	0.7192695	0.3990685	0.3635301	0.2751817
0.1034665	0.1855453	0.476988	0.9270413	0.1905461
0.221685	0.5406334	0.227440549	0.56042896	0.4115351
0.5459643	0.3181751	1.520066	0.5220552	0.4673249
0.6450766	0.7440759	0.5707885	0.2770374	0.2391895
0.3587126	0.2584132	0.694773465	0.7871887	0.6506636
0.2036393	0.7253535	0.2762221	0.710937336	0.3742496
0.4649452	0.3811402	0.8713845	0.526943	1.130259
0.4023217	2.006323	0.7724217	0.2331564	0.1980926
0.2518727	2.849906	0.9880834	0.6055015	1.674665
0.8466693	0.3305319	0.213376737	0.840965	0.6753803
1.007498	0.4007657	0.8456178	0.2756918	0.2552123
0.2097397	0.05158316	0.4435758	0.4135035	0.5146545
0.7816907	0.5686904	0.732561	0.2670648	0.5364334
0.8859229	0.6438679	0.1988298	0.12548	0.32867	0.3016721
0.405697	1.074019	1.929281	0.14413913	0.4452474
0.5222724	1.193326	0.9634309	0.1184626	0.7240498
0.293709225	0.967397	0.6507804	0.7240311	0.8815454
0.4318161	0.3546149	0.6096762	0.90771	0.5320512	0.5235263
0.5804668	1.435742	0.4896276	0.398474	0.324907059
0.6620361	0.8481686	0.3429638	0.317701215	0.320178
0.5628391	0.5691515	0.3292751	0.4979773	0.5660633
0.3464564
ZNF32-AS2	0.08026861	0.5679873	0.5316059	0.2453678	0.3579716
0.7316161	0.1984314	0.2116416	0.04956835	0.502838938
0.4960865	0.3784776	0.8428963	0.3461925	0.3664114
0.3805882	0.3522559	0.314270787	0.09911017	0	0.393945924
0.7034783	0.2042344	0.6338204	0.3408527	0.6309284

0.2032925	0.8251123	0.186137823	0	0.1361007	0.4317082
0.8522953	0.2528083	0.4051562	0	0.3006851	0.7930179
0.6333253	0.539876859	2.126126	0.891576	0.250637825	1.0114
0.5124657	0.7205795	1.214193	0.5866883	1.749056005	
0.2436205	0.05559659	1.393466	0.4650126	0.6758814	
0.3443094	1.124894	0.2121401	1.184902	0.4185284	
0.195875628	0.78372758	0.437867375	0.1279106	0.1690717	
0.3092908	0.7941184	0.4600028	0.4380837	0.161294312	
0.4485799	1.549348	0.1512611	0.7146418	0.7484612	
0.6995707	0.174202	0.5982833	0.6403222	2.150626	
0.204439924	0.08374052	0.9644863	1.048952	0.9904466	
0.1860093	0.4196826	0.3799203	0.5356309	0.27498026	
0.9234936	0.232054502	0.3764349	0.09599741	0.8673208	
0.1663045	0.2952101	0.264541	0.270474	0.4715259	
0.2278114	1.708475	0.3085616	0.2959164	0.365878	
0.7951281	1.186769	0.09263588	0.3397361	0.454282342	
0.5350573	0.5638824	1.147226	0.1126929	0.9379787	
0.6254137	0.3823751	0.2694362	0.9920403	1.363849	
1.175653	0.2159722	1.260778	0.9320127	0.1788274	
1.203662	1.10557	0.4698747	0.06945161	0.7583787	0.2228787
0.5087155	0.3838175	0.7840668	0.2911758	0	1.493711
0.8245744	0.2938492	0.3710222	0.5476796	0.8858134	
1.774915	0.5245228	0.8789334	0.9394456	0.3894846	
0.3751161	0.4529294	1.42896	0.5018142	1.320451	0.5450539
0.3927745	0.8136935	0.7860272	0.6048993	1.0615	0.9124252
0.9019799	0.2685558	0.169381509	0.975497	0.870110429	
0.7886312	0.1977714	0.7264024	0.4353548	0.4051125	
1.355525	1.798532	1.312013	0.572926352	1.044815	
0.3430586	0.3834305	0.3688425	0.3416556	0.7166448	
0.8957493	1.07745	1.065648	2.140206	0.3775465	1.226434
0.3376405	1.150463	0.8254195	0.2823719	0.9400163	
0.6245798	0.2753487	0.5413521	0.1627134	0.09817101	
0.2760512	2.641264	0.3179377	1.881787	0.3108069	
0.1687318	0.6669421	0.4735671	0.4249393	1.651271	
0.2126226	1.033109	0.2901629	0.5732088	0.09543366	
0.8587115	1.232055	0.3924504	0.9685791	0.7259463	
0.6624685	1.053434	0.08590104	0.8520951	0.5550073	
0.233011704	0.4644077	0.676972452	0.320162833	1.104018	
1.231894	0.4163209	0.762483	0.4486232	0.4896659	
0.3529553	0.1086715	0.1680144	0.4187794	0.4053112	
0.7887606	0.7190926	0.1618736	0.4765033	1.685838	
1.344108	1.03403	0.7399881	0.5322331	0.557006	0.8373433
0.7400431	0.9622566	1.012231	0.8186274	1.270606	
1.269946	1.159427	0.7773306	1.513392	0.2637614	

0.3922972 0.7172992 0.183048931 0.9043328 0.6509995
0.6618826 0.2473371 0.5945797 0.5638489 0.170536
0.7570581 0.6812051 0.1921125 0.599857 0.8299349
0.2634417 0.4363136 1.299561 0.83632535 0.4581596
0.9454038 0.7009206 0.8352079 0.2581771 0.095709717
1.28878 0.621925 0.03683477 0.2514927 0.3982499 0.3425216
0.4682889 1.592163 0.08471659 0.5616512 0.9326759
0.6655002 0.4759412 0.7388526 0.4920017 0.4081727
0.7233665 0.2888156 1.002031 0.2192042 0.2788859
0.77916 0.1232082 0.3056796 0.1490273 0.5771673
0.903479307 0.845969063 0.8173861 0.4315971 0.2502794
0.1817541 0.9454085 0.1877249 1.487895 0.7623425
0.7909487 0.854641 0.2784925 1.408931 0.4277143
0.306655691 0.6464491 0.2982323 0.2311234 0.5186478
0.4596621 0.948395213 0.4372529 0.8208609 0.6186367
0.6660037 0.4281577 0.5942401 0.4453925 1.489552
0.8224441 0.7380536 0.7868983 0.5809546 1.774506
0.5204471 0.3317627 1.219308 1.057034 0.5939748
0.889993192 0.247454 0.7452401 0.4900604 0.8549586
0.6257168 0.383303 0.3060526 0.1075052 0.3870479
0.7466313 0.4627018 1.058708 1.10544 0.633379 0.3470651
1.109104 0.8429994 0.2599423 0.9244645 2.509894
1.131106 0.6302901 0.8876703 1.543003 0.4501254
0.6106637 0.604384485 0.1326517 0.8378444 0.47852 0.3189258
0.4290562 2.262178 0.452105311 1.123495 0.7851179
1.294256 0.2806344 0.04636042 0.2201037 0.7123126
0.6800346 0.3694943 0.8742449 0.6839333 1.1519 0.7001945
1.710085 0.524327741 2.443036 0.6823786 0.2167424
0.280450797 0.2624488 1.718431 0.8091584 1.94367 0.3347644
0.4972901 0.4842379
AC092119.2 0.03897684 0.204989 0.1971882 0.2047817 0.1789364
0.3848631 0.1505537 0.04671317 0.03008676 0.231960176
0.1589203 0.1148633 0.8125684 0.1461776 0.4448052
0.1443797 0.3498719 0.066030428 0.03609445 0.0416232
0.102478029 0.1067485 0.0619826 0.2215016 0.1001076
0.2374339 0.2930596 0.1849191 0 0 0.2202927 0.09607992
1.175732 0.4296554 0.7298263 0.3631314 0.03041806
0.5075972 0.3165752 0.275951037 0.4091847 1.073739
0.101420627 0.2818904 0.521768 0.5951153 0.997764
0.3770526 0.426637798 0.08560987 0.2024745 1.581277
0.491326 0.4248948 0.3989757 0.3713381 0.2718347
0.6293051 0.2540364 0.105681463 0.453577463 0.212619704
0.1261627 0.09121982 0.1206847 0.2575123 0.1063658
0.187119 0.078321316 1.23886 0.0915449 0.3460596 0.703671

0.5905866	0.2426412	0.1586044	0.1437441	0.7614557	
0.9956261	0.276816066	0.08471403	0.3618955	0.5323126	
0.5009804	0.08211127	0.1509552	0.220576	0.2408257	
0.43395611	0.9342295	0.27231256	0.4243323	0.06659205	
0.5017652	0.05607927	0.3285062	0.4344831	0.461731	
0.08177273	0.1481527	0.8418014	0.7321307	0.3367761	
0.3389625	0.3016394	0.3528192	0.2249107	0.09704064	
0.163719502	0.3637385	0.3835701	0.3677289	0.1539041	
1.160144	0.1725503	0.4022932	0.1526383	0.3628303	
0.3599229	0.3771844	0.6756166	1.14265	0.7812166	0
0.4121294	0.9262433	0.3297651	0	0.1083099	0.171357
0.2456186	0.7321841	0.1784659	0.1865552	0.04356731	
0.4595326	0.2409797	0.8288455	0.2895445	0.7543557	
0.670424	0.7900417	0.25773	0.5334908	0.2943072	0.535857
0.7463047	0.2999093	0.6011288	0.3997728	0.2881729	
0.1470374	0.1412767	0.2614721	0.5554287	0.4589486	
0.2176702	0.8191913	0.930715	0.5542228	0.073435981	
0.753585	0.817115206	0.203882	0.04801693	0.3248798	
0.2642495	0.464465	0.8625817	0.7197785	0.3941578	
0.291663033	1.050031	0.2578062	0.4553468	0.3396773	
0.2545075	0.4349856	0.2156042	0.3495439	1.176039	
0.8660344	0.04364978	0.517459	0.3385956	0.4936275	
0.2201404	0.8305955	0.3053737	0.1179436	0.08356491	
0.174562	0.09876298	0.1489684	0.3351125	0.6566223	
0.1182783	0.9776438	0.2382974	0.5348392	0.7946414	
0.5110106	0.1623989	0.7795517	0.4609166	0.2190973	
0.5166235	0.1571268	0.5709835	0.9692799	1.470726	
0.2589213	0.3979654	0.300919	0.5891261	0.2301869	
0.08342368	0.4322871	0.1122919	0.101023087	0.2273258	
0.462268664	0.071595593	0.5514461	1.486961	0.3158705	
0.5131135	0.1134597	0.0118886	0.467422	0.1209284	
0.2946105	0.4575397	0.2383259	1.085964	0.7501852	
0.2105427	0.2479077	0.3080788	0.4775651	1.167685	
0.4491544	0.3876627	0.1931936	0.4269272	0.2901005	
0.4867216	0.3608377	0.4259027	0.4724628	0.4817662	
1.176571	0.580702	0.2649782	0.2645075	0.2857376	
0.499884	0.141407838	0.1738207	0.1505297	0.2142647	
0.08188773	1.082685	0.09059354	0.1610173	0.5929232	
0.2447094	0.1802116	0.2427322	0.3526249	0.373106	
0.1853821	0.2448004	0.730272915	0.2713088	1.420003	
1.211583	0.5381474	0.2402842	0	0.3963438	0.2814039
0.0111789	0.2065262	0.1848507	0.5859059	0.7067583	
0.6551888	0.2228238	0.1506339	0.7705402	0.4039421	
0.2006142	0.5065995	0.05972656	0.1486504	0.3269637	

0.2191299 0.3159522 0.415786 0.2418241 0.806132
0.3240654 0.265057 0.4193864 0.3992089 0.516751662
0.878324863 0.3934848 0.1375336 0.03797835 0.07354681
0.3146862 0.01266048 0.4515578 0.1867128 0.3093889
0.5471703 0.6463218 0.88434 1.025467 0.837595776 0.6478808
0.2413595 0.01002044 0.1748924 0.1530019 0.127922747
0.5175333 0.3647845 0.4940752 0.1861667 0.2502559
0.5209952 0.1431223 0.4194375 0.7200046 0.7993373
1.257428 0.208963 1.131617 1.197782 0.5739094
0.130604 0.2624699 0.4554037 0.171493264 0.1727282
0.5040385 0.2809292 0.7694607 0.2998378 0.1163277
0.4179742 0.4567708 0.6218695 0.7794811 0.7863759
1.107947 0.5503501 0.832965 0.08102305 0.4097729
0.2274132 1.043766 0.6359439 0.1917621 0.9658292
0.5738556 2.011497 0.8429082 0.130668 0.5745194
0.424769497 0.1163014 0.07265011 0.2904497 0.7743202
0.5880598 0.9219291 0.265564553 0.2324774 0.9647173
0.2715589 0.8220666 0.09848869 0.01335975 1.181773
0.2975742 0.1525062 0.2412022 0.4388523 1.500667
0.7555568 0.5147004 1.040445532 1.282239 0.3365268
0.1315573 0.212783342 0.539169 1.177632 0.2889053
0.5268108 0.2586101 0.262472 0.2165729
AC104971.3 0.4667829 0.1115877 0.2146824 0.9475359 0.6122642
0.3545449 1.08181 0.2797162 0.5404742 4.093795566 0.1001692
0 3.243747 0.05470651 3.043968 0 1.047508
0.131795673 0.5043076 1.246189 0.477270497 0 0 0.7678815
0 0.137588 2.216623 0.09227391 0.676525018 3.574713
1.813765 0.104604 0.05632181 0.1837683 0 0 0
0.05240462 0 0.361458455 0.09423761 0 0 0.03750991
0.2403324 0.128802 2.308354 0.3135796 0.475290526
0.1398076 0 1.651502 0.9389478 0.2631982 0.5688207 0
0.8567015 1.435523 0.05850606 0.711917444 0 0.318289136 0
0 0.2408848 0.03953772 0.07961417 0.9435449 0.175869099
0.1630381 0.1494999 0.04228955 0.4617587 0 0 0.1055239
0.6342243 0 0 0 0 0.06373567 0 0 0.06145979 0
0.2401452 0.1442053 0.59965575 0.3290659 0.056227396
0.7166589 0.598125 3.103056 0 0.7153017 0.6786949
0.4300835 0.04896512 0.2365684 0.9496907 0.05097632
1.075519 0.06998935 0 0.7922491 0.3366886 0.217903
0.051597078 0.2333624 0 0.2810991 0.4095867 5.274486
1.033223 0 0.3917104 0.04622575 0 0 0 0.1883155 0
0 1.682598 6.847983 0 0.1893184 0.8647392 0.05400407
0 0.3321427 0.1187384 0.1175877 0.5217579 0 0.2774951
0.06282388 0.3852841 0.8421611 0.2384832 0.6143808

0.4992947 0.4300311 0.1101436 0.09437311 0 0.04988452 0
0.1709871 0.05392391 0.1650849 0.05287237 0.3479294
0.4263949 0 0.05213595 0.3454421 0 0.1952153
0.527677336 2.062825 0.298344297 0 0.07188077 0.3705456
0.5584636 0 0.6357043 0.2155 0.0963346 0 0.3735168 0
0 0.1386799 0.6208801 0 0.2806582 0.2700712 0
0.05892923 0.3920592 0.59796 0.5869061 0.2162794 0.1818193
0 0 0.05044576 0 0 0 0.2676047 0.9029868 0.1531879
0.1491041 0.753496 0.2378189 0 0 0.1434331 0.1716064
0 0 0.1809578 0 0.1344096 0.09910208 0.1095095
0.5970601 0.06201634 0.2707952 0 0.3919621 0.05104993 0
0.09244693 0.5501442 0.060492138 0.1633459 0.492096441
0.122488846 0 2.035166 0 0.1204902 0.5435127 0.07118838
0 0.06582847 0 0.07610349 0 0.2097644 0.08167405
3.782158 0.4453379 0.2635373 0 0.3933022 0.2338709
0.2321306 0 0 0.3362144 1.360084 0.4671738 0
0.2912293 1.206368 0.06584348 0 0 0 0.2138729 0
0.181444997 0.3286832 0 2.031427 0 1.043252 0
0.06886884 0.1775198 0 0.3173819 0.04844896 0.3619716
0.5851321 0.2378698 0 0.159981915 0.1624584 0 0.1107618
0.4203131 1.000912 0 1.249099 0.04109837 0 0.5376822
0.04257219 0 0.2760022 0 0.4105409 0 0.235406
0.06046965 0.2402537 0.09945881 1.072919 0 0.7831371
0.1312139 0.09459534 0.04979413 0.6516139 0.2574443
0.0497561 0 0 0 0 0.080913338 0.2561052 0.0392164
0.18193 0.07339913 0 0.9855345 0.2253255 0.04860987
1.341542 0.08510206 0.05954068 0.2909552 0.2331816
0.139318976 0 0.1204374 0.2400077 0.8377978 0.3233535 0
0.07946062 0.0532759 0 0.03185021 0.5763539 0.539947
0.09522329 0 0 0 0.4085732 0.1173057 0.2449181
0.4728962 0 0 0 0 0 0 0 0.3958096 0.107151
0.5984715 0.04975462 0.06952248 0.2930487 0 0.7598248
0.4204269 0.5307467 0.1805737 0.9975508 0.3881297 0
0.08510871 0.2180239 0.112 0 0.9058242 0.04090738
0.05377119 0.1246245 0.3556518 0.05548701 0.173420405
0.05356976 0 0.1630499 1.014254 0 0 0.053006258 0
0.7655842 0.05807434 0.0443468 0 0.1599951 0 0.05747935
0.2148706 0.2407179 0.1420447 1.429579 0.1413824
0.3595668 0.95284459 0 0 0.7877587 0 0.07337541
3.962328 0 0.04442982 0.1659155 0.2357504 0.1389459
AL035461.2 0.6931592 0.742356 1.122166 0.3708027 0.6546203
0.47735 0.6854215 0.465215 0.2996331 0.972667076 0.5949935
0.3268342 0.6850662 0.3119524 0.9040411 0.451903
0.4424589 0.417520643 0.6846924 0.3553064 0.453589137

1.24259 0.2469131 0.7961241 0.3987876 0.544838 0.8777659
0.7892589 0.535797485 0.6920511 0.3134126 1.068697
1.177599 0.785925 2.70869 11.85211 0.6635666 1.070794
2.209079 1.570393115 1.276254 1.344305 0.923469646
1.925032 1.598853 1.938175 3.484298 1.400695
0.621097686 0.3321764 2.688583 2.668247 1.398029
5.711502 1.67585 1.90872 2.035484 0.3694966 2.668948
0.52623923 0.472177432 0.604992327 0.4970573 0.9409002
0.9157301 1.127278 0.6431427 0.7846368 0.779999591
1.878749 0.8524921 5.988491 0.7954102 0.6980401
1.402408 2.106048 0.71039 2.234369 2.644109
0.950621637 0.4338846 0.4240125 1.692847 1.76762 0.4672821
2.416112 0.9985045 0.7195127 10.63816765 1.157132
0.988593512 2.693439 2.439585 1.427733 1.978656
2.351008 1.478169 1.284622 2.815401 1.742435
1.24971 0.7993748 4.216385 8.746932 2.317385 0.6274492
0.999946 0.5177276 0.44133226 2.47658 3.391792 10.79737
1.167791 2.047902 2.062108 0.9685863 1.861372
7.490423 0.839788 2.697623 1.26248 3.967204 4.956787
1.235411 2.31871 1.425349 0.7608002 1.64931 0.79615
0.3336091 1.01838 4.293007 1.072044 0.726396 0.6198364
0.2473768 1.977947 2.14944 2.013918 2.801311 1.756538
3.532572 0.6255044 11.76454 3.061844 2.73556 8.062293
1.019301 2.419085 1.814617 0.5637313 0.549128
0.8039829 1.15733 1.317023 0.870601 2.180158 1.83849
1.79148 2.597406 1.002989745 1.470354 1.928078488 3.682564
1.263812 1.188539 0.8845893 0.3368779 2.983049
4.198548 0.6408825 0.446871268 2.780704 1.269635
3.109567 1.998948 0.5364301 5.988951 1.573721
2.983798 9.704345 2.324218 0.4657573 3.048102
1.825478 1.061998 0.3887949 1.538081 1.966495
2.732735 1.379108 1.022622 0.5339421 1.398796
1.764041 1.528663 0.5845364 3.911056 2.486206
6.929238 9.897594 6.770373 0.84264 0.8148313 2.518082
1.698292 1.236145 3.097706 0.9653942 1.769288
1.702305 0.9430271 2.470644 2.110022 2.075433
1.018855 0.385735 3.18492 1.829964 0.776123363 0.2069879
1.344578396 0.960392027 1.787577 0.797851 4.60627 9.647606
0.8393849 1.014842 0.3602116 0.7820271 0.9672583
1.591202 1.028144 2.824214 1.901727 1.278042
4.491057 1.84715 5.97338 2.242723 2.083749 1.103063
1.099434 1.73541 0.958596 5.262724 0.9989852 0.9291019
8.856927 3.115516 2.28404 2.220333 2.111128 4.812902
0.3048913 3.257709 1.465757011 1.587904 1.044031

4.013681 1.203859 1.805924 2.549125 0.6872427
1.940183 2.377028 1.146209 1.8418 0.5446844 0.4549899
0.4521341 2.670146 1.900548517 2.45106 1.909553 1.710573
0.9098763 1.486325 0.958741038 0.6825943 3.652029
0.06361731 2.197314 2.40736 2.204938 1.661281 6.478398
1.194897 0.7669984 4.832472 0.9195079 0.4566653
1.394226 1.06217 0.6579577 4.146698 1.745846 1.460902
1.892937 2.305105 2.110281 3.475616 0.9804581
1.825093 3.801845 0.390099605 1.307275415 1.168309
1.677175 1.123869 1.325387 1.711822 0.7204877
1.284872 0.739167 1.153551 2.183745 1.018555
0.3318222 2.511597 0.893741867 1.038588 1.945846
0.1710742 2.886325 0.8707094 0.873585413 1.208287
0.810119 3.177224 0.8626908 1.040737 1.225872
0.5429908 1.485214 2.103348 6.898332 3.91074 0.3716172
1.008651 7.003647 2.320598 3.034924 0.6223643
1.079846 0.317180719 11.71015 3.530341 3.197446
2.087605 1.308186 1.60772 3.667046 1.856721 1.76948
1.573198 1.75809 1.639339 2.531305 2.734771 0.4149803
1.610157 1.536829 2.072059 7.131667 0.7881521
2.74819 0.5637256 3.653876 6.78074 1.757626 3.084929
2.307418066 1.501896 0.8268803 0.723145 1.445893
1.171294 5.74888 1.309780593 0.7055955 2.645808 2.814832
3.708882 1.161001 0.4561691 8.939704 3.05913 1.786829
1.738682 1.383716 15.62442 1.578815 2.025945
1.21903121 2.903916 0.7611375 0.1069531 0.92836885
1.534162 3.574246 1.397497 1.921251 2.260124
1.008238 1.012396
AC010226.1 0.322063 1.82498 0.4059666 1.025513 1.126506
2.53683 1.105791 1.10078 1.270647 1.868100325 0.4505146
0.2343475 1.436783 0.1342061 0.8945405 2.297629
0.9041709 1.706418523 0.4418458 0.4076203 0.599359044
1.865086 0.4704266 1.983665 1.985238 0.1500138
1.246168 1.990139 0.645420419 1.10972 0.6741715 1.988764
1.04778 0.4883891 0.5729465 0.3660777 0.4716548 0.578516
0.4705742 0.548928464 0.2889799 0.22082 0.347627941 0.3450727
0.589584 0.4388573 0.6384595 0.3461733 0.558700501
0.2572322 0.302936 1.012867 0.3071238 0.5560008
1.418689 0.3812514 0.3677908 1.186104 0.4545028
0.646843436 0.636496212 0.50428445 0.5346018 0.7677013
0.5581092 0.3637275 0.3906193 1.42258 0.191752187 0.4666262
0.407504 0.4149793 0.5309941 0.3559185 0.5662063
0.6471789 0.4630596 0.252451 1.245868 0.268751864
0.3318453 0.6123962 0.3449774 0.8217806 0.2764177

0.1732406	0.7364058	0.5159067	1.38935018	0.511268	
0.229895265	0.5993554	0.6215737	1.470124	0.2334058	
0.377765	1.179358	0.2763309	0.2502527	0.5561688	
1.627861	0.5210633	0.7145846	0.2432388	0.6031054	
1.250708	0.1376609	1.633378	0.875498206	0.5406803	
0.216712	0.6033938	0.5233328	2.261762	1.140617	
0.7102822	0.3336614	0.4914047	0.4934667	0.2994978	
0.37032	0.7442947	0.6045681	0.7750901	0.2293196	1.538796
0.9710093	0.4773373	1.359009	0.4526499	0.2319462	
0.4753076	0.485482	0.5649135	0.3466606	0.5017171	
0.3517216	0.166963	0.3938249	0.1878174	0.5200421	
0.47728	0.4454076	0.3516511	0.4728577	0.6752559	0.4025945
1.019807	0.1261482	0.4777252	0.4299303	0.5512319	
0.2486043	0.8179769	0.8281094	0.262181	0.3943584	
1.892614	0.4691332	0.6385367	0.719166156	2.029482	
0.451337604	0.1627694	0.7935212	0.5113255	0.9894618	
0.6020144	0.8284903	0.6755163	1.230876	0.370767928	
0.6490545	0.3277284	0.2601234	0.4725135	0.5192537	
0.5324823	0.367206	0.42789	0.515869	0.3734604	0.3873918
1.155752	0.4908407	0.3831945	1.180146	0.2905039	
0.2262592	0.3403225	0.4705569	0.2262579	0.4715081	
0.6747242	0.3897127	0.5741402	0.8230106	0.7228022	
0.2187816	0.3064632	0.4496678	0.7135151	0.4209853	
0.3213388	0.2031212	0.6843859	0.3018361	0.6182512	
0.2735071	0.8954961	0.274633	0.4817722	0.6643153	
0.3288985	0.7326188	0.3235258	1.008456	0.1889925	
0.6873038	0.234965767	0.4897695	0.829958221	0.287969466	
0.2734364	1.809844	0.4156693	0.3448511	0.0555561	
0.3201724	0.4649036	0.3902688	0.5270949	0.3267202	
0.2540994	0.2858858	1.035208	0.635742	0.7687989	
0.6061034	0.4384542	0.7861739	0.9084097	0.9174684	
0.1773717	0.3981849	0.3780341	1.032745	2.017564	
0.4518485	0.6208861	0.9328745	0.349976	0.5331445	
0.9082358	0.519774	0.2186139	0.5823176	0.8036911	
0.7167345	0.518257	0.6229377	0.2840186	0.4265514	
1.256855	0.4364521	0.6713833	0.1859326	0.3893009	
0.5447524	0.4686611	0.869968	0.8591043	0.6492837	
0.30525285	0.2324835	0.3095565	0.5547641	1.374817	
0.7929019	0.568914902	0.9831269	0.932609	0.615803	
0.5386092	0.4264559	0.948594	0.1974844	0.6015311	
0.5140609	0.5531901	0.5678735	0.8158934	0.6188603	
0.5184842	0.7494139	0.7683119	1.029211	0.3218943	
0.4737922	0.2443101	0.888078	0.6184052	0.3458417	
0.3677285	0.7348477	0.2692764	0.697123811	0.363910726	

0.5340362 0.5371488 0.7066593 0.1350472 0.3059094
1.317345 0.905927 0.7850615 0.6529909 0.3914485
0.2921306 0.4877442 0.1906805 0.327536854 0.1787257
0.443186 0.9567793 0.4281848 0.5067995 0.281871155
0.3736215 0.2940672 0.3507934 0.41672 0.8601301 1.226481
0.9538747 0.1597398 0.5523345 0.5591423 0.8949218
0.3277431 0.2837269 3.206415 0.2588321 0.3464009
1.285198 0.2903526 0.545821665 0.2482158 0.5220876
0.4855004 0.4600104 0.4404514 0.6713198 0.3979563
0.6190594 0.3552519 0.6102393 0.2922277 1.302029
0.4706702 0.5490498 0.7141204 0.3105258 0.5741693
0.8617134 0.3777933 0.4303627 0.5270531 0.5352217
0.7035285 0.6496744 0.3199114 0.1247774 0.10635882
0.361398 0.1482229 0.1999972 0.1777265 0.4113522
0.5882963 0.628502775 0.322529 0.6260442 0.1424681
0.4804965 0.2411275 0.7850003 0.7409688 0.2232634
0.1976703 0.7873728 0.3629836 0.7097591 0.332388
0.5460557 0.839109825 0.4805014 0.7710957 0.713196
0.32993647 0.4050106 1.455313 0.424392 0.3996496
0.4522492 0.9639057 1.268765
AL356417.2 0 0.05070315 0.01625788 0 0.02318336 0.1252984 0
0 0 0.044288977 0 0 0.0272943 0 0.05762987 0.06547146
0 0.013307836 0 0 0 0 0 0 0.1452657 0 0
0.08385476 0 0 0.04994774 0 0.1023659 0 1.107971 0
0 0.1428695 0.4785215 0.078209207 0.4710161 0.04674292
0.147170953 0 0.1274025 0.01300555 0.04113211 0.2849683
0.377934027 0 0.4692794 0.2501362 0.2085789 0.2391838
0.9649193 0.02172769 0.07785353 0.5254417 0.1417811 0
0.501664587 0.361560195 0.3520654 0.3929693 0 0.5150004
0.6029176 0.5895003 0.035516144 0.04938745 0.06038197
0.02562068 0.1223913 0.46146 0.9214996 0 0.02744558
0.4316189 0.06688648 0 0.1229281 0.559897 0.07572903
0.1272198 0.7819301 0.6503057 0.200048 0.04368261
0.54494263 0.6977639 0.119226705 0.03947093 0.3170717
0.07460133 0 0.216679 0.7024327 0.01861154 0 0.2149837
0.5753601 0 0.0407245 0.1060058 0.4559452 0.3733144
0.3314663 0.08800949 0.203187034 0.1413801 0.1391492
0.1277257 0.1240719 0.09742353 0.6259671 0.1683939
0.05932841 0.224043 0.6659099 0.01849089 0 0.2662076
0.6515054 2.953263 0.2888256 0.03428742 0.1939948
0.210277 0.3438048 0.5071258 0.2418536 0.08049005 0
0.1780982 0 0.04505577 0.1176822 0.07612237 0.2723237
0.04638312 0.06020104 0.186108 0.01374966 0 0.3002819
0.1000562 0.06883214 1.344879 0.04673009 0.8459912

0.09800769 0.3667215 0.04804825 0.158092 0.05166537
0.2219927 0.3316532 0.7952733 0.04965279 0.4730764
1.225469628 0.2147992 0.343422596 0 0.4572562 0.1964297
0.1127798 0.181711 0.07221276 0.2175973 0.1750899
0.12208579 0.0565728 0.01798564 0 0.9942097 0.5642305
0.7126512 0.01700338 0.2658822 0.3199772 0.08925413
0.01979374 0.01646671 0.7273041 0.1091921 0.5369974
0.5261112 0.2623773 0.04584304 0.4395697 0.09312722
0.2687146 0 0.2127475 0.2320182 0.7452484 0 0.01801
0.2270512 0 0.2172436 0.01732766 0.04328585 0.2675335 0
0.3833538 0.1017883 0.1651098 0.3151394 0.4747609
0.9392986 0.06562336 0.1364569 0.06784745 0.1855684
0.3215541 0.08401199 0.5554984 0.25653965 0.03298713
0.708062123 0.018552143 0.3418579 0.3236582 0.2005317
0.05474827 0 0 0.2649507 0.2392889 1.171536 0.02305325
0 0.02118059 0.07422204 0.02545978 0.05995627 0.8980948
0.2165601 0.039713 0.3719315 1.3829 0.7008556 1.014082
0.3904095 0.8239924 0.01768952 0.3347637 0.07561636
0.04766512 0.03989058 0.197497 1.185568 0.01262584
0.2591453 0.4533619 0.256495239 0.1991292 0.8532536
0.1295493 0.123778 0.2708757 0.2738746 0.08344692
0.02688712 0.01530589 0.1730542 1.056682 0 0.386723
0.1200924 0.07400595 0.032307783 0 0.2822683 0.1509838
0.07073396 0.05684922 0.273971975 0.4351331 0.07469703
0.6083115 0.358324 0.1160636 0 0.01393442 0.1485533
0.2798121 1.524337 0.15688 0.5678409 0.538554 0.06025607
0.2708403 1.168407 1.660594 0.1192419 0.04298219
0.07541811 0.08772742 0.4484134 0.3315863 1.121817 0
0.1182225 0.076515887 0.220592202 0.1551587 0.1187942
0.1377755 0.533617 0.3189761 0.7348624 0.06825554
0.3386724 0 0.5929191 0.05410813 0.6345797 0.1412706
0.189911086 0.0993102 0.4742773 1.254128 0.1268927
0.3428255 0.023203505 0.02407019 0.2743514 0.071695
0.9551575 0.4364724 0.0545202 0.5769002 0.0507203 0
0.3027249 0.05304208 0.04737893 0.2720319 0.358124
0.602682 0.1381908 0.1851445 0 0.015553312 0.01362201
0.1406553 0.4795941 0 0.1812888 0.09042994 0.6317919 0
0 0.08220211 0.3820667 0.4019341 0.8068151 0.2324445
1.175721 0.07078803 0.02578111 0 0.831212 0.05797188
0.08442831 0.07434991 0.3257671 0.7550245 0.02154678
0.1176568 0.420259669 0.1622734 0.2855185 0.01646368 0
0.09142827 0.1067399 0.883115213 0.2811223 0.2529933
0.1759186 0.1343352 0.2041663 0.9208474 0.2091299
0.05223488 0.03254427 0.8458511 0.0645422 0 0.8137229

0.01555998 0.266432682 0.4271903 0.1878201 0 0.05146147
0.1778149 0.08137405 0 0 0.1005182 0.1190224 0.154328
AL121821.2 0.05482497 0.2097006 0.1008602 0.1309303 0.1438243
0.7495614 0 0.2956811 0.08464029 0.103034545 0.1411819
0.0538557 0.1269958 0 0 0.4467872 0.1640437
0.495352793 0.06769411 0 0.096097321 0.5187096 0.03487399
0.951093 1.051395 0.0430936 0 0.390162 0.211892741
0.248806 0.07746612 0.2457207 0.02646063 0 0.178535
0.060092 0.9698197 0.07386085 0.1484319 0.121298053
0.0442739 0 0.028531727 0 0 0.161367 0.03189679
0.02946465 0.139560544 0.04378879 0.0316446 0.5172627
0.4705368 0.08243567 0.5344768 0 0.08049762 0.4777178 0
0 0.591320568 0.07476792 0.4095252 0.03207754 0.03772347
0.05572581 0 0.332466 0.055083529 0 0.02341226 0.1390768
0.1355872 0.1533639 0.02133125 0.09915268 0.02128327
0.06694165 0 0.107412558 0 0.2096062 0 0.08456171 0
0.02654177 0.2820573 0.03387463 0.46954177 0.1545989
0.052832534 0 0.02341716 0 0.09465757 0.05600947 0.292287
0.05773089 0 0.1389281 0.4118542 0.095797 0 0.03288179
0.2545722 0.4342422 0 0.1023733 0.048481783 0.07309085
0.1494086 0.02201059 0.2405356 0.09065903 0.6795876 0 0
0.02171738 0.162006 0.05735665 0 0.4128729 0.1768284
0.2035708 0.05270025 0.2924782 0.05014583 0 0.7109625
0.1776021 0.05922652 0.1248355 0.1673539 0.1657321 0
0.1746974 0.05214814 0.05903074 0.3620217 0.0719376 0
0.2597787 0 0 0.4398469 0.1773502 0 0.2577994
0.02415857 0 0 0.02585292 0.04968008 0.1362176 0.2403902
0 0.1959525 0.4219608 0.3080347 0.4585717 0.206590655
0.1211423 0.280331057 0 0.0675408 0.08704326 0.08745751
0.1281016 0.03733264 0 0.2941841 0.220906174 0.1754824 0
0 0.08687116 0.1325893 0.118423 0 0.253765 0.02757036
0 0.09209692 0 0.1253342 0.03387016 1.11047 0.07417897
0.1582515 0.02369999 0.1175427 0.02888701 0.1111363 0 0
0.119949 0.2451778 0 0.1955275 0.2561044 0.1265372
0.3818569 0 0 0 0 0 0.2525887 0.02327964 0.02572439
0.2103792 0.2913598 0.2035562 0.1511692 0.07892067 0
0.08800804 0.02171631 0.1435911 0.028419891 0.1534835
0.202293419 0 0.05891135 0.0478072 0 0.02830382 0 0
0.1095796 0.03092696 0.1275078 0 0 0.0328499 0.191857
0.07897336 0.06974159 0.1238128 0 0.06159261 0.05493759
0.1090576 0 0.2573646 0 0.365133 0.5487089 0.4792611
0.01954612 0.07392591 0 0.1531533 0.3478714 0.03916392
0.06698661 0.04536379 0.028414971 0.02573652 0.2911366
0.05023092 0 0.1050282 0.1132704 0.03235536 0 0

0.02982192 0.1593331 0.1700584 0.07497318 0 0 0 0
0.02736138 0.1040743 0.2852314 0 0.424915026 0.5281567
0.05792543 0.2515884 0.1768264 0.08000358 0.1063403
0.0216115 0 0 0.4237645 0.02211928 0.4545492 0.1580234
0.07009031 0.0420058 0.2323247 0.07884143 0.06164579 0
0.1871508 0.1360603 0.2419004 0.02337598 0.07456604
0.06940123 0.1375173 0.059335905 0.114042006 0.1443854
0.05527293 0.04273639 0.03448374 0.05207515 0.03561656
0.07057366 0.5024243 0.2701164 0.03998191 0.05594577
0.3007269 0.1095514 0.032726816 0 0.05658286 0.7329291
0.5904103 0.1012767 0 0.4106465 0.07508886 0.05559744
0.2094902 0.1353888 0.05637183 0.3131589 0.07866432 0
0.123555 0.08226527 0.03674102 0.1917755 0.07405733
0.02832502 0 1.066552 0.05338105 0.048244614 0.0633809
0.3272227 0.09297791 0.1006815 0.1968181 0.07012586 0
0.2294627 0 0.05099633 0.3950426 0.0623377 0 0.2163049
0.1367608 0 0.07997007 0.03414336 0.05261889 0 0
0.01921875 0 0.2342001 0.1336714 0.182479 0.13579145
0.02516768 0 0.02553423 0 0.07090004 0.1379563 0.02490294
0.08720096 0.4250765 0.02728398 0.02083463 0.03958131
0.1127512 0 0 0.05047431 0.113092 0.1334684 0.6236585
0.1328461 0.02413265 0.344351673 0.1963105 0.1456492
0.1586134 0 0.0344726 0 0.04063753 0 0 0.03691941
0.1305567
Z84723.1 0.03759021 0 0.1383077 0.05386267 0.1972235 0 0
0.04505131 0.0580328 1.200958924 0.01613333 0 1.219028 0
0.428981 0 0.4499 0.056605645 0 0 0 0 0.07173304 0
0.02574567 0.02954671 0.1487546 0 0.036320554 0 0.2124556
0.0168476 0.1088548 0.1183915 0.1071095 0 0 0.1350453
0.02907741 0.083166841 0.09106791 0.06627457 0.019562504
0.07249651 0.1741866 0.05531987 0.5686128 0.2424258
0.22965202 0.1050818 0 0.7979765 0.1209821 0.08478183
0.1832294 0.161735 0.08278862 0.8670245 0.09423033
0.178363103 0 0.179423662 0.0561574 0.02199365 0.05172947
0.06367977 0.05129089 0.3039362 0.018883745 0.2494609
0.1605239 0 0.2231137 0.1226779 0.07312783 0.3399154 0
0.2753874 0.01422528 0.036823193 0 0.2053064 0.08052944
0.03865262 0.03959505 0.07279245 0.03867798 0.1625807
0.12877471 0.02119985 0.108672354 0.2308513 0.01605575
0.5711779 0.02163368 0.1152071 0.1639667 0.1187479
0.04731816 0.09525474 0.9412797 0.1806263 0 0 0.07757556
0.6947126 0.08134101 0.2105738 0.049861601 0 0.06829373
0.07545675 0.06596837 0.5179957 0.199694 0.07461179 0
0.05956125 0.05553894 0.03932603 0 0.08088067 0.06928039

0.06978819 0.1084003 1.476666 0.154719 0 0.08704724
0.03479176 0.05414413 0.1497865 0.07649638 0 0.04201737
0.2156032 0.1430195 0.2226062 0 0.14797 0.05121372
0.1979053 0.1754549 0.3759892 0.05321944 0.04559943
0.1219923 0.06427552 0.08282046 0 0.1563305 0.08862904
0.01703133 0.2241512 0.2334967 0 0.1511471 0.08901949
0.211201 0 0.651575701 0.8306008 0.115323772 0.06839263
0.02315435 0.1790411 0.0899466 0.08783163 0.4863385
0.04627813 0.170673 0.064912367 0.2205827 0 0.01951767 0
0.127272 0.1623912 0.09040606 0.04349789 0.0756134
0.09491189 0.2104845 0.1751053 0.3265498 0.185782
0.1610618 0 0.04650155 0.01624967 0.01611841 0.1584488
0.01904988 0.1724025 0 0.131587 0 0.4480935 0.0383033
0 0.01445984 0.1540097 0.03685209 0.07671619 0 0.218589
0.0679424 0.04329625 0.01596146 0.1234638 0.02404075
0.3396056 0.1919038 0.04145909 0.2525189 0.04933286
0.02011396 0.02977915 0.09845185 0.077943298 0.1052345
0.099071809 0.019728152 0 0.7702958 0.1980116 0 0.0437693
0.06879395 0.0751322 0.04240954 0.02185612 0.04902916
0.04448657 0.06756965 0.1841628 0.8392835 0.1115745
0.0636683 0.1535251 0.07038397 0.03766743 0.02492476 0
0.07842646 0.451258 0.2503499 0.5267037 0.3286008
0.2278273 0.2703285 0.02120961 0.2450189 0 0.2148189
0.09185744 0.07775811 0.214306963 0.1764599 0.03629363
0.1549816 0 0.07201159 0.01941569 0.1774731 0.02859148
0.1464851 0.04089422 0.1248516 0.1165989 0.0856744
0.025541 0.05246476 0.017177877 0.07849703 0.1313205
0.1248757 0.1203484 0.3627171 0 1.388148 0.03971602 0
0.08659953 0.01371342 0.5103785 0.05927086 0 0.1322441
0.2293816 0.07582935 0 0.03095636 0.08009458 0.02880088
0.03185824 0.01801896 0 0.0304712 0.01603976 0.3731536
0.04146418 0.1282201 0.08520915 0.11103 0.06285825
0.081366183 0.026063934 0.06599763 0.1010596 0.07325449
0.09457378 0 0.8302851 0.3871052 0.06263316 0.2675151
0.06853303 0 0.01874459 0.05007522 0.067316478 0.1056054
0.07759099 0.05798374 0 0.1388788 0.049348723 0.2559598
0.01716132 0 0.06155784 0.2042217 0.0773016 0.06134696
0.1078709 0.01851706 0.03388573 0.1128088 0.01259556 0
0.1015534 0.05826233 0.1469507 0.1125033 0.0732004
0.033078455 0.07242751 0.09971425 0.3506217 0 0.0963903
0.04808112 0.04478939 0 0.1332769 0.6118899 0.08125714
0.2137062 0.1454167 0.2718969 0.2500499 0.05645643
0.02741535 0.04682013 0.1082329 0.1232934 0.1571153
0.09224006 0.06928345 0.1405049 0.02291262 0.03574713 0

0.1035359 0.02335518 0.1400584 0.09334665 0.1296318
0.1135061 0.034148922 0.07473562 0.0896768 0.18707 0.214276
0.08141562 0.05153786 0.05559663 0 0.01730361 0.1240647
0.06863349 0.2960345 0.136627 0.1654632 0.212491265
0.06729921 0.1997259 0 0.082085372 0.0236358 0.8004238 0
0.08587084 0.01781499 0.1265672 0.05967662
MIR31HG 1.696631 0 1.470935 1.434434 1.099918 0.03949963
0.9039284 0.5141897 0.4516042 1.197229139 1.138298
1.685792 1.144382 0.841085 1.144552 0.01444766
1.205923 0.528597947 0.6501361 3.040533 0.865950804 0
4.043978 0 0.09349651 1.011687 1.389109 0
1.884281089 1.77003 1.157313 0.4020584 0.01882434 2.395401
0.5556753 1.83825 0 0.0700604 0.04525538 0.051775545
1.196881 3.04287 0.385657038 0.1128318 0.7229328 0
0.4765252 0.8384571 4.030958089 2.585596 0.5853189
0.1655935 0.3765874 0.3225503 0.3041855 0.09589349
1.202602 0.03998269 0.7626206 1.374781069 1.06274781
0.079785901 0.09711335 2.555869 0.161021 0.1585754
0.7450604 0.9854978 0.764145333 0.5721652 0 0.3109559
0.3858319 0.5273387 0.2731545 0.458498 2.543708
0.8889612 0 0.019103576 0.5086253 0.2556271 1.002673
0.4612113 0.2670407 0.09441034 0.5417772 1.470023
0.20042185 1.297801 0.902053474 0.02177524 0.7330042
0.7243439 0.04489352 1.294984 8.014976 0.5955193
7.069905 1.265085 1.098739 3.475695 0.04493361
0.2573165 0.382333 1.147432 2.588209 0.6311875
0.448375147 0.7279653 0.141721 2.771562 0.3080147
0.2149856 1.795727 0.0154832 0.2836615 0.03089989
0.2160987 0.4284426 2.583193 0.2517614 0.5211606
0.724111 0.3749145 0.3972278 2.96096 0.06327555 0.09031882
0.07219873 0.5898803 0.02220229 0.3571714 1.866804
0.6975448 0 0.111296 0 0.8799476 0.05117708 0.1859848
0.3901524 0.1972199 0.08213059 0.1656589 1.119747
0.7341479 2.300848 0 0.5333881 0.5406867 1.379401
3.410587 0.05814391 0.4845449 1.653324 0.4182073
0.09236519 2.848804 0.1957393 0 0.5817271 0.498575369
0.1951485 0.3363441 0.5418297 0.5288538 0.3827573
0.05311758 1.608584 0 1.077632419 0 1.766165 2.085875
1.328721 0.2829759 1.03905 0.1876078 0.06769906 0
0.07878324 1.354051 0.9811067 0.01783278 0.02409555
1.762305 1.794235 0.1608308 1.180228 1.204142
1.541287 2.114946 1.69938 0.2012024 2.901317 0.2865501
0.348701 0.9935721 0.1138719 0.07501648 0.0958788
2.447177 0 0.1106942 0.2721653 0.2349865 0.2021557

0.2152976	0.1464046	0.17461	0.8912872	0.5973488	0.02150864
0.6363089	0.03412465	0.6052276	0.169941	0.04086082
0.040436355	1.346672	0.555094693	0	0.09779021	0.5101574	0
0.2416272	3.99646	1.80828	7.093992	0.9020709	0.4989062
0.8393857	1.784797	0.6543524	0	0.08427358	0.09922964
0.220204	1.214626	0.2482995	3.654272	0.8017073
1.546586	0.1830916	4.68218	1.396205	0.117107	0.8239646
0	0	0.1320405	1.271138	0.1767707	2.201065	0.0476549
0.6454445	0.424508229	0.4943484	0.1506308	0.1965411
0.4097134	1.145677	0.443199	0.06905373	0.4153248
0.01688784	1.633601	0.04857902	0	0.3555775	0.7420259
0.5988025	0.231705351	2.09048	0.1946515	0.09254933	0.1092626
0.0209083	1.767225323	0.4592324	0.7280203	0.223728
1.455639	0	0	1.706583	0.3278143	0.1715179	0.507739
0.1416228	0.02021066	0.01605991	0.1994516	24.56409
0.1983336	1.327429	0.5262647	4.758273	1.997112
0.7259591	0.5808046	4.041064	0.6896108	1.003911	0
0.147742421	0.027043518	0.7190198	0.7602192	0.06080615
4.906412	0.148187	0.1520277	0.1255169	0.08123395
0.747302	0.4693179	1.21391	3.734225	0.07793586
0.023282164	0.01826241	1.328368	1.844999	0.8400469
0.4322954	0.07680516	0.3718117	0.07122523	0.03955254
0.1490333	0.2311605	0.1002586	0.1273052	0.1865418	0
0.4043318	0.7022914	0.7449303	0.1364309	0.02634254
0.5239179	0.1306917	0	1.291176	0.755076785	0.3156284
0.05173095	0.06614536	0.1432516	0.5400703	0.2993292
0.185891	0.9141547	0	0.9432609	0.3091407	0.04434762
0.2112349	0.4616441	0	36.31853	0.312903	0.4129284
0.1497343	0.213212	3.842621	0.9160508	0.2695777
0.5206629	1.402652	1.094174	2.260517526	2.64987	0.1696307
1.344232	0.8716948	0.5716415	0.05888603	0.44290464
0.09305337	0	0.1746907	0.6818098	7.124104	0.05347486
4.557207	0.07684487	2.746954	0.2091822	0.7358677
0.3412896	0.7560639	0.6523914	0.171482511	0.03491429
1.070701	0.8274865	2.782235028	0.3433378	0.1346768	0
1.143427	0.05545364	1.891067	0.3250774
AL009178.2	0.1442709	0.07664213	0.2064315	0.1276077	0.2242792
0.1082281	0.09906975	0.1280789	0.04949543	0.167366399
0.050453	0.07348461	0.264049	0.03005941	0.08711246
0.0712553	0.06395231	0.148857856	0.07917149	0.05706159
0.143609948	0.1436816	0.1971361	0.2045698	0.2049427
0.05460002	0.1607031	0.1140782	0.07228044	0.121246
0.04530016	0.05268671	0.05157827	0.2019491	0.3784595	0
0.0444804	0.2879459	1.864114	0.165507825	0.3452029

0.2213885	0.094546106	0.1167925	0.3411412	0.2398398	
0.1430018	0.3848068	0.223071027	0.02987429	0.4009402	
0.236944	0.1490437	0.6829214	0.3125481	0.3547072	
0.5962567	0.3231775	0.5304277	0.137635627	0.242658796	
0.218611608	0.1436878	0.4877107	0.1102985	0.4888049	
0.794707	0.2160194	0.091265649	0.1642373	0.3879081	
0.6002815	0.1691474	0.4434337	0.9729686	0.2415912	
0.09956718	0.2392239	1.136416	0.303591581	0.1579438	
0.128409	0.04578835	0.7582253	0.208249	0.3621551	
0.1154578	0.06602999	0.6406767	0.1988919	0.077237707	
0.2028565	0.2099707	0.4014479	0.1414584	0.3602816	
0.1191273	0.06189244	0.02242058	0.1787314	0.1404909	
0.07002447	0	1.294713	0.6836863	0.2902097	0.1387494
0.3059778	0.0614269	0.2279553	0.2135718	0.1844875	
0.1500362	0.1119206	0.5109494	0.04666598	1.129967	
0.2074293	0.1736843	0.2795056	0.320717	0.1437127	
2.895329	0.3968096	5.737233	0.0725597	0.1954931	
0.3178516	0.1336346	0.3907003	0.1924118	0.03650027	
0.07611657	0.1023	0.1075082	2.887679	0.2693713	0.1150654
0.3410735	0.2383809	0.4258753	0.1631645	0.2078378	
0.2025324	0.4488583	0.0216062	0.7144467	0.3883067	
3.781407	0.4697587	1.071596	0.5845671	0.2711483	
0.1327609	0.1327642	0.2013365	0.2769207	0.06959661	
0.03002177	0.2145286	0.539612664	0.1121647	0.278681444	
0.4666495	0.1184883	0.2078442	0.179	0.154815	0.4075148
0.8551837	0.0485216	0.221451679	0.1767302	0.2229319	
0.2053052	0.1312333	0.2429423	0.2616137	0.07710617	
0.6986939	0.2794553	0.2158643	0.1914875	0.1543232	
0.2882824	0.7988575	0.3246869	0	0.7050768	0.09701393
0.6873591	0.3547397	0.433264	0.1470399	0.06431719	
1.851776	0.307229	0.5785678	0.04900258	0.5990527	
0.2096544	0.5385469	0.633852	0.793887	0.7076994	
0.004142928	0.3283675	0.2338698	0.5808351	0.91762	0.2597179
0.3975523	0.1785515	1.025438	0.09230148	0.3179027	
0.3373804	0.1269913	0.3246775	1.21874193	0.4088754	
0.197159824	0.319691833	0.08421067	0.2888828	0.7101685	
0.3586122	0	0.1108277	0.1121387	0.2170233	0.6710688
0.0627245	0.1475523	0.3137594	0.2617835	0.2770893	
0.0271887	0.09653666	0.4037304	1.212597	0.3319693	
0.3188701	0.2913358	0.3734631	1.016062	0.4270405	
0.1283482	0.2880443	0.3124215	0.3218228	0.1989834	
0.09951116	0.697461	0.06107209	0.1240447	0.4377046	
0.343404177	0.2608674	0.5365424	0.2447811	0.2993615	
0.2320226	0.2042325	0.6117651	0.3088805	0.1249352	

0.4941072	0.4880533	0.2265151	0.4920086	0.2323583
0.2311903	0.50789404	0.3645007	0.2826706	0.1065049
0.1411349	0.6416288	0.254850441	0.3717665	0.1881849
0.2206835	0.3643741	0.17544	0.2487401	0.1432288	0.8532937
0.305471	0.3217135	0.5777533	0.1162912	0.3080266
0.8880512	0.09006761	0.7064585	0.2817491	0.2403256
0.1472681	0.1140009	0.1193467	0.7721197	0.1230269
0.4844919	0.05862137	0.3931473	0.092528223	0.363083352
0.3283497	0.1580195	0.1416164	0.2083736	0.2588436
0.8122776	0.2269829	0.2715467	0.2749622	0.3857762
0.3816822	0.1865153	0.4128489	0.344480131	0.1701313
0.2095584	0.1428659	0.2877138	0.2566373	0.196414815
0.1091524	0.2097922	0.03793058	0.8837818	0.2111249
0.2362475	0.2180085	0.6389005	0.7054191	0.4190602
0.1443197	0.0501321	0.829875	0.9744028	0.2153285
0.3342195	0.1999012	0.06243168	0.164571092	0.197672
0.3685283	0.4168447	1.520961	0.564509	0.1777003
0.3820029	0.07156468	0.06062408	0.2236599	0.284913
0.08505806	0.2232433	0.4005499	0.4798448	0.1177023
0.1013229	1.197969	0.4359104	0.04089374	0.3126704
0.1236248	0.3250003	2.020072	0.156335	0.2998013
0.407624057	0.1275508	0.3120695	0.1642492	0.54403	0.1474151
0.2151286	0.461148597	0.4546861	0.3505525	2.254967
0.2193038	0.1543074	0.2637359	0.4267588	0.5105916
0.3640314	0.2865774	0.03902441	0.2057277	0.1747911
0.1128971	0.281914978	0.3922239	0.3454191	0	0.544518756
0.4905277	0.2214069	0.2376377	0.1993705	0.2127185
0.6908641	0.1569337
AP002478.1	0.02995591	0.009548222	0	0	0	0	0	0.01196724	0
0	0.03428471	0	0	0.009362145	0	0.0147952	0.01991825	0
0	0	0	0.03977806	0.01270324	0.02389284	0	0.007848662
0.01580582	0	0	0	0	0.02685195	0.03855431	0.188694
0.1544543	0	0.01039022	0.05380928	0.378476	0.123715678
0.05644542	0.9594667	0.031179001	0	0.1336694	0.3526784
0.1161876	0.3327176	0.071171081	0.2711594	0.04610759
0.07536749	11.62295	0.8257731	0.1557513	0.0491001
0.3372048	0.02047224	1.381708	0.473796146	0.102029268
0.040852575	0.09944941	0	0.08244718	0.04059749	0.2724939
0.07064427	0.120388744	0.1395068	0.144979	0.03618591
1.412529	0.2607011	0.01554029	0	0.09303202	0	0.4987939
0.498859355	0	0.06544405	0.03208725	0.05133758	0.02103572
0.05800883	0.04109701	0.01233921	0.58152183	0.1013659
0.057734522	0.02229904	0.2047191	0.3118797	0.02298672
0.03060311	0.07743185	0.09463105	0.1173144	0.01012123

0.03750563 0.2442659 0.02300724 0.5389901 0.04121368
0.09791944 0.1872614 0 0.035320062 0.2529295 0.4112018
0.08017604 0 0.06604713 0.3536391 0.3646799 0.17876
0.2768779 0.007376564 0.6163376 2.118332 0.3007871
0.1564285 0.03707647 0.998226 0 0.1735287 0 0.02774743
0.04620967 0.1797827 0.1591545 0 0.03018489 0 0
0.04748885 0.1612696 0.2087949 0.1572244 0.8842726
0.3259385 0.1553569 0.1261594 0.2356164 0.07267703
0.6481102 0.136591 0.01760006 0.009753906 0.5906058
0.01883443 0.2714478 0.02977128 0 0.4389504 0.02676671
0.3192312 4.600408 0.5011196 0.150505907 0 3.124679777
0.4632719 0.1230125 0.00792662 0.1433582 0.06532742
0.01359883 3.220799 0 0.068972178 0.01065357 0.5994965
1.099133 0.2135956 0.09659428 0.2013056 0.2401508
0.1155459 0.02008562 0.0100848 0 0.04651422 0.09130874
0.2344141 0.007778867 0.05404104 0.1152897 0.01726597
0.6936232 0.9996295 0.1518099 0 0 0.4980976 0
0.2380593 0.1729703 0.6063779 1.113905 0.1636419
0.0881031 0.4809341 0.4723207 0.06967807 0.3368947
0.06900618 1.255021 0.3935567 0.08940514 0.06367863
0.06487918 0.04405206 0.4407986 0.01747276 0.0748018
0.1186561 0.02092186 0.890294597 0.2236323 0.021053611
0.167696069 0.1072956 0.1044858 0.04046074 1.257818
0.1627737 0.02436551 0.07983119 0.01126549 0.08128072
0.2214062 0.0315126 0.3589783 0.08386325 0 0.02540415
0.0451002 0.07136808 0.09722179 0.06003489 0.03972544
0.07424012 0.1354137 0.2493301 0.2161311 0.06995567
0.2182203 0.06407902 0.03590445 1.318363 0.3905146
0.5068642 0 1.817846 0.1900291 0.010350471 0.3562429
0.2699448 0.009148588 0.02797127 0.1402782 0.03094501
0.1060722 0.1974679 0.1815878 0.04345187 0.2321554
0.07226993 0.2730982 0.05427681 0.1672382 0.054756697
0.2015656 0.07973352 0.2558941 0.007992209 0.03211688
0.845337163 0.1710106 0.2250665 0.2176543 0.03680629
0.06556995 0.01936782 0.6848839 0.05035498 0.02634657
0.04874556 0.2175443 0 0.06578492 1.540381 0.04590325
0.1184776 0.229751 0.04491033 0.1456963 0 0.0743422
0.05507184 0.2809937 0 0.2106681 0.3673428 0.108068831
0.969291737 0.03506266 0.09395765 0.04670163 0.5275657
0.04742239 0.05189492 0.2570724 0.05823162 0.5247623
0.1165109 0.02037887 0.09958468 0.09311238 0.154974408
0.3366309 0.02061094 0 0 0.009222796 0.026217569 0.2175747
0 0.3442838 0.05450654 0.009863376 0.0308011 0 0.2578892
0.2754525 0.01800252 0.05993209 0.01338333 1.42507 3.021336

0.2785781 0.02230591 0.04482734 0.009722321 0.184523216
0.03078294 0.6489479 0.2370777 0.1650347 0.08193507
0.1617795 0.3926228 0.2006024 0.07080621 0.01857598
0.1870683 0.1589504 0.08498133 0.5909361 0.01660555
2.299516 0.5243398 0.0497484 0.2970888 0 0.03577323
0.09100838 3.294345 0.1492925 0.2191108 1.414862
0.187961498 0 0.2357509 0.07440903 0.71909 0.01721742
0.05025211 0.018142348 1.405554 0.9647652 0.05963097
0.2883914 0 0.08214179 1.092865 0.01967334 0.1195079 0
0.04861735 2.74357 0.108879 0.03516235 0.050173577 0.8044684
0.07958152 0 0.625071177 0.5022812 0 0.5328963 0 0
0.6993119 0.05548285
AC024075.1 2.501264 6.386554 2.814758 1.957241 2.338463
7.087326 4.35795 3.12529 2.75236 2.233673189 4.522474
2.352488 2.465485 2.020834 3.643981 5.835162
3.821678 3.005231003 1.314208 2.04594 1.55468804 6.042112
1.963418 7.003754 4.720204 3.660193 2.358714
7.280006 1.388363154 0.724545 2.331078 8.133535
1.592484 2.933224 1.581392 2.974884 0.8860236
1.075446 2.737564 1.295178845 2.922402 0.7506262
0.830868981 1.351387 3.726473 1.762181 5.789909 2.3453
1.354708573 0.8076064 2.395948 7.832836 1.484432
1.32033 4.046753 3.401936 3.750658 6.382952 3.895475
1.984067847 3.051223251 1.778134499 1.192573 0.934126
0.6957424 8.672881 1.815378 4.813958 1.443672712
1.69152 1.159035 1.697159 4.264291 0.8684069 3.209455
2.743043 4.834342 7.126139 5.296674 0.234596109
1.526807 4.12741 1.083092 5.827944 3.671716 1.15938
6.926935 1.907154 1.64081628 6.332892 0.74362276
3.862512 1.341126 3.459206 2.358349 2.636856
2.347153 1.877311 2.121373 4.396338 10.69426
1.18562 1.778005 2.968394 6.013065 6.202324 1.42029
4.074307 1.388301604 3.54745 2.127112 3.268936 0.2334869
9.47492 0.753912 1.33096 1.45887 2.150259 2.319565
0.6124345 1.046391 1.631723 1.422215 2.173659
4.297097 4.568328 1.3386 1.352621 4.510482 0.8866163
2.031347 3.968557 2.165996 1.313817 1.07075 5.290835
3.847119 0.4870577 1.786349 0.9426988 1.993911
2.297514 1.697403 2.101212 2.134788 9.489943
1.278059 7.916837 1.219433 3.249069 3.885484
2.609914 1.253831 4.204784 7.583738 2.924326
1.450353 13.2961 0.822272 1.869562 2.767406687 5.321055
2.394622798 1.646058 1.344012 3.464194 1.443209
1.143999 3.950012 2.031067 5.293904 2.634459292

7.494964 1.218477 0.6908039 2.1503 3.217598 2.337373
4.070161 2.925153 2.836818 1.424339 1.698564
3.17319 1.435604 6.148117 6.757784 1.296094 4.388974
1.955466 1.711474 0.8412151 0.485458 2.196711
2.242031 4.936803 0.9859713 5.392305 2.304687
2.921045 2.27234 7.238908 1.278248 1.238166 2.064197
1.506076 1.699348 2.697055 1.852991 4.569613
3.437607 3.987787 3.902427 4.079354 2.528063 1.8392
0.8542905 0.7799575 3.846979 2.758707383 3.451505
2.384437081 0.97755521 1.696489 8.910001 2.027038
2.06058 1.487194 1.460926 1.595527 2.641819 4.765194
2.047686 1.490572 2.136447 6.406476 3.411331
2.00837 1.382123 7.607367 3.826413 3.626283 2.681831
2.637836 0.8605006 2.325483 5.139281 9.427542
3.876807 3.263414 4.496934 2.252064 2.923751
6.029954 0.9504064 1.755638 5.306151 2.592736024
1.324064 3.005891 4.875896 0.5963106 0.9515377
2.473901 1.978655 2.30727 1.221275 2.865854 3.954979
5.144836 4.342311 0.578555 2.525419 0.924144617
2.833872 2.310684 4.142006 7.028313 2.881402
0.761470714 8.886445 1.443185 2.22847 1.422199 2.465678
3.045111 1.531408 4.830758 3.253029 3.875317
2.383292 3.72287 2.739156 3.061637 1.59022 4.645643
1.938787 1.555822 1.876574 1.74854 3.235792 2.759039
3.539791 4.053334 2.290495 2.380526 1.180741043
1.033201082 3.106754 1.144602 0.912649 0.870304
0.8087862 2.66211 1.781145 9.06681 2.738543 1.765868
3.041154 2.016863 1.098858 2.477887505 1.121332
2.471614 0.3557257 1.719327 1.376325 0.978117001
2.681578 2.308134 1.673015 4.401104 4.783738
2.845435 4.299175 3.919769 2.516693 5.469008
2.79491 1.355246 2.531717 1.976897 1.897154 0.9213445
7.366544 4.844868 2.037142101 5.783217 1.517583
2.481965 0.8795803 3.193273 3.880041 1.07798 2.361028
2.264243 5.39571 0.8052791 4.901387 1.502879 3.079506
2.566561 2.610989 1.203213 2.58514 2.860309 2.792843
1.588829 3.637835 1.4468 3.779479 3.535804 2.935324
0.896323048 1.783402 1.851646 1.685446 1.784101
2.61525 5.383327 1.595430122 1.904526 3.90402 2.595476
2.083082 0.653164 2.59025 2.361329 2.175688 1.298373
4.193517 1.230794 5.727852 1.869821 2.693929
1.738156065 3.644418 8.24723 2.206837 0.968437007 1.171184
11.11762 1.183401 4.761559 1.58896 3.332889 3.252758
NFE2L1-DT 0.029721 0.03789339 0.05012063 0.04258695 0.04548144

0.1003315 0 0.04749358 0.0152947 0.055855754 0.1658273
0.1265141 0.1376906 0.04644364 0.08075649 0.09908447
0.07904823 0.033566781 0.0428137 0.03173893 0.034730024
0.07399899 0.03781087 0.2014966 0.08820941 0.202465
0.04704562 0.03525159 0.028717138 0.05619979 0.06999155
0.02664138 0.3825198 0.1248095 0.1371122 0.5212207
0.09793309 0.08008097 0.1456052 0.206037129 0.4600229
0.2620026 0.061869003 0.1687754 0.07651224 0.1312173
0.1210403 0.1490813 0.221926483 0.1305602 0.02287301
0.1308588 0.5792472 0.1564113 0.2317949 0.2983798
0.26183 0.137104 0.1589418 0.147739658 0.050614587
0.236437932 0.01973391 0.1623016 0.1090675 0.1107676
0.2230446 0.0300387 0.054745478 0.2491431 0.09307429
0.1220673 0.4410166 0.08775855 0.1541843 0.2060469
0.01922968 0.1814477 0.307427 0.135867913 0.1550327
0.05951995 0.1326484 0.1069635 0.0626123 0.06714625
0.07135579 0.08569715 0.42423651 0.2123165 0.085922669
0.08296566 0.07616764 0.2634371 0.1197339 0.08096834
0.115237 0.06259265 0.1288652 0.1004186 0.1240384
0.1774351 0.2739219 0.1960799 0.1380054 0.08594178
0.1429176 0.03083183 0.262823167 0.07264229 0.1139936
0.07556995 0.01738614 0.2293522 0.2631495 0.05899241
0.08313663 0.07848763 0.2451771 0.02072896 0.1332798
0.3517192 0.2373674 0.009196432 0.3190225 0.2546481
0.1223298 0.01607238 0.09176614 0.0458473 0.01783729
0.07331373 0.09072372 0.03993092 0.03322137 0.1830957
0.07067468 0.02133399 0.2180606 0.3509809 0.276699
0.2868716 0.06936239 0.1199546 0.3272762 0.1201785
0.1414661 0.09316992 0.3492409 0.1354839 0.2609417
0.07007527 0.1481256 0.2018418 0.09773792 0.1555387
0.1460624 0.1994213 0.8627701 0.04971899 0.037331417
0.1258716 0.111444259 0.04956891 0.02440956 0.03539007
0.1027246 0.1018524 0.06746095 0.2256394 0.1594793
0.091241749 0.2853908 0.1865036 0.2983481 0.0706402
0.06229393 0.3495223 0.06671493 0.0802479 0.2789936
0.1951114 0.05547377 0.1061438 0.09059271 0.14689 0.07331974
0.08042589 0.2206009 0.07280494 0.05522464 0.08351921
0.100413 0.04543714 0.08517776 0.08236527 0.03164586
0.7184189 0.1918038 0.09255735 0.1257607 0.07712038
0.08741221 0.3598939 0.1499574 0.04992843 0.1372824
0.1654572 0.2397811 0.274259 0.2977921 0.1000339
0.07816407 0.3824329 0.385051 0.09968052 0.121925
0.5611589 0.07784174 0.154066236 0.1941438 0.161885971
0.088389922 0.1490359 0.1598192 0.3050902 0.1585516

	0.1614972	0.04230527	0.1930626	0.1229486	0.1267252		
	0.06460883	0.05862278	0.2255701	0.1802788	0.05708269		
	0.07141399	0.212546	0.1921943	0.4377773	0.3276026		
	0.01970696	0.1104869	0.08784539	0.1569879	0.1896936		
	0.05453414	0.1154715	0.09536479	0.1959259	0.4751372		
	0.4797028	0.1526629	0.2972345	0.07262782	0.1557495		
	0.087289087	0.06975973	0.1434793	0.06807635	0.1179457		
	0.3163141	0.1381606	0.1169338	0.1959194	0.1630054		
	0.03772224	0.1521856	0.163893	0.1038667	0.134628		
	0.1382723	0.104127336	0.196537	0.1730493	0.1645567		
	0.1110135	0.2071227	0.059063958	0.1431587	0.09071627		
	0.04546262	0.0639059	0.1084263	0.07686378	0.1718309		
	0.158207	0.06534991	0.02821193	0.1478892	0.08727174		
	0.06526904	0.2153132	0.1214488	0.1511338	0.508137		
	0.1670931	0.1284923	0.06340984	0.09219903	0.1639199		
	0.3337035	0.0673713	0.1713932	0.2609218	0.166193134		
	0.212945808	0.04348463	0.05326906	0.06178054	0.1183946		
	0.1787919	0.04505197	0.2359273	0.05777498	0.1247384		
	0.3034427	0.2021906	0.09386357	0.05278983	0.183328193		
	0.157718	0.1840438	0.03056354	0.08890712	0.09150473		
	1.020970064	0.2293603	0.07688949	0.1858619	0.05137516		
	0.07828824	0.1986372	0.04042039	0.3079879	0.1708078		
	0.08484141	0.2923554	0.07635067	2.744619	0.1739703		
	0.1330784	0.2323754	0.04447581	0.2073907	0.296409121		
	0.2710562	0.3022195	0.1680133	0.07277355	0.3353318		
	0.07603144	0.08853272	0.09951467	0.3231544	0.1520501		
	0.1856013	0.3266725	0.2836047	0.3713247	0.03295066		
	0.1537522	0.2095363	0.3023195	0.3327925	0.06498859		
	0.3667579	0.2361554	0.2875925	0.5342963	0.1207737		
	0.8008064	0.07852107	0.08186146	0.0307766	0.2537753		
	0.3444246	0.1665534	0.1096877	0.072000318	0.2048464		
	0.3427011	0.4782371	0.08282707	0.04291461	0.03395735		
	0.4249266	0.04879767	0.4104372	0.04904637	0.2954461		
	1.404372	0.07801822	0.1482681	0.273790693	0.2305795		
	0.07018441	0.02866182	0.064901459	0.5045717	0.188148		
	0.1835824	0.1244732	0.07981821	0.06671424	0.1022316		
AC110995.1	0.02977176	0.2419829	0.09584823	0.2559581	0.29288		
	0.5125644	0.1839964	0.3924911	0.206831	0.223804544	0	0
	1.195356	0.01395689	0.3882931	0.1102819	0.08908112		
	0.280200841	0.1102804	0.1271725	0.260919981	0.2668513		
	0.1136263	0.3739989	0.4893787	0.03510186	0.5890744		
	0.2942649	0.258895588	0.5742168	0.08413329	0.2668688		
	0.1436899	0.375068	0.06059399	0.06526385	0.09293715		
	0.0668481	0.1151477	0.026347528	0.03606327	0.026245		

0.247898614 0.09569639 0.04598574 0.0219069 0.069284
0.176003 0.318300994 0.02377875 0.1890246 1.446587
0.01596981 0.2238264 0.3192623 0.03659869 0.02185642
0.4425339 0.02985248 0.141265039 0.963319368 0.081202864
3.572995 0 0.06145525 0.2219136 0.203114 0.4513499
0.074780406 0.1767777 0.05085449 0.07552325 0.1030796
0.152683 0.1158357 0.1615295 0.2080352 0.1332889
0.1239319 0.204149893 0.07764874 0.4065108 0.3029549
0.5816506 0.0156798 0.259435 0.2756995 0.01839503
0.61194378 0.4029709 1.018487844 0.1994576 0.1271628
0.2010567 0.1542065 0.1976974 0.3751597 0.01567488 0
0.09053104 2.217866 0.01300523 0.2057923 0.1071353
0.7065655 0.2021209 0.1288455 0.6671047 0.078981593
0.09922682 0.1081784 0.02390495 0 0.4102569 0 0.2009167
0.3664259 0 0.1319619 0.03114654 0 0.416378 0.05487064
0.9672743 0.1144719 0.2598958 0.1089233 0.08049912
0.2895569 0.2617759 0.2251336 0.05084233 0.1211715
0.3449923 0.1996686 0.2276794 0.1699089 0.4648066
0.3276494 0.05859671 0.06084253 0.1724169 0.1389617
0.03134595 0.05620031 0.1444605 0 0.1654469 0.03935669
0.2617365 0.08254336 0.1263509 0.1079116 0.05917652
0.3263494 0 0.1064086 0.7579198 0.08363646 0.09960779
0.650675895 0.3618134 0.822036356 0.1218769 0.03667687
0.2717872 0.02374615 0.9877999 0.1824556 0.09163164
0.2334831 0.445563097 0.1905854 0.01514774 0 0.02358694
0.1872009 0.1714869 0.08592281 0.01722534 0.05988643 0
0.183376 0.2496327 0.02722422 0.2391039 0.5334422
0.02014081 0.04910614 0.03860961 0.1276591 0.04705978
0.1961396 0.1137868 0.07679089 0.1954088 1.131686
0.04436172 0.1061778 0.121689 0.09161849 0.07318617
0.277278 0.07291182 0 0 0.4304875 0.08572746 0.02528321
0.1257227 0.1332833 0.1898615 0.1519894 0.1149258
0.0142855 0.1953601 0.2708171 0.09434134 0.3898734
0.123463462 0.583426 0.345249014 0.156248579 0.06398159
0.5192175 0.0120636 0.0153699 0.06933129 0.01816179
0.1487633 0.05038306 0.2423431 0.03883149 0.01174458
0.05351572 0.4167385 0.9220302 0.05049596 0.4202151
0.1519915 0.1226383 1.282815 0.3750716 0.3320267
0.4348008 0.185848 0.2974188 0.2234752 0.2819424
0.05307091 0.3211535 0.3863582 0.1663345 0.4047977
0.180772 0.2182555 0.07390207 0.44747757 0.1397577
0.2299588 0.01363852 0.1042474 0.1140675 0.03075477
0.07028009 0.09057874 0.02578164 0.3238856 0.1606857
0.292433 0.2035645 0.1213721 0.06232878 0.081630117

0.08289372 0.2080138 0.09890261 0.01191461 0.3830333
0.7809756 0.8763528 0.09436623 0.4610951 0.2469151
0.1086114 0.5485896 0.0234715 0 0.07855381 0.2180065
0.02402299 0.5090974 0.1838827 0.08880979 0.5702632
0.2018559 0.7706427 0.1004271 0.02413344 0.03811087
0.05541389 0.2791397 0.1269392 0.06748634 0.03768714
0.02489213 0.257770735 0.247714194 0.117609 0.02001001
0.1508472 0.187258 0.08483554 1.257162 0.03832381
0.1736209 0.1466821 0.1194132 0.4253254 0.1484587
0.2776199 0.21326081 0.01394006 0.2150844 0.6735461
0.1068707 0.1237423 0.097711483 0.1013611 0.04077571
0.01509562 0.06500575 0.529348 0.367341 0.2915238
0.05695641 0 0.06709444 0.1638 0.1496368 0.4373896
0.06032334 0.01538142 0.03325318 0.5791729 0 0.026198407
0.06883582 0.0789745 0.2524503 0 0.3053676 0.2665645
0.01773678 0.02492115 0.04222256 0.1384634 0.1716169
0.5077712 0.08061986 0.2740749 0.8664319 0.07452326
0.2605582 0 0.2428772 0 0.4977464 0.08349129 0.0411548
0.09538373 0.09073492 0.1698721 0.442435256 0.2460038
0.1294824 0.06932962 0.07393131 0.1796715 0.07491478
0.595016972 0.09470596 0.1598057 0.05926438 0.06788333
0.4943608 0.3061381 0.132099 0.1173144 0.0137046
0.2702157 0.01811943 0.1302565 0.3967689 0.0655241
0.635780446 0.1998807 0.2504589 0.1435538 0.216707625
0.09359876 0.4797401 0.1986076 0.0340052 0 0.2004845
0.1063448

AC084876.1 0 0.04334684 0.354427 0.04330301 0 0.2295415 0
0 0 0.113589826 0.1167338 0.08905935 0.2100086 0.1062552
0 0.2182927 0 0.102393518 0 0.04840888 0.052970962
0.1128648 0.02883496 0.2169366 0.1241897 0.1068936
0.03587748 0.07168862 0.131399945 0 0.06405155 0.121902
0.4156919 0.0713858 0.1845233 0 0.0943387 0.06107059
0.0525979 0.100293242 0.2196427 0.1198836 0.141545854
0.07285466 0.1867169 0.2668472 0.4747197 0.1461741
0.484651747 0.05430903 0 1.133378 0.2917916 0.2385619
0.3535385 0.02786297 0.2662324 0.185879 0.06818101
0.184365516 0.205862485 0.216372061 0.1128696 0.0795683 0
0.09215188 0 0.3436174 0.113862209 0.1424994 0.01935803
0.377835 0.1569511 0.2536128 0.1587364 0.04099136
0.2991611 0.3136476 0.3945572 0.111015319 0.1182296
0.2475848 0.7283455 0.2563675 0 0.04389123 0.2565358
0.1120347 0.31058613 0.2812205 0.087367374 0.3543147
0.01936208 0.9757994 0.1304433 0.2547076 0.2636428
0.07160071 0.1141246 0.4135334 1.106738 0.1386142

0.05222393	0.435004	0.1403258	0.3077535	0.1307886		
0.05643042	0.060129512	0.3323866	0.08235734	0.2729863		
0.07955312	0.4497593	0.1605444	0.1979482	0	0.1436532	
0.100464	0.1659853	0.7036657	0.5608336	0.1879812		
0.2103989	0.1307229	1.011292	0.08292446	0.04902786		
0.08397818	0.08391272	0.1305879	0.2580453	0.1844983		
0.3654208	0	0.1444456	0.1293534	0.3660643	0.09977717	
0.2974019	0.2779203	0.4773192	0.2468503	0.310232		
0.4492517	0.1283092	0.3236506	0.1744012	0.07990043		
0.1992627	0.08378817	0.1068803	0.08215426	0.3829395		
0.2815803	0.4270155	0.24303	0.2683778	0.5093865	0.07583245	
0.170815986	0.6761103	0.185429591	0.1649532	0.05584497		
0.2339033	0.03615638	0.1482861	0.6790929	0.1674243		
0.1871085	0.078279656	0.2901894	0.04612853	0.1882953		
0.3411827	0.1973326	0.2937483	0.1308279	0.1311383		
0.2279608	0.2289139	0	0.380096	0.2279869	0.2240398	
0.2472005	0.2760013	0.1682326	0.07838373	0.1360635		
0.1194237	0.04594558	0.1039525	0.2728213	0.3372046		
0.188241	0.1801229	0.2078596	0.02646945	0.08718764		
0.1114348	0	0.1665256	0	0.5096319	0.1092449	0.2088487
0.2117321	0.2765071	0.2029399	0.2649965	0.2524605		
0.09999337	0.2827683	0.1983063	0.09702395	0.2513807		
0.142471	0.070495518	0.04230174	0.334525779	0.023790735		
0.1948394	1.24515	0.3673658	0.1638177	0.1055653	0	
0.09060404	0.02557143	0.5271382	0.08868847	0.2145905		
0.353098	0.1586337	0.09794668	0.2498802	0.7933871	0	
0.1358048	0.2271211	0.1202299	0.1123446	0.543816		
0.1523717	0.150952	0.09073813	0.1981345	0.3070661		
0.3056219	0.3325045	0.3798966	0.3287218	0.1619101		
0.02769337	0.0937707	0.093977752	0.4043163	0.1313026		
0.1245978	0.0952376	0.4920979	0.1170697	0.2942773		
0.1379171	0.1962783	0.0493155	0.2446634	0.5155695		
0.1859709	0	0.1265375	0.290014018	0.09466179	0.3619728	
0.08605211	0.1451315	0.2916072	0.08107703	0.2668706		
0.3512275	0	0.1879791	0.2149862	0.4396285	0.1072146	
0.0381001	0.05980384	0.1106471	0.365779	0.1644284		
0.2239869	0.1545413	0.06946358	0.1152562	0.49978	0.1529123	
0.1837304	0.1353996	0.506246	0.2750155	0.3672326		
0.2055122	0.153022	0.1326545	0.049060889	0.37717481		
0.2387652	0.182806	0.08833966	0	0.1722298	0.0588979	
0.3209395	0.169945	0.1737098	0.1983502	0.1156445		
0.4068834	0.2717421	0.189417298	0.148578	0.1871383		
0.02330808	0.1627236	0.04186948	0.119022038	0.2160684		
0.2069532	0.09193961	0.08660673	0.02238879	0.06991514		

0.07398003 0.260169 0.8485495 0.02043188 0.317425
0.1063254 0.1902796 0.2449322 0.07026019 0.3544239
0.4748481 0.4855094 0.039890243 0.1397478 0.4809929
0.2690702 0.4994809 0.2092316 0 0.4591087 0.1517819
0.09643342 0.4216545 0.2939708 0.2577144 0.3156518
0.4173121 0 0.2723296 0.2975485 0.08469258 0.1522746
0.07434148 0.2977382 0.2701419 0.2088772 0.1694388 0
0.08621694 0.314375212 0.06242841 0.084494 0.06333764 0
0.1367858 0.7300276 0.041181149 0.09012579 0.2162876
0.1353558 0.4823493 0.06545426 0 0.06704554 0.04465634
0.1460684 0.3179279 0.1379451 0.5553277 0.3020643
0.3990735 0.313193488 0.1420265 0.1204276 0.0437156
0.032996356 0.05700617 0.4695829 0.2352032 0.2071081
0.1503852 0.06105237 0.2698715
AL357054.4 0.1755412 6.635302 0.1187276 0.7052743 0.602718
4.507155 0.733893 0.6497139 0.8050453 1.229042721
0.3057937 0.08114718 1.42716 0.06292998 0.5218637 4.008585
0.442852 1.496634942 0.3378692 1.069623 2.346882812
2.390977 0.09195628 6.522899 3.949869 0.1095717
0.7273541 4.707101 0.947832225 0.6912016 1.02132 1.841945
0.6080114 0.4553064 0.487577 0.2603132 0.06984048
0.1020158 0.2076751 0.114228799 0.2126374 0.1137843
0.198829841 0.1062114 0.3296244 0.07598127 0.3003784
0.6270923 0.50993657 0.06597884 0.0596007 1.037558
0.1107785 0.3027621 1.197919 0.08250964 0.3259667
1.937115 0.07765463 0.29397586 0.381008371 0.471749969
1.810022 0.03624967 0.04262994 1.252477 0.28179 0.2400356
0.197118386 0.674444 0.7452153 0.194586 0.9652997
0.2984802 0.04821135 0.4108459 0.5050805 0.1176752
0.2422745 0.080922041 0.07181726 0.6654874 0.3318191
1.613905 0.3208621 0.1999593 1.391844 0.3445245
0.42448978 1.257888 0.119408287 0.2594225 1.592184
0.6668309 0.5289028 0.342844 0.30528 0.1304792 0.04332734
0.3296951 0.5429919 0.06766046 0.1189607 0.3653922
0.8790312 0.4167238 0.2532344 1.722471 0.13696879
0.1170129 0.1532079 0.3648095 0.9694935 2.248219
0.9325441 0.08198282 0.1501974 0.2495106 0.5492316
0.1404361 0.4648345 0.4610184 0.5852088 0.115024
0.7990249 0.5959358 0.8594645 0.2680329 1.372531
0.4969756 0.1301412 0.1410721 0.3362144 0.4578149
0.4385992 1.447741 0.1178615 0.1167399 0.4943384
0.2235587 0.1969562 0.4240406 0.2168869 0.3098503
0.5604053 0.2379953 0.1541507 1.553759 0.2457066
0.1966899 0.7443569 0.3116316 0.09356944 1.883134

1.777085	0.2269626	0.3967446	2.634862	0.1015288	
0.6909538	3.112808733	1.18075	0.559665912	0.2254478	0.2289763
0.2500096	0.6506478	0.4149873	0.3375061	0.4703627	
3.550361	0.374457161	1.377128	0.078807	0.06433756	
0.2004303	0.644288	0.8996055	0.4321179	0.2389756	
0.2752137	0.1355749	0.3295721	0.4425298	0.1463568	
0.1913773	0.4102565	0.1536828	0.5663086	0.2008688	
0.7261417	0.1414581	0.3610745	0.3788689	0.2219495	
0.686787	0.6299966	0.2102794	0.05260918	0.1567661	
0.1191626	0.5034432	0.1872791	0.2528853	0.05861208	
0.2041562	0.3608303	0.7968571	0.1490757	0.3003921	
0.2773656	1.289583	0.6373791	0.2163858	0.1833255	
0.2936187	0.464122	0.2944898	0.5895705	0.23016678	
0.4817949	0.239490432	0.211352022	0.1035589	0.904922	
0.1673642	0.2025725	0.04809334	0.06929103	0.1496303	
0.4135684	0.3242068	0.2020231	0.08146913	0.7795723	
0.1662215	0.6619	0.3196283	0.349791	0.4111871	1.666619
1.008849	0.3834196	0.3198868	0.4470295	0.7140075	
0.4040273	2.025582	1.083191	0.06994648	1.995702	
0.0815673	0.1923034	0.3369574	0.306116	0.1577066	
0.1708799	0.288996616	0.1114883	0.3638972	0.2932815	
0.2964865	0.9561027	0.6186803	0.195006	0.6832998	
0.04918118	0.2752222	0.5401697	0.4003684	0.6401406	
0.2104818	0.2954452	0.231217644	0.1006272	0.1133738	
0.2989271	1.49594	0.1107085	0.289339929	1.591602	0.2545645
0.8173942	0.2331294	0.07534097	1.051499	0.2401533	
0.3558311	0.1135224	0.8023357	0.1333131	3.354905	
2.185437	0.4268352	0.6329233	0.3500557	1.088949	
0.7082475	0.09207414	0.2687715	0.7303448	0.728968	
0.1320818	0.1638474	0.2962829	0.1985709	0.32409128	
0.322186905	0.3943147	0.06246195	0.4266046	0.1558753	
0.06865629	0.5634859	0.431994	1.028011	0.5991922	
0.2146151	0.210741	0.6745323	0.4470557	0.215736474	
0.1208733	0.2344549	0.5787179	0.3892009	0.1192179	
0.094891948	0.2320284	0.04714179	0.07853583	0.7440317	
0.6782911	0.5839512	1.171206	0.1185276	0.2746764	
0.2140918	0.4493309	0.134939	0.07223952	0.5858267	
0.1867197	0.2018352	2.147857	0.226215	0.236251168	
0.115395	0.2670652	0.3239689	0.1896276	0.3389212	
0.2553505	0.5352045	0.2160896	0.07322178	0.3601819	
0.2976156	4.050621	0.6391308	0.4956667	0.4121292	
0.2843218	0.5422278	0.1800595	0.2675822	0.05080265	
0.3206115	0.2027052	0.06185407	0.1268168	0.7741682	
0.05891798	0.06649631	1.109202	0.2245467	0.3174085	

0.2564213 0.2804255 0.8210703 0.384606176 0.3038399
0.2832914 0.0205551 0.1412668 0.2609215 0.9839355
0.1221782 0.06611963 0.2281568 1.350433 0.05027594
0.2439602 0.2814837 0.3681644 0.175112738 0.3558739
2.770647 0.3784025 0.172873233 0.5129238 0.8022458
0.2143074 0.1336684 0.09787483 0.4728413 2.840095
LINC02195 0.1482512 0.09450791 0.04545572 0 0.3889127 0
0.7635223 0.1184512 0.2288743 0.495313458 0.04241858 0
0.5341885 0.09266613 0.3222568 0.1830529 0.2957251
0.037207622 0.1830504 0.1055445 0.230982223 0.0984304
0.1257361 0 0.03384592 0.1165286 0.8604494 0.1953759
0.095495878 1.12132 0.1396498 0.04429659 0.763218 0.9338428
0.5632356 2.058253 0 0.621369 0 0.481066428 0.319254
0.3049419 2.468867828 0.6671405 0.4579804 0.03636247
0.6325111 0.584282 1.056672687 0.4341643 0.2852319
1.864961 0 2.006215 0.6744589 0.3644931 4.063213
0.05065835 0 0.334972494 1.514822293 4.447928281 0.3445212
0.2313074 0.3400245 0.2344022 1.078853 0.7991247
0.099300239 1.139187 2.068083 2.542983 0.04888517
0.691181 0.1922715 2.949286 0.3069424 0.2413543
0.2992146 0.242043604 1.976257 0.4318417 6.140233
0.3556961 0.7287375 0.3349319 0.5084707 0.9159976
4.57084536 0.9475756 0.809559754 2.814116 18.15228
1.376623 2.559618 0.8077556 5.364921 0.156109
0.91235 2.003589 0.1856146 0.08634764 6.831743 1.956131
0.5099142 0.8200946 7.057578 0 0.524394795 0.5270497
0.9576609 0.6745423 7.371516 0.2723882 2.275199
0.431581 0.2764623 0.3523534 0.1825323 0.5169902
0.7670914 0.3721474 0.8652391 1.100944 0.2375096
0.04793242 1.807979 1.068941 0.3204168 0.7775484
0.3203067 0.3375654 0.3016924 1.742818 0.8837939
0.4409029 0.3290299 0.106416 0.9789362 0.1296834
0.841586 5.359532 0.4997579 0.4682701 0.8862062
0.1598565 1.347143 0.8449826 0 0.5309907 0.5937133
0.2796337 0.2238983 3.683436 0.3611304 0.3103367
0.2649358 2.282033 0.1388251 1.984025 0.744850661
0.5459646 0.909645673 0.4045982 0.06087861 0.2353721
1.497784 0.3694903 1.413307 0.6692227 0.2855633
0.341341902 0.2108972 0.1005727 0.2565843 0.35236 0.3824345
0.4269674 0.2377003 0.5146511 0.5467183 0.2495473
0.2213667 6.307424 1.897917 2.014929 0.2694822
2.607618 0.1222643 2.862539 0.847587 0.4166018
0.4507824 0 6.458096 0.8649385 0.6629807 0.3436273
0.1510635 0.1154212 0.3801856 0.04049303 0.2422336

0.9278484	0.9350023	0.957876	0.2381836	0.626102
0.2098335	0.09274781	0.4424642	1.208053	0.4128245
0.05450319	1.659839	1.297085	0.2644233	0.704672
0.7247931	0.153699411	0.2305733	0.885648059	0.207481106
0.7080049	0.3878237	0.9211011	0.4081911	0.690483
0.5426298	0.4444683	0.05575268	0.3447914	0	0.389888
0.4737537	0.2766917	0.4982843	3.352657	2.1204	0.2018281
0.2220686	2.42641	0.9174684	0.9797675	0.7217102	0.6644236
4.278515	0	0.2159935	2.889368	2.443243	0.3903578
0.9203089	0.806291	2.753463	0.1811372	0.2453348
0.358569876	3.247703	0.04771255	0.6338664	0.7613609
2.145815	1.786706	0.4666216	0.1503484	0.2139701
0.8601696	0.5744662	0.1532839	2.072388	4.029223
1.103544	0.767804363	0.06879615	0.6905492	0.8442765
0.8701721	0.1589458	1.767699873	0.5289551	0.6265404
0.9070876	2.231381	0.7211207	3.92989	0.311676	0.4153429
1.260423	0.2412408	0.8772476	0.3584986	0.4069603
0.5896486	0.4543485	0.3350533	8.48037	10.33511	2.643843
0.295208	0.1226393	1.199217	1.13779	0.2688436	0.5838516
0.5371281	0.748762612	2.604088441	0.4338109	0.03321389
0.5778135	0.5594811	3.379571	0.5778605	1.526695
0.1235088	0.1623149	0.3243431	0.3025638	0.7885481
0.5924716	0.471978572	0.2776632	1.63205	0.6606334	1.064345
0.6846514	0.129750214	0	2.030464	0.6013601	0.5934539
0.7322047	1.727586	0	0.1418098	0	0.9800352	6.525259
0	0	1.602057	3.165851	0.1655873	0.2218496	0.04811557
0.391372128	0	1.638587	1.759939	1.361255	1.013737
0.1264174	5.358197	2.8956	0.0700837	0.2757965	1.637949
1.011397	0.6117394	0.7798749	0.2465416	0.5442732
1.729965	0.5539586	0.6165717	0.1620846	1.593369	0
0.6375727	0.9499441	0.9638888	0.0469941	0.24479411
0.7712952	0.1228133	0.09206219	1.227159	0.08520866
0.447654	0.538716664	1.768489	0.5894572	0	1.201889
1.569789	0.406518	1.461775	0.5354964	0.2274778
0.366972	0.9022735	0.1729665	0.478969	3.393346
0.993232037	0.6193129	0.2188041	0	0	0.2485779	0
4.835037	0.3762934	1.264683	0.6655539	0.3530359
AC005391.1	0.04260592	0.08148189	0.8230018	0.0813995	0.05588482
0.3883358	0	0.1021251	0.1973285	0.106761112	0.4388644
0.1674107	0.06579448	0	0	0.09469363	0	0	0	0
0.049786491	0.2970231	0	0.7136323	0.08754281	0.03348916
0.06744125	0.03368945	0	0	0	0.03819119	0.08225297
0.1341886	0.1734303	0.09339812	0	0.03826613	1.351247
0.565583297	0.8257536	0.03755883	0.399109499	0.2465094

0.87746 0.6583632 1.14024 0.183182 0.260294738 0.3062647
0.2459184 1.366726 2.14829 0.1921887 0.5814983 0.1571275
0.8757952 0.4367611 0.1708857 0.173281959 0.145114941 0
0.551638 0.1994263 0.9381086 0.6062837 0.05813469
0.3014294 0.085613696 0.4464422 0.3638855 0.09264016
1.475157 0.1191831 1.060933 0 0.4961935 0.3814954
0.4192076 0.041736551 0.0740813 0.4654013 0.1825492
0.4819108 0 0.2475157 0.482227 0.05264972 1.02170085
0.2883428 0.28740279 1.09419 0.03639617 0.6833569 0.09808116
1.262266 0.3716899 0.5383702 0.1430183 0.6477881
1.226907 0.1488927 0 1.482091 1.099082 0.8998958
0.2458518 0.1060759 0.075352918 0.4544065 1.36751 10.22879
0 0.516659 0.6035718 0.1014808 0.190686 1.383926
0.4406469 0.5348802 0.3086362 2.520998 10.48303
0.0791001 1.474371 0.5372366 0.3507267 0.04608043
0.2762537 0.1183022 0.3068432 1.891752 0.693627
0.08586318 0 2.063581 0.9320906 0.1834973 0.3282259
0.1118092 0.4934014 1.659909 0.397732 4.261578
1.166198 0.03445587 2.267625 0.7285187 0.6383247
0.87399 0.3937552 0.04018197 0.1544307 0.5504631 1.556779
0.1337815 0.3806995 0.6053848 0.5984544 1.425472 0
1.318 0.087141032 0.93022 0.2624386 0.03382179 0.2038965
0.1592818 0.7543163 1.993217 0.2110321 0.147147389
0.6364026 0.3034878 0.04424386 0.2362844 0.7006626
0.1227062 0.3279009 3.303225 0.4285129 0.6454565 0
0.6747973 1.168805 2.158354 0.3651049 0.1729392
2.635315 0.7735509 0.7307641 0.3142839 0.1295504 0
0.51284 1.938883 0.2994107 2.835681 0.2170707 0.4975636
3.671188 1.361563 0.08353862 0.3478101 0.0806131
0.5946134 0.05133869 0.4416598 0.3980067 2.358949
0.8719529 0.2264233 1.344605 0.5638922 2.003489
0.4100462 0.1367867 7.72935 0.3570828 0.088343357 0.4771041
0 0 0.915631 1.077414 0.448865 2.375529 0.3968759
0.1039643 0.1277358 0.09613659 1.436799 0.333427
0.2016899 0.05105703 0.2981941 0.1841168 0.108396
1.010291 3.175685 5.520456 0.4696278 0.3390059
0.1055908 0.08889099 0.8183517 0.7803246 0.3837744
0.3103719 0.5164531 1.723493 0.5288719 2.935813
1.23584 0.1521765 0.4164563 0.5993075 0.132492095 0.2800073
2.344771 0.2732503 0 0.2176538 0.440127 0.1508652
4.148028 0.3689572 0.04635078 1.132086 0.7929407
0.03884241 0.4631834 0.5946519 0.311519038 0.7414248
0.3402119 0.2021972 0.2728132 0.4111149 0.203207727
0.3192345 0.9303176 0.09775797 0.1570474 0.1865186

1.652797 0.4030766 1.002669 0.3372515 0.4853114
3.23162 0.1324658 0.350869 0.8351915 0.3917257 0.3610912
5.024118 0.1916262 1.416017 0.1090798 0.2643399
0.5639615 0.3269895 1.04305 0.3595567 2.20861 0.092222961
0.236333385 0.4488226 0.2290882 0 0 0.4451586 0
0.4936017 0.7099037 0.139943 0.3107099 0.1739077
0.1699656 0.4540545 0.203462904 0.1196965 0.3517761 0
1.835293 0.196762 0.279666884 0.2901128 0.116707
0.3024436 0.3488578 0.08417133 0.08761604 0.06953255
0.1630189 1.259269 0.3456642 1.022888 0.142762
0.1788405 0.2877594 0.08804846 2.855288 0.3187868
0.5807733 1.424701665 1.444812 0.2260385 0.2890222
0.8606646 1.048817 0.2543176 1.726033 0.2139857 0
0.5944586 0.2455983 1.550216 1.845982 0.7844489 0
0.6825543 1.305083 1.963494 0.2862406 0.186326
0.559678 0.1792247 1.099392 5.369088 1.038795
0.2431014 0.253264991 0.2738185 0.05294297 0.1984332
0.846416 0.404054 1.329397 0.270938146 0.8470767
1.016425 0.2120309 1.619114 0.1230387 0.1752438 0
0.08394344 0.3137993 0.7382486 0.3111653 3.504474
0.2580955 0.675148 0.374646542 1.945115 0.6036682
0.2465252 0 0.3214747 2.844274 0.3158049 0.1297716
0.4442254 0.1147641 0.202918
MRPL20-AS1 2.514458 3.218696 3.392599 5.80978 3.28304
2.354178 3.182663 2.572198 4.485935 6.329517498
2.553481 3.063483 6.459767 3.317814 5.684611
1.696585 5.46336 3.240555467 2.671304 2.961684
5.076348917 2.051484 2.408308 2.788011 3.361563
2.283961 6.903794 2.456861 4.781223342 3.825454
6.406525 1.402895 5.770722 4.802356 4.01916 5.486816
1.508784 4.893927 2.910862 4.333390961 5.036879
6.411389 3.461548249 6.188422 3.792017 4.974817
3.923368 5.461054 3.292633941 3.589207 8.621618
3.903234 10.23464 3.071341 2.89443 3.953993 4.460684
3.20875 17.17583 8.916025878 4.167777735 6.269697215 2.865325
3.595479 4.362925 3.563656 3.320999 4.22199
4.509214041 9.011615 3.322037 4.207869 3.739628
5.933919 4.020751 6.295705 2.635754 6.725802
5.513311 4.813571487 5.062349 4.242524 2.027723
4.011407 5.206216 6.345534 3.570008 4.536383
5.44036194 3.050344 4.424750914 5.615252 2.565779
5.648937 6.185794 9.669674 3.998991 5.289407
3.29796 3.592654 4.17835 5.313509 3.977292 2.753866
5.27225 3.463619 4.656604 4.462395 5.632623139 5.216226

5.432426 6.948548 4.129973 4.091325 2.954812
3.554145 13.35244 3.291237 11.21322 3.449236
7.294204 3.21889 11.64926 1.84806 6.543075 5.179207
4.652341 3.827231 3.864036 4.090022 4.135614
6.740747 6.123937 3.774426 3.138606 4.722987
5.72473 5.619142 8.149789 4.00144 5.957829 3.635188
3.630138 5.924868 10.46523 4.327521 4.511463
3.379562 6.074948 3.310514 6.779997 4.406362
3.832252 5.015075 3.044308 7.010045 5.506362
3.529173 7.32238 4.389212 4.639903386 3.426409
6.178385464 4.412604 3.792316 4.257019 5.149256
2.791042 6.692078 5.504082 2.417731 2.470774628
2.664321 3.261305 8.765489 4.444621 5.794337
4.076567 5.56857 11.51201 5.237276 3.841229 3.537616
4.894273 5.845585 4.25141 3.361857 3.970411 3.682358
5.521862 8.497152 6.117982 9.272729 5.06679 5.323033
4.733355 3.275256 10.48846 3.365396 4.152695
4.023884 4.276353 4.20813 5.951299 7.468317 3.430691
7.945521 7.747345 4.993921 3.882295 5.90992 4.641794
3.957557 4.35978 4.025423 7.1033 7.112096 17.43084
4.412483 5.738679487 7.839309 3.239212642 8.19566802
4.435111 5.087633 4.565077 10.52151 3.028281
5.65123 3.829644 5.569792 3.111316 4.690537 3.704384
3.94407 5.702369 5.528292 4.49412 4.060693 7.34962
4.559398 4.60105 4.585889 6.759232 3.043188 6.150326
7.070205 2.591427 4.635863 4.849449 3.858591
4.791893 8.615404 4.454132 3.670473 7.184943
5.137067 4.115388117 4.494564 4.283245 4.639149
4.924079 4.019635 3.209702 5.05961 5.321797 5.421231
6.128198 5.981729 2.522476 5.145981 3.534227
6.561118 5.463905123 4.690045 4.462452 3.391484
2.597452 5.626114 2.270916771 4.884026 2.849167
3.452366 3.573745 3.455125 5.814604 4.46823 7.089681
2.894679 3.937145 5.808216 2.492595 2.738835
4.854968 6.480539 5.052073 2.912605 4.51589 3.507483
3.461826 3.305725 4.182602 2.840945 2.582495
4.60815 7.557821 5.000548092 8.880823636 10.55681 3.673917
3.789999 4.458714 7.553062 5.741531 3.792251
3.715046 6.255647 11.5729 4.010259 2.272424 5.005232
5.268822977 4.526185 5.166407 2.136045 7.311735
5.931007 12.584588911 3.204891 3.667112 2.643246
2.936715 4.319573 5.454784 3.981503 8.987879
5.464712 3.40777 5.324165 2.965255 9.507962 7.98998
2.336562 14.00389 3.711082 3.602322 4.040510347

4.567609 8.837388 4.420409 7.121872 4.73321 6.638489
3.270255 6.559997 3.264096 4.056884 4.502547
2.81322 6.642037 5.016989 6.650275 5.749642 6.898963
3.834198 4.622231 2.994426 2.879072 5.80901 5.907079
6.679455 2.693544 5.055932 7.861061126 8.1935 5.955885
3.274753 2.600508 4.062783 5.345492 5.389193499
8.360154 6.231952 6.374078 6.970896 4.735648
4.946318 3.676379 7.5898 5.043034 4.287105 2.213197
4.994588 5.813925 4.898307 3.288728951 4.2854 5.36537
5.659829 6.835224562 6.38924 6.563115 4.375817 4.420829
4.014039 7.981903 3.402661
HLA-DQB1-AS1 0.02631996 0.2768465 2.1547 1.860539 2.968982
0.5864121 2.765278 1.040955 1.300271 1.384993337
0.7907377 0.1034186 3.739319 0.4935479 3.003644
0.5654742 0.7350268 0.515244678 0.9424443 0.7307812
0.707383058 0.4980364 0.5022612 0.4408489 0.2703995
0.5172012 2.333076 0.5202945 0.915514834 0 0.8925445
0.7785607 1.930859 1.8237 3.149834 3.461822 0.08216183
4.018637 0.3053918 1.048173393 1.870412 0.904882
1.780648558 7.647945 0.6504649 0.1355688 7.96264 4.300131
2.331571255 5.970189 0.1215336 12.01886 0.3106012
1.503854 4.875165 0.2911983 0.6956044 1.861694
0.7653483 3.782276374 2.480186097 5.707149084 0.5766985
1.047169 0.86928 1.052262 0.86191 4.788234 1.110651591
2.518894 1.86577 1.43072 0.7811 4.343923 1.67945 2.713227
2.717858 4.734828 1.135471 0.928184605 5.079803
2.731281 9.275365 2.165107 0.1663423 0.5861308
1.110345 2.797112 2.1639751 1.306249 0.913084019
3.644196 10.47748 7.37645 7.210209 6.910371 4.056493
0.8591653 3.467741 5.735819 6.689523 0.09197901
4.669595 2.651988 1.412241 4.804684 2.430014
2.326272 0.325846766 1.438645 3.79356 0.824201 6.374194
7.631016 1.025361 3.761407 3.475011 0.6255549
0.3110986 0.7434544 1.552526 1.330831 0.412325
4.495522 0.1517996 19.88726 3.274014 2.988967
1.121459 1.242387 0.7961246 0.02996504 0.6427359
1.723874 2.58894 1.576707 1.12657 0.1133561 2.056594
0.138141 2.294968 4.351085 4.954957 1.607275
0.7204228 0.2341376 0.5125002 1.080108 1.322157
1.182661 2.481086 0.7943218 0.3100505 5.257705
0.5770236 0.578508 1.293482 2.960664 0.5915158
7.044725 2.181926665 4.361783 1.66878234 0.8380265
0.03242448 0.8566349 1.259578 3.591486 12.54566
0.42124 1.759939 1.242311377 1.067094 0.05356591 0.3279817

0.3753401 0.5346809 0.3790109 5.11469 0.8527793 0.2911869
0.5050619 0.7958375 10.17625 4.308135 1.918696
1.865871 0.9971159 0.195357 4.02771 3.002024 2.357536
0.4801813 0.8047533 5.793075 2.280335 2.471765
1.202696 3.352406 0.6762178 0.4657273 1.509686
0.2064251 0.5586384 1.493971 7.468934 1.42716 1.182293
0.2011664 6.100689 4.982532 3.664694 1.392535
0.08708662 1.136629 3.569334 0.1971678 0.4587177
7.610328 0.272872143 2.701717 3.995609145 0.165759362
1.09356 8.468889 0.3199477 1.739249 0.5516359 1.34871
0.6575769 1.128386 2.203667 0.5492687 0.1453604
0.283866 6.557893 6.748502 1.473165 3.714945
0.1074954 0.3745396 3.639615 2.26874 2.217789 3.486959
4.70152 2.410242 1.475152 1.763947 1.764108 6.74306
1.752367 1.764592 2.958348 15.56766 2.25109 0.2831128
1.418689508 3.014716 0.8385999 0.9886938 0.2580502
6.084153 2.202309 4.411349 0.4404227 0.7977363
0.4008671 1.180153 1.061324 0.6958562 2.360599
1.028575 3.247458988 1.923675 0.998294 0.7494484
1.369317 6.998221 1.192556943 3.070814 0.6674017
0.7246841 3.68663 0.6913353 1.633634 0.5395044 0.2212152
1.550963 1.499014 1.274263 1.773009 1.062078
1.615125 2.90388 1.15994 6.232567 3.965656 1.493475
0.9209207 4.376358 1.712914 1.907768 3.603575
0.08884699 0.8142251 1.224877969 3.029412955 5.244868
0.07076003 1.107895 2.748079 6.349961 3.077735
7.284301 0.6797457 6.397323 0.4222727 0.3222963
2.966162 1.612839 2.073884108 1.626744 2.227438
0.2165287 1.322719 0.5105118 0.172765231 0.2867492
4.590124 1.227777 0.3017115 3.223823 1.623754
1.159758 1.711994 0.648265 0.2609876 3.212121
4.039182 0.4787439 2.488702 2.937182 1.087716
1.851158 1.486353 0.856953705 0.3042743 1.117089
1.205176 3.673416 0.3239552 0.2917676 2.602937
5.463872 2.799539 6.414266 3.261964 2.154716
6.047992 4.084453 3.063904 0.2108252 0.7678286
0.4917386 0.4799567 0.3165349 2.954532 1.383959
0.6791535 0.3654073 0.3850317 0.4004715 1.147339349
0.5799509 0.3270571 0.7354969 0.7189552 0.363063
3.523383 3.73002734 5.065396 0.9104551 0.4191452
0.3200682 4.978496 1.118661 5.917012 1.42605 0.2665446
2.91007 0.4485215 1.013358 2.200264 2.224401
4.099781154 1.272284 0.116537 0.2538198 0 0.3640856
11.48148 0.6633047 8.517729 0.6486335 2.126879

0.4178445
ZNF582-AS1 0.1069854 2.018767 0.1443336 0.1771448 0.1964611
1.076253 0.1763185 0.615458 0.6056123 0.839990348
0.3550917 0.1681503 0.958235 0.1872429 0.09302259
1.00396 0.2845465 1.047183586 0.1232917 0.2894314
0.566739888 0.9944515 0.4083184 1.126377 1.485033
0.1906104 0.4854644 0.953112 0.275658206 0.7282798
0.4635798 0.9270316 0.1101552 0.4941986 0.1916162
0.04690539 0.4898249 0.8968213 0.2758571 0.10099239
0.09215578 0.01886239 0.111353575 0.04126643 0.2276787
0.07872286 0.4481522 0.2223228 0.101672973 0.02848313
0.08233499 0.841155 0.5968334 0.2681081 1.26548 0.0350715
0.104722 1.067481 0.04291019 0.135370327 1.028390239
0.184809266 0.4120047 0.1251921 0.0490757 0.7781186
0.4184727 0.8650319 0.071659967 0.05978863 0.01218311
0.05169415 0.2822237 0.2061667 0.1221023 0.2450829
0.2381177 0.1335328 1.07964 0.020960477 0.07440861 0.4674575
0.1451566 0.5793813 0.04507655 0.1657395 0.3155663
0.2115294 0.61084226 0.6918624 0.137463148 0.143351
0.2741773 0.4696258 0.1559813 0.1530156 0.5254304
0.07510401 0.01197085 0.3036368 0.6340237 0.08723782
0.09860247 0.1197756 0.6108456 0.4519359 0.08231268
0.6659039 0.050457231 0.04754314 0.2893962 0.1317178
0.1877524 1.242315 0.3788991 0.05096458 0.1037446
0.03955399 0.20549 0.08207886 0.08119043 0.3683108 0.210324
0.6885631 0.2879498 0.5465284 0.1043782 0.1079961
0.6804727 0.1452304 0.07191307 0.05684091 0.1451441
0.1006163 0.07972368 0.2545419 0.2306601 0.03071801
0.2433324 0.1497376 0.1651938 0.06759093 0.1664539
0.1051328 1.393503 0.1153604 0.04629358 0.3475753
0.03142868 0.5085965 0.1120569 0.1950709 0.05170435
0.1913868 0.6410995 0.3583269 0.248548 0.9120893 0
0.3102169 0.365514345 0.6303921 0.612682309 0.09083763
0.2899579 0.220813 0.216175 0.4332941 0.3788245
0.5619714 1.583846 0.147797524 0.3348266 0.05806261
0.0518459 0.192123 0.07589551 0.2156846 0.03430726
0.1980788 0.3801921 0.1656788 0.2875486 0.2192813
0.1304411 0.08812561 0.5834151 0.1737033 0.08234982
0.06166418 0.1773816 0.09019213 0.1373518 0.1199426
0.1349087 0.318333 0.2916204 0.2975741 0.05814128
0.1249405 0.1865653 0.2513073 0.04195386 0.03493468
0.06747439 0.05530006 0.1976678 0.2875258 0.06662754
0.1740217 0.3010581 0.4017818 0.2317114 0.125863
0.1984963 0.1248054 0.2366179 1.282616 0.5828233

0.088733681 0.1331145 0.278208426 0.119782906 0.04598383
0.771205 0.1156021 0.1251926 0.1328765 0.03480787
0.09978899 0.217263 0.2239367 0.09302783 0.02813625
0.170942 0.4392837 0.523969 0.09072912 0.2577154
0.08738949 0.2403835 0.3502035 0.2459194 0.05302865
0.5877846 0.2054919 0.2968834 1.591848 0.2805689
0.09154146 0.2692834 0.0563403 0.2258078 0.4137667
0.09170932 1.803903 0.08262133 0.280941346 0.04017777
0.5577941 0.07841647 0.06992818 0.3643591 0.1178858
0.07576587 0.08679907 0.03705874 0.1008704 0.2901943
0.1474897 0.2730982 0.2326149 0.1294105 0.071705198
0.06454072 0.3630723 0.365563 0.7820947 0.1529375
0.144574766 0.435161 0.06028567 0.3191172 0.1183059
0.08846739 0.6225372 0.1012144 0.1798392 0.1066409
0.3655917 0.2129401 0.5174207 0.8164378 0.1154982
0.03278804 0.4473134 0.4239452 0.1764335 0.028908
0.03652058 0.203556 0.3068288 0.09123173 0.1228735
0.07824816 0.1133038 0.231576066 0.09890732 0.3067982
0.05273134 0.2390679 0.1166387 0.1354925 0.8247585
0.05508695 0.4753602 0.632526 0.1612427 0.02911267
0.2845277 0.2945392 0.238422165 0.1068669 0.3091641
0.4620766 0.8192898 0.1185788 0.224722021 0.09713154
0.05209897 0.296547 0.1829862 0.3311276 0.1980071
1.548115 0.2387863 0.007026849 0.2443199 0.2283127
0.3059046 0.2594665 0.2890308 0.1621354 0.05576478
0.8752004 0.3680593 0.037657799 0.06046648 0.08513893
0.2298202 0.03929397 0.1316814 0.05473741 0.2464516
0.1791093 0.1112669 0.3715196 0.2261265 1.119141
0.7560027 0.3845775 0.6286386 0.06427219 0.4057392
0.2043238 0.09583509 0.007797887 0.1618313 0.1150106
0.1971874 0.1142544 0.3130154 0.1085225 0.077728439
0.111321 0.2127075 0.1062986 0.1948273 0.01844724
0.3876592 0.311011683 0.09642619 0.2041831 0.1277806
0.03794624 0.06179114 0.6649573 0.1687823 0.02107858
0.1444601 0.5885003 0.07813503 0.5991417 0.2938006
0.02511596 0.474857069 0.2170788 0.4421196 0.2063454
0.103832421 0.233202 0.6977915 0.07401338 0.01629312
0.06760426 0.2305424 0.537844
AC007686.3 0.204159 0.4940322 0.4637344 0.3502489 0.5082522
0.886108 0.4634998 0.5243174 0.4245365 0.589880402
0.4077147 0.3028707 0.6755868 0.2890801 0.8966263
1.080364 0.5402262 0.580362075 0.2263589 0.3559518
0.467396205 0.9460836 0.4770545 1.05678 0.6363654 0.3635211
0.5737825 0.9290636 0.297907704 0.283626 0.4121011

1.027066	0.5670784	0.6823721	0.3731213	0.2648735	
0.2297798	0.3330481	0.3513013	0.398227735	0.7065794	
0.404024	0.169128421	0.3482064	0.4762366	0.315781	
0.4702649	0.380666	0.288486492	0.3294519	0.1875807	
0.7311695	0.6168459	0.3069765	0.6417707	0.6248752	
0.3486999	0.9524699	0.4929811	0.361505159	0.316933473	
0.477296786	0.2448304	0.2974094	0.4070933	0.5307828	
0.3809014	0.4126825	0.083723173	0.6461442	0.3024727	
0.3321798	0.5729112	0.1864821	0.366369	0.4747216	
0.4496525	0.6817029	0.283812	0.163259695	0.5795635	
0.6235202	0.4239805	0.5912287	0.3379319	0.2057425	
0.5230234	0.2522868	0.25692162	0.4182639	0.176663995	
0.4698818	0.2954175	0.5275792	0.5227385	0.3703178	
0.3432888	0.4387349	0.3776226	0.4561082	1.23633	0.4076936
0.4032041	0.3098639	0.5159097	0.4965851	0.3666449	
0.4616144	0.331600379	0.7332142	0.3254969	0.5988371	
0.1754868	1.208007	0.6049998	0.4432715	0.4988214	
0.5578508	0.4216826	0.3923017	0.2457684	0.4213472	
0.5183363	0.4873265	0.3444332	1.010333	0.2705746	
0.3424779	0.9725526	0.2506613	0.4380985	0.4316616	
0.5256905	0.3946456	0.4843505	0.7009939	0.5270895	
0.4261827	0.3393196	0.4920309	0.5080505	0.6361404	
0.3176419	0.2500484	0.5112342	0.2695601	0.3028851	
0.6625614	0.2827391	0.366296	0.3196001	0.412594	
0.3360208	0.7950392	0.8464557	0.3663165	0.3909075	
1.188967	0.9832021	0.6272977	0.395644507	0.7180993	
0.587995798	0.3449199	0.3182378	0.3869768	0.3456164	
0.2453291	0.5674304	0.5385954	1.004344	0.273406347	
0.3334015	0.2755872	0.70525	0.4291241	0.5279998	0.4259879
0.4529326	0.5399876	0.448384	0.3871382	0.284628	
0.7452947	0.3619487	0.4375827	0.7887397	0.4622624	
0.381414	0.2413497	0.3680331	0.1536719	0.2195954	
0.5605348	0.3725544	0.4703708	0.2156066	0.4759736	
0.4160636	0.4817106	0.2019443	0.5667398	0.2532511	
0.343531	0.3468652	0.4942607	0.3464147	0.5374843	
0.399833	0.5513002	0.9752738	0.5092738	0.3712698	
0.3400549	0.4158405	0.4775445	0.2942856	0.3630802	
0.4495921	0.302373949	0.2449486	0.281117324	0.297387602	
0.3402566	1.002758	0.270125	0.2839311	0.3493008	
0.2440044	0.1873726	0.329048	0.3003947	0.3478019	
0.1906616	0.4593522	0.74652	0.5581584	0.3038736	0.3340318
0.6040952	0.7114857	0.555284	0.3867733	0.4130365	
0.343366	0.5521938	0.3884839	0.8465097	0.5099107	
0.4188934	0.5355936	0.8463163	0.5431591	0.672256	

0.2440585 0.2901728 0.3964615 0.354148165 0.3051183
0.7120349 0.5000764 0.2450996 0.3831256 0.2195081
0.4573547 0.3485996 0.2164859 0.4895345 0.3217472
0.7366594 0.5089949 0.4586173 0.3198364 0.479808108
0.2523183 0.8650183 0.6406526 0.6336238 0.3841683
0.119232294 1.132786 0.2347805 0.2389982 0.4300224
0.2310399 0.6222754 0.4434478 0.3641963 0.4324101
0.4712048 0.3832655 0.7297457 0.4597822 0.390619
0.3256143 0.5084893 0.6431067 0.3841592 0.3917826
0.2026751 0.4136045 0.3860556 0.2593681 0.5062308
0.5133608 0.3622957 0.482497732 0.340894134 0.3877053
0.2464324 0.2013648 0.1991691 0.1939191 0.676684
0.3915249 0.7324102 0.3603791 0.3403106 0.2721079
0.3448908 0.2608665 0.641678353 0.4409008 0.3440082
0.1199694 0.5533862 0.315564 0.32818953 0.5390424
0.4603239 0.3422424 0.4025621 0.4197946 0.6169054
0.5779756 0.1793465 0.3037606 0.4093934 0.6835386
0.1842846 0.9910508 0.438992 0.2669229 0.2885312
0.7606635 0.6247435 0.557296551 0.4142388 0.2763088
0.5087521 0.4820413 0.6752242 0.2913364 0.1886498
0.2580879 0.4490824 0.7441047 0.3182319 1.042239
0.4964352 0.6630167 0.3533739 0.2795082 0.4558088
0.3840271 0.2239359 0.4281972 0.5970754 0.4264832
0.3455728 0.4627584 0.360629 0.3367888 0.177498545
0.340453 0.2329825 0.5937972 0.7759925 0.4166843
0.6542142 0.291450814 0.3479161 0.6063278 0.2529656
0.373673 0.3308847 0.5026974 0.2095197 0.435076
0.2723471 0.6875682 0.2789363 1.108335 0.600703
0.5318595 0.607133378 0.7198381 0.6678199 0.5062711
0.200164015 0.3615322 1.688062 0.395304 0.4822426
0.2882943 0.4601426 0.6316917
AC245041.1 0.01758913 0 0.01617915 0.05040659 0 0.01781309 0
0.02108031 0 0.132223508 10.25164 4.69966 0 4.056892 0
0 0.03508603 0.079460345 0.08687138 0 0.041107006 0
3.244632 0.02104364 0.2891245 7.534858 0 0
0.033990058 0 0.09941173 0 0.3565457 0.9417567 2.735034
0 0 0.03159503 0.6666859 0 17.76932 0.5426932
0.439374783 0.04522987 0.3803566 1.682534 1.61685 0.510459
1.379047405 0.2388238 0 0.01659499 0.1509592 3.30591
9.568171 0.2810922 0 0.1622784 0.3350995 0.858437984
0.499235219 7.987766428 0 0.06174731 0.1452308 0 0.7199974
0.1422171 1.784879728 0.8232337 1.517262 0.07648982
1.444184 0.08200453 1.779326 0.2862944 0.1775323
8.01787 0 0 0 0.2497729 0.5275361 0.7596222 0.8151979

0.9877653 0.488649 5.368678 0.66281562 2.202192 0
0.01963989 7.663024 0.04454404 13.05839 0.1437531
1.756102 0.2222569 0.04428201 1.60457 0.02202207 0.2151373
0.3647456 0.2320833 0.07259795 0.06634047 0.8627137
0.2627499 0.124432663 0.1641447 0.1491273 0 3.333719 0
2.055688 0.321192 0.5116895 3.553394 0.02598765 0
0.9283106 0.6244506 0.1620876 0.4898269 0.2028896
0.01706069 0.01608795 0.1331646 0 0.01627968 0.0126675 0
0.4653221 0.0708943 0 0.06725637 0.08365167 1.32569
5.574959 0.02307925 0.08387331 3.796739 0.8757166
0.5926129 0.2490231 1.109512 0.02283294 0.5112861
1.96866 0.1030891 17.00327 0.5142416 0.2868934 2.045244
0 2.706241 0.5500785 0 7.6095 0.1176964 0.397674506
0.0971632 0.089936719 1.024068 0.6067224 0.1535902
0.3928176 4.520777 0.4311784 0 0.01452017 4.49529928
0.05629884 1.968839 0.2739799 5.211744 1.088966
4.787101 0.2876577 2.13711 0.2476659 0.4263451 6.973052
0.1966437 0.1769245 2.781782 1.164711 6.282761
8.282886 1.201354 0.1960943 0 1.7471 0.08067018
0.03024532 1.046722 2.258629 1.712312 0.5735292 0
0.05412811 0.01441277 0.08621873 0.1723049 1.597428
0.05455022 0.2755259 0.02025908 2.43478 3.350711 0.04499636
0.4860699 1.191827 0.1551955 2.970831 4.786024
0.2070569 1.449156 0.1474156 0.16411971 2.44564
0.760262192 0.129236113 1.38601 0.2607402 0.01425433 0.8535692
3.399751 0.02145994 0.7910029 1.111274 0.2045373
0.09176644 0.3330569 0 0.7386261 0 0.1491648 0.2979152
0 1.040711 0.687385 0 2.964212 4.128427 0
2.811436 0.01760386 5.253402 0.6772516 4.996413
0.0198487 0.9171893 0.7971803 0.0125647 0.08596344
1.921096 0.164091299 0.3302749 0.8491216 0.1611525
7.144357 2.26883 0.01816989 0.0207607 4.548676 0.6854296
0.1913513 2.322207 8.820319 0.08017713 1.0995 1.301107
0 0.01224338 0 0.3005052 0 0.01885797 4.676914409
1.430868 0.3097304 0 0.03241716 0.3336711 0 0.1248025
0.1182671 0.2011079 0.2432852 2.157299 0.09114372
0.2897005 0.02998213 4.770659 0.1192563 1.281571
2.768836 0.1425801 0.1651164 0.9603284 1.144707
8.729485 0.03189669 0.08906211 0.7353122 1.085071243
0.341481714 0.3860182 0 3.043804 9.293076 0.06682768 0
0.06792501 0.3663396 0.192577 9.543382 1.543588
0.9998856 2.999178 0.776964726 0.06588619 0.07261239 0
1.136504 0.1949516 0.115455699 0 2.601747 0.2318804
0.3840533 0.05212305 0.1265978 0 0.4206224 0.01732892

0.412249 0.1583557 0.5422191 0.02461041 0.760298
0.5634142 0.1571676 0.6317073 4.966505 2.012139188 0
0.1399742 0.1193179 0.1615049 1.100507 0.4949561
0.3562817 0.2650211 0 3.517573 2.078517 0.03999877
0.1496947 0.7170883 0 118.3656 0.692719 0.04381594
0.01688136 0.03846076 1.995462 2.010058 0.04862843
1.840852 6.58283 0.05018014 0.034852043 0.2906776 0
1.097725 0.04367855 0.1516425 0.1770383 3.451438969
0.7973187 0.04196136 0.01750667 0.6817919 5.384208
0.04823095 5.09887 0.01732731 0.08096667 16.8351 0.02140988
0.06156435 6.094678 4.81572 0.486094493 0.1417072 1.012432
0 0.102424524 0.2211922 0 3.207216 0.2276894 0
0.04737842 0.9075217

LINC02762 0.2229381 2.494478 0.229036 0.4646487 0.6000324
1.809147 0.7873196 0.8466738 0.7554036 0.652949088
0.4423773 0.3754229 0.8763331 0.2497443 0.77411 3.181005
0.3465252 1.127039482 0.3574918 0.5627219 0.639435941
2.419234 0.4641062 3.020554 1.288951 0.3049529
0.6003723 1.95284 0.391651091 1.694985 0.6136465 2.828873
0.7070756 0.2644461 0.4337071 0.4887115 0.09038129
0.5304792 0.2620357 0.179360613 0.4606056 0.1352732
0.192864924 0.21591 0.411434 0.3621754 0.4649125 0.2645242
0.775342533 0.2959976 0.1971954 0.3960905 0.3354614
0.1697833 0.7620928 0.05338829 0.1275321 1.11301 0.7490128
0.639798798 0.539086486 0.236909112 0.6949468 0.1456849
0.1713268 1.053549 0.3950564 0.4272323 0.095995508
0.2588855 0.4253211 0.6505242 0.7446757 0.3590615
0.1869991 1.089138 0.4316033 0.1225529 1.097861
0.609787813 0.1610951 0.2972892 0.114748 0.8693218
0.2958227 0.2382836 0.7864779 0.3112713 0.26780162
1.254044 0.184145282 0.04526021 0.2893778 0.6256878
0.1699611 0.7453791 0.1627759 0.1067067 0.1093372
0.3844472 0.2392497 0.4274879 0.1267507 0.6807015
0.4720314 0.5460066 0.1253021 1.00918 0.140817454 0.3937118
0.7749982 0.6114102 0.4115661 1.203307 0.676763
0.2183785 0.145779 0.2775479 0.3101376 0.3392478
0.3325795 1.168059 0.3121656 0.4192715 0.4592105
0.3678894 0.2330412 0.1471764 1.00301 0.3671265 0.4983542
0.240628 0.2003266 0.7672845 0.1877049 0.9115039
0.1817598 0.09663961 0.8443926 0.3305144 0.02169541
0.1371888 0.6261491 0.2804519 0.3525231 0.2060486
0.6126307 0.6658658 0.3878467 0.3026179 0.8830067
0.3822812 0.1679103 0.2647259 1.434533 0.5272879
0.227661 1.559859 0.1382718 0.4940292 0.680785786

0.5086023	0.595130878	0.3871823	0.1676406	0.3907233	
0.2124566	0.3247215	0.5362568	0.8412143	1.328911	
0.153324959	0.9359879	0.1149031	0.2946476	0.5275804	
0.2352681	0.4336042	0.6183429	0.3316822	0.230046	
0.6754784	0.1750906	0.3884281	0.1906238	0.2003321	
0.7037255	0.1175215	0.4512911	0.01501912	1.201756	
0.2471342	0.03814912	0.2080363	0.4032669	0.4940905	
0.2922506	0.4314173	0.07670582	0.4530823	0.1670604	
0.9679593	0.2441066	0.2599911	0.2191237	0.1414251	
0.3907388	0.0600263	0.3688185	0.1766053	0.2036852	
0.2677282	0.3493679	0.3800017	0.3223092	0.4154394	
0.294354	0.1123899	0.1941255	0.246139591	0.4106759	
0.903484358	0.26135673	0.3795544	0.6210744	0.7320668	
0.445427	0.2764409	0.2719998	0.6163034	0.5977692	
0.6194977	0.3436487	0.3198045	0.5030919	0.4660694	
0.3419871	0.1178574	0.3073114	0.5528151	1.01701	0.2059886
0.5183386	0.3013693	0.4711683	0.1946398	0.4097559	
1.521306	0.4218287	0.06399814	0.6064242	0.7841384	
0.6470406	0.3621721	0.119958	0.08843887	0.1844655	
0.288113539	0.1658154	0.4947919	0.2758797	0.04460743	
0.7987001	0.5951888	0.4818486	0.3083071	0.7496714	
0.3181282	0.2716637	0.5717752	0.6942017	0.1416411	
0.8162777	0.068800513	0.3909782	0.3178893	0.07145	1.698647
0.2762704	0.38320126	0.533045	0.2712345	1.052944	
0.2507969	0.1774492	0.8929153	0.3401076	0.2287449	
0.6162409	0.2355679	0.1658726	1.149225	0.6413882	
0.2319582	0.09760615	0.4171474	0.5884564	0.2018414	
0.2675551	0.2322601	0.2586718	0.4886336	0.9727724	
0.8085666	0.5302136	0.3655343	0.291417573	0.224841686	
0.9632885	0.08951469	0.1286435	0.1384023	0.266758	
0.3310395	0.1602604	0.8852364	0.2535968	0.7031096	
0.3781747	0.5197534	0.5168286	0.117524358	0.4527936	
0.2749084	0.592498	0.498872	0.323579	0.106427238	
0.3312073	0.504932	0.2759929	0.273418	0.323174	
0.4554794	1.554561	0.1412446	1.283611	0.7334018	
0.7819955	0.09507415	0.6927306	0.09386302	0.1196673	
0.3880642	1.221808	0.2593523	0.955422193	0.3123996	
0.5376184	0.3388005	0.3721886	0.1603636	0.320956	
0.05519681	0.1648034	0.08622893	0.3527974	0.2002767	
1.514341	0.4144145	0.9062277	0.2214845	0.2464109	
0.3716425	0.2848909	0.5807687	0.5571216	0.2074534	
0.7145177	0.413571	1.830476	0.2153047	0.9664227	
0.17497559	0.7363227	0.3453848	0.4881424	0.3954397	
0.7613253	0.9092248	0.370864232	0.1657826	0.6250972	

2.008564	0.2552637	0.2550147	0.6668905	0.4196559
0.2367329	0.2079123	0.4491226	0.2396473	0.780314
0.392873	0.3568439	0.349155304	0.2099345	0.6896907
0.8097144	0.459430531	0.2803561	0.8664439	0.339078
0.6768332	0.04939773	0.2534622	2.141948
MAFA-AS1	0	0	0	0	0	0.03528444	0	0	0	0	0	0	0
0.03266648	0	0.1032471	0	0.052465424	0	0	0	0
0.04432425	0.1667342	0.07158777	0.3012418	0	0	0	0	0
0.06246137	0	0	0	0.4582555	0	0	0.08085193	0
1.350513	0	0	0.6719401	0.03587696	0.05127369	0.1216208
0	0.035475822	0	0	0.06574323	0.2616444	0.05238717
0.1358622	0	0	0.03571596	0.1397411	0	0	0.047514385	0
0	0	0	0	0	0	0.1460305	0.02975658	0	0.1378632
0.2598973	0.1084466	0	0	0.1134423	0.02636963	0
0.06057967	0	0	0	0	0.03373415	0.07169803	0.08610817
0.11935594	0	0	0.07780601	0	0.2941117	0	0	0.1350879
0.0366875	0	0	0	0.06087819	0.8830478	0	0.1797539
0.02628164	0	0.04337158	0.030809766	0.09289725	0.04219908
0.587477	0	0.1920435	0.1233921	0.05532366	1.208478
0.5520485	0.2831221	0.1457987	0	0.4872721	0.3852787	0
0	0	0	0	0	0.03224703	0.1003679	0	0.212704	0	0
0.08881497	0	0.3376219	0.03834362	0	0.04746787	0.2568023
0.08131091	1.137174	0.5261535	0.05635229	0.09045566	0
0.06141028	0	2.221743	0	0	0	0	0.2187985	0.1867892
0.1237627	0.8808905	0.8159718	0	0.03849247	0	0
0.04292161	0	0.05557851	0	0	0.3002529	0.1438088
0.04010972	0.03717254	0.0354537	0.07236047	0.2208236	0
0.1505137	0.03351745	0.4434803	0.07008293	0	0.1170536	0
0.2230167	0.3443873	0	0	0	0.03012229	0	0.1468596	0
0	0	0.2744157	0.04451669	0.6921989	0	0	0.08041335
0.2283923	0	0	0	0.2431213	0	0	0.02958803	0.03269527
0	0.1851567	0.03233957	0	0.8024547	0.3353136	0
1.959675	0.03650037	0	0	0.22038132	0.036570411	1.77205
0.09114322	0.1694111	0.3597368	0	0	0.2089108	0	0
0.1363293	0	0.04175167	0.0487694	0	0	0.236046
10.49434	0.5740766	0.03491238	0	0	0.03634512	0
0.05800974	0.10461	0	0.04968557	0.3758343	0.03931661
0.2595404	0.1263254	0	0.08513885	0	0	0	0.4373078
0.1276853	0	0.2669782	0	0.1233694	0	0.2715419	0
0.08678986	0.03602355	0	0.04734578	0.1458822	0	0	0	0
0	0.224125	0	0	0	0.07994117	0	0	0	0	0
0.06128578	0	0.477925	0	0	0.02969454	0.05338867
0.05905616	0.4342266	0	2.005217	0.2378655	0.04323258	0
0	0.06318146	0.0588052	0	0.150829879	0.434836427	0	0	0

0	0.2647466	0	0	0	0.4958972	0.07622451	0.3555306
0.3127245	0	0.041595234	0	0	0	0.6253347	0.06436052
0.091478568	0	0.06362446	0.07066342	0	0	0	0	0.1999621
0.0686508	0	0.2439681	0	0	0	0	0.7004699	0
0.03392321	0.030659048	0	0.092421	0	0	0.1786803	0
0.04151343	0	0	0.09722321	0.2510462	0.1584603	0.5930352
0.9622197	0.1738207	0	0.9655859	0	0.06687773	0.1904592
0.04160675	0.1954137	0	0.3720804	0	0.03313253
0.828425534	0	0.1731756	0	0.1730383	0.03003759	0.4558843
0.031651219	0.05541548	0.4155885	0.06935496	0.3442461	0	0
0.2061208	0	0.032076	0.05749521	0.08481804	0.6097381	0
0.797477	0.087533039	0.1559421	0.1234118	0	0	0.2190705
0.1203048	0	0	0	0	0.02765589
AL450263.1	0.4477232	0.1070313	1.87384	0.3421538	0.4698114
0.3627393	0.06917582	0.1878064	0.1382415	0.336568929
0.6149061	0.5277693	0.3802698	0.1889018	0.4379515
0.480958	0.2679296	0.421380618	0.3316907	0.1912487
0.444703063	0.06688414	0.2847953	0.2142625	0.3986413
0.35192	0.1417409	0.1239087	0.216300372	0.3047778	0.06326201
0.1003328	1.015615	0.2115176	0.6925454	0	0.04658791
0.281483	0.2770643	0.237736708	3.199795	0.7696377
0.233001779	0.7195661	2.604866	0.6918393	1.146122
0.6977991	0.319118408	0.9476326	0.05168455	0.6969896
0.624422	0.6058831	0.4801233	0.4678317	0.03286883
0.7343509	1.032555	0.364185811	0.787883945	0.213704917
0.1560697	0.2357625	0	0.2427092	0.3665441	0.4072581
0.427342941	0.6724379	0.2294329	0.1622511	0.5757754
0.7097119	0.8013188	0	0.66047	3.863156	1.21991
0.416658112	0.07784814	0.6357854	1.031093	1.219999
0.04716021	0.5418775	0.9213583	0.4149512	0.53682587
0.4040058	0.819759048	0.7248906	0.6884428	0.9259761
0.1546025	0.5946152	0.6726818	0.7543266	0.7514523
0.4084359	0.4484494	0.9387811	0.2063207	0.08055785
0.3695896	1.030087	0.5167055	0.1672045	0.17816495
0.2984449	0.528724	1.43798	0	0.4195366	0.7135446
0.2843755	0.2003819	1.081853	0.4630527	0.5854974
0.4401615	1.396843	0.8664319	0.2493664	0.6670763
0.7382626	0.4299869	0.04842351	0.4769223	0.4143918
0.6771353	0.5352154	0.5011161	0.8120619	0.3503174
2.824768	0.4045699	0.1205173	0.2956417	1.527427
0.8387331	1.037158	0.7314224	0.8249442	0.2746801
0.3801826	0.1453005	0.07655621	1.400751	0.3936129
0.8896199	0.3589135	0.1825685	0.8454309	1.423266
1.194964	0.5800828	0.2915766	0.8175496	0	0.202452476

1.236622 1.465150915 1.425549 0.02757829 0.9063094
0.2142642 0.8578268 1.859729 0.1378004 0.4250449
0.902005179 0.9553743 0.1594597 0.1859742 0.2305633
0.8229118 0.1934182 1.206009 3.315759 0.1125754
0.9269777 0.3008405 1.647636 0.2661178 1.687246
0.4011091 0.06057759 2.547769 1.180617 0.2111785
0.5661676 0.1815169 0.3080134 0.5004226 2.272563
0.2717302 0.6004212 1.916109 0.2352886 0.06889033
0.9905506 1.009543 1.224409 0 2.325823 0.1888219
0.4383328 1.406822 1.638592 0.8876555 0.5234599
0.914278 0.5184941 0.7733982 0.6267579 0.143742
0.5852361 0.2345247 0.394550415 0.3133523 1.132807523
0.258472225 0.978225 2.166793 0.3446956 0.6240796
0.364924 0.3004391 0.223718 0.2525622 0.4946083
0.496373 1.783873 0.9121036 0.9400695 0.1934786
0.2847692 1.718881 1.25715 2.179626 1.009446 0.5937392
0.1109598 0.2335272 0.3869833 0.4100011 0.3360736
0.3261536 0.4469405 0.6640802 2.071483 1.00057 0.7305071
1.071426 0.27352 0.4630741 0.208843471 1.219015 0.8645602
0.5947992 0.03135455 0.7147529 0.4162557 1.453249
1.464333 0.6785061 0.3409532 0.8364723 1.064721
0.224496 0.2737885 0.6561329 0.63425804 0.4674746
2.569609 1.232375 0.4300276 0.408018 0.720698592
0.6469716 1.087999 0.02568218 0.9076805 0.5716743
0.9986814 1.05893 1.016022 0.511911 0.4917744 0.7406039
0.3016028 0.2027904 1.354647 0.1715183 0.3035614
1.051624 0.3523973 0.3992244 0.5540268 0.6388962
0.7654901 0.6490526 0.974299 0.1322437 0.3556237
0.654157783 1.024445141 1.198762 0.2557828 0.1047009
0.05632167 0.9781142 0.05817189 1.2103 0.8206006 0.6862743
1.485614 0.5482513 0.6251279 1.192855 0.721603603
0.3144568 1.155197 0 0.7232299 0.4342104 0.499608298
0.2743779 1.124212 0.9761677 0.6843132 0.4422562
0.5984621 0.2192043 2.740928 1.27919 0.6861207 2.418523
0.5850823 0.7830586 0.483826 0.5088902 0.8001258
0.6364929 0.8064733 1.083459563 0.5693539 0.2078404 0
0.9866479 0.9643783 0.3245167 0.5067964 0.7120768
1.460419 1.415953 0.5806943 0.9163354 0.9179626
1.472029 0.3350534 0.2689727 0.9142957 0.7807203
0.6875319 1.150326 1.149539 1.993242 0.2888234
0.8128438 0.573098 0.4044823 1.752107239 0.4110592
0.3059924 0.2502276 0.05559088 0.7333984 1.442058
0.447408755 0.9969663 0.6141617 1.069498 1.293095
0.8080929 0.214847 0.1324382 0.6395352 0.4946327

0.7942578 0.2179915 0.3525958 0.9492664 1.340124
2.418420535 1.142246 0.4361247 0.3022371 0.260716971
0.2533656 2.293206 0.6969118 1.483025 0.8275324
0.3919487 0.5330897
AL390728.5 2.367022 1.427176 2.488318 2.2834 1.468255
2.562483 1.008879 1.495281 1.980148 3.915202444
2.021794 3.161321 6.194186 0.7106138 3.117563
3.273973 1.442075 1.053519183 1.094189 0.8716319
1.594166635 3.42571 1.171889 2.650552 1.173959 2.758717
1.513477 3.291535 0.653449325 2.566436 0.6260705
2.884753 10.62502 3.745864 9.692081 28.09132
2.402343 4.283257 4.554897 5.799331957 9.651603
5.273097 2.111714196 7.136155 6.976053 7.842036
9.999361 3.158345 3.43121805 2.318943 1.561404
19.42807 8.731461 5.154545 5.138013 3.72684 7.036435
2.737258 4.068754 1.32784602 3.163917223 1.71738191
2.043901 0.736805 4.412677 6.676512 2.131947
3.594378 1.89786188 5.188595 3.176814 14.96694
4.221701 3.805379 8.302239 2.214224 5.332262
6.065043 7.307225 4.066332554 2.311265 3.247911
7.294055 4.460189 2.505547 3.443401 2.963413
3.746327 3.31543215 10.33753 2.921478028 5.390158
5.58798 11.16204 2.737928 4.729114 8.216919 4.272827
5.420981 5.803093 6.452714 4.584186 6.340471
11.70681 5.269877 6.561594 2.607243 1.306369
1.556981174 14.59676 8.572544 16.48728 2.496474
16.85196 7.020276 7.100587 3.887868 5.570351
6.787741 4.135341 2.413321 7.551664 5.017977
2.402904 4.685077 12.16945 3.903406 1.046721
2.613737 1.05763 4.593462 12.30597 8.020271 4.499996
0.625607 4.607188 1.974179 4.017502 4.414379
11.62779 6.243244 6.77732 3.528541 16.53677 6.207112
9.231713 13.50171 7.995055 12.1361 6.811217 3.631555
1.616534 2.123763 2.503081 4.243485 10.14173
6.136686 10.78295 3.668727 24.92849 1.177533573
13.26878 5.34203901 8.889038 2.944746 3.091572
2.659873 10.67993 7.304733 8.61308 3.744408
2.147769268 6.145641 2.634102 3.789952 14.72522
3.41774 11.60248 5.305791 7.9742 34.46671 8.361235
2.193769 5.692356 8.839177 5.47468 2.652431 7.288709
5.510135 4.939717 4.989811 5.406461 1.323646
1.907382 2.566434 6.561385 3.076529 10.71422
4.895137 14.22988 32.07015 8.971708 3.029281
5.216258 2.845979 6.102423 4.467949 3.169522

4.871909 4.442516 8.874142 1.98293 6.699508 2.392016
4.397642 4.203138 0.7487041 11.15863 2.809591
1.60779946 2.31765 7.695128474 1.750184721 3.366206 7.65625
13.33324 12.4246 2.145152 0.9673849 4.497952 2.631025
4.081323 4.592962 2.612688 5.659094 6.52868 2.838541
14.51625 10.13804 5.286082 7.885931 5.199451
6.370736 4.594724 6.738665 2.945114 9.221733
4.726339 4.264052 18.20801 6.21567 9.237006 11.22674
7.715631 3.92315 5.442258 12.96691 1.788820503 6.218074
4.604529 5.23474 1.714812 6.16514 4.878309 2.518577
7.27247 5.119415 1.065547 4.531183 3.460082 3.986335
1.061632 8.788058 4.358672617 10.80293 5.353696
6.91706 5.553013 4.462983 1.571068089 3.082798 9.141154
1.056783 3.567344 5.96383 8.119331 4.973246 20.20807
5.425046 1.83099 8.524266 3.697772 3.034369 7.701871
1.822499 1.166098 20.73648 3.146607 9.622077
4.527629 7.610529 8.218743 8.859421 4.77877 6.681493
8.237975 7.003874997 10.089880229 8.096667 3.213164
1.745127 1.598817 6.545546 0.9695937 5.94382 1.563982
6.651262 8.137731 5.711317 4.756207 6.617043
3.466258364 4.072914 5.126516 2.39815 7.199279 1.852405
1.74506749 4.303309 4.482231 5.072709 2.475957
2.107762 3.524823 0.8182631 4.673351 8.420494
8.167127 11.20925 5.055723 5.661224 8.631305
7.626614 7.3584 2.913957 6.868633 4.853307547 7.809348
12.60423 7.197953 8.372485 2.71488 4.653295 6.988349
4.587412 7.01152 7.24508 7.830467 5.966108 6.973572
8.03534 1.551273 6.257752 6.37803 13.33235 15.8241
5.583725 10.05354 8.518256 8.843635 18.5417 6.993609
4.779142 5.394822098 2.076839 2.028489 4.920155
6.254375 4.584034 12.05313 2.033806733 2.438792
6.258843 5.907223 11.08667 3.990215 0.6554532
6.173947 2.561521 2.726666 5.089503 5.861737
29.30325 3.800135 5.871633 7.499459877 7.26475 1.817303
1.32687 3.259171376 2.668721 8.213554 4.960975 8.426013
7.482311 1.397663 2.822965
LINC01569 0.7680326 0.263165 0.57106 0.9415448 0.7891331
0.3824243 0.3164413 0.3298371 0.5236885 0.858016011
0.2142606 0.08801967 0.7215075 0.1800254 0.9390877
0.1137992 1.18733 0.409611662 1.019438 0.615134 0.42629957
0.2358427 0.2198444 0.4900664 0.385754 0.3974293
0.7800903 0.2631636 0.544200301 0.2614107 0.2532153
0.3843851 1.118224 0.4434725 1.860166 1.206605
0.8857552 2.115392 0.4703303 0.725009685 1.162921

10.97388	0.253140523	0.3209328	1.001773	1.191503	
1.288377	1.05255	0.619107786	0.7770099	1.677164	0.8393556
1.112343	0.5870364	0.6613857	0.4012636	0.4792622	
0.4264674	3.741476	2.455540213	0.719373758	1.283076789	
1.01353	0.5017944	0.3523073	0.5985001	1.283752	0.6015878
1.08674707	1.779449	0.4154386	0.2504957	1.171306	
1.241325	0.3486297	1.713114	0.2484611	0.69291	0.4359702
1.410677042	0.7010963	0.6781532	0.9460791	1.072729	
0.862928	1.747548	0.6190351	2.000991	0.94280677	
1.220037	0.622935147	1.436446	0.5248745	1.280474	
3.572941	0.9873211	0.5707614	0.714388	0.4296851	
1.141781	1.322197	1.196619	1.238742	1.274423	
1.030251	0.7048806	1.468044	0.5338144	0.735769789	
0.7252738	0.3798475	0.1901444	0.4268182	0.8607948	
1.178693	1.925885	2.821523	0.4715637	1.390076	
0.3950534	0.8014246	0.7987233	0.4718391	0.3208258	
0.3937428	0.9746539	1.71523	0.5191661	0.5217006	0.2961897
0.5393005	0.7869586	0.3125905	0.5094864	0.5580166	
0.5710372	0.4991978	0.813168	0.4296682	1.578824	
1.443138	2.102644	1.483727	1.138838	0.8517689	
1.713246	0.9222294	0.826259	1.145031	1.369178	
0.6388202	0.5432531	1.061337	0.4961443	0.5425547	
1.467059	0.8120806	1.614204	1.56426	1.284809	
0.424466438	0.9050987	2.532982511	1.176127	0.252311	
1.097435	1.03119	0.4366741	1.133136	0.9691802	0.4385346
0.346304141	0.6896898	0.2670273	1.568533	0.1926856	
0.4024407	1.078328	1.299163	0.6739586	0.83039	0.749829
0.2365309	1.174679	0.5150295	1.447166	0.5584336	
0.4849409	0.3061454	0.8189546	1.081289	0.4316508	
0.9406195	0.381602	2.201113	0.6945418	0.2821319	
0.6993648	0.5869516	0.9268273	0.2511234	1.494674	
1.386686	2.199646	1.816458	0.932929	2.969163	
0.4054493	1.934979	1.339368	1.776484	0.6598593	
0.3089213	1.009437	1.105585	0.6831695	1.767139	
0.9075886	0.945424	1.088218916	0.7824052	2.523511738	
1.545140129	0.5410156	1.663143	1.410814	0.4229364	
0.2831893	0.304542	0.2942236	0.9387082	0.4019022	
0.8264437	0.4948658	2.492685	0.5912913	1.124762	
1.807444	0.8455543	3.293621	0.8053166	0.6349285	
0.5516936	0.9358506	0.5341291	0.9711708	1.044329	
0.4227717	0.5781395	0.6891082	1.438353	1.69006	4.028764
0.8006079	0.4434839	0.1798605	0.5507603	1.598874133	
2.349506	1.069055	1.032057	0.8605787	1.038098	
0.5950439	0.7932053	0.8665234	1.424419	2.868685	

0.4464134 0.4566104 0.4784632 0.8958364 0.4109123
1.181610922 1.118225 3.251665 1.621978 0.2202786
0.7342315 0.511309135 0.7946909 0.5094206 0.4405569
0.8257093 1.022689 0.7945074 0.8577955 1.441661
0.5066202 0.1458072 0.2892071 0.3117517 0.5165348
3.49659 0.4413385 0.6834645 0.3129345 0.4605782 0.4046764
0.6008199 0.9291957 0.7483462 0.7531802 0.1973099
0.7723817 0.8187426 0.561077182 0.914178138 1.033808
0.2365941 0.7683138 0.1851792 1.793381 0.7401027
0.6096694 0.5971948 1.135203 1.19021 0.2220579 0.5297949
0.4945094 0.764106279 2.649176 0.7926586 0.2632683
0.5513964 0.537949 1.504016472 0.8018872 0.2045372
0.519236 0.1886599 0.7333659 1.868962 0.2402396
2.04481 0.7377541 0.3288632 1.139634 0.6476616 1.226856
3.017265 0.4827732 1.029413 0.4501494 0.4611464
0.782859196 0.2417037 2.2156 1.823514 0.5171571 0.7483794
0.4475286 0.8007336 1.232225 0.335846 0.660818
1.475754 0.6112939 0.6536449 1.237321 0.2554475
0.185842 1.204307 0.7732644 0.7248426 0.8396984
0.9783269 1.938469 0.731383 0.6971837 0.3901202
0.608646 0.811638056 9.736788 1.033904 0.8882988
1.271488 0.3862542 1.359264 0.482590457 1.216492
1.129888 0.9619106 1.182067 0.5452456 0.8160805
0.2271869 1.853671 0.235695 1.214618 0.8257989
2.710621 2.047118 1.171975 0.90851197 0.9510682
0.9011643 0.4690857 0.540413448 2.656061 0.972402
1.062663 1.013705 1.146571 2.594605 1.183733
AC010478.1 0.1036017 4.762977 0.2989705 0.2503274 0.3330664
2.526331 0.3954687 0.7206446 0.7652183 0.791535422
0.003487434 0 0.3984006 0.009523155 0.2516956 2.746561
0.275547 1.49891779 0.2608571 0.4729139 1.001730371
2.458076 0 4.853459 1.775321 0.007983643 0.4533896
3.236651 0.353302704 0.571572 0.9873898 1.657034
0.1745173 0.3071027 0.5093691 0.1959376 0.02113783
0.353951 0.2687038 0.048539573 0.6447015 0.1934028
0.076116521 0.02481255 0.1547946 0.2077724 0.2623725
0.4956504 0.707403359 0.4932361 0.1055262 0.6439748
0.05012448 0.1160692 1.307047 0.1248613 0.1789586
2.171977 0.2036914 0.228579384 0.795677071 0.074799282
1.048015 3.1259 0.02236403 2.64843 0.3437035 1.024507
0.087762418 0.1674491 0.08327849 0.08981208 0.823912
0.301174 0.1628178 0.4592328 0.6892363 0.3207933
0.007687456 0.017909602 0.02825696 0.4105123 0.08703756
1.169739 0.3102637 0.06490711 0.902962 0.3012341

0.31315915	1.624542	0.052854696	0.03402381	0.3904497	
0.2898061	0.2431732	0.2220566	0.3229305	0.09625851	
0.001704742	0.7206703	1.096195	0.04614383	0.04212526	
0.2461077	1.203175	0.8121513	0.09377569	2.068555	
0.149099107	0.1137446	0.1353238	0.2789178	0.5490077	
1.388449	0.6474978	0.04032083	0.04545857	0.1577182	
0.4156898	0.06800677	0.0273287	0.3649667	0.4605083	
0.2225133	0.1171608	0.4472754	0.2192478	0.1603859	
1.104524	0.6787433	0.2121345	0.1040732	0.06200892	
0.2231158	0.1316979	1.224689	0.2183412	0.1881027	
0.6215077	0.1839176	0.04289829	0.05561383	0.09639754	
0.08341388	0.5982127	0.5749872	0.2452434	1.479712	
0.0143222	0.9246981	0.2853624	0.5977404	0.2687531	
0.4926081	1.889784	0.09567846	0.7696176	2.636257	
0.02853369	0.4077903	0.998174871	0.9650566	0.847578127	
0.009239985	0.250256	0.4950646	0.2884068	0.3949083	
0.2600548	0.2876045	2.218626	0.163702842	0.5505091	
0.01240285	0	0.152893	0.7880092	0.3685818	0.02931371
0.1363384	0.09806906	0.272869	0	0.09841349	0.11517
0.01003981	1.516063	0.1181864	0.4154797	0.02107549	
0.4146208	0.04923555	0.06794512	0.1894411	0.1117791	
0.5333304	0.5463658	0.1816151	0.05381849	0.3724561	
0.1187761	0.4361154	0.05377098	0.02155819	0.1422113	
0.05670111	0.4503907	0.3533045	0.008625705	0.01525048	
0.1481067	0.9888796	0.3016913	0.3584773	0.08577694	
0.1101942	0.7239252	0.04988799	0.4958631	0.103196948	
0.2369566	0.169183954	0.341160208	0.007276058	0.7209515	
0.07902063	0.03355934	0.03311461	0.05700445	1.678895	
0.343777	0.3165409	0.01589746	0.04006819	0.4162733	
0.2388555	0.2896904	0.1567691	0.1972661	2.111491	
1.19129	0.4050802	0.3879229	0.4178602	0.3729643	0.02145999
0.4464609	2.756894	1.180898	0.05649024	1.095658	
0.01833896	0.151326	0.4271959	0.03918035	0.1389935	
0.3277648	0.673822585	0.07056671	0.2392832	0.1768126	
0.1906306	0.1271245	0.4259915	0.06473787	0.5964114	
0.04046045	0.2143659	0.546514	0.2583451	0.3555775	
0.09661796	0.08222193	0.11511003	0.1569556	0.143961	
0.2294452	1.412935	0.02395743	0.477170215	1.99827	0.03290972
1.386647	0.205916	0.05632243	0.3506762	0.1313249	
0.9834428	0.1018388	1.148693	0.02130898	2.376858	
3.086514	0.2198816	0.05914408	0.9193587	0.3544484	
0.2466866	0.03622716	0.083213	0.5948831	1.335492	
0.2321256	0.1252498	0.09257379	0.1273842	0.529850053	
0.061974729	0.1908113	0.07645883	0.2454415	0.05366388	

0.01736566 0.6677607 0.1124422 0.8614184 0.1623603
0.1466618 0.1844912 0.3991115 0.2597862 0.147938753
0.5687981 0.1635302 0.8376862 0.3062671 0.08443269
0.106673834 0.1051253 0.2504012 0.1050614 0.1607874
0.6180332 0.3216631 1.229954 0.1632241 0.03202168
0.1410034 0.2641721 0.4683041 0.09095392 0.1399448
0.2413884 0.04537906 2.1188 0.04944763 0.191271818 0.2927704
0.210157 0.148138 0.06714917 0.3708816 0.2477087
0.111341 0.1224314 0.05473819 0.5101772 0.3805709
3.071997 0.02829038 0.1789939 0.3445791 0.01220381
0.005926194 0.1467515 0.2593055 0.01332575 0.1843682
0.2292975 0.1422771 0.2342983 0.07676946 0.03863609
0.130816986 0.1230936 0.2069898 0.6982284 0.171514
0.09807574 0.6849591 0.501958592 0.02261714 0.3852736
0.01010942 0.06021418 0.07626264 1.278383 0.07811668
0.2301344 0.07854853 1.014065 0.04203545 0.1102081
0.3322546 0.06616908 0.104624746 0.7110149 1.421125
0.2350814 0.174481151 0.0791925 1.734899 0.4065458
0.2304797 0.073168 0.5471836 1.538313
ASAP1-IT2 0.07868774 0.1231255 0.14476 0.03416695 0.04691463
0.22458 0.2652591 0.1200259 0.06626197 0.0268874 0.2026318
0.1124314 0.09942061 0.02682801 0.1399461 0.2755797
0.05707741 0.059246405 0.1236559 0.06111294 0.008359032
0.1638568 0.1183071 0.3594514 0.107787 0.22491 0.02264645
0.1527222 0.055294512 0.01623181 0 0.1346564 0.186436
0.3154191 0.08153188 0.07840659 0.007443514 0.1477702
0.3873409 0.196250814 0.4043726 0.1198147 0.134019044
0.05518445 0.5303643 0.2158115 0.4910943 0.06151162
0.152960005 0.1199826 0.04128911 1.160849 0.2302294
0.1936081 0.4742133 0.05276267 0.2100627 0.454653
0.1291109 0.087280868 0.300494865 0.087799847 0.470218
0.09207879 0.09844136 0.3974796 0.1561708 0.2602762
0.043122999 0.1549103 0.1099719 0.1140631 0.9128602
0.1467441 0.6735466 0.06468602 0.7720031 0.5240632
0.3086065 0.042044797 0.1368188 0.320373 0.2451965
0.5001812 0.1808389 0.09696699 0.3753821 0.2475133
0.36758809 0.6293569 0.117188857 0.2635865 0.3238739
0.4106269 0.09880554 0.2777033 0.4645768 0.1506518
0.3181648 0.1957717 1.155362 0.2437376 0.1153761
0.4032917 0.5535996 0.6906987 0.06191689 0.07123958
0.01897736 0.1430508 0.5371813 0.2757014 0 0.9778617
0.228011 0.09655081 0.2000983 0.2040215 0.137398
0.217029 0.2590963 0.5540983 0.3296021 0.2921757
0.5363437 0.5550814 0.1897443 0.04642076 0.2451638

0.05296706	0.08242917	0.5212236	0.4076041	0.07928921	
0.04797552	0.3647055	0.1973207	0.1463416	0.558956	
0.7978308	0.5409023	0.3540176	0.3450209	0.6477236	
0.6211642	0.1851219	0.5664502	0.7400151	0.3278239	
0.4891361	0.09916583	0.1686614	0.0518571	0.3057016	
0.2613795	0.112308	0.3323763	0.9486646	0.1607665	
1.244533	0.064693085	0.395159	0.124361537	0.2472877	
0.02643768	0.06814317	0.09128996	0.1270294	0.4871073	
0.4403372	0.1889695	0.041176153	0.3816088	0.1383061	
0.1708541	0.1246821	0.3113988	0.4223425	0.2752689	
0.6042694	0.3165636	0.296213	0.05607739	0.4065357	
0.3663139	0.6363788	0.1504642	0.3000395	0.2418798	
0.1298773	0.2392524	0.5276751	0.09425518	0.06561652	
0.1722092	0.4006569	0.301622	0.6395427	0.276987	
0.4761765	0.440274	0.3927286	0.0420778	0.07007576	
0.2842296	0.2551317	0.07757677	0.3295716	0.3098221	
0.5370331	0.4391964	0.3725561	0.3452739	0.3313665	
0.3981653	0.1189154	0.06889847	0.2153454	0.07494168	
0.12607741	0.1802354	0.71642918	0.060068385	0.1537322	
0.6986301	0.04637745	0.2289669	0.09995175	0.02618302	
0.07148842	0.1775521	0.623884	0.1586145	0.1241652	
0.3343217	0.5907796	0.3297361	0.1091966	0.1373158	
0.4528462	0.3803923	0.3082294	0.1328093	0.1240991	
0.1044721	0.1648791	0.2501188	0.4438841	0.1563321	
0.3417442	0.3986879	0.2663889	0.9858317	0.57061	0.2350606
0.06118176	0.3255427	0.088980412	0.261927	0.4005881	
0.5374288	0.08015401	0.5207455	0.05911701	0.3546186	
0.1414652	0.3345139	0.05447529	0.3266905	0.2958507	
0.1695602	0.08748829	0.7388206	0.058841207	0.2838222	
1.106715	0.5363851	0.2462002	0.1994061	0.068236174	
0.4517605	0.2267391	0.1148933	0.1252474	0.08872874	
0.3330007	0.1466304	0.6493337	0.6417355	0.2153469	
0.4675434	0.2446475	0.1944032	0.1890011	0.208271	
0.2061295	0.9944139	0.3217358	0.2145512	0.1770376	
0.3106743	0.426095	0.3355046	1.122101	0.2052536	
0.2452199	0.139356114	0.30751849	0.1255937	0.1346217	
0.05576142	0.07198969	0.06794641	0.08364894	0.2854567	
0.1907058	0.2584571	0.386039	0.2481888	0.1997577	
0.4288199	0.179344886	0.1205805	0.2362495	0.3457422	
0.4108551	0.1321435	0.009391079	0.1850951	0.1894167	
0.07254221	0.179622	0.0777269	0.1618157	0.05837174	
0.07526883	0.4087612	0.7415736	0.5366887	0.278045	
0.3002688	0.2898847	0.1404397	0.4154765	0.36396	0.3482515
0.094422621	3.897827	0.3225862	0.5944452	0.2758704	

0.2934897 0.1158981 0.332414 0.1317349 0.3144967
0.7984654 0.4329713 0.3578816 0.4815076 0.7337966
0.03568844 0.4154222 0.3234634 0.5435031 0.1098494
0.1485975 0.4527577 0.43131 0.257101 0.4812863 0.1569701
0.1428565 0.333093551 0.02627055 0.09777896 0.3131746
0.5862087 0.1911846 0.7488085 0.058486964 0.3356443
0.8191452 0.2563163 0.7339822 0.04131579 0.1274997
0.2116011 0.0281878 0.1251296 0.4958006 0.4789025
2.566394 0.3033353 0.3022819 0.835701328 0.7364061
0.4814336 0 0.062483498 0.2069035 0.6175173 0.1590686
0.1579654 0.16951 0.04817155 0.2044167
ZNF790-AS1 0.1543545 1.033186 0.1774765 0.4146996 0.3163467
1.338489 0.2682975 0.6937184 0.4021257 1.01529392
0.5299788 0.3980172 0.953452 0.3527589 0.3145535
1.215004 0.2886559 0.966064033 0.3097028 0.1648345
0.755293465 1.124107 0.5891064 1.939033 0.7928845
0.5383838 0.6719047 1.495131 0.260996703 1.44476 0.3816724
1.253893 0.1862434 0.273456 0.1570776 0.2749229
0.9837597 0.1299676 0.6641562 0.247589784 0.05453392
0.1360697 0.080328369 0.1550459 0.4867689 0.06388784
0.4714625 1.161369 0.284866607 0.07705199 0.1113655
0.919292 3.218733 0.2320898 1.222625 0.07115603
0.2124688 1.582316 0.01934663 0.156943288 0.514775821
0.171032857 0.4900162 0.1580447 0 0.980567 0.3554091
0.5752663 0.164775099 0.1482607 0.2471812 0.2027708
0.5535126 1.754113 0.1951824 0.2268134 0.4344274
0.1334981 0.7958692 0.085052773 0.1341924 1.001107
0.3720075 0.5455896 0.3963055 0.2708812 0.4665371
0.1430562 0.56182886 0.9358059 0.167338107 0.3877901
0.2225097 0.4397615 0.1776663 0.4040789 0.4395067
0.1523774 0.05667078 0.3031327 1.558127 0.08428355
0.08891246 0.06943167 0.9257685 0.6986101 0.06958455
0.4803705 0.102371892 0.1800578 0.9990339 0.2943515
0.06772052 2.254637 0.6149939 0.7889132 0.1619122
0.07642902 0.1639155 0.1513896 0.1098173 0.6642311
1.102368 0.4656716 0.1576458 1.431671 0.09706181
0.05216942 1.376133 0.1250054 0.1181122 0.2526137
0.3337447 0.1652553 0.2372335 0.4057712 0.412926
0.04154886 0.6051724 0.1645585 0.1774372 0.4672721
0.1500958 0.3148748 8.941596 2.223502 0.05009298
1.10521 0.01700404 0.348673 0.2853026 0.1728678 0.06119292
0.4985602 0.9164943 1.211673 0.1810218 1.667756
0.1355066 0.4518728 0.610718192 1.300309 0.394622736
0.3861504 0.2020394 0.3369603 1.431202 1.262303

0.1707981	0.09501459	1.576855	0.277651945	0.4528829	
0.1276192	0.1703066	0.2216484	0.3266311	0.3473009	
0.05568437	0.1562862	0.3784059	0.1071761	0.3133089	
0.2426708	0.1323249	0.8820636	1.014584	0.1827384	
0.3500687	0.06672507	0.3474769	0.08132863	0.1075572	
0.1622331	0.09953241	0.3123772	0.1972214	0.5654106	
0.1376223	0.2703892	0.7793047	0.577066	0.04728863	
0.05512766	0.0547591	0.1795159	0.1743675	0.4333506	
0.09011972	0.1901145	0.3208306	0.5947144	0.4387745	
0.3724023	0.7221293	0.07596474	0.09291686	2.713096	
0.4851205	0.130021946	0.4321182	0.274598636	0.05063035	
0.1243945	1.001062	0.1719984	0.3884727	0.3819206	
0.01177019	0.09640968	0.1414919	0.3365494	0.1132456	
0.0380568	0.1849716	0.4861395	0.555855	0.1390818	
0.3159035	0.2758049	0.2312109	0.5413505	0.332628	
0.1195433	0.4025475	0.3798597	0.5139987	1.911737	
0.6465464	0.185727	0.5376727	0.06531883	2.048146	
0.9269312	0.1998504	0.4950604	0.3272759	0.289998744	
0.09963074	0.2701177	0.2032918	0.09458413	1.638654	
0.159451	0.1821869	0.2494825	0.1002505	0.08396078	
0.3284299	1.605919	0.4221597	0.06554844	0.1615747	
0.088170644	0.1611639	0.1829543	0.3204812	0.9111436	
0.2068617	0.184047371	1.208167	1.630836	0.2434862	
0.2933688	0.08446593	0.1871194	0.06845076	0.2432486	
0.313937	0.3532111	2.490989	0.7998375	0.5720143	
0.2384436	0.5026193	0.6213837	0.7676482	0.04338947	
0.07820133	0.1481921	0.15562	0.372448	0.1809853	0.41112
0.1628271	0.1048577	0.240140997	0.120402915	0.516598	
0.4473951	0.07520016	0.0970857	0.1191228	0.5640471	
0.1614384	1.117156	0.295745	0.2040246	0.2559537	
0.2693944	0.8353352	0.368556973	0.1355129	0.3783462	
0.5952381	0.6233416	0.1871199	1.671761726	0.3284478	
0.1057028	0.2739263	0.3686249	0.3907037	0.2380643	
1.023366	0.1476479	0.02851333	0.1304466	0.3763663	
1.118454	2.051717	1.785293	0.1794294	0.02155053	
1.227096	0.8453772	0.067914066	0.1040917	0.07677203	
0.3272131	0.2125945	0.9993997	0.6005244	0.1954108	
0.5168243	0.6430389	0.5204605	0.1668307	1.425985	
0.04478371	0.8500429	0.6738148	0.1159119	1.013167	
0.636844	0.2036973	0.1160207	0.2995355	0.08116285	
0.2133709	0.4842234	0.5292268	0.1284381	0.200711664	
0.2834289	0.095902	0.9345596	0.2874779	0.07485462	
0.3884028	0.245391522	0.1764574	0.7249619	0.1152232	
0.1466448	0.1392968	0.2645337	0.3995131	0.05702134	

0.4796065 1.392999 0.1056846 2.009099 0.3973916
0.1783509 0.412033011 0.1727168 0.7688653 0.4651677
0.070221329 0.1698451 1.365771 0.07150695 0.07345967
0.01828817 0.1689075 0.6355897
MAGI2-AS3 0.187466 3.206367 0.5690469 0.5527569 0.6491581
5.082999 0.3552989 1.998112 1.111354 1.453089906
1.185422 0.4468749 1.264131 0.3105212 0.4604738
4.515581 0.3864141 3.079869308 0.5107776 0.4537734
1.004757768 4.389945 1.442088 4.521574 3.551437
1.123807 0.5143519 4.825497 0.562723325 0.5756783
0.7981843 2.091553 0.2955623 0.9289426 1.715434
0.1561617 2.360324 0.3648037 0.9097077 0.237796355
1.354419 0.112376 0.589263002 0.1004298 1.118352
0.2666894 0.5089767 0.9900375 1.309456062 0.3952856
0.4486864 2.245076 0.2989933 0.6915377 3.426077
0.1551722 0.7266598 3.173448 0.1766959 0.230426498
2.739899078 0.640849507 2.351279 0.6800438 0.2063839
4.064988 1.091382 1.975539 0.391768274 0.3413597
0.3170171 0.6177658 1.938552 2.064993 1.348887
0.5650637 1.595229 0.8280994 0.2601237 0.184865097
0.2499009 2.346739 0.5274454 2.108842 1.425693
0.9075574 1.977774 0.6517333 0.69358892 2.530369
0.620242593 0.3823638 1.209615 0.7184599 0.4243645
0.5783791 1.324097 0.2539911 0.1195633 1.435268
1.794769 0.227111 0.1871751 0.626635 2.378003
2.349328 0.3209186 1.289742 0.467489502 0.4581932
0.4965008 0.62618 0.5000658 2.74587 1.496053 0.4038484
0.2838679 0.3633761 0.7967736 0.2052751 0.2431465
1.169743 1.134418 2.06504 0.4709249 1.121309 0.523544
0.4122663 3.219382 1.457483 0.4734386 0.3614055
0.6968639 1.018795 0.1927811 2.158433 0.7833884
0.3014249 0.8898986 0.2804174 0.6487357 0.3855774
0.4627832 0.2513284 0.7808966 1.098637 0.1703484
2.342139 0.1288665 1.891037 0.3511247 1.422656
0.4982965 1.217229 3.213026 0.4826836 1.300977
3.38585 0.5371727 1.095523 1.26964841 2.420476
2.278796087 0.3569977 0.6928379 0.8512269 0.6957856
1.305898 0.902085 0.6262197 3.63266 0.589897531 0.8000489
0.1551545 0.1531428 0.8960806 1.489471 1.097811
0.2344492 1.123688 0.6058601 0.407699 0.60883 0.9012116
1.260544 0.6902521 2.921813 0.5563263 0.8482669
0.3587312 1.441554 0.4174887 0.5155529 0.3496471
0.3180585 1.220601 2.006451 0.5040458 0.1693731
0.7823027 0.3422946 1.766541 0.4692643 0.2295546

0.2577469	0.2500019	0.784592	1.684196	0.4181724
0.6239354	1.130196	1.559483	1.017365	0.6450927
0.3430192	0.7785411	0.4801517	0.2742513	2.16166	0.51050681
0.5364771	1.35839254	0.297782237	0.3984012	2.068438
0.2258597	0.3819616	0.2997736	0.07929008	0.4109117
0.5766914	2.219211	0.09617517	0.2169287	0.2980369
1.011156	1.06755	0.3200814	1.679328	0.4045156	1.296403
1.506566	1.456821	1.093357	2.86952	1.153439	1.146557
3.962598	1.915533	0.5239872	1.038079	0.342709
1.061336	2.816273	0.1830176	0.5970595	0.9162119
0.770809565	0.4939861	2.100805	0.6606933	0.2905764
0.9704457	1.109003	0.8408219	0.9619969	0.1991385
0.5411299	1.305484	0.5130033	1.11545	0.4228864	0.4918961
0.255860969	0.1922564	0.4765235	1.2313	3.68116	0.4703154
1.54681722	3.483487	0.3521202	1.405108	1.018086
0.5899791	1.127207	0.3093837	0.5273086	1.024445
3.097303	0.345898	3.936176	3.702089	0.7658585
0.4979269	2.448872	1.652268	0.9921127	0.3596454
0.4095583	1.468602	2.11473	0.619197	3.384839	0.3037535
0.3782661	0.626255387	1.223576714	0.8689205	0.3637157
0.5543232	0.3128602	0.4000492	1.156152	0.3893238
2.487408	1.317704	1.099167	0.7078042	2.265982
0.6809304	0.869871734	0.3265319	0.4514461	3.006088
0.3768469	0.6568178	0.39869311	0.3999689	0.5762734
0.1761662	2.374776	1.260438	0.7761613	2.029423
0.5558945	0.07387711	0.5745708	0.9839044	0.3995052
0.5245987	0.5874897	0.8458522	0.3503755	3.852645
1.012426	0.305735978	0.3534973	0.4110886	0.6930753
0.4521358	1.320344	0.5786816	1.618681	0.1820305
0.1985132	1.404296	0.7348302	3.029639	0.7049014
0.8941224	1.695951	0.3628914	0.2698001	0.2101469
0.8852195	0.08744902	0.6223619	0.467896	0.589693
0.5712467	0.8455793	0.3185169	0.926161931	0.4819205
0.7889208	0.6554116	0.4810464	0.3932792	1.244087
1.323829587	0.9352631	0.7066185	0.2848565	0.4122479
0.2887307	1.567848	0.5951972	0.1317352	0.2554326
2.867779	0.415302	0.2405908	1.294607	0.4411967
1.020465819	1.122115	1.913528	0.4845043	0.565837125
0.1996001	1.083178	0.5502584	0.2360112	0.234551
0.6581341	2.345191
AL161725.1	0.02894147	0.006918657	0.1131413	0.05529328	0.1613368
0.06594747	0.02235812	0.08237906	0.03909555	0.054390746
0.1148977	0.0568596	0.1508391	0.04748678	0.07077454
0.01608094	0.07938038	0.038134107	0.03126807	0.0463597

0.021136944	0.007205811	0.03681915	0.1038768	0.02973317
0.2075811	0.1145292	0.07723575	0.048936882	0.008208864
0.05111684	0.03567111	0.08031749	0.250668	0.06773973
0.03172187	0.02258632	0.03899029	0.03637934	0.083241393
0.07011504	0.0669718	0.075307846	0.0651193	0.1601867
0.06122584	0.4335771	0.09332428	0.202599096	0.08957293
0.02088102	1.153666	0.3143698	0.03263763	0.2750905
0.03113076	1.32793	0.4932377	0.1160798	0.053949255
0.209469949	0.113473723	0.1405193	0.07196678	0.04978446
0.0563826	0.1085972	0.1937868	0.039982193	0.1263586
0.1390394	0.05506277	0.2147247	0.1113187	0.2026894
0.1112258	0.2162774	0.09128912	0.167023	0.088596638
0.02516108	0.1146004	0.2673804	0.1227577	0.03429567
0.07706103	0.1972856	0.1788203	0.57009105	0.1917858
0.080182843	0.4322244	0.08653153	0.4916778	0.09577322
0.2402299	0.2910563	0.1028547	0.139653	0.1613448
1.100654	0.07901583	0.0583488	0.07811062	0.08585756
0.6112838	0.1043768	0.05854515	0.051185946	0.07716763
0.03286296	0.1394296	0.07618558	0.6620336	0.2946846
0.04882836	0.0890517	0.02006262	0.1336267	0.0189237
0.07487545	0.2490867	0.1166821	0.03358209	0.04520726
1.368509	0.1191213	0.02347624	0.1038801	0.04687704
0.08076791	0.08649273	0.0515341	0.1057148	0.04852504
0.382716	0.1204367	0.07790412	0.1074977	0.1044313
0.1897592	0.2437941	0.0928718	0.04189867	0.1297533
0.0146283	0.07513947	0.2721787	0.09245948	0.1660909
0.1671695	0.08870849	0.04917288	0.3199894	0.317248
0.06815661	0.06141813	0.2827189	0.08130397	0.09682989
0.049075569	0.4836192	0.166481467	0.1513885	0.008913502
0.1780529	0.05193878	0.04395518	0.6010778	0.04453809
0.1194587	0.045812571	0.3512416	0.07362646	0.02254057
0.02292914	0.1224866	0.005209513	0.1183293	0.08372477
0.0400237	0.03288357	0.04456549	0.2628934	0.08601119
0.3933527	0.1324596	0.03426344	0.1551441	0.0500438
0.06515192	0.06099647	0.05500085	0.05530675	0.09953241
0.1298054	0.184895	0.1689044	0.07372621	0.00422483
0.02504908	0.08300264	0.1702391	0.1476634	0.02053466
0.1879307	0.03487349	0.1583396	0.058373	0.07129295
0.1388209	0.2076374	0.2316998	0.03990024	0.1249839
0.1171123	0.02710075	0.07451462	0.1061201	0.157526588
0.1012777	0.255529287	0.053161867	0.07774656	1.419574
0.05277225	0.07097098	0.05054831	0.02206911	0.03253827
0.03673347	0.2566189	0.1226828	0.03995964	0.09104069
0.111407	0.1823899	0.06135963	0.204248	0.09973304

0.08128507 0.2646334 0.1775082 0.107589 0.1018948
0.1285501 0.2228666 0.1484492 0.2002888 0.1470339
0.2439039 0.08573097 0.1347464 0.2361051 0.1860676
0.09282382 0.0658543 0.048749789 0.1392566 0.08033672
0.09612168 0 0.2864565 0.07474268 0.3202504 0.1761053
0.03759395 0.05509926 0.05106684 0.258094 0.1418193
0.03932906 0.07573816 0.062821584 0.09065469 0.3719268
0.1201805 0.104241 0.1318743 0.030195272 0.5343816
0.1630836 0.03320266 0.08334342 0.06598901 0.1754245
0.06274653 0.04864973 0.232271 0.0794725 0.05546343
0.04124162 0.04170937 0.1079163 0.07761033 0.08584907
0.322551 0.1464394 0.117302 0.05557203 0.1840506
0.1556301 0.1758437 0.5149934 0.1038023 0.06654429
0.093968216 0.406359838 0.1714936 0.06078738 0.01974004
0.04095803 0.06185221 0.0658055 0.2328439 0.1265842
0.1822001 0.04221198 0.1033659 0.274205 0.09638483
0.090699568 0.03726605 0.1120104 0.01488095 0.09090398
0.04009712 0.109234431 0.1133145 0.1519477 0.04769253
0.04739463 0.1643814 0.1301914 0.0531363 0.09343347
0.05346249 0.05870095 0.3257016 0.0945514 0.06580344
0.1075085 0.01869056 0.1333439 0.2815103 0.1514634
0.10505457 0.03903439 0.04798252 0.1533811 0.1793766
0.1150299 0.03393374 0.04741586 0.1877526 0.03078378
0.7032947 0.1720442 0.2138978 0.07277354 0.4948011
0.09625925 0.01086674 0.1319227 0.2207926 0.07638649
0.05932879 0.1684887 0.2561702 0.04334097 0.1390854
0.03087156 0.09632854 0.139781338 0.122893 0.04495406
0.104464 0.2515432 0.1029251 0.1238034 0.085448833
0.1524823 0.1769252 0.01800362 0.1154828 0.04701266
0.03968005 0.01070124 0.04989368 0.1731912 0.2537248
0.1100882 0.683769 0.1709368 0.1719812 0.236313051
0.2461214 0.2146416 0.02093255 0.031599598 0.08643901 1.016
0.06971927 0.09090634 0.03771934 0.04385099 0.06317608
AC079793.1 0.01563899 0 0.1870094 0.1045751 0.06153948
0.04751436 0.1449788 0.03748618 0.04828779 0.156751514
0.0671209 0.03072499 0.1932049 0.01466299 0.4079376
0.1390335 0.1247839 0.035325213 0.01930995 0.06680322
0.054824091 0.0467253 0 0.1309734 0.02142239 0.02458515
0.02475508 0.02473219 0.030221516 0.03548633 0 0.04205553
0.03019189 0.04925543 0 0 0 0.1264141 0.07258397
0.055361006 0.01262926 0 0.162775193 0.02010757 0.08052041
0.05753802 0.7278922 0.05042927 0.127392186 0.1249088
0.01805343 2.021445 0.1174443 0.1646051 0.2439378
0.01922515 0.1148109 0.7534952 0.03136278 0.106008518

0.213064348 0.063983311 0.4672731 0.01830042 0.06456439
0.2331406 0.021339 0.4583785 0.01571274 0.1201726
0.06678415 0.07934412 0.2630012 0.05833002 0.1947137
0.05656719 0.3521247 0.02546041 0.08285572 0.015319875
0.05438476 0.05124925 0.1507652 0.09648592 0 0.04542672
0.1770069 0.2512338 0.48217744 0.1234794 0.015070637
0.3492476 0.04007888 0.6204839 0.0540027 0.5272366
0.3335029 0.06587163 0.02624825 0.2694817 2.271331
0.04098957 0.1441358 0.3189073 0.2420583 0.7314162
0.02256068 0.1168091 0.013829572 0.1250963 0.04735473
0.1130146 0 2.465392 0.4430957 0.02483312 0 0
0.03465953 0.03272231 0.06473624 0.1514227 0.01441166
0.05806918 0.0300658 1.031501 0.1001297 0 0.1593461
0.01447472 0.07884119 0.0356097 0.06365092 0.01575853 0
0.1395322 0.08925246 0 0.1549016 0.06156124 0.191762
0.1646726 0.145992 0.0987954 0.2066526 0.02529484
0.04060281 0.24067 0.02756522 0.1680423 0.05781293 0.02949849
0 0.3574797 0.3085741 0 0.1117921 0.7036756 0
0.366265 0.047144477 0.6738461 0.063972211 0.05690797 0
0.04965869 0.2494751 0.04384964 0.5750592 0.01925349
0.1161926 0.05401212 0.3003413 0 0.01624021 0.03717038
0.07564299 0 0.3159444 0.05429042 0.04718716 0.01579481
0.08756973 0.3059725 0.2145116 0.2705238 0.07309958 0
0.03869289 0.06760492 0.04023529 0.09888126 0.01585098
0.04781742 0.05378393 0.1916086 0.2897418 0.3417778
0.1912279 0 0 0.07688881 0.0306638 0.01276676 0
0.1576319 0.07537779 0.1801292 0.01328117 0.05870367 0
0.199467 0.3048414 0.03449717 0.2401318 0.04104876
0.01673637 0.02477856 0.1146874 0.016213716 0.04378162
0.148383763 0.082076756 0.05601546 1.609183 0.06336961
0.08073748 0.1456778 0 0 0 0.1818598 0.04079606
0.03701628 0.1499285 0.06567332 0.292856 0.07957598
0.08829493 0 0.08199088 0.3134222 0.02073933 0.07751659
0.04894267 0.2553275 0.3124658 0.4695625 0.1139255
0.4237446 0.1546423 0.07059215 0.2912495 0.2268145
0.178746 0 0.05176062 0.064843637 0.2202425 0.07549779
0.2579134 0 0.2197038 0.03231072 0.2215071 0.07137098
0.09480095 0.01701358 0.1298578 0.2263784 0.08554529
0.02125209 0.1309642 0.014293325 0.06531561 0.1248787
0.1632813 0.2253132 0.2515073 0.037294852 0.5356738
0.165234 0 0.201761 0.03423189 0.3640067 0.06164743 0
0.3026027 0.1017937 0.1766685 0.01620769 0.07727426
0.03998694 0.09585824 0.05301705 0.4348019 0.2110157
0.0380316 0.1334633 0.2716811 0.1897578 0.09335294

0.2694223	0.06598969	0	0.050777228	0.108436066	0.05491514
0.02102236	0.01219068	0	0.02970918	0.2438335	0.1610508
0.03908673	0.2910835	0.02280992	0.1915043	0.2495513
0.1249994	0.056012527	0.02929063	0.09684257	0.09649393
0.2245551	0.01444475	0	0.2129784	0.1427955	0.01585933
0.08536815	0.03089604	0.08040116	0.05104542	0	0.0462229
0.12688	0.5475496	0.08384387	0.1312909	0.1690006	0
0.01746776	0.07020859	0.1218168	0.027523838	0.0602653	0
0.02652222	0.1148788	0.06416336	0.01333574	0.03726824
0.1832737	0	0.7709832	0.2929864	0.2133842	0.06049899
0.8226883	0.05201523	0	0	0.1753109	0.01500969	0
0.2988162	0.04385764	0.0576492	0	0.2097159	0.01487219
0.046481896	0	0.05829995	0.05826972	0.2330149	0.06741482
0.04722292	0.02841455	0.1741203	0.2425087	0.01556567
0.09509024	0.02258137	0.0428835	0.0462607	0	0.04319384
0.2580786	0.2284335	0.6294938	0.151579	0.2065173
0.550073071	0.05599816	0.2077343	0.0301633	0	0.05900046
1.062023	0.06955186	0.08335973	0.07411727	0.02106274
0.07448336

LINC00501	0.3053424	0	0.4049691	0	0.1117696	0.09348825	0
0	0	0.017793519	0.01828602	0.01395089	0.03289724	0	0
0.01578227	0.04249422	0.021386199	0	0.07583112	0.008297748	0
0.03613531	0.2208862	0.00486349	0.005581527	0.01124021
0.04491926	0.109778253	0.06445122	0	0.0190956	0.1028162
0.6038485	0.4624808	0	0	0.2614852	0.2691508
0.301644425	0.7340032	5.928035	0.059127333	0.1004298
0.1901163	0.5590862	1.090664	0.2671404	0.021691228
0.2211912	0.4098641	0.09379495	0.9751106	0.5552119
0.06922598	0.05237584	0.02085227	0.05823481	0.3204108
0.279176489	0.314415706	0.174312305	0.1626625	0.04985658
0.830617	0.04330598	0.2712952	0.1363609	0.064210272
0.7936751	0.01819428	0.03602673	0.6251854	0.01324257
0.06078259	0	0.05513261	0.3236931	0.03762119	0.11129747
0.06173442	0.03878344	0.06845593	0.4234974	0.02243913
0.1375087	0.2411135	0.008774953	0.63248148	0.09611428
0.102643854	0.1902939	0.006066028	0.299718	0	0.09430724
0.08948091	0.1196378	0.005959098	0.223127	0.1867032
0.1240773	0.5399281	0.2129441	0.06594491	0.112487
0.09219443	0.008839662	0.163264655	0.1514688	1.234199
0.3991168	0	0.1174225	0.3269347	0.06201604	0.02383575
1.648334	0.01573739	0.341729	1.146363	2.650868
0.2421174	0.06591675	0.02047737	0.04132589	0.2208279
0.02304022	0.02630988	0.09858514	0.3937821	0.2667856
0.1589562	0.007155265	0.09524759	0.6516571	0.04052568

1.093338 0.39856 1.779629 0.8126611 0.9570647 0.1657217
0.2093407 0.2412823 0.09762497 0.1935778 0.1092778
0.1314198 0.7491343 0.603758 0.1339399 0.8879767
0.09880107 0.1037853 0 0.05075993 0.05044873 0.05984544
0.3801259 0.010703133 0.1176786 0.341302376 0.9560594
0.06123567 0.03382179 0.1246034 0.1725552 0.174073
0.008742178 0 0.01634971 0.1060671 0.2745842 0.331829
0.1462713 0.226685 0.06135309 0.05465016 0.3122451
1.299822 0.136263 0.1431418 0.2117011 0.09090708
0.2544401 0.05531893 0.009607736 0.4743567 0.1657609
0.286216 0.1870737 0.1079586 0.04342364 0.06105238
0.3915052 0.04990178 0.4373438 0.1157711 0.3897582
0.07102 0.1861967 0.80754 0.6376518 0.04030655 0.04404544
0.4534917 0.5888797 0.1990034 0.4597952 0.2634024
0.2717079 0.4613842 0.01566367 0.4429483 0.006212822
0.1215882 1.558246 0 1.14110169 0.3512016 0.262012493
0.089442002 0.04578155 0.1114567 0.1093389 2.764118
0.06614598 0.1039643 0.06386788 0.0480683 0.1156045
0.3519507 0.3081373 0.4850418 0.3081339 0.07160097
0.283034 0.04810908 0.1667597 0.5531093 0.1565426
0.3390059 0.1407877 0.03703791 0.8592693 0.2482851
0.01421387 0.1655317 0.2075939 0.1723493 0.5368851
3.398666 0.01287333 0.06594316 0 0.1586402 0.161934783
0.2133389 0.8364388 0.07807153 0.02983739 0.199516
0.146709 0.4442141 0.5401079 1.069976 0.3012801
0.0294814 0.1248147 0.2718969 0.2122924 0.4162563
0.05191984 0.3756552 0.3898262 0.1078385 0.01705083
0.01522648 0.254009659 0.08360903 0.2250768 0.08146498
0.01963092 0.04662965 0.02754661 0.19594 0.5132713 0.4059509
0.04622013 0.3495192 0.02943684 0.05263035 0.5083774 0
0.03610912 0.3540029 0.0958131 0.06331785 0.4605592
0.2202833 0.1096592 1.774221 0.1416488 1.07867 1.7752
0.061481974 3.092028458 0.07480376 0.009545342 0.02767631
0.08932739 0.01348965 0.03690474 0.01828154 0.1538125
0.862982 0.1087485 0.007246153 0.0212457 0.463514
0.194985283 0.2061439 0.04397202 0.25558 0.5352938 0.2754668
0.009322229 0.2997833 0 0.5616809 0.02713339 0.007014278
0.4526829 0 0.4618869 1.60207 0.4800892 0.3196524
0.09041594 0.1390981 0.01918396 0.1907717 0.8089982
0.02125245 0 1.505934655 0 0.103601 0.03612778 0.2868882
0.3131884 0.04844145 0.4484302 0.2972024 0.3021204
0.2840191 0.4297971 0.09688851 0.3571096 0.7284169 0
0.01421988 0.6007527 0.2122696 0.1771966 0.04658151
0.4409584 0.0696985 0.5300641 0.265421 0.1991024

0.06752818 0.379897486 0.04563641 0.008823828 0.9987803
0.7758813 0.1591728 0.05717836 0.012901816 0.5251876
0.8809014 0.03533849 0.4425577 0.07177255 0.08762192
0.04200996 0.09093873 0.01307497 0.09960497 0.0432174
3.690882 0.421556 0.1750384 0.660091526 1.277673
0.1949345 0.01369585 0.754642468 0.6518792 0.0326928
0.1157951 0.03244289 0.07403757 0.267783 0.0958224
FAM66D 0.04747914 0.8474827 0.04367314 0.04535495 0.1660717
0.2564464 0 0.1327738 0.3420648 0.575033026 0.5977416
0.2176521 0.21996 0.3709673 0.1032064 0.4572732 0.4104071
1.203532817 0.1954133 0.06760369 0.850709037 0.3467581
0.2617448 0.4354978 0.7912866 0.1741582 0.1252585
0.1126285 0.397587415 0.07182309 0.2683467 0.2695433
0.183322 0 0.3865342 0 0.3622997 0.09950027 0.1836343
0.420182798 2.722266 0 0.065890263 0.1933109 0.1629705
0.6637932 0.2209843 0.06804472 0.193378008 0.3918572
0.09134879 0.119455 0.05093638 0.02379679 0.6171519
0.5058434 0.02323733 1.216794 0.3491244 0.085823018
0.305457993 0.151083323 0.2994857 0.129638 0.2831315
0.4611442 0.1727576 0.3678977 0.015901019 0.01105569
0.02703376 0 0.8454259 0.05902897 0.1970468 0 0.1105895
0.103062 0.04791345 0.04651034 0 0.3457556 0.01695241
0.5858524 0.1000228 0.04597105 0.276834 0.1369007
0.59638965 0.2677694 0.076256109 0.05301488 0.09463797
0.1335998 0.0364332 0.04850493 0.1687496 0.01666524
0.01328139 0.1283345 0.3566712 0.1106153 0.07293146
0.07593616 0.2122976 0.3581518 0.04566202 0.630447
0.055981144 0.04219842 0.009584433 0.01270764 0.1944198
0.5059656 0.2802532 0.1005227 0 0.2507671 0.3273652
0.04967161 0.2129138 0.1702635 0.4812836 0.3232075
0.03042607 0.1228072 0.1447565 0.01711702 0.9968521
0.454093 0.1367758 0.05405459 0.3220681 0.03189471
0.03538064 0.1613762 0.04516097 0 0.08708764 0 0.1724976
0.09998746 0.1969885 0.3166009 0.1493777 0.01279897
0.06163401 0.3653307 0.3207985 0.3864906 0.05850568
0.2089637 0.1577532 0.2516614 0.65924 0 0.155556
1.592872 0.133381 0.1059011 0.286256333 0.122396
0.161846906 0.01439747 0.1754736 0.2135784 0.1262322
0.07395844 0.1508759 0.0389684 0.4050146 0.036439549
0.8949349 0.09662871 0.1643481 0.07523156 0.627705
0.0683706 0.182703 0.2014502 0.1114227 0.2717292
0.2481333 0.1032129 0.04341641 0 1.195937 0.02141332
0.1044174 0 0.1900146 0.4336198 0.1443682 0.2177568
0.1088568 0.5817136 0.2022163 0.4244808 0 0.05544745 0

0.2982722 0.09309369 0.2454751 0 0.04908332 0.09535126
0.601549 0 0.326739 0.2429215 0.1682143 0.146902
0.2269185 0.3037615 0.08308126 0.3218014 0.02507548
0.3813454 0.147671983 0.1329187 0.300323563 0.116284345
0.1587227 0.6624265 0.08978052 0.1634099 0.2211351
0.2317112 0.9331575 0.4642413 0.2944624 0.02064245
0.06243305 0.5879345 0.265841 0.02279732 0.5100202
0.732694 0.1777538 0.3081867 0.1268711 0.1888906
0.6667862 0.1485874 0.2279885 0.02635083 1.108774
0.3228139 0.09027836 0.3272174 0.5536447 0.3831613
0.631214 0.02261098 0.03867418 0.1833329 0.114836104
0.4160456 0.1986464 0.05800085 0.2438345 0.4446729
0.04904683 0.07472045 0.04815079 0.01370527 0.08608724
0.06570692 10.35818 1.110986 0.04301349 0.1104446
0.043393788 0 0.04739063 0.1051514 0.06333695 0.06787227 0
0.6606753 0 0.1997223 0.1604261 0.161663 0.06139474
0.06238613 0.4256586 0.1670338 0.6824644 0.02554078
0.2624304 0.1824672 0.1888417 0.1455103 0.3487401
0.09103695 0.1423628 0 0 0.09819163 0.209489 0.02699187
0.1291503 0.01335608 0.03969725 0.171285565 0.087788327
0.2917591 0.09573416 0.2467352 0.1393624 0 0.2467553 0
0.3428108 0.3985378 0.06924972 0.2260989 0.1894062
0.1475801 0.132261969 0.0148208 0.03266766 0.09765018
0.3976802 0.07308915 0.166215961 0.08621218 0.04335196
0.1765431 0.4924292 0.5158933 0.2766395 0.2324569
0.3027748 0.03118451 0.08560022 0.07915866 0.5939398
0.3764482 0.1710257 0.03270645 0.2828331 0.6868154
0.3081912 0.292463268 0.02439497 0.6087403 0 0 0.4220593
0.02699107 0.03771481 0.05299138 0.04489021 0.1324905 0
0.2879214 0.2081613 0.2289502 0.0526385 0.03169268
0.2539355 0.0197124 0.01518955 0.1903347 0.1511984
0.05547896 0.102095 0.06760677 0.1929353 0.5267639
0.156796227 0.04359108 0.216328 0.08845191 0.3537106
0.3547576 0.06371837 0.158152659 0.1132758 0.2831706
0.1890263 0.02405742 0.1142598 0.6292616 0.1872601
0.0155908 0.524537 0.3134053 0.1541138 0.2492753
0.1342209 0.3622526 0.05964261 0.1983421 0.5325663
0.3662967 0.115199696 0.2985372 0.07286435 0.02346176
0.265127 0.1350097 0.1278908 0.351754
UBE2Q1-AS1 0.03363106 0 0.03093514 0.1285057 0.2205639
0.1021779 0 0 0.05192055 0 0.288682 0.1982189
0.4154799 0.09459667 0 0.02491553 0 0.050643708 0.04152532
0 0.157196235 0 0.1711409 0 0.1382042 0.5022599
0.1064698 0 0.064990251 0 0.2851184 0.03014629 0.486949

0 0.3559336 0.4423439 0.1749743 0.3322598 0.3121783
0.089288845 1.140668 0.4150598 0.245029417 0.6269886
0.2077874 0.7176537 0.5478568 0.5060826 0.582148378
0.295473 0 0.6346048 0.5411991 0.1517045 0.327862
0.2894008 0.09875865 0.1723791 0.1686112 0.27356087
0.305457993 0.321052062 0.06699023 0.07870877 0.1388433
0.1595237 0 0.373896 0.168948324 0.5168534 0.3446804
0.1950018 0.432498 0.8466968 0.3140434 0.06082283
0.2350028 0.5748924 0.8399827 0.197668944 0.05847621
0.551048 0.4322865 0.1037447 0 0.3256282 0.5190638 0
1.15211637 0.18967 0.486132695 0.6383875 0.02872938 0.7949186
0.03871027 0.4122919 0.3585928 0.3187226 0.536236
0.1704442 0.6737122 0.9402299 0 0.08068217 0.2776199
0.1268454 0.0485159 0.1255969 0.148699913 0.1345075
0.244403 0.8371158 0 0.2224504 0.3573229 0.4005202
0.5268143 0.7193882 0.4472042 0.7740492 0.4176386
1.411059 0.9607442 0.1873134 0.646554 0.5871721
0.2768468 0 0.09345489 0.2178914 0.3390901 0.3828867
0.6159552 0.4405457 0.07518386 0.4286556 0.3518792
1.376018 0.03701225 0.9708245 0.6185657 0.8144812
0.2877879 0.4957303 0.8253119 0 0.3492594 0.1150115
0.7113357 0.4270721 0.3108114 0.09515313 0.2133253
0.300814 0.3686539 0.316802 0.3906576 0.1194654
0.2834347 0.2250398 0.202764902 0.07431185 0.791026754
0.5201085 0.1242938 0.3737621 0.0536487 0.8172408
1.007636 0.3726353 0.08328929 0.232302127 0.3947001
0.06844534 0.2793918 0.1865116 0.2602679 0.04842917
0.5176584 0.7004973 0.1691237 0.44156 0.414294 0.5326525
0.3075329 0.5817514 0.3405955 0.0455033 0.7488686
0.3779929 0.3172565 1.134083 0.1363478 0 0.05783018
0.441479 0.1933694 1.603594 0.2741523 0 0.07762122
0.7440594 0.8902084 0.4941801 0 0.3129061 0.1215729
0.5035694 0.4569707 0.8521205 0.5592256 0.2859642
0.4370334 0.4080169 0.3872959 0.3530954 0.1439638
0.5594965 0.211398 0.383536957 0.4079866 0.815461897
0.211803629 0.3613775 1.085068 0.272548 0.3819705
0.1566374 0.1230966 0.1680474 0.1138284 0.1173249
0.2631912 0.1857383 0.6448314 0.2353801 0.04844431
0.05704173 0.8354499 0.2747105 0.6297084 0.3370014
0.3567933 0.1666966 0.1403325 0.5167739 0.5599552
0.03365922 0.4409868 0.4796038 0.5441768 0.6831261
1.377907 0.3658172 0.6967007 0.7807351 0.5843738
0.03486096 0.3473238 0.1298842 0.2465036 0 0.3436109
0.0347415 0.2381714 0.6650828 0.9319586 0.03658708

1.172868 0.7650002 0.06132066 0.274211 0.5163285
0.706957121 1.053441 0.5370938 0.1276838 0.1884274
0.03605714 0.040100599 0.107995 0.2605751 0.1157482
0.06198281 0.3189958 0.7827829 0.5833103 1.130656
0.2366312 0.3283555 0.4341933 0.1045621 0.2769591
1.060549 0.1030698 0.3990392 1.031752 0.07563025
0.3544041 0.1148031 0.7094346 0.890328 0.4588618
0.1219753 0.08514503 0.3655455 0.764361833 1.026026074
0.4428487 0.3842663 0.02621561 0 0.0638885 0 1.168876
0.2801819 0.3682143 0.7848301 0 0.5031101 0.492812
0.361358595 0.5354015 0.06941879 0.03458444 0.2414487
0.2485031 0.044151115 0.1374007 0.337784 0.2728393
0.2202973 0.2325428 0.1383198 0.1646569 1.286793
0.4307361 0.121267 1.042915 0.5409094 0.7058403
0.2725721 0.2780048 0.9015306 0.3522889 0.1637266
0.651078941 0.4147145 0.1784239 0.2851753 1.296974
0.4829333 0.2007461 0.2003599 0 0.6677419 0.6569318
0.387727 0.2294374 0.8066252 0.4422902 0.3355705
0.9428572 0.6377243 0.2932219 0.1291112 0.4044612
0.522107 0.7309353 0.2479449 0.6464898 0.4099874
0.06396419 0.932937551 0.09263104 0.3761157 0.06265344
0.083515 0.2609515 0.8801099 0.427731055 0.4279306
0.5215059 0.2343138 0.4600981 0.2913624 0.09221937
0.2984458 0.1656523 0.9288676 0.3607425 0.3684284
0.5885666 0.774167 0.6513581 0.633702732 0.8128482
0.17869 0 0.244799355 0.3383422 0.6580561 0.2492812
0.6146126 0.09563186 0.1811786 0.2135649
AC004585.1 0.1091465 0.03478964 0.0780867 0.1390178 0.2545139
0.02456363 0 0.029069 0.6740137 3.813768916 0.02081981 0
5.674527 0.0227411 3.123847 0.008984565 0.60478
0.200883889 0.05989629 0.02590158 0.09919896 0.09662283
0.1388555 0.05803687 0.008306093 0.02859717 0.9598275 0
0.046871099 0 0.06854265 0.02174156 0.2926572 0.3055646
0.07898462 0.1063398 0.01261918 0.3812239 11.47294
0.364906664 0.1664891 0.9728614 0.151470605 0.2572779
0.2372731 0.1338552 1.368793 0.6126581 0.58037615
0.09686168 0.02799939 3.135096 0.07806277 0.1276445
1.064046 0.1938081 0.9259242 0.3978245 0.4134493
0.361703187 0.137685573 0.826940681 0.6039186 0.1277211
0.03337803 0.1068311 0.1489277 0.5393091 0.219322566
0.4151143 0.2900149 0.1406357 0.4798745 0.327936
0.2925479 1.03084 0.6496904 0.2171785 0.3671498
0.201958921 0.3162987 0.2914391 1.143142 0.1995224
0.2171614 0.1996172 0.112305 1.903257 0.70627234 7.373

0.222046587	0.7583163	0.8391474	2.508174	1.702993	
0.7433644	1.387132	0.08939132	1.139849	0.9833978	
0.7440077	0.01059524	1.648628	0.3200348	0.2002201	
0.6312201	0.2449283	0.3472261	0.289555223	0.3395247	
0.07344331	0.1460636	0.9577252	4.879797	1.073758	
0.0288856	0.1085541	1.74863	0.4748278	0.1141867	0.9161555
0.5088292	0.2123375	0.3152119	5.51395	8.316474	0.4880637
0.2229785	0.3032993	0.2132665	0.1921484	0.5108565	
0.1480761	0.2199617	0.4066706	0.170031	0.3460582	
0.6790014	0.4537862	25.65134	0.9252669	0.4213996	
0.5000146	0.2042981	0.3548407	0.04903778	0.3306011	
0.3836278	0.5450803	0.3080052	0.2129499	0.1258118	
0.1098933	0.9160011	0.5849227	0.03807971	0.3034156	
0.4307934	0.2384826	0.8520707	4.313916383	2.331333	
0.458872456	1.312862	0.3137434	0.539116	0.5029902	
0.4080432	1.668127	0.8808876	0.3604104	0.460726089	
0.2587804	2.159627	0.03778086	0.3843215	0.1525109	
0.1047817	0.1750014	0.1263	0.512284	0.1347305	0.3802778
0.4745383	0.2439727	0.7941677	0.2172953	0.03281707	
0.1100174	0.2096994	0.4576121	0.02555946	0.1597932	
0.5005861	2.544142	0.5094329	0.6740489	0.5662106	
0.9144512	1.982779	0.08397087	0.1589977	0.1070033	
0.1485015	0.1835664	0.3761142	0.1753572	0.1117462	
0.5458473	0.1138057	1.147895	0.7476127	0.2138785	
0.05350229	0.4771692	0.2546531	0.06489185	0.2978288	
0.3811516	0.515496022	0.04526781	0.204560958	0.076376601	
0.1824383	2.421686	0.2162184	0.07513034	0.02824179	
0.059185	0.09695691	0.4651952	0.08461492	0.03163567	
0.06697742	0.4941195	0.1188296	1.589685	0.6479331	
0.4792837	0.04953044	0.2543223	0.559006	0.5307226	
0.8716087	0.7084568	0.8269246	1.171138	0.4490894	
0.2650338	0.9857899	1.002957	0.164224	0.2032669	
1.275169	0.3378623	0.3852569	0.09031104	0.289130634	
0.8425594	0.1756363	0.5555591	0.4586191	0.4956257	
0.3633068	0.3292253	0.3689685	0.04200815	0.2110934	
0.5034971	0.1003127	0.3316844	0.2142416	0.1523361	
0.21059389	0.2532479	0.2784101	0.4143861	0.4173894	
0.1040179	0.390428536	1.5058	0.05125289	0.05565187	0.9163936
0.2566058	0.61159	0.2390253	0.1019289	0.2026575	0.2960133
0.5969251	0.125684	0.4194612	0.2790741	2.843281	
0.1850065	0.8603675	0.5181745	0.07864521	0.1241944	
0.4063068	0.869519	0.7549388	0.417852	0.1432825	
0.1318161	0.525008843	0.840877341	0.57489	0.04890593	0.3686817
1.662876	0.4722842	1.181768	0.5776083	0.07072372	

0.5842252	0.3006987	0.1361285	0.423318	1.970949
0.680488305	1.624029	1.026332	0.4240202	0.3047332
0.1120131	0.095525617	0.3963745	0.4097086	0.1106844
0.5362161	0.6229241	0.5735998	0.1979182	0.06960282
0.5018155	0.09839024	0.6672322	0.1381622	0.4072427
1.261386	0.9774261	0.3386384	0.2540715	0.01180799
0.032015416	0	0.1930195	0.2468028	0.3563353	0.2363413
0.2998983	0.289	0.2233335	0.103195	1.364939	1.013657
0.7170396	0.1125948	0.6220121	1.21007	5.099929	0.3361013
0.6797322	0.4306579	0	0.8110191	0.3230932	0.06705689
0.3367359	0.1034893	1.637654	0.19223898	0.2115514
0.1506974	0.1129645	0.3011559	0.01045549	0.06103241
0.683063951	0.7040495	0.231453	0.7242325	0.5161702
0.4377732	0.3824256	0.4663527	0.08362813	1.105337
0.3602329	0.2066643	0.551818	0.9109607	0.1494693
0.396090656	0.2714017	0.1718288	0	0.088274892	0.2135114
3.657145	0.2516949	0.06464206	0.1494348	0.2613327
0.2310352
AC092718.3	2.434964	1.90151	4.740854	4.05116	5.296492
2.157722	6.207564	2.188701	4.636302	5.415069718
2.542982	4.7041	5.138953	3.59573	6.946975	1.969301
5.666687	1.329185131	5.937889	4.593841	3.959764382
2.465434	4.388472	1.626532	1.11181	3.317485	5.203337
1.829111	2.195871705	5.248904	1.892294	2.037148
10.45934	8.947153	7.846752	23.53072	2.174764
32.12981	13.7498	12.588230631	4.915887	14.47111
5.512281888	8.622512	4.972968	9.04816	11.78176	11.79944
5.743812985	3.662724	18.83296	5.781639	15.02054
12.26518	5.301473	21.75148	11.11283	1.476875
23.0321	5.859398742	2.55714415	10.432526554	2.425121
7.31332	14.10152	4.358702	8.527612	5.824347
13.700104544	45.84114	9.843611	25.61932	9.89597
3.538152	7.31072	15.1561	4.757821	5.863628	11.0115
7.036572674	5.503951	4.167031	9.75905	5.299673	9.575367
7.701563	4.259223	8.801253	11.8172383	8.30818
5.162240377	17.53667	18.18329	13.10379	10.04304
9.162555	9.893447	5.961359	15.01902	10.30435
3.937829	10.81396	16.92478	14.09279	10.17578
3.642944	13.08466	5.910771	9.007725671	26.34844
4.620446	17.57988	13.8523	6.129192	11.57007	8.26459
23.04445	7.185844	9.593654	12.10017	5.690618
4.846232	4.56254	5.311748	15.56497	8.246652	6.774239
12.59708	2.255448	3.850053	6.020812	11.18115
5.904912	5.172957	5.398117	2.120768	12.0243	8.040053

12.59492 9.824606 8.017164 7.584942 4.561928
21.21473 13.9044 10.20693 22.1526 12.68147 6.616615
8.820406 10.7829 6.984984 8.936169 9.639491 3.395735
3.376862 9.536947 5.30987 8.721554 30.88955
3.670158006 5.604525 2.635346846 22.83418 12.99877
8.569384 12.13839 2.560243 8.7325 4.846341 2.931411
7.475196951 7.620578 17.38588 21.02931 21.18819
7.459075 19.66495 7.359281 5.071757 18.57149
9.877889 4.067614 8.204564 16.95932 3.484937 4.7739
17.29634 4.334234 17.87661 4.942048 2.908066
7.486178 9.523513 22.71466 6.499361 3.72047 9.937356
10.27612 28.34139 38.40299 9.976216 6.047449
8.000749 24.26409 8.417001 20.34274 9.839405
8.39205 9.044854 4.56801 9.036629 4.95351 21.68603
6.698704 13.95417 3.561294 14.01733 9.119639
9.235273847 9.183644 5.262373497 9.690903464 6.512063
3.751129 20.88418 10.43367 4.536358 8.169761
10.22029 7.394394 5.238325 13.04782 6.131588
4.060817 7.299675 9.294811 18.06853 6.461265
10.23488 3.90574 8.987207 11.16723 10.56055 5.503544
6.703626 11.68961 3.736738 9.815036 18.5052 8.682508
5.243661 14.58664 10.59439 19.20591 6.594813
7.034872 5.72109254 12.51636 6.857013 13.77597
28.87526 5.105213 12.80739 5.795948 18.18103
10.70125 9.227302 9.704948 2.391743 11.41386
6.424809 12.12126 4.766165135 24.40688 6.663414
17.50695 4.076529 11.59549 6.943900366 5.429916
8.618431 4.679071 13.51918 14.21293 7.202477
7.694719 13.71188 10.04757 6.653327 5.861609
1.934691 2.673661 18.24534 9.514667 5.50311 11.73042
17.61382 11.53039 7.307636 9.945606 5.752315
7.855765 6.770652 13.16846 15.55744 4.963166701
4.783605568 8.496757 7.009987 19.24958 13.96254
16.99934 3.875539 18.88486 3.53311 3.754552 11.92701
8.965778 2.651025 6.946923 4.990382378 10.69818
25.17226 1.77366 7.283927 7.459258 9.403447449 12.96018
5.058011 14.01312 4.862539 10.50284 10.03552
3.742036 5.143577 2.578863 17.7795 7.875712 2.012546
1.959003 36.7013 19.43627 7.229873 2.61138 3.161112
4.571109842 4.832351 25.56744 45.87148 5.738566
6.951464 4.498763 15.1593 24.11922 11.97139 5.228192
7.368979 3.968369 5.133677 6.164388 4.251809
29.98782 7.399465 13.42034 14.78143 7.67593 10.68625
10.52737 29.58302 19.09131 9.944142 25.35555

2.914964496 13.73009 9.253706 17.63467 20.15558
8.677003 6.249651 8.792894273 7.60055 11.66631 10.96656
6.770382 11.10433 3.227163 13.44507 13.97254
10.25594 5.006058 3.803663 14.70167 3.908849
3.804937 5.964594207 4.79535 3.414077 2.074232
12.406814219 16.66288 7.940789 12.30307 6.365876
16.55981 11.99726 8.391671
SAP30-DT 0.3260932 3.379898 0.262459 0.2558783 0.6110373
2.960414 0.5938031 0.9491292 0.7731272 1.247564568
0.6647902 0.3203281 0.7913269 0.436775 0.3607468
3.774778 0.336854 1.170630824 0.3523078 0.4352919
0.734884524 5.143985 0.362997 2.737933 3.473771
0.4439763 0.4055665 4.539987 0.213803598 2.589777
0.4772162 2.834307 0.1854895 0.62356 0.4029561 0.8807873
0.2544876 0.4445466 0.4279157 0.463800972 0.4138146
0.3285306 0.581841396 0.6700818 0.3957538 0.1499678
0.2642511 0.3129512 0.741153187 0.2046402 0.410048
0.9614453 0.3373438 0.4115175 1.782518 0.6084625
0.2051963 1.707237 0.3386551 0.173675518 0.495833616
0.277944872 1.101915 0.415658 0.1762949 1.452072
0.4846732 0.8180619 0.093608883 0.9762681 0.5023078
0.430489 0.9101459 0.4723855 0.3353153 0.6318748
0.4159407 0.2180411 0.3702106 0.09697266 0.334123
0.6360795 0.3492945 1.101731 0.3680198 0.2480778
0.3954455 0.2950283 0.25933008 1.366171 0.336688296
0.1625506 0.5372337 0.4620678 0.4892854 0.2914962
0.5023566 0.2636647 0.7525516 0.4839933 0.7509393
0.391731 0.1744216 0.3702002 0.6188866 0.4919671
0.168007 0.7466344 0.40680005 0.295 0.1763228 0.7060151
0.5007382 2.092728 0.2680996 0.1109577 0.2997095
0.2906386 0.4947028 0.2375876 0.4820839 0.3194948
0.4292885 0.3675698 0.5877292 0.8867593 0.4101105
0.2078329 1.407779 0.4042188 0.1803303 0.6032882
0.6162021 0.504614 0.2473383 0.4972749 0.2658614
0.2946805 0.6664875 0.5195653 0.210238 0.3801527
0.1313685 0.1471438 0.3187762 0.8853295 0.3401604
1.115174 0.2565941 0.7906528 0.2798424 0.3569672
0.2110657 0.4456146 1.344708 0.548531 0.6295808
2.227084 0.2780954 0.526025 0.491511875 0.7141086
0.583582969 0.1854071 0.4088993 0.489989 0.1857815
0.304774 0.2220506 0.9391307 3.658186 0.160889009
0.5156628 0.2310956 0.5381792 0.7704375 0.5126079
0.6624412 0.3137072 0.2897448 0.3221146 0.4469646
0.2543278 0.3743323 0.4739088 0.2374305 0.7711835

0.1260597 0.1968969 0.2869646 0.6741629 0.5338597
0.5488886 0.2670684 0.7710072 0.4841229 0.5803406
0.2892254 0.2432758 0.5100276 0.4390343 1.336027
0.2968551 0.57519 0.2203532 0.3973093 0.4771308 0.4225419
0.113739 0.6393465 0.2234489 0.6870018 0.3513283
0.2376295 0.7879414 0.3770124 0.2866581 0.3782728
0.6649505 0.21733528 0.6466402 0.810331955 0.244486754
0.3462272 0.4671501 0.7172983 0.4449202 0.1491665
0.298392 0.4597293 0.3547614 0.5688022 0.4329206
0.1837709 0.4745132 0.3341925 0.4781133 0.350618
1.170391 0.3389013 1.037982 0.2859177 0.4401642
0.4473748 0.7471653 0.1845473 0.6398975 3.141276
0.5853901 0.2449728 0.8479998 0.4599807 0.9434734
0.6333989 0.3785322 0.149411 0.3035465 0.36216276
0.2788209 0.4947571 0.3521198 0.5872285 0.8924242
0.547398 0.2336848 0.3188953 0.5446075 0.3104109
0.4835186 0.1926646 0.5998856 0.2453062 0.4551292
0.468339844 1.244369 0.244114 0.3703086 2.316411
0.2746991 0.569348143 0.841452 0.1476576 1.02879 0.3273289
0.3653871 0.9939303 0.2938124 0.6166719 0.5633625
0.4453527 0.06578159 1.961325 1.831859 0.3374823
0.3123077 0.5626076 0.7034109 0.3666627 1.354723
0.298166 0.325154 0.616628 0.3922855 0.6019079
0.216224 0.1363231 0.403341463 0.944788885 0.2913749
0.1056721 0.2133394 0.4468374 0.4811994 0.5674382
0.412269 1.368051 0.3570277 0.2845206 0.2733382
0.6504336 0.4266429 0.264175582 0.1308745 0.7452156
0.8383434 0.4598654 0.3657328 0.107024425 0.1665329
0.4040854 0.3188775 0.4609025 0.2070716 0.3831929
0.9598279 0.8522231 0.2753736 2.451391 0.8155087
0.1717035 0.5866254 0.7944467 0.685931 0.3772337
1.46394 0.4989363 0.676391429 0.5340593 0.3089343 0.434518
0.1390172 0.2866906 0.1390338 0.3399775 0.4386922
0.2147174 0.5199786 0.3356674 1.642017 0.7073314
0.9419451 0.3098815 0.4955871 0.2208392 0.4061623
1.402784 0.2037264 0.9179167 0.1918794 0.3112479
0.9701233 0.4401244 0.2768791 0.242302017 0.2085034
0.2966716 0.5044418 0.6796347 0.4066451 0.4395801
0.423200265 0.1713438 0.5417809 0.3709309 0.3938956
0.4204026 0.6866 0.947371 0.5736416 0.123303 0.8552384
0.467808 1.049654 0.4021324 0.5177638 0.512041967
0.4587136 0.4176834 0.314472 0.262793824 0.446693
0.7841751 0.3625617 0.5897325 0.3642827 0.274491
1.469875

MIR155HG 0.3087331 0.05209749 0.5178543 0.4684032 1.04812
0.2758802 0.4208917 0.3264812 0.9112056 3.504033142
0.1948604 0.0713588 5.917473 0.0340548 2.990342
0.2892693 1.612071 0.232454618 0.7063458 0.5818143
0.509315802 0.2441689 0.3350082 0.1738204 0.06841106
0.1998466 1.078006 0.3805434 0.368494721 0.659336
0.590195 0.2197664 1.262172 1.000963 0.2587363
1.353572 0.03779444 0.52195 0.1966723 0.74734763 0.3813089
2.041205 0.425301059 0.4202985 0.9537442 0.387533
2.419571 1.054098 4.604451233 0.3263633 0.3354327
6.074119 0.5357871 2.116278 1.9475 1.518114 3.213113
1.331111 0.4097253 0.726304109 1.237104872 3.430211964
0.3979219 0.4781558 0.4123647 1.002948 0.284969
1.092116 0.282819494 1.059314 1.109009 0.9016397
0.3952366 0.9737013 1.116424 2.266259 2.016324
0.6061009 0.5566795 1.352055574 0.5841773 0.7439148
3.793314 0.6909397 0.4017166 0.949532 0.8408834
1.795355 2.98628563 1.17785 1.960087028 2.271157 2.32708
5.396362 3.752187 2.105781 2.851791 0.6501942
0.1905049 1.583086 3.626677 0.1586638 2.55251 1.87344
0.9557066 2.027497 1.716007 0.3504153 0.786919939
1.924802 0.5884003 0.7218099 1.450129 7.777978
2.090339 0.4109338 0.7213593 0.3740819 0.3018628
3.296394 2.198867 2.129613 0.5773763 1.095784
0.4713378 14.89362 2.292291 0.5303282 0.7906284
0.61352 0.8501474 0.8373732 0.7391463 1.80251 0.7104875
0.7870117 0.9587109 1.29056 1.528976 0.5004159 2.499005
1.405511 1.094902 1.49144 1.002754 0.5140386 1.838851
1.684631 0.07202274 2.23523 1.174867 0.7793041 1.143729
2.292202 1.002001 0.4561949 0.9736389 4.322258
0.9948557 3.615265 5.734698347 4.604734 1.300035275
0.5617172 0.2796611 1.412821 1.43403 1.68886 2.831914
0.5365949 1.326798 0.480865404 0.939745 0.2032826
0.1885895 1.201401 0.3601457 1.124525 0.4542453
0.6514625 0.6392877 0.2109298 0.8236918 2.360277
2.117364 3.107799 0.6790951 2.899471 0.4118778
0.8949708 0.4360835 0.3349088 0.349732 1.471491
2.545106 0.8820751 1.862147 1.064386 0.2960845
2.311743 0.4470982 0.6473317 0.4540063 0.5114764
0.6356847 1.147583 0.7440259 0.5647725 0.5320853
0.4516238 2.566845 0.9940831 1.036706 0.2403591
0.9846997 0.9136343 0.1943511 0.6761915 1.398398
0.508360803 0.2965749 1.340193901 0.219216756 1.457074
3.127637 2.347456 0.600041 0.2748986 0.6536428

0.5353991 0.1331792 0.4329287 0.7224599 0.5086576
1.001101 0.4957104 2.053554 2.495005 3.455345
0.1205292 0.1564196 2.074581 1.420929 2.880516
2.226025 1.517378 3.280777 1.045119 0.7143986
3.891265 3.926236 1.711231 0.473499 6.667018
1.881092 0.6434901 0.7513377 0.715346902 3.000878
0.4821949 1.64726 0.3179547 2.272986 3.339353 0.675216
1.049808 0.8571107 1.017487 0.6710483 0.9764184
2.044737 5.589791 0.709717 2.448223249 0.2401846
0.6163152 0.6636365 1.068383 2.72592 1.115197657 2.216057
0.4732991 0.31252 3.38891 1.23893 2.007838 0.3078288
0.2900132 1.541357 0.4876079 1.875715 0.5646353
0.6356211 1.586523 1.474929 0.4001793 6.868567
1.939916 1.15563 0.4184574 1.307029 1.26204 1.509942
0.6751334 0.5364137 0.2960918 0.707580669 2.077702801
1.052208 0.09764885 0.8493858 1.29077 1.285117 1.40396
1.683182 0.7564912 1.849172 0.4370523 0.9358678
2.743962 1.741867 2.309081419 0.2465996 1.64939 0.7656995
1.792756 0.7883761 0.119208009 1.075847 0.8456883
0.5893329 0.2428777 1.955353 1.624566 0.3852972
0.234518 0.152083 0.4338326 2.861289 0.3590286
0.2413974 2.588072 1.238512 0.2434133 0.6794143
0.4066969 0.127848228 0.2169475 1.011663 1.878735
1.067224 1.220096 0.1780905 0.8222772 2.143468
0.1931682 3.158904 0.6019461 1.899741 0.7306463
1.862926 0.9362416 0.5909694 1.430465 1.798288
2.170036 0.3871797 8.631633 0.4710996 1.146435
0.8533665 0.4538561 0.103622 0.665717581 0.8753634
0.1128347 0.7274064 1.352943 0.516684 0.484399
0.932148178 0.9965434 0.747358 0.1355673 2.091146
1.193129 0.2987908 0.4834822 1.914278 0.3427521
1.798163 2.431627 3.098803 2.211265 3.189582
2.190076642 0.8047198 0.8362692 0.280217 0.502328276
0.5138572 5.842377 1.548036 0.477093 1.402919
0.9905938 0.4757159
LINC02185 0.05278335 0.2145098 0.4491075 0.1008437 0.1038514
0.6548293 0.2854367 0.3479304 0.1426047 0.396790617
0.339811 0.07777526 0.1426443 0.08660622 0.08605224
0.4692538 0.1052899 0.317937609 0 0.02818356 0.431754237
0.7490902 0.1678766 1.184063 1.066469 0.3837718 0
0.3547643 0.127501309 0.3293686 0.07458137 0.2010848
0.101901 0.2909247 0 0.08678136 0.0549238 0.1540724
0.1224896 0.105102945 0.05328149 0.02326532 0.054938476
0.0254495 1.046297 0.1165184 0.1228359 0.09928596

0.214981546 0 0.01523309 0.5104505 0.1132536 0.01984146
0.5917596 0.04866529 0.07749997 1.041602 0.05292646
0.125226774 0.284650228 0.143967293 0.3154199 0.030883 0
0.2146026 0.07202154 0.1867165 0.079548421 0.06452665
0.04508082 0.07651298 0.4568832 0.08613082 0.1026845
0.07159526 0.1844163 0.07519029 0 0.051706318 0.04588869
0.3315297 0.1696165 0.515614 0.06949813 0.03833008
0.2579761 0.08153294 0.76849615 0.3572204 0.114446498
0.04420314 0.07890795 0.1113938 0.03037754 0.215695
0.1151192 0.06947632 0.01107385 0.347762 0.1652155
0.04611482 0 0.1266292 0.2587085 0.238898 0.03807242
0.1314147 0.046676376 0.1231458 0.08790518 0.04238188
0.2084205 0.1163773 0.4206089 0.04190729 0 0.05227162
0.08773488 0.06902594 0.01365576 0.02839271 0.1216025 0
0.1522133 0.1663921 0.1931138 0 0.7578249 0.1465615
0.0475175 0.1201868 0.1342682 0 0.02949993 0.8073208
0.1506186 0.07104063 0.1887927 0.1385174 0.242707
0.2362101 0.03079623 0 0.08718449 0.06402972 0.03425977
0.2369175 0 0.2706908 0.0487813 0.1368961 0.01195751
0.4590077 0.183222 0.0414346 0.1532825 0.5312467
0.4819162 0 0.556912865 0.3498934 0.175429698 0.03601328
0.01625641 0.1257028 0.02105017 0.03699937 0.1437696 0
0.5773514 0.060765679 0.1407897 0.01342797 0.09592198
0.07318165 0.1276518 0.07600873 0.05077848 0.1221576 0
0.02665464 0.07388944 0.0737637 0.1448002 0.2119577
0.3597991 0 0.09794467 0.04563485 0.101849 0.1112452 0
0.2219102 0.09076344 0.2078681 0.05058164 0.1179756
0.05378465 0 0.07106466 0.1081285 0.1164305 0.01077232
0.02496736 0.06138758 0.01590054 0.3191771 0.05603183
0.04953289 0.1350299 0.2244079 0.08573952 0.1018779
0.202618 0.1269989 0.04236536 0.04181523 0.1935415
0.04104235 0.1970239 0.208671686 0 0.02835878 0.2646547
0.01069398 0.04087473 0 0.1287986 0.03956212 0.02977527
0.3529336 0.03442283 0.02082236 0.06325322 0.1108275
0.1710731 0 0.05960108 0.175156 0.04941581 0.22479
0.08749699 0 0.02753118 0.1140564 0.1757679 0.6075169
0.230707 0.06586384 0.2728298 0.05956413 0.5160752
0.3827624 0.009426374 0.09673815 0.1419421 0.082070489 0
0.2165916 0.06045048 0.07392952 0.2527927 0.08178926
0.1090267 0.04014753 0.01142729 0.1292012 0.1533997
0.4229577 0.2887251 0.05379616 0.0552524 0.048241595
0.04592659 0.05268495 0.08767389 0.1689906 0 0.141608655
0.7909824 0.05576836 0.06055489 0.06080058 0.04814021
0.05119016 0.06242013 0.0887272 0.1160589 0.1932556

0.01064779 0.6017306 0.6085553 0.07872693 0.04044156
0.1789386 0.2909709 0 0.03209025 0.06756805 0.2292387
0.2183366 0.04501097 0.203403 0.08908908 0.02206604
0.057126303 0.603873657 0.231681 0.03547642 0 0 0.01253398
0.1543061 0.01698639 0.2528508 0.1011335 0.04811629
0.2154496 0.105283 0.08789316 0.04726216 0.0123574
0.02723789 0 0 0 0.034647196 0.1976772 0.1204877 0
0.1440636 0.3258677 0.05427261 0.4307103 0.05048997
0.01300063 0.1665356 0.132003 0.06190244 0.1477072
0.05347464 0.1363512 0 0.8293668 0.2184211 0.02322402
0.091531 0.05250621 0.04475776 0.02423306 0.3519078
0.04500963 0.04716921 0.1546424 0 0.1718405 0.228199
0.1200326 0.2756578 0.5032708 0.2194466 0.03963744
0.07699208 0.0986157 0.1646431 0.01442715 0.1103089
0.1480245 0.1459294 0.01409242 0.160867 0.02509767
0.104587783 0.08480662 0.09838448 0.04916673 0.196613
0.04550653 0.4117387 0.047951165 0.1049421 0.3777666
0.2889475 0.0702057 0.01905367 0.2713813 0.1951689 0
0.04859471 0.2286492 0.1606227 0.7389959 0.2398108
0.08131892 0.298375564 0.1181251 0.467418 0.2799625
0.057631238 0.06637776 0.1366951 0 0.01004816 0.03752313 0
0.07332203
AC087491.1 1.037758 0 0.9545701 0.02253022 1.577748
0.1672001 0.07288167 0.02826677 0 0.206849654 0.02024523 0
0.1821097 0.02211351 0 0.01747323 0.4234245 0.159823649
1.368718 0.755603 0.358284926 0.02348907 0.1200209 0 0
0.07415457 0.03733355 0.05594855 0.592508518 1.498491
0.1333021 0 0.04553289 0 0.3456218 0.05170253 0.02454185
1.376897 1.35007 2.98479852 0.4190208 60.04583
1.251968487 1.773987 0.7286052 1.162772 0.7958663
1.41966 0.312198748 0.7535084 4.76467 0.2892809 0.1771197
0.4964875 0.04598583 3.131327 0.9003711 0.1692449 1.8683
0.319746471 0.294548779 0.932778348 0.5402718 0.303591
0.6815946 0.07990984 0.3861804 0.8104759 0.047393296 0
0.5841662 0 0.3733049 2.463118 0.3670637 0.1279649
0.4944214 0.1151918 0.1606579 3.858395094 0.1640372
0.2833961 0.0757905 1.915909 0.6459265 0.8449419
0.1698754 0.4663261 0.40398886 0.2128245 0.954587838 0 0
0.3782869 0.4072119 0.1204749 0.1143094 0.4967106
0.3958543 0.6932875 1.092595 0.4327194 0 0.3394938
0.1946945 0.3380348 1.088772 0.05872061 1.668528892
1.006186 1.471179 0.01893768 0.08278173 0.5460146
0.7517702 0.03745124 0.5805708 2.092766 0.0871177
19.22146 3.441451 0.7104631 0.1304068 0.788176

0.1360282	0.2058915	0.7981815	1.020352	0.06553979		
1.069649	1.341891	1.530552	0.1439895	1.592302		
1.370885	1.382832	1.188994	0.05078944	0.2595664		
0.8665211	0.09639985	0.7947055	0.1284343	0.2731575		
0.2003504	0.09536893	0.4592534	0.4032872	0.06235735		
1.244098	0.08718866	0.8674999	0.192349	0.1172002		
0.2930081	0.3702881	0.8429774	1.703546	0.5300596		
0.5523705	0.071099381	0.4690332	0.361790893	4.763225		
0.5230026	0.9923073	0.3386139	0.3086084	0.6102916		
0.08710956	0.01947022	0	1.384013	0.5280069	0.4408585	
0.1868576	0.09126278	0	0.2495853	0.1910447	1.850257	
0.8575351	0	0.197761	0	0.02914154	0.1286165	0.159557
0	0.04078245	0.3033976	0.1988327	0	1.225942	0.1622249
0.4334521	0.1958807	0.1640039	27.87808	43.32624	0	
0.6377652	1.387338	1.925377	1.695754	0.6400353		
0.8525889	0.3803182	2.423577	0.088532	1.176553		
0.2005463	0.6786585	2.133057	0.3168784	0.8873241		
0.1009616	0	0.09883543	9.854228168	0.0660278	0.29837341	
0.470369098	0.4392849	0.555293	7.148545	0	0.05492479	
2.474721	0.7778195	1.649773	0.466252	1.076691		
12.30008	0.8479114	0.1650717	2.07241	3.060252	0.07989544	
0.1444906	0.05299364	0.02363387	0.5942693	0.3507122		
0.1230188	0.4303653	0	0.1180259	0.3092635	1.143575	
0.02120169	1.277535	5.27086	0.3848207	0.5054434	0	
0.4878816	0.048895892	1.195745	0	0.1944817	1.717781	
1.114504	0.6578326	1.113529	0.1076358	0.3676395		
2.591505	0.01958407	0.2438607	0.1290123	1.314076		
0.5266917	2.974728666	2.823769	4.072671	0.738742		
0.1698993	2.933272	0.196857486	0.05049117	0.4153203		
0.4329282	0.4346846	0.6367168	0.04574705	1.190036		
1.347977	2.987085	0.537309	0.0190312	0.07332926		
0.07769241	1.990064	0	0.0799559	0.2487259	0.0530394	
1.548615	0.1610226	0.702389	0.1560965	1.568771		
0.2566234	3.363782	4.239743	0.510519963	3.924822292		
0.04140922	0.07926043	0.1103098	0.2966945	0	0.3983735	
0.4554063	0.3340351	0.07746846	0.2064001	0.4091488		
1.575976	0.8797306	0.309735938	2.473727	0	0.2667942	
0.3386553	0.3703342	0.092889358	2.826528	4.199369		
1.435064	0.5536042	0.1164871	0.4365156	0.7313334		
2.391429	0.9062239	0.2763942	0.04718679	0.3477275		
1.287013	0	0.658005	2.002101	0.141177	0.1377855	
0.186791243	0.03635485	0.1251284	0.3599875	0.909566		
0.1209573	0.1810067	0.196717	0	0.5351846	0.7897808	
18.69398	0.5899818	0.2554707	0.1861065	0.07844504		

0.02361516 0 0.3525191 3.259638 1.108806 0.2253249
3.522086 0.02173543 0.1511275 0.02875237 0.22429
1.051501971 0.02165401 0.9964623 0.6590816 0.2928448
0.8133554 0.2848708 0.064278693 0.8628082 1.85679 0.2816982
0.5557029 0.03405534 0.4203766 0 3.392215 0.2171379
0.1167638 0.8612611 1.403387 0.2285989 0.04152696
0.503670223 1.097873 1.294922 0.04548977 3.193200932
1.898231 0.5972274 0.7692104 3.196784 2.056706
2.25532 0.4118753
ADORA2A-AS1 0.3212539 0.02761275 0.1460907 0.02758482 0.02840754
0.05483334 0.03346219 0.04326045 0.02786295 0.036179418
0.06816482 0.007091558 0.07803797 0.02030597 0.02353876
0.02139331 0.1440053 0.065226579 0.3520935 0.2544082
0.012653809 0.0287588 0.009184205 0.09068892 0.04944447
0.08227933 0.05713656 0.03995862 0.013950702 0.008190509
0.04080203 0.003235577 0.6306504 0.01136852 0.00293862
0.01582547 0.003755969 0.2236928 0.08655677 0.047916498
0.04663884 0.06045805 0.015027891 0.4478543 0.02973554
0.1885789 0.08400148 0.03491835 0.051455366 0.04612782
0.3000144 0.1634679 6.269465 0.03799209 0.1548323
0.02218654 0.3974882 0.09990707 0.02533567 0.327865251
0.05737298 0.073839126 0.06471006 1.605072 0.04470583
0.06604027 0.024626 0.04377814 0.859508333 0.03025825
0.09556854 0 0.1214053 0.08414378 0.1179711 2.702619
0.08687804 0.1057761 0.03278352 0.088398534 0.04393343
0.03942903 0.0850611 0.0965019 0.007604218 0.05591907
0.1077074 0.03122357 0.28440816 0.02442855 0.027827322
0.1370352 0.04933603 0.09445919 0.06232113 0.05900128
0.1679454 0.136833 0.1090493 0.03658729 0.2304849
0.01261426 0.02495071 1.082444 0.05586886 0.1334193
0.06248606 0.02696042 0.035111651 0.02887316 0.04153333
0.1767956 0.006334602 0.1790657 0.3323768 0.1404259
0.3150228 0.02001776 0.03733183 0.07552553 0.02241241
2.128036 1.081054 0 0.06939412 0.1120368 0.01980916
0.02342374 0.08693046 0.03006786 0.0285955 0.1109563
0.08814664 0.0763809 0.04841654 0.1058167 0.02060013
0.05052445 0.09533987 0.1089341 0.3098207 0.1596321
0.05335208 0.2166258 0.1737532 0.005838237 0.07965717
0.06789252 0.03181129 0.02820757 0.04336688 0.09191437
0.03270862 0.1399065 0.08177248 0.1133404 0.05805549
0.1709617 0.06084163 0.02415334 0.043525186 0.187432
0.151343947 0.3612065 0.008893571 0.03725019 0.06909686
0.03373608 0.1474755 0.0177754 0.04767665 0.0373992
0.08857684 0.09550037 0.2623853 0.005719467 0.1082456

0.01559359 0.05555986 0.2673202 0.0217823 0.05832896
0.05659289 0.09079808 0.07921742 0.2765152 0.05061579
0.03418683 2.232652 0.06553562 0.4581392 0.07227135
0.04756081 0.03862816 0.006206866 0.1137206 0.07148662
1.104388 0.05517102 0 3.001945 0.03253527 0.003538717
0.03830663 0.03414791 0.02798664 0.4436428 0.09978037
0.02452315 0.0914574 0.1200425 0.08056731 0.04690644
0.1393386 0.1212404 0.0473718 0.3669735 6.871471
0.09831976 0.392935886 0.06399912 0.121770945 0.261426193
0.03361484 0.3210506 0.01462618 0.3876041 0.03362352
0.004403952 0.003607279 0 0.09654159 0.04708017 0.04556604
0.06488366 0.02021052 0.03119693 0.003061121 0.05298573
1.109353 1.63288 0.06510616 0.03829433 0.05367422 0.03012356
0.08666395 0.0781294 0.08670282 0.04733076 0.03603284
0.1005882 0.07739268 0.08738934 0.1177886 0.01547096
0.251387 0.01493342 0.041157586 0.0440558 4.676991
0.04960694 0.01011135 0.1613477 0.02983022 0.1320742
0.6424452 0.04688736 0.03141489 0.07193316 0.09703537
0.04607048 0.05395655 0.06045505 0.019794036 0.2462304
0.02522006 0.1130596 0.06067129 1.296445 0.021519825
0.1313648 0.03813724 0.01242316 0.04656795 0.01580195
0.0560103 0.03699459 0.09101427 0.09206571 0.03524213
1.057274 0.04489026 0.03269837 0.3937827 0.09403039
0.07342038 0.1799478 0.05682134 0.2311534 0.006160863
0.08062204 0.05972387 0.03385887 0.1734625 0.01827709
0.06337752 0.074225163 0.155172684 0.1774475 0.03153876
0.08441102 0.0272443 0.0342855 0.07503812 0.07434343
0.02706453 0.2924491 0.08423518 0.02946709 0.100797
0.09136084 0.043093696 0.1284494 0.01490132 0 1.969501
0.0666791 0.071080552 0.07865121 0.02307078 0 0.03546649
0.007131037 0.1002088 0.04712666 0.3487289 0.02133718
0.01626936 0.2166489 0.04112255 0.01515145 0.06338572
0.05221656 0.3668838 0.06481866 0.0738051 0.289048169
0.1669162 0.004787523 0.07345832 0.01988616 0.1740102
0.006155976 0.05591162 0.06042992 0.01535747 0.1074407
0.1508523 0.147752 0.06143992 0.2895709 0.04201926
0.06144049 0.07897665 0.1618523 0.06582264 0.01183923
0.09483239 0.1012267 0.242832 0.37392 0.008800724 0.05148919
0.997738218 0.02982605 0.01794142 0.006724543 0.1882355
0.01555984 0.1416922 0.095095203 0.04018827 0.1119456
0.09700218 0.01920409 0.005211948 0.04948915 0 0 0.0930483
0.06850144 0.2152903 0.7138253 0.06559793 0.04766573
0.26299057 0.1615599 0.166215 0.05569531 0.01576447
0.8896928 0.2285044 0.03210618 0.1071945 0.02737091

0.01458431 0.02865219
Z99289.1 0.03851191 0.4050876 0.1416989 0.04598613 0.07577226
1.647847 0.08925469 0.2653967 0.02972787 0.012062803
0.1735536 0.05674656 0.08920842 0.009027118 0 0.8060149
0.03841094 0.420453525 0.02377593 0.06169005 0.02250125
1.112283 0.0244973 1.750875 0.5209445 0.04540677
0.09144122 1.553063 0.037211118 0 0.08162434 0.4315174
0.06505561 0.09097077 0.3370455 0.168847 0.02003682
0.09512011 0.4840936 0.127809082 0.4043037 0.02546235
0.150316257 0.01856852 0.6444301 0.1416907 0.1680447
0.1138362 0.71565241 0.1461077 0.111144 1.02647 0.21691
0.2026748 1.314055 0.07101457 0.07068213 1.924614
0.03861633 0.039157818 1.082159332 0.091911525 0.498631
0.168997 0.2384903 1.689746 0.1182342 0.4865446
0.048366916 0.1546917 0.09045292 0.1744549 0.9524347
0.3591027 0.7117485 0.0870625 0.8447018 0.3605119
0.1384537 0.084883682 0.0167407 0.4522319 0.216573
0.8019073 0.2839614 0.1398324 0.5547688 0.08328355
0.69264412 0.4995522 0.185561586 0.09675478 0.4688087
0.1462957 0.08865653 0.1967198 0.4666308 0.04055318
0.04039865 0.5465059 1.121068 0.042058 0.06655177
0.4504086 0.4868008 1.060357 0.1388924 0.2277224
0.025542092 0.08984992 0.1982434 0.3324197 0 0.7005197
0.7842633 0.06879711 0 0.183065 0.312954 0.05036286
0.06974482 0.7043425 0.2395545 0.2681226 0.09254848
0.1867744 0.1497064 0.03123942 0.6599439 0.258425
0.1733491 0.1205751 0.1175581 0.2813456 0.02152381
0.809929 0.1373684 0.2073313 0.3708583 0.1768645
0.2492299 0.3548263 0.1947367 0.07095941 0.1090487
0.2725188 0.1624785 1.5969 0.05939583 0.1880972 0.07118385
0.5629736 0.0523468 0.4114513 1.83638 0.1813896 0.2580885
1.561838 0.1352372 0.7408866 0.101584007 0.8190555
0.393838197 0.09634568 0 0.1605022 0.03071735 0.3329453
0.4458145 0.1422385 1.065043 0.099755983 0.5341612
0.02939203 0.1099793 0.08390637 0.3446092 0.4713901
0.1204096 0.5904788 0.1452514 0.1847543 0.07547594
0.4574669 0.1936907 0.3806746 0.6900459 0.09118754
0.4128951 0.09988862 0.2972452 0.1116045 0.02927547
0.05887658 0.03311151 0.9352685 0.3813567 0.1243343
0.2158336 0.1124382 0.1629583 0.2840147 0.08495031
0.05501806 0.2003842 0.1045094 0.08120971 0.1552525
0.1308226 0.117456 0.5172345 0.624232 0.3485344
0.3398052 0.3049089 0.101085 0.08242856 0.08390065
0.3530304 0.209617885 0.2605524 0.284202815 0 0.07586773

0.4113827 0.03121027 0.1192924 0.0448425 0 0.144327
0.09776116 0.7837207 0.05023133 0.1367321 0.06922643
0.7681907 0.166425 0.04899011 0.3913765 0.1277977
0.4470805 0.5981608 0.4085744 0.1193057 0.251092
0.1202039 0.2404579 0.6937949 0.5049869 0.2814692
0.3375411 0.2172966 0.3586095 0.8029069 0.08253231
0.02352744 0.3744241 0.099800761 0.2621407 0.5112736
0.1058544 0.01348516 0.172147 0.4475645 0.3409213
0.2489865 0.1417392 0.05237116 0.1918694 0.438012
0.430098 0.1177526 0.2418803 0.017599071 0.05361449
0.1441504 0.4020897 1.471885 0.06193513 0.160721287
0.8038435 0.04747147 0.2650932 0.212935 0.252894
0.3921696 0.2428966 0.194212 0.7367087 0.5483472
0.06215093 2.674129 0.5153751 0.1887345 0.02950706
0.4406317 0.2030685 0.1299096 0.1560917 0.07394873
0.2030986 0.3823277 0.09031276 0.8642545 0.03250069
0.4024969 0.052100778 0.16021806 0.1521357 0.1747196
0.03752533 0.07266952 0.04572536 0.1751325 0.1363302
0.8341957 0.2740744 0.1334055 0.2456201 0.8833933
0.3719471 0.195406636 0.04508117 0.1192402 0.08910822
0.1728062 0.1156058 0.075838097 0.1442297 0.158239 0
0.2522688 0.2092291 0.1682936 0.298543 0.07367721
0.05691328 0.173583 0.3467244 0.0709742 0.1885983
0.06502715 0.2785575 0.04301538 1.066172 0.04687203
0.194864762 0.08162374 0.0510796 0.1959375 0.03536197
0.4147657 0.02463002 0.1720785 0.2256611 0.05461792
0.7164494 0.4162477 1.401256 0.08938936 0.5951133
0.06404526 0.04820059 0.126394 0.2638248 0.1570895
0.05263186 0.1724654 0.3645067 0.2750562 0.3907214
0.2347443 0.04577954 0.133541757 0.07955589 0.1076753
0.05380971 0.5020862 0.2490193 0.455466 0.183677749
0.3292428 0.6660984 0.1149941 0.1024473 0.02780397
0.1848054 0.08543975 0.03793865 0.1418231 1.366397
0.1406327 1.061526 0.6882204 0.07628412 0.362835759
0.4998828 0.8014406 0.07427886 0.014016345 0.03632302
0.5319242 0.07136479 0.1759527 0.1095108 0.1296705
0.5731862
AC083862.1 0.5202387 0.2426666 0.7819972 0.4242371 0.3162251
0.282707 0.9018232 0.2129019 0.8031588 0.47692846
0.1960517 0.09971535 0.3918937 0.09517499 0.4964725
0.4042191 0.4302864 0.248397434 0.3446783 0.4065078
0.222408647 0.4296552 0.5004189 0.258074 0.3910755
0.159578 0.4418727 0.2207321 0.711089833 0.4606714
0.3944345 0.2388534 0.9308594 0.2397815 0.5784847

0.2781551	0.09242309	0.3988701	0.6674345	0.460402916	
0.4098719	0.3579405	0.435825249	0.3589158	0.7839664	
0.1867348	0.915396	0.8455968	0.994841185	0.4965916	
0.3222498	1.161244	0.4219935	0.7059229	1.261739	
0.06239358	0.8011095	1.443829	0.419864	0.825699883	
1.080440937	1.245914329	0.9478093	0.8760389	0.3492304	
0.6104693	0.3116426	0.5257996	0.254971794	0.1595495	
0.7152495	0.6253683	0.4769826	0.4495997	0.5233143	
0.780232	0.5615437	0.4234766	0.3265229	0.34803545	
1.059007	0.8732066	1.182464	1.056837	0.6014471	
0.5159999	0.5091812	1.034877	1.39099185	0.2719321	
0.635836091	1.473489	1.831856	2.377913	1.650376	
0.8944394	1.143853	0.5745358	0.4046357	1.003195	
1.795193	1.563081	1.520287	0.77624	0.6284637	1.043301
1.281328	0.5844368	0.359061589	0.456738	0.3995829	
0.5501687	0.578966	0.8952414	1.977291	0.594379	
0.5253025	0.6333129	0.6749073	0.2256696	0.3939299	
0.3276197	0.7717352	0.8716209	1.085533	1.329214	
0.2321161	0.5077702	1.034289	0.4345323	0.5574362	
0.2455825	1.084512	0.5114296	0.6807915	0.5498772	
0.4827688	0.5328234	0.2234311	0.266389	0.9508084	
2.137723	0.7403199	0.2404741	1.077868	0.3591536	
0.4282621	0.3362957	0.2683817	0.5453669	0.6566943	
0.9214479	0.1379761	3.417457	0.3152716	0.2390541	
0.6916096	0.7512234	0.1782297	1.103776	1.530033847	
0.9672872	0.570944999	0.473268	0.4064246	0.6950157	
0.6072377	0.628537	1.209643	0.1562139	0.7856108	
0.540482854	0.2842969	0.1807674	0.8696527	0.673536	
0.6751055	0.3471674	0.7324076	0.6460481	0.2297129	
0.2691187	0.2699901	1.548619	1.171902	0.8936399	
0.622751	1.012915	0.5964788	0.7020992	1.207867	
0.6551927	1.106025	0.3297731	1.243677	1.632354	
1.248372	0.7184661	0.284448	0.3111837	1.181198	
0.6238402	0.3109898	0.1760921	0.6242059	0.5902858	
0.6574491	0.7015126	0.3879262	1.333624	0.9088872	
0.62038	0.5653352	0.3498676	1.522123	0.677203	0.7875887
0.4121357	1.209677	0.289411293	0.5209949	0.989887448	
0.053274625	0.9907741	1.239229	0.4627366	0.5633573	
0.1181962	0.2786605	0.6593917	0.3722039	0.5902106	
0.1820502	0.8909877	0.3649351	0.4795586	0.4752196	
0.9146604	1.045921	0.2979829	0.5987125	1.678355	
0.6394234	1.100634	0.502991	0.950503	0.8873211	
0.7365638	0.6285506	0.6152332	1.55126	0.9164033	0.7561805
1.288188	0.9245421	0.4651035	1.039405	0.499805696	

0.6552135 0.6493074 0.8021594 0.2310378 1.134366
0.8257858 0.7488359 0.5790711 0.5054553 0.4003169
0.9377094 0.9708426 1.376582 1.069064 0.8500666
0.54505629 0.1854792 0.265967 0.8310041 0.2843691
0.6121842 0.605186332 1.045807 0.8758798 0.2911395
1.087433 0.2777417 0.8860145 0.4401573 0.319941
0.781193 0.7639643 0.9112371 0.2630034 0.6165181
0.6164274 0.9916305 1.354991 1.143488 1.426737
0.5759989 0.238229 0.5983086 1.049732 0.8980867
0.6557904 0.4069114 0.3500985 0.247189753 0.950184052
0.7017507 0.1961505 0.5143297 0.5746286 0.6749303
1.055121 0.6206784 0.2008501 0.9030094 0.5459539
0.7639407 1.202191 0.7437373 0.621094947 0.1425904
0.942883 0.1565814 0.455484 0.5508305 0.44976297
0.622083 0.5213594 0.218748 0.7619015 0.9400358
1.213348 0.7040702 0.2306113 0.1125094 0.4232165
1.827798 0.4081622 1.881912 0.6855963 0.2360006
0.4676936 1.101304 0.3335733 0.602952344 0.2835996
0.6563505 0.5379726 0.8155643 0.4034587 0.23804 1.012964
0.4460995 0.1799529 1.664173 0.5120028 0.4616801
0.4908608 1.084673 0.5697371 0.3430283 0.9069095
0.5215436 0.5236614 0.1526002 1.424368 0.4714888
0.5495932 0.3116702 0.4331186 0.6033304 2.237653336
0.4776364 0.2995786 0.4609542 0.2520767 0.2297285
0.7790614 0.714681758 0.6256383 1.029208 0.5304292
0.7811622 0.806145 0.6958737 1.538885 0.4749953
0.7009097 1.853128 0.7568066 0.9992781 0.5380558
3.429366 0.733213432 1.044989 0.4382196 0.6118275
0.22166635 0.5265727 1.767167 0.583122 0.4637775
0.1683789 0.5810373 0.4733868
AC067945.2 0.02793965 0 0.1027999 0.02668964 0.03664755
0.1980679 0.08633675 0.1004558 0.21567 0.210031957 0.1918625
0 0.8629199 0.026196 0.09109953 0 0.2229312
0.147256319 0.1379918 0 0.065296898 0.3060807 0.07108928
0.2339891 0.01913596 0.02196116 0.1769036 0.132555
0.431935204 0 0 0.05008922 0.2696948 0.1759935 0.02274605
0.1837428 0.1162906 0.2509375 0.06483703 0.173082991
0.3158772 0.09851969 0.145402072 0.1436918 0.2589351
0.04111756 0.5851834 0.1801877 0.45518208 0.1338926 0
2.056119 0.4196374 0.3360839 0.5992308 0.03434647
0.9845478 0.6301119 0.05603081 0 0.444088929 0.647748996
0.1113068 0.03269441 0.1537957 0.1893249 0.2668606
0.4518128 0.084214242 0.2732455 0.2147624 0.2430023
0.1105557 0.2344699 0.3043805 0.8590055 0.3687736

0.204687	0.1480249	0.082108638	0.1943209	0.7019504		
1.047464	0.1149172	0.3531574	0.1082088	0.2874815		
0.2416825	1.05285711	0.5672591	0.053848545	0.717536		
0.3818798	0.5424664	0.09647791	0.6564956	0.2437428		
0.1765233	0.2344676	0.6513592	1.084418	0.1708687		
0.5150083	0.3016272	0.1153191	0.4847447	0.1612221		
0.208684	0.024707062	0.2234893	0.1015213	0.1570371		
0.2941935	0.8932239	0.1979019	0.06654798	0.125046		
0.08854008	0.08256077	0.175379	0.1734807	0.8115675		
0.2832169	0.1037428	0.05371371	0.8130075	0.1533305	0	
0.3623174	0.05171928	0.120731	0	0.3980018	0.3378386	
0.1249209	0.1424456	0.106302	0.1203319	0.1537432		
0.1833025	0.3806558	0.7060653	0.2608204	0.2353357		
0.2109673	0.0451902	0.1813462	0.2388701	0	0.3820905	
0.07746377	0.1054004	0.1012709	0.9163256	0.2245953		
0.1754596	0.0748953	0.8601508	1.020365	0.5608685		
0.252676263	0.7717	0.485727465	0.2541707	0.1376793	0.155255	
0.2228485	0.1827911	0.799062	0.2407798	0.253712		
0.03216491	0.2384761	0.1421557	0.08704129	0.04427088		
0.2162226	0.2011673	0.1881489	0.1616532	0.05620112		
0.02821804	0.09386801	0.8589925	0.9964066	0.4833012		
0.2611904	0.151211	0.09216845	0.5072709	0.1198032		
0.2355402	0.08495515	0.4271383	0.5284788	0.1956092		
0.2855917	0.1665271	0.2562269	0.2284008	0.04299021		
0.2518355	0.1369551	0.06842494	0.05286359	0.5848938		
0.1683317	0.1287231	0.04745465	0.07865727	0.07147499		
0.207874	0.4408754	0.1232611	0.1876895	0.09778025		
0.1495008	0.5754822	0.7610328	0.086899283	0.1042901		
0.618551031	0.058653313	0.2001476	1.607977	0.249067		
0.230785	0.1951942	0	0.1116869	0.09456513	0.4548594	0
0.1102183	0.3682979	0.2737651	0.4427064	0.3554139		
0.8202599	0.1141105	0.1883313	0.3359645	0.4075677		
0.1384864	0.3497519	0.4293199	0.6512709	0.1677783		
0.1628259	0.378518	0.7283597	0.4729335	0.6243942		
0.2026064	0.4590463	0.06827481	0.1155904	0.086884239		
0.3672403	0.1348797	0.281583	0.1565315	1.534355		
0.2308974	0.4946637	0	0.1935606	0.06079084	0.2551956	
0.5199861	0.2547166	0.07593535	0.350959	0.127677875		
0.09724071	0.3346508	0.450822	0.08945126	0.02995517		
0.199886063	0.1495315	0.2164777	0.1602667	0.5149341		
0.1426987	0.2167706	0.1101355	0.3287599	0.2703057		
0.2045907	0.3156251	0.3764235	0.2991158	0.4524419		
0.5993905	0.09471699	0.3750022	0.06283128	0.1585382		
0.0953749	0.1386768	0.3390095	0.2859062	0.3293334		

0.04715725	0.09344121	0.151192451	0.387450406	0.392432
0.1314504	0.1306747	0	0.5838426	0.2541105	0.5035159
0.0465533	0.275311	0.04075079	0.2565974	0.4179684
0.3349743	0.266849424	0.1046577	0.230684	0.05746338
0.1002941	0.02580609	0	0.1521977	0.4081754	0.05666662
0.2135189	0.2207879	0.2010957	0.1823892	0.1336285
0.02752633	0.1007449	1.425404	0.1310665	0.1954635
0.4906298	0.1154789	0.03120683	0.4181011	0.2176304
0.245861978	0	0.07411453	0.6633616	0.2565443	0.1719455	0
0.3661963	0.4677508	0.07924849	0.7536669	0.4026396
0.508292	0.02161675	0.698138	0.1393908	0	1.059603
0.03479996	0.1608924	0.03054672	0.8675008	0.3330018
0.2317332	0.2685419	0.06812099	0.05313948	0.055361129
0.1795617	0.1735918	0.07807582	0.06938169	0.04817567
0.08436557	0.152291057	0.2888532	0.09998101	0.1112347	0.361
0.1613699	0.1149195	0.2479396	0.1100951	0.1543349
0.5763342	0.1700439	0.2444815	0.8124052	0.5411283
0.737045024	0.35015	0.04948338	0	0.040674354	0.1756777
1.382811	0.4141902	0.06382515	0.07944801	0.07525879
0.06653369
AC022509.2	0.08313944	0.09937527	0.133831	0.01985496	0.08178852
0.1262971	0	0.1992828	0.1283529	0.390617373	0.3925084
0.4083479	0.385166	0.1948773	0.06777075	0.2001797
0.2073037	0.046948673	0	0	0.048575661	0.3311993	0.2115389
0.1740691	0.09964936	0.1797108	0.2961047	0.1972206
0.160662594	0	0.1174737	0.09315591	0.1404419	0.2618501
0.6091646	0	0.06488316	1.642761	0.09646711	0.055182805
0.3021266	0.2015496	0.064900495	0.1469809	0.107015
0.1376468	0.8464741	0.1117043	0.359782689	0.1826099
0.3359126	0.4902526	0.1783866	0.9063206	0.8105085
0.1277551	1.068119	0.383525	0.06252365	0.253601493
0.235976132	0.028345492	0.1863076	0.07296607	0.08580876
0.3098535	0.3119646	0.4621548	0	0.07259743	0.6213124
0.3615484	1.110302	0.2325691	0.711652	8.194636
0.2582005	1.116652	0.5033997	0.081442998	0.1445592
0.5903072	0.8905474	0.1282338	0.1532538	0.3421193
0.2780222	0.4880079	0.28481503	0.632993	0.340501527
0.2088741	0.4438875	3.298595	0.2392394	0.4459123
0.3827975	0.08754613	0.03488502	0.3792202	0.4163715
0.1815895	0	0.2493183	0.7506462	0.50172	0.08995222
0.2069923	0.165420676	0.02770969	0.1006982	0.2336461
0.03647607	1.260234	0.8097273	0.6930891	0.06976817
0.6586673	0.07677325	0.1522126	0.1935836	0.2012471
0.3639203	0.8489398	0.5794012	1.391069	0.1520875

0.1573591	0.4235552	0.038475	1.901067	0.3786141	
0.2537841	0.2094374	0.1858622	0.5563324	0.03954008	
0.2237932	0.4117414	0	0.3681306	0.04377133	0.1131839
0.1531871	0.1765606	0.2017073	0.2967955	0.3376304	
0.09158829	0.2639415	0.1344626	0.1568189	0.1506749	
0.3511648	0.8505938	0.06526394	0.2228644	0.6891051	0
0.2086206	1.378453241	0.5281563	0.425108612	0.3781652	
0.05121119	0.3134927	0.2652502	0.2136859	0.3679855	
0.2302982	0.446116	0.119640581	0.2661107	0.1903544	
0.1079196	1.53143	1.105859	0.2095133	0.6598479	0.2405141
0.1045228	0.1469437	0.3025979	0.1936429	0.4181391	
0.744756	1.084868	0.02812221	0.1028489	0.1078197	
2.317223	9.790588	0.04213322	0.03177567	0.1072216	
0.2728454	0.3452432	0.1445299	0.06353744	0.5340089	
0.7835412	2.197051	0.2037673	0.1357405	0.1179788	
0.2256147	0.1001802	0.3112189	0.05295369	0.2145539	
0.63806	0.1546416	0.3665614	0.1833927	0.07978627	0.218222
0.3558933	0.06586348	0.1524243	0.237035716	0.01939586	
0.328680294	0	0.3871237	1.395574	0.3031951	0.08584277
0.4356266	0	0.394659	0.6096903	0.5559083	0.162659
0.2131834	0.6475989	0.6400721	0.5089771	0.2467728	
0.2581648	0.2122227	0.9028884	1.645377	0.1653806	
0.3605797	0.5854216	0.09980613	1.66112	0.4992544	0.3028235
0.3260482	0.4297362	1.336934	0	0.7536148	0.07423775
0.152373	0.08599001	0.236994682	0.1756269	0.2006794	
0.3237344	0.02911173	0.2654504	0.2576537	0.4170555	
0.2213283	0.1439936	0.06783527	2.554279	6.017325	
0.1326421	0.02824491	0.4931612	0.056989266	0.20255	0.2489534
0.3748314	0.5989011	0.3119794	0.346964839	0.04449579	
0.4831263	0.8584241	0.3830697	0.5156168	0.16126	0.9995704
0.2096323	0.6580989	0.2536652	0.1341713	0.3015697	
0.1711678	0.230292	3.407939	0.1056928	0.9166209	
0.09348288	0.2527279	0.1241648	0.9800621	0.2521959	
0.1063457	0.3203815	0.1578655	0.1042692	0.224950141	
0.144116031	0.3466764	0	0.08100962	0.2876112	0.09871184
0.3510702	0.2140431	0.1385277	0.3413489	0.1667343	
0.9968591	0.518225	0.1938176	0.148885945	0.1167854	
0.1716104	0.7053444	0.3730537	0.057593	0	0.39628
0.1518249	0.3372434	0.4878686	0.3900902	3.120209	
0.06784149	0.2982267	0.02047738	0.8056708	0.6861324	
0.208935	0.3780638	0.1403805	0.2147677	0.06964614	
0.3421371	0.5261737	0.054870484	0.4485325	0	1.022225
0.03816968	0.4690167	0.3367513	0.2228897	0.3479692	
0.2358181	0.3866674	0.2396253	0.3308625	0.3216227	

0.1366734 0.1382608 0.1872997 0.2122239 1.44975 0.2393821
0.1363459 0.4467815 0.1457216 0.07661818 0.2663649
0.1013531 0.1185946 0.411842451 0.05724837 0.1291384
0.2129679 0.2064577 0.1075165 0.5230102 0.245466749
0.1322361 1.487557 0.1241245 0.0789868 0.1200463
0.3989576 0.3688944 0.1638038 0.2104902 0.7545938
0.5818954 0.4728741 1.007274 0.18298 0.156657843 0.5023611
0.5521751 0.08017654 0.211809227 0.05227604 1.339703
0.09243731 0.1424424 0.1576079 0.111973 0.04949574
BVES-AS1 0.006190293 0.6452067 0.03416442 0.1005268 0.0811961
1.34786 0.1339011 0.4673954 0.2580321 0.395543778 0.4410304
0.0121617 0.2772227 0.1218834 0.08073584 1.036452
0.1358294 0.652520545 0.06114679 0.1850964 0.267642163
1.189846 0.0472515 0.9776011 1.153213 0.07298554
0.1273826 1.409703 0.059812075 0.08427814 0.2973881
0.7823906 0 0.03899301 0.04031682 0.02713997 0.00644132
0.06115733 0.1340757 0.01643493 0.00499897 0.01091398 0 0
0.01274878 0.02732995 0.02881175 0.07984455 0.1764873
0.01483259 0.007145989 0.05256377 0.8434127 0.0465391
1.255235 0.007609784 0.01817799 0.704382 0.006207077
0.016784306 0.077308073 0.025326159 0.3329246 0.07243755 0
0.6040326 0.06757198 0.1251292 0 0.02594577 0.01057392
0.008973241 0.2020809 0.06349324 0.04817027 0.01119534
0.07689907 0.02519463 0.1452408 0 0 0.04057141 0.01326145
0.3373581 0.1564907 0.005993669 0.1082802 0.0611966
0.36050885 0.5097087 0.01789598 0.006912038 0.1163375
0.04180472 0 0.02529614 0.09600626 0.0456288 0 0.1443148
0.06200334 0.01081645 0 0.007425372 0.1533001 0.04202599
0.0267902 0.4931831 0.021896331 0.04951617 0.02249299
0.3429601 0.04345424 0.5937065 0.4384703 0.01474433
0.01385256 0 0.2057863 0 0.02562419 0.206449 0.501995
0 0.07735507 0.06004316 0.0566197 0.01339022 0.6479327
0.1375067 0.03566545 0.03523797 0.03779189 0.02495044 0
0.197251 0.04710441 0.04665617 0.1703163 0.008122481
0.4933766 0.01955443 0 0.006517606 0.6310143 0.0600739 0
0.2487424 0 0.0302342 0 0.02919056 0 0.1230428
0.696657 0.1749363 0.2433752 0.4837661 0 0
0.149287495 0.1778165 0.139269711 0.01126278 0.0991385
0.02948419 0.02962451 0.06942703 0.05901324 0.1143151
1.062923 0.22804606 0.07265037 0 0.02571309 0 0.06587096
0.2139385 0.005955167 0.2363843 0.01867783 0.02500789
0.02772979 0.01153442 0.08490895 0.06118854 0.3809723 0
0.03573638 0.005351936 0.1274089 0.1891748 0.02509682
0.02839096 0 0.07042597 0.1463247 0.1537319 0.01892317

0.07952114 0.1762104 0.2333309 0 0 0.01171243 0.01919833
0.06713184 0.1140792 0 0.005809087 0.02375394 0.1513283
0.04596711 0.08192895 0.0891094 0.04332828 0.09936997
0.5884777 0.168614 0.025671142 0.02888306 0.006525991
0.038985585 0.004434458 0.06477499 0 0.0255663 0.05766283 0
0.006186322 0.04888752 0.07918303 0 0 0.02225451
0.008665035 0.02675067 0.0367478 0.06290873 0.01264113
0.3662665 0.0744361 0.0738822 0.07670741 0.06457569
0.04755994 0.0206136 0.7806301 0.1352835 0.004413908
0.08903447 0.006985525 0.01152837 0.06733406 0 0.01512693
0.03073218 0.109083465 0.01743548 0.1255123 0.04537263 0
0.06324665 0.1918406 0.03653247 0.5555908 0 0.006734392
0.07196125 0.06400435 0.1354437 0.05047258 0.1036777
0.005657648 0.426583 0 0.03525313 0.9612104 0.0398211
0.036905536 0.1590246 0.004360244 0.3550859 0.03993098
0.01354982 0.4082344 0.01952125 0.02081139 0.02177774
0.1863526 0.08491466 0.9174021 0.6882085 0.0738631
0.009485759 0.06295633 0.005934662 0.1113669 0.03512562
0.02113121 0.09217546 0.07511076 0.02639384 0.08980543
0.0156722 0.3157174 0.200988686 0.231776894 0.02173676
0.04160581 0.08685667 0.0311485 0.02351924 0.008042939
0.007968479 0.732317 0.02033258 0.07222978 0.08843579
0.1234731 0.05772412 0.014780752 0.01739092 0.02555511
0.3628493 0.222211 0.01143517 0.048759987 0.05901145
0.005652194 0 0.1757122 0.006114705 0.05091959 0.4445106
0.1243479 0 0.01674073 0.03096193 0.004148432 0.008661355
0.008361821 0.04477453 0.06222751 0.6854935 0.06027259
0.06536765 0.9541792 0.01642077 0.1049814 0.02273592
0.09524035 0.04222889 0.08113425 0.01036346 0.01755825
0.063338 0.2765466 0.8446267 0.04789396 0.1383971
0.0205889 0.0247924 0.1715591 0.01542052 0.02376482
0.006767914 0.01478485 0.06943971 0.02281896 0.1388286
0.1584749 0.01177355 0.049063123 0.05115033 0.09230619
0.005766145 0.03074434 0.01601066 0.05607596 0.011247174
0.009845869 0.08860694 0.06777401 0.009409761 0.03575303
0.2461279 0.03662223 0 0.01139812 0.3932922 0.02260489
0.1191678 0.007499835 0.00544964 0.163299281 0.01108272
0.3069783 0.2029693 0.009011787 0.03892304 0.04987498
0.01835355 0 0.005867485 0.02501144 0.7517996
MIR193BHG 0.1372474 1.743614 0.1262454 0.2996732 0.3857637
1.926073 0.1817616 0.8929401 0.4540421 1.228257056
1.279083 2.619378 0.7872252 1.10299 0.383577 1.147527
0.3519966 1.136715446 0.4115546 0.5443876 0.389490267
2.167461 1.571447 2.017349 3.075183 1.356198

0.2482857	2.976674	0.341001477	0.2669376	0.6094838	
2.495673	0.2649633	1.049785	1.308896	0.3008655	
0.1020093	0.3169737	0.4549967	0.173516783	0.2375017	
0.05185247	0.265295008	0.1008364	0.302848	0.1875538	
0.136885	0.8640578	0.838493306	0.5324386	0.4074082	
0.1479888	0.7782749	0.5011777	1.070396	0.1446167	
0.4030312	2.271217	0.2359192	0.292388946	0.178080773	
0.34760524	1.093541	0.3900386	0.0809451	1.899891	0.4548
0.4755925	0.157593902	0.7943981	0.3516578	0.1421066	
1.842595	0.146258	0.5340009	1.312	0.6241369	0.30324
0.4897065	0.076826796	0.7500107	0.4925968	0.1470124	
0.3427355	1.094582	0.2467919	0.2219155	0.1696017	
0.53734334	5.440379	0.132259584	0.3721773	0.8542048	
0.2317172	0.7898776	0.2403645	0.6841904	0.10323	0.1974464
0.5167152	0.564609	0.1370375	0.1807047	0.1646308	
0.3641654	0.5768129	0.1414229	0.5857797	0.242736052	
0.496643	0.4274579	0.04722921	0.3096774	1.253648	
0.6943926	0.1245342	0.5484475	0.2330002	0.5069521	
0.2666581	0.3855126	0.5484277	0.1084084	0.9100248	
1.168509	0.3993721	0.1793339	0.9118453	0.726451	
0.8166202	0.1129647	0.647342	0.5585991	0.276593	
0.04383189	0.4748185	0.5035357	0.3799955	0.3668258	
0.4630801	0.08013806	0.5780651	0.1220212	0.1032174	
0.3331063	0.3964053	0.3054252	1.273974	0.4838235	
0.1723717	0.5617256	0.3883172	1.474647	0.4092044	
0.7880538	0.4309534	0.5431004	1.369741	5.508042	
1.836762	0.325080572	0.6498526	0.240608032	0.231875	
1.932341	0.2179018	0.1876619	0.4581232	0.2136172	
0.3620748	2.444052	0.045143733	0.3347033	1.496376	
0.3461291	0.7456148	1.934623	0.2823401	0.4149651	
0.4764516	0.2957953	0.613866	0.8343823	0.2192006	
0.9502393	1.16283	1.084475	0.9019602	1.293592	0.745862
0.5380633	0.5165356	1.033373	0.3596954	0.4720066	
0.4804436	0.7014533	0.2531991	0.2797019	0.1831786	
1.297249	0.3534533	0.05766532	0.2400875	0.8903341	
0.1520192	0.141753	0.3613279	0.5494749	0.2943898	
0.3009473	0.541827	0.1819919	0.6919919	1.204224	
0.4631696	0.3567038	0.1553258	0.6573023	0.325237081	
0.4208196	0.454740942	0.123480658	0.1966362	0.2906497	
0.7468036	0.4251303	1.598081	0.119608	0.2743187	
0.08848199	0.2507997	0.1278662	0.06187696	0.2349588	
0.4940123	0.1412142	0.182903	0.243532	0.5205041	
0.6461267	0.6287055	0.9100395	0.7774673	0.9203997	
0.4895753	1.07729	5.278639	1.028374	0.237666	0.1938768

0.3097576 2.0448 0.8530798 0.1260539 0.8624186 0.3893572
0.264209382 0.1104482 1.116899 0.3772403 0.2196934
0.4256879 0.4253373 0.3008481 0.6263502 0.4414541 0
6.088877 0.445992 0.3217473 0.3996597 0.08209567
0.161277315 0.0682391 0.1369915 0.1674881 1.17699 0.2312329
0.070135461 0.6296065 0.04143115 1.214653 0.3432894
0.3433352 0.7225688 0.06183046 0.6921261 0.6897752
0.8614347 2.468046 1.971017 1.017236 0.2005283
3.004464 1.661702 0.6578986 1.23458 0.4450194 0.1673244
0.3892682 0.3460384 0.5350292 1.457778 0.09927841
0.2622911 0.509279833 1.957644155 0.240967 0.1713138
0.4890751 1.109902 0.5400776 0.382121 0.22715 1.796794
0.1073337 0.6148365 0.3401291 1.134136 4.5708
0.234078442 0.3488589 0.4451796 0.5645525 0.351909
0.1810954 0.72071779 0.02670134 0.4117558 0.1789472
1.07027 0.3873472 0.2822395 0.7999528 0.4876268 0.2511174
0.3358163 0.1176804 0.131395 0.2743347 0.9534507
0.04051892 0.1532967 1.907128 0.7636155 0.069013888
0.1359995 0.3380663 0.7315266 0.1080186 0.9653079
0.6186091 0.607407 0.5908432 0.05561298 0.1823755
0.8759176 0.9809144 0.5916164 0.7735601 0.3260604
11.70037 0.1715957 0.1953682 0.7715307 0.6216526
0.07024298 0.3574013 0.7046856 0.7747408 1.410224
0.7831082 0.310799323 0.1620106 0.2436377 0.1278435
1.265912 0.06761497 0.217081 0.231553654 0.5145563
0.7016211 0.4878714 0.04470589 0.7077629 0.7526893
0.2319902 0.1545194 0.1083052 0.5662231 1.02623 1.955852
0.7126361 0.3279565 1.108338382 0.4036817 0.6597784
0.1512642 0.256890659 1.010917 0.4287787 0.2034619
0.02985972 0.09292165 0.3432857 0.99606
LINC01081 0.187466 1.404205 0.3736167 0.5372367 0.614733
1.613751 0.3861945 0.4119046 0.8200097 0.587186115
0.2950144 0.1227678 0.4342436 0.02929445 0.1018747
1.342545 0.4985988 1.340914685 0.1543135 0.3336569
0.76671196 1.089085 0.4769861 1.682133 1.476555
0.02455872 0.7913107 2.371737 0.905670588 0.5671707
0.1765894 1.456357 0.09047827 0 0.4069831 0 0
0.08418548 0.2658551 0.055301478 0.07569408 0 0 0.1004298
0.2573883 0.06897139 0.1454219 0.4030003 0.190882808
0.0998196 0.1442721 0.4716546 0.1340777 0.1879179
1.035621 0.1536358 0.09174997 1.313195 0 0.254146873
0.957758611 0.852193493 0.5912428 0.219369 0.04299665
0.6986698 0 1.326289 0.031391689 0.1091303 0 0
0.3090804 0.4078711 0.1458782 0.452051 0.7277505

0.1017321 0 0 0.05432629 0.6143297 0 0.578293
0.6911253 0.2722672 0.3857816 0.07721959 0.74924729
0.5638704 0.030108864 0 0.05338105 0.3428773 0.03596309
0.06383875 0.4240018 0.164502 0.02622003 0.348366
0.6650191 0.05459399 0.07199041 0.07495634 0.2579179
0.9663167 0.04507283 0.8556793 0.248664628 0.04165393
0.1513722 0.2006987 0 0.9644301 0.2213096 0.04961283
0.0349591 0.1237657 0.2077336 0.06537425 0 0.9075593
0.259131 0 0.03003347 0.1818339 0 0.03379232 1.186576
0.2024282 0.2475202 0.1067142 0 0.1259327 0.06984823
0.4380584 0.1485942 0 0.240699 0.1639868 0.04256796
0.0657982 0.2673643 0.2631711 0.2064304 0.6822263
0.04055915 1.763015 0 0.427284 0.2598785 0.3241345
0.0283123 0.4968282 1.552628 0 0.05583592 0.6659232 0
0.2090693 0.470937837 0.3797096 0.447323966 0.02842339
0.269437 0.09921059 0.2990483 0.3796215 0.2127559
0.3461903 2.089218 0.395662978 0.3666891 0.09538189 0
0.07426083 0.3022466 0.1349768 0.09017276 0.1446188 0
0.1577783 0.1749511 0.1746534 0.3142788 0.0386047
1.143995 0 0.2576752 0.02701289 0.5626884 0 0.03166787
0.23883 0 0.3828051 0.3193714 0.4034849 0.03183704 0
0.02403754 0.4352348 0.06126165 0.07651821 0.05911627 0
0.03764837 0.359871 0.02653378 0.1466014 0.07992902
0.2988787 0.2320103 0.1378403 0.0299842 0.05467283
0.1671837 0 0.4909889 0.064785128 0.2040945 0.09881614
0.163977003 0 0.1634698 0 0.06452054 0.07276058 0.03812023
0.2810187 0.07050017 0.7993229 0 0.07395295 0.1497673
0.3498811 0.1800253 0.05299361 0.24696 0.2552149 0.9126303
0.3130852 0.3729065 0 0.2281535 0.4500934 0.2601082
1.469714 0.409691 0 0.1685193 0.03525812 0 0
0.08927689 0 0.07755744 0.129547826 0.05866819 0.4826663
0.4293934 0.08752301 0.0399032 0.5164156 0 0.5703539
0.05411372 0.237934 0.1297182 0.19383 0.4272665 0.1273754
0.1744312 0 0 0.09355828 0.3262115 1.075339 0
0.149019 0.7357595 0.08803005 0.2150675 0.4030882
0.02279672 0.4242178 0.2216921 0.1575623 0.219838
0.8897375 0.02521122 2.039973 0.7976421 0.3728101 0
0.1059201 0.2695868 0.07026294 0 0.02666395 0.814167
0.4135717 0 0.453276 0.10547 0.1044934 0.101445257
0.216638937 0.1645683 0.02099975 0.2435515 0.0786081 0
0.8119044 0.2413164 2.134444 0.581541 0.2506393
0.06376614 0.1869622 0 0.186507662 0.0877774 0.4514461
0.2570405 0.1121568 0.02885843 0.04101781 0 0 0
0.4775476 0.4320795 0.257007 2.141603 0.149434 0

0.1689914 0.312549 0.2093843 0.04371656 0.1266142
0.1937066 0.03489796 0.8883525 0 0.164965456 0 0 0
0.1147553 0.4807078 0.1864996 0 0.1569229 0.3101769
0.4068739 0.49529 2.69996 0 0.04109018 0.2078372 0
0.1822974 0 0.1499355 0.03415977 0.1492475 0.2628629
0.08638082 0.5672426 0.1523566 0.0594248 0.12381844
0.2008002 0.2717739 0.0873106 0 0.3232432 0.2201367
0.056767993 0.1987807 0.07453781 0 0.23747 0.1353425
0.8567477 0.3696877 0 0.2301195 0.696063 0.03803131
0.1640392 0.227124 0.05501206 0.196243427 0.4754725
0.7470395 0.662879 0.181941198 0.03929135 0 0 0.1427487
0.05923006 0.3787217 1.909684
LINC01798 0.04520416 0.7002524 0.09147716 0.0172727 0.08301
0.9339069 0.05587444 0.249212 0.04187245 0.226543492
0.1784906 0.05328597 0.1116909 0.07628954 0 1.044871 0
0.44246276 0 0 0.084516209 0.8463667 0.1495219 1.060013
0.38391 0.1918698 0.04293242 1.515543 0.034941865 0.041029
0.2299399 0.8590285 0.1047228 0.1993205 0.08832319
0.03963753 1.260599 0.08119944 0.1468617 0.04800595
0.094912 0.007969857 0.028229898 0.005812052 0.1210261
0.06652498 0.01051979 0.04858825 0.073644892 0.01444185
0.02087323 0.2814849 0.3976651 0.1087515 0.7579793 0
0.02654868 0.6024151 0 0.012256616 0.174493116 0.012329495
0.3061458 0.0105794 0.03732441 0.5207317 0.09868789
0.2375743 0 0.06947127 0.1081014 0.006552644 0.3309103
0.1095911 0.05628158 0.08175315 0.1263487 0.01471855
0.07526914 0 0.06287921 0.3653994 0.009684079 0.3625577
0.08570715 0.04376833 0.2325613 0.122893 0.61943282
0.3875074 0.017424545 0.06056958 0.09267774 0.1221104
0.08324997 0.05541696 0.1577427 0 0 0.1099668 0.6678429
0.03949316 0.06249323 0.04337859 0.2612081 0.2659729
0.01304223 0.2701075 0.015989636 0 0.06570137 0.06533324
0.03173214 0.6079665 0.2561518 0.01435592 0.02023147
0.01432509 0.08014611 0 0 0.07781052 0.04165661 0.01678478
0.03476183 0.07892294 0.02480767 0.009778113 0.4689609
0.04183887 0.04557776 0.01029291 0.07359266 0.08198945
0.02021122 0.2650356 0.04299706 0.009734379 0.05969866
0.1067649 0.02463485 0.1237555 0 0.01903774 0.03413286
0.2120312 0.02347232 0.7729461 0.03187067 0.1324698
0.01671071 0.1193707 0.01638484 0.1976724 0.3699845
0.05677597 0.1211747 0.845699 0 0.1512402 0.027254025
0.2097564 0.138682757 0.01644913 0.06682631 0.03588435
0.01442205 0.01689953 0.1108131 0.06678219 0.5149735
0.01040806 0.09645892 0.009199871 0.009388399 0.1289283

0.1399325 0.1822649 0.06957948 0.05230845 0.0636503
0.02739274 0 0.04211462 0.1074738 0.1005356 0.5282321 0
0.06710459 0.01563285 0.09303942 0.0476357 0.009163385 0
0.1243691 0.1107681 0.1097404 0.02694277 0.110548
0.04223253 0.01391096 0.3185519 0.02658992 0.01476082
0.03421166 0.02102916 0.06536338 0.03123958 0.01535558
0.05938861 0.02312819 0.2017943 0.008391786 0.03988534
0.07808579 0.06328031 0.04837613 0.02148658 0.0663003 0
0.05061995 0.276402334 0.009489646 0.01295292 0.1103701
0.03663371 0 0 0 0.1535956 0.05099965 0.1156457
0.05896008 0.007132985 0.01083413 0.1518619 0.06511494 0
0.04083434 0.03692437 0.1286533 0.2264851 0.1198932
0.04481202 0.1603301 0.09550832 0.1806352 0.9048404
0.1975796 0.01933934 0.1544148 0 0.2020442 0.2622411
0.03229134 0 0.07480649 0.028114377 0 0.1920378 0.09111583
0.01266279 0.05773175 0.1400902 0.02134206 0.06876544
0.03914574 0.02950644 0.08257728 0.09347742 0.107149 0
0.1261831 0.008262912 0.0881036 0.03609591 0.1115545
0.5065379 0.06785115 0 0.3774113 0.01273615 0.04148783
0.03332492 0.02638575 0.08767948 0.01425525 0.2583552
0.05566061 0.3457234 0.007295095 0.7495677 0.2829222
0.05393799 0.02770762 0.1072713 0.0693399 0.06099365
0.0586291 0.03086182 0.01121841 0.1396158 0.123353
0.1065671 0.02288902 0.007559028 0.029354104 0.012537288 0
0.01215294 0.02114215 0.01137298 0.04293689 0.02349319
0.01163785 0.376598 0.02969544 0.04615211 0.03690262
0.1442646 0.120436 0.043174144 0.05079837 0.07464572
0.1580511 0 0.05010268 0.023737749 0.08618535 0.05778463
0.1375232 0.152988 0.1250261 0.08366323 0.1180366
0.06053612 0.03562835 0.08149866 0.0904389 0.01817617
0.03794935 0.04884927 0.06539255 0 0.8929204 0.08802724
0.119335621 0.1045174 0.05995575 0.1073267 0.01660274
0.1854629 0.06938402 0.08617846 0.03027137 0.01282178
0.08409475 0.2996621 0.8840549 0.006994843 0.1783472
0.09020936 0.04526117 0.05274939 0.04504289 0.06941633
0.1186133 0.03238958 0.01267696 0.04165845 0.03862042
0.1763431 0 0.035827987 0.5727331 0.01123432 0.04210685
0.1571558 0.01558889 0.09099792 0.106771162 0.01437975
0.3666592 0.00899845 0.006871409 0.0130542 0.1239539 0 0
0.02497018 0.3431466 0.05502353 0.617061 0.1204874
0.01591824 0.238496212 0.1456754 0.3122349 0 0.026323166
0.01136931 0.1769012 0.05361019 0.1101484 0.03427751 0
0.444938
AC009093.1 0.02076506 0.08604317 0.1337035 0.02644805 0

0.1682357	0.04277762	0.08295537	0.0854871	0.026016345	
0.05347288	0.01359863	0.03206658	0	0.04513745	0.1179422
0.02761416	0.057327036	0.008546422	0.04434983	0.016176459	
0.04136046	0.04402861	0.09937329	0.03792549	0.01088118	
0.06573835	0.1532477	0.093630547	0.01570596	0.136922	
0.05584029	0.4409684	0.02180005	0.4113578	0	0.01440474
0.08081646	0.2784172	0.073506967	0.5030645	0.01830523	
0.158494493	0.004449723	0.4133969	0.1731678	0.2899432	
0.6100684	0.288961468	0.04422689	0.1677963	0.6791692	
0.1410882	0.2810039	0.6342934	0.05956224	0.5792837	
0.4115417	0.1318686	0.056302189	0.840846253	0.623006629	
0.2688545	0.3887816	0.08572699	0.2438944	0.3022228	
0.1119308	0.062588955	0.1547269	0.1064092	0.06020066	
0.2328043	0.8132161	0.7917664	0.1251809	0.1880918	
0.4056683	0.104775	0.033902249	0.09628098	0.6880356	
0.2891521	0.2206366	0.3135064	0.4155134	0.09258575	
0.3592421	0.5690873	0.4450151	0.50026042	0.3091482	
0.2187758	0.134389	0.1991762	0.5232709	0.5770038	
0.06559703	0.01742589	0.4279702	0.9792739	0.06651936	
0.04784498	0.1328431	0.2571192	0.3759314	0.3894215	
0.1550963	0.153021349	0.1199609	0.09641053	0.2445388	
0.1700595	0.4654607	0.1470829	0.02747733	0.2013604	
0.4332095	0.2198736	0.05793049	0.1146069	0.2382878	
1.505322	1.040888	0.2528306	0.2752631	0.08863317	
0.1122922	0.2564555	0.6278263	0.3738692	0.2206478	
0.1549425	0.2441108	0.01547376	0.1588007	0.2304307	
0.2832013	0.1523515	0.09990381	0.5563849	0.1749185	
0.1023069	0.1311781	0.2155907	0.078367	0.1617342	
0.3668972	0.268403	0.1757934	0.1727158	0.2806995	
0.3637841	0.3370722	0.2175041	0.1086696	0.2412064	
0.401595	0.5055643	0.1852639	0.344284951	0.4282402	
0.332684451	0.04407724	0.1023248	0.2033014	0.1214572	
0.4463722	0.4241941	0.2300788	0.05713987	0.07171595	
0.4800198	0.05634757	0.07187783	0.3564449	0.3347896	
0.2691176	0.08656413	0.5846933	0.1322694	0.2167102	
0.03100611	0.2514959	0.3101408	0.367747	0.3289249	
0.1123817	0.1141677	0.1376383	0.2493093	0.4814043	
0.2665895	0.03174539	0.08331513	0.4421942	0.397978	
0.1100131	0.07052989	0.3314167	0.159754	0.102091	
0.1017866	0.1808148	0.05238507	0.08586657	0.1334463	
0.1036408	0.2527599	0.4741671	0.265605	0.279561	
0.2184423	0.2213884	0.3387686	0.4723639	0.2370361	
0.1041845	0.2755526	0.265514086	0.1291826	0.824566315	
0.029061193	0.5057564	0.8027464	0.06170311	0.07861423	

	0.06447578	0.05066958	0.1590966	0.1952274	0.5312317
	0.04513999	0.06007136	0.01658928	0.1356435	0.1196452
	0.05869945	0.4376804	0.4028389	0.2851238	0.3745388
	0.330446	0.2058492	0.5704227	0.4453759	0.4609826
	0.2147519	0.3932955	0.1480625	0.2177744	0.1562175
	0.1417951	0.5270277	0.04944468	1.37005	0.503994
	0.229594051	0.2664385	0.6348792	0.1395171	0.2035879
	0.2386778	0.1430046	0.7679582	0.1684704	0.2277726
	0.04518042	0.7873936	0.2647963	0.3596855	0.3292099
	0.1352486	0.139174358	0.01445407	0.3523479	0.2168009
	0.2825449	0.1855251	0.057772373	0.7482988	0.1218853
	0.2938095	0.3189206	0.1868592	0.0939787	0.09276779
	0.2559731	0.2495958	0.5124787	0.2178206	0.222375
	0.1596044	0.1238852	0.2015238	0.9503287	0.8427532
	0.2334845	0.1963788	0.04134881	0.08588844	0.6031665
	0.3069281	1.117129	0.04088909	0.109957	0.082403127
	0.470330459	0.09721994	0.07443457	0.1079099	0.4005306
	0.07231971	0.2518103	0.1247395	0.2075936	0.1060962
	0.9439274	0.2048324	0.7110175	0.2858391	0.314015282
	0.2074206	0.1857341	0.2633624	0.09938621	0.4858775
	0.018173684	0.1413937	0.6004009	0.04913447	0.4571772
	0.3623698	0.1708074	0.1016653	0.03310472	0.02045787
	0.2870197	0.2838851	0.09741008	0.04842354	0.1963454
	0.1716504	0.1314285	0.2693057	0.121309	0.121818226
	0.08535335	0.2295114	0.1878163	0.06355551	0.4472712
	0.08263202	0.7092684	0.01158792	0.009816393	0.3219158
	0.8777888	0.1259227	0.3695136	0.291292	1.035968
	0.3326599	0.09086642	0.1120761	0.1527928	0.03783777
	0.1487853	0.3688096	0.5485737	0.288287	0.0928185
	1.764059	0.192010015	0.1525169	0.2580308	0.2901341
	0.05156524	0.08951172	0.4667773	0.565921984	0.2422018
	0.1816393	1.274509	0.04734692	0.03997731	0.4839869
	0.1637968	0.1022798	0.06372412	0.6453625	0.1937803
	0.7631457	0.9979282	0.1035899	0.104339049	0.4956864
	0.3126011	0.02670005	0.171301235	0.2524268	0.5815783
	0.410441	0.02108247	0.03280369	0.08389975	0.1043914
AL354696.1	0.9646768	0.1677183	1.210018	0.9215177	0.2300611
	0.7105165	0.4516611	0.5955929	0.1805205	0.476128781
	0.5771317	0.8614745	0.9479965	0.3288995	0.5718924
	0.3465113	0.4664959	0.220101426	0.2526611	0.312174
	0.580708829	0.4366983	0.5578434	0.5595829	0.2803014
	0.7123016	0.5089982	0.5778723	0.112981039	0.8623107
	0.330439	0.3668506	1.015832	0.5524141	1.499317
	1.537968	0.09125419	0.892671	0.4070252	1.681576631

1.440015 11.64792 0.395540444 1.296696 1.685712
0.6668159 2.006866 0.6284211 0.5357777 1.074015
0.6411692 1.572083 1.097643 2.197732 0.8549479
1.14995 0.3862914 0.2697022 0.4983021 0.27741384
0.365074543 0.51826053 0.4949459 0.2736595 1.206847
0.653685 0.3190975 0.6499923 0.26433444 0.7147268
0.5243026 1.864484 1.243473 0.8177353 0.5231951
0.2643407 1.929198 1.023206 1.084126 1.059537356
0.609941 0.3512515 0.5949376 0.6312353 1.262461
1.868071 0.8722779 1.661697 2.10301805 0.9891842
1.042299799 0.4896142 1.123741 1.579327 0.4037707
0.9257903 0.8784114 1.015808 0.5642318 1.688941
1.390802 1.021578 2.357432 1.823385 0.9049181
0.9261503 0.4217076 0.1819512 0.568709848 2.260375
2.708595 2.229846 0.2565068 2.062478 1.345892
0.3017199 0.4906232 1.13481 0.4535028 1.834951 2.662131
1.00637 0.7812166 0.9226223 0.7586927 1.247593 2.21923
0.4110152 0.4061621 0.1623382 1.957931 0.7987463
0.7138636 1.826277 0.1307019 0.931485 0.6673283
0.6924532 0.6434322 1.150712 2.230321 2.400906
1.387194 0.8002361 0.4138695 0.8983485 4.971148
0.7497733 2.164064 1.42776 1.242745 0.3859732 0.4768088
0.4067344 0.897231 2.570112 1.567226 1.730686
2.217292 3.325337 0.352492707 1.8409 1.106094974 1.489225
0.3241143 0.6729652 0.1398968 4.917865 1.871139
3.167026 0.1930569 0.471147977 1.403506 0.6841779
1.153545 1.20431 0.2262289 0.3788584 1.321748 0.9809781
3.439915 2.332388 0.1636867 1.388969 1.149443
1.047454 0.4554629 1.661191 0.699145 0.9098506
0.2757642 0.7085164 0.5036912 0.5362863 0.7540031
0.818646 0.2614572 1.248674 1.221274 1.092437
2.271475 2.060012 0.7451241 1.193191 1.93585 0.6119613
0.7044866 0.6060603 0.9185387 1.673379 0.9347835
0.8078346 1.058226 0.902757 0.8416087 1.432274
0.4379743 1.505285 0.3062506 0.363683114 0.8183728
1.849074655 0.153419128 1.235518 0.9176673 3.316641
2.716483 0.340379 0.5706529 0.6719192 1.154316
1.631689 0.6863095 1.060935 1.261119 3.887323
0.6737365 2.726985 1.881481 2.745999 1.357433
0.6444389 1.240521 1.956085 0.8843491 0.4772621
0.4380494 0.8484563 1.022167 2.48043 0.8671791 1.616411
3.048685 0.7949347 0.5638186 0.7143441 1.257773
0.757541991 0.9605883 0.7902811 1.04454 0.2047193 1.941367
2.687608 0.4830519 0.7559771 1.265739 0.2862184

0.6068305	0.6044999	1.838879	0.5958709	1.795203	
1.255713183	0.8139263	1.196304	1.498294	0.4679543	
0.376097	0.104568161	0.2190321	0.7412591	0.3018302	
0.8889607	1.812958	0.793808	0.7835798	2.260401	
1.311232	0.2854116	0.8019908	0.3029566	0.4333267	
0.7474419	0.1343848	0.6937019	2.354138	0.1972169	
1.374392	1.521777	0.9068402	0.8383773	1.420902	
0.3710798	2.392969	1.344275	0.664394994	0.851299483	
1.334427	1.670051	0.2278701	0.5148277	0.8052264	
0.03798144	3.198534	0.462723	1.216218	2.217106	
1.849475	0.7288516	1.635555	0.802695952	0.3832535	
1.749856	0	1.993773	0.3240041	0.345391416	1.313738
1.414652	1.067202	0.5265858	0.3753838	0.9918947	
0.09541489	2.209038	0.3744024	2.872344	1.579096	
0.6660695	0.3681163	2.527188	1.963374	0.8815772	
0.2624699	1.821615	0.848891657	1.960089	2.093698	
1.388121	3.113632	0.7495946	0.2492737	2.856157	
0.538337	1.243739	2.012149	1.011055	1.396013	
0.2714055	0.9995579	0.1944553	0.6439289	1.065999	
2.330267	5.274594	0.9268502	2.05966	0.8402886	1.293105
1.935567	0.8909184	0.8339798	5.850234441	0.2147102	
0.5085508	0.7352009	1.016295	1.335736	1.471164	
0.265564553	0.6044412	2.196766	0.6110074	1.355299	
0.3376755	0.2003962	2.075309	0.633545	0.1614772	
1.133651	0.9963133	3.529978	1.664586	0.9779307	
2.019688386	1.98878	0.5695069	0.6201986	0.936246707	0.4778998
2.018797	1.34341	2.359519	2.853947	0.07874159	0.5569018
AL031282.2	0.2460053	0.3993559	0.3946834	0.3716266	0.3752062
0.7908675	0.3712531	0.3976827	0.4769453	0.071678557	
0.7857335	0.2135566	0.5035823	0.1501925	0.5409651	
0.7904684	0.4850147	0.47813882	0.1094915	0.2688175	
0.391087149	0.6381406	0.1492049	0.9103413	0.6014698	
0.3979731	0.5478821	0.6695173	0.315085633	0.1882334	
0.4284354	0.4641068	0.6847774	0.4414563	0.4680877	
0.3198044	0.06250705	0.1849793	0.4115664	0.258215111	
0.750756	0.4967689	0.208412499	0.8716568	0.5360989	
0.3872935	0.8421028	0.2828699	0.489327458	0.3769767	
0.3599347	1.039058	0.4173594	0.2709713	0.4908053	
0.446592	0.2688009	0.8767814	1.101421	0.775600812	
0.45466872	0.366699917	0.1680889	0.2543972	0.1180947	
0.5175401	0.3395711	0.3874079	0.23566944	0.3996503	
0.422656	0.1762273	1.669546	0.9468867	0.6677828	
0.3207479	0.6707226	0.9593345	0.8811632	0.165327362	
0.2934518	0.6436814	0.3339816	0.5470955	0.4158065	

0.4154501	0.9742335	0.4206483	0.44097543	0.9615023	
0.454834134	0.6547791	0.2394738	0.6978595	0.1876751	
0.5260192	0.4796899	0.3614574	0.175239	0.8321465	
1.321558	0.3898649	0.2965937	1.029377	0.6965916	
0.634392	0.2929867	0.3632146	0.442674283	0.2707628	
0.6963971	1.028981	0.07530057	1.81324	0.3748408	0.2793469
0.7073385	0.3376691	0.8579558	0.2992619	0.6186874	
0.7139602	1.652796	0.1487001	0.9898806	0.7852083	
0.617468	0.2908175	0.2543651	0.5559899	0.1895311	
0.7783478	0.53555	0.5159483	0.2174248	1.243278	0.5006385
0.2648771	0.5194395	0.2927644	0.3273685	0.9849325	
0.4762108	0.3674369	1.117765	0.483488	0.2599331	
0.6456406	1.096625	0.5225225	0.7481505	0.5368592	
0.1918146	0.4918211	1.028486	0.7993965	0.3297222	
1.47677	0.5866244	0.9570507	0.379420679	0.5435782	1.09113303
0.4553954	0.1585793	0.8220181	0.3102942	0.09357294	
1.024571	0.8804143	0.4085259	0.197587161	0.8850709	
0.1950268	0.8168868	0.5575045	0.5921749	0.5396149	
0.8971115	1.741106	0.6329403	0.5344704	0.201819	
0.3997531	0.4420626	0.565503	0.7665837	0.3289784	
1.056879	0.1656993	1.035228	0.5968503	0.5421697	
0.2623885	0.3246415	1.031387	0.1553351	1.50035	0.2156945
0.293973	0.3257065	0.7360021	0.415045	0.6585176	
0.3680363	0.3859114	0.3343433	1.41015	0.4712763	0.4778183
0.3402767	0.355724	0.3637581	0.5584243	0.700015	
0.5831392	0.4898	0.713829	0.1198713	0.803691468	0.5552281
0.585036687	0.198167128	0.5983541	1.329499	0.556365	
0.2540044	0.3530586	0.2094023	0.7318252	0.6680443	
0.5488558	0.3134043	0.6815786	0.7027253	0.2963036	
0.407928	0.2838273	0.6815312	1.708625	0.7027144	
0.5990783	0.6448833	0.404089	0.3252598	0.4285591	
0.4143602	0.4494789	1.171103	0.6282158	0.6762835	
0.4809725	1.41172	0.7571314	0.08582288	0.5836764	0.428406
0.127500924	0.3303334	0.4529451	0.5896867	0.1923128	
0.4164734	0.4787063	0.6955224	0.8354859	0.7158961	
0.3858824	0.7814505	0.1656275	0.756276	0.1710377	
0.5509575	0.577780719	1.001547	0.6766811	0.5240089	
0.4830967	0.3864269	0.133021243	1.224751	0.4190917	
0.1772116	0.4771175	0.2734125	0.8821912	0.4803548	
0.6731843	0.3497054	0.5492633	0.8078611	0.6225552	
0.3580673	0.626567	0.6662705	0.5333542	0.4991169	
0.3409392	0.262023	0.2856177	0.4720854	0.5521822	
0.3171106	0.9259461	0.3186526	0.3802776	0.352931225	
0.706091938	0.4469816	0.2749318	0.3144015	0.1511337	

0.5542789	0.2675975	0.5302404	1.32978	0.5042339	1.005491
0.4641218	0.3708691	0.4801365	0.577149226	0.3187742
0.7144436	0.02353294	0.6777106	0.6658063	1.119086019
0.08570295	0.6007306	0.1537441	0.3763141	0.7487835
0.4823626	0.7376009	1.381801	0.5918242	0.7323294
0.9700466	0.2281213	1.817091	0.4984544	0.5290795
0.9361435	0.9502972	1.036091	0.244167713	1.09129	0.364225
0.7034212	0.8194869	0.6337506	0.6683518	0.2760778
0.6081958	0.2434094	0.7982296	0.5152898	0.8066221
0.9405991	1.185014	0.3234796	1.165703	0.358834
0.5094951	0.6451745	0.4628619	0.2801145	0.5936282
0.2794323	1.072264	0.5091303	0.8351237	0.555464295
0.3781842	0.3270185	0.3383952	0.1704831	0.3674592
1.620979	0.873147388	0.3799053	1.027041	0.6946967
0.7305042	0.2932555	0.5725989	0.5923082	1.437153
0.9849398	0.795407	0.5362123	0.9811993	0.557972
0.7907375	0.283874868	1.67979	0.9043212	0.6013714
0.403940624	0.4136848	2.808462	0.5597562	0.7362282
0.401281	0.7589595	1.001346
LRRC3-DT	0.092657	2.782077	0.06818358	0	0.09722819
1.651528	0	0.5774498	0.40053	0.371485093	0.01590697	0
0.02861724	0	0	1.468999	0.07393127	1.046464367	0
0.197896	0.303164168	1.273443	0	1.485455	0.1523066	0
0	1.523932	0.035810954	0.2102474	0.1047374	0.8970059	0
0.2918259	0	0	0.07713152	0.04993144	0.02866944
0.049199976	0.05986012	0	0.05786409	0.02382645	0.2289902
0.01363592	0.02156288	0.03983741	0.018869155	0	0.02139239
0.01748401	0.1988078	0	0.2529221	0.1822466	0.05441803
0.3419438	0.05574493	0	0	0	0.09228245	0.02168507	0
0.5022904	0.02528562	0	0	0.02589061	0	0	0.01833194
0.0691181	0.05768144	0	0.02877585	0.04525392	0.04207706	0
0.09666476	0	0.01984989	0.4763787	0.07807902	0	0.2097442
0.02289994	0.6983236	0.2508288	0.017857936	0	0	0.01564344
0	0	0.05388872	0.03902726	0.01555142	0.09391826	0.06960547
0.01619018	0	0.04445752	0	0	0	0.06920644	0.049162012	0
0	0.01487961	0.06504279	0.4902988	0.3281537	0	0.04146935
0.05872557	0.02737985	0.01938713	0	0.2392376	0.1366167
0.06880902	0.01781322	0.07189862	0.1186486	0.06012791
0.5321207	0.1200626	0.04003834	0.02109784	0.07542309
0.0373461	0	0.118099	0.07050641	0	0.1427615	0
0.02524758	0.2146414	0	0.05853376	0.06996365	0.1948251
0.04811226	0.3010251	0	0.05430587	0.03425269	0.0174771
0.01679237	0.01841718	0.555238	0.05818813	0.3311697
0.08777049	0	0	0.335182798	0.122842	0.094754758	0	0

0.04413226 0 0.06927944 0.02523762 0.06844323 0.8566899
0.021333869 0.1384013 0 0 0 0.07170647 0.08005639
0.07131009 0.06433138 0 0 0.04150626 0 0.05083707 0
0.1876751 0 0.01528303 0 0.1112458 0.03905642 0
0.0283306 0 0.1297408 0 0.05522581 0.05664882 0
0.04277088 0.06073954 0.01816752 0.03025593 0 0.201154
0.02232971 0.02134439 0 0.01739021 0.07111032 0.07878606
0.03440204 0.1430709 0.106704 0.03242713 0.01983174
0.1174453 0.07765641 0 0.05187899 0.078145417 0 0.01327509
0.09695593 0.06007181 0.01913396 0 0 0.01851951 0.06272177
0 0 0.0438624 0.04441441 0.05187969 0.0266938 0.03143116
0.188325 0.3027422 0.05551715 0.0557084 0.04915009 0 0
0 0.1542734 0.3338446 0.02699919 0 0.08329237 0.0836481
0.1035348 0 0 0.06792647 0.06133369 0 0.03479682
0.3041675 0.0509357 0.07786645 0 0.03828656 0.08749154
0.2819032 0.03209551 0 0 0.03832097 0.03378893 0
0.05172865 0.067747444 0.01289928 0.01849685 0.01758909
0.6674615 0 0.022096248 0.2380298 0 0.1913388 0
0.02704203 0.323497 0 0.03115072 0.0814929 0.1206204
0.01495308 0.3649004 0.5036133 0.0789708 0 0.0628225
0.05329842 0 0.07510918 0 0.06898463 0.08176482 0.01580264
0.01680272 0.07819441 0.1239526 0 0.025698241 0 0
0.02889067 0.02331171 0 0.02407752 0 0.4168421 0
0.02702859 0.05673072 0.05544479 0.04937263 0.022123996
0.0347079 0.07650234 0.03811346 0.1330431 0.119814 0 0
0.06768214 0.05637751 0.01011569 0 0 0.1814587 0
0.05477178 0.03341029 0.055613 0.01241884 0.07778648
0.1001285 0.09574146 0.02069841 0.9983231 0.01804334 0 0
0.0491576 0 0 0.09503789 0.01580217 0.02208048 0
0.05256278 0.1551355 0.08011706 0.4214156 0.04301293
0.2924531 0 0 0 0.09232644 0.03557144 0.1013029 0
0.05196907 0 0.0197905 0 0.03524557 0.055078675 0.03402773
0.06908247 0.1208316 0 0.07988312 0.01865225 0.016834896
0.02947481 0.1768372 0 0.02816927 0 0.1016295 0.1644497
0.01825556 0.06824333 0.152905 0.02255684 1.037799
0.02245167 0.01631416 0.023278876 0.01658874 0.4266679
0.07148393 0.026977888 0.09321672 0.1493069 0.0274718
0.014111 0 0 0.3530359

AL662890.1 0.0204917 0.05878419 0.07539623 0 0.2150265
0.06225788 0 0.049118 0 0 0.03517931 0 0.09493336 0
0 0.04554376 0 0.046286492 0.07590525 0.087532
0.023945266 0.183672 0.05213882 0.04903256 0.02806968
0.03221383 0 0.01620325 0.039599137 0 0 0 0.1186809 0
0.1501432 0.4492068 0.3838082 0.2024489 3.249472

0.235752775 0.4964422 3.938009 0.063985115 0.2239484
0.08440447 0.07539185 0.1907509 0.1101288 0.020865187
0.09820053 0 0.1740016 1.384981 0.770291 0.1198615 0
0.2707853 0.04201285 0.04109453 0.19446358 0.023264784
0.223565571 0.02040887 0.3117267 0.2537954 0.06942801
0.2516437 0 0.041176686 0.4437556 0.0175014 0.1188163
0.3243383 0.1146444 0.04783736 0 0.01590992 0.03336067 0
0.080294296 0 0 0.08779868 0.08428342 0.02158461 0
0.1265081 0 0.07019947 0.1386811 0.059240994 0.06864267
0.03501014 0 0.07075954 0.04186886 0.3773984 0.04315567
0.08598247 0.08308255 0.02565618 0.08951413 0.0944303
0.04916036 0 0.03091522 0.05912233 0 0.434900055 0.05463773
0.04963898 0.3455259 2.661158 0.09036067 0.21772 0.03253874
0 0.08117215 0.2270712 0.08575183 0.8906483 0.2645448
1.189664 0.1521757 0.177278 0.01987607 0.3935988
0.2437908 0.03796193 0.01896617 0.0147579 0.2566259
0.2919054 0.02064834 0.04581019 0.1044735 0.01949119
0.08825471 0.3382784 0.1075513 0.1256325 0.4315397
0.1115875 0.08630083 0.5608933 0.4308685 0.05320171
0.08759693 0.0902965 0.1000842 0.03787603 0.03865176
0.259962 0 0 0.2573737 0 0.04852755 0.9210627
0.2742375 0 0.09055774 0.020955636 0.01864157 0.07573334
0.162669 0.06537726 0.05745601 0.05581466 0.1513667
0.05074891 0.070771875 0.1749049 0.4587479 0.06383846
0.09740842 0.07929178 0 0.1182802 0.260834 0.08243887 0
0.02294846 0.03818234 0.1124295 0.2278712 0.04789105
0.1386278 0.06759886 0.05314948 0.01757338 0.1511577
0.08307791 0.03132749 0.1761822 0.2331308 0.2094608
0.3053388 0.6055324 0.04786155 0.0630604 0.06716474
0.04017866 0.1840107 0.387716 0.09532825 0.07407543
0.2360226 0.1044136 0.2307576 0.07863257 0.02178007
0.03804119 0.06780227 0.3933047 0.03585736 0 0.04870089 0
0.021244771 0.4206905 0.129617786 0.064526917 0.01467937
0.0357374 0.09963956 0.190422 0.1431608 0.02500128
0.1228713 0.1618322 0.02382902 0.2405471 0.04850229 0
0.1147353 0.1180702 0.5039625 0.06941551 0.1464606
0.352992 0.2464056 0.1086987 0.2539242 0.5130352
0.09839842 0.4094228 0.08203546 0.119421 0.1168907
0.05526196 0.09249663 0.114487 0 0.219572 0.02503731
0.1695544 0 0.2501052 0.2967734 0.2628445 0.229609
0.4187303 0.06350492 0.07255998 0.1870342 0.07098133 0
0.08507609 0.2118734 0.1120897 0.1949257 0.02860032
0.112370936 0.2282208 0.1022675 0.1166983 0 0 0 0
0.0721685 0.02350879 0 0.0149513 0 0.1453975 0.06889186

0.234295	0.06668998	0.3472321	0	0	0.05239473	0.03140067
0.06946804	0.5304282	0	0.03322178	0.1399011	0	0.09041413
0.05242286	0.05574048	0.1210525	0.1370647	1.108886802	0
0.05396632	0.1652731	0.01597341	0	0.3503505	0.02662451
0.3429143	0.01707173	0.06730687	0.2839336	0.2091062
0.06130989	0.1091908	0.097857334	0.2686558	0.2537849
0.04214521	1.029818	0.03785379	0.053803333	0.2232518
0.2058148	0.06234128	0.1230433	0	0.1053493	0.1672116
0.1568109	0.5652797	0.1293058	0	0.8376848	0.02867165
0.1384005	0.4234771	0.04577589	0.03066468	0.01995201
0.162289712	0.2211031	0.5163975	0.3127677	0.07526263
0.1261095	0.03494754	0.04883244	0.3430613	0.05812301
0.09530343	0.3248376	0	0.7292987	0.6467792	0.06815535
0.1231053	0.5081314	0.204186	0.03933429	0.1792304
0.04894222	0.689598	0.09442191	0.10942	0.3996944	0.09743487
0.081206734	0.03762728	0.3564876	0.3054024	0.3053186
0.1766668	0.1443774	0.409546237	0.3911129	0.366644
0.02039564	0.06229819	0.4438242	0.05619008	0	0	0.5093705
0.01690797	0.04988593	1.470337	0.04965335	0.1623593
0.180189663	0.2201225	0.09073117	0.03952285	0.447475266
0.3607711	0	0	0.03120741	0.174808	0.05519686	0.08132939
AC093702.1	0.03958751	4.315432	0.1820706	0	0.2596284
3.688426	0	0.2846703	0.6722792	0.495988272	0	0
0.06113329	0.03711695	0	2.580896	0.07896746	0.924006175	0
0	1.063965799	1.655884	0	5.115152	0.2711362	0
0.1253268	6.041427	0.229502356	0	0.2237441	0	0	0	0
0	0.2471571	0.1066655	0.4287135	0.035034315	0.4475645	0
0	0	0	0	0.04606348	0.1702045	0.120927119	0	0.1370979
0.3361503	0.2123506	0	1.312162	0	0.05812498	2.840732
0	0.107337235	0.089889546	0.053987735	0.4731299	0.09264901
0.05447804	2.575231	0.1080323	0.240064	0	0.02765428	0
0.0860771	0.7049055	0	0.09241604	0	0.1229442	0.09667323
0	0.038779738	0	0.08648601	0	1.34331	0.1667955	0.03833008
0.1221992	0	0.4068509	1.071661	0	0.04420314	0
0.03341814	0.1366989	0	0.49885	0	0	0.9630332	0	0	0
0	0.1225461	0	0	0.8870493	0.035007282	0	0.1917931	0
0.555788	0.7419054	0.140203	0.03143047	0.04429423
0.03136297	0.1462248	0	0	0	0.3648075	0.07349628
0.03805331	0.1151945	0.03620884	0.08563176	0.3300205
0.03664036	0.08553149	0.04507004	0	0	0	0.7568633	0	0
0	0	0	0.1250524	0	0.1250421	0.336283	0.1600743	0
1.726113	0	0	0	0.07467057	0	0.03934352	0.144649	0
0.672085	1.687489	0	0	1.193384711	0.08747335	0	0
0.3413847	0.1571285	0	0	0	0.3411593	5.130802

0.045574259 0.1267107 0.1208517 0 0.1254543 0.03829555
0.8550982 0 0.2748545 0.07963101 0.1999098 0 0
0.2534004 0 0.5551186 0.1071249 0.06529645 0 0.03394967
0 0.04012414 0 0 0.2425127 0.5816888 0.6685283 0
0.1386942 0.7614071 0 0 0.06463391 0 0 0.2385081 0
0 0 0 0.5890707 0 0.5239435 0.1139726 0.2078163
0.2965575 0.5331442 0.08294635 0 0 0.041734337 0
0.02835878 0.03452018 0.09624584 0 0 0 0.2373727
0.5359549 0.04603482 0 0 0 0.05541373 0.2851218 0 0
0.1616825 1.690021 0.03966883 0.3674874 0 0.1238903
0.03801879 0.06591298 0 0 0.02822736 0.284692 0 0
0.3588398 0 0.1451072 0.06551174 0.041035245 0.03716718
0.5733306 0.07254057 0 0 0.1635785 0 0.1204426 0 0
0.1643568 0 0.1443625 0 0.2210096 0 0.02755595 0 0
1.235744 0 0 0.6779849 0 2.497889 0.2188821 0 0
0 0.2661816 0.1392707 1.159533 0 2.871896 4.98798
0.06748023 0.06066233 0.6039176 0.3036219 0 0 0
0.04912257 0.1746693 0 0.07178929 0 0 0.471291998 0
0.03475216 0 0 0 0 0.7715304 0.1019184 0.9234552
0.04334294 0 0.04039681 0.1184434 0.1054718 0 0.0370722
0.08171367 0.2849682 0 0.07312898 2.442627333 0 0 0 0
0 0.04070445 0.839885 0.03786747 0 0 0 0 0.05539019
0.05347464 0 0.2653006 1.066329 0.0770898 0 0.1525517
0.05250621 0.4028198 0 0.08120949 0.03375722 0.09433841 0
0.05614335 0.03682296 0.2852487 4.411199 0 0.104125
0.7241738 0 0 0.1479236 0.1899728 0.04328146 0 0 0
0.2113863 0 0.0376465 0.117661256 0.1453828 0.09838448 0
0.09830648 0 0.1992284 0.107890122 0 0.09444164 0.1576077
0 0 0.43421 0 0 0 1.894522 0 0.06928086 0 0 0
0.03543753 0.8413523 0 0 0 0 0.05868636 0 0 0
0.4085084
AP003071.4 0.1697268 8.720784 0.3122426 0.1188978 0.5788257
11.04667 0.1748252 1.586638 1.519768 0.77971365
0.3399433 0.1556111 0.2621018 0.1803525 0.2582572
5.599707 0.2482801 3.518554416 0.2654516 0.3625011
0.925547927 9.702524 0.6477761 10.04476 3.704388
0.08893928 0.4119484 11.72967 0.634110482 0.1283754
2.781903 6.734737 0.03276666 0.4989222 1.160686
0.1240216 0.02354794 0.2134141 0.9452876 0.150205533
0.5299765 0.01994947 0.376867675 0.05091883 1.293331
0.1581937 0.0658306 1.350004 1.267348861 0.1355611
0.4963569 0.8220207 0.2549199 0.2381903 4.985953
0.09736849 0.2824313 9.476662 0.07942061 0.214758066
2.183881914 0.24689718 2.670844 0.6090735 0.1245697

10.41228 1.513041 1.841208 0.352423046 0.1738946
0.03865575 0.1886233 2.205087 0.886262 1.100619
0.5525214 0.9751516 0.5802653 0.06851176 0.133010878
0.05902272 1.928153 0.01212018 3.61846 1.263372 0.1424243
1.373821 1.020724 0.46515353 5.717752 0.218078218
0.0884407 1.072922 0.2674493 0.3125764 0.2196321
0.4825922 0.1310635 0 2.213545 1.03418 0.1482839
0.07821388 0.2307359 2.591984 2.091171 0.3427852
4.12709 0.250149386 0.1206796 0.4454074 0.4088416 0.1390011
3.816999 0.6011039 0.07186905 0.1266042 0.1434294
0.9194852 0 0.03512848 0.4017105 0.3232411 0.08402845
0.04350644 0.3621809 0.1552412 0.5262285 8.500028
0.4817466 0.7660087 0.2189969 0.3684218 0.3990565
0.2276595 4.167975 1.119317 0.1218314 0.2615071
0.02969387 0.2929034 0.3097742 0.2112564 0.1787012
0.2349558 0.4026292 0.08813087 9.925387 0.05983198
0.5415925 0.09411486 2.539788 0.2050657 1.731789
8.037345 0.1421168 1.708671 4.434734 0.1589353
0.6057147 1.569059243 0.4875413 0.717420445 0.08234813
0.5854588 0.9700849 0.1444002 0.4124393 0.4314769
1.086563 8.005113 0.247499463 1.448687 0.09211335
0.04700049 0.2061837 1.401068 1.710862 0.08708273
1.008192 0.4665918 0.4685425 0.3041207 0.189752
0.4759574 0.7409758 3.411342 0.2602619 0.6345554
0.01956541 0.9024425 0.3934843 0.1376221 0.5881468
0.2529395 1.010038 1.69153 0.2922438 0.149887 0.1585693
0.2524503 0.7973721 0.09983646 0.1385552 0.04281786
0.1140499 0.8180604 0.7037663 0.2786667 0.392878
0.6657639 1.888166 0.3255869 1.722199 0.228034
0.4454941 0.6902188 0.05378338 2.28784 0.398852367 0.4857123
0.322075971 0.427566204 0.03242266 0.6216053 0.1283778
0.1285135 0.05270042 0.1518575 0.2600809 0.9319035
1.236845 0.08855032 0.1339101 0.04067862 0.9344809
1.108333 0.1343413 0.1660962 0.2310649 1.093221
0.9184076 0.6752396 0.8132299 0.6846129 0.5976708
0.4898299 11.29063 1.86286 0.2581792 1.637616 0.178762
0.4214499 0.6153934 0.04849737 0.1106009 0.908159
0.551259295 0.616153 1.933696 0.4872478 0.3645088
0.1011565 1.098741 0.2537527 0.4819579 0.3037575
0.1969547 0.4227964 0.2105863 1.031562 0.338279
0.2684733 0.155122652 0.05513336 0.1807045 0.354412
5.932067 0.3396785 0.377770129 3.81515 0.08767011 4.231839
0.4587886 0.321977 0.5267324 0.3300634 0.1141223
0.9852263 3.176149 0.3012977 12.524 9.858707 0.3086012

0.4854876 1.994663 0.1844136 0.330794 0.2293053
0.2896901 1.951628 0.5741368 0.250873 1.754393
0.2864693 0.3027379 0.19593788 0.329513896 0.1589289
0.2129414 0.07938185 0.1565734 0.1719616 1.058511
0.2184815 5.495755 0.6318144 0.9819545 0.03463932
1.794269 0.09043959 0.243157185 0.2225198 0.2335585
1.221768 0.04061753 0.4389448 0.089127484 0.2157319
0.382267 0.05737275 1.068545 0.4247244 0.1977843
4.302624 0.7468209 0.08918187 0.1224003 0.3169306
0.06824558 0 0.4432481 0.7248911 0.03791484 9.854683
0.2533937 0.199140543 0.0784857 0.7503808 0.5373023
0.1870136 1.810516 0.1833249 0.4314292 0 0.04814161
0.8735702 0.6359447 8.465595 0.3676869 0.5357084
0.7338644 0.03398818 0.3300945 0.04228033 0.477832
0.01237095 0.5810364 0.3411178 0.8550625 0.61628 1.241457
0.02152066 0.08968158 0.5402035 0.5764764 0.2424161
0.1404925 0.2633903 1.04778 0.298098279 0.3239475 0.7963181
0.0563104 0.06879972 0.04901423 3.366438 0.7028817
0.1894938 0.2083441 3.659812 0.1515033 0.3960448
0.2741762 0.2589937 0.270063968 0.5064475 4.7595 0.5019464
0.230614719 0.4268803 0.3646622 0.3019331 0.08616064
0.3003019 0.6248121 4.760303
ZBTB40-IT1 0.05321838 1.323111 0.2937139 0.1525122 0.1396097
0.2155841 0 0.1913443 0 0.266707246 0.6395417 0.4182201
0.1643657 0.09979429 0 0.4731213 0.1061577 0.080139493 0
0.6819801 0.124375043 0 0.06770408 0.4456934 0.5102923
0.4601386 0.2527194 0.5049715 0 0 0 0 0.5137044
0.1676128 0.04332581 0.6999727 0.1661294 0.2867857
0.4939964 0.235487063 1.71906 0.5160555 0.110782531 0.513185
1.150823 0.5090745 0.9907866 0.1144049 1.137955201
0.2550336 0.06143457 0.9037887 0.7993094 1.520379
0.8301032 0.4579529 0.546971 0.4909963 0.2134507
0.288591687 0.664622886 0.072576918 0.1060065 0.1245501
0.07323604 0.2163713 0 0.4302979 0.213877439 0.3717627
0.1363571 0.1157154 0.8949747 1.290205 0.6211848 0
0.7024258 0.9097199 0.7250201 0.573457155 0.09253378
0.5231928 0.5130434 0.4925023 0 0.5152799 0.4928254
0.7234033 0.54693876 0.4201919 0.30770597 0.7130792
0.1818475 0.8086456 0 0.2174727 0.1031716 0.6164306
0.5805864 0.8091419 1.066094 0.4184539 0.3678631
0.2553458 0.8237075 0.6824563 0.3070896 0.0662489
0.329427499 0.496643 0.2578318 1.324667 0.2801843
0.4693459 0.376956 0.2112634 0.1786372 0.4637814
0.2752026 0.3897311 0.3855126 1.717603 0.9317959

0.1976054 0.6650269 1.393727 0.2920581 0 0.3450642
0.2955388 0.5365822 1.090596 0.5414991 1.018878
0.1189723 0.4748185 0.6580598 1.088717 0.4099818
0.8379545 0.9788292 0.3362216 0.4968007 0.7844524
0.5525334 0.1291149 0.6908427 0.04549906 0.9380252 0
0.6885669 0.2007626 0.0964485 0.8462459 0.7000374
0.6684175 0.1426577 1.07125 0.74752 0.8902675 0.24064406
1.469905 0.70750219 0.4357212 0 0.3379702 0.1697893
1.044521 1.522023 0.1965549 0.1757313 0.12253299
0.1703401 0.05415455 0.1657929 0 0.2574078 0 1.02394
2.216958 0 0.591235 0.1787962 0.2974866 0.4866455
0.6575526 0.1658352 0.1440105 0.4827871 0.3680877
0.456393 0.7851341 0.05393978 0.1627193 0.183023
0.6986042 0.6119822 0.8987175 0.4338234 0 0.04094306
0.348863 0.1565202 0.2606664 0.4027702 1.23787 0
0.4290769 0.7231185 0.2497056 0.8849284 0.2828218
0.5433759 0.4695659 0.4085759 0.4656203 0.0569527
1.517755 0.05575332 0.386219034 0.2483097 1.122088038
0.223441191 0.7624669 3.016407 0.2156424 1.648464
0.6196642 0 0.1595527 0.1801241 0.4950851 0.1388261
0.7977708 0.7015199 0.4469635 0.1533182 0.1805277
2.10323 0.2716917 0.4783016 0.2666385 0.4940215 0.1318918
0.666194 0.3577666 0.5316497 0.8522072 0.8528975
0.2656267 0.9089547 1.261156 0.7928816 0.2894378
0.4561951 0.7802835 0.4843789 0.275822981 0.4996466
0.3596792 0.7801433 0 1.495274 0.6047312 0.56533
0.3238273 1.33649 0.05789603 0.6186559 0.9904498 0.1940698
0.1446388 0.2228311 0.389113523 1.00019 1.27486 0.3535859
0.51115 0.05705746 0.190367679 0.5696439 0.07497065 0
0.2942481 0.6601028 0.5161206 0.5873894 0.8945847
0.3744494 0.4762959 1.116497 0.2757682 0.08765298
0.6350062 0.2446492 0.7216533 0.5102071 0.2393573
0.9922116 0.3179163 0.2641463 0.4109206 0.09076387
0.9168255 0 0.5784456 0.691165488 3.394803555 0.5606172
0.3576881 0.08296809 0.5355717 0.2021966 0.0691457
0.4110334 0.2216824 0.6992025 0.7373953 0.2715316
0.4246028 1.063411 0.063535577 0.2491849 0.8787961 0
0.1910363 0.7864714 0.0698655 0.6522757 1.020438
0.1079364 0.1743011 0.2102742 0.4924792 0.3474081
0.9672158 0.8913287 0.1439213 1.277673 0.07132871
0.4467736 0.3594358 0.05498997 0.9510653 0.8760214
1.45087 0.374646823 1.72266 0.07058527 0 0.2931934 0.382101
0.1361418 0.2536425 0 0.603798 1.188046 0.6902392
0.9681752 0.7823206 0.9798428 1.150527 0.3729984

0.7762663 0.5965708 0.5618464 0.4654739 0.381319
0.9700893 0.7847049 0.9093482 0.1297543 0.4048722
0.474523966 0 0.3306511 0 0.2643112 0.1376448 0.5892198
0.386770939 0.9311018 0.12696 0.9534402 0.6067228 0
0.1459296 0.3148439 0.4194097 0.09799043 1.009957
1.101236 1.024494 0.7737192 0.5153603 0.334260782
0.4763946 0.5183973 0.3079308 0.077474961 0.5353986
1.776369 0.07889338 0.851002 0.1008864 0.2867002
1.013847
CD44-AS1 0.09900251 0.1656706 0.2504324 0.04728659 0.162323
0.2757236 0.0764823 0.2076428 0.2674749 0.093029487
2.549451 1.215652 0.1911066 0.9978579 0 0.4767483
0.04937148 0.335439072 0.03056038 0.1585865 0.05784393
0.09859809 0.6927269 0.08883498 0.2034215 3.034905
0.1567119 0.05871262 0.047829281 0 0.1398877 0.3106043
0.4061503 0.5456696 1.12839 0 0 0.1778364 0.593511
0.963771924 2.358509 0.8727469 0.721313173 0.1591136
0.8410611 2.094404 1.497573 0.6384844 4.133084308
0.9686498 0.2285743 2.288469 0.1593172 0.6326621
0.6756079 0.3346879 1.380939 0.7357974 0.4963547
0.268434516 1.967003772 0.776338109 1.158577 0.08688804
0.9196301 0.4528315 0.2026296 0.5753494 0.298408215
1.521504 0.5707485 0.4664098 1.90977 1.684739 2.869725
0.1342869 0.5764974 4.795014 0.2809916 0.727367833
13.7713 0.9732991 0.477209 0.6617038 0.1303534 0.5272182
1.629878 0.6117054 7.37668512 1.814628 0.453171739
1.354182 0.5074383 0.8357382 0.3703524 0.278139
0.7437316 0.260625 1.163151 0.9282388 0.7747116
0.7784527 1.311648 0.8906651 7.253117 1.269578
0.2142303 0.2464865 0.284531077 0.8249181 0.3897125
1.073157 0.08687146 4.010928 1.490164 0.825331
0.02769333 0.6862988 0.1462746 1.812548 0.9220778
0.2396451 0.09123295 0.2757049 0.2854971 1.752514
0.5206794 0.02676904 0.3209626 0.4352526 1.497311
2.507875 0.2518386 1.795667 0 1.041042 0.5885544
1.492362 0.326868 3.052752 2.647082 1.198827
3.234706 1.172669 1.214769 0.1000805 2.47397 0.7617799
0.1090634 0.4593641 0 1.003728 0.807407 0.6641481
0.6511421 0.3885817 1.150011 1.05504 0.6953082 0.7452767
0.26114185 1.859442 0.885884344 0.5854153 0.09147349
0.8841489 0.7106854 2.498309 1.88762 0 0.326914
0.455897872 0.8186191 0.2518602 0.1028086 0.4706138
0.2633716 0.5346185 0.3809684 1.059696 0.1742522
0.5999337 2.106567 2.144488 0.7921461 1.743131

0.6170086 0.3348795 0.8368958 0.5991615 0.3183866
1.72141 0.1756029 0.07567697 0.3830387 0.4765266 1.24916
0.3196261 0.5548431 0.6069937 0.09520831 1.318265
0.8249972 2.788287 0.5619571 1.324121 0.268413
0.08552299 1.219108 2.787174 2.849258 2.367608
1.148688 0.1364901 0.736324 1.14771 0.05297475 0.03921514
1.529844 0.205281667 0.5543186 2.739749031 0.051958641
2.695002 3.992756 0.6017415 0.3833311 2.766637
0.1509873 0.7173077 0.4467813 0.6331944 0.4519536
0.351497 1.423682 0.4503899 0.1782618 1.196419
1.369429 0.05054298 0.5931918 0.9176543 0.4595157
1.042776 3.304867 1.022103 0.1648386 0.6192839
1.586651 1.95894 2.002422 3.966088 0.2304692 0.9422753
1.750367 0.03024097 1.474516 0.615738389 0.9759708
0.5974229 0.3628264 0.6586591 0.9799056 1.329528
2.570791 1.054232 1.993312 0.296187 0.6987545
0.3582717 0.6994942 0.1009022 0.1036335 1.244152374
1.274897 0.8399525 0.8222227 0.9310855 0.291897
1.328033465 0.7947843 1.098314 0.08518433 0.1140399
1.318287 20.30701 0.604901 1.705807 1.13196 0.5437165
1.018542 0.153904 0.2853575 0.7594112 0.4551225
0.4614831 0.8542274 0.8905558 6.380109 0.2534665
0.6449534 1.119356 1.435213 2.580807 0.4177473
1.945233 1.312566828 5.903503286 0.5431874 0.4491514
0.2508124 0.06227047 0.3526384 0.1929483 2.516967
0.1855788 1.490421 0.3790449 1.41437 2.813988 0.8902214
0.768271765 0.6489846 0.2043538 0.07635678 0.08884653
0.845842 0 0.5393034 1.378548 0.2760929 0.2567007
0.9045909 1.654182 0.2423571 1.870344 2.316526
0.08924589 3.614813 0.09951996 0.103892 0.4680625
1.278728 1.575759 0.481493 2.506272 0.239579304
0.1907546 0.2954481 2.392558 1.181764 0.4315724
0.2743709 2.064356 0 0.8424371 0.9439098 1.747744
0.2814223 4.021379 1.334556 0.04116026 3.122512
1.119172 0.7090419 0.8076596 0.9471045 0.3842423
1.683196 0.9579883 0.5550781 1.086222 0.04707416
0.613027839 0.2726856 0.03075563 0.9913546 0.6760874
1.002907 37.64209 0.179878137 2.007702 0.3837999
0.123173 0.8841408 0.178689 0.3393421 1.464266
0.5120264 0.1367192 1.327434 1.054446 0.9962517
1.469342 0.4793641 0.342005154 0.77546 1.183555 0.04773714
0.288254368 0.2801266 52.13276 1.687808 1.620818
0.2345995 0.03333439 4.793751
AC246787.2 0.3894269 0 0.275546 0.7440082 1.257351 0

1.943912 0.502624 1.71113 2.402014954 0 0 2.4055
0.02808643 2.441843 0.0443856 1.613378 0.563868085
0.6287876 0.7677549 0.490063315 0 0 0.1075178 0 0
2.276038 0 1.562982025 0.06797278 1.693074 0 0.3469888
0.283041 0.02438752 0 0 0.08071391 0 0.159063014 0 0
0.031179001 0 0.4010083 0.0220424 0.8365508 0.5151757
0.213513244 0.09570333 0 0.8196215 0 0 0.1168135 0
0.9676311 0.429917 0 1.015277455 0.102029268 0.367673172
0.1491741 0.2453766 0.4534595 0 0 0.7871791
0.240777488 0.2092602 0.05116905 0.06513463 0.08890045 0
0.06993132 0.6501145 0.209322 0.07315273 1.315002 0 0
0.09816608 0.1925235 0 0 0 0.09246828 0.4442116
1.02621499 0.03378863 0.057734522 1.337942 0.1279494
0.9609267 0.1379203 2.172821 0.7549606 0.5046989 0
0.1214547 1.950293 0.1046854 0 0.107798 0 1.062049
0.1728566 0.1491625 0.132450232 0.03993624 0.01814126
0.09621125 0.473136 1.585131 0.3182752 0 0.1005525 0
0.02212969 0.0940176 0 0.8701342 0.05521004 0 0.02879498
2.93465 0.0821978 0.4859823 0.2497269 0.0831774 0
0.03410455 0.1828816 0.1207396 1.339358 0.3054503
0.4843863 0 0.06593522 0 0.1224377 0.1577122 0.5825885
0.03153984 0.2827397 0.07267703 0 0.1536649 0 0.204832
0.02768465 0.02825165 0 0.8038246 0.0218912 0 0.2409004
0.1773507 0 0.1002239 0 1.323824 0.091902346 0
0.07380747 0.6182763 0.6690049 0 0.3671684 0 0.2225627
0.034486089 0.223725 0 0 0.3085269 0.1448914 0
0.1729086 0.03466378 0 0 0.06709466 0.1395427 0.3561041
0.1850638 0.07000981 0.2431847 0 0.1294948 0 0.06313449
0 2.106628 0.2575529 0.02621566 0.07655035 0.6844205 0
0 0 0.1472777 0.1174708 0 0 0.2554863 0.07219172
2.001179 0 0.2248895 0.3065319 0.8915008 0.4170804
0.03303905 0 0.8124834 0.03205791 0.07119367 0.6590387
0.062113577 0.02795404 0.347384575 0.062886026 0 1.358316 0
0.0618599 0 0 0 0 0 0 0.07090334 0 0 3.106828
0.6859121 0.1014755 0.06117264 0 0.2101221 0.07945087 0
0.06249862 0.7479903 1.94518 0.359772 0.1745762 0.04271935
0.1884984 0 0 0.2715344 0 0.2928074 0 0.434719767
0.1687466 0 0.1372288 0.08391382 0.1912885 0 0
0.09113903 0 0.2933001 0.4726021 0.3716739 0.7373651
0.8548598 0.08361909 0.191648439 0.08340647 0.3588008
0.2274614 0.0959065 0.03211688 0.107155415 0.3527094
0.1054999 0 0.7177226 0.0437133 0.2324139 0.09446675 0
0.2107726 0.07311834 0.4350885 0.03104524 0.04933869
0.1531871 0.5967423 0 0 0.269462 0.04856544 0.05112881

0.1858555 0.4295603 0.02554488 0.08148454 0 0
0.162103246 0.540033968 0.2629699 0.02013378 1.027436
0.03768326 0 0.8951874 0.1928043 0.1247821 0.6887505
0.08738315 0 0.08962621 0 0.143053299 0.08415772 0.06183282
0.03080511 0.4301271 0.08300517 0.078652707 0.08159049
0.02735196 0 0.03270392 0.236721 0.7084254 0.04888783
0.05730871 0.02951276 0.243034 0.3895586 0.1003749 0
0.4451075 0 0 0 0.08750089 0.026360459 0 0 0.9144425
0.3300693 0.5530617 0.02554413 0.07138597 0.1003012 0
1.393199 0.3885264 0.6812161 0.509888 0.6303319
0.697433 0 0.08738997 0.1119339 0.02875053 0 0.4292788
0.0630058 0 0 0.07303694 0 0.059356262 0.1375141 0
0.08371016 0 0.05165226 0.09045381 0.190494656 0.02382294 0
0 0 0.08650759 0.4107089 0 0 0 0.04943403 0.03646301
0.2096996 0.2540511 0.2109741 0.037630183 0.05363122
0.3713804 0.1733301 0.087219234 0.1130133 1.310206 0 0
0 1.089313 0.1664486
PTOV1-AS2 0.4828671 0.7876583 1.998719 1.940021 1.285351
1.625257 0.3949716 0.9021052 1.271673 1.138785193
1.25564 0.5859374 3.509039 0.665783 1.250281 0.8627641
2.351347 1.956837219 0.3156412 0.3336569 0.796583854
0.9617892 0.9937211 1.410273 1.118603 1.752599
0.5170496 1.448646 0.439113012 0.1611281 1.163885
0.9165886 3.578004 0.9840494 2.138974 1.899095
0.1477789 2.308723 2.219121 1.281988807 4.449895
4.982804 0.502582332 2.282495 3.524464 4.075582
8.097356 3.114094 1.966671355 2.200569 0.7705444
3.979586 1.112235 2.519808 1.744495 1.76332 3.982783
2.314834 1.594934 0.712381385 1.144795646 1.685018952
0.6365054 0.6481355 1.426707 1.279932 0.8526421
1.133949 1.854963417 5.119204 0.8248071 3.005659
2.599085 2.171781 4.829453 1.874984 2.017854
9.942002 2.945202 0.556487349 0.9383631 2.559707
3.240247 2.161297 0.9873218 3.011441 2.016586
1.491742 2.18935897 1.409676 1.327527173 4.138892
0.4488861 3.308886 1.389483 3.322517 1.96858 2.990945
4.052186 2.274456 4.996534 4.504004 1.505254
1.345807 1.187008 3.438888 1.864376 0.8662869
1.230764323 3.067244 2.184576 4.470108 0.3987765
5.244871 2.363989 2.469366 2.733166 2.689091
1.563247 2.198952 1.175757 2.36821 2.251037 1.001935
2.034085 6.722346 0.8183622 0.3840036 2.012706
0.920128 4.490139 4.963828 2.31209 1.788816 0.6667331
1.900666 1.999267 3.379409 3.454187 2.180279

1.973605 2.796423 3.56854 2.556947 5.388637 1.424176
6.452592 1.578457 2.503234 1.734107 1.90315 1.446551
0.7592845 2.794659 2.656903 2.764818 2.994836
3.968633 4.149284 2.328271 1.412813512 4.032453
1.641156107 1.705403 0.7348281 2.32243 1.540552 1.499903
4.874044 2.325419 1.664809 2.19086112 2.757744
0.7370419 1.135592 2.036547 1.840957 2.740438
3.238022 3.927715 4.513669 1.69253 1.3678 4.101709
1.948009 3.404233 1.825525 2.152133 1.241526
1.854066 1.899987 2.514271 1.12277 1.389556 1.929255
2.286887 0.7712093 5.149371 1.345839 0.9785418
2.753391 3.083883 1.768234 2.005705 1.236067
4.569714 2.036435 2.453666 2.617196 3.451796
5.358877 2.732174 3.163777 1.425394 2.180669
2.186913 0.4863527 2.171418 3.035204 1.531284849
1.828899 3.937673464 0.253419005 2.278904 7.839119
1.507726 4.179171 1.752868 0.4331844 2.512137
0.7851155 2.890114 2.445131 1.826414 2.92727 4.671708
1.166073 2.432888 5.227854 2.842166 2.85064 1.906973
2.354208 1.689452 2.740806 1.977683 2.033573
2.089438 2.524358 2.308849 2.719288 2.516148
3.887968 3.4758 3.347883 2.620204 2.50299 0.780231225
3.146748 2.331059 2.264074 0.7558806 2.013298
0.7775577 2.648522 2.290057 3.689572 0.6334607
3.726449 3.024922 1.760856 2.277318 4.777037
2.063813624 4.725347 4.507808 7.818292 1.216292
2.207839 0.423349432 3.207546 1.26043 0.4236179 2.054703
1.844462 4.848204 1.847434 3.724201 1.373988
1.005288 3.437894 1.48656 1.672475 2.638721 1.066364
2.407275 4.602038 1.181695 2.693887 1.599837
2.784381 3.80674 1.307958 4.094937 1.62999 2.29173
2.93576426 3.387445189 2.954749 3.121327 1.516662
0.9290048 2.3472 0.6089283 4.515541 2.082384 4.758063
4.194583 1.434738 1.727984 3.102706 3.051943555
5.200146 2.491748 0.1168366 3.415684 2.977665
1.081378619 1.005724 2.878773 1.987486 1.643508
1.837741 1.679307 1.135698 3.423397 1.553098
1.203424 10.15784 1.35148 2.881319 3.472297 2.362634
2.28423 1.615186 3.097457 2.749424267 7.180278 1.205538
1.926815 3.83387 3.117317 0.6055182 1.912174 2.139857
2.094701 4.636777 6.324157 2.325324 3.417264
3.324569 2.031136 2.787097 3.231635 4.68762 2.412599
1.832206 3.968626 3.006992 2.02864 4.231569 2.510422
1.566654 1.407027727 1.721145 1.094155 0.7143594

0.7758813 2.203931 9.305778 2.748086914 2.913944
4.285924 5.117013 2.838846 1.948112 0.6620323
1.848438 4.728814 3.647916 2.742066 2.489322
4.821758 2.615368 4.138407 1.766190841 8.301699
1.634935 1.040884 1.137132486 2.268182 10.10208
2.884351 3.438947 1.184601 1.472806 1.025863
AL109741.1 0.3605116 0.7584084 0.5969024 0.5165737 1.229466
1.095306 0.8912181 0.6481017 0.4452541 1.761558345
0.216619 0.07082759 1.670168 0.1690065 0.3526433
1.549091 0.4314798 0.868608703 0.9347835 0.307991
0.842540615 1.184828 1.192465 1.984055 1.432111
0.3967177 0.4565254 1.339803 0.696669683 0.4908208
0.9169067 0.9048375 0.2783947 0.5677208 0.4108964
1.659613 0.3376179 0.5504435 0.9760413 0.350951687
0.5822622 0.349586 0.150092461 0.0695283 0.4083564
0.3183295 0.5453322 0.2712502 0.256957626 0.05758823
0.1664679 2.312922 0.8508777 1.572005 0.913783
0.1329541 0.9527881 2.550013 0.2530423 0.439869587
1.064176234 0.393320074 1.831177 0.5906087 0.2976691
1.074877 1.623299 1.238842 0.796865941 0.327391
0.5850159 0.7054905 0.855915 1.008473 0.6171771
0.8475955 0.9516737 0.3228038 0.4638569 0.070631087
0.4387892 1.417684 0.4247778 0.6301911 0.7594783
0.7679332 0.8902653 0.178199 1.11152071 0.7319472
0.20844598 0.9661073 0.3387643 0.6999567 0.2904711
0.8102611 1.362863 0.151848 0.09076164 0.5481284
1.760345 0.2204757 0.4153293 1.297321 3.385172
0.7614503 0.5720783 0.8526874 0.255040645 0.1441867
0.5676457 0.4052571 2.087825 2.305094 1.021429
0.2862279 0.9277608 0.3427358 0.2663251 0.2262955
0.559615 0.4266305 0.5315508 0.9370319 0.5891182
1.853307 0.3627173 0.272938 1.235552 0.4337746
0.1298183 0.6977469 0.4401864 0.3632673 2.014853
1.010904 0.6858192 0.2717172 1.110919 0.5203427
0.221026 0.683289 0.3365424 0.1897869 0.9867825
0.4664795 0 1.202056 0 0.9508243 0.1999065 1.292005
0.4246845 1.3615 1.132856 0.3395992 0.8375389 1.280622
0 1.447403 2.662610081 0.7169342 0.626745117 0.8527016
0.3553015 0.257566 0.5175835 0.3369422 1.17834 0
1.041633 1.203590179 1.230844 0.07337068 0.1497485
0.399866 0.7323668 0.5191415 0.2427728 0.3754526
0.07251757 0.4005141 0.8882135 1.041203 0.5933935
1.069053 1.376165 0.1463332 0.4459764 0.4051933
0.8965914 0.1139711 0.3288587 0.7716046 0.3099583

1.261995 0.5297265 0.7162455 0.4775556 0.2105077
0.1941494 0.9453062 0.6008355 0.08829024 0.1364222
0.2795191 0.608166 1.370278 0.3980067 0.405973
0.7378063 2.375703 0.8031127 0.3976163 0.691943
0.8516345 0.1929043 0.3427189 1.510735 0.373760355
0.4709874 0.456074493 0.378408468 0.3357314 1.88619 0
0.2605637 0.5037271 0.04398488 0.2521962 0.3660586
0.9222956 0.4231958 0.3697647 0.08640421 0.2523181
2.233006 0.3668788 0.2035384 0.3312886 0.4860162
0.6502539 0.8605535 0.3573842 0.82737 1.765751 0.7803246
1.515417 0.9454407 0.5655278 0.8750039 0.2034123
1.074227 2.287492 0.3347883 0.3523861 0.4772766
0.896869565 0.1692352 1.079038 2.246058 0.1009881
0.8748008 0.2234491 1.148896 0.7677841 0.09365837
1.608016 1.257268 0.8200498 1.2818 0.538896 0.2515835
0.230643903 0.1505663 0.2518877 0.17109 1.789025 0.4638219
0.47284875 4.090411 0.2539328 0.9926194 0.7640958
0.2367352 3.006819 0.1136883 0.4242063 1.109759
2.023908 0.2036291 0.6725185 1.573512 0.7067005
0.7181638 3.177602 1.451621 0.3242905 0.08767087
1.26141 0.8052201 0.9146298 0.1229704 1.242151 0.1216961
0.3014233 0.273121846 0.449942407 0.7278981 0.3392268
0.4496335 0.272105 0.2397008 1.171016 0.278442
1.201376 0.7894222 0.9990517 0.4414579 0.6831311
0.7203749 0.559522985 0.1350422 0.2976567 0.1482926
0.2588234 0.4661746 0.378625936 0.3436721 0.1316695 0
0.7281289 1.13955 0.5930932 1.823892 0.3793325 0
0.9099537 0.3606335 0.1449584 0.6053062 0.5356753
0.409764 0 1.996096 0.2457118 0.253793009 0.1389242
0.2390791 1.528483 0.2648199 1.146303 0.1844501
1.374579 0.7846143 0.1533842 1.542544 0.1558605
1.065774 0.1394629 3.081764 2.158309 0.1804831
1.104301 0.4041286 0.5882087 0.03941512 0.559678
0.3033034 0.6976912 0.4235069 0.1757961 0.4799695
0.392885435 0.6619787 0.6719684 0.201486 1.163822
0.5905404 1.451451 0.753267594 0.888779 1.290078
0.3588216 0.4110057 0.2082193 1.285122 0.4265627
0.2130872 0.3650934 0.7734033 0.7021166 1.766576
0.4804547 0.5712791 1.358608339 0.4840784 1.436614
0.4867293 0.367381265 0.3626894 1.784272 0.1068878
0.1647101 0.1366847 0.7283109 0.5437162
AF001548.1 0.07924701 8.512413 0.3644722 0.2018708 0.9008633
18.13787 0.08162747 3.92569 6.402653 1.456221566 9.840803
0.7265623 0.4895109 2.055672 0.1722609 18.29796

0.6850068 1.929249023 0.09784877 0.1128367 5.09315802
15.20589 0.9073577 17.69808 12.7188 2.055565 0.1254407
21.01278 1.837687957 0 1.940878 12.17077 0.4334732
0.1663938 0.301075 0.1737205 0.02748687 0.1898 1.430344
0.023377443 0.4479714 0.2328647 0.05498842 0.05094528
0.9248428 0.2332487 0.4303167 1.334481 0.64553095
0.1898841 0.1524694 0.6479891 0.1700349 0.556066
6.489521 0 0.03878521 16.32875 0 0.214869629
0.179942527 0.036024543 1.157591 0.5254884 0 15.94873
0.9371312 1.041223 0.106160983 0.2214354 0.1128045
0.1340194 7.447434 0.2955741 0.4111114 0.1910943
1.579219 0.731084 0.01999298 0 0.09186081 0.1442744
0.1980658 8.09435 2.086839 0.2557662 4.131371 1.697427
0.36197402 6.495403 0.050911351 0.02949555 0.7446656
0.3344852 0.3648619 0.1619183 0.6401326 0.2781579
0.1108392 2.972052 1.025267 0.2077053 0 0 1.253833
3.686359 0.1524281 11.80519 0.093437618 0 0.2079648
0.1696817 0.5099355 6.406571 0.2806609 0.08389078
0.177338 0.08371062 1.209891 0.02763548 0.02733635
0.3410223 0.4868522 0.04904206 0.1777436 0.486819
0.5798681 0.08570961 8.147907 2.175971 1.883403 0
0.2687805 0.2927934 0 2.89553 0.9045331 0 0.5814288
0.1039825 0.07197855 0.4172204 0.5959351 0.0278124
0.3739875 0.833143 0.03429092 4.178055 0.04656016
0.5160703 0.146477 0.6477333 0 2.073976 12.31599
0.08294453 2.761594 7.100154 0.6678751 0.3535171
3.38433055 0.8171601 0.24312348 0.07209205 0.6183053
0.4822988 0.1264159 0.148132 0.3597509 0.6178972
21.41413 0.182462762 1.550095 0.02688035 0.2468808
0.1046403 0.8688217 1.978024 0.7369574 0.3668058
0.1062712 0.6402929 0.1183306 0.5168153 0.5797275
0.3590237 6.153013 0.03574078 0.5010612 0.137029
1.381875 0.1670194 0.3212849 0.3230719 0.04542297
0.7397583 0.7594143 0.3673688 0.1076671 0.09254683 0
2.66238 0.05179394 0.04312845 0.09996024 0.08192452 1.273199
1.217018 0.08973243 0.7436688 0.03378818 3.20072 0.2451927
1.922872 0.5323558 0.4391222 0.7632698 0 2.712044
0.219091525 0.2465038 0 0.249543185 0.1135382 1.658475
0.08562963 0.1636475 0.1845473 0.09668676 0.5015757
1.460315 0.3686134 0.1378165 0.2709367 0 0.554641
0.3424572 0.3584295 0.2087934 0.3506305 4.392113
0.7940979 1.7165 0.2618651 0.137781 0.6849604 0.04398193
17.31675 1.347014 0.07534139 2.042147 0.8048468
0.1475841 0.5267768 0.2264386 0 0.8087126 0.629779091 0

4.157238 0.2178196 0.2219902 0.3373634 1.882863
0.155894 0.6831284 0.1830028 0.08621246 0.4825516
0.9559341 1.709843 0.2871737 0.1843421 0.168999078
0.09193667 0.8437256 0.3008695 14.01783 0.3115337 0
3.336456 0.0372127 2.757752 0.2190811 0.01927359
2.510598 0.1041281 0.1332118 0.4414248 2.72953 0.1065747
27.04774 20.16584 0.09005543 0.2833482 2.686518
0.02532482 0.2376165 0.5781495 0.2028884 1.540573
2.447593 0.6757783 0.742497 0.02229252 0.419636
0.257302061 0.402948422 0.04637833 0.01775434 0.3500499
0.06645958 0.02509075 0.2059285 0.3740404 4.533445
0.3759803 0.3660162 0.3234682 0.4215148 0.1055677
0.126147 0.2226354 0.0545253 0.08149351 0.189647
0.09759395 0.208072162 0.107922 0.09647781 0.05357572
3.085764 0.5479554 0.6518633 4.440349 1.642416
0.02602489 0.2857491 0.4492182 0.01770249 0.2587226
0.07136434 0.3548353 0.08851392 14.50735 1.363158
0.046490265 0.1221523 0.4905035 0.1343953 0 0.8399276
0.405455 0.5350703 0.04422372 0.1123888 1.105693
0.2284064 10.33219 0.02043766 0.3821387 0.790726
0.1322449 0 0.03290178 0.07605821 0.1444027 0.5678187
0.8704347 0.2677805 0 0.8050662 0.05024096 0.104682863
0.6305648 0.2625971 0.4182971 0.1311945 0.6832185
1.03693 0.215976408 0.3151125 1.764513 0.02629184 0.08030803
0.07628397 5.649862 0.2344156 0.05204494 0.07295833
12.53265 0.128615 0.4160631 0.09601152 0.232551
0.099548938 0.3074044 10.08201 1.171817 0.115367259
0.2657524 0.5168732 0.5482373 0.1206876 0 0.1067306
14.32128
AL359921.1 0.05882031 0.1687368 0.7574726 0.05618872 0.1543055
0.6552621 0 0.2114858 0.227021 0.257933982 0.6311267
0.1733412 0.3179179 0.1930232 0.6712597 0.2832502
0.05866611 0.465019955 0.1089409 0 0.034366788 0.2636102
0.2993233 0.6333538 0.2820036 1.109617 0.1862143
0.2790632 0 0 0.2493343 0.3163529 0.5677786 1.204166
0.4309778 0.6447117 0.1836167 0.3698026 0.2274984
0.31233021 1.47251 0.5185247 0.489775399 0.9642477 0.8782593
0.8007103 0.6159825 0.7902967 0.59892379 0.6342283
0.1697534 1.58163 0.4101719 0.6191019 0.9174825 0.2169251
0.6477288 0.844169 0.2359192 0.318969759 0.968314205
0.40108297 0.2636215 0.1376607 0.4047255 0.0597868
0.04012941 0.6539396 0.17729314 0.6574329 0.3265394
0.3197398 1.541961 0.7404312 0.8925444 0.3191352
0.4338512 1.197 0.7568192 0.432150727 0.3579596 0.7067693

0.5040426 0.6653101 0.3407659 0.2562839 0.8170527
0.4361187 0.60451126 0.9620183 0.311754733 1.018014
0.2009894 0.8689394 0.1692595 0.6309569 0.1995555
0.371628 1.135317 0.4471574 1.141492 1.181949
0.6098783 0.7408387 0.4248597 0.7764789 0.2545611
0.2196674 0.182052039 0.3528779 0.5165117 0.4722921
0.0516129 0.389063 0.93743 0.5370538 0.2961616 1.304801
0.9342403 0.2461459 0.4869633 0.9492017 1.382207
0.1638045 0.4805964 0.884324 0.3766013 0.06361712
0.7627735 0.2722067 0.5507028 0.971013 0.7181988
0.6816042 0 0.8996561 0.5035357 0.7916573 0.1294679
0.6174401 0.581001 0.681291 0.4118217 0.9289568
0.2498297 0.4756863 0.4963159 0.5783171 2.229044
0.4883864 0.7882279 0.2496325 0.4530541 0.6430355
0.4513399 0.7387772 0.7095344 0.5571827 0.3304825
1.180776 0.310304182 0.7798232 0.631596085 0.5083414
0.362314 0.5369723 0.1876619 0.9070838 1.842449
1.484507 1.068261 0.2031468 0.8785962 0.5386953
0.4581121 1.258225 0.3698544 0.4658612 0.7073268
0.8508064 0.7690679 0.5049543 0.2964253 0.6028018
0.3765099 1.344522 0.4582288 0.2387542 0.6063714
0.3559796 0.4539909 1.45663 0.2682794 0.1798477 0.354005
0.4890229 0.3194118 0.5843055 0.5394252 0.3778059
1.131321 0.8916663 0.8073145 0.6242275 0.5564588
0.7981007 0.2835059 0.8129878 0.9241169 1.600745
1.053316 0.3438517 0.382183 0.4541197 0.5927038
0.5403646 0.3147386 0.908656 0.4313546 0.365891716
0.1646685 1.302212697 0 0.6741812 1.641316 2.359582
1.062826 0.2054676 0.179412 0.2645216 0.1990845
0.2393997 0.7671971 0.8585428 1.233534 0.9468568
0.4236425 0.3491785 1.560818 0.9309015 0.903109
1.090411 0.4680203 0.1457751 0.4908798 0.1977131
0.6365806 0.412087 0.514187 0.5242632 0.9517586
1.227968 0.8763428 0.7464448 0.4832066 0.6827481
1.143737 0.548742563 0.2761205 0.3975402 0.5389148
0.16477 0.5634105 0.6987684 1.145537 0.3579144 0.9168661
0.1279807 0.6837776 1.611653 0.2681227 0.4396257
0.9030523 0.752627472 0.8802843 0.8219493 1.060758
0.3295573 0.252254 0.315609572 0.5666458 0.8286229
0.03374035 0.5149341 0.6222951 0.1711347 0.4173556
0.4449382 0.362132 0.5025036 0.7593988 0.2438371
0.484398 0.8021131 0.1352009 0.1495531 0.9022609
0.06613819 0.73905 0.4266772 0.2554573 0.7461454 0.2758744
0.6933334 0.6453097 0.7376938 1.082219646 0.978822078

0.5938116 0.751145 0.1604778 0.2959738 0.3352206
0.1528484 1.59005 0.3430243 0.6118022 1.587136 0.6602506
0.6452845 0.3526046 0.386229429 0.3580394 0.1214126
0.1814633 1.1613 0.6791077 0.193049408 0.3604681 0.7250483
0.387719 0.3852972 0.4648166 0.3326394 0.2399858
0.6470432 1.245929 0.6893071 1.235644 0.6898238
0.6584033 0.7945422 0.151946 1.675314 1.144277
0.7158896 0.491723955 0.7933305 0.7411453 1.09729 0.7561305
0.7541468 0.1504725 0.7358969 1.329397 0.3336779
0.8480462 0.5933636 0.7356858 0.8874247 1.005628
0.3912724 1.325126 0.6863828 0.7692624 1.270203
0.7717067 1.229252 1.257778 0.4336527 1.727462
0.3226784 0.4474904 0.553611294 0.1890123 0.2192739
0.3013453 0.5112335 0.456401 1.006466 0.347330481
0.584723 0.4560537 0.8488963 0.5141177 0.2123289
0.1209679 0.9569598 0.4056134 0.7852128 1.383202
0.5369806 0.4632281 0.8907951 0.7767391 0.295556902
1.790241 0.5208777 0.3970686 0.214075549 0.6657259
1.624845 0.6975836 1.321293 0.5575299 0.1584396
0.4202128
AP001610.2 0.03905542 0.01244862 0.1317238 0.07461621 0.1024555
0.02636848 0 0.0312049 0.1205897 0.114175078 0.1899714
0.2301897 0.2010387 0.02441204 0.08489562 0.08680249
0.2337182 0.127426103 0.01607432 0 0.015212539 0.01296529
0.08281009 0.01557531 0.008916398 0.153492 0.02060705
0.04117599 0.150945098 0 0.07357893 0 0.0502657 0.2460124
0.1589778 0 0 0.03507728 0.5035134 0.023042282 0.4205227
0.02295262 0.040650042 0.1171681 0.2278959 0.613079
0.4544435 0.1819099 0.384416766 0.1247745 0.01502835
0.4176109 0.1256978 0.2740469 0.1269143 0.04801119
0.01911458 0.1067638 0.1827528 0.035298177 0.014780225
0.390588684 0.609395 0.2437433 0.1433222 0.02646476
0.01776338 0.2105221 0.078479221 0.05456516 0.3335617
0.03774229 0.09014846 0.02427804 0.07091303 0.02354432
0.2122606 0.1165681 0.04926585 0.02550567 0 0.09954416
0.8366836 0.1740234 0.0137128 0 0.1205568 0.3378357
0.22299027 0.04405238 0.238361838 0.8285712 0.1445737
0.6593795 0.3446463 0.0664987 0.1892865 0.2330445
0.04370005 0.541023 0.3585888 0.6710511 0.119984
0.1717749 0.1074658 0.08838262 0.5821908 0.01620605
0.034536754 0.1214906 0.09460762 0.1672489 0.2513123
0.08610983 1.244867 0.1136961 0.1165303 0.701339
0.163494 0.08171781 0.06736107 0.09803882 0.05998403
0.07250842 0.2878208 0.1010189 0.2619625 0.02816026

0.06029348 0.2891831 0.1406365 0.01482142 0.529854
0.5509554 0 0.03318624 0.02476569 0.5046177 0.07163661
0.1366557 0.1862348 0.4797785 0.3240779 0.04112049
0.3071881 0.0631691 0.3379929 0.1558221 0.05736579
0.07630072 0.469225 0.02455565 0 0.2458265 0.1427048
0.2861438 0.2791796 0.04624467 0.9874498 0.3484488
0.353203378 0.1006806 0.146445346 0.1658031 0.03207583
0.1550166 0.1038362 0.1825104 0.3900525 0.01602733
0.2041931 0.07493617 0.6667074 0.05298994 0.1892654
0.02062801 0.1637169 0.2624549 0.3256239 0.4669982
0.1047476 0 0.2041097 0.7762374 0.119045 0.3056206
0.1217016 0.3170553 0.18252 0.2588735 0.4130847 0.4389997
0.1583394 0.039805 0.02238587 0.4671133 0.3659464
0.5172884 0.06632717 0.04561 0 0.2560204 0.5105138
0.09564776 0.09852712 0.2422499 0.2353023 0.2549086
0.0773902 0.7330069 0.1665188 0.1245328 0.8821226
0.07179183 0.6996312 0.2164133 0.5154832 0.0928197
0.1500244 0.067484509 0.08503939 0.288213742 0.0273295
0.7926991 1.010334 0.03165074 0.120976 0.4547536
0.03176686 0.2732126 0.07343767 1.695533 0.01698008
0.09244119 1.014049 0.1822297 0.2250316 0.264968 2.793
0.03987733 0.1170039 0.3783113 0.1553777 0.1935831
0.2580308 0.2250467 0.2167568 0.3517932 0.3414091
0.1113918 0.6553527 0.2938177 1.624395 0.8260388
0.1394951 0 0.9048368 0.067472826 0.5622368 0.01256943
0.2385519 0.05470188 0.2826476 0.2017249 0.3995133
0.1386277 0.06764216 0.04248822 0.1621477 0.3365103
0.07121109 0.2299834 0.3088886 0.237965931 0.08155673
0.07796523 0.3336254 0.07293964 0 0.18627375 0.1254135
0.4584898 0.04480574 0.3239102 0.0569918 0.02525106
0.07184467 0.5908588 0.1259489 0.04236845 0.420187
0.06745942 0.117931 0.06657323 0.2792859 0.1765335
0.8986228 0.2927623 0.3060363 0.01110998 0.5007773
0.05744052 0.1443194 0.03541219 0.06591873 0.03265419
0.225433905 1.353993353 0.2057103 0.008749897 0.09133181 0
0.2596758 0.05074402 0.5530167 0.05422876 0.1995484
0.6171043 0.3453999 0.4933725 0.3641895 0.124338441
0.158487 0.2149743 0.0535501 0.046732 0.2404869 0
0.07091647 0.1307551 0.0132019 0.1634464 0.1157356
0.1606294 0.1912145 0.149434 0.02565178 0.2112393
0.4948693 0.06979476 0 0.0527559 0.01345185 0.3053572
0.2922212 0.4563219 0.217662754 0.03010025 0.03453365
0.6402646 0.1673515 0.2537069 0 0.2171643 0.08717937
0.1107775 0.3390616 0.2438928 0.08881446 0.624484

0.9245291 0.02164971 0.117314 0.1329252 0.03243007
0.1999141 0.02846648 0.994983 0.1095262 0.17996 0.2224481
0.1110934 0.1857025 0.361137117 0.02390479 0.1294161
0.1697706 0 0.03367116 0.2358607 0.082786656 0.2795353
0.4348039 0.06478723 0.4056779 0.2443685 0.05354673
0.1155274 0.1025975 0.3355909 0.4081851 0.332774
0.8429793 0.0473175 1.982726 0.327072378 0.3729196
0.3112664 0.1255453 0.037904416 0.5566274 0.3296524
0.3666846 0.3469587 0.2097731 0.05260023 0.04133515
SNHG14 0.1295034 3.254271 0.2758813 0.4272912 0.3782895
2.773914 0.2970666 1.032657 0.7972707 0.755524682
1.208131 0.2614519 0.8085347 0.2070869 0.4870199
2.549244 0.275761 1.67915608 0.1456776 0.170537
0.529188212 3.04079 0.4967872 3.711382 1.708104 0.5233257
0.3948926 3.130149 0.306298075 0.2388703 0.5141532
2.889296 0.07899191 0.4328978 0.3311683 0.1349782
3.130781 0.3446557 0.2682618 0.094921124 0.1533419
0.02381303 0.075251426 0.0288578 0.3820657 0.06196921
0.42202 0.2442376 0.508848587 0.1342111 0.06007395 0.932121
4.241729 0.169039 1.721029 0.03369566 0.169731
1.748638 0.04023102 0.116865311 0.615175511 0.148981821
0.6678471 0.1427101 0.04373377 1.796539 0.2661403
0.519536 0.071443065 0.1029536 0.04648139 0.1281244
1.280702 1.15458 0.2707919 0.2090657 0.4836149 0.2189168
0.4197252 0.038525257 0.04006179 0.5788648 0.1642483
0.9190737 0.4661386 0.1650071 1.161245 0.3303733
0.52393841 1.226832 0.094554823 0.263922 0.3983984
0.6569348 0.08779112 0.205757 0.3188324 0.1510083
0.05667277 0.5600962 1.032038 0.07843604 0.1006838
0.1315164 0.6005117 0.9307423 0.1461327 0.7472143
0.07939119 0.07626246 0.8995058 0.1744755 0.1617382
1.767487 0.6556146 0.06591779 0.06667207 0.08151217
0.2030779 0.05070244 0.0797525 0.309843 0.3096989
0.220517 0.08629912 1.046516 0.1344379 0.04726101
1.823624 0.4151062 0.1893949 0.5562865 0.2295875
0.2237601 0.07903834 0.7736687 0.2399373 0.05817045
0.5246261 1.103995 0.2016051 0.244699 0.1863461
0.1049819 0.2110942 1.081041 0.03712904 0.8551853
0.02870783 0.4326691 0.0752617 0.2847335 0.04211661
0.6601079 1.964477 0.1359616 0.6651899 1.437465
0.06919052 0.361511 0.409554832 1.20913 0.724738525 0.06179656
0.1526884 0.3011576 0.1324429 0.4459055 0.4030512
0.2210564 2.787141 0.60412654 0.6870379 0.05861449
0.02598887 0.1296666 0.5333881 0.4347542 0.1062408

1.445239 0.1242562 0.09628991 0.23356 0.4256141 0.2826144
0.1673736 1.142564 0.08546007 0.2493157 0.05666923
0.3795126 0.1440046 0.04912144 0.3164091 0.09290019
1.210517 0.4329596 2.174342 0.07771884 0.1368562
0.05745664 0.5195172 0.08918392 0.04021218 1.082335
0.2171426 0.2182747 0.6698541 0.1217864 0.08835044
1.114817 0.4973821 0.4292029 0.2094289 0.6168269
0.1824707 0.1351897 0.7836112 0.7099893 0.072897219
0.1000897 0.963222978 0.068383158 0.05833738 1.331217
0.05150936 0.1164873 0.13969 0.01890221 0.3295058 0.1877874
0.4841202 0.03782389 0.07647446 0.05474534 0.5026796
0.3404731 0.3971912 0.2736218 2.320493 0.6542563
0.4939997 0.5368142 0.1368468 0.3369086 0.2956688
0.300284 2.981074 0.9080906 0.1311465 0.5149392
0.06948375 0.226382 0.4709923 0.07775386 1.873044
0.2330456 0.22153624 0.1413527 0.6405246 0.2271125
0.03171462 0.6894766 0.5942105 0.167859 0.2797928
0.06088959 0.09464441 0.282356 0.2152253 0.315441
0.08799224 0.1275218 0.051192638 0.05779193 0.1526561
0.4351157 2.098838 1.367586 0.142100392 1.131489
0.08953922 0.4771585 0.2441688 0.1118799 0.6765155
0.124021 0.6791168 0.2452688 0.7246392 0.1288583
2.562805 2.017169 0.2237966 0.1795753 0.5413732
0.2951551 0.2939102 0.06569143 0.1278081 0.5289155
0.860166 0.1744586 0.5510951 0.08481677 0.1285382
1.312161796 0.225861774 0.390227 1.713053 0.1408947
0.0619657 0.06640924 0.2503278 0.1488062 1.47968 0.4875615
0.8650449 0.2213325 0.3965784 0.1963296 0.17927133
0.1153229 0.1188963 0.3713366 0.3351088 0.06824612
0.033376808 0.1211822 0.1269514 0.04874448 0.4083206
0.4842205 0.3304425 0.7254576 0.2355936 0.5357899
0.1636522 0.401358 0.05989892 1.417355 0.1175161
0.1617267 0.03948957 2.397465 0.7256133 0.088091635
0.07194826 0.1427859 0.1717929 0.1422557 1.542634
0.1216093 0.47333 0.1436518 0.06084537 0.5997119 0.8850518
1.706467 0.1303164 0.4957887 0.437995 0.06960639
0.0747485 0.1123175 0.1300114 0.03083655 0.2158493
0.1523448 0.1219081 0.1573933 0.3414152 0.0721545
0.157820013 0.1747004 1.435971 0.07844657 0.3067948
0.1164446 0.4346266 0.20065071 0.153221 0.2663026
0.04230647 0.09993764 0.05965389 0.5163256 0.1680364
0.01800149 0.09801454 0.923345 0.09864246 0.4713554
0.3229436 0.2427056 0.574371398 0.381565 1.386368
0.5072148 0.06534944 0.06893954 0.7114539 0.1242582

0.06201078	0.0406657	0.1086091	1.634978
AC019080.1	0.09927517	0.1607992	0.1695889	0.162572	0.05580689
0.344706	0.1126917	0.1384051	0.08757927	0.137072871
1.933876	2.272814	0.1814647	1.607049	0.09909027
0.2566671	0.07274565	0.140341899	0.08005105	0.08221612
0.158621107	0.1735263	0.6649936	0.4120691	0.3288685
1.103601	0.1379014	0.3155984	0.129201089	0.1746951
0.1488611	0.1906897	0.2640159	0.8422947	0.1649414
0.1554465	0.2044937	0.1419331	0.3275456	0.139853898
0.4106649	0.4536495	0.179243385	0.3855293	0.2023689
0.1207552	0.37955	0.09146326	0.379582881	0.3916016	0.09121376
0.4606747	0.4129747	0.1645035	0.3436721	0.1444554
0.08329275	0.436152	0.1157965	0.131840241	0.418636335
0.243144315	0.4237452	0.189665	0.1171001	0.2594735
0.1382229	0.2375309	0.132312619	0.2759836	0.2682076
0.09985312	0.6654	0.3060435	0.3436916	0.09893145	0.2170769
0.1533749	0.07973772	0.224269213	0.2501161	0.2766382
0.2408893	0.4062406	0.2902349	0.1059302	0.2960206
0.1902761	0.33315137	0.6307273	0.363145394	0.1312102
0.2682638	0.2599634	0.1749007	0.1800723	0.2160258
0.08960324	0.3366446	0.3018796	0.5885	0.1858563	0.03734546
0.2527464	0.4222951	0.4111129	0.1461363	0.1412374
0.112871756	0.2079792	0.1963131	0.2456432	0.1848881
0.2970555	0.5094504	0.1319019	0.2085554	0.3097853
0.2454603	0.2331539	0.660441	0.5863228	0.3173942
0.206878	0.4635064	0.3478325	0.1000679	0.05916365
0.2270754	0.1293884	0.13278	0.207595	0.2473785	0.3164341
0.02717565	0.3098802	0.3622954	0.5693573	0.2541876
0.3376182	0.4733912	0.484265	0.2710893	0.1301222
0.1587183	0.3850399	0.1788426	0.336037	0.346393
0.2612377	0.2359237	0.2044512	0.249682	0.360423
0.3819894	0.05089331	0.2009463	0.4534054	0.4951708
0.2643614	0.213764142	0.3849998	0.271418007	0.1750946
0.1647314	0.3747375	0.2230038	0.2310152	0.3311052
0.4764079	0.4800139	0.118953202	0.3912525	0.3958395
0.1598974	0.2728731	0.3135846	0.1108651	0.2475318
0.124255	0.2587869	0.2312218	1.654046	0.2812453
0.2278771	0.4556012	0.3866925	0.1206145	0.4594927
0.2452293	0.2866856	0.3565457	0.4784626	0.136284
0.2473528	0.7411381	0.2316873	0.2817638	0.216768
0.1845516	0.1714573	0.2755852	0.04965603	0.1769704
0.513671	0.1947864	0.06347355	0.6650662	0.160013
0.4696109	0.2824712	0.2993233	0.4475768	0.3888112
0.3324771	0.1258558	0.1561097	0.4044666	0.1761699

0.243655684 0.2420007 0.254171483 0.104203487 0.1567464
0.4840721 0.3053936 0.1527088 1.561699 0.1087631
0.4413899 0.1760046 0.2638705 0.1506257 0.2717418
0.04855794 0.3091222 0.1400838 0.05154517 0.3889114
0.1117077 0.2154725 0.3045279 0.2767374 0.1857815
0.2789821 0.1751165 0.3069719 0.2494096 0.3601197
0.1126811 0.6374404 0.5761466 0.2867575 0.5289071
0.3169541 0.2574468 0.1776975 0.170109429 0.2035306
0.1819211 0.1726316 0.03405232 0.5640757 0.1632478
0.2367433 0.2619723 0.2000118 0.03967384 0.667874
0.4524771 0.2899883 0.2450347 0.2516677 0.188872807
0.2369251 0.483317 0.1519172 0.327568 0.1477081
0.207755885 0.3252952 0.1783833 0.09064867 0.2203065
0.2572141 0.3183091 0.1277824 0.3065116 0.1977922
0.3560581 0.1389588 0.2918582 0.298657 0.2279328
0.3508196 0.3090751 0.2699878 0.164022 0.1855828
0.0795345 0.1734669 0.1318545 0.3835473 0.3251541
0.1863668 0.1710895 0.164454206 0.216336832 0.3325904
0.1620445 0.1989915 0.1835029 0.317527 0.1763693
0.2164646 0.2633108 0.3305148 0.1876435 0.3597349
0.3293543 0.1916245 0.249136368 0.1366052 0.1589149
0.22293 0.1018185 0.05613932 0.090432516 0.2014162 0.3181844
0.1212196 0.2156585 0.2481593 0.1437399 0.1388712
0.3023272 0.07185788 0.3451822 0.3100865 0.1384897
0.2296171 0.1313637 0.2051599 0.2534494 0.43052 0.641116
0.324478839 0.1436551 0.09136417 0.4020053 0.03720625
0.3969141 0.105386 0.22692 0.3290106 0.0948197 0.3411015
0.2919712 0.317904 0.1003215 0.5169077 0.0673855
0.3428298 0.08274689 0.25992 0.11667 0.09081803 0.1814602
0.1690317 0.4985177 0.5517386 0.5285528 0.7726011
0.122441433 0.1450888 0.1460195 0.4170722 0.427649
0.1956318 0.258983 0.244792672 0.2014037 0.3069189
0.3004624 0.07853299 0.09068761 0.1638884 0.1857847
0.2055742 0.1977163 0.1822152 0.2737396 0.6134028
0.4344685 0.301431 0.24432594 0.6257038 0.4521198
0.0742452 0.235957766 0.04840874 0.4104221 0.1051215
0.1866722 0.04992967 0.1009608 0.3087772
AL049552.1 0.2014366 0.5056255 0.4632229 0.1443183 0.132109
0.8160062 0.3112313 0.3017735 0.03887292 0.094641783
0.4538862 0.3462819 0.2721862 0.02360818 0 0.7648247
0.2511357 0.398128003 0.09327005 0 0.294232121 0.727225
0.2242331 0.9037458 0.3621572 0.4354171 0.2789987
1.015413 0.048658212 0.1142697 0 0.1579937 0.4861051
0.396519 0.4099808 0 0 0.3392223 1.051776

0.623938685	0.7726831	1.176431	0.340699481	1.084537	
1.400134	0.7411137	1.611423	0.1623875	1.205009754	
0.2212207	0.1453348	1.686707	0.2431175	0.2271624	
0.9818812	0.2476279	0.6654656	1.677783	0.6059484	
0.341358451	0.943372866	0.377727012	0.7021783	0.0883939	
0.1386027	0.7848614	0.1030707	0.4835259	0.075894984	
0.6859891	0.3440831	0.5474921	2.291588	0.8452293	
0.6074046	0	1.094779	1.147792	1.753284	0.369986931
0.1313434	1.237709	0.8361036	1.294561	0.2387026	
0.1950383	0.9845121	0.5289609	1.29388459	0.766832	
0.533819196	1.180844	0.279626	0.9139891	0.2028767	
0.2572359	0.2928856	0.3711986	1.267832	1.020893	
0.9142403	1.165918	0.05801653	0.6644743	0.6755258	
0.8927105	0.07264772	0.2507584	0.333994952	0.1342744	
0.3659692	0.6874019	0	0.9992919	0.6242313	0.05997391
0.08451984	1.017367	0.9114595	0.7902693	0.6774866	
2.221275	1.949096	0.4207248	0.9439464	1.294424	
1.105468	0	0.7929898	0.1165252	0.6346921	0.6020014
1.178535	0.8119056	0	0.9307101	0.3353026	0.4066676
0.4156662	0.5947008	0.3258994	1.007499	0.568049	
0.5302191	0.5703795	0.9570611	0.719099	0.904146	
0.6657215	0.6149018	0.4421387	0.4036999	0.2509834	
0.7507316	1.380056	1.027822	0.8774562	1.580173	0
0.3369747	0.151810117	1.947305	1.441980201	0.6184673	
0.09305882	0.6796012	0.200834	0.8001355	1.097334	
0.7439791	1.060098	0.318861846	1.826801	0.1537351	
0.4183614	0.1196925	0.7307349	0.2538125	1.792516	
1.748208	0.8863607	0.381457	0.02819836	0.8210526	
0.9209997	1.524451	0.6669334	0.61323	0.7683366	0.9796269
0.2159364	1.06136	0.0765627	0.1539771	0.1731898	1.145856
0.2412928	0.8754456	0.3078868	1.411459	0.3680618	
0.9490948	1.061462	1.007202	0.2382068	1.581342	
0.06068108	1.276076	0.6842681	0.6852407	0.9984201	
0.588779	0.5842981	0.7775914	0.5074448	0.5507553	
0.188625	0.4787373	0.580337	0.469888668	0.2114721	
1.990879773	0.23786612	1.352817	1.031956	0.4081135	
0.3899746	0	0.3993705	1.207844	0.2272622	0.6441683
0.5254702	1.092632	2.082012	0.9163913	0.2901621	
0.5765466	1.194139	1.491149	1.225803	0.8830956	
0.8013936	0.5616258	0.420268	0.5078177	0.9223248	
1.083629	0.8070746	0.7361128	0.950659	1.619613	
0.5158196	1.369437	0.503633	0.1845905	1.020882	
0.130502034	0.7801242	0.4375993	0.5536719	0.1410683	
2.09025	0.4681975	0.8915949	0.3830366	0.3488788	0.2739275

0.5436023 1.900508 0.5738848 0.4448206 0.6325777
0.529298919 1.437208 0.7288466 0.5735813 0.9673758
0.2969559 0.090069976 1.212838 0.7626347 0.08666066
0.2320327 1.157418 0.5372305 0.6749379 0.3386088
1.040849 0.9218994 0.2438104 1.174285 0.290303
1.22324 0.3472577 0.3841209 0.7966127 0.05662438 1.020548
0.6016718 0.6561311 0.7776869 0.3650218 1.963443
0.9987202 0.1894735 0.872042419 0.7681859 1.016787
0.6430947 0.1177658 0.1583742 0.2630833 0.09814615
1.328913 0.5034534 0.909751 1.065029 0.5138866
1.58205 0.6373092 3.246590908 0.4244349 0.6236863 0
0.813477 0.2558247 0.033055947 0.377197 0.6437428
0.2808779 0.2061708 0.3482097 0.2330101 0.08218579
0.4094544 0.8930552 0.4993594 1.989859 0.2193636
0.7750787 0.6802493 0.8325691 0.7874722 1.243434
0.7600092 0.930611021 1.37783 0.7347229 0.2989151 0.6011228
0.4907052 0.1717699 0.4800305 0.3372346 0.9641671
1.241323 0.7257282 0.7443791 0.5259952 0.9271996
0.04187361 0.4285927 1.028385 0.7840547 0.3866622
1.321397 0.4209709 1.129809 0.8353639 1.989889
0.399045 0.38312 1.172466207 0.2542939 0.3128865 0.2345432
0.4376939 1.432746 1.87544 1.280969261 0.9411507 0.9611115
0.3508616 0.8229138 0.03635718 0.06904466 0.8937856
0.4712908 0.4404474 0.914144 1.930897 2.952421
1.037213 1.485183 0.980536914 3.719098 0.8473064
0.1456934 0.403218902 0.1266584 1.275189 0.8212021
2.530883 0.7159959 0.2373847 1.219208
AP000442.1 0.3058656 0.1462386 0.474773 0.8217601 0.1253732
0.4452804 0.1772175 0.2291096 0.2065891 0.670628353
0.5415067 0.6760307 0.8561074 0.2509301 0.249325
0.4673629 0.3813297 0.215902122 0.1652271 0.04082907
0.536121897 0.5140398 0.2675202 0.3201955 0.3273257
0.796381 0.3328581 0.3174344 0.33247644 0.9976795
0.1620673 0.3084441 1.679205 0.3010414 0.6536497
0.796219 0.09945906 0.7039457 0.4288344 0.727469113
1.651824 1.011123 0.577016642 0.7373659 1.062997
1.125323 1.735017 0.4931452 0.992716182 0.9924498
0.3089512 1.533071 0.9844126 0.7760884 1.118182
0.5405049 0.7859109 0.4899195 0.709232 0.310995515
0.390664697 0.469267075 0.5711798 0.4250273 0.5524503
0.4792908 0.1304206 0.328456 0.345721623 0.627643
0.506136 0.6512035 1.248116 0.8734346 1.026426
0.2074379 0.2968456 1.633905 1.186425 0.449436756
0.8642168 0.7726273 0.839546 0.4324516 0.2819064

0.8699416	0.7081123	0.7086929	0.85135336	0.8624992	
0.957937269	1.067273	0.5389028	1.113484	0.3080523	
0.7811847	0.6670856	0.9259731	1.090891	0.8719569	
1.388202	0.8183711	0.924982	0.6420602	0.4142382	
0.8075381	0.3309295	0.2141757	0.777622281	0.7900544	
0.6251572	2.532666	0.7716129	0.6533017	1.083252	
0.7892357	0.4705679	1.423631	0.4801561	1.419954	
0.9495784	1.357358	1.321227	0.2129458	1.249551	
1.186706	0.297246	0.1033778	0.3541448	0.1592409	
0.7159136	1.458194	0.894756	0.7897718	0	0.779702
0.6545964	0.7409912	0.7994646	1.053507	1.028772	
0.6038716	0.6394675	1.569937	1.172812	0.3401157	
2.65529	0.5393435	1.095083	0.5602079	0.6890199	0.3605803
0.3810985	0.8359462	0.5727718	0.7803334	0.9053134	
0.7469731	0.8592546	1.087298	0.288139598	1.182732	
1.036118339	0.7304063	0.02355041	0.698064	0.3354456	
0.4645369	1.926561	1.011999	0.1735924	1.100378499	
0.7546514	0.5641337	1.191091	0.5300855	0.2034199	
0.5230351	1.47124	1.349379	0.7882946	0.6757477	0.128451
1.353564	0.6467902	1.204619	0.5212353	0.6724909	
0.9301737	0.6280497	0.6229764	0.8662302	0.4650177	
0.3214778	0.2301032	0.8030271	0.622853	1.709094	
0.564898	0.6474219	0.661823	0.9242001	0.5060132	
1.373301	0.831906	1.689693	0.5067669	0.264221	
0.5519725	1.004605	1.418214	0.2641406	0.9404432	
0.5271032	0.990662	0.8530041	0.3682442	2.241352	
0.6208425	0.237829615	0.4638164	1.471190297	0.401312139	
1.164016	1.766979	0.3098441	1.20403	0.4006619	0.06997068
0.5349215	0.4313497	0.9114288	0.8228189	0.9200331	
0.9621563	0.401385	0.2753676	0.7619574	1.748449	
0.5855671	2.219225	0.7853907	0.3802665	0.5211461	
0.6580858	0.6425676	0.8593838	0.248724	1.030516	
0.7224347	0.7904884	1.294344	1.317253	1.074347	
0.3823635	3.853933	1.439405	0.336866962	0.7897047	
0.9044039	0.3327799	0.2945265	0.9277493	0.5134429	
0.7220355	0.639772	0.9932717	0.3743436	0.9841513	
0.8696844	0.6099792	0.3896682	1.040563	0.594038112	
1.011303	0.7632386	0.7257816	0.3978227	0.3894171	
0.136764148	0.4501686	1.063745	0.08772492	0.5108688	
0.5858157	1.297772	0.4973488	1.188974	0.5043981	
0.2177516	1.480828	0.1386824	0.3148587	0.5050806	
0.3222287	0.2268223	1.081303	0.4298983	0.8677878	
0.3099684	0.9488413	1.075423	1.07591	0.9880001	0.6614424
0.7352348	0.496547838	1.033880819	0.8558633	0.5139413	

0.1937196 0.2885745 0.8171002 0.07451359 1.328828
0.4618578 0.8790632 1.087403 0.7802962 0.8198047
1.808078 0.798792683 0.590765 0.2367546 0.3341949
0.9607116 0.8122128 0.476832038 0.4165409 1.413844
0.2132455 0.4174053 0.5476121 0.6879588 0 1.609168
1.111194 0.534213 2.141783 0.4483854 0.6686908
1.291131 0.6518482 1.921684 0.7437799 1.042335
0.639241142 1.134463 0.3549696 0.7780783 1.228712
0.6666658 0.4075297 0.9338882 1.504188 0.4880039
1.013552 1.322353 0.7390298 1.242395 1.458161
0.06358177 1.33985 1.226909 1.142904 0.3302528 0.647912
0.6392111 1.246437 0.8103885 0.8982805 0.466091
0.9271441 1.003784689 0.4914321 0.7126402 0.534203
0.6646036 1.170162 1.885644 0.503629197 0.8057483
0.9805155 0.9513493 1.598236 0.552055 0.1572583
1.017857 0.6026256 0.7215834 1.798163 1.140189
2.141143 0.6021775 1.430495 0.888517936 1.129431
0.5417128 0.2580946 0.834894641 0.721203 1.188168
1.133573 0.9752931 0.65231 0.2317179 0.3490101
LINC01094 0.01969314 0.1129868 0.09962982 0.1740118 0.1226966
0.06980365 0.1369217 0.05310437 0.1824169 0.450288776
0.6803935 1.063974 0.4409635 0.4200592 0.2568445
0.1313068 0.1964152 0.074137857 0.03039468 0.1261813
0.086295459 0.1078698 0.5010698 0.3593035 0.134879
0.5262939 0.210414 0.1090027 0.161737852 0.0335142
0.180868 0.1412207 0.6415648 0.2170844 0.665347
0.3777386 0.2766384 0.2034031 0.3427508 0.331134231
0.3419187 0.3124854 2.244444312 0.4779177 0.2230669
0.1521531 0.2807047 0.8202375 1.14296822 0.1140348
0.4546699 1.635048 0.2746526 0.9623547 1.526275
0.2541941 0.983102 0.4441317 0.3060714 0.186885365
2.280532194 1.101122937 3.123456 2.223796 0.1287277
0.4570485 0.6448991 0.8409292 0.217646204 0.288894
0.6895945 0.4781543 0.3019583 1.395572 1.36387 0.4540998
0.3898927 0.3807198 0.3838028 0.34724358 0.6848315
0.9357546 2.12525 0.3999321 0.5341441 0.6721338 0.5673644
0.7848216 1.33241028 1.005129 3.121792284 1.121451
2.014542 0.4446957 0.9973634 1.5944 2.257276 0.2851331
0.9089492 0.8633223 1.010911 0.1892567 0.6239087
0.36024 0.4724525 0.6462025 0.6107972 0.643519
0.740123456 1.063297 0.2265966 0.1778897 1.200966
0.6241576 0.5928339 0.4729695 0.4957772 0.1287147
0.5200965 0.1751215 0.2088901 0.7521135 0.2359196
4.487905 0.5584333 0.6351248 1.999376 1.182101

0.7250909	1.198429	0.9006071	0.4652248	0.2604921	
0.7044505	0.4292435	0.4894605	0.7726795	1.065494	
0.7314654	0.2713201	0.6271598	0.6946604	0.1914981	
0.0881213	0.4460983	0.2866691	0.1597764	0.5471914	
0.1171498	0.5578666	0.3822001	1.091149	1.374072	
1.644028	0.7591448	0.123672	0.3915232	0.6703988	
0.4979076	1.976629	1.254105386	0.4460222	1.293930641	
0.3179933	0.3760404	0.6917594	0.5261972	1.964803	
0.5900353	0.1454679	0.2601132	0.935191215	0.2048583	
1.001978	0.2147274	0.6981927	0.7143918	0.5175406	
0.1468248	0.3019426	0.3070017	0.1740319	1.031032	
1.440254	1.05347	1.131454	1.004874	0.5262409	0.3085816
0.6257086	0.6966527	0.3683559	0.9131743	0.2784866	
0.4571543	0.6161258	2.582277	0.1907359	0.3160509	
0.5692054	0.1098428	0.2864288	0.4295685	0.3456414	
0.1583579	0.2557539	1.441572	0.7598631	0.2132323	
0.8454791	1.158713	0.7901567	0.7083254	0.1520401	
0.4346743	0.74089	0.6322503	0.2535164	0.809774	
0.551255361	0.7626498	1.406564543	0.180869246	0.8111707	
0.6439634	0.3750468	0.1779179	0.5388622	0.09610793	
0.4329713	0.4499134	2.003786	0.1348509	0.1825643	
0.4424885	0.3859244	0.758824	0.3715928	2.262594	
0.2412909	0.276551	1.613222	1.162147	1.305554	
1.807822	1.80617	1.926352	0.3350638	1.757372	0.6002929
1.172811	1.544499	0.467608	0.6961807	0.6400799	
0.4631859	0.6762294	2.061746408	0.5962743	0.3422498	
0.3743915	0.4275311	1.251251	0.6204731	0.8309769	
0.4343851	0.3325491	0.3909895	1.598422	0.5395842	
1.526055	0.9567185	0.2405005	0.589455563	0.1302254	
0.5602083	0.2243012	0.4137613	0.5753503	3.469388046	
0.8273639	1.081955	1.253893	1.320228	0.4633891	
0.8594431	0.1203243	0.1241384	0.649513	0.7370474	
0.2900017	0.4132878	0.6000561	0.3063136	1.976593	
0.4256006	2.954706	0.7528682	0.4350071	0.1848677	
0.27491	0.9177831	0.8186742	1.232071	0.1828122	0.3210755
0.463568042	1.324500065	0.8298122	0.1290513	0.8519763	
1.368716	0.916565	1.791087	0.5830518	0.4634822	
0.5444231	0.5816413	1.738281	1.703789	1.455982	
1.034482347	0.2812387	0.3760047	3.346545	0.07069185	
0.9958651	0.122803236	0.3218276	0.6293453	0.1797355	
0.7632393	2.266237	0.4049755	0.6749205	0.2401781	
0.053355	0.221905	0.5959191	0.9535109	0.6681923	
0.5320283	0.2543589	0.27495	0.6114959	0.2396811	
0.099644502	0.2542252	0.8031795	0.8850388	0.3797307	

1.100855 0.3442528 0.5103577 1.153923 0.1536094
0.5083216 1.191955 0.873277 0.8380063 1.042432
0.6959306 0.4436545 0.7396757 0.3863256 0.9261353
0.06459219 0.7760767 0.1346159 0.3448207 0.3259833
0.3721147 0.4775531 1.195019974 0.7322592 0.4098909
0.2659852 0.4156789 0.123092 0.2428143 1.820336571
0.4111092 0.9748515 0.2352099 0.3629645 1.514176
0.452253 0.4514612 0.4898502 0.5257812 1.088688
0.5992735 0.9305375 1.473305 0.2383825 2.121304327
0.5024179 0.4621356 0.1234436 0.802736155 0.2228862
0.3513341 0.591179 0.243679 0.2473271 0.5039355
0.218848
EDIL3-DT 0.127258 0.1622501 0.1560757 0.2026075 0.1391002
0.6014323 0.1310806 0.3177433 0.3274405 0.132866931
0.4369424 0.7917181 0.2128956 0.2187462 0.4149351
0.6285261 0.1057703 0.391250366 0.1047529 0.0226497
0.334587023 0.1478611 0.4991817 0.4060039 0.1888454
0.2834112 0.2517971 0.2431788 0.122959656 0.09625343
0.2996865 0.6749248 0.02047318 0.3674024 0.3021738
0.02324727 0.03310462 0.1333449 0.1148452 0.215857411
0.8649569 0.01869717 0.364248109 0.04772246 0.2402447
0.1014433 0.1110567 0.1481835 0.06478869 0.1185813
0.0856945 0.1600872 0.2502947 0.07972793 0.4962442
0.03910985 0.04671212 0.2391666 0.01063358 0.014376922
0.120399501 0.057849631 0.2429251 0.2978294 0.07296876
0.07186053 0.3183405 0.4716003 0.149167805 0.1037136
0.009057296 0.1614103 0.4615902 0.346095 0.1567925
0.1917917 0.3705153 0.1294857 0.1846067 0.041553763
0.01843921 0.1158408 0.2158277 0.6433686 0.1340452
0.1026799 0.4146449 0.09173349 0.50861312 0.1196167
0.143072046 0.1302541 0.2627166 0.1879954 0.0488258
0.2275129 0.2261491 0.1228362 0.0266985 0.05374592
0.2788283 0.04632519 0.0244347 0.330738 0.9848415
0.8559567 0.04589533 0.3300356 0.178179399 0.1131041
0.1926681 0.6812037 0.428048 0.3390339 0.6384864
0.1178757 0.02373136 0.09241775 0.3055351 0.1442289
0.1207189 0.5932626 0.9870307 0.2165726 0.13252 0.2468694
0.1648956 0.08028759 0.4027428 0.2355681 0.03818742
0.2414701 0.1079046 0.203032 0.1422457 0.2838514
0.1008705 0.1256019 0.4668406 0.2504689 0.1083619
0.4019934 0.1484963 0.1004901 0.4203947 0.3258972
0.05506571 0.3989305 0.009346017 0.2486178 0.07840615
0.5400808 0.1153158 0.3688814 0.4184895 0.1997934
0.05685483 0.226025 0.8937502 0.7805761 0.095906319

0.363206	0.390417267	0.3376574	0.2090314	0.2441341	
0.1184187	0.485664	0.1733106	0.03916751	0.04377247	
0.305214474	0.1244602	0.1834535	0.01101252	0.4536957	
0.2462097	0.4428618	0.08161622	0.3558731	0.6186225	
0.09639446	0.2018962	0.1383204	0.1939478	0.07861834	
0.16523	0.1434849	0.4023119	0.1925408	0.4729164	0.01117527
0.128983	0.1297004	0.182355	0.3248255	0.1287247	
0.2106912	0.3025681	0.2848461	0.08158727	0.5300744	
0.1351557	0.07791453	0.3009752	0.1151129	0.1150062	
0.8183781	0.07204793	0.09951772	0.5018901	0.3156043	
0.4921752	0.08187413	0.3256678	0.08350576	0.04539588	
0.042006	0.4666186	0.219891129	0.2078189	0.279498207	
0.133575427	0.05317789	0.1387107	0.01718843	0.3832379	
0.6174027	0.09057033	0.381529	0.07178667	0.2343071	
0.1521514	0.1004033	0.2795838	0.3117326	0.1374828	
0.01798688	0.4071363	0.3789801	0.1350242	0.2337855	
0.3515846	0.1839746	0.3097559	0.2138765	0.2295407	
1.209963	0.6798278	0.09073963	0.05719815	0.08377021	
0.3554945	0.3076069	0.1439345	0.03887179	0.2105939	
0.274816328	0.03982585	0.3993227	0.09716201	0.07426679	
0.4469449	0.1205048	0.1877556	0.4517036	0.06428473	
0.1038325	0.2377535	0.3728043	0.4447314	0.1008776	
0.2072167	0.048461675	0.07381777	0.1058506	0.1509838	
0.3225469	0.02273969	0.556373858	0.3291877	0.1045758	
0.158161	0.1172697	0.07737573	0.1645558	0.1337704	0
0.3544287	0.215708	0.2481557	0.05495234	0.04366654	
0.4248053	0.6662673	0.3595098	0.3660084	0.3338772	
0.1203501	0.2715052	0.4605689	0.6199803	0.03617305	
0.2500049	0.178991	0.3635341	0.447617941	0.088236881	
0.2234285	0.08553184	0.140531	0.1334043	0.08058345	
0.248016	0.1365111	0.265048	0.301882	0.1933432	
0.2380758	0.09518696	0.24016	0.164589608	0.03972408	0.1094486
0.5779893	0.1142035	0.2742604	0.027844207	0.08665268	
0.06778094	0.05377125	0.3531188	0.4713902	0.3162172	
0.4326752	0.2231693	0.0417918	0.143396	0.3076441	
0.1563505	0.1928953	0.04297488	0.2629885	0.03553478	
0.09521719	0.3407425	0.102651862	0.02451962	0.09845872	
0.1079087	0.1168494	0.4350932	0.2532038	0.1263584	
0.4260971	0.01503986	0.3452488	0.4126321	0.1688123	
0.164098	0.71128	0.5996179	0.1486549	0.139218	0.2905924
0.06106864	0.02318875	0.08864972	0.1710048	0.09773012	
1.970614	0.5300509	0.151273	0.325701243	0.07789121	
0.2767333	0.02963462	0.5530278	0.1554281	0.2988717	
0.664744906	0.3204794	0.6577826	0.04222047	0.1047815	

0.2296871 0.4216512 0.09410845 0.03134093 0.2147922
0.1050022 0.2839857 1.020755 0.2055721 0.1773838
1.198947067 0.1803692 0.1033011 0.2454456 0.030876882
0.2133778 0.06103054 0.06288436 0.08882722 0.1306736
0.3856326 0.5050733
AL390208.1 0.1353598 0.4530216 0.9960731 0.04310129 0.5326414
1.439375 0.4879903 0.3244534 0.06965738 0.452242722
0.3098401 0.1772889 0.9754746 0.1903685 0.2206759
0.5181192 0.2700098 0.900262679 0.1392775 0 0.210896812
1.145858 0.02870064 1.484491 0.4326391 0.4255827
0.2499725 1.105997 0.043595944 0.1023814 0.06375318
0.7886718 0.3048724 0.7105326 1.965202 0.09890919 0
0.5268129 0.453725 0.539060602 0.491894 0.1988752
0.140886479 0.1740367 1.208006 0.3320051 1.207521
0.9699543 0.666163216 0.05405604 0.1302146 2.469046
0.3388377 0.7801947 0.5718238 0.2773317 0.1987441
3.214602 0.2940747 0.214091115 0.742775182 0.153831512
0.494313 0.1583953 0.65196 1.008949 0.2154775 1.322465
0.067999077 0.157595 0.1156071 0.8175542 1.182809
0.4417548 1.018203 0.204002 0.7356608 1.26711 2.680735
2.276262245 0.03922628 2.045381 0.555797 0.6959271
0.1188159 0.1310603 0.9517245 0.4181728 0.54099377
0.6870529 0.173920766 0.3274739 0.173447 0.6475025
0.1558028 0.2765685 0.1530752 0.2375572 0.2082538
0.5030752 3.700186 0.374486 0.155942 1.217749
0.302623 0.8679064 0.06508965 0.3089215 0.19949802
0.5413717 0.7241009 9.220182 0.1583651 1.989618
0.2396948 0.1253802 0 0.5719359 0.6833058 1.321697
0.09338504 2.427048 1.559212 0.04188375 0.5204559
3.216682 0.2476145 0.0731992 1.003044 0.1252827
0.2437116 2.594117 0.2754582 0.3637186 0 0.8338816
0.2360432 0.121453 0.7448428 0.769643 4.34917 0.4988505
0.2457004 3.657936 1.277597 0.4561123 0.4685716
0.8679441 1.630329 0.4627804 0.2293441 0.0851059
0.1022144 1.322834 0.7913466 0.2833509 0.4837957
0.8548083 0.8238971 0.754792 0.068008104 2.018882
1.384243416 0.5951698 0.4446786 0.4835361 0.1799397
0.8012318 1.198239 0.2221925 1.638885 0.103886666
2.38291 0.252525 0.1874181 0.3395934 0.371003 0.2923799
0.3472491 0.9659031 0.5899372 0.364556 0.05052937
1.63941 0.7632928 3.233444 1.001771 0 3.981562
0.7606813 0.6771483 0.6418837 0.0914631 0.3448943
0.2327575 0.6712675 0.2594272 0.9860609 0.3678068
1.001154 0.2603445 0.9982412 0.4644566 2.007415

2.433038 0.6821743 0.108736 0.3897671 2.011664
0.9315106 4.905581 0.4316106 1.444886 1.492913
0.281451 0.5921475 0.120715 0.6970125 1.016286
0.187112327 0.1052617 0.808635184 0.213119179 0.6949222
1.967222 0.1645445 0.8851536 0.1050735 0.1100988
0.4509099 0.4326893 0.3672778 0.2059757 0.1245946
2.514242 0.1578947 0.2599744 0.4209042 0.9170608
0.7140766 4.207235 1.831111 1.136863 0.4472852
0.823691 0.1949939 0.4883087 0.9708909 0.8545831
0.5308409 1.541271 3.284097 0.2100705 1.881346
0.9024729 0.5512873 0.6720039 0.046769984 0.1059034
0.6752363 0.4340607 0.1263919 0.633866 0.2097437
0.2396507 1.201153 0.2149004 0.07362865 0.6369082
1.072987 0.5964481 0.4598569 0.8186621 0.556707255
0.3768833 0.7881268 0.7708612 1.336213 0.1451244
0.322797368 0.9176221 0.2701388 0.1035263 0.1871034
0.3292073 5.338482 0.2312162 1.59275 0.2579427 1.229804
0.7827615 1.215778 0.4087297 0.6152855 0.13828 0.2294387
0.8002487 0.3044 1.00581 0.1732743 0.6158628 0.6718935
0.2693319 0.6750311 0.475966 0.2074859 0.195329377
0.531841861 1.366504 0.1364658 0.3165413 0.1135179
0.1714275 0.05862353 1.45202 1.090099 0.3952014 0.5100177
0.3222963 1.01247 0.3005291 1.050408834 0.2746451 0.3259665
0.7655835 1.943587 0.2708838 0 0.245785 0.1235935
0.3202896 0.3571279 0.3788364 0.1623754 0.1472708
0.5610737 1.066859 0.5287542 1.579732 0.257016
1.294187 2.255069 0.8158838 0.7055457 1.080311
0.8127347 0.893349422 0.4868388 0.8677385 0.5356336
0.4142951 1.596636 0.3655111 0.1344029 0.4909931
0.0959842 1.762699 0.6502254 4.668552 0.5061811
0.7417288 0.3001375 0.2032957 0.4936041 0.8991792
0.6062611 0.2713156 0.7812896 0.727567 1.663233
5.204042 0.1925158 0.1501768 1.16223986 0.4556757
0.3924685 0.1681136 0.5042024 0.4473455 1.95281
0.266430524 0.376765 0.45747 0.1571795 2.314779 0.4560455
0.09279216 1.334664 0.2666899 0.1246183 1.824223
0.823815 3.316445 0.6013144 0.5958216 0.878524533
1.272284 0.4794663 0.2175598 0.065685293 0.5674061
2.362943 0.1337757 0.532537 0.8767244 0.1215359
0.6983972
AL023653.1 0.2228316 0.08523106 0.3279505 0.1702897 0.1169124
0.09026756 0.1377151 0.3738844 0.5504215 1.060897613
0.07650959 0 2.752873 0 0.2906243 0.0330169 0.4444947
0.10066602 0 0 0.156231841 0.08876852 0.05669697 0.05331915

0.06104724 0.07006014 0.4938106 0.03523958 0.344488199 0
0.1259419 0.07989691 0.2581128 0.4210886 0.2176923
1.074651 0 0.04002684 0.4826315 0.236642827 0.03598952
0.07857399 0.092771874 0.2865021 0.3212419 0.1639657
2.38541 0.3832212 0.363028653 0.3559518 0 3.447889
0.0956229 0.06701059 0.2606804 0.2191431 1.439574
1.873116 0.31281 0.604183516 0.657765372 0.486220827 0.3107031
0.3129036 0.1226591 0.6039811 0.5472864 0.4504269
0.134329465 0.3735873 0.1903144 0.2584073 0.3086064
0.04155567 0.6242335 0.4029979 1.418667 0.5078804
0.5734182 0.218284723 0.1549799 0.2434077 1.145692
0.1833035 0.4224889 0.08630147 0.04585595 0.8260838 0
0.7540254 0.085893384 1.244061 0.8756293 1.768189
0.7181587 0.6374084 1.123178 0.04692848 0.1869987
0.3162107 2.23193 0.0778718 0.6161142 0.5345812 0.367889
1.882604 0.257164 0.3328702 0.197050191 0.178243
0.188925 0.2504886 0.4692658 4.863885 0.7891793
0.2830671 0.3989198 0.2118444 0.2633841 0.1864971
0.2767176 0.7671258 0 0.1654793 0.3427133 4.668558
0.2445763 0.1446021 0.7017717 0.1237455 0.09628852
0.3044301 0.0906928 0.4041627 0.697411 0.2272138
0.3391229 0.1919405 0.09809381 0.2339075 0.8196944
0.4692663 0.6240488 0.609996 0.4206403 0.3964539
0.3471167 0.8763455 0.2749334 0.4788689 0.1235618
0.252185 0.1211523 0.9744158 0.6187958 0.2798742
0.6769678 1.31925 0 0.7455307 0.806083783 1.674068
0.683629417 0.3648831 0.1098056 0.9198283 0.7820204
0.124958 1.213883 0.5486655 0.2575325 0.359141939
0.4279403 0.1360509 0.1851185 0.1765403 0.1724475
0.6417608 0.7288504 0.2578518 0.2241149 0.04501036
0.1497281 0.8719277 0.937315 0.9361048 0.1735928
0.4823909 0.4042972 0.3853065 0 0.09392708 0.09034086
0.6131924 0.9196062 0.6630311 0.7117891 0.88542 0.1362352
1.040915 0.1714334 0.292146 0.08738242 0.3274322 0
0.3800946 0.3759063 0.2566564 0.3027781 0.1672875
1.197096 0.4263148 0.66187 0.04915318 0.5559953 0.5458898
0.09538704 0.03530565 0.6069587 0.18481647 0.1247642
0.469831341 0.046778716 0.255403 2.526009 0.5778687
0.5982004 0.1037843 0.05437395 0.1781509 0.4022402
0.2591224 0.05812813 0 0.5340628 0.3742976 0.7703536
0.944863 0.5032269 0.04550419 0.1001352 0.6698679
0.4728058 0.6626955 0.2789432 0.2996021 0.8904318
1.070487 0.1947917 0.9215454 0.9214282 0.5532065
0.3319887 0.8079398 0.8913996 0.1633569 0.2581283

0.369568952 0.5857821 0.1290873 0.7758058 0.06242055
0.853757 0 0.3156137 0.5423611 0.2315603 0.2909009
0.2960439 0.3225544 0.6907038 0.2422477 0.3732079
0.366584328 0.3722589 0.5337988 0.2115008 0.285366
3.631375 0.26569722 1.765023 0.376693 0 0.9445724
0.3251679 1.210192 0.3162173 0.1498292 0.5095556
0.1087804 0.2876863 0.1847478 0.3303119 0.7976535
0.6829165 0.3777058 1.538133 0.5011084 0.07225227 0
0.6636068 0.4915922 0.2280234 0.686953 0.3008806
0.1117855 0.241165872 0.370811431 0.4694739 0.2695827
0.2084382 0.1121251 0.4656413 0.8106591 0.6310497
0.2598988 0.7807047 0.1950038 0.4547739 0.6222515
0.2968416 0.532061734 0.2086733 0.3679622 0.1833187
0.3199565 0.2058155 0 0.5462308 0.244154 0.09038848
0.1946184 0.7483761 0.2749424 0.0727319 0.2984096
0.6146984 0.2812204 1.337441 0.1493308 0.1870693
0.4213999 0 0.09955553 0.4668368 0.1735703 0.156868992
0.3778228 0.2955488 0.3023208 0.245527 0.3656918
0.07600553 0.1062031 0.3730528 0.3160216 1.575255
0.3853483 1.013466 0.1724036 1.054984 0.7411372
0.3569801 0.3900381 0.3330549 0.7271414 0.1461745
1.117639 0.2812069 0.3696357 0.4759434 0 0.6357177
0.309071336 0.1227503 0.221516 0.290589 0.1106702
0.1921116 0.3588556 0.283404626 0.03544211 0.3189578
0.5322887 0.2371058 0 0.305512 0.6591441 0.04390294
0.04102973 0.4044947 0.1627414 2.729794 0.7019247
0.7062131 0.727786205 0.199472 0.3157222 0 0 0.280222
3.38551 0.5946044 0.305421 0.2534538 0.180067 0.3183821
AC100814.1 0.5130491 1.360277 1.372865 0.5123726 0.9788366
2.031093 0.5044394 0.8105259 0.7560565 0.847319932
0.5204617 0.7788762 0.9723431 0.3935707 0.3041525
2.67792 0.3256299 0.474083632 0.5470945 0.7969206
0.327008541 2.926369 0.3263489 2.762155 0.7826393
1.118151 0.5537111 2.341876 0.135196412 1.693319
0.3295108 2.445769 1.350639 0.8079314 2.563038
13.18937 0.4125236 1.675602 1.56941 1.754246321 1.016949
1.377378 0.194180616 0.8095638 1.82506 2.179297 2.496448
1.153047 1.685927208 0.2421386 0.4038115 2.772296
1.501111 1.718182 2.09158 1.519404 2.191396 0.645467
1.379635 0.980076824 1.403243622 0.98590443 1.556152
0.6822269 1.123227 2.149126 0.8273229 1.296333
0.468607872 0.3421052 1.055618 4.867847 1.15347 1.739602
0.7440258 1.897907 0.5431845 2.296933 1.923883
1.484896719 0.3243881 1.120848 1.024165 2.158156

0.9825675 1.264462 1.319739 1.901982 2.7961476
3.23541 1.460739014 0.8853399 1.653484 2.027671 1.315279
0.6432548 0.9946202 1.080485 0.2739846 2.245596
1.138714 0.7334698 1.558251 7.748596 2.189766
1.759141 1.009255 1.509582 0.309334008 2.860281
3.332983 2.041014 1.186848 4.858916 2.890702
2.12925 1.174188 1.21938 0.8097052 1.024686 1.206661
2.734656 0.9025912 0.8226156 1.300165 2.510797
1.578426 0.6053327 2.030506 0.4964387 1.326814
2.92051 0.6169439 0.8459522 0.2085357 1.218675 0.7098173
0.652844 1.89921 1.529973 1.874562 0.9331093 1.378762
9.109343 1.826916 4.262241 2.815378 1.555148
1.93191 4.259858 0.7112244 0.8137654 0.3592436 0.718477
1.414495 0.5858041 1.125233 4.528563 0.6551299
6.007805 0.210901536 2.421875 1.383206529 2.291208
0.9480647 2.036365 1.004428 1.874437 1.715024
5.196558 2.733707 0.510095201 2.040253 1.139071
2.542779 2.586613 1.060289 3.123099 2.46781 0.9714761
2.88493 1.460272 0.3656282 1.520858 1.514069 1.728846
0.8175302 1.199008 1.78863 0.5242148 0.5799781 0.5406461
0.6854594 0.7843438 0.8822119 1.591876 1.370657
2.409252 0.9267492 4.657052 12.97159 1.738925
0.5944249 1.218396 1.897319 0.7413314 0.5058056
0.7252295 0.5941353 0.700298 3.788289 0.6196657
1.342066 0.720177 1.230892 2.713677 0.1746976
4.988128 0.5619183 0.942919984 1.479814 2.0651463
0.293737296 0.501172 1.464143 5.726396 3.082072
0.7059995 0.1991675 1.468244 0.4472743 1.274566
1.216679 0.6623715 2.431315 1.338379 0.9741738
1.91836 2.211937 0.5952795 3.178825 0.7945228 1.051481
2.658583 2.335422 0.2911519 2.213216 3.594351
1.427013 2.145053 1.865749 2.657942 2.171515
2.452091 0.9162345 0.56987 3.453983 0.386772001 1.926727
2.206571 1.581105 0.8492401 1.429598 1.927233
0.385356 1.383543 0.848187 0.9386964 1.278026
0.337569 2.742598 0.3485957 1.692515 1.278828152
1.396021 2.350971 2.41267 1.530578 0.9751055
1.418132258 0.7987816 0.9527169 1.203929 1.031521
3.743346 2.08072 0.5331761 5.488127 1.189613 0.9676737
1.317216 1.885139 2.938345 1.967704 1.572352
0.5138739 3.756041 0.5244345 1.096425 1.393117
1.388994 3.086852 1.451712 0.63435 3.109502 3.568164
1.287198648 1.13187759 1.740118 0.8463945 0.5817089
1.056099 1.395497 0.302998 0.5403473 1.010274

0.791513 1.785709 1.499221 0.883794 2.205681
0.501145788 0.4367736 2.21427 1.079168 1.674251 0.6031087
0.765380478 2.223112 0.7665573 4.446011 1.667611
0.6219627 0.9351571 0.4186462 1.271508 2.435395
2.29142 1.259728 0.6876409 0.6525907 3.528125 2.024125
2.187984 0.9073399 0.8401303 1.026069249 2.318541
3.711675 3.598976 3.768689 1.243822 1.710185
2.834241 1.015087 1.951319 2.255953 0.9073932
3.128892 2.183188 1.257439 1.124673 1.961385
0.850404 3.107978 5.058339 2.192696 1.169664
2.305325 13.60394 5.802842 1.023455 3.614849
2.102478848 0.8350176 0.6375251 0.3475606 0.7528415
1.568222 3.403512 0.720306551 1.613498 3.922208
1.531932 2.073765 1.380581 0.8313065 1.379653
1.447317 0.9876114 1.423906 1.873485 13.38644
2.768872 1.662945 1.347559197 1.503052 1.218419
0.4048079 0.509245527 0.821145 2.496276 1.002566
1.651463 1.812554 0.5339388 0.8700286
AP001347.1 0.09029084 0.9497244 0.1434552 0.07056923 0.1291981
2.643463 0.02536447 0.6295985 0.0760328 0.606758986
1.120282 2.451195 0.1901349 0.8773441 0.4014556
4.013512 0.09824087 0.704548394 0.06080997 0.1753113
0.287749041 1.422403 2.025844 0.4517367 0.6465144
1.000039 0.03897876 4.024081 0.126896218 0.2607543
0.6262939 1.810004 0.03961619 0.6980079 0.2806631
0.2878984 0.1537401 0.1474435 0.3809633 0.021792532
0.1856002 0.02170773 0 0.01055364 0.1098808 0.1993156
0.1432652 0.07940473 0.21730444 0.01966785 0.1989855
0.4878927 0.1144774 0.1727889 1.056271 0.02018098
0.06025951 4.737328 0.2057628 0.166918497 0.223657378
0.179105073 0.3842287 0.09605138 0.01129573 1.435018
0.3359988 0.4479839 0.032987876 0.02866983 0 0.113035
0.3653959 0.09184507 0.217169 0.1187591 0.1338321
0.06013403 0.2485003 0.144733871 0.08563296 0.6814316
0.04396134 1.03815 0.3112574 0.2781638 0.1435783 0.243438
0.1687167 1.435062 0.039549779 0.0549917 0.09816684
0.3533827 0.01889586 0.1425552 0.1511726 0.05185995 0
0.3411196 1.613484 0.007171245 0.1323891 2.087343
0.4404276 0.4210408 0.1302528 0.4904664 0.014517144
0.0547149 0.4076145 0.4415797 0 1.284934 0.3779144
0.0325847 0.1102101 0.006502944 0.2728703 0.1631586
0.01698869 0.3620552 2.435636 0.3352593 0.4655189
0.4776993 0.0900925 0.08877643 0.7983265 0.2431098
0.02364599 0.01869006 0.05011161 0.04962601 0.07339984

0.512643	0.07807489	0.05302762	0.126469	0.1723251	
0.1174226	0.1555737	0.05108347	0.4839672	0.6507805	
0.3916484	0.02131074	1.775466	0.2170177	0.2164871	
0.04551544	0.2322381	0.02975191	0.195784	0.8517781	
0.1288686	0.2420345	2.021597	0.02305909	0.1373124	
0.34641868	0.353674	0.352551939	0.1792119	0.4247057	
0.0390957	0.3142541	0.1687757	0.2012166	0	4.702598
0.056697468	0.1839096	0.2505795	0.05114291	0.2146012	
0.2064498	0.5437201	0.04737891	0.3989273	0.1651106	
0.03316018	1.847662	0.09176705	0.2476943	0.1419868	
1.419577	0.09995302	0.2572385	0.1703186	1.330424	
0.2508437	0.01663905	0.06274348	0.0846869	0.2011349	
0.4404889	0.6686172	0.03345587	0.06710081	1.079856	
0.4708173	0.01609416	0.01340149	0.1863666	0.03182096	
0.1582508	0.08508813	0.02788296	0.1232445	0.07349405	
0.5932546	0.06095187	0.3711759	0.3387198	0.035908	
0.1581161	0.3771521	0.2751757	0.18721804	0.08425695	
0.354789023	0.10338889	0.01764012	0.5010302	0.7117661	
0.5593602	0.9939835	0.3505123	0.1476539	0.2778185	
0.1909015	0.07494259	0.01295221	0.1868918	0.1608563	
0.1773554	0.292363	0.09268473	0.8213378	0.454927	
0.2632038	0.03265566	0.1017132	0.3510692	0.1340109	
0.273334	0.7557922	0.1793845	0.1989949	0.154952	
0.03705091	0.9171893	0.6398701	0.07622582	0.7220929	
0.2309196	0.085084377	0.04623849	0.5626846	0.07520449	
0.02299333	0.1257965	0.2204613	0.1550133	0.3246506	0
0.1160865	0.1158669	0.3479631	0.336744	0.1003891	
0.04582515	0.210055351	0.7427652	0.1310873	0.04674525	
1.110307	0.2552112	0.156596237	1.06311	0.02890817	0.3201712
0.1664081	0.04192278	0.2706571	0.0323562	0.5243176	
0.1804814	0.6611633	0.3112945	1.641802	1.324897	
0.1119333	0.1509361	0.6539218	0.1023008	0.1292124	
0.306072	0.02101481	0.09166772	0.2897339	0.02099876	
0.2679322	0.07619759	0.2264762	0.497482956	0.147975409	
0.06485106	0.1875741	0.4286919	0.113582	0.05457594	
0.2452915	0.07396279	1.073619	0.11683	0.05985992	0.05863243
0.2619583	0.1530831	0.058797331	0.03074689	0	0.2954332
0.1178597	0.0303258	0.344827688	0.03353508	0.05246322	
0.04161955	0.1792249	0.4297256	0.168797	0.3750829	
0.1099226	0.01617366	0.04439605	0.1067434	0.02750387	
0.09187887	0.04435072	0.1781116	0.2658751	2.039011	
1.086923	0.072230638	0.2973292	0.3810408	0.6960211	
0.04522136	0.6314382	0.1119901	0.07824225	0.06870916	
0.01164102	0.2519564	0.1419475	0.615981	0.3937425	

0.3454361 0.2866568 0.04109305 0.0598646 0.5214096
0.3702646 0.008974178 0.1666386 0.1899073 0.1815461
0.1402554 0.570369 0.234174 0.634307178 0.07536085
0.5405866 0.06116675 0.04076664 0.007076651 0.2230683
0.260988441 0.04569429 0.0587459 0.1388863 0.2682606
0.04740812 0.3938861 0.09712134 0.04043039 0.068012
0.2844551 0.04995639 0.3447604 0.1093917 0.224011
0.216533001 0.06613003 0.4506622 0.1266518 0.047798111
1.476089 0.3590113 0.0608415 0.03750178 0.01556044
0.1326596 0.4756345
AL391244.1 0 1.151326 0.1230569 0.9968066 0.3158573
0.9754881 0.6614381 0.6092713 0.991367 1.374426432
0.5052733 0.7359287 0.7850505 0.3512097 1.395853
1.784007 0.1601163 1.510917244 0.5616243 0.05714566
0.594045441 0.5329381 0.7488594 1.312456 0.6780403
0.3575261 0.7623469 2.45418 0.568754458 0.182137 0.6805035
0.7434974 0.1549628 0.589886 0.1960433 0.2346133
0.3340945 0.3844935 0.5381195 0.118394048 0.4537463
0.14152 0.111394589 0 0.08265576 0.05906389 0.1245326 0 0
0.08548088 0.09266098 0.1009758 0.5166806 0.1206931
0.8868575 0.2631332 0.03928521 0.8502766 0.02682875
0.217639587 0.212639266 0.510845271 0.3997207 0.2817861
0.110461 0.2900889 0.4015897 0.2433798 0.080647156
0.3738166 0.04570348 0.2521018 0.4234914 0.2245384
0.1041028 0.1451683 0.1454161 0.1742373 0.3240114
0.183471727 0.04652251 0.2338148 0.02865988 0.3851737
0.253649 0.1295317 0.1651827 0.2975727 0.54996052
1.086463 0.103135334 0.02987579 0.1371392 0.3839702
0.2771741 0.1093371 0.3371601 0.225395 0 0.1084816
0.5694915 0.09350352 0.06164924 0.03209456 0.3865206
0.3229303 0.07719656 1.132454 0.047321077 0.1070115
0.06481406 0.9023127 0.04695538 0.7079084 0.4737983
0.2974029 0.7484339 0.04239488 0.5534461 0.02799173
0.0553775 0.4029883 2.243734 0.7451142 0.4372277
0.5190471 0.04894528 0.05787635 0.8674266 0.1733501
0.05780858 0.0304617 0.05444908 0.1348036 0.2392591
0.3069269 0.152699 0 0.1766773 0.210646 0.2187194
0.05634652 0.1248864 0.3098804 0.277793 0.2163803 0
0.3202531 0.8253067 0.02613616 0.1978203 0.3028077 0
0.186139 0.3323996 2.184359 0.4303376 1.267257
0.9019799 0.1790372 0.362960377 0.5616498 0.191533742
0.3651071 0.2307333 0.04247967 1.323137 0.1750486
0.1821943 1.087025 1.281091 0.15401246 0.3425624
0.5989912 0.4167723 0.2119784 0.25883 0.1155879 0.2573992

0.06192241 0.2152824 0 1.018776 0.09971005 0.07340012
0.2314149 0.8754449 0 0.4633883 0.1156629 1.422635
1.381576 0.6237346 0.1636183 0.1840341 0.2107392
0.2051211 0.3986836 0.05452753 0.09373989 0.1440924
0.6138833 0.1049233 0.04368443 0.05062443 0.3111774
0.2256822 0.4930829 0.1590561 0.3013023 0.03422375
0.4265765 0.09934144 0.5311802 0.333802 0.6320602
0.7444757 0.7206775 0.4765214 0.083218466 0.1997453
0.225657484 0.421266886 0.09583492 0.653277 0 1.353684
0.8723229 0.09793319 0.2406513 0.03018654 0.3422515
0.5234743 0 0.03206344 0.4119801 0.231248 0.04538127
0.0604243 8.223101 1.282518 0.2949228 0.2483754
0.06631023 0.3070259 0.3854391 0.04454892 0.615908
0.4677871 0.2861724 0.1443121 0.1811605 0.3488022
0.3880492 0.03822629 0.2288401 0.2878052 0.416020298
0.1758425 0.5683329 0.2451417 0.07495065 0.3758837
0.02763965 0.8842607 0.3663185 0.02317024 0.02910795
0.4221216 0.4149674 0.1219637 0.2545163 0.2614059
0.024453958 0.1303706 0.05341265 0.152374 0.7067143
0.1147454 0.191419434 0.7159889 0.05653864 0.2762608
0.2219053 0.1561764 0.259486 0.06328228 0.2698582
0.1647267 0.5660051 0.3454355 1.109167 0.5728921
0.02280409 0.1230004 0.4081727 0.436072 0.1805098
0.04337797 0.1370026 0.03320071 0.2065955 0.136898
0.2426028 0.09031959 0.5368998 0.550195732 1.187325
0.1409286 0.1078993 0.08342648 0.3702398 0.02541421
0.5562218 0.1377681 0.8916286 0.146472 2.224408
0.08190955 0.18679 0.1425714 0.031943301 0.4760671 0.1104564
0.1926028 0.3841835 0.07413905 3.09106588 0.07287549
0.04886077 0.2170655 0.3505282 0.3964423 0.5502223
0.8733186 0.2559367 0 0 0.1873567 0.0717228 0.224621
0.1807108 0.02764689 0.7172398 0.5605481 0.5210305
0.211903141 0.3093175 0.2484134 0.1361279 0.2456778
0.08233116 0.9354419 0.1594024 0.3135567 0.03794587
0.04977542 0.3855848 0.3650716 0.4140226 0.3166895
0.4894508 1.607397 0.468333 0.3665853 0.179757
0.02925285 0.223665 0.09379316 0.8136978 0.2285931
0.3587956 1.246772 0.265080914 0.07369542 0.1329912
0.9221478 0.1993286 0.02306754 0.4308911 0.583361692
1.191586 0.09574609 1.0386 0.02033583 0.1545347 0.6236271
0.1582917 0 0.8128874 0.3532302 0 0.1873008 0.06483282
0.6124271 0.168053763 0.09580532 0.3080181 0.9805015
0.077902999 2.422605 1.139462 0 0.1018695 0.02536094
0.1441421 0.4460086

AC138207.5 0.8170127 0.5095109 1.045593 1.323389 0.8386823
1.834705 2.7442 0.681167 0.767764 1.468683119 0.609832
0.5583085 2.139366 0.03330543 1.853174 1.921115
0.5668666 0.748882992 0.7456283 0.3034728 0.664144485
2.476403 0.3615298 1.784953 1.265126 0.4467405
1.293256 2.499856 0.755094549 1.209051 0.4015359
1.178138 4.286104 1.230663 1.966505 4.204971
0.5174789 1.97805 1.291456 1.383213006 1.004011 0.6262866
0.887343694 0.9819526 0.8778891 1.019394 1.611997
1.412718 0.868073405 0.3404605 0.8611354 9.51814 0.9146132
1.121649 5.471527 1.179033 2.607808 3.568627
0.4986605 0.674203806 1.653506105 1.404869101 1.485905
1.953671 0.7821395 4.092009 1.841832 3.41069
0.820865794 1.8859 1.061852 2.909294 2.389517 0.7286975
3.068258 1.541836 1.958165 1.937325 2.285261
0.208784801 2.717643 3.725031 1.445887 2.958626
2.656596 1.135002 2.521965 2.414292 2.67719168
3.966653 2.361963062 5.513288 4.885541 1.799187
1.799034 3.447527 7.024243 1.047343 1.162592
3.132507 9.9179 0.3724138 4.583448 3.067895 3.95863
4.742841 1.281104 2.166776 1.193671281 2.557286
0.7529287 2.167694 2.181876 4.855836 1.635473
1.720376 1.19237 0.8442697 1.994378 0.7432524 1.470415
0.802528 1.734932 10.55184 1.434117 1.722754
1.202151 2.22831 4.014219 2.137056 1.765211 0.8088364
0.9397459 0.3579382 2.144119 2.671289 1.588032
0.6119569 2.775647 0.5127093 2.274628 1.944988
1.24352 1.38382 2.011669 1.752363 2.028949 5.162864
0.3756688 2.463636 1.674278 1.909577 1.480685
4.236402 3.322753 0.4461564 1.174397 9.884374
2.993735 5.229287 1.606254971 3.571329 1.816325917
2.294372 0.8752231 2.171293 1.756634 1.294797
2.563995 0.4810549 1.671488 1.922030316 2.652974
2.42186 0.4795429 3.827428 2.302321 1.278814 1.298576
2.342984 1.500529 1.004534 0.4773728 5.692249
1.624135 3.555125 4.759754 2.258922 1.494075
2.057669 2.467535 1.160424 1.080114 0.6516731
2.99303 3.481748 0.7942801 2.540658 0.5791383 1.037098
0.847191 3.114498 1.009919 0.840952 0.470473
0.4682116 2.910615 2.536694 0.5430019 1.5334 0.8178556
3.020453 3.165324 2.272343 1.056778 1.429648
0.7983126 2.532684 2.939931 0.662899173 2.419836
2.172022249 0.559285866 1.094205 3.593138 1.209074
1.136997 0.0827229 0.2600377 1.41998 0.561071 3.635062

0.7876432 0.3363141 0.5959566 1.839763 1.995574
2.199105 1.484089 1.124365 1.037587 6.193575
2.638002 2.376954 1.000513 3.820842 6.742463
1.56429 3.260499 1.36775 4.119245 1.322826 2.381553
7.083795 3.146519 1.345464 3.115572 2.577494949
2.03438 2.675169 1.334374 0.2985198 0.9980677 0.9907699
0.8804772 1.459004 0.8305597 0.7728908 4.394877
1.983321 5.019598 1.061981 1.487357 0.357123444
1.78029 2.091908 1.314925 1.450029 1.409138
0.931824187 5.627358 2.076724 1.956116 2.618736
1.036721 4.960815 0.5601024 0.537407 3.374165
1.849709 0.7452413 1.141235 1.872216 2.755058
3.647008 2.709508 3.882314 6.230897 1.065412
1.000387 1.498659 3.722398 2.847411 3.285281
0.8993314 0.8910042 0.84579053 0.935943767 3.305448
0.6923757 2.187497 0.670283 3.070397 2.676902
1.32606 1.834815 2.566877 1.321163 1.812424 0.4251219
0.9937299 0.932994317 0.5322444 4.765965 1.424642
1.020106 0.5577649 0.559607282 0.8707641 0.8432963
0.9365926 1.473676 4.280802 2.73934 3.362383 0.9853876
0.4899552 2.465663 3.233622 0.6665485 1.242555
2.974968 1.982059 1.309313 3.13627 0.3458674
0.625174711 0.7118083 4.287408 1.626545 0.782805
2.73263 1.908315 2.327898 3.270825 1.561718 3.337205
1.330975 5.250694 3.517876 2.616108 2.717382
1.10258 1.917131 1.327334 3.307014 0.2718584 3.054282
1.394652 1.931424 1.858856 1.082607 1.553908
1.548487336 2.41339 1.103518 1.488977 3.352035 1.071879
1.823451 2.8397876 1.553733 3.008394 0.4242692
1.376919 2.205521 1.704593 1.786297 0.6648772
0.7194749 3.10685 0.2161927 4.72465 1.506293 1.438518
2.766600485 2.384885 3.334381 0.3425636 1.085975426
1.65283 4.865432 0.8425581 0.6221262 0.673398 1.62662
1.776401
AL356740.1 0.07841197 0.06426829 0.06182258 0.2247116 0.1616222
0.02268869 0.2076875 0.1745261 0.138348 0.168414213
0.2307676 0.132044 0.03459663 0.09452374 0.3287169 0
0.357515 0.109643432 0.06915551 0.1196226 0.235612553
0.01115595 0.1567582 0 0.03068836 0.2289244 0.4255506 0
0.25976057 0.4829369 0.3798647 0 1.189402 0.2116806
0.0273584 0 0.4545823 0.1710322 0.2512831 0.218093338
0.2442404 0.01974951 0.839453132 0.7777305 0.4498589
1.153955 0.7299126 0.1565238 0 0.06262776 0.6594861 0
0.09613898 0.08421531 0.1310437 1.074088 0.04934125

0.09186463 0.4380512 0.607444337 0.228916862 0.137487655
0.1115643 0.6554007 0.2003969 0.06072408 0.3821112
0.1132145 0.011254538 2.370997 0 0.02435643 0.7424365
0.1671198 0.4881353 0.02025864 0.4348548 0.1094189
0.3052128 0.647415322 0.07790817 0.2569575 0.1079883
0.2073294 0 0.1409968 0.08068101 0.09689659 0.69073685
0.4295871 0.021589239 0.050031 0.05741447 0.08510419
0.1547217 0.3776429 0 0.8728626 0.996442 0.4882301
0.2945218 0.4697525 0.4129596 0.2956065 0.7050733
0.04224921 0.1131165 0.06972224 0.485378528 0.2240063
0.1424585 0.1978747 0.3931654 0.1358371 0.9917982
0.2845948 0.2256035 0.2041132 0.5047856 0.5625109
0.7303041 0.3012758 0.7948416 0.1247793 0.5383796
0.2173035 0.2356506 0.1211521 0.02075174 0.04147113
0.2581552 0.2168018 0.3875247 0.2257467 0.07512589
0.17133 0.149167 0.01206102 0.5917394 0.7496042 1.625345
1.026163 0.05228474 0.3538201 0.2431736 0.335181
0.974264 0.1053459 3.504576 0.8753698 0 0.6021718 0
0.2894494 0.1146039 0.07034617 0.5304843 0.1459007 0
0.1124332 0.033768095 0.3465213 1.168442322 0.4076137
0.05519911 0.2045215 0.1786911 0.115163 0.5339402
1.089463 0.04623615 0.322393084 0.4541527 0.4673492
0.9887497 0.07099724 0.1408698 0.09678367 0.8082177
0.8814276 0.2253244 3.111164 0.08781279 0.824453
0.1843775 0.09688381 0.2617946 0.2576533 0.4341937 0
0.5571724 0.1298468 0.02270711 0.2740008 1.117188
0.2254709 0.03578154 1.179513 0.4337398 0.2877966
0.6894344 0.954599 0.02196351 0.009144428 0.5934417
0.6253298 0 0.5418874 1.132033 0.4414996 0.5731225
0.02381198 0.2495408 1.161334 0.5267463 0.04900321
0.01198773 0.4259544 0.1525586 0.325174391 0.1045313
0.224374351 0.282187101 0.2407326 0.1269821 1.915443
1.885248 0.1304305 0.2870039 0.2910576 1.213233
0.2344688 0.3506511 0.1148922 0.4564034 0.2195187
0.2258991 0.08549663 0.3288629 0.04574978 1.258446
0.02244944 0.2673886 0 0.292134 0.182883 0.8392851
0.123322 0.2121633 0.2955276 0.08055663 0.51827 0.1668903
0.2233826 0.04801128 0.3558502 0.3244018 0.185781771 0
0.4217981 0.06157839 0.06275744 0.01430608 0.1967167
0.145437 0.7156908 1.018544 0.463079 0.5673795
0.5327708 0.01021223 0.09133319 0.5784675 0.849742032
0.187134 0 0.3827561 0.1344872 0 0.40069681 0.2997548
0.1972531 0.05140396 0.0103225 0.008173072 0.108636
0.2031181 2.071271 0.1576325 0.1184814 0.4880908

0.1160905 0.0368994 0.1909425 0.1201554 0.1898722
0.09665218 0.2770971 0.009080278 0.1147146 0.3057946
0.7166557 0 0.2640761 0.4537567 1.554712 0.860759487
0.248541818 0.4523407 0.03764412 0.2008314 0.01409127 0
0.05821674 0.5623583 0.3359591 0.09811477 1.053802
0.1143071 0.2010889 0.1342997 0.280839987 0.1678396
0.8555049 0.1036733 0.04021042 0.1138094 1.720565873 0
0.2659277 0.5452579 0.2140128 0.1327791 1.209365 0
0.8250555 0.8387359 0.2221514 0.8180013 0.07506839
1.567325 1.694697 0.6481778 0.8507891 0.05028819
0.3490141 0.453432421 5.723828 0 0.2089673 0.7199855
0.4710714 0.7641574 0.427105 0.4875861 0.2541818
0.1354528 0.2744282 0.2037871 3.440681 0.2799011
0.1117706 0.01121584 0.3758038 0.1813784 0.5160465
0.2694329 0.1337704 0.1570692 0.08258459 0.6340285
0.09558998 0.3941417 0.122076199 0.02056881 0.2923089
0.3547624 0.4728868 0.3476671 0.6652109 0.325639063
0.1336252 0.2672327 0.4794155 0.289468 0.1779175
0.2150126 0 0.01103497 0.4228245 0.1478828 0.3817793
0.4312827 0.02714281 0.1479218 0.239214231 5.45492 0.3571044
0.1728401 0.47291385 1.056504 0.4641521 0.3487246
0.2388317 0.2123514 0.1659523 0.05335007
LINC00476 0.5127747 1.154998 1.100563 0.3646538 0.8855772
2.397453 0.695434 1.765133 0.8224215 1.499051612
0.7042483 0.5484833 1.332957 0.3365416 0.7988178
1.376041 0.5625735 1.47569806 0.8195633 0.4867466
0.995329574 1.140519 0.4457716 2.532325 1.061408
0.3806601 1.149873 1.702942 0.72666746 1.719445
0.5796289 1.687908 0.4234738 0.8792771 0.373392
1.06787 0.5898897 0.7215191 0.5927715 0.87986278 0.5383185
0.9568001 0.198659144 0.6318213 0.7333673 0.2242922
0.7325629 0.3062012 0.675856138 0.2298053 0.4998606
1.677148 1.97735 0.6253796 1.288613 1.040092 0.5479362
1.661653 1.356824 1.100680206 0.61450961 0.536129965
0.6894476 0.6833773 0.3449848 0.8976225 0.3964786
0.8493638 0.303391437 0.8019795 0.4914738 0.6152881
0.783428 0.3745388 0.3059152 0.7264392 0.9753997
0.6516092 0.9918085 0.700445715 0.3615894 0.8837514
0.4302522 0.9080712 0.7171635 1.122613 1.222304
0.7568655 0.81000558 1.108565 0.269031553 0.5153046
0.4429057 0.9234416 0.4688952 0.7537667 0.7759412
0.7229379 0.7291482 0.493767 2.100793 0.6545659
0.6038725 1.069561 1.025887 0.8251818 0.2917803
0.6596052 0.707881562 0.7177952 0.6676516 0.5581196

0.4049488	1.645609	0.7667728	0.3777141	0.8861105	
0.4197842	0.974379	1.120591	0.4539978	0.6037543	
1.283715	0.3596418	2.724655	1.605398	1.14126	0.480646
1.960574	0.8358242	0.3959661	0.37622	0.2492807	0.7951351
0.8215385	1.463279	1.045928	0.1809697	0.8245458	
0.792442	0.4211098	0.4919383	0.4055501	0.7708398	
0.6937101	1.520517	0.9577925	0.9620376	2.294685	
0.9572598	0.5344494	0.3949414	0.7305407	1.019229	
1.326113	0.5456449	0.7916384	1.177226	1.344481	
1.229512	0.605432143	1.139332	0.626347527	0.6660268	
0.6562714	0.8028034	0.3862707	0.4739257	1.241493	
1.136319	1.573286	0.337794623	1.644316	0.5044019	
0.7188587	0.593577	0.4491918	0.5455974	0.5672927	
0.5603978	0.7564009	1.004119	0.2392714	0.5732741	
0.3803277	0.5314959	1.065247	0.4432557	0.7588536	
0.3743669	1.038294	0.180119	0.1934534	0.4006725	
0.7886675	1.146807	0.5296046	0.5008873	0.5254048	
0.9647856	0.5785977	0.8706954	0.9858622	1.016267	
0.6575816	0.4329149	0.600713	1.663556	0.4693358	
0.4758508	0.5028476	1.305006	0.5870203	1.910305	
0.3800056	0.7601935	0.2621834	0.4919808	0.7520795	
0.496177864	0.4067313	0.348375333	0.565141918	0.3489631	
1.634537	0.9442346	0.3088446	0.4444815	0.6325716	
0.2249068	0.7070752	0.3842734	0.8211567	0.3641095	
0.9012467	0.4226872	0.808393	0.6160507	0.5886571	
1.180932	1.000659	0.8563801	1.114454	0.5859874	
0.4487339	0.774249	0.6687841	1.60804	0.5686136	0.8125052
0.701668	0.5400715	1.076978	0.5371057	0.4069976	
0.1601111	0.7778555	0.510856611	0.2300138	0.5335947	
0.8821929	0.6703233	1.335231	0.7533593	0.4909133	
0.54603	0.584667	0.440078	0.8988706	0.7216365	0.7843273
0.3484092	1.061594	0.598834268	0.8407699	0.9212739	
0.5624096	1.589251	1.102585	0.224185937	1.869203	
0.8547977	0.6078821	0.5407818	0.2057739	1.436637	
0.6939833	0.9577319	0.5812776	0.9201681	0.4206567	
0.8148463	0.7319539	0.558432	0.6198739	0.4538987	
0.6991637	0.6726643	0.4664663	0.4230157	0.5019556	
0.7918682	0.8356683	0.3487059	0.8077759	0.7026413	
0.206570391	0.367394149	0.3551125	0.3637898	0.4685501	
0.2150163	0.6331726	0.799487	0.8910962	1.834565	
0.8764367	1.163395	0.4011641	0.2073997	0.4174408	
0.680204413	0.4081649	0.5644972	0.3632587	0.828311	
0.452568	1.193015059	0.8573801	0.4499933	0.5691107	
0.8008454	0.7850957	0.705919	1.008863	1.242588	

0.7746233 0.6522737 0.6126881 0.208091 1.933172
0.9427789 0.6181519 0.4963722 1.956713 0.59635
0.428667589 0.9792142 0.6763314 0.7005247 1.109076
0.6428288 0.5392816 0.183294 0.9681525 0.408052
0.5432109 0.3859024 1.742644 0.4275879 0.5994332
0.8527437 0.5961393 0.810285 0.918992 0.2679436
0.5606222 0.5885398 0.7329881 0.5749406 0.9826644
0.6320559 2.039931 0.375370168 0.6957136 0.725682
0.3927265 0.6791243 0.5697953 0.7627089 0.515002568
0.5255995 1.049997 0.6181159 0.5759346 0.4360152
0.5507124 0.7331233 0.3283423 0.7763147 0.5899854
0.4958612 2.113854 0.5039036 0.594373 0.926846161
0.6757797 1.14782 0.620873 0.725758087 1.239526 2.829687
0.5490051 0.6247356 0.9909711 1.085793 1.065061
BNC2-AS1 0.1018361 1.817732 0.06244851 0.2269867 0.3116754
1.271971 0.5244756 0.1627321 0.1572174 0.893126544
0.174828 0.2000714 0.9435666 0.1273077 0.4427267
1.936426 0.4062765 0.84343072 0.08382682 0.07250022
0.555328756 1.994596 0.3022955 3.127143 1.139216
0.3468632 0.4298592 1.744688 0.262390544 1.540505
0.3837107 0.7911287 0.06553332 0 0.4145308 0.223239
0.2119315 0.2134141 0.1838059 0.210287746 0.1370629
0.05984841 0.070662689 0.02182235 0.2097293 0.07493387 0
0.3283794 0.172820299 0 0.4702328 1.120937 0.546257
0.1531223 1.65463 0 0.1993633 0.9047486 0.2042244 0
0.616625482 0.324052549 0.7437793 0.5561105 0 2.438221
0.7410815 1.029247 0 0.09485159 0 0.1722212 0.80592
0.2215655 0.1584892 0 0.4480426 0.3039485 0.3083029 0
0.05902272 1.112396 0.0727211 0.7329999 0.4648256
0.1643357 0.4191319 0.1677902 0.69773029 0.5743277 0
0.6064505 0.5799577 0.08596583 0.1172161 0.2080726
1.020024 0.07148919 0.2848671 0.7569636 1.61502 0.1186271
0 0.2443086 0.3502682 0.5121236 0.04896932 0.8451378
0.060035853 0.2715291 0.2055726 0.02725611 0.1787157
1.010382 0.6011039 0.1078036 0.1899063 0.1075721
0.376153 0 0 0.1825957 0.4066581 0.9453201 0.1305193
0.2634043 0.1241929 0.2569953 1.352039 0.2827643
0.4156004 0.1932325 0.2072373 0.1026145 0 0.3893956
0.6457596 0.03654941 0.1120745 0.1336224 0.06937185
0.4289182 0.1584423 0 0.2883548 0.4941359 0 1.886404
0.08974797 0.6631745 0.06274324 0.4481979 0.06151972
0.2698892 1.041878 0 0.9706058 1.768534 0.2860836
1.817144 0.818639605 0.637554 0.208283355 0 0.1254555
0.1616808 0 0.1586305 0.2311483 0.334327 1.849489 0

0.2535202 0.03454251 0.03525037 0.564764 0.5254007
0.2444089 0 0.2749615 0.1024226 0.1371344 0.1140452
0.06325065 0.310407 0 0.8197799 0.1377857 0.1399754
0.02934812 0.3784436 0 0.3440551 0.2075812 0 0.089121
0.4987846 0.1686022 0.03458931 0.475708 0.3395022
1.001351 0 0.02771103 0 0.07895763 0.4090302 0.1563925
0.08648277 0.2229848 0.2170969 0.721592 0.1575421
0.07487821 0.2606103 0.148498 0.3269458 0.2689169
0.4978719 0.140771423 0.1900613 0.393648409 0.071261034
0.145902 0.2072018 0.1375476 0.07009825 0.3952531
0.04141567 0.3731595 0.2297844 0.355264 0.1328255 0
0.04067862 0.04751598 0.1955882 0.08636225 0.1533196
0.1386389 0.5338986 0.2381057 0.6302236 0.5888906
0.5665762 0.1630011 0.3956319 1.223057 0.3956517
0.2904516 0.1525729 0.03830614 0.126435 0.5538541 0
0.0829507 0.3932235 0.281494108 0.1593499 0.9176862
0.3732111 0.09508925 0.08670554 0.3155957 0.1201987 0
0.05879178 0.03692901 0.8174063 0.03509771 0.5570437
0.0922579 0.1895106 0.062049061 0.04725717 0.2032925
0.09665783 1.22264 0 0.040475371 1.816738 0.04782006
1.363016 0.4692157 0.1238373 0.3950493 0.1873333
0.05706113 0.08956602 0.7733233 0.0273907 1.020213
2.432063 0.1157254 0.3120992 2.071381 0.9763074
0.1526741 0.1375832 0.08690704 0.1263644 0.1497748
0.1447344 0.4924611 0.05729385 0.1702901 0.110215057
0.094146828 0.3277908 0.02281515 0.02646062 0.2989128
0.1934568 0.8379878 0.04369631 0.8484013 0.07433111
0.2970619 0.1731966 0.2031248 0.1356594 0.040526198
0.2225198 0.1401351 0.5585225 0 0.2508256 0 0.2773696
0.4029301 0.03442365 0.6670684 0.5029631 0.1047094
1.384965 0.09741142 0.06688641 0.367201 0.03395685
0.09099411 0.6174453 0.2292663 0.3507538 0.1137445
0.914352 0.1983081 0 0 0.09004569 0.2878405 0.06233786
0.2089057 0.1157841 0.08089298 0.05682954 0.1444248
0.1262993 0.3913506 1.312296 0.5515303 1.026775
1.01612 0 0 0.2959623 0.5538507 0 0.2026871 0.2379892
0.406676 0.761287 0.3310553 0.3550909 0.201783556
0.1246623 0.421812 0.3478144 0.168591 0.2633903
0.7175016 0.431728542 0.1889694 0.3644168 0.1689312
0.02579989 0.09802847 0.8842717 0.1004117 0.06688017
0.06250324 0.5881841 0.2065953 1.188134 0.4112643 0
0.127925038 0.4254159 0.84168 0.1964138 0.04941744 0.04268803
0.6642062 0.1006444 0.129241 0.06435041 0.2285898
0.970024

AC232271.1 0.08561985 0.3172542 0.2608807 0.3015979 0.2597049
0.4335503 0.1653595 0.1090275 0.148705 0.341930478
0.5971412 0.3259113 0.586722 0.3361579 0.3140669
0.3131923 0.3842792 0.189368295 0.1255395 0.0342874
0.306402385 0.3464098 0.1361563 0.6338204 0.2565558
0.7360831 0.2032925 0.5035301 0.093068911 0.1092822
0.1965899 0.2206508 1.621944 0.4213471 0.9017992
0.9619146 0.09466011 0.5430971 0.1945509 0.293617239
1.348275 0.6274053 0.211649719 0.2889714 0.8981926
0.6142645 1.01494 0.5521773 0.550326033 0.1666877 0.2100315
1.060246 0.6142759 0.3620793 0.6364507 0.3815431
0.180712 0.7131352 0.4185284 0.210384934 0.486032608
0.532738639 0.1812067 0.2943099 0.4197518 0.3843678
0.1752392 0.3461402 0.27420033 0.990614 0.2239471
0.4460263 0.5558325 0.424128 0.7037348 0.07742309
0.2742132 1.067204 0.3483123 0.503236736 0.3256576
0.3799491 0.9343121 0.6658002 0.07891302 0.2487008
0.3138472 0.3438618 0.51329649 0.89935 0.216584202 0.4660623
0.3245627 0.8492517 0.4003626 0.3662792 0.4045921
0.4733295 0.2874063 0.3905339 0.9178863 1.033214
0.8754193 0.3016889 0.4859116 0.597421 0.4477401
0.1665373 0.241337494 0.292498 0.5055497 0.4812334
0.1408662 1.309631 0.4548463 0.5608168 0.3352984
0.1483821 0.7273862 0.6214165 0.7365207 1.070797
1.513904 0.139088 0.5452486 1.115951 0.2006757
0.03472581 0.3172303 0.3714645 0.3738288 0.4995719
0.3593639 0.4583323 0.0837407 0.491083 0.2035986
0.6568393 0.4181362 0.8987563 1.341479 0.6986969
0.5370114 1.476157 0.4242657 0.5020024 1.146188
0.416329 0.622517 0.4861325 0.7566627 0.2573866
0.286095 0.5371441 0.5592134 0.5544911 0.5307498
0.5385843 1.713762 0.6087265 0.306499874 0.8809034
1.028809815 0.8081036 0.1318476 0.4502845 0.4438911
0.4201167 0.8089425 1.014556 0.5610293 0.314185419
0.7479279 0.2395965 0.6612787 0.4239568 0.1087086
0.8861736 0.8494174 1.275602 0.91495 0.5404559 0.1857768
0.9472455 0.3474272 0.5157247 0.3001525 0.1882479
0.4678016 0.1526751 0.3350076 0.3834578 0.146442
0.3108749 0.4048751 0.4214783 0.1982837 1.339577
0.2726376 0.3312143 0.5681359 0.8682063 0.3357545
1.030953 0.3239964 0.4232012 0.812456 0.4561016
0.3999125 0.8386246 0.9240413 0.199069 0.4470365
0.6787303 0.780583 0.4166915 0.1718021 0.729156
0.3195497 0.266299091 0.3845096 0.942119996 0.050552026

0.8433473 1.777847 0.1691304 0.7072306 0.4361615
0.2285108 0.2834338 0.5976934 0.8338491 0.3280439
0.1899896 1.064506 0.8464319 0.3854133 0.6534902
0.4229701 0.9834939 0.6733218 0.4986876 0.2625683
0.570268 0.5749758 0.3032121 0.3207522 0.519505
0.5925303 0.4616915 0.6878876 1.050731 0.6378097
0.8925131 0.3593271 0.4903716 0.5357605 0.332816238
0.4169979 0.4236663 0.205919 0.2248519 0.7859386
0.2653406 0.3789689 0.8791643 0.287311 0.2037557
0.4576686 0.5200925 0.5659117 0.2836038 0.8514364
0.77274506 0.4991332 0.7638008 0.8939277 0.509691
0.4647188 0.140374251 0.6300703 0.9573876 0.1166434
0.581885 0.3084484 0.6331459 0.5020394 1.250343
0.2259109 0.2046326 0.846317 0.2717459 0.3569559
0.7844607 0.5084017 0.4263137 0.733627 0.2406797
0.5509003 0.3607736 0.4780902 1.936095 0.205347
0.3881645 0.5148217 0.460839 0.480697323 0.467509219
0.6623646 0.4711602 0.4421603 0.1211694 0.4879528
0.1738193 0.5510724 0.2853212 1.007727 1.022447
0.2894137 0.5336858 0.4562286 0.54303612 0.2856403
0.3313692 0.04952644 0.7299487 0.4695473 0.238855091
0.2696393 0.4104305 0.3635847 0.4177128 0.3065821
0.5117067 0.2707288 0.731979 1.692337 0.4727402
2.066277 0.2689605 2.515755 2.031189 0.3483508
0.215172 0.5285168 0.9795373 0.588619836 0.3134417
1.682113 0.3902333 1.621473 0.4171446 0.2920404
0.4463267 0.304598 0.8348092 0.8959575 0.5552422
1.070877 1.142702 0.8867307 0.1156884 0.492935
0.6556662 0.7664965 0.6214456 0.2925285 1.003297
0.8103729 0.7545197 0.4343268 0.2870365 0.748063
0.349906807 0.3389989 0.2659824 0.5283658 0.5049658
0.4982588 1.093386 0.272235456 0.2170388 0.8042671
0.964034 1.008657 0.1004476 0.1467358 1.187188
0.3479247 0.3399347 0.3223226 0.5927422 0.5244421
0.8428267 0.843265 0.6386043 1.336484 0.3127568
0.2167424 0.490788894 0.5854628 1.56445 0.6267011 0.8149559
0.2181041 0.3747694 0.4884856
CENATAC-DT 0.3472976 0.8486886 0.5501772 0.6174417 0.5314624
1.123549 0.6558383 0.439355 0.4021257 1.148249076
0.7618445 0.5496428 1.057736 0.6060217 0.9436605
0.6932672 0.923699 0.806263967 0.4883775 0.5151078
0.811658649 1.076068 0.7609291 1.234979 1.037357
0.8113672 0.626093 1.273913 0.391495055 0.3502449
0.7633448 0.5793852 1.126773 0.7899839 0.5811873

0.6978812 0.2911126 0.4245609 1.402937 0.606168093
0.8959144 0.382696 0.301231383 0.3535048 0.506637
0.8305419 1.257233 0.8191798 1.198404349 2.041878
0.6013734 1.656546 2.815098 0.5367076 0.9780997
0.770857 1.317307 1.493311 3.172847 1.373253769
0.755734716 1.013040771 0.7398283 2.054581 0.4912067
0.5818031 0.9609209 0.633768 0.688178353 1.30739 1.013443
0.4405021 1.775058 0.7106406 0.4354068 1.709824
0.5467793 0.5811093 0.3504745 0.850527725 0.5032216
1.254018 0.49601 0.9225425 1.381988 0.6258289 0.8735162
1.072922 1.45414528 1.828086 0.98543552 1.680424
0.6922522 1.57174 2.020954 2.109095 0.720043 0.5282415
0.5019412 0.8311703 2.572722 0.4719879 0.6668434
1.342346 1.423493 1.033361 0.8628485 0.9247132
1.040780904 1.286127 0.4732266 0.6429256 0.7787859
2.09511 0.8883246 0.5821107 0.7016195 0.5579319 2.031127
0.9487079 0.3594022 2.065344 2.018044 0.6447761
1.697011 2.264472 1.888293 0.4590909 1.259966
0.883967 0.7086733 0.7248914 0.3926408 0.9332062
0.6470005 0.3934751 1.477357 0.2804548 1.550091
1.164568 0.7294639 1.3815 0.5178307 0.8430519 2.886442
1.014229 0.4007438 0.7258098 1.266801 0.650228
3.022424 0.5458984 2.98097 1.773724 0.8248449 0.2423346
0.8016682 1.473565 2.954044 0.3227663 1.177813655
1.183068 0.828707745 0.8863907 0.5110408 1.493347
0.7848527 0.1983619 1.602874 0.5938412 1.13884
0.755213291 1.935045 1.236924 0.5109197 1.360462
0.7465853 1.347527 1.364267 1.205637 0.7956227
0.6722863 0.6914403 1.096513 0.7057329 0.9416625
1.300171 0.5743206 0.9467768 0.5838443 1.06725 0.4168092
0.4693406 0.8259141 1.277333 1.241066 0.5300324
1.849564 0.4915081 0.2703892 0.4824267 0.8142164
0.7377026 2.654003 2.792714 1.196773 0.4649799
1.35561 0.4342132 1.330802 0.4935856 1.527801 0.5820478
0.4575228 0.9720972 1.173233 0.3819915 3.355069
0.6771474 0.710119857 2.457672 0.478005034 0.870842012
0.532132 2.212432 0.8599922 1.90252 0.8312389 0.6120499
2.853726 0.2285638 0.8750284 0.4278168 0.1217818
0.8670542 0.8507442 0.4585804 0.3436139 0.67538 10.02748
0.8525901 0.8506936 0.8827435 0.7172601 0.7950314
0.9635466 1.172559 1.41932 1.026041 0.6053326 1.881854
0.8164854 0.467121 1.976287 0.5375287 1.119779
0.9658631 0.659997142 0.6340138 1.890824 0.9634265
0.4188726 1.342957 0.8670151 1.036188 1.306114

1.044276 0.6611912 1.209583 1.316654 1.556714
0.7865813 0.3231495 0.546657992 0.2014549 0.5392337
0.2746982 0.6177245 8.005548 0.655668758 2.29242 0.6591294
0.3873643 1.155695 0.9080088 0.973021 1.080001
1.037861 0.4921175 0.808461 1.5413 0.7698436 0.6514607
1.085329 0.5765339 1.308176 0.9063798 1.345073
0.3440858 0.3457815 0.7421879 0.7874615 0.501823
0.5860647 0.4070679 0.7501356 0.626454775 1.150516743
0.6859743 0.2528755 1.150562 1.007264 0.5406341
1.052888 1.043141 1.189489 0.5598031 0.7527803
0.8072387 0.3848492 0.6168629 0.978979459 0.8853511
0.4380851 0.2480159 1.142793 0.3118665 1.152502402
0.9722055 0.3082998 0.3130586 0.5634695 1.238816
1.319273 0.4565786 0.8951157 0.2661244 1.565359
0.7430822 0.4331585 0.6479108 0.7036922 1.066608
1.055976 1.313715 0.4602609 2.215696389 2.587422
2.200798 3.25577 0.6554996 2.07796 0.1892063 0.5287586
1.889639 0.1915435 0.9152926 0.8619588 1.074973
1.127057 2.296385 1.026765 0.9272949 1.899687
0.5287007 0.8333071 0.959808 0.8525241 0.6290121
0.4445228 1.01996 0.7644387 1.733914 0.630808088 0.6820007
1.042934 0.8267258 1.197825 0.4657621 1.000137
0.832578378 0.7058298 1.058675 1.104222 2.045695
0.43182 0.7406942 0.6848796 2.613478 1.243424 0.4377997
0.223112 1.48572 1.180487 0.4586165 0.375677158 0.6563237
1.691504 0.1674604 1.516780702 2.596203 2.909203
1.115508 0.6244072 0.6400858 1.663089 2.710828
AC120498.10 0.4603126 0.4676738 0.8203632 0.08244721 0.4528331
0.9323477 0 0.8619967 0.3553216 0.144180353 0.4692083 0
0.1332827 0.1348705 0 1.300147 0.1721647 0.888120148
0.6038851 0.4915659 0.403418457 1.002821 0 1.135856
0.9064004 0.02261347 0.1821581 1.933641 0.05559562
0.06528079 0.3252043 1.237848 0.05554111 0 0.1171084 0
0.2694257 0.1033564 0.1335258 0.025460581 0.5575881 0
0.029944189 0.7582944 0.2073759 0.06350831 0.06695167
0.1855398 0.087881689 0.1148914 0.06642232 0.05428698
0.06172884 0 0.3926553 0.03536666 0 0.1769531 0.05769509
0.039002738 0.032662835 0 0.2578792 1.178294 0 1.072216
0.5495743 0.1163083 0.578104364 0.08038907 0 0.04170336
0.2845988 0.6169988 0.4029705 0.2601531 0.2010321
0.1405112 0.06532359 0.028182503 0.05002321 0.1885566
0.06163293 0.5916529 0.06060788 0 0.1480103 0 0.39422913
0.5516579 0.499032058 0.1284955 0.02457642 0.02428606
0.06622906 0.08817332 0.1394348 0.06058886 0.09657278

0.1166446 0 0.02513486 0 0 0.1484305 0.1519131
0.04150271 0.143255 0.050881871 0 0.01742279 0.207902 0
0.4123041 0.1018901 1.644592 0.06438013 0.1139625
0.2550392 0.03009804 0.02977226 0.4023613 0.2651175
0.1602364 0.3042004 0.02790521 0 1.866942 0.9060498
0.3461607 0.06215853 0.09826161 0 0.08696835 0.1286314
0.1466766 0.05472973 0.1239061 0.1899718 0.1509977
0.07839248 0.1514661 0.08952251 0.03029074 0.02715421
0.06979873 0.1867327 0.7378957 0.02535454 0.1124114
0.07976468 0.4883899 0 0 0.5466299 0.09033563 0.2570664
0.5450461 0.08082097 0 0 0.03178487 0.029420883 0.05234406
0.177211 0.3197332 0.4130419 0.1882206 0.07836157
0.3187692 2.327482 0.132481213 0.06138989 0.02927563
0.1195022 0.1139646 0.4174594 0 0.1107071 0.06658192
0.3472227 0.2615058 0.03221872 0.08040974 0.2630776
0.0355469 0.4930724 0.0389256 0.1660857 0.04974651
0.2467233 0.1515853 0.1457976 0.08796511 0 0.3776613
0.1286576 0.05715781 0 0 0 0.2593158 0.2538416
0.04697158 0.05443379 0 0.06933264 0.0994099 0.09772839
0.1079915 0.110397 0.1528918 0.3471541 0.3490363
0.05521842 0.02517115 0.215518 0 0.1506996 0.029826816
0.2147756 0 0.483163922 0.04121851 0.1254347 0.489615
0.029705 0 0.07020161 0.4025152 0.421954 1.572385
0.03752429 0.06809529 0.3447613 0.3221677 0.04144147
0.02439805 0.1949132 1.498124 1.551402 0.08648591
0.03815215 0.1425998 0.03001171 0.1381475 0.2874067
0.7774281 0.4610713 0.02051375 0 0.0324654 0.1607351 0
0 0.03515139 0.1190238 0.119286612 0.1620636 0.9999794
0.2108704 0 0.07348509 0.05943893 0.6451852 0.2625887
0.07474123 0.03129825 0.1433322 0.05949236 0.1573695
0.07819085 0.04015372 0 0.1201549 1.636807 0.2730663
1.358596 0 0.068607757 0.2771515 0 0.1650271 1.033947
0.1259462 0.167407 0.2948578 0.04836072 0.4048503
0.2340752 0.06964288 1.103182 0.9476936 0.3432806
0.0440853 0.1462956 0.02758149 0.06469756 0 0.04910391
0.2855918 0.1586723 0.1962656 0.3652013 0 0.2886501
0.560459936 0.039895883 0.5051105 0.01933641 0.1345562
0.1085726 0 0.07475951 0.0370337 0.7190411 0.06299746
0.04196121 0.2055038 0.1147689 0.07664979 0.068693911 0
0.237536 0.05917021 0 0.1062904 0.037768874 0.3134367
0.07880613 0 0.1413392 0.1705095 0 0.9390337 0.1375977
0 0.07780297 0.316572 0.1542395 0 0.07772354 0.237818
0.06426753 0.43052 0.2801184 0.430380175 0.5543214 0.4197378
0.09758078 0 0.1770528 0.8341043 0.4113528 0.04816444

0.1632048 0.1070418 0.2902194 0.9159321 0.02225884
0.1513421 0.2870622 0 0.04196449 0.2150018 0.1656714
0.03145405 0 0.06051052 0.2121034 0.0921728 0.175361
0.02735894 0.02850276 0.0264136 0.07149922 0.1339915
0.07144253 0.02480331 0.3185287 0.026135759 0.1372768 0 0
0.06559815 0.1661631 0.07888865 0 0.0283413 0.1854056
0.6171912 0.03501893 0.5034867 0.06971133 0.05065467 0
0.1545216 0.3057191 0 0.083765007 0.217075 0.2317952
0.2558958 0.04381397 0.2181543 0.1549884 0.5024063
BARX1-DT 0.09378802 6.285941 0.1725397 0.08959199 0.5591762
3.704307 0.2107752 1.440809 0.908244 1.335297064
0.007318699 0 0.4213318 0 0.3614042 5.242789 0.4592069
3.639927325 0.2526611 0.2913624 0.797051333 3.252189 0
2.876606 0.5138859 0 0.2024425 4.038364 0.21419322
0.5223613 0.9155914 3.752576 0.01646025 0.2148277
0.2429449 0.05607176 0.008871935 0.1378389 0.7188895
0.037727681 0.3924632 0.3607763 0.088743048 0.06029322
0.1492557 0.1505713 0.009920949 0.2566053 0.147586913
0.04766913 0.05905506 0.04826571 0.1372054 0.0641005
3.607405 0.03144395 0.125187 3.094083 0.0170986
0.03467673 0.18392013 0.04651056 0.8321884 0.1097488
0.02346648 2.992755 0.3373791 0.4136315 0.017132788
0.03573634 0.05097387 0.04325745 0.5988433 0.6280661
0.1525986 0.06167949 0.8009953 0.1943298 0 0.033408836
0.01482495 0.1490158 0.02739844 1.166032 1.086691
0.04127682 0.6053308 0.2844753 0.37971159 1.134814
0.008216327 0.03808111 0.3131907 0.07197452 0.03925549
0.06097275 0.07438161 0.09875912 0.02146534 0.6568105
0.2775504 0.9907175 0 0.1022732 0.7742077 0.3601696
0.02459961 1.252431 0.037698569 0.1250351 0.03614411
0.1163823 0.2543692 2.227637 0.2717667 0.02030807
0.01907979 0.03377409 0.6172677 0.0178398 0.0176467
0.2568341 0.6128509 0.2532689 0.1393278 0.1819406
0.2261565 0.1383224 2.787851 0.5129436 0.1657926
0.05824192 0.2255611 0.0601395 0.1524856 1.021529
0.1621978 0.0183605 0.0750671 0.02237496 0.09873818
0.08977747 0.08622583 0.09874709 1.14274 0.1447996 0.02213616
2.58047 0.2329374 0.2165436 0.07091754 0.2814388 0.06953461
0.6439961 3.115385 2.222076 0.7923196 3.614249
0.1916178 0.2282094 0.629713534 0.1978161 0.42724164
0.007756382 0.1890667 0.1421349 0.05440432 0.167344
0.1277284 0.2309289 4.835471 0.13741816 0.3729688
0.02602851 0 0.04052968 0.5443632 1.154115 0.01640467
0.246654 0.008575288 0.07750024 0.01909678 0.0158869

0.3508474 0 1.580836 0.1499686 0.1617275 0.03685738
0.6873214 0.7367543 0.01728352 0.05213895 0.1172894
0.350696 0.3486096 0.3641965 0.0347517 0 0.00655954
1.1877 0.02507629 0.04872198 0.04839624 0.02644277 0.3801292
0.2356901 0 0.008001131 0.03271742 1.069345 0.08705494
0.3949562 0.1718284 0.3879076 0.1459914 0.07429934
0.4108862 0.079555681 0.06365122 0.13482836 0.017898898 0
0.06691324 0.020729 0.04401709 0 0.05201264 0.06816572
0.5002036 0.1982955 0.03336227 0.01345388 0.0102174
0.5728687 0.03684497 0.02892257 0.2310591 0.3395189
1.226069 0.5126219 0.531421 0.0845222 0.1423091
0.1228248 0.1135684 5.393061 0.8695514 0.02431794
0.5365123 0 2.223 0.1854848 0.0365438 0.03125255
0.1834252 0.256301707 0.09605883 0.5844787 0.06249387
0.01194196 0.1088908 0.7310395 0.06038149 0.05188078
0.0590678 0.02782679 0.1203547 0.211575 0.3186947
0.1390366 0.05950009 0.007792546 1.192911 0.05106174
0.09711162 3.105072 0.4022149 0.152495236 1.697499 0
1.516138 0.1335686 0.03110468 1.339551 0.04033131
0.2149838 0.2249663 1.553908 0.233914 2.907121
5.132715 0.05813437 0.1567822 1.011649 0.03269637
0.2109125 0.02073437 0.04365753 0.4231921 0.2445267
0.07997767 1.522974 0.05036748 0.2209907 0.821266034
0.130059643 0.03742383 0.04584453 0.03323106 0.05362788
0.06478833 0.1550909 0.02195073 3.679459 0.1867002
1.119212 0.1305073 0.4591765 0.1476543 0.061074692
0.2395334 0.1759913 0.4383943 0.2142432 0.05512569 0
0.09289059 0.03892517 0 0.05585001 0.1684415 0.2367021
2.518555 0 0.01680011 0.04611561 0.09381974 0.01142766
0.0119297 0.02303426 0.08810013 0.5142534 3.585266
0.01660326 0.157559436 0.1708507 0.9838443 1.041091
0.04697289 0.3498108 0.1744916 0.1219091 0.04282226
0.03627572 0.3013691 0.196594 2.09402 0.2044965 0.1569819
0 0.08536936 0.2487332 0.05309854 0.1145634 0 0.08145548
0.07770962 0.01571482 0.2822702 0.05197024 0.356758
0.143601052 0.1487315 0.328439 0.07941985 0.1270369
0.04410448 0.2145447 0.077456328 0.149173 0.193234
0.03394486 0.04536178 0.06155543 1.005321 0.05044154
0.008399271 0.1098942 1.202996 0.05189132 0.1641355
0.03098963 0.03753024 0.074973281 0.07632378 1.88002 0.4768952
0.07447417 0.2251643 0 0.05055843 0.03895432 0.0484894
0.2641124 2.267248
PSMA3-AS1 2.111934 3.26081 2.900429 1.908801 2.605542
3.777202 1.417932 1.819024 2.061629 2.596885873

2.669606 2.149721 4.254618 1.522331 1.751905
3.54619 1.838453 2.564598995 1.491482 1.499362 2.41287416
4.220285 1.693899 4.354309 2.322143 2.860731
2.113818 4.141547 1.428610091 2.104642 1.618121
3.345005 3.895487 4.335582 2.962973 4.685537
2.540374 1.868636 2.586247 2.793997265 3.962063
3.514343 2.085908407 3.590259 4.599873 4.357606
6.261052 2.02554 4.494531388 3.000853 1.958087 8.27184
4.35472 2.344059 4.232459 3.321812 3.719905 4.57923
2.925793 2.118776752 4.143163122 3.123520919 2.501812
1.636089 3.53508 4.657356 1.934499 2.669623 1.50916528
5.305378 3.235688 4.82517 5.493614 4.673574 3.726304
2.188144 4.4137 3.128582 5.010522 2.16872899 2.246926
3.919886 4.261835 4.724339 2.267948 1.949919
3.470421 2.457134 3.64099252 5.357064 2.55295449
4.208361 2.805855 5.464297 2.500864 3.279415
3.592484 2.12063 4.645553 4.239624 7.05611 3.911237
3.555844 3.403614 4.933084 4.555958 2.098998
1.56229 3.48546655 3.861267 3.605382 6.538979 2.178358
9.974328 3.514101 2.047308 2.685561 2.645686
4.389349 3.140836 2.914057 6.636871 5.128409
2.348181 3.49846 7.982814 4.057081 1.448545 3.768973
2.036381 2.680448 5.500917 3.763072 4.090177
1.529939 5.248702 4.019503 2.977033 2.818466
5.35187 4.318689 3.185982 2.884666 3.87737 4.44942
2.757767 5.602763 6.070634 4.301426 4.319451
5.248313 2.422687 2.857292 3.556661 3.877748
6.502117 4.093077 9.206365 4.291344 6.088377
1.560597352 8.119921 5.055455994 3.346586 2.79261 3.549166
1.942562 5.831779 5.082106 5.226652 4.540722
5.693393105 4.196909 2.252848 3.435286 5.240204
3.207735 4.269559 3.018335 4.81508 3.297577 3.475743
2.022552 4.178163 2.470956 4.038956 3.299693
4.383057 2.938468 3.32462 2.722632 2.401181 1.548281
1.960604 2.306401 4.947521 3.056745 4.382883
2.839358 3.373231 3.626752 4.489463 2.326149
4.331763 3.320652 4.05946 2.265991 4.372658 2.924267
3.441145 6.503848 2.942934 3.016013 3.743749
2.105794 4.19654 1.246533 4.340389 3.482118
2.344056051 2.216006 5.296379729 1.25761442 3.683212
6.956015 4.41786 3.324562 2.411145 2.311139 1.85974
1.74333 4.827405 3.084549 1.799315 5.613924 6.197946
2.82184 3.768311 6.403541 3.750137 5.134464 4.411357
3.47682 3.392159 4.999467 3.429373 4.714597 5.124307

4.10548 4.879802 4.004977 5.549835 6.040277 5.368018
2.936052 3.16742 5.860617 1.670841731 4.024539 3.483679
4.811362 1.722079 3.638871 3.759679 3.04592 3.833184
3.012482 1.644774 4.103219 5.758128 3.275114
1.98258 5.733788 3.77619076 4.341957 5.094816 6.257454
4.300518 3.027457 2.445719781 5.642035 2.900572
1.583761 2.927389 3.243525 6.340446 3.282095
3.932466 4.372041 2.61018 5.076 4.950562 2.591879
3.710274 2.695368 2.512832 4.412275 2.914883
4.088373 2.321103 3.615816 4.449357 5.335964
5.510077 3.486798 3.413524 7.094801144 5.025298176
3.559908 2.588505 1.334438 1.497336 3.723314
1.230596 4.649631 2.741298 3.806567 3.020256
2.79944 5.221643 4.357213 5.176172887 2.47356 3.715728
1.810845 5.131526 1.54133 2.52542707 2.342024 3.686607
3.434262 2.136547 2.066776 2.393311 1.638524
3.222523 4.968931 3.23311 8.51925 2.838515 7.888187
5.268526 3.252083 5.079186 4.431003 4.971808
3.868750548 4.186328 5.386383 4.339869 7.22022 3.32875
2.892806 3.009407 2.563949 3.996332 5.623396
2.611152 4.704565 6.495005 3.948268 1.959887
4.822146 4.482113 5.59317 5.877599 3.031501 5.774753
5.647703 3.62691 6.35792 3.621657 3.587865 3.79032076
2.934587 1.720314 5.041365 3.958942 3.800474
5.161304 2.657882164 3.098671 7.042888 3.376526
3.492001 2.443527 1.521713 3.967569 3.844188
3.188214 5.07728 3.974193 7.467559 5.452402 2.96927
5.591706008 5.445173 3.70394 1.384255 2.831888099 2.446256
10.06637 3.476761 5.369502 3.356783 1.856634
2.932547
AC015908.3 4.245877 0.5019666 1.263968 2.330309 1.174591
0.3909036 1.28817 0.8141792 1.31098 0.696386509 0.2915658
0.2426654 1.430558 0.2171401 1.258548 0.2173291
1.601504 0.918367935 0.5909747 0.6265375 2.056748191
0.4766709 0.2553473 0.4433252 0.7719459 0.594655
1.661879 0.3662519 1.969183872 4.484326 1.090782
0.5259102 0.9538198 0.8752916 0.2765301 0.101537
0.8514795 1.275754 0.1910889 0.3962489 0.2743006
0.884688 0.096419652 0.8039717 2.384808 0.1704128
0.6647141 0.1161677 0.047162862 0.1356475 0.3386409
0.3496058 0.3478397 0.7893149 0.3913438 0.1328599
0.02266936 0.2848926 0.2322212 0.104656656 0.455752415
0.252669484 0.5228228 0.1806705 0.5311753 0.5440323
0.3581369 0.4213239 0.403321483 1.348183 0 0.3804711

0.4734744 0.2735343 0.2643171 0.05584583 0.2277604
0.552986 0.08179911 0.03024902 0 0.4722265 0.03307612
0.4127738 0.2602081 0.538169 0.3494993 0.09539614
0.68759676 0.2786395 0.119027602 0.01723969 0 0.7689724
0.6219971 0.1735044 0.314284 0.7153477 0.01295676
0.109548 0.9085449 0.7958481 0.07114886 0.2592804
0.7487773 0.8502014 0.2227297 0.3843985 0.409596359
0.3910866 0.112202 0.04958816 0.2980494 0.52764 1.038932
0.1470986 0.1036513 0.3547249 0.05702923 0.08076255
0.2875981 0.5481363 0.1849625 0.5446223 0.02968239
0.4193195 0.4377768 0.1001919 0.715064 0.1571914
0.06671638 0.03515559 0.879749 0.8401087 0.759349
0.4132586 0.1174857 0.3989751 0.5097542 0 0.8308895
0.2763734 0.924836 0.1463033 0.5100432 1.448392
0.04008503 0.6468023 0.1224616 0.5127797 0.3995298
0.3349066 0.1958694 0.06137756 0.2030927 1.793753
0.1931413 0.5118857 0.04337364 0.5165633 0.442161083
1.29639 1.468386586 0.04213669 0.05706157 1.066303 0.2955525
0.4473346 0.6938872 0.1900797 0.548063 0.551007809
0.2306198 0.1099781 0.5290927 0.195714 0.1941637
0.08893264 0.6535369 0.1965263 0.2018701 0 0.5360086
0.08630588 0.1270657 0.3433808 0.6615344 0.02088993
0.05093262 0.02669712 0.397222 0.2928609 0.06259537
0.8497374 0.1592942 0.1216061 0.4537296 0.7975367
0.1258595 0.1081844 0.09502619 0.3921928 0.1665002 0
0.02921261 0.9217597 0.6697489 0.3200978 0.2360125
0.492618 0.1382407 0.1641027 0.6162389 0.1192004
0.3259706 0.05403372 0.08261471 0.5137141 0.4367244
0.272118193 0.2017087 1.383530974 0.421356422 0.5640715
1.171302 0 0.2072405 0.2516851 0 0.262304 0.03483802
0.7181627 0.3020686 0.414168 0.1480166 0.8428658
0.6004829 0.07856119 0.50558 0.03152894 0.5087991 0.1237701
0.3275977 0.2295842 0.3221236 0.2372437 0.3084811
0.8189815 0.2699345 0.2972422 0.2775822 0.01742298
0.316289 0.2799026 0.02205832 0.05659336 0.281053
0.176045996 0.1304603 1.431072 0.4243739 0.1297498
0.1183102 0.1275947 0.6924942 0.3523041 0 0.4199154
0.1153816 0.6545108 1.224589 0.1258864 2.241098 0
0.5266086 0.8321815 0.5275607 0.4572376 0.03310667
0.165686641 0.8593693 0.01087513 0.2125535 0.1565049
0.02253023 0.1197882 0.2191006 0.1816739 0.3394816
0.4271064 0.1868738 0.528033 0.2161517 0.4342471
0.1182948 0.4710686 0.2516335 0.1041624 0.3379195
0.2635226 0.708858 0.1873379 0.1184946 0.475976

0.9641928 0.2194528 4.311153914 0.406802339 0.5014873
0.02075427 0.1444227 0.0776892 0.1319864 0.8425341
0.3776178 0.5273732 0.9128271 0.3828236 0.3781244
1.231844 0.1234054 0.073730978 0.289171 0.0318692
0.4286853 1.274726 0.02852108 0.10134581 0.2102624
0.05638979 0.1252567 0.1601311 0.3660246 0.3651289
0.3527613 0.3987554 0.2433784 0.9464237 0.1698925
0.3310986 0.1512193 0.8342271 0.9891182 0.2069401
0.3465663 0.4209218 0.176623482 0.05949677 0.3276477
0.6807841 0.2551811 0.2058717 0.6846152 0.05518947
0.07754424 0.1094825 0.3590336 0.4005001 0.3159945
0.5375473 0.7309773 0.2310836 0 1.058477 0.1538447
0.07409142 0.3544848 0.2581299 1.277301 0.05691403
0.4946574 0.1317537 0.08809521 0.397705882 0.08505125
0.2494115 0.5896481 0.4217463 1.397657 0.4817474
0.252469765 0.4665854 0.2762493 0.1997732 0.04693881
0.0222934 0.7620593 0.3653661 0.623599 0.02842868
0.8025806 0.01879337 0.6484866 0.2805863 0.7747581
0.581848205 0.1658521 0.314464 3.752109 0.494489493
0.2524083 0.2843336 0.1373298 0.6466168 0.2341507
0.145559 0.4289455
AL139407.1 0.1483774 0.2364709 0.4094496 0.1181159 0.3405883
0.6511556 0.1146258 0.1778281 0.09544544 0.232375782
0.5412972 0.4129701 0.5346431 0.05796562 0 0.3114547
0.02466473 0.409632168 0.1374046 0.07922577 0.072243376
0.5048851 0.4876422 0.5917292 0.2794665 0.8163945
0.01957231 0.5964036 0.167260015 0.08417055 0.1397687
0.2105879 0.5490298 0.6230917 0.3724545 0.2168426
0.1157957 0.1887904 0.6121352 0.186024759 0.9985177
0.1635008 0.398958535 0.2702629 0.7894153 0.3821308
0.7769243 0.1993566 1.120522696 0.8098131 0.0856424
1.31825 0.596931 0.2602862 0.8196828 0.2432022 0.4901791
1.292887 0.2107712 0.050288636 0.968626794 0.32038849
0.7142583 0.04340705 0.1701569 0.5194752 0.1012286
0.8872835 0.248461876 0.5787158 0.3273731 0.6990193
1.308791 0.7263577 0.8948233 0.02236209 0.6240073
1.036694 0.1965267 0.327036959 0.2149934 1.337147
0.8741386 0.6229979 0.09116972 0.1556364 0.9669165
0.2750332 0.42358661 0.8925949 0.369378103 0.7731599
0.1056263 0.6053946 0.04269661 0.3157981 0.3595639
0.1953024 0.8819972 0.4762575 1.068192 0.1728423
0.1424491 0.2966357 0.472072 1.165898 0.214048
0.07696149 0.229617976 0.5110139 0.5017022 0.8836082
0.02169938 0.8723842 0.788239 0.3632292 0.3043673

0.773878	0.7764226	0.4915587	0.2303233	1.237113	
0.6836648	0.2295586	0.2852541	1.463183	0.4297604	
0.01337313	0.4581278	0.1029984	0.4363441	0.5630878	
0.7548728	1.171174	0.1934945	1.071675	0.3057878	
0.505906	0.5171005	0.3731572	0.5053813	0.5858839	
0.4520887	0.2994271	0.4434802	0.3299846	0.2568171	
0.8668443	0.5230588	0.1328607	0.4113821	0.4081462	
0.2240889	0.5406971	0.7409474	0.8541522	0.4419354	
1.18591	1.076811	0.4136902	0.149096915	1.502681	0.5690124
0.4949298	0.1218608	0.4122517	0.1380712	0.7280532	
0.8082913	0.5936795	0.5716126	0.213520253	1.015807	
0.1761521	0.1027211	0.2057183	0.3707987	0.9971052	
0.5352808	1.01587	0.5471836	0.3746395	0.3877215	0.7257407
0.5992573	1.573593	0.4045673	0.5018918	0.8463809	
0.4489887	0.3393233	0.7557333	0.2631802	0.1512251	
0.1700945	0.7899281	0.4423633	0.4790311	0.3779802	
0.1732792	0.08561455	0.4863299	0.3030497	0.5248824	
0.1871596	1.42845	0.1340923	0.2421089	0.7770446	0.7542173
1.407601	0.3811234	0.5853325	0.6682323	0.7475634	
0.3353655	0.2514159	0.2350906	0.466335	0.282022069	
0.219231	0.756048596	0.051914421	1.204633	1.196807	
0.4609425	0.3574712	0.1727677	0.1056011	0.2100664	
0.1813504	0.5607629	0.4354415	0.6048407	0.7260527	
0.8827053	0.2137322	0.2726352	0.9773309	0.7196245	
1.194635	1.040775	0.5739058	0.2451503	0.8900069	
0.3918686	0.5970313	0.7053828	0.738606	0.3614784	
1.489424	0.8790525	0.9901741	0.717313	0.6447881	
0.196399	0.4194702	0.140986623	0.5688318	0.6446676	
0.1699301	0.01731838	0.9632801	0.3704184	0.919443	
0.5642863	0.2783978	0.09416112	0.3901481	1.904895	
0.4621751	0.235238	0.4141814	0.350326356	0.4733761	
0.6047448	1.290965	0.9896735	0.4374729	0.235893906	
0.5294053	0.7490046	0.04255592	0.2962515	0.5773876	
1.343056	0.6336307	0.1039241	0.9569971	0.4929525	
0.528792	0.5894633	0.1731053	0.3793824	0.2273676	
0.2515038	0.4267502	0.3892866	0.5612925	0.3165635	
0.3068593	0.4637283	0.358498	1.098712	0.3026091	
0.2274399	0.468374911	1.38888605	0.3690531	0.2742478	
0.1638532	0.07777184	0.3875721	0.06426137	0.6684977	
0.5150578	0.3519815	0.459877	0.3532916	2.725272	
0.95535	0.487142139	0.2084246	0.3062698	0.127153	0.2663128
0.159888	0.09739548	0.4883278	0.6661074	0.3260139	
0.4994655	0.3908415	0.4195503	0.2421508	0.3903089	
0.4872746	0.5015809	0.977148	0.3314509	0.6747232	

0.1503206 0.1916459 0.3866991 0.7031216 0.7584652
0.544035014 0.514599 0.6887921 1.027505 0.4087241
0.5199826 0.2530499 0.4567183 0.1656037 0.2805734
0.8395948 0.3563788 0.4780108 0.5452941 0.7154937
0.1439383 1.039951 0.4689309 0.6314343 0.4272206
0.9192613 0.502043 0.372762 0.3532424 0.6734498
0.467281 0.5879262 1.800767972 0.2156924 0.2919298
0.2303514 0.337756 0.3304646 1.79214 0.449312622 0.8358304
0.501465 0.8245567 0.5450694 0.2142443 0.1186687
0.4389058 0.2557953 0.3073564 0.9488178 0.8879907
2.099004 0.7789871 0.6313512 1.149401926 1.018309
0.6131728 0.2623308 0.144004522 0.1399441 0.9392989
0.3299422 1.035688 0.4101997 0.09991805 0.3435215
TNFRSF10A-AS1 0.7982757 0.6106665 1.699345 2.701646
1.555651 0.4388676 0.7047898 1.175401 1.936628
2.343213665 1.624958 1.792372 2.00761 1.582452 1.041137
0.5914016 2.092824 1.116228658 2.337413 1.558812
2.052188213 0.2044324 2.524395 1.33708 0.3280451 2.151297
1.191392 0.4869368 1.454475685 0.9315579 1.675798
0.3680024 1.409018 3.735371 1.559729 2.599899
0.1661294 4.547602 4.0049 5.611320295 2.633845 2.372515
1.804172644 9.061381 2.2077 5.555066 5.812025 1.936711
4.57504438 3.206137 2.843543 4.605019 1.517057
4.046724 1.067276 3.420628 6.094819 2.314697
2.652887 1.484185818 2.149235048 4.572345855 1.090353
0.6672329 2.605111 1.066401 2.209575 2.812304
5.224720291 4.970998 15.44731 4.612084 2.955673
1.042088 1.561836 3.382398 6.410373 2.023663
3.745937 0.916041949 4.045048 2.890018 2.052173
3.470969 8.336437 1.037921 4.599704 6.059677
5.31311939 7.614906 2.219878782 5.628232 8.436426
2.984287 3.990379 1.864051 6.057645 2.353644
3.349537 5.617757 3.997853 6.097472 1.839315
1.997169 7.836989 5.058206 3.553466 1.675151
5.082595703 5.777275 2.582922 1.373502 4.202765
3.142941 3.150275 2.589486 1.122862 3.758436
0.9267025 2.290664 9.346713 0.5643553 1.155987
2.371265 7.125289 3.58387 8.532269 2.318779 1.668984
1.562133 4.287182 2.778423 3.20258 1.930506 1.223715
3.110546 4.859519 2.750443 2.685799 2.394156
1.444938 1.633076 3.832463 1.512872 1.915928
5.164595 4.204271 2.339952 1.246233 3.408738
2.150015 3.506176 5.187551 3.218757 1.916769
3.007879 4.524288 7.345715 4.100681 2.594494

2.406440598 2.746622 1.07291541 3.714004 1.854456
7.513801 2.947057 1.428222 2.919799 0.4773476
0.6589922 3.2558766 4.842525 2.924346 5.068527
3.758507 2.890322 2.79171 9.171575 2.005819 0.6652377
3.846867 2.554232 2.124904 0.8342494 2.620817
1.847878 3.51797 2.181947 9.682022 3.735903 2.187159
5.917964 2.092106 2.510029 2.055892 1.952515
2.605526 4.276259 6.259381 0.5264108 1.887598
4.114244 2.141189 4.444857 3.943502 2.363514
1.077071 2.55674 3.745584 1.692061 3.296894 2.815675
2.918017 8.207997 10.11726 0.7078407 1.21059 3.990345
2.530128771 5.512475 2.717056036 0.670323573 4.051967
5.170983 4.010948 1.695563 2.071449 4.062767
4.35351 4.811885 1.856569 2.766607 4.318759 5.357061
3.607634 2.135504 3.210814 3.270738 0.4657572
1.486151 4.342398 3.11536 11.13543 3.164422 3.066571
4.670923 2.510968 5.582602 5.968469 3.464963
2.676739 6.838603 7.1119 4.008 0.724549 8.964155
1.229382431 5.695972 1.277228 4.910723 8.27381 2.650712
3.534144 2.718968 1.804181 7.189395 2.10907 4.639919
4.40986 6.903339 1.239761 4.583954 4.189918829 6.667935
2.344832 2.576126 1.204854 2.616492 1.33257375
3.24697 3.245158 2.564267 4.392703 2.596035 3.450635
2.805084 3.067148 4.894589 3.767068 4.085643
0.7327554 1.089401 5.326277 2.096993 0.7732 4.045213
2.000343 7.044086 4.866067 4.443318 1.383712
5.484731 3.288164 4.523246 2.002312 1.826651648
1.138629764 5.546106 3.403147 1.653436 3.299504
1.884761 0.9186501 9.130813 1.235088 2.322351
0.8649148 2.02485 2.911562 0.9722611 1.960526378 0.7689135
5.131541 0.7270876 1.473709 2.527944 1.736656715
2.267434 5.123017 1.225849 3.324172 4.528405
2.837618 1.116669 4.581548 3.909862 2.734504
1.619907 3.118084 1.372233 2.156615 2.898757
3.64292 2.90111 3.020177 1.786262532 3.638681 1.45204
3.055718 1.9267 3.766425 3.870316 3.777461 3.665639
6.922113 7.55258 1.939244 1.970928 1.92345 2.639577
1.251673 3.19713 3.193781 4.374853 2.626814 8.179041
1.007772 1.934848 2.333096 1.510168 3.197516
2.646129 2.169252418 2.93162 1.332052 3.590426 2.09561
2.22198 6.060547 2.382785247 2.684475 1.958812 4.222378
5.321825 2.305285 1.00066 4.385326 1.977217 2.225783
1.599621 3.970004 2.91382 4.255456 5.240611
0.888178649 1.817105 5.210903 1.099753 6.109454035

0.6883696 3.937763 3.009219 5.435993 4.691216
1.105843 2.987227
YEATS2-AS1 0.04885808 0.05946111 0.1634238 0.04242927 0.08156348
0.2429028 0 0.06920218 0.09599984 0.038954261 0.3126344
0.2617871 0.1508989 0.1041113 0.1158588 0.1809823
0.06201999 0.177245235 0.02193695 0.0379457 0.02076086
0.141552 0.0734582 0.2125594 0.1399365 0.384035
0.147645 0.165069 0.042916216 0 0.06275916 0.09157246
0.3301309 0.2797817 0.2205762 0.2336809 0.08781344
0.1555797 0.3848068 0.25157016 1.373763 0.2192673
0.208034759 0.236997 0.4390787 0.490243 0.5788423
0.1861921 0.737183452 0.2731612 0.06665585 0.7668804
0.4097957 0.1001779 0.3290848 0.1856452 0.4043338
0.3551505 0.1157959 0.060215172 0.267264071 0.127203752
0.1548291 0.1351356 0.1039097 0.288936 0.04242357
0.2379234 0.026775546 0.5926267 0.2389897 0.2156884
0.4613529 0.4514336 0.5564646 0.08836138 0.3827865
1.106348 0.2084257 0.147934446 0.2703028 0.2377374
0.3520671 0.2648963 0.1169634 0.275236 0.4570174
0.2579691 0.79123027 0.2805561 0.24825315 0.4612343
0.1935083 0.5511695 0.2760725 0.4174598 0.2497124
0.1449891 0.3839218 0.4457083 0.6506441 0.2638737
0.266085 0.08524536 0.5178957 0.4858207 0.08970472
0.1050544 0.176748781 0.1835651 0.6912922 0.7418061
0.07794795 0.357444 0.3460712 0.2433243 0.05466702
0.3202168 0.4856219 0.1905167 0.1103148 0.6450857
0.7081013 0.04947686 0.6190789 0.5471417 0.2478164
0.09127335 0.2797647 0.1397733 0.345473 0.4399393
0.4067451 0.353572 0.07943608 0.4698831 0.1985647
0.4495432 0.1955279 0.4778979 0.4901627 0.4068887
0.3040645 0.4395907 0.3353806 0.1688243 0.2306329
0.296196 0.5167025 0.1561937 0.3160756 0.1926916
0.2052664 0.3045845 0.269407 0.794959 0.3333768
0.5417031 1.385026 0.1783257 0.140590278 0.3336877
0.390629458 0.3919404 0.1039645 0.2115542 0.09210979
0.6808066 0.9375959 0.1531097 0.2713331 0.235213894
0.4265012 0.1355933 0.1291472 0.1970602 0.2105377
0.5436623 0.5512089 0.8788874 0.1965579 0.2287809
0.129328 0.4220835 0.3127415 0.4280625 0.2422127
0.2463939 0.5055035 0.3072086 0.1409363 0.458695
0.1440593 0.1086454 0.1374771 0.5441894 0.3319971
0.5074044 0.1900879 0.02593537 0.1161827 0.1819772
0.1698227 0.2066764 0.1176542 0.8092895 0.1284487
0.5269342 0.3130759 0.5626965 0.2272516 0.287975

0.3256993 0.2155467 0.541338 0.2564826 0.1425994
0.7424441 0.2559266 0.170380332 0.1492135 0.688329487
0.051283544 0.4295432 0.5228684 0.2987613 0.3622993
0.3103057 0.1246395 0.3861751 0.06514422 0.521667
0.2433171 0.2628258 0.3512961 0.4289947 0.1535526
0.1393695 1.173591 0.3038528 0.5056457 0.3605122
0.3416315 0.1651167 0.4494417 0.3156571 0.2662312
0.2756126 0.2394352 0.4687243 0.4352084 0.4962115
0.6948318 0.4750815 0.3521879 0.3473228 0.3087123
0.09668561 0.3169266 0.2487296 0.2360285 0.09953702
0.3119915 0.1697671 0.4403732 0.2770253 0.7423473
0.05798453 0.3983153 0.3306546 0.2389087 0.2414331
0.4215466 0.243567697 0.3091725 0.4211696 0.4721685
0.2097497 0.3333439 0.180066523 0.641829 0.1626847
0.01528682 0.2906048 0.2981485 0.7236733 0.26613 0.418111
0.3203311 0.2927193 0.4945901 0.3590466 0.1426538
0.2195635 0.2314108 0.1731604 0.4896958 0.1997693
0.4140534 0.1326676 0.2369928 0.313561 0.2916459
0.6645063 0.187418 0.1485461 0.40860326 0.71449124
0.5341803 0.2627056 0.06924576 0.1173354 0.3206339
0.05193855 0.4574019 0.1480142 0.1702048 0.362783
0.1948951 0.5005569 0.3431783 0.397704354 0.1538989
0.1191855 0.04567557 0.2710485 0.2625579 0.320706354
0.10283 0.4988325 0.1081014 0.145473 0.214983 0.2283481
0.1377259 0.1656977 0.2494284 0.1521491 0.7642211
0.2738447 0.1802256 0.1979919 0.1835801 0.5159478
0.1595201 0.3675965 0.273597554 0.4518626 0.2297528
0.3389672 0.2039178 0.3006812 0.1401374 0.2751994
0.1561553 0.4220449 0.6899549 0.4160568 0.404023
0.5360902 0.5140355 0.1181832 0.4625135 0.5247807
0.2876769 0.3197189 0.4419049 0.3182516 0.9528881
0.3724859 0.564468 0.1299526 0.1985217 0.413642924
0.1875842 0.1710976 0.3930445 0.2316258 0.1952948
0.6392972 0.149295829 0.5333761 0.731136 0.2254619
0.5772677 0.1282671 0.05480723 0.2233553 0.1662701
0.1881021 0.4874261 0.3784529 0.7384522 0.6511328
0.3597395 0.373828429 0.6520678 0.2713946 0.01713342
0.14872066 0.2904509 0.4549956 0.4214079 0.4701194
0.1557709 0.0598205 0.1762841
AC120193.1 0.0325025 0 0.07972547 0.04139781 0.05684332
0.03291636 0.200873 0.1168613 0.08363041 0.298628584
0.02789947 0 0.3513455 0.01015803 0.03532569 0.008026479
0.1945037 0.048944254 0.05350914 0.1388371 0.050640398
0.03236973 0.01378316 0.038886 0 0.008515886 0.2572424

0.08566817 0.041872913 0.04916749 0.03061674 0.03884622
0.1987013 0 0.07056195 0 0 0.07784489 0.08380625
0.239701596 0.60369 0.2005658 0.011276522 0.02089478 0.1561892
0.1036371 0.5672918 0.08151666 0.110316313 0.07787939
0.1125613 0.6746402 0.09298454 0.7656497 0.2534879
0.07991124 0.07953717 0.8885043 0.05431771 0.190941815
0.123003219 0.059100667 0.06474223 0.02535584 0.1490935
0.05139019 0.05913178 0.2299495 0.054426306 0.2270497
0.9715824 0.2748348 0.2250686 0.03030682 0.109599
1.371575 0.3196458 0.07937154 0.08199954 0.063678586
0.05651392 0.3313685 0.4525953 0.4233341 0.03423599
0.04196015 0.03344304 0.2008227 0.92787896 0.5865766
0.135725638 0.4596995 0.04627552 0.7042232 0.08729296
0.2545695 0.1995343 0.1140842 0.009091957 0.153743
1.424291 0.04732701 0.09985247 0 0.2794832 0.5067007
0.07814641 0.1078953 0.08622599 0.2022126 0.03280578
0.1565854 0.01901325 0.7524496 1.035996 0.09461954
0.02424457 0.08583304 0.184084 0.03400338 0.1121178
0.1981456 0.1098231 0.06034258 0.05207146 1.240023
0.6342082 0.1288946 0.1806376 0.05013801 0.1014344
0.1356807 0.1322857 0.04366793 0.09688122 0.1933266
0.0824415 0.04666114 0.1430809 0.199022 0.1549874
0.1711195 0.2444178 0.0456281 0.05112929 0.1577108
0.1406414 0.2037788 0.01909626 0.08466478 0.07008902
0.03065336 0.009817465 0.4737652 0.3008612 0.136076
0.106488 0.307884 0 0.1087441 0.228620829 0.4428787
0.177271242 0.1675517 0.06673494 0.3268179 0.172828
0.06075512 0.5311758 0.5335271 0.1162696 0.137198572
0.3005404 0 0.02250135 0.1201685 0.1467282 0 0.2918343
0.06268432 0.03268969 0.1094212 0.2183954 0.3330918
0.1287923 0.4952975 0.09284174 0.1759054 0.1250907
0.2341721 0.04645611 0.06850163 0.1098103 0.09937893
0.1490388 0.2939243 0.1315085 0.1937228 0.08831752
0.1012195 0 0.2574523 0.09559285 0.1680434 0 0.1344026
0.05221922 0.2370962 0.1012083 0.1118368 0.3880223
0.1381841 0.2614658 0.01194924 0.6342295 0.132707
0.173916 0.145909 0.1702534 0.415595873 0.202203
0.319807642 0.250184152 0.1164169 0.8880517 0.2107217
0.01118645 0.1009207 0.06609212 0.03248165 0.04889273
0.4661497 0.1271797 0.07693092 0.5323105 1.046414
0.5930363 0.2021345 0.2324376 0.04424867 0.2353165
0.4776826 0.2011452 0.02685045 0.03390585 0.3641696
0.4509706 0.3686702 0.2525562 0.07725161 0.3993065
0.0855818 0.4035359 0.9624184 0.07739327 0.03971242

0.5020124 0.123534141 0.274638 0.01046047 0.3573475 0
0.345917 0.05595947 0.3708441 0.04944343 0.0281464
0.2239424 0.08096503 0.3472606 0.2765603 0.2208411
0.07560638 0.25745039 0.1432868 0.3136051 0.1028326
0.2081193 0.1045415 0.284202903 0.5450486 0.1297311
0.01242933 0.2396114 0.01580981 0.2311574 0.1281218
0.03642381 0.2191618 0.1145939 0.08742146 0.06736887
0.03568869 0.1662097 0.08300923 0.165278 0.35315 0.1949129
0.02634702 0.1386883 0.2016553 0.16731 0.2402095 0.6974696
0.1645756 0.03623373 0.058627945 0.060096707 0.3138578 0
0.07600768 0.09540245 0.01029076 0.3096861 0.1394632
0.1173379 0.3439962 0.03950487 0.01105565 0.226906
0.1587582 0.206952573 0.06087471 0.2907203 0.3230976
0.1166732 0.2501709 0 0.1622988 0.03956957 0.02197363
0.03548412 0.2675468 0.7686494 0.03536256 0.238359
0.03202168 0.3515929 0.4443511 0.1452106 0.07579493
0.05853898 0.01119483 0.2178195 0.06485088 0.1160371
0.200209754 0.04174979 0 0.05512113 0.09948025 0.1778009
0.08314699 0.1807273 0.2902078 0.06146043 0.6348871
0.2498107 0.1971006 0.008382336 0.6126758 0.03603441 0
0.1422286 0.2833823 0.06238929 0.02369022 0.1552574
0.07595777 0.249609 0.2082652 0.0528306 0.01030296
0.139538119 0.08952262 0.01346275 0.08073463 0.05380832
0.102746 0.1744771 0.108265579 0.2584816 0.05169289 0
0.1399851 0.04693085 0.02970825 0.2243351 0.1921122
0.0598465 0.3307582 0.09231315 0.3981712 0.09188276
0.8011824 0.313023631 0.3588413 0.2398523 0.06268837
0.299673841 0.01362452 1.234537 0.01606107 0.09074817
0.07188435 0.1313241 0.2235982
WASHC5-AS1 0.04950125 0.02366723 0.1138329 0.1891464 0
0.07519734 0 0.02966326 0 0.093029487 0.1912088 0.0972522
0.1911066 0.09282399 0 0.2383741 0 0.037271008 0 0
0.05784393 0.07394856 0.06297517 0.08883498 0.1356143
0.1556362 0.1175339 0.01957087 0 0.112323 0 0.06655807
0.5972799 0.3118112 0.2619477 0 0 0.1111478 0.2106007
0.219039074 0.4197347 0.2836427 0.231850663 0.2545817
0.254867 0.3642441 0.6335886 0.2128281 0.176412135
0.2767571 0.08571535 0.4670345 0.2124229 0.4093696
0.3378039 0.182557 0.2543834 0.2283509 0.1240887
0.033554315 0.281000539 0.135015323 0.3944092 0.08688804
0.3406038 0.1006292 0 0.2251367 0.049734703 0.3630861
0.253666 0.3228991 0.759011 1.223167 0.5200174
0.08952457 0.4419813 0.8260319 0.7493109 0.581894267
0.0430353 0.324433 0.4241858 0.4326525 0.1042827

0.1677512	0.4074695	0.2752674	1.27184226	0.7258511	
0.095404577	0.2763638	0.1057163	0.3760822	0.0569773	
0.3539951	0.2878961	0.2866875	0.3323288	0.3010504	
0.3718616	0.3892263	0.1710845	0.4750214	0.5107829	
0.4294161	0.1428202	0.06162163	0.240757065	0.1979803	
0.7194692	0.2186061	0.1737429	0.4092784	0.7012537	
0.3144118	0.5538666	0.3137366	0.2376963	0.284829	
0.2305195	0.3994086	0.3421236	0.09190165	0.5234113	
0.7442181	0.2716588	0.02676904	0.0917036	0.3665285	
0.267377	0.05635675	0.2014709	0.2493982	0	0.3785606
0.2118796	0.506337	0.408585	0.7144739	0.9104612	
0.4169835	0.5776261	0.4430085	0.5139408	0.1601288	
0.2891653	0.1481239	0.8506945	0.7011347	0.1601182	
0.07002751	0.06728392	0.1967846	0.1808728	0.233149	
0.486543	0.5568268	0.4171849	0.6624682	0.111917936	
0.3007921	0.329042756	0.5628993	0.1524558	0.2947163	
0.1184476	0.3932523	0.5730273	0.3961234	0.2656176	
0.142468085	0.3696989	0.05037204	0.4369364	0.2549158	
0.3112574	0.2494886	0.500021	0.9451343	0.2489317	
0.3249641	0.02771798	0.5072982	0.1584292	0.7951122	
0.05784456	0.1339518	0.4286539	0.1925876	0.1485804	
0.9128689	0.1756029	0.03783848	0.04255986	0.4765266	
0.1581215	0.7375986	0.1513209	0.5780892	0.1332916	
0.101405	0.1698524	0.8284041	0.187319	0.5948948	
0.2087657	0.1140307	0.4624201	0.6038878	0.5065348	
0.3946014	0.2527114	0.2456822	0.308781	0.2815139	
0.1324369	0.411759	0.1555774	0.256602084	0.2078695	
0.834971133	0	0.3723358	0.6258915	0.1404063	0.689996
0.1152766	0.1811847	0.2473475	0.05584766	0.9210101	
0.1291296	0.1952761	0.4152407	0.3118084	0.3565237	
0.1889082	0.9502159	0.3285294	0.4263566	0.3472205	
0.1969353	0	0.3614698	0.142619	0.2472579	0.3963417
0.3245422	0.3353141	0.3337371	0.4748134	0.9218768	
0.4038323	0.3005681	0.1209639	0.2867115	0.102623065	
0.348561	0.2628661	0.09070661	0.1733313	0.3793183	
0.07670356	0.4674165	0.2635579	0.9002053	0.08077828	
0.411032	0.7421343	0.3835936	0.03363406	0.2072671	
0.226209523	0.7235902	0.7658391	0.5403178	0.2773446	
0.1061444	0.118047419	0.7682915	0.4009716	0.02839478	
0.3421198	0.2167048	0.1920285	0.5268493	0.832101	
0.2394531	0.1812388	0.339514	0.307808	0.1019134	
0.4008004	0.1137806	0.8810133	0.4745708	0.222639	
0.341075	0.3801998	0.2149845	0.4368219	0.2321669	
0.5161613	0.1670989	0.7035948	0.750038187	0.68645387	

0.43455 0.1996229 0.2122259 0.03113524 0.3291292 0.06431611
0.3823241 0.08247945 0.2438871 0.3970946 0.3535925
0.468998 1.186962 1.122858733 0.3013143 0.4087075 0 0
0.2514665 0.227449694 0.3033582 0.3841855 0.3011923
0.1621268 0.07334521 0.2290406 0.2827499 0.2604276
0.3901517 0.1784918 0.7427699 0.2487999 0.415568
0.2674643 0.230171 1.631049 0.4074171 0.5301729
0.457378671 5.818014 0.3282756 0.5876457 0.4999772
0.1523197 0.1055273 0.4718528 0.1243086 0.4212186
0.483466 0.6776966 0.05628446 0.9574712 0.7486533
0.04116026 0.6691097 0.324921 0.2774512 0.3563204
0.3247215 0.3251281 0.3296981 0.9123698 0.8722656
0.181037 0.7767236 0.539464498 0.1363428 0.49209 0.2536023
0.6760874 0.4054306 0.6228009 0.269817205 0.2952503
0.413323 0.5912304 0.7524603 0.03573781 0.03393421
0.1464266 0.1950577 0.4785171 0.4492854 0.4217783
1.169513 0.6597044 0.4139962 0.186548266 0.99702 0.2191768
0.09547428 0.108095388 0.6536287 0.5127813 0.3669147
0.3957812 0.1642196 0.2333407 0.01964652
AL161785.1 0.224284 0.6910587 0.6189151 0.4284992 0.5556849
1.716167 0.9240846 0.8064031 0.53862 0.936677679 0.89843
0.8812751 0.9236055 1.752391 0.8938067 0.664642
0.447392 0.975694896 0.3077005 0.2128994 0.640648807
0.5956474 1.553478 1.66963 1.467856 1.27322 0.7494886
1.083783 0.818676501 0.6220151 0.56339 0.6701473 0.6735432
0.6279012 0.7100826 0.3277745 0.3371031 0.5819339
0.3855375 0.330812872 0.201245 0.175747 0.415006942
0.4325542 0.1539694 0.07334864 0.2899708 0.5625069
0.608991463 0.119424 0.316446 2.022027 0.2940855
0.1124123 1.894963 0 0.7683863 1.481691 0.09995204
0.641906123 1.92391381 0.543766688 0.7197715 1.195617
0.06858813 0.4559384 0.7140695 1.536396 0.150227807
0.2089013 0.319258 0.1444953 0.6409575 0.4182653
0.7756818 0.7661798 0.5611043 0.1825681 0.4526712 0
0.9966031 2.259391 0.5338702 1.537486 0.761237
0.1689022 1.410287 0.2771561 1.19519722 2.501691
0.264162672 0.7234757 0.8728214 0.7573251 1.979204
2.240379 2.512219 0.5248263 0.2300437 0.6567512
2.402483 0.1741764 0.1722584 0.1793552 1.440005
1.485071 0.3954503 1.178844 0.396669133 0.8305779
0.1811014 0.2000963 0.524805 0.5219762 1.941679
0.4352824 1.226867 0.07897229 0.6443422 0.07821361
0.128945 0.2144794 0.3215062 2.452103 3.066196
0.4109197 0.5470454 1.024203 2.123654 0.5766301

0.5384229 0.1986019 0.3042798 0.3013312 0.8356628
1.715211 0.8770369 0.2414894 0.7404989 0 0.4074258
0.3673639 0.1938631 0.02623812 1.058455 1.189053
0.03234992 1.576625 0.8345689 0.7302882 0.1381858
0.8695984 0.5193826 1.585078 1.092683 0.4694973
0.3785446 1.475387 0.2100236 0.5002601 1.765411052
0.5781793 0.917447094 0.3627273 0.1842022 0.5341291
0.9143285 0.349368 0.4751426 0.2761209 1.275483
0.430336703 1.196471 0.4818176 0.1552709 0.533073
1.253572 0.5382865 0.215765 2.249281 0.1754477
0.20135 0.8093366 0.9983405 0.6836409 0.4926569 2.640278
0.2697417 0.3493864 0.387818 0.8548562 0.1313046
0.7324893 0.4571772 0.4713705 0.4143693 0.8437937
0.2723084 0.1777523 0.4074389 0.4026185 0.5105039
0.2931733 0.2848105 0.04715106 0.1738964 1.111047
0.6601749 0.1269799 0.5846453 0.5100102 2.357365
0.9021242 1.401751 0.6217992 0.2180349 0.1333455
0.4145841 0.9659764 0.284198912 0.9069479 0.315262471
0.104630266 0.1963712 0.6953774 0.060587 0.07719224
0.4062361 0.03040464 0.5977069 0.1686925 1.072224
0.1300156 0.1376311 0.1194542 0.7674278 1.687158
0.2958734 0.4502284 0.4834527 0.4479463 0.8989788
2.082008 0.7411277 0.3119571 1.914634 2.987452
0.8729459 1.633845 0.4086915 1.276902 0.3937056
0.3712807 1.67159 0.5340534 0.3653815 0.639217 0.74912197
0.3743493 0.769948 1.073115 0.03490412 0.3182673
0.283176 0.6176933 0.8340081 0.2589662 0.3795517
1.282942 6.570437 1.385868 0.7111612 0.4869414
0.296090029 0.1561189 0.4477317 0.2365326 0.4587637
0.6412358 0.059428675 2.56076 0.4037227 1.372301 0.9874723
0.0545479 2.416825 0.1178809 0.251343 1.052055
1.094894 0.321735 0.9555864 0.8414235 0.5734662
1.45111 0.8025762 0.7167403 1.961457 0.3636161 0.2339384
0.2783051 0.9071267 0.5525231 0.58749 0.1892761 0.1458516
1.80704802 2.280840124 1.268841 0.2009925 0.7575997
0.4702328 0.4260694 2.687431 0.1603947 0.5813174
0.4092673 0.8723326 0.6866071 0.1988277 0.3983686
0.446274765 0.1400223 0.9259014 0.2818958 0.4472806
0.3222442 0.52345198 0.2036264 0.4778382 0.1263578
0.5849403 1.895443 1.024942 1.423449 0.2622144
0.04910357 0.2471101 0.4985774 0.434212 0.3138144
0.4376115 0.3090002 0.1391728 2.088354 1.795544
0.087717481 4.436663 4.131599 0.4648896 0.274586
0.97131 0.6375078 0.207852 1.043012 0.1060271 0.6722243

1.256954	1.076742	1.368938	0.8848838	1.782035
72.28556	1.417651	0.2483154	1.171966	0.02724579
0.4761583	0.6639205	1.607602	0.3992046	0.9721555
3.744374	0.320961608	0.549113	0.3096664	0.3481941
0.1856526	0.5371215	0.9782354	1.471537372	0.5747336
0.802592	1.240181	0.1894057	0.8635921	1.298347
1.990321	0.1227475	0.527686	0.6579898	0.1820023
0.8722496	0.3623076	0.7459183	1.220883205	0.5130843
1.809581	0.09612934	0.616743211	0.5954358	2.352031
0.3324889	0.2087363	0.25983	0.8055143	0.4747516
AC092794.2	0	0.04935227	0.07121127	0	0.03384853	0.1045372	0
0.1546388	0	0.06466348	0.06645327	0.1013979	0.1594026
0.09678096	0.4207083	0.1911813	0.1029522	0.038859825	0	0
0.120619491	0.2056024	0	0.03087397	0.07069769	0.08113537
0.08169615	0.1224309	0	0	0	0	0.04981929	0.487655
0.1260527	0	0.02685218	0.0927087	0.09980834	0.045675288
0.4376274	0.04549755	0.053718705	0.06635856	0.159439
0.05696571	0.360326	0	0.131380263	0.1236664	0	0.2191246
0.1937935	0.07760374	0.1509446	0.1903392	0	0.05290783
0.05175137	0.034984694	0.117191699	0	0.0257014	0.09059197
0.2840986	0.1398919	0	0.1564893	0.025927416	0.1802686
0.08815983	0.0748142	1.786957	0.02406243	0.3413758
0.09334089	0.1202146	0.4201193	0.1367193	0.176954081	0
0.1127544	0.1934924	0.1061402	0.02718204	0.1499162
0.2655247	0.03188908	0.53042374	0.1455372	0.149207335
0.05762896	0.06613372	1.154558	0	0.1581795	0.1750985
0.05434702	0.1299358	0	0.0969284	2.59273	0	0.1857266
0.5325569	0.1557292	0.07445424	0	0.114100111	0.1720167
0.2187906	0.0828818	0	0.1706902	0	0.1024421	0.08662158
0.02044442	0.1143826	0.02699736	0.08011541	0.1665739
0.0951221	0	0.2232506	0.2002434	0.259636	0.08373052
0.09561282	0	0.05575499	0.08813875	0.05251483	0.1040119	0
0.1973491	0.07363725	0.08335602	0.2272013	0.3047445
0.08789566	0.3804141	0.1605999	0.0271702	0.2192111
0.0626081	0.1004974	0.0661878	0.04548506	0.1260385
0.2384912	0.02433757	0.07015215	0.07693999	0.1508666
0.08102929	0.2075252	0.3361158	0.1449897	0.08633855	0
0.3421249	0.026389957	0.117379	0	0.06145594	0.08233122
0.04823719	0.1405776	8.037804	0.08521249	0.23766612
0.2477949	0	0.02679779	2.698715	0	0.03716057	0.2482554
0.1194454	0.3633607	0.1824399	0	0.04808398	0.1415853
0.1275395	0.08041387	0.418986	0.1064112	0.06693247
0.0663918	0.5982638	0.05231105	0.1183545	0.04437413
0.2935875	0.0659448	0.1025389	0.1840667	0.1808197

0.2382406 0 0.101196 0.2738618 0 0.480197 0.09328504
0.1486146 0.1095756 0.169516 0.1980479 0.1371409
0.215578 0.0853851 0.2476492 0.0677341 0.05523299
0.1022171 0.05406982 0.053508143 0.04816238 0.217641232
0.027086787 0.03697219 0.315035 0.5646537 0.2131584
0.1201906 0 0.2063133 0.02911419 0.4201188 0.1682928
0.2036005 0.1236977 0.4334672 0.1858609 0.2626149
0.2622501 0.07904635 0.1932746 0.2585872 0 0 0.2153594
0.1486987 0.1718655 0.3874099 0.1127924 0.05520129
0.1855807 0.2038458 0.192235 0.1871321 0.1290392
0.09459032 3.202861 0 0.1211399 0.07474683 0.1655033
0.03614405 0.2307014 0 0.1827532 0.1177685 0.06704143
0.05614784 0.8571076 5.389712 0.1646835 0 0
0.070755767 0.2155531 0.2833337 0.1959481 0.1032741
0.1936711 1.169256541 0.0828665 1.017896 0.1184208 0
0.2447712 0.2502681 0.8748242 0 0.5447142 0.1259765
0.04164554 0.1604647 0.08500626 0.395892 0.6722421
0.3499313 0.07422017 0 0.1045925 0.04404525 0.1280851
0.05693039 0.1760464 0.2105862 0.06533331 0.1078806
0.167573871 0.071571656 0.1132686 0.05203313 0 0.06492498
0.1470684 0 0.2989666 0.04299772 0.113015 0.07527678
0.1316663 0.2573636 0.1718834 0.061617096 0 0.1597988
0.07961178 0.2779019 0.1430107 0 0.21086 0.1178126
0.1570159 0.07043252 0.02549061 0.05306762 0.04211474
0.1234224 0.2033917 0.2093632 0.23233 0.190231 0.1083208
0.0697165 0.1866533 0.08647008 0.0772336 0.4020171
0.090833555 0.3579951 1.57444 0.5689323 0.1895602 0.3176257
0.1320309 0.09224386 0.1728103 0.07319577 0.2160324
0.3718873 0.5281542 0.09982871 0.5430035 0 2.842208
0.03764133 0.6106992 0.09906931 0 0.06163416 0.144738
0.2140342 0.5787404 0 0.07362131 0.17896493 0.02369249 0
0.2403755 0.06408256 0.2002328 0.181818 0.070329795
0.7593298 5.294435 0.05136948 0.07845367 0.07452253
0.07076158 0.6106741 0.02542159 0 0.06387789 0.1570565
0.8580736 0.09379456 0.181745 0.097250295 0.1155024
0.11426 0 0.112703362 0.032452 0.475236 0.2677891
0.2751019 0.2201401 0 0.3482285
LINC02416 0 0.05145431 0.02474811 0.3084137 0.2470316
0.190732 0.3325564 0.03224506 0.4569008 0.876429646
0.06928369 0 0.249288 0 0.08772547 0.01993242 0.1073372
0 0 0 0.220074729 0 0 0.4184566 0.03685444 0.04229557
0.7239943 0.02127426 0.103984401 0 0 0 0.4155298
0.1694752 0.7885298 0 0 0.2174792 0.9989706 0.16667251
0.8473532 0.877555 0.112013448 0.5188871 0.1385249

0.5741229 1.377469 0.4337851 0.465718703 0.365312 0
1.243825 0.7504627 0.2427272 0.1573737 0.09922313
0.07900692 0.6343551 0.1888446 0.583596522 0 0.25684165
0.2679609 0 0.2591742 0.05469385 0.2936878 0.435079
0.027031732 0.07517867 0.3217017 0.05850055 0.6387662
0.02508731 0.2512347 0.194633 0.2506696 0.1533047
0.183269 0.026355859 0 0.2938923 0.2305528 0.0553305
0.3967571 0.07815078 0.3598843 0.2327313 1.10603172
0.1213888 0.18148954 1.171629 0.04596701 1.317294
0.5574279 0.3848058 0.651987 0.1416545 0.04515672
0.2727108 1.111625 0.4231034 0.1859752 0.7745488
0.05552399 0.4262007 0.4657526 0.0334925 0.11895993 0
0.2281095 0.3456478 0.2360812 0.7118412 0.0952861
0.1495276 0 0.4476193 0 0.05629449 0.3062683 1.273571
0.867769 0 0.2586216 1.539696 0.615215 0.1454947
0.3239769 0.09960751 0.8138163 0.490095 0.5475158
0.08133151 0 0.03429245 0.1279561 0.2027815 0.5329764
0.6001461 0.3848853 0.7932338 0.5860409 0.2266196
0.6094611 0 0.5238891 0.3910392 0.2134007 0.1314068
0.2486491 0.0761225 0.04876008 0.6149975 0.07864618
0.3379222 0.1682833 0.03185744 0.3023303 0.6301115
1.094930472 0.7133938 0.13756987 0.318184 0.03314502
0.3203675 0.5150275 0.201167 1.099239 1.291803
0.06663143 0.216815319 0.2870546 0.1642688 0.1117567
0.2984185 0.4164287 0.5036634 0.2588292 0.1867993
1.055332 1.141263 0.1506524 0.350924 0.3198342
0.4321582 0.3982348 0.07280529 0.2884531 0.1628277
0.0230732 0.2268165 0.3817738 0.1645273 0.2313207
0.3767288 0.1718839 0.3741719 0.3289828 0.5341438
0.04139798 0.2425083 0.1055062 0.3074899 0.3054341
0.2503249 0.09725829 0.3408778 0.1370912 1.009921
0.3097249 0.8292963 0.5494134 0.1186958 0.5680339
0.3295557 0.115171 0.1491991 0.140932 0.111574387
0.0753206 0.567277842 0.084721452 0.1349143 2.041102
0.130823 0.08333903 0.1253099 0.03282575 0.02688759
0.06070848 0 0.3509216 0.6368171 0.4191404 0.1129824
0.1937772 0.1825335 0.33418 0.3571236 0.1612054 0.3774416
0.07135866 0.4667504 0.2245321 0.3100644 0.4479641
0.2962012 0.3135906 1.170233 1.789737 0.2428893
0.4008457 1.073064 0.115316 0.1643653 0.1335711
0.557775362 0.2778591 0.05195366 0.2465036 0.1130506
1.340082 0.1111728 0.3810743 0.2046409 0.1397938
0.175618 1.630846 0.3894546 0.5641501 0.3290532
1.389393 0.098359252 0.9551199 0.2685469 0.2809044

0.2153456	8.855634	0.288724313	0.604772	0.20846	0.03086617
0.3718969	0.1374136	0.4696697	0.2545354	0.4522623
0.3549468	0.1094518	0.4341933	0.08364968	0.1550971
0.02293078	1.154382	0.04560448	1.109133	0.605042
0.2399043	0.2296062	0.1001555	0.2670984	0.1147155
0.07318519	0.4086961	0.3374266	0.232948368	0.522340547
0.4251347	0	0.3984773	0.1353806	0.07666619	0.7340969
0.2078002	0.1120728	0.4418572	0.2550698	0.3020035
0.3756555	1.075226	0.289086876	0.02519537	0	0.3320106
0.1931589	0.1739522	0	0.5129625	0.3684916	1.446048
0.2056108	0.3189158	0.3596314	0.1756341	0.180151
0.05301367	0.6790951	0.5651929	0.1803031	0.5646722
0.2180577	0.3336058	0.03005102	0.08052318	0.20957
0.260431576	0.08294291	1.034858	1.003817	0.2964512
0.6623085	0.1147121	0.1282304	0.5405121	0.11447	0.7507792
0.8142267	0.3059165	0.1873452	1.026113	0.1342282
4.471837	0.4709349	1.206399	0.1549334	0.1176614
1.349446	0.5847483	0.7686293	3.878939	0.131196
0.4349565	0.319864303	0.02470161	0	0.05012275	1.469864
0.1855655	0.5686864	0.146650648	0.6204994	3.401823
0.05355744	0.3271809	0.1553933	0.1106632	1.750882
0.1060175	0.09907921	0.3329931	1.080723	4.708533
0.1629825	0.4263435	2.061646222	0.8670381	0.214428	0
0.078335793	0.3383422	1.455465	0	0.1024354	0.153011
0.3985929	0.04271299
AL133415.1	0.3086817	2.046511	1.249324	0.7863238	1.187671
1.583902	1.653361	1.430475	3.304088	1.392279825
0.5475963	0	1.843177	0.3087121	1.073581	0.9757288
1.724086	2.293170036	1.702421	0.8350875	0.649269436
1.844524	1.047208	2.314323	3.298107	0.3882086
1.726439	1.350584	5.289064922	0.1400856	0.7560079
1.438823	0.4966052	1.037015	0.5863713	0.09022311
0.2783727	0.7023406	1.241638	0.455297225	2.908218
0.3083973	1.070950103	0.2778177	2.182651	0.5299855
0.8141361	2.278293	1.110554014	0.4930891	0.9502344
3.844303	0.5740083	1.113932	2.688282	0.4300606
0.3323662	3.248736	1.134902	0.502174061	3.270909955
5.416438032	1.496178	4.864336	0.2265546	2.091691
4.155725	2.100674	0.55824738	0.4743923	0.4042428
1.073891	1.547153	0.7867324	2.610208	1.377043
1.517865	1.139083	1.401774	0.221747597	1.21657	1.775842
0.37473	1.565869	1.170522	0.617679	0.8257967	0.7374689
0.91647104	0.9052576	0.475941812	0.4595624	0.8613932
1.094421	1.23171	1.17731	1.336481	0.8667811	0.120887

1.397546 1.056373 0.4494725 0.1422473 0.469008
2.187142 2.328494 0.8906034 2.433662 2.2019382
0.1097398 0.5857364 0.4461354 1.805721 1.066249
1.967806 1.09468 1.151274 0.4401922 0.4560722 0.3014068
0.8092488 0.6198942 0.8533706 1.719251 0.2571563
2.614829 0.6211406 2.604067 2.249271 2.076097
0.4297978 0.1171438 0.7538036 0.6842906 0.5980631
0.8131099 0.9199788 3.766764 1.879761 0.2700207
0.7990537 0.4333734 0.7684227 0.4983384 0.7575101
0.7821874 0.2938528 1.952915 0.3264487 2.492634
0.4944807 8.403665 0.3543048 1.022597 0.812083
0.5815516 0.4413094 2.363587 1.67652 0.06885071
5.955429196 2.523656 0.673429847 0.6365067 0.6337929
0.9311533 1.936825 0.5770016 0.9809071 0.4813646
0.9683265 0.947634741 0.4830324 0.3350525 0.1923292
0.2445558 1.41341 0.3852383 0.6335083 1.762157 0.7864972
0.3741088 1.451896 2.08978 0.8279866 0.9407846 2.164255
0.7517713 0.5940035 0.711671 1.588325 1.301143
2.044058 1.447188 0.2477032 0.4142149 2.313859
0.6950422 0.3355065 0.2643571 0.5699552 1.19724 0.5648915
0.6551736 0.4282999 0.6701333 3.000404 0.9243994
0.6815724 0.6179684 0.3685113 1.793555 0.8404624
0.7036006 0.4937199 0.3240875 1.7398 0.5542888 1.271981
1.920154119 0.7873457 0.889485228 1.900832567 0.5454446
3.60687 0.2668345 0.6799331 0.8146917 0.2510752 3.043705
1.601981 1.340096 0.3220924 0.5520284 0.2959283
0.6337258 4.476089 0.3664891 0.4182628 0.693391
0.4315554 1.237263 0.9551548 0.2040026 0.9445612
1.403195 0.4454261 3.398342 0.4797137 0.6456309
1.516912 0.557338 0.3832448 0.7834499 0.4557105
0.8045983 0.2383848 2.858401218 1.197878 1.450434
0.6599034 0.7782234 0.6307644 0.5314564 1.408787
0.4382677 0.5168014 0.9402775 1.572052 1.1277 1.050616
4.89385 0.5457153 0.695898671 0.2148662 0.9653995 0.8984888
1.152984 6.001216 0.318987201 2.092594 0.5363162
1.699826 1.384341 0.4504451 0.5188986 0.8436443
0.2075538 2.24431 2.444528 0.6808101 1.002374 2.37259
0.4033999 9.239492 14.47587 0.8878036 1.15695 0.5838516
0.4566107 1.404446 2.769349 0.9476211 3.209368
0.2952338 0.7914709 0.334079636 0.62782332 0.3974347
0.345782 0.5935274 0.9578274 0.312746 6.951788
1.298016 1.114378 1.261734 1.125553 0.8189795
1.251932 0.274137 0.589638953 0.5974085 1.06193 1.07927
0.5170969 2.299886 0.864511343 0.3923509 0.8455474

0.1460811 1.280595 1.361942 0.9310135 2.04865 0.84644
0.0810974 0.3895659 1.482173 0.6067992 0.3167283
0.7505386 0.6379148 0.4367188 0.6774896 0.6411775
0.615699684 0.1586019 0.1364715 0.3489964 1.360484
1.414207 0.1579322 3.604435 0.1722595 0.46696 1.780176
0.7414043 1.684707 0.5891025 1.082546 6.912941
0.04120946 0.3602051 0.4101072 0.5925215 0.179992
0.3932015 0.4761132 0.9482314 0.2417469 0.8780467
0.4305352 0.346595173 1.79489 0.7415765 0.6134002 1.022053
0.6741864 1.843455 3.851137402 1.0474 0.3436552 0.1024115
0.4379404 1.069704 4.570737 1.217456 1.500162
1.174636 2.037578 0.3256364 0.3601427 0.6981008
0.7608963 0.930627468 1.197397 1.093401 1.746395
0.838836542 0.8540025 1.065873 1.037236 0.4544303
0.6046751 1.219491 0.7350748

NRIR 0.1493178 0.04283452 0.2884313 0.1141099 0.1175132
0.3478038 0 0 0.04610417 0.448989177 0.01281713 0
0.4611699 0 0.1460589 0.05531094 0.05957052 0.022485182
0.03687346 0.1275646 0.017448297 0.05948312 0 0.2858301
0.01022682 0.01173669 0.1181781 0.1771033 0.02885487 0 0
0 0.4900519 0.04702805 0.4376219 0.1636627 0 0.02682168
0.3118575 0.132143819 0.78378 0.6318221 0.093248605 0.03839658
0.292141 0.03296166 0.7297251 0.1123472 0.836215795
0.09540825 0.05171111 1.549662 0.1601905 0.4041296
0.17468 0.3120481 0.350781 0.7194214 0.2545284
0.323887073 0.067809791 0.733079089 0.2230713 0.1747286
0.2671272 0.1720074 0.1018701 0.03018277 0.240035111
0.3859378 0.4335959 0.0432892 0.6203845 0.02784614
0.1394314 0 0.03477944 0.04861812 0.1808204 0 0
0.228348 0.5278072 0.506675 0.01572815 0.02891498
0.07681931 0.221421 1.02305092 0.9263223 0.230226049
0.5335269 0.3061318 0.7436825 0.1890557 0.07627189
0.4631587 0.3144643 0.01253064 1.104857 0.2991199
2.804745 0.6192803 1.020924 0.2927421 0.02252712
0.2369451 0 0.039612556 0.03981311 0.1898959 0.3716691
0.4454729 0.4444435 0.9518817 0.04742028 0.1002424
0.7334358 0.07721511 0.2655619 0.2472352 0.5461731
0.302719 0.2217729 0.2727089 1.477284 0.1229165
0.09689678 0.02766188 0.2073024 0.4516555 0.2209961
0.5773393 0.1805509 0.1669035 0.171286 0.04260819
0.2893902 0.2136288 0.3526643 0.3356657 0.4874003
0.4065546 0.7231797 0.4228019 0.08452844 2.442303
0.08936147 0.09211542 0.08751449 0.4139893 0.07041136
0.02706109 0.3858333 0.1309422 0.0468854 0.747157

1.644276 1.006732 1.648596 0.315087906 0.4124193
0.122158652 0.5705126 0.05518493 0.1422392 0.2143743
0.2232891 0.2846949 0.0551485 0.5793442 0.378177359
1.258556 0 0.7907965 0.2484265 0.07222234 0.473043
0.2729278 0.2073414 0.1802132 0 0.317717 0.5286272
0.505201 1.033162 0.1046909 1.353595 0.2093446
0.2840101 0.5762372 1.211592 0.3783546 0 0.1797312
3.240719 1.364119 0.3263223 0.07607515 0.3487548 0
0.2447061 0.1171086 0.4997669 0 0.8104044 0.2159074
0.3439671 0.05072234 3.110721 0.2482893 0.111094
1.677038 0.03293718 0.3725683 0.391925 1.134547
0.08280319 0.4380045 0.046441549 0.3065463 0.456504162
0.015673033 1.026862 0.8723708 0.1331088 0.09250375
0.3129527 0.09108893 0.3730549 0 0.5382715 0.09737805
0.3416429 2.433526 0.1881105 0.06452602 1.684167
0.8767423 0.3811502 0.4696991 0.6433852 0.2970222
0.4070617 0.4984473 0.1434008 0.5718103 0.4483284
0.3915854 0.5323454 1.355688 0.6065992 1.362587
1.488835 0.5013223 0.07297616 2.026218 0.046433509
0.1542075 0.08650045 1.053404 0.1045689 1.258612
0.2776464 0.1586178 0.1135725 0.02586113 0.01624421
0.6571232 0.2624573 0.1361281 0.5681494 0.2292435
0.109175737 0.2494479 0.1192315 0.2692776 0.1792702
0.03201785 0.373887599 0.3196563 0.588978 0.2569538
0.3990342 0.05447315 1.969427 0.1412632 0.5019972
0.09192868 0.01214882 0.1807279 0.1237981 0.1106698
0.1908934 0.06864258 0.2530978 2.104329 0.1343156
0.6657159 0.1783991 0.4632062 0.3129416 0.02546612
0.1624666 0.07560669 0.2996265 0.064641377 0.310597448
0.288375 0.01003585 0.1396729 0.05635062 0.2978399
0.1552047 2.095087 0.09951785 0.3269652 0.1306706
1.234199 0.9828486 0.7956453 0.338704047 0.1118643
0.4006741 0.2149709 0.3216007 0.1103323 0 0.305021
0.2454086 0.0757108 0.717268 0.07374724 0.1228245
0.2436855 0.2142451 0.01471088 0.1884437 0.507852
0.1200786 0.2089229 1.109338 0.3085768 0.5003356
0.3128239 0.2326163 0.065697959 0.09206407 0.554526
1.24082 0.3016307 0.1837855 0.1018615 0.3558294 0.2499799
0.02117637 0.2222245 0.9252847 0.6791157 0.6122896
2.061899 0.1241576 0.2093085 0.1524609 0.2231776
0.2579569 0.08162536 1.907967 0.1884346 0.3027324
0.9886709 0.2184352 0.5821859 1.079911857 0.0959631
0.03710905 0.1390868 0.296637 0.1158593 0.7063702
0.162777697 0.2493696 0.6055719 0.05944711 0.3858582

0.08624087	0	0.1325062	0.4118664	0.05498743	0.3819325
0.4725577	3.945876	0.1989961	2.366141	1.256724349
0.7485221	0.1983404	0.1151971	0.043475158	3.398722
0.1890504	0.4869822	0.4093206	0.9341061	0.1608821
0.1422303
NADK2-AS1	0.06053591	0.1736583	0.2041719	0.134931	0.2117414
1.042214	0.1247086	0.1692866	0.06230466	0.202252995
0.1905301	0	0.4985759	0.09459667	0.3947646	0.5531248
0.08050294	0.33424847	0	0	0.094317741	0.1406735	0.07701339
0.9415274	0.3040492	0.3172168	0.1277637	0.4467595
0.03899415	0.1373617	0.2280947	0.235141	0.4285151
0.5719787	0.1314216	0	0.2729599	0.05436979	0.3902229
0.196435458	1.368801	0.8182608	0.2940353	0.3243044
0.9142646	0.1633281	0.9861423	0.2602711	0.780763707
0.8058353	0.06988182	0.8186401	0.2381276	0.4551136
0.5114646	0.5457272	0.2666483	0.8067342	0.1214001
0.109424348	0.481096339	0.30270623	0.4019414	0.188901
0.249918	0.3691835	0.2202659	0.7749845	0.101368994
0.253728	0.1378722	0.1316262	0.4790747	0.6773574
1.287578	0.1459748	0.4073381	0.8869769	0.9010724
0.830209563	0.385943	0.4628803	0.7997301	0.5394724
0.06376454	0.5275178	0.7474519	0.3490969	0.62214284
0.2731247	0.525023311	1.104035	0	0.3236454	0.02322616
0.3916773	0.2738345	0.3399708	0.5249468	0.3886129
0.7073978	0.2644396	0.09298761	0.7745488	0.2706794
0.7610726	0.1746572	0.1507162	0.214127875	0.5649314
0.8187502	3.208044	0	0.4004107	0.2143937	0.2403121
0.4289773	0.7993202	0.5664586	0.3799878	0.3758748
1.367642	0.3161158	0.1873134	0.4267256	1.154772
0.3322161	0.02182421	0.6168023	0.2241169	0.2761162
0.5283836	0.2463821	0.9759782	0.1353309	0.6944221
0.2303209	0.6083446	0.6218058	1.562145	1.072181
0.63742	0.2197653	0.4249117	0.3047305	0.3263737	0.3929168
0.5003001	1.031437	0.2759543	0.8951369	0.0761225
0.1279952	0.300814	0.560354	0.5702436	0.3786374
0.5495408	0.7369301	0	0.091244206	1.047797	0.7428773
0.881125	0.02485877	0.6567535	0.1287569	0.3771881
0.7145052	0.1738965	0.7496036	0.232302127	0.8611638
0.0616008	0.2095439	0.3676942	0.1756809	0.0290575
0.504717	2.148192	0.2435382	0.1630375	0.158185
0.526386	0.07380789	0.3490508	0.691671	0	1.9637
0.6106039	0.5364519	0.5316012	0.08180867	0.1542444
0.03469811	0.5827523	0.4769778	1.523414	0.2672985
0.02356517	0.2018152	0.2149505	0.2571713	0.2306174

0.03817926 0.9387184 0.170202 0.69725 0.771138 0.6627604
0.8001228 0.6648668 0.63682 0.4451094 0.3292015 0.4413692
0.151162 0.4795685 0.2325378 0.125521186 0.376603
0.616914653 0.317705443 0.7082999 0.7214239 0.1798817
0.2916866 0.4229208 0.04923863 0.2218226 0.06829704
0.5396943 0.07895737 0.6527375 0.5561671 0.141228
0.1453329 0.4791505 1.3671 1.483437 1.375283 0.4246218
0.4281519 0.2000359 0.6314964 0.2713063 0.2015839
0.3635196 0.2939912 0.3309266 1.324164 0.8652931
0.3382135 0.914543 0.6054089 0.3205123 0.2504459
0.125499456 0.6251829 0.5455135 0.4437065 0.02826264
0.412333 0.8337961 0.5716114 1.166453 0.4543298
0.08780898 1.625261 1.126637 0.6070745 0.0274211
0.225307 0.258193036 0.3932847 1.208461 0.4788142
0.3553203 0.3677829 0.096241438 0.561574 0.5685274
0.02314963 0.3904917 0.8539271 0.821922 0.8431485
0.101759 0.4081889 0.44328 0.5535964 1.003796 0.1661754
0.3611598 0.1855256 0.1368134 0.3482163 0.5445378
0.9814267 0.1033228 0.375583 0.4006476 0.5678415
0.9697038 0.2894931 0.3036839 1.026428748 0.671580703
0.4605626 0.2169974 0.2831286 0.1269193 0.3258313
0.05243549 1.013026 0.9582222 0.5744143 0.5297603
0.4530053 1.549579 0.4838518 1.252709794 0.1889652
0.2082564 0.02075066 0.5794768 0.3541169 0.344378694
0.0824404 0.6080112 0.1841665 0.8811891 0.2192546
0.2489756 0.1975883 0.2509246 0.5367634 0.1819005
1.069829 0.1217046 0.1976353 0.2180577 0.3961569
1.17199 0.3623543 0.4715326 0.461674158 1.492972 0.08029074
0.7186418 0.5558459 0.4553371 0.2925157 0.5289502
0.3378201 0.5437327 0.506776 0.4071133 0.4588747
0.7649929 0.6634352 0.03355705 0.3030612 0.206034
0.3518663 0.3873335 0.6397841 0.3373614 0.7073568
0.2975339 0.4956421 0.1475955 0.5181099 2.019143415
0.3149456 0.1253719 0.07518413 0.450981 0.3131418
0.9139603 0.256638633 0.4493272 0.144417 0.5824372
0.7054838 0 0.1106632 0.1790675 0.2385393 0.2043509
0.6659862 1.178971 0.4590819 0.8556582 0.6572795
0.126740546 1.101861 0.482463 0.07783806 0.058751845
0.07612699 1.323854 0.2991374 1.137033 0.3634011
0.08153036 0.3363648
AL683807.1 0.05834786 0.1487835 0.3935845 0.1114748 0.02551101
0.1969694 0.1803016 0.2564065 0.2101844 1.340230691
0.1669486 0 0.6006939 0.0182355 1.078072 0.02881796
0.1551864 0.102507746 0.02401465 0.1246189 0.045454333

0.07747935 0.04948651 0.4886518 0.01332088 0.04586266
0.3694372 0.01537899 0.263093062 0 0.1648877 0.08716999
0.2440612 0.3675365 0.2058411 0.04263555 0 0.3144277
0.07522369 0.206547689 0.1570627 0.01714533 0.121460365
0.06251652 0.1802493 0.171736 1.289962 0.08362122
0.554505613 0.1398076 0 2.734153 0.2295206 0.233954
0.492978 0.04781838 0.05711344 1.415585 0.05850606
0.237305815 0.331219511 0.344813231 0.1937067 0.06827749
0.02676498 0.3558395 0.05307612 0.3538293 0.097705055
0.08151904 0.1162777 0.04228955 1.019717 0.03627081
0.2875578 0.5276197 0.5587214 0.1108226 0.1913652
0.095262141 0.3043581 0.5736211 0.8124904 0.3799806
0.5121649 0.2071466 0.400242 0.3605131 0.93279783
0.2193773 0.299879446 0.2388863 0.1827604 0.6731497
0.2238666 1.311386 0.5467264 0.3072025 0.06528682
0.3351386 2.581211 0.03398421 0.2240665 0.3032872
0.2608958 0.1467128 0.3928034 0.1694801 0.120393182
0.3630081 0.08244922 0.2186327 0.03413223 3.473442
0.8954597 0.07720872 0.1740935 0.06163433 0.2442561
0.02034741 0.06038149 0.4603268 0.3943047 0.469412
0.6543438 2.81088 0.355787 0.1893184 0.5584774 0.1440109
0.2241146 0.4428569 0.1978973 0.2351755 0 0 0.3884932
0.04188259 0.5351168 0.2041603 0.3709738 0.5324633
0.6808564 0.04095535 0.5507179 0.2044751 0.05049533
0.1163972 1.148422 0.2279829 0.3415181 0.1283994
0.08812062 1.063118 0.6111661 0 0.2954371 1.612063
25.18812 2.407655 0.351784891 1.869436 0.218785818
0.08846637 0.04792051 0.8182882 0.4033348 0.2908438
1.509798 0.4309999 0.3692826 0.313467931 0.3527659
0.07916569 0.100985 0.2003154 0.2634037 0.0840217
0.5238953 0.1350356 0.1173678 0.07857231 0.1524675
0.61608 0.5513361 0.7930243 0.2575774 0.07894549 0.1443602
0.5044576 0.1334354 0.1024773 0.4928233 0.2973386
0.5685473 0.3574385 0.2485068 0.1159225 0.07927295 0
0.01496313 0.3187403 0.1334717 0.03175455 0.4783907
0.2563568 0.0703072 0.2016144 0.08258507 0.4380378
1.19412 0.3514259 1.426188 0.2574126 0.802589 0.2212164
0 0.09244693 0.6112713 0.141148322 0.2540936 0.451088404
0.020414808 0.222922 2.272602 0.1103327 0.1004085
0.2717564 0 0.05831043 0.02194282 0.5201873 0.1014713
0.04603497 0.2097644 0.2994715 1.148655 0.06597598
0.7027662 0.2581617 0.058267 0.3118278 0.5158457
0.3856113 0.4057808 0.2428215 1.068637 0.6812951
1.105124 0.2080209 1.36372 0.1097391 0.6519779 1.304601

0.3751243 0.9030189 0.06437164 0.181444997 0.6938868
0.1126706 0.3563908 0.0272411 0.6706621 0.04018294
0.3902568 0.05917326 0.0673705 0.1904291 0.2099455
0.6837241 0.904295 0.6607493 0.08143626 0.10665461
0.06769099 0.2135433 0.3507458 0.3113431 6.422516
0.139144247 0.3122747 0.08219673 0 0.5735277 0.0993351
0.1886224 0.07666729 0.3923239 0.3079057 0.06329745
0.5178932 0.1612524 0.176186 0.2154941 0.5066564
0.2307697 1.49169 0.8310215 0.2522542 0.06639217 0.2413385
0.686518 0.2156098 1.058099 0.098481 0.1300922
0.126297308 3.910811324 0.5634315 0.09150494 0.272895
0.195731 0.03694756 0.2021609 0.07510851 0.5185053
0.9156558 0.2694899 0.2778565 0.5431164 0.4922723
0.766254369 0.3642704 0.6423329 0 0.2094495 0.07185632 0
0.1854081 0.195345 0.1183399 0.1274008 0.8261073
0.2999706 0.317411 0.1488339 0.708978 0.1928583
1.381366 0.2997811 0.1360656 0.8144324 0.1406772
0.1086181 0.6694096 0.1514964 0.08557445 0.05995873
0.2837585 1.682191 0.03571701 0.3191848 0.0663395
0.2085674 0.2279268 0.05516629 1.772924 0.6446545
0.79612 0.782486 1.841632 0.09703242 0.1557905 2.212826
0.387598 0.3173335 0 0.8593716 0.1090863 0.03584746
0.3738736 0.1659708 0.05548701 2.158120601 0.3392752
0.04833615 0.4529164 0.6278718 0 0.1957612 0.176687527
0.2938801 0.6495866 0.9098313 0.5469439 0.05616624
0.1333292 0.5753172 0.1341185 0.01790588 0.6419144
0.2604152 2.042255 0.5890934 0.4109335 0.830685027
0.174104 0.1550082 0.112537 0.028314142 0.5136279
2.37292 0.3171577 0.02961988 0 0.07858348 0.2315764
AC084125.4 0.2579045 0.1644102 0.5139993 0.164244 0.3946659
0.2611884 0 0.2575789 0.26544 0.161563044 0.1106899 0
0.1991354 0.2015077 0 0.1910682 0.08574277 0.356004288
0.1061476 0.18361 0.050228383 0.171234 0 0.2571308
0.4121592 0.2365048 0.3401992 0.6797693 0.415322311
0.09753491 0 0.4238318 0.5808812 0 0.3499392 1.601861
0.04472715 0.7335096 1.097242 0.304321743 0.5553885
0.3789219 0.313173693 0.6355599 0.4868865 0.7590934
0.7502351 1.801877 0.481442585 0.2059887 0.5954427
0.4461009 0.737824 0.6463151 0.4190425 1.690903
0.1893361 0.1321913 1.163717 0.582733214 0.292805887
0.937917099 0.4281032 0.5029909 0.4732175 0.582538
0.2346027 0.3041048 0.388681115 1.651484 0.4405383
0.685391 0.8504284 0 0.6689682 0.3886903 0.200239
0.8047522 0.5205272 0.252641963 0.8221271 0.3756256

0.5525082	0.6187849	0.1358298	0.7907564	0.6191909	
0.4780532	0.73626372	0.09696736	0.207109787	0.5759486	0
0.3628538	0.4452826	0.351302	0.2916581	1.267347	
0.7214387	0.479261	0.9687104	1.126607	0.3961602	
0.3609214	0.5322418	0.6484879	0.9301272	0.6956134	
0.456130384	0.3438298	2.525016	1.449573	0.3771712	
0.6160165	0.4566967	1.160324	2.308542	0.1362155	
0.3175414	0.5845966	0.5782689	0.277459	0.9902689	
0.1596043	0.9090013	0.7504685	0.3145241	0.325426	
0.2388906	1.07417	0.4953066	1.125551	0.5248376	0.08662528
0.4804649	0.438294	0.8585928	0.555378	0.09461119	
0.2256031	0.7905928	0.6336483	0.6353317	3.439522	
1.663396	0.5214254	1.115977	0.1837462	1.174335	
0.1259639	0.8739502	0.6080792	0.7400568	0.299034	
0.942358	0	1.843577	1.272412	0.2415065	0.2876249
0.518310283	0.2849354	0.043957237	0.9775795	0.5295359	
0.5459518	0.06856876	0.2008694	0.9951688	0.1058373	
0.7806523	0.247422384	0.5503295	0.612363	0.3570925	
0.4086543	0.4989752	0.6808741	0.7856768	0.3481762	
0.7349377	0.7380102	0.1444123	0.6407403	0.3930598	
0.6904303	0.5692612	0.872371	0.2481458	0.8175795	
0.14745	0.9059239	0.8277682	0.3285679	1.18261	0.0752343
0.02746074	1.665271	0.7007917	0.7027718	0.09920819	
0.422661	0.6321007	0.5965251	0.2439858	0.9998185	
1.398448	0.4455799	0.7665751	1.169775	0.3298846	
0.2741196	0.3989823	0.948162	0.3300036	0.5641169	
0.6900039	1.566401	0.4503153	1.24778457	0.3610041	
0.271890563	0.045117933	0.6774225	0.2998558	1.254043	
0.9763981	0	1.206201	0.9020865	0.5334443	0.4998455
1.121288	0.2373933	0.1545306	0.361009	0.8668378	
0.1822635	0.3882887	1.009439	0.1931603	0.04307237	
0.3990173	0.8522238	0.3587199	0.4128076	0	0.2151004
0.4383774	0.7049324	0.4636773	0.339542	0.7204549	0
1.903737	0.1575573	0.8535908	0.31189214	2.138872	
0.4980174	0.5513513	0.4816355	0.05489641	0.6216468	
0.6088169	0.2615528	1.302812	0.2338109	0.5353753	
0.6666489	0.2743102	0.5257063	0.2999649	0.746424499	
0.4488033	0.4719434	0.5711772	0.3440433	0.2765092	
0.205011346	0.5981261	0.696362	0.2958769	0.7129857	
0.5958847	0.7503599	1.25385	1.806373	0.5670748	0.3847006
1.248627	0.04454717	0.2477882	0.7326995	0.7904051	
0.5100146	0.3296723	0	0.7665581	0.5869225	0.5333723
1.32759	0.2199279	0.3117949	0.8343205	1.437557	0.46520754
0.238431019	0.3773385	1.271168	0.8041523	0.1081443	

0.4082815 0.2233938 1.16196 0.5371535 0.6588639 1.692725
0.3509024 0.04286855 0.3435634 0.205268787 1.489359
1.774492 0 0.7714928 1.19105 1.467175501 0.7609883
0.4317239 0.3923074 0.04692723 0.3396737 0.6187559
0.1402994 0.534514 0.846964 0.8524567 0.6449792
0.4320874 0.2405704 1.045129 1.288034 1.776389
0.4502628 0.5022241 0.907798071 0.6294336 1.710335
0.2186906 0.4736202 0.4849744 0.5864569 0.2560813
0.647655 0.6705642 0.9995582 2.291948 0.3909938
1.130723 0.9609997 0.2144474 0.602536 0.9404764
1.391999 1.361397 1.127879 0.2053256 0.8136696
0.6734126 0.1836184 0.2620038 0.3678887 0.383269358
0.9076746 1.816038 0.1201167 1.280893 1.334095
1.124874 0.39048989 0.2734705 0.5639954 0.4278257
1.470136 0.4965229 0.4714647 0.254297 1.905491
0.7914611 0.3546672 0.6801754 1.805402 0.5728498
0.4919348 0.107991945 0.3078242 0.4948338 0.2487133
1.001214875 1.513531 0.4947446 0.446051 1.178311
0.9778217 1.099936 0.443558
AL049796.1 0.04113425 0.7237401 0.1362127 0.1257406 0.1618635
0.8581552 0.2033754 0.2070549 0.3175209 0.319527665
0.3813349 0.3717446 0.1651568 0.2005492 0.08047297
0.5485367 0.1312845 0.514122531 0.0812636 0.1581375
0.153813757 0.4752082 0.1151276 0.8563068 0.6536134
0.1681285 0.1041788 0.8131443 0.079489774 0.2800125
0.3254805 0.383469 0.301765 0.1295535 0.2478109
0.0180344 0.3167371 0.2216662 0.1272754 0.116490089
0.3188924 0.3263535 0.085627494 0.1163529 0.1270727
0.1150174 0.2201709 0.2652819 0.259680604 0.1642699
0.08547267 0.2328561 0.2294733 0.2102903 0.4330897
0.01011334 0.0362376 0.6493792 0.1154882 0.345746241
0.186803303 0.269266121 0.1392911 0.182911 0.05660657
0.529595 0.1683799 0.2494437 0.041328242 0.09195126
0.2459215 0.1550298 0.3580853 0.1994484 0.1024287
0.1487852 0.14691 0.1339338 0.3611416 0.128943728 0.2288718
0.4583122 0.09693401 0.1945654 0.1126531 0.1991384
0.4571054 0.1321609 0.33819769 0.510369 0.126845949
0.1469767 0.1686672 0.1319506 0.08522397 0.1933056
0.2153108 0.1299435 0.324484 0.1667767 0.2163051
0.1437498 0.07582229 0.03947304 0.2631573 0.2172031
0.1305479 0.3994066 0.356475623 0.3180652 0.1395007
0.03963402 0.05775034 0.5804368 0.320498 0.2416729
0.7548078 0.05214139 0.2127132 0.07746071 0.1702717
0.09735678 0.09855587 0.07636788 0.2530567 0.127675

0.1730684	0.177955	0.7391721	0.137059	0.05332399	
0.1217606	0.2009005	0.07460762	0.09195761	0.52429	0.1173776
0.07972159	0.2444569	0.1187418	0.09527172	0.2338891	
0.1087982	0.1559132	0.1863584	0.1330629	0.05339752	
0.3165096	0.2972626	0.2009051	0.2509019	0.1706937	
0.2609189	0.2371082	0.3306613	0.1808247	0.1984768	
0.3799081	0.1386679	0.08257407	0.148801423	0.1908712	
0.353350471	0.1871019	0.06080967	0.1175528	0.1312358	
0.169158	0.1792643	0.4253867	0.590854	0.179948647	
0.3423199	0.1255735	0.2135781	0.1042847	0.1432505	
0.09477419	0.3007461	0.1808759	0.1820331	0.1911029	
0.1289844	0.2069435	0.09027466	0.2134626	0.7498502	
0.05565523	0.2171125	0.1991551	0.218712	0.1127019	
0.1500906	0.07546271	0.2829291	0.1727918	0.1366506	
0.2778594	0.2179557	0.07686034	0.1075973	0.3168362	
0.1451756	0.2820688	0.2023544	0.05102886	0.08921768	
0.3221721	0.118771	0.401452	0.147321	0.3235314	
0.09927113	0.3357224	0.2605365	0.1439573	0.695526	
0.07820822	0.3447487	0.324109064	0.3301141	0.173459816	
0.233151899	0.04125352	0.3156466	0.1600101	0.06795481	
0.1915836	0.02007464	0.1479883	0.1670686	0.03826677	
0.3111797	0.03894465	0.1084456	0.2878941	0.1422057	
0.1465126	0.1579209	0.7055984	0.2895946	0.1896062	
0.1963777	0.1631097	0.1373129	0.1896203	0.3698367	
0.444622	0.3475955	0.05279443	0.3475829	0.2785112	
0.3370645	0.149144	0.1469201	0.2010357	0.1361427	
0.127915641	0.08496256	0.1985771	0.0452249	0.09218165	
0.1365882	0.1869667	0.04855137	0.0876039	0.0854911	
0.08054958	0.2527521	0.5188738	0.4200083	0.07825727	
0.2870559	0.112784533	0.0858978	0.08211514	0.1015108	
0.3424068	0.2381487	0.215807527	0.5811916	0.0579473	
0.15101	0.03032455	0.06602787	0.5425403	0.4864422	0.1106327
0.1374766	0.3681449	0.1394041	0.4945088	0.5487742	
0.1682802	0.277343	0.2231159	0.0394356	0.1665065	
0.1000322	0.04212482	0.1531256	0.2903901	0.2595713	
0.1566478	0.08331292	0.07566304	0.240401133	0.171127586	
0.3972097	0.09399946	0.1667349	0.1345374	0.3047546	
0.149646	0.1800308	0.5277447	0.2071675	0.1559883	
0.008395033	0.1640948	0.04383708	0.284830783	0.09244959	
0.06792507	0.126901	0.08859498	0.1899656	0.151203817	
0.07842574	0.1051641	0.1084559	0.2604647	0.1706543	
0.2791459	0.7250127	0.3934701	0.09726177	0.1334898	
0.2633482	0.2095029	0.4719458	0.266707	0.1870157	
0.220533	0.7140391	0.2723461	0.173746177	0.2536193	

0.3055231 0.2371832 0.1510793 0.2109561 0.2595636
0.03920974 0.2065944 0.210013 0.2448748 0.09484601
0.3928758 0.2418726 0.4003152 0.342031 0.1482701
0.5040016 0.05123438 0.04737488 0.05396703 0.05894683
0.173034 0.1743758 0.07028654 0.07020396 0.3520566
0.252667442 0.1586162 0.204457 0.1685895 0.04085898
0.1276682 0.182171 0.254105879 0.157021 0.3630869
0.3357196 0.1625715 0.190062 0.3947783 0.07300599
0.2350274 0.1363321 0.2443709 0.06008345 0.6046972
0.0498361 0.2679735 0.2686957 0.4271366 0.3205492
0.1745406 0.107789342 0.1448396 0.2840719 0.03658759
0.01879336 0.1091698 0.2105201 0.4701815
Z98884.2 0.147874 0.3181526 0.9861462 0.3178309 0.242452
0.4867098 0.2284738 0.3987557 0.4565838 0.185269919
1.586649 0.9441877 0.2854442 0.9011958 0.2410776
0.3834329 0.2212294 0.501024772 0.04564616 0.7106129
0.431989654 0.2577224 0.04703108 0.4422912 0.4304374
1.191379 0.2340709 0.7892589 0.214318994 0.251655
0.4178834 0.2319653 1.03486 1.630067 1.565021 0.8914411
0.1538705 0.2988263 1.000877 1.308660929 3.851153
1.857585 0.307823215 1.734905 1.82726 1.523337 2.451913
0.5563045 2.484390745 2.066875 1.536333 2.022984
0.5155851 0.8893823 0.6487162 0.772577 0.8141934
0.90953 1.149131 1.052478461 1.259139819 0.857072463 0.9204764
0.2162989 1.068352 1.152328 0.2017703 0.8220004
1.26285648 0.5681441 1.547115 0.8306185 0.8411235
1.378845 0.9205547 0.9360215 0.7749709 2.407401
2.853959 0.506998206 0.4499544 0.848025 2.375926
0.9883464 0.6230428 0.9306505 1.141148 1.461867
0.75986912 0.8339687 1.923749537 2.022659 0.4737059
2.309338 0.340414 0.566508 1.254204 0.817487
2.26473 0.9367928 0.971997 1.065833 0.2555385 0.443444
1.144388 1.143352 1.17327 0.2761214 0.523060457 1.281415
0.5149255 2.166895 1.232668 1.304137 1.963912
1.174044 0.7859127 1.493691 1.174338 1.972456
0.5355976 0.5965721 4.428745 0.4118036 2.807327
1.577745 0.8453336 0.8396488 1.403964 0.5474611
0.5591104 2.188592 1.805548 0.8567747 0.4958691
0.9423879 0.8790876 1.7912 0.732334 1.746271 1.88875
1.751689 0.4026235 2.491085 2.093569 0.2690714
1.151756 0.8849742 3.420928 0.7222358 1.60578 0.4183832
0.8039829 1.800291 0.7564441 2.321603 1.321308
1.619622 1.350101 0.4947441 0.390051568 1.225295
3.100047765 1.109814 0.5465133 1.173866 0.471781

2.591368 1.611098 0.637178 1.159692 0.893742536
0.7888523 1.203802 1.650758 1.142256 1.7881 1.064702
1.884909 2.866157 2.453972 0.7094 0.952215 2.307603
1.352206 0.7308371 0.4895936 0.6502457 1.646368
1.725938 1.268145 2.649078 1.161558 0.6216873
0.9535357 2.976445 1.039176 3.672353 1.092424
0.9066293 0.8532409 1.423747 0.7973368 1.508947
1.049201 2.837653 0.8909156 0.9367633 1.852301
1.561137 2.695316 0.4322207 1.441209 1.590162
2.057694 1.035028 0.2373754 1.903631 0.5809411
0.919849912 0.4484738 1.480984899 0.271626028 2.992537
2.159839 1.198379 1.488651 0.2582723 0.2255204
0.2955582 0.3336649 0.5158711 0.6268371 2.012536
0.8860279 1.241944 0.1597553 0.5643212 5.510143
0.6039436 2.464227 1.111333 0.4902503 1.557531
2.468139 0.7810782 1.231046 0.6289907 1.938995
1.687034 1.395751 1.293246 2.409664 0.3350997
1.95421 1.98744 1.927095 0.421524892 1.318915 0.8566406
0.372578 0.7249043 3.918737 0.9165392 2.007185
1.855827 3.585551 0.2010893 1.626923 1.032034
0.7077621 0.2009485 1.599508 0.743325642 1.85277 1.143887
1.122838 0.7693263 0.475624 0.396720429 0.7914137
1.249892 0.2968808 1.192341 1.024982 2.007748
0.7577772 1.92643 1.333083 0.6617241 2.058275 0.6896309
1.035108 1.22881 0.5664905 0.2506506 2.410047 1.828982
1.828001 1.325056 1.0092 1.182573 1.260994 1.474877
0.6239636 0.8963699 1.480377989 2.101893804 5.03022 0.8944933
0.6916119 0.2790288 3.406086 0.6244226 2.379392
0.4311807 2.833283 2.561182 1.131727 2.175279
1.280422 1.412332827 0.415435 1.29723 0.1900824 1.061637
0.9219276 0.582390275 0.2013811 1.147669 0.5623405
0.7870162 0.474722 1.786542 0.6033232 2.05101 1.602551
0.966433 4.178852 0.8671067 3.413896 0.7989901
0.9549785 3.34461 1.051104 2.807599 1.626567791 1.595543
1.912268 1.253901 2.172466 0.7204501 1.324005
1.497652 0.9283605 1.10101 2.131984 0.799132 1.092892
1.458718 1.361308 0.4303499 1.332544 1.40202 1.19719
1.52568 0.6062708 1.368576 1.036737 0.6132379 0.6711649
0.4506732 1.265612 1.574904394 0.5769995 0.5053157
0.3443548 0.8262247 0.3824633 2.232575 0.839602944
2.498984 2.33713 2.612458 2.135417 1.014208 0.3547982
1.530959 0.9832917 1.157185 1.799688 1.574961
1.48804 1.522829 1.757446 1.532496378 1.687746 1.047587
0.07130204 0.538184841 0.8368157 1.191415 5.206361

0.6756047 0.6307323 0.9460011 0.5282064
TMPO-AS1 0.4890479 0.07449149 0.9195948 0.8599201 0.7266192
0.2848927 0.668679 0.202288 0.4476581 1.339316081
0.3566345 0.8332633 0.9423486 0.5843183 1.114796
0.211615 0.8201371 0.348667671 0.4114681 0.369736
1.102480834 0.1336155 0.6056459 0.346916 0.2934523
0.7109738 0.9111299 0.0923976 0.535254825 0.7954456
0.7215864 0.09698538 1.595835 1.771992 1.589039
3.016951 0.3467587 0.7035519 1.034457 1.463092274
0.6710325 2.388303 0.536042907 0.4062065 0.9893577
1.359807 1.908581 1.088533 0.374572683 0.7881199
3.03758 1.033062 3.352242 2.401243 0.7341295 1.223663
2.243119 0.8207648 2.907526 1.273194826 0.437304473
0.855810936 0.3534498 2.435962 3.013608 0.2258137
1.127903 0.5642613 0.921832786 1.206285 1.079321
1.969884 1.361445 0.7263886 1.195554 1.244504
0.3528191 0.9864106 0.7926915 0.818232839 1.076088
1.33788 1.075501 0.6719764 0.797769 2.082624 0.7792915
0.6524675 3.39519119 0.4783954 0.583880556 1.744516
0.9945142 3.682626 1.165667 1.940978 0.7970672
1.17577 1.460027 0.8378728 1.175835 0.6011925 2.084119
2.08173 1.36205 0.4668456 0.8865532 0.6842167 1.622702749
1.19434 1.284263 1.803531 1.526616 1.083029 1.48677
1.03083 1.898226 0.5211653 2.065372 2.318186 2.494635
1.312992 3.75291 0.4981633 1.926141 2.38437 1.36172
0.5991417 0.5010986 1.626299 0.8477902 1.823074
0.8014574 0.8198565 0.6095335 0.468878 0.6133659
0.5591831 1.709906 1.470789 1.724687 1.599531
0.8113894 3.722819 2.307946 1.074496 1.66858 0.7363204
0.9726038 0.7778718 1.13192 0.8081638 2.078513 1.350571
0.3257596 0.7610043 1.067319 0.6508142 1.264438
1.129422 0.893687665 1.300557 2.044738234 1.133887
0.9223735 0.9860106 1.332444 0.6229165 1.273113
1.438592 0.464455 1.923187586 3.449273 1.598648
2.633624 1.21379 1.029907 1.707615 1.278186 0.9915886
2.746606 0.7561795 0.4895192 2.314405 0.7400577
0.5293916 1.028319 1.668861 0.6567379 1.298381
1.651621 0.7023407 0.9080104 0.886595 2.001885
1.090796 0.7575794 1.177975 1.609634 1.415173
1.694765 1.326319 1.493492 2.38125 1.55583 1.301958
1.627056 3.35976 1.032778 2.063162 1.533401 0.9981915
0.8837749 1.322202 1.727776 1.05266 2.209246 3.809119
1.677582 1.175569284 2.483763 1.245577888 1.848881007
1.078901 1.335953 0.8382496 2.725834 0.7256551

0.9662916 0.6660625 0.7861178 0.6391531 1.422503
0.324384 1.566266 0.9814024 0.9226497 4.863075
0.5766498 8.373882 2.508839 1.665311 0.7690665
1.630319 0.7765318 1.072341 3.285871 0.3638445
0.6116278 1.684914 2.602712 1.470034 2.506629
1.114124 1.026405 3.474144 1.253349 0.964517717
1.22304 1.629646 0.9119973 1.097166 1.962175 1.824069
1.721475 1.797328 1.611564 2.081049 1.444636
1.145543 2.055639 1.07626 1.528222 0.708028393 1.467098
1.101546 0.5052581 0.3394724 3.159882 0.882428194
0.8940712 0.9511021 0.5362274 1.108711 1.468341
1.754441 1.460337 0.7202246 0.8526337 0.6443867
0.7578011 0.2780847 0.25305 1.468062 1.087621 0.5281806
1.365066 1.265235 0.8314499 1.148647 1.176088
0.5299003 1.081339 0.5846967 1.42441 2.619805
1.840791731 0.780209932 1.937611 0.5119511 1.22124 1.110629
2.55691 0.7534966 2.01115 0.6706338 1.847982 1.151996
0.5829559 0.725126 0.7379569 0.888703174 0.8916323
1.170256 0.4628566 2.703189 1.814806 1.437456128
1.237711 0.3912136 3.348682 1.927749 1.342354
3.453173 0.2542692 0.5298965 1.671423 1.806325
1.562885 1.592275 0.7448224 2.022734 1.744052
2.146271 0.3885837 0.539376 4.253990799 1.304183
2.187008 1.504625 1.049103 1.242938 0.8709505
0.8405448 2.977888 1.595829 0.8172021 1.62783 1.092442
3.545998 1.912401 1.245119 2.547989 4.198012
2.156209 2.309463 1.012582 1.659028 0.6584297
1.021028 1.395817 0.7280849 1.720345 0.896134576
2.415857 0.4786316 1.310179 1.816283 0.9925019
0.9888305 2.355059997 1.56602 1.770689 0.4393721 0.5164283
1.468529 0.7061086 2.061116 1.236397 1.155481
1.164137 0.7480512 2.090437 1.069655 1.88597
0.788305812 1.077018 0.5250516 0.2671118 1.474309035
2.498108 2.689925 1.071441 0.8568325 0.9804189
1.754469 0.7386026
PIK3CD-AS1 0.03187848 0.007620769 0.0806384 0.06851757 0.08362797
0.1049243 0 0 0.06151859 0.309536948 0.03420478 0
0.1969142 0.007472255 0.3378128 0.005904283 0.1589745
0.036003426 0.06888238 0 0 0.03174826 0.0304167 0.09534857
0.00545842 0.01879287 0.0504607 0.01890527 0.046202614 0
0.02252169 0 0.1000075 0.05020109 0.04541724 0.01747052 0
0.05726275 0.05548314 0.232748541 0.0193076 0.0351277
0.082950167 0.03074043 0.0492398 0.01172854 0.5007603
0.08566244 0.064919002 0.05728813 0.00920002 1.323375

0.2564981 0.2276816 0.2486212 0.01959425 0.07020911
0.6209052 0.04794737 0.054021894 0.081433124 0.282584183
0.1666855 0.2051695 0 0.09720684 0.06524606 0.1933154
0.032028821 0.1002107 0.05445308 0.05198622 0.3626566
0.007431239 0.01860488 0.2306129 0.3403221 0.03892384
0.06031891 0.039035008 0.08314336 0.1741106 0.2134163
0.1393127 0 0.06173183 0.1066031 0.2560573 0.40952902
0.242711 0.061439919 0.3114588 0.231474 0.9216845
0.1742918 0.3826648 0.4171572 0.05035223 0.0133761
0.1211716 2.03555 0.04177653 0.1469031 0.05735825 0.1562469
0.4869529 0.06898146 0.08928883 0.049332807 0.06374902
0.00482639 0.07678956 0.1398616 1.432078 0.1411259
0.03163736 0.1248402 0 0.03532496 0 0.04948434 0.09431271
0.0660976 0.1479601 0.04596456 1.375973 0.1968148
0.02585863 0.08120271 0.06638679 0.06313597 0.04536672
0.04865472 0.02409162 0.1603482 0.1422111 0.08338553
0.02574297 0.131563 0.07320035 0.2605916 0.2937097
0.1735941 0.03356406 0.1278765 0.02578048 0.05172794
0.1567135 0 0.05449453 0.03682681 0.09019452 0.01444347
0.6890835 0.2154897 0.05004881 0.07121148 1.264511
0.08955478 0.2133126 0.31232246 0.4314408 0.146700948
0.05800055 0.02945416 0.09489768 0.1525589 0.04469151
0.3798795 0.08830415 0.03289539 0.073398807 0.2635926
0.02432944 0.02482802 0.05051203 0.1002239 0.02295272
0.1610051 0.04611063 0.04809311 0.008049028 0.0446255
0.2078979 0.05101368 0.1083176 0.08071162 0.09704709
0.07887152 0.08957373 0.06834629 0.03359323 0.0161553
0.08528707 0.1096331 0.09764364 0.2953046 0.2850052
0.07308723 0 0.006131352 0.02612167 0.01562626 0.02602375
0.01507903 0.0556125 0.1440468 0.07343499 0.02030424
0.03739421 0.1325208 0.1694138 0.0961672 0.06152911
0.5277251 0.09761934 0.008528847 0.02525429 0.08349235
0.033050011 0.1338666 0.016803622 0.008365256 0.03996364
1.605331 0 0.02468627 0.167034 0 0.02389352
0.008991382 0.1019433 0.09355344 0.03143913 0.0764035
0.111557 0.2410789 0.1554489 0.0809911 0.04068669
0.04775145 0.3194396 0.08455003 0.09875605 0.08313721
0.2602296 0.3317343 0.4386962 0.09289025 0.1363832
0.2507452 0.008993433 0.1484206 0.3034095 0.1707916
0.06816245 0.09232016 0.041305361 0.1571297 0.06155782
0.1387343 0.02232485 0.1323175 0.01646553 0.3104198
0.01212354 0.02760601 0.09537125 0.1191159 0.1812836
0.2760943 0.1299607 0.1334786 0.007283872 0.06102215
0.135231 0.03025749 0.229639 0.2477915 0.00950272

0.7080396 0.06736254 0.1371454 0.1468819 0.03488911
0.2627883 0.0188493 0.05358675 0.1471968 0.0583583
0.1093224 0.04955658 0.1050105 0.03396221 0.3419452
0.2296484 0.1604507 0.3046783 0.09044415 0.03400641
0.1186702 0.07911856 0.1631061 0.07226185 0.08743362
0.006663389 0.017250702 0.132621532 0.1749046 0.01606948
0.06212367 0.01002544 0.03027957 0.486675 0.1538839
0.04647669 0.1570617 0.02324785 0.2195787 0.07153381
0.1380159 0.104661184 0.02238974 0.1974037 0.05736881
0.02860829 0.05888829 0 0.1085337 0.07276851 0.03232764
0.05655465 0.2204245 0.2376399 0.1950954 0.02287004
0.03925861 0.09339487 0.302948 0.00534085 0.04460384
0.1830106 0.04940953 0.008901564 0.1669652 0.07759724
0.014026136 0.0368534 0.01057037 0.2027357 0.0292711
0.1961857 0.02718355 0.00949594 0.2668463 0.03390775
0.4521973 0.1607908 0.2718512 0.1418192 1.00618 0.0397604
0.1117154 0.0348745 0.1588238 0.07648934 0.01742654
0.3426224 0.06146205 0.04406701 0.05957777 0.03886222
0.04547317 0.078957179 0.05853591 0.00990321 0.133624
0.138535 0.02061274 0.05615116 0.137560399 0.1014076
0.199633 0.0396613 0.03028622 0.1150746 0.07648694
0.04714887 0 0.06603469 0.1578201 0.06790561 1.018159
0.1931115 0.1543536 0.280316996 0.07847575 0.2470098 0
0.023204239 0.0100222 1.669485 0.04725813 0.01820575
0.01510805 1.427564 0.05060892
LINC02106 0.1438477 5.685446 0.2866861 0.160314 0.4402544
2.767907 0.5926747 0.8619967 0.2590887 1.381728383 0 0
0.4442756 0 0.2345136 4.245014 0.3825882 1.516302692
0.2960221 1.126505 0.812439948 2.554805 0 4.847468
2.348102 0.05653368 0.3415465 3.658752 0.185318734
2.774434 0.9485126 3.782314 0 0.3775437 0.1366264 0
0 0.1291955 0.1112715 0 0 0 0 0.03082498 0.09875045
0.0176412 0.08368959 0.2061553 0.219704222 0.01914856
0.08302791 0.2714349 0.07716105 0.2523402 1.40234 0 0
1.671224 0.04807924 0.130009126 0.272190291 0.098086627
0.405921 1.991877 0.03299248 1.998221 0.09813826
0.9207737 0.048175364 0.06699089 0 0.0173764 0.2608822
0.1564852 0.05596813 0.04335885 0.4281238 0.1170927 0 0
0.08337202 0.05237684 0.05136078 1.060045 0.176773
0.1160655 0.8140565 0.2073843 0.90344175 1.568439 0 0
0.696332 0.1416687 0.1379772 0.02449259 0.1859131
0.1262268 0 0.631825 0.3001688 0 0 0 0.7174139
0.6329713 0.03458559 0.9550336 0.106003898 0.03196217
0.05807597 0.1347513 0.715256 1.955801 0.424542

0.01903462 0.1073002 0 0.2125327 0.0250817 0 0.1031696
0.2430244 0.1335304 0.04609098 0.2557977 0.2192846
0.2592975 1.53229 0.5769346 0.1899288 0.02729489 0.04878853
0.02415788 0.1071928 0.6111525 0.1596284 0.05162755
0.3693896 0.1258314 0.03266353 0.05048872 0.03730105 0
0.2715421 0.7561529 0.03112212 1.578277 0 0.6557329
0.04431371 0.3617703 0.04344957 0.4050564 1.138812 0
0.9211545 2.611679 0.06735081 0 0.867271661 0.8211092
0.269691429 0.04362005 0.08860551 0.1141904 0.1529785
0.2016649 0.1959039 0.1180627 2.038526 0.055200506
0.1790539 0 0 0.01899411 0.1623453 0.5869041 0.06919197
0.1664548 0.09645076 0.2663485 0 0.08934415 0.6576941 0
0.7657563 0.064876 0.2174932 0 0.3906453 0.02526422
0.04859921 0.03665213 0 0.2517742 0.2450622 0.07144726 0
0 0 0.589354 0.02350385 0 0 0.03717697 0.722215
0.4418218 0 0.02249823 0 0.5606031 0.1112673 0.185095
0 0.2936635 0.3335398 0.01899281 0.7283812 0.074567041
0.02237246 0.050549511 0.176153513 0 0.1881521 0.03885833
0.0742625 0 0 0 0.3516283 0.1951542 0 0 0.08619033
0.4027097 0.1381382 0.1219902 0.1353564 0.318229
0.7182417 0.2162148 0.6040758 0.2376664 0.07502928
0.04604916 0.1197528 6.070658 0.5588743 0.03418958
0.1293093 0 0.04464865 0.08692686 0.01712614 0.05858564
0.2182103 0.173959643 0.04501767 0.138886 0.2855537
0.1678969 0.06123758 0.3467271 0.02829759 0.0729413 0
0.02608188 0.1194435 0.1239424 0.3497099 0 0.06692287
0.043823429 0 0.1196496 0.1137776 2.053246 0
0.200105959 0.9495006 0.01688695 1.870307 0.06627865
0.01749253 0.6045254 0.03780229 0.2418036 0.2108595
0.4486442 0 1.962869 2.231029 0.1430336 0.03673775
0.6502025 0.160892 0.1617439 0.01943419 0.06137989
0.5057355 0.819807 0 0.04347635 0.06069745 0.08018058 0
0.132986278 0.1262776 0.01611367 0.1308185 0.1507952
0.09108855 0.4049474 0.06172284 1.018642 0.1049958
0.1748384 0.0733942 0.2151916 0.1277497 0.028622463
0.08980527 0.09897332 0.3698138 0 0.06643153 0.220318433
0.03264966 0.06567178 0.07293724 0.05234787 0.2841824
0.1232552 1.134666 0 0 0.06483581 0.1438963 0
0.03354489 0.1943088 0.09909084 0.02677814 2.080847
0.0233432 0.042194135 0.0923869 0.3179831 0.4472452
0.0880548 0.2213159 0.1022187 0.02856617 0.04013704
0.034001 0.1561026 0.06909986 2.126271 0 0.06305919
0.3986976 0 0 0.02986136 0.1610694 0.02621171 0.1145216
0.1344678 0.06628231 0 0 0 0.095009199 0.04402267

0.2085394 0.06699576 0.1190709 0.08267771 0.3860955
0.130678795 0.01906622 0.1143897 0 0.05466512 0.03461731
1.051849 0 0.07085325 0.0441442 0.9495249 0 0
0.1742783 0.08442445 0.180699393 0.128768 1.210138
0.1849617 0 0.1808958 0.5518933 0.1066232 0 0
0.5489173 1.389229
AC005180.1 0.2475063 1.798709 0.6146976 0.2837195 0.2921815
2.180723 0.4588938 0.9492243 0.5731604 0.806255552
3.590477 0 1.261304 1.647626 0 2.842153 0.3456003
2.124447456 0.1833623 0.3700352 0.751971088 2.514251
0.8501648 0.8587381 7.034992 0.8559989 0.7835593
3.483615 0.33480497 0.3931305 0.4196631 4.570321
0.04778239 0 0.04029965 0 0 0.3556729 0.8232573
0.087615629 0.1399116 0 0 0.04773408 0.4077872 0.01821221
0.2591954 0.2128281 1.61291095 0.1186102 0.05714357
0.3035724 0.1593172 0.3349388 2.798947 0.03042617
0.07268098 1.85218 0.0744532 0.100662944 0.281000539
0.135015323 2.514359 0.4923656 0.03406038 4.427686
0.6078888 1.275775 0.174071459 0.08644907 0 0.07175536
0.7345268 0.3231006 0.07703961 0.04476229 1.441243
0.04029424 0 0 0 0.08110826 0.02651161 1.476109
1.486029 0.1677512 0.5602706 0.2140969 0.33915794
1.619206 0.023851144 0.1105455 0.1480028 0.1462542
0.2848865 0.2022829 0.3838615 0.05212499 0 0.8780637 0
0.06487105 0 0.08906651 1.583427 0.354735 0.1428202
3.943784 0.043774012 0.03299672 0.04496683 0.1589862
0.477793 4.965911 0.7889104 0.03930147 0.05538666
0.01960854 0.4936768 0.05178709 0.05122655 0.4260358
0.1596577 0.1378525 0.0951657 0.2880844 0.1811059
0.02676904 2.29259 0.5268847 0.2852021 0.1127135 0
0.02493982 0 1.798163 0.1883374 0.07994794 0.381346
0.06495218 0.2023247 0.1042459 0.1732878 0 0.1635266
0.08006441 0 1.184991 0.02181268 0.5077182 0.04574805
1.377208 0 0.8363346 3.762154 0.07771634 0.5307742
2.666907 0 0.2484256 0.335753807 0.5742395 0.683396494
0.06754791 0.7012967 0.4518983 0.1579301 0.3238548
0.2359524 0.1828262 2.30883 0.28493617 0.9506544 0.02518602
0 0.01960891 0.383086 0.4989773 0.02381052 0.3723256
0.02489317 0.09998895 0.6097956 0.1614131 0.04526549
0.4892998 3.586363 0 0.224533 0.02139863 1.273546 0
0.1003445 0.03783848 0.1276796 0.649809 1.438905
0.1229331 0.05044029 0.02890446 0.01904166 1.62248 0.04852925
0.08081992 0.09365951 0.07676062 1.192947 1.026276
0.02101909 0.2090381 0.03165843 1.236418 0.2297377

0.4913643 0.1425143 0.06496473 0.1324369 0 1.063112
0.051320417 0.09238643 0.156557087 0.36371049 0 0.1079123 0
0.05111082 0.2881914 0.1811847 0.02473475 0.6143243 0
0.0322824 0.05858283 0.1186402 0.5543261 0.1782618
0.1259388 0.05589505 0.101086 1.297607 0.5704338
0.7220961 0.4293785 1.161867 0.7606349 0.1648386
16.86929 0.576964 0 0.2669896 0.0558604 0.1843754
0.6730538 0 0.06048194 0.2252733 0.333524961 0.0697122
0.5257321 0.4308564 0.03466627 0.3793183 1.303961
0.2921353 0.1506045 0.02143346 0.08077828 0.2877224
0.3582717 0.5866726 0.1345362 0.2763561 0.045241905
0.03445667 0.04940897 0.1879366 2.931929 0.02653609
0.118047419 3.099659 0 0.9654224 0.06842395 0.05417619
2.496371 0.2146423 0.7072858 0.3265269 1.047158
0.03994282 7.643899 1.222961 0.1476633 0 0.7551542
0.1898283 0.1113195 0.02006324 0.06336663 0.4913931
0.6552329 0.1266365 0.4488359 0.1462116 0.0620819
0.080361234 0.137290774 0.08690999 0.01663524 0.1929326
0.09340571 0.04701845 0.2572644 0.191162 5.031246
0.3522813 0.1263483 0.126283 1.332942 0.03297116
0.236391312 0.02317802 0.1021769 0.585402 0 0.06858178 0
0.1348259 0.158194 0.02509936 1.67531 0.3911744 0.7380196
3.110249 1.965045 0 0 0.3466259 0.06634664 0.1731533
0.4011965 0.0511491 0.05528978 3.592678 0.6747655
0.087119747 0.1335282 0.1969654 0.1678988 0.1363574
0.3554126 0.4854254 0.0294908 0 0.0702031 0.5295104
0.2140094 3.095646 0.1148965 0.455702 0.1234808
0.02478184 0.1805117 0.09248372 0 0.05412026 0.0591142
0.2949931 0.02280924 0.1585937 1.719852 0.02353708
0.049042227 0.113619 0.3998231 0 0.5531624 0.02133846
0.2740324 0.224847671 0.1181001 0.5314152 0.123173
0.01881151 0.03573781 1.832448 0.1464266 0.02438221
0.02278653 1.592921 0.03012702 1.039567 0.4198119
0.06536783 0.155456888 0.11078 1.819168 0.5728457
0.072063592 0.186751 0.3988299 0.03669147 0.07538689
0.07037984 0.2000063 2.848745
AP000787.1 0.1283543 0.1096789 0.1331374 0.1747871 0.1325374
0.2627431 0.05907251 0.1505578 0.0843221 0.475598679
0.1101767 0.0697492 0.4596833 0.05633125 0.2760384
0.178043 0.07626594 0.183002751 0.1079034 0.06415997
0.14360408 0.1495883 0.1285487 0.2352457 0.1627276
0.09015634 0.129685 0.1727534 0.116102884 0.1797062
0.1543498 0.1272944 0.3796005 0.3698509 0.3846306
0.05986609 0.07956725 0.193769 0.200686 0.227183045

0.3396274	0.7800093	0.071061163	0.1000711	0.1349837	
0.09846573	0.2828142	0.0968678	0.820311691	0.1963088	
0.06305127	0.4792459	0.3046991	0.3202905	0.4898701	
0.5505765	0.1243022	0.3583411	0.2656197	0.233246636	
0.257342618	0.115454598	0.2502312	0.09906652	0.1465685	
0.1813533	0.283199	0.3781385	0.096033784	0.08012463	
0.1889263	0.08313236	0.6375655	0.1069512	0.06800338	
0.1382919	0.2289952	0.231192	0.6324846	0.040128282	
0.02374222	0.3639403	0.1696643	0.3453996	0.1323234	
0.1956707	0.1629782	0.1653617	0.45842104	0.4497317	
0.057897307	0.1677144	0.1236444	0.5025662	0.07858476	
0.06695869	0.1667714	0.06038955	0.1650088	0.2242175	
0.6770048	0.1908737	0.2265262	0.2489621	0.2930665	
0.313127	0.1181891	0.1801796	0.330851716	0.03640804	
0.1604239	0.1710372	0.1725346	0.5087925	0.5319543	
0.08889742	0.1863938	0.1795766	0.1170128	0.2599917	
0.2034811	0.522965	0.541074	0.111543	0.3123877	
0.7363933	0.09741675	0.04430478	0.2428422	0.05813558	
0.1612772	0.2394049	0.2278571	0.1843718	0.06715671	
0.2958691	0.1220874	0.0499875	0.2644845	0.2150015	
0.1953365	0.09776969	0.1465889	0.2099	0.1701221	0.8149526
0.2765189	0.273174	0.1347794	0.2507597	0.3079136	
0.1416565	0.0791893	0.2795534	0.2195293	0.1029011	
0.2025364	0.6273281	0.1764541	0.1187804	0.440441957	
0.5129194	0.374231422	0.1316719	0.107659	0.1994469	
0.1481188	0.1812204	0.6248304	1.707959	0.1645746	
0.075454526	0.4137471	0.2306556	0.1503044	0.1492894	
0.1268072	0.06685411	0.233822	0.08216359	0.04944008	
0.1682474	0.1162175	0.2264421	0.04495067	0.2125798	
0.2787013	0.05173007	0.1689177	0.05666614	0.1709672	
0.1295029	0.1688457	0.1419512	0.1878394	0.3011352	
0.1971495	0.355383	0.03895848	0.1913562	0.2521231	
0.2125884	0.03748246	0.1984152	0.1085095	0.1799799	
0.1316278	0.09436466	0.2272828	0.03331599	0.1921226	
0.3570253	0.2205352	0.0662644	0.1074528	0.2054856	
0.1899672	0.1709139	0.1630785	0.178372212	0.1911331	
0.282146084	0.137592733	0.1017291	0.6786915	0.1682011	
0.2030214	0.1144748	0.08329843	0.570401	0.2033506	
0.1333798	0.1424795	0.06033004	0.2683565	0.3708032	
0.2124265	0.1296949	0.2096905	0.5493177	0.4172552	
0.1559835	0.148485	0.1895077	0.1994199	0.2071953	
0.2091622	0.2869892	0.2228144	0.1187831	0.257768	
0.1879881	0.1169761	0.1980364	0.02145918	0.1201226	
0.2350049	0.133048296	0.1435827	0.2636746	0.2026705	

0.06502532 0.331339 0.1297713 0.08380769 0.3323491
0.314536 0.1010133 0.3288063 0.1637717 0.1966872
0.07793365 0.38497 0.094846421 0.0494246 0.1144858 0.2151424
0.1945397 0.07612666 0.221427668 0.4794003 0.1038738
0.1253213 0.105697 0.03785884 0.2436632 0.258363
0.1836253 0.2113668 0.1622027 0.1718818 0.2037782
0.2226501 0.1419812 0.1339132 0.3980962 0.2827621
0.1228281 0.08190854 0.06059533 0.2643201 0.2349663
0.0465762 0.06685715 0.1405853 0.168967 0.339898817
0.238587881 0.2876851 0.02753257 0.1426287 0.07557903
0.005187938 0.1880581 0.33748 0.1842881 0.3348812 0.2688633
0.1003238 0.8170811 0.1818992 0.156498158 0.2838741
0.07328119 0.1235679 0.2058668 0.1538664 0.30474228
0.1376073 0.2443681 0.04984959 0.1833602 0.2023197
0.3594234 0.147077 0.6373979 0.05112032 0.091087
0.2895779 0.3678591 0.3859301 0.6271208 0.2144609
0.08845846 0.3555431 0.2180395 0.841106766 0.07577115
0.1485077 0.1296797 0.3811515 0.1624648 0.09547838
0.07158725 0.4480561 0.03873047 0.3327702 0.2676191
0.4906164 0.1056458 0.5279548 0.09537265 0.2761731
0.211124 0.319741 0.2070632 0.1283881 0.1532802
0.3044293 0.2114057 0.4578897 0.1431565 0.4051386
0.162337146 0.1454238 0.1289539 0.4655198 0.1288517
0.2307361 0.3628354 0.297711841 0.8339821 0.2996924
1.022021 0.5645715 0.05126225 0.153514 0.2665816
0.02421263 0.1684533 0.1532269 0.09307664 0.3441123
0.1786681 0.1226138 0.161236846 0.1906832 0.3409889
0.07900856 0.151076173 0.2438353 0.5783676 0.1295512
0.3951081 0.1061298 0.1397663 0.1864277
AL157932.1 0.4402611 0.4209898 0.2208923 0.2676317 0.05249786
1.378134 0 0.2878072 0.4325282 0.150436112 0.4122666
0.1572646 0.1236139 0.2626817 0 1.008152 0.2395129
0.331486086 0.04941857 0.7693412 0.093538256 1.355248
0.4073435 0.9098039 0.8771967 0.5662712 0.1900617
0.9494299 0.309375077 0 0.2262096 0.3946423 0.4636076 0
1.270771 0.3509505 0.08329354 0.6110985 0.8668753
0.106261104 1.325168 7.23292 0.374921044 0.1543796 0.5769964
1.178024 0.6519949 0.6022801 0.407532166 1.310648
0.32342 1.17061 0.6011335 0.2407212 0.4682194 0.2460078
0.117531 0.8205815 0.8829097 0.379820051 0.363520256
0.382078487 0.5182054 0.3746798 0.7710969 0.6237811
0.5461138 0.4854187 0.241274962 0.6150982 0.4101982
0.5511622 1.583718 0.2239198 0.622896 0.2895368
0.5904201 0.521272 0.5149706 1.097797769 0 0.4371951

0.3858425	1.440422	0.2951086	0.3487721	1.029548	
0.3462118	0.68555685	1.67035	0.192846028	0.7150436	0.3419034
0.9122325	0.2303421	0.6133268	0.3879592	0.7164674	
0.4366393	0.8519395	1.553436	0.5594756	0.3688765	
0.8161568	0.8672755	0.392487	0.1732138	0.5978826	
0.24775126	0.96045	2.108734	0.6748702	0	1.279549
0.7087396	0.3813217	0.8956465	0.5073371	0.7687477	
1.004926	0.8283742	0.6028172	0.9958341	0.3715308	
0.6155621	0.3105704	0.439294	0.6060275	0.4819492	
0.4074853	0.3170713	1.139167	0.2443459	0.120989	0.3579
1.173312	0.266487	0.4740348	0.8809527	0.4726479	
0.9269965	1.09573	0.4981693	1.0535	0.5666477	0.6149851
0.6754272	1.847788	2.998192	1.016502	0.8137608	
0.8304273	0.8704278	0.4375476	0.4387287	0.3770206	
0.5364402	1.279563	1.124369	1.472988	0.241306991	
0.3979676	1.146036606	1.165124	0.1972266	0.7307557	
0.319232	0.3366637	1.308185	0.5912891	1.321615	
0.737223281	1.366475	0.8145561	0.4571866	0.76102	0.9679386
0.403443	0.4235387	1.43573	2.213983	1.657324	0.3585775
0.3728826	0.4757864	0.8901415	0.9665725	0.2707635	
0.9572315	0.3460329	1.167008	0.4639429	0.3245302	
0.3059393	1.376454	0.8756609	0.6136681	1.033722	
0.2446979	0.1402225	0.4926702	0.9182882	1.098659	
0.6534613	0.6058196	0.5585763	0.2893635	1.10638	1.325593
1.91551	0.255971	0.4254013	1.151663	1.765723	1.229104
0.5953013	1.499127	0.6658471	0.6708829	0.912881352	
1.157821	0.421942196	0.420106371	0.831467	0.5235086	
0.9406285	1.776981	0.1864114	0.0976634	0.9199555	
0.9934114	0.6515893	0.7830483	0.536821	0.6235147	
0.2801217	0.05765273	0.06788437	0.5423206	3.718808	
0.6295003	0.842225	1.167687	0.595148	0.250511	
0.4612528	0.7996715	0.9613742	0.4081861	0.2853841	
0.7195726	1.083969	2.459735	0.7255878	0.1715444	
0.2934124	0.4305186	0.290411965	0.563651	0.7342212	
1.063429	0.448465	0.306694	1.116322	0.8030904	
1.765662	0.4852346	0.4354165	1.39581	1.572532	0.2919066
0.2719448	0.4468899	0.548698305	1.699435	0.5193387	
0.7597713	0.6406977	0.4291098	0.238615134	1.242389	
0.9021261	0.3214164	0.221294	0.8760723	0.5434195	
0.4733096	2.960262	0.105604	0.358206	1.162633	
1.161417	0.5273667	0.8527976	0.2453232	1.153303	
0.5755642	0.360025	0.1297754	0.2049378	0.9436135	
0.706376	1.058037	0.6532232	0.6417543	0.7696673	
0.086633691	0.333015225	0.9135126	0.4304081	0.3431862	

0.9062669 0.3041304 0.5720235 0.6697693 0.4668155
0.6134876 2.072341 0.4084195 0.2794132 0.6931211
0.668961365 0.4497685 0.4956846 0.5762168 0.4310158
0.2957392 0.630521703 0.3815423 0.2558124 0.3247013
0.5461916 0.3558152 1.316896 0.3919107 1.225112
0.8674964 0.8659064 0.3202982 0.5096171 0 0.1081278
0.4962731 0.849376 1.197865 0.4676356 1.796214779
0.9253961 0.9555262 0.6108879 1.396505 0.2463131
0.2730336 0.5245787 0.2680225 0.4540961 0.7073457
0.7498191 0.2730494 0.4954584 0.7895428 0.8652726
0.4408163 0.4086625 1.545387 1.267637 0.3500671
1.577274 0.6734504 0.1844219 0.4274312 0.3903351
0.9515334 0.475831195 0.3307158 0.09946861 0.7083463
0.2981693 0.1380239 1.248827 0.908991617 0.7320797
0.6206351 0.4780334 0.4867153 0.520118 0.164623
1.302309 0.6702757 0.7369528 0.6935063 0.5846136
1.190753 0.6788693 0.98658 0.351940691 1.325637 1.134165
0.3087791 0.466130341 0.6039827 3.961773 0.4746643
0.2133366 0.9484152 0.592948 0.8577898
AC087521.1 0.02131338 0.8220108 0.1176292 0.04750626 0.3261542
1.877874 0.0658608 1.081353 0.9213168 0.382747722
0.07317984 0.05583085 0.7679776 0.03330543 0.2084821
0.9368726 0.042515 3.851398245 0.2456187 0.2427782
0.116225285 0.2900929 0.1446119 0.2464935 0.06812215
0.0893481 0.02249141 0.5617654 0.137289918 0 0.04015359
2.795689 0.02743106 0 0.1330282 0.03114793 0 0.1148545
0.2143268 0.025149327 0.1893277 0.006262866 0.073945308
0.01370166 0.05852594 0.03136596 0.04133324 0.1832716
0.137444956 0.1361842 0.08201289 0.1474641 0.08383954
0.1281885 0.2077795 0.02620073 0.1981934 0.3495797
0.02849488 0.009631483 0.419425939 0.03875501 0.1061361
0.04988096 0.009776743 0.52474 0.1163262 0.1651492
0.085655561 0.0992579 0.03033862 0.04119354 0.2248957
0.1722376 0.2211357 0.141335 0.6233032 0.1561426
0.06452501 0.013918987 0.02470584 0.2017725 0 0.3433467
0.1721175 0.08942436 0.0511703 0.04389622 0.14602864
0.09616128 0.027385079 0.06346231 0.006068995 0.02398916
0.01635485 0.07983746 0.07575164 0.05236714 0.02384805
0.1584256 0.01778996 0 0.01636946 0.127829 0.249247
0.7127659 0.05124417 0.1680357 0.050259843 0.01894286
0.04732694 0.1312025 0.02493572 0.1018159 0.3522557
0.04512463 0.04769482 0.09005544 0.0419869 0.0297301
0.02205622 0.12229 0.1571259 0.09232858 0.1229243 0.1240383
0.03249057 0.09988976 0.3750991 0.3419289 0.02046621

0.04044182	0.04337289	0.2935093	0.0794118	0.1358283		
0.0405455	0.06119569	0.02345617	0.08389789	0.1161512		
0.07480724	0.05526756	0.09724141	0.1341112	0.03447272		
0.03688999	0.2125885	0.02504458	0.1665557	0.01313159		
0.2881116	0.04506432	0.1059101	0.1920966	0.06692346		
0.1713985	0.2187181	0.05987471	0.3803118	0.074958565		
0.204076	0.174367288	0.0323151	0.227558	0.09023555		
0.0453325	0.1062398	0.08707906	0.01749291	0.222865		
0.073609672	0.03789963	0.0433766	0.03688791	0.0394	0.274904	
0.03069155	0.04100766	0.3123978	0.04287224	0.03587623		
0.02386864	0.03971337	0.1169377	0.201896	0.1438995		
0.03844974	0.06445031	0.07370754	0.1766877	0.3368974		
0.02160229	0.02172244	0.0244329	0.1927396	0.1724722		
0.02822953	0.02895692	0.09956138	0.04372599	0.1106083		
0.1323342	0.01739901	0.01344209	0.01101674	0.06848506		
0.1718406	0.06033354	0.1000044	0.1090474	0.2189829		
0.09232196	0.070521	0.0613613	0.1429648	0.1368536		
0.03939731	0.1265287	0.007365546	0.03314843	0.067407587		
0.074571449	0.04580394	0.1300964	0.03454496	0.1173674		
0.01654458	0.008667925	0.07809891	0.0320612	0.1982761		
0.01853278	0.02802618	0.025541	0.14917	0.05116856	0.03614967	
0.04813261	0.04352381	0.005320959	0.08542862	0.4993361		
0.1232495	0.1482241	0.1773962	0.08280217	0.4692869		
0.1759634	0.0607889	0.03831856	0.01603426	0.1455393		
0.1803148	0.0659754	0.07812374	0.05878437	0.213563867		
0.1133917	0.4595803	0.2343291	0.01990132	0.09980677		
0.2495272	0.1677099	0.06484464	0.04921835	0.2009516		
0.395244	0.09549324	0.09067661	0.1255068	0.03966285		
0.045452075	0.05439774	0.09218579	0.2090393	0.2274555		
0.06093569	0.016942258	0.2281361	0.03502907	0.1630096		
0.04582788	0.04146885	0.4685214	0.02800512	0	0.4186463	
0.3005778	0.1203851	0.04417685	0.3802939	0.2119275		
0.05443295	0.3612678	0.1498437	0.04792998	0.1497336		
0.09094428	0.1498659	0.1645692	0.07875819	1.513806		
0.02398217	0.02376011	0.030756019	0.098520397	0.08731373		
0.1146001	0.02215187	0.05362264	0.02024437	0.1938447		
0.06401669	0.4735006	0.132233	0.1191637	0.06524726		
0.1417073	0.07571275	0.050890599	0.06653055	0.1319806		
0.09497614	0.05100529	0.04593358	0.111921456	0.05805094		
0.05838205	0	0.08531806	0.09824791	0.06574416	0.3246439	
0.1155282	0.04899552	0.0640432	0.2913813	0.07617697		
0.02982132	0.1343534	0.07340959	0.0714171	0.3402055		
0.02075205	0.031258736	0.03832814	0	0.08433938	0	0.1894623
0.03634886	0.2878129	0.03568173	0.02015121	0.07929991		

0.1433357 0.1615598 0.3023174 0.1401487 0.2953676
0.007113418 0.08290296 0.08848891 0.1022788 0.02330214
0.07635704 0.03486629 0.03273601 0.03034866 0.1645563
0.09458568 0.042231473 0.04565873 0.3354694 0.01323535
0.08821144 0.006125024 0.1287142 0.225892195 0.1468984
0.04237174 0.091925 0.02699842 0.04103294 0.4285833 0
0.05598966 0.03924409 0.01758594 0.06918166 0.1243329
0.1635404 0.03127214 0.23203746 0.03815816 0.1446996
0.08221526 0.031027869 0.09827641 0.3270879 0.06319186
0.02163917 0.07407378 0.162662 0.2424928
ZNF667-AS1 0.2762399 8.127342 0.5376526 0.8222381 1.375823
5.992483 0.7546436 2.768508 2.286851 3.215203994
2.745765 1.470829 2.324581 1.523974 0.6396297
5.427727 0.5749893 4.515481728 0.9441548 1.026071
2.587045453 8.265306 2.643369 7.472028 7.189511
1.378306 0.8491762 6.245805 1.11405718 2.216561
2.341915 6.409322 0.3362082 1.412215 1.675273
0.438809 8.264996 0.7335202 1.049827 0.251553912
0.8211838 0.1729321 0.550035723 0.1544222 0.5194399
0.2150486 0.3773296 0.7573608 1.402296958 0.2238308
0.5453439 1.627974 7.941444 1.149761 6.346121
0.2903691 0.7817962 3.853692 0.2007166 0.455909656
2.713517439 0.44770099 3.684259 1.10561 0.2093554 3.591799
2.141356 2.379207 0.249386162 0.2544971 0.2256712
0.6789868 1.853461 1.806784 1.049869 1.035379
1.504432 0.8733712 4.675404 0.172558615 0.2645207
2.212817 0.4717153 2.502909 2.125367 0.9186862
1.721879 0.82619 1.00118881 5.27434 0.536259688 0.2950368
2.045146 0.6184613 0.7234735 0.740285 1.781223
0.4763713 0.09071162 1.152464 2.631543 0.2483357
0.3413048 0.7011278 2.846274 1.673059 0.4793568
4.186332 0.506259896 0.5017066 2.582092 0.5689759
0.4496538 4.051552 2.183519 0.3305699 0.403152
0.3584056 0.9966898 0.1800989 0.5385916 0.9690803
1.999596 2.274395 0.4194673 1.35912 0.3149141 0.6191844
5.321443 1.734142 0.5276367 0.2917071 0.9858 0.7745431
0.4564483 1.694117 0.4569608 0.2715675 1.057435
0.231136 0.6163498 0.3793958 0.3768444 0.2318339
16.99278 1.142892 0.1870929 3.491229 0.109376
2.311224 0.3847929 1.676415 0.431706 1.221492
4.496745 0.9805236 1.663423 3.616947 0.528599
0.7500914 2.624936651 2.689233 2.133034297 0.1638907
1.129433 0.6673956 0.5364575 1.418119 0.7469613
0.7886028 5.829929 0.64063921 1.076395 0.4114642

0.2328137	0.6787632	1.785371	1.164542	0.130948	
1.088676	0.724776	0.5013775	1.223983	0.6266154	
0.878617	0.7320958	3.281014	0.7366796	0.5976091	
0.2422898	1.500362	0.3923507	0.6167774	0.2631803	
0.5369649	0.9319599	1.769897	2.322538	0.2202888	
0.9865032	0.3357241	2.109364	0.376787	0.06863234	
0.272694	0.2017637	1.307319	1.115907	0.3535888	
0.5672976	0.6247414	2.238228	0.9364484	0.9316756	
0.3150447	0.5113996	1.028256	7.640322	2.176776	
0.402608599	0.5603903	0.772366992	0.638737645	0.3298105	
2.241234	0.3341774	0.5497758	0.5500651	0.1172284	
0.5161172	0.8491492	1.652699	0.09399169	0.2495326	
0.1871059	1.350559	2.347113	0.5194586	1.283846	
0.5845451	3.625122	1.287756	1.300737	0.7342182	
2.05058	1.503329	1.006531	7.200273	2.082323	0.3967932
0.9428999	0.2981736	1.103458	1.901564	0.2316488	
9.739085	0.7155203	1.037471395	0.3345251	5.214407	
0.7262653	0.3308341	0.6340089	1.100087	0.5245153	
0.5907461	0.2773533	0.4703793	1.635543	0.5008654	
1.219046	0.6582877	0.5755293	0.215880804	0.1616302	
0.575426	0.5661856	5.107768	0.3734284	1.28410317	
3.981025	0.3045515	3.913174	1.099396	0.4177095	
1.157026	0.2525014	0.5316481	0.8908393	2.684028	
0.3133514	7.107341	5.914728	0.7336081	0.3374123	
2.727975	2.061087	0.9183167	0.2888299	0.3006583	
1.500262	1.766427	0.3413962	1.462897	0.2871797	
0.3815924	6.308670607	0.499660485	0.801301	0.3578754	
0.5804566	0.2568464	0.4449132	2.070256	0.2576746	
2.601546	0.7495378	1.039375	0.6822485	1.764781	
0.597315	0.77429717	0.3074265	0.7602591	3.149481	
4.153263	0.7617384	2.68571145	0.512498	0.4313454	
0.2354733	1.833524	0.8937377	0.8603345	3.077347	
0.8884509	0.09466205	0.6315641	0.8610386	0.4239037	
0.4929407	0.7084322	0.8976745	0.1878085	3.702424	
1.812587	0.193762815	0.1419332	0.5787837	1.059164	
0.2205616	1.831432	0.7408078	1.340429	0.3552277	
0.2271106	1.132065	0.5884817	5.162056	1.387666	
0.8792665	2.103858	0.3006394	4.420611	0.4088924	
0.6186236	0.0787868	0.6071789	0.4069884	0.715754	
0.889302	1.063956	0.4987409	0.503726782	0.5660474	
1.049683	0.3766457	1.014055	0.3244457	1.196787	
1.043809927	0.7004431	0.6208061	0.2231437	0.191697	
0.3873056	2.728006	0.8289704	0.2011381	0.2137752	
2.936652	0.3752304	0.5324833	0.9797814	0.3348247	

0.865006849 0.8601095 2.669566 1.521156 0.594479954
0.3926969 1.672698 0.4629252 0.1188917 0.1973247
0.9651541 4.808124
LINC00954 0.04343383 0.03559939 0.102734 0.05038147 0.09359485
0.2670632 0.1054551 0.1041096 0.08142313 0.252654286
0.09853286 0.03047568 0.2826648 0.03781443 0.2326608
0.06895265 0.08045136 0.177528743 0.05745977 0.08613949
0.021751689 0.08033333 0.01184062 0.1633162 0.08074452
0.05120988 0.09330597 0.0588756 0.047962055 0.01407935
0.105207 0.05561903 0.08385131 0.1856519 0.05809156
0.1156158 0 0.07244661 0.158389 0.115314741 0.9620568
0.01367452 0.029061785 0.05384991 0.1373713 0.1438193
0.5414893 0.1533949 0.189537702 0.1486743 0.01790694
0.9073913 0.7821557 0.205253 0.3205948 0.03432446
0.2641999 0.5947244 0.127544 0.067294922 0.102145466
0.160775774 0.1575837 0.1742585 0.2262764 0.1345448
0.02539901 0.2916089 0.018702287 0.1495386 0.04769441
0.06520881 0.2209697 0.05496387 0.1665778 0.09538391
0.2119692 0.1565736 0.08687958 0.042547575 0.04854911
0.3388892 0.2658521 0.2137367 0.02287517 0.02703486
0.1372643 0.1380158 0.44638051 1.760176 0.020927686
0.4364812 0.08480782 0.8171068 0.1392677 0.2947584
0.2766663 0.09473882 0.049467 0.2169807 1.336203
0.05420928 0.05003818 2.076545 0.2272891 0.8892994
0.08055939 0.06565474 0.043895528 0.00413604 1.253172
2.986776 0.1197798 1.008936 0.4175252 0.03941057
0.2464606 0.01228936 0.04125394 1.382659 0.1220008
3.320956 5.043178 0.1382352 0.325058 1.615946
0.09931722 0 0.215527 0.03158598 0.07820144 0.3708678
0.08207475 0.1375498 0.02080681 0.1581714 0.07377352
0.06346782 0.2151026 0.4111488 0.8263393 0.2548047
0.2847879 4.703699 1.578313 0.08530459 0.8578222
0.1034441 0.213264 0.2000144 0.09748448 0.07314782
0.03935791 0.6135757 0.3446123 0.05844906 0.07761945
0.4591879 0 0.7577254 0.163666737 0.997426 0.222085889
0.4910818 0.04204173 0.1650067 0.1534195 0.1942724
0.6802464 1.443753 0.2074496 0.035715894 0.4667166
0.1831055 0.02577349 0.0835692 0.2130829 0.1295581
0.268612 1.317527 0.3775544 0.02819996 0.05906422
0.4798027 0.161706 0.1916633 0.1595141 0.008395229
2.325762 0.06973859 0.1489929 0.0915403 0.07232284
0.1233165 0.170712 0.1547581 0.1645064 2.172711
0.1201282 0.06883866 0.8926689 0.05084327 0.01520749
0.04812004 0.02347988 0.2381374 0.01869154 0.1608008

0.07640573 0.07278415 0.4404801 0.1912535 0.3484428
0.3216421 0.1548191 0.0624307 0.1261643 0.5185852
0.1072564 0.157605077 0.05211163 0.124285088 0 0.0422263
2.27786 0.05531276 1.326164 0.101147 0 0.5425746
0.02100101 0.1623457 0.06879053 0.09301364 0.2342213
0.1042247 0.3306999 0.08419228 0.1681507 3.436961
1.698543 0.2673559 0.2386241 0.07688759 0.06796374
0.1817482 0.3822474 0.4936974 0.2124421 0.1659103
0.4099628 0.1295358 0.4217739 0.303715 0.137404
0.1743683 0.1052482 0.070749213 0.1194223 0.9884842
0.1961291 0.01303594 0.2456564 0.02884368 0.1867532
1.316728 0.01074648 0.04387637 0.3967166 0.785895
0.1329334 0.07167077 0.1472217 0.121925145 0.207314
0.3034701 0.1472331 0.225968 4.66336 0.022195335 0.6276301
0.3277865 0.0249144 0.1515224 0.04300855 0.3249477
0.06603897 0.6623136 0.1500736 0.09844355 1.214128
0.08038086 0.1175257 0.4600846 0.1901608 0.1367258
0.7346524 0.2581409 0.0603568 0.05559972 0.2425289
0.2395501 0.07143076 0.1237725 0.03141802 0.4046525
0.315621908 0.468944886 0.2178781 0.05421461 0.1112442
0.01561083 0.03830854 0.2257313 0.1317887 0.08270841
0.3498626 0.06334952 0.2279405 0.1206691 0.8472315
0.229639799 0.3166772 0.1408832 0.02233255 1.380944
0.03152057 0.297319998 0.1436501 0.1218076 0.0220229
0.3539433 0.131775 0.188207 0.1113887 0.08606097
0.4187435 0.1817839 0.5803481 0.070689 0.1128621
0.2514439 0.03205693 0.4851286 0.1485629 0.08760055
0.09555174 1.5231 0.1399044 0.4893104 0.6608905 0.4327704
0.01058202 0.08502141 0.2389195 0.04399874 0.6608396
0.3174344 1.284026 0.3456465 0.5793689 0.04643384
0.04348861 0.4027534 0.9699106 0.06550675 0.03731096
0.244523 0.147906 0.1686852 3.604771 0.2458349
0.04425453 0.193639825 0.05127048 0.2660037 0.1502719
0.2157159 0.05081951 0.1967269 0.090188739 0.4342358
0.4958842 0.7719683 0.1815634 0.06271477 0.04678907
0.06423945 0.01222495 0.2741974 0.2201466 0.1548296
1.775428 0.233041 0.2021103 0.440384833 0.1582997
0.1511024 0.04188595 0.049681253 2.403291 1.103398
0.08738415 0.08740807 0.02940634 0.05431875 0.09850529
LINC02542 3.269823 0.1352084 1.788367 0.5571707 1.993769
0.4474943 1.42004 0.4024748 0.9550349 0.376456305 0.9861547
1.562601 0.1910601 0.3811486 0.8068182 0.1571315
1.128216 0.359311561 2.531529 2.717964 0.598952079
0.05280755 3.777591 0.3806287 0.3752697 0.2778541

1.902467 0.2375885 1.468684786 0.5614783 0.3496342
0.8238518 1.859648 6.17904 4.172877 0.6199272 4.616256
2.635149 7.587983 0.10949289 0.727934 3.661529
0.88302572 0.1477124 1.492429 2.601111 3.228871
1.253861 0.125978009 0.2258692 0.9385588 0.3501907
0.5878133 0.6378234 2.274453 0.4128262 4.204091
0.3080175 2.995126 0.455269185 0.521731171 0.385664208
0.7393374 12.28546 2.310677 0.2515118 0.9164347
1.32191 2.965598027 4.333748 0.2868144 0.4867929 1.940777
3.44447 2.090566 16.27033 0.3567925 0.2733587 2.019972
0.813761198 0.1843921 0.2896019 0.8898161 0.5997504
0.5957562 0.3251529 0.6001439 1.070224 0.9687869
2.711311 2.997700008 0.2762965 0.6945381 3.640545
0.6510107 0.1986224 0.4625776 0.9119655 0.08899499
0.3762215 1.549046 4.694286 0.325796 4.60065 0.7842257
0.4266451 0.2804715 0.5060545 1.984981026 0.1885068
0.4602626 1.348216 4.931858 0.03896941 4.819946
0.7437398 1.107464 12.89648 5.562306 5.528776
1.188898 19.75641 8.371844 1.246933 0.7645383
0.7200358 1.018473 0.1529287 0.5075214 0.2290246
0.7637484 0.8853905 6.330401 0.3027669 4.188345
0.2478067 0.2521762 0.8563767 4.493341 10.41301
2.697007 0.186108 0.3299917 0.7257616 2.00188 2.48711
2.592677 2.11554 1.417479 1.329415 0.1796808 0.3000449
1.393399 0.6148023 0.7362315 0.3884871 0.9159944
0.3139237 0.7447918 0.6504801 1.491876069 0.48818
0.957968294 1.270235 3.462082 1.655622 6.879565
1.22242 1.444255 0.3481556 0.2188624 1.892329739 0.6411584
1.906477 2.147442 3.108656 2.633076 2.036146
0.8331656 0.2045247 2.737582 2.284906 2.771124
0.2470007 1.034388 5.918214 0.7297656 0.3826263
7.375718 1.161357 1.091346 2.458559 8.079352
0.9727529 0.182355 2.072696 0.4177908 2.106912
0.6843802 0.2683333 13.29872 0.4055214 0.1732766
0.04328585 10.46725 0.3837097 2.960344 1.30289 1.305869
0.7297966 1.763398 1.822239 2.772587 2.417236
0.5258177 0.01546403 0.9457474 6.944991 0.3147824
4.58106518 5.261447 0.186332138 1.242993559 0.1139526
0.8014394 0 4.288614 1.893368 0.8625745 0.141307
1.156563 2.507498 0.5302247 0.9900879 0.1482642
0.1731848 0.3818967 0.2847923 0.5388569 26.13158
1.535569 0.5490417 1.640728 0.1752139 0.8850169
1.154254 4.620243 0.1945847 1.107295 7.460814
0.4448745 0.4188511 0.2633293 1.025356 0.4166526

0.3671225 6.712681 0.219853062 0.3650703 3.49834 1.004007
0.1485336 0.880346 0.237358 0.9179161 5.189214
0.9795769 1.999737 4.314786 6.798197 0.563971
0.3842957 2.022829 0.775386801 3.887736 9.244287
1.241422 0.5517249 508.8384 1.201261738 0.6243214
2.77624 1.054407 0.1628745 0.03868786 0.1028474 1.992622
1.515244 1.507876 2.013275 3.879216 0.2014919
0.1892217 0.873713 3.845933 0.8388561 4.625939
0.3577256 0.4154945 1.297192 0.2193185 0.4094209
0.1808652 0.6730901 1.148526 5.408677 6.102142019
0.612756116 0.2792856 4.751769 0.6613223 1.222872
0.2686115 1.630476 0.7053073 3.578148 1.702924
3.737968 3.408812 0.1762722 9.347405 2.489945352
5.660682 0.2553801 5.161917 1.078588 2.089603 0
6.739653 0.1775215 3.15458 4.196904 0.9602393 2.67149
2.019151 1.386355 10.97035 5.273786 0.3889753
0.4145656 0.7913655 1.957744 0.5478928 1.678031
0.5289844 4.147422 2.690723055 3.269283 5.063591
1.348858 2.856318 1.812888 0.482293 1.158285
0.9468825 1.077857 0.739819 6.673432 2.934119
0.8068151 0.7438223 6.378288 2.920006 2.397643
0.06604373 0.4410513 0.1545917 0.548784 1.214382
4.984236 3.22773 4.460184 6.941749 0.840519337 0.2920921
1.317778 1.399413 0.482802 1.721899 1.316459
3.32372453 6.409589 2.382354 4.802578 3.801686
0.7656238 0.5815879 4.809987 0.1567046 16.80911
0.6125128 1.247816 1.824986 0.6209991 0.6068393
0.177621788 1.835336 0.3756403 0.5113449 5.609300249
3.622978 4.190764 0.2096145 0.2153387 2.864768
3.308823 1.178504
AC084125.2 0.409675 0.09083882 0.4942543 0.2297033 0.2375276
0.2194715 0.0825615 0.1245263 0.1558253 0.111582159
0.2242447 0.05249115 0.1650374 0.1002021 0.1451934
0.07917579 0.4204444 0.239165789 0.2455883 0.3170218
0.176918035 0.1123482 0.1133013 0.1527233 0.07523006
0.2753447 0.1456725 0.2511702 0.23520787 0.02694467
0.159396 0.08781477 0.6189645 0.1589479 0.3794417
1.008696 0.02471235 0.242631 0.4225315 0.26272157
0.6784473 0.5024625 0.194661703 0.5668105 0.4340863
0.5111549 0.8635726 0.7753886 0.235775761 0.5263781
0.1199443 0.4677455 0.2866342 0.1964037 0.1389159
0.5875534 0.2440917 0.2191124 0.5209239 0.358189222
0.141556749 0.404852855 0.06800316 0.1389546 0.1307295
0.1488602 0.1458238 0.07500979 0.262474029 0.460381

0.2839705	0.1441595	0.5814678	0.2823476	0.4227458
0.1503296	0.2881113	0.9376026	0.3819664	0.130863788
0.1806621	0.1880814	0.1748932	0.3174664	0.07192077
0.3161801	0.3482203	0.2091035	0.42713526	0.1372879
0.145899408	0.4839584	0.05325561	0.5839025	0.09225922
0.2820501	0.1208587	0.8158882	0.3662178	0.261789
0.3716843	1.076346	0.1846832	0.1816091	0.3400192
0.344862	0.2098456	0.1478214	0.286145545	0.6371927
0.8719405	0.5696938	0.005209803	0.3043579	0.2313034
0.6481651	1.062917	0.2587093	0.2061489	0.3540548
0.2027604	0.2523059	0.7824055	0.1047179	0.3966517
0.5327021	0.2443764	0.09311185	0.2337327	0.197831
0.532361	0.439373	0.4108046	0.2064032	0.1128219
0.5940567	0.2880188	0.3643888	0.1437533	0.4051083
0.3235652	0.3344697	0.2032274	1.487799	0.5828112
0.1032339	0.4046385	0.2690339	0.6174406	0.2522881
0.3017932	0.2463793	0.2313463	0.2507808	0.2928742
0.2889669	0.3315757	0.3866636	0.2335122	0.4469528
0.093966244	0.3607784	0.203403266	0.7831822	0.2194317
0.2498003	0.213104	0.130405	1.301836	0.3398946
0.1543932	0.136704153	0.4370925	0.229587	0.5579844
0.1152454	0.2326138	0.1923709	0.9681503	0.676737
0.2090029	0.7405649	0.1263337	0.6222965	0.2578903
0.4474959	0.2474567	0.1405823	0.3917253	0.369592
0.2825925	1.501605	0.5054953	0.1996919	0.2143994
0.4494534	0.0929311	0.5485117	0.1028491	0.2392148
0.1849967	0.2992049	0.5704319	0.3223177	0.2190588
0.7227403	0.8728206	0.2701238	0.794143	0.7438207
0.4670556	0.3123753	0.2893314	0.5369689	0.3960009
0.3012927	0.2605115	0.595002	0.2643555	0.332397746
0.6205408	0.447539793	0.068552707	0.5529209	0.5979807
0.3079447	0.7019278	0.235052	0.3078672	0.3916116
0.2310991	0.1104685	0.3175074	0.3677248	0.4482461
0.3781472	0.1838785	0.1435018	0.5329846	1.000274
0.4758094	0.2320311	0.2440833	0.2427898	0.226069
0.5530981	0.296568	0.1990667	0.2897862	0.2603619
0.3629328	0.4288033	0.8182363	0.3175299	0.3901987
0.1088156	0.4249495	0.061544482	0.7441713	0.4901309
0.165914	0.1704767	0.1744032	0.1134671	0.7848853
0.4380502	1.653017	0.1711683	0.2021313	0.4788321
0.2300464	0.3267671	0.3024657	0.415122669	0.7728391
1.306738	0.8340408	0.1069248	0.2641735	0.024778139
0.0889734	0.2300131	0.02724599	0.1696105	0.153787
0.2475987	0.6576625	0.5339541	0.4856404	0.1690756

1.073151 0.1569072 0.1417958 0.880497 0.2001584
0.1710867 0.3756809 0.1401957 0.4018756 0.4458884
0.2541747 0.3667555 0.1670805 0.2853235 0.4409309
1.260903 0.517279606 0.489894383 0.367454 0.7043327
0.1781847 0.09336117 0.1635462 0.04242839 0.9477116
0.2126955 0.3770322 0.6819555 0.2059956 0.2753433
0.4073293 0.233915821 0.8256708 0.3921722 0.01221126
0.596764 0.526457 0.183172095 0.09298549 0.3523786
0.1655768 0.06481979 0.07037783 0.1587257 0.1162759
0.4685462 0.5966475 0.2221163 0.7780517 0.1909872
0.6562844 0.3528846 0.1595091 0.7626352 0.2621034
0.5202852 0.616513729 0.1830371 0.2362458 0.125864
1.76635 0.2862244 0.3392139 0.1556372 0.1540691 0.7788828
0.590928 0.7829011 0.2700366 0.7464715 0.4880194
0.2468438 0.3507435 0.5542674 0.3105972 0.3789442
0.7789601 0.3935128 0.6826701 0.9493241 0.3455695
0.2641881 0.1242165 0.391170383 0.4142845 0.2914243
0.1603846 0.7224542 0.325043 0.7709089 0.355988903
0.4957839 0.446176 0.4077545 1.180047 0.1157353
0.08547345 0.3336934 0.5995164 0.4017627 0.2669932
0.1987435 1.766421 0.5179207 0.5096259 0.231209562
0.2471431 0.3102064 0.1431431 0.177191915 0.8026466
0.4578662 0.3432684 0.5289637 0.41645 0.5037756 0.2592104
AC007622.2 0.135623 0.06484323 0.350863 0.1133609 0.200129
0.1115968 0.2095454 0.0812711 0.1570339 0.116820575
0.312868 0.3164101 0.261796 0.1192117 0.1381907
0.08163688 0.08454217 0.114879075 0.1465258 0.07241562
0.039620055 0.03376726 0.1833224 0.3042365 0.06386097
0.6129649 0.2146788 0.03351255 0.098281639 0.01923384
0.1197697 0.1823551 0.3436485 0.4805422 0.2829322
0.5017016 0.06174121 0.05329127 0.2491598 0.217543883
0.3627928 0.3362545 0.202918177 0.3868948 0.349141
0.1808789 0.7002778 0.2095531 0.379761012 0.1015522
0.1565614 0.4398544 0.5001513 0.4078497 0.3470669
0.2396638 0.4355982 0.3128168 0.1784879 0.114914718
0.336823575 0.184956814 0.1772857 0.1091085 0.26829 0.2297524
0.04626355 0.3855171 0.110713394 0.2368523 0.1737479
0.2027382 0.327023 0.2687299 0.5012968 0.1226393
0.4080328 0.4622906 0.3079432 0.18267655 0.2210769
0.2777745 0.4812151 0.3312083 0.1339278 0.0902792
0.4099211 0.2304423 0.92922151 0.2199021 0.187872986
0.3312657 0.1520612 0.4651049 0.126836 0.1731915
0.3943874 0.3391774 0.8038106 0.240571 0.7428915
0.1777331 0.2929598 0.203353 0.1924227 0.422009

0.09782435	0.1371746	0.127427323	0.3051138	0.1693995	
0.3743342	0.0148756	0.4765682	0.8105433	0.148057	
0.08535814	0.3357704	0.2254285	0.3281108	0.692978	
0.3830035	0.5311631	0.1573695	0.5459119	0.5837463	
0.271355	0.1100123	0.2669517	0.07845399	0.2258717	
0.6369233	0.4139909	0.3758142	0.05684847	0.410549	
0.3708785	0.3650676	0.1959015	0.6562102	0.3811	0.437343
0.1846336	0.5444026	0.376024	0.05483992	0.2640841	
0.2971243	0.3361622	0.2235602	0.3055164	0.1518897	
0.3226017	0.2779984	0.4150261	0.3460055	0.3862745	
0.4014706	0.4286247	0.7373534	0.127763182	0.383959	
0.398743942	0.239045	0.0939818	0.3297132	0.1081738	
0.4278026	0.3809497	0.3026303	0.1329519	0.175649683	
0.5064487	0.1035066	0.2376623	0.05372429	0.1639961	
0.3661856	0.3017157	0.3923439	0.4603639	0.1969006	
0.1423902	0.3632669	0.2015291	0.3246714	0.2377229	
0.3555311	0.496333	0.454365	0.05088489	0.2769041	
0.09450476	0.1036695	0.160332	0.2522149	0.2978385	
0.4546947	0.3886757	0.3662637	0.339106	0.1597514	
0.1495799	0.3321444	0.3367972	0.4797651	0.08171058	
0.1269206	0.5614821	0.4295403	0.6071621	0.3333469	
0.2989806	0.1682794	0.4311321	0.1038273	0.05442738	
0.1074412	0.2042445	0.325154064	0.1977495	0.509358575	
0.106766823	0.2853913	0.6060971	0.3022516	0.3500823	
0.5329691	0.08273469	0.2541301	0.105195	0.4337049	
0.1437264	0.3945739	0.4266271	0.177977	0.20757	0.2444075
0.7465609	0.4673604	0.5840638	0.3737295	0.3597076	
0.1050363	0.3713818	0.2279353	0.4092834	0.2969238	
0.2593435	0.2901127	0.3505083	0.5069641	0.4262201	
0.3073377	0.4117467	0.2589185	0.3506827	0.140582822	
0.3342444	0.2618901	0.4271387	0.0949783	0.4221963	
0.07880687	0.5202531	0.4513083	0.1541483	0.06455036	
0.3026505	0.3418034	0.1854646	0.1382254	0.3785789	
0.317630372	0.1947084	0.4061106	0.2574534	0.1763975	
0.2453737	0.161712567	0.4264353	0.2328508	0.07779573	
0.5389684	0.3772631	0.2959413	0.2940374	0.2279782	
0.2460192	0.1931052	0.6087319	0.1493396	0.1116885	
0.3323224	0.2467902	0.2298849	0.3006769	0.09531	0.5015926
0.1736113	0.3365785	0.6171002	0.310816	0.3612291	
0.1216074	0.1063071	0.458693549	0.270356292	0.2083507	
0.1880054	0.151971	0.138619	0.1288207	0.1211462	
0.6765024	0.2189146	0.2412942	0.1545392	0.3719382	
0.321239	0.3500445	0.313711545	0.1984466	0.2274539	
0.1481844	0.8519729	0.1878997	0.077895549	0.4271124	

0.3560223 0.3782183 0.1897076 0.2176963 0.1917432
0.1383349 0.3000014 0.5762221 0.2674383 0.4324446
0.3805932 0.130461 0.1946489 0.31531 0.3881736 0.2156368
0.5529641 0.350575655 0.8492696 0.1573961 0.2731294
0.3735907 0.3390757 0.05782451 0.3736935 0.3831518
0.3846835 0.5440302 0.3908939 0.3469671 0.2754434
0.5908212 0.1550594 0.4328436 0.2843743 0.1900395
0.2847376 0.4077649 0.3340435 0.3209105 0.4843172
0.3168328 0.1653345 0.4433457 0.394698367 0.1245169
0.1053299 0.1894956 0.4209858 0.4969345 0.6484106
0.19251106 0.1685258 0.5358764 0.2362277 0.6442447
0.2325459 0.04648631 0.2758099 0.1419545 0.1950947
0.1468744 0.7531931 0.6378771 0.4724021 0.4178863
0.37267932 0.4856223 0.2477056 0.08174359 0.14807933
0.1705529 0.7902636 0.276449 0.419543 0.3053078
0.07991307 0.1883957
LUCAT1 0.02001187 0.02391987 0.1242519 0.06690792 0.2428026
0.354667 0 0.2098594 0 0.068949885 0.0300611 0.01965807
0.2781312 0.01407223 0 0.1779092 0.01995941 0.052736423
0.03706393 0.04274118 0.029230705 0.1444934 0.1782128
0.05386997 0.0205593 0.1219056 0.007919238 0.06725129
0.019335942 0 0.0565524 0.3856732 0.1207312 0.01575699
0.08960566 0.592229 0 0.06290719 0.4876173 0.057558098
0.2060474 0.07056507 0.177046049 0.0225137 0.3194087
0.07362648 0.1397132 0.05377501 0.193577779 0.1558392
0.2136882 0.08496361 0.1449161 0.06770285 0.1268094
0.06765213 0.9255563 0.1692449 0.5016532 0.155997521
1.499521057 0.068228301 0.2889992 0.1112331 0.06884793
0.9797431 0.2525776 0.03539512 0.331753073 0.4298698
1.486968 0.05801707 0.2425068 0.9609892 2.257998
0.02714407 2.563666 0.6027208 1.052661 0.480286524
0.2522693 0.1256936 0.4126371 0.169764 0.6639943
0.009688113 0.1338413 0.1978353 1.21686341 0.1410768
0.337480549 0.3910395 0.2692489 0.3209707 0.103654
0.1022211 0.3782602 0.06848585 0.4324409 0.4614672
0.06890238 0.227287 0.7146982 0.1620311 0.7537037
0.5509914 0.5340758 0.04359561 0.075210204 0.06669792
0.2938896 0.2731618 0.3775349 0.165459 0.5138362
0.4806243 0.3134763 0.2338507 0.01108771 0.09421185
3.168531 0.06996985 0.147531 0.4179721 0.1250361
0.05337928 0.1418554 0.1785617 0.01853651 0.01389154
0.07926782 0.170875 0.1018108 1.164519 0.03355313
0.07014364 0.2331762 0.6787316 0.2642858 0.5382934
0.2487895 0.2475929 0.1206504 0.07374501 0.1558281

0.02022977	0.2013293	1.621092	0.2601372	0.07330544	
0.2912894	0.8257089	0.4760152	0.1044148	0.01828036	
0.03141838	0.13411	0.799727	1.714663	0.200862	
0.037704217	0.09396457	0.127905871	0.3322424	0.2280432	
0.04368648	0.2314432	0.2057389	0.1090154	0.03695557	
0.09499109	0.115191138	0.05337792	0.412369	0.394843	
0.3567281	0.1935877	1.707429	0.4090999	0.1099954	
0.2364937	0.07073943	0.06163051	0.1305089	0.5444095	
3.863461	0.08574433	0.7175232	0.3177018	0.1989689	
0.2617187	0.2161552	0.2079022	0.06883633	0.04301418	
0.1751322	0.5689215	0.06460761	0.1376426	0.338871	
0.3656537	0.03279601	0.3825689	0.5350214	0.4922285	
0.1357651	0.2833351	0.5935268	0.1784452	0.1737105	
0.5823341	0.5530217	0.2972033	0.08828616	0.9218282	
0.2101063	0.01606207	0.0158535	0.2777874	0.046681433	
0.1353903	0.26898815	0.042010637	0.9174808	0.04362571	
0.1946128	0.1343067	0.1281578	0.01831189	0.144993	
0.2991523	4.584396	0.1435589	0.351302	0.665482	
0.07003043	0.1729582	0.1103121	0.1751244	0.03575777	
0.07494051	0.0451192	0.6302856	1.351482	0.5532117	
0.1297273	0.2498973	0.01001431	0.08746845	0.4708837	
0.1304225	0.4403623	0.3447356	0.1541874	0.02144305	
0.01833828	0.07451283	0.145205983	0.2771284	0.323637	
2.053518	0.315327	0.6517247	0.1757174	1.464459	
0.09893794	0.4765697	0.1632812	0.294948	0.3258866	
0.2098079	0.1495696	0.1466358	0.15546452	0.6790777	
0.1797711	0.2849143	0.1161265	0.06973034	0.208788243	
0.6265495	0.6307835	0.07461451	0.1982425	0.5584961	0
0.2839858	0.07568852	1.720465	0.2564429	0.1937722	
0.04666408	0.1854024	0.4263988	0.48298	0.1187224	0.2973748
0.1800125	0.7624306	0.07258214	0.3290231	0.1821126	
0.1706511	4.078109	0.498204	0.3513698	0.157023564	
0.894978417	0.1844593	0.2051164	0.07019718	0.5286557	
0.1948335	0.1235051	0.09660134	0.08335991	0.1698046	
0.08756369	2.644516	1.202475	0.1666156	0.645069888	
0.1499228	0.1239211	0.2006469	0.1795899	0.2541509	
0.045975541	0.347476	0.1416104	0.04566112	0.387796	
0.2569776	0.01028825	0	0.01435678	0.4140324	0.04509929
0.08507921	0.7174873	0.02800028	0.06757987	0.1395768	0.4191
0.1048132	0.4871204	0.017609949	0.2544839	0.2189748	
0.4836196	0.05512521	0.5080207	0.0255969	0.6557234	
0.07538133	0.3902388	0.6747706	0.3821194	0.1706559	
0.9328553	0.4671461	0.02495979	4.007421	0.1897362	
0.2741815	0.4801655	0.1750336	1.433886	0.08067375	

2.959971 0.1389152 0.1829696 0.199822 0.094174924
0.0183731 0.03108394 0.0699026 1.739321 0.2286022
0.1007119 0.018179832 0.08753126 0.5728939 0.1294674
0.3041971 0.05056703 0.03429646 0.813943 0.05421347
0.3224168 0.1320964 0.4810883 0.7879982 0.1151653
0.3391369 0.307948105 0.01791401 1.484161 0 0.058266293
0.1761616 0.1497182 0.1260827 0.06095331 0.1375202
0.04716632 0.07148248
AL139147.1 0 0 0 0.02401144 0.04945517 0.06364019 0
0.03012514 0.01940284 0.031492681 0.05394059 0.07407488 0 0
0.04097903 0.1955309 0 0.018925676 0 0.08052793
0.014686153 0 0 0.04510911 0.04303936 0.01975745 0
0.1391293 0.024287014 0 0 0.1464546 0.04852641 0.0395833
0.2046358 0 0 0.01128785 0.1749927 0.055612429 0.8017942
0.0110792 0.235460794 0.008079557 0.0517671 0.03699157
0.1023677 0.04052664 0.102376592 0.0803047 0.02901667
0.1541497 0.08089896 0.01889745 0.4655867 0.0463499
0.03690635 0.1674881 0.02520417 0 0.313913578 0.051419114
0.4631366 0.04412049 0 0.570595 0.06859491 0.1524282
0.01262728 0.02633855 0.05366996 0.05465449 0.2362219
0.4921974 0.4400954 0 0.292737 0.2659908 0.04756115
0.024623121 0.0218527 0.3843989 0.1076977 0.3747732
0.3574344 0.1095193 0.465541 0.1397768 0.21527434
0.2693445 0.145335173 0.0842001 0.03220872 0.06365636
0.02893224 0.308149 0.2071017 0.02646831 0.06328191
0.2038254 0.3776518 0 0 0.1206045 0.2723365 0.5024659
0.1269136 0.1877434 0.077797325 0.134042 0.05327817
0.01009136 0.02205603 0.1246954 0.08902161 0.03991343
0.09843589 0.1294401 0.1392677 0.05259347 0.03901814
0.1622511 0.1737254 0.139999 0.0604047 0.0487617
0.05747683 0.09515051 0.4307333 0.1163236 0.09956477
0.02861714 0.12788 0.2279534 0.02809639 0.2402845 0.1434525
0.02706427 0.0829894 0.04947266 0.1969136 0.1720372
0.05866203 0.01323254 0.09489879 0.2134416 0 0.4620359
0.03322849 0.1473211 0.0116151 0.1422358 0.1480518
0.2373204 0.2663493 0.1183897 0.1122998 0.2529855
0.2118404 0.1681959 0.075773735 0.2082789 0.16708313
0.01143328 0.01548297 0.05986106 0.02004865 0.1761951
0.2225103 0.09283649 0.08300109 0.028937289 0.06704562
0.01278909 0 0.2489279 0.2066834 0.0723924 0.01209064
0.3054071 0.05056156 0.0380797 0.01407479 0.1873445
0.1838813 0.04658612 0.156654 0.1020282 0.1451095
0.03259773 0.1401158 0.2781249 0.1273837 0 0 0.2859684
0.4094881 0.04993891 0.06403211 0 0 0.1750728 0.06160612

0.04103918 0.09511788 0.01948896 0.0605761 0.2026609
0.2561566 0.2358811 0.3054381 0.3072384 0.09332596
0.1247538 0.1688556 0.1209565 0 0.03982575 0.1184999
0.026059762 0.1759218 0.185493966 0.013191922 0.1080381
0.208226 0.02037037 0.09083665 0 0 0.01255995 0.04253795
0.4969053 0 0.11899 0.04518282 0.1055545 0.01810375
0.02131663 0.3405923 0.1411575 0.16002 0.2518765 0.08333407
0.2491796 0.4195409 0.2293296 0.3348106 0.3018848
0.3845283 0.03584582 0.1355735 0.0283651 0.4447098
0.2734135 0.05386733 0.03071185 0.1039914 0.091193377
0.0825973 0.2548246 0 0.01760303 0.1605103 0.1168469
0.2966841 0.1147122 0.0217672 0.02734536 0.2191519
0.03898397 0.1489519 0.05123665 0.05262361 0 0.0437415
0.1003566 0.2385787 0.1810692 0.05389857 0.119885528
0.3094113 0.01770501 0.1153476 0.1158156 0.1925692
0.07313196 0.03963354 0.1056322 0.2984501 0.4601522
0.08112954 0.1302504 0.1345504 0.04284644 0.1155523
0.2556376 0.4699113 0.08478962 0.03056346 0.01072555
0.2651174 0.4574869 0.0535868 1.093979 0.0106063
0.05254047 0.040806267 0.10457139 0.1544607 0.09291845 0
0.03162004 0.02387529 0.01632939 0.1132475 0.1779979
0.0963218 0.2749621 0.1923741 0.2882873 0.06696911
0.120036073 0.03530839 0.02594197 0.5298958 0.04511498
0.05804139 0 0.06846261 0.1950838 0 0.219535 0.2855345
0.09045825 0.0410218 0.04808776 0 0 0.1885839 0.05053479
0.0351699 0.03395362 0.2467413 0 0.357339 0.012237
0.055297677 0.2130973 0.2000323 0.149199 0.02308011
0.2191462 0.06430226 0.344425 0.04208139 0.03564811
0.1870453 0.1630063 0.4287065 0.0777904 0.1818134
0.04180116 0.176174 0.1099934 0.06261585 0.04824916 0
0.04502599 0.04405682 0.03474661 0.0402658 0.1532133
0.02390357 0.04980586 0.01153882 0.04685178 0.01170687
0.2496781 0.02167071 0.2150495 0.068504628 0.2898527
0.04497416 0.07505455 0.04776105 0.01814714 0.0344626
0.03717664 0.02476186 0.04628268 0.373321 0.1835768
0.1979539 0.2131744 0.04425711 0.078938749 0.2137594
0.278237 0 0 0 0 0.1117884 0.01914018 0.09530096
0.03385344 0.4190011
AC108010.1 0.8793292 1.581279 1.629754 1.40416 1.222247
3.2564 0.4055565 1.148239 1.681719 1.068816641 4.427397
1.830705 1.13497 1.796568 1.840095 3.179465 1.282811
3.013918211 0.3078951 0.6447084 1.34958719 0.8103836
1.035193 2.669329 2.606778 3.796285 0.7686597
3.289727 0.913033764 0.08934092 1.55772 2.047006 2.407032

1.446739 1.421063 0.2013923 0.5326046 0.6600993
2.071037 1.289243805 2.501255 2.279213 1.243076067
1.113709 3.338113 1.583787 4.291227 2.369948
2.592523051 2.484331 1.181741 4.024323 1.239038
1.46031 2.686869 0.9680305 4.027426 5.300867 3.842691
1.458991306 2.637369014 1.897227518 1.294055 1.351487
1.047533 3.38835 1.647517 2.082543 1.41092454 1.622759
2.006432 0.7324459 5.089357 1.590903 4.320012
1.28173 4.024979 4.038269 1.927056 1.259938632 1.551759
3.698742 2.164947 3.913621 1.437726 1.651968
4.915492 2.205676 0.62944894 2.486989 1.37856166
5.070463 1.726566 4.952315 1.435113 3.070402
2.569783 2.031533 3.788759 2.687199 5.422562
2.339108 0.725757 1.385372 2.410554 5.29657 1.760772
1.568429 1.566789326 2.169616 3.171278 2.697739
0.5988402 2.647702 1.440912 0.9482235 1.174777
3.06731 2.026356 1.784947 1.16803 1.764926 4.740982
0.6578813 2.914224 3.793545 1.032361 0.5961734
4.096819 2.004297 2.287381 2.136694 2.857765
1.758877 0.6161409 6.206097 2.009847 4.0415 2.152127
4.75295 3.397362 3.897072 1.572305 0.9396422 2.378385
1.846797 1.073334 2.771497 3.284867 1.525601
2.862497 1.621471 0.9870942 4.695633 4.814681
0.4533102 2.497863 4.755305 1.548521 1.009935
2.255141351 4.262963 2.576918547 3.605688 0.5658906
2.250387 1.109612 2.183398 4.200343 0.4847289
3.174473 1.495799065 3.122594 0.8680872 0.831361
1.268539 4.875263 1.228447 2.828183 3.325938
0.5675957 2.690782 2.13118 5.233291 1.728185 4.070233
3.57849 1.118715 4.134675 3.018269 1.55322 2.378807
1.928822 1.284116 1.263801 7.33932 1.534378 4.510923
1.925753 0.735693 0.4543681 3.505884 2.714854
2.035647 1.837571 3.246083 1.186077 1.768634
4.35794 5.997943 3.626048 1.966876 3.971369 3.937228
3.791091 1.745382 1.587113 2.828024 2.667396
2.258701015 2.559676 2.393635771 1.005636742 2.745294
7.942387 0.3509887 3.685889 2.322817 1.761382
1.337821 1.702799 3.357589 1.609104 2.557623
2.264774 3.380287 0.9452548 2.32619 2.815707 3.510934
3.027515 3.853324 2.105951 1.040837 1.273261
2.281368 2.906304 3.612209 4.30231 2.086861 5.238255
2.947254 2.957459 4.85377 3.196971 2.64588 4.571826
0.965903676 2.316519 3.370652 1.827734 0.3860263
1.944335 1.748938 4.120559 3.713483 2.545838

0.9137846 2.898797 3.54173 2.763916 1.391119 1.740176
1.415413622 1.45255 4.218152 4.073425 2.069419 1.632245
0.391225356 4.523498 2.412775 0.6624935 1.112667
3.51434 6.593196 2.069394 2.713574 3.081977 2.829731
5.31622 2.720315 1.599608 3.568253 2.332897 3.603866
2.755533 3.659766 3.872513 1.668845 1.872826
2.490023 3.503018 7.84212 1.36232 1.229001 1.88915445
2.457002573 3.848103 2.584563 1.06397 0.9410604 1.595654
1.074288 2.686198 3.411386 1.896753 2.737334
2.022285 3.547131 2.150453 2.616667113 2.91283 2.329762
0.05398547 1.69603 2.666863 1.964186172 1.000902 5.092974
0.8784094 3.438787 2.009429 1.740805 2.206135
6.013403 2.275709 1.644499 8.756879 1.310496
1.285433 0.5850329 1.261193 3.48885 5.361665 4.996456
2.529246795 2.468057 0.5222162 0.6899851 3.133224
2.813465 1.768244 1.423044 2.15326 3.126987 5.493507
1.3996 1.939958 1.949609 4.090645 1.942489 3.114385
3.560727 3.416495 2.040586 2.037552 3.307004
4.186632 1.572334 1.289475 0.9439711 2.833146
2.262454828 2.349666 1.696088 0.9535548 0.9125541
1.482261 3.936552 2.313026474 3.590442 3.256232
2.743186 3.640885 1.061222 1.727426 0.8152665
4.188983 3.11737 5.966803 1.214116 0.7809274 2.353309
5.661472 2.027848743 6.379427 3.672588 1.493478
1.280121503 1.782486 5.981985 1.128453 5.096787
1.331071 1.891328 2.51069
ZKSCAN7-AS1 0.031861 0.6397937 0.1465349 0.1826133 0.3343285
0.6453339 0.09845419 0.3436645 0.2459394 0.518938606
0.4922788 0.06259544 0.344411 0.1493632 0 0.5901046
0.2542198 0.287869496 0.1180193 0.2041454 0.148922749
0.222116 0 0.6861333 0.4146125 0.525912 0.3025982
0.6550234 0.307848555 0.1445912 0.09003738 0.2284771
0.4613201 0.1003471 0.1556309 0.2793751 0.3646832
0.08584704 0.3696848 0.676715454 1.749596 0.6459954
0.397942512 0.4915772 0.5577451 0.2578864 0.6302413
0.2397233 1.03813457 0.4071589 0.07355981 0.5410841
1.401421 0.1916268 0.3727273 0.3525032 0.2339021
0.75121 0.2875265 0.518325859 0.361726571 0.34760524 0.1903933
0.1491324 0.2630716 0.4965625 0.6086294 0.1610079
0.352123875 0.2225684 0.6258671 0.3232925 0.8509879
0.4753385 0.5206509 0.4033514 0.1484228 0.9077249
1.977376 0.124843543 0.2215941 0.2784243 0.4777904
0.09828445 0.1678014 0.2467919 0.7212255 0.4724619
0.21829573 0.3593747 0.245624941 0.5692124 0.1088692

0.2689574	0.8068036	0.1627468	0.4014867	0.3690472	
0.6684383	0.7750728	0.5185811	0.08350726	0.2936451	
0.3439608	0.6246449	0.5287452	0.2298121	0.4362839	
0.394446085	0.4247604	0.1543598	0.5116497	0.5591398	
0.5268562	0.7898716	0.1770721	0.1425963	0.2776586	
0.3295189	0.1333291	0.09891441	0.9254717	1.556112	
0.5323645	1.010666	0.6798835	0.49541	0.06891854	0.5902414
0.2948906	0.435973	0.7254695	0.1296748	1.027345	0
0.4467043	0.6667185	0.1029154	0.1753212	1.170564	
0.3255609	0.7716137	0.2974268	0.7380046	0.330793	
0.1545981	0	0.5992706	0.7300551	0.06224532	0.2944529
0.2403868	0.5485508	0.5382987	0.4889516	0.6002565	
0.4555036	0.3772591	0.1790114	0.1065978	0.144069799	
0.3872039	0.456152728	0.4927344	0.2747548	0.7587652	
0.1016502	0.178668	0.347128	0.509922	0.657547	
0.293434266	0.5098996	0.03242147	0.8933185	0.2524217	
0.2773908	0.09176054	0.2145558	1.06918	0.320445	0.1930708
0.3211274	0.3858845	0.0291347	0.2755665	0.4467731	
0.1724336	1.313805	0.1377302	0.4918235	0.2350237	
0.2583432	0.2435438	0	0.6134235	0.3867388	0.3164988
0.324654	0	0.5392632	0.4177176	0.40606	0.3381232
0.3014152	0.5681717	0.07678286	1.211013	0.2705748	
0.3886865	0.4890394	0.2370493	0.6506211	0.4216826	
0.5197918	0.557519	0.2045801	0.5805302	0.5340581	
0.297287019	0.5054409	0.167944098	0.066885356	0.2282384	
0.5556538	0.5164068	0.6579397	0.2967866	0.194363	
0.3502462	0.07189162	0.2222997	0.2493391	0.4524753	
0.5345313	0.4905816	0.3212622	0.1080791	0.7195262	
1.07354	0.2863516	0.5746761	0.4647702	0.1579231	0.6314964
0.336583	0.5304838	0.8609675	0.5570359	0.318053	
0.257768	0.7550342	0.3560143	0.3465637	0.1820779	
0.6617865	0.07908818	0.396314073	0.1495653	0.5229546	
0.1459561	0.2677513	0.4882891	0.09873901	0.5264842	
0.7754812	0.3310906	0.2079686	0.3439238	0.7906222	
0.6680724	0.08659294	0.1778739	0.378553699	0.1552439	
0.1908097	0.2419272	0.3825218	0.7515068	0.189950206	
0.750281	0.2693025	0	0.4991247	0.3951931	0.4325905
0.9042705	0	0.5043981	0.4147649	0.2313793	0.8254902
0.341097	0.1357744	0.4882254	0.3780371	0.213817	
0.3582485	0.3615782	0.4894238	0.2767454	0.6326015	
0.2173556	0.3466667	0.2151032	0.5061399	0.482754842	
0.530195292	0.3076633	0.3426275	0.1986867	0.1202394	
0.1815778	0.08279288	0.7382376	0.530871	0.4186015	
0.3949967	0.2600987	0.4448552	0.297102	0.266264228	

0.02983662 0.394591 0.2293494 0.4574817 0.235424
0.501928461 0.08677936 1.076383 0.03230992 0.5043648
0.4091354 0.3931193 0.5719662 0.3961968 0 0.1436056
0.286846 0.7686608 0.9807465 0.1721508 0.4938244
0.4626276 0.6674948 0.2791969 0.224295138 0.7121085
0.04225829 0.1620996 0.3510606 1.503266 0.2173491
0.2277776 0 0.2711133 0.4149043 0.2754902 0.2898156
0.3697603 0.7123199 0.1059696 0.350913 0.4647383
0.07936834 0.06115792 0.9753515 0.3804828 0.3797389
0.4991523 0.9527217 0.2330455 0.8483672 0.568180012
0.08775573 0.3563201 0.3858133 0.3955974 0.02746858
0.3527566 0.115776827 0.5067599 0.7600895 0.6976562
0.2421569 0.04600459 0.3494629 0.1884921 0.2824807
0.8799798 0.2628893 0.3102555 0.7248662 0.3474101
0.7573207 0.600349957 0.7700667 0.8746405 0 0.324681249
0.4407352 0.4400623 0.42509 0.6793086 0.2415963 0.2574643
0.303487

SENCR 0.2350547 0.6957034 0.4530163 0.5773846 1.145165
1.654998 0.5534066 0.7914697 0.846729 1.486512769
0.1729423 0.1759231 1.745784 0.1679127 1.569326
1.003378 0.8037889 1.179865731 0.7877655 0.9084311
0.889406126 0.5016311 0.7119882 1.031138 0.7896164
0.228748 2.055244 0.787705 0.713791226 0.330176
0.9172992 0.7424627 0.5078075 1.163347 0.4647344
0.8097142 0.2329396 0.7740784 0.4155964 0.307076581
0.4429095 0.197343 0.466003558 0.08634793 0.6454536
0.181196 0.3386269 0.3368685 0.478677612 0.1609187
0.3359496 1.393979 0.4803246 0.4712424 1.571313
0.2063963 0.4108603 1.204794 0.078564 0.63732517
1.347027391 0.580056203 1.805949 0.8644624 0.2618555
0.7432969 1.14545 1.527218 0.314884272 0.2502094 0.3059105
0.5841041 0.7086467 0.7514597 0.7403489 0.9514227
0.6691604 0.2368916 0.1948468 0.350869989 0.4865509
1.381611 0.5395256 0.6099996 0.5187623 0.3359641
0.4376452 0.3596243 1.15034115 0.8206367 0.388306917
0.5749132 0.6884428 0.7653476 0.7987796 0.5488755
0.8679765 0.1650089 0.09393154 0.7034174 2.298303
0.1271266 0.7479127 0.7518733 1.201166 1.274943
0.678176 1.421238 0.366227952 0.2835227 0.1830198
0.4224067 1.178587 0.9624664 1.109958 0.2843755
0.7764799 0.1418824 0.3142143 0.234199 0.3243295
0.3732942 0.4744746 0.9766851 0.3335382 1.020539
0.3583225 0.5205527 1.192306 0.4869103 0.298262
0.06371612 0.5922282 0.3834737 1.301179 0.5563936

0.5216823	1.144914	0.5666467	0.08812079	0.396492	
0.1650024	0.4353705	0.4006872	0.4648432	0.3530267	
0.1598306	1.043078	0.07891552	0.8090933	0.372399	
0.9500651	0.1622831	0.7008178	0.8915859	0.07029197	
0.7000999	1.391615	0.5659959	0.1123465	1.518393567	
0.9274669	0.618110542	0.4480297	0.5239876	0.4264985	
0.4820944	0.2510713	0.6249909	0.2756009	0.8962903	
0.541203107	0.871779	0.1822397	0.3138315	0.195092	
0.4114559	0.4190728	0.2045907	0.5310395	0.270181	
0.3278336	0.664356	0.6673967	0.4810592	0.8021331	
0.7673391	0.227166	0.2123141	0.3483789	0.6527334	
0.1887225	0.2155513	0.5475794	0.7313869	0.352639	
1.458762	0.3113295	0.2167028	0.1307159	0.3616742	
0.7337412	0.3072522	0.1736103	0.1906022	0.312424	
1.105957	0.8379892	0.1615944	0.2625949	0.4581448	
1.035023	0.5194762	0.07407059	0.3544742	0.3525513	
0.4671613	0.4256262	1.020183	0.185670784	0.6580398	
0.660804389	0.4699495	0.2886566	1.141959	0.1269931	
0.2080265	0.182462	0.04096897	0.3691347	0.3157028	
1.015249	0.1313929	0.1854521	0.3889853	0.3290243	
1.064133	0.5505538	0.1769436	0.2057154	0.192813	
0.8075572	0.6382696	0.4438392	0.7005816	0.8922114	
0.950457	0.963411	0.4076919	0.4469405	0.6137711	
0.4420847	0.1250713	1.055177	0.2638586	0.355576	
0.2593215	0.719349735	0.2101749	1.069893	0.7076059	
0.2665136	0.5432122	0.404693	0.5020316	0.3575697	
0.07754355	1.193336	0.6320013	0.4744951	0.591853	
0.7909445	0.515533	0.347818925	0.1402424	0.2793053	
0.265598	0.7704661	1.176052	0.627274701	2.216477	
0.2522895	1.155698	0.8457932	0.25317	0.7381558	0.211786
0.09407613	0.7186443	0.9653349	0.2980479	0.638006	
1.004734	0.5914657	2.075372	0.9865745	0.8799303	
0.8054796	0.2359053	0.3343265	0.8333429	0.8766096	
0.1908978	0.7002774	0.09445982	0.4211333	0.254394693	
0.419091194	0.5895551	0.1730295	0.3926283	0.6195384	
0.3402136	2.704993	0.2881667	0.4942254	0.5392155	
0.2775324	0.696736	0.5358239	0.2833031	0.534521188	
0.1467465	0.7624301	0.7136438	0.5223327	0.330827	
0.132249255	0.4572965	0.1635217	0.2043142	0.5193449	
0.3648613	0.402811	1.205624	0.342616	0.02205499	
0.3228803	0.6830088	0.2100295	0.3758681	1.194446	
0.7055069	0.01250197	1.105488	0.1416777	0.275789707	
0.1466518	0.4453724	1.689433	0.3083275	0.5051505	
0.1813476	0.8268783	0.3560384	0.126993	0.8016795	

0.3548688 0.5090752 0.3290809 0.662413 1.898636
0.1232791 0.4244944 0.3903602 0.3652513 0.07342509
0.9089377 0.1883378 0.6395375 0.2988396 0.6140336
0.2448183 0.166339295 0.6063123 0.2642662 0.5108813
0.4169316 0.2798494 0.8111574 0.64060799 0.3204534
0.3204322 0.2896558 0.1871585 0.7272836 1.258389
0.6290814 0.2315559 0.2164018 0.4525422 0.1907426
0.3917731 0.2983409 0.3645927 0.787392732 0.2504925
0.6145394 0.3885906 0.293306592 0.4645037 1.571748
0.2157108 0.1875089 0.190969 0.8894221 0.3465083
LINC01619 0.0598378 0.0381457 0.06237992 0.1143214 0.07848733
0.09291957 0.01232705 0.09083864 0.1416482 0.139944412
0.04793929 0 0.2648941 0.04114254 0.0910495 0.008866152
0.1352768 0.051060872 0.1034371 0.09372077 0.06526104
0.08343071 0.04567515 0.100226 0.03278649 0.05957613
0.1452339 0.04731508 0.084797823 0.1176739 0.06763928
0.01072751 0.04235734 0.02512811 0.06820068 0.3323045
1.199626 0.04299423 0.0339436 0.162396844 0.1159728
0.09846559 0.170234606 0.007693548 0.1478816 0.1203496
0.3063563 0.05145392 0.394003709 0.0732819 0.06907593
0.7414525 0.2610594 0.119964 0.2255604 0.2059656
0.1230009 0.3516874 0.05200003 0.027040613 0.221922529
0.201290349 0.0516498 0.07002098 0.1043041 0.08650098
0.06531772 0.1330503 0.148295992 0.07802728 0.0306635
0.4828464 0.1775812 0.1190304 0.09312655 5.865445
0.1486673 0.09092203 0.1328474 0.007815576 0.09017089
0.1568717 0.1880126 0.08614057 0.05882725 0.1004244
0.1518711 0.1478876 0.57397033 0.1169892 0.038442124
0.2004434 0.07497091 0.3502207 0.1056083 0.09780896
0.2822771 0.2016301 0.2008618 0.1496091 0.2747014
0.06970403 0.3400865 0.009570208 0.05351159 0.2377255
0.1611335 0.07448908 0.10230162 0.01595476 0.09421797
0.1697632 0.1050114 0.3298276 0.4238426 0.1108524
0.01339043 0.07584982 0.07072751 0.04590737 0.0866927
0.1373328 0.1360165 0.05184293 1.483985 0.8357771
0.1204078 0.03451604 0.09976733 0.0627675 0.0833161
0.02270827 0.1623605 0.2090233 0.1070162 0.0966064
0.0531218 0.06442812 0.1273173 0.09421811 0.2010933
0.2856314 0.124132 0.1344037 0.146843 0.01935659
0.1501758 0.3240028 0.3199253 0.1870436 1.883917
0.1015803 0.05060766 0.4004245 0.03206743 0.1878892
0.1318859 0.1605993 0.01120664 0.333667 0.216460719
0.3085105 0.285565134 0.0471772 0.04422977 0.2755055
0.1718173 0.2162462 0.402028 0.7366761 0.1416055

0.064294493	0.3958232	0.02435617	0.09942113	0.1422211	
0.06560295	0.1091452	0.1573443	0.4846933	0.1885716	
0.02417361	0.09828392	0.2304255	0.1823918	0.1675838	
0.07769256	0.03238456	0.06908838	0.1241614	0.08552669	
0.04203766	0.04447588	0.1097752	0.1097535	0.202484	
0.06371314	0.1703984	0.09755666	0.05590418	0.03682852	
0.1405582	0.06648454	0.1335182	0.09812132	0.402087	
0.07210252	0.3308204	0.03048981	0.07487055	0.09184595	
0.1441599	0.1333008	0.1319929	0.570416	0.07329492	
0.0597675	0.05688456	0.1588097	0.070308407	0.223356	
0.513073369	0.041872217	0.04000753	0.9287749	0.1357801	
0.05766456	0.03715944	0.0243354	0.08371918	0.1035145	
0.08349973	0.02081249	0.06924205	0.07170695	0.1842712	
0.1723883	0.3586017	0.5360293	0.3910212	0.04481615	
0.2678245	0.1058036	0.05931871	0.1165199	0.1340888	
0.3719504	0.1716787	0.1394884	0.2076439	0.2044025	
0.130548	0.1485837	0.2892788	0.1567309	0.1072301	
0.03960924	0.095106672	0.1498117	0.03466431	0.2266046	
0.04469874	0.2496415	0.03296723	0.2683843	0.1335054	
0.07254538	0.06075745	0.1821824	0.189732	0.1818405	
0.04878877	0.2839536	0.03645936	0.09163375	0.047781	
0.166599	0.1117527	1.573921	0.166480246	0.2946289	
0.07586609	0.1006837	0.06249338	0.07276548	0.3791403	
0.135235	0.06705701	0.301734	0.08114222	0.1126614	
0.09508777	0.06898882	0.09179868	0.1039188	0.03380892	
0.4398119	0.1614774	0.1325815	0.06127877	0.1534507	
0.2508173	0.1632853	0.1880871	0.1211949	0.02001212	
0.224505429	0.099575393	0.210116	0.0643485	0.06841109	
0.04516404	0.04546924	0.2487878	0.2721601	0.05649797	
0.5066435	0.04363759	0.1302636	0.1869879	0.1381675	
0.300040744	0.03735722	0.4281778	0.03281818	0.2291177	
0.3647715	0.026185175	0.1792773	0.1019878	0.5178103	
0.1328316	0.3388809	0.2871218	0.07161356	0.2442151	
0.03144139	0.1546304	0.4668927	0.04544708	0	0.07544006
0.09892759	0.08911341	0.191027	0.1048713	0.122863481	
0.1229796	0.3174593	0.121775	0.08790973	0.1473009	
0.05782848	0.03802548	0.2270685	0.07920497	0.4155878	
0.2357022	0.1723615	0.2283945	0.3987169	0.08624216	
0.1358034	0.6633432	0.129186	0.149318	0.218071	
0.2429574	0.08110714	0.05882055	0.03834206	0.1458934	
1.062206	0.071139507	0.1098754	0.0346993	0.07060124	
0.3368117	0.07566304	0.2087905	0.086975672	0.08565662	
0.7042411	0.6432187	0.1334057	0.08640071	0.02734677	
0.1180016	0.03536826	0.09916082	0.1415358	0.1213932	

0.5096386 0.2416545 0.9095796 0.195435175 0.7963324
0.08831466 0.2385153 0.226489815 0.05518266 0.6290402
0.3016015 0.2369347 0.07562322 0.06984479 0.07599675
MYO16-AS1 0.02504934 0.02395287 0 0 0 0.0253683 0
0.03002126 0.07734371 0 0.08600734 0.1968519 0 0 0
0.03711555 0 0.018860415 0.06185841 0 0 0.02494702
0.2549409 0 0 0.1378252 0 0 0 0 0 0.02245379 0
0.2366808 0 0.8236748 0 0 0 0.04433653 0.04045718 0
0.026072096 0.01610339 0.0257943 0.09216004 0.08744119
0.02692459 0.433600171 0.1800625 0 0 0.1881133 0
1.172163 0.03079338 0 0.05135708 0.05023452 0.03395928
0.028439192 0.375773251 0 0 0.03447145 0.01697395 0.06835838
0.0253171 0.025167474 0.01749848 1.069698 0.03631068
0.0991189 0 0 0.4983277 0.0194485 0.06117082 0.66356 0
0 0.02736238 0.1073263 0 0.02638533 0.04850738 0.05154841
0 0.60068964 0.4520685 0 0 0 0.02114562 0.9514718 0
0.6313032 0 0 0.6347579 0 0.06565398 0.05771645
0.03004715 0 0.05668679 0.6865836 0.03118267 0 0.03339496
0 0 0.3956396 0.1104581 1.064576 0.0198879 0
0.1190711 0.03701 0.05241211 0.07776719 0.0269486 0
0.1860216 1.854049 0.02429678 0.06873437 0.1625527
0.02320259 0.06955352 0 0.3992584 0 0.1262041 0
0.03192745 0.5003524 0 0.1929742 0.03286804 0.05119164
3.085992 0 0 0.04728577 1.74216 0.03251725 0.342655
4.1282 0.02446885 1.458456 0.02362422 0.02269866 0.2240545
0 0 0.0223825 0.05932075 2.25184 0.5866556 0 0.1937233
0 0 0.03085916 0.07953953 0 0 0 0 0 0 0.2672577
0.07646996 0.05202468 0.03969113 0.07269552 0.03607138
0.04819578 0.4347914 0.1007744 0.07589679 0.02805251
0.02333731 0 0.03095032 0 0.3389212 0.02065845 0.2815395
0.06444584 0.02639675 0.3300557 0 0 1.337225 0.03200597
0.07465007 0.2041962 0 0 0.06157733 0 0.02044883
0.4265545 0.01942176 0.03018361 0.2885172 0 1.292872
0.128162 0.07987277 0 0 1.27407 0.3068277 0 0 0
0.051939801 0.09350144 0.052815523 0 0.2512197 0.02184294
0.9947063 0.07759151 0 0 0.3254325 0 0.1165157 0
0.03952658 0.3301981 0.07012703 0 0.33989 0.02828482
0.102306 0 0 0.2325308 4.097286 0.1045235 0 0
0.1754922 0.07299092 0.839472 0.2251766 0.3109402
0.1399504 0.2724707 0.01789386 0 0.02072656 0 0
0.02418533 0 0.1403386 0.3519049 0.02587643 0.05913221 0
0.02169214 0 0.02079964 0.02589969 0.13702 0 0.03496143
0.045787928 0.03487253 0.350037 0.04755121 0.04009884 0
0.059736065 0 0 0.1724248 0 0.2193202 0.04858652

0.01974844 0 0.6389043 0.1426648 0 0.02596024 0.02062868
0.02134935 0.1151538 0 4.202611 0.675978 0.8325206
0.1282628 0.1554137 0.7460335 0.02136081 0.06813794
0.06341837 0 1.030194077 0 0 0.4209002 0 1.386484 0
0 0.3224486 0.06260617 0 0.2374788 0.1022457 0.2748022
0.4004291 0.119622155 0 0.3619352 0.02575944 0.08991882
0.09254599 0 0 0 0 0.01367362 0 0 0 0.07188291 0
0 0 0 0.1401945 0 3.235401 0.02797854 0 0.2195066
0 0.1351397 0 0.5097754 0 0.231237 0.02136017 0
0.04193628 0 0.04660009 0.4331846 0.05696376 0.07752215
0.4941449 0 14.87299 0.0730761 0.09359991 1.370359 0
0.02991382 0.01756196 0 0.0802539 0.152685 0 0
0.02299805 0 0 0.06220428 0 0.0252127 0 0.21913
0.2091557 0 0.8757729 0.07233825 0 1.259647 1.505265
0.04612308 0 0.09147187 0.8767613 0 0.1323135
0.188799711 0.112117 0.04436441 0 1.166933199 0.1575041
0.02883167 2.153789 0 0 0 0
PSMD6-AS2 0.1064801 0.2602046 0.4461916 0.09606533 0.2405368
0.8866509 0.1462381 0.2694091 0.09132605 0.096350165
0.4722349 0.1162195 0.2009732 0.144206 0.1735936
0.1490062 0.2006018 0.236062364 0.05843303 0.1010752
0.082950456 0.4241805 0.09030886 0.4034106 0.206631
0.6184177 0.1498206 0.5613079 0.02286302 0 0.2841896
0.2068016 0.3940004 0.2235747 0.2552448 0.1296774
0.1415753 0.09563416 0.2974337 0.141349767 0.3439519
0.2607402 0.270912003 0.1521167 0.6517891 0.3961081
1.004957 0.2988454 0.674618393 0.1606421 0.08877495
0.9543877 0.3109691 0.3113155 0.3575511 0.2108895
0.07817053 0.9520716 0.0355896 0.168413695 0.4969911
0.153280324 0.3004741 0.04845589 0.4558765 0.332706
0.08071632 0.3108973 0.106982261 0.371914 0.1717788
0.1586375 0.4974106 0.1985738 0.285401 0.1604772
0.3169093 0.5248669 0.4029529 0.075333115 0.08228574
0.7495689 0.4435513 0.4501236 0.04361743 0.2405618
0.4625924 0.2193024 0.50663098 0.5404667 0.159616207
0.5614483 0.08590741 0.88388 0.1225615 0.2840382 0.2809703
0.3612882 0.1985719 0.4017376 0.4443867 0.4599699
0.1363011 0.1135332 0.2197448 0.5265532 0.1450736
0.1251878 0.109853877 0.1577286 0.3080911 0.6554783
0.1141956 0.4173662 0.3352084 0.4931487 0.1853288
0.4639702 0.1616927 0.2413603 0.1101913 0.4391218
0.239858 0.120808 0.2729427 1.0615 0.1352671 0.0255919
0.3561636 0.1095034 0.1959748 0.3232711 0.2407643
0.3457252 0 0.5730302 0.1856822 0.3821616 0.2018192

0.1319539 0.5278964 0.2740699 0.193279 0.4110709
0.1954196 0.1435194 0.2303747 0.4602334 0.2867355
0.2889237 0.2569509 0.1617913 0.1125691 0.4056579
0.6787075 0.3529195 0.2907174 0.504323 0.09970992
0.1187507 0.098080091 0.7058416 1.0284132 0.22064 0.06558828
0.2817565 0.2264779 0.4091323 0.9828698 0.2330488
0.4004397 0.190684482 0.5869659 0.07825509 0.282577
0.1405997 0.1373401 0.2470361 0.7341223 0.383334
0.2379852 0.1911841 0.09937167 0.782598 0.2380124
0.5262563 0.20277 0.2080998 1.083054 0.1636612 0.1319005
0.1932466 0.05396174 0.09043644 0.08137666 0.4659258
0.366583 0.7521744 0.2893334 0.1588922 0.09102162
0.2859903 0.1681827 0.2607726 0.05596308 0.7843038
0.1568171 0.1294568 0.1054977 0.2220509 0.5826259
0.2640745 0.6808691 0.274025 0.2781718 0.1190403
0.1329437 0.4405157 0.2912748 0.208520537 0.1711274
0.455254326 0.018627681 0.1779814 1.181262 0.05273405
0.537496 0.2066389 0.1948695 0.08276472 0.1067836
0.2820382 0.3935009 0.3127041 0.4466035 0.3146579
0.1619018 0.08528389 0.3406617 0.4167635 0.3544417
0.391229 0.3059473 0.1466061 0.3579165 0.1647533
0.3644268 0.4203566 0.4395503 0.4175833 0.7551118
0.80106 0.2644015 0.2359342 0.4479293 0.2457448 0.6118385
0.122637905 0.1610628 0.319845 0.2493139 0.05799825
0.3475282 0.07944148 0.279289 0.3059614 0.3432233
0.09009717 0.4224291 0.4831938 0.1779697 0.1366589
0.3715364 0.237888418 1.06648 0.6022624 0.5053284 0.2746183
0.1839267 0.19044519 0.4369048 0.3666724 0.08143844
0.212599 0.1165362 0.5622268 0.2005399 0.2287092
0.2341262 0.2117733 0.1861586 0.9441242 0.2289649
0.2823399 0.199425 0.1002704 0.1361106 0.2394547
0.2397622 0.1766921 0.477125 0.4893904 0.09080083
0.4612813 0.1198132 0.09397386 0.345723454 0.311727199
0.2129136 0.1669891 0.07377944 0.05209067 0.1292337
0.09223181 0.5939566 0.2759838 0.6217664 0.2157005
0.2354232 0.7079595 0.1891277 0.423742977 0.1495718
0.1343152 0.1764144 0.2972886 0.08742129 0.069893947
0.1611213 0.3942981 0.1379749 0.1097898 0.1752998
0.2797934 0.251008 0.1980487 0.227294 0.1173169
1.307781 0.1704658 0.5297251 0.1917771 0.09168727
0.3303655 0.7612981 0.7833278 0.484115849 0.1003015
0.203996 0.2407734 0.3802203 0.3276485 0.06557643
0.1903093 0.227781 0.2936324 1.006949 0.2216485
0.4708324 0.2608797 0.6768336 0.1278884 0.3376122

0.4314346	0.1399933	0.2214235	0.4462607	0.7417556
0.3566737	0.1799014	0.2590171	0.3677868	0.1687655
0.375085177 0.1031916	0.07350795	0.09918427	0.4260081
0.2907017	0.2917528	0.225708244 0.1176112	0.3175293
0.1825227	0.3956544	0.05124941	0.113547	0.3149721
0.09324011	0.1633839	0.9957253	0.4104316	0.6729214
0.5446908	0.588479	0.371552091 2.239966	0.2881158
0.1369138	0.094730174 0.07439119	0.8647153	0.052617
0.7387363	0.1738195	0.09560563	0.3897386
AL355922.1 0 0 0.1189672 0.04118283 0 0 0 0 0.06655691
0.054014153 0.03700612 0 0.2663017 0.08084226 0 0.09581759
0 0 0.1596938 0 0.050377428 0.08587103 0.2193853
0.1031575 0 0.06777331 0 0.102268 0 0 0.121831
0.0386445 0.4161463 0 0 0 2.332713 0.07744065
0.03334842 0.076306194 0.06962957 0 0.134615568 0 0
0.03172274 0.05016409 0 0.175589527 0 0.04976747 0.04067496
1.11002 0.1944699 0.4202859 0.05299752 0 0.1325836 0
0.058446239 0.39156633 0.176381294 0.5581856 0.5549318
0.05932772 0.6134799 0.411773 0.2178623 0.086629882
0.09034825 0.2209228 0.03124658 0 0.160797 0.402572
0.3118749 0 0.03509305 0.03262949 0.04223194 0.0749606
0.2354627 0.1847159 0.04433008 0.1362328 0.04174226
0.04435916 0.3729225 0.14768969 0.2431377 0.166179485 0
0.1473127 0.1455722 0.148868 0 0.2089454 0.04539669
0.4703266 0.3495877 0 0.07532999 0.8940055 0.1551396 0
0.06504122 0.06219248 0.322005 0.076247314 0 0 0.9000177
0.3026323 0.1425795 0.6107358 0.03422844 0.09647468
0.03415493 0.09554511 0 0.08922843 0.3246627 0.07945659
0.9604677 0.04144085 0.1672656 0.2365931 0.09325476 0
0.03990212 0 0.04908221 0.4386625 0.5647351 0.2891344 0
0.0410067 0.1392565 0.04744597 0 0.0293681 0.1361847 0
0.1815646 0.2034551 0 0.2238576 0.2211498 0 0
0.03984289 0.2032945 0 0.1713836 0.09451543 0 0.42374
0.05104753 0 0 0 0.0952603 0.484964409 0.1176576
0.1062214 0 0.2063167 0.3223447 0.05871305 0 0.07117898
0.248156575 0.04599688 0 0 0.1366223 0.08340931 0.1862439
0 0.09977409 0 0.08708237 0.1448409 0.1204953 0.2365357
0.3196057 0 0 0.03555459 0.03727298 0.07394379 0.04543061
0.04369602 0.1977257 0.07413245 0.07545754 8.124957
0.2997817 0.1317883 0 0 0.03532626 0.04226509 0.03519384
0 0.03342618 0.2597402 0.04965578 0.07322379 0 0
0.4582216 0.1200499 0.04754878 0.04137285 0 0.04613676 0
0.1806606 0 0 0.27269736 0.045251814 0.1544165 0
0.1048137 0 0.2007933 0.05259913 0 0.04863884 1.05279

0.05623077 0.06802794 0 0.0603467 0 0.1096826 0.1460403
0.1320567 0.3874669 0.2592011 0 0 0.04497304 0.2070163
0.0717806 0.04314773 0.1256223 0 0.271281 0 0 0
0.09238966 0 0.1783591 0.625635273 0.08095165 0.208123 0
0 0.1101186 0.3562808 0.1526559 0.1311645 0 0.1407028
0.1073928 0.1783006 0 0.05858513 0.06017101 0 0.06001801
0.08606251 0.2045972 0.1380257 0.09244324 0 0.1384387 0
0.1483775 0.1589114 0.09436624 0 0 0 0.4170889
0.1052297 0.069574 0.04467935 0.1065101 0.07348737
0.1321251 0.2192263 0.1239939 0 0 0.03679151 0.0534955
0.2377731 0.2573439 0.5863502 0.03638245 0.1081367
0.046658798 0.179353897 0 0 0.06721154 0.1626978 0.1228479
0.3920992 0 0.1077495 0.04720136 0 0.5279155 0.2149788
0.2871524 0.30881684 0.0403724 0.2669636 0.3546701
0.1547564 0.159278 0 0.5284013 0.07872818 0 0.1647327
0.0425852 0.132984 0.2110736 0.08247693 0 0.116589
0.08625241 0 0 0 0.08909354 0.04815297 0.1290282
0.1259286 0.07587432 0 0 0 0.1583419 0.1768776 0 0
0.2887008 0 0.1604039 0.1863851 0.6862696 0 0.3401828
0.2150837 0 0.1886534 0.1610918 0.2896381 0 0.05148373
0 0.03973005 0 0 0 0 0.1187437 0.1071427 0
0.535289 0.1115045 0.2169639 0.117494584 0 0 0.04290952
0 0 0.4137557 0.1275258 0 0.03969048 0.2134318 0
0.1508966 0.05223179 0.1138603 0.270780989 0.0771844
0.1527084 0 0.062761615 0.3795055 0.1488638 0.06391066 0
0 0.1741895 0
AC010761.3 0.2911547 0.3340921 0.8302273 0.19469 0.3055186
0.6486963 0 0.1744723 0.08989851 0.291827969 0.4248659 0
0.4496176 0.2183875 0.3797335 0.1294209 0.5227044
0.263063107 0.323548 0.1243691 0.136089727 0.1159861
0.296324 0.4528388 0.3788844 1.304467 0.1843484
0.6906675 0.112528209 0.1321315 0.4936719 0.6524647
1.854895 0.9169999 0.5214726 0.2553007 0.1211846
0.9413927 2.27471 0.36073409 2.116097 0.7443271
0.242433915 0.2620432 1.888825 0.8141112 2.066579
0.2816561 0.59292255 1.441783 0.201663 3.543613
0.7184173 0.2188923 1.305666 0.393711 0.08549847
1.432646 0.5838882 0.671019047 0.859444935 0.238238461
0.2899777 0 0.5208712 0.9272744 0.07945463 1.147641
0.819077727 2.135586 0.3730009 0.5064576 2.678594
0.8416064 0.9968833 0.5265624 1.085063 2.915114
2.181598 0.370777818 0.0506247 1.84463 0.4678051 1.077781
0 0.2819066 0.6590758 0.4317488 1.49613509 1.247945
0.28057358 1.527976 0.02487193 0.7619203 0.0670254

0.8625907 0.9877798 1.257006 0.7085713 0.2656061
0.9842409 1.017483 0.7379377 0.5587927 0.4206026
1.888802 0.04200174 0.07248877 0.231721548 0.1940789
1.340053 2.174152 0 2.054211 0.4124609 1.225158
0.4560797 0.9457287 1.333547 0.4264392 0.2410419
5.136976 0.804916 0.1621631 0.4477937 2.118056
0.3728277 0 0.6202858 0.2425313 0.9855262 0.563511
1.185004 0.7041125 0.1952671 2.375044 0.4154085
1.316658 0.7049387 1.451727 1.150359 1.042354
0.8153904 1.072923 1.566401 0.8947479 1.020481
1.219721 1.796158 0.5403742 0.5919743 0.4942623
0.6068138 1.128505 0.5319255 2.285546 0.5723462
2.757999 0.8179277 0.8767083 0.482734877 2.605531
1.667379912 2.595699 0.07173672 0.6009299 0.371563
0.7075111 3.291108 0.1792234 0.6489554 0.335185434
2.050226 0.3851593 0.5442261 0.6689427 0.8167927
1.090093 1.260431 4.514628 0.9663438 0.4998947
0.09781837 1.790285 0.878595 2.626125 0.6124104
0.3545428 2.401182 0.679653 0.24969 1.779532 0.3246115
0.1780456 0.5507194 1.68169 0.3162113 2.660873 0.9197013
0.06800368 0.2463968 1.025878 0.6279627 1.093336
0.3856182 0.8804013 0.4209978 0.503026 1.063212
1.284157 1.713108 0.6498666 1.59449 0.5780178 0.5308825
0.2292643 0.1557925 0.3229158 0.5185394 0.362225466
0.6792439 1.135692513 0.519534352 0.6048547 1.98031 0.1179767
1.1123 0.5424235 0.03552286 0.2327743 0.1642414 1.11729
0.5696323 0.5513126 0.6978135 1.467187 0.3355181
0.3950626 0.2958853 1.010758 1.918958 1.575465
0.386109 0.3607861 0.637824 0.5592343 0.9695416
1.631832 1.781622 1.141822 1.38716 0.985673 1.192897
1.847413 1.185513 0.1778702 1.806824 0.24144184
0.9840736 0.843336 1.653896 0.04077976 0.8180555
0.2706913 0.8935008 1.151566 0.5042662 0.2217221
0.9186854 2.679243 0.3450669 0.2373931 1.137823
0.505594149 2.553592 2.673629 2.127893 0.7923338
0.3745896 0.138865401 1.27776 0.348636 0 0.5902651
0.1699475 3.162506 1.492016 0.4894221 1.869339
0.5685354 1.691526 0.7241816 0.1198861 0.7692611
0.3123077 0.5428669 0.7536546 0.2619019 0.5192317
0.5714849 0.4696669 0.8671331 0.7448458 2.349553
0.6142728 0.7546455 2.07972942 0.403755934 0.6645392
0.3913781 0.3404352 0.2197561 0.7466888 0.07565842
1.049412 0.6064058 1.115711 2.059589 0.7130563
1.597047 1.241143 1.042798349 0.3271863 0.3004901 0

0.731604	0.4840582	0.573345136	0.3568562	0.797537
0.3247827	0.4926889	0.7477586	0.7184866	0.2850974
0.8076624	0.8605425	1.181077	6.320193	0.8780293
1.548039	1.022555	0.3610163	0.9756043	0.8278235
0.4819271	1.588495022	0.6058655	1.004037	1.777574
1.924853	0.4479541	0.7696512	0.6244484	0.6336665
1.486505	2.220742	0.6713349	1.78768	1.937277	1.416747
0.09683797	1.253544	1.698763	0.3263804	0.558878
1.050464	1.460322	1.306413	1.529408	0.5285928
1.45525	0.6921977	1.269201043	0.3475056	0.4341536	0.3796874
0.2892063	1.255077	1.230824	0.899300564	0.6483278
1.180804	1.043243	0.8187718	0.08408053	0.2395116
1.29187	0.3155029	0.6969299	1.129107	0.5316	2.038162
0.9876936	1.435385	0.841211803	3.049403	0.8766194
0.2807786	0.254316604	0.5492127	1.373982	0.1726484
2.660447	0.3311661	0.39213	0.6933371
AL683807.2	0.02651208	0	0.1219341	0	0	0	0	0.09532299	0
0	0	0.05208672	0	0.02485752	0.08644481	0.01964144	0
0.139732274	0	0.2831213	0	0.05280755	0	0.06343812	0
0.02083906	0.04196618	0	0.10246638	0	0	0.02376496
0.07677444	0	0	0	0	0.04762317	0	0.305015907	0.02140982
0	0	0	0.05460107	0.03901666	0.2467927	0	0	0	0
0.3752043	0.1706555	0.07972793	0.0516921	0.06518308
0.03892677	0.3261362	0.07975188	0	0	0.03615602	0	0	0
0.1437211	0.1085252	0.08038641	0.026637108	0.01852029
0.09057296	0	0.4458542	0	0.08252236	0.04794792	0.2058418
0	0.1404616	0.025971102	0	0.1158408	0.05679677	0.02726138
0	0.1026799	0.1909549	0.06552392	0.45411886	0.3887542	0
0	0.2038318	0.1566628	0	0.1354244	0.2312888	0.05583462
0.02224875	0.05374592	0.3319385	0	0	0.03180173	0.2462104
0.09999494	0.03824611	0	0	0.07069006	0	0	0.09305392
0.5553141	0.1877901	0.02104924	0	0.04200807	0.01958558	0
0	0.02852224	0.1465887	0.09844209	0.1019384	0.4114491
0.1454961	0	0	0.04907669	0.01909371	0.2112864	0.05395228
0	0	0	0	0	0.3209529	0.104362	0.1444825	0.1116648
0.1031224	0.05582782	0.02502349	0.06432182	0	0.06799951
0.1168252	0.1553861	0.04900385	0.02500374	0	0.2107894
0.07749806	0	0.02368951	0.2197466	4.841147	0.4435092
0.079921932	0.8787241	0.02711231	0	0.03266115	0.02104604
0.08458482	0.04955756	0.2527447	0.09791878	0.06565871	0
0.0565728	0	0	0.04200886	0	0.03817774	0.05101014
0.03067871	0.02666476	0.05355248	0	0.1976006	0.2424347
0.2293035	0.08261498	0.322841	0	0.02292152	0	0
0.02687146	0.08106274	0.04558875	0.1624127	0.1185622	0

0.05403001 0.03096153 0 0 0.02599149 0 0.1504876
0.02055588 0 0.06107299 0 0.09951772 0.06782298 0.1690737
0.1230438 0.02924076 0.992269 0.04639209 0 0 0.05554984
0 0.04948069 0.027949821 0 0 0.3930136 0 0.02737413 0
0 0 0.05982222 0 0.1037396 0 0.03177089 0.03711102
0.4200864 0 0 0.10828 0.0595695 0.1593992 0.03515846 0
0.02765678 0.05092298 0.5297094 0.07960284 0.1931329
0.1134245 0.1906605 0.1495897 0.5431166 0.1441907
0.05681627 0.06478632 0.04387374 0 0.07467347 0 0.09716201
0 0.1354379 0 0.2816334 0.04033068 0.02295883 0.02884237
0.04402843 0.2741208 0.265872 0 0 0 0.01845444
0.1058506 0.05032792 0.04244038 1.79075 0 0 0.03734852 0
0.02443118 0 0 0 0.04456599 0.02331768 0 0.04278547 0
0.02183327 0.1355761 0 0.04493872 0.2795897 0.1192419
0.1074555 0 0.03289778 0.1462218 0 0.360584 0.08949551
0.02216671 0 0.183826835 0.1629166 0 0.04133264 0
0.05036465 0.06889335 0 0.02208733 0.08708134 0.03866864
0.02705407 0.02644082 0.1412706 0.063303695 0 0.0547243 0
0 0 0 0.03610528 0 0 0.04341625 0.2095068 0.0545202
0 0 0.1305994 0 0.1856473 0.0355342 0.03709526 0
0.08218391 0 0 0.05162766 0 0 0 0 0 0.08157997 0
0.0315896 0 0.0751993 0.271267 0.1910334 0.2411604
0.1435857 1.534133 0 0 0.116015 0.2311531 0 0
0.1266425 0.01858748 0 0 0.1939211 0.1008487
0.630389503 0 0 0 0.7900397 0 0.1334249 0 0.04216835
0.2213691 0.1847145 0.06045085 0 0.145397 0.07842371 0
0 0.08750184 0.0322711 2.923071 0.2248445 0.1166999
0.06660817 0.0237328 0.04695503 0 0.038596103 0.1000209
0.2746374 0 0.04037601 0 0 0.04208944
AC005726.2 0.01917969 0.009170074 0.1146744 0.0183216 0.02515739
0.07769564 0 0.01149329 0.08883041 0 0.07408553 0.03768121
0.04442756 0.008991367 0 0.01420925 0.03825882 0.036102445
0.03552265 0 0.056030341 0.02865202 0.07320084 0.1262063
0.02627248 0.17337 0 0.02274872 0.074127494 0 0.02710036
0.04298085 0.1666233 0.2114245 0.1795662 0.1051114
0.05987238 0.1205825 0.2077068 0.118816047 0.6195423
0.1352614 0.059888378 0.06164995 0.2468761 0.162299
0.3347584 0.1443087 0.195292642 0.1685074 0.03321116
0.4342958 0.07201698 0.1441944 0.2430723 0.08252221 0
0.1966146 0.1827011 0.039002738 0.163314175 0.104625736
0.1050619 0.07855291 0.07918194 0.116969 0.0130851
0.1260006 0.038540291 0.2344681 0.06552341 0.01390112
0.4648447 0.3129704 0.2014853 0.03468708 0.1265757
0.4449521 0.2250035 0.093941676 0.1167208 0.1676059

0.1027216	0.2070785	0	0.03714096	0.1578776	0.05925265
0.06570485	0.1406187	0.120137347	0.2141591	0.01638428	
0.1133349	0.06622906	0.1371585	0.03718262	0.2019629	
0.209241	0.2332892	0.3722093	0.1256743	0.04419213	
0.0230064	0.07916291	0.1880829	0.1106739	0.07162752	
0.152645614	0.03835461	0.1800355	0.5852057	0.03365911	
0.3488727	0.1698168	0.1294354	0.04292009	0.2507175	
0.2408704	0.1705556	0.1885576	0.6396513	0.1502333	
0.0712162	0.2488913	0.3069573	0.2192846	0.0207438	
0.222071	0.106511	0.2071951	0.04367183	0.1756387	
0.2222525	0	0.3177993	0.1094595	0.1652082	0.1688638
0.3774943	0.2155793	0.1918571	0.2163461	0.05048456	
0.2534393	0.03877707	0.2987724	0.0983861	0.2704484	
0.06557329	0.2481568	0.1085311	0.1390386	0.1238996	
0.1331534	0.2107831	0.06855104	0.07948589	0.2694032	
0.1604244	0.043363583	0.1907092	0.284401871	0.2181003	
0.05907034	0.1065777	0.09178709	0.2151092	0.3918079	
0.2007066	0.08708267	0.121441112	0.4297292	0.03903417	
0.1394193	0.07597642	0.1298763	0.01380951	0.2490911	
0.3994915	0.07716061	0.08716859	0	0.1965571	0.06138478
0.2014325	0.216653	0.0519008	0.2609918	0.1575306	
0.04934467	0.2829592	0.08747857	0.1466085	0.03298038	
0.2517742	0.1225311	0.3715257	0.1856635	0.06719572	
0.03688929	0.1964513	0.103417	0.1878863	0.1270122	
0.1635787	0.03466632	0.121501	0.4316337	0.647949	
0.4293217	0.1732773	0.2848444	0.2115371	0.1840614	
0.09229423	0.06157659	0.09116549	0.110513	0.238614532	
0.1968776	0.323516868	0.050329575	0.1854833	0.3595796	
0.023315	0.1287217	0.2233245	0.03510081	0.1437554	
0.08655466	0.1895784	0.07504859	0.2194182	0.2183488	
0.1342366	0.08288293	0.08945951	0.2923699	0.1174999	
0.3160263	0.2498482	0.08902169	0.04753328	0.1300507	
0.1565672	0.2075715	0.2015554	0.2235497	0.06154124	
0.4224105	0.2597232	0.1071568	0.1912391	0.3356723	
0.1171713	0.2221778	0.069583857	0.135053	0.06481348	
0.2108704	0.0268635	0.2204553	0.07925191	0.2376998	
0.33553	0.1827008	0.041731	0.06370318	0.555262	0.09617022
0.1303181	0.1606149	0.122705601	0.2002581	0.4307386	
0.2912708	0.122811	0.1130981	0.045738505	0.1026487	
0.08105737	0.0110018	0.1237201	0.1189492	0.1860078	
0.2494951	0.1773226	0.2277283	0.1950627	0.5339288	
0.09938575	0.06317957	0.2370271	0.0587804	0.09753037	
0.1471013	0.1940927	0.3031734	0.1309438	0.08329762	
0.2115631	0.155377	0.2434676	0.03237197	0.04009029	

0.342503294 0.199479417 0.1852072 0.1740276 0 0
0.009108855 0.07475951 0.4690936 0.09587215 0.1784928
0.2447737 0.1076448 0.3156144 0.2810492 0.274775644
0.1077663 0.05938399 0 0.1721218 0.08857537 0.088127373
0.287317 0.2977121 0.009724965 0.2617393 0.1420912
0.1479063 0 0.4311393 0.1228237 0.1210268 0.326165
0.1735195 0.09392568 0.1424932 0.1585453 0.2035138
0.1004547 0.2427693 0.177215366 0.3325929 0.1017546
0.1301077 0.2289425 0.1672165 0.1553724 0.1256911
0.08027407 0.4216124 0.2854448 0.2625795 0.2835028
0.2522668 0.3279078 0.1435311 0.0480097 0.3217278
0.2508354 0.1196515 0.1677549 0.217591 0.275659
0.1502399 0.1433799 0.05845365 0.1732733 0.361034956
0.07924081 0.1310819 0.09826044 0.09525671 0.1984265
0.5115765 0.034847679 0.2211681 0.3317302 0.1527183
0.2842586 0.02769385 0.06574054 0.226937 0.0850239
0.2825229 0.2294685 0.1284028 0.4699209 0.5112164
0.2448309 0.144559514 0.2832897 0.2462737 0.03699234
0.041882503 0.1447167 0.3090603 0.1848136 0.1022326
0.05453857 0.0387471 0.1522443
AC103563.2 0.02433604 0.09308317 0 0.1162363 0 0.1971673
0.07520119 0.08749916 0 0 9.421189 9.179826 0
9.537626 0.3173987 0.2343813 0.1941779 0.256526921
0.03004848 0 0.05687502 0.0727099 4.644023 0.1164626
1.050074 14.8247 0.1540869 0.1347014 0.047028121 0
0.1375445 0 0.04698201 2.069471 0 0 0 0.0218572 0
0 0 0 0 0 0.4260164 0 0.05663416 0.0261579 0
0.1360606 0 0.1377634 0.05221619 0 0.4270443 0 0
0.2494733 0 0 0 0.03318844 0.04847534 0 0 0.01649061
0 0 0.024450813 0 0.02078475 0.01763836 0.1685187 0 0
0 0.05668408 0 0 0.023839471 0.1269433 0 0.1303376
0.1251192 0 0 0.0751208 0.06014591 0.25010767 0 0
0.05434691 0 0.02054348 0.02801145 0 0 0.410015 0 0
0.06093879 0.02126148 0 0 0 0 0 0.06058944
0.021520389 0 0 0 0 0.2682819 1.034261 0.01932158 0
0 0 0 0.05036848 0.05236244 0.04485238 0.1355434
0.02339291 0.2360491 0 0 0 0.02252431 0 0 0.6438125
0.02452206 0 0 0.02314783 0 0.02678273 0 0.01657797
0.1281246 0.09465843 0 0.04593928 0 0 0 0 0 0 0
0.0220523 0 0.1244902 0 0.02174515 0 0.2050985 0 0
0.0806601 0.398192188 0.02213882 0 0.03863729 0 0
0.09942863 0 0.1406292 0 0 0.4457545 0.02527162 0 0
0 0 0 0 0 28.69814 0.02267276 0.02225364 0
0.1327098 0 0 0 0 0.02564508 0 0.1860234 0

0.02129748 0 0 0.04959539 0 0 0 0 0 0 0.07547485
0 0 0.02066702 0.0228374 0 0 0.02258895 0 0.02335452
0 0 0.01927913 0.02549524 0 0 0 0 0 0.424419 0
0.02512734 5.667281 0 0 0 0 0 0.01920051 0
0.03406505 0.1051655 0.1031911 0.02747939 0.09939273
0.09113368 0 0 0 0.02538679 0.04674337 0 0.07306927
0.07091245 0 0 0 0.181287 0.08823731 0 0 0 0 0
0.1174832 0.04459362 0 1.149974 0 0 0 0 0
0.02020735 0.1006487 0 0 0.1358635 0 0 0 0.02309858
0 0 0 0.05209809 0 0 0.02242594 0 0 0.01918609 0
0 0 0 0 0.08016504 0.06222423 0.03729159 0 0 0 0
0 0 0.02684406 0 0 0.04107499 0 0.105353533 0 0 0
0 0.03061371 0.02311544 0 0.06265333 0.06082341 0 0 0
0.02427064 0.03241888 0 0 0 0 0.08735831 0 0 0
0.0222206 0 0 0.04807776 0.07506801 0.3574462 0.6052453
0 0 0.02434427 0 0 0.06574604 0 0 0 18.12676 0
0.01875593 0 0 0 0 0 0 0 0 0.06790968 0.1402831
0.3320501 0 0.2560393 0 0 0 0 0 0 0 0.136495
0 0 1.572374 0 0 0 0 0.06800574 0.06043296 0 0
0 0 0 0 0 0 0 0 0 0 0.04015997 0 0
0.02948427 0.6641532 0 0 0.02155055 0.7040628 0
0.336642 0.1400533 0.03607687 0 0 0.06555205 0
LINC00893 0.1693124 0.219723 0.4301392 0.2426062 0.1533421
0.3429366 0.1245702 0.2077498 0.1555888 0.282839927
0.3010495 0.3326381 0.2925883 0.1852037 0.3154612
0.2598294 0.225158 0.376370995 0.1493253 0.09470895
0.127187631 0.3452711 0.1794985 0.5980503 0.3423659
0.53867 0.08933533 0.4335133 0.148013312 0.08232553 0.1025287
0.2782425 0.957252 0.1777514 1.142099 0.609757
0.04194722 0.1448252 0.5020492 0.128433233 0.7454943
0.2523132 0.062937634 0.414649 0.5396476 0.4241817
0.6566986 0.3466429 0.336587193 0.4217891 0.04188256
0.973671 0.09514532 0.8910326 0.4637373 0.2874277
0.822733 0.3553956 0.476977 0.17488464 0.201378522
0.159431593 0.1124187 0.08491112 0.1386892 0.4152119
0.1210118 0.1629736 0.105306747 0.9011428 0.05853064
0.1723851 0.4426522 0.5600791 0.3105579 0.1312315
0.2441316 1.283048 2.102203 0.075030831 0.3154211
0.4007177 0.1079517 0.8995045 0.02123125 0.1405153
0.3359801 0.15941 0.29001109 0.6593189 0.163159298 0.3195897
0.103311 0.4321826 0.2366439 0.2553373 0.1094122
0.1655518 0.3552145 0.2778563 0.7318488 0.274712
0.2229226 0.2466134 0.3494129 0.3223366 0.08723169
0.08531108 0.139028642 0.3493313 0.0830048 0.9678187

0.02122373	0.6399461	0.3997574	0.5729084	0.1127635	
0.3768604	0.1995295	0.253044	0.2795073	0.5724708	
1.315068	0.09729488	0.4068769	0.8133083	0.4424632	
0.008719993	0.5526389	0.1007407	0.3454881	0.7480951	
0.6562897	0.259972	0.1261687	0.4367434	0.3297603	
0.1562579	0.2528828	0.1692649	0.3103126	0.4456988	
0.2916499	0.5687498	0.3652708	0.6063813	0.9210214	
0.4894053	0.8135755	0.4489124	0.7898267	0.1254627	
0.1242002	0.488781	0.5774078	0.6455588	0.3421964	
1.050129	1.17778	0.6204206	0.121523915	0.9130213	
0.247350932	1.030506	0.01489871	0.2592097	0.2443666	
0.2637386	0.6643013	0.4516291	0.475886	0.11602215	
0.6279518	0.15178	0.2260562	0.2523096	0.2417805	0.4760142
0.4576192	0.3731839	0.340575	0.2442849	0.1896113	
0.3943513	0.283845	0.5678233	0.3737159	0.4036209	
0.495367	0.4356615	0.3802853	0.3228548	0.1021473	
0.1047698	0.173298	0.6808871	0.09013889	0.2642993	
0.1971706	0.6779235	0.1302589	0.7068977	0.2568859	
0.8819552	0.1067833	0.3656939	0.3448837	0.5432514	
0.3115363	0.6241948	0.4898535	0.4713173	0.4265701	
0.2578769	0.4410273	0.1869328	0.0862824	0.1469044	
0.3463079	0.325992869	0.4965643	0.577981197	0.059239195	
0.5169183	0.8682631	0.8232705	0.1664931	0.4412271	
0.03442879	0.3464649	0.1819233	0.567222	0.289189	
0.3975689	0.9806662	0.953642	0.1451716	0.1196547	
0.4096748	0.8191007	0.5585605	0.3675971	0.3314495	
0.1898232	0.3364243	0.5962115	0.4765523	0.5487089	
0.3641445	0.4052945	0.4493535	1.13728	1.2988	0.7308224
0.1843008	0.2610507	0.296866	0.117002823	0.2157326	
0.214071	0.4469075	0.04517003	0.3089065	0.09578013	
0.3616188	0.3740771	0.3909882	0.08332595	0.6092147	
1.421321	0.1764075	0.208169	0.4557403	0.331594037	
0.7183509	0.933506	0.5165454	0.3646057	0.7217832	
0.04326058	0.4746506	0.394688	0.1017453	0.3603396	
0.1500067	1.430903	0.2892127	0.786063	0.2694602	
0.1869549	0.2829967	0.3760058	0.1892297	0.8074129	
0.1853197	0.2049924	0.4908266	0.1903767	0.2516199	
0.1926552	0.2651174	1.071655	0.3609526	0.424003	
0.1769051	0.1954902	0.126525092	0.329827371	0.5768337	
0.224885	0.1005562	0.07099594	1.164033	0.05761502	
1.193525	0.2720346	0.4369531	0.593849	0.3126381	
0.4824496	0.2040663	0.389834133	0.5247403	0.2413093	
0.05803737	0.3762419	0.2085109	0.042338028	0.2415568	
0.3901676	0.4169808	0.321277	0.2628135	0.4103538	

0.1842111 0.7172335 0.4726219 0.3452273 1.00412 0.1512865
0.8855515 1.04007 0.299912 0.3016776 0.4343426 0.7732402
0.365382007 0.1957351 0.5025969 0.8409032 0.2294947
0.2108765 0.09968872 0.144099 0.485921 0.5316949
0.472466 0.6158017 0.7242174 0.6799321 0.3870162
0.1340792 0.754794 0.2116855 0.5573402 0.429463
0.2424074 0.288846 0.7009191 0.4272303 2.02342 0.5209252
0.3718584 0.599079924 0.1517463 0.04007448 0.3492182
0.1601708 0.7194268 1.083363 0.380868496 0.8655977
0.4712386 0.6620378 0.7384038 0.0873117 0.1326485
0.798947 0.2025334 0.4787637 0.3292973 0.2306255
0.8748151 0.4249122 0.4436149 0.26332775 0.3175611
0.4747878 0.1710537 0.058686638 0.395421 1.424464
0.3107576 0.644627 0.1910514 0.1303038 0.236794
AC008764.2 2.627516 5.579234 2.55906 1.365715 2.179359
7.23941 3.343253 1.759757 1.193068 2.03328809 2.985095
1.518272 1.90944 0.9781698 1.385876 4.980985 2.158164
2.298362306 0.4770995 0.8252685 1.038498532 5.272048
2.507031 6.055978 5.398784 2.976438 1.957231
7.516152 1.941410888 0.6137436 2.948245 6.546234
1.417332 1.825464 0.9751764 1.524675 0.9247576
0.6940824 1.703696 0.957480376 1.965832 0.5109399
0.402175943 2.111429 1.949665 1.307888 4.091442
1.578239 2.006553985 0.277756 1.739611 4.84865 1.699595
2.207785 4.821663 2.470023 3.006886 6.100019
2.402172 1.361983905 2.193448355 1.89703711 0.4233212
1.039961 0.3190443 4.555881 1.687143 3.827191
1.475242387 1.187663 0.8910356 0.7001395 4.051749
0.8647116 2.495643 2.935025 2.880034 4.749415
4.357512 0.264960499 2.553046 4.811704 0.6622262
3.655345 4.76199 1.084966 6.440809 2.291943 1.32370817
5.186464 0.930892395 3.322172 1.254313 2.381132
0.845037 2.526385 1.797819 1.057888 1.686171
2.898277 11.41727 0.8777146 1.157399 2.78096 4.266192
5.159101 0.7246417 2.789854 0.922572225 2.524161
1.123214 2.73025 0.4068634 6.858098 2.3264 1.012379
0.691744 1.775511 1.741242 0.3638181 0.3998668
1.413368 1.068226 0.4304224 3.342822 3.597991
2.014502 1.002985 4.688652 1.359007 2.254073
3.387325 1.022224 0.9344472 0 4.875729 4.116374
0.4160412 1.913612 1.216817 1.105522 1.952946
1.533013 0.6916116 1.495287 6.374703 0.4012767
5.682059 1.191866 2.302415 3.463894 2.004289
1.08543 3.724973 5.110956 1.941255 1.622732 10.29436

0.6512967 1.292782 1.688988494 2.988285 2.2918555
1.12484 0.5236208 3.128696 1.232779 1.408436 2.68378
1.664966 4.561418 2.090719147 6.802278 1.061631
0.4413796 2.387802 3.40148 1.557977 3.568539 2.41448
1.243599 1.404898 0.9519947 2.62793 0.4946699 5.012964
4.966786 1.672973 4.556945 1.169241 1.259208
0.8143678 0.5091284 1.772169 0.4651023 4.734158
0.5924509 3.339399 0.9449505 0.7219966 0.8323637
2.501306 1.515248 1.38791 1.535294 1.497955 0.6983973
2.225266 1.082875 2.864575 2.12524 5.831845 2.510617
4.048601 1.297853 1.217052 0.4962156 0.9489333
3.602762 2.44365713 3.389427 2.158974977 0.73004655
1.577759 6.401842 1.878842 1.19689 0.8998316 1.037154
2.162449 1.91813 4.31356 0.9575681 0.9755496 1.15761
3.894288 1.89242 1.769507 1.178033 8.008979 2.054725
2.710352 2.869529 2.872856 1.209248 2.263635
4.117458 8.971974 3.265179 1.653102 2.639836
1.831359 1.942929 6.93496 1.021282 1.274705 3.197182
1.642173739 1.015771 2.536886 1.84091 0.4329596 0.7402255
2.554609 2.325972 3.115331 0.9703771 1.849594
2.149665 4.59444 4.368118 0.8401358 1.402177
0.776933451 1.291026 2.352642 3.997589 5.907114
1.988513 0.829314515 6.328047 0.8165021 1.684505
1.602322 1.888914 3.972207 1.066205 2.013529
2.752726 4.086978 1.839548 4.845452 1.90925 1.97595
1.776309 4.060739 1.518816 2.346147 1.91065 0.7914088
1.678137 1.875372 2.405363 3.994043 0.5217398
0.9692041 0.669107015 0.803753501 2.374423 1.246581
0.6927615 0.9235406 1.321268 1.706943 2.238274
6.856707 1.099942 1.859797 2.010923 1.734124
0.9779984 1.891366261 0.9769905 1.993944 0.635765
1.52575 1.284814 0.862355813 1.36816 1.728775 1.293084
4.745766 5.305368 2.70165 2.963825 3.43738 1.522733
2.647232 2.125915 1.191152 2.162575 0.8873093
1.836606 0.906326 8.557729 3.085027 1.462094101
4.437225 0.5637435 1.441652 1.206304 2.457235
3.426717 0.8287228 1.358468 1.424787 4.38487 0.6125261
3.778394 1.19582 2.032653 2.313294 1.934433 0.7890664
2.887657 1.66883 2.027782 1.615028 2.465214 0.8902278
1.361755 2.873401 2.351705 0.765632641 1.596407
1.056325 1.367711 1.631208 1.165954 3.461382
1.474307042 1.32135 1.75144 2.038317 1.908918 0.6695136
2.648854 2.057371 2.283887 0.9960623 2.965056
1.269902 2.231544 2.013007 2.41519 1.45616798 2.9055

5.166802	2.086722	0.731272966	0.1943668	9.784364
1.145633	3.29537	1.941123	2.810195	3.189842
MCCC1-AS1	0.03798331	0.09080172	0.1746926	0.1269939	0.099643
0.1346344	0	0.2276122	0.05863968	0.07138341	0.8151022
0.2238707	0.205296	0.1958708	0	0.267329	0
0.128694598	0	0.2433733	0.02219241	0.1134845	0.3140938
0.4089893	0.1690969	0.4627633	0.1202482	0.3003425	0	0
0.05366934	0.03404757	0.5316337	0.05981477	0.6184547
0.5412207	0	0.3070294	0.2497426	0.352953551	2.453874
0.2678705	0.217437869	0.3174368	0.762702	0.8105265
0.9060355	0.2857878	0.812187634	0.318542	0.08769483
1.218441	0.6316111	0.3712299	0.5554367	0.1167331
0.1673088	0.5256548	0.0761726	0.077240716	0.30186437
0.051799997	0.1891489	0.06667096	0.1568113	0.3603359
0.05182727	0.1535573	0.07632489	0.4112715	0.1784228
0.1927077	0.54483	0.4427173	0.4285769	0.03434701	0.2064338
0.5101567	0.5605874	0.297666174	0.1320874	0.4149067
0.6306298	0.2929262	0.1600365	0.4229336	0.2931184
0.3989679	0.84578896	0.6212249	0.201316128	0.5937666
0.01622365	0.4649272	0.1967393	0.3686375	0.1840904
0.4199639	0.4143793	0.5005045	0.6420081	0.4811765
0.04375888	0.1139042	0.3135472	0.3724779	0.1369861
0.1654923	0.067177372	0.1772334	0.7130818	0.8234551	0
0.3140476	1.008912	0.6483716	0.08499861	0.2858745
1.473143	0.1192119	0.2554966	0.4699272	0.5775404
0.211554	0.3286015	1.086844	0.2605617	0.02054043
0.08795754	0.07031118	0.3556172	0.4756804	0.5797226
0.4784207	0	0.6293673	0.2167726	0.2453827	0.2508124
0.5980695	0.6209914	0.4399448	0.5170949	0.5598837
0.2330292	0.3532515	0.6163401	0.1623692	1.690468
0.3153763	0.1228619	0.1791118	0.03441888	0.2453698
0.4302408	1.0734	0.4921225	1.933934	0.6935813	0.3177033
0.114502533	0.7343759	0.796286779	0.5528628	0	0.1356851
0.09088721	0.656751	1.888104	0.2104294	0.1567798
0.174909837	0.6686683	0.2512346	0.177496	0.240741
0.2204622	0.4649201	0.676001	0.571386	0.4011217
0.517884	0.1914171	0.6900522	0.3125981	0.6335713
0.2515167	0.2055679	0.3289149	0.2627136	0.1954436
0.9806479	0.1154946	0.02903424	0.09797112	0.5983339
0.3518564	0.4905111	0.2322231	0.1996108	0.3652763
0.3423646	0.2792811	0.4806144	0.03593342	1.32525	0.4805704
0.2187451	0.3225675	0.4990196	0.5830117	0.06055713
0.3878212	0.3770338	0.8383816	0.1827788	0.1219458
0.7673095	0.2785479	0.157516782	0.2835599	0.880949119

0.019934459 0.5305872 0.8280332 0.3539918 0.4510112
0.2653621 0.185369 0.1518358 0.1499856 0.3091855
0.2724803 0.224759 0.5689689 0.6380184 0.02735678
0.161059 1.308127 0.3102612 0.7680961 0.342552
0.2266687 0.1412018 0.2575515 0.2735862 0.09486299
0.5322117 0.5810649 0.988548 0.478022 0.9001192
2.369705 0.06885971 0.4883971 0.3480677 0.3299993
0.275606649 0.2852884 0.3850683 0.1566023 0.02660013
0.5336074 0.2746622 0.2689936 0.1444524 0.9703334
0.04132188 0.3942415 0.3338183 0.4155376 0.1032324
0.5036274 0.20829018 0.5684451 0.5876438 0.5948563
0.3496199 0.2647019 0.203805397 0.4878833 0.2942965 0
0.2450144 0.1108546 0.3683684 0.4491801 1.021581
0.2672541 0.2781364 0.2911649 0.1771405 0.2658807
0.372288 0.1746124 0.06438279 0.4005626 0.3843796
0.8005363 0.1782825 0.2120939 0.5656202 0.6801952
0.5510414 0.2724641 0.5875193 0.596073766 0.684748952
0.7002219 0.5361113 0 0.09556279 0.2345084 0.07402658
0.8067538 0.2056865 0.3326924 0.6093975 0.05813972
0.4356342 0.3288927 0.340102207 0.2134196 0.196006
0.1953004 0.1363475 0.2104968 0.349053518 0.1551819
0.8150168 0.1925924 0.06220158 0.1688378 0.4100774
0.03099425 0.4178292 0.5613212 0.4108811 2.203777
0.3690911 0.983194 0.5643846 0.1962387 0.8272869
0.539979 1.201946 0.334243734 0.1756438 0.7556776 0
0.3487661 0.2337559 0.09716785 0.5204644 0.2543586
0.9157604 0.8302735 0.5200103 0.4318821 0.8081558
0.7992443 0.1263324 0.4183434 0.9695718 0.2602037
0.1458197 0.5190946 0.5443143 1.34481 0.17502 0.9735375
0.0694567 0.1986653 0.809068532 0.1569279 0.1179971
0.7076153 0.5187755 0.1801077 0.860198 0.241542243
0.4228961 0.5436876 0.3591499 0.7938948 0.1645341 0
0.1123561 0.1683807 0.122392 1.17527 0.6935122 1.296231
1.403567 0.3845451 1.001995849 0.9690426 0.4877186
0.07325935 0.248831344 0.4776595 0.4809047 0.2815411
0.8243039 0.07200517 0.0511563 0.1959772
AC012073.1 0.3933741 0.1504621 0.8684176 0.2755682 0.3439842
0.1327943 0.3241524 0.3300171 0.4858414 0.377855325
0.8666714 0.4637034 0.3037353 0.4302953 0.4275429 0
0.836998 0.276437855 0.129523 0.3080605 0.214513274
0.1175302 0.2335424 0.2196287 0.1257309 0.8348412
0.2075584 0.09331491 0.126695795 0.05950686 0.4446611
0.04701515 1.46823 0.825962 0.9180536 1.437218 0.08186522
0.9892554 1.369302 1.404123492 3.007269 2.936029

1.132771374 1.416168 0.9586726 1.553412 2.761605
1.804045 1.054765335 1.047295 1.120127 1.175279
5.387764 1.695586 0.588021 1.885956 0.6353354
0.6452081 1.774983 0.906602449 0.684799328 1.108697581
0.3395461 1.24286 1.082677 0.5331169 1.019823 0.5566106
0.592843939 3.746381 0.5711492 1.273496 1.984614
0.9903601 1.714201 0.6402865 0.9366175 2.380215
0.9031124 0.667935492 1.162769 0.7877798 0.716316
1.132577 0.7320261 2.14562 0.9444338 1.263878 1.52728206
1.552966 0.593889185 2.591509 0.3136378 1.272936
0.8301047 1.352972 0.7244823 1.408363 2.916032
1.927189 0.9357789 0.5613376 1.359566 1.352664
1.204189 1.40455 1.153873 0.8161529 0.962415898 1.363527
1.079961 2.095178 0.8974496 0.9395919 1.393172
0.6142274 1.672547 3.521626 2.40231 1.070002 0.7056133
2.567414 4.386284 0.2190959 3.642643 1.437194
0.9114954 0.9501802 0.9595152 0.7281776 1.322084
2.851331 0.5603636 0.9777398 0.2345085 0.8189334
1.49667 1.55302 2.496523 1.840966 1.321989 3.078946
1.040457 5.039115 4.220298 0.5408142 1.821318
0.7735252 1.779623 0.486727 1.47843 0.865653 1.817941
1.016469 0.5845228 1.029321 1.42941 1.024728 1.068252
0.7457999 0.494102199 0.7967733 1.005700316 2.409575
0.9853764 1.176223 1.066776 0.5759948 2.125063
1.662734 0.3896856 1.147253828 1.706783 2.068186
1.252724 1.267394 0.6215424 2.03927 1.652489 2.898086
1.806754 1.138908 0.3964822 1.832442 1.127103
1.863165 0.5822567 0.887068 2.703497 1.190347
0.6522136 1.506139 0.6645108 0.501155 1.53323 2.37538
0.4858667 5.601001 1.015452 0.8422207 1.361874
1.192644 1.324065 1.969584 2.257676 1.931659
0.7268038 2.643005 0.8017609 1.476599 0.7044286
1.644552 1.545732 2.096995 1.4597 1.181658 0.5893679
4.736821 0.9203826 1.957586726 0.6362824 1.755591804
0.977202302 0.5541992 1.063365 1.72148 2.5453 0.7328592
1.167862 2.358731 0.458601 0.9453757 1.419522
0.9414317 0.8799504 0.7525364 0.4533128 0.6338428
1.49542 3.655038 1.532033 1.195686 1.095497 1.299872
0.5745022 0.4659361 1.8994 0.4855677 0.8023731 1.963432
4.007656 1.21335 1.440765 2.068122 3.315855 1.538032
2.886005 0.788335936 1.661956 2.01297 1.321509 0.7162589
0.9043044 0.7449983 1.036948 3.33114 1.907655 1.241055
1.371875 1.545566 1.518184 1.104763 0.7686466
0.503336604 1.652039 2.264229 1.891748 0.3358459

0.9278531 0.375237374 1.403545 1.662481 0.4362494
1.06333 0.8514822 1.704036 2.232928 1.520875 1.187855
1.258894 4.898782 0.1766609 0.2591617 1.274024
1.085024 1.311335 1.118818 1.739769 0.7334127
2.696837 0.7972664 0.9401478 0.6373541 1.426715
0.5311574 1.688351 1.461708962 1.127382769 1.32375 1.313148
1.114116 1.055676 1.656488 0.5622145 1.822941
1.005014 0.8757385 1.357869 1.498619 0.7584793
1.816628 2.45776026 1.10514 2.111133 0.1213577 1.270874
1.392784 1.84191437 1.249999 1.700117 0.8643194
1.152385 0.8548556 0.6471567 0.4493886 1.27936 1.511464
0.8037768 2.085588 0.4130048 0.6421363 3.719576
0.8400363 3.925076 0.5101739 1.161809 2.953894086
1.091433 1.426103 1.890187 1.228078 1.667726
0.525522 1.343641 1.580559 0.7810411 1.805123
1.738473 0.41746 1.237696 1.414051 0.7632108 1.549223
3.347123 1.584215 0.9690329 1.433601 1.691157
2.620029 2.114693 3.598871 0.7193281 2.069946
1.0392703 1.408527 1.824908 0.6717715 1.237349
1.051343 2.507617 1.870192825 1.313917 1.345112
1.500865 1.584598 1.135998 0.5752891 0.3878718
1.976345 1.847005 1.731083 0.7661182 3.143838
1.334456 2.135565 0.428264085 3.063587 0.9289299
0.1517421 0.591760462 2.572378 1.463962 1.419013
1.867135 1.242869 1.62472 0.7702216
AC037459.2 0.5388796 0.4900321 1.389854 0.9033716 0.8731445
2.054555 0.8489257 0.7471474 0.8890308 0.582488626
1.546412 0.6642856 0.9463878 0.698433 1.033566
0.7084346 1.148704 0.795566607 0.8088831 0.4851978
0.784037659 0.752402 1.075387 1.687638 0.6766476
1.694284 0.8028206 1.119568 0.847378336 0.2637344
1.030159 1.354412 1.835886 1.031642 1.255913
1.35502 0.09895272 0.526695 1.961615 1.06133591 2.734758
1.248155 0.769837877 1.54534 2.867013 4.190701 5.268305
0.8177243 1.710657019 0.9452009 0.9453104 3.205054
1.28093 1.167739 1.071286 1.97436 1.194585 1.814306
0.8634856 0.931101724 1.355567036 1.556260262 0.8103138
1.038612 1.446798 1.81862 1.066888 0.9130727
1.512794011 2.775323 1.44841 1.508676 1.803063 0.3891726
1.611557 1.251667 2.379082 5.513233 2.187232
0.222539291 0.8175612 1.973674 1.228008 2.37398 1.274363
0.6343001 1.723216 1.468927 1.30310646 2.186984
0.712758919 1.946706 0.9838613 1.989072 0.6445894
0.8851533 1.633619 2.720385 2.394127 1.692195

2.883978 2.843255 1.119909 0.5196518 2.665757
2.785692 0.9526758 1.164077 1.065188845 0.6268537
1.510144 1.64167 0.3615906 2.189883 1.55299 2.31958
0.4847239 2.712224 0.8352148 0.9340791 1.886208
1.204945 1.358318 0.3040608 2.589978 2.193248
0.9616146 0.7085327 1.722561 0.6112279 1.289846
1.780381 0.7203317 1.054056 0.3661317 1.158212
1.804041 1.58138 2.866444 1.594399 1.284817 1.296165
1.557651 0.9344967 0.9025565 1.764554 2.503237
2.615237 1.922935 1.39339 0.942335 1.723967 0.8425741
1.848201 2.026926 1.808191 2.445309 4.760544
1.855209 1.23731 1.090948907 2.556544 1.258839352 2.047414
0.4751188 1.635622 1.205164 0.8986677 5.705649
1.482953 1.447962 0.894067533 3.235471 1.338641
1.804973 1.406365 2.238493 2.974643 3.984652
2.787724 0.9245594 1.392637 0.6626513 2.269065
0.5217547 1.468733 1.650407 1.158001 2.335381
2.457387 0.9197398 1.49204 0.6854079 1.599206 0.9811362
1.276083 0.9821758 2.865477 1.911091 2.029861
0.5446469 1.922108 1.973349 0.4701001 1.099563
2.261117 0.8275796 2.859993 0.8748912 2.2905 1.648863
1.291518 1.328945 1.49751 2.251104 1.825824 0.6162697
6.139871 1.970383 2.075892195 1.9671 1.414687796
0.737538745 2.388088 4.316642 0.8306074 2.116508
1.033465 1.443856 1.742316 0.8940703 1.867641
2.019012 2.196672 2.393145 2.277726 1.369829
0.6272516 1.282588 1.256875 1.657924 2.594053
1.485298 1.322419 1.206962 0.8016573 2.111133
2.009272 2.563176 1.631141 1.4485 1.687197 2.68603
3.03615 1.758674 1.34911 3.667621 0.69001883 1.949331
1.611886 1.766759 1.043354 1.302223 0.9550755
1.446697 0.988527 2.054206 1.138004 3.162906
1.851781 2.85616 0.54563 1.283021 1.052622828 2.636744
1.940569 2.141188 2.177732 1.285785 0.308672538
2.44862 1.265232 0.5939774 1.027247 1.175689 2.623319
0.7622178 2.104746 1.714587 2.295384 2.233806
1.270258 0.7396318 1.918181 0.7933751 1.459675
1.904427 1.033632 1.546015 2.547377 1.30457 1.421935
2.02283 3.396325 2.006327 2.071677 1.526658897 1.30408762
1.77629 0.6817665 0.2594488 1.448819 0.6925048 0.9335424
2.910714 1.897005 1.191568 1.325364 1.407087
1.454226 0.5208005 0.889336352 0.3859014 1.755715
0.2281818 1.213741 1.561503 1.414890701 1.295064
2.127336 1.714423 1.63805 1.419465 1.814353 1.008778

1.450379 2.045557 0.6715104 4.545031 1.49055 0.9831457
1.612834 1.550357 2.094829 2.110879 1.784965
2.761521733 1.551334 0.3924028 1.765059 1.591134
3.560209 2.89675 1.082731 0.7871822 2.884645 4.319574
1.035443 1.838169 1.635013 1.736994 1.159731
1.089698 1.888021 2.671625 1.191579 2.177592
1.375383 2.35573 1.158759 1.557217 2.382996 2.019687
1.167213921 1.401794 0.4070255 1.510791 1.403781
1.849245 4.679477 2.423735248 1.130204 2.520733
1.177874 1.104235 1.067976 1.057538 1.343983
0.6921976 3.555502 2.244979 1.093227 2.62582 2.214666
2.525504 0.4844715 2.322086 2.741559 1.03935
1.330569059 1.215817 2.882328 1.895335 3.503962
0.4657098 0.5194225 1.455193
LINC01588 0.1522614 0.03148033 0.2157618 0.1140008 0.1565345
0.08126765 0.1462381 0.0739796 0.19377 0.37379997 0.2525651
0.3153087 0.5910042 0.1929177 0.3354465 0.02134105
0.2832025 0.07281333 0.1448123 0.1494156 0.096174442
0.03483608 0.3926472 0.1156998 0.05355142 0.2506826
0.1628485 0.05694428 0.163023271 0.1680789 0.08140471
0.02951015 0.1688219 0.3499431 0.1943127 0.0270631
0.00428205 0.2439364 0.1257379 0.320483852 0.1877612
1.946256 0.119929424 0.8095256 0.2500158 0.1635152
0.615304 0.24549 0.152941437 0.4437173 0.3539122 1.038591
0.337734 0.1856291 0.2808255 0.1821175 0.253771
0.5610658 0.4394542 0.223156863 0.165858369 0.381622408
0.2110747 0.05537816 0.1670603 0.09062711 0.2470621
0.257868 0.093028053 0.2371626 0.2864445 0.1715 0.3032805
0.1688357 0.2145511 0.2642059 0.3578459 0.1289661
0.08097991 0.304355864 0.1860373 0.1595784 0.2512539
0.1438703 0.1257049 0.1852774 0.1609017 0.3813954
0.6978291 0.2599346 0.146727942 0.2113688 0.2618968
0.4776556 0.7105015 0.1807755 0.372965 0.3098303
0.07942875 0.5026283 1.246859 0.1887517 0.1848953
0.348004 0.1592353 0.6006664 0.207778 0.112701
0.174674488 0.2331641 0.1956332 0.1833852 0.151659
1.204464 0.6922783 0.1502929 0.1105066 0.7938639
0.07296123 0.1592924 0.2278352 0.2811265 0.3318193
0.1489806 0.1562498 1.173513 0.2277193 0.151326
0.1562831 0.3008962 0.3615717 0.4005751 0.1591138
0.09537246 0.1241955 0.228163 0.2994401 0.2060349
0.1698339 0.5885618 0.3251828 0.3184843 0.5314171
0.1624786 0.3748173 0.1647353 0.3712705 0.2726658
0.1668279 0.3014856 0.1711422 0.1358368 0.2442492

0.4294299	0.202992	0.3165777	0.2831334	0.1510533
1.156057	0.4887708	0.179878112	0.5523977	0.313521364
0.6869556	0.3219212	0.4426435	0.5809651	0.2596139
0.5716477	0.1874523	0.08662747	0.279512658	0.1778185
0.2659103	0.4508413	0.1336716	0.2109862	0.1392587
0.1227249	0.8547634	0.2710962	0.1953403	0.2649911
0.2990454	0.1429956	0.3203299	0.1234252	0.2505549
0.1476314	0.3219855	0.1835138	0.4401577	0.3816425
0.2107562	0.2087488	0.4807814	0.07492687	0.4639771
0.4717392	0.3364068	0.08864715	0.3962135	0.2864401
0.3510561	0.5995349	0.2329181	0.1735525	0.1374553
0.1957059	0.332111	0.2552895	0.4264556	0.2635622
0.1588551	0.3337074	0.1764227	0.1167042	0.1401826
0.1508916	0.313580756	0.1728075	0.156180162	0.090708706
0.5144141	1.645289	0.5469334	0.2931796	0.229998
0.2158941	0.1151509	0.278566	0.1794517	0.1288187
0.131494	0.2712292	0.2016115	0.4801482	0.2198623
0.3229461	0.1365577	0.2296166	0.2680357	0.2619482
0.1988744	0.3284032	0.2687429	0.2124039	0.3892095
0.2218362	0.3682509	0.3347832	0.4736702	0.3601991
0.3319865	0.2028359	0.09050449	0.1923833	0.121571489
0.245337	0.2264741	0.1790913	0.1700321	0.2312475
0.1721675	0.3497184	0.2441431	0.1585821	0.2260823
0.1008021	0.2404002	0.2626167	0.1957263	0.3848184
0.163607054	0.1188758	0.4148576	0.3827801	0.1646886
0.5934186	0.203632216	0.6056652	0.1231903	0.1038635
0.191505	0.5899995	0.2873493	0.2400801	0.1348909
0.2461152	0.1640615	0.7189001	0.1194149	0.07116762
0.3261815	0.611675	0.2127477	0.4892092	0.2406115
0.3302464	0.2370528	0.3676575	0.1588746	0.4895349
0.1567144	0.3108198	0.344069	0.118024754	0.3509606
0.1535326	0.1880788	0.1443511	0.3002493	0.1172631
0.2753587	0.4449709	0.1131362	0.2252777	0.1455509
0.1532744	0.2729235	0.1918687	0.257930508	0.246637
0.3142869	0.1206072	0.2585118	0.155838	0.018908507
0.2718046	0.1239961	0.3568047	0.1572438	0.1869865
0.5542977	0.08059119	0.1987868	0.2270406	0.198465
0.4775215	0.1020381	0.2015259	0.3390842	0.2678863
0.4159732	0.2124553	0.3465875	0.780379358	0.03647327
0.1419103	0.572273	0.3551872	0.2068248	0.05439115
0.1446473	0.2273504	0.1925937	0.7177114	0.465536
0.350931	0.2626707	0.5926099	0.188197	0.2472217
0.2070886	0.3306021	0.2962188	0.1304757	0.3145167
0.4991268	0.5043876	0.3142264	0.7023379	0.2348041

0.267044338 0.1133455 0.04090877 0.6823111 0.5569401
0.138366 0.0869822 0.059815021 0.2896304 0.3976019
0.1146847 0.2940041 0.1960847 0.1579784 0.334753
0.1682376 0.1041868 0.2393815 0.2830131 0.6337614
0.329058 0.3423548 0.279148701 0.3278561 0.2259374
0.07937035 0.359450462 0.5149164 1.409122 0.4544889
0.1472772 0.3315491 0.1995248 0.1437276
AC068790.7 0.2476469 0.3420542 0.5568326 0.02628525 0.03609228
0.5294671 0 0.1978674 0.1699218 0.137899769 0.4723888 0
0.1699691 0.1289955 0 0.3057815 0.1646651 0.165743043
0.2038516 0 0.032153775 0.1918274 0.1400243 0.6584107
0.2449983 0.3676831 0.04355581 0.4133976 0.106347683
0.499497 0.1555191 0.4439726 0.6905822 0.6933075
1.097669 0 0 0.2471354 0.5959768 0.462678559 0.7110656
0.4608781 0.14319901 0.1945827 1.133386 0.4656875
0.6083346 0.3253388 0.280178364 0.1538413 0.1270579
1.194211 0.9741583 0.6619834 0.8584022 0.1353043
0.4848152 0.9026396 0.1655456 0.111911265 0.437360308
0.26267896 0.5206968 0.1931942 0.41653 0.3169757 0.07509064
0.2781045 0 0.3075491 0.09400376 0.2792072 1.034366
0.2565748 0.83507 0.2985848 0.7050083 1.455896 0.5206505
0.13477428 0.1913767 0.4508575 0.3242149 0.3678221
0.1159355 0.3996347 0.3680634 0.5100441 0.8483766
0.310369 0.344712275 0.3072453 0.09402344 0.7433005
0.2533763 0.2248865 0.7734936 0.405647 0.9698431
0.4741449 1.653657 0.4567594 0.06340064 0.09901902
0.1419647 1.141609 0 0.3425369 0.097330852 0.9537803
0.2332938 0.662819 0 0.8796902 0.6821618 0.1747725
0.1539392 0.5667907 0.3455669 0.4893782 0.1708522
0.9472842 0.8367772 0.3575984 0.6876983 0.667241
0.4278541 0 1.146946 0.1018713 0.5152408 0.2506167
1.007927 0.1940866 0 0.6663646 0.3664197 0.3851532
0.242262 0.5415757 0.7310322 1.014077 0.5351428
1.448563 0.4674844 0.4895605 0.3929168 0.6351773
0.4607516 0.4569372 0.05086005 0.1557051 0.1496048
0.6563214 1.085853 0.777605 0.2704553 0.7167924
1.159505 0.5523705 0.207373195 1.124811 0.84417875
0.4255433 0.1694916 0.2621189 0.08778879 0.6429342
1.161695 1.355038 0.7496036 0.190065377 1.262388
0.1400018 0.5429091 0.5668014 0.4525113 0.316991
0.5558946 0.9552235 0.138374 0.8337147 0.09244577
0.6921634 0.1761325 0.7479661 0.6002103 0.1861499
0.7261757 0.2378976 0.4483543 0.8408965 0.08366795
0.04206665 0.0946312 1.493002 0.3515807 1.585371

0.4766511 0.09640295 0.3387108 0.6087759 0.2427841
0.4043292 0.4685636 0.5120282 0.09946871 0.2218523
0.6542989 1.110339 1.091077 0.175478 0.3320318
0.2731353 1.082668 0.1685228 0.1177885 0.4141728
0.259443 0.427913134 0.4108397 0.43512789 0.202176191
0.2956725 2.039502 0.3344908 0.3977544 0.5767102
0.06714359 0.2199894 0.2173088 0.607956 0.2153383
0.1736774 0.6594867 1.116985 0.158545 0.186682
0.2174932 0.3371447 1.298344 0.4963112 0.2919218
0.4773583 0.3444526 0.1849816 0.3207016 0.826181
0.4409868 0.6474651 0.6925887 1.05574 1.281112 0.3990733
0.1179368 0.2353412 1.024551 0.142613019 0.232506
0.8767181 0.2773166 0.03853996 0.8082664 0.5969222
0.5521247 0.7534505 0.1429709 0.08980464 0.6168984
0.9957647 0.275943 0.1495696 0.8449012 0.251486723
0.5362973 0.1647902 0.7835142 0.4184557 0.1180052
0.131238324 2.091176 0.4070139 0.06313536 0.5071321
0.2409199 0.3736009 0.4338672 1.110098 0.1452055
0.4701454 1.243372 0.2281355 0.475866 0.4924906
0.2108246 0.3264866 0.7650207 0.2475172 0.4014927
0.258307 0.6828782 0.6070418 0.3050388 0.6486869
0.185771 0.1380382 0.327583643 0.572369918 0.6280399
0.1479528 0.1286948 0.1038431 0.3659068 0.1072544
0.5313074 0.4584795 0.8435455 0.4816003 0.1403942
0.7135016 0.3665544 0.558463282 0.3349838 0 0.1414818
1.185294 0.279566 0.108370918 0.03747291 0.2261199
0.2511362 0.2853851 0.1902623 0.08487804 0.1796258
0.6316983 1.545228 0.1488277 1.624012 0.6638434
0.4235042 0.4088582 0.05686463 0.276606 1.029416
0.9912901 0.65376935 0.6786238 0.2919663 0.2799903
0.3537201 0.3669038 0.1642468 0.3934341 0.04606637
0.6243821 1.151764 0.4361929 0.5631644 0.4470738
0.7961223 0.1372788 0.4683674 0.6823204 0.7882719
0.264091 0.3910906 0.9200766 0.5401634 1.166468
0.9991205 0.4360775 0.3401732 0.272611622 0.1768411
0.1367693 0.2306786 0 0.3321201 2.243357 0.274969964
0.4814219 0.2625764 0.2738733 0.9620233 0.07946247
0.1131783 0.6511544 0.1355337 0.5319878 1.1125 0.4689088
1.396508 0.3333733 1.041635 0.311090432 1.280852
0.3898691 0.2122856 0.040058076 0.2422222 0.8867922
0.4894976 0.4400067 0.3651398 0.03705925 0.502363
AC002401.4 0.07476133 0.09531844 0.04584556 0 0 0.1514265
0.07700705 0.05973356 0.2308372 0.093667768 0 0 0 0 0
0.01846228 0.09942043 0.131343544 0.2153904 0.05322486

0.0291204 0.09927457 0 0.2087038 0.0170681 0.058764
0.03944677 0 0 0.2261873 0 0.2457207 0.04811023
0.2354629 0.1825927 0 0 0.1790566 0.01927688
0.022054191 0.1408715 0.02196837 0 0.1602053 0.0769847
0.1283601 0.02899708 0 0.076123933 0.6170238 0.1438391 0
0.0534701 0.3372368 1.068954 0.3982541 0 0 0.07496403
0 0.226342801 0.237897926 0.1737379 0 0.1371763 0.05065982
0.1700165 0.1259341 0.325493581 0.1392675 0.08513548
0.05418576 0.04930443 0.1161848 0.8920341 0.0450694
0.6578465 0.0405707 0.0754452 0.024411945 0.2599834 0
0.02669351 0.02562476 1.811219 0.2412889 0.05128315
0.2771561 0.93908353 0.3091978 0.048029577 0.02782599
0.1703066 0.2103681 0.4876299 0.02545885 0.7488344
0.1574479 0.1045653 0.3031159 0 0.3701248 0.1722584 0
0.05142874 0.2631771 0 0.03102221 0.11018587 0.4651236
0.1660096 0.160077 0 0.05494486 0.8825813 1.760915
0.02788333 0.03948614 0.03681955 0 0.103156 0.3217192
0.06889418 0.5089271 0.04790932 0 0 0.08085812 0.0230832
0.0230652 0.125632 0.02837171 0.1521399 0.1255547
0.1114217 0.03176316 0 0.5634753 0.246833 0.1961935 0
0.05248055 0.1163178 0.07871435 0.0940849 0.5642962
0.06469985 0.1278344 0.04392468 0.07302882 0 0.2115239
0.02258185 0.02476684 0 0 0.4230792 0.2065542 0.2800315
0.08337668 0.037561937 0.08259705 0 0.1586932 0 0.1384779
0.03975341 0.7686096 0 0 0.02057231 0.315580249 0.05317649
0.3803823 1.70798 0.7699943 0.4339287 0.1794288 0.07192167
0.2306955 0.1503838 0 0.1116326 0.04643444 0.1823042
0.3079106 0.03882762 0.06743543 0 0.02154544 0.213714
0.8403493 0.2273243 0.0380981 0.04285186 0.1744713
0.1273651 0.07426593 0.1777523 0 0.05751692 0.04084031
0.04886221 0 0.2829063 0.07728728 0 0.05740652 0.2116331
0.02338581 0.1593782 0 0.04626278 0 0.07174606 0.1090174
0.08000731 0 0 0.077508794 0.1860406 0.052543745 0
0.8033367 0.08692218 0.1009783 0.02573075 0.5223036
0.03040464 0.5977069 0.2530388 0.4636646 0.06500778
0.1376311 0.05972709 0.1395323 0.1076909 0.06340144
0.1406964 0.05088976 0.01866443 0.0749149 0.03304775
0.1852819 0.3899463 0.1435976 0.08298478 0.02494131
0.108923 0.1599228 0.08960716 0.08436549 0.1856404
0.406603 0.1068107 0 0 0.154990752 0.02339683 0.1203044
0.3653158 0.907507 0.4455743 0 0.0882419 0 0.1294831
0.162665 0.1241556 0.1288321 0.09087658 0.1354593
0.2086892 0.068328468 1.144872 0.02487399 0.1655728
0.0398925 0 0.059428675 0.2133966 0.01755316 0.3716647

0.06889341 0.1454611 0.1450095 0.05894045 0 0.1095891
0.08110325 0.04021687 0.1291333 0.02052252 0.06371846
0.6873677 0.08448171 0.1194567 0 0.181808 0.02126713
0.6493785 0.3023756 4.143924 0 0.2733988 0.812602
0.053941732 0.034558184 0 0.01674937 0.1748307 1.473396
0.02367052 0.03237869 0.03207894 0.06228401 0.3274139
0.05452079 0.2797288 0.1739743 0.09959214 0.178509906
0.1166852 0 0 0 0.04603488 0 0.2375641 1.388006
0.176901 0 0 0.02562356 0.1626799 0.02383767 0.3928286
0 0 0.03340092 0 0 0.7467506 0.05566913 0.03729204
0.02426411 0.065788111 0.03841267 0.4627391 0.3381015 0
0.02556079 0.04250052 0.02969314 0 0.1413695 0.162261
0.2154778 0.05667064 0.1542465 0.06554695 0 7.660224
0.07270006 0.2172759 0.1674237 0 0.3273588 0.08735796 0
0.07984093 0.3037986 0.1421914 0.123446772 0.06863913
0.03096664 0.02321294 0.1237684 0.1933637 0.05016592 0 0
0.08917689 0.04960724 0 0.215898 0 2.137752 0.09819799
0 0.08224872 0.6673419 0.7850247 0.2113461 0.1316326
0.156523488 0.02230801 0.2427487 0 0 0.1566936 0.1434165
0.5910914 0.01897603 0 0.0335631 0
AC104958.2 0.3183308 0.1948138 0.2928129 1.763715 0.6012639
0.3610702 2.046052 1.114023 0.4128161 0.973154201
0.1530192 0.3001948 1.435427 0.3820346 1.28705 0.1698012
1.803379 0.862851601 0.4244973 0.6254971 0.862994932
0.1394934 0.7289611 0.1218723 0.776172 0.2301976
1.390732 0.2114375 0.688976398 1.386859 0.8276179
0.1027246 0.417897 1.764562 0.2591575 19.00877
10.86466 3.385127 2.235864 1.802992971 0.2673507
0.05612428 0.145784373 0.06548619 0.5113646 0.4216261
0.1481621 3.407931 0.220410254 5.054516 1.220023
0.5285963 0.8742665 0.7849812 0.8937614 0.8765724
0.317828 0.1696899 5.541206 0.880381695 0.216845727
0.434125739 0.7355421 1.683719 2.698501 0.3106189
0.6949669 0.604859 0.58849099 0.6137505 0.03262532 1.5043
1.38558 6.26897 1.010664 1.888333 0.4844229 0.6218944
19.22638 6.199286133 0.2213998 0.2086352 0.3000622
0.2356759 1.971615 1.750687 0.2358306 0.6136622
0.69793412 0.1148991 0.331303053 0.2417032 0.4024632
1.429599 2.345008 6.296034 0.3702783 2.66822 2.821009
1.148684 0.6855214 0.2113663 0.8508244 5.193075
4.900807 0.2209178 4.592215 0.3011683 5.404805246
0.6620455 11.15043 3.108104 0.7150717 0.3088181
2.841046 0.414491 6.040786 0.4943035 0.1034723
1.598546 3.900401 4.575358 4.353295 1.631154 75.299

0.6792876 2.597167 0.4131488 0.03538345 2.687046
0.8436708 5.653703 1.217875 0.8724783 1.252493
0.8926255 0.1211153 1.028252 0.7427103 5.647196
2.50679 2.252478 0.4754657 2.842179 1.862836 2.821516
4.859633 0.3592472 17.29275 0.3855828 0.2471237
0.3002202 0.09230654 0.5441543 0.4001236 0.4398023
0.5916357 0.4824685 0.2861672 3.40814 0.690928957 1.195763
1.471430936 4.077423 1.505905 0.9299363 0.6906154
1.463794 1.075154 7.853755 0.1997191 0.513059913
2.119323 5.662307 0.1322275 0.4337848 0.4064833
0.5684167 3.050147 2.165955 2.177116 3.613689
0.5276134 0.3440259 1.385596 2.076737 0.1983918
1.429944 1.344157 2.476971 1.364979 0.5367262
2.361768 1.57678 4.55424 0.9360439 0.4636798 2.023817
1.933242 10.29019 7.278571 0.2086757 0.124832
17.31753 34.11439 0.4343938 1.856517 0.1319947
2.400598 7.754968 3.192257 0.3248113 2.080163
5.111931 0.1221968 0.2450934 0.5723222 5.850651
0.1867565 3.089075286 0.5584683 1.275256498 1.162785216
1.559783 1.210264 0.5469114 14.31737 0.504095
0.2951729 1.857859 32.30851 1.332629 0.2325125
0.3315244 0.2288841 0.81989 0.3668351 2.267671 0.2731803
17.81164 7.505374 0.854879 0.5572354 0.8204802
2.590187 1.00275 0.2756098 0.8283532 0.7606152 1.652426
0.2747115 0.05747599 1.493949 0.6463519 0.1637265
3.282696 1.738415 0.554353427 0.9922431 5.495432
0.7699727 0.9095566 5.756762 0.1052293 0.3156137
3.874008 3.782151 1.994749 0.4017739 0.8952532
0.3366456 0.2595511 3.038979 2.304244344 0.6647481
1.067598 1.643663 0.2751743 0.04095535 0.167009681
0.4770331 1.901403 0.9641284 0.4341513 0.2508438
0.4198625 3.915071 3.63871 0.1231893 0.3315211 5.681805
0.1187664 0.1782636 1.291439 0.3317023 0.9712435
0.8301038 1.603547 0.02064351 0.3042635 0.9006092
1.15173 0.4234719 0.2539994 1.203522 6.259988
1.116253465 2.030634028 0.2570929 0.8130272 2.332523
0.7688575 0.2539862 0.4136016 0.7375906 0.7319599
0.3903523 4.317941 0.9225413 0.1904852 5.478848
0.866500539 3.58918 0.4730942 0.536862 7.450416 1.869981
3.744447904 1.630022 0.337165 4.777677 3.551787
0.402488 2.526851 0.5818552 1.047479 6.887131
2.62855 0.3439135 3.891135 29.04029 20.48519 0.1973566
7.068443 0.1905457 0.7438726 0.257713344 5.564299
0.202662 2.202623 5.565279 1.776217 0.7057656

0.7585933   4.412682   2.997691   1.137026   0.8440963
1.73737 16.81674   0.3851529   2.244587   0.08924503   0.4457578
11.29215   1.454283   0.8352828   0.7602987   0.6249041
3.273916   3.589973   0.372546   21.15426   1.665200668
0.1285956   9.414429   5.040829   2.434744   1.009958
0.2563254   1.133618503 1.468316   11.36097   30.6066 3.416259
0.7721997   2.025108   1.657276   8.441907   12.78955
0.4202542   0.5889687   1.805006   0.1079884   0.4035504
1.727492531 0.05699199   0.3495496   2.406772   1.038069411
6.37305 17.71897   1.396848   0.1648304   1.496584   0.6859697
0.4649389
AC103740.1   0.05617392   0.5810991   0.1612759   0.01951299   0.2009496
1.465328   0.01052024   0.8629662   0.6990386   0.371093369
0.1972576   0.1872803   0.01840087   0.03351615   0.03330176
2.725242   0.05772442   1.950699493 0.01471266   0.0618057
0.161119234 4.636608   0.09745103   0.7148327   0.7927916
0.1016878   0.04580632   2.352806   0.088816202 0.04248797
0.2501425   2.593958   0.07065467   0.04289003   0.05266104
0.09702024   0   0.06115407   0.09743913   0.058751746 0.1512108
0.08103201   0.06201096   0.1247518   0.1910622   0.09895192
0.2971054   0.2012641   0.256523235 0.06390034   0.01572034
0.09154369   0.08765713   0.05374979   1.360771   0.06486995
0.03249142   0.6299441   0.005120566 0.05307754   0.046382374
0.039464497 0.2865157   0.01195156   0.02811028   0.321819
0.08361587   0.1101079   0.013682142 0.1331812   0.04070743
0.0629215   0.5220146   0.1539629   0.06358137   0.2616771
0.154631   0.04572582   0.04380418   0.026680096 0.05031626
0.07251753   0.04376046   1.412532   0.2241284   0.07581587
0.1488774   0.03786338   0.30906686   0.1190421   0.114826422
0.1102411   0.04653251   0.06322627   0.03526785   0.03478034
0.09075108   0.103963   0.0557118   0.1087009   0.1555813
0.06394885   0.003922149 0.04900485   0.07552824   0.05521455
0.03192329   0.9514459   0.048169364 0.06581174   0.02474097
0.02870274   0   1.717051   0.2532031   0.02838133   0.005713878
0.07821819   0.1031163   0.05876788   0.06165491   0.1172035
0.04392223   0.01580148   0.03436167   0.1651099   0.04826573
0.03313904   1.996157   0.06144505   0.09071917   0.0794574
0.1108503   0.1252134   0.02663808   0.2733753   0.1441024
0.02199388   0.1030359   0.09380966   0.05797921   0.0860349
0.09799227   0.05376739   0.0176733   0.0481817   0.06408203
0.334726   0.01650199   0.06817449   0.1211345   0.05618884
0.02313747   0.07443692   2.821311   0.04810491   0.04258838
0.5542858   0.004782026 0.2164179   0.05901207   0.1128391
0.130558401 0.0588449   0.01048524   0.09864392   0.05702414

0.05091045	0.06259286	0.1404095	0.2023535	0.017636998	
0.2106749	0.005196553	0.02651521	0.0364126	0.09880118	
0.2941498	0.1244564	0.05712315	0.008560217	0.09455604	
0.02287586	0.139559	0.115187	0.04416812	0.8022897	0
0.0463272	0.04562282	0.1124058	0.03408227	0.07246321	
0.05204731	0.008781243	0.218986	0.1294114	0.1031486	
0.07285031	0.02385507	0.07071852	0.1896981	0.1118106	
0.02084415	0.02898671	0.1623372	0.0594831	0.03529139	
0.04481365	0.1054293	0.1110437	0.2297765	0.1169226	
0.06195547	0.1257861	0.05361589	0.0127518	0.03371307	
0.06241628	0.056473504	0.07465874	0.116645883	0.008933721	
0.05731232	0.1202322	0.05931871	0.07733393	0.007928217	
0.07684341	0.01360918	0.05953485	0.06334305	0.1420955	
0.05372095	0.09791464	0.1048413	0.04168424	0.1212613	
0.28255	0.04866576	0.2626316	0.129636	0.3814992	0.02953078
0.03196326	0.08173929	0.07652392	0.3748064	0.2132857	
0.0424816	0.1576104	0.08259944	0.2187392	0.08949342	
0.01580781	0.01455889	0.2366252	0.03528983	0.07671201	
0.2185889	0.05458604	0.01192097	0.05000172	0.6699676	
0.2873126	0.09581077	0.1046611	0.02407417	0.04099003	
0.1724825	0.1924326	0.04857727	0.09028119	0.062230809	
0.02606757	0.07475893	0.04847047	5.556144	0.02737555	
0.038564232	0.1457648	0.01678605	0.06444467	0.05647081	
0.1726381	0.247629	0.07783684	0.08870381	0.08833123	
0.06924897	0.08241278	1.63183	0.07289524	0.03046691	0.04695201
0.1384966	0.05711808	0.01914017	0.08969101	0.1394586	
0.08237743	0.0544524	0.3527349	0.1173021	0.06895401	
0.07400851	0.023949876	0.047211297	0.07471612	0.004576396	
0.03317266	0.01927211	0.01616862	0.03317536	0.4272869	
1.966966	0.06150279	0.01986213	0.03300374	0.05941827	
0.1201835	0.191031046	0.02869349	0.08081372	0.08227328	
0.09776762	0.07546808	0.022347137	0.06027277	0.02331406	
0.02589336	0.09756575	0.0403549	0.09976526	1.247341	
0.1823673	0.03354116	0.1519141	0.3916476	0.02053364	
0.04763492	0.006898135	0.0299014	0.1007686	2.310407	
0.07126855	0.134813598	0.04329357	0.01128868	0.1125866	
0.07815057	0.09602891	0.02903082	0.03042372	0.0940434	
0.05311088	0.1440863	0.1962485	0.336777	0.04609548	
0.2708775	0.03113905	0.05454037	0.06207401	0.07844772	
0.09475716	0.2047185	0.08334513	0.3377411	0.08471089	
0.09634856	0.04980375	0.03237555	0.075890476	0.05626237	
0.02326761	0.09513633	0.07186106	0.09245664	0.2090276	
0.0386601	0.05956433	0.1238585	0.02710816	0.1022081	
0.01720523	0.1820401	0.005035289	0.07546058	0.01097011	

0.3384942 0.1326083 0.5809121 0.1340524 0.09141284
0.147544706 0.1630458 0.2426918 0.09192825 0.054518408
0.02140653 0.08620787 0.1034626 0.2877553 0.03065598
0.05272965 2.9794
AC005381.1 0 0.006948068 0.02339282 0 0.009530745 0.03679323
0.02245317 0.01306252 0.01682646 0.013655491 0 0 0.02805191
0 0 0.00269155 0 0 0 0 0.008490719 0 0 0.1086649
0.002488297 0.008566994 0 0 0 0 0 0 0.00350691
0.01144243 0.002957726 0 0.003780389 0.03589303 0
0.141468897 0.01173552 0 0.162600166 0 0.1496443 0.08019929
0.02959167 0.007810084 0.014797117 0.002901733 0.0293577
0.01713861 0.1325187 0 0.08500307 0.04912773 0.2560464
0.02607024 0.007285826 0.044327943 0.098993138 0.024773067
0.07960429 0.02550802 0.01999844 0.004923677 0.01982889 0
0.003650196 0.007613744 0.009308699 0.01843228 0.07906709 0 0
0 0.1156503 0 0.01374861 0.163710813 0.02526804 0.03968538
0.003891552 0.02241446 0 0 0.07476388 0.01346853 0.5102847
0 0.063018552 0 0.009310648 0.01226753 0 0.01484622
0.05986737 0.01147688 0.003048841 0.007365033 0.004548695
0.009522208 0 0.004357926 0.04123687 0.008221639 0 0.08592974
0.038552656 0.2373305 0.09020727 0 0 0.008010217 0.5275404
0 0.004065012 0.002878272 0.1744532 0.01140248 0 0.0156341
0.1439617 0.04046982 0.2130281 0.07400218 0.06645984
0.04715207 0.1110522 0.003362594 0.07064529 1.455946
0.1330796 0.01464333 0 0 0.1451385 0.003911765 0.05997484
0.05243764 0.01484929 0.1071133 0.01695756 0 0.6926733
0.005876195 0.009432361 0.08075828 0.02561449 0.1845413
0.003357603 0.01370548 0 0.1119308 0.002654972 0 0.01947765
0.05162196 0 0.1215519 0 0.0280969 0.003715315
0.003305045 0 0.1182452 0.05215958 0.01018662 0 0 0 0
0.03100965 0 0.4640461 0.005756653 0.01757248 0 0
2.492993 0 0.01467705 0.01220589 0 0.003322186 0
0.002830271 0 0.002996224 0.003141034 0.1152795 0.007656972
0.02209386 0.005554186 0.006247221 0.04133277 0.01160506
0.05413483 0.5219794 0.00424279 0.1201877 0.01488491 0
0.005931644 0.1443537 0.02535174 0 18.98947 0.006170647
0.09546136 0.02323518 0 0.003372243 0 0.156894 0.02225057
0 0.5238197 0.098959 0.13559678 0.01695137 0.026810581
0.003813419 0.06506422 0.04435226 0.5299658 0.1425454
0.0253816 0 0 0 0.004224751 2.047087 0 0.09142771
0.01017096 0.03139976 0 0.0164093 0.007419038 0.1605402
0.01092158 0.01445374 0 0.0151597 0.006978193 0.06653931
0.007272211 0.02117266 0.005181016 0.006531755 0.3607808
0.04736174 0.2436952 0.002595258 0.3551178 0 0.176998483

0.3206285 0.03858524 0.003328631 0 0.1252775 0.01125906
0.1029158 0.1492205 0.00943844 0 0.009050105 0.03380755
0.2186015 0.0148111 0.05070675 0.009961365 0.1416179
0.00362629 0.01034497 0.008723678 0.003895145 0 0.06222067 0
0 0.110481 0 0.02114042 0.008592718 0 0.009585961
0.04433908 0.005863075 0.007530353 0 0.2724858 0 0
0.1079741 0 0.02061511 0.02170322 0.004508123 0.0360673
0.03098092 0 0.3771165 0 0.019659934 0.055419263 0.01275723
0 0.002831994 0.01371072 0 0.01416112 0.004676674
0.003026721 0.03182167 0 0 0.007246597 0.04355755
0.060723425 0.00680445 0.02249731 0 0.3129957 0 0
0.03463362 0.003317254 0.01473701 0.007932685 0.1758463
0.01494227 0.005929132 0.00347521 0.8017671 0 0.4179435
0.02434701 0.06608317 0.004907525 0.01501602 0.004057902 0 0
0.00639401 0 0.009637299 0.01232265 0 0.06334908 0.009294
0 0.02432913 0 0.05406962 0.2513099 0.2230689 0.05621762
0.2771198 0.006041779 0.01455058 0.03179605 0.09955279
0.04184248 2.196553 0 0.1426388 0.006696187 0.1590762
0.03543177 0.02418451 0.269952994 0.006671103 0.410821
0.09475569 0.08119688 0 0.01828378 0 0 0.1473422
0.01446413 0 0 0.004981091 0.02149347 0.1932651
0.003344759 0.03597225 0.1061339 1.392426 0.0308114
0.0127935 0.155169221 0.204889 0.01930334 0 0 0.08223771
0.008363275 0.177732 2.666856 0.02066165 0.004893045
0.008651543
AC092115.3 0.07362475 0.02346734 0.2708915 0.1406616 0.2253329
0.1491245 0.227509 0.1470636 0.2273278 0 0.1685279
0.09643081 0.03789851 0.06903 0.3200794 0.1454528 0.1468635
0.092390548 0.03030226 0 0.258099224 0 0 0.1468078
0.1344689 0.1929021 0.1165412 0.09702789 0.142275954
0.6682459 0.2774125 0.04399728 0.521167 1.082122
0.03995928 0 0 0.9918813 0.3986601 0.086875633 0.3567339
1.449504 0.127718036 0.1104388 0.1769001 0.2889342
0.5996811 0.2110306 0.074966643 0.5292378 0.5666094
0.046309 0.6845437 0.4059121 0.09570025 0.09050758
0.1081007 0.07547409 0.590595 1.36410758 0.139313612
0.100406243 0.09776952 0.2297445 0.5065905 0.03325977
0.5022955 0.2232352 0.295887876 1.920104 0.1048015
0.1245113 0.6554907 0.6865109 0.248264 0.08876845
0.2095971 0.1398387 0.3714911 0.264448992 0.1280155
0.1608464 0 0.252352 0.02585049 0.2376206 0.1262588 0
0.08407336 0.9965362 0.118248493 0.08220888 0 0.5386418
0.02824804 0.2256463 0.02378871 0.5426899 0.1029756
0.07462694 0.1536337 0.5789084 0.05654652 0.7065141

0.4558219 0.09256288 0.2478244 0.09165177 0.39063869 0
0.1783482 0.01970543 0.1292066 0.08116433 0.4345819
0.837845 0.2471349 0.5249589 1.214703 0.513497
0.6857175 0.07920704 0.1809248 0.4100645 0.1887239
0.1666299 0 0 0.04546454 0.24986 0.2297696 0.2514634
0.4494809 0.04945835 0.1645917 0.1251211 0.02334334
0.3963635 0.1350447 0.9982557 0.8860535 0.2067308
0.1718242 0.1291961 0.09265456 0.1786234 0.5734461
0.251782 0.3460552 0 0.2041275 0.06943607 0.5114866
0.09756129 0 1.001779 0.04385761 0.1452958 0.5515485
0.2463274 0.073981789 0.2169103 0.57723574 0.08930321
1.058177 0.1363727 0.4306396 0.3669938 0.5347648
0.09064103 0.04051912 0.141264807 0.1047361 0.499466
0.4842162 0.9721645 0.2374066 0.1413608 0.2360942
0.02839854 0.1727805 0.9914445 0.1099355 0.09145702
0.4039486 0.03032295 0.114712 0.06641023 0.1619175 0
0.294651 0.4396486 1.268587 0 0.3798036 0.1503415
0.156786 0.4144424 0.05001427 0.8884704 0.1510467
0.563072 0.2405969 0.4006866 1.114422 0.09514038
1.00544 0.05653378 0.2084157 1.013332 0.1883463 0.2086766
0.1138987 2.517274 0.2590695 0.2147202 0.6828552
0.1944196 0.1028423 0.178104385 1.122175 0.232852222
0.33487871 0.01758053 0.4066035 0.1988864 0.07601871
0.05715147 0.05988482 0.2452584 0.110752 0.2853848
0.128039 0.1549014 0.3235049 0.8244664 0.03535125
0.1665002 0 0.1002322 1.507216 0.172144 0.3254533
0.1824651 0 0.5185198 0.2451696 0.4175563 0.07151137
0.2274877 0.2426734 0.3046373 2.468045 0.08898256
0.01753115 0.05997112 0.2233707 0.737733291 0.06912341
0.3080361 0.179881 0.03437348 0.09402864 0 0.2027676 0
0.2125243 0.05339736 1.324572 0.1776229 0.7607076
0.1667499 0.6508023 0 0.4270707 0.8328583 0.419286
0.01964301 0.02631197 0.029262599 0.1050762 0.1210041
0.05630991 0.1130767 0.05371862 0 0.09674065 0.8663267
0.107923 0.03993513 0.09901367 0.07630207 0.06063158
0.460165 0.4888851 0.124796 0.4705626 0 0.01989378
0.1466067 0.2436214 0.1624247 0.1883504 0.1335135
0.4763519 0.4924604 0.239047523 0.238229641 0.1938959
0.1979369 0.3252153 0.5248286 0.209796 0.0637729
0.0631825 0.02044571 0.698612 0.5190219 0.1252165
0.02447563 0.425005 0.146496728 0.4826275 0.05065695
0.07571188 0.3523846 0.3853477 0 0.5347485 0 1.119932
0.04018937 0.290903 0.4794462 0.04005169 0.5164561
0.3868565 0.6858139 0.07364965 0.09867942 1.270513

0.6630133 0.5578881 0.4659939 0.1469004 0.2150571
1.058203242 0.01891435 1.367113 0.5826825 0.04506859
0.125861 0.1674176 0.526351 0 0.3132457 0.02282775
0.2475689 0.1116182 0.4367169 0.8714311 0.1224379
0.3194429 0.4653664 0.1222701 0.2355406 0.2683158
0.2051522 0.1720598 2.058111 0.2882995 0.538524
0.5367806 0 0 0.3354545 1.051562 0.8532073 0.275057
0.1482098 0.379012653 0.2146882 0.2634635 0.4396777
0.05595788 0 0.1009428 0.943734 0.02417628 0.3840993
0.1214975 0 0.2147473 0.1189332 0.06481573 0.061657563
0.3075642 0.1303954 0.3313377 0.500184627 0.5555213
0.6496866 0.4365789 0.07475018 0.1628326 0.4957927
0.1948059
FMR1-IT1 0.2541449 0.2835237 0.7013168 0.1213873 0.05555896
0.1715873 0 0.2030593 0.1961779 0.265346495 0.03635879 0
0.2616433 0.1588562 0.1381101 0.3138049 0.08449288
0.127568989 0.2615006 0 0.098992381 0.5062135 0.2155477
0.2533826 0.2320863 0.3995267 0.268192 0.2679441 0 0
0.4787994 0 1.144827 0 0.4482895 0.5571211 0.0881503
0.076086 0.9829521 0.299885566 1.470849 0.4854177
0.176347702 0.1089209 0.6542577 0.6233566 0.936445
0.5008131 0.474424472 0.7781127 0.1466907 1.598538
0.2726507 1.01903 0.9910416 0.05207045 0.8706885 0.7815859
0.254834 0 0.240447983 0.173295907 0.3374901 0.09913174
0.5246092 0.6888554 0.2889786 0.3852923 0.170228982
0.7693211 0.289411 0.307 0.3352126 0.4739527 0.560334
0.1532097 0.4275269 1.068855 2.500579 0.248959136
0.3682467 1.295525 0.6351965 1.350193 0.1784663
0.2870845 0.5229984 0.5757699 1.01574341 0.5733231
0.408181389 0.3783686 0.07236789 0.4648337 0.04875463
0.4759988 0.4516388 0.1784103 0.355461 0.5581428
0.8485238 0.5920981 0.09759633 1.016172 0.5681901
0.6390347 0.06110457 0.05272871 0.074913542 0.3388177
0.3591228 2.210685 0.1486692 0.7938171 0.7500655
0.5717047 0.2843612 0.3691321 0.4380776 0.7090151
0.5260055 0.9569503 2.498134 0.4718332 0.5293072
2.054246 0.2711968 0.04581174 0.66699 0.2352247 0.5185918
1.06092 1.465363 0.4694939 0.1893844 1.295715 0.2820256
0.3648547 0.5593921 0.6669433 0.5770875 0.6690123
0.09885321 0.9811373 0.2798546 0.6850993 0.6048398
0.7966979 0.7465915 0.3310072 0.3914593 0.3994767
0.1535303 0.5893498 1.021483 0.2660029 0.2270878
2.908965 1.070937 1.558876 0 1.5443 0.606430449
0.8862628 0 0.1681229 0.3378461 0.4354708 0.922976

0.4171778	0.7343056	0.292578773	0.4519227	0	0.3079012
0.4362553	0.327801	0.3659721	0.325989	0.8332446	
0.8520286	0.5133544	0.2846144	0.1183875	0.7746601	
1.046716	0.4619695	0.1719309	1.013047	0.9887663	
0.4359019	0.4017231	0.1717266	0.1295113	0.218507	
1.371545	0.432967	0.2945377	0.388449	0.2473312	
0.782096	0.9371244	0.08305151	1.279393	0.2404292	
0.6568292	0	0.5366589	0.5755433	0.7154831	0.4334343
0.4051855	0.3931662	0.4671702	0.4471404	0.4447149	
0.0453297	0.3355581	0.488126	0.17565647	0.2766879	
0.893090479	0.044460237	0.4854891	1.292746	0.2402872	
0.743491	0.1972809	0.05167903	0.2116516	0	0.4925591
0.2209886	0.5012846	0.9644271	0.5929107	0.1830432	
0.2155279	1.291371	1.16772	1.681377	0.9337789	0.1123431
0.4199004	0.5302361	0.569506	0.5641997	0.2967507	
0.5554119	0.936276	0.5711477	1.816359	1.814323	
1.151844	0.7261881	0.103507	0.3504782	0.219532577	
0.4374457	0.3271718	0.6597385	0.2373073	0.9196349	
0.04375606	0.04999517	0.4510452	0.4768476	0.04608052	
0.6682566	0.5693402	0.2703115	0.4604826	0.8867768	
0.1548513	0.8255538	1.14152	0.9246838	0.5424449	0.04541308
0	0.6347461	1.432092	0.09718795	0.5854936	0.1545259
0.5751058	0.467514	1.922444	0.4843006	0.4135556	
0.4784987	0.5706713	0.1395292	1.371836	0.1298139	
0.1435943	0.7309497	0.3810177	0.824055	0.3614792	
0.6307166	0.9811778	0.5056844	0.4224685	0.2144761	
0.6020556	0.41258348	0.528649533	0.892411	0.3416286	
0.03301791	0.2664196	1.046058	0.4402747	1.254071	
0.2823057	0.3710054	0.4324574	0.6051277	0.8871166	
0.2821293	0.404553062	0.07933235	0.1748625	0.04355824	0
0.1173688	0.055607235	1.038316	0.5027816	0.3865886	
0.3468237	0.1673611	0.3484206	0.06912711	0.5267222	
1.085003	0.6872974	1.483013	0.340631	0.5926589	
0.6293794	0.1750701	0.3784852	0.8873983	0.7835964	
0.596376576	0.4570324	0.2247205	0.3591712	0.4667161	
0.3041212	0.1083577	0.6561058	0.212738	0.5406457	
0.5121934	0.6104157	0.9632356	0.4588046	0.5570535	0
1.611614	0.2471378	0.3693057	0.6911023	0.2778594	
1.062246	0.7424153	0.9758766	0.9047087	0.3614583	
0.2819646	0.503576454	0.1944442	0.2105371	0.5523732	
0.4207403	0.511252	1.406913	0.153919047	0.606339	
0.9094482	0.4215892	2.961798	0.06116062	0.05807401	
1.00236	0.166908	0.1559848	0.5591954	0.5671434	1.037799
0.3079087	0.5593428	0.425670873	0.9858451	0.3750927	

0.08169592	0.061663744	0.1598001	1.803871	0.1255854
1.257895	0.3211892	0.05704748	0.6052045
AP001189.3	0.1115869	0.9389818	0.6569085	0.3411027	0.204911
3.209421	0.4137798	0.4012058	0.4479045	0.223689949
0.4022924	0.04384565	0.1378551	0.2929445	0.7276767
1.091231	0.3116243	1.00820653	0.4960076	0.3336569
0.312943657	0.3333933	1.220857	2.216144	0.5349839
0.3508388	0.3885901	1.111752	0.51752605	0.1519207
0.4414736	0.5601375	0.7324431	0.4920247	2.4528	0
0.1625567	0.861899	0.984009	0.375260029	1.009254
0.09836836	0.348428927	0.129124	0.6204896	0.2463264
0.1558092	1.103453	1.158931334	0.3030238	0.5925463
1.747649	0.3351943	0.3020109	0.7832426	0.1646098
0.8847319	2.951257	0.2909126	0.09076674	4.535417761
0.730451565	1.711492	1.227422	0.1228476	0.8015043
2.070702	2.27815	0.829637484	0.327391	0.343092	0.3396806
0.7727011	2.143404	1.302484	0.8475955	1.056971
0.9991547	0.6756459	0.109310015	0.3880449	3.97364	0.4781049
1.97354	3.385103	2.938757	3.605221	1.378921	0.45872283
1.032084	0.752721593	0.9967774	0.9151037	0.4144671
0.3853188	0.9119821	1.687354	0.2585031	0.3371147
1.063195	3.129502	0.2339742	0.05142172	0.4818622
2.026498	3.484801	0.3863386	1.972508	1.026234975
0.3570337	0.9595916	0.4300687	0.1958278	0.5658646
1.343666	0.3898151	0.2497079	0.5834669	1.005694
0.09339178	0.1847618	0.7683042	1.665842	2.361703
0.8151943	0.5411724	0.30619	0.3861979	3.162824	3.635444
0.7714914	0.1016326	0.2724963	1.371767	0.6485907
1.706721	1.719447	0.7208824	0.7613948	0.497817
0.7449393	0.5874839	0.5034782	0.1879794	1.137473
0.4151112	0.02897082	1.52642	0.1376779	2.071019	1.381893
2.946677	0.566246	2.240163	3.13135	0.6306842	1.316132
0.3699574	0.7523427	0.07466759	3.599310614	0.6657246
2.6246049	0.1827218	0.3024292	1.027538	0.4628128
1.168066	0.8510235	0.4670821	0.9395953	1.130465653
1.119116	0	0.1390521	0.4420287	3.195179	0.3213733
0.1073485	1.575312	0.1571214	0.6536528	0.5748394
1.850494	0.6734545	1.682062	3.042541	0.120783
2.061402	0.4051933	1.43543	0.7525709	0.5428778	0.4435414
0.4221336	2.675729	0.9267473	0.1995255	0.4093334
0.2866914	0.1201877	1.060656	0.7001332	0.1821862	0
0.2076428	0.7798591	1.002498	1.004493	1.047153
1.027659	3.320875	0.9736148	0.6645873	0.4283457
0.8005664	1.146403	0.3712788	3.062835	1.480802931

1.187081 1.717518626 0.327954006 0.2557953 3.814295
0.2170336 0.3686888 0.05197184 0.1361437 0.981335
1.032324 1.505218 0.203761 0.4401961 0.2406974
1.468251 1.253748 0.132484 2.4948 2.483788 0.4847304
2.750677 1.716554 1.327427 0.884677 2.079003
1.449174 0.9157791 3.414091 0.270523 1.424389
0.5288719 2.078117 2.95351 0.1913076 1.090719 1.606547
2.012618011 0.6285877 3.447616 0.265815 0.281324
1.225598 0.4149769 0.6321969 0.9166402 0.1932633
0.7283695 1.649274 0.4153499 2.360139 0.879497
0.4672265 0.142779559 0.2485538 1.202892 1.016763
0.7680991 0.3110552 2.581221965 5.255425 0.4715895
1.766626 0.8431947 0.3093841 2.164377 0.2463246
0.712782 0.8440211 6.7729 0.7023126 0.5088367 0.9924579
1.597758 0.5813707 5.069032 1.733058 1.204508
0.2351806 0.6665988 0.5815479 3.471049 0.3615916
2.26638 0.03766785 0.5411265 0.45891902 0.804658907 1.6065
0.6899919 0.9220164 0.6176351 0.3815645 3.3926 0.402194
2.584388 1.099552 2.213438 0.660435 1.268672
0.8918928 1.092402018 0.3970883 0.7370548 1.032752
0.240336 1.504761 0.26368592 0.273535 1.589439
0.1810546 3.54506 4.364885 1.307982 3.314385 0.6831269
0.06596171 0.824839 1.071597 0.4337246 0.3747134
0.1205849 0.6456887 0.2492711 3.439861 0.06518884
0.41241364 0.5676047 0.2368022 0.5298741 0.4918084
3.181828 1.465354 0.3722817 0.3362633 0.3481577
1.266291 0.7397188 0.9135202 0.7252072 1.085955
3.674264 0.7374025 0.1302124 0.1667832 0.235613
0.04879968 0.6662833 0.234699 0.5141715 0.3575059
0.9250223 0.594248 0.552760893 1.639185 0.9706211
0.4573412 0.5542009 0.57722 1.639794 1.703039778 2.200786
0.2928271 0.3109787 0.6445614 0.5155906 4.191944
0.7261722 0.08794075 0.8834943 1.767779 0.2716522
0.5077404 3.352783 0.5697678 1.009251909 2.657052
3.932827 0.5595731 1.104642986 0.3648482 0.9247392
0.8932766 0.2719023 0.2749967 1.172234 1.027474
AL137798.1 0.300001 0.04098131 0.1576871 0.04093987 0.05621453
0.3472239 0 0 0 0.161086456 0.0367878 0 0.3309132
0.08036531 0 0.03175076 0 0.032268557 0.2116689 0
0.100160433 0.04268221 0 0.1538234 0 0.06737347 0.2713565
0.3388821 0.082819434 0.486236 0 0.03841651 0.1654765
0.1349802 0.0697814 0.2818474 0 0.07698377 0.1989101
0.303424038 0.2076563 0.1133412 0.044607125 0.2204122
0.1765273 0.06307118 0.4986813 0 0 0.03423017 0.09894771

0.5660899 0.6436916 0.06444086 0.3342451 0.1053697

0.1887776 0.1757351 0.2578409 0.058101424 0.048657025

0.116893798 0.1707362 0.1504522 0 0.2323278 0.1169553

0.1299462 0.043059396 0.05987682 0.1464129 0.3106224

0.5511479 0.1198863 0.1000492 0 0.2329231 0.1395441

0.09731095 0 0 0.2340735 0.4131588 0.1322056 0 0

0.1322924 0.1059207 2.34909391 0.290044 0.041299769

0.09570827 0.695607 0.03617834 0 0.1751329 0.4154253

0.1805155 0.1078966 0.04344066 0.4829264 0.1123283

0.2962437 0.1542243 0 0.25863 0 0 0.037898739 0.1714078

0.05190861 0.3785302 0 0.8504298 0.910699 0.06805299

0.09590551 0.3734877 0.06332095 0.08967264 0.177404

0.6454945 0.2369635 0 0.2059818 0.1247091 0 0.04635229

0.0793953 0 0.06173069 0.3415485 0 0.08636975 0.2874286

0.1092503 0.1222943 0.1845799 0.1886642 0 0.2627536

0.541525 0 0.4512337 0.04045096 0.1039775 0.1112685

0.2564859 0.03777004 0.5442334 0.03960783 0 0 0.4259313

0.1879156 0.1345708 0.2680618 0.1014927 0.2407941 0

0.064597668 0.331444 0.175310277 0 0 0.102064 0.06836649

0.08011074 0.2334666 0.5276253 0.2122771 0 0.2286275 0

0.2225244 0.2037244 0.207293 0.1234301 0.08245886 0

1.293123 0.2164215 0.2399772 0 0.07838006 0.2647668

0.1001615 0.6958381 0.1413793 0.07410616 0.07350754 0 0

0.06551974 0.1473902 0.2250371 0.1916581 0.3405863

0.1310108 0.550549 0.428634 0.456532 0 0 0.3243548

0.1329159 0 12.4888 0.1091877 0.2413085 0.3289114

0.09110365 0.2386832 0.2363413 0.699189 0.07499371 0

0.03395175 0.1795948 0.044432278 0.2799527 0.180725684 0

0.2149077 0.2989713 0.2778543 0.08850162 0 0 0.04282979

0.09670377 0.0498371 0.2235961 0.0338133 0.2567913

0.05999067 0.185203 0.2544162 0.3871433 0.4375919

0.1604921 0.3865078 0 0 0.04470771 0.04115899 0.5708569

0.257359 0.06244061 0.06111765 0.4237837 0.4836291

1.117402 0 0.1224594 0 0.07092273 0.088849173 0.2011852

0.2068951 0 0 0.1094689 0.04427236 0.3035105 0.195586

0 0.04662424 0.2491048 0.3544973 0.1953578 0 0.4187121

0.274187294 0.2088237 0.1711095 0.2440681 0.3773313

0.09189785 0.051101648 0.5046138 0.0301873 0 0.1184804 0

0.6650191 0.2027273 0 0.3769347 0.1394785 0.1383271

0.2220788 0 0.07305381 0.1313456 0.2905772 0.1232624

0.1927567 0.1737038 0.1097233 0.3190794 0.1890962

0.03654652 0.2331564 0.0723356 0.1433317 0 0.297159603

0.4138479 0 0 0.2156505 0.1221231 0 0.2206728

0.0714092 0.09384577 0 0.3936007 0.2991947 0.1141833

0.920984737 0.1204026 0.2653886 0 0.4615302 0
0.056263367 0 0.1956593 0.1738445 0.1871552 0.1270019
0.04406647 0.2797711 0.5739324 0.2533397 0.4636047
0.2572306 0.05744171 0.05996519 0.4631314 0.4428396
0.09573777 0.384801 0.2920998 0 0.06606072 0 0.4360911
0.07870385 0.1758341 0.07309086 0.1021304 0.5022457 0
0.3587795 0.3088091 0.3898405 0.03315844 1.296341
0.1425432 0.128734 0.06251348 0.1601414 0.4524604
0.04685633 0.10236 0.1201881 0.3554609 0.4119227 0.05224619
0.2852916 0.169839453 0.0393477 0 0 0.1064262 0.07389778
0.7764619 0 0.1704148 0.7156949 0.04265637 0.1954399
0.3094114 0.05875924 0.3803203 0.08443869 0.2367379
0.2121726 0.5216685 1.875079 0.2076945 0.2641066 0
0.3836452 0.03795186 0 0 0.05389521 0.6905992 0.1270672
0.326343 0.04062238 0 0.06803839
AC036108.3 0.08683522 8.215198 0.1747252 0.08295025 0.2064419
8.706149 0.0838534 1.372433 2.010879 0.33998449
1.411555 0.3625251 0.2514298 0.2951331 0.07078335
10.95248 0.03247784 3.126024001 0.100517 0.03477411
0.944939042 15.02599 0.3797447 7.350196 3.691089
0.5161734 0.06013532 18.51743 0.136341146 0 0.5674676
6.003222 0.3300412 0.136745 0.2032445 0.2736352
0.01694185 0.2290965 0.7388725 0.144089581 0.6267312
0.1770189 0.090380668 0.02791174 0.4862085 0.3154855
0.3410108 0.5600154 1.061014637 0.151715 0.01879526
0.2662637 0.09315821 0.06528338 2.042273 0.0800605
0.03187429 7.01004 0.1033964 0.088291592 0.197172735
0.066612435 1.081053 0.0254032 0.1568406 7.873707
0.05183695 0.230379 0.032716777 0.265386 0.05098747
0.1770095 2.791769 0.5566628 0.1647056 0.009815264
0.4466545 0.1722929 0.1355519 0.058481062 0.009436579
0.2312055 0.1453335 3.337192 0.8460651 0.07356741
1.34022 0.3822759 0.1859224 4.774837 0.067989571 0.1272595
0.2179012 0.1511868 0.1874057 0.1441559 0.2735569
0.09715266 0.1594064 1.303755 0.1155151 0.1185385
0.1125438 0.1692608 0.4144077 0.3152325 0.02348771
2.074108 0.06718992 0.07958903 0.1807688 0.3311866
0.1142926 3.691496 0.2306514 0.09910519 0.01821739
0.1074917 0.5212092 0.1249119 0.1123272 0.3094509
0.1250321 0.04030352 0.08346995 0.210566 0.1241002
0.05282811 3.287713 0.3265057 0.5980175 0.173007
0.1987992 0.1312485 0.01213277 3.029828 0.3458686
0.1110272 0.1194568 0.1922725 0.2883713 0.1600098
0.2068766 0.6628432 0.1946542 0.2852874 0.2958985

2.918552	0.1195746	0.1431387	0.1304084	0.1586715	
0.1327833	0.7389438	4.29528	0.3067429	0.5091873	2.390557
0.07623196	0.2360522	0.613520997	0.233845	0.32745432	
0.05430916	0.4412733	0.1507894	0.1038905	0.2485468	
0.2069542	0.8886455	11.49187	0.11246304	0.9901614	
0.05522672	0.180347	0.04299749	0.3255051	1.109764	
0.0783159	0.5714927	0.04366766	0.2247324	0.1823361	
0.2275322	0.3176193	0.2682284	2.059017	0.09546005	
0.3580694	0.098536	0.3304544	0.2116079	0.05500769	
0.05807932	0.01866465	0.9166668	1.171918	0.3558218	
0.1106031	0.06338035	0.0125261	0.360217	0.3777649	
0.1949399	0.07188026	0.1220299	0.385836	0.2812962	
0.0737435	0.2291844	0.2499088	0.6633686	0.2166159	
1.735877	0.151041	0.3276387	0.4123699	0.1160853	
0.4491714	0.112533002	0.1620645	0.114430345	0.022786494	
0.1516247	0.2176951	0.07476999	0.02801835	0.2274957	
0.07283712	0.08135579	0.3857494	0.1830215	0.1344959	
0.1027661	0.1886078	0.2734875	0.03127074	0.1288713	
0.3247947	0.5430586	3.613571	0.5492733	0.8060836	
0.2555557	0.3000611	0.2866677	0.1626526	3.383978	
0.8618763	0.1006147	0.8147406	0.2817226	0.2830027	
0.3738797	0.108553	0.06631098	0.7274813	0.180021633	
0.1120985	1.351921	0.2038696	0.03800728	0.3257693	
1.558579	0.1345219	0.222911	0.06109777	0.04132959	
0.2478552	0.5948125	0.6333185	0.05162582	0.189369	
0.074403244	0.2153317	0.2871054	0.09787342	10.41671	
0.04073097	0.058240989	1.231554	0.2331876	2.204101	
0.02000491	0.09107617	0.6210799	0.1326399	0.1368444	
0.1575184	1.126001	0.1357564	27.99343	6.163327	
0.1942735	0.01663286	0.5427563	0.06243699	0.1464577	
0.1363805	0.1621053	0.2155007	0.5747056	0.4628042	
0.2362047	0.04122075	0.1769693	0.193833579	0.180626861	
0.09528608	0.2261572	0.09730232	0.0614447	0.04639493	
0.0846176	0.4610883	4.797234	0.1485512	0.09103091	
0.332289	0.7252904	0.2168929	0.097190208	0.1728005	
0.08961947	0.1618505	0.136373	0.09022975	0.028499487	
0.09608294	0.1982174	0.03852568	0.6043568	0.1179405	
0.2455344	2.125717	0.3218665	0.1176321	0.1223089	
0.3474583	0.09820022	0.1366857	0.0366552	0.168236	
0.1030513	9.128567	0.6446803	0.071637035	0.08783831	
0.3815085	0.2761204	0.1594657	0.6290322	0.07867438	
0.09699906	0.0545156	0.2924823	0.4190004	0.1720656	
4.726907	0.2813327	0.0999242	0.08122886	0.5760088	
0.158327	0.06759799	0.1198028	0.124606	0.9980958	

0.4718178 0.07502256 0.1738785 0.251414 0.0877387
0.107537495 0.0946726 0.2158064 0.1769369 0.1482493
0.1356909 0.6391235 0.138049918 0.1251661 0.3301577
0.08642815 0.1361217 0.09403702 0.7812982 0.06421545
0.02138567 0.1948644 3.421233 0.1387283 0.1899595
0.3090397 0.1242241 0.177256857 0.2429132 4.834843
0.1884164 0.079008832 0.1774495 0.2186339 0.3057303
0.2272942 0.1337489 0.05847526 4.092597
FAM66C 0.05299002 0.640833 0.08888295 0.1042162 0.1226565
0.7134255 0.06742454 0.2465595 0.2454223 0.304616182
0.2782645 0.2143367 0.3995239 0.1285914 0.07114402
0.6419771 0.1057022 0.706425938 0.08082337 0.1264904
0.185762039 0.5246313 0.1705167 0.7943307 0.5806888
0.193556 0.1628227 0.5693524 0.120471093 0.05658322
0.264259 0.4610228 0.1504412 0.2257972 0.6242598
0.01366606 0.5157105 0.114782 0.1109134 0.121376125
0.624261 0.3077551 0.012977314 0.07013492 0.1155514
0.08027672 0.2031102 0.06700821 0.111085522 0.3684609
0.02518804 0.3587879 0.2909308 0.2296561 0.644216
0.007663662 0.05492006 0.7125644 0.1031419 0.08028991
0.162788598 0.089269142 0.1800587 0.1021306 0.0986589
0.354847 0.340252 0.3465417 0.062635208 0.0391942
0.02395976 0.07229418 0.2528478 0.267397 0.2207265
0.08455952 0.2105496 0.1573126 0.05662032 0.0213742
0.01083962 0.425611 0.06677672 0.2948744 0.1641653
0.1056318 0.333555 0.1001486 0.44848744 0.2742381
0.024030245 0.09049267 0.07721985 0.1315647 0.1327494
0.1050853 0.1873291 0.06564551 0.00523163 0.1769314
0.4488042 0.1252698 0.06463846 0.05608458 0.3152038
0.4044249 0.04946311 0.3647456 0.184680123 0.06233343
0.1396888 0.08759847 0.01094047 0.3676803 0.331181
0.1311637 0.05929022 0.1531073 0.3131677 0.02608798
0.03225701 0.3588136 0.4021414 0.1793965 0.08389527
0.2902477 0.04846749 0.0235988 0.5197062 0.3000406
0.05387695 0.03548746 0.2410432 0.05967684 0.02787336
0.2224852 0.08005147 0.06376739 0.178383 0.09406987
0.1061688 0.08205364 0.4534466 0.21004 0.6707864 0.2268721
0.02427806 0.3331164 0.1208706 0.210093 0.09794461
0.243997 0.04801732 0.1982623 0.3553505 0.2055373
0.1838237 0.586844 0.2014022 0.250291 0.244309738
0.2410631 0.184882572 0.06805514 0.05376022 0.1187717
0.4325966 0.148577 0.5985557 0.287811 0.6870428
0.186599263 0.4157087 0.09832868 0.06797475 0.128415
0.262334 0.1436355 0.1229453 0.2055954 0.1285355

0.02518495	0.1570844	0.1742412	0.1282652	0.1078381	
1.070875	0.05060913	0.1593816	0.02964405	0.1737544	
0.1872295	0.524446	0.09054122	0.03215958	0.4446426	
0.1732483	0.3313154	0.06034762	0.07280377	0.08633092	
0.2222121	0.05806122	0.1679428	0.1651349	0.06041955	
0.07887499	0.16515	0.05294232	0.1257796	0.1913769	0.3809993
0.2083165	0.1203256	0.2632382	0.03272628	0.2034828	
0.2074254	0.2971636	0.468583702	0.1570728	0.437051004	
0.183220818	0.04465854	0.6414637	0.05304775	0.06758668	
0.07984775	0.08746961	0.2242844	0.3657357	0.1486132	
0.04065602	0.03934849	0.2652096	0.2225228	0.1661304	
0.3912272	0.1337476	2.138746	0.3245032	0.2405067	
0.1488106	0.08497555	0.1203108	0.2035608	0.1764562	
0.6052161	0.2406931	0.08890317	0.2521823	0.2673293	
0.2902498	0.2938467	0.04675975	0.1675743	0.1418531	
0.32310523	0.08194162	0.3460977	0.09709947	0.02619494	
0.1671979	0.1062594	0.1250898	0.1280268	0.0134965	
0.07460278	0.1527062	0.9539711	0.1278774	0.04235827	
0.2479779	0.02848852	0.02169713	0.1337837	0.195265	
0.481513	0.05012879	0.126367217	0.5671996	0.04171559	
0.189528	0.15511	0.03184009	0.3325274	0.04423379	0.1047937
0.07676173	0.2510747	0.05281862	0.5168656	0.3516744	
0.284261	0.2531523	0.3909794	0.1553937	0.1542137	
0.03032084	0.07182277	0.1508456	0.254434	0.04784528	
0.1243567	0.04208842	0.0260617	0.178796994	0.069160853	
0.1176623	0.03352031	0.1166288	0.0941071	0.1154681	
0.1700975	0.08024896	0.2908458	0.1160337	0.154575	
0.1654007	0.1896297	0.1162656	0.078148351	0.04086612	
0.03860405	0.1795037	0.4363792	0.07485483	0.040921007	
0.05942925	0.1166903	0.03160979	0.2722403	0.2863469	
0.6153612	0.3662654	0.1788976	0.01535474	0.1320642	
0.1559057	0.2715572	0.2660417	0.1094733	0.06441648	
0.05918652	0.7696434	0.1335385	0.06857339	0.04324207	
0.2149814	0.1004385	0.02862112	0.2557724	0.1568215	
0.07056659	0.06262101	0.1281986	0.2348501	0.1482363	
0.4323917	0.56915	0.2623565	0.2228977	0.05929883	0.1636804
0.1708267	0.1106905	0.02044749	0.186119	0.1092677	
0.2326778	0.1231673	0.1519971	0.5187398	0.083380204	
0.0629597	0.1007064	0.4268123	0.1161075	0.2472349	
0.3106014	0.178397162	0.2206216	0.08923428	0.06825385	
0.05685829	0.05851002	0.2692386	0.1751872	0.0245653	
0.2152278	0.2623365	0.0607065	0.556417	0.07552934	
0.1673908	0.309332859	0.1897402	0.311912	0.1142271	
0.05899134	0.254791	0.3372458	0.05082961	0.1257971	

0.03840868	0.1511311	0.4305205
AC008687.2	0	0.05343332	0	0	0.03664755	0.2546587	0	0	0
0	0.2398282	0.2744569	0	0.026196	0	0.04139811	0
0.042073234	0	0	0.032648449	0.08347655	0.4620803	0.100281
0	0.2415728	0	0	0	0	0	0	0.08090845	0.5279804	0
0.1224952	0	0.02509375	0.08644938	0.074178425	0.6543171
0.07388977	0	0.01796148	0.08631169	0.04111756	0.09753056	0
0.08534664	0	0	0.05272101	0.4795856	0.08402097	0.1634266
0.1030394	0	0.0572829	0.05603081	0	0.063441276
0.152411529	0	0	0	0.1514599	0.03812294	0.0282383
0.056142828	0.07807016	0	0	0.2211114	0.05210442	0.1087073
0	0.1952331	0.09097199	0	0	0	0.09155875	0	0.0287293
0	0	0.1437408	0	0	3.750213	0.080772817	0	0.1909399
0	0	0.05708657	0.1895777	0.02942055	0	0.02831997	0
0.1464589	0	0.2010848	0.1441488	0.06322757	0	0	0	0
0.01692021	0.02243387	0	0.03080082	0.5937057	0.02218266
0.03126151	0.9296709	0.7017666	0	0	0.1803483	0.2317229
0.05187141	0	0.08130075	0	0	0.1552789	0	0.06036549
0.1590452	0.1137148	0.05630643	0	0	0	0.06016595
0.1537432	0.1833025	0.01903279	0.1765163	0.04347007
0.02941696	0.6856437	0	0.07253848	0.04777402	0.04924632
0.05458436	0.07746377	0	0	0.05553489	0.02041776	0.1754596
0	0.03308272	3.453542	0.3739123	0	0.246944	0	0
0.03441983	0	0	0.02611302	0	0	0.1153236	0.09649473
0.02980952	0.05686228	0	0.5312505	0.3513617	0.04023347
0.02687842	0.3556371	0	0	0.187736	0.02603008	0
0.2416506	0.04353174	0.07560549	0.06912634	0	0.0718819	0
0.368139	0	0	0.4645718	0.01784948	0.4163177	0.05693932
0	0	0	0	0.02280831	0.4229087	0.04332547	0	0.1287231
0.1423639	0.05243818	0.14295	0.02969628	0.07780154	0.1232611
0.8311965	0.04889013	0.02990017	0.06640179	0.02927049	0
0.1825076	0	0	0.04002951	0.09745314	0.0452849	0
0.9109064	0	0.2512955	0.09456513	0.1624498	0.07288373	0
0.1674082	0.1955465	0	0.09477703	0	0	0.1464799	0	0
0	0.02914599	0	0.1860774	0	0	0.03984401	0.05023171	0
0.05203285	0.4052129	0	0	0.1387085	0.057922826	0.02623145
0	0.3071814	0	0.107048	0.02886217	0.4287086	0	0	0
0.2087964	0.5777624	0.02547166	0	0.3119635	0.025535575
0.01944814	0	0.07955683	0.04472563	0.02995517	0	0	0	0
0	0.1223132	0	0.0440542	0.1878628	0.02457324	0.04546461
0	0	0.02300891	0	0.08562722	0.1420755	0.4285739	0
0.06794492	0	0.0693384	0.09245714	0.1906041	0.1013333
0.02357862	0.0233603	0.514054332	0.116235122	0	0.03755725	0
0.8083785	0	0.07260299	0.07193084	0.1163833	0	0.04075079

0.1425541 0.02786456 0.1488775 0.033356178 0 0 0
0.3008822 0.02580609 0.073358775 0.07609883 0.2295986
0.1133332 0.1067594 0 0 0.04559731 0.0267257 0
0.02518622 0.08384729 0.09361894 0.03909269 0.03774076
0.2598276 0.1248273 0.08362023 0.136019 0 0.02153326
0.07411453 0.3316808 0 0.2865758 0 0.06658115 0 0
0.1299426 0.1207919 0.127073 0.1080838 0.4041852 0
4.056358 0.2852778 0.6263994 0.08044619 0.03054672 0
0.01958834 0.05149626 0.3282179 0.204363 1.089359
0.166083388 0.02565167 0 0 0.4856718 0 0.1124874
0.025381843 0.2666337 0.33327 0.05561734 0.0212353 0
0.03830651 0.9091118 0 0.07716746 0.1383202 0.1700439
1.466889 0.1015506 0.4181446 0.245681675 0.02501071
0.09896676 0 0.040674354 0 0 0.04141902 0 0.02648267 0
0.08871159

FTX 0.3933057 0.6094466 0.6036893 0.2715972 0.584257
1.230933 0.3514294 0.6133503 0.303598 0.427462299
1.004681 0.5120331 0.3292943 0.2799025 0.4712455
1.521846 0.3591912 0.674324436 0.2164843 0.3795266
0.357152307 0.7857578 0.379792 1.119685 0.5906808
0.7952146 0.4087938 1.189652 0.27929274 0.198919
0.3749502 1.057657 1.463706 0.5745905 0.5863822
0.8517933 0.2810546 0.268125 0.5278318 0.345972431
0.692628 0.3508921 0.28606174 0.2254265 0.7392515
0.5230218 1.064163 0.6315775 0.552461291 0.3330576
0.2680399 1.683255 0.3253549 0.320628 1.510603
0.2155336 0.6574891 1.583594 1.140353 0.411144321
0.825812559 0.361890387 0.8613029 0.4408307 0.4434292
1.377514 0.4590669 1.613986 0.223765608 0.9417563
0.4148307 0.2610201 2.514908 0.5854524 0.8757571
0.329943 1.19019 0.6248761 0.774679 0.299404623 0.2512989
1.203463 0.6192441 1.690775 0.4018039 0.2913449
1.211623 0.439177 0.49511694 2.477364 0.39956112
0.9338814 0.5282608 1.000037 0.4799771 0.5542946
0.7234376 0.2844178 0.5209371 0.6051938 3.129598
0.8031513 0.8134141 1.079973 1.59645 1.422655 0.4238258
0.4748594 0.414846567 0.5401357 0.7231651 1.29174 0.1164236
2.188807 0.7803764 0.2483069 0.2412418 0.5199492
0.8681537 0.4907866 0.6497509 0.8207633 1.216138
0.5410462 0.7515715 1.061736 0.4919312 0.2152524
1.529661 0.8530476 0.4897231 0.7310064 0.6991273
0.4631599 0.1536046 1.168695 0.6805961 0.3826602
0.5684386 0.3730619 0.6052502 0.5239069 0.8791842
0.6485932 0.684302 0.8890649 0.8919442 2.499644

0.8206114	1.235892	0.490486	0.6480095	0.358544	
1.043136	1.32802	0.5058913	0.4149451	5.20972	0.53248
1.12564	0.43568662	1.146526	0.6711579	0.8039619	0.2305882
0.402497	0.5442561	0.5314587	0.7421475	2.120594	
1.136385	0.229119907	2.186614	0.3833475	0.383822	
0.503065	1.010767	0.7881351	0.5994606	0.6991264	
0.438464	0.3685095	0.374125	0.5937846	0.3964827	
0.8928754	1.376943	0.445594	1.436186	0.2724416	
0.8107228	0.8164396	0.26896	0.409306	0.3544503	1.843326
0.416255	1.106197	0.3862811	0.6668342	0.3627387	
1.279413	0.415779	0.905191	0.4617374	0.495996	
0.2797841	1.07794	0.6700299	1.280682	0.6333905	1.201218
1.136995	0.5147938	0.7935397	0.4498338	0.3397658	
0.2364995	0.6230242	0.456887291	0.7782638	0.901705336	
0.134294277	0.3411517	1.875957	0.5414692	0.5455363	
0.5462383	0.1878969	0.4285703	0.9222105	1.170953	
0.5222621	0.3925585	1.013622	1.170728	0.4914585	
0.4762036	0.8026026	1.25797	1.062938	1.115864	0.7006694
0.4756234	0.3410822	0.7463389	0.4970561	2.127052	
0.9113152	0.6352169	1.437649	1.114926	1.592891	2.0145
0.3909686	0.4805513	1.225274	0.319274547	0.3470149	
0.7343157	0.9464581	0.08296274	0.5325632	0.4797183	
1.303185	0.6812018	0.3200758	0.3943672	1.656509	
2.079831	0.7797684	0.2318183	0.6150749	0.422261464	
0.7009197	0.8560918	0.5059876	1.89639	0.5232872	
0.505691311	1.87924	0.5190177	0.3424877	0.5327368	0.3128972
1.617649	0.3343579	0.7288415	1.544123	0.9619339	
0.4970706	1.038664	0.6146215	1.122756	0.6244699	
1.269072	0.6269257	0.7086451	0.7029397	0.3760869	
0.5468371	1.155161	0.5960265	2.143997	0.2879268	
0.2773369	1.225714139	0.617696537	0.6759725	0.406074	
0.3841536	0.223537	0.4275903	0.4432903	0.6862287	
1.134984	0.635548	0.5339072	0.698729	1.023155	
0.6501858	0.760903812	0.5657864	0.5966484	0.64323	0.6804033
0.4245464	0.292382332	0.5162608	0.5256955	0.2426721	
0.9156779	0.9221099	0.4823597	0.7230728	0.5643254	
1.283844	0.8628703	0.8816819	0.4048882	0.7160619	
0.5440774	0.3647794	0.8600708	1.620312	1.192673	
0.913201631	0.5989419	0.4148093	0.703172	0.4612107	
0.9113222	0.3576053	0.4545151	0.4244236	0.3864204	
1.038015	0.4029022	1.772639	0.6159294	1.025144	
0.4964604	0.8136987	0.5806046	1.507998	0.3979454	
0.3315704	0.5347608	0.6727503	0.9454417	2.267142	
0.5747847	0.7908528	0.525804334	0.2697358	0.3415219	

0.7923024 0.9708006 0.6087167 1.705105 0.647874436
0.859211 1.170032 0.8513118 1.258742 0.1881597
0.548985 0.932132 0.2940895 1.526906 1.006799
0.4845085 2.263966 0.5654951 0.5798605 0.473230847
0.9565416 1.837954 0.2696152 0.407008868 0.6405989
1.77532 0.1966931 0.9309396 0.3099146 0.4563147 1.064481
POLH-AS1 0.4549365 0.3367918 0.8639342 0.6168273 0.2309906
0.3418314 0.7255775 0.6331757 0.4078123 0.625145622
0.6172547 0.5478049 0.6345512 0.1926332 0.2871016
0.2065724 0.3512855 0.254139333 0.507364 0.3761224
0.514460406 0.3069239 0.5974372 0.8603234 0.2110755
0.6575038 0.4645953 0.2552912 0.311953203 0.6326962
0.3317741 0.3157139 1.544071 0.92441 0.8004772 1.608523
0.04581145 0.6722083 1.646031 3.727403405 0.628108
0.4398556 0.824826297 1.858559 0.9822677 0.7774956 1.2124
0.9148922 1.94256999 1.172124 0.9148166 1.536897
1.763325 0.8605784 0.5722681 1.443246 1.034272
0.4362758 1.39794 0.378011384 0.316565557 0.780531767 0.2046247
0.7212585 1.070356 0.6563261 0.4605559 0.4301349
0.457082011 0.6458531 2.995586 0.6807336 0.8274926
0.2599958 0.5938275 0.4511948 0.6494622 1.218473
1.277329 0.100631462 0.8930912 0.4167927 0.6444999
0.7846871 1.221187 0.6820432 1.177803 1.305713
1.30712839 0.5959085 0.650533934 1.458391 0.7271146
1.226446 0.4054021 1.709137 1.436742 0.3399708
3.177391 1.606514 1.304552 0.6410658 1.623056
1.443477 1.40829 0.4649462 0.7197995 0.6211336
1.077128096 1.604307 0.5599051 0.9426759 1.030173 1.2619
0.9875105 1.200104 2.085364 4.011134 0.8130985
1.090066 2.186908 0.236821 2.921113 1.416771
2.257061 0.6405514 1.208059 0.5713974 1.169036
0.7063078 1.067473 1.236376 0.8959349 0.5323517
0.3608825 0.3553945 3.713053 0.4424324 1.970398
0.4043765 1.249628 1.205303 0.9018941 0.8343721
1.108111 0.973187 1.162081 0.9159099 0.9700033
0.9174583 0.9629503 0.7473846 0.9574706 1.86687 0.6112953
4.469789 0.734327 1.89407 1.566621 0.7855936 0.70783384
0.9403827 0.240121956 1.508593 0.3073448 0.3611416
0.749131 0.1508752 1.03928 0.542015 0.3270998
0.709577408 1.174314 0.791477 0.579103 0.7673619
0.9369645 1.035504 0.9176672 3.328423 3.011017
0.4150046 0.4601745 1.914131 0.9527928 0.5439754
0.6402244 1.191359 0.7019698 3.210032 1.321465
0.9433505 0.4611034 0.6955019 0.7822846 5.124368

0.2156362	0.6560157	3.005706	0.394181	0.6774215
3.751863	5.208169	1.593357	1.19397 1.615734	0.4951331
1.656497	0.5483648	1.583739	0.5443651	0.6863141
0.7219564	1.974667	0.9154266	1.065706	0.3298079
1.883396	0.922464	0.517299433 1.465328	0.139240925
0.385097507 1.177434	0.8957813	1.534198	1.681933
0.3759296	1.414492	0.4839766	0.2152391	0.4095704
1.282459	1.146271	0.5451756	1.170909	1.26836 1.568129
1.474811	1.977915	0.9892147	2.338177	1.459609
2.109469	0.5051971	0.7187856	2.101359	0.7637584
0.962153	3.003192	1.213679	0.1656063	3.716933
0.9843808	3.27078 1.488253	1.359997	0.197756719 0.8680225
0.623444	1.398348	0.5755301	0.4873027	1.091515
0.8487563	0.8706539	1.372521	0.5587844	0.9748517
1.138017	2.046995	0.4985654	1.515702	0.61698076
0.7967845	0.3808484	0.6964571	0.7400059	0.9755096
0.244978205 1.225252	0.6305486	0.3367219	1.000741
0.952972	2.647229	0.8445948	1.455462	1.006758
3.462658	1.065747	0.2433445	0.3988211	3.427109
1.10191 0.5472537	0.2954563	3.201222	1.915269	0.5134831
1.383966	0.4532579	5.581428	1.011286	1.263243
0.5521526	0.428836769 0.345965817 1.30117 0.720037	0.1830326
0.2769149	1.087266	0.4766863	3.173676	0.5868538
0.755174	1.701654	0.7038429	0.7317965	0.6451358
0.332887917 0.4397737	2.48393 0.09054835	0.9482348	0.5286339
0.886233282 0.6994943	1.540965	0.6994607	0.4405945
1.536594	0.9053657	0.2155509	0.5334341	0.9253295
1.389058	1.981845	0.658926	1.190945	0.8722308
1.152456	5.802579	0.6149043	1.185976	1.033116997
1.662628	1.167865	1.393729	1.697857	2.272922
0.6257021	2.308146	2.309461	1.623393	1.160295
1.036288	1.067927	1.578241	1.466795	0.3660769
0.5436735	1.626866	2.796652	1.605673	1.155221
1.857679	1.214081	0.6762135	2.131457	0.4293687
0.8652611	0.305325017 0.6736803	0.6382569	2.241854
3.607848	0.6452619	1.595276	0.639930098 0.8986543
1.697994	0.4528038	1.026158	0.1695199	0.6639795
0.6077441	0.795131	0.5134104	1.235102	0.7681173
1.900535	0.5867371	1.937925	1.142969291 2.70621 0.9226901
0.4245712	2.029609195 0.7197461	1.216172	1.43586 2.73782
0.6815944	0.2767091	0.7455358
AL731566.3	0.0458316	0.6573815	0.4215767	0	0.1803476
0.4641518	0	0	0	0.057421986 0.3540681	0.09004265
0.3538788	0.08594272	0	0.23768 0	0.103524046 0.4527177

0.2936602 0.214223261 0.04564438 0.1166133 0.7676612
0.4080729 0.7925416 0.2901888 0.5798411 0 0.1039962 0
0 0.265441 0.8660876 1.268613 0 0.04769015 0.4527957
0.2836194 0.36504209 0.6662035 0.2424144 0 0.1473181
0.3303623 0.1686209 0.9599222 0 0.56000319 0.03660577
0.3174442 18.20455 0 0 0.2680814 0.3380473 0
0.7047423 0.1378676 0 0.052033854 0.250012602 0.04564634
0.3217873 0.2522832 0.8385239 0.06253605 0.463215
0.046047745 0.1600808 0 0.1993079 1.042781 0.08547097
0.3209781 0.2486637 0.3202561 0.2611499 1.318149
0.089792832 0 0.150191 0 0.9425386 0 0.266255
0.2357892 0.2831791 0.78503828 0.3618687 0 0.1535257
0.1566069 0.2321349 0 0.6086834 0 0.09652168 0.3076925
0.4180992 0.7459716 0.6807027 0 0.1099517 1.182293
0.6914477 0.0661163 0 0.040528935 0.4277083 0.2775555
0.2576003 0.4021573 1.8189 0.162317 0.1455518 0.3589649
0.4720276 0.1015732 0.2397399 0.04742899 0.1479198
0.08446963 0 0.3524433 0.9335481 0.2934401 0 0.2547162
0.0424196 0.2640594 0.05217888 0.279803 0.04618193 0
0.6425777 0.1743755 0.1480424 0.3026364 0.1202742
0.1873259 0.5791072 0.1426147 2.605768 0.1297748
0.3335807 0.1189906 0.1959187 0.2423478 0.179078
0.1694265 0.1296724 0.124592 0.1821965 0.6028713
0.2878201 0.08190443 0.8140229 0.2575053 1.073376
0.069080787 0.5063521 0.374953842 0.2084681 0.05646155
0.1455297 0.07311117 0.08567048 0.5617561 0.6206671
0.643193 0.105525257 0.9290789 0.4197405 0.09518711
0.0363105 0.2216793 0.06599812 0.2204539 0.106069
0.2304778 0.3703061 0 0.2134959 0.2095492 0.5662835
0.2499299 0 0.6803602 0.1188738 0.235827 0.04829689
0.1393586 0.07006685 0.3940479 0.4010914 0.1463995
0.6373907 0.3269071 0.5887574 1.480925 0.9764313
0.134795 0.2993141 0.1734326 0.1421403 0.1104511
0.2111546 0.194609 0.8601847 0.9379683 1.120402
0.2127066 0.6571331 0.1319494 0.04009916 0.3433332
0.7987765 0.09602937 0.142547719 0.0427688 0.09663408
0.096213636 0.1969907 0.7193701 0 0.09464369 0.1067308
0.1677531 0.2748132 0.775613 0.1065917 0.05977845
0.1084799 0.05492255 0.2566163 0 0.1166027 0.1035028
0.3275727 0.7208473 0.1377772 0.2431147 0 0.2868627
0.08803089 0.3815467 0.8256597 0.06677402 0.2287574
0.6179926 0.620632 0.1707071 1.080142 0.4583537
0.2239931 0.7205258 0.285046084 0.258177 0.08850151
0.04199115 0.256771 0.3511985 1.136277 0.1622871

0.6274793 0.03968909 0.19944 0.2283367 1.563786 0.2924821
0.1245627 0.1919019 0.167552038 0.06380463 0.4574616
0.5655144 0.6603027 0 0.218592539 0.7849226 0.2582585 0
0.08446869 0.2340799 0.8000683 0.1806639 0.9244983
0.08061884 0.3356063 0.2218906 0.8074701 0.3019465
0.8202999 0.1404611 0.4661152 0.9666573 0.4122684
0.1114554 0.2737892 0.2843531 0.556104 0.07816573
0.2077813 0.03867787 0.1915987 0.148807712 0.1906696
0.2414014 0.03080405 0.2858081 0.1153084 0.5659271 0
0.3539814 0.2290951 0.2007174 0.4345037 1.075676
0.09141685 0.2442154 0.054716759 0.386276 0.09460226 0 0
0 0.180504241 0.1248309 0.1255429 0.5112509 0.2001439
0.3621757 0.09424943 0.2243905 0.7452845 0.0903072
0.4131493 0.3667768 0.1535705 0.1923804 2.476365
0.5209302 0.2047641 0.4800909 0.669368 0.241984029
0.6358084 0.8510312 0 0.4208297 0.2820557 0.1563268
0.1638274 0.1534577 0.2599951 0.383679 0.1981444
0.9380152 0.07091932 0.5424682 0 0.8260092 0.06685195
0.6279363 0.3958866 0.2004327 0.2736596 0.6747782
0.2956567 0.244728 1.117442 0.3922599 0.227033023
0.08415691 0 0.0426913 0.9104979 0.03951316 1.291654
0.041635831 0.2915868 0.1640067 0.182467 0.452841
0.06617695 0.1256743 0.1355716 0.5869421 0.1265838
0.4159788 0.6694471 2.727087 0.1110543 0.08069593
0.115145985 0.4923245 0.7305433 0 0 0 3.323373
0.06794288 0.3489914 0.1737664 0.06172645 0.4001816
AC138956.2 0.1650979 0.2368068 0.8352488 0.2365673 0.4691997
0.6688006 0 0.1319116 0.2973631 0.206849654 1.724219
0.5946567 0.8498453 0.6191782 0.08971923 0.3465524
0.1646651 0.372921847 0.1359011 0.4113838 0.257230203
0.2192314 0.6301095 0.5925697 0.5088426 2.033071
0.1306674 0.3698821 0 0 0.2332787 0.2466514 1.009312
1.039961 0.6720424 0 0.05726431 0.5684114 0.4895523
0.657490583 2.7776 1.018783 0.400957228 1.149807 1.388398
0.4454402 1.696933 0.4140676 2.045302057 0.4835012
0.3494091 1.531705 0.6494389 0.7033573 0.8047521
0.3382607 0.2424076 1.325752 0.3586821 0.410341305
0.874720617 0.450306789 0.1918356 0.1287962 0.7194609
0.7271795 0.1501813 0.6396403 0.193522626 0.7496509
0.9165366 0.2393204 1.116026 1.436819 1.798612
0.6469337 0.5768249 1.747076 0.4789984 0.350413127
0.09568834 1.322515 1.149489 0.9619963 0.2608549
0.6394155 1.047565 0.7140618 0.5655844 0.6517749
0.45077759 1.167532 0.1410352 1.904708 0.3800645

0.6465487	0.6401326	0.66642	2.678614	1.143526	2.135974
1.177958	0.3804039	0.3960761	0.567859	1.639766	
0.2778638	0.1370148	0.316325269	0.7336771	0.7998645	
0.9721345	0.1931574	0.7280195	0.3898068	1.33264	0.1539392
1.482376	0.5488415	0.6908869	0.6834087	1.598542	
2.256763	0.102171	0.7141483	1.921654	0.6795329	
0.02976028	0.7391432	0.5602922	0.9313969	0.9398128	
0.2239837	0.9981595	0	1.648376	0.8637035	1.836885
0.7873515	1.371992	1.012198	1.158946	1.04888	0.5504538
0.6233125	0.267033	0.8929927	0.09410034	1.576255	
0.6182092	0.4323104	0.3114102	0.199473	0.7657083	
0.8244443	0.5184033	1.647318	0.9122812	1.468707	
0.736494	0.248847835	1.672017	1.294407416	0.8510867	
0.101695	0.633454	0.1755776	2.391715	2.660657	
0.4065113	0.8404647	0.380130754	1.027525	0.2800036	
1.257263	0.1526004	0.5057479	0.2377432	1.694155	
1.114427	0.1937235	0.6391813	0.6779356	1.615048	
0.7296917	0.4079815	0.6859547	0.4095297	0.9531055	
1.213278	0.6135374	1.420825	0.4183398	0.3365332	0
1.661567	0.6855823	2.487393	0.280383	0.4498804	
0.08467769	0.5185869	0.4855682	2.021646	0.6247515	
2.154786	0.1989374	1.711432	1.705851	1.368557	
1.935781	0.2924634	1.098259	0.5159222	1.716425	
0.8666886	0.20613	0.4577699	0.778329	0.285275423	0.5649045
1.624477455	0.202176191	2.345669	2.95128	0.289892	0.7670979
1.089342	0.167859	0.5224747	0.4035734	1.087921	
0.5742354	1.237451	1.22005	2.311004	0.3567263	1.143427
2.143861	0.7585755	1.339562	0.7168939	0.4013924	
0.4091643	1.320402	0.6342226	0.9621048	1.129114	
0.8819736	0.7063255	0.6678534	1.83202	1.02489	0.9976833
0.8648698	0.4706824	1.001784	0.427839056	1.188364	
0.7704492	0.3025272	0.1541599	1.757101	0.7106217	
1.753808	0.9208839	0.881654	0.3592186	0.5483541	
1.422521	0.275943	0.3739241	0.8064966	1.056244237	
0.4405299	1.373251	1.305857	0.6827435	0.3245143	
0.426524553	0.7657827	0.4457772	0.06313536	0.9128377	
1.184523	1.654518	0.8026543	0.5087951	0.8954341	
0.8955151	1.243372	0.8269911	0.2945837	1.008433	
0.5481439	0.5130504	0.8969208	0.1856379	1.115258	
0.3522369	1.229181	1.517605	0.4692905	2.99394	0.4179847
0.4601272	1.489016559	1.52631978	0.8454383	1.257599	
0.1072457	0.3115293	0.4181792	0.1072544	1.877286	
0.7564912	0.964052	1.685601	0.533498	1.481888	
1.063008	1.182628128	0.7472717	0.6815663	0	0.5926468

1.067434 0.288989114 0.1498916 1.256222 0.6138884
0.4656283 0.4892459 0.4526829 0.5388773 0.894906
2.846473 0.3472645 3.137921 0.7560439 1.848018
0.5575339 0.5117816 1.290828 0.9470624 1.875414
1.113829263 0.9331077 0.3284621 0.6066456 0.8085031
0.8184778 0.3754213 1.081944 0.2303319 1.40486 1.945201
0.8327318 1.439198 1.277354 1.194183 0.3203173
0.6887755 0.6823204 0.8225446 1.18841 0.842349 1.182956
1.041744 0.3296541 0.7934192 0.5367108 0.5756777
1.47210276 0.4547342 0.7522313 0.4613571 1.298279
0.5930716 2.187966 0.44995085 1.225438 1.083128
0.6025212 0.7947149 0.516506 0.1131783 0.6511544
0.4879213 0.4559895 1.384949 1.842142 1.107575
2.066915 1.138531 1.278927332 2.51244 0.7797381 0.4245712
0.560813067 0.3806349 1.330188 0.6934549 0.984777
0.4173027 0.3705925 0.5460467
ABALON 0.4886135 0.2774152 0.3511293 0.3500649 0.5007022
0.4097853 0.02359175 0.155549 0.341808 0.277393756
0.1507275 0.2099891 0.2240055 0.1431626 0.5974368
0.2714915 0.6548531 0.241429173 0.6033065 1.027271
0.294402198 0.1064476 0.1554027 0.2100825 0.2091582
0.1800284 0.2296121 0.4587997 0.472109944 0.2598539
0.453073 0.2600533 0.4569081 1.03395 0.6464038 0.217569
0.1906604 0.7062644 1.210655 1.074281338 1.004945
0.1951756 1.183999213 0.8883522 0.2279874 0.9156921
0.6129614 0.6072535 4.244459284 1.280529 1.066405
0.3313417 0.5323829 1.124989 1.354588 0.6194276
0.5156416 0.7904619 0.7042863 0.879764857 0.4853941
0.780878824 0.3953937 0.7236409 0.4727823 0.175894
1.020885 0.4861202 0.797741493 0.3039936 3.944897
0.381806 1.049785 0.8827359 0.8257873 1.974457
0.503842 1.392067 1.612151 0.508558669 1.977928
1.034105 0.3925334 0.4160691 2.388406 1.38232 0.5027527
1.349111 0.99385929 0.8094711 0.588570536 0.6223049
0.5217486 0.554253 0.6854342 1.6691 3.263566 0.6431397
1.473585 0.6113422 0.4397008 0.2534619 0.5980916
2.142932 0.4490353 0.3513012 0.8260195 0.4751957
2.45071209 0.2646327 1.049533 0.8214358 2.224101
0.3114072 0.9193131 0.4061187 0.6662988 0.6592816
0.5019591 1.238005 1.129797 1.47842 4.727813 0.3685242
3.662016 0.2443722 0.7890787 0.4954312 0.6152406
1.059933 3.72238 0.3389845 0.8545044 0.4692696 0.699767
0.7687419 4.865424 0.5260964 1.403159 0.9516706
0.4316633 0.7717371 1.009657 0.7475588 1.441185

0.2284444 0.4955335 0.3459411 1.190917 0.1789839
2.229611 1.044034 0.3528251 1.563031 0.1896931
0.3356137 1.077842 0.4158375 0.1930275 0.4342334
1.024159874 0.1855649 0.374756809 1.812719 0.4702657
0.4302992 0.7185487 0.3639082 0.5718583 0.3007714
0.264705 0.984384843 0.8634264 1.180871 1.363632
0.9254312 1.233366 1.15436 0.683048 0.4858941 0.1228569
1.125756 0.3932953 0.9744522 1.040214 0.6886162
0.6839721 0.8780249 0.9003739 4.079188 2.448693
0.9493397 2.182138 0.4668669 2.625605 1.322906
3.077652 0.5536318 11.83249 0.03566352 0.2525647
0.2877707 0.4266264 1.913357 1.401177 0.7872804
0.5703639 0.9760767 0.3306614 2.665171 0.8105256
0.3813858 0.9070714 1.103067 1.809687 0.7080462
2.794244 1.167294 1.295715 0.728194373 2.771387
0.185117992 0.448761025 1.022719 0.7389641 0.2412974
2.183544 0.1955703 0.8848981 2.037127 0.7665921
1.038723 0.6771331 0.6204198 0.2470216 1.17554 0.2749335
0.6150795 0.5258629 3.07133 1.063549 1.820764 0.4353515
1.286621 1.99902 0.6085604 1.118616 0.3209212 0.5227883
1.453479 1.47554 0.4221526 0.8815235 1.370229 2.874113
1.902941 1.983563 0.458999589 0.3368873 0.678155
1.189124 2.438042 0.7410294 0.9621735 1.306626
1.289141 2.770165 0.4152816 1.007957 1.878714
0.7795425 0.8092322 0.7032704 1.262957601 0.8237101
0.2133699 1.072463 0.2994243 0.7857918 0.974046288
0.86623 0.6291743 0.7882796 0.9919855 0.3230833 2.976467
1.510761 0.4876733 0.7856206 0.6149548 2.470316
0.4035229 0.1383194 0.435962 1.392173 0.6211603
0.7099734 1.425012 1.101591 0.3844065 0.4357789
0.2273775 8.346318 0.4153442 0.167516 2.042646
0.504027802 0.614056975 0.7640361 0.1898585 1.225948
5.551103 2.037717 0.4166183 1.316905 0.3052996
1.086648 0.4676812 0.459649 3.510085 0.9356672
0.264325362 0.3360265 0.9612842 0.1491692 1.123631
0.8955514 2.826413761 1.445198 1.268711 0.4258186
0.7334753 0.957753 2.017448 1.856479 1.716175
0.699513 0.6538094 1.046292 0.9004735 0.5875202
0.9384617 0.4969903 1.483759 0.5598117 0.1561038
1.860954244 0.9885156 0.637938 0.8545369 0.1402029
0.8770454 0.2734282 0.2547088 3.757738 0.42227 0.3692745
0.4840984 0.329869 0.9214667 1.014083 0.4062818
0.8332193 2.583584 0.7036798 0.2857679 0.2754502
0.7840785 0.272981 0.4362163 0.7419515 0.6887272

2.591911 0.355498162 0.8761738 1.185861 1.144948
0.1516699 0.2830289 0.3611656 1.033413129 1.098947
0.7740691 1.671735 1.82782 2.502379 0.6489762 2.326089
1.105579 1.440892 0.3212692 2.025872 0.8551072
1.008213 0.7662085 0.853550627 1.031971 1.122282
0.3092253 1.066980435 1.161707 0.474518 0.5432574
0.6685486 0.4631336 0.6272225 0.5575358
SNHG1 1.694232 3.465768 3.383692 4.197579 4.195929
3.878189 2.294958 3.050403 3.200827 9.397010169
6.839827 7.303068 13.82831 5.240595 4.515199
3.616493 5.14653 3.291031991 1.767148 2.090153
4.501939464 4.803828 4.241887 4.26221 2.943351 8.862813
2.773656 3.076952 1.360434893 6.108864 1.7599 2.770376
6.078617 9.855821 11.41857 18.60388 3.731145
3.623495 5.996217 8.445070148 14.31588 15.91056
5.233855187 9.372294 6.333201 13.63645 13.93472
9.109487 24.828994848 9.642219 5.608407 9.937545
16.63227 14.95332 6.878188 17.55584 22.83123
4.817803 11.66682 4.714336523 4.782418197 11.589525202
2.115008 2.693198 12.45598 3.347159 3.557329
3.244849 4.543144581 11.62988 8.341707 23.81327
6.221848 9.50753 7.389552 19.02103 3.456718 9.187784
16.21316 12.329995063 5.730295 4.888659 23.6708
5.830916 3.475473 6.906232 5.914343 7.447188
13.82097939 7.962502 9.2219217 13.52743 5.703288
8.607325 10.85025 4.832904 8.717461 5.869384
10.0077 6.873096 6.731741 6.397243 18.52919 12.48589
7.332093 4.963263 6.344577 3.90514 8.072552458 13.63467
11.48538 20.76268 9.747968 10.24693 13.17871
5.881121 9.015233 18.09579 10.28141 19.89372
12.1769 29.75395 23.27292 3.36087 23.37633 13.37927
15.83599 5.066284 5.417398 3.343853 4.955885
14.76597 22.38692 25.5025 2.810387 4.723155 7.443119
27.28388 12.94128 21.59105 13.88644 15.24513
8.202834 26.86718 10.67896 3.450385 38.07652
4.669541 10.40755 7.87811 12.81576 3.837741 11.39864
4.1249 5.192721 15.08504 12.98876 7.401511 25.09076
14.40399 6.873924528 10.28637 9.545063496 7.544457
5.970869 8.112566 2.992672 13.24979 12.32166
27.27738 4.716356 11.444405129 9.789203 6.691461
12.15087 10.84236 2.072997 11.51903 16.00482
16.47176 21.03143 11.84496 4.907994 12.33559
8.839262 6.060198 3.444292 15.37634 5.247663
16.20622 4.521389 5.987902 10.94616 7.066659

7.555984 7.271287 5.743009 34.50545 7.871149
12.89233 13.95396 5.629178 6.480793 10.62566
31.11915 12.15835 3.495705 4.263695 8.875889
32.09563 13.81393 4.4361 8.538008 5.204793 9.502978
8.826256 1.77172 19.20112 9.328528 6.191843804 6.295166
19.068147513 1.534730448 20.39693 10.48659 19.48866
12.56076 9.106986 10.79317 14.34534 3.83598 5.127808
8.884596 6.262374 15.03714 7.15796 6.691815 10.5693
28.97553 8.602036 22.31316 4.899333 6.401761
6.978887 20.55894 3.744547 14.05935 5.497315
6.582397 9.835401 11.40859 22.47114 9.645749
10.7431 11.31791 10.27939 15.54205 2.89490759 14.75889
8.466538 9.363199 10.15288 14.07432 14.91243
8.975943 10.59164 11.34875 4.275428 6.356294
9.058686 6.746056 8.715178 15.62392 19.17887565
8.053271 10.93406 6.219384 4.470658 8.070349
4.137151891 4.980881 8.146471 1.650798 14.61091
15.79347 8.68814 10.69781 12.20458 5.60317 5.970196
30.21007 2.629753 3.825752 5.950647 7.45659 4.209308
14.08811 5.532365 15.63071 9.424493 7.266871
10.55593 6.438725 4.454242 8.533001 11.92097
14.752776454 16.714420698 8.411012 10.53963 5.566085
12.70004 9.163202 3.317006 17.85057 5.578216
4.582327 7.791257 4.310326 9.856457 24.68726
22.89604999 12.67093 15.65715 2.195725 17.28711
11.42916 14.193243298 5.273025 10.11527 12.89754
7.867357 6.113403 6.36757 4.014894 15.09248 12.94939
12.94341 10.9698 4.176063 12.94597 10.9412 9.972203
23.17049 3.901387 13.47182 2.488207001 16.05363
12.04618 18.54496 16.18169 8.240503 3.961414
7.355834 13.54734 18.15795 8.203403 12.70952
6.350943 17.33392 8.734453 2.566632 12.09534
27.39848 17.32994 12.08035 8.496153 8.420948
16.10604 6.109902 39.48751 3.805417 22.28406
14.872923657 21.81949 12.71343 6.211698 12.70815
7.143235 17.65238 7.407497565 8.477948 7.903717
24.34725 16.89868 5.428835 2.370598 12.00267
28.38084 8.838783 10.47492 25.90063 15.50205
12.63453 8.986585 8.736594344 13.67732 4.206351
4.110745 10.175519244 13.18237 11.54004 19.58824
11.99084 20.28984 7.585188 5.014
AL450998.2 0.2653629 0.2174977 0.4445943 0.2082245 0.223758
0.8062253 0.08785736 0.1590167 0.2340997 0.225605102
0.569455 0.2979102 0.4097884 0.2399164 0.5562241

0.3861663	0.4537152	0.306835458	0.1989315	0.1821728	
0.088595921	0.2076477	0.4099343	0.4081889	0.2077119	
0.8045263	0.09000965	0.404669	0.256399893	0.04300952	
0.2142572	0.3567999	0.7501492	0.4775817	0.3626311	
0.2077545	0	0.263869	0.6231348	0.293552366	1.017895
0.6349475	0.305790136	0.2985378	0.722173	0.4114438	
1.202007	0.2954153	0.472848965	0.3103491	0.3500929	
1.278648	0.6303751	0.3705034	0.4065232	0.174757	
0.2643871	0.5926333	0.5701765	0.308358241	0.581027729	
0.400664357	0.3869964	0.2661619	0.2999668	0.5458681	
0.2198348	0.5363988	0.228526578	0.5428753	0.1376024	
0.274758	0.9187733	0.8483537	0.5531097	0.2228187	
0.7137461	0.8485972	0.624047	0.1299741	0.1647862	
0.9834751	0.5481833	0.6821569	0.2196195	0.2936389	
0.5168291	0.2927846	0.71426706	0.5451805	0.237453465	
0.7195914	0.2023988	0.8400314	0.1418114	0.6486941	
0.3674604	0.5089582	0.7078382	0.422675	1.340832	
0.463675	0.1091831	0.2387308	0.5182963	0.8364352	
0.273436	0.2123595	0.142472558	0.1895213	0.3386249	
1.019695	0.01663195	1.17015	0.7384206	0.5267115	0.4241613
1.156277	0.4900868	0.6048077	0.2059588	0.7340989	
1.135353	0.158355	0.5465975	1.075525	0.3120621	
0.05125058	0.5003769	0.2719226	0.4573024	0.5286983	
0.6557332	0.3246894	0.04237368	0.9301239	0.5408711	
0.6224605	0.4172026	1.10675	1.045872	0.3692296	0.3170224
0.4889395	0.6619392	0.2375949	0.7873714	0.6238981	
0.3758525	0.4351082	0.3678645	0.3396464	0.3177516	
0.7252501	0.5471373	0.3570997	0.541969	0.7294169	
1.490944	0.5073305	0.185702494	0.8376444	0.407056055	
0.3707277	0.1167535	0.3836887	0.1209458	0.4605976	
1.122901	0.5133763	0.4928901	0.294582737	0.5662447	
0.1543034	0.6200202	0.2853205	0.4217257	0.3548314	
0.738499	0.8554017	0.5909732	0.4211537	0.3290177	
0.7328494	0.6239728	0.9133676	0.3691537	0.2308113	
0.7815981	0.3031681	0.7233516	1.188456	0.2497486	
0.1448871	0.1466691	0.6137509	0.5267518	1.054418	
0.2607398	0.05533894	0.2770667	1.009552	0.371646	
0.3945704	0.3227679	0.4335373	0.2055557	0.5785401	
0.5392433	0.4624685	1.284965	0.2518276	0.5454059	
0.6167073	0.9367861	0.2321723	0.1622762	0.3753961	
0.4666476	0.284940461	0.4333514	0.75933081	0.169111312	
0.3190872	1.371845	0.1075258	0.3718454	0.5738261	
0.0925031	0.227308	0.1389998	0.5069536	0.2966696	
0.4561156	0.5451412	0.8224944	0.436852	0.4179339	

0.7383944	0.3386841	0.7878872	0.7882272	0.2890654
0.1409255	0.425117	0.6462203	0.6943006	0.6829332
0.5108888	0.6081864	0.7582268	0.8769682	1.288433
0.7559745	0.4874413	0.3937047	0.8233838	0.147357483
0.435993	0.4575176	0.5209861	0.1460144	0.6899114
0.1468525	0.5369344	0.2306715	0.8781567	0.1546538
0.668899	0.5683403	0.4579249	0.3348486	0.8994643
0.216544341	0.8378052	0.7756854	0.4947435	0.4551336
0.7011033	0.192106196	1.176744	0.42723	0.1957073	0.4104693
0.1728714	0.8088246	0.3138108	0.4460669	0.7501812
0.3547011	0.7800184	0.4518065	0.3512123	0.6704224
0.4647218	0.3694766	0.5814963	0.3196895	0.3994848
0.3396914	0.4233577	0.7108686	0.3151869	1.486619
0.255935	0.3407282	0.49233706	1.35367539	0.8486055
0.2356821	0.206852	0.1430637	0.3150666	0.2093315
0.5245837	0.4184635	0.5603198	0.9468645	0.2998007
0.5954618	0.6059984	0.65624418	0.4615042	0.567305
0.2339018	0.5443227	0.4201697	0.261277829	0.3355695
0.4845917	0.2402694	0.3724791	0.3931839	0.3118285
0.3866699	1.296362	0.6722673	0.4613366	1.441028
0.2540475	0.5967182	0.4480638	0.3231602	0.8574244
0.5389223	1.015041	1.284320103	0.7158087	0.3142495
0.32145	0.5569337	0.3110646	0.2020369	0.5081536	0.2379946
0.3360178	0.9785107	0.3824157	0.6681072	0.5132745
1.233912	0.2048884	0.5408842	0.5806046	0.4603674
0.2819727	0.2901241	0.4187545	0.7707559	0.5764356
0.6679972	0.5314593	0.2523519	0.338017072	0.278437
0.2237557	0.2913201	0.6825022	0.2859743	1.220999
0.473529488	0.5803443	0.7913261	0.3018502	0.7635287
0.3421085	0.285862	0.3644426	0.1773873	0.2792056
0.7819799	0.3114698	1.360038	0.6889281	0.6007188
0.642879641	0.8738251	0.6378289	0.237627	0.220750573
0.1668537	1.88713	0.2950396	0.4402117	0.2066097	0.3701574
0.5115528
NR2F2-AS1	0.06230316	0.8478122	0.1256386	0.2334849	0.1697284
1.07992	0.2961907	0.548533	0.1405785	0.402304325	0.141927
0.08003296	0.3737469	0.05392151	0.3906627	0.8290547
0.1577395	0.683802817	0.08284518	0.07165122	0.09800458
0.6419649	0.121941	1.161096	0.4562584	0.1205455
0.3072398	0.7806528	0.092613519	0.1250596	0.1151195
0.8570436	0.03700909	0.166037	0.3687089	1.712468
0.02742793	0.1398926	0.1278989	0.095429938	0.5282855
0.2577144	0.042399944	0.02464778	0.1110393	0.03526493
0.1868143	0.1107536	0.131757307	0.09569544	0.0802206

0.8342493	0.2776423	0.1477261	0.6681181	0.0324034	
0.03870205	0.5846389	0.03363881	0.042232059	0.587640118	
0.078430475	0.4916373	0.06449368	0.01978569	0.5861795	
0.3760105	0.5352376	0.019260611	0.2728528	0.02660571	
0.1406792	0.7277384	0.3977226	0.1584979	0.1083435	
0.282794	0.930426	0.05985033	0.014084279	0.02499923	
0.3716916	0.0564689	0.6061442	0.161541	0.171692	
0.7816007	0.1006797	0.53358779	0.713557	0.025401095	
0.04816196	0.1514796	0.0829363	0.0386145	0.2717332	
0.3298325	0.05803559	0.02010937	0.2647494	0.9300629	
0.5380385	0.0331277	0.06898505	0.8233815	0.7067694	
0.06568011	0.4713145	0.112308534	0.1118122	0.1073874	
0.1115958	0.06728491	0.9034502	0.3903854	0.06278318	
0.07239181	0.04746087	0.3611268	0.02256236	0.04463629	
0.3609148	0.2406959	0.3603536	0.09213634	0.246374	
0.100821	0.04924215	0.8101582	0.1663411	0.05695046	
0.1227662	0.1121581	0.23663	0.02678495	0.5344939	0.09345036
0.051602	0.2769051	0.1352019	0.08488317	0.2094245	
0.3113096	0.05298256	0.1311804	0.6433809	0.02488538	
0.5736349	0.1161508	0.4049488	0.0398626	0.2440741	
0.008685615	0.3476999	0.9123501	0.2257273	0.1905632	
1.543533	0.107708	0.3527591	0.256440915	0.7942286	
0.436193829	0.06103785	0.1357946	0.1179384	0.6765948	
0.3672964	0.2055973	0.03540128	0.597408	0.548973261	
0.7772205	0.02194586	0.04479116	0.1196037	0.2039903	
0.1759841	0.02535783	0.1996469	0.0795325	0.079865	
0.06977269	0.1585074	0.08984039	0.1361957	0.6739055	
0.02593756	0.1521699	0.03936317	0.203446	0.4545309	
0.592617	0.1392092	0.02884355	0.2726204	0.1837057	
0.1475846	0.1074362	0.1063406	0.06821142	0.4103801	
0.07517511	0.03521125	0.1677545	0.3790174	0.1501464	
0.4305631	0.09361003	0.1079381	0.2022944	0.5297625	
0.2046306	0.2140754	0.2092662	0.0754761	0.1000124	
0.2771578	0.6125416	0.032296396	0.1431129	0.128837471	
0.12576168	0.1785251	0.9611904	0.04272301	0.02474192	
0.07254456	0.1403337	0.07663162	0.1081398	0.1950576	
0.07813693	0.0831864	0.08614769	0.3052371	0.272688	
0.0345468	0.1677589	0.2104165	0.3625326	0.3673832	
0.2637552	0.08314207	0.2074783	0.1334765	0.1795403	
1.426978	0.4678257	0.1623201	0.1960221	0.05137812	
0.3793258	0.5734334	0.01882943	0.1990926	0.1705164	
0.15648621	0.06074378	0.3701795	0.2283299	0.01006883	
0.1836218	0.09654047	0.1329329	0.09477677	0.02697654	
0.1068828	0.4616195	0.2428069	0.2315682	0.06187046	

0.09364566 0.008760349 0.5020655 0.09088871 0.3093221
0.8228099 0.05138277 0.140004862 0.9285112 0.03206933
0.1264582 0.1280753 0.03496775 0.4647894 0.05667537
0.2134875 0.1369909 0.4328249 0.03673778 0.5637351
0.3927036 0.0714814 0.04039153 0.6255102 0.2687865
0.06466555 0.05827373 0.04498965 0.2319285 0.5762242
0.08991058 0.2954929 0.1112236 0.1602818 0.466818848
0.12295144 0.1451474 0.1513936 0.04669778 0.189908
0.03641739 0.2646423 0.296123 0.5410103 0.1311798
0.838809 0.1442705 0.3847635 0.2042983 0.148763162
0.04712444 0.1780638 0.2661342 0.3956843 0.06418537
0.031458488 0.09463717 0.06345131 0.03402054 0.2367569
0.2461696 0.1626164 0.5240344 0.1123158 0.02360826
0.1684897 0.1821785 0.3532907 0.117349 0.06473757
0.3540745 0.04550031 1.140313 0.1889863 0.158149714
0.04063012 0.1175955 0.1300433 0.0572073 0.2556163
0.1266884 0.1056425 0.2045976 0.08835887 0.2808457
0.4765531 0.7302034 0.1724206 0.4695585 0.7332405
0.4078805 0.1852515 0.08058577 0.1057932 0.01833912
0.1974519 0.2654425 0.07949942 0.2635861 0.1489831
0.09343013 0.061725422 0.06820138 0.1220841 0.3839189
0.4343937 0.1983283 0.4027877 0.293881802 0.1143407
0.3344244 0.04054571 0.04917427 0.0380602 0.4172459
0.6875624 0.01180303 0.03309177 0.2926253 0.14584 0.7255008
0.2032241 0.316435 0.550860445 0.3946933 0.8721416
0.3974709 0.04186178 0.08136284 1.014981 0.03907584
0.1970653 0.006813948 0.07745586 0.5535147
AC096586.2 0.230613 0.5985501 0.3333419 0.09441234 0.3889127
0.5671914 0.7126208 0.3948375 0.1525828 0.123828364
0.7635345 0.4530718 0.3052506 0.1235548 0.1074189
0.6833974 0.19715 0.173635569 0.04067787 0.07036303
0.153988149 0.8530635 0.4191205 0.9853766 0.4512789
0.7768574 0.1564453 0.9638544 0.254655676 0 0.09309987
0.4134348 0.9540225 1.141363 0.8046222 0.2166582
0.1714034 0.177534 0.2038715 0.553955281 2.873286
0.2904209 0.240028817 0.5082976 1.492677 0.339383
1.418358 0.3541103 0.670903293 0.7893897 0.1521237
1.460886 0.1060308 1.04026 1.027747 0.2834947 0.4353442
0.9456225 0.2642723 0.223314996 0.86026945 0.224642842
0.4921731 0.07710247 0.226683 1.004581 0.08990444
0.5660467 0.198600479 0.184111 0.4501949 0.3820444
0.6518023 1.013732 0.9485393 0.4170708 0.5115706
2.735348 1.047251 0.419542248 0 0.5398021 0.5646191
1.016275 0.1735089 0.6379655 0.5084707 0.4885321

0.22572076	0.9289957	0.47621162	1.066789	0.1407154	
0.4171585	0.1137608	0.2692519	0.4470768	0.3122181	
1.520583	0.9350084	0.9074491	0.2590429	0.3795413	
0.1185534	0.2719542	1.168013	0.04752578	0.2460673	
0.233064353	0.6148913	0.2793177	1.40199 0	0.9079608	
0.3500306	0.4708156	0.1474466	0.7830075	0.3163894	
0.2757281	0.6477661	0.7797373	0.6982631	0.1834907	
0.6650269	1.022558	0.6026596	0.1425254	0.3967065	
0.09147628	0.3796228	0.8251599	0.8715557	0.6971273	0
1.511667	0.6267236	0.3547199	0.7613948	0.6916449	
1.009903	0.4856534	0.2819147	0.9018534	0.4664243	
0.4795695	1.32576	0.7041522	0.9581257	0.7079876	0.3349152
0.3417745	0.2985311	0.5893498	0.5055826	0.4137822	
0.4121223	0.8191913	2.036102	0.4408944	0.446910397	
2.365847	0.808573932	0.4495536	0.162343	0.3922868	
0.05255383	1.170053	1.525474	0.7300611	0.7615021	
0.18963439	0.2108972	0.2011455	0.3763236	0.2871082	
0.5736518	0.4269674	0.8240277	0.9149352	0.4638822	
0.5323675	0.3320501	1.197029	0.3615081	0.6512902	
0.2566497	0.7577693	0.5977364	1.3387	0.4237935	0.6596195
0.4340868	0.3021931	0.2832498	1.326239	0.3577991	
1.079971	0.201418	1.692845	0.1013828	0.7828652	
0.3229781	0.3765182	1.122003	1.200538	0.158789	
0.4932925	1.007201	0.494655	0.8006495	0.5602564	
0.5504327	0.472361	0.5058558	0.5764822	0.1410257	
0.2870886	0.4831954	0.273243398	0.3381741	0.764088521	
0.138320737	0.8024056	1.637478	1.334929	0.5782708	
0.3068813	0.3617532	0.7572423	0.1486738	0.996064	
0.73049	1.013709	0.9869868	0.9223056	0.5694678	0.7822866
1.1904	0.5718463	1.060994	0.3301238	0.4368897	0.2449419
0.5155073	0.1265569	0.6033803	0.5935014	0.767977	
0.6812331	0.5034561	0.2974155	0.9816629	1.075055	
1.106092	0.2817691	0.7905231	0.17074756	0.8041932	
0.6679757	0.3923935	0.09228617	1.220169	0.4764549	
0.8554729	0.6013936	0.7132336	0.0716808	0.410333	
0.6812617	0.2402769	0.04476914	0.7356963	0.8430793	
0.7108936	1.545515	1.657284	1.107492	0.5298193	
0.157128878	1.34002	0.7657716	0.4535438	0.6071785	1.009569
0.1278013	0.909055	0.6091696	1.043109	0.1876317	
0.3987489	0.5121409	0.949574	0.3088636	0.2019327	
0.2233689	0.6948534	0.2222603	0.1869384	0.3092656	
0.7358361	0.6904585	0.6742459	1.493575	0.361432	
0.5784456	0.534830437	0.593914908	1.098988	0.5314223	
0.0770418	0.08288609	0.4693849	0.2568269	1.65392	0.631267

0.6131895 0.9129656 0.5378913 1.609952 0.7021886
0.629304763 0.4010691 0.5440166 0.1693932 0.4730423
0.2434316 0.389250643 0.1794621 1.143076 0.2004534
0.2697518 0.1627122 0.1016227 0.4838898 0.5672393
0.3245735 0.6533568 2.471689 0.5298705 1.014105
0.9790346 0.7148696 0.7359434 1.232498 0.8340032
0.463848448 0.6093765 0.4369564 0.3352265 1.331005
0.6420337 0.4213912 0.7458296 0.7170058 1.308229
1.562847 0.6171981 1.723121 1.325435 0.3032847
0.3835091 0.8246564 0.3844366 0.5744756 0.7904765
1.008527 0.8261913 1.039381 0.7893757 1.512874
0.2811342 0.4072822 0.718062722 0.241975 0.3684398
0.3682488 0.5726743 0.1704173 1.459021 0.359144443
0.4453972 0.5501601 0.6885957 0.9014167 0.09513874
0.3613494 1.16942 0.6490865 0.4549555 0.7883102 1.283233
0.8648325 1.396993 0.7830799 0.620770023 1.209135
0.7293469 0.3812476 0.383685519 0.124289 0.568788
1.367485 1.153966 0.468401 0.3105918 0.6014686
AC078795.1 0.06009291 0.1193452 0.09354362 0.04415722 0.05456905
0.1544859 0.04285247 0.03324022 0.06422752 0.046332217
0.1111008 0.0544897 0.09993727 0.06934481 0.09043288
0.1130117 0.0368833 0.059167577 0.01141517 0.02961829
0.048614299 0.05064014 0.02940377 0.2820503 0.06648575
0.1707702 0.175609 0.1242747 0.035731197 0.02097792
0.06531506 0.1035889 0.2275631 0.1892644 0.2145062
0.2735974 0.0577198 0.07888204 0.1609064 0.122725997
0.5412741 0.09372379 0.178016917 0.2912239 0.2380002
0.2653083 0.4087822 0.1093089 0.315354432 0.2252134
0.04802569 0.327095 0.1140599 0.07645561 0.09012813
0.06819025 0.1425292 0.2085003 0.1344169 0.09400119
0.372614046 0.12608017 0.05524616 0.02163677 0.2035603
0.04698485 0.0693806 0.1354863 0.065020647 0.2195801
0.09475148 0.1273127 0.2835122 0.1508591 0.3633032
0.2424398 0.3373632 0.2069519 0.1889251 0.131318089
0.09644947 0.1514812 0.3020367 0.1425953 0.0827742
0.1835039 0.2520837 0.08568352 0.58591881 0.1772747
0.169272549 0.3148507 0.07502729 0.2731505 0.05320661
0.2691769 0.129941 2.346155 0.3103357 0.2951836
0.2720142 0.2261577 0.362128 0.06653776 0.2146409
0.2196771 0.06668427 0.02301743 0.08584191 0.4437078
0.1791615 0.4119895 0.04056121 0.4025765 0.2619382
0.1578124 0.05689336 0.2160682 0.2970927 0.1402437
0.1291584 0.1690825 0.3705994 0.04290985 0.1777355
0.2510847 0.1733485 0.019998 0.2868765 0.08128961

0.1231766	0.2262966	0.2069516	0.2561826	0.02066779	
0.1649704	0.1626829	0.2588112	0.1831417	0.3821184	
0.2361689	0.3261124	0.3380237	0.2628153	0.4406619	
0.1345785	0.2580274	0.1541293	0.3299801	0.1354623	
0.3118597	0.1656627	0.1717385	0.2434842	0.08445143	
0.7257316	0.2602153	0.4378751	0.402562	0.1701224	
0.076641649	0.3217422	0.264722423	0.1093346	0.07972522	
0.09540694	0.05899138	0.5054776	0.3273584	0.3755994	
0.1297437	0.111753362	0.4488025	0.07526156	0.1488075	
0.1428275	0.2101686	0.2729168	0.3690972	0.3316385	
0.2464051	0.2240923	0.0931811	0.1981035	0.1902144	
0.1941904	0.1692516	0.08130657	0.3202289	0.2517794	
0.1625328	0.735548	0.04685192	0.04946813	0.07153793	
0.3195842	0.1063132	0.3994954	0.1789884	0.1133647	
0.07468233	0.1477229	0.04531764	0.1811312	0.1136996	
0.3763236	0.07240993	0.4472338	0.1727271	0.4771658	
0.2424191	0.1031764	0.1716271	0.1631454	0.3149628	
0.1375083	0.09399099	0.2050716	0.1694951	0.100640595	
0.1854857	0.336251549	0.063076105	0.08278454	0.3506825	
0.1386067	0.2529603	0.03229431	0.04511844	0.1616848	
0.2503283	0.322522	0.1085256	0.2297647	0.3157479	
0.2847028	0.04661011	0.1724864	0.4541051	0.448382	
0.3531336	0.1435928	0.2635934	0.1832973	0.2218174	
0.2264072	0.1616263	0.2359466	0.343473	0.276867	
0.2160778	0.1930054	0.5251295	0.1257024	0.4061559	
0.1807338	0.4475037	0.095831593	0.203976	0.09372487	
0.1524668	0.01942326	0.4014442	0.14803	0.3328186	0.2179884
0.1801352	0.05028831	0.2610047	0.3489001	0.1685684	
0.2072941	0.2129054	0.156317042	0.1190526	0.10612	0.2895737
0.26639	0.1238998	0.06062935	0.3067704	0.1693101	0.02121252
0.119272	0.2630726	0.3496746	0.1931489	0.2875024	
0.2805243	0.1241127	0.2200668	0.1580907	0.05710133	
0.1772886	0.03541692	0.1880477	0.124086	0.1975097	
0.5208462	0.0749526	0.2237007	0.3365287	0.1616163	
0.2011836	0.4447155	0.143001	0.185106125	0.37820485	
0.3489811	0.1988395	0.02161975	0.1046691	0.180018	
0.07807767	0.6069379	0.08472316	0.2024417	0.1752945	
0.3679284	0.1475235	0.1662615	0.226265897	0.1385224	
0.1431224	0.02376786	0.2654937	0.08966037	0.182054866	
0.2203307	0.2954499	0.1359422	0.156444	0.1278504	
0.1663534	0.03771969	0.1238072	0.1001913	0.3041899	
0.4994023	0.2788015	0.2069686	0.06868505	0.1241867	
0.3252739	0.172934	0.2475434	0.252198223	0.2458203	
0.1348823	0.2665386	0.2207113	0.2228416	0.08277662	

0.1982817 0.2012088 0.2950071 0.5116669 0.3397391
0.1471669 0.2324678 0.4377026 0.06918538 0.166621
0.2292492 0.2015143 0.07542097 0.2577471 0.237369
0.3564916 0.1192786 0.1974643 0.09579853 0.2241901
0.366373367 0.06790389 0.1550894 0.1636206 0.2181009
0.1315136 0.3303401 0.163774665 0.1102844 0.2591511
0.2392448 0.1616126 0.06674544 0.06337698 0.09571535
0.1275043 0.1659726 0.2441031 0.4107455 0.7927953
0.3528263 0.240098 0.540028443 0.1779318 0.1719244
0.08024032 0.067294509 0.06394376 0.2713461 0.1096425
0.2886313 0.1708777 0.03112836 0.1247551
AC004637.1 0.06028472 3.487577 0.08317831 0.1151752 0.1976839
1.587361 0.2794301 0.1445007 0.1861384 0.339885739
0.491598 0.2960946 0.1396426 0.113045 0 2.456402 0
0.817023798 0 0.3218889 0.59878068 2.401538 0.4985099
1.767054 0.7432058 0.4501582 0.04771259 2.883943 0
0.2051875 0.425903 1.45903 0.08728712 0 0.04907862
0.06607626 0 0.1082884 0.2564784 0.133377527 1.387463 0
0.062746122 0 0.06207756 0.04435919 0.1052197 0.29159
0.184150427 0.096299 0.03479593 0.5118969 0.09701162 0
1.234176 0.07410856 0 1.266879 0.2115686 0.040863864
0.239550044 0.246641063 0.2401641 0.03527198 0 1.062105
0.2056424 0.2437165 0 0.1052813 0.0772313 0.02184668
0.8349019 0.02810612 0.1172776 0.2180536 0.4212498
0.4907203 0.1140678 0 0.1048204 0.1975541 0.09686088
0.7438624 0 0 0.310146 0.1117441 0.929342 0.3399893
0.029046933 0.1682837 0.2574916 0.1017797 0.1387787
0.2771423 0.175306 0.06348002 0.101181 0.3055266
0.7547813 0.05266848 0 0.07231265 0.3732318 0.4774862
0.04348313 0.6754089 0 0.08036961 0.0547625 0.1452151 0
1.295935 0.4270082 0 0.06745221 0.1194005 0.2004069
0.03153426 0.03119293 0.1297111 0.2499915 0 0.144871
0.08771035 0.0551396 0.06520095 1.479767 0.1673902
0.1736656 0.4461186 0.1226799 0 0 0.8836353 0.1433533
0.06490932 0.09951841 0 0.0615999 0.09521627 0.1641401
0.03173614 0.1706997 0.1462587 0 1.597753 0.132822
0.05888769 0.05571392 0.2558474 0.02731374 0.3295224
0.5286589 0 0.1077332 1.855927 0.4233879 0
0.318029598 0.1998095 0 0.05484181 0 0.02392787 0.04808348
0 0.2873529 0.1113267 2.289246 0 0.7075121 0.1840356
0.1252046 0.02388056 0.2041105 0.3038378 0.2029822
0.06975906 0.06063191 0.09132808 0.03375613 0.112329
0.02756311 0.2234588 0.63401 0.2039152 0.3231632 0.05212031
0.2584964 0.09529117 0.09165286 0.04608131 0.1036624

0.1846518 0.1155402 0.3892541 0.09214247 0 0.02318974
0.6421758 0 0.02460648 0 0.1168529 0.1089616 0.208307
0.05119589 0.3111477 0.07710995 0.3203749 0.1119137
0.5319149 0.1157066 0.0791168 0 0.07163678 0.2210469
0.062500176 0.1687682 0.063553949 0.063277433 0.02159268
0.3416925 0.07328262 0.3112246 0.07019433 0 0.3313533
0.3060614 0.3505141 0.03931487 0.0951262 0.03612126
0.1687704 0.1736759 0 0.03403568 0.2769902 0.4063579
0.06040855 0.1598908 0.07470219 0.06288761 0.2026354
0.2509343 2.021229 0.2195785 0.06447785 0.1896716
0.1360583 0.2806751 0.2185796 0.1076601 0.03682874
0.6235168 0.031244678 0 0.4074374 0.2761659 0.04221805
0.07699164 0.2179633 0.4980846 0.09170628 0 0.09837521
0.1752003 0.5298153 0.137399 0 0.1682791 0 0.08392559
0.06017234 0.1430482 2.050698 0.03231678 0 0.5484891 0
0.864509 0 0 0.8185116 0.09505472 0.1013368 0.1325528
0.5150141 0.1216101 2.499077 1.787248 0.1027603
0.3233227 1.43058 0.08669286 0 0.09773544 0.1543411
0.1496098 0.1662438 0.07711163 0.6832642 0.07631256
0.05040397 0 0 0.05292133 0.1418137 0.02349615 0.03791781
0 0.1958172 0 0.5022348 0.1320071 0.175854 0.1230343
0.0601227 0.2007684 0.107957754 0.1411359 0.4977414
0.1239874 0 0.0556812 0.039571123 0.2873441 0.05504439 0
0.3619822 0.2084201 0.03099281 1.524956 0.1729962
0.05939291 0 0.03015255 0 3.120927 0 0.1557289
0.06733424 1.894467 0.293485 0.185671701 0.1161545 0 0
0 0.1236676 0.05140623 0.1795757 0.05046275 0 0.420561
0.304068 0.6169104 0.1166049 0.1585638 0.2506335
0.09054112 0.04396695 0.2252612 0.08678842 0 0.07199177
0.1267959 0.2500026 0.1609518 0.03674576 0.1146578 0
0.08302201 0.0374555 0.1403853 0.1497032 0.1299344
0.5461008 0.054765802 0.02397122 0.32359 0.06000211 0.0916378
0 0.2479591 0.4458111 0.05938737 0.2220032 0.4725462
0.03668996 0.263756 0.07303781 0.1061436 0 0.1888776
1.334614 0.1744089 0.087762092 0.1516222 0.242856
0.1340533 0.1606664 0.05714103 0.1217881 0.9809809
ADPGK-AS1 0.04629897 0.03099064 0.1447978 0.0840324 0.08502044
0.07502161 0.01430692 0.08878181 0.05003433 0.116015102
0.08345836 0.02728826 0.2073428 0.02604574 0.1207693
0.07546118 0.06464863 0.111551723 0.01715004 0.1186619
0.032461173 0.01383295 0.07657163 0.2049508 0.04122342
0.04367038 0.07328702 0.03660963 0.03578813 0.1050567
0.0523353 0.07885301 0.1787652 0.08749196 0.03769263
0.05074691 0.04335883 0.07484942 0.1002791 0.106532006

0.1121662	0.1346875	0.077102947	0.04762252	0.08581659	
0.04769532	0.2101041	0.09953004	0.141428467	0.1035414	
0.08551504	0.3887712	0.2284833	0.1531549	0.2437337	
0.05122418	0.2107355	0.441396	0.06499431	0.056490581	
0.173462761	0.189421594	0.09222364	0.08668499	0.1529135	
0.05647166	0.06949115	0.2433281	0.032562124	0.1196678	
0.1423537	0.06040209	0.2473234	0.07339118	0.1116866	
0.1507197	0.1401928	0.1017564	0.09110885	0.045354251	
0.09660315	0.3186174	0.2281283	0.2142338	0.1267975	
0.05379403	0.1953189	0.0629347	0.57099152	0.2558912	
0.156157348	0.3205221	0.1898446	0.1094343	0.1438867	
0.2885255	0.3814685	0.1267578	0.04273915	0.08447256	
0.6376435	0.06067449	0.1386813	0.1388412	0.1767637	
0.2025646	0.05343242	0.07492586	0.077790241	0.2222074	
0.1205659	0.1412662	0.1056272	0.4338421	0.4427251	
0.09557347	0.09324656	0.1357163	0.09918869	0.1017176	
0.1533205	0.2341042	0.12373	0.2578693	0.1201626	0.359264
0.1651552	0.06509706	0.1415225	0.09856001	0.1067009	
0.06852427	0.09421877	0.1166322	0.09315323	0.1239249	
0.1012884	0.07976111	0.1579567	0.2308514	0.1356192	
0.2145046	0.0828397	0.1267423	0.2010173	0.1123275	
0.05409179	0.1504166	0.07344576	0.06783907	0.1625966	
0.1135288	0.1426442	0.4187238	0.1048866	0.2035286	
0.06205474	0.3179653	0.2991509	0.1858838	0.048849671	
0.1739153	0.151510985	0.1305682	0.04562986	0.1212864	
0.1624848	0.147125	0.2396046	0.0170999	0.1528828	
0.085281087	0.4346983	0.04711342	0.07211834	0.06969348	
0.2284187	0.05333691	0.106897	0.1232232	0.01396968	
0.0327322	0.03110986	0.2803749	0.1651152	0.2917497	
0.3137948	0.04385022	0.1450963	0.180129	0.127057	
0.1024577	0.07977518	0.05662509	0.05572927	0.2025908	
0.2514172	0.1609726	0.1509672	0.04866229	0.04986755	
0.1062265	0.1316306	0.09070993	0.08760059	0.0789744	
0.1004196	0.2613021	0.0786374	0.4735813	0.1835846	
0.3543113	0.1719006	0.122554	0.2266014	0.08911776	
0.05945731	0.08802786	0.1600643	0.100800952	0.306755	
0.195239028	0.048597391	0.08954977	0.2745334	0.03376886	
0.1242915	0.07547345	0.03389275	0.0508962	0.0313409	
0.3445716	0.1690865	0.03287583	0.1331583	0.2527523	
0.100038	0.1217181	0.1986608	0.3214585	0.2011214	
0.227331	0.2210345	0.08032033	0.05795761	0.2401072	
0.3623113	0.2131536	0.1484009	0.1551605	0.1789645	
0.1097182	0.2672943	0.2182313	0.1719818	0.08485397	
0.1992075	0.110381931	0.0999771	0.08046361	0.09332259	

0.03890841 0.2246939 0.062176 0.3169547 0.1197325
0.05613135 0.1208843 0.119177 0.5935965 0.2574764
0.1698745 0.02584784 0.13540853 0.05800976 0.1525018
0.1625954 0.166759 1.513986 0.066246496 0.5401815
0.1663187 0.05842737 0.1279951 0.1114773 0.1616454
0.03650125 0.06226173 0.3176197 0.1695143 0.1569073
0.08157043 0.03812821 0.08286649 0.1347987 0.2118908
0.2308126 0.3227662 0.1801472 0.08297434 0.08617583
0.1685325 0.1421331 0.3736223 0.03125783 0.07742102
0.095205954 0.186193503 0.1422535 0.03734182 0.1299252
0.08153899 0.08355589 0.1263264 0.1251569 0.1735735
0.1571423 0.1451861 0.2173292 0.09696653 0.191197
0.243208844 0.03468578 0.1337938 0.09998408 0.1495781
0.09835592 0.072937979 0.1702402 0.05918413 0.07042697
0.1794391 0.4298961 0.2761106 0.1208953 0.1550056
0.03192984 0.1961604 0.2130473 0.09928739 0.1166054
0.137589 0.04784022 0.08274086 0.2424933 0.1217149
0.17519037 0.04995605 0.1473788 0.2826671 0.05101454
0.3134251 0.07106444 0.06619923 0.1937782 0.04596312
0.2454747 0.1934928 0.1895161 0.2722416 0.3653334
0.1693893 0.07417178 0.2296145 0.2191355 0.1421951
0.1063003 0.2045732 0.07790392 0.1450691 0.06427814
0.1693256 0.1056693 0.105500177 0.08926586 0.08054485
0.1768193 0.160962 0.07184899 0.1631032 0.210302076
0.1178241 0.2209056 0.142854 0.07741612 0.1470739
0.08886914 0.1506495 0.05017079 0.1832871 0.1031451
0.1239835 0.6076978 0.1907176 0.1182023 0.465280782
0.1284808 0.2418977 0.05357878 0.026960694 0.06404565
0.6821106 0.05490859 0.1445456 0.07021536 0.1060051
0.09555296
AC005519.1 0.1381653 0.2235835 0.6647774 0.1319839 0.3485136
0.6350425 0.2955786 0.1655891 0.08203979 0.213053631
0.5200078 0.5011298 0.6400883 0.2092614 0.1386152
0.5354196 0.1696038 0.456126795 0.1968428 0.1588959
0.111773768 0.2328631 0.3245041 0.5849115 0.5677795
0.7685603 0.1682331 0.4454056 0 0.1205808 0.1501721
0.1619564 0.5745072 0.3012617 0.268227 0.6290538
0.05529545 0.1145464 0.6412558 0.263359635 1.141502
0.6933134 0.132744539 0.5465965 1.094418 0.6021752
1.261405 0.4683737 0.984784933 0.348036 0.1104204
1.614412 0.7639339 0.607262 0.7459999 0.4442177
0.3120976 0.7299694 0.1811676 0.072042365 0.675716877
0.507295139 0.3175543 0.1368047 0.3363929 0.9722461
0.3190391 0.5478272 0.160173393 0.7201644 0.3721641

0.2542014	0.9672612	0.5549673	0.7360608	0.1153276	
0.9902091	0.9862508	0.8204872	0.270692218	0.1293578	
0.6849603	0.4098342	0.8305647	0.4366028	0.1440673	
0.535847	0.3808728	1.05586788	0.9470455	0.184353467	
1.293532	0.2996094	1.372686	0.3547639	0.6188903	
0.5254051	0.4028915	0.7581175	0.5817298	1.477041	
0.5849784	0.5142548	1.46609	0.8773359	0.8337837	0.1993162
0.1984559	0.272554866	0.6517758	0.9010909	1.655544	
0.03730325	1.511426	0.6775281	0.3459651	0.2378344	
1.145127	0.5731541	0.4447553	0.890881	1.257733	
1.860865	0.2959744	0.8581621	1.659717	0.2916308	
0.04597931	0.9844553	0.1672269	0.5587599	0.9317003	
0.7353613	0.4497924	0.09503856	1.137897	0.6469879	
0.8239258	0.4678651	0.6275464	0.5502384	0.6378863	
0.5043418	0.8616352	0.8927914	0.2578519	0.8140017	
0.8086984	0.6556569	0.4048906	0.2455569	0.2505861	
0.2118763	0.760507	0.7456141	0.4338357	0.6932518	
0.9564223	1.731709	1.77793	0.096116793	0.8923946	
0.608648556	0.5221004	0.0785588	0.3965339	0.1186786	
1.023126	1.172414	0.7327314	0.5790647	0.220236691	
0.8958119	0.2054862	0.750495	0.1684042	0.658	1.010104
0.490772	0.9715748	1.122378	0.719178	0.2499485	
0.5742993	0.4956521	0.9717541	0.4719388	0.4313993	
0.5434365	0.6983435	0.5012978	0.7615858	0.1508104	
0.1949775	0.2924083	1.264949	0.2444347	1.224691	
0.4548481	0.3475302	0.9811958	1.567587	0.3646794	
0.6940935	0.5630533	0.6097893	0.1536781	0.3060351	
0.9838074	1.535936	0.9651999	0.3840748	0.5721762	
0.5157668	0.6119671	0.2882622	0.1706081	1.077713	
0.5010457	0.220373699	0.3173714	1.042015114	0.011155714	
0.9136216	1.288206	0.5770761	0.7901052	1.113764	
0.1296701	0.4567153	0.239814	1.087594	0.7624267	
0.5031181	0.7387022	0.981881	0.2755691	0.5407907	
0.6960508	0.9549555	1.082564	0.4259974	0.6624297	
0.3687567	0.3991316	0.5103474	0.5485676	0.9466934	
0.4800207	0.6971972	0.6401143	0.8275471	0.9896519	
0.8477753	0.7668049	0.3506139	0.8266364	0.132201329	
0.7284168	0.5951678	0.7108392	0.1042017	0.4614993	
0.5160143	0.840482	0.5820364	0.6994803	0.1503095	
0.811901	1.428557	0.3778824	0.2744116	0.4005092	
0.534248313	0.4660725	0.8698798	1.008767	0.5529357	
0.4899762	0.050690443	0.7508299	0.6288321	0.03657879	
0.4211385	0.3256912	1.360565	0.444087	0.6967552	
0.5982425	0.4755991	1.646568	0.4295689	0.5251484	

0.7065431 0.2605773 0.3602987 1.344976 0.5497163
0.6116866 0.2902411 0.7649035 0.7620218 0.5256603
0.6842039 0.5291823 0.6309162 0.85119027 1.355934704
0.4198477 0.6000368 0.09113138 0.2406546 0.262471
0.1795158 1.682771 0.5578223 0.4421804 0.5115462
0.520578 0.5723751 0.5804806 0.507540967 0.3184901
0.3729419 0.03278817 1.10639 0.4515594 0.153479222 0.5210571
0.7666351 0.2263349 0.4293138 0.08398661 0.3278391
0.1040699 0.7828069 1.350743 0.766457 1.520333
0.3133872 0.5650853 0.4163355 0.5490952 0.8072228
0.6520787 1.303872 0.215106749 1.900345 0.0563856
0.8291154 0.4098703 0.7957876 0.0453142 0.4052342
0.3558602 0.4672589 0.9490465 0.4288538 0.6525622
1.200544 0.9225001 0.1237217 1.702641 1.147193
0.7148376 0.6630288 0.7436685 0.6219099 0.871803
0.8423236 1.078271 0.2461724 0.3537449 0.336945572
0.2049132 0.2509272 0.7226916 0.818166 0.8338232
1.668797 0.289653803 1.090331 1.939646 0.8462625
0.8158579 0.1227686 0.04371481 0.6916432 0.3140971
0.4696659 1.201406 0.724459 1.227594 0.682452
0.7859441 0.734297826 0.8752835 0.7905758 0.08199474
0.170195543 0.5747114 1.284454 0.693246 1.011615
0.322364 0.2433386 0.3458909
LINC01152 0.0216899 0.01382698 0.0232764 0.04489226 0.03793327
0.03294912 0.01117072 0.01299751 0.05022819 0.135875364
0.08067869 0.04261287 0.05582463 0.01355752 0.01178695
0.01874709 0.0576881 0.013609158 0 0 0.021121166 0.0432026
0.2115521 0.129749 0.0173314 0.01704872 0.04577749 0
0.013971529 0.01640547 0 0.02592326 0.9944955 0.06831296
0.01765804 0 0.293403 0.09415619 0.2964101 0.044788831
0.5575823 0.5513083 0 0.004647906 0.02605744 0.446881
0.5762691 0.003885609 0.025766092 0.04619668 0.008346174
0.09208795 0.6554178 0.1413241 0.1762085 0 0 0.05929258
0.0942444 0.156826137 0.012312564 0.034509702 0.07200741
0.03384144 0.5770687 0.01469753 0.3354136 0.05115074
0.050848443 0.002525285 0 0.005240159 0.1037061 0 0.04219543
0.006537811 0.06736083 0.03531135 0.3228525 0.017706101 0
0.06318062 0 0.02230292 0.01142336 0.01400064 0.07439183
0.01340151 0.5696655 0.1834876 0.055737732 0 0.003088105
0.1892004 0.004160945 0.01846543 0.1086266 0.01141977
0.7584171 0.02931353 0.1629381 0.02842447 0.0416466
0.2731831 0.02984121 0.03544981 0.01042989 0.03150078
0.003196734 0.01927751 0.3218166 0.1044942 0.3362351
0.01594071 0.3968871 0.0459218 0.008089566 0.6243409

0.04806972 0.1588404 0.03740978 0.03500167 0.23319 0
0.2918904 0.2910293 0.07604847 0 0.03013624 0.04015033
0.002603472 0.07408129 0.4928868 0.07285231 0.00808147
0.01843038 0 0.02335379 0.186986 0.2229368 0.3250587
0.2207735 0.129361 0.1674696 0.2251928 0.002923477
0.5725108 0.009271892 0.7773542 0.02824968 0.01336358
0.006818637 0 0 0 0.2383701 0.00969036 0.05564549
0.3351285 0.1330417 0.005448771 0.06789578 0.01109048
0.1414097 0.008906849 0.01434838 0.2998667 0.2533983
0.1427725 0.008900969 0.01790544 0.074910066 0.2429857
0.121393 0.2777929 0.05728006 0.3636886 0.1457575
0.006955351 0.2300713 0.07635185 0.06206704 0.04048384
0.01347165 0.04297349 0.008933161 0.05632372 0 0.05962627
0.009376208 0.1953099 0.3504679 0.3480787 0.02763273
0.006216132 0.0284726 0.02078516 0.6535673 0.0626205
0.02955174 0.1140273 0.008886502 0.003544 0.002951063
0.2325524 0.002802842 0.02613559 0.2206773 0.07060931
0.02035421 0.1387173 0.0115268 0.02013277 0.01594818
0.1873359 0.003162835 0.03094917 0.04009331 0 0.258600502
0.05734786 0.087653531 0.015177767 0 0.1513082 0 0.3209973
0.01683686 0.03528421 0.0505773 0.4404725 0.02101863
0.01886018 0.07700749 0.2209338 0.01518052 0.02603625
0.05824814 0.01632764 0.1144228 0.1705712 0.01811204
0.009587876 0.02687718 0.04525279 0.07984987 0.1083407
0.01447204 0.1264038 0.002577617 0.02924663 0.00407938
0.4241344 0 0.002582343 0.6801999 0.02991143 0.348488165
0.02715174 0.08376704 0.08280166 0 0.1292709 0.003734344
0.08960318 0.03299509 0.05634883 0.007865448 0.1981115
0.06727862 0.05273058 0 0.2825447 0.016519655 0.1987882
0.01443298 0.03431164 0.06365529 0.05813642 0 0.05417216
0.01018511 0.008294468 0.009993745 0 0.01402348 0.03704982
0.4435974 0.003179419 0.0205886 0.03792034 0.0187322
0.008931051 0.04005327 1.058036 0.02451 0.09010823
0.008129452 0.03516433 0.0308503 0.02691413 0.03190028
0.01541337 0.009833293 0.04271022 0.2538885 0.395154392
0.065169563 0.3554249 0.01943744 0.06481173 0.08185491
0.006867338 0.01878754 0.02792041 0.009034978 0.277054
0.06590698 0.01844442 0.02163161 0.02407822 0.004315803
0.03723824 0.007461786 0.03717461 0.1038127 0.0100168
0.740341358 0.07384559 0.01650373 0 0.084852 0.04642094
0.1263773 0.005899627 0.1590641 0.01780753 0.006517459
0.003616206 0.1332422 13.6769 0.004883104 0 0.04037709
0.005409619 0.6335596 4.386730703 0.1476627 0.2013761
0.1042213 0.01327724 0.5042707 0.02466066 0.05599509

0.1755084 0.0102536 0.006725069 0.1667062 0.09042766
0.05034407 0.2757408 0.01803514 0.4379646 0.5800268
0.07204173 0 0.007904601 0.133827 0 0.003331432 0.173727
0.03966238 0.2543925 0.193398919 0.003318953 0.004492051
0.02357104 0.08976979 0.04051599 0.02910847 0.315267876
0.6152235 0.5174434 0.4029801 0.0219803 0 0.01982521
0.2138651 0.003561174 0.03328115 0.01193108 0.04840263
1.625902 0.01313917 0.01272984 0.213431206 0.02265215
0.04161578 0.006972307 0.073677357 0.5409771 0.04160828
0.02143607 0.07982776 0.06167648 0.1557982 0
LINC01480 0.1687412 0.07260974 0.2095398 0.2579069 0.3873318
0.09398939 0.4953584 0.131452 1.002953 2.991512072
0.07966417 0 3.752348 0.03955262 1.072879 0.01250117
1.194922 0.247748312 0.04167001 0.1982178 0.345064906
0.1344415 0.05366786 0.04037641 0.02311429 0.05305367
1.44235 0.08672783 1.043467158 0.55519 0.1430559 0.06806549
0.6108071 0.2923004 0.04808108 0.05548564 0.02633759
0.4622379 0.1435803 0.082133293 0.4088008 0.2305658
0.096596963 0.005423907 0.5212785 0.03724935 2.287418
0.3627471 0.781765972 0.05390954 0.02921888 2.746269
0.08146272 1.68725 0.4441558 0.07260229 0.7061071 0.588131
0.03383974 0.37745681 0.545992859 0.563798744 0.3949389
0.2073304 0.1625483 0.2458364 0.09209723 0.5713255
0.084768497 0.3005837 0.3963225 0.8316454 0.3255037
0.2438801 0.1772649 0.7781931 0.3799348 0.1442239
0.07662814 0.057854325 0.3667498 0.4608067 9.724188
0.2255634 0.213289 0.35127 0.2604362 0.5734294 0.86709804
0.7898729 0.430913441 0.9703385 18.33555 2.257743
0.2719161 1.353237 1.537508 0.6307821 0.07080322
0.17959 2.144377 0.06634026 0.1943992 0.2530103 0.5397628
1.031028 0.4746782 0.2205604 0.156678933 0.1237281
0.1532841 0.2574293 0.3997752 6.082894 1.225107
0.07368456 0.1699232 0.3943684 0.1059578 0.08826662
0.1484291 1.216286 0.2410229 0.3132768 0.05677059
4.656459 0.2315095 0.1642519 0.7815043 0.5310087
0.1640595 0.1536883 0.3948981 0.4675854 0.207476
0.2150746 0.1524773 0.2089387 0.2507032 0.04428215
0.4482985 0.3820078 0.5775814 0.3197942 0.4937272
1.08488 0.05476192 0.4039429 0.08922678 0.4615263 0.3664759
0.2227976 0.1452609 0.4024828 0.3391103 0.1324608
0.4146352 0.3996055 0.1185093 0.5645598 0.292490138
2.693869 0.388263397 0.04605183 0.1766965 0.4956209
0.3633905 1.340529 0.8158113 0.1350316 0.4178975
1.690058683 0.5581061 0.01717096 0.01752283 0.6283277

0.3917622	0.3766339	0.2434979	0.341706	0.4582251	
0.06816901	0.5480177	1.462038	1.172697	0.2084923	
0.4272279	0.7762515	0.06262316	5.901187	0.1953585	
0.2400541	0.2907486	1.147965	2.031109	0.3913319	
0.2748944	0.5363938	1.779602	0.2660318	0.1103465	
0.124442	0.1240709	0.04821269	0.2075249	0.5364105	
0.2338266	0.2235087	0.09314559	0.2137724	1.402935	
0.3138632	0.9867513	0.01861084	0.291484	0.2805078	
0.04514543	0.3609296	1.263969	0.139953936	0.09447881	
0.604832821	0.035423603	0.5620869	2.302769	0.09572418	
0.3136103	0.1571831	0.03088137	0.1854963	0.06663125	
0.5101791	0.08803609	0.06656624	0.3437603	0.5314505	
1.203174	0.8156769	0.7811999	0.0603023	0.7140499	
3.88902	0.3132819	0.3763741	0.4136632	0.4537527	1.208098
0.3884298	0.5531542	0.3970518	0.7205129	0.3808369	
0.1885511	0.4435693	1.72974	0.2680242	0.06981071	
0.262368202	0.3406128	0.366572	0.7420875	0.3545139	
1.077525	0.2527535	0.1593342	0.1796847	0.1169007	
0.1927514	0.2732217	0.2529807	0.4538156	0.4012844	
0.2826151	0.215910552	0.1820581	0.1178986	0.4644665	
0.4929689	0.3165993	0.241441934	0.9663106	0.1842266	
0.2129443	0.4431663	0.1169623	1.996505	0.1263808	
0.1560068	0.2448762	0.1922081	0.2314689	0.3672425	
0.2431836	0.1510076	0.672288	0.3003222	1.164765	
1.195327	0.1231058	0.2304063	0.3978301	0.7352186	
0.4100978	0.2601007	0.06408128	0.09170479	0.20088753	
0.620100649	0.5554896	0.5670665	0.427488	0.0849077	
0.3365833	0.9098563	0.7059415	0.2811582	0.5727208	
0.2091969	0.6026678	0.4627912	0.4383327	0.402908537	
0.03950493	0.6617763	0.294992	0.4542937	0.1480628	
0.033228713	0.1493694	0.3620719	0.05133562	0.13356	0.7417293
0.5725571	0.3304613	0.2098324	0.1080592	0.2890126	
1.763942	0.1639691	0.1770749	0.535647	0.4707678	
0.028271	0.2272605	0.2053713	0.12621486	0.07152742	
0.1230938	0.4006365	0.2324094	0.3201927	0.2014456	
0.6835974	3.474721	0.1196551	2.393599	0.3769193	
0.5180328	0.7572141	0.7323215	0.6313869	0.1013724	
0.7260929	0.3572958	0.372488	0.009224329	0.9773238	
0.2366071	0.05442694	0.0901031	0.1748518	0.1363975	
0.208970581	0.309846	0.1258087	0.09430761	0.08380599	
0.1745738	0.07642874	0.314251388	0.09393599	0.3924922	
0.09237259	0.1346629	0.7065792	0.1735138	0.4741856	
0.1080492	0.05437274	0.5638838	0.06161868	0.3986667	
0.8688615	0.4753656	0.731297369	0.2039201	0.2540262	

0.3254553 0.061413079 0.1697605 3.175502 0.437762
0.1220659 0.06397672 0.2613517 0.1138517
AC020558.2 0.1279249 0.06990019 0.1344803 0.1222016 0.1917658
0.3701538 0.05647184 0.08760922 0.1128537 0.137379393
0.4706062 0.0359038 0.2822128 0.1199414 0.05958711
0.1489291 0.1822708 0.178877398 0.1805176 0.3122525
0.170839682 0.2730051 0.06974797 0.1967779 0.2378155
0.4883941 0.05785526 0.1445044 0.105946371 0.124403
0.3615086 0.06552552 0.3351679 0.1726728 0.2231688
0.1602453 0.05704822 0.2626163 0.2544548 0.210249959
0.8264461 0.1449912 0.133148061 0.4816839 0.5269175
0.3899703 0.8080519 0.392862 0.986227841 0.3503103
0.0632892 0.6379574 0.4313254 0.1099142 0.3919497
0.4493123 0.348823 0.4121487 0.1832454 0.173427268
0.331969442 0.124613199 0.1638101 0.3207754 0.1508939
0.1981362 0 0.1292924 0.183611764 0.1531943 0.1404735
0.01324541 0.4338789 0.698658 0.7821458 0.03305089
0.184455 1.026439 1.078866 0.429650232 0.0635515
0.2595124 0.2936286 0.2254979 0.1539974 0.3361958
0.1692344 0.3161632 0.18781671 0.2267451 0.211330138
0.2448687 0 0.4628098 0.4417353 0.2240379 0.1062862
0.1924363 0.7361397 0.3334275 0.9609932 0.1596617
0.2526456 0.1972907 0.1697148 0.5100622 0.1054534
0.06824886 0.193927132 0.2192726 0.28775 0.7923813 0.09621424
0.5842471 0.2588905 0.2321506 0.55209 0.4198693 0.1755065
0.6882798 0.09455969 2.044704 0.5220648 0 0.8080705
0.3722449 0.1002917 0.09882659 0.1523491 0.2706317
0.3685206 0.3328947 0.5206565 0.7918313 0 0.3726877
0.243358 0.2951537 0.2011232 1.462731 0.4606175
0.5388003 0.4264988 1.173718 0.5002181 0.3251421
0.7116983 0.3437326 1.046871 0.1963664 0.3040088
0.1723528 0.1656003 0.4722212 0.5208454 0.4590641
0.52254 0.8222826 0.7187475 0.3057145 0.275454207 0.363427
0.691483273 0.665 0.0450272 0.275637 0.23322 0.3928449
0.821318 0.5849672 0.1508636 0.147270783 0.2729727
0.1487717 0.4554614 0.1158282 0.1944643 0.2894785
0.4571022 0.8458835 0.3492245 0.7382832 0.3888537
0.442675 0.5681815 0.6774033 0.1566047 0.1978106
0.527505 0.3317998 0.1880684 0.3851601 0.259318
0.139693 0.0628494 0.3358573 0.3035534 1.016618
0.4096766 0.02134204 0.2249551 0.134773 0.4658198
0.04475594 0.06915488 0.5951121 0.08808298 0.8209132
0.5897509 0.4973382 0.8648923 0.2525118 0.2883713
0.2821825 0.6839791 0.3677522 0.07822937 0.5935791

0.3254731 0.037893188 0.1193761 0.847705756 0.21100437
0.7331191 0.7330437 0.3258234 1.283107 0.2127904
0.3344511 0.4931082 0.1649438 0.1700104 0.1430171
0.201859 0.5474984 0.9464943 0.1579467 0.1704794
1.052409 0.8583407 0.5201154 0.1281877 0.2423502
0.317038 0.5147292 0.1404065 0.243422 0.1463224
0.5058867 0.3257686 0.4599834 0.4536989 1.225226
0.7620041 0.2349835 0.3126042 0.4385166 0.075773257
0.7377801 0.7587195 0.1506928 0.1791745 0.2100564
0.283176 0.2157024 0.3892038 0.5855513 0.1590502
0.2427933 0.2078491 0.08330353 0.4470156 0.3825968
0.701505606 0.1526496 0.8938052 0.4683345 0.1462725
0.2351198 0.152533599 0.4890339 0.1544678 0.04193141
0.2357686 0.4000179 0.5671484 0.5186759 0.3686364
0.1125115 0.2676407 0.8257864 0.1893955 0.2407976
0.3270881 0.1960271 0.6814858 0.8760159 0.1232916
0.2814658 0.1403631 0.1814137 0.2620588 0.1714238
0.6628093 0.154225 0.2444751 1.068046292 0.937678718
0.4652418 0.7246896 0.1282092 0.2528808 0.1735838
0.189955 0.5175402 0.3653995 0.2401035 0.5064375
0.1305401 0.3645175 0.4868949 1.199983257 0.3593905
0.6035505 0 0.3936069 0.3544686 0.76772957 0.1244383
0.5005924 0.1111949 0.1596116 0.1263629 0.1691155
0.2982465 1.188705 0.5581439 0.148266 1.133433
0.2449401 0.4858312 0.3702867 0.2832502 0.5307124
0.08204249 0.4448421 0.723669217 1.676073 0.2666259
0.1239705 0.4362866 0.2811687 0.09350114 0.3701744
0.2447602 0.207342 0.4759657 0.3950426 0.1662339
1.173559 0.2163049 0.3343041 0.2561725 0.5331338
0.2959091 0.5086493 0.159842 0.6110698 0.6021875
0.1684154 0.2146834 0.4010141 0.1042737 0.796643171
0.3187906 0.2725064 0.1191597 0.1815269 0.5829559
1.434745 0.199223521 0.3778708 0.4359759 1.000413
0.7778262 0.07916261 0.07516749 0.1081162 0.7381216
0.4542688 0.6483941 0.7118314 0.6076672 0.7306537
0.4182993 0.505049121 3.190046 0.2751152 0.1409897
0.319255309 0.6205068 1.030687 0.3251003 0.4035569
0.207864 0.8122269 0.07253149
AL133406.2 0.1576561 0.06957928 1.137835 0.1042634 0.4294921
0.1719454 0.03747505 0.1598795 0.1497808 0.075971492
0.322707 0.07147793 0.3745562 0.1478172 0.1581695
0.09883021 0.2902944 0.210014975 0.194663 0.5957364
0.09919896 0.1932457 0.07714196 0.1596014 0.09967312
0.343166 0.2303586 0.1630198 0.187484396 0.2201456

0.2398993	0.07609547	0.491664	0.5729336	0.4837808	0
0.03785754	0.2069501	1.116337	0.461499604	0.8030649	
0.4383222	1.060294238	0.7874263	1.685888	0.60681	1.312348
0.2476703	0.938480583	0.406819	0.2659942	0.8924725	
0.2341883	0.4558734	0.2837456	0.3130746	0.5697995	
0.3108004	0.1824041	0.065764216	0.771039208	0.297698645	
0.1811756	0.09933861	0.5507375	0.3369287	0.04964256	
0.7354215	0.158399631	0.4151143	0.383234	0.5010147	
1.535598	0.4410173	0.7360884	0.1535294	0.6779378	
1.855889	0.7434784	0.320758286	0.06325974	0.6623616	
0.6884831	0.6733879	0.0127742	0.4814297	0.5240898	
1.12397	1.03863579	0.369334	0.71288641	0.4739477	0.1035984
0.7063837	0.2233434	0.2106199	0.1763304	0.472497	
0.6106334	0.8358882	1.078052	0.476786	0.2235428	
0.2472996	0.2878163	1.07033	0.3498976	0.1358711	
0.353900828	0.6467137	0.8299095	0.983495	0.04256557	
0.7219425	0.6872052	0.1829421	0.1085541	0.7686284	
0.2239754	0.8500562	0.2133513	1.004611	0.4805532	
0.1801211	0.3613794	1.0822	0.1220159	0.1049311	0.3032993
0.5387785	0.6987214	0.6765397	0.5182662	0.9287271	
0.05422275	0.7574108	0.4383404	0.6659437	0.2535864	
1.273019	5.601169	0.8555688	0.8962526	0.4596708	
0.5150913	0.2844191	1.070518	0.5702575	0.6092074	
0.3909297	0.3698604	0.5032473	0.1868187	0.5664744	
0.4785731	1.104312	1.040282	1.407258	0.8857925	
0.202874	0.036558613	1.179065	0.595293997	0.3751033	
0.3137434	0.5776243	0.1160747	0.7367446	1.816772	
0.4777695	0.2703078	0.181498156	1.268024	0.1727702	
0.2518724	0.2305929	0.3636799	0.06985444	1.201676	
0.9683001	0.2927337	1.065596	0.2308829	1.15245	1.497105
0.5094661	0.2834287	0.213311	0.5500871	0.8912224	
0.1976052	1.201294	0.0860425	0.2224827	0.437926	
0.6898571	0.5035997	0.9155745	0.4572256	0.1133016	
0.09330096	0.4869304	0.4636812	0.673207	0.229458	
2.049823	0.0146131	0.2653973	0.8754155	0.7852596	
1.380577	0.3995861	0.4615272	0.2675115	0.4189778	
0.6366327	0.07787022	0.3554731	0.5463174	0.113157663	
0.1697543	1.380786466	0.089106034	1.329194	1.480507	
0.4226087	0.3380865	0.3389015	0.3107212	0.31511	0.1505043
0.6064069	0.5378065	0.708047	0.8574427	0.2716105	
0.2270978	0.2159777	0.9585675	0.1609739	0.7266352	
0.7655952	0.4824751	0.3005547	0.961477	0.2212897	
0.1211522	0.2670261	0.212027	0.9512008	1.199188	
1.259051	0.429119	1.187226	1.013587	0.2370811	

0.822834 0.276559737 1.138594 0.6908363 0.6000038
0.1358871 0.5420906 0.7767249 0.9161051 0.9408697
0.9451834 0.2110934 0.5034971 0.9655094 0.6412564
0.263682 0.8970905 0.598530004 0.5233791 0.9199637
0.8978366 0.4465096 0.1560269 0.260285691 0.5452035
0.4100231 0.09739077 0.357617 0.5397571 1.340793
0.6788319 0.9377458 0.6826357 0.6018938 1.614634
0.2136628 0.2496793 0.3410906 0.09291768 0.2672316
1.069646 0.3272681 0.6881456 0.5692242 0.5567908
0.3478076 0.2378574 0.8467001 0.4605507 0.2737718
0.538134064 0.487708858 1.415933 0.2526807 0.1607074
0.04576724 0.3455738 0.1260553 1.96699 0.3940321 0.5576695
0.4068276 0.3960101 1.439281 0.9531639 1.027971695
0.3293484 0.4505847 0.02494237 0.3917999 1.10893
0.031841872 0.2972809 1.41737 0.184474 0.1125392 0.1796896
0.4863129 0.03958364 0.4756193 1.111163 0.5028834
1.86825 0.2681972 0.2545267 0.2293428 0.3383398 0.4605482
0.6896226 0.6966717 0.71501096 0.7009991 0.1125947
0.3290704 1.87076 0.4975606 0.1964851 0.3757 0.1218183
0.601971 1.195732 0.6815969 0.2206276 0.3565501
0.4465728 0 0.4735648 0.7783398 0.1661568 0.6401672
3.341271 0.926879 1.24986 0.2011707 0.4273956 0.5617991
0.0461311 0.636791622 0.3674313 0.4068831 0.2598183
0.3915027 0.7109731 2.612187 0.374583457 0.8680062
0.4918376 0.2172698 2.525547 0.1050656 0.09976319
0.1793664 0.6809719 0.1563103 0.9306017 1.077607
0.4669229 0.9550395 0.5124663 0.853118336 3.495655
0.5369649 0.0467808 0.370754548 0.167759 0.6141771
0.521368 1.846916 1.011558 0.2939993 0.3561793
AC135012.3 0.05035916 1.307059 0.0661747 0.1065207 0.264218
0.4298604 0.2111926 0.2112415 0.1388319 0.22984433
0.2501006 0.1201386 0.5110434 0.09106013 0.1172859
0.6848779 0.2296098 0.292502206 0.06662147 0.3534004
0.311045385 0.4728753 0.1784712 0.6369256 0.8893792
0.07916675 0.307468 0.6314332 0.118169943 0.6937801
0.1423121 0.6255261 0.05555487 0.1359494 0.155207
0.01577062 0.0149718 0.1066127 0.08347427 0.035016967
0.1510508 0.01268389 0.097342709 0.02774935 0.1555705
0.05028986 0.04185515 0.08506011 0.139180458 0.07757088 0
0.3020461 0.01157702 0.05949492 0.1893628 0.008843854 0
0.7743616 0.003606836 0.082901338 0.657502301 0.014716637
0.5194687 0.1431139 0.03465068 0.2705578 0 0.6180406
0.065052843 0.03769171 0 0.03649947 0.1743599 0.06037352
0.1595487 0.1626359 0.5641463 0.02049629 0.00272249

0.024665795 0 0.5029422 0.03467711 0.3698746 0.1326126
0.0766222 0.3145997 0.06223069 0.54219926 0.64917
0.034663584 0.06426364 0.2826988 0.167008 0.06210511
0.05879677 0.03835407 0.07196708 0.006037293 0.2989758
0.6530288 0.04399689 0.02486423 0.1337578 0.5678871
0.7299061 0.02594561 0.3671819 0.085884416 0 0.08277874
0.08953554 0.03156323 0.3727517 0.3439642 0.03712671
0.02414853 0.02279814 0.09566338 0.04139506 0.003722454
0.1857512 0.142536 0 0.006915358 0.24772 0.05264132
0.0311234 0.3665079 0.2197333 0.09326088 0.01228574
0.1683621 0.1304848 0.07237307 0.3897062 0.02395018
0.01161907 0.08709196 0.03303893 0.0514578 0.04545109
0.09234295 0.07195831 0.03395116 0.229811 0.004669474
0.4151691 0.003170102 0.4497567 0.01662176 0.4376243
0.03259525 0.2109198 0.4232173 0.09035799 0.09642376
0.2044429 0 0.192557 0.276511285 0.4252279 0.345780967
0.003272314 0.1462353 0.2455702 0.06311925 0.1445623
0.2008512 0.07971204 0.5701368 0.115949762 0.2225935
0.01464144 0.003735369 0 0.247058 0.1761148 0.01384182
0.1331968 0.09768057 0.06902555 0.05639673 0.1072395
0.02960356 0.08000034 0.2550039 0.009733809 0.1038293
0.01865956 0.1203074 0.03790571 0.03645843 0.07698853
0.04329746 0.1731376 0.2114185 0.1107707 0.03298787 0
0.0304412 0.1562175 0.03526451 0.02642805 0.04083545
0.04183444 0.01300309 0.1905828 0.06109536 0.08101368
0.1012223 0.382324 0.1335539 0.07141146 0.01726003
0.0472076 0.01539796 0.01994739 0.1507369 0.085773328
0.06377729 0.003792152 0.067961749 0 0.009409934 0.005830193
0.04456853 0.1005209 0.05705293 0.07908506 0.1298641
0.2426089 0.01876679 0.03121807 0.04310584 0.1460184
0.1813513 0.06711124 0.05280207 0.09916511 0.2047496
0.191037 0.2480504 0.08914696 0.09756221 0.2176362
0.0838477 1.396837 0.2777594 0.03334309 0.08407188
0.01623672 0.1339791 0 0.03597379 0.004395011 0.1547692
0.246089551 0.03714877 0.1076633 0.1021656 0.005038153
0.1378187 0.07431706 0.04670259 0.229822 0.01245996
0.05869875 0.0119473 0.1413293 0.2557887 0.07821021
0.09036824 0.006575142 0.02003077 0.08616905 0.2048505
0.4347433 0.03085256 0.364568951 0.9933721 0.04813973
0.4952038 0.01657376 0.01049812 0.1953566 0.1276143
0.01209317 0.2404394 0.4653397 0.03773257 1.487428
0.3525108 0.116499 0.01102405 0.05487442 0.01034562
0.1375164 0.0116634 0.1043716 0.3660051 0.2340996
0.04907856 0.3587687 0.07589059 0.05714281 0.081753977

0.029929321 0.1042048 0.03626474 0.1345895 0 0.01708333
0.3365011 0.02315179 0.4315315 0.2796204 0.1180451
0.07341237 0.1650211 0.04791798 0.120244108 0.04715944 0
0.1479621 0.154948 0.09967196 0.028333669 0.1077706
0.01642201 0.04012541 0.09032242 0.3837413 0.1479429
0.3052615 0.1238686 0.01063161 0.103763 0.1115473
0.0674965 0.05536276 0.08746063 0.1003543 0 0.5867287
0.07004693 0.03481878 0.02772293 0.05725114 0.04880247
0.01981723 0.1623385 0.134962 0.1157216 0.03613228
0.05101413 0.3613555 0.1710645 1.006139 0.005566087
0.170302 0 0.003601619 0.01049372 0.05376375 0.03797575
0.007865459 0.1503468 0.1134853 0.05303898 0.1805498
0.3069577 0 0.003563727 0.02311763 0.0625773 0.1072197
0.02679758 0.08373191 0.3656746 0.003267779 0.1230075
0.02145338 0.00358022 0.04100899 0.05713271 0.103567
0.07448214 0.0177177 0.09603741 0.498624 0.01313535
0.05036113 0.1263831 0.04116711 0.122002289 0.2608198
0.5319555 0.1387556 0.109968792 0.04523515 0 0.02132993
0.04656386 0.02386653 0.1889389 0.4911095
LINC00243 0.03346066 0.0063992 0.08002384 0.05753456 0.07022284
0.1016602 0.06203838 0.1042655 0.1756354 0.352149388
0.04021071 0.1051811 0.3203655 0.05019593 0.3491239
0.05453643 0.08009504 0.045348396 0.1156818 0.02858599
0.062559902 0.07997754 0.01702737 0.1521229 0.01375039
0.03156095 0.1165233 0.04233294 0.064660959 0 0.05673475
0.02399483 0.03229878 0.1686165 0.0490334 0.02934017 0
0.06010479 0.07764914 0.088836437 0.06485076 0.02949691
0.062688318 0.01290645 0.04823807 0.06401536 0.1245899
0.122283 0.122653854 0.05345016 0.03862652 0.4482864
0.3517919 0.08049913 0.3914409 0 0.08843243 0.3567318
0.05368221 0.072579945 0.159552908 0.118643705 0.0333254
0.1252959 0 0.1813891 0.1095749 0.3314196 0.073960611
0.1028469 0.1771826 0.02910207 0.3839659 0.05616045
0.08852813 0.2904703 0.2234204 0.04902682 0.07597516
0.059000219 0.1163598 0.1242714 0.03584135 0.178913
0.1057358 0.0777548 0.08262942 0.173664 0.38973481
0.2792892 0.058040348 0.2017544 0.120052 0.3671994
0.1001368 0.2939788 0.2335261 0.08456206 0.1516317
0.2984619 1.005448 0.09354659 0.06167764 0.03210935
0.2002546 0.2877422 0.08688614 0.0333228 0.017753578
0.06245211 0.05268568 0.1988152 0.1056983 0.6123277
0.4977173 0.03719248 0.06739007 0.06362162 0.05932511
0.04200694 0.06232838 0.1223921 0.1171719 0.2484858
0.1479539 0.7919115 0.09793565 0.1809469 0.1239752

0.05574533	0.0433764	0.09904614	0.0680927	0.06743285	
0.1346453	0.06823739	0.1527693	0.06484953	0.2135833	
0.1229334	0.05926378	0.1902571	0.1301499	0.3029771	
0.2147571	0.02706	0.03474897	0.2975148	0.01769329	0.06537049
0.1298794	0.2272104	0.1394749	0.1795739	0.254305	
0.2101312	0	0.3645043	0.3007985	0.4030193	0.383301317
0.3622831	0.095810983	0.1217584	0.07419844	0.09031089	
0.256209	0.1125831	0.2734173	0.01647766	0.1160142	
0.0770417	0.1642201	0.1021478	0.1459375	0.2226799	
0.1488958	0.2505557	0.1030071	0.2710353	0.08749874	
0.04731168	0.05995562	0.09975598	0.1101509	0.529192	
0.1616148	0.04527275	0.06622882	0.121502	0.1033033	
0.01410421	0.06104562	0.1534629	0.345223	0.1698403	
0.0684052	0.01994336	0.1159244	0.007815255	0.1081191	
0.1206398	0.03936435	0.05463069	0.05064775	0.1037736	
0.1612758	0.04624781	0.07388149	0.1444405	0.1540778	
0.2133865	0.1055989	0.05904721	0.3853335	0.06440616	
0.05013202	0.0530154	0.1472288	0.055504532	0.06869406	
0.253981604	0.028097395	0.004793953	1.540577	0.04338673	
0.03454865	0.124675	0.02449457	0.06019055	0.0755011	
0.3190631	0.0872859	0.01055984	0.1042542	0.04683749	
0.3470317	0.06810325	0.1133478	0.04783075	0.105255	
0.2145881	0.3106123	0.3482891	0.1186783	0.23137	0.1448507
0.254514	0.1950011	0.2767608	0.2285994	0.1283811	
0.5234443	0.3154352	0.04780487	0.09811949	0.03876086	
0.048558058	0.0691128	0.09045824	0.13489	0.04686573	0.3162296
0.1106095	0.1500776	0.0916218	0.08692843	0.0364017	
0.1722605	0.1868214	0.1708279	0.1364108	0.1587838	
0.061163054	0.06521533	0.1068745	0.1016295	0.1392654	0
0.023938451	0.4011385	0.2026901	0.03838723	0.06783563	
0.03906209	0.09086195	0.04748357	0.01124927	0.1412594	
0.07622809	0.1025984	0.03467743	0.08817785	0.05703649	
0.06152854	0.1247769	0.3079573	0.1504941	0.2061403	
0.07995501	0.016608	0.1402543	0.09701408	0.224511	
0.1185991	0.0615481	0.202797059	0.037120997	0.08224624	
0.0179915	0.09911458	0.0505105	0.05085183	0.2434591	
0.1895184	0.06690295	0.03663486	0.102487	0.1570656	
0.2135727	0.2228705	0.047937022	0.1128047	0.1105073	
0.02752737	0.02402253	0.0803543	0.008785482	0.1184772	
0.04277287	0.02714569	0.09863148	0.2247541	0.2752379	
0.2511947	0.05761228	0.07252446	0.211142	0.2008318	
0.1121185	0.1685433	0.180794	0.04840434	0.09717097	
0.04005759	0.08470645	0.176667289	0.05157666	0.08875992	
0.1361906	0.03686864	0.1716023	0.07418504	0.191371	

0.0896289 0.04745419 0.3112397 0.2989659 0.07609162
0.06213199 0.7040787 0.1891931 0.1206103 0 0.3000716
0.1605712 0.00731658 0.1438509 0.1126036 0.770902
0.06432141 0.1794805 0.07636812 0.072930829 0.03072057
0.04157889 0.06233599 0.7976817 0.03461724 0.03367887
0.085112766 0.03725421 0.4310558 0.00666076 0.1424164
0.01932574 0.1651538 0.2375469 0.02637007 0.06161075
0.1711747 0.1058956 0.8198182 0.1216175 0.06480578
0.596865659 0.0599059 0.1007446 0.03872183 0.029227081
0.6900871 1.201607 0.06944507 0.005095821 0 0.09013029
0.01593621
AC022762.2 0.1932854 0.1848248 0.3358281 0.1025766 0.2676111
0.09787325 0 0.03860828 0.1989328 0.107629088 0.2765203
0.08438587 0.2818999 0.1812229 0.1750619 0.0875082
0.3212977 0.088935291 0.4242765 0.6880287 0.050191259
0.149719 0.05464364 0.1541645 0.1176727 0.23633 0.305953
0.1188717 0.041501535 0.04873141 0.06069038 0.1636322
1.430399 0.5072982 0.8916856 0.4943266 0.07821466
0.2411077 1.005062 0.190060512 1.985781 1.202188
0.145294605 1.042376 0.7629622 1.216812 1.162005
0.5540138 0.229609586 0.08576518 0.1363548 0.6686567
0.4377592 0.3713565 0.4606061 0.8052254 0.3153269
0.0880624 0.3014813 0.131018066 0.45107544 0.248950095
0.128336 0.0628274 0.3250966 0.509344 0.2197775
0.45582 0.280506663 0.3300527 0.2476196 0.7860606 0.276185
0.7108999 0.2841014 0.4078231 0.4752162 0.9003067
0.4632519 0.178822459 0.09335448 0.3049703 0.3105562
0.9054082 0.1357294 0.8837449 0.46405 0.3848138 1.17715127
1.332316 0.465652604 0.203831 0.08255717 0.8339469
0.2842759 0.1974613 0.1457213 1.096802 0.08110186
0.5659807 0.2151099 0.8724749 0.7422514 1.120606
0.4653674 0.4698062 0.185888 0.2005094 0.075965543
0.5439947 0.9169178 0.6035425 0.2826693 0.994368
0.7605986 0.3921724 0.1201477 0.6890814 0.2538454
0.4718252 0.2778084 1.721285 0.5145592 0.1794222
0.7638225 0.6561746 0.1866107 0.301958 0.3580711
0.4373012 0.7037436 0.6112603 0.1966686 0.5085474
0.04801098 1.327597 0.8171031 0.6590239 0.5436123
1.070823 0.4681532 0.5314189 0.5596811 1.628043
0.4054065 0.547092 0.3903032 0.4773871 0.8517102
1.132837 0.4763478 0.1924161 0.1751471 0.1707503
0.2746513 0.236021 0.5181227 0.4450152 0.15083 0.3233388
0.22659315 0.3677695 1.164005821 0.4981971 0.3704011
0.4262096 0.2912018 1.515443 0.6580808 2.458898

0.1063739	0.679908666	1.202956	0.2185394	0.3010741	
1.029388	0.1350392	0.9277784	1.301607	0.4100472	
1.846786	0.9760562	0.04810186	0.2501042	0.4320462	
0.5970461	0.3011508	0.4068056	0.7704547	0.1856762	
0.03683527	0.1810509	0.11972	0.2462438	0.295434	1.090091
0.2881247	1.600038	0.2516609	1.341804	0.5452417	
1.319839	0.1473811	0.473361	0.3453911	0.4412601	
0.1035121	0.3339379	0.2644553	2.095977	1.098802	
0.1369585	1.225964	0.3789845	0.4328096	0.4227749	
0.1723735	0.2552027	0.2699895	0.345113682	0.230471	
0.701864824	0.090169172	0.6384603	1.47008	1.22701	0.4213148
0.4751217	0.05240475	0.515097	0.6178535	0.7367272	
0.4061665	0.8217905	0.810686	0.4509277	0.2320171	
0.6192379	0.5456274	0.7894119	0.6273069	0.2582432	
0.327522	0.6120831	0.6048924	0.2062512	0.1251518	
0.2579297	0.2346715	1.822282	0.8108355	2.36292	0.3199655
0.7592126	0.3758636	0.06560033	0.8707299	0.222615444	
0.5544859	0.3421339	0.6001352	0.7068793	0.4662746	
0.3327789	0.3422064	0.539054	0.4277504	0.5490495	
0.2050755	0.6550536	0.5188462	0.3210275	0.464602	
0.726244585	0.5904876	1.178986	2.486861	0.593036	
0.03453811	0.102429911	0.2528663	0.6504659	0.04927638	
0.4057057	0.9715143	0.2499351	0.4740792	1.353778	
0.3022164	0.2533652	0.6498458	0.2114426	0.2033899	
0.7413099	0.1810006	0.2366175	0.5867947	0.1448881	
1.027125	0.3757193	0.6928715	0.675149	0.485315	
0.0876275	0.4984099	0.691313	0.313783002	0.25314572	
0.3582066	0.173213	0.05859277	0.05403217	0.2345884	
0.1116144	0.7049542	0.1789188	0.3644587	0.3837141	
1.468318	0.6104254	1.087147	0.628171038	0.251395	
0.1994828	0.06625489	0.6938303	0.4959042	0.01409703	
0.3217186	0.2941397	0.642473	0.3810019	0.4560991	
0.253944	0.1401957	1.674257	2.45438	0.5420715	0.8271115
0.07915748	0.585957	1.000841	1.209414	0.6716503	
0.3695853	1.118714	0.642548376	0.5958648	0.5554482	
0.3460043	1.005699	0.6498259	0.6501197	0.2303029	
0.05393136	1.659943	0.6592208	0.3094938	0.6348954	
0.0913879	1.878213	0.1071444	0.225784	0.3602495	1.1636
0.4637696	0.9157243	0.8078722	0.9335227	8.559873	
0.6421894	0.4188963	0.459521	0.308516568	0.1380223	
0.2268369	0.1100255	0.3733176	0.7035855	1.221316	
0.214610704	0.2305702	0.8453679	0.2351302	0.6447494	
0.09302923	0.2061134	0.9846726	0.486599	0.3361224	
0.1949228	0.5097545	2.029576	0.9366977	1.124942	

0.188846224 0.413333 0.4088871 0.103554 0.28138356
0.1080294 0.7662873 0.4138841 0.4497165 0.7328242
0.1446215 0.1704731
LYPLAL1-DT 0.02006715 0.2494539 0 0.1725242 0.05264289
0.4267761 0 0.3126524 0.1858813 0.175993628 0.2928288
0.1182743 0.2169218 0.03762962 0 0.4608685 0.04002908
0.574148553 0 0.08571849 0.211042459 0.5995554 0.2042344
0.4561586 1.443126 0.09463926 0.1270578 0.3649535
0.155114852 0 0.1134173 0.6295744 0.01937035 0.06320207
0.01633694 0.04399 0.668189 0.1622082 0.07761339
0.035518214 0.1296418 0.01769 0.020886485 0.01290051 0.02066394
0.05906389 0.09339943 0.04313885 0.061298692 0.08013832 0
0.0946648 0.1291702 0 0.5086389 0.04933747 0.1178556
0.2468545 0.06036468 0.136024742 0.136696671 0 0.1798743 0
0 0.3671438 0.2464301 0.1216899 0.060485367 0 0.05141642
0.1599877 0.1588093 0.09355765 0.04684625 0.471797
0.04674088 0.065339 0.06075214 0.019657685 0.06978376
0.02192014 0.02149491 0.1444401 0.1268245 0 0 0.07439318
0.06874507 0.2037118 0.019337875 0 0.05142718 0.1863385 0
0.1435049 0.07780618 0.02113078 0.235763 0.02034031 0
0.01753191 0.1387108 0 0.1656517 0.06054942 0 0.3996895
0.035490808 0.0802586 0 0 0.3169488 0.3097099 0.2132092
0.0159323 0.04490603 0.04769424 0.1482445 0.0209938 0
0.2806526 0.09246157 0.1117671 0.1350262 0.4476782
0.03670896 0 0.3717543 0.2600251 0.01445214 0.1827702
0.04083681 0.1617643 0 0.1534634 0.07634948 0.1296393
0.02208466 0.07899225 0.04100988 0 0.04683239 0.02112821 0
0.09737116 0 0.1200949 0.07074057 0.01960212 0.03709131
0.03785097 0.03636801 0.2193781 0.1319822 0 0.03586147
0.04752215 0 0 0 0.1330223 0.020521472 0.07302141
0.02472143 0.07964939 0.1920683 0.2250625 0.02732914
0.1235255 0.5301064 0.046203738 0.06423045 0.04084031
0.08335446 0.07949191 0.07764899 0.05779393 0.03860988
0.0232209 0.06054816 0 0.06741901 0.0560869 0.1835003
0.09917783 0.2501271 0 0.06619833 0.01734944 0.01720929
0.04229313 0.1627134 0.03067844 0.06901281 0.08780799
0.06410035 0.05980254 0.08179129 0.1406098 0.1080693
0.164433 0.01967311 0.08190831 0.1898416 0 0.09672095
0.02311326 0.0681669 0.03766278 0.2823459 0.06398647
0.07450608 0.02213251 0.1348046 0 0.1288516 0.03179459
0.2312531 0.020804616 0 0.23270928 0 0.01437524 0.2624774
0.08131267 0.02071965 0 0.07344989 0.04010856 0.1358394
0.02333533 0.05234743 0.03166494 0.09619032 0.05617911
0.08671798 0.01701797 0 0.1024473 0.2404721 0.1809753

0.1330583 0 0.1465351 0.01927196 0.4677636 0.9640299
0.2923669 0.04292586 0.1262731 0.02264507 0.112115
0.03637961 0.04300458 0.04903716 0 0.166408119 0.01884027
0.1549999 0.03677126 0.02810649 0.2819128 0.02072974
0.04737111 0 0 0.04366193 0.06665077 0.1037419 0.03658912
0.0818088 0.05601555 0 0.01396828 0.02002974 0.05714026
0.4336656 0.06454428 0.095709717 0.128878 0 0.1611521
0.07396843 0.04392462 0.2335374 0.03164114 0.1686613 0
0.1142895 0 0.3327501 0.4131434 0.01710307 0.1230004
0.1360576 0.09619235 0.2256372 0 0.01712533 0.02490053
0.1549466 0.068449 0.07278085 0.03386985 0 0.152027768
0.166967578 0.07046432 0.09441187 0 0 0.1906066 0.1303645
0.05166304 0.3343607 0.0219708 0.07317132 0.02047739
0.2401586 0.08019643 0.071872428 0.05637637 0.08284231
0.1857242 0.07203441 0.09267382 0 0 0.09161394 0.02034989
0.2519422 0.1783991 0.06190001 0.2619956 0.1343668
0.01977029 0.01808955 0.04014787 0.2151684 0.3369315
0.02710662 0 0 0.330323 0.07815457 0.07063438 0 0
0.03403197 0.07370333 0.06174837 0.1368939 0.04782071
0.2015722 0 0.09332891 0.3181075 0.1369019 0 0.2375171
0 0.02009246 0.02927081 0.02499445 0.01925968 0.02193963
0.02396411 0.02813795 0.1109588 0.1285836 0.3669501
0.1144994 0 0.03684771 0.02493585 0.09346093 0.09966431
0.01730065 0.08079207 0.109380317 0.2872573 0.1196826
0.1198385 0.01525187 0.02897526 0.1100518 0.05935938 0
0.5727161 0.08278834 0 0.07023778 0.1215615 0.01766617
0.100832258 0.08981748 0.2132433 0.116112 0.058427249 0
0.2540691 0.05949694 0.09168254 0 0 0.2707909
AL157871.2 0.02631996 0.3020144 0.435782 0.1257121 0.1035692
2.398959 0 0.4416171 0.1219004 0.032976032 0.2033326
0.1034186 0.4064478 0 0.429092 0.5459751 0.2625096
0.217988133 0.2924827 0.3372836 0.06151157 0.3669742
0.2009045 0.9446763 0.1622397 0.2896327 0.2916345
0.6659769 0.10172387 0 0.1487574 0.8493389 0.8892112
0.4973728 0.2999842 0.2307881 0 0.1418342 0.447908
0.302805647 0.4888576 0.464042 0.438313491 0.1522821
1.219622 0.4454402 1.041268 0.509226 1.768778193
0.9880242 0.0607668 1.465109 0.48002 0.2374506 0.8210804
0.1617768 0.6956044 0.6205647 0.1847392 0.321136673
0.597635204 1.363972737 0.3145628 0.4003881 0.68818 0.5172135
0.1436517 0.2926143 0.052888171 0.8089879 4.54079 0.6867456
1.17165 0.2699613 0.3072164 0.1904019 0.7765308 0.5570386
0.3187286 0.077348717 0.4576399 0.4025046 10.17753
0.487149 0.1663423 0.1783876 0.4874686 1.756326

1.89347821 0.6531244 0.20290756 10.69748 3.395064
0.6443284 1.93888 0.7259923 4.770844 0.6651603 0.7730634
2.614467 1.482899 0.9887743 4.730239 1.136566
0.8419126 0.9927034 2.581889 0.1965864 0.325846766
1.017578 0.1275146 0.6128674 0.2771388 1.131595
2.516796 0.2716572 0.4122894 0.250222 0.2138803
0.2753535 1.035017 0.4813645 0.7276324 0.4886437
1.062597 0.8424643 0.3129572 0.1138654 0.3900726
0.1948843 0.3411963 0.6292659 0.8034199 0.5304229 0
0.3690166 0.3504884 0.9918662 0.7820849 1.139664
1.452285 1.662835 0.5937759 0.5819443 0.8694757
0.02128524 0.9225003 0.7425744 0.1391744 0.2056802
0.8027043 0.446806 0.3816006 5.179232 0.2885118
0.8264401 1.058303 1.495915 0.3696974 4.491012
0.317371151 1.831949 0.484485196 0.4549287 0.1945469
0.4387642 0.2099297 0.6149805 1.971461 0.2916277
0.6518292 0.393903607 0.4773843 0.1339148 0.136659
0.3753401 0.178227 0.6443186 0.9621695 1.035518
0.3176585 0.2392399 0.05895093 3.996937 0.7220337
1.073169 0.2050408 5.484137 0.5643648 9.853098
0.2031445 1.331314 0.106707 0.1609507 0.9956848
0.9213476 2.286803 0.8628033 0.7777581 0.03073717
0.2834862 0.3882049 0.1548189 0.3652636 0.9959807
1.979471 0.1268586 0.424413 0.7152585 0.6174786
1.009973 0.3356971 0.4153174 0.2902887 2.096449
0.6908388 0.1126673 0.6880765 0.8272095 0.1910105
0.2947328 2.27527743 0.165759362 3.092135 0.7803313
0.1706388 0.2445819 0.06129288 0.0642243 0.5260615
0.02969436 0.3672778 0.2746343 0.2076577 0.47311 0.8105261
0.2274776 2.566878 0.6835499 0.3224862 0.354827
0.9758389 0.453748 1.304582 0.6314964 0.6824786
0.6573387 0.9746541 0.3067734 5.855336 1.561551
0.2376091 0.539181 6.346133 7.520607 0.1607921
0.8711162 0.109129962 2.668765 0.6861272 0.3376028
1.032201 1.14288 0.6253471 0.6523826 0.4404227 0.3874719
0.2290669 0.3933845 0.8980437 0.6478661 0.5722664
0.1102045 0.336773525 0.2931314 0.3415216 0.8243932
0.3581291 0.2257491 0.156915387 1.042386 2.688146
0.09058551 1.28547 0.2496489 2.297298 0.8092566 0.5751596
0.9722457 0.5353623 0.6796069 0.1363853 0.3034508
1.144047 0.24199 0.4015177 1.791548 5.504567 0.7894084
0.2021533 0.3265939 0.4064541 1.750658 0.8591305
0.2221175 0.2860791 0.284855342 0.29199161 0.2772618
0.1592101 0.1230994 0.6621878 1.449991 0.2051824

14.53472	0.1973455	0.4322516	0.268719	1.235469
0.6037321	0.6311111	0.816984649	0.2218288	1.358194	0
0.4723996	0.2187908	0.310977416	0.5734984	0.6008016
0.3736712	0.3304459	0.36398	0.8118769	0.04295399	0.07552915
0.3111672	0.3558922	6.4769	0.4233204	0.3682645	0.319976
1.033453	0.5291593	0.7089541	0.7431764	0.416896397
0.5476937	0.3141812	0.7141781	0.8216853	0.6479105
0.2244366	0.6585744	1.01346	0.2986175	1.39547	0.6448068
0.2992661	0.5294538	0.9691923	0.3501605	1.50213	3.186489
1.049042	0.202087	0.4028626	1.602991	0.9041865
0.2668103	0.3654073	0.2887738	0.1501768	0.36506252
0.2174816	0.06541142	0.8335631	1.56863	0.4311373	0.5298321
0.358656475	1.339609	1.601145	0.183376	2.360503
1.558155	0.07217168	0.1557108	0.1555691	0.5815519
2.171694	0.8009312	1.289729	1.339291	2.525622
0.925757035	0.9895544	0.3729182	0.2030558	0.114949262
0.430283	0.9694126	6.672065	0.4008343	0.04989489
0.2481359	0.3342756
TFAP2E-AS1	0.1410545	0.1483682	0.2854443	0.5793985	0.3700335
0.3999817	0.08717497	0.1014311	0.3484222	1.961657691
0.0847548	0.1385608	1.546553	0.05290066	1.563728
0.1254001	0.7034237	0.223028988	0.1741645	0.2711367
0.329654241	0.168574	0.08972433	0.1687577	0.1738957
0.2217438	1.250348	0.2007629	0.572418983	0.2880594
0.2391673	0.101151	0.5990891	0.5331064	0.5282384
0.247369	0.01467745	0.405398	0.250955	0.162280308
0.4556343	0.3357322	0.337671607	0.3264455	0.2033492
0.2283422	0.5252131	0.5154883	0.272888286	0.2027889
0.09769886	1.384054	0.4539767	0.4029744	1.017582
0.2774394	0.2278161	1.113402	0.0424311	0.305963225
0.432386169	0.692510634	0.4776468	0.2971065	0.2523444
0.2293955	0.2502049	0.86963	0.155891735	0.2463379	0.3975537
0.1226808	0.3209335	0.184136	0.2963594	0.5612234
0.2847412	0.3100113	0.4377103	0.02763527	0.2943113
0.3697907	0.8763245	0.6236768	0.1485776	0.2048613
0.4934634	0.5403497	1.01475725	0.3182036	0.108742692
0.5670018	0.3494386	0.8335069	0.6494306	0.5475877
1.339928	0.2673557	0.236744	0.4289238	1.006644
0.2834382	1.007516	0.3553149	0.2328773	0.7554569
0.3662715	0.4565385	0.349257116	0.3196838	0.1793871
0.1925393	0.3218039	1.803792	0.6993815	0.1567862
0.5997353	0.1564495	0.177145	0.1180545	0.1459708
0.4097234	0.4939423	0.3404376	0.33897	1.655483	0.5160639
0.2440921	0.5618181	0.2480517	0.375868	0.1766483

0.4592754	0.2416257	0.7568022	0.3236142	0.3220021	
0.1670627	0.3104717	0.1480657	0.451613	0.5049852	
0.2743257	0.5049436	0.3727772	0.1140724	0.1647962	
0.3617838	0.09944888	0.1240072	0.5735829	0.2660592	
0.166163	1.1355	0.5050917	0.4429077	0.340301	0.7181842
0.2377549	0.8022779	0.510258861	1.075285	0.331770445	
0.3207979	0.5560641	0.3807086	0.5625301	0.05273309	
1.11418	0.295214	0.3027525	0.373487692	0.3461377	0.1148287
0.2197159	0.257029	0.3274828	0.4468649	0.3663815	
0.244834	0.411414	0.21369	0.2053553	0.9856048	0.8899942
0.8539885	0.3955894	0.3053581	0.267557	0.3414639	
0.2782225	0.475654	0.1143733	0.6037994	0.4850997	
0.370328	0.6758541	0.4623956	0.0431191	0.08236369	
0.108519	0.4970028	0.05531392	0.3109017	0.1601305	
0.2952862	0.4079183	0.3736718	0.05989422	0.7015516	
0.5051824	0.5846997	0.3796917	0.3267016	0.5279256	
0.5553539	0.07547615	1.061569	0.5763161	0.131614447	
0.4080475	0.446111701	0.103639834	0.2222998	1.463689	
0.1943294	0.3641025	0.2299375	0.1032577	0.1550605	
0.1750526	0.328054	0.0551935	0.1446749	0.1014201	
0.09872251	1.178467	0.1794322	0.4300392	0.07201148	
0.4859637	0.9752756	0.168351	0.3495772	0.3090041	
0.406395	0.7045649	0.6211597	0.2671609	0.3520199	
1.204586	0.286515	0.8668774	1.048439	0.1007615	
0.2585163	0.1050414	0.307047214	0.4105349	0.4358055	
0.3618577	0.09878204	0.4863928	0.1311407	0.266382	
0.3218623	0.4030947	0.2608694	0.562196	0.7146303	
0.4243628	0.5750444	0.5315495	0.154700959	0.4123761	
0.3660581	0.2543759	0.2257993	4.476407	0.151370028	
1.238063	0.3974172	0.3074631	0.5589285	0.1337924	
0.8207821	0.1668072	0.2608192	0.7319486	0.2065771	
0.5577065	0.2046575	0.1974745	0.3967248	0.9942733	
0.4542737	1.135929	0.634413	0.2286819	0.3490907	
0.2275377	0.3889783	0.09622733	0.5371651	0.1190377	
0.235871	0.244256541	0.469454472	0.3590944	0.1042852	
0.3518491	0.301649	0.562715	1.20958	0.4720898	0.3055343
0.3552017	0.1851589	0.4893897	0.2110132	0.3194362	
0.252600183	0.1717199	0.3785009	0.2901064	0.7088746	
0.5081044	0.111106494	0.1536753	0.321983	0.07152098	
0.184793	0.3343972	0.5076201	0.29926	0.1214333	0.08338072
0.3178843	0.691401	0.1134334	0.1381528	0.5906612	
0.4664004	0.5199118	0.5277005	0.4669546	0.148949392	
0.03261347	0.3180449	1.052546	0.155421	0.4919087	
0.04811224	0.5546274	0.7320528	0.1200268	0.7085024	

0.467531 0.5453036 0.5893189 0.5750631 0.5864338
0.52256 0.6378195 0.3513783 0.1489166 0.2930113 0.623253
0.3955704 1.23491 0.6628089 0.2751294 0.06706925
0.237569118 0.2590072 0.1577495 0.2365015 0.4553595
0.1945736 0.3691335 0.615078444 0.2692224 0.4374574
0.3790619 0.2251354 0.3055062 0.2514097 0.6258669
0.06947746 0.1428472 0.6052069 0.2060337 0.7405653
0.5810406 0.4842931 0.620167334 0.3661763 0.3372557
0.1904387 0.123207753 0.4966732 1.55859 0.4182115 0.2685201
0.04010967 0.5129288 0.2127356
CEP250-AS1 0.1904825 0.06623439 0.3663547 0.3804626 0.2271362
0.2455192 1.444777 0.3735661 0.5346765 1.86583812
0.2080988 0.2381462 1.550993 0.1461231 1.072781
0.08980281 0.4835933 0.182534651 0.2993387 0.3328616
0.283290474 0.1897044 0.1542104 0.4143515 0.1423223
0.2858354 1.315707 0.09584829 0.301170553 0.5501015
0.2936141 0.1862674 0.4345976 0.3272345 0.1550744
0.7212472 0.1441504 0.2332911 0.2679003 0.367797672
0.2517122 0.1526527 0.198259797 0.1447194 0.3566315
0.1146783 1.611947 0.372259 0.264483032 0.1659694
0.1399302 2.630395 0.5944804 0.5988622 1.114184
0.1702995 0.6356337 1.739652 0.3472708 0.610376577
0.27523986 0.82654643 0.379425 0.4863245 0.07149025
0.2581497 0.1417683 0.7700743 0.226177303 0.4959642
0.1774755 0.1506093 0.7708591 0.3390824 0.3368761
0.7829399 0.7797946 0.3382987 0.484931 0.169632466
0.2107653 0.7377081 0.7975918 0.3739261 0.1641614
0.2514983 0.3919891 0.8773489 1.30509106 0.5664327
0.283683633 1.063457 0.2514763 2.163456 0.6378198
0.7253193 1.040692 0.3282197 0.1598516 0.6845398
3.490616 0.2420616 1.436376 0.2492589 0.9291499
1.593626 0.2997693 0.3449049 0.107191546 0.484804
0.4089891 0.7786345 0.6077896 4.333408 0.9199254
0.1924789 0.3487577 0.3155362 0.3198123 0.3079758
0.4480033 0.4843682 1.053202 1.028773 0.3162643
3.594417 0.3009347 0.3933039 0.3689185 0.2724663
0.2244819 0.276007 0.4933515 0.2442854 0.3096966
0.5517855 0.2470666 0.03728998 0.5336116 0.4998763
0.7549598 0.7840494 0.4041317 0.9480748 0.3922634
0.4061193 0.4945413 0.7254362 0.3052215 0.7781036
0.2560582 0.2776339 0.1569156 1.342369 0.2657472
0.2174946 0.2630411 1.865881 0.2918803 1.390473
0.574218603 2.257521 0.584385003 0.4568407 0.08533164
0.5086169 0.6077202 0.2589515 1.155576 0.3411012

0.2001326	0.219288778	0.6466426	0.01762121	0.1978054	
0.3018229	0.6533066	0.2742973	0.5330833	0.5009516	
0.3134937	0.2273587	0.1939268	0.8873184	0.6175583	
0.8986291	0.5261166	0.2811551	0.3855915	0.3443419	
0.3415604	0.1824805	0.1053079	0.6088884	0.6848637	
0.4849429	0.2765712	0.6708719	0.211741	0.3640102	
0.5328942	0.6527148	0.1867422	0.268589	0.1638204	
0.4967711	0.3129885	0.4986293	0.1323527	0.5850076	
0.7530857	0.7546184	0.8840382	0.9740415	0.6148726	
0.2575618	0.1482535	0.4527035	0.3084042	0.233388617	
0.5332591	0.419880262	0.254467292	0.1860728	3.835402	
0.3368031	0.5721487	0.1613047	0.02112742	0.2941932	
0.2344404	0.7047881	0.1806891	0.1092988	0.2490171	
0.38783	0.823149	0.5874142	0.3324057	0.7072403	0.5317471
0.9717209	0.3444607	0.4720751	0.2348354	0.7483675	
0.8072965	1.126521	0.7316491	0.6544105	1.198616	
0.3517408	1.064224	1.067366	0.4948004	0.3385259	
0.5874583	0.251298077	0.6015412	0.3343859	0.9202018	
0.04850799	0.5086587	0.1431068	0.6949269	0.4741619	
0.1799491	0.3202568	0.3019542	0.6087508	0.7893482	
0.376509	0.507545	0.189918936	0.1566978	0.3284018	
0.4109014	0.5821262	5.346943	0.144534221	1.89062	0.3781148
0.3377252	0.7021271	0.1768852	1.007635	0.2866936	
0.9605857	0.2741425	0.2395156	0.5589139	0.1256241	
0.3279935	0.4575224	0.8491281	0.2054648	1.162105	
1.168257	0.4351498	0.1921139	0.7305738	0.6112383	
0.4430013	0.6437505	0.1315232	0.1882189	0.3186034	
0.384217804	0.380037	0.1629421	0.2159742	0.1960518	
0.5098896	0.6974733	0.4903986	0.1875449	0.9100467	
0.2778242	0.4417648	0.3281309	0.5767006	0.454820829	
0.1783796	0.3931801	0.08903741	0.9324121	0.3518733	
0.159133457	0.3301546	0.2213585	0.08780287	0.217409	
0.4447336	0.6765963	0.2260844	0.2815914	0.3582688	
0.4995217	1.195249	0.2204899	0.2180617	0.8888645	
0.447326	0.6189293	0.3109596	0.354071	0.167619847	
0.20019	0.1607729	0.5873456	0.4770072	0.6216543	0.2214941
0.6808894	0.7537535	0.0736756	1.417443	0.4242352	
1.535781	0.3885348	1.639689	0.3167721	0.1387074	
0.5051745	0.9921516	0.5484532	0.07572966	0.723779	
0.2913737	0.4947078	0.09246576	0.422204	0.3952209	
0.274496184	0.3974634	0.2582153	0.2419513	1.075044	
0.104505	0.7668988	0.393282428	0.3718253	1.652447	
0.3447082	0.3685172	0.2000295	0.3561272	0.461008	
0.2047059	0.1434818	0.5429494	0.1686251	3.909373	

0.482537 0.6555222 1.022383812 0.4805396 0.5213744
0.1669946 0.100837493 0.3048709 3.268735 0.2053673
0.2109755 0.09848144 0.3731544 0.4673483
AC107959.1 0.1281183 2.021419 0.2415887 0.691483 0.4369267
1.926783 0.5740556 0.5450951 0.8010599 1.059420451
0.4508938 0.1258533 0.7518208 0.1921964 0.6057235
1.974259 0.6516904 1.471079127 0.3717505 0.7251301
0.883293131 1.071798 0.2281878 1.724409 1.974345
0.1309149 0.4562989 2.132492 0.755124816 0.23257 1.04996
0.6431208 0.2349722 0.1008781 0.3181238 0.1825546
0.02666275 0.1841093 0.2477606 0.17007399 0.2741741
0.0621178 0.213358948 0.1111901 0.2902427 0.2781055
0.3726917 0.3236175 0.260906836 0.1944238 0.1109235
0.8098765 0.1168303 0.1637446 0.8118486 0.1732467
0.6395798 1.635139 0.1413123 0.390801243 0.661825652
0.471749969 0.6124821 0.7870877 0.176309 0.5512792
0.4020726 0.7898756 0.225264432 0.1834718 0.4869295
0.139287 0.4435877 0.2269804 0.2492408 0.6603621
0.4376771 0.6830922 0.08727093 0.062752046 0.1782132
0.706741 0.3979781 0.9221751 0.1551941 0.1798708
0.5602594 0.3245572 0.48279162 0.3901782 0.172847181
0.4434736 0.4651424 0.3352718 0.4645233 0.4188362
0.4098204 0.3170363 0.1128918 0.3311488 0.689753
0.2854269 0.3247186 0.1152602 0.4230399 0.6861734
0.3234393 0.8612357 0.203931309 0.1537228 0.2793177
0.2520349 0.1573875 0.8333062 0.1134358 0.1169773
0.1003456 0.1421014 0.1892928 0.1005259 0.1325837
0.3514725 0.3896106 0.142715 0.3263558 0.7207615
0.2578044 0.3187027 0.9671839 0.3023799 0.313716
0.1458616 0.2085774 0.1807367 0.4296221 0.5307165
0.1949807 0.1586386 0.2537983 0.184919 0.1789149
0.1146682 0.2541504 0.2495513 0.2720808 0.2175825
0.2078925 0.5312437 0.09032835 0.3128733 0.2012874
0.3504052 0.1451193 0.7958042 1.315451 0.2614874
0.3491592 0.8116062 0.05398756 0.3429178 0.69519395
0.7289639 0.537177589 0.2622396 0.4182586 0.818717
0.3372204 0.1975747 0.3925853 0.4968471 1.401375
0.213865451 0.410078 0.05214883 0.1330437 0.3400347
0.4833547 0.2675135 0.4560324 0.2965068 0.1288562
0.1229251 0.272609 0.4237347 0.2870307 0.2216196
1.33244 0.2080151 0.5652836 0.2492261 0.4559704 0.2227662
0.25971 0.4211116 0.2203054 0.2410616 0.7366453 0.1399963
0.1697133 0.03740502 0.1527783 0.4514223 0.1821238
0.08890013 0.1575652 0.2036373 0.347351 0.3320238

0.1305631 0.222423 0.5899523 0.2995816 0.2378413
0.2331525 0.2459021 0.4932126 0.2193734 0.2689643
0.8388809 0.17931598 0.3706252 0.418705074 0.161374193
0.1560233 0.7541016 0.04153113 0.2579541 0.1342606
0.1094192 0.230465 0.3035424 0.2681711 0.09190805
0.08591976 0.1458547 0.1703703 0.4152369 0.2879249
0.2459333 0.1373552 0.5757334 0.6419075 0.331308
0.4127724 0.5145526 0.4121887 0.7039436 1.057861
0.7093121 0.1415973 0.5931239 0.2168654 0.4771972
0.522596 0.2333776 0.289596 0.371031 0.245686767
0.1503566 0.4824269 0.08216787 0.2153344 0.2290739
0.4499852 0.2873179 0.4385161 0.1497791 0.2926966
0.4680836 0.3046754 0.5139255 0.1828073 0.2771634
0.193205673 0.3121307 0.1982132 0.2979289 0.9280371
0.2815891 0.534674653 0.4936914 0.1173152 0.2278218 0.3778
0.1402179 0.8821839 0.5201815 0.2045948 0.2535335
0.708831 0.3669967 0.5111925 0.6224985 0.2893646
0.4711762 0.9663806 0.595714 0.302521 0.2025166
0.2132058 0.2941073 0.5794198 0.1256407 0.365926
0.167587 0.2999348 0.741829625 0.373100391 0.2924207
0.06888808 0.3745087 0.1692258 0.1034385 0.8656018
0.214397 1.259484 0.3506803 0.2429236 0.2614749
0.6260926 0.2816069 0.229434028 0.1619702 0.5289051
0.2766755 0.4369071 0.183419 0.3027505 0.09596236
0.3509444 0.07795409 0.4790591 0.5505095 0.5269324
0.919988 0.2389758 0.1199119 0.1559147 0.4934223
0.317677 0.1075566 0.3634295 0.3971498 0.2718902
1.524189 0.2432509 0.208570743 0.08393032 0.2463949
0.119502 0.3293902 0.4073723 0.251274 0.3587397
0.2788356 0.2089533 0.3694311 0.3046426 0.5098608
0.133818 0.2443127 0.4687333 0.2180758 0.5512928
0.2154284 0.09837041 0.1890988 0.1835981 0.23354 0.4604692
0.2804876 0.4841756 0.2680404 0.418869921 0.3705242
0.2547238 0.1432079 0.1431686 0.1712063 0.4384431
0.564488604 0.34642 0.3667734 0.2741631 0.2385694 0.2034912
0.9221937 0.1326426 0.145143 0.4600106 0.681843
0.3040996 0.3139022 0.3570093 0.2988916 0.249457583
0.3956722 0.5275609 0.3212364 0.289096381 0.1772269
0.6193469 0.1044607 0.8194834 0.01821559 0.3105918
0.7678168
AC124312.2 0.2721989 2.928196 0.5946502 0.2925235 0.2231467
3.066772 1.156548 1.019457 0.5252852 0.511553243
0.8761872 0.1336933 0.788147 0.09570436 0.6656453
3.680263 0.3393566 1.101589594 0.2520694 0.07267001

0.07951847 3.253044 0.3462897 3.09376 1.631262 0.695351
0.4308659 4.573717 0.197253781 0 0.1923047 3.659915 0
0.3214869 0.05540022 0.5966981 0.4248556 0.5500644
0.2368753 0 0.1923374 0.1199771 0.070828175 0 0.6306615
0.1502187 0.9105898 0.07314409 0.277160049 0.05435142
0.3142227 2.118718 2.044135 0.2046412 1.061443 0 0
2.546205 0.06823424 0.230636799 0.502181525 0.046401636
0.6777466 0.1592608 0.04682304 5.994548 0.696391
0.7221598 0.034185328 0.5229055 0.1162388 0.1233033
1.649273 0.2220844 0.07943018 0.3692102 0.8981838
0.138482 0.4120333 0.033330595 0.1183219 0.7061674
0.2551199 0.69973 0.6809515 0.1317765 1.29535 0.1681832
0.93248576 1.304858 0.065576682 0.2279516 0.7266448
1.551009 0.2741449 0.2433198 0.1978864 0.3224547
0.02855343 0.6207843 2.130003 0.1486312 0 0.530575
1.966095 1.026646 0.098168 0.296491 0 0.04536083
3.296865 1.147437 0.1194228 3.450846 1.084521
0.08104203 0.07614044 0.026956 0.2010848 0 0 0.2196279
0.2194826 0.2526757 0.03270624 2.838228 0 0.1471984
1.418238 0.2204428 0.147026 0.5035812 0.06924087
0.1028548 0.07606423 0.5637775 0.1294544 0 0.5991304
1.339353 0.3476716 0.07165377 0.5823156 0.3582394
0.1605723 1.898624 0 3.98527 0.08995815 0.6314912
0.09433527 0.4171584 0.0308319 0.3719667 2.237824 0
0.4864394 6.083502 0.09558453 1.024537 0.256423998
2.25546 0.521927845 0.1857172 0.2514985 0.1350495 0.1085538
0.2862036 0.7414069 0.0837775 3.511024 0.391703821
2.831555 0 0 0.4043476 0.59246 1.224907 0 0.748072
0.376429 0.3436388 0.3048329 0.2852945 0.5911546
0.2522415 1.378335 0.5524336 0.1964246 0.1765011
0.02917922 0.1075652 0 0.98832 0.6435808 1.220839
0.282582 2.83915 0.06934063 0.3576166 0.1308836 0.7806525
0 0.05555186 0.7725265 0.3693318 0.1639952 0.6662248
0.0577902 0.06385914 0.4787318 0.397805 1.010631
0.5629018 0.4244835 0.08930749 0.1092372 0.5390934
0.570329 0.070550549 0.06350215 0.609790467 0 0 1.424139
0.3308873 0.1053936 0.07923576 0 0.2380213 0.3454838
1.424384 0.04437885 0.02684475 0 0.8096633 1.127266
1.038774 0.1536787 1.493859 0.2548328 0.4432318
0.8121852 0.337297 0.1774697 0.228736 0.1133024
3.098856 0.8427289 0.4609587 0.3670326 0 0.1900966
1.048619 0.07291643 3.076364 0.1126127 0.211615009
0.06388924 0.3942152 2.525074 0 0.3910887 0.4569281
0.1204802 0.5175928 0.02946473 0.0370155 0.5650487

0.2110795 0.1550967 0.1849479 0.04748859 0.031097187
0.307891 0.3396144 0.3229473 2.260373 1.057901
0.081140322 1.456794 0.09586411 0.6635866 0.1254172
0.02482547 0.4619702 0.08047372 0.6291424 0.1496263
0.8305011 0.1098194 2.468351 1.485071 0.1159965
0.05213835 0.9227698 0.4566771 0 0.08274323 0.05807371
0.4644211 0.7506279 0.6093083 0.5861671 0.02871401
0.3698259 0.589190252 0.23591828 0.3285584 0.4802402
0.07956776 0.04280183 0 0.486287 0.3503891 1.332275
0.4470311 0.9428989 0.2430431 0.2036005 0.1813028
0.081242213 0 0.3511583 0.3149047 0.3664139 0.06285325 0
0.2780191 0.06213442 0 0.03714614 0.1344376 0.1049546
1.055038 0.3254652 0.4357798 1.349557 0.3403637
0.0456036 0.9997476 0.04596064 0.5273628 0.07600727
2.494899 0.6957038 0.059882074 0.05244634 0.4061546
0.692435 0 0.663084 0.05802764 0.3243297 0.2278505
0.04825435 0.6329755 0.3922671 1.237995 0.05264971
0.4922161 0.2263332 0.1703389 0 0.04237935 0.7510823
0.07439951 0.2437941 0.07156396 0.5330526 0.2543566
0.4977459 0 0.168546764 0.03123857 0.8033197 0.09508063
0.1689859 0.3520095 0.2739741 0.216369808 0.1082354
0.4870269 0.03386536 0.1293016 0.04912902 0.2332481
0.4025873 0.0670368 0.06264962 0.6176359 0 0.1190917
0.2061137 0.3294926 0.17096617 0.2132061 1.627041
0.06562459 0.148599515 0.171152 0.430787 0.1513201
0.07772618 0.09675167 0.04582502 1.269386
AC005104.1 0.09861053 0.4086077 0.7558813 0.1883975 0.3018033
0.5659082 0 0.3939442 0.1522376 0.041182737 1.241463
0.1937343 0.6598799 0.6163765 0.2143518 0.5113884 0
0.544477146 0 0 0.153639759 0.2946231 0.3345378 0.5898884
0.225129 1.08514 0.2601523 0.4938324 0.254079532 0.2237566
0.2786677 0.1767855 1.364339 0.8282045 0.8295619
0.3602801 0.06840623 0.2952206 0.7882149 0.436343675
1.566114 0.6374804 0.342122521 0.3169672 2.335493
0.8465379 3.862975 0.2826473 1.941217696 0.7350969
0.1517795 2.294915 0.6347457 0.8402097 1.025422
0.4444837 0.7721943 1.111962 0.1318372 0.311933661
0.634412755 0.179307681 0.2291611 0.07692803 0.4523403
0.4009232 0 0.3654368 0.099075578 0.7807016 0.5614704
0.476475 0.9104586 1.501833 1.995099 0.1188935
0.7911405 1.899709 1.144394 0.289795193 0.5715322
0.6822024 1.126683 0.8449794 0 0.5092177 0.9469979
0.9748536 1.01344535 1.668409 0.570161062 1.174483
0.0280794 0.8879246 0 0.5037051 0.3504799 0.5191862

1.020624 1.565928 1.399248 1.579459 0.6058921
0.1182852 0.2713389 1.93402 0.4267643 0.04091844
0.319738455 0.7449645 0.7564329 1.741924 0 1.014615
1.979019 1.017793 0.2942259 2.135378 0.9713032
0.6533727 0.8844112 1.980295 1.605401 0.1220504
0.7899076 1.944841 0.4810369 0.03555074 0.2435747
0.273808 0.9942552 0.4490689 0.9364749 1.126129
0.07348287 0.7960193 0.4064487 1.061752 0.2170492
1.68207 1.141967 1.038331 0.8949719 1.003638 0.8997135
0.6379793 1.066742 0.7868662 0.8980211 0.9953618
0.3341574 0.4650017 0.3872124 0.6860192 1.465274
1.34175 0.7342677 0.8173379 0.5540442 0 0.148633096
1.561558 0.806744579 1.10639 0.2429635 0.5740522 0.1573048
1.136684 2.730688 1.618687 0.7597792 0.71898034
0.5961903 0.2341388 0.4437399 0.2604169 0.794936
0.04733349 1.296488 2.852704 0.3967138 1.128721
0.3681099 1.347439 1.532933 1.827609 0.5121381
0.489212 0.8403594 0.6536264 0.2537008 2.286126
0.133263 0.1005031 0 1.553367 0.8189762 2.35097
0.6698744 0 0.3540371 0.781094 0.4833711 1.153832
0.4353472 2.523071 0.1584298 0.3407375 1.088665
1.202994 1.891985 0.2794944 0.9153123 0.5437988
0.9778782 1.121597 0.21106 1.145756 0.7920251 0.2726252
0.7054916 1.76728866 0.103505846 1.860195 1.9204 0.5061254
1.153925 0.6123741 0.1203116 0.4270382 0.1483375
0.6115757 0.5144734 1.063283 0.9847538 0.4140985
0.5208311 0.8362679 2.22695 0.2684953 1.6002 0.8893178
0.5230816 0.6517006 0.9258138 0.6313528 0.3831005
0.4276701 0.7183495 1.031257 1.743336 1.335342
0.6733663 0.2979507 1.361876 0.2409699 1.223899
0.238505754 1.265282 0.634728 0.3915057 0.4143482
0.8395921 0.543288 0.6207545 0.5500302 0.9962679
0.1787966 1.173627 0.9855946 0.1797999 0.4913464
0.9634168 0.781088175 1.281289 1.542018 1.74713 0.3946379
0.4933792 0.235160074 1.372172 1.041871 0 0.6663853
1.1272 1.657658 0.4923705 0.5525376 0.6071036 0.5883655
1.379202 1.158226 0.7579405 0.7003745 0.1511069
0.2228635 1.165973 1.034868 0.8792872 0.504926
0.9788951 0.7614118 0.5045404 2.324706 0.1109582
0.659585 1.280688993 0.729318411 0.8656589 0.7732375
0.1281125 0.2067464 0.593209 0.04270764 1.734803
0.4107644 0.9716859 0.9348712 0.7379272 1.507964
0.9633249 1.216519431 0.277035 0.4749376 0 0.5899651
0.3946814 0.302065544 0.1790561 1.290554 0.3999997

0.2153132 0.3246881 0.3379759 0.05364389 0.9432599
1.198205 0.5333552 3.189485 0.6608396 0.5518968
1.065621 0.1018932 2.055979 0.934579 1.728242
0.838823218 0.9119967 0.3923711 0.5574467 1.509084
0.7417256 0.2522627 1.174961 0.2751476 1.258653
2.568276 1.136865 0.6727394 0.3560407 0.8645672
0.05466306 1.21773 0.9589171 0.8597638 0.5363079 0.7546837
0.9813358 1.08312 0.8178817 0.9126914 0.480854 0.1875511
0.716438145 0.4526766 0.4492966 0.5205055 0.163251
0.08501588 2.48134 0.447914874 1.019482 0.8625813 0.9487665
0.7744638 0.4746175 0.3154654 0.3889248 0.5180944
0.5447115 1.328959 0.9202374 0.4026754 1.951365
1.157494 1.486477359 1.235824 0.8441283 0 0.526373997
0.4133592 1.097169 1.023294 1.226444 0.6231216
0.1770795 0.6783828
AC133644.1 0.2421436 0 0.3217255 0.1542068 0.2470316
0.05449486 0.4156955 0.3224506 0.3322915 1.011264976
0.3233239 0.1057167 2.326688 0.2522578 0.4386274 0
0.05366862 0.162059864 0.3322026 0.05746314 0.094317741
0.02679494 0.06845634 0.1609448 0.01842722 0.06344335
0.1277637 0 0.0519922 0.3052481 0.1520631 0.2894044
0.4415004 0.8473759 0.2847469 0 0 0.02416435 0.4370496
0.16667251 0.3259051 0.237177 0.224026896 0.1210737
0.1939349 0.2375681 0.5635099 0.6362181 0.136976089
0.5372236 0.09317576 2.995334 0.3175034 0.2022727
1.416364 0 0.9085796 1.158388 0.0269778 0.364747826
0.183274796 1.100749928 0.5359218 0.2833516 0 0.2370067
0.1101329 1.196467 0.135158659 0.6578134 0.2068083
0.1755016 0.825073 0.1254366 0.3349796 0.1459748
0.4595609 0.02190066 0.8348919 0.105423437 0.09356194
0.3232815 0.1152764 0.08299576 0.141699 0.3126031
0.2491506 0.6316991 0.55301586 0.3945135 0.155562463
1.171629 3.217691 0.5223751 1.20776 0.5222364 0.6780664
0.283309 0.1354702 0.4908794 1.953765 0.09402299
0.3099587 0.5809116 0.222096 0.6494486 0.6986289
0.200955 0.095167944 0.6815045 0.1629354 1.036943
0.1416487 2.016884 0.6670027 0.08544432 0.4816588
0.3623585 0.09937871 0.1970307 0.6682218 1.186737
0.495868 0.8491542 0.155173 3.053295 0.3199118
0.465583 0.9220882 0.199215 0.135636 0.0918928
0.7117705 0.08133151 0.3007354 0.308632 0.1791385
0.05793758 0.7698548 0.03530271 0.3482296 4.929382
0.5441808 0.1416372 0.6602495 0.4786814 0.2444816
0.6900691 0.09484477 0.6833153 0.1740544 0.152245

0.3413205 0.7219535 0.2949232 0.08448054 0.1442428
1.051295 0.3779129 0.9001593 3.203685455 1.248439
0.247625767 0.3916111 0.3977403 0.1067892 0.8583792
0.1005835 1.648858 0.06624628 0.3553676 0.371683404
0.4592873 0.1368907 0.1676351 0.3197341 0.5725894
0.6586368 0.3882438 0.03113321 0.0270598 0.3260751
0.4820876 0.852244 1.648376 0.6648588 0.1676778
1.092079 0.3550192 0.3721776 0.5076104 0.9639702
0.2726956 0.4524502 2.637056 0.1648188 0.7219124
0.4008985 0.1919066 0.3142022 0.2483879 0.1543234
0.1055062 0.08785424 0 0.2294645 0.4862914 0.2169222
0.2284853 0.1262401 0.2408972 0.3145606 0.5494134
0.08902187 0.1032789 0.1412381 0.08637827 0.1918274
0.5919144 0.251042372 0.4017099 0.255275029 0.084721452
0.3469224 1.73611 0.04360768 0.6111529 0.1879648 0.03282575
0.457089 0.03035424 0.1877198 0.07018433 0.1273634
0.06448314 0.2636257 0.5813317 0.8214009 0.6075999 0
0.2015067 1.455846 0.3924726 0.7334648 0.1964656
1.240257 0.2687785 0.2962012 0.4703859 1.553916
1.426953 0.182167 0.35074 1.414493 0.3843866 0.558842
0.2894042 0.250998913 0.6062379 1.428726 0.3697554
0.07536704 0.4466941 0.2501388 0.8574171 1.80084 0.2329896
0.2048876 0.5585088 0.3894546 0.466037 0.5118605
0.3004093 0.319667568 0.2247341 0.2685469 0.9193233
0.4737604 1.096137 0.224563354 0.7487653 0.1137055
0.09259852 1.016518 0.1963051 1.51338 0.2121128 0.2713574
0.8045462 0.08756147 0.5644512 0.250949 0.3766643
0.4815464 0.2473675 0.8664851 2.501999 1.270588
0.6979034 0.2525669 0.1335406 1.127749 0.3441464
0.9758025 0.3178748 0.1799609 0.087355638 0.41041043
0.4487533 0.05424936 0.5452847 0.7107482 0.6644404
0.4893979 0.3809671 0.4482911 0.2651143 0.1177245
0.3843681 0.3756555 1.003545 0.481811459 0.277149
0.9996305 0.3873457 0.2897384 0.1739522 0 0.4030419
0.4176239 0.4911107 0.1909243 0.4252211 0.7469267
0.1317256 0.2573586 0.0795205 0.2182806 0.511365
0.1442425 0.07528963 0.5814872 0.2780048 0.2704592
0.5234007 0.3143551 0.071026794 0.08294291 0.5352716
1.186329 0.4940853 0.9382703 0.02294241 1.154073
0.8107681 0 0.5255455 0.2326362 0.3059165 0.624484
1.946077 0.3579418 0.3502041 0.9418697 0.2680886
0.2582223 0.05883072 0.6104636 0.9997309 0.07438348
0.1723973 0.09839698 0.1790997 0.346519662 0.321121
0.06686501 0.3759206 1.135804 0.02319569 0.2166424

0.268859521 0.4921202 0.3530194 0 0.4703225 0.116545
0.1106632 1.034612 0 0.07430941 0.8879816 0.3602411
0.6591946 0.6519301 2.013289 0.135189916 0.2408439
0.4526813 0.05189204 0.039167897 0.4060106 4.583167
0.1994249 0.1434096 0.306022 0.3261214 0.1067825
AP4B1-AS1 0.1173014 0.1281907 0.4161794 0.216103 0.2747509
0.2800168 0.2848017 0.1305423 0.1681579 0.220448766
0.4315247 0.576138 0.4657976 0.2278176 0.3005129
0.1862199 0.2005609 0.119862616 0.06207246 0.1610559
0.146861535 0.1502 0.1065929 0.3308001 0.137726 0.4807645
0.1061013 0.1391293 0.080956713 0.07604798 0.1657435
0.1652309 0.792598 0.2111109 0.1159603 0.05510166
0.08718441 0.1806055 0.3110981 0.519049339 1.197617
0.4653266 0.174415403 0.377046 0.5694381 0.3884115
0.9749306 0.2611716 0.460694666 0.5019044 0.1257389
1.509876 0.197753 0.5165303 0.4737548 0.1235997
0.3321571 0.4724022 0.2184361 0.147666075 0.380501306
0.411352914 0.1919305 0.09804552 0.334377 0.3576864
0.2515147 0.3471976 0.159945541 0.7960094 0.3864237
0.4311632 0.5636173 0.3046936 0.3977159 0.2121433
0.5204213 0.8116129 0.6721976 0.213400382 0.3642117
0.7504931 0.8615815 0.4910822 0.1588597 0.1947009
0.5258889 0.5901687 0.86109736 0.6520971 0.411782989
1.029112 0.2791423 0.9619183 0.1639494 0.5906189
0.4629333 0.1852782 0.5273492 0.4840852 1.384723
0.3952869 0.3861077 0.3015112 0.414989 0.7015562
0.3384362 0.1355925 0.177822456 1.083507 0.1674457
0.6122093 0.1323362 1.0068 1.038585 0.4390478 0.1312478
0.6637953 0.2723458 0.2892641 0.6416317 0.3785859
0.4941522 0.1244435 0.3543742 1.227169 0.4061696
0.07249563 0.4035699 0.1861178 0.5672175 0.6963503
0.3580639 0.3545941 0.2435021 0.8863826 0.9563499
0.5322639 0.5255995 0.5496962 0.9531762 0.9439992
0.6126923 0.3352245 0.3558704 1.036716 0.2719147
0.5659044 0.3987418 0.3846718 0.2942492 0.2291577
0.2961036 1.174111 0.6429121 1.262823 0.5839588
0.9623372 0.6119835 0.392457 0.113660603 0.9627113
0.634059056 0.4954423 0.06193188 0.4855397 0.227218
0.3288976 1.255186 0.4538673 0.4150055 0.318310182
0.8403051 0.2046255 0.1914172 0.3850086 0.3728406
0.2171772 1.604025 0.3296458 0.3960655 0.43157 0.1782807
0.772796 0.383086 0.8592552 0.5548163 0.4081127
0.3869587 1.216982 0.1437085 0.6710316 0.169845
0.1793291 0.4898556 0.5352742 0.2569337 0.7490837

0.5207945 0.2739756 0.09024474 1.002378 0.3039235
2.47603 0.3170596 0.889996 0.2423044 0.2219619 0.8253934
1.100778 0.7394817 0.2048256 0.6143959 0.3511589
0.5950151 0.2858973 0.08069942 0.159303 0.4037775
0.121612222 0.3674812 1.40445431 0.096740758 0.6002118
1.205519 0.5771605 0.1989755 0.3121907 0.1328932
0.226079 0.1985104 0.5163917 0.4480626 0.8659831
1.044225 1.1611 0.2413834 0.2913273 1.163691 0.2053199
0.382793 0.9403389 0.2222242 0.2284147 0.3758387
0.3218661 0.6138194 0.4108988 0.3906319 0.926017
1.069524 0.9076832 0.9206274 0.5012582 1.143184
0.1228474 0.4021 0.156331503 0.346122 0.1941521 0.3300931
0.1290889 0.6527417 0.3894895 0.7515998 0.3313907
0.515157 0.1002663 0.445261 1.152193 0.5117835
0.2504903 0.5730126 0.735141812 0.8748299 0.8864836
0.6521152 0.3353132 0.1347464 0.199809213 0.6995385
0.4485268 0.07689843 0.6099623 0.4707247 0.861332
0.4293634 0.1971801 0.5232088 0.2045121 0.5138204
0.3994344 0.2415006 0.6069913 0.1155523 0.1988292
0.6426137 0.8667384 1.15462 0.214511 0.436664 0.5914982
0.385825 0.2734948 0.4101103 0.3782914 0.389926551
0.824952075 0.8311457 0.5011965 0.09143715 0.04216005
0.4536304 0.1088626 0.8089109 0.1186653 0.5779308
0.2444108 0.6412469 0.4261638 0.5469144 0.650195395
0.1961577 0.4669554 0.06892954 0.4812264 0.3250318
0.087996685 0.3879548 0.5049228 0.246405 0.2378296
0.36416 0.4565988 0.1640872 0.8735943 0.627359 0.377648
1.743353 0.3425136 0.1992961 0.362172 0.2857005
0.4024133 0.3761463 0.7097463 0.486619554 0.445567
0.1444678 0.1847225 0.5077624 0.4984501 0.1071704 0.4193
0.8837092 1.330863 1.356079 0.4950563 0.4763405
0.388952 0.8705005 0.05573488 0.3104018 0.3299803
0.407003 0.4262009 0.5404691 0.7504332 0.7871486
0.5482243 0.2773866 0.5004968 0.2629393 0.356941999
0.3000092 0.1145266 0.8116764 0.5201626 0.5128736
1.416797 0.388192891 0.5197358 0.8195291 1.617842
0.4585061 0.4355313 0.0689252 0.4213352 0.3136503
0.2468409 0.5876349 0.7547045 1.099744 0.7308835
0.9736564 1.031466316 0.750033 0.3709827 0.01616015
0.195161838 0.1580491 1.581585 0.5216791 0.6954267
0.4447378 0.1579827 0.2992865
ATP2C2-AS1 0.1259987 0.009267954 0.7667127 0.06481007 0.2987545
0.04417028 0.02995004 0.0348479 0.08977856 0.054644739
0.445098 0.3522716 0.2095416 0.2135525 0.04740336

0.02872184	0.2996707	0.102165825	0.1196727	0.09315239	
0.124582473	0.009652615	0.4130671	0.06377668	0.01991468	
0.3885323	0.06903842	0.04981499	0.074918714	0	0.1095585
0.03475169	0.5426282	0.2136812	0.2327715	0.1168567	
0.01008524	0.3612562	0.4798258	0.403140027	0.3796066	
0.4741959	0.247154422	0.4673099	0.8932502	0.6382959	
1.353326	0.1093868	0.16777058	0.406412	0.05034848	
0.4572202	0.2599488	0.2186003	0.05669234	0.4825461	
0.2988454	0.08445311	0.32071	0.105117452	0.165057355	
0.099133583	0.07239771	0.1247579	0.1600542	0.1477719	
0.04628669	0.2399973	0.141199773	0.3791533	0.05794494	
0.03161137	0.3835154	0.08133711	0.2224911	0.131465	
0.4778442	0.1656795	0.2567477	0.132922145	0.01685238	
0.2593859	0.1349634	0.4335269	0.1939735	0.07038262	
0.4487703	0.1616898	0.1162108	0.5466139	0.172789526	
0.2218561	0.07451624	0.5768141	0.09482596	0.01980321	
0.2865437	0.6889001	0.1423386	0.2357793	0.7584318	
0.8171344	0.1004936	0.1511378	0.1000099	0.8371291	
0.0978733	0.07239206	0.167131163	0.5620779	0.311088	
0.5797787	0.03401838	0.2030101	0.3089329	0.4386218	
0.09217869	0.8868775	0.2362813	0.6388054	0.1153452	
0.2763175	0.4644421	0.05398226	0.1024825	0.1786194	
0.3235727	0.04717173	0.08079889	0.06279459	0.4851254	
0.2924141	0.138066	0.3222876	0.08666959	0.4447266	
0.3042265	0.08348578	0.08533311	0.7185362	0.3697364	
0.1428773	0.1093276	0.2194007	0.2195524	0.1685212	
0.6542497	0.7333423	0.3630232	0.4781138	0.1254028	
0.1919567	0.105392	0.09632469	0.347061	0.2282496	
0.2511499	0.2410029	0.9393619	0.2269915	0.021913218	
0.1766827	0.148674576	0.9169813	0.07164101	0.7617011	
0.01546113	0.4665155	1.623559	0.01193229	0.09201272	
0.072526615	0.5067021	0.2613616	0.08555092	0.103663	
0.2156456	0.06280608	0.7039671	0.1682315	0.1754645	
0.8956732	0.1085421	0.2076849	0.2215714	0.2095702	
0.08305589	0.1442506	0.6794272	0.09636519	0.1038987	
0.1736304	0.1522656	0.2074427	0.166662	0.7294565	
0.02786377	0.6065629	0.08394657	0.2263765	0.04846795	
0.7544832	0.7268947	0.5696753	0.1925518	0.2028984	
0.1226272	0.240014	0.2921991	0.7367232	0.6570549	
0.06696027	0.3868451	0.4222452	0.306943	0.08055945	
0.005186153	0.09981678	0.05076937	0.336621192	0.162801	
0.122613756	0.05595346	0.2221785	0.2915793	0.7029884	
0.155114	0.3949895	0.1773773	0.2760507	0.1312178	
0.3493917	0.353965	0.711162	0.5400839	0.08818509	

0.2373416	0.06164617	1.740111	0.3711065	0.31577 0.2088105
0.1735172	0.04804064	0.3892613	0.2559738	0.0726186
0.1115537	0.4024482	0.3800996	0.1873217	0.3609312
0.3790519	0.1405675	0.2146308	0.0118422	0.3608827
0.10548987	0.5186792	0.04678949	0.1243207	0.05430047
0.1237824	0.2452996	0.869429	0.2137876	0.2937628
0.4692128	0.1730297	0.2304883	0.1237049	0.1251236
0.1420381	0.589072836 0.6679055	0.6917009	0.8785395
0.1047278	0.01558708	0.005778338 0.09855714 0.2423542	0
0.1741638	0.3217622	0.3383878	0.3476726	0.3665768
0.5626109	0.2760026	0.2111587	0.1155136	0.07582655
0.6443259	0.05198183	0.06571426	0.6318535	0.05449011
0.2278429	0.1199344	0.3187069	0.1443294	0.3512635
0.5712253	0.1717669	0.3403529	0.136365706 0.658588148
0.1148629	0.5699983	0.13977 0.08534669	0.08745777	0.05037165
0.4117189	0.5732982	0.2493737	0.7068183	0.1335197
0.1788239	0.419618	0.624844214 0.2269096	0.05001487
0.1046531	0.1217713	0.5505529	0.095430028 0.2573856
0.2389419	0.1032021	0.1322665	0.1148863	0.179382
0.02372642	0.6118919	0.4153739	0.7994398	0.2908645
0.2500669	0	0.1963829	0.2053044	0.2219245	0.09427497
1.038064	0.14925577	0.05975873	0.3085221	0.1725891
0.4894707	0.173972	0.06611822	0.1559038	0.1135833
0.8522253	0.5995215	0.2025285	0.1432644	0.4011862
0.2931684	0.120886	5.099678	0.3110244	0.7665743
0.7441764	0.7152701	0.2719983	0.6455397	0.8440714
0.155261	0.3544655	0.1566888	0.292871085 0	0.06021865
0.1444498	0.2527178	0.4052669	1.175527	0.030817181
0.2505064	0.2774656	0.08199755	0.2578268	0.09796307
0.04650957	0.1290146	0.6731295	0.2989231	0.07997166
0.1592672	0.636076	0.3287919	0.2090477	0.152190116
1.648471	0.5063876	0.009346798 0.366856084 0.2254862
1.377727	0.2586271	0.435436	0.4042185	0.1631695
0.08847487
PPP1R14B-AS1	2.238916	0	2.037056	0.9298904	1.500278
0.295751	0.7520119	0.379163	0.7138423	1.250106453
5.055272	15.77783	1.540825	6.525744	0.8728464
0.2884692	2.427224	0.219880218 3.125042	2.910696
0.483437668 0.1696564	9.535728	0.02911565	0.6000422
7.36453 0.8089561	0.05772916	1.034618662 2.098389	0.4126336
0.3926592	1.62088 9.427589	7.092805	3.627664	0.1266145
2.57915 3.313169	3.661291317 6.563958	7.058103
6.839012012 4.521358	3.383071	3.670964	1.302586
3.322053	9.143670184 11.50684	1.292287	0.5740143

3.785674 4.574006 0.6168418 5.50464 0.786099 0.149684
4.733993 3.728126025 1.934055635 2.953771164 2.520718
1.139102 1.908921 0.8575116 3.287385 0.9346547
2.469532085 5.355064 0.8937442 4.039184 1.564817
3.925723 2.689095 3.300937 5.819566 4.437361
4.217949 3.075291705 2.962011 0.478498 1.094836
0.97593 4.306509 7.021789 1.953141 8.901594 3.16803054
6.066389 2.087194849 0.9510709 1.683917 1.910544
2.801145 0.5469601 3.231758 3.28012 0.9598639 1.776046
1.310184 7.654132 6.953043 1.021704 1.757795
1.101452 7.548001 0.6361892 4.045833207 2.335969
2.903361 1.934499 23.74574 0.8853302 9.30835 3.613135
3.267534 1.677367 0.5573198 5.67754 3.425057 1.204336
1.547407 8.40369 2.152148 1.15664 6.878042 5.527336
0.1803351 0.9234967 1.962974 1.91174 3.862875 2.550295
3.264264 1.395823 2.800888 2.358263 4.579847
2.42684 1.425706 1.383746 1.79851 1.255518 0.8958159
5.766482 9.098294 7.927126 2.080389 1.71159 1.551871
3.282066 2.337543 0.3627907 0.3556862 4.202801
5.414541 0.7780259 13.26303 6.432294 0.440173054
1.478769 0.87104541 1.638273 2.248532 0.7341085
0.7376023 0.9325454 0.6628575 4.404213 0.02008988
1.456849523 0.6750832 11.44103 17.5385 4.646588 0.3766692
4.135219 4.471632 1.070106 1.199334 5.33354 5.5325
1.541748 2.470154 1.20276 0.625632 20.74402 0.4616142
4.3974 2.984443 1.846446 3.206571 4.018105 6.360738
0.7667093 2.487566 1.66806 3.76925 4.092523 3.819432
0.9970645 1.908655 4.112373 5.939848 7.056898
5.512932 0.3083316 3.9474 2.329415 0.8093315 1.215707
0.9261468 1.288357 2.405516 3.385457 1.093835
1.677285 0.8158475 0.782142063 2.043876 2.847791778
1.890267562 3.103125 1.145931 2.879422 1.733787
18.81537 3.177005 5.107291 6.067797 2.433754
2.031466 3.148884 1.837283 1.124147 1.226931
3.322752 2.885336 0.6460528 2.041394 0.9510553
1.839547 5.307484 1.472434 2.687744 0.9319455
0.803762 1.87918 1.127912 0.5031585 2.032214 6.979549
1.455916 1.512436 0.1784065 2.416362 1.463109241
1.530827 1.550778 2.363462 6.169493 1.522939
3.796077 1.43621 1.406777 6.533077 3.547659 2.465297
1.15746 1.419928 4.067693 5.536438 2.869223397 6.538746
1.530313 3.349294 0.5648764 2.556977 2.52452414
0.6512261 1.491313 8.989967 2.422002 5.735265
0.09440598 5.352918 1.636326 7.726782 1.386023

2.336789 0.3783151 0.4409077 1.451899 0.7458317
1.031257 3.522993 1.422913 8.107867 5.379017
3.231146 3.194443 4.025994 1.456349 5.360286
12.67639 1.764671684 2.801065245 0.8759057 4.203644
1.650398 4.714511 1.386926 1.169918 7.42442 0.4865873
3.357226 7.027976 1.713515 1.626133 2.236903
0.697294976 3.09941 1.85861 0.5255444 2.795466 1.011495
3.482391432 2.916484 4.4219 1.85092 0.8900452 1.394255
0.8507708 0.1985812 2.723604 2.157843 7.305269
1.752788 2.022295 13.89264 2.695588 16.11869
3.289001 0.2185051 5.876386 1.327737045 0.2438271
5.293541 2.228666 1.653572 1.248069 1.411131
4.726481 1.91488 1.380543 2.920116 5.962033 1.106834
3.219702 1.600246 2.306836 5.774916 2.094359
0.9699689 13.19659 2.155156 4.475549 4.913819
0.6728154 1.715309 6.942179 3.425138 1.494843227
1.094815 0.8467328 7.525969 4.592905 2.937344
1.371706 1.72443475 4.93518 2.78674 5.595273 1.960619
3.795032 0.9342425 23.75568 2.037773 1.702768
1.325279 4.917315 1.320282 4.098314 1.478276
0.611411681 0.8496102 1.012876 0.1408126 3.082257603
3.794873 0.42016 5.3033 2.909372 3.044842 3.408706
0.8692845
TSPOAP1-AS1 0.1694535 1.219128 0.3117396 0.269787 0.412781
0.7436483 0.4612938 0.5125541 0.5792724 2.234274965
0.405197 0.2060904 1.563848 0.2383174 1.223431
0.5709054 0.5472679 1.151318101 0.1345047 0.2843635
0.287589224 0.5705738 0.1334528 0.5985486 1.331918
0.1807633 0.4278895 0.5646748 0.389833216 0.1922529
0.3876533 0.7124603 0.2531432 0.3430919 0.114962
0.07959979 0.06297325 0.4058484 0.2122223 0.203522032
0.2117795 0.1564934 0.20996689 0.08559259 0.2201935
0.08015697 0.6854133 0.3902982 0.402597869 0.2126815
0.1443823 1.74341 0.1255233 0.1516624 0.6765191 0.2430292
1.871944 1.844633 0.2912793 0.393818259 0.229029875
0.434675478 0.7112386 0.1416364 0.06662629 0.4428971
0.2477303 0.8481685 0.145930816 0.1493761 0.1791841
0.1345139 0.4310475 0.142958 0.1758155 0.7807482
0.3727675 0.1215149 2.641394 0.482178284 0.1543343
0.4627518 0.5013131 0.4148635 0.2167392 0.1523517
0.510617 0.5484285 0.24878803 0.4869372 0.291598618
1.112738 0.3170843 1.392992 0.6130004 0.7089452
0.6570203 0.3441248 1.293381 0.6216079 2.192324
0.09869672 0.2231083 0.1839043 0.2955831 0.9799893

0.4307015 0.482158 0.242610866 0.1990158 0.2027982
0.1749356 0.3327821 2.272812 0.4286684 0.1089112
0.2979436 0.04794589 0.1520072 0.2490339 0.2254622
0.3472411 0.6134662 0.2546755 1.904219 2.942872
0.4465221 0.1701815 0.7100599 0.351019 0.2237373
2.664151 0.09852547 0.2479922 0.2705867 0.6993717
0.5334287 0.07819402 0.2042509 0.328224 0.5689224
0.3908424 0.5147358 0.475769 0.4493527 0.17293 0.1152236
0.4553192 0.9067012 0.2364665 0.3318544 0.2435244
0.05118386 1.122727 0.9611825 0.05067425 0.421793
1.060558 0.1020081 0.3914606 0.936513705 1.19906
0.552877302 0.2972971 0.1789334 0.5028374 0.7144023 0.181
0.9121148 0.27319 0.659468 0.185790094 0.6930443 0.1847511
0.03351771 0.1022865 0.2497878 0.3544031 0.1824239
0.1820787 0.129851 0.1874411 0.3659836 0.5563106
0.1401961 0.8424745 0.7794853 0.1310131 0.4358869
0.3313786 0.2249012 0.09778746 0.05316087 0.4441005
0.4509501 0.3389617 0.3995191 0.7294327 0.3576694
0.09894621 0.3942063 0.1818307 0.2215015 0.7048345
0.1679421 0.3284602 0.1020928 0.3438811 0.1062162
0.1476599 0.6347598 0.6260877 0.280872 0.4182866
0.3252382 0.2223883 0.08635428 0.1853816 0.5959768
0.267704168 0.1694243 0.242443932 0.139751575 0.1300598
3.39151 0.1438654 0.3207661 0.1315388 0.01476749 0.217729
0.6509176 0.2017427 0.6788448 0.07958003 0.4448101
0.2484923 1.150717 0.1744992 0.3143456 0.152422
0.3051979 1.018809 0.4601355 0.3499656 0.2020227
0.5540861 0.69863 1.877663 0.5349154 0.6415316 0.6854724
0.1138227 0.1878442 0.5266305 1.193189 1.252116
0.360542 0.221654136 0.4583401 0.237622 0.2994187
0.135623 0.5771059 0.2042233 0.3523958 0.2455008
0.02795099 0.3204138 0.1507555 0.358755 0.4303515
0.3673407 0.07883555 0.608428862 0.29769 0.1530292 0.2948677
0.7685704 4.939899 0.091403976 1.774945 0.1847201
0.1851456 0.5576904 0.1089191 0.5164932 0.1176899
0.6985512 0.3300089 0.3971998 0.4101987 1.116413
0.4684846 1.004089 0.4822327 0.4855528 0.4757635
0.7802874 0.1439026 0.09296471 0.4205361 0.3827347
0.1823469 0.2085199 0.09533595 0.1113199 0.139730207
0.469975943 0.4356421 0.04067572 0.38998 0.07105509 1.069196
1.45206 0.2752588 1.287358 0.4549865 0.2618643 0.4569966
0.4627328 0.2794812 0.264922713 0.147352 0.3081338
0.2779817 0.2751751 0.1490605 0.429029659 0.2472525
0.1878785 0.09819487 0.2576762 0.6934493 0.5019613

0.7967183 0.3550562 0.453141 0.141843 0.7506906
0.1757467 0.5419348 0.8883384 0.1542498 0.2027881
0.7546952 0.1885607 0.106510604 0.03109495 0.2033467
0.3010615 0.3556426 0.4593489 0.08256974 0.04807303
0.5808891 0.0457753 0.9419663 0.220943 0.7890453
0.1997794 0.9709995 0.7783073 0.06059542 0.235402
1.718641 0.2052297 0.09263266 0.7323528 0.09334517
0.2305248 0.275759 0.2262502 0.3184532 0.135904578
0.225957 0.3910517 0.2705877 0.1001902 0.1460923
0.296447 0.425168775 0.3689877 0.1732523 0.8513269
0.3833086 0.2446764 0.5476309 0.1074106 0.2503967
0.6240257 0.4261128 0.1129533 2.27359 0.7038889 0.3196688
0.613253896 0.2455926 0.5073387 0.1789778 0.093976847
0.3348657 3.236737 0.3169976 0.0983107 0.2829917
0.3695016 0.5732626
AC016876.2 0.6146491 0.3140399 0.9007246 0.3223569 0.3517313
0.2654675 0.3631085 0.2166611 0.316304 0.486967498
0.3310444 0.3847633 0.5443804 0.2824952 0.2848986
0.2924139 0.4808139 0.412879601 0.6584804 0.5984651
0.292224606 0.3420773 0.5021362 0.4974538 0.2022332
0.5257562 0.3195417 0.2072719 0.67540262 0.5059194
0.2298922 0.4375275 0.3606371 0.6832429 1.040035
3.104296 0.3417332 0.5736903 0.5034215 0.706607696
0.4014675 0.3771617 0.508032788 0.5132915 0.5615689
0.4988339 0.3821396 0.7383882 0.555290043 0.409101
0.5738947 0.7675269 0.6432435 0.9604388 0.6990762
0.8259677 0.5751022 0.3088667 0.5347444 0.494247677
0.277078652 0.883430573 0.543146 0.6769411 0.924624
0.4777488 0.3576694 0.5207279 0.329964276 0.5745977
0.4554757 0.9586714 0.3844917 0.4101791 0.5838003
0.5013153 0.5450582 0.6205037 0.5518593 0.330568719
0.4243467 0.5134277 0.9262514 0.6506096 0.6632993
0.5630357 0.4340243 0.5771051 1.74437395 0.5267648
0.493593048 0.7468699 0.8931252 0.4959704 0.7560304
0.8372382 0.8849283 0.5520471 0.7838277 0.5466658
0.320649 0.4632904 2.686652 1.380599 0.6435578
0.3795577 0.4303044 0.4200801 0.636788412 0.8997663
0.3667566 0.6725509 0.7032499 0.7008433 1.195127
0.6650197 0.6337878 0.3126996 0.1958721 0.750204
0.6017738 0.7520124 0.4886696 1.605411 0.3909895
0.4529822 0.67518 0.7951216 0.404674 0.2844453 0.512101
0.4665151 0.858403 0.6405995 0.3098411 0.2803418
0.6333587 0.6001622 0.5438083 0.8381282 0.6875803
0.6376844 0.6117542 1.094443 0.5801382 0.9113014

1.752236	0.7392985	0.5045144	1.262617	0.5680462	
0.4603343	0.5023643	0.8174754	0.2862382	2.790906	
0.7295898	0.6849812	1.582814	1.54233	0.35877025	0.6124949
0.449855188	0.6605697	0.7126661	0.3587691	0.3028003	
0.4449286	0.5006073	0.8791165	0.2362915	0.263616184	
0.5400579	0.5610757	0.5162549	1.255596	0.5275526	
0.9718777	0.5768102	0.3416776	0.9333441	0.6816335	
0.4858867	0.8280825	1.55667	0.3611085	0.467095	1.259673
0.3081202	0.9013073	0.5787919	0.6223108	1.010817	
0.419166	0.4196585	0.3085043	0.8045963	0.6405064	
0.9824454	0.918367	1.379217	0.5727807	0.4607964	
0.3172919	1.214262	0.5653335	0.7333703	0.5136109	
0.4145147	0.5145945	0.416221	0.4451363	0.5509464	
0.2492317	0.346976	0.5087737	0.3643579	0.7136838	
0.3693108	0.665350791	0.5623272	0.603512096	0.262492428	
0.7511137	0.3704513	1.694568	0.9550632	0.5402722	
0.2720276	0.7858848	0.5914733	0.5500789	0.4990918	
0.3137863	0.4585497	0.6494967	0.5511921	0.8559808	
0.8471369	0.4214658	0.6047708	0.5253364	0.4754777	
0.5450998	0.8140568	0.842035	0.4063449	0.4191558	
0.4697031	0.9646182	0.5254432	0.3128043	0.7799532	
0.5680712	0.6693684	0.4012667	0.4736762	0.312317187	
0.7354815	0.6545386	0.623876	0.9832726	0.8080769	
0.9026163	0.5014343	0.4950085	0.7957983	0.6227846	
0.565412	0.8099696	1.002597	0.7042366	1.732556	
0.952791507	0.6438618	0.4090022	0.5662361	0.3569146	
0.4683989	0.323332909	0.3741079	0.6706312	0.4389884	
0.8273972	0.8199869	0.5435	0.4631995	0.6280274	0.7128381
0.4584175	0.9238538	0.215456	0.2605322	0.7498394	
0.6556111	0.531138	0.8810118	0.7724261	1.038008	
0.4165482	0.7813871	0.5517006	0.6937168	0.4726227	
0.6483877	1.080717	0.665221699	0.793863544	0.4417098	
0.4475403	0.6129952	0.3486991	0.8700071	0.7594556	
0.7718171	0.2585436	0.4420404	0.3735349	0.4888583	
0.5348701	0.4736169	0.507190993	0.4373397	1.038604	
0.5979909	1.178902	0.3784751	0.288749388	0.4677663	
0.4759365	0.7699704	0.3799237	0.580358	0.6134031	
0.2999474	0.4870706	0.9855133	0.5703723	0.8348793	
0.3674864	0.3962777	1.102951	1.207955	0.4408563	
0.2028944	0.4195095	0.453381316	0.6641281	0.939112	
1.757749	0.8410311	0.6953415	0.2466476	1.116498	
0.5851251	0.405939	0.6193695	0.5774897	0.5001754	
0.7436307	0.3486956	0.7215252	0.3077121	1.103113	
0.4841107	1.596245	0.5501197	0.6476572	0.66329	0.5969807

1.20342 0.9072439 0.4383842 0.680590575 0.7358232 0.3444477
0.3704635 1.301878 0.5506932 0.6884081 0.459841931
0.3330617 0.765512 0.4618247 0.7808882 0.8265942
0.1817613 1.755767 0.4956789 0.4327267 0.5643342
0.8948637 1.323506 0.6205639 0.5304979 0.446614551
0.7282257 0.3815413 0.2498824 0.807076363 0.458467
0.7317336 0.7369872 0.4863877 0.8053544 0.3246335
0.2798223
LINC02716 0.559654 0.8776567 0.2470998 0.2352308 0.411085
0.5894513 0.6917582 0.1609769 0.2419226 0.617043036
0.03843163 0.7476731 0.4148398 0.3778037 0.1459838
0.1658476 0.8484439 0.303394045 0.5804588 0.4781216
0.392385055 0.4458943 0.3702338 0.6963531 1.073265
0.193556 0.6732694 0.4956346 0.30282052 0.6095556
0.189786 0.4013312 0.2160883 0.1410117 0.1093493
0.09814718 0.3261154 0.06031779 0.1731652 0.079245569
0.05423382 0.0592029 0.093200712 0.04317396 0.09220766
0.03294473 0.1562893 0.04812408 0.045588344 0.03575972
0.1292114 0.1689672 0.1681136 0.03366017 0.4364757
0.02751951 0.09860648 0.1835877 0.02244686 0.030348818
0.177909278 0.183175643 0.4459134 0.7858749 0 0.652281
0.4276348 0.1810036 0.022491734 0.06255236 0.03823882
0.08112557 0.2214521 0.521847 0.1741997 0.1619443
0.08690395 0.01822243 0 0 0.07784814 0.2445329 0.1438735
0.2301885 0.212221 0.216751 0.06910187 0.05532682
0.07668941 0.2020029 0.086290426 0.1749736 0.4780853
0.1511798 0.6699442 0.1829585 0.4122888 0.07071812 0
0.2495997 0 0.1760215 0 0.05370523 0.3233909 0.1182067
0.1614705 0.6688178 0.079184422 0 0.1084562 0.0359495
0.5107212 0.3208221 0.3171309 0.05332041 0.100191
0.3369707 0.2976767 0 0 0.2167515 0.1650346 0.2493664
0.02151859 0.08685442 0.3071335 0.193694 0.4976581
0.3107938 0.2257118 0 0.136668 0.06767183 0.2502267
0.1997311 0.06387946 0.07231039 0.04927362 0.0293736
0.03049939 0.2828613 0.1044889 0.04713967 0.1056462
0.4707023 0.0290601 0.4784763 0 0.02186739 0.04137767
0.1477879 0 0.08899273 0.6870937 0 0.3600514 0.4506183
0.06288843 0.2995908 0.202452476 0.07419735 0.137357898
0.06109495 0.3033612 0.1421662 0.1785535 0.209226
0.06097472 0.330721 0.4250449 0.025771577 0.07165307
0.1822397 0 0.05320691 0.04331115 0.09670911 0.02153587
0.0777131 0.04503017 0.04521842 0.4512607 0.06256844
0.0204706 0.05531953 0.36623 0 0.1292346 0 0.1343863 0
0.06806884 0.1026711 0.1924702 0.1567285 0.1430159

0.08895129 0.04562164 0.1830022 0 0.1100612 0.04389317
0.1096486 0.04235603 0 0.2697456 0.2062743 0.07604443
0.06302278 0.2863405 0.2379363 0.08311618 0.0246902
0.1933496 0.1175171 0.1676989 0.01773443 0.2110723
0.069626544 0.02089015 0.118000784 0.117487375 0.1122553
0.07808264 0.03628374 0.06934218 0.156396 0 0.111859
0.1010249 0.2603202 0 0.03532421 0.05365315 0.03133565
0.1934786 0.1328923 0.1769436 0.1371436 0.3185607
0.2243214 0.1781218 0.2773995 0.2568799 0.1289944
0.2981826 0.5601227 0.2935382 0.0798108 0.1811128
0.05052397 0.2084521 0.3246698 0.06396572 0.08205601
0.1111378 0.139228981 0.126105 0.2161401 0.1435722
0.03135455 0.2001308 0.2775038 0.1056909 0 0.03877177
0.1704766 0.1487062 0.1157305 0.1836785 0.3042094
0.1874665 0.06137981 0.3116497 0 0.04249569 0.2687673
0.432019 0.266925405 0.09584764 0.01576809 0.8988763
0.1650328 0.01633355 0.3039465 0 0 0.03937777 0.3642773
0.03612702 0.208802 0.3502743 0.1144772 0.4116439
0.4932873 0.1073086 0.3523973 0.1088794 0.1337306
0.1666686 0.1728526 0.1527183 0.1217874 0.1700277
0.07486815 0.072684198 0.031043792 0.07860734 0.03009209
0.1396012 0.1971259 0.2338969 0.2326875 0.05763334
0.3357002 0.07352939 0.0489763 0.04568761 0.178608
0.2087496 0 0 0.09241577 0.5064569 0 0.1033834
0.029388723 0.1524322 0.02044021 0.2497173 0.09775903
0.1990153 0.1611244 0.730681 0.06424051 0 0.06054006
0.06718119 0.1500211 0.09396703 0.2721521 0.3007079
0.02500393 0.1674981 0.06538973 0 0.01725315 0.2969149
0.3037183 0 0 0.1145353 0.0266735 0.03747773 0
0.08329138 0.161304 0.6108902 0.08660025 0.559371
0.1489126 0.02241439 0 0 0.02148537 0.07342509 0.3208015
0.1255586 4.332351 0 0.02729038 0.06386563 0.133071436
0.4316121 0.1947224 0 0 0.3087993 0.0450643
0.162694093 0 0.4005403 0.04456243 0.08507207 0.1292949
0.1534621 0.3973146 0 0.08243879 0.1292978 0.02724894
0.6660143 0.0542438 0.03941542 0.196848183 0 0.3766532
0.08635347 0.065179243 0.1970622 0.07729908 0.1327451
0.03409253 0.04243756 0.1205996 0.5863987
RFX5-AS1 0.1410545 0.7418412 0.3243685 0.3144023 0.3700335
0.3333181 0.9443955 0.3944541 0.2540578 3.092703568
0.08071886 0.09237385 2.359765 0.04408389 0.45992 0.5399171
0.9378983 0.566422827 0.2612467 0.2510525 0.412067801
1.194066 0.5682541 0.6187783 0.2737248 0.03695729
0.8931055 0.5204965 0.363440624 0.6934764 0.199306

0.632194	0.5673192	1.332766	0.7081457	7.936422	
0.1467745	0.9712661	0.4909989	0.457713689	0.322741	
0.1657937	0.391503313	0.2569247	0.2904989	0.5016608	
0.9300649	0.758071	0.263313259	0.225321	0.2985243	
1.175559	3.631813	0.565578	3.025243	0.2889994	
0.586799	1.108582	0.2357283	0.669294555	0.320286051	
1.090063036	0.6555937	1.072885	0.3558703	0.9239543	
1.154792	1.092978	0.283439518	0.7225911	0.381491	
0.9882617	0.3488408	0.2849724	0.3841696	2.338431	
0.4015581	0.8994156	2.028414	0.506646613	1.062791	
0.7190374	1.183542	0.6285116	0.4457327	0.6828709	
0.4354089	1.162042	0.96643548	1.060679	0.475749279	
0.8137526	2.02835	2.103613	0.7847286	1.032731	0.7520005
0.3713273	1.736122	0.9293348	2.26642	0.041078	2.491707
1.832973	1.334193	0.656148	0.8139366	0.6145711	
0.873142789	0.5641478	0.4555862	0.5662919	3.300553	
2.850821	1.165636	0.3359704	0.4734752	0.1303746	
0.9204591	0.5656776	0.7055255	1.213995	0.6932523	
2.095001	0.6779401	3.283603	1.010625	0.5848039	
0.7186046	0.3916606	0.4571368	0.4817681	0.7176178	
0.1658215	0.6306685	0.3595713	0.3354188	0.02531254	
1.526486	0.3701643	0.7847176	0.8663962	0.3657675	
1.435624	1.087267	0.5893738	0.4577671	0.8441622	
0.2279037	0.4592859	0.3041727	0.5099469	0.1065147	
1.331759	0.5669397	0.9596333	1.78553	1.614523	1.254818
0.6292376	1.02760465	0.7532192	0.745281434	0.4705036	
0.8688501	0.4852169	0.7875421	0.4833866	0.8964665	
1.302415	0.8345102	0.866058417	0.5518137	0.5502208	
1.122992	0.9498897	0.2729023	0.338534	0.7011005	
0.244834	0.6620455	0.1662033	0.4475692	0.9856048	
0.7094157	0.4066612	0.67763	1.176901	0.3489873	1.158538
0.7661199	0.2477365	0.1667944	0.6109874	1.738274	
0.2674591	0.6908731	1.588025	1.030067	0.2470911	
1.591612	0.5586465	0.1152373	0.1343402	0.4003262	
0.3098682	0.5382255	1.001874	0.1197884	0.5515343	
0.3007038	0.9495123	0.5673554	0.2074296	1.71463	0.6170599
0.3019046	0.6518406	0.911269	0.389968733	0.5045749	
0.768303484	0.27143766	0.3536588	1.024993	0.5906089	
0.5340171	0.2737352	0.4015578	0.7283143	0.2652312	
0.6287701	0.1839783	0.2225768	0.5071004	0.8555951	
2.472071	0.956972	0.3981844	0.9121455	0.5634362	
1.154312	0.6235222	1.106995	0.3188137	0.6547476	
1.369987	1.505842	0.4110168	0.7040397	1.37365	0.31835
0.6567253	0.8097701	0.6213628	0.919169	0.1945211	

0.536114183 0.2648612 1.021419 0.861566 0.3951282
0.7806305 0.3399945 0.221985 0.4291498 0.2035832
0.5115086 0.7222657 0.2916859 0.7501362 0.8945135
1.34528 0.365266153 0.5727446 0.351979 0.3347051 1.279529
8.670518 0.392440813 0.9562275 0.5795668 0.8630542
0.8882197 0.3259046 1.550366 0.2965461 0.3951806
0.4342068 0.3060401 0.5690883 0.7309195 0.948652
0.1602928 1.332903 1.11576 1.983368 1.427429 0.2286819
0.5416924 0.4375724 0.7260928 1.242936 0.4902697
0.416632 0.7076131 1.90825423 0.58681809 0.6604034
0.09480471 0.5680898 0.4140281 1.875717 1.679972
1.694681 0.5288094 0.4890458 0.9600834 0.2878763
0.1875673 0.4071245 0.617467113 0.1761229 3.445329
0.5560372 1.097068 0.2605664 1.203653688 0.7363608
0.686897 0.5244872 0.2694899 0.5805507 1.136102 1.0359
0.1574135 0.1621292 0.5298072 0.6819942 0.1417918
0.4934029 1.651311 0.5344171 0.1312909 0.3518003
0.320459 0.206874156 0.3442533 0.8107027 3.388879
0.17269 0.8680742 0.1804209 0.5042067 1.18073 0.166704
0.4592145 0.5420649 0.9623004 1.800697 1.051191
1.837493 0.2824648 2.606144 0.4685044 1.037903
0.1542165 1.122978 0.5274272 0.5199622 0.4770216
0.7164829 0.3129898 0.465821801 1.489291 1.256153
0.9416264 1.459485 0.3445574 0.6862097 0.683420493
0.7665359 0.8973484 0.3509832 0.3573577 1.25597 1.031424
1.599438 0.2779098 0.4978009 0.8534969 0.3433895
3.661684 0.5696476 0.3725331 0.324849556 0.2525354
0.5204563 0.09068512 0.479141261 1.83296 2.462355 0.1394038
0.4833361 0.4233799 0.6649078 0.5038474
AC010609.1 0 0 0.1174339 0.04878248 0 0.1034349 0.1578035
0.2142115 0.1182584 0 0.02191751 0 0.1577217 0 0 0
0 0.076900111 0 0 0 0.07628789 0.1299347 0.09164522 0
0.06020986 0 0.1817099 0.049342334 0 0 0 0.04929396
0.2412564 0 0 0 0.06879833 0 0.067790476 0.06185896 0
0 0.04924412 0.1051718 0.01878834 0.2971054 0
0.077996929 0.1427561 0 2.119959 0.02739285 0.268749
0.4978431 0.06277737 0.3374109 1.046999 0 0
0.028988984 0.104464844 0 0 0 0.4152503 0 0.1806457 0
0 0.0872302 0.0740253 0.3536228 0 0.07947671 0
0.3370163 0.04156892 0.01932537 0 0 0.2510222 0.2735029
0.105021 0.08068623 0.02472257 0.02627248 0.03155282
0.26241526 0.115202 0.024605662 0.05702127 0.6979877
0.2370985 0.1175595 0.7303871 0.3712544 0.1075479
0.06428285 0.2588116 1.246782 0.1115387 0.1176645

0.3675363 0.2371235 0.693392 0.03683456 0.1589275
0.022579389 0 0.04638933 0.06150587 0 2.561503 0.09042969
0.02027238 0 0.04045768 0.09431371 0.05342535 0.1585412 0
0.2588274 0 0.0490881 1.659355 0.1167719 0 0.3311161
0.02363271 0.1103341 0.08720934 0.1039221 0 0 0.06508936
0.291443 0 0.1405034 0 0.1217563 0.1882013 0.07945319
0.08065108 0.1927996 0.1445451 0.06629176 0.2837894 0
0.2743608 0.09439054 0.02408093 0 0.4060196 0.3731892 0
0.1368912 1.572156 0 0.4271406 0.115458398 1.043762
0.02611168 0 0 0.0810772 0.04073153 0.1193214 0.9388928
0 0.4848052 0.058789994 0.245182 0 0 0.1416046
0.09880118 0 0.07369127 0.05909292 0.07704195 0 0
0.3806155 0.2801846 0.4416812 0.1193489 0 0 0.1766045
0.06569172 0.1345353 0 0 0.1317186 0.2458006 0.03262471
0.05072881 0.2861976 0 0 0.1255355 0 0.06253246 0
0.3761473 0 0.08822847 0 0 0.03265992 0.2985286
0.2607058 0 0.3430529 0.1116998 0 0.04045569 0.02674983
0 0.07148177 0.026918281 0 0.109747 1.937074 0.144848
0.05272768 0 0 0 0 0.1187682 0.03330364 0.02014536 0
0.2859309 0.03678021 0.4330761 0.02883163 0.02607094
0.03824732 0.7675814 0.541774 0.253121 0.1065442
0.07356536 0.1700532 0.4344347 0.186005 0.2002704
0.2983887 0.02881376 0 1.157242 0.2188774 0 0.04225449
0 0.335615 0.2711817 0.1637581 0 0.1304393 0.02637668
0.2712392 0.1553688 0 0 0.2332191 0.2112031 0.3957284
0 0 0 0.1244134 0.12743 0.2181171 0.1021851 1.560406
0 0.9019195 0.0359701 0 0.47059 0.11178 0.5447839
0.04026043 0 0.2245709 0.1454228 0.0206032 0.1058484
0.2523297 0.02176208 0.11738 0.1298405 2.227605 0.5167846
0.1241876 0.0217904 0.1584181 0.1408252 0 0.1389104
0.1292888 0.06404582 0 0.070816946 0.1569038 0.01716148 0
0 0 0.06635071 0.03286822 0.1063608 0.2236465 0.1675867
0.02605559 0.02546497 0.03401419 0 0 0.1581138 0 0 0
0.033520706 0.139091 0.1865125 0.07768008 0.04181389
0.1513309 0.4988263 0.04167065 0 0 0.1150864 0.4597602
0.03422274 0 0 0.05276718 0 0.2292576 0.04972224
0.067406799 0 0.06773209 0.2598152 0.09378068 0.1833279 0
0.1825423 0.128241 0 0.5937621 0.2575761 0.4064549
0.01975521 0.5036977 0 0 0.1489776 0.3498344 0.02450616
0 0.4573818 0.03580294 0.0235308 0.3272215 0.2178914 0
0.025296825 0.04688531 0.03172856 0.3329771 0.1268136
0.02201349 0.07710034 0.04639212 0.2233662 0.09137105 0
0.07762639 0.0737367 0.0350077 0 0 0.02350737 0.3370897
0 0.3574844 0.03093517 0.1348714 0 0.2514258 0.2034993

0.1477418 0 0 2.791959 0.1892609 0.05832879 0 0
0.02026803
CARD8-AS1 1.807776 0.4938986 1.126674 1.874917 1.693716
0.4483579 0.8892378 1.070032 2.118801 4.807232187
0.836049 0.8407944 6.70001 0.6364737 1.924703 0.8199727
1.663209 1.072237117 1.375714 1.891118 1.569247942
0.507049 0.5350651 0.8739575 0.7327839 0.4581841
1.775325 1.009366 1.240524924 3.382069 0.7715138
0.7540093 1.794861 0.6739414 1.459721 2.038062
0.5220969 1.868841 1.489704 1.234172268 0.7090794
0.4488175 1.113592301 0.7684483 1.838744 1.786285
2.919147 1.530695 1.059355959 0.9429379 0.9539987
4.455447 0.878672 1.020712 1.870262 0.3265444
1.722584 2.367538 1.620312 1.230398711 2.194829704
1.911917092 1.580003 1.277888 0.5077054 1.599982
1.369251 2.736921 1.838542168 0.8659483 0.875968
3.834475 1.28467 0.9357096 1.77421 2.789015 2.503513
1.063111 3.121809 0.664986837 1.205994 2.538907
2.813705 1.426397 1.041477 1.185955 1.229936
2.224818 1.59248345 2.163914 1.080799638 3.402699
3.252476 3.513029 1.384369 1.944829 3.433114
0.8469054 0.8235539 1.652885 5.256598 1.985513
1.139086 2.088804 2.619131 3.283937 1.341197
1.515582 0.906971292 1.278812 0.6524034 1.113832
1.191317 5.938036 1.358879 0.7674366 0.9411793
0.65472 1.155599 0.872293 1.007932 1.31773 1.067539
2.383609 0.8014774 6.520026 4.778243 0.6065112
2.248613 1.987344 0.9193296 0.7224483 0.8408774
1.204483 1.418604 1.60821 1.228222 0.9692554 1.908321
0.8519984 1.844702 1.188728 1.331698 0.9400282
1.880384 1.038296 0.6034436 2.283642 0.5592421
2.551521 1.32293 2.491282 0.7889771 3.057944 1.466677
1.204781 1.819701 2.559921 0.456029 1.826834
2.413406931 3.888521 1.592149419 1.456608 0.9726356
1.915372 1.600797 2.358527 1.909292 0.4723707
1.017238 1.936755574 1.298962 0.8034071 0.6283123
1.566681 2.005742 1.668187 1.043467 2.322421
1.558446 0.9613336 1.941878 2.454151 1.126798
2.15159 2.161332 0.5490902 1.204884 1.467257 1.632584
0.5676173 0.5609025 2.684745 1.370302 1.356051
1.310473 2.909925 0.8721635 0.6548938 1.606511
1.124592 1.265913 0.7830584 1.577585 0.5091677
1.440351 1.376794 0.6078244 0.8378391 1.340201
2.188084 2.123177 0.447596 1.338325 1.368626

0.8765047	1.315221	2.357694	1.08627781	2.678491
1.345736312	0.619598223	0.4651474	4.864243	1.387296
0.9827995	0.6014111	1.233341	1.511659	0.7492209
1.398603	1.953684	0.4482623	2.767633	1.012194
2.827238	1.132607	0.9414867	1.506792	1.635796
3.194137	1.976588	0.8411869	1.778068	2.565235
2.506233	1.720672	1.956557	2.220256	3.654746
1.473804	0.7145598	2.768989	2.097825	1.415428
1.446964	1.430273135	1.24696	1.916402	2.386415	0.7854401
1.781052	0.9832781	1.193155	2.256142	0.8626178
1.452929	1.507208	1.319845	2.011342	1.293485
2.471626	1.045285597	0.7344662	0.8027766	1.183588
1.11621	8.156203	1.319714989	4.067501	1.278537	1.371342
1.890271	0.7967853	2.032294	1.029647	0.5705533
2.530957	1.620932	2.095771	0.8488169	1.768262
2.081588	1.888235	2.163723	3.360137	1.725706
0.9869095	1.385334	1.272671	2.791714	0.9186558
2.809955	0.616469	1.480633	0.830521862	1.504149772
2.714039	0.8530016	1.984345	1.345899	1.513856
2.415921	1.120791	1.045026	1.688437	1.592779
1.807085	1.537999	1.120551	1.585646651	1.133216
1.751511	1.403752	1.562733	0.9132395	0.387471438
0.9948115	1.104913	0.7707121	1.655418	2.551003
2.306407	1.673831	0.8046213	0.2689724	1.716091
1.896962	0.7219451	0.9291712	1.98345	0.8615468	0.4368005
1.545849	1.422501	0.772673397	2.303151	0.7437802
1.514137	1.802194	2.406713	1.252103	2.127621
2.754718	0.5755477	2.958124	0.7549747	2.550495
1.158895	2.455081	1.975588	1.433269	1.528326
1.259091	1.3597	0.3226876	2.264578	0.8225313	2.692765
1.686324	1.978934	1.936663	0.899161462	1.293918
1.136943	1.360875	1.154365	1.291372	1.641322
3.324776489	1.461136	0.9505559	0.7564411	1.121621
2.045604	1.517475	1.440543	1.046714	3.077293
1.296796	0.7903715	1.407538	1.314124	1.305663
2.187481325	1.109668	1.391767	1.280829	1.697209218
1.271232	4.688038	0.6672481	1.477692	0.6854022
1.798719	1.598971
AC116025.2	0	0.06272082	0.150835	0.0313287	0.04301744
0.03321357	0	0	0.1012627	0.041089773	0.2252111	0.193297
0	0.09224777	0.213868	0.1943749	0.39252	0.049386189
0.1619769	0.1400907	0.038323236	0	0	0.03923712	0.06738623
0.1288917	0.155739	0.1296626	0.253505989	0.2976686
0.09267956	0.05879547	0.09497154	0	0.3203958	0	1.33091

0.7952962 0.6088534 0.116095654 0.8475004 1.272083
0.307215212 0.2530014 0.06754263 1.616857 0.4960922
1.128037 0.100181158 0.2357478 0.6057476 0.4331929
2.885092 0.6903755 0.2557767 0.8063279 1.733515
0.3698174 2.367713 0.889227207 0.2606391 0.313080115
0.2939707 0.8059209 0.9929022 0.3555717 0.4474928
0.1988792 0.527210301 0.2749197 0.4201522 0.2376997
0.9084034 1.070316 0.2296841 0.4745004 0.2546305
0.6140096 0.7943034 0.417648152 0.5132178 0.1432974
0.2459057 0.1686144 0.3454512 0.3175427 0.3374501
0.2431633 0.33705255 0.1849603 0.063208224 0.8056346
0.05603203 0.1937951 0.4529888 0.8711179 0.1907393
0.4144114 0.2752215 0.5651208 0.4106147 0.5730521
0.7556555 0.5900908 0.5414529 0.3710873 0.1419336 0
0.464024288 0.04372252 0.3972239 5.08231 0.4028836 0.5784714
0.9292008 0.4426517 2.348494 0.2858066 0.1938221
0.7891394 0.4072684 1.234891 1.903999 0.1217749
1.103374 0.2226747 0.7799204 0.1064115 0.1215125
0.1821266 0.2125738 1.306828 0.2669603 0.03304666 0
0.71062 1.310178 0.2118711 0.5053049 0.4303265 0.8712979
0.1726646 0.2041032 0.9323119 4.643186 0.0795674
1.149481 0.5888175 0.4335455 0.0961079 0.1212375
0.3402315 0.3863383 0.4889076 0.3834667 0.1029785
0.1172176 0.7378269 0.5527936 0.4389039 0.148297581 0
0.301846307 0.3878541 0.3232201 0.1562063 0.2615828
0.2452148 0.401979 0.2018792 0.1624423 0.264289554
0.9097622 0.634085 0.3405679 0.1818804 0.8248672
0.4722664 0.4732543 0.986705 0.1979091 0.4968413
0.3672789 0.6416443 0.4198553 0.891481 0.255491
0.04437343 1.703972 2.154933 2.109401 0.2419205
0.9972162 0.1504144 0.9587016 0.3444134 0.2304719
1.726669 0.7017804 0 1.312026 0.8062043 0.385824
2.730819 2.233874 0.1779963 1.304095 0.2644199
0.2785149 0.7078563 0.1677968 0.1394318 0.4870646
1.085142 0.1573663 0.1434699 1.298599 2.28634 0.3435814
0.374013466 2.723784 0.17287248 0.929449784 0.3289106
0.4861658 1.0897 3.250786 0 0.2800933 0.3277494
0.851015 0.9152927 0.64164 0.1552511 0.1965062 0.3672567
0.2834484 0.1946887 0.2221923 3.04724 1.670277 0.3943601
0.08698346 0 0.034212 0.2834674 0.4368409 0.2297641
0.334473 0.3273864 0.1179254 0.03700909 1.282616
0.7134674 0.6091183 0.9216329 1.004045 0.203972029
0.2155361 0.6966247 0.390622 0.5512172 0.1675394
0.3387885 1.316126 1.845925 0.2556049 0.6065361

0.3267844 0.5425489 0.2989901 0.5348043 0.7323749
0.209818268 0.2511137 0.7201665 0.4980533 0.07874941 0
0.039104873 0.1053134 0.6468122 0.07524936 0.3324405
0.04785767 0.6997337 0.07756722 2.370549 0.3749777
0.1867846 1.508405 0.03398858 0 1.481442 0.402042
1.167392 1.383433 0.1475046 0.07975476 0.08396436
0.04069522 0.1808793 0.6152684 0.921836 0.1937386
0.5758341 0.106483171 0.272877035 0.3166918 0.6833215
1.354927 1.072655 2.055969 0.2130562 0.1266502 0
0.3590713 0.430505 0.3011979 0.06541567 0.4805752
0.313231829 1.044214 0.2707803 0.06745137 1.294993
0.4846652 3.056891619 0.4466297 0 0.1662903 0.2864362
0.3239551 0.3709343 0.4817052 1.003873 0.1938649
0.3252036 0.8529837 0.3076957 1.51429 0.5759086 0.3049895
2.930483 0.09815467 0.3193223 1.010087583 1.036318
0.6959739 0.2224754 0.2409084 1.177354 0.3915228
0.3907697 2.306022 0.8837191 0.4270798 0.2835746
0.1491602 3.171759 1.552708 0.4363173 0.06567467
0.6218891 0.7761256 0.4406684 0.1434248 0.3524834
0.1379585 6.830519 1.260874 0.1599229 0.9044508
0.097475578 0.2709929 1.630118 0.2749397 0.7329714
0.1979226 0.3961182 0.357523023 0.5477126 2.268944
0.5222757 0.7976419 0.9470922 0.539577 0.09701172
0.6784639 2.566442 0.4329662 0.11976 1.43488 0.2781372
0.2021041 0.24718698 0.7339487 0.7260541 0.1265088
3.007882273 4.825386 1.434424 0.2917096 0.1997838
0.7149723 1.501778 0.104131
LRP1-AS 0.1322286 0 0.0760181 0 0.04336001 0.08369518
0.3064513 0 0.1275863 0.020708498 0.1560656 0 0.05104873
0 0.4311423 0.0489807 0.1978229 0.02488974 0.02040835 0
0.019314213 0.01646106 0.02102755 0.1384236 0.01132048
0.07795088 0.02616322 0.07841708 0.095821803 0.1125147 0
0.01481592 0.1595464 0 0.05382456 0 0 0.08906996
0.0639273 0.07313762 0.1067812 0.2914121 0.034406861
0.07437972 0.06808051 0.1094597 0.05769726 0.1776594
0.016829827 0.05280559 0.0954018 0.327482 0.1773212
0.1739683 0.1289068 0.04063746 0.1698784 0.06777499
0.1657339 0.089630865 0.1501227 0.135245725 0.04938529
0.07736561 0.02274567 0.02240021 0.2029754 0.200463
0.049819574 0.1154621 0.0141166 0 0.1144547 0.046236
0.05144738 0 0.1026633 0.05381733 0 0.080956564 0.05747832
0.05416445 0.08852284 0.11897 0.05223034 0.112025 0.1190481
0.06127493 0.05662278 0.09321663 0.143351075 0.1107341 0
0.05581096 0.01902484 0.1013141 0.1602152 0.0348093

0.08322398 0.08376782 0.2276365 0.04332117 0 0.2577423
0.1534963 0.1371489 0.04768798 0.06172679 0.043848712
0.06610606 0.1401355 0.663573 0 0 0.5853753 0.2099656
0.03698745 0.039284 0.09768282 0.1210428 0.03420931
1.19138 0.5787947 0.06137232 0.04766405 0 0.1209433
0.2145178 0.04593005 0.07649041 0.1071333 0.07527056
0.1681789 0.04996475 0.03695043 0.1474692 0.06288626
0.1601687 0.07276128 0.08675069 0.09007555 0.1218277
0.07714824 0.4002581 0.06240216 0 0.1501934 0.07065554
0.1310994 0.06458218 0.01527537 0.04676468 0.04493249
0.0492801 0.1328664 0 0.04430666 0.156569 0.2321649
0.1105998 0.049826188 0.01826093 0.033805565 0.09021758
0.06108639 0.1443294 0.1845662 0.2935115 0.1800801 0
0.05457865 0.095140803 0.08817376 0.01681933 0.034328
0.09166439 0.1758807 0.09520548 0.1590077 0.1530096
0.0498713 0.1168528 0.01851019 0.06159563 0.06045698
0.1633783 0.1287628 0.0670902 0.4498324 0 0.1842706
0.0696706 0.05025788 0.07580611 0.05684331 0.1301836 0
0.1477715 0.03368424 0.1351177 0.2288899 0.2166999
0.09722413 0.1214368 0.09381934 0.1665985 0.1194982
0.01903754 0.04210993 0.1240858 0.232558 0.07027107
0.1227358 0.03645947 0.09517168 0 0.1591954 0.4321026
0.08657938 0.291311967 0.1542401 0.1045495 0.104094616
0.03552107 0.07206432 0.2009228 0.2730562 0 0.02016599
0.1486617 0.09323827 0.03844091 0.3880499 0.1173656
0.178264 0.09254534 0.1666616 0 0.3172791 2.953372
0.1733095 0.06625011 0.04383808 0.1638519 0.01724222
0.1269888 0.02751998 0.08271209 0.1444871 0.07071291 0
0.05595573 0.2462533 0.1797873 0.04722839 0.1211703
0.1777909 0.085665158 0.1396623 0.1117092 0.0454307 0
0.0211092 0.2048919 0.6633033 0.2011487 0.04294007
0.1078882 0.1372445 0.08544836 0.1506856 0.0673829
0.04613795 0.181276437 0.04602063 0.04949329 0 0.05291769
0.01772092 0.039416288 0.2123042 0.3026972 0.09481077
0.07615635 0.0241194 0.2564749 0.07818494 0.3056243 0
0.01344801 0.3067509 0.01712963 0.04083492 0.07043585
0.1266387 0.3081804 0.3803045 0 0.01339832 0.09873852
0.0410193 0.09115987 0.1127579 0.1049071 0.1255381
0.05527808 0 0.252129274 0.1886263 0.03332725 0.03865237
0.06237673 0.06279825 0.1073764 0.02127647 0.05508013
0.1266758 0.06026852 0.03373294 0.03296831 0.154128
0.098664463 0.2166973 0.1023513 0.3739369 0.2966605
0.06106561 0 0.2025839 0.03018362 0 0.2165379 0.04898025
0.1189644 0.1078981 0.1422938 0.1628406 0.148997

0.06613666 0.1772263 0 0.1116336 0 0.4430731 0.04946817
0.1126528 0.189081566 0.02547734 0.08768955 0 0 0.08476644
0.05637725 0.03938816 0.2490414 0.0703229 0.2152405
0.3334717 0.187935 0.191821 0.173897 0.137435
0.06619768 0.04821859 0.7823064 0.07931744 0.05421261
0.05921507 0.1854096 0.137089 0.3177288 0.1611964
0.03143633 0.327506113 0.04552515 0.06161622 0.01539607
0.04104491 0.05699965 0.08318183 0.045046274 0.105157
0.1182938 0.03290218 0.01256241 0.04773172 0.1359685 0
0.179108 0.04565083 0.04091383 0.04023791 0.6942274
0.08010062 0.04365292 0.124577738 0.1035711 0.1317305
0.06375814 0.120310946 0.1870697 0.03804862 0.02450272 0
0.1879998 0.1558259 0.03936009
MIR100HG 0.2225122 22.04828 0.482583 0.3412614 1.729752
13.61376 0.4415706 6.172729 3.369021 6.117890236
3.632502 0.8983769 2.509342 0.8498149 0.4193369
21.75085 0.3624159 8.613514812 0.5696502 1.281839
2.991344741 17.70183 1.786771 24.45014 16.72618
1.229109 0.4847014 13.19429 0.800812844 1.686092
1.932571 15.63808 0.1871986 1.82596 2.514498 0.528053
0.4014697 0.9534003 1.902809 0.160787668 1.989775
0.09177822 0.652296943 0.03149631 0.8114132 0.1652328
0.7387308 1.845339 16.194396044 0.436965 1.086374
2.738796 0.3285064 1.141845 17.53688 0.07528522
1.03707 10.02809 0.2742888 0.238006029 5.270341398
1.378064052 10.17995 1.586164 0.08708678 15.27846
3.303524 4.718578 0.590694806 0.5361907 0.1726066
0.1745892 4.52351 3.923096 1.540873 0.8454518 1.45816
1.090079 0.2672941 0.093987875 0.2591128 3.984827
0.6384988 5.47863 3.489892 0.5850602 4.887792 1.712865
0.92311766 11.16523 0.802620995 0.1299263 6.270942
0.7651285 0.5498305 1.003126 2.06188 0.1268263 0.10964
3.894204 4.971195 0.2479052 0.1834405 0.7762357
4.75843 4.068407 0.5654212 5.636453 0.563225209 0.4463297
0.4413472 0.9998629 1.515405 10.44657 4.149906
0.07617594 0.2923656 0.284642 2.488308 0.1195971
0.1668911 1.743764 2.026043 3.323794 0.9183483
0.9029101 1.33876 0.7418451 9.929093 4.05092 0.8556514
0.5066564 0.7269951 1.061412 0.2418729 4.243745
1.254354 0.2989276 1.379433 0.345537 0.763451
0.2536425 0.3398447 0.2471736 1.296721 3.856502
0.1960995 9.14184 0.2590673 4.07193 0.3584572 2.997624
0.364416 2.639487 8.818107 0.7115025 3.653597
16.10079 2.012916 3.162257 4.464645538 2.562083

4.375638107 0.08356883 3.591231 1.323964 0.986708
1.623652 1.239945 1.905011 10.62358 0.467672458
2.01031 0.311596 0.07419566 1.538066 3.007581 4.14195
0.03338559 0.9094555 0.9957792 0.797892 0.9716093
0.4811742 1.582976 0.4666249 7.53967 0.8949005 1.220581
0.3600453 3.31227 0.7163507 1.460762 0.4868548 0.6423805
2.13488 4.45763 0.4684378 0.1580892 1.306431 0.2245857
5.975057 0.1781167 0.1483165 0.5407432 0.240582
2.152337 3.545717 0.7090526 0.8237444 1.569296
5.42219 0.9834238 1.445464 0.5779236 0.9751921 1.926853
0.4059183 9.360799 0.459262566 0.5619677 2.483524377
0.724245203 0.8116142 3.211284 0.511197 0.223424
1.316837 0.07720993 0.5100217 2.026744 2.627866
0.02928867 0.3076264 0.2739877 1.957388 1.858432
0.5557181 3.750352 0.537764 5.663139 2.896555
2.349812 1.290101 5.238661 1.380194 1.315379
19.58113 4.794407 0.2547284 2.460838 0.1773805
1.592935 3.019881 0.1093695 0.7582466 1.23474
1.163828035 0.557727 4.728389 0.6490073 0.4145875
1.525174 4.742691 0.363833 1.21111 0.2174395 0.6107271
2.466322 0.4981242 1.868518 0.6907481 0.5498922
0.12127335 0.1307291 0.7742845 1.683768 11.94238
0.3282988 2.4073206 7.669663 0.3695388 6.327978
1.358205 0.516842 4.276324 0.2671602 1.468693
1.172417 5.728528 0.7478349 14.63805 17.12067
0.4332265 1.144905 3.664378 2.953258 2.359638
0.6668792 0.268288 4.037742 6.766593 1.230745
3.172558 0.2411864 0.4932677 0.673854789 1.876877924
0.9677086 0.5254954 1.005689 1.050308 0.3335093
2.111271 0.3127076 8.452463 1.113055 1.436613
1.068645 4.145113 2.357732 1.620706244 0.9654034
1.049212 11.33184 0.5715789 0.5958209 0.297475657
0.6282938 0.7698094 0.3871197 7.152898 1.754328
1.796738 7.825797 1.220437 0.06033592 0.6679993
0.9332336 0.3447471 0.7426348 0.882403 2.18107 0.2895741
9.802965 1.470848 0.29101325 0.5947136 1.43772 1.602912
0.2024382 1.555732 1.086227 1.950752 0.3588478
0.07527326 1.691864 0.9678738 11.88883 1.013986
1.038975 2.647975 2.738921 0.1191068 0.1906981
0.8013346 0.03571009 0.550949 0.5095116 0.8973677
1.390902 3.538807 1.077424 1.179097934 1.073932
2.701564 0.2890316 0.9378276 0.2886343 3.234112
1.937964508 0.7743879 3.840027 0.5871979 0.437536
0.5954158 7.559682 1.739096 0.09250645 0.3721213

5.659526 0.5441786 0.5966214 2.478195 0.4888243
1.274493105 1.761609 6.605479 0.8465176 0.490355431
0.4030441 2.086478 0.7202499 0.0979304 0.203169
0.7588269 10.07413
LINC01550 0.2582865 0.3348488 0.4820171 0.1233655 0.4560584
0.5382415 0.2762776 0.1994233 0.1840384 0.600535817
0.3410889 0.2000486 0.6443135 0.1699829 0.2672253
0.5501349 0.3467819 0.360894852 0.1471913 0.3129531
0.162516723 0.2498113 0.2780381 0.5972292 0.4575621
0.1971624 0.2122843 0.6559019 0.273558962 0.4620987
0.2316038 0.4697255 0.04075389 0.4693159 0.06469988
0.09799618 0.02842659 0.1271417 0.08260718 0.131872755
0.5334816 0.01532529 0.038773886 0.03831782 0.1687873
0.04751362 0.4392487 0.101439 0.146669781 0.04562268
0.01720168 1.040361 0.1998273 0.1381678 0.6391795
0.2442415 0.138566 1.227127 0.03237335 0.437697392
0.056392245 0.179506911 0.6084774 0.03487404 0.0375945
0.4106246 0.1423256 0.2836381 0.029942841 0.07807016
0.07423886 0.06840064 0.4004573 0.03705203 0.1198196
0.2694919 0.3374399 0.141512 0.02255618 0.021895637
0.05613716 0.2414438 0.3484895 0.1762064 0.08894335
0.09378093 0.2172082 0.2056219 0.41688888 0.310942
0.038292298 0.3466357 0.3797583 0.7589289 0.2001023
0.3120733 0.6764615 0.1046064 0.01667325 0.3801169
1.402358 0.4144244 0.3318942 0.1400146 0.1973237
0.5039471 0.06807153 0.2225963 0.109809166 0.07284097
0.0481286 0.06181778 1.385978 1.212868 0.4749645
0.02168971 0.04168201 0.01770802 0.0458671 0.07015159
0.06939227 0.2564954 0.1304514 0.1152698 0.05490735
1.435711 0.2294279 0.02954662 0.3381629 0.119529
0.06617847 0.1555109 0.1465658 0.04754765 0.2387377
0.1171219 0.1535473 0.02139234 0.2350562 0.3812693
0.1218099 0.3948735 0.1275122 0.08367492 0.1429889
0.5563416 0.03223933 0.5817814 0.08973774 0.2013556
0.0803328 0.03981792 0.00900186 0.6985055 0.3230543
0.07798204 0.1686531 0.6028408 0.03488427 0.5068589
0.565246158 0.5844341 0.19302111 0.04970449 0.1040244
0.2838949 0.3288253 0.06499241 0.470136 0.04892033
0.6458124 0.034309237 0.2225777 0.06318031 0.02321101
0.04328708 0.1994053 0.06794986 0.07884336 0.07759354
0.0274761 0.01254135 0.3365342 0.333185 0.0931115
0.1135374 0.3908183 0.08736635 0.04301194 0.1180948
0.115011 0.02617114 0.01762033 0.2050264 0.3117491
0.1869154 0.3490565 0.1578923 0.1265318 0.1653187

0.01146406	0.6837715	0.06573847	0.04865773	0.01409696
0.1174602	0.08977688	0.1287231	0.05694558	0.0489423
0.3081366	0.3695538	0.1313982	0.04656529	0.3098367
0.09343447	0.02657793	0.04918651	0.2107475	0.141613646
0.03708091	0.162328736	0.028674953	0.09784991	2.620407
0.07648117	0.1076997	0.4684662	0.02121049	0.02978317
0.04483087	0.1559518	0.0971783	0.04310762	0.4166603
0.09733871	1.123312	0.1495371	0.08132491	0.04057262
0.1636854	0.3409417	0.2371303	0.0738594	0.06476886
0.2766728	0.3266692	1.014127	0.2930866	0.2018763
0.3996211	0.103695	0.2451325	0.333175	0.1295087
0.06068872	0.05137352	0.105548261	0.1189159	0.103108
0.1774826	0.03478479	0.3774433	0.08209685	0.1846745
0.3060168	0.01505472	0.2431633	0.1629127	0.2285371
0.3396221	0.104622	0.06932523	0.079444011	0.06742023
0.8775287	0.2734398	0.2743172	0.02130145	0.04145785
1.116502	0.07871918	0.1396101	0.2654771	0.05979755
0.4287242	0.06461283	0.02504837	0.2337188	0.189941
0.09418654	0.3860754	0.3599616	0.08043412	0.3158693
0.1515472	0.423812	0.3239302	0.06844822	0.110211
0.2372914	0.2218971	0.1101268	0.6507852	0.2703682
0.06852356	0.206965666	0.082656087	0.220198	0.600916
0.1471301	0.04686252	0.07548672	0.3259067	0.07033238
0.3000102	0.315418	0.2046595	0.1393862	0.1114582
0.162111	0.281674392	0.009302904	0.1230315	0.5567563
0.3922612	0.05734687	0.01630195	0.2063124	0.1723407
0.0755555	0.04202594	0.296837	0.2196092	0.174283
0.01900494	0.05382926	0.3716367	0.5142634	0.08987418
0.05212359	0.1442536	0.1154789	0.01109576	0.3381974
0.2248848	0.019668958	0.03445321	0.04611571	0.1095073
0.05929023	0.2751127	0.09318148	0.07101989	0.2619405
0.1303197	0.616794	0.196846	0.6325412	0.02882234
0.9700444	0.6277749	0.007459969	0.1775062	0.2443731
0.05958977	0.01357632	0.2491284	0.1706362	0.02288723
0.1405701	0.272484	0.01889404	0.103340775	0.0980464
0.01234431	0.2429026	0.6290607	0.1691501	0.1624818
0.099271208	0.08690283	0.6191417	0.01977506	0.1132549
0.1685419	0.1668461	0.2350834	0.02446557	0.03201021
0.2909847	0.2448632	1.512526	0.2166414	0.185842
0.517881371	0.1578454	0.2727084	0.03353024	0.086771956
0.2998232	1.889485	0.06627044	0.1399426	0.04001826
0.0568622	0.3548464			

LINC00528 0.08744075 0.03859073 0.1979849 0.3341148 0.2470316
0.047683 0.2078477 0.1128577 0.1453775 1.146100306

0.04041548 0 1.474954 0.05045156 0.4605587 0.05481416
0.1610059 0.167124235 0.02491519 0.2298526 0.149336423
0.06698736 0.0598993 0.07242518 0.02303403 0.02643473
0.3832911 0.02127426 0.142978551 0 0.171071 0.01205852
0.1753016 0.105922 0.09856622 0.1769375 0.006998971
0.5618212 0.1300743 0.065478486 0.03259051 0.01778828
0.042005043 0.04324059 0.1593037 0.1484801 0.6182956
0.1518248 0.171220111 0.1719115 0.06211717 0.8249862
0.1298878 0.1921591 0.301633 0.008268594 0.3456553
0.7308874 0.09442228 0.182373913 0.282548644 0.403608307
0.1942717 0.188901 0.1295871 0.1048299 0.1743772
0.2651263 0.108126927 0.1080693 0.3044677 0.214502
0.1729992 0.03763097 0.1099152 0.3284433 0.3290039
0.1916308 0.8552552 0.342626169 0.1637334 0.2498084
0.5475629 0.110661 0.04250969 0.1367639 0.08997106
0.2992259 0.73735448 0.1820832 0.110190078 0.6383875
0.0746964 1.095852 0.3251663 0.322962 0.4563909
0.1699854 0.04515672 0.1499909 1.052675 0.07639368
0.2944608 0.185569 0.2359769 0.471865 0.1746572
0.09210437 0.142751916 0.02690149 0.04073384 0.1674232
0.07082437 1.349532 0.2620368 0.2296316 0.2935108
0.04795921 0.07950297 0.112589 0.1252916 0.3256289
0.2603307 0.06243781 0.0969831 1.95724 0.1476516 0.06547262
0.1557581 0.1369603 0.06781802 0.03063093 0.05475158
0.216884 0.2105148 0.1886085 0.1343539 0.2462347
0.0592196 0.1412108 0.4765247 0.3895345 0.2197653
0.09914607 0.3618675 0.02175825 0.06112039 0.2530254
0.04149459 0.1576881 0.2672978 0.3108335 0.09142514
0.7219535 0.3047539 0.04224027 0.1682833 0.4778616
0.8125127 0.1350239 0.364976824 0.5499077 0.343924676
0.1407352 0.2651602 0.2936702 0.579406 0.08172408
0.5312987 0.06624628 0.09439453 0.271019149 0.2152909
0.0616008 0.05587836 0.1385515 0.1952009 0.2130884
0.1229439 0.1400995 0.04735464 0.05434585 0.1958481
0.350924 0.1230132 0.9557345 0.151958 0.009100661
0.1053963 0.1104902 0.2480369 0.09923223 0.3408694
0.4318843 0.3122829 0.1589325 0.3437678 0.2873106
0.1302223 0 0.103495 0.1433003 0.09231791 0.04392712 0
0.1408078 0.28367 0.1007139 0.05712134 0.1325521 0.4731909
0.2287714 0.3121667 0.07418489 0.1807381 0.2177421
0.04318913 0.09058515 0.2043514 0.118547787 0.1067042
0.333275732 0.07060121 0.0722755 1.278621 0.03270576
0.1666781 0.03132747 0.02461931 0.08066276 0.0758856
0.273758 0.07018433 0.2865677 0.08866432 0.2259649

0.5328874 0.2167586 0.174685 0.05494209 0.09571568
0.5796424 0.1516371 0.3333931 0.4911639 0.3746611
0.2463803 0.4577655 0.2841915 0.3021504 0.6409194
0.3263825 0.1377907 0.4389806 0.1537546 0.1479288
0.06122011 0.146416033 0.1705044 0.188332 0.3944058
0.1130506 0.5411871 0.0416898 0.3572571 0.1637127
0.1281443 0.0951264 0.2680842 0.153 0.2698109 0.274211
0.1971436 0.141391424 0.03277372 0.1678418 0.1468364
0.1668929 1.298057 0.192482875 0.7487653 0.2463619
0.1003151 0.2293364 0.05889153 0.7827829 0.09014796
0.0791459 0.3667784 0.08756147 0.2116692 0.05576645
0.1274012 0.1891789 0.2267536 0.1710168 0.257938
0.3327731 0.1090474 0.04592125 0.3588904 0.296776
0.1491301 0.1646667 0.1135267 0.2924364 0.101914911
0.513013037 0.3247557 0.03616624 0.1258349 0.2199935
0.07666619 0.3233522 0.2337752 0.08405458 0.3019357
0.1079141 0.3020035 0.597024 0.1075226 0.200754775
0.1700687 0.2776752 0.1590884 0.09657947 0.3106289
0.035320892 0.1374007 0.1351136 0.01364196 0.09913377
0.3587803 0.3803794 0.1317256 0.08364154 0.02650683
0.07882354 0.5248219 0.1848107 0.07528963 0.6723446
0.0973017 0.2479209 0.2415695 0.05894157 0.094702391
0.03110359 0.08029074 0.4220594 0.1235213 0.1241828
0.02294241 0.3045471 0.3603414 0 0.6193929 0.2714089
0.3365081 0.2185694 0.4069069 0.1677852 0.1683674
0.1766006 0.08377769 0.1291112 0.04412304 0.4417828
0.1037457 1.072362 1.034384 0.09019724 0.08954987
0.086629915 0.1482097 0.05014876 0.05638809 0.133624
0.1101795 0.05416061 0.116098429 0.1016335 0.144417
1.533082 0.1584782 0.2039537 0.0737755 0.2188602
0.01987827 0.1176566 0.1498469 0.2128697 0.3413686
0.3341142 0.284229 0.751993909 0.120422 0.160821
0.07783806 0.029375923 0.1437954 1.803848 0.1096837
0.06658303 0.01912637 0.25365 0.1067825
PRDM16-DT 2.508781 0.3349124 1.940164 21.17232 2.123464
0.5885445 8.481969 10.22042 2.383153 8.470788839
0.06235532 0.04587351 12.58815 0.0170274 12.62119
0.4574491 4.445487 2.664437807 2.39613 1.418519 3.12562257
0.5038406 0.05610975 0.5183574 0.3873665 0.05302052
8.607623 0.3528488 2.356360798 2.631457 3.761119
0.3046498 5.549549 2.827209 0.3210443 0.04549823
0.3833436 1.174387 0.503722 0.408687797 0.4378768
0.06175074 0.002700324 0.0617105 1.883444 0.5994352
0.766776 0.3011708 0.044908589 0.1222567 1.650209

1.054985	1.422271	1.166391	0.1618701	1.712662	
0.03809262	0.401594	1.758567	1.00943961	0.094255609	
0.23705436	0.1653702	0.1457237	0.999672	0.2496383	
1.040756	0.7079746	0.823695486	0.06705669	0.09971113	
0.2726543	1.506519	0.3870614	0.04441471	0.7319593	
0.2618602	6.168713	0.3613017	0.139780182	0.6405651	
1.014558	0.2167608	0.5175378	0.02459489	0.05275178	
2.367821	0.1763295	0.86211222	1.079818	0.165007326	
0.02317507	0.08200187	0.827851	0.1612559	1.033675	
0.3394989	2.26202	0.481161	0.1262261	0.6983749	0.441992
0.5977775	0.818463	0.4631296	0.8630563	1.366069	
1.769839	0.500141535	0.8024331	0.02199627	11.92184	
0.6556313	0.2145057	1.166914	2.092775	3.483425	
0.4460207	1.284115	1.452096	0.4215167	0.293066	
0.9826156	0.2119318	0.03740777	2.486254	0.01661081	
0.1346865	0.6320221	0.2233165	0.1251865	0.01181479	
3.880518	0.3659918	0.5973895	0.4662548	0.3997713	
0.0139671	0.6367164	0.02042514	2.175584	0.0874176	
0.2583364	2.297255	0.5730023	0.4657819	4.853084	
0.1707921	0.04344229	0.3928124	0.2205873	0.5431884	
0.0587733	0.2629974	0.9441754	2.215804	0.6606836	
0.3532762	0.1239014	0.1128414	0.32065821	5.491858	
2.846811938	0.05664375	1.102664	1.326322	0.3269505	
2.238072	0.6995869	5.611888	0.4176363	0.576441136	
0.4650284	0.155762	6.290806	0.9639984	0.8583264	
0.02988772	1.574883	0.390277	0.9811109	1.186972	
0.1481774	0.9329923	0.3582319	0.7340753	0.3435898	
0.6739689	8.66334	0.3431843	1.05016	1.120919	0.5259129
3.898857	0.4193514	0.3405695	0.07624278	0.5747166	
0.2114889	0.3666066	1.504965	2.508543	0.2874101	
0.1948482	0.0638139	0.8709966	1.09103	0.08068178	0.4648861
0.2872863	1.144876	0.05515024	2.709161	2.025883	
0.1917115	0.8625615	1.041167	1.92581	0.9920606	
0.669745185	1.35335	1.654729204	0.631779967	0.7229616	0.9773157
0.4625529	1.433134	0.03020863	0.01266136	0.2385313	
0.1024456	0.08145697	0.5245025	0.1330493	0.3171189	
1.97921	0.4409816	0.6226512	0.3017385	1.091385	1.435951
0.7149244	2.504689	0.1414539	0.2381644	0.490843	
0.7818574	0.4440133	0.6728198	0.4125278	0.2542083	
0.4391525	0.9228398	0.3386422	0.07227841	2.079456	
0.4143091	2.390764646	0.2776786	1.292533	0.8747357	
0.1780546	0.559963	0.1179226	0.5940676	2.794225	
0.1280611	0.6491593	3.483419	0.6464748	0.9224379	
0.5852446	2.339169	0.303508548	0.303391	0.2641351	

0.1674482 0.2574918 0.0111262 0.151580264 0.5581799
0.3746189 0.3422838 0.1147567 0.005678826 1.338559
0.2720347 0.5102488 0.1620079 0.1857554 0.4061258
0.1075496 0.2692042 0.7739138 0.08746209 0.4925284
0.09949038 0.297551 0.3785503 0.8169882 0.4635767
0.1545355 0.08628241 1.013876 0.6852959 0.6746122
1.541515028 0.287820301 3.653122 0.01743729 1.069822
0.2545639 0.4263188 0.4280983 0.4675505 0.503607
1.332199 0.3613722 1.734069 0.1552454 0.1382434
0.126991735 0.05102062 0.0589068 0.1227254 4.051164
2.767703 0.023841602 0.5370403 0.1255504 0.3472854
0.1770246 1.137846 1.147066 1.354891 4.536496
0.3834024 0.2783077 0.9317031 0.9997165 3.263402
1.114431 0.3833488 0.1130133 0.5978846 0.02273461
0.956578709 0.009997583 0.8843452 0.3431881 0.4859681
0.1596636 4.030818 0.3678608 0.4430269 0.5298328
0.5815858 0.1719846 0.1769945 1.812565 2.41225 0.1510067
0.7325423 0.4011356 0.08401706 0.4407302 1.01546 0.5514827
0.1000405 0.4805704 2.138958 0.4174843 0.5847241
1.300591105 0.05478465 1.295987 2.184636 0.7086844
0.2863011 6.058251 1.01110384 0.6829314 0.8479342
6.073414 0.03154958 0.4757532 0.9924122 2.946939
0.1482351 0.07404402 0.4045866 0 1.266763 0.1194429
0.4910563 0.482338308 4.798127 0.4571911 15.50201
0.623189214 1.177793 0.5613938 1.738416 0.2469426
0.2483696 0.6219601 0.4201128
LINC02560 0 0.09080172 0.02911543 0.03023663 0 0 0 0 0
0 22.11644 23.6308 0 22.22836 0 0.02344991 0
0.023832333 0.03908266 0 0.036987349 0 20.93959 0.03786938
1.864401 22.36689 0 0 0 0 0.0894489 0 0.1222147
5.183947 0.1546137 0 0 0.7960021 1.224229
0.056024373 0.4601013 0.02790318 0.032945132 0.06104554
0.4563174 0.3260739 0 0.1020671 0 0.3033733 0 0
0.1697879 0.1427807 0.1234304 0.03891103 0 0.03244783
0.03173858 0.214557545 0.215617407 0.129499992 0.1260992 0
0.1306761 0.1072428 0.08637878 0.0319911 0.031802037
0.1105569 0 0.02294139 0.09393621 0.4427173 0.02463086
0.05724501 0.1474527 0.1803584 0.8863989 0 0.1651093
0.03457556 0 1.432084 1.033569 0.1225895 0.03256871
0.07822899 0.10843448 2.07075 0.030502444 0.1060298 0.02703942
0 0 0 0.03068174 0.03333047 0.02656277 0.03208362 0
0.1659229 0 0.03796808 0.5225787 0.07163036 0 0.1182088
0 0 1.150132 0 0.1666456 0.1046825 2.690431
0.07539204 0.6020735 0 0.1870658 0 0.4258276 0.4426851

0.2333496 0.35259 0.6085214 0.06140362 0.05790259 0.1369362
0.1172767 0.2050743 0.04559195 0.07207279 3.671576
0.03189471 0 0.1210322 0.2709658 0.06816186 0.06967011
0.2907282 0.06468661 0.4999373 0.172365 0.09997923
0.5078842 0.02559794 0.123268 0.02706153 3.291671 0
0.05850568 0.1194078 0 0.03145767 0 0.9938887 0.1414145
0.7121075 5.602003 0.7413077 0.238546944 0.03497028 0
0.1439747 0.1949707 0.1256343 0.1514787 0.1183335
0.2586444 1.324926 0 0 0.03377113 14.55873 0.03286962
0.401235 0.2755778 0.1823216 0.5176584 0.3662731
1.177897 0.09590444 11.02421 0.05897882 1.360381
0.3910934 0 0.2141332 0.3132522 0 0.2985944 0.03335537
1.122864 0 0.1088568 0.02770065 0.0606649 1.414936 0
0.03696497 0.02435175 0.4668608 0.1241249 0 0.8983355
0.07362498 0.152562 0.1458301 2.392376 0.653478 0
0.1345714 0.2056628 0.3491054 0.3037615 0.05538751
0.1016215 0.9528681 0 0.590687934 0.177225 0.033369285
0.265792789 0.02267467 0.05520221 0 0.2941377 15.55317 0
1.265298 0.4642413 0 0.7431282 0.04994644 0.03793126
0.1329205 0.1823786 0 0 0.1939133 0.21336 0 0
0.07844544 0.09905826 0.1215939 0.2108066 0.1900756
0.2766976 0.06770877 0.1991758 0.1785951 0.2947395
0.05738309 0.09044391 0.03867418 0.1309521 0.032810315
0.1485877 0.1222439 0.957014 0.1773342 0.6063721
0.1961873 0.1494409 0 0.4659793 0 1.077593 0.9490911
0 0.1290405 0.574312 0.17357515 0.3965896 0.09478127
0.360519 0.1266739 0 0 0.03388078 0.08918077 0 0
0.09237886 0 0.09981781 1.276976 0 0.02575337 0.2043262
0 0.02606674 0.05395478 0 0.05365233 1.122789 0
0.1026329 0 0 0.1047445 0.02699187 0 0.2404095
0.2381835 2.055426779 0.438941636 0.05557316 0.1063713
0.04934703 1.552895 0 0.08225175 0 0.05274013 0.06931093
1.246495 0.1291994 0 0.1686629 0 0.05928321 0.06533533
0 2.045212 0 0.166215961 0.3879548 0.1156052 0.3209874
0.08639108 0.06253252 0.06509165 0 1.241377 0.09355353
0.3424009 0.06332693 0 1.860097 0.08551283 0 0.2121249
0 14.39253 0.139268223 0.2927397 3.316585 2.254562
0.2325107 0.1298644 0.1619464 0.07542962 0 0.04489021
0.5299618 0.5473793 0.215941 2.86528 0.2081365 0.421108
0.3169268 1.292762 0.07884959 0.09113729 0.06921262
0.3023968 0.1109579 0.7292498 0.3042305 6.28969 0.1204032
0.282233209 0 0.1179971 0 0.3144094 0.1091562 2.007129
0.287550289 0.1510343 0.2265365 0.2835394 0 0 0.04339735
2.528011 0.06236321 3.380349 0.3395224 0 1.440257

0.3067906 1.588338 0 0.2266766 0.1121192 0 0
0.1194149 0.07286435 0.04692351 0.04820491 0.3300237 0
0.07537586
DSCR4 0 0 0 0 0 0.03581697 0 0 0 0 0 0 0 0
0 0.0157208 0 0 0 0 0 0 0 0.01692507 0 0 0
0 0 0 0 0 0.006827717 0.02227765 0 0.01550572 0 0
0.03282888 1.158059796 0 0 0 0 0 0.6662085 0
0.2204828 0 0 0 0 7.269668 0 0.1792865 0 0
0.007251 0 0.239732018 0 0.057877796 0.007044737 0.04138533
0.4964291 0 0 0 0 0.296469 0 0 0.02798878 0 0
0 0 0 0 0 0 0 0 0.06705521 0 0.02183404 0
0.65424953 0 0 0 0 0.04179708 0 0 0.06856338 0 0
0 0 0.0061797 0.01629773 0.186661 0.03649337 0 0 0
0.006254953 0.01885986 2.266023 1.431224 0 0 0.5260683
0 0.9813739 0 0 0 0 1.346905 2.522553 0.013132
0.1631809 0 0.03881785 0 0 0 0 0.02415877 0.6765312
0 0 0 0 0.4188769 0.1089825 0.01856228 0 0.1564069
0 0.5659973 1.041484 0 0.2570984 0.006047344 0.5236318
0 0 0 0 0 0 3.331511 0 0.008375373 0 0.3076494
0 0.007814684 0.238704488 0 0 0 0 0 0 0
0.005839171 0 0.007546713 0 1.924457 0 0.1847471
0.02037138 0 0.908532 0 0 0 0.01976968 0.02587229
0.008739623 0 0 0.01166689 0 1.844059 0 0 0 0
0.006190164 0.004518856 1.208551 0.03603755 0 0.05985979
0.02318393 0 0 0.3881124 0 0.5454798 0.2444109
0.006006918 0.006637745 0.01809493 0 0.006565531 0.023404
0.06788048 0 1.264134 1.412089 0 0 0.01320127 0
0.126215989 0 0.0308396 0 1.811224 0 0.1208192 0 0
0.06580245 0.009225788 0 0 0.02970327 0 0 1.549469
1.25666 1.070124 0.01417572 0 0 0 0.006793036 0.0235541
0 0.08244349 0 0.006358444 0 1.42267 0 0 1.322284
0.005852679 0 0 0.8195223 0 0 0.04516793 0
0.008348755 0.6671252 0 0 0.07047979 0.0146269 0.03869113
0 0.05923358 0 1.575546 0 0 0 0 0 0.007571217 0
0.008114768 0 0 0 0 0 1.300204 0 0.5193831 0 0
0.5124259 0 0 0.0610311 0 0 0.006036386 0 0 0 0
0.08953908 0.6387121 0.007655314 0.431590325 0 0 0.6726717
0.2847343 0 0 0.7284136 0.01178565 0.2865403 0 0 0
0.02826788 0 0.1523498 0 0 1.447281 0 0.232148022
0.009632763 0 0 0 0 0.0145458 0 0.020298
0.006968691 1.115845 0.007075721 0.023701 0.04948442
0.8216975 0 0.01580093 0 0.006887039 0.678454571 0.01635437
0.02814476 0.1679397 0 1.574353 0.006031598 0.008427993
0.01184179 0 0 0.09174066 0.08042595 4.1537 0.1488369 0

0.007082249 1.475397 0.05286071 0 0.01546669 0.01689388 0
0.5410366 4.381274 0 0.006726517 0.007007738 0 0 0
0.7025994 0 0 0.102812528 0.005625183 0.9196565 0.00704017
0 0 0 0.08369268 0 0 0.03501777 0.03443926 1.634003
0.008569675 0 0.079968719 0.5382053 0 0 0.02059461
4.269634 0 0 0 0 0 0
AC106873.1 0.3165276 0 0.05823086 0 0 0.2243906 0.0978107
0 0 0 0.1086803 0 0 0 0 0 0 0 0 0 0
0.06304692 0 0.4544325 0.04335817 0.04975949 0 0.05005709
0 0.1436462 0 0.1418649 0.03055366 0 0.05153789 0 0
0.08528594 0.04896915 0.252109679 0.05112237 0 0 0
0.1303764 0.04658198 0.3314764 0.1020671 0.032229668 0 0
0.2090463 0.3735335 0.1903743 0 0 0.04647466 0.06489566
0.2221701 0.042911509 0.035936234 0.043166664 0.09457444
0.03703942 0.04355869 0.0643457 0 0.06398221 0 0.04422275
0.02703376 0.06882417 0.5323052 0 0.3202011 0.2289801
0.1720282 0.05153098 0.04791345 0 0 0.06915112 0.03390483
0.2278315 0 0.03064736 0.3256871 0 0.75904137 0.3927284
0 0.03534325 0.2433548 0.05343991 0 0.1616831 0.2454539
0 0.05312555 0.09625087 0.2377808 0.08296146 0 0.1139042
0.5225787 0.09550715 0 0.1182088 0 0.2531905 0
0.3812292 0 0.1744709 0.2242026 0.02513068 0 0
0.02338323 0 0 0.2043162 0.1458435 0 0.2434086 0
0.2316104 0.0684681 0.2931918 0.02929633 0 0.3243275
0.06441362 0.03189471 0.07076128 0.1210322 0 0.8861042
0.3483506 0.2491956 0.1293732 0.2999624 0.04924713
0.09997923 0.2688799 0.2047835 0.2054467 0.2164923 0
0.06183849 0.02925284 0 0.1147296 0.09437301 0 0
0.05656581 0.6371488 0 0.3177033 0 0.1398811
0.032369381 0.1439747 0 0 0 0 0.0431074 0 0.07838992
0 0.3377113 0.09662871 0 0.1755403 0.244958 0.0455804
0.2740545 0 0.1910103 0.03196815 0 0.05897882 0
0.07821868 0.1726095 0.04282664 0.1566261 0.054732
0.08143483 0.1334215 0 0 0 0.1385032 0.04044327
0.1257721 0.3870385 0.2957197 0.09740702 0.1037468 0
0.02583948 0.3593342 0.1227083 0.03814051 0.03645751
0.1344031 0.1485177 0.4858431 0.1009286 0.08814118
0.1396422 0.1822569 0 0 0.05015095 0.03316047
0.098447989 2.924212 0.033369285 0 0 0.1656066 0.05130316
0.06536394 0 0 0.09489737 0.1071326 0.1104234 0.2477094
0.07491966 0.1137938 0.3101479 0 0.3758043 0.07148234
0.2908699 0.04741334 0.03171778 0 0 0 0.0911954
0.1054033 0.2217549 0.2766976 0.1805567 0.1422684
0.107157 0.7073747 0.2295324 0.02261098 0 0.4714276

0.032810315 0.05943509 0.03056098 0.1450021 0 0.1616992
0.03269789 0.4109624 0 0.08223164 0 0.02628277 0.2945455
0.2885678 0 0.1325335 0.086787575 0.1101638 0.03159376
0.06008649 0.3040173 0 0 0.1694039 0 0 0.05833676
0.06928415 0.6753421 0 0.319244 0.3619066 0.07726012
0.07662234 0 0.02606674 0.08093217 0.1455103 0.160957
0.4248391 0 0.05131643 0.0270125 0.07855331 0.06982965
0.2429268 0.3444009 0.160273 0 0 0 0.02778658
0.02127426 0 0.07963566 0.06013035 0.04112588 0.04074514
0.05274013 0.1386219 0.06924972 0.09689953 0.06313538
2.023955 0.037789134 0.7706818 0 0 0.2272458 0.08770698
0 0.3017426 0.02890131 0 0.05183465 0.06253252 0.09763748
0.05165708 0.03027748 0.06236902 0.2568007 0.1899808
0.04242427 0.04428802 0.1710257 0.09811936 0.1060624
0.09473315 0.09245737 0.278536445 0.04878995 0 0 0
0.2597288 0 0.2640037 0.1059828 0 0.1472116 0.4105345
0.1439607 0.02448957 0.9158008 0.1579155 0 0.09234017
0.5519472 0.1215164 0.03460631 0.2267976 0.08876634
0.11668 0.1014102 0 0.150504 0.156796227 0 0.03933236
0.08845191 0.3930118 0.02728905 0.2548735 0.057510058 0
0.906146 0.09451313 0.07217225 0 0 0.09363005 0.03118161
0.2039866 0.05223421 0.5008699 1.384863 0.3067906 0
0.795234801 0.1133383 0.420447 0 0.046079879 0.03980496
0.1821609 0 0.1446147 0 0 0.07537586
LINC02884 0.07968163 0.7619376 0.3664712 0.2029779 0.278709
0.7531648 0 0.5411511 0.3280392 0.632271704 0.7523677 0
0.6152445 0.4233504 0 0.7674165 0.1059636 0.899921094
0.4263368 0.1701833 0.372443 0.2116164 0.9461242
1.207528 1.309781 0.271403 0.0420429 0.2100201
0.359287966 0.482147 0.1501172 0.4999765 0.05127653 0
0.3027262 0 0 0.04771024 0.08218222 0.023505656 0
0.07024256 0.055290002 0 0.05470089 0.03908799 0.03090548
0.08564678 0.135223196 0.02121395 0 1.077552 0.02849458
0.03993684 0.1035732 0.03265112 0.1559917 1.633662 0
0.144032048 0.874492171 0.036222118 1.190393 0.03108061
0.03655108 0.1979777 0.2174471 1.154312 0 0 0
0.03850127 0.5780426 0 0.06200491 0.04803558 0.2887054
0.1513428 0 0.052037162 0 1.421643 0.2276024 0.2731122
0.3357255 0.2057351 0.9018615 0.1312874 0.45494906
0.2696295 0.179167004 0.1186293 0.1134469 0.1345279 0
0.05426877 0.2059659 0 0 0.296143 1.596218 0.04640988
0 0.1592993 0.5207277 1.061884 0.03831603 0.4298306
0.070462555 0.03540965 0.03217005 0.04265307 0 0.08784147
0.3762669 0 0.0891553 0.0631273 0.1765925 0.02778704 0

0.1142975 0.6853313 0.1479331 0.1531872 0.1288129
0.07288105 0.05745312 1.451541 0.4916641 0.05738584
0.09071685 0.3243055 0.2141086 0.1187548 1.117169 0
0.02859808 0.233847 0.1394038 0.09046664 0.1398362
0.04132438 0.05592988 0.100277 0.107399 0 0.09083177 0
0.9859113 0.02454672 0.0500989 0.07220413 0.1319842
2.115683 0 0.07119846 0.5031961 0 0 0.560476293
0.6162314 0.488913762 0 0 0.1475916 0.2542184 0.04964815
0.3617239 0.03269926 0.2850412 0.061154491 0.1133525 0 0
0 0.05138745 0 0 0.2151436 0.05342702 0.1341259 0
0.09898091 0.07286337 0 1.303565 0 0.5038092 0
0.3872248 0 0.02692058 0.04060547 0 0.1394654 0.3224001
0.07915363 0.02706439 0 0 0.3046971 0 0 0 0.02059346
0.09601367 0.2447386 0.09022456 0.07477474 0 0.4516876
0.098615 0.08788265 0.1529358 0.02323845 0.1136972
0.1262484 0.7234681 0.110146562 0.1239279 0.112003669 0
0.0761073 0.6948213 0 0.02742418 0 0.06481136 0.02654351
0.05993159 0.1853175 0 0 0.03182897 0.07435773 0.8417087
0 0.1199649 0.433912 0.1392496 0.3726114 0.4578956
0.1316507 0.4156101 0.1785563 0.0884463 0.372159
0.6191551 0 0.3103897 0 0.296787 0.8185745 0.03794676
0.03245238 0.1318618 0.08259562 0.04987332 0.3846661 0 0
0.2374495 0.02743753 0.156749 0.4040441 0.02300081
0.08668529 0.286708 0.274622 0.3147868 0.07218717 0
0.024275135 0.03697636 0.1590662 0.05041993 0.4676976 0
0.253359543 1.876388 0.0187084 0.2742406 0.5384686
0.0193793 0.3606239 0.08375937 0.08929493 0.2102427
1.188563 0 0.6606336 0.1312391 0 0.2035016 1.080501
0.5602018 0.2389197 0.02153038 0 0.09887377 0.1464891 0
0.5539062 0.02241478 0 0 0.03683258 0.3264288 0.03570342
0.08281641 0.03341204 0 1.035289 0 0.8629808 0.1454009
0.1355877 0.1626211 0.1324458 0.1769111 0.063419424
0.04974588 0 1.338361 0.09534354 0 0.104606663 0.03617129
0.1212587 0.02693478 0.8699124 0.5247244 0.2184795
0.8669324 0.1524391 0 0.02394302 0.07970858 0.1067975 0
0.03587787 0.08233416 0.02966646 1.788586 0.1293051
0.023372619 0.02047037 0 0.2252207 0 0.1089721 0.2264881
0.1582367 0.08893252 0.03766839 0.2223514 0.1913826
0.4832026 0.02054975 0.2095825 0.3975314 0.02659404 0
0.03308223 0.02549178 0 0 0.05586437 0.04895439 0.1134607
0.09713779 0 0.13157124 0.04877101 0.1320187 0.04948133
0.065957 0.02289885 0.4812076 0.096515965 0.08449087
0.2217738 0.02643603 0 0.1150535 0.8011452 0.3142683
0.05233037 0.02445282 0.1314927 0 0.1859311 0.3217936

0.1870611 0.200189821 0.6181807 0.5644905 0.1024559
0.038666662 0.1002037 0.3057106 0.07874915 0 0.05035096
0.035772 0.3162479
AC022730.4 0.2946234 0.3932439 0.0790434 0.005863377 0.3703461
0.006216144 0.01896709 0.02942515 0.1516159 0.192255699
0.4056916 0.01205894 0.180094 0.09207889 0.1601073
0.004547321 0.2081445 0 0.007578766 0.01310946 0.064552066 0
0.4997573 0.01468698 0.2774593 0 0.0680111 0.1067758
0.272810786 0.01392767 0.0173456 0.07152579 0.01777456 0
0.01499106 0.02691065 0 0.01653834 0 0.043456168 0.03965385
3.576594 0 0 0.05056423 1.901448 0.04285246 0.05937743
0.00624986 0 0.05668487 0 0.03292466 0.06460421 0.1077083
0 0.009012196 0.1698884 0.04308241 0 0.027874494
0.092077941 0.03056588 0.007182549 0.03378697 0.02911459 0
0.1116648 0 0.02143878 0.03669602 0.01779484 0.03035961
2.70714 0.04776326 0.01110074 0.004765582 0.7244706
0.004645601 0.006012744 0 0.1609144 0.0131494 0.006311464
0.03232676 0.01188605 0.08841848 0 0.29438107 0.05538651 0
0 0 0.02072574 0.02119498 0.1191414 0.02974845 0.03231662
0 0 0.03073972 0.01608758 0.0282852 0 0.06966888
0.0324107 0.008854612 0 0.010855659 0.008182964 0.06319166
0.01478532 0.02154354 0.1217979 0.4130272 0.01461975 0
0.02917671 0.009068779 0.01926427 0 0 0.04525031 0.1025594
0.005900112 0.01786075 0.05614129 0.006638542 0.4264101
0.01136207 0.008841024 0.01397609 0.1124177 0 0 0
0.0700596 0.00660885 0.06079578 0.00805385 0.07526274
0.09048294 0.08117351 0.1551009 3.649815 0.009927717 0
0.04722907 1.135973 0.01798724 0.005672604 0.06367662
0.005561986 0.006100157 0 0.07709251 0 0.05814285 0.05172952
0.02053595 0.055509783 0.01356262 0.087877332 0 0.0151232
0.08283267 0.04895699 0 0.03343692 0 0.1013406 0
0.04584142 0.01873789 0.02549583 0.09725753 0.05343904
0.04419392 0.03542909 0.0426158 0.04321336 0.006199146
0.1649729 0.005718479 0.1627703 0.00758394 0 0.008304786
0.005062061 0 0 0.03234076 0.05599072 0.02815107 0.02110911
0.03760126 0.1176392 1.682859 0.04378097 0.02867244
0.1369439 0.04023642 0 0 0.01161346 0.004759029
0.007396068 1.94417 0 0.01728001 0.03925538 0 0.03988137
0.006769724 0.01767129 0.02685136 0.1510799 0.004862545
0.6108839 0.031817791 0 0.0258834 0.03221348 0.01319097
0.1177507 0.00497426 0.5513685 0.08576342 0 0.03067025
0.006924921 0 0.07205235 0.004842715 0.007355491 1.271589
0.1149401 0 0.06237718 0.006267159 0.01379133 0.01845179 0
0.106483 0 0.12379 0.143076 0.1965801 0.02682809

0.02188306 0.06069399 0.0346325 0.08001672 0.03338256
0.05700029 0.03749778 0.01523625 0 1.682714 1.333412
0.01124731 0 0.1254245 0 0.07244758 0.1027095
0.005315353 0.02670996 0 0.006346355 0.07834122 0 1.293588
0 0.008545022 0.03675927 0.02330351 0.01473848 0.006580771 0
0.006570038 0.01729361 0 0.01131245 0.004478443 0.1666762
0.0193563 0.03095331 0.005398431 0.08489801 0.02476387 0
0.1465882 0.073239 0.009405609 0.3537382 0.01765355
0.09662268 0 1.220493 0.007616385 0.006770555 0.005234165
0.03339248 2.465645 0.005131963 0.019929043 0.229818492
0.8782884 0 0.02870759 0 0 0.01594996 0.007901149 0
0.04032157 4.131549 1.509496 0 2.273102 0.007327931
0.9196788 0.1266959 0.2903515 2.159267 0 0 0.04179488
0 0.006224471 0.04020635 0.01212608 0.006311168 0.0200343
0.005871298 0.02418875 0.02766547 0.04298045 0 0.05152903
0.05803817 0 0.006855746 0.009185154 0.01195266 0.010802554
0.03784467 0 0.02081888 0.02254381 2.184611 0.01570203 0
0.0102759 0 0.03425609 0.1680642 0.167498 0.8453092
0.1937332 0.01020747 0.006145729 3.652879 0.0611609 0 0
0.1979089 1.575012 0.01131308 0.05244023 0.02993064 0
0.00608107 0.0112707 0.4118681 0.0228697 0.01524228 0
0.006178017 0.01115214 0.9420982 0.2123241 0.01221843
0.04198614 0 0.1935555 2.051673 0 0.01130181 0.0151936
0 2.191348 0.01487293 0.01080719 0.038552257 0.03846177
0.01630631 0.2367698 0.026806926 0.1775332 0.3814989 0 0
0 0.008266703 0.02923322
AC002451.1 0.5989746 0.719275 0.4612688 0.1596773 0.2009818
0.3526753 0.3013095 0.3839728 0.1075248 0.523569595
0.1913107 0.02736675 0.4947518 0.09142229 0.0908375
0.9184586 0.333435 0.398546071 0.5503798 0.3570097
0.130218166 1.082074 0.0177212 1.299904 0.6869131
0.07664298 0.04409869 1.575071 0.242264711 0.4425074
0.314915 0.7242046 0.1075676 0.1754872 0.1247434
0.03053572 0.6667476 0.5629853 0.3448029 0.246550213
0.4387064 0.5648588 0.014498385 0.08059416 0.2581903
0.05124911 0.2431251 0.04491734 0.042550578 0.1335075
0.1125613 0.4468396 0.3138228 0.2932275 0.4345507 0
0.02045241 0.9710083 0.05586964 0.264380975 0.173961695
0.075986572 0.4023267 0.6031069 0.09584583 0.5569019
0.1140399 0.2534137 0.125958022 0.4573429 0.01189693
0.1110557 0.7716639 0.8832273 0.162592 0.07557659
0.1838564 0.544262 0.2213987 0.532171039 0.0484405
0.6542837 0.0596829 0.12891 0.08803541 0.5799492 0.2006582
0.06885351 0.62035336 0.3613731 0 0.1088762 0.09519535

0.5761826	0.0481002	0.09961416	0.2430417	0.07333982	
0.01168966	0.04235776	0.6976119	0.4016035	0	0.4845571
0.9055254	0.2942133	0.04018958	0.2254241	0.073907991	
0.01857054	0.3374308	0.2236935	0.04889123	0.2917662	
0.3453322	0.4976742	0.1870295	0.06621406	0.6277152	
0.2477389	0.1297363	0.1348722	0.1283647	0.3620555	
0.0133898	0.1621338	0.1783711	0.1054592	1.096729	
0.2836379	0.08025582	0.01585878	0.3401632	0.01403612	
0.1245616	1.242814	0.02649905	0.07499112	0.1073107	
0.2924405	0.4839402	0.2493455	0.09752632	0.07333088	
0.223508	0.3041565	0.4520615	0.2858196	0.07365701	
0.0680342	0.02574699	0.1970573	0.02524491	0.193813	
0.3053854	0.1749549	0.1120198	0.989627	0	0.6990691
0.293941066	0.3231817	0.740740548	0.2280956	0.05148124	
0.1879817	1.088817	0.1692464	0.01897056	0.8746034	
0.7014508	0.449013508	3.180444	0.01417468	0.01446516	
0.3421124	0.1078003	0.02005887	0.1608066	0.3384953	
0.02801973	0.2110265	0	0.05191041	0.2165409	0.3098
0.6619495	0	0.2182704	0.01204314	0.4539426	0.1321103
0.0423554	0.1277729	0	1.243422	0.06229349	0.1106987
0.08516332	0.3253484	0.2143328	0.319596	0.341403	
0.02274271	0.02635577	0.1944038	0.05035424	4.267731	
0.1419545	0.1699339	0.4097991	0.2072761	0.310311	
0.4301728	0.1470462	0.01218738	0.1043496	0.1103513	
0.1605247	0.072207777	0.4679554	0.117480358	0.146211518	0
0.4494243	0.03386599	0.1294432	0	0.1019707	0.1809692
0.03143104	0.129586	0.2543593	0.08792105	0.100156	
0.4289649	0.2608468	0.3189524	0.06291543	0.3413469	
0.4903394	0.1116661	0.1847252	0.2416541	0.04359323	
0.428085	0.2551205	0.7110067	0.2232416	0.06952645	
0.3756542	1.257528	0.8041894	0.3282885	0.01990113	
0.03403921	0.1613611	0.490927883	0.013078	0.4303736	
0.1659113	0.03902033	0.1601101	0.07194789	0.2630618	
0.04238008	0.2653803	0.1970019	0.4857902	0.2448427	
0.3555775	0.1135754	0.5443659	0.050924253	0.1260493	
0.08342205	0.1850987	0.5686115	0.0149345	0.066437042	
0.2385623	0.3826522	0.2397085	0.02567265	0.01016345	
0.756515	0.4722205	0.1404918	0.1837692	0.29467	0.0112399
0.4186494	0.5162113	0.1662097	0.08538093	0.4013894	
0.06677206	0.03132528	0.04516633	0.1188757	0.0864237	
0.3994952	0.02375699	0.1136721	0.3996837	0.04658622	
0.663333321	0.057950396	0.09782582	0.05617383	0.08686592	
0.2803664	0.03969295	0.2171825	0.3765507	0.696291	
0.3050212	0.2945935	0.05685763	0.1944909	0.1484493	

0.315972232 0.2739362 0.08625767 0.2721659 0.1500084
0.0385978 0.31087803 0.3983697 0.03815637 0.02825182
0.3649796 0.399027 0.5442611 0.59106 0.09327089 0.08234146
0.5901737 0.1114748 0.04667483 0.5457235 0.0564483
0.4749805 0.1244683 0.4377435 0.09493945 0.612887001
0.7837031 0.8683409 0.3543501 0.1279032 0.07143787
0.1306596 0.08298705 0.1166014 0.05926541 0.323922
0.3211852 0.6652144 0.1293275 0.2198305 0.3938046 0
0.2031838 0.08674967 0.05347653 0.1218354 0.1330778
0.625024 0.0385111 0.1190087 0 0.05298664 0.069002366
0.02557789 0.03461851 0.1427273 0.2421374 0.7565843
0.7010242 1.075625555 0.4320335 0.3655426 0.4159303
0.08469687 0.04022645 0.1527853 0.206022 0 0.051297
0.1149353 0.05086643 0.5363123 0.2193935 0.2207339
0.437455386 0.4364286 0.1850289 0.1880651 0.223065491
0.3853792 2.324778 0.08259978 0.06364157 0 0.2063664
0.6965944
AL356215.1 0.01196756 0.1258808 0.1100822 0.01143214 0
0.2666388 0 0.01434294 0.01847585 0.134946397 0.06163623
0.07053588 0.07392394 0.03366208 0 0.11526 0.02387237
0.162193357 0 0.02556021 0.083907051 0.1549428 0.09135031
0.08590796 0.04917974 0.0658473 0.03788709 0.2081864 0
0.05431104 0 0.1501851 0.11552 0.1507687 0.4968907 0
0.1120758 0.1182341 0.1296029 0.275368562 0.2029524
0.8545407 0.024912381 0.0153871 0.03697041 0.8277703
0.2506551 0.29586 0.243713635 0.2867552 0.06907593 0.1806585
0.1412289 0.1979407 0.2100045 0.1029828 0.1230009
0.1840225 0.5280003 0.421833565 0.122283842 0.114245874
0.08343429 0.0140042 0.1317525 0.08109466 0.4408946
0.06047742 0.048095997 0.3427627 0.0919905 0.04336943
0.4025174 0.4575231 0.2141911 0 0.1207922 1.110548
0.01811555 0.105510276 1.331755 0.5098329 0.07691424
0.07383477 0.02521168 0.1853989 0.3078468 0.01478876
5.78070118 0.10799 0.023065275 0.3340723 0.1737962 0.4546134
0.09642493 0.07335672 0.03480128 0.06300941 0.07030164
0.1091742 0.1348534 0.02091121 0.6617899 0.2727509
4.149206 0.0541653 0.1035858 0.02979563 0 0.1435928
0.03623768 0.02882771 0.02100229 0.8443586 0.04238426
0.5605964 0.01339043 0.03792491 0.06188657 0.8012922
0.111462 0.01287495 0.121312 0.06665519 0.05751881
0.1625122 0.06567699 0 0.1441084 0.03322985 0.2327105
0.1362496 0 0.1326494 0.1337703 0.1067755 0.09106594
0.3865687 0.03951225 1.067805 0.4809934 0.2520277
1.936459 0.3024083 2.055802 0 0.7767712 0.153475

0.07382892 0.2104241 0.02212035 0.2708807 0.2711124
0.1903008 0.2011502 0.1127335 0.04277381 0.26924 0.2017196
0 0.180383933 0.2247719 0.354916667 0.1197575 0.01474326
0.3895078 1.374538 0.3243693 0.3585655 0.07366761
0.2074685 0.123996523 0.1532219 0.2557397 0.3355463
0.06636985 0.02315398 0.2068014 0.126643 0.04154514
0.01203648 0.03626041 0.3618635 0.2787405 0.06566106
0.3548834 0.1398466 0.04857684 0.07895814 0.09312104
0.3900017 0.466618 0.06064893 0.05488762 0.2469453
0.04189323 0.06116461 0.02377652 0.04877833 0.3633772
0.00920713 0.1863213 0.4458375 1.709684 0.1132169
0.04639465 0.08652303 0.1240577 0.07114288 0.4716845
0.4592297 0.5215196 0.2554933 0.01319929 0.1378186
0.2198848 0.06403661 0.02844228 0.1128385 0.173703124
0.3126984 0.706527262 0.012561665 0.1886069 1.095746 0
0.01235669 0.05573916 0 0.2631174 0.2430339 0.05566649
0.1248749 0.03776839 0.1290725 0.08375963 0.103433
0.4161133 0.06756671 0.02443882 0.3137131 0.05996071
0.1428349 0.1186374 0.1373271 0.05746663 0 0.02395516
0.3661571 0.5290654 0.3012247 0.405149 0.08915019
0.1084796 0.05984272 0 0.3960924 0.161267835 0.03370763
0.04621907 0.07675318 0.2849545 0.1069892 0.1359898
0.4378901 0.2730792 0.2590906 0.07811672 0.1589956
0.06186912 0.3491338 0 0.1336252 0.109378081 0.3082226
0.05972625 0.0567951 0.1341032 0.1283088 0.156967089
0.2818189 0.1685913 0.0274592 0.02205649 0.08731857
0.9981449 0.2830501 0.04023421 0.04210243 0.07789653
0.0772535 0.198444 0.06898882 0.2039971 0.01833861
0.1419975 0.1491536 0.02691291 0.1358152 0.08170502
0.222751 0.29042 0.0714373 0.1519165 0.1211949 4.762885
0.181331308 0.829794938 0.1155638 0.008043562 0.09328785
0.1505468 0.1932443 0.0777462 0.6932379 0.04985115
3.223301 0.07854766 0.1953954 0.4058035 0.1434816
0.171451854 0.1793146 0 0.08614773 0.1718383 0.2542347
0.015711105 0.3096608 0.05463632 0.08495326 0.07185973
0.6028928 1.242827 0.3710885 0.2632944 0.2122294
0.04315267 0.09577285 0.05614052 0.05023443 0.2263202
2.028016 1.243132 0.1969966 0.06991422 0.294872355 0
0.1269837 1.075679 0.0659323 0.06137537 0.1428704
0.1853742 0 0.03394499 0.07792272 0.4656556 0.4898696
2.879622 0.07869413 0 0.2156877 0.4538664 0.2384973
0.04594401 0.02616853 0.2858322 0.3188349 0.3418944
0.1533682 0.2042507 0 0.035569754 0.06592522 0.02974226
0.3232794 0.3863429 0.9182742 8.588517 0.032615877

0.3235917 0.1284764 0.01191146 0.1546293 0.120961
0.1312645 0.1770024 0.1061048 0.03305361 0.1283697
0.07283592 0.2932167 0.02899854 0.03160701 0.075167375
0.03213897 0.264944 0 0.087111467 0.2106974 21.10269
0.3016015 0.07290297 0.01134348 0.09670817 1.614931
LINC02528 0 0.02424548 0.06996849 0.02422097 0.09977343 0 0
0 0.1174329 0.254139889 0 0 0.07831036 0 0.1653464
0.03756897 0.05057776 0.038181643 0 0 0 0 0 0 0 0
0.08027039 0 0.146993656 0 0.07165278 0 0.5629232 0 0
0.2779124 0 0 0.0196133 0.224390814 0.04095142 0
0.36946844 0 0 0 0.05900627 0.2725351 0.516349655
0.04050272 0.02926987 0.5023677 0.217613 0.1906235
0.444931 0 0.670111 0.07797672 0.0508482 0.034374141
0.086359851 0.760727699 0.5050572 0.1186813 0.06978513 0 0
0.2818902 0.101899722 0.1771225 0.9528339 0.07350854
0.07524733 0.02364249 0.03946095 0.09171191 0 0.02063937
0.1343333 0.049675965 1.410777 0.02769665 2.308546
0.02607197 0 0.07364995 0.1565344 0.5326534 0.78175017
0.2287956 0.073301684 1.58545 1.516189 0.363867 0.9047259
0.3626442 1.450074 0.05339855 0.1276682 0.05140099
0.158728 0.04430402 0.4673723 0.06082844 0.1308157
0.1912643 0.2194646 0.06312722 0.22421767 0.5408468
0.03071033 0.1017942 1.201419 0.2515669 1.077581
0.04026172 0.1418498 0.06026288 0.03746213 0.0530524
0.07871723 0.2727782 0.2803861 0.1882941 0.3655907
0.0245936 0.5565924 0.02742309 0.1174302 0.07040321
0.2556491 0.0577337 0.05159834 0.0766475 0.453465
0.06463498 0.385878 0.1365022 0.08371357 0.03326957
0.4836255 0.2936804 0.05917391 0.05339205 0.3111123 0
0.131658 0.1300654 0 0 0.4452252 0 0.0459519
1.259954 0.1111752 0.1592303 0.06796781 0.2702045 0
0.5089906 0.305739748 0.2521156 0.0259294 0.2075949 0
0.1610224 0.5662607 0.04739536 0.2762485 0 0.1046567
0.350277553 0.05410453 0 0 0.08035203 0.02452787 0
0.04878456 0.1760412 0 0.05121598 0.2555569 0.7795394
0.2318573 0.1879705 0.07901049 0 0.1254649 0.2849789
0.08697751 0 0.05139811 0 0.8283946 0.4437904 0.9557104
0 0.2066907 0.02961068 0.03901381 0 0 0.06209593
0.04797394 0.09829512 0.03055234 1.460209 0 0.1427636
0.06486386 0.2425456 0.305956 0.02796499 0.26766 0.532416
0.05426907 0.3012996 0.3187572 0.026287159 0.07098277
0.721719976 0.026614069 0.1271443 0.2653174 0 0.0523596 0
0 0.02533909 0.02860609 0.02948478 0.1653557 0 0 0
0.2191407 0.2150264 0.629868 0.05177789 0.1519213

0.7114082 0.1008735 0.9425774 0.3174013 0.4139603
1.562011 0 0.0738826 0.2711896 0.3874767 0 0.3777603
0.1378997 0.7063858 0.2788186 0.04195952 0.262826087
0.07141546 0.04896157 0.2090764 0 1.003847 0.2357329
0.1197092 0.1157132 0.02195714 0.05516795 0.1052687
0.02621609 0.6010067 0.206735 0.03538853 0.046347291
0.05294782 0.02530809 0.09626422 0.02029435 0 0.181397474
0.4342417 0.5179255 0.02908854 1.051436 0.1479996
1.229502 0.03997938 0.04262158 0.2453036 0.061889
0.08183747 0 0.02088068 0.02161016 0.2719748 0.1719122
0.4618576 1.368472 0.06166031 0.04327657 0 0.1678105
0.1945958 0 0.0213977 0.02119958 0.027441562 0.879032334
0.1335502 0.06816673 0.454587 0.1594798 0.7465923
0.3294376 0.1631939 0.02112366 0.1110426 0.07396305 0
0.5310314 0.3039907 0.060541754 0 0.2093467 0.4432602 0
0.1405149 0.066573408 0.2071801 0.06945392 0.05142521
0.2768133 0.300549 0.3128489 0.08275951 0.04850738 0
0.0228566 0.6594621 0.1019515 0 0.4452487 0 0.02832033
0.1138286 0.02468757 0 0.03908305 0.1008889 0.04300026
0.3725041 0.1560413 0.04324224 0.09063402 0.6791775
0.03595918 0.2358469 0.4384766 0.4036176 0.2746422
0.6002178 0.2529955 0.07616199 0.5917506 0.1579056 0 0
0.696423 0 0.07009962 0.1895467 0 0 0.125601166
0.3491851 0 0.0472361 0.1259284 0.02185981 0.02552071
0.345511997 0.4637782 0.4839104 0.02523649 0.2119824
0.1830549 0.1390533 0 0 0.02334327 0.1046052 0.2160418
2.484922 0.1843153 0.1116082 0.73257363 0.068092
0.06735958 0 0 0.1275426 0.3210228 0.07517589 0.0193072
0.168232 0.1365954 0.04025308
AC007552.2 0.02409389 0.3225494 0.04432498 0 0.06320638
0.7808219 0 0.02887617 0 0.060374028 0.1654536 0.1420076
0.03720716 0.0451805 0 0.4640982 0.04806145 0.126987207
0.08924846 0 0.084463649 0.3839275 0 1.008908 0.1815219
0 0.03813842 0.5905989 0.093120359 0 0 0 0.02325727
0.4553064 0.2157669 0 0 0.1298383 0.2422876
0.021322709 0.2140272 0 0.150465825 0 0.07443131 0.3013924
0.4205298 0.1294881 0.122665154 0.07697532 0.02781366
0.3409817 0.2067865 0.4347354 0.5637268 0.4442827
0.03537623 0.3457873 0 0 0.328253366 0.164291034 0.575916
0.05638837 0.3978794 0.3102039 0.263004 0.3409201 0
0.1346484 0.04115587 0.1397028 0.5481949 0.089865
0.03749772 0.08714913 0 0 0.2005929 0.188818095 0.209467
0.4737368 0.3096978 0.3468479 0 0.3032717 0.2479111
0.1786423 0.33015872 0.2989425 0.046436556 0.1076123

0.02058225 0.2440688 0 0.2461444 0.4670951 0.2283386
0.02021943 0.2197968 1.779807 0.02104992 0 0.08670322
0.2734764 0.4725466 0.06951531 0.3299261 0.063918769
0.09636356 0.4231455 0.07738384 0.1691328 0.1328062
0.7679775 0.01912932 0.05391703 0.03817649 0.1601925
0.2772714 0.2244029 0.4924935 0.1998274 0.08946313
0.06948043 0.1635902 0.3085257 0.02605875 0.4017165
0.02230019 0.1214651 0.05486138 0.04903126 0.07283419 0
0.1842579 0.09167003 0.129711 0.1590974 0.4426012
0.1641302 0.1268498 0.4123529 0.05073573 0.8869023
0.136395 0.1251078 0.2883871 0.1911051 0.07060663
0.1336025 0.340847 0.04366574 0.119727 0.7923309
0.1513084 0.08611511 0.7702843 0 0.6448903 0.980534751
0.1863342 0.172475658 0.1534297 0.05936422 0.05737926
0.1921745 0.1576308 0.09843929 0.1186501 0.03977996
0.027737567 0.1799447 0.367766 0 0.2290632 0.2563833
0.2775642 0.02317874 0.1951634 0.04846531 0.1216698 0
0.269366 0 0.08930939 1.182504 0 0.2185753 0.1666467
0 0.1269495 0.07326149 0.07366896 0.08286115 0.2741124
0.1077481 0.1914739 0.2700605 0 0.1668277 0.07897148
0.02362079 0.05900658 0.273523 0.03736193 0.02903233
0.08325373 0.163691 0.565254 0.1849104 0.3585223
0.1118209 0.1328685 0.5549314 0.04216064 0.5930448
0.381746 0.3281402 0.099917362 0.382224 0.254005004
0.075869957 0.1208188 0.3361568 0.07810331 0.2985279
0.0561089 0 0.2407844 0.1902803 0 0.03142582 0.1140568
0.1154922 0.2023566 0.2082382 0.04086572 0.2176477
0.4674178 0.2165445 0.2414339 0 0.0597122 0.5529521
0.02313913 0.4813943 0.1929126 0.1755171 0.03435967
0.2382466 0.0543782 0.3140955 0.655195 0 0.02943856
0.4186558 0.19980013 0.1357249 0.3256797 0.0441499 0
0.1538556 0.07466831 0 0.3298689 0.6259423 0.1572698
0.3801194 0.1494709 0.4832434 0.1964497 0.5716745 0
0.0335424 0.04809796 0.3201624 0.790672 0 0.632033321
0.6963259 3.920293 0.08292405 0.2442308 0.1054774
0.4205998 0.03799036 0.1215033 0.1907177 0.2744464
0.4471543 0.04994011 0.01984184 0.02053503 0.03692052
0.1633593 0.04619785 0 0.156247 0 0.1494858 0.07973087
0.0410921 0.2403096 0.04066628 0.04028974 0.02607631
0.033411975 0.1692078 0.09716303 0.03756267 0.1515455
0.160198 0.09391431 0.03101496 0.3412365 0.07913859
0.6852621 0 0.1201457 0.417252 0.057529726 0.1128151
0.09946573 0 0.08648908 0.4005722 0.094891948 0.1640605
0.0659985 0.4886674 0.2630415 0.309396 0.9661739

0.07864212 0.06914111 0 0.6950227 0.1446122 0.1614655
0.1348471 0.06509185 0.04979191 0.02691136 1.514317
0.140756 0.678464894 0.4085248 0.03195652 0.04086094 0
0.222417 0.04109088 0.1148332 0.2420203 0.06834033
0.1120566 0.8680455 0.3835371 0 0.1901188 0 0.02412428
0.175722 0.7802579 0.02312439 0.1843948 0.1150913
0.1351368 0.02220402 0.3345022 0.1468612 0 0.143222822
0.287571 0.08981867 0.06732907 0 0.1038613 0.2425102
0.065664469 0.05748322 0.4023559 0.04796189 0.3296225
0.2435268 0.165169 0 0.0474705 0.04436382 0.3379631
0.3226039 0.5903235 0.291909 0.2333223 0.030266399
0.6039071 0.7467641 0.09294097 0.350757284 0.1514965
0.3605154 0.1428716 0 0.04567492 0.5516482 0.2295026
AC010976.1 0.09228704 0.07311928 0.2498167 0.0503761 0.07262998
0.1602208 0.08147933 0.06636279 0.04070722 0.072678935
0.2353885 0.07252437 0.08550894 0.04449992 0.1289612
0.1484622 0.06837636 0.077426968 0.01302282 0.02815798
0.036973924 0.07615405 0.04360831 0.1924328 0.07946112
0.2549248 0.05843276 0.1605415 0.040763395 0.05983085
0.06706232 0.07327025 0.2061624 0.2325286 0.2082234
0.2543277 0.03292438 0.1326189 0.2957479 0.207681639
0.6813841 0.2580107 0.134477125 0.2288377 0.4480062
0.2270047 0.5860094 0.1133667 0.300701048 0.2716733
0.05478938 0.6468131 0.6279874 0.1665171 0.3341693
0.1296564 0.2206745 0.2594884 0.1480596 0.100090511
0.248468734 0.140240735 0.1444363 0.06788096 0.1741715
0.192967 0.08634742 0.1758872 0.042387325 0.5194287
0.1080958 0.1586204 0.6755735 0.2630753 0.3570178
0.06676161 0.2640902 0.5473179 0.3472465 0.170476011
0.0962788 0.3398696 0.2739645 0.238594 0.05554805
0.2067946 0.3445604 0.127076 0.49681098 0.3836634
0.215980551 0.379802 0.05405921 0.398428 0.05159496
0.1373808 0.1942471 0.1693684 0.2279144 0.2619211
0.561223 0.3271181 0.1397346 0.5218716 0.2802399
0.3261976 0.09889847 0.07549489 0.088604638 0.1933392
0.2954127 0.9506099 0.02313682 0.6278672 0.7003788
0.1905052 0.2478226 0.1963629 0.2941321 0.2758532
0.3110688 0.7091737 0.6439084 0.0832202 0.385257
0.3989786 0.2122324 0.01996261 0.1831784 0.05369043
0.2202811 0.4262759 0.1878049 0.2975759 0.0353678
0.2823059 0.1881024 0.4797989 0.2814812 0.6400619
0.5424518 0.422016 0.2522999 0.5247004 0.3633538
0.2111063 0.6126935 0.371961 0.246321 0.1777211
0.1559585 0.08952338 0.1242453 0.2070212 0.237009

0.4388082	0.2144008	0.5120317	0.4962936	0.6439978
0.063589428	0.3350102	0.183359914	0.2062888	0.08445665
0.07535324	0.1009491	0.401695	0.5530109	0.3343574
0.1523695	0.042497376	0.2869504	0.09122732	0.2300038
0.2778379	0.2499706	0.2695861	0.3906395	0.546159
0.3474065	0.3089132	0.08563398	0.3586576	0.2387036
0.4170149	0.158168	0.2247582	0.3457571	0.1572974
0.1989907	0.6279648	0.1015556	0.0524039	0.1496238
0.3968985	0.173506	0.5972005	0.2337509	0.08929957
0.2150291	0.4105155	0.1111549	0.2303184	0.1596461
0.4886106	0.1493294	0.2368878	0.2328809	0.5542649
0.4856667	0.1737583	0.323067	0.447856	0.4200508
0.1361115	0.08747563	0.3571958	0.1657422	0.125748992
0.1599367	0.4447623	0.044282719	0.1586651	0.6276972
0.06410567	0.4709941	0.09824659	0.05468973	0.1739154
0.08924477	0.3280837	0.2029104	0.2350791	0.3286182
0.4798165	0.1063489	0.0961531	0.6669264	0.492684
0.7761116	0.2166595	0.1573517	0.08495171	0.2475558
0.2025829	0.3292658	0.3193167	0.226656	0.4023449
0.3484312	0.4879827	0.9133607	0.3632943	0.2994867
0.1224238	0.6239785	0.087462416	0.2846898	0.3869652
0.2778199	0.04801058	0.4748191	0.2396975	0.4823944
0.3609999	0.4795105	0.04589653	0.256164	0.5425304
0.1610585	0.09316206	0.3606548	0.187971492	0.4349889
0.5421626	0.4254582	0.1983837	0.1300413	0.031440034
0.2765926	0.2823033	0.04839995	0.1287803	0.3078178
0.5370089	0.268163	0.6205268	0.2666933	0.1566095
0.6021181	0.1065736	0.1150863	0.2292246	0.07676923
0.2279396	0.3943502	0.1067334	0.2735883	0.1440144
0.1897683	0.2675832	0.191123	0.6096552	0.1335124
0.2755753	0.379545186	0.610638716	0.2013796	0.2622873
0.06988292	0.06633896	0.1527758	0.03083321	0.5634365
0.1164255	0.2915776	0.4288078	0.2098728	0.268228
0.8746214	0.308499243	0.1382773	0.1687217	0.04067281
0.3123496	0.2021399	0.128078297	0.2334066	0.2600173
0.106957	0.1698408	0.1119968	0.1518254	0.09036708
0.4085976	0.6156705	0.3208843	0.6646914	0.1979081
0.1660198	0.3348037	0.2315866	0.6921007	0.2604216
0.4672537	0.299317994	0.333277	0.2308177	0.2683029
0.4406416	0.1974304	0.07195006	0.3330266	0.2251316
0.1832352	0.4733588	0.4103854	0.2518398	0.5140943
0.776761	0.03069465	0.4012949	0.2846122	0.5648836
0.2252299	0.1729687	0.2330129	0.4640055	0.2891641
0.5519216	0.1767931	0.1880617	0.313478628	0.07020458

0.1539958 0.1768396 0.2815562 0.1773144 0.6502222
0.126955279 0.2600203 1.097674 0.3096806 0.316641
0.05330187 0.07230266 0.1793925 0.1064985 0.1893474
0.4721129 0.4493355 1.255153 0.3034847 0.3458721
0.6657674 0.6089723 0.3245607 0.05594169 0.118996478
0.1226874 0.7647979 0.3048921 0.2971557 0.1074687
0.0568197 0.1088367
AC079414.3 0.3198962 0.7392426 0.3555555 0.3565149 0.3496647
0.9989058 0.08237635 0.750807 0.2880876 0.300596195
0.4118883 0.6808546 0.7204193 0.3624181 0.6084446 1.0961
0.2392931 0.321146153 0.3620704 0.3700837 0.218055695
1.380554 0.2373991 0.9887034 0.7120684 0.4714598
0.3586761 0.7799262 0.33484885 0.7258745 0.2636691
1.493486 0.5275132 1.427323 0.8898088 2.016114
0.1386952 1.268961 0.7114228 0.802125107 0.6996494
0.7755036 0.263591278 0.6255208 0.6588195 0.3530829
0.7599629 0.5444202 1.302906506 0.2448557 0.6000861
0.8551445 1.630151 1.242585 0.883603 1.032285
1.50693 0.4509058 0.4811453 0.126490531 0.605311253
0.363550435 0.1327513 0.07798667 0.2384539 0.4877286
0.600174 0.8891179 0.241053609 0.7448896 0.6033489
1.149616 0.7120192 0.5095716 0.3422785 0.8437075
0.3415086 0.6835418 1.089526 0.23502656 0.3244625
0.4659136 0.5996488 0.7401089 0.5756358 0.4387915
0.6994504 0.4447211 0.50228046 1.217786 0.38533821
0.8036861 1.104473 0.7876258 0.874497 0.8851038
1.13697 0.3789583 1.442944 0.7430744 1.151492 0.1979658
0.3378265 1.726746 0.508885 0.7842539 0.2884248
0.2654818 0.436113653 2.665469 0.7749146 1.29499 0.4444392
1.557561 0.8025013 0.3280591 1.521202 0.3801538
0.324941 0.2649456 0.2482843 1.190189 0.6632803
0.5691579 1.422181 0.9308563 0.3413597 0.2306558
0.555585 0.2590709 0.5471661 2.959117 0.596742
0.2820487 0.2681788 0.5436454 0.6339108 0.7175756
0.7187847 1.399153 0.9897051 1.263144 0.3110702
0.3508445 0.5661279 2.026511 0.7267158 1.014212
0.6108351 0.5989256 0.7267855 0.3394086 0.2536419
0.7020833 0.4772885 0.3766747 0.6431376 1.041651
0.5242241 0.891901 0.160723739 0.9571912 0.408923284
1.139804 0.2134661 0.78299 0.4677773 0.8097432 0.7079501
1.050216 0.5061544 0.337584009 0.6826105 0.4069044
0.2076214 0.7286418 0.6833824 1.45874 0.333391 0.4627138
1.018839 0.2826981 0.2239054 0.5712288 1.340731
0.4117245 0.6126393 0.8836825 0.5166507 0.8181932

0.5486765 0.1685517 0.3647616 0.2649041 0.6188359
0.5249145 0.5960744 0.4766646 0.9099844 0.5915073
8.511273 1.343403 0.2221438 0.7725531 1.160089
0.4340496 0.5139521 0.3223982 0.5093756 0.5378522
1.824252 0.3400088 0.4206518 0.4998292 1.560554
0.9329492 0.142643 1.67893 0.586484 0.635666923 0.5472837
0.983628551 0.209860476 1.546828 0.8833367 1.188209
0.9633686 0.5587211 0.2601968 0.5727796 0.4060227
0.6664913 0.6954044 0.07361372 0.9264275 1.306035
0.364799 0.9042946 0.9180891 2.926045 1.23788 0.5075427
0.9014759 0.5615686 1.056743 0.4992321 1.264985
1.667521 0.6796861 0.760326 0.5152206 0.2857849
1.588673 0.8699066 0.9807148 0.8142867 0.4411524
0.621740427 0.5130775 0.7850246 0.7815748 0.6347426
1.310765 0.9087613 0.4877051 0.4258032 0.4732469
0.4060159 0.4980464 0.4547891 0.8141583 0.3260343
1.692903 0.536012803 0.6216269 1.210678 0.7084694
0.6721154 0.4430067 0.524471687 0.8988354 0.8074132
0.397579 0.7615356 1.040597 0.9307217 0.4938921
1.971698 0.4454746 0.2494297 0.5377623 0.4696661
0.5488363 0.6361714 0.5923204 0.4518609 0.7283791
0.539542 1.123689 0.5687494 0.2977098 0.5292959
1.193462 0.3021407 0.5511774 0.2117429 0.50489956
0.443612942 0.4797392 0.2687583 0.3428713 0.3688811
1.126782 0.1904996 0.7549439 0.4441783 0.4523969
0.6901465 0.3808418 0.6114865 1.100874 0.302347741
0.1872316 0.8804086 0.09594802 1.387555 0.2216009
0.174984234 0.7805366 0.5111554 1.081345 0.6184492
0.4476525 1.192342 0.4350569 0.9434907 0.2363729
1.76627 1.146682 0.3930278 1.174933 1.476391 0.5233632
0.5359558 0.7579521 0.4931625 0.22285471 4.961736
0.7601839 0.7007464 0.7098509 0.6699038 0.2500512
0.3017531 1.361198 0.302453 0.5083258 0.3457529
0.3334209 0.3403151 1.367285 0.3768241 0.9475529
0.3110762 1.842802 1.368815 0.4663279 0.5093583
0.6447979 3.402532 2.092492 0.5524675 3.650536
0.396162211 0.8688622 0.5962641 0.819438 0.9598862
0.4826406 0.6305488 0.290610458 0.4770052 1.717105
1.379718 0.4254852 0.5003948 0.3837679 0.6308432
0.7484444 1.092144 0.7258638 1.281725 2.822528
1.001221 0.3402925 1.473446059 0.6204493 0.2124608
0.1028319 0.426894141 0.536381 0.6750313 1.817877 1.1672
0.6822325 0.1615647 0.4337916
LINC01537 0.02965024 0.7655141 0.2018234 0.03965318 0.2333476

1.213122	0.01832453	0.1208203	0.1739444	0.11887523	
0.1068948	0.05825208	0.1465203	0.1111994	0.2126895	
0.571125	0.08280302	0.227710646	0.1610844	0.1646495	
0.097012534	0.939026	0.2187809	1.255764	0.398028	
0.2050903	0.09386721	0.8065116	0.171892581	0.1076467	
0.1005479	0.6059773	0.05724135	0.2427991	0.06758827	
0.01299949	0.01234104	0.1810847	0.1376133	0.036735982	
0.07662095	0.0209103	0.067893865	0.02668562	0.2137242	
0.05236195	0.08970158	0.03186993	0.102648204	0.01894535	
0.07530123	0.2573646	0.05725665	0.09808162	0.4162375	
0.01457973	0.008706885	0.5714261	0.005946126	0.040196699	
0.403952611	0.056609996	0.2480552	0.08327066	0.01632118	
0.1928795	0.1375538	0.1678162	0.017874043	0.01242749	
0.02532346	0.292264	0.5162274	0.3041197	0.09229031	
0.08579743	0.2532275	0.1399855	0.03590576	0.005809047	
0.02062181	0.2202393	0.03811179	0.31098	0.1624044	0.07464197
0.1647445	0.03663991	0.40629736	0.2140406	0.034287237	
0.0529716	0.03039452	0.05506492	0.07508213	0.0545235	
0.132207	0.03122181	0.004976455	0.1262261	0.2004638	
0.05698944	0.04099046	0.05690563	0.3304244	0.2460284	
0.02566392	0.1476404	0.015731844	0.06324596	0.07182456	
0.1047525	0.06244108	0.3464778	0.3990348	0.07062234	
0.04644567	0.03288632	0.02628465	0.01240776	0.04295712	
0.09569503	0.07104068	0.01100944	0.06270254	0.1150378	
0.01627181	0.01924093	0.3460501	0.08781723	0.02562454	
0.006751308	0.07240617	0.1673105	0.05302764	0.1209334	
0.09588872	0.08938941	0.03915745	0.03890503	0.04443574	
0.05619703	0.0276789	0.0187308	0.05597092	0.07673112	
0.007697962	0.3701024	0.01045228	0.2374977	0.02192172	
0.1454095	0.01612068	0.08250897	0.3206838	0.2234424	
0.1430653	0.3440603	0.03331803	0.05952074	0.125134911	
0.2489599	0.103093176	0.07013036	0.0657489	0.07531906	
0.01891938	0.07759297	0.05653228	0.05840489	0.8175269	
0.040961028	0.3289997	0.01206873	0.07389628	0.06577388	
0.02868259	0.1537083	0.01711442	0.08234417	0.0357852	
0.07785874	0.139461	0.08287126	0.0271131	0.1831754	
0.1201121	0.03209376	0.07335856	0.01025387	0.09153939	
0.02499611	0.0360626	0.1178553	0.05098497	0.06227557	
0.2197308	0.1060336	0.01812762	0.1592813	0.1596779	
0.2623948	0.0930177	0.01452284	0.04488011	0.02298902	
0.05716406	0.1502645	0.04028803	0.01669461	0.0530957	
0.2773269	0.1210952	0.07848459	0.04552702	0.04150672	
0.01903847	0.02348907	0.2609255	0.012295953	0.1106752	
0.068767967	0.049795465	0.0424803	0.1499585	0.01922298	

0.03061434 0 0.05064545 0.04740995 0.08697418 0.09654159
0.0309384 0.03742925 0 0.199218 0.2050084 0.02011594
0.013392 0.0847678 0.07106195 0.06536452 0.1258242
0.07348256 0.3093044 0.07973084 0.03949398 0.842772
0.1209564 0.08033852 0.04264569 0.05353478 0.07730595
0.3117658 0.04236097 0.01449098 0.1422942 0.049175297
0.01113498 0.2633733 0.1847268 0.04152878 0.0833081
0.1837755 0.06999323 0.02706271 0.04108226 0.07741527
0.05416396 0.04905085 0.1784056 0 0.1489785 0.005419796
0.03714992 0.07102792 0.09568467 0.2420661 0.07629397
0.176769987 0.2602459 0.02088468 0.1972915 0.04371685
0.06057414 0.299055 0.03272598 0.04984115 0.2242685
0.2171167 0.04306488 0.4855096 0.2392926 0.1263533
0.7178706 0.1909804 0.01137033 0.04000686 0.02884193
0.04048567 0.07358361 0.104659 0.02528422 0.2204517
0.04003554 0.05949726 0.038507791 0.008223437 0.07808597
0.11957 0.03235756 0.1417352 0.0619588 0.1849154 0.03816739
0.3655859 0.02597038 0.08649148 0.2238972 0.159681
0.03159849 0.0991155 0.01665979 0.07344224 0.2439262
0.1915821 0.005477212 0.070065116 0 0.01624371 0.006013601
0.05826638 0.04100346 0.04877889 0.1258113 0.1021031
0.005842323 0.06949341 0.0889808 0.05166237 0.08297225
0.08811311 0.1776962 0.00662349 0.8164064 0.1385728 0
0.03199226 0.2280913 0.1508519 0.02178008 0.0729891
0.06068033 0.07065754 0.05956664 0.01682009 0.237185
0.188008 0.1887942 0.03670437 0.1325787 0.1183399
0.01781258 0.04324912 0.03693057 0.0739886 0.03241693
0.1062246 0.1621435 0.120228 0.08866145 0.1156667
0.01691788 0.088125879 0.05444437 0.04421278 0.07733224
0.08835546 0.06646276 0.6684967 0.021548667 0.07073955
0.04244092 0.02951124 0.04957792 0.01712497 0.1707376
0.175413 0 0.02729733 0.1027522 0.0649637 0.1452919
0.05747628 0.05742586 0.059593922 0.1220931 0.2520623
0.1143743 0.034531697 0.0596587 0.4027019 0.06153684
0.01354656 0.005620812 0.07986647 0.2306501
LINC02773 0 0.3818279 0.1977756 0.05868336 0 0.186642 0
0.1104376 0.1422601 0.346352909 0.07909766 0 0.4268991 0
0 0.1820467 0.06127073 0.161888554 0.2275553 0.1312055
0.143570346 0.1835425 0.1172297 0.2572395 0.1472619
0.0482867 0.1458613 0.09715097 0.296784231 0.278789
0.1736027 0.1651991 0.177896 0 0.1500374 0.2020006
0.06392295 0.1931105 0.2851184 0.135915366 0.2232415
0.1624638 0.063940023 0.1184774 0.1265175 0.1808129
0.07148117 0.1980921 0.250205795 0.04906566 0.141832

0.173879 0.13181 0.4618489 1.077993 0.03775933 0.6764863
0.5037989 0.06159835 0.208207004 0.697451655 0.377001032
0.336509 0.4313174 0.1268083 0.04162745 0.08382211
0.6829729 0.185164506 0.1072846 0.07870082 0.02226236
0.1215411 0.2577681 0.3824292 0.05555066 0.405417
0.3500402 0.02324765 0 0.1068149 0.738148 0.3619143
0.5369281 0.1941246 0.2974025 0.2844426 0.2277406
1.47315007 0.3118126 0.118398492 0.5144573 0.3148692
0.1296454 0.4949677 0.1882771 0.5954722 0.09703183
0.02577656 0.217938 0.4230286 0.1610119 0.2830912
0.25791 0.697278 0.6719322 0.177242 0.3823668
0.217296786 0.04094942 0.260421 0.09865212 0.4312351
0.270891 0.6526997 0.09754742 0.2749426 0.09733793
0.3857491 0.06426855 0.0635729 0.09913439 0.3396643
0.2281026 0.2657296 0.2085516 0.1404719 0.1993247
0.3983194 0.3411504 0.1106063 0.1049093 0.06250709
0.2166548 0.2060006 0.3914994 0.05843237 0.165361
0.16902 0 0.334784 0.388112 0.1433685 0.09702001
0.1449564 0.1987222 0.07974632 0.3151267 0.02706986
0.5100695 0.227096 0.2317471 0.05566689 1.25159 0.6060577
0.2893414 0.2195662 0.3273301 0.08628878 0 0.60186453
0.3393522 0.565403442 0.1955986 0.03783998 0.2682147
0.3429887 0.05741552 0.4601464 0.113445 0.2282091
0.318249003 0.1966294 0.06251244 0.06379348 0.2190145
0.2674212 0.1326939 0.1181969 0.2843456 0.06178558
0.09306583 0.2063906 0.5150983 0.3089633 0.6451801
0.6460736 0.1662362 0.3039805 0.106224 0.263415
0.09710432 0.124529 0.1408744 0.3169045 0.3494498
0.2551004 0.1525618 0.09389571 0.2869669 0.3780957
0.2265213 0.09033827 0.1002987 0.05811642 0.04763053
0.1480464 0.8490824 0.05217002 0.1152975 0.07857715
0.8814711 0.1995755 0.1016317 0.3832018 0.05374813
0.1314849 0.2433328 0.7079375 0.19106819 0.2006426
0.226671273 0.290166494 0.06601061 0.508899 0.074677
0.2220025 0.07152995 0.03747549 0.2148733 0.03465389
0.2500284 0.04006294 0.07270216 0.07361711 0.3439634
0.1769805 0.2083892 0.06936659 0.1568115 0.3680799
0.6463588 1.059065 0.6089887 0.0640842 0.2359898
0.1534253 0.1844497 0.447513 0.2847204 0.3865611
0.1039854 0 0.3341079 0.3291259 0.3752949 0.2541523
0.159195928 0.02883796 0.1779386 0.478415 0 0.2353698
0.380761 0.5075619 0.2336281 0.106397 0.1670784
0.5611065 0.2858275 0.672064 0.2087018 0.171481 0
0.1282837 0.2146104 0.2332321 0.1475095 0.1317267

0.732492971 1.052095 0.151447 0.1409534 0.1981353
0.1344669 0.4766204 0.09686337 0 0.4052247 0.9746536
0.1982785 0.2546629 0.1011808 0.261789 0.6118816
0.6247717 0.8834241 1.036118 0.1493926 0.1310649
0.03811413 0.2032884 0.2619296 0.5291614 0.02592153
0.05136303 0.066486321 0.127784912 0.3235698 0.06193373
0.4070348 0.1159179 0.2625777 1.237167 0.1186175
0.3838433 0.3699269 0.1568001 0.1567191 0.1225334
0.2455062 0.110011919 0.0862928 0.1902045 0.347453
0.1102599 0.2837033 0.16129625 0.1673209 0.224367 0
0.4024035 0.8798841 0.6316505 0.3508968 0.08814395
0.0302615 0.1107555 0.2458102 0.1852586 0.2148858
0.3734181 0.317384 0 0.5056107 0 0.054058448 0.07101877
0.1222185 0.1562737 0.282036 0.3465568 0.1047687
0.2195911 0.3599605 0.04356154 0.4285632 0.3983833
0.6286487 0.1901178 0.4039521 0.8172879 0 0.4480353
0.3825789 0.02947993 0.1007461 0.3301272 0.1076738
0.05661322 0.1968172 0.2621146 0.204469 0.06086213
0.5358109 0.1908409 0.05722259 0.3051034 0.02648134
0.1236648 0.139519644 0.293128 0.1831928 0.0305719
0.1167268 0.1330534 0.4632415 0.09085876 0 0.2827842
0.4308494 0.1121642 0.4300394 0.2977101 0.4867346
0.308678878 0.3024552 0.7888049 0.1777275 0.044715992
0.0386268 1.237384 0.1821386 0 0.08734242 0.3309477
0.3657243
BX284668.2 0.009806694 1.289397 0.0721646 1.400508 0.1414944
0.4717493 0.3939497 1.22233 0.2573773 0.430035056 0.09259649
0.08669986 0.287737 0.04137607 0.7354373 0.3233049
0.1467148 0.417182456 0.1089777 0.01047253 0.613081233
0.2343992 0.006238006 0.7156967 0.6582306 0.08864512
0.3958389 0.3411924 0.246361962 0.2002708 0.52655 0.2461353
0.1041279 0.1389891 0.003991882 0.01074882 0.005102186
0.1012897 0.08723714 0.039054452 0.1108715 0.004322498
0.005103549 0 0.1009834 0.2742096 0.1198148 0.05797465
0.02496358 0.07832622 0.05094321 0.06014074 0.4260915
0.05160923 0.3537324 0.02411092 0.01439883 0.5931284
0.03441649 0.226013165 0.005566902 0.033434861 0.1074376
0.6196821 0.02024311 0.2624862 0.4281915 0.07929216
0.06404413 0.03767814 0 0 0.2328274 0.07315369 0.0190779
0.04433929 0.04949096 0.115749 0.00371115 0.038426362 0
0.03213672 0 0.06050315 0.07747294 0.009495201 0.4338904
0.04241474 0.33595293 0.3429038 0.023625754 0 0.1005286
0.1076193 0.02257555 0.02003714 0.07129388 0.129081
0.008229709 0.01988037 0.03069563 0.4969288 0.06778712

0.05293491 0.333931 0.007397533 0.07073529 0.3967556
0.021680176 0 0.02672514 0.02362257 0.1721011 0.2973012
0.2083881 0.07396715 0.01645898 0.03884648 0.4636555
0.4001222 0.2181926 0.1213278 0.2801495 0.009103319
0.2309527 0.1521931 0.06278806 0 0.3724318 0.09530452
0.0282507 0.1842198 0.1496753 0.01482248 0.09865503
0.07499665 0.06063123 0.1372669 0.3669496 0.199449
0.1169075 0.1032608 0.007628901 0.2632933 1.453204
0.06741164 0.06365172 0.1509162 0.1858164 0.01436915
0.04078417 0.05086823 0 0.05360438 0.3153279 0.04618916
0.1533462 0.1335367 0.08264852 0.1804572 0.051734818
0.487554 0.230660518 0.0223032 0.01812181 0.7006351
0.2346565 0.1099867 0.04006675 0.07847602 0.5343108
0.429010206 0.02615752 0.2145529 0.4837259 0 0.1992196
0.04236531 0.04717103 0.6071134 0.09370014 0.01485661
0.07687694 0.004568221 0.03138641 0 0.1833534 0 0.00404384
0.004239282 0.2270721 0.08267359 0.5069211 0.2548702 0
0.05149347 0.1816876 0.4676022 0.004996363 0.01717879
0.3734621 0.04017871 0.07691305 0 0.03710978 0.02661234
0.05908367 2.665693 0.2165329 0.04141254 0.01881556
0.0156349 0.1456427 0.03785608 0.2776296 0.03432044
0.5299888 0.007768912 0.0719167 0.259260769 0.1189674
0.020677019 0.036027347 0.007025091 0.05558414 0.0119211
0.8505475 0.1027685 0.06580661 0.04900202 0.03319194
0.07412491 0.0191864 0.02708031 0.005875953 0.006863597
0.07063092 0.00415829 0.02214674 0.420549 0.6022758
0.1326623 0.1300495 0 0.0358054 0.1224351 0.01632809
0.530005 0.1643096 0.104888 0.0969711 0.1770641
0.05478991 0.008889247 0.003502679 0.03594624 0.04868608
0.208389243 0 0.1893687 0.03144731 0.01373547 0.1127201
0.0101305 0.04051245 0.08204973 0.08492366 0.005334329
0.4112193 0.0152094 0.1072853 0.02665297 0.1094978
0.013444312 0.02389174 0.04894206 0.0279241 0.282573
0.2155397 0 0.08922435 0.01036128 0.0562529 0.009036981 0
0.02853208 0.03865708 0.07418139 0.02156275 0.1875051
0.7280028 0.4929205 0.1978626 0.01671633 0.03005478
0.2992072 0.06581206 0.06616052 0.04769674 0.01255356
0.08518116 0.04867808 0.01254398 0.02222972 0.04965596
0.192685 1.013596638 0.441978526 0.03873992 0.02636486
0.007644377 0.5119608 0.07451861 0.04459583 0 0.2736951
0.09663295 0.1609127 0.1250897 0.009780338 0.06531918
0.005853936 0.1515293 0 0.05546583 0.03520278 0.02264456
0.405540579 0.04006553 0.04029406 0.004972434 0.08297393
0.092026 0.9528799 0.1200334 0.2063733 0.00483081

0.008840252 0.03924004 0.06243369 0.01372136 0.01987026
0.005066575 0.3669409 0.4696053 0.05251629 0.133759537
0.3212183 0.09755204 0.01663122 0.009004591 0.09052816
0.2341473 0.005842423 0.0328357 0.01390794 0.01824371
0.2119871 0.4906227 0.2579711 0.1354187 0.0815426
0.004909531 0.3933729 0.01832195 0.009412086 0 0.005855557
0.06875422 0.03614992 0.1413855 0.06575296 0.1632026
0.009715757 0.02250906 0.012186 0.08221281 0.02435267
0.02959153 0.08883583 0.022272289 0.4211427 0.1052787
0.3953092 0.01118024 0.007080017 0.1815131 0.2320685 0
0.7448496 0.06473305 0.09549544 0.3604101 0.005940638
0.0820168 0.006159514 0.201909 0.3169742 1.494239 0
0.413136 0.4007047 0.02907579 0.007467449 0.05112416
0.02641551 0.1634712
AC092666.1 0.02105597 0 0.05810427 0 0.05523688 0.02132408 0
0 0.01625339 0 0.03614801 0.06205113 0 0.009870957 0
0.1637925 0 0.023780524 0 0 0.024604628 0.07339484
0.01339363 0.1007655 0 0.0413761 0.01666483 0.02497413
0.040689548 0 0.1190059 0.0188742 0.02032483 0
0.008570976 0 0 0.02836685 0.008143782 0.02795129 0.07651685
0.07424673 0.010957837 0.006768092 0.03252325 0.03873393
0.1960034 0.05658066 0.064319207 0.05886102 0.01215336
0.178793 0.1016513 0.03166008 0.02052701 0 0.04637363
0.02158486 0.01055653 0.114181928 0.011952704 0.014357608
0.05242713 0.01231963 0.086928 0.04993786 0.02873033
0.07448364 0.010577634 0.01470887 0.008991664 0.01526101
0.124976 0 0.07373193 0 0.03269604 0.0685586 0.04780929
0 0.09152798 0.0345004 0.02255408 0.07577873 0 0.06116148
0.06499582 0.01300982 0.28853001 0.1899998 0.020290756
0.01175547 0.008993546 0.01777458 0.012118 0.09679897
0.04082005 0.05543002 0 0.04268517 0.09226928 0.07358321
0.1212882 0.07577108 0.1520874 0.06353302 0 0
0.009309908 0.2386046 0.006375731 9.704438 0 0.02321221 0
0.01671737 0.0235594 0.08340732 0.02333239 0.2092686
0.01089492 13.42158 12.05929 0.05863725 0.1113197
0.06127013 0.009629452 0 0.009751814 0.01948843 0.0379107
0.03595805 0.02142453 0.01060846 0 0.06709392 0.05006977 0
0.04634577 0.0138141 0.02151533 0.06651339 0.09828015
3.203465 0.0298106 0 0.02733334 0.07200721 0.09278292
0.04113604 0.009729749 0.009929022 0.02862004 0.05231547
0.04616189 0 0 0.1495915 0 0.1056709 0 0.04652568
0.064598026 0.01915489 0 0 0 0 0.1003653 0.06480615
0.04345528 0.012120111 0.2021863 0 0.01093272 0.03336356
0.03055319 0 0.1721777 0.04873024 0.03176585 0.03189865

0.01179019 0.0294253 0.009627117 0.1691054 0.06561306 0
0.3993966 0.02730651 0.09028644 0.05547143 0.02134139 0 0
0.1382021 0.02017767 0.06274933 0.0107277 0.03688461
0.575072 0.1811623 0.0722488 0.03437775 0.03983923
0.04897661 0.01268586 0.02425217 0.08046658 0.2173525
0.0269326 0.02237981 0.0390887 0.08128084 0.1010337
0.02763355 0.02253346 0.3753144 0.03308838 0.021829771
0.04912213 0.088791314 0.011050624 0.04525075 0.04590184
0.08531938 0.6304778 0 0 0 0.1187775 0.06121297
0.0823903 0.06645048 0.0378488 0.1915789 0.07582587
0.05356963 0.02377565 6.32073 2.67303 0.1265953 0.0558459
0.02609163 0.01098255 0.03033238 0.1402322 0.09483121 0
0.05254789 0.07571156 0.07128273 0.05881975 0.2290334
0.007520607 0 0.1045339 0 0.01976863 0.2337915 0.01929159
0 0.06722821 0.108756 0.4100691 2.466367 0.01823397
0.02290669 0.07867689 0.05442689 0.03839206 0.02861332
0.05877574 0.038488403 0.03664143 0.01050836 0.04996322
0.05055941 0 0 0.2141118 0.08157132 0.01207807 0
0.02304451 0.04084085 0.0249002 0.2123666 0.2407466
0.02569739 0.008495086 0.04364331 0.04335012 0.2332958
0.03226533 0.08922615 0 0.1657289 0.119478 0.02695378
0.0914463 0.02322595 0.134666 0.07636716 0 0
0.056971068 0.029199161 0.1201468 0.02830401 0.131306 0
0.04999969 0.01367882 0.02710438 0.02631274 0 0.03071074
0.02148642 0.02099938 0.07012345 0.050275978 0.009859056
0.0434622 0 2.569854 0.009724035 0 0 0.0384513
0.0533816 0.01724066 0.1351926 0.02165005 0.03436319
0.02014111 0.1140946 0.07592368 0.1369101 0.02116602
0.08838348 0.01422115 0 0.03527729 0.04726361 0.05125354
0.037057458 0.01622796 0.04189082 0.03570891 0.05800131
0.09718657 0.02693239 0.01254427 0.1057524 0.0597235
0.03917109 0.09103155 0.1915303 0.09774532 0.1661473
0.01750802 0.1159539 0.07678286 0.1835824 0.04041741
0.06906215 0.03771743 0.02952446 0.009702193 0.1236763 0
0.0700825 0.073012504 0 0.03924685 0.09806625 0.02614383
0.09076575 0.2013362 0.038256691 0.03349022 0.025116
0.08382904 0.07201535 0 0.02886867 0.06228434 0.07259891
0.1163104 0.03474711 0 1.068632 0.02551031 0.1204884
0.052900402 0.1130919 0.04661478 0.04061116 0 2.462543
0.2060002 0.07803584 0.07215017 0.01995795 0.01417919
0.04178445

AL034550.1 0.2062887 0.04226976 1.23339 0.08445404 0.4445281
0.1193802 0.04553257 0.1059574 0.2047334 0.092306134
0.07588883 0 0.1820358 0 0.2402219 0.1637451 0.4702808

0.144226705	0.07277461	0.06294137	0.15496424	0.102723	
0.07498261	0.1762885	0.09082789	0.1042376	0.046648	
0.0582561	0.199321012	0.06686978	0.4164	0.05283244	1.30854
0.5568961	0.3958643	0.1292039	0.1839892	0.4234888	
1.014421	0.169522025	1.927666	0.6364812	0.245384429	
0.7578067	1.16833	1.019182	2.143166	1.18785	0.960221833
1.176879	0.2551466	0.8619297	0.300349	0.4874239	
0.3734833	0.5615264	0.3677908	0.3776256	1.06379	
0.239712567	0.953549395	0.261232742	0.3522082	0.3103648	
0.689429	0.4293411	0.4423189	0.6850509	0.414523109	
0.5764206	0.1761855	0.523301	0.6267813	1.607522	
1.009016	0.346431	0.8351416	1.715182	0.8475725	
0.750580654	0.1537225	0.6438208	0.6786811	0.7424164	
0.2172909	0.5564082	0.667097	0.4916288	0.85812806	
0.5318453	0.369184749	0.773286	0.0755239	0.684123	
0.1017617	0.4064379	0.3427891	1.411948	0.4451555	
0.373387	0.6641463	1.016993	0	0.5125691	0.3649041
0.789169	0.1487952	0.3301695	0.130300938	0.2553732	
0.9637312	3.561207	0.1034352	0.2599015	0.5218508	
0.596637	0.4781172	0.9572386	0.2612471	0.9403349	
0.8539143	3.867917	3.652626	0.218849	0.5523905	
0.4287666	0.1617279	0.1115558	0.5732405	0.3545866	
0.4669244	0.5535936	1.319369	0.386036	0.1317624	
3.96276	0.5185725	1.665852	0.04864896	2.14609	0.9033819
0.418913	0.6189855	1.877196	0.6536563	0.3455721	
0.8607508	0.2645498	2.22058	0.2159016	0.2042655	0.4030015
0.1201694	0.6882721	0.6353111	1.66562	0.4081515	0.6978912
0.2483647	0.2957927	0.199885887	0.4720988	0.572603193	
1.045554	0.1815245	0.3275157	0.3995903	0.8676091	
1.444841	0.6167757	0.1702953	0.254448578	0.9747046	
0.1799293	0.2907262	0.256825	0.8124794	0.4455877	
1.431698	1.636862	0.444593	1.889979	0.06600591	
1.015859	0.5254883	1.693165	0.5165531	0.0996827	
1.61622	0.6751852	0.834005	0.714265	0.3584315	0.360425
0.3293857	2.553235	0.2447515	1.44909	0.9308944	0.4301957
0.1473701	1.714506	0.6211596	0.9743267	0.5018288	
1.370948	0.3551013	0.2036592	1.038612	1.037063	
3.920251	0.3288879	2.366135	0.9263331	0.5514842	
0.3480818	0.3469147	1.470806	0.3550457	0.381910302	
0.9900153	0.4815533	0.603189239	0.9816568	3.045163	
0.3462963	2.388601	0.03431406	0.485395	0.2208818	
0.3823526	1.045214	0.4804708	0.9416626	1.236036	
0.5568911	0.2971513	0.5623181	0.5989727	1.850534	
2.317531	0.8120846	0.5471313	0.4016947	0.3381644	

0.3396243	0.4416036	0.2507032	0.5581658	0.6619116
1.549745	1.59627 0.9330029	0.9616614	1.610447	0.4140805
1.243593	0.290201583 0.470357	0.7824664	0.7965117
0.0619141	0.1881844	0.31965 1.42613 1.860447	0.6635243
0.4007509	0.4404629	0.7312854	0.2014999	0.460541
0.5552698	0.565615575 0.5948844	2.632631	1.020952
0.4009884	0.04739357	0.122986015 0.362758	0.6331066
0.1859484	0.2579893	0.924585	1.514756	1.254607
1.486136	0.1943928	0.4076138	1.533768	0.1374366
0.37617 1.53213 0.1580543	0.8741629	0.2118963	0.198817
0.4180519	0.3269444	0.3473952	0.8289261	0.4523465
1.843732	0.1865246	0.5051132	1.036572988 0.674305169
1.177097	1.14881 0.6317271	0.2038947	0.6578053	0.2105928
0.7776702	0.6383374	0.451716	1.40768 0.225542	0.4555545
2.021774	1.477685449 1.034901	0.5170503	0	1.216549
0.4082911	0.154752791 0.2006663	0.4978001	0.4931031
0.6112914	0.1455498	0.5151219	0.4088034	3.326347
1.683964	0.8766643	3.139596	0.3653607	1.546263
0.4776923	0.4263117	0.9874778	0.551249	0.7029948
1.115106252 0.4656075	0.3517809	0.5497578	1.028259
0.5289736	0.05025924	0.4213655	0.1726788	1.25383 1.74065
0.4883944	1.306816	0.2622073	1.065803	0.2695454
0.4721116	0.8167329	1.303058	0.3959758	0.6927228
0.8270292	2.128096	0.7875898	0.5664981	0.05388882
0.2942612	0.890494435 0.2435088	0.5126769	0.1784289
0.146363	0.8003219	2.061509	0.374806929 1.1601	1.195175
1.187932	2.419015	0.2765874	0.3636399	0.5666232
0.3628894	0.5154932	1.045585	0.3945845	1.160419
1.249643	0.7523709	0.296156409 3.35032 0.587176	0.1420979
0.300313691 3.48362 1.848619	0.3058119	0.9424891	0.2513974
1.012101	0.3859764
AC002398.2	0	5.76756 0.1214907	0.04205641	0.4042336	4.726191
0.06802289	1.899527	1.665234	0.772238709 0.5857621	0
0.2719505	0.0825571	0	6.31133 0.2195535	2.204382474
0.5707845	0.3291071	0.668798528 11.29041	0.3360584
3.529082	2.216292	0.3287519	0.1045339	4.055648
0.170156292 0.0499497	0.8086994	9.727933	0	0.1386615
0.1971324	0.3377899	0.04581145	0.07908333	0.3916419
0.019481202 0.01777664	0	0	0	0.1360063	0.08098913
0.05122817	0.3785761	0.089657076 0.123073	0.1270579
0.7892175	0.09446384	0.06619834	2.575207	0	0	1.150866
0.02207274	0.029843004 0.124960088 0.060040905 1.710078
0.2318331	0	4.340702	0.7809427	0.8899344	0	0.04613237
0	0	1.415448	0.1847338	0.0856482	0	0.4956422

0.0716749 0.03332163 0.064691654 0 0.4568689 0 2.625684
0.5101163 0.06394155 0.8607022 0.3264283 0 1.315965 0
0 1.015453 0.01858251 0 0.06746595 0.08535102 0
0.0184732 1.271824 1.295365 0 0 0 1.521862
0.979708 0.06351172 2.384057 0 0 0.07998645 0
0.1545259 3.130484 0.1559227 0 0.04926056 0.01743971
0.3252394 0 0 0.07104631 0.3245681 0.3269471 0
0.2135171 0.04026862 0.04761645 2.63033 0.5908536 0.09512139
0.05012335 0.04479674 0 0 0.8978386 0.2303209 0
0.0484524 0 0.02999106 0.1622524 0.05137371 0.06953101
0.08310833 0.4628572 0 2.84183 0 1.311679 0.02034402
0.2076068 0 0.3500381 4.198633 0.06912044 0.6687621
4.457145 0 0.5155458 0.431336247 0.2188822 0.112557167 0
0.2169492 0.06989837 0.1404621 0.04114779 0.1798754
0.7588211 4.579397 0.02534205 0.3757806 0.02240029 0
0.08720021 0.2768304 1.077769 0 0.05094526 0.02213983
0.111162 0.09860882 0.04101709 0.2415531 0 0.9260388
0.2680558 0.1452351 0 0.2265369 0.02319714 0.02231145
0.03365332 0.07570496 0.1348518 0.1968852 0.109336 0
0.1542447 0.6266149 1.010117 0 0 0 0 0.2917749
0.1014182 0.07477702 0 0 0.3041619 0.06129819 0.1699508
0.06337569 0.03851949 0.2355771 0.1395108 0.57654
0.068466101 0.06162595 0.069620462 0.02310585 0.0315384
0.3455157 0.01783951 0.02272883 0.1537894 0 0.1319936
0.4470351 0 0 0 0.02637947 0.03081339 0.412217
0.05600461 0.2237072 0.04495262 1.7641 0.2205827 0.4378827
0 0.1837081 0 0 4.604582 0.962153 0.03139225
0.395765 0 0 1.755923 0.01572491 0.08068841 0.6374987
0.136908498 0 1.870332 0.1613478 0.06166394 0 0.6139771
0 0.1339467 0 0.263427 0.1645062 0 0.1404801
0.1196557 0 0 0 0.1098601 0.2716183 6.836245 0
0.078742994 1.225252 0.04651588 1.793044 0.141997 0
0.7258532 0.03470937 0.4070361 0.03872147 0.3761163
0.2131494 4.768032 6.34488 0.1125693 0 1.268634
0.1688322 0 0.03568824 0.03757193 0.4916723 0.5827602
0.0938581 0.09979799 0 0.09202544 0.04764853 0.030526396
0.09662152 0.0739764 0.01715931 0 0.02090896 0.6864283
0.02833639 2.292398 0.0723039 0.2889602 0.02246307
0.1317234 0.02932435 0 0.1649151 0 0.3169192 0 0
0.20229238 0.05995665 0.04019909 0 0.2042756 0 0.02263414
3.125488 0.1473963 0 0.1587495 0.0660615 0 0.0308003
0.08920542 0.2956961 0.0245872 2.832952 0.2357663 0
0.1017936 0.1167865 0.2986563 0 0 0.07508425 0.2622894
0 0.0312191 0.2661854 0.06344623 2.553012 0.05109415

0.1447495 0.5125076 0 0 0 0.04225456 0 0.1051516
0.1697656 0.101432 0.3526308 0.1073422 0.06280121 0
0.101052 0.3008925 0 0.1093287 0.132848 1.683903
0.159982525 0 0.4201222 0.04381973 0 0 2.0523 0.06511544
0.06505617 0.08106481 0.5448978 0 0.1926218 0.0800096
0.0387585 0.055304966 0.1182325 0.7797381 0.2547427 0
0.08304762 0 0.2284322 0.03352432 0 0.1185896 2.690918
LINC00345 0 0.1462386 0 0 0.0668657 0.2581336 0
0.0610959 0 0.063869367 0 0 0 0.04779621 0 0.3021336
0 0.115147798 0 0 0 0 0.3242669 0.6098963 0.03491474
0 0 0.04030913 0 0 0.1440598 0.04569543 0.09841496 0
0.2075078 0 0.1591345 0.09157017 0.03943305 0.315800545
0.2881687 0 0.053059002 0.03277182 0 0 0 0.05479391 0
0.2442953 0 0.1923854 0 0 0.09939394 0 0.07484866
0.3135485 0.05111582 0.069110114 0.115752503 0 0.2538577
0.05965296 0 0.2072609 0.2782306 0.1545675 0 0
0.08707716 0.07389543 0.3530024 0.1901354 0 0 0
0.5809439 0.1543318 0.149812252 0 0 0 0.1572551 0
0.04935839 0.1049055 0 0.34927317 0.5174995 0.049124988
0.1138425 0 0.2581991 0 0 0.2470687 0 0.0855601
0.103343 0.1914761 0.08907441 0 0.2445944 0 0.07690839
0 0.3172974 0.270477315 0 0.3087196 0 0.2683871
0.05619799 0.7221683 0 0 0 0.0753186 0.1066632 0
0.2193711 0.09395394 0 0.2940119 0 0 0 0 0.0471825
0 0.1160751 0 0.05136727 0 0.06497516 0 0 0.2244111
0 0.1041795 0.7514847 0.0793138 0 0.0962307 0 0
0.04358331 0.04492647 0.1493888 0 0.384619 0.04619376
0.253317 0.1117604 0 0.09110072 0.1207229 0.1432091
0.5116695 0.38418613 0 0.729844365 0 0 0.08093496 0 0
0.0694256 0.5648367 0.08416602 0 1.359732 0 0
0.04038747 0.1479418 0 0.09808265 0.1769677 0 0.2574277
0.171268 0.1899739 0.09323102 0.2519465 0.5559843 0
0.5465427 0.08814732 0 0 0 0.077934 0 0.1338379
0.03256747 0.2025592 0.2077786 0.3571983 0 0.1253153
0.2498831 0.3329213 0 0.03952499 0 0.2348631 0.08658392
1.052444 0 0.4334616 0 0.1124487 0.1467647 0
0.1091094 0.04038471 0.1068116 0.105702051 0 0.053742111 0
0.1095544 0.1333569 0 0.1579055 0 0.4975693 0
0.05751329 0.4149595 0 0.04022003 0.06108929 0.1427147 0
0 0 2.758672 0.1527209 0.153247 0 0.2526769 0 0
0.4243871 0.1020406 0.07427146 0.03634892 0 0.1150528
0.3797485 0.09241698 0.07283114 0.06228579 0 0 0
0.196877 0.0934119 0 0 0.1053216 0.1203392 0.1550962
0.08829081 0.05545831 0.1269873 0.1581244 0.09294921 0

0.2134487 0 0 0.1017651 0.09677088 0.244814 0 0
0.3273954 0.07181399 0 0.04697644 0 0 0.1607592 0 0
0.08295297 0.0822682 0.1056627 0 0.04344779 0 0.2592255
0 0 0.2892626 0.08700868 0.06325608 0.05623124 0
0.09244445 0.08604129 0.55409 0.055171982 0 0.08950204 0 0
0 0 0.1987029 0 0.1698787 0.1116271 0.3345855 0
0.1525218 0 0 0.04773859 0.2104485 0.2096909 0 0
0.803085537 0 0.2327316 0 0.2504432 0.1007103 0.1572477
0 0.04876268 0 0 0.1019897 0 0.2139811 0 0
0.1138776 0.6865661 0 0 0.07857749 0 0.4322657
0.09361615 0.3660126 0.1738793 0 0.08534401 0 0
0.1469281 0.92741 0.0394411 0.536335 0.5086542 0.6635446
0 0.2539787 0 0 0.1217545 0 0 0.4899712 0
0.04847812 0.30302934 0 0 0.2374236 0.5063646 0
0.1026201 0.185242923 0.405408 0.2432287 0 0.1549804 0
0 0 0 0.09386451 0.1682491 0 1.427429 0 0 0
0.1369008 0.2708564 0 0 0.1282139 0 0.1511431 0
0.193277 0.2059714 0.1213948

AP001001.1 0.03495257 0.05570434 0.1071691 0.05564801 0.1375385
0.1415905 0.07200499 0.01396338 0.03597382 0.072986245
0.2400205 0.1144488 0.1799191 0.04369501 0.03798858
0.0776837 0.06972187 0.078950575 0.04315703 0 0.081686576
0.01160326 0.04446643 0.1115127 0.1037362 0.3296816
0.0553267 0.1934644 0.067543997 0 0.03292466 0.03133087
0.3148963 0.03669471 0.09485122 0.1277015 0 0.04185645
0.1802473 0.12372985 0.7715091 0.1643312 0.194024897
0.292108 0.3479231 0.1886066 0.6507251 0.187846
0.75924517 0.2419445 0.06724794 0.5496176 0.1999876
0.1927025 0.4088941 0.11458 0.2052786 0.2985877 0.1518718
0.126360113 0.304233222 0.079444662 0.1276414 0.0136336
0.01603323 0.2526356 0.158973 0.2943849 0.081940615
0.1546378 0.1890629 0.2111086 0.5186454 0.4019603
0.3445159 0 0.1537789 0.5310955 0.5379025 0.182609962
0.020258 0.2799872 0.6489498 0.2276227 0.04908898
0.05640393 0.3356641 0.2447557 0.83817159 0.07884916
0.123501875 0.4032872 0.1492914 0.5114289 0.1072836
0.2380515 0.1129344 0.1104155 0.4595339 0.3070457
0.4376158 0.3155463 0.1073794 0.1537296 0.2404407
0.2636595 0.1008446 0.02900714 0.123634378 0.4504436
0.2469509 0.495812 0.1431254 0.411007 0.4126262
0.2405049 0.06518037 0.1846064 0.1721395 0.5972543
0.3014232 0.9400675 0.6227179 0.08652168 0.3807773
0.6893504 0.1705039 0 0.107919 0.07548444 0.2181613
0.2254948 0.4979013 0.3052378 0 0.3415495 0.3435421

0.4139726	0.03846662	0.397474	0.2698474	0.2453582
0.1450164	0.5888111	0.3518941	0.1224883	0.499102
0.1294917	0.2053576	0.09104689	0.3660945	0.07691608
0.1900353	0.3010552	0.2894835	0.2926671	0.2498512
0.2207284	0.1963814	0.03898044	0.035122068	0.3861594
0.405097485	0.1377862	0.04305929	0.2589659	0.1115136
0.3375637	0.5553492	0.2008107	0.07694408	0.174366312
0.2237507	0.08299066	0.1209876	0.3507588	0.0450825
0.2013287	0.3810829	0.2966019	0.03515387	0.1412033
0.03914304	0.6404192	0.09588516	0.2447235	0.2178332
0.07881888	0.3939517	0.1510945	0.2098237	0.1718858
0.1062791	0.0534351	0.1202051	0.2956883	0.1042055
0.2893413	0.0831031	0.2313052	0.1434156	0.2864062
0.1027987	0.4279988	0.02204416	0.4155353	0.04211666
0.1207747	0.2770408	0.2842679	0.4470769	0.1238337
0.2595464	0.2184498	0.08944764	0.2650339	0.04987358
0.3691946	0.1952931	0.144948282	0.1413394	0.589568631
0.085604674	0.3672314	1.066748	0.2926995	0.312772
0.2713205	0.04264452	0.1164338	0.05257831	0.3793535
0.106374	0.1103067	0.5863984	0.03261722	0.134261
0.1284468	0.6840981	0.2022327	0.4275756	0.2685201
0.3244616	0.02887446	0.3524631	0.05594586	0.1163916
0.303176	0.1867209	0.2326097	0.4294066	0.683674
0.08679096	0.2745829	0.2330363	0.2277652	0.3663299
0.132846257	0.2844019	0.1687348	0.2561898	0.08159222
0.5059098	0.2647821	0.3712953	0.3012996	0.5145577
0.05069965	0.1354395	0.1325101	0.1274604	0.1266604
0.2601781	0.202318749	0.08109891	0.3605032	0.1216426
0.1119037	0.2498266	0.236165837	0.1496514	0.3200531
0.02673253	0.1395732	0.4335399	0.3163774	0.2847449
0.2741863	0.2459295	0.170629	0.7332887	0.2294161
0.1247315	0.2085285	0.08926655	0.03949706	0.290413
0.1310035	0.1983316	0.07954282	0.1445708	0.3212892
0.4868277	0.3380487	0.1081554	0.2045666	0.252189493
0.630111807	0.3375167	0.156614	0.06357339	0.04396884
0.3319947	0.09082651	0.7798757	0.02911915	0.3061464
0.3398623	0.03566711	0.4183036	0.4656152	0.347738824
0.3818704	0.1442931	0.1797171	0.4182271	0.1937009
0.321202014	0.01586664	0.5319045	0.05907507	0.1017572
0.1150859	0.1557345	0.1140847	0.5349426	0.321398
0.1365348	0.8041809	0.1639645	0.1304135	0.3304964
0.1565031	0.4814911	0.1569137	0.4537603	0.102524642
0.3950929	0.04635873	0.1975874	0.4279167	0.2151042
0.0298049	0.3609371	0.3315892	0.1652334	0.5310243

0.4533328 0.1854634 0.2343693 0.2451571 0.1162522
0.3266354 0.2549166 0.1596277 0.2012767 0.2292841
0.2365279 0.784162 0.2254768 0.5972383 0.170439
0.2105125 0.357827348 0.08557415 0.2026862 0.1844935
0.1446611 0.2611608 0.7505175 0.158763732 0.1389831
0.3196399 0.8117367 0.4604671 0.05046854 0.01597384
0.03446367 0.1147743 0.1930734 0.3268513 0.4112689
0.101949 0.2681958 0.4205313 0.160992001 0.3441731
0.2063461 0.1348278 0.152651145 0.05860618 0.2145613
0.3454353 0.372612 0.2760824 0.09414893 0.2866942
AC103739.1 0.1532555 0.1172377 0.1127762 0.2049584 0.04020406
0.3724965 0.09471542 0.07346976 0.18928 0.192012337 0.105241
0 0.3313321 0.08621469 0.1998808 0.3179095 0.1222829
0.161547017 0.3406128 0.1309287 0.035816864 0.03052588 0
0.2200259 0.2309234 0.1686469 0.2911072 0.2666015
0.059231621 0 0.08661824 0.5220269 0.7692562 0.193073
0.1247674 0 0 0.137645 0.1659682 0.271257249 0.6188071
0.8106051 0.159512821 0.1970457 0.4103144 0.7442798
0.6776385 0.329457 0.187258451 0.3916972 0.1061496
5.494553 0.4932447 0.5530494 0.4183352 0.1883983
0.1800158 0.377052 0.2766078 0.415535498 0.452387154
0.167202521 0.1831632 0.251071 0.2530815 0.9969511
0.1672905 0.3097873 0.061591288 1.263287 0.1570696
0.1555078 1.334132 0.2000634 0.7155419 0.2217339
0.9043144 0.3742519 0.5103693 0.090076987 0.3197686
0.3013326 0.1969913 0.3466911 0.3551442 0.2077426
0.2838425 0.1515068 0.31500903 0.6223095 0.265834588
0.7871703 0.1309187 0.4916133 0.1764012 0.4696997
0.5050836 0.4841356 0.2057774 0.2485465 0.9977763
0.535574 0.3531175 0.330899 0.6325518 0.601151
0.2210851 0.3052481 0.135523971 0.2860412 0.4640564
0.639886 0 1.115068 0.8684303 0.3163603 0.1371813
0.1942652 0.3170055 0.3527313 0.09515817 0.395701
0.6496498 0 0.1767793 0.6243349 0.252316 0.2320548
0.3406963 0.1134769 0.4194171 0.244272 0.1871256
0.1853123 0.34261 0.3516061 0.6414001 0.396029 0.101198
0.4826193 0.5428762 0.225921 0.3815094 0.7099791
0.4339517 0.594909 0.3978904 0.4716928 0.5132422
0.3293487 0.4532339 0.2601655 0.305522 0.517857
0.1343954 3.94599 0.1369394 1.125092 0.4305337 0.512749
0.092399196 0.6772726 0.407485439 0.5576741 0 0.3893074
0.4400552 0.4583551 0.9183514 0.3773522 0.4554554
0.317577592 0.7521557 0.06238056 0.8275656 0.2914032
0.266857 0.2648279 0.3538425 2.695584 0.2466209

0.185739 0.03432586 0.3997868 0.1681699 0.3029736
0.1193908 0.3732423 0.3286175 0.1589999 0.1577156
0.6136966 0.2174661 0.2342953 0 1.180258 0.1958171
1.004784 0.3435579 0.2505663 0.3772981 0.4520867
0.3004923 0.5004356 0.05799381 0.2614153 0.2585347
0.4942531 0.4164796 0.6040348 0.7841138 0.5864077
0.2560557 0.4394751 0.2647339 0.214539 0.1312075
0.5584848 0.2568887 0.285997639 0.3146304 0.258506358
0.096518109 0.197614 1.496749 0.273238 0.5380114
0.07137905 0.1121893 0.21442 0.2074847 1.354434 0.07995683
0.338561 0.1469236 0.5148567 0.1766071 0.3379181
0.276881 0.8762916 1.4233 0.4299968 0.04064734 0.4557779
0.127898 0.2943649 0.2041356 0.6135352 0.4912258
0.5245287 0.4959586 0.4842413 0.5708245 0.1111343
0.4598042 0.03745032 0.3043393 0.031772014 0.4316568
0.6510649 0.9267288 0.04293059 0.3914554 0.3166314
0.578847 0.3730163 0.1858019 0.03334518 0.3308634
0.887365 0.1117744 0.1249569 0.5133577 0.140068555
0.9600982 0.8872246 0.7854978 0.3925287 0.2300354 0
0.3608947 0.6260998 0.246148 0.254208 0.1565471
0.1783556 0.5074598 0.1545707 0.4313278 0.2244455
0.6430457 0.3811884 0.2019347 0.4441025 0.4696852
0.4675902 0.1175414 0.06892883 0.2733087 0.2877344
0.3803372 0.2704794 0.6273048 0.6670043 0.1810679
0.1025093 0.132692109 0.340040862 0.2421653 0.8240409
0.1194635 0.1542311 0.08734123 0.03982442 0.2367344
0.2298201 0.738293 0.4917606 0.7819424 0.3973933
0.3674824 2.159003248 0.2583322 0.253071 0.1576 0.880218
0.08493145 0.040238991 0.2921938 0.2238937 0.1243318
0.3680916 0.2119377 0.2521272 0.1500671 0.5570673
0.181186 0.4973481 0.3985989 0.6573077 0.257319
0.1242101 0.6017573 0.5477655 0.4586763 0.4775014
0.134861 0.2834758 0.3252283 0.8317011 0.2251528
0.1571935 0.1306846 0.365213 0 0.6520446 0.9123393
0.6184 0.5576199 0.4742917 1.04806 0.1529182 0.2762077
0.223545 0.03817718 0.5589369 0.2680894 0.5124478
0.601701 1.158123 0.5892059 0.2615616 0.4080753
0.364402371 0.05628215 0.1904383 0.6852224 0.4566896
0.2642547 0.8638275 0.640436372 4.265764 0.5849803
0.5491333 0.3261455 0 0.2941681 0.1813341 0.1207794
0.8183441 0.3034874 0.1119276 0.8582642 0.2228115
0.4317402 0.192517286 0.9328891 0.4342845 0.1773525
0.178486618 0.3854531 1.552287 0.1817544 0.3267561
0.1162109 0.04128119 0.1946415

AC003070.1	1.462399	0.0911991	3.479899	0.8199619	1.584585
0.5151374	0.2947163	0.1905069	0.9325249	0.557634079
3.165522	2.186046	2.25832	2.29516	0.932923	0.04710507
1.458565	0.646289065	0.6280592	1.222192	0.780133614
0.03166142	2.386235	0.1901755	0.4354792	4.298044
0.6541949	0.2011046	0.430044896	0.2885497	0.7187229
0.2849715	3.958672	0.8010205	1.837602	0	0.1323219
4.597043	2.975599	1.772491025	5.05759	2.298074
0.529428987	5.191142	5.303353	2.900722	5.770736
1.674391	3.042847387	8.201535	0.5137919	4.589151
0.341062	0.6214242	0.9917645	2.07131	1.773766	1.564312
1.051957	1.120581944	1.62420771	2.557979045	0.2216394
0.2976121	1.093733	0.8832398	1.08446	1.992129
1.756766818	2.309647	0.78741	0.5299612	2.201437	1.393252
1.929615	0.747442	3.899914	2.743094	1.419633
1.245703627	0.5527729	2.500335	0.5789045	2.942082
0.5357895	2.89346	1.668273	1.924998	0.87127225	1.757083
1.072257674	1.952386	0.217262	5.072172	0.4757043	1.0393
1.201825	2.644631	1.547383	2.48125	3.383303	3.582955
0.2197519	0.07626848	0.5248657	3.789042	2.705849
0.6332061	1.349427305	1.059577	4.8902	6.228468	0.2231665
1.962626	2.477022	1.438718	0.6047084	3.55129	1.338677
2.195116	1.48047	2.77034	3.164007	0.4131552	1.222369
5.118804	0.9014179	0.2063032	1.236795	2.030293
4.235712	2.461199	1.682083	2.658846	1.208206	3.9305
2.358648	1.916885	0.5598001	1.168002	2.468849
3.782676	2.398939	4.083615	0.4801006	0.5656192
3.507879	2.745177	3.586253	1.708001	1.792233
0.9894259	0.6337743	1.105837	1.835364	1.796829
2.442974	1.091658	2.054126	0.7445519	1.67713639
2.950359	1.170397497	3.817566	0.391648	1.716104
1.014277	1.961048	3.939941	0.9784736	0.5511309
0.512386312	3.120541	0.9705159	1.122458	0.9319165
1.260904	1.373396	2.691371	4.30415	3.485207	6.196854
2.100562	3.28765	1.715186	3.063879	1.386919	0.7742532
6.030277	2.033946	0.9814946	4.72369	1.611112	0.6318282
0.9293322	4.785362	0.4468229	4.04232	1.587324	1.002422
0.6114582	1.094109	2.119358	3.295976	0.3609068
7.517964	1.0343	0.4394048	3.131799	4.027528	3.903754
2.263936	3.186969	0.876583	1.586472	1.335118
0.3742428	2.997041	1.398835	1.878697838	0.979003
0.971944311	0.467172993	5.055806	5.682989	1.751941
3.74205	2.5912	1.357564	7.275534	0.932546	2.994479
1.534226	7.023103	0.9524315	3.60456	2.106532	1.132347

1.93847 0.9088889 2.095317 2.516671 2.192288 1.26062
1.525541 2.534115 0.68812 1.336352 1.574815 1.677459
4.858296 2.260146 4.854881 2.247735 4.882635
0.3107475 1.867658 0.724985919 6.835101 1.012926
1.543749 0.6679136 1.299255 1.050911 2.814279
2.901691 3.523905 1.037568 1.293492 1.479173
0.9274582 1.684867 1.286764 2.411631105 2.854664
5.870424 4.616733 1.017826 0.5794391 0.265348384
2.075773 2.015349 0.9847458 1.142545 1.438139
3.514815 5.163114 4.435535 1.621726 1.370902
5.540952 1.087263 0.6545203 3.5766 0.4384413 0.7544198
2.346841 0.5719434 1.340066 1.410797 2.564155
1.578043 2.711 1.527761 1.690231 3.747872
1.410688423 13.402223225 1.869848 1.965797 0.8177894
0.8798259 0.4529513 1.032646 5.115432 1.059419
3.27187 1.947478 1.751825 4.153466 1.397559
1.594089555 5.090897 1.57491 0 1.483562 3.024452
0.333886766 1.601905 3.512362 1.192851 1.874214
1.004899 1.274842 0.2594158 4.987238 2.975494
2.206688 5.056524 2.386156 0.6227457 1.674798
2.430869 3.373342 0.9514774 11.73157 1.818410251
0.2940208 1.602301 1.779194 3.96998 1.695626 0.2982011
0.7575973 1.224136 4.59884 3.932966 2.61143 1.156726
4.15685 3.010283 0.4229509 3.628777 0.5100936 2.257048
1.678163 3.614807 2.809412 4.502318 20.0103 2.172885
1.937796 0.3627903 2.141760981 0.7296975 0.4345494
2.457879 0.7894635 2.987524 14.27138 0.808664402
3.286732 2.540728 1.55047 3.841869 0.8262706 0.6973964
1.786756 2.098311 1.024397 1.757504 2.51531 6.453884
4.044249 3.246554 1.118201057 4.8095 1.632843 1.165016
1.527290853 2.078916 2.744371 2.733474 5.132082
1.838141 1.75549 0.5551754
AC009974.1 0.9898619 0.2442666 0.7342847 0.2135171 0.4188291
0.4203889 0 0.2296132 0.147888 0.280042609 0.05481786 0
0.2465485 0.02993829 0.5205687 0.02365606 0.1273893
0.456795112 0.1971312 0.3409901 0.149250052 0.4452083
0.2437347 0.4584275 0.4373934 0.3011816 0.05054388
0.3029829 0.308525145 0.144909 0 0.7155602 0.0924668
0.2011354 0.1039819 0 0.03322588 0.7743214 0.07409946
0.169550685 0.257859 0 0.132939037 0.0410548 0.1972839
0.09398299 0.2600815 0.1029644 0 0.02550336 0.1105822
0.7531573 0.3768173 0.09602396 0.7470929 0.07850621
0.04688323 0.1963985 0.09605281 0.086577506 0.181260787
0.087092302 0.03180195 0.03736504 0.04394163 0.3894683

0.1742763 0.3227234 0.096244847 0.06691728 0 0.02314307
0.1579367 0 0.1739317 0.3464896 0.2231235 0.181944
0.8941914 0.156397406 0.1665608 0.1743976 0.2736231
0.8865041 0.06726808 0 0.2956953 0.03945836 1.75020403
0.2881316 0.184623582 0.03565396 0.4091569 0.4312777 0
0.2609672 0.09285441 0 0 0.2589254 0.1599141 0.08369079
0 0.8043391 0.1317932 0.3372137 0.2303172 0.1589974
0.084709928 0.212847 0.5027719 0.6922452 0 0.7392198
0.7916076 0.07605483 0.2858195 0.1011887 0.09435517
0.1002166 0.1321757 2.301588 1.08873 0.4742529 0.3990162
0.1238869 0.1752349 0.06907001 0.1478847 0.05910775
0.183971 0.1090596 0 0.09652531 0 0.6104809 0.2429759
0.03438054 0 0.5027727 0.543794 0.06724431 0.1987203
0.605149 0.06027637 0.02582297 0.08290112 0.3275933
0.8160819 0.8109676 0 0.150572 0.02893455 0.1269369
0.1866766 0.2005252 0.2853154 0.8317942 0 0.1068321
0.048128812 0.1411109 0.326539472 0.2614327 0.1966847
0.1520866 0 0.2685911 0 0.1965549 0.764431
0.073519794 0.2044081 0.09747819 0.1326343 0.1517859
0.06177788 0 0.1228728 0.1477972 0.3853791 0.1934951
0.1072777 0.2379893 0.2043911 0.3550784 0.3980045 0
1.264024 0 0.2464522 0.0336486 0.06472774 0 0
0.1397208 0.2243935 0.4123527 0 0.3356094 0.5895801
0.1569884 0.1878242 0 0.3624932 0 0.192379 5.737371
0.2169355 0.02996468 0.3675856 0.3054475 0.2963868
0.5986966 0.3064319 0.02793722 0.3075446 0.303551
0.3010679 0.264835909 0.2085801 0.134650565 0 0 0.2505938
0 0.3626621 0.07435971 0.03895804 0.2233738 0.07204962
0.2970511 0.1665914 0 0.3443825 0.4469635 0.7359276
0.02707915 0.1081661 0.847678 0.9566032 0.1279865
0.4657917 0.07913507 0.3997164 0.06133141 1.116464
0.415451 0.4186951 0.34152 0.172223 0 0 0.05788756 0
0.1560567 0.2642067 0.066197515 0.1199152 0.7399115
0.1462769 0.0447233 0.4485821 0.1319414 0.03768866
0.3885928 0.05530304 0.06947524 0.2121106 0.5282399
0.0873314 0.08678325 0.04456622 0.408569199 0.1111322
1.784804 0.1515368 0.153345 0.03423447 0 0.5468582
0.7422094 0.4029562 0.147124 0.02329775 0.1238689 0
0.2683754 0.1965859 0.3637169 0.2834185 0.5294749
0.1577754 0.3810037 0.04892984 0.162372 0.5204112 0
0.1294189 0 0.07924389 0.1761088 0 0.3474286 0.053894
0.7742272 0.31102447 0.221400232 0.2242469 0 0.3982468 0
0 0.08297484 0.04110334 0.3724264 0.1398405 0.09314467
0.2280866 0.03184521 0.3402914 0.571820194 0.05980439

0.6590971 0.09850864 0.4584872 0.2064487 0 0 0.02915538
0.1295237 0.313742 0.1577056 0.2626556 0.05211121
0.06108731 0.4404213 0.4029795 0.3194183 0 0.04467736
0.1293969 0.03299398 1.034284 0.2866979 0.1865404
0.056197023 0.02460943 0.1270535 0.05415197 0 0.2620121
0.1089134 0.03804637 0 0.04528485 0.4455174 0.2300797
0.7987446 0.1482292 0.2939528 0.2655063 0 0 0.9942847
0.2145232 0 0.07626381 0.1119334 0 1.875531 0.272484
0.1821925 0.126539724 0.1172648 0.5158158 0 0 0.1376448
0.7392031 0 0.02539369 0.533232 0.2542507 0.1456135
0.0461057 0.08755773 0 0.2516458 0.2645741 0.2371204 0
0.7823408 0.3868596 0.1405528 0.120333881 0.08575102
0.1979335 0.3079308 0.232424882 0.3212392 0 0.1420081
0.2188291 0.1210636 0.3870452 0.05069234
AC015574.1 0 0 0.01934006 0 0 0.09581947 0 0 0 0 0
0 0 0 0 0.007788357 0 0.00791537 0 0 0 0.04187922
0 0.1131971 0 0 0.01664071 0 0 0 0 0.1036579 0
0 0 0 0 0.009441931 0 0.130250587 0 0.00926741 0 0
0.02165079 0.618846 0.03669746 0.01129976 0.021408709
0.008396548 0 0.02975571 3.936107 0.01580713 0.1024865 0
0 0.01077681 0.01054125 0 0 0.028673659 0 0.02460361
0.6365495 0.0641129 0.01434438 0 0 0 0 0.007619463
0.07279716 0.009802567 0.008180581 0 0 0.008557423 0.01591337
0 0 0.03445047 0.06756432 0.01080987 0 0 0.1081696
0.01299099 0.68426709 0.0118578 0 0 0.008980531 0.08874427
0 0.03221963 0.2038049 0 0 0.01065585 0 0.02755377 0
0.01261024 0.7159461 0 0.01516561 0 0.009296434 0
1.808087 0 0 0.04635724 0.1116957 0.01669317 0 0
0.03106483 0 0 0.067859 0.08718952 0 0.05052665 0
0.07692413 0 0 0 0 0.1675614 0 0.02118621 0 0 0
0.01131921 0.02313935 0.06897056 0 0.1771124 0 0.01106861
0.9426361 0.008501775 0 0 0.01852973 0 0.009715668 0
0.009526209 0 0.007682514 0 0 0.01244791 0 0.2462086 0
0 0.010750757 0.009563586 0.01295102 0.008345319 0
0.009825449 0 0 0.01735696 0 0.06729789 0 0.6331803 0
0 0.0151385 0 0.7785556 0 0.0106175 0 0.01958848
0.09613184 0.02597856 0 0 0 0 0.4778256 0.01107823 0
0 0 0.2024031 0.02014847 0.7519023 0.08569739 0.09821661
0 0 0 0.008581999 0 0.008150956 0.01266751 18.33233 0
0.00986536 0.1613618 0 0.009758032 0 0.1412425 0 0 0
0.0110135 0 0.02943063 0.055414261 0.022069264 0.01506175
0.06416958 0.008519591 0.07598204 0.07344501 0.01282627 0 0
0.03667463 0.02742369 0 0.07558805 0 0.01514323 0 0
0.07513789 0.03936817 0.01053434 0.04182381 0 0 0.03028849

0.03500733 0.08417242 0.09189884 0.01499196 0 0 0.2936731
0 0 0.06422377 0 0 0.01974002 0.1218016 0.03852734 0
0.1208357 0 0.06204176 0 0 0 0.02618768 0.06521775
0.1437619 0 0.02934534 0 0.09512984 0 0 0 0 0 0
0.007404843 0 0 0 0.1835178 0.01657611 0 0 0.0342136
0.008482792 0.02179007 0.008657476 0 0.01610932 0.01781941
0.04031448 0.02364129 0 0.07177272 0 0 0 0 0.1330776
0.1933733 0 0.014578452 0 0.02119729 0 0 0 0 0
0.008758219 0.03453008 0.01533315 0 0 0.1260395 0 0
0.02169965 0 0.2264236 0 0.04140365 0.04295013 0 0
0.005738568 0 0.01080936 0 0.01005598 0.03107169 0 0
0.0704513 0.05883705 0 0.03258812 0.2465836 0.01573174 0
0.018501914 0 0 0.07131446 0.03861158 0.7548016 0
0.01252612 0.1231992 0 0 0.3029994 0.09562657 0.1545395
0.3179881 0.01748269 0.01052601 3.971596 0.03928216
0.02017946 0 0 0.0221113 0 0.05613514 0.02563163
0.05998379 0.083322105 0 0 0.0293773 0.1566359
0.009063439 0.07406914 0.019100663 0 0.2633364 0.0313904
0.007990126 0 0 0 0 0 0 0.07677812 0.607135
0.03821008 0 0.633886148 0 0 0 0 1.639319 0.06050061
0.01558458 0.008005084 0 0 0.02503439
UFL1-AS1 0.02651208 0 0.02438683 0.05065188 0.03477504
0.1610979 0 0.06354866 0 0 0.04551483 0 0.08188291 0
0 0.1374901 0 0 0.03273529 0.226497 0 0.07921133 0
0.3806287 0.05447463 0 0.08393236 0.04192738 0 0.06015839
0 0 0.02559148 0 0.06475153 0.1743545 0.08276155
0.02381159 0.04101613 0.281553145 0.06422947 0.2103431
0.027594554 0.3067873 0.1092021 0.01950833 0.1233963
0.05699366 0.10798115 0.06352571 0 0.325177 0.3697535
0.03986396 0 0.1303662 0.07785353 0.1902461 0.1860877
0.071884608 0.030099875 0.216936117 0.02640491 0.5274062 0
0.08982566 0 0.08038641 0 0.09260146 0.04528648 0.05764652
0.6556679 0.04944215 0.06189177 0 0 0.06474284 0.02006594
0.155826612 0 0.1448009 0 0.02726138 0 0.05133993
0.2182341 0 0.90823772 0.2990417 0.127742898 0 0 0
0.06103225 0.1354244 0.5396739 0.05583462 0 0.1074918
0.0663877 0.09265039 0 0.5406294 0.191497 0.03999798
0.03824611 0 0.093778631 0.4241403 0.03211135 0 0.1861078
0.5845412 0.2816852 0.04209848 0 0.4410847 0.1566847
0.08320901 0.1920528 0.5134004 0.1221573 0 0.4332384
0.2057245 0.3637403 0 0 0.04907669 0.07637484 0.2716539
0.1079046 0.05342946 0 0 0.02521762 0.05709178 0.758616
0.5218101 0.07224125 0.5862403 0.1443714 0.02791391
0.100094 0.2358467 0.03441607 0.1586655 0.1168252

0.05179538 0.07350577 0 0 0.02634867 0.05812354 0.1664945
0.1658266 0.06278473 0 2.749757 0.039960966 0.292908 0
0.07235516 0.3266115 0.04209209 0 0.04955756 0 0.163198
0.1313174 0 0.3677232 0.1079138 0.08259393 0.08401772
0.05129368 0 0.07651521 0.03067871 0.1333238 0.02677624 0
0.04940014 0.3151651 0.03275764 0.02065374 0.03587122
0.06559433 0.1833722 0.02273637 0.1117527 0.05374292 0
0.1367663 0.4176327 0.06774986 0 0.5132851 0 0.1427777
0.1303462 0.05198297 0.02164292 0 0.1438911 0.03194615
4.946912 0.1350899 0.07463829 0.3052034 0.02817896
0.02460876 0 0.1526568 0 0 0.021003 0 0.24737752
0.1979228 0.223598565 0.027828214 0.3418579 0.2774213
0.1503988 0.1094965 0.3704416 0 0 0 0 0.1728994
0.02091735 0.3177089 0.07422204 0 0 0.119746 0.5414002
0.0992825 0.1062661 0.03515846 0.3942313 0 0.07638447
0.3972821 0 0.2317595 0.1890409 0.07149768 0.02991793
0.7406136 0.04806357 0.03787751 0 0.08774747 0.082444898
0.07467347 0 0.0242905 0.03713339 0.9142055 0 0.1877556
0.2419841 0 0.05768473 0.2641706 0.3563571 0.3625528
0.1080832 0.3700297 0.024230838 0.2029989 0.1323133
0.1258198 0 0.02842461 0 0.02837825 0.05602277 0
0.02443118 0.03868786 0.5142369 0.02090163 0 0.1632237
0.0647124 0 0.02747617 0 0.0903841 0.04062605 0.04493872
0.7879347 0.05962093 0.04298219 0.7240139 0 0.2047105
0.2034734 0.02403893 0.357982 0.08866684 0.086080373
0.110296101 0.116369 0.01781913 0 0.03335106 0.07554698 0
0.03412777 0.1104367 0.08708134 0 0.02705407 0.1057633
0.4238118 0.094955543 0.0496551 0.1641729 0 0.09516955
0.1714127 0.034805258 0.8665268 0.04841496 0.05377125
0.04341625 0 0.1635606 0 0.02536015 0.1044795 0.02389933
0.07956312 0.07106839 0.1112858 0.179062 0.08218391
0.08883696 0.03967383 0.02581383 0.139979812 0.1225981
0.3516383 0.1348858 0.04868723 0.5710598 0.02260748
0.0315896 0.4438512 0.03759965 0 0.3820667 0.1808703
0.02051225 1.080867 0 0.2389096 0.03867166 0.5283498 0
0.02898594 0.1266425 0.05576243 0.04886506 0.9909697
0.3878421 0.2773338 0.262662293 0.024341 0.06588888 0
0.7242031 0.02285707 0.1334249 0.02408496 0.126505
1.201718 0.1055512 1.309768 0.03828119 0.07269848
0.1568474 0.07835232 0.1464492 0 0.5163376 1.623928
0.2890858 0.04667994 0.566169448 0.5221215 0 0
0.077192205 0.03334029 0.06103054 0.07860545 0.2624441
0.1256477 0.03570673 0.06313416

AC005180.2 0.2068131 1.755123 0.1188966 0.543291 0.4069052

2.068284	0.5591918	0.8055528	0.7582987	0.647785394	
4.970673	0.3555243	1.357333	1.211915	0.4214569	
2.087584	0.4641094	2.763956941	0.2872784	0.717778	
0.7250047	3.192508	0.5262125	1.639232	7.046937	
0.6095981	0.6547321	4.170058	0.69939686	0.4106185	
1.022773	6.511598	0.02495397	0.5699432	0.08418467	
0.3400223	0	0.278621	0.5799184	0.183026244	0.1252589
0.04557851	0.05381429	0	0.7187521	0.09511179	0.300806
0.1945086	1.000266672	0.02064774	0.1790566	0.2195145	
0.3328085	0.3109673	4.838822	0.03177965	0.2277424	
1.696063	0.07776517	0.210281665	0.117400225	0.105766007	
1.287357	0.3932637	0.03557552	4.659682	1.340408	
1.332526	0.103894201	0.09029467	0.06623752	0.112421	
1.074082	0.3374734	0.1207	0.2337675	1.28457	0.1473034
0.1760947	0.075972405	0.04494968	0.112955	0.1107638	
1.116455	1.198138	0.050061	0.5851937	0	0.17712252
1.720394	0.024912138	0.0577315	0.08833519	0.2182288	
0.5653628	0.4489727	0.4259957	0.08166558	0	0.8647164
0.2589356	0.112928	0.2382601	0	1.120359	0.4290174
0.2610535	3.990494	0.274327526	0.1033936	0.07827855	
0.2283305	0.3175755	5.215314	1.739565	0.08209952	
0.1157009	0.06144241	0.4965399	0	0.05350531	0.7231048
0.3096969	0.0959898	0.1987981	0.1755248	0.1891622	0
1.867767	0.669958	0.3537432	0.02943186	0.1052166	
0.02604924	0.05779258	1.285052	0.3196625	0.08350434	
0.2276056	0.2035245	0.1232729	0.3266494	0.1206642	
0.08165563	0.4636025	0.3554105	0.06711746	1.326112	0
1.111112	0.09556622	1.243424	0	0.7450766	3.967294
0.1623469	0.3464907	4.37728	0.07262383	0.8649218	
0.311723978	1.085323	0.660922865	0.09407029	0.8598833	
0.4104353	0.577344	0.3624227	0.4224832	0.7956627	
2.432877	0.119044507	0.8550345	0	0.02684548	0.06144357
0.3751193	0.5211737	0.09947884	0.5085461	0.02600052	
0.2088737	0.4921668	0.1445086	0.07091862	0.574949	
2.215316	0.03497763	0.213201	0.02235052	1.263689	
0.0272422	0.1310103	0.1185651	0	0.5203482	1.023963
0.154082	0.02634204	0.3019025	0.09944355	2.436066	
0.101376	0.02110378	0.04891293	0.1403067	0.7787585	
0.6252918	0.1097705	0.1455579	0.09920016	1.758527	
0.2159616	0.6842963	0.4961798	0.2940367	0.1383282	
0.06143938	1.137487	0.08040503	0.09649615	0.054507124	
0.217079876	0	0.1803403	0.04190062	0.08007664	0.06020226
0.2207853	0.1291752	0.7583158	0.4809901	0.1011554	0
0.03097944	0.7237309	0.1861916	0.04384702	0	0.1847697

0.8519216	0.8030468	1.337023	0.1281369	0.8360024
0.5213716	0.473471	16.84351	0.9792732	0.1658985
0.3718219	0.02917265	0.6258756	0.6561276	0.05540092
0.06317242	0.5133695	0.348361481	0.04854218	0.6988785
0.7342474	0.03620836	0.1650799	1.148324	0.3356437
0.19663	0.02238691	0.02812387	0.2790556	0.3742091	0.3063851
0.1405209	0.1082436	0.047254445	0.1259631	0.1032138
0.3435194	3.703791	0.1385826	0.092473979	2.407404
0.01820906	1.54221	0.166758	0.03772411	1.955564	0.06114285
0.1303674	0.477473	0.9044383	0.08343928	6.296052
2.214096	0.2643976	0.07922803	0.701108	0.2478408
0.2325428	0.1047786	0.08824724	0.9944262	0.7984437
0.1322698	0.3750415	0.1308991	0.1296871	0.111914697
0.035849504	0.1815522	0	0.1410605	0.03252025	0	0.3694743
0.06655523	4.47972	0.1981281	0.1885268	0.07914036	0.6187717
0.3788164	0.24690694	0.1452544	0.1067221	0.5316896
0.1855976	0.04775505	0.067876447	0.246441	0.02360445
0.05243175	1.354711	0.3064316	0.7442686	4.640865
2.47284	0.0764076	0.1864318	0.2844641	0.0173245	0.1085135
0.3142825	0.05342442	0.1154986	2.940099	1.434734
0.022748795	0.07969603	0.1371514	0.4822609	0.04747438
0.6894136	0.7935951	0.184816	0.1298383	0.109989
1.10613	0.2235294	2.998199	0.2600165	0.7139602	0.558886
0.05176847	0.1508332	0.257594	0.2977368	0	0.1543596
1.758071	0.04764778	0.6073783	1.481207	0.245841
0.230507194	0.2373464	0.2569901	0.2408032	0.3851795
0.06686302	0.5984651	0.211364812	0.2261484	1.264291	0
0.07859327	0	1.807631	0.2294103	0	0.04760033	2.45301
0.2832047	1.945412	0.09396145	0.0910342	0.519591137
0.1851327	1.419345	0.6980495	0.075269268	0.3250975
0.1785306	0.1532946	0.0787404	0.02450354	0.3829896
3.03703
AC004908.2	0.1518671	0.6050811	0.4190796	0.4593966	0.1659993
0.435769	0.07821448	0.2730157	0.2735326	0.6025307
0.6952509	0.5967286	0.5863044	0.6407524	0.08252919
1.443885	0.2524482	0.647957123	0.2187676	0.1081188
0.325346912	0.4033246	0.06440144	0.6359284	0.7107643
1.730876	0.2804569	0.4202964	0.146737569	0.05743345
0.3576398	0.06806549	1.465937	0.9566195	0.576973
0.776799	0	0.8865875	0.4894782	0.559999722	4.292408
1.271831	0.737649534	1.448183	0.9383013	1.247853
1.855459	0.5169146	2.087572869	0.6469145	0.6135965
2.220896	1.004707	1.902914	0.9376623	1.77357	1.26356
0.4151513	0.532976	0.240199788	0.833357521	1.070067003

0.4033419	0.3850422	0.5921402	0.5145414	0.3453646	
0.5883799	0.737485925	0.9194325	0.9511739	1.027327	
0.9013948	0.8496469	0.8863246	0.4577607	1.041545	
1.194998	2.030646	0.247947107	0.3960897	0.5806164	
1.464051	1.457486	0.2666113	0.1225361	0.9115267	
0.938339	1.64748627	0.9421376	0.756131133	1.413114	
0.367576	1.816165	0.1748032	0.5430186	0.7851104	
0.8795412	0.9346024	0.9236059	1.07747	0.8845368	1.166395
0.2732511	0.235058	0.9928417	0.9128427	0.3465948	
1.522023916	0.8773449	0.7970775	0.8332581	0.7995504	
0.7812892	0.7171358	0.04019158	0.1132821	1.122947	
0.9723185	0.5560797	1.335862	0.8169088	2.052582	
0.6578813	1.338164	1.374842	0.7408303	0	0.8440246
0.5856713	0.5650939	0.6339643	0.8756437	1.351747	
0.1131687	0.7420073	0.6500347	1.144621	0.2785591	
2.225178	1.206957	1.225979	0.6103985	0.3464437	
0.5255806	0.5117357	1.314286	0.3895164	1.204562	
0.9395357	0.8421149	0.5251657	0.6422059	0.7798104	
0.7953677	1.033194	1.311755	1.288728	3.981911	
0.8468398	0.45781065	1.426166	1.656590495	0.6447256	
0.2182721	1.044822	0.6460276	1.230133	1.413307	
0.9659955	0.6895309	0.291389428	0.594113	0.2833208	
0.8936644	0.6818024	0.3427919	1.385041	1.534037	
1.435165	1.527417	1.405986	0.4818776	0.9904126	
0.5091973	0.7192984	0.374646	1.095884	1.315086	
1.312996	0.3690104	0.5067808	0.5643943	0.5804318	
1.04457	0.6202242	0.5174484	2.740637	0.7221573	0.3842682
0.7594438	1.119978	1.017381	0.8058407	0.3831229	
1.72698	0.274492	0.378993	1.074757	0.7838321	0.453256
0.5649537	1.621091	0.418744	1.384549	0.686506	
0.2979599	1.58408	0.3182019	0.734758162	0.6141122	
1.120719638	0.265677026	1.414283	1.964343	2.789676	
1.777125	0.2357747	0.6176274	0.7082584	0.3997875	
0.7947021	0.3631489	1.098343	1.728912	0.7086006	
0.3645983	0.7298162	1.457605	0.3618138	1.478652	
0.9638006	0.6713184	0.9409353	1.874686	0.9237109	
0.6742875	0.9626304	0.9588005	1.137012	1.615466	
2.427835	3.063955	0.6424107	1.21142	0.9277762	2.450356
0.787104605	1.045602	0.7087059	0.7652778	0.5672223	
1.745591	0.6536729	1.224882	0.5775578	0.9205932	
0.3855029	0.735597	1.099158	1.015316	0.8254993	
1.059806	1.202930221	0.7752152	1.162144	0.7687721	
0.8913958	0.5970158	0.060360483	0.6502277	0.4991948	
0.05807573	0.3965172	1.791369	2.01287	0.7582849	2.765576

0.8014131 0.5354369 1.327542 0.5508637 0.8129279
1.229633 0.6593594 0.514838 2.232467 0.1138407
1.723481 0.3240088 0.6281528 2.540692 0.6475228
0.6655517 1.00394 0.4867408 2.081925312 2.351702463 1.955323
0.5613958 0.3156835 0.6686482 1.29825 0.1973182 2.182988
0.8434745 0.9976426 1.199806 0.6198869 0.7572946
0.8429474 1.541125156 0.3555444 1.358383 0.3644019
1.181164 0.818242 0.398744561 0.241289 1.871989
0.4106849 0.6079283 0.4250359 0.728709 0.3304613
2.784315 1.321647 1.140839 2.506654 0.4070956
1.452014 1.367609 1.150766 1.667989 0.9469189
1.306162 1.113660526 1.209464 0.3357104 0.2575521
0.836674 0.8307702 0.5180028 1.417459 0.593245
1.543551 1.271354 0.8024733 1.093625 2.310808
1.098482 0.08418492 0.4308327 0.8860795 0.6620481
0.9960004 1.245284 0.6045302 1.100223 1.142966
1.702949 0.06171239 0.7221045 0.852599972 0.6041997
0.345974 0.6129995 0.6285449 1.0038 2.85334 0.781796135
0.5032285 0.7547928 0.9825085 0.5771265 0.4385664
0.2082166 1.123071 0.3989507 1.677787 0.8353834
0.9242802 1.727556 1.042634 1.225552 0.540524142
2.922855 0.6724223 0.2929098 0.921196178 0.5411117
1.689717 1.013106 1.888809 0.4078516 0.4431615
0.5424698
LINC01170 0.01437054 0.04122452 0 0 0 0.04366057 0 0 0
0 0.03700612 0.1693977 0 0.2155794 0 0.0212928
0.02866573 0 0 0 0.016792476 0.02862368 0.164539 0
0.0295273 0.225911 0 0 0 0.1956487 0 0 0.05548618
0.09052087 0 0 0 0.01290677 0 0.012717699 0.04641972 0
0 0.04619173 0.01479792 0 0.01672136 0 0.029264921
0.01147777 0 0.01355832 0.04625084 0.2376854 0.2241525
0.05299752 0.06329931 0 0.02881901 0.01948208 0.032630527
0.019597922 0 0 0 0 0.01960824 0.01452415 0 0.04015478
0.3068372 0 0.0426476 0 0.07827788 0.02598958 0.0223148
0.01169768 0.2284064 0.04223194 0 0 0.01539299 0 0 0
0.01478639 0.01775821 0.78767837 0.03241837 0.01384829 0 0
0.03639305 0 0 0.01392969 0.03026446 1.362741 0.01456616
0 0.138105 0.1324453 0.01723773 0.02965673 0.0108402
0.02073083 0 0.063539429 0 0.1044333 0 0 0.06336866
0.7125251 0 0 0 0.04246449 0.09020482 0.02974281
0.09276076 0.06621383 0.08003897 18.52406 0.02787761
0.09200842 0.01554246 0.01331108 0.01330071 0 0 0 0 0
0 0.05467559 0 0.03163065 0 0.05873621 0.07565816
0.01117925 0 0.06781837 0.04648646 0 0.1965776 0.1013179

0 0.03984289 0.01355297 0 0 0 0 0.08988424 0
0.2422231 0.04807973 0 0 0.029391782 0.01307307 0.1593322
0.06844648 0.04584815 0.02686206 0.09785508 0.08845943 0 0
0.1839875 0.05849334 0.134307 0.05692596 0 0.04138754
0.04147412 0.01662902 0.05781321 0.04354118 0.09656057
0.01338837 0.0919861 0 0.04478042 0.0388871 0.01185153
0.01242433 0.01232397 0.04543061 0 0.02196953 0 0.03772877
0.009180742 0.01427532 0.102502 0.2013878 0 0.01177542
0.01408836 0.5161763 1.957678 0.01114206 0.08658008 0
0.01220397 0.01348559 0.0367626 0.03054811 0.04001662 0
0.1241186 0.02514626 0.01537892 0 0 0.014898657 0.0134102
0.348446627 0 0 0.1629039 0.06987581 0.01483781 0.2342588
0 0.02872265 3.680339 0.2005315 0 0.06802794 0.03444203
0 0.06210071 0.02437391 0.0162267 0.02934593 0 0.04320018
0 0.03561469 0 0.02760217 0.0717806 0.01438258 0
0.112714 0.06459072 0.01621664 0.02676269 0 0.01026552 0
0.01189061 0.044688234 0.09444359 0.05549946 0.01316637 0
0.09176551 0.01484503 0.06784705 0 0 0.01563365 0.178988
0.01485838 0.0524046 0.05858513 0.180513 0.013134025
0.120036 0 0.4228342 0.03450642 0.07703603 0 0 0 0
0.03972785 0 0.1393679 0.02265894 0 0.0884734 0.01169218
0.01159567 0.02978624 0 0 0 0.02435848 0.137771 0
0.01164898 0 0 0.04755461 0.03676341 0.01303 0.1576573
0.01201519 0.124423461 0 0.06307637 0.06761048 0.02240385
0.01807753 0.6688386 0.09335696 0 0.01197217 0.04720136
0.01047993 0.0733216 0.02866384 0.01914349 0.051469473
0.02691493 0.02966263 0 0.05158547 0 0.094328791 0.05871126
0.05248546 0 0.09413296 0 0.014776 0.04690524 0.01374616
1.543217 0.03886301 0.0143754 0.009630435 0.06032107
0.2523516 0.05939569 0.01605099 0 0.05596826 0.177040079 0
0.1334208 0 0.07917093 0.0147398 0 0.0342455 0.0481168
0.02038042 0.1069359 0.3520607 0.09803852 0.01111841
0.07559618 0 0.172664 0 0.03579818 0.1103383 0
0.01716124 0.01007511 0.4900039 0.01534694 0.1751875
0.7926245 0.042711851 0.01319374 0.05357137 0 0.07137187
0.1362833 0.1157141 0.013054954 0.03428524 0.05142446
0.01430317 0.02184442 0 0.01970265 0.3400688 0 0.330754
0 0.0349843 0.4023909 0 0.05060457 0.09026033 0.1157766
0 0 0.167364307 0 0 0.04260711 0.04377063 0.08172693 0
0
AP001527.2 0.1611733 0.6825238 0.5506553 0.659839 0.2114058
0.8394454 0.6403423 0.8830343 0.2132774 0.43271243
0.8696146 0.2261769 0.1422245 0.3238175 0.1501482
0.9040665 0.413359 0.83212989 0.08528815 0.6884649

0.645725264 0.3210297 0.6444226 0.303015 0.8988746
0.5067425 0.4373528 0.9467216 0.177976471 0.2612266
0.5205331 0.9700321 0.7112079 0.8702053 1.087196
1.211363 0 0.4756279 2.244122 0.855814473 1.097024
1.54259 0.886699163 0.2072259 0.426771 0.4235565 0.5626169
0.8414468 0.304777225 0.8091557 0.584748 0.3475743
1.111559 0.4500648 1.032531 0.5660907 0.5409031
1.250964 1.8008 0.686720155 0.365968689 0.942004693 0.710882
0.4041464 0.9188744 1.310572 0.502667 0.3956047
0.601466744 0.5790306 0.5899443 0.3838229 1.024962
0.3005706 0.4120887 0.6662558 0.5362979 2.174101
1.394122 0.631538178 1.120964 1.559352 0.2466294
0.6392383 1.261143 0.6910956 1.066096 0.7682186
0.78877064 0.753149 0.155315929 0.8226968 0.1180136 1.0107
0.3710297 0.7997549 0.9373733 0.3636772 3.188164
0.5134397 1.066622 0.6235914 0.2652578 1.657117
2.518378 0.6947351 0.7971677 0.7738837 0.814432486
0.3376543 1.575644 0.7210126 0.8485456 0.4061218
1.141621 0.7129387 2.035218 0.4195481 1.83701 1.90295
0.7386446 0.5449511 1.697425 0.8121863 2.478833
0.2903291 1.095102 0.2241218 1.066363 1.065532
0.3150613 0.8126178 1.593089 0.2552082 0.926513
0.6749797 1.795846 0.1983283 0.7601882 0.3021151
0.7842354 0.7515697 0.1253812 0.9939298 0.9779388
0.3910277 0.8966722 1.062991 0.9942918 0.38235 0.9149973
0.9771668 1.001475 1.487384 0.9927366 0.5060799
0.7817914 1.499471 1.61706 1.694753 1.075842936 0.5850733
0.518012697 0.9006745 0.5672999 0.5666088 0.6611289
0.06455834 0.4703557 0.2551164 0.5131993 0.742188731
1.400244 0.9840508 1.434595 0.8938367 1.13594 0.8620547
0.7088065 0.8259429 0.4168324 1.441758 0.541489
0.7293371 0.9264002 0.6543222 1.793701 0.3738338
2.050784 0.9555114 1.125504 0.1698428 0.8634639
0.4927998 1.385725 0.8865963 0.397158 1.280842
3.190768 0.4840009 0.5845579 0.566004 0.564316
1.334521 1.176236 0.7140806 0.3884166 0.5303962 1.4274
1.16677 0.2650578 2.691961 0.4701794 0.8380189 0.552403
0.8057961 1.749468 0.8390548 1.905597 1.002578094
0.7305263 0.315553918 0.580026419 0.5772877 0.1806976
0.2985502 1.545272 0.5361912 0.730386 0.9203992
0.8312539 0.7764637 0.7207519 0.290655 0.2483265
0.9346566 0.431162 0.07810468 0.3639822 0.7522975
0.7242745 0.7383055 0.7328116 0.9130005 0.1441133
0.6633707 0.8050576 0.8987173 1.174101 0.3447675

0.6416284	0.1558957	0.9433531	0.6261213	0.9210658
2.785093	0.9525676	0.787603562 0.2377875	2.445363
0.6328619	0.6127304	1.058605	0.6659798	0.5163526
1.961439	1.0767	1.001941	0.7647411	0.6665785	0.9235999
0.6257747	1.606785	0.27356654	0.2884859	0.09192731
0.5026411	0.9030182	0.3456	0.494171565 1.0844	0.4541011
0.4490515	1.039661	0.3695887	3.03685 0.7805484	0.6579664
1.174531	1.517408	1.411988	1.216964	0.3033821
0.3924761	0.4233866	1.639159	0.684292	0.6731212
1.119853	0.3929869	0.399987	0.5841455	0.7853736
0.9603382	0.5440646	2.19461 0.523303189 0.510869632 1.010621
0.247604	0.7538131	0.8689247	2.055772	0.7478913
0.6816901	1.496198	0.9075228	0.9067213	0.4229181
0.2755544	0.3987368	0.30237296	2.15618 0.4277341	0.2130971
1.570373	0.8081274	2.025213726 0.5330536	1.051165
0.3969358	1.734448	1.046206	0.3787901	1.127287
0.9029982	0.3856303	0.6849374	0.644911	0.3240313
1.385281	0.9952554	1.260935	0.9001019	1.688307
0.6949688	1.965337267 0.01774531	0.9161543	0.4685733
1.818171	1.889311	0.8835191	1.042507	3.199386
0.3591929	0.4711705	0.895889	0.5759569	1.549829
0.6661689	0.2680309	1.267958	1.108302	1.462591
0.4419653	0.5286374	0.852378	0.9201243	1.18825 2.188434
0.5613774	3.569019	0.159678693 0.6764562	2.203048
0.8578854	0.8004734	0.7940205	0.5793726	1.108595228
5.346759	0.3295729	4.147943	0.9974868	1.196848
0.6629272	1.77081 0.4309585	1.653414	0.7789189	0.22421
1.410321	0.2231647	1.236464	0.173540146 1.030552
1.304918	0.4884924	0.703904832 0.8686439	0.636033
0.5119943	0.5084434	0.5019536	0.8682789	0.822445
AC147067.2	0.1005441	0.480715	0.4161794	0.1440687	0.4615816
0.661858	0	0.421752	0.3104454	0.188956085 0.6904395	0
0.5434305	0.282808	0.3278322	0.6703915	0.1002805
0.340662172 0.2482898	0	0.469956911 1.101467	0.5756018
0.5413093	0.4476093	0.3951489	0.5570321	0.7950244
0.097148056 0.6844318	0.2841318	0.7210074	0.242632
0.4749996	0.4911258	1.542846	0.1046213	1.399693
1.011069	0.13346983	0.8931378	0.3102177	0.313947726
0.420137	0.8282737	0.3699157	0.5264625	0.2161421
0.153564889 0.1606094	1.2187	0.5217373	0.3235958	0.4535388
0.5881095	0.1235997	0.3690635	0.9276262	0.05040834
0.272614292 2.168857446 0.342794095 1.101514	0.1764819
0.2075444	1.873595	1.714873	0.762141	0.353563828
0.03511806	0.3864237	1.457453	0.5967712	0.4687595

1.017109	0.4545928	0.9367584	0.6547465	0.3424403	
0.049246242	0.7866973	1.153197	0.7000349	0.5169286	
0.211813	1.508932	0.5689945	0.7454763	0.51665841	
0.1134082	0.38756046	0.6736008	0.8159543	0.08487515	
0.1735935	0.5135816	0.6822175	0.2646831	0.1265638	
1.070083	0.6923617	0.3513662	0.6949939	0.7839292	
0.9855988	1.516878	0.4351322	0.6883925	0.311189299	
0.4021261	0.3044467	0.2018272	0.5293448	0.9421428	
0.7121729	0.6785284	1.181231	0.4381049	0.6684851	
0.1051869	0.8844112	0.2704185	0.3242874	0.6533285	
0.2416188	0.5363787	0.1379444	0.978691	1.53667	0.5583535
1.086161	0.1144685	0.9207359	0.3545941	0.3371567	
0.448531	0.8128974	0.1623856	0.9405465	0.06596354	
1.232851	1.217494	0.9777004	0.6880923	0.5219433	
0.1626222	0.1305191	1.504303	0.0886093	1.227676	
0.5575247	0.806003	0.1366632	1.898563	1.212348	
0.1578529	1.212837	0.178578	0.9885887	0.5045876	
0.985058556	0.2777052	0.102820388	0.685997	0.3096594	
0.5986106	0.1603892	0.2349268	0.4792529	0.8045829	
0.913012	0.347247471	0.8045475	1.176597	0.7830704	
0.1991424	0.4376824	0.5067468	0.5319881	1.221628	
0.7078618	0.9646858	0.4503932	3.091184	1.149258	
2.049789	0.626616	0.06801878	0.7048175	0.1738546	
0.5604632	0.4238094	0.3566745	0.3842767	0.08644511	
1.099879	1.124085	0.5493281	0.05122569	0.1761272	
0.3867632	1.359389	0.492849	0.2051959	0.760943	
0.2338676	1.09037	0.1737093	0.6830842	0.5661146	0.1286055
2.083878	0.4666298	0.9980307	0.9648893	1.495463	
0.4303969	0.1194773	1.737999	0.312717141	1.032075	
0.158994828	0.949818349	0.864305	1.008252	2.688889	
0.15572	0.7024291	0.1226706	0.3014387	0.05671726	0.7015133
0.3934208	0.4759602	0.2409751	0.7036968	1.59313	0.2984329
0.2270616	0.05132998	0.6400801	0.4030024	0.5333381	
0.9967185	0.05244261	0.8207586	0.5859185	1.056597	
0.2929739	0.9319913	0.6778673	0.1701906	0.9362312	
2.916411	0.2513809	0.3685422	0.4575621	0.990099518	
0.4719846	0.5824563	1.381785	0.9153574	0.7062451	
0.1557958	1.602094	0.3823739	0.2612064	0.8203608	
0.6678915	0.5197862	1.787423	1.093049	2.034779	
0.091892726	0.2449524	0.5017832	0.5248732	0.885227	
0.4311885	0.539484875	0.6995385	0.10623	0.4037167	0.4169363
0.5868775	0.3900371	0.1189006	0.338023	2.520245	
1.104365	0.1622591	0.729402	0.7038019	1.92809	0.6162789
4.090201	0.1445881	1.356634	0.1222538	0.6006309	

0.3742834 0.5545296 0.1714778 2.552618 0.08485041
0.4203238 1.19698383 0.557714079 0.2647898 0.2365197
0.1567494 0.758881 0.8595103 1.306351 0.2588515
0.2931731 0.8256154 0.476601 1.231194 0.6016431 0
0.420126255 0.2353892 1.45275 0.05169715 0 0.5107642
0.131995028 0.1369252 0.3213145 0.05098035 0.87814 0.6455563
0.4135235 1.066567 0.3366143 0.148585 0.3625421
1.005781 0.4042783 0.4220388 1.290238 0.3636188
0.1123014 1.203668 0.3915842 0.176952565 0.3099597
0.7334518 0.1705131 0.1846409 0.7218933 1.328913 3.2346
0.3366511 0.4277773 2.852442 0.5795781 0.6859304
0.8556944 0.5950256 0.6688186 0.9563732 0.5133027
0.7513902 1.013232 0.2748148 0.5403119 0.2467182
1.019234 0.590565 0.5515679 0.1434214 0.199223442
0.4153973 0.4372833 0.09365497 0.9987122 0.2167071
1.264997 0.319688263 1.23937 0.2398622 0.3502546 0.955221
0 1.72313 1.487065 0.7923796 0.2314134 0.9955226
0.06119225 0.351918 0.3045348 0.2655427 0.442056992
0.6300277 1.157466 0.0969609 0.439114136 1.074734
2.083063 0.894307 0.1148411 0.09530096 0.9478962
0.3591438
UCKL1-AS1 0.157306 0.331696 0.285682 0.1194439 0.2909827
0.4860966 0.07478364 0.09668147 0.1432211 0.121284417
0.9071124 1.141107 0.07474486 1.554306 0.2761818
0.3018085 0.4505661 0.270287239 0.2490136 0.2756699
0.075412297 0.1847824 0.4310355 0.2798885 0.2845425
2.384146 0.159616 0.5485712 0.132506496 0.03660944
0.3419521 0.278397 0.4165969 0.1778501 0.2758322
0.4067304 0.07134986 0.311547 0.6458503 0.192756817
0.459271 0.4302373 0.142737847 0.1503937 0.2782805
0.934905 0.9480483 0.9147783 0.316239077 0.531556
0.2234977 0.7763249 0.2553041 0.1880092 0.2595225
0.2627953 0.2901892 0.4796369 0.6835074 0.524945234
0.160276404 0.418052721 0.08034364 0.3067938 0.2886338
0.4263751 0.2806831 0.142681 0.688926425 0.8396552
0.1998037 0.1695574 2.150649 0.4099498 0.536716
0.4449759 0.4697445 0.7584367 1.260801 0.193608005
0.2735167 0.5331183 0.2073823 0.5847952 0.2676623
0.06248591 0.6557324 0.2043575 0.64943284 0.4686038
0.167136793 0.9322788 0.1171508 0.7388638 0.1624929
0.7746784 0.2111265 1.04483 0.4671125 0.3025409 0.9999071
0.736496 0.2602207 0.3144857 1.140384 0.4290066
0.3374831 0.2560753 0.142672654 0.2849975 0.366401
0.7837531 0.1203346 0.7025529 0.4999742 0.2722025

0.9657913	0.4313932	0.4469559	1.223725	0.3214032	
1.037095	1.479344	0.02995351	0.9615381	0.6337937	
0.276693	0.1963088	0.4819595	0.5450483	0.2178655	
0.8587207	0.6566541	0.1341225	0.3065798	2.13352	0.5793192
1.077041	0.3861933	0.5927562	0.2472889	0.4841702	
0.2353321	0.3907017	0.7461753	0.2772636	0.3979346	
0.6517533	0.9313324	0.2048808	0.4212263	0.2244367	
0.299708	0.5291386	0.4657199	0.3292912	0.1477668	
1.117575	0.5438924	1.133571	0.13375053	0.3163927	
0.490851742	0.8476119	0.1490698	0.278565	0.6498612	
0.17718	1.202998	0.2184914	0.3629397	0.246103531	0.7530999
0.205222	0.3811581	0.4537704	0.944248	0.6447184	
0.6014438	0.4760737	0.9452148	0.6965985	0.3929844	
0.7177421	0.2950677	0.7026978	0.3425012	0.1964651	
1.087751	0.8194985	0.4081689	0.454798	0.3311411	
0.2343213	0.3953388	2.908614	0.0773048	1.951292	
0.4233301	0.2496521	0.3599625	1.110512	0.3677485	
0.4741495	0.5647393	0.3596423	0.2818924	0.3437857	
0.3733661	1.252868	0.5778322	0.681646	0.3968556	
2.019674	0.2980522	0.1658627	0.07338076	0.5847487	
0.5070735	0.372172756	1.11789	0.187097745	0.228620908	0.2918309
0.8687452	0.313801	1.111958	0.4978302	0.2706621	
0.556264	0.3230931	0.6894871	0.6470909	0.3023202	
0.4253524	0.7170401	0.2091632	0.3523222	0.2504958	
2.182736	0.7280422	0.3475924	0.3102378	0.1699361	
0.09677569	0.6662682	0.5036798	0.7912254	0.5641497	
0.4544119	0.6599013	0.6235755	1.006566	0.987158	
1.466581	0.6800944	1.688737	0.163058705	0.3635408	
0.4712177	0.8388788	0.1751311	0.2472624	0.1625	1.218761
1.527818	0.7754247	0.2939968	0.7569174	0.6630954	
0.3640425	0.3562763	0.410957	0.27279537	1.08374	0.8937647
0.7082516	0.3712649	0.3589297	0.1009974	0.4619619	
0.6534435	0.1712122	0.2824847	0.1942341	0.8136422	
0.4928885	1.640801	0.4576272	0.233003	1.220491	
0.3009716	0.1594398	2.268887	0.2595917	0.6973611	
0.5259014	0.3356119	0.356387	0.5369803	0.2902896	
0.3648318	0.4952956	0.8338479	0.2076385	0.6812233	
0.449631741	0.313230311	0.9843485	1.059986	0.1100444	
0.2435498	0.3716246	0.188663	0.8411236	0.3629141	
0.2163898	0.7236038	0.3416233	0.1689511	0.4137323	
0.365973391	0.5250317	0.7493066	0.0290348	0.709465	
0.7972521	0.783688413	0.2581698	0.2136065	0.4008511	
0.5328234	0.3785023	0.2239536	0.2501396	1.601168	
0.5960726	0.4617707	0.944155	0.2135406	0.7336663	

0.354147 0.1792136 0.2973399 0.4406196 1.205668
1.164192641 0.5035984 0.3209847 0.6566791 0.7407164
0.3888902 2.455768 0.456567 0.2836113 1.779021
0.7466106 0.2208817 0.321034 0.6927924 1.188216
0.3152611 0.7229045 0.5118573 1.271039 0.4761557
0.3175095 0.6261805 0.4637684 0.6876652 0.951966
0.796573 0.6252224 0.25574936 0.281442 0.4510882
0.4170402 1.041688 0.354697 0.8078983 0.769488406
0.3785083 1.477047 0.7908718 0.4690395 0.3843848
0.2212036 0.3221425 0.266221 1.3294 0.4160103 0.1865667
0.981184 0.4446952 0.5006754 0.192538665 0.9748763
0.6607859 0.1633692 0.381674952 0.6644732 0.9331471
0.1554651 0.3869909 0.1796881 0.7713922 0.2977577
AC100861.1 0.6239284 0.03409242 0.6121742 0.8968593 1.091183
0.186553 0.4957742 0.1851618 0.7705901 1.19862818
0.3927493 0.9572878 1.532433 0.6462752 1.084997
0.06163154 1.173466 0.398190035 0.9758197 0.8376222
1.284572916 0.02958951 0.7937576 0.4692085 0.07325669
0.2895819 0.8747503 0.08457498 0.838254618 0.4045006
0.6884821 0.06391753 0.8947912 0.9731826 1.257778
2.253511 0.1174797 1.179458 1.452491 3.454985281
1.257234 1.529574 3.754168498 2.062815 0.972904
2.247423 1.472731 0.6897981 2.849774927 1.101078
1.440509 1.087627 0.9689787 1.876296 0.3128165
2.826946 2.146274 0.7248805 2.204566 1.554765581
0.877020496 2.552659343 0.7101786 0.4450184 1.839887
0.1691147 1.467538 0.642609 2.053748265 1.930201
8.759535 1.623659 0.6995078 0.3213638 0.3467964
2.160069 0.9919135 0.8996739 1.960865 0.308509075
2.830966 0.9476674 1.062948 1.252574 1.583551
0.3969868 1.15557 3.216219 1.03817701 1.843173
1.099435318 1.665383 0.8730913 1.379438 1.921929
0.6859728 2.321234 1.051198 1.500976 2.035794
1.309399 1.848157 1.314377 1.218845 2.054047
1.120598 1.354397 0.9394338 4.082879963 1.093224
1.111959 1.316855 3.482995 1.565614 1.136418
0.6274653 1.256597 1.158083 0.49604 2.921788 4.464378
0.3899556 0.6406736 0.9818441 2.598909 1.948115
2.934915 1.298205 0.5724255 0.6159777 1.206816
0.6765114 1.971057 1.862142 1.846479 0.802822
1.034325 1.445951 1.307917 1.450227 0.9633939
0.9760739 0.799707 0.9822499 0.6898501 1.177348
2.869498 1.066855 0.8588397 1.404682 1.345451
1.759691 2.309971 1.482293 0.6947883 2.015094

3.206969 1.885648 1.736081 0.7952328 1.316603512
0.6893222 0.808201889 1.40007 0.8491633 1.929281 1.118526
0.3110066 1.83701 0.3584615 0.1373507 0.588308701 2.174571
1.148874 3.042114 1.365245 1.276111 1.232181
2.709608 1.017655 0.3406546 2.166499 0.7320041
1.389548 0.581407 0.5873599 0.5832719 1.382854
0.9948161 3.575645 2.089328 1.133387 2.36693 0.9447705
3.596682 0.6656312 1.837365 1.109726 2.452233
1.693222 0.649161 0.8180089 2.137957 0.9798713
1.315428 2.188423 1.253021 0.3148319 0.4642598
1.789976 0.7752624 1.035787 0.6287765 1.271429
2.959606 2.521491 1.647016 1.030925 1.213917
1.724953721 4.264163 1.572368889 1.397124305 0.8939105
1.741003 2.032172 1.877429 0.5396781 3.987423
2.090304 1.441361 0.4767852 1.829098 0.8954507
2.599106 1.38906 1.934444 1.662959 3.247491 0.2487562
1.330686 1.953037 0.9692519 3.47547 1.518691 0.9758468
2.968106 0.5828208 1.844028 1.910883 0.8867077
1.481923 1.405419 1.432747 1.935611 0.1960283
3.102456 0.813051693 2.248287 0.5392982 1.371951
3.179287 1.3736 3.253339 1.662232 0.6327546 1.960544
1.848837 1.899615 0.8785769 3.255788 0.7671178
1.169385 2.134335418 3.056659 0.8659402 0.7388427
0.3995124 1.019333 1.084044658 1.310251 0.7031603
0.79078 1.807008 1.786256 1.913256 1.770817 2.127575
1.965055 2.078913 1.342536 0.1724312 0.2055274
1.14457 0.7648664 0.4230305 1.691947 1.149209 2.268721
1.769799 1.659017 0.734111 1.013437 1.260761
2.432118 2.444376 1.086854197 1.021791499 2.509077
0.5950789 0.7503777 1.794001 1.083674 1.297055
2.914302 0.5247481 0.7937164 0.4853428 0.3698827
1.274134 0.5936832 0.666850707 0.645496 4.513669
0.2994206 0.5545913 1.212939 1.591390359 1.416154
3.841356 1.018377 0.8271284 2.306759 2.15677 0.5042745
2.267913 1.972889 1.226656 1.295832 1.660559
0.8896186 2.713012 1.436756 3.398162 0.7825075
0.5785676 1.845825138 0.5495604 1.363465 1.219361
1.200354 1.420103 0.714452 1.663848 3.631047
2.831554 1.906887 0.6508104 0.4999764 1.057409
1.500422 0.751019 0.7437086 2.132208 1.76149 2.30404
2.267329 0.6315548 0.6499003 1.018552 0.9518868
0.9634453 2.26033 0.753545353 3.246065 1.070661 1.809954
1.209994 0.9323814 2.039496 1.873169621 0.8222569
1.743636 2.08184 1.169722 1.561559 0.8309927 2.285033

1.627335 2.303136 0.8089894 2.220515 1.091917
1.857401 1.229334 0.791234233 1.388325 1.21553 0.2062947
4.429096152 0.8668202 1.484153 1.7706 2.832497 1.960043
1.496557 0.9292041
AL589986.2 0 0.06998823 0 0.06991745 0.1440055 0 0
0.1315793 0 0.18340322 0.9423978 8.483968 0.2825682
2.573411 0 0 0 0 0 0 0.042763711 0 1.024259
0.04378349 0.3007768 2.272455 0 0 0.07071987 0.1660796
0 0.09841206 0 0 0.05958663 0 0 0.2300787 0 0
0.1477655 0.06452171 0 0.02352637 0 0 0.04258261 0
0.037263017 0.02922929 0.04224594 0.03452761 0.6674334 0
0.21406 0 0 0.03751525 0 0 0.083096885 0 0 0.2997674
0 0.1239911 0.09986866 0 0 0 0 0.03620212
0.2047428 0 0 0 0 0.08309424 0 0 0.03997527 0 0
0 0 0.03765501 0.1356692 0.25073767 0.1238349 0 0
0.1250488 0.1235714 0 0 0 0.03853573 0.03071112
0.03709416 0 0 0 0.0438976 0 0 0 0.09111309 0 0
1.263262 0 0 0 0.5184332 0.02905537 0.2866294
0.05798595 0.02703501 1.072006 0 0.314966 0.1686201 0
0.140711 0.1064896 0.06694531 0 0.06779599 0 0
0.166657 0 0 0 0 0 0 0 0 0 0 0.02846906
0.5009035 0.1036237 0 0 0.06257554 0.032252 0.07149589
0.03382129 0 0.03316176 0 0 0 0 0 0.2056151 0
0.055160225 0 0.037424549 0.1997513 0 0 0 0.03420345
0.09967908 0 0.06042145 0.042130361 0 0.9682343 0.1140087
0.05798704 0.07080337 0.1580962 0 0.04234744 0.03680678 0
1.639341 0 0 0 0 0.04951493 0 0.06327956 0.06276841
0 0 0 0 0 0.02337967 0.07270703 0.1118707 0
0.1689288 0 0 1.194995 0.4846939 0 0 0.2107557
0.06215722 0 0 0 0 0.04036256 0.1053601 0.03201872
0.0783279 0.02899154 0 0 0 0.038580609 0 0 0 0
0.4912174 2.130584 0 0.03657254 0.5367424 0 0.8114511
0 0.04385503 0.1536789 0 0 0.04132292 0.03736616
0.02740897 0.03667119 0 0 0 0 0 0.1465066 0 0
0.06579468 0 0 0 0 0 0 0 0.03435857 0.1413349
0.03352946 0.05125718 0.420642 0 0 0 0 0.1592506
0.09116214 0.07567668 0 0.04973096 0.1021543 0 0.02547364
0.1461112 0 0.02929134 0 0 0 0 0.04198422 0.1348946
0 0.07098283 0 0.06151678 0.03218661 0 0 0 0.03013761
0 0.1682348 0 0 0 0 0 0.09082108 0.04036752
0.1248289 0 0.09265152 0.06119576 0.475285084 0.456742544
0.03212605 0 0 0.04603623 0.03476049 0 0 0.03048831
0.04006765 0.02668818 0 0 0.04875081 0.043690712 0 0 0
0.5254702 0 0 0 0 0 0 0.0722983 0 0.1194489

0.07001191 0 0 0 0.04904972 0 0 0 0 0.1095278
0.4632184 0 0 0.09707697 0.18619 0 0 0 0 0 0 0
0.158216 0.08322161 0.2831414 0.0962566 0 0.1832108
0.05338053 0.3646545 0.07024688 0 0 0 0.06745102 0
0.8030384 0 0 0 0.04547494 0 0.09087778 0 0 0 0
0.087305 0 0 0 0 0 0.1081538 0.03369192 0 0
1.473047 0 0.09665218 0 0 0.06481451 0 0.053276232
1.01247 0.04212183 0 0 0 0 0.08714741
AC005165.1 0.1027968 0.6272294 0.1981183 0.1449586 0.4815547
1.34346 0.04537915 4.071482 0.9068603 2.545198575 1.43703
0.1634905 0.2343374 0.477318 0.06384334 1.258402
0.1074096 5.650117797 0.09670588 0.1568233 0.223083067
1.784283 0.3113749 1.118588 0.3453242 0.3578308
0.1549694 0.9250864 0.283784921 0.2443631 0.1106659
4.769614 0 0.0925033 0.06376252 0.08584571 0.01528077
0.09672254 0.2915628 0.043320733 0.05138935 0.04315216 0
0.003146889 0.1663419 0.003601942 0.3645347 0.0947078
0.722724243 0.08210398 0.03955575 0.4248944 0.06301833
0.2502511 11.28128 0.05415818 0.04312372 0.5369307
0.03435851 0.039817485 0.138938103 0.053405657 0.4144005
0.3207754 0.006736334 1.857527 0.8682988 0.192949
0.034427206 0.04445369 0.00836152 0.01064363 0.5423487
0.6983537 0.02666406 0.1416467 0.3382519 0.05976933
0.003704898 0.01438561 0 0.06951226 0.01048674 0.6744803
0.6857695 0.08057324 0.8562455 0.3205992 0.85523679
1.639853 0.004717191 0.005465819 0.756876 0.214876
0.07888131 0.02500423 0.3368892 0.03092726 0.01643169
0.3225118 0.2757953 0.03848987 0 0.08807622 0.8435231
0.26217 0.1129858 1.139512 0.021643653 0.05220775 0.09486263
0.06288741 0.008590557 2.169341 0.3987376 0.04275095 0
0.02326862 0.1952751 0.03072678 0.01519709 0.3949677
0.3022322 0.01817597 0.04234841 0.3940858 0.04925037 0
9.036659 0.6342931 0.1128124 0.05573014 0.0996154
0.01973002 0.0109432 0.4617002 0.1210582 0.03162361
0.118519 0.012846 0.03334586 0.09277812 0.06092839
0.0670009 0.1894299 0.3562839 0.01906335 0.2552884
0.03019822 0.2056109 0.0226197 0.3462445 0 0.3600038
2.235772 0.01537045 0.1006006 0.8752209 0 0.1801532
0.479585449 0.3785698 0.375443379 0 0.6211341 0.2176081
0.05466094 0.1235265 0.2999944 0.09642316 0.6788863
0.118342593 0.06789499 0.009962394 0 0.03878177 0.3646206
0.2326167 0.06121904 0.04531519 0.02953967 0.1334842
0.137049 0.1322552 0.05819086 0.02419297 1.708415 0
0.1130368 0.004232141 1.427304 0 0.02480721 0.05986844

0.07575597 0.2955887 0.2157815 0.1507422 0.01995178
0.005716617 0.01882995 4.44029 0.004798967 0.02397639 0.07409452
0.06072572 0.4777715 1.527936 0.03741371 0.02296821
0.06261286 0.603531 0.09996065 0.1187758 0.03758127
0.05567676 0.08905576 0.03877912 1.020522 0.065974747
0.05481553 0.134174206 0.041104747 0 0.247573 0.04363707
0.05054254 0.09119587 0.04777872 0.01467584 0.2871789
0.09676928 0.01276939 0.007724196 0.09385686 0.3014895
0.2256382 0.02905899 0.4034971 0.5198026 1.092536
0.5935222 0.3245761 0.01213156 0.2246834 0.2209522
0.09780349 6.192572 1.069779 0.03839416 0.6644531
0.01657179 0.2005579 0.2041082 0.04196134 0.01794284
0.1377115 0.111630244 0.004595806 1.540755 0.1524867
0.006856166 0.1187819 1.208555 0.01155549 0.1563765
0.00423903 0.0372774 0.07722777 0.1973892 0.258836
0.02660807 0.02732834 0.013421663 0.05111035 0.05863154
0.1486776 5.140433 0.04723389 0.128408387 0.4506098
0.01723971 0.6458185 0.04961966 0 1.984386 0.08490231
0.08228492 0.2970647 0.3385337 0.03949871 0.5631134
0.3668401 0.07509676 0.007501037 0.3650817 0.004692942
0.1651226 0.01587213 0.05430714 0.7957095 0.2591791
0.04174282 0.1331536 0.0165241 0.07776273 0.095361276
0.027152859 0.07734931 0.009870166 0.06105199 0.03694687
0.0139487 0.0826813 0.09451829 2.932168 0.1232674
0.07496608 0.09990315 0.1025205 0.1956274 1.624652657
0.1237697 0.121249 0.8406536 0.08785868 0 0.038557847
0.09332875 0.02234788 0.05956869 0.7187866 0.1353888
0.4076891 3.562981 0.7398192 0.009645344 0.01323804
0.1224186 0.02624358 0 0.1190207 0.04552236 0.03827253
1.025531 0.2669052 0.038767994 0.03772673 0.2077604
0.0249048 0.008989422 0.1004174 0.2212304 0.06999096
0.1311215 0.04165352 0.4188984 0.126978 1.1577 0.06817144
0.32832 0.07326471 0.00980252 0.02856074 0.0853584 0.04698115
0.005351851 0.03507416 0.1544364 0.01804451 0.1097813
0.3640158 0.08379137 0.048496946 0.2966191 0.5596115
0.0273581 0.04862329 0.1392679 0.5813872 0.084492118
0.1635018 1.103564 0 0.08557081 0 0.8120773 0.0868791
0.004822223 0.03605308 0.977438 0.01787523 0.6253718
0.09489009 0.03878462 0.079938781 0.1621315 0.7239112
0.5570352 0.34205926 0.1477397 0.5521536 0.02177011
0.02236461 0 0.1252623 2.77433
AC004148.1 0.1349407 0.3942706 0.6550971 0.1647101 0.2654962
0.6605182 0.3938168 0.3234489 0.3240614 0.169065971
1.299874 0.7364169 0.6251494 0.4568756 0.4888726

0.6442554 0.3439444 0.321736496 0.1203334 0.1280912
0.315365822 0.6047527 0.438714 0.57402 0.4107616 1.64992
0.5933299 0.5453588 0.043460972 0.1360859 0.2965937
0.2217572 1.295315 1.133332 0.9032694 2.136398
0.06240568 0.5184488 0.8872436 0.610370802 1.991754
0.601387 0.48377325 1.007251 2.408533 1.086709
2.486073 0.5801708 1.862535551 0.7185157 0.1644278
2.199701 0.5629811 0.4508865 1.16934 0.8109878 0.9466154
0.8453514 0.2931643 0.19310179 1.251148375 0.327157873
0.4554506 0.1403599 0.7634234 1.203942 0.2557266
0.7425304 0.158173292 0.5341642 0.4417885 1.314904
1.193979 1.887374 1.3359 0.7728077 1.111716 3.081688
1.236924 0.492030435 0.2085591 0.6878717 1.292845
1.757557 0.1737238 0.9363576 0.9719189 0.7967005
1.10431959 0.9977932 0.989724026 1.146795 0.608387
1.651715 0.1898361 0.9879689 0.6322053 0.7657237
1.906229 1.101819 2.243274 0.8383474 1.157307
1.240957 1.400133 1.628648 0.2054791 0.2053101
0.397760758 1.049408 0.9987988 2.058638 0.1184061
2.842957 1.247864 0.7618564 0.6039375 1.377899
0.2547541 0.956832 0.4111736 3.855124 2.031063
0.3618687 1.41241 1.985114 0.5279802 0.1378371 0.6180175
0.2775441 1.160795 1.570428 1.754424 1.518356
0.1173148 0.8695206 0.8271588 1.251129 0.5940294
3.029694 1.281816 1.231424 0.9214398 4.246486
1.84678 0.9821524 2.092312 1.499779 1.400649 1.52318
0.7828984 0.6292479 0.3260736 0.7152481 1.232651
1.741921 1.440195 2.822786 2.021775 1.329337
0.32768817 2.236258 1.525620048 1.445809 0.2770637
0.5713056 0.2989711 1.78668 2.052139 2.362716 1.144905
0.379738462 1.767652 0.7018296 0.7162118 0.677084
0.5511556 1.06874 1.882321 2.420294 1.455197 1.271996
0.4785428 1.704178 0.9048894 0.6761799 0.5489761
1.217178 0.6862697 1.011102 0.4628927 1.358792
0.3115315 0.3782091 0.7347834 0.859449 0.6178241
2.680931 0.7638918 0.7266534 1.459183 1.535726
0.4997661 0.6670667 0.695028 1.859999 0.487797
0.4749071 1.107765 1.020082 2.205472 0.7569641
1.523915 0.405146 0.5323819 0.7477314 0.192546
1.508488 0.5654733 0.256482919 0.3917604 2.481621022
0.165246175 1.799056 2.399117 1.834763 2.260216
2.147338 0.4756177 0.5319247 0.3890605 1.377386
1.32003 0.4258591 1.527232 3.095649 0.8315023 1.163439
3.419866 0.5587764 1.998847 1.029159 0.735679

0.6316922 2.228811 0.7631615 0.8113282 1.23049 0.8519373
0.8606141 1.024333 2.698666 1.424057 1.535753
0.369511 0.5037821 1.104133 0.155417284 1.175407
1.708198 1.655314 0.3780019 1.09147 1.06096 0.8229081
0.9579473 1.311378 0.3425366 0.7469839 1.464976
0.6697811 0.3056221 3.798132 1.431994988 1.132368
0.9727551 1.223867 1.116064 0.3616878 0.321797995
1.155513 0.5333245 0.1376077 0.5526641 1.411209
2.152047 1.217515 1.663429 0.8769275 0.6282468
2.044607 0.9556263 0.5679805 0.8945134 0.1608272
0.4193353 1.358367 0.5394802 2.370009 0.2942941
1.255819 1.165971 0.8182799 1.291503 0.9869442
1.147041 2.093289904 1.351478195 0.7699808 0.7054096
0.2103742 0.3489299 1.01826 0.2629892 2.287089 0.680764
1.321468 2.394582 0.6502468 1.831757 1.358181
1.047156794 0.4493044 1.129613 0.07709225 1.749195
0.8309083 0.423194585 0.3062801 1.280024 0.8514613
0.3110079 0.3406377 0.832488 0.3303334 0.7314402
1.122642 0.9528656 4.417053 0.4973687 1.363605
1.762014 1.185179 1.264376 1.133058 2.999998
1.504096805 2.079992 0.5269857 0.6611123 1.734652
0.7535553 0.2173491 0.9379078 0.5898777 0.9993979
2.071035 1.447674 1.244502 1.258635 1.104219
0.1870052 0.7881469 1.869888 1.129832 0.9209663
1.163865 2.041178 2.638463 0.8359506 2.129613
0.4386739 0.313682 1.166068784 0.6469834 0.4098846
0.3701014 0.9494337 0.827289 4.353809 0.517590521
0.5067599 1.430757 1.022234 2.119585 0.3896859
0.2055664 0.7761438 0.7237415 0.7937073 1.546408
2.272165 2.177879 1.834689 1.682935 1.280746574
2.798413 0.8563842 0.347018 0.512941806 0.6033594
2.329742 1.555885 1.409994 0.7958466 0.3735756
0.4046494
AC004908.1 0.08056535 0.6676696 0.2470237 0.1539218 0.5636
0.5167442 0 0.2896691 0.08291932 0.571990911 0.7607118
1.002453 0.5391255 0.3776873 0.5253799 1.153944
0.05356942 0.444840848 0.1657943 0 0.251049071 0.1604725
0.3074841 0.7068484 1.085196 1.160978 0.2125459
0.3185241 0.103792193 0.06093678 0.2276731 0.8184634
0.6999104 0.6766477 0.4153992 0.1177403 0.3353296
0.2411968 0.4570152 0.617925017 3.621703 0.4734773
0.726741593 1.657369 1.299727 1.264688 1.906143
0.2020589 1.722708521 0.2359414 0.1240047 2.863111
1.065992 1.292152 1.623189 0.7262913 0.5914566

0.7433012	0.2423514	0.291258893	0.945169465	0.659229162		
0.1604794	0.2199772	0.1847823	0.6915081	0.1832156		
0.2714218	0.377744719	0.6003177	0.8715764	0.6617807		
1.514266	0.4507369	0.5015407	0.04856832	0.8131703		
2.798103	1.402465	0.078921427	0.373556	0.5866981		
0.9780383	1.159793	0.1131496	0.3640295	0.8013348		
0.8960184	0.73599153	1.060188	0.750495429	0.8696008		
1.101169	1.292189	0.2163768	0.3840945	0.7288756		
0.6221277	0.9916115	1.252151	1.378557	0.6804066		
0.4331401	0.2577057	0.4987922	1.35727	0.3099278	0.3343059	
0.474960165	1.038268	0.7806441	0.4312611	0	1.332235	
0.5706598	0.08528638	0.09014409	1.57441	0.7935601	0.6180948	
1.361764	1.386782	0.8414174	0.9971584	1.290718		
1.875478	0.7860196	0.08713546	0.9701343	0.2237026		
0.3094522	0.5197634	0.6011541	0.757691	0.06003591		
0.6845812	0.7918613	0.8674573	0.3251057	1.550448		
1.207404	1.215929	0.605849	0.9330779	0.6843764		
0.760131	1.115564	0.4591957	1.301705	0.7345178		
0.2481895	0.3545817	0.6083743	0.9341358	1.177512		
2.276758	1.103817	1.717122	1.659743	0.08984954		
0.445258233	1.39448	0.85135661	0.1954436	0.06616751	0.7035058	
0.2570378	0.8031805	1.280075	7.009126	0.6650827		
0.216414551	1.260706	0.08198258	0.5298632	0.2765907		
0.4676163	0.5027324	1.73095	1.86454	0.4051467	0.7594355	
0.5112712	0.9257276	0.4174716	0.8959003	0.5230246		
0.7630425	1.195975	0.673325	0.4836416	0.6225925		
0.3266298	0.1642232	0.6926794	0.7285628	0.5490118		
1.867402	0.7114784	0.4077079	0.8264292	0.4181036		
0.8424893	0.4823052	0	1.728214	0	0.6186321	0.5473512
1.008054	0.7213557	0.7421327	1.969247	0.5331439		
0.7989319	0.5404121	0.2586558	1.723255	0.5064174		
0.445472601	0.7518138	0.990901221	0.1691297	2.212352		
2.950614	1.284049	2.162809	0.5628521	0.3604158		
0.3757304	0.4241738	0.5308895	0.8056279	0.8898959		
1.673463	1.165327	0.3481543	0.4554903	2.243964		
0.712927	0.7844235	0.8342187	0.1780669	0.1996661		
1.904995	0.4900278	0.7601314	2.499617	0.5477692		
0.7276502	1.665724	2.303183	0.6501702	0.7789675		
0.7098008	0.8203073	1.533229	0.167023306	1.185021		
0.8815797	0.2952576	0.2256832	1.371903	0.6380621		
0.4437649	0.6127878	0.6046534	0.02921556	0.4682802		
0.9718369	0.3672439	0.3649388	0.7121534	1.276306741		
0.4486374	0.8309566	0.9940927	0.5158742	0.143962		
0.256169446	0.5461632	0.2837383	0.246473	0.1484838		

1.25403 1.927295 0.6775064 1.399422 0.8502978 0.5680975
1.040138 1.168931 0.6855889 0.5035447 0.3292137
0.3641615 1.544767 0.3623542 1.393227 0.3208546
0.599822 2.192083 0.3893116 0.9009489 0.4985942
0.2694423 1.482300846 1.303437594 1.438071 0.3068448
0.1046686 0.1351304 1.045835 0.1395695 1.659329
0.4922087 1.293719 0.9400553 0.4384659 2.089069
0.8228164 0.833596166 0.2011904 0.443459 0.4418626
1.253212 0.6945226 0.387811639 0.4022969 2.133301
0.1906345 0.996835 0.3979079 0.607482 0.1753094
1.669739 1.217061 0.6052141 3.143111 0.3779367
1.014531 1.088273 1.054466 2.039692 1.768235
1.385835 1.630596628 0.620922 0.3561881 0.1821748
0.7890752 0.9640812 0.4122 0.7999583 0.1798377 1.294929
2.098296 0.5418144 0.6717722 0.8934392 0.3531781
0.1786401 0.4571127 1.253505 0.3344913 0.5670391
0.9395517 0.4810541 0.7719472 0.1979893 0.7169942
0.3928604 0.5873828 0.984425259 0.4438071 0.3003364
0.3001806 0.133377 0.6019732 2.486783 0.585518297
0.4698536 0.4164335 0.9087936 0.2449321 0.1938825
0.2209174 1.668207 0.3174645 1.137305 0.5096456
1.111414 0.8929676 1.887103 1.44216 0.168675034 4.351217
0.6183136 0.4661651 0.547336967 0.3039451 1.792786
1.194338 1.963125 0.4581845 0.1446749 1.065851
FGF14-AS2 0.2164412 4.268695 0.4603979 0.5815062 0.6032855
2.712565 0.376216 1.037606 0.772717 1.593166052
0.7199311 1.408572 1.274293 0.431234 0.1764311
3.657989 0.7825425 1.874100108 0.5010877 0.6067337
1.232980526 4.634477 1.170259 3.155958 4.632542
0.7124086 0.471084 3.615436 0.575109256 1.350595
0.955704 3.407373 0.1175207 0.6390824 0.3964675 0
0.6334264 0.2672928 0.8894466 0.059858443 0.7209967
0.0119251 0.084479416 0.03478573 0.2228781 0.04976985
0.157405 0.5525307 0.192837846 0.07563147 0.2030086
0.536046 0.870756 0.3051041 2.45292 0.01662957 0.2184828
1.983034 0.08138553 0.293428419 0.399313801 0.110689937
1.468546 4.068232 0.2233904 2.062477 0.4429928
1.052758 0.122322362 0.170097 0.0346606 0.09804561
0.6958626 0.9460299 0.2421117 0.4159058 0.2730758
0.1871956 1.371958 0.490307324 0.2116904 0.857049
0.05796021 1.335351 1.168423 0.1833705 0.6820325
0.3176141 0.13902633 1.937812 0.065179803 0.07552399
0.5200179 0.7765207 0.1401355 0.1658381 0.6949671
0.2279134 0.03405674 0.5621803 0.2201797 0.05909266

0.06233806 0.1622658 0.9770981 0.5000119 0.0780591
2.374412 0.083737158 0.2344488 0.3604603 0.05430919
0.09496005 2.609753 0.7665474 0.02148041 0.1059515
0.1286057 0.3497686 0.3821106 0.04199718 0.1600857
2.331134 0.5776369 0.07801992 0.5773312 0.1732225
0.2194613 1.466041 0.4131764 0.1461362 0.07700514
0.1651724 0.2453576 0.1512078 0.5517444 0.1930064
0.05826126 0.2233141 0.1597497 0.1290118 0.1424402
0.1157583 0.0569714 2.860041 0.7220334 0.01756053
1.491937 0.03576548 0.4096368 0.1250191 0.2551592
0.08580684 0.6856542 1.482854 0.0849525 0.9549035
2.466727 0.07600483 0.3620753 0.917539502 0.5828706
0.705525927 0.03691865 0.2666415 0.3006802 0.8415953
0.214934 0.18423 4.413335 3.16034 0.077866635 0.7072127
0.02753108 0.1826192 0.2464989 0.4318412 1.071394
0.05205504 1.033136 1.714291 0.1229613 0.302988
0.1386332 0.2968809 0.05014298 1.433224 0.05490902
0.1115633 0.0935642 0.5220473 0.08553137 0.3016409
0.2688505 0.1860904 0.6392814 0.8555785 0.5643934
0.06892097 0.1105851 0 0.9754522 0.03978585 0 0.2303553
0.08390779 0.6357106 2.259246 0.09190472 0.1523344
0.03460614 0.7332826 0.2511285 0.6564741 1.168379
0.355068 0.3908737 1.382443 0.8928318 0.112197708
0.08836496 0.270962321 0.383376401 0.1065963 0.6251882
0.1096283 1.704027 0.4725373 0.03300914 0.797615
0.488381 0.1887685 0.07057642 0.03201874 0.210741
0.5112613 0.4676635 0.8489339 0.4582458 0.4005555
7.06174 0.3253287 0.6099371 0.06705113 0.5503544 0.4676949
0.09009334 2.179764 0.6701028 0.1639763 0.3769716
0.01526539 0.277121 0.7847698 0.0579801 1.371853
0.4253383 0.715136568 0.07620309 1.972208 0.7436422
0.0947351 2.280501 0.8524284 0.1117676 0.2469409
0.05857282 0.2796152 0.6739548 0.0279736 0.5303038
0.3492743 0.2454462 0 0.01883247 0.1485259 0.3595119
3.475606 0.07251716 0.225817898 1.679654 0.1524543
1.551931 0.274248 0.04935044 0.7346789 0.05332446
0.2728733 0.2974415 0.8034621 0.08732379 2.888017
2.528837 0.1383533 0.1451039 1.398707 0.4539133
0.3954744 0.07675963 0.0230889 0.5371466 0.7162416
0.2422483 0.4415643 0.2054897 1.617386 2.327856948
0.112555425 0.4987614 0.1000128 0.2214414 0.2212225
0.1413399 0.5800127 0.01741343 2.253977 1.170057
0.1578429 0.1242371 0.4991752 0.1802055 0.306851991
0.1266806 0.3071491 1.349373 0.5827141 0.08746199

0.621568764 0.07368974 0.1111651 0.01371818 0.4282874
0.4676838 0.2642758 2.759614 0.6081714 0.03998238
0.32925 0.1488537 0.09972073 2.895917 0.3106409 0.2096685
0.04532836 2.489921 0.3556251 0.63090839 2.835813
0.8612192 0.1835318 0.1490537 1.706647 0.1268882
0.4351952 0.1811772 0.05755477 0.1761605 0.4093877
1.968803 1.224547 0.2490662 1.327284 0.1354464
0.9076677 0.3201338 0.1687822 0.02957969 0.2261641
0.1138094 0.2243971 0.1300203 0.428769 0.1415074
0.147423492 0.2732357 0.420241 0.9198505 0.1343705
0.05831319 0.7761004 0.344096491 0.1506125 1.549054
0.08078497 0.03084457 0.09766337 1.965973 0.3201206 0
0.01245409 1.62962 0.01646607 0.2130677 0.3113968 0.1071813
0.152938313 0.1453137 1.054171 0.5479104 0.196933559
4.457044 0.6695175 0.1203234 0.02060154 0.0512886
0.255067 1.900609
AC009950.1 0.2995992 0.1637057 0.3757949 0.1932748 0.2653856
0.44527 0.2284432 0.3683916 0.3964539 0.531360976 0.2337907
0.1452402 0.6068633 0.1021462 0.4440309 0.1758366
0.2095577 0.275380246 0.1056927 0.09141163 0.177319374
0.2790004 0.1336494 0.4748175 0.2211859 0.3088913
0.2956289 0.3261218 0.195492901 0.09711698 0.1099546
0.2790187 0.4957647 0.6862519 0.3230987 1.467056
0.04048682 0.1852126 0.6501067 0.564714878 0.3204937
0.2812587 0.121492893 0.3626929 0.4647671 0.1603298
1.856232 0.2174734 0.495226889 0.1895677 0.1077984
2.661465 0.1377492 0.9536177 0.9103569 0.1817585
0.4684556 1.360124 0.05852173 0.184620619 0.388734972
0.392661731 0.5618998 0.2413117 0.4765442 0.8726993
0.2335975 0.3893165 0.105549719 0.4348846 0.3655422
0.4089085 0.7428608 0.1015855 0.3512173 0.2673989
0.7914822 0.1805309 0.3445496 0.080041629 0.1488371
0.3995172 0.4959602 0.5721239 0.2336098 0.1996675
0.4403841 0.9423933 1.43955922 0.4915368 0.262464787
1.142617 1.176628 1.691536 0.4075464 0.6240691
1.014543 0.2786049 0.2938694 1.305419 2.918036
0.7172589 4.348055 0.8914364 0.786912 1.737545
0.269423 0.2373357 0.178917774 0.3890414 0.1837932
0.6685703 0.2867875 2.414903 1.253979 0.2533124
0.2263822 0.5363623 0.3650451 0.1831757 0.2013255
0.2218532 0.5378322 0.8162732 0.4076718 2.573871
0.3558823 0.1094133 0.4937542 0.1548531 0.6052716
0.2923638 0.5225887 0.2626829 0.1826642 0.4165789
0.3145787 0.3267717 0.3811052 0.1123182 0.771302

0.8644604	0.7234145	0.2703777	0.7197974	0.6041497	
0.5555974	0.5089583	0.1028713	0.6613274	0.589726	
0.2495285	0.1586596	1.17941	0.7847768	0.1832599	0.5284525
0.7141049	0.7979286	2.395282	0.439848877	1.39278	
0.318319143	0.4920047	0.3642934	0.3953545	0.366202	
0.2291008	1.107482	0.1293346	0.8672436	0.25980013	
0.7804441	0.1306584	0.4404125	0.2897644	0.2785299	
1.434354	0.2957058	0.2701436	0.610476	0.188624	
0.3573051	0.7249941	0.530136	0.7595842	0.4395143	
0.7370219	0.3369307	0.5382322	0.4271076	0.21321	0.4968989
0.196296	0.6824394	0.9704499	0.420089	0.6068233	
0.2735649	0.3953192	0.1107568	0.4941798	0.1411364	
0.187402	0.5374135	0.2986603	0.3750721	0.3540402	
0.1519972	0.573253	0.3085637	0.3473845	1.24238	0.3046865
0.4779484	0.5855293	0.3705891	0.1479547	0.5992063	
0.080677784	0.2941013	0.426597798	0.130689759	0.3149617	
3.056959	0.3689252	0.2089055	2.029658	0.08070177	
0.1322057	0.09657428	0.7510801	0.1725474	0.2148874	
0.2937488	0.5337467	0.8014717	0.8348196	0.6941675	
0.1787752	0.3992358	1.239848	0.7172176	0.6846434	
0.2354159	0.5978745	0.680224	1.27339	0.5668818	1.695131
1.126245	0.2810074	0.623168	0.8394006	0.6086979	
0.08081807	0.8692498	0.185526781	0.3616484	0.341859	
1.907201	0.2615846	0.4671046	0.3738015	0.330659	
0.2781888	0.2055351	0.1439183	0.995085	0.748275	
0.2660403	0.1216104	0.5376233	0.188473606	0.4875057	
0.4621539	0.517027	0.4920538	0.5256193	0.153099662	
1.470166	0.4987937	0.1651597	0.6525624	0.1447842	
1.215053	0.2300635	0.3270241	0.5885996	0.6236469	
0.3327962	0.2540405	0.4550021	0.4045741	0.2623394	
0.2835929	0.9288255	0.7612446	0.7475033	0.1892684	
1.139423	0.6309073	0.4446069	0.6173898	0.2364177	
0.4131533	0.09264283	0.399279368	0.519179	0.1830589	
0.1850117	0.1762051	0.5358832	0.4145409	0.7462777	
0.2787713	0.5538006	0.2440246	0.4288077	0.3375985	
0.2643433	0.353035927	0.1639656	0.3935343	0.1640492	
0.6424837	0.1976578	0.045972023	1.197527	0.2522401	
0.1262631	0.3249591	0.4804236	0.5880998	0.3238459	
0.2977475	0.08050014	0.4384318	1.167664	0.143412	
0.1633224	0.4887903	0.3658585	0.3476712	0.4483334	
0.5000721	0.082173473	0.149937	0.6502389	1.616655	
0.3572657	0.4469784	0.1426681	0.4682434	0.2996411	
0.0882897	1.491103	0.4822186	1.123715	0.3823167	
1.606743	0.1747051	0.6311206	0.5845697	0.2762375	

0.4033082 0.2594917 1.510111 0.4746527 0.6812842
1.267355 1.522597 0.1332045 0.300675872 0.15718 0.1208727
0.3624302 0.5410806 0.2851318 0.3955436 0.24035939
0.2506385 0.8075595 0.1200524 0.3075535 0.3089971
0.1493687 1.116414 0.1111565 0.08238911 0.8154496
0.208388 0.680934 0.3252667 1.051586 0.268823012
0.7627799 0.6787731 0.1200716 0.20391642 0.3865473 2.1362
0.4614439 0.4325661 0.2655359 0.157209 0.5034276
AC125807.2 0.2232628 0.549539 0.6579308 0.3120121 0.5965395
1.071908 0.6388035 0.1635193 0.7914855 0.347064758
0.5784813 0.162456 0.3766987 0.4574236 0.8492944
0.7933263 0.8742151 0.423366407 0.8270086 0.7770769
0.497624052 0.4776424 0.5575471 0.8705861 0.356798
0.8059508 0.4253942 1.193272 0.455412615 0.09381559
0.5608247 0.8524004 0.3392291 0.7031725 1.130959
0.7069446 2.499553 1.42593 2.161972 0.819607562 2.367217
2.394584 1.127466035 1.371492 1.111195 0.9552753
1.982067 1.662061 1.477657821 0.5514721 1.651391
1.123435 3.153678 0.8579033 1.338169 2.302406
1.13519 2.216656 2.429383 0.947264845 2.121690011
1.714087538 0.6711986 0.8273156 0.7624135 3.737361
1.71499 0.781415 0.847414792 2.371209 1.363028 1.579215
1.423318 2.135777 3.355635 0.7701686 2.291979
2.77317 0.8010846 0.947730894 1.588742 0.7090548 0.8945895
1.254148 2.129598 1.573246 2.199389 1.481654
3.20100812 1.100582 1.043871049 5.050504 1.271484
0.4188204 1.622791 2.192163 0.8456163 0.770594
2.453035 2.258828 0.8334169 0.1950562 1.781425
1.140663 2.500002 1.78707 1.61635 1.940354 1.129746953
0.6614369 1.867868 0.4880038 0.8416693 1.043757
0.6882075 1.460748 1.239785 1.496917 0.7696901
0.7915511 0.5990036 3.34043 1.920254 0.4605544 1.764573
0.3970179 0.733636 0.7244096 1.348048 1.044688
1.777638 2.598312 0.4543415 1.220667 0.5360862
1.844415 2.756773 4.193467 2.293285 1.209776
1.782821 2.316044 1.576006 1.305932 7.679834
0.8459338 1.110991 2.431934 1.537651 1.07429 4.527905
3.045333 2.892302 2.510607 1.661775 0.3505197
2.124236 1.160249 0.8362694 1.493947 1.265059111
0.813074 1.818083318 2.71559 1.044153 1.62463 1.642255
0.5139375 1.447091 1.54738 1.040998 2.755893879 1.230724
0.8035802 0.6869508 4.137072 0.9678945 3.149523
1.885314 3.406402 0.5655297 1.11491 0.638965 2.272632
1.500943 3.095737 1.14342 0.6321256 2.114051 1.894515

0.6488599 1.067438 0.8171554 1.441136 0.6611796
1.892355 1.592739 2.764078 4.116029 0.8497945
1.851246 1.229794 1.264633 1.485072 2.127782
1.394454 1.115952 1.123854 2.075094 1.447198
0.9307614 2.096149 1.677065 1.755611 1.559323
1.674842 0.8141287 0.5895662 2.364962 1.517398969
1.651309 1.756559532 0.911346291 0.9063027 0.5912638
1.276586 0.9434338 0.7798882 0.9281636 1.342849
1.851833 1.581782 1.477586 1.148228 0.9265093
0.9722791 1.328096 0.7643657 0.4154988 1.950335
2.158312 1.740055 1.573586 1.516493 0.6124473
1.552528 2.46444 1.03449 1.34329 1.132051 2.382361
1.017108 0.4465882 2.503464 2.460227 1.409407
1.166562 1.217137586 1.304257 2.742426 2.822095
1.40718 0.5438712 0.9780613 0.6441608 2.39 2.427495
1.26391 1.380092 0.825046 1.73387 1.269767 0.9290548
0.888004342 0.8806455 3.676968 1.985676 2.47531 0.3368891
1.153582626 2.456164 1.43863 1.010309 1.649723 1.007558
0.7217463 1.307083 0.4586974 1.541807 2.112539
2.515454 2.043871 1.862451 1.335519 15.73747
9.34178 0.8204983 1.44115 0.807708 1.192593 1.744313
1.427464 1.212837 2.354256 1.496847 0.943719
1.646681124 0.355475273 0.7803412 0.8503286 0.9539678
3.72393 1.205627 1.450407 1.719053 2.183792 1.104517
1.694509 1.641198 0.9195146 1.75145 3.28246054 1.525488
1.433733 0.5229592 1.677086 1.386215 0.369090251
1.829923 1.264658 1.241054 2.778228 1.756124
0.7694578 2.273896 1.150863 1.0387 1.099478 1.402068
0.7758063 0.7346845 1.223084 2.033533 0.7157856
2.023162 0.6682505 2.022868953 5.770716 1.272222
3.533901 0.8124145 2.039793 1.798048 0.7192431
2.034996 2.644473 2.165167 2.001969 1.410315
1.020429 1.478966 1.09323 1.1467 1.966026 3.187653
1.539634 1.143633 2.236641 1.344984 0.4457929
1.483578 1.073574 1.340736 0.356365608 1.734736
0.8322924 1.344071 1.283384 1.675318 1.36912
2.482711162 1.292195 0.7792107 0.3744753 1.360654
1.552164 1.904642 1.406449 1.722859 0.9858579
1.84217 0.8102488 3.364578 1.39254 3.363188 1.090675619
4.06748 2.60682 0.7176873 1.288059803 1.221845 1.965378
1.857138 1.659139 1.434315 1.37539 1.391515
AP001107.5 0.06554264 11.08279 0.1306254 0.1669608 0.8597019
5.951822 0.4725801 2.435108 5.312292 3.791103136
0.6938787 0.2360742 0.607288 0.2355671 0.2849429

7.955285 0.1743221 8.84170595 0.3506861 0.5366107
1.91472106 7.343426 0.458606 5.09699 5.31951 0.3863843
0.3285352 7.19518 0.48552115 0.2478707 2.376987 9.517704
0.04217784 0.3096426 0.6047371 0.3352501 0.07956725
0.2354662 0.4224969 0.058004182 0.1411439 0.01925949
0.147807219 0.04915772 0.2587187 0.04019009 0.08897525
0.5283698 0.645126482 0.06979867 0.1765435 0.1752082
0.1289112 0.328503 2.854026 0.01342869 0.09623399
3.079494 0.07667373 0.044427931 0.186030808 0.223460512
0.8703693 0.2556555 0.06013065 2.649979 0.6260188
0.8942837 0.109752896 0.08394009 0.009329696 0.1029258
0.5403098 0.1324157 0.2465123 0.1975599 0.5003968
0.26676 0.05787441 0.085607001 0.07597511 0.2505818 0.1170099
1.246808 0.2761532 0.06346078 0.280997 0.2294815
0.33679913 1.700356 0.084214265 0.06098704 0.1119798
0.06454978 0.1886034 0.04463913 0.3070711 0.05751386 0
0.6089858 0.2598604 0.02863106 0.02516958 0.06551635
0.5635894 0.8899399 0.173344 2.134958 0.096599041
0.2184482 0.05292336 0.2455919 0.3067281 2.08334 0.5803138
0.0433646 0.1100029 0.06923435 0.2017462 0.09142564
0.02260901 0.1762803 0.2617288 0.2230861 0.1050043
0.1059559 0.1398805 0.2008483 2.863514 0.7582902
0.173078 0.1243662 0.2667596 0.1541018 0.2442062
0.9189345 0.2701509 0.09409412 0.09617619 0.04300029
0.08185531 0.1955394 0.05098744 0.09201096 0.1237252
0.1855177 0.01418046 1.16741 0.0577626 0.2134125 0.03028659
0.7623697 0.0791893 0.097708 2.482676 0.2058022
0.4294748 1.565086 1.074068 0.1461913 0.345767517
0.6396407 0.357474791 0.03975 0.3768066 0.1214024 0.139406
0.2246112 0.2826614 0.2824184 5.996829 0.050303017
0.4895036 0.1000434 0.01134373 0.1730891 0.4438253
0.6134848 0.05254428 0.3160138 0.208747 0.07722831
0.3058356 0.07124021 0.1198685 0.06748566 0.714837
0.2808204 0.2522505 0 0.2342017 0.06906819 0.07750295
0.1670014 0.09391968 0.2581159 0.8723431 0.06510833
0.04452398 0.153085 0.05042461 1.002522 0.0428371 0
0.02066847 0.0423482 0.355395 0.3774586 0.02783055
0.1127618 0.1816432 0.220601 0.1926514 0.337346
0.2096639 0.1815919 0.2571864 0.04326934 0.789643
0.090601758 0.0611626 0.310936501 0.160524856 0.09390381
0.1524079 0.1062323 0.04511586 0.0508777 0.06663875
0.1746682 0.3204312 0.2540568 0.02849589 0.008618577 0
0.07645428 0.2202941 0.2686537 0.1973558 0.3457634
0.4908885 0.07662348 0.4780493 0.2165802 0.5127941

0.2727634	0.1273161	4.296638	0.4456288	0.03115621
0.1963946	0.01232709	0.2441241	0.09901819	0.007803337
0.04004087	0.1626957	0.226465185	0.1230709	1.909004
0.3102609	0.1530008	0.3348268	0.7334898	0.03868047
0.2326444	0.03783892	0.08318746	0.226763	0.05647301
0.2390122	0.118756	0.1524634	0.049919169	0.03041514
0.1417443	0.2177345	3.847077	0.1522533	0.065125785
1.227733	0.01538871	2.030205	0.2315268	0.1115839
0.8263362	0.0947331	0.01836253	0.1921517	0.3555127
0.0881445	4.075563	3.445454	0.2793073	0.1841307
0.6295475	0.2408714	0.1228281	0.07083982	0.2423813
0.4202011	0.3253379	0.1024676	0.2377143	0.04609355
0.1370003	0.070935405	0.181781243	0.02876851	0.05873615
0.2043635	0.08244985	0.06225526	0.1135445	0	1.965739
0.2033207	0.2310233	0.05573544	0.5120375	0.08731161
0.117373619	0.1534455	0.1578364	0.5392052	0.2352763
0.1210752	0.200771384	0.1041352	0.18951	0.01107769	0.7394038
0.1618558	0.1235518	2.6563	0.4075167	0.05381087	0.08862519
0.1857669	0.08052637	0.01528436	0.5312082	0.1241616
0.0366035	2.811652	0.2552658	0.115351785	0.1094472
0.2318169	0.2037824	0.04012121	0.4369742	0.1024646
0.1171428	0	0.06196875	0.3251495	0.2676191	2.583499
0.2620016	0.4166174	0.1998284	0.03281265	0	0.108848
0.1677474	0.01194308	0.06522563	0.1608306	0.06040162
0.2099876	0.3728728	0.04155268	0.119047241	0.2005845
0.4750935	0.101753	0.1627601	0.1224314	0.4068154
0.129008464	0.08687313	0.3127226	0.03261769	0.02490757
0.015773	1.497698	0.3554422	0.1076117	0.07039838
0.5768542	0.1462633	0.5161685	0.1852854	0.02885031
0.123500563	0.04889313	1.218854	0.4424479	0.079513775
0.1785836	0.1383053	0.1943268	0.02495419	0	0.1618347
2.679357
AL022328.1	0.1215784	0.9591169	0.5591624	0.4355214	0.6777496
0.9850117	0.4696141	0.3278473	0.187696	0.342729971
1.539315	0.3582869	0.6571189	0.3989684	0.09910409
0.6530162	0.7881877	0.526355522	0.2627041	0.06491652
0.426205692	0.7264905	0.4253459	1.018195	0.7702425
1.48123	0.7697897	1.994796	0.176207876	0	0.2576802
0.4086777	0.792158	0.5743719	1.286722	0.1998876
0.1265086	0.4367782	1.105033	0.349683509	1.546345
1.018174	0.474533958	0.4494127	2.097001	1.744485
2.086637	0.6860702	0.588022876	0.8739436	0.2456097
2.580903	0.684764	0.1371053	0.6518828	0.7099228
0.3570187	1.744851	0.5790631	0.659293142	0.862695169

0.787565848 0.4843477 0.3201043 0.3346182 1.091588
1.161234 0.4915119 0.244303518 1.698598 0.4932247
0.5947964 1.65371 0.9352634 1.490064 0.6046654 1.439515
2.869995 0.9431834 0.416841622 0.3699415 0.8632317
0.7488143 1.03137 0.192094 0.7651582 1.43861 0.7511947
0.52062162 1.234204 0.673670078 1.629043 0.2336817
1.693419 0.5597602 0.9625895 0.7365543 1.728303
1.300854 1.139908 2.816061 3.05378 0.9104226 0.4739655
0.8781618 2.865964 0.657705 0.4162039 0.268779759
2.350223 0.7362769 1.488708 0.05334053 2.278488
0.3229361 2.340781 0.7821917 0.794638 1.324772
0.667761 0.6290791 1.14447 2.856947 0.1692875 1.256317
2.682811 0.3058056 0.230113 1.154305 0.4501092
1.269612 1.349555 0.9277987 0.6431655 0.4756402
1.123472 1.01187 0.7527037 0.9366112 0.6779893 1.055961
1.376187 1.702426 1.088058 1.176209 0.7128329
0.8285777 1.013449 2.303656 0.7125657 0.4213511
0.8026306 0.3305076 1.510363 2.021273 0.3817531
0.7332845 1.7275 0.9392479 0.7118415 0.229064952 2.081976
1.025730163 1.216616 0.3369975 0.9892521 0.7272879
0.6817793 2.483636 0.8606473 1.254567 0.594848962
1.135007 0.2474342 1.32565 0.9632157 0.764469 0.3063804
1.257325 0.6682568 0.3056964 0.8595285 0.7148108
1.387545 1.083957 2.966828 0.876099 0.3289946
1.278403 0.7095122 0.7559128 4.035721 0.06161322
0.5111362 0.2090597 1.409777 0.6019532 3.321251
0.7123371 0.6744173 1.052271 1.917743 0.3575733
1.637618 0.4025595 1.861726 0.5493669 1.470351
1.032486 0.9127315 1.205206 0.6138061 1.21314 1.441484
0.9042305 1.648763 0.2276917 1.781828 0.7323746
0.504185517 1.24799 1.473972969 0.223324328 1.524136 2.888935
0.9360143 1.349465 1.132508 0.2966679 0.7593775
0.342914 0.777584 1.070384 0.9352418 0.8377412
1.106188 0.3064761 0.7990616 1.029615 1.365506
1.502447 1.218281 1.047987 1.054582 1.014622
0.8757048 1.518205 2.068564 1.10708 0.8018814 0.9289713
1.097575 1.924562 1.983678 0.5862298 0.5570539
0.9556764 0.378073676 1.08438 0.7923477 0.7797353 0.3831421
0.5822694 0.3767782 0.7892542 0.7397896 1.447655
0.462926 0.90857 1.885584 0.4156463 0.08260749 1.060545
0.611144304 2.877349 1.607911 1.644396 0.9731099
1.075378 0 1.724305 0.3853617 0.1394789 0.4201345
0.3991811 2.888764 0.7428387 1.583864 0.7485071
0.5440535 1.790366 0.7559971 0.7759489 1.554304

0.3726038 0.8243153 1.04902 0.7518723 0.7884264 0.8819186
0.8674553 1.374607 0.6998123 1.791353 0.3078046
0.635322 0.625013143 1.138034246 1.067284 0.9192883
0.3790843 0.1911756 0.6640127 0.710841 1.721525
1.240772 1.564063 1.551599 1.30267 0.9093873 0.526366
0.943463443 0.3984874 0.9410748 0.06251246 0.5455326
0.5895451 0.758142989 0 1.054595 0.3082285 0.9291199
0.8406568 0.5312868 0.3968301 1.540921 1.287633
1.397361 2.523605 0.1425828 1.020663 0.6569102
1.22485 1.663494 1.409998 2.012398 0.668662282 1.124414
0.2821934 1.701029 0.7814403 1.340551 0.5442823
0.5432353 0.2035401 1.379388 2.176946 1.883477
1.313265 1.434484 1.478989 0.2021847 1.095585
2.039404 0.6814386 0.9918331 1.063384 0.689645
2.770234 0.7842945 0.6816545 0.6299054 0.4624691
0.813044202 0.6418285 0.1888447 0.7361124 0.9812139
1.126786 2.355647 0.717913212 1.160247 0.688851
1.179832 0.8085406 0.1755489 0.0833447 0.1798167
1.556993 0.5316698 1.906002 1.183904 1.22343 1.252033
1.632236 0.229087515 1.523665 1.130457 0.293114
0.265489509 0.8409006 2.938659 1.306692 1.481242
0.2592864 0.163743 1.254582
AC011472.1 0.5617392 11.4999 1.033419 0.2926949 1.473631
8.533378 1.420232 3.029562 3.547752 3.455007229
1.775318 1.053452 0.9069 1.173065 0.416272 7.944945
0.8149331 6.709534721 0.6620699 0.6544132 1.849887506
8.595102 1.104445 8.309167 11.38472 0.7425883
0.9295988 8.843214 0.641450346 0.8111339 2.597634
7.324117 1.133761 1.206282 1.309597 0.6716786
0.4516735 0.5733194 1.441836 1.084647617 2.350641
1.125445 0.265761256 0.7714912 1.998265 0.8830519
1.693501 0.6586825 0.727971341 1.244017 0.4126589
1.252703 0.9587498 1.382138 2.389647 0.6277737
1.462114 4.187994 0.5120566 0.484621014 0.89865849
0.591967448 0.7629115 0.5079413 0.5973436 4.79268 1.010358
1.729038 0.461772291 0.5707765 0.1526528 0.7957719
2.626912 1.023774 1.470319 0.4617832 1.804028
1.600404 2.029164 0.250125904 0.7547439 1.283002
1.230763 3.229395 0.5648036 0.5191739 2.022981
1.00969 1.48701979 3.542461 0.762775519 0.7127659 0.4144302
0.9699484 0.3232886 0.7303871 0.6187574 0.860383
0.5999733 1.734038 1.886157 1.003848 0.2353289
1.133237 2.476624 1.791263 0.6261876 2.797124
0.338690838 0.9871761 1.14427 2.460235 0.3136686 3.82818

1.266016 0.9730741 0.7142348 2.690436 0.9619998
0.9349436 0.8455531 2.032749 1.388256 0.521449
0.8590418 1.040192 0.4904421 0.414238 3.973393
0.8035122 1.30562 2.64535 1.766677 0.9005071 0.3995712
1.985225 1.311494 0.8522629 0.9835241 0.9380966
1.043626 1.720698 0.8143952 1.048464 0.8675984
0.6607777 1.12696 2.619595 1.305157 1.32192 0.3539645
0.722428 0.4164745 0.9896727 4.459611 2.80612 1.232021
6.288625 1.86499 1.879419 1.077611712 1.636167
1.253360647 1.045271 0.5032917 0.5270018 0.3258522
1.694363 2.225524 1.194529 7.904432 0.235159974
2.751486 0.5716208 0.6363652 0.8496274 0.8892106
1.617824 1.203624 1.565962 1.104268 1.366765
0.4003276 1.165635 1.354226 1.356592 3.401443
1.589177 0.9054862 0.9713246 1.554704 1.910401
0.6987524 0.2732484 1.404999 1.832332 0.6688066
1.547229 0.4943414 0.3578261 0.7071852 4.728504
0.8010311 0.7712336 1.014535 1.464995 0.5845753
0.8822847 1.517882 2.444043 2.122895 1.329809
1.564235 2.224765 2.033814 0.6478586 0.191277
0.5663796 1.257242 0.661798871 1.048399 1.776606527
0.455619757 1.518167 1.558565 1.510558 0.9490982
0.4162314 0.4984438 2.117929 0.9794443 2.197212
0.4995545 1.631774 1.28513 4.253223 1.066626 0.7795369
2.162372 3.415293 3.423135 1.202544 0.8465218
0.4429617 1.198622 0.68661 1.020319 7.513165 1.562442
0.9467328 1.147649 3.054258 3.423743 2.545933
0.7843108 0.2807787 1.204253 0.23820635 1.318488
1.79966 1.286671 0.2145775 1.076124 0.8704304 1.235645
0.2330532 1.525693 0.3888904 1.46292 2.930443 1.606192
1.24913 2.993534 0.933462142 0.7287072 1.503674 1.672231
4.577892 0.301131 0.274009014 3.607678 0.8273123
2.987889 1.670595 0.6520503 3.714435 0.9662504
2.618193 0.8084554 1.537327 1.771875 5.160112
3.364396 1.740966 0.7434068 1.471526 1.591147
0.229682 2.421657 0.5665504 1.077243 1.154766
0.5878915 1.574318 0.4956069 1.238219 0.967206538
1.487155863 1.187986 0.9095587 0.2587467 0.8351247
0.5335644 0.5308057 1.281861 3.509906 1.062321
1.042762 0.755612 1.604293 1.360568 1.524183879
0.7173373 0.8432736 0.735208 2.199771 1.24994
0.569851996 1.634319 0.6527936 0.9580543 0.9617195
0.680989 1.260193 3.20864 1.831814 1.861534 0.8516395
2.86073 0.5646752 1.107512 1.828006 1.108111 0.6559467

4.012007 2.187779 0.966164116 1.161055 1.083713
1.299076 0.6564648 1.257106 0.4136892 0.6388982
0.470217 1.520903 1.781286 1.030304 3.425835
1.718703 1.544673 0.5520104 0.9970662 0.5959105
1.144913 0.4656171 1.144563 1.58559 2.076571 1.388317
2.154208 2.054405 0.4856332 1.618996825 0.4922957
0.7297569 1.355693 1.711984 1.342823 2.801313
0.579901506 1.58387 2.406104 1.092798 1.746594 0.4424202
1.120247 1.888233 0.5533776 0.6111916 1.917197
1.243203 2.278963 1.701434 1.5735 1.250922511 1.782838
3.685599 0.886451 0.446059703 3.2752 2.057233 1.135566
0.7777171 0.5808501 1.341167 3.790121
CR936218.1 0.286749 0.6397937 0.7033674 0.27392 0.5014928
0.8712007 0 0.05727741 0.442691 0.479020252 1.312744
0.3755726 0.5904189 0.4480895 0 1.133001 0.09533242
0.539755305 0 0 0.670152371 0.4283665 0.3648002 0.9720222
0.5564536 1.38991 0 0.5668471 0.184709133 0 0.2701121
0.1285184 2.168205 0.7526036 0.7003389 0 0.1491886
0.3863117 1.109054 0.380652443 2.238453 0.8426026
0.596913768 0.7066423 1.624023 1.019824 2.057553
0.359585 1.995164876 1.488675 0.2206794 1.893794
0.7690723 0.4311602 1.397727 0.3525032 0.2806825
1.175807 0.09584217 0.129581465 1.085179713 0.325879913
0.7615731 0.2796233 0.3946074 0.3238451 0.1956309
1.449071 0.144050676 0.5675495 0.2857219 0.4156618
2.458409 0.9803857 1.487574 0 0.9647482 3.89025
1.880918 0.468163287 0.3323911 1.044091 0.7678774
1.130271 0.1510213 0.8329228 1.376885 1.240213
1.3097744 0.9164054 0.598710793 1.067273 0 1.492714
0.495084 0.3417683 0.833857 0.805194 1.163083
1.356377 2.034434 2.254696 0.2202338 0.573268
0.2465703 1.658337 0.3447182 0.1784798 0.338096644
0.2548563 1.012986 2.379171 0 0.6322274 0.6770328
0.7968245 0.3743154 1.211601 1.129779 0.5999807
0.3956577 1.336792 1.012941 0.3549097 1.102545
2.271429 0.8742529 0 1.239507 0.221168 0.894892
0.6529226 1.069817 0.9149795 0 1.279199 0.7273293
2.521428 0.7889453 1.316884 1.009239 1.308388
1.412777 0.4528664 1.127704 0.1932476 0.8065134
0.6128904 2.948299 0.5135239 1.015863 0.4507253
0.2165332 1.139927 1.012828 0.7503206 1.195697 1.0186
1.611102 0.3197934 0.216104698 1.161612 1.71057273
0.5217188 0.2355041 0.8346418 0.4574258 1.697346
1.757336 0.6472088 1.025773 0.605208174 1.223759

0.4863221 0.3970305 0.3786325 0.7397088 0.6193836
1.379287 3.096935 0.5768009 0.8205508 0.4816911
2.092681 0.9177429 2.007698 0.819084 0.06466259
1.458323 0.4958287 0.4918235 2.064851 0.3390754
0.07306313 0.4930783 1.882095 0.9159602 1.946468
0.6817735 0 0.03676795 0.9007035 0.655943 1.01437
0.09042456 2.519718 0.2879357 0.7706447 0.9740691
1.928483 1.833898 0.2031851 1.020292 0.4743929
1.100736 1.087162 0.1534351 1.70373 0.7510192
0.545026202 0.7135636 2.116095633 0.150492052 1.711788
3.417271 0.232383 1.480364 0.5564748 0.2915445
0.4298476 0.3235123 0.8336239 0.3116738 1.282013
1.202695 1.33795 0.1376838 0.1621186 1.888756 0.6831616
2.398195 1.293021 0.507022 0.9475383 1.246375
0.6425676 0.8752983 0.9087991 1.32296 0.9882362 1.031072
1.833654 0.9790392 0.9530501 1.911818 0.5255364
1.146779 0.297235555 1.660175 0.784432 0.1313605
0.2008135 0.9155421 0.5924341 0.6769082 1.235923
1.820998 0.2599608 0.912721 1.729486 0.1307098
0.2597788 0.8004328 1.179340369 1.530262 2.098906
2.268067 0.841548 0.512391 0.968746049 1.125422
0.875233 0.1644842 0.528485 1.359929 1.575866
1.394084 0.7230246 0.7986304 0.7387999 1.658218
1.089647 0.3148587 0.52952 0.1464676 1.134111 1.237084
0.5373728 0.73607 0.7749211 1.008144 1.47607 0.4890501
2.296667 0.4033186 0.679293 1.293093327 1.32548823
0.3775867 0.8672759 0.3352839 0.06011968 0.7263113
0.186284 2.460792 0.6768605 1.255805 1.638075
0.2926111 1.858859 1.0823 1.027019163 0.7160788 0.4932388
0 0.8577782 0.529704 0.376446346 0.2603381 1.789124
0.4846488 0.5739324 0.2832476 0.3931193 0.389977
1.37145 1.177113 0.560062 4.111459 0.8647434 0.4012145
0.3873393 0.4938244 2.722385 0.6436557 2.885035
1.051383457 0.92083 0.1901623 0.3241993 0.9654166 0.539215
0.3260237 0.569444 0.8801101 1.22001 2.489426 1.446324
0.8694468 1.405089 1.571294 0.3179088 1.291998
2.230744 0.5357363 0.3210791 1.358525 0.9131588
0.06701275 0.6606428 0.918696 0.2330455 0.4544824
1.751888372 0.3510229 0.4750935 0.2225846 0.7120753
1.318492 1.731714 0.607828342 1.52028 1.311154 1.094052
1.235 0.2760275 0.2620972 0.2827381 0.8003621 1.583964
2.050536 1.221631 1.672768 1.447542 1.346348
1.320769905 2.823578 0.6348197 0.1843533 0.20872366
0.1803008 3.795537 0.7793316 1.710402 0.6341903

0.3218304 0.8725252
AC013437.1 0 0 0.4519221 0.03352322 0 0.03554013 0
0.08411755 0.05417796 0 0.03012334 0 0.05419304 0 0 0
0 0.052845608 0 0 0 0.03494993 0.1339363 0.04198561 0
0.02758407 0 0.05549808 0 0 0.09917161 0 0.06774943 0
0 0 0 0 0 0.062113979 0.08501872 0 0.073052249
0.02256031 0.03613694 0.02582262 0.6941789 0 0 0.1121162
0 1.489942 0 0.05276679 0.3421168 0 0.3091575
0.7914449 0 0 0 0 0.2796114 0.1642618 0 0.09511973
0 0.0354684 0 0.04902957 0.08991664 0 0.2430089 0
0.1092325 0 0.1634802 0.08569825 0 0 0 0.1916689
0.1127704 0.1082553 0 0.0339786 0.07221758 0.3469286
0.60110419 0 0 0.2742944 0 0.6517345 0 0.3585147
0.3061504 0.03695335 0 0.1778549 2.855954 0.06131934
0.1617176 0.04209504 0.0362113 1.058884 0 0.04368587 0
0 0.04250487 0 0 0.6963664 1.118576 0 0 0 0 0
0 0.1132622 0.03233922 0 0.03373325 1.940221 0.1604909
0 0.03250605 0 0 0 0.0714151 0.03536153 0 0.08945856
0.1001395 0 0.1158644 0 0.1673414 0.07390377 0.1911003
0.03694884 0.1324915 0 0 0.03000301 0 0.03428003
0.06486499 0 0 0.3836468 0.153873 0 0 0.04155318 0
0 0 1.163142 0 0.03192482 0 0 0.1119625 0
0.4301369 0.0432041 0.08691057 0 0.1497676 0 0 0
0.03394799 0 0.202562 0.08121707 0 0 0.03930062
0.3269478 0.03209039 0.1300811 0.08201632 0 0.08682535
0.06068113 0.06019096 0.07396191 0 0 0 0.1228463 0
0.139443 0.143036 0 0 0 0 0 0 0.3809292 0 0
0.02980244 0.06586438 0 0.3729968 0.4886087 0 0.1683895
0.1842237 0 0 0.07352974 0 0.06549618 0 0 0.05027861
1.866675 0 0.1449374 0 0 0 0 0.08161729 0
0.0276877 0 0 0 0 0 0 0 0.3516537 0.09307651
0.2609163 0 0.3370265 0.4090107 0.210736 0.1022578
0.1501368 0.2523719 0.1584061 0 0.2544815 0.1504121 0
0.02903721 0 0.06589543 0.1355313 0.2250685 0 0 0
0.2071056 0.373692 0 0 0.08741877 0.2177076 0.3839206
0 0.09795956 0 0.04885524 0.07005572 0.1332353 0.1404428
0.2633739 0 0.3005078 0.09887433 0 0.5174217 0.02560501
0.6126127 0.08300068 0 0.3703793 0.08565796 0.1132678 0
0.05780015 0.1196389 0.05377555 0.0594841 0 0.7891852
0.0568943 0.05989728 0.304821 0.1935496 0.05985154
0.1909179 0 0 0.113942137 0.048665268 0.3080686 0.09434671
0.02735542 0.04414585 0.03333313 0.04559608 0.2258698
0.1169455 0.3842236 0.1023691 0.0358107 0 0 0
0.03286352 0 0 0 0 0 0 0.09612825 0 0.01915628

0.2079886	0.1082503	0.05727198	0	0	0	0.3159463	0
0.09820387	0.04740385	0	0	0.05251512	0.06833806	0	0	0
0	0.0644459	0.2159702	0	0.04181425	0.2937566	0.04976959
0.6528515	0.4045847	0.3990215	0	1.338408	0.05836008	0
0	0	0	0	0.1257248	0.09841486	0	0.03747767	0.2139065
0	0	0.03221949	0	0.09806625	0	0	0	0	0.3628107
0.04186	0	0.08001706	0	0	0	0	0	0.4343388	0
0.1228313	0	0.3089446	0	0.1256577	0.1553826	0	0	0
1.332942	0.0520239	0.08016686	0	0	0
NALT1	0.08012105	12.13054	0.2702276	0.1913412	0.4904304
8.06003	0	2.656661	1.917243	3.195522967	0.9055276
0.6558713	1.196032	0.4131649	0.2612413	13.41481
0.133185	3.951326336	0.148392	0.2852031	2.106545137
8.391644	0.6795299	14.84184	6.923419	0.5667917
0.04227475	10.33718	0.309659429	0.5454066	2.566065
7.612824	0.1160084	0.3364581	0.1956829	0.9367282
0.06947508	0.1799	0.506142	0.118176412	1.024444	0.953504
0.125088547	0.4034729	0.1375064	0.05895532	0.2330694
0.9186037	0.557472539	0.04266187	0.1541511	0.3779631
0.07162929	0.2409425	4.113703	0.2626495	0.4313429
4.777436	0.374912	0.217239514	0.257730182	0.400640599
0.7314742	0.2031381	0.01837632	3.284647	0.2733079
0.4453762	0.053665938	1.249984	0.1482633	0.2613168
1.730481	0.4607026	0.1246937	0.1449014	0.6116983
1.532644	0.3032023	0.104648264	0.139311	0.3646641
0.0286072	4.462548	0.8580081	0.1422229	1.291553
0.2475214	0.27447478	2.15387	0.090077529	0.2385669	0.3422177
0.2367221	0.1998133	0.5047545	0.3883158	0.08436775
0.2241234	0.825652	0.5517257	0.06999873	0.4615194
0.224249	0.3444733	0.3424827	0.1926366	4.072663
0.307021585	0.2136295	0.1617373	0.5682698	0.304649
4.460457	0.3783419	0.2862542	1.150469	0.1586886
0.8976966	0.2514625	0.1520082	0.3447836	0.5783607
0.02479148	0.5262759	0.3756174	0.1709936	0.04332747
6.37005	0.60561	0.6443425	1.413865	0.217396	0.161467
0.08955725	1.787116	0.6731807	0.1725347	0.02939208
0.2102588	0.5003105	0.2952755	0.2804778	0.2811916
0.8822622	0.334773	0.5027024	2.831313	0.3177474
0.4304539	0.296185	0.3148449	0.1331042	0.5441194
3.356919	0.08385936	1.109662	3.320435	0.9753487
0.08935405	0.925860326	0.5163581	0.682791636	0.3887316
0.1645065	0.4134155	0.3408271	0.06240244	0.1636734
2.087854	13.54798	0.461188047	0.6838653	0.1766494
0.2912043	0.1057944	0.3616959	2.4998	0.128463	0.04635644

0.3223299	0.7282743	0.2392714	0.323462	0.1221086	
0.1484933	1.747671	0.5239572	0.3193704	0.09236025	
0.8588829	0.1266462	0.09474165	0.1837323	0.7577454	
0.6544278	0.4777361	0.3979507	0.1496751	0.9668649	
0.4109359	1.291162	0.523652	0.7848743	0.1515941	
0.1656562	1.142427	0.4614154	0.7938186	0.4385914	
1.127308	0.6528816	0.1859228	0.7805778	0.1537792	
0.4673321	0.1857768	0.3279402	1.147145	0.083065491	
0.06230565	0.323786342	0.070082083	0.8609288	0.2911054	
0.2813657	0.09651395	0.5286511	0.1629219	0.1334494	
0.662883	0.2484526	0.2264219	0.04214231	0.0320045	
0.3738389	0.2308229	0.2717871	0.6785238	0.490843	
2.72034	0.5352376	0.7437566	0.2647534	0.208951	0.5001498
0.2001016	3.04715	0.8949438	0.1142585	0.6962262	0.2411033
0.1740805	0.7504632	0.1526241	0.1631567	0.4530124	
0.027683704	0.2131305	0.7735748	0.1835183	0.1122193	
0.1193795	0.9931983	0.1576134	0.08125446	0.2197127	
0.04358167	0.1884967	0.2209091	0.2800009	0.1996095	
0.05591258	0.427157596	0.6227696	0.4265157	0.3675603	
4.307308	0.01431681	0.015922297	1.086303	0.06584049	
2.098786	0.08613787	0.04871542	2.227478	0.463217	
0.3367026	0.1526793	0.956094	0.1293002	7.915966	
5.817362	0.3755758	0.1841615	0.6564027	0.6016973	
0.1201186	0.08659647	0.1253549	0.4970951	0.3682423	
0.2619055	0.1452941	0.2028455	0.1116485	0.245687732	
0.388874856	0.09377971	0.3590031	0.1040914	0.08399073	
0.3297774	0.1040999	0.6016275	2.825717	0.160823	
0.3895297	0.09538547	0.7457867	0.2846187	0.398557273	
0.9003638	0.2480701	0.2059808	0.09586933	0.3700138	
0.420734151	0.03637076	0.07315643	0.1218749	0.3936194	
0.1582854	0.2883357	3.900917	0.983545	0.4604588	
0.1564879	0.3606667	0.08053982	2.017873	1.01012	0.7588919
0.6115162	4.855814	0.2600364	0.329021176	0	0.2302455
0.1811702	0.07356784	0.3698092	0.1252554	0.238664	
0.1341344	0.1893806	0.2981035	0.288657	4.858674	
1.993987	0.2634228	0.2442756	0.1470738	0.07791202	
1.147631	0.3332207	0.218993	0.542188	0.1685173	
0.07383654	0.9269522	0.4069728	0.4825534	0.23813427	
0.4781397	0.5475802	0.4477878	1.326415	0.2417639	
0.4435396	0.303275696	0.26549	0.780489	0.731	0.1522383
0.1349693	1.025262	0.1185005	1.065534	0.1721137	
1.443378	0.2925755	0.5608693	0.9059911	0.340918	
0.301940714	0.2988413	2.861668	0.6696362	0.136079641	
0.5877451	0.7531213	0.4948964	0.3253831	0.2151717	

0.1798464	5.723855
DSG1-AS1	0	0	0	0	0.01658549	0.03841674	0	0	0	0
8.683073	14.03576	0.03905294	8.061711	0	0.02810316	0
0	0	0	0	0	6.820611	0.1210238	0.7707679	6.659067
0	0	0	0	0.03573285	0	0.646892	4.778934	0.1029412
0	0.01315733	0	0.3618986	0.055951234	0	0.06688021
0.026321698	0.00812878	0.05208248	0.4093863	0.4119655
0.2446412	0.012875037	0	0.3065312	0	0.2441755	0.05703773
0.2711923	0.03108819	0.01856559	0.01296219	0.01267886
0.068568782	0	0.017244124	0.1385281	0.2663357	0
0.008568226	0	0.1533568	0.050816833	0.114829	0	0
0.3377289	0.7663748	0.03935792	0.2286811	0.1865296
0.02058548	0.02871054	0.136252222	0.04397167	0	0	0
0.3063361	0.04897177	0.1170943	0.1093776	0.51980603
0.01426239	0.024370099	0	0	0.08539223	0.2765308	0.05167105
0.09805339	0.01331478	0.03183372	0	0	0.05523544	0.058269
1.365066	0.6784654	0.114459	0.01824096	0	0	0
2.940494	0.09137556	0.3328561	0	2.642137	1.204698
0.6649532	0.04007036	0	0.9127644	0.183194	0.7345781
0.05826125	0.02347531	0.2673999	0	0.0925232	0	0
0.02340646	0.1639167	0.2879148	0.2058545	0.02548246
0.1978729	0.06446622	0.03608161	0.6943434	0.1809058
0.282053	0.06890897	0.05325703	0.3737896	0.1464448
0.05967308	0	1.083342	0.3243145	0.02228728	0.4076012	0
0.0119252	0.01145798	0	0.02772124	1.151406	0.1694759
0.01497216	0.4262621	0.6768822	0	0.0279397	0.491372278
0.1380353	1.417534	0.6323704	0.584953	0.0118179
0.1722045	0.9962888	0	0	0	2.907944	0.1575682
0.2404267	0.02446383	0.3095421	0	4.63828	0.9156526
0.02554113	10.66288	0.01178036	0.2890649	0	0.04925262	0
0.1355654	0	0.3903769	0	0.01281598	0	0	0.05532897
0.01615619	0.7536473	0.07730665	0.3839338	0.7685109
0.03108342	0.5082478	0.0412892	0.02392434	0	0.8075231
0.02912794	0.4832197	0	0.04852088	0.3359893	0.02347363
0.4323255	0.1092114	0	0.3247642	0.05008548	0
0.026218524	0.02359915	0.053321155	0.371624122	0	0.1102603	0
0.05222289	9.157764	0	0	0.0285314	0.0294078	0.0494772
0.1795724	0.09091618	0	0	0	0.02855561	0.6842658
0.1420543	0	0	0.1880233	0.01319053	0	0.04210629	0
0.1842242	0	0.09093294	0	0.753548	0.02292327	0.01806517
0	0	0.589814678	0.023743	0.09766746	0.1737754	0.05313081
0.95278	0.02612416	0.05969832	0.1346462	0.4708459	0.3438994
0.6824598	0.03922147	0.06916586	0	0.6353304	0.12712227
0.5104926	0.5427032	0.08401127	0	0.01355673	0.120615901	0

1.229088	0	0	0.04612913	0	0.1794375	0.3613375	0	0
0.408119	0	0	0.5172897	0	0	0.1575914	0	0
0.1726543	0.01569015	0.04184309	0.02156531	0.1375805
0.2667729	0.4757451	0.711617731	0.122743209	0	0.1699719
0.5519653	0.2385951	0	0.04928662	0.2115981	0.03160276
0.9137098	0.1106549	0.1161276	0	0.1179099	0.649124577
0.5210112	0	0	0	0.011679	0.082999484	0.1377594
0.01154543	0.3333911	0.3520155	0	0	0	0.3507603
0.6602486	0	0	0.008473777	0.1238446	0.5124071	0
0.04236959	0	2.573115	0.411695775	0.1851598	0.3186473
0.04288798	1.579009	0.01296949	0	0.2561259	0.1058444	0
0	0.4919982	0.5175819	0.009783039	0.2993253	0.252335	0
0.2951028	0.1732426	0.07281465	0.01382445	0.3926026
0.4166572	5.39523	0.135037	6.366259	0.6974268
2.217336097	0	0.0157124	0	0.4709984	0	0.2672678
0.367583869	0.1910604	1.704347	0.2642913	0.2402601	0	0
0	0.02491272	0.5587757	0.1669313	0.07695632	2.810367	0
1.803333	0.222375058	0.2150617	0.01119728	0.07316371
0.552236664	0.6201467	0.7567999	0.0187449	0	0.3835262
0.1192089	0.09033295
AL031667.3	0.2883542	0.2121017	0.0816121	0.3178309	0.2327539
0.1797082	0.2056265	0.372172	0.2054627	0.305695367
0.3046367	0.392201	0.2397732	0.3119524	0.1446466
0.3122239	0.2212294	0.217110734	0.1917139	0	0.285113172
0.2209049	0.1975305	0.1592248	0.1215353	0.3661311
0.2457744	0.3332427	0.128591396	0	0	0.2187102	0.4710395
0.20958	0.4875642	0.9238571	0.3231281	0.3785133	1.098105
0.255188881	0.7164936	0.05866058	0.115433706	0.1140759
0.2512483	0.4243583	0.6194307	0.3576244	0.271024445
0.6909269	0.23045	0.4185485	0.3331473	0.7003885	0.5189729
0.1636045	0.3582451	0	0.845167	0.541274637	0.251827964
0.302496163	0.4418287	0.4931615	0.3968164	0.1503037
0.2421243	0.2017637	0.245142729	0.7282575	0.1515541
0.6430595	0.5046741	0.7445761	0.3279476	0.2406913
0.2066589	0.4152767	1.443768	0.282470429	0.1928376
0.5572736	0.2138333	0.3193119	0.3504616	0.1288593
0.2054066	0.2741001	0.83585603	0.4753621	0.213749949
0.1486035	0.4358094	0.8987695	0.4595589	0.4532064
0.1290038	0.04671354	0.3722844	0.2697963	0.1388567
0.3294393	0.1533231	1.277119	0.2288775	0.2342477
0.351981	0.2485092	0.176532904	0.4731377	0.4567165
0.7480241	0.2335582	0.4156936	0.4713389	0.2817706
0.9430952	0.2460198	0.2621777	0.3248751	0.5049921
1.07383	1.103779	0.04118036	0.4690724	0.3442352	0.2028801

0.2878796	0.1643665	0.1847681	0.1118221	0.6313246	
0.6319419	0.4023116	0.2479346	0.1413582	0.08439241	
0.429888	0.2929336	0.3492543	1.28435	0.3503377	0.2760847
1.704837	0.8583631	0.3946381	0.8350232	0.284456	
0.3127705	0.281672	0.3484884	0.125515	0.02009957	
0.1543107	0.437657	1.392962	0.2972943	0.709132	
0.6231235	1.706867	0.200597949	0.3185766	0.362932421	
0.5649962	0.3005823	0.2465118	0.1061507	0.3524261	
0.362497	0.3549992	0.2014202	0.229819509	0.1893246	
0.315998	0.3455074	0.509622	0.3647725	2.299757	
0.3414175	0.5133415	2.186266	0.3360316	0.4222866	
0.3719718	1.034437	0.1918447	0.1036786	0.3601361	
0.1280508	0.1150625	0.6657763	0.4674844	0.08992708	
0.1017306	0.4958386	0.4852899	0.2834116	0.198307	
0.3842318	0.6993997	2.849825	0.7633709	0.3044377	
0.199181	0.8813288	0.2407705	0.5078219	0.562058	
0.2260435	0.4787486	0.5958068	0.3300595	0.3705967	
0.1957122	0.2341514	0.0582203	0.2848505	0.3514395	
0.3718023	0.321947469	0.3518795	0.257223693	0.23282231	
0.3495707	0.3481532	0.6471247	0.8931908	0.4132356	
0.1353123	0.3990036	0.375373	0.3353162	0.2314476	
0.1050019	0.5050359	0.9935554	0.4473148	0.451457	
0.3756916	1.200338	2.04337	0.3333999	0.5294703	0.3848018
0.7635804	0.4686469	0.4431767	0.5105925	0.2908493	
0.1739754	0.2990895	0.650795	0.5783193	0.3216957	
0.1742944	0.8130435	0.385419	0.114961334	0.3331995	
0.5782324	0.5487057	0.09320199	0.3965951	0.2978752	
0.4974328	0.5061347	0.3265412	0.2171764	0.699884	
0.4128134	0.2022177	0.09042683	0.5882061	0.32436028	
0.4631924	0.4870744	0.5684366	0.1775368	0.5231864	
0.502512544	0.1661969	0.3124731	0.2290223	0.2861619	
0.1942072	0.04302318	0.4371791	0.9321436	0.4877132	
0.05414106	1.628722	0.0229877	0.2557326	0.3402858	
0.3059049	0.1879879	0.3402419	0.09976265	0.3596067	
0.7193161	0.1100946	0.2691373	0.1513193	0.3217914	
0.2433458	0.2967293	0.432110332	0.584429009	0.6620418	
0.268348	0.3630962	0.7533776	0.14748	0.115278	0.3711851
0.2771876	0.5585614	0.7602667	0.09053819	0.3318222	
1.004639	0.450181089	0.3531198	0.5951999	0.1596692	
0.6369822	0.2868219	0.582390275	0.5739362	0.3037946	
0.3823916	0.3753462	0.1314615	0.29649	0.4343927	0.3819123
0.6992948	0.9197777	0.4659605	0.2229703	0.3103542	
1.378258	0.1375169	0.5945973	0.4646987	0.4319383	
0.312301016	0.9744209	0.9708438	2.821276	1.181279	

0.1137553 0.07565741 0.5285832 0.1856721 0.1258297
0.1650568 0.2237569 0.5044119 0.8580696 0.1750254
0.3319842 0.1776725 0.1294172 0.4696667 0.3406169
0.1940067 0.6357258 0.4509804 0.4497078 0.6869571
0.4596867 0.7171802 0.703213125 0.3665644 0.4961282
0.1859516 0.2754082 0.2486011 0.6027952 0.32240753
1.199512 1.402278 0.9272388 0.3708882 0.2241933
0.334524 0.5249001 0.2185093 0.7351526 0.7503783
0.1889953 0.7375503 0.6180894 0.2538534 0.501544269
0.3971167 0.3928452 0.1711249 0.226037633 1.840995
0.9446223 0.4274696 0.2195715 0.2522929 0.1493686
0.1056413
LINC01778 0.03287018 1.466797 0.05039209 0.1465314 0.1580875
1.187298 0.0677151 0.3414183 0.4397976 0.617741049
0.05643015 0.0645781 0.13536 0.02054588 0.1071759 2.857281
0.065568 0.857963988 0.02705723 0.09360511 0.307279519
2.520665 0.2090861 1.271537 0.6378654 0.03444888
0.1040609 2.373861 0.14821306 0.2237566 0.5882985
1.473212 0 0.03450852 0.3122007 0.07205602 0.03420312
0.1377696 0.2796892 0.106661787 0.09732912 0.5505512
0.034212252 0.1126995 0.09026059 0.05643586 0.05099637
0.1884315 0.290067012 0.04375577 0.07588976 0.07236217
0.1763182 0.0329494 0.854518 0 0.03217476 1.15689
0.09887789 0.653575291 0.049757863 0 0.8293451 0.02564268
0.04523403 1.425505 0.05980069 0.2768461 0.055041988
0.0612315 0 0 0.4769069 0.3269297 0 0.03963116
0.08506887 0.1337823 0.03317085 0.053665777 0.1714597
0.09574771 0.02347257 0.7999138 0.2885273 0.04243481
0.2029281 0.02707927 0.60056021 1.371803 0.031675615
0.04893681 0.2246352 0 0.05044596 0.1231279 0.106206
0.04614988 0.2206754 0.166588 0.06859061 0.05743486
0.3786826 0.0131428 0.1808926 0.08265042 0.01580608
1.213914 0.145335661 0.2191072 0.006635376 0.02639279
0.03845667 1.038773 0.1940215 0.07829174 0.147113
0.0694432 0.1780723 0.03438804 0.1020475 0.3064743
0.03029058 0 0.0315963 0.03188265 0.09019442 0.01185025
0.9032563 0.1115514 0.07890914 0.03742241 0.1337821
0.04416191 0.02449429 0.3630965 0.1459047 0 0.1326412
0.02875334 0.01492768 0.09229611 0.05114125 0.04614426
0.01034153 0.1949381 0.1137859 0.7962336 0.202779
0.06421689 0.01012598 0.05166686 0.009928522 0.06533516
0.9528287 0.3440384 0.2545461 1.128705 0 0.1466323
0.297266191 0.121051 0.145662216 0.1794146 0.05399189
0.06958209 0.1573048 0.0409616 0.08953076 0.215825

1.474334 0.01261369 0.4442202 0.08919573 0.2275589
0.01736113 0.1165906 0.3313344 0.2529733 0.01267868
0.2093767 0.03319769 0.06135164 0.1122866 0.2604985
0.08122709 0.9474555 0.04447382 0.1807224 0.1136741
0.3006824 0.1616453 0.01110525 0.1005031 0.2260872
0.1630076 0.4689864 0.06530473 0.08931658 0.1919335
0.278172 0.5925541 0.128899 0.2414998 0.1451157
0.07645671 0.2508472 0.05047964 0.1581822 0.05140998
0.4344558 0.3144313 0.07119095 0.2416883 0.1261778
0.0671041 0.2696878 0.008679972 0.1951366 0.3407815
0.05112258 0.127059969 0.069003897 0.02354677 0.009554229
0.07103514 0.05656495 0 0.06683977 0.1313964 0.3090364
0.01274116 0.02858185 0.04322288 0.09191036 0.09202189
0.03156552 0.08362679 0.02474389 0.1566223 0.689317
0.1097923 0.05812017 0 0.1257278 0.06313528 0.2371575
0.6031246 0.1755965 0.0156251 0.1280416 0.03709282
0.1224302 0 0.04696126 0 0.1722524 0.045429667 0
0.211576 0.03011583 0 0.05597281 0.498014 0.05172954
0.03333516 0 0 0.03639152 0.07930072 0.1897888 0.1340036
0.229385 0.050069755 0.05338706 0.0874904 0.24959 1.999499
0 0 0.5512143 0.1234811 0.8547556 0 0.0319773
0.2337723 0.06046656 0.1289254 0.0289097 0.1961218
0.01768208 2.997762 1.398581 0.1120599 0.08394825
0.4271551 0.2941194 0.02463972 0 0.05610288 0.1087661
0.06042951 0.08409006 0.01986928 0.07397215 0.1282526
0.035574694 1.747325359 0.1635133 0.03682083 0.05978583
0.04134928 0.4579157 0 0.01410409 0.7941446 0.09596897
0.07990352 0.1006264 0.2294728 0.07297916 0 0.07182389
0.5201697 0.3943565 0.03933101 0.1214404 0.071920368 0 0
0 0.06579013 0.1082294 0.1013928 1.251691 0.125768
0.01079464 0.0197539 0.09864387 0.07342662 0.4292531
0.2960059 0.2264293 0 1.180521 0.04267263 0.1253414
0.008444414 0.2615807 0.1114893 0.02012112 0.05619133
0.2335766 0.1566615 0.1467454 0.0776946 0.06114944
0.4105345 1.0714 0.1441117 0.2737796 0.01822102 0
0.01598195 0.1364705 0 0.01197911 0 0.1382706 0.131265
0.02340234 0.1602847 0.02083901 0.369074196 0.502974
0.1361505 0.1122659 0.05441701 0.06612346 0.1433663
0.119443966 0.06099464 0.2744577 0.04362145 0.0249827
0.3638734 0.6309307 0.0972312 0.04317453 0.0605235
0.4791484 0.02667355 1.016276 0.03982378 0.00964578
0.096345755 0.01961625 0.6403732 0.06339751 0.255211635
0.05511456 0 0.06497102 0.02502947 0.08308289 0.07378313
1.522013

LINC02570 0 0.03477513 0.05017765 0 0 0.07366014 0 0 0
0 0.01560834 0.1428962 0 0.06819485 0.05928881 0.02694245
0 0 0 0 0 0 0.1156647 0.2393022 0 0.114341 0
0.0143781 0 0 0 0 0.0175521 0 0.01480344 0 0
0.0326627 0.02813121 0.096552643 0.02936817 0 0.037851853 0
0.05617281 0.01337993 0.08463228 0.01954476 0 0.02904638
0.02099078 0.03431155 0.2731057 0.4647968 0.1063602
0.04470629 0 0.1677622 0 0.123256462 0 0.074393612
0.01811 0 0.02502308 0.03696455 0.02481093 0.01837787
0.018269256 0.07621367 0.01553003 0.0131791 0.1978656
0.03391026 0 0 0.02823563 0.0296029 0.04128713
0.284999528 0 0.05958767 0.01947724 0.1682768 0 0.1056356
0.03741937 0 0.43604504 0 0.01752268 0.02030357 0
0.01534976 0 0 0.3172622 0.03829458 0 0 0.04553249 0
0 0.0436229 0.1125768 0 0 0 0 0.04848329 0.0330357
0 0 0.1804103 0.1287972 0.1010574 0.1627633 0.04321731
4.983612 0.01902317 0.01881726 0.2347463 0.1340519 0
0.1398304 0 0.2328423 0 0 0 0.03928668 0.1449123
0.03700357 0.03664499 0 0.09270549 0.01729569 0 0.5002907
0.6919155 0.02477359 0.2297584 0.02829091 0.01914496
0.2059506 0.05882079 0 0 0.06410034 0.05328636 0.01680482
0 0.2636339 0 0 0.05709573 0 0.02153068 0.1021642
0.2433472 0.054815042 0.1205359 0.037190353 0.03308354
0.3360134 0 0.08701967 0 0.02476383 0.0223861 0.04503251
0.020933358 0.1746039 0.05551011 0.4909463 0.01440604
0.07036029 0.3665828 0.01749284 0.04208244 0.07315289
0.01836468 0 0.1185851 0.01662756 0.5392096 0.07082761 0
0.01499612 0 0.03118781 0.03832319 0 0 0 0.04773941
0.05808342 0.09031506 0.1111706 0.04247039 0.2098396
0.08939888 0.01782645 0 0.7568954 0.0281968 0.04382101
0.06283104 0.09265238 0.06825496 0.2791013 0 0 0.04010998
0.4013529 0 0.0583783 0.4177467 0 1.055697584 0.03393669
0.153356713 0.057258553 0.03907762 0.04756786 0.01473601
0.1877475 0 0.04437023 0 0 0 0.1185843 0.01434632
0.1089515 0.1018115 0 0 0.04106433 0.111397 0.02723745
0 0 0 0.0189686 0.0349259 0.2422034 0.09099365
0.1324616 0.03889653 0.04903721 0.0615583 4.23296 0
0.02597857 0.02221708 0.07522781 0.018848479 0 0.01755631
0.01665982 0 0.3483414 0.07513557 0.2575471 0 0.01574648
0 0.1509861 0.1504062 0.0497319 0 0.02537876 0
0.03797134 0.03629921 0.01725889 0 0 0 0.09731714
0.01280788 0.02086074 0 0 0.03526932 0.02867107 0
0.0319852 0 0.01467236 0 0.01497451 0.01549765 0
0.5547879 0.2091913 0 0.02947965 0.04655346 0.04512637

0.02005745 0 0.04946183 0.3069057 0.09121921 0.039359236
0.050431592 0.0159625 0.02444277 0 0.04574814 0 0
0.02340678 0 0.07963383 0.02652117 0.01855523 0 0.557126
0 0.1021689 0 0 0 0.03358991 0 0.02476307 0 0 0
0 0.01869654 0 0 0.05374352 0 0.01818965 0.158414
0.4070728 0.02456219 0 0.06092948 0 0 0.128008239 0
0.192939 1.079312 0 0.1305552 0.01550551 0 0.1826511
0 0 0.2096346 0.1654017 0.1547532 0.6695797 0
0.4005415 0 0.3850209 0.01745182 0 0 0.2549671
0.3183874 0.3883793 0.3103384 0.2766711 0.090074428
0.01669446 0 0.0338752 0.9482454 0 0.07320834
0.016518846 0.02892147 0.4771726 0.03619652 0.05528087 0 0
0 0 0 0.01500344 0.6861344 1.527474 0.1542112
0.01600789 0.913674027 0.01627731 0 0 0 0.1143334 0 0
0.01384609 0.01723528 0.1224486 0
LRRK2-DT 0.07009421 0.3412233 0.07033674 0.109568 0.23403
0.3742936 0.01969084 0.1756507 0.2164267 0.351281518
0.4758695 0.3380154 0.4920157 0.1493632 0.3947646
0.4532004 0.1652429 0.37902817 0.05507569 0.04082907
0.104245924 0.3871164 0.4620803 0.3506904 1.029985
0.350608 0.07060626 0.5240187 0.073883653 0.1445912
0.1440598 0.4398185 0.190679 0.280972 0.06225235
0.04190626 0.07293664 0.03433881 0.1528031 0.140982386
0.07204218 0.0224694 0.059691377 0 0.06561707 0.02813307
0.3410718 0.1232863 0.077860093 0.02544743 0.03677991
0.3547107 0.09570678 0.05748803 0.5590909 0 0.09356082
0.4376615 0.01277896 0.034555057 0.238739537 0.095591441
0.1396217 0.09693606 0 0.2115788 0.09564176 0.1674482
0.057620271 0.03115958 0.07075019 0.03232925 0.1323759
0.04753385 0.06446154 0.1959135 0.1781074 0.09336599
0.06269728 0.143570075 0.07755792 0.194897 0.1638138
0.2620919 0.05369645 0.08020738 0.1966979 0.2519797
0.87318293 0.1653123 0.018421871 0.1209576 0.07620847
0.1613745 0.07334578 0.104158 0.2100084 0.07380945
0.01069501 0.2260629 0.4707121 0.0222686 0.1321403
0.04586144 0.2235571 0.341281 0.08273237 0.2141757
0.056349441 0.08495209 0.00771799 0.04093198 0.08946237
0.3723117 0.1354066 0.09612486 0.05703854 0.005048338
0.1223927 0.02666581 0.01978288 0.05484277 0.2701176
0.1774548 0.04287674 0.6118951 0.1282238 0.05513483
0.3305352 0.1061606 0.05507028 0.08705634 0.1945122
0.04494636 0.1139629 0.2274131 0.03030539 0.04802721
0.1542826 0.05016701 0.138906 0.2080002 0.1487134
0.08721872 0.1563749 0.05668596 0.008271932 0.1852291

0.01123162	0.199185	0.1354483	0.2223578	0.09816173	
0.1899878	0.3585645	0.06002565	0.1480387	0.2867169	
0.07160455	0.2558348	0.211302372	0.4576046	0.104263481	
0.1275313	0.04710082	0.1719868	0.2337954	0.08337841	
0.1822422	0	0.3103622	0.183396416	0.1291746	0.03890577
0.07940609	0.02524217	0.1171206	0.165169	0.04291116	
0.05161559	0.0256356	0.04504985	0.2426296	0.1602905	
0.1515004	0.1023533	0.3872034	0.06035175	0.06306262	
0.04407366	0.09289999	0.04028978	0.01937574	0.1363845	
0.04382919	0.1003784	0.3582422	0.05063981	0.1558339	0
0.04412153	0.1096509	0.01249415	0.005201896	0.01205661	
0.0642281	0.09981772	0.1981658	0.02705748	0.06577772	
0.1304105	0.2776863	0.1478684	0.08433651	0.1467647	
0.08362785	0.1091094	0.0454328	0.2603533	0.085882917	
0.03567818	0.134355278	0.066885356	0.01369431	0.4500796	0
0.006579397	0.1780719	0.00777452	0.0509449	0.1365941	
0.3038096	0.04986781	0.03519252	0.04581697	0.03567866	
0.1927573	0.02701977	0.09353841	0.07156931	0.4533901	
0.1596323	0.1690073	0.1105461	0.1395939	0.06119692	
0.2334129	0.3188769	0.2228144	0.1726574	0.2864088	
0.04314481	0.1898743	0.08086485	0.01365584	0.05450007	
0.03690782	0.059447111	0.04187828	0.1538102	0.09925014	
0.01785009	0.3336642	0.09873901	0.2782845	0.1550962	
0.01655453	0.05545831	0.1216961	0.1185933	0.2439917	
0.1125708	0.2312361	0.034943418	0.06209758	0.03816193	
0.04233726	0.2601148	0.02732752	0.068382074	0.3956027	
0.3276513	0.1827602	0.1526734	0.05114264	0.2966335	
0.03014235	0.09640328	0.1232973	0.2384898	0.05655939	
0.2839686	0.3096111	0.01086195	0.04882254	0.2808276	
0.1405083	0.1146395	0.01033081	0.01631413	0.1423262	
0.1265203	0.1032439	0.3235556	0.06990855	0.02663894	0
0.123712235	0.1174714	0.008565688	0.0794747	0.09619149	
0.01815778	0.2069822	0.06562112	0.2760529	0.181394	
0.05111722	0.09753703	0.4130799	0.05093177	0.038037747	
0.05967324	0.05261213	0.2555608	0.1372445	0.1589112	
0.008365474	0.04338968	0.06981947	0.01938595	0.2156594	
0.2203037	0.2031117	0.2183871	0.1340974	0.006277935	
0.1895594	0.1274871	0.08113641	0.01783175	0.111898	
0.03292163	0.07829082	0.2574623	0.4839413	0.123362326	
0.03437765	0.03380663	0.1837129	0.04680808	0.08496721	
0.005433729	0.1138888	0.09601202	0.009037109	0.231161	
0.1285621	0.3912511	0.1035329	0.3687303	0.1907453	0
0.120832	0.3174734	0.1284316	0.01393359	0.1902414	
0.01787007	0.0587238	0.1224928	0.1165227	0.01211953	

0.063131112 0.0643542 0 0.04748471 0.07911947 0.0329623
0.1731714 0.075254938 0.0405408 0.4560537 0.03171164
0.09686276 0.09200917 0.183468 0.07539683 0.00627735
0.1290637 0.2471159 0.05429471 0.5352858 0.1312438
0.2131717 0.360209974 0.1197882 0.2257137 0 0.027829821
0.06410694 0.374053 0.05667866 0.01940882 0.006039907
0.09440357 0.1416273
AL391152.1 0 0 0.01694861 0.008800643 0 0 0 0.01104143 0
0.023085313 0.007908094 0.07239954 0.02845392 0 0 0
0.110264 0.013873229 0.03412608 0 0.0322965 0 0.02344098
0.02204444 0.01892968 0.01448295 0.04374914 0 0 0.1045238
0 0 0.3646096 0.1740962 0.0900034 1.070377 0.5560121
0.2068603 0.04275873 0.048919189 0.1785555 0.04060736
0.009588976 0.005922617 0.03794722 0.1016858 0.2787176
0.01980503 0 0.01471659 0 0.008692113 3.538351 0.02770508
0.3053669 0 0 0.0283327 0.0184756 0 0 0.037692135 0
0.2910777 0.3930226 0.05618518 0 0.08380168 0 0.1673286
0 0.3472195 0.1458185 0.02577137 0.007169031 0.03332335
0.01430581 0.2099797 0.0348641 0.009024834 0 0.03019058
0.07894643 0.094732 0.0873376 0.00892019 0.05687649 0
0.50497326 0.06234934 0.03551204 0 0 0.4044083 0
0.1600017 0.01786039 0.01940227 0 0.009338226 0.09227764
0.07244003 0 0.0884076 0.1330886 0.03474777 0.01329034 0
0.114056699 0 0.351494 0.2958938 0.5497085 0 0.6851898
0.1316612 0.09277353 0.0145976 0.4900246 0.07710596
0.01906784 0.7136167 0.6282462 0.05131224 0.05313468
0.008936037 0.03370614 0.02989238 0.05973527 0.01705392
0.006634973 0.08390973 0.3937114 0.01856648 0.08238282 0
0.008763003 0.01983912 0.02027811 0.02417687 0.01882762
0.06790524 0.1075036 0.08729955 0.6173837 0 0.0597971
0.007876503 0.202981 0.2069844 0 0 0 0.01831207 0
0.3182082 0.04115996 0 2.096374 0.06164693 0.027772491
0.09160573 0.047106994 0.01676205 0.2383415 0.1170144
0.08817846 0.06888406 0 0.1361052 0.007605363 0.042424231
0.05897634 0.1406233 0.019134 0.1021852 0.2138917
0.1989988 0.09749179 0.2025534 0.09265878 0 0.09285612
0.02574947 0 0.02276625 0.179427 0.04986031 0.06838116 0
0.0395039 0.1456257 0.05602623 0.04225337 0.1108931
0.08062521 0.04708551 0.604017 0 0.01075898 0.03543898
0.03019645 0 0.007520813 0.2440374 0.01428614 0.01110114
0.3926176 0.06259079 0.2074916 0.01178408 0.02937616
0.008551427 0.1016103 0.00884125 0 0.1478892 0.8393208 0
0.124168072 0.008597152 0.242810744 0.019340344 0.01319933
0.05623485 0.3733061 0.7514781 0.1072723 0 0.01841382

0.02078794 0.02142648 0.03604902 0.02907472 0 0.03868771
0.02654146 0.007812945 0.07281952 0.6960958 1.035006
0.01846349 0.4031741 0 0.2979286 0.01769549 0.01533929 0
0.120803 0 0.1490706 0.2703048 0.1029439 0.1503168 0
0.06753881 0.2058198 0 0.4238284 0.4536474 0.01688167
0.02580736 0.141192 0.009517013 0.03262208 0.09810304
0.279233 0.01002259 0.2830439 0.4953296 0.05039415
0.01251946 0.3086006 0.008420101 0.02565132 0.06436952
0.01748871 0 0.009877417 0.032955216 0.09861306 0.01297843 0
0.04244859 0.006721924 0.03573898 0.1452643 0 0.2106723
0.1274278 0.2007146 0.01909568 0.007586957 0.03926006
0.0705868 0.01561599 0.211977 0.04143598 0 0 0.05715911
0 0 0.07518073 0.1477215 0.1001367 0.189445962 0.02555158
0 0.05572857 0.05745159 0.04635734 0.008750739 0 0.04743695
0.0537267 0.2925167 0.3628035 0.2162267 0.2480776
0.03681815 0.142985265 0.008627456 0.03803287 0.1326358
0.5622064 0.0170186 0.42331316 0.02509283 0.1598277
0.009342627 0.05531878 0 0 0 0.03525013 0.1179949
0.2408422 0 0.08026177 0 0.0995571 0.02855852 0.6791493
0 0.02691055 0.980953433 0.3195169 0.09775411 0.2031128
1.082789 0.4819271 0 0.1646585 0 0.0130657
0.008569452 0.2389795 0.3142579 0.3777792 0.06058001
0.01532091 0.05534663 0.6853491 0.1491742 0 0.01007248
0.01100191 0.09042684 0.02547056 0.6887145 0.04492443
0.2015055 0.073019118 0.01691677 0.02289607 0.0343263
0.1601454 0.01588544 0.1669122 0.30966814 0.4322723
0.8351827 0.0275089 0.01400425 0.02660506 0.08841832
0.05450373 0.03630274 0.04240867 0.03040647 0.04485621
0.6449227 0 0.0567737 0.046292045 0.008247033 0 0
0.469418674 0.2664686 0.9119363 0.02731502 0.03507618
0.06112677 0.03722375 0.02193882
DLGAP1-AS5 0.09591513 0.03861755 0.1439484 0.3423842 0.2913467
0.01022489 1.185555 0.2843571 0.07793495 0.328889513
0.1689963 0.2082745 1.099188 0.1277943 0.2304394
0.01869963 0.1510479 0.209050468 0.2742576 0.2156365
0.359836274 0 0.2183562 0.06643589 0.05186258 0.4007645
0.1678064 0.007983393 0.253638115 0.8018332 0.1141266
0.05882611 2.44131 0.09539603 0 17.88311 0.0210115
0.1994944 0 0.201039511 0.183449 0.03560128 0.010508558
0.05192477 0.01559488 0.2860228 0.1820932 0.04340865
0.030841037 0.008063953 0.02331013 2.852943 0.04874176
0.1594004 0.09842694 0.06205755 4.78077 0.005174967 0.02024741
0.006843774 0 0.075729147 0.005027758 1.246432 0.9934197
0.06841482 0 0.2500041 0.050719719 0.01763225 0.0215575

0.8378709 0.07990127 0.009414279 0.01178481 0.812549
0.06271095 0.1191676 0.01528303 0.103848312 0.01755505
0.1047719 70.78204 0 0 0 0.005194245 0.02495282
0.39775637 0.04555242 0.00972941 11.31858 18.06897
0.06392178 10.6043 0.01031446 5.867067 0.2126294 0.4998933
5.106647 0.01264088 0 0.05815764 0.6479243 0.1250158
0.01142402 0.2111908 0.0816947 0.004464097 0.6864648
0.0703147 0 0.7530319 0.1224327 0.2502998 0.1082156
0.03389017 0 0.01864646 0.4911587 0.07836166 0.0271546
0.0651279 0.103094 0.0194101 0.09793002 3.75388 0
0.004675992 0.05139581 0 0.01149458 0.1746419 6.216011
1.117256 0.006434302 0.01920675 0 0.1111139 0.2583305
0.05846069 0.05847077 0.007854216 0.02126035 0.2048831
0.02449504 0.5701253 0.04315929 0 0.04438061 0.6904809
0.009521925 0.004574434 14.3287 0.02213461 0.04755333 0.3879219
0.8906357 0 1.114721 0.022826904 0.2788631 0.263285214
0.009184764 0.01865703 0.9257041 5.97525 0.01887251 0.0412501
0.1056534 0.02083679 0.371941697 0.1023343 0 0.4927702
0.01599782 0.117202 0.0290777 0.05827704 0.09930608 0
0.005098464 0.3448568 8.522091 0.009232398 0 0.03146144
0.2800398 0 0.02182243 0.0952431 3.149266 0 0.5556656
8.932291 0.1325358 2.673577 0.1353973 0.1903253
0.01768616 0.1592341 0.02068268 0.004949039 0.004121029
0.02865434 0.5323101 0 2.965372 0 0.1184323 0.01937126
0.5043622 0.9184078 0.0167032 17.76986 0.6315966 0
0.04799017 1.34331 0.036635787 0.2355405 2.980278306 0.00529877
0.2278261 0.07043173 0.004091061 0.09382156 0.07053579
0.1355003 0.01008985 0.01708612 0.01174064 0.03950612
0.8364032 0.9134738 0 0 6.858327 0.1026037 0.2216392
0.2117291 4.330105 0.02008357 4.378825 0.06319353
0.2036211 2.218963 0.02526196 0.2647759 0.2087726
3.22195 0.5183973 0.1316189 4.630803 15.42702 0.01233596
0.213027 0.245940561 0.05213478 0.04874041 0.2960099
0.1272703 0.4190667 3.697327 1.870945 0.2150222
0.01311478 0.1043456 0.01676691 0.04175628 0.05522694
0.02744015 0.07045737 0.083048291 0.05622257 0.08062006
0.5174787 0.07272965 0.00541233 0.01805781 0.4971222 0
0.02316572 3.312182 0 0.1762485 0.5213642 0 0.07991854
0.004107296 0.07739442 0 0.05404455 0.05163011 0.007735607 0
0.07743518 22.34156 0 0.3446487 0.4196925 0.0334105
1.446419 1.364021 1.235461 0.06753222 0.234931424
0.05600395 1.068007 0.5801929 0.04328582 0.02540151
8.367217 0 0.2794256 0 3.106204 0.01472579 0.04636228
0.09565735 0.9213023 0.036160971 0.02836448 2.657114

0.06229525 0.3261822 0.009325343 0.053018156 0.391864
0.05070281 0.1484595 0.04960133 0.428841 0.02595298
0.06590855 0.01448648 0 0.02275336 12.71053 0.06766066
0.2330892 34.55207 0.1408378 0 0.06043435 0.1179651
0.093287449 0 0.006695545 0.01712241 1.158817 0 0.03874222
0 5.899057 0.02147805 0.2253903 0.1454987 0.0573992
0.1093606 0.1327793 0.092346 0 0.01472694 0.006287693
0.004845033 0.1434995 0.03014249 0.01061773 0.01395659
0.07547622 0.2092389 0.04320583 0.020005412 0.1529474 0
0.01880909 0 2.058597 0.828217 0 0.008029275 0.6683928
0 0.01151045 12.09994 1.10048 0 1.795261 0.5112322
0.01666123 0.00614474 0.3180468 0.02446437 0.07999496
0.215608657 2.273038 0.01788142 0.1655208 0 0.09522486
0.02324165 23.25913 14.5995 0.009569832 0 0.2404275
C2-AS1 0.05719928 0.05469552 0.08769017 0.03642681 0.02500882
0.05792761 0 0.04570166 0 0.023888149 0 0.07491738
0.02944346 0 0.2486706 0.01412534 0.07606577 0.014355697
0.02354192 0.04072191 0 0 0 0.1596776 0 0.01498662 0
0 0 0 0 0.01709081 0.1288306 0 0.2638781 0.08359254
0 0.1027461 0.3097202 0.033746965 0.07698545 0.05042346
0.019844902 0.3186866 0.1963346 0.4068588 0.1109271
0.2049378 0.077655736 0.07614192 0.1320601 0.3058095
0.2454572 0.08600572 0 0.04687707 0.5039024 0.01954535
2.733892 0.077544813 0.129879777 0.156011801 0.1329255
0.06693344 0.1574287 0.1291981 0 0.2312428 0.057469036
0.03995717 0.08142058 0.1105522 0.2074733 0.03555682
0.1632037 0.1724112 0.07401662 0.2483225 0.4762107
0.317515468 0 0.2290971 0.1429609 0.2548689 0.04016664
0.01846081 0.03923632 0.04712219 0.1959505 0.2150588
0.073494077 0.04257888 0.04886257 0.03219018 0.3511357
0.09739179 0.1663337 0.1806931 0.04800124 0.09662981
0.1432302 0.04997285 0.04393115 0.297317 0.137717
0.1006773 0.02750508 0.02373484 0.033720925 0.0762562
0.230932 0.9185523 0 0.1261136 0.4726791 0.3481688
0.2986664 0.01510526 0.01408517 0.3789905 0.07892384
0.3692171 0.2635519 0.03539781 0.5131704 0.03698722 0 0
0 0.035294 0.01373143 0.2170696 0.1552013 0.07684867 0
0.1215087 0.01813551 0.3695232 0.02098332 0.6754771
0.2467769 0.6625153 0.1186584 0.3613422 0.2159508
0.06167692 0.5197639 0.211911 0.8401603 0.1117475
0.1233456 0.05394508 0.05183158 0.1705402 0.09753364
0.0598681 0.153329 0.8804694 0 0.9568623 0.028738333
0.1053239 0.038996184 0.3295546 0 0.3632514 0.1216601
0.01781991 0.285629 0.07041928 0.01573971 0.065849422

0.5492462	0.2522238	0.1583954	0.07552774	0.1659977
0.3294709	0.2934756	0.5074468	0.2301148	0.1155384	0
0.1598698	0.4010035	0.2826959	0.1336801	0.2063772
0.6918681	0.03296849	0.2616174	0.08036806	0	0	0.03278561
0.08342911	0.07308449	0.2651612	0.1359968	0.0445326
0.4693945	0.1249864	0.05607612	0.2179062	0.1803745
0.07391484	0.2527184	0.1317638	0.1781106	0.1431383
0.1707145	0.2026518	0.1061858	0.3364606	0.3110565
0.06672668	0.02040431	0.1812542	0.05992384	0.118602695
0.1423382	0.080401584	0.040025883	0.02731673	0.06650345
0.07725771	0.5315324	0.04440114	0	0.0190542	0.1075544
0.2217163	0.1740792	0.01504292	0.1370901	0	0	0.1455238
0.06458739	0.4088211	0.25704	0.07642237	0.3286993	0.04725257
0.09944826	0.09155444	0.3809459	0.0190824	0.0277787
0.4894224	0.1542548	1.032758	0.4260958	0.345654
0.17706	0	0.06310448	0.039527388	0.2685109	0.07363511
0.1222813	0.08011457	0.0974015	0.01969597	0.09001754
0.3190464	0.04953323	0	0.04749524	0.1774231	0.1216756	0
0.1064443	0.261387775	0.1725321	0.4377103	0.4705198
0.04578214	0	0	0.163268	0.308885	0	0.1932692
0.01391138	0	0.09018971	0	0.06707657	0.21718	1.230784
0.03951953	0.06280647	0.6500064	0.1752998	0.0969544
0.3107442	0.128631	0.1236443	0.08135655	0.07097632
0.1261882	0.03251777	0.1555906	0.1448136	0.1275313
0.165081521	0.211521481	0.05021276	0.01281481	0.01486239
0.02398475	0.01811012	0	0.22089	0.03176866	0.146126
0.02780894	0.2918431	0.01901518	0.3555866	0.227627461
0.1964048	0.1574221	0	0.06844214	0.05283137	0	0.597206
0.05222716	0.03867014	0.1144852	0.07533445	0.09802188
0.06223255	0.01823801	0.7701592	0.1203121	0.4958947
0.06388694	0.08003229	0.1802839	0.2758158	1.0648	0.08559551
0.0742571	0.033555965	0.1469461	0.3287495	0.8083709
0.7352922	0.1955635	0	0.02271798	0.5107201	0.05408034
0.1596145	0.5495343	0.2167912	0.9588534	0.351046
0.06341489	0.1336333	0.1946778	1.42488	0	0	0.3415358
0.1203063	0.5798397	0.7330278	0.02324338	0.1631842
0.132227369	0.05251524	0	0.1953603	0.04734709	0.03287578
0.24948	0.086604713	0.6065158	0.1137142	0.03795409	0.1449128
0	0.05228185	0.0563992	0.1314783	0.4212817	0.6135463
0.3713294	0.3336755	0.207899	0.1007111	1.077793623
0.1365414	0.01688409	0	0	0.143862	0.3072351	0.2826495
0.2032577	0.07228865	0.0513577	0.0605381
AL390719.2	1.810557	0	3.96722	3.810643	4.575931	0.2385185
2.274322	1.199634	1.477132	1.881137281	2.35017	1.388135

5.432811 2.774069 6.911381 0.06543166 8.016037
1.119395264 1.944748 2.137838 2.803731027 0.01465985
1.516859 0.01761099 0.1613082 3.181809 3.75136 0.04655766
1.393833458 1.035431 3.036641 0.3034787 13.04372
11.49753 8.101005 7.260355 0.07658448 3.265492
3.87139 4.285864542 9.866306 29.76752 2.482000104 8.17602
13.42976 3.715161 12.07521 6.202204 2.083368844
2.080966 3.670389 6.930115 1.326513 4.847167
0.3157042 8.776271 1.966768 0.2112561 5.195481
2.514431825 4.361828729 9.234254716 0.9089495 0.8268009
10.61455 1.895167 2.370034 1.472855 2.351514181
18.89978 1.873218 7.670862 3.4074 2.717665 3.665431
2.848505 5.520065 14.24675 9.882041 2.263880181
5.553996 3.199763 5.881199 4.616481 0.5116668
4.518018 4.422613 6.512029 9.32899092 4.366675
2.127754047 4.322727 1.068838 5.405309 6.523093
3.173019 2.539777 17.62371 1.951758 3.789768
4.091419 13.34898 3.629061 3.937486 2.916275
3.13127 6.030718 2.821921 6.391294005 8.772013 2.790206
29.2645 1.446625 4.446303 5.004692 6.977087 13.85135
2.717203 3.425394 5.836489 12.30828 2.69213 7.840442
1.585047 7.230399 2.39866 1.602177 1.512437 2.290639
1.812009 14.73562 6.887771 10.39448 8.172705
1.678268 8.292727 4.62042 2.171339 3.191378 9.657277
5.495019 8.447268 2.977281 14.5684 3.54284 7.559172
22.12754 10.62161 8.406302 7.333218 6.094549
5.691834 2.254227 1.623847 2.129901 24.58923
23.91186 4.79314 2.108961 9.258782 0.510301942 6.407534
9.348060856 4.097633 2.448097 6.648844 2.489039
4.553772 15.03518 20.29673 0.1944261 2.728314348
4.507368 2.336711 14.65915 5.44617 2.520406 2.988772
15.6336 13.54153 19.08333 12.19065 1.945201 6.239834
3.768914 3.128272 1.376079 20.31467 3.435539
5.434189 1.729438 6.67006 1.163722 4.028169 3.670195
3.761562 1.429405 7.428198 2.159887 9.918858
1.970494 22.50723 5.772375 6.392798 8.940213
12.94233 4.416531 0.7290395 6.08785 4.88998 11.56055
2.127782 7.296161 5.032848 6.144937 4.932603
1.764322 3.76658 4.626339 2.808011941 3.475279
9.171279147 1.266959093 29.56749 6.584751 30.02568
8.055261 4.627705 7.004158 13.00411 4.334475
3.782924 6.546982 4.877748 8.766963 20.11019
6.488298 5.580034 10.60437 4.103122 4.87291 3.171296
3.728436 7.551506 2.763997 2.488055 2.818498

1.222781 2.509201 8.386214 5.187047 20.29861
24.17867 3.522519 3.448964 1.726585 9.171344
1.647895599 9.065929 2.572417 6.500503 3.834785
3.816274 2.204871 3.613835 4.030616 13.47374
4.996305 5.573544 2.83086 3.703842 9.481532 22.35265
13.614833802 15.13369 11.37202 6.147451 1.979347
0.5208011 0.438790749 2.725807 1.721135 1.013236
2.794313 4.564541 11.70605 6.255072 8.388159
7.444174 2.095886 8.694423 0.3051052 0.8364332
5.331929 0 0.8483265 2.638965 3.93921 17.02731
6.356394 7.580155 5.406916 1.092063 3.590301
8.062121 5.919837 4.906784779 10.492172689 8.024579
4.432288 1.308071 2.703497 7.00482 0.937145 21.48743
2.35455 6.043639 13.63314 7.855931 3.743506 7.76516
8.43536172 4.673013 10.26976 0.03027452 10.77933
12.05958 21.585466514 3.62838 14.85167 5.911242
1.341871 1.919311 2.451918 1.177122 12.2922 12.23987
6.38255 15.07835 1.676984 9.329964 14.07765 4.775937
8.401498 1.497878 12.4691 3.66575974 3.562259 3.533769
1.722494 11.13719 1.192759 3.891144 3.069344
3.524007 8.037259 12.17221 4.13654 5.255441 6.024657
3.368396 2.10522 3.994144 5.110144 6.508686 12.81379
14.96697 4.728627 11.30051 4.802143 26.22115
2.512263 1.889763 3.252115789 3.865164 1.701095
4.73043 6.76243 7.741297 12.65284 5.014649802 2.774395
15.74979 3.999715 8.42441 5.079801 1.816357 12.80142
17.95208 3.577693 3.801587 8.439137 22.95302
14.10666 5.040961 1.830611175 21.38606 2.398465
3.889537 9.814593943 4.979492 14.92645 13.63848
14.21268 3.753187 8.643705 2.313512
AC004918.3 0.5811447 0.7058975 1.618125 2.175567 0.8858802
1.82926 0.4368173 1.016504 1.915616 1.259434868 4.206456
2.777207 1.770618 2.194234 1.485169 1.210167
1.127911 1.714768566 0.9114921 0.2348223 1.835372259
0.5474859 1.858312 1.07111 1.549082 3.888906 2.038694
1.577285 1.244440343 1.033554 2.396844 0.9855392
2.516763 4.897375 1.700667 0.5508974 0.9806126
0.8040851 1.652351 1.501210931 3.716375 1.509215
1.618442951 1.060213 4.221341 1.571802 6.798671
2.144071 3.038647943 3.487481 0.5620765 4.015916
1.280623 2.125503 1.837445 1.737746 1.130012
3.010913 2.535621 1.958991569 4.921671276 1.499399902
1.45481 0.9924963 1.62109 1.521967 0.9429761 1.365207
2.019927898 1.909132 1.542678 1.514057 2.67246 5.858226

5.438892 2.414502 3.329163 3.01732 6.181613
4.969717786 0.955849 2.745113 1.817005 6.557113
1.025747 1.657641 3.830186 2.135017 1.50659455
1.612174 1.680074595 3.156821 0.4427742 3.619673
1.15704 2.583399 2.253267 3.208589 1.370811 2.435823
5.211255 5.598688 0.3980875 1.243465 1.669321
4.182361 1.178228 1.290457 1.333380062 2.073015
1.284107 5.246373 0.5237175 2.389478 2.225059
1.284436 3.110604 2.277155 2.192993 1.856805
3.153285 4.224375 4.197456 0.8748043 1.826847 5.4388
1.422244 0.8493745 2.284139 2.136985 1.843812
1.823948 6.041023 3.561001 0.983161 9.909591
1.105541 3.399214 2.575572 2.246397 2.118504
1.207308 1.58842 4.200425 1.482473 1.524253 1.5088
1.705403 3.169866 0.7517889 1.582215 2.133076
0.5977722 2.934644 4.602714 1.82478 1.473616 1.896981
2.426814 0.3678489 1.089011929 4.3035 5.894831763 4.186535
0.6578839 3.304115 0.6514403 4.873678 8.256768
0.3673983 2.217206 2.459137764 1.20656 1.294616 2.104047
0.6097416 4.284714 0.8142384 4.200588 3.998514
0.8056506 3.045718 1.794148 3.629015 2.384194
5.68616 2.214707 1.402584 6.567376 1.588796 3.165394
2.565479 2.67448 1.776895 1.728528 5.484458 2.077101
3.885032 1.696484 0.7520472 2.851762 0.5534193
3.741763 3.346534 3.328406 4.834654 1.154482
1.429174 4.241676 3.832239 8.156648 1.285448
4.665289 4.209532 1.477167 1.827698 1.428743
3.695529 2.41885 2.621696554 3.341792 3.046741964
1.500256623 2.734124 7.72461 0.7128088 2.351512 2.377501
1.667193 0.7063442 1.010058 4.200864 2.171541
1.908386 3.557378 1.780847 1.855234 1.092241
3.351981 3.864954 3.78789 2.691348 1.208083 0.6228144
2.621564 1.734684 2.850505 1.32046 3.089291 1.198335
3.670987 3.58032 1.140785 1.73694 2.816203 1.496879
3.340001 2.035126321 1.592603 2.305058 1.49661 0.9019601
2.407133 1.638747 4.801536 3.321155 1.115328
1.179014 3.025729 2.306053 1.016663 1.707519
2.23602 2.268111176 2.055406 5.236229 3.160485 1.257153
0.841983 1.666797653 2.521829 1.637359 0.8469011
1.302644 3.552592 1.919299 2.266053 5.016444
2.458918 1.942445 2.382665 2.099833 1.008909
1.82058 1.275614 1.863621 1.80696 1.836711 1.897071
1.581677 3.527638 2.252289 2.035858 5.468714
1.590601 0.945524 4.997686885 3.811674262 3.998564

2.396355 2.705791 0.8496048 2.834577 1.550957
2.587955 2.617051 1.857233 1.34015 3.413901 6.516391
2.029562 2.643972938 1.117857 0.648409 1.195238
3.608418 2.074531 10.330501662 0.8769876 2.997331
0.4778375 1.989106 2.110016 1.921822 0.8458917
3.440536 1.578375 3.001653 6.049091 3.115638
3.999711 1.591232 0.551802 2.666581 2.773479
6.086594 2.335821686 3.776817 1.374925 0.8790182
2.826702 2.964278 3.643007 2.002473 1.41994 3.274462
5.42022 1.177013 3.143168 1.190908 3.862699 1.175404
0.8335003 2.061931 2.445403 2.125519 5.168836
2.307083 2.587779 1.983023 1.341903 1.436064
1.926809 4.35707051 1.110371 3.903471 2.516433
1.053102 2.356181 2.466211 2.397143558 2.885409
1.836048 3.486155 4.225939 1.020556 0.9690511
1.997812 4.193134 3.629512 4.224872 2.198621
1.044531 5.175972 2.959054 3.847431939 4.260204
2.600954 2.696142 1.120413565 1.441886 4.320698
2.258568 4.09033 1.384543 1.417306 1.496109
PTPRN2-AS1 0.1745179 0.1281391 0.2923908 0.4673836 0.2534356
0.2019887 0.2696403 0.1792776 0.6158298 0.484157749
0.01070018 0.01224518 0.4716259 0.002921905 0.7519326
0.08542467 0.6402936 0.260453354 0.3463116 0.3860458
0.415143725 0.1427684 0.08722237 0.1603234 0.07897381
0.01714685 0.4094358 0.135531 0.566092684 0.01414277
0.6781194 0.1620217 0.7279794 0.1374123 0.2993775
0.5806831 0.2269936 0.3330762 0.2868665 0.358534357
0.1988147 0.0906584 0.603315674 0.2524316 0.2759802
0.249951 0.2900952 0.7536807 0.666370163 0.1070299
0.3021917 1.514231 0.04680641 0.3045805 0.6015444
0.0421411 0.649748 0.6708808 0.1562421 0.591482962
1.372791908 0.871249943 0.7821562 2.425081 0.1200807
0.2090614 0.5145207 0.7307312 1.017603805 0.2198758
0.5642645 0.2213534 0.1880538 0.3225511 0.4631837
0.2987129 0.5129532 0.1724994 0.1957699 0.070214451
1.056636 1.399109 1.218414 1.02543 0.08534764 0.3047579
0.5675632 0.8549311 0.97151434 0.3093305 0.507530042
1.151794 0.4419223 0.562976 1.151444 2.212686
1.253627 0.5184883 0.4445932 0.4580278 0.7296381
0.4737452 0.4667332 0.3775552 0.5530961 1.217716
0.5979256 0.7603702 0.782655294 0.3905391 0.2245866
0.2552322 0.514092 0.254229 0.507702 0.2424771
0.4777069 0.1802328 0.3338204 0.1467134 0.04837513
0.7275313 0.4738507 1.307578 0.242645 0.5773481

0.7183051	0.5763613	0.5715542	0.5855306	0.7428935	
0.05321977	0.4502736	0.2731985	0.9892919	1.04069	0.5276336
0.2818585	0.4287133	0.2126341	0.7366531	0.2690782	
0.3369816	0.6398285	0.3382627	0.378039	0.2427285	
0.5728373	0.1318306	0.4474934	0.4953782	1.113916	
0.3304013	2.663578	0.4304284	0.1174247	0.2394765	
0.6494489	0.01750948	0.2919436	1.103855259	0.3615171	
1.182357264	0.2863373	0.1113366	1.088507	0.6214135	
0.4310721	0.9888904	0.1611396	0.1826576	0.663720364	
0.4023198	0.06659546	0.03559818	0.21974	0.5004379	0.1884811
0.4586973	0.198339	0.2068664	0.3588085	0.2303411	
1.553318	0.5670954	0.4235587	0.9201239	0.1264957	
1.655157	0.5119238	0.8258246	0.1247929	0.1895181	
0.4621376	0.7448705	0.6736391	1.604692	0.191936	
0.1460734	0.07642756	0.03836107	0.3472916	0.2169191	
0.1450104	0.06486052	0.2778703	0.7622947	1.008634	
0.1243878	0.821779	0.4743535	0.6558407	1.304592	
0.3196538	0.2272933	0.3626385	0.536946	0.2419444	
0.8717107	0.277858996	0.7357573	0.614368473	0.461224891	
0.1540389	0.6929583	0.1793134	0.1351444	0.0508013	
0.09885748	0.6415648	0.2074402	0.384137	0.1097477	
0.5630545	0.1717891	0.6368894	0.8978095	0.377929	
0.5454324	1.600533	0.1820562	1.399011	0.652973	
0.4247866	0.2340682	1.58923	0.9910558	0.1154031	0.9625658
0.3822016	0.7984061	0.3129896	0.2031313	0.4519749	
0.4452354	0.4264613	0.5105615	0.93357382	0.2633273	
0.3430145	0.2683939	0.1309466	0.493526	0.08370154	
1.324196	0.4835529	0.1349361	1.217121	0.4399065	
1.466094	1.159174	0.7241711	0.1783318	0.233555752	
0.3210487	0.5599048	0.3164981	0.2170085	0.330779	
0.824926607	1.417695	0.8297427	0.7293474	0.603076	
0.1250592	0.5863316	0.1621558	0.2881208	0.4906215	
0.4868283	0.2011706	0.2422288	0.2361103	0.1965495	
1.384876	0.5546491	0.5527243	1.303527	0.1338883	
0.2074435	0.3209615	0.6118857	0.2870101	0.3221279	
0.07889887	0.8494291	0.107929746	0.652565548	1.058733	
0.1466199	0.9765569	0.1960144	0.4055316	2.364658	
0.2246489	0.6334924	0.4537992	0.1613599	0.5183564	
0.5656589	0.5936577	0.532038909	0.1400823	1.408746	
0.4999388	0.2125494	0.6159807	0.163648919	0.3819632	
0.2617856	0.1643356	0.6515355	1.659224	0.7786495	
0.6306552	0.3279086	0.1197413	0.1376544	0.8198882	
0.2401721	0.1918577	0.8419216	0.04508187	0.1949255	
0.6948622	0.08496083	0.312627586	0.4395334	0.2232021	

0.2906806 0.2403658 0.415541 0.5633735 0.111397
0.9547656 0.4375496 0.947895 0.3413195 0.481907
0.4798159 0.5737818 1.938276 0.1060909 0.6409442
0.1164478 0.3529371 0.06814369 0.6252258 0.2272281
0.2326279 0.2329693 0.102576 0.3082136 1.043572727
1.293258 0.3911216 0.3628577 0.1315603 0.3143509
0.2603473 1.415546791 0.280055 0.7546114 0.07444278
0.2818619 1.205948 0.9613602 0.2120233 0.4758506
0.5652105 0.4062773 0.1138003 0.1908865 1.332807
0.3923238 1.053072031 0.6974245 0.7754732 0.4087249
0.385629871 0.4350114 0.7532598 0.314152 0.1233971
0.1595095 0.7512965 0.2053192
AL109615.3 0.05695016 0.09076217 0.06402604 0.04231285 0.03319987
0.03845021 0.0977681 0.1289241 0.01953805 0.364689634
0.02715824 0.01243185 0.1074891 0.01779868 0.2269553
0.06563115 0.08835688 0.061937078 0.2187676 0.09460395
0.081336728 0.1071331 0.05635126 0.04542346 0.1950268
0.0547116 0.02003264 0.0700494 0.012228131 0.1005085
0.1430559 0.06239336 1.942367 0.4583802 0.8397018
0.2496854 0.2107007 1.079818 1.233485 0.963199523
0.6438613 1.177001 0.546650994 1.981082 0.1433516
1.559817 0.2356139 1.197065 0.19973691 1.061344
0.8400428 0.07761194 3.10916 1.056117 0.3701299 3.982754
0.8083068 0.1686552 5.386864 0.943642025 0.114945865
1.510175206 0.03151108 0.562754 0.470229 0.1200597
0.4058034 0.05755891 0.216159668 0.4597163 0.2594111
0.2339003 0.4819959 1.303972 0.3151376 3.524757
0.1179108 0.5047836 1.068005 0.737642643 0.8361894
0.1313299 0.3660126 0.1496526 0.6865241 1.029303
0.09115267 0.7741297 1.10554999 0.228397 0.414652557
0.098918 0.7892072 0.2617415 1.82815 0.5430186 0.509095
0.31317 2.819738 0.4040776 0.06338059 0.248776 0.247859
0.8501145 0.2677049 0.03341294 0.02738528 0.1575431
0.738629254 5.027861 0.5671513 1.173675 0.3664606
0.1604433 0.2016944 0.8339752 2.386005 3.027947
0.3879925 0.9201795 6.364987 2.838758 3.603682
0.199714 9.032607 0.06751456 4.045628 0.2805969
0.2051449 0.4216833 14.59674 8.594539 0.798381
0.9564246 0.1414609 0.1048489 4.171056 0.252089
4.192314 1.079377 1.069019 0.1599103 0.6350113
2.678276 1.988845 0.2098117 0.9528574 0.2542676
1.723192 0.3028766 2.280728 0.1014525 0.8084913
0.2012414 0.06473923 7.510526 7.384277 0.2097929 1.2799
0.2963939 0.162141272 0.1328292 0.381792341 0.5583784

0.5144985	0.1054869	0.7166869	0.242478	0.9565675	
1.043898	0.3238706	0.225826807	0.1080205	0.5859588	
1.084225	0.3358879	0.2632152	0.6742961	0.1460987	
40.33107	3.054834	0.3451056	0.04251861	0.1650688	
1.238275	0.9382153	0.08873194	0.5393806	0.996752	
0.7330893	1.980718	2.44055	0.7503878	0.145108	0.4569994
6.252082	0.307235	1.068597	0.7995312	0.5025046	
1.48481	0.1555525	1.600514	1.776982	1.029643	0.7800848
1.280963	0.6267961	1.628257	2.173353	0.849855	
0.1412384	0.1996997	0.3210371	0.3461372	1.456057	
1.313732	4.075496	0.3182019	0.492025555	0.3838202	
1.200771041	0.358663985	1.006317	0.3586581	4.307588	
1.443914	0	0.6639495	0.2213308	0.7353235	0.06622518
0.1238008	0.4892619	0.2502373	0.1860077	0.01822991	
0.3112451	0.6430609	0.2842823	4.127903	0.1585198	
0.3272677	0.5959257	1.082565	0.3524686	0.3476795	
0.1013295	0.09219236	0.464731	0.3242308	0.3141904	
9.403986	0.2638472	0.09040451	0.84273	0.4293358	
0.236131381	0.5168601	1.270783	0.4116267	0.992639	
0.4444792	0.2810793	0.7170039	0.539054	1.490484	
0.1583315	0.3047473	0.3729285	0.513427	0.8254993	
0.8478452	1.058347262	0.9514004	0.183164	0.564567	
0.101295	0.3731349	0.188626511	0.1761033	0.508109	
0.1161515	1.836808	1.486652	0.2209248	0.3591876	
0.8828569	3.823408	0.05663275	2.634661	0.3082214	
0.07295507	2.572522	0.4945196	0.2681448	1.286095	
0.2134512	1.64141	0.1296035	0.2983726	0.3071166	1.154749
0.1606504	0.3257465	0.8200524	0.321876611	1.000351048	
0.7054718	0.6507088	0.2466277	0.7004886	32.81086	
0.04932956	0.6190564	0.0685323	0.7620881	1.139815	
0.5940583	0.3218502	0.8513769	0.287072333	0.6696086	
0.7967439	0.06507177	1.726316	2.95736	0.913789619	0.4050208
0.6239963	0.8277868	0.3557762	0.2062674	0.4944811	
0.0619615	0.3813301	1.346584	0.8670378	0.6076738	
0.6276057	1.496283	2.248008	0.7649977	19.42217	
0.09469189	0.1109005	1.436622079	0.5705937	2.089797	
0.9121635	0.8018126	1.200723	0.4370649	0.8972216	
1.483112	1.310223	0.176577	2.763061	0.3165756	
10.71197	0.2912642	0	2.268207	1.744469	1.678765
4.560953	0.08301896	0.4231711	6.233119	3.860425	
2.304387	0.5245554	1.053069	6.425845379	0.1742884	
0.9357024	0.7426725	4.541237	0.1418412	0.210179	
0.511616588	1.746203	0.8151762	0.333801	1.346629	
1.132963	0.138811	0.4492285	1.876315	2.574937	

0.1879613 0.7086148 0.5315556 1.257294 3.615378
1.065150516 1.789965 0.06163871 1.159435 1.372582305
11.53045 0.1456652 1.650988 0.3806529 0.887677
0.4346392 0.381738
AP001065.3 2.616956 11.13139 0.871564 0.4310129 0.2663207
5.049237 0.0697129 0.5137179 1.184176 0.763159594
0.1742851 0 0.2438687 0.02115205 0.8827035 2.674164
0.5400197 3.244342861 0.5292544 0.6263839 0.764500945
4.605901 0.1148026 3.832687 0.7571185 0.2659892
0.3213932 4.28128 0.610343222 0.1023814 0.8925445 4.024249
0.1742128 0.4263195 0.2754956 0 0.8920427 0.2634064
0.3315683 0.119791245 0.1275281 0.1392126 0.375697278
0.2320489 0.9292356 0.01660026 0.341256 0.2909863
0.114855727 0.07207471 0.1302146 0.532122 0.2420269
0.1696075 1.099661 1.026127 0.06624804 0.8788122
0.09048453 0.030584445 0.204903499 0.092298907 0.6515944
0.02639921 0.06209143 0.7184937 0.1539125 0.1596078
0.22666359 0.5831016 0.0385357 0.06540434 0.4463429
0.2313954 0.5793223 0.204002 0.1926731 1.671116
0.01707475 0.088398534 0 0.1478588 0.07249526 1.020693 0
0.1747471 0.5803198 0.05575638 0.38642412 0.6616065
0.065220287 0.0755709 0.1927188 0.5903699 0.05193428
0.06914212 0.04373577 1.116519 0.227186 0.3887399
0.564914 0.1379685 0.2079226 0.2164888 0.1629508
0.3403554 0.09763448 0.7301781 0.09974901 0.4511431 0
1.032516 0.3563214 1.815527 0.7989828 0.1074688
0.4543598 0.07149199 0.1999919 0.3068224 0.2101163
0.8980077 0.8731589 0.04188375 0.06505698 0.3282328
0.1857109 0.1219987 1.525462 1.211066 0.5199181
0.1027373 0.4131874 0.2045917 0.1513017 0.3162999
0.06437542 0.0728718 0 0.0592033 0.3534661 0.3800765
0.08775013 0.4275509 0.02129328 0.7662687 0.08785717
0.7522182 0.09941027 0.132223 0.1459462 0.1276589
0.02044289 0.4035765 1.59918 0 0.9474333 0.5075424
1.964678 0.2264376 0.272032415 0.2492447 0.346060854
0.205231 0.1945469 0.2686312 0 0.2319355 0.2150684
0.08332219 2.793554 0.077914999 0.4573261 0.1147841 0
0.08936668 0.4801216 0.3898398 0.2387338 0.3132659
0.1134495 1.162022 0.1515881 0.1050904 0.4125907
0.3623687 0.4042233 0 0.09302716 0.1365325 0.1934709 0
0.04573155 0.1379577 0.1551717 0.4146064 0.230602
0.04482095 0 1.317307 0.7289645 0.2957752 0.08846792
0.2209998 0 0.1749165 0.217472 0.07795341 0.1149523
0.2117069 0.2019945 0.3596755 0.670092 0.3732284

0.7361027 0.157906 0.144858 0.303826 0.2363456
0.163723286 0.08420937 0.21405049 0.023679909 0.04848295
0.7475442 0.2376754 0.2795222 0.1050735 0.0550494
0.09018197 0.2036185 0.2623413 0.05885021 0.03559847 0
0.03157894 0.4224584 0.8609404 0.4840043 0.8292503
0.6927575 0.1582441 0.5085966 0.2795532 0.5883507
0.2599919 0.1126866 1.603099 0.4272915 0.1608609
0.4258775 0.2800393 0.1680564 0.3271905 0.03223117
0.4134655 0.0746671 0.116924959 0.08472269 0.8494908
0.4340607 0.1263919 0.1152484 0.7690604 0.37279 1.235472
0.2149004 0.3436004 0.09366297 0.6997743 0.1851046
0.09197138 0.346357 0.247425447 0.04711041 0.247697
0.3211922 2.257116 0.0483748 0.134498903 0.0965918
0.03178103 0.1552894 0.2910497 0.3950487 0.4375805
0.1067152 0.7963749 0.2777845 0.1101317 0.2730563
1.987329 0.3158366 0.1345937 0.31113 0.3823978 0.2162834
0.1014667 0.4571863 0.3272959 0.1399688 0.07465484
0.019238 0.3068323 0.4950046 0.1697612 0.097664689
0.031284815 0.05941324 0 0.615497 0 0.08571376 0.02931177
0.145202 1.860686 0.2470009 0.0987131 0.3683386
1.057469 0.2704762 0.215468479 0.0422531 0.2328332
0.092798 0.8098279 0.9793492 0.08885069 0.2765082
0.2677859 0.2974118 0.09851803 0.1559914 0.06958945
0.5522655 0.06473927 0.488977 0.5694276 0.112838
0.04535576 0.06313106 0.09142171 1.118926 0.07559418
2.599498 0.1317948 0.079408838 0.1390968 0.02992202
0.1530382 0.2485771 0.09255864 0.1731368 0.8332982 0
1.311784 1.133164 0.3251127 0.872147 0.5934538
0.5043756 0 0 0.1645347 0.02809935 0.3897393 1.430573
0.2155282 0.3954168 0.02079041 0.5300413 0.1650136
0.1072691 0.335261498 0.2692629 0.9531379 0.3782555
0.3361349 0.7001929 0.4087276 0.14346259 0.4305886
0.21528 0.06736262 0.3943698 0.1302987 0.4639608 0.3336661
1.777933 0 0.6328937 0.5217495 1.026519 0.2733247
0.357493 0.79350603 0.04038997 0.8590438 0.9572631
0.098527939 1.446886 0.07789923 0.2675515 0.1374289
0.1283011 0.09115195 1.020734
AC025165.1 0.03911551 0.9831731 0.08223989 0.04270343 0.05863608
0.7356806 0 0.1071528 0.1380288 0.224034087 0.30698
0.1756524 0.2675052 0.09430561 0.01821991 0.7907039
0.0557328 0.357622489 0.02759837 0.0238693 0.156712554
0.6399869 0.02132678 0.748765 1.029515 0.1273747
0.02653554 0.9499777 0.064790281 0.03803861 0.1737029
0.3606424 0.04315117 0.05279804 0.06823815 0.01224952

0.01162906 0.020075 0.1210291 0.02967137 0.4738159
0.01477795 0.017448249 0 0.08631169 0.06578809 0.03901222
0.1021063 0.19914216 0.008926176 0.02580252 0.1476188
0.2038239 0.04201048 0.7190769 0.02747717 0.04102282
0.7274928 0.01680924 0.030302127 0.253765102 0.076205764
0.1001762 0.05884994 0 0.8254563 0.06862129 0.1524868
0.011228566 0.02732455 0.0143175 0.03645034 0.3758894
0.1979968 0.1391454 0.03031784 0.2125871 0.3002076
0.04229284 0 0.009716047 0.1525979 0.02992753 0.5171274
0.06474553 0.03787307 0.574963 0.1035782 0.4402857
0.3781727 0.016154563 0.03743666 0.02864099 0.05660519
0.0321593 0.06279523 0.09749713 0.04118877 0.02813611
0.1529278 0.3498121 0.01464589 0.1673777 0.02010848
0.1326169 0.3034923 0.06448882 0.208684 0.014824237
0.05214751 0.09813722 0.05384129 0.02941935 0.4250514
0.2572725 0.02218266 0.03751381 0.03984304 0.2889627
0.01169193 0.01734807 0.2524877 0.5921808 0.1348657
0.1127988 0.0542005 0.0306661 0.04834901 0.7453387
0.09826664 0.1006092 0.04453267 0.06822889 0.08445965 0
0.4059698 0.1435077 0.02406638 0.05534755 0.08065312
0.09897051 0.07060653 0.05216408 0.1059011 0.2109673
0.1129755 0.06528464 0.3153085 0.08864338 0.1692115
0.01549275 0.1475605 0.02025419 0.1554977 1.106642
0.03509192 0.2246859 0.5227071 0.5180314 0.1682605
0.117915589 0.3025064 0.097145493 0 0 0.08428131 0.04456969
0.1723459 0.0532708 0.2545386 1.273173 0.057896838
0.1430857 0 0.005802753 0.2257815 0.1621669 0.1528872
0.01075137 0.1228564 0.03934078 0.01693082 0.05006294
0.0676782 0.08175644 0.08975593 0.500615 0.1890137
0.1059937 0.004831151 0.1006347 0.1177701 0.09061883
0.08542766 0 0.1467069 0.08924741 0.2775451 0.0170818
0.01305148 0.1289706 0.2289414 0.01095641 0.01824665
0.05286359 0.01299764 0.1346653 0.3990415 0.03321825
0.1101202 0.157245 0.3029021 0.03630739 0.1664024
0.2627653 0.07333519 0.06578037 0.1150964 0.3219754
0.081105997 0.05735954 0.217965601 0 0.02001476 0.3069774
0.01358547 0.1673191 0.1301295 0.006817656 0.09493387
0.08826079 0.2209317 0.007288373 0.0132262 0.08705224
0.1407935 0.03219683 0.07582162 0.2145295 0.02852763
0.3850328 0.2351751 0.08151354 0.04154591 0.2331679
0.08586398 0.1581658 1.118522 0.358217 0.019922
0.1858573 0.01891734 0.07284599 0.344431 0.02395024
0.09558473 0.1017196 0.086884239 0.03147774 0.1942268 0 0
0.07136533 0.1269936 0.05935965 0.0765042 0.03871213 0

0.2041564	0.09244198	0.1120753	0.113903	0.06239271
0.015321345	0.01166889	0.0446201	0.100772	0.5859057
0.08387446	0.026651475	0.2452317	0.01180788	0.2051413
0.0875388	0.01223132	0.3143174	0.03083794	0.2442216
0.03440254	0.1818584	0.1037054	0.8976254	0.506196
0.01428764	0.1626917	0.3125661	0.05357174	0.06283128
0.04982628	0	0.09707376	0.1972419	0.06671145	0.2482667	0
0.04204855	0.223764827	0.116235122	0.0392432	0.01877862
0.0174233	0.0632644	0.01061532	0.07986329	0.06473776
0.4469117	0.06729824	0.1100271	0.04561732	0.3232289
0.08932649	0.046698649	0.05232884	0.0230684	0.03447803
0.2808234	0.07741828	0.300770978	0.03043953	0.07143069	0
0.2440216	0.04415758	0.08043826	0.4924509	0.229841
0.005505266	0.02014898	0.1006168	0.03370282	0.03909269
0.05283706	0.01154789	0.1622755	0.9281845	0.09249293
0.019668958	0.04306651	0.07411453	0.07581276	0.05130885
0.1432879	0.04764962	0.1198461	0	0.0316994	0.1819196
0.1610558	0.7116088	0.2204909	0.09553467	0.1765617
0.0727347	0.08965875	0.02783997	0.02145232	0	0.03336542
0.0744357	0.05664588	0.0119352	0.08855729	0.1647324
0.138402824	0.03591234	0.1388735	0.01561516	0.08325803
0.04817567	0.1349849	0.111680109	0.07110232	0.2199582
0.1835372	0.008494119	0.07261647	0.1455647	0.03305861
0.01100951	0.08745645	0.4979528	0.06121579	0.1466889
0.1827912	0.05903217	0.070194764	0.14006	0.4057637	0
0.032539483	0.1827048	0.160792	0.09940566	0.00851002
0.01059307	0.02257764	0.5988033
AC027097.2	0.235947	0.3555209	0.7168671	0.252711	0.4314022
0.9630465	0.4197869	0.5227127	0.585026	1.522869501
0.6382828	0.8006795	1.19246	0.315074	1.072392	0.6727193
0.4706569	0.565255556	0.2383619	0.4123093	0.33419672
0.5411735	0.4366113	0.7442115	0.4211418	0.6856378
0.6337884	0.6388561	0.317786973	0.178462	0.2828733
0.3845432	0.9110164	0.8557162	0.4132796	1.598707
0.06695866	0.3082382	0.6968705	0.664393371	1.29913	0.4222965
0.558137873	0.5929409	1.524047	0.7207713	2.545778
0.3688881	0.960989727	0.7595157	0.1733289	2.887202
3.121909	0.4945329	1.087366	0.4746303	0.640376
2.572656	0.2796025	0.591279203	0.673751341	0.721554579
0.9684571	0.5940343	0.3443752	1.153093	0.4975494
1.112856	0.15803985	0.4645021	0.348073	0.5493063
1.584341	0.4333487	1.029296	0.3491326	1.1158	1.542315
0.2435168	0.133076336	0.3605266	0.6951015	0.6126896
1.132206	0.3539685	0.5538244	0.8975308	0.7245036

0.56335769	1.532294	0.502975088	0.6147325	0.8062325	
2.372014	0.5678523	0.8107867	0.9425575	0.760417	
0.5580144	0.7827014	4.010444	0.443509	0.4942255	
1.174974	1.379629	1.893093	0.3094321	0.4361277	
0.284520306	1.210567	0.3680479	0.9587389	0.401524	
4.492797	0.4811198	0.2213899	0.3039997	0.226579	
0.2957886	0.5460455	0.5031395	0.338449	0.8038333	
0.5309672	1.821297	4.320569	0.62781	0.2474556	1.039778
0.317646	0.4634359	0.659349	0.7566064	0.6051833	
0.511487	0.9477677	0.9317117	0.2078568	0.8734308	
0.4315548	0.8572249	0.4968865	1.145796	0.9936912	
0.7085885	0.54931	1.401506	1.34482	0.3717709	1.054617
0.6806036	0.4045881	0.3304263	0.9308653	1.081578	
1.616439	0.8177545	1.870997	0.3816325	1.339607	
0.9806956	2.109111	0.687307738	0.6764542	0.3259043	
0.45974	0.3763858	0.4610901	1.830622	1.998147	0.9030658
0.312784754	1.472285	0.3637843	0.5642834	0.7647184	
0.850738	0.7516055	1.2381	2.25869	0.575287	0.4043845
0.7446616	0.7926875	0.5622767	1.024772	0.9858911	
0.3966312	0.5306943	0.3152612	0.5763758	0.7082435	
0.3478479	0.5465352	0.3688381	0.7008045	0.7582516	
1.576762	0.4371326	0.5093416	0.3630473	0.9901263	
0.231314	0.3969006	0.2840898	1.663084	0.2929236	
1.721164	0.3461013	1.086957	0.5761616	1.443894	
1.015402	0.8910949	2.072185	0.6505852	0.3366712	
0.611733	0.6666527	0.185316712	0.527096	0.851754279	
0.135087354	0.4456041	3.859278	0.7764399	0.8711225	
0.349659	0.1395741	0.3858475	0.3871958	0.8397503	
0.9698701	0.3271836	0.4712474	2.121757	2.008341	
0.7518729	0.6539474	0.5402279	1.21023	1.117677	0.9671345
0.1949168	0.6936516	1.10552	1.119028	2.025119	0.8646119
0.8208022	1.214757	0.3065999	1.158448	2.035038	
0.4903199	0.6289884	0.8696586	0.348335106	0.8055327	
0.614393	0.6419769	0.2002864	0.4474381	0.9158626	
0.6666924	0.8374968	0.321966	0.2800208	0.7599238	
0.6727292	0.8473839	0.3692132	0.9979149	0.215644914	
0.9754698	0.8706629	0.5157621	0.8927517	0.4292787	
0.221658429	2.785761	0.9065102	0.3773208	0.7840581	
0.5060266	1.414556	0.8962602	0.4807512	1.326858	
0.7678865	0.6692388	0.6817119	1.02453	0.4753172	0.8217179
0.5332492	0.5826454	0.7074704	0.4868495	0.3722062	
0.9936684	0.8517705	0.4694752	1.491076	0.4947798	
1.064089	0.294051459	0.475923333	1.14234	0.2594999	0.4402984
0.3867542	0.3123997	1.096191	1.297729	0.7803177	

0.8767559 0.6674146 0.7004234 0.5918476 0.3524118
0.478017668 0.1941729 0.5755747 0.3382195 0.6416451
0.4094431 0.290980181 0.8081755 0.7442371 0.1232611
0.6283676 1.052328 0.6028346 0.1983662 0.7386394
0.7678112 0.6767557 1.223048 0.3929047 0.5802341
0.4732453 0.9161026 0.303468 1.027146 1.037279
0.421546816 0.4794116 0.3508769 0.8002872 0.4858181
0.8873695 0.3475233 0.8434049 0.4428901 0.3954625
1.935326 0.535796 1.186932 0.7357356 2.294337
0.8917719 0.5941909 0.6048917 0.4452751 0.8440515
0.6410001 0.845301 0.6015317 0.3426326 0.6795778
0.9239244 1.856221 0.53835429 0.2888338 0.9328853
0.5661009 1.029799 0.7766904 1.14425 0.357244442 0.8699712
1.134299 1.629654 1.065109 0.3097159 0.6077765
0.5287425 0.2394784 0.138233 1.1386 0.6005092 1.576617
0.6410218 0.484672 0.664639401 0.7936471 0.9180726
0.2344335 0.197767038 0.3057068 3.637444 1.049336
1.165102 0.3930696 0.4237011 0.6697027
GK-IT1 0.9149004 0 2.255382 0 0.1920073 0.4818059 0
0.08771956 0.2824896 0.412657245 0.2198928 0.07189804
0.3390819 0.03431214 0 0.1626727 0.2190004 0.055108518
1.581519 0.3126458 0.042763711 0.4738048 0.1862288
0.1751339 0.02506474 0.2876525 0 0.08681185 0 0 0
0.1640201 0.6358548 0.576301 0.5958663 1.203354 0
0.1972103 2.745911 0.356255743 1.004806 0.06452171
0.418992368 0.1411582 1.281268 0.861708 0.9794001
0.118007 0.55894525 0.4676687 0.4224594 0.897718
0.5889118 1.595763 0.64218 0.04498782 4.02995 0.1875762
0.4770381 0.148839163 1.288001714 0.199632229 1.202787
0.2569435 0.4028905 1.016727 0.599212 0.1479488
0.367686025 1.201533 2.281663 0.3713385 0.5792339
1.876809 5.09747 0 2.301488 1.578834 1.744979
1.147176942 0.7635785 0.7595302 0.8231955 1.279431
1.117887 0.2480352 0.3388951 0.4974536 5.76696638
0.1651132 0.282128396 0.8581199 1.438061 0.6796426
0.4633532 1.00944 0.7449403 1.310215 0.4299556 1.669237
0.3207345 0.5755059 0.5902488 2.765549 1.661524
1.049015 0.7918968 0.09111309 0.226533268 0.5854633
1.130287 1.880603 0.6422361 0.9682475 2.980991
1.394658 1.965459 0.9857611 0.1081401 1.148578
2.651006 0.9055273 0.5733083 1.42679 0.2462442 0.6389379
0.4351445 0.5541259 0.271184 0.1693579 0.2899166
0.9999419 0.9681513 1.2169 0.8999338 0.1865786 0.4177105
0.4334373 1.288809 1.776696 0.7728176 1.040424

0.5693812 0.8862139 0.6217424 0.4143384 2.850379
2.064993 0.290268 0.4647233 1.386673 0.8628495
0.4311029 0.4364455 0.4011549 0.5745528 0.3923986
0.4333254 3.495456 4.652713 0.220640901 0.6064746
0.187122746 0.8322969 0.1803356 0 0.2918922 0.684069
1.295828 0.1351624 0.1510536 0.084260721 0.3904521
0.2606785 2.128163 0.1739611 0.7434353 1.106674
0.3520599 0.4658219 1.656305 0.850095 0.2049176
0.170474 4.316925 3.436499 0.3706229 2.079627
1.056339 0.727715 1.129831 1.311194 0.1483684
0.05594758 0.4404999 0.5764804 0.8650479 0.1090605
1.155997 0.7265431 0.7883344 1.169504 0.4664064
0.5676225 0.06924198 0.6242359 0.6173574 0.8008715
1.212066 0.5494782 0.9830061 1.283598 0.2717501
0.645801 1.896481 0.8965242 0.1174918 0.318907
0.843462 0.15176365 0.2732032 0.69445096 0.192063744
0.9962004 0.4148509 0.08897285 1.209151 0.4261167
0.04464964 0.1462902 0.2890152 11.70293 0.4773242
1.357046 0.7893906 0.8196207 0.6325826 0.4034589
0.7438125 0.6352247 1.205995 0.3300407 2.135367
1.360445 1.488868 0.6677709 0.7921179 0.1831333
0.2665915 2.452865 0.4605627 1.321514 2.11277 1.194204
0.5751276 0.1341419 0.6056123 0.265540412 1.133833
1.024678 5.56589 0.6663433 1.589092 0.529261 2.375715
0.2783528 1.711329 0.3981264 0.1823243 0.7567668
0.6672676 0.5470405 1.583392 0.46825941 1.477471
1.534167 1.042054 0.4686615 0.117708 0.218179581
0.2350319 1.959057 0.5038106 1.348946 0.7209391
1.206708 0.5770324 0.4306175 4.956739 0.2977531
0.8268265 0.189634 1.084954 1.746667 0.05607828
0.3721877 2.420849 0.822979 0.9492893 1.186781
0.4995159 0.4036752 0.5617301 13.83698 0.8029798
1.223915 0.594106355 1.928468518 0.5782689 1.106851
0.1141072 0.3682898 0.6604493 0.3803885 0.6595171
0.1829299 0.6811501 0.2135054 2.203305 0.9124415
0.5362589 0.480597828 0.4797909 0.3776942 0.9784696
1.57641 0.47322 0 0.8472464 0.3341486 0.4453397 0.7990631
0.2168949 0.3762855 0.1194489 0.03500595 2.307497
0.8247377 2.96528 1.079094 0.3584317 0.6920723 1.474756
1.021886 0.05476388 1.354023 0.225425088 0.479481
0.8736927 1.737774 0.7392611 0.7131911 0.2496504
3.968035 1.102808 0.8304124 0.7829285 1.951331
0.4993297 1.387393 1.395721 0.1217182 0.07328433
0.9074689 0.6837273 1.404938 0.1600433 2.447358

0.7184037 5.935689 1.485135 1.338397 0.1740084
0.362566591 0 0.1364248 0.3749737 2.544578 0.3155081
0.5156854 0.066491477 0.8440042 2.881065 0.3642446
1.056951 0.1585246 0.1003495 2.706309 0.1081538
0.2695354 0.4831336 0.6681824 2.177548 0.5763916
0.7732174 0.965399024 0.4913944 1.393512 0.07058363
0.266381162 0.782363 0.4212183 0.5967678 0.1114663
0.7631278 0.1971515 0.02904914
KANSL1L-AS1 0.1123176 0.1453075 0.2856331 0.1072926 0.1299913
0.5018285 0.1837453 0.1583659 0.1427992 0.115888483
0.3232609 0.116822 0.3162862 0.06814058 0.3446785
0.1859997 0.2504048 0.203956651 0.03263109 0.3104421
0.208451215 0.2368775 0.07564753 0.5058894 0.1221778
0.3998749 0.1254978 0.370921 0.076605152 0 0.1867078
0.2132038 1.109686 0.4785997 0.4518179 0.4924319
0.1031226 0.1305468 0.2657562 0.122787388 1.184463
0.7280332 0.185670312 0.2548422 0.5646828 0.6174178
1.037842 0.2059444 0.464186364 0.2744018 0.06864244
1.296568 0.2906084 0.5662532 0.6183306 0.3573657
0.3007221 0.6772876 0.1258718 0.152268533 0.532572054
0.126143239 0.3224304 0.09277541 0.2273015 0.5372383
0.1262096 0.3739424 0.099571181 0.5307635 0.214426
0.2394293 0.8431191 0.4189205 0.4164396 0.04779529
0.4206325 0.6615017 1.210125 0.090609502 0.1263661
0.512407 0.4246198 0.4415872 0.118308 0.3454414
0.6390221 0.2286037 1.04114855 0.6334407 0.26103933
0.2950896 0.05643972 0.6246564 0.09125696 0.2699865
0.3394246 0.1182709 0.1607899 0.3817207 1.273895
0.8196547 0.1826769 0.2932296 0.6749232 0.7077041
0.0953109 0.09047087 0.192802725 0.4580227 0.2000571
1.538439 0.1971101 1.260046 0.6317741 0.1678579
0.199596 0.3454634 0.3318951 0.1866243 0.5059524
1.76275 0.724522 0.06133045 0.5461749 1.185559 0.3807115
0.02143715 0.6181027 0.0733807 0.4948561 0.3535303
0.7125921 0.6590848 0.04431027 0.7578958 0.1885301
0.2560952 0.3635582 0.5461565 0.9631509 0.633072
0.6784414 0.528676 0.2369665 0.9510698 1.440874
0.8190452 0.8093506 0.4646745 0.3785709 0.162007
0.1077644 0.4202367 0.5214467 0.5601302 0.6021596
1.423806 0.3340894 0.2873633 0.099584399 1.328489
0.722945622 0.4507802 0.219761 0.2569933 0.1791706
0.506348 0.6748396 0.9109994 0.2836154 0.182545737
0.9798234 0.1344629 0.2744367 0.3925794 0.4921301
0.5137589 0.8199201 1.269114 0.4119882 0.3336375

0.1479805	0.6340022	0.2899955	0.9714376	0.329408	
0.1966637	0.6810993	0.2284856	0.2096419	0.3829269	
0.2745557	0.06060352	0.2385791	0.578199	0.185719	
0.4987987	0.1211805	0.3549242	0.2643145	1.136898	
0.2396559	0.382939	0.4875278	0.7222881	0.2308723	
0.5631269	0.3703146	0.8060075	0.9634009	0.2808926	
0.6132606	0.5319452	0.3994484	0.2948081	0.1696926	
0.2250635	0.2491785	0.219191157	0.2836083	0.536321343	
0.090153796	0.3549684	1.964574	0.6103886	0.4638789	
0.1384734	0.1450961	0.2509019	0.08944769	0.2689028	
0.3877852	0.3440376	0.9500926	1.498212	0.1427554	
0.6219339	1.298092	1.005148	0.79668	0.5627418	0.2453258
0.1473661	0.3446092	0.3299457	0.3080135	0.8265567	
0.5871802	0.4569649	0.9027544	1.394211	0.8982115	
0.7665692	0.283177	0.1937402	0.9129488	0.13012222	
0.2543222	0.2806774	0.2845049	0.1202994	0.6075302	
0.3003031	0.5848684	0.402023	0.8010012	0.1150022	
0.795475	1.024681	0.289119	0.1616087	1.355531	
0.313998157	1.099143	0.4484331	0.5831997	0.5129515	
0.07083532	0.252092169	0.7779176	0.2419926	0.07579688	
0.2800642	0.3036971	0.961125	0.5208774	1.232769	
0.5345993	0.2472746	0.1492724	0.4039834	0.2394015	
0.230872	0.1518627	0.1567848	0.2723657	0.2971557	
0.6801702	0.2255341	0.3361289	0.6194643	0.3774811	
0.7787783	0.1561186	0.3093461	1.351450117	0.687156245	
0.3769953	0.2220301	0.07210188	0.0997347	0.4204613	
0.1030111	0.3572009	0.2807171	0.3689176	0.9058203	
0.4449711	0.6589164	0.5016745	0.717787331	0.06187129	
0.286388	0.08153057	0.4031831	0.1952767	0.095409776	
0.3329107	0.3740215	0.3283005	0.2380292	0.2610498	
0.3804265	0.3019085	0.8468607	0.2733856	0.607493	
1.302003	0.3320726	0.8227421	0.6693449	0.1160566	
0.3394576	0.6723081	0.5339319	0.453486213	0.697603	
0.5695933	0.19048	0.3639919	0.4133781	0.1521148	0.3857407
0.2765239	0.4310195	0.7743371	0.5141482	0.465761	
0.4907269	0.7298693	0.0219746	0.2844558	0.4047598	
0.9875024	0.5009443	0.3611709	0.741656	0.8245097	
0.6819331	1.792179	0.3543902	0.4272424	1.086578602	
0.1698446	0.1395682	0.3138655	0.3773556	0.5582155	
0.7980008	0.18606426	0.5044094	1.410676	0.6773226	
0.3967012	0.03815933	0.0543503	0.7621971	0.5857718	
0.2858834	0.6051116	0.3055995	1.237194	0.9045196	
0.8259314	0.622463802	1.354377	0.7020835	0.03822879	
0.182747895	0.2409476	2.783259	0.3232153	1.287919	

0.2317083 0.06228786 0.3356437
CFLAR-AS1 0.3151108 0.1730973 0.3453544 0.09606794 0.298998
0.4073912 0.3625586 0.08436986 0.1863102 0.142799761
0.3337894 0.1909926 0.2640129 0.08800503 0.2732566
0.7276668 0.2006072 0.103484235 0.2524862 0.2863876
0.074427013 0.1502347 0.2814702 0.3449137 0.1423497
0.3478113 0.2600083 0.310131 0.116604566 0.03042621
0.2557776 0.1832983 0.385065 0.4328773 0.3220345
0.3159891 0.01046454 0.2649492 0.282646 0.166134112
0.5874413 0.09160922 0.11862984 0.1443881 0.707647
0.1554005 1.326212 0.1477311 0.307200643 0.249002
0.06965608 1.692084 0.201395 0.6653437 0.8170072
0.07417696 0.531574 0.9622047 0.09075594 0.104526258
0.582301324 0.288013479 0.3205144 0.12945 0.3229213 0.9495074
0.3430537 0.6200177 0.13472206 0.7868261 0.3464301
0.3109951 1.309883 0.2250565 0.7773553 0.4122592
0.9812205 0.6767275 0.3120731 0.151056655 0.1748619
0.7360191 0.4703884 0.5239418 0.2153929 0.2174662
0.4725469 0.1988395 0.90723183 1.009566 0.206746605
0.9844327 0.3150024 0.7838613 0.2816722 0.6232895
0.4159238 0.3106332 0.1237798 0.5742406 1.364057
0.3163025 0.8805287 0.6594566 0.8682189 0.9786122
0.2804832 0.1585755 0.091896134 0.4469099 0.3674504
1.472333 0.1176593 1.807114 0.7360818 0.3353502
0.2888119 0.8100171 0.1758274 0.2384787 0.305279
0.5301425 0.2564006 0.4356528 0.157894 0.9787105
0.1441085 0.1305222 0.3912426 0.1520313 0.4562929
0.2366229 0.4502414 0.3208973 0.2622935 0.4486345
0.3284226 0.3537214 0.2582488 0.3738797 0.4430152
0.4059251 0.8918685 0.2823594 1.094752 0.6804598
0.4003508 1.283968 0.1418076 0.2324936 0.3469843
0.3066679 0.170109 0.8362267 0.3233675 0.1894672
0.1737304 3.1953 0.2260153 0.9196828 0.212215412 0.6333139
0.212544497 0.3507016 0.0743354 0.1995829 0.4171081
0.3603042 0.8080698 0.0453972 0.5174929 0.138931037
1.040788 0.160327 0.2924138 0.4647727 0.6647822
0.6806456 0.9932735 0.2831729 0.7147682 0.1862104
0.1351491 0.6496107 0.3678474 1.180454 0.3551643
0.4082069 0.6220413 0.3593824 0.2443609 0.6535236
0.1970656 0.1844954 0.599491 0.9387796 0.3897727
0.4229137 0.2083659 0.1526788 0.162478 1.005356
0.22019 0.1669312 0.3551887 0.4366535 0.1696522 0.2200826
0.2960715 1.009804 0.5574191 0.4845688 0.5040765
0.5656812 0.3892618 0.2551672 0.1399113 0.2151087

0.3722634 0.229378817 0.3128221 0.37107353 0.158339584
0.2209289 1.74804 0.2282008 0.2353645 0.507427 0.06543943
0.1440539 0.1285888 1.103189 0.249224 0.507808
0.2410307 1.919189 0.3669874 0.3383019 0.4315165
0.3765067 0.5925245 0.5408196 0.480115 0.257545
0.18534 0.6760748 0.6418683 1.241368 0.3272297 1.147331
0.638849 0.3707228 1.311026 1.501083 0.3280678
0.09420474 0.8182543 0.152892739 0.3934112 0.3074784
1.302249 0.08920925 0.4452512 0.190461 1.321538
0.3059697 0.4209296 0.1385818 0.6123751 1.837002
0.3942406 0.1776614 0.4959457 0.150126084 0.4223487
0.7662324 0.6268114 0.8451843 0.2587728 0.07594502
1.040579 0.6894739 0.1230659 0.2471304 0.1540909
0.8712839 0.4122839 0.4508013 1.385718 0.2127416
0.4354956 0.239716 0.2070483 0.3799928 0.5136843
0.7386789 0.9962811 0.5277016 0.3315202 0.2717767
0.2953361 0.3069105 0.4459451 2.01521 0.294216 0.3195198
0.192287214 0.678708694 0.4119903 0.15321 0.09929753 0.0969905
0.08278675 0.1219543 0.4013124 0.265313 0.3046306
0.3887028 0.8586139 0.5482902 0.6341205 0.324172177
0.1224306 0.5051202 0.1309963 0.5655715 0.1764865
0.017603398 0.6984798 0.3397537 0.3263495 0.2726522
0.5099416 0.7893227 0.2735418 0.3783777 0.1684352
0.6134419 0.5432469 0.498725 0.2298297 0.1539586
0.2390046 0.4904959 0.601973 0.7343897 0.660775338
0.1808515 0.2978946 0.6367292 0.2216202 0.4022905
0.08289759 0.5631899 0.1571401 0.3755801 1.069523
0.3236724 0.3735372 0.5835626 0.9037659 0.1170703
0.3087952 0.4302958 0.2880994 0.3667782 0.2015774
0.5204204 0.2726278 0.6858246 0.4152809 0.3310171
0.07013333 0.235802134 0.09540955 0.07914571 0.7681479
0.6493127 0.4364044 0.8367777 0.18576651 0.2106082
0.6037924 0.08341318 0.4764468 0.06776495 0.1470744
0.8825291 0.1188844 0.3240535 0.8712833 0.3060319
0.8389315 0.3695875 0.5813775 0.661132966 0.3661005
1.02118 0.1681043 0.122004293 0.2276432 1.481631 0.3130797
0.3624711 0.1080327 0.06320766 0.2687548
AC020928.1 0.007087131 0.01355382 0.006519015 0.02031017 0.02788789
0.06459635 0 0.08493821 0.02188261 0.106552797 0.01216689 0
0.07661045 0.0332242 0.1155409 0 0.09895971 0.026680587 0
0.01513663 0.016563116 0.02823272 0.0270486 0.1271857
0.02427 0.03342382 0.07852793 0.09526719 0 0.03216273
0.02002783 0.03811667 0.09577456 0.06696336 0.01153946 0
0.3318536 0.03819146 0.02741078 0.043903978 0.02289284

0.09996144	0.014752991	0.02733649	0.03648949	0.08343855		
0.09071135	0.3504137	0.014432603	0.01698151	0.03272515		
0.08692537	1.733527	0.02131264	0.08290909	0.02613682		
0.04162316	0.1525681	0.02131904	0.019215983	0.040231053		
0.086986092	0.02823393	0.02487965	0.0195058	0.02401193		
0.04835105	0.05730309	0.014241108	0.03465553	0.006052925		
0.07191287	0.07711934	0.05286692	0.03860436	0.03845189		
0.03851752	0.01730687	0.697316	0	0.04929117	0.05419087	
0.03036549	0.05101203	0.0447907	0.05489616	0.07292214		
0.06130482	0.14567247	0.1358962	0.020488715	0.02374032		
0.03632515	0.0478613	0	0.1375647	0.08930631	0.01492555	0
0.0215508	0.2218321	0.01238352	0.03265906	0.06800916		
0.0950679	0.08019106	0.01022384	0.02646724	0.050137258		
0.08503497	0.03433565	0.1024298	0.0248749	0.07812892		
0.3262968	0.01125364	0	0.05053263	0.03664893	0.05931517	
0.02933657	1.944243	0.5159446	0.06578813	0.04087483		
0.0687421	0.07778719	0.02299526	0.006564636	0.03935711		
0.01020815	0.008068636	0.01442237	0.03570652	0.01584362		
0.0270994	0.1887508	0.01526161	0.1559931	0.0185985		
0.0482783	0.2387993	0.02205311	0.1268517	9.358201		
0.05158296	0.02760001	0.127242	0.0249835	0.04846026		
0.02619913	0.01336785	0.01284412	0.04226069	0.02589569		
0.08901364	0.01266522	0.04195858	0.1393669	0.02371151		
0.085457988	0.05480952	0.014495167	0.02578902	0	0.03938177	
0.05652741	0.0662379	0.04825926	0.148327	0.0994596		
0.024476712	0.09073726	0.0216354	0.007359589	0.03368906		
0.08227006	0.0816445	0.0681794	0.3362387	0.04276768		
0.01431549	0	0.026411	0.05832624	0.02627003	0.03864769	
0.1054789	1.583948	0.01225463	0.04254473	0.02240505		
0.0430992	0.02166945	0.09749321	0.09923587	0.01358302		
0.1408034	0.01444315	0.09104201	0.2180967	0.0813021		
0.02779187	0.06364076	0.0536372	0.02747469	0.04269876		
0.04081463	0.03009319	0.05985627	0.0453256	0.08285988		
0.02631337	0.05471588	0.1292246	0.0124014	0.0910132		
1.29694	0.03712355	0.04408549	0.03306758	0.067243178		
0.029755827	0.03553839	0.1297791	0.06317796	0.2341623		
0.01650423	0	0.04957809	0.03997875	0.08241355	0.06470653	
0.02795782	0.04246451	0.03968163	0.04083501	0	0.02400761	
0.1013079	0.09023568	0.09942364	0.009398457	0.01756412	0	
0.01361258	0.1888005	0.1205821	0.0929299	0.02526693		
0.04459595	0.05598302	0.1055886	0.1927232	0.116441		
0.5108954	0.0117282	0.051424168	0.01330766	0.3695046		
0.0389596	0.02977917	0.2443827	0.01464227	0.04182522		
0.02156216	0.01841186	0	0.1176955	0.1172435	0.08399434	

0.02889247 0.01978305 0.01943195 0.04439869 0.03536959
0.04708729 0.07374274 0.06838545 0.025351403 0.1137898
0.5291465 0.08130602 0.0457161 0.02068385 0.05498572
0.02793681 0.047653 0.0498657 0.03459746 2.99085 0.05141395
0.02918203 0.01208061 0.1194604 0.06006444 0.1834505
0.1115638 0.01723481 0.03628899 0.03517655 0.09381017
0.01208709 0.07068618 0.04784735 0.142213 0.023010753
0.029484031 0.0746578 0.009526717 0.03867123 0.008915309
0.08077999 0.009208184 0.05473762 0.02361729 0.02327833
0.05685233 0.03616007 0.04947658 0.01888202 0.033844317
0.03318414 0 0.01457608 0.3561662 0.02618374 0.297729272
0.1158187 0.03235536 0.01437397 0.04255498 0.02100177
0.05829672 0.06939688 0.02711681 0.02792915 0 0.120522
0.2802175 0.01983239 0.1340257 0 0.01583176 0.02121098
0.0897062 0.018709497 0.114704 0.07519914 0.08413367
0.05205971 0.8868472 0.0362602 0.01688888 0.0355947
0.02010206 0.09229091 0.173626 0.09669945 0.04386619
0.3821387 0.02357178 0.08515282 0.2274271 0.09710039
0.04761368 0.1007297 0.04231711 0.0099375 0.01306247
0.3027464 0.03455894 0.1010946 0.007021412 0.0195203
0.03522644 0.0792184 0.2111911 0 0.09986688 0.051506569
0.04508928 0.1521662 0.007053907 0.02154606 0.05116608
0.1166013 0.08385599 0.02094491 0.4893546 0.02923842
0.008626615 0.8558045 0.08586396 0.09358759 0.089027506
0.08247436 0.03137971 0 0.061904383 0.2317231 0.5057497
0.04202516 0.02698299 0.02015267 0 0.07875859
ANKRD10-IT1 1.634469 2.570143 4.326589 1.960354 2.167702
5.149764 0.9540211 2.002418 0.7289645 2.252592734
1.590061 1.78397 4.655453 0.8173152 1.184294 4.857033
2.535842 2.625369804 1.143607 2.249682 1.33695398
3.346018 1.871425 3.715412 3.395676 1.584498
1.523582 4.638321 0.526421029 0.6181275 1.488318
2.42557 6.118027 6.062974 11.29569 4.737503 0.510225
3.148011 11.01365 1.97685502 23.05453 8.100782
3.402408476 6.129354 9.443937 7.75066 13.48197 4.626318
11.002604352 5.598944 1.907774 17.51128 4.071982
5.48867 10.83518 5.715252 9.412687 9.196769 3.077492
1.871156349 10.123641539 2.848190439 4.576102 1.806366
5.198294 9.771056 3.518747 11.10471 1.8246419
7.028266 4.668697 4.106738 17.5521 7.823478 6.712678
1.510839 13.55026 13.80727 13.9376 3.985005903 3.189293
9.95854 5.135564 8.459342 3.768484 5.486185 6.857871
5.049437 4.6660713 13.02805 4.270189596 8.800735
3.350995 9.765575 3.658121 5.213431 7.129477

3.652561 7.025821 8.578118 17.16703 7.599995
4.058909 9.650394 6.689947 9.699109 1.938695
1.605128 2.473176951 6.416006 10.45923 22.71878
1.147355 12.85318 9.068854 4.383293 1.280159
6.172351 6.090921 12.5206 11.38901 14.28208 10.91158
3.304209 19.58671 11.59078 9.302051 0.8838803
6.308205 1.848964 4.538479 8.084269 11.97417
6.020565 1.502174 20.9484 4.473984 10.03117 5.896051
8.197289 16.87447 10.51743 5.410936 10.97547
5.999383 6.22721 33.57081 12.79388 14.00442 6.510549
5.521876 5.754861 5.299001 5.90197 9.674954 11.29082
9.42807 14.92362 16.73398 13.30661 1.970874849 15.4695
14.151812563 7.004944 2.17017 5.435035 1.998951
6.755438 17.73347 12.87828 5.532075 4.076902337
10.40501 2.938358 4.582724 7.553719 6.640736
5.021137 8.962609 17.02208 5.570936 19.68067
1.728736 6.547866 16.10857 8.437058 5.418613
16.95453 8.896559 6.296198 4.874791 17.56836
4.472889 1.499255 1.780013 16.84684 6.218759
12.70046 4.404257 7.06248 9.319204 11.19065 5.626117
11.62312 6.666099 9.78614 1.881948 3.744232 7.896454
22.80274 11.21979 6.813813 5.141385 6.790143
8.243592 7.499746 1.613114 5.682886 6.430727
2.541804015 4.423203 10.051454256 1.868108006 9.783212
9.802567 6.843681 15.50731 1.987728 1.794748
5.608079 5.839397 13.05122 5.305935 10.47405
10.38098 17.79473 2.537513 5.174826 13.30872
3.374818 27.92429 8.443908 9.657502 9.631727
9.093549 10.06353 7.317494 8.270071 6.932312
10.64433 6.970905 15.98515 12.17569 12.34633
4.968677 5.014785 10.45862 2.371939727 10.28142
6.03182 5.174727 2.187528 8.187388 7.316561 5.723259
11.65839 14.04403 1.85716 27.36972 12.43056 3.360985
4.812403 7.730846 10.274238582 9.417763 7.577051
15.06541 8.299958 5.841257 3.746197956 11.72178
11.83248 1.666773 10.54328 7.248075 13.98442
6.815185 19.69037 6.948084 6.501439 21.84915
9.42941 5.354173 5.355484 1.391442 2.862821 17.02778
5.431765 14.50058 5.641426 8.270338 13.18131
5.71455 14.9682 5.164897 11.47925 5.621335312 14.682433125
6.377021 6.847625 1.698772 3.632431 6.658459
1.746102 17.41626 4.312 5.408332 4.06591 7.579602
13.6202 10.64474 3.425679476 4.829952 3.336267 2.577232
9.974245 3.757367 2.574893004 2.967854 13.96215

4.143747	5.858806	3.874827	3.41719 1.926486	10.05822
9.554389	3.994534	14.5517 4.868185	14.73795	8.046329
10.84636	16.8158 8.941093	6.312643	3.627693481 33.22797
3.396298	10.44084	19.51019	5.271561	4.227713
12.70202	4.07411 7.263124	8.919257	6.412035	10.65507
27.89102	7.523356	1.479866	36.531	4.370864	11.49095
7.616913	7.564896	11.86307	6.340746	9.506209
13.40173	7.793041	7.685298	5.487671954 2.817836
5.054995	3.55245 6.17844 5.54261 15.22658	3.514116142
18.24108	10.98291	13.52058	12.96096	1.783138
1.120465	27.39732	3.935898	2.725297	11.08867
10.8539 14.27039	15.07181	6.379164	7.209002281 21.42668
8.459184	1.015787	6.688897566 6.417505	8.444796
5.330628	12.90225	10.24217	1.540924	4.194949
NNT-AS1 1.53235 2.423826	1.84821 2.345444	1.401741	3.618028
2.708717	3.07759 1.615755	2.088615657 1.730407	1.075804
2.103793	0.7287107	1.994221	3.786858	1.198001
1.858639463 1.494003	0.9525956	2.002173568 4.217638
0.8258457	3.753782	2.646463	1.058675	1.747528
4.167492	1.211781741 3.131345	1.472563	3.150901
6.541042	4.109902	2.01147 7.236154	3.453194	2.338066
3.945402	3.982336749 4.236568	0.1187327	1.875288967
1.726049	2.637447	1.806673	3.008526	1.566373
1.87053548	3.343653	1.858134	3.87892 1.568128	0.7602748
4.623631	0.1954304	5.511273	2.763682	4.841981
1.421851329 1.942768543 1.674210529 2.001155	7.141493
1.774494	3.987331	2.292707	2.352105	1.650496746
3.235996	2.583532	4.124022	3.872642	1.566775
4.202645	2.024568	1.925165	2.897278	4.156139
5.798849775 1.66236 2.264757	2.026885	3.351111	2.321107
0.4746056	4.048509	2.122776	2.37511066	6.672047
2.210736455 1.910711	2.827391	3.403481	0.5337076
3.836947	2.163804	1.699596	5.095572	3.327983
4.760422	3.674827	3.897001	13.29564	4.128352
2.338459	1.401505	2.435281	2.448477398 3.379286
5.422182	11.67448	1.790199	3.329859	2.502346
3.074827	6.010761	1.896211	4.552511	4.603743
2.931595	2.87662 3.347109	2.795696	12.66452	3.212492
3.794736	0.8191042	3.11486 1.822908	1.392993	2.076386
3.401421	1.437269	1.164916	2.031906	2.253505
1.416917	5.9486	8.320709	8.85771 4.463878	2.464845
5.97227 2.271586	0.8999581	5.250993	3.894381	3.234091
6.759501	2.589503	1.553454	3.987581	2.604738
3.435272	1.837259	1.736175	4.922991	1.166131

3.161775	1.887007835	3.234666	4.118565942	4.60582	2.344733
2.904341	2.986847	2.286516	2.056813	5.529048	
3.543415	3.068082688	5.557255	2.768104	4.594934	
5.498066	2.593028	2.747127	2.657287	4.197874	
4.303741	3.525625	0.9890854	2.534332	1.631401	
1.426819	2.339335	2.088228	6.424337	2.966533	
6.699364	1.973097	2.199882	1.610143	2.179336	
2.399928	2.185015	2.583785	2.605363	4.517636	
5.736515	4.472498	2.523971	3.174168	3.438222	
2.170751	1.215876	8.036389	2.023241	3.319385	
2.143598	2.999235	3.516914	15.41512	2.199464	
2.805015	2.017943	5.097128	1.982386	1.156015785	
3.968411	2.877081479	1.937520369	2.630389	1.940769	
4.627319	4.399997	1.285474	1.931531	3.120104	
0.4857601	3.663959	2.767588	1.071362	2.540126	
3.532676	2.229557	0.6272857	2.909554	5.338568	
6.796717	3.522351	2.802183	2.019207	1.893338	
2.567927	3.591746	5.089275	2.557455	3.556532	
1.23457	4.076332	2.027225	3.426451	2.383255	1.235587
3.80556	1.3663801	2.622348	1.75237	4.047979	0.473163
2.791708	2.274058	0.4221932	3.315182	2.256246	
2.142646	2.333922	3.011213	3.413976	0.5760931	
2.859824	2.0038829	6.933115	2.029774	3.170827	
4.391676	2.019288	2.980266764	3.637298	5.250497	
1.324806	1.346967	0.5445229	4.629602	4.616452	
4.049328	2.209949	2.119839	1.751356	3.329461	
3.683943	2.388077	1.898113	1.493304	2.529596	
2.145049	2.792144	2.155647	3.320656	4.544732	
3.615106	4.82088	2.034785	3.0553	2.678817729	2.572011204
3.940572	1.972269	3.063641	2.410927	2.736913	
1.440143	1.986734	3.002051	3.311914	2.753466	
2.284679	2.433279	3.011941	2.512156162	1.757549	
2.169411	3.16066	1.941864	1.472438	6.113305579	3.557215
2.889019	3.869176	4.62102	2.621604	2.794732	2.180078
0.4646535	3.419623	5.47161	1.901728	2.004982	0.4232467
4.840682	3.588798	2.419336	3.37353	3.923466	
3.143745191	24.18819	7.379915	5.219904	2.558581	
2.488941	2.848694	8.321429	1.485998	2.03541	2.706913
0.9800418	4.057066	5.097612	1.161516	1.193366	
3.974984	1.294713	3.360682	3.047337	3.809334	
2.352008	2.558869	4.291407	7.859272	3.017408	
6.185823	3.03409278	3.245522	5.262418	4.002355	
25.82517	1.960706	5.347085	4.80804623	1.784041	
4.519506	6.650076	3.248936	1.520752	1.955611	

3.840426	3.158289	2.917039	3.075277	1.771144
5.63006 2.046446	1.949647	1.445073367 3.284994	3.03849
2.010067	13.27220253 2.862745	8.963494	0.3567827
3.339084	2.067741	1.436322	3.512332
AC087277.1 0	0.02596759 0 0 0 0.02750208 0 0 0 0
0.0699312 0 0 0.02546153 0 0 0 0 0 0 0 0 0
0 0 0 0 0 0 0 0 0 0 0.06632493 0 0 0
0 0.024032885 0 0 0 0 0.02796391 0.3596826 0.03159869
0 0 0 0 0.07686428 1.136215 0 0.2117927 0 0
0.02783842 0.02722992 0 0 0 0 0 0 0 0 0 0 0
0 0 0.13432 0 0 0 0 0 0 0 0 0 0 0
0.05588426 0 0.37212282 0.03063081 0 0 0 0 0 0
0.1842625 0 0 0 0 0 0 0.1401073 0 0 0 0
0.03620388 0.526266 0 0 0.5089332 0.09617662 0 0
0.02151441 0 0.0284103 0 0.05843061 0.2752763 0 0 0
0.02483859 0 0 0.0251346 0.01955767 0.03091721 0 0 0
0 0 0 0 0.1068979 0 0.1143782 0 0.4860672
0.9227355 0 0 0 0 0 0 0 0 0 0 0 0 0
0.0908572 0 0 0 0 0 0 0 0 0 0 0 0 0
0.3666038 0 0.05254007 0 0 0 0 0 0 0 0 0
0 0.06718821 0 0 0 0 0 0 0.0237656 0 0.1888344
0.05534289 0 0.04178488 0 0 0 0 0 0 0.3440635
0.02306207 0 0 0.2886368 0.02520673 0.02995128 0.2866713
0 0 0.1290801 0 0 0 0 0 0 0.0236802 0
0.5888253 0 0 0 0 0 0.03542013 0 0.09762868 0 0
0 0 0 0.04067799 0 0 0 0 0 0.04521507 0.08153711
0 0 0 0 0.101148 0.1476944 0 0 0 0 0
0.05243921 0 0 0.03468222 0 0.06410595 0.04131068 0 0
0.09019656 0 0.0247575 0 0.03790211 0 0.01890287 0 0
0 0 0 0.02906781 0.05738407 0.09346393 0 0 0.1053465
0.02140952 0 0.04776855 0 0.04382511 0 0 0 0
0.09206138 0 0 0 0.02317521 0 0.02995496 0 0.02462305
0.06875262 0 0 42.930953367 0 0.01825212 0.02116849 0
0 0 0 0 0 0 0 0.02708331 0.1085275 0 0.152585
0 0 0.09748208 0 0 0.03698261 0.0495914 0 0 0 0
0.08863776 0 0.08026369 0 0 0.01819882 0 0 0
0.03033187 0 0 0.119484326 0.06278856 0 0 0 0.1671246
0 0 0.04546363 0 0 0.1565402 0.1852653 0.02101068
0.1785695 0 0 0.3168907 0 0 0 0 0 0 0.05800282
0 0.02582479 0.026904474 0 0 0 0.1348728 0 0.1640004
0 0 0.2915334 0 0.02063991 0 0 0 0 0 0 0
1.045558 0.06580229 0 0.068226687 0 0 0 0 0
0.03125676 0 0 0 0 0
AC008870.2 0.09931672 0.1628046 0.3523706 0.1355333 0.1302706

0.6753318	0	0.1700423	0.1533279	0.124432995	0.3897207	
0.2229963	0.3286511	0.0931186	0.09252296	0.2312473		
0.1415091	0.149557199	0.210222	0.06060565	0.149213614		
0.2119522	0.2166001	0.4922649	0.2137846	0.3011081		
0.1572092	0.6394744	0	0	0.1603791	0.1907695	0.6573812
0.4468584	0.5659857	0.7464553	0.07381727	0.2676013		
0.3512006	0.351574826	1.180133	0.6628913	0.221510968		
0.2645108	0.7889428	0.5637603	0.9079994	0.2440041		
1.343542811	0.2606374	0.06551421	1.137826	0.7153975		
0.5973366	0.5809304	0.2965066	0.3541423	0.538146		
0.298758	0.153877989	0.596001045	0.193491198	0.3108765		
0.2490395	0.5271708	0.7018331	0.1548744	0.1433976		
0.171060178	0.515385	0.3392948	0.5758648	1.136867		
0.8863857	0.6513947	0.3335752	0.3745356	1.25886	0.7624275	
0.333566342	0.0986786	0.5734343	0.6686932	0.452262		
0.2092274	0.4121232	0.4963548	0.5435158	1.40954237		
0.9281974	0.232432195	1.029752	0.290885	0.5629195		
0.2449634	0.3043874	0.5638668	0.4780839	0.5119819		
0.6902992	0.9059587	0.7933189	0.3922915	0.323359		
0.4538435	0.6849653	0.286547	0.282593	0.213291438		
0.3972174	0.5241299	0.7518853	0.124496	1.79872	1.205965	
0.2928804	0.1746248	0.4046558	0.3563658	0.489828		
0.5873043	0.9463591	1.085195	0.0526819	0.5728064		
1.348661	0.1167947	0.107416	0.6045363	0.2101096		
0.5517783	0.3230606	1.183789	0.6004553	0.03171819		
0.6510207	0.5398147	0.4888484	0.2966763	0.6329666		
0.4542592	0.6872185	0.9050603	1.060619	0.4151359		
0.6884445	0.4788673	1.237272	0.3876224	0.2633269		
0.3933707	0.2542373	0.2442765	0.5358249	0.5495248		
0.9801063	0.5451364	1.663182	1.833468	0.9968561		
0.106926804	0.8151081	0.435279668	0.5291913	0.06991528		
0.3153618	0.2036974	0.384555	1.217321	0.3842802		
0.5622027	0.24500615	0.9839469	0.1443769	0.4420066		
0.2810163	0.3294016	0.9806908	0.4913711	0.8044773		
0.7563002	0.759462	0.2224476	0.8459775	0.7265465		
0.5609746	0.3647484	0.7486716	0.6318579	0.637863		
0.3406902	0.6728078	0.2157064	0.3036686	0.3171624		
1.055411	0.3172466	0.7187985	0.404803	0.4308007		
1.124295	0.4534112	0.4033757	0.5559526	0.5368958		
0.6930382	0.3590199	0.1470762	0.6145006	0.9719499		
0.7622137	0.4674845	0.6453071	0.3129675	0.7761046		
0.2730972	0.1062858	0.8767111	0.4459176	0.132385626		
0.4236784	0.568385806	0.104247099	1.290795	0.8289227		
0.5634079	0.7471213	0.429529	0.1731046	0.439549		

0.1600712 0.6764511 0.2960901 0.447762 0.5440765
0.714967 0.2043744 0.5053541 0.8170558 0.7098476
1.147644 0.7250796 0.6020887 0.316463 0.784437
0.3951504 0.4960853 0.5679994 0.6201377 0.5058321
1.135119 0.7044738 1.453261 0.643038 0.8412211
0.4160496 0.6456808 0.102948773 0.7193155 0.4794552
0.6109631 0.3378269 0.8154047 0.5716063 0.4354071
0.8633287 0.3194506 0.1234814 0.4358987 0.7628269
0.3363055 0.2313655 0.6930831 0.350116672 0.5135525
0.8072143 1.077332 0.5110252 0.1977509 0.067669761
0.5619115 0.3197967 0.1464938 0.5883524 0.403729
0.9631899 0.4362399 1.097089 0.4741868 0.2424187
1.213536 0.5440497 0.3271579 0.4836962 0.2391541
0.3366893 0.748121 0.5743172 1.035098 0.6901641
0.58098 0.5477604 0.6291426 0.7332813 0.2274969 0.4151929
0.353176115 1.062461659 0.523115 0.3814408 0.1437762
0.2677205 0.6873005 0.05530306 0.8949208 0.4373465
0.4660212 0.7760161 0.3329975 1.146148 0.5292129
0.592853944 0.3055922 0.1757163 0.04377093 1.069542
0.4062444 0.167636263 0.1352538 0.8679705 0.3453122
0.2478344 0.294312 0.2334146 0.2547037 0.4885792
0.78278 0.4860153 1.887656 0.2947534 0.655108 0.8049396
0.9822476 0.6338887 0.7431094 0.8150514 0.524377499
0.3280457 0.5080899 0.3368633 0.6513819 0.3347116
0.1935766 0.6931201 0.4038005 0.7042591 0.9370077
1.267685 1.161527 0.5049539 0.5224552 0 0.4403859
0.8899013 0.4241246 0.5719221 0.4653602 0.7624519
1.243401 0.5491593 0.8030615 0.4842977 0.4047734
0.323300346 0.1563149 0.1410434 0.3832628 0.317096
0.5626767 1.685114 0.399565729 0.3723498 0.6092593
0.4942557 1.110706 0.1843778 0.05835757 1.217099
0.02795382 0.2481818 1.018491 0.6217229 0.9435458
0.3781704 1.049205 0.659446906 1.371681 0.540258
0.1641897 0.247859347 0.4282143 1.273774 0.5468606
0.9183176 0.3093093 0.2293041 0.3829153
AC023511.1 0 0.1946433 0 0.4618103 0.0667486 0.1030726
0.9435049 0.3049445 0.2749698 1.051998942 0 0 1.414524
0 0.5807396 0.4147061 0.5075492 0.555573003 0.1256668 0
1.040633571 0 0 0.3044141 0 0.05999897 1.248549
0.4828624 0.098339013 0.3464112 0.3595188 0.0684231
0.07368195 0 0 0.05577716 0 3.2679 0 0.045035354
0.04109486 0 0 0.03271442 0.1572052 0.2433929 0 0 0
0 0.1174896 0.02400606 0.6005319 0.07651647 0.04960993 0
0 0.1304164 0.02551315 0.206967243 0.028887446 0.069399295 0

0.1488712	0.6652808	0	0	0.1285807	0.02556416	0	0	0
0.0503406	0.0237253	0	0	0	0	0	0	0	0.05558733	0
0	0	0	0.0523609	0	0.26149611	0.1147985	0	0.05682155
0.08694286	0.04295782	0	0.02599389	0.0246636	0.05358559	0
0	0	0	0	0.122083	1.023936	0.03838685	0	0.03167417
0.022500302	0	0.0308179	0.04086029	0.6251398	0	0.6307906
0.1414096	0	0	0	0	0	0.05474672	0.1406841	0
0.02445808	0.07403921	0.02327258	0	0	0	0	0.05793592
0.05177907	0.02563866	0	0.1945841	0	0	0.05600452	0	0
0.02679174	0.03958745	0.02678953	0.04803108	0	0	0.02175349
0	0	0	0	0.02305643	0	0.09297052	0.1597881	0
0.03012788	0	0.0851289	0.03835133	0.4778864	0.026020221	0
0	0.06059491	0	0.1189034	0.6237362	0	0.2520559
0.117168148	0.02714702	0	0	0	0	0	0.04895544	0.02944297
0	0	0	1.232668	0.02326694	0	0	0	0.1678725	0
0.02182054	0	0	0.2333925	0	0	0.04876566	0.3285823
0.02592684	0	10.9817	0	0	0	0	0.05918365	0.06131871
0.9671138	0	0	0.03254553	0	0	0	0.09767181	0	0
0.5845528	0.02665614	0	0	0	0.827925919	0	0	0
0.05254299	0	0	0	0.02870628	0.02958805	0	0	0.03049115
0	0	0	0	0.02597962	0.8766071	0.05099286	0.03374227	0
0	0.04887179	0.2118219	0.1018619	0.03707069	0	0	0
0.04738543	0	0	2.145096	0	0.026374667	0	0.1228327	0
0	0.09748679	0	0.03003212	0	0.04406809	0	0.04225496
0.02630792	0.09278642	0	0.07102497	0	0	0.07619019
0.0483007	0.04073087	0	0.030338807	0.02723517	0	0	0	0
0.04935234	0.06017912	0	0	0.02070192	0.02053103	0	0	0
0	0.04312858	0.07318031	0	0	0	0	0	0	0	0.5368163
0.02127383	0.027537679	0	1.407188	0	0.03966776	0.03200768
0	0.06611831	0	0	0.02785789	0.05566658	0	0	0.1016851
0	0	0.5777199	0	0	0	0	0	0.0232324	0.02580267
0.01388914	0.427269	0.9679961	0	0	0	0	0.02545277
0.08525718	0	0	0.07887353	0.02841953	0.07615152	0
0.134341396	0	0	0	0	0	0.0867874	0	0.04259728	0	0
0.2200062	0.1735848	0.01968601	0.1673112	0	0	0.07422791
0.03169174	0	0	0.2734679	0.01783877	0	0	0.03101831	0
0.327706861	0.9344217	0	0	0.06318473	0	0	0	0.1416443
0.2124523	0	0	0.03673922	0	0	0.02506542	0	0.3149147
0	0.2671742	0	0	0.287663307	0	0	0.1962991	0	0	0
0	0	0.02411732	0	0

AP001528.1	0.0834978	1.197643	0.4388828	0.6608864	0.4067938
2.57307	0.6266148	1.715501	0.3498882	1.016246577	0.3890808
0.02343475	0.9210146	0.212493	0.4667168	1.51997	0.2617337
2.245287531	1.045702	0.3566678	0.613297627	0.7365309

0.3793763	1.783871	2.042426	0.2250208	0.7930161	
1.697749	0.414913127	0.7849238	0.7752972	1.047843	
0.2533094	0.4508207	0.8739862	0.3399293	0.5585386	
0.5892292	0.4152125	0.200570016	0.6068577	0.1682438	
0.211059822	0.09968767	0.343924	0.3510859	0.3192297	
0.435922	0.340078566	0.1524339	0.1239284	1.76689	0.4094984
0.4125167	0.8837737	0.3079338	0.1926523	2.090967	
0.09568479	0.258737374	1.381332206	0.227741364	1.496885	
0.54437	0.03282995	0.8244491	0.4719983	1.603416	
0.263659256	0.1166566	0.101876	0.3631068	1.628382	
0.5338778	0.3063083	0.172581	0.6390223	0.7282241	
0.4604294	0.058424318	0.04148066	2.749268	0.3960852	
0.8831076	0.5151421	0.3926788	2.417866	0.2948038	
0.53122213	0.766902	0.275874318	0.3729322	0.6623326	
0.2718722	0.164757	0.3290211	0.4856179	0.1884075	
0.09009099	0.5440782	1.762272	0.2501104	0.2198722	
0.3863205	1.058511	2.672377	0.1204533	1.06912	
0.232059765	0.3657543	0.1878171	0.2969081	0.08373325	
0.8415857	0.8871464	0.2935833	0.1334646	0.2268022	
1.057429	0.1871861	0.172816	0.6288007	0.5386152	
0.5757812	0.1719897	0.7173334	0.250935	0.05160403	
2.552285	0.8169778	0.1889932	0.1358022	0.2184669	
0.2283693	0.1599972	1.231487	0.2042255	0.2697094	
0.3675699	0.2660746	0.260021	0.51496	0.1948658	0.0753538
0.6980281	0.4437396	0.2167817	1.203372	0.07358646	
1.037013	0.2755963	0.731226	0.281031	0.723139	
2.519196	0.2996354	0.3943584	1.454771	0.03350952	
0.4789025	0.934916002	1.489166	1.390607753	0.2495797	
0.5143193	0.4261046	0.3234779	0.6577565	0.4386125	
0.1615365	1.004395	1.002446619	0.9926715	0.04855236	
0.5326336	0.2362567	0.6231045	0.3778907	0.2639293	
0.3174667	0.1679574	0.216848	0.2938834	0.6445543	
0.4799331	0.176859	2.555435	0.1129737	0.8656872	
0.2475073	0.4910159	0.1885482	0.2176191	0.1823579	
0.3692015	0.7516018	0.7468059	0.2962304	0.182318	
0.3203934	2.193277	1.07516	0.1637165	0.02921262	0.1354141
0.1572238	0.3737018	0.5358177	0.1823381	0.2798425	
0.1220591	1.2805	0.4428769	0.2631188	0.274732	0.4696342
0.3701926	0.2929384	1.412097	0.395731818	0.6678674	
0.628756228	0.475775639	0.4016097	1.653823	0.03866691	
0.1354772	0.0555561	0.05821316	0.1907297	0.3229811	
0.5270949	0.06223241	0.1505774	0.1429429	0.1168784	
0.9965686	0.0809262	0.377131	0.474992	0.6342994	
1.111607	0.6169184	0.2660576	0.75904	0.790435	0.3177677

1.313207 1.372924 0.1956216 0.5897483 0.1480668
0.3332153 1.535351 0.1533759 0.9036042 0.5527082
0.544037053 0.1567857 1.566288 0.6229377 0.1503628
0.5027213 0.2587643 0.5350057 0.4354919 0.09296631
0.1816738 0.7329396 0.6906585 0.6850998 0.3241903
0.4828007 0.076313213 0.1079388 0.3571806 0.2151126
1.327081 0.1918311 0.881818097 1.68536 0.1344301 0.3147437
0.2857927 0.2088763 1.272504 0.1410603 0.3809697
0.3147312 2.106004 0.2983742 0.9889617 1.100196
0.3558225 0.2193407 2.891279 0.7090119 0.1877717
0.212723 0.1934122 0.3848338 0.8552412 0.162749
0.6597483 0.1610625 0.2094372 0.219464904 0.281203743
0.7225196 0.2084458 0.437013 0.2550891 0.2379296
1.146865 0.2303204 1.460811 0.4440338 0.3827497
0.4625401 0.5472252 0.1747905 0.484184915 0.1340443
0.3447002 1.5333 1.113281 0.3305214 0.234892629 0.1137109
0.1089138 0.09677059 0.6967037 0.954391 0.4660627
1.070676 0.3537096 0.05875899 0.4301095 0.4295625
0.423668 0.2503473 0.6767308 0.3204588 0.05329245
3.677093 0.3135808 0.45135253 0.2666022 0.1740292
0.1416043 0.1533368 0.4771557 0.457718 0.6964235
0.1397876 0.2875848 0.599144 0.4297467 0.949396
0.3599243 0.8314183 3.074685 0.2508093 0.06959628
0.6388565 0.3205519 0.09128905 0.398851 0.7610183
0.2418379 0.4713324 0.4653256 0.1928379 0.472705867
0.2518834 0.3853796 0.4666601 0.7701482 0.524474
1.716865 0.910245399 0.5881411 0.1707393 0.1662128
0.1903854 0.1550105 1.177501 0.2469896 0.09400563
0.1317802 0.9743739 0.2613482 1.064639 0.5347111
0.1890193 0.509459536 0.7581245 1.827391 0.9202529
0.260476161 0.1800047 0.7825738 0.300611 0.1725759
0.04522492 0.2731066 0.9752449
PVT1 0.1851453 0.2857948 0.6179595 0.4573146 1.061598
0.6562598 0.5312547 0.4564675 0.9514136 1.630395527
1.323617 1.231534 1.993215 0.9051522 1.310851
0.7877172 0.7228117 0.76670867 0.3690334 0.3276435
0.726313572 0.3793145 1.349312 0.7910998 0.1431104
1.51973 1.109472 0.3869104 0.311782829 0.5941586 0.3886704
0.5761217 1.051874 7.572236 1.658023 7.427318
0.7706131 1.259027 2.469465 2.53500344 3.801929
2.350268 2.282174587 1.923085 1.50887 2.6488 4.145537
3.360354 6.420445584 2.484314 1.099179 3.094348
1.549289 1.718048 1.155163 2.497106 2.481534
1.106239 5.357251 1.104403031 1.129076842 1.633991428

1.274973 0.8882785 2.176602 0.8710391 1.559064
1.283139 0.616518363 2.958082 5.215951 2.010936
4.913721 9.647996 2.44923 2.334321 1.201325 1.786979
1.911763 3.427845056 0.777213 1.057434 1.592212
2.559225 1.794183 0.845104 2.106418 2.908914
2.91760414 1.49167 1.037366285 2.197265 1.760102 2.24614
1.19949 4.163901 1.699795 1.439909 2.208511 2.573699
2.467089 1.760813 4.98507 3.052086 5.237328 2.198673
1.774235 1.468475 2.051230431 2.86675 6.77868 0.3865177
1.104721 2.787494 2.547901 0.3527903 6.131876
3.14734 1.219248 3.386893 5.230633 1.556474 5.506003
1.713749 3.193292 4.294598 2.673212 0.8467458
1.518961 1.304798 1.638173 2.565571 4.505119
1.828293 1.082056 0.8933642 1.456645 1.609026
2.060881 4.706 2.879208 1.924445 5.81057 7.226516
4.061687 0.7400893 8.195673 1.383907 4.967307
2.16509 4.935231 1.404379 128.733 2.796863 1.252497
4.816913 4.941754 2.96269 5.780767 10.33585
2.399956833 2.311419 1.211756892 2.971571 3.356137
1.242977 0.7341463 3.139457 4.942072 5.037395
0.9563485 0.749050661 1.938677 2.355028 0.7388395
2.097568 0.5014884 4.71141 3.877773 2.647428 0.9017962
3.15479 1.552106 2.597226 1.884097 3.947758 1.003457
5.360791 1.760312 6.663524 1.038861 2.812289 2.3859
1.15241 3.201852 1.305486 1.532592 3.796603 5.02907
4.386132 0.7223733 1.935394 4.418468 3.627406
5.194548 3.422657 1.029419 1.556722 3.820734
5.167638 2.676051 2.400788 1.492672 2.695452
2.644865 2.679745 0.7699035 1.322153 2.854046
1.653506829 1.246431 4.687245475 0.591338149 2.076608
3.127442 3.185195 1.1388 2.371375 3.762681 2.595658
1.289102 1.436348 4.084567 0.454919 5.594291
2.380591 3.086043 4.838235 5.157797 2.125368
2.038398 2.780234 2.227279 3.421695 3.622741
1.24212 3.62873 2.191839 5.140591 2.876051 17.10932
2.999636 1.270841 4.468924 7.461215 0.6334033
3.849563 0.444148814 2.828393 0.9755145 1.972567
2.778413 1.952445 4.554685 2.456899 1.299598
5.70093 0.8603458 3.623749 1.854141 2.594557 4.320283
3.924107 1.588198403 2.408128 5.404085 1.385763
1.450143 1.87442 1.863856567 2.024243 1.917033 0.5825969
1.169921 2.418058 4.976293 2.441788 5.241895
2.700775 1.59031 3.017774 0.7592887 0.7057238 1.580233
3.720645 8.101208 4.394385 4.681062 5.12636 2.354248

1.470333 1.522942 1.957744 1.935351 3.017789
2.218059 0.898842284 4.339049546 2.02235 2.019463 1.486515
10.4541 1.562634 1.006899 4.126497 1.52703 0.7529188
2.345489 1.214552 4.217882 4.224565 11.778198209
4.554981 5.939913 0.495024 4.225022 1.380271
1.097243211 1.2715 4.699627 2.636602 7.337307 1.711277
2.224599 0.9582656 3.023366 3.048793 5.727034
3.376076 1.722875 8.034321 3.354826 2.238311
5.237839 1.432794 2.176106 0.530664411 58.39185
3.936044 4.57975 5.464351 1.166546 0.6315115 9.864143
2.448688 2.561978 3.894539 10.0665 3.04346 1.909264
3.262753 1.869371 2.719772 3.371904 2.691501
4.721619 2.95246 3.145934 1.858057 2.186411 4.019464
1.908826 3.242157 1.430742023 5.942147 2.20534 2.269074
4.380911 0.9622855 3.663162 2.880952372 1.165299
7.521358 5.989015 6.696174 1.829329 1.338113
5.515787 9.864661 1.563298 1.90739 4.536236 12.21081
4.277957 1.643902 3.565071056 6.409263 2.3258 0.2652701
1.659555122 3.479141 3.41571 2.893676 4.722883 2.562161
3.729646 1.375167
AL022322.1 0.790811 0.1163377 2.844392 0.1259051 0.8245059
0.4312434 0.1253178 0.3159251 0.2817405 0.114323047
0.2871913 0.1792684 0.3131321 0.1615999 0.03305775
0.2253351 0.2224645 0.366415883 0.6008856 0.346463
0.201404248 0.1413606 0.1676774 0.2183369 0.3263663
0.3745504 0.2086302 0.1443027 0.17633084 0.02300544
0.3151622 0.28173 1.076521 0.8302273 2.434923 1.555766
0.1160471 0.7284717 2.093969 0.601157553 3.045723
1.939456 0.253261389 2.118276 1.482497 5.378854
2.654356 0.6647537 1.76529966 3.36865 0.3862275 1.061778
1.1747 1.265298 0.2569815 0.835053 1.339754 0.3741577
0.7522908 0.384856046 0.552510201 0.49775712 0.3635143
0.2610077 1.367311 0.5633504 0.8438658 0.5533367
0.254660419 3.017115 0.7879784 1.653365 1.72507 5.908561
1.041406 0.3667196 1.235855 2.880251 2.079521
0.844196744 0.9695707 1.251452 2.139411 2.074604
0.4912495 1.482302 0.9388789 0.6264324 1.52822247
1.463781 1.113796836 1.573576 0.2425057 1.369374
0.4201131 0.8182542 0.6289649 1.601398 1.676124
0.791299 0.9012605 1.727254 1.168023 1.155337
1.443701 1.124242 0.6435381 0.2397997 0.430347717
1.649007 2.118273 2.727132 0.2313036 0.9500324
1.508087 2.036532 1.361283 4.208898 1.265778
1.633444 1.196084 2.236 3.288716 0.4705711 0.4288101

0.6588784	0.306019	0.2741352	0.7043355	0.1970602	
1.431135	2.666366	2.083846	0.9705296	0.2946494	
2.481117	1.311527	4.606705	1.696003	4.230413	
1.76807	0.7045871	1.845582	2.786092	0.7368397	0.6641352
4.738034	1.412884	2.483967	0.5447009	0.6933719	
0.6119548	0.6431022	1.03784	0.9483642	2.737806	2.30104
1.380563	1.139277	1.356835	0.198661495	0.9633056	
1.378962748	2.425707	0.4246634	0.4346089	0.4690237	
2.065717	3.037673	2.209288	0.3013061	0.443530091	
0.8761876	0.2991916	2.116203	0.8514343	0.8826951	
1.270176	2.097004	4.305638	1.131866	2.344873	
0.04541648	1.794677	1.029087	2.217278	1.058371	
1.906757	1.95657	1.718042	1.799806	1.709434	0.4624216
0.2479965	0.7147858	0.7896714	0.6477132	3.001283	
1.807913	1.314255	1.201206	3.888009	1.182803	
2.234674	0.8248638	2.884938	0.5008836	1.389634	
2.436646	2.311964	4.487013	0.5603542	0.7716813	
1.44249	2.490802	1.419281	0.3472008	1.228873	0.839101
0.777828424	1.211017	2.372828599	0.446960344	2.295063	
1.927301	1.355703	1.402748	0.2833245	0.470052	
0.303963	0.6519912	1.685941	1.467847	3.375619	
1.543005	3.831784	0.6717971	1.212326	3.457341	
0.57971	2.991805	1.503595	0.9277123	0.3266461	1.745098
2.19079	1.18165	0.6291198	1.004453	1.163901	1.795442
4.702272	3.832929	1.341755	1.839584	0.3468532	
1.728129	0.346809384	1.760967	1.487912	1.699894	
0.7242178	1.02292	1.036863	1.555677	1.310958	2.563699
0.4081004	1.043901	2.421522	0.5915539	0.4271032	
1.995217	0.787629039	5.532881	2.863876	4.61907	0.705997
0.2826196	0.338490471	0.7596578	0.8426742	0.03489407	
1.130483	0.4290492	4.326337	1.262908	2.812043	
1.480225	0.8084008	2.609705	0.2626822	0.2504808	
3.058931	0.1087519	0.6358531	1.409389	0.9119963	
2.473771	1.315149	1.182575	0.7828425	2.092256	
2.077581	1.086624	3.043197	2.446933226	1.096649578	
1.361736	0.9948871	0.3161241	0.1402932	0.6741061	
0.1975936	2.101204	0.7601865	1.476352	2.432535	
0.7862859	2.91207	1.37761	1.65826526	1.936861	0.8161673
0.3440584	1.346586	1.610675	0.173030467	1.76732	0.7035542
0.8019533	0.9131652	0.4706957	0.8756713	0.4301993	
3.530105	3.176381	1.407476	6.987859	1.12787	4.440144
1.889935	2.493314	1.755248	0.4248118	1.530095	
1.106293114	0.4219496	1.169903	1.34114	1.43364	1.039912
0.9077702	1.667084	0.8995962	1.653545	1.631491	

2.381558 0.737786 1.372732 2.426701 0.3540701
0.1421194 1.153511 1.654273 0.45734 1.263649 1.985626
5.771794 1.298726 2.382042 1.409006 1.166621
3.565828504 0.4840344 0.4283467 2.021 1.888267 2.255146
3.82677 1.427617958 2.289862 1.19726 1.110018 1.579681
0.4830963 0.1251043 0.8397302 0.9088796 0.9614071
1.61454 1.345162 2.12919 2.763765 1.856513 1.146236903
3.02223 1.454459 0.07821829 1.06269911 1.402479 3.197438
1.382754 0.8337801 0.9417703 0.3959883 0.5552987
AC061975.6 0 0 0 0 0 0 0 0 0 0 0 0 0 0 0
0 0 0 0 0 0 0.009316979 0 0.04477024 0 0 0 0
0.018078421 0.06368345 0 0 0.1444856 0.02946445 0 0 0
3.579374 0.02170976 0.024837592 0 0.0412349 0 0.7758247
0 0.006883815 0 0.01005555 0 0.01494402 0.01079951 0
12.16412 0 0.1824036 0 0 0 0 0.025365593 0
0.012758209 0 0 0.01287408 0 0 0 0 0 0 0
0.08329051 0 0 0 0 0.00761517 0.007080578 0 0 0 0
0 0 0.009058043 0.01925182 0 0.03204857 0 0.00901521 0
0 0.01579454 0 0 0.0181364 0 0 0.009482539 0
0.01634656 0 0 0 0.007056951 0.02699146 0 0.024818427
0.02494408 0.005665492 0 0 0 0.1656616 0.007427548
0.02093494 0 0.09675506 0 0 0 0.3793246 0 0 0
0.02567027 0.01011811 0.01733098 0.008658734 0.00673751
0.01065081 0.09518948 0.009426705 0 0 0 0 0.03088723 0
0.01911858 0.04925332 0.02911067 0.009849853 3.267073 0
0.02428848 0 0.3215439 0 0 0 0 0 0 0.4406261
0.008359209 0 0.07884339 0 0 0 0.009566997 0.1106371
0.03457498 0.007426421 0 0 0 0 0.021539927 0.01996259
0.05711858 0 0 0 0 0.008999857 0.5629238 0.01881815 0
0 0 0 0 0.01457598 0 0 0 0.01604575 0.04929206
0.5594385 0 0 0 0.005976645 0.009293204 0.009532662 0 0
0 0.01834299 0 0 0.02176038 0 0.01077527 0.007944758 0
0.02393238 0 0 0 0.05386729 0 0.1501747 3.461045 0
0 0 0 0 0 0.0163154 0.3184231 0.1159126 0.02178601
0 0.03739677 0 0.0108788 0 0 0 0 0 0 0 0
0.5675404 0 0 0 0.009759121 0 0 0 0.01362998 0
0.03363875 0.2744821 0 0 0 0.01143043 0 0.009697318
0.01756646 0.6232432 0 0 0 0 0.0441683 0 0
0.02035495 0.03884028 0.02901833 0 0 0.01305707 0
0.0195358 0.009337755 0.01775898 0.007487871 0 0 0 0 0
0.01724184 0 0 0.01475092 0 0.016456 0.00761159
0.1585239 0 0 1.778058 0 0.04757196 0 0 0
0.007983733 0 0.3714955 0 0.01696502 0.007894967 0.0234656
2.024985816 0.012973227 0 0 0 0.07061062 0 0 0.06021255

0.01558771	0.02048533	0	0	0	0	0	2.163914	0	0
0.03358204	0	0.012281572	0	0	0.009487007	0	0.009240961	0
0	0	0	0.1770982	0.009358358	0.006269394	0	0.07582174
0.009666621	3.657207	0	0	0	0	0.0496324	0.3173103
0.01718004	0.1823161	0.0079774	0	0	0	0	0.1752636
0.04254859	0.1737135	0.04921297	0	0	0.1773966	0.01165227
0	0	0.08937549	0	0.01724279	0.1298807	0.01140467
0.0088965	0	0	0	0	0.04646295	0	0	0	0.01487975
0.02231815	0.05586803	0	0	0.07695834	0.02767302	0	0
0.007719093	0	0.4584225	0	0	0.01175186	0.01674897	0	0
0.01361923	0.3294098	0	0	0.007123649	0.01773469	0	0
AC055717.2	0	0	0.04632229	0	0	0.02550019	0	0	0	0	0
0	0	0	0	0	0	0	0	0	0.02507672	0	0	0	0
0	0	0.048658212	0	0	0	0	0	0	0	0	0.02261482
0.01947733	0	0	0	0.131038262	0	0	0.01852784	0.1171944
0	0	0	0	0.04751287	0.02701306	0	0.04909406	0.06190698
5.323724	0	1.388632	0.034135845	0	0.034338819	0
0.0883939	0	0	0	0	0	0	0	0	0	0.02347859
0.2351244	0	0	0	0.133402	0.813971248	0	0	0.02697108
0	0	0	0	0	0.17251795	0	0	0	0	0.08502224	0	0
0	0.1060567	0.8663516	0.1276116	0	0.08799378	0
1.872609	0	0.3798768	0	0	0	0	0	0.0404354	0	0
0.1783518	0	0.8451984	0	0	0	0	0.08126615	1.067362
0	0.1452225	0	0.02303058	0	0	0	0	1.347336	0
0.0507441	0.2251607	0	0.07185055	0	0	0.06607786
1.234987	0.05302628	0	0.6627739	0.07129744	0	0.5883537
0	0	0.5411136	0	0	0	0	0	0	0.02249888	0	0
0.252731	0	0	0	0	0	0	0.200834	0	0	1.146968
0	0	0.3223767	0.3074702	0	0	0	0	0	0.0291368	0
1.220662	0.02819836	0	0	0	0	1.192392	0.02076585	0
0	0	0	0	0	0.04407139	0	0	0	0.2646487	0	0	0
0.6166546	0.4764136	0	0	0.2900173	0	0.07088697
0.0322071	0	0.5842981	1.805123	0.02416404	0	0	0	0
0.13052463	0.0234969	0.026545064	0	0	0	0	0.3639763	0
0	0	0	0	0	0	0.2112186	0	0	0	0	1.002669
0.4714628	0	0	0.06240287	0	0	0	0	0.07337042	0	0
0	0	0	0	0	0.08333729	0	0	0	0	0	0.09647306	0
0.05943966	0.1532147	0	0	0	0	0	0	0	0	0
0.351857	0.2389922	0	0	0	0	0.3724495	0	0	0	0
0.01985111	0.2539566	0	0.04097331	0	0	0	0.02146035	0
0	0	0	0	0	0.3749321	0	0.02147187	0	0	0.02105261
0	0	0	0.1353883	0.372925	0.06334968	0	0	0	0	0
0	0	0	0.972735	0.180366162	0.02357972	0	0	0
0.1860543	1.256125992	0.1371625	0	0	0.192426	0	0	0

0	0.7690198	0	0.02518809	0.05062237	0	0.06802493	0	0
0	0	0	7.91767	0	0	0	0	0	0	0	0	0.04684238
0.07257282	0.6298592	0	0.03311427	0	2.142963	0
0.8154169	0.1933311	0	0	0.01765327	0.1624319	0	0
0.311285	0	0	0.4693298	0	0	0	0.1267189	0	0
1.261459	0.6766616	0	0	0.03452233	0	0.04960956
0.8113505	0	0	0.3084619	0	0.04433381	0.126520892	0	0
0	0.109968792	0	0	0.03732737	0	0.04773306	0	0
AC011899.2	0.04709438	0.02251647	0.2599154	0.06748111	0.1853166
0.1669292	0	0.1975467	0.1454112	0.059004115	0	0
0.2545404	0	0	0.05233473	0.2818255	0.070917607	0.1162978
0	0.055031421	0.023451	0.1497829	0	0	0	0.2609113	0
0.273022252	0	0	0.2110729	0.3182137	0	0.3642317
0.5678061	0	0.2326357	0.07285847	0.1667111	0.01901553
0.06227339	0.24508615	0.1362394	0.02424748	0.1386135
0.1917943	0.3037199	0.383621851	0.09403589	0.05436511
0.8442197	0.0252618	0.2832473	0.4132017	0	0.1037206
0.2655253	0.04722207	0.127691297	0.294071391	0.256901118
0.4455882	0.5786434	0	0.1116925	0.2570364	0.07139668
0.165607693	0.08224571	0.08044406	0.1365329	0.04658749
0.1097824	0.0916172	0.04258584	0.3107978	0.1150051
0.08910971	0.023066716	0.2865998	0.6430382	0.2774481
0.6295302	0.09921224	0	0.1938287	0.2618833	0.56466773
0.1327997	0.158840116	0.4732673	0.5028806	0.3975513
0.08131039	0.6735661	1.004292	0.1487717	0.2371277
0.2386772	0.1768904	0.1851506	0.162766	0.3671889
0.2186763	0.3907744	0.169845	0.2051891	0.187405271
0.5650622	0.02852029	0.1323489	0.578533	0.233627
0.2501839	0.09347636	0.1317341	0.09327561	0.1043718
0.02463454	0.04873579	0.2533256	0.1518947	0.4808825
0.02263463	0.2055578	0.08615004	0.1782723	0.2835455
0.08717675	0.3222102	0	0.1437562	0.1660903	0.4211271
0.1200514	0.44795	0.07606068	0.05182915	0.09269107	0.240609
0.1983544	0.03663604	0.1239613	0.1778006	0.4189432
0.09170181	0.2617118	0.0207521	0	0.2611421	0.2442829
0.1066874	0.4446391	0.1376627	0	0.1683222	0.390344	0
1.10295	0.141968295	0.1040607	0.529766632	0.08568477	0.08702583
0.09346217	0.03756279	0.1100387	0.09620566	0.2319157
0.058316	0.13554095	0.3517233	0	0	0.1678993	0.2505659
0.06781654	0.1585696	0.1362393	0.09473122	0.02378181
0.05274054	0.7897618	0.4737103	0.1745658	0.1834401
0.3823163	0.07767843	0.02035817	0.3836808	0.0248138
0.09546554	0.07199738	0.161962	0.1236428	0.5265163
0.02339116	0	0	0.09057906	0.05788469	0	0.03844513	0

0.09128542 0.5390978 0.1356078 0.05999129 0.5082339
0.3011912 0.1751936 0.4589913 0.1558243 0.09038996
0.041204 0.05039899 0.1492336 0.3453633 0.048825097
0.1318416 0.52130719 0.123580723 0.05060457 0.2874631
0.1144967 0.02431284 0.05483577 0.1149167 0.258853
0.1062644 0.3012035 0 0.1672032 0.05643581 0.6592168
0.2035132 0.09984614 0.2392979 0.2404273 0.1234514
0.7078668 0.437173 0.1167146 0.3438943 0.6558088
0.3920594 0 0.2744553 0.1679002 0.2116733 0.1062887
0.04385265 0.1707542 0.1009248 0.2301647 0.2532873
0.048816644 0.2210754 0.2500849 0.2157406 0.03298071
0.2105104 0.1216234 0.8615861 0.1074613 0.08156526
0.1537013 0.3323897 0.4382379 0.2790736 0.06399738
0.2300541 0.043042136 0.08195327 0.1645231 0.2234984
0.0188471 0.1009834 0.280769186 0.9577773 0.2819601
0.2971557 0.4773781 0.08590336 0.04567291 0.09282085
0.03958211 0.1035501 0.07663403 0.03800071 0.09761389
0.2520911 0.04013816 0.2164967 0.1596526 0.112874
0.4236275 0 0.0602856 0.2337502 0.1038957 0.2007985
0.34161 0.09935887 0.17719 0.076453881 0.195923058 0.3100658
0.06330557 0.1284862 0.1481068 0.1565631 0.5507001
0.2121785 0.1569382 0.3093716 0 0.3604286 0.1878713
0.1254721 0.084336528 0.06615315 0.6804616 0.09685885
0.0845266 0.1304943 0.030912936 0.09620273 0.1075016
0.02387897 0.2056584 0.4419336 0.2421156 0.2305731
0.1801927 0.0463977 0.1910397 0.1648861 0.1262414
0.1317874 0.286267 0.02433106 0.0789022 0.03523705 0
0.165767557 0.1270357 0.09369422 0.1996689 0.1297274
0.2656748 0.1003963 0.05611377 0.6701649 0.1001845
0.2409308 0.1696698 0.1606433 0.3461484 0.3096748
0.3132716 0 0.2060817 0.02932898 0.1355981 0.0772332
0.5061593 0 0.1302012 0.3017651 0 0.2015339
0.163301873 0.4539972 0.1170409 0.02193378 0.05847404
0.3654167 0.2607083 0.342264072 0.0561789 0.4494014 0
0.1073811 0.680003 0.258274 0.1393069 0.09278676
0.08671438 0.05828728 0.2292974 0.1236276 0.4849853
0.1865685 0.798650315 0.2529447 0.1876679 0.09083209
0.102839534 0.08883538 0.3252324 0.1396298 0.0717214 0
0.09514077 0.03738252
AC012313.5 0.1385327 0.5961095 0.3185693 0.3308371 0.3407043
1.192522 0.3745718 0.6018566 0.2851608 0.325436881
1.050403 0.4876326 0.3922051 0.3680138 0.4516973
0.6884882 0.2302825 0.495451195 0.08552534 0.01232821
0.438425733 0.931276 0.4552877 1.132561 0.6523105

0.5625966	0.4294322	1.6066	0.423869667	0.09168359	0.6524759
0.9158148	0.7243294	0.5817503	0.1738713	0.1518415	
1.507573	0.461398	0.1607402	0.332039565	0.4941016	
0.02544211	0.150196816	0.1113226	0.5290034	0.3780136	
0.570898	0.2605813	0.517211263	0.1890207	0.1599203	
0.7896631	0.2415077	0.2603749	0.742789	0.4328446	
0.3220544	1.236692	0.2141503	0.344314992	0.655333001	
0.283387347	0.5633886	0.3309708	0.07943361	0.9778399	
0.1102642	0.4258744	0.278372066	0.2661271	0.4929875	
0.6986597	0.5253262	0.4628744	0.3548428	0.6785479	
0.3809341	0.5779269	0.4412435	0.15266922	0.4817492	
0.5863834	0.7666774	0.5994687	0.4438437	0.3632754	
0.7839782	0.3994434	0.37570803	0.6380506	0.272558784	
0.8958819	0.3599562	0.4482838	0.1793868	0.9670924	
0.3468975	0.3039072	1.133493	0.5265691	0.7226948	
0.1714603	0.1329978	0.1315533	0.667082	0.975334	
0.1165769	0.4383166	0.199070014	0.3847651	0.24819	0.1251377
0.05064913	0.909952	0.2657562	0.2795526	0.1614619	
0.1326167	0.3198123	0.1570073	0.07765391	0.6520341	
0.4095787	0.1928949	0.3772978	0.8118231	0.3537303	
0.08740087	0.962396	0.2510964	0.3782936	0.4665832	
0.1644505	0.2617343	0.01290402	0.838714	0.5490368	
0.1553749	0.6416045	0.2120687	0.2516533	0.3585963	
0.3457571	0.1823221	1.89594	0.5088162	0.1273818	0.493494
0.7732279	0.3044753	0.5281201	0.4082852	0.2196819	
0.9178591	0.9997157	0.4349892	0.5621859	0.7586551	
0.2432336	0.2510575	0.530717193	0.7971474	0.607996519	
0.5513595	0.04977679	0.4398849	0.4603941	0.4639548	
0.8568559	0.2487197	1.334217	0.392061755	0.5665821	
0.1879595	0.2517523	0.1006076	0.396451	0.2826093	
0.7385425	1.041979	0.185774	0.221529	0.2456406	
1.123937	0.1847397	0.4350506	0.6430314	0.06247891	
0.3808311	0.2545136	0.262358	0.05474415	0.1404106	
0.2647341	0.2382135	0.4899944	0.2765712	0.5275232	
0.2705579	0.01348186	0.3419404	0.4635221	0.1980599	
0.1979077	0.07644953	0.5460007	0.1738825	0.392255	
0.2794112	0.3575047	0.5094403	0.4294576	0.444698	
0.3628782	0.6647271	0.3636167	0.1235446	0.2926568	
0.4293471	0.359059411	1.012654	0.292090617	0.133292391	
0.169533	1.429468	0.09355641	0.2503151	0.4839142	
0.2183167	0.1153699	0.1302446	0.4362974	0.2860911	
0.09563641	0.5464543	0.5251865	0.2328098	0.1468536	
0.3193702	0.5775796	0.2896509	0.4222359	0.4133529	
0.185969	0.2890282	0.7816283	0.5670297	1.051419	

0.4877661 0.1605158 0.4981261 0.4494465 0.3762407
0.4499682 0.2968802 0.578315 0.3152215 0.179498627
0.2113523 0.6074678 0.338465 0.12127 0.4496838 0.1311813
0.4224065 0.4039157 0.1949449 0.1632682 0.4026056
0.3819613 0.3420509 0.1882545 0.1691817 0.263776301
0.1406262 0.1382745 0.4382948 0.7392078 0.09282888
0.309716187 1.229521 0.2073533 0.2847487 0.4308507
0.2569046 1.097202 0.2047812 0.1455433 0.3249096
0.6997612 0.3586364 1.244276 1.088558 0.3246933
0.212282 0.4109295 0.1051428 0.3504772 0.1918403
0.3940799 0.2363621 0.681276 1.363459 0.5286082
0.1363944 0.04343513 0.768396435 0.168095289 0.2786938
1.206547 0.6524219 0.1307012 1.699632 0.3224876
0.2600599 0.7069 0.6888548 0.5093445 0.4064237 0.2878341
0.2614376 0.22051919 0.1729741 0.2025473 0.2196256
0.4144054 0.1492796 0.348578049 0.2043814 0.3636604
0.1931663 0.608115 0.3135942 0.2848827 0.7818752
0.3423268 0.07392848 0.1456938 0.5196733 0.4719257
0.8964761 0.06237646 0.292253 0.08381334 1.304303
1.140895 0.167619847 1.485854 0.2832665 0.07831274
0.2968045 0.5210055 0.4085335 0.1513088 0.4058672
0.417495 0.7033522 0.6321937 0.905717 0.1116479
0.6452481 0.431962 0.2022816 0.6230485 0.1581691
0.1661979 0.4165131 0.2826185 0.198296 0.1542637
0.06780822 0.2533224 0.2305455 0.137248092 0.3232702
0.1291076 0.03226017 0.1146714 0.2786801 0.8424267
0.251700754 0.3396922 0.2822931 0.4825915 0.1974199
0.4083935 0.4590084 0.08537186 0.4150982 0.6217545
0.433407 0.3091461 0.6667148 0.2937182 0.304894
0.398802196 0.5942156 0.8229537 0.489851 0.126046867
0.1016236 0.8171836 0.2481521 0.3296492 0.1203662
0.2487696 0.4810939
SOX9-AS1 0.05838603 0.00129838 0.06369748 0.1115477 0.09083112
0.05775437 0.06293707 0.1383223 0.01676985 0.068047737
0.029138 0.01333809 0.1153248 0.00381923 0.0796908
0.02313651 0.04333616 0.047027652 0.068738 0.06380019
0.034906379 0.01757949 0.1433745 0.09422042 0.003719888
0.02134543 0.1096141 0.04187252 0.060349825 0.2279947
0.02302264 0.007302727 0.1638333 0.1625061 0.1227011
0.0297652 0.5792793 0.05731694 0.1375919 0.272773248
0.09429927 0.06224235 0.103167526 0.2199693 0.0964755
0.1238907 0.1311345 0.04524338 0.218444858 0.0574779
0.01724187 0.07045892 0.1879124 0.1469974 0.1376652
0.07344366 0.01993633 0.03479802 0.2940832 0.044178802

0.049330039	0.0962899	0.1081861	0.006355549	0.03737092	
0.0165615	0.06669733	0.09880767	0.06957513	0.1934972	
0.02551279	0.03936485	0.3720664	0.05190963	0.1479232	
0.03192346	0.08433744	0.1492111	0.5282257	0.139661422	
0.04013545	0.07415973	0.001454422	0.02233904	0.04004651	
0.06047556	0.1159598	0.02013483	0.65121494	0.09495552	
0.081125097	0.01667739	0.01971856	0.07564993	0.02188035	
0.04993741	0.0868666	0.07434875	0.3179117	0.03165484	
0.234603	0.1625192	0.05005688	0.1042383	0.04203218	
0.1188127	0.03329914	0.02873469	0.068440872	0.08326892	
0.1907714	0.9179857	0.1310582	0.06586195	0.4905008	
0.05066768	0.05621228	0.1075721	0.1384243	0.1690413	
0.1194368	0.143155	0.1814321	0.06554219	0.4359345	
0.03292554	0.05961261	0.03230798	0.02892734	0.03770191	
0.04302687	0.1700446	0.3398691	0.1409239	0.02428368	
0.205947	0.06199293	0.04824522	0.1748362	0.6859284	
0.1822167	0.1872943	0.06020807	0.2559001	0.1781392	
0.1262792	0.1709739	0.04527369	0.6413988	0.01458984	
0.1003892	0.01920848	0.008612761	0.02159113	0.01389171	
0.09379708	0.03275795	0.09968103	0.3623725	0.258943	
0.008186396	0.1515128	0.244385803	0.144521	0.0635641	
0.1864719	0.2555884	0.1027926	0.1571809	0.3543867	
0.05940784	0.032826245	0.268007	0.08152031	0.1240813	
0.08821076	0.1405447	0.0273738	0.07706821	0.07541802	
0.1734356	0.2125583	0.01520603	0.01644517	0.08567234	
0.1711235	0.05077347	0.02572	0.2060439	0.03404381	0.1467197
0.1659788	0.07569213	0.03944043	0.04436169	0.1627944	
0.01387922	0.07418496	0.07332933	0.1775977	0.08461438	
0.1301756	0.02928536	0.2194714	0.1104701	0.172654	
0.04908362	0.1532647	0.1395255	0.2510172	1.043802	
0.03463641	0.1335957	0.2141517	0.1459418	0.01663178	
0.425756	0.3108679	0.01137993	0.128101995	0.06588793	
0.247640635	0.052733165	0.1283937	0.1255051	0.1606556	
0.1373926	0.03162025	0.1606928	0.08413051	0.2742094	
0.07894756	0.0832373	0.1360527	0.3026489	0.05891981	
0.0547647	0.07484728	0.2836413	0.07209224	0.2755934	
0.0353757	0.1062377	0.1278734	0.1118988	0.06128842	
0.08590863	0.02174323	0.07913034	0.05712215	0.05248509	
0.05669309	0.3160874	0.05415462	0.006789632	0.7880317	
0.135943	0.119634996	0.08286196	0.06817098	0.1306239	
0.009508925	0.1630064	0.08556151	0.1746887	0.1693738	
0.1117044	0.1240815	0.3438744	0.1291596	0.07055887	
0.02029674	0.1914057	0.107965365	0.2447921	0.1870291	
0.2100697	0.05542634	0.01455765	0.048570445	0.05668223	

0.08320691 0.02025052 0.02752732 0.1060047 0.02370296
0.1295276 0.5089853 0.1182272 0.07512283 0.1216147
0.009850336 0.01789104 0.09489486 0.02080661 0.04372915
0.1601144 0.07023011 0.1309792 0.06025555 0.04549117
0.05092344 0.06252528 0.08002493 0.09739955 0.0999035
0.135197137 0.086615081 0.1180047 0.060232 0.03916171
0.07173907 0.01547654 0.05292554 0.2044987 0.07239691
0.07135787 0.3713273 0.05542291 0.3317705 0.1971583
0.126441736 0.03687471 0.005605405 0.1256676 0.1364749
0.2044228 0.638152783 0.09985304 0.1425753 0.126679
0.04076529 0.03218964 0.09493648 0.008863776 0.1324795
0.03879412 0.2178726 0.09507917 0.1328514 3.600181
0.0605263 0.2651699 0.3412336 0.02641461 0.3252253
0.424169356 0.03244075 0.1152585 0.06217355 0.08228597
0.1336997 0.02084114 0.07118582 0.07956136 0.07702657
0.04420476 0.1878483 0.1358612 0.08614378 0.3267822
0.04967697 0.4418463 0.1208146 0.309492 0.1003447
0.04898895 0.280519 0.01903914 0.1876966 0.3117652
0.05462412 0.2918202 0.274425636 0.08227714 0.1990952
0.2453673 0.4046185 0.07609054 0.1476003 0.161589826
0.07990704 0.3401223 0.2838044 0.2724469 0.01568455
0.05026387 0.1486093 0.3156744 0.2112609 0.05825823
0.1388321 0.7413959 0.05593194 0.2187501 0.28314075
0.1580116 0.042084 0.1309425 0.100811577 0.07171589
0.3625784 0.05434795 0.2512444 0.04890631 0.09509335
0.0732907
LINC02057 0.1262139 0.06704953 0.07739758 0.9377442 0.09197261
0.01420233 0.3900154 0.2857236 0.7361091 0.19327265
0.2166787 0.02755166 0.151594 0.06574286 0.6858845 0
0.3356879 0.190060555 0.2597337 0 1.655112484 0.01396648
0.9277288 0 0.03841969 0.2645514 0.6881481 0
0.379402544 0.6364247 1.188911 0.1634185 0.1353681
0.3975142 0.3767589 0 0.08755469 0.2141204 0.1301747
0.037232414 0.2717973 0.1236251 0.029192694 0.009015413
1.155266 0.5675493 0.2447678 0.03014723 0 0 0.1942661
0.01323114 0.03008983 0.1476044 0.6562303 0 0.02059061
0.04312805 0.01406179 0.24715538 0.159215556 0.076499995
0.4329793 0 0.2508829 0.02850838 0.1530805 0.07086833
0.887663627 0.08816803 0 0.162627 0.09710973 0.09153478
0.03273812 0.05072483 0.07621711 0.0114154 0.0318421
0.04121283 0.0243839 0.06127484 0.09012924 0.2307218
0.2215756 0.02715664 0.1154366 0.01732969 0.19216767
0.1423623 0.067570568 0.07829417 0.07187892 0.2604422 0 0
0.2990581 0.6792853 0.01176864 0.02842931 0 1.715284 0

0 0.02894107 0.1163648 0.0606917 0.1396598 0.012401228 0
0.3566963 0.03378073 0 0.03091975 0.595998 0.1113415
0.1882932 0.3777482 0.352238 0.1026994 0.04353762
0.3319152 0 0.2603584 0.02696055 0.2176391 0.05130751 0
0.07793921 0.05191897 0.01009976 0 0.1712308 0.3956668
0.4075604 0.6434799 0 0.1509956 0.07716839 0.2760154
0.009553138 0.07383244 0.1745516 0.05906109 0.02647273
0.8732701 0.01820464 0.03596885 0.01235912 0.2328792
0.01296048 0.06612959 0.06353871 0.1672479 0.02049659
0.04403426 0.01253075 0.04981569 0.7879265 0 0.042275308
0.7436924 0.774428073 0.0255152 0.05182909 0.5900205
0.02237089 0.3538869 0.09549371 0 0.01157689 0.209879142
0.0149623 0.1141636 0.2330063 0.03333136 0.09496262 0
0.08094659 0.1785051 0.02820905 0.2549429 0.424037
0.03919587 0.08976636 0.0519822 0.2731238 0.01897435
0.01156554 0.1212451 0.252558 0.2660059 1.321889
0.6431812 0.2170306 0.1840916 0 0.01393084 0.05715917
0.1801507 0 0.06894759 0.01374839 0.06868913 0.2388046
0.293576 0.2703705 0.01615251 0.08336628 0.3421642
0.03587548 0.05962188 0.1692209 0.4640136 0.1211234
0.02453945 0.03001561 0 0.02938351 0.130852202 0.287905 0
0 0 0.1712015 0 0 0.2286059 0.0342199 0.05605906
0.1265737 0.0489231 0.2012041 1.836689 0.1176382
0.1963015 0.06060214 0.547072 0 0.01431889 0.03150973
0.1405257 0.055792 0 0.0731463 0.08080828 0.07004844
0.01403551 0.22475 0.03999784 0.03781924 0.07912658 0.2872856
0.2033887 0.08014238 0 0.04641469 0.014536617 0.01316636 0
0.6038863 0.01964199 0.6805883 0 0.4138113 0.1493325
0.06072124 0.04576916 0.1630242 0.3189962 0.012785
0.0571714 0.19573 0 1.298295 0.6718857 1.091475
0.08979663 0 0.016721485 0.03002177 0.009877889 0.3217709 0
0.02046424 0.02720095 0.1105607 0.3064557 0.04933624
0.06846022 0.1357902 0.1744047 0.01154887 0.1792851
0.1504262 0.0950827 0 0.1261478 0 0.1555826 0.1566138
0 2.021029 0.1017246 0.2248618 0.1993292 0.227664308
0.466735624 0.08617595 0.3393203 0.03279482 0.5292389
0.01332038 0.109325 0.1624693 0.01168326 0.1688952
0.02045407 2.046395 0.1258746 0.1681338 0.351592146
0.4596452 0 1.658451 0.4027252 0.01295287 0.05523151
0.4010614 0 0.1279922 0.1531023 0.290903 0.230711 0
0.04024333 0.0138163 0.6700118 0.08417103 0 0 0.01894324
0.072453 0 0 0.3823237 0.222130127 0.0108082 0.111601
0.02378296 0.2317813 0.1006888 0.1913344 0.01670955
0.07043353 0.07955447 0.1304443 0.9094368 0.1275636

0.01085011 0.9037064 0 0.09829013 0 0 0.01345947
0.01533233 0.06698848 0.1671438 0.2197041 0.0149766
0.290632 0 0 0.02575072 0.03485242 0.01306288 0.03482479
0.3989838 0.09880652 0 0.0334579 0.03345568 0 0.1279037
0.06074737 0.0192272 1.700795 0 0.0645545 0.08099832
0.06828016 0.2208829 0.03398091 0.2963005 0.317096039
0.07532184 0.223535 3.435093 0.142909894 0.0176356
0.08070641 0 0.03203579 0 0.03777468 0.1335812
LINC01711 0 0.02873353 0.01382004 0.02870448 0 0.03043146 0
0.0360131 0 0 0 0 0.02320157 0 0 0 0
0.011312348 0 0 0 0 0.01911397 0 0.07203195 0 0 0
0 0 0 0 0.05801088 1.041037 0.3547161 0 0.04690107
0.01349405 0.3719022 0 0.09706377 0 0.109465055 0.05795223
0.04641373 0.03316618 0 0.1776411 0.198877383 0.01200003
0.1907839 0.07087623 0.03223688 0.2936824 0.3808217
0.01846966 0.5735559 0.16942 0.09039096 0.020368545
0.153518702 0.245875889 0.4938018 0.0703251 0 0.1628938
0.8405182 0.4859207 0.045285838 0.1469366 0.01283195
0.03266835 0.1337644 0.2381608 0.2338276 0.05434428
0.1283159 0.6237273 0.01137139 0.029435706 0.1044952
0.3118237 0 0 0.5855523 0.145472 0 0.07426496
0.10293987 0.9659634 0.057913637 0.01677617 0.03850391
0.08878098 0.2766964 0.2455844 0.5679771 0.03164154
0.05043356 0.2132051 1.034605 0 0 0.03604415 1.333265
0.4420045 0.1300447 0.2805473 0.239150056 0.04006014
0.05459261 0.02412743 0.02636688 0.04968902 0.2128417
0.03578588 0.03362148 0.1785452 0.2885785 0 0.03109616
0.4849076 0.6368853 0.7252342 0.2455167 0.05829216
0.01374213 0.2274953 0.0974173 0.3615536 0.1731268
0.4618397 0 0.1211141 0 0.01914987 0.200072 0.1941239
0.04960483 0.03942805 0.204696 0.110741 0.02337584
0.04745653 0.1276276 0.145805 0.0780145 0.6037213
0.105928 0.7338125 0.02777054 1.048554 0.108916
0.2090455 0.09881603 0.04717631 0.2416477 0.1423207
0.1266223 0.4021394 0.158521475 0.1825904 0.445572734 0
0.07403645 0.1908291 0.04793431 0.2106324 0.3273844
1.276286 0.02480591 0.086482695 0.09617961 0.03057744
0.3120405 0.7737103 0.7267047 0.1514476 0.01445375
0.156471 0.2568862 0.1669154 0.03365141 0.02799512
0.2885148 0.03712758 0.210681 0.7318174 0.09912636
0.06494826 0.1803861 0.2533214 0.4416145 0.04593835
0.0258352 0.4601974 0.3743407 0.0447746 0.03061887
0.7369293 0.1502657 0.1477346 0 0.1103856 0.1137087 0
0.1810392 0.08652533 0.02551853 0.3665792 0.1729591

0.1437214 0.1534039 0.08285386 0.02883692 0.1314523
0.3215737 0.02380485 0.2675813 0.046729707 0.04206114
0.300944708 0.047310842 0.2152569 0.06550624 0.2678693
0.1551295 0.03498828 0.05499247 0.3303253 0.2203586
0.3843693 0 0.03556166 0.0360092 0.02103086 0.1947792
0.02548297 0.1866155 0.4141964 0.1912959 0.3312163
0.3984868 0.5585283 0.1567313 0.1875777 0.9756075 0
0.2188973 0.06427782 0.09454158 0.01695453 0.4197066
0.8988435 0.02146523 0.0917862 0.1989064 0.249181961
0.3103286 0.8123468 0 0.1893918 0.03837639 0.1862461
0.08866775 0.02285544 0.09107558 0 0.6861515 0.01553446
0.2328536 0.2245864 0.1887266 0.027463286 0.04183261
0.02999283 0.02852089 0.03607652 0.01610826 0.08957321
0.2733937 0.06349634 0.2930212 0.470736 0.2082823
0.02914187 0.07106987 0.3283228 0.09249907 0.7945728
0.01212329 0.09342466 0.1113564 0.06402596 0.2992968
1.196941 0.3168877 0 0.02435806 0.05128744 0.09321605
0.2983105 0.1409327 0.1634747 0.0507172 0.1507428
0.03252123 0.062504926 0.03956786 0.01009812 0.03513488
1.946709 0.01427085 0.2537726 0.01934025 0.150203
0.2138461 0.4382712 0.01533157 0.2097766 0.04002911
0.035874276 0.0562792 0.03101232 0.1545034 0.1617981
0.1942796 0.039448359 0.04092181 0.370397 0 0.1476242
0.5639566 0.03089666 0.1225988 0.05748651 0.01480216
0.2167005 0.1052065 0.09061771 0.1261315 0.1420645
0.07762286 0.06712534 0.224832 0 0.092547839 0.06947648
0.07970953 0.4076798 0.02759111 0.2003363 0.3074805
0.3938409 0 0.02130776 0.1816777 0.4113835 0.1366659
0.6044644 0.2173488 0.2248704 40.88788 0.04383055
0.09356764 0.519115 0 0 0.03160063 0.0969216 0.5134479
0 0.4000573 0.07442551 0.0551763 0.2613751 0.05597991
0.0746194 0.103625 0.2722033 0.668799747 0.2389685
0.2867432 0.3140338 0.02283838 0.02169399 0.5561772
1.866598 0.02960157 0.09682508 0.6570329 0.0548642
0.3155253 0.3640561 0.05290715 0.113240778 0.4169315
0.1330474 0 0.196852097 0.1889398 0 0.1113645 0.01144057
0.01424094 0.2225855 0.08348247

AP001363.2 0.07431518 1.776555 0.1025369 0.2839614 0.3899074
0.8655066 0.2296425 0.8906564 1.204663 0.558652775
0.2232671 0.07300133 0.401666 0.104516 0.2423108
1.431464 0.5929628 0.811335511 0.3670371 0.7936086
0.43419932 2.29436 0.6145314 1.644848 1.094323 0.02920666
0.3529023 1.645355 0.43083051 0.4215703 0.3150157
1.965137 0.03586734 0.5851445 0.6050101 0 0 0.5005914

0.2874276 0.032883871 0.150033 0.360315 0.03867472

0.1194369 0.3061011 0.0546832 0.2161803 0.1198179

0.075669655 0.1780669 0.04289421 0.315517 0.318906

0.6145779 2.535689 0.1827127 0.1091144 1.104637

0.111775 0.251871901 0.126558043 0.304043458 0.1480296

0.08696212 0 0.7553626 0.608407 0.4882121 0.037332827

0.5450934 0.1269411 0.269312 0.6983953 0.2771795

0.1156579 0.3360033 0.2019461 0.1814787 0.02812311 0

0.3230399 0.4870644 0.07960264 0.1528311 0.1565574

0.107932 0.4205612 0.1377511 0.76375592 0.5448524

0.035807216 0.2489394 0.5078709 0.09410071 0.213847

0.1898019 0.3601769 0.1956354 0.03118239 0.03766338

0.8374019 0.1947791 0 0.2228561 0.306731 0.4484684

0.214413 0.4625562 0.098575492 0.1981491 0.2475284

0.5668713 0.3912548 1.105994 0.789583 0.0885037

0.04157541 0 0.1921491 0.03887343 0.07690532 0.1199247

0.1712076 0.620865 0.2143054 0.2162475 0.1359452

0.1205634 0.826036 0.06878268 0.02676049 0.211518

0.3780799 0.03744162 0.1661352 0.568325 0.1413735

0.1200241 0.4498266 0.09751132 0.0506243 0.2347532

0.2601533 0 0.1753565 0.360597 0.2411765 0.9530367

0.03274691 0.3266685 0.06868058 0.1051308 0.03367064 0.2585

1.0047 0.2333478 0.19921 1.143935 0.730696 0.4972747

0.392046716 0.6157811 0.265991872 0.03380275 0.1831029

0.2949676 0.2963714 0.1041849 0.151813 0.1829821

0.8282066 0.042776862 0.1982218 0.2268674 0.2701026

0.1766306 0.2516145 0.1070149 0.1072387 0.3009809

0.2616011 0.1501113 0.2912869 0.207708 0.3058025

0.1836439 0.5499918 0.3016485 0.09193272 0.1285012

0.3823896 0.07831261 0.1883064 0.2840306 0.3833653

0.5528085 0.3085997 0.1845569 0.3028997 0.3037556

0.02858684 0.8829759 0.1457119 0.09099992 0.07030452

0.05761955 0.4029627 0.3423836 0.1262221 0.5230407

0.04752812 0.03949378 0.06898006 0.04098194 0.6418612

0.2275704 0.3976493 0.1177457 0.4282026 0.077046252

0.2427211 0 0.195011014 0.1597085 0.3564143 0.3312399

0.07673158 0.2595934 0.0906696 0.1114013 0.2934502

0.04320915 0 0.2638475 0.044528 0.4681114 0.749338

0.09453463 0.2936992 0.03793955 0.4731027 0.3723392

0.1971032 0.09208812 0 0.1427406 0.4949373 1.971002

0.05413649 0.0794842 0.1336086 0 0.3459985 0.3368138

0.02654332 0.09080026 0.1537264 0.077032914 0.1046574

0.8610224 0.2383078 0.1561312 0 0.2303069 0.3947189

0.1695745 0.09653279 0.282965 0.4628052 1.190988

0.2710028 0.1009882 0.5704704 0.033960355 0.4396971
0.2225299 0.2821453 1.695227 0.1195142 0.310138648
0.7556888 0.02617262 1.023083 0.239688 0.05422238
1.009009 0.1171774 0.4372254 0.1307221 0.1511611
0.2698439 1.694387 0.7956021 0.1900147 0.3416329
0.8817643 0.8905788 0.3342431 0 0.2219723 0.2305369
0.1639479 0.2534889 0.168457 0.09407328 0.2485393
0.040214872 0.051527931 0.2609522 0.02497413 0.02896456
0.3739413 0.0705878 0.482782 0.191325 0.8048602
0.1220475 0.02709771 0.03791721 0.03705773 0.09899782
0.133083472 0.1739833 0.306792 0.03821094 0.1333834
0.2059207 0.292684627 0.1518084 0.2035657 0.1884057
0.3650962 0.2569271 0 1.030896 0.2488019 0.07321581
0.133983 0.2230209 0.0498024 0 0.4015385 0.1919727 0
0.6116467 0.1808949 1.209816995 0.08591273 0.1971333
0.44111 0.2729474 0.2667867 0.03168517 0.1328217 0.06220727
0.05269721 0.1728136 0.5890277 2.53496 0.6037211 0.6352689
0.4325512 0.2976356 0.2167987 0.04628128 0.7845732
0.08124959 0.2218672 0.2084079 0.2397012 0.2380934
0.7247655 0.1766786 0.110439082 0.2046885 0.1846911
0.1730581 0.1845447 0.1281399 0.4487989 0.405070842
0.0295502 0.1772894 0.184917 0.02824132 0.2146096
0.5094472 0.9892218 0.1098135 0.06841787 0.3679105
0.09045811 0.195085 0.1350546 0.1962707 0 0.199574
0.3290455 0.1433335 0.108187541 0.2803654 0.7698277
0.1101682 0.02829419 0 0.05004421 0.4129286
MBNL1-AS1 0.2559331 10.08458 0.3815381 0.2212991 0.6742926
9.94314 0.3068003 1.776931 2.108424 1.379374642 2.31619
0.6762018 0.5110687 0.7447064 0.1582655 13.98357
0.2948718 4.290894839 0.2887665 0.6078788 1.678383129
15.02739 0.9150383 13.48329 12.03604 0.9607549
0.2339909 9.720836 0.558531247 0.1652095 1.708372
7.908848 0.3450128 0.6045515 0.7831412 0.4159443
0.17448 0.3170539 1.175895 0.218686482 0.3224901 0.192551
0.314607246 0.1489289 0.490738 0.4383375 0.5391211
1.413408 3.695546238 0.5727121 0.3336499 1.171952
0.194092 0.3549679 5.75149 0.21698 0.3790172 11.72486
0.3274207 0.224332376 0.59616494 0.37310157 3.588357
0.469886 0.2125347 11.42964 0.650261 1.16847
0.396793804 0.2527647 0.2129324 0.2718476 4.36951 0.7653059
0.5287947 0.3870493 0.7502958 0.4579683 0.1552985
0.142645834 0.3145727 0.8989489 0.22215 4.437532 1.770882
0.4934714 3.071534 1.597689 0.54419784 7.418541
0.429479965 0.2340383 1.688738 0.527083 1.295163

0.3335902	0.7014726	0.1184864	0.09627947	3.828636	
1.251355	0.196613	0.2795983	0.2672987	2.661357	
1.579425	0.3628414	5.501315	0.253636662	0.3059049	
0.4435999	0.5846093	0.3910667	5.40448	0.6954366	0.3188105
0.1530556	0.1852821	1.261544	0.1985054	0.2191889	
0.4581058	0.9677897	0.2539612	0.1929942	0.6077699	
0.581189	0.2696458	5.352347	1.82765	1.399881	0.4822802
0.4265385	0.4379674	0.2811431	4.949361	1.531975	
0.1757924	0.2646671	0.2287037	0.3261443	0.2485793	
0.6247543	0.3716768	0.5226927	0.5459882	0.1976219	
6.345492	0.2002758	1.090527	0.2976993	0.7532486	
0.2639042	1.859428	7.931082	0.2216893	1.516028	
5.974685	0.1859435	0.7307899	1.629509282	0.894586	
0.875433234	0.2830037	0.633304	0.6392755	0.3977092	
0.4495326	0.9495019	4.378593	12.22315	0.231137971	
1.711342	0.1167469	0.1237216	0.4090266	1.240037	
2.303419	0.2249869	0.8169809	0.5902611	0.4813136	
0.6152382	0.3288842	0.9885887	0.376198	3.729781	
0.1880665	1.062632	0.08774574	0.9517301	0.2464498	
0.3354342	0.2563478	0.2655709	2.160612	3.277148	
0.2169783	0.04945982	0.2602367	0.08996282	1.40473	0.5429122
0.3043879	0.1252349	0.3096274	1.504734	1.138471	
0.2079789	0.3457655	0.7393889	2.670997	0.5324611	
1.635245	0.3493603	0.7296768	0.5619506	0.14682	1.613366
0.43003158	0.2738307	0.290746046	0.210742198	0.1849199	
0.7349313	0.2610501	0.2369182	0.3596593	0.2018896	
0.2381297	1.46613	0.5182605	0.1352527	0.412553	0.3014109
0.9018006	0.5371028	0.2881452	0.6801244	0.551924	
4.208782	1.180595	1.574117	0.5085194	0.8630931	
1.144201	0.4114331	6.779075	1.581525	0.4090288	
2.356199	0.333627	0.2999494	0.8319633	0.1796723	
0.2453124	0.8233338	0.551168456	0.3832142	2.415668	
0.5356821	0.2132252	0.5353754	4.054642	0.3958311	
0.7283164	0.2693976	0.3960405	0.6760119	0.2600589	
1.894766	0.3328009	0.3664446	0.181482902	0.2242222	
0.4778789	0.5486622	13.85021	0.3027814	0.284120508	
5.245162	0.1476358	6.193765	0.341569	0.6133304	
1.283835	0.2383008	0.3263706	0.5394545	3.148623	
0.2563619	33.58258	14.88811	0.5189983	0.2434232	
1.959642	0.3045901	0.6053176	0.4095612	0.470719	
0.722762	1.187645	0.8071369	0.7021777	0.4059032	
0.339425	0.24356105	0.446700604	0.4319136	0.2787849	
0.3508473	0.1054674	0.1802267	0.5016607	0.2669688	
7.181439	1.038717	0.4489085	0.4390277	2.334614	

0.8523434 0.729631568 0.3429784 0.3096805 1.81963 0.2771985
0.2323136 0.85445984 0.2584009 0.4532699 0.2998125
2.77966 0.4816428 0.4401026 6.888136 0.7808693 0.1543314
0.5389889 0.5959079 0.1922138 0.1450912 0.2920682
0.6748108 0.2833969 7.547535 0.6573529 0.574686038
0.156439 0.6642733 0.639832 0.5712931 0.9527285
0.4590572 0.5257737 0.0849552 0.5381917 1.015861
0.5055465 15.54864 0.1792369 0.4381393 0.6567702
0.322529 0.1963123 0.1951123 0.4764467 0.5499802
0.5770165 0.4810671 0.2826233 0.4194084 1.500834
0.1049071 0.404384256 0.4071546 0.6607299 0.1726323
0.2355922 0.2929315 0.9637875 0.438949774 0.2842473
1.147441 0.04391954 0.2833953 0.1784014 3.094538
0.4372673 0.3694914 0.1624989 10.48223 0.4511779
0.3629539 0.8981488 0.3457364 0.310413293 0.4463568
4.991923 0.6383075 0.179868975 0.2025429 0.4698008
1.197095 0.1545632 0.2614083 0.2198905 12.53427
PGM5P4-AS1 0.05062236 0.3969332 0.06519015 0.04835754 0.1859193
0.4408957 0.09385737 0.1092063 0.1094131 0.177587996
0.07821573 0.01989095 0.2970609 0.01898526 0 0.202519
0.1009793 0.320167049 0.137511 0.1513663 0.165631155
0.4940726 0.2833663 0.4118394 0.6518229 0.1114127
0.1762872 0.2641863 0.019565009 0.3675741 0.1430559
0.4265437 0.03909166 0.06377463 0.04121236 0.08877703
0.1474905 0.1000253 0.007831651 0.053759973 0.01635203
0.008925129 0.031613551 0.006508688 0.02085114 0.007449871
0.04712278 0.01088241 0.11339902 0.04851859 0.03506266
0.5731343 0.1194786 0 0.3355844 0.02489221 0.7581361
0.1971969 0 0.054902809 0.367826768 0 1.986459 0.0236949
0 0.3224459 0.05525834 0.6958232 0.01017222 0.06365302
0.008647035 0.05870438 0.20031 0 0.007878441 0.3478981
0.04716432 0.0247241 0 0.009917884 0 0.3760183 0.06506891
0.2810867 0.07465116 0.03921154 0.3541932 0.02502237
0.24278745 0.2398168 0 0.04521966 0.04324423 0.02563998
0.01165355 0.03102964 0.2159054 0.01066111 0 0.06157373
0.861976 0.03538147 0.0233279 0.01214449 0.2925166
0.4964209 0.01460548 0.2142586 0.008953082 0.02699523
0.02452546 0.04877609 0.03553557 0.2790318 0.2868543
0.02411495 0.06796927 0.02406316 0.08975248 0.08473596
0.01047735 0.07624482 0.05597952 0.1503729 0.291963
0.0883827 0.0463019 0.01095012 0.2813415 0.1499319
0.01458307 0.04610649 0 0 0 0.1419492 0.009630144 0
0.07799654 0.05313858 0.0551752 0.04264273 0.01575222
0.02131961 0.04778005 0.1473799 0 0.05193552 0 0.5538315

0.0280705 0.2196147 0 0.2012414 0.4883188 0.09537176
0.108559 0.4915148 0.1422111 0.06774718 0.106822485
0.380311 0.269195955 0 0.01247269 0.07233386 0.06460276
0.01892512 0.1378836 0.04985783 0.5181929 0.011655577
0.3348637 0 0.0105137 0.06416963 0.07835244 0.0583175
0.009739915 0.0351469 0.01018278 0.05112675 0.09070637
0.132055 0.03703253 0 0.3785896 0 0.2254434 0.05251983
0.05209559 0.03200721 0 0.04643454 0.0174095 0.06202242
0.1681707 0 0.1031653 0 0.007789168 0.1327381 0 0
0.01915614 0 0.07319788 0.2215651 0 0.01900199 0.07770103
0.1398933 0.08457868 0.05583253 0.0582968 0.02657442
0.03250471 0.03208263 0.4773028 0.031489635 0.01889576
0.064041122 0.021254162 0 0.5561959 0.00820493 0 0 0 0
0.0114225 0 0 0 0.04853086 0.1133761 0.6708608
0.04293036 0.03429658 0.7546402 0.3639759 0.2231959
0.14769 0 0.1056161 0.03889309 0.06742875 0.6586418
0.1917601 0.02887649 0.2093279 0 0.01885511 0.3670918
0.05062653 0.03711105 0.05026371 0.094452553 0.02851642
0.2052803 0.05565656 0 0.1422333 0.0627526 0 0.09240926
0 0.1872442 0.2437978 0.09421351 0.1476824 0 0.04239226
0.018506619 0.01409482 0.1111616 0.04804826 0.3889727
0.01085483 0.156937257 0.249254 0.06418219 0.1045363
0.1119578 0.00738709 0.7069593 0.007981947 0.03403786
0.06233213 0.2635997 0.02450847 0.2833013 0.2834826
0.1466931 0.04654302 0.8065796 0.2038339 0.02276813
0.02462116 0.04320118 0.03768917 0.1675182 0.008633637 0
0.01708834 0 0.010957502 0.014040015 0.09776617 0.006804798
0.02367626 0 0 0.3420183 0.02606553 0.3036508 0.1662738
0.04430051 0.04132579 0.04038905 0.1078973 0.036261768
0.01896237 0.0835928 0.2290526 0 0.01870267 0 0.1654553
0 0 0.1326389 0.06000507 0.06246077 0.247846 0.04842287
0.02992409 0.009126714 0.08102317 0.02035478 0.1133279 0
0.03138452 0 0.560576 0.02957347 0.017818568 0.1404538
0.05371367 0.06868055 0.09296378 0.1142309 0.02590014
0.01206348 0.06779943 0.04307584 0.1318442 0.04377127
0.4604736 0.007833249 0.3328734 2.491874 0 0 0.1387148
0.009717077 0.0110692 0.06045302 0.02129464 0.027991
0.1189361 0.01234248 0.1251648 0.030091764 0.1115446
0.1383896 0.1037384 0.1508508 0.02618608 0.142667
0.211544837 0.05636159 0.2053036 0.08061608 0.01539004
0.05847552 0.3747898 0.05989713 0 0.02796312 0.100246
0.07394242 0.7441779 0.04906512 0.01782621 0.241646087
0.2175148 0.3675908 0.03905464 0.044217417 0 0.8157252 0
0 0.009596508 0.09545017 0.1928782

AC116407.1 1.308393 0.1650785 0.6268284 8.479929 0.6197309
0.230044 3.930779 4.355806 0.3647102 1.866950725
0.07799289 0.3213154 2.862368 0.1192663 1.866429
0.2221362 1.504333 0.129982349 1.144323 0.6015841
0.435312651 0.1447832 0.4854878 0.076094 0.2302538
0.2499644 1.423858 0.129322 0.842800397 2.164799
0.6419175 0.07330129 0.2104935 0.5723364 0.2219127
0.6572915 0.1607268 1.640274 0.2530226 0.14473839
0.2568108 0.120146 0.283711359 0.08761696 0.2245507
0.1872019 0.126869 0.3515857 0.148026693 0.1523981
0.3566202 0.08572522 0.4483931 0.1092956 0.1948718
0.08935666 0.2134521 0.1304001 0.4373136 0.270994633
0.144419164 0.334561892 0.3348254 0.77616 0.175052 0.06156906
0.2851472 0.3122284 0.219093962 1.161532 0.1629634
0.2436608 0.3415542 0.09319489 0.2828158 1.28173 0.1975258
0.3328244 0.1925528 0.720953896 0.3949613 0.2282766
0.06812771 0.2802859 0.3636852 0.2903162 0.2617718
0.2357878 0.24901277 0.4714348 0.19262894 0.2536359
0.108665 0.5369056 0.4706239 0.2227769 0.1849539
0.2391915 0.2363738 0.1013072 0.2388961 0.2063919
0.209353 0.228877 0.2437639 0.08224725 0.4718694
0.2488373 0.192835609 0.3876238 0.5447482 0.372074
0.3826908 0.3806281 0.5470459 0.2957688 0.7624758
0.08638057 0.7450606 0.418248 0.7898307 0.5767237
0.2595632 0.2361625 0.3056878 0.3348974 0.5484994
0.1867137 0.168324 0.117735 0.07198054 0.4241206
0.6656477 0.1556438 0.3656221 0.4053329 0.1728487
0.2054447 0.2599885 0.4172741 0.2228229 0.2009129
0.1837759 0.1626304 0.1372145 0.124916 0.2594873
0.2175077 0.2482335 0.3195182 0.747347 0.1371062
0.1729016 0.2618718 0.1327984 0.2567701 0.3491055
0.4626202 0.3828761 0.3647925 0.068475952 0.6324176
0.687591247 2.521042 0.1455147 0.1442556 0.3768494
0.4246019 0.1856125 0.1230466 0.7200695 0.1882824
0.3877661 0.4900326 0.1887074 0.1439703 0.2812652
0.4186897 0.2971923 0.1471964 0.2467366 0.1743553
0.1221047 0.2116266 0.09970297 0.3031157 0.191115
0.06146788 0.5769895 0.117833 0.2805147 0.296819
0.11972 0.6389711 0.5937087 0.2385478 0.2089695 0.3700602
0.3795955 0.5942136 0.5662126 0.1935764 0.178153
0.4227882 0.1203399 0.1408958 0.8320784 0.07325703
0.7870527 0.2728491 0.3835243 0.1255453 0.6240991
0.270573 0.1918313 0.20669 0.184749 0.3095151 0.09518859
0.301439246 0.2882815 0.086209203 0.228890976 0.2603545

0.2059985 0.1914484 0.3846417 0.1904334 0.1884555
0.1816047 0.7278183 0.1267901 0.3081263 0.2867469
0.3919801 0.1271847 0.1963221 0.1849308 0.1025966
0.2504865 0.197348 0.4552364 0.4940215 0.4278441
0.2464376 0.3054105 0.2874366 0.1545925 0.1720924
0.1360527 0.1796907 0.1127863 0.4737951 0.6094665
0.3894348 0.1221175 0.1653977 0.480335631 0.07677497
0.4561785 0.3662868 0.267249 0.2436865 0.1501772
0.3968034 0.262616 0.196708 0.7710057 0.1433471
0.4133584 0.1408189 0.1975554 0.3297176 0.365387089
0.5881877 0.2085899 0.2673454 0.3345332 0.1753473
0.18417686 0.2139638 0.4863949 0.1980531 0.1674582
0.1922538 0.1586127 0.05730628 0.8247635 0.1758086
0.1478524 0.6231855 0.197746 0.104756 0.4491508
0.5151556 0.2618193 0.1916387 0.06129881 0.2356897
0.09304869 0.07892176 0.2004491 0.2556885 0.238916
0.199364 0.5165856 0.393346213 0.289800303 0.1994065
0.1221374 0.3541321 0.1828781 0.1208248 0.1770805
0.3391861 0.2346589 0.1890121 0.9740434 0.1390772
0.2265411 0.2904927 0.119322913 0.5360515 0.5813986
0.10278 0.3913915 0.1594523 0.906547464 0.4578303 0.13274
0.221138 0.2678286 0.1795023 0.4017242 1.631123
0.121678 0.3491144 0.2293379 0.2544958 0.1887601
0.2542614 0.1350076 0.4882036 0.2740112 0.1495646
0.3361771 0.383784551 0.1260483 2.036643 0.3081819
0.1001148 0.3168643 0.1472102 0.1190882 0.5476107
0.05154374 0.1521279 0.8249201 0.227285 0.4288202
0.4301749 0.09066069 0.5367539 0.4771197 0.4639995
0.1220864 0.1788101 0.2821141 0.3376202 0.7536038
0.1067375 0.3876804 0.3283415 0.126025335 0.2919703
0.4177495 0.2200511 0.06768946 0.2193364 0.2103423
0.198102188 0.1589686 0.3143034 0.5064344 0.4695936
0.2755096 0.3986368 0.05375384 0.2685245 0.225856
0.08996436 0.1327172 1.606025 0.110082 0.3439544
0.136965394 0.2277398 0.3701196 4.556374 0.238093781
1.039783 0.1987023 0.9563417 0.2144802 0.2842043
0.1101348 0.2235821
LINC01943 0.1772984 0.01883751 0.2083875 0.3010954 0.2583963
0.1596053 0.334811 0.03541491 0.3649575 1.431539844
0.06763967 0.01935154 2.205565 0.0369408 1.798521
0.1094594 0.6287416 0.318900852 0.4013458 0.2103735
0.207179309 0.05885804 0.1002479 0.2828265 0.0539699
0.0387112 0.6392516 0.09346252 0.114206596 0.4693578
0.05567057 0.309025 1.388151 0.4032935 1.964642

1.058027 0.04099736 0.3804042 0.655257 0.470718682
0.7238407 0.2778589 0.727897557 0.3609343 0.2738568
0.181196 0.3552978 0.9528568 0.682001626 0.3146855
0.7163479 0.9479059 0.4543871 1.125596 1.017861
0.351149 1.229294 0.6361315 0.2666686 0.614260068
0.81634943 1.477616853 0.6082258 0.9681979 0.3388719
0.4939132 0.9542366 0.7765054 0.316683611 0.516057
1.110455 1.363559 0.4189873 0.6061775 0.797138
1.175716 0.5353283 0.4490007 0.6113107 0.318414515
1.55852 0.6240479 0.9601157 0.3747469 1.971297 1.230279
0.172294 0.8885488 2.56449388 0.5443978 0.522057078
1.319801 2.793548 1.213974 2.959092 2.032669
2.482413 0.3630197 0.347171 0.5491194 0.9125946
0.02581648 1.883708 1.642306 0.9452253 0.5869805
0.7815171 0.2697565 0.609720049 0.9454735 0.3877309
0.363809 1.071729 0.9989908 0.9767632 0.4379383
0.6281973 0.4448014 0.2692321 0.3812759 1.365891
0.7417717 0.9803058 1.298368 0.3124499 1.987229
1.306341 1.51275 0.6386612 0.8022625 0.4610969 0.3700632
0.4610268 0.24813 1.034938 0.3138631 0.8619468 0.1696884
1.203262 0.3618827 0.99306 0.6637811 0.4520887 0.3733462
1.059843 0.6292927 0.2685153 0.6231675 0.04340354
0.548434 0.6190099 0.8174782 0.3570229 1.693532
0.403095 0.4329986 0.4224603 0.6764576 1.162178
1.680704 2.256670261 1.164404 0.211531163 0.5645174
1.395462 0.4613293 1.272729 0.4602973 1.167056
0.388046 0.5285341 0.532956203 0.4308738 0.3608345
0.4091432 0.97546 0.6669917 0.5815441 0.2653219 0.433121
0.9114106 0.4277663 0.2647396 1.147088 1.87347 1.849885
0.2915889 0.7329888 0.2518229 0.7494016 0.9376323
0.4878477 0.8485916 0.5872789 2.743856 0.7758059
0.8746851 0.1272003 0.3713602 0.6211618 0.3334292
0.4842692 0.4248859 0.2733905 0.4286431 0.3054813
0.5340964 0.6807051 0.2760413 0.6377905 0.4661623
0.9317587 0.3017117 0.1955464 0.6711378 0.7756129
0.4321841 0.9129683 0.9493562 0.255297327 0.27575
0.581507862 0.248133336 0.9102304 0.37792 0.4310509 0.5898708
0.06881424 0.01201756 0.4035873 0.5445242 1.431761
0.08993111 0.2098258 0.2832886 0.8961996 0.7377986
1.804298 0.6339636 0.3117731 0.3098422 1.628574
0.6139263 0.7323347 0.6987133 1.229747 2.722407
0.246454 0.3587689 1.706674 1.071383 0.1667291
0.8621579 1.571402 0.5417897 0.7341278 0.4401056
0.500296138 1.266935 0.4374675 0.6587913 0.717392

0.9560535 0.295079 1.057966 1.018903 0.2729533
0.8679695 0.9487455 0.42774 1.400855 1.057432 0.3711837
0.567149448 0.2742518 0.717703 0.5796412 0.1892121
0.4752209 0.645959479 0.8856322 0.3260842 0.7910111
1.633825 0.6611822 1.528417 0.1320133 0.3145906
1.611338 0.4968743 0.8186383 0.2347862 0.3812459
1.015795 1.101834 1.101928 3.031253 5.803481
1.006046 0.218554 0.440005 1.499373 0.5207693
1.473627 0.2410615 0.4529523 0.202546632 0.46441513
0.6139233 0.2184686 1.1901 0.854963 1.319178 1.574131
0.5578907 0.2215622 0.5823528 0.7398687 0.6131277
0.6483469 0.5248562 0.435100247 0.2398257 2.033147
0.5267151 0.8839477 0.6277441 0.219827651 0.7243576
0.6295586 0.6292877 0.5161677 0.9243154 1.225466
0.6269243 0.1319072 0.07763358 0.3640476 0.9557644
0.2904409 0.2342911 2.767485 0.8549356 0.286045
0.4864146 0.1246764 0.225359589 0.1746019 0.6532128
1.586928 0.8320731 0.8789619 0.2939739 1.959968
1.335706 0.09778458 0.6779918 0.8658798 0.3583889
0.7392197 0.6995081 1.064725 0.5720152 0.8620503
0.7851816 0.8697278 0.0753831 1.117459 0.1795487
0.6626434 0.5785535 0.4562952 0.5713845 0.09758572
0.6420744 0.5752657 0.6514258 2.666139 0.4076151
0.2478536 0.680061308 0.9321634 0.7401984 0.1372523
0.4042625 1.237352 0.5942053 1.981275 0.3008021
0.3173893 0.6095467 0.4196337 0.6550447 0.6324827
0.9625246 1.026985092 0.3879628 0.2965648 0.1519821
0.415843568 0.4459235 2.040696 0.3504471 0.2175103
0.2800879 0.7428936 0.3674765
AC103563.7 0.1215784 1.366015 0.08387436 0.3484171 0.5980144
0.9234485 0.2817685 0.692122 0.6569362 0.266567755
4.565766 9.076601 0.3754965 6.525983 0.8919368
1.53121 0.3031491 0.572125567 0.9757583 0.3245826
1.172065653 1.422711 5.142819 1.054559 0.7494251
9.054614 1.058461 0.8652127 0.998511297 2.138014
1.116614 0.3541873 0.08801756 1.531658 0.04948932 0 0
0 0.07053401 0.026898731 0.07363546 0 0.253084777 0
1.189344 0.0670956 0.1414669 0.03267001 0 0.07282863
0.03508711 0.6022107 0.2608625 0 0.5926208 0.03736436 0
0.6231612 0 0.041205821 0 0.082901668 0.1210869 0.03556714
0 0.02059601 0.124418 0.06143898 0.152689699 0.02123247 0
0 0.270607 0.08502394 0 0.05496958 0.0235986 0
0.09201789 0 0 0.06640244 0 0.1562682 0.1280627 0
0.1876448 0.2629182 1.04124325 0.1028503 0 0.1018152 0

0.3078944 0.104955 0.09315382 0.05892434 2.016354 0
0.03080833 0.608878 0.1062184 0 0 0.06272584 0.1146386
0.043847 0.4162039 0 0 0 0 0 0.5026076 0.7535177
0 0 0 0 0.09539443 0 0.1307966 0.2520835 0
0.05843333 0.795999 0 0 0.2815377 0 0.02188986 0
2.10301 0.2756424 0.6115373 0 0 0 0.1338016 0
0.06211538 0.1920261 0 0.06400344 0.02868802 0 0 0 0
0 0 0.02866538 0 0.06041452 0.1554825 0.6680679
0.08147606 0.03598958 0 0 0.137438971 0.2350618
0.341910054 0 0 0.241281 0 0.1420373 0.08278785 0
0.1003653 0 0.09728628 1.237171 0.06312618 0 0.02940265
0.1750745 0 0 0 0 4.322903 0 0 0.1126644
0.3788536 0 0 0 0.1042638 0.03202953 0.03080661
0.7899377 0.1045299 0.05319915 0.1165071 0.06038639
0.06194236 0 0 0.02490575 0 0 0 0.1178308 0
0.1050251 0 0.02852286 0.2332656 0.1292223 0.08463766 0
0.1166749 0 0.03252738 0.02407875 0.06368475 0.031511595 0
0.064085781 0 0 0.9806477 0.02463195 0 5.520975 0 0
0 0 0.03964387 0 0.03642353 0 0.3064761 0.2319856 0
0.124137 0.02276435 0.1218281 0.2418431 0.07532731
0.0951208 0.08757048 0.2530341 0.2129404 0.04428319 0
0.1639361 0 0.2264191 0.1102043 0 0 0 0.063012279
0.0285363 0.05869242 0.4177153 0 0.5046335 0 0.1076256
0 0 0.03306614 0.05047611 0.0314264 0.08312926 0 0
0.055558573 0.04231395 0 0 0.8514712 0 0 0.585613 0
0 0.02800897 0 0.2358174 0.07188761 0 0.1069296
0.07418911 0.09810224 0.5039981 0 0 0.09315095 0.1545591
0 0 0.02463833 0 0.0754309 0.100581 0.07775692
0.08267783 0.05130077 0.02541288 0.098686286 0.042149417 0 0
0.02369277 0 0 0.07898233 0.07825113 0.3291844 0
0.06649711 0.3411755 0 0.04048969 0.036287056 0 0
0.1562811 0.218213 0 0 0 0.05550502 0 0.01659143 0
0.03125216 0.04960377 0.3488878 0.02994496 0.1095969
0.09121463 0 0.04252762 0.08211378 0.09421922 0.03394885
0.9551599 6.125978 0.026746491 0.0468506 0 0.1030927 0
0 0.0259182 0 0 0 0.1413601 0.4380179 0.1382384 0
0.3997267 0.1516385 0 0 0 0.08751468 0 0.03629711
0.4048804 0 0.06491947 2.556675 0 0.030112748 0.08371676
0 0.05662403 0 0.05240867 0.03059281 0.027612047 0.07251544
0.03625532 0 0.02310116 0 0.2500341 0.3596334 0 0
0.07523693 0.07399398 0.5319263 0.1841225 0.6154331
0.114543757 0.05441662 0.1076626 4.044974 0 0 1.469329
0 0 0 0.04093574 0.3860253

AL161729.4 0.06104461 0.02918627 0.05615118 0.05831351 0.04003514

0.1545548 0 0.03658053 0 0.15296445 0.2095977 0
0.1885371 0.02861748 0 0.1130621 0.1217691 0.045962357
0.1507474 0.1955678 0.035666373 0.3951691 0 0.2556183
0.08361933 0.263903 0.09662801 0.04826934 0.058982748 0
0.0862543 0.2188772 0.02946246 0.769047 0.198789
2.274911 0 0.2741334 0.188881 0.027011751 0.4436691
0.4305062 0.127074079 0.1569742 0.1885802 0.1796734
0.2841226 0.2952655 0.621572169 0.02437821 0.1057036
0.3743635 2.816054 0.04589381 0.4760886 0.5628203
1.613335 0.03128898 1.377228 0.041378955 0.27722238 0
0.06079785 0.3571658 0.4620333 0.2688731 0.04164691
0.1233943 0.061332501 0.3837903 0 0.3760749 0.1509689
0.2276832 0.2375118 0.1104011 0.2606753 0.2732982
1.108854 0.80728661 0.2122834 0.03334072 0.09808182
0.3766194 0.06430037 0.1182113 0.2198388 0.1131526
0.10456182 0.2409924 0.117652283 0 0.02607373 0.4637821
0.1756601 0.2494539 0.02958596 0.2892609 0.3073693
0.1237511 0.1146443 0.3999927 0.2109803 1.098362
1.07082 0.2072164 0.04403123 0.2279741 0.107963634 0.4476047
0.6099807 0.8332581 0 0.7066071 0.2161953 0.1211658
0.2390586 0.07254335 0.3156735 0.8621572 0.7896529
0.6238941 1.631364 0.1133325 7.510893 0.3848691 0
0.1320456 0.0848162 0.1130001 0.08792733 0.3127444 0
0.2460449 0 0.5057416 0.1161282 0.5586838 0.4366823
1.281577 0.4574267 0.2571106 0.2611857 1.285447
1.325194 0.2468372 1.109412 0.2609505 8.63466 0.6857447
0.2256648 0.1727149 0.05531605 0.1820051 0.2230511
0.4791963 0.272728 1.228787 0.6002146 0.2042378 0
0.2023281 0.780333298 0.1388327 0.1128045 0.09691791
0.04868958 0.1141073 0.290975 3.306747 0.2267708
0.070276274 0.4233452 0.3416515 0.03169571 0.3869051 0
0.4395253 0.3229928 0.3885111 0.9823388 0.9864456 0
0.2274897 0.1395527 0.03771258 0.142667 0.7846452
0.2768926 0.2638864 0.1832284 0.06432821 0.1237442
0.04666217 1.417082 0.2671134 0.1949943 0.7580014
0.404317 1.283212 1.526333 0.3251352 0.05984594
1.071416 0.8085019 0.2129865 0.1471134 0.5976428
0.1814442 0.2577843 0.3904096 0.03244132 0.3966353
0.6059472 0.08787385 0.1068188 0.0653281 0.3385189
0.1598808 0.063287993 0.1139303 0.193065148 0 0.6778106
0.3726149 0.7173281 0.7248394 0 0 0.1830164 0.4132258
0.3904256 0.2786731 0.1204066 0.256036 0.1281733 0
0.02588448 0.4135764 0.4363049 0.8458201 0.24468 0.2023827
0.1512876 0.1910409 0 0.3049168 0.3360265 0.1334078

0.1305812　0.2194999　0.1722167　0.5684261　0.1660011
0.1526241　0.03729296　0.3283156　0　0.1146248　0.4125732
0.3076117　0.04275021　1.247394　0.1891806　0.2161556
0.04643112　0.4493372　0.06641016　0.07603229　0.1577922　0
0.08295458　0.3408005　0.195272044　0.1912128　1.096755
0.1158811　0.1221498　0.1636207　0.072787642　0　0.3869808
0.03501625　0.4500264　0　0.2368083　0.2406322　0.1539212　0
0.2235025　0.566458　0.09489669　0.226222　0.2601391　0
0.2586809　0.1463094　0.2059177　0.1731929　0.0781433
0.1893696　0.8080288　0.7287805　0.1937255　0.3348561
0.8931881　0.297302802　0.126979545　0.05358841　0.02051446
0.1189616　0.07679153　0.898734　0.07931419　0.1178699
0.1017131　0.4678488　0.267106　0.1868777　0.2130819
0.5692374　0.473713788　0.4001617　0.6930212　0　0.8765196
0.05638306　0.560978868　0.4156659　0　0.7119042　0.2332559　0
0　0.3486853　0.3795498　2.70637　0.08254307　0.5495873
0.1227273　3.843567　0.7833586　0.1576918　0.4431894　0
0.5052135　0.859483888　0.3528559　0.7286891　0.2070516
1.289189　0.2817594　0　0.1454714　0　0.3462959　0.3122989
0.3518867　0.2776385　0.4486839　0.08028124　0.4060684
0.5195336　0.5342538　1.482654　0.4101178　0.06674074
0.3644961　0.2567883　0.2812821　2.379517　0.03720894
1.219082　0.241914179　0.5604567　0.1517105　0　0.4547708
0.3684021　0.430099　0.3050087　0.0970935　1.055822
0.4556883　0.4871627　0.04407162　0.1255423　0.4514306
0.3307477　0.4215029　0.07555306　0.4086768　0.9080742
0.1479169　0.1612223　0.038341678　0.1912584　0.1621724　0
0.31103918　0.9212005　0.7026205　0.3619814　0.3253831
0.08679194　0.2055387　0.2180516
AC126696.3　0.09057742　0　0　0.05191502　0　0.2751923　0
0.06513341　0.1398359　0.04539344　0.03109991　0　0.1398748
0.01698494　0.5906703　0　0　0.027279404　0.1118388　0　0　0
0.0460928　0.4117941　0　0.01423916　0.08602544　0
0.035007217　0　0　0.0324768　0.0349729　0.1711657　0.01474806
0.1985583　0.05655029　0.01627026　0.2662468　0.160319123
0.04388747　0.1596953　0.037710263　0.454188　0.1305796
0.1332988　0.5902099　0.1557732　0.202902237　0.1157509
0.08364906　0.2221908　0.7579486　0.1361936　0.07064158
0.06680859　0.3989751　0.1671346　0.1998106　0　0
0.296461327　0.05412677　0.06359511　0.1246474　0　0　0.01830912
0　0.2910596　0.263023　0.1575576　0.250887　0.2702673
0.1409671　0.3603866　0.182845　0.176953　0　0.088729202
0.1889904　0.05936477　0.1552351　0.03725495　0　0.0701603
0.1491176　0.1567018　0.31029568　0　0　0.1213657　0.2476029

0.9175406 0.2085142 0.09253435 0.07023899 0.1335297

0.6080954 0.4039656 0.1134054 0.1266145 0.2504404

0.1086493 0.07477046 0.01366514 0.104533 0.04510212

0.192234501 0.2415097 0.02194142 0.3054587 0.09537447

0.1198237 0.3207886 0.02876554 0.1621544 0.1435188

0.05353067 0.01895201 0.01874687 0.1559121 0.1168567 0

0.0696537 1.054274 0.1822632 0.3330776 0.01677993 0

0.4174904 0.6806026 0.1843258 0.03650791 0.1619922 0

0.5341608 0.01950517 0.1794309 0.2139291 1.209365

0.3051986 0.183203 0.07629338 0.08549174 0.04395057

0.3057108 0.06195134 0 0.01769568 0.1506777 0 0.01641549

0.108023 0 0 0.04856054 0.02145015 0 1.030357 0

0.6804816 0.037051237 0.09887937 0.1562197 0.07190294

0.05779611 0.1523802 0.07401358 0.2899307 0.01495469 0

0.1352951 0.05530247 0.07524767 0.2152823 0.1752427

0.05217308 0.05228221 0.02096251 0 0.2012558 0 0.06750942

0.1325229 0.2238303 0 0.1960841 0.05976009 0.9867115

0.01553557 0.4390681 0.03672209 0.0553895 0 0.1585361

0.02314646 0.2159453 0.1107548 0 0.2508656 0 0.03551954

0.3549224 1.096821 0.2247306 0.02182854 0.125192

0.0769215 0.1189994 0.1853715 0.2310534 0.01681496

0.4994993 0.6780092 0.3645423 0.09693322 0.1578629

0.03795675 0.169031023 0.2028585 0.305566119 0 0.5969488

0.3317295 0.02936178 0.1122271 0.04218662 0 0.1991425

0.08175206 0.2317239 0.07088442 0.0285853 0.1085439

0.1014306 0.02609469 0.5223372 0.1227322 0.3699343

0.4884402 0.2904431 0 0.3142708 0.1511812 0.1217834

0.5429191 0.01813065 0.1847526 0.1808381 0.2768381

0.04088536 0.5060552 0.4269388 0.1035256 0 0.2098499

0.169001762 0.3231505 0.01749063 0.33195 0.02537294 0.1619512

0.2807044 0.6842231 0.08267287 0.09412549 0.01970775

0.06016853 0.05619135 0.3303058 0.09846978 0.2781221

0.099340391 0.2143661 0.01808171 0.08597163 0.02899916

1.534362 0 0.09695311 0.0765598 0.06234813 0.1836299

0.01321755 0.3162365 0.04284574 0.1827094 0.01593278

0.01473915 0.0730874 0.01877424 0 0.1543968 0.05551889 0

0.1215718 0.3259079 0.1909009 0.03091954 0.1123939

0.01998242 0.1390317 0.04927681 0.07643943 0.04543899

0.039212007 0.075364418 0 0.1948107 0.07060564 0.02278851

0.2408963 0 0.1165961 0 0.05950196 0.1981647 0.07394328

0.0180668 0.09652904 0.216274471 0.05089338 0.1495707 0 0

0.06692852 0 0.02467044 0.03308154 0.05511218 0 0.0894714

0.2421458 0 0.01732838 0.05354248 0.1143115 0.1087297

0.1578215 0.1013875 0.1468219 0.05615559 0.06070156 0

0.03527675	0.095647054	0.04188513	0.2162444	0.491554	0
0.2601336	0.04634252	0.02158491	0.6672157	0.1027661
0.1853545	0.2871694	0.1647829	0.07007925	0.5241304
0.03012603	0.7436715	0.02642403	0.4061442	0.226025	0
0.2812347	0.114306	11.30221	0.3095412	0.2208411
0.6374087	0.017947498	0.03326402	0.04502133	0.3712333
0.7647558	0.1874166	0.05470087	0.098742331	0.2881328
1.253295	0.2524278	0.1376852	0	0	0.05358627	0.1427667
0.1167455	0.02989464	0.1764046	1.268134	0.1536344
0.06379204	0.364102517	0.01621642	0	0.2096387	0.052744799
0.06834343	0.9382875	0.6445255	0.05517719	0.06868323
0.3171755	0.07189842
SNHG32	8.09924	27.39963	10.78171	16.2752	21.78667	15.19562
14.18396	18.43716	16.91255	40.397817862	18.91087
12.09256	42.26822	13.39501	21.33176	20.03292
21.20879	25.595290472	14.52247	16.33344	20.106803731
23.61198	14.17286	28.60654	22.896	20.67105	27.27517
21.57926	18.62885449	40.23377	18.35143	32.29406
7.436449	20.9355	41.71633	74.80303	89.78331	25.19184
15.0012	14.292561043	17.5435	15.64499	23.089945853
21.20551	13.63159	31.57308	15.75074	17.84468
40.273251843	27.42185	29.66	34.45513	38.0533	24.97489
39.10139	20.88479	44.03769	21.55885	349.3249
42.553373012	24.764994077	33.642242668	26.52143
40.84605	24.30074	18.71131	23.37925	51.10427
9.280244199	13.581	23.05321	33.6016	17.81966	31.97608
16.2082	40.93778	12.0693	42.98464	106.2248	20.783226629
33.2428	26.76166	19.19151	20.42221	31.80508	26.44716
18.50991	26.39701	14.14934521	32.4474	17.638762062
19.05091	21.21808	26.42204	33.31568	15.52653
27.36043	9.904646	11.40702	15.495	37.12786	23.37053
28.25254	60.79735	34.78589	27.38949	13.81353
20.62662	19.503351768	38.58759	16.7372	78.21337
17.63963	37.84003	23.25594	17.95256	29.41602
35.15695	62.04939	53.81859	33.81829	88.69546
86.56765	23.4536	39.00433	42.33116	37.73355	28.72216
34.14066	35.71933	13.79815	29.47203	38.70976
39.56207	16.42907	29.92068	21.1394	30.71303	28.24094
68.6327	34.48856	30.65562	16.95019	33.55788	22.39287
29.74223	60.68503	18.61462	32.71039	35.23949
45.20168	16.84416	15.51171	13.14829	26.14516
52.99055	25.30776	27.2994	30.18603	59.32355
31.151714259	29.65179	30.292564632	23.61572	90.89282
16.25923	33.94721	22.1965	29.65198	151.9707	15.057

17.988775072 22.85981 12.55959 19.03277 49.2948
21.20266 20.29411 12.3393 22.28211 61.04546 50.86391
13.41567 18.34564 29.73544 16.01752 27.37496
21.85129 20.01862 21.11865 33.36608 10.68563
77.53608 15.30488 25.5375 33.61849 21.44435 35.98871
10.55448 37.05213 52.34351 25.05923 16.37865
19.55208 38.02069 12.70944 16.38864 56.19502
25.96436 26.78336 17.7993 23.51036 20.3819 44.15702
23.48806 22.29618 15.2891 97.64344 26.98476
46.826859746 21.79321 23.072553679 29.619108686
72.57633 63.94246 40.36265 39.38081 15.44213
29.12324 26.98284 35.02148 21.38817 13.40478
5.594514 22.14733 21.47733 44.27288 49.11303
24.19334 41.55434 41.83977 12.34357 18.4752 22.99135
42.26152 19.01963 36.06348 33.9581 23.71779 50.9344
27.45479 66.87278 25.23926 40.27605 14.15294
46.5224 48.93999 22.844942702 25.28881 27.57987
33.98383 14.72231 25.42682 68.79998 15.47255
21.78215 21.4115 25.38026 29.72073 34.66446 27.52749
27.61416 50.76726 44.27395133 22.51708 36.29812
15.35183 24.36144 23.17043 22.315615941 32.1042
18.83054 23.22007 39.39174 19.54113 22.70375
17.76307 38.24014 12.7639 23.88445 41.0931 15.24923
25.99534 10.95254 29.7047 30.80695 36.07838 14.24083
32.32986 11.08382 13.94391 23.76746 23.3408 13.59757
23.0717 45.02144 83.998193756 18.827379072 28.87895
34.06053 17.33671 57.03125 15.36304 50.95996
25.88791 20.26943 19.81954 31.03848 9.919424
16.26758 32.86216 40.334119993 19.9512 30.35524
36.16077 47.34593 28.26147 75.25526842 11.97468
24.85201 74.88552 40.72356 18.35935 15.07125
36.78368 36.32914 60.57687 24.59383 17.05935
21.31081 21.71652 53.88483 23.90626 125.4414
18.53017 18.51309 40.71834748 76.54074 85.7671 20.87692
61.87945 17.64072 50.14719 25.28901 24.7147 30.07747
18.03871 8.660886 21.48176 45.56202 32.89873
59.17518 35.70098 28.00507 48.60092 60.23563
24.0778 21.3383 26.70062 42.21892 72.1685 16.49201
68.94324 44.955942132 42.94213 40.63803 18.28966
24.8603 16.63599 27.70401 20.761530042 17.84121
67.03002 26.36775 21.94982 22.18949 41.84066
38.6571 75.55113 32.76711 28.41516 42.92017 20.01898
21.32582 26.7453 29.691488811 26.4372 15.99459 13.17845
30.205168476 15.71403 43.714 30.75817 17.07674

29.95821 34.10423 22.04047
AP002449.1 0.06130218 0.2344753 0.2537465 0.3513573 0.04020406
0.3724965 0 0.1102046 0.09463999 0.537634544 0.2104821
0.3613104 0.09466632 0.2873823 0 0.04541565 0
0.115390726 0.07569173 0 0.214901182 0.2442071 0.2339647
0.2200259 0.06297911 0.6023103 0 0.4120205 0.059231621
0.2086506 0 0.3022261 0.2071074 0 1.297581 0.4031488
0.09568213 0.4404641 0.8061313 0.488263049 0.86633 0.5133832
0.574246156 0.4335006 0.4103144 0.9021574 0.96296 0.4941856
0.592985094 0.7589133 0.176916 1.33026 2.038745 0.6913118
0.1792865 1.13039 0.7200631 0.408473 0.6454181
0.332428399 0.417588143 0.125401891 0.1221088 0.1434691
0.9701458 0.2907774 0.08364527 0.1548936 0.95466496
0.6851727 0.3141391 0.9330467 0.7277084 0.6859316
0.3816224 0.2771673 0.2141797 0.5738529 0.7655539
0.720615896 0.1598843 0.4352582 0.2626551 0.3782085
0.2260009 0.6825827 0.3153806 0.2272602 0.84002409
0.0691455 0.206760235 0.7529455 0.104735 1.707709
0.1764012 0.501013 0.08913239 0.2904814 0.9517207
0.4660248 0.153504 0.3481231 0.847482 0.4411987
0.5692966 0.5549086 0.1326511 0.152624 0.18973356
0.2451782 1.373607 1.304383 0.1075813 0.6420087
0.5427689 0.3406957 0.102886 0.169982 0.2037892
1.18646 0.9198623 0.3297508 0.3389477 0.05690535 0.5598012
0.8027163 0.4485618 0 0.1419568 0.08510768 0.1765967
1.884384 0.6861274 0.3706246 0.137044 0.117202
0.6122455 0.8910654 2.968476 0.7641473 0.4593568
1.065056 0.5961085 3.420808 7.57969 0.2478787 1.193671
0.3668722 0.5132422 0.4491118 0.3965796 0.1445364
0.3332967 0.2741596 0.1119961 0.3849746 0.438206
0.3266396 0.9471741 0.3076494 0.508195578 0.8804543
0.626900675 1.812441 0.1888008 0.510966 0.1955801
0.7734743 0.5844054 0.6037636 0.3795461 0.811587179
0.228917 0.561425 1.050372 0.2185524 0.1186031
1.191726 0.7666587 1.631538 1.356415 0.3714779
0.3432586 0.5996802 0.7567645 0.6438189 0.1671472
0.2073568 0.7836263 0.3179998 0.4468607 0.5490969
0.3417324 0.1405772 0.1581179 1.743563 0.3720525
2.344495 0.4372556 0.2505663 3.089128 0.8790575
0.5709354 0.2502178 0.5219443 1.021896 0.9233382
1.482759 0.5466295 0.4026898 0.9017309 0.4560948
0.3983089 0.4394751 0.7353719 0.6167995 0.1968112
0.7284584 0.353222 0.444885216 0.4576442 0.355446243
0.193036219 0.6587135 0.5612807 0.7948742 0.5380114

0.3568952 0.261775 0.1837886 0.1383231 0.2851439
0.2798489 0.2901951 0.2938472 0.6435708 0 0.259937
0.7614227 0.8762916 0.4132163 0.2457124 0.6910047
0.6077039 1.119108 0.8536582 0.6124067 0.4294747
0.401912 0.786793 0.6061716 0.830128 0.5137421
0.666806 0.4816997 0.7864567 1.293442 0.190632086
0.8345364 0.6510649 0.449323 0.214653 0.6263286
1.171536 0.4703132 1.212303 0.5308625 0.2334163
0.6108248 0.3169161 0.2514923 0.1666092 0.7700366
0.756370195 0.3627038 0.3977214 0.6400352 0.1226652
0.4272087 0.584758101 0.6233635 1.532865 0.07032799
0.5931519 0.872191 0.1783556 0.9424254 1.700277
0.2695799 0.1995072 3.833542 0.1905942 0.3281439
0.8620812 0.1409055 0.3117268 2.615296 0.06892883
0.6460024 0.4185228 0.4183709 1.183347 0.5750294
0.5002532 0.1552011 0.2562734 0.630287515 0.467556186
1.022476 0.3502174 0.5017469 0.4241355 0.1746825
0.1194733 1.025849 0.178749 0.5033816 0.737641
0.312777 0.4585307 0.8982903 0.365932754 1.291661
0.1265355 0.5988799 0.1100272 0.4812782 0.321911924
0.04174197 0.4198006 0.4973273 0.4517488 0.2119377
0.315159 0.05002236 0.8209413 1.691069 0.3315654
0.8278593 0.4108173 0.3002055 0.8280672 0.6651002
0.8558835 0.3210734 0.6864082 1.726220805 0.5905745
0.1219606 0.5717945 1.182052 0.3143869 0.3920538
1.205203 0.5131444 1.043271 0.4276591 1.325143
0.4182149 0.9485833 1.6124 0.1529182 0.6137949 1.520106
0.3817718 0.3824305 0.6032011 0.2562239 0.4297864
0.1412345 0.6546732 0.635221 1.399115 1.093207113
0.4502572 0.342789 0.6281205 0.7611494 0.5285094
0.4010628 0.361985776 0.6337707 0.402174 0.1830444
0.6988832 0.3540606 0.126072 0 0.2717536 0.1693126
0.7840091 1.044658 4.398604 0.4827583 0.4317402
0.423538029 0.329255 0.1899995 0.118235 0.312351582
0.4625437 0.5644681 0.6361403 0.8869093 0.4938962
0.3302495 0.6082546
RHOQ-AS1 0.03825837 0.06402149 0.1935534 0.02741004 0.2760026
0.5036918 0 0.2292605 0.04429824 0.179750852 0.1970412
0.01879099 0.2067826 0.01793538 0.1247446 0.6164748
0.2671065 0.302461959 0.0118097 0.0817119 0.111765592
0.1428828 0.2433603 0.2860771 0.2161772 0.1879494
0.03027974 0.1890734 0.036966146 0 0.027029 0.1971913
0.1846495 0.3916115 0.5061333 0.3354708 0.08957208
0.1374457 0.3033411 0.110038655 0.8882469 0.1939262

0.228967581	0.1721653	0.2757731	0.1196442	0.6232368	
0.1336481	0.39929567	0.2291777	0.01104124	0.8392324	
0.430964	0.1006703	0.4102699	0.1175784	0.2527805	
0.4902421	0.08631474	0.155600126	1.216201411	0.182612891	
0.3810372	0.1119229	0.07897343	0.4083135	0.09135451	
0.2803381	0.019219401	0.4877457	0.114364	0.07625483	
0.6812385	0.7759064	1.101529	0.01729787	0.4678396	
0.3892811	0.3329969	0.290452325	0.2827184	0.5223892	
0.4712749	0.3048824	0.3324655	0.2129982	0.1771446	
0.437315	0.55702058	0.2804968	0.221207978	0.3844713	0
0.7266631	0.02201822	0.4885619	0.2225083	0.201431	
0.2006635	0.4071811	1.880096	0.1587682	0.176303	
0.2868227	0.4441188	0.7647803	0.1241803	0.03571945	
0.042289903	0.2295216	0.2838229	0.913896	0.03357047	
1.265287	0.440361	0.113907	0.2033336	0.2727897	
0.2261045	0.1200751	0.2474488	0.730575	0.3878151	
0.2486003	0.2390419	1.567854	0.1049794	0.1034459	
0.3986752	0.1062305	0.1653196	0.2286733	0.5060646	
0.520436	0.1282927	0.3047716	0.2911232	0.4222311	
0.1157882	0.8534006	0.6710969	0.4330596	0.2678603	
0.3625317	0.2527719	0.2784597	0.4842266	0.4661033	
0.2528777	0.1121153	0.2563427	0.1984497	0.07800328	
0.3326975	0.4752942	0.4504887	0.2820284	0.8946928	
0.7523427	0.3840048	0.086498786	0.929901	0.459713405	
0.2871342	0.04713183	0.1670382	0.1525756	0.4022677	
0.8336557	0.2590539	0.228977	0.110110291	0.2551176	
0.04866423	0.2483074	0.1591303	0.703186	0.289236	
0.2484352	0.8743534	0.09619677	0.2414973	0.07497906	
0.481188	0.8921086	0.4727106	0.3576538	0.2329386	
0.2918566	0.165385	0.4019203	0.7760888	0.07755397	
0.2485782	0.03289353	0.292963	0.7515818	0.7220924	
0.09746033	0.1340375	0.206036	0.2037714	0.1406518	
0.1795836	0.2714523	0.3262958	0.06915007	0.2313456	
0.1299614	0.5385356	0.6117017	0.3761399	0.4350194	
0.2637251	0.4314053	0.200839	0.08188591	0.1666966	
0.3206458	0.069412637	0.1606575	0.584830217	0.070275858	
0.2603631	1.000835	0.04650721	0.3555213	0.02227365	
0.01166946	0.05735075	0.08632673	1.290187	0.02495032	
0.1282857	0.1146178	0.7229686	0.1928842	0.1297803	
0.5939999	0.1757858	0.5086087	0.1916848	0.3805169	
0.09481621	0.6684965	0.3674232	0.1910999	0.6892275	
0.3762468	0.1022986	0.2923293	0.4101455	0.7303097	
0.381471	0.03416207	0.1051765	0.1741085	0.138801242	
0.2963342	0.618724	0.3680515	0.01339638	0.5863327	

0.09880401 0.88056 0.1309486 0.289895 0.0936475 0.5241673
0.6823606 0.2092734 0.1559699 0.6808158 0.131124085
0.1997308 0.6682734 0.4085209 0.3062189 0.1845822
0.114045153 0.6961731 0.2425318 0.05486417 0.220347
0.1046788 0.3339324 0.2563787 0.2894002 0.740271
0.4591357 0.7177481 0.2180729 0.2599295 0.1548847
0.2491589 0.7295516 0.5593468 0.2365997 0.1007917
0.1224365 0.2254982 0.2004557 0.1141868 7.440896
0.09686018 0.2958869 0.16562491 1.114143102 0.4030243
0.1671409 0.08201224 0.08422296 0.1726125 0.2236879
0.3816739 0.1912394 0.4817087 0.3417809 0.2049626
0.7154166 0.4969117 0.342565093 0.1253963 0.1974254
0.1868789 0.5150057 0.1590158 0.175790613 0.1563057
0.3231276 0.02909807 0.2871551 0.1133736 0.1278479
0.1873122 0.2470236 0.1319234 0.2241723 0.4783914
0.1474236 0.1739741 0.2971557 0.03953196 0.4593711
0.3721348 0.1676284 0.227248332 0.1916587 0.07611499
0.5190604 0.2810334 0.3139316 0.1060275 1.435944
0.1921504 0.230598 0.6138695 0.3721566 0.6525144
0.8954089 0.4528306 0.1590591 0.1053432 0.3906372
0.2382618 0.2386729 0.07319952 0.388348 0.234699
0.237988 0.3779348 0.1982191 0.100052 0.312704734
0.0702508 0.2852437 0.2316404 0.3800235 0.131936
0.2214203 0.156401612 0.3042561 0.376492 0.3807902
0.370744 0.1381046 0.1573618 0.1697545 0.01884445
0.1585006 0.4419448 0.3958361 0.9038895 0.4171666
0.4462713 0.648804799 0.4537823 0.3133839 0.09223733
0.041772214 0.1683915 1.387109 0.2552219 0.1019634
0.04532913 0.1030535 0.1746199
AC068790.3 0.1050895 0.02512236 0.4833266 0.1756787 0.1033819
0.6385655 0 0 0.12168 0.065832801 0.6539979 0.1032315
0.04057128 0.2216949 0.5996425 0.4087408 0.1048139
0.257156476 0 0.1122246 0.030700169 0.05233009 0 0.3457549
0.1799403 0.2684583 0.08317348 0.1869673 0.152309882
0.05961447 0 0.02355008 0.7608028 0.6619645 0.4063851
0.1727781 0 0.09438517 0.2438717 0.139503728 0.4243249
0.4632029 0.16407033 0.3884616 0.9198256 0.7346139
0.9171047 0.2823917 0.374516902 0.1678702 0.03032846
0.4957491 0.7328207 0.7900706 0.4610225 0.226078
0.4628977 0.565578 0.4214976 0.178086642 0.477243592
0.143316446 0.5494895 0.1229736 0.433854 0.3204486
0.07169595 0.2920851 0.105585065 0.348704 0.04487702
0.1523342 0.8056771 0.3184682 0.4293252 0.237572
0.3671652 1.390078 0.8351497 0.154417692 0.09136247

0.3156818	0.590974	0.8104468	0.193715	0.3052544	
0.567685	0.1623287	0.81002323	0.7408447	0.151905479	
0.6453818	0.02244321	0.7762316	0.2116815	0.3220798	
0.1782648	0.4426383	0.7055227	0.4527098	0.7894494	
0.275438	0.06053443	0.2205993	0.5692966	0.7530903	
0.1137009	0.1635258	0.209094127	0.3502545	0.3182101	
0.5484737	0.09221256	0.2606652	0.7443688	0.2920249	0
0.666052	0.1746765	0.7695955	0.3806327	1.215367	
0.5084216	0.04877601	0.6061005	0.433212	0.09612039	
0.08524464	0.5597154	0.3890637	0.3405793	0.3888412	
0.5346447	0.07941956	0.05873314	0.5357807	0.64973	0.3394535
0.02891373	0.9307659	0.5548076	0.4979484	0.4905121	
0.5532304	1.215065	0.1487272	0.7162028	0.2920003	
0.5093833	0.384953	0.04856077	0.1734437	0.1904553	
0.8094235	0.7295748	0.6599565	0.5164571	0.4044109	
0.3690289	0.2636995	0.395996554	0.6675973	0.859749497	
0.7409098	0.1294634	0.2711248	0.2514601	0.4174306	
0.8229382	0.3234448	0.1735068	0.393191304	0.9250114	
0.05346905	0.2455414	0.3746613	0.3558093	0.4161581	
0.4043914	1.36806	0.3963551	0.2918755	0.1176886	0.8077325
0.3363398	0.3246146	0.470741	0.4621095	0.9316847	
0.249857	0.3379619	0.5813968	0.0798855	0.1204947	0
0.9656655	0.1007059	1.070029	0.321249	0.06136318	
0.3233984	0.6673661	0.2060519	0.450392	0.09941796	
0.6111006	0.1899439	0.1815624	0.5577852	0.8135978	
0.7393073	0.3909384	0.7072015	0.3477165	0.6807443	
0.3218084	0.02811589	0.645206	0.4128568	0.408568055	
0.2206499	0.498547977	0.193036219	0.5269708	1.924391	
0.6387382	0.434026	0.1223641	0.09616224	0.1312776	
0.1482033	0.2749602	0.8566803	0.2072822	0.5037381	
0.3309793	0	0.08912126	0.8009771	0.5096798	1.259326
0.6055056	0.1045217	0.1953334	0.5481343	0.3784692	0
0.3418268	0.6124373	0.337197	0.614044	1.334134	
0.9296285	0.571548	0.2627453	0.7704065	1.021711	
0.245098396	0.4193237	0.456591	0.3129214	0.1103929	
0.7717264	0.1085593	0.5581739	0.5595244	0.2502637	
0.05716317	0.6108248	1.032241	0.1197582	0.1071059	
0.6233629	0.336164531	0.219451	0.3933508	0.6982203	
0.4836515	0.3380113	0.156631634	0.7592849	0.4256245	
0.03014057	0.04842057	0.4025497	0.3567112	0.7456553	
0.5299565	0.2541753	0.4488911	1.123563	0.2722774	
0.2596303	0.403051	0.3220698	0.5789212	0.4533739	
0.2363274	0.7027938	0.156946	0.3586037	0.927358	
0.4480748	0.7146474	0.2882306	0.1757303	0.710850581	

0.546494243 0.6457742 0.1765802 0.04095893 0 0.4491835
0.1365409 0.4396496 0.3502021 0.8629398 0.4598281
0.3217134 0.7336492 0.6299698 1.191895256 0.1968246
0.2711475 0 0.8487816 0.3154597 0.172452817 0.3220095
0.3358405 0.1598552 0.2581422 0.1557094 0.2971499
0.3001342 0.5528788 0.9059298 0.2368324 1.340344
0.193671 0.5881577 0.354886 0.2171756 0.3814795
0.7863023 0.9720563 1.01723726 0.8099308 0.4181507
0.4455542 0.3377292 0.4850541 0.08961231 0.1878239
0.4838219 0.2980775 0.9530688 0.7193633 0.4182149
0.56915 0.4491684 0.04369091 0.3682769 0.7664401 0.556296
0.3025824 0.1723432 0.7216101 0.5710019 0.6295022
1.150354 0.2882516 0.5246683 0.312344889 0.2653302
0.1305863 0.1468334 0.3914482 0.09060161 0.9255294
0.286406328 0.3969772 0.7834558 0.5491333 0.8586279
0.1138052 0.1440823 0.07771463 0.1552878 0.7740003
0.4769087 0.4477104 0.9655472 0.4456231 0.7169972
0.066005927 0.9642467 0.5118353 0.05067215 0.076494265
0.5616602 0.8467022 0.3894736 0.3200876 0.09960932
0.03538388 0.3545255
SNHG11 1.828344 1.634721 1.691282 2.289256 2.012032
1.595326 1.611947 1.200851 1.921633 2.588378183
1.454145 1.552492 3.070682 1.326842 2.778649
0.9680653 2.709569 2.064923815 2.047067 1.908356
1.973530836 1.795156 1.668943 1.81668 1.574142 1.920216
2.69789 1.384453 1.397301938 2.285292 1.912006 1.597228
4.7013 2.309875 2.522847 2.819176 1.757381 5.148991
5.812975 2.129981738 2.811149 2.617969 1.591204185
1.998813 2.71239 3.564788 3.155095 3.969296
1.614496697 2.710219 3.54154 2.290262 2.521106 3.952839
3.061319 3.053942 1.478747 1.286574 6.846395
4.180142349 1.741534054 2.317327079 1.147097 2.629035
1.890599 1.420912 2.931658 3.001512 4.358902797
2.34155 1.914428 3.881874 2.948021 4.103153 3.162985
2.904966 1.37142 6.15492 3.510321 2.585366432 1.939755
2.922415 1.54904 2.97935 2.361079 1.940295 1.477367
2.227441 5.52032893 2.417635 1.433408826 2.433671
2.408979 3.121704 2.425495 2.933678 2.312943
3.496998 1.967761 1.476303 2.030477 2.689331
5.866847 7.905189 2.797912 1.535024 1.408186
1.639501 1.749256508 2.079439 5.201536 3.802833
1.912485 2.362893 2.963252 2.599779 3.918079
1.939509 3.701 7.483585 4.297239 10.27936 9.994922
1.639675 2.34828 2.194224 1.681748 2.150611 1.650494

4.101506 1.532492 3.175251 3.794267 1.889158 2.4247
3.548226 2.466145 3.442271 1.199339 3.80185 3.117237
3.279326 2.639729 9.070423 5.48905 3.015679 4.780371
1.823667 4.770223 2.623464 2.252957 1.388228
1.249596 1.98133 1.498415 2.497477 3.027407 2.807033
2.669707 4.561917 1.876133976 2.099858 3.031721082
3.307743 2.258763 1.71379 5.38829 2.838364 3.509905
7.763744 2.451601 1.831330245 3.499147 3.731524
4.633951 3.723601 5.081192 3.279893 3.507858
2.62369 3.210236 7.068051 1.365029 3.719534 2.644805
2.431355 1.536997 1.6981 2.163812 3.340072 3.007472
2.089969 3.292704 2.04504 4.946793 4.104107 1.531007
4.70988 2.347204 4.011021 8.996004 4.820383 2.850692
4.814962 3.67433 3.087459 4.617781 1.564537 2.02201
4.076123 4.875443 1.734694 3.077766 3.987471
2.58234 1.875309 3.714296 7.176649 2.532267
3.218125819 7.166837 1.981943344 2.412438062 2.20141 2.990457
2.624367 10.67083 2.261191 4.618944 3.927901
3.437917 2.786768 2.741755 1.462891 2.939909
3.831683 2.469988 3.784343 1.883711 9.334101
7.771296 2.649816 1.541073 1.625603 2.726233
1.785653 2.820597 1.922919 1.678039 3.771087
3.389256 3.642692 2.414265 3.22096 3.220895 4.953746
4.487193 2.173598834 2.104484 3.849706 2.7777 1.331047
2.902309 3.019806 1.999522 3.119155 3.099516
2.286842 3.370828 1.708846 1.732761 3.29872 3.784485
3.403411931 2.660379 4.175699 5.956156 1.099618
1.179465 1.730498368 1.691678 3.714325 1.747949
1.956127 2.031161 3.796761 4.185854 6.806604
1.589879 1.294279 5.251068 1.306041 1.548214
2.614745 2.342649 1.663356 4.382104 1.823514
1.557444 2.887015 1.666291 2.364291 2.60734 1.723351
2.297009 7.578604 2.973756098 1.812050316 4.221121
2.940227 1.928455 4.950801 3.507622 1.751454
1.981231 1.665195 6.570838 4.693078 0.9750235
1.684355 4.788637 2.552761461 3.317942 4.051116
1.582745 5.710308 4.078702 4.732723248 2.243739
2.258894 4.404808 2.858774 1.193809 2.58623 2.494965
5.567838 6.452101 5.14003 4.536239 1.204499 4.408177
8.804477 2.695056 7.332107 1.638516 2.423875
4.676709102 2.23707 5.528159 2.79413 3.319663 3.390417
1.541456 2.535433 3.908289 4.980851 2.195489
1.719341 2.854061 3.396589 2.975058 3.512932
1.835821 3.95515 6.954055 3.390579 1.123702 9.424396

2.799051	4.317045	3.408722	1.416665	5.859511
2.737551761 3.954717	6.283124	3.290675	1.821239
2.101713	3.649343	4.002264562 6.325402	11.02765
5.47988 2.736054	3.273865	2.209331	3.941649	5.622167
4.174852	2.190439	2.181494	6.544074	3.572985
2.787222	3.198565543 3.689458	1.948375	2.022185
2.849662903 5.494783	3.37063 3.062622	1.891687	2.69741
7.456854	2.127754
AC066613.1	0.3911108	0.1759957	1.005208	0.08790888	0.2263269
0.3028931	0.03554641	0.1378649	0.0355181	0.187360257
0.2369798	0.02259978	0.2842238	0.04314148	0.1500293
0.1363548	0.114731	0.147239663 0	0.09827425	0.08065175
0.08019389	0.04390312	0.4816877	0.1654511	0.1808362
0.1456688	0.2092053	0.066688333 0.02610198	0.1625378
0.1340472	0.9216175	0.3260686	0.496343	0	0.1077276
0.09298396	0.4093169	0.366487229 0.5480779	0.6591381
0.215512809 0.6877411	0.947629	1.108839	1.632974
0.2225596	0.761339782 0.2113164	0.06639603	1.172135
0.1974541	0.363226	0.5831426	0.2403981	0.1351187
0.4127271	0.1384134	0.124759352 0.6138182	0.203939417
0.3666163	0.1076871	0.4749036	0.2104609	0.06278362
0.1976458	0.127132611 0.2973218	0.4617572	0.4668927
0.5120751	0.3754158	0.6624003	0.1664321	0.4286986
0.7865512	0.6790964	0.157759537 0.2400164	0.4146604
0.9857365	0.8634713	0.04846711	0.178206	0.5444622
0.2843001	0.59110959	0.3243762	0.454493655 0.7706676
0.1965336	0.8545308	0.1191651	0.4935751	0.4348645
0.2664854	0.868811	0.5480127	0.144024	0.6029978
0.1325239	0.1931773	0.3679618	0.7462489	0.09956707
0.2004777	0.132240493 0.3680585	0.7523666	1.773395
0.06056241	0.393119	0.5296187	0.5571132	0.2316767
0.5741435	0.3994033	1.636685	0.3928382	0.5073933
0.8586408	0.2349204	0.2543215	0.5244102	0.3051233
0.06220676	0.1598278	0.2022908	0.4639333	0.4452763
0.3745476	0.5679683	0.02571609	0.6451216	0.4923726
0.7802995	0.2911747	0.4679077	0.6895807	0.5450623
0.2505638	1.041589	0.3582936	0.2883872	1.343943
0.5015704	0.3446853	0.3820473	0.2232527	0.2169763
0.4482221	0.7774005	0.3782862	0.2889596	0.6372722
0.4086227	0.7432586	0.03848662	0.034677133 0.6481547
0.811694912 0.2406874	0.05668507	0.3378698	0.2018517
0.5053066	0.6423105	0.3965336	0.3133735	0.357557669
0.3681935	0.1053505	0.6450566	0.2369526	0.4228581
0.1656485	0.5533166	0.3061556	0.1041256	0.3485363

0.09017671	0.6644574	0.6311364	0.8954274	0.4211859	
0.202333	0.2751188	0.4674321	0.2268956	0.9940059	
0.244843	0.1231024	0.09890196	1.197971	0.4335883	
0.7770384	0.2930369	0.1612059	0.4513462	0.1696668	
0.2819346	0.6573417	0.4135331	0.58865	0.1108883	0.4637292
0.3712208	0.8420003	0.7356887	0.195624	0.6833559	
1.192597	0.2207862	0.402579	0.1477253	0.6379058	
0.2651262	0.107334031	0.2146906	0.545718589	0.012074316	
0.6097924	1.133481	0.4008592	0.8195334	0.1071533	
0.1403476	0.09196705	0.02595612	0.454808	0.2100529	
0.5808498	0.4824748	0.2576321	0.0662801	0.2243732	
1.194995	1.36246	0.5083138	0.7838285	0.335606	0.2565781
0.6719962	0.3203753	0.3447515	0.3453868	0.536307	
0.5331462	0.5583952	0.8567473	0.5355717	0.4170837	
0.4601683	0.1967698	0.9137409	0.071543633	0.3563988	
0.3776205	0.274023	0.06444687	0.4260448	0.4040247	
0.841803	0.3499796	0.5379238	0.1126291	1.165307	
0.6303694	0.36705	0.09379188	1.011473	0.252323021	1.441288
0.7233544	0.5349981	0.1473148	0.147997	0.233174029	
0.381702	0.7697402	0.05278776	0.5300193	0.6966266	
0.8032356	0.4806547	0.4834157	0.7082075	0.2246233	
1.076717	0.3576471	0.2368296	0.3921654	0.2996614	
0.05849506	0.2536493	0.4397692	1.025719	0.1963379	
0.2854788	0.2537752	0.7847518	0.594521	0.3494789	
0.9329324	0.373492039	1.036882321	0.716973	0.603057	
0.1524366	0.07235306	0.3496416	0.0896759	0.8144189	
0.2012518	0.5793471	0.3439457	0.4695369	0.8604258	
0.5976317	0.412000298	0.4739841	0.3324187	0.1064642	
0.5781004	0.3293699	0.211421965	0.3603096	0.451643	
0.2333068	0.1758192	0.1363536	0.4494571	0.1689591	
0.2750864	0.4419905	0.248871	1.311815	0.1464695	
0.7403778	0.09323132	0.309041	0.7966019	0.4475635	
0.5152138	0.455516253	0.4166847	0.09154289	0.3121349	
0.6971179	0.4483561	0.1667548	0.3426583	0.4621956	
0.4567926	0.6740963	0.3481254	0.209273	1.326101	
0.2571796	0.1530393	0.4491929	0.7550618	0.05731111	
0.4416154	0.5785254	0.7830174	0.580671	1.282718	
0.8845085	0.1682799	0.1859671	0.421673844	0.1372964	
0.157236	0.1071508	0.8855367	0.5454569	1.296767	
0.365755772	0.512297	0.3018693	0.7098587	0.5945211	
0.08304866	0.03154297	0.476379	0.2266407	0.1376754	
0.8162681	0.6720973	0.7851283	0.7525844	0.9418039	
0.534658457	1.67847	0.4380239	0.06655986	0.184210061	0.1591253
1.549103	0.3581122	1.778143	0.3925222	0.1239416	

0.2465382
TRIM31-AS1 0.2646378 0 1.92305 0.6067154 1.180198 0.08040225
0.4906569 0.1268658 0.4085551 0.795749489 0.09086385
0.1559755 0.8173376 0.1240612 0.4314368 0.01960566
0.7918322 0.398507863 0.784216 0.3391267 0.896791637 0
0.3030035 0.1583064 0.05437541 0.0416022 0.4188974 0
0.306839216 0.1801464 0.6730663 0.09488668 1.04734 2.917196
5.407677 0.6961477 0.4956648 0.9031921 2.702133
0.819700869 5.278594 8.3984 1.322125941 1.309977 2.425322
2.920919 6.066199 1.365356 1.212575214 7.376696
0.8248346 2.047381 1.334366 6.287033 0.6191754
0.4229183 2.875334 0.2712851 1.565597 1.578580757
1.712567765 2.778942442 0.2899249 0.1238695 2.002986
0.1793241 0.7944015 0.4546932 1.728258266 1.922602
0.7458659 1.822148 4.686031 2.196168 2.285824
1.052933 3.944964 0.9693737 1.942851 3.551560036
0.4141266 2.514947 3.600026 2.394632 0.7247554
1.716755 1.443168 2.223755 2.35711677 0.8954909
2.065665486 4.107349 0.9720893 2.010567 0.5482897
3.055016 0.6156467 1.699854 0.02220822 1.877681
0.9608682 2.982532 1.036583 2.698223 1.583799
0.9382403 1.06894 0.4282647 1.521126977 2.504926 2.900784
5.822182 0.9752864 0.7001717 5.435994 2.269177
0.6218136 2.243338 0.9579456 3.211554 3.450654
8.199443 1.316895 0.5895767 0.2289437 0.6673869
1.597542 0.6010601 1.568816 0.3429111 5.546149
1.114765 2.746555 1.199973 1.893154 2.259929
1.082383 0.8833107 1.66009 7.430931 4.470799 2.647209
1.544019 1.253838 1.67352 3.959287 1.889436 0.7466322
2.868665 0.6462629 1.002749 0.9234533 1.606684
1.472838 0.07735689 4.73645 1.442428 1.410083 2.453336
4.515553 0.598322662 1.315685 1.461397967 1.853733
0.2282116 0.9663546 1.519753 4.303654 8.613707
0.03258014 0.02184637 0.700714614 3.52936 1.534971 1.209171
2.390144 1.331206 2.172168 8.172214 0.9799306
1.596972 4.22294 0.3556384 2.292923 0.7743779 1.471409
1.133887 3.222529 4.27769 4.095479 2.746089 1.394364
0.6437403 0.9305235 3.549446 2.663349 0.3719455
3.706668 0.8898714 2.317885 0.183237 12.79403
0.8821009 4.62315 2.653771 4.001095 1.179855 0.8534644
2.674402 8.5181 5.991401 0.9563394 1.179069 0.58375
1.396805 0.9956132 0.7363393 3.480147 0.8317298
1.399252564 2.000318 0.725371666 2.055536857 0.8530879
1.292275 0.4503744 3.934695 0.7395337 1.35608 1.137213

0.5971326 1.32327 2.450699 8.706646 2.029633 2.889387
0.4193212 0.8752633 7.350963 0.4863726 7.591187
1.485016 1.50906 0.9181974 1.104256 3.151474 1.057489
0.1589157 3.277279 2.641752 6.518227 8.988895
6.653381 1.391305 2.684405 0 7.160289 0.548631504
1.565286 1.04759 1.309298 0.5189206 2.467239 1.01149
2.530083 2.214147 5.843839 0.2591085 1.757929
6.649003 1.737084 1.941953 4.321462 0.943281348
2.836807 5.626278 2.93882 0.4024492 0.02837283
2.082601599 0.8781232 1.733543 0.1214407 0.9510805
2.799761 4.260392 7.197927 1.690423 0.5818801
0.5598192 4.441584 0.2742612 0.305109 1.75928 0
1.031708 3.678788 1.071222 0.7508182 1.716401
1.904596 1.693083 0.857545 1.967602 1.831316
3.872108 0.544182664 5.578156661 1.719124 2.561281
1.113949 0.8322579 1.407642 0.2063036 5.007645
0.1763768 1.535635 1.736919 1.134201 3.27269 5.852051
4.833911691 3.915608 1.474865 0.5442797 1.804928
1.759891 0 2.342569 0.1691437 3.435091 1.112321
0.9410631 2.067994 0.2159435 1.746663 7.169882
2.719561 6.221092 2.908497 0.4073046 2.252071 1.0391
6.118585 0.4752188 1.77791 2.25888491 0.04079159 1.965588
2.378643 5.102848 1.872921 1.196014 1.828859
1.905084 3.002493 3.667741 4.004393 0.3009015
8.04663 2.505827 0.6161294 0.02649716 2.586282 2.439167
1.473137 1.909587 7.932339 6.66075 1.438894 2.684875
3.258392 0.9059846 4.430907153 0.6317134 0.6576886
1.109274 4.468737 2.760667 3.063182 0.192328718
2.04144 5.839814 0.05267945 2.011358 0.7260177 0.6168115
2.661549 1.042795 0.1949099 3.624712 1.063006
1.574657 2.180225 2.516125 5.352190944 2.984885
1.42952 0.8166616 2.041867404 1.431021 0.5178147 2.157712
3.425709 0.6772617 0.9623255 1.28139
AL139287.1 1.242084 2.087499 1.557977 1.905613 2.221639
2.973217 2.209863 1.082632 2.091887 1.697667628
3.101612 2.440249 2.789959 2.117397 1.472697
1.756738 2.853057 2.210479757 2.649014 1.527388
1.67132992 2.361565 2.250547 3.602496 2.268553
5.147787 1.906526 4.226193 0.945557635 0.5978435
1.276385 2.496675 3.560531 2.845075 2.359461
6.600779 0.5874784 1.791668 2.533011 2.531546932
5.987873 4.379787 0.979392192 2.564862 4.302168
4.51441 6.043863 2.427398 1.877920889 2.825852 4.127734
4.758549 6.985674 1.867616 2.421762 1.804524

1.713181 3.009578 4.679879 2.245183512 1.239241496
2.566515377 0.6372749 1.84986 4.143718 4.386843 1.386648
2.510722 1.701738559 7.230591 1.318001 4.555768
7.409523 2.491833 3.192503 2.314419 3.302215
6.188924 3.931262 2.101640789 1.897901 3.4125 1.169189
3.057512 2.259842 2.441706 3.330615 1.860472
2.83671347 5.901193 2.502701794 3.992597 0.9002866
4.066971 1.343026 4.345087 2.006633 3.131078
2.590077 3.853276 5.183744 6.182133 4.422934
4.560006 5.320809 2.29978 1.954713 1.780482
1.164944395 5.870938 3.53308 9.761641 0.4623767 4.149334
2.26613 3.675655 5.685383 3.816857 12.45684 2.835142
4.206681 3.198925 6.971698 1.46745 3.835104 4.198433
1.067223 1.139834 2.545072 1.672167 2.846249
5.956368 3.906369 2.996202 2.10359 4.893337 2.506083
3.323156 4.183726 4.79056 5.461326 3.210219 1.083373
11.49243 6.039349 2.678632 5.081452 4.955626
3.61565 2.022167 3.235016 1.597389 1.80766 1.832939
3.685775 13.11856 2.556012 8.467806 4.123754
3.148226 1.191375125 3.326707 5.850639875 3.115895
3.292177 2.689095 1.981388 2.708718 5.638582
3.753384 2.299302 0.953281269 3.212632 2.489566
7.504493 2.952156 4.187212 5.365852 3.657136
7.055786 6.889733 2.50892 2.402632 3.155978 3.889255
5.022612 1.905923 2.546299 5.49429 2.73348 3.034186
7.972365 2.708587 2.35922 1.74749 4.216239 0.9618373
8.861311 1.534116 4.219752 2.577185 6.630996
2.28779 5.807257 3.988051 2.918296 3.310813 4.46529
4.443034 2.825684 4.140365 2.880386 4.262288
6.683559 3.323119 2.832081 1.168121 6.023181
2.720791 2.419372081 2.739641 3.253764615 1.540789093
3.37036 4.857509 3.782345 6.839845 2.1913 1.102129
2.144041 1.868437 2.976282 2.552819 2.76174 2.345449
4.636378 1.463871 3.51117 3.017536 6.187391 3.974798
2.753284 2.595532 2.89171 1.649089 2.313434 2.005394
3.842384 2.028996 3.8915 3.248143 3.143088 12.26681
3.548237 2.016536 2.805281 4.609243 1.677661128
2.544315 2.689208 4.965834 2.266869 2.643845
1.788557 3.554061 3.320908 4.204678 1.02368 5.438082
2.724165 2.504933 1.329852 3.939954 2.167216154
10.92522 7.138061 3.108075 2.771599 3.187975
0.493673695 3.505081 3.764653 1.813588 1.699549
2.169527 3.285255 3.560858 4.17581 1.953127 1.592439
6.620885 2.262442 2.045766 4.394868 1.845644

2.55196 2.598091 1.86215 3.539238 4.818162 4.483644
2.657145 1.412248 3.481037 2.191712 7.017774
4.806823195 6.420033148 4.791026 3.313989 1.819065
2.083318 1.787554 0.7824571 3.53691 2.665358 4.69788
6.807283 2.227685 2.102114 3.860787 3.594862702
3.700977 4.894564 0.4257649 4.728891 2.989763
3.211822016 2.101589 2.302595 1.030569 1.273843
2.044862 2.399454 1.351381 8.928876 5.970218
5.089443 6.325475 1.235964 7.372922 4.270767
2.139053 10.63619 3.886391 5.423846 2.351612491
7.020064 6.589665 5.042727 4.009003 2.16194 2.40717
3.229013 2.205457 2.562231 4.726408 2.278147
1.711864 7.018165 4.405485 2.190771 5.91676 3.349006
2.156516 6.357889 1.769494 2.517101 3.37772 5.827348
6.069634 3.533115 2.970855 4.996843909 1.485936
3.320731 2.769736 5.608054 3.309893 9.357438
4.137371896 3.472225 8.844607 4.495495 5.378142
1.684769 0.5676508 5.455527 6.414606 2.5296 5.962819
3.115415 13.43762 3.328897 3.843718 0.992910395
5.694149 2.56647 1.234116 2.958901215 3.691962 11.74031
4.575407 2.292855 2.747062 1.672851 2.927942
LINC01235 0.01127998 0.06471737 0.1919516 0.03771363 0.01479559
0.04569445 0.03485645 0.0878728 0.2873367 0.247320239
0.1984913 0.02216112 0.008709595 0.05288013 0.05516897
0.07103247 0.01125041 0.025479132 0.09053037 0.1084126
0.092267355 0.02808476 0.7103409 0.2496644 0.03090279
0.04433154 0.008927588 0.008919334 0.065393917 0 0.2071978
0.03538912 0.7621811 0.07105326 0.1882554 2.324366
0.1115053 1.909697 1.212842 0.648869244 3.516131
0.3629472 0.622248617 0.007251528 0.8305043 0.261454
0.2034411 0.6122836 2.084631442 0.04955137 0.527369
1.553796 0.4053951 0.5257834 1.594509 0.2981316
0.09109105 1.248838 0.1809691 0.282906117 3.822730895
0.52302714 6.336194 2.263733 0.1319443 5.037099
4.209507 0.6042295 0.186997462 0.2127533 1.175339
3.535922 1.807689 4.154599 4.340529 0.0306003
0.2671149 0.5371444 0.4482121 0.309394868 0.2353577
1.49091 0.09061907 0.7075259 1.960462 0.3167291 1.236081
0.7457416 1.37180564 1.27232 1.217445359 0.1448442 1.744106
0.1475925 0.4024906 0.8815621 1.820501 0.2138015
0.0804617 2.098052 1.207504 0.69477 0.4288404 0.6088747
2.095082 1.408221 0.5369896 0.4212566 0.284284678
0.293243 0.2527522 0.1992574 0.9897816 0.3295305
1.258398 0.3358399 0.2145588 1.170681 0.3999839

0.05900432	1.645911	0.06674382	0.556119	0.7853203	
0.6126199	0.1805281	0.5003876	0.2988967	1.337392	
0.3601879	0.1502888	0.23758	0.941149	1.528755	0.4160796
0.9201452	1.115841	0.4736667	3.339378	0.6882384	
0.1921011	0.6116857	0.5352758	0.2909722	0.7186483	
1.058181	0.3148215	2.864216	0.4026116	0.4407433	
0.5368737	0.3297854	0.9608158	0.8015478	0.9562105	
0.5667018	3.618389	1.783077	0.4119485	1.566193	
0.790594207	0.5732629	1.049717924	0.3335003	0.5697444	
0.1656559	0.2069307	0.3742596	0.1152152	0.3957804	
0.1955488	0.032464583	1.281717	1.021579	2.085026	
0.6479084	0.676535	2.647662	0.2061792	2.016649	
0.1361393	0.4272141	0.1768528	0.7303781	1.139782	
1.804875	1.28297	0.3281325	0.6744469	0.04876162	1.209193
0.04754694	0.8346008	0.241426	0.009698228	0.2813401	
3.754489	0.1960917	0.1896504	0.8694229	1.496981	
0.3743404	0.1990528	0.197979	1.333903	0.2405102	
2.086372	0.7405574	2.117037	0.2011216	0.6709104	
2.05015	0.2094037	0.3545669	0.552077	1.026389	0.6820396
0.9784984	1.282175	0.204654107	0.736832	0.434046827	
0.40255845	0.6343186	1.647548	0.2925236	0.09317407	
0.4465624	0.0275247	0.1014547	0.4517785	1.528138	
0.1250567	0.0489479	0.2027614	0.07105262	0.9992767	
0.2678481	0.7260064	0.1670018	0.549137	0.3164883	
0.6133077	1.244012	1.429692	1.48412	0.2253733	0.5757609
1.462652	1.343188	1.526061	0.3054974	0.3046023	
0.8793246	0.03626007	0.8544953	1.204007	0.140309951	
0.1006082	0.8494908	3.736023	0.6556581	0.316933	
1.240984	0.4260458	0.9266037	0.1221025	0.374279	
1.559488	0.02915726	0.4164853	0.7281068	0.7635598	
0.675265282	0.08244321	0.4672466	0.5674395	1.164672	
1.263792	0.659044627	1.50321	0.429044	0.9964406	0.7432162
1.271563	2.767697	0.1289475	0.5972812	2.574467	
4.350201	0.1183244	0.339015	1.096139	0.235539	
1.31366	3.42246	0.3460535	0.3551333	0.3977521	0.07219762
1.581648	0.6407873	0.7743293	6.576439	0.08091422	
0.608311	0.195329377	1.118432149	0.3168706	0.6520032	
0.06594611	0.6172536	0.6642816	1.260406	0.09438131	
0.1926468	0.2593509	0.5429221	1.364004	2.216184	
0.7362963	0.316469328	0.3961228	0.7450662	2.134354	
0.1417199	0.7032561	0.385019658	0.3149121	0.2368875	
0.06863349	2.087351	0.08913797	0.1391789	0.5338567	
1.877439	0.02778278	0.1779461	0.502129	0.09449116	
0.1183707	0.1904619	1.614284	0.3149758	1.780825	

1.367371	0.129039361 0.0869355	0.5460768	2.104275
0.05178689	1.67184 0.1154245	2.600697	0.2266122	0.0479921
0.2780448	0.4389021	0.8593213	1.060362	0.771398
0.9848263	0.4574154	0.2879357	0.4987635	1.921631	0
1.650137	0.03163335	3.12376 0.7830155	1.065713	0.3968958
0.558769163 0.233016	0.5606693	0.1260852	0.2801124
0.1312862	3.474185	0.486748073 1.184119	0.2489175
0.6848533	1.86897 0.1384424	1.879041	0.5171824	0.3389184
0.06750156	2.03829 0.4530982	0.6810556	0.3963208	0.193642
0.758081653 0.2069986	2.282459	0.02175598	0.065685293
0.3120734	0.2791389	0.6354347	0.01717861	0.3047152
0.5013357	0.1208764
AC125257.1	1.333856	2.185842	1.623747	2.28784 1.450392
2.586181	1.808062	1.824432	1.867864	2.429106792
4.307412	2.591323	2.534569	2.082806	1.972476
2.920474	1.543019	1.456049806 0.9734783	1.376762
1.749867715 3.501267	2.510689	3.399287	1.976555
3.457121	2.04858 3.101395	1.475655764 2.45282 1.611454
2.564636	5.475634	2.514364	5.502215	6.706718
3.854259	5.125976	5.603867	6.187442666 6.551026
4.436749	3.654008647 6.968317	3.385304	6.096906
5.077359	12.84478	4.523857401 4.293095	2.593021
4.69235 5.080252	3.862097	4.234589	7.034344	4.470228
2.490731	3.942809	1.653689463 3.079391878 4.36167463
2.770515	3.783179	4.162457	3.501153	2.821358
2.450661	1.962891218 5.278928	7.462037	4.154537
4.846339	5.858101	5.517732	3.52432 2.710312	6.228011
6.837871	5.911448298 3.215912	2.551145	5.035179
3.125513	4.737292	3.317541	4.959203	6.489015
5.01111639	4.038145	5.09851989	5.558854	4.10032 3.3361
3.635648	3.839803	3.734617	2.36007 14.4893 6.212222
4.73691 5.029588	3.770283	39.32157	4.175101	4.406221
3.555139	2.376481	4.617269321 5.526467	3.26716 4.144677
3.846271	4.837727	4.197141	7.55876 1.769849	3.826261
6.963564	3.631283	2.832531	9.516811	7.530424
2.909048	9.184862	3.833252	5.905056	3.030064
3.265626	2.891337	4.731721	8.631651	4.601375
5.830887	0.8757252	3.905828	4.834381	4.297861
4.272906	6.083392	6.458417	6.761438	6.66175 7.02192
5.723838	5.866706	5.941224	3.900186	8.93224 5.313499
5.641196	2.843292	5.845717	3.439751	3.022107
4.437391	5.804173	5.542542	8.789764	5.773308
2.212282728 3.933405	4.132730371 4.555988	2.284631
4.963619	4.287209	5.881044	2.99158 6.495301	2.857192

4.172875622 4.248212 42.35949 8.3726 4.364534 3.794227
6.155034 4.469707 16.80046 10.80212 2.964724
2.282306 4.545757 4.439054 3.651504 3.519088
5.434306 10.50563 5.628868 4.703153 6.892164
2.32694 2.630472 2.302154 5.172628 3.807727 11.36363
5.517479 12.69913 8.041664 3.502412 2.396575
6.788314 6.689319 3.940695 2.622986 7.493176
7.474512 3.555058 3.856234 4.174102 6.70598 6.710366
5.910161 6.164828 3.236974 7.20433 3.506465
1.454846237 4.460625 4.924281441 4.36676915 6.315646
4.241706 7.345737 6.76838 2.528865 2.492517 2.91377
3.272663 3.292464 2.890972 2.754179 5.145887
2.922119 2.349929 5.298456 5.403077 4.72877 3.977453
4.307913 3.524582 5.308802 6.693431 5.409724
4.755453 4.387049 4.002294 5.186792 6.458803
4.095959 3.195137 4.422767 9.135151 6.179686
5.846587 2.097083068 6.024102 2.714535 4.938317
4.49347 6.548074 3.96978 3.400405 4.759681 5.513505
2.184735 3.256812 3.552943 4.095634 1.886721
4.643785 4.676932062 4.176372 19.60924 4.546415
2.97662 2.232836 3.441041978 3.540172 3.433285 2.508693
4.477994 7.366064 4.404957 4.953016 5.534579
3.549953 4.1514 4.905347 3.016527 3.842566 4.88543
4.103093 2.025586 3.555847 3.42334 6.113652 2.775963
4.922316 7.176105 9.044589 3.637279 3.494143
3.528123 7.888840219 6.821242911 3.8828 5.938916 4.124208
4.684493 6.706853 1.307845 6.382942 2.672763
5.461535 8.172412 3.41546 4.965027 6.123285
4.499099498 7.67573 3.500413 4.706505 3.951518 2.33118
7.29081068 3.362893 7.198792 4.399883 3.662198
3.24739 2.584927 2.346783 4.373163 344.8671 2.735584
5.481076 3.655292 5.231216 5.860766 4.723995
8.700848 3.435564 5.011442 3.128572688 7.616452
6.017695 4.658733 6.31957 4.094219 7.255753 6.061839
5.030636 5.668025 4.390905 14.71325 2.954339
5.432372 4.167007 2.102755 4.056263 4.650683
4.249159 5.363445 4.927926 4.654305 3.837978
56.68174 10.46387 3.524157 8.681141 6.229166262
2.549409 13.66032 3.48259 3.533967 2.693233 10.78036
2.630702401 2.681504 4.518823 4.441795 32.37332
3.071536 2.291069 4.150943 3.390026 3.948797
3.162639 6.168472 7.324099 5.196528 4.374022
4.248100882 6.591561 2.397495 1.692779 4.09297837
9.14771 4.080714 6.65984 4.523381 5.602395 2.170433

3.928137
CYMP-AS1 0 0 0.01705315 0.2007116 0.04052898 0 0.09548088
0.03703176 0.04770242 0.015485129 0 0 0.01908628 0 0 0
0.03698136 0 0.06104276 0 0 0 0.007861851 0 0
0.0194297 0.05869196 0.004886475 0.011942062 0 0.0349273
0.02769714 0.1312339 0.03892667 0.02012411 0 0.00643036 0
0.03824217 0.032813935 0.06986651 0.2342514 0 0 0.07636253
0.07730311 0.05752547 0.02656957 0 0 0.02140149 0.03498289
4.117072 0.1951316 0.04819613 0 0 0.006334987 0.05576865
0.008377874 0.063144443 0 0 0 0 0.0125626 0
0.006245817 0.124178096 0.01726775 0.005277965 0 0.01833974
0.005762292 0.009617663 0.03352887 0.0335861 0.2012141
0.009354434 0 0 0.2970175 0.01985834 0.03812651 0.02603741
0 0.09537881 0.122185 0.61393934 0 0 0.01380055
0.2850698 0.03130019 0 0.006313275 0.2815386 0.05205848
0.02074405 0.05637492 0.05416061 0.04319218 0 0.01482548
0.2741955 0.167818 0 0.01538575 0.054647692 0.09886385 0
0.248099 0 0.0136252 0.1094311 0.04906415 0.03457249
0.1664599 1.351314 0.08404664 0.1087175 0.3457124
0.01138957 0.01147305 0 0.0659351 0.02260935 0.02005116
0.1144832 0 0 0 0.03772759 0.006226998 0.01381515 0
0.05878033 0.01330765 0.02040318 0 0.05472636 0.05856347
0.01442222 0.03253259 0.1283212 0.00499764 0.01604424
0.04755049 0 0.006036552 0 0.08742269 0.9631722
0.006141672 0.00451605 0.03880858 0.02208737 0 0.01736052 0
0.027943781 0.04096476 0.101114732 0.3822831 1.004924
0.04415104 0.01971607 0.1097391 0.0420806 0 0.005101513
0.014228629 0.0461534 0.02515388 0.0705907 0.3916777
0.005978081 0 0 0.2860389 0.0124307 0.01248267 0.01384131
0.005757397 0 0.3054221 0.01925692 0.04180652 0.7797662
0.03739981 0.1483908 1.634555 0.01252706 0.2267412 0 0
0 0.03683287 0.08815786 0.01443379 2.015841 0.005063782
0.02423366 0.005044799 0.0116925 0.0287485 0.04467841
0.007117822 0.1154575 1.032258 0.1264721 0.03284149
0.1204584 0 0.03558312 0.00540682 0.01322679 0.1664517
0.00647412 0.307529575 0.06920141 0.071663768 0.02594617 0
0.01616619 0.05508925 0.0063807 0.02878236 0.01507946
0.1852739 0 0 0.008060302 0.01462702 0.2073554 0.06920234
0.1068206 0.1152966 0.09769152 0.006309811 0.1342235
0.01238491 0 0.01531538 0.01933975 0.02967439 0 0.01236987
0.1530605 0.004406398 0 0.2092092 0.05754378 0
0.008828956 0.2642708 0.05624646 0.038434542 0.005801937
0.3400965 0 0.02596653 0 0.006383807 0.02188219
0.009400771 0.05351527 0.5243888 0.6722061 0.2555818

0.02816942 0.008397784 2.242528 0 0.01720635 0
0.005865525 0 0 0.110528235 0.01984425 0.1305848
0.007089636 0 0 0 0.1120562 0.01038799 0 0.01005598
0.01994593 0.006404484 0 0.005266959 0 0.324721 0.1303404
0 0.02504704 0.06328577 0.03067288 0 0.3899642 0.00560329
0.03650625 0.00516689 0.013376448 0.119976136 0.02169977 0 0
0.1632514 0 0 0.07954921 0.03603871 0.02706399 0.0225334
0.08828532 0.03081576 0.008232273 0.531201745 0.2025489
0.1020465 0 0.4880324 0.03995499 0 0 0.005642577
0.06893515 0.003373331 0.04883441 0.01270824 0 0.04729005
0.006088341 0.066849 0.04327295 0.008282747 0.008646619
0.04173797 0.1021677 0.01380481 0.05548599 0.04211903
0.135950902 0 0.008196418 0 0.0567431 0 0.2634815
0.2871684 0 0.4206811 0.05748203 0.2671704 0.0702658
0.02390624 0.06501722 0 0.111376 0.1442251 0 0.04744877
0.0135128 0.04427907 0.02599559 1.321248 0 0.03766792
0.01175352 0.667347685 0.113474 0.1075073 0.138152
0.6138406 0.01065561 0.006220062 0.028070094 0.1720095
0.02211405 0 0.1080281 0.2498454 0.01694546 0.1279597 0
0.005689363 0 0 0.1405951 0 0.01088074 0.007762926
0.2710647 0.1039761 0.04767622 0.017992909 0.04662819 0 0
0.009411337 0.03514502 0.1414904 0.1177287
THORLNC 0.1335624 0.042572 0.09555451 0.01417632 0 0.07514613
0.8713045 0.1245008 0.0229108 0.148745819 0.02547715 0
0.2291718 0.04174235 0.3387153 0.02198878 0 0.06704213
0.1099424 0.2535656 0.017341362 0.01477964 0 0.08877448
0.1016415 0.02332953 0.1879262 0.02346905 1.089765018 0
0.08387547 0.0798153 0.3008241 0.6076178 0.108735
0.03253193 0.9265236 1.106278 0.02295898 0.144467344
0.4074646 0.4055509 0.185354224 0.3625319 0.1222529
0.1091988 0.1899472 0.1276099 0.015110743 0.4859706
0.1541826 0.1260134 0.620914 0.3123967 0.5497632
0.07297309 0.196105 0.03042605 0.2827295 0.060356637
0.640244946 0.121431044 0.162583 0.05209733 0.02042231
0.1307289 0.3847341 0.07499447 0 0.4872407 0 0.02151195
0.1174443 0.1522151 0.1385769 0.1610345 0.02304419
0.1208004 0.02246402 0.101762153 0.1806252 0.09726363
0.06358454 0.1220775 0.2501081 0.2730088 0.2443152
0.03667733 1.37265427 0.7365161 0.128708473 0.01657053 0
0.2505507 2.118112 0.01516089 0.04315501 0.1093879
0.01245384 0.1203382 0.3530279 0.09075763 0 0.3204211
0.3215741 0.1455289 0.08563378 0.2586346 0.196848914
0.1187073 0.4942983 0.1310743 0.1041748 0.1635999
0.5255821 0.08247689 0.5811639 0.2704135 0.4275619

0.170781	0.43001	1.213375	0.3829175	0.4132758	0.1711816
0.1151553	0.3936371	0.1123534	0.1237156	0.1236191	
0.2030681	0.3548057	0.1208002	0.1345833	0.06635225	
0.05674542	0.07057843	0.0798935	0.4409713	0.506282	
0.5964515	0.6250493	0.2886164	0.2031243	0.2661334	
0.2040252	0.7127888	0.1776276	0.1177083	0.1014745	
0.02743014	0.0419879	0.107581	0.05899518	0.08675983	
0.2329903	0.05304128	0.1757203	6.253513	0.2482564	
0.201315102	0.1147697	0.121409978	0.4185119	0.5119027	
0.2591737	0.497141	0.06935021	0.2627394	2.009719	
0.09800742	0.017084528	0.1583345	0.2114185	0.1232863	
0.3292053	0.4450342	0.1282211	0.1427659	0.1202079	
0.3582175	0.7044421	0.1994336	0.05530394	0.7328016	
0.5684237	0.0693662	0.3011863	0.2692561	0.01283043	
0.3308966	0.4847942	0.4813258	0	0.9441854	0.2597466
0.05688497	0.1179354	0.04536534	0.5545878	1.3929	0.8633825
0.04364658	0.3270978	0.4211808	0.05753126	0.1609381	
0.1025577	0.5041148	2.283927	0.07592849	0.2208263	
0.1515236	0.09820595	0.08545036	0.0259682	0.09528961	
0.1645914	0.01554715	0.076928204	0.08309117	0.14080543	
0.218077689	0.03189277	0.3623385	0.4089055	0.3524245	
0.1036782	0.01810614	0.9343368	0.2511433	0.0862859	
0.4064813	0.02341718	0.391246	0.04154614	0.149638	
0.2013649	0.1340568	1.424342	0.6891159	0.3271564	
0.2755217	0.2574517	0.1548102	0.02850439	0.1976716	
0.04455807	0.1729713	0.1163982	0.9338274	0.117227	
0.1105499	0.3497496	0.01060106	0.7252891	2.468133	
0.246127637	0.04179884	0.7737328	0.149564	0.06235679	
0.227436	0.7205225	0.05254855	0.1580271	0.2313237	
0.1130126	0.5175477	0.2761928	0.1623525	0.2016669	
1.180618	0.067816644	0.2582491	0.2370016	0.239456	
0.1069029	0.8432731	0.07078022	0.2700426	0.2195131	0
0.1504302	0.2706965	0.2878466	0.08189852	1.94579	0.1305218
0.0603718	0.1796203	0	0.01222128	0.1011859	0.181925
0.226392	2.290616	0.3003579	0.09623796	0.08865287	
0.1841469	0.049109	0.2531005	0.01345591	0.1502866	
0.1985268	0.160613022	0.020579593	0.2475248	0.1296665	
0.2197934	0.3920368	0.3805901	0.05784508	0.05730956	
0.1854524	0.0974884	0.4870115	0.1211492	0.118403	
0.2174615	0.053151826	0	0.03063219	0.3357411	2.024321
0.1507772	0.17534171	0.9296649	0.3387564	0.3461352	
0.08100819	0.1319315	1.113905	0.09687681	0.05678187	
0.07310363	2.354487	0.1336075	0.01989044	0.1038212	
0.9221235	0.1533428	0.1657564	0.08883047	0.1444942	

0.4962444 0.1143748 0.4920781 0.7046947 0.1362646
0.1978807 0.07592789 0.1945067 0 0.04209317 0.02760782
0.8126821 0.2024861 0.04592732 0.1951679 0.02467934
0.2228847 0.0432933 0.7023977 0.2706191 0.1784752
0.1417775 0.07283101 0.4649917 1.156946 0.2170965
0.2540273 0.352863583 0.013625 0.7007508 1.285579
0.3316714 0.396625 0.1045593 0.55274861 0.4720787
1.29223 0.2067897 0.7331492 0.1499966 0.8749065 0.3072864
0.321626 0.2459269 0.09795915 0.05419171 1.40246 0.2157562
0.07838797 0.279631849 0.1859837 0.1576999 0.3148506
0.324065336 0.6905083 0.6832428 0.06599967 0.3164093
0.04219915 0.1399091 0.07067931
AC008966.2 0.6414401 0.3373494 0.2655096 0.09191135 0.2944748
0.8120095 0 0.07687564 0.04951364 0.321461714 0.6331887
0.1890299 0.1485822 0.2405637 0 0.7128151 0.1919275
0.169035951 0.1584012 0.2739964 0.187386241 1.054053
0.04080179 1.112758 0.2196623 0.5798134 0 0.9129617
0.061977457 0.1455488 0.271901 1.264946 0.2167084
1.111129 0.7571975 0.6327568 0.2669806 0.1440259
0.2728976 0.425748133 1.087789 0.4523642 0.333814911
0.8865753 0.6935421 0.4719896 0.5970966 0.2068379
0.587817918 0.5123192 0.1110704 0.7262232 0.1376294
0.5304635 0.4377283 0.5125433 0.659263 0.3616536
0.3215896 0.26087924 0.291297689 0.131215223 0.2235965
0.03753001 0.04413563 0.5867817 0.30633 0.2917334
0.612241874 0.2016382 0.2191346 0.836829 1.015258
0.4485811 1.073155 0.2320129 1.29485 0.6526688 0.5340288
0.251340644 0.1672962 0.8057709 1.030617 0.5276551
0.439173 0.6521191 0.4620012 0.5152234 1.6480605
1.374668 1.452603127 0.4655479 1.260288 0.4873295
0.4799048 0.09829476 1.64767 0.1013158 1.103499 0.45512
0.5621704 0.5604019 0.2955898 0.8463613 0.3640314
0.6048259 0.1388004 0.07984967 0.255251771 0.5986028
0.3690324 0.3862786 0.2251371 0.9899779 0.5679304
0.3819531 0.1435407 0.9401276 0.4027799 0.4361891
0.9625049 0.9661044 0.5319908 0.2381734 0.1541453
0.1555423 0.1760085 0.1040624 0.2970751 0.1187374
0.3002728 0.7302739 1.044268 0.7109775 0 0.6540546
0.5796156 0.310791 0.8118183 0.6312405 0.4151081
0.2363942 0.4490947 0.4389816 0.2118987 1.400597
0.2498014 1.233898 0.16959 0.5325891 0.3260432 0.3327209
0.08718689 0.2549946 0.5390648 0.7049369 0.8024103
0.6835636 0.6306891 0.5365188 0.096682602 1.452772
0.426375492 0.3792922 0.1975531 0.4582741 0.05116168

0.8992563	0.480462	1.895258	0.9001863	0.14768877	
0.2053106	0.4242704	0.2331349	0.609824	0.3102532	
0.6465776	0.493661	0.1855622	0.7419043	0.3563072	
0.2514198	0.50796	0.1759658	0.8321742	0.1748957	0.8678776
0.5290021	1.275509	0.4125672	0.3717688	0.2925608	
0.1961253	0.05514931	0.6455535	0.4097894	0.1911569	
0.5882473	1.123637	0.838926	0.4993246	0.3458646	
0.1570904	0.4247759	0.3978674	0.03864568	0.2585828	
0.8443366	0.8427152	0.3281854	0.1363538	0.1190782	
0.3891022	1.169582	0.5050901	0	0.127038	0.06719936
0.166253226	0.2693585	0.304301359	0.100992459	0.8500748	
0.531367	0.857714	0.3311484	0.5228161	0.3521703	
0.2884629	0.253287	1.417222	0.3764855	0.8856396	
0.9224078	0.85298	0	0.4895767	1.158866	0.2947225
0.6725786	0.4820683	0.5529114	0.4769067	0.6691353	
0.2772099	0.3203982	0.6740761	0.3738166	0.3430279	
0.461289	2.099143	0.7764726	0.8140038	1.122613	
0.07837285	0.4776717	0.166224446	0.8732235	0.1857946	
0.4113835	0.0898415	1.351688	0.6957504	0.2271304	
0.6830397	0.3055096	0.2791279	1.624484	1.260109	
0.3216289	0.1743328	0.6266817	0.615559555	0.4911407	
0.9283542	0.4261764	0.8727915	0.1375425	0.841315878	
0.446284	0.2710859	0.2575588	0.5319849	0.9360243	
1.057534	0.1769948	0.3773844	0.282077	0.2609448	
0.750493	0.3656211	0.6602998	0.6286982	0.3931669	
0.1087259	0.6764467	0.2884968	0.9619281	0.3558137	
0.4377656	0.5306591	0.9025295	0.4071229	0.5142534	
0.375415	0.277686797	0.711608692	0.4504739	0.3233406	
0.1250016	0.2017261	0.9748284	0.08334118	0.7844114	
0.240474	0.1404579	0.2105008	0.5890978	0.6397161	
0.769036	0.344606872	0.2102395	0	0.2968307	0.9210228
0.2073602	0.084208748	0.5241244	1.464205	0.4228106	
0.1925777	0.1900823	0.1648845	0.05234128	0.245428	
0.8531339	0.520404	0.641657	0.9242029	0.4936208	
0.2599363	0.4308155	0.6448034	0.4319455	0.9992744	
0.084667701	0.1235904	0.1701526	0.7614747	0.1766927	
0.296065	0.3555314	0.2292861	0.3758528	1.091635	
0.7159749	0.3235338	0.9481385	0.0744418	0.7170373	
0.0533357	1.477173	0	0.5592577	0.461722	0.210388
0.5362036	1.146761	0.5615707	1.23304	1.133846	0.1524974
0.730815792	0.2061194	0.03985332	0.2688704	0.07964344	
0.9401167	0.613342	0.174815341	0.510116	1.032916	
0.06384331	1.291931	0.09261851	0.1758886	1.138442	
0.2527569	0.08858075	0.4234084	1.288279	1.459333	

0.9325622 0.3952854 1.128074797 0.6316172 0.4544169 0
0.23345104 0.04033218 0.5537208 0.4279051 1.050133
0.5471916 0.2159745 0.5855357
AC008735.2 0.2330794 0.8419796 1.738985 0.4700422 1.256861
1.96706 0.4001347 0.6207605 0.6397056 0.973410137 1.978475
1.526391 1.839665 0.777008 1.942158 1.170364
1.549789 0.838464807 0.6075577 0.8296817 0.605247536
1.289596 0.2306283 1.084441 1.206145 2.402026
0.6149055 1.351541 0.350321778 0.05876435 0.8050401
1.114284 3.799765 2.120705 2.129452 1.021885
0.2694791 2.023603 1.262068 2.475258666 5.793094
1.209983 0.889518556 1.331903 3.093477 4.173322
6.177515 1.196968 4.904769477 2.564883 0.4484395
2.443398 1.333607 2.336412 2.676195 1.655487
1.559014 1.539797 1.350332 0.386203581 3.087249236
1.836545335 0.7479978 1.030369 2.851114 1.245967
0.777409 0.9161089 1.092833651 6.639442 1.039571
2.815534 1.921422 5.81972 2.297387 2.997557 1.689004
6.282094 1.43087 2.79062038 1.395924 1.838791 5.659024
3.222188 0.7638105 1.379131 1.412298 1.120097
2.4841354 2.132418 2.171219397 3.20981 0.5088327 3.169958
0.8346514 2.01075 1.481091 2.045279 4.216237 2.415022
2.075178 3.914272 1.730465 1.180462 1.950763 2.6373
1.270234 1.32179 0.595435801 3.901436 1.803616 6.134325
0.5908343 5.538658 3.485331 1.048635 3.129494
4.390704 1.43488 5.743844 1.58122 20.53378 2.816106
0.2404023 3.808791 3.69259 1.184371 0.5882032 1.583236
1.270395 2.611175 2.152356 3.636442 5.871526
1.042121 3.003798 1.822861 10.51242 2.764637
6.150601 3.404868 3.054162 2.115388 6.734964
3.593213 1.298513 13.34656 1.859865 2.510597
1.770829 2.178007 1.147944 0.6805551 1.595762
3.44445 7.887862 2.800007 4.139763 4.21969 2.599391
1.092978724 3.805403 4.449318587 1.931878 0.5423731
1.233501 0.9501845 5.058758 4.867218 3.283974
1.496535 1.788850607 3.426234 0.6588321 1.102626
2.215911 1.778729 2.013825 4.384871 4.015685
3.464233 2.720199 0.8120726 3.426133 3.149663
2.271897 1.654361 2.768158 1.110622 2.46294 1.088265
6.577072 1.391185 0.9898035 2.048487 2.810356
1.240873 4.707878 1.451394 2.721966 2.550293
4.965701 1.371016 1.923867 2.989007 2.851295
1.185823 1.312471 3.32098 3.402406 8.844552 1.651558
4.254815 1.228216 2.460468 1.064951 0.4711542

4.205841 1.68214 0.671236288 1.474189 5.678845559
0.135916767 4.378272 5.623098 1.553086 6.096673
1.628358 0.2843728 3.700997 0.7012316 3.071778
1.85782 1.55288 5.524171 4.205122 1.492188 2.240184
9.386931 2.088975 5.353397 2.880551 1.408093
1.540383 4.241495 0.9948589 1.250467 1.632922
1.735649 1.920467 2.840196 7.393836 3.279646
2.30054 5.272469 3.006039 2.999994 0.778499302 3.185181
2.350417 2.467673 0.6166394 4.332804 1.471405
2.720503 2.639539 2.130547 0.6761761 3.376141
2.784794 0.8027432 2.533885 5.783281 3.952779552
2.505725 3.412126 5.825659 1.969203 1.138403
1.080786197 1.912724 1.167457 0.5942151 1.789878
3.155578 3.717172 3.22593 4.39686 1.776632 0.9271215
4.869002 2.147157 1.322294 2.582472 0.9127464
2.107073 4.146319 0.9318294 2.79208 1.635484 1.510366
2.742401 1.236718 3.733619 1.639156 1.537366
6.362480166 10.019840439 1.409538 2.872025 1.049746
0.7818774 4.009601 0.7066173 3.200346 1.143502
2.268358 5.628113 2.457724 3.383483 10.10828
6.369186778 2.255457 2.993546 0.1065275 4.462281
2.128888 2.481906507 0.458492 2.482885 1.785857
1.173348 1.253493 1.517819 1.98645 5.623354 1.683964
1.867641 10.98434 1.909092 7.754429 3.043479
3.719616 2.574424 1.433915 5.370933 2.939825573
5.668505 2.404429 2.283842 4.80346 1.832859 0.331254
1.573736 1.604194 2.497528 4.215605 3.582846
2.414614 5.670448 3.031225 2.4118 2.022569 4.079757
2.935355 2.684408 1.812112 4.236992 3.032177
1.694511 4.397513 2.620412 1.18214 2.078262719 1.046186
1.126336 0.8443137 1.093287 2.32205 9.774977
2.187996292 2.718618 4.201222 2.526078 3.483951
3.103711 0.4260831 3.217469 2.194051 3.337963
2.756542 8.511286 4.260341 8.189218 3.260274
3.480959607 7.093948 0.6650708 0.6493442 1.168753282
1.563249 6.140477 2.034774 2.307262 1.693758
1.778844 1.336208
AC022031.2 0 0 0 0.01127426 0 0 0 0 0 0 0.02026168
0 0 0 0 0 0 0 0 0 0 0 0 0.01412026
0.008083428 0 0 0.027997 0 0 0.0333526 0 0
0.5204031 0 0 0 0 0.009129503 0.020889673 0.7052893 0
0.012284172 0.007587301 0 0.382116 0 0.2790884 0 0 0
0 4.684823 0 0.4832435 0 0 0.01209874 0 0 0
0.01609545 0.01175458 0.0138108 0.08120812 0 0 0 0 0

0	0	0	0.04401998	0.03673619	0.1494137	0	0.1537138	0
0	0	0	0	0.01213585	0.01243175	0	0.3278831	0.1895989
0.52561215	0	0	0	0	0.01992601	0	0	0	0.06213926	0
0.01196294	0	0.01031121	0.05438755	0.0849424	0.1826744
0.00890288	0	0	0	0.01573442	0.7933668	0.06633574
0.5385184	0	0.3343915	0.0562225	0	0	0.340035
0.01234729	0	1.142743	1.674914	0	1.951322	0
0.010795	0	0	0.7209609	0	0.0134368	0.6004438	0	0
0	0	0.5718454	0	0.1858339	0	0.08699152	0	0.01242634
0.9245886	0	1.149066	0	0.4264547	0	0	0	0	0	0
0.1482356	0	0	0	0.4343579	0.035578569	0.01303929	0
0.09663028	0	0	0	0	0	0.01453005	0	0.013587127
0.02518432	0	0.2451203	0.00935047	0.0799198	0.18695	0
0.9559995	0.01187026	0	0.01321727	0	0	0	0	0
0.2336032	0.01020389	0.1315791	0	0.01196227	0	0	0	0
1.793785	0	0	0.5538788	0	0	0	0.2009761	0
0.01422136	1.495321	0	0.01107549	0	0.01254433	0	0
0.02265256	0	0	0.9349832	0	0.012236021	0	0	0	0
0.01029156	0	1.328279	0	0	0	0	0	0.0153938	0	0
0	0	0	0	0.4579252	2.139147	0.01182653	0	0
0.381668	0.1246806	0.1179044	0	0.1719526	0	0.01060946
0	0.637413	0	0	0	0.2343734	0	0	0.5355743
0.03243996	0	0	0.02438396	0.05572166	0.8797396	0.0102205
0	0.01959998	0.07321765	0	0	0.8400968	0	0.0410765	0
0	0	0	0	0	0	0	0.02175189	0	0	0.2698365	0	0
0.04801299	0.3047465	0	0	0.09053095	0.2351097	0	0	0
0.009567116	0	0	0.01301861	0.01006439	0.02140266	0
1.263088	0.715308556	0	0	0.007932481	0.3771982	0.04454033
0	0	0.1215403	0.02949763	1.098361	0.008606991	0.02408713
0	0.2043891	0	0.2542051	0	0	0.3812967	0
8.196397985	0	0	0.01196858	0.9148332	0.01165817	0	0
0.02257898	0	0.4468457	0	0	0.03302713	0.01594248	0
0.1054593	0	0	0.778929093	0.07276876	0.04696128	0.06004666
0	1.367928	0.0100641	0	0	0.01673811	0	0.3741833
0.1341957	0.5296193	0.2017792	0	1.997102	1.256719
0.02940046	0.0566369	0	0	0.008274523	0	0.02520837
0.04316358	0.1010125	0	0	0	0	0.02930827	0	0
0.010721818	0.009385968	0.0703901	1.609334	0.4305711
0.0170415	0	0	0	0	0.05842933	0	0.4750607	0
0.01039017	0	0	0.02090281	0	0.223362011	0.1632618
0.8965688	0	0	0.01118683	0.01589544	0
THBS4-AS1	0.04044465	0.2019657	0.08267233	0.008585595	0.01178888
0.1956961	0.02777303	0.1508028	0.06243956	0.050672727
0.09257827	0.05297282	0.1110345	0.0463474	0.02930514

0.06658527 0.02689241 0.138725851 0.01664609 0.06718548
0.042009758 0.1566423 0.05717047 0.2311871 0.7109841
0.0741775 0.03556676 0.1563491 0.034736515 0 0.03809809
0.2336361 0.2342416 0.1132281 0.1353647 0.2068741
0.03273258 0.2986723 0.1842364 0.103401619 0.1197573
0.07923019 0.014031997 0.07222369 0.1527074 0.03306701
0.09935065 0.08694486 0.905999594 0.02512471 0.1348786
1.441552 0.2121273 0.1081124 1.796193 0.1381083
0.158356 1.598535 0.09913276 0.030461464 1.188763523
0.098056288 1.096543 0.2734478 0.04328923 0.4872202
0.1165031 0.517775 0.031605304 0.06592376 0.1074661
0.009771202 0.2578379 0.8715765 0.143374 0.01625454
0.04884685 0.4535937 0.187067 0.189292623 0 2.405317
0.03369508 0.6885079 0.2556104 0.1261822 1.340928
0.4553639 0.33868586 1.561196 0.034644285 0.02508901
0.6142205 0.0834575 0.09310581 0.1974104 0.470447
0.0709806 0.1734755 0.6695881 1.77795 0.2434186 0.07248029
0.07007594 2.921321 0.2169516 0.07131071 0.8615021
0.139087082 0.05991054 0.09525141 0.2958801 0.1971603
0.8124634 0.6366161 0.08206143 0.07542213 0.04984316
2.151228 0.1739505 0.1023105 0.309413 0.1656472
0.3921241 0.2419028 0.326913 0.1274197 0.04860324
0.9657101 0.3285846 0.2038946 0.4348778 0.1280303
0.06339481 0 0.4295837 0.2436429 0.05806303 0.2373913
0.1651027 0.1132666 0.12776 0.05943015 0.2791564 0.4877762
0.1490033 0.186675 0.3188875 0.2336649 1.435435
0.05399063 0.2246238 0.02443282 0.4600137 0.9129573
0.1128846 0.2971411 0.9045818 0.05049749 0.2104919
1.110848113 0.9631809 1.796876117 0.1022029 0.1605478
0.3388979 0.09319243 0.09660121 1.73811 0.2544939 0.2411345
0.06208136 0.4842548 0.03658322 0.05133273 0.1424117
0.3129977 0.1941361 0.09078637 0.1768035 0.04067758
0.2133154 0.0553588 0.1004811 0.2506683 0.0777348
1.613892 0.006080244 0.3076083 0.07381963 0.4354862
0.09944708 0.0683215 0.06870149 0.06954647 0.6371063
0.5856706 0.1919551 0.06868647 0.06822454 0.09680444
0.592854 0.07048975 0.1687519 0.07652385 0.1567918
0.4115352 0.6055917 0.06869402 0.2488097 0.1034652
0.6639182 0.1543357 0.09912736 0.185442 0.2005206
0.1827489 0.09612136 1.153437 0.074543977 0.05870953
0.293728149 0.056603259 0.048288 0.5446886 0.1056134
0.1252792 0.0837208 0.07675926 0.05389161 0.2433597
0.0940631 0.04689086 0.03900086 0.4362036 0.1132271
0.4337062 0.0724093 0.4820581 0.3028358 0.8178691

0.3917681 0.5482676 0.1670575 0.3047122 0.1683152
0.3441828 1.124403 1.086849 0.1121498 0.6382674
0.1115653 0.3096542 0.2281124 0.05457267 0.1317769
0.1710438 0.172353104 0.05062921 0.5857442 0.1111668 0
0.2123521 0.0928446 0.1644289 0.2255923 0.03891568
0.03911071 0.361951 0.07434245 0.8316697 0.07328125
0.1505299 0.05750046 0.1219945 0.161477 0.08530684
0.5790952 0.01927211 0.037508262 1.827865 0.07280246
0.6547486 0.2194801 0.0557402 1.342329 0.4180585
0.0604322 0.08695264 0.5265063 0.1051572 0.8196789
0.8622825 0.06128115 0.02754479 0.6931655 0.5040682
0.2728584 0.06192732 0.04218562 0.1728634 0.837729
0.210767 0.1915084 0.07964076 0.1390202 0.063226784
0.22434493 0.2485322 0.006040758 0.08757458 0.02826536
0.04268455 0.3328105 0.1041251 0.6888679 0.127924
0.127811 0.1421573 0.4616226 0.2394565 0.193141866
0.07154144 0.1484141 0.8318237 0.1290514 0.4731782
0.076694296 0.1162784 0.1641287 0.01822867 0.780069
1.313937 0.1062748 1.444784 0.2278256 0.6862425
0.2390081 0.4810046 0.06023118 0.1697685 0.267092
0.08822548 0.2359093 1.123041 0.1575178 0.071180579
0.07965906 0.4589462 0.3353302 0.02475775 0.2166382
0.07280827 0.128508 0.1128505 0.0764786 0.1337609
0.1942832 0.6846928 0.1251672 0.3309583 1.457287
0.07649179 0.07210416 0.1063482 0.06900838 0.07369763
0.05366538 0.3465676 0.1408063 0.2015656 0.2739168
0.04700864 0.097947931 0.2062925 0.2233659 0.1130204
0.1897105 0.08910934 1.035803 0.114308578 0.0357381
0.477072 0.08498289 0.08880335 0.06488739 2.489153
0.2525666 0.2523366 0.04964687 0.734171 0.09846029
0.7078091 0.3266708 0.07912344 0.412092986 0.9694843
1.014769 0.05200439 0.045794819 0.1525836 0.2534476
0.2131805 0.2018925 0.0553736 0.1089425 0.5564711
AC133065.1 0.07832139 0.01872327 0.1440861 0 0.02568292
0.118978 0.3025277 0.1173338 0.06045735 0.907685275
0.1344589 0 0.4535563 0 0.3192167 0.02901215 0.07811606
0.014742643 0 0 0 0 0 0.1171297 0 0 0.09298167
0.06193047 0.113513969 0 0.05533294 0 0.1512036 0.1850066
0.07970314 0 0 0.1758592 0.0908767 0.138626346 0.1739332
0.03452173 0.081519221 0.1636382 0.120976 0.01440777
1.025254 0.2104618 0.099686103 0.09383312 0 1.66263
0.08402444 0.1177652 0.5344768 0 0 1.465269 0.03926687
0.371629861 0.244531062 0.160216971 0.3120192 0.02291253
0.08083601 0.1592166 0.02671689 0.2968447 0 0.2051709

0.03344608 0.01419151 0.2130656 0.07303045 0.1218929
0.07082335 0.410463 0.3665852 0.07409796 0.076723256 0
0.1283303 0.5662837 0.1409362 0 0 0.2216165 0.3145501
0.13415479 0.1545989 0.037737525 0.9619842 0.1672654
1.173553 0.1126876 0.6001015 0.2846951 0.06185453
0.1807486 0.2580094 3.309543 0.05131982 0.1353458
0.1878959 0.2424498 0.9600592 0.02824645 0.09749838
0.034629845 0.1044155 0 0.0314437 0 3.15148 0 0.1088206
0.1095417 0 0.07232413 0 0.02026279 0.1263897 0.01804371
0.03635193 0 1.994169 0.3760937 0.02117713 0.05441039
0.03624532 0.05640621 0.02229205 0 0.09865008 0 0.2495677
0.111746 0.04216482 0.1077446 0.282612 0.3734736
0.1649389 0.1218568 0 0.03696193 0.1741833 0.0508356
0.2678435 0.01725612 0.09563297 0.03619152 0 0.01774288
0.6810882 0.3147967 0.06148179 0.1399661 0.6491704 0
0.3930615 0.029512951 0.6057117 0.300354703 0.07125 0.02412171
0.1243475 0.09370447 0.05490069 0.4533249 0 0.03232792
0.157790124 0.2089077 0 0.06099929 0.03102541 0.1325893 0
0.3578959 0.04531519 0.05907934 0.03955089 0.04385568
0.4195683 0.1074293 0.4838595 0.106776 0 0.08074055
0.1692856 0.1343345 0.06190073 0.01984577 0.1496711
0.3030239 0.1713557 0.1501088 0.2528578 0.2394214 0
0.04519187 0.01604441 0 0.06393705 0.07409452 0.1214514
0.02359366 0.1127628 0.1330265 0.716608 0.1001806
0.1456799 0.09087332 0.0215956 0.3946033 0.1370505
0.0209543 0.01551165 0.08205205 0.040599845 0.1827184
0.33027497 0.041104747 0.02805303 1.28055 0.1428122 0
0.04559794 0 0 0 0.1366155 0.1021551 0 0.1173211
0.1370407 0.5923002 0.03321028 0.06632829 0.03998481
0.1906438 0.274688 0.02596609 0.3396835 0 0.2068489
0.3260116 1.175805 0.3138019 0.1116921 0.4224338
0.0883829 0.2187904 0.3904679 0.8252397 0.07177136
0.06480541 0.081185632 0.2389819 0.03780994 0.1973358
0.02742466 0.4001075 0.06068058 0.3235536 0.119144
0.1017367 0.06390411 0.08129239 0.1822054 0.2320598
0.1064323 0.02732834 0.035791102 0.1499237 0.01954385
0.07433881 0.2664249 7.536429 0.070040938 1.446143
0.1103342 0 0.2886962 0.01428635 0.1519147 0.1543678 0
0.2755382 0.111517 0.03159897 0.06087712 0.1128739
0.1501935 0.09001244 0.03318924 0.05631531 0.3082289 0
0.06683956 0.1700753 0.1295895 0.1669713 0.3728302
0.0165241 0.0163711 0.042382789 0.651668607 0.2750198
0.01316022 0.015263 0.09852498 0.2603758 0.2035232
1.109015 0.09787487 0.08575125 0.07139627 0.1398644

0.07811089 0.2608366 0 0 0.2829143 0.08054166 0.1405739
0 0.025705231 0 0.0536349 0 0.1068828 0.3868251
0.06039839 0.1917299 0.2622144 0.01929069 0.07060288
0.3721524 0.1312179 0.109586 0.2909395 0.2023216 0
0.3516107 0.114388 0.051690658 0.03018138 0.02597005
0.2324448 0.1078731 0.08033391 0 0.04666064 0.1966823
0.1666141 0.710306 0.3386079 0.3116885 1.181638
0.7725176 0 0.01960504 0 0.04877623 0.1315472 0.1284444
0.1870622 0.05491071 0.09022255 0.1672858 0.1193494
0.03724061 0.193987785 0.01797691 0 0.1276712 0.2917397
0.06752384 0.1773724 0 0.2647172 0.1167793 0 0.0744094
0.02827236 0.1342277 0 0.01928889 0.03605308 0.290808
0.2383364 0.4454703 0.09489009 0.1551385 0.344351673
0.192805 0.3294446 0 0.028504938 0 2.38891 0.1741609
0.0745487 0 0.026371 0.09325477
RAD51-AS1 0.8480738 1.523606 2.584544 0.9983437 0.9438472
2.194896 0.9000199 1.365472 0.8199528 1.041078212
1.911838 1.447372 2.367942 0.891529 1.005533
1.459674 1.367031 1.231932077 0.6029007 0.4939933
0.850861697 1.407682 0.8500534 2.326488 1.584133
2.087346 1.288184 1.916942 0.446961251 0.5637012
0.8230775 1.090386 3.125661 2.778953 2.120104
2.10322 0.1961032 0.9540359 1.954796 0.90215427 1.653353
1.01947 0.454724403 1.233581 4.057735 2.26292 5.831131
0.8194855 2.163188425 1.16314 0.623003 5.164545 2.435396
2.653398 2.271527 1.558552 1.559641 3.108678
1.477416 0.778100021 1.575558092 1.074788845 0.8105185
0.52126 1.284956 2.002646 0.9000251 1.246748
0.370092461 2.639011 0.9218576 1.58324 3.813389 1.805221
1.813156 0.6661821 1.935447 4.630131 3.689152
1.090908793 0.5660056 2.245781 1.816827 3.884554
0.4240943 1.25244 2.221201 2.011306 2.43574791 6.869989
1.172221858 2.888686 0.636654 3.767561 1.104338
2.284136 1.278756 1.560538 0.9417412 2.040507
4.998023 3.427737 1.500083 2.496961 3.208671
3.431289 0.4943129 0.7251442 0.848431201 2.009998
1.976552 3.342859 0.4660377 5.458429 1.122533
0.8977699 1.773206 1.072296 1.822568 2.607935
2.216243 3.879899 1.634084 0.6838707 3.417282
4.960496 2.303574 0.2872148 1.967843 0.2220026
1.536188 2.077341 1.394616 1.536475 0.3447109
4.083551 1.556296 1.94617 1.536707 2.753861 2.707684
2.985651 1.25284 2.669728 1.503888 3.77562 5.504542
2.680528 3.102867 2.309536 2.88175 0.9694847 1.218714

2.205023 3.217723 2.286354 2.250392 6.633711
1.660499 2.72277 0.490652806 4.467124 4.283808958 3.615938
0.633195 2.155681 0.6559313 1.961556 5.436565
2.83695 2.849908 0.769226856 5.438127 0.9937488 1.245402
1.540606 1.301033 1.887367 3.181035 3.181976
1.654222 2.75991 0.7482875 2.785341 1.316009 2.794288
1.968682 1.773341 2.069986 1.599749 1.219506
1.056181 0.6338242 0.7857733 1.222617 3.051203
1.089071 4.952609 1.160944 1.020416 3.044802
2.990276 1.410896 2.076955 1.215613 2.391075
1.269634 3.137625 2.146092 7.218907 3.703551
1.921154 2.464939 3.061177 1.792157 1.926202
0.4767102 2.612743 1.471809 1.17232051 1.390944
2.474482 0.332691549 2.638738 6.364333 1.513413
2.626948 1.516131 0.7107085 1.215649 0.8988148
2.45054 3.229059 1.791048 4.31155 4.904344 1.19694
1.917894 3.956206 2.597763 5.524088 2.163168
1.851707 1.422425 2.582582 1.242269 1.811402
2.966457 1.996921 1.966828 3.62699 4.485432 10.91217
2.702216 1.156559 1.119932 2.374511 0.443983926
2.412798 2.605341 2.472303 0.6959008 3.205549
1.637111 1.15266 4.313385 1.765556 1.164919 3.065739
2.462303 1.108978 0.4190772 2.845564 2.654133934
4.967074 3.719438 3.42307 2.152948 1.23989 0.868215798
4.03453 2.238577 0.4324176 0.7815097 1.325059 3.555754
1.904513 2.620775 1.567124 1.163958 2.073682
2.46806 1.326268 2.270634 0.4463117 0.7840959 2.430944
1.078801 2.159602 1.089276 2.306647 2.900916
1.497524 2.291352 2.183247 4.304577 2.262181383
3.349983933 3.015549 1.295459 0.4473966 0.4202706
1.960955 0.6010295 4.201338 2.148149 2.701164
1.761703 1.582216 2.614274 2.202439 2.301104258
1.187272 1.290797 0.3523698 2.82905 0.838698
1.248310288 1.901573 1.533064 0.9903294 1.594557
1.023878 2.642429 0.8807593 3.720167 2.903249
2.702766 5.150149 0.4362996 6.136815 2.187006
1.814572 2.382465 3.256061 4.420618 3.957566032
1.927836 1.567942 2.644059 3.099103 1.84517 1.48287
1.735193 1.591897 1.83449 3.09962 1.987558 3.019482
4.71235 3.00799 0.512853 3.516654 2.611523 1.739177
1.9321 1.95744 3.028069 3.038965 2.352553 4.775486
1.148738 1.401178 2.096280502 1.274114 0.8409379
1.468218 1.680542 3.028022 4.888826 2.731541248
1.546454 3.463965 1.637039 2.845229 0.4452897

0.3523476	2.280576	0.7173056	0.5914959	2.417341
2.043735	5.996716	3.725919	1.862739	2.162958948
5.061131	2.685407	0.7600226	1.633689278	1.314268
10.26414	1.104833	3.522426	1.177355	0.6807016
1.312367
WWOX-AS1	0.03279598	0	0.211169	0	0.1290523	0.232495	0
0	0	0.041089773	0.1407569	0.1288647	0	0.1537463	0	0
0	0.049386189	0	0.1400907	0	0.03266201	0	0.667031	0
0.1031133	0.155739	0.02593251	0	0	0.09267956	0
0.06331436	0.1032919	0.05339931	0	0	0.2061879	0.4312712
0.058047827	0	0.05782194	0	0	0.06754263	0.1447932
0.1908047	0.03525116	0	0	0.03785923	0.3094235	0.3166564
0	0.3197209	0	0.2889192	0.03361976	0.0328849
0.133384081	0.037234157	0.089451461	0.03266341	0.07675437
0.2707915	0.1555626	0.04474928	0.1988792	0.032950644
0.2749197	0	0.02376997	0.8759605	0	0.1531227	0.05931255
0.1018522	0.1067843	0.07446594	0.160633905	0	0.07164868
0.1405175	0.1348915	0.03454512	0.03175427	0.06749002	0
0.67410511	0.03699206	0.063208224	0.3295778	0.02801601
0.1937951	0	0.1005136	0.09536965	0.1036028	0.1376108
0.1994544	0.4516761	0.05730521	0.07556555	0.07867877
0.169204	0	0	0.04082607	0.029001518	0.1311676	0.05958358
0.4739979	0	0.1807723	0.2323002	0.05207667	0.2568666
0.02598242	0.09691106	0.06862082	0.1357561	0.176413
0.2115554	0	0.1891499	0.09543203	0.02999694	0	0
0.03035443	0	0.1120138	0	0	0	0.08360236	0.03119471
0.07062369	0.07218642	0.1290979	0.02234097	0.3453291
0.05102581	0.1035902	0.3714548	0.3182696	0.340587
0.5327396	0	0.03203597	0	0.06186027	0.02971833	0.09778152
0.04793334	0	0	1.009658	0	0.2194519	0	0.5797331
0.067076957	0.0895048	0	0.02603438	0	0.09195556	0.2233216
0	0.02707372	0	0.3848994	0	0.06811358	0.1558974
0.2220796	0.09445329	0.03155029	0.1138506	0.09895456	0	0
0.03055449	0.2399173	0.1620875	0	0	0	0	0.08437604
0.06912016	0	0	0.05639421	0.1435056	0	0.1303146
0.1336724	0.03830004	0.8578634	0.4299756	0	0.02677274
0.1861562	0.05085608	0.1580722	0.1510971	0	0.4000927
0.1258476	0	0.09132461	0.2531999	0.251786	0	0
0.05196227	0.03435814	0.068002448	0.1836259	0.069148992
0.068848132	0.02349362	0.4003718	0.02657805	0.06772472	0	0
0.09832481	0	0.2669604	0.299432	0	0.07860247	0.2295354
0.0944828	0	0	0.8036677	0.1473774	0.09859003	0.1304752
0	0.205272	0.06299276	0	0.4595282	0.09556373	0.07015423
0.1474068	0.03700909	0.4275386	0.3567337	0.1639934

0.04007099 0.1628181 0.16997669 0.03079087 0.09499428
0.1502392 0 0.04188484 0 0.3096766 0.1496696 0
0.03567859 0.1361602 0.2373651 0.08969703 0 0.3661874
0.119896153 0.02282852 0.1636742 0.09338499 0.1049992
0.03516182 0.039104873 0.1755223 0.04620087 0.07524936
0.03022187 0.04785767 0.06361215 0 0.2205161 0.08653331
0.02668352 0.02646325 0.03398858 0.0270082 0.1677105
0.1005105 0.2223604 0.2200916 0.2950092 0.02658492
0.05597624 0.04069522 0.07235171 0.08390024 0.3865764
0.1107078 0.05484134 0.03549439 0.045479506 0 0 0.02556466
0.3713035 0 0.04261123 0 0.02732248 0.07181426 0.2391694
0.06693286 0 0.04368865 0 0 0.3384754 0 0.1177267
0.03029158 0 0.2679778 0.02994514 0 0.05370678 0.1295821
0.1686065 0.0535228 0.09411307 0.1292433 0.1478198
0.03280707 0.02197826 0 0 0.03388772 0.07326208 0.294464
0.1277289 0 0.4044169 0.1304951 0 0 0.3027482 0 0
0.1098106 0.04651153 0.1830342 0.09452486 0 0 0.388177
0.1090793 0.09851201 0 0.2450923 0.03147631 0 0.03916482
0.09197235 2.538777 0.3152185 0.1599229 0.2495037
0.162459296 0.03011032 0.08150588 0.2749397 0.7329714
0.05654931 0.1320394 0 0.1043262 0.3520776 0.0979267
0.07477892 0 0.04496475 0 0 0.2415475 0.05412077
0.15968 0.9183233 0 0.08661606 0.04119783 0.0587159
0.2904217 0.0632544 0.047744163 0.3299409 0.113244 0
0.02497297 0.03108575 0 0

CYP1B1-AS1 0.0546262 0.1287221 0.13818 0.03168208 0.2354262
0.5611207 0.2592305 0.4138556 0.1475835 0.266429688
0.09712927 0.06899199 0.3524912 0.03475446 0.04452831
0.833966 0.06226627 0.572878465 0.0915375 0.01666717
0.148182835 0.343905 0.1042426 0.3081012 0.1630167
0.1226782 0.06793934 0.4921048 0.173423695 0.1150983
0.1598838 0.421457 0.03766384 0.08602339 0.0190594
0.03849051 0.02436059 0.03153988 0.1780762 0.037984028
0.1118589 0.1341466 0.016244733 0.007525152 0.04620598
0.02296885 0.1066938 0.0817825 0.119189614 0.1994516
0.0472948 0.3092324 0.3244142 0.04106839 0.483089
0.04077115 0.02005144 0.6179815 0.01956225 0.044962872
0.230354786 0.130369469 0.2506534 0.1324108 0.01610859
0.3675144 0.1038181 0.1676021 0.013720954 0.02589409
0.02665985 0.005656022 0.2122931 0.1036911 0.05009851
0.09526487 0.3302093 0.04128983 0.07382931 0.007644509
0.02374541 0.3644154 0.02298717 0.1965953 0.09863938
0.06800283 0.3613303 0.1301856 0.48788964 0.204651
0.035720668 0.01960561 0.03499838 0.05764147 0.1032967

0.06577183	0.2552966	0.02054342	0.02946981	0.1483119	
0.3834921	0.02215796	0.008990342	0.05382423	0.2435838	
0.1765991	0.04221606	0.2088615	0.029328647	0.06242216	
0.03780748	0.07519128	0.02054259	0.2580867	0.2487393	
0.02168521	0.06985249	0.03709481	0.1253877	0.01224615	
0.03432185	0.2329731	0.2265265	0.2100768	0.08626508	
0.1343549	0.06423945	0.02321038	0.6957351	0.09931324	
0.06322692	0.04442246	0.09925423	0.03145357	0.09595104	
0.2237966	0.1243306	0.03781075	0.08373605	0.1484733	
0.06910792	0.1540696	0.05008368	0.09243413	0.07733862	
0.104131	0.02532564	0.1367721	0.03094841	0.2477442	
0.06130252	0.07359769	0.01767856	0.1861355	0.3749607	
0.055133	0.1795529	0.2633468	0.03288405	0.1109636	
0.241128773	0.2306292	0.225446851	0.02484709	0.03124454	
0.04181513	0.09647689	0.05834842	0.15676	0.09126989	0.2915069
0.060641243	0.2227207	0.02580831	0.02025938	0.08964749	
0.1094614	0.05618747	0.01313783	0.1444829	0.06082731	
0.05517049	0.05899051	0.0472575	0.08206383	0.02892631	
0.5836187	0.06863074	0.09814634	0.02530078	0.1907327	
0.01850289	0.04152501	0.04473853	0.03019252	0.1382949	
0.2492743	0.1492266	0.109337	0.01594849	0.02851775	
0.2637729	0.03251466	0.007963155	0.07013459	0.02420222	
0.07522596	0.112354	0.01491122	0.1775871	0.1372489	
0.1700349	0.07243508	0.07315884	0.1179518	0.06656988	
0.1962562	0.01700095	0.2636585	0.058656335	0.04369344	
0.106950193	0.018430073	0.04332454	0.4474181	0.08379562	
0.0282012	0.02725957	0.02142247	0.05069179	0.03301589	
0.06806007	0.06870468	0.02924553	0.0981924	0.06554104	
0.2444918	0.0264719	0.09472586	0.1155355	0.100821	
0.1505306	0.2095628	0.04351526	0.1098992	0.1199121	
0.1169387	0.3319368	0.2728706	0.04729703	0.1350394	
0.03742654	0.152598	0.2794103	0.03762828	0.05244151	
0.09362715	0.107180951	0.01465326	0.3070338	0.06434847	
0.01366262	0.1420215	0.07053734	0.05756795	0.08903403	
0.02196304	0.03820345	0.137696	0.1835619	0.1298381	
0.06097669	0.09257948	0.024962936	0.06382605	0.3972489	
0.02407252	0.4013115	0.02719174	0.053503364	0.5053592	
0.05084453	0.07385997	0.1420269	0.01138765	0.3746258	
0.0415282	0.07214819	0.06005546	0.436514	0.03305863	
0.3659603	0.2747351	0.03824363	0.1554559	0.3406098	
0.1384075	0.08774612	0.02055896	0.06659722	0.08957102	
0.1054477	0.1081378	0.09729198	0.02304987	0.07992756	
0.050674951	0.108217651	0.06336774	0.01049001	0.05930987	
0.05890066	0.0463271	0.2281335	0.05022699	0.6923921	

0.02776811 0.04695069 0.06768784 0.06809923 0.1117531
0.069874631 0.03471256 0.02416188 0.5276414 0.1820835
0.07928608 0.005122407 0.0664217 0.04097101 0.009892119
0.1916915 0.1715131 0.1103289 0.2515291 0.03918956
0.0384415 0.06682955 0.09953136 0.04053007 0.1173778
0.02371784 0.02620645 0.05665588 0.4379198 0.09687725
0.053220023 0.01353238 0.03105107 0.09264081 0.007165464
0.1540821 0.02329054 0.01859659 0.1045169 0.016601
0.1052523 0.1771246 0.3593605 0.1101883 0.2847952
0.3503956 0.02734754 0.03699435 0.08018899 0.0973665
0.006398937 0.06290459 0.0300914 0.019777 0.05625424
0.07610671 0.0630795 0.110172142 0.08776751 0.08727379
0.08177674 0.0823599 0.03868544 0.1138922 0.12583559
0.04033937 0.1512626 0.04466117 0.03410422 0.01126794
0.5510129 0.1500444 0.0461255 0.02873789 0.255949
0.08074058 0.6657829 0.1063643 0.05152533 0.294088444
0.1012922 0.2885146 0.08278198 0.059643339 0.02208061
0.3480552 0.09833334 0.01337011 0.02034119 0.0867088
0.2663611
AC002546.1 0.09951108 1.712794 0.1601847 0.02376475 0.5873648
1.209338 0 0.1192626 0.2688489 0.436367764 0.1921911 0
0.3841767 0.3498781 0 1.253285 0.0496251 0.262237281
3.686084 0.05313372 0.116282147 0.8671651 0.4430907
1.607244 1.669809 0.2933169 0.03937922 0.7081707
0.09614996 0.28225 0 0.2229999 0 0 0.02025333 0
0.2329794 0.06703125 0.0577316 0.044032855 0.02009004 0 0
0 0.1280881 0 0.2315794 0.08022053 0.126655973 0
0.1435928 0.2347168 0.3469605 0.1496264 0.3395392 0 0
0.7140745 0.02494522 0 0.141222017 0.033927224 0.2229949 0
0 0.2360077 0.03394508 0.1508622 0 0 0 0.03606198
4.23292 0 0.0967942 0.2249611 0.03863059 0.02025061
0.07531601 0 0 0.1630498 0 0.02558088 0.1048184
0.04817514 0.691137 0.09222716 0.76702542 0.3367291
0.023973667 0.05555669 0 0.04200157 0 0.07624577 0.5305209
0 0 0 0.2491812 0 0 0.02984135 0.154022 0.431622
0 0.1238764 0 0.06633245 0.03013188 0.1997537 0
0.6856348 0 0.01975168 0.02783559 0 0.05513476 0.07807968
0 0.2141122 0.0229254 0.6004294 0.04782728 0.3136947
0.1365272 0.05381311 0.3456551 0.1151285 0.0179167 0
0.1518794 0.05013586 0 0.1268351 0.02366311 0 0.3285471
0.2937863 0.05084101 0.3143441 0.01935311 0.02619319
0.1643666 0.1810703 0 0.02126925 0.04384946 0.5832301 0
0.02346241 0 0.02472444 1.381695 0.1562311 0.0444584
0.1178289 0 1.082041 0.112492857 0.2748521 0.025441003

0.02263164 0 0.05924605 0 0.02325132 0.06776129 0.1225103
0.9241689 0.057279977 0.4512262 0 0.05166835 0.03941927
0.04813174 0 0.04786568 0.05757512 0 0 0.02786037
0.06953239 0.113745 0 0.05814171 0 0.0410339 0
0.08533924 0.02621595 0 0.1521314 0.08555697 0.06531471 0
0.04942584 0.1013988 0 0 0.06115557 0 0.02030877
0.04707032 0.09644367 0.02997687 0.1432705 0.02112707
0.02334577 0.09546317 0.0264419 0.09236713 0.1371912
0.3342416 0.04353231 0 0.01970829 0.2085021 0 0.09286102
0.262268865 0 0 0.9545067 0 0.02568669 0.1158687
0.09105774 0.1243091 0 0.1157177 0.0973447 0.01962792
0.05962482 0.0696467 0.7883813 0.04219525 0.05618218
0.2032105 0.05589741 0.1495732 0.03299116 0 0.4411824
0.07167584 0.124264 0.2738846 0.2174729 0.1241714
0.1118172 0.1122947 0.2779837 5.9984 0.1243991 0.668719
0.102923 0.128937798 0 0.1200984 0 0 0.2541779 0
0.146818 0.1135336 0 0.05412883 0.1446002 0.2057784
0.1587617 0.1352273 0.2777758 0 0.03463368 0.09932557
0.07083827 0.1393847 0.02667241 0.029663457 0.1065156 0
0.08562192 0.7106796 0.0181515 1.254597 0.01961317
0.08363754 0.06564085 0.04048219 0.020074 0.3351717
0.04097477 0.04240624 0.03812171 3.71083 0.0715513 0.05594566
0.0604989 0.08492286 0 0.1646497 0.0212145 0.1353425
0.06298399 0.04160054 0 0 0.02183911 0.01672069 0 0 0
0.5494952 0.16012 0.3108868 0.1089511 0.03628495 0.1015454
0.04962182 0 0.059401413 0 0.05135088 0.6139922 0 0 0
0.1016388 0 0.07568487 0.08147981 0 0.05115937 0.2436021
0.2855623 0.07352923 0.2018348 0.09954473 0.08335933
0.3480856 0 0.02570593 0 0.9679328 0.1211128 0.02189182
0 0.0329962 0 0 0.1020681 0.02121387 0 0.2498943 0
0.1851236 0.1792573 0.1697208 0 0.8179338 0.413717
0.04981829 0 0.1549314 0.04775344 0 0.05941787 0.03488335
0.04585283 0.02656807 0.3639341 0.02365799 0.147882469
0.4111296 0 0.02317319 0.1235564 0.08579228 0.3004801
0.135601626 0 0.5044704 0 0.01890814 0 0.3751939
0.3679469 0.07352238 0.02290358 0.2257967 0.1514089
1.349672 0.0602812 0.06570362 0.718775154 0.1781585
0.1321816 0.1919295 0 0.03128506 0.744489 0 0.03788708
0.07074138 0.03350563 0.2172219
AC130456.3 0.7115121 0.02194733 3.46239 0.9647062 1.926746
0.3021753 0.2836974 0.5226451 1.204754 0.862690501
0.03940305 0.0901849 0.9215384 0 0.2993476 0.03400793
0.6409712 0.656688076 1.048564 0.784331 0.858246744 0
1.255574 0.1647587 0.07859953 0.1443261 1.017265

0.108892 0.354828287 2.13529 1.556665 0.1028688 2.038262
1.734912 5.474863 0.5031399 0 0.9688645 3.213513
3.249944676 8.618722 4.633383 0.692784594 2.685425
4.396033 1.688873 6.569831 1.184172 1.051664759
4.89459 1.11281 1.472524 0.4185957 2.933433 0.8055149
0.5925173 0.8087912 0.4235134 1.518939 1.306868989
1.563481671 1.064232158 0.8457913 0.1880058 1.137068
0.6532156 1.785093 1.113472 1.660337653 4.232809
0.01960268 2.711542 9.422559 8.838819 2.303977
0.2490565 2.174054 8.407364 2.258291 0.85437951
2.035305 4.387491 1.180081 3.469301 1.06375 1.355601
2.149072 2.666089 2.75197465 2.873635 1.592487768
3.921091 0.7450578 2.90627 0.3698574 2.297892 1.290378
2.151 0.6934017 1.535452 1.4943 2.306014 4.706671
1.706944 1.278894 2.441203 1.225084 0.571436
0.649487392 3.48827 1.84866 3.169803 0.4027926 2.30252
2.601175 5.102362 2.362639 3.945838 1.458182
1.824902 4.679136 0.2963069 0.4230154 0.4261159
0.4412501 1.224435 0.7347592 0.4964748 1.658271
0.3611362 4.744048 1.123618 6.725881 2.150852
0.6670341 1.433457 1.681015 2.075868 2.22284 2.891141
3.017584 3.310959 7.052726 0.7249679 0.281623
3.730869 2.592134 3.041537 1.638432 2.219591
1.972695 0.8874946 1.372677 1.779222 1.375376
5.261016 2.153388 5.027738 0.1934341 0.6911176
0.276759677 2.358257 0.680677463 2.42205 0.4806814 1.020317
1.061787 1.651762 10.09633 0.480364 1.212629
0.502036825 3.403842 1.051009 7.57933 2.091152 1.776236
2.908505 8.942488 1.301398 2.100661 1.3213 0.8739265
3.100581 4.869222 2.750814 0.5185297 3.353874
0.7192925 3.611534 3.562677 2.999138 0.7676832
0.912308 1.420813 2.269746 0.4105663 0.9119966
1.613731 0.6432955 0.4944214 3.968317 1.282575
6.05195 1.780492 6.068303 0.9403171 0.07930805 3.703412
6.181575 0.8513766 1.268543 1.107822 1.113828
5.506576 2.188857 0.3930006 0.05454807 0.6492223
1.189774274 2.356 4.355403334 1.325027441 2.285413 2.821984
1.246229 1.137518 0.5344971 1.008108 1.628547
0.6732599 2.989282 2.993644 6.33799 1.155196 13.04385
0.3636767 1.381978 2.332493 0.6561805 1.358021
3.081886 1.156619 1.137645 0.4788599 2.380589
1.299308 1.516101 1.070072 3.1422 3.094844 3.211664
14.44754 3.370373 2.524928 0.05608673 1.519293
1.23715104 4.309748 0.4875273 4.500151 0.8036769

2.550211 2.157596 3.575958 0.3142353 5.426119
0.8739283 2.420381 19.38835 2.176157 2.55757 2.530699
1.783052785 5.368056 3.092744 10.17355 0.6613458
0.02460772 0.602079135 1.375785 1.584332 0.2106507
1.459387 1.205741 6.677769 3.419943 2.276315
2.846304 1.49394 1.426047 0.04757328 0.4158324 4.381844
0.03517074 0.8947988 2.288436 1.806523 4.353627
6.953478 2.933463 2.70896 0.8807537 2.538936 3.195965
1.189789 1.092980496 8.084431215 3.908821 1.234109
0.1968035 0.1154906 2.528894 0.1491057 3.368136
0.4015498 3.065777 2.761784 3.396076 1.213187
3.271542 0.931654386 3.438988 1.137021 0.3304371
1.565411 1.950337 0.120526265 1.719136 0.8801886
0.8146387 1.227818 1.473663 1.392375 1.123725
2.941938 8.117875 2.02763 10.14822 3.583845 1.348791
1.395156 0.7589137 4.717013 1.23647 2.212404
4.867523388 0.6898806 2.130939 2.062993 1.264484
0.8945873 2.642183 4.266242 1.844401 6.542696
3.159678 3.60531 0.3131657 5.238563 4.497525 0.1145075
0.09192379 0.8704483 2.572888 1.101422 3.312365
3.535785 18.50526 0.9095231 1.397153 4.644711
0.3273996 0.932305908 1.095768 0.6559743 2.950353
2.393833 3.601378 11.45843 2.043378691 4.818783
3.613848 0.6624878 4.029674 1.060504 0.1888093
4.345144 1.537504 0.08452255 2.575567 0.05587539
2.249384 0.8898382 3.89973 3.315676854 3.266802 2.987764
0.7525575 0.835334291 1.212259 2.562511 3.13031 5.924749
2.393063 0.5255037 0.7287524
AL133346.1 0.1761045 2.245279 0.8909321 1.177579 2.502398
2.586029 0.453486 1.829174 1.404687 2.096076496
0.4031051 0.05766368 1.586378 0.2476713 1.052706
1.696068 0.7025711 7.513684624 0.761046 0.5641835
2.126436345 3.44924 0.7841363 2.282491 3.055568 0.299914
1.486705 3.458035 0.397031349 1.398592 1.244153
4.419994 0.198321 0.647087 1.266427 0.5147274
0.6413602 0.7644722 1.748198 2.415669105 1.825068
1.604179 0.244392977 0.3585038 0.9067087 0.7990927
0.6147381 0.9148922 0.597713843 0.5626196 1.897397
1.107674 1.007614 1.544628 1.144536 0.1443246
1.163556 6.408741 0.824049 1.671208223 0.199936141
0.600409052 0.9646593 0.7556042 1.130942 1.292764
1.161402 2.195171 1.651393073 0.5125818 0.150406
0.3190939 0.5806966 0.1642079 1.575927 0.9554713
2.050933 0.7884239 0.4665028 0.057503693 3.470297

2.244268	0.3458292	1.569374	1.236646	0.5115324	
0.785202	0.2538886	0.40219335	1.522877	0.056568168	
0.1638642	0.300875	0.8424073	0.3378351	0.449773	
1.052663	2.441608	0.1477856	1.13051	2.021137	0.7436363
0.3381368	1.020996	1.66572	0.8856118	0.8044818	1.936475
0.389323408	0.2739061	0.3554953	0.9898097	0.3090518	1.2619
1.039485	0.3961509	0.2298826	0.2092766	1.409371	
2.333662	0.1518686	0.8525557	0.9466571	0.8173677	
0.6771183	1.480385	0.4026862	1.174539	2.093389	
2.906728	1.226009	0.2673245	0.7764769	0.1774506	
1.640375	1.010068	0.809613	0.2212162	0.4199208	
0.5391687	0.7797676	0.2472417	0.570819	1.205204	
0.6925694	0.1661539	0.4572123	1.104111	0.206934	
0.9174583	0.2441283	1.051875	0.8244885	0.4958873	
1.222591	0.2764818	0.9965866	1.424896	0.4947223	
0.3927968	1.327188451	1.426788	0.510259155	0.4272121	
1.554803	1.048476	2.575138	0.3017505	2.318394	
1.047896	3.052931	0.405472804	0.9081363	0.2986706	
0.7619776	0.6278415	1.731965	0.3803892	0.9600211	
0.9170146	0.265678	1.126441	0.4601745	1.203168	
1.476158	0.5077103	2.240785	0.5559677	0.7503815	
0.4567634	1.736783	1.917631	0.7734637	0.2692266	
0.6561096	0.950384	1.125058	0.4956563	0.1196301	
1.096851	0.1129036	1.106319	0.9495556	0.7427677	
0.3332008	0.5006498	0.6012331	3.042545	0.9222499	
1.735227	1.351527	0.967079	1.362182	0.9064045	
0.5915064	0.9244679	2.355771	0.04650361	2.890387	
0.699875703	0.6299542	1.144869826	0.616156011	1.05128	1.407656
0.11893	0.1818306	0.1367017	0.2506694	1.789247	0.5629331
2.047852	0.6125178	0.4862967	0.7386251	0.4108452	
0.9724093	1.144983	0.530269	1.048895	1.406883	
1.264674	1.47907	1.091105	0.6429782	1.042944	1.172852
2.056273	0.8124847	0.7743421	0.8179142	0.4305765	
0.4372862	1.809132	0.230632	0.3944767	0.4371419	
1.916718971	1.267588	1.700302	1.344565	0.3699836	
0.1499393	1.455353	1.697513	1.026925	0.2795876	
0.5747497	1.096708	2.033257	3.371521	3.549786	
1.065088	0.187776753	1.021519	0.5859205	1.365056	
1.245089	0.4090847	0.104990659	2.104921	0.6408854	
1.313215	2.028528	0.2998114	2.675694	0.8561645	
2.269534	2.529803	2.125356	0.615765	2.889716	
3.021372	4.777941	0.9894701	3.681525	1.463212	
1.848128	0.5472197	1.052014	0.2549412	1.100769	
0.2753171	2.980633	0.4953893	0.2944814	0.063531373	

1.058248381 2.293151 0.5720842 0.5033398 1.033816
0.362422 1.754206 1.057892 3.032078 1.606753
0.3852802 0.3594092 0.4390779 0.3127931 1.156347503
1.099434 0.7875877 0.6338384 0.8428754 1.274143
0.269723173 1.07922 0.616386 1.160807 2.130875 2.986212
1.297691 1.389106 2.442567 0.318082 1.05833 2.759903
0.3147109 0.1642683 3.251042 0.1819668 0.5573099
1.976478 0.3143551 2.350341169 0.113104 2.141087
0.8461929 1.078004 1.685876 0.3754213 0.419663
0.2948248 0.4162547 0.7644298 0.2537849 0.6674541
0.7947978 0.9649967 1.95241 0.02938776 0.5137471 0.3290178
0.7887518 0.03208949 0.5958592 0.6379072 1.10899 0.3447945
1.645913 0.3628514 0.145392865 1.104836 2.662443
0.7108317 0.3644291 0.4048702 0.9453489 2.319746607
2.567584 1.050306 1.343805 0.2900012 1.398539
2.494953 0.3472824 0.6650186 0.6214969 0.9444895
0.2858111 1.027316 0.391158 0.904365 0.995489383
2.968949 0.961677 0.7359235 0.854572293 0.9596614
1.790476 0.1740436 0.2234955 0.1947412 1.818374
1.607562
AL590609.2 0 0 0.127276 0.06608864 0.09074631 0.4203889 0
0.1658317 0 0.086679855 0 0 0.2136754 0 0 0 0
0.104181341 0 0 0 0 0 0.8277164 0 0 0 0.05470525
0 0 0 0 0 0 0 0 0 0.0621369 0.1605488
0.367359818 0.1676083 0 0 0 0.07124139 0 1.288023 0
0 0 0 1.044378 0.9648806 0.3120779 0.1348918 0 0
1.347512 0.2081144 0.093792298 0.078546341 0 0.1378085 0
0 0.3281631 0.09439966 0.3496171 0 0 0 0.05014333
0.7528316 0 0 0 0.1611447 0 0.05236256 0.067772209 0
0.2267169 0.07410626 0 0.07287375 0.06698638 0.4983013
0.2564793 0 0.3121426 0 0 0 0.642424 0.1592651
0.1413572 0.7376767 0.07285089 0 0.2103769 7.276092
0.2417734 0.3188147 0.08298737 0.2855519 0.7828175 0
0.1722471 0.122358785 0.1844674 0 0.1666516 0 6.787915
0.4900428 0 0 0 0.05110905 0 0 0.2232884 0.06375446
0 0.06650269 1.74474 0.4429548 0 0 0 0.04982549
0.3938263 0 0 0 0.08818058 0 0.07449118 0.5329764
0.7262272 0.2356441 0.218544 0.1076402 0.218526
0.326497 0 0 0.1182976 0.1219433 0.1351613 0 0 0
0 0.8594903 0.2172357 0.2472734 0.08191913 0 0
0.208558185 0.9172206 0.070750219 0.0629375 0.1704601 0 0
0.06466081 0.6595432 0.08517379 0.1142253 0 0.1476281 0
0.2873744 0.2192463 0 0.09962573 0.1331122 0.8005683
0.3479117 0 0.07747836 0.06445543 0.1265278 0 0.1616893

0 0.1141133 0.05981425 0.05933109 0 0 0.3173027 0
0.4238199 0 0.3436273 0.1409926 0.08079485 0 0.05669024
0 0.1129555 0.1309003 0.05364105 0 0.1593714 0
0.06492346 0.1769857 0 0.3853029 0.07630446 1.128691
0.06053063 0 0.05480782 0.1449586 0 0.06456052 0.29174289
0 0 2.835422 0 0 0.3222254 0 0 0 0 0.09023699
0 0 0 0.4982843 0.05867149 0.07811999 0.1412797
0.103632 0.207978 0.3669874 0 0 0.3322118 0.1151908
1.246353 0.4031879 0.09866135 0.3109582 0 0.6442163 0
0 0 0.1717343 0 0.06495406 0.06679757 0.3169332 0
0.5301425 0 0 0 0 0 0.2297865 0.07153248 0.3784361
0.1880304 0 0 0.04815731 0.2071648 0.06566595 0
0.1483494 0 1.481074 0.09746184 0 0.3187687 0 0
0.0545433 0 0.1216961 0.2251581 0.05582485 0 0.05697444
0.4127541 0 0 0.1989808 0 0 0.05904161 0.3433902
0.3052553 0.2949826 0.1254603 0.4086961 0 0
0.191880201 0.1214671 0.04649945 0 0.0870304 0 0
0.08905723 0 0.1514939 0 0 0 0.368649 0 0.1295762
0 0 0 0.0639008 0.09082515 0.3768704 0.06317 0 0 0
0.07113588 0.1129076 0 0 0.1870976 0.2768292 0.04636366
0.3872038 0 0.07148696 0.07727406 0.1035298 0.0673618
0.060880109 0 0 0 0 0.4967314 0 0 0.115824 0
0.7078776 0.7976098 0.4719854 0.0535272 1.18281 0 0
0.2018292 0.08617134 0.3984002 0 0.1652383 0.09700893 0
0.07388454 0.2530208 0 0.342711754 0.06351843 0 0
0.8590114 0.05964606 0.1392701 0 0.1100393 0.8252401
0.06885957 0.05258264 0 0 0.2046485 0 0 0.05708453
0.6736976 1.573995 0.4190979 0.1218124 1.651248262 0
0.428856 0 0 0.08700227 3.742625 0 0 0 0
0.1647501
MYOSLID 0 0.05769378 0.08879722 0.06916253 0 0.06110304 0
0.01446207 0.0186293 0.045355738 0.3625309 0.4741449
0.09317241 0.3507305 0 0.08045812 0 0.018171164 0 0
0.014100659 0.02403533 0.3070301 0.04331074 0.09091171
0.1422733 0.01910089 0.0763329 0.023318761 0 0 0.03244982
0.1747192 0.6840941 0.2455969 0 0.02511259 0.1192161
0.1680162 0.064074387 0.2436168 2.616827 0.226073653
0.07757448 0.1739615 0.1687049 0.1684913 0.1037625
0.294885401 0.1156548 0.1810891 0.2390837 0.0517825
0.1088644 0.4234974 0.08900414 0.3366325 0.2968815
0.1451961 0.065436487 0.315098694 0.213933126 0.09614544
0.4094948 0.04981754 0.1880669 0.296371 0.1097635
0.096990932 0.101154 0.3091818 0.122443 0.09549661
0.4613232 0.5164501 0.08729389 0.1030577 0.2946767

0.1552611 0.330980557 0.1049075 0.3031679 0.1163296
0.09926403 0.3686056 0.2219898 0.1117453 0.1043811
0.28936876 0.4219369 0.302339005 0.02694776 0.2989383
0.1629828 0.2361198 0.406813 1.181374 0.1270655
0.03037952 0.1834682 0.1510816 0.03162733 0.2224288
0.15922 0.1245139 0.1456405 0.1392616 0.07510776 0.14939154
0.06434909 0.1461546 0.06782334 0.1058836 0.2527509
0.8119895 0.0958055 0.1350165 0.1816395 0.1961161
0.08836926 0.7367645 0.4284022 0.07783974 0.2016264
0.2087875 0.04681771 0.07725956 0.1566122 0.1564826
0.2233723 0.05214295 0.2885007 0.1227818 0.2553431 0
0.1538033 0.3328558 0.1169338 0.06640071 0.1900013
0.4685481 0.1270605 0.1126467 0.038115 0.1252837
0.04879341 0.06265783 0.1856997 0.1276151 0.1768098
0.1561285 0.2389892 0.09841112 0.1079333 0.1322745
0.6062391 0.08625815 0.05715288 8.339194 0 0.400140704
0.2266388 0.271483399 0.2854142 0.1635228 0.1532655
0.05774811 0.2030049 0.246507 0.1634148 0.1394611
0.027783643 0.14162 0.589403 0.3759258 0.1529625 0.2217898
0.208519 0.09286895 0.139634 0.08495517 0.1218719
0.3378417 0.10118 0.2979289 1.013854 0.2162125 0.5224565
0.2189383 0.06259631 0.1966205 0.1780246 0.3179938
0.01844783 0.2489964 0.179525 0.7709078 0.191792
0.0368876 0.3241189 0.1021196 0.06921483 0.2366002
0.2068661 0.0913258 0.018712 0.1308625 0.2223787
0.1537152 0.2944204 0.1389132 0.141082 0.2464146
0.02661784 0.1505436 0.1161344 0.1291369 0.05735703
0.1517009 0.162635424 0.03378167 0.394361755 0.037997993
0.2333947 0.1262682 0.1173496 0.1370526 0.4496168
0.01472251 0.1929475 0.1361403 0.5612883 0 0.0476026
0.1735261 0.03378212 0.06952804 0.051167 0.2725116
0.4681943 0.2349796 0.1813762 0.1120165 0.2093399
0.4531669 0.1970093 0.1808227 0.06038532 0.03516174
0.1290628 0.1084738 0.3268112 0.4269805 0.3062656
0.1379194 0.05897492 0.2396293 0.162607269 0.1925964
0.1631103 0.05527905 0.08450623 0.2311668 0.1246539
0.5269839 0.1101389 0.2821419 0.02625518 0.3607113
0.07485958 0.2530241 0.147582 0.06736754 0.198515765
0.142792 0.831068 0.09162691 0.06760851 80.03105
0.474812408 0.2066615 0.1954903 0.2214981 0.2668761
0.1408701 0.09362187 0.04756683 0.08113675 0.3183909
0.2552664 0.07789514 0.375173 0.09937402 0.2776834
2.237402 0.2045384 0.5900011 0.2442279 0.07825329
0.03089386 0.07486704 0.1464161 2.428461 0.7768328

0.07128421 0.1513375 0.039179439 0.502012154 0.08474446
0.1216555 0.04703133 0.5920091 0.2177727 0.2508542
0.09319943 0.2412729 0.2510218 0.2024001 0.1477637
0.2647596 0.1768229 0.187282195 0.1017022 0.2241696
0.5832246 0 0.2340553 0.063366384 0.0492999 0.08814418
0.06118489 0.3030002 0.2503118 0.08685195 0.07877276
0.3001092 0.04755379 0.1522888 0.03621312 0.06469349
0.3883294 0.2607999 0.07481194 0.06739016 0.05417257
0.234983 0.084948989 0.1023008 0.0640192 0.1841797
0.04431994 0.3465568 0.08231828 0.5751195 0 0.06845385
0.2132612 0.139118 0.3018511 0.4387986 0.2856518
0.240808 28.38103 0.1584125 0.1502989 0.2663722
0.06596468 0.1008722 0.07614073 0.611625 0.3737183
0.05884205 0.1032777 0.179325918 0.1772607 0.1949304
0.2023227 0.1198621 0.09363045 0.09716522 0.14250935
0.4702261 0.2015121 0.2522182 0.1100567 0.01742366
0.2812538 0.9994463 0.2377468 0.09998442 0.1692623
0.1762581 0.126708 0.2777742 0.1593476 0.075791689
0.04320788 0.2778303 0.02327384 0.070267988 0.04552444
0.01388901 0.1609975 0.06431992 0.08006389 0.1787709
0.1245204

AP000654.1 0.02437688 0.04661967 0.05605697 0.01164313 0
0.2839036 0 0.01460766 0 0.01527078 0.09416072 0 0
0.01142779 0 0.4153703 0 0.018354095 0 0 0.042727836
0.1456638 0 0.3937208 0.03339161 0.04790186 0.03858635
0.3469561 0 0 0 0.284063 0.02353042 0.2687148
0.009922774 0.106875 0.0507308 0.04378775 0.2168487
0.010786572 0.1476416 0.03223379 0.03805826 0 0.5271385
0.05381157 0.07091148 0.05240357 0.124105852 0.02920477
0.0422105 0.5289793 0.03922785 0 0.09505796 0 0.1968545
1.099524 0.04888594 0 0.138378621 0.066488251 0.5826801
0.01426266 0 0.718545 0 0.02463744 0 0.02554309
0.01040981 0.09717373 0.7475494 0.0795554 0.1138144 0
0.3217487 0.1686645 0.1106994 0.011939735 0 0.3328478 0
0.2255925 0.05135399 0.04720517 0.1881171 0.07530853
0.20877277 0.4261846 0.035236464 0.01360952 0.03123597
0.06173385 0.02805845 0.09961416 0.09451623 0 0.03068535
0.1853152 0.8087938 0.05324288 0.05616701 0.1023418
0.1131907 0.3493783 0 0.1062094 0.010778249 0.03249845
0.0516691 0.166372 0 0.4971542 0.1294996 0.029031
0.01363757 0.09656217 0.1170534 0.05100507 0.100906
0.07867545 0.4043487 0 0.01171608 0.1182226 0.1003337 0
0.2935362 0.03384315 0.02633394 0.1526407 0 0.03684481 0
0.4039144 0.08115335 0.07874067 0.09389684 0.04797852

0.1494521 0.05133584 0.09481726 0.1154961 0.06902454
0.08871231 0.01582215 0.4168203 0.02148329 0.1666838
0.04505723 0.08046506 0.08835718 0.0847932 0.6324023
0.2296283 0.08712653 0.6927389 0.479366 0.2446742
0.05511395 0.3366477 0.124643842 0.05543991 0 0 0.01944315
0 0.08299621 0.04501635 0.6137694 0.056126689 0.2210706
0.04961138 0.02531402 0.1158768 0.2122319 0.08775756
0.08207839 0.05641589 0.1593622 0.04923953 0 0.05677701
0.1560368 0.4969708 0.2088939 0 0.2512982 0 0.1254315
0.1284406 0.1111829 0 0 0.5759968 0.124587 0.1089691
0.03725895 0.09963795 0.2062953 0.1697854 0.04779643
0.1890488 0.06918389 0.0661513 0.05874662 0.07019295
0.1449118 0.1258164 0.04677055 0.1684118 0.09050739
0.2016435 0.1286654 0 0.0130437 0.06759019 0.07661404
0.050545444 0.04549567 0.089945899 0 0.04365635 0.1700525
0.0493879 0.100678 0.1419197 0.01487073 0.01218062
0.08250649 0.5811123 0.03179491 0.06731456 0.2921216 0
0.05267099 0.09302779 0.0825765 0.03733481 0.3286317
0.2442695 0.1616345 0.03020676 0.02542939 0.0936436
0.2841115 0.2195756 0.1598207 0.04345403 0.1753053
0.08252531 0.2496878 0.7733719 0.0609472 0.01489216
0.1613611 0 0.05721625 0.2118245 0.08933688 0 0.03113253
0.05036352 0.1007034 0.09270643 0.0949941 0 0.4048252
0.08821539 0 0 0.03402287 0 0.008484089 0.1338229
0.138824 0.7024025 0 0.130797927 0.4696695 0.08585145
0.013983 0.04492714 0.03557206 0.3073342 0.1057005
0.2253723 0.1500782 0.2280859 0.1081841 0.8336897
0.271011 0.1350454 0.09338539 0.2065975 0.105166
0.05481924 0.01976027 0.03120488 0.1209932 0.1075564
0.03118105 0.2320806 0.03085793 0.01019074 0.039573863
0.016902199 0.106997 0.07372816 0.00950096 0 0.02315422
0.03167244 0.09413768 0.1624679 0.0667234 0.07110877
0.06218804 0.0607784 0.04870992 0.029102706 0.1369681 0 0
0.04375244 0.02251538 0.016001075 0.1493886 0.04451577
0.02472034 0.04657291 0.06019804 0 0.05967433 0.03497659
0.1681138 0 0.03657768 0.04900857 0.03410772 0.1317127
0.05037672 0 0.52894 0.04746972 0.032176568 0.02818111 0
0.144693 0.06714917 0.1500195 0.02078675 0.1161819
0.1224314 0.03457149 0.09069816 0.1580833 0.6374971
0.1131615 0.08014654 0.04053871 0.951897 0 0.6831537
0.1871679 0 0.1018877 0.102543 0.07862683 0.4425635 0
0.03477248 0.072452485 0.06714197 0.06058239 0 0.2118703
0.03152435 0.2330906 0.044290464 0.2326334 0.01453863
0.0727878 0.06484604 0 0.06684357 0.1081616 0 0

0.4324441 0.08901625 0.319959 0.05906749 0.07511085
0.107176569 0.06546429 0.334594 0 0.017743846 0.06131033
0.1262595 0.05420611 0.01856212 0.1039756 0.01641551
0.3386223
AP000688.1 0.5955272 0.311591 0.6511453 0.06440192 0.3242443
0.1365532 0.4166599 0.06733307 0.121429 0.098545543
0.3279321 0.2428296 0.104111 0.2949836 0.1099113
0.04994668 0.47069 0.135363228 0.4994601 0.911944 0.1313008
0.145476 0.2287172 0.1613183 0.08465406 0.423937
0.08893065 0.1510423 0.56455568 0.2294673 0.1587666
0.1007208 0.07592337 0.2477247 0.07318142 0 0.1403042
0.2119286 0.07822565 0.099440013 0.08166531 0.3070645
0.035085419 0.1589167 0.196699 0.1074844 0.02614895
0.04831017 0.011441158 0.0628215 0.1426821 0.1696206
0.6750548 0.1182662 0.2190818 0.1381296 0.1814776
0.2418907 0.1915361 0.21326323 0.025513893 0.183883979
0.1007185 0.03944569 0.07731415 0.04568395 0.03066346
0.1135647 0.022578709 0.2904234 0.1247567 0.07329535
0.08892337 0.1885914 0.08743668 0.1422492 0.04362 0.1737825
0.05102616 0.16510631 0 0.04909569 0.08425074 0.1964169
0.1065208 0.07615621 0.2312303 0.08331115 0.65438071
0.012674 0.064968082 0.08782511 0.01919736 0.07588219
0.09053354 0.1377495 0.2505082 0.1893109 0.08486529
0.05694657 0.05627294 0.09816785 0.07766947 0.02695645
0.104349 0.07628384 0.1134664 0.06993793 0.039745313
0.1947394 0.03402362 0.04511065 0.2563481 0.06193514
0.4377412 0.1248954 0.0754338 0.06231359 0.3569333
0.09404184 0.2674438 0.08461819 0.2899275 0.08344357
0.05400451 0.1089879 0.2261022 0.04861076 0.09367172
0.05199928 0.07283076 0.025585 0.1143304 0.09057802
0.1507166 0.1432167 0.05343873 0.07259001 0.1360265
0.08846155 0.05358032 0.5679102 0.03496432 0.07098293
0.04242189 0.05452182 0.1312761 0.1344914 0.118831
0.1207357 0.1349974 0.0953739 0.06109151 0.100504
0.08211318 0.2116914 0.1907619 0.07982838 0.2209607
0.1503746 0.033872559 0.07448427 0.160870568 0.09199681
0.04152741 0.3835491 0.03584879 0.1260211 0.06121051
0.04149999 0.1020342 0.103484938 0.167837 0.2629825
0.3267134 0.02670633 0.1195662 0 0.05404786 0.2990522
0.01130108 0.07943824 0.07550095 0.2303048 0.07192417
0.1110669 0.02626045 0.1216237 0.0648672 0 0.06745298
0.1302485 0.03416603 0.1030682 0 0.1475011 0.1004982
0.1116191 0.1259447 0.0787328 0.01728918 0.05524339
0.06609436 0.5687084 0.3401586 0.06969603 0.08123662

	0.103536	0.2099309	0.01054441	0.258703	0.2507993	
	0.1564455	0.09914269	0.420544	0.1179715	0.0721489	
	0.124621	0.02354316	0.104843682	0.1992239	0.059228545	
	0.035382509	0.1207387	0.2155576	0.0728481	0.09281376	
	0.2093344	0.2330552	0.08983324	0.1521233	0.1567961	
	0.2344906	0.07092162	0.1077213	0.1572841	0.06474227	
	0.0762321	0.1649401	0.1376739	0.1514807	0.1463728	
	0.1639097	0.05569444	0.08205058	0.2266132	0.2057933	
	0.1349493	0.1964489	0.0560836	0.05050365	0.1014387	
	0.5022196	0.02037033	0.05618644	0.02745777	0.04648648	
	0.058236407	0.09494446	0.06509276	0.06176889	0.0157379	
	0.3157074	0.04642948	0.119362	0.2563946	0.1751477	
	0.1711358	0.1679414	0.1394137	0.1946326	0.2290393	
	0.2195567	0.071886692	0.1798916	0.07850792	0.1493101	
	0.0179871	0.0361408	0.04019364	0.1082456	0.05540174	
	0.1160167	0.2070887	0.1065786	0.06538326	0.06200979	
	0.1511039	0.3360054	0.0365686	0.1088002	0.05822483	
	0.04626694	0.3543364	0.1721816	0.09522977	0.1292682	0
	0.09108368	0.1342477	0.06971378	0.04957743	0.1245634	
	0.09169374	0.1043076	2.931149	0.109447899	0.14023727	
	0.08877523	0.128386	0.06131169	0.05653947	0.08538231	
	0.04379763	0.07232026	0.0842496	0.1599297	0.06555426	
	0.09172856	0.2129171	0.07484174	0.093902929	0.08417941	
	0.1159664	0	0.4033481	0.07264824	0.191765398	0.3366476
	0.01025963	0.2278936	0.1533392	0.2552807	0.05776696	
	0.01833766	0.139726	0.08856112	0.2430967	0.1236418	
	0.06777055	0.0943304	0.07589015	0.08127288	0.07530186	
	0.201775	0.2953916	0.059326324	0.1818586	0.08941893	
	0.07622319	0.0412693	0.2420269	0.2012119	0.08032992	
	0.1504906	0.1115485	0.1672269	0.2752772	0	0.1130157
	0.4728708	0.1308024	4.038941	0.1311188	0.1959348	
	0.1078423	0.06142419	0.04025526	0.1260441	0.07248506	
	0.191997	0.1780734	0.1923378	0.089057347	0.07221353	
	0.06981265	0.1465305	0.4464468	0.02906189	0.1357157	
	0.06124621	0.1251027	0.3350743	0.07828582	0.162262	
	0.06489742	0.04621667	0.1329503	0.1438984	0	0.009271269
	0.177803	2.14342	0.1633607	0.1582714	0.183494434	0.1307598
	0.1293533	0.4984525	0.016357822	0.2684757	0.09053099	
	0.1332584	0.02566827	0.1171546	0.121066	0.1783837	
FAM66B	0.04351188	0.2538043	0.2121267	0.2369215	0.108439	
	0.5772636	0.0537827	0.2607417	0.2485469	0.332545356	
	0.2614479	0.06838819	0.3561257	0.2733359	0.1418741	
	0.444853	0.1909504	0.884558559	0.107451	0.120812	
	0.391507605	0.3900073	0.1383888	0.2498766	0.5870883	

0.08550317	0.2548387	0.5160872	0.252253264	0.1579721		
0.3934787	0.3939332	0.3570084	0.1507461	0.5455237	0	
0.05885927	0.0625277	0.2389721	0.127074151	1.690138		
0.3106955	0.013586537	0.2349677	0.1164954	0.3906106		
0.1164486	0.1122463	0.14177579	0.2954006	0.100459		
0.0821052	0.2614094	0.1570203	0.4326717	0.1337239		
0.0319435	0.6021652	0.2225123	0.407023181	0.232181008		
0.118679238	0.2990184	0.2138502	0.1137692	0.2830517		
0.3384139	0.3430205	0.043717087	0.01519784	0.02601362		
0.01576834	0.6327401	0.0770877	0.1354365	0.06295409		
0.1587799	0.09563093	0.108677	0.004262403	0.03782828		
0.6891813	0.02330386	0.3310882	0.2291628	0.1263894		
0.3984619	0.1666847	0.26830958	0.2257635	0.020965291		
0.07287759	0.07434019	0.1873278	0.02003335	0.06667794		
0.1223135	0.02290908	0.003651487	0.08379793	0.5720198		
0.04181615	0.03007686	0	1.288576	0.1444192	0.06276989	
0.5795737	0.415558139	0.03480517	0.1370238	0.07686238		
0.145085	0.2254484	0.1386914	0.1105479	0.04868519		
0.1930434	0.1928643	0.07283384	0.144091	0.1451141		
0.3408257	0.2019552	0.1087465	0.09285021	0.1472536		
0.01882411	0.5723167	0.4309166	0.0720746	0.06935306		
0.1682393	0.03069114	0.2140004	0.2273839	0.3269605		
0.009369959	0.05267511	0.01141867	0.08892229	0.009163271		
0.4231138	0.05955636	0.2135578	0.1126033	0.05648399		
0.1674022	0.08436327	0.05100425	0.1407448	0.2174929		
0.06308581	0.2248672	0.5024028	0.08197573	0.1594058		
0.4430846	0.04889439	0.0436735	0.360713842	0.1346026		
0.160189175	0.04354167	0.2680191	0.1174393	0.5899911		
0.1382684	0.1422196	0.1499916	0.5711266	0.495911819		
0.4920936	0.02656638	0.06777699	0.08273443	0.4840563		
0.1127838	0.2595269	0.1460156	0.02625748	0.01318363		
0.2144055	0.06891461	0.08753497	0.02150487	1.29487	0.0294361	
0.1327734	0.03385713	0.3059842	0.1329719	0.07056273		
0.04656434	0.02992828	0.2437059	0.1111917	0.1296707		
0.02660238	0.05589581	0	0.07843933	0.1151752	0.09945763	0
0.02024191	0.1782632	0.1503504	0.07020844	0.2776601		
0.1057462	0.2173636	0.1292421	0.04319121	0.2463661		
0.06471829	0.6053463	0.4377732	0.3646758	0.11728844		
0.316712	0.123853114	0.146150213	0.1153291	0.6222524		
0.02468359	0.0449267	0.09119587	0.1167924	0.5131108		
0.05890849	0.1821539	0.05675283	0.0137319	0.2554992		
0.3898046	0.2381736	0.4095935	0.142483	0.5642301		
0.3812876	0.1962057	0.2077287	0.1725377	0.1361717		
0.2590834	0.09418114	0.3396769	0.2979533	0.04653837		

0.1642798	0.1129337	0.4294775	0.4732945	0.009324742
0.09569515	0.1116093	0.369845658	0.1225548	0.3444906
0.1435169	0.04266059	0.1944967	0.02247429	0.2105666
0.2316689	0.04521632	0.07573821	0.0722599	6.49191	0.337181
0.4612066	0.09109447	0.039767892	0.01211505	0.1346354
0.1982368	0.4527482	0.06064598	0.046693959	0.5169787
0.05210224	0.07487751	0.1884545	0.06032016	0.1181559
0.02744317	0.2998828	0.145423	0.4708494	0.03862096
0.3291874	0.1791649	0.1112545	0.04000553	0.3982709
0.08343008	0.1761308	0.00352714	0.05569963	0.1781741
0.2447802	0.03339426	0.04734352	0.04039225	0.06548441
0.051801187	0.102577466	0.3208564	0.1783941	0.1797642
0.08210415	0.01239885	0.2826711	0.1120217	0.6198742
0.3715888	0.1554852	0.2131267	0.1301848	0.04637094
0.051947327	0.0366725	0.04490703	0.0492199	0.2030512
0.08037837	0.3027505	0.0177769	0.0357566	0.03088747
0.5700414	1.001447	0.299755	0.3692576	0.2663765
0.008573639	0.2353429	0.0435266	0.4198973	0.1278503
0.05877566	0.03596828	0.1117801	0.3841672	0.1270977
0.336946514	0.02347441	0.3635807	0.09592959	0.01598119
0.3748916	0.2300425	0.05184516	0.04370718	0.1049052
0.1011832	0.08151672	0.3364257	0.2457538	0.1258918
0.1664285	0.008713351	0.06346831	0.1083916	0.01252831
0.02854321	0.150689	0.05186012	0.1002473	0.1579921
0.2705254	0.4427494	0.060351755	0.04394357	0.1946475
0.3323503	0.1728828	0.1125397	0.04379564	0.391331917
0.0622864	0.05709208	0.1126006	0.03637793	0.03141373
0.5786703	0.01287098	0.00428642	0.04406488	0.32312	0.0741491
0.04568927	0.06326006	0.2604794	0.163976987	0.04284555
0.5972388	0.2433751	0.076013169	0.1805708	0.1051721
0.05160322	0.07289206	0.05773999	0.08204313	0.5422592
CASK-AS1	0	0	0.1297474	0.05389753	0	0.02857012	0	0
0.1742111	0.035345184	0.09686263	0	0	0	0	0.1045001	0
0.084963424	0	0.1205052	0	0	0	0.06750308	0.05796524
0.06652313	0	0.04461399	0	0	0	0	0.2995446	0
0.1378013	0	0	0.02533738	0.04364434	0.099864805	0.5011977
0.0994762	0.058725497	0.03627172	0.4647982	0.1037919
0.8862971	0.03032284	0.172350496	0.06759629	0	0.2129315	0
0.1272551	0.05500441	0.03467993	0.414211	0.02891952
0.141437	0.038245403	0.06405721	0.038472813	0.3371625
0.0990355	0.3493999	0.0191163	0.1154792	0.2280997
0.085031856	0.5517968	0.2409417	0.1226808	0.1674436
0.1052206	0.241478	0	0	0.8496607	0.2989241
0.331623237	0	0.1848953	0.2719628	0.5221481	0	0.2185187

0.1741636 0.1743063 0 0.2227425 0.081557019 0.1890006

0.04819842 0.1190724 0.1948292 0.144102 0.1367274

0.08911856 0.118372 0.1429746 0.07064167 0.2218212

0.3250052 0 0.1455483 0.1702438 0.08139366 0

0.024946937 0.3384887 0.08542242 0.5209886 0.1485249

0.09329958 0.09991164 0.1567862 0 0.2681991 0.1875652

0.4427042 0.2043591 0.242799 0.1039879 0.05237502

0.1084704 0.4651771 0.02580319 0 0.2090486 0.05222141

0.08126876 0.2248251 0.05740942 0.1421327 0 0.3955284

0.1878346 0.4556256 0.1552358 0.1850822 0.2882636

0.1485251 0.416975 0.4455385 0.07988082 0.1825158

0.695806 0 0 0.1377858 0.02607195 0.1330296 0.05112707

0.3084073 0.5153997 0.1771631 0.02520748 0.2338274 0

0.09438564 0.042521572 0.03116769 0.317345643 0.4362852

0.139016 0.06718387 0.09000481 0.2372989 0.34578 0.06946212

0 0.064954381 0.3009893 0 0.02929545 0.06705104 0.08187069

0.1624963 0.4070906 0.3917344 0 0.08547599 0 0.1839796

0.2579693 0.10457 0.04395438 0.152679 0.3722531 0.1219514

0.2419326 0.3864689 0.02859332 0.04312853 0.09701994

0.370328 0.01802278 0.1681438 0.2299685 0.06589095

0.1736304 0.2773969 0.2212557 0.1151488 0.05337683

0.415588 0.1019796 0.1299728 0.383323 0.3176837

0.3608446 0.0599692 0.1571138 0.28003 0.1353655 0.3208711

0.06038092 0.1340929 0.177328 0.05849531 0.1053026

0.505593261 0.148056906 0.6062722 0.7871943 0.1143114

0.4951795 0.1313929 0.03441924 0 0.1909665 0.2296378

0.07359134 0.3116076 0.3042603 0.197445 0 0.0239243

0.4459665 0.144023 0.3803194 0.3392263 0.07482267

0.06991545 0.08828688 0.08127901 0.140913 0 0.08220336

0.241385 0.253597 0.7640401 0.1576141 0.1022867

0.2216754 0.1723442 0.2100828 0 0.2383751 0.08171353

0.02584698 0.03951282 0.1441164 0.08742716 0.266382

0.1287449 0.2687298 0.03069052 0.2108235 0.350023

0.1543137 0.03833629 0.1968702 0.103133973 0.03927392

1.013699 0.1071056 0.1354796 0 0 0.1207866 0.1987086

0.06472906 0.1819767 0.2675848 0.4924692 0.2668915

0.1896867 0.1240591 0.06885902 0.2276353 0.05847356

0.04646459 0.3606589 0 0.09563658 0.2163674 0 0

0.09630087 0.175029 0.4667739 0.1924547 0.2046343

0.1666528 0.0235871 0.122128271 0.19560603 0.5448328 0

0.04398114 0 0.1339798 0.1099618 0.1452584 0.1410158

0.1853226 0.3703179 0 0.2532158 0.03758072 0.23576017

0.02641844 0.05823091 0 0 0.2084531 0.074070996 0

0.1287932 0.1716504 0.03079884 0 0.1160275 0 0.9714662

0.5002843 0.02543075 1.044157 0.2268669 0.1973612
0.1905359 0.08745007 0.2205687 0.1688641 0.3021471
0.148949392 0.06522695 0.2619193 0.287058 0.414456
0.1157432 0.1683928 0.2016827 0 0.1200268 0.4985757
0.2845841 0.4490735 0.1091331 0.07420169 0 0.3389578
0.3291972 0.07027566 0.02707574 0.1542165 0.1347574
0.4746845 0.02599811 0.2410214 0.1375647 0.0804831
0.195645156 0 0.03505544 0.02627795 0.1401106 0.1216085
0.8802414 0.128141343 0.3140928 0.1682528 0.1403933
0.4931537 0 0 0 0.05558197 0.05194444 0.5353753
0.1373558 0.2962261 0.5810406 0.07450663 0.070876267
0.8586203 0.0249819 0 0.041069251 0.2128599 0.1948237
0.5018538 0.1718528 0.1604387 0 0
AL355488.1 0.2905724 0.268104 0.9612688 0.2142663 0.3878211
0.6453339 0.1575267 0.2016165 0.03935031 0.217155847
1.041443 0.630962 0.5038241 0.3871493 0.249325
0.868634 0.1525319 0.180398218 0.1321817 0.130653
0.244233309 0.5432322 0.2788695 0.7257766 0.4050109
0.8695078 0.169455 0.5764206 0.068958076 0.1388076
0.1296538 0.1919208 3.651195 0.9954437 1.112242
2.391451 0.05834932 0.2472395 0.6506453 0.694761199
2.251833 0.8897884 0.413860212 1.192894 2.293973
1.357889 2.965842 0.6739651 1.339193595 0.3745862
0.4237045 3.323458 0.8914403 1.816622 1.451152
1.81735 1.302367 0.6375486 0.4140382 0.331728549 1.0591354
0.56312049 0.4315581 0.4056401 1.059302 0.8739501
0.5842842 0.8449693 0.261211893 1.794792 0.7880483
1.374455 0.7816481 1.245387 1.832691 0.1843892
1.159677 2.971113 3.715537 0.71909881 0.4343244
0.9856219 1.29959 1.415296 0.1933072 1.668313 1.059546
0.8000356 1.27484708 1.460499 0.943199773 0.9961216
0.4877342 3.356589 0.399001 0.6301557 0.8103855
0.7085707 1.625642 1.431301 1.927526 2.030896
1.09236 1.186283 1.057294 1.265143 0.5221332 0.2094163
0.320064823 3.167014 0.6977063 3.368702 0.2504946
1.877013 1.083252 1.667513 0.7586126 2.346468
1.05446 1.087965 1.767271 1.903044 4.537975 0.4164273
2.675508 3.490893 0.9138857 0.159891 0.6563484
0.283095 1.207875 2.05453 0.8714146 1.484514 0.273511
1.598389 0.8873417 2.382149 0.8471519 1.264209
2.892717 1.803563 1.685418 5.141879 1.058538
0.7791742 4.188907 1.294424 2.282265 1.653236
1.262614 0.5384665 0.7160032 0.8106145 1.13623 1.392595
1.443946 1.684085 1.475054 1.978455 0.291981459

2.044436 2.194746268 2.031225 0.2072436 0.5989187
0.3659406 1.181591 2.159136 2.290727 0.6354535
1.132656268 1.359732 0.4461195 1.942802 2.520178
0.6805321 1.791166 2.540341 3.910987 3.455678
1.333475 0.5651842 1.500794 1.603574 1.31642 0.6989517
1.441545 1.34954 0.7272154 0.4983811 0.902491 0.4856851
0.5299512 0.6399061 1.311611 0.4005799 4.810782
0.6545025 1.607392 2.117834 3.479587 0.5697334
2.621756 0.6172983 2.067157 0.429984 1.268261 1.8832
1.990075 2.621251 0.5472452 1.93057 1.697975 1.183902
1.387107 0.3927938 2.633083 0.5020146 0.502084744
0.5470654 2.70860241 0.165875684 2.45402 2.471548 2.710103
2.526488 0.8191309 0.3856162 0.4279372 0.5176196
1.50571 0.7380436 1.339327 2.639057 1.370061 0.3891863
1.085114 5.312982 0.8744468 3.325497 0.8530748
0.8585573 0.6695937 1.86657 1.042795 0.7978477 1.443874
0.9283932 1.722939 3.322343 3.0489 3.569636 1.681989
2.170368 1.283087 1.189486 0.253641007 0.5647585
0.7481328 0.6772362 0.3641418 1.62763 0.8583711 1.046951
1.364847 2.569263 0.3216582 1.109022 1.138496
0.9109022 0.3948638 1.999303 1.462964448 1.930348
1.149946 1.969287 1.011898 0.2295512 0.510586153
0.9767295 2.046699 0.1696015 0.845576 2.134973
1.166758 0.5787331 1.653852 0.8563559 0.377436
1.661818 1.07776 0.7304723 1.38164 0.5390008 0.2246621
2.253021 0.9171163 2.074426 1.387789 1.669961
2.924025 1.486712 1.872 1.092724 1.201949
2.206879278 2.580283755 2.063022 1.439036 0.2503453
0.2693362 1.51557 0.1258452 2.493603 0.535118 1.384176
2.490803 0.608631 1.260847 1.317435 1.710177096
0.4200996 1.104855 0.2254177 1.866525 1.186537
0.90347123 0.3054634 1.759451 1.664607 0.4480151
0.2467401 0.807205 0.4159754 2.530783 4.53518 0.7306655
2.932204 0.4338663 1.597725 2.547832 1.548633
5.523061 0.9154214 3.122042 1.583523671 5.571144
0.9533469 1.097955 2.228064 0.9359464 0.3694936
2.235258 0.4437889 2.609917 2.783415 1.652941
1.495449 2.401963 1.541963 0.09325326 2.70522 1.390497
1.142904 1.418864 1.220583 2.069827 0.9649836 2.2409
4.518623 0.4288037 0.8435194 3.373726652 0.3557032
0.6207888 1.092148 1.367185 1.151483 3.709717
0.991049639 1.471631 2.286349 2.694221 1.530432
0.4122011 0.2236562 1.236508 0.833632 0.5584939
1.934865 1.414765 2.016243 1.17965 2.163132

1.504877225 4.732203 0.7132552 0.167147 0.578860284 1.0193
2.077094 1.96486 1.688567 0.7876039 0.4943314 0.7364619
PROX1-AS1 0.01553108 0.01215102 0.01038988 0.02157996 0.02777948
0.04146693 0.01308895 0.01184512 0.01089877 0.019458743
0.0121196 0.005547817 0.02398397 0.01059041 0.02762202
0.01464423 0.03661358 0.030829172 0.01220336 0 0.011549111
0.01546763 0.01796231 0.03716278 0.01353837 0.00887837
0.01787947 0.01451364 0.002728454 0.01281508 0.02393997
0.01265617 0.012266 0.02223435 0 0.006190235 0.2203758
0.0532602 0.1168621 0.038735219 0.3409176 0.216571
0.138139033 0.08259835 0.01599297 0.01662284 0.05092947
0.04552846 0.012938849 0.08457747 0.003259793 0.009324805
0.1514726 0.01486085 0.1046099 0.04859908 0.07048431
0.01447381 0.2378451 0.005742386 0.014426879 0.026957141
0.05765456 0.1817415 0 0.01722139 0.1599015 0.02140513 0
0.005917854 0.001205879 0.07367999 0.1634161 0.06977637
0.0747112 0.0510699 0.01096223 0.02873265 0.1773915
0.347159684 0 0.01079604 0.007561864 0.004355462 0.004461658
0.001367069 0.04213043 0.005234272 0.47401359 0.1274051
0.040818139 0 0.007236789 0.03337268 0.5915561 0.09231493
0.01231742 0.07433764 0.1895792 0.07871244 0.03535516
0.01356891 0.01301284 0.04742136 0.01602587 0.03088668
0.006110457 0.01230337 0.001248559 0.0188232 0.01795614
0.02607474 0.4633525 0.0389126 0.3200279 0.006725937
0.1864146 0.07382642 0.003129125 0.09158112 0.1724129
0.6774601 0.4098501 0.007863887 2.308594 0.004108493
0.1807979 0.006108232 0.01046259 0.007840824 0.002033693
0.1366336 0 0.2091382 0.006312814 0.02159524 0.002685958
0.592889 0.06526241 0.26863 0.005770875 0.2973388
0.003295107 0.01486572 0.005330564 0.01027649 0.1301322
0.102605 0.8249836 0.04689269 0.00913405 0.01331589
0.001279419 0.02806428 0.005159006 0.01330014 0.03280157
0.01671819 0.0237986 0.1700593 0.002128145 0.03899747
0.031765404 0.1014707 0.003478778 0.3250376 0.009009228
0.0475059 0.003845733 1.091615 0.008158951 0.004876313
0.05272431 0.3405106 0.001466196 0.01342324 0.0109267
0.006099534 0.03531547 0.01960575 0.002840095 0.4677229
0.006324764 0.002630834 0.0296953 0.2023652 0.006599564 0
0.02212401 0.02075188 0.01453006 0 0.01431055 0.02158522
0.2840591 0.08525823 0.01172619 0.01262304 0.1611344
0.1088257 0.4507915 0.006941662 0.04844672 0.75611 0.1175431
0.003284146 0.03913016 0.1918962 0.02398097 0.007949812
0.02167172 0.02701236 0.0655277 1.336107 0.07316843
0.004941276 0 0.07494131 0.01035419 0.011710431 0.01185805

0.0937745 0.004446024 0 0.01354305 0.009153799 0.04810824
0.006576029 0.001722634 0.07760557 0.3823041 0.02298609
0.064455 0.004455863 0.09475074 0.003952738 0.00610144
0.003592132 0.03188571 0.7698301 0.08988491 0.1372372
0.09174684 0.1119734 0.06333375 0.0081358 0.0658233
0.007065493 0.01439957 0.007047239 0.007615302 0.01911957
0.09203089 0.01023862 0.06959303 0.1690614 0.1109848
0.008781303 0.04241898 0.0954251 0.06209304 0.02966341
0.2055654 0.6067507 0.03166361 0.324323 0 0.01075212
0.04923996 0.03795601 0.02831834 0.01534942 0.08473645
0.091620353 0.1218675 0.124017 0.2197799 0.07345609
0.01665146 0.006734095 0 0.111385 0.04697418 0.005204387
0.003090517 0.002738599 0.06010894 0.04984115 0.04222108
0.1826537 0.01481067 0.01316934 0.006976462 0.03249084
0.004327129 0.08137009 0.03519387 0.006350295 0.002289042
0.003614792 0.02627986 0.009344551 0.001204011 0.02304373
0.02144761 0.1263136 0.053483044 0.01566369 0.0123946
0.01518349 0.01981075 0.01776131 0.1113113 0.005503434
0.005452484 0.0223492 0.004637568 0.03603812 0.1354333
0.02816243 0.08275794 0.037084031 0.05156603 0.01748625
0.06533737 0.01520493 0.005216392 0.52826873 0.1134457
0.005156734 0.03436343 0.2751468 0.004184027 0.004355258
0.009216948 0.1107467 0.06259632 0.01145496 0.001412394
0.02649352 0.5590749 0.6503587 0.05543887 0.04573362
0.02535424 0.002749461 0.004969805 0.3525678 0.02621739
0.00478895 0.05185734 0.04344589 0.01083577 0.01682322
0.3592908 0.006007174 0.009193215 0.2380621 0.1412746
0.03058697 0.1411202 0.1009646 0.05089308 0.008237928
0.3394096 0.03252246 0 0 0.002969661 0.005204675 0.1734025
0.06712797 0.9600223 0.013988235 0.07777767 0.5859948
0.01841244 0.1262221 0.007303599 0.09947868 0.030783809
0.3211351 0.1785213 2.197885 0.00429246 0.01019344
0.007743201 0.004176501 0.1975077 1.156887 0.00116499
0.03780956 0.3879392 0.005131811 0.009943871 0.06562426
0.01011123 0.01500371 0.01633918 0.061663744 0.02308224
1.811996 0.006279269 0.008600992 0.01873604 0.02281899
0.002241498

AC243571.2 0.09287701 0.02537473 0.3051137 0 0.08701701
0.2284305 0 0.01590167 0.02048372 0.049870602 0.0113891
0.02606714 0.04097884 0.01244011 0.04326188 0.009829688
0.07940016 0.029969975 0 0.02833799 0.031008572 0 0.03375929
0.1587401 0.02726219 0.06257427 0.02100225 0.07343992
0.102559957 0.03010667 0 0 0.01280743 0.0417884 0.05400889
0 0 0.04766666 0.102634 0.070452568 0.0857175 0.07017841

0.027619754	0.0170593	0.06831367	0.05857844	0.123509		
0.1426143	0.01350997	0.04238915	0.01531656	0.1502188		
0.2419827	0.1596015	0.1552179	0.1631065	0.01948116		
0.02720284	0.05321647	0.161888076	0.015063682	0.054283558		
0.07928706	0.01552611	0.1095531	0.05394462	0.03620809		
0.1072798	0.013330717	0.1575663	0.03399588	0.02884959		
0.2625067	0.03711547	0.04129886	0.09598342	0.04120596		
0.1728052	0.1707163	0.06498705	0.1384204	0.1449332		
0.09948512	0.1773608	0.02795158	0.07708022	0.1911293		
0.09837564	0.22726679	0.1945546	0.012785956	0.01481512		
0.06800599	0.06720251	0.030544	0.04066441	0.2829445		
0.04191421	0.1781525	0.0268975	0.1328967	0.02318375		
0.09171381	0.1909846	0.1369085	0.0400345	0.01914052		
0.01651685	0	0.1238206	0.04821101	0.2663382	0	0.117015
0.09398081	0.08427385	0.01484565	0.03153482	0.06861215		
0.0416425	0.04119176	0.6137882	0.1711763	0	0.2040631	
0.0514781	0.1577647	0.02870032	0.03686987	0.02456075		
0.03822229	0.362536	0.2160062	0.08021738	0.1186463		
0.1691134	0.1262033	0.02857196	0.4526649	21.17002		
0.02711521	0.3353004	0.04128664	0.0838182	0.06261587		
0.03219028	0.068895	0.06806161	0.08185233	0.09072469		
0.0735729	0.05005315	0.02404606	0.05274546	0.1260494	0	
0.02371115	0.03142104	0	0.2219571	0.03999746	0.263858	
0.108548281	0.1931233	0.01634549	0.01053263	0.04233103		
0.02480141	0.07227871	0	0.02190622	0.091647963	0.1698734	0
0.0551129	0.07358265	0.1155162	0.0191063	0.05105672		
0.1995937	0.06672279	0.09380242	0	0.07416789	0.2547889	
0.09836267	0.03100891	0	0.1094237	0.05735613	0.03413569	
0.1118547	0	0.02028419	0.04563039	0.1973956	0.05933526	
0.1845231	0.419115	0.07747452	0.183739	0.1522094		
0.07804567	0.02166269	0	0.07201128	0.01598766	0.1222575	
0.05633885	0.1369618	0.2715397	0.08461407	0.09852494		
0.0878024	0.3819904	0.02321723	0.01419917	0.2732883		
0.06950071	0.275114928	0.1361962	0.209815092	0.06963407		
0.07603779	0.06941867	0.02150516	0.1095965	0.09269498		
0.04856413	0.0397789	0.01496921	0.01542902	0.1730573		
0.03140468	0.1907994	0.3900215	0.03822455	0.09001654		
0.05992766	0.1083789	0.2285583	0.132954	0.05278586		
0.06576522	0.02768204	0.07645423	0.2209138	0.07967553		
0.1933093	0	0.08349014	0.01497263	0.593032	0.1443224	
0.06634618	0.01621137	0.05489225	0.02750673	0.09965555		
0.1793469	0.01215634	0.01858365	0.3219587	0	0.1566059	
0.06055127	0.0689394	0.07217177	0.03305148	0.123467		
0.1088652	0.1622728	0.2037022	0.024252966	0.07388518		

0.09270387 0.07556082 0.09557806 0.05690114 0 0.01420208
0.02803697 0.03044335 0.04890698 0.009680799 0.1286766
0.02092072 0.3122468 0.03500846 0.043181 0.04282454
0.08250379 0.02185321 0.2600916 0.1219895 0.06746964
0.3561665 0.05967537 0.07528753 0.237784 0.01646391
0.01463553 3.473521 0.09624354 0.1343659 0.1109348
0.043079493 0.036798943 0.02329505 0.02675311 0.04137039
0.05007228 0.06301331 0.01723907 0.05123841 0.0221075
0.1743217 0.04837994 0.02707877 0.1058599 0.4065239
0.063361507 0 0 0.08186563 0.3333976 0.08578464
0.017418522 0.3613826 0.02422959 0 0.0434559 0.07863678
0.10914 0.08661406 0.1522999 0.06535932 0.09568463 0.1194537
0.08891662 0.2227748 0.0358451 0.0959688 0.05927873
0.03971006 0.0387561 0.175134559 0.07158087 0.1055878
0.2025135 0 0.2313543 0.04525626 0.03161844 0.0888513
0.09408497 0.03702473 0.172087 0.09051775 0.08212393
0.3315359 0.06619472 0.01328488 0.3677163 0.2644162
0.0254685 0.04351862 0.1426029 0.08372003 0.1345016
0.05667855 0.06469939 0.1261759 0.105160867 0.01218162
0.01648726 0.08651324 0.6260193 0.06863383 0.2670934
0.036160434 0.1371723 0.3007039 0.1320594 0.09075908
0.01915807 0.03638244 0.07849533 0.03921194 0.07329147
0.06568631 0.2422543 1.764732 0.2250498 0.03504193
0.733358997 0.04750894 0.3407349 0 0 0.1334829 0.1527157
0.05900792 0.1111354 0.1131862 0.107218 0.03159591
LINC01614 0 0 0.08249371 0 0 0.06811858 0 0.02687088 0
0.028090694 0.03849094 0 0.1384933 0.02102148 0 0.1328828
0.04472385 0.016881236 0 0 0.078598118 0.04465824 0.02852348
0.4291863 0.1228481 0.01762315 0 0.07091421 0 0
0.06335964 0 0.605981 0.4236879 0.8943972 0 0.2099691
0.1610957 1.196683 0.178577689 0.778551 0.1976475
4.247176561 0.49006 0.09234996 0.09898671 0.02608842 3.084694
3.835330493 0 3.72703 0.6134513 0.8178119 1.213636
4.371493 0.1929339 1.152184 1.402017 1.124075 0
0.610915986 4.494728873 0.2679609 2.728571 0.1542704
0.4405893 2.967471 0.498528 0.157685102 0.09397334
2.10638 0.3412532 0.3992289 3.073196 2.669369 0.0810971
0.01740761 1.295789 0.7466513 0.461227535 1.637334
1.812336 2.017337 0.2305438 7.321113 6.469148
0.4844596 0.5818281 1.30573189 0.6322332 12.099302641
0.1001392 2.413268 0.1324864 4.206516 5.42851 3.651127
0.2596999 0.0752612 2.136235 0 0.03917624 0 0.2958346
0.3470249 0.4059054 1.487821 0.1674625 2.399025261
4.483582 0.1222015 0.1080149 3.737953 0.3954674

0.5558356 0.1246063 0.6271599 0.1421014 1.308486
0.1407362 0.8120751 0.9889471 0.867769 15.23483
3.211218 0 3.916869 1.939929 0.3115163 5.021878
2.373631 0.229732 0.09125263 3.185484 0.1002451
0.05715408 5.054265 2.1968 0.1233742 0.411865 1.252405
0.472163 0.01744169 0.07081862 0.1269711 0.09065936
0.1746297 1.130947 1.600505 0.7665396 0.5387398
1.332144 3.677322 0.9358657 0.5406925 0 0.8614501
0.0796436 5.038839 1.125199 5.102916706 0.1486237
1.719623379 0.04079282 1.325801 0.3025693 0.357658
5.82546 1.129773 0.8832838 0.2221048 0.154868085 0
0.1368907 0 3.907861 1.865253 0.3874334 0.1078455
1.089662 0.2029485 0.3170174 0.02510873 0.6475383
4.038932 0.249322 0.716124 3.973955 0.4252834
0.5621432 2.55728 0.3780275 3.726839 0.1371061 0.2698742
1.000686 8.909315 0.1113607 0.2969983 1.361543
0.4484782 0.3858086 0.3956482 1.262905 0.7211638
0.01738367 2.431457 1.730213 0.1904045 3.366402
1.405234 0.405116 0.02081111 0.4698377 1.290986
3.295557 0.8877766 0.1420944 0.8221033 1.743349805
0.3556806 11.93647125 0.141202419 4.641694 0.1368555
0.3452275 0.1388984 0 0.136774 1.52363 3.035424
2.059703 0.02924347 0.08844682 0.4030196 0 0.0322962
0.2852087 13.34188 0.3204956 0.7220657 0.9660707
8.860366 4.111848 10.87577 1.873306 4.031677
0.1570764 2.188601 0.1598679 0.4030939 3.946951
1.169133 3.048477 0 2.492873 1.539779 0.952866244
0.9261968 2.272973 0.04108394 2.35522 1.259906 1.760236
2.037689 0.7503498 0.5048109 0.09756554 2.680842
0.1159091 2.105343 2.955385 0.1251706 1.290965178
0.0936392 0.4699571 0.234087 0.07178187 4.134552
3.020911789 1.51193 0.4579804 4.552761 7.148684 1.799463
0.6088311 0.01767607 0.7160819 0.512701 1.769471
0.5427416 0.7900247 0.2400312 0.4395066 4.878637
5.852575 16.74448 5.092437 0.672459 0.09566927
0.1391048 0.1978507 2.160474 3.1307 0.1324478 0.1874592
0.24265455 6.280523243 0.2361859 0.2561775 0.5068352
5.161386 3.918494 2.330466 0.404056 0.5790426
0.5400476 3.891449 0.5719764 17.73184 0.5674805
0.642415279 0.2729498 0.4165127 4.449864 0 2.174402
0.559247452 0.1221339 2.354252 0.04547322 4.002067
1.616726 0.1613731 0.256133 0.2788051 0.02208903
1.374359 0.2242829 0.9315662 1.160715 0.6057158
0.1158354 0.3255527 0.5703725 0.02183021 0.374863633

0.1036786 0.7731701 1.596982 0.1646951 2.207695
0.5353229 1.095301 0.1126067 0.09539171 0.3128247
2.294051 0.2039443 1.457129 1.120468 2.013423
2.42449 0.1308152 0.6702215 5.229002 0.0490256 0.05354943
0.03143808 1.198401 0.6225457 0 1.663069 5.975242889
0.8028024 0.7243709 0 0.66812 0.01932974 0 7.556581978
0.7488786 1.043012 1.182727 0.204488 0.485604
1.598469 0.6632128 1.612349 0.3096225 3.348431
5.321743 0.6670421 8.17629 0.01973812 2.393988099 0.1404923
0.03970889 0.04324337 1.207676816 0.620294 0 2.791949
0.01707257 0.06375458 0.42275 0.2491591
PRKAR1B-AS1 0.1987499 0.1520401 0.3473538 0.9492896 0.6778042
0.2616648 0.9826563 0.3334783 0.9205066 2.116601113
0.1876631 0 2.731596 0.07453855 2.332946 0.2503142
0.9118527 0.433970157 0.6871277 0.7216301 0.348369222
0.0395876 0.151709 0.1426707 0.1361245 0.1562217
1.13257 0.1414404 0.614517937 0.6313751 0.3369935 0.08907795
0.8441338 0.6885654 0.5986786 0.522825 0.02068095
0.589068 0.6303354 0.404548089 0.8025023 0.4029738
2.296199063 0.472748 0.5116516 1.126092 0.8556717
0.9186037 1.01186167 0.6349674 0.6424156 1.800159
0.5756996 0.9861832 1.588807 0.1710276 1.400725
0.7130977 0.3786487 0.565833153 1.218489067 1.978639406
0.9897325 0.8140059 0.136754 0.7003207 1.491541
1.185159 0.878623733 0.8746848 1.256127 1.31086 0.7864427
1.074876 1.175407 1.58156 0.786986 0.6633115 0.9025558
0.467266668 1.69333 0.4993354 2.001173 0.9809622 0.8583352
0.6735293 0.4090024 1.080652 1.70216919 1.053641
1.053398344 1.597844 0.7130861 1.660987 3.042596
3.106572 2.273303 0.481354 1.217563 0.684948
1.16955 0.2083683 1.282237 1.71651 1.066425 1.12443
1.175532 0.7175 0.913924717 1.934258 0.6740308 0.5745036
2.267155 1.270797 0.8446702 0.3787135 1.00071 0.3464085
0.3670623 0.3326843 1.131224 7.740258 1.501849
1.992543 1.375541 3.45074 1.327047 1.612185 0.589111
0.901373 0.6011776 0.2036475 0.4853491 1.021372
1.821692 0.6079755 0.6049469 6.248699 0.8968002
0.2868647 0.6904919 1.004525 0.7266815 0.9207389
0.7503625 0.4339743 0.5160056 1.002535 0.3678316
0.3494594 0.4408335 1.537029 0.3601975 1.323417
0.4647763 0.1248139 0.4262168 1.20021 1.00501 1.396417
1.378024671 2.086018 0.731698572 0.867866 1.150781
0.615316 0.8560307 0.6501463 0.7849557 22.73127
0.5906589 1.121151718 0.5937436 0.2426954 0.4127813

1.480137	1.115129	0.4865443	0.2676811	0.7589519	
0.3198312	0.2208032	0.4228983	1.814627	2.453532	
3.437984	0.2632152	0.4033672	1.03264	0.910715	0.9033584
0.4817136	2.336751	1.002694	0.6493435	2.156785	
1.891899	0.809475	0.1822681	0.371369	1.406739	
0.7980063	0.6235112	0.2595967	0.8649091	0.4622964	
1.748254	0.8698933	0.6076272	0.6714383	1.067725	
1.394222	0.8117188	0.6576171	0.6866422	0.5564512	
0.4466619	1.054919	1.207658	0.288475503	0.4822167	
0.691442622	0.43809451	0.512553	1.334481	0.3221361	
0.3693823	0	0.2424885	0.6355917	0.5605776	1.432936
0.1036923	0.2038512	0.238173	0.8346171	1.460089	
1.061866	0.9201274	0.730557	1.14619	1.573349	0.8697751
1.280667	0.7878587	3.569346	2.647339	0.3381578	
0.4053941	1.1479	1.4829	1.233552	0.2961103	1.369187
0.7098795	1.117054	0.5097996	0.618054779	1.044952	
0.9594726	1.128993	0.6124216	0.8122566	0.3284997	
1.31369	1.390774	0.6884509	0.6702793	1.056201	0.7808881
0.9965655	1.674525	0.6934894	0.544945238	0.4703737	
0.4562726	0.7923033	0.4295129	25.40001	1.208613401	
1.595549	0.5879709	1.413679	2.087913	0.4205387	
1.233606	0.1880289	0.7015943	1.590706	0.2749023	
1.154681	0.3501614	0.540127	1.084115	1.735972	
0.8422168	3.90612	1.653726	0.4349962	0.08480669	0.4685796
0.9646235	0.5084515	1.063238	0.134182	1.578658	
0.344164484	2.094670319	0.7502377	0.3473146	1.56476	0.9000681
1.283713	2.091681	0.7419406	0.3973907	1.370906	
0.5942592	0.8315332	0.9117924	1.773903	0.877938254	
0.1488974	1.682005	1.185428	1.426892	0.5140037	
0.313104484	1.082665	0.7621879	0.3023254	0.6292486	
0.7067629	1.021788	0.5838451	0.3612174	0.1762288	
0.2687449	1.471247	0.3063427	0.05561751	1.718211	
0.3901956	0.5105796	0.6245779	0.1741639	0.244852968	
0.2910377	0.7644645	2.662781	0.5109828	0.6727278	
0.2203225	2.320777	0.8318415	0.1973082	1.774756	
0.6301227	0.3615757	1.506963	0.5488996	1.5865	0.6169017
0.724763	0.8664287	1.56417	0.1086477	1.803832	0.2647508
1.685076	0.8702407	1.162996	4.422717	0.669483426	
0.4014434	0.5433354	0.6109353	0.9870993	0.3084297	
0.4801105	1.480549629	1.090608	1.019414	2.690327	
0.5438095	2.295824	1.06273	1.175819	0.5090578	0.365956
0.9347494	0.4354612	0.7304345	1.107657	0.5948979	
2.047267267	0.409204	0.492803	0.1533335	0.405074281	
0.9997525	2.562114	0.3240996	0.3178161	0.3579327	

1.043945 0.504843
AC012085.2 0.1341791 3.286053 0.1097094 0.02136267 0.4008854
0.9285656 0.09213978 1.00954 0.4373154 1.186121978 0.3199349
0 0.115115 0.08387022 0.07291702 1.651251 0.1486971
0.780157351 0.09204177 0.0955262 0.331007362 1.648116
0.1422515 1.685586 0.4135488 0.1054677 0.05899812
0.5835414 0.086431364 0.05074418 0.1685256 1.897685
0.0143911 0.1173892 0.06068726 0.424868 0.007756684
0.0535608 0.08649373 0.131940417 0.03611878 0.01971405
0.054311292 0.01916874 0.1151413 0.04388123 0.1734766
0.1522365 0.151805209 0.01190767 0.0172105 0.2320915
0.3358822 0.07845981 1.293549 0.02749127 0 0.5119898
0.03737304 0.030317675 0.135410829 0.071161873 0.6607594
0.09595281 0 0.1919476 0.091542 0.2561586 0 0.1093543
0.02546638 0.1026536 1.084002 0.07645902 0.09281119
0.08088906 0.1446913 0.07888284 0.05641938 0 0.02592275
0.08957004 0.03193908 0.4139142 0.4318583 0.04330572
0.2607836 0.1197518 0.15322143 1.311671 0.01436698
0.09155879 0.5922174 0.1887808 0.02574064 0.1218472
0.2890286 0.07849505 0.03127838 0.113338 0.2426608
0.04558836 0.08587876 0.2682506 0.3153671 0.2249245
0.0215073 0.5382171 0.046143513 0.05962774 0.07222994
0.1616068 0.1046558 0.6820747 0.4752082 0.01775523
0.03336272 0.04724561 0.07158938 0.007798621 0.01542842
0.03207838 0.0549551 0.06919737 0.1433101 0.1373784
0.109091 0.008062303 0.9942948 0.1517877 0.03758002
0.1357882 0.09101853 0.06009101 0 0.2185286 0.04963304
0.03210497 0.1804846 0.1858419 0.08124824 0.3061186
0.06378881 0.09418259 0.189968 0.1808536 0.1064444
0.369642 0.02627818 0.2548577 0.02755681 0.05624238
0.1013229 0.08890141 0.5883331 0.09362646 0.3063957
0.6708206 0 0.4738653 0.438196905 0.2635421 0.205825419
0.0542509 0.1102 0.01775254 0.03567405 0.006967045 0.06091215
0.05506361 0.5046075 0.00858171 0.5964962 0.04551316
0.03870487 0.08858718 0.1298001 0.3327673 0.1147401
0.05175557 0.1349519 0.04517202 0.06678484 0.06944915
0.06134879 0.119736 0.2090596 0.07060133 0.1106589
0.05800356 0.11507 0.01571074 0.02266633 0.05698099 0.1538182
0.1043784 0.1428691 0.16291 0.1139371 0.2002253 0.3957129
0.6657976 0.02192407 0.006085338 0.01410419 0.1271532
0.02694688 0.2489923 0.02532213 0.1119255 0.09534888
0.09507687 0.1037886 0.1808756 0.393456 0.08478637
0.1116846 0.04133787 0.265522 0.069555095 0.1530372
0.062869171 0.05477118 0.04806006 0.279509 0.1872736

0.0769678 0.0520785 0.05456924 0 0.1513818 0.06067904
0.1458422 0.02940664 0.09826317 0.2399935 0.3758224
0.08850395 0.08417239 0.1141691 0.2791524 0.1568639
0.177939 0 0.2410638 0.07874903 0.2234075 1.216084
0.380122 0.2232409 0.09381396 0.0841202 0.3887113
0.2027104 0.04260005 0.05464787 0.1665356 0.030908024
0.04199185 0.2950883 0.3824664 0.03132237 0.1142431
1.216683 0.4839194 0.01133977 0.01291066 0.06487683
0.1485536 0.3699576 0.190286 0.1012991 0.1560618
0.013625961 0.1193431 0.074405 0.07075346 1.348424
0.01598429 0.017776766 0.2473523 0.0420051 0.8209864
0.05495449 0.02175572 0.5494341 0.03526145 0.03759184
0.3540363 0.1334313 0.03608995 0.3321952 0.472692
0.06988653 0.09138256 0.214802 0.114345 0.2346905
0.09668236 0.1908477 0.1017485 0.222011 0.1398481
0.2095297 0.132108 0.03739566 0.040338673 0.072361184
0.07198279 0.01002041 0.01162149 0.05626388 0.02832204
0.04842683 0.1247441 0.5278748 0.1305848 0.04348982
0.03042715 0.04460617 0.03972103 0.017799089 0.04886531
0.1846419 0.42928 0.2943469 0.03442578 0 0.2639446
0.06125772 0.09827254 0.04476025 0.1030872 0.1456296
0.7177657 0.2566983 0.08078533 0.3561479 0.1864229
0.02997343 0 0.02013873 0.084728 0.1082392 0.925872
0.3483873 0.065596833 0.05170633 0.08898307 0.03792583
0.02737876 0.1987945 0.03813926 0.06217429 0.2495951
0.06343132 0.1456103 0.1933662 0.355987 0.1441856
0.5686004 0.0495866 0.03731898 0.06523982 0.1671255
0.1430888 0.06519978 0.01780402 0.03135743 0.1923513
0.1751393 0.2635363 0.0779779 0.096008526 0.05475165
0.1111558 0.05554907 0.2406466 0.0706939 0.4576836
0.067719642 0.005928233 0.5868562 0.02225835 0.03399391 0
0.592778 0.1984534 0.06609093 0.09607989 0.1783709
0.02722098 1.76117 0.153533 0.1246874 0.168553872 0.07340241
0.6139088 0.2300396 0.021704119 0.01874857 0.1372796
0.02210148 0.07379135 0.01413134 0.01003965 0.7869797
LINC01891 0 0 0.07452034 0 0 0.1367436 0 0 0.0833817
0.575180451 0.02318042 0 0.5421318 0 0 0.02000652 0
0.040665579 0.06668751 0 0 0 0.1374217 0.06461726 0
0.0636792 0 0 0 0 0 0 0.05213432 0.0850526 0
0.05919844 0 0.04850836 0.02088925 0.095595492 0.02180778
0.02380587 0 0 0.02780798 0 0.2513799 0.02902651
0.137485294 0.06470648 0 1.299406 0.23177 0.04060493
0.1053058 0 0 1.328793 0.05415617 0.073220753 0
0.147312258 0 0 0 0.07319622 0 0.05458705 0 0

0.02306412 0.01957267 0.3739988 0 0.02101406 0.0976783
0.2306347 0.06594624 0 0.026453836 0 0.02949848 0.1446312
0.05553619 0.02844515 0.0784413 0.1389316 0.2002255
1.11014336 0.12184 0 0.1809207 0.06920684 0.6610954
0.06216668 0.1103532 1.073234 0.02843622 0.7478557
0.2189797 0.8452709 0 0 0.03239284 0.0557304 0.2240779
0 0 0.047760864 0.18001 0 0 0 2.203003 0.286921 0
0 0 0.1396474 0 0 0.6101003 0.174199 0.1504078 0
0.8120001 0.04940016 0 0.1750976 0.0499889 0.01944861
0.0307448 0.1099102 0 0.181112 0.03441993 0.0513727
0.05815296 0.4160782 0.3189055 0.110376 0.3696562
0.2520941 0.02843275 0.05097723 0 0 0.1847025 0
0.02637906 0.02495735 0 0 0.1341921 0 0.1695892 0
0.03197587 0 0.5421034 0.366333615 0.3580229 0.138081283
0.04913337 0 0 0 0.02523935 0.9194376 0.06649255
0.02229304 0 0.02881217 0.02747991 0 0 0.07837064 0
0.07793742 0.03124895 0 0 0.06048496 0.1509551 0.1728586
0.2335656 0.08415057 0 0.02227119 0.1400854 0.1157949 0
0 0.04128474 0.1393084 0.30723 0.03450457 0.1073037
0.2751714 0 0.02077594 0 0.0529492 0 0 0.06281387 0
0 0 0.07602562 0.2417927 0.02870273 0.1754632 0
0.440567 0.0472544 0 0.06418017 0.05658236 0.027997291
0.0504004 0.227754672 0 0.01934512 5.062853 0 0.05576589
0.06288786 0 0 0 0.06280587 0.1056679 0 0.1941686
0.1134024 0.1166986 0.1145076 0.06098587 0.08271951
0.08090232 0.08118101 0 0 0 0.0518695 0.08992588
0.7027145 0.2754118 0 0.1213777 0.03047403 0.6537957
0.2447847 0 0.09898578 0.04468923 0 0.1014153 0.130367
0.07422599 0 0.3103994 0 0.127497 0 0.02338558
0.02937847 0.1345404 0.2233728 0.1969556 0.07339476
0.03769076 0 0 0.1617271 0.02563169 0.1296877 0.6080119
0 0.3468687 0.03804273 0.03098092 0.3483941 0 0.2095181
0.02129014 0 0.1900087 0.04394349 0.04358074 0.05597376
0.02223909 0.04603205 0.08276237 0 0 0.06072912 0.08756223
0.02304598 0.1675463 0.08936378 0 0.07345726 0.02278975 0
0 0.112346214 0.07111919 0.01815034 0.04210091 0.06794194 0
0.1052608 0 0.08999152 0.2956665 0 0.1102277 0.02693229
0.1438965 0 0 0.05574148 0 0.387754 0.04988538 0
0.1103292 0.04931487 0 0.04422324 0.1600507 0.1110672 0
0.05166306 0.02660537 0.04868712 0.324168 0 0.151139
0.07295611 0.02790383 0 0.04041126 0.2629364 0 0 0
0.137393 0 0.2215902 0 0 0.1356304 0 0.3265471
0.1556673 0.8597504 0 0.710295 0.2245453 0.02703892 0
0.1681783 0.1036729 0 0.09674731 0.03786594 0 0.1730381

0.1975255	0.05136158	0.160526695	0	0	0.02515454	0.2011811
0.04656384	0.05436195	0.073597909	0.02147607	0.6442395
0.05375654	0.06157447	0.1169782	0	0.1597628	0.02660289
0.02486188	0.02228207	0.0986128	1.22877	0.1308707	0.0475476
0.441000563	0.1692175	0.07174171	0	0	0.06791999	7.211132
0	0.08225298	0	0	0
AC111149.2	0.03720517	0	0	0.01777031	0.04880083	0	0.05748414
0	0.2871918	0.093228013	0	0	0.2298173	0	0.06065526
0.06890851	0.1484305	0.09804518	0	0	0	0	0	0	0
0.02924406	0.1177847	0	0.071897025	0	0	0.1167252
0.01795663	0	0.06057847	0	0	0.5012324	0.2877956
0.032925976	0	0.01639892	0.038724239	0	0.01915582	1.163506
0.06493712	0.05998565	0.037883272	0.02971582	0.2147457	0
0.07982859	0	0.1450821	0	0.9286601	0	0.01865302
0.52960824	0.147840104	0	0	0.04353673	0.4863961	0
0.2538275	0	1.252251035	0.01299503	0	0.08089704	0
0.7805655	0.115806	0.1009301	0.1588751	0.07571292	0
0.838258055	0	0.08128135	0	0	0	0	0.05742268	0
0.25491128	0.02098269	0.017926532	0	0.04767386	0	0.02141208
0	0.1081914	1.998036	0.3746678	0	0	0.7801139	0
0.2677698	0	0.01403258	0.2415234	0.06947228	0.016450285
0.09920141	0.1013913	0.1344309	0.1305853	0	0.1976485
0.3101596	0.04162864	0.01473779	0.01374251	0.3697705
0.1155057	0	0	0.2762933	0	0.1082622	0.1701491	0
0.05169336	0	0.01339738	0	0.5299897	0	0.08317395	0	0
0.06008891	0	4.686537	0.1647396	0.03917562	0	0
0.0175581	0.04513234	0.09659414	0.2862771	0.03278884
0.09085745	0	0.1052654	0.05057063	0	0	0	0	0	0	0
0	0.02055232	0.038047493	0.05076905	0	0.02953452	0.1187003
0.01738639	0.05066914	0	0	0	0	0.5111052	0.2124952
0.02947613	0.01799548	0	0.6800481	0.1722093	0.01870972
0.4509105	0	0.01733116	0.01701078	0	0	0	0.01534174
0.03216644	2.169649	0.07841288	0.01885475	0.08531827	0	0
0	0.03695863	0.07582189	0.1303477	0.3005462	0.04572964
0.1276611	0.212605	0	0.01442333	0.5828026	0.1499846
0.03159593	0.1571131	0.09517796	0.45476	0.01726709	0.04103441
0.03570461	0	0	0	0.01948868	0.115717355	0.1909536
0.078445591	0.097630354	0	0	0	0.7875058	0.08664192	0
0.2230878	0	0.06489672	0.04852694	0.01467696	0.6018977
0.07811846	0.08038902	0	0	0.2659169	0.3204498	0	0
0.1383091	0	0.714617	0	0	0.1355145	0	0	0.08396941
0	0	0	0	0.09235393	0.019282887	0.01746524	0	0.01704378
0	0.07127395	0	0.06587071	0.05659722	0.161094	0	0
0.01923409	0.03391872	0.07583812	0.5971645	0	0.2460277

0.4084939	1.094713	0.01488945	0	0	0	0.1310306
0.06402459	0.01714249	0.09501066	0	0.3226505	0	0.03272237
0	0.1050737	0.03855811	0	0.06341932	0	0	0.03566876	0
0	0.06350186	0.06924962	0	0.01586334	0.253009	0.04709687
0.4821614	0.040266363	1.444629426	0	0.4376069	0.0580033	0
0	0	0	0	0.1833057	0.2170593	0.05694864	0	0.2973737
0.19988081	2.874401	0	0.1530395	0	0.1030922	0
0.1520028	0	0.1697822	0.7108183	0.0551263	0.01912745	0
0.03558864	0	0.3353863	0.03721775	0.5360613	0.07808528	0
0.3459932	0.1246675	0	0	0.147328304	0.1720455	0.04934642
0.09464461	0	0.32437	0.1110401	0	0	0.05276468	0.2076419
0.1608496	0.1692137	1.252166	0.2201823	1.701484
0.03725209	0.3527489	0.02317027	0.0892702	0	0.1332908
0.29997	12.49757	0	0.1360675	0.1061429	0.092150407	0
0.6010145	0.1906076	0.09239047	0.06415197	0.0561717	0
0.04438205	0.1109478	0	0.01413874	0.4029083	0.02550497
0.1650814	0	0.06850547	0	0.09057394	1.334788	0.2253792
0.2128988	0	0.06660984	0.03294668	0.03587926	0.622874875
0.538055	0	0	0	0.2292226	0.02505414	0
AC025280.1	0.1676379	4.274665	0.1027999	0.2135171	0.3664755
6.111808	0.8633675	2.276997	0.6901439	1.260191739
0.6715188	0.2195655	0.345168	0.104784	0	5.423152
0.1114656	3.365858723	0.7589552	0.5967326	0.261187591
3.339062	0.497625	2.941577	0.3444473	0.08784464
0.08845179	4.860351	0.215967602	0	0.4737351	12.0715
0.05393897	0.5279804	0.1364763	0.1224952	0.1162906
0.301125	0.3457975	0.098904566	0.2707519	0	0.058160829
0.0718459	0.2877056	0.1233527	0.1950611	0.06006255
0.05689776	0.1338926	0.3225315	0.4744891	0.1798446
0.7561887	0.3268531	0	0.1640913	1.947619	0.1120616	0
0.951619133	0.228617293	0.6121876	0	0.07689785	5.225366
0.3049835	0.282383	0.112285655	0.3513157	0.09544997
0.2025019	1.105557	0.1563132	0.3478635	0.4042379
1.431709	0.045486	0	0	0	0.4272742	0.05985506
1.091713	0.824034	0.1082088	0.2299852	0.1381043
0.38285713	0.882403	0.053848545	0.3119722	0.1909399
0.047171	0.1929558	0.3425194	0.1624952	0.1176822	0
0.7363191	0	0.09763925	0.2575042	1.072452	0.8072334
0.590124	0.08061103	1.04342	0.148242375	0.1489929	0.2368829
1.480636	0.09806452	2.279261	0.5937057	0.04436532
0.125046	0.1328101	0.2064019	0.175379	0.2313076
0.3606967	0.5664338	0.4149713	0.1074274	0.542005
0.1533305	0.1813088	2.225664	0.3103157	0.04024366
0.1908543	0.3411444	0.394145	0	0.5697822	0.2657549

0.06016595 0.2459891 0.2932841 0.3045246 0.05883877
0.2608204 0 0.3691928 0.225951 0 4.681854 0.04924632
0.1091687 0.05164251 0.4743017 0.05063546 0.7774884
1.061723 0.1754596 0.499302 2.117294 0 0.7478247
0.084225421 0.370416 0.514299669 0.1525024 0.3441983
0.08871717 0.1782788 0.3655823 0.3044046 0.06879422
3.921004 0.12865964 0.1192381 0.1137246 0.1160551
0.1770835 0.4865008 1.126537 0.2150273 0.2586451
0.05620112 0.05643607 0.187736 0.3123609 0.2554889 0
0.4353174 1.134082 0.2304211 0.1932461 0.6229764
0.05888506 0.05663677 0 0 0.3423161 0.5354844 0.2775451
0.2846966 0.06525738 0.1289706 0.5494593 0.1643462 0
0.1057272 0.3032783 0 0.2574461 0.1423639 0.1573145
0.214425 0.2969628 0.6224123 0.5546747 0.05362558
0.1955605 0.05980034 0.04426786 0.5268689 0.057932855
0.2085801 0.29454811 0.058653313 0.08005902 0.7308985
0.0905698 0 0.3903885 0 0.1116869 0.2521737 1.234618
0.1457675 0.08817467 0.1339265 0.6257489 0 0.1895541
0.5047753 0.1141105 0.1255542 0.1679822 0.5928258
0.2769727 0.116584 0.2683249 0.2791161 1.957413
0.2442388 0.239064 0.1506951 0.0630578 1.352854
0.8104258 0.1995853 0.06827481 0.3236532 0.057922826 0
1.186941 0.4095752 0.07826577 0.428192 0.5772435
0.9893274 0 0.04839016 0.1823725 0.2783952 0.8666435
0.4584898 0.227806 0.07799088 0.05107115 0.03889628
0.1115503 0.2121515 1.520671 0.05991033 0 1.794378
0.07871918 0.19232 0.6694143 0.2038553 1.192239 0.1762168
0.6575198 0.6880508 0.9547568 0.0901786 0.6949358
1.88673 0.04762547 0 1.420755 0.6428609 0 0.3623729
0.4291871 0.346692 0.3081905 0.2382552 3.850667
0.09431449 0.3737648 0.241907921 1.007371055 0.049054 0 0
0.07029378 0.0530766 0.3630149 0.2157925 1.675919
0.3059011 0.1222524 0.6272381 0.2229165 0 0.066712356
0.1046577 0.9227359 0.5746338 0.4011763 0.05161219
0.220076325 0.3043953 0 0.05666662 0.9455837 0.3311818
0.2298236 1.367919 0.1069028 0.3303159 0.6548417
0.2235928 0.03744758 0 0 0.8083525 0.187241 4.181011
0.3808532 0 0.172266 0 0.09476595 0 0 0.1429489
0.1997434 0.2806505 0.07924849 0.2598851 0.2415837
0.508292 0.2161675 0.9553467 0.3717088 0.1118995
0.2445239 0 0.8580927 0.06109344 0.1334617 0.2350601
0.205985 0.2983799 0.4087259 0.05313948 0.110722259
0.15391 0.4166204 0.6246066 0.6938169 0.3372297 0.2812186
0.101527371 0.3555116 0.7998481 0 0.04247059 0 0.4596781

0.165293	0	0.05144497	0.1844269	0	0.6845482	0.3385022
0.2951609	0.140389528	0.3001286	0.9401843	0.6466546
0.244046126	0	1.222019	0	0.0851002	0	0	0.221779
SLCO4A1-AS1	0.5777351	0.06339546	0.4181689	0.4161775	1.490748
0.3644832	0.2926669	0.3178262	0.4386508	0.083063486
0.2032442	1.730474	0.4095213	1.66056	0.2779308	0.0491164
0.6612366	0.335159661	0.8185957	0.7686725	0.398421749
0.02829714	3.446023	0.1926302	0.2010898	1.086891
0.1948938	0.1048458	0.402651461	0.7091945	0.4014705
0.04244849	1.737018	0.6562468	1.634631	0	0.07884108
13.56764	3.75835	0.444232374	6.179874	4.182912
0.788621405	2.301504	0.8679933	1.219589	1.410611
0.2850426	0.36646015	1.422137	2.033588	2.162455
0.03048214	1.153508	0.9602465	1.373859	0.347651
0.572829	16.55283	2.953173405	0.505379652	1.278706892
1.245127	0.3657341	0.2346036	0.2695472	3.954447
2.546233	2.064914171	0.8203983	0.1456016	0.2677144
6.408483	4.035884	3.766064	2.809111	0.2720762
20.08322	53.25313	2.59778742	0.3128897	8.255806
0.2840579	1.684803	0.4688813	0.4126606	0.4872568
1.065041	0.97336559	1.602427	0.438089855	0.6556703
0.02427202	0.2958181	0.7631021	0.3580006	0.6977195
3.979254	19.02764	0.6719992	3.08309	1.721099	3.38247
0.7838898	0.8111425	0.2714856	8.279709	0.4833924
1.247916035	0.6818319	1.663343	2.585599	0.4155276
1.879372	2.482159	1.797171	23.83292	1.343108
0.2168969	5.102835	2.028842	0.3158643	0.637129
0.9495106	6.026861	0.3950206	0.6756938	0.1024343
0.7193711	1.867154	0.6138864	1.164535	2.177928
1.698736	13.91068	2.752338	1.333279	2.630986
1.313332	11.07272	0.4258184	3.719807	1.643021
0.2792119	1.421347	22.8785	25.20405	0.5911022	14.99091
0.4348246	1.225416	2.241991	0.2059748	0.7624281
0.06229146	3.479453	0.4908393	3.510133	0.9578392
1.901249	0.385438367	0.6696786	3.099357705	4.928326
1.365126	0.4135122	6.013131	4.39938	1.483327	6.926062
0.1641896	0.621491481	1.343954	8.355864	0.6294511
0.8929211	3.197529	0.3273231	2.642285	1.380902
2.581441	11.45939	0.8909506	1.164736	2.953278
4.048964	0.9370391	1.858101	0.5389511	0.7615205
5.344439	1.197662	0.2303869	0.6660461	1.791454
4.392918	0.1996722	0.8091146	2.682904	4.258321
0.1311566	1.226194	4.029731	22.69234	10.08888
1.644899	1.848795	0.3381708	3.89289	2.29306	11.36331

16.96212 1.116474 13.92432 1.48152 0.6049117 0.0202713
0.007503027 0.4167324 1.040827565 0.6451843 0.698927719
4.83144236 5.800887 8.630384 0.7291637 1.232157
0.1543909 0.231107 2.432503 30.83785 26.57568
0.7411904 1.681297 4.437735 0.2916626 1.555271
3.847305 0.7593019 0.657586 2.021635 1.138863
1.595103 3.262645 1.432599 9.623315 1.908082
0.199059 0.6071474 5.254006 0.9961203 0.6840168
6.138113 1.287753 2.367962 0.150436 18.22018
0.166896278 2.258572 0.2377541 3.01975 2.347973 8.358211
0.9294598 1.83333 2.49251 1.320477 2.462544 0.9751695
0.4896291 0.0863446 0.5405567 3.357574 3.133518011
1.872296 2.43898 22.76134 3.964662 0.3757089
0.632407882 0.5372997 1.31421 6.182491 0.1920093 0.2211312
1.818669 5.002766 20.02808 4.040008 1.163586
0.5808113 0.3533572 0.2027904 1.727432 0.07256544
0.1926447 0.8171961 0.489871 2.441411 0.2263133
1.045952 1.149185 0.2745992 10.64859 0.7992754
2.494405 8.272020855 7.919748971 4.015777 4.519601
0.08859305 0.8578224 3.031663 0.07383355 6.132409
0.1183559 2.063536 7.010518 0.8504924 2.606989
1.299524 0.667123559 1.064315 0.3714403 2.756294
0.101994 1.65334 0.087035835 6.229785 0.8215394 4.91751
21.50699 0.514548 1.898966 12.24172 24.86849
2.015487 0.315895 1.771689 10.33934 152.753 70.58161
40.64271 4.125649 0.1417292 0.7100654 3.950460252
0.02919763 0.4522243 1.477706 3.791637 0.291433
13.12384 1.263913 9.608712 3.975857 1.004302
2.306647 0.5169071 4.059553 2.528493 3.244577
1.488833 0.2348534 1.993625 2.46338 0.983708 2.137649
2.231079 1.5798 0.7383638 0.7504855 124.6526
0.656826959 0.1565187 53.57221 1.70267 2.422479 3.143666
3.355557 5.016140467 5.212161 51.42526 12.019 2.749791
0.1914559 1.28554 4.482523 0.9236789 13.71575 1.054985
0.2536247 0.7624508 0.3901381 2.518039 5.008812834
0.4154322 0.9896676 0.4749441 6.232138355 4.240085
13.43022 8.49441 0.5625268 0.7451056 2.742481 1.075064
AP002954.1 0 0.1634431 0.09433399 0.3265556 0.1345181
0.1731013 0.2112711 0.04097019 0 0.642450691 0 0
0.8974367 0.1602579 0.7802407 0.1266295 0 0.102955679
0.04220927 0.07301199 0.119839012 0 0 0.4089893 0.09365365
0.05374025 0.4328935 0.4054624 0.198182035 0 0.09660481
0.1532141 0.7919509 0.1076666 0.9462357 1.423827
0.2845699 0.52195 0 0.544556907 0.08281823 0.09040631

2.206006018 0.1977876 0 0.1006171 0.03977717 1.212557
0.522120622 0 0.1973134 0.2580228 0.4400903 0.1542032
2.266182 0.08404783 0 0.5957422 0.1713883 0.092688859
0.038811133 0.512819967 1.566153 0.08000515 0.1881736
0.3011379 0.9328908 0.6219071 0 0.2626831 0.9050903
0.1734369 0.4396215 0.446259 0.3192159 0.3709477
0.5839127 0.08348018 0 0.234412112 1.248226 0 4.503917
2.109069 0.2520575 0.5957848 0.3869163 1.182822
1.52242013 0.1927939 2.009500987 1.259633 1.752154
1.010014 1.416523 3.701896 1.226042 0.1439876
0.143439 0.3811534 0.2568032 0 0.07876598 1.271171
0.4938369 0.386804 0.2958899 0.3829966 1.058043615
0.2734458 0.04140475 0.329382 1.859765 0.2261143
0.9685552 0.1899879 0.3442444 0 0.02525388 0.5364534
0.1768822 0.3309922 0.3465242 2.030918 0.09858046
0.8289488 0.9380219 0.480646 0.5066354 0 0.640111
0.2335158 0.4174003 0.2755703 0.6877996 1.220004
0.7803815 0.8465703 0.7900591 0.2691313 0.9314871
0.6119232 0.2393411 0.03599252 0.387187 0.08293731
0.1775059 0.02922646 0.09038148 0.06678557 0.5686752
0.2901611 0.3097699 0.8833314 0.6245432 0 0 0.6071653
0.09603433 1.715598 1.442731916 0.3776791 1.258521543
0.2176897 0.2105684 0.4070552 0.1090647 2.108703
0.7448959 0.2525152 0.2539833 0.826448981 0.4376738 0
0.1419968 0.3250003 0.3306934 0.1476805 0.1973192
0.237345 0.2062912 0 0.1914171 1.178397 1.844329
0.2956666 0.3728365 0.3237694 0.2537343 0.5024397
0.5570142 0.2521666 0 0.1045233 1.528349 0.2393336
2.031061 0.06791692 0.1393339 0.3193773 0.0262999
0.08403494 0.2010824 0.08371993 0 0.3180599 0.9886019
1.023727 0.1161243 0.384958 1.180599 0.7630198
0.6028857 0.3770338 0.5905123 0 0 0.5145488 0.3581331
0.106323828 1.27602 0.937009517 0.394702292 0.2693751 0.5961839
0.1662222 0.2470757 0.07960863 0.2502481 0.4441197
0.154271 1.431087 0.1783508 0.2157686 0.3277261
0.8134735 0.09848442 0.608803 2.586231 0.2443307
0.4352545 0.8563801 0.5893385 0.4236054 0.4992536
1.510196 0.9676025 0.06842722 0.8965002 0.1218758
0.6145997 1.041566 0.8276284 0.1239475 0.02441985
0.1253044 0.3111422 2.515894986 0.3209495 0.1320234
0.407166 0.3351617 1.047811 0 0.3631414 0.05200286
0.3256373 0.1115691 0.8515617 0.7776002 0.2804879 0
0.09542414 0 0.1665676 0.8871526 0.4218072 0.3009772
2.015806 1.589682097 0.6952337 0.3371033 0.7451458

0.03150185 0.1496538 0 0.05390162 0 0.09019826 0.2503228
0.08275213 0.4251372 0.6193456 0.3787625 0.05238371
0.2897226 3.015144 2.229402 0.1108435 0.116694
0.08483757 0.4147881 0.08745366 0.1239843 0.05769828
0.02858202 0.480969866 1.801416475 1.950618 0.1378572
1.172486 0.6880521 1.850812 0.9327349 0.4400475
0.1139187 0.1871395 0.2742289 0.6627928 0.647769
2.140333 0 0.1920776 0.7056216 0.9843138 0.1227127
0.2525961 0 0.5586549 0.156067 0.1386666 0.2985676
0.03376756 1.300531 0.5021068 0.1634984 0 0 0.7181274
0.4810912 0.04783106 1.015892 0.5298445 0.1145474
0.6138708 0.03328465 0.180491617 0.1053863 0.2267033
0.05797446 1.19278 0.7012678 0 0.448052 2.575381 0
0.03179771 0.1477924 0.3109551 0.899257 0.8541924 0
0.1369124 0.2493185 0.2128939 0.3280943 0 1.796237
1.461982 0.03150359 0.3285689 0.2500441 0.6501772
1.151511492 1.63205 0.2548737 0.5413257 0.2546716 0.2357774
0.06881584 0.031055431 0.02718618 1.18252 0.03402473 0.1818741
1.826328 0.2812148 0.1011205 0.03367613 0.09441666
0.7897813 0.3328859 2.572521 0.3313339 0.1504742
3.864841133 0.4284188 0.1816331 0 0.746494032 0.3439149
1.57387 0.2027096 0.1822146 0.2268163 0.6906101 0.1085412
AC245060.6 0.03278429 0.01484964 0.1777632 0.0576902 0.07695114
0.1782409 0.0266598 0.03308757 0.01331929 0.069179172
0.5287613 0.2508575 0.06661496 0.2766447 0.168783
0.06902963 0.07916441 0.040274378 0.006391546 0.07370569
0.040325875 0.02233973 0.07902561 0.1506994 0.114634
0.5045329 0.1311019 0.1323451 0.026675333 0.007830594
0.06826587 0.00773349 0.3680918 0.1630343 0.1643551
0.06808523 0.02872737 0.1208792 0.07474483 0.174081434
0.09893271 0.1992625 0.050286322 0.226289 0.326932
0.4024833 0.5742179 0.170629 0.245971314 0.03169747
0.02788633 0.5909512 0.2702653 0.1323181 0.1883999
0.1357542 0.1317408 0.3254648 0.1833977 0.063159422
0.201776408 0.094125885 0.1409182 0.04644004 0.08310812
0.1122458 0.05179649 0.06626946 0.045074289 0.07232152
0.06189511 0.09754723 0.3413834 0.1303229 0.108759
0.1778743 0.3576954 0.2556291 0.3264886 0.065920949
0.04500307 0.1960213 0.1718878 0.2466216 0.05452549
0.1119357 0.1988471 0.2430766 0.37831014 0.2140883
0.076487957 0.2350536 0.04569405 0.4821301 0.1211512
0.1604119 0.2341578 0.1653421 0.09846524 0.2466057
0.5033638 0.1899443 0.06361148 0.2111151 0.1281932
0.3787647 0.06222942 0.04081152 0.102232073 0.2438387

0.0721018	0.2050488	0.01211248	0.6695704	0.2444394	
0.04109851	0.09460133	0.2378595	0.2166975	0.05776537	
0.1928479	0.6497109	0.4404509	0.05766228	0.4179718	
1.009211	0.1751828	0.01492962	0.155033	0.08304569	
0.1242678	0.2298411	0.1720578	0.1338782	0.01542966	
0.2815076	0.1936665	0.2693891	0.2012904	0.1856537	
0.2491894	0.4469511	0.1449691	0.1925728	0.265463	
0.2358263	0.2284704	0.1829257	0.7360044	0.2275429	
0.1514929	0.1106579	0.07505114	0.1869191	0.2685832	
0.08668787	0.3098993	0.1695784	0.271451	0.1039139	
0.070221194	0.5604632	0.255860353	0.204061	0.0212569	
0.1164281	0.1045959	0.151592	0.3759866	0.3165188	
0.1125296	0.141036636	0.230121	0.08603627	0.1308031	
0.04511213	0.08178966	0.1167822	0.2606121	0.4173034	
0.09371302	0.1655547	0.1004826	0.2588169	0.1356943	
0.3261914	0.1720589	0.114396	0.2945669	0.05818037	
0.07694721	0.03454776	0.08919282	0.08705098	0.1186824	
0.5239863	0.01763749	0.4885058	0.1547235	0.05037684	
0.1513337	0.4651697	0.1082629	0.164805	0.111001	
0.1913113	0.0894037	0.1808544	0.1011088	0.1716385	
0.3597518	0.1320462	0.1953971	0.140828	0.4023829	
0.2128637	0.03139163	0.533107	0.1572681	0.127011936	
0.1835604	0.201915878	0.019922622	0.3349738	1.18865	0.1342412
0.2832735	0.06027376	0.029473	0.05518023	0.107069	
0.1163773	0.1372845	0.07215243	0.2977559	0.1666558	
0.1739853	0.1141377	0.0993664	0.2448904	0.2636339	
0.2386066	0.1761932	0.07697342	0.1511991	0.09445548	
0.2470719	0.1467894	0.2740193	0.1808596	0.4544717	
0.3095973	0.2570744	0.1814314	0.1614698	0.06746394	
0.3069598	0.119835586	0.07289975	0.1366098	0.1011777	
0.04350164	0.1762944	0.05882124	0.1873691	0.2152375	
0.1598829	0.06945461	0.1074566	0.2658256	0.1179804	
0.05627513	0.2336022	0.159278907	0.24622	0.2790081	0.3619426
0.2403073	0.2275454	0.03909094	0.337991	0.1968914	
0.02375449	0.1033538	0.1007171	0.2744388	0.1020258	
0.3799648	0.1426532	0.1684674	0.1434075	0.3093647	
0.1065733	0.2132399	0.07138991	0.1257644	0.1588065	
0.1125292	0.1608514	0.09865979	0.1755694	0.2912071	
0.2383684	0.1736627	0.1106683	0.09377414	0.119517453	
0.212959677	0.1332964	0.0695835	0.05380116	0.0260471	
0.177006	0.03138657	0.2087874	0.1552514	0.1473557	
0.4039265	0.1831194	0.220269	0.1264221	0.261620189	
0.1098781	0.05698606	0	0.2477573	0.1498102	0.038509001
0.1433405	0.1244644	0.1242359	0.1177361	0.0784033	

0.1649981 0.07603161 0.4670966 0.1954958 0.1213246
0.5799604 0.05781692 0.2921273 0.3682637 0.0802318
0.1773082 0.1239407 0.3528094 0.097176801 0.3364507
0.1075629 0.1726496 0.1520985 0.2530852 0.03825552
0.07195825 0.1242151 0.2104509 0.5649569 0.1840091
[truncated: 612,671 more chars]
