# Supplementary material for: Anoikis-Related Long Non-Coding RNA Signatures to Predict Prognosis and Immune Infiltration of Gastric Cancer
Source: Bioengineering (Basel). 2024 Sep 5;11(9):893. doi: 10.3390/bioengineering11090893 (PMC11428253; doi:10.3390/bioengineering11090893)
Supplement: Supplementary file 1 [file bioengineering-11-00893-s001.zip › Supplementary Table S3.pdf]

ID	StromalScore	ImmuneScore	ESTIMATEScore
TCGA-KB-A93J-01A-11R-A39E-31	-197.400603342598	1632.01635452424	1434.61575118164
TCGA-IN-A7NR-01A-11R-A354-31	-402.764837782419	1073.36073231096	670.59589452854
TCGA-B7-A5TN-01A-21R-A31P-31	559.791108827383	608.843801276057	1168.63491010344
TCGA-BR-8676-01A-11R-2402-13	-494.257227268533	1577.16099233249	1082.90376506396
TCGA-BR-4294-01A-01R-1131-13	-705.823922107237	-82.6508427901611	-788.474764897398
TCGA-FP-7735-01A-11R-2055-13	-12.8260924682414	1226.36141970227	1213.53532723402
TCGA-EQ-8122-01A-11R-2343-13	157.819559361208	270.134730905907	427.954290267115
TCGA-BR-8368-01A-11R-2343-13	-870.545588820288	678.718504865002	-191.827083955285
TCGA-VQ-A91Q-01A-12R-A414-31	95.0322460868916	-35.2059191811332	59.8263269057584
TCGA-IN-A6RO-01A-12R-A33Y-31	-1390.17986238844	96.0856210995959	-1294.09424128884
TCGA-BR-4257-01A-01R-1131-13	859.400674323186	1712.67873445352	2572.07940877671
TCGA-HF-A5NB-01A-11R-A31P-31	-1015.00509811915	598.059338428262	-416.945759690893
TCGA-VQ-A927-01A-12R-A414-31	-218.343209971186	737.237447081278	518.894237110092
TCGA-VQ-A8PJ-01A-11R-A414-31	-1071.3882010173	-72.9072286663541	-1144.29542968365
TCGA-FP-A9TM-01A-11R-A39E-31	-963.334168073141	1520.07685281434	556.742684741203
TCGA-BR-6565-01A-11R-1802-13	542.156583219649	1498.41150028296	2040.56808350261
TCGA-BR-4368-01A-01R-1157-13	659.374897464252	1698.68283934251	2358.05773680677
TCGA-VQ-AA68-01A-11R-A414-31	-947.954546586524	755.367967508348	-192.586579078175
TCGA-CD-5799-01A-11R-1602-13	-141.823800463189	330.068255965941	188.244455502751
TCGA-HU-A4GY-01A-21R-A24K-31	1608.42931926462	3030.87744239402	4639.30676165864
TCGA-BR-8295-01A-11R-2343-13	-1186.4465310528	-1032.02630617135	-2218.47283722415
TCGA-F1-A72C-01A-21R-A33Y-31	421.386838129243	1174.13199083787	

1595.51882896711
TCGA-RD-A7BW-01A-11R-A32D-31 1581.89539663212 2149.5942454236
3731.48964205572
TCGA-VQ-A8P2-01A-11R-A36D-31 -1136.58976482154 -272.025953594153
-1408.61571841569
TCGA-HU-8604-01A-11R-2402-13 640.423556078139 2293.36122862443
2933.78478470257
TCGA-FP-8209-01A-11R-2343-13 1636.22169994997 2509.63442783329
4145.85612778326
TCGA-D7-6528-01A-11R-1802-13 -917.947894516428 57.0223218047956
-860.925572711633
TCGA-CG-5732-01A-11R-1602-13 -663.580177052332 1707.15689068675
1043.57671363441
TCGA-CG-4462-01A-01R-1157-13 2062.96507962445 2332.77946490416
4395.74454452861
TCGA-BR-6852-01A-11R-1884-13 738.731553722829 2354.89663096495
3093.62818468778
TCGA-BR-4187-01A-01R-1131-13 2048.6945250654 2175.72082445824
4224.41534952364
TCGA-D7-6818-01A-11R-1884-13 1167.37386638665 1541.65773788438
2709.03160427103
TCGA-R5-A7ZR-01A-11R-A354-31 -1040.78990840372 354.535051570105
-686.254856833618
TCGA-BR-A4IV-01A-31R-A251-31 1475.95750953189 819.337113239094
2295.29462277098
TCGA-CG-5719-01A-11R-1602-13 1160.43161282672 1423.0813764614
2583.51298928813
TCGA-BR-6563-01A-13R-2055-13 1528.79562863246 2425.40807680458
3954.20370543704
TCGA-HF-7131-01A-11R-2055-13 -219.885035767095 1160.45062076131
940.565584994212
TCGA-HU-A4H2-01A-11R-A251-31 -464.563388658145 901.983780423621
437.420391765476
TCGA-HU-8602-01A-11R-2402-13 13.0961383209088 1693.44336411911
1706.53950244002
TCGA-HU-A4G2-01A-11R-A251-31 -73.7568254160935 1002.26428301786
928.507457601767
TCGA-BR-8369-01A-11R-2343-13 78.2186462405434 172.831587812361
251.050234052904
TCGA-CG-4475-01A-01R-1157-13 907.000531146458 719.200942099759
1626.20147324622
TCGA-VQ-A91A-01A-11R-A414-31 1194.41216365098 1071.98830801199
2266.40047166297
TCGA-CG-5722-01A-21R-1602-13 426.063059185188 2550.81043259111

2976.87349177629
TCGA-D7-A4Z0-01A-22R-A251-31 572.205812835688 1678.83017983301
2251.0359926687
TCGA-VQ-A928-01A-11R-A414-31 212.058275941824 293.586055922996
505.644331864821
TCGA-HU-A4H5-01A-21R-A251-31 -837.362958725081 552.397012531261
-284.96594619382
TCGA-CG-4436-01A-01R-1157-13 -613.779568865628 1183.23112746121
569.451558595586
TCGA-B7-5818-01A-11R-1602-13 -58.1663143211964 1745.58492971307
1687.41861539187
TCGA-BR-8291-01A-11R-2343-13 1672.99865526368 1839.36450191881
3512.3631571825
TCGA-BR-4253-01A-01R-1131-13 541.601466591251 2847.08587907985
3388.6873456711
TCGA-ZA-A8F6-01A-23R-A36D-31 817.544881281307 1395.49033297023
2213.03521425154
TCGA-BR-8059-01A-11R-2343-13 689.338121310324 290.714012784899
980.052134095223
TCGA-B7-A5TI-01A-11R-A31P-31 419.779387415464 701.380468628536
1121.159856044
TCGA-BR-8367-01A-11R-2343-13 810.250608282909 673.224041588787
1483.4746498717
TCGA-D7-A6EY-01A-21R-A31P-31 318.85128864145 2064.90962821849
2383.76091685994
TCGA-RD-A7C1-01A-11R-A32D-31 222.644922953467 2010.39494028101
2233.03986323448
TCGA-CD-A489-01A-11R-A24K-31 786.785428771213 1309.44533860127
2096.23076737249
TCGA-CG-4465-01A-01R-1157-13 692.068659821291 1456.46462398828
2148.53328380957
TCGA-HU-8608-01A-11R-2402-13 399.638965342147 2549.51637445487
2949.15533979702
TCGA-B7-A5TK-01A-12R-A36D-31 1024.04148287549 2411.47057662708
3435.51205950257
TCGA-HU-A4HB-01A-12R-A251-31 -198.29252387678 2416.83119784831
2218.53867397153
TCGA-CG-5721-01A-11R-1602-13 498.904264141373 2616.75445428253
3115.6587184239
TCGA-D7-8570-01A-11R-2343-13 837.124567404341 2652.98014397004
3490.10471137439
TCGA-BR-8366-01A-11R-2343-13 1154.63675492398 2559.34805652162
3713.98481144561
TCGA-FP-A8CX-01A-11R-A36D-31 -720.503583934114 886.546390867667

			166.042806933553
TCGA-VQ-AA69-01A-11R-A414-31	-968.061353187866	661.403033443067	-306.658319744799
TCGA-RD-A8NB-01A-12R-A39E-31	495.418373001042	1703.66251596184	2199.08088896288
TCGA-D7-8574-01A-13R-2343-13	1546.75577690075	2713.43645693855	4260.1922338393
TCGA-VQ-A91V-01A-11R-A414-31	-1305.7499344999	-37.3618354011962	-1343.11176990109
TCGA-R5-A7ZI-01A-11R-A354-31	-135.014860027553	2192.37471450951	2057.35985448195
TCGA-BR-8077-01A-11R-2343-13	152.460611550828	1623.84549086375	1776.30610241458
TCGA-CD-8530-01A-11R-2343-13	1191.68320381542	1045.39974510071	2237.08294891613
TCGA-MX-A5UG-01A-21R-A31P-31	1515.88339240902	2384.0912290902	3899.97462149922
TCGA-BR-6802-01A-11R-1884-13	51.1735368928943	1711.75209498902	1762.92563188191
TCGA-BR-6803-01A-11R-1884-13	1153.88657348635	1592.44491778702	2746.33149127337
TCGA-BR-6452-01A-12R-1802-13	169.595662802334	1277.25856871246	1446.8542315148
TCGA-BR-8487-01A-11R-2402-13	-207.241648379723	1092.20923196793	884.967583588207
TCGA-HU-A4HD-01A-11R-A251-31	-269.810813288959	159.674296120266	-110.136517168693
TCGA-CD-A4MH-01A-11R-A251-31	-249.125406190915	547.022712041545	297.89730585063
TCGA-CG-5723-01A-11R-1602-13	246.716500023648	1955.5325511411	2202.24905116475
TCGA-BR-A44T-01A-32R-A24K-31	831.122396518164	2770.09915594705	3601.22155246522
TCGA-RD-A7BS-01A-11R-A32D-31	271.611859705483	1464.36175132664	1735.97361103213
TCGA-VQ-A8E0-01A-11R-A414-31	-796.933259155855	780.108078523512	-16.8251806323423
TCGA-CG-5718-01A-11R-1602-13	3.82723754380921	1484.54492137652	1488.37215892033
TCGA-VQ-A92D-01A-11R-A414-31	-592.59908532691	-326.898248117844	-919.497333444754
TCGA-BR-8289-01A-11R-2343-13	309.342272103219	402.340716255841	711.68298835906
TCGA-RD-A7BT-01A-11R-A33Y-31	-1172.30987492952	480.839713480896	

-691.470161448623
TCGA-IN-A6RS-01A-12R-A354-31 -496.122079206 1281.02125797394
784.899178767936
TCGA-BR-4369-01A-01R-1157-13 335.917092391166 1138.83966333685
1474.75675572801
TCGA-VQ-A8E2-01A-11R-A36D-31 317.595950865585 283.309311595204
600.905262460789
TCGA-D7-A748-01A-12R-A32D-31 1750.60604862507 2240.36404200475
3990.97009062983
TCGA-BR-8678-01A-11R-2402-13 -272.878811174533 19.2655899431184
-253.613221231415
TCGA-HU-A4GJ-01A-11R-A251-31 281.136462075241 3108.40919160574
3389.54565368098
TCGA-BR-8372-01A-11R-2343-13 16.1361159344268 1842.07555551012
1858.21167144454
TCGA-BR-6566-01A-11R-1802-13 683.072684703134 1677.73447965871
2360.80716436185
TCGA-BR-8365-01A-21R-2343-13 1344.92101237443 1368.84380129583
2713.76481367026
TCGA-BR-6456-01A-11R-1802-13 1117.02809201029 1214.52599605766
2331.55408806795
TCGA-VQ-A91K-01A-11R-A414-31 9.82851649230938 1156.46399209214
1166.29250858445
TCGA-BR-8680-01A-11R-2402-13 -1041.18431855118 -643.945775713892
-1685.13009426508
TCGA-IN-A6RL-01A-11R-A32D-31 -605.268008417589 560.970579701478
-44.2974287161111
TCGA-CG-4306-01A-01R-1157-13 499.634493353912 1468.75142297314
1968.38591632706
TCGA-CG-5734-01A-11R-1602-13 307.147628138126 2398.56537928822
2705.71300742635
TCGA-BR-8682-01A-11R-2402-13 872.152290260249 1128.87364826706
2001.02593852731
TCGA-BR-7851-01A-11R-2203-13 326.469078061381 1044.77400468618
1371.24308274756
TCGA-CG-4440-01A-01R-1157-13 -676.356577120462 659.594552996013
-16.7620241244482
TCGA-BR-8286-01A-12R-2343-13 321.955664041943 992.301532872484
1314.25719691443
TCGA-CG-4466-01A-01R-1157-13 -990.757755579825 58.0027400953587
-932.755015484466
TCGA-VQ-AA6J-01A-11R-A414-31 56.1015494378914 1761.89241716672
1817.99396660461
TCGA-VQ-A8E3-01A-11R-A39E-31 -602.252939075077 1346.91670963871

744.663770563628
TCGA-VQ-AA6F-01A-31R-A414-31 -550.710233786741 1087.28462355048
536.574389763739
TCGA-VQ-A8PH-01A-12R-A414-31 -1108.16539870854 586.228092851016
-521.937305857522
TCGA-CD-8526-01A-11R-2343-13 100.571831155775 823.521190331337
924.093021487112
TCGA-BR-A4J6-01A-11R-A251-31 -33.8770768019651 426.620998470787
392.743921668822
TCGA-KB-A93H-01A-11R-A39E-31 -1412.2911698268 190.557167435228
-1221.73400239157
TCGA-BR-A4J5-01A-21R-A251-31 1231.52373601225 1257.5453635606
2489.06909957285
TCGA-VQ-A91Z-01A-11R-A414-31 -1689.40494342478 -732.433760056884
-2421.83870348166
TCGA-3M-AB47-01A-22R-A414-31 1011.89820831112 1229.13908855709
2241.03729686821
TCGA-BR-8361-01A-11R-2343-13 -374.373393952786 1154.24252368062
779.869129727832
TCGA-D7-6524-01A-11R-1802-13 1245.61661895961 1605.41286748106
2851.02948644067
TCGA-CD-8528-01A-11R-2343-13 -10.7126441919824 539.149743098674
528.437098906692
TCGA-FP-7998-01A-11R-2203-13 1277.67736037839 2846.57731183398
4124.25467221236
TCGA-BR-8592-01A-11R-2402-13 1284.49677769651 1490.56603382475
2775.06281152125
TCGA-IN-A6RN-01A-12R-A33Y-31 -1010.04901250197 217.219086050572
-792.829926451402
TCGA-MX-A5UJ-01A-11R-A31P-31 359.889350363991 713.027021401008
1072.916371765
TCGA-BR-A4J7-01A-31R-A251-31 1171.59934701834 2009.87063022148
3181.46997723983
TCGA-IN-A7NT-01A-21R-A354-31 -285.143035787147 217.423439985345
-67.7195958018028
TCGA-BR-8690-01A-11R-2402-13 627.420965177383 1920.67805633823
2548.09902151561
TCGA-CD-5803-01A-11R-1602-13 1604.61172156749 2570.96520995544
4175.57693152293
TCGA-RD-A8N0-01A-12R-A36D-31 920.436108905373 2544.23755257332
3464.67366147869
TCGA-CG-4476-01A-01R-1157-13 1244.13619327318 1790.42454844715
3034.56074172032
TCGA-HU-A4GF-01A-11R-A24K-31 -693.775674518835 729.280247177208

35.5045726583737
TCGA-BR-7717-01A-11R-2055-13 196.169482624689 656.750804670651
852.920287295339
TCGA-HF-7132-01A-11R-2055-13 371.714937084189 1883.25315353924
2254.96809062343
TCGA-BR-6454-01A-11R-1802-13 -4.33534137058292 2008.43329623067
2004.09795486008
TCGA-CG-4301-01A-01R-1157-13 868.30332641385 1313.11440058884
2181.41772700269
TCGA-RD-A8N1-01A-12R-A36D-31 536.572113350537 2394.35820309985
2930.93031645039
TCGA-D7-A6EX-01A-11R-A31P-31 -534.18746406707 -207.736556313864
-741.924020380934
TCGA-RD-A8N4-01A-21R-A36D-31 752.469708825689 1174.58275721613
1927.05246604182
TCGA-HF-7133-01A-11R-2055-13 293.498515899666 1625.96104020718
1919.45955610685
TCGA-CD-8534-01A-11R-2343-13 9.06546254731603 952.113901042462
961.179363589778
TCGA-R5-A7ZE-01B-11R-A354-31 -1316.08759945044 -443.08083728178
-1759.16843673222
TCGA-IN-A6RI-01A-11R-A32D-31 -1325.34394445543 -40.7237981658161
-1366.06774262124
TCGA-CD-A48C-01A-11R-A24K-31 200.464564078088 760.237178128544
960.701742206632
TCGA-D7-8578-01A-21R-2343-13 884.42364763485 865.953491424885
1750.37713905974
TCGA-CD-A487-01A-21R-A24K-31 198.398847951856 453.490977730874
651.88982568273
TCGA-VQ-A8PX-01A-12R-A414-31 -1370.0380297282 676.56521231614 -
693.472817412059
TCGA-VQ-A922-01A-11R-A414-31 305.348944707785 -131.551712906748
173.797231801038
TCGA-3M-AB46-01A-11R-A414-31 -213.812254957643 199.955215053934
-13.8570399037085
TCGA-B7-A5TJ-01A-11R-A31P-31 -453.85540707785 154.923681956227
-298.931725121623
TCGA-CG-5717-01A-11R-1602-13 -109.441718629566 1470.56742416638
1361.12570553681
TCGA-VQ-A8PF-01A-11R-A414-31 731.760713545114 2453.09031726345
3184.85103080856
TCGA-RD-A8MW-01A-11R-A36D-31 989.427367770511 1974.69290609936
2964.12027386987
TCGA-HU-8610-01A-22R-2402-13 372.4143549971 1914.5347120817

2286.9490670788
TCGA-BR-8364-01A-11R-2343-13 1590.6975807533 1543.04900404123
3133.74658479453
TCGA-SW-A7EA-01A-12R-A354-31 -153.279148942093 900.874302430321
747.595153488229
TCGA-BR-8373-01A-11R-2343-13 489.055290560752 443.866242280144
932.921532840896
TCGA-D7-A4YX-01A-11R-A251-31 -390.246178112873 1409.09437419366
1018.84819608078
TCGA-BR-7901-01A-11R-2203-13 936.024903953136 1278.7552852892
2214.78018924234
TCGA-VQ-A8PK-01A-12R-A414-31 -364.849736579317 459.978415516896
95.1286789375786
TCGA-D7-6525-01A-11R-1802-13 244.89703489764 741.649779468015
986.546814365655
TCGA-CG-5716-01A-21R-1802-13 -512.730098527115 1576.18761408431
1063.45751555719
TCGA-CD-5801-01A-11R-1602-13 153.831720438677 2168.95186789292
2322.7835883316
TCGA-BR-8683-01A-11R-2402-13 584.238458095576 778.188474597533
1362.42693269311
TCGA-BR-4256-01A-01R-1131-13 1670.38041549146 2493.95409342054
4164.334508912
TCGA-MX-A666-01A-11R-A31P-31 -866.830204266475 535.927635137176
-330.902569129299
TCGA-D7-8573-01A-11R-2343-13 -923.426313696321 344.105694694783
-579.320619001538
TCGA-BR-A44U-01A-11R-A36D-31 67.1994255471055 148.110596540368
215.310022087473
TCGA-VQ-A8DV-01A-12R-A36D-31 -1174.70610130127 -414.56098273052
-1589.26708403179
TCGA-BR-A4J8-01A-11R-A251-31 486.493838102421 619.942749117116
1106.43658721954
TCGA-HF-7134-01A-11R-2055-13 -389.108711411492 738.039123380277
348.930411968785
TCGA-HU-A4GU-01A-11R-A251-31 -799.3820644181 213.239623473414 -
586.142440944686
TCGA-R5-A7ZF-01A-11R-A354-31 -1255.50031345081 -158.465593672844
-1413.96590712366
TCGA-VQ-A91E-01A-31R-A414-31 -1238.3150507971 989.154740265822
-249.160310531275
TCGA-CD-5804-01A-12R-2055-13 721.946412543694 1441.13425860427
2163.08067114796
TCGA-BR-8060-01A-11R-2343-13 864.098386372367 1255.64179550768

2119.74018188004
TCGA-VQ-A8E7-01B-11R-A414-31 -419.977222868562 35.9839286294834
-383.993294239079
TCGA-FP-8099-01A-11R-2343-13 -3.03716078466298 628.735081183736
625.697920399073
TCGA-CG-4304-01A-01R-1157-13 812.840303702293 1681.41669650144
2494.25700020374
TCGA-BR-8080-01A-11R-2343-13 1406.7393047736 1785.11527628847
3191.85458106207
TCGA-VQ-A8PC-01A-11R-A39E-31 211.732937812115 1513.28459953512
1725.01753734723
TCGA-BR-7197-01A-11R-2203-13 -321.502817146526 -239.649863294864
-561.15268044139
TCGA-HJ-7597-01A-21R-2203-13 -927.303776789864 1008.21570660872
80.9119298188543
TCGA-BR-8591-01A-11R-2402-13 112.52260951638 1142.57129755711
1255.09390707349
TCGA-D7-6520-01A-11R-1802-13 433.93159941846 633.993641735507
1067.92524115397
TCGA-D7-A6F0-01A-11R-A31P-31 -517.591115244571 877.219708344512
359.628593099941
TCGA-BR-7196-01A-11R-2055-13 1279.30717310236 1827.3631275155
3106.67030061785
TCGA-D7-6815-01A-11R-1884-13 61.0301495326694 463.076804222926
524.106953755595
TCGA-D7-8576-01A-11R-2343-13 -8.16830158364726 645.54436539512
637.376063811472
TCGA-BR-4363-01A-01R-1157-13 837.239882740303 1666.67609852761
2503.91598126791
TCGA-D7-6527-01A-11R-1802-13 -74.6775760620813 744.215278989047
669.537702926966
TCGA-VQ-A91D-01A-11R-A414-31 -50.96626082065 934.543025570528
883.576764749879
TCGA-VQ-A8PQ-01A-11R-A414-31 788.840870736724 2887.7670657366
3676.60793647333
TCGA-BR-A4QL-01A-31R-A251-31 -762.771451235655 573.063907341866
-189.707543893789
TCGA-VQ-A940-01A-11R-A414-31 -601.41219691704 454.466390311951
-146.945806605089
TCGA-IN-A6RJ-01A-21R-A33Y-31 -1145.65009747473 343.757455567325
-801.892641907409
TCGA-CG-5726-01A-11R-1602-13 -1164.14459306742 110.025717978031
-1054.11887508938
TCGA-D7-6519-01A-11R-1802-13 -417.100337222027 431.654291053665

14.5539538316377
TCGA-BR-6801-01A-11R-1884-13 209.599297292336 318.170337138056
527.769634430392
TCGA-RD-A8N5-01A-12R-A36D-31 1385.62981458722 1682.89843795066
3068.52825253787
TCGA-HU-8244-01A-11R-2343-13 -1751.09086935474 -35.0709306434479
-1786.16179999818
TCGA-VQ-AA6G-01A-11R-A414-31 -825.468787893948 199.988059984287
-625.480727909661
TCGA-BR-4371-01A-01R-1157-13 -819.481648787075 739.308807212534
-80.1728415745405
TCGA-BR-7722-01A-31R-2203-13 99.0310281029202 897.510260613407
996.541288716327
TCGA-BR-6453-01A-11R-1802-13 968.221220656916 2663.0535622908
3631.27478294771
TCGA-HU-A4GX-01A-12R-A251-31 -499.918385181178 1592.57520284704
1092.65681766586
TCGA-BR-4361-01A-01R-1157-13 1056.84597104504 1593.27063178698
2650.11660283202
TCGA-CD-8533-01A-11R-2343-13 -472.933674731384 -653.902557881721
-1126.83623261311
TCGA-RD-A8N6-01A-11R-A36D-31 -129.494406352342 83.6022417423987
-45.8921646099429
TCGA-FP-7916-01A-11R-2203-13 963.13955952961 2287.54750121757
3250.68706074718
TCGA-BR-8588-01A-11R-2402-13 921.220375423012 1850.68340242944
2771.90377785245
TCGA-BR-8382-01A-11R-2402-13 529.971301222515 1463.39709081041
1993.36839203293
TCGA-BR-4201-01A-01R-1131-13 1435.64585970116 1754.36230062635
3190.00816032751
TCGA-CD-8529-01A-11R-2343-13 1146.05300987136 2196.40423421124
3342.4572440826
TCGA-BR-8381-01A-11R-2402-13 970.703071914437 2298.98961466954
3269.69268658398
TCGA-BR-8371-01A-11R-2343-13 602.41188734041 1365.54089578569
1967.9527831261
TCGA-BR-8590-01A-11R-2402-13 1161.78496333646 1610.40954929673
2772.19451263319
TCGA-HU-A4H4-01A-21R-A251-31 -174.532903815337 1698.41580568774
1523.8829018724
TCGA-D7-A4YU-01A-21R-A251-31 515.019783154172 2127.77403031086
2642.79381346503
TCGA-CG-4469-01A-01R-1157-13 -194.216083975915 1171.54249305637

977.326409080455
TCGA-FP-8631-01A-11R-2402-13	-155.675308866382	89.5111312187945	-66.1641776475875
TCGA-BR-8677-01A-11R-2402-13	1140.37255200202	1672.38303625524	2812.75558825726
TCGA-HU-A4H0-01A-11R-A251-31	-605.423756345266	1582.09211576116	976.668359415897
TCGA-BR-7723-01A-11R-2055-13	70.8222800847247	905.827907317087	976.650187401811
TCGA-BR-8679-01A-11R-2402-13	314.240992623365	143.658515851584	457.89950847495
TCGA-D7-6521-01A-11R-1802-13	1132.9452613431	1847.5951594891	2980.5404208322
TCGA-VQ-A8PO-01A-11R-A414-31	3.30879477750836	1568.30339670835	1571.61219148586
TCGA-MX-A663-01A-11R-A31P-31	1016.05141748146	456.034726595782	1472.08614407724
TCGA-HU-A4H6-01A-11R-A251-31	-76.2926793354804	1389.66069215788	1313.3680128224
TCGA-BR-7707-01A-11R-2055-13	-162.544210581348	1012.10394677181	849.559736190463
TCGA-CG-4477-01A-01R-1157-13	523.566362758065	2044.76618127078	2568.33254402885
TCGA-HU-A4GQ-01A-11R-A36D-31	217.858955763169	849.260566000298	1067.11952176347
TCGA-F1-6874-01A-11R-1884-13	235.142621945551	1531.40685351974	1766.54947546529
TCGA-BR-8485-01A-11R-2402-13	373.158667913579	1147.91516563105	1521.07383354463
TCGA-D7-A6EV-01A-11R-A31P-31	-983.317412414999	26.0476212314426	-957.269791183556
TCGA-CG-5720-01A-11R-1602-13	486.872151953884	1690.49219374472	2177.3643456986
TCGA-CD-8524-01A-11R-2343-13	559.938445222575	945.166700003384	1505.10514522596
TCGA-D7-8575-01A-11R-2343-13	-202.524651842654	1425.51039889096	1222.9857470483
TCGA-BR-8284-01A-11R-2343-13	1003.7496597721	2000.66629166875	3004.41595144086
TCGA-CG-4437-01A-01R-1802-13	573.549315721653	1866.60784542216	2440.15716114381
TCGA-IN-A6RR-01A-12R-A32D-31	-809.604690132896	467.300156978375	-342.304533154521
TCGA-BR-4280-01A-01R-1131-13	-403.226823500979	1236.07791525384

832.851091752864
TCGA-HU-A4GH-01A-11R-A24K-31 -1658.98261523239 438.526520985499
-1220.45609424689
TCGA-D7-A6F2-01A-12R-A31P-31 27.4935162540766 1333.90954313687
1361.40305939095
TCGA-VQ-AA6K-01A-11R-A414-31 -113.259129796703 788.992769483505
675.733639686802
TCGA-BR-A4J9-01A-12R-A251-31 785.692461606469 989.645810325478
1775.33827193195
TCGA-CD-8531-01A-11R-2343-13 361.492704623779 2180.85700550185
2542.34971012563
TCGA-BR-4367-01A-01R-1157-13 1167.95507804719 1881.0389887804
3048.99406682759
TCGA-FP-8210-01A-11R-2343-13 1554.16988114023 2467.64933174371
4021.81921288395
TCGA-BR-A4PF-01A-11R-A251-31 -163.136677351107 1543.47026640585
1380.33358905474
TCGA-CD-5798-01A-11R-1602-13 1074.10009994383 1333.92633316845
2408.02643311228
TCGA-BR-8081-01A-11R-2343-13 1039.35270486358 2281.65982401803
3321.01252888161
TCGA-VQ-A8PB-01A-11R-A39E-31 -223.968670454282 430.72008586705
206.751415412768
TCGA-BR-8686-01A-11R-2402-13 753.3416461945 2108.17979133429
2861.52143752879
TCGA-VQ-A91N-01A-11R-A414-31 -996.425807521342 115.046425874112
-881.37938164723
TCGA-BR-8483-01A-31R-2402-13 -651.028939551076 -311.056610241785
-962.085549792861
TCGA-F1-A448-01A-11R-A24K-31 726.439558597408 1807.1541865845
2533.59374518191
TCGA-BR-7957-01A-11R-2203-13 1309.91408116039 745.568269067743
2055.48235022813
TCGA-VQ-A8PE-01A-11R-A414-31 -465.865415405639 835.797609036635
369.932193630996
TCGA-RD-A8N2-01A-12R-A36D-31 977.413937490134 1073.50138300699
2050.91532049713
TCGA-VQ-A94P-01A-13R-A414-31 1272.45363413199 1336.32157738574
2608.77521151773
TCGA-BR-8486-01A-31R-2402-13 353.076049890409 1281.31381060903
1634.38986049944
TCGA-BR-6709-01A-11R-1884-13 1086.05561568676 2538.60774237873
3624.66335806549
TCGA-BR-6705-01A-12R-1884-13 1581.37673349377 1204.13328498288

2785.51001847665
TCGA-FP-A4BF-01A-12R-A36D-31 1233.60165185633 2508.11145560586
3741.71310746219
TCGA-BR-7958-01A-21R-2343-13 770.639414897488 2482.60668514649
3253.24610004398
TCGA-HU-A4H3-01A-21R-A251-31 -612.055234014367 579.453533416072
-32.6017005982952
TCGA-HU-A4GP-01A-11R-A251-31 -551.293476423219 366.48470808066 -
184.808768342559
TCGA-VQ-A8P8-01A-11R-A39E-31 156.723668209887 1183.80811076894
1340.53177897883
TCGA-RD-A8N9-01A-12R-A39E-31 1345.13945147753 1550.81441275774
2895.95386423527
TCGA-BR-A4J4-01A-12R-A251-31 142.266457166234 1076.96970912768
1219.23616629391
TCGA-KB-A93G-01A-11R-A39E-31 1650.23057750916 1499.25960094321
3149.49017845237
TCGA-VQ-A8DT-01A-11R-A36D-31 -1160.91653620774 238.648601029233
-922.267935178508
TCGA-VQ-A8DZ-01A-11R-A36D-31 -439.31157971535 399.849817111991
-39.4617626033586
TCGA-CG-4460-01A-01R-1157-13 -356.153808534768 541.46324118729
185.309432652522
TCGA-BR-4357-01A-01R-1157-13 714.47351333193 1964.06837537656
2678.54188870849
TCGA-BR-8484-01A-11R-2402-13 549.275993712167 1860.85121800885
2410.12721172101
TCGA-VQ-A94T-01A-11R-A414-31 -767.486653949062 -303.98387859683
-1071.47053254589
TCGA-BR-7716-01A-21R-2055-13 325.928928513137 1818.62307394759
2144.55200246072
TCGA-CG-5724-01A-11R-1602-13 93.2099111724504 1083.29999185951
1176.50990303196
TCGA-D7-A6EZ-01A-11R-A31P-31 290.948732460416 1808.44542004651
2099.39415250693
TCGA-D7-6522-01A-11R-1802-13 1881.61108782879 2974.15478514661
4855.76587297539
TCGA-HU-A4G8-01A-11R-A251-31 -385.732783331894 1098.10868752726
712.375904195369
TCGA-BR-8380-01A-11R-2343-13 918.162211823896 680.72607638963
1598.88828821353
TCGA-VQ-A8PM-01A-21R-A414-31 269.503994538942 1642.69313262506
1912.19712716401
TCGA-VQ-AA64-01A-11R-A414-31 329.49279009003 198.649859101593

528.142649191623
TCGA-R5-A805-01A-11R-A36D-31 701.84692923091 2071.53423243264
2773.38116166355
TCGA-BR-4370-01A-01R-1157-13 1293.05717872005 1832.26311204538
3125.32029076544
TCGA-CG-4438-01A-01R-1157-13 100.875315128249 1666.52742863497
1767.40274376322
TCGA-CG-4444-01A-01R-1157-13 542.960318133823 1381.32913090424
1924.28944903807
TCGA-D7-6526-01A-11R-1802-13 -593.587045170582 177.940145904712
-415.64689926587
TCGA-BR-6707-01A-11R-1884-13 151.8505403994 1870.66329262806
2022.51383302746
TCGA-BR-4279-01A-01R-1131-13 2020.47604760657 2143.41166819453
4163.88771580109
TCGA-SW-A7EB-01A-11R-A354-31 -553.472632311299 1038.92471063307
485.452078321771
TCGA-CG-4441-01A-01R-1802-13 521.704816742852 1076.56350700942
1598.26832375227
TCGA-CG-5725-01A-11R-1602-13 -903.794847754209 -390.357770856286
-1294.15261861049
TCGA-HU-8238-01A-11R-2343-13 -75.4879896000385 1170.38704690887
1094.89905730884
TCGA-VQ-A8PP-01A-21R-A414-31 -25.796974491776 887.614026010736
861.81705151896
TCGA-D7-A74A-01A-11R-A32D-31 -1169.15681265969 616.334518260724
-552.822294398963
TCGA-BR-7959-01A-11R-2343-13 1123.42368454946 774.360802360269
1897.78448690973
TCGA-BR-8058-01A-31R-2343-13 1305.53750402072 2358.92303960813
3664.46054362885
TCGA-CD-8532-01A-11R-2343-13 426.295579617444 1978.07130158174
2404.36688119919
TCGA-BR-6564-01A-12R-1884-13 1251.79568170826 1387.11897814803
2638.91465985629
TCGA-IN-7806-01A-11R-2055-13 -783.656819716285 124.309783223792
-659.347036492493
TCGA-VQ-A91X-01A-12R-A414-31 -1826.11652230734 -532.845237466734
-2358.96175977407
TCGA-HU-A4GC-01A-12R-A251-31 -123.063268135156 589.632452862778
466.569184727622
TCGA-VQ-A923-01A-11R-A414-31 49.0449430255381 2239.13174018208
2288.17668320762
TCGA-BR-4267-01A-01R-1131-13 -119.813483399035 945.08396802035

825.270484621315
TCGA-CG-4443-01A-01R-1157-13 -254.975494726866 -211.817725222033
-466.7932199489
TCGA-BR-7704-01A-11R-2055-13 161.447243624483 2102.71436378042
2264.16160740491
TCGA-VQ-A8P3-01A-11R-A36D-31 32.4484390515273 585.278617776836
617.727056828363
TCGA-VQ-AA6D-01A-11R-A414-31 -1384.85362768514 -253.546479512718
-1638.40010719785
TCGA-BR-8384-01A-21R-2402-13 1408.70440977989 1475.78818516175
2884.49259494164
TCGA-IN-AB1V-01A-21R-A414-31 -1383.16079590249 159.661776246887
-1223.49901965561
TCGA-VQ-AA6A-01A-11R-A414-31 -1040.29054867896 -19.7070464046319
-1059.9975950836
TCGA-BR-A4CR-01A-11R-A24K-31 -1046.33853507949 -718.798880354183
-1765.13741543367
TCGA-CD-8527-01A-11R-2343-13 -412.156724165522 635.334780716633
223.178056551111
TCGA-CD-8525-01A-11R-2343-13 240.473574272587 1478.69525714214
1719.16883141472
TCGA-VQ-A91U-01A-11R-A414-31 -403.139992402585 1184.98964798021
781.84965557763
TCGA-D7-8572-01A-11R-2343-13 918.149044253133 1240.85775362025
2159.00679787338
TCGA-BR-8687-01A-11R-2402-13 -98.0434874723289 195.257721841519
97.2142343691899
TCGA-ZQ-A9CR-01A-11R-A39E-31 934.752377203382 1439.55664852114
2374.30902572452
TCGA-BR-8589-01A-11R-2402-13 -495.652191714695 2153.7399150849
1658.08772337021
TCGA-VQ-A8PU-01A-12R-A414-31 -1077.48192490639 -38.9528083649143
-1116.43473327131
TCGA-VQ-A8PD-01A-11R-A414-31 534.800593497587 2255.81642423688
2790.61701773447
TCGA-VQ-A8P5-01A-11R-A39E-31 135.237869671379 755.349628421647
890.587498093026
TCGA-D7-A747-01A-22R-A33Y-31 874.267785429349 1669.78853637035
2544.0563217997
TCGA-CD-A486-01A-11R-A24K-31 141.811792576645 835.746373435221
977.558166011866
TCGA-BR-8296-01A-11R-2343-13 142.471237373755 1889.43416977625
2031.90540715
TCGA-CD-5813-01A-11R-1602-13 1713.15749662036 2381.01281299795

4094.17030961831
TCGA-IN-8663-01A-11R-2402-13 -817.933716526274 -90.3634407573918
-908.297157283666
TCGA-KB-A6F7-01A-12R-A32D-31 -662.070293924385 1449.30974518349
787.239451259101
TCGA-HU-8249-01A-11R-A36D-31 -672.690515562559 889.604805878524
216.914290315966
TCGA-CD-A4MG-01A-11R-A251-31 490.93001395807 1183.49690117992
1674.42691513799
TCGA-HU-A4G9-01A-11R-A24K-31 -1686.75063187156 -359.157718133111
-2045.90835000467
TCGA-RD-A8MV-01A-11R-A36D-31 114.573750391259 2327.04103882608
2441.61478921734
TCGA-VQ-A925-01A-11R-A414-31 -883.589269022791 158.833296667045
-724.755972355746
TCGA-BR-A4CS-01A-11R-A24K-31 230.70050966753 855.770699177623
1086.47120884515
TCGA-VQ-A8DU-01A-11R-A36D-31 -253.22208488852 432.852218222014
179.630133333494
TCGA-IN-8462-01A-11R-2343-13 116.028440104236 729.951975495778
845.980415600013
TCGA-CD-8535-01A-11R-2343-13 -718.653983289736 -427.034575348646
-1145.68855863838
TCGA-BR-4366-01A-01R-1157-13 77.147190742398 626.802390428149
703.949581170547
TCGA-BR-6455-01A-11R-1802-13 262.02983110186 1393.05168483525
1655.08151593711
TCGA-BR-7715-01A-11R-2055-13 326.557255293805 83.1057040136369
409.662959307442
TCGA-FP-8211-01A-11R-2343-13 -834.044502030538 927.369358842621
93.3248568120827
TCGA-R5-A707-01A-11R-A33Y-31 -64.044541322564 1008.08583507973
944.041293757162
TCGA-HU-A4G3-01A-11R-A24K-31 -494.684612486707 445.340594475233
-49.3440180114741
TCGA-VQ-A94U-01A-12R-A414-31 268.855550365892 63.4425126648275
332.298063030719
TCGA-BR-6458-01A-11R-1802-13 876.87578450093 1750.94695506655
2627.82273956748
TCGA-IP-7968-01A-11R-2203-13 408.859635016867 888.040420577256
1296.90005559412
TCGA-CD-A48A-01A-12R-A36D-31 -385.018981354849 653.500971956026
268.481990601177
TCGA-F1-6875-01A-11R-2055-13 -938.082925638927 -612.772436586272

-1550.8553622252
TCGA-VQ-A91S-01A-11R-A414-31 -516.333962143943 1043.66176181891
527.327799674969
TCGA-D7-5577-01A-01R-1602-13 120.754320916062 2090.56881154597
2211.32313246203
TCGA-BR-6457-01A-21R-1802-13 1466.97428322543 1586.10652236016
3053.08080558559
TCGA-IN-A7NU-01A-22R-A354-31 191.772309511303 1350.9279720802
1542.7002815915
TCGA-F1-6177-01A-11R-1802-13 -762.523470381982 369.619148745122
-392.90432163686
TCGA-D7-6822-01A-11R-1884-13 -462.011841176937 430.403283348985
-31.6085578279516
TCGA-VQ-A91Y-01A-11R-A414-31 952.798869226074 1683.73387814613
2636.53274737221
TCGA-CG-4442-01A-01R-1157-13 -256.098032819304 436.825796384338
180.727763565034
TCGA-HU-A4GD-01A-11R-A36D-31 -1579.35550340095 -80.4405278324647
-1659.79603123342
TCGA-CG-4305-01A-01R-1157-13 839.169694937958 1934.37882687399
2773.54852181195
TCGA-IN-AB1X-01A-11R-A39E-31 -700.280318125795 1541.87113484466
841.590816718861
TCGA-BR-4191-01A-02R-1131-13 1034.39730530566 2315.46535961253
3349.86266491819
TCGA-VQ-A94R-01A-11R-A414-31 503.953661537759 746.690720485165
1250.64438202292
TCGA-D7-8579-01A-11R-2343-13 1020.9165734393 1417.33460598432
2438.25117942362
TCGA-BR-6710-01A-11R-1884-13 -326.231948331222 585.622082996117
259.390134664895
TCGA-FP-7829-01A-11R-2055-13 140.39618082529 411.944090650264
552.340271475554
TCGA-CD-5800-01A-11R-1602-13 -639.589499667237 368.583938536586
-271.005561130651
TCGA-IN-7808-01A-11R-2203-13 -6.25890758906046 2874.42237973054
2868.16347214148
TCGA-VQ-A924-01A-11R-A414-31 -114.758691446874 1030.03110589034
915.272414443469
TCGA-HU-A4H8-01A-11R-A251-31 -1286.26447956691 234.08485567725 -
1052.17962388966
TCGA-HU-A4GT-01A-21R-A251-31 -674.719595550741 518.308943195983
-156.410652354757
TCGA-D7-5578-01A-01R-1602-13 461.279640468294 1468.62408820523

1929.90372867352
TCGA-BR-8297-01A-12R-2343-13 613.797905196809 693.254889466619
1307.05279466343
