# Supplementary figures and images for: Anoikis-Related Long Non-Coding RNA Signatures to Predict Prognosis and Immune Infiltration of Gastric Cancer
Source: Bioengineering (Basel). 2024 Sep 5;11(9):893. doi: 10.3390/bioengineering11090893 (PMC11428253; doi:10.3390/bioengineering11090893)

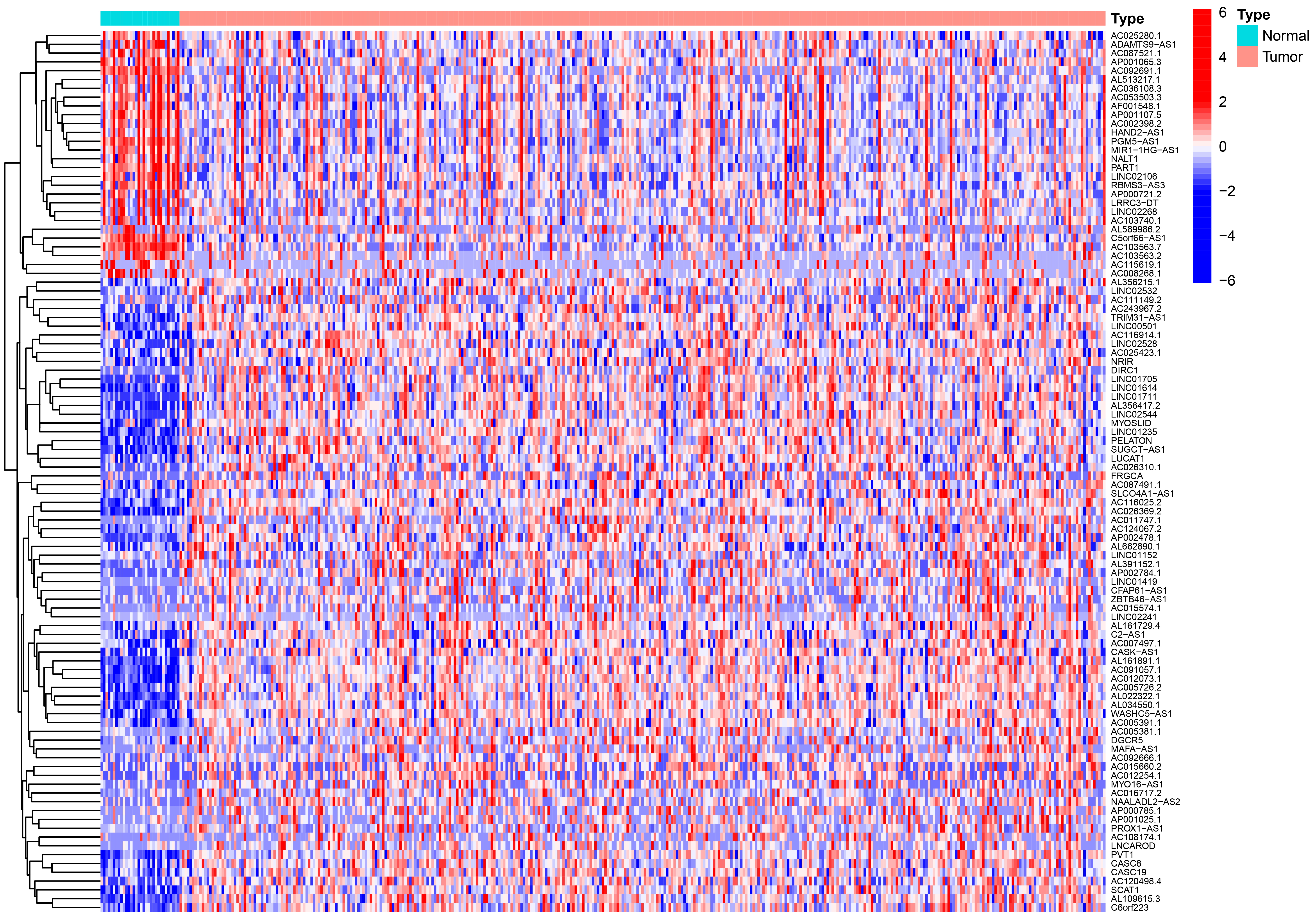

Supplement: Supplementary file 1 [file bioengineering-11-00893-s001.zip › Supplementary Figure S1.png]

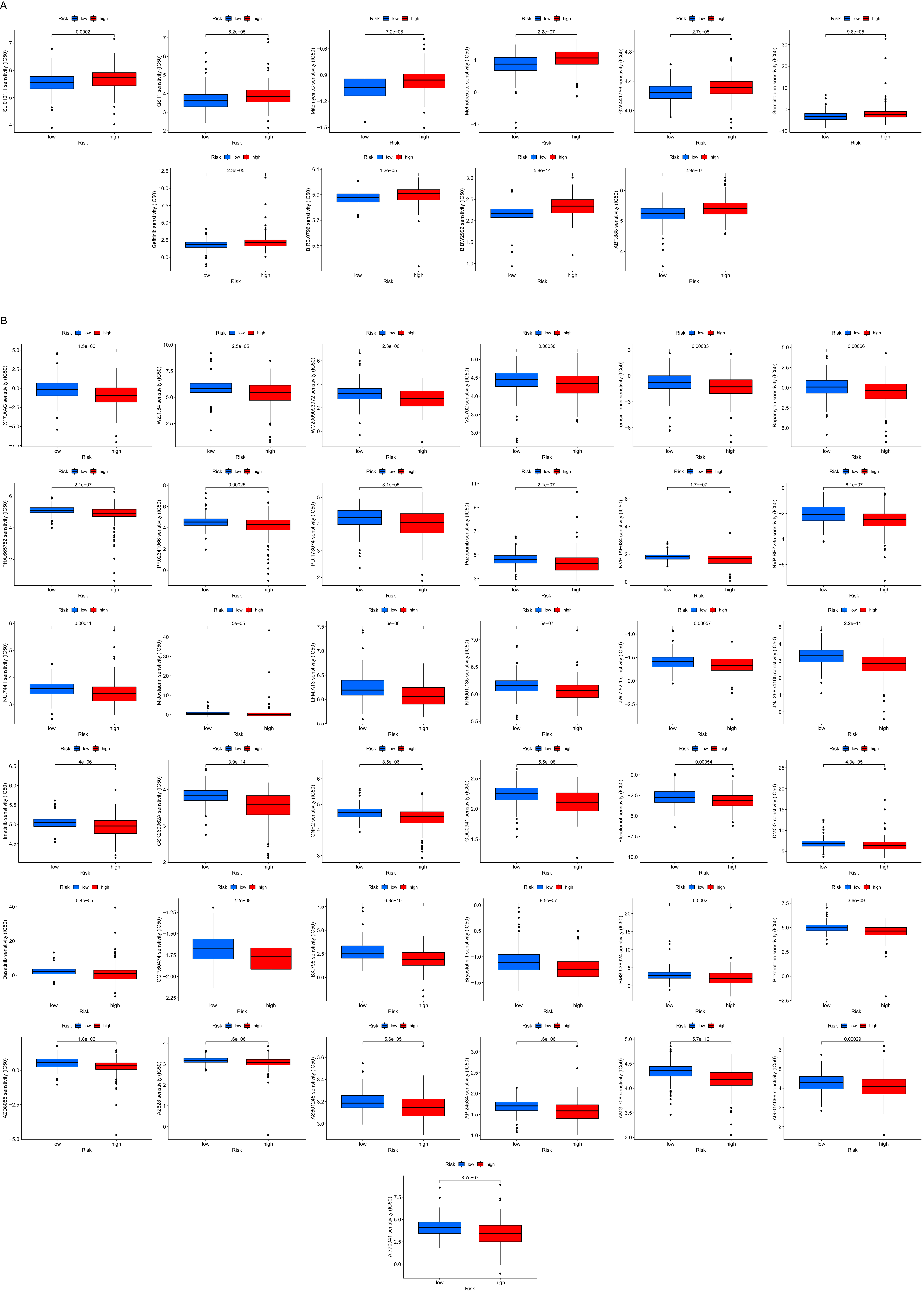

Supplement: Supplementary file 1 [file bioengineering-11-00893-s001.zip › Supplementary Figure S2.tif]

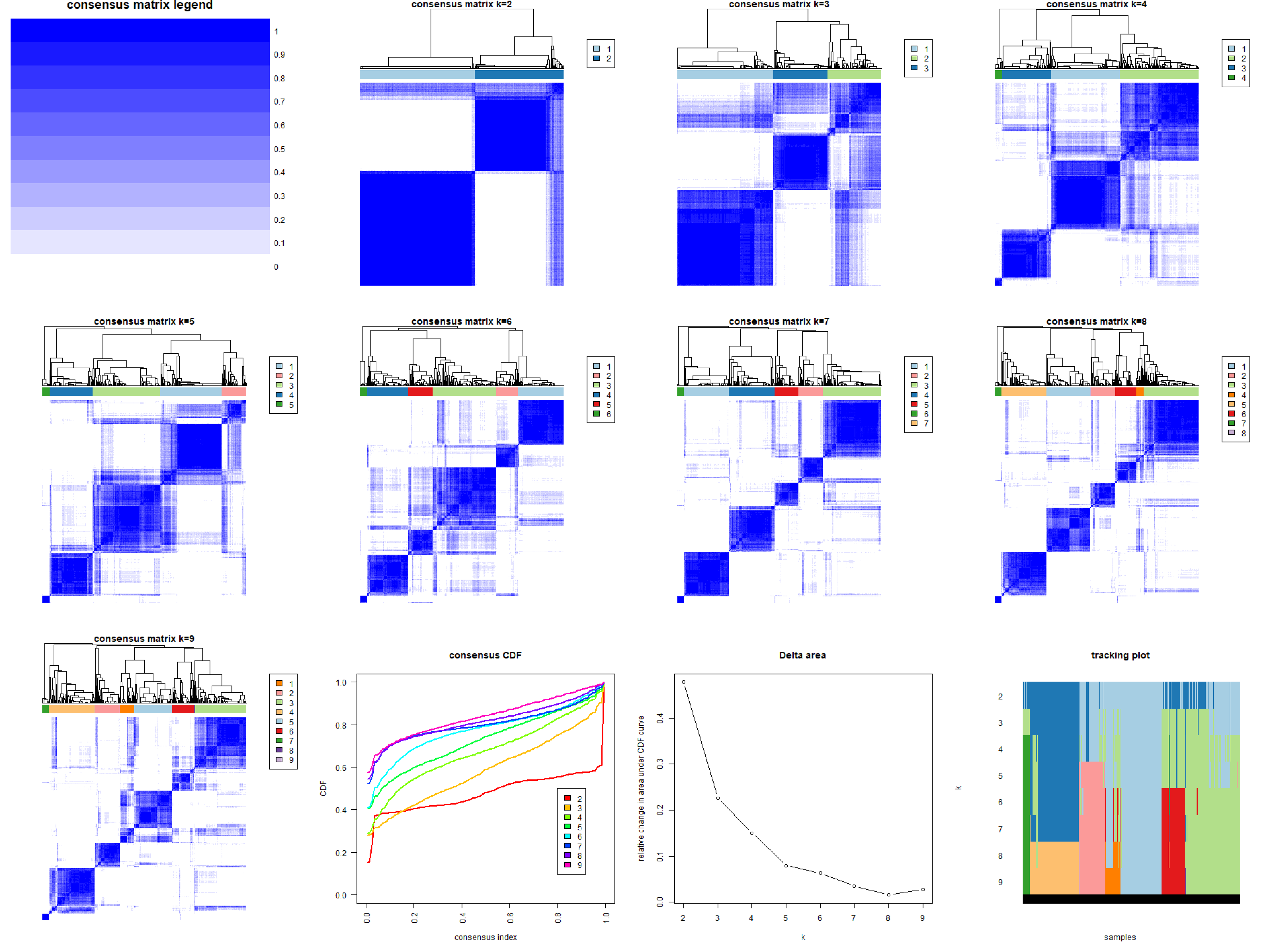

Supplement: Supplementary file 1 [file bioengineering-11-00893-s001.zip › Supplementary Figure S3.tif]

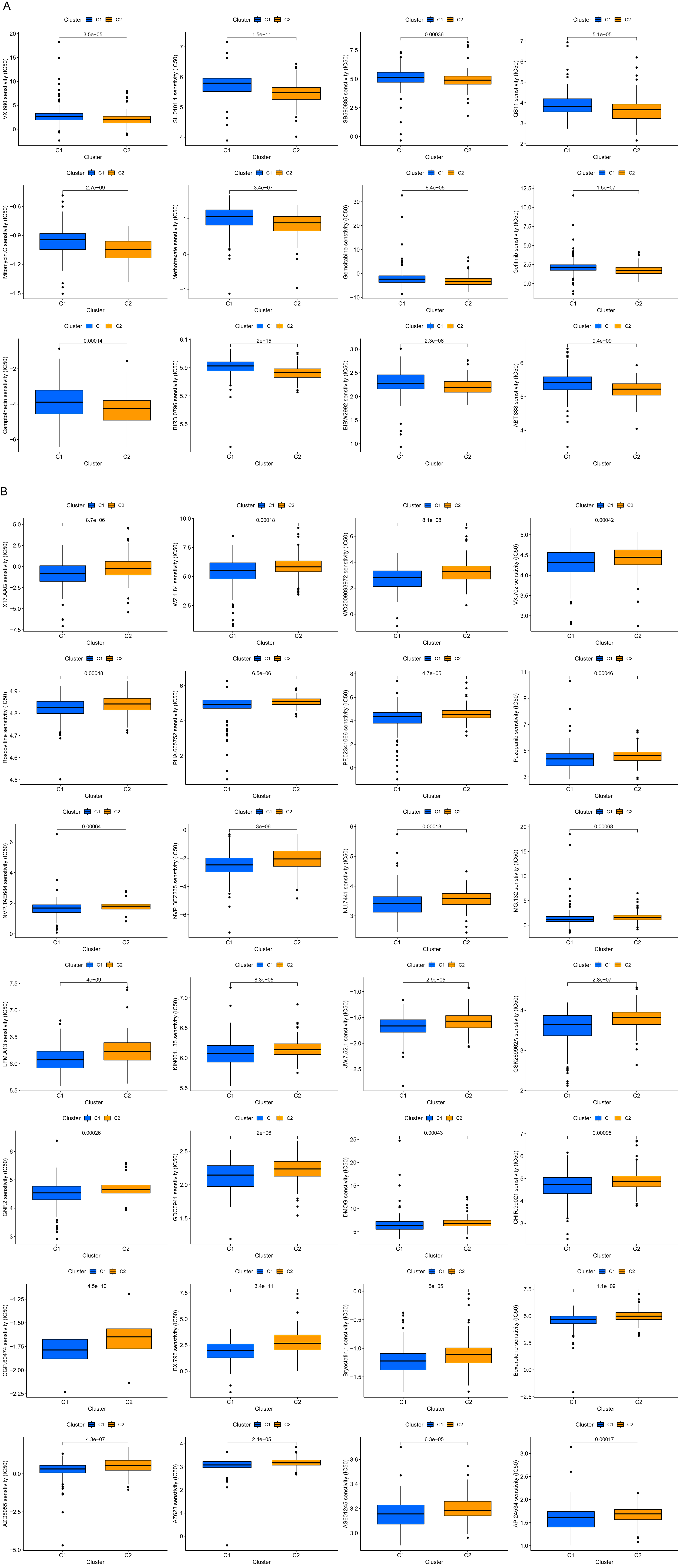

Supplement: Supplementary file 1 [file bioengineering-11-00893-s001.zip › Supplementary Figure S4.tif]
